# Supplementary material for: Bacterial death and TRADD-N domains help define novel apoptosis and immunity mechanisms shared by prokaryotes and metazoans
Source: eLife. 2021 Jun 1;10:e70394. doi: 10.7554/eLife.70394 (PMC8195603; doi:10.7554/eLife.70394)
Supplement: Figure 2—source data 1. [file elife-70394-fig2-data1.pdf]

Bacterial Death and TRADD-N domains help define novel apoptosis and immunity mechanisms shared by prokaryotes and metazoans

Figure 2-Source data 1

Gurmeet Kaur†, Lakshminarayan M. Iyer†, A. Maxwell Burroughs, L. Aravind*

Computational Biology Branch, National Center for Biotechnology Information, National Library of Medicine, National Institutes of Health, Bethesda, MD 20894, USA

†These authors contributed equally to the manuscript

Index.

1. Source data. Gene neighborhoods and domain architectures of the bacterial Death-like domain 3 (bDLD3)
2. Source data. Gene neighborhoods and domain architectures of the iSTAND2 systems
3. Source data. Gene neighborhoods and domain architectures of the TERNS3 systems
4. Source data. Gene neighborhoods and domain architectures of the bacterial NPCBM domain described in this study
5. Source data. Gene neighborhoods and domain architectures of the FGS domains described in this study
6. Source data. Gene neighborhoods and domain architectures of the bacterial and prophage/ phage SPRY domain.
7. Source data. Gene neighborhoods and domain architectures of TPR-GREAB-C-PIN systems.
8. Source data. Gene neighborhoods and domain architectures of the novel STAND1 (nSTAND1) domain.

1. Source data. Gene neighborhoods and domain architectures of the bacterial Death-like domain 3 (bDLD3)

acc	operon	architecture	len	gen.name	taxend	species	defline	gca
ADE14635.1	<-NTP_transferase<-?||?→?→||<-?||HKD→ TIR+bDLD3→ *bDLD3+nSTAND1+FGS*→	TIR+bDLD3	799	Nhal_1492	Gammaproteobacteria	Nitrosococcus halophilus Nc 4	protein of unknown function DUF323 [Nitrosococcus halophilus Nc 4].	GCA_000024725.1
ADE14636.1	*bDLD3→		246	Nhal_1493	Gammaproteobacteria	Nitrosococcus halophilus Nc 4	TIR protein [Nitrosococcus halophilus Nc 4].	GCA_000024725.1
AMV23994.1	<-HEPN<-?||?→?→ bDLD3→*?*→ *McrA-NTD+HNH2*→*?*→*||<-?||?*→*?*→*?*→ *PNPase+TRADDN+CASPASE+bDLD3*→ APATPase+TPR+TPR→	bDLD3	816	VT84_06335	Planctomycetes	Gemmata sp. SH-PL17	5'-methylthioadenosine/S-adenosylhomocysteine nucleosidase [Gemmata sp. SH-PL17].	GCA_001610855.1
AMV24002.1	*bDLD3→		518	VT84_06375	Planctomycetes	Gemmata sp. SH-PL17	hypothetical protein VT84_06375 [Gemmata sp. SH-PL17].	GCA_001610855.1
APA97850.1	<-SIG+NPCBM<-TM+TM+TM+TM||PNPase+bDLD3→ *bDLD3+NPCBM*→ TM+NPCBM→||<-LD-peptidase<-?<-HD||?→ GNTR-HTH→	PNPase+bDLD3	349	mtnN	Actinobacteria	Nocardia seriolae	Adenosylhomocysteine nucleosidase [Nocardia seriolae].	GCA_001865855.1
APA97851.1	*bDLD3→		217	NS506_03802	Actinobacteria	Nocardia seriolae	hypothetical protein NS506_03802 [Nocardia seriolae].	GCA_001865855.1
BAW07945.1	<-SIG+NPCBM||?→ PNPase+bDLD3*→ bDLD3+NPCBM→ TM+NPCBM→||<-LD-peptidase<-?<-HD||?→ GNTR-HTH→	PNPase+bDLD3	245	NSERUTF1_4813	Actinobacteria	Nocardia seriolae	conserved hypothetical protein [Nocardia seriolae].	GCA_002356035.1
BAY07541.1	Uma2→||<-?<-?||EAD9+CASPASE+bDLD3*→ APATPase+BetaPropeller→?→?→||<-?<-?||Pkinase→	EAD9+CASPASE+bDLD3	391	NIES2098_06580	Cyanobacteria	Calothrix sp. NIES-2098	hypothetical protein NIES2098_06580 [Calothrix sp. NIES-2098].	GCA_002368175.1
ETW99001.1	bDLD3+nSTAND1+FGS*→	bDLD3+nSTAND1+FGS	783	ETSY1_16630	Nitrospinae/Tectomicrobia group	Candidatus Entotheonella factor	hypothetical protein ETSY1_16630 [Candidatus Entotheonella factor].	GCA_000522425.1
ETW99008.1	bDLD3+NACHT*→	bDLD3+NACHT	326	ETSY1_16605	Nitrospinae/Tectomicrobia group	Candidatus Entotheonella factor	hypothetical protein ETSY1_16605 [Candidatus Entotheonella factor].	GCA_000522425.1
ETX07101.1	TIR→ bDLD3*→||<-FGS	bDLD3	125	ETSY2_13175	Nitrospinae/Tectomicrobia group	Candidatus Entotheonella gemina	hypothetical protein ETSY2_13175 [Candidatus Entotheonella gemina].	GCA_000522445.1
ETX07918.1	bDLD3+NACHT+FGS*→	bDLD3+NACHT+FGS	922	ETSY2_08400	Nitrospinae/Tectomicrobia group	Candidatus Entotheonella gemina	hypothetical protein ETSY2_08400 [Candidatus Entotheonella gemina].	GCA_000522445.1
GAX40011.1	SIG+CASPASE→?→||<-?||?→||<-?||?→ CASPASE+bDLD3*→ APATPase+BetaPropeller→||<-?<-?||Calcineurin→ Calcineurin→||<-BetaPropeller<-DNAJ+TPR	CASPASE+bDLD3	292	NIES4075_09730	Cyanobacteria	Tolypothrix sp. NIES-4075	hypothetical protein NIES4075_09730 [Tolypothrix sp. NIES-4075].	GCA_002218085.1
HID99200.1	Trypsin+bDLD3→ *bDLD3+iSTAND*→ MoxR-AAA→	Trypsin+bDLD3	390	EYP59_02780	Gammaproteobacteria	Thiotrichaceae bacterium	TPA: hypothetical protein EYP59_02780 [Thiotrichaceae bacterium].	-
HID99201.1	*bDLD3→		317	EYP59_02785	Gammaproteobacteria	Thiotrichaceae bacterium	TPA: serine protease [Thiotrichaceae bacterium].	-
HIE01287.1	bDLD3→ *bDLD3+ClpABC*→	bDLD3	679	EYP59_13525	Gammaproteobacteria	Thiotrichaceae bacterium	TPA: AAA family ATPase, partial [Thiotrichaceae bacterium].	-
HIE01288.1	*bDLD3→		232	EYP59_13530	Gammaproteobacteria	Thiotrichaceae bacterium	TPA: hypothetical protein EYP59_13530 [Thiotrichaceae bacterium].	-
NER21298.1	<-HAD||?→ PNPase+bDLD3*→	PNPase+bDLD3	346	F6J96_11435	Cyanobacteria	Symploca sp. SIO1C2	5'-methylthioadenosine/S-adenosylhomocysteine nucleosidase [Symploca sp. SIO1C2].	-
NES00173.1	PNPase+bDLD3→ bDLD3*→	bDLD3	58	F6J86_41320	Cyanobacteria	Symploca sp. SIO1B1	hypothetical protein F6J86_41320 [Symploca sp. SIO1B1].	-
NES19675.1	bDLD3→ *bDLD3+APATPase+TPR*→	bDLD3	911	F6K41_12275	Cyanobacteria	Symploca sp. SIO3E6	hypothetical protein F6K41_12275 [Symploca sp. SIO3E6].	-
NES19676.1	bDLD3*→	bDLD3	72	F6K41_12280	Cyanobacteria	Symploca sp. SIO3E6	hypothetical protein F6K41_12280, partial [Symploca sp. SIO3E6].	-

<table>
<tr><th>acc</th><th>operon</th><th>architecture</th><th>len</th><th>gen.name</th><th>taxend</th><th>species</th><th>defline</th><th>gca</th></tr>
<tr><td>NET43317.1</td><td>Trypco2→ TCAD4+CASPASE+bDLD3→ SIG+bDLD3+APATPase→||<-?||?→||<-?<-?<-?<-?<-Pkinase</td><td>TCAD4+CASPASE+bDLD3</td><td>537</td><td>F6K15_16045</td><td>Cyanobacteria</td><td>Okeania sp. SIO2B3</td><td>CHAT domain-containing protein [Okeania sp. SIO2B3].</td><td>-</td></tr>
<tr><td>NET43318.1</td><td>SIG+bDLD3+APATPase*→</td><td>SIG+bDLD3+APATPase</td><td>241</td><td>F6K15_16050</td><td>Cyanobacteria</td><td>Okeania sp. SIO2B3</td><td>hypothetical protein F6K15_16050 [Okeania sp. SIO2B3].</td><td>-</td></tr>
<tr><td>NET60851.1</td><td>PNPase+bDLD3→ bDLD3+FGS→||<-HAD</td><td>PNPase+bDLD3</td><td>361</td><td>F6K47_33365</td><td>Cyanobacteria</td><td>Symploca sp. SIO2E6</td><td>5'-methylthioadenosine/S-adenosylhomocysteine nucleosidase [Symploca sp. SIO2E6].</td><td>-</td></tr>
<tr><td>NET60852.1</td><td>bDLD3+FGS*→</td><td>bDLD3+FGS</td><td>414</td><td>F6K47_33370</td><td>Cyanobacteria</td><td>Symploca sp. SIO2E6</td><td>formylglycine-generating enzyme family protein [Symploca sp. SIO2E6].</td><td>-</td></tr>
<tr><td>NJL79331.1</td><td>PNPase+bDLD3*→ TM+TM+TM+TM→||<-PSE<-PSE<-PsaD</td><td>PNPase+bDLD3</td><td>392</td><td>HC917_11650</td><td>Cyanobacteria</td><td>Richelia sp. SM2_1_7</td><td>5'-methylthioadenosine/S-adenosylhomocysteine nucleosidase [Richelia sp. SM2_1_7].</td><td>-</td></tr>
<tr><td>NJM20757.1</td><td>SIG+Band_7→||<-?||?→?→?→?→ PNPase+bDLD3*→ TM+TM+TM+TM→||<-PSE<-?<-PsaD</td><td>PNPase+bDLD3</td><td>687</td><td>HC907_19595</td><td>Cyanobacteria</td><td>Richelia sp. SM1_7_0</td><td>5'-methylthioadenosine/S-adenosylhomocysteine nucleosidase [Richelia sp. SM1_7_0].</td><td>-</td></tr>
<tr><td>NJN10092.1</td><td><-PP2C+TM||?→ SIG+Band_7→||<-?||?→?→?→ PNPase+bDLD3*→ TM+TM+TM+TM→||<-PSE<-?<-PsaD</td><td>PNPase+bDLD3</td><td>687</td><td>HC815_19700</td><td>Cyanobacteria</td><td>Richelia sp. RM1_1_1</td><td>5'-methylthioadenosine/S-adenosylhomocysteine nucleosidase [Richelia sp. RM1_1_1].</td><td>-</td></tr>
<tr><td>NJO29889.1</td><td>SIG+Band_7→||<-?||?→?→?→?→?→ PNPase+bDLD3*→ TM+TM+TM+TM→||<-PSE<-PSE<-PsaD</td><td>PNPase+bDLD3</td><td>560</td><td>HC874_21920</td><td>Cyanobacteria</td><td>Richelia sp. SL_2_1</td><td>5'-methylthioadenosine/S-adenosylhomocysteine nucleosidase [Richelia sp. SL_2_1].</td><td>-</td></tr>
<tr><td>NJS15985.1</td><td>PNPase+bDLD3*→</td><td>PNPase+bDLD3</td><td>359</td><td>HC787_01195</td><td>Cyanobacteria</td><td>Nostocaceae cyanobacterium CSU_2_110</td><td>5'-methylthioadenosine/S-adenosylhomocysteine nucleosidase [Nostocaceae cyanobacterium CSU_2_110].</td><td>-</td></tr>
<tr><td>OUL27313.1</td><td><-TPR+nSTAND1+BetaPropeller<-AAA_22||EAD9+CASPASE+bDLD3*→ PSE→ APATPase+BetaPropeller→ CASPASE→?→||<-CASPASE+GUN4<-CASPASE</td><td>EAD9+CASPASE+bDLD3</td><td>391</td><td>BV378_11875</td><td>Cyanobacteria</td><td>Nostoc sp. RF31YmG</td><td>hypothetical protein BV378_11875 [Nostoc sp. RF31Y].</td><td>GCA_002155185.1</td></tr>
<tr><td>OUL31312.1</td><td>Uma2→||<-?<-?<-?<-?<-TPR+nSTAND1+BetaPropeller<-AAA_22||EAD9+CASPASE+bDLD3*→ APATPase+BetaPropeller→</td><td>EAD9+CASPASE+bDLD3</td><td>398</td><td>BV372_20335</td><td>Cyanobacteria</td><td>Nostoc sp. T09</td><td>hypothetical protein BV372_20335 [Nostoc sp. T09].</td><td>GCA_002154695.1</td></tr>
<tr><td>OUL35760.1</td><td>EAD9+CASPASE+bDLD3*→ PSE→ APATPase+BetaPropeller→||<-?||?→ RelE-ParE→?→?→ RelE-ParE→</td><td>EAD9+CASPASE+bDLD3</td><td>391</td><td>BV375_01415</td><td>Cyanobacteria</td><td>Nostoc sp. 106C</td><td>hypothetical protein BV375_01415 [Nostoc sp. 106C].</td><td>GCA_002154725.1</td></tr>
<tr><td>PKW31742.1</td><td>Pkinase+bDLD3*→||<-TPR+TPR+Caspase<-vWA-L+TM+CASPASE<-VWA<-MoxR-AAA<-bDLD3+iSTAND2<-?||TCAD9+Pkinase+bDLD3→</td><td>Pkinase+bDLD3</td><td>501</td><td>CLT72_1056</td><td>Actinobacteria</td><td>Micromonospora sp. CNZ309</td><td>hypothetical protein CLT72_1056 [Micromonospora sp. CNZ309].</td><td>GCA_002846525.1</td></tr>
<tr><td>RKZ54270.1</td><td>CASPASE+APATPase+bDLD3→ bDLD3+EAD2+Pkinase+TM+TM+TM+TM+TM+TM+TM→</td><td>CASPASE+APATPase+bDLD3</td><td>663</td><td>DRR00_01560</td><td>Gammaproteobacteria</td><td>Gammaproteobacteria bacterium</td><td>hypothetical protein DRR00_01560 [Gammaproteobacteria bacterium].</td><td>GCA_003645185.1</td></tr>
<tr><td>RKZ54271.1</td><td>bDLD3+EAD2+Pkinase+TM+TM+TM+TM+TM+TM+TM*→</td><td>bDLD3+EAD2+Pkinase+TM+TM+TM+TM+TM+TM+TM</td><td>935</td><td>DRR00_01565</td><td>Gammaproteobacteria</td><td>Gammaproteobacteria bacterium</td><td>hypothetical protein DRR00_01565 [Gammaproteobacteria bacterium].</td><td>GCA_003645185.1</td></tr>
<tr><td>RYZ09240.1</td><td>EAD5+CASPASE+bDLD3*→</td><td>EAD5+CASPASE+bDLD3</td><td>775</td><td>EOO73_05625</td><td>Deltaproteobacteria</td><td>Myxococcales bacterium</td><td>caspase family protein [Myxococcales bacterium].</td><td>GCA_004193285.1</td></tr>
<tr><td>RZU04509.1</td><td>Pkinase+bDLD3*→ bDLD3+TPR→?→?→||<-?||?→||<-?<-BACTERIALFRINGE+TM+TM</td><td>Pkinase+bDLD3</td><td>594</td><td>EV371_0866</td><td>Actinobacteria</td><td>Plantactinospora sp. CNZ321</td><td>hypothetical protein EV371_0866 [Plantactinospora sp. CNZ321].</td><td>GCA_004217235.1</td></tr>
<tr><td>SFL94903.1</td><td><-SIG+HAD<-?<-KAP-NTPase||TIR+bDLD3*→ bDLD3+nSTAND1+FGS→||<-?<-NACHT+FGS<-ABhydrolase+bDLD3<-?<-?||LexA-protease→</td><td>TIR+bDLD3</td><td>254</td><td>SAMN05421863_1007111</td><td>Betaproteobacteria</td><td>Nitrosomonas communis</td><td>TIR domain-containing protein [Nitrosomonas communis].</td><td>GCA_900114745.1</td></tr>
<tr><td>SPT51846.1</td><td>SIG+TM+TM+NACHT+APATPase+TPR+TPR+TPR→||<-?<-?||?→?→?→?→ SIG+bDLD3*→</td><td>SIG+bDLD3</td><td>301</td><td>NCTC11373_02572</td><td>Actinobacteria</td><td>Actinomadura madurae</td><td>Uncharacterised protein [Actinomadura madurae].</td><td>GCA_900445005.1</td></tr>
<tr><td>TVR58388.1</td><td>TIR+bDLD3→ bDLD3+nSTAND1+FGS→?→||<-NACHT+FGS</td><td>TIR+bDLD3</td><td>802</td><td>EA420_17215</td><td>Gammaproteobacteria</td><td>Candidatus Competibacteraceae bacterium</td><td>hypothetical protein EA420_17215 [Candidatus Competibacteraceae bacterium].</td><td>GCA_007695245.1</td></tr>
<tr><td>TVR58389.1</td><td>TIR+bDLD3*→</td><td>TIR+bDLD3</td><td>268</td><td>EA420_17220</td><td>Gammaproteobacteria</td><td>Candidatus Competibacteraceae bacterium</td><td>toll/interleukin-1 receptor domain-containing protein [Candidatus Competibacteraceae bacterium].</td><td>GCA_007695245.1</td></tr>
<tr><td>WP_009738298.1</td><td>bDLD3+NACHT+BetaPropeller→ TPR+TPR+Caspase→||<-Pkinase+bDLD3</td><td>bDLD3+NACHT+BetaPropeller</td><td>1268</td><td>FRAQA3DRAFT_RS06645</td><td>Actinobacteria</td><td>Frankia sp. QA3</td><td>AAA family ATPase [Frankia sp. QA3].</td><td>GCF_000262465.1</td></tr>
<tr><td>WP_009738300.1</td><td>Pkinase+bDLD3*→</td><td>Pkinase+bDLD3</td><td>513</td><td>FRAQA3DRAFT_RS06655</td><td>Actinobacteria</td><td>Frankia sp. QA3</td><td>hypothetical protein [Frankia sp. QA3].</td><td>GCF_000262465.1</td></tr>
</table>

<table>
<tr><th>acc</th><th>operon</th><th>architecture</th><th>len</th><th>gen.name</th><th>taxend</th><th>species</th><th>defline</th><th>gca</th></tr>
<tr><td>WP_030438849.1</td><td>PNPase+bDLD3→ bDLD3+TM→||<-?<-?<-?<-?<-ACYC</td><td>PNPase+bDLD3</td><td>254</td><td>IH26_RS0125970</td><td>Actinobacteria</td><td>Actinoplanes subtropicus</td><td>hypothetical protein [Actinoplanes subtropicus].</td><td>GCF_000721705.1</td></tr>
<tr><td>WP_033090725.1</td><td><-SIG+NPCBM<-TM+TM+TM+TM||?→?→ PNPase+bDLD3→ bDLD3+NPCBM→ TM+NPCBM→||<-LD-peptidase<-?<-HD||?→ GNTR-HTH→</td><td>PNPase+bDLD3</td><td>279</td><td>NSERUTF1_RS23480</td><td>Actinobacteria</td><td>Nocardia seriolae</td><td>hypothetical protein [Nocardia seriolae].</td><td>GCF_002356035.1</td></tr>
<tr><td>WP_038072636.1</td><td>PNPase+bDLD3*→ TM+TM+TM+TM→ PSE→||<-?<-?<-PsaD</td><td>PNPase+bDLD3</td><td>672</td><td>HC643_RS13555</td><td>Cyanobacteria</td><td>Tolypothrix bouteillei</td><td>hypothetical protein [Tolypothrix bouteillei].</td><td>GCF_000760695.4</td></tr>
<tr><td>WP_044290893.1</td><td><-TCAD10<-?<-?||?→||<-?||?→ bDLD3+APATPase*→ APATPase+BetaPropeller→</td><td>bDLD3+APATPase</td><td>327</td><td>RIV7116_RS11765</td><td>Cyanobacteria</td><td>Rivularia sp. PCC 7116</td><td>hypothetical protein [Rivularia sp. PCC 7116].</td><td>GCF_000316665.1</td></tr>
<tr><td>WP_051808508.1</td><td>PNPase+bDLD3*→</td><td>PNPase+bDLD3</td><td>394</td><td>IH26_RS0125965</td><td>Actinobacteria</td><td>Actinoplanes subtropicus</td><td>hypothetical protein [Actinoplanes subtropicus].</td><td>GCF_000721705.1</td></tr>
<tr><td>WP_051809451.1</td><td>PNPase+bDLD3*→</td><td>PNPase+bDLD3</td><td>413</td><td>IH26_RS0141600</td><td>Actinobacteria</td><td>Actinoplanes subtropicus</td><td>hypothetical protein [Actinoplanes subtropicus].</td><td>GCF_000721705.1</td></tr>
<tr><td>WP_052086955.1</td><td>bDLD3+NPCBM*→</td><td>bDLD3+NPCBM</td><td>241</td><td>NSERUTF1_RS23475</td><td>Actinobacteria</td><td>Nocardia seriolae</td><td>NPCBM/NEW2 domain-containing protein [Nocardia seriolae].</td><td>GCF_002356035.1</td></tr>
<tr><td>WP_063628695.1</td><td>CASPASE+bDLD3*→ APATPase+BetaPropeller→||<-?||?→ Calcineurin→?→ Calcineurin→</td><td>CASPASE+bDLD3</td><td>292</td><td>TOL9009_RS39910</td><td>Cyanobacteria</td><td>[Scytonema hofmanni] UTEX B 1581</td><td>CHAT domain-containing protein [[Scytonema hofmanni] UTEX B 1581].</td><td>GCF_000582685.1</td></tr>
<tr><td>WP_074904183.1</td><td><-LexA-protease||?→?→ ABhydrolase+bDLD3→ NACHT+FGS→||<-bDLD3+nSTAND1+FGS<-TIR+bDLD3*</td><td>ABhydrolase+bDLD3</td><td>363</td><td>BM122_RS04970</td><td>Betaproteobacteria</td><td>Nitrosomonas communis</td><td>hypothetical protein [Nitrosomonas communis].</td><td>GCF_900114745.1</td></tr>
<tr><td>WP_074904186.1</td><td>SIG+bDLD3+nSTAND1+FGS*→</td><td>SIG+bDLD3+nSTAND1+FGS</td><td>823</td><td>BM122_RS04980</td><td>Betaproteobacteria</td><td>Nitrosomonas communis</td><td>SUMF1/EgtB/PvdO family nonheme iron enzyme [Nitrosomonas communis].</td><td>GCF_900114745.1</td></tr>
<tr><td>WP_074904188.1</td><td>TIR+bDLD3*→</td><td>TIR+bDLD3</td><td>277</td><td>BM122_RS04985</td><td>Betaproteobacteria</td><td>Nitrosomonas communis</td><td>TIR domain-containing protein [Nitrosomonas communis].</td><td>GCF_900114745.1</td></tr>
<tr><td>WP_081986291.1</td><td>PNPase+bDLD3*→ bDLD3+NPCBM→ TM+NPCBM→||<-LD-peptidase<-?<-HD||?→ GNTR-HTH→</td><td>PNPase+bDLD3</td><td>285</td><td>GL308_RS29360</td><td>Actinobacteria</td><td>Nocardia seriolae</td><td>hypothetical protein [Nocardia seriolae].</td><td>GCF_003667075.1</td></tr>
<tr><td>WP_082062736.1</td><td><-SIG+NPCBM<-TM+TM+TM+TM||PNPase+bDLD3*→ bDLD3+NPCBM→ TM+NPCBM→||<-PSE<-?<-HD||?→ GNTR-HTH→</td><td>PNPase+bDLD3</td><td>397</td><td>AOQ36_RS28625</td><td>Actinobacteria</td><td>Nocardia seriolae</td><td>5'-methylthioadenosine/S-adenosylhomocysteine nucleosidase [Nocardia seriolae].</td><td>GCF_000583715.2</td></tr>
<tr><td>WP_086688304.1</td><td>Uma2→||<-?<-?<-?<-?<-TPR+nSTAND1+BetaPropeller<-AAA_22||CASPASE+bDLD3*→</td><td>CASPASE+bDLD3</td><td>288</td><td>BV372_RS20170</td><td>Cyanobacteria</td><td>Nostoc sp. T09</td><td>CHAT domain-containing protein [Nostoc sp. T09].</td><td>GCF_002154695.1</td></tr>
<tr><td>WP_086756477.1</td><td>CASPASE+bDLD3*→ APATPase+BetaPropeller→||<-?||?→ RelE-ParE→?→?→ RelE-ParE→</td><td>CASPASE+bDLD3</td><td>303</td><td>BV375_RS01380</td><td>Cyanobacteria</td><td>Nostoc sp. 106C</td><td>CHAT domain-containing protein [Nostoc sp. 106C].</td><td>GCF_002154725.1</td></tr>
<tr><td>WP_086834185.1</td><td><-TPR+nSTAND1+BetaPropeller<-AAA_22||CASPASE+bDLD3*→ APATPase+BetaPropeller→ CASPASE→?→||<-CASPASE+GUN4<-CASPASE<-?<-CASPASE</td><td>CASPASE+bDLD3</td><td>325</td><td>BV378_RS11765</td><td>Cyanobacteria</td><td>Nostoc sp. RF31YmG</td><td>CHAT domain-containing protein [Nostoc sp. RF31YmG].</td><td>GCF_002155185.1</td></tr>
<tr><td>WP_089718394.1</td><td>TIR+bDLD3→ bDLD3+nSTAND1+FGS→</td><td>TIR+bDLD3</td><td>798</td><td>FLV43_RS07510</td><td>Nitrospinae/Tectomicrobia group</td><td>Candidatus Entotheonella palauensis</td><td>SUMF1/EgtB/PvdO family nonheme iron enzyme [Candidatus Entotheonella palauensis].</td><td>GCF_900079105.1</td></tr>
<tr><td>WP_089718395.1</td><td>TIR+bDLD3*→</td><td>TIR+bDLD3</td><td>256</td><td>FLV43_RS07515</td><td>Nitrospinae/Tectomicrobia group</td><td>Candidatus Entotheonella palauensis</td><td>TIR domain-containing protein [Candidatus Entotheonella palauensis].</td><td>GCF_900079105.1</td></tr>
<tr><td>WP_089941919.1</td><td>TIR+bDLD3*→||<-NACHT+FGS<-FxSC</td><td>TIR+bDLD3</td><td>239</td><td>FLV42_RS28560</td><td>Nitrospinae/Tectomicrobia group</td><td>Candidatus Entotheonella palauensis</td><td>toll/interleukin-1 receptor domain-containing protein [Candidatus Entotheonella palauensis].</td><td>GCF_900079095.1</td></tr>
<tr><td>WP_091254464.1</td><td>NUDIX→?→?→ PNPase+bDLD3*→</td><td>PNPase+bDLD3</td><td>438</td><td>GA0070216_RS32615</td><td>Actinobacteria</td><td>Micromonospora matsumotoense</td><td>hypothetical protein [Micromonospora matsumotoense].</td><td>GCF_900091525.1</td></tr>
<tr><td>WP_096595110.1</td><td>Uma2→||<-?<-?||CASPASE+bDLD3*→ APATPase+BetaPropeller→</td><td>CASPASE+bDLD3</td><td>288</td><td>CA733_RS03310</td><td>Cyanobacteria</td><td>Calothrix sp. NIES-2098</td><td>CHAT domain-containing protein [Calothrix sp. NIES-2098].</td><td>GCF_002368175.1</td></tr>
<tr><td>WP_096656445.1</td><td><-PP2C+TM||?→ SIG+Band_7→||<-?||?→?→?→ PNPase+bDLD3*→ TM+TM+TM+TM→?→||<-PsaD</td><td>PNPase+bDLD3</td><td>687</td><td>CA729_RS14610</td><td>Cyanobacteria</td><td>Calothrix parasitica</td><td>5'-methylthioadenosine/S-adenosylhomocysteine nucleosidase [Calothrix parasitica].</td><td>GCF_002368095.1</td></tr>
<tr><td>WP_096691054.1</td><td>PNPase+bDLD3*→?→||<-?<-PsaD</td><td>PNPase+bDLD3</td><td>679</td><td>CDC41_RS35135</td><td>Cyanobacteria</td><td>unclassified Calothrix</td><td>MULTISPECIES: hypothetical protein [unclassified Calothrix].</td><td>GCF_002368455.1</td></tr>
<tr><td>WP_102152967.1</td><td>SIG+CASPASE+bDLD3+APATPase+BetaPropeller*→</td><td>SIG+CASPASE+bDLD3+APATPase+BetaPropeller</td><td>955</td><td>CEN39_RS27445</td><td>Cyanobacteria</td><td>Fischerella thermalis</td><td>hypothetical protein [Fischerella thermalis].</td><td>GCF_002870785.1</td></tr>
<tr><td>WP_128137951.1</td><td>bDLD3→?→||<-?<-bDLD3</td><td>bDLD3</td><td>246</td><td>DRA43_RS16270</td><td>Actinobacteria</td><td>Micromonospora chalcea</td><td>hypothetical protein [Micromonospora chalcea].</td><td>GCF_003298855.1</td></tr>
</table>

<table>
<tr><th>acc</th><th>operon</th><th>architecture</th><th>len</th><th>gen.name</th><th>taxend</th><th>species</th><th>defline</th><th>gca</th></tr>
<tr><td>WP_128137954.1</td><td>bDLD3*→</td><td>bDLD3</td><td>161</td><td>DRA43_RS16285</td><td>Actinobacteria</td><td>Micromonospora chalcea</td><td>hypothetical protein [Micromonospora chalcea].</td><td>GCF_003298855.1</td></tr>
<tr><td>WP_130460897.1</td><td>Pkinase+bDLD3→ bDLD3+TPR→?→?→?→||<-?<-BACTERIALFRINGE+TM+TM</td><td>Pkinase+bDLD3</td><td>509</td><td>EV371_RS04300</td><td>Actinobacteria</td><td>Plantactinospora sp. CNZ321</td><td>hypothetical protein [Plantactinospora sp. CNZ321].</td><td>GCF_004217235.1</td></tr>
<tr><td>WP_130460898.1</td><td>bDLD3+TPR*→</td><td>bDLD3+TPR</td><td>351</td><td>EV371_RS04305</td><td>Actinobacteria</td><td>Plantactinospora sp. CNZ321</td><td>tetratricopeptide repeat protein [Plantactinospora sp. CNZ321].</td><td>GCF_004217235.1</td></tr>
<tr><td>WP_130472516.1</td><td>PNPase+bDLD3→ bDLD3→</td><td>PNPase+bDLD3</td><td>374</td><td>EJP87_RS14170</td><td>Alphaproteobacteria</td><td>Candidatus Magnetaquicoccus inordinatus</td><td>hypothetical protein [Candidatus Magnetaquicoccus inordinatus].</td><td>GCF_004217665.1</td></tr>
<tr><td>WP_130472517.1</td><td>bDLD3*→</td><td>bDLD3</td><td>703</td><td>EJP87_RS14175</td><td>Alphaproteobacteria</td><td>Candidatus Magnetaquicoccus inordinatus</td><td>hypothetical protein [Candidatus Magnetaquicoccus inordinatus].</td><td>GCF_004217665.1</td></tr>
<tr><td>WP_143309823.1</td><td>TIR→ TIR+bDLD3*→</td><td>TIR+bDLD3</td><td>180</td><td>FLV42_RS32380</td><td>Nitrospinae/Tectomicrobia group</td><td>Candidatus Entotheonella palauensis</td><td>hypothetical protein [Candidatus Entotheonella palauensis].</td><td>GCF_900079095.1</td></tr>
<tr><td>WP_144082196.1</td><td>Pkinase+bDLD3→//<-TPR+TPR+Caspase<-vWA-L+TM+CASPASE<-VWA<-MoxR-AAA<-bDLD3+iSTAND2<- ?||TCAD9+Pkinase+bDLD3*→||<-?||?→||<-?||?→ GNTR-HTH→</td><td>Pkinase+bDLD3</td><td>334</td><td>CLT72_RS05190</td><td>Actinobacteria</td><td>Micromonospora sp. CNZ309</td><td>hypothetical protein [Micromonospora sp. CNZ309].</td><td>GCF_002846525.1</td></tr>
<tr><td>WP_144082198.1</td><td>TCAD9+Pkinase+bDLD3*→</td><td>TCAD9+Pkinase+bDLD3</td><td>532</td><td>CLT72_RS05200</td><td>Actinobacteria</td><td>Micromonospora sp. CNZ309</td><td>hypothetical protein [Micromonospora sp. CNZ309].</td><td>GCF_002846525.1</td></tr>
<tr><td>WP_146877386.1</td><td>APATPase+TPR+TPR+TPR→||<-?||?→?→?→ Pkinase+bDLD3→ SIG+bDLD3+MgtE_N→</td><td>Pkinase+bDLD3</td><td>326</td><td>DTB52_RS46895</td><td>Actinobacteria</td><td>Actinomadura madurae</td><td>hypothetical protein [Actinomadura madurae].</td><td>GCF_900445005.1</td></tr>
<tr><td>WP_146877388.1</td><td>SIG+bDLD3+MgtE_N*→</td><td>SIG+bDLD3+MgtE_N</td><td>535</td><td>DTB52_RS46900</td><td>Actinobacteria</td><td>Actinomadura madurae</td><td>hypothetical protein [Actinomadura madurae].</td><td>GCF_900445005.1</td></tr>
<tr><td>WP_147207373.1</td><td>PNPase+bDLD3*→ bDLD3+NPCBM→ TM+NPCBM→||<-LD-peptidase<-?<-HD||?→ GNTR-HTH→</td><td>PNPase+bDLD3</td><td>281</td><td>NS2_RS30205</td><td>Actinobacteria</td><td>Nocardia seriolae</td><td>5’-methylthioadenosine/S-adenosylhomocysteine nucleosidase, partial [Nocardia seriolae].</td><td>GCF_007990715.1</td></tr>
<tr><td>WP_154788099.1</td><td><-TM+TM+TM+TM||PNPase+bDLD3→ bDLD3+APATPase→ TPR→ TPR→</td><td>PNPase+bDLD3</td><td>350</td><td>GLP40_RS12945</td><td>Actinobacteria</td><td>Nocardia sp. CT2-14</td><td>hypothetical protein [Nocardia sp. CT2-14].</td><td>GCF_009708175.1</td></tr>
<tr><td>WP_154788100.1</td><td>bDLD3+APATPase*→</td><td>bDLD3+APATPase</td><td>500</td><td>GLP40_RS12950</td><td>Actinobacteria</td><td>Nocardia sp. CT2-14</td><td>hypothetical protein [Nocardia sp. CT2-14].</td><td>GCF_009708175.1</td></tr>
<tr><td>WP_157437393.1</td><td>PNPase+bDLD3*→</td><td>PNPase+bDLD3</td><td>405</td><td>-</td><td>Actinobacteria</td><td>Actinoplanes subtropicus</td><td>hypothetical protein [Actinoplanes subtropicus].</td><td>-</td></tr>
<tr><td>WP_158241970.1</td><td>SIG+bDLD3*→</td><td>SIG+bDLD3</td><td>379</td><td>CLT72_RS05165</td><td>Actinobacteria</td><td>Micromonospora sp. CNZ309</td><td>hypothetical protein [Micromonospora sp. CNZ309].</td><td>GCF_002846525.1</td></tr>
<tr><td>WP_158660795.1</td><td><-SIG+NPCBM<-TM+TM+TM+TM||PNPase+bDLD3→ bDLD3+NPCBM*→ TM+NPCBM→||<-LD-peptidase<-?<-HD||?→ GNTR-HTH→</td><td>bDLD3+NPCBM</td><td>227</td><td>NS506_RS18405</td><td>Actinobacteria</td><td>Nocardia seriolae</td><td>NPCBM/NEW2 domain-containing protein [Nocardia seriolae].</td><td>GCF_001865855.1</td></tr>
<tr><td>WP_167727085.1</td><td>CASPASE+bDLD3*→ APATPase+BetaPropeller→</td><td>CASPASE+bDLD3</td><td>290</td><td>HCG51_RS32855</td><td>Cyanobacteria</td><td>Tolypothrix sp. PCC 7910</td><td>CHAT domain-containing protein [Tolypothrix sp. PCC 7910].</td><td>GCF_011769525.1</td></tr>
</table>

2. Source data. Gene neighborhoods and domain architectures of the iSTAND2 systems

acc	operon	architecture	len	gen.name	taxend	species	defline	gca
APR86273.1	Calcineurin+EAD8→ EAD2?+iSTAND2*→ MoxR→ vWA→ vWA-L+SLOG+PNPase→	EAD2?+iSTAND2	314	A7982_11622	deltaproteobacteria	Minicystis rosea	Hypothetical protein A7982_11622 [Minicystis rosea].	GCA_001931535.1
ARV63238.1	EAD1+iSTAND2*→	EAD1+iSTAND2	333	BZZ01_25840	cyanobacteria	Nostocales cyanobacterium HT-58-2	hypothetical protein BZZ01_25840 [Nostocales cyanobacterium HT-58-2].	GCA_002163975.1
EFC83428.1	<-EAD1+TIR||EAD1+iSTAND2*→ MoxR→ vWA→ vWA-L+EAD1+EAD1+cNMP_cyclase→	EAD1+iSTAND2	371	FrEUN1fDRAFT_3426	actinobacteria	Frankia sp. EUN1f	hypothetical protein FrEUN1fDRAFT_3426 [Frankia sp. EUN1f].	GCA_000177675.1
GAK57647.1	TIR+iSTAND2*→ MoxR→	TIR+iSTAND2	388	U27_04614	bacteria	Candidatus Vecturithrix granuli	methyltransferase type 11 [Candidatus Vecturithrix granuli].	GCA_000739535.1
GAK60733.1	TIR+iSTAND2*→	TIR+iSTAND2	382	U27_00631	bacteria	Candidatus Vecturithrix granuli	TIR protein [Candidatus Vecturithrix granuli].	GCA_000739535.1
PZN29457.1	EAD5+EAD7→ EAD8+iSTAND2*→ MoxR→	EAD8+iSTAND2	345	DIU80_09340	chloroflexi	Chloroflexi bacterium	hypothetical protein DIU80_09340 [Chloroflexi bacterium].	GCA_003242165.1
WP_006101837.1	EAD8+iSTAND2*→ MoxR→ vWA→ PSE→	EAD8+iSTAND2	375	-	cyanobacteria	Coleofasciculus chthonoplastes	hypothetical protein [Coleofasciculus chthonoplastes].	GCF_000155555.1
WP_011611104.1	??+iSTAND2*→ MoxR→	??+iSTAND2	357	-	cyanobacteria	Trichodesmium erythraeum	hypothetical protein [Trichodesmium erythraeum].	GCF_000014265.1
WP_015179963.1	Trypsin→ EAD1+iSTAND2*→ MoxR→ vWA→ vWA-L+HTH→	EAD1+iSTAND2	352	-	cyanobacteria	Crinalium epipsammum	hypothetical protein [Crinalium epipsammum].	GCF_000317495.1
WP_023806061.1	EAD5+Trypsin→??+iSTAND2*→ MoxR→ vWA→ vWA-L+REC+HNH+CASPASE→	??+iSTAND2	340	-	alphaproteobacteria	Mesorhizobium sp. L2C089B000	hypothetical protein [Mesorhizobium sp. L2C089B000].	GCF_000502355.1
WP_024968615.1	EAD8+Trypsin_2→?→ EAD8+iSTAND2*→ MoxR→ Uma2→ vWA→ HD+RelA_SpoT+TGS+ACT	EAD8+iSTAND2	387	-	cyanobacteria	Microcystis aeruginosa	hypothetical protein [Microcystis aeruginosa].	GCF_000599945.1
WP_033364276.1	Trypco1→ Trypsin+iSTAND2*→ MoxR→ vWA→ vWA-L+FtsK+FtsK+FtsK+FtsK+FtsK+FtsK→	Trypsin+iSTAND2	417	-	actinobacteria	Dactylosporangium aurantiacum	trypsin-like peptidase domain-containing protein [Dactylosporangium aurantiacum].	GCF_000716715.1
WP_038071878.1	EAD1+iSTAND2*→	EAD1+iSTAND2	200	-	cyanobacteria	Tolypothrix bouteillei	hypothetical protein [Tolypothrix bouteillei].	-
WP_073635979.1	EAD1+iSTAND2*→ MoxR→ vWA→ vWA-L+TIR+GUN4→	EAD1+iSTAND2	334	-	cyanobacteria	Scytonema sp. HK-05	hypothetical protein [Scytonema sp. HK-05].	GCF_002368235.1
WP_073644499.1	??+iSTAND2*→ MoxR→ vWA→ vWA-L+HTH→	??+iSTAND2	291	-	cyanobacteria	Nostoc calcicola	hypothetical protein, partial [Nostoc calcicola].	GCF_001904715.1
WP_077000516.1	EAD1+Trypsin→ EAD1+iSTAND2*→ MoxR→ vWA→ vWA-L+STAND+HEATs+PUA+MPTase(inactive)	EAD1+iSTAND2	320	-	betaproteobacteria	Variovorax sp. KK3	hypothetical protein [Variovorax sp. KK3].	GCF_001984055.1
WP_086923325.1	EAD1+Trypsin→ EAD1+iSTAND2*→ MoxR→ vWA→ vWA-L+STAND+HEATs+PUA+MPTase(inactive)→	EAD1+iSTAND2	320	-	betaproteobacteria	Variovorax sp. JS1663	hypothetical protein [Variovorax sp. JS1663].	GCF_002157355.1
WP_091114990.1	EAD1+Trypsin→ EAD1+iSTAND2*→ MoxR→ vWA→ vWA-L+subtilase	EAD1+iSTAND2	305	-	actinobacteria	Geodermatophilus dictyosporus	hypothetical protein [Geodermatophilus dictyosporus].	GCF_900115505.1
WP_092544601.1	EAD1+Trypsin→ EAD1+iSTAND2*→ MoxR→ vWA→ PSE→	EAD1+iSTAND2	321	-	actinobacteria	Actinoplanes derwentensis	hypothetical protein [Actinoplanes derwentensis].	GCF_900104725.1
WP_101409760.1	bDLD3+iSTAND2*→	bDLD3+iSTAND2	310	-	actinobacteria	Micromonospora sp. CNZ309	hypothetical protein [Micromonospora sp. CNZ309].	-
WP_103920108.1	EAD1+Trypsin→?→??+iSTAND2*→ MoxR→ vWA→ ParD→ ParE→	??+iSTAND2	329	-	gammaproteobacteria	Thiotrichales bacterium HS_08	hypothetical protein [Thiotrichales bacterium HS_08].	GCF_900099115.1
WP_111653783.1	EAD1+iSTAND2*→	EAD1+iSTAND2	292	-	actinobacteria	Actinoplanes lutulentus	hypothetical protein [Actinoplanes lutulentus].	-
WP_114085439.1	EAD1+iSTAND2*→ MoxR→ vWA→ pentapeptides+TIR+EAD1+CASPASE	EAD1+iSTAND2	348	-	cyanobacteria	Nostoc sp. ATCC 53789	hypothetical protein [Nostoc sp. ATCC 53789].	GCF_009873495.1
WP_118164052.1	EAD8+Trypsin_2→?→ EAD8→ iSTAND2*→ MoxR→ vWA→ vWA-L+??	iSTAND2	223	-	cyanobacteria	Nostoc sphaeroides	hypothetical protein [Nostoc sphaeroides].	GCF_003443655.1

<table>
<tr><th>acc</th><th>operon</th><th>architecture</th><th>len</th><th>gen.name</th><th>taxend</th><th>species</th><th>defline</th><th>gca</th></tr>
<tr><td>WP_127507280.1</td><td><-EAD5+Trypsin<-?||EAD2?+iSTAND2*→ MoxR→ vWA→ vWA-L+Trypsin+STAND→</td><td>EAD2?+iSTAND2</td><td>348</td><td>-</td><td>actinobacteria</td><td>Actinoplanes sp. LAM7112</td><td>hypothetical protein [Actinoplanes sp. LAM7112].</td><td>GCF_003999975.1</td></tr>
</table>

3. Source data. Gene neighborhoods and domain architectures of the TERNS3 systems

acc	operon	architecture	len	gen.name	taxend	species	defline	gca
AGZ40701.1	TIR+TERNS3-N+TERNS3*→ MoxR→||<-?||vWA→ vWA-L+??+a/b_hydrolase→ SLATT+SLATT→	TIR+TERNS3-N+TERNS3	413	AFR_12075	actinobacteria	Actinoplanes friuliensis DSM 7358	hypothetical protein AFR_12075 [Actinoplanes friuliensis DSM 7358].	GCA_000494755.1
AIS01002.1	bcl2?+TERNS3-N+TERNS3*→	bcl2?+TERNS3-N+TERNS3	294	SGLAU_25320	actinobacteria	Streptomyces glaucescens	hypothetical protein SGLAU_25320 [Streptomyces glaucescens].	GCA_000761215.1
EXG79871.1	TIR+TERNS3-N_TERNS3*→ MoxR→ vWA→	TIR+TERNS3-N_TERNS3	390	CryarDRAFT_0921	actinobacteria	Cryptosporangium arvum DSM 44712	hypothetical protein CryarDRAFT_0921 [Cryptosporangium arvum DSM 44712].	GCA_000585375.1
EXI90101.1	TIR+TERNS3-N+TERNS3*→ MoxR→	TIR+TERNS3-N+TERNS3	420	AW11_01135	betaproteobacteria	Candidatus Accumulibacter sp. BA-93	hypothetical protein AW11_01135 [Candidatus Accumulibacter sp. BA-93].	GCA_000585075.1
HAO80527.1	TIR+TERNS3-N_TERNS3*→ MoxR→	TIR+TERNS3-N_TERNS3	445	DCQ92_16470	verrucomicrobia	Verrucomicrobia subdivision 3 bacterium	TPA: hypothetical protein DCQ92_16470 [Verrucomicrobia subdivision 3 bacterium].	GCA_003455565.1
HBL29163.1	CASPASE+TIR+TERNS3-N+TERNS3*→ MoxR→ vWA→	CASPASE+TIR+TERNS3-N+TERNS3	690	DD490_20220	acidobacteria	Acidobacteria bacterium	TPA: hypothetical protein DD490_20220 [Acidobacteria bacterium].	GCA_003504885.1
KDN76873.1	SLATT+SLATT→ TIR+TERNS3-N+TERNS3*→ MoxR→ vWA→ vWA-L+?+CASPASE→	TIR+TERNS3-N+TERNS3	422	DF19_14140	actinobacteria	Streptomyces olindensis	hypothetical protein DF19_14140 [Streptomyces olindensis].	GCA_000696115.1
KFB69572.1	TIR+TERNS3-N+TERNS3*→ MoxR→ vWA→ vWA-L+TIR+FGS_fold→	TIR+TERNS3-N+TERNS3	443	CAPSK01_000631	betaproteobacteria	Candidatus Accumulibacter sp. SK-01	hypothetical protein CAPSK01_000631 [Candidatus Accumulibacter sp. SK-01].	GCA_000584955.2
KFB72205.1	TIR+TERNS3-N+TERNS3*→ MoxR→ vWA→ vWA-L+TIR+FGS_fold→	TIR+TERNS3-N+TERNS3	443	AW09_002623	betaproteobacteria	Candidatus Accumulibacter sp. BA-91	hypothetical protein AW09_002623 [Candidatus Accumulibacter sp. BA-91].	GCA_000585035.2
KNZ33320.1	TIR+TERNS3-N+TERNS3*→ MoxR→ vWA→	TIR+TERNS3-N+TERNS3	444	AD742_06935	betaproteobacteria	Methylibium sp. NZG	hypothetical protein AD742_06935 [Methylibium sp. NZG].	GCA_001263235.1
KQP26499.1	TIR+TERNS3-N+TERNS3*→ MoxR→ vWA→	TIR+TERNS3-N+TERNS3	369	ASF25_21095	alphaproteobacteria	Methylobacterium sp. Leaf100	hypothetical protein ASF25_21095 [Methylobacterium sp. Leaf100].	GCA_001422845.1
KUI28159.1	TIR+TERNS3-N+TERNS3*→	TIR+TERNS3-N+TERNS3	467	AU195_20580	actinobacteria	Mycobacterium sp. IS-1496	hypothetical protein AU195_20580 [Mycobacterium sp. IS-1496].	GCA_001499995.1
KUM86142.1	TIR+TERNS3-N+TERNS3*→ MoxR→ vWA→ vWA-L+??+S1+S1+S1+S1→?→ SLOG→	TIR+TERNS3-N+TERNS3	463	AQI94_24265	actinobacteria	Streptomyces pseudovenezuelae	hypothetical protein AQI94_24265 [Streptomyces pseudovenezuelae].	GCA_001513955.1
KYF52375.1	trypsin+TERNS3-N+TERNS3*→ MoxR→	trypsin+TERNS3-N+TERNS3	503	BE04_07830	deltaproteobacteria	Sorangium cellulosum	hypothetical protein BE04_07830 [Sorangium cellulosum].	GCA_001589195.1
NEM04743.1	TIR+TERNS3-N_TERNS3*→	TIR+TERNS3-N_TERNS3	444	GCU54_01715	actinobacteria	Geodermatophilus normandii	toll/interleukin-1 receptor domain-containing protein [Geodermatophilus normandii].	-
NMQ07203.1	TIR+TERNS3-N+TERNS3*→ MoxR→ vWA→ vWA-L+TIR+FGS_fold→?→ TIR+TIR+TIR+SLOG→	TIR+TERNS3-N+TERNS3	468	E4Q08_19150	betaproteobacteria	Candidatus Accumulibacter phosphatis	TIR domain-containing protein [Candidatus Accumulibacter phosphatis].	-

acc	operon	architecture	len	gen.name	taxend	species	defline	gca
NMQ29341.1	TIR+TERNS3-N+TERNS3*→ MoxR→ vWA→ vWA-L+TIR+FGS_fold→	TIR+TERNS3-N+TERNS3	460	E4Q23_17175	betaproteobacteria	Candidatus Accumulibacter phosphatis	TIR domain-containing protein [Candidatus Accumulibacter phosphatis].	-
OPC80455.1	TIR+TERNS3-N+TERNS3*→ SLATT+SLATT→	TIR+TERNS3-N+TERNS3	582	B4N89_05385	actinobacteria	Embleya scabrispora	hypothetical protein B4N89_05385 [Embleya scabrispora].	GCA_002024165.1
PSK62948.1	TIR+TERNS3-N+TERNS3*→ MoxR→ vWA→ vWA-L+??+S1+S1+S1+OST-HTH→	TIR+TERNS3-N+TERNS3	452	B0E53_05125	actinobacteria	Micromonospora sp. MH33	hypothetical protein B0E53_05125 [Micromonospora sp. MH33].	GCA_003013775.1
PTH87452.1	TIR+TERNS3-N+TERNS3*→ MoxR→ vWA→ vWA-L+??+S1+S1+S1+S1→?→ SLOG→	TIR+TERNS3-N+TERNS3	463	C9J60_19135	actinobacteria	Streptomyces sp. A244	hypothetical protein C9J60_19135 [Streptomyces sp. A244].	GCA_003041235.1
PUB76299.1	TIR+TERNS3-N+TERNS3*→ MoxR→	TIR+TERNS3-N+TERNS3	446	DBP03_04880	gammaproteobacteria	gamma proteobacterium symbiont of Ctena orbiculata	hypothetical protein DBP03_04880 [gamma proteobacterium symbiont of Ctena orbiculata].	GCA_003058495.1
PZN81550.1	TIR+TERNS3-N+TERNS3*→ MoxR→ vWA→ vWA-L+TIR+FGS_fold→	TIR+TERNS3-N+TERNS3	413	DM484_08310	gammaproteobacteria	Candidatus Methyloumidiphilus alinensis	hypothetical protein DM484_08310 [Candidatus Methyloumidiphilus alinensis].	GCA_003242955.1
RIK40644.1	TIR+TERNS3-N+TERNS3*→	TIR+TERNS3-N+TERNS3	425	DCC55_14310	chloroflexi	Chloroflexi bacterium	hypothetical protein DCC55_14310 [Chloroflexi bacterium].	GCA_003577005.1
RLJ15768.1	TIR+TERNS3-N+TERNS3*→ MoxR→	TIR+TERNS3-N+TERNS3	445	DJ030_18025	bacteria	bacterium endosymbiont of Escarpia laminata	hypothetical protein DJ030_18025 [bacterium endosymbiont of Escarpia laminata].	GCA_003660225.1
RSO09108.1	SLATT+SLATT→ TIR+TERNS3-N+TERNS3*→ MoxR→ vWA→ vWA-L+?+CASPASE→	TIR+TERNS3-N+TERNS3	420	DMH26_01890	actinobacteria	Streptomyces sp. WAC 05379	hypothetical protein DMH26_01890 [Streptomyces sp. WAC 05379].	GCA_003947555.1
RZL40587.1	TIR+TERNS3-N+TERNS3*→ MoxR→ vWA+vWA-L+??+VipA+VipB→	TIR+TERNS3-N+TERNS3	416	EOP35_00425	betaproteobacteria	Rubrivivax sp.	TIR domain-containing protein [Rubrivivax sp.].	GCA_004211235.1
SDH38921.1	TIR+TERNS3-N+TERNS3*→ MoxR→ vWA→ vWA-L+??+S1+S1+S1+OST-HTH→	TIR+TERNS3-N+TERNS3	446	SAMN05216553_120150	actinobacteria	Lechevalieria fradiae	TIR domain-containing protein [Lechevalieria fradiae].	GCA_900100955.1
TMC21179.1	PNPase+TIR→?→ TRANSGLUTAMINASE+TIR+TERNS3-N+TERNS3*→ MoxR→ vWA→ vWA-L+TPRs+PNPase+TIR→	TRANSGLUTAMI-NASE+TIR+TERNS3-N+TERNS3	672	E6J34_10390	chloroflexi	Chloroflexi bacterium	hypothetical protein E6J34_10390 [Chloroflexi bacterium].	GCA_005879655.1
VFJ73521.1	TIR+TERNS3-N+TERNS3*→ MoxR→	TIR+TERNS3-N+TERNS3	427	BECKFW1821C_GA0114237_105219	gammaproteobacteria	Candidatus Kentron sp. FW	TIR domain-containing protein [Candidatus Kentron sp. FW].	-
WP_003954434.1	SLATT+SLATT→?→ MoxR→ vWA→ vWA-L+Betapropeller+TIM_barrel_glycosyl_hydrolase→ TIR+TERNS3-N+TERNS3*→	TIR+TERNS3-N+TERNS3	415	-	actinobacteria	Streptomyces clavuligerus	toll/interleukin-1 receptor domain-containing protein [Streptomyces clavuligerus].	GCF_000163875.1
WP_003959455.1	TIR+TERNS3-N+TERNS3*→ MoxR→ vWA→	TIR+TERNS3-N+TERNS3	434	-	actinobacteria	Streptomyces clavuligerus	toll/interleukin-1 receptor domain-containing protein [Streptomyces clavuligerus].	GCF_000163875.1
WP_005160854.1	SLATT+SLATT→ TIR+TERNS3-N_TERNS3*→ MoxR→ vWA→ vWA-L+STAND+TPRs→	TIR+TERNS3-N_TERNS3	431	-	actinobacteria	Amycolatopsis azurea	toll/interleukin-1 receptor domain-containing protein [Amycolatopsis azurea].	GCF_000340415.1
WP_006140008.1	MoxR→ vWA→ vWA-L+Betapropeller+TIM_barrel_glycosyl_hydrolase→ TIR+TERNS3-N+TERNS3*→||<-ParA-Soj_NTPase+TIR+STAND+TPRs	TIR+TERNS3-N+TERNS3	406	-	actinobacteria	Streptomyces	MULTISPECIES: toll/interleukin-1 receptor domain-containing protein [Streptomyces].	GCF_000204605.1
WP_009737883.1	E2+EAD1?→ EAD1+TERNS3-N+TERNS3*→ MoxR→ vWA→ vWA-L+Tir+Pkinase→ EAD1+fvmYukDl-Nterm+Ub→||<-E1+ZnR?	EAD1+TERNS3-N+TERNS3	372	-	actinobacteria	Frankia sp. QA3	hypothetical protein [Frankia sp. QA3].	GCF_000262465.1
WP_015037308.1	SLATT+SLATT→?→ MoxR→ vWA→ vWA-L+Betapropeller+TIM_barrel_glycosyl_hydrolase→ TIR+TERNS3-N+TERNS3*→	TIR+TERNS3-N+TERNS3	422	-	actinobacteria	Streptomyces	MULTISPECIES: toll/interleukin-1 receptor domain-containing protein [Streptomyces].	GCF_000253235.1
WP_029084398.1	TIR+TERNS3-N+TERNS3*→ MoxR→ vWA→	TIR+TERNS3-N+TERNS3	414	-	alphaproteobacteria	Bradyrhizobium sp. th.b2	toll/interleukin-1 receptor domain-containing protein [Bradyrhizobium sp. th.b2].	GCF_000426785.1

acc	operon	architecture	len	gen.name	taxend	species	defline	gca
WP_030935396.1	SLATT+SLATT→ TIR+TERNS3-N+TERNS3*→ MoxR→ vWA→ vWA-L+TIR+STAND+TPRs→ TIR+STAND+TPRs→	TIR+TERNS3-N+TERNS3	418	-	actinobacteria	Streptomyces sp. NRRL S-646	toll/interleukin-1 receptor domain-containing protein [Streptomyces sp. NRRL S-646].	GCF_000720765.1
WP_053636820.1	TIR+TERNS3-N+TERNS3*→ MoxR→	TIR+TERNS3-N+TERNS3	427	-	actinobacteria	Streptomyces sp. XY152	toll/interleukin-1 receptor domain-containing protein [Streptomyces sp. XY152].	GCF_001279455.1
WP_058554423.1	TIR+TERNS3-N+TERNS3*→ MoxR→ vWA→ vWA-L+TIR+FGS_fold→	TIR+TERNS3-N+TERNS3	447	-	gammaproteobacteria	Thiohalocapsa sp. ML1	toll/interleukin-1 receptor domain-containing protein [Thiohalocapsa sp. ML1].	GCF_001469165.1
WP_062724228.1	TIR+TERNS3-N+TERNS3*→ MoxR→ vWA→ vWA-L+??+CASPASE+a/b_hydrolase→	TIR+TERNS3-N+TERNS3	410	-	actinobacteria	Streptomyces caeruleatus	toll/interleukin-1 receptor domain-containing protein [Streptomyces caeruleatus].	GCF_001514235.1
WP_067167006.1	vWA→ vWA-L+Betapropeller+TIM_barrel_glycosyl_hydrolase→ TIR+TERNS3-N+TERNS3*→	TIR+TERNS3-N+TERNS3	421	-	actinobacteria	Streptomyces sp. ERV7	toll/interleukin-1 receptor domain-containing protein [Streptomyces sp. ERV7].	GCF_001653515.1
WP_067805560.1	TIR+TERNS3-N+TERNS3*→ MoxR→ vWA→ vWA-L+??+S1+S1+S1→	TIR+TERNS3-N+TERNS3	469	-	actinobacteria	Nocardia beijingensis	toll/interleukin-1 receptor domain-containing protein [Nocardia beijingensis].	GCF_001612785.1
WP_070017317.1	MoxR→ vWA→ vWA-L+Betapropeller+TIM_barrel_glycosyl_hydrolase→ TIR+TERNS3-N+TERNS3*→	TIR+TERNS3-N+TERNS3	413	-	actinobacteria	Streptomyces nanshensis	toll/interleukin-1 receptor domain-containing protein [Streptomyces nanshensis].	GCF_001751255.1
WP_074954915.1	TIR+TERNS3-N+TERNS3*→ MoxR→	TIR+TERNS3-N+TERNS3	410	-	deltaproteobacteria	Myxococcus fulvus	toll/interleukin-1 receptor domain-containing protein [Myxococcus fulvus].	GCF_007991095.1
WP_077058200.1	<-SLATT+SLATT||?→ MoxR→ vWA→ vWA-L+Betapropeller+TIM_barrel_glycosyl_hydrolase→ TIR+TERNS3-N+TERNS3*→	TIR+TERNS3-N+TERNS3	436	-	actinobacteria	Streptomyces sp. MP131-18	toll/interleukin-1 receptor domain-containing protein [Streptomyces sp. MP131-18].	GCF_001984575.1
WP_078922055.1	TIR+TERNS3-N+TERNS3*→ MoxR→ vWA→ vWA-L+ZnR+GAF+REC(inactive?)+TIR→	TIR+TERNS3-N+TERNS3	406	-	gammaproteobacteria	Thiothrix eikelboomii	toll/interleukin-1 receptor domain-containing protein [Thiothrix eikelboomii].	GCF_900167255.1
WP_079308407.1	MoxR→ vWA→ vWA-L+Betapropeller+TIM_barrel_glycosyl_hydrolase→ TIR+TERNS3-N+TERNS3*→	TIR+TERNS3-N+TERNS3	416	-	actinobacteria	Streptomyces sp. GKU 895	toll/interleukin-1 receptor domain-containing protein [Streptomyces sp. GKU 895].	GCF_002027195.1
WP_086569346.1	TIR+TERNS3-N+TERNS3*→	TIR+TERNS3-N+TERNS3	428	-	actinobacteria	Streptomyces alboverticillatus	toll/interleukin-1 receptor domain-containing protein [Streptomyces alboverticillatus].	GCF_002150845.1
WP_086575434.1	TIR+TERNS3-N+TERNS3*→ MoxR→	TIR+TERNS3-N+TERNS3	444	-	actinobacteria	Streptomyces alboverticillatus	toll/interleukin-1 receptor domain-containing protein [Streptomyces alboverticillatus].	-
WP_093784682.1	vWA-L+Betapropeller+TIM_barrel_glycosyl_hydrolase→ TIR+TERNS3-N+TERNS3*→||<-ParA-Soj_NTPase+TIR+STAND+TPRs	TIR+TERNS3-N+TERNS3	407	-	actinobacteria	Streptomyces guanduensis	toll/interleukin-1 receptor domain-containing protein [Streptomyces guanduensis].	GCF_900103985.1
WP_093909564.1	TIR+TERNS3-N+TERNS3*→ MoxR→ vWA→ vWA-L+??+CASPASE+a/b_hydrolase→	TIR+TERNS3-N+TERNS3	410	-	actinobacteria	Streptomyces sp. cf386	toll/interleukin-1 receptor domain-containing protein [Streptomyces sp. cf386].	GCF_900103585.1
WP_109500779.1	TIR+TERNS3-N+TERNS3*→ MoxR→ vWA→	TIR+TERNS3-N+TERNS3	573	-	actinobacteria	Streptomyces sp. Act143	toll/interleukin-1 receptor domain-containing protein [Streptomyces sp. Act143].	GCF_003143935.1
WP_109888160.1	SLATT+SLATT→ TIR+TERNS3-N+TERNS3*→ MoxR→	TIR+TERNS3-N+TERNS3	423	-	actinobacteria	Streptomyces sp. NEAU-S7GS2	toll/interleukin-1 receptor domain-containing protein [Streptomyces sp. NEAU-S7GS2].	GCF_003173275.1

acc	operon	architecture	len	gen.name	taxend	species	defline	gca
WP_111601039.1	MoxR→ vWA→ vWA-L+Betapropeller+TIM_barrel_glycosyl_hydrolase→ TIR+TERNS3-N+TERNS3*→	TIR+TERNS3-N+TERNS3	417	-	actinobacteria	Streptomyces sp. Amel2xB2	toll/interleukin-1 receptor domain-containing protein [Streptomyces sp. Amel2xB2].	GCF_003259355.1
WP_114029261.1	TIR+TERNS3-N+TERNS3*→ MoxR→ vWA→ vWA-L+??+S1+S1+S1→	TIR+TERNS3-N+TERNS3	456	-	actinobacteria	Sphaerisporangium album	toll/interleukin-1 receptor domain-containing protein [Sphaerisporangium album].	GCF_003323745.1
WP_120606365.1	TIR+TERNS3-N+TERNS3*→ MoxR→	TIR+TERNS3-N+TERNS3	410	-	deltaproteobacteria	Corallococcus sp. CA043D	toll/interleukin-1 receptor domain-containing protein [Corallococcus sp. CA043D].	GCF_003611695.1
WP_121007100.1	SLATT+SLATT→ TIR+TERNS3-N+TERNS3*→ MoxR→ vWA→ vWA-L+STAND+TPRs→	TIR+TERNS3-N+TERNS3	404	-	actinobacteria	Saccharothrix australiensis	toll/interleukin-1 receptor domain-containing protein [Saccharothrix australiensis].	GCF_003634935.1
WP_128963913.1	TIR+TERNS3-N+TERNS3*→ MoxR→ vWA→ vWA-L+CASPASE+TPRs	TIR+TERNS3-N+TERNS3	417	-	alphaproteobacteria	Bradyrhizobium guangdongense	TIR domain-containing protein [Bradyrhizobium guangdongense].	GCF_004114975.1
WP_132123911.1	TIR+TERNS3-N_TERNS3*→ MoxR→ vWA→ vWA-L+STAND+TPRs→	TIR+TERNS3-N_TERNS3	423	-	actinobacteria	Actinocrispum wychmicini	toll/interleukin-1 receptor domain-containing protein [Actinocrispum wychmicini].	GCF_004345645.1
WP_133291404.1	TIR+TERNS3-N+TERNS3*→ MoxR→ vWA→ vWA+vWA-L+??+VipA+VipB→	TIR+TERNS3-N+TERNS3	412	-	alphaproteobacteria	Dankookia rubra	toll/interleukin-1 receptor domain-containing protein [Dankookia rubra].	GCF_004355005.1
WP_141296792.1	TIR+TERNS3-N+TERNS3*→	TIR+TERNS3-N+TERNS3	572	-	actinobacteria	Streptomyces gardneri	TIR domain-containing protein [Streptomyces gardneri].	-
WP_141982648.1	TIR+TERNS3-N_TERNS3*→ MoxR→ vWA→ vWA-L+STAND+TPRs→	TIR+TERNS3-N_TERNS3	423	-	actinobacteria	Saccharothrix saharensis	TIR domain-containing protein [Saccharothrix saharensis].	GCF_006716745.1
WP_143049943.1	TIR+TERNS3-N+TERNS3*→ MoxR→	TIR+TERNS3-N+TERNS3	427	-	actinobacteria	Asanoa ishikariensis	toll/interleukin-1 receptor domain-containing protein [Asanoa ishikariensis].	GCF_900107455.1
WP_146519555.1	TIR+TERNS3-N+TERNS3*→ MoxR→ vWA+vWA-L+??+VipA+VipB→	TIR+TERNS3-N+TERNS3	417	-	planctomycetes	Planctomycetes bacterium Pla52n	TIR domain-containing protein [Planctomycetes bacterium Pla52n].	GCF_007860045.1
WP_146520106.1	TIR+TERNS3-N+TERNS3*→ MoxR→ vWA→ vWA-L+??+S1+??+S1+??→	TIR+TERNS3-N+TERNS3	616	-	planctomycetes	Planctomycetes bacterium Pla52n	TIR domain-containing protein [Planctomycetes bacterium Pla52n].	GCF_007860045.1
WP_149224808.1	TIR+TERNS3-N+TERNS3*→ MoxR→ vWA+vWA-L+??+Calcineurin+??+REase→	TIR+TERNS3-N+TERNS3	407	-	alphaproteobacteria	Azospirillum sp. B21	TIR domain-containing protein [Azospirillum sp. B21].	GCF_008364825.1
WP_150234755.1	SLATT+SLATT→ TIR+TERNS3-N+TERNS3*→ MoxR→ vWA→ vWA-L+?+CASPASE→	TIR+TERNS3-N+TERNS3	423	-	actinobacteria	Streptomyces filamentosus	TIR domain-containing protein [Streptomyces filamentosus].	-
WP_153453600.1	SLATT+SLATT→ TIR+TERNS3-N+TERNS3*→ MoxR→ vWA→ vWA-L+TIR+STAND+TPRs→ TIR+STAND+TPRs→ TIR+STAND+TPRs→	TIR+TERNS3-N+TERNS3	424	-	actinobacteria	Streptomyces sp. RB5	TIR domain-containing protein [Streptomyces sp. RB5].	GCF_009604385.1
WP_157858102.1	TIR+TERNS3-N+TERNS3*→ MoxR→ vWA→ vWA-L+??+CASPASE+a/b_hydrolase→	TIR+TERNS3-N+TERNS3	414	-	actinobacteria	Streptomyces durhamensis	TIR domain-containing protein [Streptomyces durhamensis].	GCF_000725475.1
WP_158088688.1	SLATT+SLATT→ TIR+TERNS3-N+TERNS3*→ MoxR→ vWA→ vWA-L?+Trypsin+STAND+TPRs→	TIR+TERNS3-N+TERNS3	419	-	actinobacteria	Thermoactinospora rubra	TIR domain-containing protein [Thermoactinospora rubra].	GCF_002093975.1
WP_158625805.1	TIR+TERNS3-N+TERNS3*→ MoxR→	TIR+TERNS3-N+TERNS3	409	-	deltaproteobacteria	Corallococcus carmarthensis	TIR domain-containing protein [Corallococcus carmarthensis].	GCF_003611695.1
WP_158626622.1	<-TIR+TIR+TIR+SLOG||?→?→ TIR+TERNS3-N+TERNS3*→ MoxR→ vWA→ vWA-L+TIR+STAND+TPRs→	TIR+TERNS3-N+TERNS3	413	-	deltaproteobacteria	Corallococcus interemptor	TIR domain-containing protein [Corallococcus interemptor].	GCF_003668875.1
WP_158997559.1	TIR+TERNS3-N+TERNS3*→ MoxR→ vWA→ vWA-L?+STAND+TPRs→	TIR+TERNS3-N+TERNS3	440	-	actinobacteria	Streptomyces aureus	TIR domain-containing protein [Streptomyces aureus].	GCF_000725495.1
WP_159049469.1	TIR+TERNS3-N+TERNS3*→ MoxR→ vWA→ vWA-L?+TIR+STAND+TPRs→	TIR+TERNS3-N+TERNS3	435	-	actinobacteria	Streptomyces sp. NRRL WC-3618	TIR domain-containing protein [Streptomyces sp. NRRL WC-3618].	GCF_001280005.1
WP_159688556.1	TIR+TERNS3-N+TERNS3*→ MoxR→ vWA→ vWA-L+CASPASE+a/b_hydrolase+TIR→	TIR+TERNS3-N+TERNS3	438	-	betaproteobacteria	Azoarcus sp. HKLI-1	TIR domain-containing protein [Azoarcus sp. HKLI-1].	GCF_009800965.1

acc	operon	architecture	len	gen.name	taxend	species	defline	gca
WP_164008272.1	TIR+TERNS3-N+TERNS3*→ MoxR→	TIR+TERNS3-N+TERNS3	402	-	deltaproteobacteria	Pyxidicoccus sp. AB060A	TIR domain-containing protein [Pyxidicoccus sp. AB060A].	GCF_010894435.1
WP_168498259.1	vWA→ vWA-L+Betapropeller+TIM_barrel_glycosyl_hydrolase→ TIR+TERNS3-N+TERNS3*→	TIR+TERNS3-N+TERNS3	417	-	actinobacteria	Streptomyces sp. RLB1-33	TIR domain-containing protein [Streptomyces sp. RLB1-33].	GCF_012273535.1
WP_169849771.1	TIR+TERNS3-N+TERNS3*→ MoxR→	TIR+TERNS3-N+TERNS3	403	-	deltaproteobacteria	Corallococcus exiguus	TIR domain-containing protein [Corallococcus exiguus].	GCF_012985275.1
WP_170164844.1	TIR+TERNS3-N+TERNS3*→ MoxR→ vWA→ vWA-L+TIR+FGS_fold→	TIR+TERNS3-N+TERNS3	416	-	gammaproteobacteria	Thiocapsa rosea	toll/interleukin-1 receptor domain-containing protein [Thiocapsa rosea].	GCF_003634315.1

4. Source data. Gene neighborhoods and domain architectures of the bacterial NPCBM domain described in this study

<table>
<tr><th>acc</th><th>operon</th><th>architecture</th><th>len</th><th>gen.name</th><th>taxend</th><th>species</th><th>defline</th><th>gca</th></tr>
<tr><td>APA97851.1</td><td><-SIG+NPCBM<-?||PNPase+bDLD3→ bDLD3+NPCBM*→ DUF1707-SHOCT-bihelical+TM+NPCBM→||<-LD-peptidase</td><td>bDLD3+NPCBM</td><td>217</td><td>NS506_03802</td><td>actinobacteria</td><td>Nocardia seriolae</td><td>hypothetical protein NS506_03802 [Nocardia seriolae].</td><td>GCA_001865855.1</td></tr>
<tr><td>ATE54357.1</td><td>HTH+APATPase+TPR→?→ TIR+SWACOS→ SIG+TIR+APATPase+TPR+TPR+TPR→?→ TIR+NPCBM*→</td><td>TIR+NPCBM</td><td>345</td><td>CNX65_14540</td><td>actinobacteria</td><td>Actinosynnema pretiosum</td><td>hypothetical protein CNX65_14540 [Actinosynnema pretiosum].</td><td>GCA_002354875.1</td></tr>
<tr><td>OLO83644.1</td><td>NPCBM*→</td><td>NPCBM</td><td>151</td><td>BKH12_07845</td><td>actinobacteria</td><td>Actinomyces naeslundii</td><td>hypothetical protein BKH12_07845 [Actinomyces naeslundii].</td><td>GCA_001937595.1</td></tr>
<tr><td>ONF63682.1</td><td>TIR+NPCBM*→</td><td>TIR+NPCBM</td><td>322</td><td>AVR91_0232695</td><td>actinobacteria</td><td>Amycolatopsis keratiniphila subsp. keratiniphila</td><td>hypothetical protein AVR91_0232695 [Amycolatopsis keratiniphila subsp. keratiniphila].</td><td>GCA_001620365.2</td></tr>
<tr><td>QFU87533.1</td><td>TIR+NPCBM*→||<-?<-?<-AAA<-TIR+Pkinase</td><td>TIR+NPCBM</td><td>325</td><td>YIM_11700</td><td>actinobacteria</td><td>Amycolatopsis sp. YIM 10</td><td>SEFIR domain protein [Amycolatopsis sp. YIM 10].</td><td>-</td></tr>
<tr><td>RLK54954.1</td><td>TIR+NPCBM*→</td><td>TIR+NPCBM</td><td>326</td><td>CLV68_5345</td><td>actinobacteria</td><td>Actinokineospora cianjurensis</td><td>SEFIR domain-containing protein [Actinokineospora cianjurensis].</td><td>GCA_003663795.1</td></tr>
<tr><td>RSD09590.1</td><td><-AAA?<-?<-ParA+TIR+pIdsmall1<-TIR||TIR+NPCBM*→</td><td>TIR+NPCBM</td><td>433</td><td>EIY87_41985</td><td>actinobacteria</td><td>Amycolatopsis sp. GLM-1</td><td>TIR domain-containing protein [Amycolatopsis sp. GLM-1].</td><td>GCA_003937945.1</td></tr>
<tr><td>RSM57848.1</td><td>TIR+NPCBM*→||<-SIGMA-HTH</td><td>TIR+NPCBM</td><td>327</td><td>DMH03_26220</td><td>actinobacteria</td><td>Amycolatopsis sp. WAC 01376</td><td>hypothetical protein DMH03_26220 [Amycolatopsis sp. WAC 01376].</td><td>GCA_003947415.1</td></tr>
<tr><td>RSM82716.1</td><td>TIR+NPCBM*→||<-SIGMA-HTH</td><td>TIR+NPCBM</td><td>327</td><td>DL991_05030</td><td>actinobacteria</td><td>Amycolatopsis sp. WAC 01375</td><td>hypothetical protein DL991_05030 [Amycolatopsis sp. WAC 01375].</td><td>GCA_003947325.1</td></tr>
<tr><td>RSN39203.1</td><td><-TIR<-AAA?<-?<-ParA+TIR+pIdsmall1<-TIR||TIR+NPCBM*→</td><td>TIR+NPCBM</td><td>343</td><td>DMC64_39445</td><td>actinobacteria</td><td>Amycolatopsis sp. WAC 04197</td><td>hypothetical protein DMC64_39445 [Amycolatopsis sp. WAC 04197].</td><td>GCA_003947475.1</td></tr>
<tr><td>RSN62459.1</td><td>TIR+NPCBM*→||<-SIGMA-HTH</td><td>TIR+NPCBM</td><td>327</td><td>DMH01_07185</td><td>actinobacteria</td><td>Amycolatopsis sp. WAC 04182</td><td>hypothetical protein DMH01_07185 [Amycolatopsis sp. WAC 04182].</td><td>GCA_003947465.1</td></tr>
<tr><td>SCF21250.1</td><td>SIG+NPCBM*→</td><td>SIG+NPCBM</td><td>246</td><td>GA0070563_106214</td><td>actinobacteria</td><td>Micromonospora carbonacea</td><td>NPCBM/NEW2 domain-containing protein [Micromonospora carbonacea].</td><td>GCA_900091535.1</td></tr>
<tr><td>SDD51526.1</td><td>TIR+NPCBM*→</td><td>TIR+NPCBM</td><td>295</td><td>SAMN05216174_11273</td><td>actinobacteria</td><td>Alloactinosynnema iranicum</td><td>NPCBM/NEW2 domain-containing protein [Alloactinosynnema iranicum].</td><td>GCA_900101685.1</td></tr>
<tr><td>SDI31347.1</td><td>TIR+NPCBM*→</td><td>TIR+NPCBM</td><td>292</td><td>SAMN05421871_10490</td><td>actinobacteria</td><td>Alloactinosynnema album</td><td>NPCBM/NEW2 domain-containing protein [Alloactinosynnema album].</td><td>GCA_900099755.1</td></tr>
<tr><td>SDW33810.1</td><td>TIR+NPCBM*→||<-SIGMA-HTH</td><td>TIR+NPCBM</td><td>318</td><td>SAMN05421504_101304</td><td>actinobacteria</td><td>Amycolatopsis xylanica</td><td>NPCBM/NEW2 domain-containing protein [Amycolatopsis xylanica].</td><td>GCA_900107045.1</td></tr>
<tr><td>SER09138.1</td><td>TIR+NPCBM*→</td><td>TIR+NPCBM</td><td>326</td><td>SAMN04487818_101553</td><td>actinobacteria</td><td>Actinokineospora terrae</td><td>NPCBM/NEW2 domain-containing protein [Actinokineospora terrae].</td><td>GCA_900111175.1</td></tr>
<tr><td>SES13392.1</td><td><-TIR<-Pkinase+PASTA+NPCBM||TIR+NPCBM*→?→||<-?<-?<-?||SIG+Phytase-like→</td><td>TIR+NPCBM</td><td>369</td><td>SAMN04488000_116167</td><td>actinobacteria</td><td>Lentzea albida</td><td>NPCBM/NEW2 domain-containing protein [Lentzea albida].</td><td>GCA_900111005.1</td></tr>
<tr><td>WP_003100488.1</td><td>TIR+NPCBM*→||<-Pkinase+PASTA+NPCBM</td><td>TIR+NPCBM</td><td>330</td><td>H480_RS44040</td><td>actinobacteria</td><td>Amycolatopsis vancoresmycina</td><td>TIR domain-containing protein [Amycolatopsis vancoresmycina].</td><td>GCF_000388135.1</td></tr>
<tr><td>WP_004558432.1</td><td>TIR+NPCBM*→||<-?<-PSE||DeoR→</td><td>TIR+NPCBM</td><td>323</td><td>H480_RS29455</td><td>actinobacteria</td><td>Amycolatopsis vancoresmycina</td><td>TIR domain-containing protein [Amycolatopsis vancoresmycina].</td><td>GCF_000388135.1</td></tr>
<tr><td>WP_018681508.1</td><td>TIR+NPCBM*→</td><td>TIR+NPCBM</td><td>320</td><td>C503_RS0107190</td><td>actinobacteria</td><td>Actinokineospora enzanensis</td><td>TIR domain-containing protein [Actinokineospora enzanensis].</td><td>GCF_000374445.1</td></tr>
<tr><td>WP_019932839.1</td><td><-HTH+APATPase+TPR||?→?→ TPR+TM+TM→ TIR+NPCBM*→ DUF1707-SHOCT-bihelical+TM+NPCBM→</td><td>TIR+NPCBM</td><td>369</td><td>G361_RS0140320</td><td>actinobacteria</td><td>Nocardia sp. BMG111209</td><td>TIR domain-containing protein [Nocardia sp. BMG111209].</td><td>GCF_000381925.1</td></tr>
</table>

acc	operon	architecture	len	gen.name	taxend	species	defline	gca
WP_026425923.1	TIR+NPCBM*→	TIR+NPCBM	324	H504_RS0130665	actinobacteria	Actinokineospora inagensis	TIR domain-containing protein [Actinokineospora inagensis].	GCF_000482865.1
WP_043787644.1	TIR+NPCBM*→||<-?||DeoR→	TIR+NPCBM	323	DV20_RS35395	actinobacteria	Amycolatopsis rifamycinica	TIR domain-containing protein [Amycolatopsis rifamycinica].	GCF_000695625.1
WP_043833411.1	TIR+NPCBM*→	TIR+NPCBM	317	L943_RS22200	actinobacteria	Amycolatopsis orientalis	TIR domain-containing protein [Amycolatopsis orientalis].	GCF_000411995.1
WP_051173847.1	TIR+NPCBM*→	TIR+NPCBM	311	L324_RS40905	actinobacteria	Amycolatopsis orientalis	TIR domain-containing protein [Amycolatopsis orientalis].	GCF_000478275.1
WP_051729748.1	TIR+NPCBM*→	TIR+NPCBM	317	IF70_RS45275	actinobacteria	Streptomyces sp. NRRL F-3213	TIR domain-containing protein [Streptomyces sp. NRRL F-3213].	GCF_000720075.1
WP_051913277.1	TIR+NPCBM*→||<-?<-?<-HTH+APATPase+TPR	TIR+NPCBM	297	KALB_RS43740	actinobacteria	Kutzneria albida	TIR domain-containing protein [Kutzneria albida].	GCF_000525635.1
WP_051972565.1	TIR+NPCBM*→	TIR+NPCBM	311	AJAP_RS42550	actinobacteria	Amycolatopsis japonica	TIR domain-containing protein [Amycolatopsis japonica].	GCF_000732925.1
WP_052086955.1	PNPase+bDLD3→ bDLD3+NPCBM*→?→||<-LD-peptidase	bDLD3+NPCBM	241	FH09_RS38115	actinobacteria	Nocardia seriolae	NPCBM/NEW2 domain-containing protein [Nocardia seriolae].	GCF_002356035.1
WP_052120938.1	TIR+NPCBM*→	TIR+NPCBM	311	ED92_RS41465	actinobacteria	Amycolatopsis sp. MJM2582	TIR domain-containing protein [Amycolatopsis sp. MJM2582].	GCF_000754115.1
WP_053738304.1	<-TIR+SWACOS||TIR+NPCBM*→	TIR+NPCBM	313	ADL03_RS37120	actinobacteria	Nocardia sp. NRRL S-836	TIR domain-containing protein [Nocardia sp. NRRL S-836].	GCF_001279525.1
WP_059080926.1	SIG+NPCBM*→	SIG+NPCBM	249	AV118_RS18065	actinobacteria	Streptomyces scabiei	hypothetical protein [Streptomyces scabiei].	GCF_001485125.1
WP_060884640.1	SIG+NPCBM*→	SIG+NPCBM	249	AW217_RS20820	actinobacteria	Streptomyces	MULTISPECIES: hypothetical protein [Streptomyces].	GCF_001550315.1
WP_063021686.1	TIR+NPCBM*→	TIR+NPCBM	365	NNI01S_RS20285	actinobacteria	Nocardia niwae	TIR domain-containing protein [Nocardia niwae].	GCF_001613465.1
WP_063271509.1	TIR+NPCBM*→	TIR+NPCBM	311	AVR91_RS32695	actinobacteria	Amycolatopsis keratiniphila	TIR domain-containing protein [Amycolatopsis keratiniphila].	GCF_001620365.2
WP_065912847.1	TIR+NPCBM*→||<-SIGMA-HTH<-?<-?||TIR+APATPase+TPR→ Pkinase+PASTA+NPCBM→	TIR+NPCBM	311	SD37_RS16400	actinobacteria	Amycolatopsis orientalis	TIR domain-containing protein [Amycolatopsis orientalis].	GCF_000943515.2
WP_065917610.1	<-TIR+SWACOS||TIR+NPCBM*→	TIR+NPCBM	313	BBK82_RS27620	actinobacteria	Lentzea guizhouensis	TIR domain-containing protein [Lentzea guizhouensis].	GCF_001701025.1
WP_067504284.1	TIR+NPCBM*→ DUF1707-SHOCT-bihelical+TM+NPCBM→	TIR+NPCBM	354	DFR74_RS01985	actinobacteria	Nocardia puris	TIR domain-containing protein [Nocardia puris].	GCF_001613185.1
WP_067576374.1	TIR+NPCBM*→	TIR+NPCBM	317	BM362_RS43075	actinobacteria	Amycolatopsis	MULTISPECIES: TIR domain-containing protein [Amycolatopsis].	GCF_900115345.1
WP_072027654.1	TIR+NPCBM*→	TIR+NPCBM	311	BS330_RS33980	actinobacteria	Amycolatopsis keratiniphila	TIR domain-containing protein [Amycolatopsis keratiniphila].	GCF_900105855.1
WP_072480469.1	TIR+NPCBM*→	TIR+NPCBM	313	BT341_RS36010	actinobacteria	Amycolatopsis australiensis	TIR domain-containing protein [Amycolatopsis australiensis].	GCF_900119165.1
WP_073842247.1	TIR+NPCBM*→	TIR+NPCBM	311	AMK34_RS00365	actinobacteria	Amycolatopsis sp. CB00013	TIR domain-containing protein [Amycolatopsis sp. CB00013].	GCF_001905755.1
WP_075973728.1	TIR+NPCBM*→	TIR+NPCBM	321	BJP25_RS05870	actinobacteria	Actinokineospora bangkokensis	TIR domain-containing protein [Actinokineospora bangkokensis].	GCF_001940455.1
WP_076994098.1	TIR+NPCBM*→	TIR+NPCBM	319	ALI144C_RS48690	actinobacteria	Actinosynnema sp. ALI-1.44	TIR domain-containing protein [Actinosynnema sp. ALI-1.44].	GCF_001984155.1
WP_081378834.1	TM+NPCBM*→	TM+NPCBM	241	BKH12_RS13860	actinobacteria	Actinomyces naeslundii	NPCBM/NEW2 domain-containing protein [Actinomyces naeslundii].	GCF_001937595.1
WP_083302693.1	SIG+NPCBM*→	SIG+NPCBM	248	GA0070563_RS15405	actinobacteria	Micromonospora carbonacea	NPCBM/NEW2 domain-containing protein [Micromonospora carbonacea].	GCF_900091535.1

<table>
<tr><th>acc</th><th>operon</th><th>architecture</th><th>len</th><th>gen.name</th><th>taxend</th><th>species</th><th>defline</th><th>gca</th></tr>
<tr><td>WP_083946854.1</td><td>SIG+NPCM→*</td><td>*</td><td>237</td><td>-</td><td>actinobacteria</td><td>Actinomadura latina</td><td>NPCBM/NEW2 domain-containing protein [Actinomadura latina].</td><td>-</td></tr>
<tr><td>WP_084466056.1</td><td>TIR+NPCBM*→</td><td>TIR+NPCBM</td><td>351</td><td>NC5_RS29410</td><td>actinobacteria</td><td>Nocardia salmonicida</td><td>TIR domain-containing protein [Nocardia salmonicida].</td><td>GCF_001612865.1</td></tr>
<tr><td>WP_084468436.1</td><td>TIR+NPCM→*</td><td>*</td><td>386</td><td>-</td><td>actinobacteria</td><td>Actinokineospora inagensis</td><td>TIR domain-containing protein [Actinokineospora inagensis].</td><td>-</td></tr>
<tr><td>WP_084476879.1</td><td>TIR+NPCM→*</td><td>*</td><td>418</td><td>-</td><td>actinobacteria</td><td>Actinokineospora enzanensis</td><td>TIR domain-containing protein [Actinokineospora enzanensis].</td><td>-</td></tr>
<tr><td>WP_084477357.1</td><td>TIR+NPCBM*→</td><td>TIR+NPCBM</td><td>368</td><td>C503_RS45000</td><td>actinobacteria</td><td>Actinokineospora enzanensis</td><td>TIR domain-containing protein [Actinokineospora enzanensis].</td><td>GCF_000374445.1</td></tr>
<tr><td>WP_086672996.1</td><td>TIR+NPCBM*→||<-?<-?||DeoR→</td><td>TIR+NPCBM</td><td>324</td><td>CCN36_RS07390</td><td>actinobacteria</td><td>Amycolatopsis pretoriensis</td><td>NPCBM/NEW2 domain-containing protein [Amycolatopsis pretoriensis].</td><td>GCF_900107925.1</td></tr>
<tr><td>WP_086838076.1</td><td>TIR+NPCBM*→||<-Pkinase+PASTA+NPCBM</td><td>TIR+NPCBM</td><td>334</td><td>CCN39_RS01920</td><td>actinobacteria</td><td>Amycolatopsis kentuckyensis</td><td>TIR domain-containing protein [Amycolatopsis kentuckyensis].</td><td>GCF_002155975.1</td></tr>
<tr><td>WP_086857686.1</td><td>TIR+NPCBM*→||<-?||?→ ABhydrolase→</td><td>TIR+NPCBM</td><td>324</td><td>CCN50_RS10835</td><td>actinobacteria</td><td>Amycolatopsis lexingtonensis</td><td>TIR domain-containing protein [Amycolatopsis lexingtonensis].</td><td>GCF_002156005.1</td></tr>
<tr><td>WP_086858461.1</td><td>HTH+APATPase+TPR→ TIR+NPCBM*→</td><td>TIR+NPCBM</td><td>333</td><td>CCN50_RS15120</td><td>actinobacteria</td><td>Amycolatopsis lexingtonensis</td><td>TIR domain-containing protein [Amycolatopsis lexingtonensis].</td><td>GCF_002156005.1</td></tr>
<tr><td>WP_089922416.1</td><td><-TIR<-Pkinase+PASTA+NPCBM||TIR+NPCBM*→?→||<-?<-?<-?||SIG+Phytase-like→</td><td>TIR+NPCBM</td><td>400</td><td>BMY74_RS32975</td><td>actinobacteria</td><td>Lentzea albida</td><td>TIR domain-containing protein [Lentzea albida].</td><td>GCF_900111005.1</td></tr>
<tr><td>WP_091285568.1</td><td>TIR+NPCBM*→||<-SIGMA-HTH</td><td>TIR+NPCBM</td><td>332</td><td>BLV57_RS01490</td><td>actinobacteria</td><td>Amycolatopsis xylanica</td><td>TIR domain-containing protein [Amycolatopsis xylanica].</td><td>GCF_900107045.1</td></tr>
<tr><td>WP_091312295.1</td><td>TIR+NPCBM*→||<-?||DeoR→?→?→?→?→ TPRs+TM+TM→</td><td>TIR+NPCBM</td><td>324</td><td>BLW76_RS27015</td><td>actinobacteria</td><td>Amycolatopsis tolypomycina</td><td>TIR domain-containing protein [Amycolatopsis tolypomycina].</td><td>GCF_900105945.1</td></tr>
<tr><td>WP_091377330.1</td><td><-AAA?<-?||TIR+NPCBM*→</td><td>TIR+NPCBM</td><td>406</td><td>BLR56_RS14460</td><td>actinobacteria</td><td>Alloactinosynnema album</td><td>TIR domain-containing protein [Alloactinosynnema album].</td><td>GCF_900099755.1</td></tr>
<tr><td>WP_091509601.1</td><td>TIR+NPCBM*→</td><td>TIR+NPCBM</td><td>307</td><td>BMZ92_RS19550</td><td>actinobacteria</td><td>Amycolatopsis sacchari</td><td>TIR domain-containing protein [Amycolatopsis sacchari].</td><td>GCF_900114035.1</td></tr>
<tr><td>WP_091597157.1</td><td>TIR+NPCM→*</td><td>*</td><td>338</td><td>-</td><td>actinobacteria</td><td>Amycolatopsis lurida</td><td>TIR domain-containing protein [Amycolatopsis lurida].</td><td>-</td></tr>
<tr><td>WP_092774771.1</td><td><-AAA?<-?<-ParA+TIR+pIdsmall1<-TIR||TIR+NPCBM*→</td><td>TIR+NPCBM</td><td>370</td><td>BMY07_RS02730</td><td>actinobacteria</td><td>Actinokineospora terrae</td><td>TIR domain-containing protein [Actinokineospora terrae].</td><td>GCF_900111175.1</td></tr>
<tr><td>WP_096492220.1</td><td>TIR+NPCBM*→</td><td>TIR+NPCBM</td><td>321</td><td>CNX65_RS08180</td><td>actinobacteria</td><td>Actinosynnema pretiosum</td><td>TIR domain-containing protein [Actinosynnema pretiosum].</td><td>GCF_002354875.1</td></tr>
<tr><td>WP_101607037.1</td><td>TIR+NPCBM*→||<-SIGMA-HTH</td><td>TIR+NPCBM</td><td>311</td><td>BKN51_RS08135</td><td>actinobacteria</td><td>Amycolatopsis sp. BJA-103</td><td>TIR domain-containing protein [Amycolatopsis sp. BJA-103].</td><td>GCF_002849735.1</td></tr>
<tr><td>WP_103339180.1</td><td>TIR+NPCBM*→||<-?||DeoR→</td><td>TIR+NPCBM</td><td>324</td><td>C2L58_RS20385</td><td>actinobacteria</td><td>Amycolatopsis sp. CA-126428</td><td>TIR domain-containing protein [Amycolatopsis sp. CA-126428].</td><td>GCF_002904335.1</td></tr>
<tr><td>WP_103349374.1</td><td>TIR+NPCBM*→||<-Pkinase+PASTA+NPCBM</td><td>TIR+NPCBM</td><td>330</td><td>C2L59_RS10725</td><td>actinobacteria</td><td>Amycolatopsis sp. CA-128772</td><td>TIR domain-containing protein [Amycolatopsis sp. CA-128772].</td><td>GCF_002904295.1</td></tr>
<tr><td>WP_103354377.1</td><td>TIR+NPCBM*→||<-?||?→ HTH+APATPase+TPR→||<-?||?→ ABhydrolase→</td><td>TIR+NPCBM</td><td>324</td><td>C2L59_RS37045</td><td>actinobacteria</td><td>Amycolatopsis sp. CA-128772</td><td>NPCBM/NEW2 domain-containing protein [Amycolatopsis sp. CA-128772].</td><td>GCF_002904295.1</td></tr>
<tr><td>WP_116202815.1</td><td>HTH+APATPase+TPR→ TIR+NPCBM*→</td><td>TIR+NPCBM</td><td>332</td><td>D0D37_RS23965</td><td>actinobacteria</td><td>Amycolatopsis circi</td><td>TIR domain-containing protein [Amycolatopsis circi].</td><td>GCF_003385235.1</td></tr>
<tr><td>WP_116206775.1</td><td><-ParA+TIR+pIdsmall1<-TIR||TIR+NPCBM*→</td><td>TIR+NPCBM</td><td>302</td><td>D0D37_RS45550</td><td>actinobacteria</td><td>Amycolatopsis circi</td><td>TIR domain-containing protein, partial [Amycolatopsis circi].</td><td>GCF_003385235.1</td></tr>
<tr><td>WP_118945693.1</td><td>TIR+NPCBM*→</td><td>TIR+NPCBM</td><td>321</td><td>APASM_RS08545</td><td>actinobacteria</td><td>Actinosynnema pretiosum</td><td>TIR domain-containing protein [Actinosynnema pretiosum].</td><td>GCF_003516205.1</td></tr>
<tr><td>WP_121393603.1</td><td>TIR+NPCM→*</td><td>*</td><td>374</td><td>-</td><td>actinobacteria</td><td>Actinokineospora cianjurensis</td><td>TIR domain-containing protein [Actinokineospora cianjurensis].</td><td>-</td></tr>
<tr><td>WP_121434386.1</td><td>SIG+NPCBM*→||<-MACRODOMAIN</td><td>SIG+NPCBM</td><td>236</td><td>BZB76_2536</td><td>actinobacteria</td><td>Actinomadura pelletieri</td><td>NPCBM/NEW2 domain-containing protein [Actinomadura pelletieri].</td><td>-</td></tr>
</table>

<table>
<tr><th>acc</th><th>operon</th><th>architecture</th><th>len</th><th>gen.name</th><th>taxend</th><th>species</th><th>defline</th><th>gca</th></tr>
<tr><td>WP_125313210.1</td><td>HTH+APATPase+TPR→ TIR+NPCBM*→</td><td>TIR+NPCBM</td><td>333</td><td>EIY87_RS30160</td><td>actinobacteria</td><td>Amycolatopsis sp. GLM-1</td><td>TIR domain-containing protein [Amycolatopsis sp. GLM-1].</td><td>GCF_003937945.1</td></tr>
<tr><td>WP_125687207.1</td><td>TIR+NPCBM*→</td><td>TIR+NPCBM</td><td>311</td><td>DMC61_RS04995</td><td>actinobacteria</td><td>Amycolatopsis sp. WAC 04169</td><td>TIR domain-containing protein [Amycolatopsis sp. WAC 04169].</td><td>GCF_003947535.1</td></tr>
<tr><td>WP_132047124.1</td><td>AbiJ-NTD3+HEPN→||<-?<-AbiJ-NTD2<-?||SIG+NPCBM*→</td><td>SIG+NPCBM</td><td>233</td><td>EDF27_RS02545</td><td>actinobacteria</td><td>Curtobacterium sp. PhB136</td><td>NPCBM/NEW2 domain-containing protein [Curtobacterium sp. PhB136].</td><td>GCF_004346855.1</td></tr>
<tr><td>WP_132121932.1</td><td>TIR+NPCBM*→||<-TIR+APATPase+TPR<-HTH+APATPase+TPR</td><td>TIR+NPCBM</td><td>320</td><td>EV192_RS31600</td><td>actinobacteria</td><td>Actinocrispum wychmicini</td><td>TIR domain-containing protein [Actinocrispum wychmicini].</td><td>GCF_004345645.1</td></tr>
<tr><td>WP_132396057.1</td><td>SIG+NPCBM*→</td><td>SIG+NPCBM</td><td>248</td><td>E1258_RS01390</td><td>actinobacteria</td><td>Micromonospora sp. KC207</td><td>hypothetical protein [Micromonospora sp. KC207].</td><td>GCF_004348615.1</td></tr>
<tr><td>WP_134666532.1</td><td>TIR+NPCM→*</td><td>*</td><td>401</td><td>-</td><td>actinobacteria</td><td>Amycolatopsis sp. CFH S0078</td><td>TIR domain-containing protein [Amycolatopsis sp. CFH S0078].</td><td>-</td></tr>
<tr><td>WP_134731967.1</td><td>TIR+NPCM→*</td><td>*</td><td>401</td><td>-</td><td>actinobacteria</td><td>Amycolatopsis sp. CFH S0261</td><td>TIR domain-containing protein [Amycolatopsis sp. CFH S0261].</td><td>-</td></tr>
<tr><td>WP_134736094.1</td><td>TIR+NPCM→*</td><td>*</td><td>401</td><td>-</td><td>actinobacteria</td><td>Amycolatopsis sp. CFH S0740</td><td>TIR domain-containing protein [Amycolatopsis sp. CFH S0740].</td><td>-</td></tr>
<tr><td>WP_139190910.1</td><td><-AAA?<-?||TIR+NPCBM*→</td><td>TIR+NPCBM</td><td>428</td><td>BLS56_RS22670</td><td>actinobacteria</td><td>Alloactinosynnema iranicum</td><td>TIR domain-containing protein [Alloactinosynnema iranicum].</td><td>GCF_900101685.1</td></tr>
<tr><td>WP_145934223.1</td><td><-AAA?<-?<-ParA+TIR+pIdsmall1<-TIR||TIR+NPCBM*→</td><td>TIR+NPCBM</td><td>318</td><td>FNH07_RS09925</td><td>actinobacteria</td><td>Amycolatopsis bartoniae</td><td>TIR domain-containing protein [Amycolatopsis bartoniae].</td><td>GCF_007713755.1</td></tr>
<tr><td>WP_146108413.1</td><td>TIR+NPCBM*→</td><td>TIR+NPCBM</td><td>324</td><td>CLV40_RS36380</td><td>actinobacteria</td><td>Actinokineospora auranticolor</td><td>TIR domain-containing protein [Actinokineospora auranticolor].</td><td>GCF_002934265.1</td></tr>
<tr><td>WP_153029663.1</td><td>TIR+NPCBM*→</td><td>TIR+NPCBM</td><td>314</td><td>YIM_RS07645</td><td>actinobacteria</td><td>Amycolatopsis sp. YIM 10</td><td>TIR domain-containing protein [Amycolatopsis sp. YIM 10].</td><td>GCF_009429145.1</td></tr>
<tr><td>WP_153030365.1</td><td><-AAA?<-?<-ParA+TIR+pIdsmall1<-TIR||TIR+NPCBM*→||<-?<-?<-AAA<-TIR+Pkinase||SIG+TIR→</td><td>TIR+NPCBM</td><td>341</td><td>YIM_RS11635</td><td>actinobacteria</td><td>Amycolatopsis sp. YIM 10</td><td>TIR domain-containing protein [Amycolatopsis sp. YIM 10].</td><td>GCF_009429145.1</td></tr>
<tr><td>WP_153804578.1</td><td><-HTH+APATPase+TPR||?→?→ TPR+TM+TM→ TIR+NPCBM*→ DUF1707-SHOCT-bihelical+TM+NPCBM→</td><td>TIR+NPCBM</td><td>348</td><td>GFY24_RS09395</td><td>actinobacteria</td><td>Nocardia sp. SYP-A9097</td><td>TIR domain-containing protein [Nocardia sp. SYP-A9097].</td><td>GCF_009649815.1</td></tr>
<tr><td>WP_154760338.1</td><td><-AAA?<-?<-ParA+TIR+pIdsmall1<-TIR||TIR+NPCBM*→</td><td>TIR+NPCBM</td><td>380</td><td>GKO32_30380</td><td>actinobacteria</td><td>Amycolatopsis pithecelloba</td><td>TIR domain-containing protein [Amycolatopsis pithecelloba].</td><td>-</td></tr>
<tr><td>WP_155543819.1</td><td>TIR+NPCBM*→||<-?<-?<-?||DeoR→</td><td>TIR+NPCBM</td><td>324</td><td>AA23TX_RS18915</td><td>actinobacteria</td><td>Amycolatopsis sp. A23</td><td>TIR domain-containing protein [Amycolatopsis sp. A23].</td><td>GCF_902497555.1</td></tr>
<tr><td>WP_156077140.1</td><td>HTH+APATPase+TPR→?→ TIR+SWACOS→?→ TIR+NPCBM*→</td><td>TIR+NPCBM</td><td>345</td><td>OQ02_RS41080</td><td>actinobacteria</td><td>Saccharothrix sp. NRRL B-16314</td><td>TIR domain-containing protein [Saccharothrix sp. NRRL B-16314].</td><td>GCF_000716595.1</td></tr>
<tr><td>WP_156753285.1</td><td><-AAA?<-?<-ParA+TIR+pIdsmall1<-TIR||TIR+NPCBM*→</td><td>TIR+NPCBM</td><td>409</td><td>GCL84_RS00345</td><td>actinobacteria</td><td>Actinokineospora sp. TRM65233</td><td>TIR domain-containing protein [Actinokineospora sp. TRM65233].</td><td>GCF_009745975.1</td></tr>
<tr><td>WP_157767649.1</td><td>SIG+TIR+APATPase+TPR+TPR+TPR→?→ TIR+NPCBM*→</td><td>TIR+NPCBM</td><td>338</td><td>CNX65_RS14535</td><td>actinobacteria</td><td>Actinosynnema pretiosum</td><td>TIR domain-containing protein [Actinosynnema pretiosum].</td><td>GCF_002354875.1</td></tr>
<tr><td>WP_158005434.1</td><td>TIR+NPCBM*→||<-SIGMA-HTH</td><td>TIR+NPCBM</td><td>311</td><td>BLW75_RS08360</td><td>actinobacteria</td><td>Amycolatopsis lurida</td><td>TIR domain-containing protein [Amycolatopsis lurida].</td><td>GCF_900105055.1</td></tr>
<tr><td>WP_158104517.1</td><td>TIR+NPCBM*→||<-?||DeoR→?→?→?→?→ TPRs+TM+TM→</td><td>TIR+NPCBM</td><td>323</td><td>CCN39_RS17180</td><td>actinobacteria</td><td>Amycolatopsis kentuckyensis</td><td>TIR domain-containing protein [Amycolatopsis kentuckyensis].</td><td>GCF_002155975.1</td></tr>
<tr><td>WP_158242545.1</td><td>TIR+NPCBM*→</td><td>TIR+NPCBM</td><td>322</td><td>ATK30_RS34520</td><td>actinobacteria</td><td>Amycolatopsis niigatensis</td><td>TIR domain-containing protein [Amycolatopsis niigatensis].</td><td>GCF_002846615.1</td></tr>
<tr><td>WP_158632605.1</td><td>TIR+NPCBM*→||<-SIGMA-HTH</td><td>TIR+NPCBM</td><td>311</td><td>DL990_RS12775</td><td>actinobacteria</td><td>unclassified Amycolatopsis</td><td>MULTISPECIES: TIR domain-containing protein [unclassified Amycolatopsis].</td><td>GCF_003947325.1</td></tr>
<tr><td>WP_158633124.1</td><td>TIR+NPCBM*→||<-SIGMA-HTH</td><td>TIR+NPCBM</td><td>311</td><td>DMH03_RS26290</td><td>actinobacteria</td><td>Amycolatopsis sp. WAC 01376</td><td>TIR domain-containing protein [Amycolatopsis sp. WAC 01376].</td><td>GCF_003947415.1</td></tr>
<tr><td>WP_158633195.1</td><td>TIR+NPCBM*→||<-SIGMA-HTH</td><td>TIR+NPCBM</td><td>311</td><td>DMH01_RS07200</td><td>actinobacteria</td><td>Amycolatopsis sp. WAC 04182</td><td>TIR domain-containing protein [Amycolatopsis sp. WAC 04182].</td><td>GCF_003947465.1</td></tr>
</table>

acc	operon	architecture	len	gen.name	taxend	species	defline	gca
WP_158634321.1	TIR+NPCBM*→	TIR+NPCBM	311	DMC64_RS39520	actinobacteria	Amycolatopsis sp. WAC 04197	TIR domain-containing protein [Amycolatopsis sp. WAC 04197].	GCF_003947475.1
WP_158641456.1	TIR+NPCBM*→	TIR+NPCBM	323	EIY87_RS41990	actinobacteria	Amycolatopsis sp. GLM-1	TIR domain-containing protein [Amycolatopsis sp. GLM-1].	GCF_003937945.1
WP_158660795.1	<-SIG+NPCBM<-?||PNPase+bDLD3→ bDLD3+NPCBM*→ DUF1707-SHOCT-bihelical+TM+NPCBM→||<-LD-peptidase	bDLD3+NPCBM	227	NS506_RS18405	actinobacteria	Nocardia seriolae	NPCBM/NEW2 domain-containing protein [Nocardia seriolae].	GCF_001865855.1
WP_160694349.1	TIR+NPCBM*→	TIR+NPCBM	333	GTY80_RS02520	actinobacteria	Amycolatopsis sp. SID8362	NPCBM/NEW2 domain-containing protein [Amycolatopsis sp. SID8362].	GCF_010550475.1
WP_160695878.1	<-ParA+TIR+pIdsmall1<-TIR||TIR+NPCBM*→	TIR+NPCBM	323	GTY80_RS10210	actinobacteria	Amycolatopsis sp. SID8362	TIR domain-containing protein [Amycolatopsis sp. SID8362].	GCF_010550475.1
WP_162945306.1	HTH+APATPase+TPR→?→ TIR+SWACOS→ SIG+TIR+APATPase+TPR+TPR+TPR→?→ TIR+NPCBM*→	TIR+NPCBM	334	APASM_RS15040	actinobacteria	Actinosynnema pretiosum	TIR domain-containing protein [Actinosynnema pretiosum].	GCF_003516205.1
WP_166054137.1	TIR+NPCBM*→	TIR+NPCBM	358	G7043_RS41275	actinobacteria	Lentzea sp. NEAU-D13	TIR domain-containing protein [Lentzea sp. NEAU-D13].	GCF_011067745.1
WP_166459784.1	TIR+NPCBM*→	TIR+NPCBM	312	GKO32_RS30390	actinobacteria	Amycolatopsis pithecelloba	TIR domain-containing protein [Amycolatopsis pithecelloba].	GCF_009707865.1
WP_166658167.1	<-AAA?<-?||TIR+NPCBM*→	TIR+NPCBM	333	C8E96_RS30065	actinobacteria	Alloactinosynnema album	TIR domain-containing protein [Alloactinosynnema album].	GCF_004362515.1
WP_167754290.1	TIR+NPCBM*→	TIR+NPCBM	317	EIZ44_RS27085	actinobacteria	Amycolatopsis sp. CFH S0261	TIR domain-containing protein [Amycolatopsis sp. CFH S0261].	GCF_004522235.1
WP_167756625.1	TIR+NPCBM*→	TIR+NPCBM	317	EIZ45_RS33520	actinobacteria	Amycolatopsis sp. CFH S0740	TIR domain-containing protein [Amycolatopsis sp. CFH S0740].	GCF_004522315.1
WP_167757025.1	TIR+NPCBM*→	TIR+NPCBM	317	EIZ40_RS28840	actinobacteria	Amycolatopsis sp. CFH S0078	TIR domain-containing protein [Amycolatopsis sp. CFH S0078].	GCF_004522265.1
WP_168444669.1	GDSL+YEATS→||<-?||?→ CASPASE→ HTH+APATPase+TPR→?→ TIR+APATPase+TPR→ SIG+NPCBM*→	SIG+NPCBM	234	AL2_RS24450	actinobacteria	Actinomadura latina	NPCBM/NEW2 domain-containing protein [Actinomadura latina].	GCF_001552195.1
WP_170180607.1	SIG+NPCBM*→||<-MACRODOMAIN	SIG+NPCBM	213	BZB76_RS12335	actinobacteria	Actinomadura pelletieri	NPCBM/NEW2 domain-containing protein [Actinomadura pelletieri].	GCF_003634705.1
WP_170224594.1	TIR+NPCBM*→	TIR+NPCBM	316	CLV68_RS26440	actinobacteria	Actinokineospora cianjurensis	TIR domain-containing protein [Actinokineospora cianjurensis].	GCF_003663795.1

5. Source data. Gene neighborhoods and domain architectures of the FGS domains described in this study

<table>
<tr><th>acc</th><th>operon</th><th>architecture</th><th>len</th><th>gen.name</th><th>taxend</th><th>species</th><th>defline</th><th>gca</th></tr>
<tr><td>ABX05873.1</td><td>BACTERIALFRINGE→ BetaPropeller→||<-?||?→?→ NACHT+FGS*→</td><td>NACHT+FGS</td><td>957</td><td>Haur_3236</td><td>chloroflexi</td><td>Herpetosiphon aurantiacus DSM 785</td><td>protein of unknown function DUF323 [Herpetosiphon aurantiacus DSM 785].</td><td>GCA_000018565.1</td></tr>
<tr><td>AEQ20336.1</td><td>Pkinase+FHA→ SIGMA-HTH→?→ DrHyd+nSTAND1+FGS*→||<-RVT<-TIR+FGS<-TIR+nSTAND1+FGS</td><td>DrHyd+nSTAND1+FGS</td><td>947</td><td>THIMO_RS13755</td><td>bacteria</td><td>uncultured bacterium EC5</td><td>formylglycine-generating sulfatase-like enzyme [uncultured bacterium EC5].</td><td>-</td></tr>
<tr><td>AGA91585.1</td><td><-ABhydrolase<-?<-?<-?<-?||TIR+nSTAND1+FGS*→||<-SIG+SWC3<-TIR+nSTAND1+FGS</td><td>TIR+nSTAND1+FGS</td><td>891</td><td>Thimo_2887</td><td>gammaproteobacteria</td><td>Thioflavicoccus mobilis 8321</td><td>hypothetical protein Thimo_2887 [Thioflavicoccus mobilis 8321].</td><td>GCA_000327045.1</td></tr>
<tr><td>ASF47990.1</td><td><-SIG+TM+TM+TM<-?||TIR+NACHT→ SIG+FGS*→</td><td>SIG+FGS</td><td>257</td><td>CEK71_19030</td><td>gammaproteobacteria</td><td>Methylovulum psychrotolerans</td><td>hypothetical protein CEK71_19030 [Methylovulum psychrotolerans].</td><td>GCA_002209385.1</td></tr>
<tr><td>AUB79799.1</td><td>FGS*→||<-?<-CASPASE</td><td>FGS</td><td>289</td><td>THSYN_01695</td><td>gammaproteobacteria</td><td>Candidatus Thiodictyon syntrophicum</td><td>hypothetical protein THSYN_01695 [Candidatus Thiodictyon syntrophicum].</td><td>GCA_002813775.1</td></tr>
<tr><td>AUB81902.1</td><td>EAD8→ nSTAND1→ FGS*→?→?→?→||<-FGS</td><td>FGS</td><td>294</td><td>THSYN_13670</td><td>gammaproteobacteria</td><td>Candidatus Thiodictyon syntrophicum</td><td>hypothetical protein THSYN_13670 [Candidatus Thiodictyon syntrophicum].</td><td>GCA_002813775.1</td></tr>
<tr><td>CAB1059904.1</td><td><-REC<-?||?→ CNMP→ CNMP→ KAP-NTPase→ KAP-NTPase+FGS*→||<-?<-?<-Cluster455_2clades<-?<-?||REC+ACYC→||<-LRP-HTH</td><td>KAP-NTPase+FGS</td><td>540</td><td>D1BOALGB6SA_4669</td><td>deltaproteobacteria</td><td>Olavius sp. associated proteobacterium Delta 1</td><td>hypothetical protein D1BOALGB6SA_4669 [Olavius sp. associated proteobacterium Delta 1].</td><td>-</td></tr>
<tr><td>CAB1065856.1</td><td>KAP-NTPase+FGS*→</td><td>KAP-NTPase+FGS</td><td>661</td><td>D1BOALGB6SA_10655</td><td>deltaproteobacteria</td><td>Olavius sp. associated proteobacterium Delta 1</td><td>hypothetical protein D1BOALGB6SA_10655 [Olavius sp. associated proteobacterium Delta 1].</td><td>-</td></tr>
<tr><td>CBH39249.1</td><td><-N-OB<-?||Calcineurin+NACHT+FGS*→?→?→||<-?<-?<-LittleFinger</td><td>Calcineurin+NACHT+FGS</td><td>1107</td><td>BSM_27270</td><td>archaea</td><td>uncultured archaeon</td><td>hypothetical protein, calcineurin-like phosphoesterase, NACHT domain, and DUF323 family [uncultured archaeon].</td><td>-</td></tr>
<tr><td>CUS31463.1</td><td>DrHyd+nSTAND1+FGS*→</td><td>DrHyd+nSTAND1+FGS</td><td>911</td><td>COMA1_10121</td><td>nitrospirae</td><td>Candidatus Nitrospira nitrosa</td><td>Sigma 54 interacting domain protein [Candidatus Nitrospira nitrosa].</td><td>GCA_001458735.1</td></tr>
<tr><td>ETW99001.1</td><td>bDLD3+nSTAND1+FGS*→</td><td>bDLD3+nSTAND1+FGS</td><td>783</td><td>ETSY1_16630</td><td>nitrospinae/tectomicrobia group</td><td>Candidatus Entotheonella factor</td><td>hypothetical protein ETSY1_16630 [Candidatus Entotheonella factor].</td><td>GCA_000522425.1</td></tr>
<tr><td>EXI66627.1</td><td>LRP-HTH→?→ YBAK→||<-?<-?<-?<-?||EAD8+nSTAND1+FGS*→</td><td>EAD8+nSTAND1+FGS</td><td>826</td><td>pkn1_3</td><td>betaproteobacteria</td><td>Candidatus Accumulibacter sp. SK-12</td><td>Serine/threonine-protein kinase pkn1 [Candidatus Accumulibacter sp. SK-12].</td><td>GCA_000585015.1</td></tr>
<tr><td>EXI69278.1</td><td>SIG+TM+TM+TM+TM+TM+TM+TM+TM→||<-Patatin||?→||<-?||?→||<-?||TIR+nSTAND1+FGS*→</td><td>TIR+nSTAND1+FGS</td><td>762</td><td>pkn1_1</td><td>betaproteobacteria</td><td>Candidatus Accumulibacter sp. SK-12</td><td>Serine/threonine-protein kinase pkn1 [Candidatus Accumulibacter sp. SK-12].</td><td>GCA_000585015.1</td></tr>
</table>

<table>
<tr><th>acc</th><th>operon</th><th>architecture</th><th>len</th><th>gen.name</th><th>taxend</th><th>species</th><th>defline</th><th>gca</th></tr>
<tr><td>EXI70483.1</td><td>EAD8+nSTAND1+FGS*→</td><td>EAD8+nSTAND1+FGS</td><td>824</td><td>pkn1_6</td><td>betaproteobacteria</td><td>Candidatus Accumulibacter sp. SK-11</td><td>Serine/threonine-protein kinase pkn1 [Candidatus Accumulibacter sp. SK-11].</td><td>GCA_000584995.1</td></tr>
<tr><td>EXI75672.1</td><td>SWC3→||<-?<-?||?→ FGS*→</td><td>FGS</td><td>248</td><td>pkn1_2</td><td>betaproteobacteria</td><td>Candidatus Accumulibacter sp. SK-11</td><td>Serine/threonine-protein kinase pkn1 [Candidatus Accumulibacter sp. SK-11].</td><td>GCA_000584995.1</td></tr>
<tr><td>EXI76855.1</td><td>SIG+TM+TM+TM+TM+TM+TM+TM+TM→||<-Patatin||?→||<-?||?→||<-?||nSTAND1+FGS*→||<-fvmYukDl-Nterm</td><td>nSTAND1+FGS</td><td>765</td><td>pkn1_1</td><td>betaproteobacteria</td><td>Candidatus Accumulibacter sp. SK-11</td><td>Serine/threonine-protein kinase pkn1 [Candidatus Accumulibacter sp. SK-11].</td><td>GCA_000584995.1</td></tr>
<tr><td>EXI82219.1</td><td><-CNMP+CRP-HTH||FAD-NAD-dep-oxidoreductase→?→||<-?<-?<-?<-ABhydrolase||nSTAND1+FGS*→||<-?<-SIG+CACHE+TM+PAS+PAS+GGDEF+EAL<-?||?→?→ ABC-ATPase→</td><td>nSTAND1+FGS</td><td>784</td><td>pkn1_4</td><td>betaproteobacteria</td><td>Candidatus Accumulibacter sp. BA-92</td><td>Serine/threonine-protein kinase pkn1 [Candidatus Accumulibacter sp. BA-92].</td><td>GCA_000585055.1</td></tr>
<tr><td>EXI89946.1</td><td>nSTAND1→ nSTAND1+FGS*→</td><td>nSTAND1+FGS</td><td>409</td><td>pkn1</td><td>betaproteobacteria</td><td>Candidatus Accumulibacter sp. BA-94</td><td>Serine/threonine-protein kinase pkn1 [Candidatus Accumulibacter sp. BA-94].</td><td>GCA_000585095.1</td></tr>
<tr><td>EXI90315.1</td><td><-CNMP+CRP-HTH||FAD-NAD-dep-oxidoreductase→?→||<-?<-?<-?<-ABhydrolase||TIR+nSTAND1+FGS*→||<-ABHYDROLASE<-SIG+CACHE+TM+PAS+PAS+GGDEF+EAL<-?||?→ ABC-ATPase→</td><td>TIR+nSTAND1+FGS</td><td>778</td><td>pkn1_1</td><td>betaproteobacteria</td><td>Candidatus Accumulibacter sp. BA-93</td><td>Serine/threonine-protein kinase pkn1 [Candidatus Accumulibacter sp. BA-93].</td><td>GCA_000585075.1</td></tr>
<tr><td>GAK51596.1</td><td>FGS*→ Four-helical-protein→</td><td>FGS</td><td>347</td><td>U14_02841</td><td>bacteria</td><td>Candidatus Moduliflexus flocculans</td><td>hypothetical protein U14_02841 [Candidatus Moduliflexus flocculans].</td><td>GCA_000739515.1</td></tr>
<tr><td>GAK55793.1</td><td>SIG+TM+TM+TM+TM+TM+TM+TM+TM+TM→?→ ABC-ATPase→ ABC-ATPase→ NACHT+FGS*→</td><td>NACHT+FGS</td><td>1007</td><td>U27_02752</td><td>bacteria</td><td>Candidatus Vecturithrix granuli</td><td>hypothetical protein U27_02752 [Candidatus Vecturithrix granuli].</td><td>GCA_000739535.1</td></tr>
<tr><td>GAK57663.1</td><td>TIR+NACHT+FGS*→ MED26C→?→||<-ABC-ATPase</td><td>TIR+NACHT+FGS</td><td>1091</td><td>U27_04630</td><td>bacteria</td><td>Candidatus Vecturithrix granuli</td><td>hypothetical protein U27_04630 [Candidatus Vecturithrix granuli].</td><td>GCA_000739535.1</td></tr>
<tr><td>GAK60834.1</td><td>SIG+TM+TM+TM+TM+TM→?→?→ REase→ TIR+NACHT+FGS*→||<-?<-?<-?<-?<-?<-?||GNTR-HTH→</td><td>TIR+NACHT+FGS</td><td>1098</td><td>U27_00732</td><td>bacteria</td><td>Candidatus Vecturithrix granuli</td><td>signal transduction protein [Candidatus Vecturithrix granuli].</td><td>GCA_000739535.1</td></tr>
<tr><td>GDY22761.1</td><td>NACHT+FGS*→</td><td>NACHT+FGS</td><td>859</td><td>LBMAG56_41080</td><td>verrucomicrobia</td><td>Verrucomicrobia bacterium</td><td>hypothetical protein LBMAG56_41080 [Verrucomicrobia bacterium].</td><td>-</td></tr>
<tr><td>HAW49373.1</td><td>REase+NACHT+FGS*→</td><td>REase+NACHT+FGS</td><td>821</td><td>DCX16_00245</td><td>bacteria</td><td>bacterium</td><td>TPA: hypothetical protein DCX16_00245 [bacterium].</td><td>GCA_003498085.1</td></tr>
<tr><td>HAY28136.1</td><td>nSTAND1-C+FGS*→||<-RVT</td><td>nSTAND1-C+FGS</td><td>564</td><td>DCY47_11690</td><td>betaproteobacteria</td><td>Candidatus Accumulibacter sp.</td><td>TPA: hypothetical protein DCY47_11690, partial [Candidatus Accumulibacter sp.].</td><td>GCA_003487685.1</td></tr>
<tr><td>HBW50062.1</td><td>BACTERIALFRINGE→ BetaPropeller→||<-?||?→?→ NACHT+FGS*→</td><td>NACHT+FGS</td><td>961</td><td>DEF47_09155</td><td>chloroflexi</td><td>Herpetosiphon sp.</td><td>TPA: hypothetical protein DEF47_09155 [Herpetosiphon sp.].</td><td>GCA_003512625.1</td></tr>
<tr><td>HBY06356.1</td><td><-ABC-ATPase<-LRP-HTH||SIG+TM+TM+TM→?→?→?→ KAP-NTPase→ SIG+KAP-NTPase+FGS*→ KAP-NTPase→</td><td>SIG+KAP-NTPase+FGS</td><td>655</td><td>DEH22_00650</td><td>chloroflexi</td><td>Chloroflexi bacterium</td><td>TPA: hypothetical protein DEH22_00650 [Chloroflexi bacterium].</td><td>GCA_003512075.1</td></tr>
<tr><td>HBY99326.1</td><td>FGS*→</td><td>FGS</td><td>244</td><td>DEP84_36210</td><td>chloroflexi</td><td>Chloroflexi bacterium</td><td>TPA: hypothetical protein DEP84_36210 [Chloroflexi bacterium].</td><td>GCA_003520455.1</td></tr>
<tr><td>HCB49403.1</td><td>NACHT+FGS*→</td><td>NACHT+FGS</td><td>568</td><td>DEP47_07750</td><td>chloroflexi</td><td>Chloroflexi bacterium</td><td>TPA: hypothetical protein DEP47_07750 [Chloroflexi bacterium].</td><td>GCA_003519205.1</td></tr>
</table>

acc	operon	architecture	len	gen.name	taxend	species	defline	gca
HCQ00349.1	FGS*→	FGS	230	DIT99_06480	bacteria	Candidatus Latescibacteria bacterium	TPA: formylglycine-generating enzyme family protein [Candidatus Latescibacteria bacterium].	GCA_003533805.1
HCS89156.1	FGS*→||<-Trypsin<-Trypco1	FGS	269	DIW77_03590	gammaproteobacteria	Chromatiaceae bacterium	TPA: hypothetical protein DIW77_03590 [Chromatiaceae bacterium].	GCA_003525925.1
HCS89670.1	FGS*→||<-Four-helical-protein||RVT→	FGS	269	DIW77_06290	gammaproteobacteria	Chromatiaceae bacterium	TPA: hypothetical protein DIW77_06290 [Chromatiaceae bacterium].	GCA_003525925.1
HCZ14308.1	FGS*→||<-RVT||Four-helical-protein→||<-SIG+FGS	FGS	328	DHV85_06865	betaproteobacteria	Candidatus Accumulibacter sp.	TPA: hypothetical protein DHV85_06865 [Candidatus Accumulibacter sp.].	GCA_003538495.1
HCZ17485.1	<-ABhydrolase<-?||nSTAND1+FGS*→||<-SIG+CACHE+TM+PAS+PAS+GGDEF+EAL	nSTAND1+FGS	833	DHV85_23525	betaproteobacteria	Candidatus Accumulibacter sp.	TPA: hypothetical protein DHV85_23525 [Candidatus Accumulibacter sp.].	GCA_003538495.1
HDP89132.1	NACHT+FGS*→	NACHT+FGS	860	ENN42_04130	gammaproteobacteria	Thioalkalivibrio sp.	TPA: NACHT domain-containing protein [Thioalkalivibrio sp.].	-
HDQ70913.1	TM+NACHT+FGS*→	TM+NACHT+FGS	1091	ENN19_02320	chloroflexi	Chloroflexi bacterium	TPA: NACHT domain-containing protein [Chloroflexi bacterium].	-
HDQ71358.1	FGS→ *Four-helical-protein→ FGS→ RVT→ NACHT+FGS→*	FGS	210	ENN19_04585	chloroflexi	Chloroflexi bacterium	TPA: hypothetical protein ENN19_04585, partial [Chloroflexi bacterium].	-
HDQ71362.1	NACHT+FGS*→	NACHT+FGS	951	ENN19_04605	chloroflexi	Chloroflexi bacterium	TPA: hypothetical protein ENN19_04605 [Chloroflexi bacterium].	-
HDU42372.1	TIR+FGS*→?→ Eukglutathionesyn-ATPgrasp→	TIR+FGS	438	ENQ67_02445	chloroflexi	Chloroflexi bacterium	TPA: TIR domain-containing protein [Chloroflexi bacterium].	-
HDU42685.1	TIR+FGS*→||<-TIMbarrel	TIR+FGS	376	ENQ67_04025	chloroflexi	Chloroflexi bacterium	TPA: TIR domain-containing protein [Chloroflexi bacterium].	-
HDU45349.1	SWC3→ SWC3→ FGS*→	FGS	293	ENQ67_17585	chloroflexi	Chloroflexi bacterium	TPA: hypothetical protein ENQ67_17585 [Chloroflexi bacterium].	-
HDW29972.1	ABhydrolase→?→||<-?||TIR+FGS*→?→?→?→?→?→ SIR2→	TIR+FGS	403	ENQ61_08065	chloroflexi	Chloroflexi bacterium	TPA: TIR domain-containing protein [Chloroflexi bacterium].	-
HDX49653.1	KAP-NTPase+FGS*→	KAP-NTPase+FGS	750	ENQ23_11400	chloroflexi	Chloroflexi bacterium	TPA: hypothetical protein ENQ23_11400 [Chloroflexi bacterium].	-
HDY04216.1	<-SIG+TM+TM+TM+TM+TM+TM+TM+TM+TM<-?||?→?→?→?→?→ START+FGS*→?→?→?→?→?→ MoxR-AAA→	START+FGS	371	ENQ01_08530	chloroflexi	Chloroflexi bacterium	TPA: hypothetical protein ENQ01_08530 [Chloroflexi bacterium].	-
HEC22740.1	NACHT+FGS*→	NACHT+FGS	981	ENI95_07470	chloroflexi	Chloroflexi bacterium	TPA: NACHT domain-containing protein [Chloroflexi bacterium].	-
HET90350.1	KAP-NTPase+FGS*→||<-?<-?<-?<-TPR+TPR+TPR+TPR	KAP-NTPase+FGS	776	ENN99_06380	chloroflexi	Chloroflexi bacterium	TPA: hypothetical protein ENN99_06380 [Chloroflexi bacterium].	-
HET91868.1	NACHT+FGS*→	NACHT+FGS	1053	ENN99_14185	chloroflexi	Chloroflexi bacterium	TPA: NACHT domain-containing protein [Chloroflexi bacterium].	-

acc	operon	architecture	len	gen.name	taxend	species	defline	gca
HEU31392.1	NACHT+FGS*→ Four-helical-protein→ RVT→	NACHT+FGS	452	ENQ60_07770	bacteria	bacterium	TPA: formylglycine-generating enzyme family protein [bacterium].	-
HEY85774.1	CASPASE+FGS*→ RVT→ Four-helical-protein→	CASPASE+FGS	599	G4N96_11770	chloroflexi	Chloroflexi bacterium	TPA: SUMF1/EgtB/PvdO family nonheme iron enzyme [Chloroflexi bacterium].	-
HFG28526.1	NACHT→ NACHT+FGS*→ SIG+SWC3→ RVT→||<-?||?→?→?→ SIGMA-HTH→	NACHT+FGS	437	ENS73_16885	verrucomicrobia	Verrucomicrobia subdivision 3 bacterium	TPA: hypothetical protein ENS73_16885 [Verrucomicrobia subdivision 3 bacterium].	-
HFI29792.1	NACHT+FGS*→ FURR-HTH→	NACHT+FGS	761	ENS47_13485	chloroflexi	Chloroflexi bacterium	TPA: NACHT domain-containing protein, partial [Chloroflexi bacterium].	-
HGH57499.1	KAP-NTPase+FGS*→	KAP-NTPase+FGS	750	ENV55_04065	chloroflexi	Chloroflexi bacterium	TPA: hypothetical protein ENV55_04065 [Chloroflexi bacterium].	-
HGH59410.1	FGS*→	FGS	299	ENV55_14010	chloroflexi	Chloroflexi bacterium	TPA: formylglycine-generating enzyme family protein [Chloroflexi bacterium].	-
HGV26478.1	NACHT+FGS*→ SIG+SWC3→?→?→||<-?<-?<-SIG+PTSIIB-sorb	NACHT+FGS	1042	ENS93_08620	chloroflexi	Chloroflexi bacterium	TPA: NACHT domain-containing protein [Chloroflexi bacterium].	-
HGV28164.1	bDLD1+NACHT+FGS→?→?→ KAP-NTPase+FGS*→?→?→?→?→ SIG+TPR+TPR+TPR→	KAP-NTPase+FGS	742	ENS93_17300	chloroflexi	Chloroflexi bacterium	TPA: hypothetical protein ENS93_17300 [Chloroflexi bacterium].	-
HGY49632.1	EAD7+TIR+FGS*→	EAD7+TIR+FGS	580	ENR34_13005	chloroflexi	Anaerolineae bacterium	TPA: TIR domain-containing protein [Anaerolineae bacterium].	-
HGY52758.1	FGS*→	FGS	228	ENR34_29005	chloroflexi	Anaerolineae bacterium	TPA: formylglycine-generating enzyme family protein [Anaerolineae bacterium].	-
HHF60465.1	ZNR+NACHT+FGS→ *CASPASE*→ *NACHT*→ *FGS*→	ZNR+NACHT+FGS	925	ENL51_05545	acidobacteria	Candidatus Solibacter sp.	TPA: NACHT domain-containing protein [Candidatus Solibacter sp.].	-
HHF60468.1	FGS*→	FGS	344	ENL51_05560	acidobacteria	Candidatus Solibacter sp.	TPA: hypothetical protein ENL51_05560 [Candidatus Solibacter sp.].	-
HHH41247.1	NACHT+FGS*→ SIG+SWC3→	NACHT+FGS	1027	ENK56_04500	chloroflexi	Chloroflexi bacterium	TPA: NACHT domain-containing protein [Chloroflexi bacterium].	-
HHR86634.1	NACHT+FGS*→	NACHT+FGS	996	ENL64_01430	chloroflexi	Anaerolineae bacterium	TPA: NACHT domain-containing protein [Anaerolineae bacterium].	-
HHW76671.1	SIG+TM+TM+TM+TM+TM+TM+TM+TM+TM+TM+TM→ ABC-ATPase→ TIR+nSTAND1+FGS*→||<-MED24<-?||FGS→ REase→ RVT→||<-FGS	TIR+nSTAND1+FGS	754	GX399_06480	gammaproteobacteria	Xanthomonadaceae bacterium	TPA: SUMF1/EgtB/PvdO family nonheme iron enzyme [Xanthomonadaceae bacterium].	-
HHW77288.1	NACHT+FGS*→	NACHT+FGS	827	GX399_09735	gammaproteobacteria	Xanthomonadaceae bacterium	TPA: SUMF1/EgtB/PvdO family nonheme iron enzyme [Xanthomonadaceae bacterium].	-
HHZ92662.1	FGS*→	FGS	236	EYN65_19595	bacteria	Candidatus Poribacteria bacterium	TPA: formylglycine-generating enzyme family protein [Candidatus Poribacteria bacterium].	-

acc	operon	architecture	len	gen.name	taxend	species	defline	gca
HIC91070.1	FGS*→	FGS	259	EYP21_03210	deltaproteobacteria	Syntrophaceae bacterium	TPA: hypothetical protein EYP21_03210 [Syntrophaceae bacterium].	-
HID19936.1	NACHT+FGS*→	NACHT+FGS	740	EYP28_03210	euryarchaeota	Methanophagales archaeon	TPA: NACHT domain-containing protein [Methanophagales archaeon].	-
HID51452.1	<-PIN<-?||SIG+KAP-NTPase+FGS*→	SIG+KAP-NTPase+FGS	847	EYP41_05380	chloroflexi	Anaerolineae bacterium	TPA: hypothetical protein EYP41_05380 [Anaerolineae bacterium].	-
HID51956.1	NACHT+FGS*→	NACHT+FGS	383	EYP41_07970	chloroflexi	Anaerolineae bacterium	TPA: hypothetical protein EYP41_07970, partial [Anaerolineae bacterium].	-
HID52846.1	EAD7+NACHT+FGS*→	EAD7+NACHT+FGS	964	EYP41_12530	chloroflexi	Anaerolineae bacterium	TPA: NACHT domain-containing protein [Anaerolineae bacterium].	-
HID54715.1	FGS→?→ NACHT+FGS*→	NACHT+FGS	985	EYP41_22100	chloroflexi	Anaerolineae bacterium	TPA: NACHT domain-containing protein, partial [Anaerolineae bacterium].	-
HIE28389.1	SIG+Trypsin+PDZ+PDZ→ SIG+FGS*→	SIG+FGS	524	EYP66_14005	bacteria	Candidatus Poribacteria bacterium	TPA: PEGA domain-containing protein [Candidatus Poribacteria bacterium].	-
HIE38682.1	KAP-NTPase+FGS*→	KAP-NTPase+FGS	769	EYP77_06385	chloroflexi	Anaerolineae bacterium	TPA: hypothetical protein EYP77_06385 [Anaerolineae bacterium].	-
KAA3641003.1	TIMbarrel→?→ EAD8+Trypsin→ DADA-Ligase→?→ Cluster323_2clades→?→ EAD8+NACHT+FGS*→	EAD8+NACHT+FGS	1039	DWQ02_01180	bacteroidetes	Bacteroidetes bacterium	hypothetical protein DWQ02_01180 [Bacteroidetes bacterium].	-
KAB2859035.1	TIR+FGS*→	TIR+FGS	340	F9K46_11255	chloroflexi	Anaerolineae bacterium	formylglycine-generating enzyme family protein, partial [Anaerolineae bacterium].	-
KAB2893788.1	TIR→ ParA-Soj-PloopNTPase+TIR+NACHT+FGS*→||<-?<-?||?→||<-?<-?<-?<-SF2-DUF3427A+LPD33	ParA-Soj-PloopNTPase+TIR+NACHT+FGS	1177	F9K28_07510	bacteroidetes	Bacteriodetes bacterium	SUMF1/EgtB/PvdO family nonheme iron enzyme [Bacteriodetes bacterium].	-
KAB2903680.1	FGS*→?→||<-VWA+FHA<-?<-?<-ABC-ATPase<-ABC-ATPase	FGS	280	F9K27_12805	chloroflexi	Anaerolineae bacterium	formylglycine-generating enzyme family protein [Anaerolineae bacterium].	-
KAB2903832.1	TIR+FGS*→	TIR+FGS	549	F9K27_11930	chloroflexi	Anaerolineae bacterium	SUMF1/EgtB/PvdO family nonheme iron enzyme [Anaerolineae bacterium].	-
KAB2904272.1	TIR+FGS→ *TIR+FGS*→||<-?||ABhydrolase→	TIR+FGS	523	F9K27_09315	chloroflexi	Anaerolineae bacterium	SUMF1/EgtB/PvdO family nonheme iron enzyme [Anaerolineae bacterium].	-
KAB2904273.1	TIR+FGS*→	TIR+FGS	421	F9K27_09320	chloroflexi	Anaerolineae bacterium	SUMF1/EgtB/PvdO family nonheme iron enzyme [Anaerolineae bacterium].	-
KAB2905607.1	DrHyd+NACHT+FGS→ NUDIX→?→?→ TIR+NACHT+FGS*→ NUDIX→	TIR+NACHT+FGS	902	F9K27_02420	chloroflexi	Anaerolineae bacterium	SUMF1/EgtB/PvdO family nonheme iron enzyme [Anaerolineae bacterium].	-
KAF0106945.1	nSTAND1+Pentapeptide→ KAP-NTPase+FGS*→	KAP-NTPase+FGS	727	FD146_2055	chloroflexi	Anaerolineaceae bacterium	hypothetical protein FD146_2055 [Anaerolineaceae bacterium].	-

<table>
<tr><th>acc</th><th>operon</th><th>architecture</th><th>len</th><th>gen.name</th><th>taxend</th><th>species</th><th>defline</th><th>gca</th></tr>
<tr><td>KFB67876.1</td><td>TIR+NACHT→ FGS*→</td><td>FGS</td><td>279</td><td>pkn1_17</td><td>betaproteobacteria</td><td>Candidatus Accumulibacter sp. SK-01</td><td>Serine/threonine-protein kinase pkn1 [Candidatus Accumulibacter sp. SK-01].</td><td>GCA_000584955.2</td></tr>
<tr><td>KFB69575.1</td><td>TIR→ MoxR-AAA→ MED26C→ vWA-L+FGS*→</td><td>vWA-L+FGS</td><td>808</td><td>pkn1_4</td><td>betaproteobacteria</td><td>Candidatus Accumulibacter sp. SK-01</td><td>Serine/threonine-protein kinase pkn1 [Candidatus Accumulibacter sp. SK-01].</td><td>GCA_000584955.2</td></tr>
<tr><td>KFB72199.1</td><td>ABC-ATPase→||<-?<-?||?→?→ SIG+FGS→*//<-Four-helical-protein//RVT→//<-FGS*<-MED26C<-MoxR-AAA<-TIR</td><td>SIG+FGS</td><td>194</td><td>egtB_1</td><td>betaproteobacteria</td><td>Candidatus Accumulibacter sp. BA-91</td><td>Iron(II)-dependent oxidoreductase EgtB [Candidatus Accumulibacter sp. BA-91].</td><td>GCA_000585035.2</td></tr>
<tr><td>KFB72202.1</td><td>FGS*→</td><td>FGS</td><td>826</td><td>pkn1_7</td><td>betaproteobacteria</td><td>Candidatus Accumulibacter sp. BA-91</td><td>Serine/threonine-protein kinase pkn1 [Candidatus Accumulibacter sp. BA-91].</td><td>GCA_000585035.2</td></tr>
<tr><td>KFB75278.1</td><td>NACHT+FGS*→||<-DrHyd+NACHT+FGS<-?||?→?→?→?→ MoxR-AAA→</td><td>NACHT+FGS</td><td>959</td><td>pkn1_15</td><td>betaproteobacteria</td><td>Candidatus Accumulibacter sp. SK-02</td><td>Serine/threonine-protein kinase pkn1 [Candidatus Accumulibacter sp. SK-02].</td><td>GCA_000584975.2</td></tr>
<tr><td>KFB77125.1</td><td>SIG+drhyd→ FGS*→</td><td>FGS</td><td>250</td><td>pkn1_7</td><td>betaproteobacteria</td><td>Candidatus Accumulibacter sp. SK-02</td><td>Serine/threonine-protein kinase pkn1 [Candidatus Accumulibacter sp. SK-02].</td><td>GCA_000584975.2</td></tr>
<tr><td>KFB77789.1</td><td>TIR→ MoxR-AAA→ MED26C→
vWA-L+FGS→*//<-RVT//Four-helical-protein→//<-FGS*<-?<-SIG+SWC3||?→||<-nSTAND1+TM+TPR+TPR<-?||?→ REase→</td><td>vWA-L+FGS</td><td>789</td><td>pkn1_3</td><td>betaproteobacteria</td><td>Candidatus Accumulibacter sp. SK-02</td><td>Serine/threonine-protein kinase pkn1 [Candidatus Accumulibacter sp. SK-02].</td><td>GCA_000584975.2</td></tr>
<tr><td>KFB77792.1</td><td>FGS*→</td><td>FGS</td><td>300</td><td>pkn1_4</td><td>betaproteobacteria</td><td>Candidatus Accumulibacter sp. SK-02</td><td>Serine/threonine-protein kinase pkn1 [Candidatus Accumulibacter sp. SK-02].</td><td>GCA_000584975.2</td></tr>
<tr><td>KKO20907.1</td><td>NACHT+FGS*→</td><td>NACHT+FGS</td><td>635</td><td>BROFUL_00364</td><td>planctomycetes</td><td>Candidatus Brocadia fulgida</td><td>hypothetical protein BROFUL_00364 [Candidatus Brocadia fulgida].</td><td>GCA_000987375.1</td></tr>
<tr><td>KPA14107.1</td><td>DrHyd+nSTAND1+FGS*→</td><td>DrHyd+nSTAND1+FGS</td><td>1037</td><td>MHK_005707</td><td>deltaproteobacteria</td><td>Candidatus Magnetomorum sp. HK-1</td><td>signal transduction protein [Candidatus Magnetomorum sp. HK-1].</td><td>GCA_001292585.1</td></tr>
<tr><td>KXK13840.1</td><td>TIR+NACHT+TCAD10+TCAD10→ TIR+FGS*→||<-TRANSGLUTAMINASE</td><td>TIR+FGS</td><td>530</td><td>UZ15_CFX003003227</td><td>chloroflexi</td><td>Chloroflexi bacterium OLB15</td><td>serine/threonine protein kinase [Chloroflexi bacterium OLB15].</td><td>GCA_001567085.1</td></tr>
<tr><td>KXK49058.1</td><td>SIG+TM+TM+TM+TM+TM→?→||<-?<-?<-?<-?||FGS*→||<-ABhydrolase||?→||<-?||?→?→ ABC-ATPase→</td><td>FGS</td><td>253</td><td>UZ13_03331</td><td>chloroflexi</td><td>Chloroflexi bacterium OLB13</td><td>serine/threonine protein kinase [Chloroflexi bacterium OLB13].</td><td>GCA_001567485.1</td></tr>
<tr><td>MAF11603.1</td><td>SIG+FGS*→</td><td>SIG+FGS</td><td>549</td><td>CMK11_14230</td><td>bacteria</td><td>Candidatus Poribacteria bacterium</td><td>hypothetical protein CMK11_14230 [Candidatus Poribacteria bacterium].</td><td>GCA_002687025.1</td></tr>
<tr><td>MAS33641.1</td><td>SAM-methylase→?→?→?→?→ TIR+NACHT+FGS*→</td><td>TIR+NACHT+FGS</td><td>1028</td><td>CL610_06530</td><td>chloroflexi</td><td>Anaerolineaceae bacterium</td><td>hypothetical protein CL610_06530 [Anaerolineaceae bacterium].</td><td>GCA_002702065.1</td></tr>
<tr><td>MAS34537.1</td><td>TIR+FGS*→||<-?<-?||?→?→?→?→ Cluster323_2clades→</td><td>TIR+FGS</td><td>498</td><td>CL610_11050</td><td>chloroflexi</td><td>Anaerolineaceae bacterium</td><td>hypothetical protein CL610_11050 [Anaerolineaceae bacterium].</td><td>GCA_002702065.1</td></tr>
<tr><td>MAS34702.1</td><td>ABC-ATPase→?→?→?→ FHA+FHA+TIR→ FGS*→</td><td>FGS</td><td>304</td><td>CL610_11890</td><td>chloroflexi</td><td>Anaerolineaceae bacterium</td><td>hypothetical protein CL610_11890 [Anaerolineaceae bacterium].</td><td>GCA_002702065.1</td></tr>
<tr><td>MAS35610.1</td><td>FURR-HTH→||<-?<-?<-?<-?||?→||<-?||TIR+FGS*→?→?→ TIR+APATPase→||<-?<-?<-PSE<-?||TIR+TPR+TPR+TPR+TPR→</td><td>TIR+FGS</td><td>528</td><td>CL610_16485</td><td>chloroflexi</td><td>Anaerolineaceae bacterium</td><td>hypothetical protein CL610_16485 [Anaerolineaceae bacterium].</td><td>GCA_002702065.1</td></tr>
</table>

<table>
<thead>
<tr><th>acc</th><th>operon</th><th>architecture</th><th>len</th><th>gen.name</th><th>taxend</th><th>species</th><th>defline</th><th>gca</th></tr>
</thead>
<tbody>
<tr><td>MAS36115.1</td><td>TIR+NACHT+TCAD10+TCAD10→ TIR+FGS*→</td><td>TIR+FGS</td><td>531</td><td>CL610_19065</td><td>chloroflexi</td><td>Anaerolineaceae bacterium</td><td>hypothetical protein CL610_19065 [Anaerolineaceae bacterium].</td><td>GCA_002702065.1</td></tr>
<tr><td>MAS36626.1</td><td>TIR+FGS*→||<-?<-?||?→?→||<-?<-TIR</td><td>TIR+FGS</td><td>403</td><td>CL610_21660</td><td>chloroflexi</td><td>Anaerolineaceae bacterium</td><td>hypothetical protein CL610_21660 [Anaerolineaceae bacterium].</td><td>GCA_002702065.1</td></tr>
<tr><td>MAT95609.1</td><td>MED26C→?→ NACHT+FGS*→ RVT→ Four-helical-protein→?→?→ MED24→ ABC-ATPase→</td><td>NACHT+FGS</td><td>965</td><td>CL608_00455</td><td>chloroflexi</td><td>Anaerolineaceae bacterium</td><td>hypothetical protein CL608_00455 [Anaerolineaceae bacterium].</td><td>GCA_002699125.1</td></tr>
<tr><td>MAU09406.1</td><td><-ABC-ATPase<-?||TIR+FGS*→</td><td>TIR+FGS</td><td>395</td><td>CL607_06270</td><td>chloroflexi</td><td>Anaerolineaceae bacterium</td><td>hypothetical protein CL607_06270 [Anaerolineaceae bacterium].</td><td>GCA_002699585.1</td></tr>
<tr><td>MAU10918.1</td><td>TIR+FGS*→||<-?<-TIMbarrel</td><td>TIR+FGS</td><td>485</td><td>CL607_13935</td><td>chloroflexi</td><td>Anaerolineaceae bacterium</td><td>hypothetical protein CL607_13935 [Anaerolineaceae bacterium].</td><td>GCA_002699585.1</td></tr>
<tr><td>MAU11412.1</td><td>ABhydrolase→||<-?<-?<-?||?→ TIR+NACHT+TCAD10+TCAD10→ TIR+FGS*→</td><td>TIR+FGS</td><td>543</td><td>CL607_16440</td><td>chloroflexi</td><td>Anaerolineaceae bacterium</td><td>hypothetical protein CL607_16440 [Anaerolineaceae bacterium].</td><td>GCA_002699585.1</td></tr>
<tr><td>MQM29574.1</td><td>FGS→//<-FGS</td><td>FGS</td><td>689</td><td>CRU78_03095</td><td>betaproteobacteria</td><td>Candidatus Accumulibacter phosphatis</td><td>hypothetical protein CRU78_03095, partial [Candidatus Accumulibacter phosphatis].</td><td>GCA_009467885.1</td></tr>
<tr><td>MQM29575.1</td><td>FGS*→</td><td>FGS</td><td>253</td><td>CRU78_03100</td><td>betaproteobacteria</td><td>Candidatus Accumulibacter phosphatis</td><td>hypothetical protein CRU78_03100 [Candidatus Accumulibacter phosphatis].</td><td>GCA_009467885.1</td></tr>
<tr><td>MQM29634.1</td><td><-CNMP+CRP-HTH||FAD-NAD-dep-oxidoreductase→?→||<-?<-?<-?<-ABhydrolase||nSTAND1+FGS*→||<-SIG+CACHE+TM+PAS+PAS+GGDEF+EAL</td><td>nSTAND1+FGS</td><td>753</td><td>CRU78_03420</td><td>betaproteobacteria</td><td>Candidatus Accumulibacter phosphatis</td><td>hypothetical protein CRU78_03420 [Candidatus Accumulibacter phosphatis].</td><td>GCA_009467885.1</td></tr>
<tr><td>MQM32166.1</td><td>EAD8+nSTAND1+FGS*→</td><td>EAD8+nSTAND1+FGS</td><td>832</td><td>CRU78_17325</td><td>betaproteobacteria</td><td>Candidatus Accumulibacter phosphatis</td><td>hypothetical protein CRU78_17325 [Candidatus Accumulibacter phosphatis].</td><td>GCA_009467885.1</td></tr>
<tr><td>MQM33262.1</td><td><-CNMP+CRP-HTH||FAD-NAD-dep-oxidoreductase→?→||<-?<-?<-?<-ABhydrolase||TIR+nSTAND1+FGS*→||<-ABHYDROLASE<-SIG+CACHE+TM+PAS+PAS+GGDEF+EAL<-?||?→ ABC-ATPase→</td><td>TIR+nSTAND1+FGS</td><td>779</td><td>CRU72_02110</td><td>betaproteobacteria</td><td>Candidatus Accumulibacter phosphatis</td><td>hypothetical protein CRU72_02110 [Candidatus Accumulibacter phosphatis].</td><td>GCA_009467855.1</td></tr>
<tr><td>MSP13386.1</td><td>TPR→?→?→||<-ICP8OBfold<-MED26C||?→ TPR→ EAD10+NACHT+FGS*→||<-ABhydrolase</td><td>EAD10+NACHT+FGS</td><td>858</td><td>EXR62_10580</td><td>chloroflexi</td><td>Chloroflexi bacterium</td><td>NACHT domain-containing protein [Chloroflexi bacterium].</td><td>GCA_009692745.1</td></tr>
<tr><td>MSP38253.1</td><td>DrHyd+nSTAND1+FGS*→</td><td>DrHyd+nSTAND1+FGS</td><td>889</td><td>EXR70_07155</td><td>deltaproteobacteria</td><td>Deltaproteobacteria bacterium</td><td>DUF4062 domain-containing protein [Deltaproteobacteria bacterium].</td><td>GCA_009692615.1</td></tr>
<tr><td>MXV93275.1</td><td>TCAD10→ TIR+FGS*→</td><td>TIR+FGS</td><td>544</td><td>F4Z94_07615</td><td>chloroflexi</td><td>Chloroflexi bacterium</td><td>SUMF1/EgtB/PvdO family nonheme iron enzyme [Chloroflexi bacterium].</td><td>-</td></tr>
<tr><td>MYD08867.1</td><td>SIG+TM+TM+TM+TM+TM+TM+TM+TM+TM→?→ ABC-ATPase→ ABC-ATPase→?→||<-SIG+TM+TM+TM+TM+TM+TM+TM+TM||TIR+NACHT+TCAD10→ TIR+FGS*→</td><td>TIR+FGS</td><td>570</td><td>F4X02_02375</td><td>chloroflexi</td><td>Chloroflexi bacterium</td><td>SUMF1/EgtB/PvdO family nonheme iron enzyme [Chloroflexi bacterium].</td><td>-</td></tr>
<tr><td>MYH66053.1</td><td>SIG+TM+TM+TM+TM+TM+TM+TM+TM+TM→?→ ABC-ATPase→ ABC-ATPase→?→||<-SIG+TM+TM+TM+TM+TM+TM+TM+TM||TIR+NACHT+TCAD10→ TIR+FGS*→</td><td>TIR+FGS</td><td>544</td><td>F4136_10500</td><td>chloroflexi</td><td>Chloroflexi bacterium</td><td>SUMF1/EgtB/PvdO family nonheme iron enzyme [Chloroflexi bacterium].</td><td>-</td></tr>
<tr><td>NAS88359.1</td><td>Beta-helix+Beta-helix+Beta-helix+ABhydrolase→?→ Calcineurin+NACHT+FGS*→||<-?<-tRNA<-?<-?||?→||<-?<-ParA-Soj-PloopNTPase</td><td>Calcineurin+NACHT+FGS</td><td>1124</td><td>C4E24_01245</td><td>euryarchaeota</td><td>ANME-1 cluster archaeon AG-394-G21</td><td>hypothetical protein C4E24_01245 [ANME-1 cluster archaeon AG-394-G21].</td><td>-</td></tr>
</tbody>
</table>

acc	operon	architecture	len	gen.name	taxend	species	defline	gca
NAT10007.1	Beta-helix+Beta-helix+Beta-helix+ABhydrolase→?→ Calcineurin+NACHT+FGS*→||<-?<-tRNA<-?<-?||?→||<-?<-ParA-Soj-PloopNTPase	Calcineurin+NACHT+FGS	1123	C4E22_00360	euryarchaeota	ANME-1 cluster archaeon AG-394-G06	hypothetical protein C4E22_00360 [ANME-1 cluster archaeon AG-394-G06].	-
NBO65033.1	NACHT+FGS*→?→||<-Four-helical-protein||RVT→	NACHT+FGS	795	EBU88_09360	acidobacteria	Acidobacteria bacterium	NACHT domain-containing protein, partial [Acidobacteria bacterium].	-
NBO66226.1	FGS*→	FGS	269	EBU88_15525	acidobacteria	Acidobacteria bacterium	hypothetical protein EBU88_15525, partial [Acidobacteria bacterium].	-
NCA69346.1	SWC3→ SIG+NACHT+FGS*→||<-NACHT+FGS<-CASPASE+TCAD1+TCAD2<-?<-REase	SIG+NACHT+FGS	1164	EOM91_04440	bacteroidetes	Sphingobacteriia bacterium	NACHT domain-containing protein [Sphingobacteriia bacterium].	GCA_009928635.1
NCC35438.1	FGS*→	FGS	488	EOM24_26010	chloroflexi	Chloroflexia bacterium	hypothetical protein EOM24_26010, partial [Chloroflexia bacterium].	GCA_009929835.1
NCC37506.1	FGS*→	FGS	334	EOM24_36670	chloroflexi	Chloroflexia bacterium	formylglycine-generating enzyme family protein, partial [Chloroflexia bacterium].	GCA_009929835.1
NDD63295.1	TIR→ NACHT+FGS*→	NACHT+FGS	819	EBZ36_04855	acidobacteria	Acidobacteria bacterium	NACHT domain-containing protein [Acidobacteria bacterium].	-
NDD65503.1	FGS*→	FGS	316	EBZ36_16240	acidobacteria	Acidobacteria bacterium	hypothetical protein EBZ36_16240, partial [Acidobacteria bacterium].	-
NDD65920.1	FGS*→	FGS	346	EBZ36_18380	acidobacteria	Acidobacteria bacterium	formylglycine-generating enzyme family protein, partial [Acidobacteria bacterium].	-
NDJ33386.1	Pkinase+TCAD10+FGS*→	Pkinase+TCAD10+FGS	476	GYB64_01830	chloroflexi	Chloroflexi bacterium	SUMF1/EgtB/PvdO family nonheme iron enzyme, partial [Chloroflexi bacterium].	-
NDJ36083.1	<-ABhydrolase<-?||?→ TRANSGLUTAMINASE→ FGS*→	FGS	316	GYB64_15620	chloroflexi	Chloroflexi bacterium	formylglycine-generating enzyme family protein [Chloroflexi bacterium].	-
NDJ51790.1	DrHyd+nSTAND1+FGS*→	DrHyd+nSTAND1+FGS	1023	GYB68_01745	chloroflexi	Chloroflexi bacterium	SUMF1/EgtB/PvdO family nonheme iron enzyme [Chloroflexi bacterium].	-
NDJ54987.1	NACHT+FGS*→	NACHT+FGS	957	GYB68_18090	chloroflexi	Chloroflexi bacterium	SUMF1/EgtB/PvdO family nonheme iron enzyme [Chloroflexi bacterium].	-
NDJ55381.1	TIR+FGS*→	TIR+FGS	372	GYB68_20100	chloroflexi	Chloroflexi bacterium	formylglycine-generating enzyme family protein, partial [Chloroflexi bacterium].	-
NDJ62967.1	Pkinase+NACHT+FGS*→	Pkinase+NACHT+FGS	1135	GYB67_17740	chloroflexi	Chloroflexi bacterium	SUMF1/EgtB/PvdO family nonheme iron enzyme, partial [Chloroflexi bacterium].	-
NDJ74926.1	TIR+FGS*→ FGS→	TIR+FGS	407	GYB65_01595	chloroflexi	Chloroflexi bacterium	SUMF1/EgtB/PvdO family nonheme iron enzyme [Chloroflexi bacterium].	-
NDJ77993.1	DrHyd+nSTAND1+FGS*→	DrHyd+nSTAND1+FGS	919	GYB65_17215	chloroflexi	Chloroflexi bacterium	SUMF1/EgtB/PvdO family nonheme iron enzyme [Chloroflexi bacterium].	-

acc	operon	architecture	len	gen.name	taxend	species	defline	gca
NDJ78043.1	FGS*→?→?→?→?→?→||<-BLBD	FGS	497	GYB65_17470	chloroflexi	Chloroflexi bacterium	formylglycine-generating enzyme family protein, partial [Chloroflexi bacterium].	-
NDJ79077.1	FGS*→ Four-helical-protein→||<-?<-MED26C	FGS	315	GYB65_22730	chloroflexi	Chloroflexi bacterium	formylglycine-generating enzyme family protein, partial [Chloroflexi bacterium].	-
NDJ84742.1	TIR+FGS*→?→||<-?<-?||?→?→||<-?||REC→	TIR+FGS	401	GYB66_02545	chloroflexi	Chloroflexi bacterium	SUMF1/EgtB/PvdO family nonheme iron enzyme [Chloroflexi bacterium].	-
NDJ86912.1	TIR+FGS*→	TIR+FGS	571	GYB66_13605	chloroflexi	Chloroflexi bacterium	SUMF1/EgtB/PvdO family nonheme iron enzyme [Chloroflexi bacterium].	-
NEP63621.1	nSTAND1-C+FGS*→||<-EAD1+FGS	nSTAND1-C+FGS	517	F6K31_43145	cyanobacteria	Symploca sp. SIO2G7	formylglycine-generating enzyme family protein [Symploca sp. SIO2G7].	-
NET36060.1	ACYC→ nSTAND1+FGS*→	nSTAND1+FGS	814	F6K19_29210	cyanobacteria	Cyanothece sp. SIO1E1	SUMF1/EgtB/PvdO family nonheme iron enzyme [Cyanothece sp. SIO1E1].	-
NGZ09817.1	FGS*→	FGS	148	CV088_10590	nitrospirae	Nitrospira sp. LK70	hypothetical protein CV088_10590 [Nitrospira sp. LK70].	-
NGZ10364.1	TIR+FGS*→?→||<-?<-?||?→?→ SIG+TM+HAMP+HISKIN→ SIG+Trypsin+PDZ+PDZ→	TIR+FGS	401	CV088_13405	nitrospirae	Nitrospira sp. LK70	TIR domain-containing protein [Nitrospira sp. LK70].	-
NGZ10802.1	DrHyd+iSTAND→ MoxR-AAA→ SIG+VWA→||<-?||TIR+TIR+FGS*→	TIR+TIR+FGS	593	CV088_15685	nitrospirae	Nitrospira sp. LK70	TIR domain-containing protein [Nitrospira sp. LK70].	-
NGZ11578.1	SWC3→||<-?<-?||DrHyd+nSTAND1+FGS*→ Four-helical-protein→?→?→ FGS→	DrHyd+nSTAND1+FGS	880	CV088_19770	nitrospirae	Nitrospira sp. LK70	DUF4062 domain-containing protein [Nitrospira sp. LK70].	-
NIA29268.1	RVT→ DrHyd→ NACHT+FGS*→ Four-helical-protein→	NACHT+FGS	707	GWP06_05045	actinobacteria	Actinobacteria bacterium	SUMF1/EgtB/PvdO family nonheme iron enzyme [Actinobacteria bacterium].	-
NIA30537.1	NACHT+FGS*→?→?→ TM+TM+TM+TM+TM+TM+TM→	NACHT+FGS	847	GWP06_11575	actinobacteria	Actinobacteria bacterium	SUMF1/EgtB/PvdO family nonheme iron enzyme, partial [Actinobacteria bacterium].	-
NIM11161.1	NACHT+FGS*→?→ Four-helical-protein→ RVT→ FGS→ Calcineurin→	NACHT+FGS	519	GTO81_04210	bacteria	Candidatus Aminicenantes bacterium	SUMF1/EgtB/PvdO family nonheme iron enzyme, partial [Candidatus Aminicenantes bacterium].	-
NIM14207.1	Calcineurin→ NACHT+FGS*→	NACHT+FGS	752	GTO81_19680	bacteria	Candidatus Aminicenantes bacterium	SUMF1/EgtB/PvdO family nonheme iron enzyme [Candidatus Aminicenantes bacterium].	-
NIM14242.1	NACHT+FGS*→	NACHT+FGS	438	GTO81_19860	bacteria	Candidatus Aminicenantes bacterium	SUMF1/EgtB/PvdO family nonheme iron enzyme, partial [Candidatus Aminicenantes bacterium].	-
NIM15894.1	SWC3→?→ Calcineurin+NACHT+FGS*→	Calcineurin+NACHT+FGS	1153	GTO81_28290	bacteria	Candidatus Aminicenantes bacterium	SUMF1/EgtB/PvdO family nonheme iron enzyme [Candidatus Aminicenantes bacterium].	-

acc	operon	architecture	len	gen.name	taxend	species	defline	gca
NIM16827.1	Calcineurin→ NACHT+FGS*→?→?→?→||<-Cluster235_2clades||?→ Eukglutathionesyn-ATPgrasp→	NACHT+FGS	835	GTO81_32990	bacteria	Candidatus Aminicenantes bacterium	SUMF1/EgtB/PvdO family nonheme iron enzyme [Candidatus Aminicenantes bacterium].	-
NIM17712.1	NACHT+FGS→||<-?||Calcineurin+NACHT+FGS*→?→?→||<-?||SIG+TM→	Calcineurin+NACHT+FGS	1126	GTO81_37510	bacteria	Candidatus Aminicenantes bacterium	SUMF1/EgtB/PvdO family nonheme iron enzyme [Candidatus Aminicenantes bacterium].	-
NIM97201.1	NACHT+FGS*→	NACHT+FGS	753	GTO24_03660	bacteria	candidate division Zixibacteria bacterium	SUMF1/EgtB/PvdO family nonheme iron enzyme, partial [candidate division Zixibacteria bacterium].	-
NIO69120.1	NACHT+FGS*→?→ RVT→ Four-helical-protein→ DrHyd→	NACHT+FGS	666	GTN71_08815	chloroflexi	Anaerolineae bacterium	SUMF1/EgtB/PvdO family nonheme iron enzyme, partial [Anaerolineae bacterium].	-
NIO69566.1	KAP-NTPase+FGS*→	KAP-NTPase+FGS	725	GTN71_11185	chloroflexi	Anaerolineae bacterium	SUMF1/EgtB/PvdO family nonheme iron enzyme, partial [Anaerolineae bacterium].	-
NIO69816.1	KAP-NTPase+FGS*→ REase→	KAP-NTPase+FGS	741	GTN71_12500	chloroflexi	Anaerolineae bacterium	SUMF1/EgtB/PvdO family nonheme iron enzyme [Anaerolineae bacterium].	-
NIO71404.1	TIR+FGS*→	TIR+FGS	394	GTN71_20820	chloroflexi	Anaerolineae bacterium	SUMF1/EgtB/PvdO family nonheme iron enzyme, partial [Anaerolineae bacterium].	-
NIP46832.1	ACYC+NACHT+FGS*→	ACYC+NACHT+FGS	944	GWO29_10605	gammaproteobacteria	Gammaproteobacteria bacterium	SUMF1/EgtB/PvdO family nonheme iron enzyme [Gammaproteobacteria bacterium].	-
NIP84022.1	FGS*→	FGS	268	GTO03_00060	planctomycetes	Planctomycetales bacterium	SUMF1/EgtB/PvdO family nonheme iron enzyme, partial [Planctomycetales bacterium].	-
NIW94495.1	KAP-NTPase→ FGS*→	FGS	314	GWN20_16860	planctomycetes	Phycisphaerae bacterium	SUMF1/EgtB/PvdO family nonheme iron enzyme [Phycisphaerae bacterium].	-
NJD06933.1	DrHyd+iSTAND→ MoxR-AAA→ SIG+VWA→ vWA-L+TIR+DrHyd+FGS*→	vWA-L+TIR+DrHyd+FGS	905	FIA97_10615	gammaproteobacteria	Methylococcaceae bacterium	DUF4062 domain-containing protein [Methylococcaceae bacterium].	-
NJL17799.1	FGS*→	FGS	337	HC938_11975	nitrospirae	Nitrospira sp.	formylglycine-generating enzyme family protein, partial [Nitrospira sp.].	-
NJL55027.1	TIR→ NACHT+FGS→ *PSE*→ *Four-helical-protein*→ *TIR+FGS*→	NACHT+FGS	859	HC928_07430	bacteria	bacterium	SUMF1/EgtB/PvdO family nonheme iron enzyme [bacterium].	-
NJL55029.1	TIR+FGS*→	TIR+FGS	428	HC928_07445	bacteria	bacterium	SUMF1/EgtB/PvdO family nonheme iron enzyme [bacterium].	-
NJL55119.1	TIR→ NACHT+FGS*→	NACHT+FGS	721	HC928_07980	bacteria	bacterium	formylglycine-generating enzyme family protein [bacterium].	-
NJL55722.1	TIR+FGS*→	TIR+FGS	402	HC928_11325	bacteria	bacterium	SUMF1/EgtB/PvdO family nonheme iron enzyme [bacterium].	-

acc	operon	architecture	len	gen.name	taxend	species	defline	gca
NJL93048.1	<-TIMbarrel<-?<-?<-?||FGS*→	FGS	308	HC915_04650	chloroflexi	Anaerolineae bacterium	formylglycine-generating enzyme family protein [Anaerolineae bacterium].	-
NJL95335.1	FGS*→?→?→?→||<-SIG+TM+HAMP+HISKIN	FGS	212	HC915_17250	chloroflexi	Anaerolineae bacterium	formylglycine-generating enzyme family protein, partial [Anaerolineae bacterium].	-
NJN82052.1	FGS*→	FGS	310	HC802_07060	chloroflexi	Caldilineaceae bacterium	formylglycine-generating enzyme family protein [Caldilineaceae bacterium].	-
NJN93303.1	NACHT+FGS*→	NACHT+FGS	695	HC875_04045	chloroflexi	Anaerolineales bacterium	SUMF1/EgtB/PvdO family nonheme iron enzyme [Anaerolineales bacterium].	-
NJN94208.1	FGS*→||<-?<-?<-PSE||?→ PSE→?→?→||<-PSE<-YBAK	FGS	242	HC875_09015	chloroflexi	Anaerolineales bacterium	formylglycine-generating enzyme family protein [Anaerolineales bacterium].	-
NJN98786.1	NACHT+FGS*→ FGS→	NACHT+FGS	463	HC875_34180	chloroflexi	Anaerolineales bacterium	SUMF1/EgtB/PvdO family nonheme iron enzyme [Anaerolineales bacterium].	-
NJN99601.1	NACHT→?→ FGS*→	FGS	264	HC875_38615	chloroflexi	Anaerolineales bacterium	formylglycine-generating enzyme family protein [Anaerolineales bacterium].	-
NJP06778.1	VWA+FGS*→||<-PSE<-PSE<-?<-tRNA||SbcC+SbcC→	VWA+FGS	782	HC837_14760	chloroflexi	Chloroflexaceae bacterium	SUMF1/EgtB/PvdO family nonheme iron enzyme [Chloroflexaceae bacterium].	-
NJP06949.1	<-FGS||?→?→?→?→?→ Pkinase+FGS*→	Pkinase+FGS	514	HC837_15690	chloroflexi	Chloroflexaceae bacterium	SUMF1/EgtB/PvdO family nonheme iron enzyme [Chloroflexaceae bacterium].	-
NJP07891.1	FGS*→	FGS	408	HC837_20850	chloroflexi	Chloroflexaceae bacterium	formylglycine-generating enzyme family protein [Chloroflexaceae bacterium].	-
NKB76742.1	<-Four-helical-protein||RVT→ DrHyd→ NACHT+FGS*→||<-?<-SIG+TM+TM+TM+TM+TM+TM+TM+TM<-PAS+HISKIN+REC<-?<-REC+ACYC	NACHT+FGS	610	GKR96_06745	gammaproteobacteria	Gammaproteobacteria bacterium	SUMF1/EgtB/PvdO family nonheme iron enzyme [Gammaproteobacteria bacterium].	-
NKQ37578.1	NACHT+FGS*→?→?→||<-?<-ABC-ATPase<-?||?→||<-LittleFinger	NACHT+FGS	946	HF973_18435	chloroflexi	Chloroflexi bacterium	SUMF1/EgtB/PvdO family nonheme iron enzyme [Chloroflexi bacterium].	-
NLE52136.1	FGS*→||<-RVT	FGS	304	GX613_12100	chloroflexi	Chloroflexi bacterium	formylglycine-generating enzyme family protein, partial [Chloroflexi bacterium].	-
NLF75470.1	TIR+TM+NACHT+7xTM+FGS*→?→||<-ABhydrolase<-ABhydrolase<-?<-ABhydrolase<-?||wHTH-4stranded+TPRs+nSTAND1+TM+BetaPropeller→	TIR+TM+NACHT+7xTM+FGS	1172	GX573_07210	chloroflexi	Chloroflexi bacterium	SUMF1/EgtB/PvdO family nonheme iron enzyme [Chloroflexi bacterium].	-
NLF75680.1	SWC3→?→?→?→?→ TIR+NACHT+FGS*→	TIR+NACHT+FGS	818	GX573_08280	chloroflexi	Chloroflexi bacterium	SUMF1/EgtB/PvdO family nonheme iron enzyme [Chloroflexi bacterium].	-
NLF77023.1	<-TIR+APATPase+BetaPropeller||?→?→ TIR→?→||<-PSE||FGS*→	FGS	224	GX573_15095	chloroflexi	Chloroflexi bacterium	formylglycine-generating enzyme family protein, partial [Chloroflexi bacterium].	-
NLF78373.1	DrHyd+nSTAND1+FGS*→	DrHyd+nSTAND1+FGS	972	GX573_21985	chloroflexi	Chloroflexi bacterium	SUMF1/EgtB/PvdO family nonheme iron enzyme [Chloroflexi bacterium].	-

<table>
<thead>
<tr><th>acc</th><th>operon</th><th>architecture</th><th>len</th><th>gen.name</th><th>taxend</th><th>species</th><th>defline</th><th>gca</th></tr>
</thead>
<tbody>
<tr><td>NLF78947.1</td><td>FGS*→</td><td>FGS</td><td>250</td><td>GX573_24915</td><td>chloroflexi</td><td>Chloroflexi bacterium</td><td>formylglycine-generating enzyme family protein [Chloroflexi bacterium].</td><td>-</td></tr>
<tr><td>NLF78951.1</td><td>TIR+FGS*→</td><td>TIR+FGS</td><td>508</td><td>GX573_24935</td><td>chloroflexi</td><td>Chloroflexi bacterium</td><td>SUMF1/EgtB/PvdO family nonheme iron enzyme, partial [Chloroflexi bacterium].</td><td>-</td></tr>
<tr><td>NLF78992.1</td><td>TIR+NACHT+FGS→?→ DrHyd+nSTAND1+FGS→</td><td>TIR+NACHT+FGS</td><td>993</td><td>GX573_25145</td><td>chloroflexi</td><td>Chloroflexi bacterium</td><td>SUMF1/EgtB/PvdO family nonheme iron enzyme [Chloroflexi bacterium].</td><td>-</td></tr>
<tr><td>NLF78994.1</td><td>TIR+NACHT+FGS*→</td><td>TIR+NACHT+FGS</td><td>923</td><td>GX573_25155</td><td>chloroflexi</td><td>Chloroflexi bacterium</td><td>SUMF1/EgtB/PvdO family nonheme iron enzyme [Chloroflexi bacterium].</td><td>-</td></tr>
<tr><td>NLS76118.1</td><td>FGS→ CASPASE+TCAD1+TCAD2→ NACHT+FGS→ NACHT+FGS*→</td><td>NACHT+FGS</td><td>1049</td><td>GXY76_02550</td><td>chloroflexi</td><td>Chloroflexi bacterium</td><td>SUMF1/EgtB/PvdO family nonheme iron enzyme [Chloroflexi bacterium].</td><td>-</td></tr>
<tr><td>NLX11872.1</td><td><-DrHyd<-?<-?||FGS*→</td><td>FGS</td><td>250</td><td>GXY36_19670</td><td>chloroflexi</td><td>Chloroflexi bacterium</td><td>formylglycine-generating enzyme family protein [Chloroflexi bacterium].</td><td>-</td></tr>
<tr><td>NMB90542.1</td><td>TPR→?→ FGS*→</td><td>FGS</td><td>276</td><td>GYA17_19435</td><td>chloroflexi</td><td>Chloroflexi bacterium</td><td>formylglycine-generating enzyme family protein [Chloroflexi bacterium].</td><td>-</td></tr>
<tr><td>NMC02153.1</td><td>FGS*→</td><td>FGS</td><td>221</td><td>GYA30_07250</td><td>chloroflexi</td><td>Chloroflexi bacterium</td><td>formylglycine-generating enzyme family protein, partial [Chloroflexi bacterium].</td><td>-</td></tr>
<tr><td>NMC03023.1</td><td>NACHT+FGS*→</td><td>NACHT+FGS</td><td>839</td><td>GYA30_11700</td><td>chloroflexi</td><td>Chloroflexi bacterium</td><td>SUMF1/EgtB/PvdO family nonheme iron enzyme, partial [Chloroflexi bacterium].</td><td>-</td></tr>
<tr><td>NMC11372.1</td><td>TPR→?→ FGS*→?→?→?→?→?→?→ SIG+TM→</td><td>FGS</td><td>262</td><td>GYA34_00645</td><td>chloroflexi</td><td>Chloroflexi bacterium</td><td>formylglycine-generating enzyme family protein [Chloroflexi bacterium].</td><td>-</td></tr>
<tr><td>NMQ05699.1</td><td>FGS*→||<-TRANSGLUTAMINASE||?→||<-?||PSE→?→||<-Patatin</td><td>FGS</td><td>259</td><td>E4Q08_10675</td><td>betaproteobacteria</td><td>Candidatus Accumulibacter phosphatis</td><td>hypothetical protein E4Q08_10675 [Candidatus Accumulibacter phosphatis].</td><td>-</td></tr>
<tr><td>NMQ07713.1</td><td>TIR→ MoxR-AAA→ MED26C→ SIG+FGS*→||<-RVT||Four-helical-protein→||<-SIG+FGS<-?<-?<-ABC-ATPase</td><td>SIG+FGS</td><td>803</td><td>E4Q08_21970</td><td>betaproteobacteria</td><td>Candidatus Accumulibacter phosphatis</td><td>hypothetical protein E4Q08_21970 [Candidatus Accumulibacter phosphatis].</td><td>-</td></tr>
<tr><td>NMQ07738.1</td><td><-BLBD<-?||?→?→||<-?<-PSE<-?<-?||TIR+nSTAND1+FGS*→ FGS→||<-Four-helical-protein||RVT→||<-TIR+FGS<-?<-RVT</td><td>TIR+nSTAND1+FGS</td><td>955</td><td>E4Q08_22105</td><td>betaproteobacteria</td><td>Candidatus Accumulibacter phosphatis</td><td>TIR domain-containing protein [Candidatus Accumulibacter phosphatis].</td><td>-</td></tr>
<tr><td>NNJ84960.1</td><td>FGS*→</td><td>FGS</td><td>333</td><td>HKP13_08525</td><td>gammaproteobacteria</td><td>Gammaproteobacteria bacterium</td><td>SUMF1/EgtB/PvdO family nonheme iron enzyme [Gammaproteobacteria bacterium].</td><td>-</td></tr>
<tr><td>NNK95096.1</td><td>NACHT+FGS*→</td><td>NACHT+FGS</td><td>471</td><td>HKP41_12165</td><td>deltaproteobacteria</td><td>Desulfobacterales bacterium</td><td>formylglycine-generating enzyme family protein, partial [Desulfobacterales bacterium].</td><td>-</td></tr>
<tr><td>OAI48271.1</td><td>EAD1+nSTAND1+FGS*→?→ EAD1+Trypsin→||<-?||?→?→ VWA→</td><td>EAD1+nSTAND1+FGS</td><td>770</td><td>AYO44_07515</td><td>planctomycetes</td><td>Planctomycetaceae bacterium SCGC AG-212-F19</td><td>hypothetical protein AYO44_07515 [Planctomycetaceae bacterium SCGC AG-212-F19].</td><td>GCA_001644415.1</td></tr>
</tbody>
</table>

acc	operon	architecture	len	gen.name	taxend	species	defline	gca
OGO38189.1	<-ABhydrolase<-?<-?<-PSE<-PSE<-TIMbarrel||?→||<-?<-?||FGS*→	FGS	282	A2W35_08715	chloroflexi	Chloroflexi bacterium RBG_16_57_11	hypothetical protein A2W35_08715 [Chloroflexi bacterium RBG_16_57_11].	GCA_001795165.1
OGO41187.1	SIG+TM+NACHT+FGS*→	SIG+TM+NACHT+FGS	973	A2W36_01705	chloroflexi	Chloroflexi bacterium RBG_16_58_14	hypothetical protein A2W36_01705 [Chloroflexi bacterium RBG_16_58_14].	GCA_001796915.1
OIO90464.1	PIN→?→?→?→ CASPASE+FGS*→	CASPASE+FGS	501	AUK03_12910	chloroflexi	Anaerolineae bacterium CG2_30_64_16	hypothetical protein AUK03_12910 [Anaerolineae bacterium CG2_30_64_16].	GCA_001871755.1
OQA20201.1	NACHT+FGS*→	NACHT+FGS	1059	pkn1_2	chloroflexi	Chloroflexi bacterium ADurb.Bin360	Serine/threonine-protein kinase pkn1 [Chloroflexi bacterium ADurb.Bin360].	GCA_002068755.1
OQA95056.1	NACHT+FGS*→	NACHT+FGS	891	pkn1_5	chloroflexi	Chloroflexi bacterium ADurb.Bin222	Serine/threonine-protein kinase pkn1 [Chloroflexi bacterium ADurb.Bin222].	GCA_002069935.1
OQA95154.1	FGS*→?→ RVT→ Four-helical-protein→	FGS	256	pkn1_4	chloroflexi	Chloroflexi bacterium ADurb.Bin222	Serine/threonine-protein kinase pkn1 [Chloroflexi bacterium ADurb.Bin222].	GCA_002069935.1
OQB01206.1	<-SIG+TPR+TPR+TPR<-?<-?<-?||FGS*→	FGS	260	pkn1_3	chloroflexi	Chloroflexi bacterium ADurb.Bin222	Serine/threonine-protein kinase pkn1 [Chloroflexi bacterium ADurb.Bin222].	GCA_002069935.1
OQB02365.1	TM→?→?→ EAD7→?→?→?→ ZNR+NACHT+FGS*→ RVT→	ZNR+NACHT+FGS	965	egtB	chloroflexi	Chloroflexi bacterium ADurb.Bin222	Iron(II)-dependent oxidoreductase EgtB [Chloroflexi bacterium ADurb.Bin222].	GCA_002069935.1
OQW81078.1	ABC-ATPase→ DrHyd+nSTAND1+FGS*→	DrHyd+nSTAND1+FGS	908	BVN30_11975	proteobacteria	Proteobacteria bacterium ST_bin16	hypothetical protein BVN30_11975 [Proteobacteria bacterium ST_bin16].	GCA_002083595.1
OQX00845.1	TIR+NACHT+FGS*→	TIR+NACHT+FGS	954	BWK73_47570	gammaproteobacteria	Thiothrix lacustris	hypothetical protein BWK73_47570 [Thiothrix lacustris].	GCA_002083875.1
OQX02689.1	TIR+FGS*→||<-RVT	TIR+FGS	455	BWK73_41905	gammaproteobacteria	Thiothrix lacustris	hypothetical protein BWK73_41905 [Thiothrix lacustris].	GCA_002083875.1
OQX03012.1	FGS→ SIG+TM+NACHT+FGS*→ RVT→ Four-helical-protein→	SIG+TM+NACHT+FGS	959	BWK73_40840	gammaproteobacteria	Thiothrix lacustris	hypothetical protein BWK73_40840 [Thiothrix lacustris].	GCA_002083875.1
OQX05770.1	TIR+NACHT+FGS*→?→ MED26C→ ABhydrolase→	TIR+NACHT+FGS	934	BWK73_32675	gammaproteobacteria	Thiothrix lacustris	hypothetical protein BWK73_32675 [Thiothrix lacustris].	GCA_002083875.1
OQX14334.1	<-PAS+HISKIN+REC||?→||<-?||?→||<-?||?→||<-?||TIR+FGS*→||<-?||?→?→||<-PAS+HISKIN+REC	TIR+FGS	427	BWK73_09810	gammaproteobacteria	Thiothrix lacustris	hypothetical protein BWK73_09810 [Thiothrix lacustris].	GCA_002083875.1
OQY20877.1	ZNR+NACHT→ ZNR+NACHT+FGS*→	ZNR+NACHT+FGS	991	B6I35_09750	chloroflexi	Anaerolineaceae bacterium 4572_32.2	hypothetical protein B6I35_09750 [Anaerolineaceae bacterium 4572_32.2].	GCA_002084625.1
OQY22096.1	KAP-NTPase+FGS*→||<-?<-?<-PSE<-TPR+TPR+TPR+TPR	KAP-NTPase+FGS	762	B6I35_06850	chloroflexi	Anaerolineaceae bacterium 4572_32.2	hypothetical protein B6I35_06850 [Anaerolineaceae bacterium 4572_32.2].	GCA_002084625.1
OQY80126.1	FGS*→||<-ABhydrolase	FGS	253	B6D42_13475	chloroflexi	Anaerolineae bacterium UTCFX5	hypothetical protein B6D42_13475 [Anaerolineae bacterium UTCFX5].	GCA_002050245.1

<table>
<tr><th>acc</th><th>operon</th><th>architecture</th><th>len</th><th>gen.name</th><th>taxend</th><th>species</th><th>defline</th><th>gca</th></tr>
<tr><td>OQY84655.1</td><td>NACHT+FGS*→</td><td>NACHT+FGS</td><td>1052</td><td>B6D41_15310</td><td>chloroflexi</td><td>Chloroflexi bacterium UTCFX4</td><td>hypothetical protein B6D41_15310 [Chloroflexi bacterium UTCFX4].</td><td>GCA_002050085.1</td></tr>
<tr><td>OQY90620.1</td><td>ABC-ATPase→ ABC-ATPase→?→?→?→?→ KAP-NTPase+FGS*→||<-?||?→ SWC3→</td><td>KAP-NTPase+FGS</td><td>730</td><td>B6D38_02510</td><td>chloroflexi</td><td>Anaerolineae bacterium UTCFX1</td><td>hypothetical protein B6D38_02510 [Anaerolineae bacterium UTCFX1].</td><td>GCA_002050275.1</td></tr>
<tr><td>OQY94544.1</td><td><-LexA-HTH+LexA-protease<-?||SIG+TM+TM+TM+TM+TM+TM+TM+TM+TM+TM+TM+TM→?→ SIG+NACHT+FGS*→ SIG+SWC3→</td><td>SIG+NACHT+FGS</td><td>1100</td><td>B6D41_06985</td><td>chloroflexi</td><td>Chloroflexi bacterium UTCFX4</td><td>hypothetical protein B6D41_06985 [Chloroflexi bacterium UTCFX4].</td><td>GCA_002050085.1</td></tr>
<tr><td>OQZ00200.1</td><td>NACHT+FGS*→ Four-helical-protein→ RVT→</td><td>NACHT+FGS</td><td>612</td><td>B6D41_00255</td><td>chloroflexi</td><td>Chloroflexi bacterium UTCFX4</td><td>hypothetical protein B6D41_00255, partial [Chloroflexi bacterium UTCFX4].</td><td>GCA_002050085.1</td></tr>
<tr><td>OQZ00584.1</td><td>SIG+DrHyd+VirB11+NACHT+FGS*→?→||<-ICP8OBfold<-MED26C</td><td>SIG+DrHyd+VirB11+NACHT+FGS</td><td>983</td><td>B6D35_06300</td><td>planctomycetes</td><td>Candidatus Brocadia sp. UTAMX2</td><td>hypothetical protein B6D35_06300 [Candidatus Brocadia sp. UTAMX2].</td><td>GCA_002050315.1</td></tr>
<tr><td>PDW00941.1</td><td>MNS-STAND+FGS*→ KAP-NTPase→</td><td>MNS-STAND+FGS</td><td>742</td><td>A9Q02_21485</td><td>chloroflexi</td><td>Candidatus Chloroploca asiatica</td><td>hypothetical protein A9Q02_21485 [Candidatus Chloroploca asiatica].</td><td>GCA_002532075.1</td></tr>
<tr><td>PDW01800.1</td><td>MNS-STAND+FGS*→</td><td>MNS-STAND+FGS</td><td>619</td><td>CJ255_17210</td><td>chloroflexi</td><td>Candidatus Viridilinea mediisalina</td><td>hypothetical protein CJ255_17210 [Candidatus Viridilinea mediisalina].</td><td>GCA_002532535.1</td></tr>
<tr><td>PID85266.1</td><td>NACHT+FGS*→ RVT→ Four-helical-protein→ EAD1+Trypsin→</td><td>NACHT+FGS</td><td>946</td><td>CSB13_08890</td><td>chloroflexi</td><td>Chloroflexi bacterium</td><td>hypothetical protein CSB13_08890 [Chloroflexi bacterium].</td><td>GCA_002746795.1</td></tr>
<tr><td>PIE33434.1</td><td><-Cluster366_2clades<-?||?→?→?→?→ NACHT+FGS*→</td><td>NACHT+FGS</td><td>750</td><td>CSA56_11545</td><td>bacteria</td><td>candidate division KSB3 bacterium</td><td>hypothetical protein CSA56_11545 [candidate division KSB3 bacterium].</td><td>GCA_002748445.1</td></tr>
<tr><td>PIE79716.1</td><td>NACHT+FGS→*?*→ *NACHT+FGS*→?→ SIG+TM+HAMP+HISKIN→</td><td>NACHT+FGS</td><td>370</td><td>CSA11_10845</td><td>chloroflexi</td><td>Chloroflexi bacterium</td><td>hypothetical protein CSA11_10845, partial [Chloroflexi bacterium].</td><td>GCA_002748985.1</td></tr>
<tr><td>PIE79718.1</td><td>NACHT+FGS*→</td><td>NACHT+FGS</td><td>1071</td><td>CSA11_10855</td><td>chloroflexi</td><td>Chloroflexi bacterium</td><td>hypothetical protein CSA11_10855 [Chloroflexi bacterium].</td><td>GCA_002748985.1</td></tr>
<tr><td>PJF20835.1</td><td>TIR+FGS*→</td><td>TIR+FGS</td><td>353</td><td>CUN56_14160</td><td>chloroflexi</td><td>Candidatus Thermofonsia Clade 2 bacterium</td><td>hypothetical protein CUN56_14160 [Candidatus Thermofonsia Clade 2 bacterium].</td><td>GCA_002794505.1</td></tr>
<tr><td>PJF23219.1</td><td>TIR+FGS*→</td><td>TIR+FGS</td><td>490</td><td>CUN56_01990</td><td>chloroflexi</td><td>Candidatus Thermofonsia Clade 2 bacterium</td><td>hypothetical protein CUN56_01990 [Candidatus Thermofonsia Clade 2 bacterium].</td><td>GCA_002794505.1</td></tr>
<tr><td>PJF28084.1</td><td>TCAD10→ TIR+FGS*→</td><td>TIR+FGS</td><td>506</td><td>CUN53_01595</td><td>chloroflexi</td><td>Candidatus Thermofonsia Clade 2 bacterium</td><td>hypothetical protein CUN53_01595 [Candidatus Thermofonsia Clade 2 bacterium].</td><td>GCA_002794695.1</td></tr>
<tr><td>PJF28825.1</td><td>TIR+FGS*→</td><td>TIR+FGS</td><td>500</td><td>CUN52_11490</td><td>chloroflexi</td><td>Candidatus Thermofonsia Clade 2 bacterium</td><td>hypothetical protein CUN52_11490 [Candidatus Thermofonsia Clade 2 bacterium].</td><td>GCA_002794515.1</td></tr>
</table>

<table>
<tr><th>acc</th><th>operon</th><th>architecture</th><th>len</th><th>gen.name</th><th>taxend</th><th>species</th><th>defline</th><th>gca</th></tr>
<tr><td>PJF29540.1</td><td>DrHyd→ nSTAND1+FGS*→</td><td>nSTAND1+FGS</td><td>718</td><td>CUN52_07865</td><td>chloroflexi</td><td>Candidatus Thermofonsia Clade 2 bacterium</td><td>hypothetical protein CUN52_07865 [Candidatus Thermofonsia Clade 2 bacterium].</td><td>GCA_002794515.1</td></tr>
<tr><td>PJF30051.1</td><td>TIR+FGS*→</td><td>TIR+FGS</td><td>445</td><td>CUN52_05275</td><td>chloroflexi</td><td>Candidatus Thermofonsia Clade 2 bacterium</td><td>hypothetical protein CUN52_05275, partial [Candidatus Thermofonsia Clade 2 bacterium].</td><td>GCA_002794515.1</td></tr>
<tr><td>PJF30836.1</td><td>TIR+FGS*→</td><td>TIR+FGS</td><td>513</td><td>CUN51_06530</td><td>chloroflexi</td><td>Candidatus Thermofonsia Clade 1 bacterium</td><td>hypothetical protein CUN51_06530 [Candidatus Thermofonsia Clade 1 bacterium].</td><td>GCA_002794705.1</td></tr>
<tr><td>PJF31663.1</td><td><-ABhydrolase||DrHyd+nSTAND1+FGS*→</td><td>DrHyd+nSTAND1+FGS</td><td>1006</td><td>CUN52_02370</td><td>chloroflexi</td><td>Candidatus Thermofonsia Clade 2 bacterium</td><td>hypothetical protein CUN52_02370 [Candidatus Thermofonsia Clade 2 bacterium].</td><td>GCA_002794515.1</td></tr>
<tr><td>PJF37172.1</td><td>TIR+FGS*→</td><td>TIR+FGS</td><td>525</td><td>CUN49_01770</td><td>chloroflexi</td><td>Candidatus Thermofonsia Clade 1 bacterium</td><td>hypothetical protein CUN49_01770 [Candidatus Thermofonsia Clade 1 bacterium].</td><td>GCA_002794555.1</td></tr>
<tr><td>PJF43622.1</td><td>TIR+FGS*→</td><td>TIR+FGS</td><td>486</td><td>CUN55_08140</td><td>chloroflexi</td><td>Candidatus Thermofonsia Clade 2 bacterium</td><td>hypothetical protein CUN55_08140, partial [Candidatus Thermofonsia Clade 2 bacterium].</td><td>GCA_002794585.1</td></tr>
<tr><td>PJF44139.1</td><td>FGS*→</td><td>FGS</td><td>376</td><td>CUN55_05530</td><td>chloroflexi</td><td>Candidatus Thermofonsia Clade 2 bacterium</td><td>hypothetical protein CUN55_05530 [Candidatus Thermofonsia Clade 2 bacterium].</td><td>GCA_002794585.1</td></tr>
<tr><td>PUB73139.1</td><td>ABHYDROLASE→?→?→ drhyd+NACHT+FGS*→||<-?<-?<-SIG+CACHE+TM+PAS+PAS+GGDEF+EAL<-?||?→?→||<-CBS</td><td>drhyd+NACHT+FGS</td><td>1022</td><td>DBO99_20690</td><td>gammaproteobacteria</td><td>gamma proteobacterium symbiont of Ctena orbiculata</td><td>hypothetical protein DBO99_20690 [gamma proteobacterium symbiont of Ctena orbiculata].</td><td>GCA_003058485.1</td></tr>
<tr><td>PUB73261.1</td><td>ABHYDROLASE→?→?→ drhyd+NACHT+FGS*→||<-?<-?<-SIG+CACHE+TM+PAS+PAS+GGDEF+EAL</td><td>drhyd+NACHT+FGS</td><td>1022</td><td>DBP03_13830</td><td>gammaproteobacteria</td><td>gamma proteobacterium symbiont of Ctena orbiculata</td><td>hypothetical protein DBP03_13830 [gamma proteobacterium symbiont of Ctena orbiculata].</td><td>GCA_003058495.1</td></tr>
<tr><td>PUB76292.1</td><td>TIR+nSTAND1+FGS→ FGS→ FGS→||<-RVT<-?<-TIR+FGS*<-MoxR-AAA<-TIR<-TIR+TCAD2</td><td>TIR+nSTAND1+FGS</td><td>913</td><td>DBP03_04845</td><td>gammaproteobacteria</td><td>gamma proteobacterium symbiont of Ctena orbiculata</td><td>hypothetical protein DBP03_04845 [gamma proteobacterium symbiont of Ctena orbiculata].</td><td>GCA_003058495.1</td></tr>
<tr><td>PUB76293.1</td><td>FGS*→</td><td>FGS</td><td>255</td><td>DBP03_04850</td><td>gammaproteobacteria</td><td>gamma proteobacterium symbiont of Ctena orbiculata</td><td>hypothetical protein DBP03_04850 [gamma proteobacterium symbiont of Ctena orbiculata].</td><td>GCA_003058495.1</td></tr>
<tr><td>PUB76297.1</td><td>TIR+FGS*→</td><td>TIR+FGS</td><td>961</td><td>DBP03_04870</td><td>gammaproteobacteria</td><td>gamma proteobacterium symbiont of Ctena orbiculata</td><td>hypothetical protein DBP03_04870 [gamma proteobacterium symbiont of Ctena orbiculata].</td><td>GCA_003058495.1</td></tr>
<tr><td>PWB73128.1</td><td>nSTAND1-C+FGS*→||<-RVT||Four-helical-protein→||<-FGS</td><td>nSTAND1-C+FGS</td><td>467</td><td>C3F07_10390</td><td>chloroflexi</td><td>Anaerolineales bacterium</td><td>hypothetical protein C3F07_10390, partial [Anaerolineales bacterium].</td><td>GCA_003105325.1</td></tr>
</table>

<table>
<tr><th>acc</th><th>operon</th><th>architecture</th><th>len</th><th>gen.name</th><th>taxend</th><th>species</th><th>defline</th><th>gca</th></tr>
<tr><td>PWH19196.1</td><td>TPR→?→ FGS*→</td><td>FGS</td><td>269</td><td>DDG59_03890</td><td>chloroflexi</td><td>Anaerolineae bacterium</td><td>formylglycine-generating enzyme family protein [Anaerolineae bacterium].</td><td>GCA_003130835.1</td></tr>
<tr><td>PYR00059.1</td><td>drhyd+NACHT+FGS*→?→ ORF6N→ VWA→</td><td>drhyd+NACHT+FGS</td><td>998</td><td>DMF96_04700</td><td>acidobacteria</td><td>Acidobacteria bacterium</td><td>hypothetical protein DMF96_04700 [Acidobacteria bacterium].</td><td>GCA_003222505.1</td></tr>
<tr><td>PYR17830.1</td><td>SIG+HISKIN+REC→?→ drhyd+NACHT+FGS*→?→ ORF6N→ VWA→</td><td>drhyd+NACHT+FGS</td><td>998</td><td>DMF94_22285</td><td>acidobacteria</td><td>Acidobacteria bacterium</td><td>hypothetical protein DMF94_22285 [Acidobacteria bacterium].</td><td>GCA_003222535.1</td></tr>
<tr><td>PYR41523.1</td><td>drhyd+NACHT+FGS*→?→ ORF6N→ VWA→</td><td>drhyd+NACHT+FGS</td><td>858</td><td>DMF95_30835</td><td>acidobacteria</td><td>Acidobacteria bacterium</td><td>hypothetical protein DMF95_30835, partial [Acidobacteria bacterium].</td><td>GCA_003223755.1</td></tr>
<tr><td>PZN49102.1</td><td>TIR+FGS*→</td><td>TIR+FGS</td><td>519</td><td>DIU68_20660</td><td>chloroflexi</td><td>Chloroflexi bacterium</td><td>hypothetical protein DIU68_20660 [Chloroflexi bacterium].</td><td>GCA_003242205.1</td></tr>
<tr><td>PZN54847.1</td><td>ABC-ATPase→?→||<-Cluster235_2clades<-?<-SIG+TM+TM+TM+TM+TM<-?||FGS*→</td><td>FGS</td><td>254</td><td>DIU68_09635</td><td>chloroflexi</td><td>Chloroflexi bacterium</td><td>hypothetical protein DIU68_09635 [Chloroflexi bacterium].</td><td>GCA_003242205.1</td></tr>
<tr><td>PZN71605.1</td><td>FGS*→||<-RVT</td><td>FGS</td><td>215</td><td>DM484_25995</td><td>gammaproteobacteria</td><td>Candidatus Methyloumidiphilus alinensis</td><td>hypothetical protein DM484_25995, partial [Candidatus Methyloumidiphilus alinensis].</td><td>GCA_003242955.1</td></tr>
<tr><td>PZN73048.1</td><td>nSTAND1-C+FGS*→</td><td>nSTAND1-C+FGS</td><td>478</td><td>DM484_23385</td><td>gammaproteobacteria</td><td>Candidatus Methyloumidiphilus alinensis</td><td>hypothetical protein DM484_23385, partial [Candidatus Methyloumidiphilus alinensis].</td><td>GCA_003242955.1</td></tr>
<tr><td>PZN74864.1</td><td>CASPASE+NACHT+FGS*→?→ Trypco1→</td><td>CASPASE+NACHT+FGS</td><td>1074</td><td>DM484_20170</td><td>gammaproteobacteria</td><td>Candidatus Methyloumidiphilus alinensis</td><td>hypothetical protein DM484_20170 [Candidatus Methyloumidiphilus alinensis].</td><td>GCA_003242955.1</td></tr>
<tr><td>PZN74977.1</td><td>NACHT+FGS*→||<-?<-Four-helical-protein||RVT→||<-NACHT+FGS<-?<-TIR<-DrHyd</td><td>NACHT+FGS</td><td>914</td><td>DM484_19825</td><td>gammaproteobacteria</td><td>Candidatus Methyloumidiphilus alinensis</td><td>hypothetical protein DM484_19825 [Candidatus Methyloumidiphilus alinensis].</td><td>GCA_003242955.1</td></tr>
<tr><td>PZN77746.1</td><td>DrHyd+nSTAND1+FGS*→</td><td>DrHyd+nSTAND1+FGS</td><td>1041</td><td>DM484_14085</td><td>gammaproteobacteria</td><td>Candidatus Methyloumidiphilus alinensis</td><td>hypothetical protein DM484_14085 [Candidatus Methyloumidiphilus alinensis].</td><td>GCA_003242955.1</td></tr>
<tr><td>PZN84786.1</td><td><-SAM-methylase<-?<-?||?→?→?→ drhyd+NACHT+FGS*→</td><td>drhyd+NACHT+FGS</td><td>1034</td><td>DM484_02145</td><td>gammaproteobacteria</td><td>Candidatus Methyloumidiphilus alinensis</td><td>hypothetical protein DM484_02145 [Candidatus Methyloumidiphilus alinensis].</td><td>GCA_003242955.1</td></tr>
<tr><td>RCK71670.1</td><td>TPR→?→ FGS*→||<-?<-?<-?<-?||?→?→ SIG+TM+TM+TM+TM+TM+TM+TM+TM→</td><td>FGS</td><td>269</td><td>ANABAC_1196</td><td>chloroflexi</td><td>Anaerolineae bacterium</td><td>serine/threonine kinase [Anaerolineae bacterium].</td><td>GCA_003327455.1</td></tr>
<tr><td>RCV64808.1</td><td>TIR→ ParA-Soj-PloopNTPase+TIR+NACHT+FGS→*?→ Calcineurin+NACHT+FGS*→?→?→?→?→?→||<-?||LRP-HTH→</td><td>ParA-Soj-PloopNTPase+TIR+NACHT+FGS</td><td>1130</td><td>C5S53_06925</td><td>euryarchaeota</td><td>Methanophagales archaeon</td><td>Formylglycine-generating enzyme [Methanophagales archaeon].</td><td>GCA_003336485.1</td></tr>
<tr><td>RCV64810.1</td><td>ParA-Soj-PloopNTPase+TIR+NACHT+FGS*→</td><td>ParA-Soj-PloopNTPase+TIR+NACHT+FGS</td><td>1273</td><td>C5S53_06935</td><td>euryarchaeota</td><td>Methanophagales archaeon</td><td>Formylglycine-generating enzyme [Methanophagales archaeon].</td><td>GCA_003336485.1</td></tr>
<tr><td>RDE49756.1</td><td>TIR+nSTAND1+FGS*→||<-?<-?||?→?→ KAP-NTPase→</td><td>TIR+nSTAND1+FGS</td><td>1034</td><td>DVS81_14945</td><td>betaproteobacteria</td><td>Candidatus Accumulibacter phosphatis</td><td>TIR domain-containing protein [Candidatus Accumulibacter phosphatis].</td><td>GCA_003332265.1</td></tr>
<tr><td>RDE50257.1</td><td>TIR+NACHT+FGS*→||<-?<-FGS</td><td>TIR+NACHT+FGS</td><td>962</td><td>DVS81_12365</td><td>betaproteobacteria</td><td>Candidatus Accumulibacter phosphatis</td><td>TIR domain-containing protein [Candidatus Accumulibacter phosphatis].</td><td>GCA_003332265.1</td></tr>
</table>

acc	operon	architecture	len	gen.name	taxend	species	defline	gca
RDE50357.1	FAD-NAD-dep-oxidoreductase→?→||<-?<-?<-?<-ABhydrolase||?→ nSTAND1+FGS*→||<-SIG+CACHE+TM+PAS+PAS+GGDEF+EAL<-?||?→||<-PIN	nSTAND1+FGS	753	DVS81_11680	betaproteobacteria	Candidatus Accumulibacter phosphatis	hypothetical protein DVS81_11680 [Candidatus Accumulibacter phosphatis].	GCA_003332265.1
RDE51785.1	EAD8+nSTAND1+FGS*→ EAD8+TIR+NACHT+FGS→	EAD8+nSTAND1+FGS	830	DVS81_03940	betaproteobacteria	Candidatus Accumulibacter phosphatis	hypothetical protein DVS81_03940 [Candidatus Accumulibacter phosphatis].	GCA_003332265.1
RIK31324.1	KAP-NTPase+FGS*→||<-?<-ICP8OBfold<-MED26C	KAP-NTPase+FGS	831	DCC56_03825	chloroflexi	Anaerolineae bacterium	hypothetical protein DCC56_03825 [Anaerolineae bacterium].	GCA_003577395.1
RIK35349.1	NACHT+FGS*→	NACHT+FGS	973	DCC55_30565	chloroflexi	Chloroflexi bacterium	hypothetical protein DCC55_30565 [Chloroflexi bacterium].	GCA_003577005.1
RIL10952.1	EAD8→?→ EAD8→ KAP-NTPase+FGS*→ TIR→||<-?||ACYC+APATPase+TPR→||<-?||?→||<-ACYC+NACHT+FGS	KAP-NTPase+FGS	836	DCC79_06390	bacteria	bacterium	hypothetical protein DCC79_06390 [bacterium].	GCA_003576755.1
RIL11051.1	EAD8+NACHT+FGS*→||<-EAD8+NACHT+FGS	EAD8+NACHT+FGS	967	DCC79_06090	bacteria	bacterium	hypothetical protein DCC79_06090 [bacterium].	GCA_003576755.1
RIL11755.1	FGS→||<-?<-?||?→ PSE→ PSE→?→ TIR+NACHT+FGS→ *FGS→||<-Four-helical-protein||RVT→||<-DrHyd+nSTAND1+FGS*||?→?→ ABhydrolase+ABhydrolase+CASPASE+TPR-S→	TIR+NACHT+FGS	995	DCC79_03775	bacteria	bacterium	hypothetical protein DCC79_03775 [bacterium].	GCA_003576755.1
RIL11759.1	DrHyd+nSTAND1+FGS*→	DrHyd+nSTAND1+FGS	944	DCC79_03795	bacteria	bacterium	hypothetical protein DCC79_03795 [bacterium].	GCA_003576755.1
RKU24994.1	HSP70→ FGS→ DNAJ+FGS*→	DNAJ+FGS	445	C6499_15805	bacteria	Candidatus Poribacteria bacterium	hypothetical protein C6499_15805 [Candidatus Poribacteria bacterium].	GCA_003635255.1
RLC59981.1	ABC-ATPase→?→ KAP-NTPase+FGS*→	KAP-NTPase+FGS	780	DRI80_11660	chloroflexi	Chloroflexi bacterium	hypothetical protein DRI80_11660 [Chloroflexi bacterium].	GCA_003647875.1
RLC64729.1	<-Cluster366_2clades<-?<-?||ACYC+ACYC+SWACOS+TPR+TPR+TPR→?→?→?→ KAP-NTPase+FGS*→	KAP-NTPase+FGS	778	DRI80_00630	chloroflexi	Chloroflexi bacterium	hypothetical protein DRI80_00630 [Chloroflexi bacterium].	GCA_003647875.1
RLC71356.1	NACHT+FGS*→ Four-helical-protein→	NACHT+FGS	843	DRI81_17855	chloroflexi	Chloroflexi bacterium	hypothetical protein DRI81_17855, partial [Chloroflexi bacterium].	GCA_003648025.1
RLC71990.1	FGS*→	FGS	341	DRI81_16965	chloroflexi	Chloroflexi bacterium	hypothetical protein DRI81_16965 [Chloroflexi bacterium].	GCA_003648025.1
RLC79416.1	KAP-NTPase+FGS*→||<-?<-?<-?<-TPR+TPR+TPR+TPR	KAP-NTPase+FGS	762	DRI81_05360	chloroflexi	Chloroflexi bacterium	hypothetical protein DRI81_05360 [Chloroflexi bacterium].	GCA_003648025.1
RLC80321.1	ZNR+NACHT+FGS*→ Four-helical-protein→ RVT→ SIG+SWC3→	ZNR+NACHT+FGS	945	DRJ03_21950	chloroflexi	Chloroflexi bacterium	hypothetical protein DRJ03_21950 [Chloroflexi bacterium].	GCA_003648075.1
RLC85952.1	FGS*→||<-?<-?<-?<-TPR+TPR+TPR+TPR	FGS	288	DRJ03_10225	chloroflexi	Chloroflexi bacterium	DNA-binding protein [Chloroflexi bacterium].	GCA_003648075.1
RLD03540.1	NACHT+FGS*→ TIR+TCAD2→	NACHT+FGS	911	DRI56_12305	chloroflexi	Chloroflexi bacterium	hypothetical protein DRI56_12305 [Chloroflexi bacterium].	GCA_003648195.1
RLJ15771.1	<-Cluster393_2clades<-Pentapeptide||?→ TIR→ MoxR-AAA→||<-?||SIG+FGS*→||<-?<-PSE<-?<-Cluster187_2clades<-?<-HSP70<-?<-IES1	SIG+FGS	788	DJ030_18040	bacteria	bacterium endosymbiont of Escarpia laminata	hypothetical protein DJ030_18040 [bacterium endosymbiont of Escarpia laminata].	GCA_003660225.1

acc	operon	architecture	len	gen.name	taxend	species	defline	gca
RLJ21529.1	MalT→ Cluster440_2clades→ SIG+RFC-AAA→?→ SIG+TM+TM+TM+TM→ SIG+TM+HAMP→?→ drhyd+NACHT+FGS*→	drhyd+NACHT+FGS	953	DJ031_02685	bacteria	bacterium endosymbiont of Escarpia laminata	hypothetical protein DJ031_02685 [bacterium endosymbiont of Escarpia laminata].	GCA_003660235.1
RLJ22000.1	SIG+FGS*→||<-?<-Cluster187_2clades<-?<-HSP70<-?<-IES1	SIG+FGS	788	DJ031_01870	bacteria	bacterium endosymbiont of Escarpia laminata	hypothetical protein DJ031_01870 [bacterium endosymbiont of Escarpia laminata].	GCA_003660235.1
RLT41139.1	NACHT+FGS*→ RVT→ Four-helical-protein→	NACHT+FGS	1064	DWI57_07410	chloroflexi	Chloroflexi bacterium	NACHT domain-containing protein [Chloroflexi bacterium].	GCA_003670625.1
RLW71008.1	TIR→ TIR+FGS→ SIG+FGS*→	SIG+FGS	146	B6D71_04250	gammaproteobacteria	gamma proteobacterium symbiont of Stewartia floridana	hypothetical protein B6D71_04250, partial [gamma proteobacterium symbiont of Stewartia floridana].	GCA_003676185.1
RME02281.1	NACHT+FGS*→	NACHT+FGS	853	D6816_11190	bacteroidetes	Bacteroidetes bacterium	NACHT domain-containing protein, partial [Bacteroidetes bacterium].	GCA_003694615.1
RME06715.1	FGS*→	FGS	309	D6816_07410	bacteroidetes	Bacteroidetes bacterium	hypothetical protein D6816_07410 [Bacteroidetes bacterium].	GCA_003694615.1
RME41053.1	NACHT+FGS*→	NACHT+FGS	885	D6796_16335	chloroflexi	Caldilineae bacterium	NACHT domain-containing protein, partial [Caldilineae bacterium].	GCA_003695385.1
RMF51509.1	TIR+FGS*→	TIR+FGS	471	D6749_07615	chloroflexi	Chloroflexi bacterium	TIR domain-containing protein [Chloroflexi bacterium].	GCA_003695215.1
RMF78466.1	TIR+NACHT+TCAD10+TCAD10→ TIR+FGS*→	TIR+FGS	519	D6737_14645	chloroflexi	Chloroflexi bacterium	TIR domain-containing protein [Chloroflexi bacterium].	GCA_003695675.1
RMG71792.1	TIR→ EAD8+TIR+NACHT+FGS*→ FGS→	EAD8+TIR+NACHT+FGS	1095	D6711_14505	chloroflexi	Chloroflexi bacterium	TIR domain-containing protein [Chloroflexi bacterium].	GCA_003696565.1
RMG75601.1	TIR+FGS*→	TIR+FGS	463	D6711_06165	chloroflexi	Chloroflexi bacterium	TIR domain-containing protein [Chloroflexi bacterium].	GCA_003696565.1
RMG81795.1	TIR+FGS*→	TIR+FGS	524	D6712_15685	chloroflexi	Chloroflexi bacterium	TIR domain-containing protein [Chloroflexi bacterium].	GCA_003696545.1
RNC70126.1	<-ABhydrolase+ABhydrolase+CASPASE+TPR-S<-TIR+APATPase<-?||DrHyd+nSTAND1+FGS*→ DrHyd+NACHT+FGS→||<-?||?→?→?→ TRD+TRD→ SF2-DUF3427A+LPD33→	DrHyd+nSTAND1+FGS	908	ED859_06590	deltaproteobacteria	Desulfuromonadales bacterium	DUF4062 domain-containing protein [Desulfuromonadales bacterium].	GCA_003712145.1
RPH58975.1	NACHT+FGS*→	NACHT+FGS	477	EHM81_09025	chloroflexi	Chloroflexi bacterium	hypothetical protein EHM81_09025, partial [Chloroflexi bacterium].	GCA_003818555.1
RPI90984.1	FGS*→	FGS	257	EHM40_16975	chloroflexi	Chloroflexi bacterium	hypothetical protein EHM40_16975 [Chloroflexi bacterium].	GCA_003820075.1
RPJ19812.1	NACHT+FGS*→	NACHT+FGS	400	EHM35_20270	planctomycetes	Planctomycetaceae bacterium	hypothetical protein EHM35_20270 [Planctomycetaceae bacterium].	GCA_003820145.1
RPJ25004.1	FGS*→	FGS	258	EHM33_15980	chloroflexi	Chloroflexi bacterium	hypothetical protein EHM33_15980 [Chloroflexi bacterium].	GCA_003820175.1
RQW03777.1	FGS*→ Four-helical-protein→ FGS→	FGS	342	EH223_08950	bacteria	candidate division KSB1 bacterium	formylglycine-generating enzyme family protein, partial [candidate division KSB1 bacterium].	GCA_003854975.1

<table>
<tr><th>acc</th><th>operon</th><th>architecture</th><th>len</th><th>gen.name</th><th>taxend</th><th>species</th><th>defline</th><th>gca</th></tr>
<tr><td>RQW04566.1</td><td>SIG+TM+TM+NACHT+TCAD10+FGS→ DrHyd+NACHT+FGS*→ DrHyd→</td><td>DrHyd+NACHT+FGS</td><td>977</td><td>EH223_07060</td><td>bacteria</td><td>candidate division KSB1 bacterium</td><td>DUF4062 domain-containing protein [candidate division KSB1 bacterium].</td><td>GCA_003854975.1</td></tr>
<tr><td>RQW05893.1</td><td>TM+NACHT+FGS*→ Four-helical-protein→ FGS→</td><td>TM+NACHT+FGS</td><td>922</td><td>EH223_03525</td><td>bacteria</td><td>candidate division KSB1 bacterium</td><td>NACHT domain-containing protein [candidate division KSB1 bacterium].</td><td>GCA_003854975.1</td></tr>
<tr><td>RRR65524.1</td><td>SIG+NACHT+FGS*→?→?→?→ KAP-NTPase→</td><td>SIG+NACHT+FGS</td><td>1077</td><td>EI684_22890</td><td>chloroflexi</td><td>Candidatus Viridilinea halotolerans</td><td>hypothetical protein EI684_22890 [Candidatus Viridilinea halotolerans].</td><td>GCA_003934145.1</td></tr>
<tr><td>RRR69407.1</td><td>iSTAND→?→?→ MoxR-AAA→ SIG+VWA→ vWA-L+MNS-STAND+FGS*→</td><td>vWA-L+MNS-STAND+FGS</td><td>920</td><td>EI684_15695</td><td>chloroflexi</td><td>Candidatus Viridilinea halotolerans</td><td>hypothetical protein EI684_15695 [Candidatus Viridilinea halotolerans].</td><td>GCA_003934145.1</td></tr>
<tr><td>RRR73738.1</td><td>VWA+NACHT+FGS*→</td><td>VWA+NACHT+FGS</td><td>1417</td><td>EI684_08395</td><td>chloroflexi</td><td>Candidatus Viridilinea halotolerans</td><td>NACHT domain-containing protein [Candidatus Viridilinea halotolerans].</td><td>GCA_003934145.1</td></tr>
<tr><td>RRR76062.1</td><td>MNS-STAND+FGS→ *MNS-STAND+FGS*→ Four-helical-protein→</td><td>MNS-STAND+FGS</td><td>605</td><td>EI684_03590</td><td>chloroflexi</td><td>Candidatus Viridilinea halotolerans</td><td>hypothetical protein EI684_03590 [Candidatus Viridilinea halotolerans].</td><td>GCA_003934145.1</td></tr>
<tr><td>RRR76063.1</td><td>MNS-STAND+EAD9+FGS*→</td><td>MNS-STAND+EAD9+FGS</td><td>715</td><td>EI684_03595</td><td>chloroflexi</td><td>Candidatus Viridilinea halotolerans</td><td>hypothetical protein EI684_03595 [Candidatus Viridilinea halotolerans].</td><td>GCA_003934145.1</td></tr>
<tr><td>RRS32393.1</td><td><-Cluster393_2clades<-Pentapeptide||TIR→ MED26C→ vWA-L+FGS*→||<-Cluster187_2clades<-?<-HSP70<-?<-IES1</td><td>vWA-L+FGS</td><td>797</td><td>OI74_11720</td><td>gammaproteobacteria</td><td>Gammaproteobacteria bacterium (ex Lamellibrachia satsuma)</td><td>hypothetical protein OI74_11720 [Gammaproteobacteria bacterium (ex Lamellibrachia satsuma)].</td><td>GCA_003934985.1</td></tr>
<tr><td>RRS33515.1</td><td>SIG+TM+TM+TM+TM+TM→?→?→?→?→ TIR→?→ NACHT+FGS*→||<-?<-SIG+TM+HAMP<-SIG+TM+TM+TM+TM<-?<-SIG+RFC-AAA<-Cluster440_2clades<-MalT</td><td>NACHT+FGS</td><td>526</td><td>OI74_07790</td><td>gammaproteobacteria</td><td>Gammaproteobacteria bacterium (ex Lamellibrachia satsuma)</td><td>hypothetical protein OI74_07790 [Gammaproteobacteria bacterium (ex Lamellibrachia satsuma)].</td><td>GCA_003934985.1</td></tr>
<tr><td>RZB36777.1</td><td><-TM<-?||?→||<-?||?→ SIG+TM+TM+TM+TM+TM+TM+TM+TM+TM+TM+TM+TM+TM→||<-?||KAP-NTPase+FGS*→ Four-helical-protein→ NACHT+FGS→||<-?<-?<-?<-LexA-HTH+LexA-protease</td><td>KAP-NTPase+FGS</td><td>658</td><td>SRB2_01856</td><td>deltaproteobacteria</td><td>Desulfobacteraceae bacterium Eth-SRB2</td><td>hypothetical protein SRB2_01856 [Desulfobacteraceae bacterium Eth-SRB2].</td><td>GCA_004193595.1</td></tr>
<tr><td>TAH46387.1</td><td>PIN→ TIR+NACHT+FGS*→</td><td>TIR+NACHT+FGS</td><td>956</td><td>EYC67_09120</td><td>betaproteobacteria</td><td>Betaproteobacteria bacterium</td><td>TIR domain-containing protein [Betaproteobacteria bacterium].</td><td>GCA_004295105.1</td></tr>
<tr><td>TAH48128.1</td><td><-ABhydrolase||NACHT+FGS*→ ABC-ATPase→?→?→||<-PIN</td><td>NACHT+FGS</td><td>1067</td><td>EYC68_22510</td><td>chloroflexi</td><td>Chloroflexi bacterium</td><td>NACHT domain-containing protein [Chloroflexi bacterium].</td><td>GCA_004295045.1</td></tr>
<tr><td>TAH51040.1</td><td>TIR+FGS*→?→||<-?||?→ N-OB→</td><td>TIR+FGS</td><td>430</td><td>EYC68_12490</td><td>chloroflexi</td><td>Chloroflexi bacterium</td><td>TIR domain-containing protein [Chloroflexi bacterium].</td><td>GCA_004295045.1</td></tr>
<tr><td>TAN48986.1</td><td>SIG+TM+NACHT+FGS*→</td><td>SIG+TM+NACHT+FGS</td><td>961</td><td>EPN21_13470</td><td>gammaproteobacteria</td><td>Methylococcaceae bacterium</td><td>NACHT domain-containing protein [Methylococcaceae bacterium].</td><td>GCA_004299145.1</td></tr>
<tr><td>TEU10577.1</td><td>FGS*→?→||<-?<-?<-?<-TIR+TM+FGS</td><td>FGS</td><td>301</td><td>E3J21_25805</td><td>chloroflexi</td><td>Anaerolineales bacterium</td><td>formylglycine-generating enzyme family protein [Anaerolineales bacterium].</td><td>GCA_004377365.1</td></tr>
<tr><td>TEU11372.1</td><td>Pkinase+FGS*→</td><td>Pkinase+FGS</td><td>972</td><td>E3J25_08125</td><td>chloroflexi</td><td>Anaerolineales bacterium</td><td>hypothetical protein E3J25_08125 [Anaerolineales bacterium].</td><td>GCA_004377265.1</td></tr>
</table>

acc	operon	architecture	len	gen.name	taxend	species	defline	gca
TEU12777.1	FGS*→	FGS	245	E3J21_20265	chloroflexi	Anaerolineales bacterium	formylglycine-generating enzyme family protein [Anaerolineales bacterium].	GCA_004377365.1
TEU17403.1	FGS*→	FGS	293	E3J21_08455	chloroflexi	Anaerolineales bacterium	formylglycine-generating enzyme family protein [Anaerolineales bacterium].	GCA_004377365.1
TEU18290.1	<-RADICAL-SAM<-?<-?||?→?→ Pkinase+Pkinase+FGS*→	Pkinase+Pkinase+FGS	869	E3J21_06280	chloroflexi	Anaerolineales bacterium	hypothetical protein E3J21_06280 [Anaerolineales bacterium].	GCA_004377365.1
THI85089.1	<-SIG+TM+TM+TM+TM+TM+TM+TM+TM+TM+TM+TM<-?<-FURR-HTH||?→||<-?||?→?→ DrHyd+nSTAND1+FGS*→||<-TIR+TM+BetaPropeller||CASPASE→ CASPASE+TPR-S→	DrHyd+nSTAND1+FGS	886	CAF41_012685	nitrospirae	Nitrospira sp. CG24A	DUF4062 domain-containing protein [Nitrospira sp. CG24A].	GCA_002869925.2
THI90736.1	TIR+FGS→ DrHyd+nSTAND1+FGS*→	DrHyd+nSTAND1+FGS	877	CAF41_002995	nitrospirae	Nitrospira sp. CG24A	DUF4062 domain-containing protein [Nitrospira sp. CG24A].	GCA_002869925.2
TKB61807.1	nSTAND1+FGS*→	nSTAND1+FGS	646	E8D48_09975	nitrospirae	Nitrospira sp.	formylglycine-generating enzyme family protein [Nitrospira sp.].	GCA_005116895.1
TKB62458.1	TPR→?→?→?→?→||<-?||?→ PSE→ DrHyd+nSTAND1+FGS*→	DrHyd+nSTAND1+FGS	879	E8D49_01790	nitrospirae	Nitrospira sp.	DUF4062 domain-containing protein [Nitrospira sp.].	GCA_005116885.1
TKB70058.1	DrHyd+nSTAND1+FGS*→ Four-helical-protein→	DrHyd+nSTAND1+FGS	900	E8D52_03140	nitrospirae	Nitrospira sp.	DUF4062 domain-containing protein [Nitrospira sp.].	GCA_005116745.1
TKB79855.1	DrHyd+nSTAND1+FGS*→	DrHyd+nSTAND1+FGS	892	E8D42_00345	nitrospirae	Nitrospira sp.	DUF4062 domain-containing protein [Nitrospira sp.].	GCA_005116955.1
TKB89607.1	TPR→?→?→?→?→||<-?||?→ DrHyd+nSTAND1+FGS*→	DrHyd+nSTAND1+FGS	870	E8D41_13485	nitrospirae	Nitrospira sp.	DUF4062 domain-containing protein [Nitrospira sp.].	GCA_005116945.1
TKB92435.1	<-TIR||CASPASE→ FGS→?→ FGS*→ CASPASE+TPR-S→	FGS	279	E8D41_06385	nitrospirae	Nitrospira sp.	formylglycine-generating enzyme family protein [Nitrospira sp.].	GCA_005116945.1
TKJ29986.1	NACHT+FGS*→ KAP-NTPase+FGS→	NACHT+FGS	1038	CEE40_06785	chloroflexi	Chloroflexi bacterium B3_Chlor	hypothetical protein CEE40_06785 [Chloroflexi bacterium B3_Chlor].	GCA_005223035.1
TLD45545.1	<-PAS+HISKIN+REC||?→||<-?||?→ DrHyd+iSTAND→ MoxR-AAA→ SIG+VWA→ vWA-L+TIR+DrHyd+FGS*→	vWA-L+TIR+DrHyd+FGS	896	pkn1_9	betaproteobacteria	Candidatus Accumulibacter sp.	Serine/threonine-protein kinase pkn1 [Candidatus Accumulibacter sp.].	GCA_005524045.1
TLD45954.1	EAD8+DrHyd+nSTAND1+FGS*→||<-RVT||Four-helical-protein→||<-FGS<-TIR+NACHT+FGS	EAD8+DrHyd+nSTAND1+FGS	1062	pkn1_8	betaproteobacteria	Candidatus Accumulibacter sp.	Serine/threonine-protein kinase pkn1 [Candidatus Accumulibacter sp.].	GCA_005524045.1
TLD46242.1	SIG+TM+TM+TM+TM+TM+TM+TM+TM→||<-Patatin||?→||<-?||?→||<-?||nSTAND1+FGS*→	nSTAND1+FGS	764	pkn1_4	betaproteobacteria	Candidatus Accumulibacter sp.	Serine/threonine-protein kinase pkn1 [Candidatus Accumulibacter sp.].	GCA_005524045.1
TLD46403.1	TIR+NACHT+FGS*→	TIR+NACHT+FGS	991	pkn1_3	betaproteobacteria	Candidatus Accumulibacter sp.	Serine/threonine-protein kinase pkn1 [Candidatus Accumulibacter sp.].	GCA_005524045.1
TMQ74907.1	SIG+drhyd→ FGS*→	FGS	250	ACCUM_2481	betaproteobacteria	Candidatus Accumulibacter phosphatis	protein of unknown function DUF323 [Candidatus Accumulibacter phosphatis].	GCA_005889575.1
TSA11332.1	KAP-NTPase+FGS*→	KAP-NTPase+FGS	535	D4R73_04040	deltaproteobacteria	Deltaproteobacteria bacterium	hypothetical protein D4R73_04040 [Deltaproteobacteria bacterium].	GCA_007280345.1
TVR19569.1	TRD+TRD→?→ SWC3→ SF2-DUF3427A+LPD33→ DrHyd+nSTAND1+FGS*→	DrHyd+nSTAND1+FGS	963	EA396_12385	chloroflexi	Anaerolineaceae bacterium	hypothetical protein EA396_12385 [Anaerolineaceae bacterium].	GCA_007693385.1

acc	operon	architecture	len	gen.name	taxend	species	defline	gca
TVR20270.1	DrHyd+nSTAND1+FGS*→	DrHyd+nSTAND1+FGS	925	EA396_10895	chloroflexi	Anaerolineaceae bacterium	hypothetical protein EA396_10895, partial [Anaerolineaceae bacterium].	GCA_007693385.1
TVR25455.1	NACHT+FGS*→	NACHT+FGS	1003	EA396_00070	chloroflexi	Anaerolineaceae bacterium	TIR domain-containing protein [Anaerolineaceae bacterium].	GCA_007693385.1
TVR58137.1	<-SWC3<-FGS||?→ NACHT+FGS*→	NACHT+FGS	935	EA420_17850	gammaproteobacteria	Candidatus Competibacteraceae bacterium	NACHT domain-containing protein [Candidatus Competibacteraceae bacterium].	GCA_007695245.1
TVR58388.1	TIR+bDLD3→ bDLD3+nSTAND1+FGS*→?→||<-NACHT+FGS	bDLD3+nSTAND1+FGS	802	EA420_17215	gammaproteobacteria	Candidatus Competibacteraceae bacterium	hypothetical protein EA420_17215 [Candidatus Competibacteraceae bacterium].	GCA_007695245.1
TXG93317.1	TIR+BetaPropeller+BetaPropeller→ TIR+NACHT+FGS*→ TIR+NACHT→	TIR+NACHT+FGS	974	E6R15_09770	betaproteobacteria	Zoogloea sp.	TIR domain-containing protein [Zoogloea sp.].	-
TXH27877.1	FGS*→||<-?<-?||ABhydrolase→	FGS	242	E6Q99_02970	elusimicrobia	Elusimicrobia bacterium	hypothetical protein E6Q99_02970, partial [Elusimicrobia bacterium].	-
TXI20020.1	DrHyd+nSTAND1+FGS*→	DrHyd+nSTAND1+FGS	896	E6Q62_02620	betaproteobacteria	Nitrosomonas sp.	DUF4062 domain-containing protein [Nitrosomonas sp.].	-
VEN74385.1	SIG+PTSIIB-sorb→?→ NACHT+FGS→*?*→ *KAP-NTPase+FGS*→?→?→||<-?<-?<-?<-?||?→ NACHT+FGS*→?→?→ MED26C→	NACHT+FGS	898	EPICR_30322	deltaproteobacteria	uncultured Desulfobacteraceae bacterium	Signal transduction protein [uncultured Desulfobacteraceae bacterium].	GCA_900659855.1
VEN74387.1	KAP-NTPase+FGS*→	KAP-NTPase+FGS	669	EPICR_30324	deltaproteobacteria	uncultured Desulfobacteraceae bacterium	conserved hypothetical protein [uncultured Desulfobacteraceae bacterium].	GCA_900659855.1
VEN74395.1	TM+NACHT+FGS*→	TM+NACHT+FGS	1024	EPICR_30332	deltaproteobacteria	uncultured Desulfobacteraceae bacterium	hypothetical protein EPICR_30332 [uncultured Desulfobacteraceae bacterium].	GCA_900659855.1
WP_002710120.1	TIR+NACHT→?→?→?→ FGS*→ ABhydrolase+ABhydrolase+CASPASE+TPR-S→	FGS	266	THINI_RS24560	gammaproteobacteria	Thiothrix nivea	SUMF1/EgtB/PvdO family nonheme iron enzyme [Thiothrix nivea].	GCF_000260135.1
WP_006563305.1	MED26C→?→?→?→?→?→?→ MNS-STAND+FGS*→ KAP-NTPase→	MNS-STAND+FGS	646	OSCT_RS18055	chloroflexi	Oscillochloris trichoides	formylglycine-generating enzyme family protein [Oscillochloris trichoides].	GCF_000152145.1
WP_007042322.1	DrHyd+nSTAND1+FGS*→||<-PSE<-NACHT+FGS	DrHyd+nSTAND1+FGS	892	THIDR-DRAFT_RS24020	gammaproteobacteria	Thiorhodococcus drewsii	SUMF1/EgtB/PvdO family nonheme iron enzyme [Thiorhodococcus drewsii].	GCF_000224065.1
WP_013032524.1	TIR+bDLD3→ bDLD3+nSTAND1+FGS*→||<-?<-Four-helical-protein||RVT→	bDLD3+nSTAND1+FGS	799	NHAL_RS07270	gammaproteobacteria	Nitrosococcus halophilus	SUMF1/EgtB/PvdO family nonheme iron enzyme [Nitrosococcus halophilus].	GCF_000024725.1
WP_013964922.1	SIG+CACHE+TM+PAS+PAS+GGDEF+EAL→?→?→?→?→ TIR+NACHT+FGS*→	TIR+NACHT+FGS	954	NIT79A3_RS03700	betaproteobacteria	Nitrosomonas sp. Is79A3	SUMF1/EgtB/PvdO family nonheme iron enzyme [Nitrosomonas sp. Is79A3].	GCF_000219585.1
WP_013967247.1	DrHyd+nSTAND1+FGS*→	DrHyd+nSTAND1+FGS	932	NIT79A3_RS16330	betaproteobacteria	Nitrosomonas sp. Is79A3	SUMF1/EgtB/PvdO family nonheme iron enzyme [Nitrosomonas sp. Is79A3].	GCF_000219585.1
WP_015766782.1	<-CNMP+CRP-HTH||FAD-NAD-dep-oxidoreductase→?→||<-?<-?<-?<-?||nSTAND1+FGS*→||<-?<-ABHYDROLASE	nSTAND1+FGS	844	CAP2UW1_RS11490	betaproteobacteria	Candidatus Accumulibacter phosphatis	SUMF1/EgtB/PvdO family nonheme iron enzyme [Candidatus Accumulibacter phosphatis].	GCF_000024165.1

<table>
<thead>
<tr><th>acc</th><th>operon</th><th>architecture</th><th>len</th><th>gen.name</th><th>taxend</th><th>species</th><th>defline</th><th>gca</th></tr>
</thead>
<tbody>
<tr><td>WP_015766797.1</td><td>EAD8+TIR+NACHT+FGS*→||<-RVT<-?<-EAD8+TIR+ABhydrolase</td><td>EAD8+TIR+NACHT+FGS</td><td>1092</td><td>CAP2UW1_RS23785</td><td>betaproteobacteria</td><td>Candidatus Accumulibacter phosphatis</td><td>SUMF1/EgtB/PvdO family nonheme iron enzyme [Candidatus Accumulibacter phosphatis].</td><td>GCF_000024165.1</td></tr>
<tr><td>WP_020502879.1</td><td>NACHT+FGS*→</td><td>NACHT+FGS</td><td>728</td><td>-</td><td>gammaproteobacteria</td><td>Lamprocystis purpurea</td><td>SUMF1/EgtB/PvdO family nonheme iron enzyme [Lamprocystis purpurea].</td><td>-</td></tr>
<tr><td>WP_020504760.1</td><td>EAD8+nSTAND1+FGS*→</td><td>EAD8+nSTAND1+FGS</td><td>925</td><td>A39O_RS0111260</td><td>gammaproteobacteria</td><td>Lamprocystis purpurea</td><td>SUMF1/EgtB/PvdO family nonheme iron enzyme [Lamprocystis purpurea].</td><td>GCF_000379525.1</td></tr>
<tr><td>WP_026199145.1</td><td>EAD8+nSTAND1+FGS*→||<-Trypsin+EAD8<-Trypco1</td><td>EAD8+nSTAND1+FGS</td><td>868</td><td>A39O_RS36165</td><td>gammaproteobacteria</td><td>Lamprocystis purpurea</td><td>SUMF1/EgtB/PvdO family nonheme iron enzyme [Lamprocystis purpurea].</td><td>GCF_000379525.1</td></tr>
<tr><td>WP_026199412.1</td><td>nSTAND1+FGS*→</td><td>nSTAND1+FGS</td><td>833</td><td>A39O_RS0114350</td><td>gammaproteobacteria</td><td>Lamprocystis purpurea</td><td>SUMF1/EgtB/PvdO family nonheme iron enzyme [Lamprocystis purpurea].</td><td>GCF_000379525.1</td></tr>
<tr><td>WP_028314253.1</td><td>Calcineurin+NACHT+FGS*→||<-MED26C</td><td>Calcineurin+NACHT+FGS</td><td>1101</td><td>G491_RS0108205</td><td>deltaproteobacteria</td><td>Desulfatibacillum aliphaticivorans</td><td>SUMF1/EgtB/PvdO family nonheme iron enzyme [Desulfatibacillum aliphaticivorans].</td><td>GCF_000429905.1</td></tr>
<tr><td>WP_028315860.1</td><td>Calcineurin+NACHT+FGS*→</td><td>Calcineurin+NACHT+FGS</td><td>1088</td><td>G491_RS0120200</td><td>deltaproteobacteria</td><td>Desulfatibacillum aliphaticivorans</td><td>SUMF1/EgtB/PvdO family nonheme iron enzyme [Desulfatibacillum aliphaticivorans].</td><td>GCF_000429905.1</td></tr>
<tr><td>WP_046850450.1</td><td><-ADP_ribosyl_GH<-?<-?<-?||DrHyd+nSTAND1+FGS*→?→||<-nSTAND1<-?<-PSE<-SIG+nSTAND1<-TIR</td><td>DrHyd+nSTAND1+FGS</td><td>868</td><td>AAW31_RS12305</td><td>betaproteobacteria</td><td>Nitrosomonas communis</td><td>SUMF1/EgtB/PvdO family nonheme iron enzyme [Nitrosomonas communis].</td><td>GCF_001007935.1</td></tr>
<tr><td>WP_052347676.1</td><td>nSTAND1+FGS*→</td><td>nSTAND1+FGS</td><td>515</td><td>D779_RS20855</td><td>gammaproteobacteria</td><td>Imhoffiella purpurea</td><td>formylglycine-generating enzyme family protein [Imhoffiella purpurea].</td><td>GCF_000585215.1</td></tr>
<tr><td>WP_052515241.1</td><td>KAP-NTPase+FGS*→ Four-helical-protein→ RVT→ Calcineurin+NACHT+FGS→</td><td>KAP-NTPase+FGS</td><td>689</td><td>X474_RS14675</td><td>deltaproteobacteria</td><td>Dethiosulfatarculus sandiegensis</td><td>SUMF1/EgtB/PvdO family nonheme iron enzyme [Dethiosulfatarculus sandiegensis].</td><td>GCF_000931935.2</td></tr>
<tr><td>WP_054534535.1</td><td>BACTERIALFRINGE→ BetaPropeller→||<-?||?→?→ NACHT+FGS*→</td><td>NACHT+FGS</td><td>957</td><td>SE18_RS11130</td><td>chloroflexi</td><td>Herpetosiphon geysericola</td><td>SUMF1/EgtB/PvdO family nonheme iron enzyme [Herpetosiphon geysericola].</td><td>GCF_001306135.1</td></tr>
<tr><td>WP_058554345.1</td><td>nSTAND1+FGS*→||<-SIG+CACHE+TM+PAS+PAS+GGDEF+EAL||?→||<-?||SIR2→</td><td>nSTAND1+FGS</td><td>820</td><td>ML01_RS05180</td><td>gammaproteobacteria</td><td>Thiohalocapsa sp. ML1</td><td>SUMF1/EgtB/PvdO family nonheme iron enzyme [Thiohalocapsa sp. ML1].</td><td>GCF_001469165.1</td></tr>
<tr><td>WP_058554420.1</td><td>TIR→ MoxR-AAA→ MED26C→ FGS*→?→||<-?<-FGS||?→?→ TIR→ EAD1+fvmYukDl-Nterm→</td><td>FGS</td><td>857</td><td>ML01_RS05595</td><td>gammaproteobacteria</td><td>Thiohalocapsa sp. ML1</td><td>SUMF1/EgtB/PvdO family nonheme iron enzyme [Thiohalocapsa sp. ML1].</td><td>GCF_001469165.1</td></tr>
<tr><td>WP_058557121.1</td><td>EAD8→ EAD8+nSTAND1+FGS*→?→?→ RVT→</td><td>EAD8+nSTAND1+FGS</td><td>964</td><td>ML01_RS19795</td><td>gammaproteobacteria</td><td>Thiohalocapsa sp. ML1</td><td>SUMF1/EgtB/PvdO family nonheme iron enzyme [Thiohalocapsa sp. ML1].</td><td>GCF_001469165.1</td></tr>
<tr><td>WP_073644744.1</td><td>TIR+nSTAND1+FGS*→||<-EAD1+FGS<-EAD1+Trypsin</td><td>TIR+nSTAND1+FGS</td><td>931</td><td>FACHB389_RS31975</td><td>cyanobacteria</td><td>Nostoc calcicola</td><td>SUMF1/EgtB/PvdO family nonheme iron enzyme [Nostoc calcicola].</td><td>GCF_001904715.1</td></tr>
<tr><td>WP_074904186.1</td><td>TIR+bDLD3→ bDLD3+nSTAND1+FGS*→||<-NACHT+FGS<-ABhydrolase+bDLD3</td><td>bDLD3+nSTAND1+FGS</td><td>823</td><td>BM122_RS04980</td><td>betaproteobacteria</td><td>Nitrosomonas communis</td><td>SUMF1/EgtB/PvdO family nonheme iron enzyme [Nitrosomonas communis].</td><td>GCF_900114745.1</td></tr>
</tbody>
</table>

<table>
<tr><th>acc</th><th>operon</th><th>architecture</th><th>len</th><th>gen.name</th><th>taxend</th><th>species</th><th>defline</th><th>gca</th></tr>
<tr><td>WP_074906405.1</td><td><-ADP_ribosyl_GH<-?<-?<-?||DrHyd+nSTAND1+FGS*→ FGS→ PSE→?→||<-?<-?<-?<-SWC3</td><td>DrHyd+nSTAND1+FGS</td><td>824</td><td>BM122_RS14970</td><td>betaproteobacteria</td><td>Nitrosomonas communis</td><td>SUMF1/EgtB/PvdO family nonheme iron enzyme [Nitrosomonas communis].</td><td>GCF_900114745.1</td></tr>
<tr><td>WP_078922991.1</td><td>TIR+NACHT+FGS*→</td><td>TIR+NACHT+FGS</td><td>961</td><td>B5D50_RS12585</td><td>gammaproteobacteria</td><td>Thiothrix eikelboomii</td><td>SUMF1/EgtB/PvdO family nonheme iron enzyme [Thiothrix eikelboomii].</td><td>GCF_900167255.1</td></tr>
<tr><td>WP_081485890.1</td><td>FGS*→</td><td>FGS</td><td>285</td><td>-</td><td>gammaproteobacteria</td><td>Thiothrix nivea</td><td>SUMF1/EgtB/PvdO family nonheme iron enzyme [Thiothrix nivea].</td><td>-</td></tr>
<tr><td>WP_083884749.1</td><td><-ABhydrolase<-?<-?<-?<-?||TIR+nSTAND1+FGS*→||<-RVT<-SIG+SWC3<-FGS<-TIR+nSTAND1+FGS</td><td>TIR+nSTAND1+FGS</td><td>974</td><td>THIMO_RS13755</td><td>gammaproteobacteria</td><td>Thioflavicoccus mobilis</td><td>SUMF1/EgtB/PvdO family nonheme iron enzyme [Thioflavicoccus mobilis].</td><td>GCF_000327045.1</td></tr>
<tr><td>WP_084511416.1</td><td>Calcineurin+NACHT+FGS*→</td><td>Calcineurin+NACHT+FGS</td><td>1109</td><td>-</td><td>deltaproteobacteria</td><td>Desulfatibacillum aliphaticivorans</td><td>SUMF1/EgtB/PvdO family nonheme iron enzyme [Desulfatibacillum aliphaticivorans].</td><td>-</td></tr>
<tr><td>WP_088618636.1</td><td>DrHyd+NACHT+FGS*→?→||<-FGS<-?||?→||<-RADICAL-SAM</td><td>DrHyd+NACHT+FGS</td><td>1016</td><td>CEK71_RS06560</td><td>gammaproteobacteria</td><td>Methylovulum psychrotolerans</td><td>SUMF1/EgtB/PvdO family nonheme iron enzyme [Methylovulum psychrotolerans].</td><td>GCF_002209385.1</td></tr>
<tr><td>WP_089718394.1</td><td>TIR+bDLD3→ bDLD3+nSTAND1+FGS*→</td><td>bDLD3+nSTAND1+FGS</td><td>798</td><td>FLV43_RS07510</td><td>nitrospinae/tectomicrobia group</td><td>Candidatus Entotheonella palauensis</td><td>SUMF1/EgtB/PvdO family nonheme iron enzyme [Candidatus Entotheonella palauensis].</td><td>GCF_900079105.1</td></tr>
<tr><td>WP_089936193.1</td><td>ABC-ATPase→ ABC-ATPase→ drhyd+nSTAND1+FGS*→</td><td>drhyd+nSTAND1+FGS</td><td>929</td><td>FLV42_RS11295</td><td>nitrospinae/tectomicrobia group</td><td>Candidatus Entotheonella palauensis</td><td>SUMF1/EgtB/PvdO family nonheme iron enzyme [Candidatus Entotheonella palauensis].</td><td>GCF_900079095.1</td></tr>
<tr><td>WP_090322194.1</td><td>DrHyd→ nSTAND1→ FGS*→</td><td>FGS</td><td>263</td><td>BMY41_RS13900</td><td>betaproteobacteria</td><td>Nitrosomonas oligotropha</td><td>formylglycine-generating enzyme family protein [Nitrosomonas oligotropha].</td><td>GCF_900106555.1</td></tr>
<tr><td>WP_090742272.1</td><td>DrHyd+nSTAND1+FGS*→</td><td>DrHyd+nSTAND1+FGS</td><td>914</td><td>COMA1_RS00565</td><td>nitrospirae</td><td>Candidatus Nitrospira nitrosa</td><td>SUMF1/EgtB/PvdO family nonheme iron enzyme [Candidatus Nitrospira nitrosa].</td><td>GCF_001458735.1</td></tr>
<tr><td>WP_095042164.1</td><td>NACHT+FGS*→</td><td>NACHT+FGS</td><td>1092</td><td>CFX0092_RS03310</td><td>chloroflexi</td><td>Candidatus Promineofilum breve</td><td>SUMF1/EgtB/PvdO family nonheme iron enzyme [Candidatus Promineofilum breve].</td><td>GCF_900066015.1</td></tr>
<tr><td>WP_095043336.1</td><td><-MED26C||?→?→||<-?<-?||EAD7+TIR+FGS*→||<-ABhydrolase||?→ Cluster455_2clades→||<-ABhydrolase<-?||?→ MED26C→</td><td>EAD7+TIR+FGS</td><td>604</td><td>CFX0092_RS09700</td><td>chloroflexi</td><td>Candidatus Promineofilum breve</td><td>SUMF1/EgtB/PvdO family nonheme iron enzyme [Candidatus Promineofilum breve].</td><td>GCF_900066015.1</td></tr>
<tr><td>WP_097644514.1</td><td>CASPASE+EAD2→ EAD2→?→ MoxR-AAA→ SIG+VWA→ vWA-L+MNS-STAND+FGS*→</td><td>vWA-L+MNS-STAND+FGS</td><td>917</td><td>CJ255_RS12915</td><td>chloroflexi</td><td>Candidatus Viridilinea mediisalina</td><td>SUMF1/EgtB/PvdO family nonheme iron enzyme [Candidatus Viridilinea mediisalina].</td><td>GCF_002532535.1</td></tr>
<tr><td>WP_097646022.1</td><td>MNS-STAND+FGS→//<-?<-?//FGS→</td><td>MNS-STAND+FGS</td><td>331</td><td>CJ255_RS20935</td><td>chloroflexi</td><td>Candidatus Viridilinea mediisalina</td><td>formylglycine-generating enzyme family protein [Candidatus Viridilinea mediisalina].</td><td>GCF_002532535.1</td></tr>
</table>

<table>
<tr><th>acc</th><th>operon</th><th>architecture</th><th>len</th><th>gen.name</th><th>taxend</th><th>species</th><th>defline</th><th>gca</th></tr>
<tr><td>WP_097646178.1</td><td>FGS*→</td><td>FGS</td><td>273</td><td>CJ255_RS21825</td><td>chloroflexi</td><td>Candidatus Viridilinea mediisalina</td><td>SUMF1/EgtB/PvdO family nonheme iron enzyme [Candidatus Viridilinea mediisalina].</td><td>GCF_002532535.1</td></tr>
<tr><td>WP_097650659.1</td><td>MNS-STAND+FGS*→ KAP-NTPase→</td><td>MNS-STAND+FGS</td><td>798</td><td>A9Q02_RS01775</td><td>chloroflexi</td><td>Candidatus Chloroploca asiatica</td><td>SUMF1/EgtB/PvdO family nonheme iron enzyme [Candidatus Chloroploca asiatica].</td><td>GCF_002532075.1</td></tr>
<tr><td>WP_097650736.1</td><td>MNS-STAND+FGS*→ KAP-NTPase→</td><td>MNS-STAND+FGS</td><td>677</td><td>A9Q02_RS02220</td><td>chloroflexi</td><td>Candidatus Chloroploca asiatica</td><td>SUMF1/EgtB/PvdO family nonheme iron enzyme [Candidatus Chloroploca asiatica].</td><td>GCF_002532075.1</td></tr>
<tr><td>WP_097651198.1</td><td>Eukglutathionesyn-ATPgrasp→?→?→||<-?<-?<-?<-?||MNS-STAND+FGS*→ EAD2→||<-?||ABhydrolase→</td><td>MNS-STAND+FGS</td><td>748</td><td>A9Q02_RS04675</td><td>chloroflexi</td><td>Candidatus Chloroploca asiatica</td><td>SUMF1/EgtB/PvdO family nonheme iron enzyme [Candidatus Chloroploca asiatica].</td><td>GCF_002532075.1</td></tr>
<tr><td>WP_100918361.1</td><td>nSTAND1+FGS*→</td><td>nSTAND1+FGS</td><td>825</td><td>THSYN_RS06160</td><td>gammaproteobacteria</td><td>Candidatus Thiodictyon syntrophicum</td><td>SUMF1/EgtB/PvdO family nonheme iron enzyme [Candidatus Thiodictyon syntrophicum].</td><td>GCF_002813775.1</td></tr>
<tr><td>WP_100921702.1</td><td><-HSP70<-?<-?||?→ SbcC+SbcC→?→||<-?||PSE→ FGS→//<-SIG+SWC3<-TIR+FGS<-TIR<-FURR-HTH</td><td>FGS</td><td>290</td><td>THSYN_RS26100</td><td>gammaproteobacteria</td><td>Candidatus Thiodictyon syntrophicum</td><td>SUMF1/EgtB/PvdO family nonheme iron enzyme [Candidatus Thiodictyon syntrophicum].</td><td>GCF_002813775.1</td></tr>
<tr><td>WP_100921704.1</td><td>TIR+FGS*→</td><td>TIR+FGS</td><td>408</td><td>THSYN_RS26110</td><td>gammaproteobacteria</td><td>Candidatus Thiodictyon syntrophicum</td><td>SUMF1/EgtB/PvdO family nonheme iron enzyme [Candidatus Thiodictyon syntrophicum].</td><td>GCF_002813775.1</td></tr>
<tr><td>WP_103975510.1</td><td>SIG+drhyd+NACHT+FGS*→?→?→?→ SIG+TM+TM+TM→</td><td>SIG+drhyd+NACHT+FGS</td><td>920</td><td>AADEFJLK_RS19975</td><td>gammaproteobacteria</td><td>Methylovulum psychrotolerans</td><td>SUMF1/EgtB/PvdO family nonheme iron enzyme [Methylovulum psychrotolerans].</td><td>GCF_002923755.1</td></tr>
<tr><td>WP_106247884.1</td><td>DrHyd+nSTAND1+FGS*→</td><td>DrHyd+nSTAND1+FGS</td><td>937</td><td>bacteria>actinobacteria</td><td>Allonocardiopsis opalescens</td><td>-</td><td>1361040121</td><td>-</td></tr>
<tr><td>WP_107803916.1</td><td>DrHyd+nSTAND1→ SIG+FGS*→</td><td>SIG+FGS</td><td>232</td><td>C8R26_RS11925</td><td>betaproteobacteria</td><td>Nitrosomonas oligotropha</td><td>SUMF1/EgtB/PvdO family nonheme iron enzyme [Nitrosomonas oligotropha].</td><td>GCF_003050805.1</td></tr>
<tr><td>WP_109821479.1</td><td>SIG+CACHE+TM+PAS+PAS+GGDEF+EAL→||<-?||?→?→?→?→?→ TIR+NACHT+FGS*→||<-?||?→||<-?<-?||GNTR-HTH→</td><td>TIR+NACHT+FGS</td><td>932</td><td>DKT75_RS00505</td><td>gammaproteobacteria</td><td>Leucothrix arctica</td><td>SUMF1/EgtB/PvdO family nonheme iron enzyme [Leucothrix arctica].</td><td>GCF_003172895.1</td></tr>
<tr><td>WP_109838176.1</td><td><-SIG+CACHE+TM+PAS+PAS+GGDEF+EAL<-SIG+CACHE+TM+PAS+PAS+GGDEF+EAL<-?||TIR+NACHT+FGS→//<-RVT||Four-helical-protein→||<-?<-TIR+NACHT+FGS<-?||?→||<-?||SWC3→</td><td>TIR+NACHT+FGS</td><td>935</td><td>DKW60_RS13450</td><td>gammaproteobacteria</td><td>Leucothrix pacifica</td><td>SUMF1/EgtB/PvdO family nonheme iron enzyme [Leucothrix pacifica].</td><td>GCF_003172875.1</td></tr>
<tr><td>WP_109838180.1</td><td>TIR+NACHT+FGS*→</td><td>TIR+NACHT+FGS</td><td>1009</td><td>DKW60_RS13470</td><td>gammaproteobacteria</td><td>Leucothrix pacifica</td><td>SUMF1/EgtB/PvdO family nonheme iron enzyme [Leucothrix pacifica].</td><td>GCF_003172875.1</td></tr>
<tr><td>WP_113718124.1</td><td>CASPASE+FGS*→</td><td>CASPASE+FGS</td><td>581</td><td>bacteria>actinobacteria</td><td>Arthrobacter dokdonellae</td><td>-</td><td>1428153431</td><td>-</td></tr>
<tr><td>WP_117400917.1</td><td><-TIMbarrel||?→?→?→ EAD8+Trypsin→ DADA-Ligase→ EAD2+NACHT+FGS*→</td><td>EAD2+NACHT+FGS</td><td>982</td><td>D0T12_RS19005</td><td>actinobacteria</td><td>Actinomadura sp. LHW52907</td><td>SUMF1/EgtB/PvdO family nonheme iron enzyme [Actinomadura sp. LHW52907].</td><td>GCF_003432485.1</td></tr>
</table>

acc	operon	architecture	len	gen.name	taxend	species	defline	gca
WP_120798715.1	TIR+nSTAND1+FGS*→	TIR+nSTAND1+FGS	943	-	gammaproteobacteria	Thiocapsa rosea	SUMF1/EgtB/PvdO family nonheme iron enzyme [Thiocapsa rosea].	-
WP_120798722.1	TIR+nSTAND1+FGS→ *PSE→?→ TIR+TCAD2→ ABhydrolase+ABhydrolase+CASPASE+TPR-S→ TIR→ MoxR-AAA→ MED26C→ vWA-L+TIR+FGS*→||<-DrHyd<-TM+TM+TM+TM+TM+TM<-TIR+LDcluster2	TIR+nSTAND1+FGS	887	BDD21_RS20395	gammaproteobacteria	Thiocapsa rosea	SUMF1/EgtB/PvdO family nonheme iron enzyme [Thiocapsa rosea].	GCF_003634315.1
WP_121435660.1	<-TIMbarrel||?→?→?→ EAD8+Trypsin→ DADA-Ligase→ NACHT+FGS*→	NACHT+FGS	978	BZB76_RS18480	actinobacteria	Actinomadura pelletieri	SUMF1/EgtB/PvdO family nonheme iron enzyme [Actinomadura pelletieri].	GCF_003634705.1
WP_124681989.1	MNS-STAND+FGS*→	MNS-STAND+FGS	639	CJ255_RS17220	chloroflexi	Candidatus Viridilinea mediisalina	formylglycine-generating enzyme family protein [Candidatus Viridilinea mediisalina].	GCF_002532535.1
WP_129630085.1	MNS-STAND+FGS*→ KAP-NTPase→||<-?||?→ FAD-NAD-dep-oxidoreductase→	MNS-STAND+FGS	723	EKD03_RS09875	chloroflexi	Candidatus Oscillochloris fontis	SUMF1/EgtB/PvdO family nonheme iron enzyme [Candidatus Oscillochloris fontis].	GCF_004138135.1
WP_129675764.1	<-EAD2+CASPASE||CASPASE+EAD2→?→?→ EAD2+iSTAND→ MoxR-AAA→ SIG+VWA→ vWA-L+MNS-STAND+FGS*→||<-?||EAD2+SAVED→	vWA-L+MNS-STAND+FGS	910	EKD05_RS18730	chloroflexi	Candidatus Chloroploca sp. Khr17	SUMF1/EgtB/PvdO family nonheme iron enzyme [Candidatus Chloroploca sp. Khr17].	GCF_004138165.1
WP_129676117.1	<-ABC-ATPase<-?||?→||<-?<-?<-SIG+HISKIN+REC<-?||MNS-STAND+FGS*→	MNS-STAND+FGS	771	EKD05_RS19365	chloroflexi	Candidatus Chloroploca sp. Khr17	SUMF1/EgtB/PvdO family nonheme iron enzyme [Candidatus Chloroploca sp. Khr17].	GCF_004138165.1
WP_129680571.1	TIR+NACHT+FGS*→||<-?<-?<-?<-TM+TM+TM+TM+TM+TM+TM<-?||SIG+TM+HAMP+HISKIN→	TIR+NACHT+FGS	1113	EKD04_RS05935	chloroflexi	Chloroflexales bacterium ZM16-3	SUMF1/EgtB/PvdO family nonheme iron enzyme [Chloroflexales bacterium ZM16-3].	GCF_004138175.2
WP_129682868.1	ABhydrolase→||<-?||?→ CNMP+CRP-HTH→?→ Pkinase+FHA→?→ Pkinase+FHA+TM+TM+FGS*→	Pkinase+FHA+TM+TM+FGS	994	EKD04_RS11830	chloroflexi	Chloroflexales bacterium ZM16-3	SUMF1/EgtB/PvdO family nonheme iron enzyme [Chloroflexales bacterium ZM16-3].	GCF_004138175.2
WP_133117020.1	MNS-STAND+FGS*→	MNS-STAND+FGS	468	CJ255_RS20920	chloroflexi	Candidatus Viridilinea mediisalina	formylglycine-generating enzyme family protein, partial [Candidatus Viridilinea mediisalina].	GCF_002532535.1
WP_135477535.1	MNS-STAND+FGS*→ KAP-NTPase→	MNS-STAND+FGS	671	EYB53_RS07210	chloroflexi	Candidatus Chloroploca sp. M-50	SUMF1/EgtB/PvdO family nonheme iron enzyme [Candidatus Chloroploca sp. M-50].	GCF_004762035.1
WP_135478271.1	MNS-STAND+FGS→*?→?→ MNS-STAND+FGS*→ KAP-NTPase→	MNS-STAND+FGS	749	EYB53_RS11155	chloroflexi	Candidatus Chloroploca sp. M-50	SUMF1/EgtB/PvdO family nonheme iron enzyme [Candidatus Chloroploca sp. M-50].	GCF_004762035.1
WP_135478274.1	MNS-STAND+EAD9+FGS*→	MNS-STAND+EAD9+FGS	726	bacteria>chloroflexi	Candidatus Chloroploca sp. M-50	-	1610950059	-
WP_135480949.1	<-CASPASE<-EAD2||CASPASE+EAD2→ EAD2+iSTAND→ MoxR-AAA→ SIG+VWA→ vWA-L+MNS-STAND+FGS*→||<-?||EAD2+SAVED→||<-?||?→||<-?<-SIG+TM+HAMP+HISKIN	vWA-L+MNS-STAND+FGS	865	EYB53_RS21460	chloroflexi	Candidatus Chloroploca sp. M-50	SUMF1/EgtB/PvdO family nonheme iron enzyme [Candidatus Chloroploca sp. M-50].	GCF_004762035.1

acc	operon	architecture	len	gen.name	taxend	species	defline	gca
WP_135481914.1	FGS*→	FGS	498	bacteria>chloroflexi	Candidatus Chloroploca sp. M-50	-	1610955746	-
WP_138678219.1	NACHT+FGS*→||<-DrHyd+NACHT+FGS<-?||?→?→?→?→ MoxR-AAA→	NACHT+FGS	959	ACCUM_RS11740	betaproteobacteria	Candidatus Accumulibacter phosphatis	SUMF1/EgtB/PvdO family nonheme iron enzyme [Candidatus Accumulibacter phosphatis].	GCF_005889575.1
WP_138678880.1	FGS*→	FGS	336	-	betaproteobacteria	Candidatus Accumulibacter phosphatis	SUMF1/EgtB/PvdO family nonheme iron enzyme [Candidatus Accumulibacter phosphatis] .	-
WP_138678883.1	nSTAND1+TM+TPR+TPR→||<-?||SIG+SWC3→?→ FGS→*||<-RVT||Four-helical-protein→||<-?<-FGS*<-MED26C	FGS	785	ACCUM_RS18245	betaproteobacteria	Candidatus Accumulibacter phosphatis	SUMF1/EgtB/PvdO family nonheme iron enzyme [Candidatus Accumulibacter phosphatis].	GCF_005889575.1
WP_138679000.1	SIG+drhyd→ FGS*→	FGS	273	ACCUM_RS19505	betaproteobacteria	Candidatus Accumulibacter phosphatis	SUMF1/EgtB/PvdO family nonheme iron enzyme [Candidatus Accumulibacter phosphatis].	GCF_005889575.1
WP_141354765.1	TIR+nSTAND1+FGS*→	TIR+nSTAND1+FGS	1034	-	betaproteobacteria	Zoogloea ramigera	SUMF1/EgtB/PvdO family nonheme iron enzyme [Zoogloea ramigera].	-
WP_141354768.1	TIR+NACHT+FGS→*||<-DrHyd||TM+TM+TM+TM+TM+TM→||<-TIR+nSTAND1+FGS*	TIR+NACHT+FGS	1005	ZRA01_RS18100	betaproteobacteria	Zoogloea ramigera	SUMF1/EgtB/PvdO family nonheme iron enzyme [Zoogloea ramigera].	GCF_006539865.1
WP_141508656.1	FGS*→	FGS	438	A9Q02_RS01600	chloroflexi	Candidatus Chloroploca asiatica	SUMF1/EgtB/PvdO family nonheme iron enzyme, partial [Candidatus Chloroploca asiatica].	GCF_002532075.1
WP_144412951.1	<-ADP_ribosyl_GH<-?<-?<-?||PSE→ nSTAND1+FGS*→?→||<-nSTAND1<-PSE<-SIG+nSTAND1<-TIR	nSTAND1+FGS	811	BCL69_RS09525	betaproteobacteria	Nitrosomonas communis	SUMF1/EgtB/PvdO family nonheme iron enzyme [Nitrosomonas communis].	GCF_008124795.1
WP_155247659.1	Trypco1→ SIG+Trypsin+NACHT+FGS*→	SIG+Trypsin+NACHT+FGS	1057	K382_RS0113985	gammaproteobacteria	Teredinibacter turnerae	SUMF1/EgtB/PvdO family nonheme iron enzyme [Teredinibacter turnerae].	GCF_000426805.1
WP_155312115.1	NACHT+FGS→ Calcineurin+NACHT+FGS→?→?→ NACHT+FGS→?→ PSE→?→ NACHT+FGS*→?→||<-?||?→?→ ABC-ATPase→	NACHT+FGS	527	GN112_RS21635	deltaproteobacteria	Desulfosarcina ovata	SUMF1/EgtB/PvdO family nonheme iron enzyme [Desulfosarcina ovata].	GCF_009689005.1
WP_155342158.1	DrHyd+nSTAND1+FGS*→	DrHyd+nSTAND1+FGS	889	Acor_RS41020	actinobacteria	Acrocarpospora corrugata	SUMF1/EgtB/PvdO family nonheme iron enzyme, partial [Acrocarpospora corrugata].	GCF_009687845.1
WP_156427941.1	NACHT+FGS*→	NACHT+FGS	859	ML01_RS23445	gammaproteobacteria	Thiohalocapsa sp. ML1	SUMF1/EgtB/PvdO family nonheme iron enzyme, partial [Thiohalocapsa sp. ML1].	GCF_001469165.1
WP_156428023.1	NACHT+FGS*→	NACHT+FGS	942	ML01_RS26210	gammaproteobacteria	Thiohalocapsa sp. ML1	SUMF1/EgtB/PvdO family nonheme iron enzyme, partial [Thiohalocapsa sp. ML1].	GCF_001469165.1
WP_157468427.1	Calcineurin+NACHT+FGS*→	Calcineurin+NACHT+FGS	1121	-	deltaproteobacteria	Desulfatibacillum aliphaticivorans	SUMF1/EgtB/PvdO family nonheme iron enzyme [Desulfatibacillum aliphaticivorans].	-

<table>
<tr><th>acc</th><th>operon</th><th>architecture</th><th>len</th><th>gen.name</th><th>taxend</th><th>species</th><th>defline</th><th>gca</th></tr>
<tr><td>WP_157633685.1</td><td>NACHT+FGS*→</td><td>NACHT+FGS</td><td>720</td><td>THIMO_RS07195</td><td>gammaproteobacteria</td><td>Thioflavicoccus mobilis</td><td>SUMF1/EgtB/PvdO family nonheme iron enzyme [Thioflavicoccus mobilis].</td><td>GCF_000327045.1</td></tr>
<tr><td>WP_157679426.1</td><td><-SIG+TM+TM+TM<-?||TIR+NACHT→ SIG+FGS*→</td><td>SIG+FGS</td><td>279</td><td>CEK71_RS19030</td><td>gammaproteobacteria</td><td>Methylovulum psychrotolerans</td><td>SUMF1/EgtB/PvdO family nonheme iron enzyme [Methylovulum psychrotolerans].</td><td>GCF_002209385.1</td></tr>
<tr><td>WP_157817391.1</td><td>FGS*→||<-?<-CASPASE</td><td>FGS</td><td>207</td><td>THSYN_RS01715</td><td>gammaproteobacteria</td><td>Candidatus Thiodictyon syntrophicum</td><td>SUMF1/EgtB/PvdO family nonheme iron enzyme [Candidatus Thiodictyon syntrophicum].</td><td>GCF_002813775.1</td></tr>
<tr><td>WP_157817655.1</td><td>EAD8→ nSTAND1→ FGS*→?→?→?→||<-FGS</td><td>FGS</td><td>267</td><td>THSYN_RS13780</td><td>gammaproteobacteria</td><td>Candidatus Thiodictyon syntrophicum</td><td>SUMF1/EgtB/PvdO family nonheme iron enzyme [Candidatus Thiodictyon syntrophicum].</td><td>GCF_002813775.1</td></tr>
<tr><td>WP_159693692.1</td><td>SWC3→?→?→?→?→?→?→ DrHyd+nSTAND1+FGS*→||<-CASPASE</td><td>DrHyd+nSTAND1+FGS</td><td>981</td><td>GQB03_RS21575</td><td>betaproteobacteria</td><td>Azoarcus sp. HKLI-1</td><td>SUMF1/EgtB/PvdO family nonheme iron enzyme [Azoarcus sp. HKLI-1].</td><td>GCF_009800965.1</td></tr>
<tr><td>WP_164689583.1</td><td>BACTERIALFRINGE→ BetaPropeller→||<-?||?→?→ NACHT+FGS*→</td><td>NACHT+FGS</td><td>960</td><td>C5S61_RS17250</td><td>chloroflexi</td><td>Herpetosiphon llansteffanensis</td><td>SUMF1/EgtB/PvdO family nonheme iron enzyme [Herpetosiphon llansteffanensis].</td><td>GCF_003205875.1</td></tr>
<tr><td>WP_165360989.1</td><td>FGS*→ KAP-NTPase→</td><td>FGS</td><td>232</td><td>EKD05_RS25290</td><td>chloroflexi</td><td>Candidatus Chloroploca sp. Khr17</td><td>SUMF1/EgtB/PvdO family nonheme iron enzyme, partial [Candidatus Chloroploca sp. Khr17].</td><td>GCF_004138165.1</td></tr>
<tr><td>WP_167857354.1</td><td>MNS-STAND+EAD9+FGS*→</td><td>MNS-STAND+EAD9+FGS</td><td>719</td><td>EYB53_RS11170</td><td>chloroflexi</td><td>Candidatus Chloroploca sp. M-50</td><td>SUMF1/EgtB/PvdO family nonheme iron enzyme [Candidatus Chloroploca sp. M-50].</td><td>GCF_004762035.1</td></tr>
<tr><td>WP_167857593.1</td><td>VWA→?→?→?→?→ FGS*→</td><td>FGS</td><td>490</td><td>EYB53_RS23920</td><td>chloroflexi</td><td>Candidatus Chloroploca sp. M-50</td><td>SUMF1/EgtB/PvdO family nonheme iron enzyme [Candidatus Chloroploca sp. M-50].</td><td>GCF_004762035.1</td></tr>
<tr><td>WP_167857622.1</td><td>FGS*→ VWA→</td><td>FGS</td><td>240</td><td>EYB53_RS25075</td><td>chloroflexi</td><td>Candidatus Chloroploca sp. M-50</td><td>SUMF1/EgtB/PvdO family nonheme iron enzyme, partial [Candidatus Chloroploca sp. M-50].</td><td>GCF_004762035.1</td></tr>
<tr><td>WP_169065694.1</td><td>nSTAND1+FGS*→||<-?||PSE→ CBS→</td><td>nSTAND1+FGS</td><td>617</td><td>E4Q23_RS05470</td><td>betaproteobacteria</td><td>Candidatus Accumulibacter phosphatis</td><td>SUMF1/EgtB/PvdO family nonheme iron enzyme [Candidatus Accumulibacter phosphatis].</td><td>GCF_012939955.1</td></tr>
<tr><td>WP_169066177.1</td><td><-CNMP+CRP-HTH||FAD-NAD-dep-oxidoreductase→?→||<-?<-?<-PSE<-ABhydrolase||TIR+nSTAND1+FGS*→?→||<-PIN</td><td>TIR+nSTAND1+FGS</td><td>776</td><td>E4Q23_RS08160</td><td>betaproteobacteria</td><td>Candidatus Accumulibacter phosphatis</td><td>SUMF1/EgtB/PvdO family nonheme iron enzyme [Candidatus Accumulibacter phosphatis].</td><td>GCF_012939955.1</td></tr>
<tr><td>WP_169067799.1</td><td>FGS→//*<-FGS*<-MED26C<-MoxR-AAA<-TIR||SIG+TM+TM+TM+TM+TM+TM+TM+TM→?→||<-ABhydrolase</td><td>FGS</td><td>253</td><td>E4Q23_RS17320</td><td>betaproteobacteria</td><td>Candidatus Accumulibacter phosphatis</td><td>SUMF1/EgtB/PvdO family nonheme iron enzyme [Candidatus Accumulibacter phosphatis].</td><td>GCF_012939955.1</td></tr>
</table>

<table>
<tr><th>acc</th><th>operon</th><th>architecture</th><th>len</th><th>gen.name</th><th>taxend</th><th>species</th><th>defline</th><th>gca</th></tr>
<tr><td>WP_169067800.1</td><td>FGS*→</td><td>FGS</td><td>808</td><td>E4Q23_RS17325</td><td>betaproteobacteria</td><td>Candidatus Accumulibacter phosphatis</td><td>SUMF1/EgtB/PvdO family nonheme iron enzyme [Candidatus Accumulibacter phosphatis].</td><td>GCF_012939955.1</td></tr>
<tr><td>WP_169070388.1</td><td>FGS*→||<-TRANSGLUTAMINASE||?→||<-?||PSE→?→||<-Patatin</td><td>FGS</td><td>242</td><td>E4Q08_RS10725</td><td>betaproteobacteria</td><td>Candidatus Accumulibacter phosphatis</td><td>SUMF1/EgtB/PvdO family nonheme iron enzyme [Candidatus Accumulibacter phosphatis].</td><td>GCF_012940005.1</td></tr>
<tr><td>WP_169071960.1</td><td>ABC-ATPase→?→?→ SIG+FGS→//<-Four-helical-protein//RVT→//<-SIG+FGS<-MED26C<-MoxR-AAA<-TIR</td><td>SIG+FGS</td><td>194</td><td>E4Q08_RS22030</td><td>betaproteobacteria</td><td>Candidatus Accumulibacter phosphatis</td><td>SUMF1/EgtB/PvdO family nonheme iron enzyme [Candidatus Accumulibacter phosphatis].</td><td>GCF_012940005.1</td></tr>
<tr><td>WP_169071963.1</td><td>FGS*→</td><td>FGS</td><td>825</td><td>E4Q08_RS22045</td><td>betaproteobacteria</td><td>Candidatus Accumulibacter phosphatis</td><td>SUMF1/EgtB/PvdO family nonheme iron enzyme [Candidatus Accumulibacter phosphatis].</td><td>GCF_012940005.1</td></tr>
<tr><td>WP_169071986.1</td><td><-BLBD<-?||?→?→||<-?<-PSE<-?<-?||TIR+nSTAND1+FGS*→ FGS→||<-Four-helical-protein||RVT→||<-TIR+FGS<-?<-RVT</td><td>TIR+nSTAND1+FGS</td><td>934</td><td>E4Q08_RS22180</td><td>betaproteobacteria</td><td>Candidatus Accumulibacter phosphatis</td><td>SUMF1/EgtB/PvdO family nonheme iron enzyme [Candidatus Accumulibacter phosphatis].</td><td>GCF_012940005.1</td></tr>
<tr><td>WP_170141027.1</td><td>DrHyd+nSTAND1+FGS*→</td><td>DrHyd+nSTAND1+FGS</td><td>920</td><td>CLV72_RS18670</td><td>actinobacteria</td><td>Allonocardiopsis opalescens</td><td>SUMF1/EgtB/PvdO family nonheme iron enzyme [Allonocardiopsis opalescens].</td><td>GCF_003002095.1</td></tr>
<tr><td>WP_170164843.1</td><td>TIR+nSTAND1+FGS*→</td><td>TIR+nSTAND1+FGS</td><td>939</td><td>BDD21_RS20355</td><td>gammaproteobacteria</td><td>Thiocapsa rosea</td><td>SUMF1/EgtB/PvdO family nonheme iron enzyme [Thiocapsa rosea].</td><td>GCF_003634315.1</td></tr>
<tr><td>WP_170183022.1</td><td>TIR+nSTAND1+FGS*→</td><td>TIR+nSTAND1+FGS</td><td>893</td><td>ZRA01_RS18085</td><td>betaproteobacteria</td><td>Zoogloea ramigera</td><td>SUMF1/EgtB/PvdO family nonheme iron enzyme [Zoogloea ramigera].</td><td>GCF_006539865.1</td></tr>
<tr><td>WP_171047426.1</td><td>FGS*→</td><td>FGS</td><td>233</td><td>ACCUM_RS18225</td><td>betaproteobacteria</td><td>Candidatus Accumulibacter phosphatis</td><td>SUMF1/EgtB/PvdO family nonheme iron enzyme [Candidatus Accumulibacter phosphatis].</td><td>GCF_005889575.1</td></tr>
</table>

6. Source data. Gene neighborhoods and domain architectures of the bacterial and prophage/phage SPRY domain

acc	operon	architecture	len	gen.name	taxend	species	defline	gca
ACF52192.1	Phage_connect_1→ Phage_H_T_join→ HK97-gp10_like→ DUF3168→ Phage_TTP_11→ Phage_TAC_13→?→ SPRY+X*→?→ Phage-tail-tape→	SPRY+X	655	Smal_2491	Gammaproteobacteria	Stenotrophomonas maltophilia R551-3	hypothetical protein Smal_2491 [Stenotrophomonas maltophilia R551-3].	GCA_000020665.1
ASA22786.1	SUN→ SPRY+SUN*→ SUN→ Laminin_G_3→ Disaggr_repeat→ Phage_holin_4_1→	SPRY+SUN	358	B9T62_19455	Firmicutes	Paenibacillus donghaensis	hypothetical protein B9T62_19455 [Paenibacillus donghaensis].	GCA_002192415.1
CAA2107092.1	TM+PhageMin_Tail→?→?→?→?→ SPRY*→	SPRY	456	VVAX_04063	Betaproteobacteria	Variovorax paradoxus	hypothetical protein VVAX_04063 [Variovorax paradoxus].	-
CAA2108205.1	TM+PhageMin_Tail→?→?→?→?→ SPRY*→	SPRY	455	VVAX_04716	Betaproteobacteria	Variovorax paradoxus	hypothetical protein VVAX_04716 [Variovorax paradoxus].	-
EJP76868.1	Phage_connect_1→ Phage_H_T_join→ HK97-gp10_like→ DUF3168→ Phage_TTP_11→ Phage_TAC_13→?→ SPRY+X*→?→ Phage-tail-tape→ ConA-repeats→	SPRY+X	658	A1OC_01671	Gammaproteobacteria	Stenotrophomonas maltophilia Ab55555	hypothetical protein A1OC_01671 [Stenotrophomonas maltophilia Ab55555].	GCA_000287935.1
HAG44903.1	SPRY*→ SPRY→ SUN→	SPRY	249	DCL31_18225	Firmicutes	Clostridium sp.	TPA: hypothetical protein DCL31_18225 [Clostridium sp.].	GCA_003451415.1
HAU79614.1	Phage_H_T_join→ HK97-gp10_like→ DUF3168→ Phage_TTP_11→ Phage_TAC_13→?→ SPRY+X*→||<-?||?→?→?→ Phage-tail-tape→	SPRY+X	655	DCW96_02920	Gammaproteobacteria	Stenotrophomonas sp.	TPA: hypothetical protein DCW96_02920 [Stenotrophomonas sp.].	GCA_003484865.1
HBC50726.1	Phage_connect_1→ Phage_H_T_join→ HK97-gp10_like→ DUF3168→ Phage_TTP_11→ Phage_TAC_13→?→ SPRY*→	SPRY	169	DC051_05805	Gammaproteobacteria	Stenotrophomonas maltophilia	TPA: hypothetical protein DC051_05805, partial [Stenotrophomonas maltophilia].	GCA_003487985.1
HBJ00556.1	SPRY*→ SUN→	SPRY	221	DDY89_06200	Firmicutes	Lysinibacillus sp.	TPA: hypothetical protein DDY89_06200, partial [Lysinibacillus sp.].	GCA_003507435.1
HBJ00935.1	SPRY*→	SPRY	146	DDY89_08865	Firmicutes	Lysinibacillus sp.	TPA: hypothetical protein DDY89_08865, partial [Lysinibacillus sp.].	GCA_003507435.1

acc	operon	architecture	len	gen.name	taxend	species	defline	gca
HBS54551.1	Phage_connect_1→ Phage_H_T_join→ HK97-gp10_like→ DUF3168→ Phage_TTP_11→ Phage_TAC_13→?→ SPRY+X*→?→ Phage-tail-tape→ ConA-repeats→	SPRY+X	655	DEA38_02315	Gammaproteobacteria	Stenotrophomonas sp.	TPA: hypothetical protein DEA38_02315 [Stenotrophomonas sp.].	GCA_003510825.1
HBT70779.1	DISCOIDIN→ SUN→ SPRY*→	SPRY	169	DEB37_00445	Firmicutes	Lysinibacillus sp.	TPA: hypothetical protein DEB37_00445, partial [Lysinibacillus sp.].	GCA_003510425.1
HDO36693.1	Cluster187_2clades→?→ Cluster187_2clades→?→ SPRY+SPRY*→	SPRY+SPRY	913	ENH07_10435	Nitrospirae	Nitrospirae bacterium	TPA: hypothetical protein ENH07_10435 [Nitrospirae bacterium].	GCA_011041165.1
HHG11560.1	SPRY+LisH*→	SPRY+LisH	821	ENL98_17835	unclassified Bacteria	bacterium	TPA: hypothetical protein ENL98_17835 [bacterium].	GCA_011373825.1
HHG11879.1	SIG+SPRY*→	SIG+SPRY	553	ENL98_19440	unclassified Bacteria	bacterium	TPA: hypothetical protein ENL98_19440 [bacterium].	GCA_011373825.1
KAB2901287.1	SPRY*→	SPRY	854	F9K31_02560	Gammaproteobacteria	Dokdonella sp.	hypothetical protein F9K31_02560 [Dokdonella sp.].	GCA_008933665.1
KAF6631785.1	<-Polbetant‖ACET→ Cupin_2→ ABhydrolase→‖<-?<-DUF2935‖SIG+SPRY*→	SIG+SPRY	323	H6F38_13125	Firmicutes	Paenibacillus sp. EKM208P	hypothetical protein H6F38_13125 [Paenibacillus sp. EKM208P].	GCA_011066295.1
KGM23936.1	DUF3168→ Phage_TTP_11→ Phage_TAC_13→?→ SPRY+X*→?→?→ Phage-tail-tape→ ConA-repeats→	SPRY+X	657	LI87_0109275	Gammaproteobacteria	Stenotrophomonas maltophilia	hypothetical protein LI87_0109275 [Stenotrophomonas maltophilia].	GCA_000758465.1
KHS06610.1	Phage-tail-tape→?→?→?→?→ SPRY+X*→?→ DAM→	SPRY+X	658	RM61_15205	Gammaproteobacteria	Xanthomonas phaseoli pv. phaseoli	hypothetical protein RM61_15205 [Xanthomonas phaseoli pv. phaseoli].	GCA_000807875.2
KKW33547.1	SIG+SPRY*→	SIG+SPRY	838	UY79_C0020G0005	Bacteria	Parcubacteria group bacterium GW2011_GWA2_53_21	Filamentous hemagglutinin-like protein [Parcubacteria group bacterium GW2011_GWA2_53_21].	GCA_001004625.1
KOS00949.1	SPRY*→	SPRY	155	AM598_20185	Firmicutes	Paenibacillus polymyxa	hypothetical protein AM598_20185, partial [Paenibacillus polymyxa].	GCA_001277855.1
KZT13763.1	SPRY*→?→?→?→ DAM→	SPRY	449	A1D30_21880	Betaproteobacteria	Acidovorax sp. GW101-3H11	hypothetical protein A1D30_21880 [Acidovorax sp. GW101-3H11].	GCA_001633105.1
MAE42393.1	SIG+TM+TM+IG*→	SIG+TM+TM+IG	1722	CMO93_01365	DPANN group	Candidatus Woesearchaeota archaeon	hypothetical protein CMO93_01365, partial [Candidatus Woesearchaeota archaeon].	GCA_002686855.1

acc	operon	architecture	len	gen.name	taxend	species	defline	gca
MAH29320.1	Laminin_G_3+SPRY*→ Cluster74_6clades→	Laminin_G_3+SPRY	903	CL959_01385	Euryarchaeota	Euryarchaeota archaeon	hypothetical protein CL959_01385 [Euryarchaeota archaeon].	GCA_002689085.1
MAL42735.1	SPRY*→?→ Cluster388_2clades→	SPRY	473	CME98_01825	Alphaproteobacteria	Hyphomonas sp.	hypothetical protein CME98_01825 [Hyphomonas sp.].	GCA_002692925.1
MAN64718.1	LamG→ Laminin_G_3+SPRY*→ Cluster74_6clades→ Cluster153_2clades→	Laminin_G_3+SPRY	671	CMI60_22525	Alphaproteobacteria	Parvibaculum sp.	hypothetical protein CMI60_22525 [Parvibaculum sp.].	GCA_002695905.1
MAT11680.1	Phage_T7_tail→ Cluster93_5clades→ Laminin_G_3+Laminin_G_3+SPRY*→ Cluster74_6clades→	Laminin_G_3+Laminin_G_3+SPRY	921	CMM02_11805	Planctomycetes	Rhodopirellula sp.	hypothetical protein CMM02_11805 [Rhodopirellula sp.].	GCA_002698965.1
MAU75364.1	Laminin_G_3+SPRY*→ Cluster74_6clades→	Laminin_G_3+SPRY	630	CL831_00645	Bacteroidetes	Crocinitomicaceae bacterium	hypothetical protein CL831_00645 [Crocinitomicaceae bacterium].	GCA_002700285.1
MBA0297283.1	Phage-tail-tape→ ConA-repeats→?→ SPRY+X*→?→?→?→?→ SIG+TM+TM+TM+TM+TM+TM+TM+TM→ ABC-ATPase→	SPRY+X	604	D7Y24_02420	Gammaproteobacteria	Stenotrophomonas maltophilia	hypothetical protein D7Y24_02420 [Stenotrophomonas maltophilia].	GCA_013463875.1
MBA0313726.1	Phage_connect_1→ Phage_H_T_join→ HK97-gp10_like→ DUF3168→ Phage_TTP_11→ Phage_TAC_13→?→ SPRY*→	SPRY	249	D7Y33_22370	Gammaproteobacteria	Stenotrophomonas maltophilia	hypothetical protein D7Y33_22370, partial [Stenotrophomonas maltophilia].	GCA_013464125.1
MBA0313748.1	SPRY*→	SPRY	249	D7Y33_22510	Gammaproteobacteria	Stenotrophomonas maltophilia	hypothetical protein D7Y33_22510, partial [Stenotrophomonas maltophilia].	GCA_013464125.1
MBA0421339.1	Phage-tail-tape→ ConA-repeats→?→ SPRY+X*→?→?→?→?→?→?→||<-Ribonuc_L-PSP	SPRY+X	604	D7Y22_10225	Gammaproteobacteria	Stenotrophomonas maltophilia	hypothetical protein D7Y22_10225 [Stenotrophomonas maltophilia].	GCA_013463895.1
MBB5530851.1	Phage_connect_1→ Phage_H_T_join→ HK97-gp10_like→ DUF3168→ Phage_TTP_11→ Phage_TAC_13→?→ SPRY+X*→?→ Phage-tail-tape→ ConA-repeats→ ConA→?→||<-SRAP	SPRY+X	657	GGD72_001629	Gammaproteobacteria	Stenotrophomonas maltophilia	hypothetical protein GGD72_001629 [Stenotrophomonas maltophilia].	GCA_014200115.1
MBC17709.1	Phage_T7_tail→ SUN+SPRY*→ Phage_tail_APC→	SUN+SPRY	749	CL942_11735	Deltaproteobacteria	Desulfovibrio sp.	hypothetical protein CL942_11735 [Desulfovibrio sp.].	GCA_002711645.1
MBO71803.1	Cluster310_2clades→?→?→?→ Cluster359_2clades→ Laminin_G_3+SPRY+SPRY*→	Laminin_G_3+SPRY+SPRY	973	CL508_05750	Actinobacteria	Actinobacteria bacterium	hypothetical protein CL508_05750, partial [Actinobacteria bacterium].	GCA_002724835.1
MPS35004.1	Phage_connect_1→ Phage_H_T_join→ HK97-gp10_like→ DUF3168→ Phage_TTP_11→ Phage_TAC_13→?→ SPRY+X*→?→ Phage-tail-tape→?→?→ Cluster201_3clades→	SPRY+X	652	E2593_06880	Gammaproteobacteria	Stenotrophomonas sp.	hypothetical protein E2593_06880 [Stenotrophomonas sp.].	GCA_009360425.1
NBO27128.1	Laminin_G_3+SPRY*→ Cluster366_2clades→	Laminin_G_3+SPRY	547	EBU96_10150	Actinobacteria	Actinobacteria bacterium	hypothetical protein EBU96_10150 [Actinobacteria bacterium].	GCA_009918685.1

acc	operon	architecture	len	gen.name	taxend	species	defline	gca
NBO39260.1	IG+IG+IG+LEVANB+SPRY*→	IG+IG+IG+LEVANB+SPRY	2486	EBU99_11835	unclassified Bacteria	bacterium	hypothetical protein EBU99_11835, partial [bacterium].	GCA_009918675.1
NBQ97491.1	Laminin_G_3+SPRY*→?→ Cluster358_2clades→	Laminin_G_3+SPRY	447	EBU12_04555	Actinobacteria	Microbacteriaceae bacterium	hypothetical protein EBU12_04555 [Microbacteriaceae bacterium].	GCA_009920365.1
NBR26007.1	SPRY*→	SPRY	290	EBU08_19945	Actinobacteria	Micrococcales bacterium	hypothetical protein EBU08_19945, partial [Micrococcales bacterium].	GCA_009920425.1
NBR26025.1	Laminin_G_3+SPRY*→	Laminin_G_3+SPRY	450	EBU08_20035	Actinobacteria	Micrococcales bacterium	hypothetical protein EBU08_20035, partial [Micrococcales bacterium].	GCA_009920425.1
NBS68787.1	Laminin_G_3+SPRY*→?→?→ Cluster153_2clades→	Laminin_G_3+SPRY	775	EBT31_07700	unclassified Bacteria	bacterium	hypothetical protein EBT31_07700 [bacterium].	GCA_009921885.1
NBS71002.1	Laminin_G_3+SPRY*→	Laminin_G_3+SPRY	470	EBT31_19160	unclassified Bacteria	bacterium	hypothetical protein EBT31_19160, partial [bacterium].	GCA_009921885.1
NBS80068.1	Laminin_G_3+SPRY*→	Laminin_G_3+SPRY	347	EBS57_09835	unclassified Bacteria	bacterium	hypothetical protein EBS57_09835 [bacterium].	GCA_009923095.1
NBT48002.1	Laminin_G_3+SPRY*→	Laminin_G_3+SPRY	734	EBT07_09340	Actinobacteria	Actinobacteria bacterium	hypothetical protein EBT07_09340, partial [Actinobacteria bacterium].	GCA_009922275.1
NBT76477.1	Laminin_G_3+SPRY*→ Cluster74_6clades→	Laminin_G_3+SPRY	576	EBT15_11040	Betaproteobacteria	Betaproteobacteria bacterium	hypothetical protein EBT15_11040, partial [Betaproteobacteria bacterium].	GCA_009922135.1
NBU64721.1	Cluster359_2clades→?→ Laminin_G_3+SPRY*→	Laminin_G_3+SPRY	351	EBS29_09505	Chloroflexi	Chloroflexia bacterium	hypothetical protein EBS29_09505, partial [Chloroflexia bacterium].	GCA_009923655.1
NBU69993.1	Laminin_G_3+SPRY+Laminin_G_3*→	Laminin_G_3+SPRY+Laminin_G_3	937	EBS53_00865	Bacteroidetes	Bacteroidetes bacterium	hypothetical protein EBS53_00865 [Bacteroidetes bacterium].	GCA_009923185.1
NBU73444.1	Laminin_G_3+SPRY*→	Laminin_G_3+SPRY	374	EBS53_18720	Bacteroidetes	Bacteroidetes bacterium	hypothetical protein EBS53_18720, partial [Bacteroidetes bacterium].	GCA_009923185.1
NBW17394.1	Laminin_G_3+SPRY*→ Cluster214_3clades→	Laminin_G_3+SPRY	581	EBR82_56380	Alphaproteobacteria	Caulobacteraceae bacterium	hypothetical protein EBR82_56380, partial [Caulobacteraceae bacterium].	GCA_009924585.1
NBW17505.1	Laminin_G_3+SPRY*→	Laminin_G_3+SPRY	764	EBR82_56945	Alphaproteobacteria	Caulobacteraceae bacterium	hypothetical protein EBR82_56945, partial [Caulobacteraceae bacterium].	GCA_009924585.1

acc	operon	architecture	len	gen.name	taxend	species	defline	gca
NBW18892.1	Laminin_G_3+SPRY*→	Laminin_G_3+SPRY	722	EBR82_64050	Alphaproteobacteria	Caulobacteraceae bacterium	hypothetical protein EBR82_64050, partial [Caulobacteraceae bacterium].	GCA_009924585.1
NBW20101.1	SPRY*→	SPRY	400	EBR82_70235	Alphaproteobacteria	Caulobacteraceae bacterium	hypothetical protein EBR82_70235, partial [Caulobacteraceae bacterium].	GCA_009924585.1
NBW20127.1	Laminin_G_3+SPRY*→	Laminin_G_3+SPRY	498	EBR82_70370	Alphaproteobacteria	Caulobacteraceae bacterium	hypothetical protein EBR82_70370, partial [Caulobacteraceae bacterium].	GCA_009924585.1
NBW20301.1	Laminin_G_3+SPRY*→	Laminin_G_3+SPRY	508	EBR82_71270	Alphaproteobacteria	Caulobacteraceae bacterium	hypothetical protein EBR82_71270 [Caulobacteraceae bacterium].	GCA_009924585.1
NBW20891.1	Laminin_G_3+SPRY*→	Laminin_G_3+SPRY	494	EBR82_74310	Alphaproteobacteria	Caulobacteraceae bacterium	hypothetical protein EBR82_74310, partial [Caulobacteraceae bacterium].	GCA_009924585.1
NBW21054.1	Laminin_G_3+SPRY*→	Laminin_G_3+SPRY	448	EBR82_75135	Alphaproteobacteria	Caulobacteraceae bacterium	hypothetical protein EBR82_75135, partial [Caulobacteraceae bacterium].	GCA_009924585.1
NBW21679.1	SPRY*→	SPRY	285	EBR82_78355	Alphaproteobacteria	Caulobacteraceae bacterium	hypothetical protein EBR82_78355, partial [Caulobacteraceae bacterium].	GCA_009924585.1
NBW22642.1	SPRY*→	SPRY	394	EBR82_83325	Alphaproteobacteria	Caulobacteraceae bacterium	hypothetical protein EBR82_83325, partial [Caulobacteraceae bacterium].	GCA_009924585.1
NBW23811.1	Laminin_G_3+SPRY*→	Laminin_G_3+SPRY	335	EBR82_89280	Alphaproteobacteria	Caulobacteraceae bacterium	hypothetical protein EBR82_89280, partial [Caulobacteraceae bacterium].	GCA_009924585.1
NBX81523.1	Laminin_G_3+SPRY*→	Laminin_G_3+SPRY	455	EBQ90_00310	unclassified Bacteria	bacterium	hypothetical protein EBQ90_00310, partial [bacterium].	GCA_009927045.1
NBX88437.1	SPRY*→	SPRY	317	EBQ97_07980	Bacteroidetes	Bacteroidetes bacterium	hypothetical protein EBQ97_07980, partial [Bacteroidetes bacterium].	GCA_009926925.1
NDA10477.1	Laminin_G_3+SPRY*→ Cluster214_3clades→	Laminin_G_3+SPRY	692	EBZ07_06485	Verrucomicrobia	Verrucomicrobia bacterium	hypothetical protein EBZ07_06485, partial [Verrucomicrobia bacterium].	GCA_010025475.1
NDB59170.1	Cluster201_3clades→?→?→?→ LamG→ SPRY+TM*→?→ Cluster366_2clades→	SPRY+TM	300	EB001_12060	unclassified Bacteria	bacterium	hypothetical protein EB001_12060 [bacterium].	GCA_010026765.1

acc	operon	architecture	len	gen.name	taxend	species	defline	gca
NDB60922.1	Laminin_G_3+SPRY*→	Laminin_G_3+SPRY	799	EB001_21125	unclassified Bacteria	bacterium	hypothetical protein EB001_21125 [bacterium].	GCA_010026765.1
NDC49301.1	Cluster93_5clades→ Laminin_G_3+SPRY*→ Cluster214_3clades→	Laminin_G_3+SPRY	803	EBZ61_09545	Actinobacteria	Micrococcales bacterium	hypothetical protein EBZ61_09545 [Micrococcales bacterium].	GCA_010027415.1
NDC57204.1	SIG+SPRY*→	SIG+SPRY	239	EBZ69_10460	Alphaproteobacteria	Alphaproteobacteria bacterium	hypothetical protein EBZ69_10460, partial [Alphaproteobacteria bacterium].	GCA_010027225.1
NDD55753.1	Laminin_G_3+SPRY*→	Laminin_G_3+SPRY	430	EBZ39_18125	unclassified Bacteria	bacterium	hypothetical protein EBZ39_18125, partial [bacterium].	GCA_010027825.1
NDD86023.1	SPRY*→	SPRY	370	EBZ38_17325	unclassified Bacteria	bacterium	hypothetical protein EBZ38_17325, partial [bacterium].	GCA_010027835.1
NDG32417.1	Laminin_G_3+SPRY*→	Laminin_G_3+SPRY	801	EB118_20375	unclassified Bacteria	bacterium	hypothetical protein EB118_20375, partial [bacterium].	GCA_010031135.1
NEU24684.1	HTH_20→||<-PSE<-Polbetant<-Polbetant||ACET→ Cupin_2→||<-?||SIG+SPRY*→	SIG+SPRY	355	G3M74_00590	Firmicutes	Paenibacillus polymyxa	hypothetical protein G3M74_00590 [Paenibacillus polymyxa].	GCA_010692785.1
NEZ74825.1	Cluster171_2clades→?→?→ Cluster216_2clades→?→?→ PSE→ SUN→ SPRY*→ SUN→ TM→ Cluster104_2clades→?→?→35exo→ Cluster112_2clades→	SPRY	447	EXM56_05600	Firmicutes	Clostridium botulinum	cell adhesion protein [Clostridium botulinum].	GCA_011010615.1
NFM46879.1	Cluster171_2clades→?→?→ Cluster143_2clades→?→?→ PSE→ SUN→ SPRY*→ SUN→ TM→ Cluster104_2clades→?→?→35exo→ Cluster112_2clades→	SPRY	447	FDB72_12170	Firmicutes	Clostridium botulinum	cell adhesion protein [Clostridium botulinum].	GCA_011017125.1
NFM77397.1	Cluster216_2clades→?→?→?→ SUN→ SPRY*→ SUN→ TM→ Cluster104_2clades→?→?→35exo→ Cluster112_2clades→	SPRY	448	FDC04_00825	Firmicutes	Clostridium botulinum	cell adhesion protein [Clostridium botulinum].	GCA_011017715.1
NIT14181.1	Cluster165_3clades→?→?→ Cluster303_2clades→ SIG+HORMA→ Cluster138_2clades→ Cluster138_2clades→ SPRY*→ ThuA-GATase→	SPRY	831	GTN99_08085	Bacteria	Candidatus Dadabacteria bacterium	hypothetical protein GTN99_08085 [Candidatus Dadabacteria bacterium].	GCA_011772905.1
NSB16447.1	SPRY+Big_2*→ TM→	SPRY+Big_2	376	BCD95_004706	Firmicutes	Clostridium beijerinckii	uncharacterized protein YjdB [Clostridium beijerinckii].	GCA_013297045.1
NUH63603.1	HK97-gp10_like→ DUF3168→ Phage_TTP_11→ Phage_TAC_13→?→ SPRY*→	SPRY	353	HUF69_20240	Gammaproteobacteria	Stenotrophomonas maltophilia	hypothetical protein HUF69_20240, partial [Stenotrophomonas maltophilia].	GCA_013345715.1
OBU49329.1	Phage_connect_1→ Phage_H_T_join→ HK97-gp10_like→ DUF3168→ Phage_TTP_11→ Phage_TAC_13→?→ SPRY+X*→	SPRY+X	657	A9K69_19720	Gammaproteobacteria	Stenotrophomonas maltophilia	hypothetical protein A9K69_19720 [Stenotrophomonas maltophilia].	GCA_001676445.1

acc	operon	architecture	len	gen.name	taxend	species	defline	gca
ODV27998.1	SPRY*→	SPRY	1123	ABT19_00375	Gammaproteobacteria	Rhodanobacter sp. SCN 68-63	hypothetical protein ABT19_00375 [Rhodanobacter sp. SCN 68-63].	GCA_001725485.1
OEU67544.1	Cluster138_2clades→?→?→ SPRY*→ Cluster135_2clades→?→ ThuA-GATase→	SPRY	483	BA863_12330	Deltaproteobacteria	Desulfovibrio sp. S3730MH75	hypothetical protein BA863_12330 [Desulfovibrio sp. S3730MH75].	GCA_001750915.1
OEU68515.1	SIG+PTX*→	SIG+PTX	1668	BBJ57_07495	Deltaproteobacteria	Desulfobacterales bacterium PC51MH44	hypothetical protein BBJ57_07495 [Desulfobacterales bacterium PC51MH44].	GCA_001751165.1
OEU69113.1	SPRY*→ Cluster135_2clades→?→?→ ThuA-GATase→	SPRY	436	BA863_04090	Deltaproteobacteria	Desulfovibrio sp. S3730MH75	hypothetical protein BA863_04090 [Desulfovibrio sp. S3730MH75].	GCA_001750915.1
OUW13744.1	Laminin_G_3+Laminin_G_3+SPRY*→ Cluster74_6clades→ Cluster388_2clades→	Laminin_G_3+Laminin_G_3+SPRY	1163	CBD24_02790	Euryarchaeota	Euryarchaeota archaeon TMED164	hypothetical protein CBD24_02790 [Euryarchaeota archaeon TMED164].	GCA_002170935.1
OYV39756.1	SPRY*→	SPRY	137	B7Z80_06345	Alphaproteobacteria	Rhodospirillales bacterium 20-64-7	hypothetical protein B7Z80_06345 [Rhodospirillales bacterium 20-64-7].	GCA_002255195.1
PJO52612.1	Phage-tail-tape→ ConA-repeats→?→ SPRY+X*→?→?→?→?→?→||<-Ribonuc_L-PSP	SPRY+X	604	CR156_10635	Gammaproteobacteria	Stenotrophomonas lactitubi	hypothetical protein CR156_10635 [Stenotrophomonas lactitubi].	GCA_002803515.1
PKQ92730.1	SUN→ SUN→ SUN→ SPRY+SUN*→ Laminin_G_3→ Disaggr_repeat→	SPRY+SUN	425	CXK86_01005	Firmicutes	Paenibacillus sp. BGI2013	hypothetical protein CXK86_01005 [Paenibacillus sp. BGI2013].	GCA_002843485.1
PPC99495.1	Cluster368_2clades→?→?→ Cluster201_3clades→ TM+TM→ Laminin_G_3+SPRY*→?→?→||<-?<-?<-REC+OmpR-HTH	Laminin_G_3+SPRY	541	CTY35_04040	Betaproteobacteria	Methylotenera sp.	hypothetical protein CTY35_04040 [Methylotenera sp.].	GCA_002928435.1
PPD25805.1	SPRY*→	SPRY	389	CTY21_14065	Gammaproteobacteria	Methylomonas sp.	hypothetical protein CTY21_14065, partial [Methylomonas sp.].	GCA_002928935.1
QDL55294.1	Phage-tail_3+FN3→ Cluster93_5clades→ Laminin_G_3+SPRY*→?→?→||<-?<-REC+OmpR-HTH	Laminin_G_3+SPRY	544	EXZ61_14585	Betaproteobacteria	Rhodoferax sediminis	hypothetical protein EXZ61_14585 [Rhodoferax sediminis].	GCA_006974105.1
QDY47412.1	Phage_connect_1→ Phage_H_T_join→ HK97-gp10_like→ DUF3168→ Phage_TTP_11→ Phage_TAC_13→?→ SPRY+X*→?→?→?→ Phage-tail-tape→ ConA-repeats→	SPRY+X	657	DUW70_02065	Gammaproteobacteria	Stenotrophomonas maltophilia	hypothetical protein DUW70_02065 [Stenotrophomonas maltophilia].	GCA_007833655.1
QDY50235.1	Phage-tail-tape→ ConA-repeats→?→ SPRY+X*→	SPRY+X	604	DUW70_17765	Gammaproteobacteria	Stenotrophomonas maltophilia	hypothetical protein DUW70_17765 [Stenotrophomonas maltophilia].	GCA_007833655.1

acc	operon	architecture	len	gen.name	taxend	species	defline	gca
QDY50408.1	Phage_connect_1→ Phage_H_T_join→ HK97-gp10_like→ DUF3168→ Phage_TTP_11→ Phage_TAC_13→?→ SPRY+X*→||<-?||?→ Phage-tail-tape→ ConA-repeats→	SPRY+X	655	DUW70_18735	Gammaproteobacteria	Stenotrophomonas maltophilia	hypothetical protein DUW70_18735 [Stenotrophomonas maltophilia].	GCA_007833655.1
QGG50745.1	SUN→ SUN→ SPRY+SUN*→ SUN→ Laminin_G_3→ Disaggr_repeat→?→?→ Phage_holin_1→	SPRY+SUN	444	GDS87_07165	Firmicutes	Lysinibacillus pakistanensis	hypothetical protein GDS87_07165 [Lysinibacillus pakistanensis].	GCA_009649975.1
QIO87716.1	Phage_connect_1→ Phage_H_T_join→ HK97-gp10_like→?→ Phage_TTP_11→?→ SPRY*→?→?→||<-SRAP	SPRY	278	G9274_001401	Gammaproteobacteria	Stenotrophomonas rhizophila	hypothetical protein G9274_001401 [Stenotrophomonas rhizophila].	GCA_011604605.1
RCK36140.1	Baseplate_J→ SPRY*→	SPRY	575	TH9_05700	Alphaproteobacteria	Thalassospira xiamenensis	hypothetical protein TH9_05700 [Thalassospira xiamenensis].	GCA_003326725.1
REJ66078.1	JAB+NLPC→?→ Phage-tail_3→ Cluster93_5clades→ Laminin_G_3+SPRY*→ Cluster74_6clades→	Laminin_G_3+SPRY	1115	DWQ28_08310	Proteobacteria	Proteobacteria bacterium	hypothetical protein DWQ28_08310 [Proteobacteria bacterium].	GCA_003388625.1
REK63903.1	Phage_tail_APC→ TM+TM→ Laminin_G_3+SPRY*→ Cluster74_6clades→?→?→ Phage_tail_APC→	Laminin_G_3+SPRY	797	DWQ49_02055	Bacteroidetes	Bacteroidetes bacterium	hypothetical protein DWQ49_02055 [Bacteroidetes bacterium].	GCA_003389155.1
RLC76825.1	Cluster187_2clades→ SPRY*→ Cluster135_2clades→	SPRY	515	DRJ03_27900	Chloroflexi	Chloroflexi bacterium	hypothetical protein DRJ03_27900 [Chloroflexi bacterium].	GCA_003648075.1
RPI56186.1	Cluster138_2clades→?→?→ SPRY*→ Cluster135_2clades→	SPRY	495	EHM49_00895	Deltaproteobacteria	Deltaproteobacteria bacterium	hypothetical protein EHM49_00895 [Deltaproteobacteria bacterium].	GCA_003819885.1
RRY03816.1	SPRY*→?→ Phage_lysozyme→	SPRY	771	EGJ58_22195	Alphaproteobacteria	Ochrobactrum anthropi	hypothetical protein EGJ58_22195 [Ochrobactrum anthropi].	GCA_003937425.1
RYG67597.1	fn3+SPRY*→	fn3+SPRY	1230	EON64_07085	Archaea	archaeon	hypothetical protein EON64_07085 [archaeon].	GCA_004145555.1
RYH32433.1	SF2-helicase+SPRY*→	SF2-helicase+SPRY	535	EON65_00615	Archaea	archaeon	DEAD/DEAH box helicase [archaeon].	GCA_004146225.1
RYY37588.1	SPRY*→	SPRY	952	EON62_01040	Archaea	archaeon	hypothetical protein EON62_01040, partial [archaeon].	GCA_004173375.1
RYY72402.1	SPRY*→	SPRY	164	EON63_20910	Archaea	archaeon	hypothetical protein EON63_20910 [archaeon].	GCA_004173275.1
TDL67967.1	SUN→ SUN→ SPRY+SUN*→ Laminin_G_3→ Disaggr_repeat→	SPRY+SUN	425	E2R58_01745	Firmicutes	Paenibacillus amylolyticus	hypothetical protein E2R58_01745 [Paenibacillus amylolyticus].	GCA_004358835.1

<table>
<tr><th>acc</th><th>operon</th><th>architecture</th><th>len</th><th>gen.name</th><th>taxend</th><th>species</th><th>defline</th><th>gca</th></tr>
<tr><td>TSC88001.1</td><td>Laminin_G_3+Laminin_G_3+Laminin_G_3+Laminin_G_3→?→?→?→?→ SPRY+SUN*→ Laminin_G_3+Laminin_G_3+Laminin_G_3+Laminin_G_3→</td><td>SPRY+SUN</td><td>607</td><td>G01um10145_950</td><td>Bacteria</td><td>Microgenomates group bacterium Gr01-1014_5</td><td>hypothetical protein G01um10145_950 [Microgenomates group bacterium Gr01-1014_5].</td><td>GCA_007376395.1</td></tr>
<tr><td>VUI62768.1</td><td>Phage_connect_1→ Phage_H_T_join→ HK97-gp10_like→ DUF3168→ Phage_TTP_11→ Phage_TAC_13→?→ SPRY+X*→?→ Phage-tail-tape→</td><td>SPRY+X</td><td>655</td><td>GCPJBBEJ_01199</td><td>Gammaproteobacteria</td><td>Stenotrophomonas maltophilia</td><td>hypothetical protein GCPJBBEJ_01199 [Stenotrophomonas maltophilia].</td><td>GCA_902156415.1</td></tr>
<tr><td>VUJ81145.1</td><td>Phage-tail-tape→ ConA-repeats→?→ SPRY+X*→?→?→?→?→ SIG+TM+TM+TM+TM+TM+TM+TM+TM→ ABC-ATPase→</td><td>SPRY+X</td><td>604</td><td>IEE-HEPME_02021</td><td>Gammaproteobacteria</td><td>Stenotrophomonas maltophilia</td><td>hypothetical protein IEEHEPME_02021 [Stenotrophomonas maltophilia].</td><td>GCA_902156595.1</td></tr>
<tr><td>VUL57431.1</td><td>Terminase_LS→ SPRY*→?→||<-?||SIG+TM+TM+TM+TM+ABC-ATPase→</td><td>SPRY</td><td>217</td><td>LBG-BLGGB_01284</td><td>Gammaproteobacteria</td><td>Stenotrophomonas maltophilia</td><td>hypothetical protein LBGBLGGB_01284 [Stenotrophomonas maltophilia].</td><td>GCA_902156925.1</td></tr>
<tr><td>VUL62146.1</td><td>Phage_connect_1→ Phage_H_T_join→ HK97-gp10_like→ DUF3168→ Phage_TTP_11→ Phage_TAC_13→?→ SPRY+X*→?→?→ Phage-tail-tape→</td><td>SPRY+X</td><td>657</td><td>CCAN-PDJD_00380</td><td>Gammaproteobacteria</td><td>Stenotrophomonas maltophilia</td><td>hypothetical protein CCANPDJD_00380 [Stenotrophomonas maltophilia].</td><td>GCA_902157015.1</td></tr>
<tr><td>VUL75017.1</td><td>Phage_connect_1→ Phage_H_T_join→ HK97-gp10_like→?→ Phage_TTP_11→ Phage_TAC_13→?→ SPRY+X*→?→?→ Phage-tail-tape→</td><td>SPRY+X</td><td>655</td><td>GEILP-BKL_02464</td><td>Gammaproteobacteria</td><td>Stenotrophomonas maltophilia</td><td>hypothetical protein GEILPBKL_02464 [Stenotrophomonas maltophilia].</td><td>GCA_902156945.1</td></tr>
<tr><td>VUM07479.1</td><td>Phage_connect_1→ Phage_H_T_join→ HK97-gp10_like→ DUF3168→ Phage_TTP_11→ Phage_TAC_13→?→ SPRY+X*→?→?→ Phage-tail-tape→ ConA-repeats→?→?→ SRAP→</td><td>SPRY+X</td><td>657</td><td>IKOGN-NCB_02002</td><td>Gammaproteobacteria</td><td>Stenotrophomonas maltophilia</td><td>hypothetical protein IKOGNNCB_02002 [Stenotrophomonas maltophilia].</td><td>GCA_902157055.1</td></tr>
<tr><td>VUM45402.1</td><td>ConA-repeats→?→ SPRY+X*→</td><td>SPRY+X</td><td>604</td><td>FMOPOFGM_00942</td><td>Gammaproteobacteria</td><td>Stenotrophomonas maltophilia</td><td>hypothetical protein FMOPOFGM_00942 [Stenotrophomonas maltophilia].</td><td>GCA_902157105.1</td></tr>
<tr><td>VUM59055.1</td><td>Phage_connect_1→ Phage_H_T_join→ HK97-gp10_like→ DUF3168→ Phage_TTP_11→ Phage_TAC_13→?→ SPRY+X*→?→?→ Phage-tail-tape→ ConA-repeats→</td><td>SPRY+X</td><td>657</td><td>FMOPOFGM_03099</td><td>Gammaproteobacteria</td><td>Stenotrophomonas maltophilia</td><td>hypothetical protein FMOPOFGM_03099 [Stenotrophomonas maltophilia].</td><td>GCA_902157105.1</td></tr>
<tr><td>VUN16325.1</td><td>Phage_connect_1→ Phage_H_T_join→ HK97-gp10_like→ DUF3168→ Phage_TTP_11→ Phage_TAC_13→?→ SPRY+X*→||<-?||?→?→ Phage-tail-tape→ ConA-repeats→</td><td>SPRY+X</td><td>657</td><td>CP-DIOOCO_00061</td><td>Gammaproteobacteria</td><td>Stenotrophomonas maltophilia</td><td>hypothetical protein CPDIOOCO_00061 [Stenotrophomonas maltophilia].</td><td>GCA_902157315.1</td></tr>
<tr><td>VUN62462.1</td><td>Phage_connect_1→ Phage_H_T_join→ HK97-gp10_like→ DUF3168→ Phage_TTP_11→ Phage_TAC_13→?→ SPRY+X*→?→?→ Phage-tail-tape→ ConA-repeats→ ConA→</td><td>SPRY+X</td><td>655</td><td>FHOP-GADK_00135</td><td>Gammaproteobacteria</td><td>Stenotrophomonas maltophilia</td><td>hypothetical protein FHOPGADK_00135 [Stenotrophomonas maltophilia].</td><td>GCA_902157435.1</td></tr>
<tr><td>VUN67178.1</td><td>Phage_H_T_join→ HK97-gp10_like→ DUF3168→ Phage_TTP_11→ Phage_TAC_13→?→?→ SPRY+X*→||<-?||?→ Phage-tail-tape→</td><td>SPRY+X</td><td>601</td><td>JLECIBJP_00444</td><td>Gammaproteobacteria</td><td>Stenotrophomonas maltophilia</td><td>hypothetical protein JLECIBJP_00444 [Stenotrophomonas maltophilia].</td><td>GCA_902157425.1</td></tr>
</table>

<table>
<thead>
<tr><th>acc</th><th>operon</th><th>architecture</th><th>len</th><th>gen.name</th><th>taxend</th><th>species</th><th>defline</th><th>gca</th></tr>
</thead>
<tbody>
<tr><td>VUN71522.1</td><td>Phage_connect_1→ Phage_H_T_join→ HK97-gp10_like→ DUF3168→ Phage_TTP_11→ Phage_TAC_13→?→ SPRY+X*→?→ Phage-tail-tape→ ConA-repeats→?→?→ SRAP→</td><td>SPRY+X</td><td>655</td><td>IJ-MOMJOI_00888</td><td>Gammaproteobac-teria</td><td>Stenotrophomonas maltophilia</td><td>hypothetical protein IJMOMJOI_00888 [Stenotrophomonas maltophilia].</td><td>GCA_902157415.1</td></tr>
<tr><td>VUO23348.1</td><td>Phage_connect_1→ Phage_H_T_join→ HK97-gp10_like→ DUF3168→ Phage_TTP_11→ Phage_TAC_13→?→ SPRY+X*→?→?→ Phage-tail-tape→?→?→?→||<-SRAP</td><td>SPRY+X</td><td>656</td><td>JBID-JGIM_02130</td><td>Gammaproteobac-teria</td><td>Stenotrophomonas maltophilia</td><td>hypothetical protein JBIDJGIM_02130 [Stenotrophomonas maltophilia].</td><td>GCA_902157545.1</td></tr>
<tr><td>VUO59656.1</td><td>Phage_connect_1→ Phage_H_T_join→ HK97-gp10_like→ DUF3168→ Phage_TTP_11→ Phage_TAC_13→?→ SPRY+X*→?→?→ Phage-tail-tape→ ConA-repeats→?→?→||<-SRAP</td><td>SPRY+X</td><td>655</td><td>CMGNCPHI_00882</td><td>Gammaproteobac-teria</td><td>Stenotrophomonas maltophilia</td><td>hypothetical protein CMGNCPHI_00882 [Stenotrophomonas maltophilia].</td><td>GCA_902157585.1</td></tr>
<tr><td>VUP11864.1</td><td>Phage_connect_1→ Phage_H_T_join→ HK97-gp10_like→ DUF3168→ Phage_TTP_11→ Phage_TAC_13→?→ SPRY+X*→?→?→ Phage-tail-tape→</td><td>SPRY+X</td><td>658</td><td>ECE-FJIBE_02107</td><td>Gammaproteobac-teria</td><td>Stenotrophomonas maltophilia</td><td>hypothetical protein ECEFJIBE_02107 [Stenotrophomonas maltophilia].</td><td>GCA_902157845.1</td></tr>
<tr><td>VUP33787.1</td><td>Phage_connect_1→ Phage_H_T_join→ HK97-gp10_like→ DUF3168→ Phage_TTP_11→ Phage_TAC_13→?→ SPRY+X*→?→?→ Phage-tail-tape→</td><td>SPRY+X</td><td>657</td><td>ECE-FJIBE_03296</td><td>Gammaproteobac-teria</td><td>Stenotrophomonas maltophilia</td><td>hypothetical protein ECEFJIBE_03296 [Stenotrophomonas maltophilia].</td><td>GCA_902157845.1</td></tr>
<tr><td>VUP45028.1</td><td>Phage_connect_1→ Phage_H_T_join→ HK97-gp10_like→ DUF3168→ Phage_TTP_11→ Phage_TAC_13→?→ SPRY+X*→?→?→ Phage-tail-tape→ ConA-repeats→?→?→ SRAP→</td><td>SPRY+X</td><td>657</td><td>KEFPI-IEB_04064</td><td>Gammaproteobac-teria</td><td>Stenotrophomonas maltophilia</td><td>hypothetical protein KEFPIIEB_04064 [Stenotrophomonas maltophilia].</td><td>GCA_902157865.1</td></tr>
<tr><td>VUP91253.1</td><td>Phage_connect_1→ Phage_H_T_join→ HK97-gp10_like→ DUF3168→ Phage_TTP_11→ Phage_TAC_13→?→ SPRY+X*→||<-?||?→?→ Phage-tail-tape→ ConA-repeats→</td><td>SPRY+X</td><td>657</td><td>BLDAJIEI_03531</td><td>Gammaproteobac-teria</td><td>Stenotrophomonas maltophilia</td><td>hypothetical protein BLDAJIEI_03531 [Stenotrophomonas maltophilia].</td><td>GCA_902157935.1</td></tr>
<tr><td>VUP99062.1</td><td>Phage_connect_1→ Phage_H_T_join→ HK97-gp10_like→ DUF3168→ Phage_TTP_11→ Phage_TAC_13→?→ SPRY+X*→?→?→ Phage-tail-tape→</td><td>SPRY+X</td><td>655</td><td>BIEDFHEJ_01659</td><td>Gammaproteobac-teria</td><td>Stenotrophomonas maltophilia</td><td>hypothetical protein BIEDFHEJ_01659 [Stenotrophomonas maltophilia].</td><td>GCA_902157995.1</td></tr>
<tr><td>VUQ06316.1</td><td>Phage_connect_1→ Phage_H_T_join→ HK97-gp10_like→ DUF3168→ Phage_TTP_11→ Phage_TAC_13→?→ SPRY+X*→?→?→ Phage-tail-tape→</td><td>SPRY+X</td><td>655</td><td>JPICFAGD_01619</td><td>Gammaproteobac-teria</td><td>Stenotrophomonas maltophilia</td><td>hypothetical protein JPICFAGD_01619 [Stenotrophomonas maltophilia].</td><td>GCA_902158065.1</td></tr>
<tr><td>VUQ08603.1</td><td>Phage_connect_1→ Phage_H_T_join→ HK97-gp10_like→ DUF3168→ Phage_TTP_11→ Phage_TAC_13→?→ SPRY+X*→||<-?||?→ Phage-tail-tape→ ConA-repeats→</td><td>SPRY+X</td><td>655</td><td>MKCCK-LKB_01148</td><td>Gammaproteobac-teria</td><td>Stenotrophomonas maltophilia</td><td>hypothetical protein MKCCKLKB_01148 [Stenotrophomonas maltophilia].</td><td>GCA_902158105.1</td></tr>
<tr><td>VUQ16634.1</td><td>Phage_connect_1→ Phage_H_T_join→ HK97-gp10_like→ DUF3168→ Phage_TTP_11→ Phage_TAC_13→?→ SPRY+X*→||<-?||?→ Phage-tail-tape→</td><td>SPRY+X</td><td>657</td><td>DOOMH-BAD_02908</td><td>Gammaproteobac-teria</td><td>Stenotrophomonas maltophilia</td><td>hypothetical protein DOOMHBAD_02908 [Stenotrophomonas maltophilia].</td><td>GCA_902158025.1</td></tr>
<tr><td>VUR39033.1</td><td>Phage_connect_1→ Phage_H_T_join→ HK97-gp10_like→ DUF3168→ Phage_TTP_11→ Phage_TAC_13→?→ SPRY+X*→?→?→ Phage-tail-tape→ ConA-repeats→?→?→ SRAP→</td><td>SPRY+X</td><td>657</td><td>JHKEGKPK_04271</td><td>Gammaproteobac-teria</td><td>Stenotrophomonas maltophilia</td><td>hypothetical protein JHKEGKPK_04271 [Stenotrophomonas maltophilia].</td><td>GCA_902158305.1</td></tr>
<tr><td>VUR45784.1</td><td>Phage_connect_1→ Phage_H_T_join→ HK97-gp10_like→ DUF3168→ Phage_TTP_11→ Phage_TAC_13→?→ SPRY+X*→?→?→ Phage-tail-tape→ ConA-repeats→?→?→||<-SRAP</td><td>SPRY+X</td><td>657</td><td>PGEAM-FKH_03207</td><td>Gammaproteobac-teria</td><td>Stenotrophomonas maltophilia</td><td>hypothetical protein PGEAMFKH_03207 [Stenotrophomonas maltophilia].</td><td>GCA_902158325.1</td></tr>
</tbody>
</table>

<table>
<thead>
<tr><th>acc</th><th>operon</th><th>architecture</th><th>len</th><th>gen.name</th><th>taxend</th><th>species</th><th>defline</th><th>gca</th></tr>
</thead>
<tbody>
<tr><td>VUR81206.1</td><td>Phage_connect_1→ Phage_H_T_join→ HK97-gp10_like→ DUF3168→ Phage_TTP_11→ Phage_TAC_13→?→ SPRY+X*→?→?→ Phage-tail-tape→ ConA-repeats→ ConA→?→||<-SRAP</td><td>SPRY+X</td><td>655</td><td>GMKOK-IBD_01185</td><td>Gammaproteobacteria</td><td>Stenotrophomonas maltophilia</td><td>hypothetical protein GMKOKIBD_01185 [Stenotrophomonas maltophilia].</td><td>GCA_902158475.1</td></tr>
<tr><td>WP_007428531.1</td><td><-ABhydrolase<-?||SIG+SPRY→ SIG+SPRY→ SUN→||<-?<-Glycos_transf_2<-?||SUN→</td><td>SIG+SPRY</td><td>322</td><td>WG8_RS02665</td><td>Firmicutes</td><td>Paenibacillus sp. Aloe-11</td><td>hypothetical protein [Paenibacillus sp. Aloe-11].</td><td>GCF_000245715.1</td></tr>
<tr><td>WP_010076376.1</td><td><-SIG+IG<-?||SPRY+Big_2→ SPRY+Big_2→||<-ACET</td><td>SPRY+Big_2</td><td>292</td><td>CLO-CEL_RS05090</td><td>Firmicutes</td><td>Clostridium cellulovorans</td><td>Ig-like domain-containing protein [Clostridium cellulovorans].</td><td>GCF_000145275.1</td></tr>
<tr><td>WP_013308530.1</td><td>HTH_20→||<-ABhydrolase<-Polbetant||ACET→ Cupin_2→||<-DUF2935||SIG+SPRY*→||<-?||?→||<-Glycos_transf_2</td><td>SIG+SPRY</td><td>346</td><td>PPE_RS02405</td><td>Firmicutes</td><td>Paenibacillus polymyxa</td><td>hypothetical protein [Paenibacillus polymyxa].</td><td>GCF_000146875.3</td></tr>
<tr><td>WP_013513300.1</td><td>Phage_T7_tail→ SPRY*→ Phage_tail_APC→</td><td>SPRY</td><td>528</td><td>DAES_RS01675</td><td>Deltaproteobacteria</td><td>Pseudodesulfovibrio aespoeensis</td><td>SPla/RYanodine receptor SPRY domain-containing protein [Pseudodesulfovibrio aespoeensis].</td><td>GCF_000176915.2</td></tr>
<tr><td>WP_019226907.1</td><td>SIG+SPRY*→</td><td>SIG+SPRY</td><td>378</td><td>BN359_RS15990</td><td>Firmicutes</td><td>Dehalobacter</td><td>MULTISPECIES: hypothetical protein [Dehalobacter].</td><td>GCF_000309295.1</td></tr>
<tr><td>WP_019639008.1</td><td>SPRY+SUN→ SUN+SPRY→ Laminin_G_3→ Disaggr_repeat→ Phage_holin_4_1→</td><td>SPRY+SUN</td><td>411</td><td>G358_RS0119880</td><td>Firmicutes</td><td>Paenibacillus fonticola</td><td>hypothetical protein [Paenibacillus fonticola].</td><td>GCF_000381905.1</td></tr>
<tr><td>WP_019639009.1</td><td>-</td><td>-</td><td>413</td><td>G358_RS0119885</td><td>Firmicutes</td><td>Paenibacillus fonticola</td><td>discoidin domain-containing protein [Paenibacillus fonticola].</td><td>GCF_000381905.1</td></tr>
<tr><td>WP_019687775.1</td><td>SUN→ SPRY+Glyco_hydro_106*→ Laminin_G_3→ Disaggr_repeat→ Phage_holin_4_1→</td><td>SPRY+Glyco_hydro_106</td><td>403</td><td>H7A89_RS14065</td><td>Firmicutes</td><td>Paenibacillus polymyxa</td><td>hypothetical protein [Paenibacillus polymyxa].</td><td>GCF_903797665.1</td></tr>
<tr><td>WP_020591223.1</td><td>Phage_T7_tail→ SPRY*→</td><td>SPRY</td><td>1351</td><td>B152_RS0104640</td><td>Alphaproteobacteria</td><td>Kiloniella laminariae</td><td>hypothetical protein [Kiloniella laminariae].</td><td>GCF_000374005.1</td></tr>
<tr><td>WP_021132610.1</td><td>SPRY*→ Phage_tail_APC→</td><td>SPRY</td><td>746</td><td>K678_RS11425</td><td>Alphaproteobacteria</td><td>Phaeospirillum fulvum</td><td>hypothetical protein [Phaeospirillum fulvum].</td><td>GCF_000442515.1</td></tr>
<tr><td>WP_021203812.1</td><td>Phage_connect_1→ Phage_H_T_join→ HK97-gp10_like→ DUF3168→ Phage_TTP_11→ Phage_TAC_13→?→ SPRY+X*→?→?→ Phage-tail-tape→ ConA-repeats→?→?→||<-SRAP</td><td>SPRY+X</td><td>657</td><td>L681_RS129520</td><td>Gammaproteobacteria</td><td>Stenotrophomonas maltophilia</td><td>hypothetical protein [Stenotrophomonas maltophilia].</td><td>GCF_000455685.1</td></tr>
<tr><td>WP_023986763.1</td><td><-Trypsin<-ABhydrolase<-Polbetant||ACET→ Cupin_2→ ABhydrolase→||<-DUF2935||SIG+SPRY*→||<-PSE||?→ SIG+SPRY→ PSE→||<-Glycos_transf_2</td><td>SIG+SPRY</td><td>331</td><td>X809_RS02170</td><td>Firmicutes</td><td>Paenibacillus polymyxa</td><td>hypothetical protein [Paenibacillus polymyxa].</td><td>GCF_000507205.3</td></tr>
<tr><td>WP_024956478.1</td><td>Phage_connect_1→ Phage_H_T_join→ HK97-gp10_like→ DUF3168→ Phage_TTP_11→ Phage_TAC_13→ SPRY+X*→?→?→ Phage-tail-tape→</td><td>SPRY+X</td><td>657</td><td>BMR86_RS03335</td><td>Gammaproteobacteria</td><td>Stenotrophomonas</td><td>MULTISPECIES: hypothetical protein [Stenotrophomonas].</td><td>GCF_001591205.1</td></tr>
<tr><td>WP_025116403.1</td><td>SPRY→?→ SPRY+SUN→</td><td>SPRY</td><td>559</td><td>U863_RS0116605</td><td>Firmicutes</td><td>Lysinibacillus fusiformis</td><td>discoidin domain-containing protein [Lysinibacillus fusiformis].</td><td>GCF_000633275.1</td></tr>
</tbody>
</table>

<table>
<thead>
<tr><th>acc</th><th>operon</th><th>architecture</th><th>len</th><th>gen.name</th><th>taxend</th><th>species</th><th>defline</th><th>gca</th></tr>
</thead>
<tbody>
<tr><td>WP_025116405.1</td><td>-</td><td>-</td><td>396</td><td>U863_RS0116615</td><td>Firmicutes</td><td>Lysinibacillus fusiformis</td><td>hypothetical protein [Lysinibacillus fusiformis].</td><td>GCF_000633275.1</td></tr>
<tr><td>WP_025219035.1</td><td>SUN→ SPRY*→ SUN→ SPRY→ Laminin_G_3→ Disaggr_repeat→?→?→ Phage_holin_1→</td><td>SPRY</td><td>281</td><td>T479_RS06935</td><td>Firmicutes</td><td>Lysinibacillus varians</td><td>hypothetical protein [Lysinibacillus varians].</td><td>GCF_000600105.1</td></tr>
<tr><td>WP_025363879.1</td><td>HTH_20→||<-Trypsin<-ABhydrolase||ACET→||<-DUF2935<-?||SIG+SPRY*→</td><td>SIG+SPRY</td><td>332</td><td>LK13_RS15595</td><td>Firmicutes</td><td>Paenibacillus polymyxa</td><td>hypothetical protein [Paenibacillus polymyxa].</td><td>GCF_000597985.1</td></tr>
<tr><td>WP_025676232.1</td><td>HTH_20→||<-Trypsin<-ABhydrolase||ACET→||<-DUF2935<-?||SIG+SPRY*→</td><td>SIG+SPRY</td><td>327</td><td>L695_RS0111830</td><td>Firmicutes</td><td>Paenibacillus polymyxa</td><td>hypothetical protein [Paenibacillus polymyxa].</td><td>GCF_000520795.1</td></tr>
<tr><td>WP_025682581.1</td><td>HTH_20→||<-?||?→||<-DUF2935<-?||SIG+SPRY*→||<-PSE||SUN→||<-?||?→||<-?<-?<-Phage_holin_4_1</td><td>SIG+SPRY</td><td>332</td><td>L696_RS0104535</td><td>Firmicutes</td><td>Paenibacillus maysiensis</td><td>hypothetical protein [Paenibacillus maysiensis].</td><td>GCF_000520815.1</td></tr>
<tr><td>WP_025716755.1</td><td>HTH_20→||<-ABhydrolase<-?||SIG+SPRY*→</td><td>SIG+SPRY</td><td>314</td><td>L693_RS0107595</td><td>Firmicutes</td><td>Paenibacillus sp. 1-18</td><td>hypothetical protein [Paenibacillus sp. 1-18].</td><td>GCF_000520755.1</td></tr>
<tr><td>WP_025721904.1</td><td>HTH_20→||<-Trypsin<-ABhydrolase<-Polbetant||ACET→ Cupin_2→||<-DUF2935||SIG+SPRY*→</td><td>SIG+SPRY</td><td>218</td><td>L694_RS0112470</td><td>Firmicutes</td><td>Paenibacillus polymyxa</td><td>hypothetical protein, partial [Paenibacillus polymyxa].</td><td>GCF_000520775.1</td></tr>
<tr><td>WP_028541175.1</td><td><-ABhydrolase<-Polbetant||ACET→ Cupin_2→ ABhydrolase→||<-DUF2935<-?||SIG+SPRY*→||<-?||?→ SIG+SPRY→||<-Glycos_transf_2</td><td>SIG+SPRY</td><td>320</td><td>BM358_RS15270</td><td>Firmicutes</td><td>unclassified Paenibacillus</td><td>MULTISPECIES: hypothetical protein [unclassified Paenibacillus].</td><td>GCF_900116035.1</td></tr>
<tr><td>WP_028562231.1</td><td>SPRY+SUN*→ SUN→ Laminin_G_3→ Disaggr_repeat→</td><td>SPRY+SUN</td><td>414</td><td>H583_RS35510</td><td>Firmicutes</td><td>Paenibacillus pinihumi</td><td>hypothetical protein [Paenibacillus pinihumi].</td><td>GCF_000422505.1</td></tr>
<tr><td>WP_029516016.1</td><td><-ABhydrolase<-Polbetant||ACET→ Cupin_2→ ABhydrolase→||<-DUF2935||SUN→ SIG+SPRY*→?→||<-Glycos_transf_2</td><td>SIG+SPRY</td><td>322</td><td>B439_RS0108145</td><td>Firmicutes</td><td>Paenibacillus polymyxa</td><td>hypothetical protein [Paenibacillus polymyxa].</td><td>GCF_000463565.1</td></tr>
<tr><td>WP_029518099.1</td><td>SUN→ SUN*→ Laminin_G_3→ Disaggr_repeat→</td><td>SUN</td><td>403</td><td>B439_RS0121250</td><td>Firmicutes</td><td>Paenibacillus polymyxa</td><td>hypothetical protein [Paenibacillus polymyxa].</td><td>GCF_000463565.1</td></tr>
<tr><td>WP_031462749.1</td><td>HTH_20→||<-Trypsin<-ABhydrolase||ACET→||<-DUF2935<-?||SIG+SPRY*→</td><td>SIG+SPRY</td><td>308</td><td>FC01_RS0115670</td><td>Firmicutes</td><td>Paenibacillus polymyxa</td><td>hypothetical protein [Paenibacillus polymyxa].</td><td>GCF_000735775.1</td></tr>
<tr><td>WP_033066158.1</td><td>Cluster143_2clades→?→?→?→ SUN→ SPRY*→ PSE→ PSE→ SUN→ TM→ Cluster104_2clades→?→?→35exo→ Cluster112_2clades→</td><td>SPRY</td><td>447</td><td>N496_RS05195</td><td>Firmicutes</td><td>Clostridium botulinum</td><td>cell adhesion protein [Clostridium botulinum].</td><td>GCF_000710995.1</td></tr>
<tr><td>WP_036124491.1</td><td>SUN→ SUN→?→ SUN→ SUN→ SPRY*→ SUN→ Laminin_G_3→ Disaggr_repeat→?→?→ Phage_holin_1→</td><td>SPRY</td><td>257</td><td>HR49_RS16910</td><td>Firmicutes</td><td>Lysinibacillus</td><td>MULTISPECIES: hypothetical protein [Lysinibacillus].</td><td>GCF_000724775.3</td></tr>
<tr><td>WP_036147628.1</td><td>SUN→ SPRY+SUN→ SPRY+SUN→ SUN→ SUN→ SUN→ Laminin_G_3→ Disaggr_repeat→</td><td>SPRY+SUN</td><td>414</td><td>KQ41_RS11015</td><td>Firmicutes</td><td>Lysinibacillus fusiformis</td><td>discoidin domain-containing protein [Lysinibacillus fusiformis].</td><td>GCF_000755455.1</td></tr>
<tr><td>WP_036147629.1</td><td>-</td><td>-</td><td>433</td><td>KQ41_RS11020</td><td>Firmicutes</td><td>Lysinibacillus fusiformis</td><td>discoidin domain-containing protein [Lysinibacillus fusiformis].</td><td>GCF_000755455.1</td></tr>
</tbody>
</table>

<table>
<tr><th>acc</th><th>operon</th><th>architecture</th><th>len</th><th>gen.name</th><th>taxend</th><th>species</th><th>defline</th><th>gca</th></tr>
<tr><td>WP_036669882.1</td><td>SUN→ SPRY+SUN→ SPRY+SUN→ Laminin_G_3→ Disaggr_repeat→</td><td>SPRY+SUN</td><td>395</td><td>C161_RS09450</td><td>Firmicutes</td><td>Paenibacillus sp. FSL R5-192</td><td>hypothetical protein [Paenibacillus sp. FSL R5-192].</td><td>GCF_000517845.1</td></tr>
<tr><td>WP_040102262.1</td><td>HTH_20→||<-Trypsin<-ABhydrolase||ACET→||<-DUF2935<-?||SIG+SPRY*→</td><td>SIG+SPRY</td><td>319</td><td>RE92_RS09150</td><td>Firmicutes</td><td>Paenibacillus polymyxa</td><td>hypothetical protein [Paenibacillus polymyxa].</td><td>GCF_000819665.1</td></tr>
<tr><td>WP_044646514.1</td><td><-DUF2935||?→||<-?||SIG+SPRY*→</td><td>SIG+SPRY</td><td>326</td><td>QD47_RS12890</td><td>Firmicutes</td><td>Paenibacillus terrae</td><td>hypothetical protein [Paenibacillus terrae].</td><td>GCF_000943545.1</td></tr>
<tr><td>WP_044648488.1</td><td>SIG+SPRY*→||<-?<-?<-?<-?<-?<-Phage_holin_4_1</td><td>SIG+SPRY</td><td>327</td><td>QD47_RS23995</td><td>Firmicutes</td><td>Paenibacillus terrae</td><td>hypothetical protein [Paenibacillus terrae].</td><td>GCF_000943545.1</td></tr>
<tr><td>WP_044787083.1</td><td>HTH_20→||<-Trypsin<-ABhydrolase||ACET→||<-DUF2935<-?||SIG+SPRY*→</td><td>SIG+SPRY</td><td>357</td><td>DS866_RS12660</td><td>Firmicutes</td><td>Paenibacillus polymyxa</td><td>hypothetical protein [Paenibacillus polymyxa].</td><td>GCF_000943535.1</td></tr>
<tr><td>WP_046501577.1</td><td>Phage_T7_tail→ SPRY*→</td><td>SPRY</td><td>1158</td><td>WH95_RS00475</td><td>Alphaproteobacteria</td><td>Kiloniella litopenaei</td><td>hypothetical protein [Kiloniella litopenaei].</td><td>GCF_000982415.1</td></tr>
<tr><td>WP_048035679.1</td><td>SUN→ SUN→ SPRY*→?→?→?→||<-?<-?<-?<-ABC-ATPase</td><td>SPRY</td><td>890</td><td>AB432_RS03735</td><td>Firmicutes</td><td>Brevibacillus brevis</td><td>hypothetical protein [Brevibacillus brevis].</td><td>GCF_001039275.2</td></tr>
<tr><td>WP_049431026.1</td><td>Phage_connect_1→ Phage_H_T_join→ HK97-gp10_like→ DUF3168→ Phage_TTP_11→ Phage_TAC_13→ SPRY+X*→||<-?||?→?→ Phage-tail-tape→ ConA-repeats→</td><td>SPRY+X</td><td>657</td><td>G5A48_RS10580</td><td>Gammaproteobacteria</td><td>Stenotrophomonas maltophilia</td><td>hypothetical protein [Stenotrophomonas maltophilia].</td><td>GCF_001069295.1</td></tr>
<tr><td>WP_049432438.1</td><td>Phage_connect_1→ Phage_H_T_join→ HK97-gp10_like→ DUF3168→ Phage_TTP_11→ Phage_TAC_13→?→ SPRY+X*→?→?→ Phage-tail-tape→ ConA-repeats→</td><td>SPRY+X</td><td>655</td><td>A7X76_RS05335</td><td>Gammaproteobacteria</td><td>Stenotrophomonas maltophilia</td><td>hypothetical protein [Stenotrophomonas maltophilia].</td><td>GCF_001070785.1</td></tr>
<tr><td>WP_049448603.1</td><td>Phage_connect_1→ Phage_H_T_join→ HK97-gp10_like→ DUF3168→ Phage_TTP_11→ Phage_TAC_13→?→ SPRY+X*→?→ Phage-tail-tape→ ConA-repeats→</td><td>SPRY+X</td><td>657</td><td>ADH94_RS22020</td><td>Gammaproteobacteria</td><td>Stenotrophomonas maltophilia</td><td>hypothetical protein [Stenotrophomonas maltophilia].</td><td>GCF_001071475.1</td></tr>
<tr><td>WP_049450667.1</td><td>Phage_connect_1→ Phage_H_T_join→ HK97-gp10_like→ DUF3168→ Phage_TTP_11→ Phage_TAC_13→?→ SPRY+X*→?→ Phage-tail-tape→?→?→?→||<-SRAP</td><td>SPRY+X</td><td>656</td><td>EGS96_RS20790</td><td>Gammaproteobacteria</td><td>Stenotrophomonas maltophilia</td><td>hypothetical protein [Stenotrophomonas maltophilia].</td><td>GCF_001071195.1</td></tr>
<tr><td>WP_049454514.1</td><td>Phage_connect_1→ Phage_H_T_join→ HK97-gp10_like→ DUF3168→ Phage_TTP_11→ Phage_TAC_13→?→ SPRY+X*→?→?→ Phage-tail-tape→ ConA-repeats→?→?→||<-SRAP</td><td>SPRY+X</td><td>658</td><td>ADF89_RS18240</td><td>Gammaproteobacteria</td><td>Stenotrophomonas maltophilia</td><td>hypothetical protein [Stenotrophomonas maltophilia].</td><td>GCF_001069005.1</td></tr>
<tr><td>WP_049459324.1</td><td>Phage_connect_1→ Phage_H_T_join→ HK97-gp10_like→ DUF3168→ Phage_TTP_11→ Phage_TAC_13→?→ SPRY+X*→?→?→ Phage-tail-tape→ ConA-repeats→</td><td>SPRY+X</td><td>657</td><td>ADM04_RS16360</td><td>Gammaproteobacteria</td><td>Stenotrophomonas maltophilia</td><td>hypothetical protein [Stenotrophomonas maltophilia].</td><td>GCF_001074705.1</td></tr>
<tr><td>WP_049736558.1</td><td>SUN→ Laminin_G_3+SPRY*→</td><td>Laminin_G_3+SPRY</td><td>822</td><td>BRE01_RS08970</td><td>Firmicutes</td><td>Brevibacillus reuszeri</td><td>hypothetical protein [Brevibacillus reuszeri].</td><td>GCF_006540225.1</td></tr>
<tr><td>WP_051447145.1</td><td>SUN→ SPRY+SUN→ SUN→ SPRY+SUN→ Laminin_G_3→ Disaggr_repeat→</td><td>SPRY+SUN</td><td>421</td><td>C170_RS30045</td><td>Firmicutes</td><td>Paenibacillus sp. FSL H7-689</td><td>discoidin domain-containing protein [Paenibacillus sp. FSL H7-689].</td><td>GCF_000517905.1</td></tr>
<tr><td>WP_051447146.1</td><td>-</td><td>-</td><td>399</td><td>C170_RS30050</td><td>Firmicutes</td><td>Paenibacillus sp. FSL H7-689</td><td>discoidin domain-containing protein [Paenibacillus sp. FSL H7-689].</td><td>GCF_000517905.1</td></tr>
<tr><td>WP_051449672.1</td><td>-</td><td>-</td><td>402</td><td>C161_RS31475</td><td>Firmicutes</td><td>Paenibacillus sp. FSL R5-192</td><td>hypothetical protein [Paenibacillus sp. FSL R5-192].</td><td>GCF_000517845.1</td></tr>
<tr><td>WP_051626263.1</td><td><-REC+OmpR-HTH<-?<-OBfold<-?<-?||?→||<-?||SIG+SPRY*→</td><td>SIG+SPRY</td><td>653</td><td>EP01_RS18270</td><td>Oligoflexia</td><td>Bdellovibrio bacteriovorus</td><td>hypothetical protein [Bdellovibrio bacteriovorus].</td><td>GCF_000691605.1</td></tr>
</table>

<table>
<tr><th>acc</th><th>operon</th><th>architecture</th><th>len</th><th>gen.name</th><th>taxend</th><th>species</th><th>defline</th><th>gca</th></tr>
<tr><td>WP_052310845.1</td><td>-</td><td>-</td><td>229</td><td>WG8_RS02660</td><td>Firmicutes</td><td>Paenibacillus sp. Aloe-11</td><td>hypothetical protein, partial [Paenibacillus sp. Aloe-11].</td><td>GCF_000245715.1</td></tr>
<tr><td>WP_053324515.1</td><td><-ABhydrolase<-Polbetant||ACET→ Cupin_2→ ABhydrolase→||<-DUF2935<-?||SIG+SPRY→//<-PSE//?→ SIG+SPRY→||<-Glycos_transf_2</td><td>SIG+SPRY</td><td>373</td><td>BK119_RS14050</td><td>Firmicutes</td><td>Paenibacillus</td><td>MULTISPECIES: hypothetical protein [Paenibacillus].</td><td>GCF_001922145.1</td></tr>
<tr><td>WP_053324516.1</td><td><-ABhydrolase<-Polbetant||ACET→ Cupin_2→ ABhydrolase→||<-DUF2935<-?||SIG+SPRY*→?→ SIG+SPRY→ PSE→||<-Glycos_transf_2</td><td>SIG+SPRY</td><td>328</td><td>ABE82_RS02500</td><td>Firmicutes</td><td>Paenibacillus peoriae</td><td>hypothetical protein [Paenibacillus peoriae].</td><td>GCF_001272655.2</td></tr>
<tr><td>WP_053497216.1</td><td>Phage_connect_1→ Phage_H_T_join→ HK97-gp10_like→ DUF3168→ Phage_TTP_11→ Phage_TAC_13→?→ SPRY+X*→?→?→ Phage-tail-tape→ ConA-repeats→ ConA→?→||<-SRAP</td><td>SPRY+X</td><td>655</td><td>ABW42_RS03390</td><td>Gammaproteobacteria</td><td>Stenotrophomonas maltophilia</td><td>hypothetical protein [Stenotrophomonas maltophilia].</td><td>GCF_001276345.1</td></tr>
<tr><td>WP_053519524.1</td><td>Phage_connect_1→ Phage_H_T_join→ HK97-gp10_like→ DUF3168→ Phage_TTP_11→ Phage_TAC_13→?→ SPRY+X*→?→ Phage-tail-tape→ ConA-repeats→</td><td>SPRY+X</td><td>655</td><td>ABW43_RS14405</td><td>Gammaproteobacteria</td><td>Stenotrophomonas maltophilia</td><td>hypothetical protein [Stenotrophomonas maltophilia].</td><td>GCF_001276355.1</td></tr>
<tr><td>WP_053595626.1</td><td>SUN→ SPRY+SUN*→ SUN→ SUN→ Laminin_G_3→ Disaggr_repeat→</td><td>SPRY+SUN</td><td>413</td><td>AN161_RS17530</td><td>Firmicutes</td><td>Lysinibacillus sp. FJAT-14222</td><td>discoidin domain-containing protein [Lysinibacillus sp. FJAT-14222].</td><td>GCF_001278875.1</td></tr>
<tr><td>WP_054136806.1</td><td><-SIG+IG<-?||?→?→?→||<-?<-?||SUN+SPRY*→</td><td>SUN+SPRY</td><td>466</td><td>IP80_RS13885</td><td>Betaproteobacteria</td><td>unclassified Betaproteobacteria</td><td>MULTISPECIES: tautomerase family protein [unclassified Betaproteobacteria].</td><td>GCF_001295865.1</td></tr>
<tr><td>WP_054704414.1</td><td>SPRY+TM*→ SUN→ SUN→ Laminin_G_3→ Disaggr_repeat→</td><td>SPRY+TM</td><td>211</td><td>JCM16419_RS00705</td><td>Firmicutes</td><td>Paenibacillus pinihumi</td><td>hypothetical protein [Paenibacillus pinihumi].</td><td>GCF_001312305.1</td></tr>
<tr><td>WP_058710989.1</td><td>HTH_20→||<-Trypsin<-ABhydrolase||ACET→||<-DUF2935<-PSE||SIG+SPRY*→</td><td>SIG+SPRY</td><td>306</td><td>NS115_RS07620</td><td>Firmicutes</td><td>Paenibacillus</td><td>MULTISPECIES: hypothetical protein [Paenibacillus].</td><td>GCF_002872435.1</td></tr>
<tr><td>WP_058830612.1</td><td>HTH_20→||<-Trypsin<-ABhydrolase||ACET→||<-DUF2935<-?||SIG+SPRY*→</td><td>SIG+SPRY</td><td>339</td><td>AVK83_RS16520</td><td>Firmicutes</td><td>Paenibacillus polymyxa</td><td>hypothetical protein [Paenibacillus polymyxa].</td><td>GCF_001481575.1</td></tr>
<tr><td>WP_058981995.1</td><td>Phage_connect_1→ Phage_H_T_join→ HK97-gp10_like→ DUF3168→ Phage_TTP_11→ Phage_TAC_13→?→ SPRY+X*→?→?→?→ Phage-tail-tape→ ConA-repeats→</td><td>SPRY+X</td><td>657</td><td>BN1263_RS14155</td><td>Gammaproteobacteria</td><td>Stenotrophomonas maltophilia</td><td>hypothetical protein [Stenotrophomonas maltophilia].</td><td>GCF_001499715.1</td></tr>
<tr><td>WP_059064521.1</td><td>Phage_connect_1→ Phage_H_T_join→ HK97-gp10_like→ DUF3168→ Phage_TTP_11→ Phage_TAC_13→?→ SPRY+X*→?→ Phage-tail-tape→ ConA-repeats→?→ SRAP→</td><td>SPRY+X</td><td>655</td><td>AV528_RS10010</td><td>Gammaproteobacteria</td><td>Stenotrophomonas maltophilia</td><td>hypothetical protein [Stenotrophomonas maltophilia].</td><td>GCF_001499755.1</td></tr>
<tr><td>WP_063211944.1</td><td>HTH_20→||<-ABhydrolase<-?||ACET→ Cupin_2→ SIG+SPRY*→</td><td>SIG+SPRY</td><td>337</td><td>AV545_RS14475</td><td>Firmicutes</td><td>Paenibacillus jamilae</td><td>hypothetical protein [Paenibacillus jamilae].</td><td>GCF_001619755.1</td></tr>
<tr><td>WP_064201871.1</td><td>SPRY*→?→?→?→||<-?<-?<-?<-ABC-ATPase</td><td>SPRY</td><td>894</td><td>A4S11_RS18990</td><td>Firmicutes</td><td>Brevibacillus brevis</td><td>hypothetical protein [Brevibacillus brevis].</td><td>GCF_001649505.1</td></tr>
<tr><td>WP_064796708.1</td><td>HTH_20→||<-ABhydrolase<-Polbetant||ACET→ Cupin_2→||<-?||SIG+SPRY*→</td><td>SIG+SPRY</td><td>314</td><td>A9P44_RS12255</td><td>Firmicutes</td><td>Paenibacillus polymyxa</td><td>hypothetical protein [Paenibacillus polymyxa].</td><td>GCF_001663585.1</td></tr>
<tr><td>WP_065179322.1</td><td>Phage_connect_1→ Phage_H_T_join→ HK97-gp10_like→ DUF3168→ Phage_TTP_11→ Phage_TAC_13→?→ SPRY+X*→?→ Phage-tail-tape→</td><td>SPRY+X</td><td>655</td><td>A9K70_RS20570</td><td>Gammaproteobacteria</td><td>Stenotrophomonas maltophilia</td><td>hypothetical protein [Stenotrophomonas maltophilia].</td><td>GCF_001676395.1</td></tr>
<tr><td>WP_065181897.1</td><td>Phage_connect_1→ Phage_H_T_join→ HK97-gp10_like→ DUF3168→ Phage_TTP_11→ Phage_TAC_13→?→ SPRY+X*→||<-?||?→?→ Phage-tail-tape→ ConA-repeats→</td><td>SPRY+X</td><td>655</td><td>A9K56_RS08050</td><td>Gammaproteobacteria</td><td>Stenotrophomonas maltophilia</td><td>hypothetical protein [Stenotrophomonas maltophilia].</td><td>GCF_001676315.1</td></tr>
</table>

<table>
<tr><th>acc</th><th>operon</th><th>architecture</th><th>len</th><th>gen.name</th><th>taxend</th><th>species</th><th>defline</th><th>gca</th></tr>
<tr><td>WP_065200553.1</td><td>Phage_connect_1→ Phage_H_T_join→ HK97-gp10_like→ DUF3168→ Phage_TTP_11→ Phage_TAC_13→?→ SPRY+X*→||<-?||?→?→?→ Phage-tail-tape→ ConA-repeats→ ConA→</td><td>SPRY+X</td><td>655</td><td>A9K58_RS17510</td><td>Gammaproteobacteria</td><td>Stenotrophomonas maltophilia</td><td>hypothetical protein [Stenotrophomonas maltophilia].</td><td>GCF_001676385.1</td></tr>
<tr><td>WP_068434738.1</td><td>Cluster93_5clades→ Laminin_G_3+SPRY*→?→?→?→||<-?<-Cluster297_2clades<-EMAP</td><td>Laminin_G_3+SPRY</td><td>467</td><td>XM1_RS14675</td><td>Alphaproteobacteria</td><td>Magnetospirillum sp. XM-1</td><td>hypothetical protein [Magnetospirillum sp. XM-1].</td><td>GCF_001511835.1</td></tr>
<tr><td>WP_068499071.1</td><td><-SUN<-?<-?||SIG+SPRY*→</td><td>SIG+SPRY</td><td>378</td><td>BAZ21_RS08685</td><td>Firmicutes</td><td>Paenibacillus kribbensis</td><td>hypothetical protein [Paenibacillus kribbensis].</td><td>GCF_001705305.1</td></tr>
<tr><td>WP_068504121.1</td><td><-ABhydrolase||?→||<-?||SIG+SPRY*→||<-PSE||SUN→</td><td>SIG+SPRY</td><td>308</td><td>BAZ21_RS19160</td><td>Firmicutes</td><td>Paenibacillus kribbensis</td><td>hypothetical protein [Paenibacillus kribbensis].</td><td>GCF_001705305.1</td></tr>
<tr><td>WP_068940847.1</td><td>SUN→ SPRY+SUN→ *SPRY*→ Disaggr_repeat→</td><td>SPRY+SUN</td><td>698</td><td>A7312_RS18605</td><td>Firmicutes</td><td>Paenibacillus polymyxa</td><td>hypothetical protein [Paenibacillus polymyxa].</td><td>GCF_001707685.1</td></tr>
<tr><td>WP_068940849.1</td><td>-</td><td>-</td><td>419</td><td>A7312_RS18610</td><td>Firmicutes</td><td>Paenibacillus polymyxa</td><td>hypothetical protein [Paenibacillus polymyxa].</td><td>GCF_001707685.1</td></tr>
<tr><td>WP_068941209.1</td><td>-</td><td>-</td><td>320</td><td>BK119_RS14035</td><td>Firmicutes</td><td>Paenibacillus</td><td>MULTISPECIES: hypothetical protein [Paenibacillus].</td><td>GCF_001922145.1</td></tr>
<tr><td>WP_069010793.1</td><td>HTH_20→||<-ABhydrolase<-Polbetant||ACET→ Cupin_2→ ABhydrolase→||<-DUF2935||SIG+SPRY*→?→||<-Glycos_transf_2</td><td>SIG+SPRY</td><td>346</td><td>A7311_RS06950</td><td>Firmicutes</td><td>Paenibacillus polymyxa</td><td>hypothetical protein [Paenibacillus polymyxa].</td><td>GCF_001709075.1</td></tr>
<tr><td>WP_069481585.1</td><td>SUN+SPRY→*?*→*?*→*?*→ *SUN*→ *SPRY+SUN*→</td><td>SUN+SPRY</td><td>559</td><td>BG258_RS12185</td><td>Firmicutes</td><td>Lysinibacillus fusiformis</td><td>discoidin domain-containing protein [Lysinibacillus fusiformis].</td><td>GCF_001726065.1</td></tr>
<tr><td>WP_069481589.1</td><td>-</td><td>-</td><td>403</td><td>BG258_RS12205</td><td>Firmicutes</td><td>Lysinibacillus fusiformis</td><td>hypothetical protein [Lysinibacillus fusiformis].</td><td>GCF_001726065.1</td></tr>
<tr><td>WP_071558628.1</td><td><-SUN||SIG+SPRY*→||<-?<-?<-?<-?<-?<-Phage_holin_4_1</td><td>SIG+SPRY</td><td>334</td><td>CG775_RS21655</td><td>Firmicutes</td><td>Paenibacillus polymyxa</td><td>hypothetical protein [Paenibacillus polymyxa].</td><td>GCF_001874425.3</td></tr>
<tr><td>WP_071639359.1</td><td>HTH_20→||<-ABhydrolase<-Polbetant||ACET→ Cupin_2→ ABhydrolase→||<-DUF2935||SIG+SPRY→*?*→ *SIG+SPRY*→ PSE→||<-Glycos_transf_2</td><td>SIG+SPRY</td><td>373</td><td>PPYC1_RS02475</td><td>Firmicutes</td><td>Paenibacillus polymyxa</td><td>hypothetical protein [Paenibacillus polymyxa].</td><td>GCF_001874405.2</td></tr>
<tr><td>WP_071639361.1</td><td>-</td><td>-</td><td>323</td><td>PPYC1_RS02485</td><td>Firmicutes</td><td>Paenibacillus polymyxa</td><td>hypothetical protein [Paenibacillus polymyxa].</td><td>GCF_001874405.2</td></tr>
<tr><td>WP_072733996.1</td><td>SUN→ SUN→ SUN→ TM+SPRY+SUN→ *SPRY*→ Disaggr_repeat→</td><td>TM+SPRY+SUN</td><td>708</td><td>BUA17_RS14040</td><td>Firmicutes</td><td>Paenibacillus sp. ov031</td><td>hypothetical protein [Paenibacillus sp. ov031].</td><td>GCF_900143165.1</td></tr>
<tr><td>WP_072733997.1</td><td>-</td><td>-</td><td>422</td><td>BUA17_RS14045</td><td>Firmicutes</td><td>Paenibacillus sp. ov031</td><td>discoidin domain-containing protein [Paenibacillus sp. ov031].</td><td>GCF_900143165.1</td></tr>
<tr><td>WP_075141582.1</td><td>Cluster216_2clades→?→?→?→ SUN→ SPRY*→ PSE→ SUN→ SUN→ TM→ Cluster104_2clades→?→?→35exo→</td><td>SPRY</td><td>447</td><td>RSJ3_RS04310</td><td>Firmicutes</td><td>Clostridium botulinum</td><td>cell adhesion protein [Clostridium botulinum].</td><td>GCF_001921965.1</td></tr>
<tr><td>WP_076158833.1</td><td><-ABhydrolase<-Polbetant||ACET→ Cupin_2→ ABhydrolase→||<-DUF2935||SIG+SPRY*→||<-PSE||?→ PSE→||<-Glycos_transf_2</td><td>SIG+SPRY</td><td>301</td><td>BK134_RS12705</td><td>Firmicutes</td><td>Paenibacillus peoriae</td><td>hypothetical protein [Paenibacillus peoriae].</td><td>GCF_001956115.1</td></tr>
</table>

<table>
<tr><th>acc</th><th>operon</th><th>architecture</th><th>len</th><th>gen.name</th><th>taxend</th><th>species</th><th>defline</th><th>gca</th></tr>
<tr><td>WP_076172386.1</td><td>Cluster171_2clades→?→?→ Cluster143_2clades→?→?→ PSE→ SUN→ SPRY*→ SUN→ TM→ Cluster104_2clades→?→?→35exo→ Cluster112_2clades→</td><td>SPRY</td><td>465</td><td>N487_RS14900</td><td>Firmicutes</td><td>Clostridium botulinum</td><td>cell adhesion protein [Clostridium botulinum].</td><td>GCF_000710975.1</td></tr>
<tr><td>WP_076252374.1</td><td>SUN→ SPRY+SUN*→ SUN→ SUN→ Laminin_G_3→ Disaggr_repeat→</td><td>SPRY+SUN</td><td>403</td><td>BK136_RS15140</td><td>Firmicutes</td><td>Paenibacillus amylolyticus</td><td>hypothetical protein [Paenibacillus amylolyticus].</td><td>GCF_001956175.1</td></tr>
<tr><td>WP_076265166.1</td><td><-ABhydrolase<-Polbetant||ACET→ Cupin_2→ ABhydrolase→||<-DUF2935<-?||SIG+SPRY*→||<-?||?→ SIG+SPRY→||<-Glycos_transf_2</td><td>SIG+SPRY</td><td>320</td><td>BK143_RS19680</td><td>Firmicutes</td><td>Paenibacillus peoriae</td><td>hypothetical protein [Paenibacillus peoriae].</td><td>GCF_001956225.1</td></tr>
<tr><td>WP_076293278.1</td><td>HTH_20→||<-PSE<-ABhydrolase<-Polbetant||ACET→ Cupin_2→ ABhydrolase→||<-DUF2935||SIG+SPRY*→||<-?||?→ PSE→||<-Glycos_transf_2</td><td>SIG+SPRY</td><td>326</td><td>BK135_RS10440</td><td>Firmicutes</td><td>Paenibacillus peoriae</td><td>hypothetical protein [Paenibacillus peoriae].</td><td>GCF_001956155.1</td></tr>
<tr><td>WP_076295423.1</td><td><-ABhydrolase<-Polbetant||ACET→ Cupin_2→ ABhydrolase→||<-DUF2935<-?||SIG+SPRY→*//<-?//?→ SIG+SPRY*→||<-Glycos_transf_2</td><td>SIG+SPRY</td><td>331</td><td>BK145_RS14470</td><td>Firmicutes</td><td>Paenibacillus peoriae</td><td>hypothetical protein [Paenibacillus peoriae].</td><td>GCF_001956235.1</td></tr>
<tr><td>WP_076318343.1</td><td>SUN→ SUN→ SUN→ SPRY+SUN*→ Laminin_G_3→ Disaggr_repeat→</td><td>SPRY+SUN</td><td>412</td><td>BK124_RS08255</td><td>Firmicutes</td><td>Paenibacillus amylolyticus</td><td>hypothetical protein [Paenibacillus amylolyticus].</td><td>GCF_001955995.1</td></tr>
<tr><td>WP_076327021.1</td><td>SUN→ SPRY+SUN*→ SUN→ Laminin_G_3→ Disaggr_repeat→</td><td>SPRY+SUN</td><td>414</td><td>BK129_RS03095</td><td>Firmicutes</td><td>Paenibacillus amylolyticus</td><td>discoidin domain-containing protein [Paenibacillus amylolyticus].</td><td>GCF_001956035.1</td></tr>
<tr><td>WP_079564340.1</td><td>SUN→ SPRY+SUN→ *SUN→ SUN→ SPRY+SUN*→ SUN→ SUN→ SUN→</td><td>SPRY+SUN</td><td>485</td><td>B5X66_RS24790</td><td>Firmicutes</td><td>unclassified Lysinibacillus</td><td>MULTISPECIES: hypothetical protein [unclassified Lysinibacillus].</td><td>GCF_900167885.1</td></tr>
<tr><td>WP_079564349.1</td><td>-</td><td>-</td><td>413</td><td>B5X66_RS24805</td><td>Firmicutes</td><td>unclassified Lysinibacillus</td><td>MULTISPECIES: discoidin domain-containing protein [unclassified Lysinibacillus].</td><td>GCF_900167885.1</td></tr>
<tr><td>WP_079564406.1</td><td>SUN→ SUN→ SUN→ SPRY+SUN*→?→ SUN→ SUN→ SUN→ SUN→ SUN→</td><td>SPRY+SUN</td><td>389</td><td>B5X66_RS24915</td><td>Firmicutes</td><td>unclassified Lysinibacillus</td><td>MULTISPECIES: discoidin domain-containing protein [unclassified Lysinibacillus].</td><td>GCF_900167885.1</td></tr>
<tr><td>WP_080291782.1</td><td>DUF3168→ Phage_TTP_11→ Phage_TAC_13→?→ SPRY+X*→?→?→ Phage-tail-tape→ ConA-repeats→</td><td>SPRY+X</td><td>655</td><td>LI87_RS0109275</td><td>Gammaproteobacteria</td><td>Stenotrophomonas maltophilia</td><td>hypothetical protein [Stenotrophomonas maltophilia].</td><td>GCF_000758465.1</td></tr>
<tr><td>WP_080639352.1</td><td>RHS_repeat+RHS_repeat+RHS_repeat+RHS_repeat+Laminin_G_3+SPRY*→?→?→ PSE→ PSE→35exo→</td><td>RHS_repeat+RHS_repeat+RHS_repeat+RHS_repeat+Laminin_G_3+SPRY</td><td>2000</td><td>YUK_RS0107305</td><td>Gammaproteobacteria</td><td>Teredinibacter turnerae</td><td>hypothetical protein [Teredinibacter turnerae].</td><td>GCF_000379165.1</td></tr>
<tr><td>WP_080974705.1</td><td>Cluster143_2clades→?→?→?→?→?→ SPRY→ SPRY*→ PSE→ TM→ Cluster104_2clades→</td><td>SPRY</td><td>311</td><td>ADC50_RS20320</td><td>Firmicutes</td><td>Clostridium sporogenes</td><td>hypothetical protein [Clostridium sporogenes].</td><td>GCF_001058825.1</td></tr>
<tr><td>WP_081280966.1</td><td>Phage_connect_1→ Phage_H_T_join→ HK97-gp10_like→ DUF3168→ Phage_TTP_11→ Phage_TAC_13→?→ SPRY+X*→?→?→ Phage-tail-tape→</td><td>SPRY+X</td><td>655</td><td>A9K69_RS19730</td><td>Gammaproteobacteria</td><td>Stenotrophomonas maltophilia</td><td>hypothetical protein [Stenotrophomonas maltophilia].</td><td>GCF_001676445.1</td></tr>
<tr><td>WP_084315242.1</td><td>SIG+TM+TM+TM+TM+TM+TM+TM+TM→ SIG+TM+TM+TM+TM+TM+TM+TM+TM→ ABC-ATPase→ SIG+TM+TM+TM+TM+ABC-ATPase→||<-?||?→ SPRY+Big_2*→?→?→ ACET→</td><td>SPRY+Big_2</td><td>535</td><td>BR69_RS21395</td><td>Firmicutes</td><td>Clostridium hydrogeniformans</td><td>Ig domain-containing protein [Clostridium hydrogeniformans].</td><td>GCF_000686705.1</td></tr>
<tr><td>WP_084575678.1</td><td>EMAP→ Cluster297_2clades→?→?→?→ SIG+SPRY*→?→?→?→?→?→ SIG+METHYLASE→</td><td>SIG+SPRY</td><td>349</td><td>B9A22_RS10460</td><td>Firmicutes</td><td>Sporomusa malonica</td><td>hypothetical protein [Sporomusa malonica].</td><td>GCF_900176355.1</td></tr>
</table>

acc	operon	architecture	len	gen.name	taxend	species	defline	gca
WP_085321805.1	Cluster171_2clades→?→?→ Cluster143_2clades→?→?→ PSE→ SUN→ SPRY*→ SUN→ TM→ Cluster104_2clades→?→?→35exo→ Cluster112_2clades→	SPRY	447	B2M06_RS15395	Firmicutes	Clostridium botulinum	cell adhesion protein [Clostridium botulinum].	GCF_002866045.1
WP_085899262.1	Phage_T7_tail→ SPRY*→	SPRY	1359	BKB51_RS01170	Alphaproteobacteria	Kiloniella majae	hypothetical protein [Kiloniella majae].	GCF_002118315.1
WP_087445183.1	SUN→ SPRY+SUN*→ SUN→ Laminin_G_3→ Disaggr_repeat→ Phage_holin_4_1→?→ SIG+ConA+beta-propeller→	SPRY+SUN	430	FLT43_09110	Firmicutes	Paenibacillus thiaminolyticus	hypothetical protein [Paenibacillus thiaminolyticus].	-
WP_087803178.1	Phage_connect_1→ Phage_H_T_join→ HK97-gp10_like→ DUF3168→ Phage_TTP_11→ Phage_TAC_13→?→ SPRY+X*→?→?→ Phage-tail-tape→ ConA-repeats→?→?→||<-SRAP	SPRY+X	657	B7H27_RS11730	Gammaproteobacteria	Stenotrophomonas maltophilia	hypothetical protein [Stenotrophomonas maltophilia].	GCF_002189565.1
WP_088432438.1	Phage_connect_1→ Phage_H_T_join→ HK97-gp10_like→ DUF3168→ Phage_TTP_11→ Phage_TAC_13→?→ SPRY+X*→?→?→ Phage-tail-tape→ ConA-repeats→	SPRY+X	658	CEE60_RS02675	Gammaproteobacteria	Stenotrophomonas maltophilia	hypothetical protein [Stenotrophomonas maltophilia].	GCF_002205165.1
WP_089986629.1	SUN→ SPRY+SUN*→ DISCOIDIN→ SUN→ SUN→ SUN→ Laminin_G_3→ Disaggr_repeat→	SPRY+SUN	433	BLP61_RS17210	Firmicutes	Lysinibacillus fusiformis	discoidin domain-containing protein [Lysinibacillus fusiformis].	GCF_900102355.1
WP_090738921.1	<-SUN<-?<-PSE||SIG+SPRY*→	SIG+SPRY	373	BN1546_RS23290	Firmicutes	Paenibacillus sp. Mc5Re-14	hypothetical protein [Paenibacillus sp. Mc5Re-14].	GCF_001049835.1
WP_090920079.1	SUN→ SUN→ SPRY+SUN*→ Laminin_G_3→ Disaggr_repeat→	SPRY+SUN	412	BLQ13_RS12675	Firmicutes	Paenibacillus polysaccharolyticus	discoidin domain-containing protein [Paenibacillus polysaccharolyticus].	GCF_900102085.1
WP_092074738.1	SPRY+Big_2*→	SPRY+Big_2	341	HSX37_RS17715	Firmicutes	Dendrosporobacter quercicolus	Ig-like domain-containing protein [Dendrosporobacter quercicolus].	GCF_900104455.1
WP_093534303.1	Phage_connect_1→ Phage_H_T_join→ HK97-gp10_like→ DUF3168→ Phage_TTP_11→ Phage_TAC_13→?→ SPRY+X*→?→?→ Phage-tail-tape→	SPRY+X	655	BQ2375_RS10775	Gammaproteobacteria	Stenotrophomonas rhizophila	hypothetical protein [Stenotrophomonas rhizophila].	GCF_900095765.1
WP_094156505.1	<-ABhydrolase<-?||SIG+SPRY→ *SIG+SPRY*→ SUN→||<-PSE<-Glycos_transf_2	SIG+SPRY	358	B4V02_RS22570	Firmicutes	Paenibacillus kribbensis	hypothetical protein [Paenibacillus kribbensis].	GCF_002240415.1
WP_094936690.1	SUN→ SPRY+SUN*→ SUN→ Laminin_G_3→ Disaggr_repeat→	SPRY+SUN	423	CA599_RS04535	Firmicutes	Paenibacillus taichungensis	discoidin domain-containing protein [Paenibacillus taichungensis].	GCF_002264305.1
WP_095523199.1	Cluster180_2clades→?→?→ Cluster242_2clades→?→?→?→ VWA+Phage-tail_3+SPRY*→?→?→ DAM→	VWA+Phage-tail_3+SPRY	1093	BSR46_RS01740	Betaproteobacteria	Candidatus Dactylopiibacterium carminicum	VWA domain-containing protein [Candidatus Dactylopiibacterium carminicum].	GCF_002284975.1
WP_095529535.1	Cluster180_2clades→?→?→ Cluster242_2clades→?→?→?→ VWA+Phage-tail_3+SPRY*→?→?→ DAM→	VWA+Phage-tail_3+SPRY	1093	CGU28_RS03090	Betaproteobacteria	Candidatus Dactylopiibacterium carminicum	VWA domain-containing protein [Candidatus Dactylopiibacterium carminicum].	GCF_002284935.1

acc	operon	architecture	len	gen.name	taxend	species	defline	gca
WP_097012028.1	Phage_T7_tail→ SUN+SPRY*→ Phage_tail_APC→	SUN+SPRY	749	DPRO_RS10715	Deltaproteobacteria	Pseudodesulfovibrio profundus	hypothetical protein [Pseudodesulfovibrio profundus].	GCF_900217235.1
WP_097053084.1	SPRY*→	SPRY	749	CRO34_RS20720	Alphaproteobacteria	Thalassospira xiamenensis	hypothetical protein [Thalassospira xiamenensis].	GCF_900217835.1
WP_099470440.1	Phage_connect_1→ Phage_H_T_join→ HK97-gp10_like→ DUF3168→ Phage_TTP_11→ Phage_TAC_13→?→ SPRY+X*→?→?→ Phage-tail-tape→ ConA-repeats→?→?→ SRAP→	SPRY+X	657	BRB94_RS07965	Gammaproteobacteria	Stenotrophomonas maltophilia	hypothetical protein [Stenotrophomonas maltophilia].	GCF_002739365.1
WP_099473308.1	Phage_connect_1→ Phage_H_T_join→ HK97-gp10_like→ DUF3168→ Phage_TTP_11→ Phage_TAC_13→?→ SPRY+X*→?→?→ Phage-tail-tape→ ConA-repeats→?→?→ SRAP→	SPRY+X	657	BRB96_RS16380	Gammaproteobacteria	Stenotrophomonas maltophilia	hypothetical protein [Stenotrophomonas maltophilia].	GCF_002739315.1
WP_099475740.1	Phage_TAC_13→?→ SPRY+X*→?→ Phage-tail-tape→?→?→?→ SRAP→	SPRY+X	657	BRB49_RS05320	Gammaproteobacteria	Stenotrophomonas maltophilia	hypothetical protein [Stenotrophomonas maltophilia].	GCF_002739555.1
WP_099491760.1	Phage_connect_1→ Phage_H_T_join→ HK97-gp10_like→ DUF3168→ Phage_TTP_11→ Phage_TAC_13→?→ SPRY+X*→?→?→ Phage-tail-tape→ ConA-repeats→?→?→||<-SRAP	SPRY+X	657	BRB88_RS10470	Gammaproteobacteria	Stenotrophomonas maltophilia	hypothetical protein [Stenotrophomonas maltophilia].	GCF_002739275.1
WP_099494832.1	Phage_connect_1→ Phage_H_T_join→ HK97-gp10_like→ DUF3168→ Phage_TTP_11→ Phage_TAC_13→?→ SPRY+X*→?→ Phage-tail-tape→ ConA-repeats→ ConA→	SPRY+X	655	BRC08_RS17670	Gammaproteobacteria	Stenotrophomonas maltophilia	hypothetical protein [Stenotrophomonas maltophilia].	GCF_002739465.1
WP_099529564.1	Phage_connect_1→ Phage_H_T_join→ HK97-gp10_like→ DUF3168→ Phage_TTP_11→ Phage_TAC_13→?→ PSE→ SPRY+X*→?→ Phage-tail-tape→ ConA-repeats→	SPRY+X	657	BRB33_RS00490	Gammaproteobacteria	Stenotrophomonas maltophilia	hypothetical protein [Stenotrophomonas maltophilia].	GCF_002738715.1
WP_099539415.1	Phage_connect_1→ Phage_H_T_join→ HK97-gp10_like→ DUF3168→ Phage_TTP_11→ Phage_TAC_13→?→ SPRY+X*→?→ Phage-tail-tape→ ConA-repeats→	SPRY+X	657	BRB39_RS15215	Gammaproteobacteria	Stenotrophomonas maltophilia	hypothetical protein [Stenotrophomonas maltophilia].	GCF_002738665.1
WP_099539896.1	Phage_connect_1→ Phage_H_T_join→ HK97-gp10_like→ DUF3168→ Phage_TTP_11→ Phage_TAC_13→?→ SPRY+X*→?→ Phage-tail-tape→	SPRY+X	656	BRB48_RS14865	Gammaproteobacteria	Stenotrophomonas maltophilia	hypothetical protein [Stenotrophomonas maltophilia].	GCF_002738885.1
WP_099553167.1	Phage_connect_1→ Phage_H_T_join→ HK97-gp10_like→ DUF3168→ Phage_TTP_11→ Phage_TAC_13→?→ SPRY+X*→?→?→ Phage-tail-tape→ ConA-repeats→?→?→||<-SRAP	SPRY+X	657	BRC04_RS15490	Gammaproteobacteria	Stenotrophomonas maltophilia	hypothetical protein [Stenotrophomonas maltophilia].	GCF_002739455.1
WP_099605638.1	Phage_connect_1→ Phage_H_T_join→ HK97-gp10_like→ DUF3168→ Phage_TTP_11→ Phage_TAC_13→?→ SPRY+X*→?→ Phage-tail-tape→ ConA-repeats→?→?→?→||<-SRAP	SPRY+X	657	BRC06_RS15605	Gammaproteobacteria	Stenotrophomonas maltophilia	hypothetical protein [Stenotrophomonas maltophilia].	GCF_002740185.1
WP_099654222.1	SPRY*→	SPRY	991	BJV55_RS14285	Gammaproteobacteria	Rhodanobacter sp. TND4EH1	hypothetical protein [Rhodanobacter sp. TND4EH1].	GCF_002749725.1
WP_099854778.1	SUN→ SPRY+SUN*→ SUN→ SUN→ SUN→ Laminin_G_3→ Disaggr_repeat→	SPRY+SUN	425	CS562_RS07475	Firmicutes	Paenibacillus sp. LK1	discoidin domain-containing protein [Paenibacillus sp. LK1].	GCF_002750415.1
WP_099858289.1	SPRY+SUN*→ SUN→ Laminin_G_3→ Disaggr_repeat→ Phage_holin_4_1→	SPRY+SUN	410	CS562_RS22615	Firmicutes	Paenibacillus sp. LK1	hypothetical protein [Paenibacillus sp. LK1].	GCF_002750415.1
WP_100317531.1	Phage-tail-tape→?→?→ SPRY+X*→?→ DAM→	SPRY+X	655	CO615_RS10755	Gammaproteobacteria	Xanthomonadaceae bacterium NML75-0749	hypothetical protein [Xanthomonadaceae bacterium NML75-0749].	GCF_002798185.1

<table>
<tr><th>acc</th><th>operon</th><th>architecture</th><th>len</th><th>gen.name</th><th>taxend</th><th>species</th><th>defline</th><th>gca</th></tr>
<tr><td>WP_100438259.1</td><td>Phage_connect_1→ Phage_H_T_join→ HK97-gp10_like→ DUF3168→ Phage_TTP_11→ Phage_TAC_13→?→ SPRY+X*→?→?→ Phage-tail-tape→ ConA-repeats→</td><td>SPRY+X</td><td>658</td><td>B9Y60_RS06960</td><td>Gammaproteobacteria</td><td>Stenotrophomonas maltophilia</td><td>hypothetical protein [Stenotrophomonas maltophilia].</td><td>GCF_002799135.1</td></tr>
<tr><td>WP_100443561.1</td><td>Phage_connect_1→ Phage_H_T_join→ HK97-gp10_like→ DUF3168→ Phage_TTP_11→ Phage_TAC_13→?→ SPRY+X*→?→ Phage-tail-tape→ ConA-repeats→</td><td>SPRY+X</td><td>657</td><td>B9Y57_RS09005</td><td>Gammaproteobacteria</td><td>Stenotrophomonas maltophilia</td><td>hypothetical protein [Stenotrophomonas maltophilia].</td><td>GCF_002798925.1</td></tr>
<tr><td>WP_100474847.1</td><td>Phage_connect_1→ Phage_H_T_join→ HK97-gp10_like→ DUF3168→ Phage_TTP_11→ Phage_TAC_13→?→ SPRY+X*→?→?→ Phage-tail-tape→</td><td>SPRY+X</td><td>657</td><td>B9Y82_RS18595</td><td>Gammaproteobacteria</td><td>Stenotrophomonas maltophilia</td><td>hypothetical protein [Stenotrophomonas maltophilia].</td><td>GCF_002799155.1</td></tr>
<tr><td>WP_102039859.1</td><td>Glyco_hydro_16+SPRY*→</td><td>Glyco_hydro_16+SPRY</td><td>1003</td><td>CXF86_RS19745</td><td>Gammaproteobacteria</td><td>Shewanella sp. GutCb</td><td>DUF1833 family protein [Shewanella sp. GutCb].</td><td>GCF_002836135.1</td></tr>
<tr><td>WP_102945666.1</td><td>Phage_H_T_join→ HK97-gp10_like→ DUF3168→ Phage_TTP_11→ Phage_TAC_13→?→ SPRY+X*→?→ Phage-tail-tape→</td><td>SPRY+X</td><td>655</td><td>C2U58_RS09715</td><td>Gammaproteobacteria</td><td>Stenotrophomonas sp. VV52</td><td>hypothetical protein [Stenotrophomonas sp. VV52].</td><td>GCF_002893095.1</td></tr>
<tr><td>WP_103046829.1</td><td><-Trypsin<-ABhydrolase<-Polbetant||ACET→ Cupin_2→||<-DUF2935<-?||SIG+SPRY*→</td><td>SIG+SPRY</td><td>326</td><td>C1T21_RS00825</td><td>Firmicutes</td><td>Paenibacillus sp. F4</td><td>hypothetical protein [Paenibacillus sp. F4].</td><td>GCF_002894905.1</td></tr>
<tr><td>WP_103744628.1</td><td>-</td><td>-</td><td>320</td><td>CG775_RS10930</td><td>Firmicutes</td><td>Paenibacillus polymyxa</td><td>hypothetical protein [Paenibacillus polymyxa].</td><td>GCF_002916985.1</td></tr>
<tr><td>WP_103977131.1</td><td>SUN→ SUN→ SUN→ SUN+SPRY*→ Disaggr_repeat→?→?→ Phage_holin_1→</td><td>SUN+SPRY</td><td>980</td><td>LYSIN_RS10210</td><td>Firmicutes</td><td>Lysinibacillus sphaericus</td><td>hypothetical protein [Lysinibacillus sphaericus].</td><td>GCF_002923795.1</td></tr>
<tr><td>WP_104497237.1</td><td>HTH_20→||<-ABhydrolase<-Polbetant||ACET→ Cupin_2→||<-DUF2935||SIG+SPRY*→||<-?||?→||<-Glycos_transf_2</td><td>SIG+SPRY</td><td>332</td><td>C5G87_RS19490</td><td>Firmicutes</td><td>Paenibacillus peoriae</td><td>hypothetical protein [Paenibacillus peoriae].</td><td>GCF_002937395.1</td></tr>
<tr><td>WP_105407850.1</td><td>SUN→ SPRY+SUN*→ SUN→ Laminin_G_3→ Disaggr_repeat→</td><td>SPRY+SUN</td><td>419</td><td>C0Q44_RS13525</td><td>Firmicutes</td><td>Paenibacillus sp. PCH8</td><td>hypothetical protein [Paenibacillus sp. PCH8].</td><td>GCF_002968835.1</td></tr>
<tr><td>WP_106549337.1</td><td>Phage_connect_1→ Phage_H_T_join→ HK97-gp10_like→ DUF3168→ Phage_TTP_11→ Phage_TAC_13→ SPRY+X*→||<-?||?→ Phage-tail-tape→ ConA-repeats→</td><td>SPRY+X</td><td>657</td><td>CV100_RS06160</td><td>Gammaproteobacteria</td><td>Stenotrophomonas maltophilia</td><td>hypothetical protein [Stenotrophomonas maltophilia].</td><td>GCF_003015035.1</td></tr>
<tr><td>WP_106891819.1</td><td>SPRY*→?→?→ DAM→</td><td>SPRY</td><td>820</td><td>C7S18_RS12140</td><td>Gammaproteobacteria</td><td>Ahniella affigens</td><td>hypothetical protein [Ahniella affigens].</td><td>GCF_003015185.1</td></tr>
<tr><td>WP_107951039.1</td><td>SUN→ SPRY+SUN→ SPRY+SUN→ SUN→ SUN→ Laminin_G_3→ Disaggr_repeat→?→?→ Phage_holin_1→</td><td>SPRY+SUN</td><td>435</td><td>C9J32_RS17875</td><td>Firmicutes</td><td>Lysinibacillus parviboronicapiens</td><td>discoidin domain-containing protein [Lysinibacillus parviboronicapiens].</td><td>GCF_003049605.1</td></tr>
<tr><td>WP_107951040.1</td><td>-</td><td>-</td><td>411</td><td>C9J32_RS17880</td><td>Firmicutes</td><td>Lysinibacillus parviboronicapiens</td><td>discoidin domain-containing protein [Lysinibacillus parviboronicapiens].</td><td>GCF_003049605.1</td></tr>
<tr><td>WP_107981750.1</td><td>SPRY*→</td><td>SPRY</td><td>401</td><td>B5V90_RS01965</td><td>Firmicutes</td><td>Bacillus sporothermodurans</td><td>hypothetical protein [Bacillus sporothermodurans].</td><td>GCF_003055065.1</td></tr>
<tr><td>WP_108753440.1</td><td>Phage_H_T_join→ HK97-gp10_like→ DUF3168→||<-?||Phage_TTP_11→ Phage_TAC_13→?→ SPRY+X*→?→?→ Phage-tail-tape→ ConA-repeats→ ConA→?→||<-SRAP</td><td>SPRY+X</td><td>657</td><td>C1933_RS11930</td><td>Gammaproteobacteria</td><td>Stenotrophomonas sp. ZAC14D2_NAIMI4_6</td><td>hypothetical protein [Stenotrophomonas sp. ZAC14D2_NAIMI4_6].</td><td>GCF_003086875.1</td></tr>
<tr><td>WP_108762304.1</td><td>Phage_connect_1→ Phage_H_T_join→ HK97-gp10_like→ DUF3168→ Phage_TTP_11→ Phage_TAC_13→?→ SPRY+X*→?→?→ Phage-tail-tape→ ConA-repeats→ ConA→</td><td>SPRY+X</td><td>657</td><td>C1932_RS11845</td><td>Gammaproteobacteria</td><td>Stenotrophomonas sp. YAU14D1_LEIMI4_1</td><td>hypothetical protein [Stenotrophomonas sp. YAU14D1_LEIMI4_1].</td><td>GCF_003086815.1</td></tr>
</table>

<table>
<tr><th>acc</th><th>operon</th><th>architecture</th><th>len</th><th>gen.name</th><th>taxend</th><th>species</th><th>defline</th><th>gca</th></tr>
<tr><td>WP_108767941.1</td><td>Phage_connect_1→ Phage_H_T_join→ HK97-gp10_like→ DUF3168→ Phage_TTP_11→ Phage_TAC_13→?→ SPRY+X*→?→?→ Phage-tail-tape→ ConA-repeats→?→||<-SRAP</td><td>SPRY+X</td><td>655</td><td>C1925_RS05020</td><td>Gammaproteobacteria</td><td>Stenotrophomonas sp. SAU14A_NAIMI4_5</td><td>hypothetical protein [Stenotrophomonas sp. SAU14A_NAIMI4_5].</td><td>GCF_003086795.1</td></tr>
<tr><td>WP_109073515.1</td><td>SIG+SPRY*→ Phage_tail_APC→?→ Phage_lysozyme→</td><td>SIG+SPRY</td><td>399</td><td>TSH20_RS03980</td><td>Alphaproteobacteria</td><td>unclassified Azospirillum</td><td>MULTISPECIES: hypothetical protein [unclassified Azospirillum].</td><td>GCF_003115945.1</td></tr>
<tr><td>WP_109150419.1</td><td>SIG+SPRY*→ Phage_tail_APC→ Phage_lysozyme→ Cluster368_2clades→</td><td>SIG+SPRY</td><td>399</td><td>TSO5_RS04345</td><td>Alphaproteobacteria</td><td>Azospirillum sp. TSO5</td><td>hypothetical protein [Azospirillum sp. TSO5].</td><td>GCF_003116065.1</td></tr>
<tr><td>WP_109813956.1</td><td>Phage_connect_1→ Phage_H_T_join→ HK97-gp10_like→ DUF3168→ Phage_TTP_11→ Phage_TAC_13→?→ SPRY+X*→||<-?||?→?→ Phage-tail-tape→ ConA-repeats→</td><td>SPRY+X</td><td>657</td><td>DKY64_RS04935</td><td>Gammaproteobacteria</td><td>Stenotrophomonas maltophilia</td><td>hypothetical protein [Stenotrophomonas maltophilia].</td><td>GCF_003172845.1</td></tr>
<tr><td>WP_110895171.1</td><td>-</td><td>-</td><td>698</td><td>HUB98_RS06520</td><td>Firmicutes</td><td>Paenibacillus barcinonensis</td><td>hypothetical protein [Paenibacillus barcinonensis].</td><td>GCF_013347305.1</td></tr>
<tr><td>WP_110932422.1</td><td>SPRY+SUN*→ SUN→ Laminin_G_3→ Disaggr_repeat→ Phage_holin_4_1→</td><td>SPRY+SUN</td><td>419</td><td>CZ379_RS15410</td><td>Firmicutes</td><td>Paenibacillus bouchesdurhonensis</td><td>hypothetical protein [Paenibacillus bouchesdurhonensis].</td><td>GCF_900155685.1</td></tr>
<tr><td>WP_111008118.1</td><td>Phage_connect_1→ Phage_H_T_join→ HK97-gp10_like→ DUF3168→ Phage_TTP_11→ Phage_TAC_13→?→ SPRY+X*→?→?→ Phage-tail-tape→ ConA-repeats→ ConA→</td><td>SPRY+X</td><td>655</td><td>A7X97_RS14925</td><td>Gammaproteobacteria</td><td>Stenotrophomonas maltophilia</td><td>hypothetical protein [Stenotrophomonas maltophilia].</td><td>GCF_003244875.1</td></tr>
<tr><td>WP_111096871.1</td><td>Phage_connect_1→ Phage_H_T_join→ HK97-gp10_like→ DUF3168→ Phage_TTP_11→ Phage_TAC_13→?→ SPRY+X*→?→?→ Phage-tail-tape→ ConA-repeats→ ConA→?→||<-SRAP</td><td>SPRY+X</td><td>655</td><td>A7X84_RS18825</td><td>Gammaproteobacteria</td><td>Stenotrophomonas maltophilia</td><td>hypothetical protein [Stenotrophomonas maltophilia].</td><td>GCF_003244495.1</td></tr>
<tr><td>WP_111113613.1</td><td>Phage_connect_1→ Phage_H_T_join→ HK97-gp10_like→ DUF3168→ Phage_TTP_11→ Phage_TAC_13→?→ SPRY+X*→?→ Phage-tail-tape→ ConA-repeats→ ConA→?→ SRAP→</td><td>SPRY+X</td><td>655</td><td>A7X83_RS21130</td><td>Gammaproteobacteria</td><td>Stenotrophomonas maltophilia</td><td>hypothetical protein [Stenotrophomonas maltophilia].</td><td>GCF_003244775.1</td></tr>
<tr><td>WP_111118812.1</td><td>Phage_connect_1→ Phage_H_T_join→ HK97-gp10_like→ DUF3168→ Phage_TTP_11→ Phage_TAC_13→?→ SPRY+X*→?→?→ Phage-tail-tape→ ConA-repeats→?→?→||<-SRAP</td><td>SPRY+X</td><td>657</td><td>A7X74_RS15970</td><td>Gammaproteobacteria</td><td>Stenotrophomonas maltophilia</td><td>hypothetical protein [Stenotrophomonas maltophilia].</td><td>GCF_003244465.1</td></tr>
<tr><td>WP_111176598.1</td><td>Phage_connect_1→ Phage_H_T_join→ HK97-gp10_like→ DUF3168→ Phage_TTP_11→ Phage_TAC_13→?→ SPRY+X*→?→ Phage-tail-tape→ ConA-repeats→?→||<-SRAP</td><td>SPRY+X</td><td>655</td><td>A7X87_RS07275</td><td>Gammaproteobacteria</td><td>Stenotrophomonas maltophilia</td><td>hypothetical protein [Stenotrophomonas maltophilia].</td><td>GCF_003244795.1</td></tr>
<tr><td>WP_111187870.1</td><td>Phage_connect_1→ Phage_H_T_join→ HK97-gp10_like→ DUF3168→ Phage_TTP_11→ Phage_TAC_13→ SPRY+X*→?→ Phage-tail-tape→ ConA-repeats→?→?→ SRAP→</td><td>SPRY+X</td><td>657</td><td>A7X60_RS00295</td><td>Gammaproteobacteria</td><td>Stenotrophomonas maltophilia</td><td>hypothetical protein [Stenotrophomonas maltophilia].</td><td>GCF_003244645.1</td></tr>
<tr><td>WP_111203746.1</td><td>Phage_connect_1→ Phage_H_T_join→ HK97-gp10_like→ DUF3168→ Phage_TTP_11→ Phage_TAC_13→?→ SPRY+X*→?→?→ Phage-tail-tape→ ConA-repeats→?→?→ SRAP→</td><td>SPRY+X</td><td>657</td><td>A7X75_RS09430</td><td>Gammaproteobacteria</td><td>Stenotrophomonas maltophilia</td><td>hypothetical protein [Stenotrophomonas maltophilia].</td><td>GCF_003244735.1</td></tr>
<tr><td>WP_117310047.1</td><td>Phage_H_T_join→ HK97-gp10_like→ DUF3168→||<-?||Phage_TTP_11→ Phage_TAC_13→?→ SPRY+X*→?→ Phage-tail-tape→ ConA-repeats→?→ SRAP→</td><td>SPRY+X</td><td>655</td><td>D0Y85_RS07625</td><td>Gammaproteobacteria</td><td>Stenotrophomonas sp. G4</td><td>hypothetical protein [Stenotrophomonas sp. G4].</td><td>GCF_003428825.1</td></tr>
<tr><td>WP_119554390.1</td><td>SPRY*→</td><td>SPRY</td><td>674</td><td>D3H34_RS15355</td><td>Betaproteobacteria</td><td>Acidovorax cavernicola</td><td>hypothetical protein [Acidovorax cavernicola].</td><td>GCF_003581445.1</td></tr>
<tr><td>WP_119798183.1</td><td>SUN→ SUN→?→ SPRY*→ Disaggr_repeat→?→ Phage_holin_4_1→</td><td>SPRY</td><td>707</td><td>D3P07_RS00690</td><td>Firmicutes</td><td>Paenibacillus sp. 1011MAR3C5</td><td>hypothetical protein [Paenibacillus sp. 1011MAR3C5].</td><td>GCF_003591485.1</td></tr>
<tr><td>WP_120033984.1</td><td>SPRY*→?→?→ Phage_holin_1→</td><td>SPRY</td><td>411</td><td>CJ483_RS08545</td><td>Firmicutes</td><td>Bacillus sp. PK3_68</td><td>hypothetical protein [Bacillus sp. PK3_68].</td><td>GCF_003600835.1</td></tr>
</table>

<table>
<tr><th>acc</th><th>operon</th><th>architecture</th><th>len</th><th>gen.name</th><th>taxend</th><th>species</th><th>defline</th><th>gca</th></tr>
<tr><td>WP_120225163.1</td><td>Cluster165_3clades→?→||<-?||?→?→ SPRY*→</td><td>SPRY</td><td>554</td><td>CLY44_RS11090</td><td>Alphaproteobacteria</td><td>Thalassospira sp. DSM 26710</td><td>hypothetical protein [Thalassospira sp. DSM 26710].</td><td>GCF_003610215.1</td></tr>
<tr><td>WP_120497558.1</td><td>Phage_T7_tail→ SPRY*→</td><td>SPRY</td><td>1175</td><td>D9A03_RS12780</td><td>Alphaproteobacteria</td><td>Kiloniella sp. EL199</td><td>hypothetical protein [Kiloniella sp. EL199].</td><td>GCF_900313065.1</td></tr>
<tr><td>WP_120498928.1</td><td><-ACET||?→?→?→?→?→?→ Beta-Roll+Beta-Roll+Beta-Roll+Beta-Roll+BetaPropeller+Beta-Roll+CADHERIN+Beta-Roll+CADHERIN+Beta-Roll+Beta-Roll+BetaPropeller+Beta-Roll*→</td><td>Beta-Roll+Beta-Roll+Beta-Roll+Beta-Roll+BetaPropeller+Beta-Roll+CADHERIN+Beta-Roll+CADHERIN+Beta-Roll+Beta-Roll+BetaPropeller+Beta-Roll</td><td>4942</td><td>D9A03_RS19645</td><td>Alphaproteobacteria</td><td>Kiloniella sp. EL199</td><td>hypothetical protein [Kiloniella sp. EL199].</td><td>GCF_900313065.1</td></tr>
<tr><td>WP_121219693.1</td><td>SPRY+CARDB*→</td><td>SPRY+CARDB</td><td>601</td><td>BCL74_RS10110</td><td>Alphaproteobacteria</td><td>Oceanibaculum indicum</td><td>hypothetical protein [Oceanibaculum indicum].</td><td>GCF_003633955.1</td></tr>
<tr><td>WP_122906669.1</td><td>SUN→ SPRY+SUN*→ Laminin_G_3→ Disaggr_repeat→?→?→ Phage_holin→</td><td>SPRY+SUN</td><td>424</td><td>EDM57_RS21100</td><td>Firmicutes</td><td>Brevibacillus gelatini</td><td>discoidin domain-containing protein [Brevibacillus gelatini].</td><td>GCF_003710935.1</td></tr>
<tr><td>WP_123769372.1</td><td>Phage-tail-tape→?→?→?→?→ SPRY+X*→?→ DAM→?→||<-?||?→||<-Redoxin</td><td>SPRY+X</td><td>660</td><td>EDC50_RS05235</td><td>Gammaproteobacteria</td><td>Vulcaniibacterium tengchongense</td><td>hypothetical protein [Vulcaniibacterium tengchongense].</td><td>GCF_003814555.1</td></tr>
<tr><td>WP_123796746.1</td><td>HTH_20→||<-Trypsin<-ABhydrolase||ACET→||<-DUF2935<-?||SIG+SPRY*→</td><td>SIG+SPRY</td><td>332</td><td>EG487_RS02750</td><td>Firmicutes</td><td>Paenibacillus polymyxa</td><td>hypothetical protein [Paenibacillus polymyxa].</td><td>GCF_003813765.1</td></tr>
<tr><td>WP_123958466.1</td><td>Phage_connect_1→ Phage_H_T_join→ HK97-gp10_like→ DUF3168→ Phage_TTP_11→ Phage_TAC_13→?→ SPRY+X*→?→ Phage-tail-tape→ ConA-repeats→</td><td>SPRY+X</td><td>657</td><td>EGY09_RS07240</td><td>Gammaproteobacteria</td><td>Stenotrophomonas maltophilia</td><td>hypothetical protein [Stenotrophomonas maltophilia].</td><td>GCF_003812985.1</td></tr>
<tr><td>WP_124116383.1</td><td>SUN→ SPRY+SUN→ *SPRY*→ Disaggr_repeat→ Phage_holin_4_1→</td><td>SPRY+SUN</td><td>707</td><td>EDO6_RS18680</td><td>Firmicutes</td><td>Paenibacillus xylanexedens</td><td>hypothetical protein [Paenibacillus xylanexedens].</td><td>GCF_003833245.1</td></tr>
<tr><td>WP_124116384.1</td><td>-</td><td>-</td><td>413</td><td>EDO6_RS18685</td><td>Firmicutes</td><td>Paenibacillus xylanexedens</td><td>discoidin domain-containing protein [Paenibacillus xylanexedens].</td><td>GCF_003833245.1</td></tr>
<tr><td>WP_124650057.1</td><td>Arm-DNA-bind_3+SPRY*→</td><td>Arm-DNA-bind_3+SPRY</td><td>469</td><td>DIE19_RS04180</td><td>Betaproteobacteria</td><td>Burkholderia sp. Bp9126</td><td>integrase arm-type DNA-binding domain-containing protein [Burkholderia sp. Bp9126].</td><td>GCF_003853715.1</td></tr>
<tr><td>WP_125335565.1</td><td>SPRY*→?→ Phage_lysozyme→</td><td>SPRY</td><td>816</td><td>EGJ58_RS22040</td><td>Alphaproteobacteria</td><td>Ochrobactrum anthropi</td><td>hypothetical protein [Ochrobactrum anthropi].</td><td>GCF_003937425.1</td></tr>
<tr><td>WP_129381357.1</td><td>SUN→ SPRY+SUN→ *SPRY+SUN*→ Laminin_G_3→ Disaggr_repeat→ Phage_holin_4_1→</td><td>SPRY+SUN</td><td>423</td><td>EBB07_RS29185</td><td>Firmicutes</td><td>Paenibacillaceae bacterium</td><td>hypothetical protein [Paenibacillaceae bacterium].</td><td>GCF_004134985.1</td></tr>
<tr><td>WP_129381358.1</td><td>-</td><td>-</td><td>415</td><td>EBB07_RS29190</td><td>Firmicutes</td><td>Paenibacillaceae bacterium</td><td>hypothetical protein [Paenibacillaceae bacterium].</td><td>GCF_004134985.1</td></tr>
<tr><td>WP_130767937.1</td><td>Phage_connect_1→ Phage_H_T_join→ HK97-gp10_like→ DUF3168→ Phage_TTP_11→ Phage_TAC_13→?→ SPRY+X*→?→ Phage-tail-tape→ ConA-repeats→ ConA→?→ PSE→||<-?||SRAP→</td><td>SPRY+X</td><td>655</td><td>STNY_RS04735</td><td>Gammaproteobacteria</td><td>Stenotrophomonas sp. Y</td><td>hypothetical protein [Stenotrophomonas sp. Y].</td><td>GCF_004306035.1</td></tr>
<tr><td>WP_134709562.1</td><td>Phage_connect_1→ Phage_H_T_join→ HK97-gp10_like→ DUF3168→ Phage_TTP_11→ Phage_TAC_13→?→ SPRY+X*→?→?→ Phage-tail-tape→ ConA-repeats→ ConA→</td><td>SPRY+X</td><td>655</td><td>EUA01_RS04295</td><td>Gammaproteobacteria</td><td>Stenotrophomonas maltophilia</td><td>hypothetical protein [Stenotrophomonas maltophilia].</td><td>GCF_004522395.1</td></tr>
</table>

acc	operon	architecture	len	gen.name	taxend	species	defline	gca
WP_134729602.1	Phage_connect_1→ Phage_H_T_join→ HK97-gp10_like→ DUF3168→ Phage_TTP_11→ Phage_TAC_13→?→ SPRY+X*→?→?→ Phage-tail-tape→ ConA-repeats→?→?→||<-SRAP	SPRY+X	657	EUA00_RS03030	Gammaproteobacteria	Stenotrophomonas maltophilia	hypothetical protein [Stenotrophomonas maltophilia].	GCF_004522355.1
WP_136006293.1	Phage_connect_1→ Phage_H_T_join→ HK97-gp10_like→ DUF3168→ Phage_TTP_11→ Phage_TAC_13→?→ SPRY+X*→?→ Phage-tail-tape→	SPRY+X	656	E5352_RS15345	Gammaproteobacteria	Stenotrophomonas maltophilia	hypothetical protein [Stenotrophomonas maltophilia].	GCF_004794075.1
WP_136702221.1	SPRY*→ Phage_tail_APC→	SPRY	539	E6C67_RS08475	Alphaproteobacteria	Azospirillum sp. TSA2s	hypothetical protein [Azospirillum sp. TSA2s].	GCF_004923315.1
WP_136702953.1	SPRY*→?→?→ Cluster153_2clades→	SPRY	708	E6C67_RS14155	Alphaproteobacteria	Azospirillum sp. TSA2s	hypothetical protein [Azospirillum sp. TSA2s].	GCF_004923315.1
WP_137222083.1	Redoxin→||<-tRNA<-?||?→||<-tRNA<-tRNA<-tRNA||?→?→ SIG+SPRY+Cohesin*→	SIG+SPRY+Cohesin	509	PaeCFBP13512_RS06670	Firmicutes	Paenibacillus sp. CFBP13512	hypothetical protein [Paenibacillus sp. CFBP13512].	GCF_005233705.1
WP_140785213.1	SPRY+Laminin_G_3*→	SPRY+Laminin_G_3	473	FJ417_RS24630	Alphaproteobacteria	Mesorhizobium sp. B3-1-7	hypothetical protein [Mesorhizobium sp. B3-1-7].	GCF_006439385.1
WP_140977547.1	SPRY+Clostridium_P47+TM+TM+TM*→	SPRY+Clostridium_P47+TM+TM+TM	750	C2U70_RS08460	Alphaproteobacteria	Bradyrhizobium guangdongense	TULIP family P47-like protein [Bradyrhizobium guangdongense].	GCF_006483445.1
WP_144787026.1	SUN→ SUN→ SUN→ SUN→ SUN→ SPRY+SUN*→ SUN→ Laminin_G_3→ Disaggr_repeat→?→?→ Phage_holin_1→	SPRY+SUN	427	FS727_RS01315	Firmicutes	Lysinibacillus fusiformis	discoidin domain-containing protein [Lysinibacillus fusiformis].	GCF_007678325.1
WP_145413289.1	SUN→ SUN→ SPRY+SUN*→ Laminin_G_3→ Disaggr_repeat→ Phage_holin_4_1→	SPRY+SUN	411	FS823_RS10960	Firmicutes	Paenibacillus xylanexedens	discoidin domain-containing protein [Paenibacillus xylanexedens].	GCF_007679855.1
WP_145958251.1	Phage-tail-tape→ PSE→?→?→?→ SPRY+X*→?→ DAM→	SPRY+X	658	C0575_RS17020	Gammaproteobacteria	Xanthomonas	MULTISPECIES: hypothetical protein [Xanthomonas].	GCF_002759275.1
WP_147429703.1	<-REC+OmpR-HTH<-?<-?<-?<-?||RHS_repeat+RHS_repeat+RHS_repeat+IG+SPRY+SPRY*→	RHS_repeat+RHS_repeat+RHS_repeat+IG+SPRY+SPRY	1116	K256_RS17555	Gammaproteobacteria	Alteromonadaceae bacterium 2052S.S.stab0a.01	hypothetical protein [Alteromonadaceae bacterium 2052S.S.stab0a.01].	GCF_003634075.1
WP_149425237.1	TM+TM→ Laminin_G_3+SPRY*→ Cluster358_2clades→?→?→ Phage_holin→?→?→||<-SRAP	Laminin_G_3+SPRY	714	OTERR_RS06755	Betaproteobacteria	Oryzomicrobium terrae	hypothetical protein [Oryzomicrobium terrae].	GCF_008274805.1
WP_151150764.1	Phage_T7_tail→ SPRY*→ Phage_tail_APC→	SPRY	494	F8A88_RS08730	Deltaproteobacteria	Desulfovibrio senegalensis	hypothetical protein [Desulfovibrio senegalensis].	GCF_008830225.1
WP_152380319.1	HTH_20→||<-ABhydrolase<-?||SIG+SPRY*→	SIG+SPRY	377	GCU48_RS13350	Firmicutes	Paenibacillus brasilensis	hypothetical protein [Paenibacillus brasilensis].	GCF_009363115.1
WP_152906267.1	Phage-tail-tape→ ConA-repeats→?→ SPRY+X*→?→ PSE→||<-?||REC+OmpR-HTH→	SPRY+X	633	ADI69_RS15035	Gammaproteobacteria	Stenotrophomonas maltophilia	hypothetical protein [Stenotrophomonas maltophilia].	GCF_001073045.1
WP_153164331.1	Phage_connect_1→ Phage_H_T_join→ HK97-gp10_like→ DUF3168→ Phage_TTP_11→ Phage_TAC_13→?→ SPRY+X*→?→ Phage-tail-tape→?→?→?→||<-SRAP	SPRY+X	656	GDJ08_RS03810	Gammaproteobacteria	Stenotrophomonas sp. CPCC 101271	hypothetical protein [Stenotrophomonas sp. CPCC 101271].	GCF_009467805.1

acc	operon	architecture	len	gen.name	taxend	species	defline	gca
WP_153794576.1	SIG+SPRY*→	SIG+SPRY	333	BJH92_RS10685	Firmicutes	Paenibacillus polymyxa	hypothetical protein [Paenibacillus polymyxa].	GCF_009650715.1
WP_154081143.1	Phage_connect_1→ Phage_H_T_join→ HK97-gp10_like→ DUF3168→ Phage_TTP_11→ Phage_TAC_13→?→ SPRY+X*→?→?→ Phage-tail-tape→	SPRY+X	655	B861_RS0208195	Gammaproteobacteria	Stenotrophomonas maltophilia	hypothetical protein [Stenotrophomonas maltophilia].	GCF_000295735.1
WP_154263126.1	Phage-tail-tape→ ConA-repeats→?→ SPRY+X*→	SPRY+X	655	FEO89_RS08390	Gammaproteobacteria	Stenotrophomonas maltophilia	hypothetical protein [Stenotrophomonas maltophilia].	GCF_009676565.1
WP_154352257.1	Phage_connect_1→ Phage_H_T_join→ HK97-gp10_like→ DUF3168→ Phage_TTP_11→ Phage_TAC_13→?→ SPRY+X*→?→?→ Phage-tail-tape→ ConA-repeats→	SPRY+X	657	FEO88_RS08425	Gammaproteobacteria	Stenotrophomonas maltophilia	hypothetical protein [Stenotrophomonas maltophilia].	GCF_009676585.1
WP_154965920.1	SIG+SPRY*→?→||<-?<-PSE<-PSE<-?<-?<-?<-Phage_holin	SIG+SPRY	346	F1386_RS02215	Firmicutes	Paenibacillus azotifigens	hypothetical protein [Paenibacillus azotifigens].	GCF_008635805.1
WP_156830543.1	Cluster180_2clades→?→?→?→?→?→?→ SPRY*→	SPRY	830	METMA_RS27135	Gammaproteobacteria	Methylobacter marinus	hypothetical protein [Methylobacter marinus].	GCF_000383855.1
WP_157628481.1	Phage_connect_1→ Phage_H_T_join→ HK97-gp10_like→ DUF3168→ Phage_TTP_11→ Phage_TAC_13→?→ SPRY+X*→?→ Phage-tail-tape→	SPRY+X	657	SMAL_RS12680	Gammaproteobacteria	Stenotrophomonas maltophilia	hypothetical protein [Stenotrophomonas maltophilia].	GCF_000020665.1
WP_157793929.1	SUN→ SPRY+SUN*→ SUN→ Laminin_G_3→ Disaggr_repeat→ Phage_holin_4_1→	SPRY+SUN	409	B9T62_RS19340	Firmicutes	Paenibacillus donghaensis	discoidin domain-containing protein [Paenibacillus donghaensis].	GCF_002192415.1
WP_158702666.1	SUN→ SPRY+SUN*→	SPRY+SUN	405	C8268_RS03870	Firmicutes	Paenibacillus sp. Marseille-P3787	discoidin domain-containing protein [Paenibacillus sp. Marseille-P3787].	GCF_900289175.1
WP_159054946.1	OBfold→?→?→||<-?||?→ ConA-repeats→?→ SPRY+X*→	SPRY+X	655	C9J74_RS07540	Gammaproteobacteria	Stenotrophomonas maltophilia	hypothetical protein [Stenotrophomonas maltophilia].	GCF_003030985.1
WP_160788604.1	HTH_20→||<-PSE<-ABhydrolase<-Polbetant||ACET→ Cupin_2→ ABhydrolase→||<-DUF2935||SIG+SPRY*→?→ PSE→||<-Glycos_transf_2	SIG+SPRY	338	GRP75_RS03415	Firmicutes	Paenibacillus sp. OT2-17	hypothetical protein [Paenibacillus sp. OT2-17].	GCF_009827695.1
WP_161771818.1	Phage_TAC_13→?→ SPRY+X*→?→?→ Phage-tail-tape→	SPRY+X	657	AR275_RS25795	Gammaproteobacteria	Stenotrophomonas maltophilia	hypothetical protein [Stenotrophomonas maltophilia].	GCF_001506105.1
WP_162174107.1	Cluster165_3clades→?→?→ Cluster303_2clades→?→?→ TM+TM→ SPRY*→?→?→?→?→?→?→ SIG+HORMA→	SPRY	125	HMCN_RS19520	Betaproteobacteria	Herminiimonas sp. CN	hypothetical protein [Herminiimonas sp. CN].	GCF_000622895.1
WP_162407361.1	Phage-tail-tape→?→?→ SUN→ SPRY+X*→?→||<-?||DAM→	SPRY+X	654	CSC62_RS14285	Gammaproteobacteria	Pseudoxanthomonas jiangsuensis	hypothetical protein [Pseudoxanthomonas jiangsuensis].	GCF_010093185.1
WP_162815241.1	SPRY*→	SPRY	219	DOU47_RS11710	Actinobacteria	Microbacterium arborescens	hypothetical protein [Microbacterium arborescens].	GCF_003339645.1
WP_163369807.1	SPRY*→	SPRY	262	G7B23_RS04230	Gammaproteobacteria	Endozoicomonas acroporae	hypothetical protein [Endozoicomonas acroporae].	GCF_010994325.1

<table>
<tr><th>acc</th><th>operon</th><th>architecture</th><th>len</th><th>gen.name</th><th>taxend</th><th>species</th><th>defline</th><th>gca</th></tr>
<tr><td>WP_163387923.1</td><td>SPRY*→</td><td>SPRY</td><td>262</td><td>EO230_RS13880</td><td>Gammaproteobacteria</td><td>Endozoicomonas acroporae</td><td>hypothetical protein [Endozoicomonas acroporae].</td><td>GCF_010994335.1</td></tr>
<tr><td>WP_163679668.1</td><td>Laminin_G_3+SPRY+SPRY*→</td><td>Laminin_G_3+SPRY+SPRY</td><td>704</td><td>G4223_RS11800</td><td>Alphaproteobacteria</td><td>Magnetospirillum aberrantis</td><td>hypothetical protein [Magnetospirillum aberrantis].</td><td>GCF_011022235.1</td></tr>
<tr><td>WP_164136018.1</td><td>Phage_H_T_join→ HK97-gp10_like→ DUF3168→?→ Phage_TTP_11→ Phage_TAC_13→?→ SPRY+X*→?→?→ Phage-tail-tape→ ConA-repeats→ ConA→</td><td>SPRY+X</td><td>657</td><td>G4995_RS01655</td><td>Gammaproteobacteria</td><td>Stenotrophomonas maltophilia</td><td>hypothetical protein [Stenotrophomonas maltophilia].</td><td>GCF_010604725.1</td></tr>
<tr><td>WP_164170835.1</td><td>Phage_connect_1→ Phage_H_T_join→ HK97-gp10_like→ DUF3168→ Phage_TTP_11→ Phage_TAC_13→?→ SPRY+X*→?→ Phage-tail-tape→ ConA-repeats→</td><td>SPRY+X</td><td>657</td><td>G5A21_RS04165</td><td>Gammaproteobacteria</td><td>Stenotrophomonas maltophilia</td><td>hypothetical protein [Stenotrophomonas maltophilia].</td><td>GCF_010605085.1</td></tr>
<tr><td>WP_164171533.1</td><td>Phage_connect_1→ Phage_H_T_join→ HK97-gp10_like→ DUF3168→ Phage_TTP_11→ Phage_TAC_13→?→ SPRY+X*→?→?→ Phage-tail-tape→ ConA-repeats→?→?→ SRAP→</td><td>SPRY+X</td><td>657</td><td>G5A20_RS11530</td><td>Gammaproteobacteria</td><td>Stenotrophomonas maltophilia</td><td>hypothetical protein [Stenotrophomonas maltophilia].</td><td>GCF_010607635.1</td></tr>
<tr><td>WP_164186354.1</td><td>Phage_connect_1→ Phage_H_T_join→ HK97-gp10_like→ DUF3168→ Phage_TTP_11→ Phage_TAC_13→?→ SPRY+X*→?→?→ Phage-tail-tape→ ConA-repeats→?→?→ SRAP→</td><td>SPRY+X</td><td>657</td><td>G4996_RS10295</td><td>Gammaproteobacteria</td><td>Stenotrophomonas maltophilia</td><td>hypothetical protein [Stenotrophomonas maltophilia].</td><td>GCF_010608865.1</td></tr>
<tr><td>WP_164222458.1</td><td>Phage_connect_1→ Phage_H_T_join→ HK97-gp10_like→ DUF3168→ Phage_TTP_11→ Phage_TAC_13→?→ SPRY+X*→||<-?||?→?→ Phage-tail-tape→ ConA-repeats→</td><td>SPRY+X</td><td>658</td><td>G5A44_RS14400</td><td>Gammaproteobacteria</td><td>Stenotrophomonas maltophilia</td><td>hypothetical protein [Stenotrophomonas maltophilia].</td><td>GCF_010608905.1</td></tr>
<tr><td>WP_164227274.1</td><td>Phage_connect_1→ Phage_H_T_join→ HK97-gp10_like→ DUF3168→ Phage_TTP_11→ Phage_TAC_13→?→ SPRY+X*→?→ Phage-tail-tape→ ConA-repeats→?→?→||<-PSE||?→ SRAP→</td><td>SPRY+X</td><td>658</td><td>G5A23_RS09720</td><td>Gammaproteobacteria</td><td>Stenotrophomonas maltophilia</td><td>hypothetical protein [Stenotrophomonas maltophilia].</td><td>GCF_010605135.1</td></tr>
<tr><td>WP_164262535.1</td><td>Phage_connect_1→ Phage_H_T_join→ HK97-gp10_like→ DUF3168→ Phage_TTP_11→ Phage_TAC_13→?→ SPRY+X*→?→?→ Phage-tail-tape→ ConA-repeats→?→||<-SRAP</td><td>SPRY+X</td><td>655</td><td>G5A42_RS09935</td><td>Gammaproteobacteria</td><td>Stenotrophomonas maltophilia</td><td>hypothetical protein [Stenotrophomonas maltophilia].</td><td>GCF_010609005.1</td></tr>
<tr><td>WP_164272506.1</td><td>Phage_connect_1→ Phage_H_T_join→ HK97-gp10_like→ DUF3168→ Phage_TTP_11→ Phage_TAC_13→?→ SPRY+X*→?→?→ Phage-tail-tape→ ConA-repeats→ ConA→?→||<-SRAP</td><td>SPRY+X</td><td>657</td><td>G4G00_RS08180</td><td>Gammaproteobacteria</td><td>Stenotrophomonas sp. B1-1</td><td>hypothetical protein [Stenotrophomonas sp. B1-1].</td><td>GCF_010894375.1</td></tr>
<tr><td>WP_164283369.1</td><td>Phage_connect_1→ Phage_H_T_join→ HK97-gp10_like→ DUF3168→ Phage_TTP_11→ Phage_TAC_13→?→ SPRY+X*→?→ Phage-tail-tape→ ConA-repeats→ ConA→?→||<-SRAP</td><td>SPRY+X</td><td>655</td><td>GJV38_RS08100</td><td>Gammaproteobacteria</td><td>Stenotrophomonas maltophilia</td><td>hypothetical protein [Stenotrophomonas maltophilia].</td><td>GCF_010994225.1</td></tr>
<tr><td>WP_164687941.1</td><td>Phage_connect_1→ Phage_H_T_join→ HK97-gp10_like→ DUF3168→ Phage_TTP_11→ Phage_TAC_13→?→ SPRY+X*→?→?→ Phage-tail-tape→ ConA-repeats→ ConA→?→||<-SRAP</td><td>SPRY+X</td><td>655</td><td>G5C63_RS04670</td><td>Gammaproteobacteria</td><td>Stenotrophomonas pavanii</td><td>hypothetical protein [Stenotrophomonas pavanii].</td><td>GCF_011043715.1</td></tr>
<tr><td>WP_164776353.1</td><td>SUN→ SPRY+SUN*→ SUN→ Laminin_G_3→ Disaggr_repeat→ Phage_holin_4_1→?→ SIG+ConA+beta-propeller→</td><td>SPRY+SUN</td><td>423</td><td>FLT43_RS09110</td><td>Firmicutes</td><td>Paenibacillus thiaminolyticus</td><td>hypothetical protein [Paenibacillus thiaminolyticus].</td><td>GCF_004001005.1</td></tr>
<tr><td>WP_164965245.1</td><td>HTH_20→||<-Trypsin<-ABhydrolase||ACET→||<-DUF2935<-?||SIG+SPRY*→</td><td>SIG+SPRY</td><td>369</td><td>G9G57_RS16175</td><td>Firmicutes</td><td>Paenibacillus sp. EKM211P</td><td>hypothetical protein [Paenibacillus sp. EKM211P].</td><td>GCF_011066175.1</td></tr>
<tr><td>WP_165083476.1</td><td>HTH_20→||<-Trypsin<-ABhydrolase||ACET→||<-DUF2935<-?||SIG+SPRY*→</td><td>SIG+SPRY</td><td>312</td><td>G9G64_RS19615</td><td>Firmicutes</td><td>unclassified Paenibacillus</td><td>MULTISPECIES: hypothetical protein [unclassified Paenibacillus].</td><td>GCF_011066085.1</td></tr>
<tr><td>WP_165148029.1</td><td>HTH_20→||<-ABhydrolase<-Polbetant||ACET→ Cupin_2→ ABhydrolase→||<-DUF2935||SIG+SPRY*→?→||<-Glycos_transf_2</td><td>SIG+SPRY</td><td>345</td><td>G9G52_RS14675</td><td>Firmicutes</td><td>unclassified Paenibacillus</td><td>MULTISPECIES: hypothetical protein [unclassified Paenibacillus].</td><td>GCF_011066155.1</td></tr>
</table>

acc	operon	architecture	len	gen.name	taxend	species	defline	gca
WP_165178844.1	HTH_20→||<-ABhydrolase<-Polbetant||ACET→ Cupin_2→||<-DUF2935||SIG+SPRY*→	SIG+SPRY	330	G9G54_RS16165	Firmicutes	Paenibacillus sp. EKM212P	hypothetical protein [Paenibacillus sp. EKM212P].	GCF_011066115.1
WP_165980135.1	SUN→ SUN→ SPRY+SUN*→ Laminin_G_3→ Disaggr_repeat→	SPRY+SUN	412	E2R58_RS01745	Firmicutes	Paenibacillus amylolyticus	hypothetical protein [Paenibacillus amylolyticus].	GCF_004358835.1
WP_167440979.1	HTH_20→||<-Trypsin<-ABhydrolase||ACET→||<-DUF2935<-?||SIG+SPRY*→	SIG+SPRY	332	HFD99_RS19285	Firmicutes	Paenibacillus sp. EKM301P	hypothetical protein [Paenibacillus sp. EKM301P].	GCF_011800295.1
WP_168180444.1	SPRY+SUN*→ SUN→ SUN→ Laminin_G_3→ Disaggr_repeat→ Phage_holin_4_1→	SPRY+SUN	417	HRG14_RS16580	Firmicutes	Paenibacillus dendritiformis	hypothetical protein [Paenibacillus dendritiformis].	GCF_012272915.1
WP_168224778.1	Phage-tail_3+FN3→ Cluster93_5clades→ Laminin_G_3+SPRY*→?→?→||<-?<-REC+OmpR-HTH	Laminin_G_3+SPRY	442	EXZ61_RS14580	Betaproteobacteria	Rhodoferax sediminis	hypothetical protein [Rhodoferax sediminis].	GCF_006974105.1
WP_168759630.1	Phage_H_T_join→ HK97-gp10_like→ DUF3168→?→ Phage_TTP_11→ Phage_TAC_13→?→ SPRY+X*→?→ Phage-tail-tape→ ConA-repeats→?→?→||<-PSE||?→ SRAP→	SPRY+X	655	HGN30_RS14120	Gammaproteobacteria	Stenotrophomonas maltophilia	hypothetical protein [Stenotrophomonas maltophilia].	GCF_012647025.1
WP_170142066.1	Phage-tail_3→?→ SPRY*→?→?→?→?→?→ DAM→	SPRY	223	DFQ59_RS06730	Gammaproteobacteria	Thioalbus denitrificans	hypothetical protein [Thioalbus denitrificans].	GCF_003337735.1
WP_171365214.1	SUN→ SUN→?→ DISCOIDIN→ SUN→ SPRY+SUN*→ SUN→ Laminin_G_3→ Disaggr_repeat→?→?→ Phage_holin_1→	SPRY+SUN	425	HL291_RS06145	Firmicutes	Lysinibacillus fusiformis	discoidin domain-containing protein [Lysinibacillus fusiformis].	GCF_013112215.1
WP_180361703.1	SUN→ SUN→ SUN→ SPRY+SUN*→ Laminin_G_3→ Disaggr_repeat→	SPRY+SUN	412	CXK86_RS01005	Firmicutes	Paenibacillus sp. BGI2013	hypothetical protein [Paenibacillus sp. BGI2013].	GCF_002843485.1
WP_180832871.1	Phage_connect_1→ Phage_H_T_join→ HK97-gp10_like→ DUF3168→ Phage_TTP_11→ Phage_TAC_13→?→ SPRY+X*→?→?→ Phage-tail-tape→ ConA-repeats→?→?→||<-SRAP	SPRY+X	657	D7U76_RS15765	Gammaproteobacteria	Stenotrophomonas maltophilia	hypothetical protein [Stenotrophomonas maltophilia].	GCF_013463655.1
WP_180835450.1	Phage_connect_1→ Phage_H_T_join→ HK97-gp10_like→ DUF3168→ Phage_TTP_11→ Phage_TAC_13→?→ SPRY+X*→?→ Phage-tail-tape→?→?→?→||<-PSE||?→||<-SRAP	SPRY+X	657	D7U87_RS15230	Gammaproteobacteria	Stenotrophomonas maltophilia	hypothetical protein [Stenotrophomonas maltophilia].	GCF_013463795.1
WP_180837803.1	Phage-tail-tape→ ConA-repeats→?→ SPRY+X*→?→?→?→?→?→?→||<-Ribonuc_L-PSP	SPRY+X	633	D7Y22_RS10130	Gammaproteobacteria	Stenotrophomonas maltophilia	hypothetical protein [Stenotrophomonas maltophilia].	GCF_013463895.1
WP_180844236.1	Phage-tail-tape→ ConA-repeats→?→ SPRY+X*→?→?→?→?→ SIG+TM+TM+TM+TM+TM+TM+TM+TM→ ABC-ATPase→	SPRY+X	655	D7Y24_RS02400	Gammaproteobacteria	Stenotrophomonas maltophilia	hypothetical protein [Stenotrophomonas maltophilia].	GCF_013463875.1
WP_180874375.1	Phage_connect_1→ Phage_H_T_join→ HK97-gp10_like→ DUF3168→ Phage_TTP_11→ Phage_TAC_13→?→ SPRY+X*→?→?→ Phage-tail-tape→ ConA-repeats→?→?→||<-SRAP	SPRY+X	657	D7Y39_RS15850	Gammaproteobacteria	Stenotrophomonas maltophilia	hypothetical protein [Stenotrophomonas maltophilia].	GCF_013464195.1
WP_180877176.1	Phage_connect_1→ Phage_H_T_join→ HK97-gp10_like→ DUF3168→ Phage_TTP_11→ Phage_TAC_13→?→ SPRY+X*→?→?→ Phage-tail-tape→ ConA-repeats→?→?→||<-SRAP	SPRY+X	657	D7U95_RS06530	Gammaproteobacteria	Stenotrophomonas maltophilia	hypothetical protein [Stenotrophomonas maltophilia].	GCF_013463605.1
WP_180890353.1	Phage_connect_1→ Phage_H_T_join→ HK97-gp10_like→ DUF3168→ Phage_TTP_11→ Phage_TAC_13→?→ SPRY+X*→?→?→ Phage-tail-tape→ ConA-repeats→?→?→||<-SRAP	SPRY+X	658	D7Y45_RS11625	Gammaproteobacteria	Stenotrophomonas maltophilia	hypothetical protein [Stenotrophomonas maltophilia].	GCF_013464305.1
WP_182068260.1	Phage_connect_1→ Phage_H_T_join→ HK97-gp10_like→ DUF3168→ Phage_TTP_11→ Phage_TAC_13→?→ SPRY+X*→?→?→ Phage-tail-tape→ ConA-repeats→ ConA→	SPRY+X	655	H7A80_RS13755	Gammaproteobacteria	Stenotrophomonas pavanii	hypothetical protein [Stenotrophomonas pavanii].	GCF_903970895.1

acc	operon	architecture	len	gen.name	taxend	species	defline	gca
WP_182680994.1	Phage_connect_1→ Phage_H_T_join→ HK97-gp10_like→ DUF3168→ Phage_TTP_11→ Phage_TAC_13→?→ SPRY+X*→?→?→ Phage-tail-tape→ ConA-repeats→?→?→||<-SRAP	SPRY+X	657	H4O10_RS07755	Gammaproteobacteria	Stenotrophomonas sp. I18B00994	hypothetical protein [Stenotrophomonas sp. I18B00994].	GCF_014145825.1
WP_182852413.1	Cluster242_2clades→?→ PSE→?→ SPRY*→	SPRY	255	H7K70_RS11535	Gammaproteobacteria	Pseudomonas otitidis	hypothetical protein [Pseudomonas otitidis].	GCF_014161995.1
WP_183080221.1	Phage_connect_1→ Phage_H_T_join→ HK97-gp10_like→ DUF3168→ Phage_TTP_11→ Phage_TAC_13→?→ SPRY+X*→?→?→ Phage-tail-tape→ ConA-repeats→?→?→ SRAP→	SPRY+X	657	C7543_RS14050	Gammaproteobacteria	Stenotrophomonas sp. CF319	hypothetical protein [Stenotrophomonas sp. CF319].	GCF_003633905.1

7. Source data. Gene neighborhoods and domain architectures of TPR-GREAB-C-PIN systems

acc	operon	architecture	len	gen.name	taxend	species	defline	gca
-	-	-	NA	NA	NA	NA	NA	GCF_016236915.1
AAQ66293.1	TPR+GreAB-C+PIN*→	TPR+GreAB-C+PIN	1140	PG_1202	Bacteroidetes	Porphyromonas gingivalis W83	hypothetical protein PG_1202 [Porphyromonas gingivalis W83].	GCA_000007585.1
ABA73572.1	REase+TPR+GreAB-C+PIN*→	REase+TPR+GreAB-C+PIN	1113	Pfl01_1829	Gammaproteobacteria	Pseudomonas fluorescens Pf0-1	hypothetical protein Pfl01_1829 [Pseudomonas fluorescens Pf0-1].	GCA_000012445.1
ABP70317.1	TPR+GreAB-C+PIN*→	TPR+GreAB-C+PIN	1188	Rsph17025_1421	Alphaproteobacteria	Rhodobacter sphaeroides ATCC 17025	hypothetical protein Rsph17025_1421 [Rhodobacter sphaeroides ATCC 17025].	GCA_000016405.1
ACB93983.1	TPR+GreAB-C+PIN*→	TPR+GreAB-C+PIN	1100	Bind_0329	Alphaproteobacteria	Beijerinckia indica subsp. indica ATCC 9039	Tetratricopeptide TPR_2 repeat protein [Beijerinckia indica subsp. indica ATCC 9039].	GCA_000019845.1
ACQ93407.1	HetE-N1→ TPR+GreAB-C+PIN*→	TPR+GreAB-C+PIN	1069	Tola_1798	Gammaproteobacteria	Tolumonas auensis DSM 9187	hypothetical protein Tola_1798 [Tolumonas auensis DSM 9187].	GCA_000023065.1
ADX44119.1	REase+TPR+GreAB-C+PIN*→	REase+TPR+GreAB-C+PIN	1383	Acav_0193	Betaproteobacteria	Acidovorax avenae subsp. avenae ATCC 19860	hypothetical protein Acav_0193 [Acidovorax avenae subsp. avenae ATCC 19860].	GCA_000176855.2
AFD27801.1	TPR+GreAB-C+PIN*→	TPR+GreAB-C+PIN	1286	DGo_PC0009	Deinococci	Deinococcus gobiensis I-0	hypothetical protein DGo_PC0009 (plasmid) [Deinococcus gobiensis I-0].	GCA_000252445.1
AFJ56694.1	REase+TPR+GreAB-C+PIN*→	REase+TPR+GreAB-C+PIN	1321	PflA506_2672	Gammaproteobacteria	Pseudomonas fluorescens A506	tetratricopeptide repeat protein [Pseudomonas fluorescens A506].	GCA_000262325.2
AGO88322.1	REase+TPR+GreAB-C+PIN*→?→?→ Calcineurin→ Calcineurin→?→ Calcineurin→	REase+TPR+GreAB-C+PIN	1344	MOC_1p0084	Alphaproteobacteria	Methylobacterium oryzae CBMB20	Tetratricopeptide TPR_2 repeat protein (plasmid) [Methylobacterium oryzae CBMB20].	-
AHC85672.1	HetE-N1→ TPR+TPR+GreAB-C+PIN*→	TPR+TPR+GreAB-C+PIN	1114	X969_07375	Gammaproteobacteria	Pseudomonas monteilii SB3078	hypothetical protein X969_07375 [Pseudomonas monteilii SB3078].	GCA_000510285.1
AHG89833.1	TPR+GreAB-C+PIN*→	TPR+GreAB-C+PIN	1325	J421_2296	Gemmatimonadetes	Gemmatirosa kalamazoonesis	hypothetical protein J421_2296 [Gemmatirosa kalamazoonesis].	GCA_000522985.1
AHZ68657.1	REase+TPR+GreAB-C+PIN*→	REase+TPR+GreAB-C+PIN	1527	OU5_1578	Gammaproteobacteria	Pseudomonas mandelii JR-1	hypothetical protein OU5_1578 [Pseudomonas mandelii JR-1].	GCA_000257545.3
AIO22452.1	REase+TPR+GreAB-C+PIN*→	REase+TPR+GreAB-C+PIN	1474	DM41_7691	Betaproteobacteria	Burkholderia cepacia ATCC 25416	tetratricopeptide repeat family protein (plasmid) [Burkholderia cepacia ATCC 25416].	GCA_003546465.1
AJG23424.1	REase+TPR+GreAB-C+PIN*→	REase+TPR+GreAB-C+PIN	1348	RR42_s1836	Betaproteobacteria	Cupriavidus basilensis	hypothetical protein RR42_s1836 [Cupriavidus basilensis].	GCA_000832305.1
AJG25013.1	NACHT→ REase+TPR+GreAB-C+PIN*→	REase+TPR+GreAB-C+PIN	1361	RR42_s3437	Betaproteobacteria	Cupriavidus basilensis	hypothetical protein RR42_s3437 [Cupriavidus basilensis].	GCA_000832305.1
ALA80840.1	TPR+GreAB-C+PIN*→	TPR+GreAB-C+PIN	706	VN11_01310	Gammaproteobacteria	Stenotrophomonas maltophilia	hypothetical protein VN11_01310 [Stenotrophomonas maltophilia].	GCA_001274595.1
ALK23663.1	REase+TPR+GreAB-C+PIN*→	REase+TPR+GreAB-C+PIN	1346	APZ15_37705	Betaproteobacteria	Burkholderia cepacia ATCC 25416	hypothetical protein APZ15_37705 (plasmid) [Burkholderia cepacia ATCC 25416].	GCA_001411495.1
ALY84300.1	TPR+GreAB-C+PIN*→	TPR+GreAB-C+PIN	808	HV95_15440	Gammaproteobacteria	Pseudomonas aeruginosa	hypothetical protein HV95_15440 [Pseudomonas aeruginosa].	GCA_001516225.1
AMM82949.1	HetE-N1→ TPR+GreAB-C+PIN*→	TPR+GreAB-C+PIN	1090	AW43_10865	Gammaproteobacteria	Pasteurella multocida subsp. multocida PMTB2.1	hypothetical protein AW43_10865 [Pasteurella multocida subsp. multocida PMTB2.1].	GCA_001578435.2
ANP41882.1	REase+TPR+GreAB-C+PIN*→	REase+TPR+GreAB-C+PIN	1247	K529_013985	Alphaproteobacteria	Epibacterium mobile F1926	hypothetical protein K529_013985 [Epibacterium mobile F1926].	GCA_000376545.2
AOE80004.1	TPR+GreAB-C+PIN*→	TPR+GreAB-C+PIN	962	A7318_15795	Gammaproteobacteria	Pseudomonas lurida	hypothetical protein A7318_15795 [Pseudomonas lurida].	GCA_001708485.1

<table>
<tr><th>acc</th><th>operon</th><th>architecture</th><th>len</th><th>gen.name</th><th>taxend</th><th>species</th><th>defline</th><th>gca</th></tr>
<tr><td>APP29939.1</td><td>REase+TPR+GreAB-C+PIN*→||||<-?||HTH→</td><td>REase+TPR+GreAB-C+PIN</td><td>1216</td><td>AUO97_03535</td><td>Gammaproteobacteria</td><td>Acinetobacter baumannii</td><td>hypothetical protein AUO97_03535 [Acinetobacter baumannii].</td><td>GCA_001593425.2</td></tr>
<tr><td>APT35017.1</td><td>REase+TPR+GreAB-C+PIN*→?→?→ Calcineurin→ Calcineurin→?→ Calcineurin→</td><td>REase+TPR+GreAB-C+PIN</td><td>1340</td><td>MCBMB27_05726</td><td>Alphaproteobacteria</td><td>Methylobacterium phyllosphaerae</td><td>hypothetical protein MCBMB27_05726 (plasmid) [Methylobacterium phyllosphaerae].</td><td>GCA_001936175.1</td></tr>
<tr><td>APV35618.1</td><td>REase+TPR+GreAB-C+PIN*→</td><td>REase+TPR+GreAB-C+PIN</td><td>1211</td><td>BEN76_06135</td><td>Gammaproteobacteria</td><td>Acinetobacter soli</td><td>hypothetical protein BEN76_06135 [Acinetobacter soli].</td><td>GCA_001953195.1</td></tr>
<tr><td>ARP63802.1</td><td>REase+TPR+GreAB-C+PIN*→</td><td>REase+TPR+GreAB-C+PIN</td><td>1328</td><td>A9K65_010750</td><td>Alphaproteobacteria</td><td>Mesorhizobium sp. WSM1497</td><td>hypothetical protein A9K65_010750 [Mesorhizobium sp. WSM1497].</td><td>GCA_001672455.2</td></tr>
<tr><td>ARS41908.1</td><td>TPR+GreAB-C+PIN*→</td><td>TPR+GreAB-C+PIN</td><td>1265</td><td>CA265_20515</td><td>Bacteroidetes</td><td>Sphingobacteriaceae bacterium GW460-11-11-14-LB5</td><td>hypothetical protein CA265_20515 [Sphingobacteriaceae bacterium GW460-11-11-14-LB5].</td><td>GCA_002151545.1</td></tr>
<tr><td>AWD09427.1</td><td>HetE-N1→?→ TPR+GreAB-C+PIN*→</td><td>TPR+GreAB-C+PIN</td><td>512</td><td>C7D56_14785</td><td>Gammaproteobacteria</td><td>Salmonella enterica subsp. enterica serovar Corvallis</td><td>hypothetical protein C7D56_14785 [Salmonella enterica subsp. enterica serovar Corvallis].</td><td>GCA_003071545.1</td></tr>
<tr><td>AXQ72363.1</td><td>HetE-N1→ TPR+GreAB-C+PIN*→</td><td>TPR+GreAB-C+PIN</td><td>1090</td><td>AWY89_05050</td><td>Gammaproteobacteria</td><td>Pasteurella multocida subsp. multocida</td><td>hypothetical protein AWY89_05050 [Pasteurella multocida subsp. multocida].</td><td>GCA_003428945.1</td></tr>
<tr><td>AXW62973.1</td><td>REase+TPR+GreAB-C+PIN*→</td><td>REase+TPR+GreAB-C+PIN</td><td>1325</td><td>CJO94_14695</td><td>Betaproteobacteria</td><td>Ralstonia solanacearum</td><td>hypothetical protein CJO94_14695 [Ralstonia solanacearum].</td><td>GCA_003515385.1</td></tr>
<tr><td>AYG03885.1</td><td>X+PIN*→</td><td>X+PIN</td><td>1170</td><td>D7I44_10295</td><td>Actinobacteria</td><td>Gryllotalpicola protaetiae</td><td>hypothetical protein D7I44_10295 [Gryllotalpicola protaetiae].</td><td>GCA_003627055.1</td></tr>
<tr><td>BAE85210.1</td><td>REase+TPR+GreAB-C+PIN*→</td><td>REase+TPR+GreAB-C+PIN</td><td>1306</td><td>DSY3421</td><td>Firmicutes</td><td>Desulfitobacterium hafniense Y51</td><td>hypothetical protein DSY3421 [Desulfitobacterium hafniense Y51].</td><td>GCA_000010045.1</td></tr>
<tr><td>BBD03377.1</td><td>REase+TPR→ TPR→ TPR+GreAB-C+PIN*→</td><td>TPR+GreAB-C+PIN</td><td>656</td><td>YGS_C2P1391</td><td>Alphaproteobacteria</td><td>Sphingobium sp. YG1</td><td>hypothetical protein YGS_C2P1391 [Sphingobium sp. YG1].</td><td>GCA_003609795.1</td></tr>
<tr><td>BBZ79150.1</td><td>TPR+PIN*→</td><td>TPR+PIN</td><td>1186</td><td>MANY_44870</td><td>Actinobacteria</td><td>Mycolicibacterium anyangense</td><td>hypothetical protein MANY_44870 [Mycolicibacterium anyangense].</td><td>GCA_010731855.1</td></tr>
<tr><td>BBZ91069.1</td><td>PIN*→</td><td>PIN</td><td>190</td><td>F07S3_09020</td><td>Alphaproteobacteria</td><td>Bradyrhizobium diazoefficiens</td><td>hypothetical protein F07S3_09020 [Bradyrhizobium diazoefficiens].</td><td>GCA_014163475.1</td></tr>
<tr><td>CAA9240899.1</td><td>TPR+GreAB-C+PIN*→</td><td>TPR+GreAB-C+PIN</td><td>1079</td><td>AVDCRST_MAG77-1676</td><td>environmental samples</td><td>uncultured Chloroflexi bacterium</td><td>MAG: hypothetical protein AVDCRST_MAG77-1676 [uncultured Chloroflexi bacterium].</td><td>GCA_902805635.1</td></tr>
<tr><td>CAB3809090.1</td><td>PIN*→</td><td>PIN</td><td>270</td><td>LMG28138_06082</td><td>Betaproteobacteria</td><td>Pararobbsia alpina</td><td>hypothetical protein LMG28138_06082 [Pararobbsia alpina].</td><td>GCA_902859895.1</td></tr>
<tr><td>CAC9458388.1</td><td>TPR+GreAB-C+PIN*→?→||||<-PSE||PSE→ PSE→?→ PSE→ PSE→ PSE→?→?→ TPR→</td><td>TPR+GreAB-C+PIN</td><td>1164</td><td>SMARSOXL104_LOCUS2140</td><td>environmental samples</td><td>uncultured Gammaproteobacteria bacterium</td><td>hypothetical protein [uncultured Gammaproteobacteria bacterium].</td><td>GCA_903813475.1</td></tr>
<tr><td>CAC9474194.1</td><td>TPR+GreAB-C+PIN*→</td><td>TPR+GreAB-C+PIN</td><td>1164</td><td>SMARSOXL102_LOCUS3344</td><td>environmental samples</td><td>uncultured Gammaproteobacteria bacterium</td><td>hypothetical protein [uncultured Gammaproteobacteria bacterium].</td><td>GCA_903813445.1</td></tr>
<tr><td>CAC9479061.1</td><td>TPR+GreAB-C+PIN*→</td><td>TPR+GreAB-C+PIN</td><td>677</td><td>SMARSOXL51_LOCUS3828</td><td>environmental samples</td><td>uncultured Gammaproteobacteria bacterium</td><td>hypothetical protein [uncultured Gammaproteobacteria bacterium].</td><td>GCA_903813485.1</td></tr>
<tr><td>CAD79080.1</td><td>TRD+TRD→?→?→ REase+SNF2→?→ TM+TM+TPR+GreAB-C+PIN*→</td><td>TM+TM+TPR+GreAB-C+PIN</td><td>1274</td><td>RB11367</td><td>Planctomycetes</td><td>Rhodopirellula baltica SH 1</td><td>hypothetical protein RB11367 [Rhodopirellula baltica SH 1].</td><td>GCA_000196115.1</td></tr>
<tr><td>CAE7733776.1</td><td>IMS+HHH+GreAB+AraC-HTH+AraC-HTH*→</td><td>IMS+HHH+GreAB+AraC-HTH+AraC-HTH</td><td>771</td><td>dinB</td><td>Alveolata</td><td>Symbiodinium microadriaticum</td><td>dinB [Symbiodinium microadriaticum].</td><td>GCA_905231925.1</td></tr>
<tr><td>CAG37728.1</td><td>HetE-N1→ TPR+GreAB-C+PIN*→</td><td>TPR+GreAB-C+PIN</td><td>1114</td><td>DP2999</td><td>Deltaproteobacteria</td><td>Desulfotalea psychrophila LSv54</td><td>unknown protein [Desulfotalea psychrophila LSv54].</td><td>GCA_000025945.1</td></tr>
<tr><td>CCD86698.1</td><td>REase+TPR+TPR+GreAB-C+PIN*→</td><td>REase+TPR+TPR+GreAB-C+PIN</td><td>1326</td><td>BRAO285_2000017</td><td>Alphaproteobacteria</td><td>Bradyrhizobium sp. ORS 285</td><td>conserved hypothetical protein [Bradyrhizobium sp. ORS 285].</td><td>GCA_000239755.2</td></tr>
<tr><td>CDF45779.1</td><td>X+PIN*→?→?→ CITB-HTH+LexA-protease→</td><td>X+PIN</td><td>1212</td><td>BN450_02242</td><td>environmental samples</td><td>Roseburia sp. CAG:100</td><td>uncharacterized protein BN450_02242 [Roseburia sp. CAG:100].</td><td>GCA_000436955.1</td></tr>
<tr><td>CDH20863.1</td><td>HetE-N1→ TPR+GreAB-C+PIN*→ TPR+GreAB-C+PIN→</td><td>TPR+GreAB-C+PIN</td><td>846</td><td>XBKQ1_2810009</td><td>Gammaproteobacteria</td><td>Xenorhabdus bovienii str. kraussei Quebec</td><td>conserved hypothetical protein [Xenorhabdus bovienii str. kraussei Quebec].</td><td>GCA_000736555.1</td></tr>
<tr><td>CEL29322.1</td><td>REase+TPR+GreAB-C+PIN*→</td><td>REase+TPR+GreAB-C+PIN</td><td>1486</td><td>SRM1_02673</td><td>Gammaproteobacteria</td><td>Pseudomonas fluorescens</td><td>hypothetical protein SRM1_02673 [Pseudomonas fluorescens].</td><td>GCA_000827755.2</td></tr>
</table>

acc	operon	architecture	len	gen.name	taxend	species	defline	gca
CFK79862.1	REase+TPR+GreAB-C+PIN*→	REase+TPR+GreAB-C+PIN	1327	ERS012324_00072	Betaproteobacteria	Burkholderia pseudomallei	Uncharacterised protein [Burkholderia pseudomallei].	GCA_001327015.1
CGG41859.1	TPR+GreAB-C+PIN*→	TPR+GreAB-C+PIN	1147	ERS020510_00033	Firmicutes	Streptococcus pneumoniae	putative PEP-CTERM system TPR-repeat lipoprotein [Streptococcus pneumoniae].	GCA_001339955.1
CNG28227.1	HetE-N1→ TPR+GreAB-C+PIN*→	TPR+GreAB-C+PIN	1094	ERS137937_00749	Gammaproteobacteria	Yersinia enterocolitica	Uncharacterised protein [Yersinia enterocolitica].	GCA_001160345.1
COE44409.1	TPR+GreAB-C+PIN*→	TPR+GreAB-C+PIN	1147	ERS020529_01064	Firmicutes	Streptococcus pneumoniae	putative PEP-CTERM system TPR-repeat lipoprotein [Streptococcus pneumoniae].	GCA_002889635.1
CRI57148.1	REase+TPR+GreAB-C+PIN*→	REase+TPR+GreAB-C+PIN	1419	CCOS191_2612	Gammaproteobacteria	Pseudomonas sp. CCOS 191	hypothetical protein CCOS191_2612 [Pseudomonas sp. CCOS 191].	GCA_001007005.1
CTR52279.1	HetE-N1→ TPR+GreAB-C+PIN*→	TPR+GreAB-C+PIN	1093	ERS085432_03851	Gammaproteobacteria	Escherichia coli	Uncharacterised protein [Escherichia coli].	GCA_001277455.1
CTU76757.1	HetE-N1→ TPR+GreAB-C+PIN*→	TPR+GreAB-C+PIN	1093	ERS085409_04356	Gammaproteobacteria	Escherichia coli	Uncharacterised protein [Escherichia coli].	GCA_001284445.1
EAA6823230.1	HetE-N1→ TPR+GreAB-C+PIN*→	TPR+GreAB-C+PIN	839	DRV62_14440	Gammaproteobacteria	Salmonella enterica subsp. enterica serovar Corvallis	hypothetical protein DRV62_14440, partial [Salmonella enterica subsp. enterica serovar Corvallis].	GCA_004191475.1
EAA7528536.1	HetE-N1→ TPR+GreAB-C+PIN*→	TPR+GreAB-C+PIN	997	WB97_04605	Gammaproteobacteria	Salmonella enterica subsp. enterica	hypothetical protein WB97_04605, partial [Salmonella enterica subsp. enterica].	GCA_004218105.1
EAB9718450.1	HetE-N1→ TPR+GreAB-C+PIN*→	TPR+GreAB-C+PIN	1094	EBD60_05220	Gammaproteobacteria	Escherichia coli	hypothetical protein EBD60_05220 [Escherichia coli].	GCA_004268345.1
EAC0684623.1	HetE-N1→ TPR+GreAB-C+PIN*→	TPR+GreAB-C+PIN	1117	ECA66_22430	Gammaproteobacteria	Salmonella enterica subsp. enterica serovar Molade	hypothetical protein ECA66_22430 [Salmonella enterica subsp. enterica serovar Molade].	GCA_004273605.1
EAM8584006.1	HetE-N1→ TPR+GreAB-C+PIN*→?→||||<-Trypsin	TPR+GreAB-C+PIN	1121	BET14_04310	Gammaproteobacteria	Salmonella enterica	hypothetical protein BET14_04310 [Salmonella enterica].	GCA_005437065.1
EAM9325916.1	HetE-N1→ TPR+GreAB-C+PIN*→	TPR+GreAB-C+PIN	1106	DPR23_14355	Gammaproteobacteria	Salmonella enterica	hypothetical protein DPR23_14355 [Salmonella enterica].	GCA_005440115.1
EAN1748525.1	HetE-N1→ TPR+GreAB-C+PIN*→	TPR+GreAB-C+PIN	1107	D8T88_07580	Gammaproteobacteria	Salmonella enterica	hypothetical protein D8T88_07580 [Salmonella enterica].	GCA_005450625.1
EAO3021897.1	HetE-N1→ TPR+GreAB-C+PIN*→	TPR+GreAB-C+PIN	727	E3A68_19440	Gammaproteobacteria	Salmonella enterica	hypothetical protein E3A68_19440, partial [Salmonella enterica].	GCA_005498265.1
EAO5192492.1	HetE-N1→ TPR+GreAB-C+PIN*→	TPR+GreAB-C+PIN	867	E7928_13885	Gammaproteobacteria	Salmonella enterica	hypothetical protein E7928_13885, partial [Salmonella enterica].	GCA_005509025.1
EAO9608739.1	HetE-N1→ TPR+GreAB-C+PIN*→	TPR+GreAB-C+PIN	776	BH019_23310	Gammaproteobacteria	Salmonella enterica	hypothetical protein BH019_23310, partial [Salmonella enterica].	GCA_005533575.1
EAR5961962.1	HetE-N1→ TPR+GreAB-C+PIN*→	TPR+GreAB-C+PIN	1101	ES925_14455	Gammaproteobacteria	Salmonella enterica	hypothetical protein ES925_14455 [Salmonella enterica].	GCA_005654255.1
EAR6368406.1	HetE-N1→ TPR+GreAB-C+PIN*→	TPR+GreAB-C+PIN	1117	EU234_19825	Gammaproteobacteria	Salmonella enterica	hypothetical protein EU234_19825 [Salmonella enterica].	GCA_005655905.1
EAS2553591.1	TPR+GreAB-C+PIN*→	TPR+GreAB-C+PIN	590	DMT50_24325	Gammaproteobacteria	Salmonella enterica	hypothetical protein DMT50_24325, partial [Salmonella enterica].	GCA_005679955.1
EAS5437941.1	TPR+GreAB-C+PIN*→	TPR+GreAB-C+PIN	985	D8Z11_24970	Gammaproteobacteria	Salmonella enterica	hypothetical protein D8Z11_24970 [Salmonella enterica].	GCA_005692135.1
EAT5893226.1	HetE-N1→ TPR+GreAB-C+PIN*→	TPR+GreAB-C+PIN	1106	E3L72_21800	Gammaproteobacteria	Salmonella enterica	hypothetical protein E3L72_21800 [Salmonella enterica].	GCA_005737135.1
EAT6283696.1	HetE-N1→ TPR+GreAB-C+PIN*→	TPR+GreAB-C+PIN	843	E5797_05930	Gammaproteobacteria	Salmonella enterica	hypothetical protein E5797_05930, partial [Salmonella enterica].	GCA_005739275.1
EAV44914.1	REase+TPR+GreAB-C+PIN*→	REase+TPR+GreAB-C+PIN	1295	SIAM614_12903	Alphaproteobacteria	Labrenzia aggregata IAM 12614	hypothetical protein SIAM614_12903 [Labrenzia aggregata IAM 12614].	GCA_000168975.1
EAW0681535.1	HetE-N1→ TPR+GreAB-C+PIN+TM+TM*→	TPR+GreAB-C+PIN+TM+TM	1106	AIB54_02885	Gammaproteobacteria	Salmonella enterica	hypothetical protein AIB54_02885 [Salmonella enterica].	GCA_005896365.1
EAW0902300.1	TPR+GreAB-C+PIN*→	TPR+GreAB-C+PIN	638	B2H02_24145	Gammaproteobacteria	Salmonella enterica	hypothetical protein B2H02_24145, partial [Salmonella enterica].	GCA_005898285.1
EAY5481430.1	HetE-N1→ TPR+GreAB-C+PIN*→	TPR+GreAB-C+PIN	858	MB58_12360	Gammaproteobacteria	Salmonella enterica	hypothetical protein MB58_12360, partial [Salmonella enterica].	GCA_006012385.1

<table>
<tr><th>acc</th><th>operon</th><th>architecture</th><th>len</th><th>gen.name</th><th>taxend</th><th>species</th><th>defline</th><th>gca</th></tr>
<tr><td>EBD0849223.1</td><td>HetE-N1→ TPR+GreAB-C+PIN*→</td><td>TPR+GreAB-C+PIN</td><td>970</td><td>FHN97_07895</td><td>Gammaproteobacteria</td><td>Salmonella enterica</td><td>hypothetical protein FHN97_07895, partial [Salmonella enterica].</td><td>GCA_006217605.1</td></tr>
<tr><td>EBD3738487.1</td><td>HetE-N1→ TPR+GreAB-C+PIN*→</td><td>TPR+GreAB-C+PIN</td><td>1107</td><td>CRZ11_10865</td><td>Gammaproteobacteria</td><td>Salmonella enterica</td><td>hypothetical protein CRZ11_10865 [Salmonella enterica].</td><td>GCA_006248335.1</td></tr>
<tr><td>EBD8181391.1</td><td>TPR+GreAB-C+PIN*→</td><td>TPR+GreAB-C+PIN</td><td>606</td><td>C6600_23545</td><td>Gammaproteobacteria</td><td>Salmonella enterica</td><td>hypothetical protein C6600_23545 [Salmonella enterica].</td><td>GCA_006268595.1</td></tr>
<tr><td>EBF2432475.1</td><td>HetE-N1→ TPR+GreAB-C+PIN*→</td><td>TPR+GreAB-C+PIN</td><td>986</td><td>DKG18_08085</td><td>Gammaproteobacteria</td><td>Salmonella enterica</td><td>hypothetical protein DKG18_08085, partial [Salmonella enterica].</td><td>GCA_006332345.1</td></tr>
<tr><td>EBF2786917.1</td><td>TPR+GreAB-C+PIN*→</td><td>TPR+GreAB-C+PIN</td><td>738</td><td>C2240_24885</td><td>Gammaproteobacteria</td><td>Salmonella enterica subsp. enterica serovar Corvallis</td><td>hypothetical protein C2240_24885, partial [Salmonella enterica subsp. enterica serovar Corvallis].</td><td>GCA_006331745.1</td></tr>
<tr><td>EBF4219990.1</td><td>HetE-N1→ TPR+GreAB-C+PIN*→</td><td>TPR+GreAB-C+PIN</td><td>702</td><td>FH403_17525</td><td>Gammaproteobacteria</td><td>Salmonella enterica</td><td>hypothetical protein FH403_17525, partial [Salmonella enterica].</td><td>GCA_006342775.1</td></tr>
<tr><td>EBG7115752.1</td><td>HetE-N1→ TPR+GreAB-C+PIN*→</td><td>TPR+GreAB-C+PIN</td><td>822</td><td>FJA42_18775</td><td>Gammaproteobacteria</td><td>Salmonella enterica</td><td>hypothetical protein FJA42_18775, partial [Salmonella enterica].</td><td>GCA_006457615.1</td></tr>
<tr><td>EBI5135933.1</td><td>TPR+GreAB-C+PIN*→</td><td>TPR+GreAB-C+PIN</td><td>642</td><td>DQO15_25245</td><td>Gammaproteobacteria</td><td>Salmonella enterica</td><td>hypothetical protein DQO15_25245, partial [Salmonella enterica].</td><td>GCA_006559185.1</td></tr>
<tr><td>EBI9233829.1</td><td>HetE-N1→ TPR+GreAB-C+PIN*→</td><td>TPR+GreAB-C+PIN</td><td>670</td><td>DLA64_23180</td><td>Gammaproteobacteria</td><td>Salmonella enterica</td><td>hypothetical protein DLA64_23180, partial [Salmonella enterica].</td><td>GCA_006576485.1</td></tr>
<tr><td>EBI9496533.1</td><td>TPR+GreAB-C+PIN*→</td><td>TPR+GreAB-C+PIN</td><td>749</td><td>DLP94_24755</td><td>Gammaproteobacteria</td><td>Salmonella enterica</td><td>hypothetical protein DLP94_24755, partial [Salmonella enterica].</td><td>GCA_006578745.1</td></tr>
<tr><td>EBK5150964.1</td><td>HetE-N1→ TPR+GreAB-C+PIN*→</td><td>TPR+GreAB-C+PIN</td><td>786</td><td>DON25_22080</td><td>Gammaproteobacteria</td><td>Salmonella enterica</td><td>hypothetical protein DON25_22080, partial [Salmonella enterica].</td><td>GCA_006645025.1</td></tr>
<tr><td>EBL0898297.1</td><td>HetE-N1→ TPR+GreAB-C+PIN*→?→||||<-Trypsin</td><td>TPR+GreAB-C+PIN</td><td>1121</td><td>D0R07_07295</td><td>Gammaproteobacteria</td><td>Salmonella enterica</td><td>hypothetical protein D0R07_07295 [Salmonella enterica].</td><td>GCA_006670825.1</td></tr>
<tr><td>EBM3541219.1</td><td>HetE-N1→ TPR+GreAB-C+PIN*→</td><td>TPR+GreAB-C+PIN</td><td>1117</td><td>DYJ09_14310</td><td>Gammaproteobacteria</td><td>Salmonella enterica</td><td>hypothetical protein DYJ09_14310 [Salmonella enterica].</td><td>GCA_006729905.1</td></tr>
<tr><td>EBM4506587.1</td><td>TPR+GreAB-C+PIN*→</td><td>TPR+GreAB-C+PIN</td><td>652</td><td>DZ654_25740</td><td>Gammaproteobacteria</td><td>Salmonella enterica</td><td>hypothetical protein DZ654_25740, partial [Salmonella enterica].</td><td>GCA_006730405.1</td></tr>
<tr><td>EBM4858227.1</td><td>TPR+GreAB-C+PIN*→</td><td>TPR+GreAB-C+PIN</td><td>648</td><td>D0R05_25725</td><td>Gammaproteobacteria</td><td>Salmonella enterica</td><td>hypothetical protein D0R05_25725, partial [Salmonella enterica].</td><td>GCA_006733825.1</td></tr>
<tr><td>EBM6537845.1</td><td>HetE-N1→ TPR+GreAB-C+PIN*→</td><td>TPR+GreAB-C+PIN</td><td>1028</td><td>D2U20_21570</td><td>Gammaproteobacteria</td><td>Salmonella enterica</td><td>hypothetical protein D2U20_21570, partial [Salmonella enterica].</td><td>GCA_006740525.1</td></tr>
<tr><td>EBO1039185.1</td><td>TPR+GreAB-C+PIN*→</td><td>TPR+GreAB-C+PIN</td><td>643</td><td>D3T71_25030</td><td>Gammaproteobacteria</td><td>Salmonella enterica</td><td>hypothetical protein D3T71_25030, partial [Salmonella enterica].</td><td>GCA_006805285.1</td></tr>
<tr><td>EBQ9134585.1</td><td>TPR+GreAB-C+PIN*→</td><td>TPR+GreAB-C+PIN</td><td>707</td><td>DK746_24010</td><td>Gammaproteobacteria</td><td>Salmonella enterica subsp. enterica serovar Corvallis</td><td>hypothetical protein DK746_24010, partial [Salmonella enterica subsp. enterica serovar Corvallis].</td><td>GCA_006932075.1</td></tr>
<tr><td>EBR0236973.1</td><td>TPR+GreAB-C+PIN*→</td><td>TPR+GreAB-C+PIN</td><td>757</td><td>DNV25_24155</td><td>Gammaproteobacteria</td><td>Salmonella enterica subsp. enterica serovar Corvallis</td><td>hypothetical protein DNV25_24155, partial [Salmonella enterica subsp. enterica serovar Corvallis].</td><td>GCA_006936135.1</td></tr>
<tr><td>EBS1151706.1</td><td>TPR+GreAB-C+PIN*→</td><td>TPR+GreAB-C+PIN</td><td>1007</td><td>D6P66_23730</td><td>Gammaproteobacteria</td><td>Salmonella enterica subsp. enterica serovar Corvallis</td><td>hypothetical protein D6P66_23730 [Salmonella enterica subsp. enterica serovar Corvallis].</td><td>GCA_006989745.1</td></tr>
<tr><td>EBS2228649.1</td><td>TPR+GreAB-C+PIN*→</td><td>TPR+GreAB-C+PIN</td><td>684</td><td>DRL12_24325</td><td>Gammaproteobacteria</td><td>Salmonella enterica subsp. enterica serovar Corvallis</td><td>hypothetical protein DRL12_24325, partial [Salmonella enterica subsp. enterica serovar Corvallis].</td><td>GCA_006993385.1</td></tr>
<tr><td>EBS6357254.1</td><td>HetE-N1→ TPR+GreAB-C+PIN*→</td><td>TPR+GreAB-C+PIN</td><td>999</td><td>D4E88_23815</td><td>Gammaproteobacteria</td><td>Salmonella enterica subsp. enterica serovar Albany</td><td>hypothetical protein D4E88_23815, partial [Salmonella enterica subsp. enterica serovar Albany].</td><td>GCA_007016725.1</td></tr>
<tr><td>EBU7006421.1</td><td>TPR+GreAB-C+PIN*→</td><td>TPR+GreAB-C+PIN</td><td>620</td><td>DKU11_24275</td><td>Gammaproteobacteria</td><td>Salmonella enterica subsp. enterica serovar Kintambo</td><td>hypothetical protein DKU11_24275, partial [Salmonella enterica subsp. enterica serovar Kintambo].</td><td>GCA_007139165.1</td></tr>
</table>

acc	operon	architecture	len	gen.name	taxend	species	defline	gca
EBU7167144.1	HetE-N1→ TPR+GreAB-C+PIN*→	TPR+GreAB-C+PIN	1106	DKU92_07615	Gammaproteobacteria	Salmonella enterica subsp. enterica serovar Stockholm	hypothetical protein DKU92_07615 [Salmonella enterica subsp. enterica serovar Stockholm].	GCA_007140245.1
EBV1256929.1	TPR+GreAB-C+PIN*→	TPR+GreAB-C+PIN	719	DNW25_24775	Gammaproteobacteria	Salmonella enterica subsp. enterica serovar Corvallis	hypothetical protein DNW25_24775, partial [Salmonella enterica subsp. enterica serovar Corvallis].	GCA_007158045.1
EBV2191014.1	HetE-N1→ TPR+GreAB-C+PIN*→	TPR+GreAB-C+PIN	1106	DN155_01500	Gammaproteobacteria	Salmonella enterica subsp. enterica serovar Afula	hypothetical protein DN155_01500 [Salmonella enterica subsp. enterica serovar Afula].	GCA_007161245.1
EBV8290347.1	HetE-N1→ TPR+GreAB-C+PIN*→	TPR+GreAB-C+PIN	704	AUE34_22015	Gammaproteobacteria	Salmonella enterica subsp. arizonae serovar 18:z4,z23:-	hypothetical protein AUE34_22015, partial [Salmonella enterica subsp. arizonae serovar 18:z4,z23:-].	GCA_007188075.1
EBV8290357.1	TPR+GreAB-C+PIN*→	TPR+GreAB-C+PIN	385	AUE34_22090	Gammaproteobacteria	Salmonella enterica subsp. arizonae serovar 18:z4,z23:-	hypothetical protein AUE34_22090, partial [Salmonella enterica subsp. arizonae serovar 18:z4,z23:-].	GCA_007188075.1
EBV8849078.1	HetE-N1→ TPR+GreAB-C+PIN*→	TPR+GreAB-C+PIN	1000	AUA06_21700	Gammaproteobacteria	Salmonella enterica subsp. arizonae serovar 18:z4,z23:-	hypothetical protein AUA06_21700, partial [Salmonella enterica subsp. arizonae serovar 18:z4,z23:-].	GCA_007190395.1
EBV8917369.1	HetE-N1→ TPR+GreAB-C+PIN*→	TPR+GreAB-C+PIN	688	ASH63_21975	Gammaproteobacteria	Salmonella enterica subsp. arizonae serovar 18:z4,z23:-	hypothetical protein ASH63_21975, partial [Salmonella enterica subsp. arizonae serovar 18:z4,z23:-].	GCA_007194415.1
EBV9159081.1	TPR+GreAB-C+PIN*→	TPR+GreAB-C+PIN	620	ASA02_25470	Gammaproteobacteria	Salmonella enterica subsp. enterica serovar Alachua	hypothetical protein ASA02_25470, partial [Salmonella enterica subsp. enterica serovar Alachua].	GCA_007191635.1
EBV9391419.1	HetE-N1→ TPR+GreAB-C+PIN*→	TPR+GreAB-C+PIN	665	AUA09_21865	Gammaproteobacteria	Salmonella enterica subsp. arizonae serovar 18:z4,z23:-	hypothetical protein AUA09_21865, partial [Salmonella enterica subsp. arizonae serovar 18:z4,z23:-].	GCA_007194695.1
EBV9454977.1	TPR+GreAB-C*→	TPR+GreAB-C	257	AUA11_22355	Gammaproteobacteria	Salmonella enterica subsp. arizonae serovar 18:z4,z23:-	hypothetical protein AUA11_22355, partial [Salmonella enterica subsp. arizonae serovar 18:z4,z23:-].	GCA_007193095.1
EBV9865076.1	TPR+GreAB-C*→	TPR+GreAB-C	371	AUA58_21825	Gammaproteobacteria	Salmonella enterica subsp. arizonae serovar 18:z4,z23:-	hypothetical protein AUA58_21825, partial [Salmonella enterica subsp. arizonae serovar 18:z4,z23:-].	GCA_007194715.1
EBV9897412.1	TPR+GreAB-C*→	TPR+GreAB-C	379	AUA59_22385	Gammaproteobacteria	Salmonella enterica subsp. arizonae serovar 18:z4,z23:-	hypothetical protein AUA59_22385, partial [Salmonella enterica subsp. arizonae serovar 18:z4,z23:-].	GCA_007194855.1
EBW0217976.1	HetE-N1→ TPR+GreAB-C+PIN*→	TPR+GreAB-C+PIN	1107	AUA78_22040	Gammaproteobacteria	Salmonella enterica subsp. arizonae serovar 18:z4,z23:-	hypothetical protein AUA78_22040 [Salmonella enterica subsp. arizonae serovar 18:z4,z23:-].	GCA_007204575.1
EBW0231167.1	HetE-N1→ TPR+GreAB-C+PIN*→	TPR+GreAB-C+PIN	990	AUA77_22025	Gammaproteobacteria	Salmonella enterica subsp. arizonae serovar 18:z4,z23:-	hypothetical protein AUA77_22025, partial [Salmonella enterica subsp. arizonae serovar 18:z4,z23:-].	GCA_007204595.1
EBW0678507.1	TPR+GreAB-C+PIN*→	TPR+GreAB-C+PIN	861	AR803_21985	Gammaproteobacteria	Salmonella enterica subsp. arizonae serovar 18:z4,z23:-	hypothetical protein AR803_21985, partial [Salmonella enterica subsp. arizonae serovar 18:z4,z23:-].	GCA_007197055.1
EBW5988048.1	TPR+GreAB-C+PIN*→	TPR+GreAB-C+PIN	813	AUB18_21995	Gammaproteobacteria	Salmonella enterica subsp. arizonae serovar 18:z4,z23:-	hypothetical protein AUB18_21995, partial [Salmonella enterica subsp. arizonae serovar 18:z4,z23:-].	GCA_007217995.1
EBW8169800.1	TPR+GreAB-C*→	TPR+GreAB-C	283	AUB36_22265	Gammaproteobacteria	Salmonella enterica subsp. arizonae serovar 18:z4,z23:-	hypothetical protein AUB36_22265, partial [Salmonella enterica subsp. arizonae serovar 18:z4,z23:-].	GCA_007226835.1
EBW9751630.1	HetE-N1→ TPR+GreAB-C+PIN*→	TPR+GreAB-C+PIN	1106	DQR71_22645	Gammaproteobacteria	Salmonella enterica subsp. enterica serovar Kingston	hypothetical protein DQR71_22645 [Salmonella enterica subsp. enterica serovar Kingston].	GCA_007234155.1

<table>
<tr><th>acc</th><th>operon</th><th>architecture</th><th>len</th><th>gen.name</th><th>taxend</th><th>species</th><th>defline</th><th>gca</th></tr>
<tr><td>EBX0426906.1</td><td>HetE-N1→ TPR+GreAB-C+PIN*→</td><td>TPR+GreAB-C+PIN</td><td>864</td><td>DP854_08040</td><td>Gammaproteobacteria</td><td>Salmonella enterica subsp. enterica serovar Corvallis</td><td>hypothetical protein DP854_08040, partial [Salmonella enterica subsp. enterica serovar Corvallis].</td><td>GCA_007237735.1</td></tr>
<tr><td>EBX5460983.1</td><td>HetE-N1→ TPR+GreAB-C+PIN*→</td><td>TPR+GreAB-C+PIN</td><td>817</td><td>DSN29_17980</td><td>Gammaproteobacteria</td><td>Salmonella enterica subsp. enterica serovar Corvallis</td><td>hypothetical protein DSN29_17980, partial [Salmonella enterica subsp. enterica serovar Corvallis].</td><td>GCA_007257955.1</td></tr>
<tr><td>EBX5569113.1</td><td>HetE-N1→ TPR+GreAB-C+PIN*→</td><td>TPR+GreAB-C+PIN</td><td>1106</td><td>DSN37_03170</td><td>Gammaproteobacteria</td><td>Salmonella enterica subsp. enterica serovar Kuessel</td><td>hypothetical protein DSN37_03170 [Salmonella enterica subsp. enterica serovar Kuessel].</td><td>GCA_007259425.1</td></tr>
<tr><td>EBX6009347.1</td><td>TPR+GreAB-C+PIN*→</td><td>TPR+GreAB-C+PIN</td><td>1021</td><td>DSR61_24855</td><td>Gammaproteobacteria</td><td>Salmonella enterica subsp. enterica serovar Corvallis</td><td>hypothetical protein DSR61_24855, partial [Salmonella enterica subsp. enterica serovar Corvallis].</td><td>GCA_007258725.1</td></tr>
<tr><td>EBY0196060.1</td><td>HetE-N1→ TPR+GreAB-C+PIN*→</td><td>TPR+GreAB-C+PIN</td><td>991</td><td>DUP89_08940</td><td>Gammaproteobacteria</td><td>Salmonella enterica subsp. enterica serovar Corvallis</td><td>hypothetical protein DUP89_08940, partial [Salmonella enterica subsp. enterica serovar Corvallis].</td><td>GCA_007275795.1</td></tr>
<tr><td>EBY1533788.1</td><td>HetE-N1→?→ TPR+GreAB-C+PIN*→</td><td>TPR+GreAB-C+PIN</td><td>775</td><td>DTD21_03800</td><td>Gammaproteobacteria</td><td>Salmonella enterica subsp. enterica serovar Mgulani</td><td>hypothetical protein DTD21_03800 [Salmonella enterica subsp. enterica serovar Mgulani].</td><td>GCA_007282305.1</td></tr>
<tr><td>EBY3547778.1</td><td>HetE-N1→ TPR+GreAB-C+PIN*→</td><td>TPR+GreAB-C+PIN</td><td>781</td><td>D4F04_22510</td><td>Gammaproteobacteria</td><td>Salmonella enterica subsp. enterica serovar Corvallis</td><td>hypothetical protein D4F04_22510, partial [Salmonella enterica subsp. enterica serovar Corvallis].</td><td>GCA_007290715.1</td></tr>
<tr><td>ECB7917957.1</td><td>HetE-N1→ TPR+GreAB-C+PIN*→</td><td>TPR+GreAB-C+PIN</td><td>841</td><td>E1365_23450</td><td>Gammaproteobacteria</td><td>Salmonella enterica subsp. enterica serovar Corvallis</td><td>hypothetical protein E1365_23450, partial [Salmonella enterica subsp. enterica serovar Corvallis].</td><td>GCA_007440385.1</td></tr>
<tr><td>ECC0701089.1</td><td>HetE-N1→?→ TPR+GreAB-C+PIN*→</td><td>TPR+GreAB-C+PIN</td><td>815</td><td>FMV45_11650</td><td>Gammaproteobacteria</td><td>Salmonella enterica subsp. enterica serovar Mgulani</td><td>hypothetical protein FMV45_11650 [Salmonella enterica subsp. enterica serovar Mgulani].</td><td>GCA_007457195.1</td></tr>
<tr><td>ECC3303083.1</td><td>TPR+GreAB-C+PIN*→</td><td>TPR+GreAB-C+PIN</td><td>878</td><td>ACP91_22675</td><td>Gammaproteobacteria</td><td>Salmonella enterica subsp. arizonae</td><td>hypothetical protein ACP91_22675, partial [Salmonella enterica subsp. arizonae].</td><td>GCA_007472415.1</td></tr>
<tr><td>ECC9297450.1</td><td>TPR+GreAB-C+PIN*→?→||||<-Trypsin</td><td>TPR+GreAB-C+PIN</td><td>1011</td><td>DRF02_24595</td><td>Gammaproteobacteria</td><td>Salmonella enterica subsp. salamae</td><td>hypothetical protein DRF02_24595 [Salmonella enterica subsp. salamae].</td><td>GCA_007500125.1</td></tr>
<tr><td>ECE0143412.1</td><td>TPR+GreAB-C+PIN*→</td><td>TPR+GreAB-C+PIN</td><td>625</td><td>NC53_22885</td><td>Gammaproteobacteria</td><td>Salmonella enterica subsp. arizonae</td><td>hypothetical protein NC53_22885, partial [Salmonella enterica subsp. arizonae].</td><td>GCA_007547655.1</td></tr>
<tr><td>ECE0541238.1</td><td>TPR+GreAB-C+PIN*→</td><td>TPR+GreAB-C+PIN</td><td>879</td><td>ACK19_22505</td><td>Gammaproteobacteria</td><td>Salmonella enterica subsp. arizonae</td><td>hypothetical protein ACK19_22505, partial [Salmonella enterica subsp. arizonae].</td><td>GCA_007549055.1</td></tr>
<tr><td>ECE0545534.1</td><td>TPR+GreAB-C*→</td><td>TPR+GreAB-C</td><td>384</td><td>ACK84_23000</td><td>Gammaproteobacteria</td><td>Salmonella enterica subsp. arizonae</td><td>hypothetical protein ACK84_23000, partial [Salmonella enterica subsp. arizonae].</td><td>GCA_007549175.1</td></tr>
<tr><td>ECE0554408.1</td><td>HetE-N1→ TPR+GreAB-C+PIN*→</td><td>TPR+GreAB-C+PIN</td><td>669</td><td>ACK35_22635</td><td>Gammaproteobacteria</td><td>Salmonella enterica subsp. arizonae</td><td>hypothetical protein ACK35_22635, partial [Salmonella enterica subsp. arizonae].</td><td>GCA_007549195.1</td></tr>
<tr><td>ECE0554409.1</td><td>TPR+GreAB-C+PIN*→</td><td>TPR+GreAB-C+PIN</td><td>428</td><td>ACK35_22640</td><td>Gammaproteobacteria</td><td>Salmonella enterica subsp. arizonae</td><td>hypothetical protein ACK35_22640, partial [Salmonella enterica subsp. arizonae].</td><td>GCA_007549195.1</td></tr>
<tr><td>ECE1015908.1</td><td>HetE-N1→ TPR+GreAB-C+PIN*→</td><td>TPR+GreAB-C+PIN</td><td>1116</td><td>AHY48_24995</td><td>Gammaproteobacteria</td><td>Salmonella enterica subsp. enterica</td><td>hypothetical protein AHY48_24995 [Salmonella enterica subsp. enterica].</td><td>GCA_007551035.1</td></tr>
<tr><td>ECF6862263.1</td><td>TPR+GreAB-C+PIN*→</td><td>TPR+GreAB-C+PIN</td><td>985</td><td>C702_18880</td><td>Gammaproteobacteria</td><td>Salmonella enterica subsp. enterica serovar Labadi</td><td>hypothetical protein C702_18880 [Salmonella enterica subsp. enterica serovar Labadi].</td><td>GCA_007627255.1</td></tr>
<tr><td>ECF7216075.1</td><td>HetE-N1→ TPR+GreAB-C+PIN*→</td><td>TPR+GreAB-C+PIN</td><td>985</td><td>ACL93_22670</td><td>Gammaproteobacteria</td><td>Salmonella enterica subsp. arizonae</td><td>hypothetical protein ACL93_22670, partial [Salmonella enterica subsp. arizonae].</td><td>GCA_007627105.1</td></tr>
<tr><td>ECF7535990.1</td><td>HetE-N1→ TPR+GreAB-C+PIN*→</td><td>TPR+GreAB-C+PIN</td><td>866</td><td>AF553_23825</td><td>Gammaproteobacteria</td><td>Salmonella enterica subsp. enterica</td><td>hypothetical protein AF553_23825, partial [Salmonella enterica subsp. enterica].</td><td>GCA_007629935.1</td></tr>
<tr><td>ECG8314709.1</td><td>HetE-N1→ TPR+GreAB-C+PIN*→</td><td>TPR+GreAB-C+PIN</td><td>967</td><td>E1871_22275</td><td>Gammaproteobacteria</td><td>Salmonella enterica subsp. enterica serovar Mgulani</td><td>hypothetical protein E1871_22275 [Salmonella enterica subsp. enterica serovar Mgulani].</td><td>GCA_007694365.1</td></tr>
<tr><td>ECH7956768.1</td><td>HetE-N1→ TPR+GreAB-C+PIN*→</td><td>TPR+GreAB-C+PIN</td><td>731</td><td>SW47_20185</td><td>Gammaproteobacteria</td><td>Salmonella enterica subsp. enterica</td><td>hypothetical protein SW47_20185, partial [Salmonella enterica subsp. enterica].</td><td>GCA_007742835.1</td></tr>
<tr><td>ECH9260474.1</td><td>HetE-N1→ TPR+GreAB-C+PIN→ TPR+GreAB-C+PIN*→</td><td>TPR+GreAB-C+PIN</td><td>654</td><td>YR28_14840</td><td>Gammaproteobacteria</td><td>Salmonella enterica subsp. enterica</td><td>hypothetical protein YR28_14840 [Salmonella enterica subsp. enterica].</td><td>GCA_007749495.1</td></tr>
</table>

<table>
<tr><th>acc</th><th>operon</th><th>architecture</th><th>len</th><th>gen.name</th><th>taxend</th><th>species</th><th>defline</th><th>gca</th></tr>
<tr><td>ECI0362610.1</td><td>HetE-N1→ TPR+GreAB-C+PIN*→</td><td>TPR+GreAB-C+PIN</td><td>969</td><td>ACK10_22270</td><td>Gammaproteobacteria</td><td>Salmonella enterica subsp. arizonae</td><td>hypothetical protein ACK10_22270, partial [Salmonella enterica subsp. arizonae].</td><td>GCA_007755195.1</td></tr>
<tr><td>ECI0389037.1</td><td>TPR+GreAB-C*→</td><td>TPR+GreAB-C</td><td>376</td><td>ACL48_23155</td><td>Gammaproteobacteria</td><td>Salmonella enterica subsp. arizonae</td><td>hypothetical protein ACL48_23155, partial [Salmonella enterica subsp. arizonae].</td><td>GCA_007754635.1</td></tr>
<tr><td>ECI1216662.1</td><td>TPR+GreAB-C+PIN*→</td><td>TPR+GreAB-C+PIN</td><td>836</td><td>AF582_21855</td><td>Gammaproteobacteria</td><td>Salmonella enterica subsp. arizonae</td><td>hypothetical protein AF582_21855, partial [Salmonella enterica subsp. arizonae].</td><td>GCA_007758765.1</td></tr>
<tr><td>ECI5614625.1</td><td>HetE-N1→?→ TPR+GreAB-C+PIN*→</td><td>TPR+GreAB-C+PIN</td><td>655</td><td>AHW94_03505</td><td>Gammaproteobacteria</td><td>Salmonella enterica subsp. enterica</td><td>hypothetical protein AHW94_03505, partial [Salmonella enterica subsp. enterica].</td><td>GCA_007777085.1</td></tr>
<tr><td>ECM8537065.1</td><td>HetE-N1→ TPR+GreAB-C+PIN*→</td><td>TPR+GreAB-C+PIN</td><td>1117</td><td>YI35_19225</td><td>Gammaproteobacteria</td><td>Salmonella enterica subsp. enterica serovar Corvallis</td><td>hypothetical protein YI35_19225 [Salmonella enterica subsp. enterica serovar Corvallis].</td><td>GCA_008050935.1</td></tr>
<tr><td>ECS4963358.1</td><td>TPR+GreAB-C+PIN*→</td><td>TPR+GreAB-C+PIN</td><td>874</td><td>A9T74_22060</td><td>Gammaproteobacteria</td><td>Salmonella enterica subsp. arizonae serovar 18:z4,z23:-</td><td>hypothetical protein A9T74_22060, partial [Salmonella enterica subsp. arizonae serovar 18:z4,z23:-].</td><td>GCA_008426085.1</td></tr>
<tr><td>ECS5089259.1</td><td>TPR+GreAB-C+PIN*→</td><td>TPR+GreAB-C+PIN</td><td>688</td><td>A9T03_21360</td><td>Gammaproteobacteria</td><td>Salmonella enterica subsp. arizonae serovar 18:z4,z23:-</td><td>hypothetical protein A9T03_21360, partial [Salmonella enterica subsp. arizonae serovar 18:z4,z23:-].</td><td>GCA_008426245.1</td></tr>
<tr><td>ECS5722565.1</td><td>HetE-N1→ TPR+GreAB-C+PIN*→</td><td>TPR+GreAB-C+PIN</td><td>763</td><td>BEE21_21925</td><td>Gammaproteobacteria</td><td>Salmonella enterica</td><td>hypothetical protein BEE21_21925, partial [Salmonella enterica].</td><td>GCA_008427725.1</td></tr>
<tr><td>ECS7544753.1</td><td>HetE-N1→ TPR+GreAB-C+PIN*→?→||||<-Trypsin</td><td>TPR+GreAB-C+PIN</td><td>1121</td><td>CI474_11845</td><td>Gammaproteobacteria</td><td>Salmonella enterica subsp. enterica serovar Denver</td><td>hypothetical protein CI474_11845 [Salmonella enterica subsp. enterica serovar Denver].</td><td>GCA_008435705.1</td></tr>
<tr><td>ECU7350952.1</td><td>TPR+GreAB-C+PIN*→</td><td>TPR+GreAB-C+PIN</td><td>816</td><td>A9S02_22180</td><td>Gammaproteobacteria</td><td>Salmonella enterica subsp. enterica serovar Kentucky</td><td>hypothetical protein A9S02_22180, partial [Salmonella enterica subsp. enterica serovar Kentucky].</td><td>GCA_008522685.1</td></tr>
<tr><td>ECV0014755.1</td><td>TPR+GreAB-C+PIN*→</td><td>TPR+GreAB-C+PIN</td><td>672</td><td>C9G23_25520</td><td>Gammaproteobacteria</td><td>Salmonella enterica</td><td>hypothetical protein C9G23_25520 [Salmonella enterica].</td><td>GCA_008534115.1</td></tr>
<tr><td>ECX3800078.1</td><td>HetE-N1→ TPR+GreAB-C+PIN*→</td><td>TPR+GreAB-C+PIN</td><td>647</td><td>F6B58_20445</td><td>Gammaproteobacteria</td><td>Salmonella enterica</td><td>hypothetical protein F6B58_20445, partial [Salmonella enterica].</td><td>GCA_008659475.1</td></tr>
<tr><td>ECY0836069.1</td><td>MarR-HTH→||||<-PSE||PSE→||||<-?||?→?→||||<-?||?→ HetE-N1→ TPR+GreAB-C+PIN*→?→||||<-Trypsin</td><td>TPR+GreAB-C+PIN</td><td>1121</td><td>F6Y43_14905</td><td>Gammaproteobacteria</td><td>Salmonella enterica subsp. enterica</td><td>hypothetical protein F6Y43_14905 [Salmonella enterica subsp. enterica].</td><td>GCA_008716255.1</td></tr>
<tr><td>ECY8988972.1</td><td>HetE-N1→ TPR+GreAB-C+PIN*→</td><td>TPR+GreAB-C+PIN</td><td>1117</td><td>F7L41_18095</td><td>Gammaproteobacteria</td><td>Salmonella enterica</td><td>hypothetical protein F7L41_18095 [Salmonella enterica].</td><td>GCA_008760695.1</td></tr>
<tr><td>ECZ2850292.1</td><td>TPR+GreAB-C+PIN*→</td><td>TPR+GreAB-C+PIN</td><td>736</td><td>F7T15_22920</td><td>Gammaproteobacteria</td><td>Salmonella enterica</td><td>hypothetical protein F7T15_22920, partial [Salmonella enterica].</td><td>GCA_008785865.1</td></tr>
<tr><td>EDA5083812.1</td><td>HetE-N1→ TPR+GreAB-C+PIN*→</td><td>TPR+GreAB-C+PIN</td><td>861</td><td>F9F62_19420</td><td>Gammaproteobacteria</td><td>Salmonella enterica</td><td>hypothetical protein F9F62_19420, partial [Salmonella enterica].</td><td>GCA_008860845.1</td></tr>
<tr><td>EDB4036569.1</td><td>HetE-N1→ TPR+GreAB-C+PIN*→</td><td>TPR+GreAB-C+PIN</td><td>810</td><td>A6J23_15240</td><td>Gammaproteobacteria</td><td>Salmonella enterica</td><td>hypothetical protein A6J23_15240, partial [Salmonella enterica].</td><td>GCA_008904195.1</td></tr>
<tr><td>EDB4763898.1</td><td>TPR+GreAB-C+PIN*→</td><td>TPR+GreAB-C+PIN</td><td>985</td><td>A8H08_23565</td><td>Gammaproteobacteria</td><td>Salmonella enterica subsp. enterica serovar Corvallis</td><td>hypothetical protein A8H08_23565 [Salmonella enterica subsp. enterica serovar Corvallis].</td><td>GCA_008907365.1</td></tr>
<tr><td>EDB6166846.1</td><td>HetE-N1→ TPR+GreAB-C+PIN*→</td><td>TPR+GreAB-C+PIN</td><td>994</td><td>BBD73_10015</td><td>Gammaproteobacteria</td><td>Salmonella enterica subsp. enterica serovar Corvallis</td><td>hypothetical protein BBD73_10015, partial [Salmonella enterica subsp. enterica serovar Corvallis].</td><td>GCA_008914005.1</td></tr>
<tr><td>EDN4259744.1</td><td>HetE-N1→ TPR+GreAB-C+PIN*→</td><td>TPR+GreAB-C+PIN</td><td>840</td><td>Y652_004461</td><td>Gammaproteobacteria</td><td>Salmonella enterica subsp. enterica</td><td>hypothetical protein Y652_004461, partial [Salmonella enterica subsp. enterica].</td><td>GCA_009997765.1</td></tr>
<tr><td>EDO1617810.1</td><td>HetE-N1→ TPR+GreAB-C+PIN*→</td><td>TPR+GreAB-C+PIN</td><td>838</td><td>CVA64_22730</td><td>Gammaproteobacteria</td><td>Salmonella enterica subsp. enterica serovar Molade</td><td>hypothetical protein CVA64_22730, partial [Salmonella enterica subsp. enterica serovar Molade].</td><td>GCA_010125745.1</td></tr>
<tr><td>EDP8916875.1</td><td>HetE-N1→ TPR+GreAB-C+PIN*→</td><td>TPR+GreAB-C+PIN</td><td>775</td><td>ZT80_004634</td><td>Gammaproteobacteria</td><td>Salmonella enterica subsp. enterica serovar Chailey</td><td>hypothetical protein ZT80_004634, partial [Salmonella enterica subsp. enterica serovar Chailey].</td><td>GCA_010369345.1</td></tr>
</table>

acc	operon	architecture	len	gen.name	taxend	species	defline	gca
EDP9779517.1	HetE-N1→ TPR+GreAB-C+PIN*→	TPR+GreAB-C+PIN	844	A9223_002456	Gammaproteobacteria	Salmonella enterica subsp. enterica serovar Alachua	hypothetical protein A9223_002456, partial [Salmonella enterica subsp. enterica serovar Alachua].	GCA_010373825.1
EDR2934822.1	HetE-N1→ TPR+GreAB-C+PIN*→	TPR+GreAB-C+PIN	754	KS39_001997	Gammaproteobacteria	Salmonella enterica subsp. enterica serovar Ruiru	hypothetical protein KS39_001997, partial [Salmonella enterica subsp. enterica serovar Ruiru].	GCA_010439595.1
EDR3921234.1	<-HTH<-?<-PSE||HetE-N1→ TPR+GreAB-C+PIN*→	TPR+GreAB-C+PIN	1101	BWC65_003323	Gammaproteobacteria	Salmonella enterica	hypothetical protein BWC65_003323 [Salmonella enterica].	GCA_010444135.1
EDR7156767.1	HetE-N1→ TPR+GreAB-C+PIN→ TPR+GreAB-C+PIN*→	TPR+GreAB-C+PIN	457	GRJ98_004481	Gammaproteobacteria	Salmonella enterica	hypothetical protein GRJ98_004481 [Salmonella enterica].	GCA_010460165.1
EDR9691108.1	TPR+GreAB-C+PIN*→	TPR+GreAB-C+PIN	644	CSL67_004218	Gammaproteobacteria	Salmonella enterica subsp. arizonae	hypothetical protein CSL67_004218 [Salmonella enterica subsp. arizonae].	GCA_010471185.1
EDT9803200.1	HetE-N1→ TPR+GreAB-C+PIN*→	TPR+GreAB-C+PIN	828	GQ119_004456	Gammaproteobacteria	Salmonella enterica	hypothetical protein GQ119_004456, partial [Salmonella enterica].	GCA_010569565.1
EDU6324669.1	HetE-N1→ TPR+GreAB-C+PIN*→?→||||<-Trypsin	TPR+GreAB-C+PIN	1121	CAE39_004078	Gammaproteobacteria	Salmonella enterica subsp. enterica serovar Edinburgh	hypothetical protein CAE39_004078 [Salmonella enterica subsp. enterica serovar Edinburgh].	GCA_010619425.1
EDV9361828.1	HetE-N1→ TPR+GreAB-C+PIN*→	TPR+GreAB-C+PIN	870	QG02_001683	Gammaproteobacteria	Salmonella enterica subsp. enterica	hypothetical protein QG02_001683, partial [Salmonella enterica subsp. enterica].	GCA_010676285.1
EDW0654491.1	<-HTH<-?<-?||HetE-N1→ TPR+GreAB-C+PIN*→	TPR+GreAB-C+PIN	1106	JU54_004053	Gammaproteobacteria	Salmonella enterica subsp. enterica serovar Weslaco	hypothetical protein JU54_004053 [Salmonella enterica subsp. enterica serovar Weslaco].	GCA_010695505.1
EDW1854316.1	TPR+GreAB-C+PIN*→	TPR+GreAB-C+PIN	1107	S887_003116	Gammaproteobacteria	Salmonella enterica subsp. diarizonae	hypothetical protein S887_003116 [Salmonella enterica subsp. diarizonae].	GCA_010701205.1
EDW7381411.1	HetE-N1→ TPR+GreAB-C+PIN*→	TPR+GreAB-C+PIN	838	CJF03_003360	Gammaproteobacteria	Salmonella enterica subsp. enterica serovar Alachua	hypothetical protein CJF03_003360, partial [Salmonella enterica subsp. enterica serovar Alachua].	GCA_010728605.1
EDX0815680.1	MarR-HTH→||||<-PSE||PSE→||||<-?||?→?→||||<-?||?→ HetE-N1→ TPR+GreAB-C+PIN*→	TPR+GreAB-C+PIN	1047	GTA77_003301	Gammaproteobacteria	Salmonella enterica subsp. enterica	hypothetical protein GTA77_003301, partial [Salmonella enterica subsp. enterica].	GCA_010745015.1
EDY1906151.1	MarR-HTH→||||<-PSE||PSE→||||<-?||?→?→||||<-?||?→ HetE-N1→ TPR+GreAB-C+PIN*→	TPR+GreAB-C+PIN	1043	GTA76_003216	Gammaproteobacteria	Salmonella enterica subsp. enterica	hypothetical protein GTA76_003216, partial [Salmonella enterica subsp. enterica].	GCA_010803285.1
EDY7338112.1	HetE-N1→ TPR+GreAB-C+PIN*→	TPR+GreAB-C+PIN	873	GRN59_004759	Gammaproteobacteria	Salmonella enterica subsp. enterica serovar Alachua	hypothetical protein GRN59_004759, partial [Salmonella enterica subsp. enterica serovar Alachua].	GCA_010827805.1
EDZ6443278.1	HetE-N1→ TPR+GreAB-C+PIN*→	TPR+GreAB-C+PIN	813	GOH01_21475	Gammaproteobacteria	Salmonella enterica	hypothetical protein GOH01_21475, partial [Salmonella enterica].	GCA_010859905.1
EED4174297.1	HetE-N1→ TPR+GreAB-C+PIN*→	TPR+GreAB-C+PIN	944	S577_12620	Gammaproteobacteria	Salmonella enterica subsp. enterica serovar Rubislaw	hypothetical protein S577_12620, partial [Salmonella enterica subsp. enterica serovar Rubislaw].	GCA_011083145.1
EEG2646873.1	HetE-N1→ TPR+GreAB-C+PIN*→	TPR+GreAB-C+PIN	1117	GXG13_10075	Gammaproteobacteria	Salmonella enterica	hypothetical protein GXG13_10075 [Salmonella enterica].	GCA_011218655.1
EEH6562392.1	TPR+GreAB-C+PIN*→	TPR+GreAB-C+PIN	561	G9D83_004649	Gammaproteobacteria	Salmonella enterica	hypothetical protein G9D83_004649, partial [Salmonella enterica].	GCA_011296115.1
EEH8607740.1	HetE-N1→ TPR+GreAB-C+PIN*→	TPR+GreAB-C+PIN	751	GXG09_15375	Gammaproteobacteria	Salmonella enterica	hypothetical protein GXG09_15375, partial [Salmonella enterica].	GCA_011310075.1
EEI7790049.1	TPR+GreAB-C+PIN*→	TPR+GreAB-C+PIN	1003	G7421_004640	Gammaproteobacteria	Salmonella enterica	hypothetical protein G7421_004640, partial [Salmonella enterica].	GCA_011384105.1
EEJ3916542.1	HetE-N1→ TPR+GreAB-C+PIN*→	TPR+GreAB-C+PIN	931	C1D58_004804	Gammaproteobacteria	Salmonella enterica subsp. enterica serovar Waral	hypothetical protein C1D58_004804, partial [Salmonella enterica subsp. enterica serovar Waral].	GCA_011421105.1
EEJ8590178.1	HetE-N1→ TPR+GreAB-C+PIN*→	TPR+GreAB-C+PIN	1106	YR33_002856	Gammaproteobacteria	Salmonella enterica subsp. enterica	hypothetical protein YR33_002856 [Salmonella enterica subsp. enterica].	GCA_011444435.1

acc	operon	architecture	len	gen.name	taxend	species	defline	gca
EEM3099552.1	HetE-N1→ TPR+GreAB-C+PIN*→	TPR+GreAB-C+PIN	837	C7106_19315	Gammaproteobacteria	Salmonella enterica subsp. enterica serovar Corvallis	hypothetical protein C7106_19315, partial [Salmonella enterica subsp. enterica serovar Corvallis].	GCA_011562745.1
EEN9063089.1	TPR+GreAB-C+PIN*→	TPR+GreAB-C+PIN	1003	G5014_004598	Gammaproteobacteria	Salmonella enterica subsp. enterica	hypothetical protein G5014_004598, partial [Salmonella enterica subsp. enterica].	GCA_011642815.1
EEO4791769.1	GreAB-C*→	GreAB-C	475	GCV36_004805	Gammaproteobacteria	Salmonella enterica	hypothetical protein GCV36_004805, partial [Salmonella enterica].	GCA_011672825.1
EEP6626700.1	TPR+GreAB-C+PIN*→	TPR+GreAB-C+PIN	1098	HB923_004453	Gammaproteobacteria	Salmonella enterica	hypothetical protein HB923_004453, partial [Salmonella enterica].	GCA_011737335.1
EEQ0870631.1	GreAB-C*→	GreAB-C	401	HC115_004664	Gammaproteobacteria	Salmonella enterica subsp. enterica serovar Corvallis	hypothetical protein HC115_004664, partial [Salmonella enterica subsp. enterica serovar Corvallis].	GCA_011766905.1
EER8058271.1	HetE-N1→ TPR+GreAB-C+PIN*→	TPR+GreAB-C+PIN	1094	F0645_003838	Gammaproteobacteria	Escherichia coli	hypothetical protein F0645_003838 [Escherichia coli].	GCA_011871345.1
EES4613658.1	HetE-N1→ TPR+GreAB-C+PIN*→	TPR+GreAB-C+PIN	1094	EZQ25_003302	Gammaproteobacteria	Escherichia coli	hypothetical protein EZQ25_003302 [Escherichia coli].	GCA_011897605.1
EEU6346619.1	TPR+GreAB-C+PIN*→	TPR+GreAB-C+PIN	663	HES36_004810	Gammaproteobacteria	Salmonella enterica	hypothetical protein HES36_004810, partial [Salmonella enterica].	GCA_011985205.1
EEU8409948.1	TPR+GreAB-C+PIN*→	TPR+GreAB-C+PIN	733	HDI19_004538	Gammaproteobacteria	Salmonella enterica	hypothetical protein HDI19_004538, partial [Salmonella enterica].	GCA_012002765.1
EEU8431734.1	TPR+GreAB-C+PIN*→	TPR+GreAB-C+PIN	770	HDI14_004564	Gammaproteobacteria	Salmonella enterica	hypothetical protein HDI14_004564, partial [Salmonella enterica].	GCA_012002965.1
EEU8450206.1	TPR+GreAB-C+PIN*→	TPR+GreAB-C+PIN	686	HDI09_004415	Gammaproteobacteria	Salmonella enterica	hypothetical protein HDI09_004415, partial [Salmonella enterica].	GCA_012003165.1
EEU8461067.1	HetE-N1→ TPR+GreAB-C+PIN*→	TPR+GreAB-C+PIN	777	HDI12_003619	Gammaproteobacteria	Salmonella enterica	hypothetical protein HDI12_003619, partial [Salmonella enterica].	GCA_012003025.1
EEU8472241.1	HetE-N1→ TPR+GreAB-C+PIN*→	TPR+GreAB-C+PIN	832	HDI08_003001	Gammaproteobacteria	Salmonella enterica	hypothetical protein HDI08_003001, partial [Salmonella enterica].	GCA_012003185.1
EEU8646489.1	HetE-N1→ TPR+GreAB-C+PIN*→	TPR+GreAB-C+PIN	746	HDI02_002414	Gammaproteobacteria	Salmonella enterica	hypothetical protein HDI02_002414, partial [Salmonella enterica].	GCA_012004065.1
EEV5892122.1	HetE-N1→ TPR+GreAB-C+PIN*→	TPR+GreAB-C+PIN	1094	ELJ91_00185	Gammaproteobacteria	Escherichia coli	hypothetical protein ELJ91_00185 [Escherichia coli].	GCA_012046565.1
EEW2250301.1	TPR+GreAB-C+PIN*→	TPR+GreAB-C+PIN	649	D9F29_22745	Gammaproteobacteria	Escherichia coli	hypothetical protein D9F29_22745, partial [Escherichia coli].	GCA_012074905.1
EEW6232025.1	TPR+GreAB-C+PIN*→	TPR+GreAB-C+PIN	1025	D7V76_23725	Gammaproteobacteria	Escherichia coli	hypothetical protein D7V76_23725, partial [Escherichia coli].	GCA_012087875.1
EEZ4464859.1	HetE-N1→ TPR+GreAB-C+PIN*→	TPR+GreAB-C+PIN	1009	DED50_002664	Gammaproteobacteria	Escherichia coli	hypothetical protein DED50_002664, partial [Escherichia coli].	GCA_012207825.1
EFA5448049.1	GreAB-C*→	GreAB-C	270	EZF43_26870	Gammaproteobacteria	Escherichia coli	hypothetical protein EZF43_26870, partial [Escherichia coli].	GCA_012252965.1
EFB4141030.1	TPR+GreAB-C+PIN*→	TPR+GreAB-C+PIN	803	C0X77_005316	Gammaproteobacteria	Escherichia coli O88:H1	hypothetical protein C0X77_005316, partial [Escherichia coli O88:H1].	GCA_012307025.1
EFB7592548.1	TPR+GreAB-C+PIN*→	TPR+GreAB-C+PIN	871	E4J78_25685	Gammaproteobacteria	Escherichia coli	hypothetical protein E4J78_25685, partial [Escherichia coli].	GCA_012318985.1
EFD6843528.1	HetE-N1→ TPR+GreAB-C+PIN*→	TPR+GreAB-C+PIN	785	HG348_004580	Gammaproteobacteria	Salmonella enterica subsp. enterica serovar Corvallis	hypothetical protein HG348_004580, partial [Salmonella enterica subsp. enterica serovar Corvallis].	GCA_012407905.1
EFD8840406.1	TPR+GreAB-C+PIN*→	TPR+GreAB-C+PIN	342	CO273_005362	Gammaproteobacteria	Escherichia coli	hypothetical protein CO273_005362, partial [Escherichia coli].	GCA_012416985.1
EFE7110517.1	TPR+GreAB-C+PIN*→	TPR+GreAB-C+PIN	631	GCY86_26975	Gammaproteobacteria	Escherichia coli	hypothetical protein GCY86_26975, partial [Escherichia coli].	GCA_012449745.1

acc	operon	architecture	len	gen.name	taxend	species	defline	gca
EFE7435055.1	HetE-N1→ TPR+GreAB-C+PIN*→	TPR+GreAB-C+PIN	917	F9S89_16525	Gammaproteobacteria	Escherichia coli	hypothetical protein F9S89_16525, partial [Escherichia coli].	GCA_012452135.1
EFE7908555.1	TPR+GreAB-C+PIN*→	TPR+GreAB-C+PIN	1007	F3644_26165	Gammaproteobacteria	Escherichia coli	hypothetical protein F3644_26165, partial [Escherichia coli].	GCA_012454005.1
EFF2139424.1	TPR+GreAB-C+PIN*→	TPR+GreAB-C+PIN	639	B9T00_004906	Gammaproteobacteria	Escherichia coli	hypothetical protein B9T00_004906, partial [Escherichia coli].	GCA_012475085.1
EFG3018646.1	TPR+GreAB-C+PIN*→	TPR+GreAB-C+PIN	644	BOZ88_000259	Gammaproteobacteria	Escherichia coli	hypothetical protein BOZ88_000259, partial [Escherichia coli].	GCA_012551215.1
EFG6909290.1	HetE-N1→ TPR+GreAB-C+PIN*→	TPR+GreAB-C+PIN	747	BMM73_004701	Gammaproteobacteria	Escherichia coli	hypothetical protein BMM73_004701, partial [Escherichia coli].	GCA_012567505.1
EFH5729117.1	GreAB-C*→	GreAB-C	499	GPH08_24725	Gammaproteobacteria	Escherichia coli	hypothetical protein GPH08_24725, partial [Escherichia coli].	GCA_012607235.1
EFK2051151.1	HetE-N1→ TPR+GreAB-C+PIN*→	TPR+GreAB-C+PIN	1094	A8650_000222	Gammaproteobacteria	Escherichia coli	hypothetical protein A8650_000222 [Escherichia coli].	GCA_012745335.1
EFK2630094.1	HetE-N1→ TPR+GreAB-C+PIN*→	TPR+GreAB-C+PIN	1094	A8611_000403	Gammaproteobacteria	Escherichia coli	hypothetical protein A8611_000403 [Escherichia coli].	GCA_012750705.1
EFK4277858.1	HetE-N1→ TPR+GreAB-C+PIN*→	TPR+GreAB-C+PIN	1094	A8W36_000742	Gammaproteobacteria	Escherichia coli	hypothetical protein A8W36_000742 [Escherichia coli].	GCA_012757595.1
EFK5336626.1	GreAB-C*→	GreAB-C	296	G5Y50_005070	Gammaproteobacteria	Escherichia coli	hypothetical protein G5Y50_005070, partial [Escherichia coli].	GCA_012762195.1
EFM2410662.1	TPR+GreAB-C+PIN*→	TPR+GreAB-C+PIN	1064	HCP88_003534	Gammaproteobacteria	Escherichia coli	hypothetical protein HCP88_003534, partial [Escherichia coli].	GCA_012855135.1
EFM6245585.1	HetE-N1→ TPR+GreAB-C+PIN*→	TPR+GreAB-C+PIN	1094	G8G69_000691	Gammaproteobacteria	Escherichia coli	hypothetical protein G8G69_000691 [Escherichia coli].	GCA_012890895.1
EFN7425919.1	HetE-N1→ HetE-N1+TPR+GreAB-C+PIN*→	HetE-N1+TPR+GreAB-C+PIN	1094	EHR20_13780	Gammaproteobacteria	Escherichia coli	hypothetical protein EHR20_13780 [Escherichia coli].	GCA_013040925.1
EFO0890621.1	GreAB-C*→	GreAB-C	328	DYJ49_27190	Gammaproteobacteria	Escherichia coli	hypothetical protein DYJ49_27190, partial [Escherichia coli].	GCA_013061045.1
EFO1176558.1	TPR+GreAB-C+PIN*→	TPR+GreAB-C+PIN	1073	DW520_17505	Gammaproteobacteria	Escherichia coli	hypothetical protein DW520_17505, partial [Escherichia coli].	GCA_013062025.1
EFS2240967.1	HetE-N1→ TPR+GreAB-C+PIN*→	TPR+GreAB-C+PIN	1094	GTJ56_01995	Gammaproteobacteria	Shigella sonnei	hypothetical protein GTJ56_01995 [Shigella sonnei].	GCA_013627075.1
EFT8083545.1	<-HTH<-PSE||HetE-N1→ TPR+GreAB-C+PIN*→	TPR+GreAB-C+PIN	1107	HVC06_002423	Gammaproteobacteria	Salmonella enterica	hypothetical protein HVC06_002423 [Salmonella enterica].	GCA_013717865.1
EFU7164914.1	TPR+GreAB-C+PIN*→	TPR+GreAB-C+PIN	1012	HTZ39_004610	Gammaproteobacteria	Escherichia coli	hypothetical protein HTZ39_004610, partial [Escherichia coli].	GCA_013766175.1
EFW2864166.1	TPR+GreAB-C*→	TPR+GreAB-C	440	H3T30_004508	Gammaproteobacteria	Salmonella enterica	hypothetical protein H3T30_004508, partial [Salmonella enterica].	GCA_013859415.1
EFZ7349264.1	HetE-N1→ TPR+GreAB-C+PIN*→	TPR+GreAB-C+PIN	1094	A8I24_003489	Gammaproteobacteria	Shigella sonnei	hypothetical protein A8I24_003489 [Shigella sonnei].	GCA_014033075.1
EGB9339943.1	TPR+GreAB-C+PIN*→	TPR+GreAB-C+PIN	694	H8Q22_004544	Gammaproteobacteria	Salmonella enterica	hypothetical protein H8Q22_004544, partial [Salmonella enterica].	GCA_014249275.1
EGF6340579.1	GreAB-C*→	GreAB-C	417	IBT07_004591	Gammaproteobacteria	Salmonella enterica subsp. enterica serovar Molade	hypothetical protein IBT07_004591, partial [Salmonella enterica subsp. enterica serovar Molade].	GCA_014515725.1
EGG4494193.1	TPR+GreAB-C+PIN*→	TPR+GreAB-C+PIN	990	HKL61_004648	Gammaproteobacteria	Salmonella enterica	hypothetical protein HKL61_004648, partial [Salmonella enterica].	GCA_014566815.1
EGG4638401.1	HetE-N1→ TPR+GreAB-C+PIN*→	TPR+GreAB-C+PIN	777	HKL37_003624	Gammaproteobacteria	Salmonella enterica	hypothetical protein HKL37_003624, partial [Salmonella enterica].	GCA_014566995.1
EGG4725781.1	HetE-N1→ TPR+GreAB-C+PIN*→	TPR+GreAB-C+PIN	855	HKE56_003805	Gammaproteobacteria	Salmonella enterica	hypothetical protein HKE56_003805, partial [Salmonella enterica].	GCA_014566275.1

acc	operon	architecture	len	gen.name	taxend	species	defline	gca
EGI5600308.1	HetE-N1→ TPR+GreAB-C+PIN*→	TPR+GreAB-C+PIN	1070	VS45_000308	Gammaproteobacteria	Salmonella enterica subsp. enterica serovar Augustenborg	hypothetical protein VS45_000308, partial [Salmonella enterica subsp. enterica serovar Augustenborg].	GCA_014756925.1
EGI6298009.1	HetE-N1→ TPR+GreAB-C+PIN*→	TPR+GreAB-C+PIN	1117	WB72_004704	Gammaproteobacteria	Salmonella enterica subsp. enterica serovar Molade	hypothetical protein WB72_004704 [Salmonella enterica subsp. enterica serovar Molade].	GCA_014759725.1
EGJ1631486.1	<-HTH<-?<-?||HetE-N1→ TPR+GreAB-C+PIN*→	TPR+GreAB-C+PIN	915	IHG43_004265	Gammaproteobacteria	Salmonella enterica	hypothetical protein IHG43_004265, partial [Salmonella enterica].	GCA_014806485.1
EGJ1647992.1	<-HTH<-?<-?||HetE-N1→ TPR+GreAB-C+PIN*→	TPR+GreAB-C+PIN	1107	IHG42_002561	Gammaproteobacteria	Salmonella enterica	hypothetical protein IHG42_002561 [Salmonella enterica].	GCA_014806725.1
EGK4803692.1	TPR+GreAB-C+PIN*→	TPR+GreAB-C+PIN	879	IOX27_004405	Gammaproteobacteria	Salmonella enterica	hypothetical protein IOX27_004405, partial [Salmonella enterica].	GCA_014937925.1
EGQ7796229.1	HetE-N1→ TPR+GreAB-C+PIN*→	TPR+GreAB-C+PIN	1130	I6Z01_004778	Gammaproteobacteria	Vibrio parahaemolyticus	hypothetical protein I6Z01_004778 [Vibrio parahaemolyticus].	GCA_015745865.1
EGQ8097028.1	TPR+GreAB-C+PIN*→	TPR+GreAB-C+PIN	450	G4T96_003617	Gammaproteobacteria	Vibrio cholerae	hypothetical protein G4T96_003617, partial [Vibrio cholerae].	GCA_015960365.1
EGQ8394401.1	HetE-N1→ TPR+GreAB-C+PIN*→	TPR+GreAB-C+PIN	1089	GTW12_14360	Gammaproteobacteria	Vibrio cholerae	hypothetical protein GTW12_14360 [Vibrio cholerae].	GCA_015775695.1
EGR2511155.1	HetE-N1→ HetE-N1+TPR+GreAB-C+PIN*→	HetE-N1+TPR+GreAB-C+PIN	1088	DYC71_07390	Gammaproteobacteria	Vibrio cholerae	hypothetical protein DYC71_07390 [Vibrio cholerae].	GCA_015803535.1
EGR2571797.1	HetE-N1→ TPR+GreAB-C+PIN*→	TPR+GreAB-C+PIN	1089	DXI14_15890	Gammaproteobacteria	Vibrio cholerae	hypothetical protein DXI14_15890 [Vibrio cholerae].	GCA_015804695.1
EGR4199274.1	HetE-N1→ HetE-N1+TPR+GreAB-C+PIN*→	HetE-N1+TPR+GreAB-C+PIN	1088	DDN79_15180	Gammaproteobacteria	Vibrio cholerae	hypothetical protein DDN79_15180 [Vibrio cholerae].	GCA_015810035.1
EGR4999920.1	HetE-N1→ HetE-N1+TPR+GreAB-C+PIN*→	HetE-N1+TPR+GreAB-C+PIN	1091	C4G32_21475	Gammaproteobacteria	Vibrio parahaemolyticus	hypothetical protein C4G32_21475 [Vibrio parahaemolyticus].	GCA_015814505.1
EGS2470077.1	HetE-N1→ TPR+GreAB-C+PIN*→	TPR+GreAB-C+PIN	1117	I8K59_003334	Gammaproteobacteria	Salmonella enterica	hypothetical protein I8K59_003334 [Salmonella enterica].	GCA_015867645.1
EGS6048781.1	HetE-N1→ TPR+GreAB-C+PIN*→	TPR+GreAB-C+PIN	998	I4Y87_004789	Gammaproteobacteria	Salmonella enterica subsp. enterica serovar Alachua	hypothetical protein I4Y87_004789, partial [Salmonella enterica subsp. enterica serovar Alachua].	GCA_015906765.1
EGT0637485.1	GreAB-C*→	GreAB-C	478	JAG24_004773	Gammaproteobacteria	Citrobacter freundii	hypothetical protein JAG24_004773, partial [Citrobacter freundii].	GCA_015943305.1
EGT3640898.1	TPR+GreAB-C+PIN*→	TPR+GreAB-C+PIN	1067	DFW15_03700	Firmicutes	Clostridioides difficile	hypothetical protein DFW15_03700 [Clostridioides difficile].	GCA_016071215.1
EGT5618809.1	TPR+GreAB-C+PIN*→	TPR+GreAB-C+PIN	1233	BUL45_06960	Firmicutes	Clostridium perfringens	tetratricopeptide repeat protein [Clostridium perfringens].	GCA_016085335.1
EGY6095294.1	REase+TPR+GreAB-C+PIN*→||||<-?||HTH→	REase+TPR+GreAB-C+PIN	1216	A1Z38_19500	Gammaproteobacteria	Acinetobacter baumannii	hypothetical protein A1Z38_19500 [Acinetobacter baumannii].	GCA_016522135.1
EGY8392823.1	REase+TPR+GreAB-C+PIN*→||||<-?||HTH→	REase+TPR+GreAB-C+PIN	1216	AXE44_13945	Gammaproteobacteria	Acinetobacter baumannii	hypothetical protein AXE44_13945 [Acinetobacter baumannii].	GCA_016539145.1
EHA1126835.1	HetE-N1→ TPR+GreAB-C+PIN*→	TPR+GreAB-C+PIN	1114	FG475_17175	Gammaproteobacteria	Vibrio navarrensis	hypothetical protein FG475_17175 [Vibrio navarrensis].	GCA_016611225.1
EHB5819355.1	HetE-N1→ TPR+GreAB-C+PIN*→	TPR+GreAB-C+PIN	1106	JXL86_002072	-	Enterobacter hormaechei	hypothetical protein JXL86_002072 [Enterobacter hormaechei].	GCA_016985635.1
EHB7585965.1	HetE-N1→ TPR+GreAB-C+PIN*→	TPR+GreAB-C+PIN	759	JV429_000621	Gammaproteobacteria	Escherichia coli	hypothetical protein JV429_000621, partial [Escherichia coli].	GCA_017003995.1
EHB7691780.1	TPR+GreAB-C+PIN*→	TPR+GreAB-C+PIN	745	JV466_004427	Gammaproteobacteria	Escherichia coli	hypothetical protein JV466_004427, partial [Escherichia coli].	GCA_017004675.1
EHC1615065.1	TPR+GreAB-C+PIN*→	TPR+GreAB-C+PIN	732	JT622_005238	Gammaproteobacteria	Escherichia coli	hypothetical protein JT622_005238, partial [Escherichia coli].	GCA_017029715.1
EHD4655635.1	HetE-N1→ TPR+GreAB-C+PIN*→	TPR+GreAB-C+PIN	807	JRB69_003203	Gammaproteobacteria	Salmonella enterica	hypothetical protein JRB69_003203, partial [Salmonella enterica].	GCA_017143955.1

acc	operon	architecture	len	gen.name	taxend	species	defline	gca
EHD4703897.1	GreAB-C*→	GreAB-C	419	JRB68_004676	Gammaproteobacteria	Salmonella enterica	hypothetical protein JRB68_004676, partial [Salmonella enterica].	GCA_017144295.1
EHD5811550.1	TPR+GreAB-C+PIN*→	TPR+GreAB-C+PIN	628	JQZ13_004415	Gammaproteobacteria	Escherichia coli	hypothetical protein JQZ13_004415, partial [Escherichia coli].	GCA_017158335.1
EHD6029608.1	HetE-N1→ TPR+GreAB-C+PIN*→	TPR+GreAB-C+PIN	1085	JQ325_002365	Gammaproteobacteria	Vibrio parahaemolyticus	hypothetical protein JQ325_002365 [Vibrio parahaemolyticus].	GCA_017160295.1
EHF0016324.1	?*→	?	690	IFX66_005031	Gammaproteobacteria	Escherichia coli	hypothetical protein IFX66_005031, partial [Escherichia coli].	GCA_017278275.1
EHG1523212.1	TPR+GreAB-C+PIN*→	TPR+GreAB-C+PIN	1001	J1L78_003684	Gammaproteobacteria	Salmonella enterica subsp. enterica serovar Corvallis	hypothetical protein J1L78_003684, partial [Salmonella enterica subsp. enterica serovar Corvallis].	GCA_017373035.1
EHG6154572.1	HetE-N1→ TPR+GreAB-C+PIN*→	TPR+GreAB-C+PIN	1094	J5T29_002362	Gammaproteobacteria	Escherichia fergusonii	hypothetical protein J5T29_002362 [Escherichia fergusonii].	GCA_017528445.1
EHG8446233.1	HetE-N1→ TPR+GreAB-C+PIN*→	TPR+GreAB-C+PIN	1106	J6D68_002379	Gammaproteobacteria	Salmonella enterica subsp. enterica	hypothetical protein J6D68_002379 [Salmonella enterica subsp. enterica].	GCA_017573985.1
EHH5222655.1	TPR+GreAB-C+PIN*→	TPR+GreAB-C+PIN	630	J6876_004216	Gammaproteobacteria	Escherichia coli	hypothetical protein J6876_004216, partial [Escherichia coli].	GCA_017685995.1
EHH5972888.1	TPR+GreAB-C+PIN*→	TPR+GreAB-C+PIN	1039	J8O14_004858	Gammaproteobacteria	Salmonella enterica subsp. enterica serovar Alachua	hypothetical protein J8O14_004858, partial [Salmonella enterica subsp. enterica serovar Alachua].	GCA_017690295.1
EHH6676921.1	HetE-N1→ TPR+GreAB-C+PIN*→	TPR+GreAB-C+PIN	1094	J6H48_001187	Gammaproteobacteria	Escherichia coli	hypothetical protein J6H48_001187 [Escherichia coli].	GCA_017723775.1
EHH7564006.1	TPR+GreAB-C+PIN*→	TPR+GreAB-C+PIN	1015	J5J30_004554	Gammaproteobacteria	Escherichia coli	hypothetical protein J5J30_004554, partial [Escherichia coli].	GCA_017727775.1
EHH7810630.1	GreAB-C*→	GreAB-C	318	J5B41_005221	Gammaproteobacteria	Escherichia coli	hypothetical protein J5B41_005221, partial [Escherichia coli].	GCA_017733975.1
EHI0937798.1	TPR+GreAB-C+PIN*→	TPR+GreAB-C+PIN	636	J1A00_004749	Gammaproteobacteria	Escherichia coli	hypothetical protein J1A00_004749, partial [Escherichia coli].	GCA_017780845.1
EHI2008363.1	TPR+GreAB-C+PIN*→	TPR+GreAB-C+PIN	694	J9U59_004724	Gammaproteobacteria	Salmonella enterica	hypothetical protein J9U59_004724, partial [Salmonella enterica].	GCA_017791685.1
EHI3384480.1	TPR+GreAB-C+PIN*→	TPR+GreAB-C+PIN	747	J9U04_004755	Gammaproteobacteria	Salmonella enterica	hypothetical protein J9U04_004755, partial [Salmonella enterica].	GCA_017798465.1
EHI3712656.1	TPR+GreAB-C+PIN*→	TPR+GreAB-C+PIN	723	J9T76_004561	Gammaproteobacteria	Salmonella enterica	hypothetical protein J9T76_004561, partial [Salmonella enterica].	GCA_017800075.1
EHI5184709.1	TPR+GreAB-C+PIN*→	TPR+GreAB-C+PIN	999	J9014_004542	Gammaproteobacteria	Salmonella enterica	hypothetical protein J9014_004542, partial [Salmonella enterica].	GCA_017806615.1
EHI6070991.1	HetE-N1→ TPR+GreAB-C+PIN*→	TPR+GreAB-C+PIN	984	J9J70_003931	Gammaproteobacteria	Salmonella enterica	hypothetical protein J9J70_003931, partial [Salmonella enterica].	GCA_017810795.1
EHI8244355.1	HetE-N1→ TPR+GreAB-C+PIN*→	TPR+GreAB-C+PIN	784	J9M94_004032	Gammaproteobacteria	Salmonella enterica	hypothetical protein J9M94_004032, partial [Salmonella enterica].	GCA_017828575.1
EHI9713257.1	TPR+GreAB-C+PIN*→	TPR+GreAB-C+PIN	735	J9T07_004736	Gammaproteobacteria	Salmonella enterica	hypothetical protein J9T07_004736, partial [Salmonella enterica].	GCA_017836875.1
EHJ6284301.1	<-HTH<-?<-PSE||HetE-N1→ TPR+GreAB-C+PIN*→	TPR+GreAB-C+PIN	1106	KAM53_001182	Gammaproteobacteria	Salmonella enterica	hypothetical protein KAM53_001182 [Salmonella enterica].	GCA_017922215.1
EHJ8153220.1	TPR+GreAB-C+PIN*→	TPR+GreAB-C+PIN	707	KB264_004740	Gammaproteobacteria	Escherichia coli	hypothetical protein KB264_004740, partial [Escherichia coli].	GCA_017943665.1
EHJ9993052.1	HetE-N1→ TPR+GreAB-C+PIN*→	TPR+GreAB-C+PIN	1085	KB972_002278	Gammaproteobacteria	Vibrio parahaemolyticus	hypothetical protein KB972_002278 [Vibrio parahaemolyticus].	GCA_017968025.1
EHK2882579.1	HetE-N1→ HetE-N1+TPR+GreAB-C+PIN*→	HetE-N1+TPR+GreAB-C+PIN	1091	J7H88_002175	Gammaproteobacteria	Vibrio parahaemolyticus	hypothetical protein J7H88_002175 [Vibrio parahaemolyticus].	GCA_018017275.1
EHL5831134.1	HetE-N1→ TPR+GreAB-C+PIN*→	TPR+GreAB-C+PIN	1117	KEB97_001561	Gammaproteobacteria	Salmonella enterica	hypothetical protein KEB97_001561 [Salmonella enterica].	GCA_018132225.1

<table>
<tr><th>acc</th><th>operon</th><th>architecture</th><th>len</th><th>gen.name</th><th>taxend</th><th>species</th><th>defline</th><th>gca</th></tr>
<tr><td>EKD28835.1</td><td>TPR+GreAB-C+PIN*→</td><td>TPR+GreAB-C+PIN</td><td>1282</td><td>ACD_79C00188G0006</td><td>environmental samples</td><td>uncultured bacterium</td><td>MAG: hypothetical protein ACD_79C00188G0006 [uncultured bacterium].</td><td>GCA_000299275.1</td></tr>
<tr><td>EKM26807.1</td><td>HetE-N1→ TPR+GreAB-C+PIN*→</td><td>TPR+GreAB-C+PIN</td><td>1091</td><td>VCHENC03_4410</td><td>Gammaproteobacteria</td><td>Vibrio sp. HENC-03</td><td>hypothetical protein VCHENC03_4410 [Vibrio sp. HENC-03].</td><td>GCA_000305755.2</td></tr>
<tr><td>ENE18425.1</td><td>GreAB-C*→</td><td>GreAB-C</td><td>334</td><td>ECP03047993_5652</td><td>Gammaproteobacteria</td><td>Escherichia coli P0304799.3</td><td>hypothetical protein ECP03047993_5652, partial [Escherichia coli P0304799.3].</td><td>GCA_000357685.2</td></tr>
<tr><td>ENV19736.1</td><td>HetE-N1→?→?→ GreAB-C+PIN*→</td><td>GreAB-C+PIN</td><td>553</td><td>F963_04374</td><td>Gammaproteobacteria</td><td>Acinetobacter bereziniae NIPH 3</td><td>hypothetical protein F963_04374 [Acinetobacter bereziniae NIPH 3].</td><td>GCA_000368505.1</td></tr>
<tr><td>EOS47428.1</td><td>HNH→?→?→ TPR+GreAB-C+PIN*→</td><td>TPR+GreAB-C+PIN</td><td>1071</td><td>C809_02304</td><td>Firmicutes</td><td>Lachnospiraceae bacterium MD335</td><td>hypothetical protein C809_02304 [Lachnospiraceae bacterium MD335].</td><td>GCA_000403335.2</td></tr>
<tr><td>ERS81691.1</td><td>TPR+GreAB-C+PIN*→</td><td>TPR+GreAB-C+PIN</td><td>480</td><td>Q672_10295</td><td>Gammaproteobacteria</td><td>Marinobacter sp. EVN1</td><td>hypothetical protein Q672_10295 [Marinobacter sp. EVN1].</td><td>GCA_000475375.1</td></tr>
<tr><td>ERU60319.1</td><td>DOC+HTH→ REase+TOPC→?→ HetE-N1→ HetE-N1+TPR+GreAB-C+PIN*→</td><td>HetE-N1+TPR+GreAB-C+PIN</td><td>1126</td><td>Q088_04776</td><td>Gammaproteobacteria</td><td>Pseudomonas aeruginosa C41</td><td>hypothetical protein Q088_04776 [Pseudomonas aeruginosa C41].</td><td>GCA_000480455.1</td></tr>
<tr><td>ETK41841.1</td><td>REase+TPR+GreAB-C+PIN*→</td><td>REase+TPR+GreAB-C+PIN</td><td>1486</td><td>H098_09645</td><td>Gammaproteobacteria</td><td>Pseudomonas fluorescens FH5</td><td>hypothetical protein H098_09645 [Pseudomonas fluorescens FH5].</td><td>GCA_000511155.2</td></tr>
<tr><td>EWS54147.1</td><td>REase+TPR→ TPR+GreAB-C+PIN*→</td><td>TPR+GreAB-C+PIN</td><td>1054</td><td>X551_03051</td><td>Betaproteobacteria</td><td>Methylibium sp. T29</td><td>hypothetical protein X551_03051 [Methylibium sp. T29].</td><td>GCA_000576205.1</td></tr>
<tr><td>EXI84113.1</td><td>REase+TPR+GreAB-C+PIN*→</td><td>REase+TPR+GreAB-C+PIN</td><td>1363</td><td>AW11_03978</td><td>Betaproteobacteria</td><td>Candidatus Accumulibacter sp. BA-93</td><td>MAG: hypothetical protein AW11_03978 [Candidatus Accumulibacter sp. BA-93].</td><td>GCA_000585075.1</td></tr>
<tr><td>EXU80592.1</td><td>REase+TPR+GreAB-C+PIN*→</td><td>REase+TPR+GreAB-C+PIN</td><td>1391</td><td>AX13_15135</td><td>Betaproteobacteria</td><td>Comamonas aquatica DA1877</td><td>hypothetical protein AX13_15135 [Comamonas aquatica DA1877].</td><td>GCA_000590985.1</td></tr>
<tr><td>GAC55419.1</td><td>TPR+PIN*→</td><td>TPR+PIN</td><td>1183</td><td>GOAMI_53_00020</td><td>Actinobacteria</td><td>Gordonia amicalis NBRC 100051 = JCM 11271</td><td>hypothetical protein GOAMI_53_00020 [Gordonia amicalis NBRC 100051 = JCM 11271].</td><td>GCA_000332995.1</td></tr>
<tr><td>GAF05793.1</td><td>TPR+GreAB-C+PIN*→</td><td>TPR+GreAB-C+PIN</td><td>524</td><td>JCM21142_104546</td><td>Bacteroidetes</td><td>Saccharicrinis fermentans DSM 9555 = JCM 21142</td><td>hypothetical protein JCM21142_104546 [Saccharicrinis fermentans DSM 9555 = JCM 21142].</td><td>GCA_000583675.1</td></tr>
<tr><td>GBF59066.1</td><td>REase+TPR+GreAB-C+PIN*→</td><td>REase+TPR+GreAB-C+PIN</td><td>1327</td><td>bepA_7</td><td>Alphaproteobacteria</td><td>Candidatus Phycosocius bacilliformis</td><td>beta-barrel assembly-enhancing protease [Candidatus Phycosocius bacilliformis].</td><td>GCA_003112735.1</td></tr>
<tr><td>GBH08795.1</td><td>HetE-N1→||||<-?||HetE-N1+TPR+GreAB-C+PIN→||||<-?<-?||TPR+GreAB-C+PIN*→</td><td>TPR+GreAB-C+PIN</td><td>953</td><td>KPSA1_02176</td><td>Gammaproteobacteria</td><td>Pseudomonas syringae pv. actinidiae</td><td>ATP-dependent exoDNAse beta subunit [Pseudomonas syringae pv. actinidiae].</td><td>GCA_003202975.1</td></tr>
<tr><td>GBH17945.1</td><td>REase+TPR+GreAB-C+PIN*→?→ REase+nSTAND3→ REase+nSTAND3→</td><td>REase+TPR+GreAB-C+PIN</td><td>1491</td><td>KPSA3_03922</td><td>Gammaproteobacteria</td><td>Pseudomonas syringae pv. actinidiae</td><td>Tetratricopeptide repeat [Pseudomonas syringae pv. actinidiae].</td><td>GCA_003202995.1</td></tr>
<tr><td>GBU16512.1</td><td>TPR+GreAB-C+PIN*→</td><td>TPR+GreAB-C+PIN</td><td>1107</td><td>AwMethylo_07270</td><td>Alphaproteobacteria</td><td>Methylobacterium sp.</td><td>hypothetical protein AwMethylo_07270 [Methylobacterium sp.].</td><td>GCA_003864615.1</td></tr>
<tr><td>GCL65539.1</td><td>REase+TPR+GreAB-C+PIN*→</td><td>REase+TPR+GreAB-C+PIN</td><td>1455</td><td>AQPW35_46200</td><td>Betaproteobacteria</td><td>Aquabacterium pictum</td><td>hypothetical protein AQPW35_46200 [Aquabacterium pictum].</td><td>GCA_005403045.1</td></tr>
<tr><td>GCM29814.1</td><td>TPR+GreAB-C+PIN*→</td><td>TPR+GreAB-C+PIN</td><td>622</td><td>ExPECSC050_01644</td><td>Gammaproteobacteria</td><td>Escherichia coli</td><td>hypothetical protein ExPECSC050_01644 [Escherichia coli].</td><td>GCA_005383045.1</td></tr>
<tr><td>GFJ77328.1</td><td>TM+TPR+PIN*→ REase→</td><td>TM+TPR+PIN</td><td>1362</td><td>Phou_015080</td><td>Actinobacteria</td><td>Phytohabitans houttuyneae</td><td>hypothetical protein Phou_015080 [Phytohabitans houttuyneae].</td><td>GCA_011764425.1</td></tr>
<tr><td>GGJ71040.1</td><td>TPR+PIN*→</td><td>TPR+PIN</td><td>1223</td><td>GCM10012282_79870</td><td>Actinobacteria</td><td>Streptomyces lacrimifluminis</td><td>hypothetical protein GCM10012282_79870 [Streptomyces lacrimifluminis].</td><td>GCA_014646095.1</td></tr>
<tr><td>GGK48292.1</td><td>HetE-N1→ TPR+GreAB-C+PIN*→</td><td>TPR+GreAB-C+PIN</td><td>1120</td><td>GCM10009103_48460</td><td>Gammaproteobacteria</td><td>Pseudomonas koreensis</td><td>hypothetical protein GCM10009103_48460 [Pseudomonas koreensis].</td><td>GCA_014646955.1</td></tr>
<tr><td>GGR17482.1</td><td>REase+TPR+GreAB-C+PIN*→</td><td>REase+TPR+GreAB-C+PIN</td><td>1357</td><td>GCM10008957_32760</td><td>Deinococci</td><td>Deinococcus ruber</td><td>hypothetical protein GCM10008957_32760 [Deinococcus ruber].</td><td>GCA_014648095.1</td></tr>
<tr><td>GGZ02836.1</td><td>HetE-N1→?→ GreAB-C*→</td><td>GreAB-C</td><td>451</td><td>GCM10007169_19550</td><td>Gammaproteobacteria</td><td>Shewanella fodinae</td><td>hypothetical protein GCM10007169_19550 [Shewanella fodinae].</td><td>GCA_014651955.1</td></tr>
<tr><td>GHB08256.1</td><td>HetE-N1→ TPR+GreAB-C+PIN*→</td><td>TPR+GreAB-C+PIN</td><td>1025</td><td>GCM10007107_21780</td><td>Gammaproteobacteria</td><td>Shewanella indica</td><td>hypothetical protein GCM10007107_21780 [Shewanella indica].</td><td>GCA_014652115.1</td></tr>
</table>

acc	operon	architecture	len	gen.name	taxend	species	defline	gca
GIE51569.1	TPR+PIN→?→?→ PIN*→||||<-?<-?||AbiJ-NTD3+REase→	PIN	177	Ani05nite_51030	Actinobacteria	Actinoplanes nipponensis	hypothetical protein Ani05nite_51030 [Actinoplanes nipponensis].	GCA_016862275.1
GIG67348.1	TM+TPR+PIN*→	TM+TPR+PIN	1072	Pen01_36430	Actinobacteria	Phytomonospora endophytica	hypothetical protein Pen01_36430 [Phytomonospora endophytica].	GCA_016862855.1
GIJ10564.1	REase+TPR+GreAB-C+PIN*→	REase+TPR+GreAB-C+PIN	1377	Van01_37780	Actinobacteria	Micromonospora andamanensis	hypothetical protein Van01_37780 [Micromonospora andamanensis].	GCA_016863495.1
GIM80122.1	REase+TPR+GreAB-C+PIN*→	REase+TPR+GreAB-C+PIN	1253	Aau02nite_89080	Actinobacteria	Actinoplanes auranticolor	hypothetical protein Aau02nite_89080 [Actinoplanes auranticolor].	GCA_018332655.1
GIM97804.1	TPR+PIN*→	TPR+PIN	1137	Ato02nite_095970	Actinobacteria	Actinoplanes toevensis	hypothetical protein Ato02nite_095970 [Actinoplanes toevensis].	GCA_018332695.1
HAF5476147.1	HetE-N1→ TPR+GreAB-C+PIN*→	TPR+GreAB-C+PIN	1117	G8A91_001303	Gammaproteobacteria	Salmonella enterica	TPA: hypothetical protein G8A91_001303 [Salmonella enterica].	GCA_011304215.1
HAH9826030.1	HetE-N1→ TPR+GreAB-C+PIN*→	TPR+GreAB-C+PIN	986	HIF24_22945	Gammaproteobacteria	Escherichia coli	TPA: hypothetical protein HIF24_22945, partial [Escherichia coli].	GCA_012753875.1
HAK4911912.1	HetE-N1→ TPR+GreAB-C+PIN*→	TPR+GreAB-C+PIN	1117	H2B38_003821	Gammaproteobacteria	Salmonella enterica	TPA: hypothetical protein H2B38_003821 [Salmonella enterica].	GCA_013572415.1
HAK9873068.1	TPR+GreAB-C+PIN*→	TPR+GreAB-C+PIN	1028	H3L74_004566	Gammaproteobacteria	Escherichia coli	TPA: hypothetical protein H3L74_004566, partial [Escherichia coli].	GCA_013794255.1
HAL9130497.1	HetE-N1→ TPR+GreAB-C+PIN*→	TPR+GreAB-C+PIN	1094	H7U58_003240	Gammaproteobacteria	Escherichia coli	TPA: hypothetical protein H7U58_003240 [Escherichia coli].	GCA_014184975.1
HAL9453557.1	GreAB-C*→	GreAB-C	337	H7V29_005028	Gammaproteobacteria	Escherichia coli	TPA: hypothetical protein H7V29_005028, partial [Escherichia coli].	GCA_014186535.1
HAM4053647.1	HetE-N1→ TPR+GreAB-C+PIN*→	TPR+GreAB-C+PIN	1094	IBV36_002404	Gammaproteobacteria	Escherichia coli	TPA: hypothetical protein IBV36_002404 [Escherichia coli].	GCA_014461175.1
HAM8943229.1	HetE-N1→ TPR+GreAB-C+PIN*→	TPR+GreAB-C+PIN	618	IDX96_004717	Gammaproteobacteria	Escherichia coli	TPA: hypothetical protein IDX96_004717, partial [Escherichia coli].	GCA_014571125.1
HAM8943373.1	TPR+GreAB-C+PIN*→	TPR+GreAB-C+PIN	474	IDX96_004882	Gammaproteobacteria	Escherichia coli	TPA: hypothetical protein IDX96_004882, partial [Escherichia coli].	GCA_014571125.1
HAM8966916.1	HetE-N1→ TPR+GreAB-C+PIN*→	TPR+GreAB-C+PIN	1094	IDX85_003830	Gammaproteobacteria	Escherichia coli	TPA: hypothetical protein IDX85_003830 [Escherichia coli].	GCA_014573095.1
HAM8991159.1	HetE-N1→ TPR+GreAB-C+PIN*→	TPR+GreAB-C+PIN	1094	IDX80_003853	Gammaproteobacteria	Escherichia coli	TPA: hypothetical protein IDX80_003853 [Escherichia coli].	GCA_014573135.1
HAN3697638.1	TPR+GreAB-C+PIN*→	TPR+GreAB-C+PIN	1050	IE973_004718	Gammaproteobacteria	Escherichia coli	TPA: hypothetical protein IE973_004718, partial [Escherichia coli].	GCA_014687295.1
HAN4834938.1	HetE-N1→ TPR+GreAB-C+PIN*→	TPR+GreAB-C+PIN	1094	IFD90_003495	Gammaproteobacteria	Escherichia coli	TPA: hypothetical protein IFD90_003495 [Escherichia coli].	GCA_014683685.1
HAN6118733.1	HetE-N1→ TPR+GreAB-C+PIN*→	TPR+GreAB-C+PIN	1094	IFZ61_002838	Gammaproteobacteria	Escherichia coli	TPA: hypothetical protein IFZ61_002838 [Escherichia coli].	GCA_014708655.1
HAN7634134.1	GreAB-C*→	GreAB-C	447	IF647_005249	Gammaproteobacteria	Escherichia coli	TPA: hypothetical protein IF647_005249, partial [Escherichia coli].	GCA_014724455.1
HAO1938999.1	HetE-N1→ TPR+GreAB-C+PIN*→	TPR+GreAB-C+PIN	1094	IHJ42_000411	Gammaproteobacteria	Escherichia coli	TPA: hypothetical protein IHJ42_000411 [Escherichia coli].	GCA_014815885.1
HAS1413650.1	TPR+GreAB-C+PIN*→	TPR+GreAB-C+PIN	845	I4D28_09180	Gammaproteobacteria	Enterobacter hormaechei	TPA: hypothetical protein I4D28_09180 [Enterobacter hormaechei].	-
HAT2286137.1	HetE-N1→ TPR+GreAB-C+PIN+TM+TM*→	TPR+GreAB-C+PIN+TM+TM	1106	I8156_002891	Gammaproteobacteria	Citrobacter freundii	TPA: hypothetical protein I8156_002891 [Citrobacter freundii].	GCA_015897485.1
HAT3687084.1	HetE-N1→ TPR+GreAB-C+PIN*→	TPR+GreAB-C+PIN	958	I8574_002665	Gammaproteobacteria	Citrobacter freundii	TPA: hypothetical protein I8574_002665, partial [Citrobacter freundii].	GCA_015902705.1
HAT3994325.1	TPR+GreAB-C+PIN*→	TPR+GreAB-C+PIN	1131	I9Y74_003743	Bacteroidetes	Elizabethkingia anophelis	TPA: hypothetical protein I9Y74_003743 [Elizabethkingia anophelis].	GCA_015930145.1
HAT8488811.1	HetE-N1→ HetE-N1+TPR+GreAB-C+PIN*→	HetE-N1+TPR+GreAB-C+PIN	1088	I7187_21710	Gammaproteobacteria	Vibrio vulnificus	TPA: hypothetical protein I7187_21710 [Vibrio vulnificus].	GCA_015968635.1

acc	operon	architecture	len	gen.name	taxend	species	defline	gca
HAU8300026.1	HetE-N1→ TPR+GreAB-C+PIN*→	TPR+GreAB-C+PIN	1130	I7159_21955	Gammaproteobacteria	Vibrio vulnificus	TPA: hypothetical protein I7159_21955 [Vibrio vulnificus].	GCA_016110185.1
HAU9261412.1	HetE-N1→ TPR+GreAB-C+PIN*→	TPR+GreAB-C+PIN	1094	JEX66_004253	Gammaproteobacteria	Escherichia coli	TPA: hypothetical protein JEX66_004253 [Escherichia coli].	GCA_016116905.1
HAV13598.1	GreAB*→	GreAB	620	DCX06_08945	Verrucomicrobia	Opitutae bacterium	MAG TPA: transcription elongation factor GreAB [Opitutae bacterium].	GCA_003485085.1
HAV1378302.1	HetE-N1→ NACHT+GreAB-C+PIN*→	NACHT+GreAB-C+PIN	1085	JG804_003376	Gammaproteobacteria	Vibrio parahaemolyticus	TPA: hypothetical protein JG804_003376 [Vibrio parahaemolyticus].	GCA_016429865.1
HAV1873259.1	TPR+GreAB-C+PIN*→	TPR+GreAB-C+PIN	742	JG951_004564	Gammaproteobacteria	Enterobacter hormaechei subsp. steigerwaltii	TPA: hypothetical protein JG951_004564, partial [Enterobacter hormaechei subsp. steigerwaltii].	GCA_016431145.1
HAV7695504.1	HetE-N1→ TPR+GreAB-C+PIN*→	TPR+GreAB-C+PIN	1094	JK751_001166	Gammaproteobacteria	Escherichia coli	TPA: hypothetical protein JK751_001166 [Escherichia coli].	GCA_016656125.1
HAV9376548.1	TPR+GreAB-C+PIN*→	TPR+GreAB-C+PIN	575	JLF84_000973	Gammaproteobacteria	Escherichia coli	TPA: hypothetical protein JLF84_000973 [Escherichia coli].	GCA_016665225.1
HAW2926691.1	HetE-N1→ TPR+GreAB-C+PIN*→	TPR+GreAB-C+PIN	1094	JLR28_003506	Gammaproteobacteria	Escherichia coli	TPA: hypothetical protein JLR28_003506 [Escherichia coli].	GCA_016681635.1
HAW5283885.1	HetE-N1→ TPR+GreAB-C+PIN*→	TPR+GreAB-C+PIN	1094	JLY51_001282	Gammaproteobacteria	Escherichia coli	TPA: hypothetical protein JLY51_001282 [Escherichia coli].	GCA_016693495.1
HAW6997627.1	REase+TPR+GreAB-C+PIN*→	REase+TPR+GreAB-C+PIN	1250	JIR16_12805	Gammaproteobacteria	Acinetobacter baumannii	TPA: hypothetical protein JIR16_12805 [Acinetobacter baumannii].	GCA_016844125.1
HAY34439.1	TPR+GreAB-C+PIN*→	TPR+GreAB-C+PIN	1108	DCY06_09910	Bacteroidetes	Bacteroidetes bacterium	MAG TPA: hypothetical protein DCY06_09910 [Bacteroidetes bacterium].	GCA_003486905.1
HBA3776861.1	TPR+GreAB-C+PIN*→	TPR+GreAB-C+PIN	1117	J5F89_004850	Gammaproteobacteria	Escherichia coli	TPA: hypothetical protein J5F89_004850 [Escherichia coli].	GCA_017730375.1
HBC0128974.1	TPR+GreAB-C+PIN*→	TPR+GreAB-C+PIN	661	JES91_004961	Gammaproteobacteria	Salmonella enterica subsp. enterica	TPA: hypothetical protein JES91_004961, partial [Salmonella enterica subsp. enterica].	GCA_018032925.1
HBC3987585.1	HetE-N1→ TPR+GreAB-C+PIN*→	TPR+GreAB-C+PIN	1130	KD867_004179	Gammaproteobacteria	Vibrio parahaemolyticus	TPA: hypothetical protein KD867_004179 [Vibrio parahaemolyticus].	GCA_018126825.1
HBJ33554.1	TPR+GreAB-C+PIN*→	TPR+GreAB-C+PIN	857	DDZ51_02100	Planctomycetes	Planctomycetaceae bacterium	MAG TPA: hypothetical protein DDZ51_02100, partial [Planctomycetaceae bacterium].	GCA_003506095.1
HBX77020.1	X+PIN*→	X+PIN	940	DEG43_05200	Actinobacteria	Acidimicrobiaceae bacterium	MAG TPA: hypothetical protein DEG43_05200 [Acidimicrobiaceae bacterium].	GCA_003514205.1
HCD05045.1	HetE-N1→?→ TPR+GreAB-C+PIN*→||||<-REase+UvrD	TPR+GreAB-C+PIN	841	DEQ60_06940	Gammaproteobacteria	Methylophaga sp.	MAG TPA: hypothetical protein DEQ60_06940, partial [Methylophaga sp.].	GCA_003517125.1
HCG03150.1	REase+TPR+GreAB-C+PIN*→	REase+TPR+GreAB-C+PIN	1325	DEV93_21745	Chloroflexi	Chloroflexi bacterium	MAG TPA: hypothetical protein DEV93_21745 [Chloroflexi bacterium].	GCA_003530725.1
HCN10799.1	HTH→?→?→?→?→?→ TPR+GreAB-C+PIN*→	TPR+GreAB-C+PIN	1128	DIS75_00410	Bacteroidetes	Chryseobacterium sp.	MAG TPA: hypothetical protein DIS75_00410 [Chryseobacterium sp.].	GCA_003536155.1
HEE61010.1	GreAB*→	GreAB	217	ENP71_13075	Chloroflexi	Chloroflexi bacterium	MAG TPA: transcription elongation factor GreA [Chloroflexi bacterium].	GCA_011051445.1
HEN27530.1	PIN*→	PIN	194	ENQ77_02470	Bacteria	candidate division WOR-3 bacterium	MAG TPA: RNA ligase partner protein [candidate division WOR-3 bacterium].	GCA_011046955.1
HFX16357.1	REase+TPR+GreAB-C+PIN*→	REase+TPR+GreAB-C+PIN	1269	ENW10_10035	unclassified Bacteria	bacterium	MAG TPA: hypothetical protein ENW10_10035 [bacterium].	GCA_011334465.1
HGB35656.1	PIN*→	PIN	194	ENV38_01955	Bacteria	candidate division WOR-3 bacterium	MAG TPA: RNA ligase partner protein [candidate division WOR-3 bacterium].	GCA_011338235.1
HGZ37950.1	Calcineurin+TPR+GreAB-C+PIN*→	Calcineurin+TPR+GreAB-C+PIN	1423	ENR19_05950	Nitrospirae	Nitrospirae bacterium	MAG TPA: hypothetical protein ENR19_05950, partial [Nitrospirae bacterium].	GCA_011331925.1
HHC78382.1	TPR+GreAB-C+PIN*→	TPR+GreAB-C+PIN	1134	ENK46_00760	Bacteroidetes	Flavobacteriia bacterium	MAG TPA: hypothetical protein ENK46_00760 [Flavobacteriia bacterium].	GCA_011330545.1
HIB63801.1	TPR+GreAB-C+PIN*→	TPR+GreAB-C+PIN	1486	EYO33_01435	Planctomycetes	Phycisphaerales bacterium	MAG TPA: hypothetical protein EYO33_01435 [Phycisphaerales bacterium].	GCA_012964585.1

acc	operon	architecture	len	gen.name	taxend	species	defline	gca
HIP26815.1	TPR+GreAB-C+PIN*→	TPR+GreAB-C+PIN	1038	EYG80_04080	Bacteroidetes	Flavobacteriaceae bacterium	MAG TPA: tetratricopeptide repeat protein [Flavobacteriaceae bacterium].	GCA_015662995.1
KAA0086698.1	TPR+PIN*→	TPR+PIN	1159	CIW52_01945	Actinobacteria	Mycolicibacterium sp. P9-64	hypothetical protein CIW52_01945 [Mycolicibacterium sp. P9-64].	GCA_008329605.1
KAB1070169.1	TPR+GreAB-C+PIN*→	TPR+GreAB-C+PIN	641	F6X53_30340	Alphaproteobacteria	Methylobacterium soli	tetratricopeptide repeat protein [Methylobacterium soli].	GCA_008806385.1
KCZ93824.1	TPR+GreAB-C+PIN*→	TPR+GreAB-C+PIN	1142	HJO_00570	Alphaproteobacteria	Hyphomonas johnsonii MHS-2	hypothetical protein HJO_00570 [Hyphomonas johnsonii MHS-2].	GCA_000685275.1
KGF68042.1	REase+TPR+GreAB-C+PIN*→	REase+TPR+GreAB-C+PIN	1283	LL06_18825	Alphaproteobacteria	Hoeflea sp. BAL378	hypothetical protein LL06_18825 [Hoeflea sp. BAL378].	GCA_000759435.1
KGM47444.1	REase+TPR+GreAB-C+PIN*→	REase+TPR+GreAB-C+PIN	1329	ATO9_17580	Alphaproteobacteria	Pseudooceanicola atlanticus	hypothetical protein ATO9_17580 [Pseudooceanicola atlanticus].	GCA_000768315.1
KGS12485.1	GreAB-C+PIN*→	GreAB-C+PIN	509	OA77_21590	Gammaproteobacteria	Pseudomonas coronafaciens	hypothetical protein OA77_21590, partial [Pseudomonas coronafaciens].	GCA_000773135.1
KHO23731.1	X+PIN*→	X+PIN	762	QQ44_16085	Actinobacteria	Mycolicibacterium setense	hypothetical protein QQ44_16085 [Mycolicibacterium setense].	GCA_000805375.1
KIF65994.1	REase+TPR+TPR+GreAB-C+PIN*→	REase+TPR+TPR+GreAB-C+PIN	1274	HY68_38205	Actinobacteria	Streptomyces sp. AcH 505	hypothetical protein HY68_38205 [Streptomyces sp. AcH 505].	GCA_000818175.1
KIU53353.1	REase+TPR+TPR+GreAB-C+PIN*→	REase+TPR+TPR+GreAB-C+PIN	1254	QU41_01355	Alphaproteobacteria	Bradyrhizobium elkanii	hypothetical protein QU41_01355 [Bradyrhizobium elkanii].	GCA_000878305.1
KJS28984.1	TPR+GreAB-C+PIN*→	TPR+GreAB-C+PIN	1318	VR64_22320	Deltaproteobacteria	Desulfatitalea sp. BRH_c12	MAG: hypothetical protein VR64_22320 [Desulfatitalea sp. BRH_c12].	GCA_000961655.1
KOY46643.1	HetE-N1→ TPR+GreAB-C+PIN*→	TPR+GreAB-C+PIN	1085	ACX03_05530	Gammaproteobacteria	Vibrio parahaemolyticus	hypothetical protein ACX03_05530 [Vibrio parahaemolyticus].	GCA_001280625.1
KPC32725.1	PIN*→	PIN	407	ABJ99_1577	Gammaproteobacteria	Pseudomonas syringae pv. cilantro	Tetratricopeptide repeat protein [Pseudomonas syringae pv. cilantro].	GCA_001293775.1
KPH04186.1	GreAB-C+PIN*→	GreAB-C+PIN	532	AOG23_34735	Alphaproteobacteria	Rhizobium acidisoli	hypothetical protein AOG23_34735 [Rhizobium acidisoli].	GCA_001297245.1
KPW31206.1	REase+TPR+GreAB-C+PIN*→	REase+TPR+GreAB-C+PIN	1491	ALO87_03437	Gammaproteobacteria	Pseudomonas syringae pv. apii	Uncharacterized protein ALO87_03437 [Pseudomonas syringae pv. apii].	GCA_001401385.1
KPW71831.1	PIN*→	PIN	419	ALO76_102575	Gammaproteobacteria	Pseudomonas syringae pv. coriandricola	hypothetical protein ALO76_102575 [Pseudomonas syringae pv. coriandricola].	GCA_001400185.1
KPX07181.1	REase→ TPR+GreAB-C+PIN*→	TPR+GreAB-C+PIN	882	ALO74_05417	Gammaproteobacteria	Pseudomonas syringae pv. cunninghamiae	Uncharacterized protein ALO74_05417, partial [Pseudomonas syringae pv. cunninghamiae].	GCA_001400795.1
KQR06792.1	TPR+PIN*→	TPR+PIN	1157	ASF74_05425	Actinobacteria	Arthrobacter sp. Leaf145	hypothetical protein ASF74_05425 [Arthrobacter sp. Leaf145].	GCA_001423565.1
KQY47975.1	HTH+PNPase+TPR+GreAB-C+PIN*→	HTH+PNPase+TPR+GreAB-C+PIN	1492	ASD18_12170	Actinobacteria	Cellulomonas sp. Root137	hypothetical protein ASD18_12170 [Cellulomonas sp. Root137].	GCA_001426725.1
KRK09263.1	X+PIN*→	X+PIN	1209	FD11_GL001067	Firmicutes	Lactobacillus pobuzihii E100301 = KCTC 13174	hypothetical protein FD11_GL001067 [Lactobacillus pobuzihii E100301 = KCTC 13174].	GCA_001433815.1
KSV64106.1	REase+TPR+TPR+GreAB-C+PIN*→	REase+TPR+TPR+GreAB-C+PIN	1352	N182_36590	Alphaproteobacteria	Sinorhizobium sp. GL2	hypothetical protein N182_36590 [Sinorhizobium sp. GL2].	GCA_001461715.1
KTR52035.1	PIN*→	PIN	473	NS359_08065	Actinobacteria	Curtobacterium oceanosedimentum	hypothetical protein NS359_08065 [Curtobacterium oceanosedimentum].	GCA_001476135.1
KVD10066.1	REase+TPR+GreAB-C+PIN*→	REase+TPR+GreAB-C+PIN	1276	WI81_28275	Betaproteobacteria	Burkholderia ubonensis	hypothetical protein WI81_28275 [Burkholderia ubonensis].	GCA_001523425.1
KVK86408.1	REase+TPR+GreAB-C+PIN*→	REase+TPR+GreAB-C+PIN	1305	WJ44_35230	Betaproteobacteria	Burkholderia ubonensis	hypothetical protein WJ44_35230 [Burkholderia ubonensis].	GCA_001526225.1
KVP54755.1	PIN*→	PIN	270	WJ90_06090	Betaproteobacteria	Burkholderia ubonensis	hypothetical protein WJ90_06090 [Burkholderia ubonensis].	GCA_001527145.1
KVR94766.1	REase+TPR+GreAB-C+PIN*→	REase+TPR+GreAB-C+PIN	1283	WK29_06535	Betaproteobacteria	Burkholderia vietnamiensis	hypothetical protein WK29_06535 [Burkholderia vietnamiensis].	GCA_001528565.1

acc	operon	architecture	len	gen.name	taxend	species	defline	gca
KVS41438.1	REase+TPR+GreAB-C+PIN*→	REase+TPR+GreAB-C+PIN	1273	WK37_19925	Betaproteobacteria	Burkholderia ubonensis	hypothetical protein WK37_19925 [Burkholderia ubonensis].	GCA_001529725.1
KWO74502.1	REase+TPR+GreAB-C+PIN*→	REase+TPR+GreAB-C+PIN	1308	WM31_08005	Betaproteobacteria	Burkholderia ubonensis	hypothetical protein WM31_08005 [Burkholderia ubonensis].	GCA_001534425.1
KWW10220.1	HetE-N1→ TPR+GreAB-C+PIN→ TPR+GreAB-C+PIN*→	TPR+GreAB-C+PIN	695	VM82_06380	Gammaproteobacteria	Pasteurella multocida	hypothetical protein VM82_06380 [Pasteurella multocida].	GCA_001542975.1
KXC04457.1	X+PIN*→?→ Calcineurin→	X+PIN	1058	MhomT_16215	Actinobacteria	Microbacterium hominis	hypothetical protein MhomT_16215 [Microbacterium hominis].	GCA_001553805.1
KXF54879.1	HTH+PNPase+TPR+GreAB-C+PIN*→	HTH+PNPase+TPR+GreAB-C+PIN	1496	AXA44_39745	Actinobacteria	Rhodococcus sp. SC4	hypothetical protein AXA44_39745 [Rhodococcus sp. SC4].	GCA_001555475.1
KYK33744.1	TPR+GreAB-C+PIN*→	TPR+GreAB-C+PIN	1243	AYK18_14685	Euryarchaeota	Theionarchaea archaeon DG-70	MAG: hypothetical protein AYK18_14685 [Theionarchaea archaeon DG-70].	GCA_001595795.1
KZM34260.1	X+PIN*→	X+PIN	802	OJAG_30920	Actinobacteria	Oerskovia enterophila	hypothetical protein OJAG_30920 [Oerskovia enterophila].	GCA_001624335.1
KZM57325.1	TPR+PIN*→	TPR+PIN	1172	A3Q36_16940	Firmicutes	Geobacillus stearothermophilus	hypothetical protein A3Q36_16940 [Geobacillus stearothermophilus].	GCA_001624615.1
MAK67575.1	HetE-N1→ TPR+GreAB-C+PIN*→||||<-REase+UvrD	TPR+GreAB-C+PIN	1111	CMH19_11360	Gammaproteobacteria	Methylophaga sp.	MAG: hypothetical protein CMH19_11360 [Methylophaga sp.].	GCA_002692615.1
MAK82682.1	REase+TPR+GreAB-C+PIN*→	REase+TPR+GreAB-C+PIN	1322	CMJ17_12550	Alphaproteobacteria	Phenylobacterium sp.	MAG: hypothetical protein CMJ17_12550 [Phenylobacterium sp.].	GCA_002693985.1
MAM61427.1	REase+TOPC→||||<-?||REase+TPR+GreAB-C+PIN*→	REase+TPR+GreAB-C+PIN	1313	CMH11_08050	Alphaproteobacteria	Maritimibacter sp.	MAG: hypothetical protein CMH11_08050 [Maritimibacter sp.].	GCA_002695005.1
MAQ47157.1	TPR+GreAB-C+PIN*→	TPR+GreAB-C+PIN	928	CL812_15115	Alphaproteobacteria	Actibacterium sp.	MAG: hypothetical protein CL812_15115 [Actibacterium sp.].	GCA_002701285.1
MAX35781.1	REase+TPR+GreAB-C+PIN*→	REase+TPR+GreAB-C+PIN	1254	CME33_04335	Planctomycetes	Gimesia sp.	MAG: hypothetical protein CME33_04335 [Gimesia sp.].	GCA_002706765.1
MAX78311.1	REase+TPR+GreAB-C+PIN*→	REase+TPR+GreAB-C+PIN	1318	CMN12_16605	Alphaproteobacteria	Roseobacter sp.	MAG: hypothetical protein CMN12_16605 [Roseobacter sp.].	GCA_002706785.1
MBA2669970.1	TPR+GreAB-C+PIN*→	TPR+GreAB-C+PIN	581	H0U67_06335	Gemmatimonadetes	Gemmatimonadetes bacterium	MAG: hypothetical protein H0U67_06335 [Gemmatimonadetes bacterium].	GCA_013697765.1
MBA3005285.1	TPR+GreAB-C+PIN*→	TPR+GreAB-C+PIN	1285	FP810_01590	Deltaproteobacteria	Desulfocapsa sp.	MAG: hypothetical protein FP810_01590 [Desulfocapsa sp.].	GCA_013792135.1
MBA3178888.1	HetE-N1→ TPR+GreAB-C+PIN*→?→||||<-Trypsin	TPR+GreAB-C+PIN	1121	CBX59_021210	Gammaproteobacteria	Salmonella enterica	hypothetical protein CBX59_021210 [Salmonella enterica].	GCA_006112745.2
MBA3341438.1	REase+TPR+GreAB-C+PIN*→	REase+TPR+GreAB-C+PIN	1397	H0T48_06340	Gemmatimonadetes	Gemmatimonadaceae bacterium	MAG: hypothetical protein H0T48_06340 [Gemmatimonadaceae bacterium].	GCA_013813715.1
MBA3557151.1	TPR+GreAB-C+PIN*→	TPR+GreAB-C+PIN	877	H0W30_00995	Gemmatimonadetes	Gemmatimonadaceae bacterium	MAG: hypothetical protein H0W30_00995 [Gemmatimonadaceae bacterium].	GCA_013817545.1
MBA3619565.1	REase→ PIN*→	PIN	572	H0W56_08245	Actinobacteria	Acidothermales bacterium	MAG: hypothetical protein H0W56_08245, partial [Acidothermales bacterium].	GCA_013817005.1
MBA3973802.1	TPR+GreAB-C+PIN*→	TPR+GreAB-C+PIN	1263	C0504_06240	Acidobacteria	Candidatus Solibacter sp.	MAG: hypothetical protein C0504_06240 [Candidatus Solibacter sp.].	GCA_013822245.1
MBA4090929.1	REase+TPR+GreAB-C+PIN*→	REase+TPR+GreAB-C+PIN	1315	C0494_10085	Alphaproteobacteria	Sphingobium sp.	MAG: hypothetical protein C0494_10085 [Sphingobium sp.].	GCA_013822485.1
MBA4148880.1	TPR+PIN*→	TPR+PIN	1103	H0X66_12260	Verrucomicrobia	Verrucomicrobia bacterium	MAG: hypothetical protein H0X66_12260 [Verrucomicrobia bacterium].	GCA_013820875.1
MBB1073428.1	REase+TPR+GreAB-C+PIN*→	REase+TPR+GreAB-C+PIN	1321	HUU62_03265	Betaproteobacteria	Rhodoferax jenense	DUF4365 domain-containing protein [Rhodoferax jenense].	GCA_014145235.1
MBB1121889.1	DOC+HTH→ REase+TOPC→ HetE-N1→?→ TPR+GreAB-C+PIN*→	TPR+GreAB-C+PIN	992	H4983_26725	Gammaproteobacteria	Pseudomonas aeruginosa	hypothetical protein H4983_26725 [Pseudomonas aeruginosa].	GCA_014145725.1
MBB4577222.1	REase+TPR+GreAB-C+PIN*→	REase+TPR+GreAB-C+PIN	1268	GGE58_005472	Alphaproteobacteria	Rhizobium lentis	tetratricopeptide (TPR) repeat protein [Rhizobium lentis].	GCA_014198785.1

acc	operon	architecture	len	gen.name	taxend	species	defline	gca
MBB5991665.1	REase→ TPR+GreAB-C+PIN*→?→?→ HNH→	TPR+GreAB-C+PIN	1242	HNP04_003690	Gammaproteobacteria	Pseudomonas viridiflava	tetratricopeptide (TPR) repeat protein/transcription elongation GreA/GreB family factor [Pseudomonas viridiflava].	GCA_014204055.1
MBB6509406.1	TPR+GreAB-C+PIN*→	TPR+GreAB-C+PIN	780	F4695_002763	Alphaproteobacteria	Rhizobium soli	putative nucleic acid-binding protein [Rhizobium soli].	GCA_014207075.1
MBB9310955.1	TPR+GreAB-C+PIN*→	TPR+GreAB-C+PIN	575	FSF17_011690	Gammaproteobacteria	Escherichia coli	hypothetical protein FSF17_011690 [Escherichia coli].	GCA_012385615.2
MBC6414865.1	REase+TPR+GreAB-C+PIN*→	REase+TPR+GreAB-C+PIN	1326	GDA46_00495	Oligoflexia	Bdellovibrionales bacterium	MAG: hypothetical protein GDA46_00495 [Bdellovibrionales bacterium].	GCA_014323935.1
MBC70602.1	REase+TPR+GreAB-C+PIN*→	REase+TPR+GreAB-C+PIN	1253	CL492_17300	Gammaproteobacteria	Acinetobacter sp.	MAG: hypothetical protein CL492_17300 [Acinetobacter sp.].	GCA_002711945.1
MBC7145404.1	REase+TPR+GreAB-C+PIN*→	REase+TPR+GreAB-C+PIN	1332	H5U24_08360	Alphaproteobacteria	Thioclava marina	MAG: hypothetical protein H5U24_08360 [Thioclava marina].	GCA_014359625.1
MBC7391182.1	TPR+GreAB-C+PIN*→	TPR+GreAB-C+PIN	1270	H7329_18405	Verrucomicrobia	Cytophagales bacterium	MAG: tetratricopeptide repeat protein [Opitutaceae bacterium].	GCA_014376535.1
MBC74230.1	TPR+GreAB-C+PIN*→	TPR+GreAB-C+PIN	1131	CMH47_18575	Bacteroidetes	Muricauda sp.	MAG: hypothetical protein CMH47_18575 [Muricauda sp.].	GCA_002711825.1
MBD0373736.1	TPR+GreAB-C*→	TPR+GreAB-C	885	ICV60_23090	Acidobacteria	Pyrinomonadaceae bacterium	MAG: tetratricopeptide repeat protein, partial [Pyrinomonadaceae bacterium].	GCA_014534425.1
MBD3293793.1	TPR+PIN*→	TPR+PIN	1505	GF393_12790	Armatimonadetes	Armatimonadia bacterium	MAG: hypothetical protein GF393_12790 [Armatimonadia bacterium].	GCA_014728405.1
MBD3811358.1	REase+TPR+GreAB-C+PIN*→	REase+TPR+GreAB-C+PIN	1374	IE917_03935	Betaproteobacteria	Betaproteobacteria bacterium	MAG: hypothetical protein IE917_03935 [Betaproteobacteria bacterium].	GCA_014764035.1
MBD8566197.1	TPR+GreAB-C+PIN*→	TPR+GreAB-C+PIN	1235	IFU01_18215	Betaproteobacteria	Oxalobacteraceae sp. CFBP 8763	hypothetical protein IFU01_18215 [Oxalobacteraceae sp. CFBP 8763].	GCA_014841525.1
MBD8722144.1	REase+TPR+GreAB-C+PIN*→	REase+TPR+GreAB-C+PIN	1511	IFT43_02050	Betaproteobacteria	Oxalobacteraceae sp. CFBP 13708	hypothetical protein IFT43_02050 [Oxalobacteraceae sp. CFBP 13708].	GCA_014842315.1
MBE2259083.1	REase+TPR+GreAB-C+PIN*→	REase+TPR+GreAB-C+PIN	1335	IAE88_09505	Alphaproteobacteria	Rhodobacteraceae bacterium	MAG: hypothetical protein IAE88_09505 [Rhodobacteraceae bacterium].	GCA_014879755.1
MBE3996728.1	HetE-N1→?→ TPR+GreAB-C+PIN*→	TPR+GreAB-C+PIN	910	HJ133_08285	Gammaproteobacteria	Vibrio parahaemolyticus	hypothetical protein HJ133_08285 [Vibrio parahaemolyticus].	GCA_014919735.1
MBE7465263.1	REase+TPR+GreAB-C+PIN*→	REase+TPR+GreAB-C+PIN	1253	HS116_17430	Planctomycetes	Planctomycetes bacterium	MAG: hypothetical protein HS116_17430 [Planctomycetes bacterium].	GCA_015075375.1
MBE8523541.1	TPR+PIN*→	TPR+PIN	1310	ILP97_39665	Actinobacteria	Amycolatopsis sp. H6(2020)	hypothetical protein ILP97_39665 [Amycolatopsis sp. H6(2020)].	GCA_015163035.1
MBE8960063.1	GreAB-C*→	GreAB-C	307	IH766_22715	Gammaproteobacteria	Escherichia coli	hypothetical protein IH766_22715, partial [Escherichia coli].	GCA_015206795.1
MBE9468467.1	TPR+GreAB-C+PIN*→	TPR+GreAB-C+PIN	1221	IMY72_09155	Bacteroidetes	Bacteroidetes bacterium	MAG: hypothetical protein IMY72_09155 [Bacteroidetes bacterium].	GCA_015222055.1
MBF0231519.1	TPR+GreAB-C+PIN*→	TPR+GreAB-C+PIN	1223	HQK65_00575	Deltaproteobacteria	Desulfamplus sp.	MAG: hypothetical protein HQK65_00575 [Desulfamplus sp.].	GCA_015231525.1
MBF0529118.1	TPR+GreAB-C+PIN*→	TPR+GreAB-C+PIN	678	HQK55_07585	Deltaproteobacteria	Deltaproteobacteria bacterium	MAG: hypothetical protein HQK55_07585 [Deltaproteobacteria bacterium].	GCA_015233515.1
MBF3699451.1	TPR+GreAB-C+PIN*→||||<-HTH+HTH	TPR+GreAB-C+PIN	872	ISF48_14710	Betaproteobacteria	Burkholderia pseudomallei	hypothetical protein ISF48_14710 [Burkholderia pseudomallei].	GCA_015319855.1
MBF3842067.1	TPR+GreAB-C+PIN*→||||<-HTH+HTH	TPR+GreAB-C+PIN	872	ISF68_21105	Betaproteobacteria	Burkholderia pseudomallei	hypothetical protein ISF68_21105 [Burkholderia pseudomallei].	GCA_015320325.1
MBF4269202.1	TPR+GreAB-C*→	TPR+GreAB-C	686	EAY71_20240	Gammaproteobacteria	Vibrio anguillarum	hypothetical protein EAY71_20240, partial [Vibrio anguillarum].	GCA_015343035.1
MBF9243682.1	REase+TPR+GreAB-C+PIN*→	REase+TPR+GreAB-C+PIN	1438	I4I80_05390	Gammaproteobacteria	Pseudomonas syringae pv. tomato	hypothetical protein I4I80_05390 [Pseudomonas syringae pv. tomato].	GCA_015694365.1

acc	operon	architecture	len	gen.name	taxend	species	defline	gca
MBF9246381.1	REase+TPR+GreAB-C+PIN*→	REase+TPR+GreAB-C+PIN	1320	I4I80_19980	Gammaproteobacteria	Pseudomonas syringae pv. tomato	DUF4365 domain-containing protein [Pseudomonas syringae pv. tomato].	GCA_015694365.1
MBG2992436.1	PAIREDC-HTH→ PSE→ HetE-N1→ TPR+GreAB-C+PIN*→	TPR+GreAB-C+PIN	1092	I4607_12420	Gammaproteobacteria	Proteus mirabilis	hypothetical protein I4607_12420 [Proteus mirabilis].	GCA_015723275.1
MBG6040926.1	PAIREDC-HTH→ PSE→ HetE-N1→ TPR+GreAB-C+PIN*→	TPR+GreAB-C+PIN	1092	I5F94_05610	Gammaproteobacteria	Proteus mirabilis	hypothetical protein I5F94_05610 [Proteus mirabilis].	GCA_015740165.1
MBG6251627.1	TPR+GreAB-C+PIN*→	TPR+GreAB-C+PIN	1140	I5I20_11010	Gammaproteobacteria	Pseudomonas aeruginosa	hypothetical protein I5I20_11010 [Pseudomonas aeruginosa].	GCA_015762745.1
MBG7090225.1	TPR+GreAB-C+PIN*→	TPR+GreAB-C+PIN	1170	I5G28_04925	Gammaproteobacteria	Pseudomonas aeruginosa	hypothetical protein I5G28_04925 [Pseudomonas aeruginosa].	GCA_015765435.1
MBH04121.1	REase+TPR+GreAB-C+PIN*→	REase+TPR+GreAB-C+PIN	1338	CMP08_08385	Gammaproteobacteria	Xanthomonadales bacterium	MAG: hypothetical protein CMP08_08385 [Xanthomonadales bacterium].	GCA_002715985.1
MBH1476826.1	REase+TPR+GreAB-C+PIN*→	REase+TPR+GreAB-C+PIN	1243	I5U61_05170	Gammaproteobacteria	Stenotrophomonas maltophilia	CDC27 family protein [Stenotrophomonas maltophilia].	GCA_015997065.1
MBH7847781.1	TPR+GreAB-C+PIN*→	TPR+GreAB-C+PIN	1071	I6A75_20240	Firmicutes	Clostridioides difficile	hypothetical protein I6A75_20240 [Clostridioides difficile].	GCA_016052935.1
MBI1182363.1	REase+TPR+TPR+GreAB-C+PIN*→	REase+TPR+TPR+GreAB-C+PIN	1295	GC201_17615	Alphaproteobacteria	Alphaproteobacteria bacterium	MAG: hypothetical protein GC201_17615 [Alphaproteobacteria bacterium].	GCA_016124315.1
MBI1904375.1	TPR+GreAB-C+PIN*→	TPR+GreAB-C+PIN	1292	HYS13_25040	Planctomycetes	Planctomycetia bacterium	MAG: hypothetical protein HYS13_25040 [Planctomycetia bacterium].	GCA_016177915.1
MBI3888209.1	TPR+PIN*→	TPR+PIN	1190	HY311_00230	Bacteria	Candidatus Nomurabacteria bacterium	MAG: tetratricopeptide repeat protein [Candidatus Nomurabacteria bacterium].	GCA_016199805.1
MBI4633825.1	REase+TPR+GreAB-C+PIN*→	REase+TPR+GreAB-C+PIN	1294	HY742_08030	Deltaproteobacteria	Deltaproteobacteria bacterium	MAG: hypothetical protein HY742_08030 [Deltaproteobacteria bacterium].	GCA_016209105.1
MBI4696055.1	Cas_Cas1→ REase+TPR+GreAB-C+PIN*→	REase+TPR+GreAB-C+PIN	1313	HY749_18755	Gammaproteobacteria	Gammaproteobacteria bacterium	MAG: DUF4365 domain-containing protein [Gammaproteobacteria bacterium].	GCA_016209015.1
MBI4846278.1	TM+SIR2+TPR+PIN+PIN+TM*→	TM+SIR2+TPR+PIN+PIN+TM	2304	HY810_07390	PVC group	Candidatus Omnitrophica bacterium	MAG: SIR2 family protein [Candidatus Omnitrophica bacterium].	GCA_016213925.1
MBI5207389.1	HTH+REase+PIN*→	HTH+REase+PIN	808	HY934_06310	Bacteria	Candidatus Firestonebacteria bacterium	MAG: helix-turn-helix domain-containing protein [Candidatus Firestonebacteria bacterium].	GCA_016212735.1
MBI5354508.1	TPR+GreAB-C+PIN*→	TPR+GreAB-C+PIN	1282	HZB50_17850	Chloroflexi	Chloroflexi bacterium	MAG: hypothetical protein HZB50_17850 [Chloroflexi bacterium].	GCA_016219545.1
MBI5356660.1	TPR+PIN*→||||<-?<-?||HTH→	TPR+PIN	1074	HZB78_03530	Bacteria	Candidatus Collierbacteria bacterium	MAG: hypothetical protein HZB78_03530 [Candidatus Collierbacteria bacterium].	GCA_016219325.1
MBI5458930.1	HEPN→ REase+SNF2→ REase+TPR+GreAB-C+PIN*→	REase+TPR+GreAB-C+PIN	1256	HY987_04275	Euryarchaeota	Methanobacterium sp.	MAG: hypothetical protein HY987_04275 [Methanobacterium sp.].	GCA_016217785.1
MBI5516335.1	GreAB*→	GreAB	172	HY909_21300	Deltaproteobacteria	Deltaproteobacteria bacterium	MAG: GreA/GreB family elongation factor [Deltaproteobacteria bacterium].	GCA_016218725.1
MBI5711428.1	GreAB*→	GreAB	903	HZC42_14175	Bacteria	Candidatus Eisenbacteria bacterium	MAG: GreA/GreB family elongation factor [Candidatus Eisenbacteria bacterium].	GCA_016223045.1
MBI5910925.1	REase+TPR+GreAB-C+PIN*→	REase+TPR+GreAB-C+PIN	1318	HY848_13355	Betaproteobacteria	Betaproteobacteria bacterium	MAG: hypothetical protein HY848_13355 [Betaproteobacteria bacterium].	GCA_016234705.1
MBI5925874.1	REase+TPR+GreAB-C+PIN*→	REase+TPR+GreAB-C+PIN	1369	HY836_09780	Betaproteobacteria	Aquabacterium sp.	MAG: hypothetical protein HY836_09780 [Aquabacterium sp.].	GCA_016234775.1
MBI6873434.1	TPR+PIN*→	TPR+PIN	1232	I6U51_12055	Firmicutes	Clostridium aciditolerans	hypothetical protein I6U51_12055 [Clostridium aciditolerans].	GCA_016316925.1
MBJ3588954.1	TPR+GreAB-C+PIN*→	TPR+GreAB-C+PIN	924	JGC49_23910	Gammaproteobacteria	Salmonella enterica subsp. enterica serovar London	hypothetical protein JGC49_23910, partial [Salmonella enterica subsp. enterica serovar London].	GCA_016412075.1
MBJ7510055.1	REase+TPR+TPR+GreAB-C+PIN*→?→ HNH→	REase+TPR+TPR+GreAB-C+PIN	1324	JHC97_02180	Alphaproteobacteria	Brevundimonas sp.	MAG: hypothetical protein JHC97_02180 [Brevundimonas sp.].	GCA_016463795.1

acc	operon	architecture	len	gen.name	taxend	species	defline	gca
MBK4592903.1	TPR+GreAB-C+PIN*→	TPR+GreAB-C+PIN	444	JJQ23_24565	Gammaproteobacteria	Enterobacter hormaechei	hypothetical protein JJQ23_24565, partial [Enterobacter hormaechei].	GCA_016633275.1
MBK4660580.1	TPR+GreAB-C*→	TPR+GreAB-C	362	JJQ36_24785	Gammaproteobacteria	Enterobacter hormaechei	hypothetical protein JJQ36_24785, partial [Enterobacter hormaechei].	GCA_016633665.1
MBK4769022.1	HetE-N1→ TPR+GreAB-C+PIN*→	TPR+GreAB-C+PIN	1118	FT726_04985	Gammaproteobacteria	Pantoea sp. Morm	MAG: hypothetical protein FT726_04985 [Pantoea sp. Morm].	GCA_016643525.1
MBK5650341.1	REase+TPR+TPR+GreAB-C+PIN*→	REase+TPR+TPR+GreAB-C+PIN	1254	I4N50_01815	Alphaproteobacteria	Rhizobium sp.	MAG: hypothetical protein I4N50_01815, partial [Rhizobium sp.].	GCA_016652855.1
MBK7033605.1	TPR+GreAB-C+PIN*→	TPR+GreAB-C+PIN	852	IPH49_10150	Ignavibacteriae	Ignavibacteria bacterium	MAG: hypothetical protein IPH49_10150 [Ignavibacteria bacterium].	GCA_016708405.1
MBK7130254.1	TPR+GreAB-C+PIN*→	TPR+GreAB-C+PIN	1238	IPH66_12960	Bacteroidetes	Crocinitomicaceae bacterium	MAG: hypothetical protein IPH66_12960 [Crocinitomicaceae bacterium].	GCA_016708105.1
MBK7149038.1	TPR+GreAB-C+PIN*→?→?→?→ TRD+TRD→	TPR+GreAB-C+PIN	1255	IPH78_09510	Bacteroidetes	Bacteroidetes bacterium	MAG: tetratricopeptide repeat protein [Bacteroidetes bacterium].	GCA_016707905.1
MBK7953772.1	REase+TPR+GreAB-C+PIN*→	REase+TPR+GreAB-C+PIN	1351	IPK02_07330	Betaproteobacteria	Candidatus Accumulibacter sp.	MAG: hypothetical protein IPK02_07330 [Candidatus Accumulibacter sp.].	GCA_016713625.1
MBK8138001.1	TPR+GreAB-C+PIN*→	TPR+GreAB-C+PIN	749	IPK52_19665	Chloroflexi	Chloroflexi bacterium	MAG: hypothetical protein IPK52_19665 [Chloroflexi bacterium].	GCA_016712885.1
MBK8143858.1	TPR+PIN*→	TPR+PIN	641	IPK62_02070	Bacteroidetes	Bacteroidetes bacterium	MAG: hypothetical protein IPK62_02070 [Bacteroidetes bacterium].	GCA_016712665.1
MBK8927388.1	TPR+GreAB-C+PIN*→	TPR+GreAB-C+PIN	1238	IPM74_16180	Bacteroidetes	Crocinitomicaceae bacterium	MAG: hypothetical protein IPM74_16180 [Crocinitomicaceae bacterium].	GCA_016720025.1
MBK9927324.1	TPR+GreAB-C+PIN*→	TPR+GreAB-C+PIN	1144	IPP66_18800	Chloroflexi	Anaerolineales bacterium	MAG: tetratricopeptide repeat protein [Anaerolineales bacterium].	GCA_016721115.1
MBL0869327.1	TPR+GreAB-C+PIN*→	TPR+GreAB-C+PIN	1510	IBJ18_01985	Planctomycetes	Phycisphaerales bacterium	MAG: SIR2 family protein [Phycisphaerales bacterium].	GCA_016741915.1
MBL0919126.1	REase+TPR+GreAB-C+PIN*→	REase+TPR+GreAB-C+PIN	1320	IBJ14_10515	Betaproteobacteria	Hydrogenophaga sp.	MAG: DUF4365 domain-containing protein [Hydrogenophaga sp.].	GCA_016741995.1
MBL1279106.1	TPR+PIN*→	TPR+PIN	1234	COA33_002470	Bacteroidetes	Fluviicola sp.	MAG: hypothetical protein COA33_002470 [Fluviicola sp.].	GCA_002734065.2
MBL4609540.1	HetE-N1→ TPR+GreAB-C+PIN*→	TPR+GreAB-C+PIN	1114	JKY58_02600	Gammaproteobacteria	Pseudomonas sp.	MAG: hypothetical protein JKY58_02600 [Pseudomonas sp.].	GCA_016763285.1
MBL7760873.1	TPR+GreAB-C+PIN*→	TPR+GreAB-C+PIN	1264	JNK08_09270	Bacteroidetes	Sediminibacterium sp.	MAG: hypothetical protein JNK08_09270 [Sediminibacterium sp.].	GCA_016786865.1
MBL7768787.1	TPR+GreAB-C+PIN*→	TPR+GreAB-C+PIN	1258	JNK20_07425	Bacteroidetes	Flavipsychrobacter sp.	MAG: hypothetical protein JNK20_07425 [Flavipsychrobacter sp.].	GCA_016787265.1
MBL7772244.1	TPR+GreAB-C+PIN*→	TPR+GreAB-C+PIN	1151	JNM95_05270	Bacteroidetes	Chitinophagaceae bacterium	MAG: hypothetical protein JNM95_05270 [Chitinophagaceae bacterium].	GCA_016787065.1
MBL7856049.1	TPR+GreAB-C+PIN*→	TPR+GreAB-C+PIN	1173	JNM57_00050	Bacteroidetes	Cyclobacteriaceae bacterium	MAG: hypothetical protein JNM57_00050 [Cyclobacteriaceae bacterium].	GCA_016787565.1
MBL8263961.1	REase+TPR+GreAB-C+PIN*→	REase+TPR+GreAB-C+PIN	1239	JNM58_16225	Gammaproteobacteria	Xanthomonadaceae bacterium	MAG: hypothetical protein JNM58_16225 [Xanthomonadaceae bacterium].	GCA_016789405.1
MBL8602392.1	<-SIG+SM-MscS<-?<-?||GreAB*→	GreAB	172	JNK72_10760	Deltaproteobacteria	Myxococcales bacterium	MAG: GreA/GreB family elongation factor [Myxococcales bacterium].	GCA_016792845.1
MBL8821106.1	REase→?→ REase+TPR+GreAB-C+PIN*→	REase+TPR+GreAB-C+PIN	1262	JNJ77_00865	Planctomycetes	Planctomycetia bacterium	MAG: hypothetical protein JNJ77_00865 [Planctomycetia bacterium].	GCA_016795155.1
MBL8873833.1	MNS+TPR+PIN*→	MNS+TPR+PIN	2502	JNK90_28920	Planctomycetes	Planctomycetaceae bacterium	MAG: tetratricopeptide repeat protein [Planctomycetaceae bacterium].	GCA_016792705.1
MBL8874044.1	TPR+GreAB-C+PIN*→	TPR+GreAB-C+PIN	1287	JNK90_29995	Planctomycetes	Planctomycetaceae bacterium	MAG: hypothetical protein JNK90_29995 [Planctomycetaceae bacterium].	GCA_016792705.1
MBL9102506.1	Calcineurin+TPR+GreAB-C+PIN*→	Calcineurin+TPR+GreAB-C+PIN	1429	JNL82_16210	Deltaproteobacteria	Myxococcales bacterium	MAG: metallophosphoesterase [Myxococcales bacterium].	GCA_016793465.1

<table>
<tr><th>acc</th><th>operon</th><th>architecture</th><th>len</th><th>gen.name</th><th>taxend</th><th>species</th><th>defline</th><th>gca</th></tr>
<tr><td>MBM0224540.1</td><td>TPR+PIN*→</td><td>TPR+PIN</td><td>990</td><td>JNW87_02235</td><td>Actinobacteria</td><td>Micromonospora sp. ATA51</td><td>hypothetical protein JNW87_02235 [Micromonospora sp. ATA51].</td><td>GCA_016802855.1</td></tr>
<tr><td>MBM0258116.1</td><td>TM+TPR+PIN*→</td><td>TM+TPR+PIN</td><td>1165</td><td>JNW89_16135</td><td>Actinobacteria</td><td>Micromonospora sp. 4G55</td><td>hypothetical protein JNW89_16135 [Micromonospora sp. 4G55].</td><td>GCA_016803375.1</td></tr>
<tr><td>MBM4070384.1</td><td>TPR+GreAB-C*→</td><td>TPR+GreAB-C</td><td>409</td><td>FJ271_15725</td><td>Planctomycetes</td><td>Planctomycetes bacterium</td><td>MAG: hypothetical protein FJ271_15725 [Planctomycetes bacterium].</td><td>GCA_016873095.1</td></tr>
<tr><td>MBM4845996.1</td><td>HetE-N1→ HetE-N1+TPR+GreAB-C+PIN*→</td><td>HetE-N1+TPR+GreAB-C+PIN</td><td>1091</td><td>HYO58_20990</td><td>Gammaproteobacteria</td><td>Vibrio parahaemolyticus</td><td>hypothetical protein HYO58_20990 [Vibrio parahaemolyticus].</td><td>GCA_016876175.1</td></tr>
<tr><td>MBN1214554.1</td><td>TPR+PIN*→</td><td>TPR+PIN</td><td>1151</td><td>JXA99_03840</td><td>Asgard group</td><td>Candidatus Lokiarchaeota archaeon</td><td>MAG: hypothetical protein JXA99_03840 [Candidatus Lokiarchaeota archaeon].</td><td>GCA_016926735.1</td></tr>
<tr><td>MBN1453691.1</td><td>TPR+GreAB-C+PIN*→</td><td>TPR+GreAB-C+PIN</td><td>1287</td><td>JW963_21935</td><td>Chloroflexi</td><td>Anaerolineales bacterium</td><td>MAG: hypothetical protein JW963_21935 [Anaerolineales bacterium].</td><td>GCA_016928525.1</td></tr>
<tr><td>MBN2019675.1</td><td>TPR+PIN*→</td><td>TPR+PIN</td><td>1063</td><td>JW749_05560</td><td>Planctomycetes</td><td>Sedimentisphaerales bacterium</td><td>MAG: tetratricopeptide repeat protein [Sedimentisphaerales bacterium].</td><td>GCA_016932095.1</td></tr>
<tr><td>MBN2455312.1</td><td>TPR+GreAB-C+PIN*→</td><td>TPR+GreAB-C+PIN</td><td>958</td><td>JXB29_02055</td><td>Planctomycetes</td><td>Sedimentisphaerales bacterium</td><td>MAG: hypothetical protein JXB29_02055 [Sedimentisphaerales bacterium].</td><td>GCA_016935195.1</td></tr>
<tr><td>MBN4052768.1</td><td>TPR+GreAB-C+PIN*→</td><td>TPR+GreAB-C+PIN</td><td>1150</td><td>JYU07_00650</td><td>Chloroflexi</td><td>Roseiflexus sp. AH-315-K22</td><td>hypothetical protein JYU07_00650, partial [Roseiflexus sp. AH-315-K22].</td><td>GCA_017114345.1</td></tr>
<tr><td>MBN5297052.1</td><td>HetE-N1→ TPR+GreAB-C+PIN*→</td><td>TPR+GreAB-C+PIN</td><td>1090</td><td>JY477_14370</td><td>Gammaproteobacteria</td><td>Serratia marcescens</td><td>hypothetical protein JY477_14370 [Serratia marcescens].</td><td>GCA_017152845.1</td></tr>
<tr><td>MBN8213566.1</td><td>REase+TPR+GreAB-C+PIN*→</td><td>REase+TPR+GreAB-C+PIN</td><td>1240</td><td>J0M09_11660</td><td>Gammaproteobacteria</td><td>Xanthomonadales bacterium</td><td>MAG: hypothetical protein J0M09_11660 [Xanthomonadales bacterium].</td><td>GCA_017302135.1</td></tr>
<tr><td>MBN8506452.1</td><td>REase+TPR+GreAB-C+PIN*→</td><td>REase+TPR+GreAB-C+PIN</td><td>1310</td><td>J0L58_18450</td><td>Betaproteobacteria</td><td>Burkholderiales bacterium</td><td>MAG: DUF4365 domain-containing protein [Burkholderiales bacterium].</td><td>GCA_017302655.1</td></tr>
<tr><td>MBN8549455.1</td><td><-HNH<-?<-?||?→||||<-?||?→?→ REase+TPR+GreAB-C+PIN*→</td><td>REase+TPR+GreAB-C+PIN</td><td>1254</td><td>J0M12_09085</td><td>Deltaproteobacteria</td><td>Deltaproteobacteria bacterium</td><td>MAG: hypothetical protein J0M12_09085 [Deltaproteobacteria bacterium].</td><td>GCA_017302875.1</td></tr>
<tr><td>MBN8618419.1</td><td>TPR+PIN*→</td><td>TPR+PIN</td><td>1277</td><td>J0L63_05915</td><td>Chloroflexi</td><td>Anaerolineae bacterium</td><td>MAG: hypothetical protein J0L63_05915 [Anaerolineae bacterium].</td><td>GCA_017303775.1</td></tr>
<tr><td>MBN8865737.1</td><td>TPR+GreAB-C+PIN*→||||<-REase</td><td>TPR+GreAB-C+PIN</td><td>435</td><td>J0H92_20355</td><td>Bacteroidetes</td><td>Sphingobacteriales bacterium</td><td>MAG: hypothetical protein J0H92_20355 [Sphingobacteriales bacterium].</td><td>GCA_017304765.1</td></tr>
<tr><td>MBN8904954.1</td><td>TPR+GreAB-C+PIN*→ REase→</td><td>TPR+GreAB-C+PIN</td><td>679</td><td>J0H57_28395</td><td>Alphaproteobacteria</td><td>Rhodospirillales bacterium</td><td>MAG: hypothetical protein J0H57_28395, partial [Rhodospirillales bacterium].</td><td>GCA_017305035.1</td></tr>
<tr><td>MBN8956855.1</td><td>REase+TPR+TPR+GreAB-C+PIN*→</td><td>REase+TPR+TPR+GreAB-C+PIN</td><td>1322</td><td>J0H17_09800</td><td>Alphaproteobacteria</td><td>Rhizobiales bacterium</td><td>MAG: GreA/GreB family elongation factor [Rhizobiales bacterium].</td><td>GCA_017304555.1</td></tr>
<tr><td>MBN8979284.1</td><td>TPR+GreAB-C+PIN*→</td><td>TPR+GreAB-C+PIN</td><td>1129</td><td>J0I08_22735</td><td>Alphaproteobacteria</td><td>Rhizobiales bacterium</td><td>MAG: hypothetical protein J0I08_22735 [Rhizobiales bacterium].</td><td>GCA_017305555.1</td></tr>
<tr><td>MBN9409395.1</td><td>REase+TPR+GreAB-C+PIN*→</td><td>REase+TPR+GreAB-C+PIN</td><td>1520</td><td>J0H69_09625</td><td>Betaproteobacteria</td><td>Burkholderiales bacterium</td><td>MAG: hypothetical protein J0H69_09625 [Burkholderiales bacterium].</td><td>GCA_017307855.1</td></tr>
<tr><td>MBN9598737.1</td><td>TIR→?→?→ TPR+GreAB-C+PIN*→</td><td>TPR+GreAB-C+PIN</td><td>1117</td><td>J0G28_03540</td><td>Alphaproteobacteria</td><td>Afipia sp.</td><td>MAG: hypothetical protein J0G28_03540 [Afipia sp.].</td><td>GCA_017308625.1</td></tr>
<tr><td>MBO1224632.1</td><td>TPR+GreAB-C+PIN*→</td><td>TPR+GreAB-C+PIN</td><td>1279</td><td>JYX80_09380</td><td>Planctomycetes</td><td>Candidatus Scalindua sediminis</td><td>MAG: hypothetical protein JYX80_09380 [Candidatus Scalindua sediminis].</td><td>GCA_017368835.1</td></tr>
<tr><td>MBO5183033.1</td><td>TPR+GreAB-C+PIN*→</td><td>TPR+GreAB-C+PIN</td><td>1061</td><td>J6B64_01405</td><td>Firmicutes</td><td>Bacilli bacterium</td><td>MAG: hypothetical protein J6B64_01405 [Bacilli bacterium].</td><td>GCA_017623135.1</td></tr>
<tr><td>MBO5920175.1</td><td>TPR+GreAB-C+PIN*→</td><td>TPR+GreAB-C+PIN</td><td>507</td><td>J6Q34_06770</td><td>Bacteroidetes</td><td>Bacteroidales bacterium</td><td>MAG: hypothetical protein J6Q34_06770, partial [Bacteroidales bacterium].</td><td>GCA_017631435.1</td></tr>
<tr><td>MBO6496597.1</td><td>TPR+GreAB-C+PIN*→</td><td>TPR+GreAB-C+PIN</td><td>1166</td><td>JJ978_13575</td><td>Bacteroidetes</td><td>Roseivirga sp.</td><td>MAG: hypothetical protein JJ978_13575 [Roseivirga sp.].</td><td>GCA_017640405.1</td></tr>
<tr><td>MBO6761342.1</td><td>TPR+GreAB-C+PIN*→</td><td>TPR+GreAB-C+PIN</td><td>1095</td><td>JJ909_10290</td><td>Bacteroidetes</td><td>Roseivirga sp.</td><td>MAG: hypothetical protein JJ909_10290, partial [Roseivirga sp.].</td><td>GCA_017643085.1</td></tr>
<tr><td>MBO7636147.1</td><td>TPR+GreAB-C+PIN*→</td><td>TPR+GreAB-C+PIN</td><td>1171</td><td>J6S89_06175</td><td>Bacteroidetes</td><td>Paludibacteraceae bacterium</td><td>MAG: hypothetical protein J6S89_06175 [Paludibacteraceae bacterium].</td><td>GCA_017651825.1</td></tr>
</table>

<table>
<tr><th>acc</th><th>operon</th><th>architecture</th><th>len</th><th>gen.name</th><th>taxend</th><th>species</th><th>defline</th><th>gca</th></tr>
<tr><td>MBP8894107.1</td><td>TPR+GreAB-C+PIN*→</td><td>TPR+GreAB-C+PIN</td><td>985</td><td>KBH09_17290</td><td>Bacteroidetes</td><td>Saprospiraceae bacterium</td><td>MAG: hypothetical protein KBH09_17290 [Saprospiraceae bacterium].</td><td>GCA_018055665.1</td></tr>
<tr><td>MBR7522021.1</td><td>HetE-N1→ TPR+GreAB-C+PIN*→</td><td>TPR+GreAB-C+PIN</td><td>1105</td><td>KC233_15980</td><td>Gammaproteobacteria</td><td>Pseudomonas juntendi</td><td>hypothetical protein KC233_15980 [Pseudomonas juntendi].</td><td>GCA_018138545.1</td></tr>
<tr><td>MHJ03184.1</td><td>TPR+GreAB-C+PIN*→</td><td>TPR+GreAB-C+PIN</td><td>523</td><td>EEN62_25195</td><td>Gammaproteobacteria</td><td>Salmonella enterica</td><td>hypothetical protein EEN62_25195 [Salmonella enterica].</td><td>GCA_003786665.1</td></tr>
<tr><td>MKC77438.1</td><td>TPR+GreAB-C+PIN*→?→||||<-Trypsin</td><td>TPR+GreAB-C+PIN</td><td>1057</td><td>D1800_22940</td><td>Gammaproteobacteria</td><td>Salmonella enterica subsp. enterica serovar Denver</td><td>hypothetical protein D1800_22940, partial [Salmonella enterica subsp. enterica serovar Denver].</td><td>GCA_003891855.1</td></tr>
<tr><td>MPS34312.1</td><td>REase+TPR+GreAB-C+PIN*→</td><td>REase+TPR+GreAB-C+PIN</td><td>1237</td><td>E2593_03230</td><td>Gammaproteobacteria</td><td>Stenotrophomonas sp.</td><td>MAG: hypothetical protein E2593_03230 [Stenotrophomonas sp.].</td><td>GCA_009360425.1</td></tr>
<tr><td>MSW50706.1</td><td>TPR+PIN*→</td><td>TPR+PIN</td><td>1289</td><td>F2817_07440</td><td>Actinobacteria</td><td>Actinobacteria bacterium</td><td>MAG: hypothetical protein F2817_07440 [Actinobacteria bacterium].</td><td>GCA_009699505.1</td></tr>
<tr><td>MTI67851.1</td><td>TPR+GreAB-C+PIN*→</td><td>TPR+GreAB-C+PIN</td><td>1274</td><td>FH753_14795</td><td>Firmicutes</td><td>Firmicutes bacterium</td><td>MAG: hypothetical protein FH753_14795 [Firmicutes bacterium].</td><td>GCA_009711765.1</td></tr>
<tr><td>MVM42293.1</td><td>-</td><td>-</td><td>400</td><td>GO730_39050</td><td>Bacteroidetes</td><td>Spirosoma sp. HMF3257</td><td>hypothetical protein GO730_39050 [Spirosoma sp. HMF3257].</td><td>GCA_009754885.1</td></tr>
<tr><td>MXP30641.1</td><td>REase→?→ REase+TPR+GreAB-C+PIN*→</td><td>REase+TPR+GreAB-C+PIN</td><td>1265</td><td>GRI94_02255</td><td>Alphaproteobacteria</td><td>Parerythrobacter jejuensis</td><td>hypothetical protein GRI94_02255 [Parerythrobacter jejuensis].</td><td>GCA_009827995.1</td></tr>
<tr><td>MXW27298.1</td><td>REase+TPR+GREAB-C+PIN*→</td><td>REase+TPR+GREAB-C+PIN</td><td>1302</td><td>F4Z80_00100</td><td>Chloroflexi</td><td>Chloroflexi bacterium</td><td>MAG: hypothetical protein F4Z80_00100 [Chloroflexi bacterium].</td><td>GCA_009835825.1</td></tr>
<tr><td>MXX46821.1</td><td>TPR+GreAB-C+PIN*→</td><td>TPR+GreAB-C+PIN</td><td>1267</td><td>F4Z38_00735</td><td>Chloroflexi</td><td>Chloroflexi bacterium</td><td>MAG: tetratricopeptide repeat protein [Chloroflexi bacterium].</td><td>GCA_009836695.1</td></tr>
<tr><td>MXX90812.1</td><td>SFI-Helicase+REase+TPR+GreAB-C+PIN*→</td><td>SFI-Helicase+REase+TPR+GreAB-C+PIN</td><td>1846</td><td>F4Y68_16490</td><td>Alphaproteobacteria</td><td>Boseongicola sp. SB0665_bin_10</td><td>MAG: DUF4011 domain-containing protein [Boseongicola sp. SB0665_bin_10].</td><td>GCA_009838075.1</td></tr>
<tr><td>MXX91940.1</td><td>GreAB*→?→?→ DAGKIN→</td><td>GreAB</td><td>297</td><td>F4Y63_00605</td><td>Chloroflexi</td><td>Chloroflexi bacterium</td><td>MAG: transcription elongation factor GreA [Chloroflexi bacterium].</td><td>GCA_009838175.1</td></tr>
<tr><td>MXX97366.1</td><td>REase+TPR+GreAB-C+PIN*→</td><td>REase+TPR+GreAB-C+PIN</td><td>1278</td><td>F4Y64_07095</td><td>Bacteroidetes</td><td>Rhodothermaceae bacterium</td><td>MAG: hypothetical protein F4Y64_07095 [Rhodothermaceae bacterium].</td><td>GCA_009838195.1</td></tr>
<tr><td>MXZ48148.1</td><td>REase+TPR+GreAB-C+PIN*→</td><td>REase+TPR+GreAB-C+PIN</td><td>1281</td><td>F4Z13_02660</td><td>Bacteria</td><td>Candidatus Dadabacteria bacterium</td><td>MAG: hypothetical protein F4Z13_02660 [Candidatus Dadabacteria bacterium].</td><td>GCA_009837205.1</td></tr>
<tr><td>MXZ55165.1</td><td>REase+TPR+GreAB-C+PIN*→</td><td>REase+TPR+GreAB-C+PIN</td><td>1297</td><td>F4Z14_03240</td><td>Gammaproteobacteria</td><td>Gammaproteobacteria bacterium</td><td>MAG: hypothetical protein F4Z14_03240 [Gammaproteobacteria bacterium].</td><td>GCA_009837185.1</td></tr>
<tr><td>MXZ61096.1</td><td>REase+TPR+GreAB-C+PIN*→</td><td>REase+TPR+GreAB-C+PIN</td><td>1297</td><td>F4Z12_09585</td><td>Acidobacteria</td><td>Acidobacteria bacterium</td><td>MAG: hypothetical protein F4Z12_09585 [Acidobacteria bacterium].</td><td>GCA_009837215.1</td></tr>
<tr><td>MYA00894.1</td><td>TPR+GreAB-C+PIN*→</td><td>TPR+GreAB-C+PIN</td><td>1267</td><td>F4Y35_03835</td><td>Chloroflexi</td><td>Chloroflexi bacterium</td><td>MAG: tetratricopeptide repeat protein [Chloroflexi bacterium].</td><td>GCA_009838735.1</td></tr>
<tr><td>MYA68860.1</td><td>TPR+GreAB-C+PIN*→</td><td>TPR+GreAB-C+PIN</td><td>1308</td><td>F4Y19_01960</td><td>Bacteria</td><td>Candidatus Poribacteria bacterium</td><td>MAG: tetratricopeptide repeat protein [Candidatus Poribacteria bacterium].</td><td>GCA_009839085.1</td></tr>
<tr><td>MYB15443.1</td><td>TPR+GreAB-C+PIN*→</td><td>TPR+GreAB-C+PIN</td><td>1263</td><td>F4X41_00185</td><td>Chloroflexi</td><td>Chloroflexi bacterium</td><td>MAG: hypothetical protein F4X41_00185 [Chloroflexi bacterium].</td><td>GCA_009840625.1</td></tr>
<tr><td>MYB42498.1</td><td>TPR+GreAB-C+PIN*→</td><td>TPR+GreAB-C+PIN</td><td>1283</td><td>F4X76_09995</td><td>Chloroflexi</td><td>Chloroflexi bacterium</td><td>MAG: tetratricopeptide repeat protein [Chloroflexi bacterium].</td><td>GCA_009839925.1</td></tr>
<tr><td>MYB43588.1</td><td>TPR+PIN*→</td><td>TPR+PIN</td><td>1200</td><td>F4X74_01405</td><td>Actinobacteria</td><td>Acidimicrobiia bacterium</td><td>MAG: hypothetical protein F4X74_01405 [Acidimicrobiia bacterium].</td><td>GCA_009839965.1</td></tr>
<tr><td>MYB75895.1</td><td>TPR+GreAB-C+PIN*→</td><td>TPR+GreAB-C+PIN</td><td>1300</td><td>F4X83_02110</td><td>Chloroflexi</td><td>Chloroflexi bacterium</td><td>MAG: hypothetical protein F4X83_02110 [Chloroflexi bacterium].</td><td>GCA_009839775.1</td></tr>
<tr><td>MYC64541.1</td><td>TPR+GreAB-C+PIN*→</td><td>TPR+GreAB-C+PIN</td><td>1258</td><td>F4X16_17195</td><td>Chloroflexi</td><td>Caldilineaceae bacterium SB0661_bin_34</td><td>MAG: tetratricopeptide repeat protein [Caldilineaceae bacterium SB0661_bin_34].</td><td>GCA_009841145.1</td></tr>
<tr><td>MYD93275.1</td><td>REase+TPR+GreAB-C+PIN*→</td><td>REase+TPR+GreAB-C+PIN</td><td>1302</td><td>F4Y02_06215</td><td>Chloroflexi</td><td>Chloroflexi bacterium</td><td>MAG: hypothetical protein F4Y02_06215 [Chloroflexi bacterium].</td><td>GCA_009839425.1</td></tr>
</table>

acc	operon	architecture	len	gen.name	taxend	species	defline	gca
MYE61432.1	TPR+GreAB-C+PIN*→	TPR+GreAB-C+PIN	638	F4235_05210	Bacteria	Candidatus Dadabacteria bacterium	MAG: GreA/GreB family elongation factor [Candidatus Dadabacteria bacterium].	GCA_009841925.1
MYF79095.1	TPR+GreAB-C+PIN*→	TPR+GreAB-C+PIN	716	F4180_04215	Chloroflexi	Chloroflexi bacterium	MAG: tetratricopeptide repeat protein, partial [Chloroflexi bacterium].	GCA_009843025.1
MYH48359.1	TPR+GreAB-C+PIN*→	TPR+GreAB-C+PIN	1248	F4151_02205	Gammaproteobacteria	Gammaproteobacteria bacterium	MAG: hypothetical protein F4151_02205 [Gammaproteobacteria bacterium].	GCA_009843625.1
MYK18704.1	TPR+GreAB-C+PIN*→	TPR+GreAB-C+PIN	911	F4055_11145	Bacteria	Candidatus Poribacteria bacterium	MAG: tetratricopeptide repeat protein, partial [Candidatus Poribacteria bacterium].	GCA_009845505.1
MYK96751.1	TPR+GreAB-C+PIN*→	TPR+GreAB-C+PIN	470	F4009_22610	Bacteria	Candidatus Poribacteria bacterium	MAG: hypothetical protein F4009_22610 [Candidatus Poribacteria bacterium].	GCA_009846415.1
NAT27530.1	TPR+GreAB-C+PIN*→	TPR+GreAB-C+PIN	692	CVE22_06655	Gammaproteobacteria	Pseudomonas syringae pv. actinidiae	hypothetical protein CVE22_06655, partial [Pseudomonas syringae pv. actinidiae].	GCA_009903115.1
NBW14976.1	REase+TPR+GreAB-C+PIN*→	REase+TPR+GreAB-C+PIN	1102	EBR82_43945	Alphaproteobacteria	Caulobacteraceae bacterium	MAG: hypothetical protein EBR82_43945, partial [Caulobacteraceae bacterium].	GCA_009924585.1
NCC04365.1	REase+TPR+GreAB-C+PIN*→	REase+TPR+GreAB-C+PIN	1308	EOM37_10050	Proteobacteria	Proteobacteria bacterium	MAG: tetratricopeptide repeat protein [Proteobacteria bacterium].	GCA_009929575.1
NCN59296.1	<-CITB-HTH+LexA-protease||TPR+GreAB-C+PIN*→	TPR+GreAB-C+PIN	1274	GW940_05255	Bacteria	Candidatus Microgenomates bacterium	MAG: hypothetical protein GW940_05255 [Candidatus Microgenomates bacterium].	GCA_009992065.1
NCO24709.1	TPR+GreAB-C+PIN*→	TPR+GreAB-C+PIN	415	GW897_06315	unclassified Bacteria	bacterium	MAG: hypothetical protein GW897_06315 [bacterium].	GCA_009992775.1
NEZ58684.1	TPR+TPR→ TPR+TPR→ TPR+TPR→ TPR+TPR→ TPR+TPR→ TPR→ TPR→ PNPase+TPR+GreAB-C+PIN*→?→||||<-TPR+TPR	PNPase+TPR+GreAB-C+PIN	1392	DXZ20_24195	Cyanobacteria	Leptolyngbyaceae cyanobacterium CCMR0081	hypothetical protein DXZ20_24195 [Leptolyngbyaceae cyanobacterium CCMR0081].	GCA_011009555.1
NEZ64367.1	TPR+TPR→ TPR+TPR→ TPR+TPR→ TPR+TPR→ TPR+TPR→ TPR+TPR→ PNPase+TPR+GreAB-C+PIN*→?→||||<-TPR+TPR<-TPR+TPR<-?<-TIR	PNPase+TPR+GreAB-C+PIN	1392	D0962_16475	Cyanobacteria	Leptolyngbyaceae cyanobacterium CCMR0082	hypothetical protein D0962_16475 [Leptolyngbyaceae cyanobacterium CCMR0082].	GCA_011009535.1
NHI06604.1	PIN*→	PIN	211	STPH2_1967	Actinobacteria	Streptomyces sp. KO7888	hypothetical protein STPH2_1967 [Streptomyces sp. KO7888].	GCA_011317205.1
NHK99992.1	TPR+GreAB-C+PIN*→	TPR+GreAB-C+PIN	911	G7087_16540	Betaproteobacteria	Rubrivivax benzoatilyticus	hypothetical protein G7087_16540 [Rubrivivax benzoatilyticus].	GCA_011365705.1
NJN11600.1	TPR+GreAB-C+PIN*→	TPR+GreAB-C+PIN	298	HC815_27960	Cyanobacteria	Richelia sp. RM1_1_1	MAG: hypothetical protein HC815_27960 [Richelia sp. RM1_1_1].	GCA_012032385.1
NJO54209.1	REase+TPR+TPR+GreAB-C+PIN*→	REase+TPR+TPR+GreAB-C+PIN	1338	HC829_04580	Bacteroidetes	Bacteroidales bacterium	MAG: hypothetical protein HC829_04580 [Bacteroidales bacterium].	GCA_012033435.1
NKI94745.1	REase+TPR+GreAB-C+PIN*→	REase+TPR+GreAB-C+PIN	1340	FHT26_002685	Betaproteobacteria	Rhizobacter sp. SG703	tetratricopeptide (TPR) repeat protein [Rhizobacter sp. SG703].	GCA_012275445.1
NLD36189.1	REase+TPR+PIN*→	REase+TPR+PIN	1360	GX654_04895	Deltaproteobacteria	Desulfatiglans sp.	MAG: tetratricopeptide repeat protein [Desulfatiglans sp.].	GCA_012513605.1
NLD38959.1	TPR+PIN*→	TPR+PIN	1143	GX654_19035	Deltaproteobacteria	Desulfatiglans sp.	MAG: tetratricopeptide repeat protein [Desulfatiglans sp.].	GCA_012513605.1
NLD90744.1	PNPase+TPR+GreAB-C+PIN*→	PNPase+TPR+GreAB-C+PIN	1613	GX634_10460	Lentisphaerae	Lentisphaerae bacterium	MAG: hypothetical protein GX634_10460 [Lentisphaerae bacterium].	GCA_012514035.1
NLE27049.1	SIR2+TPR+GreAB-C+PIN*→	SIR2+TPR+GreAB-C+PIN	1448	GX625_17245	Firmicutes	Clostridiaceae bacterium	MAG: hypothetical protein GX625_17245 [Clostridiaceae bacterium].	GCA_012514215.1
NLE28033.1	TPR+GreAB-C+PIN*→	TPR+GreAB-C+PIN	758	GX625_22395	Firmicutes	Clostridiaceae bacterium	MAG: hypothetical protein GX625_22395, partial [Clostridiaceae bacterium].	GCA_012514215.1
NLG15547.1	TPR+GreAB-C+PIN*→	TPR+GreAB-C+PIN	1341	GX561_15255	Lentisphaerae	Lentisphaerae bacterium	MAG: hypothetical protein GX561_15255 [Lentisphaerae bacterium].	GCA_012515425.1
NLX50682.1	REase+TPR+GreAB-C+PIN*→ PSE→?→?→ REase+SNF2→	REase+TPR+GreAB-C+PIN	1299	GXY72_01105	Deltaproteobacteria	Deltaproteobacteria bacterium	MAG: hypothetical protein GXY72_01105 [Deltaproteobacteria bacterium].	GCA_012729055.1
NMQ30291.1	REase+TPR+GreAB-C+PIN*→	REase+TPR+GreAB-C+PIN	1072	E4Q23_22545	Betaproteobacteria	Candidatus Accumulibacter phosphatis	hypothetical protein E4Q23_22545, partial [Candidatus Accumulibacter phosphatis].	GCA_012939955.1
NOT34484.1	GreAB*→	GreAB	904	HOP12_09975	Bacteria	Candidatus Eisenbacteria bacterium	MAG: hypothetical protein HOP12_09975 [Candidatus Eisenbacteria bacterium].	GCA_013140805.1

acc	operon	architecture	len	gen.name	taxend	species	defline	gca
NOU31123.1	TPR+GreAB-C+PIN*→	TPR+GreAB-C+PIN	536	HOO96_24755	Deltaproteobacteria	Polyangiaceae bacterium	MAG: hypothetical protein HOO96_24755 [Polyangiaceae bacterium].	GCA_013141295.1
NQE54029.1	REase+TPR+GreAB-C+PIN*→	REase+TPR+GreAB-C+PIN	1291	lapB_1	Euryarchaeota	ANME-1 cluster archaeon GoMg3.2	Lipopolysaccharide assembly protein B [ANME-1 cluster archaeon GoMg3.2].	GCA_013180565.1
NQV22738.1	REase+TPR+GreAB-C+PIN*→	REase+TPR+GreAB-C+PIN	1245	HQ518_00080	Planctomycetes	Rhodopirellula sp.	MAG: hypothetical protein HQ518_00080 [Rhodopirellula sp.].	GCA_013203845.1
NQY79025.1	TPR+PIN*→	TPR+PIN	1272	HRT47_01810	Terrabacteria group	Candidatus Caenarcaniphilales bacterium	MAG: hypothetical protein HRT47_01810 [Candidatus Caenarcaniphilales bacterium].	GCA_013216135.1
NQZ35237.1	TPR+GreAB-C+PIN*→	TPR+GreAB-C+PIN	1069	HRT58_06210	Bacteroidetes	Crocinitomicaceae bacterium	MAG: hypothetical protein HRT58_06210 [Crocinitomicaceae bacterium].	GCA_013214785.1
NQZ40471.1	HetE-N1→ TPR+GreAB-C+PIN*→	TPR+GreAB-C+PIN	1088	HRT96_10385	Gammaproteobacteria	Moritella sp.	MAG: hypothetical protein HRT96_10385 [Moritella sp.].	GCA_013215705.1
NSZ07544.1	TPR+GreAB-C+PIN*→	TPR+GreAB-C+PIN	1180	G6M14_14320	Alphaproteobacteria	Agrobacterium tumefaciens	hypothetical protein G6M14_14320 [Agrobacterium tumefaciens].	GCA_013318315.1
NTV22000.1	TPR+PIN*→	TPR+PIN	942	HGB03_00315	Bacteria	Candidatus Yonathbacteria bacterium	MAG: hypothetical protein HGB03_00315 [Candidatus Yonathbacteria bacterium].	GCA_013334825.1
NTW26872.1	REase→ REase+TPR+GreAB-C+PIN*→	REase+TPR+GreAB-C+PIN	1310	HGA36_00950	Bacteria	Candidatus Moranbacteria bacterium	MAG: hypothetical protein HGA36_00950 [Candidatus Moranbacteria bacterium].	GCA_013336225.1
NTZ52560.1	HetE-N1→?→ TPR+GreAB-C+PIN*→	TPR+GreAB-C+PIN	513	FCH32_20020	Gammaproteobacteria	Citrobacter gillenii	hypothetical protein FCH32_20020 [Citrobacter gillenii].	GCA_013337685.1
NWE92806.1	PIN*→	PIN	344	HX893_32300	Gammaproteobacteria	Pseudomonas reactans	hypothetical protein HX893_32300, partial [Pseudomonas reactans].	GCA_013386215.1
NWO07476.1	HetE-N1→ TPR+GreAB-C+PIN*→	TPR+GreAB-C+PIN	1130	HLX50_17830	Gammaproteobacteria	Alteromonadaceae bacterium	MAG: hypothetical protein HLX50_17830 [Alteromonadaceae bacterium].	GCA_013393545.1
OBH13398.1	X+PIN*→	X+PIN	755	A5695_13915	Actinobacteria	Mycobacterium sp. E1747	hypothetical protein A5695_13915 [Mycobacterium sp. E1747].	GCA_001667775.1
OBR53101.1	REase+TPR+GreAB-C+PIN*→	REase+TPR+GreAB-C+PIN	1277	A6456_09045	Betaproteobacteria	Paraburkholderia tropica	hypothetical protein A6456_09045 [Paraburkholderia tropica].	GCA_001673675.1
OCX17561.1	REase+TPR+TPR+GreAB-C+PIN*→	REase+TPR+TPR+GreAB-C+PIN	1207	QV13_12440	Alphaproteobacteria	Mesorhizobium hungaricum	hypothetical protein QV13_12440 [Mesorhizobium hungaricum].	GCA_001705425.1
ODM45291.1	TPR+GreAB-C+PIN*→	TPR+GreAB-C+PIN	1187	A9320_11670	Alphaproteobacteria	Ruegeria sp. PBVC088	hypothetical protein A9320_11670 [Ruegeria sp. PBVC088].	GCA_001717995.1
ODS63626.1	GreAB-C+PIN*→	GreAB-C+PIN	516	ABS37_11570	Betaproteobacteria	Acidovorax sp. SCN 65-108	MAG: hypothetical protein ABS37_11570 [Acidovorax sp. SCN 65-108].	GCA_001724055.1
ODT73806.1	REase+TPR+TPR+GreAB-C+PIN*→	REase+TPR+TPR+GreAB-C+PIN	1296	ABS76_38455	Alphaproteobacteria	Pelagibacterium sp. SCN 64-44	MAG: hypothetical protein ABS76_38455 [Pelagibacterium sp. SCN 64-44].	GCA_001724955.1
OEJ21925.1	REase+PIN*→	REase+PIN	1260	AR457_38995	Actinobacteria	Streptomyces agglomeratus	hypothetical protein AR457_38995 [Streptomyces agglomeratus].	GCA_001746465.1
OFX87846.1	TPR+GreAB-C+PIN*→	TPR+GreAB-C+PIN	1236	A2X00_15495	Bacteroidetes	Bacteroidetes bacterium GWE2_32_14	MAG: hypothetical protein A2X00_15495 [Bacteroidetes bacterium GWE2_32_14].	GCA_001768405.1
OFY15991.1	TPR+GreAB-C+PIN*→	TPR+GreAB-C+PIN	1237	A2X02_04985	Bacteroidetes	Bacteroidetes bacterium GWF2_29_10	MAG: hypothetical protein A2X02_04985 [Bacteroidetes bacterium GWF2_29_10].	GCA_001768555.1
OGC02499.1	PIN*→	PIN	178	A2V82_02050	Bacteria	candidate division KSB1 bacterium RBG_16_48_16	MAG: hypothetical protein A2V82_02050 [candidate division KSB1 bacterium RBG_16_48_16].	GCA_001771235.1
OGH88116.1	TPR+GreAB-C+PIN*→	TPR+GreAB-C+PIN	1191	A3J93_00010	Bacteria	Candidatus Magasanikbacteria bacterium RIFOXYC2_FULL_42_28	MAG: hypothetical protein A3J93_00010 [Candidatus Magasanikbacteria bacterium RIFOXYC2_FULL_42_28].	GCA_001784435.1
OGI36016.1	TRD+TRD→?→?→?→ TPR+PIN*→	TPR+PIN	1186	A2259_01680	Bacteria	Candidatus Moranbacteria bacterium RIFOXYA2_FULL_43_15	MAG: hypothetical protein A2259_01680 [Candidatus Moranbacteria bacterium RIFOXYA2_FULL_43_15].	GCA_001784835.1

acc	operon	architecture	len	gen.name	taxend	species	defline	gca
OGS08035.1	TPR+GreAB-C+PIN*→	TPR+GreAB-C+PIN	1270	A2270_03040	Elusimicrobia	Elusimicrobia bacterium RIFOXYA12_FULL_51_18	MAG: hypothetical protein A2270_03040 [Elusimicrobia bacterium RIFOXYA12_FULL_51_18].	GCA_001800245.1
OGU66359.1	REase+TPR+GreAB-C+PIN*→	REase+TPR+GreAB-C+PIN	1303	A2W30_02620	Ignavibacteriae	Ignavibacteria bacterium RBG_16_36_9	MAG: hypothetical protein A2W30_02620 [Ignavibacteria bacterium RBG_16_36_9].	GCA_001802895.1
OGU77131.1	PIN*→	PIN	573	A2W11_11280	Ignavibacteriae	Ignavibacteria bacterium RBG_16_35_7	MAG: hypothetical protein A2W11_11280 [Ignavibacteria bacterium RBG_16_35_7].	GCA_001802885.1
OIP66815.1	TPR+GreAB-C+PIN*→	TPR+GreAB-C+PIN	402	AUK42_07760	Bacteria	Candidatus Atribacteria bacterium CG2_30_33_13	MAG: hypothetical protein AUK42_07760 [Candidatus Atribacteria bacterium CG2_30_33_13].	GCA_001873345.1
OJV65414.1	HetE-N1→ HetE-N1+TPR+GreAB-C+PIN*→	HetE-N1+TPR+GreAB-C+PIN	1117	BGO35_21125	Betaproteobacteria	Burkholderiales bacterium 64-34	MAG: hypothetical protein BGO35_21125 [Burkholderiales bacterium 64-34].	GCA_001897585.1
OJW35018.1	TPR+GreAB-C+PIN*→||||<-REase	TPR+GreAB-C+PIN	1246	BGO54_02365	Bacteroidetes	Sphingobacteriales bacterium 46-32	MAG: hypothetical protein BGO54_02365 [Sphingobacteriales bacterium 46-32].	GCA_001898485.1
OJW61617.1	TIR→?→?→ TPR+GreAB-C+PIN*→	TPR+GreAB-C+PIN	1114	BGO65_09490	Alphaproteobacteria	Afipia sp. 64-13	MAG: hypothetical protein BGO65_09490 [Afipia sp. 64-13].	GCA_001897755.1
OMR67119.1	TPR+GreAB-C+PIN*→||||<-HTH+HTH	TPR+GreAB-C+PIN	879	AQ727_25970	Betaproteobacteria	Burkholderia pseudomallei	hypothetical protein AQ727_25970 [Burkholderia pseudomallei].	GCA_001976575.1
OSN14318.1	REase+TPR+GreAB-C+PIN*→	REase+TPR+GreAB-C+PIN	1486	BV340_04459	Gammaproteobacteria	Pseudomonas syringae pv. actinidiae	hypothetical protein BV340_04459 [Pseudomonas syringae pv. actinidiae].	GCA_002111665.1
OSN30099.1	REase+TPR+GreAB-C+PIN*→	REase+TPR+GreAB-C+PIN	1336	BV342_05184	Gammaproteobacteria	Pseudomonas syringae pv. actinidiae	hypothetical protein BV342_05184 [Pseudomonas syringae pv. actinidiae].	GCA_002111745.1
OUC14796.1	TPR+TPR+PNPase→ PNPase+TPR+GreAB-C+PIN*→	PNPase+TPR+GreAB-C+PIN	1396	B0A82_10750	Cyanobacteria	Alkalinema sp. CACIAM 70d	MAG: hypothetical protein B0A82_10750 [Alkalinema sp. CACIAM 70d].	GCA_002148405.1
OUR78422.1	TPR+GreAB-C+PIN*→	TPR+GreAB-C+PIN	1191	A9Q83_07770	Alphaproteobacteria	Alphaproteobacteria bacterium 46_93_T64	hypothetical protein A9Q83_07770 [Alphaproteobacteria bacterium 46_93_T64].	GCA_002163135.1
OWZ89184.1	REase+TPR+TPR+GreAB-C+PIN*→	REase+TPR+TPR+GreAB-C+PIN	1352	B9J07_34245	Alphaproteobacteria	Sinorhizobium sp. LM21	hypothetical protein B9J07_34245 [Sinorhizobium sp. LM21].	GCA_002216665.1
OYD66813.1	REase+TPR+GreAB-C+PIN*→?→ Trypsin→	REase+TPR+GreAB-C+PIN	1243	BDB13_0310	Actinobacteria	Rhodococcus sp. OK302	uncharacterized protein DUF4365 [Rhodococcus sp. OK302].	GCA_002245895.1
OYW86349.1	TPR+GreAB-C+PIN*→	TPR+GreAB-C+PIN	629	B7Z22_06790	Alphaproteobacteria	Hyphomonas sp. 32-62-5	MAG: hypothetical protein B7Z22_06790, partial [Hyphomonas sp. 32-62-5].	GCA_002280065.1
OYX15563.1	REase+TPR+GreAB-C+PIN*→	REase+TPR+GreAB-C+PIN	1211	B7Z15_00100	Alphaproteobacteria	Rhizobiales bacterium 32-66-8	MAG: hypothetical protein B7Z15_00100 [Rhizobiales bacterium 32-66-8].	GCA_002280205.1
OZB15488.1	REase+TPR+GreAB-C+PIN*→	REase+TPR+GreAB-C+PIN	1294	B7X53_11340	Alphaproteobacteria	Hyphomonas sp. 34-62-18	MAG: hypothetical protein B7X53_11340 [Hyphomonas sp. 34-62-18].	GCA_002282565.1
PBC66280.1	X+PIN*→	X+PIN	685	BX265_8343	Actinobacteria	Streptomyces sp. TLI_235	hypothetical protein BX265_8343 [Streptomyces sp. TLI_235].	GCA_002300355.1
PCJ29538.1	REase+TPR+PIN*→	REase+TPR+PIN	1292	COA99_19375	Gammaproteobacteria	Moraxellaceae bacterium	MAG: hypothetical protein COA99_19375 [Moraxellaceae bacterium].	GCA_002402125.1
PDQ17661.1	REase+TPR+TPR+GreAB-C+PIN*→	REase+TPR+TPR+GreAB-C+PIN	1265	CN311_28810	Alphaproteobacteria	Mesorhizobium sanjuanii	hypothetical protein CN311_28810 [Mesorhizobium sanjuanii].	GCA_002529485.1
PGS40120.1	TPR+PIN*→	TPR+PIN	1006	COC58_18130	Firmicutes	Bacillus cereus	hypothetical protein COC58_18130 [Bacillus cereus].	GCA_002577165.1
PIG41510.1	TPR+PIN*→	TPR+PIN	1214	CLZ78_0083	Actinobacteria	Streptomyces sp. 61	hypothetical protein CLZ78_0083 [Streptomyces sp. 61].	GCA_002754535.1
PIP62053.1	<-CITB-HTH+LexA-protease||TPR+GreAB-C+PIN*→	TPR+GreAB-C+PIN	1272	COW99_00715	Bacteria	Candidatus Roizmanbacteria bacterium CG22_combo_CG10-13_8_21_14_all_38_20	MAG: hypothetical protein COW99_00715 [Candidatus Roizmanbacteria bacterium CG22_combo_CG10-13_8_21_14_all_38_20].	GCA_002786635.1
PIU06549.1	TPR+GreAB-C+PIN*→	TPR+GreAB-C+PIN	1132	COT56_09580	Alphaproteobacteria	Methylobacterium sp. CG09_land_8_20_14_0_10_71_15	MAG: hypothetical protein COT56_09580 [Methylobacterium sp. CG09_land_8_20_14_0_10_71_15].	GCA_002778835.1

acc	operon	architecture	len	gen.name	taxend	species	defline	gca
PKM50370.1	TPR+PIN*→	TPR+PIN	1214	CVV02_11355	Firmicutes	Firmicutes bacterium HGW-Firmicutes-7	MAG: hypothetical protein CVV02_11355 [Firmicutes bacterium HGW-Firmicutes-7].	GCA_002841375.1
PKN75416.1	TPR+GreAB-C+PIN*→	TPR+GreAB-C+PIN	1116	CVU49_03915	FCB group	Candidatus Cloacimonetes bacterium HGW-Cloacimonetes-2	MAG: hypothetical protein CVU49_03915 [Candidatus Cloacimonetes bacterium HGW-Cloacimonetes-2].	GCA_002842035.1
PKO82710.1	HetE-N1→ HetE-N1+TPR+GreAB-C+PIN*→	HetE-N1+TPR+GreAB-C+PIN	1126	CVU19_00260	Betaproteobacteria	Betaproteobacteria bacterium HGW-Betaproteobacteria-13	MAG: hypothetical protein CVU19_00260 [Betaproteobacteria bacterium HGW-Betaproteobacteria-13].	GCA_002842325.1
PLP59030.1	REase+TPR+TPR+GreAB-C+PIN*→	REase+TPR+TPR+GreAB-C+PIN	1226	CYK37_11065	Alphaproteobacteria	Mesorhizobium loti	hypothetical protein CYK37_11065 [Mesorhizobium loti].	GCA_002858745.1
PLX86308.1	TPR+GreAB-C+PIN*→	TPR+GreAB-C+PIN	1333	C0617_01645	Deltaproteobacteria	Desulfuromonas sp.	MAG: hypothetical protein C0617_01645 [Desulfuromonas sp.].	GCA_002868845.1
PLY41243.1	TPR+GreAB-C+PIN*→	TPR+GreAB-C+PIN	1106	CSZ94_17070	Betaproteobacteria	Janthinobacterium sp. ROICE36	hypothetical protein CSZ94_17070 [Janthinobacterium sp. ROICE36].	GCA_002869965.1
PMJ73057.1	HetE-N1→?→ GreAB-C*→	GreAB-C	461	BCU23_16040	Gammaproteobacteria	Vibrio splendidus	hypothetical protein BCU23_16040 [Vibrio splendidus].	GCA_002877525.1
POU10505.1	PIN*→	PIN	385	C3420_16980	Gammaproteobacteria	Acinetobacter sp. ACNIH3	hypothetical protein C3420_16980 [Acinetobacter sp. ACNIH3].	GCA_002918965.1
PQZ99140.1	TPR+PIN*→	TPR+PIN	932	CQ019_16410	Actinobacteria	Arthrobacter sp. MYb229	hypothetical protein CQ019_16410 [Arthrobacter sp. MYb229].	GCA_002975405.1
PRY64434.1	HetE-N1→ TPR+GreAB-C+PIN*→	TPR+GreAB-C+PIN	1090	B0H98_10595	Gammaproteobacteria	Halomonas songnenensis	hypothetical protein B0H98_10595 [Halomonas songnenensis].	GCA_003002925.1
PSL26440.1	HNH+TPR+GreAB-C+PIN*→	HNH+TPR+GreAB-C+PIN	1234	CLV42_111154	Bacteroidetes	Chitinophaga ginsengisoli	HNH endonuclease [Chitinophaga ginsengisoli].	GCA_003014595.1
PTH81074.1	TPR+GreAB-C+PIN*→	TPR+GreAB-C+PIN	851	DAA48_11040	Gammaproteobacteria	Aeromonas veronii	hypothetical protein DAA48_11040 [Aeromonas veronii].	GCA_003036425.1
PTL60251.1	X+PIN*→	X+PIN	1319	C7Y72_11685	Actinobacteria	Conexibacter sp. Seoho-28	hypothetical protein C7Y72_11685 [Conexibacter sp. Seoho-28].	GCA_003044185.1
PTX57421.1	TPR+GreAB-C+PIN*→	TPR+GreAB-C+PIN	1184	C8N43_2089	Alphaproteobacteria	Litoreibacter ponti	hypothetical protein C8N43_2089 [Litoreibacter ponti].	GCA_003054285.1
PWM31885.1	GreAB*→	GreAB	675	DBX55_01895	Verrucomicrobia	Verrucomicrobia bacterium	MAG: transcription elongation factor GreAB [Verrucomicrobia bacterium].	GCA_003343565.1
PWM77364.1	NACHT→?→ X+PIN*→ HTH→	X+PIN	733	DBY32_08445	Firmicutes	Phascolarctobacterium sp.	MAG: hypothetical protein DBY32_08445 [Phascolarctobacterium sp.].	GCA_003150755.1
PWM80532.1	TPR+GreAB-C+PIN*→	TPR+GreAB-C+PIN	1185	DBY41_05620	Firmicutes	Clostridium sp.	MAG: hypothetical protein DBY41_05620 [Clostridium sp.].	GCA_003150745.1
PZR80846.1	BirA-HTH→ HEPN→ REase+TPR+TPR+GreAB-C+PIN*→	REase+TPR+TPR+GreAB-C+PIN	1131	DI537_39015	Gammaproteobacteria	Pseudomonas stutzeri	MAG: hypothetical protein DI537_39015, partial [Pseudomonas stutzeri].	GCA_003243385.1
PZR82088.1	TPR+GreAB-C+PIN*→	TPR+GreAB-C+PIN	1139	DI537_37240	Gammaproteobacteria	Pseudomonas stutzeri	MAG: hypothetical protein DI537_37240 [Pseudomonas stutzeri].	GCA_003243385.1
PZU06315.1	REase+TPR+GreAB-C+PIN*→	REase+TPR+GreAB-C+PIN	1323	DI605_19340	Alphaproteobacteria	Sphingomonas sp.	MAG: hypothetical protein DI605_19340 [Sphingomonas sp.].	GCA_003248515.1
PZU12119.1	TPR+GreAB-C+PIN*→	TPR+GreAB-C+PIN	859	DI622_15520	Bacteroidetes	Chryseobacterium sp.	MAG: hypothetical protein DI622_15520, partial [Chryseobacterium sp.].	GCA_003248465.1
QDS92471.1	REase+TPR+GreAB-C+PIN*→	REase+TPR+GreAB-C+PIN	1250	FF011L_12140	Planctomycetes	Planctomycetes bacterium FF011L	hypothetical protein FF011L_12140 [Planctomycetes bacterium FF011L].	GCA_007741495.1
QDT50511.1	REase+TPR+GreAB-C+PIN→ TPR+GreAB-C+PIN*→	TPR+GreAB-C+PIN	495	Pan258_45900	Planctomycetes	Symmachiella dynata	hypothetical protein Pan258_45900 [Symmachiella dynata].	GCA_007744975.1
QDV12361.1	REase+TPR+GreAB-C+PIN*→	REase+TPR+GreAB-C+PIN	1246	CA51_22440	Planctomycetes	Rosistilla oblonga	Tetratricopeptide repeat protein [Rosistilla oblonga].	GCA_007751715.1
QDV84971.1	TPR+GreAB-C+PIN*→	TPR+GreAB-C+PIN	954	TBK1r_39230	Planctomycetes	Planctomycetes bacterium TBK1r	hypothetical protein TBK1r_39230 [Planctomycetes bacterium TBK1r].	GCA_007753675.1

acc	operon	architecture	len	gen.name	taxend	species	defline	gca
QFS96076.1	TPR+GreAB-C+PIN*→	TPR+GreAB-C+PIN	1184	FIV06_01510	Alphaproteobacteria	Labrenzia sp. THAF191b	hypothetical protein FIV06_01510 [Labrenzia sp. THAF191b].	GCA_009363315.1
QHB84489.1	HetE-N1→ TPR+GreAB-C+PIN*→	TPR+GreAB-C+PIN	1094	GIS01_21340	Gammaproteobacteria	Aeromonas veronii	hypothetical protein GIS01_21340 [Aeromonas veronii].	GCA_009833025.1
QIC10441.1	HetE-N1→ TPR+GreAB-C+PIN*→	TPR+GreAB-C+PIN	1069	PmVP161_2026	Gammaproteobacteria	Pasteurella multocida	hypothetical protein PmVP161_2026 [Pasteurella multocida].	GCA_011390865.1
QND19257.1	REase+TPR+TPR+GreAB-C+PIN*→	REase+TPR+TPR+GreAB-C+PIN	1348	HB774_02700	Alphaproteobacteria	Rhizobium leguminosarum bv. viciae	hypothetical protein HB774_02700 [Rhizobium leguminosarum bv. viciae].	GCA_014189655.1
QPL52208.1	HetE-N1→ TPR+GreAB-C+PIN*→	TPR+GreAB-C+PIN	1130	I3X05_08770	Gammaproteobacteria	Vibrio navarrensis	hypothetical protein I3X05_08770 [Vibrio navarrensis].	GCA_015767675.1
QQO85647.1	HetE-N1→ TPR+GreAB-C+PIN*→	TPR+GreAB-C+PIN	1089	D7032_21665	Gammaproteobacteria	Shewanella algae	hypothetical protein D7032_21665 [Shewanella algae].	-
QQR55448.1	X+PIN*→	X+PIN	1237	IPG41_02740	Bacteria	Candidatus Peregrinibacteria bacterium	MAG: hypothetical protein IPG41_02740 [Candidatus Peregrinibacteria bacterium].	GCA_016699145.1
RAZ30556.1	TPR+GreAB-C+PIN*→	TPR+GreAB-C+PIN	1208	DO944_13495	Actinobacteria	Microbacterium sp. SMR1	hypothetical protein DO944_13495 [Microbacterium sp. SMR1].	GCA_003289625.1
RBN51622.1	TPR+GreAB-C+PIN*→	TPR+GreAB-C+PIN	1135	DR980_00180	Bacteroidetes	Flavobacterium psychrolimnae	hypothetical protein DR980_00180 [Flavobacterium psychrolimnae].	GCA_003312425.1
RBP77372.1	TPR+GreAB-C+PIN*→	TPR+GreAB-C+PIN	1175	DFO80_1601	Alphaproteobacteria	Rhodobacter sp. 140A	hypothetical protein DFO80_1601, partial [Rhodobacter sp. 140A].	GCA_003315475.1
RCI79301.1	TPR+GreAB-C+PIN*→	TPR+GreAB-C+PIN	630	DNK03_13895	Alphaproteobacteria	Brucella anthropi	hypothetical protein DNK03_13895 [Brucella anthropi].	GCA_003325675.1
REJ78752.1	TPR+GreAB-C+PIN*→||||<-?<-?<-?<-REase+SNF2	TPR+GreAB-C+PIN	1257	DWQ32_08270	Acidobacteria	Acidobacteria bacterium	MAG: hypothetical protein DWQ32_08270 [Acidobacteria bacterium].	GCA_003385635.1
REJ87566.1	TPR+PIN*→	TPR+PIN	2085	DWQ34_24785	Planctomycetes	Planctomycetes bacterium	MAG: hypothetical protein DWQ34_24785 [Planctomycetes bacterium].	GCA_003388575.1
RGJ64387.1	X+PIN*→ HTH→?→?→ HTH→	X+PIN	1176	DXD50_08885	Firmicutes	Dorea formicigenerans	hypothetical protein DXD50_08885 [Dorea formicigenerans].	GCA_003437395.1
RGN88412.1	X+PIN*→ HTH→?→?→ HTH→	X+PIN	1176	DXB36_13695	Firmicutes	Dorea formicigenerans	hypothetical protein DXB36_13695 [Dorea formicigenerans].	GCA_003439255.1
RGR97298.1	TM+TM+TPR+PIN*→	TM+TM+TPR+PIN	1206	DWY20_07060	Bacteroidetes	Phocaeicola coprocola	hypothetical protein DWY20_07060 [Phocaeicola coprocola].	GCA_003458565.1
RGS66838.1	X+PIN*→ HTH→?→?→ HTH→	X+PIN	766	DWX78_14950	Firmicutes	Dorea formicigenerans	hypothetical protein DWX78_14950 [Dorea formicigenerans].	GCA_003458845.1
RGY02630.1	TPR+GreAB-C+PIN*→	TPR+GreAB-C+PIN	410	DXA56_15985	Firmicutes	Blautia obeum	hypothetical protein DXA56_15985 [Blautia obeum].	GCA_003463085.1
RGZ63692.1	Mbetalac→ Trypsin→?→ TPR+PIN*→	TPR+PIN	1371	DW979_12370	Firmicutes	Eubacterium sp. AM49-13BH	hypothetical protein DW979_12370, partial [Eubacterium sp. AM49-13BH].	GCA_003464165.1
RHN14952.1	X+PIN*→ HTH→?→?→ HTH→	X+PIN	1175	DWZ24_11335	Firmicutes	Dorea formicigenerans	hypothetical protein DWZ24_11335 [Dorea formicigenerans].	GCA_003475555.1
RHV44852.1	X+PIN*→	X+PIN	1229	DXB47_09745	Firmicutes	Firmicutes bacterium OM04-13BH	hypothetical protein DXB47_09745 [Firmicutes bacterium OM04-13BH].	GCA_003481745.1
RHZ93582.1	TPR+GreAB-C+PIN*→	TPR+GreAB-C+PIN	924	D1920_20895	Alphaproteobacteria	Rhodopseudomonas palustris	hypothetical protein D1920_20895 [Rhodopseudomonas palustris].	GCA_003547145.1
RJT21930.1	TPR+GreAB-C*→	TPR+GreAB-C	282	D5I55_15915	Alphaproteobacteria	Aestuariisphingobium litorale	hypothetical protein D5I55_15915 [Aestuariisphingobium litorale].	GCA_003602075.1
RKE07817.1	TPR+PIN*→	TPR+PIN	806	C8E86_2653	Actinobacteria	Catellatospora citrea	hypothetical protein C8E86_2653 [Catellatospora citrea].	GCA_003610235.1
RKG98095.1	TPR+GreAB-C+PIN*→	TPR+GreAB-C+PIN	461	D7X32_30660	Deltaproteobacteria	Corallococcus carmarthensis	hypothetical protein D7X32_30660 [Corallococcus carmarthensis].	GCA_003611695.1
RKS06034.1	TPR+PIN*→ PAIREDC-HTH→	TPR+PIN	1209	DFP74_1655	Actinobacteria	Nocardiopsis sp. Huas11	hypothetical protein DFP74_1655 [Nocardiopsis sp. Huas11].	GCA_003634495.1

acc	operon	architecture	len	gen.name	taxend	species	defline	gca
RKU09109.1	TPR+GreAB-C+PIN*→	TPR+GreAB-C+PIN	1315	C6502_13840	Bacteria	Candidatus Poribacteria bacterium	MAG: hypothetical protein C6502_13840 [Candidatus Poribacteria bacterium].	GCA_003635265.1
RKW53732.1	X+PIN*→	X+PIN	1207	D8H95_11955	Firmicutes	Lachnospiraceae bacterium	MAG: hypothetical protein D8H95_11955 [Lachnospiraceae bacterium].	GCA_003640285.1
RKY60125.1	PIN*→	PIN	174	DRP94_01435	FCB group	Candidatus Latescibacteria bacterium	MAG: DUF3368 domain-containing protein [Candidatus Latescibacteria bacterium].	GCA_003645885.1
RMB87410.1	TPR+PIN*→	TPR+PIN	746	CTZ28_00010	Actinobacteria	Streptomyces shenzhenensis	hypothetical protein CTZ28_00010 [Streptomyces shenzhenensis].	GCA_003688995.1
RMQ50424.1	REase+TPR+GreAB-C+PIN*→	REase+TPR+GreAB-C+PIN	1489	ALQ04_05433	Gammaproteobacteria	Pseudomonas cichorii	hypothetical protein ALQ04_05433 [Pseudomonas cichorii].	GCA_003700275.1
RMV01228.1	REase+TPR+GreAB-C+PIN*→	REase+TPR+GreAB-C+PIN	1486	ALP20_200053	Gammaproteobacteria	Pseudomonas coronafaciens pv. coronafaciens	hypothetical protein ALP20_200053 [Pseudomonas coronafaciens pv. coronafaciens].	GCA_003702885.1
RMV06369.1	TPR+GreAB-C+PIN*→	TPR+GreAB-C+PIN	904	ALP17_05452	Gammaproteobacteria	Pseudomonas savastanoi	hypothetical protein ALP17_05452 [Pseudomonas savastanoi].	GCA_003702965.1
RMV08832.1	REase→ TPR+GreAB-C+PIN*→	TPR+GreAB-C+PIN	881	ALP16_05399	Gammaproteobacteria	Pseudomonas savastanoi	hypothetical protein ALP16_05399, partial [Pseudomonas savastanoi].	GCA_003702985.1
RNA68542.1	<-REase||TPR+PIN*→	TPR+PIN	999	EBO34_00790	Firmicutes	Bacillus sp. KQ-3	hypothetical protein EBO34_00790 [Bacillus sp. KQ-3].	GCA_003710255.1
RND30553.1	HetE-N1→ HetE-N1+TPR+GreAB-C+PIN*→	HetE-N1+TPR+GreAB-C+PIN	1088	EC575_19215	Gammaproteobacteria	Vibrio cholerae	hypothetical protein EC575_19215 [Vibrio cholerae].	GCA_003712005.1
ROZ42670.1	HTH+PNPase+TPR+GreAB-C+PIN*→	HTH+PNPase+TPR+GreAB-C+PIN	1571	EEB13_29810	Actinobacteria	Rhodococcus sp. WS3	hypothetical protein EEB13_29810 [Rhodococcus sp. WS3].	GCA_003797085.1
RPV62916.1	TPR+GreAB-C+PIN*→	TPR+GreAB-C+PIN	808	IPC830_03545	Gammaproteobacteria	Pseudomonas aeruginosa	hypothetical protein IPC830_03545 [Pseudomonas aeruginosa].	GCA_003836445.1
RPX47920.1	DOC+HTH→ REase+TOPC→?→ HetE-N1→ HetE-N1+TPR+GreAB-C+PIN*→	HetE-N1+TPR+GreAB-C+PIN	1126	IPC713_13275	Gammaproteobacteria	Pseudomonas aeruginosa	hypothetical protein IPC713_13275 [Pseudomonas aeruginosa].	GCA_003836815.1
RQR65213.1	REase+TPR+GreAB-C+PIN*→	REase+TPR+GreAB-C+PIN	1346	DIE12_31765	Betaproteobacteria	Burkholderia sp. Bp9015	tetratricopeptide repeat protein [Burkholderia sp. Bp9015].	GCA_003853505.1
RQY09920.1	REase+TPR+GreAB-C+PIN*→	REase+TPR+GreAB-C+PIN	1135	DF117_34925	Betaproteobacteria	Burkholderia stagnalis	tetratricopeptide repeat protein [Burkholderia stagnalis].	GCA_003857615.1
RSM21562.1	TPR+GreAB-C+PIN*→	TPR+GreAB-C+PIN	851	C5B76_20720	Gammaproteobacteria	Aeromonas salmonicida	hypothetical protein C5B76_20720 [Aeromonas salmonicida].	GCA_003947355.1
RSM25744.1	MNS-Npun2340+TPR+GreAB-C+PIN*→	MNS-Npun2340+TPR+GreAB-C+PIN	851	C5B77_18585	Gammaproteobacteria	Aeromonas salmonicida	hypothetical protein C5B77_18585 [Aeromonas salmonicida].	GCA_003947375.1
RWE64146.1	REase+TPR+GreAB-C+PIN*→	REase+TPR+GreAB-C+PIN	1329	EOS62_30690	Alphaproteobacteria	Mesorhizobium sp.	MAG: hypothetical protein EOS62_30690 [Mesorhizobium sp.].	GCA_004018195.1
RWI50090.1	REase+TPR+GreAB-C+PIN*→	REase+TPR+GreAB-C+PIN	1328	EOR16_31040	Alphaproteobacteria	Mesorhizobium sp.	MAG: hypothetical protein EOR16_31040 [Mesorhizobium sp.].	GCA_004019035.1
RWN29279.1	REase+TPR+TPR+GreAB-C+PIN*→	REase+TPR+TPR+GreAB-C+PIN	1333	EOR95_21980	Alphaproteobacteria	Mesorhizobium sp.	MAG: hypothetical protein EOR95_21980 [Mesorhizobium sp.].	GCA_004020785.1
RWN66659.1	REase+TPR+TPR+GreAB-C+PIN*→	REase+TPR+TPR+GreAB-C+PIN	1333	EOR99_16025	Alphaproteobacteria	Mesorhizobium sp.	MAG: hypothetical protein EOR99_16025 [Mesorhizobium sp.].	GCA_004020565.1
RWQ61473.1	REase+TPR+TPR+GreAB-C+PIN*→	REase+TPR+TPR+GreAB-C+PIN	1333	EOS86_33015	Alphaproteobacteria	Mesorhizobium sp.	MAG: hypothetical protein EOS86_33015 [Mesorhizobium sp.].	GCA_004021745.1
RWQ70448.1	REase+TPR+TPR+GreAB-C+PIN*→	REase+TPR+TPR+GreAB-C+PIN	1002	EOS85_27065	Alphaproteobacteria	Mesorhizobium sp.	MAG: hypothetical protein EOS85_27065 [Mesorhizobium sp.].	GCA_004021925.1
RWU17258.1	REase+TPR+GreAB-C+PIN*→	REase+TPR+GreAB-C+PIN	1447	DM813_28290	Gammaproteobacteria	Pseudomonas alkylphenolica	hypothetical protein DM813_28290 [Pseudomonas alkylphenolica].	GCA_004025535.1
RXH58244.1	REase+TPR+GreAB-C+PIN*→	REase+TPR+GreAB-C+PIN	1185	GRAN_1554	Acidobacteria	Granulicella sibirica	hypothetical protein GRAN_1554 [Granulicella sibirica].	GCA_004115155.1
RXK05389.1	REase+TPR+GreAB-C+PIN*→	REase+TPR+GreAB-C+PIN	1249	CRU97_08585	Epsilonproteobacteria	Halarcobacter bivalviorum	hypothetical protein CRU97_08585 [Halarcobacter bivalviorum].	GCA_004116705.1

<table>
<tr><th>acc</th><th>operon</th><th>architecture</th><th>len</th><th>gen.name</th><th>taxend</th><th>species</th><th>defline</th><th>gca</th></tr>
<tr><td>RXO70745.1</td><td>HetE-N1→?→ TPR+GreAB-C+PIN*→</td><td>TPR+GreAB-C+PIN</td><td>686</td><td>D0516_07920</td><td>Gammaproteobacteria</td><td>Salmonella enterica</td><td>hypothetical protein D0516_07920 [Salmonella enterica].</td><td>GCA_004120835.1</td></tr>
<tr><td>RYE55646.1</td><td>PIN*→</td><td>PIN</td><td>344</td><td>EOP48_09765</td><td>Bacteroidetes</td><td>Sphingobacteriales bacterium</td><td>MAG: hypothetical protein EOP48_09765 [Sphingobacteriales bacterium].</td><td>GCA_004144425.1</td></tr>
<tr><td>RYG99407.1</td><td>REase+TPR+GreAB-C+PIN*→</td><td>REase+TPR+GreAB-C+PIN</td><td>1330</td><td>EON58_04040</td><td>Alphaproteobacteria</td><td>Alphaproteobacteria bacterium</td><td>MAG: hypothetical protein EON58_04040 [Alphaproteobacteria bacterium].</td><td>GCA_004145575.1</td></tr>
<tr><td>RYX84272.1</td><td>TPR+GreAB-C+PIN*→</td><td>TPR+GreAB-C+PIN</td><td>1192</td><td>EON83_11290</td><td>unclassified Bacteria</td><td>bacterium</td><td>MAG: hypothetical protein EON83_11290 [bacterium].</td><td>GCA_004172935.1</td></tr>
<tr><td>RYY90115.1</td><td>REase+TPR+GreAB-C+PIN*→</td><td>REase+TPR+GreAB-C+PIN</td><td>1333</td><td>EOO15_03925</td><td>Bacteroidetes</td><td>Chitinophagaceae bacterium</td><td>MAG: hypothetical protein EOO15_03925 [Chitinophagaceae bacterium].</td><td>GCA_004173495.1</td></tr>
<tr><td>RZJ58585.1</td><td>TPR+PIN*→</td><td>TPR+PIN</td><td>1309</td><td>EOO55_00010</td><td>Bacteroidetes</td><td>Hymenobacter sp.</td><td>MAG: hypothetical protein EOO55_00010 [Hymenobacter sp.].</td><td>GCA_004211105.1</td></tr>
<tr><td>RZJ92304.1</td><td>TPR+GreAB-C+PIN*→</td><td>TPR+GreAB-C+PIN</td><td>1267</td><td>EOO20_02310</td><td>Bacteroidetes</td><td>Chryseobacterium sp.</td><td>MAG: hypothetical protein EOO20_02310 [Chryseobacterium sp.].</td><td>GCA_004210875.1</td></tr>
<tr><td>RZJ92386.1</td><td>REase+nSTAND3→ TPR+GreAB-C+PIN*→</td><td>TPR+GreAB-C+PIN</td><td>1237</td><td>EOO20_01975</td><td>Bacteroidetes</td><td>Chryseobacterium sp.</td><td>MAG: tetratricopeptide repeat protein [Chryseobacterium sp.].</td><td>GCA_004210875.1</td></tr>
<tr><td>RZL63110.1</td><td>PIN*→</td><td>PIN</td><td>307</td><td>EOO93_08085</td><td>Bacteroidetes</td><td>Pedobacter sp.</td><td>MAG: hypothetical protein EOO93_08085 [Pedobacter sp.].</td><td>GCA_004295955.1</td></tr>
<tr><td>RZL84003.1</td><td>REase+TPR+GreAB-C+PIN*→</td><td>REase+TPR+GreAB-C+PIN</td><td>1323</td><td>EOP66_03300</td><td>Alphaproteobacteria</td><td>Sphingomonas sp.</td><td>MAG: tetratricopeptide repeat protein [Sphingomonas sp.].</td><td>GCA_004296035.1</td></tr>
<tr><td>SCB54124.1</td><td>REase+TPR+GreAB-C+PIN*→</td><td>REase+TPR+GreAB-C+PIN</td><td>1171</td><td>GA0061098_102379</td><td>Alphaproteobacteria</td><td>Bradyrhizobium shewense</td><td>Transcription elongation factor, GreA/GreB family [Bradyrhizobium shewense].</td><td>GCA_900094605.1</td></tr>
<tr><td>SCE01180.1</td><td>TPR+PIN*→||||<-NUDIX</td><td>TPR+PIN</td><td>1134</td><td>GA0115241_108994</td><td>Actinobacteria</td><td>Streptomyces sp. DpondAA-D4</td><td>hypothetical protein GA0115241_108994 [Streptomyces sp. DpondAA-D4].</td><td>GCA_900091745.1</td></tr>
<tr><td>SCF05211.1</td><td>REase+TPR+GreAB-C+PIN*→</td><td>REase+TPR+GreAB-C+PIN</td><td>1358</td><td>GA0070215_1072</td><td>Actinobacteria</td><td>Micromonospora marina</td><td>hypothetical protein GA0070215_1072 [Micromonospora marina].</td><td>GCA_900091565.1</td></tr>
<tr><td>SCF05420.1</td><td>SAD+HNH→ X+PIN*→</td><td>X+PIN</td><td>1233</td><td>GA0070562_5649</td><td>Actinobacteria</td><td>Micromonospora tulbaghiae</td><td>hypothetical protein GA0070562_5649 [Micromonospora tulbaghiae].</td><td>GCA_900091605.1</td></tr>
<tr><td>SCL17674.1</td><td>REase+TPR+GreAB-C+PIN*→</td><td>REase+TPR+GreAB-C+PIN</td><td>1264</td><td>GA0074694_2093</td><td>Actinobacteria</td><td>Micromonospora inyonensis</td><td>Tfp pilus assembly protein PilF [Micromonospora inyonensis].</td><td>GCA_900091415.1</td></tr>
<tr><td>SCQ18794.1</td><td>CHC2+Toprim-DNAG→?→?→?→ HTH→ SIG+TM→ TPR+GreAB-C+PIN*→</td><td>TPR+GreAB-C+PIN</td><td>1126</td><td>TFUB4_00594</td><td>Bacteroidetes</td><td>Tannerella forsythia</td><td>hypothetical protein TFUB4_00594 [Tannerella forsythia].</td><td>GCA_900096725.1</td></tr>
<tr><td>SCQ19220.1</td><td>CHC2+Toprim-DNAG→?→?→?→ HTH→ SIG+TM→ TPR+GreAB-C+PIN*→</td><td>TPR+GreAB-C+PIN</td><td>1126</td><td>TFUB20_00631</td><td>Bacteroidetes</td><td>Tannerella forsythia</td><td>hypothetical protein TFUB20_00631 [Tannerella forsythia].</td><td>GCA_900096735.1</td></tr>
<tr><td>SEP47521.1</td><td>TPR+GreAB-C+PIN*→</td><td>TPR+GreAB-C+PIN</td><td>975</td><td>SAMN02787149_1275</td><td>Gammaproteobacteria</td><td>Pseudomonas sp. Snoq117.2</td><td>hypothetical protein SAMN02787149_1275 [Pseudomonas sp. Snoq117.2].</td><td>GCA_900110545.1</td></tr>
<tr><td>SFD04359.1</td><td><-KAP_NTPase||TPR+PIN*→</td><td>TPR+PIN</td><td>1216</td><td>SAMN04487968_1267</td><td>Actinobacteria</td><td>Nocardioides terrae</td><td>hypothetical protein SAMN04487968_1267 [Nocardioides terrae].</td><td>GCA_900112345.1</td></tr>
<tr><td>SFF03357.1</td><td><-Cas_Cas1<-?||?→?→?→?→ REase+TPR+GreAB-C+PIN*→</td><td>REase+TPR+GreAB-C+PIN</td><td>1349</td><td>SAMN05428977_10446</td><td>Betaproteobacteria</td><td>Nitrosomonas sp. Nm166</td><td>hypothetical protein SAMN05428977_10446 [Nitrosomonas sp. Nm166].</td><td>GCA_900112825.1</td></tr>
<tr><td>SHH05937.1</td><td>REase+TPR+GreAB-C+PIN*→</td><td>REase+TPR+GreAB-C+PIN</td><td>1445</td><td>SAMN05428948_2561</td><td>Betaproteobacteria</td><td>Massilia sp. CF038</td><td>hypothetical protein SAMN05428948_2561 [Massilia sp. CF038].</td><td>GCA_900129765.1</td></tr>
<tr><td>SHN30683.1</td><td>PAIREDC-HTH→ REase+TPR+GreAB-C+PIN*→</td><td>REase+TPR+GreAB-C+PIN</td><td>1588</td><td>SAMN02787076_04976</td><td>Betaproteobacteria</td><td>Rhizobacter sp. OV335</td><td>hypothetical protein SAMN02787076_04976 [Rhizobacter sp. OV335].</td><td>GCA_900142965.1</td></tr>
<tr><td>SIO63359.1</td><td>REase+TPR+GreAB-C+PIN*→</td><td>REase+TPR+GreAB-C+PIN</td><td>1291</td><td>SAMN05444166_7323</td><td>Planctomycetes</td><td>Singulisphaera sp. GP187</td><td>Transcription elongation factor, GreA/GreB family [Singulisphaera sp. GP187].</td><td>GCA_900129635.1</td></tr>
<tr><td>SOZ22209.1</td><td>REase+TPR+GreAB-C+PIN→?→?→ PIN*→</td><td>PIN</td><td>271</td><td>CBM2604_U10003</td><td>Betaproteobacteria</td><td>Cupriavidus taiwanensis</td><td>hypothetical protein CBM2604_U10003 [Cupriavidus taiwanensis].</td><td>GCA_900249855.1</td></tr>
<tr><td>SPT59210.1</td><td>TPR+GreAB-C+PIN*→</td><td>TPR+GreAB-C+PIN</td><td>1218</td><td>NCTC11373_05764</td><td>Actinobacteria</td><td>Actinomadura madurae</td><td>Flp pilus assembly protein TadD, contains TPR repeats [Actinomadura madurae].</td><td>GCA_900445005.1</td></tr>
<tr><td>SPY99988.1</td><td>TPR+GreAB-C+PIN*→</td><td>TPR+GreAB-C+PIN</td><td>1146</td><td>NCTC11842_00133</td><td>Gammaproteobacteria</td><td>Pseudomonas luteola</td><td>Uncharacterised protein [Pseudomonas luteola].</td><td>GCA_900455515.1</td></tr>
</table>

acc	operon	architecture	len	gen.name	taxend	species	defline	gca
SQK85244.1	DOC+HTH→ REase+TOPC→ HetE-N1→ TPR+GreAB-C+PIN*→	TPR+GreAB-C+PIN	1088	NCTC9433_00599	Gammaproteobacteria	Pseudomonas aeruginosa	Uncharacterised protein [Pseudomonas aeruginosa].	GCA_900478745.1
SSC09307.1	TPR+GreAB-C+PIN*→	TPR+GreAB-C+PIN	1137	BTURTLESOX_2492	unclassified Bacteria	bacterium endosymbiont of Bathymodiolus sp. 5 South	hypothetical protein BTURTLESOX_2492 [bacterium endosymbiont of Bathymodiolus sp. 5 South].	GCA_900127295.1
STJ38438.1	HetE-N1→ TPR+GreAB-C+PIN*→	TPR+GreAB-C+PIN	1093	NCTC9110_01467	Gammaproteobacteria	Escherichia coli	Uncharacterised protein [Escherichia coli].	GCA_900449425.1
STR27356.1	REase+TPR+GreAB-C+PIN*→	REase+TPR+GreAB-C+PIN	1303	NCTC8661_05000	Betaproteobacteria	Janthinobacterium lividum	Putative Zn-dependent protease, contains TPR repeats [Janthinobacterium lividum].	GCA_900451225.1
SUJ03282.1	PIN*→	PIN	223	NCTC4822_01389	Firmicutes	Sporosarcina pasteurii	Uncharacterised protein [Sporosarcina pasteurii].	GCA_900457495.1
TAA79883.1	HetE-N1→ TPR+GreAB-C+PIN*→	TPR+GreAB-C+PIN	1069	PMCND_08490	Gammaproteobacteria	Pasteurella multocida	hypothetical protein PMCND_08490 [Pasteurella multocida].	GCA_004286935.1
TAJ47852.1	TPR+PIN*→	TPR+PIN	1186	EPO52_06520	Actinobacteria	Herbiconiux sp.	MAG: hypothetical protein EPO52_06520 [Herbiconiux sp.].	GCA_004297105.1
TAM51231.1	REase+TPR+GreAB-C+PIN*→	REase+TPR+GreAB-C+PIN	1301	EPN57_18445	Betaproteobacteria	Paraburkholderia sp.	MAG: hypothetical protein EPN57_18445 [Paraburkholderia sp.].	GCA_004322045.1
TAM55201.1	REase+TPR+GreAB-C+PIN*→||||<-?||AbiJ-NTD3+REase→	REase+TPR+GreAB-C+PIN	1335	EPN57_04160	Betaproteobacteria	Paraburkholderia sp.	MAG: hypothetical protein EPN57_04160 [Paraburkholderia sp.].	GCA_004322045.1
TCU73661.1	TPR+GreAB-C+PIN*→	TPR+GreAB-C+PIN	1313	EDE10_104327	Alphaproteobacteria	Bradyrhizobium sp. Y-H1	tetratricopeptide repeat protein [Bradyrhizobium sp. Y-H1].	GCA_004346395.1
TDM08142.1	REase+TPR+GreAB-C+PIN*→	REase+TPR+GreAB-C+PIN	1202	C4K60_01155	Betaproteobacteria	Ideonella sp. MAG2	MAG: hypothetical protein C4K60_01155 [Ideonella sp. MAG2].	GCA_004359425.1
TDZ78257.1	TPR+PIN*→	TPR+PIN	1185	DE4585_04094	Actinobacteria	Mycobacteroides salmoniphilum	hypothetical protein DE4585_04094 [Mycobacteroides salmoniphilum].	GCA_004366855.1
TIN45883.1	TPR+GreAB-C+PIN*→ TPR→	TPR+GreAB-C+PIN	539	E5Y32_12205	Alphaproteobacteria	Mesorhizobium sp.	MAG: GreA/GreB family elongation factor [Mesorhizobium sp.].	GCA_004961865.1
TIT34473.1	TPR+GreAB-C+PIN*→	TPR+GreAB-C+PIN	502	E5W78_10605	Alphaproteobacteria	Mesorhizobium sp.	MAG: hypothetical protein E5W78_10605, partial [Mesorhizobium sp.].	GCA_004965505.1
TIX43905.1	TPR+GreAB-C+PIN*→	TPR+GreAB-C+PIN	708	E5V40_01360	Alphaproteobacteria	Mesorhizobium sp.	MAG: GreA/GreB family elongation factor [Mesorhizobium sp.].	GCA_004964855.1
TLM83646.1	TPR+PIN*→	TPR+PIN	1179	FDW83_09305	Actinobacteria	Pseudarthrobacter sp. NamE2	tetratricopeptide repeat protein [Pseudarthrobacter sp. NamE2].	GCA_005796205.1
TMA89552.1	REase+TPR+GreAB-C+PIN*→	REase+TPR+GreAB-C+PIN	1299	E6J74_25025	Deltaproteobacteria	Deltaproteobacteria bacterium	MAG: hypothetical protein E6J74_25025 [Deltaproteobacteria bacterium].	GCA_005879255.1
TMJ19619.1	REase+TPR+GreAB-C+PIN*→	REase+TPR+GreAB-C+PIN	1331	E6G92_07565	Alphaproteobacteria	Alphaproteobacteria bacterium	MAG: hypothetical protein E6G92_07565 [Alphaproteobacteria bacterium].	GCA_005883305.1
TOF36973.1	TPR+GreAB-C+PIN*→	TPR+GreAB-C+PIN	785	CGJ28_20670	Gammaproteobacteria	Vibrio parahaemolyticus	hypothetical protein CGJ28_20670 [Vibrio parahaemolyticus].	GCA_006371865.1
TOH27190.1	GreAB-C*→	GreAB-C	599	CGI84_22675	Gammaproteobacteria	Vibrio parahaemolyticus	hypothetical protein CGI84_22675, partial [Vibrio parahaemolyticus].	GCA_006372765.1
TQE99579.1	REase+SNF2→ REase+TPR+GreAB-C+PIN*→	REase+TPR+GreAB-C+PIN	1230	FKY71_07895	Gammaproteobacteria	Spiribacter salinus	MAG: hypothetical protein FKY71_07895 [Spiribacter salinus].	GCA_006569195.1
TQS42859.1	REase+TPR+GreAB-C+PIN*→	REase+TPR+GreAB-C+PIN	1252	FL583_22685	Actinobacteria	Cryptosporangium phraense	DUF4365 domain-containing protein [Cryptosporangium phraense].	GCA_006912135.1
TSP11291.1	REase+TPR+GreAB-C+PIN*→	REase+TPR+GreAB-C+PIN	1314	FGG12_18885	Betaproteobacteria	Cupriavidus campinensis	DUF4365 domain-containing protein [Cupriavidus campinensis].	GCA_007572485.1
TVP63446.1	EAD10+SWACOS→?→ EAD10+TPR+GreAB-C+PIN*→	EAD10+TPR+GreAB-C+PIN	1213	EA343_08005	Cyanobacteria	Nodularia sp. (in: Bacteria)	MAG: hypothetical protein EA343_08005 [Nodularia sp. (in: Bacteria)].	GCA_007692755.1
TWD39045.1	HetE-N1→ TPR+GreAB-C+PIN*→	TPR+GreAB-C+PIN	1117	FBY13_107200	Gammaproteobacteria	Pantoea sp. SJZ147	hypothetical protein FBY13_107200 [Pantoea sp. SJZ147].	GCA_007828605.1
TWI95027.1	TPR+GreAB-C+PIN→ PIN*→	PIN	240	JN11_04456	Bacteroidetes	Mucilaginibacter frigoritolerans	hypothetical protein JN11_04456 [Mucilaginibacter frigoritolerans].	GCA_007830615.1

acc	operon	architecture	len	gen.name	taxend	species	defline	gca
TXH46669.1	REase+TPR+GreAB-C+PIN*→	REase+TPR+GreAB-C+PIN	1345	E6Q92_01630	Betaproteobacteria	Burkholderiaceae bacterium	MAG: tetratricopeptide repeat protein [Burkholderiaceae bacterium].	GCA_008012415.1
TXJ01161.1	X+PIN*→	X+PIN	1259	E6Q32_04910	Betaproteobacteria	Neisseriales bacterium	MAG: hypothetical protein E6Q32_04910 [Neisseriales bacterium].	GCA_008017145.1
VBE91451.1	REase+TPR+GreAB-C+PIN*→	REase+TPR+GreAB-C+PIN	1327	SAMEA2066717_00371	Betaproteobacteria	Burkholderia pseudomallei	Uncharacterised protein [Burkholderia pseudomallei].	GCA_900591745.1
VBP17170.1	REase+TPR+GreAB-C+PIN*→	REase+TPR+GreAB-C+PIN	1327	SAMEA2065229_01356	Betaproteobacteria	Burkholderia pseudomallei	Uncharacterised protein [Burkholderia pseudomallei].	GCA_900593515.1
VBP73370.1	REase+TPR+GreAB-C+PIN*→	REase+TPR+GreAB-C+PIN	1327	SAMEA2068240_03247	Betaproteobacteria	Burkholderia pseudomallei	Uncharacterised protein [Burkholderia pseudomallei].	GCA_900593615.1
VEA49048.1	HetE-N1→ TPR+GreAB-C+PIN*→	TPR+GreAB-C+PIN	1107	NCTC7306_04489	Gammaproteobacteria	Salmonella enterica subsp. arizonae	Uncharacterised protein [Salmonella enterica subsp. arizonae].	GCA_900635595.1
VTU02511.1	REase+TPR+GreAB-C+PIN*→	REase+TPR+GreAB-C+PIN	2231	GCJUQL4_63830	Planctomycetes	Gemmataceae bacterium	atpase aaa : Uncharacterized protein OS=delta proteobacterium MLMS-1 GN=MldDRAFT_0410 PE=4 SV=1: TPR_9 [Gemmataceae bacterium].	GCA_901538445.1
VUO82246.1	REase+TPR+GreAB-C+PIN*→	REase+TPR+GreAB-C+PIN	1284	MFJNGALL_01363	Gammaproteobacteria	Stenotrophomonas maltophilia	hypothetical protein MFJNGALL_01363 [Stenotrophomonas maltophilia].	GCA_902157665.1
VUP91206.1	REase+TPR→ TPR+PIN*→	TPR+PIN	777	MKCCKLKB_00143	Gammaproteobacteria	Stenotrophomonas maltophilia	hypothetical protein MKCCKLKB_00143 [Stenotrophomonas maltophilia].	GCA_902158105.1
VVH66851.1	TPR+GreAB-C+PIN*→	TPR+GreAB-C+PIN	1163	BSPLISOX_572	environmental samples	uncultured Gammaproteobacteria bacterium	hypothetical protein BSPLISOX_572 [uncultured Gammaproteobacteria bacterium].	GCA_902497535.1
VVM25671.1	TPR→ TPR+GreAB-C+PIN*→	TPR+GreAB-C+PIN	622	BSPWISOXPB_7707	environmental samples	uncultured Gammaproteobacteria bacterium	hypothetical protein BSPWISOXPB_7707 [uncultured Gammaproteobacteria bacterium].	GCA_902497575.1
WP_000012642.1	TPR+PIN*→	TPR+PIN	1218	-	Firmicutes	Bacillus cereus	hypothetical protein [Bacillus cereus].	GCF_000291235.1
WP_000989353.1	HetE-N1→ TPR+GreAB-C+PIN*→	TPR+GreAB-C+PIN	1094	-	Gammaproteobacteria	Escherichia	MULTISPECIES: hypothetical protein [Escherichia].	GCF_000503375.1
WP_002163366.1	TPR+PIN*→	TPR+PIN	1214	-	Firmicutes	Bacillus paranthracis	hypothetical protein [Bacillus paranthracis].	GCF_900176925.1
WP_002203277.1	TPR+PIN*→	TPR+PIN	1158	-	Firmicutes	Bacillus cereus	hypothetical protein [Bacillus cereus].	GCF_000293545.1
WP_002981151.1	TPR+GreAB-C+PIN*→	TPR+GreAB-C+PIN	1137	-	Bacteroidetes	Chryseobacterium	MULTISPECIES: hypothetical protein [Chryseobacterium].	GCF_003815675.1
WP_003193440.1	TPR+PIN*→	TPR+PIN	1179	-	Firmicutes	Bacillus mycoides	hypothetical protein [Bacillus mycoides].	GCF_000003925.1
WP_003320554.1	TPR+GreAB-C+PIN*→	TPR+GreAB-C+PIN	1148	-	Firmicutes	Alkalihalobacillus alcalophilus	hypothetical protein [Alkalihalobacillus alcalophilus].	GCF_000292245.2
WP_003365555.1	TPR+GreAB-C+PIN*→	TPR+GreAB-C+PIN	1052	-	Firmicutes	Clostridium botulinum	hypothetical protein [Clostridium botulinum].	GCF_000171095.1
WP_004107781.1	REase+TPR+TPR+GreAB-C+PIN*→	REase+TPR+TPR+GreAB-C+PIN	1363	-	Alphaproteobacteria	Rhizobium freirei	restriction endonuclease [Rhizobium freirei].	GCF_000359745.1
WP_004552328.1	REase+TPR+GreAB-C+PIN*→	REase+TPR+GreAB-C+PIN	1345	-	Betaproteobacteria	Burkholderia pseudomallei	hypothetical protein [Burkholderia pseudomallei].	GCF_000259815.1
WP_005336021.1	X+PIN*→ HTH→?→?→ HTH→	X+PIN	1180	-	Firmicutes	Dorea formicigenerans	hypothetical protein [Dorea formicigenerans].	GCF_000169235.1
WP_005619555.1	REase+TPR+GreAB-C+PIN*→	REase+TPR+GreAB-C+PIN	1313	-	Alphaproteobacteria	Epibacterium mobile	tetratricopeptide repeat protein [Epibacterium mobile].	GCF_000376545.2
WP_005875010.1	HTH→ SIG+TM→ TPR+GreAB-C+PIN*→	TPR+GreAB-C+PIN	1125	-	Bacteroidetes	Porphyromonas gingivalis	hypothetical protein [Porphyromonas gingivalis].	GCF_000007585.1
WP_007114289.1	TPR+GreAB-C+PIN*→	TPR+GreAB-C+PIN	1116	-	Gammaproteobacteria	Halomonas boliviensis	hypothetical protein [Halomonas boliviensis].	GCF_000236035.1
WP_007575774.1	REase+TPR+GreAB-C+PIN*→	REase+TPR+GreAB-C+PIN	1298	-	Actinobacteria	Patulibacter medicamentivorans	hypothetical protein [Patulibacter medicamentivorans].	GCF_000240225.1
WP_008074062.1	HetE-N1→ TPR+GreAB-C+PIN*→	TPR+GreAB-C+PIN	1116	-	Gammaproteobacteria	Vibrio sinaloensis	hypothetical protein [Vibrio sinaloensis].	GCF_000189275.1
WP_008371053.1	TPR+GreAB-C+PIN*→	TPR+GreAB-C+PIN	916	-	Gammaproteobacteria	Pseudomonas sp. M47T1	hypothetical protein [Pseudomonas sp. M47T1].	GCF_000263855.1
WP_008676251.1	REase+TPR+GreAB-C+PIN*→	REase+TPR+GreAB-C+PIN	1224	-	Planctomycetes	Rhodopirellula sallentina	phosphorylase family 1 protein [Rhodopirellula sallentina].	GCF_000346505.1
WP_008891423.1	REase+TPR+GreAB-C+PIN*→	REase+TPR+GreAB-C+PIN	1287	-	Alphaproteobacteria	Thalassospira profundimaris	tetratricopeptide repeat protein [Thalassospira profundimaris].	GCF_000300275.1
WP_009135826.1	X+PIN*→	X+PIN	1248	-	Bacteroidetes	Odoribacter laneus	hypothetical protein [Odoribacter laneus].	GCF_902374615.1
WP_009204273.1	X+PIN*→	X+PIN	1224	-	Firmicutes	Anaerostipes hadrus	hypothetical protein [Anaerostipes hadrus].	GCF_000332875.2

<table>
<tr><th>acc</th><th>operon</th><th>architecture</th><th>len</th><th>gen.name</th><th>taxend</th><th>species</th><th>defline</th><th>gca</th></tr>
<tr><td>WP_009805247.1</td><td>REase+TPR+GreAB-C+PIN*→</td><td>REase+TPR+GreAB-C+PIN</td><td>1334</td><td>-</td><td>Alphaproteobacteria</td><td>Pseudooceanicola batsensis</td><td>restriction endonuclease [Pseudooceanicola batsensis].</td><td>GCF_000152725.1</td></tr>
<tr><td>WP_009857784.1</td><td>REase+TPR+GreAB-C+PIN*→</td><td>REase+TPR+GreAB-C+PIN</td><td>1374</td><td>-</td><td>Betaproteobacteria</td><td>Rubrivivax benzoatilyticus</td><td>restriction endonuclease [Rubrivivax benzoatilyticus].</td><td>GCF_000190375.1</td></tr>
<tr><td>WP_009899558.1</td><td>TPR+GreAB-C+PIN*→</td><td>TPR+GreAB-C+PIN</td><td>1069</td><td>-</td><td>Firmicutes</td><td>Clostridioides difficile</td><td>hypothetical protein [Clostridioides difficile].</td><td>GCF_000211235.1</td></tr>
<tr><td>WP_010516915.1</td><td>REase+TPR+GreAB-C+PIN*→</td><td>REase+TPR+GreAB-C+PIN</td><td>1323</td><td>-</td><td>Alphaproteobacteria</td><td>Komagataeibacter oboediens</td><td>tetratricopeptide repeat protein [Komagataeibacter oboediens].</td><td>GCF_000227565.1</td></tr>
<tr><td>WP_010555470.1</td><td>HetE-N1→ TPR+GreAB-C+PIN*→</td><td>TPR+GreAB-C+PIN</td><td>1100</td><td>-</td><td>Gammaproteobacteria</td><td>Pseudoalteromonas arctica</td><td>hypothetical protein [Pseudoalteromonas arctica].</td><td>GCF_000238395.3</td></tr>
<tr><td>WP_011204815.1</td><td>REase+TPR+GreAB-C+PIN*→||||<-HTH+HTH</td><td>REase+TPR+GreAB-C+PIN</td><td>1344</td><td>-</td><td>Betaproteobacteria</td><td>Burkholderia pseudomallei</td><td>hypothetical protein [Burkholderia pseudomallei].</td><td>GCF_000350505.1</td></tr>
<tr><td>WP_011691689.1</td><td>TPR+PIN*→</td><td>TPR+PIN</td><td>1180</td><td>-</td><td>Actinobacteria</td><td>Arthrobacter sp. FB24</td><td>tetratricopeptide repeat protein [Arthrobacter sp. FB24].</td><td>GCF_000196235.1</td></tr>
<tr><td>WP_012187551.1</td><td>TPR+PIN*→</td><td>TPR+PIN</td><td>1280</td><td>-</td><td>Gammaproteobacteria</td><td>Fluoribacter dumoffii</td><td>hypothetical protein [Fluoribacter dumoffii].</td><td>-</td></tr>
<tr><td>WP_012304478.1</td><td>HetE-N1→ TPR+GreAB-C+PIN*→</td><td>TPR+GreAB-C+PIN</td><td>1090</td><td>-</td><td>Gammaproteobacteria</td><td>Yersinia pseudotuberculosis</td><td>hypothetical protein [Yersinia pseudotuberculosis].</td><td>GCF_000019465.1</td></tr>
<tr><td>WP_012365289.1</td><td>REase+TPR+GreAB-C+PIN*→</td><td>REase+TPR+GreAB-C+PIN</td><td>1351</td><td>-</td><td>Betaproteobacteria</td><td>Burkholderia ambifaria</td><td>hypothetical protein [Burkholderia ambifaria].</td><td>GCF_000019925.1</td></tr>
<tr><td>WP_012655030.1</td><td>REase+TPR+GreAB-C+PIN*→</td><td>REase+TPR+GreAB-C+PIN</td><td>1290</td><td>-</td><td>Alphaproteobacteria</td><td>Agrobacterium vitis</td><td>tetratricopeptide repeat protein [Agrobacterium vitis].</td><td>GCF_000016285.1</td></tr>
<tr><td>WP_013320645.1</td><td><-TIR+CASPASE<-?<-?||PNPase+TPR+GreAB-C+PIN*→</td><td>PNPase+TPR+GreAB-C+PIN</td><td>1389</td><td>-</td><td>Cyanobacteria</td><td>Gloeothece verrucosa</td><td>purine or other phosphorylase family 1 [Gloeothece verrucosa].</td><td>GCF_000147335.1</td></tr>
<tr><td>WP_013724217.1</td><td>HetE-N1→ MNS-Npun2340+TPR+GreAB-C+PIN*→</td><td>MNS-Npun2340+TPR+GreAB-C+PIN</td><td>1094</td><td>-</td><td>Gammaproteobacteria</td><td>Aeromonas veronii</td><td>hypothetical protein [Aeromonas veronii].</td><td>GCF_000204115.1</td></tr>
<tr><td>WP_013846680.1</td><td>REase+TPR+GreAB-C+PIN*→</td><td>REase+TPR+GreAB-C+PIN</td><td>1311</td><td>-</td><td>Alphaproteobacteria</td><td>Sphingobium chlorophenolicum</td><td>hypothetical protein [Sphingobium chlorophenolicum].</td><td>GCF_000147835.2</td></tr>
<tr><td>WP_013921742.1</td><td>Mbetalac→ Trypsin+TPR+GreAB-C+PIN*→</td><td>Trypsin+TPR+GreAB-C+PIN</td><td>1363</td><td>-</td><td>Bacteroidetes</td><td>Runella slithyformis</td><td>trypsin-like peptidase domain-containing protein [Runella slithyformis].</td><td>GCF_000218895.1</td></tr>
<tr><td>WP_014399772.1</td><td>TPR+GreAB-C+PIN*→</td><td>TPR+GreAB-C+PIN</td><td>1160</td><td>-</td><td>Deltaproteobacteria</td><td>Corallococcus coralloides</td><td>GreA/GreB family elongation factor [Corallococcus coralloides].</td><td>GCF_000255295.1</td></tr>
<tr><td>WP_015949235.1</td><td>REase+TPR+GreAB-C+PIN*→</td><td>REase+TPR+GreAB-C+PIN</td><td>1112</td><td>-</td><td>Deltaproteobacteria</td><td>Desulfatibacillum aliphaticivorans</td><td>hypothetical protein [Desulfatibacillum aliphaticivorans].</td><td>GCF_000021905.1</td></tr>
<tr><td>WP_016083038.1</td><td>TPR+PIN*→</td><td>TPR+PIN</td><td>1154</td><td>-</td><td>Firmicutes</td><td>Bacillus cereus</td><td>hypothetical protein [Bacillus cereus].</td><td>GCF_000399005.1</td></tr>
<tr><td>WP_016796932.1</td><td>TPR+GreAB-C+PIN*→</td><td>TPR+GreAB-C+PIN</td><td>1236</td><td>-</td><td>Gammaproteobacteria</td><td>Vibrio cyclitrophicus</td><td>hypothetical protein [Vibrio cyclitrophicus].</td><td>GCF_000256385.2</td></tr>
<tr><td>WP_016981456.1</td><td>REase+TPR+GreAB-C+PIN*→</td><td>REase+TPR+GreAB-C+PIN</td><td>1113</td><td>-</td><td>Gammaproteobacteria</td><td>Pseudomonas amygdali</td><td>hypothetical protein [Pseudomonas amygdali].</td><td>GCF_000275945.1</td></tr>
<tr><td>WP_017010147.1</td><td>TPR+GreAB-C+PIN*→</td><td>TPR+GreAB-C+PIN</td><td>1114</td><td>-</td><td>Gammaproteobacteria</td><td>Enterovibrio norvegicus</td><td>hypothetical protein [Enterovibrio norvegicus].</td><td>GCF_000286875.2</td></tr>
<tr><td>WP_017056082.1</td><td>HetE-N1→ TPR+GreAB-C+PIN*→</td><td>TPR+GreAB-C+PIN</td><td>1088</td><td>-</td><td>Gammaproteobacteria</td><td>Vibrio kanaloae</td><td>hypothetical protein [Vibrio kanaloae].</td><td>GCF_000272165.2</td></tr>
<tr><td>WP_017278805.1</td><td>TPR+GreAB-C+PIN*→</td><td>TPR+GreAB-C+PIN</td><td>1158</td><td>-</td><td>Gammaproteobacteria</td><td>Pseudomonas syringae</td><td>hypothetical protein [Pseudomonas syringae].</td><td>GCF_001913215.1</td></tr>
<tr><td>WP_017307658.1</td><td>EAD10→?→?→ EAD10+TPR+GreAB-C+PIN*→</td><td>EAD10+TPR+GreAB-C+PIN</td><td>1214</td><td>-</td><td>Cyanobacteria</td><td>Fischerella sp. PCC 9339</td><td>hypothetical protein [Fischerella sp. PCC 9339].</td><td>GCF_000315585.1</td></tr>
<tr><td>WP_017684618.1</td><td>REase+TPR+GreAB-C+PIN*→</td><td>REase+TPR+GreAB-C+PIN</td><td>1438</td><td>-</td><td>Gammaproteobacteria</td><td>Pseudomonas syringae</td><td>hypothetical protein [Pseudomonas syringae].</td><td>GCF_000233815.1</td></tr>
<tr><td>WP_017867504.1</td><td>X+PIN*→</td><td>X+PIN</td><td>1207</td><td>-</td><td>Firmicutes</td><td>Ligilactobacillus pobuzihii</td><td>DUF4365 domain-containing protein [Ligilactobacillus pobuzihii].</td><td>GCF_001433815.1</td></tr>
<tr><td>WP_018464656.1</td><td>TPR+PIN*→</td><td>TPR+PIN</td><td>1200</td><td>-</td><td>Bacteroidetes</td><td>Prevotella paludivivens</td><td>hypothetical protein [Prevotella paludivivens].</td><td>GCF_000613605.1</td></tr>
<tr><td>WP_019086277.1</td><td>REase+TPR+GreAB-C+PIN*→</td><td>REase+TPR+GreAB-C+PIN</td><td>1323</td><td>-</td><td>Alphaproteobacteria</td><td>Komagataeibacter europaeus</td><td>tetratricopeptide repeat protein [Komagataeibacter europaeus].</td><td>GCF_000285295.1</td></tr>
<tr><td>WP_019092358.1</td><td>REase+TPR+GreAB-C+PIN*→</td><td>REase+TPR+GreAB-C+PIN</td><td>1323</td><td>-</td><td>Alphaproteobacteria</td><td>Acetobacteraceae</td><td>MULTISPECIES: tetratricopeptide repeat protein [Acetobacteraceae].</td><td>GCF_003850965.1</td></tr>
<tr><td>WP_019865944.1</td><td>HetE-N1→ TPR+GreAB-C+PIN*→</td><td>TPR+GreAB-C+PIN</td><td>1111</td><td>-</td><td>Gammaproteobacteria</td><td>Methylovulum miyakonense</td><td>hypothetical protein [Methylovulum miyakonense].</td><td>GCF_000384075.1</td></tr>
<tr><td>WP_019949723.1</td><td>TPR+GreAB-C+PIN*→</td><td>TPR+GreAB-C+PIN</td><td>909</td><td>-</td><td>Bacteroidetes</td><td>Hymenobacter aerophilus</td><td>hypothetical protein [Hymenobacter aerophilus].</td><td>GCF_000382225.1</td></tr>
<tr><td>WP_020099407.1</td><td>TPR+PIN*→</td><td>TPR+PIN</td><td>1185</td><td>-</td><td>Actinobacteria</td><td>Mycobacterium sp. 360MFTsu5.1</td><td>hypothetical protein [Mycobacterium sp. 360MFTsu5.1].</td><td>GCF_000383495.1</td></tr>
<tr><td>WP_020795266.1</td><td>HetE-N1→ TPR+GreAB-C+PIN*→</td><td>TPR+GreAB-C+PIN</td><td>1112</td><td>-</td><td>Gammaproteobacteria</td><td>Pseudomonas sp. G5(2012)</td><td>hypothetical protein [Pseudomonas sp. G5(2012)].</td><td>GCF_000408945.1</td></tr>
<tr><td>WP_021554497.1</td><td>HetE-N1→ TPR+GreAB-C+PIN*→</td><td>TPR+GreAB-C+PIN</td><td>1093</td><td>-</td><td>Gammaproteobacteria</td><td>Enterobacteriaceae</td><td>MULTISPECIES: hypothetical protein [Enterobacteriaceae].</td><td>GCF_002110225.1</td></tr>
<tr><td>WP_021876213.1</td><td>PIN*→</td><td>PIN</td><td>1097</td><td>-</td><td>Firmicutes</td><td>Clostridium chauvoei</td><td>tetratricopeptide repeat protein [Clostridium chauvoei].</td><td>GCF_002327205.1</td></tr>
</table>

acc	operon	architecture	len	gen.name	taxend	species	defline	gca
WP_022388983.1	X+PIN*→	X+PIN	1200	-	Firmicutes	Blautia obeum	hypothetical protein [Blautia obeum].	GCF_013299585.1
WP_022619607.1	TPR+GreAB-C+PIN*→	TPR+GreAB-C+PIN	1069	-	Firmicutes	Clostridioides difficile	hypothetical protein [Clostridioides difficile].	GCF_000530295.1
WP_022674357.1	REase→?→ REase+TPR+GreAB-C+PIN*→	REase+TPR+GreAB-C+PIN	1290	-	Alphaproteobacteria	Novosphingopyxis baekryungensis	hypothetical protein [Novosphingopyxis baekryungensis].	GCF_000420305.1
WP_024552132.1	HNH→?→?→ HetE-N1→ TPR+GreAB-C+PIN*→	TPR+GreAB-C+PIN	1092	-	Gammaproteobacteria	Franconibacter helveticus	hypothetical protein [Franconibacter helveticus].	GCF_000485945.1
WP_024678816.1	REase+TPR+GreAB-C+PIN*→	REase+TPR+GreAB-C+PIN	1317	-	Gammaproteobacteria	Pseudomonas syringae	DUF4365 domain-containing protein [Pseudomonas syringae].	GCF_000452685.1
WP_024698051.1	REase+TPR+GreAB-C+PIN*→	REase+TPR+GreAB-C+PIN	1438	-	Gammaproteobacteria	Pseudomonas avellanae	hypothetical protein [Pseudomonas avellanae].	GCF_000452845.1
WP_024925158.1	REase+TPR+TPR+GreAB-C+PIN*→	REase+TPR+TPR+GreAB-C+PIN	1315	-	Alphaproteobacteria	Mesorhizobium	MULTISPECIES: GreA/GreB family elongation factor [Mesorhizobium].	GCF_000568635.1
WP_025240246.1	REase+TOPC→ HetE-N1→ HetE-N1+TPR+GreAB-C+PIN*→	HetE-N1+TPR+GreAB-C+PIN	952	-	Gammaproteobacteria	Pseudomonas stutzeri	hypothetical protein [Pseudomonas stutzeri].	GCF_000590475.1
WP_026105277.1	TPR+GreAB-C+PIN*→	TPR+GreAB-C+PIN	611	-	Alphaproteobacteria	Methylobacterium sp. MB200	hypothetical protein [Methylobacterium sp. MB200].	GCF_000333655.1
WP_026508802.1	HEPN+SNF→?→?→?→ X+PIN*→?→ CITB-HTH+LexA-protease→	X+PIN	1212	-	Firmicutes	Butyrivibrio sp. MC2013	hypothetical protein [Butyrivibrio sp. MC2013].	GCF_000424585.1
WP_026811785.1	TPR+PIN*→	TPR+PIN	1261	-	Bacteroidetes	Arenibacter certesii	hypothetical protein [Arenibacter certesii].	GCF_000429545.1
WP_027471277.1	TPR+GreAB-C+PIN*→	TPR+GreAB-C+PIN	1073	-	Bacteroidetes	Saccharicrinis fermentans	hypothetical protein [Saccharicrinis fermentans].	GCF_000517085.1
WP_028048816.1	HTH+PNPase+TPR+GreAB-C+PIN*→	HTH+PNPase+TPR+GreAB-C+PIN	1518	-	Actinobacteria	Cellulomonas sp. URHD0024	helix-turn-helix domain-containing protein [Cellulomonas sp. URHD0024].	GCF_000426185.1
WP_029071033.1	X+PIN*→	X+PIN	1212	-	Firmicutes	Kandleria vitulina	hypothetical protein [Kandleria vitulina].	GCF_000622125.1
WP_029379391.1	REase+TPR+GreAB-C+PIN*→	REase+TPR+GreAB-C+PIN	1438	-	Gammaproteobacteria	Pseudomonas fuscovaginae	hypothetical protein [Pseudomonas fuscovaginae].	GCF_000251185.1
WP_031437352.1	REase+TPR+GreAB-C+PIN*→	REase+TPR+GreAB-C+PIN	1323	-	Gammaproteobacteria	Methylobacter tundripaludum	DUF4365 domain-containing protein [Methylobacter tundripaludum].	GCF_000733835.1
WP_031445473.1	TPR+GreAB-C+PIN*→	TPR+GreAB-C+PIN	1244	-	Bacteroidetes	Arenibacter algicola	hypothetical protein [Arenibacter algicola].	GCF_000733925.1
WP_031548868.1	REase→ X+PIN*→?→?→?→?→?→?→ MACRODOMAIN→	X+PIN	1209	-	Firmicutes	Oribacterium sp. FC2011	hypothetical protein [Oribacterium sp. FC2011].	GCF_000701645.1
WP_032661435.1	TPR+GreAB-C+PIN*→	TPR+GreAB-C+PIN	1139	-	Gammaproteobacteria	Pseudomonas syringae	hypothetical protein [Pseudomonas syringae].	GCF_000800685.1
WP_032902625.1	REase+TPR+GreAB-C+PIN*→	REase+TPR+GreAB-C+PIN	1438	-	Gammaproteobacteria	Pseudomonas	MULTISPECIES: hypothetical protein [Pseudomonas].	GCF_000511155.2
WP_033132679.1	HetE-N1→ TPR+GreAB-C+PIN*→	TPR+GreAB-C+PIN	1074	-	Gammaproteobacteria	Acinetobacter sp. MN12	hypothetical protein [Acinetobacter sp. MN12].	GCF_000764915.1
WP_033469897.1	REase+TPR+GreAB-C+PIN*→	REase+TPR+GreAB-C+PIN	1282	-	Betaproteobacteria	Bordetella bronchiseptica	hypothetical protein [Bordetella bronchiseptica].	GCF_000690175.1
WP_033736921.1	HetE-N1→?→ TPR+GreAB-C+PIN*→	TPR+GreAB-C+PIN	760	-	Gammaproteobacteria	Pantoea sp. Sc1	hypothetical protein [Pantoea sp. Sc1].	GCF_000255315.1
WP_034127539.1	TPR+GreAB-C+PIN*→	TPR+GreAB-C+PIN	1158	-	Gammaproteobacteria	Pseudomonas fluorescens	hypothetical protein [Pseudomonas fluorescens].	GCF_000801795.1
WP_034129364.1	REase+TPR+GreAB-C+PIN*→	REase+TPR+GreAB-C+PIN	1244	-	Gammaproteobacteria	Acinetobacter baumannii	hypothetical protein [Acinetobacter baumannii].	GCF_000802915.1
WP_034392332.1	REase+TPR+GreAB-C+PIN*→	REase+TPR+GreAB-C+PIN	1413	-	Betaproteobacteria	Delftia acidovorans	hypothetical protein [Delftia acidovorans].	GCF_001598795.1
WP_034708052.1	HetE-N1→?→?→ GreAB-C+PIN*→	GreAB-C+PIN	575	-	Gammaproteobacteria	Acinetobacter bereziniae	hypothetical protein [Acinetobacter bereziniae].	GCF_000368505.1
WP_035460854.1	HetE-N1→ TPR+GreAB-C+PIN*→	TPR+GreAB-C+PIN	1104	-	Gammaproteobacteria	unclassified Alcanivorax	MULTISPECIES: hypothetical protein [unclassified Alcanivorax].	GCF_000524665.1
WP_035530189.1	REase+TPR+GreAB-C+PIN*→	REase+TPR+GreAB-C+PIN	1332	-	Alphaproteobacteria	Hoeflea sp. BAL378	hypothetical protein [Hoeflea sp. BAL378].	GCF_000759435.1
WP_035646445.1	REase+TPR+TPR+GreAB-C+PIN*→	REase+TPR+TPR+GreAB-C+PIN	1316	-	Alphaproteobacteria	Bradyrhizobium sp. ORS 285	hypothetical protein [Bradyrhizobium sp. ORS 285].	GCF_000239755.1
WP_036748977.1	HetE-N1→ TPR+GreAB-C+PIN*→	TPR+GreAB-C+PIN	1130	-	Gammaproteobacteria	Photobacterium galatheae	hypothetical protein [Photobacterium galatheae].	GCF_000695255.1
WP_036783497.1	HetE-N1→ TPR+GreAB-C+PIN*→	TPR+GreAB-C+PIN	1104	-	Gammaproteobacteria	Photorhabdus aegyptia	hypothetical protein [Photorhabdus aegyptia].	GCF_000612035.1
WP_038397119.1	<-HTH<-?<-?||HetE-N1→ TPR+GreAB-C+PIN*→	TPR+GreAB-C+PIN	1107	-	Gammaproteobacteria	Salmonella enterica	hypothetical protein [Salmonella enterica].	GCF_000756465.1
WP_039292971.1	TPR+PIN*→	TPR+PIN	1180	-	Firmicutes	Paenibacillus sp. IHB B 3415	hypothetical protein [Paenibacillus sp. IHB B 3415].	GCF_000802655.2
WP_039419134.1	CHC2+Toprim-DNAG→?→?→?→ HTH→ SIG+TM→ TPR+GreAB-C+PIN*→	TPR+GreAB-C+PIN	1140	-	Bacteroidetes	Porphyromonas gulae	hypothetical protein [Porphyromonas gulae].	GCF_000765945.1
WP_039490314.1	HetE-N1→ TPR+GreAB-C+PIN*→	TPR+GreAB-C+PIN	1093	-	Gammaproteobacteria	Pectobacterium odoriferum	hypothetical protein [Pectobacterium odoriferum].	GCF_000754765.1
WP_039768974.1	REase+TPR+GreAB-C+PIN*→	REase+TPR+GreAB-C+PIN	1438	-	Gammaproteobacteria	Pseudomonas fluorescens	hypothetical protein [Pseudomonas fluorescens].	GCF_000817905.1
WP_040182639.1	REase+TPR+GreAB-C+PIN*→	REase+TPR+GreAB-C+PIN	1319	-	Alphaproteobacteria	Phaeobacter sp. S60	hypothetical protein [Phaeobacter sp. S60].	GCF_000826845.1
WP_040439475.1	REase+TPR+GreAB-C+PIN*→	REase+TPR+GreAB-C+PIN	1318	-	Alphaproteobacteria	Labrenzia aggregata	hypothetical protein [Labrenzia aggregata].	GCF_000168975.1
WP_041272554.1	REase+TPR+GreAB-C+PIN*→	REase+TPR+GreAB-C+PIN	1294	-	Firmicutes	Desulfitobacterium hafniense	tetratricopeptide repeat protein [Desulfitobacterium hafniense].	GCF_000010045.1

acc	operon	architecture	len	gen.name	taxend	species	defline	gca
WP_041475188.1	REase+TPR+GreAB-C+PIN*→	REase+TPR+GreAB-C+PIN	1321	-	Gammaproteobacteria	Pseudomonas	MULTISPECIES: DUF4365 domain-containing protein [Pseudomonas].	GCF_000012445.1
WP_041609504.1	HetE-N1→ TPR+GreAB-C+PIN*→	TPR+GreAB-C+PIN	1086	-	Gammaproteobacteria	Tolumonas auensis	hypothetical protein [Tolumonas auensis].	GCF_000023065.1
WP_042089608.1	REase+TPR+GreAB-C+PIN*→	REase+TPR+GreAB-C+PIN	1245	-	Gammaproteobacteria	Acinetobacter bereziniae	hypothetical protein [Acinetobacter bereziniae].	GCF_000825165.1
WP_042562078.1	REase+TPR+GreAB-C+PIN*→	REase+TPR+GreAB-C+PIN	1438	-	Gammaproteobacteria	Pseudomonas fluorescens	hypothetical protein [Pseudomonas fluorescens].	GCF_000827755.1
WP_043355224.1	REase+TPR+GreAB-C+PIN*→	REase+TPR+GreAB-C+PIN	1332	-	Betaproteobacteria	Cupriavidus	MULTISPECIES: hypothetical protein [Cupriavidus].	GCF_001652915.1
WP_043626766.1	REase+TPR+TPR+GreAB-C+PIN*→	REase+TPR+TPR+GreAB-C+PIN	1357	-	Alphaproteobacteria	Ensifer sp. ZNC0028	hypothetical protein [Ensifer sp. ZNC0028].	GCF_000799055.1
WP_043752287.1	REase+TPR+GreAB-C+PIN*→	REase+TPR+GreAB-C+PIN	1318	-	Alphaproteobacteria	Pseudooceanicola atlanticus	restriction endonuclease [Pseudooceanicola atlanticus].	GCF_000768315.1
WP_044036524.1	TPR→ TPR→?→?→ TPR+GreAB-C+PIN*→	TPR+GreAB-C+PIN	473	-	Alphaproteobacteria	Octadecabacter arcticus	hypothetical protein [Octadecabacter arcticus].	GCF_000155735.2
WP_044439030.1	X+PIN*→	X+PIN	1051	-	Actinobacteria	Agreia bicolorata	hypothetical protein [Agreia bicolorata].	GCF_000938265.1
WP_045324066.1	TPR+PIN*→	TPR+PIN	1167	-	Actinobacteria	Streptomyces sp. NRRL F-4428	hypothetical protein [Streptomyces sp. NRRL F-4428].	GCF_000956015.1
WP_045495160.1	HetE-N1→ TPR+GreAB-C+PIN*→	TPR+GreAB-C+PIN	1086	-	Gammaproteobacteria	Vibrio hyugaensis	hypothetical protein [Vibrio hyugaensis].	GCF_000818475.1
WP_046103485.1	REase+TPR+GreAB-C+PIN*→	REase+TPR+GreAB-C+PIN	1314	-	Alphaproteobacteria	Devosia chinhatensis	hypothetical protein [Devosia chinhatensis].	GCF_000969445.1
WP_047008502.1	TPR+GreAB-C+PIN*→	TPR+GreAB-C+PIN	1236	-	-	Vibrio diabolicus	hypothetical protein [Vibrio diabolicus].	GCF_001010935.1
WP_047057492.1	HetE-N1→ TPR+GreAB-C+PIN*→	TPR+GreAB-C+PIN	1106	-	Gammaproteobacteria	Enterobacter hormaechei	hypothetical protein [Enterobacter hormaechei].	GCF_001011695.1
WP_047274590.1	REase+TPR+GreAB-C+PIN*→	REase+TPR+GreAB-C+PIN	1321	-	Gammaproteobacteria	Pseudomonas	MULTISPECIES: DUF4365 domain-containing protein [Pseudomonas].	GCF_012986325.1
WP_047583000.1	HetE-N1→ TPR+GreAB-C+PIN*→	TPR+GreAB-C+PIN	1105	-	Gammaproteobacteria	unclassified Pseudomonas	MULTISPECIES: hypothetical protein [unclassified Pseudomonas].	GCF_000763225.1
WP_047736827.1	HetE-N1→ HetE-N1+TPR+GreAB-C+PIN*→	HetE-N1+TPR+GreAB-C+PIN	1122	-	Gammaproteobacteria	Pseudomonas chlororaphis	hypothetical protein [Pseudomonas chlororaphis].	GCF_001023535.1
WP_047738516.1	HetE-N1→ TPR+GreAB-C+PIN*→	TPR+GreAB-C+PIN	1111	-	Gammaproteobacteria	Pseudomonas chlororaphis	hypothetical protein [Pseudomonas chlororaphis].	GCF_001023535.1
WP_047741525.1	HetE-N1→ TPR+GreAB-C+PIN*→	TPR+GreAB-C+PIN	1106	-	Gammaproteobacteria	Enterobacter cloacae complex	MULTISPECIES: hypothetical protein [Enterobacter cloacae complex].	GCF_014982105.1
WP_047744683.1	HetE-N1→ HetE-N1+TPR+GreAB-C+PIN*→	HetE-N1+TPR+GreAB-C+PIN	1092	-	Gammaproteobacteria	Enterobacter ludwigii	hypothetical protein [Enterobacter ludwigii].	GCF_001022445.2
WP_048126291.1	TPR+GreAB-C+PIN*→	TPR+GreAB-C+PIN	1292	-	Euryarchaeota	Methanosarcina lacustris	hypothetical protein [Methanosarcina lacustris].	GCF_000970265.1
WP_048377212.1	REase+TPR+GreAB-C+PIN*→	REase+TPR+GreAB-C+PIN	1321	-	Gammaproteobacteria	Pseudomonas	MULTISPECIES: DUF4365 domain-containing protein [Pseudomonas].	GCF_012986365.1
WP_048411963.1	TPR+GreAB-C+PIN*→	TPR+GreAB-C+PIN	1163	-	Betaproteobacteria	Chromobacterium sp. LK1	hypothetical protein [Chromobacterium sp. LK1].	GCF_001043555.1
WP_049042149.1	HetE-N1→ HetE-N1+TPR+GreAB-C+PIN*→	HetE-N1+TPR+GreAB-C+PIN	1094	-	Gammaproteobacteria	Citrobacter braakii	hypothetical protein [Citrobacter braakii].	GCF_001059745.1
WP_049582258.1	HetE-N1→ TPR+GreAB-C+PIN*→	TPR+GreAB-C+PIN	1098	-	Gammaproteobacteria	Photorhabdus luminescens	hypothetical protein [Photorhabdus luminescens].	GCF_900102985.1
WP_050091697.1	HetE-N1→ TPR+GreAB-C+PIN*→	TPR+GreAB-C+PIN	1096	-	Gammaproteobacteria	Yersinia pseudotuberculosis	hypothetical protein [Yersinia pseudotuberculosis].	GCF_001123345.1
WP_050298251.1	HetE-N1→ TPR+GreAB-C+PIN*→	TPR+GreAB-C+PIN	1095	-	Gammaproteobacteria	Yersinia enterocolitica	hypothetical protein [Yersinia enterocolitica].	GCF_001160345.1
WP_050323220.1	HetE-N1→ TPR+GreAB-C+PIN*→	TPR+GreAB-C+PIN	1121	-	Gammaproteobacteria	Yersinia enterocolitica	hypothetical protein [Yersinia enterocolitica].	GCF_001182365.1
WP_050376143.1	TPR+GreAB-C+PIN*→||||<-HTH+HTH	TPR+GreAB-C+PIN	872	-	Betaproteobacteria	Burkholderia pseudomallei	hypothetical protein [Burkholderia pseudomallei].	GCF_006538545.1
WP_050590144.1	REase+TPR+GreAB-C+PIN*→	REase+TPR+GreAB-C+PIN	1423	-	Gammaproteobacteria	Pseudomonas sp. URMO17WK12:I12	hypothetical protein [Pseudomonas sp. URMO17WK12:I12].	GCF_000514395.1
WP_051097719.1	TPR+GreAB-C+PIN*→	TPR+GreAB-C+PIN	1140	-	Gammaproteobacteria	Pseudomonas sp. 10-1B	hypothetical protein [Pseudomonas sp. 10-1B].	GCF_000935045.1
WP_051560695.1	HetE-N1→ TPR+GreAB-C+PIN*→	TPR+GreAB-C+PIN	1098	-	Gammaproteobacteria	Photorhabdus aegyptia	hypothetical protein [Photorhabdus aegyptia].	GCF_000612035.1
WP_051570263.1	REase+TPR+GreAB-C+PIN*→	REase+TPR+GreAB-C+PIN	1248	-	Actinobacteria	Cryptosporangium arvum	DUF4365 domain-containing protein [Cryptosporangium arvum].	GCF_000585375.1
WP_052241169.1	REase+TPR+GreAB-C+PIN*→	REase+TPR+GreAB-C+PIN	1236	-	Actinobacteria	Microbacterium hominis	DUF4365 domain-containing protein [Microbacterium hominis].	GCF_000813805.1
WP_052515067.1	PNPase+TPR+GreAB-C+PIN*→	PNPase+TPR+GreAB-C+PIN	1617	-	Deltaproteobacteria	Dethiosulfatarculus sandiegensis	hypothetical protein [Dethiosulfatarculus sandiegensis].	GCF_000931935.2
WP_052917795.1	HetE-N1→ TPR+GreAB-C+PIN*→	TPR+GreAB-C+PIN	1094	-	Gammaproteobacteria	Escherichia coli	hypothetical protein [Escherichia coli].	GCF_001265885.1
WP_053052653.1	HetE-N1→ TPR+GreAB-C+PIN*→	TPR+GreAB-C+PIN	1129	-	Gammaproteobacteria	Vibrio harveyi	hypothetical protein [Vibrio harveyi].	GCF_001262735.1
WP_053269091.1	REase+TPR+GreAB-C+PIN*→	REase+TPR+GreAB-C+PIN	1438	-	Gammaproteobacteria	Pseudomonas chlororaphis	hypothetical protein [Pseudomonas chlororaphis].	GCF_001269595.1
WP_053316591.1	TPR+GreAB-C+PIN*→	TPR+GreAB-C+PIN	982	-	Gammaproteobacteria	Vibrio parahaemolyticus	hypothetical protein [Vibrio parahaemolyticus].	GCF_001268015.1
WP_053446146.1	TPR+PIN*→	TPR+PIN	1160	-	Firmicutes	Bacillus toyonensis	hypothetical protein [Bacillus toyonensis].	GCF_001275045.2

<table>
<thead>
<tr><th>acc</th><th>operon</th><th>architecture</th><th>len</th><th>gen.name</th><th>taxend</th><th>species</th><th>defline</th><th>gca</th></tr>
</thead>
<tbody>
<tr><td>WP_053882512.1</td><td>HetE-N1→ TPR+GreAB-C+PIN*→</td><td>TPR+GreAB-C+PIN</td><td>1094</td><td>-</td><td>Gammaproteobacteria</td><td>Escherichia coli</td><td>hypothetical protein [Escherichia coli].</td><td>GCF_001284445.1</td></tr>
<tr><td>WP_054070021.1</td><td>TPR+GreAB-C+PIN*→</td><td>TPR+GreAB-C+PIN</td><td>1140</td><td>-</td><td>Gammaproteobacteria</td><td>Pseudomonas syringae group genomosp. 3</td><td>hypothetical protein [Pseudomonas syringae group genomosp. 3].</td><td>GCF_016803175.1</td></tr>
<tr><td>WP_054211402.1</td><td>BirA-HTH→ HEPN→ REase+TPR+TPR+GreAB-C+PIN*→</td><td>REase+TPR+TPR+GreAB-C+PIN</td><td>1333</td><td>-</td><td>Alphaproteobacteria</td><td>Bosea vaviloviae</td><td>hypothetical protein [Bosea vaviloviae].</td><td>GCF_001298265.1</td></tr>
<tr><td>WP_054464024.1</td><td>HetE-N1→ TPR+GreAB-C+PIN*→</td><td>TPR+GreAB-C+PIN</td><td>1094</td><td>-</td><td>Gammaproteobacteria</td><td>Escherichia coli</td><td>hypothetical protein [Escherichia coli].</td><td>GCF_001277455.1</td></tr>
<tr><td>WP_055843189.1</td><td>REase+TPR+GreAB-C+PIN*→</td><td>REase+TPR+GreAB-C+PIN</td><td>1293</td><td>-</td><td>Alphaproteobacteria</td><td>Sphingomonas sp. Leaf343</td><td>tetratricopeptide repeat protein [Sphingomonas sp. Leaf343].</td><td>GCF_001423765.1</td></tr>
<tr><td>WP_056497005.1</td><td>TPR+GreAB-C+PIN*→</td><td>TPR+GreAB-C+PIN</td><td>1110</td><td>-</td><td>Alphaproteobacteria</td><td>Methylobacterium sp. Leaf111</td><td>hypothetical protein [Methylobacterium sp. Leaf111].</td><td>GCF_001423285.1</td></tr>
<tr><td>WP_056734772.1</td><td>TPR+PIN*→</td><td>TPR+PIN</td><td>1204</td><td>-</td><td>Actinobacteria</td><td>Agromyces sp. Soil535</td><td>hypothetical protein [Agromyces sp. Soil535].</td><td>GCF_001428255.1</td></tr>
<tr><td>WP_056836493.1</td><td>TPR+PIN*→</td><td>TPR+PIN</td><td>1174</td><td>-</td><td>Firmicutes</td><td>Paenibacillus sp. Soil787</td><td>hypothetical protein [Paenibacillus sp. Soil787].</td><td>GCF_001429545.1</td></tr>
<tr><td>WP_057430028.1</td><td>REase+TPR+GreAB-C+PIN*→</td><td>REase+TPR+GreAB-C+PIN</td><td>1438</td><td>-</td><td>Gammaproteobacteria</td><td>Pseudomonas syringae group genomosp. 3</td><td>hypothetical protein [Pseudomonas syringae group genomosp. 3].</td><td>GCF_001401385.1</td></tr>
<tr><td>WP_057580842.1</td><td>TPR+GreAB-C+PIN*→</td><td>TPR+GreAB-C+PIN</td><td>1233</td><td>-</td><td>Firmicutes</td><td>Paeniclostridium sordellii</td><td>hypothetical protein [Paeniclostridium sordellii].</td><td>GCF_001373055.1</td></tr>
<tr><td>WP_057657430.1</td><td>REase+TPR+GreAB-C+PIN*→</td><td>REase+TPR+GreAB-C+PIN</td><td>1287</td><td>-</td><td>Gammaproteobacteria</td><td>Pseudoxanthomonas dokdonensis</td><td>tetratricopeptide repeat protein [Pseudoxanthomonas dokdonensis].</td><td>GCF_001431405.1</td></tr>
<tr><td>WP_057831910.1</td><td>HetE-N1→ TPR+GreAB-C+PIN*→</td><td>TPR+GreAB-C+PIN</td><td>1091</td><td>-</td><td>Gammaproteobacteria</td><td>Colwellia sp. TT2012</td><td>hypothetical protein [Colwellia sp. TT2012].</td><td>GCF_001440345.1</td></tr>
<tr><td>WP_057932289.1</td><td>TPR+GreAB-C+PIN*→</td><td>TPR+GreAB-C+PIN</td><td>1276</td><td>-</td><td>Bacteroidetes</td><td>Pedobacter ginsenosidimutans</td><td>tetratricopeptide repeat protein [Pedobacter ginsenosidimutans].</td><td>GCF_001442625.1</td></tr>
<tr><td>WP_058039540.1</td><td>TPR+GreAB-C+PIN*→||||<-HTH+HTH</td><td>TPR+GreAB-C+PIN</td><td>872</td><td>-</td><td>Betaproteobacteria</td><td>Burkholderia pseudomallei</td><td>hypothetical protein [Burkholderia pseudomallei].</td><td>GCF_001445875.1</td></tr>
<tr><td>WP_058297418.1</td><td>TPR+PIN*→</td><td>TPR+PIN</td><td>1225</td><td>-</td><td>Firmicutes</td><td>Bacillus enclensis</td><td>hypothetical protein [Bacillus enclensis].</td><td>GCF_900094975.1</td></tr>
<tr><td>WP_058461037.1</td><td>TPR+PIN*→</td><td>TPR+PIN</td><td>1277</td><td>-</td><td>Gammaproteobacteria</td><td>Fluoribacter bozemanae</td><td>hypothetical protein [Fluoribacter bozemanae].</td><td>GCF_900640135.1</td></tr>
<tr><td>WP_058587024.1</td><td><-MarR-HTH||?→?→?→||||<-?||?→ HetE-N1→ TPR+GreAB-C+PIN*→</td><td>TPR+GreAB-C+PIN</td><td>1117</td><td>-</td><td>Gammaproteobacteria</td><td>Citrobacter amalonaticus</td><td>hypothetical protein [Citrobacter amalonaticus].</td><td>GCF_001471655.2</td></tr>
<tr><td>WP_058721158.1</td><td>REase+TPR+GreAB-C+PIN*→</td><td>REase+TPR+GreAB-C+PIN</td><td>1509</td><td>-</td><td>Betaproteobacteria</td><td>Paucibacter sp. KCTC 42545</td><td>hypothetical protein [Paucibacter sp. KCTC 42545].</td><td>GCF_001477625.1</td></tr>
<tr><td>WP_058751880.1</td><td>REase+TPR+GreAB-C+PIN*→</td><td>REase+TPR+GreAB-C+PIN</td><td>1317</td><td>-</td><td>Alphaproteobacteria</td><td>Sphingomonas sanguinis</td><td>tetratricopeptide repeat protein [Sphingomonas sanguinis].</td><td>GCF_001476905.1</td></tr>
<tr><td>WP_058820070.1</td><td><-HTH<-?<-?||HetE-N1→ TPR+GreAB-C+PIN*→</td><td>TPR+GreAB-C+PIN</td><td>1107</td><td>-</td><td>Gammaproteobacteria</td><td>Salmonella enterica</td><td>hypothetical protein [Salmonella enterica].</td><td>GCF_001952125.1</td></tr>
<tr><td>WP_059294982.1</td><td>HetE-N1→ TPR+GreAB-C+PIN*→</td><td>TPR+GreAB-C+PIN</td><td>1106</td><td>-</td><td>Gammaproteobacteria</td><td>Enterobacter hormaechei</td><td>hypothetical protein [Enterobacter hormaechei].</td><td>GCF_001518595.1</td></tr>
<tr><td>WP_059458986.1</td><td>REase+TPR+GreAB-C+PIN*→</td><td>REase+TPR+GreAB-C+PIN</td><td>1335</td><td>-</td><td>Betaproteobacteria</td><td>Burkholderia vietnamiensis</td><td>hypothetical protein [Burkholderia vietnamiensis].</td><td>GCF_001523785.1</td></tr>
<tr><td>WP_059543415.1</td><td>TPR+GreAB-C+PIN*→||||<-HTH+HTH</td><td>TPR+GreAB-C+PIN</td><td>872</td><td>-</td><td>Betaproteobacteria</td><td>Burkholderia</td><td>MULTISPECIES: hypothetical protein [Burkholderia].</td><td>GCF_001523065.1</td></tr>
<tr><td>WP_059687512.1</td><td>REase+TPR+GreAB-C+PIN*→</td><td>REase+TPR+GreAB-C+PIN</td><td>1355</td><td>-</td><td>Betaproteobacteria</td><td>Burkholderia cepacia</td><td>hypothetical protein [Burkholderia cepacia].</td><td>GCF_001524765.1</td></tr>
<tr><td>WP_059702921.1</td><td>REase+TPR→ TPR+GreAB-C+PIN*→</td><td>TPR+GreAB-C+PIN</td><td>954</td><td>-</td><td>Betaproteobacteria</td><td>Burkholderia sp. RF2-non_BP3</td><td>hypothetical protein [Burkholderia sp. RF2-non_BP3].</td><td>GCF_001522185.1</td></tr>
<tr><td>WP_059764594.1</td><td>REase+TPR+GreAB-C+PIN*→</td><td>REase+TPR+GreAB-C+PIN</td><td>1321</td><td>-</td><td>Gammaproteobacteria</td><td>unclassified Pseudomonas</td><td>MULTISPECIES: DUF4365 domain-containing protein [unclassified Pseudomonas].</td><td>GCF_001529305.1</td></tr>
<tr><td>WP_059837999.1</td><td>TPR+GreAB-C+PIN*→||||<-HTH+HTH</td><td>TPR+GreAB-C+PIN</td><td>872</td><td>-</td><td>Betaproteobacteria</td><td>Burkholderia ubonensis</td><td>hypothetical protein [Burkholderia ubonensis].</td><td>GCF_001528175.1</td></tr>
<tr><td>WP_059992407.1</td><td>REase+TPR+GreAB-C+PIN*→</td><td>REase+TPR+GreAB-C+PIN</td><td>1345</td><td>-</td><td>Betaproteobacteria</td><td>Burkholderia ubonensis</td><td>hypothetical protein [Burkholderia ubonensis].</td><td>GCF_001527095.1</td></tr>
<tr><td>WP_060002797.1</td><td>TPR+GreAB-C+PIN*→</td><td>TPR+GreAB-C+PIN</td><td>872</td><td>-</td><td>Betaproteobacteria</td><td>Burkholderia gladioli</td><td>hypothetical protein [Burkholderia gladioli].</td><td>GCF_902831175.1</td></tr>
<tr><td>WP_060217774.1</td><td>REase+TPR+GreAB-C+PIN*→</td><td>REase+TPR+GreAB-C+PIN</td><td>1355</td><td>-</td><td>Betaproteobacteria</td><td>Burkholderia cepacia</td><td>hypothetical protein [Burkholderia cepacia].</td><td>GCF_001530905.1</td></tr>
<tr><td>WP_060322122.1</td><td>REase+TPR+GreAB-C+PIN*→</td><td>REase+TPR+GreAB-C+PIN</td><td>1351</td><td>-</td><td>Betaproteobacteria</td><td>Burkholderia ubonensis</td><td>hypothetical protein [Burkholderia ubonensis].</td><td>GCF_001718975.1</td></tr>
<tr><td>WP_060322181.1</td><td>REase+TPR+GreAB-C+PIN*→||||<-HEPN</td><td>REase+TPR+GreAB-C+PIN</td><td>1345</td><td>-</td><td>Betaproteobacteria</td><td>Burkholderia diffusa</td><td>hypothetical protein [Burkholderia diffusa].</td><td>GCF_001533185.1</td></tr>
<tr><td>WP_060360485.1</td><td>REase+TPR+GreAB-C+PIN*→||||<-HEPN</td><td>REase+TPR+GreAB-C+PIN</td><td>1351</td><td>-</td><td>Betaproteobacteria</td><td>Burkholderia territorii</td><td>hypothetical protein [Burkholderia territorii].</td><td>GCF_001533385.1</td></tr>
<tr><td>WP_061510730.1</td><td>REase+TPR+GreAB-C+PIN*→</td><td>REase+TPR+GreAB-C+PIN</td><td>1304</td><td>-</td><td>Alphaproteobacteria</td><td>Gluconobacter thailandicus</td><td>hypothetical protein [Gluconobacter thailandicus].</td><td>GCF_001580835.1</td></tr>
<tr><td>WP_061928567.1</td><td>REase+TPR+GreAB-C+PIN*→</td><td>REase+TPR+GreAB-C+PIN</td><td>1323</td><td>-</td><td>Alphaproteobacteria</td><td>Gluconobacter japonicus</td><td>hypothetical protein [Gluconobacter japonicus].</td><td>GCF_001580865.1</td></tr>
<tr><td>WP_062265952.1</td><td>REase+TPR+GreAB-C+PIN*→</td><td>REase+TPR+GreAB-C+PIN</td><td>1319</td><td>-</td><td>Euryarchaeota</td><td>Methanoculleus bourgensis</td><td>tetratricopeptide repeat protein [Methanoculleus bourgensis].</td><td>GCF_900036045.1</td></tr>
<tr><td>WP_062316067.1</td><td>TPR+PIN*→</td><td>TPR+PIN</td><td>1207</td><td>-</td><td>Actinobacteria</td><td>Demequina maris</td><td>hypothetical protein [Demequina maris].</td><td>GCF_000971375.1</td></tr>
<tr><td>WP_062477592.1</td><td>HetE-N1→ TPR+GreAB-C+PIN*→</td><td>TPR+GreAB-C+PIN</td><td>1109</td><td>-</td><td>Gammaproteobacteria</td><td>Lacimicrobium alkaliphilum</td><td>hypothetical protein [Lacimicrobium alkaliphilum].</td><td>GCF_001466725.1</td></tr>
<tr><td>WP_062692005.1</td><td>HetE-N1→ TPR+GreAB-C+PIN*→</td><td>TPR+GreAB-C+PIN</td><td>941</td><td>-</td><td>Gammaproteobacteria</td><td>Photobacterium sanguinicancri</td><td>hypothetical protein [Photobacterium sanguinicancri].</td><td>GCF_001563765.1</td></tr>
</tbody>
</table>

acc	operon	architecture	len	gen.name	taxend	species	defline	gca
WP_062794399.1	REase+TPR+GreAB-C+PIN*→	REase+TPR+GreAB-C+PIN	1127	-	Betaproteobacteria	Cupriavidus metallidurans	hypothetical protein, partial [Cupriavidus metallidurans].	GCF_001598775.1
WP_063146823.1	HetE-N1→ TPR+GreAB-C+PIN*→	TPR+GreAB-C+PIN	1106	-	Gammaproteobacteria	Enterobacter cloacae complex	MULTISPECIES: hypothetical protein [Enterobacter cloacae complex].	GCF_013635495.1
WP_063496996.1	TIR+DnaB→ REase+TPR+GreAB-C+PIN*→	REase+TPR+GreAB-C+PIN	1364	-	Betaproteobacteria	Paraburkholderia phytofirmans	hypothetical protein [Paraburkholderia phytofirmans].	GCF_001634365.1
WP_063800929.1	REase+TPR+GreAB-C+PIN*→	REase+TPR+GreAB-C+PIN	1351	-	Betaproteobacteria	Burkholderia ubonensis	hypothetical protein [Burkholderia ubonensis].	GCF_001526225.1
WP_063809712.1	HetE-N1→ TPR+GreAB-C+PIN*→	TPR+GreAB-C+PIN	1117	-	Gammaproteobacteria	Salmonella enterica	hypothetical protein [Salmonella enterica].	GCF_008727555.1
WP_063897359.1	REase+TPR+GreAB-C+PIN*→	REase+TPR+GreAB-C+PIN	1351	-	Betaproteobacteria	Burkholderia ubonensis	hypothetical protein [Burkholderia ubonensis].	GCF_001529725.1
WP_063906337.1	REase+TPR+GreAB-C+PIN*→	REase+TPR+GreAB-C+PIN	1354	-	Betaproteobacteria	Burkholderia ubonensis	hypothetical protein [Burkholderia ubonensis].	GCF_001534425.1
WP_063932878.1	HetE-N1→ TPR+GreAB-C+PIN*→	TPR+GreAB-C+PIN	1034	-	Gammaproteobacteria	Enterobacter hormaechei	hypothetical protein [Enterobacter hormaechei].	GCF_900076145.1
WP_064273010.1	REase+TPR+GreAB-C+PIN*→	REase+TPR+GreAB-C+PIN	1328	-	Alphaproteobacteria	Gluconobacter cerinus	tetratricopeptide repeat protein [Gluconobacter cerinus].	GCF_001645165.1
WP_064374215.1	HetE-N1→ TPR+GreAB-C+PIN*→	TPR+GreAB-C+PIN	1116	-	Gammaproteobacteria	Pantoea ananatis	hypothetical protein [Pantoea ananatis].	GCF_001543055.1
WP_064477497.1	TPR+GreAB-C+PIN*→	TPR+GreAB-C+PIN	879	-	Betaproteobacteria	Ralstonia	MULTISPECIES: hypothetical protein [Ralstonia].	GCF_014884725.1
WP_065176860.1	HetE-N1→ GreAB-C*→	GreAB-C	1123	-	Gammaproteobacteria	Photobacterium aquimaris	hypothetical protein [Photobacterium aquimaris].	GCF_001676065.1
WP_065192964.1	HetE-N1→ GreAB-C*→	GreAB-C	1128	-	Gammaproteobacteria	Photobacterium phosphoreum	hypothetical protein [Photobacterium phosphoreum].	GCF_001676135.1
WP_065645553.1	HetE-N1→ TPR+GreAB-C+PIN*→	TPR+GreAB-C+PIN	1085	-	Gammaproteobacteria	Vibrio diabolicus	hypothetical protein [Vibrio diabolicus].	GCF_001691185.1
WP_065663920.1	BirA-HTH→ HEPN→ REase+TPR+TPR+GreAB-C+PIN*→	REase+TPR+TPR+GreAB-C+PIN	1333	-	Alphaproteobacteria	Agrobacterium sp. B131/95	GreA/GreB family elongation factor [Agrobacterium sp. B131/95].	GCF_001692155.1
WP_065996373.1	REase+TPR+GreAB-C+PIN*→	REase+TPR+GreAB-C+PIN	1244	-	Gammaproteobacteria	Acinetobacter baumannii	hypothetical protein [Acinetobacter baumannii].	GCF_001704675.1
WP_066036869.1	TPR+PIN*→	TPR+PIN	1175	-	Firmicutes	Lysinibacillus sp. AR18-8	hypothetical protein [Lysinibacillus sp. AR18-8].	GCF_001705465.1
WP_066542710.1	REase+TPR+GreAB-C+PIN*→	REase+TPR+GreAB-C+PIN	1122	-	Alphaproteobacteria	Sphingomonas sp. CCH18-B1	tetratricopeptide repeat protein [Sphingomonas sp. CCH18-B1].	GCF_001555895.1
WP_066945120.1	HTH+PNPase+PIN*→	HTH+PNPase+PIN	1527	-	Actinobacteria	Streptomyces lushanensis	5'-methylthioadenosine/S-adenosylhomocysteine nucleosidase [Streptomyces lushanensis].	GCF_001700515.1
WP_067386574.1	REase+TPR+GreAB-C+PIN*→	REase+TPR+GreAB-C+PIN	1361	-	Gammaproteobacteria	Marinobacterium aestuarii	hypothetical protein [Marinobacterium aestuarii].	GCF_001651805.1
WP_067584887.1	GreAB-C*→	GreAB-C	1154	-	Gammaproteobacteria	Endozoicomonas ascidiicola	hypothetical protein [Endozoicomonas ascidiicola].	GCF_001646955.1
WP_067776912.1	PNPase+TPR+GreAB-C+PIN*→	PNPase+TPR+GreAB-C+PIN	1386	-	Cyanobacteria	Nostoc sp. NIES-3756	hypothetical protein [Nostoc sp. NIES-3756].	GCF_001548375.1
WP_067990948.1	TPR+PIN*→||||<-KAP_NTPase	TPR+PIN	1216	-	Actinobacteria	Mycobacterium sp. YC-RL4	hypothetical protein [Mycobacterium sp. YC-RL4].	GCF_001644575.1
WP_068215362.1	TPR+GreAB-C+PIN*→	TPR+GreAB-C+PIN	1166	-	Bacteroidetes	Roseivirga spongicola	hypothetical protein [Roseivirga spongicola].	GCF_001592965.1
WP_069047857.1	REase+TPR+GreAB-C+PIN*→	REase+TPR+GreAB-C+PIN	1383	-	Betaproteobacteria	Hydrogenophaga sp. RAC07	hypothetical protein [Hydrogenophaga sp. RAC07].	GCF_001713375.1
WP_069206296.1	REase+TPR+GreAB-C+PIN*→	REase+TPR+GreAB-C+PIN	1323	-	Alphaproteobacteria	Sphingomonas panacis	hypothetical protein [Sphingomonas panacis].	GCF_001717955.1
WP_069604736.1	TPR+GreAB-C+PIN*→	TPR+GreAB-C+PIN	1147	-	Firmicutes	Bacillus cereus group	MULTISPECIES: tetratricopeptide repeat protein [Bacillus cereus group].	GCF_001729295.1
WP_069859257.1	TPR+GreAB-C+PIN*→	TPR+GreAB-C+PIN	1244	-	Bacteroidetes	Arenibacter sp. NBRC 103722	hypothetical protein [Arenibacter sp. NBRC 103722].	GCF_001748465.2
WP_069931472.1	REase+PIN*→	REase+PIN	1282	-	Actinobacteria	Streptomyces agglomeratus	DUF4365 domain-containing protein [Streptomyces agglomeratus].	GCF_001746465.1
WP_070715046.1	HetE-N1→ TPR+GreAB-C+PIN*→	TPR+GreAB-C+PIN	1094	-	Gammaproteobacteria	Hafnia sp. HMSC23F03	hypothetical protein [Hafnia sp. HMSC23F03].	GCF_001816025.1
WP_070991866.1	HetE-N1→ TPR+GreAB-C+PIN*→	TPR+GreAB-C+PIN	1106	-	Gammaproteobacteria	Pseudoalteromonas byunsanensis	hypothetical protein [Pseudoalteromonas byunsanensis].	GCF_001854475.1
WP_071234936.1	HetE-N1→ TPR+GreAB-C+PIN*→	TPR+GreAB-C+PIN	1111	-	Gammaproteobacteria	Vibrio rotiferianus	hypothetical protein [Vibrio rotiferianus].	GCF_001858245.1
WP_071684330.1	HetE-N1→ HetE-N1+TPR+GreAB-C+PIN*→	HetE-N1+TPR+GreAB-C+PIN	1094	-	Gammaproteobacteria	Citrobacter freundii	hypothetical protein [Citrobacter freundii].	GCF_014901875.1
WP_071741873.1	TPR+PIN*→	TPR+PIN	1160	-	Firmicutes	Bacillus paranthracis	hypothetical protein [Bacillus paranthracis].	GCF_001883995.1
WP_071769547.1	PIN*→	PIN	341	-	Betaproteobacteria	Burkholderia ubonensis	hypothetical protein [Burkholderia ubonensis].	GCF_001882755.1
WP_071890709.1	TPR+GreAB-C+PIN*→||||<-HTH	TPR+GreAB-C+PIN	1157	-	Gammaproteobacteria	Pseudomonas putida	tetratricopeptide repeat protein [Pseudomonas putida].	GCF_001886975.1
WP_072702162.1	TPR+GreAB-C+PIN*→	TPR+GreAB-C+PIN	1125	-	Alphaproteobacteria	Donghicola eburneus	hypothetical protein [Donghicola eburneus].	GCF_900095785.1

acc	operon	architecture	len	gen.name	taxend	species	defline	gca
WP_073552134.1	TPR+GreAB-C+PIN*→	TPR+GreAB-C+PIN	1131	-	Bacteroidetes	Elizabethkingia meningoseptica	hypothetical protein [Elizabethkingia meningoseptica].	GCF_900143665.1
WP_073772512.1	REase+PIN*→	REase+PIN	1292	-	Actinobacteria	Streptomyces sp. MJM1172	DUF4365 domain-containing protein [Streptomyces sp. MJM1172].	GCF_001905425.1
WP_074449514.1	CHC2+Toprim-DNAG→?→?→?→ HTH→ SIG+TM→ TPR+GreAB-C+PIN*→	TPR+GreAB-C+PIN	1141	-	Bacteroidetes	Tannerella forsythia	hypothetical protein [Tannerella forsythia].	GCF_900096735.1
WP_074452850.1	CHC2+Toprim-DNAG→?→ PSE→?→ HTH→ SIG+TM→ TPR+GreAB-C+PIN*→	TPR+GreAB-C+PIN	1141	-	Bacteroidetes	Bacteroidales	MULTISPECIES: hypothetical protein [Bacteroidales].	GCF_900096725.1
WP_074647048.1	TPR+GreAB-C+PIN*→	TPR+GreAB-C+PIN	1184	-	Alphaproteobacteria	Celeribacter baekdonensis	hypothetical protein [Celeribacter baekdonensis].	GCF_900102315.1
WP_074661038.1	TPR+GreAB-C+PIN*→	TPR+GreAB-C+PIN	1127	-	Bacteroidetes	Flavobacterium frigidimaris	hypothetical protein [Flavobacterium frigidimaris].	GCF_002217275.1
WP_074804411.1	REase+TPR+GreAB-C+PIN*→	REase+TPR+GreAB-C+PIN	1335	-	Betaproteobacteria	Burkholderia cenocepacia	hypothetical protein [Burkholderia cenocepacia].	GCF_900100915.1
WP_074854446.1	TPR+GreAB-C+PIN*→	TPR+GreAB-C+PIN	1161	-	Gammaproteobacteria	Pseudomonas salomonii	hypothetical protein [Pseudomonas salomonii].	GCF_900107155.1
WP_075042282.1	REase+TPR+TPR+GreAB-C+PIN*→	REase+TPR+TPR+GreAB-C+PIN	1332	-	Alphaproteobacteria	Brucella intermedia	GreA/GreB family elongation factor [Brucella intermedia].	GCF_001917355.1
WP_075481088.1	TPR+GreAB-C+PIN*→	TPR+GreAB-C+PIN	1236	-	Gammaproteobacteria	Moritella viscosa	hypothetical protein [Moritella viscosa].	GCF_900120145.1
WP_075594884.1	REase+TPR+GreAB-C+PIN*→	REase+TPR+GreAB-C+PIN	1086	-	Alphaproteobacteria	Komagataeibacter hansenii	hypothetical protein [Komagataeibacter hansenii].	GCF_001938745.1
WP_076004144.1	REase+TPR+GreAB-C+PIN*→	REase+TPR+GreAB-C+PIN	1301	-	Chloroflexi	Dehalogenimonas formicexedens	hypothetical protein [Dehalogenimonas formicexedens].	GCF_001953175.1
WP_076215124.1	TPR+PIN*→	TPR+PIN	1179	-	Firmicutes	Paenibacillus odorifer	tetratricopeptide repeat protein [Paenibacillus odorifer].	GCF_001954655.1
WP_076272073.1	TPR+PIN*→	TPR+PIN	1180	-	Firmicutes	Paenibacillus odorifer	hypothetical protein [Paenibacillus odorifer].	GCF_001954285.1
WP_076387298.1	TPR+GreAB-C+PIN*→	TPR+GreAB-C+PIN	1130	-	Bacteroidetes	Kaistella chaponensis	hypothetical protein [Kaistella chaponensis].	GCF_900156725.1
WP_076506278.1	TPR+GreAB-C+PIN*→	TPR+GreAB-C+PIN	1137	-	Bacteroidetes	Chryseobacterium shigense	hypothetical protein [Chryseobacterium shigense].	GCF_900156575.1
WP_076822916.1	REase+TPR+GreAB-C+PIN*→	REase+TPR+GreAB-C+PIN	1162	-	Actinobacteria	Frankia asymbiotica	hypothetical protein [Frankia asymbiotica].	GCF_001983105.1
WP_076832171.1	TPR+GreAB-C+PIN*→	TPR+GreAB-C+PIN	1129	-	Alphaproteobacteria	Bradyrhizobium sp. UFLA 03-321	hypothetical protein [Bradyrhizobium sp. UFLA 03-321].	GCF_001969825.1
WP_076835387.1	REase+TPR+GreAB-C+PIN*→	REase+TPR+GreAB-C+PIN	1345	-	Betaproteobacteria	Burkholderia pseudomallei	hypothetical protein [Burkholderia pseudomallei].	GCF_001980695.1
WP_078005484.1	TPR+GreAB-C+PIN*→	TPR+GreAB-C+PIN	1131	-	Bacteroidetes	Flavobacterium sp. KBS0721	hypothetical protein [Flavobacterium sp. KBS0721].	GCF_002007065.3
WP_078350655.1	TPR+GreAB-C+PIN*→	TPR+GreAB-C+PIN	1286	-	Bacteroidetes	Mucilaginibacter pedocola	hypothetical protein [Mucilaginibacter pedocola].	GCF_002013915.1
WP_078403924.1	TPR+GreAB-C+PIN*→	TPR+GreAB-C+PIN	1140	-	Bacteroidetes	Elizabethkingia ursingii	hypothetical protein [Elizabethkingia ursingii].	GCF_002022125.1
WP_078946818.1	REase+PIN*→	REase+PIN	1277	-	Actinobacteria	Streptomyces sp. NRRL B-3648	DUF4365 domain-containing protein [Streptomyces sp. NRRL B-3648].	GCF_001279545.1
WP_079454673.1	REase+TPR+GreAB-C+PIN*→||||<-?||HTH→	REase+TPR+GreAB-C+PIN	1250	-	Gammaproteobacteria	Acinetobacter baumannii	GreA/GreB family elongation factor [Acinetobacter baumannii].	GCF_902728005.1
WP_079469502.1	TPR+GreAB-C+PIN*→	TPR+GreAB-C+PIN	1234	-	Bacteroidetes	Chitinophaga ginsengisegetis	hypothetical protein [Chitinophaga ginsengisegetis].	GCF_900168065.1
WP_079579807.1	X+PIN*→	X+PIN	1007	-	Epsilonproteobacteria	Malaciobacter marinus	hypothetical protein [Malaciobacter marinus].	GCF_900168035.1
WP_079820569.1	HetE-N1→ TPR+GreAB-C+PIN*→	TPR+GreAB-C+PIN	1117	-	Gammaproteobacteria	Salmonella enterica	hypothetical protein [Salmonella enterica].	GCF_016071195.1
WP_079943789.1	<-HTH<-?<-?||HetE-N1→ TPR+GreAB-C+PIN*→	TPR+GreAB-C+PIN	998	-	Gammaproteobacteria	Salmonella enterica	hypothetical protein [Salmonella enterica].	GCF_002045995.1
WP_079951728.1	<-HTH<-?<-?||HetE-N1→ TPR+GreAB-C+PIN*→	TPR+GreAB-C+PIN	993	-	Gammaproteobacteria	Salmonella enterica	hypothetical protein [Salmonella enterica].	GCF_002052255.1
WP_079954008.1	<-HTH<-?<-?||HetE-N1→ TPR+GreAB-C+PIN*→	TPR+GreAB-C+PIN	998	-	Gammaproteobacteria	Salmonella enterica	hypothetical protein [Salmonella enterica].	GCF_002045095.1
WP_079959318.1	HetE-N1→ TPR+GreAB-C+PIN*→	TPR+GreAB-C+PIN	1119	-	Gammaproteobacteria	Salmonella enterica	hypothetical protein [Salmonella enterica].	GCF_002046115.1
WP_079973127.1	<-HTH<-?<-?||HetE-N1→ TPR+GreAB-C+PIN*→	TPR+GreAB-C+PIN	1106	-	Gammaproteobacteria	Salmonella enterica	hypothetical protein [Salmonella enterica].	GCF_002046215.1
WP_080170163.1	HetE-N1→ TPR+GreAB-C+PIN*→	TPR+GreAB-C+PIN	1106	-	Gammaproteobacteria	Salmonella enterica	hypothetical protein [Salmonella enterica].	GCF_016863875.1
WP_080323117.1	REase+TPR+GreAB-C+PIN*→	REase+TPR+GreAB-C+PIN	1334	-	Betaproteobacteria	Burkholderia cenocepacia	hypothetical protein [Burkholderia cenocepacia].	GCF_002071875.1
WP_080865316.1	PIN+GNAT→?→?→ REase+TPR+GreAB-C+PIN*→	REase+TPR+GreAB-C+PIN	1290	-	Alphaproteobacteria	Agrobacterium tumefaciens	hypothetical protein [Agrobacterium tumefaciens].	GCF_900012605.1
WP_081143626.1	TPR+PIN*→	TPR+PIN	1157	-	Firmicutes	Bacillus cereus	hypothetical protein [Bacillus cereus].	GCF_002078195.1
WP_082055264.1	NACHT→ REase+TPR+GreAB-C+PIN*→	REase+TPR+GreAB-C+PIN	1329	-	Betaproteobacteria	Cupriavidus basilensis	DUF4365 domain-containing protein [Cupriavidus basilensis].	GCF_000832305.1
WP_082573822.1	HTH+PNPase+TPR+GreAB-C+PIN*→	HTH+PNPase+TPR+GreAB-C+PIN	1517	-	Actinobacteria	Cellulomonas sp. Root137	hypothetical protein [Cellulomonas sp. Root137].	GCF_001426725.1

<table>
<tr><th>acc</th><th>operon</th><th>architecture</th><th>len</th><th>gen.name</th><th>taxend</th><th>species</th><th>defline</th><th>gca</th></tr>
<tr><td>WP_082991752.1</td><td>REase+TPR+GreAB-C+PIN*→</td><td>REase+TPR+GreAB-C+PIN</td><td>1320</td><td>-</td><td>Betaproteobacteria</td><td>Paraburkholderia tropica</td><td>DUF4365 domain-containing protein [Paraburkholderia tropica].</td><td>GCF_001673675.1</td></tr>
<tr><td>WP_083024260.1</td><td>TPR+GreAB-C*→</td><td>TPR+GreAB-C</td><td>833</td><td>-</td><td>Gammaproteobacteria</td><td>Halomonas lionensis</td><td>hypothetical protein [Halomonas lionensis].</td><td>GCF_002087295.1</td></tr>
<tr><td>WP_083366146.1</td><td>HetE-N1→ TPR+GreAB-C+PIN*→</td><td>TPR+GreAB-C+PIN</td><td>1112</td><td>-</td><td>Gammaproteobacteria</td><td>Pseudomonas koreensis</td><td>hypothetical protein [Pseudomonas koreensis].</td><td>GCF_014646955.1</td></tr>
<tr><td>WP_083417785.1</td><td>REase+TPR+GreAB-C+PIN*→||||<-TIR</td><td>REase+TPR+GreAB-C+PIN</td><td>1329</td><td>-</td><td>Betaproteobacteria</td><td>Burkholderia contaminans</td><td>DUF4365 domain-containing protein [Burkholderia contaminans].</td><td>GCF_001865715.1</td></tr>
<tr><td>WP_083925131.1</td><td>TPR+GreAB-C+PIN*→</td><td>TPR+GreAB-C+PIN</td><td>1307</td><td>-</td><td>Gammaproteobacteria</td><td>Thioalkalivibrio sp. ALJ15</td><td>GreA/GreB family elongation factor [Thioalkalivibrio sp. ALJ15].</td><td>GCF_000383695.1</td></tr>
<tr><td>WP_084371210.1</td><td>X+PIN*→</td><td>X+PIN</td><td>1158</td><td>-</td><td>Bacteroidetes</td><td>Reichenbachiella faecimaris</td><td>hypothetical protein [Reichenbachiella faecimaris].</td><td>GCF_900176375.1</td></tr>
<tr><td>WP_084886264.1</td><td>TPR+GreAB-C+PIN*→</td><td>TPR+GreAB-C+PIN</td><td>999</td><td>-</td><td>Gammaproteobacteria</td><td>Pantoea septica</td><td>hypothetical protein [Pantoea septica].</td><td>GCF_002095575.1</td></tr>
<tr><td>WP_085383443.1</td><td>REase+TPR+GreAB-C+PIN*→</td><td>REase+TPR+GreAB-C+PIN</td><td>1301</td><td>-</td><td>Alphaproteobacteria</td><td>Bradyrhizobium canariense</td><td>hypothetical protein [Bradyrhizobium canariense].</td><td>GCF_002108905.1</td></tr>
<tr><td>WP_085905967.1</td><td>TPR+GreAB-C+PIN*→</td><td>TPR+GreAB-C+PIN</td><td>1176</td><td>-</td><td>Alphaproteobacteria</td><td>Kiloniella majae</td><td>hypothetical protein [Kiloniella majae].</td><td>GCF_002118275.1</td></tr>
<tr><td>WP_086630532.1</td><td>HetE-N1→ TPR+GreAB-C+PIN+TM+TM*→</td><td>TPR+GreAB-C+PIN+TM+TM</td><td>1106</td><td>-</td><td>Gammaproteobacteria</td><td>Klebsiella aerogenes</td><td>hypothetical protein [Klebsiella aerogenes].</td><td>GCF_002152925.1</td></tr>
<tr><td>WP_086759874.1</td><td>APATPase+BetaPropeller→ PNPase+TPR+GreAB-C+PIN*→</td><td>PNPase+TPR+GreAB-C+PIN</td><td>1399</td><td>-</td><td>Cyanobacteria</td><td>Nostoc sp. 106C</td><td>tetratricopeptide repeat protein [Nostoc sp. 106C].</td><td>GCF_002154725.1</td></tr>
<tr><td>WP_086979350.1</td><td>HetE-N1→ TPR+GreAB-C+PIN*→</td><td>TPR+GreAB-C+PIN</td><td>940</td><td>-</td><td>Gammaproteobacteria</td><td>Pseudomonas putida</td><td>hypothetical protein [Pseudomonas putida].</td><td>GCF_002157515.1</td></tr>
<tr><td>WP_087258361.1</td><td>TPR+GreAB-C+PIN*→</td><td>TPR+GreAB-C+PIN</td><td>1189</td><td>-</td><td>Firmicutes</td><td>[Clostridium] spiroforme</td><td>hypothetical protein [[Clostridium] spiroforme].</td><td>GCF_002160695.1</td></tr>
<tr><td>WP_087391814.1</td><td>TPR+GreAB-C+PIN*→</td><td>TPR+GreAB-C+PIN</td><td>1189</td><td>-</td><td>Firmicutes</td><td>[Clostridium] spiroforme</td><td>hypothetical protein [[Clostridium] spiroforme].</td><td>GCF_002160645.1</td></tr>
<tr><td>WP_088070184.1</td><td>TPR+PIN*→</td><td>TPR+PIN</td><td>1157</td><td>-</td><td>Firmicutes</td><td>Bacillus thuringiensis</td><td>hypothetical protein [Bacillus thuringiensis].</td><td>GCF_002148155.1</td></tr>
<tr><td>WP_088082523.1</td><td>TPR+PIN*→</td><td>TPR+PIN</td><td>1179</td><td>-</td><td>Firmicutes</td><td>Bacillus thuringiensis</td><td>hypothetical protein [Bacillus thuringiensis].</td><td>GCF_002147935.1</td></tr>
<tr><td>WP_088087080.1</td><td>TPR+PIN*→</td><td>TPR+PIN</td><td>1176</td><td>-</td><td>Firmicutes</td><td>Bacillus sp. OV166</td><td>hypothetical protein [Bacillus sp. OV166].</td><td>GCF_900177675.1</td></tr>
<tr><td>WP_088476259.1</td><td>REase+TPR+GreAB-C+PIN*→</td><td>REase+TPR+GreAB-C+PIN</td><td>1284</td><td>-</td><td>Gammaproteobacteria</td><td>Stenotrophomonas pavanii</td><td>GreA/GreB family elongation factor [Stenotrophomonas pavanii].</td><td>GCF_002205885.1</td></tr>
<tr><td>WP_089172847.1</td><td>BirA-HTH→ HEPN→ REase+TPR+TPR+GreAB-C+PIN*→</td><td>REase+TPR+TPR+GreAB-C+PIN</td><td>1332</td><td>-</td><td>Alphaproteobacteria</td><td>unclassified Bosea</td><td>MULTISPECIES: hypothetical protein [unclassified Bosea].</td><td>GCF_002220095.1</td></tr>
<tr><td>WP_090371407.1</td><td>REase→ TPR+GreAB-C+PIN*→</td><td>TPR+GreAB-C+PIN</td><td>1155</td><td>-</td><td>Betaproteobacteria</td><td>Nitrosospira sp. Nl5</td><td>hypothetical protein [Nitrosospira sp. Nl5].</td><td>GCF_900102495.1</td></tr>
<tr><td>WP_090455730.1</td><td>HetE-N1→ TPR+GreAB-C+PIN*→||||<-TIR</td><td>TPR+GreAB-C+PIN</td><td>1112</td><td>-</td><td>Gammaproteobacteria</td><td>Pseudomonas jessenii</td><td>hypothetical protein [Pseudomonas jessenii].</td><td>GCF_900104905.1</td></tr>
<tr><td>WP_090543864.1</td><td>TIR+TPR+GreAB-C+PIN*→</td><td>TIR+TPR+GreAB-C+PIN</td><td>1348</td><td>-</td><td>Betaproteobacteria</td><td>Nitrosomonas sp. Nm132</td><td>toll/interleukin-1 receptor domain-containing protein [Nitrosomonas sp. Nm132].</td><td>GCF_900100485.1</td></tr>
<tr><td>WP_090721286.1</td><td><-Cas_Cas1<-?||?→?→?→?→ REase+TPR+GreAB-C+PIN*→</td><td>REase+TPR+GreAB-C+PIN</td><td>1380</td><td>-</td><td>Betaproteobacteria</td><td>Nitrosomonas sp. Nm166</td><td>hypothetical protein [Nitrosomonas sp. Nm166].</td><td>GCF_900112825.1</td></tr>
<tr><td>WP_091343709.1</td><td>HetE-N1→ TPR+GreAB-C+PIN*→</td><td>TPR+GreAB-C+PIN</td><td>1098</td><td>-</td><td>Gammaproteobacteria</td><td>Alkalimonas amylolytica</td><td>hypothetical protein [Alkalimonas amylolytica].</td><td>GCF_900107845.1</td></tr>
<tr><td>WP_091456057.1</td><td>REase+TPR+GreAB-C+PIN*→</td><td>REase+TPR+GreAB-C+PIN</td><td>1259</td><td>-</td><td>Actinobacteria</td><td>Micromonospora inyonensis</td><td>hypothetical protein [Micromonospora inyonensis].</td><td>GCF_900091415.1</td></tr>
<tr><td>WP_091965577.1</td><td>TPR+GreAB-C+PIN*→</td><td>TPR+GreAB-C+PIN</td><td>1229</td><td>-</td><td>Alphaproteobacteria</td><td>Bradyrhizobium shewense</td><td>hypothetical protein [Bradyrhizobium shewense].</td><td>GCF_900094605.1</td></tr>
<tr><td>WP_092226352.1</td><td>TPR+GreAB-C+PIN*→</td><td>TPR+GreAB-C+PIN</td><td>1306</td><td>-</td><td>Alphaproteobacteria</td><td>Bradyrhizobium sp. Gha</td><td>tetratricopeptide repeat protein [Bradyrhizobium sp. Gha].</td><td>GCF_900113735.1</td></tr>
<tr><td>WP_092234200.1</td><td>REase+TPR+GreAB-C+PIN*→</td><td>REase+TPR+GreAB-C+PIN</td><td>1321</td><td>-</td><td>Gammaproteobacteria</td><td>Pseudomonas proteolytica</td><td>DUF4365 domain-containing protein [Pseudomonas proteolytica].</td><td>GCF_900105955.1</td></tr>
<tr><td>WP_092689872.1</td><td>HTH→ HetE-N1→ TPR+GreAB-C+PIN*→</td><td>TPR+GreAB-C+PIN</td><td>1077</td><td>-</td><td>Gammaproteobacteria</td><td>Acinetobacter kyonggiensis</td><td>hypothetical protein [Acinetobacter kyonggiensis].</td><td>GCF_900107285.1</td></tr>
<tr><td>WP_093257662.1</td><td>HetE-N1→ HetE-N1+TPR+GreAB-C+PIN*→</td><td>HetE-N1+TPR+GreAB-C+PIN</td><td>1120</td><td>-</td><td>Gammaproteobacteria</td><td>Pseudomonas</td><td>MULTISPECIES: hypothetical protein [Pseudomonas].</td><td>GCF_900110545.1</td></tr>
<tr><td>WP_093289087.1</td><td>REase+TPR+GreAB-C+PIN*→</td><td>REase+TPR+GreAB-C+PIN</td><td>1291</td><td>-</td><td>Gammaproteobacteria</td><td>Pseudoxanthomonas sp. CF125</td><td>hypothetical protein [Pseudoxanthomonas sp. CF125].</td><td>GCF_900104085.1</td></tr>
<tr><td>WP_095032727.1</td><td>HetE-N1→ TPR+GreAB-C+PIN*→</td><td>TPR+GreAB-C+PIN</td><td>1104</td><td>-</td><td>Gammaproteobacteria</td><td>Klebsiella quasivariicola</td><td>hypothetical protein [Klebsiella quasivariicola].</td><td>GCF_002269255.1</td></tr>
<tr><td>WP_095064594.1</td><td>HetE-N1→ TPR+GreAB-C+PIN*→</td><td>TPR+GreAB-C+PIN</td><td>1105</td><td>-</td><td>Gammaproteobacteria</td><td>Pseudomonas sp. Irchel s3f19</td><td>hypothetical protein [Pseudomonas sp. Irchel s3f19].</td><td>GCF_900187555.1</td></tr>
<tr><td>WP_095490577.1</td><td>HetE-N1→ HetE-N1+TPR+GreAB-C+PIN*→</td><td>HetE-N1+TPR+GreAB-C+PIN</td><td>1088</td><td>-</td><td>Gammaproteobacteria</td><td>Vibrio cholerae</td><td>hypothetical protein [Vibrio cholerae].</td><td>GCF_002284425.1</td></tr>
<tr><td>WP_095570744.1</td><td>HetE-N1→ TPR+GreAB-C+PIN*→</td><td>TPR+GreAB-C+PIN</td><td>1086</td><td>-</td><td>Gammaproteobacteria</td><td>Vibrio coralliilyticus</td><td>hypothetical protein [Vibrio coralliilyticus].</td><td>GCF_002286405.1</td></tr>
<tr><td>WP_095743432.1</td><td>REase+TPR+GreAB-C+PIN*→</td><td>REase+TPR+GreAB-C+PIN</td><td>1300</td><td>-</td><td>Betaproteobacteria</td><td>Variovorax boronicumulans</td><td>hypothetical protein [Variovorax boronicumulans].</td><td>GCF_002291405.1</td></tr>
<tr><td>WP_096138380.1</td><td>REase+TPR+GreAB-C+PIN*→</td><td>REase+TPR+GreAB-C+PIN</td><td>1321</td><td>-</td><td>Gammaproteobacteria</td><td>Pseudomonas syringae</td><td>DUF4365 domain-containing protein [Pseudomonas syringae].</td><td>GCF_002318715.1</td></tr>
<tr><td>WP_096145332.1</td><td>PIN*→</td><td>PIN</td><td>1088</td><td>-</td><td>Firmicutes</td><td>Clostridium chauvoei</td><td>tetratricopeptide repeat protein [Clostridium chauvoei].</td><td>GCF_002327185.1</td></tr>
<tr><td>WP_096360261.1</td><td>REase+TPR+GreAB-C+PIN*→</td><td>REase+TPR+GreAB-C+PIN</td><td>2100</td><td>-</td><td>Gammaproteobacteria</td><td>Sulfuricaulis limicola</td><td>hypothetical protein [Sulfuricaulis limicola].</td><td>GCF_002355735.1</td></tr>
</table>

acc	operon	architecture	len	gen.name	taxend	species	defline	gca
WP_096444074.1	HetE-N1→ TPR+GreAB-C+PIN*→	TPR+GreAB-C+PIN	1088	-	Gammaproteobacteria	Vibrio mediterranei	hypothetical protein [Vibrio mediterranei].	GCF_002995655.1
WP_096819326.1	REase+TPR+GreAB-C+PIN*→	REase+TPR+GreAB-C+PIN	1424	-	Gammaproteobacteria	Pseudomonas fluorescens	tetratricopeptide repeat protein [Pseudomonas fluorescens].	GCF_002417665.1
WP_096985931.1	HetE-N1→ TPR+GreAB-C+PIN*→	TPR+GreAB-C+PIN	1094	-	Gammaproteobacteria	Escherichia coli	hypothetical protein [Escherichia coli].	GCF_013372365.1
WP_097373330.1	PIN*→	PIN	245	-	Alphaproteobacteria	Celeribacter manganoxidans	hypothetical protein [Celeribacter manganoxidans].	GCF_002504165.1
WP_097730210.1	HetE-N1→ TPR+GreAB-C+PIN*→	TPR+GreAB-C+PIN	1094	-	Gammaproteobacteria	Escherichia coli	hypothetical protein [Escherichia coli].	GCF_002535535.1
WP_097759664.1	HetE-N1→ TPR+GreAB-C+PIN*→	TPR+GreAB-C+PIN	1094	-	Gammaproteobacteria	Escherichia coli	hypothetical protein [Escherichia coli].	GCF_002538945.1
WP_097979402.1	TPR+PIN*→	TPR+PIN	1217	-	Firmicutes	Bacillus toyonensis	hypothetical protein [Bacillus toyonensis].	GCF_002552775.1
WP_098090392.1	HetE-N1→ TPR+GreAB-C+PIN*→	TPR+GreAB-C+PIN	1101	-	Gammaproteobacteria	Pseudomonas putida	hypothetical protein [Pseudomonas putida].	GCF_002554535.1
WP_098280847.1	TPR+PIN*→	TPR+PIN	1183	-	Firmicutes	Bacillus cereus	hypothetical protein [Bacillus cereus].	GCF_002560075.1
WP_098365372.1	TPR+PIN*→	TPR+PIN	1217	-	Firmicutes	Bacillus cereus	hypothetical protein [Bacillus cereus].	GCF_002561575.1
WP_098420086.1	TPR+PIN*→	TPR+PIN	1183	-	Firmicutes	Bacillus cereus	hypothetical protein [Bacillus cereus].	GCF_002562505.1
WP_098433845.1	TPR+PIN*→	TPR+PIN	1184	-	Firmicutes	Priestia megaterium	hypothetical protein [Priestia megaterium].	GCF_002564705.1
WP_098573825.1	TPR+GreAB-C+PIN*→	TPR+GreAB-C+PIN	1147	-	Firmicutes	Bacillus wiedmannii	tetratricopeptide repeat protein [Bacillus wiedmannii].	GCF_002568785.1
WP_098687609.1	TPR+PIN*→	TPR+PIN	1157	-	Firmicutes	Bacillus toyonensis	hypothetical protein [Bacillus toyonensis].	GCF_002571615.1
WP_099262235.1	REase+TPR+GreAB-C+PIN*→	REase+TPR+GreAB-C+PIN	1243	-	Planctomycetes	Rhodopirellula bahusiensis	GreA/GreB family elongation factor [Rhodopirellula bahusiensis].	GCF_002727185.1
WP_099438436.1	TPR+GreAB-C+PIN*→	TPR+GreAB-C+PIN	1252	-	Bacteroidetes	Pedobacter ginsengisoli	hypothetical protein [Pedobacter ginsengisoli].	GCF_002736205.1
WP_100140263.1	HetE-N1→ TPR+GreAB-C+PIN*→	TPR+GreAB-C+PIN	1066	-	Betaproteobacteria	Snodgrassella alvi	hypothetical protein [Snodgrassella alvi].	GCF_002777775.1
WP_100151648.1	HetE-N1→ TPR+GreAB-C+PIN*→	TPR+GreAB-C+PIN	1066	-	Betaproteobacteria	Snodgrassella alvi	hypothetical protein [Snodgrassella alvi].	GCF_002777655.1
WP_100344325.1	REase+TPR+GreAB-C+PIN*→	REase+TPR+GreAB-C+PIN	1215	-	Actinobacteria	Compostimonas suwonensis	DUF4365 domain-containing protein [Compostimonas suwonensis].	GCF_002797855.1
WP_100632757.1	REase+TPR+GreAB-C+PIN*→	REase+TPR+GreAB-C+PIN	1321	-	Gammaproteobacteria	Pseudomonas qingdaonensis	DUF4365 domain-containing protein [Pseudomonas qingdaonensis].	GCF_002806685.1
WP_101291505.1	HetE-N1→ TPR+GreAB-C+PIN*→	TPR+GreAB-C+PIN	1094	-	Gammaproteobacteria	Escherichia coli	hypothetical protein [Escherichia coli].	GCF_002843705.1
WP_102155042.1	REase+TPR+GreAB-C+PIN*→	REase+TPR+GreAB-C+PIN	1270	-	Alphaproteobacteria	Tsuneonella flava	GreA/GreB family elongation factor [Tsuneonella flava].	GCF_002870965.1
WP_102488850.1	HetE-N1→ HetE-N1+TPR+GreAB-C+PIN*→	HetE-N1+TPR+GreAB-C+PIN	1088	-	Gammaproteobacteria	Vibrio splendidus	hypothetical protein [Vibrio splendidus].	GCF_002875305.1
WP_102520861.1	HetE-N1→ TPR+GreAB-C+PIN*→	TPR+GreAB-C+PIN	1088	-	Gammaproteobacteria	Shewanella sp. 10N.286.48.A6	hypothetical protein [Shewanella sp. 10N.286.48.A6].	GCF_002873135.1
WP_102570991.1	HetE-N1→ TPR+GreAB-C+PIN*→	TPR+GreAB-C+PIN	1088	-	Gammaproteobacteria	Vibrio splendidus	hypothetical protein [Vibrio splendidus].	GCF_002875585.1
WP_102574526.1	HetE-N1→ TPR+GreAB-C+PIN*→	TPR+GreAB-C+PIN	1116	-	Gammaproteobacteria	Vibrio splendidus	hypothetical protein [Vibrio splendidus].	GCF_002877195.1
WP_102576920.1	HetE-N1→ HetE-N1+TPR+GreAB-C+PIN*→	HetE-N1+TPR+GreAB-C+PIN	1091	-	Gammaproteobacteria	Vibrio splendidus	hypothetical protein [Vibrio splendidus].	GCF_002877345.1
WP_103206664.1	TPR+GreAB-C+PIN*→	TPR+GreAB-C+PIN	1213	-	Actinobacteria	Microbacterium testaceum	hypothetical protein [Microbacterium testaceum].	GCF_002899925.1
WP_103448257.1	REase+TPR+GreAB-C+PIN*→	REase+TPR+GreAB-C+PIN	1421	-	Gammaproteobacteria	Pseudomonas	MULTISPECIES: hypothetical protein [Pseudomonas].	GCF_015680835.1
WP_103712798.1	REase+TPR+GreAB-C+PIN*→||||<-KAP_NTPase	REase+TPR+GreAB-C+PIN	1321	-	Gammaproteobacteria	Pseudomonas syringae	DUF4365 domain-containing protein [Pseudomonas syringae].	GCF_002916275.1
WP_103713374.1	HetE-N1→ TPR+GreAB-C+PIN*→	TPR+GreAB-C+PIN	1107	-	Gammaproteobacteria	Pseudomonas syringae	hypothetical protein [Pseudomonas syringae].	GCF_002917155.1
WP_103715293.1	TPR+GreAB-C+PIN*→	TPR+GreAB-C+PIN	1131	-	Bacteroidetes	Flavobacterium columnare	hypothetical protein [Flavobacterium columnare].	GCF_002916795.1
WP_103949560.1	HetE-N1→ HetE-N1+TPR+GreAB-C+PIN*→	HetE-N1+TPR+GreAB-C+PIN	1094	-	Gammaproteobacteria	Lelliottia	MULTISPECIES: hypothetical protein [Lelliottia].	GCF_002922995.1
WP_104189366.1	HetE-N1→ TPR+GreAB-C+PIN*→	TPR+GreAB-C+PIN	1110	-	Gammaproteobacteria	unclassified Pantoea	MULTISPECIES: hypothetical protein [unclassified Pantoea].	GCF_002928355.1
WP_104723186.1	REase+TPR+GreAB-C+PIN*→	REase+TPR+GreAB-C+PIN	1321	-	Gammaproteobacteria	Pseudomonas syringae	DUF4365 domain-containing protein [Pseudomonas syringae].	GCF_900235865.1
WP_104925436.1	HetE-N1→ TPR+GreAB-C+PIN*→	TPR+GreAB-C+PIN	1118	-	Gammaproteobacteria	Rahnella sp. ERMR1:05	hypothetical protein [Rahnella sp. ERMR1:05].	GCF_002951615.1
WP_105080712.1	HetE-N1→ TPR+GreAB-C+PIN*→	TPR+GreAB-C+PIN	1111	-	Gammaproteobacteria	Pantoea	MULTISPECIES: hypothetical protein [Pantoea].	GCF_017920435.1
WP_105696567.1	REase+TPR+GreAB-C+PIN*→	REase+TPR+GreAB-C+PIN	1321	-	Gammaproteobacteria	Pseudomonas poae	DUF4365 domain-containing protein [Pseudomonas poae].	GCF_002980135.1
WP_105730942.1	REase+TPR+GreAB-C+PIN*→	REase+TPR+GreAB-C+PIN	1344	-	Betaproteobacteria	Malikia spinosa	hypothetical protein [Malikia spinosa].	GCF_002980625.1
WP_105731559.1	REase+TPR+GreAB-C+PIN*→	REase+TPR+GreAB-C+PIN	1278	-	Alphaproteobacteria	Ochrobactrum sp. MYb29	hypothetical protein [Ochrobactrum sp. MYb29].	GCF_002979345.1

acc	operon	architecture	len	gen.name	taxend	species	defline	gca
WP_105794455.1	REase+TPR+GreAB-C+PIN*→	REase+TPR+GreAB-C+PIN	1351	-	Betaproteobacteria	Burkholderia multivorans	hypothetical protein [Burkholderia multivorans].	GCF_002981485.1
WP_106098487.1	SIG+TM→ TPR+GreAB-C+PIN*→	TPR+GreAB-C+PIN	1129	-	Bacteroidetes	Capnocytophaga sp. oral taxon 878	hypothetical protein [Capnocytophaga sp. oral taxon 878].	GCF_002999135.1
WP_106374900.1	HetE-N1→ TPR+GreAB-C+PIN*→	TPR+GreAB-C+PIN	1145	-	Gammaproteobacteria	Halomonas songnenensis	hypothetical protein [Halomonas songnenensis].	GCF_003002925.1
WP_106946809.1	TPR+GreAB-C+PIN*→	TPR+GreAB-C+PIN	1155	-	Alphaproteobacteria	Bradyrhizobium sp. MOS002	hypothetical protein [Bradyrhizobium sp. MOS002].	GCF_003020115.1
WP_107027340.1	REase+TPR+TPR+GreAB-C+PIN*→	REase+TPR+TPR+GreAB-C+PIN	1357	-	Alphaproteobacteria	Ensifer sp. NM-2	hypothetical protein [Ensifer sp. NM-2].	GCF_003024455.1
WP_107248740.1	HetE-N1→ GreAB-C*→	GreAB-C	1128	-	Gammaproteobacteria	Photobacterium kishitanii	hypothetical protein [Photobacterium kishitanii].	GCF_003025535.1
WP_107289369.1	HetE-N1→ GreAB-C*→	GreAB-C	1164	-	Gammaproteobacteria	Photobacterium kishitanii	hypothetical protein [Photobacterium kishitanii].	GCF_003025915.1
WP_107901223.1	TPR+PIN*→	TPR+PIN	1216	-	Firmicutes	Bacillus sp. OV186	hypothetical protein [Bacillus sp. OV186].	GCF_003053645.1
WP_108393929.1	HetE-N1→ TPR+GreAB-C+PIN*→	TPR+GreAB-C+PIN	1106	-	Gammaproteobacteria	Enterobacter hormaechei	hypothetical protein [Enterobacter hormaechei].	GCF_003056805.1
WP_108462916.1	REase+TPR+TPR+GreAB-C+PIN*→	REase+TPR+TPR+GreAB-C+PIN	1323	-	Alphaproteobacteria	Devosia naphthalenivorans	GreA/GreB family elongation factor [Devosia naphthalenivorans].	GCF_003056355.1
WP_108677990.1	HetE-N1→ HetE-N1+TPR+GreAB-C+PIN*→	HetE-N1+TPR+GreAB-C+PIN	1088	-	Gammaproteobacteria	Vibrio vulnificus	hypothetical protein [Vibrio vulnificus].	GCF_011754395.1
WP_108816955.1	REase+TPR+GreAB-C+PIN*→	REase+TPR+GreAB-C+PIN	1311	-	Alphaproteobacteria	Loktanella sp. Alg231-35	hypothetical protein [Loktanella sp. Alg231-35].	GCF_900143545.1
WP_109158749.1	X+PIN*→	X+PIN	1041	-	Epsilonproteobacteria	Aliarcobacter skirrowii	hypothetical protein [Aliarcobacter skirrowii].	GCF_003122145.1
WP_109211325.1	REase+TPR+GreAB-C+PIN*→	REase+TPR+GreAB-C+PIN	1211	-	Actinobacteria	Microbacterium	MULTISPECIES: DUF4365 domain-containing protein [Microbacterium].	GCF_015278315.1
WP_109426752.1	TPR→ TPR+GreAB-C+PIN*→||||<-?||HTH→	TPR+GreAB-C+PIN	494	-	Gammaproteobacteria	Acinetobacter baumannii	GreA/GreB family elongation factor [Acinetobacter baumannii].	GCF_013416255.1
WP_109802449.1	TPR+PIN*→	TPR+PIN	1203	-	Actinobacteria	Micromonospora sp. 4G51	hypothetical protein [Micromonospora sp. 4G51].	GCF_003172935.1
WP_109937286.1	HetE-N1→ HetE-N1+TPR+GreAB-C+PIN*→	HetE-N1+TPR+GreAB-C+PIN	1123	-	Gammaproteobacteria	Pseudomonas sp. RW407	hypothetical protein [Pseudomonas sp. RW407].	GCF_003176655.1
WP_109950172.1	REase+TPR+GreAB-C+PIN*→	REase+TPR+GreAB-C+PIN	1332	-	-	Methylobacterium sp. 17Sr1-43	hypothetical protein [Methylobacterium sp. 17Sr1-43].	GCF_003173735.1
WP_109967732.1	PNPase+TPR+GreAB-C+PIN*→	PNPase+TPR+GreAB-C+PIN	1415	-	Euryarchaeota	Methanospirillum lacunae	hypothetical protein [Methanospirillum lacunae].	GCF_003173355.1
WP_110317339.1	HTH+PNPase+TPR+GreAB-C+PIN*→	HTH+PNPase+TPR+GreAB-C+PIN	1518	-	Actinobacteria	Mycolicibacterium moriokaense	hypothetical protein [Mycolicibacterium moriokaense].	GCF_003201655.1
WP_110434863.1	HetE-N1→ TPR+GreAB-C+PIN*→	TPR+GreAB-C+PIN	950	-	Gammaproteobacteria	Gilliamella apicola	hypothetical protein [Gilliamella apicola].	GCF_003202655.1
WP_110455267.1	HetE-N1→ TPR+GreAB-C+PIN*→	TPR+GreAB-C+PIN	1126	-	Gammaproteobacteria	Gilliamella apicola	hypothetical protein [Gilliamella apicola].	GCF_003202815.1
WP_110459582.1	HetE-N1→ HetE-N1+TPR+GreAB-C+PIN*→	HetE-N1+TPR+GreAB-C+PIN	1107	-	Gammaproteobacteria	Pseudomonas syringae	hypothetical protein [Pseudomonas syringae].	GCF_003202975.1
WP_110575159.1	HetE-N1→ HetE-N1+TPR+GreAB-C+PIN*→?→ MACRODOMAIN→	HetE-N1+TPR+GreAB-C+PIN	1099	-	Gammaproteobacteria	Marinomonas alcarazii	hypothetical protein [Marinomonas alcarazii].	GCF_003208215.1
WP_110766563.1	REase+TPR+GreAB-C+PIN*→||||<-KAP_NTPase	REase+TPR+GreAB-C+PIN	1321	-	Gammaproteobacteria	Pseudomonas syringae	DUF4365 domain-containing protein [Pseudomonas syringae].	GCF_003205895.1
WP_110818130.1	TPR+GreAB-C+PIN*→	TPR+GreAB-C+PIN	1140	-	Gammaproteobacteria	Pseudomonas syringae	hypothetical protein [Pseudomonas syringae].	GCF_003205965.1
WP_111087282.1	REase+TPR+GreAB-C+PIN*→	REase+TPR+GreAB-C+PIN	1208	-	Actinobacteria	Curtobacterium sp. MCLR17_055	DUF4365 domain-containing protein [Curtobacterium sp. MCLR17_055].	GCF_003234305.1
WP_112353670.1	TPR+GreAB-C+PIN*→	TPR+GreAB-C+PIN	1235	-	Gammaproteobacteria	Shewanella benthica	hypothetical protein [Shewanella benthica].	GCF_900476435.1
WP_112921055.1	HNH→?→?→ PSE→ TPR+GreAB-C+PIN*→	TPR+GreAB-C+PIN	1092	-	Gammaproteobacteria	Escherichia coli	hypothetical protein [Escherichia coli].	GCF_003292515.1
WP_114150230.1	TPR+GreAB-C+PIN*→?→||||<-?||?→ PAIREDC-HTH→	TPR+GreAB-C+PIN	900	-	Gammaproteobacteria	Acinetobacter baumannii	GreA/GreB family elongation factor [Acinetobacter baumannii].	GCF_017726575.1
WP_114915524.1	TPR+PIN*→	TPR+PIN	1187	-	Firmicutes	Oceanobacillus zhaokaii	hypothetical protein [Oceanobacillus zhaokaii].	GCF_003352005.1
WP_115015610.1	HetE-N1→ TPR+GreAB-C+PIN*→	TPR+GreAB-C+PIN	1087	-	Gammaproteobacteria	Shewanella putrefaciens	hypothetical protein [Shewanella putrefaciens].	GCF_900457065.1
WP_115596495.1	TPR+GreAB-C+PIN*→?→||||<-?||?→ PAIREDC-HTH→	TPR+GreAB-C+PIN	756	-	Gammaproteobacteria	Acinetobacter baumannii	GreA/GreB family elongation factor [Acinetobacter baumannii].	GCF_900444745.1
WP_116227219.1	HetE-N1→ TPR+GreAB-C+PIN*→	TPR+GreAB-C+PIN	1121	-	Gammaproteobacteria	Pectobacterium aquaticum	hypothetical protein [Pectobacterium aquaticum].	GCF_003382595.2
WP_116600438.1	REase+TPR+GreAB-C+PIN*→	REase+TPR+GreAB-C+PIN	1438	-	Gammaproteobacteria	Pseudomonas sp. HMWF011	hypothetical protein [Pseudomonas sp. HMWF011].	GCF_003094675.1
WP_116893902.1	TPR+GreAB-C+PIN*→	TPR+GreAB-C+PIN	1158	-	Gammaproteobacteria	Pseudomonas syringae	hypothetical protein [Pseudomonas syringae].	GCF_003412595.1
WP_116994473.1	REase+TPR+GreAB-C+PIN*→	REase+TPR+GreAB-C+PIN	1327	-	Betaproteobacteria	Duganella sp. BJB476	hypothetical protein [Duganella sp. BJB476].	GCF_003416895.1
WP_117157406.1	HetE-N1→ TPR+GreAB-C+PIN*→	TPR+GreAB-C+PIN	1117	-	Gammaproteobacteria	Enterobacteriaceae	MULTISPECIES: hypothetical protein [Enterobacteriaceae].	GCF_017814635.1
WP_117181470.1	REase+TPR+GreAB-C+PIN*→	REase+TPR+GreAB-C+PIN	1438	-	Gammaproteobacteria	Pseudomonas cichorii	hypothetical protein [Pseudomonas cichorii].	GCF_003416755.1

acc	operon	architecture	len	gen.name	taxend	species	defline	gca
WP_117831329.1	HEPN+SNF→?→?→?→ X+PIN*→	X+PIN	1212	-	Firmicutes	Roseburia sp. TF10-5	hypothetical protein [Roseburia sp. TF10-5].	GCF_003462365.1
WP_118235377.1	X+PIN*→	X+PIN	1200	-	Firmicutes	Blautia obeum	hypothetical protein [Blautia obeum].	GCF_003470555.1
WP_118282562.1	X+PIN*→?→ CITB-HTH+LexA-protease→	X+PIN	1212	-	Firmicutes	Roseburia sp. AM16-25	hypothetical protein [Roseburia sp. AM16-25].	GCF_003473825.1
WP_118371145.1	X+PIN*→	X+PIN	1200	-	Firmicutes	Lachnospira eligens	hypothetical protein [Lachnospira eligens].	GCF_003474735.1
WP_118468213.1	Mbetalac→ Trypsin+TPR+PIN*→	Trypsin+TPR+PIN	1933	-	Firmicutes	unclassified Clostridium	MULTISPECIES: trypsin-like peptidase domain-containing protein [unclassified Clostridium].	GCF_003477925.1
WP_118891177.1	REase+TPR+GreAB-C+PIN*→	REase+TPR+GreAB-C+PIN	1327	-	Betaproteobacteria	Ralstonia solanacearum	DUF4365 domain-containing protein [Ralstonia solanacearum].	GCF_003515285.1
WP_118897359.1	X+PIN*→	X+PIN	1033	-	Epsilonproteobacteria	Malaciobacter marinus	hypothetical protein [Malaciobacter marinus].	GCF_003544855.1
WP_118994196.1	HetE-N1→ HetE-N1+TPR+GreAB-C+PIN*→	HetE-N1+TPR+GreAB-C+PIN	1104	-	Betaproteobacteria	Pusillimonas	MULTISPECIES: hypothetical protein [Pusillimonas].	GCF_004022565.1
WP_119088708.1	REase+TPR+GreAB-C+PIN*→	REase+TPR+GreAB-C+PIN	1271	-	Bacteria	Candidatus Cryosericum terrychapinii	hypothetical protein [Candidatus Cryosericum terrychapinii].	GCF_003570925.1
WP_119196537.1	X+PIN*→ HTH→?→?→ HTH→	X+PIN	1109	-	Firmicutes	Dorea formicigenerans	hypothetical protein [Dorea formicigenerans].	GCF_003465045.1
WP_119369914.1	REase+TPR+GreAB-C+PIN*→||||<-?<-TPR	REase+TPR+GreAB-C+PIN	1436	-	Gammaproteobacteria	Pseudomonas monteilii	GreA/GreB family elongation factor [Pseudomonas monteilii].	GCF_003576045.1
WP_119587627.1	REase+TPR+GreAB-C+PIN*→	REase+TPR+GreAB-C+PIN	1290	-	Alphaproteobacteria	Aurantiacibacter zhengii	hypothetical protein [Aurantiacibacter zhengii].	GCF_003584125.1
WP_119956833.1	REase+TPR+GreAB-C+PIN*→	REase+TPR+GreAB-C+PIN	1511	-	Gammaproteobacteria	Pseudomonas sp. K1S02-6	tetratricopeptide repeat protein [Pseudomonas sp. K1S02-6].	GCF_003596405.1
WP_119992948.1	TM+TM+TPR+PIN*→	TM+TM+TPR+PIN	1208	-	Bacteroidetes	Bacteroides sp. AM41-16	hypothetical protein [Bacteroides sp. AM41-16].	GCF_003603495.1
WP_120172805.1	TM+TM+TPR+PIN*→	TM+TM+TPR+PIN	1208	-	Bacteroidetes	Bacteroides sp. AF20-13LB	hypothetical protein [Bacteroides sp. AF20-13LB].	GCF_003603065.1
WP_120777109.1	TPR+GreAB-C+PIN*→	TPR+GreAB-C+PIN	1192	-	Actinobacteria	Microbacterium sp. CGR2	hypothetical protein [Microbacterium sp. CGR2].	GCF_003626735.1
WP_121482750.1	REase+TPR+GreAB-C+PIN*→	REase+TPR+GreAB-C+PIN	1233	-	Alphaproteobacteria	Brevundimonas naejangsanensis	hypothetical protein [Brevundimonas naejangsanensis].	GCF_003627995.1
WP_122071999.1	PAIREDC-HTH→?→?→?→ GreAB-C+PIN*→	GreAB-C+PIN	683	-	Gammaproteobacteria	Acinetobacter wuhouensis	hypothetical protein [Acinetobacter wuhouensis].	GCF_002165345.2
WP_122252741.1	TPR+GreAB-C+PIN*→	TPR+GreAB-C+PIN	1158	-	Gammaproteobacteria	Pseudomonas savastanoi	hypothetical protein [Pseudomonas savastanoi].	GCF_003703035.1
WP_122265719.1	TPR+GreAB-C+PIN*→	TPR+GreAB-C+PIN	1140	-	Gammaproteobacteria	Pseudomonas syringae	hypothetical protein [Pseudomonas syringae].	GCF_003700575.1
WP_122311960.1	TPR+GreAB-C+PIN*→	TPR+GreAB-C+PIN	1143	-	Gammaproteobacteria	Pseudomonas coronafaciens	hypothetical protein [Pseudomonas coronafaciens].	GCF_003699785.1
WP_122314252.1	REase+TPR+GreAB-C+PIN*→	REase+TPR+GreAB-C+PIN	1436	-	Gammaproteobacteria	Pseudomonas cichorii	hypothetical protein [Pseudomonas cichorii].	GCF_003700275.1
WP_122364448.1	REase+TPR+GreAB-C+PIN*→	REase+TPR+GreAB-C+PIN	1438	-	Gammaproteobacteria	Pseudomonas	MULTISPECIES: hypothetical protein [Pseudomonas].	GCF_014842015.1
WP_122365799.1	TPR+GreAB-C+PIN*→	TPR+GreAB-C+PIN	1140	-	Gammaproteobacteria	Pseudomonas syringae group genomosp. 3	hypothetical protein [Pseudomonas syringae group genomosp. 3].	GCF_003702755.1
WP_122471279.1	HetE-N1→ TPR+GreAB-C+PIN*→	TPR+GreAB-C+PIN	1100	-	Gammaproteobacteria	Pseudomonas viridiflava	hypothetical protein [Pseudomonas viridiflava].	GCF_900582365.1
WP_122509345.1	TPR+GreAB-C+PIN*→	TPR+GreAB-C+PIN	1140	-	Gammaproteobacteria	Pseudomonas viridiflava	hypothetical protein [Pseudomonas viridiflava].	GCF_900581635.1
WP_122559800.1	REase+TPR+GreAB-C+PIN*→	REase+TPR+GreAB-C+PIN	1438	-	Gammaproteobacteria	Pseudomonas viridiflava	hypothetical protein [Pseudomonas viridiflava].	GCF_900580935.1
WP_122732542.1	HetE-N1→ HetE-N1+TPR+GreAB-C+PIN*→	HetE-N1+TPR+GreAB-C+PIN	1106	-	Gammaproteobacteria	Pseudomonas viridiflava	hypothetical protein [Pseudomonas viridiflava].	GCF_900591195.1
WP_122848883.1	REase+TPR+GreAB-C+PIN*→	REase+TPR+GreAB-C+PIN	1321	-	Gammaproteobacteria	Pseudomonas viridiflava	DUF4365 domain-containing protein [Pseudomonas viridiflava].	GCF_900585905.1
WP_122887609.1	REase+TPR+GreAB-C+PIN*→	REase+TPR+GreAB-C+PIN	1345	-	Betaproteobacteria	Burkholderia pseudomallei	hypothetical protein [Burkholderia pseudomallei].	GCF_900593895.1
WP_123094036.1	HetE-N1→ TPR+GreAB-C+PIN*→?→ MACRODOMAIN→	TPR+GreAB-C+PIN	1099	-	Gammaproteobacteria	Marinomonas hwangdonensis	hypothetical protein [Marinomonas hwangdonensis].	GCF_003721245.1
WP_123338621.1	REase+TPR+GreAB-C+PIN*→	REase+TPR+GreAB-C+PIN	1210	-	Actinobacteria	Curtobacterium sp. PhB171	DUF4365 domain-containing protein [Curtobacterium sp. PhB171].	GCF_003752665.1
WP_123718714.1	HetE-N1→ TPR+GreAB-C+PIN*→	TPR+GreAB-C+PIN	1112	-	Gammaproteobacteria	Pseudomonas poae	hypothetical protein [Pseudomonas poae].	GCF_003731985.1
WP_123788903.1	REase+TPR+GreAB-C+PIN*→	REase+TPR+GreAB-C+PIN	1351	-	Betaproteobacteria	Burkholderia multivorans	hypothetical protein [Burkholderia multivorans].	GCF_003812365.1
WP_123901658.1	TPR+GreAB-C+PIN→ GreAB-C+PIN*→	GreAB-C+PIN	535	-	Bacteroidetes	Chryseobacterium bernardetii	hypothetical protein [Chryseobacterium bernardetii].	GCF_003815955.1
WP_124017047.1	TPR+GreAB-C+PIN*→	TPR+GreAB-C+PIN	838	-	Gammaproteobacteria	Shewanella vesiculosa	hypothetical protein [Shewanella vesiculosa].	GCF_003797885.1
WP_124131211.1	DOC+HTH→ REase+TOPC→?→ HetE-N1→ HetE-N1+TPR+GreAB-C+PIN*→	HetE-N1+TPR+GreAB-C+PIN	1124	-	Gammaproteobacteria	Pseudomonas aeruginosa	hypothetical protein [Pseudomonas aeruginosa].	GCF_003836815.1
WP_124132190.1	TPR+GreAB-C+PIN*→	TPR+GreAB-C+PIN	1140	-	Gammaproteobacteria	Pseudomonas aeruginosa	hypothetical protein [Pseudomonas aeruginosa].	GCF_003836445.1

acc	operon	architecture	len	gen.name	taxend	species	defline	gca
WP_124135313.1	TPR+GreAB-C+PIN*→	TPR+GreAB-C+PIN	1158	-	Gammaproteobacteria	Pseudomonas aeruginosa	hypothetical protein [Pseudomonas aeruginosa].	GCF_003836905.1
WP_124172921.1	DOC+HTH→ REase+TOPC→?→ HetE-N1→ HetE-N1+TPR+GreAB-C+PIN*→	HetE-N1+TPR+GreAB-C+PIN	1124	-	Gammaproteobacteria	Pseudomonas aeruginosa	hypothetical protein [Pseudomonas aeruginosa].	GCF_003839465.1
WP_124325287.1	TPR+GreAB-C+PIN*→	TPR+GreAB-C+PIN	1139	-	Gammaproteobacteria	Pseudomonas chlororaphis	hypothetical protein [Pseudomonas chlororaphis].	GCF_003850385.1
WP_124410055.1	HetE-N1→ TPR+GreAB-C+PIN*→	TPR+GreAB-C+PIN	1110	-	Gammaproteobacteria	Pseudomonas sp. R4-34-07	hypothetical protein [Pseudomonas sp. R4-34-07].	GCF_003852375.1
WP_124421918.1	TPR+GreAB-C+PIN*→	TPR+GreAB-C+PIN	1157	-	Gammaproteobacteria	Pseudomonas orientalis	hypothetical protein [Pseudomonas orientalis].	GCF_003851585.1
WP_124432744.1	REase+TPR+GreAB-C+PIN*→	REase+TPR+GreAB-C+PIN	1321	-	Gammaproteobacteria	Pseudomonas orientalis	DUF4365 domain-containing protein [Pseudomonas orientalis].	GCF_003852045.1
WP_124433851.1	REase+TPR+GreAB-C+PIN*→	REase+TPR+GreAB-C+PIN	1321	-	Gammaproteobacteria	Pseudomonas orientalis	DUF4365 domain-containing protein [Pseudomonas orientalis].	GCF_003852045.1
WP_124551445.1	HEPN→?→?→ REase+TPR+GreAB-C+PIN*→	REase+TPR+GreAB-C+PIN	1498	-	Betaproteobacteria	Burkholderia sp. Bp9015	tetratricopeptide repeat protein [Burkholderia sp. Bp9015].	GCF_003853505.1
WP_124582885.1	TPR+GreAB-C+PIN*→	TPR+GreAB-C+PIN	1248	-	Bacteroidetes	Pedobacter sp. KBW06	hypothetical protein [Pedobacter sp. KBW06].	GCF_003852525.1
WP_124686982.1	REase+TPR+GreAB-C+PIN*→	REase+TPR+GreAB-C+PIN	1344	-	Betaproteobacteria	Cupriavidus pauculus	tetratricopeptide repeat protein [Cupriavidus pauculus].	GCF_003854935.1
WP_124885098.1	REase+TPR+GreAB-C+PIN*→	REase+TPR+GreAB-C+PIN	1351	-	Betaproteobacteria	Burkholderia stagnalis	hypothetical protein [Burkholderia stagnalis].	GCF_003857545.1
WP_124919377.1	REase+TPR+GreAB-C+PIN*→	REase+TPR+GreAB-C+PIN	1500	-	Betaproteobacteria	Burkholderia	MULTISPECIES: tetratricopeptide repeat protein [Burkholderia].	GCF_001528845.1
WP_124920354.1	HEPN→?→?→ REase+TPR+GreAB-C+PIN*→	REase+TPR+GreAB-C+PIN	1289	-	Betaproteobacteria	Burkholderia stagnalis	tetratricopeptide repeat protein [Burkholderia stagnalis].	GCF_003857615.1
WP_125127612.1	X+PIN*→?→?→?→?→ MACRODOMAIN→	X+PIN	1232	-	Firmicutes	Schaedlerella arabinosiphila	hypothetical protein [Schaedlerella arabinosiphila].	GCF_003885045.1
WP_125255381.1	REase+TPR+GreAB-C+PIN*→	REase+TPR+GreAB-C+PIN	1327	-	Alphaproteobacteria	Brevundimonas fluminis	DUF4365 domain-containing protein [Brevundimonas fluminis].	GCF_003934285.1
WP_125429200.1	REase+TPR+GreAB-C+PIN*→	REase+TPR+GreAB-C+PIN	1284	-	Gammaproteobacteria	Stenotrophomonas maltophilia	GreA/GreB family elongation factor [Stenotrophomonas maltophilia].	GCF_003935615.1
WP_125440526.1	<-REase+nSTAND3||TPR+GreAB-C+PIN*→	TPR+GreAB-C+PIN	1282	-	Bacteroidetes	Hymenobacter perfusus	hypothetical protein [Hymenobacter perfusus].	GCF_003944765.1
WP_125601225.1	HetE-N1→ MNS-Npun2340+TPR+GreAB-C+PIN*→	MNS-Npun2340+TPR+GreAB-C+PIN	1094	-	Gammaproteobacteria	Aeromonas salmonicida	hypothetical protein [Aeromonas salmonicida].	GCF_003947375.1
WP_125730033.1	HetE-N1→ TPR+GreAB-C+PIN*→	TPR+GreAB-C+PIN	1094	-	Gammaproteobacteria	Aeromonas salmonicida	hypothetical protein [Aeromonas salmonicida].	GCF_003947355.1
WP_126243019.1	REase+TPR+GreAB-C+PIN*→	REase+TPR+GreAB-C+PIN	1333	-	Betaproteobacteria	Burkholderia gladioli	hypothetical protein [Burkholderia gladioli].	GCF_902829535.1
WP_126550729.1	TPR+GreAB-C+PIN*→	TPR+GreAB-C+PIN	1140	-	Gammaproteobacteria	Pseudomonas aeruginosa	hypothetical protein [Pseudomonas aeruginosa].	GCF_001516225.2
WP_126566690.1	TPR+GreAB-C+PIN*→	TPR+GreAB-C+PIN	1043	-	Gammaproteobacteria	Pseudomonas aeruginosa	hypothetical protein [Pseudomonas aeruginosa].	GCF_003968275.1
WP_126570139.1	DOC+HTH→ REase+TOPC→?→ HetE-N1→ HetE-N1→ GreAB-C+PIN*→	GreAB-C+PIN	523	-	Gammaproteobacteria	Pseudomonas aeruginosa	hypothetical protein [Pseudomonas aeruginosa].	GCF_003969065.1
WP_127030837.1	HetE-N1→ TPR+GreAB-C+PIN*→	TPR+GreAB-C+PIN	1093	-	Gammaproteobacteria	Pectobacterium versatile	hypothetical protein [Pectobacterium versatile].	GCF_003990505.1
WP_127257681.1	REase+TPR+GreAB-C+PIN*→	REase+TPR+GreAB-C+PIN	1287	-	Alphaproteobacteria	Mesorhizobium sp. M7A.F.Ca.US.008.03.1.1	hypothetical protein [Mesorhizobium sp. M7A.F.Ca.US.008.03.1.1].	GCF_003997495.1
WP_127294273.1	REase+TPR+TPR+GreAB-C+PIN*→	REase+TPR+TPR+GreAB-C+PIN	1333	-	Alphaproteobacteria	Mesorhizobium sp. M6A.T.Ce.TU.016.01.1.1	GreA/GreB family elongation factor [Mesorhizobium sp. M6A.T.Ce.TU.016.01.1.1].	GCF_003997065.1
WP_127581798.1	BirA-HTH→?→ REase+TPR+TPR+GreAB-C+PIN*→	REase+TPR+TPR+GreAB-C+PIN	1322	-	Alphaproteobacteria	Sinorhizobium medicae	hypothetical protein [Sinorhizobium medicae].	GCF_004002295.1
WP_128106456.1	REase+TPR+GreAB-C+PIN*→	REase+TPR+GreAB-C+PIN	1323	-	Alphaproteobacteria	Acetobacter oryzoeni	tetratricopeptide repeat protein [Acetobacter oryzoeni].	GCF_004014775.2
WP_128141476.1	REase+TPR+GreAB-C+PIN*→	REase+TPR+GreAB-C+PIN	1616	-	Betaproteobacteria	Janthinobacterium lividum	hypothetical protein [Janthinobacterium lividum].	GCF_900451225.1
WP_128142627.1	REase+TPR+GreAB-C+PIN*→	REase+TPR+GreAB-C+PIN	1269	-	Betaproteobacteria	Janthinobacterium lividum	hypothetical protein [Janthinobacterium lividum].	GCF_900451225.1
WP_128181279.1	REase+TPR+TPR+GreAB-C+PIN*→	REase+TPR+TPR+GreAB-C+PIN	1330	-	Alphaproteobacteria	Sinirhodobacter populi	hypothetical protein [Sinirhodobacter populi].	GCF_004022225.1
WP_128291227.1	REase+TPR+GreAB-C+PIN*→	REase+TPR+GreAB-C+PIN	1320	-	Alphaproteobacteria	Afifella aestuarii	hypothetical protein [Afifella aestuarii].	GCF_004023665.1
WP_128326676.1	REase+TPR+GreAB-C+PIN*→	REase+TPR+GreAB-C+PIN	1493	-	Gammaproteobacteria	Pseudomonas alkylphenolica	hypothetical protein [Pseudomonas alkylphenolica].	GCF_004025535.1
WP_128384334.1	X+PIN*→	X+PIN	1055	-	Gammaproteobacteria	Hydrogenovibrio thermophilus	hypothetical protein [Hydrogenovibrio thermophilus].	GCF_004028275.1
WP_128449906.1	REase+TPR+TPR+GreAB-C+PIN*→	REase+TPR+TPR+GreAB-C+PIN	1385	-	Alphaproteobacteria	Rhizobium leguminosarum	hypothetical protein [Rhizobium leguminosarum].	GCF_004054135.1
WP_128549293.1	TPR+GreAB-C+PIN*→	TPR+GreAB-C+PIN	1187	-	Alphaproteobacteria	Salipiger	MULTISPECIES: tetratricopeptide repeat protein [Salipiger].	GCF_014637265.1
WP_128552168.1	DOC+HTH→ REase+TOPC→ HetE-N1→ HetE-N1+TPR+GreAB-C+PIN*→	HetE-N1+TPR+GreAB-C+PIN	1123	-	Gammaproteobacteria	Pseudomonas aeruginosa	hypothetical protein [Pseudomonas aeruginosa].	GCF_003698765.1

acc	operon	architecture	len	gen.name	taxend	species	defline	gca
WP_128664039.1	DOC+HTH→ REase+TOPC→?→ HetE-N1→ HetE-N1+TPR+GreAB-C+PIN*→	HetE-N1+TPR+GreAB-C+PIN	1124	-	Gammaproteobacteria	Pseudomonas	MULTISPECIES: hypothetical protein [Pseudomonas].	GCF_016624455.1
WP_128715623.1	SIG+HIN-HTH→?→ GreAB-C+PIN*→	GreAB-C+PIN	540	-	Alphaproteobacteria	Rhizobium acidisoli	hypothetical protein [Rhizobium acidisoli].	GCF_002531755.2
WP_128816805.1	REase→ TPR+PIN*→	TPR+PIN	875	-	Actinobacteria	Streptomyces sp. S063	tetratricopeptide repeat protein [Streptomyces sp. S063].	GCF_002832675.1
WP_128912272.1	REase+TPR+GreAB-C+PIN*→	REase+TPR+GreAB-C+PIN	1277	-	Acidobacteria	Granulicella sibirica	hypothetical protein [Granulicella sibirica].	GCF_004115155.1
WP_129013963.1	TPR+GreAB-C+PIN*→?→?→?→?→?→?→ TIR+TPR+TPR→	TPR+GreAB-C+PIN	1046	-	Epsilonproteobacteria	Arcobacter cloacae	hypothetical protein [Arcobacter cloacae].	GCF_013201935.1
WP_129107976.1	REase+TPR+GreAB-C+PIN*→	REase+TPR+GreAB-C+PIN	1200	-	Epsilonproteobacteria	Halarcobacter bivalviorum	hypothetical protein [Halarcobacter bivalviorum].	GCF_004116705.1
WP_129330071.1	HetE-N1→?→ TPR+GreAB-C+PIN*→	TPR+GreAB-C+PIN	739	-	Gammaproteobacteria	Salmonella enterica	hypothetical protein [Salmonella enterica].	GCF_004120835.1
WP_129368690.1	HetE-N1→?→ TPR+GreAB-C+PIN*→	TPR+GreAB-C+PIN	855	-	Gammaproteobacteria	Salmonella enterica	hypothetical protein [Salmonella enterica].	GCF_004120585.1
WP_129411798.1	REase+TPR+GreAB-C+PIN*→	REase+TPR+GreAB-C+PIN	1323	-	Alphaproteobacteria	Mesorhizobium sp. Pch-S	hypothetical protein [Mesorhizobium sp. Pch-S].	GCF_004136315.1
WP_129485462.1	REase+TPR+GreAB-C+PIN*→	REase+TPR+GreAB-C+PIN	1468	-	Gammaproteobacteria	Pseudomonas syringae	hypothetical protein [Pseudomonas syringae].	GCF_004124725.1
WP_129893847.1	REase+TPR+GreAB-C+PIN*→	REase+TPR+GreAB-C+PIN	1309	-	Chloroflexi	Ktedonosporobacter rubrisoli	tetratricopeptide repeat protein [Ktedonosporobacter rubrisoli].	GCF_004208415.1
WP_130007120.1	HetE-N1→ HetE-N1+TPR+GreAB-C+PIN*→	HetE-N1+TPR+GreAB-C+PIN	1104	-	Betaproteobacteria	Pusillimonas ginsengisoli	hypothetical protein [Pusillimonas ginsengisoli].	GCF_004153455.1
WP_130044922.1	HetE-N1→ TPR+GreAB-C+PIN*→	TPR+GreAB-C+PIN	1116	-	Gammaproteobacteria	Aliivibrio finisterrensis	hypothetical protein [Aliivibrio finisterrensis].	GCF_004167675.1
WP_130132480.1	REase+TPR+GreAB-C+PIN*→	REase+TPR+GreAB-C+PIN	1245	-	Gammaproteobacteria	Acinetobacter wuhouensis	hypothetical protein [Acinetobacter wuhouensis].	GCF_004209325.1
WP_130204115.1	HetE-N1→ TPR+GreAB-C+PIN*→	TPR+GreAB-C+PIN	1113	-	Gammaproteobacteria	Pseudomonas moorei	hypothetical protein [Pseudomonas moorei].	GCF_004212425.1
WP_130252287.1	HetE-N1→ TPR+GreAB-C+PIN*→	TPR+GreAB-C+PIN	1114	-	Gammaproteobacteria	Vibrio vulnificus	hypothetical protein [Vibrio vulnificus].	GCF_004214575.1
WP_130472343.1	REase+TPR+GreAB-C+PIN*→	REase+TPR+GreAB-C+PIN	1348	-	Alphaproteobacteria	Candidatus Magnetaquicoccus inordinatus	hypothetical protein [Candidatus Magnetaquicoccus inordinatus].	GCF_004217665.1
WP_130525307.1	HetE-N1→ TPR+GreAB-C+PIN*→	TPR+GreAB-C+PIN	1086	-	Gammaproteobacteria	Pasteurella multocida	hypothetical protein [Pasteurella multocida].	GCF_003428945.1
WP_130555679.1	HetE-N1→ TPR+GreAB-C+PIN*→	TPR+GreAB-C+PIN	1086	-	Gammaproteobacteria	Pasteurella multocida	hypothetical protein [Pasteurella multocida].	GCF_001578435.2
WP_130710767.1	REase+TPR+TPR+GreAB-C+PIN*→	REase+TPR+TPR+GreAB-C+PIN	1343	-	Alphaproteobacteria	Rhizobium leguminosarum	hypothetical protein [Rhizobium leguminosarum].	GCF_004304355.1
WP_130725527.1	REase+TPR+GreAB-C+PIN*→	REase+TPR+GreAB-C+PIN	1255	-	Alphaproteobacteria	Rhizobium leguminosarum	hypothetical protein [Rhizobium leguminosarum].	GCF_004303985.1
WP_130928792.1	REase+TPR+GreAB-C+PIN*→	REase+TPR+GreAB-C+PIN	1438	-	Gammaproteobacteria	Pseudomonas sp. Sample_20	hypothetical protein [Pseudomonas sp. Sample_20].	GCF_004307585.1
WP_131049955.1	HetE-N1→ TPR+GreAB-C+PIN*→	TPR+GreAB-C+PIN	1092	-	Gammaproteobacteria	Raoultella ornithinolytica	hypothetical protein [Raoultella ornithinolytica].	GCF_004312065.1
WP_131189445.1	HetE-N1→ HetE-N1+TPR+GreAB-C+PIN*→	HetE-N1+TPR+GreAB-C+PIN	1125	-	Gammaproteobacteria	Pseudomonas kirkiae	hypothetical protein [Pseudomonas kirkiae].	GCF_004327175.1
WP_131590605.1	TPR+PIN*→	TPR+PIN	1153	-	Actinobacteria	Mycolicibacterium setense	hypothetical protein [Mycolicibacterium setense].	GCF_000805375.1
WP_131623255.1	REase+TPR+TPR+GreAB-C+PIN*→	REase+TPR+TPR+GreAB-C+PIN	1361	-	Alphaproteobacteria	Rhizobium leguminosarum	hypothetical protein [Rhizobium leguminosarum].	GCF_004330075.1
WP_131714066.1	REase+TPR+TPR+GreAB-C+PIN*→	REase+TPR+TPR+GreAB-C+PIN	1337	-	Alphaproteobacteria	Rhizobium leguminosarum	hypothetical protein [Rhizobium leguminosarum].	GCF_004330185.1
WP_131718233.1	TPR+GreAB-C+PIN*→	TPR+GreAB-C+PIN	556	-	Alphaproteobacteria	Rhizobium leguminosarum	hypothetical protein [Rhizobium leguminosarum].	GCF_004330625.1
WP_131814603.1	TPR+GreAB-C+PIN*→	TPR+GreAB-C+PIN	595	-	Alphaproteobacteria	Cognatishimia maritima	hypothetical protein [Cognatishimia maritima].	GCF_900129685.1
WP_131869160.1	TPR+GreAB-C+PIN*→	TPR+GreAB-C+PIN	1306	-	Alphaproteobacteria	unclassified Bradyrhizobium	MULTISPECIES: tetratricopeptide repeat protein [unclassified Bradyrhizobium].	GCF_004346395.1
WP_132139369.1	REase+TPR+GreAB-C+PIN*→	REase+TPR+GreAB-C+PIN	1368	-	Betaproteobacteria	Massilia sp. GV090	hypothetical protein [Massilia sp. GV090].	GCF_004341465.1
WP_132251347.1	TPR+GreAB-C+PIN*→	TPR+GreAB-C+PIN	1114	-	Alphaproteobacteria	Methylobacterium segetis	tetratricopeptide repeat protein [Methylobacterium segetis].	GCF_004348265.1
WP_132403375.1	TPR+GreAB-C+PIN*→	TPR+GreAB-C+PIN	1266	-	Bacteroidetes	Pedobacter sp. CF074	hypothetical protein [Pedobacter sp. CF074].	GCF_004342125.1
WP_132537157.1	BirA-HTH→ HEPN→ REase+TPR+TPR+GreAB-C+PIN*→	REase+TPR+TPR+GreAB-C+PIN	1332	-	Alphaproteobacteria	Rhizobium sp. PP-F2F-G48	GreA/GreB family elongation factor [Rhizobium sp. PP-F2F-G48].	GCF_004342335.1
WP_132665856.1	REase+TPR+GreAB-C+PIN*→	REase+TPR+GreAB-C+PIN	1297	-	Alphaproteobacteria	Rhizobium sp. PP-CC-3G-465	tetratricopeptide repeat protein [Rhizobium sp. PP-CC-3G-465].	GCF_004343445.1
WP_132831667.1	TPR+GreAB-C+PIN*→	TPR+GreAB-C+PIN	1188	-	Alphaproteobacteria	Antarcticimicrobium sediminis	hypothetical protein [Antarcticimicrobium sediminis].	GCF_004348975.1
WP_132850706.1	TPR+PIN*→	TPR+PIN	1135	-	Actinobacteria	Streptomyces sp. BK308	tetratricopeptide repeat protein [Streptomyces sp. BK308].	GCF_004346005.1
WP_133023868.1	TPR+PIN*→	TPR+PIN	1216	-	Actinobacteria	Streptomyces sp. BK329	hypothetical protein [Streptomyces sp. BK329].	GCF_004341055.1
WP_133030467.1	REase+TPR+GreAB-C+PIN*→	REase+TPR+GreAB-C+PIN	1317	-	Alphaproteobacteria	Sphingomonas sp. PP-CE-1G-424	tetratricopeptide repeat protein [Sphingomonas sp. PP-CE-1G-424].	GCF_004341085.1

acc	operon	architecture	len	gen.name	taxend	species	defline	gca
WP_133031998.1	REase+TPR+GreAB-C+PIN*→	REase+TPR+GreAB-C+PIN	1323	-	Alphaproteobacteria	Sphingomonas sp. PP-CE-1G-424	hypothetical protein [Sphingomonas sp. PP-CE-1G-424].	GCF_004341085.1
WP_133117283.1	REase+TPR+TPR+GreAB-C+PIN*→	REase+TPR+TPR+GreAB-C+PIN	1332	-	Alphaproteobacteria	Mesorhizobium sanjuanii	hypothetical protein [Mesorhizobium sanjuanii].	GCF_002529485.1
WP_133245819.1	REase+TPR+GreAB-C+PIN*→	REase+TPR+GreAB-C+PIN	1322	-	Alphaproteobacteria	Candidatus Phycosocius bacilliformis	hypothetical protein [Candidatus Phycosocius bacilliformis].	GCF_003112735.1
WP_133303262.1	TPR+GreAB-C+PIN*→	TPR+GreAB-C+PIN	1082	-	Alphaproteobacteria	Rhodopseudomonas palustris	hypothetical protein [Rhodopseudomonas palustris].	GCF_013415845.1
WP_133404970.1	REase+TPR+GreAB-C+PIN*→	REase+TPR+GreAB-C+PIN	1260	-	Actinobacteria	Arthrobacter crusticola	DUF4365 domain-containing protein [Arthrobacter crusticola].	GCF_004357995.1
WP_133633736.1	REase+TPR+GreAB-C+PIN*→	REase+TPR+GreAB-C+PIN	1321	-	Gammaproteobacteria	Pseudomonas sp. OV184	DUF4365 domain-containing protein [Pseudomonas sp. OV184].	GCF_004363115.1
WP_133936590.1	REase+TPR+GreAB-C+PIN*→	REase+TPR+GreAB-C+PIN	1489	-	Betaproteobacteria	Comamonas kerstersii	tetratricopeptide repeat protein [Comamonas kerstersii].	GCF_002002425.1
WP_134016909.1	REase+TPR+GreAB-C+PIN*→	REase+TPR+GreAB-C+PIN	1313	-	Alphaproteobacteria	Litoreibacter halocynthiae	hypothetical protein [Litoreibacter halocynthiae].	GCF_004365635.1
WP_134268494.1	PIN*→	PIN	186	-	Firmicutes	Sporosarcina pasteurii	hypothetical protein [Sporosarcina pasteurii].	GCF_004379295.1
WP_134281591.1	TPR+GreAB-C+PIN*→	TPR+GreAB-C+PIN	1159	-	Gammaproteobacteria	Pseudomonas	MULTISPECIES: hypothetical protein [Pseudomonas].	GCF_013522725.1
WP_135194680.1	REase+TPR+GreAB-C+PIN*→	REase+TPR+GreAB-C+PIN	1327	-	Alphaproteobacteria	Brevundimonas intermedia	DUF4365 domain-containing protein [Brevundimonas intermedia].	GCF_004614235.1
WP_135269295.1	TPR+PIN*→	TPR+PIN	1211	-	Actinobacteria	Nocardioides seonyuensis	hypothetical protein [Nocardioides seonyuensis].	GCF_004683965.1
WP_135300409.1	HetE-N1→ TPR+GreAB-C+PIN*→	TPR+GreAB-C+PIN	1130	-	Gammaproteobacteria	Photobacterium damselae	hypothetical protein [Photobacterium damselae].	GCF_004683985.2
WP_135312567.1	REase+TPR+GreAB-C+PIN→ PIN*→	PIN	479	-	Alphaproteobacteria	Paracoccus liaowanqingii	hypothetical protein [Paracoccus liaowanqingii].	GCF_004683865.2
WP_135953001.1	HetE-N1→ TPR+GreAB-C+PIN*→	TPR+GreAB-C+PIN	987	-	Gammaproteobacteria	Pasteurella multocida	hypothetical protein [Pasteurella multocida].	GCF_004792575.1
WP_135961458.1	HetE-N1→ TPR+GreAB-C+PIN*→	TPR+GreAB-C+PIN	1086	-	Gammaproteobacteria	Pasteurella multocida	hypothetical protein [Pasteurella multocida].	GCF_004792555.1
WP_136247578.1	HetE-N1→ TPR+GreAB-C+PIN*→	TPR+GreAB-C+PIN	1103	-	Gammaproteobacteria	Halomonas borealis	hypothetical protein [Halomonas borealis].	GCF_004798905.1
WP_136878408.1	TPR+GreAB-C+PIN*→	TPR+GreAB-C+PIN	1248	-	Bacteroidetes	Pedobacter sp. AR-2-6	hypothetical protein [Pedobacter sp. AR-2-6].	GCF_005116475.1
WP_137004448.1	REase+TPR+GreAB-C+PIN*→	REase+TPR+GreAB-C+PIN	1290	-	Alphaproteobacteria	Agrobacterium tumefaciens	hypothetical protein [Agrobacterium tumefaciens].	GCF_005221405.1
WP_137059419.1	TPR+PIN*→	TPR+PIN	1157	-	Firmicutes	Bacillus mycoides	hypothetical protein [Bacillus mycoides].	GCF_005217805.1
WP_137213367.1	TPR+GreAB-C+PIN*→	TPR+GreAB-C+PIN	1139	-	Gammaproteobacteria	Pseudomonas sp. CFBP13508	hypothetical protein [Pseudomonas sp. CFBP13508].	GCF_005233515.1
WP_137383131.1	HetE-N1→ TPR+GreAB-C+PIN*→	TPR+GreAB-C+PIN	1091	-	Gammaproteobacteria	Citrobacter sp. wls619	hypothetical protein [Citrobacter sp. wls619].	GCF_005281345.1
WP_137387523.1	HetE-N1→ TPR+GreAB-C+PIN*→	TPR+GreAB-C+PIN	1101	-	Gammaproteobacteria	Pantoea sp. SO10	hypothetical protein [Pantoea sp. SO10].	GCF_005281435.1
WP_137434419.1	REase+TPR+GreAB-C+PIN*→	REase+TPR+GreAB-C+PIN	1288	-	Gammaproteobacteria	Marinobacter sp. PJ-16	hypothetical protein [Marinobacter sp. PJ-16].	GCF_005298175.1
WP_137735239.1	REase+TPR+GreAB-C+PIN*→	REase+TPR+GreAB-C+PIN	1437	-	Betaproteobacteria	Aquabacterium pictum	hypothetical protein [Aquabacterium pictum].	GCF_005403045.1
WP_137905827.1	TPR+GreAB-C+PIN*→	TPR+GreAB-C+PIN	1130	-	Bacteroidetes	Chryseobacterium sp. 2VB	hypothetical protein [Chryseobacterium sp. 2VB].	GCF_005503635.1
WP_138242320.1	HetE-N1→ TPR+GreAB-C+PIN*→	TPR+GreAB-C+PIN	1130	-	Gammaproteobacteria	Photobacterium damselae	hypothetical protein [Photobacterium damselae].	GCF_005819845.1
WP_138262872.1	X+PIN*→	X+PIN	1204	-	Firmicutes	Enterocloster clostridioformis	hypothetical protein [Enterocloster clostridioformis].	GCF_015548065.1
WP_138402485.1	TPR+GreAB-C+PIN*→	TPR+GreAB-C+PIN	1137	-	Bacteroidetes	Chryseobacterium indologenes	hypothetical protein [Chryseobacterium indologenes].	GCF_005862425.1
WP_138437829.1	HetE-N1→ GreAB-C*→	GreAB-C	802	-	Gammaproteobacteria	Marinobacter	MULTISPECIES: hypothetical protein [Marinobacter].	GCF_005871095.1
WP_138616099.1	HetE-N1→ TPR+GreAB-C+PIN*→	TPR+GreAB-C+PIN	1106	-	Gammaproteobacteria	Pseudoalteromonas sp. S2721	hypothetical protein [Pseudoalteromonas sp. S2721].	GCF_005887425.1
WP_139051161.1	TPR+GreAB-C+PIN*→	TPR+GreAB-C+PIN	1158	-	Gammaproteobacteria	unclassified Pseudomonas	MULTISPECIES: hypothetical protein [unclassified Pseudomonas].	GCF_901007645.1
WP_139100594.1	TPR+PIN*→	TPR+PIN	1197	-	Actinobacteria	Amycolatopsis alkalitolerans	hypothetical protein [Amycolatopsis alkalitolerans].	GCF_006152065.1
WP_139135497.1	X+PIN*→	X+PIN	1093	-	Actinobacteria	Micromonospora tulbaghiae	hypothetical protein [Micromonospora tulbaghiae].	GCF_900091605.1
WP_139156465.1	HetE-N1→?→ TPR+GreAB-C+PIN*→	TPR+GreAB-C+PIN	523	-	Gammaproteobacteria	Salmonella enterica	hypothetical protein [Salmonella enterica].	GCF_003071545.1
WP_139230170.1	TPR+PIN*→	TPR+PIN	1206	-	Actinobacteria	Nocardioides terrae	hypothetical protein [Nocardioides terrae].	GCF_900112345.1
WP_139231027.1	TPR+GreAB-C+PIN*→	TPR+GreAB-C+PIN	1155	-	Gammaproteobacteria	Pseudomonas sagittaria	hypothetical protein [Pseudomonas sagittaria].	GCF_900115715.1

acc	operon	architecture	len	gen.name	taxend	species	defline	gca
WP_139231685.1	REase+TPR+GreAB-C+PIN*→?→?→ Calcineurin→ Calcineurin→?→ Calcineurin→	REase+TPR+GreAB-C+PIN	1314	-	Alphaproteobacteria	Methylobacterium phyllosphaerae	hypothetical protein [Methylobacterium phyllosphaerae].	GCF_001936175.1
WP_139585558.1	TPR+PIN*→	TPR+PIN	1173	-	Actinobacteria	Micromonospora orduensis	hypothetical protein [Micromonospora orduensis].	GCF_006228125.1
WP_139699848.1	TPR+GreAB-C+PIN*→	TPR+GreAB-C+PIN	1163	-	unclassified Bacteria	bacterium endosymbiont of Bathymodiolus sp. 5 South	tetratricopeptide repeat protein [bacterium endosymbiont of Bathymodiolus sp. 5 South].	GCF_900127295.1
WP_139711990.1	TPR+PIN*→	TPR+PIN	816	-	Actinobacteria	Streptomyces sp. NP160	hypothetical protein [Streptomyces sp. NP160].	GCF_006335115.1
WP_139741421.1	HetE-N1→ TPR+GreAB-C+PIN*→	TPR+GreAB-C+PIN	1086	-	Gammaproteobacteria	Aeromonas veronii	hypothetical protein [Aeromonas veronii].	GCF_006243415.1
WP_139832542.1	REase+TPR+GreAB-C+PIN*→	REase+TPR+GreAB-C+PIN	1427	-	Gammaproteobacteria	Pseudomonas sp. B26(2017)	hypothetical protein [Pseudomonas sp. B26(2017)].	GCF_002113045.1
WP_139834254.1	HetE-N1→?→ TPR+GreAB-C+PIN*→	TPR+GreAB-C+PIN	686	-	Gammaproteobacteria	Pseudomonas sp. B11(2017)	hypothetical protein [Pseudomonas sp. B11(2017)].	GCF_002113285.1
WP_140034365.1	HetE-N1→ TPR+GreAB-C+PIN*→	TPR+GreAB-C+PIN	1111	-	Gammaproteobacteria	Pantoea vagans	hypothetical protein [Pantoea vagans].	GCF_006385385.1
WP_140089969.1	GreAB-C*→	GreAB-C	561	-	Gammaproteobacteria	Vibrio parahaemolyticus	hypothetical protein [Vibrio parahaemolyticus].	GCF_006372765.1
WP_140136560.1	TPR+GreAB-C+PIN*→	TPR+GreAB-C+PIN	381	-	Gammaproteobacteria	Vibrio parahaemolyticus	hypothetical protein, partial [Vibrio parahaemolyticus].	GCF_006369095.1
WP_140150658.1	HetE-N1→ TPR+GreAB-C+PIN*→	TPR+GreAB-C+PIN	1100	-	Gammaproteobacteria	Vibrio parahaemolyticus	hypothetical protein [Vibrio parahaemolyticus].	GCF_006375025.1
WP_140184851.1	HetE-N1→ TPR+GreAB-C+PIN*→	TPR+GreAB-C+PIN	1133	-	Gammaproteobacteria	Vibrio parahaemolyticus	hypothetical protein [Vibrio parahaemolyticus].	GCF_006375315.1
WP_140247351.1	HetE-N1→ TPR+GreAB-C+PIN*→	TPR+GreAB-C+PIN	1114	-	Gammaproteobacteria	Vibrio parahaemolyticus	hypothetical protein [Vibrio parahaemolyticus].	GCF_006371495.1
WP_140523105.1	REase+TPR+GreAB-C+PIN*→	REase+TPR+GreAB-C+PIN	1330	-	Alphaproteobacteria	unclassified Mesorhizobium	MULTISPECIES: tetratricopeptide repeat protein [unclassified Mesorhizobium].	GCF_006439485.1
WP_140649671.1	REase+TPR+GreAB-C+PIN*→	REase+TPR+GreAB-C+PIN	1330	-	Alphaproteobacteria	Mesorhizobium sp. B2-4-10	tetratricopeptide repeat protein [Mesorhizobium sp. B2-4-10].	GCF_006442675.1
WP_140682745.1	HetE-N1→ TPR+GreAB-C+PIN*→	TPR+GreAB-C+PIN	1113	-	Gammaproteobacteria	Pseudomonas mandelii	hypothetical protein [Pseudomonas mandelii].	GCF_006438925.1
WP_140739373.1	REase+TPR+GreAB-C+PIN*→	REase+TPR+GreAB-C+PIN	994	-	Alphaproteobacteria	unclassified Mesorhizobium	MULTISPECIES: hypothetical protein [unclassified Mesorhizobium].	GCF_006440845.1
WP_141097119.1	TPR+GreAB-C+PIN*→	TPR+GreAB-C+PIN	1209	-	Actinobacteria	Microbacterium sp. AISO3	hypothetical protein [Microbacterium sp. AISO3].	GCF_002204305.1
WP_141144329.1	X+PIN*→	X+PIN	1189	-	Actinobacteria	Schumannella sp. 10F1B-5-1	hypothetical protein [Schumannella sp. 10F1B-5-1].	GCF_006517535.1
WP_141202816.1	TPR+PIN*→	TPR+PIN	1217	-	Actinobacteria	Streptomyces griseorubiginosus	hypothetical protein [Streptomyces griseorubiginosus].	GCF_006516935.1
WP_141414632.1	HetE-N1→ TPR+GreAB-C+PIN*→	TPR+GreAB-C+PIN	1106	-	Gammaproteobacteria	Enterobacter hormaechei	hypothetical protein [Enterobacter hormaechei].	GCF_006546545.1
WP_141566570.1	TPR+PIN*→	TPR+PIN	1237	-	-	Pseudonocardia sp. N23	hypothetical protein [Pseudonocardia sp. N23].	GCF_002583555.1
WP_141708179.1	REase+TPR+GreAB-C+PIN*→	REase+TPR+GreAB-C+PIN	1338	-	Actinobacteria	Micromonospora marina	hypothetical protein [Micromonospora marina].	GCF_900091565.1
WP_141709791.1	TPR+PIN*→||||<-NUDIX	TPR+PIN	1088	-	Actinobacteria	Streptomyces	MULTISPECIES: hypothetical protein [Streptomyces].	GCF_000514055.1
WP_142342557.1	TPR+PIN*→	TPR+PIN	1016	-	Firmicutes	Bacillus cereus	hypothetical protein [Bacillus cereus].	GCF_002577165.1
WP_142621974.1	TPR+GreAB-C+PIN*→	TPR+GreAB-C+PIN	456	-	Gammaproteobacteria	Vibrio cholerae	hypothetical protein [Vibrio cholerae].	GCF_006802685.1
WP_142996755.1	<-REase||TPR+PIN*→	TPR+PIN	993	-	Firmicutes	Bacillus sp. KQ-3	hypothetical protein [Bacillus sp. KQ-3].	GCF_003710255.1
WP_143042354.1	REase+TPR+GreAB-C+PIN*→	REase+TPR+GreAB-C+PIN	1329	-	Alphaproteobacteria	Citreimonas salinaria	restriction endonuclease [Citreimonas salinaria].	GCF_900107235.1
WP_143064837.1	TPR+PIN*→	TPR+PIN	1134	-	Actinobacteria	Streptomyces sp. PAN_FS17	hypothetical protein [Streptomyces sp. PAN_FS17].	GCF_900105465.1
WP_143167506.1	REase+TPR+GreAB-C+PIN*→	REase+TPR+GreAB-C+PIN	1482	-	Betaproteobacteria	Massilia sp. CF038	hypothetical protein [Massilia sp. CF038].	GCF_900129765.1
WP_143171741.1	PAIREDC-HTH→ REase+TPR+GreAB-C+PIN*→	REase+TPR+GreAB-C+PIN	1539	-	Betaproteobacteria	Rhizobacter sp. OV335	hypothetical protein [Rhizobacter sp. OV335].	GCF_900142965.1
WP_143206624.1	REase+TPR+GreAB-C+PIN*→	REase+TPR+GreAB-C+PIN	1273	-	Planctomycetes	Singulisphaera sp. GP187	hypothetical protein [Singulisphaera sp. GP187].	GCF_900129635.1
WP_143268820.1	REase+TPR+GreAB-C+PIN→ PIN*→	PIN	279	-	Actinobacteria	Amycolatopsis vastitatis	hypothetical protein [Amycolatopsis vastitatis].	GCF_002234595.1
WP_143494315.1	DOC+HTH→ REase+TOPC→ HetE-N1→ HetE-N1+TPR+GreAB-C+PIN*→	HetE-N1+TPR+GreAB-C+PIN	1124	-	Gammaproteobacteria	Pseudomonas putida	hypothetical protein [Pseudomonas putida].	GCF_007049805.1
WP_143685467.1	TPR+PIN*→	TPR+PIN	1135	-	Actinobacteria	Streptomyces sp. 1222.2	hypothetical protein [Streptomyces sp. 1222.2].	GCF_900215595.1
WP_143686526.1	TPR+PIN*→	TPR+PIN	1189	-	Actinobacteria	Streptomyces sp. 61	hypothetical protein [Streptomyces sp. 61].	GCF_002754535.1
WP_143775988.1	REase+TPR+GreAB-C+PIN*→	REase+TPR+GreAB-C+PIN	1312	-	Alphaproteobacteria	Sphingorhabdus contaminans	hypothetical protein [Sphingorhabdus contaminans].	GCF_007280415.1
WP_144044861.1	HetE-N1→ GreAB-C*→ TPR+GreAB-C+PIN→	GreAB-C	779	-	Gammaproteobacteria	Shewanella sp. YLB-06	GreA/GreB family elongation factor [Shewanella sp. YLB-06].	GCF_007197555.1

acc	operon	architecture	len	gen.name	taxend	species	defline	gca
WP_144084411.1	HetE-N1→ TPR+TPR+GreAB-C+PIN*→	TPR+TPR+GreAB-C+PIN	1108	-	Gammaproteobacteria	Pseudomonas monteilii	hypothetical protein [Pseudomonas monteilii].	GCF_000510285.1
WP_144190111.1	HetE-N1→ TPR+GreAB-C+PIN*→	TPR+GreAB-C+PIN	1126	-	Gammaproteobacteria	Gilliamella apicola	hypothetical protein [Gilliamella apicola].	GCF_007559165.1
WP_144217154.1	TPR+GreAB-C+PIN*→	TPR+GreAB-C+PIN	1236	-	Gammaproteobacteria	Vibrio cholerae	hypothetical protein [Vibrio cholerae].	GCF_007623805.1
WP_144226215.1	HetE-N1→ TPR+GreAB-C+PIN*→	TPR+GreAB-C+PIN	1101	-	Gammaproteobacteria	Shewanella algae	hypothetical protein [Shewanella algae].	GCF_007595165.1
WP_144269797.1	REase+TPR+GreAB-C+PIN*→	REase+TPR+GreAB-C+PIN	1288	-	Gammaproteobacteria	Pseudomonas fluorescens	DUF4365 domain-containing protein [Pseudomonas fluorescens].	GCF_000262325.2
WP_144298906.1	X+PIN*→	X+PIN	676	-	Actinobacteria	Streptomyces sp. TLI_235	hypothetical protein [Streptomyces sp. TLI_235].	GCF_002300355.1
WP_144431187.1	REase+TPR+GreAB-C+PIN*→	REase+TPR+GreAB-C+PIN	1441	-	Gammaproteobacteria	Pseudomonas syringae pv. coryli	hypothetical protein [Pseudomonas syringae pv. coryli].	GCF_000972175.1
WP_144762711.1	X+PIN*→	X+PIN	1177	-	Actinobacteria	Curtobacterium sp. 9128	hypothetical protein [Curtobacterium sp. 9128].	GCF_007673775.1
WP_144916164.1	TPR+GreAB-C+PIN→ PIN*→	PIN	238	-	Bacteroidetes	Mucilaginibacter frigoritolerans	hypothetical protein [Mucilaginibacter frigoritolerans].	GCF_007830615.1
WP_145052948.1	TPR+PIN*→	TPR+PIN	1410	-	Planctomycetes	Lignipirellula cremea	hypothetical protein [Lignipirellula cremea].	GCF_007751035.1
WP_145376920.1	TPR+PIN*→	TPR+PIN	2081	-	Planctomycetes	Symmachiella dynata	hypothetical protein [Symmachiella dynata].	GCF_007747995.1
WP_145427683.1	REase+TPR+GreAB-C+PIN→ TPR+GreAB-C+PIN*→	TPR+GreAB-C+PIN	514	-	Planctomycetes	Symmachiella dynata	hypothetical protein [Symmachiella dynata].	GCF_007744975.1
WP_145441613.1	TPR+PIN*→	TPR+PIN	2077	-	Planctomycetes	Gimesia chilikensis	hypothetical protein [Gimesia chilikensis].	GCF_007746535.1
WP_145577942.1	HetE-N1→ TPR+GreAB-C+PIN*→	TPR+GreAB-C+PIN	1084	-	Gammaproteobacteria	Vibrio crassostreae	hypothetical protein [Vibrio crassostreae].	GCF_017916675.1
WP_145860820.1	TPR+GreAB-C+PIN→ PIN*→	PIN	347	-	Bacteroidetes	Pedobacter suwonensis	hypothetical protein [Pedobacter suwonensis].	GCF_007833675.1
WP_145895823.1	REase+TPR+GreAB-C+PIN*→	REase+TPR+GreAB-C+PIN	1508	-	Betaproteobacteria	Caenimonas sp. HX-9-20	hypothetical protein [Caenimonas sp. HX-9-20].	GCF_007833165.1
WP_145923949.1	TPR+GreAB-C+PIN*→	TPR+GreAB-C+PIN	1170	-	Gammaproteobacteria	Pseudomonas citronellolis	hypothetical protein [Pseudomonas citronellolis].	GCF_001586155.1
WP_145975538.1	REase+TPR+GreAB-C+PIN*→	REase+TPR+GreAB-C+PIN	1365	-	Deinococci	Deinococcus gobiensis	hypothetical protein [Deinococcus gobiensis].	GCF_000252445.1
WP_145980369.1	HetE-N1→ TPR+GreAB-C+PIN*→	TPR+GreAB-C+PIN	1113	-	Gammaproteobacteria	Pseudomonas lurida	hypothetical protein [Pseudomonas lurida].	GCF_001708485.1
WP_145994348.1	REase+TPR+TPR+GreAB-C+PIN*→	REase+TPR+TPR+GreAB-C+PIN	1339	-	Alphaproteobacteria	Mesorhizobium loti	hypothetical protein [Mesorhizobium loti].	GCF_002858745.1
WP_146048182.1	PIN*→	PIN	371	-	Gammaproteobacteria	Acinetobacter sp. ACNIH3	hypothetical protein [Acinetobacter sp. ACNIH3].	GCF_002918965.1
WP_146113387.1	TPR+PIN*→	TPR+PIN	1210	-	Actinobacteria	unclassified Arthrobacter	MULTISPECIES: hypothetical protein [unclassified Arthrobacter].	GCF_002975405.1
WP_146162727.1	TPR+GreAB-C+PIN*→	TPR+GreAB-C+PIN	1197	-	Gammaproteobacteria	Vibrio splendidus	hypothetical protein [Vibrio splendidus].	GCF_003050425.1
WP_146163497.1	HetE-N1→ TPR+GreAB-C+PIN*→	TPR+GreAB-C+PIN	1094	-	Gammaproteobacteria	Aeromonas veronii	hypothetical protein [Aeromonas veronii].	GCF_003036425.1
WP_146169670.1	REase+TPR+GreAB-C+PIN*→	REase+TPR+GreAB-C+PIN	1355	-	Actinobacteria	Actinoplanes italicus	hypothetical protein [Actinoplanes italicus].	GCF_016862235.1
WP_146174202.1	TPR+GreAB-C+PIN*→	TPR+GreAB-C+PIN	1185	-	Alphaproteobacteria	Litoreibacter ponti	hypothetical protein [Litoreibacter ponti].	GCF_003054285.1
WP_146175347.1	X+PIN*→	X+PIN	1323	-	Actinobacteria	Conexibacter sp. Seoho-28	hypothetical protein [Conexibacter sp. Seoho-28].	GCF_003044185.1
WP_146181743.1	HetE-N1→ TPR+GreAB-C+PIN*→	TPR+GreAB-C+PIN	1094	-	Gammaproteobacteria	Aeromonas sp. HMWF014	hypothetical protein [Aeromonas sp. HMWF014].	GCF_003061085.1
WP_146562359.1	TPR+GreAB-C+PIN*→	TPR+GreAB-C+PIN	1275	-	Planctomycetes	Posidoniimonas corsicana	hypothetical protein [Posidoniimonas corsicana].	GCF_007859765.1
WP_146571406.1	REase+TPR+GreAB-C+PIN*→	REase+TPR+GreAB-C+PIN	1140	-	Planctomycetes	Botrimarina hoheduenensis	hypothetical protein [Botrimarina hoheduenensis].	GCF_007859815.1
WP_146752056.1	TPR+GreAB-C+PIN*→	TPR+GreAB-C+PIN	1230	-	Actinobacteria	Microbacterium sp. SMR1	hypothetical protein [Microbacterium sp. SMR1].	GCF_003289625.1
WP_146766012.1	TPR+GreAB-C+PIN*→	TPR+GreAB-C+PIN	1147	-	Gammaproteobacteria	Pseudomonas	MULTISPECIES: hypothetical protein [Pseudomonas].	GCF_013522795.1
WP_146779203.1	TPR+GreAB-C+PIN*→	TPR+GreAB-C+PIN	1133	-	Bacteroidetes	Flavobacterium psychrolimnae	hypothetical protein [Flavobacterium psychrolimnae].	GCF_003312425.1
WP_146877508.1	TPR+GreAB-C+PIN*→	TPR+GreAB-C+PIN	1076	-	Actinobacteria	Actinomadura madurae	hypothetical protein [Actinomadura madurae].	GCF_900445005.1
WP_146915636.1	TIR→ TPR+GreAB-C+PIN*→||||<-HTH	TPR+GreAB-C+PIN	1255	-	Bacteroidetes	Algoriphagus aquimarinus	hypothetical protein [Algoriphagus aquimarinus].	GCF_007997215.1
WP_146943293.1	TPR+GreAB-C+PIN*→	TPR+GreAB-C+PIN	1126	-	Bacteroidetes	Chryseobacterium hagamense	hypothetical protein [Chryseobacterium hagamense].	GCF_007991455.1
WP_146990354.1	REase+TPR+TPR+GreAB-C+PIN*→	REase+TPR+TPR+GreAB-C+PIN	1315	-	Alphaproteobacteria	Bradyrhizobium macuxiense	hypothetical protein [Bradyrhizobium macuxiense].	GCF_007993935.1
WP_147282746.1	HetE-N1→ TPR+GreAB-C+PIN*→	TPR+GreAB-C+PIN	1118	-	Betaproteobacteria	Comamonas sp. AG1104	hypothetical protein [Comamonas sp. AG1104].	GCF_003350425.1
WP_147289649.1	HetE-N1→ TPR+GreAB-C+PIN*→	TPR+GreAB-C+PIN	1110	-	Gammaproteobacteria	Pantoea agglomerans	hypothetical protein [Pantoea agglomerans].	GCF_900454505.1
WP_147309007.1	REase+TPR+GreAB-C+PIN→?→ PIN*→	PIN	256	-	Betaproteobacteria	Cupriavidus taiwanensis	hypothetical protein [Cupriavidus taiwanensis].	GCF_900249855.1
WP_147316401.1	TPR+GreAB-C+PIN*→	TPR+GreAB-C+PIN	1139	-	Gammaproteobacteria	Pseudomonas aeruginosa	hypothetical protein [Pseudomonas aeruginosa].	GCF_003412045.2
WP_147329285.1	X+PIN*→ HTH→?→?→ HTH→	X+PIN	1180	-	Firmicutes	Dorea formicigenerans	hypothetical protein [Dorea formicigenerans].	GCF_003437395.1
WP_147334977.1	TM+TM+TPR+PIN*→	TM+TM+TPR+PIN	1154	-	Bacteroidetes	Phocaeicola coprocola	hypothetical protein [Phocaeicola coprocola].	GCF_003458565.1

<table>
<thead>
<tr><th>acc</th><th>operon</th><th>architecture</th><th>len</th><th>gen.name</th><th>taxend</th><th>species</th><th>defline</th><th>gca</th></tr>
</thead>
<tbody>
<tr><td>WP_147344604.1</td><td>Mbetalac→ Trypsin→?→ TPR+PIN*→</td><td>TPR+PIN</td><td>1394</td><td>-</td><td>Firmicutes</td><td>Eubacterium sp. AM49-13BH</td><td>hypothetical protein, partial [Eubacterium sp. AM49-13BH].</td><td>GCF_003464165.1</td></tr>
<tr><td>WP_147352805.1</td><td>Mbetalac→ Trypsin→ TPR+PIN*→</td><td>TPR+PIN</td><td>1645</td><td>-</td><td>Firmicutes</td><td>Clostridium sp. AM48-13</td><td>hypothetical protein [Clostridium sp. AM48-13].</td><td>GCF_003478455.1</td></tr>
<tr><td>WP_147353595.1</td><td>Mbetalac→ Trypsin+TPR+PIN*→</td><td>Trypsin+TPR+PIN</td><td>1710</td><td>-</td><td>Firmicutes</td><td>Clostridium sp. AF37-7</td><td>trypsin-like peptidase domain-containing protein, partial [Clostridium sp. AF37-7].</td><td>GCF_003477595.1</td></tr>
<tr><td>WP_147364185.1</td><td>X+PIN*→ HTH→?→?→ HTH→</td><td>X+PIN</td><td>1179</td><td>-</td><td>Firmicutes</td><td>Dorea formicigenerans</td><td>hypothetical protein [Dorea formicigenerans].</td><td>GCF_003475555.1</td></tr>
<tr><td>WP_147402393.1</td><td>TPR+GreAB-C+PIN*→</td><td>TPR+GreAB-C+PIN</td><td>1139</td><td>-</td><td>Gammaproteobacteria</td><td>Pseudomonas aeruginosa</td><td>hypothetical protein [Pseudomonas aeruginosa].</td><td>GCF_003585235.1</td></tr>
<tr><td>WP_147402585.1</td><td>DOC+HTH→ REase+TOPC→ HetE-N1→ HetE-N1+TPR+GreAB-C+PIN*→</td><td>HetE-N1+TPR+GreAB-C+PIN</td><td>1123</td><td>-</td><td>Gammaproteobacteria</td><td>Pseudomonas aeruginosa</td><td>hypothetical protein [Pseudomonas aeruginosa].</td><td>GCF_003585225.1</td></tr>
<tr><td>WP_147432801.1</td><td>REase+TPR+GreAB-C+PIN*→</td><td>REase+TPR+GreAB-C+PIN</td><td>1375</td><td>-</td><td>Actinobacteria</td><td>Catellatospora citrea</td><td>hypothetical protein [Catellatospora citrea].</td><td>GCF_016862615.1</td></tr>
<tr><td>WP_147432945.1</td><td><-TIR<-?||REase+TPR+GreAB-C+PIN*→</td><td>REase+TPR+GreAB-C+PIN</td><td>1245</td><td>-</td><td>Actinobacteria</td><td>Catellatospora citrea</td><td>DUF4365 domain-containing protein [Catellatospora citrea].</td><td>GCF_016862615.1</td></tr>
<tr><td>WP_147450330.1</td><td>REase+TPR+GreAB-C+PIN*→</td><td>REase+TPR+GreAB-C+PIN</td><td>1306</td><td>-</td><td>Deltaproteobacteria</td><td>Corallococcus carmarthensis</td><td>hypothetical protein [Corallococcus carmarthensis].</td><td>GCF_003611695.1</td></tr>
<tr><td>WP_147453837.1</td><td>TPR+PIN*→ PAIREDC-HTH→</td><td>TPR+PIN</td><td>1206</td><td>-</td><td>Actinobacteria</td><td>Nocardiopsis sp. Huas11</td><td>hypothetical protein [Nocardiopsis sp. Huas11].</td><td>GCF_003634495.1</td></tr>
<tr><td>WP_147481098.1</td><td>TPR+PIN*→</td><td>TPR+PIN</td><td>1134</td><td>-</td><td>Actinobacteria</td><td>Streptomyces shenzhenensis</td><td>tetratricopeptide repeat protein [Streptomyces shenzhenensis].</td><td>GCF_003688995.1</td></tr>
<tr><td>WP_147627327.1</td><td>REase+TPR+GreAB-C+PIN→ SIG+HIN-HTH→?→ PIN*→</td><td>PIN</td><td>507</td><td>-</td><td>Gammaproteobacteria</td><td>Rhodanobacter glycinis</td><td>hypothetical protein [Rhodanobacter glycinis].</td><td>GCF_008000795.1</td></tr>
<tr><td>WP_147831354.1</td><td>TPR+GreAB-C+PIN*→</td><td>TPR+GreAB-C+PIN</td><td>1107</td><td>-</td><td>Alphaproteobacteria</td><td>Methylobacterium sp. WL19</td><td>hypothetical protein [Methylobacterium sp. WL19].</td><td>GCF_008040005.1</td></tr>
<tr><td>WP_147893054.1</td><td>X+PIN*→</td><td>X+PIN</td><td>1120</td><td>-</td><td>Actinobacteria</td><td>Microbacterium hatanonis</td><td>hypothetical protein [Microbacterium hatanonis].</td><td>GCF_008017415.1</td></tr>
<tr><td>WP_148210527.1</td><td>TPR+GreAB-C+PIN*→</td><td>TPR+GreAB-C+PIN</td><td>1107</td><td>-</td><td>Alphaproteobacteria</td><td>Beijerinckia indica</td><td>hypothetical protein [Beijerinckia indica].</td><td>GCF_000019845.1</td></tr>
<tr><td>WP_148306266.1</td><td>TPR+GreAB-C+PIN*→</td><td>TPR+GreAB-C+PIN</td><td>1259</td><td>-</td><td>Gemmatimonadetes</td><td>Gemmatirosa kalamazoonesis</td><td>hypothetical protein [Gemmatirosa kalamazoonesis].</td><td>GCF_000522985.1</td></tr>
<tr><td>WP_148421250.1</td><td><-MarR-HTH||?→?→?→||||<-?||?→ HetE-N1→ TPR+GreAB-C+PIN*→</td><td>TPR+GreAB-C+PIN</td><td>1117</td><td>-</td><td>Gammaproteobacteria</td><td>Enterobacter cloacae</td><td>hypothetical protein [Enterobacter cloacae].</td><td>GCF_013376835.1</td></tr>
<tr><td>WP_148564915.1</td><td>REase+TPR+GreAB-C+PIN*→</td><td>REase+TPR+GreAB-C+PIN</td><td>1243</td><td>-</td><td>Gammaproteobacteria</td><td>Stenotrophomonas maltophilia</td><td>hypothetical protein [Stenotrophomonas maltophilia].</td><td>GCF_001274595.1</td></tr>
<tr><td>WP_149326491.1</td><td>HetE-N1→ TPR+GreAB-C+PIN*→ PhospholipaseD→</td><td>TPR+GreAB-C+PIN</td><td>1103</td><td>-</td><td>Gammaproteobacteria</td><td>Halomonas sp. L5</td><td>hypothetical protein [Halomonas sp. L5].</td><td>GCF_008297955.1</td></tr>
<tr><td>WP_149369557.1</td><td>TPR+PIN*→</td><td>TPR+PIN</td><td>1189</td><td>-</td><td>Actinobacteria</td><td>Mycolicibacterium sp. P9-64</td><td>hypothetical protein [Mycolicibacterium sp. P9-64].</td><td>GCF_008329605.1</td></tr>
<tr><td>WP_150024336.1</td><td>TPR+GreAB-C+PIN*→||||<-?||HTH→</td><td>TPR+GreAB-C+PIN</td><td>804</td><td>-</td><td>Gammaproteobacteria</td><td>Acinetobacter baumannii</td><td>GreA/GreB family elongation factor [Acinetobacter baumannii].</td><td>GCF_008630895.1</td></tr>
<tr><td>WP_150359822.1</td><td>REase+TPR+GreAB-C+PIN*→</td><td>REase+TPR+GreAB-C+PIN</td><td>1240</td><td>-</td><td>Gammaproteobacteria</td><td>Stenotrophomonas maltophilia</td><td>hypothetical protein [Stenotrophomonas maltophilia].</td><td>GCF_008693985.1</td></tr>
<tr><td>WP_150409130.1</td><td>REase+TPR+GreAB-C+PIN*→</td><td>REase+TPR+GreAB-C+PIN</td><td>1284</td><td>-</td><td>Gammaproteobacteria</td><td>Xanthomonas sacchari</td><td>GreA/GreB family elongation factor [Xanthomonas sacchari].</td><td>GCF_008705275.1</td></tr>
<tr><td>WP_150412179.1</td><td>REase+TPR+GreAB-C+PIN*→</td><td>REase+TPR+GreAB-C+PIN</td><td>1314</td><td>-</td><td>Alphaproteobacteria</td><td>Sphingobium limneticum</td><td>hypothetical protein [Sphingobium limneticum].</td><td>GCF_008710155.1</td></tr>
<tr><td>WP_150784121.1</td><td>REase+TPR+GreAB-C+PIN*→</td><td>REase+TPR+GreAB-C+PIN</td><td>1318</td><td>-</td><td>Gammaproteobacteria</td><td>Pseudomonas fluorescens</td><td>DUF4365 domain-containing protein [Pseudomonas fluorescens].</td><td>GCF_902497995.1</td></tr>
<tr><td>WP_150981548.1</td><td>REase+TPR+GreAB-C+PIN*→</td><td>REase+TPR+GreAB-C+PIN</td><td>1329</td><td>-</td><td>Betaproteobacteria</td><td>Cupriavidus pauculus</td><td>GreA/GreB family elongation factor [Cupriavidus pauculus].</td><td>GCF_008801835.1</td></tr>
<tr><td>WP_151013632.1</td><td>TPR+PIN*→</td><td>TPR+PIN</td><td>1145</td><td>-</td><td>Actinobacteria</td><td>Micromonospora aurantiaca</td><td>hypothetical protein [Micromonospora aurantiaca].</td><td>GCF_008806405.1</td></tr>
<tr><td>WP_151024479.1</td><td>REase+TPR+GreAB-C+PIN*→</td><td>REase+TPR+GreAB-C+PIN</td><td>1499</td><td>-</td><td>Betaproteobacteria</td><td>Castellaniella defragrans</td><td>hypothetical protein [Castellaniella defragrans].</td><td>GCF_014203015.1</td></tr>
<tr><td>WP_151868021.1</td><td>TPR+GreAB-C+PIN*→</td><td>TPR+GreAB-C+PIN</td><td>463</td><td>-</td><td>Gammaproteobacteria</td><td>Acinetobacter soli</td><td>hypothetical protein, partial [Acinetobacter soli].</td><td>GCF_009013695.1</td></tr>
<tr><td>WP_152091999.1</td><td>HetE-N1→ TPR+GreAB-C+PIN*→</td><td>TPR+GreAB-C+PIN</td><td>1107</td><td>-</td><td>Gammaproteobacteria</td><td>Pseudomonas sp. KUIN-1</td><td>hypothetical protein [Pseudomonas sp. KUIN-1].</td><td>GCF_009176725.1</td></tr>
<tr><td>WP_152099374.1</td><td>TPR+GreAB-C+PIN*→</td><td>TPR+GreAB-C+PIN</td><td>1188</td><td>-</td><td>Planctomycetes</td><td>Lacipirellula parvula</td><td>tetratricopeptide repeat protein [Lacipirellula parvula].</td><td>GCF_009177095.1</td></tr>
<tr><td>WP_152217660.1</td><td>REase+TPR+TPR+GreAB-C+PIN*→</td><td>REase+TPR+TPR+GreAB-C+PIN</td><td>1333</td><td>-</td><td>Alphaproteobacteria</td><td>Parvibaculum sedimenti</td><td>hypothetical protein [Parvibaculum sedimenti].</td><td>GCF_009184905.1</td></tr>
<tr><td>WP_152508433.1</td><td>TPR+GreAB-C+PIN*→</td><td>TPR+GreAB-C+PIN</td><td>1208</td><td>-</td><td>Alphaproteobacteria</td><td>unclassified Labrenzia</td><td>MULTISPECIES: hypothetical protein [unclassified Labrenzia].</td><td>GCF_009363315.1</td></tr>
<tr><td>WP_152533063.1</td><td>REase+TPR+GreAB-C+PIN*→</td><td>REase+TPR+GreAB-C+PIN</td><td>1484</td><td>-</td><td>Gammaproteobacteria</td><td>Pseudomonas syringae</td><td>hypothetical protein [Pseudomonas syringae].</td><td>GCF_000452465.1</td></tr>
<tr><td>WP_152541917.1</td><td>TPR+GreAB-C+PIN*→</td><td>TPR+GreAB-C+PIN</td><td>529</td><td>-</td><td>Bacteroidetes</td><td>Saccharicrinis fermentans</td><td>hypothetical protein [Saccharicrinis fermentans].</td><td>GCF_000583675.1</td></tr>
<tr><td>WP_152640028.1</td><td>TPR+GreAB-C+PIN*→</td><td>TPR+GreAB-C+PIN</td><td>395</td><td>-</td><td>Firmicutes</td><td>Blautia obeum</td><td>hypothetical protein [Blautia obeum].</td><td>GCF_003463085.1</td></tr>
</tbody>
</table>

acc	operon	architecture	len	gen.name	taxend	species	defline	gca
WP_152648143.1	REase+TPR+TPR+GreAB-C+PIN*→	REase+TPR+TPR+GreAB-C+PIN	1321	-	Alphaproteobacteria	Bradyrhizobium elkanii	hypothetical protein [Bradyrhizobium elkanii].	GCF_000878305.1
WP_152803033.1	REase+TPR+GreAB-C+PIN*→||||<-HTH	REase+TPR+GreAB-C+PIN	1328	-	Betaproteobacteria	Rugamonas sp. FT103W	hypothetical protein [Rugamonas sp. FT103W].	GCF_009380165.1
WP_152993715.1	TPR+GreAB-C+PIN*→	TPR+GreAB-C+PIN	677	-	Gammaproteobacteria	Pseudomonas aeruginosa	hypothetical protein [Pseudomonas aeruginosa].	GCF_001451965.1
WP_153001774.1	PIN*→	PIN	483	-	Actinobacteria	Curtobacterium oceanosedimentum	hypothetical protein [Curtobacterium oceanosedimentum].	GCF_001476135.1
WP_153041348.1	TPR+GreAB-C+PIN*→	TPR+GreAB-C+PIN	1322	-	Alphaproteobacteria	Pseudovibrio sp. Ad5	hypothetical protein [Pseudovibrio sp. Ad5].	GCF_001623095.1
WP_153065368.1	REase→ TPR+GreAB-C+PIN*→	TPR+GreAB-C+PIN	1111	-	Gammaproteobacteria	Xanthomonas arboricola	hypothetical protein [Xanthomonas arboricola].	GCF_003352905.1
WP_153226839.1	TPR+PIN*→	TPR+PIN	1140	-	Actinobacteria	Mycobacteroides salmoniphilum	hypothetical protein [Mycobacteroides salmoniphilum].	GCF_004366855.1
WP_153278476.1	TM+TPR+PIN*→	TM+TPR+PIN	1138	-	Actinobacteria	Saccharothrix syringae	hypothetical protein [Saccharothrix syringae].	GCF_009498035.1
WP_153574636.1	TPR+GreAB-C+PIN*→	TPR+GreAB-C+PIN	1139	-	Gammaproteobacteria	Pseudomonas aeruginosa	hypothetical protein [Pseudomonas aeruginosa].	GCF_000791765.1
WP_153576067.1	TPR+GreAB-C+PIN*→	TPR+GreAB-C+PIN	1170	-	Gammaproteobacteria	Pseudomonas aeruginosa	hypothetical protein [Pseudomonas aeruginosa].	GCF_000795585.1
WP_153576415.1	TPR+GreAB-C+PIN*→	TPR+GreAB-C+PIN	1158	-	Gammaproteobacteria	Pseudomonas aeruginosa	hypothetical protein [Pseudomonas aeruginosa].	GCF_000796465.1
WP_153786096.1	TPR+GreAB-C+PIN*→	TPR+GreAB-C+PIN	1139	-	Gammaproteobacteria	Pseudomonas sp. EMN2	hypothetical protein [Pseudomonas sp. EMN2].	GCF_009650555.1
WP_154691510.1	REase+TPR+GreAB-C+PIN*→	REase+TPR+GreAB-C+PIN	1325	-	Alphaproteobacteria	Novosphingobium sp. Gsoil 351	hypothetical protein [Novosphingobium sp. Gsoil 351].	GCF_009707465.1
WP_154808640.1	TPR+GreAB-C+PIN*→||||<-?<-?<-?<-?||?→?→ Cas_Cas1→	TPR+GreAB-C+PIN	1207	-	Euryarchaeota	Methanolobus vulcani	GreA/GreB family elongation factor [Methanolobus vulcani].	GCF_006546655.1
WP_154898518.1	TPR+GreAB-C+PIN*→	TPR+GreAB-C+PIN	970	-	Planctomycetes	Gimesia maris	GreA/GreB family elongation factor [Gimesia maris].	GCF_007747015.1
WP_155001003.1	TPR+GreAB-C+PIN*→	TPR+GreAB-C+PIN	1157	-	Gammaproteobacteria	Pseudomonas fluorescens	hypothetical protein [Pseudomonas fluorescens].	GCF_902497965.1
WP_155247385.1	REase+TPR+GreAB-C+PIN*→	REase+TPR+GreAB-C+PIN	1356	-	Actinobacteria	Salinispora arenicola	hypothetical protein [Salinispora arenicola].	GCF_000375205.1
WP_155347963.1	REase+TPR+GreAB-C+PIN*→	REase+TPR+GreAB-C+PIN	1228	-	Actinobacteria	Acrocarpospora pleiomorpha	DUF4365 domain-containing protein [Acrocarpospora pleiomorpha].	GCF_009687885.1
WP_155468027.1	REase+TPR+GreAB-C+PIN*→	REase+TPR+GreAB-C+PIN	1410	-	Betaproteobacteria	Duganella radicis	DUF4365 domain-containing protein [Duganella radicis].	GCF_009720825.1
WP_155543363.1	REase+TPR+GreAB-C+PIN*→	REase+TPR+GreAB-C+PIN	1179	-	Actinobacteria	Amycolatopsis sp. A23	DUF4365 domain-containing protein [Amycolatopsis sp. A23].	GCF_902497555.1
WP_155546438.1	REase+TPR+GreAB-C+PIN*→	REase+TPR+GreAB-C+PIN	1244	-	Actinobacteria	Amycolatopsis sp. A23	DUF4365 domain-containing protein [Amycolatopsis sp. A23].	GCF_902497555.1
WP_155620995.1	TPR+PIN*→	TPR+PIN	1179	-	Firmicutes	Paenibacillus macerans	hypothetical protein [Paenibacillus macerans].	GCF_009725145.1
WP_155672395.1	HetE-N1→ TPR+GreAB-C+PIN*→	TPR+GreAB-C+PIN	1088	-	Gammaproteobacteria	Aliivibrio fischeri	hypothetical protein [Aliivibrio fischeri].	GCF_009727805.1
WP_155681246.1	DOC+HTH→ REase+TOPC→ HetE-N1→ HetE-N1+TPR+GreAB-C+PIN*→	HetE-N1+TPR+GreAB-C+PIN	1123	-	Gammaproteobacteria	Pseudomonas aeruginosa	hypothetical protein [Pseudomonas aeruginosa].	GCF_009727245.1
WP_155728442.1	HetE-N1→ MNS-Npun2340+TPR+GreAB-C+PIN*→	MNS-Npun2340+TPR+GreAB-C+PIN	1094	-	Gammaproteobacteria	Aeromonas veronii	hypothetical protein [Aeromonas veronii].	GCF_001593245.1
WP_155754272.1	REase+TPR+GreAB-C+PIN*→	REase+TPR+GreAB-C+PIN	1265	-	Betaproteobacteria	Burkholderia vietnamiensis	hypothetical protein [Burkholderia vietnamiensis].	GCF_001528565.1
WP_155849001.1	TPR+PIN*→	TPR+PIN	1211	-	Actinobacteria	Amycolatopsis vancoresmycina	hypothetical protein [Amycolatopsis vancoresmycina].	GCF_000388135.1
WP_155854387.1	TPR+PIN*→	TPR+PIN	1219	-	Actinobacteria	Arthrobacter sp. MA-N2	hypothetical protein [Arthrobacter sp. MA-N2].	GCF_000517125.1
WP_155858633.1	REase+TPR+GreAB-C+PIN*→	REase+TPR+GreAB-C+PIN	1245	-	Gammaproteobacteria	Acinetobacter soli	hypothetical protein [Acinetobacter soli].	GCF_001953195.1
WP_155950759.1	HetE-N1→ TPR+TPR+GreAB-C+PIN*→	TPR+TPR+GreAB-C+PIN	1108	-	Gammaproteobacteria	Pseudomonas taiwanensis	hypothetical protein [Pseudomonas taiwanensis].	GCF_000425785.1
WP_155952204.1	REase+TPR+GreAB-C+PIN*→	REase+TPR+GreAB-C+PIN	1443	-	Gammaproteobacteria	Pseudomonas sp. CHM02	hypothetical protein [Pseudomonas sp. CHM02].	GCF_000612585.1
WP_155956985.1	HTH+PNPase+TPR+GreAB-C+PIN*→	HTH+PNPase+TPR+GreAB-C+PIN	1520	-	Actinobacteria	Rhodococcus sp. UNC23MFCrub1.1	helix-turn-helix domain-containing protein [Rhodococcus sp. UNC23MFCrub1.1].	GCF_000686025.1
WP_156093738.1	REase+TPR+GreAB-C+PIN*→	REase+TPR+GreAB-C+PIN	1179	-	Actinobacteria	Lentzea aerocolonigenes	DUF4365 domain-containing protein [Lentzea aerocolonigenes].	GCF_000974445.1
WP_156124401.1	HetE-N1→ HetE-N1+TPR+GreAB-C+PIN*→	HetE-N1+TPR+GreAB-C+PIN	1122	-	Betaproteobacteria	Achromobacter sp. RTa	hypothetical protein [Achromobacter sp. RTa].	GCF_000757485.1
WP_156177573.1	REase+TPR+GreAB-C+PIN*→	REase+TPR+GreAB-C+PIN	1471	-	Gammaproteobacteria	Pseudomonas sp. CCOS 191	hypothetical protein [Pseudomonas sp. CCOS 191].	GCF_001007005.1
WP_156289500.1	TPR+PIN*→	TPR+PIN	1076	-	Firmicutes	Oceanobacillus salinisoli	hypothetical protein [Oceanobacillus salinisoli].	GCF_009733865.1
WP_156340841.1	REase+TPR+GreAB-C+PIN*→	REase+TPR+GreAB-C+PIN	1470	-	Gammaproteobacteria	Pseudomonas sp. NBRC 111124	hypothetical protein [Pseudomonas sp. NBRC 111124].	GCF_001320245.1

acc	operon	architecture	len	gen.name	taxend	species	defline	gca
WP_156343172.1	REase+TPR+GreAB-C+PIN*→	REase+TPR+GreAB-C+PIN	1368	-	Gammaproteobacteria	Pseudomonas sp. NBRC 111134	hypothetical protein [Pseudomonas sp. NBRC 111134].	GCF_001320655.1
WP_156369768.1	TPR+PIN*→	TPR+PIN	1065	-	Actinobacteria	Arthrobacter sp. Leaf145	hypothetical protein [Arthrobacter sp. Leaf145].	GCF_001423565.1
WP_156457128.1	TPR+GreAB-C+PIN*→	TPR+GreAB-C+PIN	1119	-	Alphaproteobacteria	Bradyrhizobium sp. CCH5-F6	hypothetical protein [Bradyrhizobium sp. CCH5-F6].	GCF_001556045.1
WP_156523151.1	REase+TPR+GreAB-C+PIN*→	REase+TPR+GreAB-C+PIN	1289	-	Betaproteobacteria	Bordetella ansorpii	hypothetical protein [Bordetella ansorpii].	GCF_900078315.1
WP_156681909.1	-	-	1775	-	Actinobacteria	Rhodococcus sp. SC4	helix-turn-helix domain-containing protein [Rhodococcus sp. SC4].	-
WP_156749419.1	TPR+PIN*→	TPR+PIN	1166	-	Actinobacteria	Mycobacterium sp. E1747	hypothetical protein [Mycobacterium sp. E1747].	GCF_001667775.1
WP_156792330.1	HetE-N1→ TPR+GreAB-C+PIN*→	TPR+GreAB-C+PIN	1110	-	Deltaproteobacteria	Desulfotalea psychrophila	hypothetical protein [Desulfotalea psychrophila].	GCF_000025945.1
WP_156831299.1	REase+TPR+GreAB-C+PIN*→	REase+TPR+GreAB-C+PIN	1304	-	Gammaproteobacteria	Arhodomonas aquaeolei	hypothetical protein [Arhodomonas aquaeolei].	GCF_000374645.1
WP_156924526.1	REase+TPR+GreAB-C+PIN*→	REase+TPR+GreAB-C+PIN	1326	-	Betaproteobacteria	Derxia gummosa	hypothetical protein [Derxia gummosa].	GCF_000482785.1
WP_156945361.1	REase+TPR+GreAB-C+PIN*→	REase+TPR+GreAB-C+PIN	1305	-	Alphaproteobacteria	Hyphomonas johnsonii	hypothetical protein [Hyphomonas johnsonii].	GCF_000685275.1
WP_156971043.1	TPR+GreAB-C+PIN*→	TPR+GreAB-C+PIN	1141	-	Alphaproteobacteria	Beijerinckia mobilis	tetratricopeptide repeat protein [Beijerinckia mobilis].	GCF_000745425.1
WP_157119081.1	REase+TPR+GreAB-C+PIN*→	REase+TPR+GreAB-C+PIN	1521	-	Betaproteobacteria	Azohydromonas lata	hypothetical protein [Azohydromonas lata].	GCF_001571085.1
WP_157228354.1	TPR+PIN*→	TPR+PIN	1070	-	Actinobacteria	Gordonia amicalis	hypothetical protein [Gordonia amicalis].	GCF_000332995.1
WP_157237724.1	TPR+GreAB-C+PIN*→	TPR+GreAB-C+PIN	801	-	Deltaproteobacteria	Desulfobacter curvatus	hypothetical protein [Desulfobacter curvatus].	GCF_000373985.1
WP_157376943.1	REase+TPR+GreAB-C+PIN*→	REase+TPR+GreAB-C+PIN	1354	-	Betaproteobacteria	Burkholderia ubonensis	hypothetical protein [Burkholderia ubonensis].	GCF_001523425.1
WP_157516442.1	TPR+PIN*→	TPR+PIN	1239	-	Actinobacteria	Oerskovia enterophila	hypothetical protein [Oerskovia enterophila].	GCF_001624335.1
WP_157553798.1	X+PIN*→?→ Calcineurin→	X+PIN	1140	-	Actinobacteria	Microbacterium hominis	hypothetical protein [Microbacterium hominis].	GCF_001592125.1
WP_157561005.1	TPR+PIN*→	TPR+PIN	758	-	Actinobacteria	Humibacter albus	hypothetical protein [Humibacter albus].	GCF_000421825.1
WP_157621026.1	HetE-N1→ TPR+GreAB-C+PIN*→	TPR+GreAB-C+PIN	1092	-	Gammaproteobacteria	Vibrio sp. HENC-03	hypothetical protein [Vibrio sp. HENC-03].	GCF_000305755.2
WP_157678465.1	REase+TPR+GreAB-C+PIN*→	REase+TPR+GreAB-C+PIN	1297	-	Alphaproteobacteria	Mesorhizobium sp. WSM1497	hypothetical protein [Mesorhizobium sp. WSM1497].	GCF_001672455.2
WP_157721532.1	REase+TPR+GreAB-C+PIN*→	REase+TPR+GreAB-C+PIN	1240	-	Gammaproteobacteria	Stenotrophomonas sp. WZN-1	hypothetical protein [Stenotrophomonas sp. WZN-1].	GCF_002192255.1
WP_157768703.1	REase+TPR+GreAB-C+PIN*→	REase+TPR+GreAB-C+PIN	1380	-	Betaproteobacteria	Acidovorax avenae	hypothetical protein [Acidovorax avenae].	GCF_000176855.2
WP_157788679.1	REase+TPR+GreAB-C+PIN*→	REase+TPR+GreAB-C+PIN	1382	-	Gammaproteobacteria	Pseudomonas fluorescens	GreA/GreB family elongation factor [Pseudomonas fluorescens].	GCF_000308175.1
WP_157822505.1	TPR+GreAB-C+PIN*→	TPR+GreAB-C+PIN	769	-	Gammaproteobacteria	Psychromonas sp. Urea-02u-13	hypothetical protein, partial [Psychromonas sp. Urea-02u-13].	GCF_002835995.1
WP_157852789.1	TPR+PIN*→	TPR+PIN	1134	-	Actinobacteria	Streptomyces iakyrus	hypothetical protein [Streptomyces iakyrus].	GCF_000717055.1
WP_157874592.1	REase+TPR+TPR+GreAB-C+PIN*→	REase+TPR+TPR+GreAB-C+PIN	1330	-	Actinobacteria	Streptomyces sp. AcH 505	hypothetical protein [Streptomyces sp. AcH 505].	GCF_000818175.1
WP_157937504.1	TPR+GreAB-C+PIN*→	TPR+GreAB-C+PIN	964	-	Gammaproteobacteria	Vibrio splendidus	hypothetical protein, partial [Vibrio splendidus].	GCF_002874795.1
WP_157972098.1	TPR+GreAB-C+PIN*→	TPR+GreAB-C+PIN	1234	-	Bacteroidetes	Negadavirga sp. SW125	hypothetical protein [Negadavirga sp. SW125].	GCF_003347495.1
WP_158764527.1	TPR+GreAB-C+PIN*→	TPR+GreAB-C+PIN	761	-	Alphaproteobacteria	Terricaulis silvestris	hypothetical protein [Terricaulis silvestris].	GCF_009792355.1
WP_158793614.1	REase+TPR+GreAB-C+PIN*→	REase+TPR+GreAB-C+PIN	1358	-	Acidobacteria	Granulicella sp. L60	hypothetical protein [Granulicella sp. L60].	GCF_009765825.1
WP_158941358.1	REase+TPR+GreAB-C+PIN*→	REase+TPR+GreAB-C+PIN	1279	-	Acidobacteria	Granulicella sp. S190	hypothetical protein [Granulicella sp. S190].	GCF_009766095.1
WP_159057317.1	TPR+PIN*→	TPR+PIN	1196	-	Actinobacteria	Streptomyces scabiei	hypothetical protein [Streptomyces scabiei].	GCF_001550225.1
WP_159073521.1	TPR+PIN*→	TPR+PIN	1075	-	Actinobacteria	Streptomyces sp. RTd22	hypothetical protein [Streptomyces sp. RTd22].	GCF_001650215.1
WP_159104186.1	TPR+PIN*→	TPR+PIN	1211	-	Actinobacteria	Streptomyces sp. CdTB01	hypothetical protein [Streptomyces sp. CdTB01].	GCF_001484565.1
WP_159389385.1	REase+TPR+GreAB-C+PIN*→	REase+TPR+GreAB-C+PIN	1423	-	Gammaproteobacteria	Pseudomonas sp. R32	hypothetical protein [Pseudomonas sp. R32].	GCF_009866705.1
WP_159401920.1	TPR+PIN*→	TPR+PIN	1075	-	Actinobacteria	Streptomyces hygroscopicus	hypothetical protein [Streptomyces hygroscopicus].	GCF_001705785.1
WP_159477578.1	Mbetalac→ Trypsin+TPR+GreAB-C+PIN*→	Trypsin+TPR+GreAB-C+PIN	1359	-	Bacteroidetes	Dyadobacter sp. 3J3	hypothetical protein [Dyadobacter sp. 3J3].	GCF_009801115.1
WP_159617138.1	PIN*→	PIN	499	-	Actinobacteria	Arthrobacter zhaoguopingii	hypothetical protein [Arthrobacter zhaoguopingii].	GCF_009828605.1
WP_159719775.1	TPR+PIN*→	TPR+PIN	1170	-	Firmicutes	Anoxybacillus sp. PDR2	hypothetical protein [Anoxybacillus sp. PDR2].	GCF_009834045.1
WP_159964006.1	REase+TPR+GreAB-C+PIN*→	REase+TPR+GreAB-C+PIN	1113	-	Alphaproteobacteria	Profundibacterium mesophilum	hypothetical protein [Profundibacterium mesophilum].	GCF_009835145.1
WP_160086901.1	BTLCP+SFI-helicase+REase→?→ REase+TPR+GreAB-C+PIN*→	REase+TPR+GreAB-C+PIN	1492	-	Gammaproteobacteria	Pseudomonas sp. 9AZ	GreA/GreB family elongation factor [Pseudomonas sp. 9AZ].	GCF_902506525.1

acc	operon	architecture	len	gen.name	taxend	species	defline	gca
WP_160308988.1	TPR+GreAB-C+PIN*→	TPR+GreAB-C+PIN	1249	-	Deltaproteobacteria	Anaeromyxobacter sp. PSR-1	GreA/GreB family elongation factor [Anaeromyxobacter sp. PSR-1].	GCF_000964525.1
WP_160382728.1	REase+TPR+GreAB-C+PIN*→	REase+TPR+GreAB-C+PIN	1329	-	Alphaproteobacteria	Pseudooceanicola pacificus	hypothetical protein [Pseudooceanicola pacificus].	GCF_009789075.1
WP_160512238.1	HetE-N1→ TPR+GreAB-C+PIN*→	TPR+GreAB-C+PIN	1094	-	Gammaproteobacteria	Escherichia coli	hypothetical protein [Escherichia coli].	GCF_009820085.1
WP_160657313.1	HetE-N1→ TPR+GreAB-C+PIN*→	TPR+GreAB-C+PIN	1114	-	Gammaproteobacteria	Photobacterium alginatilyticum	hypothetical protein [Photobacterium alginatilyticum].	GCF_009910675.1
WP_160663512.1	TPR+PIN*→	TPR+PIN	1180	-	Actinobacteria	Pseudarthrobacter sp. ATCC 49987	hypothetical protein [Pseudarthrobacter sp. ATCC 49987].	GCF_009928425.1
WP_160778164.1	REase→ PSE→ REase+TPR+GreAB-C+PIN*→	REase+TPR+GreAB-C+PIN	1252	-	Alphaproteobacteria	Parerythrobacter jejuensis	hypothetical protein [Parerythrobacter jejuensis].	GCF_009827995.1
WP_160856107.1	REase+TPR+GreAB-C+PIN*→	REase+TPR+GreAB-C+PIN	1263	-	Betaproteobacteria	Delftia sp. CH05	hypothetical protein [Delftia sp. CH05].	GCF_009827015.1
WP_160933674.1	REase+TPR+GreAB-C+PIN*→	REase+TPR+GreAB-C+PIN	1351	-	Betaproteobacteria	unclassified Burkholderia	MULTISPECIES: hypothetical protein [unclassified Burkholderia].	GCF_009826925.1
WP_161036960.1	REase+TPR+GreAB-C+PIN*→	REase+TPR+GreAB-C+PIN	1075	-	Betaproteobacteria	Duganella fentianensis	hypothetical protein [Duganella fentianensis].	GCF_009857835.1
WP_161077003.1	REase+TPR+GreAB-C+PIN*→	REase+TPR+GreAB-C+PIN	1292	-	Betaproteobacteria	Duganella sp. CY15W	restriction endonuclease [Duganella sp. CY15W].	GCF_009857555.1
WP_161352699.1	REase+TPR+GreAB-C+PIN*→	REase+TPR+GreAB-C+PIN	1351	-	Alphaproteobacteria	Maritimibacter sp. DP07	hypothetical protein [Maritimibacter sp. DP07].	GCF_009882975.1
WP_161505253.1	TPR+PIN*→?→?→||||<-TIR	TPR+PIN	1268	-	Gammaproteobacteria	Legionella pneumophila	hypothetical protein [Legionella pneumophila].	GCF_009933055.1
WP_161571911.1	HetE-N1→ TPR+GreAB-C+PIN*→	TPR+GreAB-C+PIN	1117	-	Gammaproteobacteria	Cronobacter malonaticus	hypothetical protein [Cronobacter malonaticus].	GCF_009938405.1
WP_161629377.1	TPR+GreAB-C+PIN*→	TPR+GreAB-C+PIN	488	-	Gammaproteobacteria	Marinobacter sp. EVN1	hypothetical protein, partial [Marinobacter sp. EVN1].	GCF_000475375.1
WP_161722347.1	TPR+GreAB-C+PIN*→	TPR+GreAB-C+PIN	1135	-	Alphaproteobacteria	unclassified Microvirga	MULTISPECIES: tetratricopeptide repeat protein [unclassified Microvirga].	GCF_009910725.1
WP_161796830.1	REase+TPR+GreAB-C+PIN*→	REase+TPR+GreAB-C+PIN	1210	-	Alphaproteobacteria	Devosia soli	GreA/GreB family elongation factor [Devosia soli].	GCF_000970455.1
WP_161897930.1	HetE-N1→ TPR+GreAB-C+PIN*→	TPR+GreAB-C+PIN	1110	-	Gammaproteobacteria	Pseudomonas sp. MY50	hypothetical protein [Pseudomonas sp. MY50].	GCF_009932725.1
WP_161916726.1	X+PIN*→	X+PIN	1235	-	Actinobacteria	Cellulomonas sp. APG4	hypothetical protein [Cellulomonas sp. APG4].	GCF_009996735.1
WP_161984907.1	HTH→ HetE-N1→ HetE-N1+GreAB-C+PIN*→	HetE-N1+GreAB-C+PIN	1077	-	Gammaproteobacteria	Acinetobacter haemolyticus	hypothetical protein [Acinetobacter haemolyticus].	GCF_010014695.1
WP_162386033.1	Mbetalac→ Trypsin+TPR+GreAB-C+PIN*→	Trypsin+TPR+GreAB-C+PIN	1366	-	Bacteroidetes	Spirosoma endbachense	hypothetical protein [Spirosoma endbachense].	GCF_010233585.1
WP_162486659.1	REase+TPR+GreAB-C+PIN*→||||<-HTH+HTH	REase+TPR+GreAB-C+PIN	1334	-	Betaproteobacteria	Burkholderia pseudomallei	hypothetical protein [Burkholderia pseudomallei].	GCF_000011545.1
WP_162599154.1	HetE-N1→ TPR+GreAB-C+PIN*→	TPR+GreAB-C+PIN	1027	-	Gammaproteobacteria	Shewanella algidipiscicola	hypothetical protein [Shewanella algidipiscicola].	GCF_900380485.1
WP_162599389.1	TPR+GreAB-C+PIN*→	TPR+GreAB-C+PIN	1158	-	Gammaproteobacteria	Pseudomonas aeruginosa	hypothetical protein [Pseudomonas aeruginosa].	GCF_900406905.1
WP_162672520.1	HTH→ GT4+HEPN+TPR+GreAB-C+PIN*→	GT4+HEPN+TPR+GreAB-C+PIN	1685	-	Planctomycetes	Gemmata massiliana	glycosyltransferase [Gemmata massiliana].	GCF_901538265.1
WP_162916214.1	PIN*→	PIN	348	-	Betaproteobacteria	Burkholderia sp. PAMC 26561	hypothetical protein [Burkholderia sp. PAMC 26561].	GCF_001557535.2
WP_162940198.1	X+PIN*→	X+PIN	1169	-	Actinobacteria	Gryllotalpicola protaetiae	hypothetical protein [Gryllotalpicola protaetiae].	GCF_003627055.1
WP_162960625.1	DOC+HTH→ REase+TOPC→?→ HetE-N1→ HetE-N1+TPR+GreAB-C+PIN*→	HetE-N1+TPR+GreAB-C+PIN	770	-	Gammaproteobacteria	Pseudomonas aeruginosa	hypothetical protein, partial [Pseudomonas aeruginosa].	GCF_003630455.1
WP_163210080.1	TM+TM+TPR+PIN*→	TM+TM+TPR+PIN	1195	-	Bacteroidetes	Bacteroides sp. 519	hypothetical protein [Bacteroides sp. 519].	GCF_010500955.1
WP_163325035.1	TPR+GreAB-C+PIN*→	TPR+GreAB-C+PIN	1227	-	Bacteroidetes	Draconibacterium sp. GM2-18	hypothetical protein [Draconibacterium sp. GM2-18].	GCF_010586825.1
WP_163385713.1	TPR+GreAB-C+PIN*→	TPR+GreAB-C+PIN	509	-	Gammaproteobacteria	Enterobacter hormaechei	hypothetical protein, partial [Enterobacter hormaechei].	GCF_011006815.1
WP_163511264.1	REase+TPR+GreAB-C+PIN*→	REase+TPR+GreAB-C+PIN	1231	-	Actinobacteria	Fodinicola acaciae	DUF4365 domain-containing protein [Fodinicola acaciae].	GCF_010993745.1
WP_163806192.1	TPR+PIN*→	TPR+PIN	1223	-	Actinobacteria	Mycolicibacterium anyangense	hypothetical protein [Mycolicibacterium anyangense].	GCF_010731855.1
WP_163887020.1	REase+TPR+GreAB-C+PIN*→	REase+TPR+GreAB-C+PIN	1290	-	Alphaproteobacteria	Pseudoruegeria sp. M32A2M	tetratricopeptide repeat protein [Pseudoruegeria sp. M32A2M].	GCF_010374725.1
WP_164141894.1	TPR+PIN*→	TPR+PIN	1215	-	Actinobacteria	Streptomyces coelicoflavus	hypothetical protein [Streptomyces coelicoflavus].	GCF_010548435.1
WP_164363703.1	TPR+PIN*→	TPR+PIN	1135	-	Actinobacteria	Streptomyces griseus	hypothetical protein [Streptomyces griseus].	GCF_010548565.1
WP_164366948.1	TPR+PIN*→	TPR+PIN	1215	-	Actinobacteria	Streptomyces diastaticus	hypothetical protein [Streptomyces diastaticus].	GCF_010548605.1
WP_164922417.1	TRD+TRD→?→?→ REase+SNF2→?→ TPR+GreAB-C+PIN*→	TPR+GreAB-C+PIN	1273	-	Planctomycetes	Rhodopirellula baltica	hypothetical protein [Rhodopirellula baltica].	GCF_000196115.1
WP_165069081.1	TPR+PIN*→	TPR+PIN	1123	-	Actinobacteria	Marisediminicola senii	hypothetical protein [Marisediminicola senii].	GCF_011057645.1
WP_165087560.1	REase+TPR+GreAB-C+PIN*→	REase+TPR+GreAB-C+PIN	1326	-	Betaproteobacteria	Caballeronia sp. SBC2	hypothetical protein [Caballeronia sp. SBC2].	GCF_011039955.1

<table>
<tr><th>acc</th><th>operon</th><th>architecture</th><th>len</th><th>gen.name</th><th>taxend</th><th>species</th><th>defline</th><th>gca</th></tr>
<tr><td>WP_165198604.1</td><td>TPR+GreAB-C+PIN*→</td><td>TPR+GreAB-C+PIN</td><td>1240</td><td>-</td><td>Alphaproteobacteria</td><td>Pseudohalocynthiibacter aestuariivivens</td><td>hypothetical protein [Pseudohalocynthiibacter aestuariivivens].</td><td>GCF_011040495.1</td></tr>
<tr><td>WP_165399219.1</td><td>REase+TPR+GreAB-C+PIN*→</td><td>REase+TPR+GreAB-C+PIN</td><td>1316</td><td>-</td><td>Alphaproteobacteria</td><td>Komagataeibacter xylinus</td><td>GreA/GreB family elongation factor [Komagataeibacter xylinus].</td><td>GCF_004302915.1</td></tr>
<tr><td>WP_165684855.1</td><td>TPR+PIN*→</td><td>TPR+PIN</td><td>1088</td><td>-</td><td>Actinobacteria</td><td>Streptomyces sp. KO7888</td><td>hypothetical protein [Streptomyces sp. KO7888].</td><td>GCF_011317205.1</td></tr>
<tr><td>WP_165714007.1</td><td>TPR+GreAB-C+PIN*→</td><td>TPR+GreAB-C+PIN</td><td>924</td><td>-</td><td>Betaproteobacteria</td><td>Rubrivivax benzoatilyticus</td><td>hypothetical protein, partial [Rubrivivax benzoatilyticus].</td><td>GCF_011365705.1</td></tr>
<tr><td>WP_165715394.1</td><td>HetE-N1→?→?→ TPR+GreAB-C+PIN*→</td><td>TPR+GreAB-C+PIN</td><td>776</td><td>-</td><td>Gammaproteobacteria</td><td>Salmonella enterica</td><td>hypothetical protein [Salmonella enterica].</td><td>GCF_011368495.1</td></tr>
<tr><td>WP_165722314.1</td><td>HetE-N1→ TPR+GreAB-C+PIN*→</td><td>TPR+GreAB-C+PIN</td><td>1113</td><td>-</td><td>Gammaproteobacteria</td><td>unclassified Pseudoalteromonas</td><td>MULTISPECIES: hypothetical protein [unclassified Pseudoalteromonas].</td><td>GCF_011378665.1</td></tr>
<tr><td>WP_165919434.1</td><td>REase+TPR+GreAB-C+PIN*→ TPR→</td><td>REase+TPR+GreAB-C+PIN</td><td>1252</td><td>-</td><td>Actinobacteria</td><td>Promicromonospora sp. CF082</td><td>DUF4365 domain-containing protein [Promicromonospora sp. CF082].</td><td>GCF_004341335.1</td></tr>
<tr><td>WP_166221458.1</td><td>HTH→ HetE-N1→ TPR+GreAB-C+PIN*→</td><td>TPR+GreAB-C+PIN</td><td>1076</td><td>-</td><td>Gammaproteobacteria</td><td>Acinetobacter shaoyimingii</td><td>hypothetical protein [Acinetobacter shaoyimingii].</td><td>GCF_011578045.1</td></tr>
<tr><td>WP_166315930.1</td><td>X+PIN*→</td><td>X+PIN</td><td>1081</td><td>-</td><td>Actinobacteria</td><td>Microbacterium excoecariae</td><td>hypothetical protein [Microbacterium excoecariae].</td><td>GCF_011326725.1</td></tr>
<tr><td>WP_166694175.1</td><td>Mbetalac→ Trypsin+TPR+GreAB-C+PIN*→</td><td>Trypsin+TPR+GreAB-C+PIN</td><td>1365</td><td>-</td><td>Bacteroidetes</td><td>Fibrella aestuarina</td><td>trypsin-like serine protease [Fibrella aestuarina].</td><td>GCF_011742925.1</td></tr>
<tr><td>WP_166748348.1</td><td>REase+TPR+GreAB-C+PIN*→</td><td>REase+TPR+GreAB-C+PIN</td><td>1323</td><td>-</td><td>Alphaproteobacteria</td><td>Sphingomonas aerolata</td><td>hypothetical protein [Sphingomonas aerolata].</td><td>GCF_011759485.1</td></tr>
<tr><td>WP_166760128.1</td><td>X+PIN*→</td><td>X+PIN</td><td>1124</td><td>-</td><td>Actinobacteria</td><td>Frigoribacterium faeni</td><td>hypothetical protein [Frigoribacterium faeni].</td><td>GCF_011761235.1</td></tr>
<tr><td>WP_166908249.1</td><td>TPR+PIN*→</td><td>TPR+PIN</td><td>1129</td><td>-</td><td>Actinobacteria</td><td>Mycobacterium sp. DL440</td><td>hypothetical protein [Mycobacterium sp. DL440].</td><td>GCF_011745145.1</td></tr>
<tr><td>WP_167081447.1</td><td>REase+TPR+GreAB-C+PIN*→</td><td>REase+TPR+GreAB-C+PIN</td><td>1510</td><td>-</td><td>Betaproteobacteria</td><td>‘Massilia aquatica’ Holochova et al. 2020</td><td>hypothetical protein [‘Massilia aquatica’ Holochova et al. 2020].</td><td>GCF_011682045.1</td></tr>
<tr><td>WP_167302996.1</td><td>REase+TPR+GreAB-C+PIN*→</td><td>REase+TPR+GreAB-C+PIN</td><td>1316</td><td>-</td><td>Alphaproteobacteria</td><td>Sphingobium vermicomposti</td><td>hypothetical protein [Sphingobium vermicomposti].</td><td>GCF_011762025.1</td></tr>
<tr><td>WP_167344464.1</td><td>TPR+PIN*→||||<-NUDIX</td><td>TPR+PIN</td><td>1134</td><td>-</td><td>Actinobacteria</td><td>Streptomyces griseolus</td><td>hypothetical protein [Streptomyces griseolus].</td><td>GCF_000721185.1</td></tr>
<tr><td>WP_167432534.1</td><td>HetE-N1→ TPR+GreAB-C+PIN*→</td><td>TPR+GreAB-C+PIN</td><td>1111</td><td>-</td><td>Gammaproteobacteria</td><td>Pantoea</td><td>MULTISPECIES: hypothetical protein [Pantoea].</td><td>GCF_011800365.1</td></tr>
<tr><td>WP_168003144.1</td><td>TM+TPR+PIN*→</td><td>TM+TPR+PIN</td><td>1126</td><td>-</td><td>Actinobacteria</td><td>Micromonospora sp. HSS6-12</td><td>hypothetical protein, partial [Micromonospora sp. HSS6-12].</td><td>GCF_012034245.1</td></tr>
<tr><td>WP_168341898.1</td><td>REase+TPR+TPR+GreAB-C+PIN*→</td><td>REase+TPR+TPR+GreAB-C+PIN</td><td>1233</td><td>-</td><td>Alphaproteobacteria</td><td>Rhizobium leguminosarum</td><td>hypothetical protein [Rhizobium leguminosarum].</td><td>GCF_012276075.1</td></tr>
<tr><td>WP_168426515.1</td><td>TPR+GreAB-C+PIN*→</td><td>TPR+GreAB-C+PIN</td><td>461</td><td>-</td><td>Gammaproteobacteria</td><td>Pseudomonas sp. SST3</td><td>hypothetical protein, partial [Pseudomonas sp. SST3].</td><td>GCF_003325755.1</td></tr>
<tr><td>WP_168560628.1</td><td>REase+TPR+GreAB-C+PIN*→</td><td>REase+TPR+GreAB-C+PIN</td><td>1337</td><td>-</td><td>Betaproteobacteria</td><td>Rhizobacter sp. SG703</td><td>hypothetical protein [Rhizobacter sp. SG703].</td><td>GCF_012275445.1</td></tr>
<tr><td>WP_168617642.1</td><td>TPR+PIN*→</td><td>TPR+PIN</td><td>1210</td><td>-</td><td>Actinobacteria</td><td>Nocardioides sp. JQ2195</td><td>hypothetical protein [Nocardioides sp. JQ2195].</td><td>GCF_012272695.1</td></tr>
<tr><td>WP_168725201.1</td><td>TPR+PIN*→</td><td>TPR+PIN</td><td>1218</td><td>-</td><td>Actinobacteria</td><td>Streptomyces galbus</td><td>hypothetical protein [Streptomyces galbus].</td><td>GCF_014650535.1</td></tr>
<tr><td>WP_168927382.1</td><td>TPR+GreAB-C+PIN*→</td><td>TPR+GreAB-C+PIN</td><td>546</td><td>-</td><td>Gammaproteobacteria</td><td>Enterobacter hormaechei</td><td>hypothetical protein, partial [Enterobacter hormaechei].</td><td>GCF_902166805.1</td></tr>
<tr><td>WP_169321903.1</td><td>TPR+GreAB-C+PIN*→</td><td>TPR+GreAB-C+PIN</td><td>1137</td><td>-</td><td>Bacteroidetes</td><td>Chryseobacterium</td><td>MULTISPECIES: hypothetical protein [Chryseobacterium].</td><td>GCF_012952015.1</td></tr>
<tr><td>WP_169448013.1</td><td>REase+TPR+GreAB-C+PIN*→</td><td>REase+TPR+GreAB-C+PIN</td><td>1243</td><td>-</td><td>Gammaproteobacteria</td><td>Stenotrophomonas maltophilia</td><td>hypothetical protein [Stenotrophomonas maltophilia].</td><td>GCF_012971765.1</td></tr>
<tr><td>WP_169574578.1</td><td>REase+TPR+GreAB-C+PIN→ TPR+GreAB-C+PIN*→</td><td>TPR+GreAB-C+PIN</td><td>628</td><td>-</td><td>Alphaproteobacteria</td><td>Sphingobium psychrophilum</td><td>hypothetical protein [Sphingobium psychrophilum].</td><td>GCF_012927105.1</td></tr>
<tr><td>WP_169902587.1</td><td>REase+TPR+GreAB-C+PIN*→</td><td>REase+TPR+GreAB-C+PIN</td><td>1321</td><td>-</td><td>Gammaproteobacteria</td><td>Pseudomonas rhodesiae</td><td>DUF4365 domain-containing protein [Pseudomonas rhodesiae].</td><td>GCF_012985855.1</td></tr>
<tr><td>WP_169912953.1</td><td>TPR+GreAB-C+PIN*→</td><td>TPR+GreAB-C+PIN</td><td>1140</td><td>-</td><td>Gammaproteobacteria</td><td>Pseudomonas chlororaphis</td><td>hypothetical protein [Pseudomonas chlororaphis].</td><td>GCF_012986925.1</td></tr>
<tr><td>WP_169922112.1</td><td>TPR+GreAB-C+PIN*→</td><td>TPR+GreAB-C+PIN</td><td>1148</td><td>-</td><td>Gammaproteobacteria</td><td>Pseudomonas sp. WS 5503</td><td>hypothetical protein [Pseudomonas sp. WS 5503].</td><td>GCF_012985385.1</td></tr>
<tr><td>WP_170029824.1</td><td>REase+TPR+GreAB-C+PIN*→</td><td>REase+TPR+GreAB-C+PIN</td><td>1470</td><td>-</td><td>Gammaproteobacteria</td><td>Pseudomonas sp. SK</td><td>hypothetical protein [Pseudomonas sp. SK].</td><td>GCF_012975065.1</td></tr>
<tr><td>WP_170117623.1</td><td>HNH+TPR+GreAB-C+PIN*→</td><td>HNH+TPR+GreAB-C+PIN</td><td>1207</td><td>-</td><td>Bacteroidetes</td><td>Chitinophaga ginsengisoli</td><td>hypothetical protein [Chitinophaga ginsengisoli].</td><td>GCF_003014595.1</td></tr>
<tr><td>WP_170323822.1</td><td>REase+TPR+GreAB-C+PIN*→</td><td>REase+TPR+GreAB-C+PIN</td><td>1251</td><td>-</td><td>Actinobacteria</td><td>Cryptosporangium phraense</td><td>DUF4365 domain-containing protein [Cryptosporangium phraense].</td><td>GCF_006912135.1</td></tr>
<tr><td>WP_170431548.1</td><td>REase+TPR+GreAB-C+PIN*→?→||||<-?<-?<-BTLCP+SFI-helicase+REase</td><td>REase+TPR+GreAB-C+PIN</td><td>1310</td><td>-</td><td>Alphaproteobacteria</td><td>Ruegeria arenilitoris</td><td>tetratricopeptide repeat protein [Ruegeria arenilitoris].</td><td>GCF_013032225.1</td></tr>
<tr><td>WP_171027511.1</td><td>TPR+PIN*→</td><td>TPR+PIN</td><td>973</td><td>-</td><td>Actinobacteria</td><td>Pseudarthrobacter sp. NamE2</td><td>tetratricopeptide repeat protein [Pseudarthrobacter sp. NamE2].</td><td>GCF_005796205.1</td></tr>
</table>

acc	operon	architecture	len	gen.name	taxend	species	defline	gca
WP_171071268.1	REase+TPR+GreAB-C+PIN*→	REase+TPR+GreAB-C+PIN	1155	-	Gammaproteobacteria	Acinetobacter baumannii	GreA/GreB family elongation factor, partial [Acinetobacter baumannii].	GCF_000580875.1
WP_171275163.1	HetE-N1→ TPR+GreAB-C+PIN*→	TPR+GreAB-C+PIN	1094	-	Gammaproteobacteria	Aeromonas media	hypothetical protein [Aeromonas media].	GCF_013085485.1
WP_171434497.1	DOC+HTH→ REase+TOPC→?→ HetE-N1→ HetE-N1+TPR+GreAB-C+PIN*→	HetE-N1+TPR+GreAB-C+PIN	1124	-	Gammaproteobacteria	Pseudomonas aeruginosa	hypothetical protein [Pseudomonas aeruginosa].	GCF_013115755.1
WP_171451709.1	DOC+HTH→ REase+TOPC→?→ HetE-N1→ HetE-N1→ GreAB-C+PIN*→	GreAB-C+PIN	523	-	Gammaproteobacteria	Pseudomonas aeruginosa	hypothetical protein [Pseudomonas aeruginosa].	GCF_013115805.1
WP_171477953.1	TPR+GreAB-C+PIN*→||||<-?||HTH→	TPR+GreAB-C+PIN	652	-	Gammaproteobacteria	Acinetobacter baumannii	GreA/GreB family elongation factor, partial [Acinetobacter baumannii].	GCF_006491975.1
WP_171493638.1	TPR+GreAB-C+PIN*→	TPR+GreAB-C+PIN	816	-	Gammaproteobacteria	Acinetobacter soli	hypothetical protein, partial [Acinetobacter soli].	GCF_008987205.1
WP_171564538.1	TPR+PIN*→	TPR+PIN	1172	-	Firmicutes	Brevibacillus sp. MCWH	hypothetical protein [Brevibacillus sp. MCWH].	GCF_018195675.1
WP_171662370.1	REase+TPR+GreAB-C+PIN*→	REase+TPR+GreAB-C+PIN	1496	-	Betaproteobacteria	Achromobacter deleyi	hypothetical protein [Achromobacter deleyi].	GCF_013116765.1
WP_171694982.1	HetE-N1→ TPR+GreAB-C+PIN*→	TPR+GreAB-C+PIN	1120	-	Gammaproteobacteria	Methylomonas sp. ZR1	hypothetical protein [Methylomonas sp. ZR1].	GCF_013141865.1
WP_171798091.1	REase+TPR+GreAB-C+PIN*→	REase+TPR+GreAB-C+PIN	1323	-	Alphaproteobacteria	unclassified Novosphingobium	MULTISPECIES: hypothetical protein [unclassified Novosphingobium].	GCF_013149315.1
WP_172598885.1	TPR+GreAB-C+PIN*→	TPR+GreAB-C+PIN	709	-	Alphaproteobacteria	Sphingobium sp. YG1	hypothetical protein [Sphingobium sp. YG1].	GCF_003609795.1
WP_172685391.1	REase+TPR+GreAB-C+PIN*→?→?→ Calcineurin→ Calcineurin→?→ Calcineurin→	REase+TPR+GreAB-C+PIN	1311	-	Alphaproteobacteria	Methylobacterium oryzae	hypothetical protein [Methylobacterium oryzae].	-
WP_172806748.1	TPR+GreAB-C+PIN*→	TPR+GreAB-C+PIN	766	-	Gammaproteobacteria	Endozoicomonas arenosclerae	hypothetical protein [Endozoicomonas arenosclerae].	GCF_001562015.1
WP_172870236.1	TPR+GreAB-C*→	TPR+GreAB-C	233	-	Firmicutes	Bacillus velezensis	hypothetical protein, partial [Bacillus velezensis].	GCF_013267055.1
WP_172883554.1	TPR+GreAB-C+PIN*→	TPR+GreAB-C+PIN	1159	-	Gammaproteobacteria	Pseudomonas stutzeri	hypothetical protein [Pseudomonas stutzeri].	GCF_013267085.1
WP_173054693.1	TM+TPR+PIN*→ REase→	TM+TPR+PIN	1322	-	Actinobacteria	Phytohabitans houttuyneae	hypothetical protein [Phytohabitans houttuyneae].	GCF_011764425.1
WP_173520689.1	REase+TPR+TPR+GreAB-C+PIN*→	REase+TPR+TPR+GreAB-C+PIN	1357	-	Alphaproteobacteria	Ensifer sesbaniae	hypothetical protein [Ensifer sesbaniae].	GCF_013283665.1
WP_173522752.1	REase+TPR+GreAB-C+PIN*→	REase+TPR+GreAB-C+PIN	1313	-	Actinobacteria	Nonomuraea sp. NN258	hypothetical protein [Nonomuraea sp. NN258].	GCF_013283785.1
WP_173794050.1	NACHT→ X+PIN*→ HTH→?→?→ HTH→	X+PIN	1179	-	Firmicutes	Dorea formicigenerans	hypothetical protein [Dorea formicigenerans].	GCF_013301385.1
WP_173821757.1	X+PIN*→ HTH→?→?→ HTH→	X+PIN	1178	-	Firmicutes	Dorea formicigenerans	hypothetical protein [Dorea formicigenerans].	GCF_013301365.1
WP_173853982.1	TPR+GreAB-C+PIN→ PIN*→	PIN	248	-	Bacteroidetes	Flavobacterium sp. 28A	hypothetical protein [Flavobacterium sp. 28A].	GCF_013294005.1
WP_173968533.1	GreAB-C+PIN*→	GreAB-C+PIN	552	-	Bacteroidetes	Flavobacterium collinsii	hypothetical protein, partial [Flavobacterium collinsii].	GCF_902804485.1
WP_173994169.1	TPR+GreAB-C+PIN*→	TPR+GreAB-C+PIN	1181	-	Alphaproteobacteria	Agrobacterium fabrum	hypothetical protein [Agrobacterium fabrum].	GCF_013318355.1
WP_174011044.1	HEPN→ REase+TPR+TPR+GreAB-C+PIN*→	REase+TPR+TPR+GreAB-C+PIN	1333	-	Alphaproteobacteria	Agrobacterium rhizogenes	GreA/GreB family elongation factor [Agrobacterium rhizogenes].	GCF_013321105.1
WP_174166670.1	BirA-HTH→ HEPN→ REase+TPR+TPR+GreAB-C+PIN*→	REase+TPR+TPR+GreAB-C+PIN	1341	-	Alphaproteobacteria	Agrobacterium rhizogenes	hypothetical protein [Agrobacterium rhizogenes].	GCF_013322015.1
WP_174168420.1	BirA-HTH→ HEPN→ REase+TPR+TPR+GreAB-C+PIN*→	REase+TPR+TPR+GreAB-C+PIN	1333	-	Alphaproteobacteria	Agrobacterium rhizogenes	GreA/GreB family elongation factor [Agrobacterium rhizogenes].	GCF_013321875.1
WP_174361404.1	HetE-N1→?→ TPR+GreAB-C+PIN*→	TPR+GreAB-C+PIN	512	-	Gammaproteobacteria	Citrobacter gillenii	hypothetical protein [Citrobacter gillenii].	GCF_013337685.1
WP_175386063.1	PIN*→	PIN	434	-	Gammaproteobacteria	Pseudomonas corrugata	hypothetical protein [Pseudomonas corrugata].	GCF_013359545.1
WP_175666066.1	REase+TPR+GreAB-C+PIN*→	REase+TPR+GreAB-C+PIN	1351	-	Betaproteobacteria	Burkholderia ambifaria	hypothetical protein [Burkholderia ambifaria].	GCF_902829825.1
WP_175722904.1	REase+TPR+GreAB-C+PIN*→	REase+TPR+GreAB-C+PIN	1351	-	Betaproteobacteria	Burkholderia ambifaria	hypothetical protein [Burkholderia ambifaria].	GCF_902829925.1
WP_175818825.1	REase+TPR+GreAB-C+PIN*→	REase+TPR+GreAB-C+PIN	1335	-	Betaproteobacteria	Burkholderia sp. BCC0419	hypothetical protein [Burkholderia sp. BCC0419].	GCF_902830285.1
WP_175896466.1	TPR+GreAB-C+PIN*→||||<-HTH+HTH	TPR+GreAB-C+PIN	872	-	Betaproteobacteria	Burkholderia cepacia	hypothetical protein [Burkholderia cepacia].	GCF_902830675.1
WP_176170943.1	REase+TPR+GreAB-C+PIN*→	REase+TPR+GreAB-C+PIN	1244	-	Actinobacteria	Amycolatopsis sp. Hca4	DUF4365 domain-containing protein [Amycolatopsis sp. Hca4].	GCF_013364075.1
WP_176203105.1	TPR+GreAB-C+PIN*→	TPR+GreAB-C+PIN	681	-	Gammaproteobacteria	Salmonella enterica	hypothetical protein, partial [Salmonella enterica].	GCF_002044735.1
WP_176308009.1	REase+TPR+GreAB-C+PIN*→	REase+TPR+GreAB-C+PIN	1351	-	Betaproteobacteria	Burkholderia ambifaria	hypothetical protein [Burkholderia ambifaria].	GCF_902830205.1
WP_176329154.1	REase+TPR+GreAB-C+PIN*→	REase+TPR+GreAB-C+PIN	1335	-	Betaproteobacteria	Burkholderia vietnamiensis	hypothetical protein [Burkholderia vietnamiensis].	GCF_902833435.1
WP_176389972.1	REase+TPR+GreAB-C+PIN*→	REase+TPR+GreAB-C+PIN	1374	-	Betaproteobacteria	Janthinobacterium sp. BJB401	hypothetical protein [Janthinobacterium sp. BJB401].	GCF_013372745.1
WP_176422167.1	REase+TPR+TPR+GreAB-C+PIN*→	REase+TPR+TPR+GreAB-C+PIN	1325	-	Alphaproteobacteria	Bradyrhizobium sp. 2S1	hypothetical protein [Bradyrhizobium sp. 2S1].	GCF_011516625.2
WP_176457673.1	REase+TPR+GreAB-C+PIN*→	REase+TPR+GreAB-C+PIN	1516	-	Gammaproteobacteria	Pseudomonas mandelii	hypothetical protein [Pseudomonas mandelii].	GCF_000257545.3
WP_176459502.1	REase+TPR+GreAB-C+PIN*→?→ Trypsin→	REase+TPR+GreAB-C+PIN	1228	-	Actinobacteria	Rhodococcus sp. OK302	DUF4365 domain-containing protein [Rhodococcus sp. OK302].	GCF_002245895.1
WP_176508867.1	REase+TPR+GreAB-C+PIN*→	REase+TPR+GreAB-C+PIN	1548	-	Gammaproteobacteria	Pseudomonas sp. BML-PP042	hypothetical protein [Pseudomonas sp. BML-PP042].	GCF_013373915.1

<table>
<tr><th>acc</th><th>operon</th><th>architecture</th><th>len</th><th>gen.name</th><th>taxend</th><th>species</th><th>defline</th><th>gca</th></tr>
<tr><td>WP_177062188.1</td><td>REase+TPR+GreAB-C+PIN*→</td><td>REase+TPR+GreAB-C+PIN</td><td>1424</td><td>-</td><td>Gammaproteobacteria</td><td>Pseudomonas reactans</td><td>hypothetical protein [Pseudomonas reactans].</td><td>GCF_013386305.1</td></tr>
<tr><td>WP_177327801.1</td><td>REase+TPR+GreAB-C+PIN*→</td><td>REase+TPR+GreAB-C+PIN</td><td>1404</td><td>-</td><td>Gammaproteobacteria</td><td>Pseudomonas sp. 5</td><td>GreA/GreB family elongation factor [Pseudomonas sp. 5].</td><td>GCF_000955815.1</td></tr>
<tr><td>WP_179038946.1</td><td>REase+TPR+TPR+GreAB-C+PIN*→</td><td>REase+TPR+TPR+GreAB-C+PIN</td><td>1350</td><td>-</td><td>Alphaproteobacteria</td><td>Rhizobium leguminosarum</td><td>hypothetical protein [Rhizobium leguminosarum].</td><td>GCF_013391705.1</td></tr>
<tr><td>WP_179093684.1</td><td>TPR+GreAB-C+PIN*→||||<-HTH+HTH</td><td>TPR+GreAB-C+PIN</td><td>872</td><td>-</td><td>Betaproteobacteria</td><td>Burkholderia pseudomallei</td><td>hypothetical protein [Burkholderia pseudomallei].</td><td>GCF_002880985.1</td></tr>
<tr><td>WP_179103158.1</td><td>TPR+GreAB-C+PIN*→||||<-HTH+HTH</td><td>TPR+GreAB-C+PIN</td><td>872</td><td>-</td><td>Betaproteobacteria</td><td>Burkholderia pseudomallei</td><td>hypothetical protein [Burkholderia pseudomallei].</td><td>GCF_001981185.1</td></tr>
<tr><td>WP_179448733.1</td><td>-</td><td>-</td><td>914</td><td>-</td><td>Gammaproteobacteria</td><td>Pseudomonas yamanorum</td><td>hypothetical protein [Pseudomonas yamanorum].</td><td>GCF_013403585.1</td></tr>
<tr><td>WP_179622762.1</td><td>DOC+HTH→ REase+TOPC→?→ HetE-N1→ HetE-N1+TPR+GreAB-C+PIN*→</td><td>HetE-N1+TPR+GreAB-C+PIN</td><td>1124</td><td>-</td><td>Gammaproteobacteria</td><td>Pseudomonas mendocina</td><td>hypothetical protein [Pseudomonas mendocina].</td><td>GCF_013410555.1</td></tr>
<tr><td>WP_179654574.1</td><td>REase+TPR+TPR+GreAB-C+PIN*→ REase→</td><td>REase+TPR+TPR+GreAB-C+PIN</td><td>1329</td><td>-</td><td>Alphaproteobacteria</td><td>Mesorhizobium sp. YL-MPnR-2016</td><td>hypothetical protein [Mesorhizobium sp. YL-MPnR-2016].</td><td>GCF_013408905.1</td></tr>
<tr><td>WP_179833445.1</td><td>REase+TPR+PIN*→</td><td>REase+TPR+PIN</td><td>1241</td><td>-</td><td>Actinobacteria</td><td>Actinomadura citrea</td><td>DUF4365 domain-containing protein [Actinomadura citrea].</td><td>GCF_014648455.1</td></tr>
<tr><td>WP_179902819.1</td><td>HetE-N1→ TPR+GreAB-C+PIN*→</td><td>TPR+GreAB-C+PIN</td><td>1094</td><td>-</td><td>Gammaproteobacteria</td><td>Aeromonas veronii</td><td>hypothetical protein [Aeromonas veronii].</td><td>GCF_013415825.1</td></tr>
<tr><td>WP_180191279.1</td><td>HTH→ HetE-N1→ HetE-N1+GreAB-C+PIN*→</td><td>HetE-N1+GreAB-C+PIN</td><td>1077</td><td>-</td><td>Gammaproteobacteria</td><td>Acinetobacter sp. YH01006</td><td>hypothetical protein [Acinetobacter sp. YH01006].</td><td>GCF_013420445.1</td></tr>
<tr><td>WP_180269129.1</td><td>TPR+GreAB-C+PIN*→</td><td>TPR+GreAB-C+PIN</td><td>1231</td><td>-</td><td>Bacteroidetes</td><td>Sphingobacterium sp. 1.A.5</td><td>tetratricopeptide repeat protein [Sphingobacterium sp. 1.A.5].</td><td>GCF_002734235.1</td></tr>
<tr><td>WP_180491971.1</td><td>HetE-N1→ TPR+GreAB-C+PIN*→</td><td>TPR+GreAB-C+PIN</td><td>1106</td><td>-</td><td>Gammaproteobacteria</td><td>Escherichia fergusonii</td><td>hypothetical protein [Escherichia fergusonii].</td><td>GCF_903932165.1</td></tr>
<tr><td>WP_180778225.1</td><td>HetE-N1→ TPR+GreAB-C+PIN*→</td><td>TPR+GreAB-C+PIN</td><td>1117</td><td>-</td><td>Gammaproteobacteria</td><td>Pectobacterium aroidearum</td><td>hypothetical protein [Pectobacterium aroidearum].</td><td>GCF_013449715.1</td></tr>
<tr><td>WP_180799798.1</td><td>TPR+GreAB-C+PIN*→</td><td>TPR+GreAB-C+PIN</td><td>796</td><td>-</td><td>Gammaproteobacteria</td><td>Vibrio parahaemolyticus</td><td>hypothetical protein, partial [Vibrio parahaemolyticus].</td><td>GCF_006371865.1</td></tr>
<tr><td>WP_180830729.1</td><td>TPR+GreAB-C+PIN*→</td><td>TPR+GreAB-C+PIN</td><td>764</td><td>-</td><td>Gammaproteobacteria</td><td>Vibrio parahaemolyticus</td><td>hypothetical protein, partial [Vibrio parahaemolyticus].</td><td>GCF_006375165.1</td></tr>
<tr><td>WP_180977515.1</td><td>TPR+GreAB-C+PIN*→</td><td>TPR+GreAB-C+PIN</td><td>1154</td><td>-</td><td>Betaproteobacteria</td><td>Janthinobacterium sp. ROICE36</td><td>hypothetical protein, partial [Janthinobacterium sp. ROICE36].</td><td>GCF_002869965.1</td></tr>
<tr><td>WP_181464939.1</td><td>TPR+GreAB-C+PIN*→</td><td>TPR+GreAB-C+PIN</td><td>762</td><td>-</td><td>Betaproteobacteria</td><td>Herbaspirillum rubrisubalbicans</td><td>tetratricopeptide repeat protein [Herbaspirillum rubrisubalbicans].</td><td>GCF_003263475.1</td></tr>
<tr><td>WP_181641518.1</td><td>TPR+PIN*→</td><td>TPR+PIN</td><td>1210</td><td>-</td><td>Actinobacteria</td><td>Nocardioides massiliensis</td><td>hypothetical protein [Nocardioides massiliensis].</td><td>GCF_900067705.2</td></tr>
<tr><td>WP_181834083.1</td><td>TPR+GreAB-C+PIN*→</td><td>TPR+GreAB-C+PIN</td><td>678</td><td>-</td><td>Alphaproteobacteria</td><td>Brucella anthropi</td><td>hypothetical protein, partial [Brucella anthropi].</td><td>GCF_003325675.1</td></tr>
<tr><td>WP_182064218.1</td><td>TPR+PIN*→</td><td>TPR+PIN</td><td>1177</td><td>-</td><td>Actinobacteria</td><td>Curtobacterium sp. ME12</td><td>hypothetical protein [Curtobacterium sp. ME12].</td><td>GCF_903970855.1</td></tr>
<tr><td>WP_182166026.1</td><td>REase+TPR+GreAB-C+PIN*→||||<-HTH</td><td>REase+TPR+GreAB-C+PIN</td><td>1304</td><td>-</td><td>Betaproteobacteria</td><td>Duganella sp. LX20W</td><td>hypothetical protein [Duganella sp. LX20W].</td><td>GCF_014042345.1</td></tr>
<tr><td>WP_182270399.1</td><td>HetE-N1→ TPR+GreAB-C+PIN*→</td><td>TPR+GreAB-C+PIN</td><td>1092</td><td>-</td><td>Gammaproteobacteria</td><td>Citrobacter freundii</td><td>hypothetical protein [Citrobacter freundii].</td><td>GCF_014101305.1</td></tr>
<tr><td>WP_182650750.1</td><td>GreAB-C+PIN*→</td><td>GreAB-C+PIN</td><td>566</td><td>-</td><td>Bacteroidetes</td><td>Flavobacterium sp. SOK18b</td><td>hypothetical protein, partial [Flavobacterium sp. SOK18b].</td><td>GCF_014156605.1</td></tr>
<tr><td>WP_182869571.1</td><td>PIN*→</td><td>PIN</td><td>188</td><td>-</td><td>Alphaproteobacteria</td><td>Bradyrhizobium diazoefficiens</td><td>hypothetical protein [Bradyrhizobium diazoefficiens].</td><td>GCF_014163475.1</td></tr>
<tr><td>WP_182925384.1</td><td>REase+TPR+GreAB-C+PIN*→||||<-?||HTH→</td><td>REase+TPR+GreAB-C+PIN</td><td>1250</td><td>-</td><td>Gammaproteobacteria</td><td>Acinetobacter baumannii</td><td>GreA/GreB family elongation factor [Acinetobacter baumannii].</td><td>GCF_014169655.1</td></tr>
<tr><td>WP_183061688.1</td><td>CARF+PIN*→</td><td>CARF+PIN</td><td>1208</td><td>-</td><td>Actinobacteria</td><td>Motilibacter peucedani</td><td>hypothetical protein [Motilibacter peucedani].</td><td>GCF_003634695.1</td></tr>
<tr><td>WP_183296812.1</td><td>REase+TPR+GreAB-C+PIN*→</td><td>REase+TPR+GreAB-C+PIN</td><td>1234</td><td>-</td><td>Actinobacteria</td><td>Cellulomonas cellasea</td><td>DUF4365 domain-containing protein [Cellulomonas cellasea].</td><td>GCF_014190965.1</td></tr>
<tr><td>WP_183420610.1</td><td>REase+TPR+GreAB-C+PIN*→</td><td>REase+TPR+GreAB-C+PIN</td><td>1219</td><td>-</td><td>Actinobacteria</td><td>Microbacterium proteolyticum</td><td>DUF4365 domain-containing protein [Microbacterium proteolyticum].</td><td>GCF_014192415.1</td></tr>
<tr><td>WP_183438886.1</td><td>TPR+GreAB-C+PIN*→</td><td>TPR+GreAB-C+PIN</td><td>1161</td><td>-</td><td>Alphaproteobacteria</td><td>Methylobacterium sp. R2-1</td><td>hypothetical protein [Methylobacterium sp. R2-1].</td><td>GCF_014191355.1</td></tr>
<tr><td>WP_183694785.1</td><td>REase+TPR+TPR+GreAB-C+PIN*→</td><td>REase+TPR+TPR+GreAB-C+PIN</td><td>1363</td><td>-</td><td>Alphaproteobacteria</td><td>Rhizobium lusitanum</td><td>restriction endonuclease [Rhizobium lusitanum].</td><td>GCF_014189535.1</td></tr>
<tr><td>WP_183714092.1</td><td>TIR→?→ TPR+GreAB-C+PIN*→</td><td>TPR+GreAB-C+PIN</td><td>1131</td><td>-</td><td>Alphaproteobacteria</td><td>Bradyrhizobium sp. ERR14</td><td>hypothetical protein [Bradyrhizobium sp. ERR14].</td><td>GCF_014198235.1</td></tr>
<tr><td>WP_183806882.1</td><td>REase+TPR+TPR+GreAB-C+PIN*→</td><td>REase+TPR+TPR+GreAB-C+PIN</td><td>1345</td><td>-</td><td>Alphaproteobacteria</td><td>Rhizobium azooxidifex</td><td>tetratricopeptide repeat protein [Rhizobium azooxidifex].</td><td>GCF_014196765.1</td></tr>
<tr><td>WP_183919890.1</td><td>REase+TPR+GreAB-C+PIN*→</td><td>REase+TPR+GreAB-C+PIN</td><td>1290</td><td>-</td><td>Alphaproteobacteria</td><td>Rhizobium lentis</td><td>hypothetical protein [Rhizobium lentis].</td><td>GCF_014200135.1</td></tr>
<tr><td>WP_183998882.1</td><td>REase+TPR+GreAB-C+PIN*→</td><td>REase+TPR+GreAB-C+PIN</td><td>1313</td><td>-</td><td>Alphaproteobacteria</td><td>Sphingomonas kyeonggiensis</td><td>hypothetical protein [Sphingomonas kyeonggiensis].</td><td>GCF_014196745.1</td></tr>
<tr><td>WP_184249301.1</td><td>REase+TPR+GreAB-C+PIN*→</td><td>REase+TPR+GreAB-C+PIN</td><td>1318</td><td>-</td><td>Betaproteobacteria</td><td>Paraburkholderia atlantica</td><td>DUF4365 domain-containing protein [Paraburkholderia atlantica].</td><td>GCF_014200895.1</td></tr>
</table>

acc	operon	architecture	len	gen.name	taxend	species	defline	gca
WP_184301652.1	REase+TPR+GreAB-C+PIN*→?→?→ URI+RAMA→	REase+TPR+GreAB-C+PIN	1255	-	Planctomycetes	Rhodopirellula rubra	hypothetical protein [Rhodopirellula rubra].	GCF_014192335.1
WP_184659486.1	TPR+GreAB-C+PIN*→	TPR+GreAB-C+PIN	1265	-	Bacteroidetes	Pedobacter cryoconitis	hypothetical protein [Pedobacter cryoconitis].	GCF_014207145.1
WP_184790984.1	TM+TPR+PIN*→	TM+TPR+PIN	1158	-	Actinobacteria	Phytomonospora endophytica	hypothetical protein [Phytomonospora endophytica].	GCF_016862855.1
WP_184920984.1	REase+PIN*→	REase+PIN	1270	-	Actinobacteria	Saccharothrix ecbatanensis	DUF4365 domain-containing protein [Saccharothrix ecbatanensis].	GCF_014205015.1
WP_185002860.1	-	-	1209	-	Actinobacteria	unclassified Curtobacterium	MULTISPECIES: DUF4365 domain-containing protein [unclassified Curtobacterium].	GCF_003752345.1
WP_186065300.1	REase+TPR+GreAB-C+PIN*→	REase+TPR+GreAB-C+PIN	1351	-	Betaproteobacteria	Burkholderia gladioli	hypothetical protein [Burkholderia gladioli].	GCF_902831285.1
WP_186083524.1	REase+TPR+GreAB-C+PIN*→||||<-?<-?<-HEPN	REase+TPR+GreAB-C+PIN	1333	-	Betaproteobacteria	Burkholderia gladioli	hypothetical protein [Burkholderia gladioli].	GCF_902831185.1
WP_186117779.1	HEPN→?→?→ REase+TPR+GreAB-C+PIN*→	REase+TPR+GreAB-C+PIN	1498	-	Betaproteobacteria	Burkholderia gladioli	tetratricopeptide repeat protein [Burkholderia gladioli].	GCF_902831505.1
WP_186135768.1	REase+TPR+GreAB-C+PIN*→||||<-HEPN	REase+TPR+GreAB-C+PIN	1351	-	Betaproteobacteria	Burkholderia gladioli	hypothetical protein [Burkholderia gladioli].	GCF_902831685.1
WP_186296902.1	REase+TPR+GreAB-C+PIN*→	REase+TPR+GreAB-C+PIN	1297	-	Betaproteobacteria	Cupriavidus campinensis	DUF4365 domain-containing protein [Cupriavidus campinensis].	GCF_007572485.1
WP_186384760.1	HetE-N1→ TPR+GreAB-C+PIN*→	TPR+GreAB-C+PIN	1117	-	Gammaproteobacteria	Klebsiella sp. Kpp	hypothetical protein [Klebsiella sp. Kpp].	GCF_014267405.1
WP_186426553.1	TPR+GreAB-C+PIN*→	TPR+GreAB-C+PIN	1039	-	Betaproteobacteria	Cupriavidus metallidurans	hypothetical protein [Cupriavidus metallidurans].	GCF_900185755.1
WP_187028576.1	TPR+GreAB-C+PIN*→	TPR+GreAB-C+PIN	1238	-	Bacteroidetes	Pontibacter sp. KCTC 32443	hypothetical protein [Pontibacter sp. KCTC 32443].	GCF_014297265.1
WP_187110408.1	REase+TPR+GreAB-C+PIN*→	REase+TPR+GreAB-C+PIN	1301	-	Betaproteobacteria	Variovorax sp. PAMC26660	hypothetical protein [Variovorax sp. PAMC26660].	GCF_014302995.1
WP_187305744.1	X+PIN*→ HTH→?→?→ HTH→	X+PIN	762	-	Firmicutes	Dorea formicigenerans	hypothetical protein [Dorea formicigenerans].	GCF_003458845.1
WP_187328643.1	HTH+PNPase+TPR+GreAB-C+PIN*→	HTH+PNPase+TPR+GreAB-C+PIN	1523	-	Actinobacteria	Rhodococcus sp. WS3	hypothetical protein [Rhodococcus sp. WS3].	GCF_003797085.1
WP_187494340.1	HetE-N1→ TPR+GreAB-C+PIN*→	TPR+GreAB-C+PIN	1094	-	Gammaproteobacteria	Pantoea agglomerans	hypothetical protein [Pantoea agglomerans].	GCF_014353705.1
WP_187598162.1	REase+TPR+GreAB-C+PIN*→	REase+TPR+GreAB-C+PIN	1341	-	Betaproteobacteria	Diaphorobacter ruginosibacter	hypothetical protein [Diaphorobacter ruginosibacter].	GCF_014395975.1
WP_187723864.1	TPR+GreAB-C+PIN*→	TPR+GreAB-C+PIN	545	-	Betaproteobacteria	Diaphorobacter aerolatus	hypothetical protein [Diaphorobacter aerolatus].	GCF_014489535.1
WP_187993242.1	TPR+GreAB-C+PIN*→	TPR+GreAB-C+PIN	557	-	Gammaproteobacteria	Vibrio harveyi	hypothetical protein [Vibrio harveyi].	GCF_014525195.1
WP_188104481.1	REase+TPR+GreAB-C+PIN*→	REase+TPR+GreAB-C+PIN	1312	-	Alphaproteobacteria	Bradyrhizobium campsiandrae	tetratricopeptide repeat protein [Bradyrhizobium campsiandrae].	GCF_014529705.1
WP_188533557.1	TPR+PIN*→	TPR+PIN	1228	-	Firmicutes	Paenibacillus abyssi	hypothetical protein [Paenibacillus abyssi].	GCF_014640295.1
WP_189032568.1	TPR+PIN*→	TPR+PIN	531	-	Firmicutes	Paenibacillus albidus	hypothetical protein [Paenibacillus albidus].	GCF_014644435.1
WP_189091600.1	REase+TPR+GreAB-C+PIN*→	REase+TPR+GreAB-C+PIN	1377	-	Deinococci	Deinococcus ruber	tetratricopeptide repeat protein [Deinococcus ruber].	GCF_014648095.1
WP_189152287.1	TPR+PIN*→	TPR+PIN	1218	-	Actinobacteria	Streptomyces lacrimifluminis	hypothetical protein [Streptomyces lacrimifluminis].	GCF_014646095.1
WP_189484308.1	HetE-N1→ TPR+GreAB-C+PIN*→	TPR+GreAB-C+PIN	1026	-	Gammaproteobacteria	Shewanella indica	hypothetical protein [Shewanella indica].	GCF_014652115.1
WP_189568857.1	Mbetalac→ TPR+GreAB-C+PIN*→	TPR+GreAB-C+PIN	1342	-	Bacteroidetes	Persicitalea jodogahamensis	hypothetical protein [Persicitalea jodogahamensis].	GCF_014651615.1
WP_189617689.1	HetE-N1→?→ GreAB-C*→	GreAB-C	498	-	Gammaproteobacteria	Shewanella fodinae	hypothetical protein [Shewanella fodinae].	GCF_014651955.1
WP_189635482.1	REase+TPR+GreAB-C+PIN*→	REase+TPR+GreAB-C+PIN	1307	-	Alphaproteobacteria	Gemmobacter lanyuensis	restriction endonuclease [Gemmobacter lanyuensis].	GCF_014652355.1
WP_189677864.1	REase+TPR+GreAB-C+PIN*→	REase+TPR+GreAB-C+PIN	1323	-	Alphaproteobacteria	Sphingomonas glacialis	tetratricopeptide repeat protein [Sphingomonas glacialis].	GCF_014653575.1
WP_189835257.1	TPR+PIN*→	TPR+PIN	1215	-	Actinobacteria	Streptomyces zaomyceticus	hypothetical protein [Streptomyces zaomyceticus].	GCF_014656215.1
WP_190462433.1	PNPase+TPR+GreAB-C+PIN*→ TPR+TPR→ TPR+TPR→	PNPase+TPR+GreAB-C+PIN	1385	-	Cyanobacteria	unclassified Planktothrix	MULTISPECIES: hypothetical protein [unclassified Planktothrix].	GCF_014696265.1
WP_190659344.1	BTLCP+NACHT→ APATPase+BetaPropeller→ APATPase+BetaPropeller→ PNPase+TPR+GreAB-C+PIN*→	PNPase+TPR+GreAB-C+PIN	1399	-	Cyanobacteria	Nostoc linckia	tetratricopeptide repeat protein [Nostoc linckia].	GCF_014696615.1
WP_190786133.1	<-REase+nSTAND3||TPR+GreAB-C+PIN*→	TPR+GreAB-C+PIN	1282	-	Bacteroidetes	Hymenobacter sp. BT646	hypothetical protein [Hymenobacter sp. BT646].	GCF_014698995.1
WP_191117766.1	HetE-N1→ TPR+GreAB-C+PIN*→	TPR+GreAB-C+PIN	1091	-	Gammaproteobacteria	Vibrio campbellii	hypothetical protein [Vibrio campbellii].	GCF_014769105.1
WP_191340685.1	REase+SNF2→ URI+RAMA→ TPR+GreAB-C+PIN*→	TPR+GreAB-C+PIN	1182	-	Firmicutes	Massilimicrobiota sp. An134	hypothetical protein [Massilimicrobiota sp. An134].	GCF_904393125.1
WP_191375963.1	TPR+GreAB-C+PIN*→	TPR+GreAB-C+PIN	879	-	Firmicutes	[Clostridium] spiroforme	hypothetical protein [[Clostridium] spiroforme].	GCF_904395875.1

acc	operon	architecture	len	gen.name	taxend	species	defline	gca
WP_191791215.1	TPR+PIN*→	TPR+PIN	1225	-	Actinobacteria	Oerskovia sp. Sa2CUA8	hypothetical protein [Oerskovia sp. Sa2CUA8].	GCF_014836745.1
WP_191917190.1	HetE-N1→ TPR+GreAB-C+PIN*→	TPR+GreAB-C+PIN	1094	-	Gammaproteobacteria	Pantoea agglomerans	hypothetical protein [Pantoea agglomerans].	GCF_014839545.1
WP_191940182.1	X+PIN*→	X+PIN	1078	-	Actinobacteria	Plantibacter sp. CFBP 8775	hypothetical protein [Plantibacter sp. CFBP 8775].	GCF_014839505.1
WP_191950701.1	REase+TPR+GreAB-C+PIN*→	REase+TPR+GreAB-C+PIN	1485	-	Gammaproteobacteria	Pseudomonas fluorescens	hypothetical protein [Pseudomonas fluorescens].	GCF_014839025.1
WP_191971068.1	TPR+GreAB-C+PIN*→	TPR+GreAB-C+PIN	525	-	Alphaproteobacteria	Methylobacterium soli	hypothetical protein [Methylobacterium soli].	GCF_008806385.1
WP_191999584.1	REase+TPR+GreAB-C+PIN*→||||<-?<-EAD2	REase+TPR+GreAB-C+PIN	1036	-	Gammaproteobacteria	Pseudomonas coleopterorum	hypothetical protein [Pseudomonas coleopterorum].	GCF_014841305.1
WP_192069209.1	HetE-N1→ TPR+GreAB-C+PIN*→	TPR+GreAB-C+PIN	1105	-	Gammaproteobacteria	Pseudomonas coleopterorum	hypothetical protein [Pseudomonas coleopterorum].	GCF_014842375.1
WP_192163653.1	REase+TPR+GreAB-C+PIN*→	REase+TPR+GreAB-C+PIN	1250	-	Gammaproteobacteria	Rhodanobacter sp. DHG33	hypothetical protein [Rhodanobacter sp. DHG33].	GCF_014843005.1
WP_192260260.1	REase+TPR+GreAB-C+PIN*→	REase+TPR+GreAB-C+PIN	1441	-	Gammaproteobacteria	Pseudomonas sp. Pdm06	hypothetical protein [Pseudomonas sp. Pdm06].	GCF_014851795.1
WP_192264655.1	REase+TPR+GreAB-C+PIN*→	REase+TPR+GreAB-C+PIN	1316	-	Alphaproteobacteria	Komagataeibacter hansenii	hypothetical protein [Komagataeibacter hansenii].	GCF_014843995.1
WP_192393207.1	HetE-N1→ TPR+GreAB-C+PIN*→	TPR+GreAB-C+PIN	1117	-	Gammaproteobacteria	Methylomonas sp. EbB	hypothetical protein [Methylomonas sp. EbB].	GCF_903064685.1
WP_192433772.1	HEPN→ REase+TPR+TPR+GreAB-C+PIN*→	REase+TPR+TPR+GreAB-C+PIN	1339	-	Alphaproteobacteria	Ensifer sp. ENS11	hypothetical protein [Ensifer sp. ENS11].	GCF_014851895.1
WP_192504527.1	HetE-N1→ TPR+GreAB-C+PIN*→	TPR+GreAB-C+PIN	1119	-	Gammaproteobacteria	Rouxiella badensis	hypothetical protein [Rouxiella badensis].	GCF_014859005.1
WP_192836185.1	HetE-N1→ TPR+GreAB-C+PIN*→	TPR+GreAB-C+PIN	1092	-	Gammaproteobacteria	Acinetobacter oleivorans	hypothetical protein [Acinetobacter oleivorans].	GCF_014876825.1
WP_193132365.1	HetE-N1→ TPR+GreAB-C+PIN+TM+TM*→	TPR+GreAB-C+PIN+TM+TM	1086	-	Gammaproteobacteria	Enterobacter hormaechei	hypothetical protein, partial [Enterobacter hormaechei].	GCF_014901495.1
WP_193343548.1	HetE-N1→ TPR+GreAB-C+PIN*→?→ PhospholipaseD→	TPR+GreAB-C+PIN	1103	-	Gammaproteobacteria	Halomonas elongata	hypothetical protein [Halomonas elongata].	GCF_014926355.1
WP_193426153.1	TPR+PIN*→	TPR+PIN	1217	-	Firmicutes	Priestia aryabhattai	hypothetical protein [Priestia aryabhattai].	GCF_014932885.1
WP_193843312.1	HetE-N1→ TPR+GreAB-C+PIN*→	TPR+GreAB-C+PIN	1090	-	Gammaproteobacteria	Serratia marcescens	hypothetical protein [Serratia marcescens].	GCF_015160915.1
WP_194030396.1	PNPase+TPR+GreAB-C+PIN*→	PNPase+TPR+GreAB-C+PIN	1392	-	Cyanobacteria	Lusitaniella coriacea	hypothetical protein [Lusitaniella coriacea].	GCF_015207425.1
WP_194552914.1	TPR+GreAB-C+PIN*→	TPR+GreAB-C+PIN	1236	-	Gammaproteobacteria	Vibrio anguillarum	hypothetical protein [Vibrio anguillarum].	GCF_015343355.1
WP_194640651.1	TPR+GreAB-C+PIN*→	TPR+GreAB-C+PIN	1123	-	Bacteroidetes	Flavobacterium sp. HJJ	hypothetical protein [Flavobacterium sp. HJJ].	GCF_015351475.1
WP_194689942.1	REase+TPR+GreAB-C+PIN*→	REase+TPR+GreAB-C+PIN	1323	-	Alphaproteobacteria	unclassified Gluconobacter	MULTISPECIES: hypothetical protein [unclassified Gluconobacter].	GCF_015353155.1
WP_194782158.1	TPR+PIN*→	TPR+PIN	1183	-	Actinobacteria	Arthrobacter gandavensis	hypothetical protein [Arthrobacter gandavensis].	GCF_015355785.1
WP_194849029.1	HetE-N1→ TPR+GreAB-C+PIN*→	TPR+GreAB-C+PIN	1094	-	Gammaproteobacteria	Escherichia coli	hypothetical protein [Escherichia coli].	GCF_015356625.1
WP_194908878.1	TPR+PIN*→	TPR+PIN	1322	-	Actinobacteria	Catenulispora rubra	hypothetical protein [Catenulispora rubra].	GCF_015356825.1
WP_195550768.1	TM+TM+TPR+PIN*→	TM+TM+TPR+PIN	1183	-	Bacteroidetes	Bacteroides uniformis	hypothetical protein [Bacteroides uniformis].	GCF_015557045.1
WP_195789843.1	REase+TPR+GreAB-C+PIN*→	REase+TPR+GreAB-C+PIN	1310	-	Alphaproteobacteria	Bradyrhizobium genosp. L	hypothetical protein [Bradyrhizobium genosp. L].	GCF_015624485.1
WP_196103949.1	TPR+GreAB-C+PIN*→	TPR+GreAB-C+PIN	468	-	Alphaproteobacteria	Pontivivens sp. MT2928	hypothetical protein [Pontivivens sp. MT2928].	GCF_015679265.1
WP_196165874.1	TPR+GreAB-C+PIN*→	TPR+GreAB-C+PIN	1139	-	Gammaproteobacteria	Pseudomonas monteilii	hypothetical protein [Pseudomonas monteilii].	GCF_015680735.1
WP_196184162.1	REase+TPR+GreAB-C+PIN*→	REase+TPR+GreAB-C+PIN	1545	-	Gammaproteobacteria	Pseudomonas asiatica	hypothetical protein [Pseudomonas asiatica].	GCF_015680805.1
WP_196343881.1	HetE-N1→ TPR+GreAB-C+PIN*→	TPR+GreAB-C+PIN	1106	-	Gammaproteobacteria	Enterobacter hormaechei	hypothetical protein [Enterobacter hormaechei].	GCF_015704145.1
WP_196402364.1	REase+TPR+TPR+GreAB-C+PIN*→	REase+TPR+TPR+GreAB-C+PIN	1304	-	Alphaproteobacteria	Methylocystis sp. H62	hypothetical protein [Methylocystis sp. H62].	GCF_015709515.1
WP_197022618.1	REase+TPR+GreAB-C+PIN*→	REase+TPR+GreAB-C+PIN	1388	-	Betaproteobacteria	Comamonas aquatica	hypothetical protein [Comamonas aquatica].	GCF_000590985.1
WP_197265512.1	TPR+PIN*→	TPR+PIN	1231	-	Firmicutes	Fictibacillus sp. 23RED33	hypothetical protein [Fictibacillus sp. 23RED33].	GCF_015903925.1
WP_197283942.1	GreAB-C+PIN*→	GreAB-C+PIN	553	-	Alphaproteobacteria	Rhizobium acidisoli	hypothetical protein, partial [Rhizobium acidisoli].	GCF_001297245.1
WP_197310646.1	REase+TPR+TPR+GreAB-C+PIN*→	REase+TPR+TPR+GreAB-C+PIN	1326	-	Alphaproteobacteria	Methylobrevis sp. L22	hypothetical protein [Methylobrevis sp. L22].	GCF_015904235.1
WP_197451745.1	REase+TPR+GreAB-C+PIN*→	REase+TPR+GreAB-C+PIN	1195	-	Planctomycetes	Rosistilla oblonga	GreA/GreB family elongation factor [Rosistilla oblonga].	GCF_007751715.1
WP_197873110.1	TPR+GreAB-C+PIN*→	TPR+GreAB-C+PIN	1139	-	Gammaproteobacteria	Pseudomonas lutea	hypothetical protein [Pseudomonas lutea].	GCF_016009245.1
WP_198113276.1	TPR+GreAB-C+PIN*→||||<-HTH+HTH	TPR+GreAB-C+PIN	872	-	Betaproteobacteria	Burkholderia contaminans	hypothetical protein [Burkholderia contaminans].	GCF_016092105.1
WP_198215548.1	HetE-N1→ TPR+GreAB-C+PIN*→	TPR+GreAB-C+PIN	1126	-	Gammaproteobacteria	Gilliamella sp. M0320	hypothetical protein [Gilliamella sp. M0320].	GCF_016101635.1
WP_198575560.1	REase+TPR+GreAB-C+PIN*→	REase+TPR+GreAB-C+PIN	1332	-	Alphaproteobacteria	Caulobacter hibisci	DUF4365 domain-containing protein [Caulobacter hibisci].	GCF_016135805.1
WP_198597141.1	HetE-N1→?→ GreAB-C*→	GreAB-C	475	-	Gammaproteobacteria	Vibrio splendidus	hypothetical protein [Vibrio splendidus].	GCF_002877525.1
WP_198628691.1	TPR+GreAB-C+PIN*→	TPR+GreAB-C+PIN	1231	-	Bacteroidetes	Salegentibacter sp. F63223	hypothetical protein [Salegentibacter sp. F63223].	GCF_016236915.1
WP_198694995.1	REase+TPR+GreAB-C+PIN*→	REase+TPR+GreAB-C+PIN	1436	-	Gammaproteobacteria	Pseudomonas viridiflava	hypothetical protein [Pseudomonas viridiflava].	GCF_016308365.1

acc	operon	architecture	len	gen.name	taxend	species	defline	gca
WP_198710862.1	REase+TPR+GreAB-C+PIN*→	REase+TPR+GreAB-C+PIN	1321	-	Gammaproteobacteria	Pseudomonas syringae	DUF4365 domain-containing protein [Pseudomonas syringae].	GCF_016308175.1
WP_198739586.1	HetE-N1→ TPR+GreAB-C+PIN*→	TPR+GreAB-C+PIN	1106	-	Gammaproteobacteria	Enterobacter hormaechei	hypothetical protein [Enterobacter hormaechei].	GCF_016342225.1
WP_198742183.1	HetE-N1→ TPR+GreAB-C+PIN*→	TPR+GreAB-C+PIN	1106	-	Gammaproteobacteria	Enterobacter hormaechei	hypothetical protein [Enterobacter hormaechei].	GCF_016342135.1
WP_198830693.1	REase+TPR+GreAB-C+PIN*→	REase+TPR+GreAB-C+PIN	1441	-	Gammaproteobacteria	Pseudomonas sp. MF5691	hypothetical protein [Pseudomonas sp. MF5691].	GCF_016405645.1
WP_198862330.1	REase+TPR+GreAB-C+PIN*→	REase+TPR+GreAB-C+PIN	1324	-	Gammaproteobacteria	Pseudomonas carnis	DUF4365 domain-containing protein [Pseudomonas carnis].	GCF_016405215.1
WP_199041247.1	REase+TPR+GreAB-C+PIN*→	REase+TPR+GreAB-C+PIN	1250	-	Gammaproteobacteria	Dyella sp. ASV24	hypothetical protein [Dyella sp. ASV24].	GCF_016428905.1
WP_199059400.1	REase+TPR+TPR+GreAB-C+PIN*→	REase+TPR+TPR+GreAB-C+PIN	1334	-	Alphaproteobacteria	Brevundimonas sp. ASV9	hypothetical protein [Brevundimonas sp. ASV9].	GCF_016429195.1
WP_199059891.1	REase+TPR+GreAB-C+PIN*→	REase+TPR+GreAB-C+PIN	1302	-	Alphaproteobacteria	Brevundimonas sp. ASV9	hypothetical protein [Brevundimonas sp. ASV9].	GCF_016429195.1
WP_199133123.1	TPR+GreAB-C+PIN*→	TPR+GreAB-C+PIN	1258	-	Bacteroidetes	Pedobacter sp. ASV17	hypothetical protein [Pedobacter sp. ASV17].	GCF_016429065.1
WP_199161798.1	TPR+GreAB-C+PIN*→	TPR+GreAB-C+PIN	1140	-	Bacteroidetes	Elizabethkingia sp. ASV34	hypothetical protein [Elizabethkingia sp. ASV34].	GCF_016428745.1
WP_199216499.1	TPR+PIN*→	TPR+PIN	1215	-	Actinobacteria	Streptomyces sp. I5	hypothetical protein [Streptomyces sp. I5].	GCF_016432735.1
WP_199272510.1	REase+TPR+GreAB-C+PIN*→	REase+TPR+GreAB-C+PIN	1359	-	Betaproteobacteria	Paraburkholderia acidisoli	hypothetical protein [Paraburkholderia acidisoli].	GCF_009789675.1
WP_199515964.1	TM+TPR+PIN*→	TM+TPR+PIN	1145	-	Actinobacteria	Pseudosporangium sp. NEAU-24	tetratricopeptide repeat protein [Pseudosporangium sp. NEAU-24].	GCF_016464385.1
WP_199591321.1	REase+TPR+GreAB-C+PIN*→	REase+TPR+GreAB-C+PIN	1505	-	Betaproteobacteria	Pelomonas sp. OAE585	hypothetical protein [Pelomonas sp. OAE585].	GCF_016467315.1
WP_199660563.1	TPR+GreAB-C+PIN*→	TPR+GreAB-C+PIN	1035	-	Gammaproteobacteria	Klebsiella quasipneumoniae	hypothetical protein, partial [Klebsiella quasipneumoniae].	GCF_016491635.1
WP_199707330.1	REase→ PIN*→	PIN	932	-	Actinobacteria	Antrihabitans sp. YC3-6	hypothetical protein [Antrihabitans sp. YC3-6].	GCF_016482825.1
WP_199727385.1	REase+TPR+GreAB-C+PIN*→	REase+TPR+GreAB-C+PIN	1488	-	Gammaproteobacteria	Pseudomonas syringae group genomosp. 7	hypothetical protein [Pseudomonas syringae group genomosp. 7].	GCF_003703295.1
WP_199743158.1	REase+TPR+GreAB-C+PIN*→	REase+TPR+GreAB-C+PIN	1009	-	Alphaproteobacteria	Sphingomonas ginsenosidivorax	tetratricopeptide repeat protein [Sphingomonas ginsenosidivorax].	GCF_007995065.1
WP_199871858.1	HetE-N1→ HetE-N1+TPR+GreAB-C+PIN*→	HetE-N1+TPR+GreAB-C+PIN	1088	-	Gammaproteobacteria	Vibrio anguillarum	hypothetical protein [Vibrio anguillarum].	GCF_015350415.1
WP_200204777.1	HetE-N1→ TPR+GreAB-C+PIN*→	TPR+GreAB-C+PIN	1116	-	Gammaproteobacteria	Marinobacter sp. 1-3A	hypothetical protein [Marinobacter sp. 1-3A].	GCF_016597775.1
WP_200282902.1	TPR+PIN*→	TPR+PIN	1263	-	Verrucomicrobia	Haloferula rosea	hypothetical protein [Haloferula rosea].	GCF_016595525.1
WP_200597934.1	HetE-N1→ TPR+GreAB-C+PIN*→	TPR+GreAB-C+PIN	1112	-	Gammaproteobacteria	Pseudomonas stutzeri	hypothetical protein [Pseudomonas stutzeri].	GCF_016622345.1
WP_200602419.1	REase+TPR+GreAB-C+PIN*→	REase+TPR+GreAB-C+PIN	1441	-	Gammaproteobacteria	Pseudomonas sp. S44	hypothetical protein [Pseudomonas sp. S44].	GCF_016624805.1
WP_201009307.1	REase+PIN*→	REase+PIN	1247	-	Actinobacteria	Rhodococcus sp. NKCM2511	DUF4365 domain-containing protein [Rhodococcus sp. NKCM2511].	GCF_014905595.1
WP_201090676.1	HetE-N1→ TPR+GreAB-C+PIN*→	TPR+GreAB-C+PIN	1083	-	Gammaproteobacteria	Entomomonas sp. F2A	hypothetical protein [Entomomonas sp. F2A].	GCF_016653615.1
WP_201420192.1	REase+TPR+GreAB-C+PIN*→	REase+TPR+GreAB-C+PIN	1442	-	Gammaproteobacteria	Pseudomonas syringae	hypothetical protein [Pseudomonas syringae].	GCF_016694755.2
WP_201621921.1	REase+TPR+GreAB-C+PIN*→	REase+TPR+GreAB-C+PIN	1244	-	Gammaproteobacteria	Psychrobacter urativorans	hypothetical protein [Psychrobacter urativorans].	GCF_904846695.1
WP_201915404.1	REase+TPR+GreAB-C+PIN*→	REase+TPR+GreAB-C+PIN	1321	-	Gammaproteobacteria	Pseudomonas fluorescens	DUF4365 domain-containing protein [Pseudomonas fluorescens].	GCF_016728065.1
WP_201921162.1	HetE-N1→ TPR+GreAB-C+PIN*→	TPR+GreAB-C+PIN	1094	-	Gammaproteobacteria	Aeromonas caviae	hypothetical protein [Aeromonas caviae].	GCF_016729435.1
WP_201996904.1	HetE-N1→ MNS-Npun2340+TPR+GreAB-C+PIN*→	MNS-Npun2340+TPR+GreAB-C+PIN	1094	-	Gammaproteobacteria	Aeromonas caviae	hypothetical protein [Aeromonas caviae].	GCF_016728955.1
WP_202685167.1	REase+TPR+GreAB-C+PIN*→	REase+TPR+GreAB-C+PIN	1019	-	Alphaproteobacteria	Skermanella mucosa	hypothetical protein, partial [Skermanella mucosa].	GCF_016765655.1
WP_202722978.1	HetE-N1→ TPR+GreAB-C+PIN*→	TPR+GreAB-C+PIN	1141	-	Gammaproteobacteria	Shewanella schlegeliana	hypothetical protein [Shewanella schlegeliana].	GCF_016765635.1
WP_202727723.1	HetE-N1→ TPR+GreAB-C+PIN*→	TPR+GreAB-C+PIN	1094	-	Gammaproteobacteria	Serratia fonticola	hypothetical protein [Serratia fonticola].	GCF_016770915.1
WP_203042134.1	HetE-N1→ TPR+GreAB-C+PIN*→	TPR+GreAB-C+PIN	1106	-	Gammaproteobacteria	Enterobacter hormaechei	hypothetical protein [Enterobacter hormaechei].	GCF_016791625.1
WP_203341318.1	TPR+PIN*→	TPR+PIN	1141	-	Firmicutes	Planomicrobium sp. REN14	hypothetical protein [Planomicrobium sp. REN14].	GCF_016820615.1
WP_203380482.1	REase+TPR+GreAB-C+PIN*→	REase+TPR+GreAB-C+PIN	1231	-	Actinobacteria	Actinoplanes sp. LDG1-06	DUF4365 domain-containing protein [Actinoplanes sp. LDG1-06].	GCF_016834655.1
WP_203735920.1	TPR+PIN*→	TPR+PIN	1176	-	Actinobacteria	Actinoplanes durhamensis	hypothetical protein [Actinoplanes durhamensis].	GCF_016862175.1
WP_203841861.1	REase+TPR+GreAB-C+PIN*→	REase+TPR+GreAB-C+PIN	1259	-	Actinobacteria	Actinoplanes humidus	DUF4365 domain-containing protein [Actinoplanes humidus].	GCF_016862215.1
WP_204008964.1	REase+TPR+GreAB-C+PIN*→	REase+TPR+GreAB-C+PIN	1356	-	Actinobacteria	Micromonospora andamanensis	hypothetical protein [Micromonospora andamanensis].	GCF_016863495.1
WP_204028926.1	REase+TPR+GreAB-C+PIN*→	REase+TPR+GreAB-C+PIN	1338	-	Actinobacteria	Sinosporangium siamense	hypothetical protein [Sinosporangium siamense].	GCF_016863435.1

acc	operon	architecture	len	gen.name	taxend	species	defline	gca
WP_204127404.1	HetE-N1→ TPR+GreAB-C+PIN*→||||<-HNH	TPR+GreAB-C+PIN	1110	-	Gammaproteobacteria	Pseudomonas sp. RDP1	hypothetical protein [Pseudomonas sp. RDP1].	GCF_016887885.1
WP_204370319.1	TPR+GreAB-C+PIN*→	TPR+GreAB-C+PIN	860	-	Betaproteobacteria	Burkholderia cepacia	tetratricopeptide repeat protein, partial [Burkholderia cepacia].	GCF_001052915.1
WP_204511064.1	REase+TPR+GreAB-C+PIN*→	REase+TPR+GreAB-C+PIN	1349	-	Alphaproteobacteria	Bradyrhizobium canariense	tetratricopeptide repeat protein [Bradyrhizobium canariense].	GCF_016907215.1
WP_204648469.1	TPR+PIN*→	TPR+PIN	1235	-	Firmicutes	Gemmiger formicilis	hypothetical protein [Gemmiger formicilis].	GCF_016900095.1
WP_204732305.1	REase+TPR+GreAB-C+PIN*→	REase+TPR+GreAB-C+PIN	1447	-	Betaproteobacteria	Hydrogenophaga laconesensis	hypothetical protein [Hydrogenophaga laconesensis].	GCF_016909585.1
WP_204856598.1	REase+PIN*→	REase+PIN	1247	-	Actinobacteria	Rhodococcus fascians	DUF4365 domain-containing protein [Rhodococcus fascians].	GCF_016909505.1
WP_204934318.1	REase+TPR+GreAB-C+PIN*→	REase+TPR+GreAB-C+PIN	1321	-	Gammaproteobacteria	Pseudomonas stutzeri	DUF4365 domain-containing protein [Pseudomonas stutzeri].	GCF_016909445.1
WP_204944661.1	TPR+PIN*→	TPR+PIN	1203	-	Actinobacteria	Micromonospora luteifusca	hypothetical protein [Micromonospora luteifusca].	GCF_016907275.1
WP_205045164.1	TPR+PIN*→	TPR+PIN	1168	-	Actinobacteria	Streptomyces bryophytorum	hypothetical protein [Streptomyces bryophytorum].	GCF_017164735.1
WP_205483425.1	REase+TPR+GreAB-C+PIN→ TPR→||||<-?||PIN*→	PIN	365	-	Gammaproteobacteria	Pseudomonas sp. 15A4	hypothetical protein [Pseudomonas sp. 15A4].	GCF_016925575.1
WP_206013307.1	HetE-N1→ TPR+GreAB-C+PIN*→	TPR+GreAB-C+PIN	1090	-	Gammaproteobacteria	Pseudoalteromonas sp. Z9A6	hypothetical protein [Pseudoalteromonas sp. Z9A6].	GCF_011378805.1
WP_206497574.1	REase+PIN*→	REase+PIN	1021	-	Actinobacteria	Rhodococcus sp. KRD175	hypothetical protein [Rhodococcus sp. KRD175].	GCF_017168275.1
WP_206556849.1	HetE-N1→ TPR+GreAB-C+PIN*→	TPR+GreAB-C+PIN	1116	-	Gammaproteobacteria	Marinobacter daepoensis	hypothetical protein [Marinobacter daepoensis].	GCF_017255165.1
WP_206628468.1	HetE-N1→ TPR+GreAB-C+PIN*→	TPR+GreAB-C+PIN	1116	-	Gammaproteobacteria	Vibrio vulnificus	hypothetical protein [Vibrio vulnificus].	GCF_017291135.1
WP_206676035.1	HTH→ SIG+TM→ TPR+GreAB-C+PIN*→	TPR+GreAB-C+PIN	1125	-	Bacteroidetes	Capnocytophaga canis	hypothetical protein [Capnocytophaga canis].	GCF_013276255.1
WP_206820216.1	REase+TPR+GreAB-C+PIN*→	REase+TPR+GreAB-C+PIN	1215	-	Actinobacteria	Microbacterium esteraromaticum	DUF4365 domain-containing protein [Microbacterium esteraromaticum].	GCF_017303135.1
WP_206852091.1	REase+TPR+GreAB-C+PIN*→	REase+TPR+GreAB-C+PIN	1289	-	Gammaproteobacteria	Marinobacter hydrocarbonoclasticus	hypothetical protein [Marinobacter hydrocarbonoclasticus].	GCF_017303195.1
WP_207418840.1	TPR+GreAB-C+PIN*→||||<-HTH+HTH	TPR+GreAB-C+PIN	872	-	Betaproteobacteria	Burkholderia pseudomallei	hypothetical protein [Burkholderia pseudomallei].	GCF_017356705.1
WP_207505725.1	Mbetalac→ Trypsin+TPR+GreAB-C+PIN*→	Trypsin+TPR+GreAB-C+PIN	1362	-	Bacteroidetes	Telluribacter humicola	serine protease [Telluribacter humicola].	GCF_017355935.1
WP_207509935.1	Mbetalac→ TPR+GreAB-C+PIN*→	TPR+GreAB-C+PIN	1342	-	Bacteroidetes	Telluribacter humicola	hypothetical protein [Telluribacter humicola].	GCF_017355935.1
WP_207967591.1	HetE-N1→ TPR+GreAB-C+PIN*→	TPR+GreAB-C+PIN	1090	-	Gammaproteobacteria	Yersinia pseudotuberculosis	hypothetical protein [Yersinia pseudotuberculosis].	GCF_017498805.1
WP_207993663.1	HetE-N1→ TPR+GreAB-C+PIN*→	TPR+GreAB-C+PIN	1122	-	Gammaproteobacteria	Yersinia pseudotuberculosis	hypothetical protein [Yersinia pseudotuberculosis].	GCF_017498865.1
WP_208133228.1	TPR+GreAB-C+PIN*→	TPR+GreAB-C+PIN	1306	-	Bacteroidetes	Siccationidurans ginsengisoli	hypothetical protein [Siccationidurans ginsengisoli].	GCF_017571525.1
WP_208209475.1	REase+TPR+GreAB-C+PIN*→	REase+TPR+GreAB-C+PIN	1543	-	Gammaproteobacteria	Pseudomonas asiatica	hypothetical protein [Pseudomonas asiatica].	GCF_017582485.1
WP_208467197.1	TPR+PIN*→	TPR+PIN	1140	-	Actinobacteria	Actinoplanes sp. NEAU-H7	hypothetical protein [Actinoplanes sp. NEAU-H7].	GCF_017592555.1
WP_208622113.1	HetE-N1→ TPR+GreAB-C+PIN*→	TPR+GreAB-C+PIN	1022	-	Gammaproteobacteria	Shewanella indica	hypothetical protein, partial [Shewanella indica].	GCF_002836975.1
WP_208660584.1	HetE-N1→ TPR+GreAB-C+PIN*→	TPR+GreAB-C+PIN	1087	-	Gammaproteobacteria	Shewanella decolorationis	hypothetical protein [Shewanella decolorationis].	GCF_007923045.1
WP_208757658.1	TPR+PIN*→	TPR+PIN	1184	-	Firmicutes	Priestia megaterium	hypothetical protein [Priestia megaterium].	GCF_002872495.1
WP_209187630.1	REase+TPR+GreAB-C+PIN→ *PIN*→	REase+TPR+GreAB-C+PIN	359	-	Alphaproteobacteria	Rhizobium sp. L245/93	hypothetical protein [Rhizobium sp. L245/93].	GCF_017741845.1
WP_209187631.1	-	-	978	-	Alphaproteobacteria	Rhizobium sp. L245/93	hypothetical protein [Rhizobium sp. L245/93].	GCF_017741845.1
WP_209302601.1	X+PIN*→	X+PIN	1221	-	Firmicutes	Anaerostipes hadrus	hypothetical protein [Anaerostipes hadrus].	GCF_017776445.1
WP_209570872.1	TPR+GreAB-C+PIN*→?→||||<-TPR	TPR+GreAB-C+PIN	1131	-	Bacteroidetes	Flavobacterium sp. 1750	hypothetical protein [Flavobacterium sp. 1750].	GCF_017833855.1
WP_209629149.1	<-Calcineurin<-?||?→?→?→ TPR+PIN*→	TPR+PIN	1016	-	Euryarchaeota	Methanofollis sp. W23	hypothetical protein [Methanofollis sp. W23].	GCF_017875325.1
WP_209829315.1	TPR+PIN*→	TPR+PIN	1242	-	Actinobacteria	Cellulosimicrobium sp. BE325	hypothetical protein [Cellulosimicrobium sp. BE325].	GCF_017834095.1
WP_210277108.1	REase+TPR+TPR+GreAB-C+PIN*→	REase+TPR+TPR+GreAB-C+PIN	1346	-	Alphaproteobacteria	Rhizobium leguminosarum	hypothetical protein [Rhizobium leguminosarum].	GCF_014138515.1
WP_210311799.1	TPR+GreAB-C+PIN*→	TPR+GreAB-C+PIN	812	-	Alphaproteobacteria	Rhizobium soli	hypothetical protein, partial [Rhizobium soli].	GCF_014207075.1
WP_210441888.1	HetE-N1→ TPR+GreAB-C+PIN*→	TPR+GreAB-C+PIN	1089	-	Gammaproteobacteria	Vibrio crassostreae	hypothetical protein [Vibrio crassostreae].	GCF_017917835.1
WP_210504843.1	HetE-N1→ TPR+GreAB-C+PIN*→	TPR+GreAB-C+PIN	1123	-	Gammaproteobacteria	Pantoea ananatis	hypothetical protein [Pantoea ananatis].	GCF_017920595.1

Gene neighborhoods and domain architectures of the novel STAND1 (nSTAND1) domain

<table>
<thead>
<tr><th>acc</th><th>operon</th><th>architecture</th><th>len</th><th>gen.name</th><th>taxend</th><th>species</th><th>defline</th><th>gca</th></tr>
</thead>
<tbody>
<tr><td>ABW16115.1</td><td><-ABhydrolase<-?<-TIR+CASPASE+APATPase+TPR+TPR||?→||<-?||nSTAND1+BetaPropeller*→ TIR+EACC1+EAD1→</td><td>nSTAND1+BetaPropeller</td><td>1230</td><td>Franean1_6781</td><td>actinobacteria</td><td>Frankia sp. EAN1pec</td><td>WD-40 repeat protein [Frankia sp. EAN1pec].</td><td>GCA_000018005.1</td></tr>
<tr><td>AMY09307.1</td><td><-ABhydrolase<-?<-?||?→?→ SIG+TPR→ TIR+DrHyd→ nSTAND1+TM*→||<-?<-?<-?||?→?→||<-?<-ABhydrolase</td><td>nSTAND1+TM</td><td>884</td><td>LuPra_02522</td><td>acidobacteria</td><td>Luteitalea pratensis</td><td>putative family GH19 chitinase [Luteitalea pratensis].</td><td>GCA_001618865.1</td></tr>
<tr><td>AQZ62998.1</td><td>nSTAND1+BetaPropeller*→?→?→ ParB→</td><td>nSTAND1+BetaPropeller</td><td>1212</td><td>BKM31_17390</td><td>actinobacteria</td><td>Nonomuraea sp. ATCC 55076</td><td>hypothetical protein BKM31_17390 [Nonomuraea sp. ATCC 55076].</td><td>GCA_002057455.1</td></tr>
<tr><td>AUG75452.1</td><td><-ABhydrolase||?→||<-?||?→?→||<-?||EACC1→ CASPASE+nSTAND1+TM+BetaPropeller*→</td><td>CASPASE+nSTAND1+TM+BetaPropeller</td><td>1485</td><td>CFP65_0489</td><td>actinobacteria</td><td>Kitasatospora sp. MMS16-BH015</td><td>hypothetical protein CFP65_0489 [Kitasatospora sp. MMS16-BH015].</td><td>GCA_002943525.1</td></tr>
<tr><td>BAY12967.1</td><td>EAD8+Trypsin→?→?→ MoxR→?→ VWA+nSTAND1*→</td><td>VWA+nSTAND1</td><td>1139</td><td>NIES2098_61590</td><td>cyanobacteria</td><td>Calothrix sp. NIES-2098</td><td>WD-40 repeat-containing protein [Calothrix sp. NIES-2098].</td><td>GCA_002368175.1</td></tr>
<tr><td>BAY22501.1</td><td><-ACT||CASPASE+nSTAND1+BetaPropeller*→||<-?<-VWA+SidE||?→ MNS→</td><td>CASPASE+nSTAND1+BetaPropeller</td><td>1839</td><td>NIES2100_22640</td><td>cyanobacteria</td><td>Calothrix sp. NIES-2100</td><td>WD-40 repeat protein [Calothrix sp. NIES-2100].</td><td>GCA_002368195.1</td></tr>
<tr><td>BBD52759.1</td><td>CASPASE+nSTAND1+TM+BetaPropeller*→</td><td>CASPASE+nSTAND1+TM+BetaPropeller</td><td>1514</td><td>NIES204_00160</td><td>cyanobacteria</td><td>Planktothrix agardhii NIES-204</td><td>WD-40 repeat-containing protein [Planktothrix agardhii NIES-204].</td><td>GCA_003609755.1</td></tr>
<tr><td>BBD65423.1</td><td>nSTAND1+BetaPropeller*→ BetaPropeller→||<-?<-?<-?||ENOYCOADEHYD→</td><td>nSTAND1+BetaPropeller</td><td>770</td><td>NIES4070_17810</td><td>cyanobacteria</td><td>Nostoc commune HK-02</td><td>WD-40 repeat-containing protein [Nostoc commune HK-02].</td><td>GCA_003990685.1</td></tr>
<tr><td>BBD65510.1</td><td>Mbetalac→?→ Mbetalac→ CASPASE→ nSTAND1+BetaPropeller*→?→||<-?||ABC-ATPase→</td><td>nSTAND1+BetaPropeller</td><td>1279</td><td>NIES4070_18680</td><td>cyanobacteria</td><td>Nostoc commune HK-02</td><td>WD-40 repeat-containing protein [Nostoc commune HK-02].</td><td>GCA_003990685.1</td></tr>
<tr><td>CEJ42488.1</td><td>DinB_2+FGS→?→?→||<-?||nSTAND1+BetaPropeller*→</td><td>nSTAND1+BetaPropeller</td><td>490</td><td>apha_00274</td><td>cyanobacteria</td><td>Chrysosporum ovalisporum</td><td>WD-40 repeat protein [Chrysosporum ovalisporum].</td><td>GCA_001458455.1</td></tr>
<tr><td>EDM78150.1</td><td>Pkinase+TPR→||<-?||Pkinase+TIR+nSTAND1+BetaPropeller+FGS*→</td><td>Pkinase+TIR+nSTAND1+BetaPropeller+FGS</td><td>1781</td><td>PPSIR1_00415</td><td>deltaproteobacteria</td><td>Plesiocystis pacifica SIR-1</td><td>peptidase C14, caspase catalytic subunit p20, partial [Plesiocystis pacifica SIR-1].</td><td>GCA_000170895.1</td></tr>
<tr><td>EDN71045.1</td><td>nSTAND1+BetaPropeller*→</td><td>nSTAND1+BetaPropeller</td><td>1207</td><td>BGP_1542</td><td>gammaproteobacteria</td><td>Beggiatoa sp. PS</td><td>WD-40 repeat protein [Beggiatoa sp. PS].</td><td>GCA_000170715.1</td></tr>
<tr><td>ETX01284.1</td><td>TIR+DrHyd→ nSTAND1+TM*→</td><td>nSTAND1+TM</td><td>561</td><td>ETSY1_07910</td><td>nitrospinae/tectomicrobia group</td><td>Candidatus Entotheonella factor</td><td>hypothetical protein ETSY1_07910, partial [Candidatus Entotheonella factor].</td><td>GCA_000522425.1</td></tr>
</tbody>
</table>

<table>
<tr><th>acc</th><th>operon</th><th>architecture</th><th>len</th><th>gen.name</th><th>taxend</th><th>species</th><th>defline</th><th>gca</th></tr>
<tr><td>ETX06250.1</td><td>nSTAND1+TM+BetaPropeller*→</td><td>nSTAND1+TM+BetaPropeller</td><td>810</td><td>ETSY2_18270</td><td>nitrospinae/tectomicrobia group</td><td>Candidatus Entotheonella gemina</td><td>hypothetical protein ETSY2_18270, partial [Candidatus Entotheonella gemina].</td><td>GCA_000522445.1</td></tr>
<tr><td>ETX08407.1</td><td>TIR+DrHyd→ nSTAND1+TM+BetaPropeller+PDZ+BetaPropeller*→</td><td>nSTAND1+TM+BetaPropeller+PDZ+BetaPropeller</td><td>1401</td><td>ETSY2_05600</td><td>nitrospinae/tectomicrobia group</td><td>Candidatus Entotheonella gemina</td><td>hypothetical protein ETSY2_05600 [Candidatus Entotheonella gemina].</td><td>GCA_000522445.1</td></tr>
<tr><td>GAK58629.1</td><td>SIG+TIR→ nSTAND1*→ nSTAND1+TM→</td><td>nSTAND1</td><td>191</td><td>U27_05603</td><td>bacteria</td><td>Candidatus Vecturithrix granuli</td><td>peptidase C14, caspase catalytic subunit p20 [Candidatus Vecturithrix granuli].</td><td>GCA_000739535.1</td></tr>
<tr><td>GCL34384.1</td><td>nSTAND1+BetaPropeller+TPR*→ REC→</td><td>nSTAND1+BetaPropeller+TPR</td><td>1185</td><td>PA905_35650</td><td>cyanobacteria</td><td>Planktothrix agardhii CCAP 1459/11A</td><td>WD-40 repeat-containing protein [Planktothrix agardhii CCAP 1459/11A].</td><td>GCA_005402765.2</td></tr>
<tr><td>GES04627.1</td><td><-wHTH-4stranded+TPRs+APATPase+TPR+TPR+TPR||?→?→||<-APATPase+TPR||EACC1→ CASPASE+nSTAND1+BetaPropeller*→?→||<-?||REC→?→||<-?<-ABhydrolase</td><td>CASPASE+nSTAND1+BetaPropeller</td><td>1472</td><td>Acor_66950</td><td>actinobacteria</td><td>Acrocarpospora corrugata</td><td>hypothetical protein Acor_66950 [Acrocarpospora corrugata].</td><td>GCA_009687845.1</td></tr>
<tr><td>GFJ81020.1</td><td><-REC||?→?→?→?→||<-?||EACC1→ CASPASE+nSTAND1+TM+BetaPropeller*→?→||<-?<-?<-?<-?<-?||REC→</td><td>CASPASE+nSTAND1+TM+BetaPropeller</td><td>1373</td><td>Phou_052000</td><td>actinobacteria</td><td>Phytohabitans houttuyneae</td><td>hypothetical protein Phou_052000 [Phytohabitans houttuyneae].</td><td>GCA_011764425.1</td></tr>
<tr><td>HAA13664.1</td><td>FAD-NAD-dep-oxidoreductase→?→ TIR+nSTAND1+TM+BetaPropeller*→</td><td>TIR+nSTAND1+TM+BetaPropeller</td><td>1414</td><td>DCE41_19005</td><td>bacteroidetes</td><td>Cytophagales bacterium</td><td>TPA: hypothetical protein DCE41_19005, partial [Cytophagales bacterium].</td><td>GCA_003444355.1</td></tr>
<tr><td>HAA18104.1</td><td><-ABhydrolase<-?<-?<-PSE<-?||TIR+DrHyd→ nSTAND1+TM+BetaPropeller*→</td><td>nSTAND1+TM+BetaPropeller</td><td>1014</td><td>DCP28_04755</td><td>bacteroidetes</td><td>Cytophagales bacterium</td><td>TPA: hypothetical protein DCP28_04755 [Cytophagales bacterium].</td><td>GCA_003444325.1</td></tr>
<tr><td>HAA22846.1</td><td>TIR→ nSTAND1+TM+BetaPropeller*→</td><td>nSTAND1+TM+BetaPropeller</td><td>1115</td><td>DCP28_30300</td><td>bacteroidetes</td><td>Cytophagales bacterium</td><td>TPA: hypothetical protein DCP28_30300 [Cytophagales bacterium].</td><td>GCA_003444325.1</td></tr>
<tr><td>HAA23288.1</td><td><-SIG+Trypsin||BetaPropeller+ABhydrolase→||<-?<-?||NUDIX→ PSE→?→ TIR+DrHyd→ nSTAND1+TM+PspA+BetaPropeller*→</td><td>nSTAND1+TM+PspA+BetaPropeller</td><td>1043</td><td>DCP28_32665</td><td>bacteroidetes</td><td>Cytophagales bacterium</td><td>TPA: hypothetical protein DCP28_32665 [Cytophagales bacterium].</td><td>GCA_003444325.1</td></tr>
<tr><td>HAA50805.1</td><td>Pkinase+TIR+DrHyd→ TIR+DrHyd→ nSTAND1+BetaPropeller*→</td><td>nSTAND1+BetaPropeller</td><td>2457</td><td>DCE43_13910</td><td>planctomycetes</td><td>Planctomycetaceae bacterium</td><td>TPA: hypothetical protein DCE43_13910 [Planctomycetaceae bacterium].</td><td>GCA_003444155.1</td></tr>
<tr><td>HAA60996.1</td><td>Pkinase+TIR+DrHyd+TIR→ nSTAND1+BetaPropeller*→</td><td>nSTAND1+BetaPropeller</td><td>1113</td><td>DCE39_08695</td><td>planctomycetes</td><td>Planctomycetaceae bacterium</td><td>TPA: hypothetical protein DCE39_08695 [Planctomycetaceae bacterium].</td><td>GCA_003444135.1</td></tr>
<tr><td>HAB16129.1</td><td>TIR+DrHyd→ nSTAND1+TM+BetaPropeller*→</td><td>nSTAND1+TM+BetaPropeller</td><td>1225</td><td>DCE44_06740</td><td>verrucomicrobia</td><td>Verrucomicrobiales bacterium</td><td>TPA: hypothetical protein DCE44_06740, partial [Verrucomicrobiales bacterium].</td><td>GCA_003445095.1</td></tr>
<tr><td>HAB19453.1</td><td>TIR+DrHyd→ nSTAND1*→?→||<-p450<-SIG+IES1</td><td>nSTAND1</td><td>1272</td><td>DCE44_23860</td><td>verrucomicrobia</td><td>Verrucomicrobiales bacterium</td><td>TPA: hypothetical protein DCE44_23860 [Verrucomicrobiales bacterium].</td><td>GCA_003445095.1</td></tr>
</table>

<table>
<tr><th>acc</th><th>operon</th><th>architecture</th><th>len</th><th>gen.name</th><th>taxend</th><th>species</th><th>defline</th><th>gca</th></tr>
<tr><td>HAB19519.1</td><td>TIR+DrHyd→ nSTAND1+TM+BetaPropeller*→?→ Cluster515_2clades→</td><td>nSTAND1+TM+BetaPropeller</td><td>1398</td><td>DCE44_24195</td><td>verrucomicrobia</td><td>Verrucomicrobiales bacterium</td><td>TPA: hypothetical protein DCE44_24195 [Verrucomicrobiales bacterium].</td><td>GCA_003445095.1</td></tr>
<tr><td>HAC65097.1</td><td>SIG+IES1+CBS→||<-?<-?<-?||?→?→ nSTAND1+TM*→||<-?<-?<-?<-?<-?<-?||Redoxin→</td><td>nSTAND1+TM</td><td>781</td><td>DCF68_16600</td><td>cyanobacteria</td><td>Cyanothece sp. UBA12306</td><td>TPA: hypothetical protein DCF68_16600 [Cyanothece sp. UBA12306].</td><td>GCA_003448685.1</td></tr>
<tr><td>HAE30915.1</td><td>TIR+DrHyd→ nSTAND1+BetaPropeller*→</td><td>nSTAND1+BetaPropeller</td><td>1040</td><td>DCF89_07360</td><td>bacteroidetes</td><td>Flavobacteriales bacterium</td><td>TPA: hypothetical protein DCF89_07360 [Flavobacteriales bacterium].</td><td>GCA_003448535.1</td></tr>
<tr><td>HAI83309.1</td><td>TIR+DrHyd→ TIR+DrHyd→ nSTAND1+BetaPropeller*→</td><td>nSTAND1+BetaPropeller</td><td>856</td><td>DCL43_06560</td><td>bacteroidetes</td><td>Chitinophagaceae bacterium</td><td>TPA: hypothetical protein DCL43_06560, partial [Chitinophagaceae bacterium].</td><td>GCA_003450935.1</td></tr>
<tr><td>HAN37555.1</td><td>TIR→ nSTAND1+TM*→?→?→?→?→ Cluster106_3clades→</td><td>nSTAND1+TM</td><td>559</td><td>DCQ29_01515</td><td>bacteroidetes</td><td>Chitinophagaceae bacterium</td><td>TPA: hypothetical protein DCQ29_01515, partial [Chitinophagaceae bacterium].</td><td>GCA_003456175.1</td></tr>
<tr><td>HAP59714.1</td><td>SIG+IES1→||<-?<-?||PSE→?→ SIG+HISKIN+REC+AraC-HTH→ SIG+nSTAND1+TM+BetaPropeller*→</td><td>SIG+nSTAND1+TM+BetaPropeller</td><td>1024</td><td>DCR93_09475</td><td>bacteroidetes</td><td>Cytophagales bacterium</td><td>TPA: hypothetical protein DCR93_09475 [Cytophagales bacterium].</td><td>GCA_003454975.1</td></tr>
<tr><td>HAW02034.1</td><td>TIR→ nSTAND1+TM*→</td><td>nSTAND1+TM</td><td>563</td><td>DCX10_09905</td><td>verrucomicrobia</td><td>Verrucomicrobiales bacterium</td><td>TPA: hypothetical protein DCX10_09905, partial [Verrucomicrobiales bacterium].</td><td>GCA_003543825.1</td></tr>
<tr><td>HAW53197.1</td><td>TIR+DrHyd→ nSTAND1+TM*→</td><td>nSTAND1+TM</td><td>442</td><td>DCX54_12865</td><td>bacteroidetes</td><td>Flavobacteriales bacterium</td><td>TPA: High-affnity carbon uptake protein Hat/HatR, partial [Flavobacteriales bacterium].</td><td>GCA_003487525.1</td></tr>
<tr><td>HAX76296.1</td><td><-Trypco1||CASPASE+nSTAND1+TM*→ SIG+Pentapeptide→</td><td>CASPASE+nSTAND1+TM</td><td>797</td><td>DCY88_10755</td><td>cyanobacteria</td><td>Cyanobacteria bacterium UBA11372</td><td>TPA: hypothetical protein DCY88_10755, partial [Cyanobacteria bacterium UBA11372].</td><td>GCA_003486675.1</td></tr>
<tr><td>HAX87398.1</td><td>CASPASE+nSTAND1+BetaPropeller*→</td><td>CASPASE+nSTAND1+BetaPropeller</td><td>1185</td><td>DCY91_14325</td><td>cyanobacteria</td><td>Cyanobacteria bacterium UBA11370</td><td>TPA: hypothetical protein DCY91_14325 [Cyanobacteria bacterium UBA11370].</td><td>GCA_003486645.1</td></tr>
<tr><td>HAZ02032.1</td><td>TM+TM+TM+TM+TM+TM+TM+TM+TM+TM+TM+cNMP→?→ TIR+DrHyd→ nSTAND1+BetaPropeller*→</td><td>nSTAND1+BetaPropeller</td><td>1055</td><td>DCY97_07630</td><td>bacteroidetes</td><td>Marinilabiliales bacterium</td><td>TPA: hypothetical protein DCY97_07630 [Marinilabiliales bacterium].</td><td>GCA_003485835.1</td></tr>
<tr><td>HAZ49897.1</td><td>EACC2+CASPASE+nSTAND1+BetaPropeller*→||<-?<-?||SIG+TPR→ SIG+TPR→||<-?||Uma2→</td><td>EACC2+CASPASE+nSTAND1+BetaPropeller</td><td>1758</td><td>DCZ55_36965</td><td>cyanobacteria</td><td>Cyanobacteria bacterium UBA11371</td><td>TPA: hypothetical protein DCZ55_36965 [Cyanobacteria bacterium UBA11371].</td><td>GCA_003486305.1</td></tr>
<tr><td>HBB96021.1</td><td>nSTAND1+TM+BetaPropeller*→||<-?<-?<-?<-?<-Cluster533_2clades</td><td>nSTAND1+TM+BetaPropeller</td><td>1196</td><td>DC054_11585</td><td>acidobacteria</td><td>Blastocatellia bacterium</td><td>TPA: hypothetical protein DC054_11585 [Blastocatellia bacterium].</td><td>GCA_003487805.1</td></tr>
<tr><td>HBE43600.1</td><td>MORC→?→ AAA→ TM+TM+TM+TM+TM+TM+TM+TM+TM+TM+TM+cNMP→ nSTAND1+TM+BetaPropeller*→</td><td>nSTAND1+TM+BetaPropeller</td><td>953</td><td>DDW27_20875</td><td>bacteroidetes</td><td>Bacteroidales bacterium</td><td>TPA: hypothetical protein DDW27_20875, partial [Bacteroidales bacterium].</td><td>GCA_003501665.1</td></tr>
</table>

acc	operon	architecture	len	gen.name	taxend	species	defline	gca
HBH24377.1	TIR+DrHyd→ nSTAND1+BetaPropeller*→	nSTAND1+BetaPropeller	1031	DDY13_13250	bacteroidetes	Cytophagales bacterium	TPA: hypothetical protein DDY13_13250 [Cytophagales bacterium].	GCA_003500135.1
HBK97025.1	SIG+Trypsin+PDZ→?→ EACC2+CASPASE+nSTAND1+BetaPropeller*→?→?→?→||<-XisI	EACC2+CASPASE+nSTAND1+BetaPropeller	1576	DD001_06660	cyanobacteria	Microcoleaceae bacterium UBA10368	TPA: hypothetical protein DD001_06660 [Microcoleaceae bacterium UBA10368].	GCA_003506815.1
HBL28746.1	TIR+TIR→ nSTAND1+TIR+TM*→	nSTAND1+TIR+TM	926	DD490_18070	acidobacteria	Acidobacteria bacterium	TPA: hypothetical protein DD490_18070 [Acidobacteria bacterium].	GCA_003504885.1
HBL30162.1	TIR+DrHyd→ nSTAND1+TM+BetaPropeller*→?→?→ tRNA→?→?→ PSE→?→ AbiEii→	nSTAND1+TM+BetaPropeller	1255	DD490_25280	acidobacteria	Acidobacteria bacterium	TPA: hypothetical protein DD490_25280 [Acidobacteria bacterium].	GCA_003504885.1
HBR00557.1	nSTAND1*→?→||<-?<-?||GUN4→	nSTAND1	454	DD761_18925	cyanobacteria	Cyanobacteria bacterium UBA11691	TPA: hypothetical protein DD761_18925 [Cyanobacteria bacterium UBA11691].	GCA_003535175.1
HBY06929.1	wHTH-4stranded+TPRs+Pkinase+nSTAND1+TM+BetaPropeller*→	wHTH-4stranded+TPRs+Pkinase+nSTAND1+TM+BetaPropeller	1319	DEH22_03775	chloroflexi	Chloroflexi bacterium	TPA: hypothetical protein DEH22_03775, partial [Chloroflexi bacterium].	GCA_003512075.1
HBY08818.1	Pkinase+nSTAND1*→	Pkinase+nSTAND1	633	DEH22_13955	chloroflexi	Chloroflexi bacterium	TPA: hypothetical protein DEH22_13955, partial [Chloroflexi bacterium].	GCA_003512075.1
HCA49859.1	TIR+DrHyd→ nSTAND1+BetaPropeller+7TMR-DISMED1*→	nSTAND1+BetaPropeller+7TMR-DISMED1	1946	DEP12_05575	planctomycetes	Planctomycetaceae bacterium	TPA: hypothetical protein DEP12_05575, partial [Planctomycetaceae bacterium].	GCA_003519945.1
HCD01670.1	Pkinase+TIR+DrHyd→ nSTAND1+BetaPropeller+TPR+TPR*→	nSTAND1+BetaPropeller+TPR+TPR	2680	DER64_14220	planctomycetes	Planctomycetaceae bacterium	TPA: hypothetical protein DER64_14220 [Planctomycetaceae bacterium].	GCA_003517085.1
HCM75174.1	TM+TM+TM+TM+TM+TM+TM+TM+TM+TM+TM+cNMP→ TIR+DrHyd→ nSTAND1+TM+BetaPropeller*→	nSTAND1+TM+BetaPropeller	1054	DIS90_02245	bacteroidetes	Cytophagales bacterium	TPA: High-affnity carbon uptake protein Hat/HatR [Cytophagales bacterium].	GCA_003535515.1
HDN27296.1	TIR+DrHyd→ nSTAND1+TM+BetaPropeller*→	nSTAND1+TM+BetaPropeller	784	ENG03_09425	gammaproteobacteria	Thioploca sp.	TPA: hypothetical protein ENG03_09425, partial [Thioploca sp.].	-
HDV39880.1	Pkinase+nSTAND1+TM*→||<-?<-?||?→?→?→?→ REC→	Pkinase+nSTAND1+TM	922	ENQ62_18990	chloroflexi	Chloroflexi bacterium	TPA: serine/threonine protein kinase [Chloroflexi bacterium].	-
HDX76165.1	nSTAND1+TM+BetaPropeller*→	nSTAND1+TM+BetaPropeller	810	ENQ42_15745	chloroflexi	Chloroflexi bacterium	TPA: hypothetical protein ENQ42_15745 [Chloroflexi bacterium].	-
HEB93882.1	TIR+nSTAND1+BetaPropeller*→	TIR+nSTAND1+BetaPropeller	1688	ENI94_10555	gammaproteobacteria	Gammaproteobacteria bacterium	TPA: TIR domain-containing protein, partial [Gammaproteobacteria bacterium].	-
HEC44295.1	MORC→?→ AAA→||<-?||TM+TM+TM+TM+TM+TM+TM+TM+TM+TM+TM+cNMP→?→ TIR+DrHyd→ SIG+nSTAND1+TM+BetaPropeller*→?→?→?→||<-Mbetalac	SIG+nSTAND1+TM+BetaPropeller	1041	ENI20_15845	bacteroidetes	Bacteroides sp.	TPA: hypothetical protein ENI20_15845 [Bacteroides sp.].	-

<table>
<tr><th>acc</th><th>operon</th><th>architecture</th><th>len</th><th>gen.name</th><th>taxend</th><th>species</th><th>defline</th><th>gca</th></tr>
<tr><td>HEC84902.1</td><td>Pkinase+nSTAND1*→</td><td>Pkinase+nSTAND1</td><td>801</td><td>ENI48_06635</td><td>gammaproteobacteria</td><td>Thioploca sp.</td><td>TPA: hypothetical protein ENI48_06635, partial [Thioploca sp.].</td><td>-</td></tr>
<tr><td>HEP84957.1</td><td>nSTAND1+TM+TPR*→?→ CASPASE→||<-SWACOS+TPR+TPR</td><td>nSTAND1+TM+TPR</td><td>1040</td><td>ENQ97_01465</td><td>chloroflexi</td><td>Chloroflexi bacterium</td><td>TPA: ATP-binding protein [Chloroflexi bacterium].</td><td>-</td></tr>
<tr><td>HET82137.1</td><td>SIG+nSTAND1+BetaPropeller*→</td><td>SIG+nSTAND1+BetaPropeller</td><td>1664</td><td>ENQ84_03870</td><td>chloroflexi</td><td>Chloroflexi bacterium</td><td>TPA: hypothetical protein ENQ84_03870 [Chloroflexi bacterium].</td><td>-</td></tr>
<tr><td>HEY47862.1</td><td>SIG+LexA-HTH+LexA-protease→||<-?||tRNA→?→ tRNA→ tRNA→ wHTH-4stranded+TPRs+STYKIN+nSTAND1+BetaPropeller→ wHTH-4stranded+TPRs+STYKIN+nSTAND1+BetaPropeller*→ SWACOS+TPR+TPR→</td><td>wHTH-4stranded+TPRs+STYKIN+nSTAND1+BetaPropeller</td><td>1765</td><td>G4O14_13900</td><td>chloroflexi</td><td>Anaerolineae bacterium</td><td>TPA: protein kinase [Anaerolineae bacterium].</td><td>GCA_011174825.1</td></tr>
<tr><td>HFG28088.1</td><td>TIR+DrHyd→ nSTAND1+BetaPropeller*→||<-?<-?<-?||TM→?→?→ Cluster157_3clades→</td><td>nSTAND1+BetaPropeller</td><td>1281</td><td>ENS73_14620</td><td>verrucomicrobia</td><td>Verrucomicrobia subdivision 3 bacterium</td><td>TPA: hypothetical protein ENS73_14620 [Verrucomicrobia subdivision 3 bacterium].</td><td>-</td></tr>
<tr><td>HFK76611.1</td><td>TCAD9+Pkinase+nSTAND1+TM+BetaPropeller*→</td><td>TCAD9+Pkinase+nSTAND1+TM+BetaPropeller</td><td>1386</td><td>ENR96_14405</td><td>chloroflexi</td><td>Anaerolineae bacterium</td><td>TPA: hypothetical protein ENR96_14405, partial [Anaerolineae bacterium].</td><td>-</td></tr>
<tr><td>HFN01514.1</td><td>CASPASE+nSTAND1+BetaPropeller*→</td><td>CASPASE+nSTAND1+BetaPropeller</td><td>1643</td><td>ENR64_27960</td><td>cyanobacteria</td><td>Oscillatoriales cyanobacterium SpSt-418</td><td>TPA: hypothetical protein ENR64_27960 [Oscillatoriales cyanobacterium SpSt-418].</td><td>-</td></tr>
<tr><td>HGT17894.1</td><td>SIG+Pentapeptide→||<-?||PSE→?→?→ MED15→?→ CASPASE+nSTAND1+TM+BetaPropeller*→</td><td>CASPASE+nSTAND1+TM+BetaPropeller</td><td>1526</td><td>ENT48_13530</td><td>chloroflexi</td><td>Chloroflexi bacterium</td><td>TPA: CHAT domain-containing protein [Chloroflexi bacterium].</td><td>-</td></tr>
<tr><td>HGV41566.1</td><td>HxxxH→?→?→ Cluster515_2clades→?→||<-?||SIG+Trypsin→ SIG+TM+nSTAND1+TM+BetaPropeller*→?→?→ nSTAND1+TM+Tox-ODYAM1+BetaPropeller→</td><td>SIG+TM+nSTAND1+TM+BetaPropeller</td><td>1587</td><td>ENT18_00225</td><td>chloroflexi</td><td>Chloroflexi bacterium</td><td>TPA: hypothetical protein ENT18_00225 [Chloroflexi bacterium].</td><td>-</td></tr>
<tr><td>HGX16402.1</td><td>HTH+Pkinase+nSTAND1+TM+BetaPropeller*→</td><td>HTH+Pkinase+nSTAND1+TM+BetaPropeller</td><td>1240</td><td>ENR56_13340</td><td>chloroflexi</td><td>Anaerolineae bacterium</td><td>TPA: helix-turn-helix domain-containing protein, partial [Anaerolineae bacterium].</td><td>-</td></tr>
<tr><td>HIG27385.1</td><td>nSTAND1+BetaPropeller+SUN+TPR*→</td><td>nSTAND1+BetaPropeller+SUN+TPR</td><td>1629</td><td>EYQ50_06160</td><td>verrucomicrobia</td><td>Verrucomicrobiales bacterium</td><td>TPA: hypothetical protein EYQ50_06160 [Verrucomicrobiales bacterium].</td><td>-</td></tr>
<tr><td>HIG31009.1</td><td>nSTAND1+TM*→</td><td>nSTAND1+TM</td><td>482</td><td>EYQ50_25695</td><td>verrucomicrobia</td><td>Verrucomicrobiales bacterium</td><td>TPA: ATP-binding protein, partial [Verrucomicrobiales bacterium].</td><td>-</td></tr>
<tr><td>HIG83463.1</td><td>TIR+DrHyd→ nSTAND1+TM+BetaPropeller*→</td><td>nSTAND1+TM+BetaPropeller</td><td>938</td><td>EYQ23_05285</td><td>verrucomicrobia</td><td>Verrucomicrobiales bacterium</td><td>TPA: hypothetical protein EYQ23_05285, partial [Verrucomicrobiales bacterium].</td><td>-</td></tr>
<tr><td>KAA0256025.1</td><td><-REC+NtrC-AAA+FIS-HTH<-?<-?<-ABC-ATPase||TIR+DrHyd→ nSTAND1+BetaPropeller*→?→ TM+TM+TM+TM+TM+TM+TM+TM+TM+TM+TM+cNMP→ TM+TM+TM+TM+TM+TM+TM+TM+TM+TM+TM+cNMP→?→?→||<-SIG+IES1</td><td>nSTAND1+BetaPropeller</td><td>1035</td><td>EDX89_02880</td><td>acidobacteria</td><td>Acidobacteria bacterium</td><td>hypothetical protein EDX89_02880 [Acidobacteria bacterium].</td><td>-</td></tr>
<tr><td>KAA3662250.1</td><td><-HAD||?→ EAD7+nSTAND1+TM+FGS*→</td><td>EAD7+nSTAND1+TM+FGS</td><td>781</td><td>DWQ04_13990</td><td>chloroflexi</td><td>Chloroflexi bacterium</td><td>hypothetical protein DWQ04_13990, partial [Chloroflexi bacterium].</td><td>-</td></tr>
<tr><td>KAB2902286.1</td><td>Pkinase+nSTAND1+TM+EP1*→</td><td>Pkinase+nSTAND1+TM+EP1</td><td>937</td><td>F9K40_07755</td><td>deltaproteobacteria</td><td>Kofleriaceae bacterium</td><td>protein kinase [Kofleriaceae bacterium].</td><td>-</td></tr>
</table>

acc	operon	architecture	len	gen.name	taxend	species	defline	gca
KEI67755.1	CASPASE→ nSTAND1+BetaPropeller*→ REC→	nSTAND1+BetaPropeller	1315	A19Y_2900	cyanobacteria	Planktothrix agardhii NIVA-CYA 126/8	putative WD40 repeat-containing protein [Planktothrix agardhii NIVA-CYA 126/8].	GCA_000710505.1
KKD36164.1	nSTAND1+BetaPropeller*→	nSTAND1+BetaPropeller	1221	WN50_21290	cyanobacteria	Limnoraphis robusta CS-951	peptidase C14 [Limnoraphis robusta CS-951].	GCA_000972705.2
KKO17953.1	TIR→ SIG+nSTAND1+TM+BetaPropeller*→ BetaPropeller→	SIG+nSTAND1+TM+BetaPropeller	1179	BROFUL_03361	planctomycetes	Candidatus Brocadia fulgida	hypothetical protein BROFUL_03361 [Candidatus Brocadia fulgida].	GCA_000987375.1
KOX34481.1	nSTAND1+TM+BetaPropeller*→	nSTAND1+TM+BetaPropeller	1229	ADK67_03315	actinobacteria	Saccharothrix sp. NRRL B-16348	hypothetical protein ADK67_03315 [Saccharothrix sp. NRRL B-16348].	GCA_001280085.1
KPA09462.1	TIR→ nSTAND1+CarboxypepD-reg-IG+FGS*→	nSTAND1+CarboxypepD-reg-IG+FGS	1095	MHK_010294	deltaproteobacteria	Candidatus Magnetomorum sp. HK-1	Sulphatase-modifying factor domain protein [Candidatus Magnetomorum sp. HK-1].	GCA_001292585.1
KPA09563.1	TIR+DrHyd→ nSTAND1+BetaPropeller*→	nSTAND1+BetaPropeller	1143	MHK_010235	deltaproteobacteria	Candidatus Magnetomorum sp. HK-1	repeat-containing protein [Candidatus Magnetomorum sp. HK-1].	GCA_001292585.1
KPK06025.1	Pkinase+LuxR-HTH+nSTAND1+BetaPropeller*→	Pkinase+LuxR-HTH+nSTAND1+BetaPropeller	1617	AMJ56_15120	chloroflexi	Anaerolineae bacterium SG8_19	hypothetical protein AMJ56_15120 [Anaerolineae bacterium SG8_19].	GCA_001303105.1
KPK11168.1	wHTH-4stranded+TPRs+nSTAND1+TM+BetaPropeller*→	wHTH-4stranded+TPRs+nSTAND1+TM+BetaPropeller	1833	AMJ56_06610	chloroflexi	Anaerolineae bacterium SG8_19	hypothetical protein AMJ56_06610 [Anaerolineae bacterium SG8_19].	GCA_001303105.1
KPK13148.1	wHTH-4stranded+TPRs+nSTAND1+TM+BetaPropeller*→	wHTH-4stranded+TPRs+nSTAND1+TM+BetaPropeller	1822	AMJ56_03150	chloroflexi	Anaerolineae bacterium SG8_19	hypothetical protein AMJ56_03150 [Anaerolineae bacterium SG8_19].	GCA_001303105.1
KPK92042.1	nSTAND1+BetaPropeller→ wHTH-4stranded+TPRs+STYKIN+nSTAND1+BetaPropeller*→ SWACOS+TPR+TPR→	wHTH-4stranded+TPRs+STYKIN+nSTAND1+BetaPropeller	1764	AMJ88_11890	chloroflexi	Anaerolineae bacterium SM23_ 63	hypothetical protein AMJ88_11890 [Anaerolineae bacterium SM23_ 63].	GCA_001303965.1
KPL74836.1	TIR+nSTAND1+BetaPropeller*→?→?→ SIG+ApbE→	TIR+nSTAND1+BetaPropeller	1565	ADN00_13440	chloroflexi	Ornatilinea apprima	hypothetical protein ADN00_13440 [Ornatilinea apprima].	GCA_001306115.1
KPQ33209.1	CASPASE+CASPASE+nSTAND1+BetaPropeller*→||<-?<-?<-?<-?<-?||PKinase+SWACOS→	CASPASE+CASPASE+nSTAND1+BetaPropeller	1796	HLUCCA11_19215	cyanobacteria	Phormidesmis priestleyi Ana	WD-40 repeat-containing protein [Phormidesmis priestleyi Ana].	GCA_001314865.1
KWW98255.1	Cluster157_3clades→?→||<-?||CASPASE+nSTAND1+TM+BetaPropeller*→	CASPASE+nSTAND1+TM+BetaPropeller	1514	TH66_19845	actinobacteria	Streptomyces thermoautotrophicus	hypothetical protein TH66_19845 [Streptomyces thermoautotrophicus].	GCA_001543895.1
KXK14067.1	<-SIG+TPR<-?||Pkinase+LuxR-HTH+nSTAND1+BetaPropeller*→	Pkinase+LuxR-HTH+nSTAND1+BetaPropeller	1564	UZ15_CFX003003150	chloroflexi	Chloroflexi bacterium OLB15	NB-ARC domain-containing protein [Chloroflexi bacterium OLB15].	GCA_001567085.1

<table>
<thead>
<tr><th>acc</th><th>operon</th><th>architecture</th><th>len</th><th>gen.name</th><th>taxend</th><th>species</th><th>defline</th><th>gca</th></tr>
</thead>
<tbody>
<tr><td>KXK22484.1</td><td>Pkinase+LuxR-HTH+nSTAND1+BetaPropeller*→ SIG+TPR→||<-tRNA<-?<-?<-?<-?||?→ STYKIN→</td><td>Pkinase+LuxR-HTH+nSTAND1+BetaPropeller</td><td>1598</td><td>UZ15_CFX003001056</td><td>chloroflexi</td><td>Chloroflexi bacterium OLB15</td><td>WD-40 repeat-containing protein, partial [Chloroflexi bacterium OLB15].</td><td>GCA_001567085.1</td></tr>
<tr><td>KXK24442.1</td><td>TM+TM+TM+TM+TM+TM+TM+TM+TM+TM+TM+cNMP→||<-?||?→ TIR+DrHyd→ nSTAND1+TM+BetaPropeller*→?→?→?→?→?→?→ ABhydrolase→</td><td>nSTAND1+TM+BetaPropeller</td><td>1066</td><td>UZ12_BCD005001992</td><td>bacteroidetes</td><td>Bacteroidetes bacterium OLB12</td><td>WD-40 repeat-containing protein [Bacteroidetes bacterium OLB12].</td><td>GCA_001567185.1</td></tr>
<tr><td>MAJ16671.1</td><td><-SIG+BetaPropeller<-?<-?<-?||?→ TIR+DrHyd→ nSTAND1+BetaPropeller*→ SIG+IES1→</td><td>nSTAND1+BetaPropeller</td><td>1469</td><td>CMO51_06400</td><td>verrucomicrobia</td><td>Verrucomicrobiales bacterium</td><td>hypothetical protein CMO51_06400 [Verrucomicrobiales bacterium].</td><td>GCA_002691505.1</td></tr>
<tr><td>MAM90836.1</td><td>TIR+DrHyd→ nSTAND1+TM+BetaPropeller*→</td><td>nSTAND1+TM+BetaPropeller</td><td>1106</td><td>CMI15_05100</td><td>verrucomicrobia</td><td>Opitutaceae bacterium</td><td>hypothetical protein CMI15_05100, partial [Opitutaceae bacterium].</td><td>GCA_002694885.1</td></tr>
<tr><td>MAR12587.1</td><td>Pkinase+TIR+DrHyd→ nSTAND1+ANK*→||<-?<-?<-?<-?<-?<-SIG+BetaPropeller</td><td>nSTAND1+ANK</td><td>1675</td><td>CL681_21775</td><td>planctomycetes</td><td>Blastopirellula sp.</td><td>hypothetical protein CL681_21775 [Blastopirellula sp.].</td><td>GCA_002701545.1</td></tr>
<tr><td>MAT97242.1</td><td>REC→?→?→?→?→||<-TM||?→ HTH+Pkinase+nSTAND1+TM+BetaPropeller*→</td><td>HTH+Pkinase+nSTAND1+TM+BetaPropeller</td><td>1592</td><td>CL608_08880</td><td>chloroflexi</td><td>Anaerolineaceae bacterium</td><td>hypothetical protein CL608_08880 [Anaerolineaceae bacterium].</td><td>GCA_002699125.1</td></tr>
<tr><td>MAU11726.1</td><td><-Uma2||?→?→||<-?||Pkinase+LuxR-HTH+nSTAND1*→</td><td>Pkinase+LuxR-HTH+nSTAND1</td><td>757</td><td>CL607_18015</td><td>chloroflexi</td><td>Anaerolineaceae bacterium</td><td>hypothetical protein CL607_18015, partial [Anaerolineaceae bacterium].</td><td>GCA_002699585.1</td></tr>
<tr><td>MAU12933.1</td><td>SIG+ABhydrolase→?→ ABC-ATPase→?→?→ PSE→||<-PSE||Pkinase+LuxR-HTH+nSTAND1+BetaPropeller*→ SIG+ABhydrolase→||<-?<-REC<-SIG+HISKIN+REC+AraC-HTH||?→||<-REC</td><td>Pkinase+LuxR-HTH+nSTAND1+BetaPropeller</td><td>1592</td><td>CL607_24150</td><td>chloroflexi</td><td>Anaerolineaceae bacterium</td><td>hypothetical protein CL607_24150 [Anaerolineaceae bacterium].</td><td>GCA_002699585.1</td></tr>
<tr><td>MAV38177.1</td><td>Pkinase+TIR+DrHyd+TIR→ nSTAND1+TM+BetaPropeller*→</td><td>nSTAND1+TM+BetaPropeller</td><td>1393</td><td>CMJ59_22285</td><td>planctomycetes</td><td>Planctomycetaceae bacterium</td><td>hypothetical protein CMJ59_22285, partial [Planctomycetaceae bacterium].</td><td>GCA_002702965.1</td></tr>
<tr><td>MBB75929.1</td><td>Pkinase→ nSTAND1+BetaPropeller+TPR+TPR*→</td><td>nSTAND1+BetaPropeller+TPR+TPR</td><td>2720</td><td>CMJ75_15595</td><td>planctomycetes</td><td>Planctomycetaceae bacterium</td><td>hypothetical protein CMJ75_15595 [Planctomycetaceae bacterium].</td><td>GCA_002709225.1</td></tr>
<tr><td>MBE75923.1</td><td><-SIG+BetaPropeller||?→||<-?<-?<-?<-?||Pkinase+TIR+DrHyd+TIR→ nSTAND1+BetaPropeller+7TMR-DISMED1*→</td><td>nSTAND1+BetaPropeller+7TMR-DISMED1</td><td>2375</td><td>CMM04_16030</td><td>planctomycetes</td><td>Rhodopirellula sp.</td><td>hypothetical protein CMM04_16030 [Rhodopirellula sp.].</td><td>GCA_002714565.1</td></tr>
<tr><td>MBH57175.1</td><td>Pkinase+TIR+DrHyd→ nSTAND1+ANK*→||<-?<-?||SIG+BetaPropeller→||<-SIG+BetaPropeller</td><td>nSTAND1+ANK</td><td>1672</td><td>CMJ82_08335</td><td>planctomycetes</td><td>Planctomycetaceae bacterium</td><td>hypothetical protein CMJ82_08335 [Planctomycetaceae bacterium].</td><td>GCA_002717145.1</td></tr>
<tr><td>MBL58930.1</td><td>TIR+DrHyd→ nSTAND1+BetaPropeller+TPR*→||<-?<-ABC-ATPase</td><td>nSTAND1+BetaPropeller+TPR</td><td>1457</td><td>CMO75_04590</td><td>verrucomicrobia</td><td>Verrucomicrobiales bacterium</td><td>hypothetical protein CMO75_04590 [Verrucomicrobiales bacterium].</td><td>GCA_002721375.1</td></tr>
<tr><td>MBL59292.1</td><td>ABC-ATPase→ REC→?→?→||<-?||?→ TIR+DrHyd→ nSTAND1+BetaPropeller+BetaPropeller+TPR+TPR*→||<-?<-?<-SIG+BetaPropeller</td><td>nSTAND1+BetaPropeller+BetaPropeller+TPR+TPR</td><td>2167</td><td>CMO75_06445</td><td>verrucomicrobia</td><td>Verrucomicrobiales bacterium</td><td>hypothetical protein CMO75_06445 [Verrucomicrobiales bacterium].</td><td>GCA_002721375.1</td></tr>
</tbody>
</table>

<table>
<tr><th>acc</th><th>operon</th><th>architecture</th><th>len</th><th>gen.name</th><th>taxend</th><th>species</th><th>defline</th><th>gca</th></tr>
<tr><td>MBL59695.1</td><td><-ABhydrolase<-?<-?||TIR+DrHyd→ nSTAND1+BetaPropeller*→</td><td>nSTAND1+BetaPropeller</td><td>1446</td><td>CMO75_08500</td><td>verrucomicrobia</td><td>Verrucomicrobiales bacterium</td><td>hypothetical protein CMO75_08500 [Verrucomicrobiales bacterium].</td><td>GCA_002721375.1</td></tr>
<tr><td>MBO11083.1</td><td>Pkinase+DrHyd→ SIG+nSTAND1+BetaPropeller+TPR*→</td><td>SIG+nSTAND1+BetaPropeller+TPR</td><td>2094</td><td>CMJ68_09995</td><td>planctomycetes</td><td>Planctomycetaceae bacterium</td><td>hypothetical protein CMJ68_09995 [Planctomycetaceae bacterium].</td><td>GCA_002724415.1</td></tr>
<tr><td>MBO89933.1</td><td>TIR+DrHyd→ nSTAND1+TM+BetaPropeller*→</td><td>nSTAND1+TM+BetaPropeller</td><td>1371</td><td>CMP14_10460</td><td>alphaproteobacteria</td><td>Rickettsiales bacterium</td><td>hypothetical protein CMP14_10460, partial [Rickettsiales bacterium].</td><td>GCA_002724555.1</td></tr>
<tr><td>MBT32320.1</td><td>TM+TM+TM+TM+TM+TM+TM+TM+TM+TM+TM+cNMP→ TIR→ nSTAND1+BetaPropeller*→</td><td>nSTAND1+BetaPropeller</td><td>783</td><td>CMO01_21870</td><td>alphaproteobacteria</td><td>Thalassobius sp.</td><td>hypothetical protein CMO01_21870, partial [Thalassobius sp.].</td><td>GCA_002729595.1</td></tr>
<tr><td>MBV59171.1</td><td>TIR+DrHyd→ nSTAND1+BetaPropeller*→</td><td>nSTAND1+BetaPropeller</td><td>966</td><td>CMO58_06170</td><td>verrucomicrobia</td><td>Verrucomicrobiales bacterium</td><td>hypothetical protein CMO58_06170, partial [Verrucomicrobiales bacterium].</td><td>GCA_002731855.1</td></tr>
<tr><td>MRR18651.1</td><td>AAA+TPR→?→ SIG+TM→ MORC→?→ AAA→?→ nSTAND1+TM+BetaPropeller*→?→?→?→||<-SIG+IES1+CBS<-?<-?<-SIG+TPR</td><td>nSTAND1+TM+BetaPropeller</td><td>1007</td><td>EG827_00520</td><td>bacteria</td><td>bacterium</td><td>hypothetical protein EG827_00520 [bacterium].</td><td>GCA_009668865.1</td></tr>
<tr><td>MSR57801.1</td><td>Pkinase→ TIR→ TIR+DrHyd→ nSTAND1+BetaPropeller+PDZ*→</td><td>nSTAND1+BetaPropeller+PDZ</td><td>1868</td><td>EXS05_09020</td><td>planctomycetes</td><td>Planctomycetaceae bacterium</td><td>PDZ domain-containing protein [Planctomycetaceae bacterium].</td><td>GCA_009691905.1</td></tr>
<tr><td>MSV29424.1</td><td><-SIG+Metallopeptidase||TPR→?→ nSTAND1+TM+BetaPropeller*→ SIG+BetaPropeller→</td><td>nSTAND1+TM+BetaPropeller</td><td>957</td><td>EXQ52_11895</td><td>acidobacteria</td><td>Bryobacterales bacterium</td><td>hypothetical protein EXQ52_11895 [Bryobacterales bacterium].</td><td>GCA_009697455.1</td></tr>
<tr><td>MSV29798.1</td><td>TIR→ nSTAND1+FGS*→</td><td>nSTAND1+FGS</td><td>844</td><td>EXQ52_13790</td><td>acidobacteria</td><td>Bryobacterales bacterium</td><td>hypothetical protein EXQ52_13790 [Bryobacterales bacterium].</td><td>GCA_009697455.1</td></tr>
<tr><td>MZQ49612.1</td><td>nSTAND1+TM+BetaPropeller*→</td><td>nSTAND1+TM+BetaPropeller</td><td>1023</td><td>GT598_11475</td><td>bacteroidetes</td><td>Bacteroidales bacterium</td><td>hypothetical protein GT598_11475 [Bacteroidales bacterium].</td><td>-</td></tr>
<tr><td>NBC83322.1</td><td>MORC→?→ AAA→||<-?||TM+TM+TM+TM+TM+TM+TM+TM+TM+TM+TM+cNMP→ TIR+DrHyd→ nSTAND1+BetaPropeller*→</td><td>nSTAND1+BetaPropeller</td><td>1023</td><td>GVY19_08055</td><td>bacteroidetes</td><td>Bacteroidetes bacterium</td><td>hypothetical protein GVY19_08055 [Bacteroidetes bacterium].</td><td>-</td></tr>
<tr><td>NBQ24983.1</td><td>TIR→ TIR+DrHyd→ nSTAND1+BetaPropeller+BetaPropeller*→</td><td>nSTAND1+BetaPropeller+BetaPropeller</td><td>1109</td><td>EBU26_12125</td><td>verrucomicrobia</td><td>Verrucomicrobia bacterium</td><td>hypothetical protein EBU26_12125, partial [Verrucomicrobia bacterium].</td><td>GCA_009920095.1</td></tr>
<tr><td>NBW34856.1</td><td>SIG+HISKIN+REC+AraC-HTH→?→?→ SIG+ASH-IG→?→?→ TM+TM+TM+TM+TM+TM+TM+TM+TM+TM+TM+cNMP→ nSTAND1+TM+BetaPropeller*→</td><td>nSTAND1+TM+BetaPropeller</td><td>1061</td><td>EBR30_07525</td><td>bacteroidetes</td><td>Cytophagia bacterium</td><td>hypothetical protein EBR30_07525 [Cytophagia bacterium].</td><td>-</td></tr>
<tr><td>NCF64926.1</td><td>Pkinase+LuxR-HTH+nSTAND1+BetaPropeller*→ BetaPropeller→?→||<-?<-?<-?<-ABhydrolase</td><td>Pkinase+LuxR-HTH+nSTAND1+BetaPropeller</td><td>1614</td><td>GWP61_03065</td><td>chloroflexi</td><td>Chloroflexi bacterium</td><td>protein kinase [Chloroflexi bacterium].</td><td>-</td></tr>
<tr><td>NCF85778.1</td><td>TIR+DrHyd→ nSTAND1+TM+BetaPropeller*→?→?→||<-?||ABhydrolase→</td><td>nSTAND1+TM+BetaPropeller</td><td>1368</td><td>GWQ08_09620</td><td>verrucomicrobia</td><td>Verrucomicrobiaceae bacterium</td><td>hypothetical protein GWQ08_09620 [Verrucomicrobiaceae bacterium].</td><td>-</td></tr>
<tr><td>NCR43132.1</td><td>CASPASE+nSTAND1+TM+BetaPropeller*→</td><td>CASPASE+nSTAND1+TM+BetaPropeller</td><td>1425</td><td>GPJ09_01855</td><td>cyanobacteria</td><td>Microcystis aeruginosa SX13-01</td><td>hypothetical protein GPJ09_01855 [Microcystis aeruginosa SX13-01].</td><td>-</td></tr>
</table>

<table>
<thead>
<tr><th>acc</th><th>operon</th><th>architecture</th><th>len</th><th>gen.name</th><th>taxend</th><th>species</th><th>defline</th><th>gca</th></tr>
</thead>
<tbody>
<tr><td>NCS30183.1</td><td><-Trypco1||CASPASE+nSTAND1+FGS*→</td><td>CASPASE+nSTAND1+FGS</td><td>1065</td><td>GPJ18_17185</td><td>cyanobacteria</td><td>Microcystis aeruginosa F13-15</td><td>SUMF1/EgtB/PvdO family nonheme iron enzyme [Microcystis aeruginosa F13-15].</td><td>-</td></tr>
<tr><td>NDE10486.1</td><td>TIR+TIR→ nSTAND1+BetaPropeller*→</td><td>nSTAND1+BetaPropeller</td><td>1021</td><td>EBZ95_07970</td><td>bacteroidetes</td><td>Chitinophagia bacterium</td><td>hypothetical protein EBZ95_07970 [Chitinophagia bacterium].</td><td>-</td></tr>
<tr><td>NDJ26297.1</td><td>nSTAND1+TM*→</td><td>nSTAND1+TM</td><td>850</td><td>GS682_33040</td><td>cyanobacteria</td><td>Nostoc sp. B(2019)</td><td>hypothetical protein GS682_33040, partial [Nostoc sp. B(2019)].</td><td>-</td></tr>
<tr><td>NDJ53782.1</td><td>wHTH-4stranded+TPRs+nSTAND1+TM+BetaPropeller*→</td><td>wHTH-4stranded+TPRs+nSTAND1+TM+BetaPropeller</td><td>1239</td><td>GYB68_11940</td><td>chloroflexi</td><td>Chloroflexi bacterium</td><td>hypothetical protein GYB68_11940 [Chloroflexi bacterium].</td><td>-</td></tr>
<tr><td>NDJ62327.1</td><td>Pkinase+nSTAND1+TM+BetaPropeller*→</td><td>Pkinase+nSTAND1+TM+BetaPropeller</td><td>1217</td><td>GYB67_14465</td><td>chloroflexi</td><td>Chloroflexi bacterium</td><td>protein kinase, partial [Chloroflexi bacterium].</td><td>-</td></tr>
<tr><td>NDJ75022.1</td><td><-SIG+ABhydrolase<-?||Pkinase+LuxR-HTH+nSTAND1+BetaPropeller*→</td><td>Pkinase+LuxR-HTH+nSTAND1+BetaPropeller</td><td>1613</td><td>GYB65_02080</td><td>chloroflexi</td><td>Chloroflexi bacterium</td><td>protein kinase [Chloroflexi bacterium].</td><td>-</td></tr>
<tr><td>NDJ75955.1</td><td><-REC||?→?→||<-?<-SIG+ABhydrolase||Pkinase+LuxR-HTH+nSTAND1+BetaPropeller*→ ROK-HTH→ SIG+TM+TM+TM+TM+TM→ SIG+TM+TM+TM+TM+TM→</td><td>Pkinase+LuxR-HTH+nSTAND1+BetaPropeller</td><td>1599</td><td>GYB65_06825</td><td>chloroflexi</td><td>Chloroflexi bacterium</td><td>protein kinase [Chloroflexi bacterium].</td><td>-</td></tr>
<tr><td>NDJ77885.1</td><td>Pkinase+nSTAND1+BetaPropeller*→</td><td>Pkinase+nSTAND1+BetaPropeller</td><td>1485</td><td>GYB65_16675</td><td>chloroflexi</td><td>Chloroflexi bacterium</td><td>protein kinase [Chloroflexi bacterium].</td><td>-</td></tr>
<tr><td>NDJ85618.1</td><td>REC→?→ Pkinase+nSTAND1+BetaPropeller*→||<-VWA+FHA||?→?→||<-ABC-ATPase</td><td>Pkinase+nSTAND1+BetaPropeller</td><td>1487</td><td>GYB66_07010</td><td>chloroflexi</td><td>Chloroflexi bacterium</td><td>protein kinase [Chloroflexi bacterium].</td><td>-</td></tr>
<tr><td>NDQ57070.1</td><td>ROK-HTH→?→ ABhydrolase→ TIR+DrHyd→ nSTAND1+TPR*→?→||<-?<-?<-?<-?||SIG+VIT+VWA+TM→</td><td>nSTAND1+TPR</td><td>939</td><td>GZ088_08360</td><td>acidobacteria</td><td>Acidipila sp.</td><td>tetratricopeptide repeat protein [Acidipila sp.].</td><td>-</td></tr>
<tr><td>NEO05953.1</td><td><-Cluster59_2clades||APATPase+BetaPropeller→?→||<-?<-?||PSE→ nSTAND1+BetaPropeller+TPR*→</td><td>nSTAND1+BetaPropeller+TPR</td><td>1521</td><td>F6K51_10425</td><td>cyanobacteria</td><td>Moorea sp. SIO3I8</td><td>hypothetical protein F6K51_10425 [Moorea sp. SIO3I8].</td><td>-</td></tr>
<tr><td>NEO22392.1</td><td>APATPase+BetaPropeller→?→||<-?<-?||PSE→ nSTAND1+TM+BetaPropeller+TPR*→ Cluster59_2clades→</td><td>nSTAND1+TM+BetaPropeller+TPR</td><td>1415</td><td>F6K57_24570</td><td>cyanobacteria</td><td>Moorea sp. SIO4A5</td><td>hypothetical protein F6K57_24570 [Moorea sp. SIO4A5].</td><td>-</td></tr>
<tr><td>NEO24022.1</td><td>Trypsin+nSTAND1+BetaPropeller*→</td><td>Trypsin+nSTAND1+BetaPropeller</td><td>1347</td><td>F6K57_33355</td><td>cyanobacteria</td><td>Moorea sp. SIO4A5</td><td>hypothetical protein F6K57_33355 [Moorea sp. SIO4A5].</td><td>-</td></tr>
<tr><td>NEO40588.1</td><td>RVT→ RVT→ RVT→ PSE→ PSE→?→||<-?||?→?→ nSTAND1*→ BetaPropeller→</td><td>nSTAND1</td><td>417</td><td>F6J90_31350</td><td>cyanobacteria</td><td>Moorea sp. SIOASIH</td><td>hypothetical protein F6J90_31350 [Moorea sp. SIOASIH].</td><td>-</td></tr>
<tr><td>NEO55095.1</td><td><-SUA||?→ nSTAND1+BetaPropeller+TPR*→</td><td>nSTAND1+BetaPropeller+TPR</td><td>1462</td><td>F6K54_19650</td><td>cyanobacteria</td><td>Okeania sp. SIO3B5</td><td>WD40 repeat domain-containing protein [Okeania sp. SIO3B5].</td><td>-</td></tr>
<tr><td>NEO77038.1</td><td>EACC2+CASPASE+nSTAND1+BetaPropeller*→</td><td>EACC2+CASPASE+nSTAND1+BetaPropeller</td><td>1208</td><td>F6J99_12650</td><td>cyanobacteria</td><td>Moorea sp. SIO4G3</td><td>CHAT domain-containing protein, partial [Moorea sp. SIO4G3].</td><td>-</td></tr>
<tr><td>NEO88965.1</td><td>Pentapeptide→ CASPASE→ nSTAND1+BetaPropeller*→</td><td>nSTAND1+BetaPropeller</td><td>1295</td><td>F6K56_01245</td><td>cyanobacteria</td><td>Moorea sp. SIO3G5</td><td>hypothetical protein F6K56_01245 [Moorea sp. SIO3G5].</td><td>-</td></tr>
<tr><td>NEO95082.1</td><td>Trypsin+nSTAND1+ThuA-GATase*→</td><td>Trypsin+nSTAND1+ThuA-GATase</td><td>909</td><td>F6K56_34765</td><td>cyanobacteria</td><td>Moorea sp. SIO3G5</td><td>trypsin-like serine protease [Moorea sp. SIO3G5].</td><td>-</td></tr>
<tr><td>NEP00479.1</td><td><-PKinase+SWACOS+GAF+SHELIX+HISKIN+REC<-?<-?<-TPR+TPR<-?<-MNS||?→ nSTAND1+*→</td><td>nSTAND1+</td><td>776</td><td>F6K58_17710</td><td>cyanobacteria</td><td>Symploca sp. SIO2E9</td><td>hypothetical protein F6K58_17710 [Symploca sp. SIO2E9].</td><td>-</td></tr>
</tbody>
</table>

<table>
<tr><th>acc</th><th>operon</th><th>architecture</th><th>len</th><th>gen.name</th><th>taxend</th><th>species</th><th>defline</th><th>gca</th></tr>
<tr><td>NEP10746.1</td><td><-Cluster108_2clades||?→ PKinase+SWACOS+GAF+SHELIX+HISKIN+REC→?→?→?→||<-Trypco1||CASPASE+nSTAND1+TM+GUN4*→</td><td>CASPASE+nSTAND1+TM+GUN4</td><td>1012</td><td>F6K14_11125</td><td>cyanobacteria</td><td>Symploca sp. SIO2C1</td><td>hypothetical protein F6K14_11125 [Symploca sp. SIO2C1].</td><td>-</td></tr>
<tr><td>NEP16657.1</td><td>ABhydrolase→?→?→||<-?||CASPASE+nSTAND1*→</td><td>CASPASE+nSTAND1</td><td>919</td><td>F6J97_07085</td><td>cyanobacteria</td><td>Leptolyngbya sp. SIO4C1</td><td>hypothetical protein F6J97_07085 [Leptolyngbya sp. SIO4C1].</td><td>-</td></tr>
<tr><td>NEP23856.1</td><td>CASPASE+nSTAND1+TM+BetaPropeller*→?→||<-?<-?<-SIG+TM+TM+TM+TM+TM+TM+TM+TM</td><td>CASPASE+nSTAND1+TM+BetaPropeller</td><td>1358</td><td>F6K49_18200</td><td>cyanobacteria</td><td>Moorea sp. SIO3I6</td><td>CHAT domain-containing protein [Moorea sp. SIO3I6].</td><td>-</td></tr>
<tr><td>NEQ36510.1</td><td>GUN4→ CASPASE+EAD10→?→ EAD10+nSTAND1+TM+BetaPropeller*→</td><td>EAD10+nSTAND1+TM+BetaPropeller</td><td>1351</td><td>F6K40_09560</td><td>cyanobacteria</td><td>Okeania sp. SIO3I5</td><td>WD40 repeat domain-containing protein [Okeania sp. SIO3I5].</td><td>-</td></tr>
<tr><td>NEQ36637.1</td><td>RRM→||<-?||?→ Trypsin+nSTAND1+BetaPropeller*→</td><td>Trypsin+nSTAND1+BetaPropeller</td><td>1463</td><td>F6K40_10255</td><td>cyanobacteria</td><td>Okeania sp. SIO3I5</td><td>trypsin-like serine protease [Okeania sp. SIO3I5].</td><td>-</td></tr>
<tr><td>NEQ38190.1</td><td><-SIG+TPR||?→ Trypsin+nSTAND1+TM+TPR+TPR+TPR*→</td><td>Trypsin+nSTAND1+TM+TPR+TPR+TPR</td><td>1116</td><td>F6K40_18755</td><td>cyanobacteria</td><td>Okeania sp. SIO3I5</td><td>tetratricopeptide repeat protein, partial [Okeania sp. SIO3I5].</td><td>-</td></tr>
<tr><td>NEQ49185.1</td><td><-NUDIX<-?||?→?→||<-ParA-Soj-PloopNTPase||TIR+nSTAND1*→</td><td>TIR+nSTAND1</td><td>848</td><td>F6K11_03505</td><td>cyanobacteria</td><td>Leptolyngbya sp. SIO3F4</td><td>TIR domain-containing protein [Leptolyngbya sp. SIO3F4].</td><td>-</td></tr>
<tr><td>NEQ50781.1</td><td><-Pentapeptide||TIR+nSTAND1*→ TIR+TM+BetaPropeller→</td><td>TIR+nSTAND1</td><td>927</td><td>F6K11_11700</td><td>cyanobacteria</td><td>Leptolyngbya sp. SIO3F4</td><td>TIR domain-containing protein [Leptolyngbya sp. SIO3F4].</td><td>-</td></tr>
<tr><td>NEQ65990.1</td><td>CASPASE+nSTAND1+TM+BetaPropeller*→ PSE→?→||<-?<-TM</td><td>CASPASE+nSTAND1+TM+BetaPropeller</td><td>1386</td><td>F6K21_10900</td><td>cyanobacteria</td><td>Symploca sp. SIO2D2</td><td>hypothetical protein F6K21_10900 [Symploca sp. SIO2D2].</td><td>-</td></tr>
<tr><td>NEQ66888.1</td><td><-SIG+Pentapeptide<-Trypco1||CASPASE+nSTAND1+TM+BetaPropeller*→ BetaPropeller→</td><td>CASPASE+nSTAND1+TM+BetaPropeller</td><td>1422</td><td>F6K21_15565</td><td>cyanobacteria</td><td>Symploca sp. SIO2D2</td><td>peptidase C14, partial [Symploca sp. SIO2D2].</td><td>-</td></tr>
<tr><td>NEQ68332.1</td><td>nSTAND1+TM+BetaPropeller*→</td><td>nSTAND1+TM+BetaPropeller</td><td>1227</td><td>F6K21_23100</td><td>cyanobacteria</td><td>Symploca sp. SIO2D2</td><td>hypothetical protein F6K21_23100 [Symploca sp. SIO2D2].</td><td>-</td></tr>
<tr><td>NER26420.1</td><td><-ABhydrolase<-Trypco1||CASPASE+nSTAND1+GUN4*→||<-?||?→ ParA-Soj-PloopNTPase→</td><td>CASPASE+nSTAND1+GUN4</td><td>980</td><td>F6J89_02035</td><td>cyanobacteria</td><td>Symploca sp. SIO1C4</td><td>hypothetical protein F6J89_02035 [Symploca sp. SIO1C4].</td><td>-</td></tr>
<tr><td>NER33117.1</td><td>CASPASE+nSTAND1+BetaPropeller*→</td><td>CASPASE+nSTAND1+BetaPropeller</td><td>1563</td><td>F6J93_03400</td><td>cyanobacteria</td><td>Oscillatoria sp. SIO1A7</td><td>hypothetical protein F6J93_03400 [Oscillatoria sp. SIO1A7].</td><td>-</td></tr>
<tr><td>NER37564.1</td><td>EAD8+Trypsin→||<-?||HTH+nSTAND1+TM+BetaPropeller*→</td><td>HTH+nSTAND1+TM+BetaPropeller</td><td>1342</td><td>F6J93_26975</td><td>cyanobacteria</td><td>Oscillatoria sp. SIO1A7</td><td>hypothetical protein F6J93_26975 [Oscillatoria sp. SIO1A7].</td><td>-</td></tr>
<tr><td>NER38168.1</td><td><-TM<-?||CASPASE+nSTAND1+BetaPropeller*→||<-Uma2<-?<-?<-?<-SIG+TM+TM||?→ Uma2→</td><td>CASPASE+nSTAND1+BetaPropeller</td><td>1954</td><td>F6J93_30125</td><td>cyanobacteria</td><td>Oscillatoria sp. SIO1A7</td><td>hypothetical protein F6J93_30125 [Oscillatoria sp. SIO1A7].</td><td>-</td></tr>
<tr><td>NER71216.1</td><td><-ABhydrolase<-?<-?||?→||<-Trypco1||CASPASE+nSTAND1*→</td><td>CASPASE+nSTAND1</td><td>926</td><td>F6J95_03475</td><td>cyanobacteria</td><td>Leptolyngbya sp. SIO1E4</td><td>peptidase C14 [Leptolyngbya sp. SIO1E4].</td><td>-</td></tr>
<tr><td>NER94406.1</td><td><-EAD4+APATPase+BetaPropeller||?→?→||<-?<-?<-Trypco1||PASE+nSTAND1+TM+Pentapeptide*→</td><td>PASE+nSTAND1+TM+Pentapeptide</td><td>1064</td><td>F6J86_11280</td><td>cyanobacteria</td><td>Symploca sp. SIO1B1</td><td>hypothetical protein F6J86_11280 [Symploca sp. SIO1B1].</td><td>-</td></tr>
<tr><td>NES05250.1</td><td>CASPASE+EAD10→?→ EAD10+nSTAND1+TM+BetaPropeller*→</td><td>EAD10+nSTAND1+TM+BetaPropeller</td><td>1461</td><td>F6K22_21960</td><td>cyanobacteria</td><td>Okeania sp. SIO2F4</td><td>hypothetical protein F6K22_21960 [Okeania sp. SIO2F4].</td><td>-</td></tr>
</table>

acc	operon	architecture	len	gen.name	taxend	species	defline	gca
NES64500.1	Cluster59_2clades→ GUN4→ nSTAND1*→	nSTAND1	623	F6K24_04135	cyanobacteria	Okeania sp. SIO2D1	S-layer homology domain-containing protein [Okeania sp. SIO2D1].	-
NES65413.1	nSTAND1+TM+BetaPropeller*→	nSTAND1+TM+BetaPropeller	1165	F6K24_09195	cyanobacteria	Okeania sp. SIO2D1	WD40 repeat domain-containing protein, partial [Okeania sp. SIO2D1].	-
NES97446.1	nSTAND1+BetaPropeller*→	nSTAND1+BetaPropeller	1191	F6K32_19940	cyanobacteria	Desertifilum sp. SIO1I2	WD40 repeat domain-containing protein, partial [Desertifilum sp. SIO1I2].	-
NET34881.1	CASPASE+nSTAND1+TM*→	CASPASE+nSTAND1+TM	975	F6K19_23135	cyanobacteria	Cyanothece sp. SIO1E1	hypothetical protein F6K19_23135 [Cyanothece sp. SIO1E1].	-
NET36631.1	<-MNS<-?||?→||<-?||CASPASE+nSTAND1*→	CASPASE+nSTAND1	704	F6K19_32150	cyanobacteria	Cyanothece sp. SIO1E1	hypothetical protein F6K19_32150 [Cyanothece sp. SIO1E1].	-
NET37730.1	CASPASE+nSTAND1*→	CASPASE+nSTAND1	567	F6K19_37925	cyanobacteria	Cyanothece sp. SIO1E1	hypothetical protein F6K19_37925, partial [Cyanothece sp. SIO1E1].	-
NET40110.1	nSTAND1*→	nSTAND1	497	F6K19_50595	cyanobacteria	Cyanothece sp. SIO1E1	ATP-binding protein, partial [Cyanothece sp. SIO1E1].	-
NET86531.1	<-FAD-NAD-dep-oxidoreductase<-?<-TM<-?||?→?→ EACC2+CASPASE+nSTAND1+BetaPropeller*→	EACC2+CASPASE+nSTAND1+BetaPropeller	1577	F6K45_00165	cyanobacteria	Kamptonema sp. SIO1D9	CHAT domain-containing protein [Kamptonema sp. SIO1D9].	-
NET91215.1	CASPASE+nSTAND1+TM+BetaPropeller*→	CASPASE+nSTAND1+TM+BetaPropeller	1474	F6K45_24510	cyanobacteria	Kamptonema sp. SIO1D9	hypothetical protein F6K45_24510 [Kamptonema sp. SIO1D9].	-
NIM11146.1	TIR+DrHyd→ nSTAND1+BetaPropeller*→?→?→?→?→ TIR→	nSTAND1+BetaPropeller	1081	GTO81_04125	bacteria	Candidatus Aminicenantes bacterium	AAA family ATPase [Candidatus Aminicenantes bacterium].	-
NIM60309.1	TIR+DrHyd→ nSTAND1+BetaPropeller*→ BetaPropeller→	nSTAND1+BetaPropeller	1137	GTO30_01255	acidobacteria	Acidobacteria bacterium	hypothetical protein GTO30_01255 [Acidobacteria bacterium].	-
NIN64528.1	wHTH-4stranded+TPRs+nSTAND1+BetaPropeller*→ PSE→?→?→ ICLR-HTH→	wHTH-4stranded+TPRs+nSTAND1+BetaPropeller	1481	GTO63_07465	chloroflexi	Anaerolineae bacterium	hypothetical protein GTO63_07465 [Anaerolineae bacterium].	-
NIO87402.1	nSTAND1*→	nSTAND1	271	GTN68_43335	bacteria	Candidatus Aminicenantes bacterium	hypothetical protein GTN68_43335, partial [Candidatus Aminicenantes bacterium].	-
NIP95306.1	TIR+DrHyd→ nSTAND1*→ nSTAND1→	nSTAND1	157	GWO24_18410	verrucomicrobia	Akkermansiaceae bacterium	ATP-binding protein, partial [Akkermansiaceae bacterium].	-
NIQ11829.1	Pkinase+LuxR-HTH+nSTAND1*→ nSTAND1→	Pkinase+LuxR-HTH+nSTAND1	550	GWO23_20275	gammaproteobacteria	Gammaproteobacteria bacterium	protein kinase [Gammaproteobacteria bacterium].	-
NIR47522.1	nSTAND1*→	nSTAND1	215	GWO09_03595	bacteria	candidate division KSB1 bacterium	hypothetical protein GWO09_03595, partial [candidate division KSB1 bacterium].	-

acc	operon	architecture	len	gen.name	taxend	species	defline	gca
NJD08806.1	TIR→ nSTAND1+TM+TM+TM+TM*→	nSTAND1+TM+TM+TM+TM	793	FIA97_20290	gammaproteobacteria	Methylococcaceae bacterium	hypothetical protein FIA97_20290 [Methylococcaceae bacterium].	-
NJK52968.1	SIG+nSTAND1*→	SIG+nSTAND1	216	HC936_09320	cyanobacteria	Leptolyngbyaceae cyanobacterium SU_3_3	ATP-binding protein, partial [Leptolyngbyaceae cyanobacterium SU_3_3].	-
NJK55885.1	CASPASE+CASPASE+nSTAND1+BetaPropeller*→||<-?||?→?→ ABhydrolase→ HOP2→	CASPASE+CASPASE+nSTAND1+BetaPropeller	1614	HC939_07740	cyanobacteria	Pleurocapsa sp. SU_5_0	NACHT domain-containing protein [Pleurocapsa sp. SU_5_0].	-
NJL22036.1	<-SIG+TPR||CASPASE+nSTAND1*→ nSTAND1→	CASPASE+nSTAND1	461	HC895_16420	cyanobacteria	Leptolyngbyaceae cyanobacterium SM1_3_5	hypothetical protein HC895_16420 [Leptolyngbyaceae cyanobacterium SM1_3_5].	-
NJL38884.1	nSTAND1+BetaPropeller*→ SIG+TPR→	nSTAND1+BetaPropeller	1232	HC899_20695	cyanobacteria	Leptolyngbyaceae cyanobacterium SM1_4_3	hypothetical protein HC899_20695 [Leptolyngbyaceae cyanobacterium SM1_4_3].	-
NJL61102.1	<-ENOYCOADEHYD<-ENOYCOADEHYD||VWA+FHA→?→||<-Trypco1||CASPASE+nSTAND1*→ Pentapeptide→	CASPASE+nSTAND1	666	HC903_03770	verrucomicrobia	Methylacidiphilales bacterium	hypothetical protein HC903_03770 [Methylacidiphilales bacterium].	-
NJL84534.1	<-Trypco1||CASPASE+nSTAND1+TM*→	CASPASE+nSTAND1+TM	783	HC890_19295	chloroflexi	Chloroflexaceae bacterium	AAA family ATPase [Chloroflexaceae bacterium].	-
NJM08748.1	HTH+CR-Kinase+nSTAND1*→ nSTAND1→	HTH+CR-Kinase+nSTAND1	746	HC891_25000	bacteria	Candidatus Gracilibacteria bacterium	protein kinase [Candidatus Gracilibacteria bacterium].	-
NJM70522.1	<-Trypco1||CASPASE+nSTAND1*→	CASPASE+nSTAND1	957	HC862_09975	cyanobacteria	Scytonema sp. RU_4_4	hypothetical protein HC862_09975 [Scytonema sp. RU_4_4].	-
NJM71751.1	nSTAND1*→ nSTAND1→	nSTAND1	293	HC862_16985	cyanobacteria	Scytonema sp. RU_4_4	hypothetical protein HC862_16985 [Scytonema sp. RU_4_4].	-
NJM72049.1	TIR+DrHyd→ nSTAND1+TM+BetaPropeller*→?→?→||<-?<-AAA	nSTAND1+TM+BetaPropeller	1212	HC862_18715	cyanobacteria	Scytonema sp. RU_4_4	hypothetical protein HC862_18715 [Scytonema sp. RU_4_4].	-
NJM73217.1	Pkinase→?→||<-?||?→ EACC2+CASPASE+nSTAND1+BetaPropeller*→	EACC2+CASPASE+nSTAND1+BetaPropeller	1020	HC862_25525	cyanobacteria	Scytonema sp. RU_4_4	CHAT domain-containing protein, partial [Scytonema sp. RU_4_4].	-
NJM73324.1	nSTAND1+BetaPropeller*→||<-PSE<-?<-TPR	nSTAND1+BetaPropeller	1058	HC862_26150	cyanobacteria	Scytonema sp. RU_4_4	hypothetical protein HC862_26150 [Scytonema sp. RU_4_4].	-
NJM74119.1	EACC2+CASPASE+nSTAND1*→ nSTAND1→	EACC2+CASPASE+nSTAND1	595	HC862_30810	cyanobacteria	Scytonema sp. RU_4_4	CHAT domain-containing protein [Scytonema sp. RU_4_4].	-
NJN08585.1	Pentapeptide→ CASPASE+nSTAND1+TM*→	CASPASE+nSTAND1+TM	758	HC815_11555	cyanobacteria	Richelia sp. RM1_1_1	hypothetical protein HC815_11555 [Richelia sp. RM1_1_1].	-

<table>
<tr><th>acc</th><th>operon</th><th>architecture</th><th>len</th><th>gen.name</th><th>taxend</th><th>species</th><th>defline</th><th>gca</th></tr>
<tr><td>NJN41702.1</td><td>TM+TM+TM+TM+TM+TM+TM+TM+TM+TM+TM+cNMP→?→ nSTAND1+BetaPropeller*→ BetaPropeller→</td><td>nSTAND1+BetaPropeller</td><td>821</td><td>HC811_05160</td><td>bacteroidetes</td><td>Flammeovirgaceae bacterium</td><td>High-affinity carbon uptake protein Hat/HatR [Flammeovirgaceae bacterium].</td><td>-</td></tr>
<tr><td>NJN44056.1</td><td>Pkinase→?→ nSTAND1+TM+BetaPropeller*→</td><td>nSTAND1+TM+BetaPropeller</td><td>724</td><td>HC806_04550</td><td>chloroflexi</td><td>Anaerolineae bacterium</td><td>hypothetical protein HC806_04550 [Anaerolineae bacterium].</td><td>-</td></tr>
<tr><td>NJN57460.1</td><td>nSTAND1+BetaPropeller*→?→||<-?<-?||?→ REC→?→ TM+HAMP+GAF+GAF+GAF+GAF+MCPsignal→</td><td>nSTAND1+BetaPropeller</td><td>1198</td><td>HC879_08140</td><td>cyanobacteria</td><td>Leptolyngbyaceae cyanobacterium SL_5_9</td><td>hypothetical protein HC879_08140, partial [Leptolyngbyaceae cyanobacterium SL_5_9].</td><td>-</td></tr>
<tr><td>NJN73136.1</td><td>Uma2→ PSE→||<-?<-PSE||?→||<-Trypco1||CASPASE+nSTAND1*→?→?→||<-?<-PSE<-?||SIG+TM+TM+TM+TM+TM→</td><td>CASPASE+nSTAND1</td><td>902</td><td>HC799_10205</td><td>cyanobacteria</td><td>Limnothrix sp. RL_2_0</td><td>peptidase C14 [Limnothrix sp. RL_2_0].</td><td>-</td></tr>
<tr><td>NJN87470.1</td><td>SIG+nSTAND1*→ nSTAND1+BetaPropeller→||<-GGDEF+EAL</td><td>SIG+nSTAND1</td><td>393</td><td>HC881_15635</td><td>cyanobacteria</td><td>Leptolyngbyaceae cyanobacterium SL_7_1</td><td>hypothetical protein HC881_15635 [Leptolyngbyaceae cyanobacterium SL_7_1].</td><td>-</td></tr>
<tr><td>NJO16618.1</td><td>REC→ GGDEF+EAL→?→?→?→ TIR+DrHyd+TIR→ nSTAND1+BetaPropeller*→</td><td>nSTAND1+BetaPropeller</td><td>1205</td><td>HC877_13005</td><td>gammaproteobacteria</td><td>Thioploca sp.</td><td>hypothetical protein HC877_13005 [Thioploca sp.].</td><td>-</td></tr>
<tr><td>NJP11832.1</td><td><-Trypco1||CASPASE+nSTAND1*→</td><td>CASPASE+nSTAND1</td><td>1033</td><td>HC866_22115</td><td>cyanobacteria</td><td>Leptolyngbyaceae cyanobacterium RU_5_1</td><td>AAA family ATPase [Leptolyngbyaceae cyanobacterium RU_5_1].</td><td>-</td></tr>
<tr><td>NJR38187.1</td><td>SIG+TPR→?→?→ nSTAND1*→ nSTAND1→ BetaPropeller→</td><td>nSTAND1</td><td>274</td><td>HC781_04220</td><td>cyanobacteria</td><td>Leptolyngbyaceae cyanobacterium CSU_1_4</td><td>ATP-binding protein [Leptolyngbyaceae cyanobacterium CSU_1_4].</td><td>-</td></tr>
<tr><td>NJR62416.1</td><td>HTH+APATPase+BetaPropeller→||<-?||?→||<-TPR||CASPASE+nSTAND1+BetaPropeller*→</td><td>CASPASE+nSTAND1+BetaPropeller</td><td>1723</td><td>HC769_28590</td><td>cyanobacteria</td><td>Cyanobacteria bacterium CRU_2_1</td><td>hypothetical protein HC769_28590 [Cyanobacteria bacterium CRU_2_1].</td><td>-</td></tr>
<tr><td>NJR67118.1</td><td>CASPASE+nSTAND1+BetaPropeller*→</td><td>CASPASE+nSTAND1+BetaPropeller</td><td>1120</td><td>HC772_20280</td><td>cyanobacteria</td><td>Leptolyngbyaceae cyanobacterium CRU_2_3</td><td>hypothetical protein HC772_20280, partial [Leptolyngbyaceae cyanobacterium CRU_2_3].</td><td>-</td></tr>
<tr><td>NJR75219.1</td><td>TIR+DrHyd→ nSTAND1→ nSTAND1+TM+BetaPropeller*→ BetaPropeller→</td><td>nSTAND1+TM+BetaPropeller</td><td>760</td><td>HC773_19440</td><td>cyanobacteria</td><td>Scytonema sp. CRU_2_7</td><td>WD40 repeat domain-containing protein [Scytonema sp. CRU_2_7].</td><td>-</td></tr>
<tr><td>NKQ34063.1</td><td>Pkinase+nSTAND1+BetaPropeller*→</td><td>Pkinase+nSTAND1+BetaPropeller</td><td>1595</td><td>HF973_00435</td><td>chloroflexi</td><td>Chloroflexi bacterium</td><td>protein kinase [Chloroflexi bacterium].</td><td>-</td></tr>
<tr><td>NLF66968.1</td><td><-REC<-?<-?<-HTH+MED15||?→?→ bDLD1+nSTAND1*→</td><td>bDLD1+nSTAND1</td><td>1094</td><td>GX579_20460</td><td>chloroflexi</td><td>Chloroflexi bacterium</td><td>ATP-binding protein [Chloroflexi bacterium].</td><td>-</td></tr>
<tr><td>NLF75477.1</td><td>TIR+TM+NACHT+TM+TM+TM+TM+TM+TM+TM+FGS→?→||<-SIG+ABhydrolase<-SIG+ABhydrolase<-?<-SIG+ABhydrolase<-?||wHTH-4stranded+TPRs+nSTAND1+TM+BetaPropeller*→</td><td>wHTH-4stranded+TPRs+nSTAND1+TM+BetaPropeller</td><td>1465</td><td>GX573_07245</td><td>chloroflexi</td><td>Chloroflexi bacterium</td><td>hypothetical protein GX573_07245 [Chloroflexi bacterium].</td><td>-</td></tr>
<tr><td>NLF76559.1</td><td>Pkinase+nSTAND1+TM+BetaPropeller*→</td><td>Pkinase+nSTAND1+TM+BetaPropeller</td><td>1037</td><td>GX573_12755</td><td>chloroflexi</td><td>Chloroflexi bacterium</td><td>protein kinase, partial [Chloroflexi bacterium].</td><td>-</td></tr>
</table>

<table>
<tr><th>acc</th><th>operon</th><th>architecture</th><th>len</th><th>gen.name</th><th>taxend</th><th>species</th><th>defline</th><th>gca</th></tr>
<tr><td>NLF76682.1</td><td>wHTH-4stranded+TPRs+nSTAND1+TM+BetaPropeller*→</td><td>wHTH-4stranded+TPRs+nSTAND1+TM+BetaPropeller</td><td>1439</td><td>GX573_13375</td><td>chloroflexi</td><td>Chloroflexi bacterium</td><td>PQQ-binding-like beta-propeller repeat protein, partial [Chloroflexi bacterium].</td><td>-</td></tr>
<tr><td>NNE66468.1</td><td><-Pkinase+TPR||?→ TIR+TIR→ nSTAND1*→</td><td>nSTAND1</td><td>181</td><td>HKN33_07865</td><td>acidobacteria</td><td>Pyrinomonadaceae bacterium</td><td>hypothetical protein HKN33_07865, partial [Pyrinomonadaceae bacterium].</td><td>-</td></tr>
<tr><td>NNF77676.1</td><td>nSTAND1+TM+BetaPropeller*→</td><td>nSTAND1+TM+BetaPropeller</td><td>821</td><td>HKN05_06570</td><td>alphaproteobacteria</td><td>Rhizobiales bacterium</td><td>WD40 repeat domain-containing protein, partial [Rhizobiales bacterium].</td><td>-</td></tr>
<tr><td>NNL69935.1</td><td>TPRs+nSTAND1+PBPB*→</td><td>TPRs+nSTAND1+PBPB</td><td>1201</td><td>HKO70_08255</td><td>actinobacteria</td><td>Acidimicrobiia bacterium</td><td>transporter substrate-binding domain-containing protein, partial [Acidimicrobiia bacterium].</td><td>-</td></tr>
<tr><td>NNM23296.1</td><td><-Patatin||TIR→ nSTAND1+BetaPropeller*→||<-?<-?<-?||?→?→||<-TPR</td><td>nSTAND1+BetaPropeller</td><td>1191</td><td>HKO54_07065</td><td>bacteroidetes</td><td>Flavobacteriaceae bacterium</td><td>hypothetical protein HKO54_07065 [Flavobacteriaceae bacterium].</td><td>-</td></tr>
<tr><td>NOG19904.1</td><td><-SUA<-?<-?<-?||?→ CASPASE+nSTAND1+BetaPropeller*→||<-?||?→?→ HOP2→</td><td>CASPASE+nSTAND1+BetaPropeller</td><td>1739</td><td>HAV12_42195</td><td>bacteroidetes</td><td>Flavobacterium sp. CLA17</td><td>AAA family ATPase [Flavobacterium sp. CLA17].</td><td>-</td></tr>
<tr><td>OFV91558.1</td><td>TIR+DrHyd→ nSTAND1+TPR*→?→||<-?<-?||?→?→||<-S1COLD</td><td>nSTAND1+TPR</td><td>928</td><td>A3H95_01005</td><td>acidobacteria</td><td>Acidobacteria bacterium RIFC-SPLOWO2_02_FULL_64_15</td><td>hypothetical protein A3H95_01005 [Acidobacteria bacterium RIFC-SPLOWO2_02_FULL_64_15].</td><td>GCA_001767165.1</td></tr>
<tr><td>OFW18721.1</td><td>AbiEii→ TIR+DrHyd→ nSTAND1+TM+TPR*→?→ tRNA→||<-?<-?<-ACET</td><td>nSTAND1+TM+TPR</td><td>935</td><td>A3H97_17825</td><td>acidobacteria</td><td>Acidobacteria bacterium RIFC-SPLOWO2_02_FULL_65_29</td><td>hypothetical protein A3H97_17825 [Acidobacteria bacterium RIFC-SPLOWO2_02_FULL_65_29].</td><td>GCA_001767435.1</td></tr>
<tr><td>OFX23090.1</td><td><-ABhydrolase||?→ TM+TM+TM+TM+TM+TM+TM+TM+TM+TM+TM+cNMP→?→ TIR+DrHyd→ nSTAND1+BetaPropeller*→</td><td>nSTAND1+BetaPropeller</td><td>1019</td><td>A2041_06150</td><td>bacteroidetes</td><td>Bacteroidetes bacterium GWA2_31_9b</td><td>hypothetical protein A2041_06150 [Bacteroidetes bacterium GWA2_31_9b].</td><td>GCA_001769005.1</td></tr>
<tr><td>OFX91075.1</td><td>SIG+TM→?→ MORC→?→ AAA→ TM+TM+TM+TM+TM+TM+TM+TM+TM+TM+TM+cNMP→?→ nSTAND1+TM+BetaPropeller*→</td><td>nSTAND1+TM+BetaPropeller</td><td>1030</td><td>A2X06_13615</td><td>bacteroidetes</td><td>Bacteroidetes bacterium GWC2_40_22</td><td>hypothetical protein A2X06_13615 [Bacteroidetes bacterium GWC2_40_22].</td><td>GCA_001768305.1</td></tr>
<tr><td>OFX95806.1</td><td>SIG+TM→?→ MORC→?→ AAA→ TM+TM+TM+TM+TM+TM+TM+TM+TM+TM+TM+cNMP→?→ nSTAND1+TM+BetaPropeller*→</td><td>nSTAND1+TM+BetaPropeller</td><td>1030</td><td>A2X05_06820</td><td>bacteroidetes</td><td>Bacteroidetes bacterium GWE2_41_25</td><td>hypothetical protein A2X05_06820 [Bacteroidetes bacterium GWE2_41_25].</td><td>GCA_001768485.1</td></tr>
<tr><td>OFY35811.1</td><td>TM+TM+TM+TM+TM+TM+TM+TM+TM+TM+TM+cNMP→?→ TIR+DrHyd→ SIG+nSTAND1+TM+BetaPropeller*→?→?→?→?→||<-?<-?||SIG+TPR→</td><td>SIG+nSTAND1+TM+BetaPropeller</td><td>1024</td><td>A2W91_17400</td><td>bacteroidetes</td><td>Bacteroidetes bacterium GWF2_38_335</td><td>hypothetical protein A2W91_17400 [Bacteroidetes bacterium GWF2_38_335].</td><td>GCA_001769535.1</td></tr>
</table>

<table>
<tr><th>acc</th><th>operon</th><th>architecture</th><th>len</th><th>gen.name</th><th>taxend</th><th>species</th><th>defline</th><th>gca</th></tr>
<tr><td>OGA98753.1</td><td>nSTAND1+prokYEATS*→||<-?<-?||ABC-ATPase→</td><td>nSTAND1+prokYEATS</td><td>746</td><td>A3E25_24070</td><td>betaproteobacteria</td><td>Burkholderiales bacterium RIFC-SPHIGHO2_12_FULL_69_20</td><td>hypothetical protein A3E25_24070 [Burkholderiales bacterium RIFC-SPHIGHO2_12_FULL_69_20].</td><td>GCA_001770815.1</td></tr>
<tr><td>OGB06577.1</td><td>TIR+TIR→ nSTAND1+TM+TM+TM*→||<-?||?→||<-Cluster510_2clades</td><td>nSTAND1+TM+TM+TM</td><td>787</td><td>A3E25_20360</td><td>betaproteobacteria</td><td>Burkholderiales bacterium RIFC-SPHIGHO2_12_FULL_69_20</td><td>hypothetical protein A3E25_20360 [Burkholderiales bacterium RIFC-SPHIGHO2_12_FULL_69_20].</td><td>GCA_001770815.1</td></tr>
<tr><td>OGO35294.1</td><td>FGS→ PSE→?→?→?→?→||<-?||TIR+nSTAND1+BetaPropeller*→ PSE→ TIR+CASPASE→</td><td>TIR+nSTAND1+BetaPropeller</td><td>1217</td><td>A2W35_01450</td><td>chloroflexi</td><td>Chloroflexi bacterium RBG_16_57_11</td><td>hypothetical protein A2W35_01450, partial [Chloroflexi bacterium RBG_16_57_11].</td><td>GCA_001795165.1</td></tr>
<tr><td>OGO45061.1</td><td>nSTAND1→||<-?<-PSE||HTH+nSTAND1+BetaPropeller*→ PSE→ PSE→ PSE→?→ PSE→ PSE→||<-?||?→?→ NUDIX→</td><td>HTH+nSTAND1+BetaPropeller</td><td>1317</td><td>A2W37_00850</td><td>chloroflexi</td><td>Chloroflexi bacterium RBG_16_63_12</td><td>hypothetical protein A2W37_00850 [Chloroflexi bacterium RBG_16_63_12].</td><td>GCA_001796945.1</td></tr>
<tr><td>OGO66215.1</td><td>SIG+ABhydrolase→||<-PSE<-PSE<-?||?→ SIG+nSTAND1+BetaPropeller*→</td><td>SIG+nSTAND1+BetaPropeller</td><td>1671</td><td>A2Z45_02015</td><td>chloroflexi</td><td>Chloroflexi bacterium RBG_19FT_COMBO_55_16</td><td>hypothetical protein A2Z45_02015 [Chloroflexi bacterium RBG_19FT_COMBO_55_16].</td><td>GCA_001795335.1</td></tr>
<tr><td>OJJ20442.1</td><td>TM+TM+TM+TM+TM+TM+TM+TM+TM+TM+TM+cNMP→ TIR+DrHyd→ nSTAND1+BetaPropeller*→?→?→?→ TPR+TPR→?→ SIG+TPR→</td><td>nSTAND1+BetaPropeller</td><td>1084</td><td>BKI52_18460</td><td>bacteroidetes</td><td>marine bacterium AO1-C</td><td>hypothetical protein BKI52_18460 [marine bacterium AO1-C].</td><td>GCA_001890965.1</td></tr>
<tr><td>OLF10306.1</td><td><-ABhydrolase||?→?→ APATPase+TPR→||<-?||nSTAND1+TM+BetaPropeller*→?→?→?→||<-?<-?<-STYKIN</td><td>nSTAND1+TM+BetaPropeller</td><td>1150</td><td>BLA60_17405</td><td>actinobacteria</td><td>Actinophytocola xinjiangensis</td><td>hypothetical protein BLA60_17405 [Actinophytocola xinjiangensis].</td><td>GCA_001921215.1</td></tr>
<tr><td>OOO02143.1</td><td>TIR→ nSTAND1*→ nSTAND1+TM+TPR→</td><td>nSTAND1</td><td>241</td><td>USCGTAY-LOR_01645</td><td>gammaproteobacteria</td><td>Chromatiales bacterium USCg_Taylor</td><td>hypothetical protein USCGTAYLOR_01645 [Chromatiales bacterium USCg_Taylor].</td><td>GCA_002007425.1</td></tr>
<tr><td>OQW92136.1</td><td><-Trypco1||CASPASE+nSTAND1+TM+BetaPropeller*→</td><td>CASPASE+nSTAND1+TM+BetaPropeller</td><td>1686</td><td>BWK78_02590</td><td>gammaproteobacteria</td><td>Thiotrichaceae bacterium IS1</td><td>hypothetical protein BWK78_02590, partial [Thiotrichaceae bacterium IS1].</td><td>GCA_002083855.1</td></tr>
<tr><td>OQY53884.1</td><td>Uma2→ TIR+DrHyd+TIR→ nSTAND1+BetaPropeller*→||<-?<-ABhydrolase<-?<-?<-ParA-Soj-PloopNTPase</td><td>nSTAND1+BetaPropeller</td><td>1231</td><td>B6247_13565</td><td>gammaproteobacteria</td><td>Beggiatoa sp. 4572_84</td><td>hypothetical protein B6247_13565 [Beggiatoa sp. 4572_84].</td><td>GCA_002085445.1</td></tr>
<tr><td>OUC13656.1</td><td>CASPASE+nSTAND1*→?→?→?→ TPR→</td><td>CASPASE+nSTAND1</td><td>967</td><td>B0A82_16145</td><td>cyanobacteria</td><td>Alkalinema sp. CACIAM 70d</td><td>hypothetical protein B0A82_16145 [Alkalinema sp. CACIAM 70d].</td><td>GCA_002148405.1</td></tr>
<tr><td>OUC14859.1</td><td><-ABhydrolase||?→?→?→?→ CASPASE+CASPASE+nSTAND1+BetaPropeller*→||<-?<-?||?→?→?→ TM+TM+TM+TM+TM+TM+TM+TM+TM+TM+TM+TM→</td><td>CASPASE+CASPASE+nSTAND1+BetaPro-peller</td><td>1735</td><td>B0A82_11125</td><td>cyanobacteria</td><td>Alkalinema sp. CACIAM 70d</td><td>hypothetical protein B0A82_11125 [Alkalinema sp. CACIAM 70d].</td><td>GCA_002148405.1</td></tr>
<tr><td>OUV42838.1</td><td>TIR+DrHyd→ nSTAND1+MA+BetaPropeller*→</td><td>nSTAND1+MA+BetaPropeller</td><td>1144</td><td>CBC62_02045</td><td>verrucomicrobia</td><td>Opitutae bacterium TMED102</td><td>hypothetical protein CBC62_02045, partial [Opitutae bacterium TMED102].</td><td>GCA_002171405.1</td></tr>
</table>

<table>
<tr><th>acc</th><th>operon</th><th>architecture</th><th>len</th><th>gen.name</th><th>taxend</th><th>species</th><th>defline</th><th>gca</th></tr>
<tr><td>OYD95156.1</td><td>Pentapeptide→ Pentapeptide→ NACHT+TM+TM+TM→?→?→ PSE→ PSE→ Pentapeptide→ NACHT+TM+TM+TM→ nSTAND1+*→?→ TIR→?→?→ MoxR→ VWA+FGS→ FGS→</td><td>nSTAND1+</td><td>814</td><td>CDG77_10550</td><td>cyanobacteria</td><td>Nostoc sp. ‘Peltigera membranacea cyanobiont’ 213</td><td>hypothetical protein CDG77_10550 [Nostoc sp. ‘Peltigera membranacea cyanobiont’ 213].</td><td>GCA_002245975.1</td></tr>
<tr><td>PCH95772.1</td><td>TM+TM+TM+TM+TM+TM+TM+TM+TM+TM+TM+cNMP→?→ TIR+DrHyd→ nSTAND1+BetaPropeller*→</td><td>nSTAND1+BetaPropeller</td><td>1001</td><td>COB85_04230</td><td>bacteroidetes</td><td>Bacteroidetes bacterium</td><td>hypothetical protein COB85_04230, partial [Bacteroidetes bacterium].</td><td>GCA_002401055.1</td></tr>
<tr><td>PJF20968.1</td><td>Pkinase+nSTAND1*→</td><td>Pkinase+nSTAND1</td><td>712</td><td>CUN56_13480</td><td>chloroflexi</td><td>Candidatus Thermofonsia Clade 2 bacterium</td><td>hypothetical protein CUN56_13480, partial [Candidatus Thermofonsia Clade 2 bacterium].</td><td>GCA_002794505.1</td></tr>
<tr><td>PJF22587.1</td><td>TIR+nSTAND1+TM+BetaPropeller+SH3*→</td><td>TIR+nSTAND1+TM+BetaPropeller+SH3</td><td>1467</td><td>CUN56_05240</td><td>chloroflexi</td><td>Candidatus Thermofonsia Clade 2 bacterium</td><td>hypothetical protein CUN56_05240 [Candidatus Thermofonsia Clade 2 bacterium].</td><td>GCA_002794505.1</td></tr>
<tr><td>PJF29877.1</td><td>TIR+nSTAND1+TM+BetaPropeller+SH3*→||<-?<-?<-REC<-REC</td><td>TIR+nSTAND1+TM+BetaPropeller+SH3</td><td>1475</td><td>CUN52_06100</td><td>chloroflexi</td><td>Candidatus Thermofonsia Clade 2 bacterium</td><td>hypothetical protein CUN52_06100 [Candidatus Thermofonsia Clade 2 bacterium].</td><td>GCA_002794515.1</td></tr>
<tr><td>PJF40565.1</td><td><-ABC-ATPase||?→ Pkinase+nSTAND1+fvmX2+BetaPropeller*→</td><td>Pkinase+nSTAND1+fvmX2+BetaPropeller</td><td>1488</td><td>CUN54_04825</td><td>chloroflexi</td><td>Candidatus Thermofonsia Clade 2 bacterium</td><td>hypothetical protein CUN54_04825 [Candidatus Thermofonsia Clade 2 bacterium].</td><td>GCA_002794595.1</td></tr>
<tr><td>PJF43571.1</td><td><-FAD-NAD-dep-oxidoreductase<-?||?→ Pkinase+nSTAND1+TM+BetaPropeller*→</td><td>Pkinase+nSTAND1+TM+BetaPropeller</td><td>1074</td><td>CUN55_08445</td><td>chloroflexi</td><td>Candidatus Thermofonsia Clade 2 bacterium</td><td>hypothetical protein CUN55_08445, partial [Candidatus Thermofonsia Clade 2 bacterium].</td><td>GCA_002794585.1</td></tr>
<tr><td>PKP08385.1</td><td>TM+TM+TM+TM+TM+TM+TM+TM+TM+TM+TM+cNMP→?→ TIR+DrHyd→ nSTAND1+BetaPropeller*→</td><td>nSTAND1+BetaPropeller</td><td>1044</td><td>CVU09_16155</td><td>bacteroidetes</td><td>Bacteroidetes bacterium HGW-Bacteroidetes-4</td><td>hypothetical protein CVU09_16155 [Bacteroidetes bacterium HGW-Bacteroidetes-4].</td><td>GCA_002840905.1</td></tr>
<tr><td>PLX14833.1</td><td>TIR+DrHyd→ nSTAND1+BetaPropeller*→</td><td>nSTAND1+BetaPropeller</td><td>1026</td><td>C0597_09500</td><td>bacteroidetes</td><td>Marinilabiliales bacterium</td><td>hypothetical protein C0597_09500 [Marinilabiliales bacterium].</td><td>GCA_002869305.1</td></tr>
<tr><td>PSO51577.1</td><td><-REC||?→?→?→?→ CASPASE+CASPASE+nSTAND1+BetaPropeller*→||<-?<-?<-Cluster59_2clades</td><td>CASPASE+CASPASE+nSTAND1+BetaPropeller</td><td>1683</td><td>BRC33_00055</td><td>cyanobacteria</td><td>Cyanobacteria bacterium SW_9_44_58</td><td>hypothetical protein BRC33_00055 [Cyanobacteria bacterium SW_9_44_58].</td><td>GCA_003021905.1</td></tr>
<tr><td>PTQ78533.1</td><td><-SIG+Trypsin+PDZ||?→?→||<-?<-?||tRNA→ TIR→ TIR+DrHyd→ nSTAND1+BetaPropeller*→?→||<-SLATT</td><td>nSTAND1+BetaPropeller</td><td>1071</td><td>C8R26_102101</td><td>betaproteobacteria</td><td>Nitrosomonas oligotropha</td><td>WD40 repeat protein [Nitrosomonas oligotropha].</td><td>GCA_003050805.1</td></tr>
<tr><td>PWH17201.1</td><td>nSTAND1+TM+BetaPropeller+TM+TM+TM+TM+TM*→||<-?<-?||?→||<-?<-?<-?<-FAD-NAD-dep-oxidoreductase</td><td>nSTAND1+TM+BetaPropeller+TM+TM+TM+TM+TM</td><td>1488</td><td>DDG60_02865</td><td>chloroflexi</td><td>Anaerolineae bacterium</td><td>hypothetical protein DDG60_02865 [Anaerolineae bacterium].</td><td>GCA_003130845.1</td></tr>
<tr><td>PWT98015.1</td><td><-REC<-?||TIR+DrHyd→ nSTAND1+TM+TM+TM*→</td><td>nSTAND1+TM+TM+TM</td><td>748</td><td>C5B51_30205</td><td>acidobacteria</td><td>Acidobacteriia bacterium</td><td>hypothetical protein C5B51_30205 [Acidobacteriia bacterium].</td><td>GCA_003175985.1</td></tr>
<tr><td>PWU20186.1</td><td>TPRs+ACYC+nSTAND1+BetaPropeller*→||<-?<-?<-?<-SWACOS+TPR+TPR</td><td>TPRs+ACYC+nSTAND1+BetaPropeller</td><td>1499</td><td>C5B48_12805</td><td>bacteria</td><td>Candidatus Rokubacteria bacterium</td><td>hypothetical protein C5B48_12805, partial [Candidatus Rokubacteria bacterium].</td><td>GCA_003176155.1</td></tr>
</table>

acc	operon	architecture	len	gen.name	taxend	species	defline	gca
PYP60217.1	TIR→ SLATT→ TIR+TIR→ nSTAND1+BetaPropeller+BetaPropeller+TPRs*→	nSTAND1+BetaPropeller+BetaPropeller+TPRs	2017	DMD40_01135	gemmatimonadetes	Gemmatimonadetes bacterium	hypothetical protein DMD40_01135 [Gemmatimonadetes bacterium].	GCA_003223395.1
PYQ05559.1	SIGMA-HTH→ PSE→||<-PSE||?→?→ TIR+DrHyd→ nSTAND1+TM*→	nSTAND1+TM	791	DMF82_08210	acidobacteria	Acidobacteria bacterium	hypothetical protein DMF82_08210 [Acidobacteria bacterium].	GCA_003223575.1
PYS22176.1	TIR+DrHyd→ nSTAND1+TM+TPR*→	nSTAND1+TM+TPR	1292	DMF72_14520	acidobacteria	Acidobacteria bacterium	hypothetical protein DMF72_14520 [Acidobacteria bacterium].	GCA_003222895.1
PYS83976.1	TIR+DrHyd+TIR→ nSTAND1+TPR+TPR*→	nSTAND1+TPR+TPR	1090	DMF67_06825	acidobacteria	Acidobacteria bacterium	hypothetical protein DMF67_06825 [Acidobacteria bacterium].	GCA_003222975.1
PYS96660.1	nSTAND1+TM+TPR*→	nSTAND1+TM+TPR	940	DMF65_12750	acidobacteria	Acidobacteria bacterium	hypothetical protein DMF65_12750, partial [Acidobacteria bacterium].	GCA_003223015.1
PYS98866.1	TIR+TIR→ nSTAND1*→ nSTAND1+TIR→	nSTAND1	360	DMF65_09895	acidobacteria	Acidobacteria bacterium	hypothetical protein DMF65_09895 [Acidobacteria bacterium].	GCA_003223015.1
PZN54462.1	<-REC<-REC||?→||<-?<-?<-?||Pkinase+nSTAND1+BetaPropeller*→	Pkinase+nSTAND1+BetaPropeller	962	DIU68_10520	chloroflexi	Chloroflexi bacterium	hypothetical protein DIU68_10520, partial [Chloroflexi bacterium].	GCA_003242205.1
PZO11654.1	CASPASE+nSTAND1*→?→ GGDEF+EAL→	CASPASE+nSTAND1	915	DCF25_18990	cyanobacteria	Leptolyngbya foveolarum	hypothetical protein DCF25_18990 [Leptolyngbya foveolarum].	GCA_003242035.1
PZV25433.1	CASPASE+nSTAND1+TM*→	CASPASE+nSTAND1+TM	1002	DCF12_14930	cyanobacteria	Snowella sp.	hypothetical protein DCF12_14930 [Snowella sp.].	GCA_003249095.1
QDL09256.1	EACC2+CASPASE+nSTAND1+BetaPropeller*→	EACC2+CASPASE+nSTAND1+BetaPropeller	1599	DP114_16315	cyanobacteria	Brasilonema sennae CENA114	hypothetical protein DP114_16315 [Brasilonema sennae CENA114].	GCA_006968745.1
QDL10821.1	EACC2+CASPASE+nSTAND1*→	EACC2+CASPASE+nSTAND1	600	DP114_25545	cyanobacteria	Brasilonema sennae CENA114	hypothetical protein DP114_25545 [Brasilonema sennae CENA114].	GCA_006968745.1
RCJ19564.1	TPR+CASPASE→?→?→ nSTAND1+BetaPropeller*→?→?→?→||<-REC<-REC	nSTAND1+BetaPropeller	1246	A6770_05315	cyanobacteria	Nostoc minutum NIES-26	hypothetical protein A6770_05315 [Nostoc minutum NIES-26].	GCA_003326215.1
RCJ22548.1	REC→ Pentapeptide→ nSTAND1+TM*→	nSTAND1+TM	901	A6770_29780	cyanobacteria	Nostoc minutum NIES-26	hypothetical protein A6770_29780 [Nostoc minutum NIES-26].	GCA_003326215.1
REK19006.1	wHTH-4stranded+TPRs+Pkinase+nSTAND1+BetaPropeller*→	wHTH-4stranded+TPRs+Pkinase+nSTAND1+BetaPropeller	1641	DWQ40_07995	actinobacteria	Actinobacteria bacterium	hypothetical protein DWQ40_07995, partial [Actinobacteria bacterium].	GCA_003388545.1
RIK38666.1	TIR+nSTAND1+BetaPropeller*→ TIR+nSTAND1→	TIR+nSTAND1+BetaPropeller	1082	DCC58_16370	chloroflexi	Chloroflexi bacterium	hypothetical protein DCC58_16370 [Chloroflexi bacterium].	GCA_003577355.1
RIK57209.1	TIR+nSTAND1+BetaPropeller*→ TIR+CASPASE→	TIR+nSTAND1+BetaPropeller	1528	DCC57_02180	chloroflexi	Chloroflexi bacterium	hypothetical protein DCC57_02180 [Chloroflexi bacterium].	GCA_003577365.1

acc	operon	architecture	len	gen.name	taxend	species	defline	gca
RIL11133.1	SIG+ACYC+nSTAND1+BetaPropeller*→	SIG+ACYC+nSTAND1+BetaPropeller	1397	DCC79_05805	bacteria	bacterium	hypothetical protein DCC79_05805 [bacterium].	GCA_003576755.1
RKZ38698.1	TIR+TIR→ nSTAND1+BetaPropeller*→||<-?<-?||?→ ICP8OBfold→	nSTAND1+BetaPropeller	1240	DRQ49_13590	gammaproteobacteria	Gammaproteobacteria bacterium	hypothetical protein DRQ49_13590 [Gammaproteobacteria bacterium].	GCA_003646115.1
RKZ43202.1	nSTAND1+TM+BetaPropeller*→	nSTAND1+TM+BetaPropeller	1128	DRQ41_05855	gammaproteobacteria	Gammaproteobacteria bacterium	hypothetical protein DRQ41_05855, partial [Gammaproteobacteria bacterium].	GCA_003645175.1
RKZ45073.1	nSTAND1+TM+BetaPropeller*→||<-?<-ABC-ATPase	nSTAND1+TM+BetaPropeller	1431	DRQ41_01220	gammaproteobacteria	Gammaproteobacteria bacterium	hypothetical protein DRQ41_01220, partial [Gammaproteobacteria bacterium].	GCA_003645175.1
RKZ52679.1	<-TM<-?||?→?→ CASPASE+nSTAND1+TPR*→?→?→ XisI→	CASPASE+nSTAND1+TPR	990	DRR00_07525	gammaproteobacteria	Gammaproteobacteria bacterium	hypothetical protein DRR00_07525 [Gammaproteobacteria bacterium].	GCA_003645185.1
RKZ62359.1	TIR+DrHyd+TIR→?→?→ nSTAND1+BetaPropeller*→	nSTAND1+BetaPropeller	935	DRR08_06350	gammaproteobacteria	Gammaproteobacteria bacterium	hypothetical protein DRR08_06350, partial [Gammaproteobacteria bacterium].	GCA_003646135.1
RKZ80031.1	PKinase+SWACOS+GAF+SHELIX+HISKIN+REC→ nSTAND1*→	nSTAND1	253	DRR19_24185	gammaproteobacteria	Gammaproteobacteria bacterium	hypothetical protein DRR19_24185, partial [Gammaproteobacteria bacterium].	GCA_003646175.1
RLD63389.1	<-SIG+TPR||?→||<-?||TM+TM+TM+TM+TM+TM+TM+TM+TM+TM+TM+cNMP→?→ TIR+DrHyd→ nSTAND1+TM+BetaPropeller*→	nSTAND1+TM+BetaPropeller	1040	DRJ01_03910	bacteroidetes	Bacteroidetes bacterium	hypothetical protein DRJ01_03910 [Bacteroidetes bacterium].	GCA_003648455.1
RLE12060.1	wHTH-4stranded+TPRs+ACYC+CR-Kinase+nSTAND1+TM*→	wHTH-4stranded+TPRs+ACYC+CR-Kinase+nSTAND1+TM	1147	DRJ28_09685	actinobacteria	Actinobacteria bacterium	hypothetical protein DRJ28_09685, partial [Actinobacteria bacterium].	GCA_003648875.1
RLU96809.1	HTH+nSTAND1+TM+BetaPropeller*→ SIG+TM+TM+TM+TM+TM→?→ ABC-ATPase→	HTH+nSTAND1+TM+BetaPropeller	1311	CTZ27_17585	actinobacteria	Streptomyces griseocarneus	DNA-binding protein [Streptomyces griseocarneus].	GCA_003674045.1
RMD57402.1	nSTAND1+TM+BetaPropeller*→	nSTAND1+TM+BetaPropeller	733	D6828_03745	nitrospirae	Nitrospirae bacterium	hypothetical protein D6828_03745, partial [Nitrospirae bacterium].	GCA_003693935.1
RMF02938.1	nSTAND1+TM+FGS*→	nSTAND1+TM+FGS	996	D6768_07070	chloroflexi	Chloroflexi bacterium	hypothetical protein D6768_07070, partial [Chloroflexi bacterium].	GCA_003695455.1
RMF02990.1	nSTAND1+BetaPropeller*→	nSTAND1+BetaPropeller	1236	D6768_06945	chloroflexi	Chloroflexi bacterium	hypothetical protein D6768_06945, partial [Chloroflexi bacterium].	GCA_003695455.1
RMF76640.1	Pkinase+nSTAND1+TM+BetaPropeller*→	Pkinase+nSTAND1+TM+BetaPropeller	1222	D6737_19790	chloroflexi	Chloroflexi bacterium	hypothetical protein D6737_19790, partial [Chloroflexi bacterium].	GCA_003695675.1
RMH78629.1	SIG+Trypsin+BetaPropeller→||<-TM<-?||CASPASE+nSTAND1+TM*→	CASPASE+nSTAND1+TM	828	D6680_01360	cyanobacteria	Cyanobacteria bacterium J007	caspase family protein, partial [Cyanobacteria bacterium J007].	GCA_003695985.1

<table>
<tr><th>acc</th><th>operon</th><th>architecture</th><th>len</th><th>gen.name</th><th>taxend</th><th>species</th><th>defline</th><th>gca</th></tr>
<tr><td>RPH30586.1</td><td>TM+TM+TM+TM+TM+TM+TM+TM+TM+TM+TM+cNMP→?→ TIR+DrHyd→ nSTAND1+TM+PspA+BetaPropeller*→</td><td>nSTAND1+TM+PspA+BetaPropeller</td><td>1019</td><td>EHM93_15545</td><td>bacteroidetes</td><td>Bacteroidales bacterium</td><td>High-affinity carbon uptake protein Hat/HatR [Bacteroidales bacterium].</td><td>GCA_003818205.1</td></tr>
<tr><td>RPH58066.1</td><td>nSTAND1*→</td><td>nSTAND1</td><td>216</td><td>EHM81_10640</td><td>chloroflexi</td><td>Chloroflexi bacterium</td><td>ATP-binding protein, partial [Chloroflexi bacterium].</td><td>GCA_003818555.1</td></tr>
<tr><td>RPI91735.1</td><td>RRM→?→?→?→||<-?||?→ SIG+nSTAND1+TM*→</td><td>SIG+nSTAND1+TM</td><td>777</td><td>EHM40_14820</td><td>chloroflexi</td><td>Chloroflexi bacterium</td><td>ATP-binding protein, partial [Chloroflexi bacterium].</td><td>GCA_003820075.1</td></tr>
<tr><td>RPI98474.1</td><td>Pkinase+nSTAND1*→</td><td>Pkinase+nSTAND1</td><td>646</td><td>EHM39_07925</td><td>chloroflexi</td><td>Chloroflexi bacterium</td><td>serine/threonine protein kinase, partial [Chloroflexi bacterium].</td><td>GCA_003818635.1</td></tr>
<tr><td>RPJ24401.1</td><td>wHTH-4stranded+TPRs+ACYC+nSTAND1+BetaPropeller*→</td><td>wHTH-4stranded+TPRs+ACYC+nSTAND1+BetaPropeller</td><td>1323</td><td>EHM33_17965</td><td>chloroflexi</td><td>Chloroflexi bacterium</td><td>hypothetical protein EHM33_17965, partial [Chloroflexi bacterium].</td><td>GCA_003820175.1</td></tr>
<tr><td>RQW03303.1</td><td>TIR+DrHyd→ nSTAND1+TM+CHASE2+TM+TM+TM*→</td><td>nSTAND1+TM+CHASE2+TM+TM+TM</td><td>828</td><td>EH223_10290</td><td>bacteria</td><td>candidate division KSB1 bacterium</td><td>CHASE2 domain-containing protein [candidate division KSB1 bacterium].</td><td>GCA_003854975.1</td></tr>
<tr><td>RYD57998.1</td><td>TIR→ nSTAND1+BetaPropeller*→?→ Cluster106_3clades→</td><td>nSTAND1+BetaPropeller</td><td>1019</td><td>EOP56_06920</td><td>bacteroidetes</td><td>Sphingobacteriales bacterium</td><td>hypothetical protein EOP56_06920 [Sphingobacteriales bacterium].</td><td>GCA_004144175.1</td></tr>
<tr><td>RZO55510.1</td><td>TIR+DrHyd+TIR→ nSTAND1+BetaPropeller*→</td><td>nSTAND1+BetaPropeller</td><td>1385</td><td>EVA72_06320</td><td>verrucomicrobia</td><td>Verrucomicrobia subdivision 3 bacterium</td><td>hypothetical protein EVA72_06320 [Verrucomicrobia subdivision 3 bacterium].</td><td>GCA_004213515.1</td></tr>
<tr><td>SBO96141.1</td><td>ABC-ATPase→ ABC-ATPase→?→||<-?||?→ nSTAND1+BetaPropeller*→?→ SIG+HOP2→?→ AAA+TPR→</td><td>nSTAND1+BetaPropeller</td><td>1263</td><td>BN4615_P5657</td><td>actinobacteria</td><td>Nonomuraea gerenzanensis</td><td>High-affnity carbon uptake protein Hat/HatR [Nonomuraea gerenzanensis].</td><td>-</td></tr>
<tr><td>SBO97731.1</td><td>ABhydrolase→?→ SIG+nSTAND1+TM+BetaPropeller*→||<-?||?→||<-?<-?<-SIG+TM+TM+TM+TM+TM<-SIG+TM+TM+TM+TM+TM</td><td>SIG+nSTAND1+TM+BetaPropeller</td><td>1766</td><td>BN4615_P7247</td><td>actinobacteria</td><td>Nonomuraea gerenzanensis</td><td>High-affnity carbon uptake protein Hat/HatR [Nonomuraea gerenzanensis].</td><td>-</td></tr>
<tr><td>SBV24853.1</td><td>HTH+nSTAND1+TM+BetaPropeller*→||<-?<-?<-?||?→ tRNA→||<-?<-TM+TM+TM+TM+TM+TM<-ABC-ATPase</td><td>HTH+nSTAND1+TM+BetaPropeller</td><td>1258</td><td>GA0070620_0295</td><td>actinobacteria</td><td>Micromonospora krabiensis</td><td>WD40 repeat [Micromonospora krabiensis].</td><td>GCA_900091425.1</td></tr>
<tr><td>SCG38369.1</td><td>SIG+nSTAND1+TM+BetaPropeller*→</td><td>SIG+nSTAND1+TM+BetaPropeller</td><td>1396</td><td>GA0070614_0509</td><td>actinobacteria</td><td>Micromonospora coxensis</td><td>WD40 repeat [Micromonospora coxensis].</td><td>GCA_900090295.1</td></tr>
<tr><td>SEF93234.1</td><td><-SIG+HOP2<-?<-?||?→ nSTAND1+BetaPropeller*→||<-?<-?<-p450</td><td>nSTAND1+BetaPropeller</td><td>1224</td><td>SAMN05444920_1011053</td><td>actinobacteria</td><td>Nonomuraea solani</td><td>WD40 repeat [Nonomuraea solani].</td><td>GCA_900108335.1</td></tr>
<tr><td>SMD22401.1</td><td>nSTAND1+BetaPropeller*→</td><td>nSTAND1+BetaPropeller</td><td>1243</td><td>SAMN05661093_07396</td><td>actinobacteria</td><td>Kibdelosporangium aridum</td><td>WD40 repeat [Kibdelosporangium aridum].</td><td>GCA_900176515.1</td></tr>
<tr><td>SPL89441.1</td><td>nSTAND1+BetaPropeller*→</td><td>nSTAND1+BetaPropeller</td><td>1231</td><td>KIS93_02102</td><td>actinobacteria</td><td>Actinomadura parvosata subsp. kistnae</td><td>High-affnity carbon uptake protein Hat/HatR [Actinomadura parvosata subsp. kistnae].</td><td>GCA_900323885.1</td></tr>
<tr><td>TAH51625.1</td><td>wHTH-4stranded+TPRs+nSTAND1+BetaPropeller*→</td><td>wHTH-4stranded+TPRs+nSTAND1+BetaPropeller</td><td>1534</td><td>EYC68_09685</td><td>chloroflexi</td><td>Chloroflexi bacterium</td><td>hypothetical protein EYC68_09685 [Chloroflexi bacterium].</td><td>GCA_004295045.1</td></tr>
</table>

acc	operon	architecture	len	gen.name	taxend	species	defline	gca
TCS45832.1	nSTAND1+BetaPropeller*→	nSTAND1+BetaPropeller	1284	EV585_125129	actinobacteria	Streptomyces sp. BK335	WD-40 repeat-containing protein [Streptomyces sp. BK335].	GCA_004343515.1
TDI65915.1	TIR+DrHyd→ nSTAND1+TM+BetaPropeller*→	nSTAND1+TM+BetaPropeller	1178	E2O88_09730	bacteroidetes	Bacteroidetes bacterium	hypothetical protein E2O88_09730 [Bacteroidetes bacterium].	GCA_004356345.1
TDX54622.1	nSTAND1+TM+BetaPropeller*→	nSTAND1+TM+BetaPropeller	1232	CLW03_1390	actinobacteria	Streptomyces sp. 25	WD40 repeat protein [Streptomyces sp. 25].	GCA_004365455.1
TFG73542.1	TIR→ nSTAND1*→	nSTAND1	184	E4H26_09660	bacteroidetes	Flavobacteriales bacterium	hypothetical protein E4H26_09660, partial [Flavobacteriales bacterium].	GCA_004525075.1
TLU54259.1	TIR+DrHyd→ nSTAND1+TPR*→	nSTAND1+TPR	934	FDX18_11420	chlorobi	Chlorobium sp.	hypothetical protein FDX18_11420 [Chlorobium sp.].	GCA_005843805.1
TMA13290.1	SLATT→||<-?||TIR→ nSTAND1+TM+TM+TM*→	nSTAND1+TM+TM+TM	677	E6J89_01820	deltaproteobacteria	Deltaproteobacteria bacterium	ATP-binding protein, partial [Deltaproteobacteria bacterium].	GCA_005878685.1
TMC17178.1	TIR+nSTAND1+TM+Beta-helix*→	TIR+nSTAND1+TM+Beta-helix	948	E6J34_19025	chloroflexi	Chloroflexi bacterium	TIR domain-containing protein, partial [Chloroflexi bacterium].	GCA_005879655.1
TMC23062.1	nSTAND1+TM+Beta-helix→ Pkinase+TIR+nSTAND1+TM+Beta-helix*→?→||<-?<-?<-?<-?<-ABhydrolase	Pkinase+TIR+nSTAND1+TM+Beta-helix	1380	E6J34_04260	chloroflexi	Chloroflexi bacterium	TIR domain-containing protein [Chloroflexi bacterium].	GCA_005879655.1
TMD76903.1	TIR+nSTAND1+TM*→	TIR+nSTAND1+TM	914	E6I97_10225	chloroflexi	Chloroflexi bacterium	TIR domain-containing protein [Chloroflexi bacterium].	GCA_005880275.1
TMD99845.1	TIR+nSTAND1+TM*→	TIR+nSTAND1+TM	908	E6I80_27410	chloroflexi	Chloroflexi bacterium	hypothetical protein E6I80_27410 [Chloroflexi bacterium].	GCA_005881495.1
TME05606.1	TIR+nSTAND1+TM*→	TIR+nSTAND1+TM	884	E6I80_16835	chloroflexi	Chloroflexi bacterium	TIR domain-containing protein, partial [Chloroflexi bacterium].	GCA_005881495.1
TMH35459.1	TIR→ TIR+DrHyd→ nSTAND1+TM+TPR*→?→||<-?<-ABC-ATPase||?→?→ Cluster106_3clades→	nSTAND1+TM+TPR	759	E6H66_07570	betaproteobacteria	Betaproteobacteria bacterium	ATP-binding protein [Betaproteobacteria bacterium].	GCA_005882995.1
TNF40174.1	TIR→ nSTAND1+TM+prokYEATS*→	nSTAND1+TM+prokYEATS	681	EP310_09390	bacteroidetes	Bacteroidetes bacterium	hypothetical protein EP310_09390 [Bacteroidetes bacterium].	GCA_006226805.1
TQM00650.1	nSTAND1+BetaPropeller*→	nSTAND1+BetaPropeller	1393	FB559_6366	actinobacteria	Actinoallomurus bryophytorum	WD40 repeat protein [Actinoallomurus bryophytorum].	GCA_006716425.1
VFJ52061.1	nSTAND1+BetaPropeller*→?→?→ ABC-ATPase→	nSTAND1+BetaPropeller	1305	BECKFM1743C_GA0114222_101051	gammaproteobacteria	Candidatus Kentron sp. FM	WD40 repeat [Candidatus Kentron sp. FM].	-
VFJ73513.1	TIR→ MoxR→?→ TIR→ nSTAND1+BetaPropeller*→?→ ABC-ATPase→	nSTAND1+BetaPropeller	1354	BECKFW1821C_GA0114237_105215	gammaproteobacteria	Candidatus Kentron sp. FW	WD40 repeat [Candidatus Kentron sp. FW].	-
WP_006105558.1	CASPASE+nSTAND1+BetaPropeller*→	CASPASE+nSTAND1+BetaPropeller	1249	MC7420_RS35880	cyanobacteria	Coleofasciculus chthonoplastes	caspase family protein [Coleofasciculus chthonoplastes].	GCF_000155555.1
WP_006458142.1	CASPASE+CASPASE+nSTAND1+BetaPropeller*→	CASPASE+CASPASE+nSTAND1+BetaPropeller	1660	S7335_RS22355	cyanobacteria	Synechococcus sp. PCC 7335	caspase family protein [Synechococcus sp. PCC 7335].	GCF_000155595.1

<table>
<thead>
<tr><th>acc</th><th>operon</th><th>architecture</th><th>len</th><th>gen.name</th><th>taxend</th><th>species</th><th>defline</th><th>gca</th></tr>
</thead>
<tbody>
<tr><td>WP_007305744.1</td><td><-Trypco1||?→ CASPASE+nSTAND1*→</td><td>CASPASE+nSTAND1</td><td>961</td><td>CWAT-DRAFT_RS14705</td><td>cyanobacteria</td><td>Crocosphaera watsonii</td><td>pentapeptide repeat-containing protein [Crocosphaera watsonii].</td><td>GCF_000167195.1</td></tr>
<tr><td>WP_008178364.1</td><td>FAD-NAD-dep-oxidoreductase→||<-Trypco1||CASPASE+nSTAND1*→</td><td>CASPASE+nSTAND1</td><td>1005</td><td>BI334_RS09780</td><td>cyanobacteria</td><td>Moorea producens</td><td>caspase family protein [Moorea producens].</td><td>GCF_000211815.1</td></tr>
<tr><td>WP_011141629.1</td><td><-SIG+Metallopeptidase||?→||<-?<-?||CASPASE+CASPASE+nSTAND1+BetaPropeller*→||<-?<-?||?→?→||<-?<-?||DinB_2+FGS→</td><td>CASPASE+CASPASE+nSTAND1+BetaPro-peller</td><td>1730</td><td>GLL_RS08460</td><td>cyanobacteria</td><td>Gloeobacter violaceus</td><td>caspase family protein [Gloeobacter violaceus].</td><td>GCF_000011385.1</td></tr>
<tr><td>WP_011142241.1</td><td>ABhydrolase→||<-?<-?||CASPASE+CASPASE+nSTAND1+BetaPropeller*→?→?→?→?→ ABC-ATPase→</td><td>CASPASE+CASPASE+nSTAND1+BetaPro-peller</td><td>1721</td><td>GLL_RS11610</td><td>cyanobacteria</td><td>Gloeobacter violaceus</td><td>caspase family protein [Gloeobacter violaceus].</td><td>GCF_000011385.1</td></tr>
<tr><td>WP_011316937.1</td><td>CASPASE+CASPASE+nSTAND1+BetaPropeller*→</td><td>CASPASE+CASPASE+nSTAND1+BetaPro-peller</td><td>1686</td><td>AVA_RS00195</td><td>cyanobacteria</td><td>Nostocaceae</td><td>MULTISPECIES: caspase family protein [Nostocaceae].</td><td>GCF_000204075.1</td></tr>
<tr><td>WP_011318971.1</td><td>CASPASE+NACHT+BetaPropeller→ CASPASE+nSTAND1+BetaPropeller*→?→ RVe+MutnpC→</td><td>CASPASE+nSTAND1+BetaPropeller</td><td>1240</td><td>AVA_RS29445</td><td>cyanobacteria</td><td>Trichormus variabilis</td><td>caspase family protein [Trichormus variabilis].</td><td>GCF_000204075.1</td></tr>
<tr><td>WP_011319818.1</td><td><-REC<-SIG+Trypsin+PDZ<-HAD||?→||<-HSP20||?→ CASPASE+nSTAND1+BetaPropeller→ CASPASE+CASPASE+nSTAND1+BetaPropeller*→ PSE→||<-?<-?<-?||?→||<-?||?→ Na+ORC-AAA→</td><td>CASPASE+CASPASE+nSTAND1+BetaPro-peller</td><td>1760</td><td>AVA_RS15560</td><td>cyanobacteria</td><td>Nostocaceae</td><td>MULTISPECIES: caspase family protein [Nostocaceae].</td><td>GCF_000204075.1</td></tr>
<tr><td>WP_012163883.1</td><td>CASPASE+CASPASE+nSTAND1+BetaPropeller*→</td><td>CASPASE+CASPASE+nSTAND1+BetaPro-peller</td><td>1703</td><td>AM1_RS15835</td><td>cyanobacteria</td><td>Acaryochloris marina</td><td>caspase family protein [Acaryochloris marina].</td><td>GCF_000018105.1</td></tr>
<tr><td>WP_012165630.1</td><td>Redoxin→ CASPASE+nSTAND1+TM*→</td><td>CASPASE+nSTAND1+TM</td><td>1013</td><td>AM1_RS24605</td><td>cyanobacteria</td><td>Acaryochloris marina</td><td>caspase family protein [Acaryochloris marina].</td><td>GCF_000018105.1</td></tr>
<tr><td>WP_015103078.1</td><td>HTH+nSTAND1+TM+BetaPropeller*→||<-?<-PSE||NACHT→?→ NACHT→</td><td>HTH+nSTAND1+TM+BetaPropeller</td><td>1307</td><td>BN6_RS27490</td><td>actinobacteria</td><td>Saccharothrix espanaensis</td><td>helix-turn-helix domain-containing protein [Saccharothrix espanaensis].</td><td>GCF_000328705.1</td></tr>
<tr><td>WP_015104506.1</td><td>nSTAND1+TM+BetaPropeller*→</td><td>nSTAND1+TM+BetaPropeller</td><td>969</td><td>BN6_RS34550</td><td>actinobacteria</td><td>Saccharothrix espanaensis</td><td>hypothetical protein [Saccharothrix espanaensis].</td><td>GCF_000328705.1</td></tr>
<tr><td>WP_015118666.1</td><td>EACC2+CASPASE+nSTAND1+BetaPropeller*→||<-PSE<-?<-?||?→?→?→ Uma2→</td><td>EACC2+CASPASE+nSTAND1+BetaPro-peller</td><td>1548</td><td>RIV7116_RS12515</td><td>cyanobacteria</td><td>Rivularia sp. PCC 7116</td><td>WD40 repeat domain-containing protein [Rivularia sp. PCC 7116].</td><td>GCF_000316665.1</td></tr>
<tr><td>WP_015158653.1</td><td>nSTAND1+BetaPropeller*→</td><td>nSTAND1+BetaPropeller</td><td>1104</td><td>CHA6605_RS06215</td><td>cyanobacteria</td><td>Chamaesiphon minutus</td><td>WD40 repeat domain-containing protein [Chamaesiphon minutus].</td><td>GCF_000317145.1</td></tr>
<tr><td>WP_015205337.1</td><td>CASPASE+CASPASE+nSTAND1+BetaPropeller*→</td><td>CASPASE+CASPASE+nSTAND1+BetaPro-peller</td><td>1759</td><td>CRI9333_RS21890</td><td>cyanobacteria</td><td>Crinalium epipsammum</td><td>caspase family protein [Crinalium epipsammum].</td><td>GCF_000317495.1</td></tr>
<tr><td>WP_015210065.1</td><td><-Trypco1||CASPASE+nSTAND1+TM*→||<-?||?→?→||<-?||Cluster108_2clades→</td><td>CASPASE+nSTAND1+TM</td><td>895</td><td>CYLST_RS22615</td><td>cyanobacteria</td><td>Cylindrospermum stagnale</td><td>pentapeptide repeat-containing protein [Cylindrospermum stagnale].</td><td>GCF_000317535.1</td></tr>
<tr><td>WP_015691683.1</td><td>nSTAND1+TM+BetaPropeller*→</td><td>nSTAND1+TM+BetaPropeller</td><td>1119</td><td>SGRA_RS05810</td><td>bacteroidetes</td><td>Saprospira grandis</td><td>hypothetical protein [Saprospira grandis].</td><td>GCF_000250635.1</td></tr>
<tr><td>WP_015724204.1</td><td>TIR+DrHyd+TIR→ nSTAND1+TM+Ntox24+PDZ*→</td><td>nSTAND1+TM+Ntox24+PDZ</td><td>1380</td><td>DESPR_RS17200</td><td>deltaproteobacteria</td><td>Desulfobulbus propionicus</td><td>YD repeat-containing protein [Desulfobulbus propionicus].</td><td>GCF_000186885.1</td></tr>
<tr><td>WP_015795480.1</td><td><-ABhydrolase<-?||HTH+nSTAND1+TM+BetaPropeller*→||<-nSTAND1</td><td>HTH+nSTAND1+TM+BetaPropeller</td><td>1334</td><td>CACI_RS46450</td><td>actinobacteria</td><td>Catenulispora acidiphila</td><td>helix-turn-helix domain-containing protein [Catenulispora acidiphila].</td><td>GCF_000024025.1</td></tr>
<tr><td>WP_016952361.1</td><td>ABC-ATPase→ Cluster158_2clades→ SIG+TM+TM+TM+TM+TM→||<-Trypco1||CASPASE+nSTAND1+TM+BetaPropeller*→||<-?<-TM+HAMP+GAF+GAF+GAF+GAF+MCPsignal<-?<-REC</td><td>CASPASE+nSTAND1+TM+BetaPropeller</td><td>1622</td><td>ANA7108_RS0118800</td><td>cyanobacteria</td><td>Anabaena sp. PCC 7108</td><td>caspase family protein [Anabaena sp. PCC 7108].</td><td>GCF_000332135.1</td></tr>
</tbody>
</table>

acc	operon	architecture	len	gen.name	taxend	species	defline	gca
WP_016953798.1	EACC2+CASPASE+nSTAND1+BetaPropeller*→?→?→?→?→?→ ParA-Soj-PloopNTPase→ ParB→	EACC2+CASPASE+nSTAND1+BetaPropeller	1578	ANA7108_RS0126175	cyanobacteria	Anabaena sp. PCC 7108	CHAT domain-containing protein [Anabaena sp. PCC 7108].	GCF_000332135.1
WP_017315667.1	CASPASE→ nSTAND1+BetaPropeller→ nSTAND1*→	nSTAND1	312	MAS10914_RS0109350	cyanobacteria	Mastigocladopsis repens	ATP-binding protein [Mastigocladopsis repens].	GCF_000315565.1
WP_017324258.1	ABhydrolase→||<-Uma2<-Uma2||TIR+TIR→ SIG+nSTAND1+TM+BetaPropeller*→||<-?||?→?→ ABC-ATPase→	SIG+nSTAND1+TM+BetaPropeller	1203	SYN7336_RS02075	cyanobacteria	Synechococcus sp. PCC 7336	hypothetical protein [Synechococcus sp. PCC 7336].	GCF_000332275.1
WP_017652542.1	SIG+TM+TM+TM+TM+TM→?→||<-?<-?||?→||<-PSE||CASPASE+CASPASE+nSTAND1+BetaPropeller*→	CASPASE+CASPASE+nSTAND1+BetaPropeller	1714	MIC7126_RS0107600	cyanobacteria	Fortiea contorta	caspase family protein [Fortiea contorta].	GCF_000332295.1
WP_017712679.1	Pkinase+TIR+DrHyd→||<-?<-?<-?||?→?→ CASPASE+nSTAND1+BetaPropeller*→	CASPASE+nSTAND1+BetaPropeller	1866	PRO9006_RS0111975	cyanobacteria	Prochlorothrix hollandica	hypothetical protein [Prochlorothrix hollandica].	GCF_000332315.1
WP_017715273.1	<-TM<-?||CASPASE+nSTAND1+TM+BetaPropeller*→?→ PSE→||<-?<-?<-ABhydrolase<-?<-ENOYCOADEHYD	CASPASE+nSTAND1+TM+BetaPropeller	1430	OSC10802_RS38460	cyanobacteria	Oscillatoria sp. PCC 10802	caspase family protein [Oscillatoria sp. PCC 10802].	GCF_000332335.1
WP_017717001.1	<-ACET<-?||?→||<-?||?→?→ EACC2+CASPASE+nSTAND1*→||<-?||?→ PSE→||<-?||?→?→||<-Pkinase+TPR	EACC2+CASPASE+nSTAND1	829	OSC10802_RS39855	cyanobacteria	Oscillatoria sp. PCC 10802	CHAT domain-containing protein [Oscillatoria sp. PCC 10802].	GCF_000332335.1
WP_017717209.1	CASPASE+CASPASE+nSTAND1+BetaPropeller*→	CASPASE+CASPASE+nSTAND1+BetaPropeller	1766	OSC10802_RS0113050	cyanobacteria	Oscillatoria sp. PCC 10802	caspase family protein [Oscillatoria sp. PCC 10802].	GCF_000332335.1
WP_017720241.1	<-S4+PSYN<-TM<-?<-TPR+TPR+TPR+CASPASE<-?<-TPR+TPR+TPR+TPR||?→ EACC2+CASPASE+nSTAND1+IES1*→?→||<-?<-SIG+Trypsin<-?<-?||?→||<-SIG+Pentapeptide	EACC2+CASPASE+nSTAND1+IES1	1048	OSC10802_RS42290	cyanobacteria	Oscillatoria sp. PCC 10802	DUF1822 family protein [Oscillatoria sp. PCC 10802].	GCF_000332335.1
WP_018342883.1	MED15→ TM+TM+TM+TM+TM+TM+TM+TM+TM+TM+TM+cNMP→ TIR→ nSTAND1+BetaPropeller*→	nSTAND1+BetaPropeller	1044	A3EE_RS0107780	bacteroidetes	Cytophaga aurantiaca	hypothetical protein [Cytophaga aurantiaca].	GCF_000379725.1
WP_018504462.1	nSTAND1+BetaPropeller*→	nSTAND1+BetaPropeller	1541	B056_RS0124325	actinobacteria	Frankia discariae	hypothetical protein [Frankia discariae].	GCF_000373365.1
WP_019487727.1	<-AAA+TPR<-?<-?<-?<-PSE||?→?→?→ nSTAND1+Pentapeptide*→	nSTAND1+Pentapeptide	725	OS-CIL6407_RS0123130	cyanobacteria	Kamptonema formosum	pentapeptide repeat-containing protein [Kamptonema formosum].	GCF_000332155.1
WP_019492639.1	CASPASE+CASPASE+nSTAND1+BetaPropeller*→	CASPASE+CASPASE+nSTAND1+BetaPropeller	1697	CAL7103_RS0122950	cyanobacteria	Calothrix sp. PCC 7103	caspase family protein [Calothrix sp. PCC 7103].	GCF_000331305.1
WP_019493356.1	<-REC<-PKinase+SWACOS+GAF+SHELIX+HISKIN+REC||Pentapeptide→ CASPASE+nSTAND1+TM+BetaPropeller*→||<-?<-?<-?<-PSE||MNS→	CASPASE+nSTAND1+TM+BetaPropeller	1446	CAL7103_RS0126645	cyanobacteria	Calothrix sp. PCC 7103	caspase family protein [Calothrix sp. PCC 7103].	GCF_000331305.1
WP_023071899.1	<-Trypco1||CASPASE+nSTAND1+Pentapeptide*→	CASPASE+nSTAND1+Pentapeptide	969	N836_RS48210	cyanobacteria	Leptolyngbya sp. Heron Island J	pentapeptide repeat-containing protein [Leptolyngbya sp. Heron Island J].	GCF_000482245.1
WP_023173850.1	CASPASE+CASPASE+nSTAND1+BetaPropeller*→	CASPASE+CASPASE+nSTAND1+BetaPropeller	1707	GKIL_RS11810	cyanobacteria	Gloeobacter kilaueensis	caspase family protein [Gloeobacter kilaueensis].	GCF_000484535.1
WP_026073174.1	CASPASE+nSTAND1+BetaPropeller→||<-?<-?||SIG+HTH+ACYC+APATPase+BetaPropeller→?→ nSTAND1+BetaPropeller*→||<-?<-?||?→||<-Cluster510_2clades	nSTAND1+BetaPropeller	1233	LEPTO7104_RS0128640	cyanobacteria	Nodosilinea nodulosa	WD40 repeat domain-containing protein [Nodosilinea nodulosa].	GCF_000309385.1
WP_026316665.1	nSTAND1+BetaPropeller*→||<-?<-?||?→?→||<-?<-AraC-HTH+AraC-HTH	nSTAND1+BetaPropeller	1263	C503_RS0124640	actinobacteria	Actinokineospora enzanensis	hypothetical protein [Actinokineospora enzanensis].	GCF_000374445.1
WP_026735835.1	<-Trypco1||PASE+nSTAND1+TM+Pentapeptide*→	PASE+nSTAND1+TM+Pentapeptide	969	FIS9605_RS0129725	cyanobacteria	Fischerella sp. PCC 9605	pentapeptide repeat-containing protein [Fischerella sp. PCC 9605].	GCF_000517105.1
WP_027844322.1	CASPASE+CASPASE+nSTAND1+BetaPropeller*→?→?→?→?→ XisI→	CASPASE+CASPASE+nSTAND1+BetaPropeller	1804	BC008_RS42445	cyanobacteria	Mastigocoleus testarum	caspase family protein [Mastigocoleus testarum].	GCF_001456025.1

<table>
<tr><th>acc</th><th>operon</th><th>architecture</th><th>len</th><th>gen.name</th><th>taxend</th><th>species</th><th>defline</th><th>gca</th></tr>
<tr><td>WP_027930056.1</td><td>ACT→||<-ICLR-HTH<-ABhydrolase||Pkinase+TPR→ EACC1→ CASPASE+nSTAND1+TM+BetaPropeller*→</td><td>CASPASE+nSTAND1+TM+BetaPropeller</td><td>1457</td><td>AMYTH_RS0109125</td><td>actinobacteria</td><td>Amycolatopsis thermoflava</td><td>caspase family protein [Amycolatopsis thermoflava].</td><td>GCF_000473265.1</td></tr>
<tr><td>WP_028922565.1</td><td>ABhydrolase→ ABhydrolase→?→ nSTAND1+BetaPropeller+BetaPropeller*→ nSTAND1+TM+TPR→</td><td>nSTAND1+BetaPropeller+BetaPropeller</td><td>1251</td><td>N912_RS60190</td><td>actinobacteria</td><td>Pseudonocardia acaciae</td><td>hypothetical protein [Pseudonocardia acaciae].</td><td>GCF_000620785.1</td></tr>
<tr><td>WP_035149788.1</td><td><-Trypco1||CASPASE+nSTAND1+TM*→</td><td>CASPASE+nSTAND1+TM</td><td>1012</td><td>IJ00_RS02610</td><td>cyanobacteria</td><td>Calothrix sp. 336/3</td><td>pentapeptide repeat-containing protein [Calothrix sp. 336/3].</td><td>GCF_000734895.2</td></tr>
<tr><td>WP_035956063.1</td><td>SWC3+CASPASE+nSTAND1+TM+BetaPropeller*→ TIR+nSTAND1+BetaPropeller→</td><td>SWC3+CASPASE+nSTAND1+TM+BetaPropeller</td><td>1650</td><td>FREUN1FDRAFT_RS33550</td><td>actinobacteria</td><td>Frankia sp. EUN1f</td><td>hypothetical protein [Frankia sp. EUN1f].</td><td>GCF_000177675.1</td></tr>
<tr><td>WP_038089860.1</td><td>EACC2+CASPASE+nSTAND1+BetaPropeller*→</td><td>EACC2+CASPASE+nSTAND1+BetaPropeller</td><td>1523</td><td>HC643_RS24730</td><td>cyanobacteria</td><td>Tolypothrix bouteillei</td><td>CHAT domain-containing protein [Tolypothrix bouteillei].</td><td>GCF_000760695.4</td></tr>
<tr><td>WP_038091175.1</td><td>EACC2+CASPASE+nSTAND1+BetaPropeller*→</td><td>EACC2+CASPASE+nSTAND1+BetaPropeller</td><td>1527</td><td>bacteria>cyanobacteria</td><td>Tolypothrix bouteillei</td><td>-</td><td>740250270</td><td>-</td></tr>
<tr><td>WP_042222922.1</td><td>nSTAND1+BetaPropeller*→?→?→ REC→ REC→||<-?<-?<-REC</td><td>nSTAND1+BetaPropeller</td><td>1227</td><td>KALB_RS34215</td><td>actinobacteria</td><td>Kutzneria albida</td><td>hypothetical protein [Kutzneria albida].</td><td>GCF_000525635.1</td></tr>
<tr><td>WP_044290980.1</td><td>Pentapeptide→ CASPASE+nSTAND1+TM*→?→ BetaPropeller→</td><td>CASPASE+nSTAND1+TM</td><td>817</td><td>RIV7116_RS15845</td><td>cyanobacteria</td><td>Rivularia sp. PCC 7116</td><td>caspase family protein [Rivularia sp. PCC 7116].</td><td>GCF_000316665.1</td></tr>
<tr><td>WP_045225177.1</td><td>TIR+DrHyd→ nSTAND1+FGS*→</td><td>nSTAND1+FGS</td><td>817</td><td>EK23_RS09735</td><td>gammaproteobacteria</td><td>Methyloterricola oryzae</td><td>SUMF1/EgtB/PvdO family nonheme iron enzyme [Methyloterricola oryzae].</td><td>GCF_000934725.1</td></tr>
<tr><td>WP_045872156.1</td><td>nSTAND1+BetaPropeller*→||<-?<-?||CASPASE→||<-?||CASPASE→||<-?<-MNS</td><td>nSTAND1+BetaPropeller</td><td>1200</td><td>FDU-TEX481_RS24680</td><td>cyanobacteria</td><td>Nostocales</td><td>MULTISPECIES: WD40 repeat domain-containing protein [Nostocales].</td><td>GCF_002368275.1</td></tr>
<tr><td>WP_046725303.1</td><td><-ABhydrolase<-?||?→ nSTAND1+BetaPropeller*→||<-?<-SIG+TM+TM+TM+TM+TM<-SIG+TM+TM+TM+TM+TM||TRPR-HTH+PBPI→||<-TRPR-HTH+PBPI||?→ SIG+TM+TM+TM+TM+TM→</td><td>nSTAND1+BetaPropeller</td><td>1285</td><td>SXIM_RS26680</td><td>actinobacteria</td><td>Streptomyces xiamenensis</td><td>PD40 domain-containing protein [Streptomyces xiamenensis].</td><td>GCF_000993785.3</td></tr>
<tr><td>WP_048709317.1</td><td>SIG+TIR→ TIR+nSTAND1+RHS+RHS*→ PSE→?→?→?→||<-?||?→ TIR→</td><td>TIR+nSTAND1+RHS+RHS</td><td>1623</td><td>HMMV_RS27495</td><td>alphaproteobacteria</td><td>Microvirga massiliensis</td><td>TIR domain-containing protein [Microvirga massiliensis].</td><td>GCF_902385745.1</td></tr>
<tr><td>WP_049564506.1</td><td>nSTAND1+BetaPropeller*→</td><td>nSTAND1+BetaPropeller</td><td>1250</td><td>ADY01_RS01750</td><td>actinobacteria</td><td>Streptomyces sp. SBT349</td><td>hypothetical protein [Streptomyces sp. SBT349].</td><td>GCF_001083795.1</td></tr>
<tr><td>WP_049565443.1</td><td>REC→?→?→?→||<-?||?→?→ SIG+nSTAND1+BetaPropeller*→?→?→||<-?<-?<-?<-ABC-ATPase</td><td>SIG+nSTAND1+BetaPropeller</td><td>1425</td><td>ADY59_RS23235</td><td>actinobacteria</td><td>Nonomuraea sp. SBT364</td><td>hypothetical protein [Nonomuraea sp. SBT364].</td><td>GCF_001083785.1</td></tr>
<tr><td>WP_049641845.1</td><td>Patatin→?→?→ nSTAND1+TM+TPR*→||<-?||?→||<-?<-Cluster157_3clades<-FAD-NAD-dep-oxidoreductase<-?<-Cluster428_2clades</td><td>nSTAND1+TM+TPR</td><td>945</td><td>AIOL_RS04910</td><td>alphaproteobacteria</td><td>Candidatus Rhodobacter lobularis</td><td>hypothetical protein [Candidatus Rhodobacter lobularis].</td><td>GCF_001078595.1</td></tr>
<tr><td>WP_050045050.1</td><td>CASPASE+nSTAND1+Pentapeptide+Pentapeptide*→</td><td>CASPASE+nSTAND1+Pentapeptide+Pentapeptide</td><td>1134</td><td>bacteria>cyanobacteria</td><td>Tolypothrix bouteillei</td><td>-</td><td>910240059</td><td>-</td></tr>
<tr><td>WP_050366129.1</td><td>nSTAND1+TM+BetaPropeller*→</td><td>nSTAND1+TM+BetaPropeller</td><td>1266</td><td>HS99_RS15320</td><td>actinobacteria</td><td>Kitasatospora aureofaciens</td><td>WD40 repeat domain-containing protein [Kitasatospora aureofaciens].</td><td>GCF_002082605.1</td></tr>
<tr><td>WP_051169531.1</td><td>SIG+TPR→ EACC1→ CASPASE+nSTAND1+TM+BetaPropeller*→</td><td>CASPASE+nSTAND1+TM+BetaPropeller</td><td>1539</td><td>ON19_RS32425</td><td>actinobacteria</td><td>Nocardia abscessus</td><td>caspase family protein [Nocardia abscessus].</td><td>GCF_000308455.1</td></tr>
<tr><td>WP_051292932.1</td><td>TRPR-HTH+PBPI→?→ TIR+DrHyd→ nSTAND1+BetaPropeller*→</td><td>nSTAND1+BetaPropeller</td><td>1067</td><td>A37S_RS24150</td><td>bacteroidetes</td><td>Olivibacter sitiensis</td><td>hypothetical protein [Olivibacter sitiensis].</td><td>GCF_000427965.1</td></tr>
<tr><td>WP_051675149.1</td><td>TRPR-HTH+PBPI→||<-?<-?<-?||nSTAND1+BetaPropeller*→||<-?<-?<-?||ABhydrolase→</td><td>nSTAND1+BetaPropeller</td><td>1279</td><td>N545_RS24235</td><td>actinobacteria</td><td>Streptomyces sp. URHA0041</td><td>PD40 domain-containing protein [Streptomyces sp. URHA0041].</td><td>GCF_000701325.1</td></tr>
</table>

<table>
<tr><th>acc</th><th>operon</th><th>architecture</th><th>len</th><th>gen.name</th><th>taxend</th><th>species</th><th>defline</th><th>gca</th></tr>
<tr><td>WP_051737318.1</td><td>EACC1→ CASPASE+nSTAND1+TM+BetaPropeller*→||<-TM</td><td>CASPASE+nSTAND1+TM+BetaPropeller</td><td>1475</td><td>OQ00_RS16195</td><td>actinobacteria</td><td>Pseudonocardia autotrophica</td><td>WD40 repeat domain-containing protein [Pseudonocardia autotrophica].</td><td>GCF_000717175.1</td></tr>
<tr><td>WP_051766655.1</td><td>CHTH+nSTAND1+BetaPropeller*→</td><td>CHTH+nSTAND1+BetaPropeller</td><td>1272</td><td>OQ01_RS52670</td><td>actinobacteria</td><td>Saccharothrix syringae</td><td>helix-turn-helix domain-containing protein [Saccharothrix syringae].</td><td>GCF_009498035.1</td></tr>
<tr><td>WP_051945655.1</td><td>TIR+TM+BetaPropeller→||<-SIG+TIR<-?<-nSTAND1+TM<-TIR||TIR+DrHyd→ nSTAND1*→||<-?<-?||TIR→||<-TIR+nSTAND1</td><td>nSTAND1</td><td>1322</td><td>BV106_RS35440</td><td>verrucomicrobia</td><td>Verrucomicrobium sp. BvORR106</td><td>ATP-binding protein [Verrucomicrobium sp. BvORR106].</td><td>GCF_000739655.1</td></tr>
<tr><td>WP_052288432.1</td><td>ABhydrolase→?→?→||<-?||?→ nSTAND1+BetaPropeller*→||<-?||ABC-ATPase→ TM+TM+TM+TM+TM+TM→?→?→ TM+TM+TM→</td><td>nSTAND1+BetaPropeller</td><td>1340</td><td>DYY88_RS24230</td><td>bacteria</td><td>Cyanobacteria</td><td>MULTISPECIES: hypothetical protein [Cyanobacteria].</td><td>GCF_000817775.2</td></tr>
<tr><td>WP_052335365.1</td><td><-TM<-?||CASPASE+nSTAND1+TM+BetaPropeller*→ BetaPropeller→ PSE→?→?→||<-?<-RRM||ParA-Soj-PloopNTPase→ ParB→</td><td>CASPASE+nSTAND1+TM+BetaPropeller</td><td>1092</td><td>FDU-TEX481_RS45850</td><td>cyanobacteria</td><td>Tolypothrix sp. PCC 7601</td><td>caspase family protein [Tolypothrix sp. PCC 7601].</td><td>GCF_000300115.1</td></tr>
<tr><td>WP_052335392.1</td><td><-TM<-?||CASPASE+nSTAND1+Trypsin→<i>?→ CASPASE+nSTAND1+BetaPropeller+BetaPropeller</i>→ BetaPropeller→</td><td>CASPASE+nSTAND1+Trypsin</td><td>1348</td><td>FDU-TEX481_RS40400</td><td>cyanobacteria</td><td>Nostocales</td><td>MULTISPECIES: caspase family protein [Nostocales].</td><td>GCF_001548455.1</td></tr>
<tr><td>WP_052335393.1</td><td>CASPASE+nSTAND1+Trypsin*→</td><td>CASPASE+nSTAND1+Trypsin</td><td>986</td><td>FDU-TEX481_RS33325</td><td>cyanobacteria</td><td>Nostocales</td><td>MULTISPECIES: trypsin-like peptidase domain-containing protein [Nostocales].</td><td>GCF_001548455.1</td></tr>
<tr><td>WP_052490005.1</td><td>EAD4+nSTAND1+BetaPropeller*→||<-pPIWI+MID+RNaseH<-pPIWI+DnaA-AAA+AAA<-pPIWI</td><td>EAD4+nSTAND1+BetaPropeller</td><td>1298</td><td>SD81_RS16770</td><td>cyanobacteria</td><td>Tolypothrix campylonemoides</td><td>AAA family ATPase [Tolypothrix campylonemoides].</td><td>GCF_000828075.3</td></tr>
<tr><td>WP_052490177.1</td><td>ParA-Soj-PloopNTPase→||<-?<-?<-?||PSE→||<-?||nSTAND1*→ PSE→ BetaPropeller→</td><td>nSTAND1</td><td>394</td><td>SD81_RS06920</td><td>cyanobacteria</td><td>Tolypothrix campylonemoides</td><td>hypothetical protein [Tolypothrix campylonemoides].</td><td>GCF_000828075.3</td></tr>
<tr><td>WP_052490698.1</td><td><-TM<-?||CASPASE+nSTAND1+BetaPropeller*→?→?→||<-?<-?<-PSE<-PSE||?→?→ TPR+CASPASE→</td><td>CASPASE+nSTAND1+BetaPropeller</td><td>889</td><td>SD81_RS38450</td><td>cyanobacteria</td><td>Tolypothrix campylonemoides</td><td>caspase family protein [Tolypothrix campylonemoides].</td><td>GCF_000828075.3</td></tr>
<tr><td>WP_052508296.1</td><td>CASPASE→ nSTAND1+TM*→</td><td>nSTAND1+TM</td><td>800</td><td>SP67_RS33610</td><td>cyanobacteria</td><td>Mastigocladus laminosus</td><td>pentapeptide repeat-containing protein [Mastigocladus laminosus].</td><td>GCF_000934435.1</td></tr>
<tr><td>WP_052596711.1</td><td><-ABhydrolase||?→ nSTAND1+TM+BetaPropeller*→</td><td>nSTAND1+TM+BetaPropeller</td><td>1134</td><td>CG11_RS12640</td><td>bacteroidetes</td><td>Aureispira sp. CCB-QB1</td><td>hypothetical protein [Aureispira sp. CCB-QB1].</td><td>GCF_000724545.1</td></tr>
<tr><td>WP_052668847.1</td><td>wHTH-4stranded+TPRs+nSTAND1+BetaPropeller*→||<-?||?→?→?→?→||<-ABhydrolase<-TPR</td><td>wHTH-4stranded+TPRs+nSTAND1+BetaPropeller</td><td>1431</td><td>NITAL_RS24320</td><td>actinobacteria</td><td>Nitriliruptor alkaliphilus</td><td>hypothetical protein [Nitriliruptor alkaliphilus].</td><td>GCF_000969705.1</td></tr>
<tr><td>WP_052691807.1</td><td>TIR→ nSTAND1+TM+TM+TM+TM+TM*→</td><td>nSTAND1+TM+TM+TM+TM+TM</td><td>926</td><td>K380_RS22645</td><td>gammaproteobacteria</td><td>Cellvibrionaceae bacterium Bs12</td><td>RDD family protein [Cellvibrionaceae bacterium Bs12].</td><td>GCF_000966245.1</td></tr>
<tr><td>WP_053662778.1</td><td><-Pkinase+PASTA||?→ PSE→?→ nSTAND1+BetaPropeller*→</td><td>nSTAND1+BetaPropeller</td><td>1284</td><td>ADK64_RS26775</td><td>actinobacteria</td><td>Streptomyces sp. MMG1121</td><td>hypothetical protein [Streptomyces sp. MMG1121].</td><td>GCF_001280015.1</td></tr>
<tr><td>WP_054153119.1</td><td>TIR→ nSTAND1+TM+TM+TM+TM+TM*→?→?→||<-?||?→||<-Na+ORC-AAA<-?||ParA-Soj-PloopNTPase→</td><td>nSTAND1+TM+TM+TM+TM+TM</td><td>1122</td><td>D621_RS07050</td><td>betaproteobacteria</td><td>beta proteobacterium AAP51</td><td>RDD family protein [beta proteobacterium AAP51].</td><td>GCF_001295905.1</td></tr>
<tr><td>WP_054290262.1</td><td><-p450<-?<-ABC-ATPase||?→||<-AraC-HTH+AraC-HTH||PSE→||<-?||nSTAND1+TM+BetaPropeller*→ REC→||<-?<-?||?→||<-?<-REC</td><td>nSTAND1+TM+BetaPropeller</td><td>960</td><td>AOZ06_RS16850</td><td>actinobacteria</td><td>Kibdelosporangium phytohabitans</td><td>PD40 domain-containing protein [Kibdelosporangium phytohabitans].</td><td>GCF_001302585.1</td></tr>
<tr><td>WP_055568262.1</td><td>nSTAND1+BetaPropeller*→</td><td>nSTAND1+BetaPropeller</td><td>1173</td><td>AOK03_RS28690</td><td>actinobacteria</td><td>Streptomyces atriruber</td><td>hypothetical protein [Streptomyces atriruber].</td><td>GCF_001418325.1</td></tr>
</table>

<table>
<tr><th>acc</th><th>operon</th><th>architecture</th><th>len</th><th>gen.name</th><th>taxend</th><th>species</th><th>defline</th><th>gca</th></tr>
<tr><td>WP_057232519.1</td><td>nSTAND1+BetaPropeller*→?→||<-ABhydrolase</td><td>nSTAND1+BetaPropeller</td><td>1319</td><td>ASC99_RS15845</td><td>actinobacteria</td><td>unclassified Kitasatospora</td><td>MULTISPECIES: hypothetical protein [unclassified Kitasatospora].</td><td>GCF_001424875.1</td></tr>
<tr><td>WP_058184087.1</td><td>EAD10+nSTAND1+TM+BetaPropeller*→ CASPASE→</td><td>EAD10+nSTAND1+TM+BetaPropeller</td><td>1369</td><td>BC008_RS28055</td><td>cyanobacteria</td><td>Mastigocoleus testarum</td><td>WD40 repeat domain-containing protein [Mastigocoleus testarum].</td><td>GCF_001456025.1</td></tr>
<tr><td>WP_058883925.1</td><td><-REC<-?<-?||CASPASE+nSTAND1+BetaPropeller*→</td><td>CASPASE+nSTAND1+BetaPropeller</td><td>1732</td><td>AVR62_RS18820</td><td>cyanobacteria</td><td>Oscillatoriales cyanobacterium MTP1</td><td>PD40 domain-containing protein [Oscillatoriales cyanobacterium MTP1].</td><td>GCF_001482745.2</td></tr>
<tr><td>WP_058941697.1</td><td>ABhydrolase→?→ nSTAND1+BetaPropeller*→||<-?||?→ ABhydrolase→</td><td>nSTAND1+BetaPropeller</td><td>1281</td><td>ATE80_RS09355</td><td>actinobacteria</td><td>Streptomyces kanasensis</td><td>WD40 repeat domain-containing protein [Streptomyces kanasensis].</td><td>GCF_001484625.1</td></tr>
<tr><td>WP_062291516.1</td><td>RVe+MutnpC→?→?→||<-TM<-?||CASPASE+nSTAND1+TM*→?→||<-?||?→?→?→?→ REC→</td><td>CASPASE+nSTAND1+TM</td><td>853</td><td>ACX27_RS09860</td><td>cyanobacteria</td><td>Nostoc piscinale</td><td>caspase family protein [Nostoc piscinale].</td><td>GCF_001298445.1</td></tr>
<tr><td>WP_062716636.1</td><td>nSTAND1+BetaPropeller*→||<-?<-PSE<-?<-?<-?<-?<-ROK-HTH</td><td>nSTAND1+BetaPropeller</td><td>1299</td><td>AQJ67_RS04560</td><td>actinobacteria</td><td>Streptomyces caeruleatus</td><td>WD40 repeat domain-containing protein [Streptomyces caeruleatus].</td><td>GCF_001514235.1</td></tr>
<tr><td>WP_066377691.1</td><td>XisI→||<-?<-HAD||?→||<-HSP20<-PSE||?→ PSE→?→ CASPASE+CASPASE+nSTAND1+BetaPropeller*→||<-?||?→||<-SIG+TPR</td><td>CASPASE+CASPASE+nSTAND1+BetaPropeller</td><td>1750</td><td>A3776_RS06945</td><td>cyanobacteria</td><td>Anabaena sp. CA = ATCC 33047</td><td>caspase family protein [Anabaena sp. CA = ATCC 33047].</td><td>GCF_001597855.1</td></tr>
<tr><td>WP_067768577.1</td><td><-SIG+TPR||ABC-ATPase→?→||<-?<-?<-?<-Trypco1||CASPASE+nSTAND1+TM+BetaPropeller*→||<-?||?→||<-?<-?<-ABC-ATPase</td><td>CASPASE+nSTAND1+TM+BetaPropeller</td><td>1611</td><td>NOS3756_RS11575</td><td>cyanobacteria</td><td>Nostoc sp. NIES-3756</td><td>caspase family protein [Nostoc sp. NIES-3756].</td><td>GCF_001548375.1</td></tr>
<tr><td>WP_068382524.1</td><td>CASPASE+CASPASE+nSTAND1+BetaPropeller*→ Uma2→||<-?||?→?→ SIG+TM+TM+TM+TM+TM→</td><td>CASPASE+CASPASE+nSTAND1+BetaPropeller</td><td>1612</td><td>LEP3755_RS08100</td><td>cyanobacteria</td><td>Leptolyngbya sp. NIES-3755</td><td>caspase family protein [Leptolyngbya sp. NIES-3755].</td><td>GCF_001548435.1</td></tr>
<tr><td>WP_068789388.1</td><td>nSTAND1+TM+BetaPropeller*→||<-REC</td><td>nSTAND1+TM+BetaPropeller</td><td>1292</td><td>AY600_RS10555</td><td>cyanobacteria</td><td>Phormidium willei</td><td>WD40 repeat domain-containing protein [Phormidium willei].</td><td>GCF_001637315.1</td></tr>
<tr><td>WP_069068225.1</td><td>RRM→?→ Pkinase+TPR→?→?→ nSTAND1+BetaPropeller*→?→ PSE→ SIG+Pentapeptide→?→||<-?<-?<-zinc_ribbon_2+Pkinase+TM+TM+TM+TM</td><td>nSTAND1+BetaPropeller</td><td>1225</td><td>A4S05_RS02400</td><td>cyanobacteria</td><td>Nostoc sp. KVJ20</td><td>WD40 repeat domain-containing protein [Nostoc sp. KVJ20].</td><td>GCF_001712795.1</td></tr>
<tr><td>WP_069471539.1</td><td>ACT→?→?→?→ tRNA→ tRNA→?→||<-SIG+ApbE||?→ nSTAND1+TM+BetaPropeller→*?→?→ nSTAND1+BetaPropeller*→</td><td>nSTAND1+TM+BetaPropeller</td><td>1371</td><td>BHU68_RS00110</td><td>gammaproteobacteria</td><td>Candidatus Marithrix sp. Canyon 246</td><td>WD40 repeat domain-containing protein [Candidatus Marithrix sp. Canyon 246].</td><td>GCF_001723675.1</td></tr>
<tr><td>WP_069471542.1</td><td>nSTAND1+BetaPropeller*→</td><td>nSTAND1+BetaPropeller</td><td>1154</td><td>BHU68_RS00095</td><td>gammaproteobacteria</td><td>Candidatus Marithrix sp. Canyon 246</td><td>hypothetical protein [Candidatus Marithrix sp. Canyon 246].</td><td>GCF_001723675.1</td></tr>
<tr><td>WP_069472735.1</td><td>S4+PSYN→?→?→ nSTAND1+BetaPropeller*→</td><td>nSTAND1+BetaPropeller</td><td>1471</td><td>BHU65_RS01520</td><td>gammaproteobacteria</td><td>Candidatus Marithrix sp. Canyon 246</td><td>ABC transporter substrate-binding protein, partial [Candidatus Marithrix sp. Canyon 246].</td><td>GCF_001723755.1</td></tr>
<tr><td>WP_069775370.1</td><td><-AraC-HTH+AraC-HTH||?→ HTH+nSTAND1+TM+BetaPropeller*→ SIG+NLPC→</td><td>HTH+nSTAND1+TM+BetaPropeller</td><td>1301</td><td>BGM19_RS05250</td><td>actinobacteria</td><td>Streptomyces agglomeratus</td><td>helix-turn-helix domain-containing protein [Streptomyces agglomeratus].</td><td>GCF_001746475.1</td></tr>
<tr><td>WP_071926783.1</td><td>EACC1→ CASPASE+nSTAND1+BetaPropeller*→?→||<-?<-?||PSE→||<-?<-TM+TM+TM+TM+TM+TM<-ABC-ATPase</td><td>CASPASE+nSTAND1+BetaPropeller</td><td>1400</td><td>BOX37_RS06100</td><td>actinobacteria</td><td>Nocardia mangyaensis</td><td>hypothetical protein [Nocardia mangyaensis].</td><td>GCF_001886715.1</td></tr>
</table>

<table>
<tr><th>acc</th><th>operon</th><th>architecture</th><th>len</th><th>gen.name</th><th>taxend</th><th>species</th><th>defline</th><th>gca</th></tr>
<tr><td>WP_073484145.1</td><td>HTH+nSTAND1+TM+BetaPropeller*→</td><td>HTH+nSTAND1+TM+BetaPropeller</td><td>1258</td><td>BUE43_RS16570</td><td>actinobacteria</td><td>Streptoalloteichus hindustanus</td><td>helix-turn-helix domain-containing protein [Streptoalloteichus hindustanus].</td><td>GCF_900129375.1</td></tr>
<tr><td>WP_073549910.1</td><td>CASPASE+CASPASE+nSTAND1+BetaPropeller*→ SIG+TPR→</td><td>CASPASE+CASPASE+nSTAND1+BetaPropeller</td><td>1691</td><td>NIES1031_RS13280</td><td>cyanobacteria</td><td>Chroogloeocystis siderophila</td><td>caspase family protein [Chroogloeocystis siderophila].</td><td>GCF_001904655.1</td></tr>
<tr><td>WP_073619116.1</td><td>Pentapeptide→?→?→?→||<-Trypco1||CASPASE+nSTAND1+Pentapeptide+Pentapeptide+Pentapeptide*→</td><td>CASPASE+nSTAND1+Pentapeptide+Pentapeptide+Pentapeptide</td><td>1155</td><td>NIES2101_RS45020</td><td>cyanobacteria</td><td>Calothrix sp. HK-06</td><td>pentapeptide repeat-containing protein [Calothrix sp. HK-06].</td><td>GCF_001904745.1</td></tr>
<tr><td>WP_075128359.1</td><td>HTH+nSTAND1+TM+BetaPropeller*→</td><td>HTH+nSTAND1+TM+BetaPropeller</td><td>1232</td><td>BU204_RS25880</td><td>actinobacteria</td><td>Actinophytocola xanthii</td><td>helix-turn-helix domain-containing protein [Actinophytocola xanthii].</td><td>GCF_001921205.1</td></tr>
<tr><td>WP_075900997.1</td><td><-ABhydrolase||?→?→?→||<-Trypco1||CASPASE+nSTAND1+TM+BetaPropeller*→</td><td>CASPASE+nSTAND1+TM+BetaPropeller</td><td>1830</td><td>BJP37_RS36840</td><td>cyanobacteria</td><td>Moorea bouillonii</td><td>caspase family protein [Moorea bouillonii].</td><td>GCF_001942495.1</td></tr>
<tr><td>WP_075905263.1</td><td>CASPASE+nSTAND1+BetaPropeller*→||<-?||MED15→||<-?<-Uma2</td><td>CASPASE+nSTAND1+BetaPropeller</td><td>1452</td><td>BJP37_RS32685</td><td>cyanobacteria</td><td>Moorea bouillonii</td><td>caspase family protein [Moorea bouillonii].</td><td>GCF_001942495.1</td></tr>
<tr><td>WP_076082468.1</td><td>nSTAND1*→||<-?||PSE→||<-ACET||?→||<-?<-VWA<-MoxR-AAA</td><td>nSTAND1</td><td>584</td><td>AV521_RS01825</td><td>actinobacteria</td><td>Streptomyces sp. IMTB 2501</td><td>ATP-binding protein [Streptomyces sp. IMTB 2501].</td><td>GCF_001953885.1</td></tr>
<tr><td>WP_076440611.1</td><td>nSTAND1+BetaPropeller*→||<-?<-ParA-Soj-PloopNTPase</td><td>nSTAND1+BetaPropeller</td><td>1266</td><td>BXA11_RS32200</td><td>actinobacteria</td><td>Microbispora rosea</td><td>hypothetical protein [Microbispora rosea].</td><td>GCF_900156315.1</td></tr>
<tr><td>WP_076985819.1</td><td>Pkinase→||<-?||?→?→?→||<-?||PSE→||<-?||HTH+nSTAND1+BetaPropeller*→</td><td>HTH+nSTAND1+BetaPropeller</td><td>1262</td><td>ALI144C_RS05040</td><td>actinobacteria</td><td>Actinosynnema sp. ALI-1.44</td><td>PD40 domain-containing protein [Actinosynnema sp. ALI-1.44].</td><td>GCF_001984155.1</td></tr>
<tr><td>WP_078616701.1</td><td>HTH+nSTAND1+TM+BetaPropeller*→</td><td>HTH+nSTAND1+TM+BetaPropeller</td><td>1390</td><td>H295_RS0120055</td><td>actinobacteria</td><td>Streptomyces sp. 351MFTsu5.1</td><td>helix-turn-helix domain-containing protein [Streptomyces sp. 351MFTsu5.1].</td><td>GCF_000383655.1</td></tr>
<tr><td>WP_078831679.1</td><td>TIR+DrHyd→ nSTAND1+BetaPropeller*→</td><td>nSTAND1+BetaPropeller</td><td>1025</td><td>B5D42_RS11600</td><td>bacteroidetes</td><td>Sediminibacterium ginsengisoli</td><td>hypothetical protein [Sediminibacterium ginsengisoli].</td><td>GCF_900167075.1</td></tr>
<tr><td>WP_079315441.1</td><td><-ABC-ATPase<-ABC-ATPase<-?<-PSE<-?<-PSE<-?||?→ nSTAND1+BetaPropeller*→||<-PSE<-?<-tRNA<-tRNA<-?<-SIG+PDZ+LonC<-?<-?||?→ NUDIX→</td><td>nSTAND1+BetaPropeller</td><td>1250</td><td>B1L11_RS17305</td><td>actinobacteria</td><td>Microbispora sp. GKU 823</td><td>hypothetical protein [Microbispora sp. GKU 823].</td><td>GCF_002027145.1</td></tr>
<tr><td>WP_079432573.1</td><td><-ICP8OBfold||?→?→?→?→ TIR+DrHyd→ nSTAND1+BetaPropeller*→</td><td>nSTAND1+BetaPropeller</td><td>1076</td><td>B4920_RS00090</td><td>betaproteobacteria</td><td>Zoogloea sp. LCSB751</td><td>WD40 repeat domain-containing protein [Zoogloea sp. LCSB751].</td><td>GCF_002028455.1</td></tr>
<tr><td>WP_080045844.1</td><td>ENOYCOADEHYD→||<-?<-?<-?<-?<-?||nSTAND1+BetaPropeller*→||<-?<-?<-?<-?||?→ Pkinase→</td><td>nSTAND1+BetaPropeller</td><td>1207</td><td>BKM31_RS55895</td><td>actinobacteria</td><td>Nonomuraea sp. ATCC 55076</td><td>hypothetical protein [Nonomuraea sp. ATCC 55076].</td><td>GCF_002057455.1</td></tr>
<tr><td>WP_080808279.1</td><td><-NACHT||?→||<-PSE<-?<-?||PSE→ PSE→||<-?||?→ nSTAND1*→ nSTAND1+TM+TPR→?→?→?→||<-PSE||Uma2→</td><td>nSTAND1</td><td>353</td><td>XM38_RS18855</td><td>cyanobacteria</td><td>Halomicronema hongdechloris</td><td>ATP-binding protein [Halomicronema hongdechloris].</td><td>GCF_002075285.3</td></tr>
<tr><td>WP_081771813.1</td><td>TIR+TIR→ nSTAND1+BetaPropeller*→ SLATT+SLATT→</td><td>nSTAND1+BetaPropeller</td><td>1486</td><td>A3CW_RS50325</td><td>betaproteobacteria</td><td>Paraburkholderia nodosa</td><td>AAA family ATPase [Paraburkholderia nodosa].</td><td>GCF_000519185.1</td></tr>
<tr><td>WP_083582706.1</td><td><-REC<-PSE||?→||<-?<-Trypco1||CASPASE+nSTAND1*→</td><td>CASPASE+nSTAND1</td><td>995</td><td>BRW63_RS04300</td><td>cyanobacteria</td><td>Phormidesmis priestleyi</td><td>caspase family protein [Phormidesmis priestleyi].</td><td>GCF_001895925.1</td></tr>
<tr><td>WP_084556682.1</td><td>HTH+nSTAND1+BetaPropeller*→</td><td>HTH+nSTAND1+BetaPropeller</td><td>1331</td><td>bacteria>actinobacteria</td><td>Couchioplanes caeruleus</td><td>-</td><td>1181239683</td><td>-</td></tr>
<tr><td>WP_086168347.1</td><td><-S1COLD||?→ nSTAND1+BetaPropeller*→?→||<-?<-?<-CASPASE+nSTAND1+BetaPropeller<-?<-EACC1</td><td>nSTAND1+BetaPropeller</td><td>1276</td><td>OQI_RS06360</td><td>actinobacteria</td><td>Streptomyces pharetrae</td><td>hypothetical protein [Streptomyces pharetrae].</td><td>GCF_002128465.1</td></tr>
</table>

<table>
<tr><th>acc</th><th>operon</th><th>architecture</th><th>len</th><th>gen.name</th><th>taxend</th><th>species</th><th>defline</th><th>gca</th></tr>
<tr><td>WP_086476322.1</td><td>TIR→ nSTAND1+TM*→</td><td>nSTAND1+TM</td><td>707</td><td>BV214_RS04690</td><td>bacteroidetes</td><td>Arenibacter sp. AK53</td><td>hypothetical protein [Arenibacter sp. AK53].</td><td>GCF_002150785.1</td></tr>
<tr><td>WP_086667199.1</td><td><-HAD<-?||?→?→||<-?<-ABC-ATPase||?→ nSTAND1+TM+BetaPropeller*→</td><td>nSTAND1+TM+BetaPropeller</td><td>945</td><td>B0F77_RS34380</td><td>actinobacteria</td><td>Lentzea kentuckyensis</td><td>hypothetical protein [Lentzea kentuckyensis].</td><td>GCF_002150765.1</td></tr>
<tr><td>WP_086687140.1</td><td>SIG+HOP2→||<-ABhydrolase<-?<-?||?→?→ nSTAND1+BetaPropeller*→||<-?<-?<-?<-ABhydrolase</td><td>nSTAND1+BetaPropeller</td><td>1159</td><td>BV372_RS14140</td><td>cyanobacteria</td><td>Nostoc sp. T09</td><td>AAA family ATPase [Nostoc sp. T09].</td><td>GCF_002154695.1</td></tr>
<tr><td>WP_086756226.1</td><td><-Trypco1||CASPASE+nSTAND1+BetaPropeller*→</td><td>CASPASE+nSTAND1+BetaPropeller</td><td>906</td><td>BV375_RS00710</td><td>cyanobacteria</td><td>Nostoc sp. 106C</td><td>caspase family protein [Nostoc sp. 106C].</td><td>GCF_002154725.1</td></tr>
<tr><td>WP_087545319.1</td><td><-Cluster158_2clades<-?||?→ SLATT→ TIR+DrHyd→ nSTAND1+BetaPropeller*→||<-PSE||?→||<-?<-?<-HxxxH</td><td>nSTAND1+BetaPropeller</td><td>1320</td><td>BZZ01_RS31645</td><td>cyanobacteria</td><td>Nostocales cyanobacterium HT-58-2</td><td>hypothetical protein [Nostocales cyanobacterium HT-58-2].</td><td>GCF_002163975.1</td></tr>
<tr><td>WP_090879784.1</td><td><-ParA-Soj-PloopNTPase<-?||?→||<-PSE<-?||?→||<-?||TIR+DrHyd+TIR→ nSTAND1+EP1*→</td><td>nSTAND1+EP1</td><td>2283</td><td>BLT60_RS20960</td><td>alphaproteobacteria</td><td>Bauldia litoralis</td><td>hypothetical protein [Bauldia litoralis].</td><td>GCF_900104485.1</td></tr>
<tr><td>WP_090933370.1</td><td><-wHTH-4stranded+TPRs+APATPase||HTH+nSTAND1+TM+BetaPropeller*→</td><td>HTH+nSTAND1+TM+BetaPropeller</td><td>1266</td><td>BLS95_RS20045</td><td>actinobacteria</td><td>Nonomuraea jiangxiensis</td><td>hypothetical protein [Nonomuraea jiangxiensis].</td><td>GCF_900099965.1</td></tr>
<tr><td>WP_090944045.1</td><td>SIG+Trypsin+PDZ→||<-?<-?||?→?→||<-ABhydrolase<-?||nSTAND1+TM+BetaPropeller*→||<-?<-?<-?<-?||?→||<-HTH+MED15<-ACT</td><td>nSTAND1+TM+BetaPropeller</td><td>1222</td><td>BLS95_RS39570</td><td>actinobacteria</td><td>Nonomuraea jiangxiensis</td><td>hypothetical protein [Nonomuraea jiangxiensis].</td><td>GCF_900099965.1</td></tr>
<tr><td>WP_091280677.1</td><td>CASPASE+nSTAND1+TM+BetaPropeller*→</td><td>CASPASE+nSTAND1+TM+BetaPropeller</td><td>1556</td><td>AWX74_RS22975</td><td>actinobacteria</td><td>Frankia irregularis</td><td>WD40 repeat domain-containing protein [Frankia irregularis].</td><td>GCF_001536285.1</td></tr>
<tr><td>WP_091294574.1</td><td>nSTAND1+TM+BetaPropeller*→</td><td>nSTAND1+TM+BetaPropeller</td><td>1264</td><td>BLV57_RS30410</td><td>actinobacteria</td><td>Amycolatopsis xylanica</td><td>hypothetical protein [Amycolatopsis xylanica].</td><td>GCF_900107045.1</td></tr>
<tr><td>WP_092636218.1</td><td>ABC-ATPase→ ABC-ATPase→ TIR→ nSTAND1+TM+TPR+TPR+TPR+TPR+TPR*→?→||<-?<-AraC-HTH+AraC-HTH</td><td>nSTAND1+TM+TPR+TPR+TPR+TPR+TPR</td><td>1612</td><td>BLV06_RS20590</td><td>alphaproteobacteria</td><td>Rhizobiales bacterium GAS113</td><td>ATP-binding protein [Rhizobiales bacterium GAS113].</td><td>GCF_900103895.1</td></tr>
<tr><td>WP_092980844.1</td><td>SLATT→ TIR→ nSTAND1+TM*→||<-?<-?<-TPR</td><td>nSTAND1+TM</td><td>809</td><td>BM318_RS03935</td><td>bacteroidetes</td><td>Robiginitalea myxolifaciens</td><td>hypothetical protein [Robiginitalea myxolifaciens].</td><td>GCF_900115205.1</td></tr>
<tr><td>WP_093158527.1</td><td>nSTAND1+BetaPropeller*→</td><td>nSTAND1+BetaPropeller</td><td>1257</td><td>ATL45_RS01025</td><td>actinobacteria</td><td>Saccharopolyspora antimicrobica</td><td>AAA family ATPase [Saccharopolyspora antimicrobica].</td><td>GCF_900114905.1</td></tr>
<tr><td>WP_093269708.1</td><td>ACET→||<-?<-?||?→ CASPASE+nSTAND1+TM+BetaPropeller*→</td><td>CASPASE+nSTAND1+TM+BetaPropeller</td><td>1659</td><td>BLW84_RS25305</td><td>actinobacteria</td><td>Saccharopolyspora shandongensis</td><td>caspase family protein [Saccharopolyspora shandongensis].</td><td>-</td></tr>
<tr><td>WP_094341574.1</td><td>Trypsin+nSTAND1+BetaPropeller*→</td><td>Trypsin+nSTAND1+BetaPropeller</td><td>1438</td><td>CDG79_RS07440</td><td>cyanobacteria</td><td>Nostoc sp. ‘Peltigera membranacea cyanobiont’ 232</td><td>trypsin-like peptidase domain-containing protein [Nostoc sp. ‘Peltigera membranacea cyanobiont’ 232].</td><td>GCF_002245985.1</td></tr>
<tr><td>WP_094349283.1</td><td>nSTAND1+BetaPropeller→?→||<-Trypco1||CASPASE+nSTAND1+TM+BetaPropeller*→</td><td>CASPASE+nSTAND1+TM+BetaPropeller</td><td>1387</td><td>CDG76_RS12330</td><td>cyanobacteria</td><td>Nostoc sp. ‘Peltigera membranacea cyanobiont’ 210A</td><td>caspase family protein [Nostoc sp. ‘Peltigera membranacea cyanobiont’ 210A].</td><td>GCF_002246015.1</td></tr>
<tr><td>WP_094555851.1</td><td>TIR+DrHyd→ nSTAND1+BetaPropeller+BetaPropeller*→</td><td>nSTAND1+BetaPropeller+BetaPropeller</td><td>1242</td><td>CJZ82_RS11170</td><td>cyanobacteria</td><td>Synechococcus sp. 1G10</td><td>hypothetical protein [Synechococcus sp. 1G10].</td><td>GCF_002252625.1</td></tr>
<tr><td>WP_095722688.1</td><td>CASPASE+CASPASE+nSTAND1+BetaPropeller*→</td><td>CASPASE+CASPASE+nSTAND1+BetaPropeller</td><td>1706</td><td>CK510_RS16200</td><td>cyanobacteria</td><td>Calothrix elsteri</td><td>caspase family protein [Calothrix elsteri].</td><td>GCF_002289455.1</td></tr>
<tr><td>WP_096542102.1</td><td><-Trypco1||CASPASE+nSTAND1+Pentapeptide*→?→?→ XisI→</td><td>CASPASE+nSTAND1+Pentapeptide</td><td>996</td><td>CA726_RS33005</td><td>cyanobacteria</td><td>Nostoc linckia</td><td>pentapeptide repeat-containing protein [Nostoc linckia].</td><td>GCF_002368035.1</td></tr>
<tr><td>WP_096556689.1</td><td><-HAD||?→||<-HSP20||?→||<-REC<-?||CASPASE+CASPASE+nSTAND1+BetaPropeller*→</td><td>CASPASE+CASPASE+nSTAND1+BetaPropeller</td><td>1739</td><td>CDC32_RS19410</td><td>cyanobacteria</td><td>Nostoc sp. NIES-4103</td><td>caspase family protein [Nostoc sp. NIES-4103].</td><td>GCF_002368335.1</td></tr>
</table>

<table>
<tr><th>acc</th><th>operon</th><th>architecture</th><th>len</th><th>gen.name</th><th>taxend</th><th>species</th><th>defline</th><th>gca</th></tr>
<tr><td>WP_096563584.1</td><td>HSP20→||<-ABhydrolase||?→||<-PSE||?→?→ nSTAND1+BetaPropeller+TPR*→ CASPASE→?→||<-?<-?||ABhydrolase→</td><td>nSTAND1+BetaPropeller+TPR</td><td>1393</td><td>CDC39_RS16830</td><td>cyanobacteria</td><td>Scytonema sp. NIES-4073</td><td>hypothetical protein [Scytonema sp. NIES-4073].</td><td>GCF_002368435.1</td></tr>
<tr><td>WP_096586192.1</td><td>Pentapeptide→ nSTAND1+BetaPropeller*→||<-?||?→||<-TM+TM+NACHT+FGS+GUN4<-CASPASE||?→||<-PSE<-?<-ParA-Soj-PloopNTPase</td><td>nSTAND1+BetaPropeller</td><td>1298</td><td>CA723_RS30235</td><td>cyanobacteria</td><td>Anabaenopsis circularis</td><td>PD40 domain-containing protein [Anabaenopsis circularis].</td><td>GCF_002367975.1</td></tr>
<tr><td>WP_096624791.1</td><td>Redoxin→||<-?<-?<-?||?→?→?→ CASPASE+CASPASE+nSTAND1+BetaPropeller*→||<-?<-?||?→ SIG+Pentapeptide→?→ ncRNA→||<-REC</td><td>CASPASE+CASPASE+nSTAND1+BetaPro-peller</td><td>1715</td><td>CDC37_RS20940</td><td>cyanobacteria</td><td>Calothrix sp. NIES-3974</td><td>caspase family protein [Calothrix sp. NIES-3974].</td><td>GCF_002368395.1</td></tr>
<tr><td>WP_096641665.1</td><td><-Cluster108_2clades||TPR+CASPASE→ CASPASE+nSTAND1+FGS*→</td><td>CASPASE+nSTAND1+FGS</td><td>1012</td><td>CA724_RS00570</td><td>cyanobacteria</td><td>Calothrix brevissima</td><td>SUMF1/EgtB/PvdO family nonheme iron enzyme [Calothrix brevissima].</td><td>GCF_002367995.1</td></tr>
<tr><td>WP_096655823.1</td><td>Pentapeptide→ CASPASE+nSTAND1+TM*→</td><td>CASPASE+nSTAND1+TM</td><td>767</td><td>CA729_RS11090</td><td>cyanobacteria</td><td>Calothrix parasitica</td><td>caspase family protein [Calothrix parasitica].</td><td>GCF_002368095.1</td></tr>
<tr><td>WP_096661654.1</td><td>RVT→?→?→?→||<-?<-Trypco1||CASPASE+nSTAND1+Pentapeptide*→||<-NACHT+BetaPropeller</td><td>CASPASE+nSTAND1+Pentapeptide</td><td>991</td><td>CA729_RS36575</td><td>cyanobacteria</td><td>Calothrix parasitica</td><td>pentapeptide repeat-containing protein [Calothrix parasitica].</td><td>GCF_002368095.1</td></tr>
<tr><td>WP_096677027.1</td><td><-TM<-?||CASPASE+nSTAND1+BetaPropeller*→||<-?<-?<-?||?→||<-?<-?||HxxxH→</td><td>CASPASE+nSTAND1+BetaPropeller</td><td>1263</td><td>CDC30_RS18535</td><td>cyanobacteria</td><td>Fischerella sp. NIES-4106</td><td>caspase family protein [Fischerella sp. NIES-4106].</td><td>GCF_002368315.1</td></tr>
<tr><td>WP_096682926.1</td><td>APATPase+BetaPropeller→||<-Pentapeptide<-?||PSE→ PSE→||<-?||NACHT+TM+TM+TM→ FGS→ nSTAND1+TM+BetaPropeller*→ ABhydrolase→ Pentapeptide→ NACHT→?→ NACHT+HEAT→||<-?||Pentapeptide→</td><td>nSTAND1+TM+BetaPropeller</td><td>1278</td><td>CA737_RS29000</td><td>cyanobacteria</td><td>Nostoc sp. NIES-2111</td><td>peptidase C14 [Nostoc sp. NIES-2111].</td><td>GCF_002368215.1</td></tr>
<tr><td>WP_096684184.1</td><td>nSTAND1+BetaPropeller*→ CASPASE→</td><td>nSTAND1+BetaPropeller</td><td>1280</td><td>CDC41_RS02675</td><td>cyanobacteria</td><td>unclassified Calothrix</td><td>MULTISPECIES: WD40 repeat domain-containing protein [unclassified Calothrix].</td><td>GCF_002368455.1</td></tr>
<tr><td>WP_096722951.1</td><td><-ATPcone<-?<-?<-?<-?||?→||<-REC||CASPASE+CASPASE+nSTAND1+BetaPropeller*→</td><td>CASPASE+CASPASE+nSTAND1+BetaPro-peller</td><td>1589</td><td>CDC35_RS17615</td><td>cyanobacteria</td><td>Chondrocystis sp. NIES-4102</td><td>caspase family protein [Chondrocystis sp. NIES-4102].</td><td>GCF_002368355.1</td></tr>
<tr><td>WP_099198599.1</td><td><-SIG+ASH-IG<-?||?→ nSTAND1+BetaPropeller*→||<-?<-?<-?||Cluster106_3clades→</td><td>nSTAND1+BetaPropeller</td><td>1284</td><td>CYQ11_RS04175</td><td>actinobacteria</td><td>Streptomyces cinnamoneus</td><td>hypothetical protein [Streptomyces cinnamoneus].</td><td>GCF_002727115.1</td></tr>
<tr><td>WP_101833019.1</td><td>nSTAND1+BetaPropeller*→</td><td>nSTAND1+BetaPropeller</td><td>1222</td><td>bacteria>actinobacte-ria</td><td>Frankia canadensis</td><td>-</td><td>1325963764</td><td>-</td></tr>
<tr><td>WP_102179560.1</td><td>EACC2+CASPASE+nSTAND1+BetaPropeller*→||<-ABhydrolase<-NUDIX</td><td>EACC2+CASPASE+nSTAND1+BetaPro-peller</td><td>1565</td><td>CEN45_RS19405</td><td>cyanobacteria</td><td>Fischerella thermalis</td><td>CHAT domain-containing protein [Fischerella thermalis].</td><td>GCF_002870635.1</td></tr>
<tr><td>WP_102939115.1</td><td>SIG+TM+TM+TM+TM+TM→ SIG+TM+TM+TM+TM+TM→ ABC-ATPase→||<-?||?→?→ EACC2+CASPASE+nSTAND1+BetaPropeller*→</td><td>EACC2+CASPASE+nSTAND1+BetaPro-peller</td><td>1603</td><td>CEP15_RS15365</td><td>cyanobacteria</td><td>Cylindrospermopsis raciborskii</td><td>PQQ-binding-like beta-propeller repeat protein [Cylindrospermopsis raciborskii].</td><td>GCF_002893125.1</td></tr>
<tr><td>WP_103138901.1</td><td>TM+TM+TM→?→?→||<-?<-?<-?||?→ nSTAND1+BetaPropeller*→?→?→?→||<-Uma2</td><td>nSTAND1+BetaPropeller</td><td>1225</td><td>CLI64_RS20305</td><td>cyanobacteria</td><td>Nostoc sp. CENA543</td><td>hypothetical protein [Nostoc sp. CENA543].</td><td>GCF_002896875.1</td></tr>
<tr><td>WP_103964494.1</td><td>SIG+nSTAND1+BetaPropeller*→?→||<-?||PSE→||<-?||?→ PSE→||<-pPIWI+MID+RNaseH<-pPIWI+viral-AAA<-pPIWI</td><td>SIG+nSTAND1+BetaPropeller</td><td>1377</td><td>C4V41_RS57390</td><td>actinobacteria</td><td>Nonomuraea solani</td><td>AAA family ATPase [Nonomuraea solani].</td><td>GCF_900108335.1</td></tr>
<tr><td>WP_104899206.1</td><td>EACC2+CASPASE+nSTAND1+BetaPropeller*→||<-XisI<-?||?→?→ EACC2+CASPASE+nSTAND1+BetaPropeller→</td><td>EACC2+CASPASE+nSTAND1+BetaPro-peller</td><td>1559</td><td>NPM_RS08915</td><td>cyanobacteria</td><td>Nostoc sp. ‘Peltigera membranacea cyanobiont’ N6</td><td>CHAT domain-containing protein [Nostoc sp. ‘Peltigera membranacea cyanobiont’ N6].</td><td>GCF_002949735.1</td></tr>
<tr><td>WP_106172461.1</td><td><-TPR||CASPASE+nSTAND1+BetaPropeller*→ Redoxin→</td><td>CASPASE+nSTAND1+BetaPropeller</td><td>1796</td><td>C7B76_RS10855</td><td>cyanobacteria</td><td>filamentous cyanobacterium CCP2</td><td>caspase family protein [filamentous cyanobacterium CCP2].</td><td>GCF_003003615.1</td></tr>
</table>

<table>
<thead>
<tr><th>acc</th><th>operon</th><th>architecture</th><th>len</th><th>gen.name</th><th>taxend</th><th>species</th><th>defline</th><th>gca</th></tr>
</thead>
<tbody>
<tr><td>WP_106238916.1</td><td>Cluster59_2clades→ TPR→?→?→||<-?||nSTAND1+BetaPropeller*→?→?→||<-?||PSE→?→ ACT→?→ Cluster108_2clades→</td><td>nSTAND1+BetaPropeller</td><td>1232</td><td>B0I32_RS14395</td><td>actinobacteria</td><td>Nonomuraea fuscirosea</td><td>WD40 repeat domain-containing protein [Nonomuraea fuscirosea].</td><td>GCF_003001935.1</td></tr>
<tr><td>WP_106256509.1</td><td>CASPASE+nSTAND1+BetaPropeller*→</td><td>CASPASE+nSTAND1+BetaPropeller</td><td>1771</td><td>C7B82_RS11920</td><td>cyanobacteria</td><td>Leptolyngbya frigida</td><td>PD40 domain-containing protein [Leptolyngbya frigida].</td><td>GCF_003003795.1</td></tr>
<tr><td>WP_106259571.1</td><td>CASPASE+nSTAND1+TM*→?→||<-?||PSE→?→?→?→ Pentapeptide→</td><td>CASPASE+nSTAND1+TM</td><td>985</td><td>C7B82_RS25475</td><td>cyanobacteria</td><td>Leptolyngbya frigida</td><td>caspase family protein [Leptolyngbya frigida].</td><td>GCF_003003795.1</td></tr>
<tr><td>WP_106259865.1</td><td>TIR+DrHyd→ nSTAND1+TM+BetaPropeller+TPR*→</td><td>nSTAND1+TM+BetaPropeller+TPR</td><td>1061</td><td>C7B82_RS26795</td><td>cyanobacteria</td><td>Leptolyngbya frigida</td><td>hypothetical protein [Leptolyngbya frigida].</td><td>GCF_003003795.1</td></tr>
<tr><td>WP_106288913.1</td><td><-FHA+Pkinase<-?<-?<-?||?→?→||<-?||CASPASE+CASPASE+nSTAND1+BetaPropeller*→?→?→?→?→?→ Uma2→</td><td>CASPASE+CASPASE+nSTAND1+BetaPropeller</td><td>1761</td><td>C7B64_RS12100</td><td>cyanobacteria</td><td>Merismopedia glauca</td><td>caspase family protein [Merismopedia glauca].</td><td>GCF_003003775.1</td></tr>
<tr><td>WP_106409482.1</td><td>HTH+nSTAND1+TM+BetaPropeller*→?→||<-?||ABhydrolase→</td><td>HTH+nSTAND1+TM+BetaPropeller</td><td>1356</td><td>WH07_RS00620</td><td>actinobacteria</td><td>Streptomyces odonnellii</td><td>helix-turn-helix domain-containing protein [Streptomyces odonnellii].</td><td>GCF_000981895.1</td></tr>
<tr><td>WP_106859678.1</td><td><-AAA+TPR||?→ nSTAND1+TM+BetaPropeller*→</td><td>nSTAND1+TM+BetaPropeller</td><td>1282</td><td>DAA54_RS06965</td><td>acidobacteria</td><td>Candidatus Sulfopaludibacter sp. SbA4</td><td>WD40 repeat domain-containing protein [Candidatus Sulfopaludibacter sp. SbA4].</td><td>GCF_900290305.1</td></tr>
<tr><td>WP_106870381.1</td><td>TIR+DrHyd→ nSTAND1+BetaPropeller*→||<-?||ABC-ATPase→</td><td>nSTAND1+BetaPropeller</td><td>1551</td><td>C7293_RS12420</td><td>cyanobacteria</td><td>unclassified Cyanobacteria (miscellaneous)</td><td>MULTISPECIES: pre-peptidase C-terminal domain-containing protein [unclassified Cyanobacteria (miscellaneous)].</td><td>GCF_003017855.1</td></tr>
<tr><td>WP_109279715.1</td><td><-nSTAND1+BetaPropeller||?→?→?→ EACC1→ CASPASE+nSTAND1+TM+BetaPropeller*→</td><td>CASPASE+nSTAND1+TM+BetaPropeller</td><td>1552</td><td>CV755_RS09465</td><td>actinobacteria</td><td>Streptomyces orinoci</td><td>caspase family protein [Streptomyces orinoci].</td><td>GCF_003121295.1</td></tr>
<tr><td>WP_109364478.1</td><td>SWACOS+TPR+TPR→?→?→ nSTAND1+BetaPropeller*→||<-?<-?||APATPase+TPR→</td><td>nSTAND1+BetaPropeller</td><td>1274</td><td>DF268_RS38315</td><td>actinobacteria</td><td>Streptomyces sp. V2</td><td>hypothetical protein [Streptomyces sp. V2].</td><td>GCF_003129505.1</td></tr>
<tr><td>WP_111843756.1</td><td>TIR→ nSTAND1+TM*→?→?→||<-?<-?<-SIGMA-HTH</td><td>nSTAND1+TM</td><td>949</td><td>ESU54_RS03485</td><td>bacteroidetes</td><td>Aequorivita antarctica</td><td>hypothetical protein [Aequorivita antarctica].</td><td>GCF_900489835.1</td></tr>
<tr><td>WP_111882267.1</td><td>TIR→ nSTAND1+TM*→</td><td>nSTAND1+TM</td><td>744</td><td>DSY54_RS14580</td><td>bacteroidetes</td><td>Aequorivita sp. CIP111184</td><td>hypothetical protein [Aequorivita sp. CIP111184].</td><td>GCF_900489465.1</td></tr>
<tr><td>WP_112443263.1</td><td>nSTAND1+BetaPropeller*→</td><td>nSTAND1+BetaPropeller</td><td>1291</td><td>DN051_RS43725</td><td>actinobacteria</td><td>Streptomyces sp. ZFG47</td><td>hypothetical protein [Streptomyces sp. ZFG47].</td><td>GCF_003261055.1</td></tr>
<tr><td>WP_114006120.1</td><td>REC→?→?→?→?→ TIR+DrHyd→ nSTAND1+BetaPropeller*→?→?→||<-?<-?<-SIG+HISKIN+REC+AraC-HTH</td><td>nSTAND1+BetaPropeller</td><td>1022</td><td>DJ568_RS15045</td><td>bacteroidetes</td><td>Mucilaginibacter hurinus</td><td>hypothetical protein [Mucilaginibacter hurinus].</td><td>GCF_003324525.1</td></tr>
<tr><td>WP_114324839.1</td><td>TIR+DrHyd→ nSTAND1*→</td><td>nSTAND1</td><td>259</td><td>DWA31_RS01095</td><td>gammaproteobacteria</td><td>Candidatus Colwellia aromaticivorans</td><td>hypothetical protein [Candidatus Colwellia aromaticivorans].</td><td>GCF_003333305.1</td></tr>
<tr><td>WP_116215210.1</td><td>HTH+nSTAND1+TM+BetaPropeller*→||<-?<-?<-?||?→?→||<-?||ABhydrolase→</td><td>HTH+nSTAND1+TM+BetaPropeller</td><td>1291</td><td>DXZ73_RS36490</td><td>actinobacteria</td><td>Streptomyces olivoreticuli</td><td>helix-turn-helix domain-containing protein [Streptomyces olivoreticuli].</td><td>GCF_003391135.1</td></tr>
<tr><td>WP_117303390.1</td><td>Redoxin→?→?→||<-?||?→ TIR+DrHyd→ nSTAND1+TM+TPR*→||<-SIG+TPR</td><td>nSTAND1+TM+TPR</td><td>1216</td><td>D0Y96_RS19515</td><td>acidobacteria</td><td>Acidipila sp. 4G-K13</td><td>hypothetical protein [Acidipila sp. 4G-K13].</td><td>GCF_003428625.1</td></tr>
<tr><td>WP_120036984.1</td><td>p450→ NUDIX→||<-?<-PSE||?→||<-?||EACC1→ CASPASE+nSTAND1+TM+BetaPropeller*→||<-tRNA||?→||<-?||?→||<-SIG+PDZ+LonC</td><td>CASPASE+nSTAND1+TM+BetaPropeller</td><td>1417</td><td>D5S18_RS01040</td><td>actinobacteria</td><td>Nocardia panacis</td><td>caspase family protein [Nocardia panacis].</td><td>GCF_003598715.1</td></tr>
<tr><td>WP_121179107.1</td><td>EACC1→ CASPASE+nSTAND1+TM+BetaPropeller*→</td><td>CASPASE+nSTAND1+TM+BetaPropeller</td><td>1551</td><td>BX285_RS31390</td><td>actinobacteria</td><td>Streptomyces sp. 1114.5</td><td>hypothetical protein [Streptomyces sp. 1114.5].</td><td>GCF_003635085.1</td></tr>
</tbody>
</table>

<table>
<tr><th>acc</th><th>operon</th><th>architecture</th><th>len</th><th>gen.name</th><th>taxend</th><th>species</th><th>defline</th><th>gca</th></tr>
<tr><td>WP_121407767.1</td><td>ABhydrolase→||<-?||HTH+nSTAND1+TM+BetaPropeller*→ SIG+NLPC→||<-TM+TM+TM+TM+TM+TM+TM+TM+TM+TM+TM+TM</td><td>HTH+nSTAND1+TM+BetaPropeller</td><td>1288</td><td>CLZ79_RS27460</td><td>actinobacteria</td><td>Streptomyces sp. 57</td><td>helix-turn-helix domain-containing protein [Streptomyces sp. 57].</td><td>GCF_003664315.1</td></tr>
<tr><td>WP_121432864.1</td><td>ABhydrolase→||<-?||EACC1→ CASPASE+nSTAND1+TM+BetaPropeller*→</td><td>CASPASE+nSTAND1+TM+BetaPropeller</td><td>1529</td><td>BZB76_RS03830</td><td>actinobacteria</td><td>Actinomadura pelletieri</td><td>caspase family protein [Actinomadura pelletieri].</td><td>GCF_003634705.1</td></tr>
<tr><td>WP_121894653.1</td><td>CASPASE+nSTAND1+TM+BetaPropeller*→?→ PSE→||<-PSE<-PSE<-PSE||?→||<-?||REC→</td><td>CASPASE+nSTAND1+TM+BetaPropeller</td><td>1525</td><td>CTZ28_RS39810</td><td>actinobacteria</td><td>Streptomyces shenzhenensis</td><td>caspase family protein [Streptomyces shenzhenensis].</td><td>GCF_003688995.1</td></tr>
<tr><td>WP_121906839.1</td><td>TIR→ nSTAND1+TM*→?→||<-?<-REC+NtrC-AAA+FIS-HTH||?→ ABC-ATPase→</td><td>nSTAND1+TM</td><td>934</td><td>BXY75_RS06160</td><td>bacteroidetes</td><td>Ulvibacter antarcticus</td><td>hypothetical protein [Ulvibacter antarcticus].</td><td>GCF_003688405.1</td></tr>
<tr><td>WP_121971445.1</td><td>CASPASE+nSTAND1*→</td><td>CASPASE+nSTAND1</td><td>921</td><td>CKL79_RS20690</td><td>cyanobacteria</td><td>Leptolyngbya sp. BC1307</td><td>caspase family protein [Leptolyngbya sp. BC1307].</td><td>GCF_002286735.1</td></tr>
<tr><td>WP_124450359.1</td><td>TIR→ nSTAND1+BetaPropeller+BetaPropeller*→||<-?<-?<-SIG+TM+TM+TM+TM+TM<-SIG+TM+TM+TM+TM+TM<-ABC-ATPase<-Cluster158_2clades</td><td>nSTAND1+BetaPropeller+BetaPropeller</td><td>1395</td><td>DBR47_RS12145</td><td>betaproteobacteria</td><td>Paucibacter sp. KBW04</td><td>AAA family ATPase [Paucibacter sp. KBW04].</td><td>GCF_003852425.1</td></tr>
<tr><td>WP_125486544.1</td><td><-TPR<-SIG+BetaPropeller<-TRPR-HTH+PBPI<-?<-?||SIG+BetaPropeller→ TIR+DrHyd+TIR→ nSTAND1+NACHT+TPR*→?→ SLATT→||<-SIG+ASH-IG<-?<-?<-?||REC→</td><td>nSTAND1+NACHT+TPR</td><td>940</td><td>EDE15_RS18050</td><td>acidobacteria</td><td>Edaphobacter aggregans</td><td>ATP-binding protein [Edaphobacter aggregans].</td><td>GCF_003945235.1</td></tr>
<tr><td>WP_125646125.1</td><td>nSTAND1+BetaPropeller*→</td><td>nSTAND1+BetaPropeller</td><td>1367</td><td>DMB42_RS45370</td><td>actinobacteria</td><td>Nonomuraea sp. WAC 01424</td><td>hypothetical protein [Nonomuraea sp. WAC 01424].</td><td>GCF_003947315.1</td></tr>
<tr><td>WP_125647756.1</td><td>EACC1→ CASPASE+nSTAND1+BetaPropeller*→</td><td>CASPASE+nSTAND1+BetaPropeller</td><td>1501</td><td>DMB42_RS48695</td><td>actinobacteria</td><td>Nonomuraea sp. WAC 01424</td><td>caspase family protein [Nonomuraea sp. WAC 01424].</td><td>GCF_003947315.1</td></tr>
<tr><td>WP_126614131.1</td><td><-SIG+TM+TM+TM+TM+TM+TM+TM+TM||HxxxH→?→ tRNA→ tRNA→ tRNA→ tRNA→ tRNA→ tRNA→ tRNA→ tRNA→?→?→?→ TIR+DrHyd→ nSTAND1+TM+BetaPropeller*→</td><td>nSTAND1+TM+BetaPropeller</td><td>1051</td><td>EI427_RS09850</td><td>bacteroidetes</td><td>Flammeovirga pectinis</td><td>hypothetical protein [Flammeovirga pectinis].</td><td>GCF_003970675.1</td></tr>
<tr><td>WP_127056740.1</td><td>CASPASE+CASPASE+nSTAND1+BetaPropeller*→||<-?<-PSE<-?<-?||SIG+VIT+VWA+TM→||<-?<-?<-ABhydrolase</td><td>CASPASE+CASPASE+nSTAND1+BetaPropeller</td><td>1675</td><td>DSM107003_RS25415</td><td>cyanobacteria</td><td>Trichormus variabilis</td><td>caspase family protein [Trichormus variabilis].</td><td>GCF_003991935.1</td></tr>
<tr><td>WP_127150006.1</td><td><-ABC-ATPase<-?||?→ nSTAND1+BetaPropeller*→?→||<-NUDIX</td><td>nSTAND1+BetaPropeller</td><td>1287</td><td>D9V26_RS29450</td><td>actinobacteria</td><td>Streptomyces lydicus</td><td>hypothetical protein [Streptomyces lydicus].</td><td>GCF_003994375.1</td></tr>
<tr><td>WP_128433449.1</td><td>SIG+nSTAND1+TM+BetaPropeller*→?→?→?→||<-?||SIG+TM+TM→</td><td>SIG+nSTAND1+TM+BetaPropeller</td><td>1296</td><td>EHO50_RS25360</td><td>actinobacteria</td><td>Streptomyces cyaneus</td><td>AAA family ATPase [Streptomyces cyaneus].</td><td>GCF_004028635.1</td></tr>
<tr><td>WP_129567753.1</td><td>TIR→ SIG+nSTAND1+TM+BetaPropeller+TPR*→ SLATT→ PSE→||<-?<-ABhydrolase</td><td>SIG+nSTAND1+TM+BetaPropeller+TPR</td><td>1355</td><td>DIU36_RS01770</td><td>bacteroidetes</td><td>Mucilaginibacter rubeus</td><td>hypothetical protein [Mucilaginibacter rubeus].</td><td>GCF_003286445.1</td></tr>
<tr><td>WP_129958652.1</td><td><-TIR<-?<-?<-?<-?<-?||PSE→ TIR+TIR→ nSTAND1+TM+RHS+RHS+PDZ*→||<-?<-TRPR-HTH+PBPI</td><td>nSTAND1+TM+RHS+RHS+PDZ</td><td>1555</td><td>EVC45_RS14430</td><td>betaproteobacteria</td><td>Paraburkholderia sp. UYCP14C</td><td>hypothetical protein [Paraburkholderia sp. UYCP14C].</td><td>GCF_004197395.1</td></tr>
<tr><td>WP_131334817.1</td><td>HTH+nSTAND1+TM+BetaPropeller*→||<-?||?→||<-?<-?<-SIG+TM+TM+TM+TM+TM<-SIG+TM+TM+TM+TM+TM</td><td>HTH+nSTAND1+TM+BetaPropeller</td><td>1311</td><td>E0H45_RS03495</td><td>actinobacteria</td><td>Kribbella soli</td><td>helix-turn-helix domain-containing protein [Kribbella soli].</td><td>GCF_004331345.1</td></tr>
<tr><td>WP_132126505.1</td><td>ABhydrolase→||<-?||nSTAND1+TM+BetaPropeller*→</td><td>nSTAND1+TM+BetaPropeller</td><td>947</td><td>EV192_RS53305</td><td>actinobacteria</td><td>Actinocrispum wychmicini</td><td>hypothetical protein [Actinocrispum wychmicini].</td><td>GCF_004345645.1</td></tr>
<tr><td>WP_132170809.1</td><td>nSTAND1+BetaPropeller*→ SWACOS+TPR+TPR→</td><td>nSTAND1+BetaPropeller</td><td>1283</td><td>E1264_RS07080</td><td>actinobacteria</td><td>Actinomadura sp. KC216</td><td>PD40 domain-containing protein [Actinomadura sp. KC216].</td><td>GCF_004348535.1</td></tr>
<tr><td>WP_132171295.1</td><td><-APATPase||nSTAND1+BetaPropeller*→?→ NACHT+TM+TM+TM+TM+TM+TM+TM+TM→ SWACOS+TPR+TPR→</td><td>nSTAND1+BetaPropeller</td><td>1155</td><td>E1264_RS08360</td><td>actinobacteria</td><td>Actinomadura sp. KC216</td><td>WD40 repeat domain-containing protein [Actinomadura sp. KC216].</td><td>GCF_004348535.1</td></tr>
<tr><td>WP_133475184.1</td><td>TIR→ nSTAND1+TM+BetaPropeller*→</td><td>nSTAND1+TM+BetaPropeller</td><td>1045</td><td>BC659_RS12750</td><td>bacteroidetes</td><td>Sediminibacterium goheungense</td><td>hypothetical protein [Sediminibacterium goheungense].</td><td>GCF_004361915.1</td></tr>
<tr><td>WP_135106243.1</td><td>FGS→?→ PSE→?→||<-PSE||?→ nSTAND1+BetaPropeller*→||<-?<-?<-?<-PSE<-RVe+MutnpC||PSE→ RVT→</td><td>nSTAND1+BetaPropeller</td><td>1364</td><td>BLD44_RS07925</td><td>cyanobacteria</td><td>Mastigocladus laminosus</td><td>hypothetical protein [Mastigocladus laminosus].</td><td>GCF_001990805.2</td></tr>
</table>

acc	operon	architecture	len	gen.name	taxend	species	defline	gca
WP_136533593.1	nSTAND1+BetaPropeller*→||<-?||?→?→||<-SIG+LexA-HTH+LexA-protease||ATPcone→	nSTAND1+BetaPropeller	1227	FAB82_RS05815	actinobacteria	Glycomyces buryatensis	hypothetical protein [Glycomyces buryatensis].	GCF_004912275.1
WP_136810415.1	TIR→ nSTAND1+BetaPropeller*→	nSTAND1+BetaPropeller	1285	FCL46_RS25075	deltaproteobacteria	Desulfopila sp. IMCC35005	hypothetical protein [Desulfopila sp. IMCC35005].	GCF_005116655.2
WP_137953472.1	TIR+DrHyd+TIR→ SIG+nSTAND1+TM+BetaPropeller*→ SLATT+SLATT→||<-FAD-NAD-dep-oxidoreductase<-FAD-NAD-dep-oxidoreductase<-?<-SIG+TM+TM+TM+TM+TM<-SIG+TM+TM+TM+TM+TM<-ABC-ATPase	SIG+nSTAND1+TM+BetaPropeller	1430	FFS45_RS04240	betaproteobacteria	Burkholderia sp. 4M9327F10	AAA family ATPase [Burkholderia sp. 4M9327F10].	GCF_005503145.1
WP_138199695.1	<-APATPase||nSTAND1+BetaPropeller*→?→ NACHT+TM+TM+TM+TM+TM+TM+TM+TM→||<-?<-?<-?||REC→	nSTAND1+BetaPropeller	1163	FE391_RS07385	actinobacteria	unclassified Nonomuraea	MULTISPECIES: WD40 repeat domain-containing protein [unclassified Nonomuraea].	GCF_009908215.1
WP_138710239.1	<-APATPase||?→ nSTAND1+BetaPropeller*→?→ NACHT+TM+TM+TM+TM+TM+TM+TM+TM→	nSTAND1+BetaPropeller	1211	EJK15_RS10830	actinobacteria	Nonomuraea sp. 160415	AAA family ATPase [Nonomuraea sp. 160415].	GCF_005893125.1
WP_139580102.1	HTH+nSTAND1+TM+BetaPropeller*→||<-?<-ABhydrolase||?→||<-?||?→?→||<-SIG+HOP2	HTH+nSTAND1+TM+BetaPropeller	1288	FH610_RS38115	actinobacteria	Microbispora catharanthi	hypothetical protein [Microbispora catharanthi].	GCF_006334915.2
WP_141634977.1	nSTAND1+TM+BetaPropeller*→ BetaPropeller→	nSTAND1+TM+BetaPropeller	991	E6W39_RS21975	actinobacteria	Kitasatospora sp. MMS16-CNU292	hypothetical protein [Kitasatospora sp. MMS16-CNU292].	GCF_006636205.1
WP_141974623.1	nSTAND1+BetaPropeller*→	nSTAND1+BetaPropeller	1258	FHX81_RS00130	actinobacteria	Saccharothrix saharensis	hypothetical protein [Saccharothrix saharensis].	GCF_006716745.1
WP_143138532.1	ABhydrolase→ nSTAND1+TM+BetaPropeller*→||<-?<-?||?→||<-REC<-?||?→ ABC-ATPase→	nSTAND1+TM+BetaPropeller	1307	BM417_RS06445	actinobacteria	Lentzea waywayandensis	hypothetical protein [Lentzea waywayandensis].	GCF_900115955.1
WP_143176134.1	CASPASE+nSTAND1+TM+Pentapeptide*→ PSE→?→ SIG+TPR→	CASPASE+nSTAND1+TM+Pentapeptide	931	NIES2101_RS34720	cyanobacteria	Calothrix sp. HK-06	pentapeptide repeat-containing protein [Calothrix sp. HK-06].	GCF_001904745.1
WP_143227460.1	nSTAND1+BetaPropeller*→ PSE→ Trypco1→?→ Trypsin+nSTAND1+BetaPropeller→	nSTAND1+BetaPropeller	1252	CHC11_RS38105	actinobacteria	Actinomadura mexicana	hypothetical protein [Actinomadura mexicana].	GCF_900188105.1
WP_143264194.1	<-TRPR-HTH+PBPI||?→ SIG+TM+TM+TM+TM+TM→ SIG+TM+TM+TM+TM+TM→||<-?||SIG+HOP2→||<-?||nSTAND1+TM+BetaPropeller*→||<-?||?→||<-?<-?||?→||<-?||ABhydrolase→	nSTAND1+TM+BetaPropeller	983	CCN39_RS28880	actinobacteria	Amycolatopsis kentuckyensis	helix-turn-helix domain-containing protein [Amycolatopsis kentuckyensis].	GCF_002155975.1
WP_143281672.1	TIR+nSTAND1+BetaPropeller*→ SLATT+SLATT→ TIR+nSTAND1+BetaPropeller→	TIR+nSTAND1+BetaPropeller	1465	ACU09_RS37780	betaproteobacteria	Caballeronia cordobensis	TIR domain-containing protein [Caballeronia cordobensis].	GCF_001544575.2
WP_143626210.1	HTH+nSTAND1+TM+BetaPropeller*→	HTH+nSTAND1+TM+BetaPropeller	1284	B7L92_RS00625	actinobacteria	Streptomyces viridosporus	helix-turn-helix domain-containing protein [Streptomyces viridosporus].	GCF_002078235.1
WP_143654068.1	wHTH-4stranded+TPRs+APATPase+TPR+TPR+TPR→?→?→ PSE→||<-?<-?<-?||PSE→ PSE→ PSE→ EACC1→ CASPASE+nSTAND1+BetaPropeller*→	CASPASE+nSTAND1+BetaPropeller	1524	FNV64_RS04700	actinobacteria	Streptomyces sp. S1A1-7	caspase family protein [Streptomyces sp. S1A1-7].	GCF_007113565.2
WP_144869529.1	CASPASE+CASPASE+nSTAND1+BetaPropeller*→	CASPASE+CASPASE+nSTAND1+BetaPro-peller	1785	H1P_RS17045	cyanobacteria	Hyella patelloides	caspase family protein [Hyella patelloides].	GCF_900659865.1
WP_144873934.1	nSTAND1*→||<-?||Pentapeptide→ Cluster533_2clades→||<-PSE<-?<-Uma2	nSTAND1	400	H1P_RS28015	cyanobacteria	Hyella patelloides	hypothetical protein [Hyella patelloides].	GCF_900659865.1
WP_145797060.1	<-FAD-NAD-dep-oxidoreductase||PSE→?→ HTH+nSTAND1+TM+BetaPropeller*→ SIG+NLPC→?→||<-?||Pkinase→	HTH+nSTAND1+TM+BetaPropeller	1252	FB465_RS01075	actinobacteria	Kitasatospora atroaurantiaca	helix-turn-helix domain-containing protein [Kitasatospora atroaurantiaca].	GCF_007828955.1
WP_146784494.1	<-REC<-?<-?||?→?→ TIR→ SIG+nSTAND1+TM+BetaPropeller+TPR*→	SIG+nSTAND1+TM+BetaPropeller+TPR	1401	FSB75_RS06485	bacteroidetes	Flavisolibacter ginsenosidimutans	hypothetical protein [Flavisolibacter ginsenosidimutans].	GCF_007970805.1

<table>
<tr><th>acc</th><th>operon</th><th>architecture</th><th>len</th><th>gen.name</th><th>taxend</th><th>species</th><th>defline</th><th>gca</th></tr>
<tr><td>WP_149303455.1</td><td><-VWA<-MoxR-AAA||TIR→ nSTAND1+TM*→?→?→?→||<-?||?→||<-NUDIX</td><td>nSTAND1+TM</td><td>774</td><td>F2B13_RS05070</td><td>bacteroidetes</td><td>Flavobacteriaceae bacterium S2-4-21</td><td>hypothetical protein [Flavobacteriaceae bacterium S2-4-21].</td><td>GCF_008367235.1</td></tr>
<tr><td>WP_149853426.1</td><td>nSTAND1+BetaPropeller*→ nSTAND1+BetaPropeller→||<-MED15<-?<-?||?→ BetaPropeller+ABhydrolase→</td><td>nSTAND1+BetaPropeller</td><td>1270</td><td>F0L68_RS31105</td><td>actinobacteria</td><td>Goodfellowiella sp. AN110305</td><td>hypothetical protein [Goodfellowiella sp. AN110305].</td><td>GCF_008386585.1</td></tr>
<tr><td>WP_149854623.1</td><td>nSTAND1*→</td><td>nSTAND1</td><td>264</td><td>F0L68_RS37265</td><td>actinobacteria</td><td>Goodfellowiella sp. AN110305</td><td>ATP-binding protein, partial [Goodfellowiella sp. AN110305].</td><td>GCF_008386585.1</td></tr>
<tr><td>WP_150477790.1</td><td>nSTAND1+TM+BetaPropeller*→</td><td>nSTAND1+TM+BetaPropeller</td><td>1249</td><td>CP975_RS04485</td><td>actinobacteria</td><td>Streptomyces alboniger</td><td>hypothetical protein [Streptomyces alboniger].</td><td>GCF_008704395.1</td></tr>
<tr><td>WP_151694506.1</td><td>SIG+Pentapeptide→ REC→?→?→ nSTAND1+TM+BetaPropeller*→</td><td>nSTAND1+TM+BetaPropeller</td><td>1253</td><td>MiAbW_RS00775</td><td>cyanobacteria</td><td>Microcystis aeruginosa</td><td>WD40 repeat domain-containing protein [Microcystis aeruginosa].</td><td>GCF_008974145.1</td></tr>
<tr><td>WP_151729819.1</td><td>wHTH-4stranded+TPRs+SWACOS+TPR+TPR→?→?→?→ HOP2→?→||<-?||Pkinase+TIR+nSTAND1+FilH*→?→?→||<-?<-ABC-ATPase<-ABC-ATPase</td><td>Pkinase+TIR+nSTAND1+FilH</td><td>1507</td><td>KTAU_RS18765</td><td>chloroflexi</td><td>Thermogemmatispora aurantia</td><td>protein kinase [Thermogemmatispora aurantia].</td><td>GCF_008974285.1</td></tr>
<tr><td>WP_151998675.1</td><td>TM+TM+TM+TM+TM+TM+TM+TM+TM+TM+TM+cNMP→ TIR+DrHyd→ nSTAND1+TM+LPD8+BetaPropeller*→</td><td>nSTAND1+TM+LPD8+BetaPropeller</td><td>1056</td><td>GC130_RS12600</td><td>bacteroidetes</td><td>Imperialibacter sp. EC-SDR9</td><td>High-affnity carbon uptake protein Hat/HatR [Imperialibacter sp. EC-SDR9].</td><td>GCF_902498805.1</td></tr>
<tr><td>WP_152590945.1</td><td>CASPASE+nSTAND1+BetaPropeller→?→?→?→ CASPASE+ABhydrolase→ ABhydrolase→ CASPASE+nSTAND1+BetaPropeller→ CASPASE+nSTAND1+TM+BetaPropeller*→</td><td>CASPASE+nSTAND1+TM+BetaPropeller</td><td>1471</td><td>GXM_RS28955</td><td>cyanobacteria</td><td>Nostoc sphaeroides</td><td>caspase family protein [Nostoc sphaeroides].</td><td>GCF_009372195.1</td></tr>
<tr><td>WP_152780064.1</td><td>HTH+nSTAND1+TM+BetaPropeller*→</td><td>HTH+nSTAND1+TM+BetaPropeller</td><td>1251</td><td>FNH04_RS03190</td><td>actinobacteria</td><td>Streptomyces phyllanthi</td><td>hypothetical protein [Streptomyces phyllanthi].</td><td>GCF_009377205.1</td></tr>
<tr><td>WP_153167482.1</td><td><-Cluster428_2clades<-?<-?||TIR→ SIG+nSTAND1+BetaPropeller*→</td><td>SIG+nSTAND1+BetaPropeller</td><td>1296</td><td>GH664_RS07725</td><td>betaproteobacteria</td><td>Thauera sp. 2A1</td><td>hypothetical protein [Thauera sp. 2A1].</td><td>GCF_009469595.1</td></tr>
<tr><td>WP_153167675.1</td><td><-REC<-?||?→||<-PSE||?→ TIR+DrHyd→ nSTAND1+BetaPropeller*→?→?→ HOP2→</td><td>nSTAND1+BetaPropeller</td><td>1225</td><td>GH664_RS08515</td><td>betaproteobacteria</td><td>Thauera sp. 2A1</td><td>AAA family ATPase [Thauera sp. 2A1].</td><td>GCF_009469595.1</td></tr>
<tr><td>WP_155176087.1</td><td>TM+TM+TM+TM+TM+TM+TM+TM+TM+TM+TM+cNMP→ TIR+DrHyd→ nSTAND1+TM+BetaPropeller*→</td><td>nSTAND1+TM+BetaPropeller</td><td>1054</td><td>E1163_RS26420</td><td>bacteroidetes</td><td>Fulvivirga kasyanovii</td><td>High-affnity carbon uptake protein Hat/HatR [Fulvivirga kasyanovii].</td><td>GCF_009711405.1</td></tr>
<tr><td>WP_155342137.1</td><td>nSTAND1+BetaPropeller*→</td><td>nSTAND1+BetaPropeller</td><td>1430</td><td>Acor_RS40905</td><td>actinobacteria</td><td>Acrocarpospora corrugata</td><td>AAA family ATPase [Acrocarpospora corrugata].</td><td>GCF_009687845.1</td></tr>
<tr><td>WP_155349768.1</td><td>HTH+nSTAND1+TM+BetaPropeller*→</td><td>HTH+nSTAND1+TM+BetaPropeller</td><td>1306</td><td>Aple_RS38900</td><td>actinobacteria</td><td>Acrocarpospora pleiomorpha</td><td>helix-turn-helix domain-containing protein [Acrocarpospora pleiomorpha].</td><td>GCF_009687885.1</td></tr>
<tr><td>WP_155356770.1</td><td><-SIG+TM+TM+TM+TM+TM<-?||ABhydrolase→?→?→?→?→ nSTAND1+BetaPropeller*→</td><td>nSTAND1+BetaPropeller</td><td>1404</td><td>Amac_RS24660</td><td>actinobacteria</td><td>Acrocarpospora macrocephala</td><td>hypothetical protein [Acrocarpospora macrocephala].</td><td>GCF_009687865.1</td></tr>
<tr><td>WP_155743298.1</td><td>CASPASE+ABhydrolase→ CASPASE+nSTAND1+BetaPropeller→ CASPASE+nSTAND1+TM+BetaPropeller*→</td><td>CASPASE+nSTAND1+TM+BetaPropeller</td><td>1444</td><td>F7734_RS02865</td><td>cyanobacteria</td><td>Scytonema sp. UIC 10036</td><td>caspase family protein [Scytonema sp. UIC 10036].</td><td>GCF_009725235.1</td></tr>
<tr><td>WP_155744071.1</td><td>CASPASE+nSTAND1+TM+BetaPropeller*→</td><td>CASPASE+nSTAND1+TM+BetaPropeller</td><td>1426</td><td>F7734_RS07420</td><td>cyanobacteria</td><td>Scytonema sp. UIC 10036</td><td>CHAT domain-containing protein [Scytonema sp. UIC 10036].</td><td>GCF_009725235.1</td></tr>
<tr><td>WP_157357215.1</td><td>nSTAND1+BetaPropeller*→</td><td>nSTAND1+BetaPropeller</td><td>1257</td><td>bacteria>actinobacteria</td><td>Amycolatopsis nigrescens</td><td>-</td><td>1786243365</td><td>-</td></tr>
</table>

<table>
<tr><th>acc</th><th>operon</th><th>architecture</th><th>len</th><th>gen.name</th><th>taxend</th><th>species</th><th>defline</th><th>gca</th></tr>
<tr><td>WP_158887400.1</td><td><-ABhydrolase<-?||?→||<-?||?→||<-?||HTH+nSTAND1+TM+BetaPropeller*→</td><td>HTH+nSTAND1+TM+BetaPropeller</td><td>1240</td><td>FXO23_RS22610</td><td>actinobacteria</td><td>Amycolatopsis sp. EGI 650086</td><td>PD40 domain-containing protein [Amycolatopsis sp. EGI 650086].</td><td>GCF_009765355.1</td></tr>
<tr><td>WP_158891295.1</td><td><-ABC-ATPase<-ABC-ATPase<-?<-?<-?||nSTAND1+TM+BetaPropeller*→||<-?<-?<-?<-?<-?<-ABhydrolase</td><td>nSTAND1+TM+BetaPropeller</td><td>1241</td><td>FXO23_RS37595</td><td>actinobacteria</td><td>Amycolatopsis sp. EGI 650086</td><td>hypothetical protein [Amycolatopsis sp. EGI 650086].</td><td>GCF_009765355.1</td></tr>
<tr><td>WP_159585268.1</td><td>TM+TM+TM+TM+TM+TM+TM+TM+TM+TM+TM+cNMP→ TIR+DrHyd→ nSTAND1+TM+BetaPropeller*→</td><td>nSTAND1+TM+BetaPropeller</td><td>1086</td><td>GV030_RS20605</td><td>bacteroidetes</td><td>Marinoscillum sp. 108</td><td>hypothetical protein [Marinoscillum sp. 108].</td><td>GCF_902506655.1</td></tr>
<tr><td>WP_159788932.1</td><td><-TM<-?||CASPASE+nSTAND1+TM+BetaPropeller*→ SIG+TPR→</td><td>CASPASE+nSTAND1+TM+BetaPropeller</td><td>1524</td><td>L855_RS13815</td><td>cyanobacteria</td><td>Microcoleus sp. IPPAS B-353</td><td>caspase family protein [Microcoleus sp. IPPAS B-353].</td><td>GCF_009846485.1</td></tr>
<tr><td>WP_160147137.1</td><td>ABC-ATPase→ TM+TM+TM+TM+TM+TM→
HTH+nSTAND1+TM+BetaPropeller*→||<-?||?→||<-?<-?<-?<-SIG+TM+TM+TM+TM+TM<-SIG+TM+TM+TM+TM+TM</td><td>HTH+nSTAND1+TM+BetaPropeller</td><td>1267</td><td>C4U03_RS30005</td><td>actinobacteria</td><td>Actinomadura echinospora</td><td>helix-turn-helix domain-containing protein [Actinomadura echinospora].</td><td>GCF_900108175.1</td></tr>
<tr><td>WP_160846342.1</td><td>TIR→ SIG+nSTAND1+TM+BetaPropeller+TPR*→ SLATT→?→||<-?<-ABhydrolase</td><td>SIG+nSTAND1+TM+BetaPropeller+TPR</td><td>1335</td><td>GS399_RS19525</td><td>bacteroidetes</td><td>Pedobacter sp. HMF7647</td><td>hypothetical protein [Pedobacter sp. HMF7647].</td><td>GCF_009834875.1</td></tr>
<tr><td>WP_161602925.1</td><td><-ABC-ATPase<-?<-?<-?<-?<-?||?→ CASPASE+nSTAND1+TPR*→?→||<-ABhydrolase</td><td>CASPASE+nSTAND1+TPR</td><td>902</td><td>SD80_RS22720</td><td>cyanobacteria</td><td>Scytonema tolypothrichoides</td><td>tetratricopeptide repeat protein [Scytonema tolypothrichoides].</td><td>GCF_000828085.3</td></tr>
<tr><td>WP_162442111.1</td><td>TM+TM+TM+TM+TM+TM+TM+TM+TM+TM+TM+cNMP→ TIR+DrHyd→ nSTAND1+TM+fvmX2+BetaPropeller*→</td><td>nSTAND1+TM+fvmX2+BetaPropeller</td><td>1036</td><td>GXP67_RS04800</td><td>bacteroidetes</td><td>Rhodocytophaga sp. 172606-1</td><td>High-affnity carbon uptake protein Hat/HatR [Rhodocytophaga sp. 172606-1].</td><td>GCF_010119975.1</td></tr>
<tr><td>WP_162444893.1</td><td>TM+TM+TM+TM+TM+TM+TM+TM+TM+TM+TM+cNMP→ TIR+DrHyd→ nSTAND1+BetaPropeller*→</td><td>nSTAND1+BetaPropeller</td><td>1040</td><td>GXP67_RS20705</td><td>bacteroidetes</td><td>Rhodocytophaga sp. 172606-1</td><td>High-affnity carbon uptake protein Hat/HatR [Rhodocytophaga sp. 172606-1].</td><td>GCF_010119975.1</td></tr>
<tr><td>WP_162795474.1</td><td>SIG+nSTAND1*→</td><td>SIG+nSTAND1</td><td>336</td><td>DQW85_RS29495</td><td>actinobacteria</td><td>Nonomuraea sp. NEAU-YG30</td><td>AAA family ATPase, partial [Nonomuraea sp. NEAU-YG30].</td><td>GCF_003313395.1</td></tr>
<tr><td>WP_163697206.1</td><td>CASPASE+ABhydrolase→?→ CASPASE+nSTAND1+TM+BetaPropeller→ CASPASE+nSTAND1+TM+BetaPropeller→||<-?<-EAD4+APATPase+BetaPropeller</td><td>CASPASE+nSTAND1+TM+BetaPropeller</td><td>1573</td><td>DXZ20_RS06775</td><td>cyanobacteria</td><td>Leptolyngbyaceae cyanobacterium CCMR0081</td><td>PD40 domain-containing protein [Leptolyngbyaceae cyanobacterium CCMR0081].</td><td>GCF_011009555.1</td></tr>
<tr><td>WP_163697208.1</td><td>CASPASE+nSTAND1+BetaPropeller*→</td><td>CASPASE+nSTAND1+BetaPropeller</td><td>1477</td><td>DXZ20_RS06780</td><td>cyanobacteria</td><td>Leptolyngbyaceae cyanobacterium CCMR0081</td><td>caspase family protein [Leptolyngbyaceae cyanobacterium CCMR0081].</td><td>GCF_011009555.1</td></tr>
<tr><td>WP_163938808.1</td><td><-TM<-?||CASPASE+nSTAND1+TM+BetaPropeller*→</td><td>CASPASE+nSTAND1+TM+BetaPropeller</td><td>1216</td><td>G1O98_RS35625</td><td>cyanobacteria</td><td>Nostoc sp. UIC 10630</td><td>caspase family protein, partial [Nostoc sp. UIC 10630].</td><td>GCF_010747425.1</td></tr>
<tr><td>WP_164607717.1</td><td>HTH+nSTAND1+TM+BetaPropeller*→ SIG+NLPC→</td><td>HTH+nSTAND1+TM+BetaPropeller</td><td>1254</td><td>G3I60_RS24410</td><td>actinobacteria</td><td>Streptomyces sp. SID13666</td><td>helix-turn-helix domain-containing protein [Streptomyces sp. SID13666].</td><td>GCF_010548325.1</td></tr>
<tr><td>WP_164737886.1</td><td>HTH+nSTAND1+TM+BetaPropeller*→||<-?||?→?→?→ ABC-ATPase→ ABC-ATPase→</td><td>HTH+nSTAND1+TM+BetaPropeller</td><td>1245</td><td>EKH77_RS28040</td><td>actinobacteria</td><td>Streptomyces luteoverticillatus</td><td>DNA-binding protein [Streptomyces luteoverticillatus].</td><td>GCF_003970715.1</td></tr>
<tr><td>WP_165253283.1</td><td>TIR+TIR→ nSTAND1+BetaPropeller*→||<-?||TIR+nSTAND1+BetaPropeller→</td><td>nSTAND1+BetaPropeller</td><td>1322</td><td>G6O68_RS30360</td><td>planctomycetes</td><td>Paludisphaera sp. JC670</td><td>hypothetical protein [Paludisphaera sp. JC670].</td><td>GCF_011064595.1</td></tr>
</table>

<table>
<thead>
<tr><th>acc</th><th>operon</th><th>architecture</th><th>len</th><th>gen.name</th><th>taxend</th><th>species</th><th>defline</th><th>gca</th></tr>
</thead>
<tbody>
<tr><td>WP_165741954.1</td><td>TIR+nSTAND1+BetaPropeller*→</td><td>TIR+nSTAND1+BetaPropeller</td><td>1776</td><td>HBF22_RS11055</td><td>gammaproteobacteria</td><td>Candidatus Thiosymbion oneisti</td><td>hypothetical protein, partial [Candidatus Thiosymbion oneisti].</td><td>GCF_011392785.1</td></tr>
<tr><td>WP_165977045.1</td><td><-APATPase||nSTAND1+BetaPropeller*→?→ NACHT+TM+TM+TM+TM+TM+TM+TM+TM→||<-?<-?<-?||REC→</td><td>nSTAND1+BetaPropeller</td><td>1163</td><td>E1294_RS02715</td><td>actinobacteria</td><td>Nonomuraea sp. KC712</td><td>AAA family ATPase [Nonomuraea sp. KC712].</td><td>GCF_004349015.1</td></tr>
<tr><td>WP_166362185.1</td><td>TM+TM+TM+TM+TM+TM+TM+TM+TM+TM+TM+cNMP→?→ TIR+DrHyd→ nSTAND1+TM+RapA-C+BetaPropeller*→</td><td>nSTAND1+TM+RapA-C+BetaPropeller</td><td>1020</td><td>G8O23_RS04390</td><td>bacteroidetes</td><td>Bacteroidales bacterium M08MB</td><td>High-affnity carbon uptake protein Hat/HatR [Bacteroidales bacterium M08MB].</td><td>GCF_011174675.1</td></tr>
<tr><td>WP_167485984.1</td><td>nSTAND1+BetaPropeller*→||<-?||?→||<-?<-PSE||?→ PSE→?→||<-?<-ICLR-HTH</td><td>nSTAND1+BetaPropeller</td><td>1286</td><td>F6W96_RS10530</td><td>actinobacteria</td><td>Nocardia terpenica</td><td>hypothetical protein [Nocardia terpenica].</td><td>GCF_011801165.1</td></tr>
<tr><td>WP_168094374.1</td><td>HTH+nSTAND1+TM+BetaPropeller*→</td><td>HTH+nSTAND1+TM+BetaPropeller</td><td>1232</td><td>HCK01_RS21035</td><td>actinobacteria</td><td>Streptomyces sp. AA8</td><td>helix-turn-helix domain-containing protein [Streptomyces sp. AA8].</td><td>GCF_012034305.1</td></tr>
<tr><td>WP_169156509.1</td><td>CASPASE+nSTAND1+TM+BetaPropeller*→</td><td>CASPASE+nSTAND1+TM+BetaPropeller</td><td>1399</td><td>DP116_RS18170</td><td>cyanobacteria</td><td>Brasilonema bromeliae</td><td>CHAT domain-containing protein [Brasilonema bromeliae].</td><td>GCF_012912135.1</td></tr>
<tr><td>WP_169616265.1</td><td><-TPR+nSTAND1+BetaPropeller<-?||?→ nSTAND1+BetaPropeller→<i>||<-?||nSTAND1+BetaPropeller</i>→</td><td>nSTAND1+BetaPropeller</td><td>1227</td><td>E1H13_RS20825</td><td>cyanobacteria</td><td>Nodosilinea sp. P-1105</td><td>AAA family ATPase [Nodosilinea sp. P-1105].</td><td>GCF_012911975.1</td></tr>
<tr><td>WP_169616268.1</td><td>nSTAND1+BetaPropeller*→</td><td>nSTAND1+BetaPropeller</td><td>1430</td><td>E1H13_RS20835</td><td>cyanobacteria</td><td>Nodosilinea sp. P-1105</td><td>AAA family ATPase [Nodosilinea sp. P-1105].</td><td>GCF_012911975.1</td></tr>
<tr><td>WP_169682334.1</td><td>TM+TM+TM+TM+TM+TM+TM+TM+TM+TM+TM+cNMP→ TIR+DrHyd→ nSTAND1+BetaPropeller*→</td><td>nSTAND1+BetaPropeller</td><td>1042</td><td>HH304_RS13030</td><td>bacteroidetes</td><td>Flammeovirgaceae bacterium KN852</td><td>hypothetical protein [Flammeovirgaceae bacterium KN852].</td><td>GCF_012926615.1</td></tr>
</tbody>
</table>
