# Supplementary material for: Bacterial death and TRADD-N domains help define novel apoptosis and immunity mechanisms shared by prokaryotes and metazoans
Source: eLife. 2021 Jun 1;10:e70394. doi: 10.7554/eLife.70394 (PMC8195603; doi:10.7554/eLife.70394)
Supplement: Figure 3—source data 1. [file elife-70394-fig3-data1.pdf]

Bacterial Death and TRADD-N domains help define novel apoptosis and immunity mechanisms shared by prokaryotes and metazoans

Figure 3-Source data 1

Gurmeet Kaur†, Lakshminarayan M. Iyer†, A. Maxwell Burroughs, L. Aravind*

Computational Biology Branch, National Center for Biotechnology Information, National Library of Medicine, National Institutes of Health, Bethesda, MD 20894, USA

†These authors contributed equally to the manuscript

Index.

1. Gene neighborhoods and domain architectures of the bacterial TRADD-N domains

Gene neighborhoods and domain architectures of the bacterial TRADD-N domains

acc	operon	architecture	len	gen.name	taxend	species	defline	gca
AKV69521.1	ECF-HTH+TRADD-N+Pentapeptide*→||<-?<-?||ECF-HTH+TRADD-N+Pentapeptide→	ECF-HTH+TRADD-N+Pentapeptide	463	VL20_4620	cyanobacteria	Microcystis panniformis FACHB-1757	Pentapeptide repeat family protein [Microcystis panniformis FACHB-1757]	GCA_001264245.1
AKV69524.1	ECF-HTH+TRADD-N+Pentapeptide→||<-?<-?||ECF-HTH+TRADD-N+Pentapeptide*→	ECF-HTH+TRADD-N+Pentapeptide	430	VL20_4623	cyanobacteria	Microcystis panniformis FACHB-1757	Pentapeptide repeat family protein [Microcystis panniformis FACHB-1757]	GCA_001264245.1
AOW98657.1	TRADD-N+Pentapeptide→ TRADD-N-aHTH1+TRADD-N+Pentapeptide→?→||<-?||TRADD-N-aHTH1+TRADD-N+Pentapeptide*→	TRADD-N-aHTH1+TRADD-N+Pentapeptide	359	BJP34_03640	cyanobacteria	Moorea producens PAL-8-15-08-1	hypothetical protein BJP34_03640 [Moorea producens PAL-8-15-08-1]	GCA_001767235.1
AOX04112.1	TRADD-N+Pentapeptide*→ TRADD-N-aHTH1+TRADD-N+Pentapeptide→?→||<-?||TRADD-N-aHTH1+TRADD-N+Pentapeptide→	TRADD-N+Pentapeptide	345	BJP34_03620	cyanobacteria	Moorea producens PAL-8-15-08-1	hypothetical protein BJP34_03620 [Moorea producens PAL-8-15-08-1]	GCA_001767235.1
AOX04113.1	TRADD-N+Pentapeptide→ TRADD-N-aHTH1+TRADD-N+Pentapeptide*→?→||<-?||TRADD-N-aHTH1+TRADD-N+Pentapeptide→	TRADD-N-aHTH1+TRADD-N+Pentapeptide	462	BJP34_03625	cyanobacteria	Moorea producens PAL-8-15-08-1	hypothetical protein BJP34_03625 [Moorea producens PAL-8-15-08-1]	GCA_001767235.1
AOY81242.1	TRADD-N*→ TRADD-N+Pentapeptide→	TRADD-N	93	BJP36_16370	cyanobacteria	Moorea producens JHB	hypothetical protein BJP36_16370 [Moorea producens JHB]	GCA_001854205.1
AOY84499.1	TRADD-N-aHTH1+TRADD-N+Pentapeptide*→	TRADD-N-aHTH1+TRADD-N+Pentapeptide	351	BJP36_03795	cyanobacteria	Moorea producens JHB	hypothetical protein BJP36_03795 [Moorea producens JHB]	GCA_001854205.1
ASQ04924.1	NTase+helical-region+TRADD-N+X*→	NTase+helical-region+TRADD-N+X	553	CDO23_13830	alphaproteobacteria	Sinorhizobium meliloti	hypothetical protein CDO23_13830 [Sinorhizobium meliloti]	GCA_002197105.1
ASY58478.1	HNH+TRADD-N+STAND+TPRs*→	HNH+TRADD-N+STAND+TPRs	1591	SS05631_c35640	alphaproteobacteria	Sinorhizobium sp. CCBAU 05631	hypothetical protein SS05631_c35640 [Sinorhizobium sp. CCBAU 05631]	GCA_002288505.1
AUB41700.1	TRADD-N-aHTH1+TRADD-N+Pentapeptide*→	TRADD-N-aHTH1+TRADD-N+Pentapeptide	478	COO91_07762	cyanobacteria	Nostoc flagelliforme CCNUN1	putative protein YjbI, containings pentapeptide repeats [Nostoc flagelliforme CCNUN1]	GCA_002813575.1
BAU04807.1	??→ TRADD-N+TPRs*→ EACC2+CASPASE+CHASE2+TM+TM→ TM+beta_rich+TPRs→ TPRs+CASPASE→	TRADD-N+TPRs	201	FIS3754_06960	cyanobacteria	Fischerella sp. NIES-3754	hypothetical protein FIS3754_06960 [Fischerella sp. NIES-3754]	GCA_001548455.1
BAY32115.1	CASPASE+TRADD-N+??*→ Uma2_REase→	CASPASE+TRADD-N+??	303	NIES2107_40010	cyanobacteria	Nostoc carneum NIES-2107	hypothetical protein NIES2107_40010 [Nostoc carneum NIES-2107]	GCA_002368155.1
BAZ00462.1	MetJarc-HTH/ParD_antitoxin→ CASPASE+TRADD-N+PAS+HTH*→	CASPASE+TRADD-N+PAS+HTH	517	NIES37_44540	cyanobacteria	Tolypothrix tenuis PCC 7101	hypothetical protein NIES37_44540 [Tolypothrix tenuis PCC 7101]	GCA_002368295.1
BAZ15419.1	CASPASE+TRADD-N+helical-region+HTH+HTH*→	CASPASE+TRADD-N+helical-region+HTH+HTH	704	NIES4071_72910	cyanobacteria	Calothrix sp. NIES-4071	hypothetical protein NIES4071_72910 [Calothrix sp. NIES-4071]	GCA_002368455.1

acc	operon	architecture	len	gen.name	taxend	species	defline	gca
BBC23917.1	CASPASE+TRADD-N+vWA*→	CASPASE+TRADD-N+vWA	495	ABRG53_1660	cyanobacteria	Pseudanabaena sp. ABRG5-3	von Willebrand factor type A [Pseudanabaena sp. ABRG5-3]	GCA_003967015.1
BBC25632.1	TRADD-N-aHTH1+TRADD-N+Pentapeptide*→	TRADD-N-aHTH1+TRADD-N+Pentapeptide	429	ABRG53_3375	cyanobacteria	Pseudanabaena sp. ABRG5-3	pentapeptide repeat protein [Pseudanabaena sp. ABRG5-3]	GCA_003967015.1
CCI08969.1	TRADD-N+Pentapeptide*→	TRADD-N+Pentapeptide	249	MICAD_3610002	cyanobacteria	Microcystis aeruginosa PCC 7941	Genome sequencing data, contig C319 [Microcystis aeruginosa PCC 7941]	GCA_000312205.1
EDX70590.1	TRADD-N-aHTH1+TRADD-N+Pentapeptide*→ adh_short→	TRADD-N-aHTH1+TRADD-N+Pentapeptide	436	MC7420_162	cyanobacteria	Coleofasciculus chthonoplastes PCC 7420	Pentapeptide repeat protein [Coleofasciculus chthonoplastes PCC 7420]	GCA_000155555.1
EIN00431.1	NTase+helical-region+TRADD-N+X*→	NTase+helical-region+TRADD-N+X	526	WQE_15411	betaproteobacteria	Paraburkholderia hospita	hypothetical protein WQE_15411 [Paraburkholderia hospita]	GCA_000265115.1
ELP52905.1	ECF-HTH+TRADD-N+Pentapeptide*→	ECF-HTH+TRADD-N+Pentapeptide	433	O53_4634	cyanobacteria	Microcystis aeruginosa TAIHU98	pentapeptide repeats family protein [Microcystis aeruginosa TAIHU98]	GCA_000330925.1
ELS45411.1	ECF-HTH+TRADD-N+Pentapeptide*→ SIG→||<-?<-XisI	ECF-HTH+TRADD-N+Pentapeptide	403	C789_4786	cyanobacteria	Microcystis aeruginosa DIANCHI905	pentapeptide repeats family protein [Microcystis aeruginosa DIANCHI905]	GCA_000332585.1
ETD83219.1	NTase+helical-region+TRADD-N+X*→	NTase+helical-region+TRADD-N+X	511	U703_10115	alphaproteobacteria	Rhodobacter capsulatus YW1	hypothetical protein U703_10115 [Rhodobacter capsulatus YW1]	GCA_000506525.2
GAL95421.1	ECF-HTH+TRADD-N+Pentapeptide*→	ECF-HTH+TRADD-N+Pentapeptide	433	N44_04276	cyanobacteria	Microcystis aeruginosa NIES-44	hypothetical protein N44_04276 [Microcystis aeruginosa NIES-44]	GCA_000787675.1
GBE74065.1	ECF-HTH+TRADD-N+Pentapeptide*→	ECF-HTH+TRADD-N+Pentapeptide	390	myaer87_12920	cyanobacteria	Microcystis aeruginosa NIES-87	hypothetical protein myaer87_12920 [Microcystis aeruginosa NIES-87]	GCA_002933835.1
HCB01408.1	TIR+TRADD-N+TM+TM*→	TIR+TRADD-N+TM+TM	516	DEP19_03405	Chloroflexi	Anaerolineae bacterium	TPA: hypothetical protein DEP19_03405 [Anaerolineae bacterium].	GCA_003519565.1
HCK67110.1	TRADD-N*→	TRADD-N	203	DHW49_12675	Chloroflexi	Anaerolineae bacterium	TPA: hypothetical protein DHW49_12675, partial [Anaerolineae bacterium].	GCA_003526495.1
HCR71678.1	TIR+TRADD-N+TM+TM*→	TIR+TRADD-N+TM+TM	508	DIW23_09555	Chloroflexi	Anaerolineae bacterium	TPA: hypothetical protein DIW23_09555 [Anaerolineae bacterium].	GCA_003524705.1
HFK75788.1	TRADD-N+HTH+TM*→	TRADD-N+HTH+TM	519	ENR96_10195	Chloroflexi	Anaerolineae bacterium	TPA: hypothetical protein ENR96_10195, partial [Anaerolineae bacterium].	GCA_011329465.1
HFK76016.1	HTH+TRADD-N+Pentapeptide+Pentapeptide+TM+TM*→	HTH+TRADD-N+Pentapeptide+Pentapeptide+TM+TM	428	ENR96_11345	Chloroflexi	Anaerolineae bacterium	TPA: pentapeptide repeat-containing protein [Anaerolineae bacterium].	GCA_011329465.1
KAA3644872.1	Coiled-coil+TRADD-N+APATPase+TPR+TPR*→?→?→ PCRF+RF-1→	Coiled-coil+TRADD-N+APATPase+TPR+TPR	1021	DWQ07_15840	Chloroflexi	Chloroflexi bacterium	tetratricopeptide repeat protein [Chloroflexi bacterium].	GCA_008501685.1
KDR56649.1	EAD4+TRADD-N+STAND*→	EAD4+TRADD-N+STAND	541	AP-PUASWS_015575	cyanobacteria	Arthrospira platensis str. Paraca	hypothetical protein APPUASWS_015575 [Arthrospira platensis str. Paraca]	GCA_000175415.3
KEZ00677.1	ZNR+HTH+HTH+TIR→ PNPase+TRADD-N+STAND+wHTH+TPRs*→??+HEPN→??+SIR2+??+STAND→	PNPase+TRADD-N+STAND+wHTH+TPRs	1719	AI27_03035	alphaproteobacteria	Sphingomonas sp. BHC-A	hypothetical protein AI27_03035 [Sphingomonas sp. BHC-A]	GCA_000576455.2

<table>
<thead>
<tr><th>acc</th><th>operon</th><th>architecture</th><th>len</th><th>gen.name</th><th>taxend</th><th>species</th><th>defline</th><th>gca</th></tr>
</thead>
<tbody>
<tr><td>KIF37416.1</td><td>TRADD-N-aHTH1+TRADD-N+Pentapeptide*→</td><td>TRADD-N-aHTH1+TRADD-N+Pentapeptide</td><td>436</td><td>PI95_05020</td><td>cyanobacteria</td><td>Hassallia byssoidea VB512170</td><td>hypothetical protein PI95_05020 [Hassallia byssoidea VB512170]</td><td>-</td></tr>
<tr><td>KPK03089.1</td><td>EAD7→?→||||<-?||?→ EAD7+TRADD-N+TM*→</td><td>EAD7+TRADD-N+TM</td><td>234</td><td>AMJ56_20055</td><td>Chloroflexi</td><td>Anaerolineae bacterium SG8_19</td><td>hypothetical protein AMJ56_20055, partial [Anaerolineae bacterium SG8_19].</td><td>GCA_001303105.1</td></tr>
<tr><td>KST65300.1</td><td>EAD1+TRADD-N+Pentapeptide*→</td><td>EAD1+TRADD-N+Pentapeptide</td><td>705</td><td>BC008_21105</td><td>cyanobacteria</td><td>Mastigocoleus testarum BC008</td><td>hypothetical protein BC008_21105 [Mastigocoleus testarum BC008]</td><td>GCA_001456025.1</td></tr>
<tr><td>KST65646.1</td><td>EAD1+TRADD-N+Pentapeptide*→</td><td>EAD1+TRADD-N+Pentapeptide</td><td>744</td><td>BC008_21980</td><td>cyanobacteria</td><td>Mastigocoleus testarum BC008</td><td>hypothetical protein BC008_21980 [Mastigocoleus testarum BC008]</td><td>GCA_001456025.1</td></tr>
<tr><td>KVE14567.1</td><td>PNPase+TRADD-N+STAND+wHTH+TPRs*→</td><td>PNPase+TRADD-N+STAND+wHTH+TPRs</td><td>1631</td><td>WS66_12190</td><td>betaproteobacteria</td><td>Burkholderia sp. LA-2-3-30-S1-D2</td><td>hypothetical protein WS66_12190 [Burkholderia sp. LA-2-3-30-S1-D2]</td><td>GCA_001523915.1</td></tr>
<tr><td>MBC8162005.1</td><td><-Cluster22_2clades||TIR+TRADD-N+TPR*→ TPR-repeats+CASPASE+TM→?→ TPR-repeats→</td><td>TIR+TRADD-N+TPR</td><td>643</td><td>H7Z42_12380</td><td>Chloroflexi</td><td>Roseiflexaceae bacterium</td><td>toll/interleukin-1 receptor domain-containing protein [Roseiflexaceae bacterium].</td><td>GCA_014381165.1</td></tr>
<tr><td>MBC8265192.1</td><td>TM+APATPase+TPR+TPR→ REase+TRADD-N+Zf-TRC1+GNAT*→</td><td>REase+TRADD-N+Zf-TRC1+GNAT</td><td>524</td><td>H8E47_13840</td><td>Chloroflexi</td><td>Anaerolineales bacterium</td><td>zinc-ribbon domain containing protein [Anaerolineales bacterium].</td><td>GCA_014382145.1</td></tr>
<tr><td>MBD2211899.1</td><td>HTH+TRADD-N+Pentapeptide*→
SIG+NACHT→||||<-?||?→||||<-?||?→||||<-TRADDN+Pentapeptide+TM+TM+TM+TM+TM+Pentapeptide-repeats</td><td>HTH+TRADD-N+Pentapeptide</td><td>542</td><td>H6G27_18785</td><td>Cyanobacteria</td><td>Nostoc linckia FACHB-104</td><td>pentapeptide repeat-containing protein [Nostoc linckia FACHB-104].</td><td>GCA_014696615.1</td></tr>
<tr><td>MBE0669634.1</td><td>TIR+TRADD-N+TM+TM*→</td><td>TIR+TRADD-N+TM+TM</td><td>524</td><td>IH588_03520</td><td>Chloroflexi</td><td>Anaerolineales bacterium</td><td>TIR domain-containing protein [Anaerolineales bacterium].</td><td>GCA_014860805.1</td></tr>
<tr><td>MBE7472266.1</td><td>APATPase→ TPR-repeats→||||<-?||HTH+TRADD-N+TM+IG+GBD-PPC*→</td><td>HTH+TRADD-N+TM+IG+GBD-PPC</td><td>1056</td><td>HS114_24290</td><td>Chloroflexi</td><td>Anaerolineales bacterium</td><td>Ig-like domain-containing protein [Anaerolineales bacterium].</td><td>GCA_015075405.1</td></tr>
<tr><td>MBE7530003.1</td><td><-TPR-repeats<-?||?→?→ STAND+TRADD-N+EAD7*→</td><td>STAND+TRADD-N+EAD7</td><td>514</td><td>HS099_09760</td><td>Chloroflexi</td><td>Ardenticatenaceae bacterium</td><td>hypothetical protein HS099_09760 [Ardenticatenaceae bacterium].</td><td>GCA_015075765.1</td></tr>
<tr><td>MBE7552287.1</td><td>TPR-repeats→||||<-?||?→ EAD7+TRADD-N+TPR+TM*→</td><td>EAD7+TRADD-N+TPR+TM</td><td>649</td><td>HS126_14560</td><td>Chloroflexi</td><td>Anaerolineales bacterium</td><td>hypothetical protein HS126_14560 [Anaerolineales bacterium].</td><td>GCA_015075185.1</td></tr>
<tr><td>MBI1926498.1</td><td>HNH+TRADD-N+Dehydrogenase-Rossmann*→</td><td>HNH+TRADD-N+Dehydrogenase-Rossmann</td><td>411</td><td>HYR99_19920</td><td>Bacteria</td><td>Candidatus Poribacteria bacterium</td><td>nucleotide sugar dehydrogenase, partial [Candidatus Poribacteria bacterium].</td><td>GCA_016178185.1</td></tr>
<tr><td>MBI5950754.1</td><td>TIR+TRADD-N+NACHT*→</td><td>TIR+TRADD-N+NACHT</td><td>624</td><td>HY865_03780</td><td>Chloroflexi</td><td>Chloroflexi bacterium</td><td>toll/interleukin-1 receptor domain-containing protein, partial [Chloroflexi bacterium].</td><td>GCA_016234555.1</td></tr>
<tr><td>NEO19477.1</td><td><-Pkinase+GUN4+TM<-?||?→ Pentapeptide-repeats→?→||||<-?<-?||HTH+TRADD-N+Pentapeptide*→</td><td>HTH+TRADD-N+Pentapeptide</td><td>409</td><td>F6K57_08850</td><td>Cyanobacteria</td><td>Moorea sp. SIO4A5</td><td>pentapeptide repeat-containing protein [Moorea sp. SIO4A5].</td><td>GCA_010672005.1</td></tr>
<tr><td>NEO36452.1</td><td>HTH+TRADDN+Pentapeptide-repeats→?→||||<-?||?→||||<-?||HTH+TRADD-N+Pentapeptide*→</td><td>HTH+TRADD-N+Pentapeptide</td><td>493</td><td>F6J90_09010</td><td>Cyanobacteria</td><td>Moorea sp. SIOASIH</td><td>pentapeptide repeat-containing protein [Moorea sp. SIOASIH].</td><td>GCA_010671925.1</td></tr>
<tr><td>NEO62923.1</td><td>HTH+TRADD-N+Pentapeptide*→</td><td>HTH+TRADD-N+Pentapeptide</td><td>424</td><td>F6J98_21785</td><td>Cyanobacteria</td><td>Moorea sp. SIO4G2</td><td>pentapeptide repeat-containing protein [Moorea sp. SIO4G2].</td><td>GCA_010692345.1</td></tr>
<tr><td>NEO66288.1</td><td>HTH+TRADD-N+Pentapeptide*→</td><td>HTH+TRADD-N+Pentapeptide</td><td>375</td><td>F6J98_40265</td><td>Cyanobacteria</td><td>Moorea sp. SIO4G2</td><td>pentapeptide repeat-containing protein [Moorea sp. SIO4G2].</td><td>GCA_010692345.1</td></tr>
</tbody>
</table>

<table>
<tr><th>acc</th><th>operon</th><th>architecture</th><th>len</th><th>gen.name</th><th>taxend</th><th>species</th><th>defline</th><th>gca</th></tr>
<tr><td>NEO94808.1</td><td>Trypsin+TRADD-N+EAD7+TM+TM+TM*→</td><td>Trypsin+TRADD-N+EAD7+TM+TM+TM</td><td>672</td><td>F6K56_33295</td><td>Cyanobacteria</td><td>Moorea sp. SIO3G5</td><td>trypsin-like peptidase domain-containing protein [Moorea sp. SIO3G5].</td><td>GCA_010692495.1</td></tr>
<tr><td>NEP53221.1</td><td>HTH+TRADD-N+Pentapeptide*→</td><td>HTH+TRADD-N+Pentapeptide</td><td>622</td><td>F6K65_32240</td><td>Cyanobacteria</td><td>Moorea sp. SIO3C2</td><td>pentapeptide repeat-containing protein [Moorea sp. SIO3C2].</td><td>GCA_010692465.1</td></tr>
<tr><td>NEQ53750.1</td><td>CASPASE+TRADD-N*→</td><td>CASPASE+TRADD-N</td><td>293</td><td>F6K11_27065</td><td>Cyanobacteria</td><td>Leptolyngbya sp. SIO3F4</td><td>CHAT domain-containing protein, partial [Leptolyngbya sp. SIO3F4].</td><td>GCA_010672145.1</td></tr>
<tr><td>NEQ58411.1</td><td><-Pkinase+GUN4+TM<-?||?→?→||||<-?||HTH+TRADD-N+Pentapeptide*→</td><td>HTH+TRADD-N+Pentapeptide</td><td>385</td><td>F6K53_13800</td><td>Cyanobacteria</td><td>Moorea sp. SIO4A1</td><td>pentapeptide repeat-containing protein [Moorea sp. SIO4A1].</td><td>GCA_010672065.1</td></tr>
<tr><td>NEQ87655.1</td><td>HTH+TRADD-N+Pentapeptide*→</td><td>HTH+TRADD-N+Pentapeptide</td><td>376</td><td>F6K26_49000</td><td>Cyanobacteria</td><td>Moorea sp. SIO2I5</td><td>pentapeptide repeat-containing protein [Moorea sp. SIO2I5].</td><td>GCA_010672285.1</td></tr>
<tr><td>NEQ87826.1</td><td>HTH+TRADD-N*→</td><td>HTH+TRADD-N</td><td>243</td><td>F6K26_49970</td><td>Cyanobacteria</td><td>Moorea sp. SIO2I5</td><td>pentapeptide repeat-containing protein, partial [Moorea sp. SIO2I5].</td><td>GCA_010672285.1</td></tr>
<tr><td>NES20938.1</td><td>HTH+TRADD-N+Pentapeptide*→</td><td>HTH+TRADD-N+Pentapeptide</td><td>574</td><td>F6K41_18895</td><td>Cyanobacteria</td><td>Caldora sp. SIO3E6</td><td>pentapeptide repeat-containing protein [Caldora sp. SIO3E6].</td><td>GCA_010672185.1</td></tr>
<tr><td>NJM06280.1</td><td><-Cluster22_2clades||TIR+TRADD-N+TM+TPR*→ TPR-repeats→ APATPase→</td><td>TIR+TRADD-N+TM+TPR</td><td>679</td><td>HC891_08905</td><td>Bacteria</td><td>Candidatus Gracilibacteria bacterium</td><td>toll/interleukin-1 receptor domain-containing protein [Candidatus Gracilibacteria bacterium].</td><td>GCA_012031775.1</td></tr>
<tr><td>NJO84451.1</td><td>TRADD-N+TPR*→ TPR-repeats→</td><td>TRADD-N+TPR</td><td>271</td><td>HC828_17885</td><td>Alphaproteobacteria</td><td>Blastochloris sp.</td><td>tetratricopeptide repeat protein [Blastochloris sp.].</td><td>GCA_012033475.1</td></tr>
<tr><td>NJP07803.1</td><td>TRADD-N*→||||<-TCAD4+CASPASE+TM+TM+TM+TM+TM+TM</td><td>TRADD-N</td><td>500</td><td>HC837_20375</td><td>Chloroflexi</td><td>Chloroflexaceae bacterium</td><td>hypothetical protein HC837_20375 [Chloroflexaceae bacterium].</td><td>GCA_012034075.1</td></tr>
<tr><td>NQS90905.1</td><td>TRADD-N*→ HEPN→</td><td>TRADD-N</td><td>490</td><td>HQ574_00730</td><td>Chloroflexi</td><td>Chloroflexi bacterium</td><td>hypothetical protein HQ574_00730 [Chloroflexi bacterium].</td><td>GCA_013202705.1</td></tr>
<tr><td>OAE61635.1</td><td>NTase+helical-region+TRADD-N+X*→</td><td>NTase+helical-region+TRADD-N+X</td><td>526</td><td>A7J71_09945</td><td>betaproteobacteria</td><td>Achromobacter insolitus</td><td>hypothetical protein A7J71_09945 [Achromobacter insolitus]</td><td>GCA_001641515.1</td></tr>
<tr><td>OBQ35868.1</td><td>TRADD-N-aHTH1+TRADD-N+Pentapeptide*→</td><td>TRADD-N-aHTH1+TRADD-N+Pentapeptide</td><td>505</td><td>AN487_14515</td><td>cyanobacteria</td><td>Anabaena sp. CRKS33</td><td>hypothetical protein AN487_14515 [Anabaena sp. CRKS33]</td><td>GCA_001672075.1</td></tr>
<tr><td>OCQ96999.1</td><td>TRADD-N-aHTH1+TRADD-N+Pentapeptide*→</td><td>TRADD-N-aHTH1+TRADD-N+Pentapeptide</td><td>342</td><td>BCD64_15540</td><td>cyanobacteria</td><td>Nostoc sp. MBR 210</td><td>hypothetical protein BCD64_15540 [Nostoc sp. MBR 210]</td><td>GCA_001698435.1</td></tr>
<tr><td>OCY15932.1</td><td>ECF-HTH+TRADD-N+Pentapeptide*→</td><td>ECF-HTH+TRADD-N+Pentapeptide</td><td>350</td><td>BEV12_00535</td><td>cyanobacteria</td><td>Microcystis aeruginosa CACIAM 03</td><td>hypothetical protein BEV12_00535 [Microcystis aeruginosa CACIAM 03]</td><td>GCA_001706385.1</td></tr>
<tr><td>ODV36416.1</td><td>ECF-HTH+TRADD-N+Pentapeptide*→</td><td>ECF-HTH+TRADD-N+Pentapeptide</td><td>403</td><td>BFG60_4120</td><td>cyanobacteria</td><td>Microcystis aeruginosa NIES-98</td><td>Pentapeptide repeat family protein [Microcystis aeruginosa NIES-98]</td><td>GCA_001725075.1</td></tr>
<tr><td>OEU65679.1</td><td>TRADD-N+NACHT+helical-region+TPRs*→ Pentapeptide→</td><td>TRADD-N+NACHT+helical-region+TPRs</td><td>1359</td><td>BBJ57_01575</td><td>deltaproteobacteria</td><td>Desulfobacterales bacterium PC51MH44</td><td>hypothetical protein BBJ57_01575, partial [Desulfobacterales bacterium PC51MH44]</td><td>GCA_001751165.1</td></tr>
<tr><td>OGA89041.1</td><td>NTase+helical-region+TRADD-N+X*→</td><td>NTase+helical-region+TRADD-N+X</td><td>510</td><td>A2Z90_14570</td><td>betaproteobacteria</td><td>Burkholderiales bacterium GWA2_64_37</td><td>hypothetical protein A2Z90_14570 [Burkholderiales bacterium GWA2_64_37]</td><td>GCA_001770675.1</td></tr>
</table>

acc	operon	architecture	len	gen.name	taxend	species	defline	gca
OJJ27191.1	HTH+TRADD-N+TPRs*→ EACC2+CASPASE+STAND+TPRs→	HTH+TRADD-N+TPRs	327	BI308_01500	cyanobacteria	Roseofilum reptotaenium AO1-A	hypothetical protein BI308_01500 [Roseofilum reptotaenium AO1-A]	GCA_001890975.1
OKH33849.1	TRADD-N-aHTH1+TRADD-N+Pentapeptide*→	TRADD-N-aHTH1+TRADD-N+Pentapeptide	578	NIES2119_22150	cyanobacteria	Phormidium ambiguum IAM M-71	hypothetical protein NIES2119_22150 [Phormidium ambiguum IAM M-71]	GCA_001904725.1
OLT63091.1	TRADD-N-aHTH1+TRADD-N+Pentapeptide*→	TRADD-N-aHTH1+TRADD-N+Pentapeptide	377	BJP37_03960	cyanobacteria	Moorea bouillonii PNG	hypothetical protein BJP37_03960 [Moorea bouillonii PNG]	GCA_001942495.1
OMQ34815.1	NTase+helical-region+TRADD-N+X*→	NTase+helical-region+TRADD-N+X	520	BKP54_32310	alphaproteobacteria	Ensifer sp. 1H6	hypothetical protein BKP54_32310 [Ensifer sp. 1H6]	GCA_001976035.1
OQW92716.1	helical-region+TRADD-N+TPRs*→ EACC2+CASPASE+NACHT+68TM-wHTH+NACHT+68TM-wHTH→	helical-region+TRADD-N+TPRs	469	BWK78_00820	gammaproteobacteria	Thiotrichaceae bacterium IS1	hypothetical protein BWK78_00820 [Thiotrichaceae bacterium IS1]	GCA_002083855.1
OQX07180.1	TRADD-N+helical-region*→	TRADD-N+helical-region	473	BWK80_49815	deltaproteobacteria	Desulfobacteraceae bacterium IS3	hypothetical protein BWK80_49815 [Desulfobacteraceae bacterium IS3]	GCA_002083955.1
OUC12593.1	TRADD-N-aHTH1+TRADD-N+Pentapeptide*→	TRADD-N-aHTH1+TRADD-N+Pentapeptide	447	B0A82_21820	cyanobacteria	Alkalinema sp. CACIAM 70d	hypothetical protein B0A82_21820 [Alkalinema sp. CACIAM 70d]	GCA_002148405.1
OUL32398.1	TRADD-N-aHTH1+TRADD-N+Pentapeptide*→	TRADD-N-aHTH1+TRADD-N+Pentapeptide	387	BV375_10070	cyanobacteria	Nostoc sp. 106C	hypothetical protein BV375_10070 [Nostoc sp. 106C]	GCA_002154725.1
PBB89920.1	wHTH+TIR→ STAND→ PNPase→ TRADD-N+STAND+wHTH+TPRs*→	TRADD-N+STAND+wHTH+TPRs	1433	CK215_24450	alphaproteobacteria	Mesorhizobium sp. WSM3864	hypothetical protein CK215_24450 [Mesorhizobium sp. WSM3864]	GCA_002294955.1
PHJ69131.1	TRADD-N-aHTH1+TRADD-N+Pentapeptide*→||<-?||TM→ TRADD-N-aHTH1+TRADD-N+Pentapeptide→	TRADD-N-aHTH1+TRADD-N+Pentapeptide	417	VF02_00530	cyanobacteria	Nostoc linckia z1	hypothetical protein VF02_00530 [Nostoc linckia z1]	GCA_002607965.1
PHJ69278.1	TRADD-N-aHTH1+TRADD-N+Pentapeptide→||<-?||TM→ TRADD-N-aHTH1+TRADD-N+Pentapeptide*→	TRADD-N-aHTH1+TRADD-N+Pentapeptide	425	VF02_00510	cyanobacteria	Nostoc linckia z1	hypothetical protein VF02_00510 [Nostoc linckia z1]	GCA_002607965.1
PHJ73282.1	TRADD-N-aHTH1+TRADD-N+Pentapeptide*→	TRADD-N-aHTH1+TRADD-N+Pentapeptide	417	VF05_01545	cyanobacteria	Nostoc linckia z3	hypothetical protein VF05_01545 [Nostoc linckia z3]	GCA_002608015.1
PKP02884.1	Calcineurin+TRADD-N+STAND+wHTH+helical-region*→	Calcineurin+TRADD-N+STAND+wHTH+helical-region	1155	CVU11_10430	bacteroidetes	Bacteroidetes bacterium HGW-Bacteroidetes-6	hypothetical protein CVU11_10430 [Bacteroidetes bacterium HGW-Bacteroidetes-6]	GCA_002842495.1
PSB60411.1	TRADD-N-aHTH1+TRADD-N+Pentapeptide*→	TRADD-N-aHTH1+TRADD-N+Pentapeptide	530	C7B79_25840	cyanobacteria	Chroococcidiopsis cubana CCALA 043	hypothetical protein C7B79_25840, partial [Chroococcidiopsis cubana CCALA 043]	GCA_003003835.1
RAQ43000.1	EAD4+TRADD-N*→	EAD4+TRADD-N	223	B9S53_11830	cyanobacteria	Arthrospira sp. O9.13F	hypothetical protein B9S53_11830, partial [Arthrospira sp. O9.13F]	GCA_003268325.1
RKS67322.1	NTase+helical-region+TRADD-N+X*→	NTase+helical-region+TRADD-N+X	437	DFS04_2001	betaproteobacteria	Herbaspirillum sp. XFZ15 10_5	hypothetical protein DFS04_2001 [Herbaspirillum sp. XFZ15 10_5]	GCA_003634405.1
RLC20956.1	CASPASE+TRADD-N+TM+TM+TM+IG+FGS*→	CASPASE+TRADD-N+TM+TM+TM+IG+FGS	879	DRI57_03535	Deltaproteobacteria	Deltaproteobacteria bacterium	hypothetical protein DRI57_03535 [Deltaproteobacteria bacterium].	GCA_003647425.1

acc	operon	architecture	len	gen.name	taxend	species	defline	gca
RMF77178.1	DrHyd+TRADD-N+TIR+TPR*→	DrHyd+TRADD-N+TIR+TPR	580	D6737_18470	Chloroflexi	Chloroflexi bacterium	DUF4062 domain-containing protein [Chloroflexi bacterium].	GCA_003695675.1
RUS94453.1	HTH+TRADD-N+Pentapeptide+TM+TM*→	HTH+TRADD-N+Pentapeptide+TM+TM	484	DSM106972_093480	Cyanobacteria	Calothrix desertica PCC 7102	hypothetical protein DSM106972_093480 [Calothrix desertica PCC 7102].	GCA_003991905.1
RWI99965.1	Methylase_S→?→ HNH+TRADD-N+SF2-helicase*→	HNH+TRADD-N+SF2-helicase	1207	EOR23_31910	Alphaproteobacteria	Mesorhizobium sp.	HsdR family type I site-specific deoxyribonuclease [Mesorhizobium sp.].	GCA_004018865.1
SCY06844.1	NTase+helical-region+TRADD-N+NACHT+alpha-rich-tail*→	NTase+helical-region+TRADD-N+NACHT+alpha-rich-tail	987	SAMN05192588_0977	Bacteroidetes	Nonlabens sp. Hel1_33_55	hypothetical protein SAMN05192588_0977 [Nonlabens sp. Hel1_33_55]	GCA_900101765.1
SHF09466.1	NTase+helical-region+TRADD-N+X*→	NTase+helical-region+TRADD-N+X	504	SAMN02745157_1624	Alphaproteobacteria	Kaistia soli DSM 19436	hypothetical protein SAMN02745157_1624 [Kaistia soli DSM 19436]	GCA_900129325.1
SNS54311.1	TRADD-N+Calcineurin*→	TRADD-N+Calcineurin	465	SAMN04488107_2944	Actinobacteria	Geodermatophilus saharensis	3',5'-cyclic AMP phosphodiesterase CpdA [Geodermatophilus saharensis].	GCA_900188205.1
TEU17517.1	TIR+TRADD-N+TM+CBM9*→	TIR+TRADD-N+TM+CBM9	464	E3J21_08170	Chloroflexi	Anaerolineales bacterium	TIR domain-containing protein [Anaerolineales bacterium].	GCA_004377365.1
TXI20304.1	SIG+TIR+TRADD-N+NACHT+68TM-wHTH*→||||<-PCRF+RF-1	SIG+TIR+TRADD-N+NACHT+68TM-wHTH	892	E6Q62_02005	Betaproteobacteria	Nitrosomonas sp.	TIR domain-containing protein [Nitrosomonas sp.].	GCA_008015565.1
WP_002751650.1	ECF-HTH+TRADD-N+Pentapeptide*→	ECF-HTH+TRADD-N+Pentapeptide	470	-	cyanobacteria	Microcystis aeruginosa	pentapeptide repeat-containing protein [Microcystis aeruginosa]	GCF_000307995.1
WP_002759140.1	ECF-HTH+TRADD-N+Pentapeptide*→	ECF-HTH+TRADD-N+Pentapeptide	450	-	cyanobacteria	Microcystis aeruginosa	pentapeptide repeat-containing protein [Microcystis aeruginosa]	GCF_000312165.1
WP_002766355.1	ECF-HTH+TRADD-N+Pentapeptide*→	ECF-HTH+TRADD-N+Pentapeptide	432	-	cyanobacteria	Microcystis aeruginosa	hypothetical protein [Microcystis aeruginosa]	GCF_000312185.1
WP_002781357.1	ECF-HTH+TRADD-N+Pentapeptide*→	ECF-HTH+TRADD-N+Pentapeptide	405	-	cyanobacteria	Microcystis aeruginosa	pentapeptide repeat-containing protein [Microcystis aeruginosa]	GCF_000312725.1
WP_002790455.1	ECF-HTH+TRADD-N+Pentapeptide*→	ECF-HTH+TRADD-N+Pentapeptide	432	-	cyanobacteria	Microcystis aeruginosa	pentapeptide repeat-containing protein [Microcystis aeruginosa]	GCF_000312225.1
WP_002790923.1	ECF-HTH+TRADD-N+Pentapeptide*→	ECF-HTH+TRADD-N+Pentapeptide	425	-	cyanobacteria	Microcystis aeruginosa	pentapeptide repeat-containing protein [Microcystis aeruginosa]	GCF_000312245.1
WP_002798711.1	ECF-HTH+TRADD-N+Pentapeptide*→	ECF-HTH+TRADD-N+Pentapeptide	442	-	cyanobacteria	Microcystis aeruginosa	pentapeptide repeat-containing protein [Microcystis aeruginosa]	GCF_000312265.1
WP_006508060.1	TRADD-N+Pentapeptide*→	TRADD-N+Pentapeptide	393	-	cyanobacteria	Xenococcus sp. PCC 7305	pentapeptide repeat-containing protein [Xenococcus sp. PCC 7305]	GCF_000332055.1
WP_006624703.1	EAD4+TRADD-N+STAND+wHTH+HEAT_repeats*→	EAD4+TRADD-N+STAND+wHTH+HEAT_repeats	1746	-	cyanobacteria	Arthrospira platensis	NACHT domain-containing protein [Arthrospira platensis]	GCF_000307915.1
WP_006669967.1	EAD4+TRADD-N+STAND+wHTH+HEAT_repeats*→	EAD4+TRADD-N+STAND+wHTH+HEAT_repeats	1822	-	cyanobacteria	Arthrospira maxima	NACHT domain-containing protein [Arthrospira maxima]	-
WP_006669976.1	HTH+TRADD-N+STAND+wHTH+HEAT_repeats*→	HTH+TRADD-N+STAND+wHTH+HEAT_repeats	1177	-	cyanobacteria	Arthrospira maxima	NACHT domain-containing protein [Arthrospira maxima]	-
WP_007303210.1	TRADD-N*→	TRADD-N	131	-	cyanobacteria	Crocosphaera watsonii	hypothetical protein [Crocosphaera watsonii]	GCF_000167195.1
WP_007311526.1	TRADD-N+Pentapeptide*→	TRADD-N+Pentapeptide	323	-	cyanobacteria	Crocosphaera watsonii	pentapeptide repeat-containing protein [Crocosphaera watsonii]	GCF_000235665.1
WP_007686738.1	ZNR+HTH+HTH+TIR→ PNPase+TRADD-N+STAND+wHTH+TPRs*→??+HEPN→??+SIR2+??+STAND→	PNPase+TRADD-N+STAND+wHTH+TPRs	1737	-	alphaproteobacteria	Sphingomonadaceae	MULTISPECIES: hypothetical protein [Sphingomonadaceae]	GCF_000264945.2

acc	operon	architecture	len	gen.name	taxend	species	defline	gca
WP_008050000.1	EAD4+TRADD-N+STAND+wHTH+HEAT_repeats*→	EAD4+TRADD-N+STAND+wHTH+HEAT_repeats	1378	-	cyanobacteria	Arthrospira sp. PCC 8005	NACHT domain-containing protein [Arthrospira sp. PCC 8005]	GCF_000176895.2
WP_008177582.1	TRADD-N-aHTH1+TRADD-N+Pentapeptide*→	TRADD-N-aHTH1+TRADD-N+Pentapeptide	544	-	cyanobacteria	Moorea producens	hypothetical protein [Moorea producens]	GCF_000211815.1
WP_008190049.1	TRADD-N-aHTH1+TRADD-N+Pentapeptide*→	TRADD-N-aHTH1+TRADD-N+Pentapeptide	383	-	cyanobacteria	Moorea producens	low-complexity protein [Moorea producens]	GCF_000211815.1
WP_008203869.1	ECF-HTH+TRADD-N+Pentapeptide*→	ECF-HTH+TRADD-N+Pentapeptide	436	-	cyanobacteria	Microcystis sp. T1-4	pentapeptide repeat-containing protein [Microcystis sp. T1-4]	GCF_000297435.1
WP_009545863.1	preATP-grasp+BtrJ-ATPgrasp+alpha/beta-Hammerhead/Barrel-sandwich_hybrid→ TRADD-N+Pentapeptide*→ TM_region→ Aldose_epim→ tRNA→	TRADD-N+Pentapeptide	325	-	cyanobacteria	Cyanothece	MULTISPECIES: pentapeptide repeat-containing protein [Cyanothece]	GCF_000017845.1
WP_012167159.1	TPR-repeats+CASPASE→||||<-?<-SIG+Pkinase+TM+TPR<-?<-?||?→?→ PNPase+TRADD-N+HTH*→	PNPase+TRADD-N+HTH	576	-	Cyanobacteria	Acaryochloris marina	hypothetical protein [Acaryochloris marina].	GCF_000018105.1
WP_012362559.1	HTH→ TRADD-N+TPRs*→||<-Pentapeptide+TM_region+Pentapeptide+EACC2+CASPASE	TRADD-N+TPRs	348	-	cyanobacteria	Cyanothece	MULTISPECIES: DUF1822 family protein [Cyanothece]	-
WP_012599489.1	TRADD-N-aHTH1+TRADD-N+TPRs*→ EACC2+CASPASE+APATPase+GUN4→??→	TRADD-N-aHTH1+TRADD-N+TPRs	326	-	cyanobacteria	Cyanothece sp. PCC 7424	hypothetical protein [Cyanothece sp. PCC 7424]	GCF_000021825.1
WP_014276969.1	EAD4+TRADD-N+STAND→ *PSE→||<-EAD4+TRADD-N+STAND*	EAD4+TRADD-N+STAND	525	-	cyanobacteria	Arthrospira platensis	NACHT domain-containing protein [Arthrospira platensis]	-
WP_015117156.1	EAD1+TRADD-N+GUN4*→ AAA+HEAT_repeats→ STAND+HEAT_repeats+TM→	EAD1+TRADD-N+GUN4	351	-	cyanobacteria	Rivularia sp. PCC 7116	GUN4 protein [Rivularia sp. PCC 7116]	GCF_000316665.1
WP_015117657.1	TRADD-N+Pentapeptide*→	TRADD-N+Pentapeptide	312	-	cyanobacteria	Rivularia sp. PCC 7116	pentapeptide repeat-containing protein [Rivularia sp. PCC 7116]	GCF_000316665.1
WP_015121169.1	TRADD-N-aHTH1+TRADD-N+Pentapeptide*→	TRADD-N-aHTH1+TRADD-N+Pentapeptide	487	-	cyanobacteria	Rivularia sp. PCC 7116	pentapeptide repeat-containing protein [Rivularia sp. PCC 7116]	GCF_000316665.1
WP_015121187.1	ADH_N+ADH_zinc_N→ TRADD-N+Pentapeptide*→	TRADD-N+Pentapeptide	436	-	cyanobacteria	Rivularia sp. PCC 7116	putative low-complexity protein [Rivularia sp. PCC 7116]	GCF_000316665.1
WP_015128708.1	helical-region+TRADD-N+TPRs*→ EACC2+CASPASE+DZR+DZR+Pkinase→	helical-region+TRADD-N+TPRs	453	-	cyanobacteria	Calothrix sp. PCC 7507	DUF1822 family protein [Calothrix sp. PCC 7507]	GCF_000316575.1
WP_015139478.1	TRADD-N-aHTH1+TRADD-N+TPRs*→	TRADD-N-aHTH1+TRADD-N+TPRs	360	-	cyanobacteria	Nostoc sp. PCC 7524	hypothetical protein [Nostoc sp. PCC 7524]	GCF_000316645.1
WP_015148075.1	EAD4+TRADD-N+TPRs*→ EACC2+CASPASE+TM_region→ Acetyltransf→	EAD4+TRADD-N+TPRs	415	-	cyanobacteria	Oscillatoria acuminata	DUF1822 family protein [Oscillatoria acuminata]	-
WP_015150426.1	HTH+TRADD-N+TPRs*→	HTH+TRADD-N+TPRs	362	-	cyanobacteria	Oscillatoria acuminata	DUF1822 family protein [Oscillatoria acuminata]	GCF_000317105.1
WP_015195683.1	HTH+TRADD-N+TPRs*→	HTH+TRADD-N+TPRs	433	-	cyanobacteria	Stanieria cyanosphaera	DUF1822 family protein [Stanieria cyanosphaera]	GCF_000317575.1
WP_015217750.1	TRADD-N-aHTH1+TRADD-N+Pentapeptide*→	TRADD-N-aHTH1+TRADD-N+Pentapeptide	475	-	cyanobacteria	Anabaena cylindrica	transcriptional regulator, XRE family [Anabaena cylindrica]	GCF_000317695.1
WP_016516706.1	ECF-HTH+TRADD-N+Pentapeptide*→	ECF-HTH+TRADD-N+Pentapeptide	425	-	cyanobacteria	Microcystis aeruginosa	pentapeptide repeat-containing protein [Microcystis aeruginosa]	GCF_000412595.1
WP_016562538.1	TRADD-N-aHTH1+TRADD-N+Pentapeptide*→	TRADD-N-aHTH1+TRADD-N+Pentapeptide	459	-	cyanobacteria	Trichormus variabilis	pentapeptide repeat-containing protein [Trichormus variabilis]	-
WP_016867683.1	??→ TRADD-N+TPRs*→ EACC2+CASPASE+CHASE2+TM+TM→ TM+beta_rich+TPRs→ TPRs+CASPASE→	TRADD-N+TPRs	204	-	cyanobacteria	Fischerella	MULTISPECIES: DUF1822 family protein [Fischerella]	-
WP_017298126.1	HTH+HTH+TRADD-N+VMAP-M0*→	HTH+HTH+TRADD-N+VMAP-M0	427	-	cyanobacteria	Nodosilinea nodulosa	sigma-70 family RNA polymerase sigma factor [Nodosilinea nodulosa]	GCF_000309385.1

<table>
<tr><th>acc</th><th>operon</th><th>architecture</th><th>len</th><th>gen.name</th><th>taxend</th><th>species</th><th>defline</th><th>gca</th></tr>
<tr><td>WP_017307182.1</td><td>TRADD-N+helical-region*→</td><td>TRADD-N+helical-region</td><td>543</td><td>-</td><td>cyanobacteria</td><td>Spirulina subsalsa</td><td>hypothetical protein [Spirulina subsalsa]</td><td>GCF_000314005.1</td></tr>
<tr><td>WP_017719549.1</td><td>??→ HTH+TRADD-N+TPRs*→ EACC2+CASPASE+BetaPropeller→</td><td>HTH+TRADD-N+TPRs</td><td>401</td><td>-</td><td>cyanobacteria</td><td>Oscillatoria sp. PCC 10802</td><td>DUF1822 family protein [Oscillatoria sp. PCC 10802]</td><td>GCF_000332335.1</td></tr>
<tr><td>WP_019486969.1</td><td>TRADD-N-aHTH1+TRADD-N+Pentapeptide*→</td><td>TRADD-N-aHTH1+TRADD-N+Pentapeptide</td><td>490</td><td>-</td><td>cyanobacteria</td><td>Kamptonema formosum</td><td>pentapeptide repeat-containing protein [Kamptonema formosum]</td><td>GCF_000332155.1</td></tr>
<tr><td>WP_019496109.1</td><td>TRADD-N-aHTH1+TRADD-N+Pentapeptide*→</td><td>TRADD-N-aHTH1+TRADD-N+Pentapeptide</td><td>768</td><td>-</td><td>cyanobacteria</td><td>Calothrix sp. PCC 7103</td><td>hypothetical protein [Calothrix sp. PCC 7103]</td><td>GCF_000331305.1</td></tr>
<tr><td>WP_019496191.1</td><td>TRADD-N-aHTH1+TRADD-N+Pentapeptide+TPRs→||<-?||TRADD-N-aHTH1+TRADD-N+Pentapeptide→
TRADD-N-aHTH1+TRADD-N+Pentapeptide+TPRs*→||<-TM+TM+TM</td><td>TRADD-N-aHTH1+TRADD-N+Pentapeptide+TPRs</td><td>702</td><td>-</td><td>cyanobacteria</td><td>Calothrix sp. PCC 7103</td><td>hypothetical protein [Calothrix sp. PCC 7103]</td><td>-</td></tr>
<tr><td>WP_019496192.1</td><td>TRADD-N-aHTH1+TRADD-N+Pentapeptide+TPRs→||<-?||TRADD-N-aHTH1+TRADD-N+Pentapeptide*→
TRADD-N-aHTH1+TRADD-N+Pentapeptide+TPRs→||<-TM+TM+TM</td><td>TRADD-N-aHTH1+TRADD-N+Pentapeptide</td><td>324</td><td>-</td><td>cyanobacteria</td><td>Calothrix sp. PCC 7103</td><td>pentapeptide repeat-containing protein [Calothrix sp. PCC 7103]</td><td>GCF_000331305.1</td></tr>
<tr><td>WP_019496194.1</td><td>TRADD-N-aHTH1+TRADD-N+Pentapeptide+TPRs*→||<-?||TRADD-N-aHTH1+TRADD-N+Pentapeptide→
TRADD-N-aHTH1+TRADD-N+Pentapeptide+TPRs→||<-TM+TM+TM</td><td>TRADD-N-aHTH1+TRADD-N+Pentapeptide+TPRs</td><td>583</td><td>-</td><td>cyanobacteria</td><td>Calothrix sp. PCC 7103</td><td>hypothetical protein [Calothrix sp. PCC 7103]</td><td>GCF_000331305.1</td></tr>
<tr><td>WP_019496514.1</td><td>TRADD-N-aHTH1+TRADD-N+Pentapeptide*→</td><td>TRADD-N-aHTH1+TRADD-N+Pentapeptide</td><td>950</td><td>-</td><td>cyanobacteria</td><td>Calothrix sp. PCC 7103</td><td>hypothetical protein [Calothrix sp. PCC 7103]</td><td>-</td></tr>
<tr><td>WP_019606632.1</td><td>TM+helical-region→ TIR+TRADD-N*→</td><td>TIR+TRADD-N</td><td>342</td><td>-</td><td>gammaproteobacteria</td><td>Teredinibacter turnerae</td><td>toll/interleukin-1 receptor domain-containing protein [Teredinibacter turnerae]</td><td>GCF_000381665.1</td></tr>
<tr><td>WP_020471543.1</td><td>HTH+TRADD-N+TM+TM*→??→</td><td>HTH+TRADD-N+TM+TM</td><td>461</td><td>-</td><td>planctomycetes</td><td>Zavarzinella formosa</td><td>hypothetical protein [Zavarzinella formosa]</td><td>-</td></tr>
<tr><td>WP_021831880.1</td><td>TRADD-N*→</td><td>TRADD-N</td><td>113</td><td>-</td><td>cyanobacteria</td><td>Crocosphaera watsonii</td><td>hypothetical protein [Crocosphaera watsonii]</td><td>GCF_001039555.1</td></tr>
<tr><td>WP_023669251.1</td><td>HNH+TRADD-N+STAND+TPRs*→</td><td>HNH+TRADD-N+STAND+TPRs</td><td>1617</td><td>-</td><td>alphaproteobacteria</td><td>Mesorhizobium</td><td>MULTISPECIES: AAA family ATPase [Mesorhizobium]</td><td>-</td></tr>
<tr><td>WP_023692716.1</td><td>HNH+TRADD-N+STAND+TPRs*→</td><td>HNH+TRADD-N+STAND+TPRs</td><td>1617</td><td>-</td><td>alphaproteobacteria</td><td>Mesorhizobium</td><td>MULTISPECIES: AAA family ATPase [Mesorhizobium]</td><td>-</td></tr>
<tr><td>WP_026099162.1</td><td>HTH+TRADD-N+TPRs*→ Pentapeptide+TM_region+Pentapeptide+EACC2+CASPASE→</td><td>HTH+TRADD-N+TPRs</td><td>305</td><td>-</td><td>cyanobacteria</td><td>Oscillatoria sp. PCC 10802</td><td>DUF1822 family protein [Oscillatoria sp. PCC 10802]</td><td>-</td></tr>
<tr><td>WP_026724013.1</td><td>TRADD-N-aHTH1+TRADD-N+Pentapeptide*→</td><td>TRADD-N-aHTH1+TRADD-N+Pentapeptide</td><td>426</td><td>-</td><td>cyanobacteria</td><td>Fischerella sp. PCC 9431</td><td>hypothetical protein [Fischerella sp. PCC 9431]</td><td>GCF_000447295.1</td></tr>
<tr><td>WP_027029362.1</td><td>PNPase+TRADD-N+STAND+wHTH+TPRs*→ SIR2+??→</td><td>PNPase+TRADD-N+STAND+wHTH+TPRs</td><td>1737</td><td>-</td><td>alphaproteobacteria</td><td>Mesorhizobium loti</td><td>hypothetical protein [Mesorhizobium loti]</td><td>GCF_013170845.1</td></tr>
<tr><td>WP_029630437.1</td><td>HTH+TRADD-N+TM+TM*→??→</td><td>HTH+TRADD-N+TM+TM</td><td>435</td><td>-</td><td>planctomycetes</td><td>Zavarzinella formosa</td><td>XRE family transcriptional regulator [Zavarzinella formosa]</td><td>GCF_000255705.1</td></tr>
<tr><td>WP_032992391.1</td><td>NTase+helical-region+TRADD-N+X*→</td><td>NTase+helical-region+TRADD-N+X</td><td>511</td><td>-</td><td>alphaproteobacteria</td><td>Rhizobium leguminosarum</td><td>hypothetical protein [Rhizobium leguminosarum]</td><td>GCF_000427765.1</td></tr>
<tr><td>WP_035737013.1</td><td>EAD4+TRADD-N+STAND→//<-PSE<-EAD4+TRADD-N+STAND</td><td>EAD4+TRADD-N+STAND</td><td>525</td><td>-</td><td>cyanobacteria</td><td>Arthrospira platensis</td><td>NACHT domain-containing protein [Arthrospira platensis]</td><td>GCF_000175415.3</td></tr>
<tr><td>WP_036402801.1</td><td>ECF-HTH+TRADD-N+Pentapeptide*→</td><td>ECF-HTH+TRADD-N+Pentapeptide</td><td>405</td><td>-</td><td>cyanobacteria</td><td>Microcystis aeruginosa</td><td>pentapeptide repeat-containing protein [Microcystis aeruginosa]</td><td>GCF_000332585.1</td></tr>
<tr><td>WP_041005222.1</td><td>PNPase+TRADD-N+STAND+wHTH+TPRs*→</td><td>PNPase+TRADD-N+STAND+wHTH+TPRs</td><td>1731</td><td>-</td><td>alphaproteobacteria</td><td>Mesorhizobium plurifarium</td><td>AAA family ATPase [Mesorhizobium plurifarium]</td><td>-</td></tr>
<tr><td>WP_042790997.1</td><td>ECF-HTH+TRADD-N+Pentapeptide*→</td><td>ECF-HTH+TRADD-N+Pentapeptide</td><td>435</td><td>-</td><td>cyanobacteria</td><td>Microcystis aeruginosa</td><td>pentapeptide repeat-containing protein [Microcystis aeruginosa]</td><td>GCF_000330925.1</td></tr>
<tr><td>WP_045056756.1</td><td>helical-region+TRADD-N+TM*→</td><td>helical-region+TRADD-N+TM</td><td>333</td><td>-</td><td>cyanobacteria</td><td>Aliterella atlantica</td><td>hypothetical protein [Aliterella atlantica]</td><td>-</td></tr>
<tr><td>WP_045361906.1</td><td>ECF-HTH+TRADD-N+Pentapeptide*→</td><td>ECF-HTH+TRADD-N+Pentapeptide</td><td>440</td><td>-</td><td>cyanobacteria</td><td>Microcystis aeruginosa</td><td>pentapeptide repeat-containing protein [Microcystis aeruginosa]</td><td>GCF_000787675.1</td></tr>
</table>

acc	operon	architecture	len	gen.name	taxend	species	defline	gca
WP_046320853.1	HTH+TRADD-N+STAND+wHTH+HEAT_repeats*→	HTH+TRADD-N+STAND+wHTH+HEAT_repeats	1162	-	cyanobacteria	Arthrospira	MULTISPECIES: NACHT domain-containing protein [Arthrospira]	-
WP_051502925.1	helical-region+TRADD-N+TPRs*→ EACC2+CASPASE+DZR+Pkinase→	helical-region+TRADD-N+TPRs	511	-	cyanobacteria	[Scytonema hofmanni] UTEX B 1581	DUF1822 family protein [[Scytonema hofmanni] UTEX B 1581]	GCF_014698435.1
WP_051503073.1	TRADD-N-aHTH1+TRADD-N+Pentapeptide*→	TRADD-N-aHTH1+TRADD-N+Pentapeptide	383	-	cyanobacteria	[Scytonema hofmanni] UTEX B 1581	hypothetical protein [[Scytonema hofmanni] UTEX B 1581]	GCF_000582685.1
WP_052277454.1	ECF-HTH+TRADD-N+Pentapeptide*→	ECF-HTH+TRADD-N+Pentapeptide	432	-	cyanobacteria	Microcystis aeruginosa	pentapeptide repeat-containing protein [Microcystis aeruginosa]	GCF_014218745.1
WP_052490229.1	helical-region+TRADD-N+TPRs*→	helical-region+TRADD-N+TPRs	469	-	cyanobacteria	Tolypothrix campylonemoides	DUF1822 family protein [Tolypothrix campylonemoides]	-
WP_053458907.1	HTH→ TRADD-N+Pentapeptide*→	TRADD-N+Pentapeptide	252	-	cyanobacteria	Hapalosiphon sp. MRB220	pentapeptide repeat-containing protein [Hapalosiphon sp. MRB220]	-
WP_054359398.1	PNPase+TRADD-N+STAND+wHTH+TPRs*→	PNPase+TRADD-N+STAND+wHTH+TPRs	1763	-	alphaproteobacteria	Prosthecomicrobium hirschii	AAA family ATPase [Prosthecomicrobium hirschii]	GCF_001305515.1
WP_054465896.1	TM→??→ TRADD-N-aHTH1+TRADD-N+TPRs*→	TRADD-N-aHTH1+TRADD-N+TPRs	325	-	cyanobacteria	Planktothricoides sp. SR001	DUF1822 family protein [Planktothricoides sp. SR001]	GCF_001276715.1
WP_054466474.1	EAD4→ TRADD-N+TPRs*→ EACC2+CASPASE+TM_region→ TM+TM+TM→	TRADD-N+TPRs	287	-	cyanobacteria	Planktothricoides sp. SR001	DUF1822 family protein [Planktothricoides sp. SR001]	GCF_001276715.1
WP_058183652.1	TRADD-N-aHTH1+TRADD-N+Pentapeptide*→	TRADD-N-aHTH1+TRADD-N+Pentapeptide	404	-	cyanobacteria	Mastigocoleus testarum	pentapeptide repeat-containing protein [Mastigocoleus testarum]	GCF_001456025.1
WP_061431382.1	ECF-HTH+TRADD-N+Pentapeptide*→	ECF-HTH+TRADD-N+Pentapeptide	427	-	cyanobacteria	Microcystis aeruginosa	pentapeptide repeat-containing protein [Microcystis aeruginosa]	GCF_001578075.1
WP_062291418.1	TRADD-N-aHTH1+TRADD-N+Pentapeptide*→	TRADD-N-aHTH1+TRADD-N+Pentapeptide	350	-	cyanobacteria	Nostoc piscinale	pentapeptide repeat-containing protein [Nostoc piscinale]	GCF_001298445.1
WP_062587307.1	PNPase+TRADD-N+STAND+wHTH+TPRs*→ SLATT→	PNPase+TRADD-N+STAND+wHTH+TPRs	1417	-	alphaproteobacteria	Rhizobium sp. Leaf311	ATP-binding protein [Rhizobium sp. Leaf311]	GCF_001423445.1
WP_063712050.1	TRADD-N-aHTH1+TRADD-N+Pentapeptide*→	TRADD-N-aHTH1+TRADD-N+Pentapeptide	463	-	cyanobacteria	Coleofasciculus chthonoplastes	pentapeptide repeat-containing protein [Coleofasciculus chthonoplastes]	GCF_000155555.1
WP_064835079.1	NTase+helical-region+TRADD-N+X*→	NTase+helical-region+TRADD-N+X	517	-	alphaproteobacteria	Rhizobium phaseoli	hypothetical protein [Rhizobium phaseoli]	-
WP_068789484.1	EAD4+TRADD-N+STAND+wHTH+HEAT_repeats*→	EAD4+TRADD-N+STAND+wHTH+HEAT_repeats	1398	-	cyanobacteria	Phormidium willei	NACHT domain-containing protein [Phormidium willei]	GCF_001637315.1
WP_069475331.1	ECF-HTH+TRADD-N+Pentapeptide*→	ECF-HTH+TRADD-N+Pentapeptide	410	-	cyanobacteria	Microcystis aeruginosa	pentapeptide repeat-containing protein [Microcystis aeruginosa]	GCF_001725075.1
WP_070393194.1	TRADD-N-aHTH1+TRADD-N+Pentapeptide*→	TRADD-N-aHTH1+TRADD-N+Pentapeptide	514	-	cyanobacteria	Moorea producens	hypothetical protein [Moorea producens]	GCF_001767235.1
WP_071102675.1	TRADD-N-aHTH1+TRADD-N+Pentapeptide→ TRADD-N-aHTH1+TRADD-N+Pentapeptide*→||<-?<-?||TRADD-N-aHTH1+TRADD-N+Pentapeptide→	TRADD-N-aHTH1+TRADD-N+Pentapeptide	499	-	cyanobacteria	Moorea producens	hypothetical protein [Moorea producens]	GCF_001854205.1
WP_071104586.1	TRADD-N+Pentapeptide*→	TRADD-N+Pentapeptide	201	-	cyanobacteria	Moorea producens	pentapeptide repeat-containing protein [Moorea producens]	GCF_001854205.1
WP_071107626.1	TRADD-N-aHTH1+TRADD-N+Pentapeptide*→ TRADD-N-aHTH1+TRADD-N+Pentapeptide→||<-?<-?||TRADD-N-aHTH1+TRADD-N+Pentapeptide→	TRADD-N-aHTH1+TRADD-N+Pentapeptide	568	-	cyanobacteria	Moorea producens	pentapeptide repeat-containing protein [Moorea producens]	GCF_001854205.1
WP_072619436.1	TRADD-N+helical-region*→	TRADD-N+helical-region	533	-	cyanobacteria	Spirulina major	hypothetical protein [Spirulina major]	GCF_001890765.1
WP_072722590.1	HTH+TRADD-N+helical-region*→ TPR_region+CASPASE→	HTH+TRADD-N+helical-region	316	-	cyanobacteria	Planktothrix tepida	XRE family transcriptional regulator [Planktothrix tepida]	GCF_900009145.1

<table>
<tr><th>acc</th><th>operon</th><th>architecture</th><th>len</th><th>gen.name</th><th>taxend</th><th>species</th><th>defline</th><th>gca</th></tr>
<tr><td>WP_072926218.1</td><td>ECF-HTH+TRADD-N+Pentapeptide*→</td><td>ECF-HTH+TRADD-N+Pentapeptide</td><td>352</td><td>-</td><td>cyanobacteria</td><td>Microcystis aeruginosa</td><td>pentapeptide repeat-containing protein, partial [Microcystis aeruginosa]</td><td>GCF_001895325.1</td></tr>
<tr><td>WP_073555229.1</td><td>??→ TRADD-N+TPRs*→ EACC2+CASPASE+CHASE2+TM+TM→ TM+beta_rich+TPRs→ TPRs+CASPASE→</td><td>TRADD-N+TPRs</td><td>204</td><td>-</td><td>cyanobacteria</td><td>Fischerella major</td><td>DUF1822 family protein [Fischerella major]</td><td>-</td></tr>
<tr><td>WP_073619092.1</td><td>TRADD-N-aHTH1+TRADD-N+Pentapeptide+TPRs+Pentapeptide*→</td><td>TRADD-N-aHTH1+TRADD-N+Pentapeptide+TPRs+Pentapeptide</td><td>782</td><td>-</td><td>cyanobacteria</td><td>Calothrix sp. HK-06</td><td>hypothetical protein [Calothrix sp. HK-06]</td><td>GCF_001904745.1</td></tr>
<tr><td>WP_075224986.1</td><td>PNPase+TRADD-N+STAND+wHTH+TPRs*→</td><td>PNPase+TRADD-N+STAND+wHTH+TPRs</td><td>1748</td><td>-</td><td>alphaproteobacteria</td><td>Rhizobium leguminosarum</td><td>hypothetical protein [Rhizobium leguminosarum]</td><td>GCF_001927265.1</td></tr>
<tr><td>WP_077514517.1</td><td>HNH+TRADD-N+SNF2-helicase+LPD33*→</td><td>HNH+TRADD-N+SNF2-helicase+LPD33</td><td>1204</td><td>-</td><td>Gammaproteobacteria</td><td>Rhodanobacter sp. B05</td><td>DEAD/DEAH box helicase family protein [Rhodanobacter sp. B05].</td><td>GCF_002001085.1</td></tr>
<tr><td>WP_079209656.1</td><td>ECF-HTH+TRADD-N+Pentapeptide+TRADD-N+Pentapeptide*→ DDE+DDE_Tnp_1→ Heme_oxygenase→</td><td>ECF-HTH+TRADD-N+Pentapeptide+TRADD-N+Pentapeptide</td><td>620</td><td>-</td><td>cyanobacteria</td><td>Microcystis aeruginosa</td><td>pentapeptide repeat-containing protein [Microcystis aeruginosa]</td><td>-</td></tr>
<tr><td>WP_080880220.1</td><td>TIR+TRADD-N+NACHT+68TM+wHTH*→</td><td>TIR+TRADD-N+NACHT+68TM+wHTH</td><td>917</td><td>-</td><td>nitrospirae</td><td>Nitrospira sp. ND1</td><td>TIR domain-containing protein [Nitrospira sp. ND1]</td><td>GCF_900170025.1</td></tr>
<tr><td>WP_081431064.1</td><td>TRADD-N-aHTH1+TRADD-N+Pentapeptide*→</td><td>TRADD-N-aHTH1+TRADD-N+Pentapeptide</td><td>389</td><td>-</td><td>cyanobacteria</td><td>Moorea bouillonii</td><td>pentapeptide repeat-containing protein [Moorea bouillonii]</td><td>GCF_001942495.1</td></tr>
<tr><td>WP_081618935.1</td><td>alpha-helical-protein→ TRADD-N+Pentapeptide*→ ISOCOT_Eif2b→</td><td>TRADD-N+Pentapeptide</td><td>383</td><td>-</td><td>cyanobacteria</td><td>filamentous cyanobacterium ESFC-1</td><td>hypothetical protein [filamentous cyanobacterium ESFC-1]</td><td>-</td></tr>
<tr><td>WP_082241536.1</td><td>TRADD-N+AB_hydrolase*→</td><td>TRADD-N+AB_hydrolase</td><td>362</td><td>-</td><td>alphaproteobacteria</td><td>Rhodovulum sulfidophilum</td><td>hypothetical protein [Rhodovulum sulfidophilum]</td><td>GCF_001548075.1</td></tr>
<tr><td>WP_082348721.1</td><td>??→ TRADD-N+TPRs*→ BrnT_toxin→ CopG_antitoxin→</td><td>TRADD-N+TPRs</td><td>188</td><td>-</td><td>cyanobacteria</td><td>Planktothricoides sp. SR001</td><td>DUF1822 family protein [Planktothricoides sp. SR001]</td><td>-</td></tr>
<tr><td>WP_082728980.1</td><td>PNPase+TRADD-N+STAND+wHTH+TPRs*→</td><td>PNPase+TRADD-N+STAND+wHTH+TPRs</td><td>1725</td><td>-</td><td>betaproteobacteria</td><td>Burkholderia sp. LA-2-3-30-S1-D2</td><td>hypothetical protein [Burkholderia sp. LA-2-3-30-S1-D2]</td><td>GCF_001523915.2</td></tr>
<tr><td>WP_083304982.1</td><td>TRADD-N-aHTH1+TRADD-N+Pentapeptide*→ TRADD-N-aHTH1+TRADD-N+Pentapeptide→?→||<-?||TRADD-N-aHTH1+TRADD-N+Pentapeptide→</td><td>TRADD-N-aHTH1+TRADD-N+Pentapeptide</td><td>591</td><td>-</td><td>cyanobacteria</td><td>Moorea producens</td><td>pentapeptide repeat-containing protein [Moorea producens]</td><td>-</td></tr>
<tr><td>WP_083304983.1</td><td>TRADD-N-aHTH1+TRADD-N+Pentapeptide→ TRADD-N-aHTH1+TRADD-N+Pentapeptide*→?→||<-?||TRADD-N-aHTH1+TRADD-N+Pentapeptide→</td><td>TRADD-N-aHTH1+TRADD-N+Pentapeptide</td><td>487</td><td>-</td><td>cyanobacteria</td><td>Moorea producens</td><td>pentapeptide repeat-containing protein [Moorea producens]</td><td>GCF_001767235.1</td></tr>
<tr><td>WP_083304984.1</td><td>TRADD-N-aHTH1+TRADD-N+Pentapeptide→ TRADD-N-aHTH1+TRADD-N+Pentapeptide→?→||<-?||TRADD-N-aHTH1+TRADD-N+Pentapeptide*→</td><td>TRADD-N-aHTH1+TRADD-N+Pentapeptide</td><td>352</td><td>-</td><td>cyanobacteria</td><td>Moorea producens</td><td>pentapeptide repeat-containing protein [Moorea producens]</td><td>GCF_001767235.1</td></tr>
<tr><td>WP_083374064.1</td><td>TRADD-N-aHTH1+TRADD-N+Pentapeptide→ TRADD-N-aHTH1+TRADD-N+Pentapeptide→||<-?<-?||TRADD-N-aHTH1+TRADD-N+Pentapeptide*→</td><td>TRADD-N-aHTH1+TRADD-N+Pentapeptide</td><td>372</td><td>-</td><td>cyanobacteria</td><td>Moorea producens</td><td>pentapeptide repeat-containing protein [Moorea producens]</td><td>GCF_001854205.1</td></tr>
<tr><td>WP_083604910.1</td><td>TRADD-N+REC+HISKIN+REC*→</td><td>TRADD-N+REC+HISKIN+REC</td><td>599</td><td>-</td><td>cyanobacteria</td><td>Calothrix sp. HK-06</td><td>response regulator [Calothrix sp. HK-06]</td><td>-</td></tr>
<tr><td>WP_083825169.1</td><td>TM+TM+TM→ TIR→ TIR+TRADD-N+DsrC_HTH*→</td><td>TIR+TRADD-N+DsrC_HTH</td><td>276</td><td>-</td><td>gammaproteobacteria</td><td>endosymbiont of Tevnia jerichonana</td><td>TusE/DsrC/DsvC family sulfur relay protein [endosymbiont of Tevnia jerichonana]</td><td>GCF_000224925.1</td></tr>
<tr><td>WP_083853982.1</td><td>HNH+TRADD-N+STAND+TPRs*→</td><td>HNH+TRADD-N+STAND+TPRs</td><td>1629</td><td>-</td><td>alphaproteobacteria</td><td>Sinorhizobium sp. CCBAU 05631</td><td>AAA family ATPase [Sinorhizobium sp. CCBAU 05631]</td><td>-</td></tr>
<tr><td>WP_084555219.1</td><td>CASPASE+HTH+TRADD-N+Pentapeptide*→</td><td>CASPASE+HTH+TRADD-N+Pentapeptide</td><td>749</td><td>-</td><td>cyanobacteria</td><td>Phormidium ambiguum</td><td>CHAT domain-containing protein [Phormidium ambiguum]</td><td>-</td></tr>
<tr><td>WP_084613256.1</td><td>HTH+TRADD-N+STAND+HEAT_repeats*→</td><td>HTH+TRADD-N+STAND+HEAT_repeats</td><td>1228</td><td>-</td><td>cyanobacteria</td><td>Planktothrix rubescens</td><td>NACHT domain-containing protein [Planktothrix rubescens]</td><td>GCF_000464785.1</td></tr>
<tr><td>WP_086759910.1</td><td>TRADD-N-aHTH1+TRADD-N+Pentapeptide*→</td><td>TRADD-N-aHTH1+TRADD-N+Pentapeptide</td><td>415</td><td>-</td><td>cyanobacteria</td><td>Nostoc sp. 106C</td><td>hypothetical protein [Nostoc sp. 106C]</td><td>GCF_002154725.1</td></tr>
</table>

acc	operon	architecture	len	gen.name	taxend	species	defline	gca
WP_086834672.1	TRADD-N+Pentapeptide*→	TRADD-N+Pentapeptide	120	-	cyanobacteria	Nostoc sp. RF31Y	hypothetical protein [Nostoc sp. RF31Y]	GCF_002155185.1
WP_086834681.1	TM→ TRADD-N-aHTH1+TRADD-N+Pentapeptide*→	TRADD-N-aHTH1+TRADD-N+Pentapeptide	373	-	cyanobacteria	Nostoc sp. RF31Y	pentapeptide repeat-containing protein [Nostoc sp. RF31Y]	GCF_002155185.1
WP_088240080.1	TRADD-N+TPRs*→	TRADD-N+TPRs	191	-	cyanobacteria	Calothrix rhizosoleniae	DUF1822 family protein [Calothrix rhizosoleniae]	GCF_900185595.1
WP_088430566.1	TIR+TRADD-N+??*→ APATPase→ TPRs→	TIR+TRADD-N+??	347	-	cyanobacteria	Halomicronema hongdechloris	toll/interleukin-1 receptor domain-containing protein [Halomicronema hongdechloris]	GCF_002075285.3
WP_089126482.1	TRADD-N-aHTH1+TRADD-N+Pentapeptide*→	TRADD-N-aHTH1+TRADD-N+Pentapeptide	426	-	cyanobacteria	Tolypothrix sp. NIES-4075	hypothetical protein [Tolypothrix sp. NIES-4075]	GCF_002218085.1
WP_091585698.1	HNH+TRADD-N+STAND+TPRs*→	HNH+TRADD-N+STAND+TPRs	1614	-	alphaproteobacteria	Mesorhizobium qingshengii	AAA family ATPase [Mesorhizobium qingshengii]	GCF_900103325.1
WP_094530172.1	HTH+TRADD-N+TPRs*→ EACC2+CASPASE+STAND+BetaPropeller+PDZ+BetaPropeller→	HTH+TRADD-N+TPRs	358	-	cyanobacteria	Pseudanabaena sp. SR411	DUF1822 family protein [Pseudanabaena sp. SR411]	GCF_002251945.1
WP_094672516.1	TRADD-N+Pentapeptide*→	TRADD-N+Pentapeptide	457	-	cyanobacteria	Hydrocoleum sp. CS-953	hypothetical protein [Hydrocoleum sp. CS-953]	GCF_002260545.1
WP_094672636.1	helical-region+TRADD-N+TPRs*→ XisI→	helical-region+TRADD-N+TPRs	356	-	cyanobacteria	Hydrocoleum sp. CS-953	DUF1822 family protein [Hydrocoleum sp. CS-953]	GCF_002260545.1
WP_094676040.1	TRADD-N+Pentapeptide*→	TRADD-N+Pentapeptide	439	-	cyanobacteria	Hydrocoleum sp. CS-953	hypothetical protein [Hydrocoleum sp. CS-953]	-
WP_095496078.1	PNPase+TRADD-N+STAND+wHTH+TPRs*→	PNPase+TRADD-N+STAND+wHTH+TPRs	1728	-	alphaproteobacteria	Mesorhizobium temperatum	AAA family ATPase [Mesorhizobium temperatum]	GCF_002284575.1
WP_095825962.1	wHTH+TIR→ STAND→ PNPase+TRADD-N+STAND+wHTH+TPRs*→	PNPase+TRADD-N+STAND+wHTH+TPRs	1770	-	alphaproteobacteria	Mesorhizobium sp. WSM3882	hypothetical protein [Mesorhizobium sp. WSM3882]	GCF_002294765.1
WP_096606018.1	HTH→ TRADD-N+TPRs*→ CASPASE+TM_region→ SIG+TM+Abi→	TRADD-N+TPRs	196	-	cyanobacteria	Calothrix sp. NIES-2100	DUF1822 family protein [Calothrix sp. NIES-2100]	GCF_002368195.1
WP_096648825.1	TRADD-N-aHTH1+TRADD-N+Pentapeptide*→	TRADD-N-aHTH1+TRADD-N+Pentapeptide	478	-	cyanobacteria	Calothrix brevissima	pentapeptide repeat-containing protein [Calothrix brevissima]	GCF_002367995.1
WP_096655265.1	TRADD-N+Pentapeptide*→	TRADD-N+Pentapeptide	445	-	cyanobacteria	Calothrix parasitica	hypothetical protein [Calothrix parasitica]	GCF_002368095.1
WP_096660958.1	HTH→ TRADD-N+TPRs*→	TRADD-N+TPRs	201	-	cyanobacteria	Calothrix parasitica	DUF1822 family protein [Calothrix parasitica]	GCF_002368095.1
WP_096691930.1	??→ CASPASE+TRADD-N+helical-region*→	CASPASE+TRADD-N+helical-region	424	-	cyanobacteria	Calothrix	MULTISPECIES: CHAT domain-containing protein [Calothrix]	GCF_002368455.1
WP_096695037.1	EAD1+TRADD-N+helical-region+HTH+HTH*→	EAD1+TRADD-N+helical-region+HTH+HTH	514	-	cyanobacteria	Calothrix	MULTISPECIES: sigma-70 family RNA polymerase sigma factor [Calothrix]	GCF_002368455.1
WP_096829425.1	HTH+TRADD-N+TPRs*→	HTH+TRADD-N+TPRs	365	-	cyanobacteria	Tychonema bourrellyi	DUF1822 family protein [Tychonema bourrellyi]	-
WP_099065822.1	TM→ TRADD-N-aHTH1+TRADD-N+Pentapeptide*→	TRADD-N-aHTH1+TRADD-N+Pentapeptide	436	-	cyanobacteria	Nostoc linckia	hypothetical protein [Nostoc linckia]	GCF_002607965.1
WP_099065844.1	TRADD-N+Pentapeptide→?→||<-?||TM→ TRADD-N-aHTH1+TRADD-N+Pentapeptide*→	TRADD-N-aHTH1+TRADD-N+Pentapeptide	453	-	cyanobacteria	Nostoc linckia	transcriptional regulator, XRE family protein [Nostoc linckia]	GCF_002607965.1
WP_099066009.1	TRADD-N+Pentapeptide*→?→||<-?||TM→ TRADD-N-aHTH1+TRADD-N+Pentapeptide→	TRADD-N+Pentapeptide	367	-	cyanobacteria	Nostoc linckia	hypothetical protein [Nostoc linckia]	GCF_002607965.1
WP_099076546.1	TRADD-N+Pentapeptide*→?→||<-?||TM→ TRADD-N-aHTH1+TRADD-N+Pentapeptide→	TRADD-N+Pentapeptide	367	-	cyanobacteria	Nostoc linckia	hypothetical protein [Nostoc linckia]	GCF_002608015.1
WP_099148846.1	HsdM_N+METHYLASE→ TRD+TRD→ REase+SF2+Helicase_C+EcoR124_C+TRADD-N*→	REase+SF2+Helicase_C+EcoR124_C+TRADD-N	1190	-	bacteroidetes	Lewinella nigricans	type I restriction endonuclease subunit R [Lewinella nigricans]	GCF_002646595.1

acc	operon	architecture	len	gen.name	taxend	species	defline	gca
WP_099153795.1	TRADD-N+Pentapeptide*→	TRADD-N+Pentapeptide	340	-	bacteroidetes	Lewinella nigricans	pentapeptide repeat-containing protein [Lewinella nigricans]	GCF_002646595.1
WP_100903218.1	TRADD-N-aHTH1+TRADD-N+Pentapeptide*→	TRADD-N-aHTH1+TRADD-N+Pentapeptide	450	-	cyanobacteria	Nostoc flagelliforme	pentapeptide repeat-containing protein [Nostoc flagelliforme]	GCF_002813575.1
WP_102179539.1	wHTH?→ TRADD-N+TPRs*→ PSE→ TPRs+CASPASE→	TRADD-N+TPRs	204	-	cyanobacteria	Fischerella thermalis	DUF1822 family protein [Fischerella thermalis]	-
WP_103112689.1	ECF-HTH+TRADD-N+Pentapeptide*→	ECF-HTH+TRADD-N+Pentapeptide	420	-	cyanobacteria	Microcystis aeruginosa	pentapeptide repeat-containing protein [Microcystis aeruginosa]	GCF_002897315.1
WP_103127034.1	TRADD-N-aHTH1+TRADD-N+Pentapeptide*→	TRADD-N-aHTH1+TRADD-N+Pentapeptide	409	-	cyanobacteria	Nostoc cycadae	pentapeptide repeat-containing protein [Nostoc cycadae]	-
WP_103127406.1	ECF-HTH+TRADD-N+Pentapeptide*→	ECF-HTH+TRADD-N+Pentapeptide	420	-	cyanobacteria	Microcystis aeruginosa	pentapeptide repeat-containing protein [Microcystis aeruginosa]	GCF_002897275.1
WP_105530726.1	TRADD-N*→??→	TRADD-N	168	-	betaproteobacteria	Solimicrobium silvestre	hypothetical protein [Solimicrobium silvestre]	GCF_002976435.1
WP_106168212.1	TM+TRADD-N+TM+TM*→	TM+TRADD-N+TM+TM	354	-	cyanobacteria	Chroococcidiopsis cubana	hypothetical protein [Chroococcidiopsis cubana]	GCF_003003835.1
WP_106216281.1	TRADD-N+TM+TM*→ Haemagg_act→ Haemagg_act→ SIG+TPRs+CASPASE→	TRADD-N+TM+TM	556	-	cyanobacteria	Cyanosarcina burmensis	pentapeptide repeat-containing protein [Cyanosarcina burmensis]	GCF_003004015.1
WP_106303880.1	all-helical-protein→ TRADD-N+helical-region*→	TRADD-N+helical-region	295	-	cyanobacteria	Chamaesiphon polymorphus	hypothetical protein [Chamaesiphon polymorphus]	GCF_003003845.1
WP_106868049.1	SIG+TM+TM→ TM+TRADD-N*→ TM+all-helical-region→	TM+TRADD-N	190	-	cyanobacteria	filamentous cyanobacterium CCP4	hypothetical protein [filamentous cyanobacterium CCP4]	-
WP_106903024.1	SIG+TM+TM→ TM+TRADD-N*→ TM+all-helical-region→	TM+TRADD-N	177	-	cyanobacteria	filamentous cyanobacterium CCT1	hypothetical protein [filamentous cyanobacterium CCT1]	-
WP_106922102.1	HTH→ TIR+TRADD-N+??*→ APATPase+TPRs→	TIR+TRADD-N+??	349	-	cyanobacteria	filamentous cyanobacterium CCP3	toll/interleukin-1 receptor domain-containing protein [filamentous cyanobacterium CCP3]	-
WP_110578064.1	ECF-HTH+TRADD-N+Pentapeptide*→	ECF-HTH+TRADD-N+Pentapeptide	539	-	cyanobacteria	Microcystis aeruginosa	pentapeptide repeat-containing protein [Microcystis aeruginosa]	GCF_003206555.1
WP_124033433.1	Coiled-coil+TRADD-N+TM*→	Coiled-coil+TRADD-N+TM	415	-	Chloroflexi	Herpetosiphon llansteffanensis	hypothetical protein [Herpetosiphon llansteffanensis].	GCF_003205875.1
WP_153009599.1	TRADD-N+Pentapeptide*→	TRADD-N+Pentapeptide	370	-	Cyanobacteria	Mastigocoleus testarum	pentapeptide repeat-containing protein [Mastigocoleus testarum].	-
WP_153228487.1	HTH+TRADD-N+Pentapeptide*→	HTH+TRADD-N+Pentapeptide	487	-	Cyanobacteria	Nostocaceae	MULTISPECIES: pentapeptide repeat-containing protein [Nostocaceae].	GCF_009498015.1
WP_162271610.1	bDLD3→?→ McrA-NTD+HNH→?→||<-?||?→?→ PNPase+TRADD-N+CASPASE+bDLD3*→ APATPase+TPR+TPR→||<-?<-?||SIG+NPCBM→	PNPase+TRADD-N+CASPASE+bDLD3	816	VT84_RS06140	Planctomycetes	Gemmata sp. SH-PL17	caspase family protein [Gemmata sp. SH-PL17].	GCF_001610855.1
WP_162666668.1	SIG+TPR→||<-?||?→?→?→||<-?||?→ ACYC+FGS+TRADD-N+CASPASE*→?→ MoxR-AAA→ PSE→ FGS→ FGS→ FGS→ FGS→	ACYC+FGS+TRADD-N+CASPASE	622	SOIL9_RS04980	Planctomycetes	Gemmata massiliana	hypothetical protein [Gemmata massiliana].	-
WP_163926701.1	HTH+TRADD-N+Pentapeptide*→	HTH+TRADD-N+Pentapeptide	511	-	Cyanobacteria	Nostoc sp. UIC 10630	pentapeptide repeat-containing protein [Nostoc sp. UIC 10630].	GCF_010747425.1
WP_172109858.1	Methylase_S→ HNH+TRADD-N+SF2-helicase*→ PSE→?→?→||||<-?<-TPR-repeats	HNH+TRADD-N+SF2-helicase	1180	-	Alphaproteobacteria	Bradyrhizobium sp. 83012	HsdR family type I site-specific deoxyribonuclease [Bradyrhizobium sp. 83012].	GCF_013178925.1

acc	operon	architecture	len	gen.name	taxend	species	defline	gca
WP_190623519.1	HTH+TRADD-N+Pentapeptide*→	HTH+TRADD-N+Pentapeptide	386	-	Cyanobacteria	Calothrix membranacea	pentapeptide repeat-containing protein, partial [Calothrix membranacea].	GCF_014696315.1
WP_190677679.1	HTH+TRADD-N+Pentapeptide*→	HTH+TRADD-N+Pentapeptide	362	-	Cyanobacteria	Nostoc sp. FACHB-190	pentapeptide repeat-containing protein [Nostoc sp. FACHB-190].	GCF_014696905.1
WP_190734619.1	TRADD-N+TM+TM+TM+TM+TM+TM+TM+Pentapeptide*→ Pentapeptide-repeats→?→||||<-?||?→ Pentapeptide+TM+TM+TM+Pentapeptide→	TRADD-N+TM+TM+TM+TM+TM+TM+TM+Pentapeptide	884	-	Cyanobacteria	Nostoc sp. FACHB-888	pentapeptide repeat-containing protein [Nostoc sp. FACHB-888].	GCF_014696655.1
WP_190756292.1	<-Methylase_S<-?<-?<-?||HTH+TRADD-N+Pentapeptide*→||||<-?<-PSE<-?<-?<-?<-TPR-repeats+CASPASE	HTH+TRADD-N+Pentapeptide	478	-	Cyanobacteria	Nostoc sp. FACHB-145	pentapeptide repeat-containing protein [Nostoc sp. FACHB-145].	GCF_014697455.1
WP_193968417.1	TIR→ TRADD-N*→	TRADD-N	311	-	Cyanobacteria	Nodosilinea sp. LEGE 07088	hypothetical protein [Nodosilinea sp. LEGE 07088].	GCF_015207395.1
WP_198126279.1	HTH+TRADD-N+Pentapeptide*→	HTH+TRADD-N+Pentapeptide	558	-	Cyanobacteria	Nostoc sp. CENA67	pentapeptide repeat-containing protein [Nostoc sp. CENA67].	GCF_016056305.1
WP_199349019.1	HTH+TRADD-N+Pentapeptide*→	HTH+TRADD-N+Pentapeptide	1121	-	Cyanobacteria	Calothrix sp. FACHB-156	pentapeptide repeat-containing protein [Calothrix sp. FACHB-156].	GCF_014697055.1
YP_009217716.1	??→ TRADD-N+Pentapeptide*→ Pentapeptide→ SIG→??→	TRADD-N+Pentapeptide	300	AVV41_gp032	caudovirales	Microcystis phage MaMV-DC	hypothetical protein MaMVDC_32 [Microcystis phage MaMV-DC]	GCF_001505175.1
