# Supplementary material for: Bacterial death and TRADD-N domains help define novel apoptosis and immunity mechanisms shared by prokaryotes and metazoans
Source: eLife. 2021 Jun 1;10:e70394. doi: 10.7554/eLife.70394 (PMC8195603; doi:10.7554/eLife.70394)
Supplement: Figure 4—source data 1. [file elife-70394-fig4-data1.pdf]

Bacterial Death and TRADD-N domains help define novel apoptosis and immunity mechanisms shared by prokaryotes and metazoans

Figure 4-Source data 1

Gurmeet Kaur†, Lakshminarayan M. Iyer†, A. Maxwell Burroughs, L. Aravind*

Computational Biology Branch, National Center for Biotechnology Information, National Library of Medicine, National Institutes of Health, Bethesda, MD 20894, USA

†These authors contributed equally to the manuscript

Index.

1. Non-redundant counts of TRADD-N domains in eukaryotic lineages
2. Domain architectures of the Eukaryotic TRADD-N domains

Non-redundant counts of TRADD-N domains in eukaryotic lineages

GCA	taxonomy	count
Amphimedon queenslandica	GCF_000090795.1	307
Branchiostoma floridae	GCF_000003815.1	113
Stylophora pistillata	GCF_002571385.1	86
Acropora digitifera	GCF_000222465.1	83
Branchiostoma belcheri	GCF_001625305.1	77
Branchiostoma floridae	GCA_000003815.1	65
Orbicella faveolata	GCF_002042975.1	61
Stylophora pistillata	GCA_002571385.1	52
Crassostrea gigas	GCF_000297895.1	35
Crassostrea virginica	GCF_002022765.2	32
Lingula anatina	GCF_001039355.2	31
Saccoglossus kowalevskii	GCF_000003605.2	24
Exaiptasia pallida	GCA_001417965.1	17
Pocillopora damicornis	GCA_003704095.1	17
Acanthaster planci	GCF_001949145.1	15
Exaiptasia pallida	GCF_001417965.1	12
Apostichopus japonicus	GCA_002754855.1	9
Mizuhopecten yessoensis	GCF_002113885.1	9
Capitella teleta	GCA_000328365.1	8
Carassius auratus	GCF_003368295.1	8
Oncorhynchus kisutch	GCF_002021735.1	8
Oncorhynchus mykiss	GCF_002163495.1	8
Salmo salar	GCF_000233375.1	8
Sinocyclocheilus anshuiensis	GCF_001515605.1	8
Sinocyclocheilus rhinocerous	GCF_001515625.1	8
Nematostella vectensis	GCF_000209225.1	8
Limulus polyphemus	GCF_000517525.1	7
Xenopus laevis	GCF_001663975.1	7
Cyprinus carpio	GCA_001270105.1	7
Salvelinus alpinus	GCF_002910315.2	7
Sinocyclocheilus grahami	GCF_001515645.1	7
Cyprinus carpio	GCF_000951615.1	6
Heterocephalus glaber	GCA_000230445.1	5
Microtus ochrogaster	GCF_000317375.1	5

GCA	taxonomy	count
Odocoileus virginianus texanus	GCF_002102435.1	5
Phascolarctos cinereus	GCF_002099425.1	5
Rhincodon typus	GCF_001642345.1	5
Oncorhynchus tshawytscha	GCF_002872995.1	5
Paramormyrops kingsleyae	GCF_002872115.1	5
Lottia gigantea	GCF_000327385.1	5
Pogona vitticeps	GCF_900067755.1	4
Theropithecus gelada	GCF_003255815.1	4
Ailuropoda melanoleuca	GCF_000004335.2	4
Alligator mississippiensis	GCA_000281125.4	4
Alligator sinensis	GCF_000455745.1	4
Anolis carolinensis	GCF_000090745.1	4
Apteryx australis mantelli	GCF_001039765.1	4
Balaenoptera acutorostrata scammoni	GCF_000493695.1	4
Bos taurus	GCF_002263795.1	4
Bubalus bubalis	GCF_003121395.1	4
Calidris pugnax	GCF_001431845.1	4
Callithrix jacchus	GCF_000004665.1	4
Camelus ferus	GCF_000311805.1	4
Canis lupus familiaris	GCF_000002285.3	4
Cariama cristata	GCA_000690535.1	4
Carlito syrichta	GCF_000164805.1	4
Castor canadensis	GCF_001984765.1	4
Cavia porcellus	GCF_000151735.1	4
Cebus capucinus imitator	GCF_001604975.1	4
Cercocebus atys	GCF_000955945.1	4
Chaetura pelagica	GCF_000747805.1	4
Chinchilla lanigera	GCF_000276665.1	4
Chrysemys picta bellii	GCF_000241765.3	4
Chrysochloris asiatica	GCF_000296735.1	4
Dasypus novemcinctus	GCF_000208655.1	4
Desmodus rotundus	GCF_002940915.1	4
Dipodomys ordii	GCF_000151885.1	4
Echinops telfairi	GCF_000313985.2	4

GCA	taxonomy	count
Elephantulus edwardii	GCF_000299155.1	4
Erinaceus europaeus	GCF_000296755.1	4
Gekko japonicus	GCF_001447785.1	4
Heterocephalus glaber	GCF_000247695.1	4
Hipposideros armiger	GCF_001890085.1	4
Homo sapiens	GCF_000001405.39	4
Ictidomys tridecemlineatus	GCF_000236235.1	4
Jaculus jaculus	GCF_000280705.1	4
Latimeria chalumnae	GCF_000225785.1	4
Leptonychotes weddellii	GCF_000349705.1	4
Lipotes vexillifer	GCF_000442215.1	4
Loxodonta africana	GCF_000001905.1	4
Manis javanica	GCF_001685135.1	4
Marmota marmota marmota	GCF_001458135.1	4
Meriones unguiculatus	GCF_002204375.1	4
Mesocricetus auratus	GCF_000349665.1	4
Miniopterus natalensis	GCF_001595765.1	4
Monodelphis domestica	GCF_000002295.2	4
Mus caroli	GCF_900094665.1	4
Mus musculus	GCF_000001635.26	4
Mustela putorius furo	GCF_000215625.1	4
Myotis brandtii	GCF_000412655.1	4
Myotis davidii	GCF_000327345.1	4
Myotis lucifugus	GCF_000147115.1	4
Nannospalax galili	GCF_000622305.1	4
Nanorana parkeri	GCF_000935625.1	4
Neotoma lepida	GCA_001675575.1	4
Nipponia nippon	GCF_000708225.1	4
Ochotona princeps	GCF_000292845.1	4
Octodon degus	GCF_000260255.1	4
Odobenus rosmarus divergens	GCF_000321225.1	4
Orcinus orca	GCF_000331955.2	4
Orycteropus afer afer	GCF_000298275.1	4
Otolemur garnettii	GCF_000181295.1	4

GCA	taxonomy	count
Panthera pardus	GCF_001857705.1	4
Panthera tigris altaica	GCF_000464555.1	4
Pelodiscus sinensis	GCF_000230535.1	4
Peromyscus maniculatus bairdii	GCF_000500345.1	4
Pongo abelii	GCF_002880775.1	4
Protobothrops mucrosquamatus	GCF_001527695.2	4
Pseudopodoces humilis	GCF_000331425.1	4
Pteropus alecto	GCF_000325575.1	4
Python bivittatus	GCF_000186305.1	4
Rattus norvegicus	GCF_000002265.2	4
Rousettus aegyptiacus	GCF_001466805.2	4
Sorex araneus	GCF_000181275.1	4
Sus scrofa	GCF_000003025.6	4
Thamnophis sirtalis	GCF_001077635.1	4
Trichechus manatus latirostris	GCF_000243295.1	4
Tupaia chinensis	GCF_000334495.1	4
Ursus maritimus	GCF_000687225.1	4
Xenopus tropicalis	GCF_000004195.3	4
Acanthochromis polyacanthus	GCF_002109545.1	4
Amphiprion ocellaris	GCF_002776465.1	4
Austrofundulus limnaeus	GCF_001266775.1	4
Boleophthalmus pectinirostris	GCF_000788275.1	4
Clupea harengus	GCF_000966335.1	4
Cynoglossus semilaevis	GCF_000523025.1	4
Cyprinodon variegatus	GCF_000732505.1	4
Danio rerio	GCF_000002035.6	4
Esox lucius	GCF_004634155.1	4
Fundulus heteroclitus	GCF_000826765.1	4
Gambusia affinis	GCA_003097735.1	4
Hippocampus comes	GCF_001891065.1	4
Ictalurus punctatus	GCF_001660625.1	4
Kryptolebias marmoratus	GCF_001649575.1	4
Lates calcarifer	GCF_001640805.1	4
Lepisosteus oculatus	GCF_000242695.1	4

GCA	taxonomy	count
Maylandia zebra	GCF_000238955.4	4
Monopterus albus	GCF_001952655.1	4
Neolamprologus brichardi	GCF_000239395.1	4
Nothobranchius furzeri	GCF_001465895.1	4
Notothenia coriiceps	GCF_000735185.1	4
Oncorhynchus mykiss	GCA_900005705.1	4
Oreochromis niloticus	GCF_001858045.2	4
Oryzias latipes	GCF_002234675.1	4
Oryzias melastigma	GCF_002922805.1	4
Paralichthys olivaceus	GCF_001970005.1	4
Poecilia formosa	GCF_000485575.1	4
Poecilia reticulata	GCF_000633615.1	4
Pundamilia nyererei	GCF_000239375.1	4
Pygocentrus nattereri	GCF_001682695.1	4
Scophthalmus maximus	GCA_003186165.1	4
Seriola dumerili	GCF_002260705.1	4
Seriola lalandi dorsalis	GCF_002814215.1	4
Stegastes partitus	GCF_000690725.1	4
Xiphophorus maculatus	GCF_002775205.1	4
Pomacea canaliculata	GCF_003073045.1	4
Octopus bimaculoides	GCF_001194135.1	4
Aplysia californica	GCF_000002075.1	4
Cervus elaphus hippelaphus	GCA_002197005.1	3
Hirundo rustica rustica	GCA_003692655.1	3
Chelonia mydas	GCA_000344595.1	3
Alligator mississippiensis	GCF_000281125.3	3
Amazona aestiva	GCA_001420675.1	3
Aotus nancymaae	GCF_000952055.2	3
Aptenodytes forsteri	GCF_000699145.1	3
Bos indicus	GCF_000247795.1	3
Bos mutus	GCA_000298355.1	3
Bos mutus	GCF_000298355.1	3
Callipepla squamata	GCA_002218305.1	3
Callorhinchus milii	GCF_000165045.1	3

GCA	taxonomy	count
Callorhinus ursinus	GCF_003265705.1	3
Camelus ferus	GCA_000311805.2	3
Capra hircus	GCF_001704415.1	3
Cariama cristata	GCF_000690535.1	3
Ceratotherium simum simum	GCF_000283155.1	3
Charadrius vociferus	GCA_000708025.2	3
Charadrius vociferus	GCF_000708025.1	3
Columba livia	GCF_000337935.1	3
Condylura cristata	GCF_000260355.1	3
Coturnix japonica	GCF_001577835.1	3
Crocodylus porosus	GCF_001723895.1	3
Cuculus canorus	GCF_000709325.1	3
Delphinapterus leucas	GCF_002288925.2	3
Egretta garzetta	GCF_000687185.1	3
Enhydra lutris kenyoni	GCF_002288905.1	3
Eptesicus fuscus	GCF_000308155.1	3
Equus asinus	GCF_001305755.1	3
Equus caballus	GCF_002863925.1	3
Felis catus	GCF_000181335.3	3
Fukomys damarensis	GCF_000743615.1	3
Galeopterus variegatus	GCF_000696425.1	3
Gallus gallus	GCF_000002315.5	3
Macaca fascicularis	GCA_000230815.1	3
Meleagris gallopavo	GCF_000146605.2	3
Neomonachus schauinslandi	GCF_002201575.1	3
Neophocaena asiaeorientalis asiaeorientalis	GCF_003031525.1	3
Notechis scutatus	GCF_900518725.1	3
Numida meleagris	GCF_002078875.1	3
Oryctolagus cuniculus	GCF_000003625.3	3
Pan paniscus	GCF_000258655.2	3
Papio anubis	GCF_000264685.3	3
Phaethon lepturus	GCF_000687285.1	3
Piliocolobus tephrosceles	GCF_002776525.2	3
Pongo abelii	GCA_002880775.3	3

GCA	taxonomy	count
Propithecus coquereli	GCF_000956105.1	3
Pseudonaja textilis	GCF_900518735.1	3
Pteropus vampyrus	GCF_000151845.1	3
Sarcophilus harrisii	GCF_000189315.1	3
Struthio camelus australis	GCA_000698965.1	3
Struthio camelus australis	GCF_000698965.1	3
Sturnus vulgaris	GCF_001447265.1	3
Xenopus laevis	GCA_001663975.1	3
Acanthisitta chloris	GCF_000695815.1	3
Antrostomus carolinensis	GCF_000700745.1	3
Bambusicola thoracicus	GCA_002909625.1	3
Bison bison bison	GCF_000754665.1	3
Colius striatus	GCF_000690715.1	3
Colobus angolensis palliatus	GCF_000951035.1	3
Corvus brachyrhynchos	GCA_000691975.1	3
Corvus brachyrhynchos	GCF_000691975.1	3
Cyanistes caeruleus	GCF_002901205.1	3
Ficedula albicollis	GCF_000247815.1	3
Lepidothrix coronata	GCF_001604755.1	3
Parus major	GCF_001522545.3	3
Picoides pubescens	GCF_000699005.1	3
Tinamus guttatus	GCF_000705375.1	3
Anabas testudineus	GCF_900324465.1	3
Astyanax mexicanus	GCF_000372685.2	3
Electrophorus electricus	GCF_003665695.1	3
Haplochromis burtoni	GCF_000239415.1	3
Labrus bergylta	GCF_900080235.1	3
Larimichthys crocea	GCF_000972845.2	3
Mastacembelus armatus	GCF_900324485.1	3
Pangasianodon hypophthalmus	GCF_003671635.1	3
Poecilia latipinna	GCF_001443285.1	3
Scleropages formosus	GCA_001005745.2	3
Tetraodon nigroviridis	GCA_000180735.1	3
Biomphalaria glabrata	GCF_000457365.1	3

GCA	taxonomy	count
Macrostomum lignano	GCA_002269645.1	2
Centruroides sculpturatus	GCF_000671375.1	2
Parasteatoda tepidariorum	GCF_000365465.2	2
Ciona intestinalis	GCF_000224145.3	2
Acinonyx jubatus	GCF_003709585.1	2
Aptenodytes forsteri	GCA_000699145.1	2
Chaetura pelagica	GCA_000747805.1	2
Colinus virginianus	GCA_000599465.2	2
Cricetulus griseus	GCA_000448345.1	2
Cuculus canorus	GCA_000709325.1	2
Egretta garzetta	GCA_000687185.1	2
Falco peregrinus	GCF_000337955.1	2
Gorilla gorilla gorilla	GCF_008122165.1	2
Haliaeetus leucocephalus	GCF_000737465.1	2
Macaca mulatta	GCA_000230795.1	2
Macaca mulatta	GCF_003339765.1	2
Mandrillus leucophaeus	GCF_000951045.1	2
Melopsittacus undulatus	GCF_000238935.1	2
Nothoprocta perdicaria	GCF_003342845.1	2
Pan troglodytes	GCA_002880755.3	2
Physeter catodon	GCF_002837175.2	2
Puma concolor	GCF_003327715.1	2
Rhinopithecus roxellana	GCF_007565055.1	2
Tupaia chinensis	GCA_000334495.1	2
Vicugna pacos	GCF_000164845.2	2
Vulpes vulpes	GCF_003160815.1	2
Zonotrichia albicollis	GCF_000385455.1	2
Acanthisitta chloris	GCA_000695815.1	2
Apteryx rowi	GCF_003343035.1	2
Phalacrocorax carbo	GCF_000708925.1	2
Rana catesbeiana	GCA_002284835.2	2
Xenopus tropicalis	GCA_000004195.3	2
Anas platyrhynchos	GCA_000355885.1	2
Anas platyrhynchos	GCF_003850225.1	2

GCA	taxonomy	count
Anser cygnoides domesticus	GCF_000971095.1	2
Antrostomus carolinensis	GCA_000700745.1	2
Apaloderma vittatum	GCA_000703405.1	2
Apaloderma vittatum	GCF_000703405.1	2
Aquila chrysaetos canadensis	GCF_000766835.1	2
Balearica regulorum gibbericeps	GCA_000709895.1	2
Balearica regulorum gibbericeps	GCF_000709895.1	2
Buceros rhinoceros silvestris	GCA_000710305.1	2
Buceros rhinoceros silvestris	GCF_000710305.1	2
Camelus bactrianus	GCF_000767855.1	2
Cathartes aura	GCA_000699945.1	2
Chlamydotis macqueenii	GCA_000695195.1	2
Chlorocebus sabaeus	GCF_000409795.2	2
Colius striatus	GCA_000690715.1	2
Cricetulus griseus	GCF_000223135.1	2
Equus przewalskii	GCF_000696695.1	2
Eurypyga helias	GCA_000690775.1	2
Fulmarus glacialis	GCA_000690835.1	2
Gavia stellata	GCA_000690875.1	2
Gavialis gangeticus	GCF_001723915.1	2
Haliaeetus albicilla	GCF_000691405.1	2
Leptosomus discolor	GCA_000691785.1	2
Lonchura striata domestica	GCA_002197715.1	2
Macaca fascicularis	GCF_000364345.1	2
Merops nubicus	GCA_000691845.1	2
Merops nubicus	GCF_000691845.1	2
Mesitornis unicolor	GCA_000695765.1	2
Microcebus murinus	GCF_000165445.2	2
Mus pahari	GCF_900095145.1	2
Myotis davidii	GCA_000327345.1	2
Nestor notabilis	GCF_000696875.1	2
Nipponia nippon	GCA_000708225.1	2
Nomascus leucogenys	GCF_006542625.1	2
Opisthocomus hoazin	GCF_000692075.1	2

GCA	taxonomy	count
Pelecanus crispus	GCA_000687375.1	2
Phaethon lepturus	GCA_000687285.1	2
Phalacrocorax carbo	GCA_000708925.1	2
Phoenicopterus ruber ruber	GCA_000687265.1	2
Podiceps cristatus	GCA_000699545.1	2
Pterocles gutturalis	GCA_000699245.1	2
Pterocles gutturalis	GCF_000699245.1	2
Pygoscelis adeliae	GCF_000699105.1	2
Rattus norvegicus	GCF_000001895.5	2
Rhinopithecus bieti	GCF_001698545.1	2
Saimiri boliviensis boliviensis	GCF_000235385.1	2
Tauraco erythrolophus	GCA_000709365.1	2
Tauraco erythrolophus	GCF_000709365.1	2
Tursiops truncatus	GCF_001922835.1	2
Tyto alba	GCA_000687205.1	2
Astatotilapia calliptera	GCF_900246225.1	2
Poecilia mexicana	GCF_001443325.1	2
Scleropages formosus	GCF_900964775.1	2
Takifugu rubripes	GCF_901000725.2	2
Drosophila serrata	GCF_002093755.1	2
Mizuhopecten yessoensis	GCA_002113885.2	2
Pomacea canaliculata	GCA_003073045.1	2
Clonorchis sinensis	GCA_003604175.1	1
Echinococcus granulosus	GCF_000524195.1	1
Echinococcus multilocularis	GCA_000469725.3	1
Hymenolepis microstoma	GCA_000469805.2	1
Opisthorchis viverrini	GCF_000715545.1	1
Schistosoma mansoni	GCF_000237925.1	1
Stegodyphus mimosarum	GCA_000611955.2	1
Nephila clavipes	GCA_002102615.1	1
Euroglyphus maynei	GCA_002135145.1	1
Galendromus occidentalis	GCF_000255335.1	1
Priapulus caudatus	GCF_000485595.1	1
Sarcoptes scabiei	GCA_000828355.1	1

GCA	taxonomy	count
Tetranychus urticae	GCF_000239435.1	1
Varroa destructor	GCF_002443255.1	1
Varroa jacobsoni	GCF_002532875.1	1
Helobdella robusta	GCF_000326865.1	1
Canis lupus dingo	GCF_003254725.1	1
Chelonia mydas	GCF_000344595.1	1
Columba livia	GCA_000337935.2	1
Lonchura striata domestica	GCF_005870125.1	1
Mus musculus	GCA_000002165.1	1
Cricetulus griseus	GCA_003668045.1	1
Ailuropoda melanoleuca	GCA_000004335.1	1
Falco cherrug	GCF_000337975.1	1
Fukomys damarensis	GCA_000743615.1	1
Limosa lapponica baueri	GCA_002844005.1	1
Macaca nemestrina	GCF_000956065.1	1
Myotis brandtii	GCA_000412655.1	1
Pan troglodytes	GCF_002880755.1	1
Patagioenas fasciata monilis	GCA_002029285.1	1
Pteropus alecto	GCA_000325575.1	1
Vicugna pacos	GCF_000164845.1	1
Ophiophagus hannah	GCA_000516915.1	1
Gulo gulo	GCA_900006375.2	1
Athene cunicularia	GCF_003259725.1	1
Calypte anna	GCA_000699085.1	1
Chlamydotis macqueenii	GCF_000695195.1	1
Corvus cornix cornix	GCF_000738735.2	1
Cricetulus griseus	GCA_000223135.1	1
Dromaius novaehollandiae	GCF_003342905.1	1
Erythrura gouldiae	GCA_003676055.1	1
Eurypyga helias	GCF_000690775.1	1
Fulmarus glacialis	GCF_000690835.1	1
Gavia stellata	GCF_000690875.1	1
Geospiza fortis	GCF_000277835.1	1
Haliaeetus albicilla	GCA_000691405.1	1

GCA	taxonomy	count
Leptosomus discolor	GCF_000691785.1	1
Manacus vitellinus	GCA_000692015.2	1
Manacus vitellinus	GCF_001715985.3	1
Mesitornis unicolor	GCF_000695765.1	1
Nestor notabilis	GCA_000696875.1	1
Opisthocomus hoazin	GCA_000692075.1	1
Pelecanus crispus	GCF_000687375.1	1
Picoides pubescens	GCA_000699005.1	1
Pygoscelis adeliae	GCA_000699105.1	1
Serinus canaria	GCF_007115625.1	1
Terrapene mexicana triunguis	GCF_002925995.2	1
Tinamus guttatus	GCA_000705375.2	1
Tyto alba	GCF_000687205.1	1
Urocitellus parryii	GCF_003426925.1	1
Ursus arctos horribilis	GCF_003584765.1	1
Anabarilius grahami	GCA_003731715.1	1
Tachysurus fulvidraco	GCF_003724035.1	1
Nematostella vectensis	GCA_000209225.1	1
Daphnia magna	GCA_001632505.1	1
Eurytemora affinis	GCF_000591075.1	1
Hyalella azteca	GCF_000764305.1	1
Strongylocentrotus purpuratus	GCF_000002235.5	1
Acromyrmex echinatior	GCF_000204515.1	1
Acyrthosiphon pisum	GCF_005508785.1	1
Aedes aegypti	GCA_000004015.3	1
Aedes aegypti	GCF_002204515.2	1
Aedes albopictus	GCA_001444175.2	1
Amyelois transitella	GCF_001186105.1	1
Anopheles darlingi	GCA_000211455.3	1
Anopheles gambiae str. PEST	GCF_000005575.2	1
Anopheles sinensis	GCA_000441895.2	1
Anoplophora glabripennis	GCF_000390285.2	1
Apis cerana	GCF_001442555.1	1
Apis cerana cerana	GCA_002290385.1	1

GCA	taxonomy	count
Apis mellifera	GCF_003254395.2	1
Athalia rosae	GCF_000344095.2	1
Atta cephalotes	GCF_000143395.1	1
Atta colombica	GCA_001594045.1	1
Atta colombica	GCF_001594045.1	1
Bactrocera latifrons	GCF_001853355.1	1
Bactrocera oleae	GCF_001188975.1	1
Bemisia tabaci	GCF_001854935.1	1
Bicyclus anynana	GCF_900239965.1	1
Blattella germanica	GCA_003018175.1	1
Bombus impatiens	GCF_000188095.3	1
Bombus terrestris	GCF_000214255.1	1
Bombyx mori	GCF_000151625.1	1
Camponotus floridanus	GCA_000147175.1	1
Camponotus floridanus	GCF_003227725.1	1
Cephus cinctus	GCF_000341935.1	1
Ceratina calcarata	GCF_001652005.1	1
Ceratitis capitata	GCF_000347755.3	1
Ceratosolen solmsi marchali	GCF_000503995.1	1
Cimex lectularius	GCF_000648675.2	1
Clunio marinus	GCA_900005825.1	1
Copidosoma floridanum	GCF_000648655.2	1
Cryptotermes secundus	GCF_002891405.2	1
Culex quinquefasciatus	GCF_000209185.1	1
Cyphomyrmex costatus	GCA_001594065.1	1
Cyphomyrmex costatus	GCF_001594065.1	1
Danaus plexippus plexippus	GCA_000235995.2	1
Dendroctonus ponderosae	GCF_000355655.1	1
Diachasma alloeum	GCF_001412515.2	1
Diaphorina citri	GCF_000475195.1	1
Dinoponera quadriceps	GCF_001313825.1	1
Diuraphis noxia	GCF_001186385.1	1
Drosophila ananassae	GCF_000005115.1	1
Drosophila arizonae	GCF_001654025.1	1

GCA	taxonomy	count
Drosophila biarmipes	GCF_000233415.1	1
Drosophila bipectinata	GCF_000236285.1	1
Drosophila busckii	GCA_001277935.1	1
Drosophila busckii	GCF_001277935.1	1
Drosophila elegans	GCF_000224195.1	1
Drosophila eugracilis	GCF_000236325.1	1
Drosophila ficusphila	GCF_000220665.1	1
Drosophila grimshawi	GCF_000005155.2	1
Drosophila kikkawai	GCF_000224215.1	1
Drosophila miranda	GCF_000269505.1	1
Drosophila mojavensis	GCF_000005175.2	1
Drosophila obscura	GCF_002217835.1	1
Drosophila pseudoobscura pseudoobscura	GCF_000001765.3	1
Drosophila rhopaloa	GCF_000236305.1	1
Drosophila sechellia	GCF_000005215.3	1
Drosophila suzukii	GCF_000472105.1	1
Drosophila takahashii	GCF_000224235.1	1
Drosophila virilis	GCF_000005245.1	1
Drosophila willistoni	GCA_000005925.1	1
Drosophila willistoni	GCF_000005925.1	1
Drosophila yakuba	GCF_000005975.2	1
Dufourea novaeangliae	GCA_001272555.1	1
Dufourea novaeangliae	GCF_001272555.1	1
Eufriesea mexicana	GCA_001483705.1	1
Eufriesea mexicana	GCF_001483705.1	1
Folsomia candida	GCA_002217175.1	1
Folsomia candida	GCF_002217175.1	1
Fopius arisanus	GCF_000806365.1	1
Habropoda laboriosa	GCF_001263275.1	1
Halyomorpha halys	GCF_000696795.2	1
Harpegnathos saltator	GCA_000147195.1	1
Harpegnathos saltator	GCF_003227715.1	1
Helicoverpa armigera	GCA_002156985.1	1
Helicoverpa armigera	GCF_002156985.1	1

GCA	taxonomy	count
Heliothis virescens	GCA_002382865.1	1
Leptinotarsa decemlineata	GCF_000500325.1	1
Linepithema humile	GCF_000217595.1	1
Lucilia cuprina	GCA_001187945.1	1
Lucilia cuprina	GCF_000699065.1	1
Megachile rotundata	GCF_000220905.1	1
Melanaphis sacchari	GCF_002803265.2	1
Melipona quadrifasciata	GCA_001276565.1	1
Microplitis demolitor	GCF_000572035.2	1
Monomorium pharaonis	GCF_003260585.2	1
Musca domestica	GCF_000371365.1	1
Myzus persicae	GCF_001856785.1	1
Nasonia vitripennis	GCF_000002325.3	1
Neodiprion lecontei	GCF_001263575.1	1
Nicrophorus vespilloides	GCF_001412225.1	1
Nilaparvata lugens	GCF_000757685.1	1
Onthophagus taurus	GCF_000648695.1	1
Operophtera brumata	GCA_001266575.1	1
Orussus abietinus	GCF_000612105.2	1
Oryctes borbonicus	GCA_001443705.1	1
Papilio machaon	GCA_001298355.1	1
Papilio machaon	GCF_001298355.1	1
Papilio polytes	GCF_000836215.1	1
Papilio xuthus	GCA_001298345.1	1
Papilio xuthus	GCF_000836235.1	1
Pediculus humanus corporis	GCF_000006295.1	1
Pieris rapae	GCF_001856805.1	1
Plutella xylostella	GCF_000330985.1	1
Pogonomyrmex barbatus	GCF_000187915.1	1
Polistes canadensis	GCF_001313835.1	1
Polistes dominula	GCF_001465965.1	1
Pseudomyrmex gracilis	GCF_002006095.1	1
Rhagoletis zephyria	GCF_001687245.1	1
Sipha flava	GCF_003268045.1	1

GCA	taxonomy	count
Spodoptera litura	GCF_002706865.1	1
Stomoxys calcitrans	GCF_001015335.1	1
Temnothorax curvispinosus	GCF_003070985.1	1
Trachymyrmex cornetzi	GCF_001594075.1	1
Trachymyrmex septentrionalis	GCA_001594115.1	1
Trachymyrmex septentrionalis	GCF_001594115.1	1
Trachymyrmex zeteki	GCA_001594055.1	1
Trachymyrmex zeteki	GCF_001594055.1	1
Tribolium castaneum	GCF_000002335.3	1
Trichogramma pretiosum	GCF_000599845.2	1
Trichomalopsis sarcophagae	GCA_002249905.1	1
Vollenhovia emeryi	GCF_000949405.1	1
Wasmannia auropunctata	GCF_000956235.1	1
Zeugodacus cucurbitae	GCF_000806345.1	1
Zootermopsis nevadensis	GCF_000696155.1	1
Acromyrmex echinatior	GCA_000204515.1	1
Apis florea	GCF_000184785.2	1
Habropoda laboriosa	GCA_001263275.1	1
Ooceraea biroi	GCA_000611835.1	1
Trachymyrmex cornetzi	GCA_001594075.1	1
Octopus bimaculoides	GCA_001194135.1	1
Toxocara canis	GCA_000803305.1	1
Trichinella britovi	GCA_001447585.1	1
Trichinella murrelli	GCA_001447425.1	1
Trichinella nativa	GCA_001447565.1	1
Trichinella nelsoni	GCA_001447455.1	1
Trichinella papuae	GCA_001447755.1	1
Trichinella patagoniensis	GCA_001447655.1	1
Trichinella pseudospiralis	GCA_001447445.1	1
Trichinella pseudospiralis	GCA_001447675.1	1
Trichinella pseudospiralis	GCA_001447575.1	1
Trichinella pseudospiralis	GCA_001447725.1	1
Trichinella sp. T6	GCA_001447435.1	1
Trichinella sp. T8	GCA_001447745.1	1

GCA	taxonomy	count
Trichinella sp. T9	GCA_001447505.1	1
Trichinella spiralis	GCA_001447595.1	1
Trichinella spiralis	GCF_000181795.1	1
Trichinella zimbabwensis	GCA_001447665.1	1
Trichuris suis	GCA_000701005.1	1
Trichuris suis	GCA_000701025.1	1
Trichoplax adhaerens	GCA_000150275.1	1
Trichoplax adhaerens	GCF_000150275.1	1

Domain architectures of the Eukaryotic TRADD-N domains

acc	architecture	len	gen.name	taxend	species	defline	gca	clade
XP_023930852.1	ABC2_membrane_3+TM+TM+TRADDN-DEDD2+Coiled-coil	1079	LOC106158803	metazoa	Lingula anatina	ATP-binding cassette sub-family A member 17-like [Lingula anatina]	GCF_001039355.2	TRADDN-DEDD2
XP_019860785.1	Ankyrin+Death+CARD+DED+TRADDN-NCOA6	523	LOC109589107	metazoa	Amphimedon queenslandica	PREDICTED: uncharacterized protein LOC109589107 [Amphimedon queenslandica]	GCF_000090795.1	TRADDN-NCOA6
XP_013383107.1	Ankyrin+LRRs+APGTPase+COR+TBP-like+TRADDN-DEDD2+P-loop-NTPase	2305	LOC106153652	metazoa	Lingula anatina	leucine-rich repeat serine/threonine-protein kinase 1 [Lingula anatina]	GCF_001039355.2	TRADDN-DEDD2
XP_019859900.1	Ankyrin+TRADDN-NCOA6+Ankyrin	309	LOC109580158	metazoa	Amphimedon queenslandica	PREDICTED: E3 ubiquitin-protein ligase MIB1-like [Amphimedon queenslandica]	GCF_000090795.1	TRADDN-NCOA6
XP_019861957.1	Ankyrin+TRADDN-NCOA6+Ankyrin	973	LOC109590491	metazoa	Amphimedon queenslandica	PREDICTED: ankyrin repeat and KH domain-containing protein 1-like [Amphimedon queenslandica]	GCF_000090795.1	TRADDN-NCOA6
XP_019855546.1	APGTPase+COR+DED+TRADDN-NCOA6+CASPASE	1170	LOC105313777	metazoa	Amphimedon queenslandica	PREDICTED: uncharacterized protein LOC105313777 [Amphimedon queenslandica]	GCF_000090795.1	TRADDN-NCOA6
XP_019863337.1	CARD+CARD+DED+TRADDN-NCOA6	470	LOC109592291	metazoa	Amphimedon queenslandica	PREDICTED: uncharacterized protein LOC109592291, partial [Amphimedon queenslandica]	GCF_000090795.1	TRADDN-NCOA6
XP_019850626.1	CARD+CARD+DED+TRADDN-NCOA6+PDZ+KELCH	979	LOC109581195	metazoa	Amphimedon queenslandica	PREDICTED: uncharacterized protein LOC109581195 isoform X1 [Amphimedon queenslandica]	GCF_000090795.1	TRADDN-NCOA6
XP_019850627.1	CARD+CARD+DED+TRADDN-NCOA6+PDZ+KELCH	965	LOC109581195	metazoa	Amphimedon queenslandica	PREDICTED: uncharacterized protein LOC109581195 isoform X2 [Amphimedon queenslandica]	GCF_000090795.1	TRADDN-NCOA6
XP_019850628.1	CARD+CARD+DED+TRADDN-NCOA6+PDZ+KELCH	964	LOC109581195	metazoa	Amphimedon queenslandica	PREDICTED: uncharacterized protein LOC109581195 isoform X3 [Amphimedon queenslandica]	GCF_000090795.1	TRADDN-NCOA6
XP_019850632.1	CARD+CARD+DED+TRADDN-NCOA6+PDZ+KELCH	982	LOC109581197	metazoa	Amphimedon queenslandica	PREDICTED: uncharacterized protein LOC109581197 [Amphimedon queenslandica]	GCF_000090795.1	TRADDN-NCOA6
XP_019863846.1	CARD+Coiled-coil+DED+TRADDN-NCOA6	368	LOC109593016	metazoa	Amphimedon queenslandica	PREDICTED: uncharacterized protein LOC109593016 [Amphimedon queenslandica]	GCF_000090795.1	TRADDN-NCOA6
XP_019860013.1	CARD+Death+Death+TRADDN-NCOA6+Coiled-coil+LRRs	772	LOC109588286	metazoa	Amphimedon queenslandica	PREDICTED: uncharacterized protein LOC109588286 [Amphimedon queenslandica]	GCF_000090795.1	TRADDN-NCOA6
XP_011408984.2	CARD+Death+DED+TRADDN-NCOA6+Coiled-coil+Ub-like+Ub-like+Ub-like+Ub-like	1010	LOC100632060	metazoa	Amphimedon queenslandica	PREDICTED: uncharacterized protein LOC100632060 isoform X2 [Amphimedon queenslandica]	GCF_000090795.1	TRADDN-NCOA6

acc	architecture	len	gen.name	taxend	species	defline	gca	clade
XP_019849457.1	CARD+Death+DED+TRADDN-NCOA6+Coiled-coil+Ub-like+Ub-like+Ub-like+Ub-like	904	LOC100634889	metazoa	Amphimedon queenslandica	PREDICTED: ubiquitin carboxyl-terminal hydrolase 47-like [Amphimedon queenslandica]	GCF_000090795.1	TRADDN-NCOA6
XP_019852598.1	CARD+Death+DED+TRADDN-NCOA6+Coiled-coil+Ub-like+Ub-like+Ub-like+Ub-like	982	LOC100641592	metazoa	Amphimedon queenslandica	PREDICTED: ubiquitin carboxyl-terminal hydrolase 47-like [Amphimedon queenslandica]	GCF_000090795.1	TRADDN-NCOA6
XP_019849060.1	CARD+Death+TRADDN-NCOA6+CASPASE	780	LOC109580415	metazoa	Amphimedon queenslandica	PREDICTED: uncharacterized protein LOC109580415 [Amphimedon queenslandica]	GCF_000090795.1	TRADDN-NCOA6
XP_019849061.1	CARD+Death+TRADDN-NCOA6+CASPASE	776	LOC109580416	metazoa	Amphimedon queenslandica	PREDICTED: uncharacterized protein LOC109580416 isoform X1 [Amphimedon queenslandica]	GCF_000090795.1	TRADDN-NCOA6
XP_019849063.1	CARD+Death+TRADDN-NCOA6+CASPASE	760	LOC109580416	metazoa	Amphimedon queenslandica	PREDICTED: uncharacterized protein LOC109580416 isoform X2 [Amphimedon queenslandica]	GCF_000090795.1	TRADDN-NCOA6
XP_019849064.1	CARD+Death+TRADDN-NCOA6+CASPASE	770	LOC109580417	metazoa	Amphimedon queenslandica	PREDICTED: uncharacterized protein LOC109580417 isoform X1 [Amphimedon queenslandica]	GCF_000090795.1	TRADDN-NCOA6
XP_019849065.1	CARD+Death+TRADDN-NCOA6+CASPASE	764	LOC109580417	metazoa	Amphimedon queenslandica	PREDICTED: uncharacterized protein LOC109580417 isoform X2 [Amphimedon queenslandica]	GCF_000090795.1	TRADDN-NCOA6
XP_019852753.1	CARD+Death+TRADDN-NCOA6+CASPASE	756	LOC109582478	metazoa	Amphimedon queenslandica	PREDICTED: uncharacterized protein LOC109582478 [Amphimedon queenslandica]	GCF_000090795.1	TRADDN-NCOA6
XP_019851638.1	CARD+DED+TRADDN-NCOA6	666	LOC109581721	metazoa	Amphimedon queenslandica	PREDICTED: uncharacterized protein LOC109581721 [Amphimedon queenslandica]	GCF_000090795.1	TRADDN-NCOA6
XP_019852755.1	CARD+DED+TRADDN-NCOA6	390	LOC109582480	metazoa	Amphimedon queenslandica	PREDICTED: uncharacterized protein LOC109582480 [Amphimedon queenslandica]	GCF_000090795.1	TRADDN-NCOA6
XP_019853775.1	CARD+DED+TRADDN-NCOA6	593	LOC109583044	metazoa	Amphimedon queenslandica	PREDICTED: uncharacterized protein LOC109583044 [Amphimedon queenslandica]	GCF_000090795.1	TRADDN-NCOA6
XP_019856613.1	CARD+DED+TRADDN-NCOA6	519	LOC109585106	metazoa	Amphimedon queenslandica	PREDICTED: uncharacterized protein LOC109585106 [Amphimedon queenslandica]	GCF_000090795.1	TRADDN-NCOA6
XP_019857199.1	CARD+DED+TRADDN-NCOA6	709	LOC109585514	metazoa	Amphimedon queenslandica	PREDICTED: pollen-specific leucine-rich repeat extensin-like protein 2 [Amphimedon queenslandica]	GCF_000090795.1	TRADDN-NCOA6
XP_019858130.1	CARD+DED+TRADDN-NCOA6	411	LOC109586384	metazoa	Amphimedon queenslandica	PREDICTED: uncharacterized protein LOC109586384 [Amphimedon queenslandica]	GCF_000090795.1	TRADDN-NCOA6
XP_019858853.1	CARD+DED+TRADDN-NCOA6	471	LOC109587076	metazoa	Amphimedon queenslandica	PREDICTED: uncharacterized protein LOC109587076 [Amphimedon queenslandica]	GCF_000090795.1	TRADDN-NCOA6
XP_019861307.1	CARD+DED+TRADDN-NCOA6	469	LOC109589711	metazoa	Amphimedon queenslandica	PREDICTED: uncharacterized protein LOC109589711 [Amphimedon queenslandica]	GCF_000090795.1	TRADDN-NCOA6
XP_019862829.1	CARD+DED+TRADDN-NCOA6	506	LOC109591555	metazoa	Amphimedon queenslandica	PREDICTED: uncharacterized protein LOC109591555 [Amphimedon queenslandica]	GCF_000090795.1	TRADDN-NCOA6

acc	architecture	len	gen.name	taxend	species	defline	gca	clade
XP_019863176.1	CARD+DED+TRADDN-NCOA6	359	LOC109592045	metazoa	Amphimedon queenslandica	PREDICTED: uncharacterized protein LOC109592045 [Amphimedon queenslandica]	GCF_000090795.1	TRADDN-NCOA6
XP_011406623.2	CARD+DED+TRADDN-NCOA6+2OGFeDO	656	LOC100636424	metazoa	Amphimedon queenslandica	PREDICTED: uncharacterized protein LOC100636424 isoform X1 [Amphimedon queenslandica]	GCF_000090795.1	TRADDN-NCOA6
XP_019857326.1	CARD+DED+TRADDN-NCOA6+2OGFeDO	551	LOC100636424	metazoa	Amphimedon queenslandica	PREDICTED: uncharacterized protein LOC100636424 isoform X2 [Amphimedon queenslandica]	GCF_000090795.1	TRADDN-NCOA6
XP_019848629.1	CARD+DED+TRADDN-NCOA6+Ankyrin	591	LOC100640609	metazoa	Amphimedon queenslandica	PREDICTED: uncharacterized protein LOC100640609 [Amphimedon queenslandica]	GCF_000090795.1	TRADDN-NCOA6
XP_019850303.1	CARD+DED+TRADDN-NCOA6+Ankyrin	1054	LOC109581027	metazoa	Amphimedon queenslandica	PREDICTED: ankyrin repeat domain-containing protein 17-like isoform X1 [Amphimedon queenslandica]	GCF_000090795.1	TRADDN-NCOA6
XP_019850304.1	CARD+DED+TRADDN-NCOA6+Ankyrin	956	LOC109581027	metazoa	Amphimedon queenslandica	PREDICTED: ankyrin repeat domain-containing protein 17-like isoform X2 [Amphimedon queenslandica]	GCF_000090795.1	TRADDN-NCOA6
XP_019850305.1	CARD+DED+TRADDN-NCOA6+Ankyrin	956	LOC109581027	metazoa	Amphimedon queenslandica	PREDICTED: ankyrin repeat domain-containing protein 17-like isoform X2 [Amphimedon queenslandica]	GCF_000090795.1	TRADDN-NCOA6
XP_019852068.1	CARD+DED+TRADDN-NCOA6+Ankyrin	938	LOC109581980	metazoa	Amphimedon queenslandica	PREDICTED: uncharacterized protein LOC109581980 [Amphimedon queenslandica]	GCF_000090795.1	TRADDN-NCOA6
XP_019854819.1	CARD+DED+TRADDN-NCOA6+Ankyrin	377	LOC109583792	metazoa	Amphimedon queenslandica	PREDICTED: uncharacterized protein LOC109583792, partial [Amphimedon queenslandica]	GCF_000090795.1	TRADDN-NCOA6
XP_019858043.1	CARD+DED+TRADDN-NCOA6+Ankyrin	802	LOC100642029	metazoa	Amphimedon queenslandica	PREDICTED: uncharacterized protein LOC100642029 [Amphimedon queenslandica]	GCF_000090795.1	TRADDN-NCOA6
XP_019858457.1	CARD+DED+TRADDN-NCOA6+Ankyrin	642	LOC109586688	metazoa	Amphimedon queenslandica	PREDICTED: uncharacterized protein LOC109586688 [Amphimedon queenslandica]	GCF_000090795.1	TRADDN-NCOA6
XP_019859180.1	CARD+DED+TRADDN-NCOA6+Ankyrin	439	LOC109587382	metazoa	Amphimedon queenslandica	PREDICTED: uncharacterized protein LOC109587382 [Amphimedon queenslandica]	GCF_000090795.1	TRADDN-NCOA6
XP_019859911.1	CARD+DED+TRADDN-NCOA6+Ankyrin	520	LOC109588171	metazoa	Amphimedon queenslandica	PREDICTED: uncharacterized protein LOC109588171 [Amphimedon queenslandica]	GCF_000090795.1	TRADDN-NCOA6
XP_019860724.1	CARD+DED+TRADDN-NCOA6+Ankyrin	546	LOC109589044	metazoa	Amphimedon queenslandica	PREDICTED: uncharacterized protein LOC109589044 [Amphimedon queenslandica]	GCF_000090795.1	TRADDN-NCOA6
XP_019861310.1	CARD+DED+TRADDN-NCOA6+Ankyrin	880	LOC109589714	metazoa	Amphimedon queenslandica	PREDICTED: uncharacterized protein LOC109589714 [Amphimedon queenslandica]	GCF_000090795.1	TRADDN-NCOA6
XP_019861721.1	CARD+DED+TRADDN-NCOA6+Ankyrin	923	LOC109590240	metazoa	Amphimedon queenslandica	PREDICTED: ankyrin repeat and KH domain-containing protein 1-like [Amphimedon queenslandica]	GCF_000090795.1	TRADDN-NCOA6

acc	architecture	len	gen.name	taxend	species	defline	gca	clade
XP_019851555.1	CARD+DED+TRADDN-NCOA6+Ankyrin+YafQ_toxin+SF1-helicase+SF1-helicase	2856	LOC109581679	metazoa	Amphimedon queenslandica	PREDICTED: uncharacterized protein LOC109581679 [Amphimedon queenslandica]	GCF_000090795.1	TRADDN-NCOA6
XP_003385831.2	CARD+DED+TRADDN-NCOA6+Coiled-coil	544	LOC100631905	metazoa	Amphimedon queenslandica	PREDICTED: uncharacterized protein LOC100631905 [Amphimedon queenslandica]	GCF_000090795.1	TRADDN-NCOA6
XP_019856730.1	CARD+DED+TRADDN-NCOA6+Coiled-coil+NACHT+LRRs	1406	LOC109585190	metazoa	Amphimedon queenslandica	PREDICTED: uncharacterized protein LOC109585190 [Amphimedon queenslandica]	GCF_000090795.1	TRADDN-NCOA6
XP_019858706.1	CARD+DED+TRADDN-NCOA6+Coiled-coil+SH3	534	LOC109586929	metazoa	Amphimedon queenslandica	PREDICTED: uncharacterized protein LOC109586929 [Amphimedon queenslandica]	GCF_000090795.1	TRADDN-NCOA6
XP_019861548.1	CARD+DED+TRADDN-NCOA6+Coiled-coil+SH3	503	LOC109590027	metazoa	Amphimedon queenslandica	PREDICTED: uncharacterized protein LOC109590027 [Amphimedon queenslandica]	GCF_000090795.1	TRADDN-NCOA6
XP_019858709.1	CARD+DED+TRADDN-NCOA6+Coiled-coil+SH3+SH2+SH3	897	LOC109586932	metazoa	Amphimedon queenslandica	PREDICTED: uncharacterized protein LOC109586932 [Amphimedon queenslandica]	GCF_000090795.1	TRADDN-NCOA6
XP_019857393.1	CARD+DED+TRADDN-NCOA6+Death+DED+DED	748	LOC109585709	metazoa	Amphimedon queenslandica	PREDICTED: uncharacterized protein LOC109585709 [Amphimedon queenslandica]	GCF_000090795.1	TRADDN-NCOA6
XP_019864256.1	CARD+DED+TRADDN-NCOA6+SH2+P85+SH2	998	LOC100639472	metazoa	Amphimedon queenslandica	PREDICTED: uncharacterized protein LOC100639472 [Amphimedon queenslandica]	GCF_000090795.1	TRADDN-NCOA6
XP_023931052.1	CARD+TRADDN-DEDD2	369	LOC112041699	metazoa	Lingula anatina	uncharacterized protein LOC112041699 [Lingula anatina]	GCF_001039355.2	TRADDN-DEDD2
XP_023931977.1	CARD+TRADDN-DEDD2	404	LOC106162832	metazoa	Lingula anatina	uncharacterized protein LOC106162832 isoform X1 [Lingula anatina]	GCF_001039355.2	TRADDN-DEDD2
XP_023931978.1	CARD+TRADDN-DEDD2	369	LOC106162832	metazoa	Lingula anatina	uncharacterized protein LOC106162832 isoform X2 [Lingula anatina]	GCF_001039355.2	TRADDN-DEDD2
XP_013396435.2	CARD+TRADDN-DEDD2+CARD	626	LOC106163407	metazoa	Lingula anatina	uncharacterized protein LOC106163407 [Lingula anatina]	GCF_001039355.2	TRADDN-DEDD2
XP_013409313.1	CARD+TRADDN-DEDD2+Coiled-coil+vWA	858	LOC106172928	metazoa	Lingula anatina	uncharacterized protein LOC106172928 [Lingula anatina]	GCF_001039355.2	TRADDN-DEDD2
XP_023933343.1	CARD+TRADDN-DEDD2+Coiled-coil+vWA	834	LOC106161705	metazoa	Lingula anatina	uncharacterized protein LOC106161705 [Lingula anatina]	GCF_001039355.2	TRADDN-DEDD2
XP_013388646.1	CARD+TRADDN-DEDD2+Coiled-coil+vWA+MIB_HERC2+MIB_HERC2+WWE	1221	LOC106157515	metazoa	Lingula anatina	uncharacterized protein LOC106157515 [Lingula anatina]	GCF_001039355.2	TRADDN-DEDD2
XP_013417662.1	CARD+TRADDN-DEDD2+Coiled-coil+vWA+MIB_HERC2+MIB_HERC2+WWE	1192	LOC106178854	metazoa	Lingula anatina	uncharacterized protein LOC106178854 [Lingula anatina]	GCF_001039355.2	TRADDN-DEDD2
XP_013381666.1	CARD+TRADDN-DEDD2+Coiled-coil+vWA+SH3+SH3	1290	LOC106152581	metazoa	Lingula anatina	uncharacterized protein LOC106152581 [Lingula anatina]	GCF_001039355.2	TRADDN-DEDD2
XP_003389740.1	CARD+TRADDN-NCOA6+TM+TM	586	LOC100632970	metazoa	Amphimedon queenslandica	PREDICTED: uncharacterized protein LOC100632970 [Amphimedon queenslandica]	GCF_000090795.1	TRADDN-NCOA6
XP_013416937.1	Coiled-coil+CARD+TRADDN-DEDD2+Coiled-coil+vWA	1450	LOC106178346	metazoa	Lingula anatina	uncharacterized protein LOC106178346 [Lingula anatina]	GCF_001039355.2	TRADDN-DEDD2
XP_013421821.1	Coiled-coil+CARD+TRADDN-DEDD2+vWA+Coiled-coil+MIB_HERC2+MIB_HERC2+WWE	1328	LOC106181853	metazoa	Lingula anatina	uncharacterized protein LOC106181853 [Lingula anatina]	GCF_001039355.2	TRADDN-DEDD2

acc	architecture	len	gen.name	taxend	species	defline	gca	clade
XP_013421834.1	Coiled-coil+CARD+TRADDN-DEDD2+vWA+Coiled-coil+MIB_HERC2+MIB_HERC2+WWE	1356	LOC106181859	metazoa	Lingula anatina	uncharacterized protein LOC106181859 [Lingula anatina]	GCF_001039355.2	TRADDN-DEDD2
XP_023931976.1	Coiled-coil+FIIND+CARD+TRADDN-DEDD2+Coiled-coil+vWA	855	LOC106162821	metazoa	Lingula anatina	uncharacterized protein LOC106162821 [Lingula anatina]	GCF_001039355.2	TRADDN-DEDD2
XP_013399402.1	Coiled-coil+TRADDN-DEDD2+SF2-helicase+HA2	1814	LOC106165647	metazoa	Lingula anatina	uncharacterized protein LOC106165647 [Lingula anatina]	GCF_001039355.2	TRADDN-DEDD2
XP_019854810.1	Death+CARD+Death+Death+TRADDN-NCOA6	574	LOC105313548	metazoa	Amphimedon queenslandica	PREDICTED: uncharacterized protein LOC105313548 [Amphimedon queenslandica]	GCF_000090795.1	TRADDN-NCOA6
XP_019854811.1	Death+CARD+Death+Death+TRADDN-NCOA6	574	LOC105313548	metazoa	Amphimedon queenslandica	PREDICTED: uncharacterized protein LOC105313548 [Amphimedon queenslandica]	GCF_000090795.1	TRADDN-NCOA6
XP_019855266.1	Death+CARD+Death+Death+TRADDN-NCOA6	633	LOC109584104	metazoa	Amphimedon queenslandica	PREDICTED: uncharacterized protein LOC109584104 [Amphimedon queenslandica]	GCF_000090795.1	TRADDN-NCOA6
XP_019857909.1	Death+CARD+Death+Death+TRADDN-NCOA6+Coiled-coil	743	LOC109586179	metazoa	Amphimedon queenslandica	PREDICTED: uncharacterized protein LOC109586179 isoform X2 [Amphimedon queenslandica]	GCF_000090795.1	TRADDN-NCOA6
XP_019860012.1	Death+CARD+Death+Death+TRADDN-NCOA6+Coiled-coil	736	LOC109588285	metazoa	Amphimedon queenslandica	PREDICTED: uncharacterized protein LOC109588285, partial [Amphimedon queenslandica]	GCF_000090795.1	TRADDN-NCOA6
XP_011405212.2	Death+CARD+Death+Death+TRADDN-NCOA6+Coiled-coil+LRRs	960	LOC105313460	metazoa	Amphimedon queenslandica	PREDICTED: putative leucine-rich repeat-containing protein DDB_G0290503 [Amphimedon queenslandica]	GCF_000090795.1	TRADDN-NCOA6
XP_011407704.2	Death+CARD+Death+Death+TRADDN-NCOA6+Coiled-coil+LRRs	968	LOC105314951	metazoa	Amphimedon queenslandica	PREDICTED: uncharacterized protein LOC105314951 [Amphimedon queenslandica]	GCF_000090795.1	TRADDN-NCOA6
XP_019852468.1	Death+CARD+Death+Death+TRADDN-NCOA6+Coiled-coil+LRRs	902	LOC109582257	metazoa	Amphimedon queenslandica	PREDICTED: uncharacterized protein LOC109582257 [Amphimedon queenslandica]	GCF_000090795.1	TRADDN-NCOA6
XP_019854455.1	Death+CARD+Death+Death+TRADDN-NCOA6+Coiled-coil+LRRs	915	LOC109583508	metazoa	Amphimedon queenslandica	PREDICTED: uncharacterized protein LOC109583508 [Amphimedon queenslandica]	GCF_000090795.1	TRADDN-NCOA6
XP_019854600.1	Death+CARD+Death+Death+TRADDN-NCOA6+Coiled-coil+LRRs	915	LOC109583619	metazoa	Amphimedon queenslandica	PREDICTED: uncharacterized protein LOC109583619 [Amphimedon queenslandica]	GCF_000090795.1	TRADDN-NCOA6
XP_019856609.1	Death+CARD+Death+Death+TRADDN-NCOA6+Coiled-coil+LRRs	956	LOC100638228	metazoa	Amphimedon queenslandica	PREDICTED: uncharacterized protein LOC100638228 isoform X1 [Amphimedon queenslandica]	GCF_000090795.1	TRADDN-NCOA6
XP_019856610.1	Death+CARD+Death+Death+TRADDN-NCOA6+Coiled-coil+LRRs	952	LOC100638228	metazoa	Amphimedon queenslandica	PREDICTED: uncharacterized protein LOC100638228 isoform X2 [Amphimedon queenslandica]	GCF_000090795.1	TRADDN-NCOA6
XP_019856617.1	Death+CARD+Death+Death+TRADDN-NCOA6+Coiled-coil+LRRs	890	LOC109585109	metazoa	Amphimedon queenslandica	PREDICTED: uncharacterized protein LOC109585109 [Amphimedon queenslandica]	GCF_000090795.1	TRADDN-NCOA6
XP_019857908.1	Death+CARD+Death+Death+TRADDN-NCOA6+Coiled-coil+LRRs	944	LOC109586179	metazoa	Amphimedon queenslandica	PREDICTED: uncharacterized protein LOC109586179 isoform X1 [Amphimedon queenslandica]	GCF_000090795.1	TRADDN-NCOA6
XP_019860268.1	Death+CARD+Death+Death+TRADDN-NCOA6+Coiled-coil+LRRs	948	LOC109588559	metazoa	Amphimedon queenslandica	PREDICTED: uncharacterized protein LOC109588559 isoform X1 [Amphimedon queenslandica]	GCF_000090795.1	TRADDN-NCOA6

acc	architecture	len	gen.name	taxend	species	defline	gca	clade
XP_019860273.1	Death+CARD+Death+Death+TRADDN-NCOA6+Coiled-coil+LRRs	947	LOC109588559	metazoa	Amphimedon queenslandica	PREDICTED: uncharacterized protein LOC109588559 isoform X2 [Amphimedon queenslandica]	GCF_000090795.1	TRADDN-NCOA6
XP_019860406.1	Death+CARD+Death+Death+TRADDN-NCOA6+Coiled-coil+LRRs	923	LOC109588730	metazoa	Amphimedon queenslandica	PREDICTED: uncharacterized protein LOC109588730 [Amphimedon queenslandica]	GCF_000090795.1	TRADDN-NCOA6
XP_019854580.1	Death+CARD+Death+TRADDN-NCOA6+Coiled-coil+Ub-like+Ub-like+Ub-like	1165	LOC100636162	metazoa	Amphimedon queenslandica	PREDICTED: uncharacterized protein LOC100636162 [Amphimedon queenslandica]	GCF_000090795.1	TRADDN-NCOA6
XP_019850797.1	Death+CARD+Death+TRADDN-NCOA6+Coiled-coil+Ub-like+Ub-like+Ub-like+Ub-like	1219	LOC100632060	metazoa	Amphimedon queenslandica	PREDICTED: uncharacterized protein LOC100632060 isoform X1 [Amphimedon queenslandica]	GCF_000090795.1	TRADDN-NCOA6
XP_019858357.1	Death+CARD+DED+Ankyrin+TRADDN-NCOA6+Ankyrin	1827	LOC100637643	metazoa	Amphimedon queenslandica	PREDICTED: uncharacterized protein LOC100637643 [Amphimedon queenslandica]	GCF_000090795.1	TRADDN-NCOA6
XP_019851761.1	Death+CARD+DED+TRADDN-NCOA6	566	LOC109581801	metazoa	Amphimedon queenslandica	PREDICTED: uncharacterized protein LOC109581801 [Amphimedon queenslandica]	GCF_000090795.1	TRADDN-NCOA6
XP_019853174.1	Death+CARD+DED+TRADDN-NCOA6	487	LOC109582722	metazoa	Amphimedon queenslandica	PREDICTED: uncharacterized protein LOC109582722 [Amphimedon queenslandica]	GCF_000090795.1	TRADDN-NCOA6
XP_019853511.1	Death+CARD+DED+TRADDN-NCOA6	511	LOC105313190	metazoa	Amphimedon queenslandica	PREDICTED: uncharacterized protein LOC105313190 [Amphimedon queenslandica]	GCF_000090795.1	TRADDN-NCOA6
XP_019858280.1	Death+CARD+DED+TRADDN-NCOA6	602	LOC109586524	metazoa	Amphimedon queenslandica	PREDICTED: uncharacterized protein LOC109586524 [Amphimedon queenslandica]	GCF_000090795.1	TRADDN-NCOA6
XP_019860339.1	Death+CARD+DED+TRADDN-NCOA6	525	LOC109588638	metazoa	Amphimedon queenslandica	PREDICTED: uncharacterized protein LOC109588638 [Amphimedon queenslandica]	GCF_000090795.1	TRADDN-NCOA6
XP_019861958.1	Death+CARD+DED+TRADDN-NCOA6	423	LOC109590492	metazoa	Amphimedon queenslandica	PREDICTED: uncharacterized protein LOC109590492 [Amphimedon queenslandica]	GCF_000090795.1	TRADDN-NCOA6
XP_019852795.1	Death+CARD+DED+TRADDN-NCOA6+Ankyrin	1205	LOC109582504	metazoa	Amphimedon queenslandica	PREDICTED: uncharacterized protein LOC109582504 [Amphimedon queenslandica]	GCF_000090795.1	TRADDN-NCOA6
XP_019862457.1	Death+CARD+DED+TRADDN-NCOA6+Ankyrin	760	LOC109591094	metazoa	Amphimedon queenslandica	PREDICTED: uncharacterized protein LOC109591094 [Amphimedon queenslandica]	GCF_000090795.1	TRADDN-NCOA6
XP_019849880.1	Death+CARD+DED+TRADDN-NCOA6+Ankyrin+TRADDN-NCOA6+Ankyrin	1433	LOC109580779	metazoa	Amphimedon queenslandica	PREDICTED: uncharacterized protein LOC109580779 [Amphimedon queenslandica]	GCF_000090795.1	TRADDN-NCOA6
XP_019852232.1	Death+CARD+DED+TRADDN-NCOA6+Ankyrin+TRADDN-NCOA6+Ankyrin	1642	LOC109582081	metazoa	Amphimedon queenslandica	PREDICTED: uncharacterized protein LOC109582081 [Amphimedon queenslandica]	GCF_000090795.1	TRADDN-NCOA6
XP_019852233.1	Death+CARD+DED+TRADDN-NCOA6+Ankyrin+TRADDN-NCOA6+Ankyrin	1642	LOC109582081	metazoa	Amphimedon queenslandica	PREDICTED: uncharacterized protein LOC109582081 [Amphimedon queenslandica]	GCF_000090795.1	TRADDN-NCOA6
XP_019852234.1	Death+CARD+DED+TRADDN-NCOA6+Ankyrin+TRADDN-NCOA6+Ankyrin	1642	LOC109582081	metazoa	Amphimedon queenslandica	PREDICTED: uncharacterized protein LOC109582081 [Amphimedon queenslandica]	GCF_000090795.1	TRADDN-NCOA6

acc	architecture	len	gen.name	taxend	species	defline	gca	clade
XP_019852235.1	Death+CARD+DED+TRADDN-NCOA6+Ankyrin+TRADDN-NCOA6+Ankyrin	1642	LOC109582081	metazoa	Amphimedon queenslandica	PREDICTED: uncharacterized protein LOC109582081 [Amphimedon queenslandica]	GCF_000090795.1	TRADDN-NCOA6
XP_019852236.1	Death+CARD+DED+TRADDN-NCOA6+Ankyrin+TRADDN-NCOA6+Ankyrin	1642	LOC109582081	metazoa	Amphimedon queenslandica	PREDICTED: uncharacterized protein LOC109582081 [Amphimedon queenslandica]	GCF_000090795.1	TRADDN-NCOA6
XP_019852237.1	Death+CARD+DED+TRADDN-NCOA6+Ankyrin+TRADDN-NCOA6+Ankyrin	1642	LOC109582081	metazoa	Amphimedon queenslandica	PREDICTED: uncharacterized protein LOC109582081 [Amphimedon queenslandica]	GCF_000090795.1	TRADDN-NCOA6
XP_019857877.1	Death+CARD+DED+TRADDN-NCOA6+Ankyrin+TRADDN-NCOA6+Ankyrin	1053	LOC109586143	metazoa	Amphimedon queenslandica	PREDICTED: uncharacterized protein LOC109586143 [Amphimedon queenslandica]	GCF_000090795.1	TRADDN-NCOA6
XP_019858348.1	Death+CARD+DED+TRADDN-NCOA6+Ankyrin+TRADDN-NCOA6+Ankyrin	1255	LOC109586591	metazoa	Amphimedon queenslandica	PREDICTED: uncharacterized protein LOC109586591 [Amphimedon queenslandica]	GCF_000090795.1	TRADDN-NCOA6
XP_019855314.1	Death+CARD+DED+TRADDN-NCOA6+Ankyrin+YafQ_toxin+SF1-helicase+TPRs	2897	LOC100632547	metazoa	Amphimedon queenslandica	PREDICTED: LOW QUALITY PROTEIN: uncharacterized protein LOC100632547 [Amphimedon queenslandica]	GCF_000090795.1	TRADDN-NCOA6
XP_019857167.1	Death+CARD+DED+TRADDN-NCOA6+Coiled-coil	775	LOC109585498	metazoa	Amphimedon queenslandica	PREDICTED: uncharacterized protein MAL8P1.12-like isoform X1 [Amphimedon queenslandica]	GCF_000090795.1	TRADDN-NCOA6
XP_019857168.1	Death+CARD+DED+TRADDN-NCOA6+Coiled-coil	759	LOC109585498	metazoa	Amphimedon queenslandica	PREDICTED: uncharacterized protein MAL8P1.12-like isoform X2 [Amphimedon queenslandica]	GCF_000090795.1	TRADDN-NCOA6
XP_019859490.1	Death+CARD+DED+TRADDN-NCOA6+Coiled-coil	552	LOC109587707	metazoa	Amphimedon queenslandica	PREDICTED: uncharacterized protein LOC109587707 [Amphimedon queenslandica]	GCF_000090795.1	TRADDN-NCOA6
XP_019863334.1	Death+CARD+DED+TRADDN-NCOA6+Coiled-coil	754	LOC105316517	metazoa	Amphimedon queenslandica	PREDICTED: titin-like [Amphimedon queenslandica]	GCF_000090795.1	TRADDN-NCOA6
XP_019857773.1	Death+CARD+DED+TRADDN-NCOA6+Coiled-coil+Death	739	LOC109586046	metazoa	Amphimedon queenslandica	PREDICTED: uncharacterized protein LOC109586046 [Amphimedon queenslandica]	GCF_000090795.1	TRADDN-NCOA6
XP_011405220.2	Death+CARD+DED+TRADDN-NCOA6+Coiled-coil+Pkinase	905	LOC100638861	metazoa	Amphimedon queenslandica	PREDICTED: centromere-associated protein E-like [Amphimedon queenslandica]	GCF_000090795.1	TRADDN-NCOA6
XP_019856701.1	Death+CARD+DED+TRADDN-NCOA6+Coiled-coil+Pkinase	971	LOC109585159	metazoa	Amphimedon queenslandica	PREDICTED: putative leucine-rich repeat-containing protein DDB_G0290503 isoform X1 [Amphimedon queenslandica]	GCF_000090795.1	TRADDN-NCOA6
XP_019856702.1	Death+CARD+DED+TRADDN-NCOA6+Coiled-coil+Pkinase	970	LOC109585159	metazoa	Amphimedon queenslandica	PREDICTED: putative leucine-rich repeat-containing protein DDB_G0290503 isoform X2 [Amphimedon queenslandica]	GCF_000090795.1	TRADDN-NCOA6
XP_019854978.1	Death+CARD+DED+TRADDN-NCOA6+Coiled-coil+SH2	688	LOC109583908	metazoa	Amphimedon queenslandica	PREDICTED: uncharacterized protein LOC109583908 [Amphimedon queenslandica]	GCF_000090795.1	TRADDN-NCOA6
XP_019856303.1	Death+CARD+DED+TRADDN-NCOA6+Coiled-coil+SH3+SH3	665	LOC100637348	metazoa	Amphimedon queenslandica	PREDICTED: uncharacterized protein LOC100637348 [Amphimedon queenslandica]	GCF_000090795.1	TRADDN-NCOA6

acc	architecture	len	gen.name	taxend	species	defline	gca	clade
XP_019856458.1	Death+CARD+DED+TRADDN-NCOA6+Coiled-coil+SH3+SH3	774	LOC109584988	metazoa	Amphimedon queenslandica	PREDICTED: uncharacterized protein LOC109584988 [Amphimedon queenslandica]	GCF_000090795.1	TRADDN-NCOA6
XP_019858030.1	Death+CARD+DED+TRADDN-NCOA6+Death+CARD	721	LOC109586293	metazoa	Amphimedon queenslandica	PREDICTED: uncharacterized protein LOC109586293 isoform X1 [Amphimedon queenslandica]	GCF_000090795.1	TRADDN-NCOA6
XP_019858031.1	Death+CARD+DED+TRADDN-NCOA6+Death+CARD	719	LOC109586293	metazoa	Amphimedon queenslandica	PREDICTED: uncharacterized protein LOC109586293 isoform X2 [Amphimedon queenslandica]	GCF_000090795.1	TRADDN-NCOA6
XP_019849161.1	Death+CARD+DED+TRADDN-NCOA6+LRRs	723	LOC109580450	metazoa	Amphimedon queenslandica	PREDICTED: uncharacterized protein LOC109580450 [Amphimedon queenslandica]	GCF_000090795.1	TRADDN-NCOA6
XP_019855983.1	Death+CARD+DED+TRADDN-NCOA6+LRRs	908	LOC109584611	metazoa	Amphimedon queenslandica	PREDICTED: uncharacterized protein LOC109584611 [Amphimedon queenslandica]	GCF_000090795.1	TRADDN-NCOA6
XP_019852029.1	Death+CARD+DED+TRADDN-NCOA6+Ras	753	LOC109581957	metazoa	Amphimedon queenslandica	PREDICTED: uncharacterized protein LOC109581957 [Amphimedon queenslandica]	GCF_000090795.1	TRADDN-NCOA6
XP_019854903.1	Death+CARD+DED+TRADDN-NCOA6+TM	624	LOC109583850	metazoa	Amphimedon queenslandica	PREDICTED: uncharacterized protein LOC109583850 [Amphimedon queenslandica]	GCF_000090795.1	TRADDN-NCOA6
XP_019851728.1	Death+CARD+DED+TRADDN-NCOA6+TM+CASPASE	920	LOC109581770	metazoa	Amphimedon queenslandica	PREDICTED: uncharacterized protein LOC109581770 isoform X1 [Amphimedon queenslandica]	GCF_000090795.1	TRADDN-NCOA6
XP_019851729.1	Death+CARD+DED+TRADDN-NCOA6+TM+CASPASE	914	LOC109581770	metazoa	Amphimedon queenslandica	PREDICTED: uncharacterized protein LOC109581770 isoform X2 [Amphimedon queenslandica]	GCF_000090795.1	TRADDN-NCOA6
XP_019860510.1	Death+CARD+DED+TRADDN-NCOA6+TM+CASPASE	964	LOC109588840	metazoa	Amphimedon queenslandica	PREDICTED: uncharacterized protein LOC109588840 [Amphimedon queenslandica]	GCF_000090795.1	TRADDN-NCOA6
XP_019857226.1	Death+CARD+TRADDN-NCOA6+Coiled-coil+LRRs	835	LOC109585544	metazoa	Amphimedon queenslandica	PREDICTED: uncharacterized protein LOC109585544 [Amphimedon queenslandica]	GCF_000090795.1	TRADDN-NCOA6
XP_019858256.1	Death+CARD+TRADDN-NCOA6+SH3+LRRs	728	LOC109586504	metazoa	Amphimedon queenslandica	PREDICTED: uncharacterized protein LOC109586504 [Amphimedon queenslandica]	GCF_000090795.1	TRADDN-NCOA6
XP_003390803.2	Death+Death+Death+TRADDN-NCOA6	448	LOC100632849	metazoa	Amphimedon queenslandica	PREDICTED: uncharacterized protein LOC100632849 [Amphimedon queenslandica]	GCF_000090795.1	TRADDN-NCOA6
XP_019853778.1	Death+Death+Death+TRADDN-NCOA6	503	LOC109583047	metazoa	Amphimedon queenslandica	PREDICTED: uncharacterized protein LOC109583047 [Amphimedon queenslandica]	GCF_000090795.1	TRADDN-NCOA6
XP_019857771.1	Death+Death+Death+TRADDN-NCOA6	331	LOC109586044	metazoa	Amphimedon queenslandica	PREDICTED: uncharacterized protein LOC109586044 [Amphimedon queenslandica]	GCF_000090795.1	TRADDN-NCOA6
XP_019863886.1	Death+Death+Death+TRADDN-NCOA6	362	LOC109593080	metazoa	Amphimedon queenslandica	PREDICTED: uncharacterized protein LOC109593080 [Amphimedon queenslandica]	GCF_000090795.1	TRADDN-NCOA6
XP_019852724.1	Death+Death+Death+TRADDN-NCOA6+Coiled-coil	579	LOC109582449	metazoa	Amphimedon queenslandica	PREDICTED: uncharacterized protein LOC109582449 [Amphimedon queenslandica]	GCF_000090795.1	TRADDN-NCOA6

acc	architecture	len	gen.name	taxend	species	defline	gca	clade
XP_019858064.1	Death+Death+Death+TRADDN-NCOA6+Coiled-coil	752	LOC109586326	metazoa	Amphimedon queenslandica	PREDICTED: uncharacterized protein LOC109586326, partial [Amphimedon queenslandica]	GCF_000090795.1	TRADDN-NCOA6
XP_019858931.1	Death+Death+Death+TRADDN-NCOA6+Coiled-coil	626	LOC109587132	metazoa	Amphimedon queenslandica	PREDICTED: uncharacterized protein LOC109587132 [Amphimedon queenslandica]	GCF_000090795.1	TRADDN-NCOA6
XP_019858934.1	Death+Death+Death+TRADDN-NCOA6+Coiled-coil	655	LOC109587134	metazoa	Amphimedon queenslandica	PREDICTED: uncharacterized protein LOC109587134 [Amphimedon queenslandica]	GCF_000090795.1	TRADDN-NCOA6
XP_019860235.1	Death+Death+Death+TRADDN-NCOA6+Coiled-coil	542	LOC109588519	metazoa	Amphimedon queenslandica	PREDICTED: uncharacterized protein LOC109588519 [Amphimedon queenslandica]	GCF_000090795.1	TRADDN-NCOA6
XP_019861046.1	Death+Death+Death+TRADDN-NCOA6+Coiled-coil	699	LOC109589397	metazoa	Amphimedon queenslandica	PREDICTED: flagellar attachment zone protein 1-like [Amphimedon queenslandica]	GCF_000090795.1	TRADDN-NCOA6
XP_019862001.1	Death+Death+Death+TRADDN-NCOA6+Coiled-coil	565	LOC109590542	metazoa	Amphimedon queenslandica	PREDICTED: uncharacterized protein LOC109590542, partial [Amphimedon queenslandica]	GCF_000090795.1	TRADDN-NCOA6
XP_011407286.2	Death+Death+Death+TRADDN-NCOA6+Coiled-coil+HD	1081	LOC105314677	metazoa	Amphimedon queenslandica	PREDICTED: uncharacterized protein LOC105314677 [Amphimedon queenslandica]	GCF_000090795.1	TRADDN-NCOA6
XP_019855145.1	Death+Death+Death+TRADDN-NCOA6+Coiled-coil+HD	958	LOC109584024	metazoa	Amphimedon queenslandica	PREDICTED: uncharacterized protein LOC109584024 [Amphimedon queenslandica]	GCF_000090795.1	TRADDN-NCOA6
XP_019858068.1	Death+Death+Death+TRADDN-NCOA6+Coiled-coil+HD	1139	LOC109586329	metazoa	Amphimedon queenslandica	PREDICTED: uncharacterized protein LOC109586329 isoform X1 [Amphimedon queenslandica]	GCF_000090795.1	TRADDN-NCOA6
XP_019858069.1	Death+Death+Death+TRADDN-NCOA6+Coiled-coil+HD	1125	LOC109586329	metazoa	Amphimedon queenslandica	PREDICTED: uncharacterized protein LOC109586329 isoform X2 [Amphimedon queenslandica]	GCF_000090795.1	TRADDN-NCOA6
XP_019858070.1	Death+Death+Death+TRADDN-NCOA6+Coiled-coil+HD	1109	LOC109586329	metazoa	Amphimedon queenslandica	PREDICTED: uncharacterized protein LOC109586329 isoform X3 [Amphimedon queenslandica]	GCF_000090795.1	TRADDN-NCOA6
XP_019858071.1	Death+Death+Death+TRADDN-NCOA6+Coiled-coil+SH3	596	LOC100632133	metazoa	Amphimedon queenslandica	PREDICTED: uncharacterized protein LOC100632133 isoform X1 [Amphimedon queenslandica]	GCF_000090795.1	TRADDN-NCOA6
XP_019858072.1	Death+Death+Death+TRADDN-NCOA6+Coiled-coil+SH3	593	LOC100632133	metazoa	Amphimedon queenslandica	PREDICTED: uncharacterized protein LOC100632133 isoform X2 [Amphimedon queenslandica]	GCF_000090795.1	TRADDN-NCOA6
XP_019858830.1	Death+Death+Death+TRADDN-NCOA6+Coiled-coil+SH3	692	LOC109587056	metazoa	Amphimedon queenslandica	PREDICTED: uncharacterized protein LOC109587056, partial [Amphimedon queenslandica]	GCF_000090795.1	TRADDN-NCOA6
XP_019852151.1	Death+Death+Death+TRADDN-NCOA6+Coiled-coil+SH3+LRRs	929	LOC109582031	metazoa	Amphimedon queenslandica	PREDICTED: uncharacterized protein LOC109582031 isoform X1 [Amphimedon queenslandica]	GCF_000090795.1	TRADDN-NCOA6
XP_019852152.1	Death+Death+Death+TRADDN-NCOA6+Coiled-coil+SH3+LRRs	928	LOC109582031	metazoa	Amphimedon queenslandica	PREDICTED: uncharacterized protein LOC109582031 isoform X2 [Amphimedon queenslandica]	GCF_000090795.1	TRADDN-NCOA6
XP_019852153.1	Death+Death+Death+TRADDN-NCOA6+Coiled-coil+SH3+LRRs	928	LOC109582031	metazoa	Amphimedon queenslandica	PREDICTED: uncharacterized protein LOC109582031 isoform X3 [Amphimedon queenslandica]	GCF_000090795.1	TRADDN-NCOA6

acc	architecture	len	gen.name	taxend	species	defline	gca	clade
XP_019852564.1	Death+Death+Death+TRADDN-NCOA6+Coiled-coil+SH3+LRRs	937	LOC109582341	metazoa	Amphimedon queenslandica	PREDICTED: uncharacterized protein LOC109582341 isoform X1 [Amphimedon queenslandica]	GCF_000090795.1	TRADDN-NCOA6
XP_019852565.1	Death+Death+Death+TRADDN-NCOA6+Coiled-coil+SH3+LRRs	935	LOC109582341	metazoa	Amphimedon queenslandica	PREDICTED: uncharacterized protein LOC109582341 isoform X2 [Amphimedon queenslandica]	GCF_000090795.1	TRADDN-NCOA6
XP_019853828.1	Death+Death+Death+TRADDN-NCOA6+Coiled-coil+SH3+LRRs	928	LOC109583095	metazoa	Amphimedon queenslandica	PREDICTED: uncharacterized protein LOC109583095 [Amphimedon queenslandica]	GCF_000090795.1	TRADDN-NCOA6
XP_019857076.1	Death+Death+Death+TRADDN-NCOA6+Coiled-coil+SH3+LRRs	922	LOC105314204	metazoa	Amphimedon queenslandica	PREDICTED: uncharacterized protein LOC105314204 [Amphimedon queenslandica]	GCF_000090795.1	TRADDN-NCOA6
XP_019857726.1	Death+Death+Death+TRADDN-NCOA6+Coiled-coil+SH3+LRRs	900	LOC109586006	metazoa	Amphimedon queenslandica	PREDICTED: uncharacterized protein LOC109586006 isoform X1 [Amphimedon queenslandica]	GCF_000090795.1	TRADDN-NCOA6
XP_019857727.1	Death+Death+Death+TRADDN-NCOA6+Coiled-coil+SH3+LRRs	896	LOC109586006	metazoa	Amphimedon queenslandica	PREDICTED: uncharacterized protein LOC109586006 isoform X2 [Amphimedon queenslandica]	GCF_000090795.1	TRADDN-NCOA6
XP_019857728.1	Death+Death+Death+TRADDN-NCOA6+Coiled-coil+SH3+LRRs	893	LOC109586006	metazoa	Amphimedon queenslandica	PREDICTED: uncharacterized protein LOC109586006 isoform X3 [Amphimedon queenslandica]	GCF_000090795.1	TRADDN-NCOA6
XP_019857729.1	Death+Death+Death+TRADDN-NCOA6+Coiled-coil+SH3+LRRs	858	LOC109586006	metazoa	Amphimedon queenslandica	PREDICTED: uncharacterized protein LOC109586006 isoform X4 [Amphimedon queenslandica]	GCF_000090795.1	TRADDN-NCOA6
XP_019858258.1	Death+Death+Death+TRADDN-NCOA6+Coiled-coil+SH3+LRRs	928	LOC109586505	metazoa	Amphimedon queenslandica	PREDICTED: uncharacterized protein LOC109586505 isoform X1 [Amphimedon queenslandica]	GCF_000090795.1	TRADDN-NCOA6
XP_019858259.1	Death+Death+Death+TRADDN-NCOA6+Coiled-coil+SH3+LRRs	928	LOC109586505	metazoa	Amphimedon queenslandica	PREDICTED: uncharacterized protein LOC109586505 isoform X2 [Amphimedon queenslandica]	GCF_000090795.1	TRADDN-NCOA6
XP_019858260.1	Death+Death+Death+TRADDN-NCOA6+Coiled-coil+SH3+LRRs	928	LOC109586505	metazoa	Amphimedon queenslandica	PREDICTED: uncharacterized protein LOC109586505 isoform X3 [Amphimedon queenslandica]	GCF_000090795.1	TRADDN-NCOA6
XP_019858609.1	Death+Death+Death+TRADDN-NCOA6+Coiled-coil+SH3+LRRs	846	LOC109586830	metazoa	Amphimedon queenslandica	PREDICTED: uncharacterized protein LOC109586830 [Amphimedon queenslandica]	GCF_000090795.1	TRADDN-NCOA6
XP_019858845.1	Death+Death+Death+TRADDN-NCOA6+Coiled-coil+SH3+LRRs	919	LOC109587067	metazoa	Amphimedon queenslandica	PREDICTED: uncharacterized protein LOC109587067 [Amphimedon queenslandica]	GCF_000090795.1	TRADDN-NCOA6
XP_019860967.1	Death+Death+Death+TRADDN-NCOA6+Coiled-coil+SH3+LRRs	822	LOC100638444	metazoa	Amphimedon queenslandica	PREDICTED: uncharacterized protein LOC100638444 [Amphimedon queenslandica]	GCF_000090795.1	TRADDN-NCOA6
XP_019857549.1	Death+Death+Death+TRADDN-NCOA6+Coiled-coil+TM+TM	841	LOC109585848	metazoa	Amphimedon queenslandica	PREDICTED: uncharacterized protein LOC109585848 [Amphimedon queenslandica]	GCF_000090795.1	TRADDN-NCOA6
XP_019857556.1	Death+Death+Death+TRADDN-NCOA6+Coiled-coil+TM+TM	829	LOC109585852	metazoa	Amphimedon queenslandica	PREDICTED: uncharacterized protein LOC109585852 [Amphimedon queenslandica]	GCF_000090795.1	TRADDN-NCOA6
XP_011404451.1	Death+Death+DED+TRADDN-NCOA6+Coiled-coil+SH3+SH3+SH2+Pkinase	1170	LOC105313037	metazoa	Amphimedon queenslandica	PREDICTED: uncharacterized protein LOC105313037 [Amphimedon queenslandica]	GCF_000090795.1	TRADDN-NCOA6

acc	architecture	len	gen.name	taxend	species	defline	gca	clade
XP_019856453.1	Death+Death+DED+TRADDN-NCOA6+Coiled-coil+SH3+SH3+SH2+Pkinase	1199	LOC100638653	metazoa	Amphimedon queenslandica	PREDICTED: uncharacterized protein LOC100638653 isoform X1 [Amphimedon queenslandica]	GCF_000090795.1	TRADDN-NCOA6
XP_019856454.1	Death+Death+DED+TRADDN-NCOA6+Coiled-coil+SH3+SH3+SH2+Pkinase	1195	LOC100638653	metazoa	Amphimedon queenslandica	PREDICTED: uncharacterized protein LOC100638653 isoform X2 [Amphimedon queenslandica]	GCF_000090795.1	TRADDN-NCOA6
XP_019856456.1	Death+Death+DED+TRADDN-NCOA6+Coiled-coil+SH3+SH3+SH2+Pkinase	1133	LOC100638399	metazoa	Amphimedon queenslandica	PREDICTED: uncharacterized protein LOC100638399 [Amphimedon queenslandica]	GCF_000090795.1	TRADDN-NCOA6
XP_019856457.1	Death+Death+DED+TRADDN-NCOA6+Coiled-coil+SH3+SH3+SH2+Pkinase	1084	LOC100638525	metazoa	Amphimedon queenslandica	PREDICTED: uncharacterized protein LOC100638525 [Amphimedon queenslandica]	GCF_000090795.1	TRADDN-NCOA6
XP_019859170.1	Death+Death+DED+TRADDN-NCOA6+Coiled-coil+SH3+SH3+SH2+Pkinase	1121	LOC100642077	metazoa	Amphimedon queenslandica	PREDICTED: uncharacterized protein LOC100642077 isoform X1 [Amphimedon queenslandica]	GCF_000090795.1	TRADDN-NCOA6
XP_019859171.1	Death+Death+DED+TRADDN-NCOA6+Coiled-coil+SH3+SH3+SH2+Pkinase	1118	LOC100642077	metazoa	Amphimedon queenslandica	PREDICTED: uncharacterized protein LOC100642077 isoform X2 [Amphimedon queenslandica]	GCF_000090795.1	TRADDN-NCOA6
XP_019859172.1	Death+Death+DED+TRADDN-NCOA6+Coiled-coil+SH3+SH3+SH2+Pkinase	1117	LOC100642077	metazoa	Amphimedon queenslandica	PREDICTED: uncharacterized protein LOC100642077 isoform X3 [Amphimedon queenslandica]	GCF_000090795.1	TRADDN-NCOA6
XP_019856071.1	Death+Death+Ras+wHTH+wHTH+COR+TRADDN-NCOA6+TM	1211	LOC109584694	metazoa	Amphimedon queenslandica	PREDICTED: uncharacterized protein LOC109584694, partial [Amphimedon queenslandica]	GCF_000090795.1	TRADDN-NCOA6
XP_019863286.1	Death+Death+TRADDN-NCOA6	418	LOC109592211	metazoa	Amphimedon queenslandica	PREDICTED: uncharacterized protein LOC109592211, partial [Amphimedon queenslandica]	GCF_000090795.1	TRADDN-NCOA6
XP_019863443.1	Death+Death+TRADDN-NCOA6	349	LOC109592451	metazoa	Amphimedon queenslandica	PREDICTED: uncharacterized protein LOC109592451, partial [Amphimedon queenslandica]	GCF_000090795.1	TRADDN-NCOA6
XP_019852575.1	Death+Death+TRADDN-NCOA6+Coiled-coil	447	LOC109582349	metazoa	Amphimedon queenslandica	PREDICTED: uncharacterized protein LOC109582349 [Amphimedon queenslandica]	GCF_000090795.1	TRADDN-NCOA6
XP_019855219.1	Death+Death+TRADDN-NCOA6+Coiled-coil	435	LOC109584075	metazoa	Amphimedon queenslandica	PREDICTED: uncharacterized protein LOC109584075 [Amphimedon queenslandica]	GCF_000090795.1	TRADDN-NCOA6
XP_019862587.1	Death+Death+TRADDN-NCOA6+Coiled-coil	439	LOC109591263	metazoa	Amphimedon queenslandica	PREDICTED: uncharacterized protein LOC109591263, partial [Amphimedon queenslandica]	GCF_000090795.1	TRADDN-NCOA6
XP_019860407.1	Death+Death+TRADDN-NCOA6+Coiled-coil+LRRs	686	LOC109588731	metazoa	Amphimedon queenslandica	PREDICTED: uncharacterized protein LOC109588731 isoform X1 [Amphimedon queenslandica]	GCF_000090795.1	TRADDN-NCOA6
XP_019860408.1	Death+Death+TRADDN-NCOA6+Coiled-coil+LRRs	653	LOC109588731	metazoa	Amphimedon queenslandica	PREDICTED: uncharacterized protein LOC109588731 isoform X2 [Amphimedon queenslandica]	GCF_000090795.1	TRADDN-NCOA6
XP_019858939.1	Death+Death+TRADDN-NCOA6+Coiled-coil+SH3	589	LOC109587144	metazoa	Amphimedon queenslandica	PREDICTED: uncharacterized protein LOC109587144 [Amphimedon queenslandica]	GCF_000090795.1	TRADDN-NCOA6
XP_019857436.1	Death+Death+TRADDN-NCOA6+Coiled-coil+SH3+LRRs	677	LOC109585750	metazoa	Amphimedon queenslandica	PREDICTED: uncharacterized protein LOC109585750 [Amphimedon queenslandica]	GCF_000090795.1	TRADDN-NCOA6

acc	architecture	len	gen.name	taxend	species	defline	gca	clade
XP_019858625.1	Death+Death+TRADDN-NCOA6+Coiled-coil+SH3+LRRs	775	LOC109586842	metazoa	Amphimedon queenslandica	PREDICTED: uncharacterized protein LOC109586842 [Amphimedon queenslandica]	GCF_000090795.1	TRADDN-NCOA6
XP_019850243.1	Death+Death+TRADDN-NCOA6+UPA+UPA+UPA+Coiled-coil	1176	LOC109580999	metazoa	Amphimedon queenslandica	PREDICTED: uncharacterized protein LOC109580999 [Amphimedon queenslandica]	GCF_000090795.1	TRADDN-NCOA6
XP_019853792.1	Death+DED+TRADDN-NCOA6	380	LOC109583061	metazoa	Amphimedon queenslandica	PREDICTED: uncharacterized protein LOC109583061 [Amphimedon queenslandica]	GCF_000090795.1	TRADDN-NCOA6
XP_019855769.1	Death+DED+TRADDN-NCOA6	475	LOC109584462	metazoa	Amphimedon queenslandica	PREDICTED: uncharacterized protein LOC109584462 [Amphimedon queenslandica]	GCF_000090795.1	TRADDN-NCOA6
XP_019858668.1	Death+DED+TRADDN-NCOA6	411	LOC100636127	metazoa	Amphimedon queenslandica	PREDICTED: uncharacterized protein LOC100636127 [Amphimedon queenslandica]	GCF_000090795.1	TRADDN-NCOA6
XP_019859535.1	Death+DED+TRADDN-NCOA6	344	LOC109587752	metazoa	Amphimedon queenslandica	PREDICTED: uncharacterized protein LOC109587752 [Amphimedon queenslandica]	GCF_000090795.1	TRADDN-NCOA6
XP_019860511.1	Death+DED+TRADDN-NCOA6	684	LOC109588841	metazoa	Amphimedon queenslandica	PREDICTED: uncharacterized protein LOC109588841 [Amphimedon queenslandica]	GCF_000090795.1	TRADDN-NCOA6
XP_019861361.1	Death+DED+TRADDN-NCOA6	395	LOC109589786	metazoa	Amphimedon queenslandica	PREDICTED: uncharacterized protein LOC109589786 [Amphimedon queenslandica]	GCF_000090795.1	TRADDN-NCOA6
XP_019861554.1	Death+DED+TRADDN-NCOA6	316	LOC109590031	metazoa	Amphimedon queenslandica	PREDICTED: uncharacterized protein LOC109590031 [Amphimedon queenslandica]	GCF_000090795.1	TRADDN-NCOA6
XP_019864104.1	Death+DED+TRADDN-NCOA6	331	LOC109593486	metazoa	Amphimedon queenslandica	PREDICTED: uncharacterized protein LOC109593486, partial [Amphimedon queenslandica]	GCF_000090795.1	TRADDN-NCOA6
XP_011403628.1	Death+DED+TRADDN-NCOA6+Ankyrin	882	LOC105312571	metazoa	Amphimedon queenslandica	PREDICTED: ankyrin-1-like [Amphimedon queenslandica]	GCF_000090795.1	TRADDN-NCOA6
XP_011403949.1	Death+DED+TRADDN-NCOA6+Ankyrin	906	LOC105312758	metazoa	Amphimedon queenslandica	PREDICTED: ankyrin repeat domain-containing protein 50-like [Amphimedon queenslandica]	GCF_000090795.1	TRADDN-NCOA6
XP_011406422.1	Death+DED+TRADDN-NCOA6+Ankyrin	752	LOC105314137	metazoa	Amphimedon queenslandica	PREDICTED: ankyrin-1-like [Amphimedon queenslandica]	GCF_000090795.1	TRADDN-NCOA6
XP_011406888.1	Death+DED+TRADDN-NCOA6+Ankyrin	428	LOC105314419	metazoa	Amphimedon queenslandica	PREDICTED: uncharacterized protein LOC105314419 [Amphimedon queenslandica]	GCF_000090795.1	TRADDN-NCOA6
XP_011407078.1	Death+DED+TRADDN-NCOA6+Ankyrin	1103	LOC105314542	metazoa	Amphimedon queenslandica	PREDICTED: ankyrin repeat domain-containing protein 17-like, partial [Amphimedon queenslandica]	GCF_000090795.1	TRADDN-NCOA6
XP_011408079.1	Death+DED+TRADDN-NCOA6+Ankyrin	591	LOC105315218	metazoa	Amphimedon queenslandica	PREDICTED: uncharacterized protein LOC105315218 [Amphimedon queenslandica]	GCF_000090795.1	TRADDN-NCOA6
XP_011408586.1	Death+DED+TRADDN-NCOA6+Ankyrin	453	LOC105315583	metazoa	Amphimedon queenslandica	PREDICTED: uncharacterized protein LOC105315583 [Amphimedon queenslandica]	GCF_000090795.1	TRADDN-NCOA6
XP_019851760.1	Death+DED+TRADDN-NCOA6+Ankyrin	557	LOC109581800	metazoa	Amphimedon queenslandica	PREDICTED: uncharacterized protein LOC109581800 [Amphimedon queenslandica]	GCF_000090795.1	TRADDN-NCOA6

acc	architecture	len	gen.name	taxend	species	defline	gca	clade
XP_019855944.1	Death+DED+TRADDN-NCOA6+Ankyrin	486	LOC109584591	metazoa	Amphimedon queenslandica	PREDICTED: KN motif and ankyrin repeat domain-containing protein 1-like, partial [Amphimedon queenslandica]	GCF_000090795.1	TRADDN-NCOA6
XP_019857239.1	Death+DED+TRADDN-NCOA6+Ankyrin	718	LOC109585560	metazoa	Amphimedon queenslandica	PREDICTED: uncharacterized protein LOC109585560 [Amphimedon queenslandica]	GCF_000090795.1	TRADDN-NCOA6
XP_019858058.1	Death+DED+TRADDN-NCOA6+Ankyrin	881	LOC105315225	metazoa	Amphimedon queenslandica	PREDICTED: LOW QUALITY PROTEIN: uncharacterized protein LOC105315225 [Amphimedon queenslandica]	GCF_000090795.1	TRADDN-NCOA6
XP_019858242.1	Death+DED+TRADDN-NCOA6+Ankyrin	407	LOC109586486	metazoa	Amphimedon queenslandica	PREDICTED: uncharacterized protein LOC109586486 [Amphimedon queenslandica]	GCF_000090795.1	TRADDN-NCOA6
XP_019858786.1	Death+DED+TRADDN-NCOA6+Ankyrin	560	LOC109587006	metazoa	Amphimedon queenslandica	PREDICTED: uncharacterized protein LOC109587006 [Amphimedon queenslandica]	GCF_000090795.1	TRADDN-NCOA6
XP_019860877.1	Death+DED+TRADDN-NCOA6+Ankyrin	425	LOC109589205	metazoa	Amphimedon queenslandica	PREDICTED: uncharacterized protein LOC109589205 [Amphimedon queenslandica]	GCF_000090795.1	TRADDN-NCOA6
XP_019861228.1	Death+DED+TRADDN-NCOA6+Ankyrin	561	LOC109589610	metazoa	Amphimedon queenslandica	PREDICTED: uncharacterized protein LOC109589610 [Amphimedon queenslandica]	GCF_000090795.1	TRADDN-NCOA6
XP_019862298.1	Death+DED+TRADDN-NCOA6+Ankyrin	532	LOC105315602	metazoa	Amphimedon queenslandica	PREDICTED: uncharacterized protein LOC105315602, partial [Amphimedon queenslandica]	GCF_000090795.1	TRADDN-NCOA6
XP_019857240.1	Death+DED+TRADDN-NCOA6+Ankyrin+Coiled-coil	607	LOC109585562	metazoa	Amphimedon queenslandica	PREDICTED: uncharacterized protein LOC109585562 [Amphimedon queenslandica]	GCF_000090795.1	TRADDN-NCOA6
XP_019855945.1	Death+DED+TRADDN-NCOA6+Ankyrin+Death+DED+TRADDN-NCOA6+Ankyrin	1464	LOC109584592	metazoa	Amphimedon queenslandica	PREDICTED: uncharacterized protein LOC109584592 [Amphimedon queenslandica]	GCF_000090795.1	TRADDN-NCOA6
XP_019860958.1	Death+DED+TRADDN-NCOA6+Ankyrin+Death+DED+TRADDN-NCOA6+Ankyrin+Coiled-coil	980	LOC109589295	metazoa	Amphimedon queenslandica	PREDICTED: uncharacterized protein LOC109589295 [Amphimedon queenslandica]	GCF_000090795.1	TRADDN-NCOA6
XP_019852568.1	Death+DED+TRADDN-NCOA6+Ankyrin+SH3+LRRs	948	LOC109582345	metazoa	Amphimedon queenslandica	PREDICTED: uncharacterized protein LOC109582345 [Amphimedon queenslandica]	GCF_000090795.1	TRADDN-NCOA6
XP_019848838.1	Death+DED+TRADDN-NCOA6+Coiled-coil+MATH+MATH	1052	LOC109580272	metazoa	Amphimedon queenslandica	PREDICTED: uncharacterized protein LOC109580272 [Amphimedon queenslandica]	GCF_000090795.1	TRADDN-NCOA6
XP_019851434.1	Death+DED+TRADDN-NCOA6+Coiled-coil+MATH+MATH	1010	LOC109581612	metazoa	Amphimedon queenslandica	PREDICTED: uncharacterized protein LOC109581612 isoform X2 [Amphimedon queenslandica]	GCF_000090795.1	TRADDN-NCOA6
XP_019854917.1	Death+DED+TRADDN-NCOA6+Coiled-coil+MATH+MATH	930	LOC109583857	metazoa	Amphimedon queenslandica	PREDICTED: kinectin-like [Amphimedon queenslandica]	GCF_000090795.1	TRADDN-NCOA6
XP_019857635.1	Death+DED+TRADDN-NCOA6+Coiled-coil+MATH+MATH	1122	LOC109585927	metazoa	Amphimedon queenslandica	PREDICTED: WEB family protein At4g27595, chloroplastic-like [Amphimedon queenslandica]	GCF_000090795.1	TRADDN-NCOA6
XP_019859161.1	Death+DED+TRADDN-NCOA6+Coiled-coil+MATH+MATH	988	LOC109587362	metazoa	Amphimedon queenslandica	PREDICTED: uncharacterized protein LOC109587362 [Amphimedon queenslandica]	GCF_000090795.1	TRADDN-NCOA6

acc	architecture	len	gen.name	taxend	species	defline	gca	clade
XP_019858737.1	Death+DED+TRADDN-NCOA6+Coiled-coil+Ub-like+Ub-like	748	LOC100639420	metazoa	Amphimedon queenslandica	PREDICTED: uncharacterized protein LOC100639420 [Amphimedon queenslandica]	GCF_000090795.1	TRADDN-NCOA6
XP_019855003.1	Death+DED+TRADDN-NCOA6+LRRs	709	LOC109583922	metazoa	Amphimedon queenslandica	PREDICTED: uncharacterized protein LOC109583922 isoform X1 [Amphimedon queenslandica]	GCF_000090795.1	TRADDN-NCOA6
XP_019855004.1	Death+DED+TRADDN-NCOA6+LRRs	708	LOC109583922	metazoa	Amphimedon queenslandica	PREDICTED: uncharacterized protein LOC109583922 isoform X2 [Amphimedon queenslandica]	GCF_000090795.1	TRADDN-NCOA6
XP_019855005.1	Death+DED+TRADDN-NCOA6+LRRs	681	LOC109583922	metazoa	Amphimedon queenslandica	PREDICTED: uncharacterized protein LOC109583922 isoform X3 [Amphimedon queenslandica]	GCF_000090795.1	TRADDN-NCOA6
XP_019854336.1	Death+DED+TRADDN-NCOA6+Pkinase	694	LOC109583430	metazoa	Amphimedon queenslandica	PREDICTED: uncharacterized protein LOC109583430 [Amphimedon queenslandica]	GCF_000090795.1	TRADDN-NCOA6
XP_019859623.1	Death+DED+TRADDN-NCOA6+Pkinase	774	LOC109587843	metazoa	Amphimedon queenslandica	PREDICTED: uncharacterized protein LOC109587843 [Amphimedon queenslandica]	GCF_000090795.1	TRADDN-NCOA6
XP_019861886.1	Death+DED+TRADDN-NCOA6+PNPase	553	LOC109590401	metazoa	Amphimedon queenslandica	PREDICTED: uncharacterized protein LOC109590401 [Amphimedon queenslandica]	GCF_000090795.1	TRADDN-NCOA6
XP_019854783.1	Death+DED+TRADDN-NCOA6+TM	357	LOC105314141	metazoa	Amphimedon queenslandica	PREDICTED: uncharacterized protein LOC105314141 [Amphimedon queenslandica]	GCF_000090795.1	TRADDN-NCOA6
XP_019858854.1	Death+DED+TRADDN-NCOA6+TM+TM+TM+TM+TM+TM	710	LOC109587077	metazoa	Amphimedon queenslandica	PREDICTED: uncharacterized protein LOC109587077 [Amphimedon queenslandica]	GCF_000090795.1	TRADDN-NCOA6
XP_019858855.1	Death+DED+TRADDN-NCOA6+TM+TM+TM+TM+TM+TM	710	LOC109587077	metazoa	Amphimedon queenslandica	PREDICTED: uncharacterized protein LOC109587077 [Amphimedon queenslandica]	GCF_000090795.1	TRADDN-NCOA6
XP_019858856.1	Death+DED+TRADDN-NCOA6+TM+TM+TM+TM+TM+TM	659	LOC109587078	metazoa	Amphimedon queenslandica	PREDICTED: uncharacterized protein LOC109587078 [Amphimedon queenslandica]	GCF_000090795.1	TRADDN-NCOA6
XP_019858858.1	Death+DED+TRADDN-NCOA6+TM+TM+TM+TM+TM+TM	657	LOC105314661	metazoa	Amphimedon queenslandica	PREDICTED: uncharacterized protein LOC105314661 isoform X1 [Amphimedon queenslandica]	GCF_000090795.1	TRADDN-NCOA6
XP_019858859.1	Death+DED+TRADDN-NCOA6+TM+TM+TM+TM+TM+TM	656	LOC105314661	metazoa	Amphimedon queenslandica	PREDICTED: uncharacterized protein LOC105314661 isoform X2 [Amphimedon queenslandica]	GCF_000090795.1	TRADDN-NCOA6
XP_019859374.1	Death+DED+TRADDN-NCOA6+UPA+CARD	740	LOC109587585	metazoa	Amphimedon queenslandica	PREDICTED: uncharacterized protein LOC109587585 [Amphimedon queenslandica]	GCF_000090795.1	TRADDN-NCOA6
XP_019855315.1	Death+DED+TRADDN-NCOA6+UPA+UPA+UPA+RRM+RRM+RRM+KH+KH+KH+KH+KH+KH+KH+Macro+KH+KH+PARP	2382	LOC100631792	metazoa	Amphimedon queenslandica	PREDICTED: uncharacterized protein LOC100631792 isoform X1 [Amphimedon queenslandica]	GCF_000090795.1	TRADDN-NCOA6
XP_019855316.1	Death+DED+TRADDN-NCOA6+UPA+UPA+UPA+RRM+RRM+RRM+KH+KH+KH+KH+KH+KH+KH+Macro+KH+KH+PARP	2377	LOC100631792	metazoa	Amphimedon queenslandica	PREDICTED: uncharacterized protein LOC100631792 isoform X2 [Amphimedon queenslandica]	GCF_000090795.1	TRADDN-NCOA6
XP_019858095.1	Death+DED+TRADDN-NCOA6+zf-TRAF+TM+TM+TM+TM+TM+TM	703	LOC109586357	metazoa	Amphimedon queenslandica	PREDICTED: uncharacterized protein LOC109586357 [Amphimedon queenslandica]	GCF_000090795.1	TRADDN-NCOA6

acc	architecture	len	gen.name	taxend	species	defline	gca	clade
XP_019851433.1	Death+TM+DED+TRADDN-NCOA6+Coiled-coil+MATH+MATH	1040	LOC109581612	metazoa	Amphimedon queenslandica	PREDICTED: uncharacterized protein LOC109581612 isoform X1 [Amphimedon queenslandica]	GCF_000090795.1	TRADDN-NCOA6
XP_019862050.1	Death+TM+TM+DED+TRADDN-NCOA6	444	LOC109590594	metazoa	Amphimedon queenslandica	PREDICTED: uncharacterized protein LOC109590594 [Amphimedon queenslandica]	GCF_000090795.1	TRADDN-NCOA6
XP_013395730.1	Death+TRADDN-DEDD2+Coiled-coil	468	LOC106162845	metazoa	Lingula anatina	uncharacterized protein LOC106162845 [Lingula anatina]	GCF_001039355.2	TRADDN-DEDD2
XP_013413759.1	Death+TRADDN-DEDD2+Coiled-coil	590	LOC106176083	metazoa	Lingula anatina	uncharacterized protein LOC106176083 [Lingula anatina]	GCF_001039355.2	TRADDN-DEDD2
XP_019859154.1	Death+TRADDN-NCOA6+CASPASE	618	LOC109587358	metazoa	Amphimedon queenslandica	PREDICTED: uncharacterized protein LOC109587358 [Amphimedon queenslandica]	GCF_000090795.1	TRADDN-NCOA6
XP_019861377.1	Death+TRADDN-NCOA6+Coiled-coil	615	LOC109589811	metazoa	Amphimedon queenslandica	PREDICTED: myosin-14-like [Amphimedon queenslandica]	GCF_000090795.1	TRADDN-NCOA6
XP_019860764.1	Death+TRADDN-NCOA6+Coiled-coil+HD	954	LOC109589081	metazoa	Amphimedon queenslandica	PREDICTED: uncharacterized protein LOC109589081 [Amphimedon queenslandica]	GCF_000090795.1	TRADDN-NCOA6
XP_019848917.1	Death+TRADDN-NCOA6+Coiled-coil+LRRs	526	LOC109580325	metazoa	Amphimedon queenslandica	PREDICTED: uncharacterized protein LOC109580325 [Amphimedon queenslandica]	GCF_000090795.1	TRADDN-NCOA6
XP_019850826.1	Death+TRADDN-NCOA6+Coiled-coil+LRRs	581	LOC100640283	metazoa	Amphimedon queenslandica	PREDICTED: uncharacterized protein LOC100640283 [Amphimedon queenslandica]	GCF_000090795.1	TRADDN-NCOA6
XP_019854437.1	Death+TRADDN-NCOA6+Coiled-coil+LRRs	584	LOC109583492	metazoa	Amphimedon queenslandica	PREDICTED: uncharacterized protein LOC109583492 [Amphimedon queenslandica]	GCF_000090795.1	TRADDN-NCOA6
XP_019856063.1	Death+TRADDN-NCOA6+Coiled-coil+LRRs	558	LOC109584685	metazoa	Amphimedon queenslandica	PREDICTED: uncharacterized protein LOC109584685 [Amphimedon queenslandica]	GCF_000090795.1	TRADDN-NCOA6
XP_019860892.1	Death+TRADDN-NCOA6+Coiled-coil+SH2+SH2	670	LOC109589223	metazoa	Amphimedon queenslandica	PREDICTED: uncharacterized protein LOC109589223 [Amphimedon queenslandica]	GCF_000090795.1	TRADDN-NCOA6
XP_019852132.1	Death+TRADDN-NCOA6+Coiled-coil+SH3+LRRs	620	LOC109582017	metazoa	Amphimedon queenslandica	PREDICTED: uncharacterized protein LOC109582017 [Amphimedon queenslandica]	GCF_000090795.1	TRADDN-NCOA6
XP_019858261.1	Death+TRADDN-NCOA6+Coiled-coil+SH3+LRRs	522	LOC109586506	metazoa	Amphimedon queenslandica	PREDICTED: uncharacterized protein LOC109586506 [Amphimedon queenslandica]	GCF_000090795.1	TRADDN-NCOA6
XP_019860598.1	Death+TRADDN-NCOA6+Coiled-coil+SH3+LRRs	558	LOC109588939	metazoa	Amphimedon queenslandica	PREDICTED: uncharacterized protein LOC109588939, partial [Amphimedon queenslandica]	GCF_000090795.1	TRADDN-NCOA6
XP_019860600.1	Death+TRADDN-NCOA6+Coiled-coil+SH3+LRRs	731	LOC109588940	metazoa	Amphimedon queenslandica	PREDICTED: uncharacterized protein LOC109588940 isoform X1 [Amphimedon queenslandica]	GCF_000090795.1	TRADDN-NCOA6
XP_019860601.1	Death+TRADDN-NCOA6+Coiled-coil+SH3+LRRs	726	LOC109588940	metazoa	Amphimedon queenslandica	PREDICTED: uncharacterized protein LOC109588940 isoform X2 [Amphimedon queenslandica]	GCF_000090795.1	TRADDN-NCOA6
XP_019860602.1	Death+TRADDN-NCOA6+Coiled-coil+SH3+LRRs	711	LOC109588940	metazoa	Amphimedon queenslandica	PREDICTED: uncharacterized protein LOC109588940 isoform X3 [Amphimedon queenslandica]	GCF_000090795.1	TRADDN-NCOA6

acc	architecture	len	gen.name	taxend	species	defline	gca	clade
XP_019860671.1	Death+TRADDN-NCOA6+Coiled-coil+SH3+LRRs	582	LOC109588993	metazoa	Amphimedon queenslandica	PREDICTED: uncharacterized protein LOC109588993 [Amphimedon queenslandica]	GCF_000090795.1	TRADDN-NCOA6
XP_019861128.1	Death+TRADDN-NCOA6+Coiled-coil+SH3+LRRs	616	LOC109589495	metazoa	Amphimedon queenslandica	PREDICTED: uncharacterized protein LOC109589495 [Amphimedon queenslandica]	GCF_000090795.1	TRADDN-NCOA6
XP_019861670.1	Death+TRADDN-NCOA6+Coiled-coil+SH3+LRRs	662	LOC109590192	metazoa	Amphimedon queenslandica	PREDICTED: uncharacterized protein LOC109590192 [Amphimedon queenslandica]	GCF_000090795.1	TRADDN-NCOA6
XP_019859113.1	Death+TRADDN-NCOA6+HSP90-Sacsin+HSP90-Sacsin	1275	LOC109587308	metazoa	Amphimedon queenslandica	PREDICTED: sacsin-like, partial [Amphimedon queenslandica]	GCF_000090795.1	TRADDN-NCOA6
XP_019863545.1	Death+TRADDN-NCOA6+PDZ+KELCH	757	LOC109592557	metazoa	Amphimedon queenslandica	PREDICTED: uncharacterized protein LOC109592557 [Amphimedon queenslandica]	GCF_000090795.1	TRADDN-NCOA6
XP_019854545.1	DED+DED+DED+TRADDN-NCOA6+PNPase	809	LOC109583571	metazoa	Amphimedon queenslandica	PREDICTED: uncharacterized protein LOC109583571 [Amphimedon queenslandica]	GCF_000090795.1	TRADDN-NCOA6
XP_019859896.1	DED+DED+DED+TRADDN-NCOA6+PNPase	815	LOC109588155	metazoa	Amphimedon queenslandica	PREDICTED: uncharacterized protein LOC109588155 [Amphimedon queenslandica]	GCF_000090795.1	TRADDN-NCOA6
RJW68759.1	DED+low-complexity+TRADDN-DEDD2+low-complexity	775	Dedd	metazoa	Clonorchis sinensis	Death effector domain-containing protein [Clonorchis sinensis]	GCA_003604175.1	TRADDN-DEDD2
CDS15143.1	DED+low-complexity+TRADDN-DEDD2+low-complexity	654	EgrG_000754700	metazoa	Echinococcus granulosus	DNA binding death effector domain containing protein [Echinococcus granulosus]	-	TRADDN-DEDD2
XP_024352569.1	DED+low-complexity+TRADDN-DEDD2+low-complexity	681	EGR_03859	metazoa	Echinococcus granulosus	DNA-binding death effector domain-containing protein [Echinococcus granulosus]	GCF_000524195.1	TRADDN-DEDD2
CDS39986.1	DED+low-complexity+TRADDN-DEDD2+low-complexity	654	EmuJ_000754700	metazoa	Echinococcus multilocularis	DNA binding death effector domain containing protein [Echinococcus multilocularis]	GCA_000469725.3	TRADDN-DEDD2
CDS28814.1	DED+low-complexity+TRADDN-DEDD2+low-complexity	630	HmN_000830400	metazoa	Hymenolepis microstoma	DNA binding death effector domain containing protein [Hymenolepis microstoma]	GCA_000469805.2	TRADDN-DEDD2
XP_009176246.1	DED+low-complexity+TRADDN-DEDD2+low-complexity	793	T265_11351	metazoa	Opisthorchis viverrini	hypothetical protein T265_11351 [Opisthorchis viverrini]	GCF_000715545.1	TRADDN-DEDD2
XP_013411363.1	DED+TRADDN-DEDD2	314	LOC106174367	metazoa	Lingula anatina	uncharacterized protein LOC106174367 [Lingula anatina]	GCF_001039355.2	TRADDN-DEDD2
PAA59450.1	DED+TRADDN-DEDD2	458	BOX15_Mlig006619g1	metazoa	Macrostomum lignano	hypothetical protein BOX15_Mlig006619g1 [Macrostomum lignano]	GCA_002269645.1	TRADDN-DEDD2
XP_018652160.1	DED+TRADDN-DEDD2	682	Smp_169670.1	metazoa	Schistosoma mansoni	hypothetical protein Smp_169670.1 [Schistosoma mansoni]	GCF_000237925.1	TRADDN-DEDD2
XP_023932635.1	DED+TRADDN-DEDD2+Death+Coiled-coil+SUFU+SUFU_C	1311	LOC106174700	metazoa	Lingula anatina	uncharacterized protein LOC106174700 [Lingula anatina]	GCF_001039355.2	TRADDN-DEDD2
XP_013414794.1	DED+TRADDN-DEDD2+HEPN	539	LOC106176808	metazoa	Lingula anatina	uncharacterized protein LOC106176808 [Lingula anatina]	GCF_001039355.2	TRADDN-DEDD2
XP_019853509.1	DED+TRADDN-NCOA6	324	LOC109582892	metazoa	Amphimedon queenslandica	PREDICTED: uncharacterized protein LOC109582892 [Amphimedon queenslandica]	GCF_000090795.1	TRADDN-NCOA6

acc	architecture	len	gen.name	taxend	species	defline	gca	clade
XP_019853796.1	DED+TRADDN-NCOA6	234	LOC109583065	metazoa	Amphimedon queenslandica	PREDICTED: uncharacterized protein LOC109583065 [Amphimedon queenslandica]	GCF_000090795.1	TRADDN-NCOA6
XP_019856848.1	DED+TRADDN-NCOA6	572	LOC109585287	metazoa	Amphimedon queenslandica	PREDICTED: uncharacterized protein LOC109585287 [Amphimedon queenslandica]	GCF_000090795.1	TRADDN-NCOA6
XP_019857234.1	DED+TRADDN-NCOA6	378	LOC105314236	metazoa	Amphimedon queenslandica	PREDICTED: uncharacterized protein LOC105314236 [Amphimedon queenslandica]	GCF_000090795.1	TRADDN-NCOA6
XP_019859169.1	DED+TRADDN-NCOA6	260	LOC109587370	metazoa	Amphimedon queenslandica	PREDICTED: uncharacterized protein LOC109587370 [Amphimedon queenslandica]	GCF_000090795.1	TRADDN-NCOA6
XP_019860177.1	DED+TRADDN-NCOA6	324	LOC109588444	metazoa	Amphimedon queenslandica	PREDICTED: uncharacterized protein LOC109588444 [Amphimedon queenslandica]	GCF_000090795.1	TRADDN-NCOA6
XP_019860545.1	DED+TRADDN-NCOA6	295	LOC109588878	metazoa	Amphimedon queenslandica	PREDICTED: uncharacterized protein LOC109588878 [Amphimedon queenslandica]	GCF_000090795.1	TRADDN-NCOA6
XP_019861949.1	DED+TRADDN-NCOA6	219	LOC109590475	metazoa	Amphimedon queenslandica	PREDICTED: uncharacterized protein LOC109590475 [Amphimedon queenslandica]	GCF_000090795.1	TRADDN-NCOA6
XP_019862149.1	DED+TRADDN-NCOA6	329	LOC109590706	metazoa	Amphimedon queenslandica	PREDICTED: uncharacterized protein LOC109590706, partial [Amphimedon queenslandica]	GCF_000090795.1	TRADDN-NCOA6
XP_019862203.1	DED+TRADDN-NCOA6	248	LOC109590774	metazoa	Amphimedon queenslandica	PREDICTED: uncharacterized protein LOC109590774 [Amphimedon queenslandica]	GCF_000090795.1	TRADDN-NCOA6
XP_019862479.1	DED+TRADDN-NCOA6	370	LOC109591126	metazoa	Amphimedon queenslandica	PREDICTED: uncharacterized protein LOC109591126 [Amphimedon queenslandica]	GCF_000090795.1	TRADDN-NCOA6
XP_019863047.1	DED+TRADDN-NCOA6	211	LOC109591881	metazoa	Amphimedon queenslandica	PREDICTED: uncharacterized protein LOC109591881 [Amphimedon queenslandica]	GCF_000090795.1	TRADDN-NCOA6
XP_019863194.1	DED+TRADDN-NCOA6	258	LOC109592070	metazoa	Amphimedon queenslandica	PREDICTED: uncharacterized protein LOC109592070, partial [Amphimedon queenslandica]	GCF_000090795.1	TRADDN-NCOA6
XP_019863290.1	DED+TRADDN-NCOA6	246	LOC109592220	metazoa	Amphimedon queenslandica	PREDICTED: uncharacterized protein LOC109592220 [Amphimedon queenslandica]	GCF_000090795.1	TRADDN-NCOA6
XP_019863533.1	DED+TRADDN-NCOA6	285	LOC109592548	metazoa	Amphimedon queenslandica	PREDICTED: uncharacterized protein LOC109592548, partial [Amphimedon queenslandica]	GCF_000090795.1	TRADDN-NCOA6
XP_019863571.1	DED+TRADDN-NCOA6	230	LOC109592599	metazoa	Amphimedon queenslandica	PREDICTED: uncharacterized protein LOC109592599, partial [Amphimedon queenslandica]	GCF_000090795.1	TRADDN-NCOA6
XP_019863629.1	DED+TRADDN-NCOA6	231	LOC105316165	metazoa	Amphimedon queenslandica	PREDICTED: uncharacterized protein LOC105316165 [Amphimedon queenslandica]	GCF_000090795.1	TRADDN-NCOA6
XP_019864031.1	DED+TRADDN-NCOA6	281	LOC109593366	metazoa	Amphimedon queenslandica	PREDICTED: uncharacterized protein LOC109593366, partial [Amphimedon queenslandica]	GCF_000090795.1	TRADDN-NCOA6

acc	architecture	len	gen.name	taxend	species	defline	gca	clade
XP_011407589.1	DED+TRADDN-NCOA6+Ankyrin	379	LOC100639125	metazoa	Amphimedon queenslandica	PREDICTED: uncharacterized protein LOC100639125 [Amphimedon queenslandica]	GCF_000090795.1	TRADDN-NCOA6
XP_011409020.1	DED+TRADDN-NCOA6+Ankyrin	361	LOC105315940	metazoa	Amphimedon queenslandica	PREDICTED: uncharacterized protein LOC105315940 [Amphimedon queenslandica]	GCF_000090795.1	TRADDN-NCOA6
XP_011409201.1	DED+TRADDN-NCOA6+Ankyrin	481	LOC105316094	metazoa	Amphimedon queenslandica	PREDICTED: ankyrin-1-like, partial [Amphimedon queenslandica]	GCF_000090795.1	TRADDN-NCOA6
XP_011410102.1	DED+TRADDN-NCOA6+Ankyrin	1078	LOC105316704	metazoa	Amphimedon queenslandica	PREDICTED: uncharacterized protein LOC105316704 [Amphimedon queenslandica]	GCF_000090795.1	TRADDN-NCOA6
XP_019851849.1	DED+TRADDN-NCOA6+Ankyrin	511	LOC109581842	metazoa	Amphimedon queenslandica	PREDICTED: ankyrin repeat domain-containing protein 29-like [Amphimedon queenslandica]	GCF_000090795.1	TRADDN-NCOA6
XP_019854767.1	DED+TRADDN-NCOA6+Ankyrin	799	LOC109583761	metazoa	Amphimedon queenslandica	PREDICTED: ankyrin-2-like [Amphimedon queenslandica]	GCF_000090795.1	TRADDN-NCOA6
XP_019859676.1	DED+TRADDN-NCOA6+Ankyrin	275	LOC109587894	metazoa	Amphimedon queenslandica	PREDICTED: uncharacterized protein LOC109587894 [Amphimedon queenslandica]	GCF_000090795.1	TRADDN-NCOA6
XP_019859682.1	DED+TRADDN-NCOA6+Ankyrin	525	LOC109587903	metazoa	Amphimedon queenslandica	PREDICTED: serine/threonine-protein phosphatase 6 regulatory ankyrin repeat subunit A-like [Amphimedon queenslandica]	GCF_000090795.1	TRADDN-NCOA6
XP_019862955.1	DED+TRADDN-NCOA6+Ankyrin	355	LOC109591735	metazoa	Amphimedon queenslandica	PREDICTED: uncharacterized protein LOC109591735 [Amphimedon queenslandica]	GCF_000090795.1	TRADDN-NCOA6
XP_019863772.1	DED+TRADDN-NCOA6+Ankyrin	371	LOC109592895	metazoa	Amphimedon queenslandica	PREDICTED: uncharacterized protein LOC109592895, partial [Amphimedon queenslandica]	GCF_000090795.1	TRADDN-NCOA6
XP_019861208.1	DED+TRADDN-NCOA6+Ankyrin+APGTPase+COR+Death	1184	LOC109589593	metazoa	Amphimedon queenslandica	PREDICTED: uncharacterized protein LOC109589593 [Amphimedon queenslandica]	GCF_000090795.1	TRADDN-NCOA6
XP_019849449.1	DED+TRADDN-NCOA6+Ankyrin+HSP70	1281	LOC109580573	metazoa	Amphimedon queenslandica	PREDICTED: uncharacterized protein LOC109580573 [Amphimedon queenslandica]	GCF_000090795.1	TRADDN-NCOA6
XP_019857397.1	DED+TRADDN-NCOA6+Ankyrin+LRRs	655	LOC109585711	metazoa	Amphimedon queenslandica	PREDICTED: uncharacterized protein LOC109585711 [Amphimedon queenslandica]	GCF_000090795.1	TRADDN-NCOA6
XP_019860269.1	DED+TRADDN-NCOA6+Ankyrin+LRRs	555	LOC109588565	metazoa	Amphimedon queenslandica	PREDICTED: uncharacterized protein LOC109588565 [Amphimedon queenslandica]	GCF_000090795.1	TRADDN-NCOA6
XP_019864332.1	DED+TRADDN-NCOA6+Ankyrin+Ras+wHTH+wHTH+COR	1100	LOC109593741	metazoa	Amphimedon queenslandica	PREDICTED: uncharacterized protein LOC109593741 isoform X2 [Amphimedon queenslandica]	GCF_000090795.1	TRADDN-NCOA6
XP_019856065.1	DED+TRADDN-NCOA6+Coiled-coil	293	LOC109584687	metazoa	Amphimedon queenslandica	PREDICTED: uncharacterized protein LOC109584687 [Amphimedon queenslandica]	GCF_000090795.1	TRADDN-NCOA6
XP_019858669.1	DED+TRADDN-NCOA6+Coiled-coil	391	LOC109586882	metazoa	Amphimedon queenslandica	PREDICTED: uncharacterized protein LOC109586882 [Amphimedon queenslandica]	GCF_000090795.1	TRADDN-NCOA6

acc	architecture	len	gen.name	taxend	species	defline	gca	clade
XP_019859555.1	DED+TRADDN-NCOA6+Coiled-coil	302	LOC109587776	metazoa	Amphimedon queenslandica	PREDICTED: uncharacterized protein LOC109587776 [Amphimedon queenslandica]	GCF_000090795.1	TRADDN-NCOA6
XP_019861077.1	DED+TRADDN-NCOA6+Coiled-coil	301	LOC109589427	metazoa	Amphimedon queenslandica	PREDICTED: uncharacterized protein LOC109589427 [Amphimedon queenslandica]	GCF_000090795.1	TRADDN-NCOA6
XP_019862666.1	DED+TRADDN-NCOA6+Coiled-coil	346	LOC109591361	metazoa	Amphimedon queenslandica	PREDICTED: uncharacterized protein LOC109591361 [Amphimedon queenslandica]	GCF_000090795.1	TRADDN-NCOA6
XP_019863296.1	DED+TRADDN-NCOA6+Coiled-coil	414	LOC109592229	metazoa	Amphimedon queenslandica	PREDICTED: uncharacterized protein LOC109592229, partial [Amphimedon queenslandica]	GCF_000090795.1	TRADDN-NCOA6
XP_019861674.1	DED+TRADDN-NCOA6+Coiled-coil+Death+CARD	671	LOC109590195	metazoa	Amphimedon queenslandica	PREDICTED: uncharacterized protein LOC109590195 isoform X2 [Amphimedon queenslandica]	GCF_000090795.1	TRADDN-NCOA6
XP_019861673.1	DED+TRADDN-NCOA6+Coiled-coil+Death+CARD+DED	792	LOC109590195	metazoa	Amphimedon queenslandica	PREDICTED: uncharacterized protein LOC109590195 isoform X1 [Amphimedon queenslandica]	GCF_000090795.1	TRADDN-NCOA6
XP_019860830.1	DED+TRADDN-NCOA6+Coiled-coil+HD	826	LOC109589155	metazoa	Amphimedon queenslandica	PREDICTED: uncharacterized protein LOC109589155 [Amphimedon queenslandica]	GCF_000090795.1	TRADDN-NCOA6
XP_019860219.1	DED+TRADDN-NCOA6+Coiled-coil+MATH+MATH	822	LOC109588500	metazoa	Amphimedon queenslandica	PREDICTED: polyamine-modulated factor 1-binding protein 1-like [Amphimedon queenslandica]	GCF_000090795.1	TRADDN-NCOA6
XP_019856595.1	DED+TRADDN-NCOA6+Coiled-coil+SH3+Beta-propeller	832	LOC109585094	metazoa	Amphimedon queenslandica	PREDICTED: E3 ubiquitin-protein ligase TRIM71-like isoform X1 [Amphimedon queenslandica]	GCF_000090795.1	TRADDN-NCOA6
XP_019856596.1	DED+TRADDN-NCOA6+Coiled-coil+SH3+Beta-propeller	831	LOC109585094	metazoa	Amphimedon queenslandica	PREDICTED: E3 ubiquitin-protein ligase TRIM71-like isoform X2 [Amphimedon queenslandica]	GCF_000090795.1	TRADDN-NCOA6
XP_019856597.1	DED+TRADDN-NCOA6+Coiled-coil+SH3+Beta-propeller	812	LOC109585094	metazoa	Amphimedon queenslandica	PREDICTED: E3 ubiquitin-protein ligase TRIM71-like isoform X3 [Amphimedon queenslandica]	GCF_000090795.1	TRADDN-NCOA6
XP_019852391.1	DED+TRADDN-NCOA6+Coiled-coil+SH3+SH3+SH2+Pkinase	889	LOC100637712	metazoa	Amphimedon queenslandica	PREDICTED: uncharacterized protein LOC100637712 [Amphimedon queenslandica]	GCF_000090795.1	TRADDN-NCOA6
XP_019852393.1	DED+TRADDN-NCOA6+Coiled-coil+SH3+SH3+SH2+Pkinase	988	LOC100637961	metazoa	Amphimedon queenslandica	PREDICTED: uncharacterized protein LOC100637961 [Amphimedon queenslandica]	GCF_000090795.1	TRADDN-NCOA6
XP_019859739.1	DED+TRADDN-NCOA6+Coiled-coil+SH3+SH3+SH2+Pkinase	955	LOC109587969	metazoa	Amphimedon queenslandica	PREDICTED: uncharacterized protein LOC109587969 [Amphimedon queenslandica]	GCF_000090795.1	TRADDN-NCOA6
XP_019862004.1	DED+TRADDN-NCOA6+Coiled-coil+SH3+SH3+SH2+Pkinase	834	LOC100638702	metazoa	Amphimedon queenslandica	PREDICTED: tyrosine-protein kinase STK-like [Amphimedon queenslandica]	GCF_000090795.1	TRADDN-NCOA6
XP_019857552.1	DED+TRADDN-NCOA6+Coiled-coil+TM+TM	652	LOC109585850	metazoa	Amphimedon queenslandica	PREDICTED: uncharacterized protein LOC109585850 [Amphimedon queenslandica]	GCF_000090795.1	TRADDN-NCOA6
XP_019857558.1	DED+TRADDN-NCOA6+Coiled-coil+TM+TM	632	LOC109585853	metazoa	Amphimedon queenslandica	PREDICTED: uncharacterized protein LOC109585853 [Amphimedon queenslandica]	GCF_000090795.1	TRADDN-NCOA6

acc	architecture	len	gen.name	taxend	species	defline	gca	clade
XP_019862678.1	DED+TRADDN-NCOA6+SH3	512	LOC109591376	metazoa	Amphimedon queenslandica	PREDICTED: uncharacterized protein LOC109591376, partial [Amphimedon queenslandica]	GCF_000090795.1	TRADDN-NCOA6
XP_019854924.1	DED+TRADDN-NCOA6+SH3+LRRs	555	LOC109583863	metazoa	Amphimedon queenslandica	PREDICTED: uncharacterized protein LOC109583863 [Amphimedon queenslandica]	GCF_000090795.1	TRADDN-NCOA6
XP_011408461.2	DED+TRADDN-NCOA6+UPA+CARD	475	LOC105315485	metazoa	Amphimedon queenslandica	PREDICTED: uncharacterized protein LOC105315485, partial [Amphimedon queenslandica]	GCF_000090795.1	TRADDN-NCOA6
XP_019855800.1	DED+TRADDN-NCOA6+UPA+Death+Coiled-coil+Death+UPA+CARD	1316	LOC100638991	metazoa	Amphimedon queenslandica	PREDICTED: uncharacterized protein LOC100638991, partial [Amphimedon queenslandica]	GCF_000090795.1	TRADDN-NCOA6
XP_011405642.2	DED+TRADDN-NCOA6+UPA+UPA+UPA+RRM+RRM+RRM+KH+KH+KH+KH+KH+KH+KH+Macro+KH+KH+PARP	2244	LOC100631792	metazoa	Amphimedon queenslandica	PREDICTED: uncharacterized protein LOC100631792 isoform X3 [Amphimedon queenslandica]	GCF_000090795.1	TRADDN-NCOA6
XP_019855317.1	DED+TRADDN-NCOA6+UPA+UPA+UPA+RRM+RRM+RRM+KH+KH+KH+KH+KH+KH+KH+Macro+KH+KH+PARP	2244	LOC100631792	metazoa	Amphimedon queenslandica	PREDICTED: uncharacterized protein LOC100631792 isoform X3 [Amphimedon queenslandica]	GCF_000090795.1	TRADDN-NCOA6
XP_013416013.1	HEPN+Ankyrin+TRADDN-DEDD2+SF2-helicase	1078	LOC106177707	metazoa	Lingula anatina	uncharacterized protein LOC106177707 isoform X1 [Lingula anatina]	GCF_001039355.2	TRADDN-DEDD2
XP_013416018.1	HEPN+Ankyrin+TRADDN-DEDD2+SF2-helicase	1070	LOC106177707	metazoa	Lingula anatina	uncharacterized protein LOC106177707 isoform X2 [Lingula anatina]	GCF_001039355.2	TRADDN-DEDD2
XP_013399072.1	HEPN+Coiled-coil+TRADDN-DEDD2+GTPase-AIG	1112	LOC106165415	metazoa	Lingula anatina	uncharacterized protein LOC106165415 [Lingula anatina]	GCF_001039355.2	TRADDN-DEDD2
XP_013399073.1	HEPN+Coiled-coil+TRADDN-DEDD2+RNA-receptor_SF2-helicase+RIG-I_C-RD	1529	LOC106165417	metazoa	Lingula anatina	uncharacterized protein LOC106165417 [Lingula anatina]	GCF_001039355.2	TRADDN-DEDD2
XP_023230968.1	low-complexity+DED+TRADDN-DEDD2	303	LOC111631013	metazoa	Centruroides sculpturatus	death effector domain-containing protein-like isoform X1 [Centruroides sculpturatus]	GCF_000671375.1	TRADDN-DEDD2
XP_013782170.1	low-complexity+DED+TRADDN-DEDD2	325	LOC106466432	metazoa	Limulus polyphemus	death effector domain-containing protein-like [Limulus polyphemus]	GCF_000517525.1	TRADDN-DEDD2
XP_013782555.1	low-complexity+DED+TRADDN-DEDD2	324	LOC106466800	metazoa	Limulus polyphemus	death effector domain-containing protein-like [Limulus polyphemus]	GCF_000517525.1	TRADDN-DEDD2
XP_013785157.1	low-complexity+DED+TRADDN-DEDD2	322	LOC106469233	metazoa	Limulus polyphemus	death effector domain-containing protein-like [Limulus polyphemus]	GCF_000517525.1	TRADDN-DEDD2
XP_013414953.1	low-complexity+DED+TRADDN-DEDD2	341	LOC106176911	metazoa	Lingula anatina	death effector domain-containing protein [Lingula anatina]	GCF_001039355.2	TRADDN-DEDD2
XP_015919266.1	low-complexity+DED+TRADDN-DEDD2	324	LOC107448539	metazoa	Parasteatoda tepidariorum	death effector domain-containing protein [Parasteatoda tepidariorum]	GCF_000365465.2	TRADDN-DEDD2
KFM80261.1	low-complexity+DED+TRADDN-DEDD2	326	X975_03323	metazoa	Stegodyphus mimosarum	Death effector domain-containing protein, partial [Stegodyphus mimosarum]	GCA_000611955.2	TRADDN-DEDD2
XP_013390221.1	N-ter+TRADDN-Bac	130	LOC106158693	metazoa	Lingula anatina	PREDICTED: uncharacterized protein LOC106158693 [Lingula anatina]	-	TRADDN-Bacterial-like
XP_013391308.1	N-ter+TRADDN-Bac+FAM124	405	LOC106159546	metazoa	Lingula anatina	uncharacterized protein LOC106159546 [Lingula anatina]	GCF_001039355.2	TRADDN-Bacterial-like
XP_019862936.1	PNPase+DED+TRADDN-NCOA6	550	LOC109591708	metazoa	Amphimedon queenslandica	PREDICTED: uncharacterized protein LOC109591708, partial [Amphimedon queenslandica]	GCF_000090795.1	TRADDN-NCOA6

acc	architecture	len	gen.name	taxend	species	defline	gca	clade
XP_019851861.1	TM+CARD+CARD+DED+TRADDN-NCOA6+Ankyrin	1649	LOC100639136	metazoa	Amphimedon queenslandica	PREDICTED: uncharacterized protein LOC100639136 [Amphimedon queenslandica]	GCF_000090795.1	TRADDN-NCOA6
XP_019854237.1	TM+CARD+CARD+TRADDN-NCOA6+Ankyrin	2115	LOC100635111	metazoa	Amphimedon queenslandica	PREDICTED: uncharacterized protein LOC100635111 [Amphimedon queenslandica]	GCF_000090795.1	TRADDN-NCOA6
XP_011403406.2	TM+CARD+DED+TRADDN-NCOA6+Ankyrin	1822	LOC105312446	metazoa	Amphimedon queenslandica	PREDICTED: uncharacterized protein LOC105312446 [Amphimedon queenslandica]	GCF_000090795.1	TRADDN-NCOA6
XP_019859923.1	TM+CARD+DED+TRADDN-NCOA6+Ankyrin	1026	LOC109588182	metazoa	Amphimedon queenslandica	PREDICTED: uncharacterized protein LOC109588182 [Amphimedon queenslandica]	GCF_000090795.1	TRADDN-NCOA6
XP_019861853.1	TM+CARD+DED+TRADDN-NCOA6+Ankyrin	1624	LOC100640644	metazoa	Amphimedon queenslandica	PREDICTED: uncharacterized protein LOC100640644, partial [Amphimedon queenslandica]	GCF_000090795.1	TRADDN-NCOA6
XP_011408342.2	TM+Death+CARD+DED+TRADDN-NCOA6+Ankyrin	676	LOC100639549	metazoa	Amphimedon queenslandica	PREDICTED: uncharacterized protein LOC100639549, partial [Amphimedon queenslandica]	GCF_000090795.1	TRADDN-NCOA6
XP_019850302.1	TM+Death+CARD+DED+TRADDN-NCOA6+Ankyrin	1134	LOC109581026	metazoa	Amphimedon queenslandica	PREDICTED: uncharacterized protein LOC109581026 [Amphimedon queenslandica]	GCF_000090795.1	TRADDN-NCOA6
XP_019856195.1	TM+Death+CARD+DED+TRADDN-NCOA6+Ankyrin	1369	LOC100641054	metazoa	Amphimedon queenslandica	PREDICTED: ankyrin repeat and KH domain-containing protein 1-like [Amphimedon queenslandica]	GCF_000090795.1	TRADDN-NCOA6
XP_019857996.1	TM+Death+CARD+DED+TRADDN-NCOA6+Ankyrin	550	LOC109586265	metazoa	Amphimedon queenslandica	PREDICTED: uncharacterized protein LOC109586265 [Amphimedon queenslandica]	GCF_000090795.1	TRADDN-NCOA6
XP_019859824.1	TM+Death+CARD+DED+TRADDN-NCOA6+Ankyrin	556	LOC109588077	metazoa	Amphimedon queenslandica	PREDICTED: uncharacterized protein LOC109588077, partial [Amphimedon queenslandica]	GCF_000090795.1	TRADDN-NCOA6
XP_019861870.1	TM+Death+CARD+DED+TRADDN-NCOA6+Ankyrin	648	LOC109590384	metazoa	Amphimedon queenslandica	PREDICTED: uncharacterized protein LOC109590384, partial [Amphimedon queenslandica]	GCF_000090795.1	TRADDN-NCOA6
XP_019860025.1	TM+Death+CARD+DED+TRADDN-NCOA6+Ankyrin+Death+CARD+DED+TRADDN-NCOA6+Ankyrin	2373	LOC105314953	metazoa	Amphimedon queenslandica	PREDICTED: uncharacterized protein LOC105314953 [Amphimedon queenslandica]	GCF_000090795.1	TRADDN-NCOA6
XP_019860782.1	TM+Death+DED+TRADDN-NCOA6+Ankyrin	609	LOC109589105	metazoa	Amphimedon queenslandica	PREDICTED: uncharacterized protein LOC109589105 [Amphimedon queenslandica]	GCF_000090795.1	TRADDN-NCOA6
XP_019852427.1	TM+Death+DED+TRADDN-NCOA6+Ankyrin+Coiled-coil	1201	LOC109582217	metazoa	Amphimedon queenslandica	PREDICTED: uncharacterized protein LOC109582217 [Amphimedon queenslandica]	GCF_000090795.1	TRADDN-NCOA6
XP_019852657.1	TM+TM+Death+CARD+DED+TRADDN-NCOA6	885	LOC109582413	metazoa	Amphimedon queenslandica	PREDICTED: uncharacterized protein LOC109582413 [Amphimedon queenslandica]	GCF_000090795.1	TRADDN-NCOA6
XP_019852670.1	TM+TM+Death+CARD+DED+TRADDN-NCOA6	638	LOC109582420	metazoa	Amphimedon queenslandica	PREDICTED: uncharacterized protein LOC109582420 isoform X1 [Amphimedon queenslandica]	GCF_000090795.1	TRADDN-NCOA6
XP_019850895.1	TM+TM+Death+DED+TRADDN-NCOA6+Ankyrin	897	LOC105312422	metazoa	Amphimedon queenslandica	PREDICTED: uncharacterized protein LOC105312422 isoform X1 [Amphimedon queenslandica]	GCF_000090795.1	TRADDN-NCOA6

acc	architecture	len	gen.name	taxend	species	defline	gca	clade
XP_019850896.1	TM+TM+Death+DED+TRADDN-NCOA6+Ankyrin	882	LOC105312422	metazoa	Amphimedon queenslandica	PREDICTED: uncharacterized protein LOC105312422 isoform X2 [Amphimedon queenslandica]	GCF_000090795.1	TRADDN-NCOA6
XP_019854303.1	TM+TM+Death+DED+TRADDN-NCOA6+Ankyrin	974	LOC109583413	metazoa	Amphimedon queenslandica	PREDICTED: uncharacterized protein LOC109583413 [Amphimedon queenslandica]	GCF_000090795.1	TRADDN-NCOA6
XP_023230971.1	TRADDN-DEDD2	249	LOC111631013	metazoa	Centruroides sculpturatus	death effector domain-containing protein-like isoform X3 [Centruroides sculpturatus]	GCF_000671375.1	TRADDN-DEDD2
PIS85829.1	TRADDN-DEDD2	200	D915_06944	metazoa	Fasciola hepatica	hypothetical protein D915_06944 [Fasciola hepatica]	-	TRADDN-DEDD2
XP_013380659.1	TRADDN-DEDD2	393	LOC106151793	metazoa	Lingula anatina	uncharacterized protein LOC106151793 isoform X1 [Lingula anatina]	GCF_001039355.2	TRADDN-DEDD2
XP_013380662.1	TRADDN-DEDD2	326	LOC106151793	metazoa	Lingula anatina	uncharacterized protein LOC106151793 isoform X2 [Lingula anatina]	GCF_001039355.2	TRADDN-DEDD2
XP_013392589.1	TRADDN-DEDD2	170	LOC106160515	metazoa	Lingula anatina	uncharacterized protein LOC106160515 [Lingula anatina]	GCF_001039355.2	TRADDN-DEDD2
XP_013421807.1	TRADDN-DEDD2	387	LOC106181845	metazoa	Lingula anatina	uncharacterized protein LOC106181845 [Lingula anatina]	GCF_001039355.2	TRADDN-DEDD2
PAA67295.1	TRADDN-DEDD2	311	BOX15_Mlig007289g1	metazoa	Macrostomum lignano	hypothetical protein BOX15_Mlig007289g1 [Macrostomum lignano]	GCA_002269645.1	TRADDN-DEDD2
PRD34936.1	TRADDN-DEDD2	240	NCL1_13077	metazoa	Nephila clavipes	Dedd [Nephila clavipes]	GCA_002102615.1	TRADDN-DEDD2
XP_018652159.1	TRADDN-DEDD2	522	Smp_169670.1	metazoa	Schistosoma mansoni	hypothetical protein Smp_169670.1 [Schistosoma mansoni]	GCF_000237925.1	TRADDN-DEDD2
XP_013393640.1	TRADDN-DEDD2+Death	1097	LOC106161282	metazoa	Lingula anatina	uncharacterized protein LOC106161282 [Lingula anatina]	GCF_001039355.2	TRADDN-DEDD2
XP_013419526.1	TRADDN-DEDD2+Death	1322	LOC106180164	metazoa	Lingula anatina	uncharacterized protein LOC106180164 isoform X1 [Lingula anatina]	GCF_001039355.2	TRADDN-DEDD2
XP_013419534.1	TRADDN-DEDD2+Death	1277	LOC106180164	metazoa	Lingula anatina	uncharacterized protein LOC106180164 isoform X2 [Lingula anatina]	GCF_001039355.2	TRADDN-DEDD2
XP_019853513.1	TRADDN-NCOA6	162	LOC109582894	metazoa	Amphimedon queenslandica	PREDICTED: uncharacterized protein LOC109582894 [Amphimedon queenslandica]	GCF_000090795.1	TRADDN-NCOA6
XP_019860989.1	TRADDN-NCOA6	129	LOC109589327	metazoa	Amphimedon queenslandica	PREDICTED: uncharacterized protein LOC109589327 [Amphimedon queenslandica]	GCF_000090795.1	TRADDN-NCOA6
XP_019861561.1	TRADDN-NCOA6	186	LOC109590040	metazoa	Amphimedon queenslandica	PREDICTED: uncharacterized protein LOC109590040 [Amphimedon queenslandica]	GCF_000090795.1	TRADDN-NCOA6
XP_019863799.1	TRADDN-NCOA6	288	LOC109592940	metazoa	Amphimedon queenslandica	PREDICTED: uncharacterized protein LOC109592940, partial [Amphimedon queenslandica]	GCF_000090795.1	TRADDN-NCOA6
XP_023215973.1	TRADDN-NCOA6	2564	LOC111618641	metazoa	Centruroides sculpturatus	uncharacterized protein LOC111618641 [Centruroides sculpturatus]	GCF_000671375.1	TRADDN-NCOA6
OTF82418.1	TRADDN-NCOA6	1068	BLA29_000798	metazoa	Euroglyphus maynei	hypothetical protein BLA29_000798 [Euroglyphus maynei]	GCA_002135145.1	TRADDN-NCOA6

acc	architecture	len	gen.name	taxend	species	defline	gca	clade
XP_018495753.1	TRADDN-NCOA6	1106	LOC108864486	metazoa	Galendromus occidentalis	PREDICTED: putative uncharacterized protein DDB_G0271606 [Galendromus occidentalis]	GCF_000255335.1	TRADDN-NCOA6
XP_002409522.1	TRADDN-NCOA6	1417	IscW_ISCW016558	metazoa	Ixodes scapularis	hypothetical protein IscW_ISCW016558 [Ixodes scapularis]	-	TRADDN-NCOA6
XP_013776014.2	TRADDN-NCOA6	2652	LOC106460818	metazoa	Limulus polyphemus	uncharacterized protein LOC106460818 isoform X1 [Limulus polyphemus]	GCF_000517525.1	TRADDN-NCOA6
XP_013786660.1	TRADDN-NCOA6	1027	LOC106470641	metazoa	Limulus polyphemus	pheromone-regulated protein PRM7-like, partial [Limulus polyphemus]	GCF_000517525.1	TRADDN-NCOA6
XP_022243330.1	TRADDN-NCOA6	2647	LOC106460818	metazoa	Limulus polyphemus	uncharacterized protein LOC106460818 isoform X2 [Limulus polyphemus]	GCF_000517525.1	TRADDN-NCOA6
XP_022243331.1	TRADDN-NCOA6	2592	LOC106460818	metazoa	Limulus polyphemus	uncharacterized protein LOC106460818 isoform X3 [Limulus polyphemus]	GCF_000517525.1	TRADDN-NCOA6
XP_022257147.1	TRADDN-NCOA6	1944	LOC106472893	metazoa	Limulus polyphemus	uncharacterized protein LOC106472893, partial [Limulus polyphemus]	GCF_000517525.1	TRADDN-NCOA6
XP_022257455.1	TRADDN-NCOA6	2874	LOC106473285	metazoa	Limulus polyphemus	uncharacterized protein LOC106473285, partial [Limulus polyphemus]	GCF_000517525.1	TRADDN-NCOA6
XP_013399320.1	TRADDN-NCOA6	2286	LOC106165614	metazoa	Lingula anatina	mucin-12 isoform X1 [Lingula anatina]	GCF_001039355.2	TRADDN-NCOA6
XP_013399322.1	TRADDN-NCOA6	2285	LOC106165614	metazoa	Lingula anatina	mucin-12 isoform X2 [Lingula anatina]	GCF_001039355.2	TRADDN-NCOA6
XP_013399323.1	TRADDN-NCOA6	2281	LOC106165614	metazoa	Lingula anatina	mucin-12 isoform X3 [Lingula anatina]	GCF_001039355.2	TRADDN-NCOA6
XP_015923751.1	TRADDN-NCOA6	2522	LOC107452001	metazoa	Parasteatoda tepidariorum	uncharacterized threonine-rich GPI-anchored glycoprotein PJ4664.02-like isoform X1 [Parasteatoda tepidariorum]	GCF_000365465.2	TRADDN-NCOA6
XP_015923752.1	TRADDN-NCOA6	2513	LOC107452001	metazoa	Parasteatoda tepidariorum	uncharacterized threonine-rich GPI-anchored glycoprotein PJ4664.02-like isoform X2 [Parasteatoda tepidariorum]	GCF_000365465.2	TRADDN-NCOA6
XP_014662554.1	TRADDN-NCOA6	2048	LOC106805474	metazoa	Priapulus caudatus	PREDICTED: serine-rich adhesin for platelets-like [Priapulus caudatus]	GCF_000485595.1	TRADDN-NCOA6
KPM06795.1	TRADDN-NCOA6	2407	QR98_0052740	metazoa	Sarcoptes scabiei	hypothetical protein QR98_0052740 [Sarcoptes scabiei]	GCA_000828355.1	TRADDN-NCOA6
XP_015793616.1	TRADDN-NCOA6	288	LOC107370147	metazoa	Tetranychus urticae	uncharacterized protein LOC107370147 isoform X2 [Tetranychus urticae]	GCF_000239435.1	TRADDN-NCOA6
XP_022666252.1	TRADDN-NCOA6	1692	LOC111252497	metazoa	Varroa destructor	histone-lysine N-methyltransferase 2D-like isoform X1 [Varroa destructor]	GCF_002443255.1	TRADDN-NCOA6

acc	architecture	len	gen.name	taxend	species	defline	gca	clade
XP_022666261.1	TRADDN-NCOA6	1689	LOC111252497	metazoa	Varroa destructor	mediator of RNA polymerase II transcription subunit 12-like isoform X2 [Varroa destructor]	GCF_002443255.1	TRADDN-NCOA6
XP_022666270.1	TRADDN-NCOA6	1601	LOC111252497	metazoa	Varroa destructor	histone-lysine N-methyltransferase 2D-like isoform X3 [Varroa destructor]	GCF_002443255.1	TRADDN-NCOA6
XP_022708482.1	TRADDN-NCOA6	1694	LOC111271766	metazoa	Varroa jacobsoni	histone-lysine N-methyltransferase 2D-like isoform X1 [Varroa jacobsoni]	GCF_002532875.1	TRADDN-NCOA6
XP_022708484.1	TRADDN-NCOA6	1603	LOC111271766	metazoa	Varroa jacobsoni	histone-lysine N-methyltransferase 2D-like isoform X2 [Varroa jacobsoni]	GCF_002532875.1	TRADDN-NCOA6
XP_011409118.1	TRADDN-NCOA6+Ankyrin	348	LOC105316026	metazoa	Amphimedon queenslandica	PREDICTED: ankyrin repeat domain-containing protein 29-like, partial [Amphimedon queenslandica]	GCF_000090795.1	TRADDN-NCOA6
XP_011409199.1	TRADDN-NCOA6+Ankyrin	203	LOC105316093	metazoa	Amphimedon queenslandica	PREDICTED: ankyrin-3-like [Amphimedon queenslandica]	GCF_000090795.1	TRADDN-NCOA6
XP_019850301.1	TRADDN-NCOA6+Ankyrin	636	LOC105312201	metazoa	Amphimedon queenslandica	PREDICTED: ankyrin repeat domain-containing protein 17-like [Amphimedon queenslandica]	GCF_000090795.1	TRADDN-NCOA6
XP_019857878.1	TRADDN-NCOA6+Ankyrin	658	LOC109586144	metazoa	Amphimedon queenslandica	PREDICTED: ankyrin repeat domain-containing protein 50-like [Amphimedon queenslandica]	GCF_000090795.1	TRADDN-NCOA6
XP_019858083.1	TRADDN-NCOA6+Ankyrin	2099	LOC100633272	metazoa	Amphimedon queenslandica	PREDICTED: uncharacterized protein LOC100633272 [Amphimedon queenslandica]	GCF_000090795.1	TRADDN-NCOA6
XP_019858350.1	TRADDN-NCOA6+Ankyrin	611	LOC109586592	metazoa	Amphimedon queenslandica	PREDICTED: uncharacterized protein LOC109586592, partial [Amphimedon queenslandica]	GCF_000090795.1	TRADDN-NCOA6
XP_019858351.1	TRADDN-NCOA6+Ankyrin	668	LOC109586593	metazoa	Amphimedon queenslandica	PREDICTED: ankyrin repeat domain-containing protein 17-like [Amphimedon queenslandica]	GCF_000090795.1	TRADDN-NCOA6
XP_019860272.1	TRADDN-NCOA6+Ankyrin	737	LOC105315012	metazoa	Amphimedon queenslandica	PREDICTED: LOW QUALITY PROTEIN: ankyrin repeat domain-containing protein 50-like [Amphimedon queenslandica]	GCF_000090795.1	TRADDN-NCOA6
XP_019861354.1	TRADDN-NCOA6+Ankyrin	934	LOC109580161	metazoa	Amphimedon queenslandica	PREDICTED: ankyrin repeat domain-containing protein 50-like [Amphimedon queenslandica]	GCF_000090795.1	TRADDN-NCOA6
XP_019854640.1	TRADDN-NCOA6+Ankyrin+LRRs	478	LOC105313507	metazoa	Amphimedon queenslandica	PREDICTED: tankyrase-1-like [Amphimedon queenslandica]	GCF_000090795.1	TRADDN-NCOA6
XP_011409104.1	TRADDN-NCOA6+Ankyrin+NACHT	425	LOC105316014	metazoa	Amphimedon queenslandica	PREDICTED: uncharacterized protein LOC105316014, partial [Amphimedon queenslandica]	GCF_000090795.1	TRADDN-NCOA6
XP_019852240.1	TRADDN-NCOA6+Ankyrin+TRADDN-NCOA6+Ankyrin	1414	LOC109582083	metazoa	Amphimedon queenslandica	PREDICTED: serine/threonine-protein phosphatase 6 regulatory ankyrin repeat subunit B-like [Amphimedon queenslandica]	GCF_000090795.1	TRADDN-NCOA6
XP_011408474.1	TRADDN-NCOA6+CASPASE	366	LOC105315492	metazoa	Amphimedon queenslandica	PREDICTED: caspase-3-like [Amphimedon queenslandica]	GCF_000090795.1	TRADDN-NCOA6

acc	architecture	len	gen.name	taxend	species	defline	gca	clade
XP_019858304.1	TRADDN-NCOA6+Coiled-coil	368	LOC109586544	metazoa	Amphimedon queenslandica	PREDICTED: uncharacterized protein LOC109586544 [Amphimedon queenslandica]	GCF_000090795.1	TRADDN-NCOA6
XP_019858663.1	TRADDN-NCOA6+Coiled-coil	202	LOC109586878	metazoa	Amphimedon queenslandica	PREDICTED: uncharacterized protein LOC109586878 [Amphimedon queenslandica]	GCF_000090795.1	TRADDN-NCOA6
XP_019859162.1	TRADDN-NCOA6+Coiled-coil	195	LOC109587363	metazoa	Amphimedon queenslandica	PREDICTED: uncharacterized protein LOC109587363, partial [Amphimedon queenslandica]	GCF_000090795.1	TRADDN-NCOA6
XP_019860361.1	TRADDN-NCOA6+Coiled-coil	395	LOC109588658	metazoa	Amphimedon queenslandica	PREDICTED: uncharacterized protein LOC109588658 [Amphimedon queenslandica]	GCF_000090795.1	TRADDN-NCOA6
XP_019861027.1	TRADDN-NCOA6+Coiled-coil	356	LOC109589378	metazoa	Amphimedon queenslandica	PREDICTED: histone-lysine N-methyltransferase, H3 lysine-79 specific-like [Amphimedon queenslandica]	GCF_000090795.1	TRADDN-NCOA6
XP_019863404.1	TRADDN-NCOA6+Coiled-coil	280	LOC109592390	metazoa	Amphimedon queenslandica	PREDICTED: protein NETWORKED 1D-like [Amphimedon queenslandica]	GCF_000090795.1	TRADDN-NCOA6
XP_019863811.1	TRADDN-NCOA6+Coiled-coil	186	LOC109592958	metazoa	Amphimedon queenslandica	PREDICTED: uncharacterized protein LOC109592958 [Amphimedon queenslandica]	GCF_000090795.1	TRADDN-NCOA6
XP_019862385.1	TRADDN-NCOA6+Death+CARD+DED	613	LOC109591010	metazoa	Amphimedon queenslandica	PREDICTED: uncharacterized protein LOC109591010 [Amphimedon queenslandica]	GCF_000090795.1	TRADDN-NCOA6
XP_019861074.1	TRADDN-NCOA6+DED	260	LOC109589425	metazoa	Amphimedon queenslandica	PREDICTED: uncharacterized protein LOC109589425 [Amphimedon queenslandica]	GCF_000090795.1	TRADDN-NCOA6
XP_019853789.1	TRADDN-NCOA6+HSP70	394	LOC109583059	metazoa	Amphimedon queenslandica	PREDICTED: heat shock 70 kDa protein 12A-like [Amphimedon queenslandica]	GCF_000090795.1	TRADDN-NCOA6
XP_019859366.1	TRADDN-NCOA6+LRRs	285	LOC109587575	metazoa	Amphimedon queenslandica	PREDICTED: uncharacterized protein LOC109587575 [Amphimedon queenslandica]	GCF_000090795.1	TRADDN-NCOA6
XP_019860334.1	TRADDN-NCOA6+LRRs	405	LOC109588630	metazoa	Amphimedon queenslandica	PREDICTED: uncharacterized protein LOC109588630 [Amphimedon queenslandica]	GCF_000090795.1	TRADDN-NCOA6
XP_019863044.1	TRADDN-NCOA6+LRRs	280	LOC109591879	metazoa	Amphimedon queenslandica	PREDICTED: ARF GTPase-activating protein GIT1-like [Amphimedon queenslandica]	GCF_000090795.1	TRADDN-NCOA6
XP_019861299.1	TRADDN-NCOA6+TM+TM+TM+TM+TM+TM+TM+TM	386	LOC109589697	metazoa	Amphimedon queenslandica	PREDICTED: uncharacterized protein LOC109589697 [Amphimedon queenslandica]	GCF_000090795.1	TRADDN-NCOA6
XP_019858034.1	TRADDN-NCOA6+UPA+UPA+UPA	664	LOC109586295	metazoa	Amphimedon queenslandica	PREDICTED: uncharacterized protein LOC109586295 [Amphimedon queenslandica]	GCF_000090795.1	TRADDN-NCOA6
XP_019860681.1	TRADDN-NCOA6+UPA+UPA+UPA	516	LOC109589000	metazoa	Amphimedon queenslandica	PREDICTED: uncharacterized protein LOC109589000 [Amphimedon queenslandica]	GCF_000090795.1	TRADDN-NCOA6

acc	architecture	len	gen.name	taxend	species	defline	gca	clade
XP_019850766.1	TRADDN-NCOA6+ZnR+RING+IBR+IBR	589	LOC100634100	metazoa	Amphimedon queenslandica	PREDICTED: ranBP-type and C3HC4-type zinc finger-containing protein 1-like [Amphimedon queenslandica]	GCF_000090795.1	TRADDN-NCOA6
ELT91616.1	CARD+TRADDN-DEDD2	240	CAPTEDRAFT_211634	annelida	Capitella teleta	hypothetical protein CAPTEDRAFT_211634 [Capitella teleta]	GCA_000328365.1	TRADDN-DEDD2
ELT98186.1	CARD+TRADDN-DEDD2	225	CAPTEDRAFT_192039	annelida	Capitella teleta	hypothetical protein CAPTEDRAFT_192039, partial [Capitella teleta]	GCA_000328365.1	TRADDN-DEDD2
ELT98437.1	CARD+TRADDN-DEDD2	299	CAPTEDRAFT_205544	annelida	Capitella teleta	hypothetical protein CAPTEDRAFT_205544 [Capitella teleta]	GCA_000328365.1	TRADDN-DEDD2
ELU00167.1	CARD+TRADDN-DEDD2	422	CAPTEDRAFT_220800	annelida	Capitella teleta	hypothetical protein CAPTEDRAFT_220800 [Capitella teleta]	GCA_000328365.1	TRADDN-DEDD2
ELU13510.1	CARD+TRADDN-DEDD2	244	CAPTEDRAFT_187038	annelida	Capitella teleta	hypothetical protein CAPTEDRAFT_187038 [Capitella teleta]	GCA_000328365.1	TRADDN-DEDD2
ELU15725.1	CARD+TRADDN-DEDD2	372	CAPTEDRAFT_201143	annelida	Capitella teleta	hypothetical protein CAPTEDRAFT_201143 [Capitella teleta]	GCA_000328365.1	TRADDN-DEDD2
ELT99737.1	low-complexity+DED+TRADDN-DEDD2	327	CAPTEDRAFT_161793	annelida	Capitella teleta	hypothetical protein CAPTEDRAFT_161793, partial [Capitella teleta]	GCA_000328365.1	TRADDN-DEDD2
ELT87317.1	TRADDN-DEDD2+Coiled-coil+7TM_GPCR_Srsx	827	CAPTEDRAFT_189113	annelida	Capitella teleta	hypothetical protein CAPTEDRAFT_189113 [Capitella teleta]	GCA_000328365.1	TRADDN-DEDD2
XP_009027296.1	TRADDN-NCOA6	1503	HELRODRAFT_163970	annelida	Helobdella robusta	hypothetical protein HELRODRAFT_163970 [Helobdella robusta]	GCF_000326865.1	TRADDN-NCOA6
EEN43952.1	Beta-propeller+DED+low-complexity+TRADDN-NCOA6+domain1+Pro-rich+Beta-propeller	1101	BRAFLDRAFT_87329	chordata	Branchiostoma floridae	hypothetical protein BRAFLDRAFT_87329 [Branchiostoma floridae]	GCA_000003815.1	TRADDN-NCOA6
XP_002587941.1	Beta-propeller+DED+low-complexity+TRADDN-NCOA6+domain1+Pro-rich+Beta-propeller	1101	BRAFLDRAFT_87329	chordata	Branchiostoma floridae	hypothetical protein BRAFLDRAFT_87329 [Branchiostoma floridae]	GCF_000003815.1	TRADDN-NCOA6
EEN43902.1	Beta-propeller+DED+TRADDN-NCOA6+domain1+Pro-rich+Beta-propeller	946	BRAFLDRAFT_87279	chordata	Branchiostoma floridae	hypothetical protein BRAFLDRAFT_87279 [Branchiostoma floridae]	GCA_000003815.1	TRADDN-NCOA6
XP_002587891.1	Beta-propeller+DED+TRADDN-NCOA6+domain1+Pro-rich+Beta-propeller	946	BRAFLDRAFT_87279	chordata	Branchiostoma floridae	hypothetical protein BRAFLDRAFT_87279 [Branchiostoma floridae]	GCF_000003815.1	TRADDN-NCOA6
XP_019633890.1	CARD+Death+FN3+TRADDN-DEDD2+Death+Death+zf-TAZ+LRRs	1789	LOC109477248	chordata	Branchiostoma belcheri	PREDICTED: uncharacterized protein LOC109477248 [Branchiostoma belcheri]	GCF_001625305.1	TRADDN-DEDD2
XP_019633519.1	CARD+Death+TRADDN-DEDD2	505	LOC109476942	chordata	Branchiostoma belcheri	PREDICTED: uncharacterized protein LOC109476942 [Branchiostoma belcheri]	GCF_001625305.1	TRADDN-DEDD2
XP_002605446.1	CARD+FN3+TRADDN-DEDD2+NTP_transf+HEPN+TPRs	1299	BRAFLDRAFT_74261	chordata	Branchiostoma floridae	hypothetical protein BRAFLDRAFT_74261 [Branchiostoma floridae]	GCF_000003815.1	TRADDN-DEDD2

acc	architecture	len	gen.name	taxend	species	defline	gca	clade
XP_019613615.1	CARD+FN3+TRADDN-DEDD2+Nucleotidyltransferase+HEPN+TPRs	1361	LOC109461677	chordata	Branchiostoma belcheri	PREDICTED: uncharacterized protein LOC109461677 [Branchiostoma belcheri]	GCF_001625305.1	TRADDN-DEDD2
XP_019630679.1	CARD+FN3+TRADDN-DEDD2+STAND+LRRs+Death+GT4-GTase	2024	LOC109474759	chordata	Branchiostoma belcheri	PREDICTED: uncharacterized protein LOC109474759 isoform X1 [Branchiostoma belcheri]	GCF_001625305.1	TRADDN-DEDD2
XP_019630680.1	CARD+FN3+TRADDN-DEDD2+STAND+LRRs+Death+GT4-GTase	2001	LOC109474759	chordata	Branchiostoma belcheri	PREDICTED: uncharacterized protein LOC109474759 isoform X2 [Branchiostoma belcheri]	GCF_001625305.1	TRADDN-DEDD2
XP_019631927.1	CARD+GT4-GTase+Death+TRADDN-DEDD2+Death+helical-region+LRRs	2067	LOC109475631	chordata	Branchiostoma belcheri	PREDICTED: LOW QUALITY PROTEIN: uncharacterized protein LOC109475631 [Branchiostoma belcheri]	GCF_001625305.1	TRADDN-DEDD2
XP_002589318.1	CARD+TIR+Death+TRADDN-DEDD2+Death+LRRs+Death+Death+TRADDN-DEDD2+Death+LRRs	2945	BRAFLDRAFT_77771	chordata	Branchiostoma floridae	hypothetical protein BRAFLDRAFT_77771 [Branchiostoma floridae]	GCF_000003815.1	TRADDN-DEDD2
XP_019620289.1	CARD+TIR+TRADDN-DEDD2	435	LOC109466871	chordata	Branchiostoma belcheri	PREDICTED: uncharacterized protein LOC109466871 [Branchiostoma belcheri]	GCF_001625305.1	TRADDN-DEDD2
XP_019637190.1	CARD+TIR+TRADDN-DEDD2	552	LOC109479645	chordata	Branchiostoma belcheri	PREDICTED: uncharacterized protein LOC109479645 [Branchiostoma belcheri]	GCF_001625305.1	TRADDN-DEDD2
XP_019640463.1	CARD+TIR+TRADDN-DEDD2	450	LOC109482212	chordata	Branchiostoma belcheri	PREDICTED: uncharacterized protein LOC109482212 [Branchiostoma belcheri]	GCF_001625305.1	TRADDN-DEDD2
XP_002603309.1	CARD+TIR+TRADDN-DEDD2	492	BRAFLDRAFT_71397	chordata	Branchiostoma floridae	hypothetical protein BRAFLDRAFT_71397 [Branchiostoma floridae]	GCF_000003815.1	TRADDN-DEDD2
XP_002610849.1	CARD+TIR+TRADDN-DEDD2	531	BRAFLDRAFT_94911	chordata	Branchiostoma floridae	hypothetical protein BRAFLDRAFT_94911, partial [Branchiostoma floridae]	GCF_000003815.1	TRADDN-DEDD2
XP_002599430.1	CARD+TIR+TRADDN-DEDD2+Coiled-coil+STAND+Death+FIIND	1426	BRAFLDRAFT_106573	chordata	Branchiostoma floridae	hypothetical protein BRAFLDRAFT_106573 [Branchiostoma floridae]	GCF_000003815.1	TRADDN-DEDD2
XP_002589337.1	CARD+TIR+TRADDN-DEDD2+Death+LRRs	1627	BRAFLDRAFT_121797	chordata	Branchiostoma floridae	hypothetical protein BRAFLDRAFT_121797 [Branchiostoma floridae]	GCF_000003815.1	TRADDN-DEDD2
XP_002592322.1	CARD+TIR+TRADDN-DEDD2+Dynamin-like_sGTPase+helical-region+Pkinase	1265	BRAFLDRAFT_101263	chordata	Branchiostoma floridae	hypothetical protein BRAFLDRAFT_101263 [Branchiostoma floridae]	GCF_000003815.1	TRADDN-DEDD2
XP_002603295.1	CARD+TIR+TRADDN-DEDD2+LRRs	903	BRAFLDRAFT_71410	chordata	Branchiostoma floridae	hypothetical protein BRAFLDRAFT_71410 [Branchiostoma floridae]	GCF_000003815.1	TRADDN-DEDD2
XP_019637202.1	CARD+TIR+TRADDN-DEDD2+STAND	751	LOC109479656	chordata	Branchiostoma belcheri	PREDICTED: uncharacterized protein LOC109479656 [Branchiostoma belcheri]	GCF_001625305.1	TRADDN-DEDD2
XP_019637612.1	CARD+TRADDN-DEDD2+LRRs	858	LOC109479969	chordata	Branchiostoma belcheri	PREDICTED: uncharacterized protein LOC109479969 [Branchiostoma belcheri]	GCF_001625305.1	TRADDN-DEDD2
XP_002614043.1	CARD+TRADDN-DEDD2+LRRs	981	BRAFLDRAFT_67373	chordata	Branchiostoma floridae	hypothetical protein BRAFLDRAFT_67373 [Branchiostoma floridae]	GCF_000003815.1	TRADDN-DEDD2

acc	architecture	len	gen.name	taxend	species	defline	gca	clade
XP_019616599.1	Death+Death+TRADDN-DEDD2+Death	941	LOC109464106	chordata	Branchiostoma belcheri	PREDICTED: uncharacterized protein LOC109464106 [Branchiostoma belcheri]	GCF_001625305.1	TRADDN-DEDD2
XP_019632346.1	Death+Death+TRADDN-DEDD2+Death	692	LOC109475980	chordata	Branchiostoma belcheri	PREDICTED: uncharacterized protein LOC109475980 [Branchiostoma belcheri]	GCF_001625305.1	TRADDN-DEDD2
XP_019633949.1	Death+Death+TRADDN-DEDD2+Death+LRRs	973	LOC109477286	chordata	Branchiostoma belcheri	PREDICTED: uncharacterized protein LOC109477286 [Branchiostoma belcheri]	GCF_001625305.1	TRADDN-DEDD2
XP_019633976.1	Death+Death+TRADDN-DEDD2+Death+LRRs	1224	LOC109477298	chordata	Branchiostoma belcheri	PREDICTED: uncharacterized protein LOC109477298 [Branchiostoma belcheri]	GCF_001625305.1	TRADDN-DEDD2
XP_002589336.1	Death+Death+TRADDN-DEDD2+Death+LRRs	1470	BRAFLDRAFT_77788	chordata	Branchiostoma floridae	hypothetical protein BRAFLDRAFT_77788 [Branchiostoma floridae]	GCF_000003815.1	TRADDN-DEDD2
XP_002607774.1	Death+Death+TRADDN-DEDD2+Death+LRRs	753	BRAFLDRAFT_64173	chordata	Branchiostoma floridae	hypothetical protein BRAFLDRAFT_64173 [Branchiostoma floridae]	GCF_000003815.1	TRADDN-DEDD2
XP_019620287.1	Death+FN3+FN3+TRADDN-DEDD2	526	LOC109466869	chordata	Branchiostoma belcheri	PREDICTED: uncharacterized protein LOC109466869 [Branchiostoma belcheri]	GCF_001625305.1	TRADDN-DEDD2
XP_019626208.1	Death+FN3+FN3+TRADDN-DEDD2+Coiled-coil+Death+FN3+FN3+Death	1298	LOC109471359	chordata	Branchiostoma belcheri	PREDICTED: uncharacterized protein LOC109471359 [Branchiostoma belcheri]	GCF_001625305.1	TRADDN-DEDD2
XP_002587196.1	Death+FN3+FN3+TRADDN-DEDD2+p450	811	BRAFLDRAFT_102082	chordata	Branchiostoma floridae	hypothetical protein BRAFLDRAFT_102082, partial [Branchiostoma floridae]	GCF_000003815.1	TRADDN-DEDD2
XP_019621042.1	Death+TRADDN-DEDD2+CARD+Kinesin+SIGMA-HTH	862	LOC109467451	chordata	Branchiostoma belcheri	PREDICTED: kinesin-like protein KIF3A [Branchiostoma belcheri]	GCF_001625305.1	TRADDN-DEDD2
XP_018669464.1	Death+TRADDN-DEDD2+Coiled-coil	712	LOC100180910	chordata	Ciona intestinalis	uncharacterized protein LOC100180910 isoform X1 [Ciona intestinalis]	GCF_000224145.3	TRADDN-DEDD2
XP_026692598.1	Death+TRADDN-DEDD2+Coiled-coil	711	LOC100180910	chordata	Ciona intestinalis	uncharacterized protein LOC100180910 isoform X2 [Ciona intestinalis]	GCF_000224145.3	TRADDN-DEDD2
XP_019632108.1	Death+TRADDN-DEDD2+Coiled-coil+Beta-propeller	707	LOC109475790	chordata	Branchiostoma belcheri	PREDICTED: uncharacterized protein LOC109475790 [Branchiostoma belcheri]	GCF_001625305.1	TRADDN-DEDD2
XP_019640973.1	Death+TRADDN-DEDD2+Coiled-coil+Beta-propeller	700	LOC109482613	chordata	Branchiostoma belcheri	PREDICTED: uncharacterized protein LOC109482613 [Branchiostoma belcheri]	GCF_001625305.1	TRADDN-DEDD2
XP_019642611.1	Death+TRADDN-DEDD2+Coiled-coil+Beta-propeller	708	LOC109483912	chordata	Branchiostoma belcheri	PREDICTED: uncharacterized protein LOC109483912 [Branchiostoma belcheri]	GCF_001625305.1	TRADDN-DEDD2
XP_002607640.1	Death+TRADDN-DEDD2+Coiled-coil+Beta-propeller	735	BRAFLDRAFT_84671	chordata	Branchiostoma floridae	hypothetical protein BRAFLDRAFT_84671 [Branchiostoma floridae]	GCF_000003815.1	TRADDN-DEDD2
XP_002607641.1	Death+TRADDN-DEDD2+Coiled-coil+Beta-propeller	798	BRAFLDRAFT_84670	chordata	Branchiostoma floridae	hypothetical protein BRAFLDRAFT_84670 [Branchiostoma floridae]	GCF_000003815.1	TRADDN-DEDD2

acc	architecture	len	gen.name	taxend	species	defline	gca	clade
XP_002592893.1	Death+TRADDN-DEDD2+Coiled-coil+GT4-GTase	873	BRAFLDRAFT_65479	chordata	Branchiostoma floridae	hypothetical protein BRAFLDRAFT_65479 [Branchiostoma floridae]	GCF_000003815.1	TRADDN-DEDD2
XP_019628563.1	Death+TRADDN-DEDD2+DUF4561	564	LOC109473095	chordata	Branchiostoma belcheri	PREDICTED: uncharacterized protein LOC109473095 [Branchiostoma belcheri]	GCF_001625305.1	TRADDN-DEDD2
XP_002588465.1	Death+TRADDN-DEDD2+DUF4561	564	BRAFLDRAFT_63414	chordata	Branchiostoma floridae	hypothetical protein BRAFLDRAFT_63414 [Branchiostoma floridae]	GCF_000003815.1	TRADDN-DEDD2
XP_019643637.1	Death+TRADDN-DEDD2+RING+NYN+CCCH-ZF	851	LOC109484719	chordata	Branchiostoma belcheri	PREDICTED: uncharacterized protein LOC109484719 isoform X1 [Branchiostoma belcheri]	GCF_001625305.1	TRADDN-DEDD2
XP_019643638.1	Death+TRADDN-DEDD2+RING+NYN+CCCH-ZF	850	LOC109484719	chordata	Branchiostoma belcheri	PREDICTED: uncharacterized protein LOC109484719 isoform X2 [Branchiostoma belcheri]	GCF_001625305.1	TRADDN-DEDD2
XP_002614092.1	Death+TRADDN-DEDD2+STAND+wHTH+helical-region+LRRs+Ig+TM+SH3	1642	BRAFLDRAFT_67322	chordata	Branchiostoma floridae	hypothetical protein BRAFLDRAFT_67322 [Branchiostoma floridae]	GCF_000003815.1	TRADDN-DEDD2
XP_002602467.1	DED+CARD+TRADDN-DEDD2	350	BRAFLDRAFT_86851	chordata	Branchiostoma floridae	hypothetical protein BRAFLDRAFT_86851 [Branchiostoma floridae]	GCF_000003815.1	TRADDN-DEDD2
XP_002602468.1	DED+CARD+TRADDN-DEDD2	457	BRAFLDRAFT_86852	chordata	Branchiostoma floridae	hypothetical protein BRAFLDRAFT_86852 [Branchiostoma floridae]	GCF_000003815.1	TRADDN-DEDD2
XP_019619676.1	DED+Death+TRADDN-DEDD2+Coiled-coil+Ankyrin	910	LOC109466407	chordata	Branchiostoma belcheri	PREDICTED: uncharacterized protein LOC109466407 [Branchiostoma belcheri]	GCF_001625305.1	TRADDN-DEDD2
XP_002589319.1	DED+Death+TRADDN-DEDD2+Death+LRRs	1288	BRAFLDRAFT_77772	chordata	Branchiostoma floridae	hypothetical protein BRAFLDRAFT_77772 [Branchiostoma floridae]	GCF_000003815.1	TRADDN-DEDD2
XP_019640446.1	DED+Death+TRADDN-DEDD2+GT4-GTase+GT4-GTase	1614	LOC109482191	chordata	Branchiostoma belcheri	PREDICTED: uncharacterized protein LOC109482191 [Branchiostoma belcheri]	GCF_001625305.1	TRADDN-DEDD2
ACN39567.1	DED+Death+TRADDN-NCOA6+domain1	593	-	chordata	Branchiostoma floridae	DED, DD and repetition domain-containing protein 1 [Branchiostoma floridae]	-	TRADDN-NCOA6
EEN43918.1	DED+Death+TRADDN-NCOA6+domain1+Pro-rich+Beta-propeller	841	BRAFLDRAFT_87295	chordata	Branchiostoma floridae	hypothetical protein BRAFLDRAFT_87295 [Branchiostoma floridae]	GCA_000003815.1	TRADDN-NCOA6
EEN43936.1	DED+Death+TRADDN-NCOA6+domain1+Pro-rich+Beta-propeller	830	BRAFLDRAFT_87313	chordata	Branchiostoma floridae	hypothetical protein BRAFLDRAFT_87313 [Branchiostoma floridae]	GCA_000003815.1	TRADDN-NCOA6
EEN43954.1	DED+Death+TRADDN-NCOA6+domain1+Pro-rich+Beta-propeller	931	BRAFLDRAFT_87331	chordata	Branchiostoma floridae	hypothetical protein BRAFLDRAFT_87331 [Branchiostoma floridae]	GCA_000003815.1	TRADDN-NCOA6
XP_002587907.1	DED+Death+TRADDN-NCOA6+domain1+Pro-rich+Beta-propeller	841	BRAFLDRAFT_87295	chordata	Branchiostoma floridae	hypothetical protein BRAFLDRAFT_87295 [Branchiostoma floridae]	GCF_000003815.1	TRADDN-NCOA6
XP_002587925.1	DED+Death+TRADDN-NCOA6+domain1+Pro-rich+Beta-propeller	830	BRAFLDRAFT_87313	chordata	Branchiostoma floridae	hypothetical protein BRAFLDRAFT_87313 [Branchiostoma floridae]	GCF_000003815.1	TRADDN-NCOA6

acc	architecture	len	gen.name	taxend	species	defline	gca	clade
XP_002587943.1	DED+Death+TRADDN-NCOA6+domain1+Pro-rich+Beta-propeller	931	BRAFLDRAFT_87331	chordata	Branchiostoma floridae	hypothetical protein BRAFLDRAFT_87331 [Branchiostoma floridae]	GCF_000003815.1	TRADDN-NCOA6
EEN43913.1	DED+Death+TRADDN-NCOA6+domain1+Pro-rich+Beta-propeller+DED+TRADDN-NCOA6+domain1+Pro-rich+Beta-propeller	1664	BRAFLDRAFT_87290	chordata	Branchiostoma floridae	hypothetical protein BRAFLDRAFT_87290 [Branchiostoma floridae]	GCA_000003815.1	TRADDN-NCOA6
XP_002587902.1	DED+Death+TRADDN-NCOA6+domain1+Pro-rich+Beta-propeller+DED+TRADDN-NCOA6+domain1+Pro-rich+Beta-propeller	1664	BRAFLDRAFT_87290	chordata	Branchiostoma floridae	hypothetical protein BRAFLDRAFT_87290 [Branchiostoma floridae]	GCF_000003815.1	TRADDN-NCOA6
EEN61843.1	DED+DED+TRADDN-NCOA6+domain1+Pro-rich+Beta-propeller	684	BRAFLDRAFT_84318	chordata	Branchiostoma floridae	hypothetical protein BRAFLDRAFT_84318 [Branchiostoma floridae]	GCA_000003815.1	TRADDN-NCOA6
XP_002605833.1	DED+DED+TRADDN-NCOA6+domain1+Pro-rich+Beta-propeller	684	BRAFLDRAFT_84318	chordata	Branchiostoma floridae	hypothetical protein BRAFLDRAFT_84318 [Branchiostoma floridae]	GCF_000003815.1	TRADDN-NCOA6
XP_019631370.1	DED+FN3+FN3+TRADDN-DEDD2	530	LOC109475213	chordata	Branchiostoma belcheri	PREDICTED: uncharacterized protein LOC109475213 [Branchiostoma belcheri]	GCF_001625305.1	TRADDN-DEDD2
EEN43934.1	DED+low-complexity+Death+TRADDN-NCOA6+domain1+Pro-rich+Beta-propeller	908	BRAFLDRAFT_87311	chordata	Branchiostoma floridae	hypothetical protein BRAFLDRAFT_87311 [Branchiostoma floridae]	GCA_000003815.1	TRADDN-NCOA6
EEN43958.1	DED+low-complexity+Death+TRADDN-NCOA6+domain1+Pro-rich+Beta-propeller	932	BRAFLDRAFT_87335	chordata	Branchiostoma floridae	hypothetical protein BRAFLDRAFT_87335 [Branchiostoma floridae]	GCA_000003815.1	TRADDN-NCOA6
XP_002587923.1	DED+low-complexity+Death+TRADDN-NCOA6+domain1+Pro-rich+Beta-propeller	908	BRAFLDRAFT_87311	chordata	Branchiostoma floridae	hypothetical protein BRAFLDRAFT_87311 [Branchiostoma floridae]	GCF_000003815.1	TRADDN-NCOA6
XP_002587947.1	DED+low-complexity+Death+TRADDN-NCOA6+domain1+Pro-rich+Beta-propeller	932	BRAFLDRAFT_87335	chordata	Branchiostoma floridae	hypothetical protein BRAFLDRAFT_87335 [Branchiostoma floridae]	GCF_000003815.1	TRADDN-NCOA6
EEN43928.1	DED+low-complexity+Death+TRADDN-NCOA6+Ig+Ig	503	BRAFLDRAFT_87305	chordata	Branchiostoma floridae	hypothetical protein BRAFLDRAFT_87305 [Branchiostoma floridae]	GCA_000003815.1	TRADDN-NCOA6
XP_002587917.1	DED+low-complexity+Death+TRADDN-NCOA6+Ig+Ig	503	BRAFLDRAFT_87305	chordata	Branchiostoma floridae	hypothetical protein BRAFLDRAFT_87305 [Branchiostoma floridae]	GCF_000003815.1	TRADDN-NCOA6
EEN42177.1	DED+low-complexity+TRADDN-NCOA6+Beta-propeller	649	BRAFLDRAFT_109861	chordata	Branchiostoma floridae	hypothetical protein BRAFLDRAFT_109861 [Branchiostoma floridae]	GCA_000003815.1	TRADDN-NCOA6
XP_002586166.1	DED+low-complexity+TRADDN-NCOA6+Beta-propeller	649	BRAFLDRAFT_109861	chordata	Branchiostoma floridae	hypothetical protein BRAFLDRAFT_109861 [Branchiostoma floridae]	GCF_000003815.1	TRADDN-NCOA6
EEN43907.1	DED+low-complexity+TRADDN-NCOA6+domain1+DED+Death+TRADDN-NCOA6+domain1+Pro-rich+Beta-propeller	1288	BRAFLDRAFT_87284	chordata	Branchiostoma floridae	hypothetical protein BRAFLDRAFT_87284 [Branchiostoma floridae]	GCA_000003815.1	TRADDN-NCOA6
XP_002587896.1	DED+low-complexity+TRADDN-NCOA6+domain1+DED+Death+TRADDN-NCOA6+domain1+Pro-rich+Beta-propeller	1288	BRAFLDRAFT_87284	chordata	Branchiostoma floridae	hypothetical protein BRAFLDRAFT_87284 [Branchiostoma floridae]	GCF_000003815.1	TRADDN-NCOA6
XP_019618785.1	DED+low-complexity+TRADDN-NCOA6+domain1+Pro-rich+Beta-propeller	820	LOC109465788	chordata	Branchiostoma belcheri	PREDICTED: uncharacterized protein LOC109465788 [Branchiostoma belcheri]	GCF_001625305.1	TRADDN-NCOA6

acc	architecture	len	gen.name	taxend	species	defline	gca	clade
EEN43903.1	DED+low-complexity+TRADDN-NCOA6+domain1+Pro-rich+Beta-propeller	856	BRAFLDRAFT_87280	chordata	Branchiostoma floridae	hypothetical protein BRAFLDRAFT_87280 [Branchiostoma floridae]	GCA_000003815.1	TRADDN-NCOA6
EEN43909.1	DED+low-complexity+TRADDN-NCOA6+domain1+Pro-rich+Beta-propeller	851	BRAFLDRAFT_87286	chordata	Branchiostoma floridae	hypothetical protein BRAFLDRAFT_87286 [Branchiostoma floridae]	GCA_000003815.1	TRADDN-NCOA6
EEN43949.1	DED+low-complexity+TRADDN-NCOA6+domain1+Pro-rich+Beta-propeller	866	BRAFLDRAFT_87326	chordata	Branchiostoma floridae	hypothetical protein BRAFLDRAFT_87326 [Branchiostoma floridae]	GCA_000003815.1	TRADDN-NCOA6
EEN43955.1	DED+low-complexity+TRADDN-NCOA6+domain1+Pro-rich+Beta-propeller	854	BRAFLDRAFT_87332	chordata	Branchiostoma floridae	hypothetical protein BRAFLDRAFT_87332 [Branchiostoma floridae]	GCA_000003815.1	TRADDN-NCOA6
XP_002587892.1	DED+low-complexity+TRADDN-NCOA6+domain1+Pro-rich+Beta-propeller	856	BRAFLDRAFT_87280	chordata	Branchiostoma floridae	hypothetical protein BRAFLDRAFT_87280 [Branchiostoma floridae]	GCF_000003815.1	TRADDN-NCOA6
XP_002587898.1	DED+low-complexity+TRADDN-NCOA6+domain1+Pro-rich+Beta-propeller	851	BRAFLDRAFT_87286	chordata	Branchiostoma floridae	hypothetical protein BRAFLDRAFT_87286 [Branchiostoma floridae]	GCF_000003815.1	TRADDN-NCOA6
XP_002587938.1	DED+low-complexity+TRADDN-NCOA6+domain1+Pro-rich+Beta-propeller	866	BRAFLDRAFT_87326	chordata	Branchiostoma floridae	hypothetical protein BRAFLDRAFT_87326 [Branchiostoma floridae]	GCF_000003815.1	TRADDN-NCOA6
XP_002587944.1	DED+low-complexity+TRADDN-NCOA6+domain1+Pro-rich+Beta-propeller	854	BRAFLDRAFT_87332	chordata	Branchiostoma floridae	hypothetical protein BRAFLDRAFT_87332 [Branchiostoma floridae]	GCF_000003815.1	TRADDN-NCOA6
EEN43939.1	DED+low-complexity+TRADDN-NCOA6+domain1+Pro-rich+DED+Beta-propeller	968	BRAFLDRAFT_87316	chordata	Branchiostoma floridae	hypothetical protein BRAFLDRAFT_87316 [Branchiostoma floridae]	GCA_000003815.1	TRADDN-NCOA6
XP_002587928.1	DED+low-complexity+TRADDN-NCOA6+domain1+Pro-rich+DED+Beta-propeller	968	BRAFLDRAFT_87316	chordata	Branchiostoma floridae	hypothetical protein BRAFLDRAFT_87316 [Branchiostoma floridae]	GCF_000003815.1	TRADDN-NCOA6
XP_019620964.1	DED+TRADDN-DEDD2	561	LOC109467429	chordata	Branchiostoma belcheri	PREDICTED: uncharacterized protein LOC109467429 [Branchiostoma belcheri]	GCF_001625305.1	TRADDN-DEDD2
XP_002605016.1	DED+TRADDN-DEDD2+Coiled-coil+C2H2-ZF+C2H2-ZF	893	BRAFLDRAFT_85156	chordata	Branchiostoma floridae	hypothetical protein BRAFLDRAFT_85156 [Branchiostoma floridae]	GCF_000003815.1	TRADDN-DEDD2
XP_002599373.1	DED+TRADDN-DEDD2+Coiled-coil+Death+Coiled-coil	1029	BRAFLDRAFT_64269	chordata	Branchiostoma floridae	hypothetical protein BRAFLDRAFT_64269 [Branchiostoma floridae]	GCF_000003815.1	TRADDN-DEDD2
XP_002589317.1	DED+TRADDN-DEDD2+STAND+STAND+wHTH+helical-region+LRRs	2458	BRAFLDRAFT_77770	chordata	Branchiostoma floridae	hypothetical protein BRAFLDRAFT_77770 [Branchiostoma floridae]	GCF_000003815.1	TRADDN-DEDD2
XP_019634615.1	DED+TRADDN-NCOA6+domain1+Beta-propeller	696	LOC109477711	chordata	Branchiostoma belcheri	PREDICTED: uncharacterized protein LOC109477711 [Branchiostoma belcheri]	GCF_001625305.1	TRADDN-NCOA6
XP_019645067.1	DED+TRADDN-NCOA6+domain1+Beta-propeller	631	LOC109485833	chordata	Branchiostoma belcheri	PREDICTED: uncharacterized protein LOC109485833 [Branchiostoma belcheri]	GCF_001625305.1	TRADDN-NCOA6
EEN43951.1	DED+TRADDN-NCOA6+domain1+Beta-propeller	631	BRAFLDRAFT_87328	chordata	Branchiostoma floridae	hypothetical protein BRAFLDRAFT_87328 [Branchiostoma floridae]	GCA_000003815.1	TRADDN-NCOA6

acc	architecture	len	gen.name	taxend	species	defline	gca	clade
EEN59587.1	DED+TRADDN-NCOA6+domain1+Beta-propeller	757	BRAFLDRAFT_93221	chordata	Branchiostoma floridae	hypothetical protein BRAFLDRAFT_93221 [Branchiostoma floridae]	GCA_000003815.1	TRADDN-NCOA6
XP_002587940.1	DED+TRADDN-NCOA6+domain1+Beta-propeller	631	BRAFLDRAFT_87328	chordata	Branchiostoma floridae	hypothetical protein BRAFLDRAFT_87328 [Branchiostoma floridae]	GCF_000003815.1	TRADDN-NCOA6
XP_002603576.1	DED+TRADDN-NCOA6+domain1+Beta-propeller	757	BRAFLDRAFT_93221	chordata	Branchiostoma floridae	hypothetical protein BRAFLDRAFT_93221 [Branchiostoma floridae]	GCF_000003815.1	TRADDN-NCOA6
EEN43962.1	DED+TRADDN-NCOA6+domain1+Pro-rich	716	BRAFLDRAFT_124881	chordata	Branchiostoma floridae	hypothetical protein BRAFLDRAFT_124881 [Branchiostoma floridae]	GCA_000003815.1	TRADDN-NCOA6
XP_002587951.1	DED+TRADDN-NCOA6+domain1+Pro-rich	716	BRAFLDRAFT_124881	chordata	Branchiostoma floridae	hypothetical protein BRAFLDRAFT_124881 [Branchiostoma floridae]	GCF_000003815.1	TRADDN-NCOA6
XP_019616863.1	DED+TRADDN-NCOA6+domain1+Pro-rich+Beta-propeller	742	LOC109464356	chordata	Branchiostoma belcheri	PREDICTED: uncharacterized protein LOC109464356 [Branchiostoma belcheri]	GCF_001625305.1	TRADDN-NCOA6
XP_019626565.1	DED+TRADDN-NCOA6+domain1+Pro-rich+Beta-propeller	722	LOC109471670	chordata	Branchiostoma belcheri	PREDICTED: protein lin-41-like [Branchiostoma belcheri]	GCF_001625305.1	TRADDN-NCOA6
XP_019630271.1	DED+TRADDN-NCOA6+domain1+Pro-rich+Beta-propeller	726	LOC109474417	chordata	Branchiostoma belcheri	PREDICTED: uncharacterized protein LOC109474417 [Branchiostoma belcheri]	GCF_001625305.1	TRADDN-NCOA6
XP_019640714.1	DED+TRADDN-NCOA6+domain1+Pro-rich+Beta-propeller	763	LOC109482446	chordata	Branchiostoma belcheri	PREDICTED: uncharacterized protein LOC109482446 [Branchiostoma belcheri]	GCF_001625305.1	TRADDN-NCOA6
XP_019642983.1	DED+TRADDN-NCOA6+domain1+Pro-rich+Beta-propeller	689	LOC109484188	chordata	Branchiostoma belcheri	PREDICTED: uncharacterized protein LOC109484188 [Branchiostoma belcheri]	GCF_001625305.1	TRADDN-NCOA6
XP_019647870.1	DED+TRADDN-NCOA6+domain1+Pro-rich+Beta-propeller	716	LOC109488153	chordata	Branchiostoma belcheri	PREDICTED: uncharacterized protein LOC109488153 [Branchiostoma belcheri]	GCF_001625305.1	TRADDN-NCOA6
EEN43900.1	DED+TRADDN-NCOA6+domain1+Pro-rich+Beta-propeller	811	BRAFLDRAFT_87276	chordata	Branchiostoma floridae	hypothetical protein BRAFLDRAFT_87276 [Branchiostoma floridae]	GCA_000003815.1	TRADDN-NCOA6
EEN43905.1	DED+TRADDN-NCOA6+domain1+Pro-rich+Beta-propeller	779	BRAFLDRAFT_87282	chordata	Branchiostoma floridae	hypothetical protein BRAFLDRAFT_87282 [Branchiostoma floridae]	GCA_000003815.1	TRADDN-NCOA6
EEN43906.1	DED+TRADDN-NCOA6+domain1+Pro-rich+Beta-propeller	854	BRAFLDRAFT_87283	chordata	Branchiostoma floridae	hypothetical protein BRAFLDRAFT_87283 [Branchiostoma floridae]	GCA_000003815.1	TRADDN-NCOA6
EEN43908.1	DED+TRADDN-NCOA6+domain1+Pro-rich+Beta-propeller	1220	BRAFLDRAFT_87285	chordata	Branchiostoma floridae	hypothetical protein BRAFLDRAFT_87285 [Branchiostoma floridae]	GCA_000003815.1	TRADDN-NCOA6
EEN43910.1	DED+TRADDN-NCOA6+domain1+Pro-rich+Beta-propeller	779	BRAFLDRAFT_87287	chordata	Branchiostoma floridae	hypothetical protein BRAFLDRAFT_87287 [Branchiostoma floridae]	GCA_000003815.1	TRADDN-NCOA6
EEN43911.1	DED+TRADDN-NCOA6+domain1+Pro-rich+Beta-propeller	793	BRAFLDRAFT_87288	chordata	Branchiostoma floridae	hypothetical protein BRAFLDRAFT_87288 [Branchiostoma floridae]	GCA_000003815.1	TRADDN-NCOA6

acc	architecture	len	gen.name	taxend	species	defline	gca	clade
EEN43912.1	DED+TRADDN-NCOA6+domain1+Pro-rich+Beta-propeller	783	BRAFLDRAFT_87289	chordata	Branchiostoma floridae	hypothetical protein BRAFLDRAFT_87289 [Branchiostoma floridae]	GCA_000003815.1	TRADDN-NCOA6
EEN43914.1	DED+TRADDN-NCOA6+domain1+Pro-rich+Beta-propeller	828	BRAFLDRAFT_87291	chordata	Branchiostoma floridae	hypothetical protein BRAFLDRAFT_87291 [Branchiostoma floridae]	GCA_000003815.1	TRADDN-NCOA6
EEN43915.1	DED+TRADDN-NCOA6+domain1+Pro-rich+Beta-propeller	792	BRAFLDRAFT_87292	chordata	Branchiostoma floridae	hypothetical protein BRAFLDRAFT_87292 [Branchiostoma floridae]	GCA_000003815.1	TRADDN-NCOA6
EEN43917.1	DED+TRADDN-NCOA6+domain1+Pro-rich+Beta-propeller	809	BRAFLDRAFT_87294	chordata	Branchiostoma floridae	hypothetical protein BRAFLDRAFT_87294 [Branchiostoma floridae]	GCA_000003815.1	TRADDN-NCOA6
EEN43920.1	DED+TRADDN-NCOA6+domain1+Pro-rich+Beta-propeller	792	BRAFLDRAFT_87297	chordata	Branchiostoma floridae	hypothetical protein BRAFLDRAFT_87297 [Branchiostoma floridae]	GCA_000003815.1	TRADDN-NCOA6
EEN43921.1	DED+TRADDN-NCOA6+domain1+Pro-rich+Beta-propeller	792	BRAFLDRAFT_87298	chordata	Branchiostoma floridae	hypothetical protein BRAFLDRAFT_87298 [Branchiostoma floridae]	GCA_000003815.1	TRADDN-NCOA6
EEN43922.1	DED+TRADDN-NCOA6+domain1+Pro-rich+Beta-propeller	729	BRAFLDRAFT_87299	chordata	Branchiostoma floridae	hypothetical protein BRAFLDRAFT_87299 [Branchiostoma floridae]	GCA_000003815.1	TRADDN-NCOA6
EEN43925.1	DED+TRADDN-NCOA6+domain1+Pro-rich+Beta-propeller	785	BRAFLDRAFT_87302	chordata	Branchiostoma floridae	hypothetical protein BRAFLDRAFT_87302 [Branchiostoma floridae]	GCA_000003815.1	TRADDN-NCOA6
EEN43929.1	DED+TRADDN-NCOA6+domain1+Pro-rich+Beta-propeller	764	BRAFLDRAFT_87306	chordata	Branchiostoma floridae	hypothetical protein BRAFLDRAFT_87306 [Branchiostoma floridae]	GCA_000003815.1	TRADDN-NCOA6
EEN43930.1	DED+TRADDN-NCOA6+domain1+Pro-rich+Beta-propeller	835	BRAFLDRAFT_87307	chordata	Branchiostoma floridae	hypothetical protein BRAFLDRAFT_87307 [Branchiostoma floridae]	GCA_000003815.1	TRADDN-NCOA6
EEN43931.1	DED+TRADDN-NCOA6+domain1+Pro-rich+Beta-propeller	800	BRAFLDRAFT_87308	chordata	Branchiostoma floridae	hypothetical protein BRAFLDRAFT_87308 [Branchiostoma floridae]	GCA_000003815.1	TRADDN-NCOA6
EEN43932.1	DED+TRADDN-NCOA6+domain1+Pro-rich+Beta-propeller	798	BRAFLDRAFT_87309	chordata	Branchiostoma floridae	hypothetical protein BRAFLDRAFT_87309 [Branchiostoma floridae]	GCA_000003815.1	TRADDN-NCOA6
EEN43935.1	DED+TRADDN-NCOA6+domain1+Pro-rich+Beta-propeller	724	BRAFLDRAFT_87312	chordata	Branchiostoma floridae	hypothetical protein BRAFLDRAFT_87312 [Branchiostoma floridae]	GCA_000003815.1	TRADDN-NCOA6
EEN43937.1	DED+TRADDN-NCOA6+domain1+Pro-rich+Beta-propeller	752	BRAFLDRAFT_87314	chordata	Branchiostoma floridae	hypothetical protein BRAFLDRAFT_87314 [Branchiostoma floridae]	GCA_000003815.1	TRADDN-NCOA6
EEN43938.1	DED+TRADDN-NCOA6+domain1+Pro-rich+Beta-propeller	791	BRAFLDRAFT_87315	chordata	Branchiostoma floridae	hypothetical protein BRAFLDRAFT_87315 [Branchiostoma floridae]	GCA_000003815.1	TRADDN-NCOA6
EEN43940.1	DED+TRADDN-NCOA6+domain1+Pro-rich+Beta-propeller	809	BRAFLDRAFT_87317	chordata	Branchiostoma floridae	hypothetical protein BRAFLDRAFT_87317 [Branchiostoma floridae]	GCA_000003815.1	TRADDN-NCOA6
EEN43942.1	DED+TRADDN-NCOA6+domain1+Pro-rich+Beta-propeller	815	BRAFLDRAFT_87319	chordata	Branchiostoma floridae	hypothetical protein BRAFLDRAFT_87319 [Branchiostoma floridae]	GCA_000003815.1	TRADDN-NCOA6

acc	architecture	len	gen.name	taxend	species	defline	gca	clade
EEN43944.1	DED+TRADDN-NCOA6+domain1+Pro-rich+Beta-propeller	816	BRAFLDRAFT_87321	chordata	Branchiostoma floridae	hypothetical protein BRAFLDRAFT_87321 [Branchiostoma floridae]	GCA_000003815.1	TRADDN-NCOA6
EEN43945.1	DED+TRADDN-NCOA6+domain1+Pro-rich+Beta-propeller	818	BRAFLDRAFT_87322	chordata	Branchiostoma floridae	hypothetical protein BRAFLDRAFT_87322 [Branchiostoma floridae]	GCA_000003815.1	TRADDN-NCOA6
EEN43946.1	DED+TRADDN-NCOA6+domain1+Pro-rich+Beta-propeller	766	BRAFLDRAFT_87323	chordata	Branchiostoma floridae	hypothetical protein BRAFLDRAFT_87323 [Branchiostoma floridae]	GCA_000003815.1	TRADDN-NCOA6
EEN43947.1	DED+TRADDN-NCOA6+domain1+Pro-rich+Beta-propeller	705	BRAFLDRAFT_87324	chordata	Branchiostoma floridae	hypothetical protein BRAFLDRAFT_87324 [Branchiostoma floridae]	GCA_000003815.1	TRADDN-NCOA6
EEN43948.1	DED+TRADDN-NCOA6+domain1+Pro-rich+Beta-propeller	755	BRAFLDRAFT_87325	chordata	Branchiostoma floridae	hypothetical protein BRAFLDRAFT_87325 [Branchiostoma floridae]	GCA_000003815.1	TRADDN-NCOA6
EEN43950.1	DED+TRADDN-NCOA6+domain1+Pro-rich+Beta-propeller	857	BRAFLDRAFT_87327	chordata	Branchiostoma floridae	hypothetical protein BRAFLDRAFT_87327 [Branchiostoma floridae]	GCA_000003815.1	TRADDN-NCOA6
EEN43957.1	DED+TRADDN-NCOA6+domain1+Pro-rich+Beta-propeller	786	BRAFLDRAFT_87334	chordata	Branchiostoma floridae	hypothetical protein BRAFLDRAFT_87334 [Branchiostoma floridae]	GCA_000003815.1	TRADDN-NCOA6
EEN43959.1	DED+TRADDN-NCOA6+domain1+Pro-rich+Beta-propeller	774	BRAFLDRAFT_87336	chordata	Branchiostoma floridae	hypothetical protein BRAFLDRAFT_87336 [Branchiostoma floridae]	GCA_000003815.1	TRADDN-NCOA6
EEN43961.1	DED+TRADDN-NCOA6+domain1+Pro-rich+Beta-propeller	797	BRAFLDRAFT_87338	chordata	Branchiostoma floridae	hypothetical protein BRAFLDRAFT_87338 [Branchiostoma floridae]	GCA_000003815.1	TRADDN-NCOA6
EEN56723.1	DED+TRADDN-NCOA6+domain1+Pro-rich+Beta-propeller	740	BRAFLDRAFT_83452	chordata	Branchiostoma floridae	hypothetical protein BRAFLDRAFT_83452 [Branchiostoma floridae]	GCA_000003815.1	TRADDN-NCOA6
XP_002587889.1	DED+TRADDN-NCOA6+domain1+Pro-rich+Beta-propeller	811	BRAFLDRAFT_87276	chordata	Branchiostoma floridae	hypothetical protein BRAFLDRAFT_87276 [Branchiostoma floridae]	GCF_000003815.1	TRADDN-NCOA6
XP_002587890.1	DED+TRADDN-NCOA6+domain1+Pro-rich+Beta-propeller	817	BRAFLDRAFT_87278	chordata	Branchiostoma floridae	hypothetical protein BRAFLDRAFT_87278 [Branchiostoma floridae]	GCF_000003815.1	TRADDN-NCOA6
XP_002587894.1	DED+TRADDN-NCOA6+domain1+Pro-rich+Beta-propeller	779	BRAFLDRAFT_87282	chordata	Branchiostoma floridae	hypothetical protein BRAFLDRAFT_87282 [Branchiostoma floridae]	GCF_000003815.1	TRADDN-NCOA6
XP_002587895.1	DED+TRADDN-NCOA6+domain1+Pro-rich+Beta-propeller	854	BRAFLDRAFT_87283	chordata	Branchiostoma floridae	hypothetical protein BRAFLDRAFT_87283 [Branchiostoma floridae]	GCF_000003815.1	TRADDN-NCOA6
XP_002587897.1	DED+TRADDN-NCOA6+domain1+Pro-rich+Beta-propeller	1220	BRAFLDRAFT_87285	chordata	Branchiostoma floridae	hypothetical protein BRAFLDRAFT_87285 [Branchiostoma floridae]	GCF_000003815.1	TRADDN-NCOA6
XP_002587899.1	DED+TRADDN-NCOA6+domain1+Pro-rich+Beta-propeller	779	BRAFLDRAFT_87287	chordata	Branchiostoma floridae	hypothetical protein BRAFLDRAFT_87287 [Branchiostoma floridae]	GCF_000003815.1	TRADDN-NCOA6
XP_002587900.1	DED+TRADDN-NCOA6+domain1+Pro-rich+Beta-propeller	793	BRAFLDRAFT_87288	chordata	Branchiostoma floridae	hypothetical protein BRAFLDRAFT_87288 [Branchiostoma floridae]	GCF_000003815.1	TRADDN-NCOA6

acc	architecture	len	gen.name	taxend	species	defline	gca	clade
XP_002587901.1	DED+TRADDN-NCOA6+domain1+Pro-rich+Beta-propeller	783	BRAFLDRAFT_87289	chordata	Branchiostoma floridae	hypothetical protein BRAFLDRAFT_87289 [Branchiostoma floridae]	GCF_000003815.1	TRADDN-NCOA6
XP_002587903.1	DED+TRADDN-NCOA6+domain1+Pro-rich+Beta-propeller	828	BRAFLDRAFT_87291	chordata	Branchiostoma floridae	hypothetical protein BRAFLDRAFT_87291 [Branchiostoma floridae]	GCF_000003815.1	TRADDN-NCOA6
XP_002587904.1	DED+TRADDN-NCOA6+domain1+Pro-rich+Beta-propeller	792	BRAFLDRAFT_87292	chordata	Branchiostoma floridae	hypothetical protein BRAFLDRAFT_87292 [Branchiostoma floridae]	GCF_000003815.1	TRADDN-NCOA6
XP_002587906.1	DED+TRADDN-NCOA6+domain1+Pro-rich+Beta-propeller	809	BRAFLDRAFT_87294	chordata	Branchiostoma floridae	hypothetical protein BRAFLDRAFT_87294 [Branchiostoma floridae]	GCF_000003815.1	TRADDN-NCOA6
XP_002587909.1	DED+TRADDN-NCOA6+domain1+Pro-rich+Beta-propeller	792	BRAFLDRAFT_87297	chordata	Branchiostoma floridae	hypothetical protein BRAFLDRAFT_87297 [Branchiostoma floridae]	GCF_000003815.1	TRADDN-NCOA6
XP_002587910.1	DED+TRADDN-NCOA6+domain1+Pro-rich+Beta-propeller	792	BRAFLDRAFT_87298	chordata	Branchiostoma floridae	hypothetical protein BRAFLDRAFT_87298 [Branchiostoma floridae]	GCF_000003815.1	TRADDN-NCOA6
XP_002587911.1	DED+TRADDN-NCOA6+domain1+Pro-rich+Beta-propeller	729	BRAFLDRAFT_87299	chordata	Branchiostoma floridae	hypothetical protein BRAFLDRAFT_87299 [Branchiostoma floridae]	GCF_000003815.1	TRADDN-NCOA6
XP_002587914.1	DED+TRADDN-NCOA6+domain1+Pro-rich+Beta-propeller	785	BRAFLDRAFT_87302	chordata	Branchiostoma floridae	hypothetical protein BRAFLDRAFT_87302 [Branchiostoma floridae]	GCF_000003815.1	TRADDN-NCOA6
XP_002587915.1	DED+TRADDN-NCOA6+domain1+Pro-rich+Beta-propeller	965	BRAFLDRAFT_87303	chordata	Branchiostoma floridae	hypothetical protein BRAFLDRAFT_87303 [Branchiostoma floridae]	GCF_000003815.1	TRADDN-NCOA6
XP_002587918.1	DED+TRADDN-NCOA6+domain1+Pro-rich+Beta-propeller	764	BRAFLDRAFT_87306	chordata	Branchiostoma floridae	hypothetical protein BRAFLDRAFT_87306 [Branchiostoma floridae]	GCF_000003815.1	TRADDN-NCOA6
XP_002587919.1	DED+TRADDN-NCOA6+domain1+Pro-rich+Beta-propeller	835	BRAFLDRAFT_87307	chordata	Branchiostoma floridae	hypothetical protein BRAFLDRAFT_87307 [Branchiostoma floridae]	GCF_000003815.1	TRADDN-NCOA6
XP_002587920.1	DED+TRADDN-NCOA6+domain1+Pro-rich+Beta-propeller	800	BRAFLDRAFT_87308	chordata	Branchiostoma floridae	hypothetical protein BRAFLDRAFT_87308 [Branchiostoma floridae]	GCF_000003815.1	TRADDN-NCOA6
XP_002587921.1	DED+TRADDN-NCOA6+domain1+Pro-rich+Beta-propeller	798	BRAFLDRAFT_87309	chordata	Branchiostoma floridae	hypothetical protein BRAFLDRAFT_87309 [Branchiostoma floridae]	GCF_000003815.1	TRADDN-NCOA6
XP_002587924.1	DED+TRADDN-NCOA6+domain1+Pro-rich+Beta-propeller	724	BRAFLDRAFT_87312	chordata	Branchiostoma floridae	hypothetical protein BRAFLDRAFT_87312 [Branchiostoma floridae]	GCF_000003815.1	TRADDN-NCOA6
XP_002587926.1	DED+TRADDN-NCOA6+domain1+Pro-rich+Beta-propeller	752	BRAFLDRAFT_87314	chordata	Branchiostoma floridae	hypothetical protein BRAFLDRAFT_87314 [Branchiostoma floridae]	GCF_000003815.1	TRADDN-NCOA6
XP_002587927.1	DED+TRADDN-NCOA6+domain1+Pro-rich+Beta-propeller	791	BRAFLDRAFT_87315	chordata	Branchiostoma floridae	hypothetical protein BRAFLDRAFT_87315 [Branchiostoma floridae]	GCF_000003815.1	TRADDN-NCOA6
XP_002587929.1	DED+TRADDN-NCOA6+domain1+Pro-rich+Beta-propeller	809	BRAFLDRAFT_87317	chordata	Branchiostoma floridae	hypothetical protein BRAFLDRAFT_87317 [Branchiostoma floridae]	GCF_000003815.1	TRADDN-NCOA6

acc	architecture	len	gen.name	taxend	species	defline	gca	clade
XP_002587931.1	DED+TRADDN-NCOA6+domain1+Pro-rich+Beta-propeller	815	BRAFLDRAFT_87319	chordata	Branchiostoma floridae	hypothetical protein BRAFLDRAFT_87319 [Branchiostoma floridae]	GCF_000003815.1	TRADDN-NCOA6
XP_002587933.1	DED+TRADDN-NCOA6+domain1+Pro-rich+Beta-propeller	816	BRAFLDRAFT_87321	chordata	Branchiostoma floridae	hypothetical protein BRAFLDRAFT_87321 [Branchiostoma floridae]	GCF_000003815.1	TRADDN-NCOA6
XP_002587934.1	DED+TRADDN-NCOA6+domain1+Pro-rich+Beta-propeller	818	BRAFLDRAFT_87322	chordata	Branchiostoma floridae	hypothetical protein BRAFLDRAFT_87322 [Branchiostoma floridae]	GCF_000003815.1	TRADDN-NCOA6
XP_002587935.1	DED+TRADDN-NCOA6+domain1+Pro-rich+Beta-propeller	766	BRAFLDRAFT_87323	chordata	Branchiostoma floridae	hypothetical protein BRAFLDRAFT_87323 [Branchiostoma floridae]	GCF_000003815.1	TRADDN-NCOA6
XP_002587936.1	DED+TRADDN-NCOA6+domain1+Pro-rich+Beta-propeller	705	BRAFLDRAFT_87324	chordata	Branchiostoma floridae	hypothetical protein BRAFLDRAFT_87324 [Branchiostoma floridae]	GCF_000003815.1	TRADDN-NCOA6
XP_002587937.1	DED+TRADDN-NCOA6+domain1+Pro-rich+Beta-propeller	755	BRAFLDRAFT_87325	chordata	Branchiostoma floridae	hypothetical protein BRAFLDRAFT_87325 [Branchiostoma floridae]	GCF_000003815.1	TRADDN-NCOA6
XP_002587939.1	DED+TRADDN-NCOA6+domain1+Pro-rich+Beta-propeller	857	BRAFLDRAFT_87327	chordata	Branchiostoma floridae	hypothetical protein BRAFLDRAFT_87327 [Branchiostoma floridae]	GCF_000003815.1	TRADDN-NCOA6
XP_002587946.1	DED+TRADDN-NCOA6+domain1+Pro-rich+Beta-propeller	786	BRAFLDRAFT_87334	chordata	Branchiostoma floridae	hypothetical protein BRAFLDRAFT_87334 [Branchiostoma floridae]	GCF_000003815.1	TRADDN-NCOA6
XP_002587948.1	DED+TRADDN-NCOA6+domain1+Pro-rich+Beta-propeller	774	BRAFLDRAFT_87336	chordata	Branchiostoma floridae	hypothetical protein BRAFLDRAFT_87336 [Branchiostoma floridae]	GCF_000003815.1	TRADDN-NCOA6
XP_002587950.1	DED+TRADDN-NCOA6+domain1+Pro-rich+Beta-propeller	797	BRAFLDRAFT_87338	chordata	Branchiostoma floridae	hypothetical protein BRAFLDRAFT_87338 [Branchiostoma floridae]	GCF_000003815.1	TRADDN-NCOA6
XP_002600711.1	DED+TRADDN-NCOA6+domain1+Pro-rich+Beta-propeller	740	BRAFLDRAFT_83452	chordata	Branchiostoma floridae	hypothetical protein BRAFLDRAFT_83452 [Branchiostoma floridae]	GCF_000003815.1	TRADDN-NCOA6
EEN43923.1	DED+TRADDN-NCOA6+domain1+Pro-rich+Beta-propeller+DED	898	BRAFLDRAFT_87300	chordata	Branchiostoma floridae	hypothetical protein BRAFLDRAFT_87300 [Branchiostoma floridae]	GCA_000003815.1	TRADDN-NCOA6
XP_002587912.1	DED+TRADDN-NCOA6+domain1+Pro-rich+Beta-propeller+DED	898	BRAFLDRAFT_87300	chordata	Branchiostoma floridae	hypothetical protein BRAFLDRAFT_87300 [Branchiostoma floridae]	GCF_000003815.1	TRADDN-NCOA6
EEN43916.1	DED+TRADDN-NCOA6+domain1+Pro-rich+Beta-propeller+DED+Death+TRADDN-NCOA6+domain1+Pro-rich+Beta-propeller	1751	BRAFLDRAFT_87293	chordata	Branchiostoma floridae	hypothetical protein BRAFLDRAFT_87293 [Branchiostoma floridae]	GCA_000003815.1	TRADDN-NCOA6
EEN43933.1	DED+TRADDN-NCOA6+domain1+Pro-rich+Beta-propeller+DED+Death+TRADDN-NCOA6+domain1+Pro-rich+Beta-propeller	1494	BRAFLDRAFT_87310	chordata	Branchiostoma floridae	hypothetical protein BRAFLDRAFT_87310 [Branchiostoma floridae]	GCA_000003815.1	TRADDN-NCOA6
XP_002587905.1	DED+TRADDN-NCOA6+domain1+Pro-rich+Beta-propeller+DED+Death+TRADDN-NCOA6+domain1+Pro-rich+Beta-propeller	1751	BRAFLDRAFT_87293	chordata	Branchiostoma floridae	hypothetical protein BRAFLDRAFT_87293 [Branchiostoma floridae]	GCF_000003815.1	TRADDN-NCOA6
XP_002587922.1	DED+TRADDN-NCOA6+domain1+Pro-rich+Beta-propeller+DED+Death+TRADDN-NCOA6+domain1+Pro-rich+Beta-propeller	1494	BRAFLDRAFT_87310	chordata	Branchiostoma floridae	hypothetical protein BRAFLDRAFT_87310 [Branchiostoma floridae]	GCF_000003815.1	TRADDN-NCOA6

acc	architecture	len	gen.name	taxend	species	defline	gca	clade
XP_019628362.1	DED+TRADDN-NCOA6+domain1+Pro-rich+Beta-propeller+DED+low-complexity+TRADDN-NCOA6+domain1+Pro-rich+Beta-propeller	1544	LOC109472937	chordata	Branchiostoma belcheri	PREDICTED: uncharacterized protein LOC109472937 [Branchiostoma belcheri]	GCF_001625305.1	TRADDN-NCOA6
XP_019628363.1	DED+TRADDN-NCOA6+domain1+Pro-rich+Beta-propeller+DED+TRADDN-NCOA6+domain1+Pro-rich+Beta-propeller	1566	LOC109472938	chordata	Branchiostoma belcheri	PREDICTED: uncharacterized protein LOC109472938 [Branchiostoma belcheri]	GCF_001625305.1	TRADDN-NCOA6
EEN43919.1	DED+TRADDN-NCOA6+domain1+Pro-rich+DED+low-complexity+TRADDN-NCOA6+domain1+Pro-rich+Beta-propeller	1403	BRAFLDRAFT_87296	chordata	Branchiostoma floridae	hypothetical protein BRAFLDRAFT_87296 [Branchiostoma floridae]	GCA_000003815.1	TRADDN-NCOA6
XP_002587908.1	DED+TRADDN-NCOA6+domain1+Pro-rich+DED+low-complexity+TRADDN-NCOA6+domain1+Pro-rich+Beta-propeller	1403	BRAFLDRAFT_87296	chordata	Branchiostoma floridae	hypothetical protein BRAFLDRAFT_87296 [Branchiostoma floridae]	GCF_000003815.1	TRADDN-NCOA6
EEN43964.1	DED+TRADDN-NCOA6+domain1+Pro-rich+DED+TRADDN-NCOA6	740	BRAFLDRAFT_87341	chordata	Branchiostoma floridae	hypothetical protein BRAFLDRAFT_87341 [Branchiostoma floridae]	GCA_000003815.1	TRADDN-NCOA6
XP_002587953.1	DED+TRADDN-NCOA6+domain1+Pro-rich+DED+TRADDN-NCOA6	740	BRAFLDRAFT_87341	chordata	Branchiostoma floridae	hypothetical protein BRAFLDRAFT_87341 [Branchiostoma floridae]	GCF_000003815.1	TRADDN-NCOA6
XP_019618784.1	DED+TRADDN-NCOA6+domain1+Pro-rich+DED+TRADDN-NCOA6+domain1+Pro-rich+Beta-propeller	1516	LOC109465787	chordata	Branchiostoma belcheri	PREDICTED: uncharacterized protein LOC109465787 [Branchiostoma belcheri]	GCF_001625305.1	TRADDN-NCOA6
EEN43953.1	DED+TRADDN-NCOA6+domain1+Pro-rich+Pro-rich	513	BRAFLDRAFT_87330	chordata	Branchiostoma floridae	hypothetical protein BRAFLDRAFT_87330 [Branchiostoma floridae]	GCA_000003815.1	TRADDN-NCOA6
XP_002587942.1	DED+TRADDN-NCOA6+domain1+Pro-rich+Pro-rich	513	BRAFLDRAFT_87330	chordata	Branchiostoma floridae	hypothetical protein BRAFLDRAFT_87330 [Branchiostoma floridae]	GCF_000003815.1	TRADDN-NCOA6
XP_002601333.1	DUF1720+TRADDN-DEDD2+LRRs+ACAS_N+AMP-binding	1738	BRAFLDRAFT_82757	chordata	Branchiostoma floridae	hypothetical protein BRAFLDRAFT_82757 [Branchiostoma floridae]	GCF_000003815.1	TRADDN-DEDD2
XP_019635466.1	FN3+FN3+TRADDN-DEDD2	454	LOC109478379	chordata	Branchiostoma belcheri	PREDICTED: titin-like, partial [Branchiostoma belcheri]	GCF_001625305.1	TRADDN-DEDD2
XP_019626729.1	FN3+FN3+TRADDN-DEDD2+Coiled-coil+Beta-propeller	910	LOC109471815	chordata	Branchiostoma belcheri	PREDICTED: uncharacterized protein LOC109471815 [Branchiostoma belcheri]	GCF_001625305.1	TRADDN-DEDD2
XP_002598013.1	FN3+FN3+TRADDN-DEDD2+Coiled-coil+Death+Trypsin	864	BRAFLDRAFT_79761	chordata	Branchiostoma floridae	hypothetical protein BRAFLDRAFT_79761 [Branchiostoma floridae]	GCF_000003815.1	TRADDN-DEDD2
XP_002607644.1	GT4-GTase+Coiled-coil+Death+TRADDN-DEDD2+Beta-propeller+Death+Death+Death+zf-C2H2+zf-C2H2+zf-C2H2+zf-C2H2+zf-C2H2+zf-C2H2+zf-C2H2+zf-C2H2	3703	BRAFLDRAFT_84667	chordata	Branchiostoma floridae	hypothetical protein BRAFLDRAFT_84667 [Branchiostoma floridae]	GCF_000003815.1	TRADDN-DEDD2
XP_019616577.1	GT4-GTase+TRADDN-DEDD2	575	LOC109464089	chordata	Branchiostoma belcheri	PREDICTED: uncharacterized protein LOC109464089 [Branchiostoma belcheri]	GCF_001625305.1	TRADDN-DEDD2
XP_019626675.1	GT4-GTase+TRADDN-DEDD2	569	LOC109471761	chordata	Branchiostoma belcheri	PREDICTED: uncharacterized protein LOC109471761 [Branchiostoma belcheri]	GCF_001625305.1	TRADDN-DEDD2
XP_019635472.1	GT4-GTase+TRADDN-DEDD2	642	LOC109478386	chordata	Branchiostoma belcheri	PREDICTED: uncharacterized protein LOC109478386 [Branchiostoma belcheri]	GCF_001625305.1	TRADDN-DEDD2

acc	architecture	len	gen.name	taxend	species	defline	gca	clade
XP_002606956.1	GT4-GTase+TRADDN-DEDD2	718	BRAFLDRAFT_64952	chordata	Branchiostoma floridae	hypothetical protein BRAFLDRAFT_64952 [Branchiostoma floridae]	GCF_000003815.1	TRADDN-DEDD2
XP_019626189.1	GT4-GTase+TRADDN-DEDD2+Ankyrin	856	LOC109471342	chordata	Branchiostoma belcheri	PREDICTED: uncharacterized protein LOC109471342 [Branchiostoma belcheri]	GCF_001625305.1	TRADDN-DEDD2
XP_019641871.1	GT4-GTase+TRADDN-DEDD2+Ankyrin	812	LOC109483324	chordata	Branchiostoma belcheri	PREDICTED: uncharacterized protein LOC109483324 [Branchiostoma belcheri]	GCF_001625305.1	TRADDN-DEDD2
XP_019644604.1	GT4-GTase+TRADDN-DEDD2+Beta-propeller	1071	LOC109485438	chordata	Branchiostoma belcheri	PREDICTED: uncharacterized protein LOC109485438 [Branchiostoma belcheri]	GCF_001625305.1	TRADDN-DEDD2
XP_002606063.1	GT4-GTase+TRADDN-DEDD2+Coiled-coil+GTPase-AIG	1278	BRAFLDRAFT_92082	chordata	Branchiostoma floridae	hypothetical protein BRAFLDRAFT_92082 [Branchiostoma floridae]	GCF_000003815.1	TRADDN-DEDD2
XP_002606060.1	GT4-GTase+TRADDN-DEDD2+DED+DED+DED+DED	1082	BRAFLDRAFT_92079	chordata	Branchiostoma floridae	hypothetical protein BRAFLDRAFT_92079 [Branchiostoma floridae]	GCF_000003815.1	TRADDN-DEDD2
XP_019616531.1	GT4-GTase+TRADDN-DEDD2+P-loop-NTPase	960	LOC109464048	chordata	Branchiostoma belcheri	PREDICTED: uncharacterized protein LOC109464048 [Branchiostoma belcheri]	GCF_001625305.1	TRADDN-DEDD2
XP_019620789.1	GT4-GTase+TRADDN-DEDD2+TPRs	1532	LOC109467286	chordata	Branchiostoma belcheri	PREDICTED: uncharacterized protein LOC109467286 [Branchiostoma belcheri]	GCF_001625305.1	TRADDN-DEDD2
XP_019633153.1	GT4-GTase+TRADDN-DEDD2+TPRs	1184	LOC109476602	chordata	Branchiostoma belcheri	PREDICTED: LOW QUALITY PROTEIN: uncharacterized protein LOC109476602 [Branchiostoma belcheri]	GCF_001625305.1	TRADDN-DEDD2
XP_019636955.1	GT4-GTase+TRADDN-DEDD2+TPRs	1131	LOC109479430	chordata	Branchiostoma belcheri	PREDICTED: uncharacterized protein LOC109479430 [Branchiostoma belcheri]	GCF_001625305.1	TRADDN-DEDD2
XP_019640556.1	GT4-GTase+TRADDN-DEDD2+TPRs	1537	LOC109482313	chordata	Branchiostoma belcheri	PREDICTED: uncharacterized protein LOC109482313 [Branchiostoma belcheri]	GCF_001625305.1	TRADDN-DEDD2
XP_019640557.1	GT4-GTase+TRADDN-DEDD2+TPRs	1507	LOC109482314	chordata	Branchiostoma belcheri	PREDICTED: uncharacterized protein LOC109482314 [Branchiostoma belcheri]	GCF_001625305.1	TRADDN-DEDD2
XP_019640558.1	GT4-GTase+TRADDN-DEDD2+TPRs	1607	LOC109482316	chordata	Branchiostoma belcheri	PREDICTED: LOW QUALITY PROTEIN: uncharacterized protein LOC109482316 [Branchiostoma belcheri]	GCF_001625305.1	TRADDN-DEDD2
XP_019646023.1	GT4-GTase+TRADDN-DEDD2+TPRs	1345	LOC109486599	chordata	Branchiostoma belcheri	PREDICTED: tetratricopeptide repeat protein 28-like, partial [Branchiostoma belcheri]	GCF_001625305.1	TRADDN-DEDD2
XP_002599992.1	GT4-GTase+TRADDN-DEDD2+TPRs	1404	BRAFLDRAFT_74113	chordata	Branchiostoma floridae	hypothetical protein BRAFLDRAFT_74113 [Branchiostoma floridae]	GCF_000003815.1	TRADDN-DEDD2
XP_002589321.1	HEPN+Death+Death+TRADDN-DEDD2+Death+LRRs	1432	BRAFLDRAFT_77774	chordata	Branchiostoma floridae	hypothetical protein BRAFLDRAFT_77774 [Branchiostoma floridae]	GCF_000003815.1	TRADDN-DEDD2

acc	architecture	len	gen.name	taxend	species	defline	gca	clade
XP_002610714.1	low-complexity+DED+TRADDN-DEDD2	299	BRAFLDRAFT_202645	chordata	Branchiostoma floridae	hypothetical protein BRAFLDRAFT_202645 [Branchiostoma floridae]	GCF_000003815.1	TRADDN-DEDD2
XP_002606802.1	LRRs+CARD+Death+TRADDN-DEDD2	692	BRAFLDRAFT_82441	chordata	Branchiostoma floridae	hypothetical protein BRAFLDRAFT_82441 [Branchiostoma floridae]	GCF_000003815.1	TRADDN-DEDD2
XP_019614048.1	N-ter+TRADDN-Bac+FAM124	417	LOC109462003	chordata	Branchiostoma belcheri	PREDICTED: uncharacterized protein LOC109462003 [Branchiostoma belcheri]	GCF_001625305.1	TRADDN-Bacterial-like
EEN44785.1	N-ter+TRADDN-Bac+FAM124	416	BRAFLDRAFT_89797	chordata	Branchiostoma floridae	hypothetical protein BRAFLDRAFT_89797 [Branchiostoma floridae]	GCA_000003815.1	TRADDN-Bacterial-like
EEN44794.1	N-ter+TRADDN-Bac+FAM124	392	BRAFLDRAFT_89787	chordata	Branchiostoma floridae	hypothetical protein BRAFLDRAFT_89787 [Branchiostoma floridae]	GCA_000003815.1	TRADDN-Bacterial-like
XP_002588774.1	N-ter+TRADDN-Bac+FAM124	416	BRAFLDRAFT_89797	chordata	Branchiostoma floridae	hypothetical protein BRAFLDRAFT_89797 [Branchiostoma floridae]	GCF_000003815.1	TRADDN-Bacterial-like
XP_002588783.1	N-ter+TRADDN-Bac+FAM124	392	BRAFLDRAFT_89787	chordata	Branchiostoma floridae	hypothetical protein BRAFLDRAFT_89787 [Branchiostoma floridae]	GCF_000003815.1	TRADDN-Bacterial-like
XP_002611490.1	RING+TIR+TRADDN-DEDD2+Coiled-coil+GTPase-AIG	899	BRAFLDRAFT_63875	chordata	Branchiostoma floridae	hypothetical protein BRAFLDRAFT_63875 [Branchiostoma floridae]	GCF_000003815.1	TRADDN-DEDD2
XP_002592407.1	RRM+RRM+FN3+TRADDN-DEDD2+Coiled-coil+DnaJ_CXXCXGXG+DnaJ_C+SH2+Pkinase	1558	BRAFLDRAFT_118420	chordata	Branchiostoma floridae	hypothetical protein BRAFLDRAFT_118420 [Branchiostoma floridae]	GCF_000003815.1	TRADDN-DEDD2
XP_019632330.1	TIR+TRADDN-DEDD2+CARD+STAND+wHTH+helical-repeat+LRRs	1367	LOC109475966	chordata	Branchiostoma belcheri	PREDICTED: uncharacterized protein LOC109475966 [Branchiostoma belcheri]	GCF_001625305.1	TRADDN-DEDD2
XP_019616027.1	TIR+TRADDN-DEDD2+Coiled-coil	536	LOC109463615	chordata	Branchiostoma belcheri	PREDICTED: uncharacterized protein LOC109463615 [Branchiostoma belcheri]	GCF_001625305.1	TRADDN-DEDD2
XP_002600337.1	TIR+TRADDN-DEDD2+Coiled-coil+NTP_transf	1092	BRAFLDRAFT_66570	chordata	Branchiostoma floridae	hypothetical protein BRAFLDRAFT_66570 [Branchiostoma floridae]	GCF_000003815.1	TRADDN-DEDD2
XP_019643131.1	TIR+TRADDN-DEDD2+DEAD+RNA-receptor_SF2-helicase+RIG-I_C-RD	1021	LOC109484316	chordata	Branchiostoma belcheri	PREDICTED: probable ATP-dependent RNA helicase DHX58 [Branchiostoma belcheri]	GCF_001625305.1	TRADDN-DEDD2
XP_019635553.1	TIR+TRADDN-DEDD2+DED+STAND+wHTH+TPRs	1266	LOC109478460	chordata	Branchiostoma belcheri	PREDICTED: LOW QUALITY PROTEIN: uncharacterized protein LOC109478460 [Branchiostoma belcheri]	GCF_001625305.1	TRADDN-DEDD2
XP_002607394.1	TM+TM+TRADDN-DEDD2	576	BRAFLDRAFT_69805	chordata	Branchiostoma floridae	hypothetical protein BRAFLDRAFT_69805 [Branchiostoma floridae]	GCF_000003815.1	TRADDN-DEDD2
XP_019613650.1	TRADDN-DEDD2	279	LOC109461702	chordata	Branchiostoma belcheri	PREDICTED: uncharacterized protein LOC109461702 isoform X3 [Branchiostoma belcheri]	GCF_001625305.1	TRADDN-DEDD2
XP_019623524.1	TRADDN-DEDD2	264	LOC109469171	chordata	Branchiostoma belcheri	PREDICTED: uncharacterized protein LOC109469171 [Branchiostoma belcheri]	GCF_001625305.1	TRADDN-DEDD2

acc	architecture	len	gen.name	taxend	species	defline	gca	clade
XP_019633455.1	TRADDN-DEDD2	454	LOC109476880	chordata	Branchiostoma belcheri	PREDICTED: uncharacterized protein LOC109476880 [Branchiostoma belcheri]	GCF_001625305.1	TRADDN-DEDD2
XP_019633520.1	TRADDN-DEDD2	293	LOC109476943	chordata	Branchiostoma belcheri	PREDICTED: uncharacterized protein LOC109476943 [Branchiostoma belcheri]	GCF_001625305.1	TRADDN-DEDD2
XP_019642703.1	TRADDN-DEDD2	205	LOC109483987	chordata	Branchiostoma belcheri	PREDICTED: death effector domain-containing protein-like [Branchiostoma belcheri]	GCF_001625305.1	TRADDN-DEDD2
XP_019645723.1	TRADDN-DEDD2	225	LOC109486339	chordata	Branchiostoma belcheri	PREDICTED: uncharacterized protein LOC109486339 [Branchiostoma belcheri]	GCF_001625305.1	TRADDN-DEDD2
XP_002586220.1	TRADDN-DEDD2	247	BRAFLDRAFT_132418	chordata	Branchiostoma floridae	hypothetical protein BRAFLDRAFT_132418, partial [Branchiostoma floridae]	GCF_000003815.1	TRADDN-DEDD2
XP_002592365.1	TRADDN-DEDD2	234	BRAFLDRAFT_131341	chordata	Branchiostoma floridae	hypothetical protein BRAFLDRAFT_131341 [Branchiostoma floridae]	GCF_000003815.1	TRADDN-DEDD2
XP_002601760.1	TRADDN-DEDD2	496	BRAFLDRAFT_76020	chordata	Branchiostoma floridae	hypothetical protein BRAFLDRAFT_76020 [Branchiostoma floridae]	GCF_000003815.1	TRADDN-DEDD2
XP_002604763.1	TRADDN-DEDD2	347	BRAFLDRAFT_119471	chordata	Branchiostoma floridae	hypothetical protein BRAFLDRAFT_119471 [Branchiostoma floridae]	GCF_000003815.1	TRADDN-DEDD2
XP_002586223.1	TRADDN-DEDD2+ADH_zinc_N+ADH_zinc_N	496	BRAFLDRAFT_109550	chordata	Branchiostoma floridae	hypothetical protein BRAFLDRAFT_109550 [Branchiostoma floridae]	GCF_000003815.1	TRADDN-DEDD2
XP_019616149.1	TRADDN-DEDD2+Ankyrin	405	LOC109463743	chordata	Branchiostoma belcheri	PREDICTED: ankyrin repeat domain-containing protein 2-like [Branchiostoma belcheri]	GCF_001625305.1	TRADDN-DEDD2
XP_002598015.1	TRADDN-DEDD2+Coiled-coil	482	BRAFLDRAFT_79759	chordata	Branchiostoma floridae	hypothetical protein BRAFLDRAFT_79759 [Branchiostoma floridae]	GCF_000003815.1	TRADDN-DEDD2
XP_002605447.1	TRADDN-DEDD2+Coiled-coil+GT4-GTase	734	BRAFLDRAFT_74262	chordata	Branchiostoma floridae	hypothetical protein BRAFLDRAFT_74262 [Branchiostoma floridae]	GCF_000003815.1	TRADDN-DEDD2
XP_002592356.1	TRADDN-DEDD2+DED+STAND+wHTH+helical-region+TPRs+Coiled-coil	1531	BRAFLDRAFT_101242	chordata	Branchiostoma floridae	hypothetical protein BRAFLDRAFT_101242 [Branchiostoma floridae]	GCF_000003815.1	TRADDN-DEDD2
XP_019621030.1	TRADDN-DEDD2+FN3+Pkinase+CASPASE	1165	LOC109467467	chordata	Branchiostoma belcheri	PREDICTED: uncharacterized protein LOC109467467 isoform X1 [Branchiostoma belcheri]	GCF_001625305.1	TRADDN-DEDD2
XP_019621031.1	TRADDN-DEDD2+FN3+Pkinase+CASPASE	1163	LOC109467467	chordata	Branchiostoma belcheri	PREDICTED: uncharacterized protein LOC109467467 isoform X2 [Branchiostoma belcheri]	GCF_001625305.1	TRADDN-DEDD2
XP_019621032.1	TRADDN-DEDD2+FN3+Pkinase+CASPASE	1161	LOC109467467	chordata	Branchiostoma belcheri	PREDICTED: uncharacterized protein LOC109467467 isoform X3 [Branchiostoma belcheri]	GCF_001625305.1	TRADDN-DEDD2
XP_019621033.1	TRADDN-DEDD2+FN3+Pkinase+CASPASE	1157	LOC109467467	chordata	Branchiostoma belcheri	PREDICTED: uncharacterized protein LOC109467467 isoform X4 [Branchiostoma belcheri]	GCF_001625305.1	TRADDN-DEDD2

acc	architecture	len	gen.name	taxend	species	defline	gca	clade
XP_019614641.1	TRADDN-DEDD2+GTPase-AIG	620	LOC109462531	chordata	Branchiostoma belcheri	PREDICTED: uncharacterized protein LOC109462531 [Branchiostoma belcheri]	GCF_001625305.1	TRADDN-DEDD2
XP_019624508.1	TRADDN-DEDD2+GTPase-AIG+GTPase-AIG	762	LOC109470168	chordata	Branchiostoma belcheri	PREDICTED: GTPase IMAP family member 8-like [Branchiostoma belcheri]	GCF_001625305.1	TRADDN-DEDD2
XP_019622962.1	TRADDN-DEDD2+LRRs	650	LOC109469014	chordata	Branchiostoma belcheri	PREDICTED: uncharacterized protein LOC109469014 [Branchiostoma belcheri]	GCF_001625305.1	TRADDN-DEDD2
XP_002603206.1	TRADDN-DEDD2+LRRs	637	BRAFLDRAFT_93392	chordata	Branchiostoma floridae	hypothetical protein BRAFLDRAFT_93392 [Branchiostoma floridae]	GCF_000003815.1	TRADDN-DEDD2
XP_019637544.1	TRADDN-DEDD2+NYN+ZBD+ZBD+ZBD	713	LOC109479923	chordata	Branchiostoma belcheri	PREDICTED: uncharacterized protein LOC109479923 [Branchiostoma belcheri]	GCF_001625305.1	TRADDN-DEDD2
XP_019624418.1	TRADDN-DEDD2+PARP	688	LOC109470081	chordata	Branchiostoma belcheri	PREDICTED: poly [ADP-ribose] polymerase 14-like [Branchiostoma belcheri]	GCF_001625305.1	TRADDN-DEDD2
XP_019621996.1	TRADDN-DEDD2+TM	535	LOC109468182	chordata	Branchiostoma belcheri	PREDICTED: uncharacterized protein LOC109468182 [Branchiostoma belcheri]	GCF_001625305.1	TRADDN-DEDD2
XP_019633154.1	TRADDN-DEDD2+TPRs	878	LOC109476604	chordata	Branchiostoma belcheri	PREDICTED: tetratricopeptide repeat protein 28-like [Branchiostoma belcheri]	GCF_001625305.1	TRADDN-DEDD2
XP_019633157.1	TRADDN-DEDD2+TPRs	1175	LOC109476607	chordata	Branchiostoma belcheri	PREDICTED: tetratricopeptide repeat protein 28-like [Branchiostoma belcheri]	GCF_001625305.1	TRADDN-DEDD2
XP_019635475.1	TRADDN-DEDD2+TPRs	828	LOC109478387	chordata	Branchiostoma belcheri	PREDICTED: tetratricopeptide repeat protein 28-like isoform X1 [Branchiostoma belcheri]	GCF_001625305.1	TRADDN-DEDD2
XP_019635476.1	TRADDN-DEDD2+TPRs	763	LOC109478387	chordata	Branchiostoma belcheri	PREDICTED: tetratricopeptide repeat protein 28-like isoform X2 [Branchiostoma belcheri]	GCF_001625305.1	TRADDN-DEDD2
XP_019613648.1	TRADDN-DEDD2+Trypsin	864	LOC109461702	chordata	Branchiostoma belcheri	PREDICTED: uncharacterized protein LOC109461702 isoform X1 [Branchiostoma belcheri]	GCF_001625305.1	TRADDN-DEDD2
XP_019613649.1	TRADDN-DEDD2+Trypsin	863	LOC109461702	chordata	Branchiostoma belcheri	PREDICTED: uncharacterized protein LOC109461702 isoform X2 [Branchiostoma belcheri]	GCF_001625305.1	TRADDN-DEDD2
EEN51213.1	TRADDN-NCOA6	72	BRAFLDRAFT_238710	chordata	Branchiostoma floridae	hypothetical protein BRAFLDRAFT_238710, partial [Branchiostoma floridae]	GCA_000003815.1	TRADDN-NCOA6
XP_002595201.1	TRADDN-NCOA6	72	BRAFLDRAFT_238710	chordata	Branchiostoma floridae	hypothetical protein BRAFLDRAFT_238710, partial [Branchiostoma floridae]	GCF_000003815.1	TRADDN-NCOA6
XP_009860299.2	TRADDN-NCOA6	650	LOC100179823	chordata	Ciona intestinalis	nuclear receptor coactivator 6 [Ciona intestinalis]	GCF_000224145.3	TRADDN-NCOA6
EEN43927.1	TRADDN-NCOA6+Coiled-coil	180	BRAFLDRAFT_87304	chordata	Branchiostoma floridae	hypothetical protein BRAFLDRAFT_87304 [Branchiostoma floridae]	GCA_000003815.1	TRADDN-NCOA6

acc	architecture	len	gen.name	taxend	species	defline	gca	clade
XP_002587916.1	TRADDN-NCOA6+Coiled-coil	180	BRAFLDRAFT_87304	chordata	Branchiostoma floridae	hypothetical protein BRAFLDRAFT_87304 [Branchiostoma floridae]	GCF_000003815.1	TRADDN-NCOA6
EEN61321.1	TRADDN-NCOA6+domain1+Pro-rich+Beta-propeller	599	BRAFLDRAFT_89047	chordata	Branchiostoma floridae	hypothetical protein BRAFLDRAFT_89047 [Branchiostoma floridae]	GCA_000003815.1	TRADDN-NCOA6
EEN61323.1	TRADDN-NCOA6+domain1+Pro-rich+Beta-propeller	535	BRAFLDRAFT_89045	chordata	Branchiostoma floridae	hypothetical protein BRAFLDRAFT_89045 [Branchiostoma floridae]	GCA_000003815.1	TRADDN-NCOA6
XP_002605311.1	TRADDN-NCOA6+domain1+Pro-rich+Beta-propeller	599	BRAFLDRAFT_89047	chordata	Branchiostoma floridae	hypothetical protein BRAFLDRAFT_89047 [Branchiostoma floridae]	GCF_000003815.1	TRADDN-NCOA6
XP_002605313.1	TRADDN-NCOA6+domain1+Pro-rich+Beta-propeller	535	BRAFLDRAFT_89045	chordata	Branchiostoma floridae	hypothetical protein BRAFLDRAFT_89045 [Branchiostoma floridae]	GCF_000003815.1	TRADDN-NCOA6
XP_019620330.1	TRADDN-NCOA6+low-complexity	690	LOC109466905	chordata	Branchiostoma belcheri	PREDICTED: extensin-like, partial [Branchiostoma belcheri]	GCF_001625305.1	TRADDN-NCOA6
XP_005031341.1	DED+TRADDN-DEDD2	266	LOC101796850	vertebrata	Anas platyrhynchos	LOW QUALITY PROTEIN: death effector domain-containing protein-like, partial [Anas platyrhynchos]	-	TRADDN-DEDD2
OWK05416.1	DED+TRADDN-DEDD2	216	Celaphus_00001791	vertebrata	Cervus elaphus hippelaphus	DEDD [Cervus elaphus hippelaphus]	GCA_002197005.1	TRADDN-DEDD2
RMB93944.1	DED+TRADDN-DEDD2	307	DUI87_29680	vertebrata	Hirundo rustica rustica	hypothetical protein DUI87_29680 [Hirundo rustica rustica]	GCA_003692655.1	TRADDN-DEDD2
XP_020665928.1	DED+TRADDN-DEDD2	250	DEDD	vertebrata	Pogona vitticeps	death effector domain-containing protein [Pogona vitticeps]	GCF_900067755.1	TRADDN-DEDD2
XP_025214527.1	DED+TRADDN-DEDD2	319	DEDD	vertebrata	Theropithecus gelada	death effector domain-containing protein isoform X1 [Theropithecus gelada]	GCF_003255815.1	TRADDN-DEDD2
XP_014166987.1	ERM+TRADDN-DEDD2	363	LOC102033576	vertebrata	Geospiza fortis	PREDICTED: uncharacterized protein LOC102033576 [Geospiza fortis]	-	TRADDN-DEDD2
EMP27985.1	Ig+DED+TRADDN-DEDD2	451	UY3_14962	vertebrata	Chelonia mydas	Death effector domain-containing protein [Chelonia mydas]	GCA_000344595.1	TRADDN-DEDD2
XP_026893437.1	low-complexity+DED+TRADDN-DEDD2	327	DEDD2	vertebrata	Acinonyx jubatus	DNA-binding death effector domain-containing protein 2 [Acinonyx jubatus]	GCF_003709585.1	TRADDN-DEDD2
XP_002923987.1	low-complexity+DED+TRADDN-DEDD2	327	DEDD2	vertebrata	Ailuropoda melanoleuca	PREDICTED: DNA-binding death effector domain-containing protein 2 isoform X3 [Ailuropoda melanoleuca]	GCF_000004335.2	TRADDN-DEDD2
XP_002928789.1	low-complexity+DED+TRADDN-DEDD2	318	DEDD	vertebrata	Ailuropoda melanoleuca	PREDICTED: death effector domain-containing protein [Ailuropoda melanoleuca]	GCF_000004335.2	TRADDN-DEDD2
XP_019660119.1	low-complexity+DED+TRADDN-DEDD2	358	DEDD2	vertebrata	Ailuropoda melanoleuca	PREDICTED: DNA-binding death effector domain-containing protein 2 isoform X1 [Ailuropoda melanoleuca]	GCF_000004335.2	TRADDN-DEDD2
XP_019660120.1	low-complexity+DED+TRADDN-DEDD2	335	DEDD2	vertebrata	Ailuropoda melanoleuca	PREDICTED: DNA-binding death effector domain-containing protein 2 isoform X2 [Ailuropoda melanoleuca]	GCF_000004335.2	TRADDN-DEDD2
KYO27160.1	low-complexity+DED+TRADDN-DEDD2	362	DEDD	vertebrata	Alligator mississippiensis	death effector domain-containing protein isoform B [Alligator mississippiensis]	GCA_000281125.4	TRADDN-DEDD2

acc	architecture	len	gen.name	taxend	species	defline	gca	clade
KYO30372.1	low-complexity+DED+TRADDN-DEDD2	335	DEDD2	vertebrata	Alligator mississippiensis	DNA-binding death effector domain-containing protein 2 [Alligator mississippiensis]	GCA_000281125.4	TRADDN-DEDD2
XP_006270333.1	low-complexity+DED+TRADDN-DEDD2	323	DEDD2	vertebrata	Alligator mississippiensis	PREDICTED: DNA-binding death effector domain-containing protein 2 [Alligator mississippiensis]	GCF_000281125.3	TRADDN-DEDD2
XP_006032777.1	low-complexity+DED+TRADDN-DEDD2	318	DEDD	vertebrata	Alligator sinensis	death effector domain-containing protein isoform X1 [Alligator sinensis]	GCF_000455745.1	TRADDN-DEDD2
XP_006038909.1	low-complexity+DED+TRADDN-DEDD2	323	DEDD2	vertebrata	Alligator sinensis	DNA-binding death effector domain-containing protein 2 isoform X1 [Alligator sinensis]	GCF_000455745.1	TRADDN-DEDD2
XP_025070243.1	low-complexity+DED+TRADDN-DEDD2	282	DEDD	vertebrata	Alligator sinensis	death effector domain-containing protein isoform X2 [Alligator sinensis]	GCF_000455745.1	TRADDN-DEDD2
KQK85160.1	low-complexity+DED+TRADDN-DEDD2	391	AAES_42378	vertebrata	Amazona aestiva	death effector domain-containing protein [Amazona aestiva]	GCA_001420675.1	TRADDN-DEDD2
XP_003229451.2	low-complexity+DED+TRADDN-DEDD2	439	dedd2	vertebrata	Anolis carolinensis	PREDICTED: DNA-binding death effector domain-containing protein 2 [Anolis carolinensis]	GCF_000090745.1	TRADDN-DEDD2
XP_008122491.1	low-complexity+DED+TRADDN-DEDD2	401	dedd	vertebrata	Anolis carolinensis	PREDICTED: death effector domain-containing protein [Anolis carolinensis]	GCF_000090745.1	TRADDN-DEDD2
XP_021526089.1	low-complexity+DED+TRADDN-DEDD2	326	DEDD2	vertebrata	Aotus nancymaae	DNA-binding death effector domain-containing protein 2 [Aotus nancymaae]	GCF_000952055.2	TRADDN-DEDD2
KFM08834.1	low-complexity+DED+TRADDN-DEDD2	323	AS27_07638	vertebrata	Aptenodytes forsteri	Death effector domain-containing protein [Aptenodytes forsteri]	GCA_000699145.1	TRADDN-DEDD2
XP_009279519.1	low-complexity+DED+TRADDN-DEDD2	325	DEDD	vertebrata	Aptenodytes forsteri	PREDICTED: death effector domain-containing protein [Aptenodytes forsteri]	GCF_000699145.1	TRADDN-DEDD2
XP_013808421.1	low-complexity+DED+TRADDN-DEDD2	318	DEDD	vertebrata	Apteryx australis mantelli	PREDICTED: death effector domain-containing protein [Apteryx australis mantelli]	GCF_001039765.1	TRADDN-DEDD2
XP_007168099.1	low-complexity+DED+TRADDN-DEDD2	327	DEDD2	vertebrata	Balaenoptera acutorostrata scammoni	PREDICTED: DNA-binding death effector domain-containing protein 2 [Balaenoptera acutorostrata scammoni]	GCF_000493695.1	TRADDN-DEDD2
XP_007171792.1	low-complexity+DED+TRADDN-DEDD2	275	DEDD	vertebrata	Balaenoptera acutorostrata scammoni	PREDICTED: death effector domain-containing protein isoform X6 [Balaenoptera acutorostrata scammoni]	GCF_000493695.1	TRADDN-DEDD2
XP_019835475.1	low-complexity+DED+TRADDN-DEDD2	327	DEDD2	vertebrata	Bos indicus	PREDICTED: DNA-binding death effector domain-containing protein 2 [Bos indicus]	GCF_000247795.1	TRADDN-DEDD2
ELR46493.1	low-complexity+DED+TRADDN-DEDD2	354	M91_20757	vertebrata	Bos mutus	Death effector domain-containing protein [Bos mutus]	GCA_000298355.1	TRADDN-DEDD2
XP_005909260.1	low-complexity+DED+TRADDN-DEDD2	275	DEDD	vertebrata	Bos mutus	PREDICTED: death effector domain-containing protein isoform X3 [Bos mutus]	GCF_000298355.1	TRADDN-DEDD2
ABM06141.1	low-complexity+DED+TRADDN-DEDD2	318	DEDD	vertebrata	Bos taurus	death effector domain-containing protein [Bos taurus]	-	TRADDN-DEDD2

acc	architecture	len	gen.name	taxend	species	defline	gca	clade
NP_001029815.1	low-complexity+DED+TRADDN-DEDD2	312	DEDD	vertebrata	Bos taurus	death effector domain-containing protein [Bos taurus]	GCF_002263795.1	TRADDN-DEDD2
NP_001069485.1	low-complexity+DED+TRADDN-DEDD2	327	DEDD2	vertebrata	Bos taurus	DNA-binding death effector domain-containing protein 2 [Bos taurus]	GCF_002263795.1	TRADDN-DEDD2
XP_005203591.1	low-complexity+DED+TRADDN-DEDD2	318	DEDD	vertebrata	Bos taurus	death effector domain-containing protein isoform X1 [Bos taurus]	GCF_002263795.1	TRADDN-DEDD2
XP_006041310.1	low-complexity+DED+TRADDN-DEDD2	326	DEDD2	vertebrata	Bubalus bubalis	DNA-binding death effector domain-containing protein 2 isoform X2 [Bubalus bubalis]	GCF_003121395.1	TRADDN-DEDD2
XP_006048553.1	low-complexity+DED+TRADDN-DEDD2	318	DEDD	vertebrata	Bubalus bubalis	death effector domain-containing protein [Bubalus bubalis]	GCF_003121395.1	TRADDN-DEDD2
XP_025124957.1	low-complexity+DED+TRADDN-DEDD2	364	DEDD2	vertebrata	Bubalus bubalis	DNA-binding death effector domain-containing protein 2 isoform X1 [Bubalus bubalis]	GCF_003121395.1	TRADDN-DEDD2
XP_014817780.1	low-complexity+DED+TRADDN-DEDD2	318	DEDD	vertebrata	Calidris pugnax	PREDICTED: death effector domain-containing protein isoform X2 [Calidris pugnax]	GCF_001431845.1	TRADDN-DEDD2
OXB53792.1	low-complexity+DED+TRADDN-DEDD2	324	ASZ78_000038	vertebrata	Callipepla squamata	hypothetical protein ASZ78_000038, partial [Callipepla squamata]	GCA_002218305.1	TRADDN-DEDD2
XP_002760226.1	low-complexity+DED+TRADDN-DEDD2	318	DEDD	vertebrata	Callithrix jacchus	PREDICTED: death effector domain-containing protein [Callithrix jacchus]	GCF_000004665.1	TRADDN-DEDD2
XP_002762234.2	low-complexity+DED+TRADDN-DEDD2	326	DEDD2	vertebrata	Callithrix jacchus	PREDICTED: DNA-binding death effector domain-containing protein 2 [Callithrix jacchus]	GCF_000004665.1	TRADDN-DEDD2
XP_007907831.1	low-complexity+DED+TRADDN-DEDD2	325	dedd	vertebrata	Callorhinchus milii	PREDICTED: death effector domain-containing protein isoform X1 [Callorhinchus milii]	GCF_000165045.1	TRADDN-DEDD2
XP_007907832.1	low-complexity+DED+TRADDN-DEDD2	278	dedd	vertebrata	Callorhinchus milii	PREDICTED: death effector domain-containing protein isoform X2 [Callorhinchus milii]	GCF_000165045.1	TRADDN-DEDD2
XP_025705346.1	low-complexity+DED+TRADDN-DEDD2	327	DEDD2	vertebrata	Callorhinus ursinus	DNA-binding death effector domain-containing protein 2 [Callorhinus ursinus]	GCF_003265705.1	TRADDN-DEDD2
XP_025716367.1	low-complexity+DED+TRADDN-DEDD2	320	DEDD	vertebrata	Callorhinus ursinus	death effector domain-containing protein isoform X1 [Callorhinus ursinus]	GCF_003265705.1	TRADDN-DEDD2
EPY88999.1	low-complexity+DED+TRADDN-DEDD2	290	CB1_000141020	vertebrata	Camelus ferus	death effector domain-containing protein [Camelus ferus]	GCA_000311805.2	TRADDN-DEDD2
XP_006174018.1	low-complexity+DED+TRADDN-DEDD2	319	DEDD	vertebrata	Camelus ferus	PREDICTED: death effector domain-containing protein [Camelus ferus]	GCF_000311805.1	TRADDN-DEDD2
XP_006175117.1	low-complexity+DED+TRADDN-DEDD2	326	DEDD2	vertebrata	Camelus ferus	PREDICTED: DNA-binding death effector domain-containing protein 2 [Camelus ferus]	GCF_000311805.1	TRADDN-DEDD2
XP_025280606.1	low-complexity+DED+TRADDN-DEDD2	329	DEDD2	vertebrata	Canis lupus dingo	DNA-binding death effector domain-containing protein 2 [Canis lupus dingo]	GCF_003254725.1	TRADDN-DEDD2
XP_022271143.1	low-complexity+DED+TRADDN-DEDD2	320	DEDD	vertebrata	Canis lupus familiaris	death effector domain-containing protein isoform X1 [Canis lupus familiaris]	GCF_000002285.3	TRADDN-DEDD2

acc	architecture	len	gen.name	taxend	species	defline	gca	clade
XP_855274.1	low-complexity+DED+TRADDN-DEDD2	329	DEDD2	vertebrata	Canis lupus familiaris	DNA-binding death effector domain-containing protein 2 [Canis lupus familiaris]	GCF_000002285.3	TRADDN-DEDD2
XP_017901892.1	low-complexity+DED+TRADDN-DEDD2	318	DEDD	vertebrata	Capra hircus	PREDICTED: death effector domain-containing protein [Capra hircus]	GCF_001704415.1	TRADDN-DEDD2
KFP64974.1	low-complexity+DED+TRADDN-DEDD2	286	N322_06189	vertebrata	Cariama cristata	Death effector domain-containing protein, partial [Cariama cristata]	GCA_000690535.1	TRADDN-DEDD2
XP_009698398.1	low-complexity+DED+TRADDN-DEDD2	253	DEDD	vertebrata	Cariama cristata	PREDICTED: death effector domain-containing protein, partial [Cariama cristata]	GCF_000690535.1	TRADDN-DEDD2
XP_008046461.1	low-complexity+DED+TRADDN-DEDD2	326	DEDD2	vertebrata	Carlito syrichta	DNA-binding death effector domain-containing protein 2 isoform X1 [Carlito syrichta]	GCF_000164805.1	TRADDN-DEDD2
XP_008046462.1	low-complexity+DED+TRADDN-DEDD2	321	DEDD2	vertebrata	Carlito syrichta	DNA-binding death effector domain-containing protein 2 isoform X2 [Carlito syrichta]	GCF_000164805.1	TRADDN-DEDD2
XP_008064100.1	low-complexity+DED+TRADDN-DEDD2	318	LOC103268351	vertebrata	Carlito syrichta	death effector domain-containing protein isoform X1 [Carlito syrichta]	GCF_000164805.1	TRADDN-DEDD2
XP_008064101.1	low-complexity+DED+TRADDN-DEDD2	317	LOC103268351	vertebrata	Carlito syrichta	death effector domain-containing protein isoform X2 [Carlito syrichta]	GCF_000164805.1	TRADDN-DEDD2
XP_021572145.1	low-complexity+DED+TRADDN-DEDD2	275	LOC103268351	vertebrata	Carlito syrichta	death effector domain-containing protein isoform X4 [Carlito syrichta]	GCF_000164805.1	TRADDN-DEDD2
XP_020027681.1	low-complexity+DED+TRADDN-DEDD2	327	Dedd2	vertebrata	Castor canadensis	DNA-binding death effector domain-containing protein 2 [Castor canadensis]	GCF_001984765.1	TRADDN-DEDD2
XP_020029941.1	low-complexity+DED+TRADDN-DEDD2	318	Dedd	vertebrata	Castor canadensis	death effector domain-containing protein [Castor canadensis]	GCF_001984765.1	TRADDN-DEDD2
XP_003466629.1	low-complexity+DED+TRADDN-DEDD2	318	Dedd	vertebrata	Cavia porcellus	death effector domain-containing protein [Cavia porcellus]	GCF_000151735.1	TRADDN-DEDD2
XP_005001360.1	low-complexity+DED+TRADDN-DEDD2	330	Dedd2	vertebrata	Cavia porcellus	DNA-binding death effector domain-containing protein 2 [Cavia porcellus]	GCF_000151735.1	TRADDN-DEDD2
XP_017358636.1	low-complexity+DED+TRADDN-DEDD2	326	DEDD2	vertebrata	Cebus capucinus imitator	PREDICTED: DNA-binding death effector domain-containing protein 2 [Cebus capucinus imitator]	GCF_001604975.1	TRADDN-DEDD2
XP_017370555.1	low-complexity+DED+TRADDN-DEDD2	318	DEDD	vertebrata	Cebus capucinus imitator	PREDICTED: death effector domain-containing protein [Cebus capucinus imitator]	GCF_001604975.1	TRADDN-DEDD2
XP_004440150.1	low-complexity+DED+TRADDN-DEDD2	326	LOC101405113	vertebrata	Ceratotherium simum simum	PREDICTED: DNA-binding death effector domain-containing protein 2 [Ceratotherium simum simum]	GCF_000283155.1	TRADDN-DEDD2
XP_011922952.1	low-complexity+DED+TRADDN-DEDD2	282	DEDD	vertebrata	Cercocebus atys	PREDICTED: death effector domain-containing protein isoform X2 [Cercocebus atys]	GCF_000955945.1	TRADDN-DEDD2
XP_011942305.1	low-complexity+DED+TRADDN-DEDD2	326	DEDD2	vertebrata	Cercocebus atys	PREDICTED: DNA-binding death effector domain-containing protein 2 [Cercocebus atys]	GCF_000955945.1	TRADDN-DEDD2
KFU91941.1	low-complexity+DED+TRADDN-DEDD2	293	M959_03872	vertebrata	Chaetura pelagica	Death effector domain-containing protein, partial [Chaetura pelagica]	GCA_000747805.1	TRADDN-DEDD2

acc	architecture	len	gen.name	taxend	species	defline	gca	clade
XP_009999021.1	low-complexity+DED+TRADDN-DEDD2	317	DEDD	vertebrata	Chaetura pelagica	PREDICTED: death effector domain-containing protein [Chaetura pelagica]	GCF_000747805.1	TRADDN-DEDD2
KGL94642.1	low-complexity+DED+TRADDN-DEDD2	313	N301_05719	vertebrata	Charadrius vociferus	Death effector domain-containing protein, partial [Charadrius vociferus]	GCA_000708025.2	TRADDN-DEDD2
XP_009884968.1	low-complexity+DED+TRADDN-DEDD2	296	DEDD2	vertebrata	Charadrius vociferus	PREDICTED: DNA-binding death effector domain-containing protein 2, partial [Charadrius vociferus]	GCF_000708025.1	TRADDN-DEDD2
XP_007068191.1	low-complexity+DED+TRADDN-DEDD2	318	DEDD	vertebrata	Chelonia mydas	PREDICTED: death effector domain-containing protein [Chelonia mydas]	GCF_000344595.1	TRADDN-DEDD2
XP_007069738.1	low-complexity+DED+TRADDN-DEDD2	377	DEDD2	vertebrata	Chelonia mydas	PREDICTED: DNA-binding death effector domain-containing protein 2, partial [Chelonia mydas]	-	TRADDN-DEDD2
XP_005400295.1	low-complexity+DED+TRADDN-DEDD2	318	Dedd	vertebrata	Chinchilla lanigera	PREDICTED: death effector domain-containing protein [Chinchilla lanigera]	GCF_000276665.1	TRADDN-DEDD2
XP_005412443.1	low-complexity+DED+TRADDN-DEDD2	330	Dedd2	vertebrata	Chinchilla lanigera	PREDICTED: DNA-binding death effector domain-containing protein 2 [Chinchilla lanigera]	GCF_000276665.1	TRADDN-DEDD2
XP_005293799.1	low-complexity+DED+TRADDN-DEDD2	318	DEDD	vertebrata	Chrysemys picta bellii	death effector domain-containing protein [Chrysemys picta bellii]	GCF_000241765.3	TRADDN-DEDD2
XP_005314656.1	low-complexity+DED+TRADDN-DEDD2	359	DEDD2	vertebrata	Chrysemys picta bellii	DNA-binding death effector domain-containing protein 2 [Chrysemys picta bellii]	GCF_000241765.3	TRADDN-DEDD2
XP_006861723.1	low-complexity+DED+TRADDN-DEDD2	318	DEDD	vertebrata	Chrysochloris asiatica	PREDICTED: death effector domain-containing protein isoform X1 [Chrysochloris asiatica]	GCF_000296735.1	TRADDN-DEDD2
XP_006861724.1	low-complexity+DED+TRADDN-DEDD2	275	DEDD	vertebrata	Chrysochloris asiatica	PREDICTED: death effector domain-containing protein isoform X2 [Chrysochloris asiatica]	GCF_000296735.1	TRADDN-DEDD2
XP_006871437.1	low-complexity+DED+TRADDN-DEDD2	328	DEDD2	vertebrata	Chrysochloris asiatica	PREDICTED: DNA-binding death effector domain-containing protein 2 isoform X1 [Chrysochloris asiatica]	GCF_000296735.1	TRADDN-DEDD2
XP_006871438.1	low-complexity+DED+TRADDN-DEDD2	323	DEDD2	vertebrata	Chrysochloris asiatica	PREDICTED: DNA-binding death effector domain-containing protein 2 isoform X2 [Chrysochloris asiatica]	GCF_000296735.1	TRADDN-DEDD2
OXB71086.1	low-complexity+DED+TRADDN-DEDD2	318	H355_010466	vertebrata	Colinus virginianus	hypothetical protein H355_010466 [Colinus virginianus]	GCA_000599465.2	TRADDN-DEDD2
PKK17218.1	low-complexity+DED+TRADDN-DEDD2	300	DEDD	vertebrata	Columba livia	death effector domain containing [Columba livia]	GCA_000337935.2	TRADDN-DEDD2
XP_021137301.1	low-complexity+DED+TRADDN-DEDD2	325	DEDD	vertebrata	Columba livia	death effector domain-containing protein [Columba livia]	GCF_000337935.1	TRADDN-DEDD2
XP_004694216.1	low-complexity+DED+TRADDN-DEDD2	328	DEDD2	vertebrata	Condylura cristata	PREDICTED: DNA-binding death effector domain-containing protein 2 [Condylura cristata]	GCF_000260355.1	TRADDN-DEDD2
XP_015740188.1	low-complexity+DED+TRADDN-DEDD2	318	DEDD	vertebrata	Coturnix japonica	PREDICTED: death effector domain-containing protein [Coturnix japonica]	GCF_001577835.1	TRADDN-DEDD2
ERE52693.1	low-complexity+DED+TRADDN-DEDD2	326	H671_21275	vertebrata	Cricetulus griseus	death effector domain-containing protein [Cricetulus griseus]	GCA_000448345.1	TRADDN-DEDD2

acc	architecture	len	gen.name	taxend	species	defline	gca	clade
XP_007614869.1	low-complexity+DED+TRADDN-DEDD2	327	Dedd2	vertebrata	Cricetulus griseus	PREDICTED: LOW QUALITY PROTEIN: DNA-binding death effector domain-containing protein 2 isoform X2 [Cricetulus griseus]	-	TRADDN-DEDD2
XP_007625798.1	low-complexity+DED+TRADDN-DEDD2	318	Dedd	vertebrata	Cricetulus griseus	PREDICTED: death effector domain-containing protein [Cricetulus griseus]	-	TRADDN-DEDD2
XP_019397245.1	low-complexity+DED+TRADDN-DEDD2	317	DEDD2	vertebrata	Crocodylus porosus	PREDICTED: DNA-binding death effector domain-containing protein 2, partial [Crocodylus porosus]	GCF_001723895.1	TRADDN-DEDD2
KFO69894.1	low-complexity+DED+TRADDN-DEDD2	296	N303_13441	vertebrata	Cuculus canorus	Death effector domain-containing protein [Cuculus canorus]	GCA_000709325.1	TRADDN-DEDD2
XP_009558864.1	low-complexity+DED+TRADDN-DEDD2	269	DEDD	vertebrata	Cuculus canorus	PREDICTED: death effector domain-containing protein [Cuculus canorus]	GCF_000709325.1	TRADDN-DEDD2
XP_004448423.1	low-complexity+DED+TRADDN-DEDD2	318	DEDD	vertebrata	Dasypus novemcinctus	death effector domain-containing protein isoform X1 [Dasypus novemcinctus]	GCF_000208655.1	TRADDN-DEDD2
XP_004448424.1	low-complexity+DED+TRADDN-DEDD2	275	DEDD	vertebrata	Dasypus novemcinctus	death effector domain-containing protein isoform X2 [Dasypus novemcinctus]	GCF_000208655.1	TRADDN-DEDD2
XP_004463327.1	low-complexity+DED+TRADDN-DEDD2	394	DEDD2	vertebrata	Dasypus novemcinctus	DNA-binding death effector domain-containing protein 2 [Dasypus novemcinctus]	GCF_000208655.1	TRADDN-DEDD2
XP_022441259.1	low-complexity+DED+TRADDN-DEDD2	322	DEDD2	vertebrata	Delphinapterus leucas	DNA-binding death effector domain-containing protein 2 [Delphinapterus leucas]	GCF_002288925.2	TRADDN-DEDD2
XP_024409848.1	low-complexity+DED+TRADDN-DEDD2	318	DEDD	vertebrata	Desmodus rotundus	death effector domain-containing protein [Desmodus rotundus]	GCF_002940915.1	TRADDN-DEDD2
XP_024433329.1	low-complexity+DED+TRADDN-DEDD2	327	DEDD2	vertebrata	Desmodus rotundus	DNA-binding death effector domain-containing protein 2 [Desmodus rotundus]	GCF_002940915.1	TRADDN-DEDD2
XP_012886054.1	low-complexity+DED+TRADDN-DEDD2	318	Dedd	vertebrata	Dipodomys ordii	PREDICTED: death effector domain-containing protein [Dipodomys ordii]	GCF_000151885.1	TRADDN-DEDD2
XP_012888216.1	low-complexity+DED+TRADDN-DEDD2	327	Dedd2	vertebrata	Dipodomys ordii	PREDICTED: DNA-binding death effector domain-containing protein 2 [Dipodomys ordii]	GCF_000151885.1	TRADDN-DEDD2
XP_004710418.1	low-complexity+DED+TRADDN-DEDD2	327	DEDD2	vertebrata	Echinops telfairi	PREDICTED: DNA-binding death effector domain-containing protein 2 isoform X1 [Echinops telfairi]	GCF_000313985.2	TRADDN-DEDD2
XP_004710419.1	low-complexity+DED+TRADDN-DEDD2	322	DEDD2	vertebrata	Echinops telfairi	PREDICTED: DNA-binding death effector domain-containing protein 2 isoform X2 [Echinops telfairi]	GCF_000313985.2	TRADDN-DEDD2
XP_004716583.1	low-complexity+DED+TRADDN-DEDD2	318	DEDD	vertebrata	Echinops telfairi	PREDICTED: death effector domain-containing protein isoform X1 [Echinops telfairi]	GCF_000313985.2	TRADDN-DEDD2
XP_004716584.1	low-complexity+DED+TRADDN-DEDD2	275	DEDD	vertebrata	Echinops telfairi	PREDICTED: death effector domain-containing protein isoform X2 [Echinops telfairi]	GCF_000313985.2	TRADDN-DEDD2
KFP11278.1	low-complexity+DED+TRADDN-DEDD2	302	Z169_01825	vertebrata	Egretta garzetta	Death effector domain-containing protein [Egretta garzetta]	GCA_000687185.1	TRADDN-DEDD2

acc	architecture	len	gen.name	taxend	species	defline	gca	clade
XP_009647307.1	low-complexity+DED+TRADDN-DEDD2	275	DEDD	vertebrata	Egretta garzetta	PREDICTED: death effector domain-containing protein [Egretta garzetta]	GCF_000687185.1	TRADDN-DEDD2
XP_006895541.1	low-complexity+DED+TRADDN-DEDD2	318	DEDD	vertebrata	Elephantulus edwardii	PREDICTED: death effector domain-containing protein isoform X1 [Elephantulus edwardii]	GCF_000299155.1	TRADDN-DEDD2
XP_006895544.1	low-complexity+DED+TRADDN-DEDD2	275	DEDD	vertebrata	Elephantulus edwardii	PREDICTED: death effector domain-containing protein isoform X4 [Elephantulus edwardii]	GCF_000299155.1	TRADDN-DEDD2
XP_006900997.1	low-complexity+DED+TRADDN-DEDD2	329	DEDD2	vertebrata	Elephantulus edwardii	PREDICTED: DNA-binding death effector domain-containing protein 2 [Elephantulus edwardii]	GCF_000299155.1	TRADDN-DEDD2
XP_022347502.1	low-complexity+DED+TRADDN-DEDD2	327	LOC111139511	vertebrata	Enhydra lutris kenyoni	DNA-binding death effector domain-containing protein 2 [Enhydra lutris kenyoni]	GCF_002288905.1	TRADDN-DEDD2
XP_008151292.1	low-complexity+DED+TRADDN-DEDD2	317	DEDD	vertebrata	Eptesicus fuscus	PREDICTED: death effector domain-containing protein [Eptesicus fuscus]	GCF_000308155.1	TRADDN-DEDD2
XP_008158450.1	low-complexity+DED+TRADDN-DEDD2	327	DEDD2	vertebrata	Eptesicus fuscus	PREDICTED: DNA-binding death effector domain-containing protein 2 isoform X1 [Eptesicus fuscus]	GCF_000308155.1	TRADDN-DEDD2
XP_008158451.1	low-complexity+DED+TRADDN-DEDD2	322	DEDD2	vertebrata	Eptesicus fuscus	PREDICTED: DNA-binding death effector domain-containing protein 2 isoform X2 [Eptesicus fuscus]	-	TRADDN-DEDD2
XP_014723042.1	low-complexity+DED+TRADDN-DEDD2	327	DEDD2	vertebrata	Equus asinus	PREDICTED: DNA-binding death effector domain-containing protein 2 [Equus asinus]	GCF_001305755.1	TRADDN-DEDD2
XP_001501404.1	low-complexity+DED+TRADDN-DEDD2	327	DEDD2	vertebrata	Equus caballus	DNA-binding death effector domain-containing protein 2 [Equus caballus]	GCF_002863925.1	TRADDN-DEDD2
XP_007532985.1	low-complexity+DED+TRADDN-DEDD2	322	DEDD2	vertebrata	Erinaceus europaeus	PREDICTED: DNA-binding death effector domain-containing protein 2 [Erinaceus europaeus]	GCF_000296755.1	TRADDN-DEDD2
XP_007537472.1	low-complexity+DED+TRADDN-DEDD2	318	DEDD	vertebrata	Erinaceus europaeus	PREDICTED: death effector domain-containing protein isoform X2 [Erinaceus europaeus]	GCF_000296755.1	TRADDN-DEDD2
XP_007537473.1	low-complexity+DED+TRADDN-DEDD2	275	DEDD	vertebrata	Erinaceus europaeus	PREDICTED: death effector domain-containing protein isoform X3 [Erinaceus europaeus]	GCF_000296755.1	TRADDN-DEDD2
XP_016049926.1	low-complexity+DED+TRADDN-DEDD2	319	DEDD	vertebrata	Erinaceus europaeus	PREDICTED: death effector domain-containing protein isoform X1 [Erinaceus europaeus]	GCF_000296755.1	TRADDN-DEDD2
XP_005442631.2	low-complexity+DED+TRADDN-DEDD2	306	DEDD	vertebrata	Falco cherrug	PREDICTED: death effector domain-containing protein isoform X2 [Falco cherrug]	-	TRADDN-DEDD2
XP_005244355.1	low-complexity+DED+TRADDN-DEDD2	318	DEDD	vertebrata	Falco peregrinus	PREDICTED: death effector domain-containing protein isoform X1 [Falco peregrinus]	-	TRADDN-DEDD2
XP_013160233.1	low-complexity+DED+TRADDN-DEDD2	317	DEDD	vertebrata	Falco peregrinus	PREDICTED: death effector domain-containing protein isoform X2 [Falco peregrinus]	-	TRADDN-DEDD2

acc	architecture	len	gen.name	taxend	species	defline	gca	clade
XP_013160581.1	low-complexity+DED+TRADDN-DEDD2	303	LOC101912306	vertebrata	Falco peregrinus	PREDICTED: uncharacterized protein LOC101912306 [Falco peregrinus]	GCF_000337955.1	TRADDN-DEDD2
XP_003997805.2	low-complexity+DED+TRADDN-DEDD2	327	DEDD2	vertebrata	Felis catus	DNA-binding death effector domain-containing protein 2 [Felis catus]	GCF_000181335.3	TRADDN-DEDD2
XP_010621607.1	low-complexity+DED+TRADDN-DEDD2	326	Dedd2	vertebrata	Fukomys damarensis	PREDICTED: DNA-binding death effector domain-containing protein 2 [Fukomys damarensis]	GCF_000743615.1	TRADDN-DEDD2
XP_008565461.1	low-complexity+DED+TRADDN-DEDD2	324	DEDD2	vertebrata	Galeopterus variegatus	PREDICTED: DNA-binding death effector domain-containing protein 2 [Galeopterus variegatus]	GCF_000696425.1	TRADDN-DEDD2
XP_423146.1	low-complexity+DED+TRADDN-DEDD2	318	DEDD	vertebrata	Gallus gallus	death effector domain-containing protein [Gallus gallus]	GCF_000002315.5	TRADDN-DEDD2
XP_015263851.1	low-complexity+DED+TRADDN-DEDD2	318	DEDD	vertebrata	Gekko japonicus	PREDICTED: death effector domain-containing protein [Gekko japonicus]	GCF_001447785.1	TRADDN-DEDD2
XP_015264891.1	low-complexity+DED+TRADDN-DEDD2	363	DEDD2	vertebrata	Gekko japonicus	PREDICTED: DNA-binding death effector domain-containing protein 2 [Gekko japonicus]	GCF_001447785.1	TRADDN-DEDD2
XP_004060880.1	low-complexity+DED+TRADDN-DEDD2	326	DEDD2	vertebrata	Gorilla gorilla gorilla	PREDICTED: DNA-binding death effector domain-containing protein 2 [Gorilla gorilla gorilla]	GCF_008122165.1	TRADDN-DEDD2
XP_010565157.1	low-complexity+DED+TRADDN-DEDD2	349	DEDD	vertebrata	Haliaeetus leucocephalus	PREDICTED: death effector domain-containing protein isoform X1 [Haliaeetus leucocephalus]	GCF_000737465.1	TRADDN-DEDD2
XP_010565161.1	low-complexity+DED+TRADDN-DEDD2	318	DEDD	vertebrata	Haliaeetus leucocephalus	PREDICTED: death effector domain-containing protein isoform X2 [Haliaeetus leucocephalus]	GCF_000737465.1	TRADDN-DEDD2
EHB02521.1	low-complexity+DED+TRADDN-DEDD2	327	GW7_15428	vertebrata	Heterocephalus glaber	DNA-binding death effector domain-containing protein 2 [Heterocephalus glaber]	GCA_000230445.1	TRADDN-DEDD2
EHB06402.1	low-complexity+DED+TRADDN-DEDD2	279	GW7_06456	vertebrata	Heterocephalus glaber	Death effector domain-containing protein [Heterocephalus glaber]	GCA_000230445.1	TRADDN-DEDD2
XP_004858739.1	low-complexity+DED+TRADDN-DEDD2	318	Dedd	vertebrata	Heterocephalus glaber	death effector domain-containing protein isoform X2 [Heterocephalus glaber]	GCF_000247695.1	TRADDN-DEDD2
XP_004873097.1	low-complexity+DED+TRADDN-DEDD2	327	Dedd2	vertebrata	Heterocephalus glaber	DNA-binding death effector domain-containing protein 2 isoform X1 [Heterocephalus glaber]	GCF_000247695.1	TRADDN-DEDD2
XP_012932953.1	low-complexity+DED+TRADDN-DEDD2	335	Dedd	vertebrata	Heterocephalus glaber	death effector domain-containing protein isoform X1 [Heterocephalus glaber]	GCF_000247695.1	TRADDN-DEDD2
XP_019484602.1	low-complexity+DED+TRADDN-DEDD2	327	DEDD2	vertebrata	Hipposideros armiger	PREDICTED: DNA-binding death effector domain-containing protein 2 isoform X1 [Hipposideros armiger]	GCF_001890085.1	TRADDN-DEDD2
XP_019484607.1	low-complexity+DED+TRADDN-DEDD2	318	DEDD2	vertebrata	Hipposideros armiger	PREDICTED: DNA-binding death effector domain-containing protein 2 isoform X2 [Hipposideros armiger]	GCF_001890085.1	TRADDN-DEDD2
XP_019484609.1	low-complexity+DED+TRADDN-DEDD2	295	DEDD2	vertebrata	Hipposideros armiger	PREDICTED: DNA-binding death effector domain-containing protein 2 isoform X4 [Hipposideros armiger]	GCF_001890085.1	TRADDN-DEDD2

acc	architecture	len	gen.name	taxend	species	defline	gca	clade
XP_019488629.1	low-complexity+DED+TRADDN-DEDD2	318	DEDD	vertebrata	Hipposideros armiger	PREDICTED: death effector domain-containing protein [Hipposideros armiger]	GCF_001890085.1	TRADDN-DEDD2
RMB88258.1	low-complexity+DED+TRADDN-DEDD2	276	DUI87_35370	vertebrata	Hirundo rustica rustica	hypothetical protein DUI87_35370 [Hirundo rustica rustica]	GCA_003692655.1	TRADDN-DEDD2
AAC17110.3	low-complexity+DED+TRADDN-DEDD2	348	-	vertebrata	Homo sapiens	KE05 protein [Homo sapiens]	-	TRADDN-DEDD2
AAM10835.1	low-complexity+DED+TRADDN-DEDD2	326	FLAME-3	vertebrata	Homo sapiens	death effector domain-containing protein FLAME-3 [Homo sapiens]	-	TRADDN-DEDD2
BAC11551.1	low-complexity+DED+TRADDN-DEDD2	326	-	vertebrata	Homo sapiens	unnamed protein product [Homo sapiens]	-	TRADDN-DEDD2
BAG51086.1	low-complexity+DED+TRADDN-DEDD2	318	-	vertebrata	Homo sapiens	unnamed protein product [Homo sapiens]	-	TRADDN-DEDD2
BAG59233.1	low-complexity+DED+TRADDN-DEDD2	275	-	vertebrata	Homo sapiens	unnamed protein product [Homo sapiens]	-	TRADDN-DEDD2
NP_001257544.1	low-complexity+DED+TRADDN-DEDD2	321	FLAME-3,FLAME3	vertebrata	Homo sapiens	DNA-binding death effector domain-containing protein 2 isoform 2 [Homo sapiens]	GCF_000001405.39	TRADDN-DEDD2
NP_001317694.1	low-complexity+DED+TRADDN-DEDD2	348	CASP8IP1,DEDD1,DEFT,FLDED1,KE05	vertebrata	Homo sapiens	death effector domain-containing protein isoform a [Homo sapiens]	GCF_000001405.39	TRADDN-DEDD2
NP_127491.1	low-complexity+DED+TRADDN-DEDD2	318	CASP8IP1,DEDD1,DEFT,FLDED1,KE05	vertebrata	Homo sapiens	death effector domain-containing protein isoform b [Homo sapiens]	GCF_000001405.39	TRADDN-DEDD2
NP_579874.1	low-complexity+DED+TRADDN-DEDD2	326	FLAME-3,FLAME3	vertebrata	Homo sapiens	DNA-binding death effector domain-containing protein 2 isoform 1 [Homo sapiens]	GCF_000001405.39	TRADDN-DEDD2
XP_011524871.1	low-complexity+DED+TRADDN-DEDD2	366	FLAME-3,FLAME3	vertebrata	Homo sapiens	DNA-binding death effector domain-containing protein 2 isoform X1 [Homo sapiens]	GCF_000001405.39	TRADDN-DEDD2
XP_005338219.1	low-complexity+DED+TRADDN-DEDD2	327	Dedd2	vertebrata	Ictidomys tridecemlineatus	DNA-binding death effector domain-containing protein 2 [Ictidomys tridecemlineatus]	GCF_000236235.1	TRADDN-DEDD2
XP_005341892.1	low-complexity+DED+TRADDN-DEDD2	318	Dedd	vertebrata	Ictidomys tridecemlineatus	death effector domain-containing protein [Ictidomys tridecemlineatus]	GCF_000236235.1	TRADDN-DEDD2
XP_004671188.1	low-complexity+DED+TRADDN-DEDD2	318	Dedd	vertebrata	Jaculus jaculus	PREDICTED: death effector domain-containing protein isoform X1 [Jaculus jaculus]	GCF_000280705.1	TRADDN-DEDD2
XP_004671190.1	low-complexity+DED+TRADDN-DEDD2	275	Dedd	vertebrata	Jaculus jaculus	PREDICTED: death effector domain-containing protein isoform X2 [Jaculus jaculus]	GCF_000280705.1	TRADDN-DEDD2
XP_005998029.1	low-complexity+DED+TRADDN-DEDD2	372	DEDD2	vertebrata	Latimeria chalumnae	PREDICTED: DNA-binding death effector domain-containing protein 2 [Latimeria chalumnae]	GCF_000225785.1	TRADDN-DEDD2
XP_006004949.1	low-complexity+DED+TRADDN-DEDD2	315	DEDD	vertebrata	Latimeria chalumnae	PREDICTED: death effector domain-containing protein [Latimeria chalumnae]	GCF_000225785.1	TRADDN-DEDD2
XP_006747447.1	low-complexity+DED+TRADDN-DEDD2	318	DEDD	vertebrata	Leptonychotes weddellii	PREDICTED: death effector domain-containing protein isoform X1 [Leptonychotes weddellii]	GCF_000349705.1	TRADDN-DEDD2
XP_006747448.1	low-complexity+DED+TRADDN-DEDD2	275	DEDD	vertebrata	Leptonychotes weddellii	PREDICTED: death effector domain-containing protein isoform X2 [Leptonychotes weddellii]	-	TRADDN-DEDD2

acc	architecture	len	gen.name	taxend	species	defline	gca	clade
XP_006748342.1	low-complexity+DED+TRADDN-DEDD2	328	DEDD2	vertebrata	Leptonychotes weddellii	PREDICTED: DNA-binding death effector domain-containing protein 2-like [Leptonychotes weddellii]	GCF_000349705.1	TRADDN-DEDD2
XP_007458662.1	low-complexity+DED+TRADDN-DEDD2	323	DEDD2	vertebrata	Lipotes vexillifer	PREDICTED: DNA-binding death effector domain-containing protein 2 [Lipotes vexillifer]	GCF_000442215.1	TRADDN-DEDD2
XP_007467180.1	low-complexity+DED+TRADDN-DEDD2	318	DEDD	vertebrata	Lipotes vexillifer	PREDICTED: death effector domain-containing protein isoform X1 [Lipotes vexillifer]	GCF_000442215.1	TRADDN-DEDD2
XP_007467181.1	low-complexity+DED+TRADDN-DEDD2	275	DEDD	vertebrata	Lipotes vexillifer	PREDICTED: death effector domain-containing protein isoform X2 [Lipotes vexillifer]	GCF_000442215.1	TRADDN-DEDD2
XP_021401768.1	low-complexity+DED+TRADDN-DEDD2	324	DEDD	vertebrata	Lonchura striata domestica	death effector domain-containing protein isoform X1 [Lonchura striata domestica]	GCF_005870125.1	TRADDN-DEDD2
XP_021401769.1	low-complexity+DED+TRADDN-DEDD2	275	DEDD	vertebrata	Lonchura striata domestica	death effector domain-containing protein isoform X2 [Lonchura striata domestica]	GCF_005870125.1	TRADDN-DEDD2
XP_003415242.1	low-complexity+DED+TRADDN-DEDD2	318	DEDD	vertebrata	Loxodonta africana	death effector domain-containing protein isoform X2 [Loxodonta africana]	GCF_000001905.1	TRADDN-DEDD2
XP_010593191.1	low-complexity+DED+TRADDN-DEDD2	320	DEDD	vertebrata	Loxodonta africana	death effector domain-containing protein isoform X1 [Loxodonta africana]	GCF_000001905.1	TRADDN-DEDD2
XP_023399782.1	low-complexity+DED+TRADDN-DEDD2	386	DEDD2	vertebrata	Loxodonta africana	LOW QUALITY PROTEIN: DNA-binding death effector domain-containing protein 2 [Loxodonta africana]	GCF_000001905.1	TRADDN-DEDD2
XP_023409960.1	low-complexity+DED+TRADDN-DEDD2	277	DEDD	vertebrata	Loxodonta africana	death effector domain-containing protein isoform X3 [Loxodonta africana]	GCF_000001905.1	TRADDN-DEDD2
XP_023409961.1	low-complexity+DED+TRADDN-DEDD2	275	DEDD	vertebrata	Loxodonta africana	death effector domain-containing protein isoform X4 [Loxodonta africana]	GCF_000001905.1	TRADDN-DEDD2
EHH59630.1	low-complexity+DED+TRADDN-DEDD2	326	EGM_09786	vertebrata	Macaca fascicularis	hypothetical protein EGM_09786 [Macaca fascicularis]	GCA_000230815.1	TRADDN-DEDD2
EHH30085.1	low-complexity+DED+TRADDN-DEDD2	326	EGK_10673	vertebrata	Macaca mulatta	hypothetical protein EGK_10673 [Macaca mulatta]	GCA_000230795.1	TRADDN-DEDD2
NP_001244370.1	low-complexity+DED+TRADDN-DEDD2	318	DEDD	vertebrata	Macaca mulatta	death effector domain-containing protein [Macaca mulatta]	GCF_003339765.1	TRADDN-DEDD2
XP_017926376.1	low-complexity+DED+TRADDN-DEDD2	314	LOC103754474	vertebrata	Manacus vitellinus	LOW QUALITY PROTEIN: death effector domain-containing protein [Manacus vitellinus]	-	TRADDN-DEDD2
XP_011831689.1	low-complexity+DED+TRADDN-DEDD2	318	DEDD	vertebrata	Mandrillus leucophaeus	PREDICTED: death effector domain-containing protein isoform X1 [Mandrillus leucophaeus]	GCF_000951045.1	TRADDN-DEDD2
XP_011831699.1	low-complexity+DED+TRADDN-DEDD2	275	DEDD	vertebrata	Mandrillus leucophaeus	PREDICTED: death effector domain-containing protein isoform X2 [Mandrillus leucophaeus]	GCF_000951045.1	TRADDN-DEDD2
XP_017495548.1	low-complexity+DED+TRADDN-DEDD2	318	DEDD	vertebrata	Manis javanica	PREDICTED: death effector domain-containing protein [Manis javanica]	GCF_001685135.1	TRADDN-DEDD2

acc	architecture	len	gen.name	taxend	species	defline	gca	clade
XP_017509902.1	low-complexity+DED+TRADDN-DEDD2	327	DEDD2	vertebrata	Manis javanica	PREDICTED: DNA-binding death effector domain-containing protein 2 [Manis javanica]	GCF_001685135.1	TRADDN-DEDD2
XP_015347449.1	low-complexity+DED+TRADDN-DEDD2	318	Dedd	vertebrata	Marmota marmota marmota	PREDICTED: death effector domain-containing protein [Marmota marmota marmota]	GCF_001458135.1	TRADDN-DEDD2
XP_015352266.1	low-complexity+DED+TRADDN-DEDD2	327	Dedd2	vertebrata	Marmota marmota marmota	PREDICTED: DNA-binding death effector domain-containing protein 2 [Marmota marmota marmota]	GCF_001458135.1	TRADDN-DEDD2
XP_010725554.1	low-complexity+DED+TRADDN-DEDD2	318	DEDD	vertebrata	Meleagris gallopavo	PREDICTED: death effector domain-containing protein [Meleagris gallopavo]	GCF_000146605.2	TRADDN-DEDD2
XP_005139706.1	low-complexity+DED+TRADDN-DEDD2	318	LOC101872591	vertebrata	Melopsittacus undulatus	PREDICTED: death effector domain-containing protein [Melopsittacus undulatus]	GCF_000238935.1	TRADDN-DEDD2
XP_021502769.1	low-complexity+DED+TRADDN-DEDD2	318	Dedd	vertebrata	Meriones unguiculatus	death effector domain-containing protein isoform X1 [Meriones unguiculatus]	GCF_002204375.1	TRADDN-DEDD2
XP_021502775.1	low-complexity+DED+TRADDN-DEDD2	305	Dedd	vertebrata	Meriones unguiculatus	death effector domain-containing protein isoform X2 [Meriones unguiculatus]	GCF_002204375.1	TRADDN-DEDD2
XP_021516819.1	low-complexity+DED+TRADDN-DEDD2	333	Dedd2	vertebrata	Meriones unguiculatus	DNA-binding death effector domain-containing protein 2 isoform X1 [Meriones unguiculatus]	GCF_002204375.1	TRADDN-DEDD2
XP_021516822.1	low-complexity+DED+TRADDN-DEDD2	325	Dedd2	vertebrata	Meriones unguiculatus	DNA-binding death effector domain-containing protein 2 isoform X2 [Meriones unguiculatus]	GCF_002204375.1	TRADDN-DEDD2
XP_005087104.1	low-complexity+DED+TRADDN-DEDD2	325	Dedd2	vertebrata	Mesocricetus auratus	DNA-binding death effector domain-containing protein 2 [Mesocricetus auratus]	GCF_000349665.1	TRADDN-DEDD2
XP_005087819.1	low-complexity+DED+TRADDN-DEDD2	318	Dedd	vertebrata	Mesocricetus auratus	death effector domain-containing protein [Mesocricetus auratus]	GCF_000349665.1	TRADDN-DEDD2
XP_005348865.1	low-complexity+DED+TRADDN-DEDD2	318	Dedd	vertebrata	Microtus ochrogaster	death effector domain-containing protein [Microtus ochrogaster]	GCF_000317375.1	TRADDN-DEDD2
XP_005361193.1	low-complexity+DED+TRADDN-DEDD2	328	Dedd2	vertebrata	Microtus ochrogaster	DNA-binding death effector domain-containing protein 2 isoform X2 [Microtus ochrogaster]	GCF_000317375.1	TRADDN-DEDD2
XP_026642138.1	low-complexity+DED+TRADDN-DEDD2	287	Dedd2	vertebrata	Microtus ochrogaster	DNA-binding death effector domain-containing protein 2 isoform X1 [Microtus ochrogaster]	GCF_000317375.1	TRADDN-DEDD2
XP_016064748.1	low-complexity+DED+TRADDN-DEDD2	327	DEDD2	vertebrata	Miniopterus natalensis	PREDICTED: DNA-binding death effector domain-containing protein 2 [Miniopterus natalensis]	GCF_001595765.1	TRADDN-DEDD2
XP_016065904.1	low-complexity+DED+TRADDN-DEDD2	318	DEDD	vertebrata	Miniopterus natalensis	PREDICTED: death effector domain-containing protein [Miniopterus natalensis]	GCF_001595765.1	TRADDN-DEDD2
XP_001371905.1	low-complexity+DED+TRADDN-DEDD2	318	DEDD	vertebrata	Monodelphis domestica	PREDICTED: death effector domain-containing protein isoform X1 [Monodelphis domestica]	GCF_000002295.2	TRADDN-DEDD2

acc	architecture	len	gen.name	taxend	species	defline	gca	clade
XP_003342303.2	low-complexity+DED+TRADDN-DEDD2	378	DEDD2	vertebrata	Monodelphis domestica	PREDICTED: LOW QUALITY PROTEIN: DNA-binding death effector domain-containing protein 2 [Monodelphis domestica]	GCF_000002295.2	TRADDN-DEDD2
XP_007485030.1	low-complexity+DED+TRADDN-DEDD2	282	DEDD	vertebrata	Monodelphis domestica	PREDICTED: death effector domain-containing protein isoform X2 [Monodelphis domestica]	GCF_000002295.2	TRADDN-DEDD2
XP_007485031.1	low-complexity+DED+TRADDN-DEDD2	275	DEDD	vertebrata	Monodelphis domestica	PREDICTED: death effector domain-containing protein isoform X3 [Monodelphis domestica]	GCF_000002295.2	TRADDN-DEDD2
XP_021023161.1	low-complexity+DED+TRADDN-DEDD2	413	Dedd2	vertebrata	Mus caroli	DNA-binding death effector domain-containing protein 2 isoform X1 [Mus caroli]	GCF_900094665.1	TRADDN-DEDD2
XP_021023162.1	low-complexity+DED+TRADDN-DEDD2	369	Dedd2	vertebrata	Mus caroli	DNA-binding death effector domain-containing protein 2 isoform X2 [Mus caroli]	-	TRADDN-DEDD2
AAD16415.1	low-complexity+DED+TRADDN-DEDD2	318	Dedpro1	vertebrata	Mus musculus	death effector domain-containing protein DEDPRO1 [Mus musculus]	-	TRADDN-DEDD2
AAH54445.1	low-complexity+DED+TRADDN-DEDD2	318	CASP8IP1	vertebrata	Mus musculus	Death effector domain-containing [Mus musculus]	-	TRADDN-DEDD2
BAE22179.1	low-complexity+DED+TRADDN-DEDD2	318	-	vertebrata	Mus musculus	unnamed protein product [Mus musculus]	-	TRADDN-DEDD2
EDL24288.1	low-complexity+DED+TRADDN-DEDD2	313	mCG_7275	vertebrata	Mus musculus	mCG7275, isoform CRA_b, partial [Mus musculus]	GCA_000002165.1	TRADDN-DEDD2
NP_035745.3	low-complexity+DED+TRADDN-DEDD2	318	CASP8IP1,Ded-pro1,DEFT,FLDED1,KE05	vertebrata	Mus musculus	death effector domain-containing protein [Mus musculus]	GCF_000001635.26	TRADDN-DEDD2
NP_997560.3	low-complexity+DED+TRADDN-DEDD2	330	2410050E11Rik,FLAME-3	vertebrata	Mus musculus	DNA-binding death effector domain-containing protein 2 [Mus musculus]	GCF_000001635.26	TRADDN-DEDD2
XP_021053693.1	low-complexity+DED+TRADDN-DEDD2	275	Dedd	vertebrata	Mus pahari	death effector domain-containing protein isoform X2 [Mus pahari]	-	TRADDN-DEDD2
XP_004775959.1	low-complexity+DED+TRADDN-DEDD2	318	DEDD	vertebrata	Mustela putorius furo	PREDICTED: death effector domain-containing protein [Mustela putorius furo]	GCF_000215625.1	TRADDN-DEDD2
XP_012919430.1	low-complexity+DED+TRADDN-DEDD2	440	DEDD2	vertebrata	Mustela putorius furo	PREDICTED: DNA-binding death effector domain-containing protein 2 [Mustela putorius furo]	GCF_000215625.1	TRADDN-DEDD2
XP_005880525.1	low-complexity+DED+TRADDN-DEDD2	318	DEDD	vertebrata	Myotis brandtii	PREDICTED: death effector domain-containing protein [Myotis brandtii]	GCF_000412655.1	TRADDN-DEDD2
XP_005883974.1	low-complexity+DED+TRADDN-DEDD2	292	DEDD2	vertebrata	Myotis brandtii	PREDICTED: DNA-binding death effector domain-containing protein 2 [Myotis brandtii]	GCF_000412655.1	TRADDN-DEDD2
XP_006761133.1	low-complexity+DED+TRADDN-DEDD2	327	DEDD2	vertebrata	Myotis davidii	PREDICTED: DNA-binding death effector domain-containing protein 2 [Myotis davidii]	GCF_000327345.1	TRADDN-DEDD2
XP_006764795.1	low-complexity+DED+TRADDN-DEDD2	318	DEDD	vertebrata	Myotis davidii	PREDICTED: death effector domain-containing protein [Myotis davidii]	GCF_000327345.1	TRADDN-DEDD2
XP_006103322.1	low-complexity+DED+TRADDN-DEDD2	318	DEDD	vertebrata	Myotis lucifugus	death effector domain-containing protein isoform X1 [Myotis lucifugus]	GCF_000147115.1	TRADDN-DEDD2

acc	architecture	len	gen.name	taxend	species	defline	gca	clade
XP_023600366.1	low-complexity+DED+TRADDN-DEDD2	282	DEDD	vertebrata	Myotis lucifugus	death effector domain-containing protein isoform X2 [Myotis lucifugus]	GCF_000147115.1	TRADDN-DEDD2
XP_023600627.1	low-complexity+DED+TRADDN-DEDD2	335	DEDD2	vertebrata	Myotis lucifugus	DNA-binding death effector domain-containing protein 2 isoform X1 [Myotis lucifugus]	GCF_000147115.1	TRADDN-DEDD2
XP_023600630.1	low-complexity+DED+TRADDN-DEDD2	327	DEDD2	vertebrata	Myotis lucifugus	DNA-binding death effector domain-containing protein 2 isoform X2 [Myotis lucifugus]	GCF_000147115.1	TRADDN-DEDD2
XP_008844653.1	low-complexity+DED+TRADDN-DEDD2	329	Dedd2	vertebrata	Nannospalax galili	PREDICTED: DNA-binding death effector domain-containing protein 2 isoform X1 [Nannospalax galili]	GCF_000622305.1	TRADDN-DEDD2
XP_008844655.1	low-complexity+DED+TRADDN-DEDD2	334	Dedd2	vertebrata	Nannospalax galili	PREDICTED: DNA-binding death effector domain-containing protein 2 isoform X4 [Nannospalax galili]	GCF_000622305.1	TRADDN-DEDD2
XP_008846226.1	low-complexity+DED+TRADDN-DEDD2	318	Dedd	vertebrata	Nannospalax galili	PREDICTED: death effector domain-containing protein [Nannospalax galili]	GCF_000622305.1	TRADDN-DEDD2
XP_017656942.1	low-complexity+DED+TRADDN-DEDD2	280	Dedd2	vertebrata	Nannospalax galili	PREDICTED: DNA-binding death effector domain-containing protein 2 isoform X2 [Nannospalax galili]	GCF_000622305.1	TRADDN-DEDD2
XP_017656943.1	low-complexity+DED+TRADDN-DEDD2	275	Dedd2	vertebrata	Nannospalax galili	PREDICTED: DNA-binding death effector domain-containing protein 2 isoform X3 [Nannospalax galili]	GCF_000622305.1	TRADDN-DEDD2
XP_018410288.1	low-complexity+DED+TRADDN-DEDD2	353	DEDD2	vertebrata	Nanorana parkeri	PREDICTED: DNA-binding death effector domain-containing protein 2 [Nanorana parkeri]	GCF_000935625.1	TRADDN-DEDD2
XP_018427058.1	low-complexity+DED+TRADDN-DEDD2	318	DEDD	vertebrata	Nanorana parkeri	PREDICTED: death effector domain-containing protein [Nanorana parkeri]	GCF_000935625.1	TRADDN-DEDD2
XP_021547923.1	low-complexity+DED+TRADDN-DEDD2	327	DEDD2	vertebrata	Neomonachus schauinslandi	DNA-binding death effector domain-containing protein 2 isoform X1 [Neomonachus schauinslandi]	GCF_002201575.1	TRADDN-DEDD2
XP_024624548.1	low-complexity+DED+TRADDN-DEDD2	323	DEDD2	vertebrata	Neophocaena asiaeorientalis asiaeorientalis	DNA-binding death effector domain-containing protein 2 isoform X1 [Neophocaena asiaeorientalis asiaeorientalis]	GCF_003031525.1	TRADDN-DEDD2
XP_024624549.1	low-complexity+DED+TRADDN-DEDD2	318	DEDD2	vertebrata	Neophocaena asiaeorientalis asiaeorientalis	DNA-binding death effector domain-containing protein 2 isoform X2 [Neophocaena asiaeorientalis asiaeorientalis]	GCF_003031525.1	TRADDN-DEDD2
OBS72595.1	low-complexity+DED+TRADDN-DEDD2	335	A6R68_12808	vertebrata	Neotoma lepida	hypothetical protein A6R68_12808 [Neotoma lepida]	GCA_001675575.1	TRADDN-DEDD2
OBS82753.1	low-complexity+DED+TRADDN-DEDD2	318	A6R68_23240	vertebrata	Neotoma lepida	hypothetical protein A6R68_23240 [Neotoma lepida]	GCA_001675575.1	TRADDN-DEDD2
XP_009465339.1	low-complexity+DED+TRADDN-DEDD2	318	DEDD	vertebrata	Nipponia nippon	PREDICTED: death effector domain-containing protein [Nipponia nippon]	GCF_000708225.1	TRADDN-DEDD2
XP_003258780.1	low-complexity+DED+TRADDN-DEDD2	318	DEDD	vertebrata	Nomascus leucogenys	PREDICTED: LOW QUALITY PROTEIN: death effector domain-containing protein [Nomascus leucogenys]	-	TRADDN-DEDD2

acc	architecture	len	gen.name	taxend	species	defline	gca	clade
XP_003281023.1	low-complexity+DED+TRADDN-DEDD2	344	DEDD2	vertebrata	Nomascus leucogenys	PREDICTED: DNA-binding death effector domain-containing protein 2 isoform X2 [Nomascus leucogenys]	-	TRADDN-DEDD2
XP_012352165.1	low-complexity+DED+TRADDN-DEDD2	361	DEDD2	vertebrata	Nomascus leucogenys	PREDICTED: DNA-binding death effector domain-containing protein 2 isoform X1 [Nomascus leucogenys]	-	TRADDN-DEDD2
XP_026537206.1	low-complexity+DED+TRADDN-DEDD2	362	DEDD2	vertebrata	Notechis scutatus	DNA-binding death effector domain-containing protein 2 [Notechis scutatus]	GCF_900518725.1	TRADDN-DEDD2
XP_026547675.1	low-complexity+DED+TRADDN-DEDD2	331	DEDD	vertebrata	Notechis scutatus	death effector domain-containing protein [Notechis scutatus]	GCF_900518725.1	TRADDN-DEDD2
XP_025904294.1	low-complexity+DED+TRADDN-DEDD2	318	DEDD	vertebrata	Nothoprocta perdicaria	death effector domain-containing protein [Nothoprocta perdicaria]	GCF_003342845.1	TRADDN-DEDD2
XP_021232088.1	low-complexity+DED+TRADDN-DEDD2	318	DEDD	vertebrata	Numida meleagris	death effector domain-containing protein [Numida meleagris]	GCF_002078875.1	TRADDN-DEDD2
XP_004589156.1	low-complexity+DED+TRADDN-DEDD2	318	DEDD	vertebrata	Ochotona princeps	PREDICTED: death effector domain-containing protein isoform X1 [Ochotona princeps]	GCF_000292845.1	TRADDN-DEDD2
XP_004589158.1	low-complexity+DED+TRADDN-DEDD2	275	DEDD	vertebrata	Ochotona princeps	PREDICTED: death effector domain-containing protein isoform X2 [Ochotona princeps]	GCF_000292845.1	TRADDN-DEDD2
XP_004598037.1	low-complexity+DED+TRADDN-DEDD2	333	DEDD2	vertebrata	Ochotona princeps	PREDICTED: DNA-binding death effector domain-containing protein 2 isoform X1 [Ochotona princeps]	GCF_000292845.1	TRADDN-DEDD2
XP_004598038.1	low-complexity+DED+TRADDN-DEDD2	328	DEDD2	vertebrata	Ochotona princeps	PREDICTED: DNA-binding death effector domain-containing protein 2 isoform X2 [Ochotona princeps]	GCF_000292845.1	TRADDN-DEDD2
XP_004639969.1	low-complexity+DED+TRADDN-DEDD2	318	Dedd	vertebrata	Octodon degus	death effector domain-containing protein [Octodon degus]	GCF_000260255.1	TRADDN-DEDD2
XP_004644421.1	low-complexity+DED+TRADDN-DEDD2	331	Dedd2	vertebrata	Octodon degus	DNA-binding death effector domain-containing protein 2 [Octodon degus]	GCF_000260255.1	TRADDN-DEDD2
XP_004407955.1	low-complexity+DED+TRADDN-DEDD2	318	DEDD	vertebrata	Odobenus rosmarus divergens	PREDICTED: death effector domain-containing protein isoform X1 [Odobenus rosmarus divergens]	GCF_000321225.1	TRADDN-DEDD2
XP_004407956.1	low-complexity+DED+TRADDN-DEDD2	312	DEDD	vertebrata	Odobenus rosmarus divergens	PREDICTED: death effector domain-containing protein isoform X2 [Odobenus rosmarus divergens]	GCF_000321225.1	TRADDN-DEDD2
XP_004407957.1	low-complexity+DED+TRADDN-DEDD2	275	DEDD	vertebrata	Odobenus rosmarus divergens	PREDICTED: death effector domain-containing protein isoform X3 [Odobenus rosmarus divergens]	GCF_000321225.1	TRADDN-DEDD2
XP_004408213.1	low-complexity+DED+TRADDN-DEDD2	327	DEDD2	vertebrata	Odobenus rosmarus divergens	PREDICTED: DNA-binding death effector domain-containing protein 2 [Odobenus rosmarus divergens]	GCF_000321225.1	TRADDN-DEDD2
XP_020726806.1	low-complexity+DED+TRADDN-DEDD2	327	DEDD2	vertebrata	Odocoileus virginianus texanus	DNA-binding death effector domain-containing protein 2 [Odocoileus virginianus texanus]	GCF_002102435.1	TRADDN-DEDD2
XP_020744941.1	low-complexity+DED+TRADDN-DEDD2	318	DEDD	vertebrata	Odocoileus virginianus texanus	death effector domain-containing protein isoform X1 [Odocoileus virginianus texanus]	GCF_002102435.1	TRADDN-DEDD2

acc	architecture	len	gen.name	taxend	species	defline	gca	clade
XP_020744947.1	low-complexity+DED+TRADDN-DEDD2	312	DEDD	vertebrata	Odocoileus virginianus texanus	death effector domain-containing protein isoform X2 [Odocoileus virginianus texanus]	GCF_002102435.1	TRADDN-DEDD2
XP_004284454.1	low-complexity+DED+TRADDN-DEDD2	318	DEDD	vertebrata	Orcinus orca	PREDICTED: death effector domain-containing protein isoform X1 [Orcinus orca]	GCF_000331955.2	TRADDN-DEDD2
XP_012389054.1	low-complexity+DED+TRADDN-DEDD2	323	DEDD2	vertebrata	Orcinus orca	PREDICTED: DNA-binding death effector domain-containing protein 2 [Orcinus orca]	GCF_000331955.2	TRADDN-DEDD2
XP_012393971.1	low-complexity+DED+TRADDN-DEDD2	275	DEDD	vertebrata	Orcinus orca	PREDICTED: death effector domain-containing protein isoform X2 [Orcinus orca]	GCF_000331955.2	TRADDN-DEDD2
XP_007941131.1	low-complexity+DED+TRADDN-DEDD2	329	DEDD2	vertebrata	Orycteropus afer afer	PREDICTED: DNA-binding death effector domain-containing protein 2 isoform X1 [Orycteropus afer afer]	GCF_000298275.1	TRADDN-DEDD2
XP_007941132.1	low-complexity+DED+TRADDN-DEDD2	324	DEDD2	vertebrata	Orycteropus afer afer	PREDICTED: DNA-binding death effector domain-containing protein 2 isoform X2 [Orycteropus afer afer]	GCF_000298275.1	TRADDN-DEDD2
XP_007945916.1	low-complexity+DED+TRADDN-DEDD2	318	DEDD	vertebrata	Orycteropus afer afer	PREDICTED: death effector domain-containing protein isoform X1 [Orycteropus afer afer]	GCF_000298275.1	TRADDN-DEDD2
XP_007945918.1	low-complexity+DED+TRADDN-DEDD2	275	DEDD	vertebrata	Orycteropus afer afer	PREDICTED: death effector domain-containing protein isoform X2 [Orycteropus afer afer]	GCF_000298275.1	TRADDN-DEDD2
XP_002722349.3	low-complexity+DED+TRADDN-DEDD2	338	DEDD2	vertebrata	Oryctolagus cuniculus	PREDICTED: DNA-binding death effector domain-containing protein 2 isoform X2 [Oryctolagus cuniculus]	GCF_000003625.3	TRADDN-DEDD2
XP_008247900.2	low-complexity+DED+TRADDN-DEDD2	346	DEDD2	vertebrata	Oryctolagus cuniculus	PREDICTED: DNA-binding death effector domain-containing protein 2 isoform X1 [Oryctolagus cuniculus]	GCF_000003625.3	TRADDN-DEDD2
XP_003785827.1	low-complexity+DED+TRADDN-DEDD2	318	DEDD	vertebrata	Otolemur garnettii	death effector domain-containing protein [Otolemur garnettii]	GCF_000181295.1	TRADDN-DEDD2
XP_003799518.1	low-complexity+DED+TRADDN-DEDD2	326	DEDD2	vertebrata	Otolemur garnettii	DNA-binding death effector domain-containing protein 2 [Otolemur garnettii]	GCF_000181295.1	TRADDN-DEDD2
XP_004015335.2	low-complexity+DED+TRADDN-DEDD2	286	DEDD2	vertebrata	Ovis aries	PREDICTED: LOW QUALITY PROTEIN: DNA-binding death effector domain-containing protein 2 [Ovis aries]	-	TRADDN-DEDD2
XP_012040120.1	low-complexity+DED+TRADDN-DEDD2	358	DEDD	vertebrata	Ovis aries	PREDICTED: death effector domain-containing protein isoform X1 [Ovis aries]	-	TRADDN-DEDD2
XP_012017925.1	low-complexity+DED+TRADDN-DEDD2	326	DEDD2	vertebrata	Ovis aries musimon	PREDICTED: DNA-binding death effector domain-containing protein 2 [Ovis aries musimon]	-	TRADDN-DEDD2
XP_012023522.1	low-complexity+DED+TRADDN-DEDD2	318	DEDD	vertebrata	Ovis aries musimon	PREDICTED: death effector domain-containing protein isoform X2 [Ovis aries musimon]	-	TRADDN-DEDD2
XP_003811820.1	low-complexity+DED+TRADDN-DEDD2	326	DEDD2	vertebrata	Pan paniscus	DNA-binding death effector domain-containing protein 2 isoform X1 [Pan paniscus]	GCF_000258655.2	TRADDN-DEDD2

acc	architecture	len	gen.name	taxend	species	defline	gca	clade
XP_008962420.1	low-complexity+DED+TRADDN-DEDD2	321	DEDD2	vertebrata	Pan paniscus	DNA-binding death effector domain-containing protein 2 isoform X2 [Pan paniscus]	GCF_000258655.2	TRADDN-DEDD2
PNI19827.1	low-complexity+DED+TRADDN-DEDD2	287	CK820_G0049338	vertebrata	Pan troglodytes	DEDD isoform 9, partial [Pan troglodytes]	GCA_002880755.3	TRADDN-DEDD2
XP_019288765.1	low-complexity+DED+TRADDN-DEDD2	318	DEDD	vertebrata	Panthera pardus	PREDICTED: death effector domain-containing protein [Panthera pardus]	GCF_001857705.1	TRADDN-DEDD2
XP_019289993.1	low-complexity+DED+TRADDN-DEDD2	327	DEDD2	vertebrata	Panthera pardus	PREDICTED: DNA-binding death effector domain-containing protein 2 [Panthera pardus]	GCF_001857705.1	TRADDN-DEDD2
XP_007086659.1	low-complexity+DED+TRADDN-DEDD2	295	DEDD2	vertebrata	Panthera tigris altaica	PREDICTED: LOW QUALITY PROTEIN: DNA-binding death effector domain-containing protein 2 [Panthera tigris altaica]	GCF_000464555.1	TRADDN-DEDD2
XP_007093104.1	low-complexity+DED+TRADDN-DEDD2	302	DEDD	vertebrata	Panthera tigris altaica	PREDICTED: death effector domain-containing protein, partial [Panthera tigris altaica]	GCF_000464555.1	TRADDN-DEDD2
XP_005958871.1	low-complexity+DED+TRADDN-DEDD2	318	DEDD	vertebrata	Pantholops hodgsonii	PREDICTED: death effector domain-containing protein isoform X1 [Pantholops hodgsonii]	-	TRADDN-DEDD2
XP_005958872.1	low-complexity+DED+TRADDN-DEDD2	312	DEDD	vertebrata	Pantholops hodgsonii	PREDICTED: death effector domain-containing protein isoform X2 [Pantholops hodgsonii]	-	TRADDN-DEDD2
XP_005958873.1	low-complexity+DED+TRADDN-DEDD2	275	DEDD	vertebrata	Pantholops hodgsonii	PREDICTED: death effector domain-containing protein isoform X3 [Pantholops hodgsonii]	-	TRADDN-DEDD2
XP_005971433.1	low-complexity+DED+TRADDN-DEDD2	326	DEDD2	vertebrata	Pantholops hodgsonii	PREDICTED: DNA-binding death effector domain-containing protein 2 [Pantholops hodgsonii]	-	TRADDN-DEDD2
XP_003915662.1	low-complexity+DED+TRADDN-DEDD2	326	DEDD2	vertebrata	Papio anubis	DNA-binding death effector domain-containing protein 2 isoform X1 [Papio anubis]	GCF_000264685.3	TRADDN-DEDD2
XP_009192863.1	low-complexity+DED+TRADDN-DEDD2	279	DEDD2	vertebrata	Papio anubis	DNA-binding death effector domain-containing protein 2 isoform X2 [Papio anubis]	GCF_000264685.3	TRADDN-DEDD2
XP_006113986.1	low-complexity+DED+TRADDN-DEDD2	318	DEDD	vertebrata	Pelodiscus sinensis	death effector domain-containing protein [Pelodiscus sinensis]	GCF_000230535.1	TRADDN-DEDD2
XP_006127950.1	low-complexity+DED+TRADDN-DEDD2	359	DEDD2	vertebrata	Pelodiscus sinensis	DNA-binding death effector domain-containing protein 2 [Pelodiscus sinensis]	GCF_000230535.1	TRADDN-DEDD2
XP_006988136.1	low-complexity+DED+TRADDN-DEDD2	329	Dedd2	vertebrata	Peromyscus maniculatus bairdii	PREDICTED: DNA-binding death effector domain-containing protein 2 [Peromyscus maniculatus bairdii]	GCF_000500345.1	TRADDN-DEDD2
XP_006990684.1	low-complexity+DED+TRADDN-DEDD2	318	Dedd	vertebrata	Peromyscus maniculatus bairdii	PREDICTED: death effector domain-containing protein isoform X1 [Peromyscus maniculatus bairdii]	GCF_000500345.1	TRADDN-DEDD2
XP_015864395.1	low-complexity+DED+TRADDN-DEDD2	305	Dedd	vertebrata	Peromyscus maniculatus bairdii	PREDICTED: death effector domain-containing protein isoform X2 [Peromyscus maniculatus bairdii]	GCF_000500345.1	TRADDN-DEDD2

acc	architecture	len	gen.name	taxend	species	defline	gca	clade
XP_010292665.1	low-complexity+DED+TRADDN-DEDD2	318	DEDD	vertebrata	Phaethon lepturus	PREDICTED: death effector domain-containing protein [Phaethon lepturus]	GCF_000687285.1	TRADDN-DEDD2
XP_020827647.1	low-complexity+DED+TRADDN-DEDD2	334	DEDD	vertebrata	Phascolarctos cinereus	death effector domain-containing protein isoform X1 [Phascolarctos cinereus]	GCF_002099425.1	TRADDN-DEDD2
XP_020827649.1	low-complexity+DED+TRADDN-DEDD2	318	DEDD	vertebrata	Phascolarctos cinereus	death effector domain-containing protein isoform X3 [Phascolarctos cinereus]	GCF_002099425.1	TRADDN-DEDD2
XP_020859458.1	low-complexity+DED+TRADDN-DEDD2	393	DEDD2	vertebrata	Phascolarctos cinereus	LOW QUALITY PROTEIN: DNA-binding death effector domain-containing protein 2 [Phascolarctos cinereus]	GCF_002099425.1	TRADDN-DEDD2
XP_007102243.2	low-complexity+DED+TRADDN-DEDD2	327	DEDD2	vertebrata	Physeter catodon	DNA-binding death effector domain-containing protein 2 [Physeter catodon]	GCF_002837175.2	TRADDN-DEDD2
XP_023070073.1	low-complexity+DED+TRADDN-DEDD2	312	DEDD	vertebrata	Piliocolobus tephrosceles	death effector domain-containing protein isoform X2 [Piliocolobus tephrosceles]	GCF_002776525.2	TRADDN-DEDD2
XP_020663564.1	low-complexity+DED+TRADDN-DEDD2	363	DEDD2	vertebrata	Pogona vitticeps	DNA-binding death effector domain-containing protein 2 [Pogona vitticeps]	GCF_900067755.1	TRADDN-DEDD2
NP_001126047.1	low-complexity+DED+TRADDN-DEDD2	318	DEDD	vertebrata	Pongo abelii	death effector domain-containing protein [Pongo abelii]	GCF_002880775.1	TRADDN-DEDD2
PNJ19230.1	low-complexity+DED+TRADDN-DEDD2	321	CR201_G0044088	vertebrata	Pongo abelii	DEDD2 isoform 1 [Pongo abelii]	GCA_002880775.3	TRADDN-DEDD2
PNJ57085.1	low-complexity+DED+TRADDN-DEDD2	348	CR201_G0019390	vertebrata	Pongo abelii	DEDD isoform 8 [Pongo abelii]	GCA_002880775.3	TRADDN-DEDD2
PNJ57086.1	low-complexity+DED+TRADDN-DEDD2	287	CR201_G0019390	vertebrata	Pongo abelii	DEDD isoform 9, partial [Pongo abelii]	GCA_002880775.3	TRADDN-DEDD2
XP_009230944.2	low-complexity+DED+TRADDN-DEDD2	326	DEDD2	vertebrata	Pongo abelii	DNA-binding death effector domain-containing protein 2 [Pongo abelii]	GCF_002880775.1	TRADDN-DEDD2
XP_024113149.1	low-complexity+DED+TRADDN-DEDD2	318	DEDD	vertebrata	Pongo abelii	death effector domain-containing protein isoform X1 [Pongo abelii]	GCF_002880775.1	TRADDN-DEDD2
XP_012508808.1	low-complexity+DED+TRADDN-DEDD2	326	DEDD2	vertebrata	Propithecus coquereli	PREDICTED: DNA-binding death effector domain-containing protein 2 [Propithecus coquereli]	GCF_000956105.1	TRADDN-DEDD2
XP_015678489.1	low-complexity+DED+TRADDN-DEDD2	318	DEDD	vertebrata	Protobothrops mucrosquamatus	PREDICTED: death effector domain-containing protein [Protobothrops mucrosquamatus]	GCF_001527695.2	TRADDN-DEDD2
XP_015681195.1	low-complexity+DED+TRADDN-DEDD2	363	DEDD2	vertebrata	Protobothrops mucrosquamatus	PREDICTED: DNA-binding death effector domain-containing protein 2 [Protobothrops mucrosquamatus]	GCF_001527695.2	TRADDN-DEDD2
XP_026571203.1	low-complexity+DED+TRADDN-DEDD2	362	DEDD2	vertebrata	Pseudonaja textilis	DNA-binding death effector domain-containing protein 2 [Pseudonaja textilis]	GCF_900518735.1	TRADDN-DEDD2
XP_026573488.1	low-complexity+DED+TRADDN-DEDD2	355	DEDD	vertebrata	Pseudonaja textilis	death effector domain-containing protein isoform X1 [Pseudonaja textilis]	GCF_900518735.1	TRADDN-DEDD2
XP_026573489.1	low-complexity+DED+TRADDN-DEDD2	362	DEDD	vertebrata	Pseudonaja textilis	death effector domain-containing protein isoform X2 [Pseudonaja textilis]	GCF_900518735.1	TRADDN-DEDD2

acc	architecture	len	gen.name	taxend	species	defline	gca	clade
XP_014117322.1	low-complexity+DED+TRADDN-DEDD2	322	DEDD	vertebrata	Pseudopodoces humilis	PREDICTED: death effector domain-containing protein isoform X1 [Pseudopodoces humilis]	GCF_000331425.1	TRADDN-DEDD2
XP_014117323.1	low-complexity+DED+TRADDN-DEDD2	323	DEDD	vertebrata	Pseudopodoces humilis	PREDICTED: death effector domain-containing protein isoform X2 [Pseudopodoces humilis]	GCF_000331425.1	TRADDN-DEDD2
XP_014118337.1	low-complexity+DED+TRADDN-DEDD2	357	LOC102110117	vertebrata	Pseudopodoces humilis	PREDICTED: DNA-binding death effector domain-containing protein 2-like [Pseudopodoces humilis]	GCF_000331425.1	TRADDN-DEDD2
XP_006903926.1	low-complexity+DED+TRADDN-DEDD2	326	DEDD2	vertebrata	Pteropus alecto	DNA-binding death effector domain-containing protein 2 [Pteropus alecto]	GCF_000325575.1	TRADDN-DEDD2
XP_006907932.1	low-complexity+DED+TRADDN-DEDD2	318	DEDD	vertebrata	Pteropus alecto	death effector domain-containing protein [Pteropus alecto]	GCF_000325575.1	TRADDN-DEDD2
XP_011381121.1	low-complexity+DED+TRADDN-DEDD2	326	DEDD2	vertebrata	Pteropus vampyrus	DNA-binding death effector domain-containing protein 2 [Pteropus vampyrus]	GCF_000151845.1	TRADDN-DEDD2
XP_025769996.1	low-complexity+DED+TRADDN-DEDD2	327	DEDD2	vertebrata	Puma concolor	DNA-binding death effector domain-containing protein 2 [Puma concolor]	GCF_003327715.1	TRADDN-DEDD2
XP_025771039.1	low-complexity+DED+TRADDN-DEDD2	319	DEDD	vertebrata	Puma concolor	LOW QUALITY PROTEIN: death effector domain-containing protein [Puma concolor]	GCF_003327715.1	TRADDN-DEDD2
XP_007438974.1	low-complexity+DED+TRADDN-DEDD2	355	DEDD	vertebrata	Python bivittatus	death effector domain-containing protein [Python bivittatus]	GCF_000186305.1	TRADDN-DEDD2
XP_007442354.1	low-complexity+DED+TRADDN-DEDD2	362	DEDD2	vertebrata	Python bivittatus	DNA-binding death effector domain-containing protein 2 [Python bivittatus]	GCF_000186305.1	TRADDN-DEDD2
NP_113988.1	low-complexity+DED+TRADDN-DEDD2	318	Deft	vertebrata	Rattus norvegicus	death effector domain-containing protein [Rattus norvegicus]	GCF_000002265.2	TRADDN-DEDD2
XP_003753272.1	low-complexity+DED+TRADDN-DEDD2	331	Dedd2	vertebrata	Rattus norvegicus	PREDICTED: DNA-binding death effector domain-containing protein 2 [Rattus norvegicus]	GCF_000002265.2	TRADDN-DEDD2
XP_020380645.1	low-complexity+DED+TRADDN-DEDD2	314	dedd	vertebrata	Rhincodon typus	death effector domain-containing protein [Rhincodon typus]	GCF_001642345.1	TRADDN-DEDD2
XP_020381127.1	low-complexity+DED+TRADDN-DEDD2	346	LOC109925611	vertebrata	Rhincodon typus	DNA-binding death effector domain-containing protein 2-like [Rhincodon typus]	GCF_001642345.1	TRADDN-DEDD2
XP_019568501.1	low-complexity+DED+TRADDN-DEDD2	318	DEDD	vertebrata	Rhinolophus sinicus	PREDICTED: death effector domain-containing protein [Rhinolophus sinicus]	-	TRADDN-DEDD2
XP_019569316.1	low-complexity+DED+TRADDN-DEDD2	331	DEDD2	vertebrata	Rhinolophus sinicus	PREDICTED: DNA-binding death effector domain-containing protein 2 [Rhinolophus sinicus]	-	TRADDN-DEDD2
XP_010357226.1	low-complexity+DED+TRADDN-DEDD2	326	DEDD2	vertebrata	Rhinopithecus roxellana	PREDICTED: DNA-binding death effector domain-containing protein 2 [Rhinopithecus roxellana]	GCF_007565055.1	TRADDN-DEDD2
XP_010365416.1	low-complexity+DED+TRADDN-DEDD2	282	DEDD	vertebrata	Rhinopithecus roxellana	PREDICTED: death effector domain-containing protein isoform X2 [Rhinopithecus roxellana]	-	TRADDN-DEDD2

acc	architecture	len	gen.name	taxend	species	defline	gca	clade
XP_015985433.1	low-complexity+DED+TRADDN-DEDD2	326	DEDD2	vertebrata	Rousettus aegyptiacus	PREDICTED: DNA-binding death effector domain-containing protein 2 [Rousettus aegyptiacus]	GCF_001466805.2	TRADDN-DEDD2
XP_015989540.1	low-complexity+DED+TRADDN-DEDD2	318	DEDD	vertebrata	Rousettus aegyptiacus	PREDICTED: death effector domain-containing protein [Rousettus aegyptiacus]	GCF_001466805.2	TRADDN-DEDD2
XP_003769678.1	low-complexity+DED+TRADDN-DEDD2	318	DEDD	vertebrata	Sarcophilus harrisii	death effector domain-containing protein [Sarcophilus harrisii]	GCF_000189315.1	TRADDN-DEDD2
XP_004613810.1	low-complexity+DED+TRADDN-DEDD2	318	DEDD	vertebrata	Sorex araneus	PREDICTED: death effector domain-containing protein [Sorex araneus]	GCF_000181275.1	TRADDN-DEDD2
XP_004620755.1	low-complexity+DED+TRADDN-DEDD2	327	DEDD2	vertebrata	Sorex araneus	PREDICTED: DNA-binding death effector domain-containing protein 2 isoform X1 [Sorex araneus]	GCF_000181275.1	TRADDN-DEDD2
XP_004620756.1	low-complexity+DED+TRADDN-DEDD2	322	DEDD2	vertebrata	Sorex araneus	PREDICTED: DNA-binding death effector domain-containing protein 2 isoform X2 [Sorex araneus]	GCF_000181275.1	TRADDN-DEDD2
KFV72542.1	low-complexity+DED+TRADDN-DEDD2	319	N308_07951	vertebrata	Struthio camelus australis	Death effector domain-containing protein [Struthio camelus australis]	GCA_000698965.1	TRADDN-DEDD2
XP_009665721.1	low-complexity+DED+TRADDN-DEDD2	318	DEDD	vertebrata	Struthio camelus australis	PREDICTED: death effector domain-containing protein [Struthio camelus australis]	GCF_000698965.1	TRADDN-DEDD2
XP_014748917.1	low-complexity+DED+TRADDN-DEDD2	319	DEDD	vertebrata	Sturnus vulgaris	PREDICTED: death effector domain-containing protein [Sturnus vulgaris]	GCF_001447265.1	TRADDN-DEDD2
XP_001927399.1	low-complexity+DED+TRADDN-DEDD2	318	DEDD	vertebrata	Sus scrofa	death effector domain-containing protein [Sus scrofa]	GCF_000003025.6	TRADDN-DEDD2
XP_013844083.2	low-complexity+DED+TRADDN-DEDD2	326	DEDD2	vertebrata	Sus scrofa	LOW QUALITY PROTEIN: DNA-binding death effector domain-containing protein 2 [Sus scrofa]	GCF_000003025.6	TRADDN-DEDD2
XP_004174339.1	low-complexity+DED+TRADDN-DEDD2	332	LOC100225852	vertebrata	Taeniopygia guttata	PREDICTED: LOW QUALITY PROTEIN: death effector domain-containing protein-like [Taeniopygia guttata]	-	TRADDN-DEDD2
XP_026515566.1	low-complexity+DED+TRADDN-DEDD2	333	DEDD	vertebrata	Terrapene mexicana triunguis	death effector domain-containing protein [Terrapene mexicana triunguis]	-	TRADDN-DEDD2
XP_013927334.1	low-complexity+DED+TRADDN-DEDD2	363	DEDD2	vertebrata	Thamnophis sirtalis	PREDICTED: DNA-binding death effector domain-containing protein 2 [Thamnophis sirtalis]	GCF_001077635.1	TRADDN-DEDD2
XP_025214868.1	low-complexity+DED+TRADDN-DEDD2	275	DEDD	vertebrata	Theropithecus gelada	death effector domain-containing protein isoform X3 [Theropithecus gelada]	GCF_003255815.1	TRADDN-DEDD2
XP_025222042.1	low-complexity+DED+TRADDN-DEDD2	326	DEDD2	vertebrata	Theropithecus gelada	DNA-binding death effector domain-containing protein 2 [Theropithecus gelada]	GCF_003255815.1	TRADDN-DEDD2
XP_004388812.1	low-complexity+DED+TRADDN-DEDD2	327	LOC101346351	vertebrata	Trichechus manatus latirostris	DNA-binding death effector domain-containing protein 2 isoform X1 [Trichechus manatus latirostris]	GCF_000243295.1	TRADDN-DEDD2

acc	architecture	len	gen.name	taxend	species	defline	gca	clade
XP_004390231.1	low-complexity+DED+TRADDN-DEDD2	318	LOC101349768	vertebrata	Trichechus manatus latirostris	death effector domain-containing protein [Trichechus manatus latirostris]	GCF_000243295.1	TRADDN-DEDD2
ELW71049.1	low-complexity+DED+TRADDN-DEDD2	282	TREES_T100011471	vertebrata	Tupaia chinensis	DNA-binding death effector domain-containing protein 2 [Tupaia chinensis]	GCA_000334495.1	TRADDN-DEDD2
XP_006142066.1	low-complexity+DED+TRADDN-DEDD2	326	DEDD2	vertebrata	Tupaia chinensis	PREDICTED: DNA-binding death effector domain-containing protein 2 isoform X1 [Tupaia chinensis]	GCF_000334495.1	TRADDN-DEDD2
XP_006163170.1	low-complexity+DED+TRADDN-DEDD2	318	DEDD	vertebrata	Tupaia chinensis	PREDICTED: death effector domain-containing protein [Tupaia chinensis]	GCF_000334495.1	TRADDN-DEDD2
XP_004321849.1	low-complexity+DED+TRADDN-DEDD2	318	DEDD	vertebrata	Tursiops truncatus	PREDICTED: death effector domain-containing protein [Tursiops truncatus]	-	TRADDN-DEDD2
XP_008698884.1	low-complexity+DED+TRADDN-DEDD2	318	DEDD	vertebrata	Ursus maritimus	PREDICTED: death effector domain-containing protein [Ursus maritimus]	GCF_000687225.1	TRADDN-DEDD2
XP_008707915.1	low-complexity+DED+TRADDN-DEDD2	330	DEDD2	vertebrata	Ursus maritimus	PREDICTED: DNA-binding death effector domain-containing protein 2 [Ursus maritimus]	GCF_000687225.1	TRADDN-DEDD2
XP_006208797.1	low-complexity+DED+TRADDN-DEDD2	303	DEDD2	vertebrata	Vicugna pacos	PREDICTED: DNA-binding death effector domain-containing protein 2 [Vicugna pacos]	GCF_000164845.2	TRADDN-DEDD2
XP_025869648.1	low-complexity+DED+TRADDN-DEDD2	329	DEDD2	vertebrata	Vulpes vulpes	DNA-binding death effector domain-containing protein 2 [Vulpes vulpes]	GCF_003160815.1	TRADDN-DEDD2
NP_001088238.1	low-complexity+DED+TRADDN-DEDD2	318	dedd	vertebrata	Xenopus laevis	death effector domain containing L homeolog [Xenopus laevis]	GCF_001663975.1	TRADDN-DEDD2
NP_001088715.1	low-complexity+DED+TRADDN-DEDD2	357	dedd2	vertebrata	Xenopus laevis	death effector domain containing 2 L homeolog [Xenopus laevis]	GCF_001663975.1	TRADDN-DEDD2
NP_001088842.1	low-complexity+DED+TRADDN-DEDD2	318	dedd.S	vertebrata	Xenopus laevis	uncharacterized protein LOC496151 [Xenopus laevis]	GCF_001663975.1	TRADDN-DEDD2
OCT69466.1	low-complexity+DED+TRADDN-DEDD2	318	XELAEV_18040777mg	vertebrata	Xenopus laevis	hypothetical protein XELAEV_18040777mg [Xenopus laevis]	GCA_001663975.1	TRADDN-DEDD2
OCT72981.1	low-complexity+DED+TRADDN-DEDD2	357	XELAEV_18035962mg	vertebrata	Xenopus laevis	hypothetical protein XELAEV_18035962mg [Xenopus laevis]	GCA_001663975.1	TRADDN-DEDD2
XP_018082413.1	low-complexity+DED+TRADDN-DEDD2	355	MGC80767	vertebrata	Xenopus laevis	PREDICTED: MGC80767 protein isoform X1 [Xenopus laevis]	GCF_001663975.1	TRADDN-DEDD2
XP_002941793.3	low-complexity+DED+TRADDN-DEDD2	318	dedd	vertebrata	Xenopus tropicalis	PREDICTED: death effector domain-containing protein [Xenopus tropicalis]	GCF_000004195.3	TRADDN-DEDD2
XP_012822197.1	low-complexity+DED+TRADDN-DEDD2	356	dedd2	vertebrata	Xenopus tropicalis	PREDICTED: DNA-binding death effector domain-containing protein 2 isoform X1 [Xenopus tropicalis]	GCF_000004195.3	TRADDN-DEDD2
XP_014130289.1	low-complexity+DED+TRADDN-DEDD2	329	DEDD	vertebrata	Zonotrichia albicollis	death effector domain-containing protein [Zonotrichia albicollis]	GCF_000385455.1	TRADDN-DEDD2
KFP69481.1	TRADDN-DEDD2	175	N310_09166	vertebrata	Acanthisitta chloris	Death effector domain-containing protein, partial [Acanthisitta chloris]	GCA_000695815.1	TRADDN-DEDD2

acc	architecture	len	gen.name	taxend	species	defline	gca	clade
XP_009081974.1	TRADDN-DEDD2	173	LOC103810805	vertebrata	Acanthisitta chloris	PREDICTED: death effector domain-containing protein-like, partial [Acanthisitta chloris]	GCF_000695815.1	TRADDN-DEDD2
XP_010174856.1	TRADDN-DEDD2	124	LOC104531626	vertebrata	Antrostomus carolinensis	PREDICTED: death effector domain-containing protein, partial [Antrostomus carolinensis]	GCF_000700745.1	TRADDN-DEDD2
XP_013799486.1	TRADDN-DEDD2	136	LOC106485618	vertebrata	Apteryx australis mantelli	PREDICTED: DNA-binding death effector domain-containing protein 2-like, partial [Apteryx australis mantelli]	GCF_001039765.1	TRADDN-DEDD2
XP_025920279.1	TRADDN-DEDD2	218	DEDD2	vertebrata	Apteryx rowi	DNA-binding death effector domain-containing protein 2, partial [Apteryx rowi]	GCF_003343035.1	TRADDN-DEDD2
POI23242.1	TRADDN-DEDD2	209	CIB84_013009	vertebrata	Bambusicola thoracicus	hypothetical protein CIB84_013009, partial [Bambusicola thoracicus]	GCA_002909625.1	TRADDN-DEDD2
XP_010834143.1	TRADDN-DEDD2	114	LOC104985576	vertebrata	Bison bison bison	PREDICTED: DNA-binding death effector domain-containing protein 2, partial [Bison bison bison]	GCF_000754665.1	TRADDN-DEDD2
XP_014817779.1	TRADDN-DEDD2	320	DEDD	vertebrata	Calidris pugnax	PREDICTED: death effector domain-containing protein isoform X1 [Calidris pugnax]	GCF_001431845.1	TRADDN-DEDD2
XP_014818092.1	TRADDN-DEDD2	141	LOC106900518	vertebrata	Calidris pugnax	PREDICTED: DNA-binding death effector domain-containing protein 2-like, partial [Calidris pugnax]	GCF_001431845.1	TRADDN-DEDD2
XP_008499335.1	TRADDN-DEDD2	153	DEDD	vertebrata	Calypte anna	PREDICTED: death effector domain-containing protein, partial [Calypte anna]	-	TRADDN-DEDD2
XP_010007410.1	TRADDN-DEDD2	121	LOC104398947	vertebrata	Chaetura pelagica	PREDICTED: DNA-binding death effector domain-containing protein 2-like, partial [Chaetura pelagica]	GCF_000747805.1	TRADDN-DEDD2
XP_010195668.1	TRADDN-DEDD2	130	LOC104554364	vertebrata	Colius striatus	PREDICTED: LOW QUALITY PROTEIN: death effector domain-containing protein-like, partial [Colius striatus]	GCF_000690715.1	TRADDN-DEDD2
XP_011810531.1	TRADDN-DEDD2	204	LOC105521110	vertebrata	Colobus angolensis palliatus	PREDICTED: death effector domain-containing protein, partial [Colobus angolensis palliatus]	GCF_000951035.1	TRADDN-DEDD2
KFO62040.1	TRADDN-DEDD2	186	N302_10476	vertebrata	Corvus brachyrhynchos	Death effector domain-containing protein, partial [Corvus brachyrhynchos]	GCA_000691975.1	TRADDN-DEDD2
XP_017594516.1	TRADDN-DEDD2	265	LOC103619000	vertebrata	Corvus brachyrhynchos	PREDICTED: death effector domain-containing protein, partial [Corvus brachyrhynchos]	GCF_000691975.1	TRADDN-DEDD2
RLQ59557.1	TRADDN-DEDD2	216	CgPICR_023308	vertebrata	Cricetulus griseus	DEDD2, partial [Cricetulus griseus]	GCA_003668045.1	TRADDN-DEDD2
XP_023802693.1	TRADDN-DEDD2	142	LOC111944134	vertebrata	Cyanistes caeruleus	death effector domain-containing protein-like [Cyanistes caeruleus]	GCF_002901205.1	TRADDN-DEDD2
XP_014142490.1	TRADDN-DEDD2	246	LOC106631721	vertebrata	Falco cherrug	PREDICTED: DNA-binding death effector domain-containing protein 2-like, partial [Falco cherrug]	-	TRADDN-DEDD2
XP_005062991.1	TRADDN-DEDD2	206	DEDD2	vertebrata	Ficedula albicollis	PREDICTED: DNA-binding death effector domain-containing protein 2, partial [Ficedula albicollis]	GCF_000247815.1	TRADDN-DEDD2

acc	architecture	len	gen.name	taxend	species	defline	gca	clade
XP_012807084.1	TRADDN-DEDD2	255	Dedd2	vertebrata	Jaculus jaculus	PREDICTED: DNA-binding death effector domain-containing protein 2 [Jaculus jaculus]	GCF_000280705.1	TRADDN-DEDD2
XP_017678799.1	TRADDN-DEDD2	234	LOC108501432	vertebrata	Lepidothrix coronata	PREDICTED: DNA-binding death effector domain-containing protein 2-like, partial [Lepidothrix coronata]	GCF_001604755.1	TRADDN-DEDD2
BAE87720.1	TRADDN-DEDD2	210	-	vertebrata	Macaca fascicularis	unnamed protein product [Macaca fascicularis]	-	TRADDN-DEDD2
XP_026644834.1	TRADDN-DEDD2	108	LOC113458262	vertebrata	Microtus ochrogaster	DNA-binding death effector domain-containing protein 2-like, partial [Microtus ochrogaster]	GCF_000317375.1	TRADDN-DEDD2
XP_009466332.1	TRADDN-DEDD2	281	LOC104015334	vertebrata	Nipponia nippon	PREDICTED: uncharacterized protein LOC104015334, partial [Nipponia nippon]	GCF_000708225.1	TRADDN-DEDD2
XP_001520660.2	TRADDN-DEDD2	120	LOC100091904	vertebrata	Ornithorhynchus anatinus	PREDICTED: DNA-binding death effector domain-containing protein 2-like, partial [Ornithorhynchus anatinus]	-	TRADDN-DEDD2
XP_015472045.1	TRADDN-DEDD2	225	LOC107199218	vertebrata	Parus major	PREDICTED: death effector domain-containing protein-like [Parus major]	GCF_001522545.3	TRADDN-DEDD2
XP_009498737.1	TRADDN-DEDD2	136	LOC104041205	vertebrata	Phalacrocorax carbo	PREDICTED: LOW QUALITY PROTEIN: death effector domain-containing protein-like, partial [Phalacrocorax carbo]	GCF_000708925.1	TRADDN-DEDD2
XP_020841292.1	TRADDN-DEDD2	202	LOC110207827	vertebrata	Phascolarctos cinereus	death effector domain-containing protein-like [Phascolarctos cinereus]	GCF_002099425.1	TRADDN-DEDD2
XP_009894949.1	TRADDN-DEDD2	129	DEDD	vertebrata	Picoides pubescens	PREDICTED: death effector domain-containing protein [Picoides pubescens]	GCF_000699005.1	TRADDN-DEDD2
PIO15121.1	TRADDN-DEDD2	193	AB205_0121290	vertebrata	Rana catesbeiana	hypothetical protein AB205_0121290 [Rana catesbeiana]	GCA_002284835.2	TRADDN-DEDD2
XP_012402660.1	TRADDN-DEDD2	214	DEDD2	vertebrata	Sarcophilus harrisii	DNA-binding death effector domain-containing protein 2, partial [Sarcophilus harrisii]	GCF_000189315.1	TRADDN-DEDD2
XP_009098808.2	TRADDN-DEDD2	128	LOC103825280	vertebrata	Serinus canaria	PREDICTED: death effector domain-containing protein, partial [Serinus canaria]	-	TRADDN-DEDD2
XP_013927609.1	TRADDN-DEDD2	226	DEDD	vertebrata	Thamnophis sirtalis	PREDICTED: death effector domain-containing protein [Thamnophis sirtalis]	GCF_001077635.1	TRADDN-DEDD2
XP_010213717.1	TRADDN-DEDD2	151	LOC104568514	vertebrata	Tinamus guttatus	PREDICTED: death effector domain-containing protein-like [Tinamus guttatus]	GCF_000705375.1	TRADDN-DEDD2
OCA14460.1	TRADDN-DEDD2	221	XENTR_v90026734mg	vertebrata	Xenopus tropicalis	hypothetical protein XENTR_v90026734mg [Xenopus tropicalis]	GCA_000004195.3	TRADDN-DEDD2
XP_009079859.1	TRADDN-NCOA6	876	NCOA6	vertebrata	Acanthisitta chloris	PREDICTED: nuclear receptor coactivator 6, partial [Acanthisitta chloris]	GCF_000695815.1	TRADDN-NCOA6

acc	architecture	len	gen.name	taxend	species	defline	gca	clade
XP_014926674.1	TRADDN-NCOA6	2067	NCOA6	vertebrata	Acinonyx jubatus	PREDICTED: LOW QUALITY PROTEIN: nuclear receptor coactivator 6 [Acinonyx jubatus]	-	TRADDN-NCOA6
EFB21630.1	TRADDN-NCOA6	1958	PANDA_004518	vertebrata	Ailuropoda melanoleuca	hypothetical protein PANDA_004518, partial [Ailuropoda melanoleuca]	GCA_000004335.1	TRADDN-NCOA6
XP_002916443.1	TRADDN-NCOA6	2057	NCOA6	vertebrata	Ailuropoda melanoleuca	PREDICTED: nuclear receptor coactivator 6 isoform X1 [Ailuropoda melanoleuca]	GCF_000004335.2	TRADDN-NCOA6
XP_011219545.1	TRADDN-NCOA6	1064	NCOA6	vertebrata	Ailuropoda melanoleuca	PREDICTED: nuclear receptor coactivator 6 isoform X8 [Ailuropoda melanoleuca]	GCF_000004335.2	TRADDN-NCOA6
XP_019652147.1	TRADDN-NCOA6	2056	NCOA6	vertebrata	Ailuropoda melanoleuca	PREDICTED: nuclear receptor coactivator 6 isoform X2 [Ailuropoda melanoleuca]	GCF_000004335.2	TRADDN-NCOA6
XP_019652148.1	TRADDN-NCOA6	2046	NCOA6	vertebrata	Ailuropoda melanoleuca	PREDICTED: nuclear receptor coactivator 6 isoform X3 [Ailuropoda melanoleuca]	GCF_000004335.2	TRADDN-NCOA6
XP_019652149.1	TRADDN-NCOA6	2045	NCOA6	vertebrata	Ailuropoda melanoleuca	PREDICTED: nuclear receptor coactivator 6 isoform X4 [Ailuropoda melanoleuca]	GCF_000004335.2	TRADDN-NCOA6
XP_019652150.1	TRADDN-NCOA6	2034	NCOA6	vertebrata	Ailuropoda melanoleuca	PREDICTED: nuclear receptor coactivator 6 isoform X5 [Ailuropoda melanoleuca]	GCF_000004335.2	TRADDN-NCOA6
XP_019652151.1	TRADDN-NCOA6	2033	NCOA6	vertebrata	Ailuropoda melanoleuca	PREDICTED: nuclear receptor coactivator 6 isoform X6 [Ailuropoda melanoleuca]	GCF_000004335.2	TRADDN-NCOA6
XP_019652152.1	TRADDN-NCOA6	1957	NCOA6	vertebrata	Ailuropoda melanoleuca	PREDICTED: nuclear receptor coactivator 6 isoform X7 [Ailuropoda melanoleuca]	GCF_000004335.2	TRADDN-NCOA6
KYO42234.1	TRADDN-NCOA6	935	NCOA6	vertebrata	Alligator mississippiensis	nuclear receptor coactivator 6 [Alligator mississippiensis]	GCA_000281125.4	TRADDN-NCOA6
XP_014465041.1	TRADDN-NCOA6	2037	NCOA6	vertebrata	Alligator mississippiensis	PREDICTED: nuclear receptor coactivator 6 isoform X1 [Alligator mississippiensis]	GCF_000281125.3	TRADDN-NCOA6
XP_019340209.1	TRADDN-NCOA6	1975	NCOA6	vertebrata	Alligator mississippiensis	PREDICTED: nuclear receptor coactivator 6 isoform X2 [Alligator mississippiensis]	GCF_000281125.3	TRADDN-NCOA6
XP_006024750.1	TRADDN-NCOA6	2007	NCOA6	vertebrata	Alligator sinensis	LOW QUALITY PROTEIN: nuclear receptor coactivator 6 [Alligator sinensis]	GCF_000455745.1	TRADDN-NCOA6
KQK78778.1	TRADDN-NCOA6	824	AAES_110940	vertebrata	Amazona aestiva	nuclear receptor coactivator 6 [Amazona aestiva]	GCA_001420675.1	TRADDN-NCOA6
EOB02387.1	TRADDN-NCOA6	2026	Anapl_04446	vertebrata	Anas platyrhynchos	Nuclear receptor coactivator 6, partial [Anas platyrhynchos]	GCA_000355885.1	TRADDN-NCOA6
XP_005018068.1	TRADDN-NCOA6	2051	NCOA6	vertebrata	Anas platyrhynchos	nuclear receptor coactivator 6 isoform X1 [Anas platyrhynchos]	GCF_003850225.1	TRADDN-NCOA6
XP_005018070.1	TRADDN-NCOA6	1989	NCOA6	vertebrata	Anas platyrhynchos	nuclear receptor coactivator 6 isoform X3 [Anas platyrhynchos]	-	TRADDN-NCOA6
XP_012953507.1	TRADDN-NCOA6	2028	NCOA6	vertebrata	Anas platyrhynchos	nuclear receptor coactivator 6 isoform X2 [Anas platyrhynchos]	-	TRADDN-NCOA6

acc	architecture	len	gen.name	taxend	species	defline	gca	clade
XP_012953508.1	TRADDN-NCOA6	1966	NCOA6	vertebrata	Anas platyrhynchos	nuclear receptor coactivator 6 isoform X4 [Anas platyrhynchos]	-	TRADDN-NCOA6
XP_003220610.1	TRADDN-NCOA6	2035	ncoa6	vertebrata	Anolis carolinensis	PREDICTED: nuclear receptor coactivator 6 isoform X2 [Anolis carolinensis]	GCF_000090745.1	TRADDN-NCOA6
XP_008108217.1	TRADDN-NCOA6	2036	ncoa6	vertebrata	Anolis carolinensis	PREDICTED: nuclear receptor coactivator 6 isoform X1 [Anolis carolinensis]	GCF_000090745.1	TRADDN-NCOA6
XP_013051372.1	TRADDN-NCOA6	2052	NCOA6	vertebrata	Anser cygnoides domesticus	PREDICTED: nuclear receptor coactivator 6 isoform X1 [Anser cygnoides domesticus]	GCF_000971095.1	TRADDN-NCOA6
XP_013051373.1	TRADDN-NCOA6	1990	NCOA6	vertebrata	Anser cygnoides domesticus	PREDICTED: nuclear receptor coactivator 6 isoform X2 [Anser cygnoides domesticus]	GCF_000971095.1	TRADDN-NCOA6
KFZ64822.1	TRADDN-NCOA6	170	N321_09632	vertebrata	Antrostomus carolinensis	Nuclear receptor coactivator 6, partial [Antrostomus carolinensis]	GCA_000700745.1	TRADDN-NCOA6
XP_010172959.1	TRADDN-NCOA6	909	LOC104530153	vertebrata	Antrostomus carolinensis	PREDICTED: nuclear receptor coactivator 6-like [Antrostomus carolinensis]	GCF_000700745.1	TRADDN-NCOA6
XP_012310564.1	TRADDN-NCOA6	2098	NCOA6	vertebrata	Aotus nancymaae	nuclear receptor coactivator 6 isoform X1 [Aotus nancymaae]	GCF_000952055.2	TRADDN-NCOA6
XP_012310567.1	TRADDN-NCOA6	2086	NCOA6	vertebrata	Aotus nancymaae	nuclear receptor coactivator 6 isoform X3 [Aotus nancymaae]	GCF_000952055.2	TRADDN-NCOA6
XP_021521524.1	TRADDN-NCOA6	2097	NCOA6	vertebrata	Aotus nancymaae	nuclear receptor coactivator 6 isoform X2 [Aotus nancymaae]	GCF_000952055.2	TRADDN-NCOA6
KFP76911.1	TRADDN-NCOA6	131	N311_08707	vertebrata	Apaloderma vittatum	Nuclear receptor coactivator 6, partial [Apaloderma vittatum]	GCA_000703405.1	TRADDN-NCOA6
XP_009877278.1	TRADDN-NCOA6	778	LOC104269611	vertebrata	Apaloderma vittatum	PREDICTED: nuclear receptor coactivator 6-like, partial [Apaloderma vittatum]	GCF_000703405.1	TRADDN-NCOA6
XP_019328674.1	TRADDN-NCOA6	2049	NCOA6	vertebrata	Aptenodytes forsteri	PREDICTED: LOW QUALITY PROTEIN: nuclear receptor coactivator 6 [Aptenodytes forsteri]	GCF_000699145.1	TRADDN-NCOA6
XP_013817000.1	TRADDN-NCOA6	2044	NCOA6	vertebrata	Apteryx australis mantelli	PREDICTED: nuclear receptor coactivator 6 [Apteryx australis mantelli]	GCF_001039765.1	TRADDN-NCOA6
XP_011596090.1	TRADDN-NCOA6	2054	NCOA6	vertebrata	Aquila chrysaetos canadensis	PREDICTED: nuclear receptor coactivator 6 isoform X1 [Aquila chrysaetos canadensis]	GCF_000766835.1	TRADDN-NCOA6
XP_011596097.1	TRADDN-NCOA6	2046	NCOA6	vertebrata	Aquila chrysaetos canadensis	PREDICTED: nuclear receptor coactivator 6 isoform X2 [Aquila chrysaetos canadensis]	GCF_000766835.1	TRADDN-NCOA6
XP_011596098.1	TRADDN-NCOA6	2045	NCOA6	vertebrata	Aquila chrysaetos canadensis	PREDICTED: nuclear receptor coactivator 6 isoform X3 [Aquila chrysaetos canadensis]	GCF_000766835.1	TRADDN-NCOA6
XP_011596099.1	TRADDN-NCOA6	2043	NCOA6	vertebrata	Aquila chrysaetos canadensis	PREDICTED: nuclear receptor coactivator 6 isoform X4 [Aquila chrysaetos canadensis]	GCF_000766835.1	TRADDN-NCOA6
XP_011596100.1	TRADDN-NCOA6	2005	NCOA6	vertebrata	Aquila chrysaetos canadensis	PREDICTED: nuclear receptor coactivator 6 isoform X5 [Aquila chrysaetos canadensis]	GCF_000766835.1	TRADDN-NCOA6

acc	architecture	len	gen.name	taxend	species	defline	gca	clade
XP_011596101.1	TRADDN-NCOA6	1986	NCOA6	vertebrata	Aquila chrysaetos canadensis	PREDICTED: nuclear receptor coactivator 6 isoform X6 [Aquila chrysaetos canadensis]	GCF_000766835.1	TRADDN-NCOA6
XP_011596102.1	TRADDN-NCOA6	1984	NCOA6	vertebrata	Aquila chrysaetos canadensis	PREDICTED: nuclear receptor coactivator 6 isoform X7 [Aquila chrysaetos canadensis]	GCF_000766835.1	TRADDN-NCOA6
XP_011596104.1	TRADDN-NCOA6	1781	NCOA6	vertebrata	Aquila chrysaetos canadensis	PREDICTED: nuclear receptor coactivator 6 isoform X9 [Aquila chrysaetos canadensis]	GCF_000766835.1	TRADDN-NCOA6
XP_011596105.1	TRADDN-NCOA6	1732	NCOA6	vertebrata	Aquila chrysaetos canadensis	PREDICTED: nuclear receptor coactivator 6 isoform X10 [Aquila chrysaetos canadensis]	GCF_000766835.1	TRADDN-NCOA6
XP_011596106.1	TRADDN-NCOA6	445	NCOA6	vertebrata	Aquila chrysaetos canadensis	PREDICTED: nuclear receptor coactivator 6 isoform X11 [Aquila chrysaetos canadensis]	GCF_000766835.1	TRADDN-NCOA6
XP_011596107.1	TRADDN-NCOA6	222	NCOA6	vertebrata	Aquila chrysaetos canadensis	PREDICTED: nuclear receptor coactivator 6 isoform X12 [Aquila chrysaetos canadensis]	GCF_000766835.1	TRADDN-NCOA6
XP_007193310.1	TRADDN-NCOA6	2043	NCOA6	vertebrata	Balaenoptera acutorostrata scammoni	PREDICTED: nuclear receptor coactivator 6 isoform X1 [Balaenoptera acutorostrata scammoni]	GCF_000493695.1	TRADDN-NCOA6
XP_007193311.1	TRADDN-NCOA6	2032	NCOA6	vertebrata	Balaenoptera acutorostrata scammoni	PREDICTED: nuclear receptor coactivator 6 isoform X2 [Balaenoptera acutorostrata scammoni]	GCF_000493695.1	TRADDN-NCOA6
XP_007193312.1	TRADDN-NCOA6	2031	NCOA6	vertebrata	Balaenoptera acutorostrata scammoni	PREDICTED: nuclear receptor coactivator 6 isoform X3 [Balaenoptera acutorostrata scammoni]	GCF_000493695.1	TRADDN-NCOA6
XP_007193313.1	TRADDN-NCOA6	1058	NCOA6	vertebrata	Balaenoptera acutorostrata scammoni	PREDICTED: nuclear receptor coactivator 6 isoform X4 [Balaenoptera acutorostrata scammoni]	GCF_000493695.1	TRADDN-NCOA6
KFO09167.1	TRADDN-NCOA6	2036	N312_03074	vertebrata	Balearica regulorum gibbericeps	Nuclear receptor coactivator 6 [Balearica regulorum gibbericeps]	GCA_000709895.1	TRADDN-NCOA6
XP_010297219.1	TRADDN-NCOA6	2075	NCOA6	vertebrata	Balearica regulorum gibbericeps	PREDICTED: nuclear receptor coactivator 6 [Balearica regulorum gibbericeps]	GCF_000709895.1	TRADDN-NCOA6
POI28073.1	TRADDN-NCOA6	101	CIB84_008176	vertebrata	Bambusicola thoracicus	hypothetical protein CIB84_008176, partial [Bambusicola thoracicus]	GCA_002909625.1	TRADDN-NCOA6
XP_010853183.1	TRADDN-NCOA6	2048	NCOA6	vertebrata	Bison bison bison	PREDICTED: nuclear receptor coactivator 6 isoform X1 [Bison bison bison]	GCF_000754665.1	TRADDN-NCOA6
XP_010853186.1	TRADDN-NCOA6	2047	NCOA6	vertebrata	Bison bison bison	PREDICTED: nuclear receptor coactivator 6 isoform X2 [Bison bison bison]	GCF_000754665.1	TRADDN-NCOA6
XP_019827851.1	TRADDN-NCOA6	2037	NCOA6	vertebrata	Bos indicus	PREDICTED: nuclear receptor coactivator 6 isoform X2 [Bos indicus]	GCF_000247795.1	TRADDN-NCOA6

acc	architecture	len	gen.name	taxend	species	defline	gca	clade
XP_019827852.1	TRADDN-NCOA6	2036	NCOA6	vertebrata	Bos indicus	PREDICTED: nuclear receptor coactivator 6 isoform X3 [Bos indicus]	GCF_000247795.1	TRADDN-NCOA6
XP_019827853.1	TRADDN-NCOA6	1719	NCOA6	vertebrata	Bos indicus	PREDICTED: nuclear receptor coactivator 6 isoform X4 [Bos indicus]	GCF_000247795.1	TRADDN-NCOA6
XP_019827854.1	TRADDN-NCOA6	1670	NCOA6	vertebrata	Bos indicus	PREDICTED: nuclear receptor coactivator 6 isoform X5 [Bos indicus]	GCF_000247795.1	TRADDN-NCOA6
XP_019827855.1	TRADDN-NCOA6	1061	NCOA6	vertebrata	Bos indicus	PREDICTED: nuclear receptor coactivator 6 isoform X6 [Bos indicus]	GCF_000247795.1	TRADDN-NCOA6
ELR53021.1	TRADDN-NCOA6	2049	M91_20163	vertebrata	Bos mutus	Nuclear receptor coactivator 6, partial [Bos mutus]	GCA_000298355.1	TRADDN-NCOA6
XP_005900492.1	TRADDN-NCOA6	2048	NCOA6	vertebrata	Bos mutus	PREDICTED: nuclear receptor coactivator 6 isoform X1 [Bos mutus]	GCF_000298355.1	TRADDN-NCOA6
XP_005900493.1	TRADDN-NCOA6	1061	NCOA6	vertebrata	Bos mutus	PREDICTED: nuclear receptor coactivator 6 isoform X2 [Bos mutus]	GCF_000298355.1	TRADDN-NCOA6
XP_002692491.1	TRADDN-NCOA6	2048	NCOA6	vertebrata	Bos taurus	nuclear receptor coactivator 6 isoform X1 [Bos taurus]	GCF_002263795.1	TRADDN-NCOA6
XP_024856968.1	TRADDN-NCOA6	2047	NCOA6	vertebrata	Bos taurus	nuclear receptor coactivator 6 isoform X2 [Bos taurus]	GCF_002263795.1	TRADDN-NCOA6
XP_025119384.1	TRADDN-NCOA6	2049	NCOA6	vertebrata	Bubalus bubalis	nuclear receptor coactivator 6 isoform X1 [Bubalus bubalis]	GCF_003121395.1	TRADDN-NCOA6
XP_025119388.1	TRADDN-NCOA6	2048	NCOA6	vertebrata	Bubalus bubalis	nuclear receptor coactivator 6 isoform X2 [Bubalus bubalis]	GCF_003121395.1	TRADDN-NCOA6
XP_025119390.1	TRADDN-NCOA6	2038	NCOA6	vertebrata	Bubalus bubalis	nuclear receptor coactivator 6 isoform X3 [Bubalus bubalis]	GCF_003121395.1	TRADDN-NCOA6
XP_025119391.1	TRADDN-NCOA6	2037	NCOA6	vertebrata	Bubalus bubalis	nuclear receptor coactivator 6 isoform X4 [Bubalus bubalis]	GCF_003121395.1	TRADDN-NCOA6
XP_025119392.1	TRADDN-NCOA6	2006	NCOA6	vertebrata	Bubalus bubalis	nuclear receptor coactivator 6 isoform X5 [Bubalus bubalis]	GCF_003121395.1	TRADDN-NCOA6
XP_025119396.1	TRADDN-NCOA6	1720	NCOA6	vertebrata	Bubalus bubalis	nuclear receptor coactivator 6 isoform X9 [Bubalus bubalis]	GCF_003121395.1	TRADDN-NCOA6
XP_025119397.1	TRADDN-NCOA6	1671	NCOA6	vertebrata	Bubalus bubalis	nuclear receptor coactivator 6 isoform X10 [Bubalus bubalis]	GCF_003121395.1	TRADDN-NCOA6
XP_025119398.1	TRADDN-NCOA6	1061	NCOA6	vertebrata	Bubalus bubalis	nuclear receptor coactivator 6 isoform X11 [Bubalus bubalis]	GCF_003121395.1	TRADDN-NCOA6
KFO87084.1	TRADDN-NCOA6	1872	N320_06575	vertebrata	Buceros rhinoceros silvestris	Nuclear receptor coactivator 6, partial [Buceros rhinoceros silvestris]	GCA_000710305.1	TRADDN-NCOA6
XP_010130159.1	TRADDN-NCOA6	1814	NCOA6	vertebrata	Buceros rhinoceros silvestris	PREDICTED: nuclear receptor coactivator 6, partial [Buceros rhinoceros silvestris]	GCF_000710305.1	TRADDN-NCOA6
XP_014793052.1	TRADDN-NCOA6	2041	NCOA6	vertebrata	Calidris pugnax	PREDICTED: nuclear receptor coactivator 6 isoform X1 [Calidris pugnax]	GCF_001431845.1	TRADDN-NCOA6
XP_014793053.1	TRADDN-NCOA6	1979	NCOA6	vertebrata	Calidris pugnax	PREDICTED: nuclear receptor coactivator 6 isoform X2 [Calidris pugnax]	GCF_001431845.1	TRADDN-NCOA6
OXB57330.1	TRADDN-NCOA6	142	ASZ78_011750	vertebrata	Callipepla squamata	hypothetical protein ASZ78_011750 [Callipepla squamata]	GCA_002218305.1	TRADDN-NCOA6

acc	architecture	len	gen.name	taxend	species	defline	gca	clade
XP_008993731.1	TRADDN-NCOA6	2065	NCOA6	vertebrata	Callithrix jacchus	PREDICTED: nuclear receptor coactivator 6 isoform X1 [Callithrix jacchus]	GCF_000004665.1	TRADDN-NCOA6
XP_008993734.1	TRADDN-NCOA6	2064	NCOA6	vertebrata	Callithrix jacchus	PREDICTED: nuclear receptor coactivator 6 isoform X2 [Callithrix jacchus]	GCF_000004665.1	TRADDN-NCOA6
XP_008993735.1	TRADDN-NCOA6	2053	NCOA6	vertebrata	Callithrix jacchus	PREDICTED: nuclear receptor coactivator 6 isoform X3 [Callithrix jacchus]	GCF_000004665.1	TRADDN-NCOA6
XP_017827305.1	TRADDN-NCOA6	2050	NCOA6	vertebrata	Callithrix jacchus	PREDICTED: nuclear receptor coactivator 6 isoform X4 [Callithrix jacchus]	GCF_000004665.1	TRADDN-NCOA6
XP_017827306.1	TRADDN-NCOA6	1977	NCOA6	vertebrata	Callithrix jacchus	PREDICTED: nuclear receptor coactivator 6 isoform X5 [Callithrix jacchus]	GCF_000004665.1	TRADDN-NCOA6
XP_017827307.1	TRADDN-NCOA6	1070	NCOA6	vertebrata	Callithrix jacchus	PREDICTED: nuclear receptor coactivator 6 isoform X6 [Callithrix jacchus]	GCF_000004665.1	TRADDN-NCOA6
XP_007903405.1	TRADDN-NCOA6	2032	ncoa6	vertebrata	Callorhinchus milii	PREDICTED: nuclear receptor coactivator 6 [Callorhinchus milii]	GCF_000165045.1	TRADDN-NCOA6
XP_008489371.1	TRADDN-NCOA6	1049	NCOA6	vertebrata	Calypte anna	PREDICTED: nuclear receptor coactivator 6 [Calypte anna]	-	TRADDN-NCOA6
XP_010959132.1	TRADDN-NCOA6	2049	NCOA6	vertebrata	Camelus bactrianus	PREDICTED: nuclear receptor coactivator 6 [Camelus bactrianus]	GCF_000767855.1	TRADDN-NCOA6
XP_010973454.1	TRADDN-NCOA6	2046	NCOA6	vertebrata	Camelus dromedarius	PREDICTED: nuclear receptor coactivator 6 isoform X1 [Camelus dromedarius]	-	TRADDN-NCOA6
XP_010973455.1	TRADDN-NCOA6	2046	NCOA6	vertebrata	Camelus dromedarius	PREDICTED: nuclear receptor coactivator 6 isoform X2 [Camelus dromedarius]	-	TRADDN-NCOA6
XP_010973456.1	TRADDN-NCOA6	2045	NCOA6	vertebrata	Camelus dromedarius	PREDICTED: nuclear receptor coactivator 6 isoform X3 [Camelus dromedarius]	-	TRADDN-NCOA6
EPY78264.1	TRADDN-NCOA6	593	CB1_001108014	vertebrata	Camelus ferus	hypothetical protein CB1_001108014 [Camelus ferus]	GCA_000311805.2	TRADDN-NCOA6
XP_006188620.1	TRADDN-NCOA6	2049	NCOA6	vertebrata	Camelus ferus	PREDICTED: nuclear receptor coactivator 6 isoform X1 [Camelus ferus]	GCF_000311805.1	TRADDN-NCOA6
XP_014417993.1	TRADDN-NCOA6	2048	NCOA6	vertebrata	Camelus ferus	PREDICTED: nuclear receptor coactivator 6 isoform X2 [Camelus ferus]	GCF_000311805.1	TRADDN-NCOA6
XP_005635015.1	TRADDN-NCOA6	2060	NCOA6	vertebrata	Canis lupus familiaris	nuclear receptor coactivator 6 isoform X1 [Canis lupus familiaris]	GCF_000002285.3	TRADDN-NCOA6
XP_013962381.1	TRADDN-NCOA6	2059	NCOA6	vertebrata	Canis lupus familiaris	nuclear receptor coactivator 6 isoform X2 [Canis lupus familiaris]	GCF_000002285.3	TRADDN-NCOA6
XP_013962382.1	TRADDN-NCOA6	2049	NCOA6	vertebrata	Canis lupus familiaris	nuclear receptor coactivator 6 isoform X4 [Canis lupus familiaris]	GCF_000002285.3	TRADDN-NCOA6
XP_013962383.1	TRADDN-NCOA6	2048	NCOA6	vertebrata	Canis lupus familiaris	nuclear receptor coactivator 6 isoform X5 [Canis lupus familiaris]	GCF_000002285.3	TRADDN-NCOA6
XP_013962384.1	TRADDN-NCOA6	1063	NCOA6	vertebrata	Canis lupus familiaris	nuclear receptor coactivator 6 isoform X8 [Canis lupus familiaris]	GCF_000002285.3	TRADDN-NCOA6

acc	architecture	len	gen.name	taxend	species	defline	gca	clade
XP_022265089.1	TRADDN-NCOA6	2053	NCOA6	vertebrata	Canis lupus familiaris	nuclear receptor coactivator 6 isoform X3 [Canis lupus familiaris]	GCF_000002285.3	TRADDN-NCOA6
XP_022265090.1	TRADDN-NCOA6	2031	NCOA6	vertebrata	Canis lupus familiaris	nuclear receptor coactivator 6 isoform X6 [Canis lupus familiaris]	GCF_000002285.3	TRADDN-NCOA6
XP_022265091.1	TRADDN-NCOA6	1953	NCOA6	vertebrata	Canis lupus familiaris	nuclear receptor coactivator 6 isoform X7 [Canis lupus familiaris]	GCF_000002285.3	TRADDN-NCOA6
XP_017913233.1	TRADDN-NCOA6	2043	NCOA6	vertebrata	Capra hircus	PREDICTED: nuclear receptor coactivator 6 isoform X1 [Capra hircus]	GCF_001704415.1	TRADDN-NCOA6
XP_017913236.1	TRADDN-NCOA6	2032	NCOA6	vertebrata	Capra hircus	PREDICTED: nuclear receptor coactivator 6 isoform X2 [Capra hircus]	GCF_001704415.1	TRADDN-NCOA6
XP_017913237.1	TRADDN-NCOA6	2031	NCOA6	vertebrata	Capra hircus	PREDICTED: nuclear receptor coactivator 6 isoform X3 [Capra hircus]	GCF_001704415.1	TRADDN-NCOA6
KFP58253.1	TRADDN-NCOA6	131	N322_11703	vertebrata	Cariama cristata	Nuclear receptor coactivator 6, partial [Cariama cristata]	GCA_000690535.1	TRADDN-NCOA6
KFP60844.1	TRADDN-NCOA6	1914	N322_05085	vertebrata	Cariama cristata	Nuclear receptor coactivator 6, partial [Cariama cristata]	GCA_000690535.1	TRADDN-NCOA6
XP_009696492.1	TRADDN-NCOA6	1914	NCOA6	vertebrata	Cariama cristata	PREDICTED: nuclear receptor coactivator 6, partial [Cariama cristata]	GCF_000690535.1	TRADDN-NCOA6
XP_021574354.1	TRADDN-NCOA6	1962	NCOA6	vertebrata	Carlito syrichta	nuclear receptor coactivator 6 [Carlito syrichta]	GCF_000164805.1	TRADDN-NCOA6
XP_020018573.1	TRADDN-NCOA6	2095	Ncoa6	vertebrata	Castor canadensis	nuclear receptor coactivator 6 isoform X1 [Castor canadensis]	GCF_001984765.1	TRADDN-NCOA6
XP_020018574.1	TRADDN-NCOA6	2084	Ncoa6	vertebrata	Castor canadensis	nuclear receptor coactivator 6 isoform X2 [Castor canadensis]	GCF_001984765.1	TRADDN-NCOA6
XP_020018575.1	TRADDN-NCOA6	2083	Ncoa6	vertebrata	Castor canadensis	nuclear receptor coactivator 6 isoform X3 [Castor canadensis]	GCF_001984765.1	TRADDN-NCOA6
APL97873.1	TRADDN-NCOA6	2060	NCOA6	vertebrata	Castor fiber	nuclear receptor coactivator 6 [Castor fiber]	-	TRADDN-NCOA6
KFP49468.1	TRADDN-NCOA6	170	N323_00992	vertebrata	Cathartes aura	Nuclear receptor coactivator 6, partial [Cathartes aura]	GCA_000699945.1	TRADDN-NCOA6
XP_003467778.1	TRADDN-NCOA6	2063	Ncoa6	vertebrata	Cavia porcellus	nuclear receptor coactivator 6 isoform X1 [Cavia porcellus]	GCF_000151735.1	TRADDN-NCOA6
XP_003467780.2	TRADDN-NCOA6	1073	Ncoa6	vertebrata	Cavia porcellus	nuclear receptor coactivator 6 isoform X5 [Cavia porcellus]	GCF_000151735.1	TRADDN-NCOA6
XP_013005709.1	TRADDN-NCOA6	2052	Ncoa6	vertebrata	Cavia porcellus	nuclear receptor coactivator 6 isoform X2 [Cavia porcellus]	GCF_000151735.1	TRADDN-NCOA6
XP_013005710.1	TRADDN-NCOA6	2051	Ncoa6	vertebrata	Cavia porcellus	nuclear receptor coactivator 6 isoform X3 [Cavia porcellus]	GCF_000151735.1	TRADDN-NCOA6
XP_013005711.1	TRADDN-NCOA6	1972	Ncoa6	vertebrata	Cavia porcellus	nuclear receptor coactivator 6 isoform X4 [Cavia porcellus]	GCF_000151735.1	TRADDN-NCOA6
XP_017369161.1	TRADDN-NCOA6	2076	NCOA6	vertebrata	Cebus capucinus imitator	PREDICTED: nuclear receptor coactivator 6 isoform X1 [Cebus capucinus imitator]	GCF_001604975.1	TRADDN-NCOA6
XP_017369188.1	TRADDN-NCOA6	2075	NCOA6	vertebrata	Cebus capucinus imitator	PREDICTED: nuclear receptor coactivator 6 isoform X2 [Cebus capucinus imitator]	GCF_001604975.1	TRADDN-NCOA6

acc	architecture	len	gen.name	taxend	species	defline	gca	clade
XP_017369198.1	TRADDN-NCOA6	2064	NCOA6	vertebrata	Cebus capucinus imitator	PREDICTED: nuclear receptor coactivator 6 isoform X3 [Cebus capucinus imitator]	GCF_001604975.1	TRADDN-NCOA6
XP_017369215.1	TRADDN-NCOA6	1928	NCOA6	vertebrata	Cebus capucinus imitator	PREDICTED: nuclear receptor coactivator 6 isoform X4 [Cebus capucinus imitator]	GCF_001604975.1	TRADDN-NCOA6
XP_017369223.1	TRADDN-NCOA6	1075	NCOA6	vertebrata	Cebus capucinus imitator	PREDICTED: nuclear receptor coactivator 6 isoform X5 [Cebus capucinus imitator]	GCF_001604975.1	TRADDN-NCOA6
XP_014643006.1	TRADDN-NCOA6	2053	LOC101398778	vertebrata	Ceratotherium simum simum	PREDICTED: nuclear receptor coactivator 6 isoform X1 [Ceratotherium simum simum]	GCF_000283155.1	TRADDN-NCOA6
XP_014643008.1	TRADDN-NCOA6	2052	LOC101398778	vertebrata	Ceratotherium simum simum	PREDICTED: nuclear receptor coactivator 6 isoform X2 [Ceratotherium simum simum]	GCF_000283155.1	TRADDN-NCOA6
XP_014643009.1	TRADDN-NCOA6	2042	LOC101398778	vertebrata	Ceratotherium simum simum	PREDICTED: nuclear receptor coactivator 6 isoform X3 [Ceratotherium simum simum]	GCF_000283155.1	TRADDN-NCOA6
XP_014643010.1	TRADDN-NCOA6	2041	LOC101398778	vertebrata	Ceratotherium simum simum	PREDICTED: nuclear receptor coactivator 6 isoform X4 [Ceratotherium simum simum]	GCF_000283155.1	TRADDN-NCOA6
XP_014643011.1	TRADDN-NCOA6	1953	LOC101398778	vertebrata	Ceratotherium simum simum	PREDICTED: nuclear receptor coactivator 6 isoform X5 [Ceratotherium simum simum]	GCF_000283155.1	TRADDN-NCOA6
XP_014643012.1	TRADDN-NCOA6	1061	LOC101398778	vertebrata	Ceratotherium simum simum	PREDICTED: nuclear receptor coactivator 6 isoform X6 [Ceratotherium simum simum]	GCF_000283155.1	TRADDN-NCOA6
XP_011907991.1	TRADDN-NCOA6	2068	NCOA6	vertebrata	Cercocebus atys	PREDICTED: nuclear receptor coactivator 6 isoform X1 [Cercocebus atys]	GCF_000955945.1	TRADDN-NCOA6
XP_011907994.1	TRADDN-NCOA6	2067	NCOA6	vertebrata	Cercocebus atys	PREDICTED: nuclear receptor coactivator 6 isoform X2 [Cercocebus atys]	GCF_000955945.1	TRADDN-NCOA6
XP_011907995.1	TRADDN-NCOA6	2057	NCOA6	vertebrata	Cercocebus atys	PREDICTED: nuclear receptor coactivator 6 isoform X3 [Cercocebus atys]	GCF_000955945.1	TRADDN-NCOA6
XP_011907996.1	TRADDN-NCOA6	2056	NCOA6	vertebrata	Cercocebus atys	PREDICTED: nuclear receptor coactivator 6 isoform X4 [Cercocebus atys]	GCF_000955945.1	TRADDN-NCOA6
XP_011907997.1	TRADDN-NCOA6	2053	NCOA6	vertebrata	Cercocebus atys	PREDICTED: nuclear receptor coactivator 6 isoform X5 [Cercocebus atys]	GCF_000955945.1	TRADDN-NCOA6
XP_011907998.1	TRADDN-NCOA6	2039	NCOA6	vertebrata	Cercocebus atys	PREDICTED: nuclear receptor coactivator 6 isoform X6 [Cercocebus atys]	GCF_000955945.1	TRADDN-NCOA6
XP_011907999.1	TRADDN-NCOA6	2025	NCOA6	vertebrata	Cercocebus atys	PREDICTED: nuclear receptor coactivator 6 isoform X7 [Cercocebus atys]	GCF_000955945.1	TRADDN-NCOA6
XP_011908002.1	TRADDN-NCOA6	1072	NCOA6	vertebrata	Cercocebus atys	PREDICTED: nuclear receptor coactivator 6 isoform X9 [Cercocebus atys]	GCF_000955945.1	TRADDN-NCOA6

acc	architecture	len	gen.name	taxend	species	defline	gca	clade
OWK02943.1	TRADDN-NCOA6	1896	Celaphus_00007913	vertebrata	Cervus elaphus hippelaphus	hypothetical protein Celaphus_00007913 [Cervus elaphus hippelaphus]	GCA_002197005.1	TRADDN-NCOA6
XP_009998148.1	TRADDN-NCOA6	1046	NCOA6	vertebrata	Chaetura pelagica	PREDICTED: nuclear receptor coactivator 6 [Chaetura pelagica]	GCF_000747805.1	TRADDN-NCOA6
KGL94362.1	TRADDN-NCOA6	2047	N301_05407	vertebrata	Charadrius vociferus	Nuclear receptor coactivator 6 [Charadrius vociferus]	GCA_000708025.2	TRADDN-NCOA6
XP_009884631.1	TRADDN-NCOA6	1049	NCOA6	vertebrata	Charadrius vociferus	PREDICTED: nuclear receptor coactivator 6 [Charadrius vociferus]	GCF_000708025.1	TRADDN-NCOA6
EMP38212.1	TRADDN-NCOA6	2031	UY3_04555	vertebrata	Chelonia mydas	Nuclear receptor coactivator 6, partial [Chelonia mydas]	GCA_000344595.1	TRADDN-NCOA6
XP_007057222.1	TRADDN-NCOA6	2026	NCOA6	vertebrata	Chelonia mydas	PREDICTED: nuclear receptor coactivator 6 isoform X1 [Chelonia mydas]	-	TRADDN-NCOA6
XP_007057223.1	TRADDN-NCOA6	1044	NCOA6	vertebrata	Chelonia mydas	PREDICTED: nuclear receptor coactivator 6 isoform X2 [Chelonia mydas]	-	TRADDN-NCOA6
XP_005384876.1	TRADDN-NCOA6	2063	Ncoa6	vertebrata	Chinchilla lanigera	PREDICTED: nuclear receptor coactivator 6 isoform X2 [Chinchilla lanigera]	GCF_000276665.1	TRADDN-NCOA6
XP_005384877.1	TRADDN-NCOA6	2062	Ncoa6	vertebrata	Chinchilla lanigera	PREDICTED: nuclear receptor coactivator 6 isoform X3 [Chinchilla lanigera]	GCF_000276665.1	TRADDN-NCOA6
XP_005384880.1	TRADDN-NCOA6	1075	Ncoa6	vertebrata	Chinchilla lanigera	PREDICTED: nuclear receptor coactivator 6 isoform X6 [Chinchilla lanigera]	GCF_000276665.1	TRADDN-NCOA6
XP_013369541.1	TRADDN-NCOA6	2074	Ncoa6	vertebrata	Chinchilla lanigera	PREDICTED: nuclear receptor coactivator 6 isoform X1 [Chinchilla lanigera]	GCF_000276665.1	TRADDN-NCOA6
XP_013369542.1	TRADDN-NCOA6	1983	Ncoa6	vertebrata	Chinchilla lanigera	PREDICTED: nuclear receptor coactivator 6 isoform X4 [Chinchilla lanigera]	GCF_000276665.1	TRADDN-NCOA6
KFP43437.1	TRADDN-NCOA6	131	N324_07749	vertebrata	Chlamydotis macqueenii	Nuclear receptor coactivator 6, partial [Chlamydotis macqueenii]	GCA_000695195.1	TRADDN-NCOA6
XP_008019663.1	TRADDN-NCOA6	2067	NCOA6	vertebrata	Chlorocebus sabaeus	PREDICTED: nuclear receptor coactivator 6 isoform X1 [Chlorocebus sabaeus]	GCF_000409795.2	TRADDN-NCOA6
XP_008019687.1	TRADDN-NCOA6	2056	NCOA6	vertebrata	Chlorocebus sabaeus	PREDICTED: nuclear receptor coactivator 6 isoform X2 [Chlorocebus sabaeus]	GCF_000409795.2	TRADDN-NCOA6
XP_008019692.1	TRADDN-NCOA6	2055	NCOA6	vertebrata	Chlorocebus sabaeus	PREDICTED: nuclear receptor coactivator 6 isoform X3 [Chlorocebus sabaeus]	GCF_000409795.2	TRADDN-NCOA6
XP_008019700.1	TRADDN-NCOA6	2015	NCOA6	vertebrata	Chlorocebus sabaeus	PREDICTED: nuclear receptor coactivator 6 isoform X4 [Chlorocebus sabaeus]	GCF_000409795.2	TRADDN-NCOA6
XP_005295438.1	TRADDN-NCOA6	2020	NCOA6	vertebrata	Chrysemys picta bellii	nuclear receptor coactivator 6 [Chrysemys picta bellii]	GCF_000241765.3	TRADDN-NCOA6
XP_006876936.1	TRADDN-NCOA6	2081	NCOA6	vertebrata	Chrysochloris asiatica	PREDICTED: nuclear receptor coactivator 6 isoform X1 [Chrysochloris asiatica]	GCF_000296735.1	TRADDN-NCOA6

acc	architecture	len	gen.name	taxend	species	defline	gca	clade
XP_006876937.1	TRADDN-NCOA6	2055	NCOA6	vertebrata	Chrysochloris asiatica	PREDICTED: nuclear receptor coactivator 6 isoform X2 [Chrysochloris asiatica]	GCF_000296735.1	TRADDN-NCOA6
XP_006876938.1	TRADDN-NCOA6	1084	NCOA6	vertebrata	Chrysochloris asiatica	PREDICTED: nuclear receptor coactivator 6 isoform X3 [Chrysochloris asiatica]	GCF_000296735.1	TRADDN-NCOA6
KFP32585.1	TRADDN-NCOA6	1786	N325_05462	vertebrata	Colius striatus	Nuclear receptor coactivator 6, partial [Colius striatus]	GCA_000690715.1	TRADDN-NCOA6
XP_010202326.1	TRADDN-NCOA6	1784	NCOA6	vertebrata	Colius striatus	PREDICTED: nuclear receptor coactivator 6, partial [Colius striatus]	GCF_000690715.1	TRADDN-NCOA6
XP_011784569.1	TRADDN-NCOA6	2062	NCOA6	vertebrata	Colobus angolensis palliatus	PREDICTED: nuclear receptor coactivator 6 [Colobus angolensis palliatus]	GCF_000951035.1	TRADDN-NCOA6
XP_013225784.1	TRADDN-NCOA6	2050	NCOA6	vertebrata	Columba livia	nuclear receptor coactivator 6 [Columba livia]	GCF_000337935.1	TRADDN-NCOA6
XP_012583760.1	TRADDN-NCOA6	2066	NCOA6	vertebrata	Condylura cristata	PREDICTED: nuclear receptor coactivator 6 isoform X1 [Condylura cristata]	GCF_000260355.1	TRADDN-NCOA6
XP_012583761.1	TRADDN-NCOA6	2055	NCOA6	vertebrata	Condylura cristata	PREDICTED: nuclear receptor coactivator 6 isoform X2 [Condylura cristata]	GCF_000260355.1	TRADDN-NCOA6
XP_012583762.1	TRADDN-NCOA6	2041	NCOA6	vertebrata	Condylura cristata	PREDICTED: nuclear receptor coactivator 6 isoform X3 [Condylura cristata]	GCF_000260355.1	TRADDN-NCOA6
XP_012583763.1	TRADDN-NCOA6	2037	NCOA6	vertebrata	Condylura cristata	PREDICTED: nuclear receptor coactivator 6 isoform X4 [Condylura cristata]	GCF_000260355.1	TRADDN-NCOA6
XP_012583764.1	TRADDN-NCOA6	2032	NCOA6	vertebrata	Condylura cristata	PREDICTED: nuclear receptor coactivator 6 isoform X5 [Condylura cristata]	GCF_000260355.1	TRADDN-NCOA6
KFO54056.1	TRADDN-NCOA6	1974	N302_14124	vertebrata	Corvus brachyrhynchos	Nuclear receptor coactivator 6 [Corvus brachyrhynchos]	GCA_000691975.1	TRADDN-NCOA6
XP_017602427.1	TRADDN-NCOA6	1035	NCOA6	vertebrata	Corvus brachyrhynchos	PREDICTED: nuclear receptor coactivator 6 [Corvus brachyrhynchos]	GCF_000691975.1	TRADDN-NCOA6
XP_019149598.1	TRADDN-NCOA6	1048	NCOA6	vertebrata	Corvus cornix cornix	nuclear receptor coactivator 6 [Corvus cornix cornix]	-	TRADDN-NCOA6
XP_015736734.1	TRADDN-NCOA6	2049	NCOA6	vertebrata	Coturnix japonica	PREDICTED: nuclear receptor coactivator 6 isoform X1 [Coturnix japonica]	GCF_001577835.1	TRADDN-NCOA6
XP_015736736.1	TRADDN-NCOA6	2043	NCOA6	vertebrata	Coturnix japonica	PREDICTED: nuclear receptor coactivator 6 isoform X2 [Coturnix japonica]	GCF_001577835.1	TRADDN-NCOA6
XP_015736737.1	TRADDN-NCOA6	1981	NCOA6	vertebrata	Coturnix japonica	PREDICTED: nuclear receptor coactivator 6 isoform X3 [Coturnix japonica]	GCF_001577835.1	TRADDN-NCOA6
XP_015736738.1	TRADDN-NCOA6	1777	NCOA6	vertebrata	Coturnix japonica	PREDICTED: nuclear receptor coactivator 6 isoform X4 [Coturnix japonica]	GCF_001577835.1	TRADDN-NCOA6
ERE71670.1	TRADDN-NCOA6	2011	H671_6g15530	vertebrata	Cricetulus griseus	nuclear receptor coactivator 6 isoform 1 [Cricetulus griseus]	GCA_000448345.1	TRADDN-NCOA6

acc	architecture	len	gen.name	taxend	species	defline	gca	clade
XP_003501176.1	TRADDN-NCOA6	2060	Ncoa6	vertebrata	Cricetulus griseus	nuclear receptor coactivator 6 isoform X1 [Cricetulus griseus]	GCF_000223135.1	TRADDN-NCOA6
XP_007640016.1	TRADDN-NCOA6	2059	Ncoa6	vertebrata	Cricetulus griseus	nuclear receptor coactivator 6 isoform X2 [Cricetulus griseus]	GCF_000223135.1	TRADDN-NCOA6
XP_007640017.1	TRADDN-NCOA6	2017	Ncoa6	vertebrata	Cricetulus griseus	nuclear receptor coactivator 6 isoform X5 [Cricetulus griseus]	GCF_000223135.1	TRADDN-NCOA6
XP_016830462.1	TRADDN-NCOA6	2049	Ncoa6	vertebrata	Cricetulus griseus	nuclear receptor coactivator 6 isoform X3 [Cricetulus griseus]	GCF_000223135.1	TRADDN-NCOA6
XP_016830463.1	TRADDN-NCOA6	2048	Ncoa6	vertebrata	Cricetulus griseus	nuclear receptor coactivator 6 isoform X4 [Cricetulus griseus]	GCF_000223135.1	TRADDN-NCOA6
XP_019395281.1	TRADDN-NCOA6	2038	NCOA6	vertebrata	Crocodylus porosus	PREDICTED: nuclear receptor coactivator 6 isoform X1 [Crocodylus porosus]	GCF_001723895.1	TRADDN-NCOA6
XP_019395282.1	TRADDN-NCOA6	1976	NCOA6	vertebrata	Crocodylus porosus	PREDICTED: nuclear receptor coactivator 6 isoform X2 [Crocodylus porosus]	GCF_001723895.1	TRADDN-NCOA6
KFO79464.1	TRADDN-NCOA6	2048	N303_12473	vertebrata	Cuculus canorus	Nuclear receptor coactivator 6 [Cuculus canorus]	GCA_000709325.1	TRADDN-NCOA6
XP_009563652.1	TRADDN-NCOA6	2040	NCOA6	vertebrata	Cuculus canorus	PREDICTED: LOW QUALITY PROTEIN: nuclear receptor coactivator 6 [Cuculus canorus]	GCF_000709325.1	TRADDN-NCOA6
XP_023795796.1	TRADDN-NCOA6	2029	NCOA6	vertebrata	Cyanistes caeruleus	LOW QUALITY PROTEIN: nuclear receptor coactivator 6 [Cyanistes caeruleus]	GCF_002901205.1	TRADDN-NCOA6
XP_012379285.1	TRADDN-NCOA6	2082	NCOA6	vertebrata	Dasypus novemcinctus	nuclear receptor coactivator 6 isoform X2 [Dasypus novemcinctus]	GCF_000208655.1	TRADDN-NCOA6
XP_012379286.1	TRADDN-NCOA6	2072	NCOA6	vertebrata	Dasypus novemcinctus	nuclear receptor coactivator 6 isoform X3 [Dasypus novemcinctus]	GCF_000208655.1	TRADDN-NCOA6
XP_012379287.1	TRADDN-NCOA6	2071	NCOA6	vertebrata	Dasypus novemcinctus	nuclear receptor coactivator 6 isoform X4 [Dasypus novemcinctus]	GCF_000208655.1	TRADDN-NCOA6
XP_023443533.1	TRADDN-NCOA6	2083	NCOA6	vertebrata	Dasypus novemcinctus	nuclear receptor coactivator 6 isoform X1 [Dasypus novemcinctus]	GCF_000208655.1	TRADDN-NCOA6
XP_022448316.1	TRADDN-NCOA6	2037	NCOA6	vertebrata	Delphinapterus leucas	nuclear receptor coactivator 6 isoform X1 [Delphinapterus leucas]	GCF_002288925.2	TRADDN-NCOA6
XP_022448319.1	TRADDN-NCOA6	2036	NCOA6	vertebrata	Delphinapterus leucas	nuclear receptor coactivator 6 isoform X2 [Delphinapterus leucas]	GCF_002288925.2	TRADDN-NCOA6
XP_022448320.1	TRADDN-NCOA6	2026	NCOA6	vertebrata	Delphinapterus leucas	nuclear receptor coactivator 6 isoform X3 [Delphinapterus leucas]	GCF_002288925.2	TRADDN-NCOA6
XP_022448321.1	TRADDN-NCOA6	2025	NCOA6	vertebrata	Delphinapterus leucas	nuclear receptor coactivator 6 isoform X4 [Delphinapterus leucas]	GCF_002288925.2	TRADDN-NCOA6
XP_024415488.1	TRADDN-NCOA6	2066	NCOA6	vertebrata	Desmodus rotundus	nuclear receptor coactivator 6 isoform X1 [Desmodus rotundus]	GCF_002940915.1	TRADDN-NCOA6
XP_024415490.1	TRADDN-NCOA6	2065	NCOA6	vertebrata	Desmodus rotundus	nuclear receptor coactivator 6 isoform X2 [Desmodus rotundus]	GCF_002940915.1	TRADDN-NCOA6
XP_024415491.1	TRADDN-NCOA6	2055	NCOA6	vertebrata	Desmodus rotundus	nuclear receptor coactivator 6 isoform X3 [Desmodus rotundus]	GCF_002940915.1	TRADDN-NCOA6
XP_024415492.1	TRADDN-NCOA6	2054	NCOA6	vertebrata	Desmodus rotundus	nuclear receptor coactivator 6 isoform X4 [Desmodus rotundus]	GCF_002940915.1	TRADDN-NCOA6
XP_012866403.1	TRADDN-NCOA6	2056	Ncoa6	vertebrata	Dipodomys ordii	PREDICTED: nuclear receptor coactivator 6 isoform X1 [Dipodomys ordii]	GCF_000151885.1	TRADDN-NCOA6

acc	architecture	len	gen.name	taxend	species	defline	gca	clade
XP_012866405.1	TRADDN-NCOA6	2055	Ncoa6	vertebrata	Dipodomys ordii	PREDICTED: nuclear receptor coactivator 6 isoform X2 [Dipodomys ordii]	GCF_000151885.1	TRADDN-NCOA6
XP_012866406.1	TRADDN-NCOA6	2033	Ncoa6	vertebrata	Dipodomys ordii	PREDICTED: nuclear receptor coactivator 6 isoform X3 [Dipodomys ordii]	GCF_000151885.1	TRADDN-NCOA6
XP_012866407.1	TRADDN-NCOA6	2032	Ncoa6	vertebrata	Dipodomys ordii	PREDICTED: nuclear receptor coactivator 6 isoform X4 [Dipodomys ordii]	GCF_000151885.1	TRADDN-NCOA6
XP_004698295.1	TRADDN-NCOA6	2050	NCOA6	vertebrata	Echinops telfairi	PREDICTED: nuclear receptor coactivator 6 [Echinops telfairi]	GCF_000313985.2	TRADDN-NCOA6
XP_009636267.1	TRADDN-NCOA6	1048	NCOA6	vertebrata	Egretta garzetta	PREDICTED: nuclear receptor coactivator 6 [Egretta garzetta]	GCF_000687185.1	TRADDN-NCOA6
XP_006903722.1	TRADDN-NCOA6	2060	NCOA6	vertebrata	Elephantulus edwardii	PREDICTED: LOW QUALITY PROTEIN: nuclear receptor coactivator 6 [Elephantulus edwardii]	GCF_000299155.1	TRADDN-NCOA6
XP_022349547.1	TRADDN-NCOA6	2064	LOC111141360	vertebrata	Enhydra lutris kenyoni	nuclear receptor coactivator 6 isoform X1 [Enhydra lutris kenyoni]	GCF_002288905.1	TRADDN-NCOA6
XP_008147929.1	TRADDN-NCOA6	2059	NCOA6	vertebrata	Eptesicus fuscus	PREDICTED: nuclear receptor coactivator 6 isoform X1 [Eptesicus fuscus]	-	TRADDN-NCOA6
XP_008147930.1	TRADDN-NCOA6	1971	NCOA6	vertebrata	Eptesicus fuscus	PREDICTED: nuclear receptor coactivator 6 isoform X2 [Eptesicus fuscus]	-	TRADDN-NCOA6
XP_008147931.1	TRADDN-NCOA6	1064	NCOA6	vertebrata	Eptesicus fuscus	PREDICTED: nuclear receptor coactivator 6 isoform X3 [Eptesicus fuscus]	-	TRADDN-NCOA6
XP_014686953.1	TRADDN-NCOA6	2062	NCOA6	vertebrata	Equus asinus	PREDICTED: nuclear receptor coactivator 6 isoform X1 [Equus asinus]	GCF_001305755.1	TRADDN-NCOA6
XP_014686954.1	TRADDN-NCOA6	2061	NCOA6	vertebrata	Equus asinus	PREDICTED: nuclear receptor coactivator 6 isoform X2 [Equus asinus]	GCF_001305755.1	TRADDN-NCOA6
XP_014686955.1	TRADDN-NCOA6	2051	NCOA6	vertebrata	Equus asinus	PREDICTED: nuclear receptor coactivator 6 isoform X3 [Equus asinus]	GCF_001305755.1	TRADDN-NCOA6
XP_014686956.1	TRADDN-NCOA6	2050	NCOA6	vertebrata	Equus asinus	PREDICTED: nuclear receptor coactivator 6 isoform X4 [Equus asinus]	GCF_001305755.1	TRADDN-NCOA6
XP_014686957.1	TRADDN-NCOA6	1962	NCOA6	vertebrata	Equus asinus	PREDICTED: nuclear receptor coactivator 6 isoform X5 [Equus asinus]	GCF_001305755.1	TRADDN-NCOA6
XP_023482163.1	TRADDN-NCOA6	2062	NCOA6	vertebrata	Equus caballus	nuclear receptor coactivator 6 isoform X1 [Equus caballus]	GCF_002863925.1	TRADDN-NCOA6
XP_023482168.1	TRADDN-NCOA6	2061	NCOA6	vertebrata	Equus caballus	nuclear receptor coactivator 6 isoform X2 [Equus caballus]	GCF_002863925.1	TRADDN-NCOA6
XP_023482169.1	TRADDN-NCOA6	2051	NCOA6	vertebrata	Equus caballus	nuclear receptor coactivator 6 isoform X3 [Equus caballus]	GCF_002863925.1	TRADDN-NCOA6
XP_023482170.1	TRADDN-NCOA6	2050	NCOA6	vertebrata	Equus caballus	nuclear receptor coactivator 6 isoform X4 [Equus caballus]	GCF_002863925.1	TRADDN-NCOA6
XP_023482171.1	TRADDN-NCOA6	1962	NCOA6	vertebrata	Equus caballus	nuclear receptor coactivator 6 isoform X5 [Equus caballus]	GCF_002863925.1	TRADDN-NCOA6

acc	architecture	len	gen.name	taxend	species	defline	gca	clade
XP_023482172.1	TRADDN-NCOA6	1066	NCOA6	vertebrata	Equus caballus	nuclear receptor coactivator 6 isoform X6 [Equus caballus]	GCF_002863925.1	TRADDN-NCOA6
XP_008522723.1	TRADDN-NCOA6	2061	NCOA6	vertebrata	Equus przewalskii	PREDICTED: nuclear receptor coactivator 6 isoform X1 [Equus przewalskii]	GCF_000696695.1	TRADDN-NCOA6
XP_008522724.1	TRADDN-NCOA6	2060	NCOA6	vertebrata	Equus przewalskii	PREDICTED: nuclear receptor coactivator 6 isoform X2 [Equus przewalskii]	GCF_000696695.1	TRADDN-NCOA6
XP_008522725.1	TRADDN-NCOA6	2050	NCOA6	vertebrata	Equus przewalskii	PREDICTED: nuclear receptor coactivator 6 isoform X3 [Equus przewalskii]	GCF_000696695.1	TRADDN-NCOA6
XP_008522726.1	TRADDN-NCOA6	2049	NCOA6	vertebrata	Equus przewalskii	PREDICTED: nuclear receptor coactivator 6 isoform X4 [Equus przewalskii]	GCF_000696695.1	TRADDN-NCOA6
XP_008522727.1	TRADDN-NCOA6	1065	NCOA6	vertebrata	Equus przewalskii	PREDICTED: nuclear receptor coactivator 6 isoform X5 [Equus przewalskii]	GCF_000696695.1	TRADDN-NCOA6
XP_007522722.1	TRADDN-NCOA6	2050	NCOA6	vertebrata	Erinaceus europaeus	PREDICTED: nuclear receptor coactivator 6 isoform X1 [Erinaceus europaeus]	GCF_000296755.1	TRADDN-NCOA6
XP_016043446.1	TRADDN-NCOA6	2049	NCOA6	vertebrata	Erinaceus europaeus	PREDICTED: nuclear receptor coactivator 6 isoform X2 [Erinaceus europaeus]	GCF_000296755.1	TRADDN-NCOA6
XP_016043447.1	TRADDN-NCOA6	2039	NCOA6	vertebrata	Erinaceus europaeus	PREDICTED: nuclear receptor coactivator 6 isoform X3 [Erinaceus europaeus]	GCF_000296755.1	TRADDN-NCOA6
KFV93685.1	TRADDN-NCOA6	170	N326_02781	vertebrata	Eurypyga helias	Nuclear receptor coactivator 6, partial [Eurypyga helias]	GCA_000690775.1	TRADDN-NCOA6
XP_014132069.1	TRADDN-NCOA6	2040	NCOA6	vertebrata	Falco cherrug	PREDICTED: nuclear receptor coactivator 6 isoform X1 [Falco cherrug]	-	TRADDN-NCOA6
XP_014132070.1	TRADDN-NCOA6	2039	NCOA6	vertebrata	Falco cherrug	PREDICTED: nuclear receptor coactivator 6 isoform X2 [Falco cherrug]	GCF_000337975.1	TRADDN-NCOA6
XP_014132071.1	TRADDN-NCOA6	1978	NCOA6	vertebrata	Falco cherrug	PREDICTED: nuclear receptor coactivator 6 isoform X3 [Falco cherrug]	-	TRADDN-NCOA6
XP_014132072.1	TRADDN-NCOA6	1978	NCOA6	vertebrata	Falco cherrug	PREDICTED: nuclear receptor coactivator 6 isoform X4 [Falco cherrug]	-	TRADDN-NCOA6
XP_013153127.1	TRADDN-NCOA6	2033	NCOA6	vertebrata	Falco peregrinus	PREDICTED: LOW QUALITY PROTEIN: nuclear receptor coactivator 6 [Falco peregrinus]	-	TRADDN-NCOA6
XP_011279160.2	TRADDN-NCOA6	2080	NCOA6	vertebrata	Felis catus	nuclear receptor coactivator 6 isoform X1 [Felis catus]	GCF_000181335.3	TRADDN-NCOA6
XP_011279164.2	TRADDN-NCOA6	2079	NCOA6	vertebrata	Felis catus	nuclear receptor coactivator 6 isoform X2 [Felis catus]	GCF_000181335.3	TRADDN-NCOA6
XP_011279167.2	TRADDN-NCOA6	1078	NCOA6	vertebrata	Felis catus	nuclear receptor coactivator 6 isoform X3 [Felis catus]	GCF_000181335.3	TRADDN-NCOA6
XP_005057019.1	TRADDN-NCOA6	1049	NCOA6	vertebrata	Ficedula albicollis	PREDICTED: nuclear receptor coactivator 6 [Ficedula albicollis]	GCF_000247815.1	TRADDN-NCOA6

acc	architecture	len	gen.name	taxend	species	defline	gca	clade
KFO35537.1	TRADDN-NCOA6	96	H920_03038	vertebrata	Fukomys damarensis	Nuclear receptor coactivator 6 [Fukomys damarensis]	GCA_000743615.1	TRADDN-NCOA6
XP_010638228.1	TRADDN-NCOA6	2063	Ncoa6	vertebrata	Fukomys damarensis	PREDICTED: nuclear receptor coactivator 6 isoform X1 [Fukomys damarensis]	GCF_000743615.1	TRADDN-NCOA6
XP_010638232.1	TRADDN-NCOA6	2052	Ncoa6	vertebrata	Fukomys damarensis	PREDICTED: nuclear receptor coactivator 6 isoform X2 [Fukomys damarensis]	GCF_000743615.1	TRADDN-NCOA6
XP_010638233.1	TRADDN-NCOA6	2051	Ncoa6	vertebrata	Fukomys damarensis	PREDICTED: nuclear receptor coactivator 6 isoform X3 [Fukomys damarensis]	GCF_000743615.1	TRADDN-NCOA6
XP_010638234.1	TRADDN-NCOA6	1077	Ncoa6	vertebrata	Fukomys damarensis	PREDICTED: nuclear receptor coactivator 6 isoform X4 [Fukomys damarensis]	GCF_000743615.1	TRADDN-NCOA6
KFV90230.1	TRADDN-NCOA6	170	N327_10484	vertebrata	Fulmarus glacialis	Nuclear receptor coactivator 6, partial [Fulmarus glacialis]	GCA_000690835.1	TRADDN-NCOA6
XP_008569466.1	TRADDN-NCOA6	2059	NCOA6	vertebrata	Galeopterus variegatus	PREDICTED: nuclear receptor coactivator 6 [Galeopterus variegatus]	GCF_000696425.1	TRADDN-NCOA6
XP_004946924.1	TRADDN-NCOA6	1969	NCOA6	vertebrata	Gallus gallus	nuclear receptor coactivator 6 isoform X5 [Gallus gallus]	GCF_000002315.5	TRADDN-NCOA6
XP_015151765.1	TRADDN-NCOA6	2054	NCOA6	vertebrata	Gallus gallus	nuclear receptor coactivator 6 isoform X1 [Gallus gallus]	GCF_000002315.5	TRADDN-NCOA6
XP_015151766.1	TRADDN-NCOA6	2031	NCOA6	vertebrata	Gallus gallus	nuclear receptor coactivator 6 isoform X2 [Gallus gallus]	GCF_000002315.5	TRADDN-NCOA6
XP_015151767.1	TRADDN-NCOA6	1994	NCOA6	vertebrata	Gallus gallus	nuclear receptor coactivator 6 isoform X3 [Gallus gallus]	GCF_000002315.5	TRADDN-NCOA6
XP_015151768.1	TRADDN-NCOA6	1992	NCOA6	vertebrata	Gallus gallus	nuclear receptor coactivator 6 isoform X4 [Gallus gallus]	GCF_000002315.5	TRADDN-NCOA6
KFV42278.1	TRADDN-NCOA6	170	N328_08716	vertebrata	Gavia stellata	Nuclear receptor coactivator 6, partial [Gavia stellata]	GCA_000690875.1	TRADDN-NCOA6
XP_019368618.1	TRADDN-NCOA6	2038	NCOA6	vertebrata	Gavialis gangeticus	PREDICTED: nuclear receptor coactivator 6 isoform X5 [Gavialis gangeticus]	GCF_001723915.1	TRADDN-NCOA6
XP_015268900.1	TRADDN-NCOA6	2038	NCOA6	vertebrata	Gekko japonicus	PREDICTED: nuclear receptor coactivator 6 [Gekko japonicus]	GCF_001447785.1	TRADDN-NCOA6
XP_014165548.1	TRADDN-NCOA6	1966	NCOA6	vertebrata	Geospiza fortis	PREDICTED: nuclear receptor coactivator 6 [Geospiza fortis]	-	TRADDN-NCOA6
XP_018872830.1	TRADDN-NCOA6	2070	NCOA6	vertebrata	Gorilla gorilla gorilla	PREDICTED: nuclear receptor coactivator 6 isoform X1 [Gorilla gorilla gorilla]	-	TRADDN-NCOA6
XP_018872831.1	TRADDN-NCOA6	2069	NCOA6	vertebrata	Gorilla gorilla gorilla	PREDICTED: nuclear receptor coactivator 6 isoform X2 [Gorilla gorilla gorilla]	-	TRADDN-NCOA6
XP_018872832.1	TRADDN-NCOA6	2059	NCOA6	vertebrata	Gorilla gorilla gorilla	PREDICTED: nuclear receptor coactivator 6 isoform X3 [Gorilla gorilla gorilla]	-	TRADDN-NCOA6
XP_018872833.1	TRADDN-NCOA6	2058	NCOA6	vertebrata	Gorilla gorilla gorilla	PREDICTED: nuclear receptor coactivator 6 isoform X4 [Gorilla gorilla gorilla]	-	TRADDN-NCOA6

acc	architecture	len	gen.name	taxend	species	defline	gca	clade
XP_018872834.1	TRADDN-NCOA6	1982	NCOA6	vertebrata	Gorilla gorilla gorilla	PREDICTED: nuclear receptor coactivator 6 isoform X5 [Gorilla gorilla gorilla]	-	TRADDN-NCOA6
XP_018872835.1	TRADDN-NCOA6	1076	NCOA6	vertebrata	Gorilla gorilla gorilla	PREDICTED: nuclear receptor coactivator 6 isoform X6 [Gorilla gorilla gorilla]	-	TRADDN-NCOA6
XP_009920176.1	TRADDN-NCOA6	2045	LOC104319919	vertebrata	Haliaeetus albicilla	PREDICTED: LOW QUALITY PROTEIN: nuclear receptor coactivator 6-like [Haliaeetus albicilla]	GCF_000691405.1	TRADDN-NCOA6
XP_010562854.1	TRADDN-NCOA6	2061	NCOA6	vertebrata	Haliaeetus leucocephalus	PREDICTED: nuclear receptor coactivator 6 isoform X1 [Haliaeetus leucocephalus]	GCF_000737465.1	TRADDN-NCOA6
XP_010562861.1	TRADDN-NCOA6	2053	NCOA6	vertebrata	Haliaeetus leucocephalus	PREDICTED: nuclear receptor coactivator 6 isoform X2 [Haliaeetus leucocephalus]	GCF_000737465.1	TRADDN-NCOA6
XP_010562862.1	TRADDN-NCOA6	2052	NCOA6	vertebrata	Haliaeetus leucocephalus	PREDICTED: nuclear receptor coactivator 6 isoform X3 [Haliaeetus leucocephalus]	GCF_000737465.1	TRADDN-NCOA6
XP_010562863.1	TRADDN-NCOA6	2050	NCOA6	vertebrata	Haliaeetus leucocephalus	PREDICTED: nuclear receptor coactivator 6 isoform X4 [Haliaeetus leucocephalus]	GCF_000737465.1	TRADDN-NCOA6
XP_010562864.1	TRADDN-NCOA6	2012	NCOA6	vertebrata	Haliaeetus leucocephalus	PREDICTED: nuclear receptor coactivator 6 isoform X5 [Haliaeetus leucocephalus]	GCF_000737465.1	TRADDN-NCOA6
XP_010562865.1	TRADDN-NCOA6	1993	NCOA6	vertebrata	Haliaeetus leucocephalus	PREDICTED: nuclear receptor coactivator 6 isoform X6 [Haliaeetus leucocephalus]	GCF_000737465.1	TRADDN-NCOA6
XP_010562867.1	TRADDN-NCOA6	1991	NCOA6	vertebrata	Haliaeetus leucocephalus	PREDICTED: nuclear receptor coactivator 6 isoform X7 [Haliaeetus leucocephalus]	GCF_000737465.1	TRADDN-NCOA6
XP_010562869.1	TRADDN-NCOA6	1739	NCOA6	vertebrata	Haliaeetus leucocephalus	PREDICTED: nuclear receptor coactivator 6 isoform X9 [Haliaeetus leucocephalus]	GCF_000737465.1	TRADDN-NCOA6
XP_010562870.1	TRADDN-NCOA6	455	NCOA6	vertebrata	Haliaeetus leucocephalus	PREDICTED: nuclear receptor coactivator 6 isoform X10 [Haliaeetus leucocephalus]	GCF_000737465.1	TRADDN-NCOA6
XP_010562871.1	TRADDN-NCOA6	232	NCOA6	vertebrata	Haliaeetus leucocephalus	PREDICTED: nuclear receptor coactivator 6 isoform X11 [Haliaeetus leucocephalus]	GCF_000737465.1	TRADDN-NCOA6
EHB08380.1	TRADDN-NCOA6	117	GW7_03039	vertebrata	Heterocephalus glaber	Nuclear receptor coactivator 6 [Heterocephalus glaber]	GCA_000230445.1	TRADDN-NCOA6
EHB17144.1	TRADDN-NCOA6	380	GW7_21530	vertebrata	Heterocephalus glaber	Nuclear receptor coactivator 6 [Heterocephalus glaber]	GCA_000230445.1	TRADDN-NCOA6
XP_004867960.1	TRADDN-NCOA6	2056	Ncoa6	vertebrata	Heterocephalus glaber	nuclear receptor coactivator 6 isoform X1 [Heterocephalus glaber]	GCF_000247695.1	TRADDN-NCOA6
XP_004867962.1	TRADDN-NCOA6	2045	Ncoa6	vertebrata	Heterocephalus glaber	nuclear receptor coactivator 6 isoform X2 [Heterocephalus glaber]	GCF_000247695.1	TRADDN-NCOA6
XP_004867963.1	TRADDN-NCOA6	2044	Ncoa6	vertebrata	Heterocephalus glaber	nuclear receptor coactivator 6 isoform X3 [Heterocephalus glaber]	GCF_000247695.1	TRADDN-NCOA6
XP_021102557.1	TRADDN-NCOA6	1956	Ncoa6	vertebrata	Heterocephalus glaber	nuclear receptor coactivator 6 isoform X4 [Heterocephalus glaber]	GCF_000247695.1	TRADDN-NCOA6

acc	architecture	len	gen.name	taxend	species	defline	gca	clade
XP_021102558.1	TRADDN-NCOA6	1949	Ncoa6	vertebrata	Heterocephalus glaber	nuclear receptor coactivator 6 isoform X5 [Heterocephalus glaber]	GCF_000247695.1	TRADDN-NCOA6
XP_021102559.1	TRADDN-NCOA6	1945	Ncoa6	vertebrata	Heterocephalus glaber	nuclear receptor coactivator 6 isoform X6 [Heterocephalus glaber]	GCF_000247695.1	TRADDN-NCOA6
XP_021102560.1	TRADDN-NCOA6	1944	Ncoa6	vertebrata	Heterocephalus glaber	nuclear receptor coactivator 6 isoform X7 [Heterocephalus glaber]	GCF_000247695.1	TRADDN-NCOA6
XP_021102561.1	TRADDN-NCOA6	1938	Ncoa6	vertebrata	Heterocephalus glaber	nuclear receptor coactivator 6 isoform X8 [Heterocephalus glaber]	GCF_000247695.1	TRADDN-NCOA6
XP_021102562.1	TRADDN-NCOA6	1937	Ncoa6	vertebrata	Heterocephalus glaber	nuclear receptor coactivator 6 isoform X9 [Heterocephalus glaber]	GCF_000247695.1	TRADDN-NCOA6
XP_021102563.1	TRADDN-NCOA6	1825	Ncoa6	vertebrata	Heterocephalus glaber	nuclear receptor coactivator 6 isoform X10 [Heterocephalus glaber]	GCF_000247695.1	TRADDN-NCOA6
XP_021102564.1	TRADDN-NCOA6	1066	Ncoa6	vertebrata	Heterocephalus glaber	nuclear receptor coactivator 6 isoform X11 [Heterocephalus glaber]	GCF_000247695.1	TRADDN-NCOA6
XP_019510722.1	TRADDN-NCOA6	2019	NCOA6	vertebrata	Hipposideros armiger	PREDICTED: nuclear receptor coactivator 6 isoform X1 [Hipposideros armiger]	GCF_001890085.1	TRADDN-NCOA6
XP_019510725.1	TRADDN-NCOA6	2018	NCOA6	vertebrata	Hipposideros armiger	PREDICTED: nuclear receptor coactivator 6 isoform X2 [Hipposideros armiger]	GCF_001890085.1	TRADDN-NCOA6
XP_019510726.1	TRADDN-NCOA6	2008	NCOA6	vertebrata	Hipposideros armiger	PREDICTED: nuclear receptor coactivator 6 isoform X3 [Hipposideros armiger]	GCF_001890085.1	TRADDN-NCOA6
XP_019510727.1	TRADDN-NCOA6	2007	NCOA6	vertebrata	Hipposideros armiger	PREDICTED: nuclear receptor coactivator 6 isoform X4 [Hipposideros armiger]	GCF_001890085.1	TRADDN-NCOA6
XP_019510728.1	TRADDN-NCOA6	1996	NCOA6	vertebrata	Hipposideros armiger	PREDICTED: nuclear receptor coactivator 6 isoform X5 [Hipposideros armiger]	GCF_001890085.1	TRADDN-NCOA6
XP_019510730.1	TRADDN-NCOA6	1995	NCOA6	vertebrata	Hipposideros armiger	PREDICTED: nuclear receptor coactivator 6 isoform X6 [Hipposideros armiger]	GCF_001890085.1	TRADDN-NCOA6
XP_019510731.1	TRADDN-NCOA6	1947	NCOA6	vertebrata	Hipposideros armiger	PREDICTED: nuclear receptor coactivator 6 isoform X7 [Hipposideros armiger]	GCF_001890085.1	TRADDN-NCOA6
XP_019510732.1	TRADDN-NCOA6	1919	NCOA6	vertebrata	Hipposideros armiger	PREDICTED: nuclear receptor coactivator 6 isoform X8 [Hipposideros armiger]	GCF_001890085.1	TRADDN-NCOA6
XP_019510733.1	TRADDN-NCOA6	1020	NCOA6	vertebrata	Hipposideros armiger	PREDICTED: nuclear receptor coactivator 6 isoform X9 [Hipposideros armiger]	GCF_001890085.1	TRADDN-NCOA6
AAF13595.1	TRADDN-NCOA6	2063	ASC2	vertebrata	Homo sapiens	cancer-amplified transcriptional coactivator ASC-2 [Homo sapiens]	-	TRADDN-NCOA6
AAF16403.1	TRADDN-NCOA6	2001	AIB3	vertebrata	Homo sapiens	transcriptional coactivator AIB3 [Homo sapiens]	-	TRADDN-NCOA6
AAI14614.1	TRADDN-NCOA6	357	AIB3	vertebrata	Homo sapiens	NCOA6 protein, partial [Homo sapiens]	-	TRADDN-NCOA6
AEG79566.1	TRADDN-NCOA6	982	NCOA6	vertebrata	Homo sapiens	nuclear receptor coactivator-6 delta [Homo sapiens]	-	TRADDN-NCOA6
NP_001229468.1	TRADDN-NCOA6	1070	AIB3,ASC2,NRC,PRIP,RAP250,TRBP	vertebrata	Homo sapiens	nuclear receptor coactivator 6 isoform 2 [Homo sapiens]	GCF_000001405.39	TRADDN-NCOA6

acc	architecture	len	gen.name	taxend	species	defline	gca	clade
NP_054790.2	TRADDN-NCOA6	2063	AIB3,ASC2,NRC,PRIP,RAP250,TRBP	vertebrata	Homo sapiens	nuclear receptor coactivator 6 isoform 1 [Homo sapiens]	GCF_000001405.39	TRADDN-NCOA6
XP_006723818.1	TRADDN-NCOA6	1975	AIB3,ASC2,NRC,PRIP,RAP250,TRBP	vertebrata	Homo sapiens	nuclear receptor coactivator 6 isoform X11 [Homo sapiens]	GCF_000001405.39	TRADDN-NCOA6
XP_011527023.1	TRADDN-NCOA6	2068	AIB3,ASC2,NRC,PRIP,RAP250,TRBP	vertebrata	Homo sapiens	nuclear receptor coactivator 6 isoform X4 [Homo sapiens]	GCF_000001405.39	TRADDN-NCOA6
XP_011527028.1	TRADDN-NCOA6	2057	AIB3,ASC2,NRC,PRIP,RAP250,TRBP	vertebrata	Homo sapiens	nuclear receptor coactivator 6 isoform X6 [Homo sapiens]	GCF_000001405.39	TRADDN-NCOA6
XP_011527029.1	TRADDN-NCOA6	2056	AIB3,ASC2,NRC,PRIP,RAP250,TRBP	vertebrata	Homo sapiens	nuclear receptor coactivator 6 isoform X7 [Homo sapiens]	GCF_000001405.39	TRADDN-NCOA6
XP_016883228.1	TRADDN-NCOA6	2087	AIB3,ASC2,NRC,PRIP,RAP250,TRBP	vertebrata	Homo sapiens	nuclear receptor coactivator 6 isoform X1 [Homo sapiens]	GCF_000001405.39	TRADDN-NCOA6
XP_016883230.1	TRADDN-NCOA6	2086	AIB3,ASC2,NRC,PRIP,RAP250,TRBP	vertebrata	Homo sapiens	nuclear receptor coactivator 6 isoform X2 [Homo sapiens]	GCF_000001405.39	TRADDN-NCOA6
XP_016883231.1	TRADDN-NCOA6	2082	AIB3,ASC2,NRC,PRIP,RAP250,TRBP	vertebrata	Homo sapiens	nuclear receptor coactivator 6 isoform X3 [Homo sapiens]	GCF_000001405.39	TRADDN-NCOA6
XP_016883232.1	TRADDN-NCOA6	2058	AIB3,ASC2,NRC,PRIP,RAP250,TRBP	vertebrata	Homo sapiens	nuclear receptor coactivator 6 isoform X5 [Homo sapiens]	GCF_000001405.39	TRADDN-NCOA6
XP_016883233.1	TRADDN-NCOA6	2051	AIB3,ASC2,NRC,PRIP,RAP250,TRBP	vertebrata	Homo sapiens	nuclear receptor coactivator 6 isoform X8 [Homo sapiens]	GCF_000001405.39	TRADDN-NCOA6
XP_016883234.1	TRADDN-NCOA6	2039	AIB3,ASC2,NRC,PRIP,RAP250,TRBP	vertebrata	Homo sapiens	nuclear receptor coactivator 6 isoform X9 [Homo sapiens]	GCF_000001405.39	TRADDN-NCOA6
XP_016883235.1	TRADDN-NCOA6	1999	AIB3,ASC2,NRC,PRIP,RAP250,TRBP	vertebrata	Homo sapiens	nuclear receptor coactivator 6 isoform X10 [Homo sapiens]	GCF_000001405.39	TRADDN-NCOA6
XP_016883237.1	TRADDN-NCOA6	1651	AIB3,ASC2,NRC,PRIP,RAP250,TRBP	vertebrata	Homo sapiens	nuclear receptor coactivator 6 isoform X13 [Homo sapiens]	GCF_000001405.39	TRADDN-NCOA6
XP_016883238.1	TRADDN-NCOA6	1650	AIB3,ASC2,NRC,PRIP,RAP250,TRBP	vertebrata	Homo sapiens	nuclear receptor coactivator 6 isoform X14 [Homo sapiens]	GCF_000001405.39	TRADDN-NCOA6
XP_016883239.1	TRADDN-NCOA6	1094	AIB3,ASC2,NRC,PRIP,RAP250,TRBP	vertebrata	Homo sapiens	nuclear receptor coactivator 6 isoform X15 [Homo sapiens]	GCF_000001405.39	TRADDN-NCOA6
XP_005329963.1	TRADDN-NCOA6	2064	Ncoa6	vertebrata	Ictidomys tridecemlineatus	nuclear receptor coactivator 6 isoform X1 [Ictidomys tridecemlineatus]	GCF_000236235.1	TRADDN-NCOA6
XP_013215998.1	TRADDN-NCOA6	2063	Ncoa6	vertebrata	Ictidomys tridecemlineatus	nuclear receptor coactivator 6 isoform X2 [Ictidomys tridecemlineatus]	GCF_000236235.1	TRADDN-NCOA6
XP_013215999.1	TRADDN-NCOA6	2053	Ncoa6	vertebrata	Ictidomys tridecemlineatus	nuclear receptor coactivator 6 isoform X3 [Ictidomys tridecemlineatus]	GCF_000236235.1	TRADDN-NCOA6
XP_021585249.1	TRADDN-NCOA6	2052	Ncoa6	vertebrata	Ictidomys tridecemlineatus	nuclear receptor coactivator 6 isoform X4 [Ictidomys tridecemlineatus]	GCF_000236235.1	TRADDN-NCOA6
XP_004668165.1	TRADDN-NCOA6	1972	Ncoa6	vertebrata	Jaculus jaculus	PREDICTED: nuclear receptor coactivator 6 [Jaculus jaculus]	GCF_000280705.1	TRADDN-NCOA6
XP_005986810.1	TRADDN-NCOA6	2049	NCOA6	vertebrata	Latimeria chalumnae	PREDICTED: nuclear receptor coactivator 6 isoform X1 [Latimeria chalumnae]	GCF_000225785.1	TRADDN-NCOA6
XP_014342664.1	TRADDN-NCOA6	2007	NCOA6	vertebrata	Latimeria chalumnae	PREDICTED: nuclear receptor coactivator 6 isoform X2 [Latimeria chalumnae]	GCF_000225785.1	TRADDN-NCOA6

acc	architecture	len	gen.name	taxend	species	defline	gca	clade
XP_014342667.1	TRADDN-NCOA6	2000	NCOA6	vertebrata	Latimeria chalumnae	PREDICTED: nuclear receptor coactivator 6 isoform X3 [Latimeria chalumnae]	GCF_000225785.1	TRADDN-NCOA6
XP_017691054.1	TRADDN-NCOA6	2058	NCOA6	vertebrata	Lepidothrix coronata	PREDICTED: nuclear receptor coactivator 6 isoform X1 [Lepidothrix coronata]	GCF_001604755.1	TRADDN-NCOA6
XP_017691055.1	TRADDN-NCOA6	2049	NCOA6	vertebrata	Lepidothrix coronata	PREDICTED: nuclear receptor coactivator 6 isoform X2 [Lepidothrix coronata]	GCF_001604755.1	TRADDN-NCOA6
XP_017691056.1	TRADDN-NCOA6	1987	NCOA6	vertebrata	Lepidothrix coronata	PREDICTED: nuclear receptor coactivator 6 isoform X3 [Lepidothrix coronata]	GCF_001604755.1	TRADDN-NCOA6
XP_017691057.1	TRADDN-NCOA6	1744	NCOA6	vertebrata	Lepidothrix coronata	PREDICTED: nuclear receptor coactivator 6 isoform X4 [Lepidothrix coronata]	GCF_001604755.1	TRADDN-NCOA6
XP_006727542.1	TRADDN-NCOA6	2046	NCOA6	vertebrata	Leptonychotes weddellii	PREDICTED: nuclear receptor coactivator 6 isoform X1 [Leptonychotes weddellii]	GCF_000349705.1	TRADDN-NCOA6
XP_006727543.1	TRADDN-NCOA6	1054	NCOA6	vertebrata	Leptonychotes weddellii	PREDICTED: nuclear receptor coactivator 6 isoform X2 [Leptonychotes weddellii]	-	TRADDN-NCOA6
KFQ00536.1	TRADDN-NCOA6	170	N330_11487	vertebrata	Leptosomus discolor	Nuclear receptor coactivator 6, partial [Leptosomus discolor]	GCA_000691785.1	TRADDN-NCOA6
PKU40219.1	TRADDN-NCOA6	897	llap_9474	vertebrata	Limosa lapponica baueri	nuclear receptor coactivator 6 [Limosa lapponica baueri]	GCA_002844005.1	TRADDN-NCOA6
XP_007447854.1	TRADDN-NCOA6	2039	NCOA6	vertebrata	Lipotes vexillifer	PREDICTED: nuclear receptor coactivator 6 isoform X1 [Lipotes vexillifer]	GCF_000442215.1	TRADDN-NCOA6
XP_007447855.1	TRADDN-NCOA6	1055	NCOA6	vertebrata	Lipotes vexillifer	PREDICTED: nuclear receptor coactivator 6 isoform X2 [Lipotes vexillifer]	GCF_000442215.1	TRADDN-NCOA6
OWK53644.1	TRADDN-NCOA6	1817	NCOA6	vertebrata	Lonchura striata domestica	Nuclear receptor coactivator 6 [Lonchura striata domestica]	GCA_002197715.1	TRADDN-NCOA6
XP_021392297.1	TRADDN-NCOA6	1047	NCOA6	vertebrata	Lonchura striata domestica	nuclear receptor coactivator 6 [Lonchura striata domestica]	-	TRADDN-NCOA6
XP_003411561.2	TRADDN-NCOA6	2070	NCOA6	vertebrata	Loxodonta africana	nuclear receptor coactivator 6 [Loxodonta africana]	GCF_000001905.1	TRADDN-NCOA6
EHH65404.1	TRADDN-NCOA6	2065	EGM_02156	vertebrata	Macaca fascicularis	hypothetical protein EGM_02156 [Macaca fascicularis]	GCA_000230815.1	TRADDN-NCOA6
XP_005568843.1	TRADDN-NCOA6	2065	NCOA6	vertebrata	Macaca fascicularis	PREDICTED: nuclear receptor coactivator 6 isoform X1 [Macaca fascicularis]	GCF_000364345.1	TRADDN-NCOA6
XP_005568845.1	TRADDN-NCOA6	1071	NCOA6	vertebrata	Macaca fascicularis	PREDICTED: nuclear receptor coactivator 6 isoform X6 [Macaca fascicularis]	GCF_000364345.1	TRADDN-NCOA6
XP_015313448.1	TRADDN-NCOA6	2064	NCOA6	vertebrata	Macaca fascicularis	PREDICTED: nuclear receptor coactivator 6 isoform X2 [Macaca fascicularis]	GCF_000364345.1	TRADDN-NCOA6
XP_015313449.1	TRADDN-NCOA6	2054	NCOA6	vertebrata	Macaca fascicularis	PREDICTED: nuclear receptor coactivator 6 isoform X3 [Macaca fascicularis]	GCF_000364345.1	TRADDN-NCOA6

acc	architecture	len	gen.name	taxend	species	defline	gca	clade
XP_015313450.1	TRADDN-NCOA6	2053	NCOA6	vertebrata	Macaca fascicularis	PREDICTED: nuclear receptor coactivator 6 isoform X4 [Macaca fascicularis]	GCF_000364345.1	TRADDN-NCOA6
EHH19760.1	TRADDN-NCOA6	2063	EGK_02479	vertebrata	Macaca mulatta	Amplified in breast cancer protein 3 [Macaca mulatta]	GCA_000230795.1	TRADDN-NCOA6
XP_015004643.1	TRADDN-NCOA6	2065	NCOA6	vertebrata	Macaca mulatta	PREDICTED: nuclear receptor coactivator 6 isoform X1 [Macaca mulatta]	GCF_003339765.1	TRADDN-NCOA6
XP_015004644.1	TRADDN-NCOA6	2064	NCOA6	vertebrata	Macaca mulatta	PREDICTED: nuclear receptor coactivator 6 isoform X2 [Macaca mulatta]	GCF_003339765.1	TRADDN-NCOA6
XP_015004645.1	TRADDN-NCOA6	2054	NCOA6	vertebrata	Macaca mulatta	PREDICTED: nuclear receptor coactivator 6 isoform X3 [Macaca mulatta]	GCF_003339765.1	TRADDN-NCOA6
XP_015004646.1	TRADDN-NCOA6	2053	NCOA6	vertebrata	Macaca mulatta	PREDICTED: nuclear receptor coactivator 6 isoform X4 [Macaca mulatta]	GCF_003339765.1	TRADDN-NCOA6
XP_015004650.1	TRADDN-NCOA6	1071	NCOA6	vertebrata	Macaca mulatta	PREDICTED: nuclear receptor coactivator 6 isoform X6 [Macaca mulatta]	GCF_003339765.1	TRADDN-NCOA6
XP_011764761.1	TRADDN-NCOA6	2066	NCOA6	vertebrata	Macaca nemestrina	nuclear receptor coactivator 6 isoform X1 [Macaca nemestrina]	GCF_000956065.1	TRADDN-NCOA6
XP_011764762.1	TRADDN-NCOA6	2065	NCOA6	vertebrata	Macaca nemestrina	nuclear receptor coactivator 6 isoform X2 [Macaca nemestrina]	GCF_000956065.1	TRADDN-NCOA6
XP_011764763.1	TRADDN-NCOA6	2055	NCOA6	vertebrata	Macaca nemestrina	nuclear receptor coactivator 6 isoform X3 [Macaca nemestrina]	GCF_000956065.1	TRADDN-NCOA6
XP_011764764.1	TRADDN-NCOA6	2054	NCOA6	vertebrata	Macaca nemestrina	nuclear receptor coactivator 6 isoform X4 [Macaca nemestrina]	GCF_000956065.1	TRADDN-NCOA6
XP_011764765.1	TRADDN-NCOA6	1070	NCOA6	vertebrata	Macaca nemestrina	nuclear receptor coactivator 6 isoform X5 [Macaca nemestrina]	GCF_000956065.1	TRADDN-NCOA6
XP_017942003.1	TRADDN-NCOA6	2067	NCOA6	vertebrata	Manacus vitellinus	nuclear receptor coactivator 6 isoform X1 [Manacus vitellinus]	-	TRADDN-NCOA6
XP_017942004.1	TRADDN-NCOA6	2044	NCOA6	vertebrata	Manacus vitellinus	nuclear receptor coactivator 6 isoform X2 [Manacus vitellinus]	-	TRADDN-NCOA6
XP_017942005.1	TRADDN-NCOA6	1982	NCOA6	vertebrata	Manacus vitellinus	nuclear receptor coactivator 6 isoform X3 [Manacus vitellinus]	-	TRADDN-NCOA6
XP_017942006.1	TRADDN-NCOA6	1753	NCOA6	vertebrata	Manacus vitellinus	nuclear receptor coactivator 6 isoform X4 [Manacus vitellinus]	-	TRADDN-NCOA6
XP_011827816.1	TRADDN-NCOA6	2066	NCOA6	vertebrata	Mandrillus leucophaeus	PREDICTED: nuclear receptor coactivator 6 [Mandrillus leucophaeus]	GCF_000951045.1	TRADDN-NCOA6
XP_017506268.1	TRADDN-NCOA6	2040	NCOA6	vertebrata	Manis javanica	PREDICTED: nuclear receptor coactivator 6 isoform X1 [Manis javanica]	GCF_001685135.1	TRADDN-NCOA6
XP_017506269.1	TRADDN-NCOA6	2039	NCOA6	vertebrata	Manis javanica	PREDICTED: nuclear receptor coactivator 6 isoform X2 [Manis javanica]	GCF_001685135.1	TRADDN-NCOA6
XP_017506270.1	TRADDN-NCOA6	2029	NCOA6	vertebrata	Manis javanica	PREDICTED: nuclear receptor coactivator 6 isoform X3 [Manis javanica]	GCF_001685135.1	TRADDN-NCOA6

acc	architecture	len	gen.name	taxend	species	defline	gca	clade
XP_017506271.1	TRADDN-NCOA6	2028	NCOA6	vertebrata	Manis javanica	PREDICTED: nuclear receptor coactivator 6 isoform X4 [Manis javanica]	GCF_001685135.1	TRADDN-NCOA6
XP_017506272.1	TRADDN-NCOA6	1053	NCOA6	vertebrata	Manis javanica	PREDICTED: nuclear receptor coactivator 6 isoform X5 [Manis javanica]	GCF_001685135.1	TRADDN-NCOA6
XP_015336937.1	TRADDN-NCOA6	2058	Ncoa6	vertebrata	Marmota marmota marmota	PREDICTED: nuclear receptor coactivator 6 isoform X1 [Marmota marmota marmota]	GCF_001458135.1	TRADDN-NCOA6
XP_015336944.1	TRADDN-NCOA6	2057	Ncoa6	vertebrata	Marmota marmota marmota	PREDICTED: nuclear receptor coactivator 6 isoform X2 [Marmota marmota marmota]	GCF_001458135.1	TRADDN-NCOA6
XP_015336952.1	TRADDN-NCOA6	2047	Ncoa6	vertebrata	Marmota marmota marmota	PREDICTED: nuclear receptor coactivator 6 isoform X3 [Marmota marmota marmota]	GCF_001458135.1	TRADDN-NCOA6
XP_015336959.1	TRADDN-NCOA6	2046	Ncoa6	vertebrata	Marmota marmota marmota	PREDICTED: nuclear receptor coactivator 6 isoform X4 [Marmota marmota marmota]	GCF_001458135.1	TRADDN-NCOA6
XP_015336968.1	TRADDN-NCOA6	2033	Ncoa6	vertebrata	Marmota marmota marmota	PREDICTED: nuclear receptor coactivator 6 isoform X5 [Marmota marmota marmota]	GCF_001458135.1	TRADDN-NCOA6
XP_015336972.1	TRADDN-NCOA6	1062	Ncoa6	vertebrata	Marmota marmota marmota	PREDICTED: nuclear receptor coactivator 6 isoform X6 [Marmota marmota marmota]	GCF_001458135.1	TRADDN-NCOA6
XP_010720521.1	TRADDN-NCOA6	2042	NCOA6	vertebrata	Meleagris gallopavo	PREDICTED: nuclear receptor coactivator 6 isoform X1 [Meleagris gallopavo]	GCF_000146605.2	TRADDN-NCOA6
XP_010720522.1	TRADDN-NCOA6	1980	NCOA6	vertebrata	Meleagris gallopavo	PREDICTED: nuclear receptor coactivator 6 isoform X2 [Meleagris gallopavo]	GCF_000146605.2	TRADDN-NCOA6
XP_019477668.1	TRADDN-NCOA6	1770	NCOA6	vertebrata	Meleagris gallopavo	PREDICTED: nuclear receptor coactivator 6 isoform X3 [Meleagris gallopavo]	GCF_000146605.2	TRADDN-NCOA6
XP_005146817.1	TRADDN-NCOA6	1046	LOC101879127	vertebrata	Melopsittacus undulatus	PREDICTED: nuclear receptor coactivator 6 [Melopsittacus undulatus]	-	TRADDN-NCOA6
XP_021496225.1	TRADDN-NCOA6	2073	Ncoa6	vertebrata	Meriones unguiculatus	nuclear receptor coactivator 6 isoform X1 [Meriones unguiculatus]	GCF_002204375.1	TRADDN-NCOA6
XP_021496227.1	TRADDN-NCOA6	2072	Ncoa6	vertebrata	Meriones unguiculatus	nuclear receptor coactivator 6 isoform X2 [Meriones unguiculatus]	GCF_002204375.1	TRADDN-NCOA6
XP_021496228.1	TRADDN-NCOA6	2062	Ncoa6	vertebrata	Meriones unguiculatus	nuclear receptor coactivator 6 isoform X3 [Meriones unguiculatus]	GCF_002204375.1	TRADDN-NCOA6
XP_021496229.1	TRADDN-NCOA6	2061	Ncoa6	vertebrata	Meriones unguiculatus	nuclear receptor coactivator 6 isoform X4 [Meriones unguiculatus]	GCF_002204375.1	TRADDN-NCOA6
KFQ18008.1	TRADDN-NCOA6	170	N331_02691	vertebrata	Merops nubicus	Nuclear receptor coactivator 6, partial [Merops nubicus]	GCA_000691845.1	TRADDN-NCOA6
XP_008935730.1	TRADDN-NCOA6	486	LOC103770229	vertebrata	Merops nubicus	PREDICTED: nuclear receptor coactivator 6-like, partial [Merops nubicus]	GCF_000691845.1	TRADDN-NCOA6
KFQ26307.1	TRADDN-NCOA6	169	N332_00124	vertebrata	Mesitornis unicolor	Nuclear receptor coactivator 6, partial [Mesitornis unicolor]	GCA_000695765.1	TRADDN-NCOA6

acc	architecture	len	gen.name	taxend	species	defline	gca	clade
XP_005084691.1	TRADDN-NCOA6	2060	Ncoa6	vertebrata	Mesocricetus auratus	nuclear receptor coactivator 6 isoform X1 [Mesocricetus auratus]	GCF_000349665.1	TRADDN-NCOA6
XP_012979529.1	TRADDN-NCOA6	2059	Ncoa6	vertebrata	Mesocricetus auratus	nuclear receptor coactivator 6 isoform X2 [Mesocricetus auratus]	GCF_000349665.1	TRADDN-NCOA6
XP_012979530.1	TRADDN-NCOA6	2049	Ncoa6	vertebrata	Mesocricetus auratus	nuclear receptor coactivator 6 isoform X3 [Mesocricetus auratus]	GCF_000349665.1	TRADDN-NCOA6
XP_012979531.1	TRADDN-NCOA6	2048	Ncoa6	vertebrata	Mesocricetus auratus	nuclear receptor coactivator 6 isoform X4 [Mesocricetus auratus]	GCF_000349665.1	TRADDN-NCOA6
XP_021091327.1	TRADDN-NCOA6	1064	Ncoa6	vertebrata	Mesocricetus auratus	nuclear receptor coactivator 6 isoform X5 [Mesocricetus auratus]	GCF_000349665.1	TRADDN-NCOA6
XP_012610105.1	TRADDN-NCOA6	2048	NCOA6	vertebrata	Microcebus murinus	nuclear receptor coactivator 6 isoform X1 [Microcebus murinus]	GCF_000165445.2	TRADDN-NCOA6
XP_012610107.1	TRADDN-NCOA6	2047	NCOA6	vertebrata	Microcebus murinus	nuclear receptor coactivator 6 isoform X2 [Microcebus murinus]	GCF_000165445.2	TRADDN-NCOA6
XP_012610108.1	TRADDN-NCOA6	2037	NCOA6	vertebrata	Microcebus murinus	nuclear receptor coactivator 6 isoform X3 [Microcebus murinus]	GCF_000165445.2	TRADDN-NCOA6
XP_012610109.1	TRADDN-NCOA6	2036	NCOA6	vertebrata	Microcebus murinus	nuclear receptor coactivator 6 isoform X4 [Microcebus murinus]	GCF_000165445.2	TRADDN-NCOA6
XP_012610112.1	TRADDN-NCOA6	1060	NCOA6	vertebrata	Microcebus murinus	nuclear receptor coactivator 6 isoform X5 [Microcebus murinus]	GCF_000165445.2	TRADDN-NCOA6
XP_005363137.1	TRADDN-NCOA6	2063	Ncoa6	vertebrata	Microtus ochrogaster	nuclear receptor coactivator 6 isoform X1 [Microtus ochrogaster]	GCF_000317375.1	TRADDN-NCOA6
XP_013207639.1	TRADDN-NCOA6	2062	Ncoa6	vertebrata	Microtus ochrogaster	nuclear receptor coactivator 6 isoform X2 [Microtus ochrogaster]	GCF_000317375.1	TRADDN-NCOA6
XP_016068639.1	TRADDN-NCOA6	2057	NCOA6	vertebrata	Miniopterus natalensis	PREDICTED: nuclear receptor coactivator 6 isoform X1 [Miniopterus natalensis]	GCF_001595765.1	TRADDN-NCOA6
XP_016068640.1	TRADDN-NCOA6	2056	NCOA6	vertebrata	Miniopterus natalensis	PREDICTED: nuclear receptor coactivator 6 isoform X2 [Miniopterus natalensis]	GCF_001595765.1	TRADDN-NCOA6
XP_001371478.2	TRADDN-NCOA6	2054	NCOA6	vertebrata	Monodelphis domestica	PREDICTED: nuclear receptor coactivator 6 isoform X1 [Monodelphis domestica]	GCF_000002295.2	TRADDN-NCOA6
XP_007474291.1	TRADDN-NCOA6	2031	NCOA6	vertebrata	Monodelphis domestica	PREDICTED: nuclear receptor coactivator 6 isoform X2 [Monodelphis domestica]	GCF_000002295.2	TRADDN-NCOA6
XP_007474292.1	TRADDN-NCOA6	1620	NCOA6	vertebrata	Monodelphis domestica	PREDICTED: nuclear receptor coactivator 6 isoform X3 [Monodelphis domestica]	GCF_000002295.2	TRADDN-NCOA6
XP_007474293.1	TRADDN-NCOA6	1053	NCOA6	vertebrata	Monodelphis domestica	PREDICTED: nuclear receptor coactivator 6 isoform X4 [Monodelphis domestica]	GCF_000002295.2	TRADDN-NCOA6
XP_021010953.1	TRADDN-NCOA6	1069	LOC110289096	vertebrata	Mus caroli	LOW QUALITY PROTEIN: nuclear receptor coactivator 6-like [Mus caroli]	GCF_900094665.1	TRADDN-NCOA6
XP_021012014.1	TRADDN-NCOA6	2069	Ncoa6	vertebrata	Mus caroli	nuclear receptor coactivator 6 isoform X1 [Mus caroli]	-	TRADDN-NCOA6
XP_021012016.1	TRADDN-NCOA6	2058	Ncoa6	vertebrata	Mus caroli	nuclear receptor coactivator 6 isoform X2 [Mus caroli]	GCF_900094665.1	TRADDN-NCOA6
XP_021012017.1	TRADDN-NCOA6	2057	Ncoa6	vertebrata	Mus caroli	nuclear receptor coactivator 6 isoform X3 [Mus caroli]	GCF_900094665.1	TRADDN-NCOA6

acc	architecture	len	gen.name	taxend	species	defline	gca	clade
XP_021012018.1	TRADDN-NCOA6	1075	Ncoa6	vertebrata	Mus caroli	nuclear receptor coactivator 6 isoform X4 [Mus caroli]	GCF_900094665.1	TRADDN-NCOA6
AAH31113.1	TRADDN-NCOA6	458	AIB3	vertebrata	Mus musculus	Ncoa6 protein [Mus musculus]	-	TRADDN-NCOA6
AEG79562.1	TRADDN-NCOA6	1973	Ncoa6	vertebrata	Mus musculus	nuclear receptor coactivator-6 gamma [Mus musculus]	-	TRADDN-NCOA6
AEG79563.1	TRADDN-NCOA6	981	Ncoa6	vertebrata	Mus musculus	nuclear receptor coactivator-6 delta [Mus musculus]	-	TRADDN-NCOA6
BAE42705.1	TRADDN-NCOA6	1516	-	vertebrata	Mus musculus	unnamed protein product, partial [Mus musculus]	-	TRADDN-NCOA6
NP_001229487.1	TRADDN-NCOA6	1077	AIB3,ASC-2,ASC2,mKIAA0181,Ncoa7,NRC,PRIP,RAP250	vertebrata	Mus musculus	nuclear receptor coactivator 6 isoform 2 [Mus musculus]	GCF_000001635.26	TRADDN-NCOA6
NP_062799.2	TRADDN-NCOA6	2069	AIB3,ASC-2,ASC2,mKIAA0181,Ncoa7,NRC,PRIP,RAP250	vertebrata	Mus musculus	nuclear receptor coactivator 6 isoform 1 [Mus musculus]	GCF_000001635.26	TRADDN-NCOA6
Q9JL19.1	TRADDN-NCOA6	2067	-	vertebrata	Mus musculus	RecName: Full=Nuclear receptor coactivator 6; AltName: Full=Activating signal cointegrator 2; Short=ASC-2; AltName: Full=Amplified in breast cancer protein 3; AltName: Full=Cancer-amplified transcriptional coactivator ASC-2; AltName: Full=Nuclear receptor coactivator RAP250; Short=NRC; AltName: Full=Nuclear receptor-activating protein, 250 kDa; AltName: Full=Peroxisome proliferator-activated receptor-interacting protein; Short=PPAR-interacting protein; AltName: Full=Thyroid hormone receptor-binding protein	-	TRADDN-NCOA6
XP_021049610.1	TRADDN-NCOA6	2070	Ncoa6	vertebrata	Mus pahari	nuclear receptor coactivator 6 isoform X1 [Mus pahari]	GCF_900095145.1	TRADDN-NCOA6
XP_021049611.1	TRADDN-NCOA6	1974	Ncoa6	vertebrata	Mus pahari	nuclear receptor coactivator 6 isoform X2 [Mus pahari]	-	TRADDN-NCOA6
XP_021049612.1	TRADDN-NCOA6	1072	Ncoa6	vertebrata	Mus pahari	nuclear receptor coactivator 6 isoform X3 [Mus pahari]	GCF_900095145.1	TRADDN-NCOA6
XP_004746634.1	TRADDN-NCOA6	2066	NCOA6	vertebrata	Mustela putorius furo	PREDICTED: nuclear receptor coactivator 6 isoform X1 [Mustela putorius furo]	GCF_000215625.1	TRADDN-NCOA6
XP_004746637.1	TRADDN-NCOA6	2065	NCOA6	vertebrata	Mustela putorius furo	PREDICTED: nuclear receptor coactivator 6 isoform X2 [Mustela putorius furo]	GCF_000215625.1	TRADDN-NCOA6
XP_004746638.1	TRADDN-NCOA6	2055	NCOA6	vertebrata	Mustela putorius furo	PREDICTED: nuclear receptor coactivator 6 isoform X3 [Mustela putorius furo]	GCF_000215625.1	TRADDN-NCOA6
XP_004746639.1	TRADDN-NCOA6	2054	NCOA6	vertebrata	Mustela putorius furo	PREDICTED: nuclear receptor coactivator 6 isoform X4 [Mustela putorius furo]	GCF_000215625.1	TRADDN-NCOA6
XP_004746640.1	TRADDN-NCOA6	1966	NCOA6	vertebrata	Mustela putorius furo	PREDICTED: nuclear receptor coactivator 6 isoform X5 [Mustela putorius furo]	GCF_000215625.1	TRADDN-NCOA6

acc	architecture	len	gen.name	taxend	species	defline	gca	clade
EPQ16132.1	TRADDN-NCOA6	1797	D623_10023707	vertebrata	Myotis brandtii	Nuclear receptor coactivator 6 [Myotis brandtii]	GCA_000412655.1	TRADDN-NCOA6
XP_005879842.1	TRADDN-NCOA6	2060	NCOA6	vertebrata	Myotis brandtii	PREDICTED: nuclear receptor coactivator 6 isoform X1 [Myotis brandtii]	GCF_000412655.1	TRADDN-NCOA6
XP_005879843.1	TRADDN-NCOA6	2059	NCOA6	vertebrata	Myotis brandtii	PREDICTED: nuclear receptor coactivator 6 isoform X2 [Myotis brandtii]	GCF_000412655.1	TRADDN-NCOA6
XP_005879844.1	TRADDN-NCOA6	2049	NCOA6	vertebrata	Myotis brandtii	PREDICTED: nuclear receptor coactivator 6 isoform X3 [Myotis brandtii]	GCF_000412655.1	TRADDN-NCOA6
XP_005879845.1	TRADDN-NCOA6	2048	NCOA6	vertebrata	Myotis brandtii	PREDICTED: nuclear receptor coactivator 6 isoform X4 [Myotis brandtii]	GCF_000412655.1	TRADDN-NCOA6
XP_005879846.1	TRADDN-NCOA6	2004	NCOA6	vertebrata	Myotis brandtii	PREDICTED: nuclear receptor coactivator 6 isoform X5 [Myotis brandtii]	GCF_000412655.1	TRADDN-NCOA6
XP_014406139.1	TRADDN-NCOA6	1064	NCOA6	vertebrata	Myotis brandtii	PREDICTED: nuclear receptor coactivator 6 isoform X6 [Myotis brandtii]	GCF_000412655.1	TRADDN-NCOA6
ELK29648.1	TRADDN-NCOA6	2029	MDA_GLEAN10022763	vertebrata	Myotis davidii	Nuclear receptor coactivator 6 [Myotis davidii]	GCA_000327345.1	TRADDN-NCOA6
XP_006764639.1	TRADDN-NCOA6	2062	NCOA6	vertebrata	Myotis davidii	PREDICTED: nuclear receptor coactivator 6 isoform X1 [Myotis davidii]	GCF_000327345.1	TRADDN-NCOA6
XP_006764640.1	TRADDN-NCOA6	2061	NCOA6	vertebrata	Myotis davidii	PREDICTED: nuclear receptor coactivator 6 isoform X2 [Myotis davidii]	GCF_000327345.1	TRADDN-NCOA6
XP_006764641.1	TRADDN-NCOA6	2006	NCOA6	vertebrata	Myotis davidii	PREDICTED: nuclear receptor coactivator 6 isoform X5 [Myotis davidii]	GCF_000327345.1	TRADDN-NCOA6
XP_015419968.1	TRADDN-NCOA6	2051	NCOA6	vertebrata	Myotis davidii	PREDICTED: nuclear receptor coactivator 6 isoform X3 [Myotis davidii]	GCF_000327345.1	TRADDN-NCOA6
XP_015419969.1	TRADDN-NCOA6	2050	NCOA6	vertebrata	Myotis davidii	PREDICTED: nuclear receptor coactivator 6 isoform X4 [Myotis davidii]	GCF_000327345.1	TRADDN-NCOA6
XP_015419970.1	TRADDN-NCOA6	1064	NCOA6	vertebrata	Myotis davidii	PREDICTED: nuclear receptor coactivator 6 isoform X6 [Myotis davidii]	GCF_000327345.1	TRADDN-NCOA6
XP_023610171.1	TRADDN-NCOA6	2061	NCOA6	vertebrata	Myotis lucifugus	nuclear receptor coactivator 6 isoform X1 [Myotis lucifugus]	GCF_000147115.1	TRADDN-NCOA6
XP_023610172.1	TRADDN-NCOA6	2060	NCOA6	vertebrata	Myotis lucifugus	nuclear receptor coactivator 6 isoform X2 [Myotis lucifugus]	GCF_000147115.1	TRADDN-NCOA6
XP_023610174.1	TRADDN-NCOA6	2050	NCOA6	vertebrata	Myotis lucifugus	nuclear receptor coactivator 6 isoform X3 [Myotis lucifugus]	GCF_000147115.1	TRADDN-NCOA6
XP_023610175.1	TRADDN-NCOA6	2049	NCOA6	vertebrata	Myotis lucifugus	nuclear receptor coactivator 6 isoform X4 [Myotis lucifugus]	GCF_000147115.1	TRADDN-NCOA6
XP_023610176.1	TRADDN-NCOA6	2038	NCOA6	vertebrata	Myotis lucifugus	nuclear receptor coactivator 6 isoform X5 [Myotis lucifugus]	GCF_000147115.1	TRADDN-NCOA6
XP_023610177.1	TRADDN-NCOA6	2037	NCOA6	vertebrata	Myotis lucifugus	nuclear receptor coactivator 6 isoform X6 [Myotis lucifugus]	GCF_000147115.1	TRADDN-NCOA6

acc	architecture	len	gen.name	taxend	species	defline	gca	clade
XP_008846104.1	TRADDN-NCOA6	2049	Ncoa6	vertebrata	Nannospalax galili	PREDICTED: nuclear receptor coactivator 6 isoform X2 [Nannospalax galili]	GCF_000622305.1	TRADDN-NCOA6
XP_008846105.1	TRADDN-NCOA6	1069	Ncoa6	vertebrata	Nannospalax galili	PREDICTED: nuclear receptor coactivator 6 isoform X5 [Nannospalax galili]	GCF_000622305.1	TRADDN-NCOA6
XP_017657374.1	TRADDN-NCOA6	2060	Ncoa6	vertebrata	Nannospalax galili	PREDICTED: nuclear receptor coactivator 6 isoform X1 [Nannospalax galili]	GCF_000622305.1	TRADDN-NCOA6
XP_017657375.1	TRADDN-NCOA6	2040	Ncoa6	vertebrata	Nannospalax galili	PREDICTED: nuclear receptor coactivator 6 isoform X3 [Nannospalax galili]	GCF_000622305.1	TRADDN-NCOA6
XP_017657376.1	TRADDN-NCOA6	2019	Ncoa6	vertebrata	Nannospalax galili	PREDICTED: nuclear receptor coactivator 6 isoform X4 [Nannospalax galili]	-	TRADDN-NCOA6
XP_018411685.1	TRADDN-NCOA6	1917	NCOA6	vertebrata	Nanorana parkeri	PREDICTED: nuclear receptor coactivator 6 [Nanorana parkeri]	GCF_000935625.1	TRADDN-NCOA6
XP_021544696.1	TRADDN-NCOA6	2053	NCOA6	vertebrata	Neomonachus schauinslandi	nuclear receptor coactivator 6 isoform X1 [Neomonachus schauinslandi]	GCF_002201575.1	TRADDN-NCOA6
XP_021544698.1	TRADDN-NCOA6	2042	NCOA6	vertebrata	Neomonachus schauinslandi	nuclear receptor coactivator 6 isoform X2 [Neomonachus schauinslandi]	GCF_002201575.1	TRADDN-NCOA6
XP_024598703.1	TRADDN-NCOA6	2039	NCOA6	vertebrata	Neophocaena asiaeorientalis asiaeorientalis	nuclear receptor coactivator 6 isoform X1 [Neophocaena asiaeorientalis asiaeorientalis]	GCF_003031525.1	TRADDN-NCOA6
XP_024598704.1	TRADDN-NCOA6	2038	NCOA6	vertebrata	Neophocaena asiaeorientalis asiaeorientalis	nuclear receptor coactivator 6 isoform X2 [Neophocaena asiaeorientalis asiaeorientalis]	GCF_003031525.1	TRADDN-NCOA6
XP_024598705.1	TRADDN-NCOA6	2028	NCOA6	vertebrata	Neophocaena asiaeorientalis asiaeorientalis	nuclear receptor coactivator 6 isoform X3 [Neophocaena asiaeorientalis asiaeorientalis]	GCF_003031525.1	TRADDN-NCOA6
XP_024598707.1	TRADDN-NCOA6	2027	NCOA6	vertebrata	Neophocaena asiaeorientalis asiaeorientalis	nuclear receptor coactivator 6 isoform X4 [Neophocaena asiaeorientalis asiaeorientalis]	GCF_003031525.1	TRADDN-NCOA6
XP_024598709.1	TRADDN-NCOA6	1714	NCOA6	vertebrata	Neophocaena asiaeorientalis asiaeorientalis	nuclear receptor coactivator 6 isoform X6 [Neophocaena asiaeorientalis asiaeorientalis]	GCF_003031525.1	TRADDN-NCOA6
OBS65359.1	TRADDN-NCOA6	1980	A6R68_06076	vertebrata	Neotoma lepida	hypothetical protein A6R68_06076, partial [Neotoma lepida]	GCA_001675575.1	TRADDN-NCOA6
XP_010018687.1	TRADDN-NCOA6	1052	NCOA6	vertebrata	Nestor notabilis	PREDICTED: nuclear receptor coactivator 6 [Nestor notabilis]	GCF_000696875.1	TRADDN-NCOA6
KFR06714.1	TRADDN-NCOA6	2045	Y956_05669	vertebrata	Nipponia nippon	Nuclear receptor coactivator 6 [Nipponia nippon]	GCA_000708225.1	TRADDN-NCOA6
XP_009471645.1	TRADDN-NCOA6	1056	NCOA6	vertebrata	Nipponia nippon	PREDICTED: LOW QUALITY PROTEIN: nuclear receptor coactivator 6 [Nipponia nippon]	GCF_000708225.1	TRADDN-NCOA6
XP_004090170.1	TRADDN-NCOA6	1080	NCOA6	vertebrata	Nomascus leucogenys	PREDICTED: nuclear receptor coactivator 6 isoform X2 [Nomascus leucogenys]	-	TRADDN-NCOA6

acc	architecture	len	gen.name	taxend	species	defline	gca	clade
XP_012367438.1	TRADDN-NCOA6	2074	NCOA6	vertebrata	Nomascus leucogenys	PREDICTED: nuclear receptor coactivator 6 isoform X1 [Nomascus leucogenys]	GCF_006542625.1	TRADDN-NCOA6
XP_021272769.1	TRADDN-NCOA6	2045	NCOA6	vertebrata	Numida meleagris	nuclear receptor coactivator 6 isoform X1 [Numida meleagris]	GCF_002078875.1	TRADDN-NCOA6
XP_021272770.1	TRADDN-NCOA6	1983	NCOA6	vertebrata	Numida meleagris	nuclear receptor coactivator 6 isoform X2 [Numida meleagris]	GCF_002078875.1	TRADDN-NCOA6
XP_004585801.1	TRADDN-NCOA6	2065	NCOA6	vertebrata	Ochotona princeps	PREDICTED: nuclear receptor coactivator 6 isoform X1 [Ochotona princeps]	GCF_000292845.1	TRADDN-NCOA6
XP_004585802.1	TRADDN-NCOA6	1061	NCOA6	vertebrata	Ochotona princeps	PREDICTED: nuclear receptor coactivator 6 isoform X2 [Ochotona princeps]	GCF_000292845.1	TRADDN-NCOA6
XP_023570540.1	TRADDN-NCOA6	2069	Ncoa6	vertebrata	Octodon degus	nuclear receptor coactivator 6 isoform X1 [Octodon degus]	GCF_000260255.1	TRADDN-NCOA6
XP_023570543.1	TRADDN-NCOA6	2058	Ncoa6	vertebrata	Octodon degus	nuclear receptor coactivator 6 isoform X2 [Octodon degus]	GCF_000260255.1	TRADDN-NCOA6
XP_023570544.1	TRADDN-NCOA6	2057	Ncoa6	vertebrata	Octodon degus	nuclear receptor coactivator 6 isoform X3 [Octodon degus]	GCF_000260255.1	TRADDN-NCOA6
XP_023570545.1	TRADDN-NCOA6	1838	Ncoa6	vertebrata	Octodon degus	nuclear receptor coactivator 6 isoform X4 [Octodon degus]	GCF_000260255.1	TRADDN-NCOA6
XP_023570546.1	TRADDN-NCOA6	1075	Ncoa6	vertebrata	Octodon degus	nuclear receptor coactivator 6 isoform X5 [Octodon degus]	GCF_000260255.1	TRADDN-NCOA6
XP_012420658.1	TRADDN-NCOA6	2053	NCOA6	vertebrata	Odobenus rosmarus divergens	PREDICTED: nuclear receptor coactivator 6 isoform X1 [Odobenus rosmarus divergens]	GCF_000321225.1	TRADDN-NCOA6
XP_012420661.1	TRADDN-NCOA6	1060	NCOA6	vertebrata	Odobenus rosmarus divergens	PREDICTED: nuclear receptor coactivator 6 isoform X2 [Odobenus rosmarus divergens]	GCF_000321225.1	TRADDN-NCOA6
XP_020744807.1	TRADDN-NCOA6	1895	LOC110134568	vertebrata	Odocoileus virginianus texanus	nuclear receptor coactivator 6 isoform X1 [Odocoileus virginianus texanus]	GCF_002102435.1	TRADDN-NCOA6
XP_020744808.1	TRADDN-NCOA6	1894	LOC110134568	vertebrata	Odocoileus virginianus texanus	nuclear receptor coactivator 6 isoform X2 [Odocoileus virginianus texanus]	GCF_002102435.1	TRADDN-NCOA6
XP_020744809.1	TRADDN-NCOA6	1884	LOC110134568	vertebrata	Odocoileus virginianus texanus	nuclear receptor coactivator 6 isoform X3 [Odocoileus virginianus texanus]	GCF_002102435.1	TRADDN-NCOA6
XP_020744810.1	TRADDN-NCOA6	1883	LOC110134568	vertebrata	Odocoileus virginianus texanus	nuclear receptor coactivator 6 isoform X4 [Odocoileus virginianus texanus]	GCF_002102435.1	TRADDN-NCOA6
XP_020744812.1	TRADDN-NCOA6	1795	LOC110134568	vertebrata	Odocoileus virginianus texanus	nuclear receptor coactivator 6 isoform X6 [Odocoileus virginianus texanus]	GCF_002102435.1	TRADDN-NCOA6
XP_020749657.1	TRADDN-NCOA6	143	LOC110137564	vertebrata	Odocoileus virginianus texanus	nuclear receptor coactivator 6-like [Odocoileus virginianus texanus]	GCF_002102435.1	TRADDN-NCOA6
XP_009942846.1	TRADDN-NCOA6	1048	NCOA6	vertebrata	Opisthocomus hoazin	PREDICTED: nuclear receptor coactivator 6 [Opisthocomus hoazin]	GCF_000692075.1	TRADDN-NCOA6
XP_004272797.1	TRADDN-NCOA6	2041	NCOA6	vertebrata	Orcinus orca	PREDICTED: nuclear receptor coactivator 6 isoform X1 [Orcinus orca]	GCF_000331955.2	TRADDN-NCOA6

acc	architecture	len	gen.name	taxend	species	defline	gca	clade
XP_004272798.1	TRADDN-NCOA6	1057	NCOA6	vertebrata	Orcinus orca	PREDICTED: nuclear receptor coactivator 6 isoform X2 [Orcinus orca]	GCF_000331955.2	TRADDN-NCOA6
XP_007655684.1	TRADDN-NCOA6	753	LOC100092044	vertebrata	Ornithorhynchus anatinus	PREDICTED: nuclear receptor coactivator 6-like [Ornithorhynchus anatinus]	-	TRADDN-NCOA6
XP_007932923.1	TRADDN-NCOA6	2109	NCOA6	vertebrata	Orycteropus afer afer	PREDICTED: nuclear receptor coactivator 6 [Orycteropus afer afer]	GCF_000298275.1	TRADDN-NCOA6
XP_008254292.1	TRADDN-NCOA6	2074	NCOA6	vertebrata	Oryctolagus cuniculus	PREDICTED: nuclear receptor coactivator 6 isoform X1 [Oryctolagus cuniculus]	GCF_000003625.3	TRADDN-NCOA6
XP_008254293.1	TRADDN-NCOA6	2073	NCOA6	vertebrata	Oryctolagus cuniculus	PREDICTED: nuclear receptor coactivator 6 isoform X2 [Oryctolagus cuniculus]	GCF_000003625.3	TRADDN-NCOA6
XP_008254295.1	TRADDN-NCOA6	2063	NCOA6	vertebrata	Oryctolagus cuniculus	PREDICTED: nuclear receptor coactivator 6 isoform X3 [Oryctolagus cuniculus]	GCF_000003625.3	TRADDN-NCOA6
XP_008254296.1	TRADDN-NCOA6	2062	NCOA6	vertebrata	Oryctolagus cuniculus	PREDICTED: nuclear receptor coactivator 6 isoform X4 [Oryctolagus cuniculus]	GCF_000003625.3	TRADDN-NCOA6
XP_008254298.1	TRADDN-NCOA6	2050	NCOA6	vertebrata	Oryctolagus cuniculus	PREDICTED: nuclear receptor coactivator 6 isoform X6 [Oryctolagus cuniculus]	GCF_000003625.3	TRADDN-NCOA6
XP_008254300.1	TRADDN-NCOA6	1631	NCOA6	vertebrata	Oryctolagus cuniculus	PREDICTED: nuclear receptor coactivator 6 isoform X8 [Oryctolagus cuniculus]	GCF_000003625.3	TRADDN-NCOA6
XP_017197155.1	TRADDN-NCOA6	2051	NCOA6	vertebrata	Oryctolagus cuniculus	PREDICTED: nuclear receptor coactivator 6 isoform X5 [Oryctolagus cuniculus]	GCF_000003625.3	TRADDN-NCOA6
XP_017197156.1	TRADDN-NCOA6	1982	NCOA6	vertebrata	Oryctolagus cuniculus	PREDICTED: nuclear receptor coactivator 6 isoform X7 [Oryctolagus cuniculus]	GCF_000003625.3	TRADDN-NCOA6
XP_017197157.1	TRADDN-NCOA6	1067	NCOA6	vertebrata	Oryctolagus cuniculus	PREDICTED: nuclear receptor coactivator 6 isoform X9 [Oryctolagus cuniculus]	GCF_000003625.3	TRADDN-NCOA6
XP_012659617.1	TRADDN-NCOA6	2054	NCOA6	vertebrata	Otolemur garnettii	nuclear receptor coactivator 6 isoform X1 [Otolemur garnettii]	GCF_000181295.1	TRADDN-NCOA6
XP_023367932.1	TRADDN-NCOA6	2053	NCOA6	vertebrata	Otolemur garnettii	nuclear receptor coactivator 6 isoform X2 [Otolemur garnettii]	GCF_000181295.1	TRADDN-NCOA6
XP_023367933.1	TRADDN-NCOA6	1954	NCOA6	vertebrata	Otolemur garnettii	nuclear receptor coactivator 6 isoform X3 [Otolemur garnettii]	GCF_000181295.1	TRADDN-NCOA6
XP_014955323.1	TRADDN-NCOA6	2043	NCOA6	vertebrata	Ovis aries	PREDICTED: nuclear receptor coactivator 6 isoform X1 [Ovis aries]	-	TRADDN-NCOA6
XP_014955325.1	TRADDN-NCOA6	2032	NCOA6	vertebrata	Ovis aries	PREDICTED: nuclear receptor coactivator 6 isoform X2 [Ovis aries]	-	TRADDN-NCOA6
XP_014955326.1	TRADDN-NCOA6	2031	NCOA6	vertebrata	Ovis aries	PREDICTED: nuclear receptor coactivator 6 isoform X3 [Ovis aries]	-	TRADDN-NCOA6
XP_011970304.1	TRADDN-NCOA6	2046	NCOA6	vertebrata	Ovis aries musimon	PREDICTED: nuclear receptor coactivator 6 isoform X1 [Ovis aries musimon]	-	TRADDN-NCOA6

acc	architecture	len	gen.name	taxend	species	defline	gca	clade
XP_014946800.1	TRADDN-NCOA6	2035	NCOA6	vertebrata	Ovis aries musimon	PREDICTED: nuclear receptor coactivator 6 isoform X2 [Ovis aries musimon]	-	TRADDN-NCOA6
XP_014946801.1	TRADDN-NCOA6	2034	NCOA6	vertebrata	Ovis aries musimon	PREDICTED: nuclear receptor coactivator 6 isoform X3 [Ovis aries musimon]	-	TRADDN-NCOA6
XP_014946805.1	TRADDN-NCOA6	1061	NCOA6	vertebrata	Ovis aries musimon	PREDICTED: nuclear receptor coactivator 6 isoform X5 [Ovis aries musimon]	-	TRADDN-NCOA6
XP_008955456.1	TRADDN-NCOA6	2070	NCOA6	vertebrata	Pan paniscus	nuclear receptor coactivator 6 isoform X1 [Pan paniscus]	GCF_000258655.2	TRADDN-NCOA6
XP_008955458.1	TRADDN-NCOA6	2069	NCOA6	vertebrata	Pan paniscus	nuclear receptor coactivator 6 isoform X2 [Pan paniscus]	GCF_000258655.2	TRADDN-NCOA6
XP_008955459.1	TRADDN-NCOA6	2059	NCOA6	vertebrata	Pan paniscus	nuclear receptor coactivator 6 isoform X3 [Pan paniscus]	GCF_000258655.2	TRADDN-NCOA6
XP_008955460.1	TRADDN-NCOA6	2058	NCOA6	vertebrata	Pan paniscus	nuclear receptor coactivator 6 isoform X4 [Pan paniscus]	GCF_000258655.2	TRADDN-NCOA6
XP_008955461.1	TRADDN-NCOA6	1077	NCOA6	vertebrata	Pan paniscus	nuclear receptor coactivator 6 isoform X6 [Pan paniscus]	GCF_000258655.2	TRADDN-NCOA6
XP_024782616.1	TRADDN-NCOA6	2041	NCOA6	vertebrata	Pan paniscus	nuclear receptor coactivator 6 isoform X5 [Pan paniscus]	GCF_000258655.2	TRADDN-NCOA6
PNI27815.1	TRADDN-NCOA6	985	CK820_G0043061	vertebrata	Pan troglodytes	NCOA6 isoform 9 [Pan troglodytes]	GCA_002880755.3	TRADDN-NCOA6
XP_001160316.2	TRADDN-NCOA6	2066	NCOA6	vertebrata	Pan troglodytes	nuclear receptor coactivator 6 isoform X1 [Pan troglodytes]	GCF_002880755.1	TRADDN-NCOA6
XP_009435351.2	TRADDN-NCOA6	2065	NCOA6	vertebrata	Pan troglodytes	nuclear receptor coactivator 6 isoform X2 [Pan troglodytes]	GCF_002880755.1	TRADDN-NCOA6
XP_009435352.2	TRADDN-NCOA6	2055	NCOA6	vertebrata	Pan troglodytes	nuclear receptor coactivator 6 isoform X3 [Pan troglodytes]	GCF_002880755.1	TRADDN-NCOA6
XP_009435353.2	TRADDN-NCOA6	2054	NCOA6	vertebrata	Pan troglodytes	nuclear receptor coactivator 6 isoform X4 [Pan troglodytes]	GCF_002880755.1	TRADDN-NCOA6
XP_009435354.2	TRADDN-NCOA6	2037	NCOA6	vertebrata	Pan troglodytes	nuclear receptor coactivator 6 isoform X5 [Pan troglodytes]	GCF_002880755.1	TRADDN-NCOA6
XP_016793230.1	TRADDN-NCOA6	1073	NCOA6	vertebrata	Pan troglodytes	nuclear receptor coactivator 6 isoform X6 [Pan troglodytes]	GCF_002880755.1	TRADDN-NCOA6
XP_019314267.1	TRADDN-NCOA6	2075	NCOA6	vertebrata	Panthera pardus	PREDICTED: nuclear receptor coactivator 6 isoform X1 [Panthera pardus]	GCF_001857705.1	TRADDN-NCOA6
XP_019314268.1	TRADDN-NCOA6	1075	NCOA6	vertebrata	Panthera pardus	PREDICTED: nuclear receptor coactivator 6 isoform X2 [Panthera pardus]	GCF_001857705.1	TRADDN-NCOA6
XP_007073664.1	TRADDN-NCOA6	2088	NCOA6	vertebrata	Panthera tigris altaica	PREDICTED: nuclear receptor coactivator 6 isoform X1 [Panthera tigris altaica]	GCF_000464555.1	TRADDN-NCOA6
XP_007073665.1	TRADDN-NCOA6	2087	NCOA6	vertebrata	Panthera tigris altaica	PREDICTED: nuclear receptor coactivator 6 isoform X2 [Panthera tigris altaica]	GCF_000464555.1	TRADDN-NCOA6
XP_007073666.1	TRADDN-NCOA6	2077	NCOA6	vertebrata	Panthera tigris altaica	PREDICTED: nuclear receptor coactivator 6 isoform X3 [Panthera tigris altaica]	GCF_000464555.1	TRADDN-NCOA6
XP_007073667.1	TRADDN-NCOA6	2066	NCOA6	vertebrata	Panthera tigris altaica	PREDICTED: nuclear receptor coactivator 6 isoform X4 [Panthera tigris altaica]	GCF_000464555.1	TRADDN-NCOA6

acc	architecture	len	gen.name	taxend	species	defline	gca	clade
XP_007073668.1	TRADDN-NCOA6	2065	NCOA6	vertebrata	Panthera tigris altaica	PREDICTED: nuclear receptor coactivator 6 isoform X5 [Panthera tigris altaica]	GCF_000464555.1	TRADDN-NCOA6
XP_007073669.1	TRADDN-NCOA6	2063	NCOA6	vertebrata	Panthera tigris altaica	PREDICTED: nuclear receptor coactivator 6 isoform X6 [Panthera tigris altaica]	GCF_000464555.1	TRADDN-NCOA6
XP_007073672.1	TRADDN-NCOA6	1094	NCOA6	vertebrata	Panthera tigris altaica	PREDICTED: nuclear receptor coactivator 6 isoform X7 [Panthera tigris altaica]	GCF_000464555.1	TRADDN-NCOA6
XP_005976899.1	TRADDN-NCOA6	2056	NCOA6	vertebrata	Pantholops hodgsonii	PREDICTED: nuclear receptor coactivator 6 [Pantholops hodgsonii]	-	TRADDN-NCOA6
XP_009214527.1	TRADDN-NCOA6	2072	NCOA6	vertebrata	Papio anubis	nuclear receptor coactivator 6 isoform X1 [Papio anubis]	GCF_000264685.3	TRADDN-NCOA6
XP_009214528.1	TRADDN-NCOA6	2071	NCOA6	vertebrata	Papio anubis	nuclear receptor coactivator 6 isoform X2 [Papio anubis]	GCF_000264685.3	TRADDN-NCOA6
XP_009214529.1	TRADDN-NCOA6	2063	NCOA6	vertebrata	Papio anubis	nuclear receptor coactivator 6 isoform X3 [Papio anubis]	GCF_000264685.3	TRADDN-NCOA6
XP_009214530.1	TRADDN-NCOA6	2061	NCOA6	vertebrata	Papio anubis	nuclear receptor coactivator 6 isoform X4 [Papio anubis]	GCF_000264685.3	TRADDN-NCOA6
XP_009214531.1	TRADDN-NCOA6	2060	NCOA6	vertebrata	Papio anubis	nuclear receptor coactivator 6 isoform X5 [Papio anubis]	GCF_000264685.3	TRADDN-NCOA6
XP_009214535.1	TRADDN-NCOA6	1077	NCOA6	vertebrata	Papio anubis	nuclear receptor coactivator 6 isoform X8 [Papio anubis]	GCF_000264685.3	TRADDN-NCOA6
XP_009214536.1	TRADDN-NCOA6	1068	NCOA6	vertebrata	Papio anubis	nuclear receptor coactivator 6 isoform X9 [Papio anubis]	GCF_000264685.3	TRADDN-NCOA6
XP_015502926.1	TRADDN-NCOA6	2033	NCOA6	vertebrata	Parus major	PREDICTED: nuclear receptor coactivator 6 [Parus major]	GCF_001522545.3	TRADDN-NCOA6
OPJ70713.1	TRADDN-NCOA6	928	NCOA6	vertebrata	Patagioenas fasciata monilis	nuclear receptor coactivator 6 [Patagioenas fasciata monilis]	GCA_002029285.1	TRADDN-NCOA6
KFQ54008.1	TRADDN-NCOA6	170	N334_04406	vertebrata	Pelecanus crispus	Nuclear receptor coactivator 6, partial [Pelecanus crispus]	GCA_000687375.1	TRADDN-NCOA6
XP_025042210.1	TRADDN-NCOA6	2025	NCOA6	vertebrata	Pelodiscus sinensis	LOW QUALITY PROTEIN: nuclear receptor coactivator 6 [Pelodiscus sinensis]	GCF_000230535.1	TRADDN-NCOA6
XP_015862217.1	TRADDN-NCOA6	1990	Ncoa6	vertebrata	Peromyscus maniculatus bairdii	PREDICTED: nuclear receptor coactivator 6 [Peromyscus maniculatus bairdii]	GCF_000500345.1	TRADDN-NCOA6
KFQ70233.1	TRADDN-NCOA6	170	N335_04326	vertebrata	Phaethon lepturus	Nuclear receptor coactivator 6, partial [Phaethon lepturus]	GCA_000687285.1	TRADDN-NCOA6
XP_010294641.1	TRADDN-NCOA6	992	LOC104627927	vertebrata	Phaethon lepturus	PREDICTED: nuclear receptor coactivator 6-like, partial [Phaethon lepturus]	GCF_000687285.1	TRADDN-NCOA6
KFW95898.1	TRADDN-NCOA6	170	N336_09921	vertebrata	Phalacrocorax carbo	Nuclear receptor coactivator 6, partial [Phalacrocorax carbo]	GCA_000708925.1	TRADDN-NCOA6
XP_020854203.1	TRADDN-NCOA6	2056	NCOA6	vertebrata	Phascolarctos cinereus	nuclear receptor coactivator 6 isoform X1 [Phascolarctos cinereus]	GCF_002099425.1	TRADDN-NCOA6
XP_020854204.1	TRADDN-NCOA6	1622	NCOA6	vertebrata	Phascolarctos cinereus	nuclear receptor coactivator 6 isoform X2 [Phascolarctos cinereus]	GCF_002099425.1	TRADDN-NCOA6
XP_020854206.1	TRADDN-NCOA6	1056	NCOA6	vertebrata	Phascolarctos cinereus	nuclear receptor coactivator 6 isoform X4 [Phascolarctos cinereus]	GCF_002099425.1	TRADDN-NCOA6
KFQ87443.1	TRADDN-NCOA6	170	N337_03435	vertebrata	Phoenicopterus ruber ruber	Nuclear receptor coactivator 6, partial [Phoenicopterus ruber ruber]	GCA_000687265.1	TRADDN-NCOA6

acc	architecture	len	gen.name	taxend	species	defline	gca	clade
XP_023984297.1	TRADDN-NCOA6	2040	NCOA6	vertebrata	Physeter catodon	nuclear receptor coactivator 6 [Physeter catodon]	-	TRADDN-NCOA6
XP_009901667.1	TRADDN-NCOA6	1866	NCOA6	vertebrata	Picoides pubescens	PREDICTED: LOW QUALITY PROTEIN: nuclear receptor coactivator 6 [Picoides pubescens]	GCF_000699005.1	TRADDN-NCOA6
XP_023076250.1	TRADDN-NCOA6	2065	NCOA6	vertebrata	Piliocolobus tephrosceles	nuclear receptor coactivator 6 isoform X1 [Piliocolobus tephrosceles]	GCF_002776525.2	TRADDN-NCOA6
XP_023076252.1	TRADDN-NCOA6	2064	NCOA6	vertebrata	Piliocolobus tephrosceles	nuclear receptor coactivator 6 isoform X2 [Piliocolobus tephrosceles]	GCF_002776525.2	TRADDN-NCOA6
XP_023076253.1	TRADDN-NCOA6	2054	NCOA6	vertebrata	Piliocolobus tephrosceles	nuclear receptor coactivator 6 isoform X3 [Piliocolobus tephrosceles]	GCF_002776525.2	TRADDN-NCOA6
XP_023076254.1	TRADDN-NCOA6	2053	NCOA6	vertebrata	Piliocolobus tephrosceles	nuclear receptor coactivator 6 isoform X4 [Piliocolobus tephrosceles]	GCF_002776525.2	TRADDN-NCOA6
XP_023076255.1	TRADDN-NCOA6	2060	NCOA6	vertebrata	Piliocolobus tephrosceles	nuclear receptor coactivator 6 isoform X5 [Piliocolobus tephrosceles]	GCF_002776525.2	TRADDN-NCOA6
XP_023076259.1	TRADDN-NCOA6	1074	NCOA6	vertebrata	Piliocolobus tephrosceles	nuclear receptor coactivator 6 isoform X7 [Piliocolobus tephrosceles]	GCF_002776525.2	TRADDN-NCOA6
XP_023076260.1	TRADDN-NCOA6	1069	NCOA6	vertebrata	Piliocolobus tephrosceles	nuclear receptor coactivator 6 isoform X8 [Piliocolobus tephrosceles]	GCF_002776525.2	TRADDN-NCOA6
KFZ66672.1	TRADDN-NCOA6	1954	N338_03763	vertebrata	Podiceps cristatus	Nuclear receptor coactivator 6, partial [Podiceps cristatus]	GCA_000699545.1	TRADDN-NCOA6
XP_020656752.1	TRADDN-NCOA6	2038	NCOA6	vertebrata	Pogona vitticeps	nuclear receptor coactivator 6 [Pogona vitticeps]	GCF_900067755.1	TRADDN-NCOA6
PNJ57496.1	TRADDN-NCOA6	980	CR201_G0018618	vertebrata	Pongo abelii	NCOA6 isoform 9 [Pongo abelii]	GCA_002880775.3	TRADDN-NCOA6
XP_024094451.1	TRADDN-NCOA6	2067	NCOA6	vertebrata	Pongo abelii	nuclear receptor coactivator 6 isoform X1 [Pongo abelii]	GCF_002880775.1	TRADDN-NCOA6
XP_024094454.1	TRADDN-NCOA6	2066	NCOA6	vertebrata	Pongo abelii	nuclear receptor coactivator 6 isoform X2 [Pongo abelii]	GCF_002880775.1	TRADDN-NCOA6
XP_024094455.1	TRADDN-NCOA6	2056	NCOA6	vertebrata	Pongo abelii	nuclear receptor coactivator 6 isoform X3 [Pongo abelii]	GCF_002880775.1	TRADDN-NCOA6
XP_024094456.1	TRADDN-NCOA6	2055	NCOA6	vertebrata	Pongo abelii	nuclear receptor coactivator 6 isoform X4 [Pongo abelii]	GCF_002880775.1	TRADDN-NCOA6
XP_024094457.1	TRADDN-NCOA6	1969	NCOA6	vertebrata	Pongo abelii	nuclear receptor coactivator 6 isoform X5 [Pongo abelii]	GCF_002880775.1	TRADDN-NCOA6
XP_024094458.1	TRADDN-NCOA6	1075	NCOA6	vertebrata	Pongo abelii	nuclear receptor coactivator 6 isoform X6 [Pongo abelii]	GCF_002880775.1	TRADDN-NCOA6
XP_012493131.1	TRADDN-NCOA6	2057	NCOA6	vertebrata	Propithecus coquereli	PREDICTED: nuclear receptor coactivator 6 isoform X1 [Propithecus coquereli]	GCF_000956105.1	TRADDN-NCOA6
XP_012493132.1	TRADDN-NCOA6	2046	NCOA6	vertebrata	Propithecus coquereli	PREDICTED: nuclear receptor coactivator 6 isoform X2 [Propithecus coquereli]	GCF_000956105.1	TRADDN-NCOA6
XP_012493133.1	TRADDN-NCOA6	2034	NCOA6	vertebrata	Propithecus coquereli	PREDICTED: nuclear receptor coactivator 6 isoform X3 [Propithecus coquereli]	GCF_000956105.1	TRADDN-NCOA6

acc	architecture	len	gen.name	taxend	species	defline	gca	clade
XP_015672481.1	TRADDN-NCOA6	2049	NCOA6	vertebrata	Protobothrops mucrosquamatus	PREDICTED: nuclear receptor coactivator 6 [Protobothrops mucrosquamatus]	GCF_001527695.2	TRADDN-NCOA6
XP_014116263.1	TRADDN-NCOA6	2033	NCOA6	vertebrata	Pseudopodoces humilis	PREDICTED: nuclear receptor coactivator 6 isoform X1 [Pseudopodoces humilis]	GCF_000331425.1	TRADDN-NCOA6
XP_014116264.1	TRADDN-NCOA6	1990	NCOA6	vertebrata	Pseudopodoces humilis	PREDICTED: nuclear receptor coactivator 6 isoform X2 [Pseudopodoces humilis]	GCF_000331425.1	TRADDN-NCOA6
XP_014116266.1	TRADDN-NCOA6	1984	NCOA6	vertebrata	Pseudopodoces humilis	PREDICTED: nuclear receptor coactivator 6 isoform X3 [Pseudopodoces humilis]	GCF_000331425.1	TRADDN-NCOA6
KFV06441.1	TRADDN-NCOA6	170	N339_08685	vertebrata	Pterocles gutturalis	Nuclear receptor coactivator 6, partial [Pterocles gutturalis]	GCA_000699245.1	TRADDN-NCOA6
XP_010071209.1	TRADDN-NCOA6	535	LOC104461352	vertebrata	Pterocles gutturalis	PREDICTED: nuclear receptor coactivator 6-like, partial [Pterocles gutturalis]	GCF_000699245.1	TRADDN-NCOA6
ELK04121.1	TRADDN-NCOA6	1475	PAL_GLEAN10024254	vertebrata	Pteropus alecto	Nuclear receptor coactivator 6 [Pteropus alecto]	GCA_000325575.1	TRADDN-NCOA6
XP_024895575.1	TRADDN-NCOA6	1061	NCOA6	vertebrata	Pteropus alecto	nuclear receptor coactivator 6 isoform X1 [Pteropus alecto]	GCF_000325575.1	TRADDN-NCOA6
XP_024895576.1	TRADDN-NCOA6	2058	NCOA6	vertebrata	Pteropus alecto	nuclear receptor coactivator 6 isoform X2 [Pteropus alecto]	GCF_000325575.1	TRADDN-NCOA6
XP_011376599.1	TRADDN-NCOA6	2058	NCOA6	vertebrata	Pteropus vampyrus	nuclear receptor coactivator 6 isoform X1 [Pteropus vampyrus]	GCF_000151845.1	TRADDN-NCOA6
XP_011376602.1	TRADDN-NCOA6	2047	NCOA6	vertebrata	Pteropus vampyrus	nuclear receptor coactivator 6 isoform X3 [Pteropus vampyrus]	GCF_000151845.1	TRADDN-NCOA6
XP_011376604.1	TRADDN-NCOA6	2046	NCOA6	vertebrata	Pteropus vampyrus	nuclear receptor coactivator 6 isoform X4 [Pteropus vampyrus]	GCF_000151845.1	TRADDN-NCOA6
XP_011376605.1	TRADDN-NCOA6	1061	NCOA6	vertebrata	Pteropus vampyrus	nuclear receptor coactivator 6 isoform X6 [Pteropus vampyrus]	GCF_000151845.1	TRADDN-NCOA6
XP_023377983.1	TRADDN-NCOA6	2047	NCOA6	vertebrata	Pteropus vampyrus	nuclear receptor coactivator 6 isoform X2 [Pteropus vampyrus]	GCF_000151845.1	TRADDN-NCOA6
XP_023377984.1	TRADDN-NCOA6	2035	NCOA6	vertebrata	Pteropus vampyrus	nuclear receptor coactivator 6 isoform X5 [Pteropus vampyrus]	GCF_000151845.1	TRADDN-NCOA6
XP_009327609.1	TRADDN-NCOA6	2045	NCOA6	vertebrata	Pygoscelis adeliae	PREDICTED: LOW QUALITY PROTEIN: nuclear receptor coactivator 6 [Pygoscelis adeliae]	GCF_000699105.1	TRADDN-NCOA6
XP_025023946.1	TRADDN-NCOA6	2043	NCOA6	vertebrata	Python bivittatus	nuclear receptor coactivator 6 [Python bivittatus]	GCF_000186305.1	TRADDN-NCOA6
PIN98361.1	TRADDN-NCOA6	912	AB205_0179540	vertebrata	Rana catesbeiana	hypothetical protein AB205_0179540, partial [Rana catesbeiana]	GCA_002284835.2	TRADDN-NCOA6
NP_001263643.1	TRADDN-NCOA6	2058	Aib3,NRC,PRIP,RAP250,Trbp	vertebrata	Rattus norvegicus	nuclear receptor coactivator 6 [Rattus norvegicus]	GCF_000002265.2	TRADDN-NCOA6
XP_008760518.1	TRADDN-NCOA6	2057	Aib3,NRC,PRIP,RAP250,Trbp	vertebrata	Rattus norvegicus	PREDICTED: nuclear receptor coactivator 6 isoform X2 [Rattus norvegicus]	GCF_000001895.5	TRADDN-NCOA6
XP_008760519.1	TRADDN-NCOA6	2047	Aib3,NRC,PRIP,RAP250,Trbp	vertebrata	Rattus norvegicus	PREDICTED: nuclear receptor coactivator 6 isoform X3 [Rattus norvegicus]	GCF_000001895.5	TRADDN-NCOA6

acc	architecture	len	gen.name	taxend	species	defline	gca	clade
XP_008760520.1	TRADDN-NCOA6	2046	Aib3,NRC,PRIP,RAP250,Trbp	vertebrata	Rattus norvegicus	PREDICTED: nuclear receptor coactivator 6 isoform X4 [Rattus norvegicus]	GCF_000001895.5	TRADDN-NCOA6
XP_008760521.1	TRADDN-NCOA6	1066	Aib3,NRC,PRIP,RAP250,Trbp	vertebrata	Rattus norvegicus	PREDICTED: nuclear receptor coactivator 6 isoform X5 [Rattus norvegicus]	GCF_000001895.5	TRADDN-NCOA6
XP_020371347.1	TRADDN-NCOA6	2026	ncoa6	vertebrata	Rhincodon typus	nuclear receptor coactivator 6 isoform X1 [Rhincodon typus]	GCF_001642345.1	TRADDN-NCOA6
XP_020371348.1	TRADDN-NCOA6	2025	ncoa6	vertebrata	Rhincodon typus	nuclear receptor coactivator 6 isoform X2 [Rhincodon typus]	GCF_001642345.1	TRADDN-NCOA6
XP_020371349.1	TRADDN-NCOA6	1857	ncoa6	vertebrata	Rhincodon typus	nuclear receptor coactivator 6 isoform X3 [Rhincodon typus]	GCF_001642345.1	TRADDN-NCOA6
XP_020371350.1	TRADDN-NCOA6	1856	ncoa6	vertebrata	Rhincodon typus	nuclear receptor coactivator 6 isoform X4 [Rhincodon typus]	GCF_001642345.1	TRADDN-NCOA6
XP_019589097.1	TRADDN-NCOA6	2067	NCOA6	vertebrata	Rhinolophus sinicus	PREDICTED: nuclear receptor coactivator 6 isoform X1 [Rhinolophus sinicus]	-	TRADDN-NCOA6
XP_019589126.1	TRADDN-NCOA6	2066	NCOA6	vertebrata	Rhinolophus sinicus	PREDICTED: nuclear receptor coactivator 6 isoform X2 [Rhinolophus sinicus]	-	TRADDN-NCOA6
XP_019589136.1	TRADDN-NCOA6	2056	NCOA6	vertebrata	Rhinolophus sinicus	PREDICTED: nuclear receptor coactivator 6 isoform X3 [Rhinolophus sinicus]	-	TRADDN-NCOA6
XP_019589146.1	TRADDN-NCOA6	2055	NCOA6	vertebrata	Rhinolophus sinicus	PREDICTED: nuclear receptor coactivator 6 isoform X4 [Rhinolophus sinicus]	-	TRADDN-NCOA6
XP_019589157.1	TRADDN-NCOA6	1967	NCOA6	vertebrata	Rhinolophus sinicus	PREDICTED: nuclear receptor coactivator 6 isoform X5 [Rhinolophus sinicus]	-	TRADDN-NCOA6
XP_017734234.1	TRADDN-NCOA6	2062	NCOA6	vertebrata	Rhinopithecus bieti	PREDICTED: nuclear receptor coactivator 6 isoform X1 [Rhinopithecus bieti]	GCF_001698545.1	TRADDN-NCOA6
XP_017734242.1	TRADDN-NCOA6	2061	NCOA6	vertebrata	Rhinopithecus bieti	PREDICTED: nuclear receptor coactivator 6 isoform X2 [Rhinopithecus bieti]	GCF_001698545.1	TRADDN-NCOA6
XP_017734251.1	TRADDN-NCOA6	2051	NCOA6	vertebrata	Rhinopithecus bieti	PREDICTED: nuclear receptor coactivator 6 isoform X3 [Rhinopithecus bieti]	GCF_001698545.1	TRADDN-NCOA6
XP_017734255.1	TRADDN-NCOA6	2050	NCOA6	vertebrata	Rhinopithecus bieti	PREDICTED: nuclear receptor coactivator 6 isoform X4 [Rhinopithecus bieti]	GCF_001698545.1	TRADDN-NCOA6
XP_017734258.1	TRADDN-NCOA6	1974	NCOA6	vertebrata	Rhinopithecus bieti	PREDICTED: nuclear receptor coactivator 6 isoform X5 [Rhinopithecus bieti]	GCF_001698545.1	TRADDN-NCOA6
XP_017734266.1	TRADDN-NCOA6	1070	NCOA6	vertebrata	Rhinopithecus bieti	PREDICTED: nuclear receptor coactivator 6 isoform X6 [Rhinopithecus bieti]	GCF_001698545.1	TRADDN-NCOA6
XP_010384811.1	TRADDN-NCOA6	2063	NCOA6	vertebrata	Rhinopithecus roxellana	PREDICTED: nuclear receptor coactivator 6 isoform X1 [Rhinopithecus roxellana]	-	TRADDN-NCOA6

acc	architecture	len	gen.name	taxend	species	defline	gca	clade
XP_010384812.1	TRADDN-NCOA6	2062	NCOA6	vertebrata	Rhinopithecus roxellana	PREDICTED: nuclear receptor coactivator 6 isoform X2 [Rhinopithecus roxellana]	-	TRADDN-NCOA6
XP_015981895.1	TRADDN-NCOA6	2060	NCOA6	vertebrata	Rousettus aegyptiacus	PREDICTED: nuclear receptor coactivator 6 isoform X1 [Rousettus aegyptiacus]	GCF_001466805.2	TRADDN-NCOA6
XP_015981903.1	TRADDN-NCOA6	2049	NCOA6	vertebrata	Rousettus aegyptiacus	PREDICTED: nuclear receptor coactivator 6 isoform X2 [Rousettus aegyptiacus]	GCF_001466805.2	TRADDN-NCOA6
XP_015981904.1	TRADDN-NCOA6	2049	NCOA6	vertebrata	Rousettus aegyptiacus	PREDICTED: nuclear receptor coactivator 6 isoform X3 [Rousettus aegyptiacus]	GCF_001466805.2	TRADDN-NCOA6
XP_015981905.1	TRADDN-NCOA6	2048	NCOA6	vertebrata	Rousettus aegyptiacus	PREDICTED: nuclear receptor coactivator 6 isoform X4 [Rousettus aegyptiacus]	GCF_001466805.2	TRADDN-NCOA6
XP_015981906.1	TRADDN-NCOA6	1960	NCOA6	vertebrata	Rousettus aegyptiacus	PREDICTED: nuclear receptor coactivator 6 isoform X5 [Rousettus aegyptiacus]	GCF_001466805.2	TRADDN-NCOA6
XP_015981908.1	TRADDN-NCOA6	1061	NCOA6	vertebrata	Rousettus aegyptiacus	PREDICTED: nuclear receptor coactivator 6 isoform X7 [Rousettus aegyptiacus]	GCF_001466805.2	TRADDN-NCOA6
XP_010340981.1	TRADDN-NCOA6	2065	NCOA6	vertebrata	Saimiri boliviensis boliviensis	PREDICTED: nuclear receptor coactivator 6 isoform X1 [Saimiri boliviensis boliviensis]	GCF_000235385.1	TRADDN-NCOA6
XP_010340983.1	TRADDN-NCOA6	2064	NCOA6	vertebrata	Saimiri boliviensis boliviensis	PREDICTED: nuclear receptor coactivator 6 isoform X2 [Saimiri boliviensis boliviensis]	GCF_000235385.1	TRADDN-NCOA6
XP_010340984.1	TRADDN-NCOA6	2053	NCOA6	vertebrata	Saimiri boliviensis boliviensis	PREDICTED: nuclear receptor coactivator 6 isoform X3 [Saimiri boliviensis boliviensis]	GCF_000235385.1	TRADDN-NCOA6
XP_003756975.1	TRADDN-NCOA6	2063	NCOA6	vertebrata	Sarcophilus harrisii	nuclear receptor coactivator 6 isoform X1 [Sarcophilus harrisii]	GCF_000189315.1	TRADDN-NCOA6
XP_012408688.1	TRADDN-NCOA6	1695	NCOA6	vertebrata	Sarcophilus harrisii	nuclear receptor coactivator 6 isoform X3 [Sarcophilus harrisii]	GCF_000189315.1	TRADDN-NCOA6
XP_023362736.1	TRADDN-NCOA6	1915	NCOA6	vertebrata	Sarcophilus harrisii	nuclear receptor coactivator 6 isoform X2 [Sarcophilus harrisii]	GCF_000189315.1	TRADDN-NCOA6
XP_018779346.1	TRADDN-NCOA6	1058	NCOA6	vertebrata	Serinus canaria	PREDICTED: nuclear receptor coactivator 6 [Serinus canaria]	-	TRADDN-NCOA6
XP_004612606.1	TRADDN-NCOA6	2057	NCOA6	vertebrata	Sorex araneus	PREDICTED: nuclear receptor coactivator 6 isoform X1 [Sorex araneus]	GCF_000181275.1	TRADDN-NCOA6
XP_004612607.1	TRADDN-NCOA6	1055	NCOA6	vertebrata	Sorex araneus	PREDICTED: nuclear receptor coactivator 6 isoform X2 [Sorex araneus]	GCF_000181275.1	TRADDN-NCOA6
KFV83564.1	TRADDN-NCOA6	2033	N308_06379	vertebrata	Struthio camelus australis	Nuclear receptor coactivator 6 [Struthio camelus australis]	GCA_000698965.1	TRADDN-NCOA6
XP_009677514.1	TRADDN-NCOA6	2055	NCOA6	vertebrata	Struthio camelus australis	PREDICTED: nuclear receptor coactivator 6 isoform X1 [Struthio camelus australis]	GCF_000698965.1	TRADDN-NCOA6
XP_009677516.1	TRADDN-NCOA6	2054	NCOA6	vertebrata	Struthio camelus australis	PREDICTED: nuclear receptor coactivator 6 isoform X2 [Struthio camelus australis]	GCF_000698965.1	TRADDN-NCOA6

acc	architecture	len	gen.name	taxend	species	defline	gca	clade
XP_009677517.1	TRADDN-NCOA6	1993	NCOA6	vertebrata	Struthio camelus australis	PREDICTED: nuclear receptor coactivator 6 isoform X3 [Struthio camelus australis]	GCF_000698965.1	TRADDN-NCOA6
XP_009677518.1	TRADDN-NCOA6	1985	NCOA6	vertebrata	Struthio camelus australis	PREDICTED: nuclear receptor coactivator 6 isoform X4 [Struthio camelus australis]	GCF_000698965.1	TRADDN-NCOA6
XP_009677519.1	TRADDN-NCOA6	1783	NCOA6	vertebrata	Struthio camelus australis	PREDICTED: nuclear receptor coactivator 6 isoform X5 [Struthio camelus australis]	GCF_000698965.1	TRADDN-NCOA6
XP_014741744.1	TRADDN-NCOA6	2051	NCOA6	vertebrata	Sturnus vulgaris	PREDICTED: nuclear receptor coactivator 6 isoform X1 [Sturnus vulgaris]	GCF_001447265.1	TRADDN-NCOA6
XP_014741745.1	TRADDN-NCOA6	2038	NCOA6	vertebrata	Sturnus vulgaris	PREDICTED: nuclear receptor coactivator 6 isoform X2 [Sturnus vulgaris]	GCF_001447265.1	TRADDN-NCOA6
XP_013840750.1	TRADDN-NCOA6	2061	NCOA6	vertebrata	Sus scrofa	nuclear receptor coactivator 6 isoform X1 [Sus scrofa]	GCF_000003025.6	TRADDN-NCOA6
XP_020933952.1	TRADDN-NCOA6	2060	NCOA6	vertebrata	Sus scrofa	nuclear receptor coactivator 6 isoform X2 [Sus scrofa]	GCF_000003025.6	TRADDN-NCOA6
XP_020933953.1	TRADDN-NCOA6	2050	NCOA6	vertebrata	Sus scrofa	nuclear receptor coactivator 6 isoform X3 [Sus scrofa]	GCF_000003025.6	TRADDN-NCOA6
XP_020933956.1	TRADDN-NCOA6	2049	NCOA6	vertebrata	Sus scrofa	nuclear receptor coactivator 6 isoform X4 [Sus scrofa]	GCF_000003025.6	TRADDN-NCOA6
XP_020933958.1	TRADDN-NCOA6	1736	NCOA6	vertebrata	Sus scrofa	nuclear receptor coactivator 6 isoform X5 [Sus scrofa]	GCF_000003025.6	TRADDN-NCOA6
XP_004175601.1	TRADDN-NCOA6	213	LOC101233431	vertebrata	Taeniopygia guttata	PREDICTED: nuclear receptor coactivator 6-like, partial [Taeniopygia guttata]	-	TRADDN-NCOA6
XP_012425613.1	TRADDN-NCOA6	1047	NCOA6	vertebrata	Taeniopygia guttata	PREDICTED: nuclear receptor coactivator 6 [Taeniopygia guttata]	-	TRADDN-NCOA6
KFV06893.1	TRADDN-NCOA6	1877	N340_13206	vertebrata	Tauraco erythrolophus	Nuclear receptor coactivator 6, partial [Tauraco erythrolophus]	GCA_000709365.1	TRADDN-NCOA6
XP_009990530.1	TRADDN-NCOA6	1864	NCOA6	vertebrata	Tauraco erythrolophus	PREDICTED: nuclear receptor coactivator 6, partial [Tauraco erythrolophus]	GCF_000709365.1	TRADDN-NCOA6
XP_024051101.1	TRADDN-NCOA6	2020	NCOA6	vertebrata	Terrapene mexicana triunguis	nuclear receptor coactivator 6 isoform X1 [Terrapene mexicana triunguis]	-	TRADDN-NCOA6
XP_024051103.1	TRADDN-NCOA6	1748	NCOA6	vertebrata	Terrapene mexicana triunguis	nuclear receptor coactivator 6 isoform X2 [Terrapene mexicana triunguis]	-	TRADDN-NCOA6
XP_013908633.1	TRADDN-NCOA6	2049	NCOA6	vertebrata	Thamnophis sirtalis	PREDICTED: nuclear receptor coactivator 6 [Thamnophis sirtalis]	GCF_001077635.1	TRADDN-NCOA6
XP_025254383.1	TRADDN-NCOA6	2065	NCOA6	vertebrata	Theropithecus gelada	nuclear receptor coactivator 6 [Theropithecus gelada]	GCF_003255815.1	TRADDN-NCOA6
XP_010223929.1	TRADDN-NCOA6	1044	NCOA6	vertebrata	Tinamus guttatus	PREDICTED: nuclear receptor coactivator 6 [Tinamus guttatus]	GCF_000705375.1	TRADDN-NCOA6
XP_004370473.1	TRADDN-NCOA6	2051	LOC101354990	vertebrata	Trichechus manatus latirostris	nuclear receptor coactivator 6 isoform X1 [Trichechus manatus latirostris]	GCF_000243295.1	TRADDN-NCOA6

acc	architecture	len	gen.name	taxend	species	defline	gca	clade
XP_023580929.1	TRADDN-NCOA6	2050	LOC101354990	vertebrata	Trichechus manatus latirostris	nuclear receptor coactivator 6 isoform X2 [Trichechus manatus latirostris]	GCF_000243295.1	TRADDN-NCOA6
ELW69582.1	TRADDN-NCOA6	2038	TREES_T100008603	vertebrata	Tupaia chinensis	Nuclear receptor coactivator 6 [Tupaia chinensis]	GCA_000334495.1	TRADDN-NCOA6
XP_006144094.2	TRADDN-NCOA6	2068	NCOA6	vertebrata	Tupaia chinensis	PREDICTED: nuclear receptor coactivator 6 isoform X3 [Tupaia chinensis]	-	TRADDN-NCOA6
XP_006144095.2	TRADDN-NCOA6	2067	NCOA6	vertebrata	Tupaia chinensis	PREDICTED: nuclear receptor coactivator 6 isoform X4 [Tupaia chinensis]	-	TRADDN-NCOA6
XP_006144096.1	TRADDN-NCOA6	2064	NCOA6	vertebrata	Tupaia chinensis	PREDICTED: nuclear receptor coactivator 6 isoform X5 [Tupaia chinensis]	-	TRADDN-NCOA6
XP_006144097.1	TRADDN-NCOA6	2075	NCOA6	vertebrata	Tupaia chinensis	PREDICTED: nuclear receptor coactivator 6 isoform X6 [Tupaia chinensis]	GCF_000334495.1	TRADDN-NCOA6
XP_006144098.1	TRADDN-NCOA6	1077	NCOA6	vertebrata	Tupaia chinensis	PREDICTED: nuclear receptor coactivator 6 isoform X7 [Tupaia chinensis]	GCF_000334495.1	TRADDN-NCOA6
XP_014441611.1	TRADDN-NCOA6	2079	NCOA6	vertebrata	Tupaia chinensis	PREDICTED: nuclear receptor coactivator 6 isoform X1 [Tupaia chinensis]	-	TRADDN-NCOA6
XP_019806240.1	TRADDN-NCOA6	2039	NCOA6	vertebrata	Tursiops truncatus	PREDICTED: nuclear receptor coactivator 6 isoform X1 [Tursiops truncatus]	GCF_001922835.1	TRADDN-NCOA6
XP_019806245.1	TRADDN-NCOA6	2038	NCOA6	vertebrata	Tursiops truncatus	PREDICTED: nuclear receptor coactivator 6 isoform X2 [Tursiops truncatus]	GCF_001922835.1	TRADDN-NCOA6
XP_019806246.1	TRADDN-NCOA6	2028	NCOA6	vertebrata	Tursiops truncatus	PREDICTED: nuclear receptor coactivator 6 isoform X3 [Tursiops truncatus]	GCF_001922835.1	TRADDN-NCOA6
XP_019806248.1	TRADDN-NCOA6	2027	NCOA6	vertebrata	Tursiops truncatus	PREDICTED: nuclear receptor coactivator 6 isoform X4 [Tursiops truncatus]	GCF_001922835.1	TRADDN-NCOA6
XP_019806249.1	TRADDN-NCOA6	1996	NCOA6	vertebrata	Tursiops truncatus	PREDICTED: nuclear receptor coactivator 6 isoform X5 [Tursiops truncatus]	GCF_001922835.1	TRADDN-NCOA6
XP_019806252.1	TRADDN-NCOA6	1759	NCOA6	vertebrata	Tursiops truncatus	PREDICTED: nuclear receptor coactivator 6 isoform X7 [Tursiops truncatus]	GCF_001922835.1	TRADDN-NCOA6
XP_019806253.1	TRADDN-NCOA6	1716	NCOA6	vertebrata	Tursiops truncatus	PREDICTED: nuclear receptor coactivator 6 isoform X8 [Tursiops truncatus]	GCF_001922835.1	TRADDN-NCOA6
XP_019806254.1	TRADDN-NCOA6	1710	NCOA6	vertebrata	Tursiops truncatus	PREDICTED: nuclear receptor coactivator 6 isoform X9 [Tursiops truncatus]	GCF_001922835.1	TRADDN-NCOA6
XP_019806255.1	TRADDN-NCOA6	1705	NCOA6	vertebrata	Tursiops truncatus	PREDICTED: nuclear receptor coactivator 6 isoform X10 [Tursiops truncatus]	GCF_001922835.1	TRADDN-NCOA6

acc	architecture	len	gen.name	taxend	species	defline	gca	clade
XP_019806256.1	TRADDN-NCOA6	1675	NCOA6	vertebrata	Tursiops truncatus	PREDICTED: nuclear receptor coactivator 6 isoform X11 [Tursiops truncatus]	GCF_001922835.1	TRADDN-NCOA6
XP_019806257.1	TRADDN-NCOA6	1667	NCOA6	vertebrata	Tursiops truncatus	PREDICTED: nuclear receptor coactivator 6 isoform X12 [Tursiops truncatus]	GCF_001922835.1	TRADDN-NCOA6
XP_019806258.1	TRADDN-NCOA6	1055	NCOA6	vertebrata	Tursiops truncatus	PREDICTED: nuclear receptor coactivator 6 isoform X13 [Tursiops truncatus]	GCF_001922835.1	TRADDN-NCOA6
KFV49966.1	TRADDN-NCOA6	170	N341_06987	vertebrata	Tyto alba	Nuclear receptor coactivator 6, partial [Tyto alba]	GCA_000687205.1	TRADDN-NCOA6
XP_008696391.1	TRADDN-NCOA6	2057	NCOA6	vertebrata	Ursus maritimus	PREDICTED: nuclear receptor coactivator 6 isoform X1 [Ursus maritimus]	GCF_000687225.1	TRADDN-NCOA6
XP_008696392.1	TRADDN-NCOA6	2045	NCOA6	vertebrata	Ursus maritimus	PREDICTED: nuclear receptor coactivator 6 isoform X2 [Ursus maritimus]	GCF_000687225.1	TRADDN-NCOA6
XP_008696393.1	TRADDN-NCOA6	1680	NCOA6	vertebrata	Ursus maritimus	PREDICTED: nuclear receptor coactivator 6 isoform X3 [Ursus maritimus]	GCF_000687225.1	TRADDN-NCOA6
XP_008696394.1	TRADDN-NCOA6	1060	NCOA6	vertebrata	Ursus maritimus	PREDICTED: nuclear receptor coactivator 6 isoform X4 [Ursus maritimus]	GCF_000687225.1	TRADDN-NCOA6
XP_006202687.1	TRADDN-NCOA6	2055	NCOA6	vertebrata	Vicugna pacos	PREDICTED: nuclear receptor coactivator 6 isoform X1 [Vicugna pacos]	GCF_000164845.1	TRADDN-NCOA6
XP_015093358.1	TRADDN-NCOA6	2054	NCOA6	vertebrata	Vicugna pacos	PREDICTED: nuclear receptor coactivator 6 isoform X2 [Vicugna pacos]	GCF_000164845.1	TRADDN-NCOA6
XP_015093359.1	TRADDN-NCOA6	2044	NCOA6	vertebrata	Vicugna pacos	PREDICTED: nuclear receptor coactivator 6 isoform X3 [Vicugna pacos]	GCF_000164845.1	TRADDN-NCOA6
XP_015093360.1	TRADDN-NCOA6	2043	NCOA6	vertebrata	Vicugna pacos	PREDICTED: nuclear receptor coactivator 6 isoform X4 [Vicugna pacos]	GCF_000164845.1	TRADDN-NCOA6
OCT62832.1	TRADDN-NCOA6	1931	XELAEV_18043923mg	vertebrata	Xenopus laevis	hypothetical protein XELAEV_18043923mg [Xenopus laevis]	GCA_001663975.1	TRADDN-NCOA6
XP_018092310.1	TRADDN-NCOA6	1917	LOC108701802	vertebrata	Xenopus laevis	PREDICTED: nuclear receptor coactivator 6-like [Xenopus laevis]	GCF_001663975.1	TRADDN-NCOA6
XP_018097668.1	TRADDN-NCOA6	1907	LOC108705342	vertebrata	Xenopus laevis	PREDICTED: nuclear receptor coactivator 6-like isoform X1 [Xenopus laevis]	GCF_001663975.1	TRADDN-NCOA6
XP_018097669.1	TRADDN-NCOA6	1906	LOC108705342	vertebrata	Xenopus laevis	PREDICTED: nuclear receptor coactivator 6-like isoform X2 [Xenopus laevis]	GCF_001663975.1	TRADDN-NCOA6
XP_018097670.1	TRADDN-NCOA6	1906	LOC108705342	vertebrata	Xenopus laevis	PREDICTED: nuclear receptor coactivator 6-like isoform X3 [Xenopus laevis]	GCF_001663975.1	TRADDN-NCOA6
XP_018097671.1	TRADDN-NCOA6	1884	LOC108705342	vertebrata	Xenopus laevis	PREDICTED: nuclear receptor coactivator 6-like isoform X4 [Xenopus laevis]	GCF_001663975.1	TRADDN-NCOA6

acc	architecture	len	gen.name	taxend	species	defline	gca	clade
XP_018097672.1	TRADDN-NCOA6	1863	LOC108705342	vertebrata	Xenopus laevis	PREDICTED: nuclear receptor coactivator 6-like isoform X5 [Xenopus laevis]	GCF_001663975.1	TRADDN-NCOA6
XP_018097673.1	TRADDN-NCOA6	1840	LOC108705342	vertebrata	Xenopus laevis	PREDICTED: nuclear receptor coactivator 6-like isoform X6 [Xenopus laevis]	GCF_001663975.1	TRADDN-NCOA6
XP_018097674.1	TRADDN-NCOA6	1905	LOC108705342	vertebrata	Xenopus laevis	PREDICTED: nuclear receptor coactivator 6-like isoform X7 [Xenopus laevis]	GCF_001663975.1	TRADDN-NCOA6
OCA21180.1	TRADDN-NCOA6	167	XENTR_v900254112mg	vertebrata	Xenopus tropicalis	hypothetical protein XENTR_v900254112mg, partial [Xenopus tropicalis]	GCA_000004195.3	TRADDN-NCOA6
XP_002932921.2	TRADDN-NCOA6	1919	ncoa6	vertebrata	Xenopus tropicalis	PREDICTED: nuclear receptor coactivator 6 isoform X3 [Xenopus tropicalis]	GCF_000004195.3	TRADDN-NCOA6
XP_004918586.1	TRADDN-NCOA6	1920	ncoa6	vertebrata	Xenopus tropicalis	PREDICTED: nuclear receptor coactivator 6 isoform X1 [Xenopus tropicalis]	GCF_000004195.3	TRADDN-NCOA6
XP_004918587.1	TRADDN-NCOA6	1919	ncoa6	vertebrata	Xenopus tropicalis	PREDICTED: nuclear receptor coactivator 6 isoform X2 [Xenopus tropicalis]	GCF_000004195.3	TRADDN-NCOA6
XP_014128366.1	TRADDN-NCOA6	2044	NCOA6	vertebrata	Zonotrichia albicollis	PREDICTED: nuclear receptor coactivator 6 [Zonotrichia albicollis]	-	TRADDN-NCOA6
BAG63082.1	TRADD_N	150	-	vertebrata	Homo sapiens	unnamed protein product [Homo sapiens]	-	TRADD-N
XP_010716234.1	TRADD_N	150	LOC100542387	vertebrata	Meleagris gallopavo	PREDICTED: tumor necrosis factor receptor type 1-associated DEATH domain protein-like [Meleagris gallopavo]	GCF_000146605.2	TRADD-N
ETE56722.1	TRADD_N	143	TRADD	vertebrata	Ophiophagus hannah	Tumor necrosis factor receptor type 1-associated DEATH domain protein, partial [Ophiophagus hannah]	GCA_000516915.1	TRADD-N
XP_001510463.1	TRADD_N	110	TRADD	vertebrata	Ornithorhynchus anatinus	PREDICTED: tumor necrosis factor receptor type 1-associated DEATH domain protein, partial [Ornithorhynchus anatinus]	-	TRADD-N
XP_020371013.1	TRADD_N	184	LOC109915754	vertebrata	Rhincodon typus	tumor necrosis factor receptor type 1-associated DEATH domain protein-like [Rhincodon typus]	GCF_001642345.1	TRADD-N
VCW85474.1	TRADD_N(fragments)	94	BN2614_LOCUS10	vertebrata	Gulo gulo	unnamed protein product [Gulo gulo]	GCA_900006375.2	TRADD-N
AAH28542.1	TRADD_N(fragments)	58	Tradd	vertebrata	Mus musculus	Tradd protein [Mus musculus]	-	TRADD-N
KFP80105.1	TRADD_N+Death	307	N310_08418	vertebrata	Acanthisitta chloris	Tumor necrosis factor receptor type 1-associated DEATH domain protein, partial [Acanthisitta chloris]	GCA_000695815.1	TRADD-N
XP_009072859.1	TRADD_N+Death	306	TRADD	vertebrata	Acanthisitta chloris	PREDICTED: tumor necrosis factor receptor type 1-associated DEATH domain protein [Acanthisitta chloris]	GCF_000695815.1	TRADD-N
XP_014923740.1	TRADD_N+Death	312	TRADD	vertebrata	Acinonyx jubatus	tumor necrosis factor receptor type 1-associated DEATH domain protein [Acinonyx jubatus]	GCF_003709585.1	TRADD-N

acc	architecture	len	gen.name	taxend	species	defline	gca	clade
XP_011222212.1	TRADD_N+Death	311	TRADD	vertebrata	Ailuropoda melanoleuca	PREDICTED: tumor necrosis factor receptor type 1-associated DEATH domain protein isoform X3 [Ailuropoda melanoleuca]	GCF_000004335.2	TRADD-N
XP_019654400.1	TRADD_N+Death	388	TRADD	vertebrata	Ailuropoda melanoleuca	PREDICTED: tumor necrosis factor receptor type 1-associated DEATH domain protein isoform X1 [Ailuropoda melanoleuca]	GCF_000004335.2	TRADD-N
XP_019654401.1	TRADD_N+Death	319	TRADD	vertebrata	Ailuropoda melanoleuca	PREDICTED: tumor necrosis factor receptor type 1-associated DEATH domain protein isoform X2 [Ailuropoda melanoleuca]	GCF_000004335.2	TRADD-N
KYO25425.1	TRADD_N+Death	336	TRADD	vertebrata	Alligator mississippiensis	tumor necrosis factor receptor type 1-associated DEATH domain protein [Alligator mississippiensis]	GCA_000281125.4	TRADD-N
XP_006268229.2	TRADD_N+Death	304	TRADD	vertebrata	Alligator mississippiensis	PREDICTED: tumor necrosis factor receptor type 1-associated DEATH domain protein [Alligator mississippiensis]	GCF_000281125.3	TRADD-N
XP_006019109.1	TRADD_N+Death	304	B3GNT9	vertebrata	Alligator sinensis	UDP-GlcNAc:betaGal beta-1,3-N-acetylglucosaminyltransferase 9 isoform X2 [Alligator sinensis]	GCF_000455745.1	TRADD-N
KQK84207.1	TRADD_N+Death	304	AAES_51189	vertebrata	Amazona aestiva	Tumor necrosis factor receptor type 1-associated DEATH domain protein [Amazona aestiva]	GCA_001420675.1	TRADD-N
EOA96918.1	TRADD_N+Death	303	Anapl_06541	vertebrata	Anas platyrhynchos	Tumor necrosis factor receptor type 1-associated DEATH domain protein, partial [Anas platyrhynchos]	GCA_000355885.1	TRADD-N
XP_027322284.1	TRADD_N+Death	429	TRADD	vertebrata	Anas platyrhynchos	tumor necrosis factor receptor type 1-associated DEATH domain protein isoform X1 [Anas platyrhynchos]	GCF_003850225.1	TRADD-N
XP_027322285.1	TRADD_N+Death	303	TRADD	vertebrata	Anas platyrhynchos	tumor necrosis factor receptor type 1-associated DEATH domain protein isoform X2 [Anas platyrhynchos]	GCF_003850225.1	TRADD-N
XP_008109942.1	TRADD_N+Death	327	tradd	vertebrata	Anolis carolinensis	PREDICTED: tumor necrosis factor receptor type 1-associated DEATH domain protein [Anolis carolinensis]	GCF_000090745.1	TRADD-N
XP_013047861.1	TRADD_N+Death	303	TRADD	vertebrata	Anser cygnoides domesticus	PREDICTED: tumor necrosis factor receptor type 1-associated DEATH domain protein isoform X1 [Anser cygnoides domesticus]	GCF_000971095.1	TRADD-N
XP_013047863.1	TRADD_N+Death	265	TRADD	vertebrata	Anser cygnoides domesticus	PREDICTED: tumor necrosis factor receptor type 1-associated DEATH domain protein isoform X2 [Anser cygnoides domesticus]	GCF_000971095.1	TRADD-N
KFZ52403.1	TRADD_N+Death	307	N321_09123	vertebrata	Antrostomus carolinensis	Tumor necrosis factor receptor type 1-associated DEATH domain protein, partial [Antrostomus carolinensis]	GCA_000700745.1	TRADD-N

acc	architecture	len	gen.name	taxend	species	defline	gca	clade
XP_010176330.1	TRADD_N+Death	306	TRADD	vertebrata	Antrostomus carolinensis	PREDICTED: tumor necrosis factor receptor type 1-associated DEATH domain protein [Antrostomus carolinensis]	GCF_000700745.1	TRADD-N
XP_012323680.1	TRADD_N+Death	350	TRADD	vertebrata	Aotus nancymaae	tumor necrosis factor receptor type 1-associated DEATH domain protein isoform X1 [Aotus nancymaae]	GCF_000952055.2	TRADD-N
XP_012323681.1	TRADD_N+Death	312	TRADD	vertebrata	Aotus nancymaae	tumor necrosis factor receptor type 1-associated DEATH domain protein isoform X2 [Aotus nancymaae]	GCF_000952055.2	TRADD-N
KFP90336.1	TRADD_N+Death	307	N311_08319	vertebrata	Apaloderma vittatum	Tumor necrosis factor receptor type 1-associated DEATH domain protein, partial [Apaloderma vittatum]	GCA_000703405.1	TRADD-N
XP_009870638.1	TRADD_N+Death	306	TRADD	vertebrata	Apaloderma vittatum	PREDICTED: tumor necrosis factor receptor type 1-associated DEATH domain protein [Apaloderma vittatum]	GCF_000703405.1	TRADD-N
KFM05840.1	TRADD_N+Death	307	AS27_09169	vertebrata	Aptenodytes forsteri	Tumor necrosis factor receptor type 1-associated DEATH domain protein, partial [Aptenodytes forsteri]	GCA_000699145.1	TRADD-N
XP_009275028.1	TRADD_N+Death	306	TRADD	vertebrata	Aptenodytes forsteri	PREDICTED: tumor necrosis factor receptor type 1-associated DEATH domain protein [Aptenodytes forsteri]	GCF_000699145.1	TRADD-N
XP_013796480.1	TRADD_N+Death	307	TRADD	vertebrata	Apteryx australis mantelli	PREDICTED: tumor necrosis factor receptor type 1-associated DEATH domain protein [Apteryx australis mantelli]	GCF_001039765.1	TRADD-N
XP_025943956.1	TRADD_N+Death	314	TRADD	vertebrata	Apteryx rowi	tumor necrosis factor receptor type 1-associated DEATH domain protein [Apteryx rowi]	GCF_003343035.1	TRADD-N
XP_011572262.1	TRADD_N+Death	306	TRADD	vertebrata	Aquila chrysaetos canadensis	PREDICTED: tumor necrosis factor receptor type 1-associated DEATH domain protein [Aquila chrysaetos canadensis]	GCF_000766835.1	TRADD-N
XP_026712680.1	TRADD_N+Death	306	TRADD	vertebrata	Athene cunicularia	tumor necrosis factor receptor type 1-associated DEATH domain protein [Athene cunicularia]	GCF_003259725.1	TRADD-N
XP_007198138.1	TRADD_N+Death	343	TRADD	vertebrata	Balaenoptera acutorostrata scammoni	PREDICTED: tumor necrosis factor receptor type 1-associated DEATH domain protein isoform X1 [Balaenoptera acutorostrata scammoni]	GCF_000493695.1	TRADD-N
XP_007198139.1	TRADD_N+Death	325	TRADD	vertebrata	Balaenoptera acutorostrata scammoni	PREDICTED: tumor necrosis factor receptor type 1-associated DEATH domain protein isoform X2 [Balaenoptera acutorostrata scammoni]	GCF_000493695.1	TRADD-N

acc	architecture	len	gen.name	taxend	species	defline	gca	clade
XP_007198140.1	TRADD_N+Death	315	TRADD	vertebrata	Balaenoptera acutorostrata scammoni	PREDICTED: tumor necrosis factor receptor type 1-associated DEATH domain protein isoform X3 [Balaenoptera acutorostrata scammoni]	GCF_000493695.1	TRADD-N
KFO08106.1	TRADD_N+Death	307	N312_06144	vertebrata	Balearica regulorum gibbericeps	Tumor necrosis factor receptor type 1-associated DEATH domain protein, partial [Balearica regulorum gibbericeps]	GCA_000709895.1	TRADD-N
XP_010311259.1	TRADD_N+Death	306	TRADD	vertebrata	Balearica regulorum gibbericeps	PREDICTED: tumor necrosis factor receptor type 1-associated DEATH domain protein [Balearica regulorum gibbericeps]	GCF_000709895.1	TRADD-N
POI29546.1	TRADD_N+Death	307	CIB84_006704	vertebrata	Bambusicola thoracicus	hypothetical protein CIB84_006704, partial [Bambusicola thoracicus]	GCA_002909625.1	TRADD-N
XP_010849371.1	TRADD_N+Death	312	TRADD	vertebrata	Bison bison bison	PREDICTED: tumor necrosis factor receptor type 1-associated DEATH domain protein [Bison bison bison]	GCF_000754665.1	TRADD-N
XP_019834938.1	TRADD_N+Death	321	TRADD	vertebrata	Bos indicus	PREDICTED: tumor necrosis factor receptor type 1-associated DEATH domain protein isoform X1 [Bos indicus]	GCF_000247795.1	TRADD-N
ELR55836.1	TRADD_N+Death	310	M91_18288	vertebrata	Bos mutus	Tumor necrosis factor receptor type 1-associated DEATH domain protein [Bos mutus]	GCA_000298355.1	TRADD-N
XP_005896623.1	TRADD_N+Death	312	TRADD	vertebrata	Bos mutus	PREDICTED: tumor necrosis factor receptor type 1-associated DEATH domain protein [Bos mutus]	GCF_000298355.1	TRADD-N
NP_001039361.1	TRADD_N+Death	312	TRADD	vertebrata	Bos taurus	tumor necrosis factor receptor type 1-associated DEATH domain protein [Bos taurus]	GCF_002263795.1	TRADD-N
XP_010812733.1	TRADD_N+Death	252	TRADD	vertebrata	Bos taurus	tumor necrosis factor receptor type 1-associated DEATH domain protein isoform X3 [Bos taurus]	GCF_002263795.1	TRADD-N
XP_006072131.1	TRADD_N+Death	312	TRADD	vertebrata	Bubalus bubalis	tumor necrosis factor receptor type 1-associated DEATH domain protein [Bubalus bubalis]	GCF_003121395.1	TRADD-N
KFO87319.1	TRADD_N+Death	307	N320_08560	vertebrata	Buceros rhinoceros silvestris	Tumor necrosis factor receptor type 1-associated DEATH domain protein, partial [Buceros rhinoceros silvestris]	GCA_000710305.1	TRADD-N
XP_010130518.1	TRADD_N+Death	306	TRADD	vertebrata	Buceros rhinoceros silvestris	PREDICTED: tumor necrosis factor receptor type 1-associated DEATH domain protein [Buceros rhinoceros silvestris]	GCF_000710305.1	TRADD-N
XP_014812854.1	TRADD_N+Death	306	TRADD	vertebrata	Calidris pugnax	PREDICTED: tumor necrosis factor receptor type 1-associated DEATH domain protein [Calidris pugnax]	GCF_001431845.1	TRADD-N
OXB57886.1	TRADD_N+Death	311	ASZ78_009492	vertebrata	Callipepla squamata	hypothetical protein ASZ78_009492 [Callipepla squamata]	GCA_002218305.1	TRADD-N

acc	architecture	len	gen.name	taxend	species	defline	gca	clade
XP_008984275.1	TRADD_N+Death	312	TRADD	vertebrata	Callithrix jacchus	PREDICTED: tumor necrosis factor receptor type 1-associated DEATH domain protein isoform X3 [Callithrix jacchus]	GCF_000004665.1	TRADD-N
XP_017822392.1	TRADD_N+Death	363	TRADD	vertebrata	Callithrix jacchus	PREDICTED: tumor necrosis factor receptor type 1-associated DEATH domain protein isoform X1 [Callithrix jacchus]	GCF_000004665.1	TRADD-N
XP_017822393.1	TRADD_N+Death	350	TRADD	vertebrata	Callithrix jacchus	PREDICTED: tumor necrosis factor receptor type 1-associated DEATH domain protein isoform X2 [Callithrix jacchus]	GCF_000004665.1	TRADD-N
XP_007902885.1	TRADD_N+Death	310	tradd	vertebrata	Callorhinchus milii	PREDICTED: tumor necrosis factor receptor type 1-associated DEATH domain protein [Callorhinchus milii]	GCF_000165045.1	TRADD-N
XP_025744570.1	TRADD_N+Death	313	TRADD	vertebrata	Callorhinus ursinus	tumor necrosis factor receptor type 1-associated DEATH domain protein [Callorhinus ursinus]	GCF_003265705.1	TRADD-N
KFO99994.1	TRADD_N+Death	307	N300_11603	vertebrata	Calypte anna	Tumor necrosis factor receptor type 1-associated DEATH domain protein, partial [Calypte anna]	GCA_000699085.1	TRADD-N
XP_008490893.1	TRADD_N+Death	306	TRADD	vertebrata	Calypte anna	PREDICTED: tumor necrosis factor receptor type 1-associated DEATH domain protein [Calypte anna]	-	TRADD-N
XP_010960749.1	TRADD_N+Death	309	TRADD	vertebrata	Camelus bactrianus	PREDICTED: tumor necrosis factor receptor type 1-associated DEATH domain protein [Camelus bactrianus]	GCF_000767855.1	TRADD-N
XP_010977614.1	TRADD_N+Death	287	TRADD	vertebrata	Camelus dromedarius	PREDICTED: LOW QUALITY PROTEIN: tumor necrosis factor receptor type 1-associated DEATH domain protein [Camelus dromedarius]	-	TRADD-N
EPY84220.1	TRADD_N+Death	271	CB1_000490069	vertebrata	Camelus ferus	TNFRSF1A-associated via death domain isoform 1-like protein [Camelus ferus]	GCA_000311805.2	TRADD-N
XP_006180698.1	TRADD_N+Death	330	TRADD	vertebrata	Camelus ferus	PREDICTED: tumor necrosis factor receptor type 1-associated DEATH domain protein isoform X1 [Camelus ferus]	GCF_000311805.1	TRADD-N
XP_014411308.1	TRADD_N+Death	315	TRADD	vertebrata	Camelus ferus	PREDICTED: tumor necrosis factor receptor type 1-associated DEATH domain protein isoform X2 [Camelus ferus]	GCF_000311805.1	TRADD-N
XP_005620913.1	TRADD_N+Death	323	TRADD	vertebrata	Canis lupus familiaris	tumor necrosis factor receptor type 1-associated DEATH domain protein isoform X2 [Canis lupus familiaris]	GCF_000002285.3	TRADD-N
XP_013969589.1	TRADD_N+Death	340	TRADD	vertebrata	Canis lupus familiaris	tumor necrosis factor receptor type 1-associated DEATH domain protein isoform X1 [Canis lupus familiaris]	GCF_000002285.3	TRADD-N
XP_022275094.1	TRADD_N+Death	252	TRADD	vertebrata	Canis lupus familiaris	tumor necrosis factor receptor type 1-associated DEATH domain protein isoform X4 [Canis lupus familiaris]	GCF_000002285.3	TRADD-N

acc	architecture	len	gen.name	taxend	species	defline	gca	clade
XP_853998.2	TRADD_N+Death	312	TRADD	vertebrata	Canis lupus familiaris	tumor necrosis factor receptor type 1-associated DEATH domain protein isoform X3 [Canis lupus familiaris]	GCF_000002285.3	TRADD-N
XP_005692184.2	TRADD_N+Death	312	TRADD	vertebrata	Capra hircus	PREDICTED: tumor necrosis factor receptor type 1-associated DEATH domain protein isoform X1 [Capra hircus]	GCF_001704415.1	TRADD-N
XP_017917599.1	TRADD_N+Death	252	TRADD	vertebrata	Capra hircus	PREDICTED: tumor necrosis factor receptor type 1-associated DEATH domain protein isoform X2 [Capra hircus]	GCF_001704415.1	TRADD-N
KFP67735.1	TRADD_N+Death	307	N322_00821	vertebrata	Cariama cristata	Tumor necrosis factor receptor type 1-associated DEATH domain protein, partial [Cariama cristata]	GCA_000690535.1	TRADD-N
XP_009703964.1	TRADD_N+Death	306	TRADD	vertebrata	Cariama cristata	PREDICTED: tumor necrosis factor receptor type 1-associated DEATH domain protein [Cariama cristata]	GCF_000690535.1	TRADD-N
XP_008065007.1	TRADD_N+Death	310	TRADD	vertebrata	Carlito syrichta	tumor necrosis factor receptor type 1-associated DEATH domain protein isoform X2 [Carlito syrichta]	GCF_000164805.1	TRADD-N
XP_021572269.1	TRADD_N+Death	337	TRADD	vertebrata	Carlito syrichta	tumor necrosis factor receptor type 1-associated DEATH domain protein isoform X1 [Carlito syrichta]	GCF_000164805.1	TRADD-N
XP_020031343.1	TRADD_N+Death	389	Tradd	vertebrata	Castor canadensis	tumor necrosis factor receptor type 1-associated DEATH domain protein isoform X1 [Castor canadensis]	GCF_001984765.1	TRADD-N
XP_020031352.1	TRADD_N+Death	321	Tradd	vertebrata	Castor canadensis	tumor necrosis factor receptor type 1-associated DEATH domain protein isoform X2 [Castor canadensis]	GCF_001984765.1	TRADD-N
XP_020031362.1	TRADD_N+Death	310	Tradd	vertebrata	Castor canadensis	tumor necrosis factor receptor type 1-associated DEATH domain protein isoform X3 [Castor canadensis]	GCF_001984765.1	TRADD-N
KFP50879.1	TRADD_N+Death	300	N323_05948	vertebrata	Cathartes aura	Tumor necrosis factor receptor type 1-associated DEATH domain protein, partial [Cathartes aura]	GCA_000699945.1	TRADD-N
XP_005004714.1	TRADD_N+Death	310	Tradd	vertebrata	Cavia porcellus	tumor necrosis factor receptor type 1-associated DEATH domain protein [Cavia porcellus]	GCF_000151735.1	TRADD-N
XP_017394762.1	TRADD_N+Death	352	TRADD	vertebrata	Cebus capucinus imitator	PREDICTED: tumor necrosis factor receptor type 1-associated DEATH domain protein isoform X1 [Cebus capucinus imitator]	GCF_001604975.1	TRADD-N
XP_017394763.1	TRADD_N+Death	350	TRADD	vertebrata	Cebus capucinus imitator	PREDICTED: tumor necrosis factor receptor type 1-associated DEATH domain protein isoform X2 [Cebus capucinus imitator]	GCF_001604975.1	TRADD-N
XP_017394764.1	TRADD_N+Death	312	TRADD	vertebrata	Cebus capucinus imitator	PREDICTED: tumor necrosis factor receptor type 1-associated DEATH domain protein isoform X3 [Cebus capucinus imitator]	GCF_001604975.1	TRADD-N

acc	architecture	len	gen.name	taxend	species	defline	gca	clade
XP_004431761.1	TRADD_N+Death	307	LOC101408131	vertebrata	Ceratotherium simum simum	PREDICTED: tumor necrosis factor receptor type 1-associated DEATH domain protein isoform X2 [Ceratotherium simum simum]	GCF_000283155.1	TRADD-N
XP_014643936.1	TRADD_N+Death	452	LOC101408131	vertebrata	Ceratotherium simum simum	PREDICTED: tumor necrosis factor receptor type 1-associated DEATH domain protein isoform X1 [Ceratotherium simum simum]	GCF_000283155.1	TRADD-N
XP_011918730.1	TRADD_N+Death	308	TRADD	vertebrata	Cercocebus atys	PREDICTED: tumor necrosis factor receptor type 1-associated DEATH domain protein isoform X1 [Cercocebus atys]	GCF_000955945.1	TRADD-N
XP_011918740.1	TRADD_N+Death	248	TRADD	vertebrata	Cercocebus atys	PREDICTED: tumor necrosis factor receptor type 1-associated DEATH domain protein isoform X2 [Cercocebus atys]	GCF_000955945.1	TRADD-N
OWK16244.1	TRADD_N+Death	312	Celaphus_00004220	vertebrata	Cervus elaphus hippelaphus	TRADD [Cervus elaphus hippelaphus]	GCA_002197005.1	TRADD-N
KFU94762.1	TRADD_N+Death	307	M959_10139	vertebrata	Chaetura pelagica	Tumor necrosis factor receptor type 1-associated DEATH domain protein, partial [Chaetura pelagica]	GCA_000747805.1	TRADD-N
XP_010002736.1	TRADD_N+Death	306	TRADD	vertebrata	Chaetura pelagica	PREDICTED: tumor necrosis factor receptor type 1-associated DEATH domain protein [Chaetura pelagica]	GCF_000747805.1	TRADD-N
KGL86877.1	TRADD_N+Death	307	N301_00711	vertebrata	Charadrius vociferus	Tumor necrosis factor receptor type 1-associated DEATH domain protein, partial [Charadrius vociferus]	GCA_000708025.2	TRADD-N
XP_009887790.1	TRADD_N+Death	306	TRADD	vertebrata	Charadrius vociferus	PREDICTED: tumor necrosis factor receptor type 1-associated DEATH domain protein [Charadrius vociferus]	GCF_000708025.1	TRADD-N
EMP41239.1	TRADD_N+Death	307	UY3_01474	vertebrata	Chelonia mydas	Tumor necrosis factor receptor type 1-associated DEATH domain protein [Chelonia mydas]	GCA_000344595.1	TRADD-N
XP_007054032.1	TRADD_N+Death	277	TRADD	vertebrata	Chelonia mydas	PREDICTED: tumor necrosis factor receptor type 1-associated DEATH domain protein [Chelonia mydas]	-	TRADD-N
XP_005403749.1	TRADD_N+Death	310	Tradd	vertebrata	Chinchilla lanigera	PREDICTED: tumor necrosis factor receptor type 1-associated DEATH domain protein [Chinchilla lanigera]	GCF_000276665.1	TRADD-N
KFP44634.1	TRADD_N+Death	307	N324_05185	vertebrata	Chlamydotis macqueenii	Tumor necrosis factor receptor type 1-associated DEATH domain protein, partial [Chlamydotis macqueenii]	GCA_000695195.1	TRADD-N
XP_010124279.1	TRADD_N+Death	306	TRADD	vertebrata	Chlamydotis macqueenii	PREDICTED: tumor necrosis factor receptor type 1-associated DEATH domain protein [Chlamydotis macqueenii]	GCF_000695195.1	TRADD-N

acc	architecture	len	gen.name	taxend	species	defline	gca	clade
XP_007992208.1	TRADD_N+Death	366	TRADD	vertebrata	Chlorocebus sabaeus	PREDICTED: tumor necrosis factor receptor type 1-associated DEATH domain protein isoform X1 [Chlorocebus sabaeus]	GCF_000409795.2	TRADD-N
XP_007992209.1	TRADD_N+Death	308	TRADD	vertebrata	Chlorocebus sabaeus	PREDICTED: tumor necrosis factor receptor type 1-associated DEATH domain protein isoform X2 [Chlorocebus sabaeus]	GCF_000409795.2	TRADD-N
XP_007992210.1	TRADD_N+Death	248	TRADD	vertebrata	Chlorocebus sabaeus	PREDICTED: tumor necrosis factor receptor type 1-associated DEATH domain protein isoform X3 [Chlorocebus sabaeus]	GCF_000409795.2	TRADD-N
XP_008165123.1	TRADD_N+Death	307	TRADD	vertebrata	Chrysemys picta bellii	tumor necrosis factor receptor type 1-associated DEATH domain protein [Chrysemys picta bellii]	GCF_000241765.3	TRADD-N
XP_006863702.1	TRADD_N+Death	309	TRADD	vertebrata	Chrysochloris asiatica	PREDICTED: tumor necrosis factor receptor type 1-associated DEATH domain protein [Chrysochloris asiatica]	GCF_000296735.1	TRADD-N
OXB72455.1	TRADD_N+Death	307	H355_007808	vertebrata	Colinus virginianus	hypothetical protein H355_007808, partial [Colinus virginianus]	GCA_000599465.2	TRADD-N
KFP26363.1	TRADD_N+Death	307	N325_10356	vertebrata	Colius striatus	Tumor necrosis factor receptor type 1-associated DEATH domain protein, partial [Colius striatus]	GCA_000690715.1	TRADD-N
XP_010203006.1	TRADD_N+Death	306	TRADD	vertebrata	Colius striatus	PREDICTED: tumor necrosis factor receptor type 1-associated DEATH domain protein [Colius striatus]	GCF_000690715.1	TRADD-N
XP_011787468.1	TRADD_N+Death	346	TRADD	vertebrata	Colobus angolensis palliatus	PREDICTED: tumor necrosis factor receptor type 1-associated DEATH domain protein [Colobus angolensis palliatus]	GCF_000951035.1	TRADD-N
XP_005504824.1	TRADD_N+Death	306	TRADD	vertebrata	Columba livia	tumor necrosis factor receptor type 1-associated DEATH domain protein [Columba livia]	GCF_000337935.1	TRADD-N
XP_004690544.1	TRADD_N+Death	306	TRADD	vertebrata	Condylura cristata	PREDICTED: tumor necrosis factor receptor type 1-associated DEATH domain protein [Condylura cristata]	GCF_000260355.1	TRADD-N
KFO63108.1	TRADD_N+Death	302	N302_07036	vertebrata	Corvus brachyrhynchos	Tumor necrosis factor receptor type 1-associated DEATH domain protein, partial [Corvus brachyrhynchos]	GCA_000691975.1	TRADD-N
XP_008636989.1	TRADD_N+Death	301	TRADD	vertebrata	Corvus brachyrhynchos	PREDICTED: tumor necrosis factor receptor type 1-associated DEATH domain protein [Corvus brachyrhynchos]	GCF_000691975.1	TRADD-N
XP_010394933.2	TRADD_N+Death	301	TRADD	vertebrata	Corvus cornix cornix	tumor necrosis factor receptor type 1-associated DEATH domain protein [Corvus cornix cornix]	GCF_000738735.2	TRADD-N
XP_015729060.1	TRADD_N+Death	304	TRADD	vertebrata	Coturnix japonica	PREDICTED: tumor necrosis factor receptor type 1-associated DEATH domain protein [Coturnix japonica]	GCF_001577835.1	TRADD-N

acc	architecture	len	gen.name	taxend	species	defline	gca	clade
EGV97026.1	TRADD_N+Death	306	I79_016510	vertebrata	Cricetulus griseus	Tumor necrosis factor receptor type 1-associated DEATH domain protein [Cricetulus griseus]	GCA_000223135.1	TRADD-N
XP_007647347.1	TRADD_N+Death	310	Tradd	vertebrata	Cricetulus griseus	tumor necrosis factor receptor type 1-associated DEATH domain protein [Cricetulus griseus]	GCF_000223135.1	TRADD-N
XP_019391196.1	TRADD_N+Death	304	TRADD	vertebrata	Crocodylus porosus	PREDICTED: tumor necrosis factor receptor type 1-associated DEATH domain protein [Crocodylus porosus]	GCF_001723895.1	TRADD-N
XP_009555419.1	TRADD_N+Death	306	TRADD	vertebrata	Cuculus canorus	PREDICTED: tumor necrosis factor receptor type 1-associated DEATH domain protein [Cuculus canorus]	GCF_000709325.1	TRADD-N
XP_023790487.1	TRADD_N+Death	357	TRADD	vertebrata	Cyanistes caeruleus	tumor necrosis factor receptor type 1-associated DEATH domain protein [Cyanistes caeruleus]	GCF_002901205.1	TRADD-N
XP_004460942.1	TRADD_N+Death	315	TRADD	vertebrata	Dasypus novemcinctus	tumor necrosis factor receptor type 1-associated DEATH domain protein [Dasypus novemcinctus]	GCF_000208655.1	TRADD-N
XP_022439217.1	TRADD_N+Death	315	TRADD	vertebrata	Delphinapterus leucas	tumor necrosis factor receptor type 1-associated DEATH domain protein [Delphinapterus leucas]	GCF_002288925.2	TRADD-N
XP_024411986.1	TRADD_N+Death	319	TRADD	vertebrata	Desmodus rotundus	tumor necrosis factor receptor type 1-associated DEATH domain protein isoform X1 [Desmodus rotundus]	GCF_002940915.1	TRADD-N
XP_024411987.1	TRADD_N+Death	310	TRADD	vertebrata	Desmodus rotundus	tumor necrosis factor receptor type 1-associated DEATH domain protein isoform X2 [Desmodus rotundus]	GCF_002940915.1	TRADD-N
XP_012868461.1	TRADD_N+Death	309	Tradd	vertebrata	Dipodomys ordii	PREDICTED: tumor necrosis factor receptor type 1-associated DEATH domain protein [Dipodomys ordii]	GCF_000151885.1	TRADD-N
XP_025957849.1	TRADD_N+Death	305	TRADD	vertebrata	Dromaius novaehollandiae	tumor necrosis factor receptor type 1-associated DEATH domain protein [Dromaius novaehollandiae]	GCF_003342905.1	TRADD-N
XP_004704900.1	TRADD_N+Death	307	TRADD	vertebrata	Echinops telfairi	PREDICTED: tumor necrosis factor receptor type 1-associated DEATH domain protein [Echinops telfairi]	GCF_000313985.2	TRADD-N
KFP19624.1	TRADD_N+Death	307	Z169_06047	vertebrata	Egretta garzetta	Tumor necrosis factor receptor type 1-associated DEATH domain protein, partial [Egretta garzetta]	GCA_000687185.1	TRADD-N
XP_009641600.1	TRADD_N+Death	306	TRADD	vertebrata	Egretta garzetta	PREDICTED: tumor necrosis factor receptor type 1-associated DEATH domain protein [Egretta garzetta]	GCF_000687185.1	TRADD-N
XP_006878963.1	TRADD_N+Death	310	TRADD	vertebrata	Elephantulus edwardii	PREDICTED: tumor necrosis factor receptor type 1-associated DEATH domain protein [Elephantulus edwardii]	GCF_000299155.1	TRADD-N
XP_022367209.1	TRADD_N+Death	314	LOC111152832	vertebrata	Enhydra lutris kenyoni	tumor necrosis factor receptor type 1-associated DEATH domain protein [Enhydra lutris kenyoni]	GCF_002288905.1	TRADD-N
XP_008138113.1	TRADD_N+Death	310	TRADD	vertebrata	Eptesicus fuscus	PREDICTED: tumor necrosis factor receptor type 1-associated DEATH domain protein [Eptesicus fuscus]	GCF_000308155.1	TRADD-N

acc	architecture	len	gen.name	taxend	species	defline	gca	clade
XP_014683141.1	TRADD_N+Death	312	TRADD	vertebrata	Equus asinus	PREDICTED: tumor necrosis factor receptor type 1-associated DEATH domain protein [Equus asinus]	GCF_001305755.1	TRADD-N
XP_023494393.1	TRADD_N+Death	312	TRADD	vertebrata	Equus caballus	tumor necrosis factor receptor type 1-associated DEATH domain protein isoform X1 [Equus caballus]	GCF_002863925.1	TRADD-N
XP_023494396.1	TRADD_N+Death	252	TRADD	vertebrata	Equus caballus	tumor necrosis factor receptor type 1-associated DEATH domain protein isoform X2 [Equus caballus]	GCF_002863925.1	TRADD-N
XP_008507841.1	TRADD_N+Death	276	TRADD	vertebrata	Equus przewalskii	PREDICTED: tumor necrosis factor receptor type 1-associated DEATH domain protein [Equus przewalskii]	GCF_000696695.1	TRADD-N
XP_007517809.2	TRADD_N+Death	314	TRADD	vertebrata	Erinaceus europaeus	PREDICTED: tumor necrosis factor receptor type 1-associated DEATH domain protein isoform X2 [Erinaceus europaeus]	GCF_000296755.1	TRADD-N
XP_016041350.1	TRADD_N+Death	332	TRADD	vertebrata	Erinaceus europaeus	PREDICTED: tumor necrosis factor receptor type 1-associated DEATH domain protein isoform X1 [Erinaceus europaeus]	GCF_000296755.1	TRADD-N
RLW01327.1	TRADD_N+Death	306	DV515_00008217	vertebrata	Erythrura gouldiae	hypothetical protein DV515_00008217 [Erythrura gouldiae]	GCA_003676055.1	TRADD-N
KFV92737.1	TRADD_N+Death	307	N326_03667	vertebrata	Eurypyga helias	Tumor necrosis factor receptor type 1-associated DEATH domain protein, partial [Eurypyga helias]	GCA_000690775.1	TRADD-N
XP_010148912.1	TRADD_N+Death	306	TRADD	vertebrata	Eurypyga helias	PREDICTED: tumor necrosis factor receptor type 1-associated DEATH domain protein [Eurypyga helias]	GCF_000690775.1	TRADD-N
XP_005233540.1	TRADD_N+Death	306	TRADD	vertebrata	Falco peregrinus	PREDICTED: tumor necrosis factor receptor type 1-associated DEATH domain protein [Falco peregrinus]	GCF_000337955.1	TRADD-N
XP_003998167.1	TRADD_N+Death	311	TRADD	vertebrata	Felis catus	tumor necrosis factor receptor type 1-associated DEATH domain protein isoform X2 [Felis catus]	GCF_000181335.3	TRADD-N
XP_019675452.1	TRADD_N+Death	319	TRADD	vertebrata	Felis catus	tumor necrosis factor receptor type 1-associated DEATH domain protein isoform X1 [Felis catus]	GCF_000181335.3	TRADD-N
XP_005052598.1	TRADD_N+Death	306	TRADD	vertebrata	Ficedula albicollis	PREDICTED: tumor necrosis factor receptor type 1-associated DEATH domain protein [Ficedula albicollis]	GCF_000247815.1	TRADD-N
XP_010610494.1	TRADD_N+Death	320	Tradd	vertebrata	Fukomys damarensis	PREDICTED: tumor necrosis factor receptor type 1-associated DEATH domain protein isoform X1 [Fukomys damarensis]	GCF_000743615.1	TRADD-N
XP_010610496.1	TRADD_N+Death	310	Tradd	vertebrata	Fukomys damarensis	PREDICTED: tumor necrosis factor receptor type 1-associated DEATH domain protein isoform X2 [Fukomys damarensis]	GCF_000743615.1	TRADD-N
KFW03688.1	TRADD_N+Death	306	N327_09538	vertebrata	Fulmarus glacialis	Tumor necrosis factor receptor type 1-associated DEATH domain protein, partial [Fulmarus glacialis]	GCA_000690835.1	TRADD-N

acc	architecture	len	gen.name	taxend	species	defline	gca	clade
XP_009574714.1	TRADD_N+Death	306	TRADD	vertebrata	Fulmarus glacialis	PREDICTED: tumor necrosis factor receptor type 1-associated DEATH domain protein [Fulmarus glacialis]	GCF_000690835.1	TRADD-N
XP_008585531.1	TRADD_N+Death	326	TRADD	vertebrata	Galeopterus variegatus	PREDICTED: tumor necrosis factor receptor type 1-associated DEATH domain protein isoform X1 [Galeopterus variegatus]	GCF_000696425.1	TRADD-N
XP_008585532.1	TRADD_N+Death	309	TRADD	vertebrata	Galeopterus variegatus	PREDICTED: tumor necrosis factor receptor type 1-associated DEATH domain protein isoform X2 [Galeopterus variegatus]	GCF_000696425.1	TRADD-N
XP_004944196.2	TRADD_N+Death	461	TRADD	vertebrata	Gallus gallus	tumor necrosis factor receptor type 1-associated DEATH domain protein [Gallus gallus]	GCF_000002315.5	TRADD-N
KFV44833.1	TRADD_N+Death	307	N328_11878	vertebrata	Gavia stellata	Tumor necrosis factor receptor type 1-associated DEATH domain protein, partial [Gavia stellata]	GCA_000690875.1	TRADD-N
XP_009815127.1	TRADD_N+Death	306	TRADD	vertebrata	Gavia stellata	PREDICTED: tumor necrosis factor receptor type 1-associated DEATH domain protein [Gavia stellata]	GCF_000690875.1	TRADD-N
XP_019361318.1	TRADD_N+Death	304	TRADD	vertebrata	Gavialis gangeticus	PREDICTED: tumor necrosis factor receptor type 1-associated DEATH domain protein [Gavialis gangeticus]	GCF_001723915.1	TRADD-N
XP_015261591.1	TRADD_N+Death	310	TRADD	vertebrata	Gekko japonicus	PREDICTED: tumor necrosis factor receptor type 1-associated DEATH domain protein [Gekko japonicus]	GCF_001447785.1	TRADD-N
XP_005422791.1	TRADD_N+Death	306	TRADD	vertebrata	Geospiza fortis	PREDICTED: tumor necrosis factor receptor type 1-associated DEATH domain protein [Geospiza fortis]	GCF_000277835.1	TRADD-N
XP_004057845.1	TRADD_N+Death	311	TRADD	vertebrata	Gorilla gorilla gorilla	PREDICTED: tumor necrosis factor receptor type 1-associated DEATH domain protein isoform X1 [Gorilla gorilla gorilla]	GCF_008122165.1	TRADD-N
XP_018868334.1	TRADD_N+Death	251	TRADD	vertebrata	Gorilla gorilla gorilla	PREDICTED: tumor necrosis factor receptor type 1-associated DEATH domain protein isoform X2 [Gorilla gorilla gorilla]	GCF_008122165.1	TRADD-N
KFP94957.1	TRADD_N+Death	307	N329_08562	vertebrata	Haliaeetus albicilla	Tumor necrosis factor receptor type 1-associated DEATH domain protein, partial [Haliaeetus albicilla]	GCA_000691405.1	TRADD-N
XP_009922991.1	TRADD_N+Death	306	TRADD	vertebrata	Haliaeetus albicilla	PREDICTED: tumor necrosis factor receptor type 1-associated DEATH domain protein [Haliaeetus albicilla]	GCF_000691405.1	TRADD-N
EHB17005.1	TRADD_N+Death	310	GW7_02340	vertebrata	Heterocephalus glaber	Tumor necrosis factor receptor type 1-associated DEATH domain protein [Heterocephalus glaber]	GCA_000230445.1	TRADD-N
XP_004843120.1	TRADD_N+Death	310	Tradd	vertebrata	Heterocephalus glaber	tumor necrosis factor receptor type 1-associated DEATH domain protein [Heterocephalus glaber]	GCF_000247695.1	TRADD-N

acc	architecture	len	gen.name	taxend	species	defline	gca	clade
XP_019511720.1	TRADD_N+Death	342	TRADD	vertebrata	Hipposideros armiger	PREDICTED: tumor necrosis factor receptor type 1-associated DEATH domain protein isoform X1 [Hipposideros armiger]	GCF_001890085.1	TRADD-N
XP_019511730.1	TRADD_N+Death	309	TRADD	vertebrata	Hipposideros armiger	PREDICTED: tumor necrosis factor receptor type 1-associated DEATH domain protein isoform X2 [Hipposideros armiger]	GCF_001890085.1	TRADD-N
XP_019511759.1	TRADD_N+Death	250	TRADD	vertebrata	Hipposideros armiger	PREDICTED: tumor necrosis factor receptor type 1-associated DEATH domain protein isoform X3 [Hipposideros armiger]	GCF_001890085.1	TRADD-N
RMC09132.1	TRADD_N+Death	361	DUI87_14139	vertebrata	Hirundo rustica rustica	hypothetical protein DUI87_14139 [Hirundo rustica rustica]	GCA_003692655.1	TRADD-N
AAA98482.1	TRADD_N+Death	328	TRADD	vertebrata	Homo sapiens	tumor necrosis factor receptor type 1 associated protein, partial [Homo sapiens]	-	TRADD-N
NP_003780.1	TRADD_N+Death	312	Hs.89862	vertebrata	Homo sapiens	tumor necrosis factor receptor type 1-associated DEATH domain protein [Homo sapiens]	GCF_000001405.39	TRADD-N
XP_005256270.1	TRADD_N+Death	252	Hs.89862	vertebrata	Homo sapiens	tumor necrosis factor receptor type 1-associated DEATH domain protein isoform X2 [Homo sapiens]	GCF_000001405.39	TRADD-N
XP_005318353.2	TRADD_N+Death	382	Tradd	vertebrata	Ictidomys tridecemlineatus	tumor necrosis factor receptor type 1-associated DEATH domain protein [Ictidomys tridecemlineatus]	GCF_000236235.1	TRADD-N
XP_004661772.1	TRADD_N+Death	309	Tradd	vertebrata	Jaculus jaculus	PREDICTED: tumor necrosis factor receptor type 1-associated DEATH domain protein [Jaculus jaculus]	GCF_000280705.1	TRADD-N
XP_005998087.1	TRADD_N+Death	294	TRADD	vertebrata	Latimeria chalumnae	PREDICTED: tumor necrosis factor receptor type 1-associated DEATH domain protein [Latimeria chalumnae]	GCF_000225785.1	TRADD-N
XP_017679908.1	TRADD_N+Death	306	TRADD	vertebrata	Lepidothrix coronata	PREDICTED: tumor necrosis factor receptor type 1-associated DEATH domain protein [Lepidothrix coronata]	GCF_001604755.1	TRADD-N
XP_006741565.1	TRADD_N+Death	312	TRADD	vertebrata	Leptonychotes weddellii	PREDICTED: tumor necrosis factor receptor type 1-associated DEATH domain protein [Leptonychotes weddellii]	GCF_000349705.1	TRADD-N
KFQ03468.1	TRADD_N+Death	307	N330_09323	vertebrata	Leptosomus discolor	Tumor necrosis factor receptor type 1-associated DEATH domain protein, partial [Leptosomus discolor]	GCA_000691785.1	TRADD-N
XP_009954005.1	TRADD_N+Death	306	TRADD	vertebrata	Leptosomus discolor	PREDICTED: tumor necrosis factor receptor type 1-associated DEATH domain protein [Leptosomus discolor]	GCF_000691785.1	TRADD-N
XP_007457361.1	TRADD_N+Death	341	TRADD	vertebrata	Lipotes vexillifer	PREDICTED: tumor necrosis factor receptor type 1-associated DEATH domain protein [Lipotes vexillifer]	GCF_000442215.1	TRADD-N

acc	architecture	len	gen.name	taxend	species	defline	gca	clade
OWK54101.1	TRADD_N+Death	306	TRADD	vertebrata	Lonchura striata domestica	Tumor necrosis factor receptor type 1-associated DEATH domain protein [Lonchura striata domestica]	GCA_002197715.1	TRADD-N
XP_021390934.1	TRADD_N+Death	400	TRADD	vertebrata	Lonchura striata domestica	tumor necrosis factor receptor type 1-associated DEATH domain protein [Lonchura striata domestica]	-	TRADD-N
XP_003417161.1	TRADD_N+Death	310	TRADD	vertebrata	Loxodonta africana	tumor necrosis factor receptor type 1-associated DEATH domain protein isoform X4 [Loxodonta africana]	GCF_000001905.1	TRADD-N
XP_023412319.1	TRADD_N+Death	351	TRADD	vertebrata	Loxodonta africana	tumor necrosis factor receptor type 1-associated DEATH domain protein isoform X1 [Loxodonta africana]	GCF_000001905.1	TRADD-N
XP_023412320.1	TRADD_N+Death	328	TRADD	vertebrata	Loxodonta africana	tumor necrosis factor receptor type 1-associated DEATH domain protein isoform X2 [Loxodonta africana]	GCF_000001905.1	TRADD-N
XP_023412321.1	TRADD_N+Death	318	TRADD	vertebrata	Loxodonta africana	tumor necrosis factor receptor type 1-associated DEATH domain protein isoform X3 [Loxodonta africana]	GCF_000001905.1	TRADD-N
EHH60464.1	TRADD_N+Death	308	EGM_11831	vertebrata	Macaca fascicularis	Tumor necrosis factor receptor type 1-associated DEATH domain protein [Macaca fascicularis]	GCA_000230815.1	TRADD-N
XP_005592259.1	TRADD_N+Death	308	TRADD	vertebrata	Macaca fascicularis	PREDICTED: tumor necrosis factor receptor type 1-associated DEATH domain protein isoform X2 [Macaca fascicularis]	GCF_000364345.1	TRADD-N
XP_015298663.1	TRADD_N+Death	248	TRADD	vertebrata	Macaca fascicularis	PREDICTED: tumor necrosis factor receptor type 1-associated DEATH domain protein isoform X3 [Macaca fascicularis]	GCF_000364345.1	TRADD-N
XP_014981851.1	TRADD_N+Death	365	TRADD	vertebrata	Macaca mulatta	PREDICTED: tumor necrosis factor receptor type 1-associated DEATH domain protein isoform X1 [Macaca mulatta]	-	TRADD-N
KFW75858.1	TRADD_N+Death	307	N305_10431	vertebrata	Manacus vitellinus	Tumor necrosis factor receptor type 1-associated DEATH domain protein, partial [Manacus vitellinus]	GCA_000692015.2	TRADD-N
XP_008931637.2	TRADD_N+Death	306	TRADD	vertebrata	Manacus vitellinus	tumor necrosis factor receptor type 1-associated DEATH domain protein [Manacus vitellinus]	GCF_001715985.3	TRADD-N
XP_017504690.1	TRADD_N+Death	325	TRADD	vertebrata	Manis javanica	PREDICTED: tumor necrosis factor receptor type 1-associated DEATH domain protein isoform X1 [Manis javanica]	GCF_001685135.1	TRADD-N
XP_017504691.1	TRADD_N+Death	314	TRADD	vertebrata	Manis javanica	PREDICTED: tumor necrosis factor receptor type 1-associated DEATH domain protein isoform X2 [Manis javanica]	GCF_001685135.1	TRADD-N
XP_017504692.1	TRADD_N+Death	311	TRADD	vertebrata	Manis javanica	PREDICTED: tumor necrosis factor receptor type 1-associated DEATH domain protein isoform X3 [Manis javanica]	GCF_001685135.1	TRADD-N

acc	architecture	len	gen.name	taxend	species	defline	gca	clade
XP_017504698.1	TRADD_N+Death	302	TRADD	vertebrata	Manis javanica	PREDICTED: tumor necrosis factor receptor type 1-associated DEATH domain protein isoform X4 [Manis javanica]	GCF_001685135.1	TRADD-N
XP_015351727.1	TRADD_N+Death	314	Tradd	vertebrata	Marmota marmota marmota	PREDICTED: tumor necrosis factor receptor type 1-associated DEATH domain protein [Marmota marmota marmota]	GCF_001458135.1	TRADD-N
XP_005152306.1	TRADD_N+Death	303	LOC101871193	vertebrata	Melopsittacus undulatus	PREDICTED: tumor necrosis factor receptor type 1-associated DEATH domain protein [Melopsittacus undulatus]	GCF_000238935.1	TRADD-N
XP_021487414.1	TRADD_N+Death	312	Tradd	vertebrata	Meriones unguiculatus	tumor necrosis factor receptor type 1-associated DEATH domain protein [Meriones unguiculatus]	GCF_002204375.1	TRADD-N
KFQ23293.1	TRADD_N+Death	306	N331_05462	vertebrata	Merops nubicus	Tumor necrosis factor receptor type 1-associated DEATH domain protein, partial [Merops nubicus]	GCA_000691845.1	TRADD-N
XP_008946956.1	TRADD_N+Death	305	TRADD	vertebrata	Merops nubicus	PREDICTED: tumor necrosis factor receptor type 1-associated DEATH domain protein [Merops nubicus]	GCF_000691845.1	TRADD-N
KFQ19613.1	TRADD_N+Death	304	N332_02934	vertebrata	Mesitornis unicolor	Tumor necrosis factor receptor type 1-associated DEATH domain protein, partial [Mesitornis unicolor]	GCA_000695765.1	TRADD-N
XP_010179850.1	TRADD_N+Death	310	TRADD	vertebrata	Mesitornis unicolor	PREDICTED: tumor necrosis factor receptor type 1-associated DEATH domain protein, partial [Mesitornis unicolor]	GCF_000695765.1	TRADD-N
XP_005076251.1	TRADD_N+Death	310	Tradd	vertebrata	Mesocricetus auratus	tumor necrosis factor receptor type 1-associated DEATH domain protein isoform X2 [Mesocricetus auratus]	GCF_000349665.1	TRADD-N
XP_021086346.1	TRADD_N+Death	338	Tradd	vertebrata	Mesocricetus auratus	tumor necrosis factor receptor type 1-associated DEATH domain protein isoform X1 [Mesocricetus auratus]	GCF_000349665.1	TRADD-N
XP_012598048.1	TRADD_N+Death	309	TRADD	vertebrata	Microcebus murinus	tumor necrosis factor receptor type 1-associated DEATH domain protein [Microcebus murinus]	GCF_000165445.2	TRADD-N
XP_005345550.1	TRADD_N+Death	303	Tradd	vertebrata	Microtus ochrogaster	tumor necrosis factor receptor type 1-associated DEATH domain protein [Microtus ochrogaster]	GCF_000317375.1	TRADD-N
XP_016069064.1	TRADD_N+Death	313	TRADD	vertebrata	Miniopterus natalensis	PREDICTED: tumor necrosis factor receptor type 1-associated DEATH domain protein [Miniopterus natalensis]	GCF_001595765.1	TRADD-N
XP_001372553.3	TRADD_N+Death	361	TRADD	vertebrata	Monodelphis domestica	PREDICTED: tumor necrosis factor receptor type 1-associated DEATH domain protein isoform X1 [Monodelphis domestica]	GCF_000002295.2	TRADD-N
XP_007477195.1	TRADD_N+Death	339	TRADD	vertebrata	Monodelphis domestica	PREDICTED: tumor necrosis factor receptor type 1-associated DEATH domain protein isoform X2 [Monodelphis domestica]	GCF_000002295.2	TRADD-N

acc	architecture	len	gen.name	taxend	species	defline	gca	clade
XP_007477196.1	TRADD_N+Death	327	TRADD	vertebrata	Monodelphis domestica	PREDICTED: tumor necrosis factor receptor type 1-associated DEATH domain protein isoform X3 [Monodelphis domestica]	GCF_000002295.2	TRADD-N
XP_021026411.1	TRADD_N+Death	310	Tradd	vertebrata	Mus caroli	tumor necrosis factor receptor type 1-associated DEATH domain protein [Mus caroli]	GCF_900094665.1	TRADD-N
NP_001028333.1	TRADD_N+Death	310	9130005N23Rik,AA930854	vertebrata	Mus musculus	tumor necrosis factor receptor type 1-associated DEATH domain protein [Mus musculus]	GCF_000001635.26	TRADD-N
XP_021076272.1	TRADD_N+Death	310	Tradd	vertebrata	Mus pahari	tumor necrosis factor receptor type 1-associated DEATH domain protein [Mus pahari]	GCF_900095145.1	TRADD-N
XP_004744405.1	TRADD_N+Death	314	TRADD	vertebrata	Mustela putorius furo	PREDICTED: tumor necrosis factor receptor type 1-associated DEATH domain protein [Mustela putorius furo]	GCF_000215625.1	TRADD-N
XP_005876426.1	TRADD_N+Death	251	TRADD	vertebrata	Myotis brandtii	PREDICTED: tumor necrosis factor receptor type 1-associated DEATH domain protein isoform X2 [Myotis brandtii]	GCF_000412655.1	TRADD-N
XP_005876427.1	TRADD_N+Death	310	TRADD	vertebrata	Myotis brandtii	PREDICTED: tumor necrosis factor receptor type 1-associated DEATH domain protein isoform X1 [Myotis brandtii]	GCF_000412655.1	TRADD-N
ELK25625.1	TRADD_N+Death	310	MDA_GLEAN10017117	vertebrata	Myotis davidii	Tumor necrosis factor receptor type 1-associated DEATH domain protein [Myotis davidii]	GCA_000327345.1	TRADD-N
XP_006769870.1	TRADD_N+Death	365	TRADD	vertebrata	Myotis davidii	PREDICTED: tumor necrosis factor receptor type 1-associated DEATH domain protein [Myotis davidii]	GCF_000327345.1	TRADD-N
XP_006099450.1	TRADD_N+Death	310	TRADD	vertebrata	Myotis lucifugus	tumor necrosis factor receptor type 1-associated DEATH domain protein isoform X1 [Myotis lucifugus]	GCF_000147115.1	TRADD-N
XP_014320401.1	TRADD_N+Death	251	TRADD	vertebrata	Myotis lucifugus	tumor necrosis factor receptor type 1-associated DEATH domain protein isoform X2 [Myotis lucifugus]	GCF_000147115.1	TRADD-N
XP_008837333.1	TRADD_N+Death	319	Tradd	vertebrata	Nannospalax galili	PREDICTED: tumor necrosis factor receptor type 1-associated DEATH domain protein isoform X1 [Nannospalax galili]	GCF_000622305.1	TRADD-N
XP_008837335.1	TRADD_N+Death	313	Tradd	vertebrata	Nannospalax galili	PREDICTED: tumor necrosis factor receptor type 1-associated DEATH domain protein isoform X2 [Nannospalax galili]	-	TRADD-N
XP_018420366.1	TRADD_N+Death	301	TRADD	vertebrata	Nanorana parkeri	PREDICTED: tumor necrosis factor receptor type 1-associated DEATH domain protein [Nanorana parkeri]	GCF_000935625.1	TRADD-N
XP_021559631.1	TRADD_N+Death	312	TRADD	vertebrata	Neomonachus schauinslandi	tumor necrosis factor receptor type 1-associated DEATH domain protein isoform X1 [Neomonachus schauinslandi]	GCF_002201575.1	TRADD-N

acc	architecture	len	gen.name	taxend	species	defline	gca	clade
XP_021559639.1	TRADD_N+Death	288	TRADD	vertebrata	Neomonachus schauinslandi	tumor necrosis factor receptor type 1-associated DEATH domain protein isoform X2 [Neomonachus schauinslandi]	GCF_002201575.1	TRADD-N
XP_024589574.1	TRADD_N+Death	311	TRADD	vertebrata	Neophocaena asiaeorientalis asiaeorientalis	tumor necrosis factor receptor type 1-associated DEATH domain protein [Neophocaena asiaeorientalis asiaeorientalis]	GCF_003031525.1	TRADD-N
OBS58325.1	TRADD_N+Death	319	A6R68_10559	vertebrata	Neotoma lepida	hypothetical protein A6R68_10559 [Neotoma lepida]	GCA_001675575.1	TRADD-N
KFQ54821.1	TRADD_N+Death	307	N333_10317	vertebrata	Nestor notabilis	Tumor necrosis factor receptor type 1-associated DEATH domain protein, partial [Nestor notabilis]	GCA_000696875.1	TRADD-N
XP_010018560.1	TRADD_N+Death	306	TRADD	vertebrata	Nestor notabilis	PREDICTED: tumor necrosis factor receptor type 1-associated DEATH domain protein [Nestor notabilis]	GCF_000696875.1	TRADD-N
KFR01638.1	TRADD_N+Death	307	Y956_12945	vertebrata	Nipponia nippon	Tumor necrosis factor receptor type 1-associated DEATH domain protein, partial [Nipponia nippon]	GCA_000708225.1	TRADD-N
XP_009467511.1	TRADD_N+Death	306	TRADD	vertebrata	Nipponia nippon	PREDICTED: tumor necrosis factor receptor type 1-associated DEATH domain protein [Nipponia nippon]	GCF_000708225.1	TRADD-N
XP_003262922.1	TRADD_N+Death	311	TRADD	vertebrata	Nomascus leucogenys	PREDICTED: tumor necrosis factor receptor type 1-associated DEATH domain protein [Nomascus leucogenys]	GCF_006542625.1	TRADD-N
XP_026527890.1	TRADD_N+Death	311	TRADD	vertebrata	Notechis scutatus	tumor necrosis factor receptor type 1-associated DEATH domain protein [Notechis scutatus]	GCF_900518725.1	TRADD-N
XP_025898916.1	TRADD_N+Death	306	TRADD	vertebrata	Nothoprocta perdicaria	tumor necrosis factor receptor type 1-associated DEATH domain protein [Nothoprocta perdicaria]	GCF_003342845.1	TRADD-N
XP_021264133.1	TRADD_N+Death	304	TRADD	vertebrata	Numida meleagris	tumor necrosis factor receptor type 1-associated DEATH domain protein [Numida meleagris]	GCF_002078875.1	TRADD-N
XP_004583984.1	TRADD_N+Death	312	TRADD	vertebrata	Ochotona princeps	PREDICTED: tumor necrosis factor receptor type 1-associated DEATH domain protein [Ochotona princeps]	GCF_000292845.1	TRADD-N
XP_004625946.1	TRADD_N+Death	309	Tradd	vertebrata	Octodon degus	tumor necrosis factor receptor type 1-associated DEATH domain protein [Octodon degus]	GCF_000260255.1	TRADD-N
XP_004393459.1	TRADD_N+Death	313	TRADD	vertebrata	Odobenus rosmarus divergens	PREDICTED: tumor necrosis factor receptor type 1-associated DEATH domain protein isoform X2 [Odobenus rosmarus divergens]	GCF_000321225.1	TRADD-N
XP_012421593.1	TRADD_N+Death	324	TRADD	vertebrata	Odobenus rosmarus divergens	PREDICTED: tumor necrosis factor receptor type 1-associated DEATH domain protein isoform X1 [Odobenus rosmarus divergens]	GCF_000321225.1	TRADD-N

acc	architecture	len	gen.name	taxend	species	defline	gca	clade
XP_020740576.1	TRADD_N+Death	367	TRADD	vertebrata	Odocoileus virginianus texanus	tumor necrosis factor receptor type 1-associated DEATH domain protein isoform X1 [Odocoileus virginianus texanus]	GCF_002102435.1	TRADD-N
XP_020740577.1	TRADD_N+Death	357	TRADD	vertebrata	Odocoileus virginianus texanus	tumor necrosis factor receptor type 1-associated DEATH domain protein isoform X2 [Odocoileus virginianus texanus]	GCF_002102435.1	TRADD-N
XP_020740578.1	TRADD_N+Death	322	TRADD	vertebrata	Odocoileus virginianus texanus	tumor necrosis factor receptor type 1-associated DEATH domain protein isoform X3 [Odocoileus virginianus texanus]	GCF_002102435.1	TRADD-N
XP_020740579.1	TRADD_N+Death	312	TRADD	vertebrata	Odocoileus virginianus texanus	tumor necrosis factor receptor type 1-associated DEATH domain protein isoform X4 [Odocoileus virginianus texanus]	GCF_002102435.1	TRADD-N
XP_020740580.1	TRADD_N+Death	297	TRADD	vertebrata	Odocoileus virginianus texanus	tumor necrosis factor receptor type 1-associated DEATH domain protein isoform X5 [Odocoileus virginianus texanus]	GCF_002102435.1	TRADD-N
KFR15328.1	TRADD_N+Death	307	N306_14394	vertebrata	Opisthocomus hoazin	Tumor necrosis factor receptor type 1-associated DEATH domain protein, partial [Opisthocomus hoazin]	GCA_000692075.1	TRADD-N
XP_009938729.1	TRADD_N+Death	306	TRADD	vertebrata	Opisthocomus hoazin	PREDICTED: tumor necrosis factor receptor type 1-associated DEATH domain protein [Opisthocomus hoazin]	GCF_000692075.1	TRADD-N
XP_004280921.1	TRADD_N+Death	314	TRADD	vertebrata	Orcinus orca	PREDICTED: tumor necrosis factor receptor type 1-associated DEATH domain protein isoform X1 [Orcinus orca]	GCF_000331955.2	TRADD-N
XP_012392583.1	TRADD_N+Death	254	TRADD	vertebrata	Orcinus orca	PREDICTED: tumor necrosis factor receptor type 1-associated DEATH domain protein isoform X2 [Orcinus orca]	GCF_000331955.2	TRADD-N
XP_007938578.1	TRADD_N+Death	312	TRADD	vertebrata	Orycteropus afer afer	PREDICTED: tumor necrosis factor receptor type 1-associated DEATH domain protein [Orycteropus afer afer]	GCF_000298275.1	TRADD-N
XP_002711696.1	TRADD_N+Death	304	TRADD	vertebrata	Oryctolagus cuniculus	PREDICTED: tumor necrosis factor receptor type 1-associated DEATH domain protein [Oryctolagus cuniculus]	GCF_000003625.3	TRADD-N
XP_003799456.1	TRADD_N+Death	309	TRADD	vertebrata	Otolemur garnettii	tumor necrosis factor receptor type 1-associated DEATH domain protein [Otolemur garnettii]	GCF_000181295.1	TRADD-N
XP_004015107.1	TRADD_N+Death	312	TRADD	vertebrata	Ovis aries	PREDICTED: tumor necrosis factor receptor type 1-associated DEATH domain protein isoform X1 [Ovis aries]	-	TRADD-N

acc	architecture	len	gen.name	taxend	species	defline	gca	clade
XP_014197275.1	TRADD_N+Death	312	TRADD	vertebrata	Pan paniscus	LOW QUALITY PROTEIN: tumor necrosis factor receptor type 1-associated DEATH domain protein [Pan paniscus]	GCF_000258655.2	TRADD-N
XP_019311196.1	TRADD_N+Death	378	TRADD	vertebrata	Panthera pardus	PREDICTED: tumor necrosis factor receptor type 1-associated DEATH domain protein isoform X1 [Panthera pardus]	GCF_001857705.1	TRADD-N
XP_019311197.1	TRADD_N+Death	317	TRADD	vertebrata	Panthera pardus	PREDICTED: tumor necrosis factor receptor type 1-associated DEATH domain protein isoform X2 [Panthera pardus]	GCF_001857705.1	TRADD-N
XP_019311198.1	TRADD_N+Death	311	TRADD	vertebrata	Panthera pardus	PREDICTED: tumor necrosis factor receptor type 1-associated DEATH domain protein isoform X3 [Panthera pardus]	GCF_001857705.1	TRADD-N
XP_015396586.1	TRADD_N+Death	277	TRADD	vertebrata	Panthera tigris altaica	PREDICTED: tumor necrosis factor receptor type 1-associated DEATH domain protein [Panthera tigris altaica]	GCF_000464555.1	TRADD-N
XP_005959242.1	TRADD_N+Death	312	TRADD	vertebrata	Pantholops hodgsonii	PREDICTED: tumor necrosis factor receptor type 1-associated DEATH domain protein [Pantholops hodgsonii]	-	TRADD-N
XP_003917062.1	TRADD_N+Death	308	TRADD	vertebrata	Papio anubis	tumor necrosis factor receptor type 1-associated DEATH domain protein isoform X2 [Papio anubis]	GCF_000264685.3	TRADD-N
XP_017808975.1	TRADD_N+Death	366	TRADD	vertebrata	Papio anubis	tumor necrosis factor receptor type 1-associated DEATH domain protein isoform X1 [Papio anubis]	GCF_000264685.3	TRADD-N
XP_015495776.1	TRADD_N+Death	364	TRADD	vertebrata	Parus major	PREDICTED: tumor necrosis factor receptor type 1-associated DEATH domain protein isoform X1 [Parus major]	GCF_001522545.3	TRADD-N
XP_015495777.1	TRADD_N+Death	306	TRADD	vertebrata	Parus major	PREDICTED: tumor necrosis factor receptor type 1-associated DEATH domain protein isoform X2 [Parus major]	GCF_001522545.3	TRADD-N
KFQ65703.1	TRADD_N+Death	307	N334_10538	vertebrata	Pelecanus crispus	Tumor necrosis factor receptor type 1-associated DEATH domain protein, partial [Pelecanus crispus]	GCA_000687375.1	TRADD-N
XP_009488746.1	TRADD_N+Death	306	TRADD	vertebrata	Pelecanus crispus	PREDICTED: tumor necrosis factor receptor type 1-associated DEATH domain protein [Pelecanus crispus]	GCF_000687375.1	TRADD-N
XP_006121021.1	TRADD_N+Death	304	TRADD	vertebrata	Pelodiscus sinensis	tumor necrosis factor receptor type 1-associated DEATH domain protein [Pelodiscus sinensis]	GCF_000230535.1	TRADD-N
XP_006986839.1	TRADD_N+Death	310	Tradd	vertebrata	Peromyscus maniculatus bairdii	PREDICTED: tumor necrosis factor receptor type 1-associated DEATH domain protein [Peromyscus maniculatus bairdii]	GCF_000500345.1	TRADD-N

acc	architecture	len	gen.name	taxend	species	defline	gca	clade
KFQ71879.1	TRADD_N+Death	307	N335_03863	vertebrata	Phaethon lepturus	Tumor necrosis factor receptor type 1-associated DEATH domain protein, partial [Phaethon lepturus]	GCA_000687285.1	TRADD-N
XP_010281617.1	TRADD_N+Death	306	TRADD	vertebrata	Phaethon lepturus	PREDICTED: tumor necrosis factor receptor type 1-associated DEATH domain protein [Phaethon lepturus]	GCF_000687285.1	TRADD-N
KFW89154.1	TRADD_N+Death	307	N336_02754	vertebrata	Phalacrocorax carbo	Tumor necrosis factor receptor type 1-associated DEATH domain protein, partial [Phalacrocorax carbo]	GCA_000708925.1	TRADD-N
XP_009499517.1	TRADD_N+Death	306	TRADD	vertebrata	Phalacrocorax carbo	PREDICTED: tumor necrosis factor receptor type 1-associated DEATH domain protein [Phalacrocorax carbo]	GCF_000708925.1	TRADD-N
XP_020845056.1	TRADD_N+Death	351	TRADD	vertebrata	Phascolarctos cinereus	tumor necrosis factor receptor type 1-associated DEATH domain protein isoform X1 [Phascolarctos cinereus]	GCF_002099425.1	TRADD-N
XP_020845057.1	TRADD_N+Death	332	TRADD	vertebrata	Phascolarctos cinereus	tumor necrosis factor receptor type 1-associated DEATH domain protein isoform X2 [Phascolarctos cinereus]	GCF_002099425.1	TRADD-N
XP_020845058.1	TRADD_N+Death	332	TRADD	vertebrata	Phascolarctos cinereus	tumor necrosis factor receptor type 1-associated DEATH domain protein isoform X3 [Phascolarctos cinereus]	GCF_002099425.1	TRADD-N
KFQ78172.1	TRADD_N+Death	306	N337_11812	vertebrata	Phoenicopterus ruber ruber	Tumor necrosis factor receptor type 1-associated DEATH domain protein, partial [Phoenicopterus ruber ruber]	GCA_000687265.1	TRADD-N
XP_007125939.1	TRADD_N+Death	313	TRADD	vertebrata	Physeter catodon	tumor necrosis factor receptor type 1-associated DEATH domain protein [Physeter catodon]	GCF_002837175.2	TRADD-N
KFV66369.1	TRADD_N+Death	306	N307_05671	vertebrata	Picoides pubescens	Tumor necrosis factor receptor type 1-associated DEATH domain protein, partial [Picoides pubescens]	GCA_000699005.1	TRADD-N
XP_009897449.1	TRADD_N+Death	305	TRADD	vertebrata	Picoides pubescens	PREDICTED: tumor necrosis factor receptor type 1-associated DEATH domain protein [Picoides pubescens]	GCF_000699005.1	TRADD-N
XP_023065900.1	TRADD_N+Death	346	TRADD	vertebrata	Piliocolobus tephrosceles	tumor necrosis factor receptor type 1-associated DEATH domain protein isoform X1 [Piliocolobus tephrosceles]	GCF_002776525.2	TRADD-N
XP_023065901.1	TRADD_N+Death	308	TRADD	vertebrata	Piliocolobus tephrosceles	tumor necrosis factor receptor type 1-associated DEATH domain protein isoform X2 [Piliocolobus tephrosceles]	GCF_002776525.2	TRADD-N
XP_023065902.1	TRADD_N+Death	248	TRADD	vertebrata	Piliocolobus tephrosceles	tumor necrosis factor receptor type 1-associated DEATH domain protein isoform X3 [Piliocolobus tephrosceles]	GCF_002776525.2	TRADD-N
KFZ64629.1	TRADD_N+Death	307	N338_09052	vertebrata	Podiceps cristatus	Tumor necrosis factor receptor type 1-associated DEATH domain protein, partial [Podiceps cristatus]	GCA_000699545.1	TRADD-N

acc	architecture	len	gen.name	taxend	species	defline	gca	clade
XP_020661796.1	TRADD_N+Death	355	TRADD	vertebrata	Pogona vitticeps	tumor necrosis factor receptor type 1-associated DEATH domain protein isoform X1 [Pogona vitticeps]	GCF_900067755.1	TRADD-N
XP_020661797.1	TRADD_N+Death	326	TRADD	vertebrata	Pogona vitticeps	tumor necrosis factor receptor type 1-associated DEATH domain protein isoform X2 [Pogona vitticeps]	GCF_900067755.1	TRADD-N
XP_024089129.1	TRADD_N+Death	311	TRADD	vertebrata	Pongo abelii	tumor necrosis factor receptor type 1-associated DEATH domain protein isoform X1 [Pongo abelii]	GCF_002880775.1	TRADD-N
XP_024089134.1	TRADD_N+Death	251	TRADD	vertebrata	Pongo abelii	tumor necrosis factor receptor type 1-associated DEATH domain protein isoform X2 [Pongo abelii]	GCF_002880775.1	TRADD-N
XP_012516046.1	TRADD_N+Death	309	TRADD	vertebrata	Propithecus coquereli	PREDICTED: tumor necrosis factor receptor type 1-associated DEATH domain protein [Propithecus coquereli]	GCF_000956105.1	TRADD-N
XP_015678437.1	TRADD_N+Death	352	TRADD	vertebrata	Protobothrops mucrosquamatus	PREDICTED: tumor necrosis factor receptor type 1-associated DEATH domain protein [Protobothrops mucrosquamatus]	GCF_001527695.2	TRADD-N
XP_026570211.1	TRADD_N+Death	311	TRADD	vertebrata	Pseudonaja textilis	tumor necrosis factor receptor type 1-associated DEATH domain protein [Pseudonaja textilis]	GCF_900518735.1	TRADD-N
XP_005526385.1	TRADD_N+Death	306	TRADD	vertebrata	Pseudopodoces humilis	PREDICTED: tumor necrosis factor receptor type 1-associated DEATH domain protein [Pseudopodoces humilis]	GCF_000331425.1	TRADD-N
KFV05659.1	TRADD_N+Death	306	N339_07494	vertebrata	Pterocles gutturalis	Tumor necrosis factor receptor type 1-associated DEATH domain protein, partial [Pterocles gutturalis]	GCA_000699245.1	TRADD-N
XP_010086149.1	TRADD_N+Death	305	TRADD	vertebrata	Pterocles gutturalis	PREDICTED: tumor necrosis factor receptor type 1-associated DEATH domain protein [Pterocles gutturalis]	GCF_000699245.1	TRADD-N
XP_006909805.1	TRADD_N+Death	310	TRADD	vertebrata	Pteropus alecto	tumor necrosis factor receptor type 1-associated DEATH domain protein [Pteropus alecto]	GCF_000325575.1	TRADD-N
XP_011378738.1	TRADD_N+Death	310	TRADD	vertebrata	Pteropus vampyrus	tumor necrosis factor receptor type 1-associated DEATH domain protein [Pteropus vampyrus]	GCF_000151845.1	TRADD-N
KFW64901.1	TRADD_N+Death	307	AS28_12927	vertebrata	Pygoscelis adeliae	Tumor necrosis factor receptor type 1-associated DEATH domain protein, partial [Pygoscelis adeliae]	GCA_000699105.1	TRADD-N
XP_009319092.1	TRADD_N+Death	306	TRADD	vertebrata	Pygoscelis adeliae	PREDICTED: tumor necrosis factor receptor type 1-associated DEATH domain protein [Pygoscelis adeliae]	GCF_000699105.1	TRADD-N
XP_007430728.1	TRADD_N+Death	316	TRADD	vertebrata	Python bivittatus	tumor necrosis factor receptor type 1-associated DEATH domain protein isoform X2 [Python bivittatus]	GCF_000186305.1	TRADD-N
XP_025023978.1	TRADD_N+Death	361	TRADD	vertebrata	Python bivittatus	tumor necrosis factor receptor type 1-associated DEATH domain protein isoform X1 [Python bivittatus]	GCF_000186305.1	TRADD-N

acc	architecture	len	gen.name	taxend	species	defline	gca	clade
NP_001093950.1	TRADD_N+Death	310	Tradd	vertebrata	Rattus norvegicus	tumor necrosis factor receptor type 1-associated DEATH domain protein [Rattus norvegicus]	GCF_000002265.2	TRADD-N
XP_006255504.1	TRADD_N+Death	358	Tradd	vertebrata	Rattus norvegicus	PREDICTED: tumor necrosis factor receptor type 1-associated DEATH domain protein isoform X1 [Rattus norvegicus]	GCF_000001895.5	TRADD-N
XP_020378542.1	TRADD_N+Death	317	tradd	vertebrata	Rhincodon typus	tumor necrosis factor receptor type 1-associated DEATH domain protein isoform X1 [Rhincodon typus]	GCF_001642345.1	TRADD-N
XP_020378550.1	TRADD_N+Death	313	tradd	vertebrata	Rhincodon typus	tumor necrosis factor receptor type 1-associated DEATH domain protein isoform X2 [Rhincodon typus]	GCF_001642345.1	TRADD-N
XP_019611094.1	TRADD_N+Death	309	TRADD	vertebrata	Rhinolophus sinicus	PREDICTED: tumor necrosis factor receptor type 1-associated DEATH domain protein isoform X1 [Rhinolophus sinicus]	-	TRADD-N
XP_019611097.1	TRADD_N+Death	253	TRADD	vertebrata	Rhinolophus sinicus	PREDICTED: tumor necrosis factor receptor type 1-associated DEATH domain protein isoform X2 [Rhinolophus sinicus]	-	TRADD-N
XP_017707803.1	TRADD_N+Death	349	TRADD	vertebrata	Rhinopithecus bieti	PREDICTED: tumor necrosis factor receptor type 1-associated DEATH domain protein isoform X1 [Rhinopithecus bieti]	GCF_001698545.1	TRADD-N
XP_017707804.1	TRADD_N+Death	308	TRADD	vertebrata	Rhinopithecus bieti	PREDICTED: tumor necrosis factor receptor type 1-associated DEATH domain protein isoform X2 [Rhinopithecus bieti]	GCF_001698545.1	TRADD-N
XP_017707805.1	TRADD_N+Death	248	TRADD	vertebrata	Rhinopithecus bieti	PREDICTED: tumor necrosis factor receptor type 1-associated DEATH domain protein isoform X3 [Rhinopithecus bieti]	GCF_001698545.1	TRADD-N
XP_010353442.1	TRADD_N+Death	308	TRADD	vertebrata	Rhinopithecus roxellana	PREDICTED: tumor necrosis factor receptor type 1-associated DEATH domain protein isoform X1 [Rhinopithecus roxellana]	GCF_007565055.1	TRADD-N
XP_010353443.1	TRADD_N+Death	248	TRADD	vertebrata	Rhinopithecus roxellana	PREDICTED: tumor necrosis factor receptor type 1-associated DEATH domain protein isoform X2 [Rhinopithecus roxellana]	GCF_007565055.1	TRADD-N
XP_016018941.1	TRADD_N+Death	310	TRADD	vertebrata	Rousettus aegyptiacus	PREDICTED: tumor necrosis factor receptor type 1-associated DEATH domain protein isoform X1 [Rousettus aegyptiacus]	GCF_001466805.2	TRADD-N
XP_016018971.1	TRADD_N+Death	251	TRADD	vertebrata	Rousettus aegyptiacus	PREDICTED: tumor necrosis factor receptor type 1-associated DEATH domain protein isoform X2 [Rousettus aegyptiacus]	GCF_001466805.2	TRADD-N

acc	architecture	len	gen.name	taxend	species	defline	gca	clade
XP_003936333.1	TRADD_N+Death	312	TRADD	vertebrata	Saimiri boliviensis boliviensis	PREDICTED: tumor necrosis factor receptor type 1-associated DEATH domain protein isoform X1 [Saimiri boliviensis boliviensis]	GCF_000235385.1	TRADD-N
XP_010345051.1	TRADD_N+Death	252	TRADD	vertebrata	Saimiri boliviensis boliviensis	PREDICTED: tumor necrosis factor receptor type 1-associated DEATH domain protein isoform X2 [Saimiri boliviensis boliviensis]	GCF_000235385.1	TRADD-N
XP_009088901.1	TRADD_N+Death	306	TRADD	vertebrata	Serinus canaria	PREDICTED: tumor necrosis factor receptor type 1-associated DEATH domain protein [Serinus canaria]	GCF_007115625.1	TRADD-N
XP_004600669.1	TRADD_N+Death	317	TRADD	vertebrata	Sorex araneus	PREDICTED: tumor necrosis factor receptor type 1-associated DEATH domain protein [Sorex araneus]	GCF_000181275.1	TRADD-N
KFV83046.1	TRADD_N+Death	305	N308_03904	vertebrata	Struthio camelus australis	Tumor necrosis factor receptor type 1-associated DEATH domain protein, partial [Struthio camelus australis]	GCA_000698965.1	TRADD-N
XP_009676546.1	TRADD_N+Death	306	TRADD	vertebrata	Struthio camelus australis	PREDICTED: tumor necrosis factor receptor type 1-associated DEATH domain protein [Struthio camelus australis]	GCF_000698965.1	TRADD-N
XP_014736314.1	TRADD_N+Death	306	TRADD	vertebrata	Sturnus vulgaris	PREDICTED: tumor necrosis factor receptor type 1-associated DEATH domain protein [Sturnus vulgaris]	GCF_001447265.1	TRADD-N
XP_020949706.1	TRADD_N+Death	304	TRADD	vertebrata	Sus scrofa	tumor necrosis factor receptor type 1-associated DEATH domain protein [Sus scrofa]	GCF_000003025.6	TRADD-N
XP_012425553.1	TRADD_N+Death	348	TRADD	vertebrata	Taeniopygia guttata	PREDICTED: tumor necrosis factor receptor type 1-associated DEATH domain protein [Taeniopygia guttata]	-	TRADD-N
KFV14431.1	TRADD_N+Death	307	N340_07016	vertebrata	Tauraco erythrolophus	Tumor necrosis factor receptor type 1-associated DEATH domain protein, partial [Tauraco erythrolophus]	GCA_000709365.1	TRADD-N
XP_009980988.1	TRADD_N+Death	306	TRADD	vertebrata	Tauraco erythrolophus	PREDICTED: tumor necrosis factor receptor type 1-associated DEATH domain protein [Tauraco erythrolophus]	GCF_000709365.1	TRADD-N
XP_024058017.1	TRADD_N+Death	307	TRADD	vertebrata	Terrapene mexicana triunguis	tumor necrosis factor receptor type 1-associated DEATH domain protein [Terrapene mexicana triunguis]	GCF_002925995.2	TRADD-N
XP_013921136.1	TRADD_N+Death	248	TRADD	vertebrata	Thamnophis sirtalis	PREDICTED: tumor necrosis factor receptor type 1-associated DEATH domain protein [Thamnophis sirtalis]	GCF_001077635.1	TRADD-N
XP_025226729.1	TRADD_N+Death	366	TRADD	vertebrata	Theropithecus gelada	tumor necrosis factor receptor type 1-associated DEATH domain protein isoform X1 [Theropithecus gelada]	GCF_003255815.1	TRADD-N
KGL82346.1	TRADD_N+Death	307	N309_01012	vertebrata	Tinamus guttatus	Tumor necrosis factor receptor type 1-associated DEATH domain protein, partial [Tinamus guttatus]	GCA_000705375.2	TRADD-N

acc	architecture	len	gen.name	taxend	species	defline	gca	clade
XP_010220336.1	TRADD_N+Death	306	TRADD	vertebrata	Tinamus guttatus	PREDICTED: tumor necrosis factor receptor type 1-associated DEATH domain protein [Tinamus guttatus]	GCF_000705375.1	TRADD-N
XP_004371564.1	TRADD_N+Death	309	LOC101341635	vertebrata	Trichechus manatus latirostris	tumor necrosis factor receptor type 1-associated DEATH domain protein [Trichechus manatus latirostris]	GCF_000243295.1	TRADD-N
XP_006155647.1	TRADD_N+Death	310	TRADD	vertebrata	Tupaia chinensis	PREDICTED: tumor necrosis factor receptor type 1-associated DEATH domain protein isoform X2 [Tupaia chinensis]	GCF_000334495.1	TRADD-N
XP_006155649.2	TRADD_N+Death	318	TRADD	vertebrata	Tupaia chinensis	PREDICTED: tumor necrosis factor receptor type 1-associated DEATH domain protein isoform X1 [Tupaia chinensis]	GCF_000334495.1	TRADD-N
XP_019791250.1	TRADD_N+Death	314	TRADD	vertebrata	Tursiops truncatus	PREDICTED: tumor necrosis factor receptor type 1-associated DEATH domain protein [Tursiops truncatus]	GCF_001922835.1	TRADD-N
KFV45478.1	TRADD_N+Death	306	N341_10343	vertebrata	Tyto alba	Tumor necrosis factor receptor type 1-associated DEATH domain protein, partial [Tyto alba]	GCA_000687205.1	TRADD-N
XP_009975372.1	TRADD_N+Death	305	TRADD	vertebrata	Tyto alba	PREDICTED: tumor necrosis factor receptor type 1-associated DEATH domain protein [Tyto alba]	GCF_000687205.1	TRADD-N
XP_026269046.1	TRADD_N+Death	314	Tradd	vertebrata	Urocitellus parryii	tumor necrosis factor receptor type 1-associated DEATH domain protein [Urocitellus parryii]	GCF_003426925.1	TRADD-N
XP_026350765.1	TRADD_N+Death	325	TRADD	vertebrata	Ursus arctos horribilis	tumor necrosis factor receptor type 1-associated DEATH domain protein isoform X1 [Ursus arctos horribilis]	GCF_003584765.1	TRADD-N
XP_026350766.1	TRADD_N+Death	314	TRADD	vertebrata	Ursus arctos horribilis	tumor necrosis factor receptor type 1-associated DEATH domain protein isoform X2 [Ursus arctos horribilis]	GCF_003584765.1	TRADD-N
XP_008700708.1	TRADD_N+Death	325	TRADD	vertebrata	Ursus maritimus	PREDICTED: tumor necrosis factor receptor type 1-associated DEATH domain protein isoform X1 [Ursus maritimus]	GCF_000687225.1	TRADD-N
XP_008700710.1	TRADD_N+Death	314	TRADD	vertebrata	Ursus maritimus	PREDICTED: tumor necrosis factor receptor type 1-associated DEATH domain protein isoform X2 [Ursus maritimus]	GCF_000687225.1	TRADD-N
XP_006203738.1	TRADD_N+Death	309	TRADD	vertebrata	Vicugna pacos	PREDICTED: tumor necrosis factor receptor type 1-associated DEATH domain protein [Vicugna pacos]	GCF_000164845.2	TRADD-N
XP_025867256.1	TRADD_N+Death	324	TRADD	vertebrata	Vulpes vulpes	tumor necrosis factor receptor type 1-associated DEATH domain protein isoform X1 [Vulpes vulpes]	GCF_003160815.1	TRADD-N
XP_025867257.1	TRADD_N+Death	313	TRADD	vertebrata	Vulpes vulpes	tumor necrosis factor receptor type 1-associated DEATH domain protein isoform X2 [Vulpes vulpes]	GCF_003160815.1	TRADD-N
AAI08638.1	TRADD_N+Death	330	tradd	vertebrata	Xenopus laevis	Tradd protein, partial [Xenopus laevis]	-	TRADD-N

acc	architecture	len	gen.name	taxend	species	defline	gca	clade
XP_018113350.1	TRADD_N+Death	303	tradd.L	vertebrata	Xenopus laevis	PREDICTED: tumor necrosis factor receptor type 1-associated DEATH domain protein [Xenopus laevis]	GCF_001663975.1	TRADD-N
XP_002937214.1	TRADD_N+Death	303	tradd	vertebrata	Xenopus tropicalis	PREDICTED: tumor necrosis factor receptor type 1-associated DEATH domain protein [Xenopus tropicalis]	GCF_000004195.3	TRADD-N
XP_026651507.1	TRADD_N+Death	363	TRADD	vertebrata	Zonotrichia albicollis	LOW QUALITY PROTEIN: tumor necrosis factor receptor type 1-associated DEATH domain protein [Zonotrichia albicollis]	GCF_000385455.1	TRADD-N
ROL48186.1	DED+TRADDN-DEDD2	515	DPX16_6681	actinopterygii	Anabarilius grahami	DNA-binding death effector domain-containing protein 2 [Anabarilius grahami]	GCA_003731715.1	TRADDN-DEDD2
KKF31408.1	EF-hand+EF-hand+NLPC+DED+TRADDN-DEDD2	705	EH28_02103	actinopterygii	Larimichthys crocea	Death effector domain-containing protein [Larimichthys crocea]	-	TRADDN-DEDD2
XP_022066547.1	low-complexity+DED+TRADDN-DEDD2	376	dedd2	actinopterygii	Acanthochromis polyacanthus	DNA-binding death effector domain-containing protein 2 [Acanthochromis polyacanthus]	GCF_002109545.1	TRADDN-DEDD2
XP_022074822.1	low-complexity+DED+TRADDN-DEDD2	404	LOC110969101	actinopterygii	Acanthochromis polyacanthus	death effector domain-containing protein-like [Acanthochromis polyacanthus]	GCF_002109545.1	TRADDN-DEDD2
XP_023131844.1	low-complexity+DED+TRADDN-DEDD2	381	dedd2	actinopterygii	Amphiprion ocellaris	DNA-binding death effector domain-containing protein 2 isoform X1 [Amphiprion ocellaris]	GCF_002776465.1	TRADDN-DEDD2
XP_023131845.1	low-complexity+DED+TRADDN-DEDD2	376	dedd2	actinopterygii	Amphiprion ocellaris	DNA-binding death effector domain-containing protein 2 isoform X2 [Amphiprion ocellaris]	GCF_002776465.1	TRADDN-DEDD2
XP_023147422.1	low-complexity+DED+TRADDN-DEDD2	404	LOC111582805	actinopterygii	Amphiprion ocellaris	death effector domain-containing protein-like [Amphiprion ocellaris]	GCF_002776465.1	TRADDN-DEDD2
XP_026223749.1	low-complexity+DED+TRADDN-DEDD2	404	dedd	actinopterygii	Anabas testudineus	death effector domain-containing protein [Anabas testudineus]	GCF_900324465.1	TRADDN-DEDD2
XP_026226393.1	low-complexity+DED+TRADDN-DEDD2	385	dedd2	actinopterygii	Anabas testudineus	DNA-binding death effector domain-containing protein 2 isoform X1 [Anabas testudineus]	GCF_900324465.1	TRADDN-DEDD2
XP_026226395.1	low-complexity+DED+TRADDN-DEDD2	379	dedd2	actinopterygii	Anabas testudineus	DNA-binding death effector domain-containing protein 2 isoform X2 [Anabas testudineus]	GCF_900324465.1	TRADDN-DEDD2
XP_026039561.1	low-complexity+DED+TRADDN-DEDD2	382	dedd2	actinopterygii	Astatotilapia calliptera	DNA-binding death effector domain-containing protein 2 isoform X1 [Astatotilapia calliptera]	GCF_900246225.1	TRADDN-DEDD2
XP_026039565.1	low-complexity+DED+TRADDN-DEDD2	379	dedd2	actinopterygii	Astatotilapia calliptera	DNA-binding death effector domain-containing protein 2 isoform X2 [Astatotilapia calliptera]	GCF_900246225.1	TRADDN-DEDD2
XP_007237013.2	low-complexity+DED+TRADDN-DEDD2	402	dedd	actinopterygii	Astyanax mexicanus	death effector domain-containing protein [Astyanax mexicanus]	GCF_000372685.2	TRADDN-DEDD2
XP_007250919.1	low-complexity+DED+TRADDN-DEDD2	364	dedd2	actinopterygii	Astyanax mexicanus	DNA-binding death effector domain-containing protein 2 [Astyanax mexicanus]	GCF_000372685.2	TRADDN-DEDD2
XP_013868863.1	low-complexity+DED+TRADDN-DEDD2	377	dedd2	actinopterygii	Austrofundulus limnaeus	PREDICTED: DNA-binding death effector domain-containing protein 2 [Austrofundulus limnaeus]	GCF_001266775.1	TRADDN-DEDD2

acc	architecture	len	gen.name	taxend	species	defline	gca	clade
XP_013877708.1	low-complexity+DED+TRADDN-DEDD2	402	LOC106527389	actinopterygii	Austrofundulus limnaeus	PREDICTED: death effector domain-containing protein-like [Austrofundulus limnaeus]	GCF_001266775.1	TRADDN-DEDD2
XP_020787371.1	low-complexity+DED+TRADDN-DEDD2	335	dedd	actinopterygii	Boleophthalmus pectinirostris	death effector domain-containing protein [Boleophthalmus pectinirostris]	GCF_000788275.1	TRADDN-DEDD2
XP_020796249.1	low-complexity+DED+TRADDN-DEDD2	384	dedd2	actinopterygii	Boleophthalmus pectinirostris	DNA-binding death effector domain-containing protein 2 [Boleophthalmus pectinirostris]	GCF_000788275.1	TRADDN-DEDD2
XP_026084519.1	low-complexity+DED+TRADDN-DEDD2	370	LOC113059990	actinopterygii	Carassius auratus	DNA-binding death effector domain-containing protein 2-like [Carassius auratus]	GCF_003368295.1	TRADDN-DEDD2
XP_026084564.1	low-complexity+DED+TRADDN-DEDD2	370	LOC113060014	actinopterygii	Carassius auratus	DNA-binding death effector domain-containing protein 2-like [Carassius auratus]	GCF_003368295.1	TRADDN-DEDD2
XP_026105718.1	low-complexity+DED+TRADDN-DEDD2	404	LOC113077620	actinopterygii	Carassius auratus	death effector domain-containing protein-like [Carassius auratus]	GCF_003368295.1	TRADDN-DEDD2
XP_026115049.1	low-complexity+DED+TRADDN-DEDD2	404	LOC113093575	actinopterygii	Carassius auratus	death effector domain-containing protein-like [Carassius auratus]	GCF_003368295.1	TRADDN-DEDD2
XP_026125202.1	low-complexity+DED+TRADDN-DEDD2	404	LOC113107143	actinopterygii	Carassius auratus	death effector domain-containing protein-like [Carassius auratus]	GCF_003368295.1	TRADDN-DEDD2
XP_026140354.1	low-complexity+DED+TRADDN-DEDD2	365	LOC113116413	actinopterygii	Carassius auratus	DNA-binding death effector domain-containing protein 2-like [Carassius auratus]	GCF_003368295.1	TRADDN-DEDD2
XP_012675412.1	low-complexity+DED+TRADDN-DEDD2	393	dedd2	actinopterygii	Clupea harengus	PREDICTED: DNA-binding death effector domain-containing protein 2 isoform X1 [Clupea harengus]	GCF_000966335.1	TRADDN-DEDD2
XP_012675413.1	low-complexity+DED+TRADDN-DEDD2	377	dedd2	actinopterygii	Clupea harengus	PREDICTED: DNA-binding death effector domain-containing protein 2 isoform X2 [Clupea harengus]	GCF_000966335.1	TRADDN-DEDD2
XP_012690051.1	low-complexity+DED+TRADDN-DEDD2	414	dedd	actinopterygii	Clupea harengus	PREDICTED: death effector domain-containing protein isoform X1 [Clupea harengus]	GCF_000966335.1	TRADDN-DEDD2
XP_012690052.1	low-complexity+DED+TRADDN-DEDD2	412	dedd	actinopterygii	Clupea harengus	PREDICTED: death effector domain-containing protein isoform X2 [Clupea harengus]	GCF_000966335.1	TRADDN-DEDD2
XP_008316760.1	low-complexity+DED+TRADDN-DEDD2	403	LOC103384854	actinopterygii	Cynoglossus semilaevis	death effector domain-containing protein [Cynoglossus semilaevis]	GCF_000523025.1	TRADDN-DEDD2
XP_008321053.1	low-complexity+DED+TRADDN-DEDD2	375	dedd2	actinopterygii	Cynoglossus semilaevis	DNA-binding death effector domain-containing protein 2 isoform X3 [Cynoglossus semilaevis]	GCF_000523025.1	TRADDN-DEDD2
XP_015226246.1	low-complexity+DED+TRADDN-DEDD2	377	dedd2	actinopterygii	Cyprinodon variegatus	PREDICTED: DNA-binding death effector domain-containing protein 2 [Cyprinodon variegatus]	GCF_000732505.1	TRADDN-DEDD2
XP_015239779.1	low-complexity+DED+TRADDN-DEDD2	401	dedd	actinopterygii	Cyprinodon variegatus	PREDICTED: death effector domain-containing protein [Cyprinodon variegatus]	GCF_000732505.1	TRADDN-DEDD2
KTF84371.1	low-complexity+DED+TRADDN-DEDD2	401	cypCar_00035271	actinopterygii	Cyprinus carpio	hypothetical protein cypCar_00035271 [Cyprinus carpio]	GCA_001270105.1	TRADDN-DEDD2
KTF94442.1	low-complexity+DED+TRADDN-DEDD2	369	cypCar_00027008	actinopterygii	Cyprinus carpio	hypothetical protein cypCar_00027008 [Cyprinus carpio]	GCA_001270105.1	TRADDN-DEDD2

acc	architecture	len	gen.name	taxend	species	defline	gca	clade
XP_018936189.1	low-complexity+DED+TRADDN-DEDD2	384	LOC109063602	actinopterygii	Cyprinus carpio	PREDICTED: death effector domain-containing protein-like [Cyprinus carpio]	GCF_000951615.1	TRADDN-DEDD2
XP_018937141.1	low-complexity+DED+TRADDN-DEDD2	404	LOC109064552	actinopterygii	Cyprinus carpio	PREDICTED: death effector domain-containing protein-like [Cyprinus carpio]	GCF_000951615.1	TRADDN-DEDD2
XP_018973526.1	low-complexity+DED+TRADDN-DEDD2	370	dedd2	actinopterygii	Cyprinus carpio	PREDICTED: DNA-binding death effector domain-containing protein 2 [Cyprinus carpio]	GCF_000951615.1	TRADDN-DEDD2
AAF66963.1	low-complexity+DED+TRADDN-DEDD2	369	dedd1	actinopterygii	Danio rerio	Dedd1 [Danio rerio]	-	TRADDN-DEDD2
NP_001002639.1	low-complexity+DED+TRADDN-DEDD2	404	fk97g03,wu:fk97g03,zgc:92202	actinopterygii	Danio rerio	death effector domain-containing protein [Danio rerio]	GCF_000002035.6	TRADDN-DEDD2
NP_571677.2	low-complexity+DED+TRADDN-DEDD2	369	dedd1	actinopterygii	Danio rerio	DNA-binding death effector domain-containing protein 2 [Danio rerio]	GCF_000002035.6	TRADDN-DEDD2
XP_026875241.1	low-complexity+DED+TRADDN-DEDD2	373	dedd2	actinopterygii	Electrophorus electricus	DNA-binding death effector domain-containing protein 2 [Electrophorus electricus]	GCF_003665695.1	TRADDN-DEDD2
XP_026881685.1	low-complexity+DED+TRADDN-DEDD2	403	LOC113587292	actinopterygii	Electrophorus electricus	death effector domain-containing protein-like [Electrophorus electricus]	GCF_003665695.1	TRADDN-DEDD2
XP_010897051.1	low-complexity+DED+TRADDN-DEDD2	413	dedd	actinopterygii	Esox lucius	PREDICTED: death effector domain-containing protein [Esox lucius]	GCF_004634155.1	TRADDN-DEDD2
XP_010901875.1	low-complexity+DED+TRADDN-DEDD2	396	dedd2	actinopterygii	Esox lucius	PREDICTED: DNA-binding death effector domain-containing protein 2 isoform X1 [Esox lucius]	GCF_004634155.1	TRADDN-DEDD2
XP_010901879.1	low-complexity+DED+TRADDN-DEDD2	385	dedd2	actinopterygii	Esox lucius	PREDICTED: DNA-binding death effector domain-containing protein 2 isoform X2 [Esox lucius]	GCF_004634155.1	TRADDN-DEDD2
XP_012711055.1	low-complexity+DED+TRADDN-DEDD2	404	LOC105920096	actinopterygii	Fundulus heteroclitus	death effector domain-containing protein [Fundulus heteroclitus]	GCF_000826765.1	TRADDN-DEDD2
XP_012725727.1	low-complexity+DED+TRADDN-DEDD2	377	dedd2	actinopterygii	Fundulus heteroclitus	DNA-binding death effector domain-containing protein 2 [Fundulus heteroclitus]	GCF_000826765.1	TRADDN-DEDD2
PWA24942.1	low-complexity+DED+TRADDN-DEDD2	372	CCH79_00015437	actinopterygii	Gambusia affinis	hypothetical protein CCH79_00015437, partial [Gambusia affinis]	GCA_003097735.1	TRADDN-DEDD2
PWA27946.1	low-complexity+DED+TRADDN-DEDD2	396	CCH79_00020622	actinopterygii	Gambusia affinis	hypothetical protein CCH79_00020622 [Gambusia affinis]	GCA_003097735.1	TRADDN-DEDD2
XP_005925214.1	low-complexity+DED+TRADDN-DEDD2	379	LOC102289080	actinopterygii	Haplochromis burtoni	PREDICTED: DNA-binding death effector domain-containing protein 2-like isoform X2 [Haplochromis burtoni]	GCF_000239415.1	TRADDN-DEDD2
XP_014189945.1	low-complexity+DED+TRADDN-DEDD2	382	LOC102289080	actinopterygii	Haplochromis burtoni	PREDICTED: DNA-binding death effector domain-containing protein 2-like isoform X1 [Haplochromis burtoni]	GCF_000239415.1	TRADDN-DEDD2
XP_019747132.1	low-complexity+DED+TRADDN-DEDD2	386	LOC109528674	actinopterygii	Hippocampus comes	PREDICTED: death effector domain-containing protein-like [Hippocampus comes]	GCF_001891065.1	TRADDN-DEDD2

acc	architecture	len	gen.name	taxend	species	defline	gca	clade
XP_019751686.1	low-complexity+DED+TRADDN-DEDD2	383	dedd2	actinopterygii	Hippocampus comes	PREDICTED: DNA-binding death effector domain-containing protein 2 [Hippocampus comes]	GCF_001891065.1	TRADDN-DEDD2
XP_017323845.1	low-complexity+DED+TRADDN-DEDD2	368	LOC108265722	actinopterygii	Ictalurus punctatus	PREDICTED: death effector domain-containing protein-like [Ictalurus punctatus]	GCF_001660625.1	TRADDN-DEDD2
XP_017325003.1	low-complexity+DED+TRADDN-DEDD2	361	dedd2	actinopterygii	Ictalurus punctatus	PREDICTED: DNA-binding death effector domain-containing protein 2 [Ictalurus punctatus]	GCF_001660625.1	TRADDN-DEDD2
XP_017295691.1	low-complexity+DED+TRADDN-DEDD2	402	LOC108250358	actinopterygii	Kryptolebias marmoratus	death effector domain-containing protein-like [Kryptolebias marmoratus]	GCF_001649575.1	TRADDN-DEDD2
XP_024861545.1	low-complexity+DED+TRADDN-DEDD2	383	dedd2	actinopterygii	Kryptolebias marmoratus	DNA-binding death effector domain-containing protein 2 [Kryptolebias marmoratus]	GCF_001649575.1	TRADDN-DEDD2
XP_020486007.1	low-complexity+DED+TRADDN-DEDD2	402	LOC109981527	actinopterygii	Labrus bergylta	death effector domain-containing protein-like isoform X1 [Labrus bergylta]	GCF_900080235.1	TRADDN-DEDD2
XP_020486008.1	low-complexity+DED+TRADDN-DEDD2	367	LOC109981527	actinopterygii	Labrus bergylta	death effector domain-containing protein-like isoform X2 [Labrus bergylta]	-	TRADDN-DEDD2
XP_020504738.1	low-complexity+DED+TRADDN-DEDD2	383	dedd2	actinopterygii	Labrus bergylta	DNA-binding death effector domain-containing protein 2 [Labrus bergylta]	GCF_900080235.1	TRADDN-DEDD2
XP_010733338.2	low-complexity+DED+TRADDN-DEDD2	388	LOC104922252	actinopterygii	Larimichthys crocea	PREDICTED: DNA-binding death effector domain-containing protein 2 isoform X1 [Larimichthys crocea]	GCF_000972845.2	TRADDN-DEDD2
XP_019112568.1	low-complexity+DED+TRADDN-DEDD2	402	LOC104934583	actinopterygii	Larimichthys crocea	PREDICTED: death effector domain-containing protein [Larimichthys crocea]	GCF_000972845.2	TRADDN-DEDD2
XP_019116818.1	low-complexity+DED+TRADDN-DEDD2	345	LOC104922252	actinopterygii	Larimichthys crocea	PREDICTED: DNA-binding death effector domain-containing protein 2 isoform X2 [Larimichthys crocea]	GCF_000972845.2	TRADDN-DEDD2
XP_018527852.1	low-complexity+DED+TRADDN-DEDD2	412	dedd2	actinopterygii	Lates calcarifer	PREDICTED: DNA-binding death effector domain-containing protein 2 isoform X1 [Lates calcarifer]	GCF_001640805.1	TRADDN-DEDD2
XP_018527853.1	low-complexity+DED+TRADDN-DEDD2	410	dedd2	actinopterygii	Lates calcarifer	PREDICTED: DNA-binding death effector domain-containing protein 2 isoform X2 [Lates calcarifer]	GCF_001640805.1	TRADDN-DEDD2
XP_018527854.1	low-complexity+DED+TRADDN-DEDD2	406	dedd2	actinopterygii	Lates calcarifer	PREDICTED: DNA-binding death effector domain-containing protein 2 isoform X3 [Lates calcarifer]	GCF_001640805.1	TRADDN-DEDD2
XP_018557076.1	low-complexity+DED+TRADDN-DEDD2	402	LOC108900491	actinopterygii	Lates calcarifer	PREDICTED: death effector domain-containing protein-like [Lates calcarifer]	GCF_001640805.1	TRADDN-DEDD2
XP_006625679.2	low-complexity+DED+TRADDN-DEDD2	407	LOC102686801	actinopterygii	Lepisosteus oculatus	PREDICTED: death effector domain-containing protein-like [Lepisosteus oculatus]	GCF_000242695.1	TRADDN-DEDD2
XP_026173316.1	low-complexity+DED+TRADDN-DEDD2	385	dedd2	actinopterygii	Mastacembelus armatus	DNA-binding death effector domain-containing protein 2 isoform X1 [Mastacembelus armatus]	GCF_900324485.1	TRADDN-DEDD2

acc	architecture	len	gen.name	taxend	species	defline	gca	clade
XP_026173317.1	low-complexity+DED+TRADDN-DEDD2	380	dedd2	actinopterygii	Mastacembelus armatus	DNA-binding death effector domain-containing protein 2 isoform X2 [Mastacembelus armatus]	GCF_900324485.1	TRADDN-DEDD2
XP_026188351.1	low-complexity+DED+TRADDN-DEDD2	404	LOC113145622	actinopterygii	Mastacembelus armatus	death effector domain-containing protein-like isoform X1 [Mastacembelus armatus]	GCF_900324485.1	TRADDN-DEDD2
XP_026188355.1	low-complexity+DED+TRADDN-DEDD2	369	LOC113145622	actinopterygii	Mastacembelus armatus	death effector domain-containing protein-like isoform X2 [Mastacembelus armatus]	GCF_900324485.1	TRADDN-DEDD2
XP_004550932.1	low-complexity+DED+TRADDN-DEDD2	382	dedd2	actinopterygii	Maylandia zebra	DNA-binding death effector domain-containing protein 2 isoform X1 [Maylandia zebra]	GCF_000238955.4	TRADDN-DEDD2
XP_004550936.1	low-complexity+DED+TRADDN-DEDD2	379	dedd2	actinopterygii	Maylandia zebra	DNA-binding death effector domain-containing protein 2 isoform X2 [Maylandia zebra]	GCF_000238955.4	TRADDN-DEDD2
XP_004570968.1	low-complexity+DED+TRADDN-DEDD2	404	LOC101472216	actinopterygii	Maylandia zebra	death effector domain-containing protein [Maylandia zebra]	GCF_000238955.4	TRADDN-DEDD2
XP_020464747.1	low-complexity+DED+TRADDN-DEDD2	322	dedd	actinopterygii	Monopterus albus	death effector domain-containing protein [Monopterus albus]	GCF_001952655.1	TRADDN-DEDD2
XP_020466449.1	low-complexity+DED+TRADDN-DEDD2	384	dedd2	actinopterygii	Monopterus albus	DNA-binding death effector domain-containing protein 2 [Monopterus albus]	GCF_001952655.1	TRADDN-DEDD2
XP_006804371.1	low-complexity+DED+TRADDN-DEDD2	404	LOC102781701	actinopterygii	Neolamprologus brichardi	PREDICTED: death effector domain-containing protein-like [Neolamprologus brichardi]	GCF_000239395.1	TRADDN-DEDD2
XP_015805772.1	low-complexity+DED+TRADDN-DEDD2	376	dedd2	actinopterygii	Nothobranchius furzeri	PREDICTED: DNA-binding death effector domain-containing protein 2 [Nothobranchius furzeri]	GCF_001465895.1	TRADDN-DEDD2
XP_015822820.1	low-complexity+DED+TRADDN-DEDD2	406	LOC107390540	actinopterygii	Nothobranchius furzeri	PREDICTED: death effector domain-containing protein-like [Nothobranchius furzeri]	GCF_001465895.1	TRADDN-DEDD2
XP_010768504.1	low-complexity+DED+TRADDN-DEDD2	361	dedd2	actinopterygii	Notothenia coriiceps	PREDICTED: DNA-binding death effector domain-containing protein 2 [Notothenia coriiceps]	GCF_000735185.1	TRADDN-DEDD2
XP_010783930.1	low-complexity+DED+TRADDN-DEDD2	412	dedd	actinopterygii	Notothenia coriiceps	PREDICTED: death effector domain-containing protein [Notothenia coriiceps]	GCF_000735185.1	TRADDN-DEDD2
XP_020314429.1	low-complexity+DED+TRADDN-DEDD2	452	LOC109869026	actinopterygii	Oncorhynchus kisutch	death effector domain-containing protein-like isoform X1 [Oncorhynchus kisutch]	GCF_002021735.1	TRADDN-DEDD2
XP_020314430.1	low-complexity+DED+TRADDN-DEDD2	416	LOC109869026	actinopterygii	Oncorhynchus kisutch	death effector domain-containing protein-like isoform X2 [Oncorhynchus kisutch]	GCF_002021735.1	TRADDN-DEDD2
XP_020351943.1	low-complexity+DED+TRADDN-DEDD2	384	LOC109900635	actinopterygii	Oncorhynchus kisutch	DNA-binding death effector domain-containing protein 2-like [Oncorhynchus kisutch]	GCF_002021735.1	TRADDN-DEDD2
XP_020359702.1	low-complexity+DED+TRADDN-DEDD2	385	LOC109906424	actinopterygii	Oncorhynchus kisutch	LOW QUALITY PROTEIN: DNA-binding death effector domain-containing protein 2-like [Oncorhynchus kisutch]	GCF_002021735.1	TRADDN-DEDD2
XP_020362029.1	low-complexity+DED+TRADDN-DEDD2	414	LOC109908047	actinopterygii	Oncorhynchus kisutch	death effector domain-containing protein-like [Oncorhynchus kisutch]	GCF_002021735.1	TRADDN-DEDD2

acc	architecture	len	gen.name	taxend	species	defline	gca	clade
CDQ74273.1	low-complexity+DED+TRADDN-DEDD2	400	GSONMT00005094001	actinopterygii	Oncorhynchus mykiss	unnamed protein product [Oncorhynchus mykiss]	GCA_900005705.1	TRADDN-DEDD2
XP_021422110.1	low-complexity+DED+TRADDN-DEDD2	416	LOC110492261	actinopterygii	Oncorhynchus mykiss	death effector domain-containing protein-like [Oncorhynchus mykiss]	GCF_002163495.1	TRADDN-DEDD2
XP_021446498.1	low-complexity+DED+TRADDN-DEDD2	396	LOC110509740	actinopterygii	Oncorhynchus mykiss	DNA-binding death effector domain-containing protein 2-like isoform X1 [Oncorhynchus mykiss]	GCF_002163495.1	TRADDN-DEDD2
XP_021446507.1	low-complexity+DED+TRADDN-DEDD2	385	LOC110509740	actinopterygii	Oncorhynchus mykiss	DNA-binding death effector domain-containing protein 2-like isoform X2 [Oncorhynchus mykiss]	GCF_002163495.1	TRADDN-DEDD2
XP_021471822.1	low-complexity+DED+TRADDN-DEDD2	414	LOC110532342	actinopterygii	Oncorhynchus mykiss	death effector domain-containing protein-like isoform X1 [Oncorhynchus mykiss]	GCF_002163495.1	TRADDN-DEDD2
XP_021471824.1	low-complexity+DED+TRADDN-DEDD2	360	LOC110532342	actinopterygii	Oncorhynchus mykiss	death effector domain-containing protein-like isoform X2 [Oncorhynchus mykiss]	GCF_002163495.1	TRADDN-DEDD2
XP_021479253.1	low-complexity+DED+TRADDN-DEDD2	385	LOC110537492	actinopterygii	Oncorhynchus mykiss	DNA-binding death effector domain-containing protein 2-like [Oncorhynchus mykiss]	GCF_002163495.1	TRADDN-DEDD2
XP_024240371.1	low-complexity+DED+TRADDN-DEDD2	416	LOC112222044	actinopterygii	Oncorhynchus tshawytscha	death effector domain-containing protein-like [Oncorhynchus tshawytscha]	GCF_002872995.1	TRADDN-DEDD2
XP_024245993.1	low-complexity+DED+TRADDN-DEDD2	396	dedd2	actinopterygii	Oncorhynchus tshawytscha	DNA-binding death effector domain-containing protein 2 isoform X1 [Oncorhynchus tshawytscha]	GCF_002872995.1	TRADDN-DEDD2
XP_024246002.1	low-complexity+DED+TRADDN-DEDD2	385	dedd2	actinopterygii	Oncorhynchus tshawytscha	DNA-binding death effector domain-containing protein 2 isoform X2 [Oncorhynchus tshawytscha]	GCF_002872995.1	TRADDN-DEDD2
XP_003450711.2	low-complexity+DED+TRADDN-DEDD2	382	dedd2	actinopterygii	Oreochromis niloticus	DNA-binding death effector domain-containing protein 2 isoform X1 [Oreochromis niloticus]	GCF_001858045.2	TRADDN-DEDD2
XP_005466163.1	low-complexity+DED+TRADDN-DEDD2	404	LOC100693850	actinopterygii	Oreochromis niloticus	death effector domain-containing protein [Oreochromis niloticus]	GCF_001858045.2	TRADDN-DEDD2
XP_025767453.1	low-complexity+DED+TRADDN-DEDD2	379	dedd2	actinopterygii	Oreochromis niloticus	DNA-binding death effector domain-containing protein 2 isoform X2 [Oreochromis niloticus]	GCF_001858045.2	TRADDN-DEDD2
XP_004084396.1	low-complexity+DED+TRADDN-DEDD2	389	LOC101170239	actinopterygii	Oryzias latipes	death effector domain-containing protein [Oryzias latipes]	GCF_002234675.1	TRADDN-DEDD2
XP_011483789.1	low-complexity+DED+TRADDN-DEDD2	377	dedd2	actinopterygii	Oryzias latipes	DNA-binding death effector domain-containing protein 2 [Oryzias latipes]	GCF_002234675.1	TRADDN-DEDD2
XP_024118697.1	low-complexity+DED+TRADDN-DEDD2	395	LOC112140028	actinopterygii	Oryzias melastigma	death effector domain-containing protein-like [Oryzias melastigma]	GCF_002922805.1	TRADDN-DEDD2
XP_024123617.1	low-complexity+DED+TRADDN-DEDD2	377	dedd2	actinopterygii	Oryzias melastigma	DNA-binding death effector domain-containing protein 2 [Oryzias melastigma]	GCF_002922805.1	TRADDN-DEDD2
XP_026795800.1	low-complexity+DED+TRADDN-DEDD2	360	dedd2	actinopterygii	Pangasianodon hypophthalmus	DNA-binding death effector domain-containing protein 2 isoform X1 [Pangasianodon hypophthalmus]	GCF_003671635.1	TRADDN-DEDD2
XP_019944930.1	low-complexity+DED+TRADDN-DEDD2	404	LOC109630882	actinopterygii	Paralichthys olivaceus	PREDICTED: death effector domain-containing protein-like [Paralichthys olivaceus]	GCF_001970005.1	TRADDN-DEDD2

acc	architecture	len	gen.name	taxend	species	defline	gca	clade
XP_019948625.1	low-complexity+DED+TRADDN-DEDD2	383	dedd2	actinopterygii	Paralichthys olivaceus	PREDICTED: DNA-binding death effector domain-containing protein 2 [Paralichthys olivaceus]	GCF_001970005.1	TRADDN-DEDD2
XP_023664528.1	low-complexity+DED+TRADDN-DEDD2	410	dedd	actinopterygii	Paramormyrops kingsleyae	death effector domain-containing protein [Paramormyrops kingsleyae]	GCF_002872115.1	TRADDN-DEDD2
XP_023665797.1	low-complexity+DED+TRADDN-DEDD2	405	dedd2	actinopterygii	Paramormyrops kingsleyae	DNA-binding death effector domain-containing protein 2 isoform X1 [Paramormyrops kingsleyae]	GCF_002872115.1	TRADDN-DEDD2
XP_023665798.1	low-complexity+DED+TRADDN-DEDD2	371	dedd2	actinopterygii	Paramormyrops kingsleyae	DNA-binding death effector domain-containing protein 2 isoform X2 [Paramormyrops kingsleyae]	GCF_002872115.1	TRADDN-DEDD2
XP_007550282.1	low-complexity+DED+TRADDN-DEDD2	377	dedd2	actinopterygii	Poecilia formosa	PREDICTED: DNA-binding death effector domain-containing protein 2 [Poecilia formosa]	GCF_000485575.1	TRADDN-DEDD2
XP_007574476.1	low-complexity+DED+TRADDN-DEDD2	399	LOC103153477	actinopterygii	Poecilia formosa	PREDICTED: death effector domain-containing protein [Poecilia formosa]	GCF_000485575.1	TRADDN-DEDD2
XP_014891523.1	low-complexity+DED+TRADDN-DEDD2	377	dedd2	actinopterygii	Poecilia latipinna	PREDICTED: DNA-binding death effector domain-containing protein 2 [Poecilia latipinna]	GCF_001443285.1	TRADDN-DEDD2
XP_014842625.1	low-complexity+DED+TRADDN-DEDD2	377	dedd2	actinopterygii	Poecilia mexicana	PREDICTED: DNA-binding death effector domain-containing protein 2 [Poecilia mexicana]	GCF_001443325.1	TRADDN-DEDD2
XP_008413127.1	low-complexity+DED+TRADDN-DEDD2	399	LOC103468063	actinopterygii	Poecilia reticulata	PREDICTED: death effector domain-containing protein [Poecilia reticulata]	GCF_000633615.1	TRADDN-DEDD2
XP_008429549.1	low-complexity+DED+TRADDN-DEDD2	377	dedd2	actinopterygii	Poecilia reticulata	PREDICTED: DNA-binding death effector domain-containing protein 2 [Poecilia reticulata]	GCF_000633615.1	TRADDN-DEDD2
XP_005731894.1	low-complexity+DED+TRADDN-DEDD2	382	LOC102204864	actinopterygii	Pundamilia nyererei	PREDICTED: DNA-binding death effector domain-containing protein 2-like [Pundamilia nyererei]	GCF_000239375.1	TRADDN-DEDD2
XP_005745959.1	low-complexity+DED+TRADDN-DEDD2	404	LOC102201469	actinopterygii	Pundamilia nyererei	PREDICTED: death effector domain-containing protein-like [Pundamilia nyererei]	GCF_000239375.1	TRADDN-DEDD2
XP_017538787.1	low-complexity+DED+TRADDN-DEDD2	393	LOC108411641	actinopterygii	Pygocentrus nattereri	PREDICTED: death effector domain-containing protein-like [Pygocentrus nattereri]	GCF_001682695.1	TRADDN-DEDD2
XP_017561563.1	low-complexity+DED+TRADDN-DEDD2	362	dedd2	actinopterygii	Pygocentrus nattereri	PREDICTED: DNA-binding death effector domain-containing protein 2 [Pygocentrus nattereri]	GCF_001682695.1	TRADDN-DEDD2
XP_013990771.1	low-complexity+DED+TRADDN-DEDD2	416	LOC106566840	actinopterygii	Salmo salar	PREDICTED: death effector domain-containing protein-like [Salmo salar]	GCF_000233375.1	TRADDN-DEDD2
XP_014001232.1	low-complexity+DED+TRADDN-DEDD2	416	LOC106572035	actinopterygii	Salmo salar	PREDICTED: death effector domain-containing protein-like [Salmo salar]	GCF_000233375.1	TRADDN-DEDD2
XP_014008769.1	low-complexity+DED+TRADDN-DEDD2	396	dedd2	actinopterygii	Salmo salar	PREDICTED: DNA-binding death effector domain-containing protein 2 isoform X1 [Salmo salar]	GCF_000233375.1	TRADDN-DEDD2

acc	architecture	len	gen.name	taxend	species	defline	gca	clade
XP_014008786.1	low-complexity+DED+TRADDN-DEDD2	385	dedd2	actinopterygii	Salmo salar	PREDICTED: DNA-binding death effector domain-containing protein 2 isoform X2 [Salmo salar]	GCF_000233375.1	TRADDN-DEDD2
XP_014057310.1	low-complexity+DED+TRADDN-DEDD2	396	LOC106605858	actinopterygii	Salmo salar	PREDICTED: DNA-binding death effector domain-containing protein 2-like isoform X1 [Salmo salar]	GCF_000233375.1	TRADDN-DEDD2
XP_014057312.1	low-complexity+DED+TRADDN-DEDD2	385	LOC106605858	actinopterygii	Salmo salar	PREDICTED: DNA-binding death effector domain-containing protein 2-like isoform X2 [Salmo salar]	GCF_000233375.1	TRADDN-DEDD2
XP_023846965.1	low-complexity+DED+TRADDN-DEDD2	360	LOC111966504	actinopterygii	Salvelinus alpinus	death effector domain-containing protein isoform X2 [Salvelinus alpinus]	GCF_002910315.2	TRADDN-DEDD2
XP_023862080.1	low-complexity+DED+TRADDN-DEDD2	416	LOC111977050	actinopterygii	Salvelinus alpinus	death effector domain-containing protein [Salvelinus alpinus]	GCF_002910315.2	TRADDN-DEDD2
XP_023996466.1	low-complexity+DED+TRADDN-DEDD2	399	LOC112073403	actinopterygii	Salvelinus alpinus	DNA-binding death effector domain-containing protein 2-like isoform X1 [Salvelinus alpinus]	GCF_002910315.2	TRADDN-DEDD2
XP_023996469.1	low-complexity+DED+TRADDN-DEDD2	385	LOC112073403	actinopterygii	Salvelinus alpinus	DNA-binding death effector domain-containing protein 2-like isoform X2 [Salvelinus alpinus]	GCF_002910315.2	TRADDN-DEDD2
KPP74621.1	low-complexity+DED+TRADDN-DEDD2	411	Z043_106207	actinopterygii	Scleropages formosus	death effector domain-containing protein-like [Scleropages formosus]	GCA_001005745.2	TRADDN-DEDD2
XP_018587534.1	low-complexity+DED+TRADDN-DEDD2	435	dedd2	actinopterygii	Scleropages formosus	PREDICTED: DNA-binding death effector domain-containing protein 2 isoform X1 [Scleropages formosus]	-	TRADDN-DEDD2
XP_018587535.1	low-complexity+DED+TRADDN-DEDD2	385	dedd2	actinopterygii	Scleropages formosus	PREDICTED: DNA-binding death effector domain-containing protein 2 isoform X2 [Scleropages formosus]	-	TRADDN-DEDD2
XP_018615687.1	low-complexity+DED+TRADDN-DEDD2	407	dedd	actinopterygii	Scleropages formosus	PREDICTED: death effector domain-containing protein [Scleropages formosus]	GCF_900964775.1	TRADDN-DEDD2
AWO96347.1	low-complexity+DED+TRADDN-DEDD2	381	SMAX5B_012179	actinopterygii	Scophthalmus maximus	putative DNA-binding death effector domain-containing protein 2 isoform 2 [Scophthalmus maximus]	GCA_003186165.1	TRADDN-DEDD2
AWP09177.1	low-complexity+DED+TRADDN-DEDD2	404	SMAX5B_000525	actinopterygii	Scophthalmus maximus	putative death effector domain-containing protein [Scophthalmus maximus]	GCA_003186165.1	TRADDN-DEDD2
XP_022612372.1	low-complexity+DED+TRADDN-DEDD2	382	dedd2	actinopterygii	Seriola dumerili	DNA-binding death effector domain-containing protein 2 isoform X1 [Seriola dumerili]	GCF_002260705.1	TRADDN-DEDD2
XP_022612374.1	low-complexity+DED+TRADDN-DEDD2	376	dedd2	actinopterygii	Seriola dumerili	DNA-binding death effector domain-containing protein 2 isoform X2 [Seriola dumerili]	GCF_002260705.1	TRADDN-DEDD2
XP_022617410.1	low-complexity+DED+TRADDN-DEDD2	404	LOC111233792	actinopterygii	Seriola dumerili	death effector domain-containing protein-like [Seriola dumerili]	GCF_002260705.1	TRADDN-DEDD2
XP_023267293.1	low-complexity+DED+TRADDN-DEDD2	404	LOC111658850	actinopterygii	Seriola lalandi dorsalis	death effector domain-containing protein-like [Seriola lalandi dorsalis]	GCF_002814215.1	TRADDN-DEDD2
XP_023285668.1	low-complexity+DED+TRADDN-DEDD2	411	dedd2	actinopterygii	Seriola lalandi dorsalis	DNA-binding death effector domain-containing protein 2 isoform X1 [Seriola lalandi dorsalis]	GCF_002814215.1	TRADDN-DEDD2

acc	architecture	len	gen.name	taxend	species	defline	gca	clade
XP_023285669.1	low-complexity+DED+TRADDN-DEDD2	405	dedd2	actinopterygii	Seriola lalandi dorsalis	DNA-binding death effector domain-containing protein 2 isoform X2 [Seriola lalandi dorsalis]	GCF_002814215.1	TRADDN-DEDD2
XP_023285670.1	low-complexity+DED+TRADDN-DEDD2	381	dedd2	actinopterygii	Seriola lalandi dorsalis	DNA-binding death effector domain-containing protein 2 isoform X3 [Seriola lalandi dorsalis]	GCF_002814215.1	TRADDN-DEDD2
XP_016311309.1	low-complexity+DED+TRADDN-DEDD2	404	LOC107665071	actinopterygii	Sinocyclocheilus anshuiensis	PREDICTED: death effector domain-containing protein-like isoform X1 [Sinocyclocheilus anshuiensis]	GCF_001515605.1	TRADDN-DEDD2
XP_016319562.1	low-complexity+DED+TRADDN-DEDD2	370	LOC107671190	actinopterygii	Sinocyclocheilus anshuiensis	PREDICTED: DNA-binding death effector domain-containing protein 2-like isoform X1 [Sinocyclocheilus anshuiensis]	GCF_001515605.1	TRADDN-DEDD2
XP_016319564.1	low-complexity+DED+TRADDN-DEDD2	367	LOC107671190	actinopterygii	Sinocyclocheilus anshuiensis	PREDICTED: DNA-binding death effector domain-containing protein 2-like isoform X2 [Sinocyclocheilus anshuiensis]	GCF_001515605.1	TRADDN-DEDD2
XP_016355877.1	low-complexity+DED+TRADDN-DEDD2	404	LOC107699191	actinopterygii	Sinocyclocheilus anshuiensis	PREDICTED: death effector domain-containing protein-like [Sinocyclocheilus anshuiensis]	GCF_001515605.1	TRADDN-DEDD2
XP_016357225.1	low-complexity+DED+TRADDN-DEDD2	370	LOC107700182	actinopterygii	Sinocyclocheilus anshuiensis	PREDICTED: DNA-binding death effector domain-containing protein 2-like [Sinocyclocheilus anshuiensis]	GCF_001515605.1	TRADDN-DEDD2
XP_016088881.1	low-complexity+DED+TRADDN-DEDD2	370	LOC107551690	actinopterygii	Sinocyclocheilus grahami	PREDICTED: DNA-binding death effector domain-containing protein 2-like [Sinocyclocheilus grahami]	GCF_001515645.1	TRADDN-DEDD2
XP_016123220.1	low-complexity+DED+TRADDN-DEDD2	370	LOC107582037	actinopterygii	Sinocyclocheilus grahami	PREDICTED: DNA-binding death effector domain-containing protein 2-like isoform X1 [Sinocyclocheilus grahami]	GCF_001515645.1	TRADDN-DEDD2
XP_016123221.1	low-complexity+DED+TRADDN-DEDD2	367	LOC107582037	actinopterygii	Sinocyclocheilus grahami	PREDICTED: DNA-binding death effector domain-containing protein 2-like isoform X2 [Sinocyclocheilus grahami]	GCF_001515645.1	TRADDN-DEDD2
XP_016151315.1	low-complexity+DED+TRADDN-DEDD2	404	LOC107603111	actinopterygii	Sinocyclocheilus grahami	PREDICTED: death effector domain-containing protein-like [Sinocyclocheilus grahami]	GCF_001515645.1	TRADDN-DEDD2
XP_016379669.1	low-complexity+DED+TRADDN-DEDD2	370	LOC107717438	actinopterygii	Sinocyclocheilus rhinocerous	PREDICTED: DNA-binding death effector domain-containing protein 2-like [Sinocyclocheilus rhinocerous]	GCF_001515625.1	TRADDN-DEDD2
XP_016387604.1	low-complexity+DED+TRADDN-DEDD2	370	LOC107723479	actinopterygii	Sinocyclocheilus rhinocerous	PREDICTED: DNA-binding death effector domain-containing protein 2-like isoform X1 [Sinocyclocheilus rhinocerous]	GCF_001515625.1	TRADDN-DEDD2
XP_016387606.1	low-complexity+DED+TRADDN-DEDD2	367	LOC107723479	actinopterygii	Sinocyclocheilus rhinocerous	PREDICTED: DNA-binding death effector domain-containing protein 2-like isoform X2 [Sinocyclocheilus rhinocerous]	GCF_001515625.1	TRADDN-DEDD2
XP_016427542.1	low-complexity+DED+TRADDN-DEDD2	404	LOC107755304	actinopterygii	Sinocyclocheilus rhinocerous	PREDICTED: death effector domain-containing protein-like [Sinocyclocheilus rhinocerous]	GCF_001515625.1	TRADDN-DEDD2

acc	architecture	len	gen.name	taxend	species	defline	gca	clade
XP_008298216.1	low-complexity+DED+TRADDN-DEDD2	382	dedd2	actinopterygii	Stegastes partitus	PREDICTED: DNA-binding death effector domain-containing protein 2 isoform X1 [Stegastes partitus]	GCF_000690725.1	TRADDN-DEDD2
XP_008298217.1	low-complexity+DED+TRADDN-DEDD2	376	dedd2	actinopterygii	Stegastes partitus	PREDICTED: DNA-binding death effector domain-containing protein 2 isoform X2 [Stegastes partitus]	GCF_000690725.1	TRADDN-DEDD2
XP_008303019.1	low-complexity+DED+TRADDN-DEDD2	404	LOC103374682	actinopterygii	Stegastes partitus	PREDICTED: death effector domain-containing protein-like [Stegastes partitus]	GCF_000690725.1	TRADDN-DEDD2
XP_003979667.2	low-complexity+DED+TRADDN-DEDD2	386	dedd	actinopterygii	Takifugu rubripes	PREDICTED: death effector domain-containing protein [Takifugu rubripes]	GCF_901000725.2	TRADDN-DEDD2
XP_011604270.1	low-complexity+DED+TRADDN-DEDD2	388	dedd2	actinopterygii	Takifugu rubripes	PREDICTED: DNA-binding death effector domain-containing protein 2 [Takifugu rubripes]	-	TRADDN-DEDD2
CAF89497.1	low-complexity+DED+TRADDN-DEDD2	358	GSTEN:00003422:G:001	actinopterygii	Tetraodon nigroviridis	unnamed protein product, partial [Tetraodon nigroviridis]	GCA_000180735.1	TRADDN-DEDD2
XP_005816364.1	low-complexity+DED+TRADDN-DEDD2	399	LOC102229203	actinopterygii	Xiphophorus maculatus	death effector domain-containing protein-like [Xiphophorus maculatus]	GCF_002775205.1	TRADDN-DEDD2
XP_014324194.1	low-complexity+DED+TRADDN-DEDD2	377	dedd2	actinopterygii	Xiphophorus maculatus	DNA-binding death effector domain-containing protein 2 [Xiphophorus maculatus]	GCF_002775205.1	TRADDN-DEDD2
KTG36075.1	TRADDN-DEDD2	156	cypCar_00002981	actinopterygii	Cyprinus carpio	hypothetical protein cypCar_00002981 [Cyprinus carpio]	GCA_001270105.1	TRADDN-DEDD2
XP_018973535.1	TRADDN-DEDD2	144	LOC109104702	actinopterygii	Cyprinus carpio	PREDICTED: death effector domain-containing protein-like [Cyprinus carpio]	GCF_000951615.1	TRADDN-DEDD2
XP_006802877.1	TRADDN-DEDD2	171	LOC102797878	actinopterygii	Neolamprologus brichardi	PREDICTED: death effector domain-containing protein-like [Neolamprologus brichardi]	GCF_000239395.1	TRADDN-DEDD2
XP_026768191.1	TRADDN-DEDD2	173	LOC113525802	actinopterygii	Pangasianodon hypophthalmus	death effector domain-containing protein-like, partial [Pangasianodon hypophthalmus]	GCF_003671635.1	TRADDN-DEDD2
KPP72408.1	TRADDN-DEDD2	259	Z043_108600	actinopterygii	Scleropages formosus	hypothetical protein Z043_108600 [Scleropages formosus]	GCA_001005745.2	TRADDN-DEDD2
XP_016431135.1	TRADDN-DEDD2	206	LOC107758014	actinopterygii	Sinocyclocheilus rhinocerous	PREDICTED: death effector domain-containing protein-like [Sinocyclocheilus rhinocerous]	GCF_001515625.1	TRADDN-DEDD2
XP_015191868.1	TRADDN-DEDD2+Coiled-coil	389	dedd2	actinopterygii	Lepisosteus oculatus	PREDICTED: DNA-binding death effector domain-containing protein 2 isoform X2 [Lepisosteus oculatus]	GCF_000242695.1	TRADDN-DEDD2
XP_022045877.1	TRADDN-NCOA6	2395	ncoa6	actinopterygii	Acanthochromis polyacanthus	nuclear receptor coactivator 6 [Acanthochromis polyacanthus]	GCF_002109545.1	TRADDN-NCOA6
XP_023137646.1	TRADDN-NCOA6	2382	ncoa6	actinopterygii	Amphiprion ocellaris	nuclear receptor coactivator 6 isoform X1 [Amphiprion ocellaris]	GCF_002776465.1	TRADDN-NCOA6
XP_023137647.1	TRADDN-NCOA6	2368	ncoa6	actinopterygii	Amphiprion ocellaris	nuclear receptor coactivator 6 isoform X2 [Amphiprion ocellaris]	GCF_002776465.1	TRADDN-NCOA6
XP_022522166.1	TRADDN-NCOA6	2330	LOC103028954	actinopterygii	Astyanax mexicanus	LOW QUALITY PROTEIN: nuclear receptor coactivator 6-like [Astyanax mexicanus]	GCF_000372685.2	TRADDN-NCOA6

acc	architecture	len	gen.name	taxend	species	defline	gca	clade
XP_013872715.1	TRADDN-NCOA6	2329	LOC106523732	actinopterygii	Austrofundulus limnaeus	PREDICTED: nuclear receptor coactivator 6-like isoform X1 [Austrofundulus limnaeus]	GCF_001266775.1	TRADDN-NCOA6
XP_013872725.1	TRADDN-NCOA6	2328	LOC106523732	actinopterygii	Austrofundulus limnaeus	PREDICTED: nuclear receptor coactivator 6-like isoform X2 [Austrofundulus limnaeus]	GCF_001266775.1	TRADDN-NCOA6
XP_013872733.1	TRADDN-NCOA6	2315	LOC106523732	actinopterygii	Austrofundulus limnaeus	PREDICTED: nuclear receptor coactivator 6-like isoform X3 [Austrofundulus limnaeus]	GCF_001266775.1	TRADDN-NCOA6
XP_020785811.1	TRADDN-NCOA6	2189	ncoa6	actinopterygii	Boleophthalmus pectinirostris	nuclear receptor coactivator 6 [Boleophthalmus pectinirostris]	GCF_000788275.1	TRADDN-NCOA6
XP_012687590.1	TRADDN-NCOA6	657	LOC105904278	actinopterygii	Clupea harengus	PREDICTED: nuclear receptor coactivator 6-like [Clupea harengus]	GCF_000966335.1	TRADDN-NCOA6
XP_024915498.1	TRADDN-NCOA6	530	LOC112487660	actinopterygii	Cynoglossus semilaevis	nuclear receptor coactivator 6-like isoform X1 [Cynoglossus semilaevis]	GCF_000523025.1	TRADDN-NCOA6
XP_024915499.1	TRADDN-NCOA6	529	LOC112487660	actinopterygii	Cynoglossus semilaevis	nuclear receptor coactivator 6-like isoform X2 [Cynoglossus semilaevis]	GCF_000523025.1	TRADDN-NCOA6
XP_024915500.1	TRADDN-NCOA6	489	LOC112487660	actinopterygii	Cynoglossus semilaevis	nuclear receptor coactivator 6-like isoform X3 [Cynoglossus semilaevis]	GCF_000523025.1	TRADDN-NCOA6
XP_024915501.1	TRADDN-NCOA6	488	LOC112487660	actinopterygii	Cynoglossus semilaevis	nuclear receptor coactivator 6-like isoform X4 [Cynoglossus semilaevis]	GCF_000523025.1	TRADDN-NCOA6
XP_015234750.1	TRADDN-NCOA6	2393	ncoa6	actinopterygii	Cyprinodon variegatus	PREDICTED: nuclear receptor coactivator 6 isoform X1 [Cyprinodon variegatus]	GCF_000732505.1	TRADDN-NCOA6
XP_015234752.1	TRADDN-NCOA6	2388	ncoa6	actinopterygii	Cyprinodon variegatus	PREDICTED: nuclear receptor coactivator 6 isoform X2 [Cyprinodon variegatus]	GCF_000732505.1	TRADDN-NCOA6
XP_015234753.1	TRADDN-NCOA6	2379	ncoa6	actinopterygii	Cyprinodon variegatus	PREDICTED: nuclear receptor coactivator 6 isoform X3 [Cyprinodon variegatus]	GCF_000732505.1	TRADDN-NCOA6
KTF94987.1	TRADDN-NCOA6	969	cypCar_00013510	actinopterygii	Cyprinus carpio	hypothetical protein cypCar_00013510 [Cyprinus carpio]	GCA_001270105.1	TRADDN-NCOA6
KTG34246.1	TRADDN-NCOA6	971	cypCar_00039879	actinopterygii	Cyprinus carpio	hypothetical protein cypCar_00039879 [Cyprinus carpio]	GCA_001270105.1	TRADDN-NCOA6
XP_018957021.1	TRADDN-NCOA6	212	LOC109087260	actinopterygii	Cyprinus carpio	PREDICTED: nuclear receptor coactivator 6-like [Cyprinus carpio]	GCF_000951615.1	TRADDN-NCOA6
XP_001918997.4	TRADDN-NCOA6	2269	ncoa6	actinopterygii	Danio rerio	nuclear receptor coactivator 6 isoform X2 [Danio rerio]	GCF_000002035.6	TRADDN-NCOA6
XP_005170622.1	TRADDN-NCOA6	2271	ncoa6	actinopterygii	Danio rerio	nuclear receptor coactivator 6 isoform X1 [Danio rerio]	GCF_000002035.6	TRADDN-NCOA6
XP_005170623.1	TRADDN-NCOA6	2257	ncoa6	actinopterygii	Danio rerio	nuclear receptor coactivator 6 isoform X3 [Danio rerio]	GCF_000002035.6	TRADDN-NCOA6
XP_010874177.1	TRADDN-NCOA6	2306	ncoa6	actinopterygii	Esox lucius	PREDICTED: nuclear receptor coactivator 6 isoform X1 [Esox lucius]	GCF_004634155.1	TRADDN-NCOA6
XP_019907388.1	TRADDN-NCOA6	2302	ncoa6	actinopterygii	Esox lucius	PREDICTED: nuclear receptor coactivator 6 isoform X2 [Esox lucius]	GCF_004634155.1	TRADDN-NCOA6
XP_019907389.1	TRADDN-NCOA6	2292	ncoa6	actinopterygii	Esox lucius	PREDICTED: nuclear receptor coactivator 6 isoform X3 [Esox lucius]	GCF_004634155.1	TRADDN-NCOA6

acc	architecture	len	gen.name	taxend	species	defline	gca	clade
XP_019907390.1	TRADDN-NCOA6	2255	ncoa6	actinopterygii	Esox lucius	PREDICTED: nuclear receptor coactivator 6 isoform X4 [Esox lucius]	GCF_004634155.1	TRADDN-NCOA6
XP_012717724.1	TRADDN-NCOA6	2344	LOC105926085	actinopterygii	Fundulus heteroclitus	nuclear receptor coactivator 6-like isoform X1 [Fundulus heteroclitus]	GCF_000826765.1	TRADDN-NCOA6
XP_012717726.1	TRADDN-NCOA6	2332	LOC105926085	actinopterygii	Fundulus heteroclitus	nuclear receptor coactivator 6-like isoform X3 [Fundulus heteroclitus]	GCF_000826765.1	TRADDN-NCOA6
XP_021171975.1	TRADDN-NCOA6	2341	LOC105926085	actinopterygii	Fundulus heteroclitus	nuclear receptor coactivator 6-like isoform X2 [Fundulus heteroclitus]	GCF_000826765.1	TRADDN-NCOA6
PWA31822.1	TRADDN-NCOA6	973	CCH79_00006734	actinopterygii	Gambusia affinis	hypothetical protein CCH79_00006734 [Gambusia affinis]	GCA_003097735.1	TRADDN-NCOA6
XP_014194868.1	TRADDN-NCOA6	2281	ncoa6	actinopterygii	Haplochromis burtoni	PREDICTED: nuclear receptor coactivator 6 [Haplochromis burtoni]	GCF_000239415.1	TRADDN-NCOA6
XP_019724052.1	TRADDN-NCOA6	2333	ncoa6	actinopterygii	Hippocampus comes	PREDICTED: nuclear receptor coactivator 6 [Hippocampus comes]	GCF_001891065.1	TRADDN-NCOA6
XP_017342455.1	TRADDN-NCOA6	2282	ncoa6	actinopterygii	Ictalurus punctatus	PREDICTED: nuclear receptor coactivator 6 isoform X1 [Ictalurus punctatus]	GCF_001660625.1	TRADDN-NCOA6
XP_017342456.1	TRADDN-NCOA6	2280	ncoa6	actinopterygii	Ictalurus punctatus	PREDICTED: nuclear receptor coactivator 6 isoform X2 [Ictalurus punctatus]	GCF_001660625.1	TRADDN-NCOA6
XP_017342457.1	TRADDN-NCOA6	1836	ncoa6	actinopterygii	Ictalurus punctatus	PREDICTED: nuclear receptor coactivator 6 isoform X3 [Ictalurus punctatus]	GCF_001660625.1	TRADDN-NCOA6
XP_017342458.1	TRADDN-NCOA6	1834	ncoa6	actinopterygii	Ictalurus punctatus	PREDICTED: nuclear receptor coactivator 6 isoform X4 [Ictalurus punctatus]	GCF_001660625.1	TRADDN-NCOA6
XP_017342459.1	TRADDN-NCOA6	1828	ncoa6	actinopterygii	Ictalurus punctatus	PREDICTED: nuclear receptor coactivator 6 isoform X5 [Ictalurus punctatus]	GCF_001660625.1	TRADDN-NCOA6
XP_017270566.1	TRADDN-NCOA6	2330	ncoa6	actinopterygii	Kryptolebias marmoratus	nuclear receptor coactivator 6 isoform X1 [Kryptolebias marmoratus]	GCF_001649575.1	TRADDN-NCOA6
XP_017270567.1	TRADDN-NCOA6	2329	ncoa6	actinopterygii	Kryptolebias marmoratus	nuclear receptor coactivator 6 isoform X2 [Kryptolebias marmoratus]	GCF_001649575.1	TRADDN-NCOA6
XP_017270568.1	TRADDN-NCOA6	2316	ncoa6	actinopterygii	Kryptolebias marmoratus	nuclear receptor coactivator 6 isoform X3 [Kryptolebias marmoratus]	GCF_001649575.1	TRADDN-NCOA6
XP_020500542.1	TRADDN-NCOA6	2387	ncoa6	actinopterygii	Labrus bergylta	nuclear receptor coactivator 6 [Labrus bergylta]	-	TRADDN-NCOA6
XP_019112018.1	TRADDN-NCOA6	2325	ncoa6	actinopterygii	Larimichthys crocea	PREDICTED: nuclear receptor coactivator 6 [Larimichthys crocea]	-	TRADDN-NCOA6
XP_018523685.1	TRADDN-NCOA6	2401	LOC108877920	actinopterygii	Lates calcarifer	PREDICTED: LOW QUALITY PROTEIN: nuclear receptor coactivator 6-like [Lates calcarifer]	GCF_001640805.1	TRADDN-NCOA6
XP_015220266.1	TRADDN-NCOA6	2104	ncoa6	actinopterygii	Lepisosteus oculatus	PREDICTED: nuclear receptor coactivator 6 [Lepisosteus oculatus]	GCF_000242695.1	TRADDN-NCOA6
XP_023010653.1	TRADDN-NCOA6	2277	ncoa6	actinopterygii	Maylandia zebra	nuclear receptor coactivator 6 [Maylandia zebra]	GCF_000238955.4	TRADDN-NCOA6
XP_020463776.1	TRADDN-NCOA6	2303	ncoa6	actinopterygii	Monopterus albus	nuclear receptor coactivator 6 [Monopterus albus]	GCF_001952655.1	TRADDN-NCOA6

acc	architecture	len	gen.name	taxend	species	defline	gca	clade
XP_006796375.1	TRADDN-NCOA6	2391	LOC102789118	actinopterygii	Neolamprologus brichardi	PREDICTED: nuclear receptor coactivator 6-like isoform X1 [Neolamprologus brichardi]	GCF_000239395.1	TRADDN-NCOA6
XP_006796376.1	TRADDN-NCOA6	2377	LOC102789118	actinopterygii	Neolamprologus brichardi	PREDICTED: nuclear receptor coactivator 6-like isoform X2 [Neolamprologus brichardi]	GCF_000239395.1	TRADDN-NCOA6
XP_015823540.1	TRADDN-NCOA6	2344	ncoa6	actinopterygii	Nothobranchius furzeri	PREDICTED: nuclear receptor coactivator 6 isoform X1 [Nothobranchius furzeri]	GCF_001465895.1	TRADDN-NCOA6
XP_015823541.1	TRADDN-NCOA6	2343	ncoa6	actinopterygii	Nothobranchius furzeri	PREDICTED: nuclear receptor coactivator 6 isoform X2 [Nothobranchius furzeri]	GCF_001465895.1	TRADDN-NCOA6
XP_015823542.1	TRADDN-NCOA6	2330	ncoa6	actinopterygii	Nothobranchius furzeri	PREDICTED: nuclear receptor coactivator 6 isoform X3 [Nothobranchius furzeri]	GCF_001465895.1	TRADDN-NCOA6
XP_010792648.1	TRADDN-NCOA6	165	LOC104965390	actinopterygii	Notothenia coriiceps	PREDICTED: nuclear receptor coactivator 6-like [Notothenia coriiceps]	GCF_000735185.1	TRADDN-NCOA6
XP_020327030.1	TRADDN-NCOA6	2292	LOC109879268	actinopterygii	Oncorhynchus kisutch	nuclear receptor coactivator 6-like isoform X1 [Oncorhynchus kisutch]	GCF_002021735.1	TRADDN-NCOA6
XP_020327031.1	TRADDN-NCOA6	2291	LOC109879268	actinopterygii	Oncorhynchus kisutch	nuclear receptor coactivator 6-like isoform X2 [Oncorhynchus kisutch]	GCF_002021735.1	TRADDN-NCOA6
XP_020327032.1	TRADDN-NCOA6	2244	LOC109879268	actinopterygii	Oncorhynchus kisutch	nuclear receptor coactivator 6-like isoform X3 [Oncorhynchus kisutch]	GCF_002021735.1	TRADDN-NCOA6
XP_020327843.1	TRADDN-NCOA6	2371	LOC109880027	actinopterygii	Oncorhynchus kisutch	nuclear receptor coactivator 6-like [Oncorhynchus kisutch]	GCF_002021735.1	TRADDN-NCOA6
CDQ74450.1	TRADDN-NCOA6	553	GSONMT00036928001	actinopterygii	Oncorhynchus mykiss	unnamed protein product [Oncorhynchus mykiss]	GCA_900005705.1	TRADDN-NCOA6
CDQ90105.1	TRADDN-NCOA6	186	GSONMT00038399001	actinopterygii	Oncorhynchus mykiss	unnamed protein product [Oncorhynchus mykiss]	GCA_900005705.1	TRADDN-NCOA6
XP_021422596.1	TRADDN-NCOA6	2411	LOC110492529	actinopterygii	Oncorhynchus mykiss	nuclear receptor coactivator 6-like isoform X1 [Oncorhynchus mykiss]	GCF_002163495.1	TRADDN-NCOA6
XP_021422597.1	TRADDN-NCOA6	2410	LOC110492529	actinopterygii	Oncorhynchus mykiss	nuclear receptor coactivator 6-like isoform X2 [Oncorhynchus mykiss]	GCF_002163495.1	TRADDN-NCOA6
XP_021422598.1	TRADDN-NCOA6	2410	LOC110492529	actinopterygii	Oncorhynchus mykiss	nuclear receptor coactivator 6-like isoform X3 [Oncorhynchus mykiss]	GCF_002163495.1	TRADDN-NCOA6
XP_021422599.1	TRADDN-NCOA6	2363	LOC110492529	actinopterygii	Oncorhynchus mykiss	nuclear receptor coactivator 6-like isoform X4 [Oncorhynchus mykiss]	GCF_002163495.1	TRADDN-NCOA6
XP_021422600.1	TRADDN-NCOA6	2360	LOC110492529	actinopterygii	Oncorhynchus mykiss	nuclear receptor coactivator 6-like isoform X5 [Oncorhynchus mykiss]	GCF_002163495.1	TRADDN-NCOA6
XP_021444583.1	TRADDN-NCOA6	2304	LOC110508399	actinopterygii	Oncorhynchus mykiss	nuclear receptor coactivator 6-like isoform X1 [Oncorhynchus mykiss]	GCF_002163495.1	TRADDN-NCOA6
XP_021444585.1	TRADDN-NCOA6	2303	LOC110508399	actinopterygii	Oncorhynchus mykiss	nuclear receptor coactivator 6-like isoform X2 [Oncorhynchus mykiss]	GCF_002163495.1	TRADDN-NCOA6
XP_021444586.1	TRADDN-NCOA6	2256	LOC110508399	actinopterygii	Oncorhynchus mykiss	nuclear receptor coactivator 6-like isoform X3 [Oncorhynchus mykiss]	GCF_002163495.1	TRADDN-NCOA6
XP_024240859.1	TRADDN-NCOA6	2425	LOC112222320	actinopterygii	Oncorhynchus tshawytscha	nuclear receptor coactivator 6-like isoform X1 [Oncorhynchus tshawytscha]	GCF_002872995.1	TRADDN-NCOA6
XP_024240861.1	TRADDN-NCOA6	2424	LOC112222320	actinopterygii	Oncorhynchus tshawytscha	nuclear receptor coactivator 6-like isoform X2 [Oncorhynchus tshawytscha]	GCF_002872995.1	TRADDN-NCOA6

acc	architecture	len	gen.name	taxend	species	defline	gca	clade
XP_024240862.1	TRADDN-NCOA6	2424	LOC112222320	actinopterygii	Oncorhynchus tshawytscha	nuclear receptor coactivator 6-like isoform X3 [Oncorhynchus tshawytscha]	GCF_002872995.1	TRADDN-NCOA6
XP_024240863.1	TRADDN-NCOA6	2413	LOC112222320	actinopterygii	Oncorhynchus tshawytscha	nuclear receptor coactivator 6-like isoform X4 [Oncorhynchus tshawytscha]	GCF_002872995.1	TRADDN-NCOA6
XP_024240864.1	TRADDN-NCOA6	2377	LOC112222320	actinopterygii	Oncorhynchus tshawytscha	nuclear receptor coactivator 6-like isoform X5 [Oncorhynchus tshawytscha]	GCF_002872995.1	TRADDN-NCOA6
XP_024240865.1	TRADDN-NCOA6	2374	LOC112222320	actinopterygii	Oncorhynchus tshawytscha	nuclear receptor coactivator 6-like isoform X6 [Oncorhynchus tshawytscha]	GCF_002872995.1	TRADDN-NCOA6
XP_024270325.1	TRADDN-NCOA6	2228	ncoa6	actinopterygii	Oncorhynchus tshawytscha	nuclear receptor coactivator 6 isoform X1 [Oncorhynchus tshawytscha]	GCF_002872995.1	TRADDN-NCOA6
XP_024270327.1	TRADDN-NCOA6	2227	ncoa6	actinopterygii	Oncorhynchus tshawytscha	nuclear receptor coactivator 6 isoform X2 [Oncorhynchus tshawytscha]	GCF_002872995.1	TRADDN-NCOA6
XP_019205106.1	TRADDN-NCOA6	2385	ncoa6	actinopterygii	Oreochromis niloticus	nuclear receptor coactivator 6 isoform X1 [Oreochromis niloticus]	GCF_001858045.2	TRADDN-NCOA6
XP_019205107.1	TRADDN-NCOA6	2384	ncoa6	actinopterygii	Oreochromis niloticus	nuclear receptor coactivator 6 isoform X2 [Oreochromis niloticus]	GCF_001858045.2	TRADDN-NCOA6
XP_019205108.1	TRADDN-NCOA6	2371	ncoa6	actinopterygii	Oreochromis niloticus	nuclear receptor coactivator 6 isoform X3 [Oreochromis niloticus]	GCF_001858045.2	TRADDN-NCOA6
XP_011475497.2	TRADDN-NCOA6	2320	ncoa6	actinopterygii	Oryzias latipes	nuclear receptor coactivator 6 isoform X1 [Oryzias latipes]	GCF_002234675.1	TRADDN-NCOA6
XP_020560371.1	TRADDN-NCOA6	2318	ncoa6	actinopterygii	Oryzias latipes	nuclear receptor coactivator 6 isoform X2 [Oryzias latipes]	GCF_002234675.1	TRADDN-NCOA6
XP_020560372.1	TRADDN-NCOA6	2306	ncoa6	actinopterygii	Oryzias latipes	nuclear receptor coactivator 6 isoform X3 [Oryzias latipes]	GCF_002234675.1	TRADDN-NCOA6
XP_024148491.1	TRADDN-NCOA6	2330	ncoa6	actinopterygii	Oryzias melastigma	nuclear receptor coactivator 6 isoform X1 [Oryzias melastigma]	GCF_002922805.1	TRADDN-NCOA6
XP_024148492.1	TRADDN-NCOA6	2316	ncoa6	actinopterygii	Oryzias melastigma	nuclear receptor coactivator 6 isoform X2 [Oryzias melastigma]	GCF_002922805.1	TRADDN-NCOA6
XP_019951700.1	TRADDN-NCOA6	2371	ncoa6	actinopterygii	Paralichthys olivaceus	PREDICTED: nuclear receptor coactivator 6 isoform X1 [Paralichthys olivaceus]	GCF_001970005.1	TRADDN-NCOA6
XP_019951701.1	TRADDN-NCOA6	2357	ncoa6	actinopterygii	Paralichthys olivaceus	PREDICTED: nuclear receptor coactivator 6 isoform X2 [Paralichthys olivaceus]	GCF_001970005.1	TRADDN-NCOA6
XP_023652499.1	TRADDN-NCOA6	2330	LOC111835928	actinopterygii	Paramormyrops kingsleyae	nuclear receptor coactivator 6-like [Paramormyrops kingsleyae]	GCF_002872115.1	TRADDN-NCOA6
XP_023654689.1	TRADDN-NCOA6	1974	ncoa6	actinopterygii	Paramormyrops kingsleyae	nuclear receptor coactivator 6 isoform X1 [Paramormyrops kingsleyae]	GCF_002872115.1	TRADDN-NCOA6
XP_023654690.1	TRADDN-NCOA6	1941	ncoa6	actinopterygii	Paramormyrops kingsleyae	nuclear receptor coactivator 6 isoform X2 [Paramormyrops kingsleyae]	GCF_002872115.1	TRADDN-NCOA6
XP_007557741.1	TRADDN-NCOA6	2361	ncoa6	actinopterygii	Poecilia formosa	PREDICTED: nuclear receptor coactivator 6 isoform X1 [Poecilia formosa]	GCF_000485575.1	TRADDN-NCOA6

acc	architecture	len	gen.name	taxend	species	defline	gca	clade
XP_007557742.1	TRADDN-NCOA6	2360	ncoa6	actinopterygii	Poecilia formosa	PREDICTED: nuclear receptor coactivator 6 isoform X2 [Poecilia formosa]	GCF_000485575.1	TRADDN-NCOA6
XP_007557743.1	TRADDN-NCOA6	2359	ncoa6	actinopterygii	Poecilia formosa	PREDICTED: nuclear receptor coactivator 6 isoform X3 [Poecilia formosa]	GCF_000485575.1	TRADDN-NCOA6
XP_007557744.1	TRADDN-NCOA6	2347	ncoa6	actinopterygii	Poecilia formosa	PREDICTED: nuclear receptor coactivator 6 isoform X4 [Poecilia formosa]	GCF_000485575.1	TRADDN-NCOA6
XP_014890771.1	TRADDN-NCOA6	2364	ncoa6	actinopterygii	Poecilia latipinna	PREDICTED: nuclear receptor coactivator 6 isoform X1 [Poecilia latipinna]	GCF_001443285.1	TRADDN-NCOA6
XP_014890772.1	TRADDN-NCOA6	2362	ncoa6	actinopterygii	Poecilia latipinna	PREDICTED: nuclear receptor coactivator 6 isoform X2 [Poecilia latipinna]	GCF_001443285.1	TRADDN-NCOA6
XP_014890773.1	TRADDN-NCOA6	2362	ncoa6	actinopterygii	Poecilia latipinna	PREDICTED: nuclear receptor coactivator 6 isoform X3 [Poecilia latipinna]	GCF_001443285.1	TRADDN-NCOA6
XP_014890774.1	TRADDN-NCOA6	2361	ncoa6	actinopterygii	Poecilia latipinna	PREDICTED: nuclear receptor coactivator 6 isoform X4 [Poecilia latipinna]	GCF_001443285.1	TRADDN-NCOA6
XP_014890775.1	TRADDN-NCOA6	2350	ncoa6	actinopterygii	Poecilia latipinna	PREDICTED: nuclear receptor coactivator 6 isoform X5 [Poecilia latipinna]	GCF_001443285.1	TRADDN-NCOA6
XP_014843508.1	TRADDN-NCOA6	2350	ncoa6	actinopterygii	Poecilia mexicana	PREDICTED: nuclear receptor coactivator 6 isoform X1 [Poecilia mexicana]	GCF_001443325.1	TRADDN-NCOA6
XP_014843509.1	TRADDN-NCOA6	2349	ncoa6	actinopterygii	Poecilia mexicana	PREDICTED: nuclear receptor coactivator 6 isoform X2 [Poecilia mexicana]	GCF_001443325.1	TRADDN-NCOA6
XP_014843510.1	TRADDN-NCOA6	2348	ncoa6	actinopterygii	Poecilia mexicana	PREDICTED: nuclear receptor coactivator 6 isoform X3 [Poecilia mexicana]	GCF_001443325.1	TRADDN-NCOA6
XP_014843511.1	TRADDN-NCOA6	2336	ncoa6	actinopterygii	Poecilia mexicana	PREDICTED: nuclear receptor coactivator 6 isoform X4 [Poecilia mexicana]	GCF_001443325.1	TRADDN-NCOA6
XP_008412102.1	TRADDN-NCOA6	2330	LOC103467472	actinopterygii	Poecilia reticulata	PREDICTED: nuclear receptor coactivator 6 isoform X1 [Poecilia reticulata]	GCF_000633615.1	TRADDN-NCOA6
XP_008412103.1	TRADDN-NCOA6	2328	LOC103467472	actinopterygii	Poecilia reticulata	PREDICTED: nuclear receptor coactivator 6 isoform X2 [Poecilia reticulata]	GCF_000633615.1	TRADDN-NCOA6
XP_008412104.1	TRADDN-NCOA6	2316	LOC103467472	actinopterygii	Poecilia reticulata	PREDICTED: nuclear receptor coactivator 6 isoform X3 [Poecilia reticulata]	GCF_000633615.1	TRADDN-NCOA6
XP_013768208.1	TRADDN-NCOA6	2304	ncoa6	actinopterygii	Pundamilia nyererei	PREDICTED: nuclear receptor coactivator 6 [Pundamilia nyererei]	GCF_000239375.1	TRADDN-NCOA6
XP_017553708.1	TRADDN-NCOA6	2363	ncoa6	actinopterygii	Pygocentrus nattereri	PREDICTED: nuclear receptor coactivator 6 isoform X1 [Pygocentrus nattereri]	GCF_001682695.1	TRADDN-NCOA6

acc	architecture	len	gen.name	taxend	species	defline	gca	clade
XP_017553709.1	TRADDN-NCOA6	2349	ncoa6	actinopterygii	Pygocentrus nattereri	PREDICTED: nuclear receptor coactivator 6 isoform X2 [Pygocentrus nattereri]	GCF_001682695.1	TRADDN-NCOA6
XP_017553710.1	TRADDN-NCOA6	2319	ncoa6	actinopterygii	Pygocentrus nattereri	PREDICTED: nuclear receptor coactivator 6 isoform X3 [Pygocentrus nattereri]	GCF_001682695.1	TRADDN-NCOA6
ACI67963.1	TRADDN-NCOA6	186	NCOA6	actinopterygii	Salmo salar	Nuclear receptor coactivator 6 [Salmo salar]	-	TRADDN-NCOA6
XP_013990145.1	TRADDN-NCOA6	1099	LOC100196047	actinopterygii	Salmo salar	PREDICTED: nuclear receptor coactivator 6, partial [Salmo salar]	GCF_000233375.1	TRADDN-NCOA6
XP_014047602.1	TRADDN-NCOA6	2324	LOC106600629	actinopterygii	Salmo salar	PREDICTED: nuclear receptor coactivator 6-like isoform X1 [Salmo salar]	GCF_000233375.1	TRADDN-NCOA6
XP_014047604.1	TRADDN-NCOA6	2323	LOC106600629	actinopterygii	Salmo salar	PREDICTED: nuclear receptor coactivator 6-like isoform X2 [Salmo salar]	GCF_000233375.1	TRADDN-NCOA6
XP_014047605.1	TRADDN-NCOA6	2276	LOC106600629	actinopterygii	Salmo salar	PREDICTED: nuclear receptor coactivator 6-like isoform X3 [Salmo salar]	GCF_000233375.1	TRADDN-NCOA6
XP_023860801.1	TRADDN-NCOA6	2401	LOC111976261	actinopterygii	Salvelinus alpinus	nuclear receptor coactivator 6 isoform X1 [Salvelinus alpinus]	GCF_002910315.2	TRADDN-NCOA6
XP_023860803.1	TRADDN-NCOA6	2400	LOC111976261	actinopterygii	Salvelinus alpinus	nuclear receptor coactivator 6 isoform X2 [Salvelinus alpinus]	GCF_002910315.2	TRADDN-NCOA6
XP_023860804.1	TRADDN-NCOA6	2400	LOC111976261	actinopterygii	Salvelinus alpinus	nuclear receptor coactivator 6 isoform X3 [Salvelinus alpinus]	GCF_002910315.2	TRADDN-NCOA6
XP_023860805.1	TRADDN-NCOA6	2355	LOC111976261	actinopterygii	Salvelinus alpinus	nuclear receptor coactivator 6 isoform X4 [Salvelinus alpinus]	GCF_002910315.2	TRADDN-NCOA6
XP_023860806.1	TRADDN-NCOA6	2350	LOC111976261	actinopterygii	Salvelinus alpinus	nuclear receptor coactivator 6 isoform X5 [Salvelinus alpinus]	GCF_002910315.2	TRADDN-NCOA6
XP_023992312.1	TRADDN-NCOA6	2336	LOC112069250	actinopterygii	Salvelinus alpinus	nuclear receptor coactivator 6-like [Salvelinus alpinus]	GCF_002910315.2	TRADDN-NCOA6
KPP72068.1	TRADDN-NCOA6	1804	Z043_108968	actinopterygii	Scleropages formosus	hypothetical protein Z043_108968 [Scleropages formosus]	GCA_001005745.2	TRADDN-NCOA6
XP_018594596.1	TRADDN-NCOA6	2220	ncoa6	actinopterygii	Scleropages formosus	PREDICTED: nuclear receptor coactivator 6 [Scleropages formosus]	-	TRADDN-NCOA6
AWP09816.1	TRADDN-NCOA6	2179	SMAX5B_014610	actinopterygii	Scophthalmus maximus	putative nuclear receptor coactivator 6-like isoform 2 [Scophthalmus maximus]	GCA_003186165.1	TRADDN-NCOA6
XP_022597681.1	TRADDN-NCOA6	2337	ncoa6	actinopterygii	Seriola dumerili	nuclear receptor coactivator 6 [Seriola dumerili]	GCF_002260705.1	TRADDN-NCOA6
XP_023280848.1	TRADDN-NCOA6	2397	ncoa6	actinopterygii	Seriola lalandi dorsalis	nuclear receptor coactivator 6 isoform X1 [Seriola lalandi dorsalis]	GCF_002814215.1	TRADDN-NCOA6
XP_023280849.1	TRADDN-NCOA6	2392	ncoa6	actinopterygii	Seriola lalandi dorsalis	nuclear receptor coactivator 6 isoform X2 [Seriola lalandi dorsalis]	GCF_002814215.1	TRADDN-NCOA6
XP_023280850.1	TRADDN-NCOA6	2383	ncoa6	actinopterygii	Seriola lalandi dorsalis	nuclear receptor coactivator 6 isoform X3 [Seriola lalandi dorsalis]	GCF_002814215.1	TRADDN-NCOA6
XP_016308855.1	TRADDN-NCOA6	2282	LOC107663200	actinopterygii	Sinocyclocheilus anshuiensis	PREDICTED: nuclear receptor coactivator 6-like isoform X1 [Sinocyclocheilus anshuiensis]	GCF_001515605.1	TRADDN-NCOA6
XP_016308857.1	TRADDN-NCOA6	2280	LOC107663200	actinopterygii	Sinocyclocheilus anshuiensis	PREDICTED: nuclear receptor coactivator 6-like isoform X2 [Sinocyclocheilus anshuiensis]	GCF_001515605.1	TRADDN-NCOA6

acc	architecture	len	gen.name	taxend	species	defline	gca	clade
XP_016308858.1	TRADDN-NCOA6	2272	LOC107663200	actinopterygii	Sinocyclocheilus anshuiensis	PREDICTED: nuclear receptor coactivator 6-like isoform X3 [Sinocyclocheilus anshuiensis]	GCF_001515605.1	TRADDN-NCOA6
XP_016308859.1	TRADDN-NCOA6	2270	LOC107663200	actinopterygii	Sinocyclocheilus anshuiensis	PREDICTED: nuclear receptor coactivator 6-like isoform X4 [Sinocyclocheilus anshuiensis]	GCF_001515605.1	TRADDN-NCOA6
XP_016308860.1	TRADDN-NCOA6	2268	LOC107663200	actinopterygii	Sinocyclocheilus anshuiensis	PREDICTED: nuclear receptor coactivator 6-like isoform X5 [Sinocyclocheilus anshuiensis]	GCF_001515605.1	TRADDN-NCOA6
XP_016308861.1	TRADDN-NCOA6	2268	LOC107663200	actinopterygii	Sinocyclocheilus anshuiensis	PREDICTED: nuclear receptor coactivator 6-like isoform X6 [Sinocyclocheilus anshuiensis]	GCF_001515605.1	TRADDN-NCOA6
XP_016308862.1	TRADDN-NCOA6	2256	LOC107663200	actinopterygii	Sinocyclocheilus anshuiensis	PREDICTED: nuclear receptor coactivator 6-like isoform X7 [Sinocyclocheilus anshuiensis]	GCF_001515605.1	TRADDN-NCOA6
XP_016308863.1	TRADDN-NCOA6	2198	LOC107663200	actinopterygii	Sinocyclocheilus anshuiensis	PREDICTED: nuclear receptor coactivator 6-like isoform X8 [Sinocyclocheilus anshuiensis]	GCF_001515605.1	TRADDN-NCOA6
XP_016361801.1	TRADDN-NCOA6	2261	LOC107703622	actinopterygii	Sinocyclocheilus anshuiensis	PREDICTED: nuclear receptor coactivator 6-like isoform X1 [Sinocyclocheilus anshuiensis]	GCF_001515605.1	TRADDN-NCOA6
XP_016361804.1	TRADDN-NCOA6	2259	LOC107703622	actinopterygii	Sinocyclocheilus anshuiensis	PREDICTED: nuclear receptor coactivator 6-like isoform X2 [Sinocyclocheilus anshuiensis]	GCF_001515605.1	TRADDN-NCOA6
XP_016361806.1	TRADDN-NCOA6	2257	LOC107703622	actinopterygii	Sinocyclocheilus anshuiensis	PREDICTED: nuclear receptor coactivator 6-like isoform X3 [Sinocyclocheilus anshuiensis]	GCF_001515605.1	TRADDN-NCOA6
XP_016361807.1	TRADDN-NCOA6	2247	LOC107703622	actinopterygii	Sinocyclocheilus anshuiensis	PREDICTED: nuclear receptor coactivator 6-like isoform X4 [Sinocyclocheilus anshuiensis]	GCF_001515605.1	TRADDN-NCOA6
XP_016361808.1	TRADDN-NCOA6	2245	LOC107703622	actinopterygii	Sinocyclocheilus anshuiensis	PREDICTED: nuclear receptor coactivator 6-like isoform X5 [Sinocyclocheilus anshuiensis]	GCF_001515605.1	TRADDN-NCOA6
XP_016361809.1	TRADDN-NCOA6	2243	LOC107703622	actinopterygii	Sinocyclocheilus anshuiensis	PREDICTED: nuclear receptor coactivator 6-like isoform X6 [Sinocyclocheilus anshuiensis]	GCF_001515605.1	TRADDN-NCOA6
XP_016361810.1	TRADDN-NCOA6	2243	LOC107703622	actinopterygii	Sinocyclocheilus anshuiensis	PREDICTED: nuclear receptor coactivator 6-like isoform X7 [Sinocyclocheilus anshuiensis]	GCF_001515605.1	TRADDN-NCOA6
XP_016361811.1	TRADDN-NCOA6	2231	LOC107703622	actinopterygii	Sinocyclocheilus anshuiensis	PREDICTED: nuclear receptor coactivator 6-like isoform X8 [Sinocyclocheilus anshuiensis]	GCF_001515605.1	TRADDN-NCOA6
XP_016361812.1	TRADDN-NCOA6	2177	LOC107703622	actinopterygii	Sinocyclocheilus anshuiensis	PREDICTED: nuclear receptor coactivator 6-like isoform X9 [Sinocyclocheilus anshuiensis]	GCF_001515605.1	TRADDN-NCOA6
XP_016096405.1	TRADDN-NCOA6	2264	LOC107557491	actinopterygii	Sinocyclocheilus grahami	PREDICTED: nuclear receptor coactivator 6-like isoform X1 [Sinocyclocheilus grahami]	GCF_001515645.1	TRADDN-NCOA6
XP_016096407.1	TRADDN-NCOA6	2180	LOC107557491	actinopterygii	Sinocyclocheilus grahami	PREDICTED: nuclear receptor coactivator 6-like isoform X2 [Sinocyclocheilus grahami]	GCF_001515645.1	TRADDN-NCOA6

acc	architecture	len	gen.name	taxend	species	defline	gca	clade
XP_016102777.1	TRADDN-NCOA6	2250	LOC107562623	actinopterygii	Sinocyclocheilus grahami	PREDICTED: nuclear receptor coactivator 6-like isoform X6 [Sinocyclocheilus grahami]	GCF_001515645.1	TRADDN-NCOA6
XP_016387002.1	TRADDN-NCOA6	2259	LOC107723017	actinopterygii	Sinocyclocheilus rhinocerous	PREDICTED: nuclear receptor coactivator 6-like [Sinocyclocheilus rhinocerous]	GCF_001515625.1	TRADDN-NCOA6
XP_016405793.1	TRADDN-NCOA6	2273	LOC107738548	actinopterygii	Sinocyclocheilus rhinocerous	PREDICTED: nuclear receptor coactivator 6-like isoform X1 [Sinocyclocheilus rhinocerous]	GCF_001515625.1	TRADDN-NCOA6
XP_016405795.1	TRADDN-NCOA6	2271	LOC107738548	actinopterygii	Sinocyclocheilus rhinocerous	PREDICTED: nuclear receptor coactivator 6-like isoform X2 [Sinocyclocheilus rhinocerous]	GCF_001515625.1	TRADDN-NCOA6
XP_016405796.1	TRADDN-NCOA6	2259	LOC107738548	actinopterygii	Sinocyclocheilus rhinocerous	PREDICTED: nuclear receptor coactivator 6-like isoform X3 [Sinocyclocheilus rhinocerous]	GCF_001515625.1	TRADDN-NCOA6
XP_016405797.1	TRADDN-NCOA6	2257	LOC107738548	actinopterygii	Sinocyclocheilus rhinocerous	PREDICTED: nuclear receptor coactivator 6-like isoform X4 [Sinocyclocheilus rhinocerous]	GCF_001515625.1	TRADDN-NCOA6
XP_016405798.1	TRADDN-NCOA6	2255	LOC107738548	actinopterygii	Sinocyclocheilus rhinocerous	PREDICTED: nuclear receptor coactivator 6-like isoform X5 [Sinocyclocheilus rhinocerous]	GCF_001515625.1	TRADDN-NCOA6
XP_016405799.1	TRADDN-NCOA6	2243	LOC107738548	actinopterygii	Sinocyclocheilus rhinocerous	PREDICTED: nuclear receptor coactivator 6-like isoform X6 [Sinocyclocheilus rhinocerous]	GCF_001515625.1	TRADDN-NCOA6
XP_008291864.1	TRADDN-NCOA6	2380	ncoa6	actinopterygii	Stegastes partitus	PREDICTED: nuclear receptor coactivator 6 [Stegastes partitus]	GCF_000690725.1	TRADDN-NCOA6
XP_011600780.1	TRADDN-NCOA6	2359	ncoa6	actinopterygii	Takifugu rubripes	PREDICTED: nuclear receptor coactivator 6 isoform X1 [Takifugu rubripes]	-	TRADDN-NCOA6
XP_011600781.1	TRADDN-NCOA6	2348	ncoa6	actinopterygii	Takifugu rubripes	PREDICTED: nuclear receptor coactivator 6 isoform X2 [Takifugu rubripes]	-	TRADDN-NCOA6
XP_011600782.1	TRADDN-NCOA6	1195	ncoa6	actinopterygii	Takifugu rubripes	PREDICTED: nuclear receptor coactivator 6 isoform X3 [Takifugu rubripes]	-	TRADDN-NCOA6
CAF91445.1	TRADDN-NCOA6	1553	GSTEN:00006210:G:001	actinopterygii	Tetraodon nigroviridis	unnamed protein product [Tetraodon nigroviridis]	GCA_000180735.1	TRADDN-NCOA6
XP_023197675.1	TRADDN-NCOA6	2317	ncoa6	actinopterygii	Xiphophorus maculatus	nuclear receptor coactivator 6 [Xiphophorus maculatus]	GCF_002775205.1	TRADDN-NCOA6
KKF08926.1	TRADDN-NCOA6+Topoisom_I_N+Topoisom_I+Topo_C_assoc	2703	EH28_23771	actinopterygii	Larimichthys crocea	DNA topoisomerase 1 [Larimichthys crocea]	-	TRADDN-NCOA6
AWP09815.1	TRADDN-NCOA6+Topoisom_I_N+Topoisom_I+Topo_C_assoc	2848	SMAX5B_014610	actinopterygii	Scophthalmus maximus	putative nuclear receptor coactivator 6-like [Scophthalmus maximus]	GCA_003186165.1	TRADDN-NCOA6
XP_022072468.1	TRADD_N+Death	292	tradd	actinopterygii	Acanthochromis polyacanthus	tumor necrosis factor receptor type 1-associated DEATH domain protein [Acanthochromis polyacanthus]	GCF_002109545.1	TRADD-N
XP_023140009.1	TRADD_N+Death	292	tradd	actinopterygii	Amphiprion ocellaris	tumor necrosis factor receptor type 1-associated DEATH domain protein [Amphiprion ocellaris]	GCF_002776465.1	TRADD-N
XP_026234968.1	TRADD_N+Death	288	tradd	actinopterygii	Anabas testudineus	tumor necrosis factor receptor type 1-associated DEATH domain protein [Anabas testudineus]	GCF_900324465.1	TRADD-N

acc	architecture	len	gen.name	taxend	species	defline	gca	clade
XP_026025056.1	TRADD_N+Death	331	tradd	actinopterygii	Astatotilapia calliptera	tumor necrosis factor receptor type 1-associated DEATH domain protein isoform X1 [Astatotilapia calliptera]	GCF_900246225.1	TRADD-N
XP_026025061.1	TRADD_N+Death	330	tradd	actinopterygii	Astatotilapia calliptera	tumor necrosis factor receptor type 1-associated DEATH domain protein isoform X2 [Astatotilapia calliptera]	GCF_900246225.1	TRADD-N
XP_013883390.1	TRADD_N+Death	292	tradd	actinopterygii	Austrofundulus limnaeus	PREDICTED: tumor necrosis factor receptor type 1-associated DEATH domain protein [Austrofundulus limnaeus]	GCF_001266775.1	TRADD-N
XP_020797264.1	TRADD_N+Death	286	tradd	actinopterygii	Boleophthalmus pectinirostris	tumor necrosis factor receptor type 1-associated DEATH domain protein [Boleophthalmus pectinirostris]	GCF_000788275.1	TRADD-N
XP_026067670.1	TRADD_N+Death	293	tradd	actinopterygii	Carassius auratus	tumor necrosis factor receptor type 1-associated DEATH domain protein [Carassius auratus]	GCF_003368295.1	TRADD-N
XP_026072544.1	TRADD_N+Death	293	LOC113052311	actinopterygii	Carassius auratus	tumor necrosis factor receptor type 1-associated DEATH domain protein-like [Carassius auratus]	GCF_003368295.1	TRADD-N
XP_012672697.1	TRADD_N+Death	289	tradd	actinopterygii	Clupea harengus	PREDICTED: tumor necrosis factor receptor type 1-associated DEATH domain protein [Clupea harengus]	GCF_000966335.1	TRADD-N
XP_008308247.1	TRADD_N+Death	288	tradd	actinopterygii	Cynoglossus semilaevis	tumor necrosis factor receptor type 1-associated DEATH domain protein [Cynoglossus semilaevis]	GCF_000523025.1	TRADD-N
XP_015259088.1	TRADD_N+Death	292	tradd	actinopterygii	Cyprinodon variegatus	PREDICTED: tumor necrosis factor receptor type 1-associated DEATH domain protein [Cyprinodon variegatus]	GCF_000732505.1	TRADD-N
KTF84358.1	TRADD_N+Death	330	cypCar_00021773	actinopterygii	Cyprinus carpio	hypothetical protein cypCar_00021773, partial [Cyprinus carpio]	GCA_001270105.1	TRADD-N
KTF92785.1	TRADD_N+Death	341	cypCar_00003717	actinopterygii	Cyprinus carpio	hypothetical protein cypCar_00003717, partial [Cyprinus carpio]	GCA_001270105.1	TRADD-N
XP_018962454.1	TRADD_N+Death	291	LOC109093186	actinopterygii	Cyprinus carpio	PREDICTED: tumor necrosis factor receptor type 1-associated DEATH domain protein isoform X1 [Cyprinus carpio]	GCF_000951615.1	TRADD-N
NP_571682.1	TRADD_N+Death	293	hm:zehn0873,wu:fc59e02,zehn0873,zgc:103556	actinopterygii	Danio rerio	tumor necrosis factor receptor type 1-associated DEATH domain protein [Danio rerio]	GCF_000002035.6	TRADD-N
XP_026868658.1	TRADD_N+Death	293	tradd	actinopterygii	Electrophorus electricus	tumor necrosis factor receptor type 1-associated DEATH domain protein [Electrophorus electricus]	GCF_003665695.1	TRADD-N
AEA39769.1	TRADD_N+Death	202	-	actinopterygii	Epinephelus coioides	tumor necrosis factor receptor type 1-associated DEATH domain protein, partial [Epinephelus coioides]	-	TRADD-N
XP_010878280.1	TRADD_N+Death	299	tradd	actinopterygii	Esox lucius	PREDICTED: tumor necrosis factor receptor type 1-associated DEATH domain protein [Esox lucius]	GCF_004634155.1	TRADD-N

acc	architecture	len	gen.name	taxend	species	defline	gca	clade
XP_012736473.1	TRADD_N+Death	292	tradd	actinopterygii	Fundulus heteroclitus	tumor necrosis factor receptor type 1-associated DEATH domain protein [Fundulus heteroclitus]	GCF_000826765.1	TRADD-N
PWA17301.1	TRADD_N+Death	292	CCH79_00010441	actinopterygii	Gambusia affinis	hypothetical protein CCH79_00010441, partial [Gambusia affinis]	GCA_003097735.1	TRADD-N
XP_005915280.1	TRADD_N+Death	330	tradd	actinopterygii	Haplochromis burtoni	PREDICTED: tumor necrosis factor receptor type 1-associated DEATH domain protein isoform X2 [Haplochromis burtoni]	GCF_000239415.1	TRADD-N
XP_014196190.1	TRADD_N+Death	331	tradd	actinopterygii	Haplochromis burtoni	PREDICTED: tumor necrosis factor receptor type 1-associated DEATH domain protein isoform X1 [Haplochromis burtoni]	GCF_000239415.1	TRADD-N
XP_019742516.1	TRADD_N+Death	288	tradd	actinopterygii	Hippocampus comes	PREDICTED: tumor necrosis factor receptor type 1-associated DEATH domain protein [Hippocampus comes]	GCF_001891065.1	TRADD-N
XP_017333644.1	TRADD_N+Death	302	tradd	actinopterygii	Ictalurus punctatus	PREDICTED: tumor necrosis factor receptor type 1-associated DEATH domain protein isoform X1 [Ictalurus punctatus]	GCF_001660625.1	TRADD-N
XP_017333645.1	TRADD_N+Death	294	tradd	actinopterygii	Ictalurus punctatus	PREDICTED: tumor necrosis factor receptor type 1-associated DEATH domain protein isoform X2 [Ictalurus punctatus]	GCF_001660625.1	TRADD-N
XP_017296772.1	TRADD_N+Death	290	tradd	actinopterygii	Kryptolebias marmoratus	tumor necrosis factor receptor type 1-associated DEATH domain protein [Kryptolebias marmoratus]	GCF_001649575.1	TRADD-N
XP_020516801.1	TRADD_N+Death	291	tradd	actinopterygii	Labrus bergylta	tumor necrosis factor receptor type 1-associated DEATH domain protein [Labrus bergylta]	GCF_900080235.1	TRADD-N
XP_010743445.1	TRADD_N+Death	293	tradd	actinopterygii	Larimichthys crocea	tumor necrosis factor receptor type 1-associated DEATH domain protein [Larimichthys crocea]	GCF_000972845.2	TRADD-N
XP_018523584.1	TRADD_N+Death	290	tradd	actinopterygii	Lates calcarifer	PREDICTED: tumor necrosis factor receptor type 1-associated DEATH domain protein [Lates calcarifer]	GCF_001640805.1	TRADD-N
XP_015224118.1	TRADD_N+Death	304	tradd	actinopterygii	Lepisosteus oculatus	PREDICTED: tumor necrosis factor receptor type 1-associated DEATH domain protein [Lepisosteus oculatus]	GCF_000242695.1	TRADD-N
XP_026176095.1	TRADD_N+Death	292	tradd	actinopterygii	Mastacembelus armatus	tumor necrosis factor receptor type 1-associated DEATH domain protein [Mastacembelus armatus]	GCF_900324485.1	TRADD-N
XP_004543104.1	TRADD_N+Death	292	tradd	actinopterygii	Maylandia zebra	tumor necrosis factor receptor type 1-associated DEATH domain protein [Maylandia zebra]	GCF_000238955.4	TRADD-N
XP_020465742.1	TRADD_N+Death	290	tradd	actinopterygii	Monopterus albus	tumor necrosis factor receptor type 1-associated DEATH domain protein [Monopterus albus]	GCF_001952655.1	TRADD-N

acc	architecture	len	gen.name	taxend	species	defline	gca	clade
XP_006780114.1	TRADD_N+Death	292	LOC102779391	actinopterygii	Neolamprologus brichardi	PREDICTED: tumor necrosis factor receptor type 1-associated DEATH domain protein-like isoform X1 [Neolamprologus brichardi]	GCF_000239395.1	TRADD-N
XP_015808523.1	TRADD_N+Death	292	tradd	actinopterygii	Nothobranchius furzeri	PREDICTED: tumor necrosis factor receptor type 1-associated DEATH domain protein [Nothobranchius furzeri]	GCF_001465895.1	TRADD-N
XP_010793143.1	TRADD_N+Death	293	tradd	actinopterygii	Notothenia coriiceps	PREDICTED: tumor necrosis factor receptor type 1-associated DEATH domain protein [Notothenia coriiceps]	GCF_000735185.1	TRADD-N
XP_020330116.1	TRADD_N+Death	299	LOC109882432	actinopterygii	Oncorhynchus kisutch	tumor necrosis factor receptor type 1-associated DEATH domain protein-like [Oncorhynchus kisutch]	GCF_002021735.1	TRADD-N
XP_020334945.1	TRADD_N+Death	300	LOC109888162	actinopterygii	Oncorhynchus kisutch	tumor necrosis factor receptor type 1-associated DEATH domain protein [Oncorhynchus kisutch]	GCF_002021735.1	TRADD-N
CDQ78991.1	TRADD_N+Death	291	GSONMT00047171001	actinopterygii	Oncorhynchus mykiss	unnamed protein product [Oncorhynchus mykiss]	GCA_900005705.1	TRADD-N
NP_001118111.1	TRADD_N+Death	292	tradd	actinopterygii	Oncorhynchus mykiss	tumor necrosis factor receptor type 1-associated DEATH domain protein [Oncorhynchus mykiss]	GCF_002163495.1	TRADD-N
XP_021441944.1	TRADD_N+Death	299	LOC110506559	actinopterygii	Oncorhynchus mykiss	tumor necrosis factor receptor type 1-associated DEATH domain protein-like [Oncorhynchus mykiss]	GCF_002163495.1	TRADD-N
XP_021460610.1	TRADD_N+Death	300	tradd	actinopterygii	Oncorhynchus mykiss	tumor necrosis factor receptor type 1-associated DEATH domain protein isoform X1 [Oncorhynchus mykiss]	GCF_002163495.1	TRADD-N
XP_024296035.1	TRADD_N+Death	299	LOC112263724	actinopterygii	Oncorhynchus tshawytscha	tumor necrosis factor receptor type 1-associated DEATH domain protein-like [Oncorhynchus tshawytscha]	GCF_002872995.1	TRADD-N
XP_003442307.1	TRADD_N+Death	291	tradd	actinopterygii	Oreochromis niloticus	tumor necrosis factor receptor type 1-associated DEATH domain protein [Oreochromis niloticus]	GCF_001858045.2	TRADD-N
XP_023809263.1	TRADD_N+Death	290	tradd	actinopterygii	Oryzias latipes	tumor necrosis factor receptor type 1-associated DEATH domain protein [Oryzias latipes]	GCF_002234675.1	TRADD-N
XP_024151861.1	TRADD_N+Death	290	tradd	actinopterygii	Oryzias melastigma	tumor necrosis factor receptor type 1-associated DEATH domain protein [Oryzias melastigma]	GCF_002922805.1	TRADD-N
ACO09635.1	TRADD_N+Death	292	TRADD	actinopterygii	Osmerus mordax	Tumor necrosis factor receptor type 1-associated DEATH domain protein [Osmerus mordax]	-	TRADD-N
XP_026786398.1	TRADD_N+Death	294	tradd	actinopterygii	Pangasianodon hypophthalmus	tumor necrosis factor receptor type 1-associated DEATH domain protein [Pangasianodon hypophthalmus]	GCF_003671635.1	TRADD-N
XP_019954258.1	TRADD_N+Death	322	tradd	actinopterygii	Paralichthys olivaceus	PREDICTED: tumor necrosis factor receptor type 1-associated DEATH domain protein [Paralichthys olivaceus]	GCF_001970005.1	TRADD-N

acc	architecture	len	gen.name	taxend	species	defline	gca	clade
XP_023683006.1	TRADD_N+Death	304	tradd	actinopterygii	Paramormyrops kingsleyae	tumor necrosis factor receptor type 1-associated DEATH domain protein [Paramormyrops kingsleyae]	GCF_002872115.1	TRADD-N
XP_007574895.1	TRADD_N+Death	292	tradd	actinopterygii	Poecilia formosa	PREDICTED: tumor necrosis factor receptor type 1-associated DEATH domain protein [Poecilia formosa]	GCF_000485575.1	TRADD-N
XP_014890432.1	TRADD_N+Death	292	tradd	actinopterygii	Poecilia latipinna	PREDICTED: tumor necrosis factor receptor type 1-associated DEATH domain protein [Poecilia latipinna]	GCF_001443285.1	TRADD-N
XP_008403636.1	TRADD_N+Death	323	tradd	actinopterygii	Poecilia reticulata	PREDICTED: tumor necrosis factor receptor type 1-associated DEATH domain protein [Poecilia reticulata]	GCF_000633615.1	TRADD-N
XP_005741721.1	TRADD_N+Death	331	tradd	actinopterygii	Pundamilia nyererei	PREDICTED: tumor necrosis factor receptor type 1-associated DEATH domain protein isoform X1 [Pundamilia nyererei]	GCF_000239375.1	TRADD-N
XP_005741722.1	TRADD_N+Death	330	tradd	actinopterygii	Pundamilia nyererei	PREDICTED: tumor necrosis factor receptor type 1-associated DEATH domain protein isoform X2 [Pundamilia nyererei]	GCF_000239375.1	TRADD-N
XP_017581097.1	TRADD_N+Death	293	tradd	actinopterygii	Pygocentrus nattereri	PREDICTED: tumor necrosis factor receptor type 1-associated DEATH domain protein [Pygocentrus nattereri]	GCF_001682695.1	TRADD-N
XP_013982430.1	TRADD_N+Death	299	LOC106562229	actinopterygii	Salmo salar	PREDICTED: tumor necrosis factor receptor type 1-associated DEATH domain protein-like [Salmo salar]	GCF_000233375.1	TRADD-N
XP_014031273.1	TRADD_N+Death	299	LOC106587422	actinopterygii	Salmo salar	PREDICTED: tumor necrosis factor receptor type 1-associated DEATH domain protein [Salmo salar]	GCF_000233375.1	TRADD-N
XP_023851343.1	TRADD_N+Death	299	LOC111969426	actinopterygii	Salvelinus alpinus	tumor necrosis factor receptor type 1-associated DEATH domain protein [Salvelinus alpinus]	GCF_002910315.2	TRADD-N
XP_023858136.1	TRADD_N+Death	299	LOC111974567	actinopterygii	Salvelinus alpinus	tumor necrosis factor receptor type 1-associated DEATH domain protein [Salvelinus alpinus]	GCF_002910315.2	TRADD-N
XP_018582130.1	TRADD_N+Death	309	tradd	actinopterygii	Scleropages formosus	PREDICTED: tumor necrosis factor receptor type 1-associated DEATH domain protein [Scleropages formosus]	GCF_900964775.1	TRADD-N
AWP01632.1	TRADD_N+Death	292	SMAX5B_012648	actinopterygii	Scophthalmus maximus	putative tumor necrosis factor receptor type 1-associated DEATH domain protein [Scophthalmus maximus]	GCA_003186165.1	TRADD-N
XP_022600264.1	TRADD_N+Death	292	tradd	actinopterygii	Seriola dumerili	tumor necrosis factor receptor type 1-associated DEATH domain protein [Seriola dumerili]	GCF_002260705.1	TRADD-N
XP_023251461.1	TRADD_N+Death	292	tradd	actinopterygii	Seriola lalandi dorsalis	tumor necrosis factor receptor type 1-associated DEATH domain protein [Seriola lalandi dorsalis]	GCF_002814215.1	TRADD-N

acc	architecture	len	gen.name	taxend	species	defline	gca	clade
XP_016323919.1	TRADD_N+Death	293	LOC107674458	actinopterygii	Sinocyclocheilus anshuiensis	PREDICTED: tumor necrosis factor receptor type 1-associated DEATH domain protein-like [Sinocyclocheilus anshuiensis]	GCF_001515605.1	TRADD-N
XP_016357491.1	TRADD_N+Death	293	LOC107700380	actinopterygii	Sinocyclocheilus anshuiensis	PREDICTED: tumor necrosis factor receptor type 1-associated DEATH domain protein [Sinocyclocheilus anshuiensis]	GCF_001515605.1	TRADD-N
XP_016128639.1	TRADD_N+Death	293	LOC107585960	actinopterygii	Sinocyclocheilus grahami	PREDICTED: tumor necrosis factor receptor type 1-associated DEATH domain protein [Sinocyclocheilus grahami]	GCF_001515645.1	TRADD-N
XP_016140213.1	TRADD_N+Death	293	LOC107594616	actinopterygii	Sinocyclocheilus grahami	PREDICTED: tumor necrosis factor receptor type 1-associated DEATH domain protein-like [Sinocyclocheilus grahami]	GCF_001515645.1	TRADD-N
XP_016409644.1	TRADD_N+Death	293	LOC107741433	actinopterygii	Sinocyclocheilus rhinocerous	PREDICTED: tumor necrosis factor receptor type 1-associated DEATH domain protein-like [Sinocyclocheilus rhinocerous]	GCF_001515625.1	TRADD-N
XP_016421433.1	TRADD_N+Death	293	LOC107750529	actinopterygii	Sinocyclocheilus rhinocerous	PREDICTED: tumor necrosis factor receptor type 1-associated DEATH domain protein [Sinocyclocheilus rhinocerous]	GCF_001515625.1	TRADD-N
AKN80430.1	TRADD_N+Death	293	TRADD	actinopterygii	Sparus aurata	Tumor necrosis factor receptor type 1-associated DEATH domain protein [Sparus aurata]	-	TRADD-N
XP_008302445.1	TRADD_N+Death	292	tradd	actinopterygii	Stegastes partitus	PREDICTED: tumor necrosis factor receptor type 1-associated DEATH domain protein [Stegastes partitus]	GCF_000690725.1	TRADD-N
XP_003970098.1	TRADD_N+Death	292	tradd	actinopterygii	Takifugu rubripes	PREDICTED: tumor necrosis factor receptor type 1-associated DEATH domain protein [Takifugu rubripes]	GCF_901000725.2	TRADD-N
CAF99560.1	TRADD_N+Death	305	GSTEN:00017689:G:001	actinopterygii	Tetraodon nigroviridis	unnamed protein product, partial [Tetraodon nigroviridis]	GCA_000180735.1	TRADD-N
XP_023187695.1	TRADD_N+Death	290	tradd	actinopterygii	Xiphophorus maculatus	tumor necrosis factor receptor type 1-associated DEATH domain protein [Xiphophorus maculatus]	GCF_002775205.1	TRADD-N
XP_027007789.1	TRADD_N+G-alpha	510	LOC113645995	actinopterygii	Tachysurus fulvidraco	guanine nucleotide-binding protein G(o) subunit alpha-like isoform X1 [Tachysurus fulvidraco]	GCF_003724035.1	TRADD-N
XP_020902163.1	Ankyrin+TRADDN-DEDD2	744	LOC110240689	cnidaria	Exaiptasia pallida	LOW QUALITY PROTEIN: uncharacterized protein LOC110240689 [Exaiptasia pallida]	-	TRADDN-DEDD2
KXJ13244.1	Ankyrin+TRADDN-DEDD2+Ankyrin	1132	AnkyrinRD50	cnidaria	Exaiptasia pallida	Ankyrinyrin repeat domain-containing protein 50 [Exaiptasia pallida]	GCA_001417965.1	TRADDN-DEDD2
PFX21248.1	CAP_GLY+CAP_GLY+Pkinase+HEPN+TRADDN-DEDD2+Thioredoxin+PITH	1887	Txnl1	cnidaria	Stylophora pistillata	Thioredoxin-like protein 1 [Stylophora pistillata]	GCA_002571385.1	TRADDN-DEDD2
XP_020622138.1	CARD+Death+TRADDN-DEDD2+CARD	543	LOC110059758	cnidaria	Orbicella faveolata	uncharacterized protein LOC110059758 isoform X1 [Orbicella faveolata]	GCF_002042975.1	TRADDN-DEDD2

acc	architecture	len	gen.name	taxend	species	defline	gca	clade
XP_020630030.1	CARD+TRADDN-DEDD2+CARD	424	LOC110067080	cnidaria	Orbicella faveolata	uncharacterized protein LOC110067080 [Orbicella faveolata]	GCF_002042975.1	TRADDN-DEDD2
XP_020627278.1	CARD+TRADDN-DEDD2+RNA-receptor_SF2-helicase+helical-insertion-in-helicase+RIG-I_C-RD	1143	LOC110064561	cnidaria	Orbicella faveolata	probable ATP-dependent RNA helicase DHX58 [Orbicella faveolata]	GCF_002042975.1	TRADDN-DEDD2
KXJ16647.1	CARD+TRADDN-DEDD2+RNA-receptor_SF2-helicase+RIG-I_C-RD	972	IFIH1	cnidaria	Exaiptasia pallida	Interferon-induced helicase C domain-containing protein 1 [Exaiptasia pallida]	GCA_001417965.1	TRADDN-DEDD2
XP_020896387.1	CARD+TRADDN-DEDD2+RNA-receptor_SF2-helicase+RIG-I_C-RD	985	LOC110235281	cnidaria	Exaiptasia pallida	probable ATP-dependent RNA helicase DHX58 [Exaiptasia pallida]	-	TRADDN-DEDD2
XP_022806512.1	CARD+TRADDN-DEDD2+RNA-receptor_SF2-helicase+RIG-I_C-RD	1226	LOC111343597	cnidaria	Stylophora pistillata	probable ATP-dependent RNA helicase DDX58 [Stylophora pistillata]	GCF_002571385.1	TRADDN-DEDD2
XP_022806513.1	CARD+TRADDN-DEDD2+RNA-receptor_SF2-helicase+RIG-I_C-RD	1207	LOC111343600	cnidaria	Stylophora pistillata	probable ATP-dependent RNA helicase DHX58 isoform X1 [Stylophora pistillata]	GCF_002571385.1	TRADDN-DEDD2
XP_022806514.1	CARD+TRADDN-DEDD2+RNA-receptor_SF2-helicase+RIG-I_C-RD	1104	LOC111343600	cnidaria	Stylophora pistillata	probable ATP-dependent RNA helicase DHX58 isoform X2 [Stylophora pistillata]	GCF_002571385.1	TRADDN-DEDD2
RMX49226.1	CARD+TRADDN-DEDD2+RNA-receptor_SF2-helicase+RIG-I_C-RD+CARD+RNA-receptor_SF2-helicase+RIG-I_C-RD	2192	pdam_00005823	cnidaria	Pocillopora damicornis	hypothetical protein pdam_00005823 [Pocillopora damicornis]	GCA_003704095.1	TRADDN-DEDD2
XP_001622723.1	Coiled-coil+TRADDN-DEDD2+Coiled-coil	1012	NEMVEDRAFT_v1g248321	cnidaria	Nematostella vectensis	predicted protein [Nematostella vectensis]	GCF_000209225.1	TRADDN-DEDD2
XP_001633002.1	Coiled-coil+TRADDN-DEDD2+Coiled-coil	1003	NEMVEDRAFT_v1g243073	cnidaria	Nematostella vectensis	predicted protein [Nematostella vectensis]	GCF_000209225.1	TRADDN-DEDD2
PFX11660.1	Coiled-coil+TRADDN-DEDD2+TPRs+CASPASE	1462	TTC28	cnidaria	Stylophora pistillata	Tetratricopeptide repeat protein 28 [Stylophora pistillata]	GCA_002571385.1	TRADDN-DEDD2
XP_020601168.1	Death+Death+TRADDN-DEDD2	403	LOC110040292	cnidaria	Orbicella faveolata	uncharacterized protein LOC110040292 [Orbicella faveolata]	GCF_002042975.1	TRADDN-DEDD2
XP_020617425.1	Death+TRADDN-DEDD2	587	LOC110055381	cnidaria	Orbicella faveolata	uncharacterized protein LOC110055381 isoform X1 [Orbicella faveolata]	GCF_002042975.1	TRADDN-DEDD2
XP_020617490.1	Death+TRADDN-DEDD2+Coiled-coil+ArgoN+ArgoL1+PAZ+PIWI	1165	LOC110055440	cnidaria	Orbicella faveolata	protein argonaute-2-like isoform X1 [Orbicella faveolata]	GCF_002042975.1	TRADDN-DEDD2
XP_020617491.1	Death+TRADDN-DEDD2+Coiled-coil+ArgoN+ArgoL1+PAZ+PIWI	1107	LOC110055440	cnidaria	Orbicella faveolata	protein argonaute-2-like isoform X2 [Orbicella faveolata]	GCF_002042975.1	TRADDN-DEDD2
PFX26681.1	Death+TRADDN-DEDD2+RVT_1	448	RTase	cnidaria	Stylophora pistillata	putative RNA-directed DNA polymerase from transposon BS [Stylophora pistillata]	GCA_002571385.1	TRADDN-DEDD2
PFX17719.1	Death+TRADDN-DEDD2+SF2-helicase	2256	DDX60	cnidaria	Stylophora pistillata	putative ATP-dependent RNA helicase DDX60 [Stylophora pistillata]	GCA_002571385.1	TRADDN-DEDD2
XP_022803051.1	Death+TRADDN-DEDD2+SF2-helicase	2273	LOC111340467	cnidaria	Stylophora pistillata	probable ATP-dependent RNA helicase DDX60-like [Stylophora pistillata]	GCF_002571385.1	TRADDN-DEDD2
XP_020619853.1	Death+TRADDN-DEDD2+TPRs	690	LOC110057601	cnidaria	Orbicella faveolata	kinesin light chain-like isoform X1 [Orbicella faveolata]	GCF_002042975.1	TRADDN-DEDD2
XP_020619854.1	Death+TRADDN-DEDD2+TPRs	659	LOC110057601	cnidaria	Orbicella faveolata	kinesin light chain 3-like isoform X2 [Orbicella faveolata]	GCF_002042975.1	TRADDN-DEDD2
XP_022778232.1	DED+DED+Coiled-coil+TRADDN-DEDD2+Coiled-coil+domain1	702	LOC111319752	cnidaria	Stylophora pistillata	uncharacterized protein LOC111319752 isoform X2 [Stylophora pistillata]	GCF_002571385.1	TRADDN-DEDD2

acc	architecture	len	gen.name	taxend	species	defline	gca	clade
XP_022804881.1	DED+DED+Coiled-coil+TRADDN-DEDD2+Coiled-coil+domain1	766	LOC111342099	cnidaria	Stylophora pistillata	uncharacterized protein LOC111342099 isoform X2 [Stylophora pistillata]	GCF_002571385.1	TRADDN-DEDD2
PFX21941.1	DED+DED+RVT_1+Coiled-coil+GIY-YIG+TRADDN-DEDD2+Death	1081	AWC38_SpisGene13552	cnidaria	Stylophora pistillata	hypothetical protein AWC38_SpisGene13552 [Stylophora pistillata]	GCA_002571385.1	TRADDN-DEDD2
XP_015775969.1	DED+DED+TRADDN-DEDD2	335	LOC107354067	cnidaria	Acropora digitifera	PREDICTED: uncharacterized protein LOC107354067 [Acropora digitifera]	GCF_000222465.1	TRADDN-DEDD2
RMX41115.1	DED+DED+TRADDN-DEDD2	326	pdam_00003220	cnidaria	Pocillopora damicornis	hypothetical protein pdam_00003220, partial [Pocillopora damicornis]	GCA_003704095.1	TRADDN-DEDD2
PFX12077.1	DED+DED+TRADDN-DEDD2	287	AWC38_SpisGene24020	cnidaria	Stylophora pistillata	hypothetical protein AWC38_SpisGene24020 [Stylophora pistillata]	GCA_002571385.1	TRADDN-DEDD2
PFX13328.1	DED+DED+TRADDN-DEDD2+Ankyrin+RVT_1+helical-region	1263	AWC38_SpisGene22591	cnidaria	Stylophora pistillata	putative ankyrin repeat protein [Stylophora pistillata]	GCA_002571385.1	TRADDN-DEDD2
XP_015778391.1	DED+DED+TRADDN-DEDD2+Coiled-coil-Znf	481	LOC107356270	cnidaria	Acropora digitifera	PREDICTED: uncharacterized protein LOC107356270 [Acropora digitifera]	GCF_000222465.1	TRADDN-DEDD2
XP_020617411.1	DED+DED+TRADDN-DEDD2+Coiled-coil+ArgoN+ArgoL1+PAZ+PIWI	1412	LOC110055369	cnidaria	Orbicella faveolata	protein argonaute-2-like [Orbicella faveolata]	GCF_002042975.1	TRADDN-DEDD2
XP_022777458.1	DED+DED+TRADDN-DEDD2+Coiled-coil+Death+Coiled-coil+domain1	798	LOC111318857	cnidaria	Stylophora pistillata	uncharacterized protein LOC111318857 [Stylophora pistillata]	GCF_002571385.1	TRADDN-DEDD2
XP_022778231.1	DED+DED+TRADDN-DEDD2+Coiled-coil+Death+Coiled-coil+domain1	798	LOC111319752	cnidaria	Stylophora pistillata	uncharacterized protein LOC111319752 isoform X1 [Stylophora pistillata]	GCF_002571385.1	TRADDN-DEDD2
XP_022789506.1	DED+DED+TRADDN-DEDD2+Coiled-coil+Death+Coiled-coil+domain1	840	LOC111329163	cnidaria	Stylophora pistillata	uncharacterized protein LOC111329163 [Stylophora pistillata]	GCF_002571385.1	TRADDN-DEDD2
XP_022804880.1	DED+DED+TRADDN-DEDD2+Coiled-coil+Death+Coiled-coil+domain1	862	LOC111342099	cnidaria	Stylophora pistillata	uncharacterized protein LOC111342099 isoform X1 [Stylophora pistillata]	GCF_002571385.1	TRADDN-DEDD2
PFX21953.1	DED+DED+TRADDN-DEDD2+Coiled-coil+DED+DED+TRADDN-DEDD2+Retrotran_gag_2	1135	AWC38_SpisGene13547	cnidaria	Stylophora pistillata	Retrovirus-related Pol polyprotein from transposon TNT 1-94 [Stylophora pistillata]	GCA_002571385.1	TRADDN-DEDD2
XP_022798487.1	DED+DED+TRADDN-DEDD2+Coiled-coil+Filamin+Beta-propeller	902	LOC111336627	cnidaria	Stylophora pistillata	uncharacterized protein LOC111336627 [Stylophora pistillata]	GCF_002571385.1	TRADDN-DEDD2
XP_015752590.1	DED+DED+TRADDN-DEDD2+Death+Coiled-coil+domain1	797	LOC107332373	cnidaria	Acropora digitifera	PREDICTED: uncharacterized protein LOC107332373 isoform X1 [Acropora digitifera]	GCF_000222465.1	TRADDN-DEDD2
XP_015752591.1	DED+DED+TRADDN-DEDD2+Death+Coiled-coil+domain1	794	LOC107332373	cnidaria	Acropora digitifera	PREDICTED: uncharacterized protein LOC107332373 isoform X2 [Acropora digitifera]	GCF_000222465.1	TRADDN-DEDD2
XP_015752592.1	DED+DED+TRADDN-DEDD2+Death+Coiled-coil+domain1	766	LOC107332373	cnidaria	Acropora digitifera	PREDICTED: uncharacterized protein LOC107332373 isoform X3 [Acropora digitifera]	GCF_000222465.1	TRADDN-DEDD2
XP_015752593.1	DED+DED+TRADDN-DEDD2+Death+Coiled-coil+domain1	783	LOC107332375	cnidaria	Acropora digitifera	PREDICTED: uncharacterized protein LOC107332375 [Acropora digitifera]	GCF_000222465.1	TRADDN-DEDD2

acc	architecture	len	gen.name	taxend	species	defline	gca	clade
XP_015752594.1	DED+DED+TRADDN-DEDD2+Death+Coiled-coil+domain1	780	LOC107332376	cnidaria	Acropora digitifera	PREDICTED: uncharacterized protein LOC107332376 [Acropora digitifera]	GCF_000222465.1	TRADDN-DEDD2
XP_015752596.1	DED+DED+TRADDN-DEDD2+Death+Coiled-coil+domain1	776	LOC107332377	cnidaria	Acropora digitifera	PREDICTED: uncharacterized protein LOC107332377 [Acropora digitifera]	GCF_000222465.1	TRADDN-DEDD2
PFX14010.1	DED+DED+TRADDN-DEDD2+Death+Coiled-coil+domain1	867	AWC38_SpisGene21867	cnidaria	Stylophora pistillata	hypothetical protein AWC38_SpisGene21867 [Stylophora pistillata]	GCA_002571385.1	TRADDN-DEDD2
XP_022789524.1	DED+DED+TRADDN-DEDD2+Death+Coiled-coil+domain1	820	LOC111329179	cnidaria	Stylophora pistillata	uncharacterized protein LOC111329179 [Stylophora pistillata]	GCF_002571385.1	TRADDN-DEDD2
XP_022808330.1	DED+DED+TRADDN-DEDD2+Death+Coiled-coil+domain1	829	LOC111345314	cnidaria	Stylophora pistillata	uncharacterized protein LOC111345314 [Stylophora pistillata]	GCF_002571385.1	TRADDN-DEDD2
XP_022796399.1	DED+DED+TRADDN-DEDD2+helical-region+Death	648	LOC111334847	cnidaria	Stylophora pistillata	uncharacterized protein LOC111334847 isoform X1 [Stylophora pistillata]	GCF_002571385.1	TRADDN-DEDD2
XP_022796400.1	DED+DED+TRADDN-DEDD2+helical-region+Death	568	LOC111334847	cnidaria	Stylophora pistillata	uncharacterized protein LOC111334847 isoform X2 [Stylophora pistillata]	GCF_002571385.1	TRADDN-DEDD2
XP_022796409.1	DED+DED+TRADDN-DEDD2+helical-region+Death	630	LOC111334858	cnidaria	Stylophora pistillata	uncharacterized protein LOC111334858 [Stylophora pistillata]	GCF_002571385.1	TRADDN-DEDD2
XP_015771254.1	DED+DED+TRADDN-DEDD2+TPRs	677	LOC107349586	cnidaria	Acropora digitifera	PREDICTED: tetratricopeptide repeat protein 28-like isoform X1 [Acropora digitifera]	GCF_000222465.1	TRADDN-DEDD2
XP_015771255.1	DED+DED+TRADDN-DEDD2+TPRs	637	LOC107349586	cnidaria	Acropora digitifera	PREDICTED: uncharacterized protein LOC107349586 isoform X2 [Acropora digitifera]	GCF_000222465.1	TRADDN-DEDD2
KXJ08000.1	DED+TRADDN-DEDD2	1584	AC249_AIPGENE7955	cnidaria	Exaiptasia pallida	hypothetical protein AC249_AIPGENE7955 [Exaiptasia pallida]	GCA_001417965.1	TRADDN-DEDD2
XP_001623934.1	DED+TRADDN-DEDD2	524	NEMVEDRAFT_v1g218706	cnidaria	Nematostella vectensis	predicted protein [Nematostella vectensis]	GCF_000209225.1	TRADDN-DEDD2
RMX42794.1	DED+TRADDN-DEDD2	361	pdam_00021837	cnidaria	Pocillopora damicornis	hypothetical protein pdam_00021837 [Pocillopora damicornis]	GCA_003704095.1	TRADDN-DEDD2
XP_022804883.1	DED+TRADDN-DEDD2	324	LOC111342102	cnidaria	Stylophora pistillata	uncharacterized protein LOC111342102 [Stylophora pistillata]	GCF_002571385.1	TRADDN-DEDD2
PFX13329.1	DED+TRADDN-DEDD2+Ankyrin	2392	Ankyrinrd28	cnidaria	Stylophora pistillata	Serine/threonine-protein phosphatase 6 regulatory ankyrin repeat subunit A [Stylophora pistillata]	GCA_002571385.1	TRADDN-DEDD2
XP_022809215.1	DED+TRADDN-DEDD2+Ankyrin	692	LOC111346175	cnidaria	Stylophora pistillata	uncharacterized protein LOC111346175, partial [Stylophora pistillata]	GCF_002571385.1	TRADDN-DEDD2
PFX33087.1	DED+TRADDN-DEDD2+Ankyrin+RVT_1+helical-region+RNaseH	1295	ankrd52	cnidaria	Stylophora pistillata	Serine/threonine-protein phosphatase 6 regulatory ankyrin repeat subunit C [Stylophora pistillata]	GCA_002571385.1	TRADDN-DEDD2

acc	architecture	len	gen.name	taxend	species	defline	gca	clade
KXJ07119.1	DED+TRADDN-DEDD2+Coiled-coil	1074	AC249_AIPGENE15612	cnidaria	Exaiptasia pallida	hypothetical protein AC249_AIPGENE15612 [Exaiptasia pallida]	GCA_001417965.1	TRADDN-DEDD2
KXJ08006.1	DED+TRADDN-DEDD2+Coiled-coil	675	AC249_AIPGENE7952	cnidaria	Exaiptasia pallida	hypothetical protein AC249_AIPGENE7952, partial [Exaiptasia pallida]	GCA_001417965.1	TRADDN-DEDD2
XP_020895892.1	DED+TRADDN-DEDD2+Coiled-coil	677	LOC110234834	cnidaria	Exaiptasia pallida	uncharacterized protein LOC110234834, partial [Exaiptasia pallida]	GCF_001417965.1	TRADDN-DEDD2
XP_020895893.1	DED+TRADDN-DEDD2+Coiled-coil	951	LOC110234835	cnidaria	Exaiptasia pallida	uncharacterized protein LOC110234835 [Exaiptasia pallida]	GCF_001417965.1	TRADDN-DEDD2
XP_022805074.1	DED+TRADDN-DEDD2+Coiled-coil+Ankyrin	927	LOC111342273	cnidaria	Stylophora pistillata	ankyrin-3-like [Stylophora pistillata]	GCF_002571385.1	TRADDN-DEDD2
PFX16507.1	DED+TRADDN-DEDD2+Coiled-coil+Death+Coiled-coil+domain1	1249	AWC38_SpisGene19211	cnidaria	Stylophora pistillata	hypothetical protein AWC38_SpisGene19211 [Stylophora pistillata]	GCA_002571385.1	TRADDN-DEDD2
XP_020616560.1	DED+TRADDN-DEDD2+Coiled-coil+Death+domain1	742	LOC110054557	cnidaria	Orbicella faveolata	uncharacterized protein LOC110054557 isoform X1 [Orbicella faveolata]	GCF_002042975.1	TRADDN-DEDD2
XP_020616561.1	DED+TRADDN-DEDD2+Coiled-coil+Death+domain1	720	LOC110054557	cnidaria	Orbicella faveolata	uncharacterized protein LOC110054557 isoform X2 [Orbicella faveolata]	GCF_002042975.1	TRADDN-DEDD2
XP_020616562.1	DED+TRADDN-DEDD2+Coiled-coil+Death+domain1	654	LOC110054557	cnidaria	Orbicella faveolata	uncharacterized protein LOC110054557 isoform X3 [Orbicella faveolata]	GCF_002042975.1	TRADDN-DEDD2
XP_020616563.1	DED+TRADDN-DEDD2+Coiled-coil+Death+domain1	741	LOC110054558	cnidaria	Orbicella faveolata	uncharacterized protein LOC110054558 [Orbicella faveolata]	GCF_002042975.1	TRADDN-DEDD2
XP_022808331.1	DED+TRADDN-DEDD2+Coiled-coil+Death+domain1	720	LOC111345316	cnidaria	Stylophora pistillata	uncharacterized protein LOC111345316 [Stylophora pistillata]	GCF_002571385.1	TRADDN-DEDD2
XP_020892738.1	DED+TRADDN-DEDD2+Death+Death	586	LOC110231998	cnidaria	Exaiptasia pallida	uncharacterized protein LOC110231998 [Exaiptasia pallida]	GCF_001417965.1	TRADDN-DEDD2
RMX47092.1	DED+TRADDN-DEDD2+DED+TRADDN-DEDD2+Ankyrin	583	pdam_00023287	cnidaria	Pocillopora damicornis	hypothetical protein pdam_00023287, partial [Pocillopora damicornis]	GCA_003704095.1	TRADDN-DEDD2
XP_020604804.1	DED+TRADDN-DEDD2+EF-hand+SH2	587	LOC110043668	cnidaria	Orbicella faveolata	uncharacterized protein LOC110043668 isoform X1 [Orbicella faveolata]	GCF_002042975.1	TRADDN-DEDD2
XP_020604805.1	DED+TRADDN-DEDD2+EF-hand+SH2	586	LOC110043668	cnidaria	Orbicella faveolata	uncharacterized protein LOC110043668 isoform X2 [Orbicella faveolata]	GCF_002042975.1	TRADDN-DEDD2
RMX43465.1	DED+TRADDN-DEDD2+EF-hand+SH2	570	pdam_00001887	cnidaria	Pocillopora damicornis	hypothetical protein pdam_00001887, partial [Pocillopora damicornis]	GCA_003704095.1	TRADDN-DEDD2
XP_020613044.1	DED+TRADDN-DEDD2+HEPN+APATPase	971	LOC110051349	cnidaria	Orbicella faveolata	uncharacterized protein LOC110051349 isoform X1 [Orbicella faveolata]	GCF_002042975.1	TRADDN-DEDD2
XP_020613045.1	DED+TRADDN-DEDD2+HEPN+APATPase	880	LOC110051349	cnidaria	Orbicella faveolata	uncharacterized protein LOC110051349 isoform X2 [Orbicella faveolata]	GCF_002042975.1	TRADDN-DEDD2
XP_020613046.1	DED+TRADDN-DEDD2+HEPN+APATPase	816	LOC110051349	cnidaria	Orbicella faveolata	uncharacterized protein LOC110051349 isoform X3 [Orbicella faveolata]	GCF_002042975.1	TRADDN-DEDD2

acc	architecture	len	gen.name	taxend	species	defline	gca	clade
XP_015750526.1	DED+TRADDN-DEDD2+TPRs	566	LOC107330421	cnidaria	Acropora digitifera	PREDICTED: tetratricopeptide repeat protein 28-like [Acropora digitifera]	GCF_000222465.1	TRADDN-DEDD2
XP_020892739.1	DED+TRADDN-DEDD2+TPRs	495	LOC110231999	cnidaria	Exaiptasia pallida	uncharacterized protein LOC110231999 isoform X1 [Exaiptasia pallida]	GCF_001417965.1	TRADDN-DEDD2
XP_020892740.1	DED+TRADDN-DEDD2+TPRs	494	LOC110231999	cnidaria	Exaiptasia pallida	uncharacterized protein LOC110231999 isoform X2 [Exaiptasia pallida]	-	TRADDN-DEDD2
KXJ18617.1	DED+TRADDN-DEDD2+TPRs+DED+TRADDN-DEDD2+TPRs	1303	TTC28	cnidaria	Exaiptasia pallida	Tetratricopeptide repeat protein 28 [Exaiptasia pallida]	GCA_001417965.1	TRADDN-DEDD2
XP_015766339.1	DED+TRADDN-NCOA6	203	LOC107345145	cnidaria	Acropora digitifera	PREDICTED: uncharacterized protein LOC107345145 [Acropora digitifera]	GCF_000222465.1	TRADDN-NCOA6
KXJ06545.1	DED+TRADDN-NCOA6	145	AC249_AIPGENE1391	cnidaria	Exaiptasia pallida	hypothetical protein AC249_AIPGENE1391, partial [Exaiptasia pallida]	GCA_001417965.1	TRADDN-NCOA6
KXJ09358.1	DED+TRADDN-NCOA6	279	AC249_AIPGENE18212	cnidaria	Exaiptasia pallida	hypothetical protein AC249_AIPGENE18212 [Exaiptasia pallida]	GCA_001417965.1	TRADDN-NCOA6
XP_020632770.1	DED+TRADDN-NCOA6+Ankyrin	1159	LOC110069584	cnidaria	Orbicella faveolata	protein fem-1 homolog C-like [Orbicella faveolata]	GCF_002042975.1	TRADDN-NCOA6
XP_022791057.1	DED+TRADDN-NCOA6+Ankyrin	1115	LOC111330464	cnidaria	Stylophora pistillata	protein fem-1 homolog C-like [Stylophora pistillata]	GCF_002571385.1	TRADDN-NCOA6
XP_022785642.1	DED+TRADDN-NCOA6+vWA	448	LOC111325991	cnidaria	Stylophora pistillata	uncharacterized protein LOC111325991 [Stylophora pistillata]	GCF_002571385.1	TRADDN-NCOA6
XP_020909160.1	DED+TRADDN-NCOA6+vWA+C-ter	659	LOC110247112	cnidaria	Exaiptasia pallida	uncharacterized protein LOC110247112 [Exaiptasia pallida]	GCF_001417965.1	TRADDN-NCOA6
XP_020914592.1	DED+TRADDN-NCOA6+vWA+C-ter	600	LOC110252162	cnidaria	Exaiptasia pallida	uncharacterized protein LOC110252162 isoform X1 [Exaiptasia pallida]	GCF_001417965.1	TRADDN-NCOA6
XP_020914601.1	DED+TRADDN-NCOA6+vWA+C-ter	478	LOC110252162	cnidaria	Exaiptasia pallida	uncharacterized protein LOC110252162 isoform X2 [Exaiptasia pallida]	GCF_001417965.1	TRADDN-NCOA6
XP_001635271.1	DED+TRADDN-NCOA6+vWA+C-ter	619	NEMVEDRAFT_v1g241726	cnidaria	Nematostella vectensis	predicted protein [Nematostella vectensis]	GCF_000209225.1	TRADDN-NCOA6
XP_020605636.1	DED+TRADDN-NCOA6+vWA+C-ter	599	LOC110044428	cnidaria	Orbicella faveolata	uncharacterized protein LOC110044428 [Orbicella faveolata]	GCF_002042975.1	TRADDN-NCOA6
XP_022799237.1	DED+TRADDN-NCOA6+vWA+C-ter	603	LOC111337234	cnidaria	Stylophora pistillata	uncharacterized protein LOC111337234 [Stylophora pistillata]	GCF_002571385.1	TRADDN-NCOA6
XP_022810130.1	FN3+TRADDN-DEDD2+HEPN	528	LOC111347144	cnidaria	Stylophora pistillata	uncharacterized protein LOC111347144, partial [Stylophora pistillata]	GCF_002571385.1	TRADDN-DEDD2
PFX12555.1	FN3+TRADDN-DEDD2+HEPN+PNPase+DUF1759+Gag-knuckle+Gag-knuckle+RVT_1+Peptidase_A17+retroviral_integrase_ZF+rve+RVT_1+Peptidase_A17+retroviral_integrase_ZF+rve+LRRs	3885	SHOC2	cnidaria	Stylophora pistillata	Leucine-rich repeat protein SHOC-2 [Stylophora pistillata]	GCA_002571385.1	TRADDN-DEDD2
PFX14009.1	GT4-GTase+Coiled-coil+TRADDN-DEDD2+LRRs	1463	NLRP3	cnidaria	Stylophora pistillata	NACHT, LRR and PYD domains-containing protein 3 [Stylophora pistillata]	GCA_002571385.1	TRADDN-DEDD2

acc	architecture	len	gen.name	taxend	species	defline	gca	clade
PFX12985.1	GT4-GTase+GT4-GTase+TRADDN-DEDD2	1190	mshA	cnidaria	Stylophora pistillata	D-inositol 3-phosphate glycosyltransferase [Stylophora pistillata]	GCA_002571385.1	TRADDN-DEDD2
XP_015754268.1	GT4-GTase+GT4-GTase+TRADDN-DEDD2+GT4-GTase+RVT_1+Ig	2108	LOC107333918	cnidaria	Acropora digitifera	PREDICTED: uncharacterized protein LOC107333918 [Acropora digitifera]	GCF_000222465.1	TRADDN-DEDD2
PFX15535.1	GT4-GTase+GT4-GTase+TRADDN-DEDD2+GT4-GTase+TRADDN-DEDD2+TRADDN-DEDD2+TRADDN-DEDD2	2524	mshA	cnidaria	Stylophora pistillata	D-inositol 3-phosphate glycosyltransferase, partial [Stylophora pistillata]	GCA_002571385.1	TRADDN-DEDD2
XP_015749857.1	GT4-GTase+PNPase+TRADDN-DEDD2	1031	LOC107329706	cnidaria	Acropora digitifera	PREDICTED: uncharacterized protein LOC107329706 isoform X1 [Acropora digitifera]	GCF_000222465.1	TRADDN-DEDD2
XP_015749858.1	GT4-GTase+PNPase+TRADDN-DEDD2	982	LOC107329706	cnidaria	Acropora digitifera	PREDICTED: uncharacterized protein LOC107329706 isoform X2 [Acropora digitifera]	GCF_000222465.1	TRADDN-DEDD2
XP_015749859.1	GT4-GTase+PNPase+TRADDN-DEDD2	961	LOC107329706	cnidaria	Acropora digitifera	PREDICTED: uncharacterized protein LOC107329706 isoform X3 [Acropora digitifera]	GCF_000222465.1	TRADDN-DEDD2
XP_015749860.1	GT4-GTase+PNPase+TRADDN-DEDD2	933	LOC107329706	cnidaria	Acropora digitifera	PREDICTED: uncharacterized protein LOC107329706 isoform X4 [Acropora digitifera]	GCF_000222465.1	TRADDN-DEDD2
PFX15539.1	GT4-GTase+TM+TRADDN-DEDD2+TRADDN-DEDD2+LRRs	1171	NLRP9	cnidaria	Stylophora pistillata	NACHT, LRR and PYD domains-containing protein 9 [Stylophora pistillata]	GCA_002571385.1	TRADDN-DEDD2
XP_015748178.1	GT4-GTase+TRADDN-DEDD2	374	LOC107327971	cnidaria	Acropora digitifera	PREDICTED: uncharacterized protein LOC107327971 [Acropora digitifera]	GCF_000222465.1	TRADDN-DEDD2
XP_015750005.1	GT4-GTase+TRADDN-DEDD2	637	LOC107329865	cnidaria	Acropora digitifera	PREDICTED: uncharacterized protein LOC107329865 [Acropora digitifera]	GCF_000222465.1	TRADDN-DEDD2
XP_015750171.1	GT4-GTase+TRADDN-DEDD2	397	LOC107330034	cnidaria	Acropora digitifera	PREDICTED: uncharacterized protein LOC107330034 [Acropora digitifera]	GCF_000222465.1	TRADDN-DEDD2
XP_015754272.1	GT4-GTase+TRADDN-DEDD2	660	LOC107333922	cnidaria	Acropora digitifera	PREDICTED: uncharacterized protein LOC107333922 [Acropora digitifera]	GCF_000222465.1	TRADDN-DEDD2
XP_015761974.1	GT4-GTase+TRADDN-DEDD2	503	LOC107341068	cnidaria	Acropora digitifera	PREDICTED: uncharacterized protein LOC107341068 [Acropora digitifera]	GCF_000222465.1	TRADDN-DEDD2
XP_015765567.1	GT4-GTase+TRADDN-DEDD2	1016	LOC107344434	cnidaria	Acropora digitifera	PREDICTED: uncharacterized protein LOC107344434 [Acropora digitifera]	GCF_000222465.1	TRADDN-DEDD2
XP_015768793.1	GT4-GTase+TRADDN-DEDD2	589	LOC107347415	cnidaria	Acropora digitifera	PREDICTED: uncharacterized protein LOC107347415 [Acropora digitifera]	GCF_000222465.1	TRADDN-DEDD2
XP_015768812.1	GT4-GTase+TRADDN-DEDD2	503	LOC107347417	cnidaria	Acropora digitifera	PREDICTED: uncharacterized protein LOC107347417 [Acropora digitifera]	GCF_000222465.1	TRADDN-DEDD2
XP_015774883.1	GT4-GTase+TRADDN-DEDD2	620	LOC107353084	cnidaria	Acropora digitifera	PREDICTED: uncharacterized protein LOC107353084 isoform X1 [Acropora digitifera]	GCF_000222465.1	TRADDN-DEDD2

acc	architecture	len	gen.name	taxend	species	defline	gca	clade
XP_015774884.1	GT4-GTase+TRADDN-DEDD2	606	LOC107353084	cnidaria	Acropora digitifera	PREDICTED: uncharacterized protein LOC107353084 isoform X2 [Acropora digitifera]	GCF_000222465.1	TRADDN-DEDD2
XP_015774885.1	GT4-GTase+TRADDN-DEDD2	570	LOC107353084	cnidaria	Acropora digitifera	PREDICTED: D-inositol 3-phosphate glycosyltransferase-like isoform X3 [Acropora digitifera]	GCF_000222465.1	TRADDN-DEDD2
XP_015775499.1	GT4-GTase+TRADDN-DEDD2	662	LOC107353672	cnidaria	Acropora digitifera	PREDICTED: uncharacterized protein LOC107353672 [Acropora digitifera]	GCF_000222465.1	TRADDN-DEDD2
XP_015777355.1	GT4-GTase+TRADDN-DEDD2	700	LOC107355327	cnidaria	Acropora digitifera	PREDICTED: uncharacterized protein LOC107355327 [Acropora digitifera]	GCF_000222465.1	TRADDN-DEDD2
XP_015778002.1	GT4-GTase+TRADDN-DEDD2	437	LOC107355902	cnidaria	Acropora digitifera	PREDICTED: uncharacterized protein LOC107355902 [Acropora digitifera]	GCF_000222465.1	TRADDN-DEDD2
XP_015780640.1	GT4-GTase+TRADDN-DEDD2	651	LOC107358547	cnidaria	Acropora digitifera	PREDICTED: D-inositol 3-phosphate glycosyltransferase-like [Acropora digitifera]	GCF_000222465.1	TRADDN-DEDD2
XP_020611284.1	GT4-GTase+TRADDN-DEDD2	404	LOC110049798	cnidaria	Orbicella faveolata	uncharacterized protein LOC110049798 [Orbicella faveolata]	GCF_002042975.1	TRADDN-DEDD2
XP_020611387.1	GT4-GTase+TRADDN-DEDD2	879	LOC110049886	cnidaria	Orbicella faveolata	uncharacterized protein LOC110049886 isoform X1 [Orbicella faveolata]	GCF_002042975.1	TRADDN-DEDD2
XP_020611388.1	GT4-GTase+TRADDN-DEDD2	745	LOC110049886	cnidaria	Orbicella faveolata	uncharacterized protein LOC110049886 isoform X2 [Orbicella faveolata]	GCF_002042975.1	TRADDN-DEDD2
XP_020617511.1	GT4-GTase+TRADDN-DEDD2	421	LOC110055458	cnidaria	Orbicella faveolata	uncharacterized protein LOC110055458 [Orbicella faveolata]	GCF_002042975.1	TRADDN-DEDD2
XP_020617752.1	GT4-GTase+TRADDN-DEDD2	647	LOC110055699	cnidaria	Orbicella faveolata	uncharacterized protein LOC110055699 isoform X1 [Orbicella faveolata]	GCF_002042975.1	TRADDN-DEDD2
XP_020617753.1	GT4-GTase+TRADDN-DEDD2	645	LOC110055699	cnidaria	Orbicella faveolata	uncharacterized protein LOC110055699 isoform X2 [Orbicella faveolata]	GCF_002042975.1	TRADDN-DEDD2
XP_020622245.1	GT4-GTase+TRADDN-DEDD2	587	LOC110059861	cnidaria	Orbicella faveolata	uncharacterized protein LOC110059861 [Orbicella faveolata]	GCF_002042975.1	TRADDN-DEDD2
XP_020622252.1	GT4-GTase+TRADDN-DEDD2	598	LOC110059874	cnidaria	Orbicella faveolata	uncharacterized protein LOC110059874 [Orbicella faveolata]	GCF_002042975.1	TRADDN-DEDD2
XP_020622253.1	GT4-GTase+TRADDN-DEDD2	648	LOC110059875	cnidaria	Orbicella faveolata	uncharacterized protein LOC110059875 [Orbicella faveolata]	GCF_002042975.1	TRADDN-DEDD2
XP_020622254.1	GT4-GTase+TRADDN-DEDD2	340	LOC110059876	cnidaria	Orbicella faveolata	uncharacterized protein LOC110059876 [Orbicella faveolata]	GCF_002042975.1	TRADDN-DEDD2
XP_020632526.1	GT4-GTase+TRADDN-DEDD2	300	LOC110069356	cnidaria	Orbicella faveolata	uncharacterized protein LOC110069356 isoform X1 [Orbicella faveolata]	GCF_002042975.1	TRADDN-DEDD2
XP_020632527.1	GT4-GTase+TRADDN-DEDD2	299	LOC110069356	cnidaria	Orbicella faveolata	uncharacterized protein LOC110069356 isoform X2 [Orbicella faveolata]	GCF_002042975.1	TRADDN-DEDD2
XP_020632528.1	GT4-GTase+TRADDN-DEDD2	276	LOC110069356	cnidaria	Orbicella faveolata	uncharacterized protein LOC110069356 isoform X3 [Orbicella faveolata]	GCF_002042975.1	TRADDN-DEDD2

acc	architecture	len	gen.name	taxend	species	defline	gca	clade
RMX54007.1	GT4-GTase+TRADDN-DEDD2	683	pdam_00022334	cnidaria	Pocillopora damicornis	hypothetical protein pdam_00022334, partial [Pocillopora damicornis]	GCA_003704095.1	TRADDN-DEDD2
RMX57318.1	GT4-GTase+TRADDN-DEDD2	570	pdam_00021309	cnidaria	Pocillopora damicornis	hypothetical protein pdam_00021309, partial [Pocillopora damicornis]	GCA_003704095.1	TRADDN-DEDD2
RMX57323.1	GT4-GTase+TRADDN-DEDD2	490	pdam_00021310	cnidaria	Pocillopora damicornis	hypothetical protein pdam_00021310 [Pocillopora damicornis]	GCA_003704095.1	TRADDN-DEDD2
PFX16627.1	GT4-GTase+TRADDN-DEDD2	784	mshA	cnidaria	Stylophora pistillata	D-inositol 3-phosphate glycosyltransferase [Stylophora pistillata]	GCA_002571385.1	TRADDN-DEDD2
PFX22046.1	GT4-GTase+TRADDN-DEDD2	659	mshA	cnidaria	Stylophora pistillata	D-inositol 3-phosphate glycosyltransferase [Stylophora pistillata]	GCA_002571385.1	TRADDN-DEDD2
PFX28903.1	GT4-GTase+TRADDN-DEDD2	693	AWC38_SpisGene6346	cnidaria	Stylophora pistillata	putative glycosyltransferase MJ1607 [Stylophora pistillata]	GCA_002571385.1	TRADDN-DEDD2
PFX32397.1	GT4-GTase+TRADDN-DEDD2	493	mshA	cnidaria	Stylophora pistillata	D-inositol 3-phosphate glycosyltransferase [Stylophora pistillata]	GCA_002571385.1	TRADDN-DEDD2
XP_022777916.1	GT4-GTase+TRADDN-DEDD2	553	LOC111319398	cnidaria	Stylophora pistillata	uncharacterized protein LOC111319398 [Stylophora pistillata]	GCF_002571385.1	TRADDN-DEDD2
XP_022779373.1	GT4-GTase+TRADDN-DEDD2	292	LOC111320905	cnidaria	Stylophora pistillata	uncharacterized protein LOC111320905 [Stylophora pistillata]	GCF_002571385.1	TRADDN-DEDD2
XP_022780314.1	GT4-GTase+TRADDN-DEDD2	666	LOC111321639	cnidaria	Stylophora pistillata	LOW QUALITY PROTEIN: uncharacterized protein LOC111321639 [Stylophora pistillata]	GCF_002571385.1	TRADDN-DEDD2
XP_022785147.1	GT4-GTase+TRADDN-DEDD2	731	LOC111325582	cnidaria	Stylophora pistillata	uncharacterized protein LOC111325582 [Stylophora pistillata]	GCF_002571385.1	TRADDN-DEDD2
XP_022785159.1	GT4-GTase+TRADDN-DEDD2	709	LOC111325594	cnidaria	Stylophora pistillata	uncharacterized protein LOC111325594 [Stylophora pistillata]	GCF_002571385.1	TRADDN-DEDD2
XP_022790555.1	GT4-GTase+TRADDN-DEDD2	676	LOC111330032	cnidaria	Stylophora pistillata	uncharacterized protein LOC111330032 isoform X1 [Stylophora pistillata]	GCF_002571385.1	TRADDN-DEDD2
XP_022790556.1	GT4-GTase+TRADDN-DEDD2	675	LOC111330032	cnidaria	Stylophora pistillata	uncharacterized protein LOC111330032 isoform X2 [Stylophora pistillata]	GCF_002571385.1	TRADDN-DEDD2
XP_022796266.1	GT4-GTase+TRADDN-DEDD2	729	LOC111334716	cnidaria	Stylophora pistillata	uncharacterized protein LOC111334716 [Stylophora pistillata]	GCF_002571385.1	TRADDN-DEDD2
XP_022804734.1	GT4-GTase+TRADDN-DEDD2	554	LOC111341951	cnidaria	Stylophora pistillata	uncharacterized protein LOC111341951 [Stylophora pistillata]	GCF_002571385.1	TRADDN-DEDD2
XP_022809575.1	GT4-GTase+TRADDN-DEDD2	431	LOC111346560	cnidaria	Stylophora pistillata	uncharacterized protein LOC111346560, partial [Stylophora pistillata]	GCF_002571385.1	TRADDN-DEDD2

acc	architecture	len	gen.name	taxend	species	defline	gca	clade
XP_022809578.1	GT4-GTase+TRADDN-DEDD2	746	LOC111346564	cnidaria	Stylophora pistillata	uncharacterized protein LOC111346564 [Stylophora pistillata]	GCF_002571385.1	TRADDN-DEDD2
XP_015754284.1	GT4-GTase+TRADDN-DEDD2+Ankyrin	1391	LOC107333936	cnidaria	Acropora digitifera	PREDICTED: uncharacterized protein LOC107333936 isoform X1 [Acropora digitifera]	GCF_000222465.1	TRADDN-DEDD2
XP_015754287.1	GT4-GTase+TRADDN-DEDD2+Ankyrin	1391	LOC107333936	cnidaria	Acropora digitifera	PREDICTED: uncharacterized protein LOC107333936 isoform X2 [Acropora digitifera]	GCF_000222465.1	TRADDN-DEDD2
XP_015754288.1	GT4-GTase+TRADDN-DEDD2+Ankyrin	1351	LOC107333936	cnidaria	Acropora digitifera	PREDICTED: uncharacterized protein LOC107333936 isoform X3 [Acropora digitifera]	GCF_000222465.1	TRADDN-DEDD2
XP_015765548.1	GT4-GTase+TRADDN-DEDD2+Ankyrin	1084	LOC107344415	cnidaria	Acropora digitifera	PREDICTED: uncharacterized protein LOC107344415 isoform X1 [Acropora digitifera]	GCF_000222465.1	TRADDN-DEDD2
XP_015765549.1	GT4-GTase+TRADDN-DEDD2+Ankyrin	1083	LOC107344415	cnidaria	Acropora digitifera	PREDICTED: uncharacterized protein LOC107344415 isoform X2 [Acropora digitifera]	GCF_000222465.1	TRADDN-DEDD2
XP_015777357.1	GT4-GTase+TRADDN-DEDD2+Ankyrin	1436	LOC107355329	cnidaria	Acropora digitifera	PREDICTED: uncharacterized protein LOC107355329 isoform X1 [Acropora digitifera]	GCF_000222465.1	TRADDN-DEDD2
XP_015777358.1	GT4-GTase+TRADDN-DEDD2+Ankyrin	1420	LOC107355329	cnidaria	Acropora digitifera	PREDICTED: uncharacterized protein LOC107355329 isoform X2 [Acropora digitifera]	GCF_000222465.1	TRADDN-DEDD2
XP_015777359.1	GT4-GTase+TRADDN-DEDD2+Ankyrin	1420	LOC107355329	cnidaria	Acropora digitifera	PREDICTED: uncharacterized protein LOC107355329 isoform X3 [Acropora digitifera]	GCF_000222465.1	TRADDN-DEDD2
XP_015777360.1	GT4-GTase+TRADDN-DEDD2+Ankyrin	1404	LOC107355329	cnidaria	Acropora digitifera	PREDICTED: uncharacterized protein LOC107355329 isoform X4 [Acropora digitifera]	GCF_000222465.1	TRADDN-DEDD2
XP_015777361.1	GT4-GTase+TRADDN-DEDD2+Ankyrin	1400	LOC107355329	cnidaria	Acropora digitifera	PREDICTED: uncharacterized protein LOC107355329 isoform X5 [Acropora digitifera]	GCF_000222465.1	TRADDN-DEDD2
XP_015777362.1	GT4-GTase+TRADDN-DEDD2+Ankyrin	1388	LOC107355329	cnidaria	Acropora digitifera	PREDICTED: uncharacterized protein LOC107355329 isoform X6 [Acropora digitifera]	GCF_000222465.1	TRADDN-DEDD2
XP_015777363.1	GT4-GTase+TRADDN-DEDD2+Ankyrin	1388	LOC107355329	cnidaria	Acropora digitifera	PREDICTED: uncharacterized protein LOC107355329 isoform X7 [Acropora digitifera]	GCF_000222465.1	TRADDN-DEDD2
XP_015777365.1	GT4-GTase+TRADDN-DEDD2+Ankyrin	1388	LOC107355329	cnidaria	Acropora digitifera	PREDICTED: uncharacterized protein LOC107355329 isoform X8 [Acropora digitifera]	GCF_000222465.1	TRADDN-DEDD2
XP_015777366.1	GT4-GTase+TRADDN-DEDD2+Ankyrin	1384	LOC107355329	cnidaria	Acropora digitifera	PREDICTED: uncharacterized protein LOC107355329 isoform X9 [Acropora digitifera]	GCF_000222465.1	TRADDN-DEDD2
XP_015777367.1	GT4-GTase+TRADDN-DEDD2+Ankyrin	1372	LOC107355329	cnidaria	Acropora digitifera	PREDICTED: uncharacterized protein LOC107355329 isoform X10 [Acropora digitifera]	GCF_000222465.1	TRADDN-DEDD2
XP_015777368.1	GT4-GTase+TRADDN-DEDD2+Ankyrin	1372	LOC107355329	cnidaria	Acropora digitifera	PREDICTED: uncharacterized protein LOC107355329 isoform X11 [Acropora digitifera]	GCF_000222465.1	TRADDN-DEDD2

acc	architecture	len	gen.name	taxend	species	defline	gca	clade
XP_015777369.1	GT4-GTase+TRADDN-DEDD2+Ankyrin	1372	LOC107355329	cnidaria	Acropora digitifera	PREDICTED: uncharacterized protein LOC107355329 isoform X12 [Acropora digitifera]	GCF_000222465.1	TRADDN-DEDD2
XP_015777370.1	GT4-GTase+TRADDN-DEDD2+Ankyrin	1372	LOC107355329	cnidaria	Acropora digitifera	PREDICTED: uncharacterized protein LOC107355329 isoform X13 [Acropora digitifera]	GCF_000222465.1	TRADDN-DEDD2
XP_015777371.1	GT4-GTase+TRADDN-DEDD2+Ankyrin	1368	LOC107355329	cnidaria	Acropora digitifera	PREDICTED: uncharacterized protein LOC107355329 isoform X14 [Acropora digitifera]	GCF_000222465.1	TRADDN-DEDD2
XP_015777372.1	GT4-GTase+TRADDN-DEDD2+Ankyrin	1356	LOC107355329	cnidaria	Acropora digitifera	PREDICTED: uncharacterized protein LOC107355329 isoform X15 [Acropora digitifera]	GCF_000222465.1	TRADDN-DEDD2
XP_015777373.1	GT4-GTase+TRADDN-DEDD2+Ankyrin	1356	LOC107355329	cnidaria	Acropora digitifera	PREDICTED: uncharacterized protein LOC107355329 isoform X16 [Acropora digitifera]	GCF_000222465.1	TRADDN-DEDD2
XP_015777374.1	GT4-GTase+TRADDN-DEDD2+Ankyrin	1352	LOC107355329	cnidaria	Acropora digitifera	PREDICTED: uncharacterized protein LOC107355329 isoform X17 [Acropora digitifera]	GCF_000222465.1	TRADDN-DEDD2
XP_015777375.1	GT4-GTase+TRADDN-DEDD2+Ankyrin	878	LOC107355332	cnidaria	Acropora digitifera	PREDICTED: ankyrin-1-like [Acropora digitifera]	GCF_000222465.1	TRADDN-DEDD2
XP_015778952.1	GT4-GTase+TRADDN-DEDD2+Ankyrin	1280	LOC107356842	cnidaria	Acropora digitifera	PREDICTED: uncharacterized protein LOC107356842 [Acropora digitifera]	GCF_000222465.1	TRADDN-DEDD2
XP_015779234.1	GT4-GTase+TRADDN-DEDD2+Ankyrin	1392	LOC107357096	cnidaria	Acropora digitifera	PREDICTED: ankyrin-3-like [Acropora digitifera]	GCF_000222465.1	TRADDN-DEDD2
XP_015779236.1	GT4-GTase+TRADDN-DEDD2+Ankyrin	1086	LOC107357098	cnidaria	Acropora digitifera	PREDICTED: uncharacterized protein LOC107357098 [Acropora digitifera]	GCF_000222465.1	TRADDN-DEDD2
XP_015780567.1	GT4-GTase+TRADDN-DEDD2+Ankyrin	1294	LOC107358478	cnidaria	Acropora digitifera	PREDICTED: uncharacterized protein LOC107358478 [Acropora digitifera]	GCF_000222465.1	TRADDN-DEDD2
PFX11667.1	GT4-GTase+TRADDN-DEDD2+Ankyrin	1115	Ankyrin3	cnidaria	Stylophora pistillata	Ankyrinyrin-3 [Stylophora pistillata]	GCA_002571385.1	TRADDN-DEDD2
XP_015754281.1	GT4-GTase+TRADDN-DEDD2+Ankyrin+DED+APATPase+TPRs	1597	LOC107333935	cnidaria	Acropora digitifera	PREDICTED: uncharacterized protein LOC107333935 isoform X1 [Acropora digitifera]	GCF_000222465.1	TRADDN-DEDD2
XP_015754282.1	GT4-GTase+TRADDN-DEDD2+Ankyrin+DED+APATPase+TPRs	1560	LOC107333935	cnidaria	Acropora digitifera	PREDICTED: uncharacterized protein LOC107333935 isoform X2 [Acropora digitifera]	GCF_000222465.1	TRADDN-DEDD2
XP_015754283.1	GT4-GTase+TRADDN-DEDD2+Ankyrin+DED+APATPase+TPRs	1546	LOC107333935	cnidaria	Acropora digitifera	PREDICTED: uncharacterized protein LOC107333935 isoform X3 [Acropora digitifera]	GCF_000222465.1	TRADDN-DEDD2
XP_020630898.1	GT4-GTase+TRADDN-DEDD2+APGTPase	866	LOC110067895	cnidaria	Orbicella faveolata	uncharacterized protein LOC110067895 [Orbicella faveolata]	GCF_002042975.1	TRADDN-DEDD2
XP_015768823.1	GT4-GTase+TRADDN-DEDD2+Death	402	LOC107347423	cnidaria	Acropora digitifera	PREDICTED: uncharacterized protein LOC107347423 [Acropora digitifera]	GCF_000222465.1	TRADDN-DEDD2
XP_022786056.1	GT4-GTase+TRADDN-DEDD2+Death	917	LOC111326347	cnidaria	Stylophora pistillata	uncharacterized protein LOC111326347 [Stylophora pistillata]	GCF_002571385.1	TRADDN-DEDD2
XP_015754270.1	GT4-GTase+TRADDN-DEDD2+DUF4062+STAND	1358	LOC107333920	cnidaria	Acropora digitifera	PREDICTED: LOW QUALITY PROTEIN: uncharacterized protein LOC107333920 [Acropora digitifera]	GCF_000222465.1	TRADDN-DEDD2

acc	architecture	len	gen.name	taxend	species	defline	gca	clade
XP_020614207.1	GT4-GTase+TRADDN-DEDD2+GBP+GBP_C	986	LOC110052421	cnidaria	Orbicella faveolata	uncharacterized protein LOC110052421 isoform X1 [Orbicella faveolata]	GCF_002042975.1	TRADDN-DEDD2
XP_015765571.1	GT4-GTase+TRADDN-DEDD2+GT4-GTase	1248	LOC107344438	cnidaria	Acropora digitifera	PREDICTED: uncharacterized protein LOC107344438 [Acropora digitifera]	GCF_000222465.1	TRADDN-DEDD2
XP_015778949.1	GT4-GTase+TRADDN-DEDD2+GT4-GTase	1007	LOC107356840	cnidaria	Acropora digitifera	PREDICTED: uncharacterized protein LOC107356840 [Acropora digitifera]	GCF_000222465.1	TRADDN-DEDD2
XP_022804733.1	GT4-GTase+TRADDN-DEDD2+helical-region+LRRs	1261	LOC111341950	cnidaria	Stylophora pistillata	uncharacterized protein LOC111341950 isoform X6 [Stylophora pistillata]	GCF_002571385.1	TRADDN-DEDD2
XP_022808343.1	GT4-GTase+TRADDN-DEDD2+helical-region+LRRs	1234	LOC111345328	cnidaria	Stylophora pistillata	uncharacterized protein LOC111345328 isoform X1 [Stylophora pistillata]	GCF_002571385.1	TRADDN-DEDD2
XP_022808344.1	GT4-GTase+TRADDN-DEDD2+helical-region+LRRs	1230	LOC111345328	cnidaria	Stylophora pistillata	uncharacterized protein LOC111345328 isoform X2 [Stylophora pistillata]	GCF_002571385.1	TRADDN-DEDD2
XP_022808345.1	GT4-GTase+TRADDN-DEDD2+helical-region+LRRs	1214	LOC111345328	cnidaria	Stylophora pistillata	uncharacterized protein LOC111345328 isoform X3 [Stylophora pistillata]	GCF_002571385.1	TRADDN-DEDD2
PFX26360.1	GT4-GTase+TRADDN-DEDD2+HSP90-Sacsin+DUF3684+HSP90-Sacsin+DUF3684+HSP90-Sacsin+DUF3684+DnaJ+HEPN	4913	SACS	cnidaria	Stylophora pistillata	Sacsin, partial [Stylophora pistillata]	GCA_002571385.1	TRADDN-DEDD2
PFX13778.1	GT4-GTase+TRADDN-DEDD2+HSP90-Sacsin+DUF3684+HSP90-Sacsin+DUF3684+HSP90-Sacsin+DUF3684+HSP90-Sacsin+DUF3684+HSP90-Sacsin+DUF3684+HSP90-Sacsin+DUF3684+HSP90-Sacsin+DUF3684+DUF3684+DnaJ+HEPN	9556	SACS	cnidaria	Stylophora pistillata	Sacsin [Stylophora pistillata]	GCA_002571385.1	TRADDN-DEDD2
XP_022809377.1	GT4-GTase+TRADDN-DEDD2+HSP90-Sacsin+DUF3684+TRADDN-DEDD2+HSP90-Sacsin+DUF3684+TRADDN-DEDD2+HSP90-Sacsin+DUF3684+DnaJ+HEPN	4937	LOC111346359	cnidaria	Stylophora pistillata	sacsin-like [Stylophora pistillata]	GCF_002571385.1	TRADDN-DEDD2
PFX19528.1	GT4-GTase+TRADDN-DEDD2+low-complexity+Rossmann-fold+Octopine_DH	1192	tadh	cnidaria	Stylophora pistillata	Tauropine dehydrogenase [Stylophora pistillata]	GCA_002571385.1	TRADDN-DEDD2
XP_022800207.1	GT4-GTase+TRADDN-DEDD2+low-complexity+Rossmann-fold+Octopine_DH	1180	LOC111338068	cnidaria	Stylophora pistillata	uncharacterized protein LOC111338068 isoform X1 [Stylophora pistillata]	GCF_002571385.1	TRADDN-DEDD2
XP_022800208.1	GT4-GTase+TRADDN-DEDD2+low-complexity+Rossmann-fold+Octopine_DH	1179	LOC111338068	cnidaria	Stylophora pistillata	uncharacterized protein LOC111338068 isoform X2 [Stylophora pistillata]	GCF_002571385.1	TRADDN-DEDD2
XP_022800209.1	GT4-GTase+TRADDN-DEDD2+low-complexity+Rossmann-fold+Octopine_DH	1179	LOC111338068	cnidaria	Stylophora pistillata	uncharacterized protein LOC111338068 isoform X3 [Stylophora pistillata]	GCF_002571385.1	TRADDN-DEDD2
PFX31900.1	GT4-GTase+TRADDN-DEDD2+P-loop-NTPase	1327	HELZ	cnidaria	Stylophora pistillata	putative helicase with zinc finger domain, partial [Stylophora pistillata]	GCA_002571385.1	TRADDN-DEDD2
PFX25676.1	GT4-GTase+TRADDN-DEDD2+PNMA+zf-C2H2+SF1-helicase	1626	MOV10	cnidaria	Stylophora pistillata	putative helicase MOV-10 [Stylophora pistillata]	GCA_002571385.1	TRADDN-DEDD2
XP_015773770.1	GT4-GTase+TRADDN-DEDD2+STAND+wHTH+helical-region+LRRs	1843	LOC107351981	cnidaria	Acropora digitifera	PREDICTED: uncharacterized protein LOC107351981 [Acropora digitifera]	GCF_000222465.1	TRADDN-DEDD2
XP_015759411.1	GT4-GTase+TRADDN-DEDD2+STAND+wHTH+helical-repeat+LRRs	1346	LOC107338694	cnidaria	Acropora digitifera	PREDICTED: uncharacterized protein LOC107338694 [Acropora digitifera]	GCF_000222465.1	TRADDN-DEDD2
PFX28922.1	GT4-GTase+TRADDN-DEDD2+TRADDN-DEDD2	1022	mshA	cnidaria	Stylophora pistillata	D-inositol 3-phosphate glycosyltransferase [Stylophora pistillata]	GCA_002571385.1	TRADDN-DEDD2

acc	architecture	len	gen.name	taxend	species	defline	gca	clade
XP_022785151.1	GT4-GTase+TRADDN-DEDD2+TRADDN-DEDD2	848	LOC111325586	cnidaria	Stylophora pistillata	uncharacterized protein LOC111325586 [Stylophora pistillata]	GCF_002571385.1	TRADDN-DEDD2
XP_015771496.1	GT4-GTase+TRADDN-DEDD2+TRADDN-DEDD2+Coiled-coil+TPRs+RVT_1	1988	LOC107349827	cnidaria	Acropora digitifera	PREDICTED: uncharacterized protein LOC107349827 [Acropora digitifera]	GCF_000222465.1	TRADDN-DEDD2
PFX16629.1	GT4-GTase+TRADDN-DEDD2+TRADDN-DEDD2+LRRs	1560	NLRP5	cnidaria	Stylophora pistillata	NACHT, LRR and PYD domains-containing protein 5 [Stylophora pistillata]	GCA_002571385.1	TRADDN-DEDD2
XP_022804727.1	GT4-GTase+TRADDN-DEDD2+TRADDN-DEDD2+LRRs	1529	LOC111341950	cnidaria	Stylophora pistillata	uncharacterized protein LOC111341950 isoform X1 [Stylophora pistillata]	GCF_002571385.1	TRADDN-DEDD2
XP_022804728.1	GT4-GTase+TRADDN-DEDD2+TRADDN-DEDD2+LRRs	1522	LOC111341950	cnidaria	Stylophora pistillata	uncharacterized protein LOC111341950 isoform X2 [Stylophora pistillata]	GCF_002571385.1	TRADDN-DEDD2
XP_022804730.1	GT4-GTase+TRADDN-DEDD2+TRADDN-DEDD2+LRRs	1521	LOC111341950	cnidaria	Stylophora pistillata	uncharacterized protein LOC111341950 isoform X3 [Stylophora pistillata]	GCF_002571385.1	TRADDN-DEDD2
XP_022804731.1	GT4-GTase+TRADDN-DEDD2+TRADDN-DEDD2+LRRs	1517	LOC111341950	cnidaria	Stylophora pistillata	uncharacterized protein LOC111341950 isoform X4 [Stylophora pistillata]	GCF_002571385.1	TRADDN-DEDD2
XP_022804732.1	GT4-GTase+TRADDN-DEDD2+TRADDN-DEDD2+LRRs	1516	LOC111341950	cnidaria	Stylophora pistillata	uncharacterized protein LOC111341950 isoform X5 [Stylophora pistillata]	GCF_002571385.1	TRADDN-DEDD2
PFX29241.1	GT4-GTase+TRADDN-DEDD2+TRADDN-DEDD2+PH+GT4-GTase+STING	1498	Tmem173	cnidaria	Stylophora pistillata	Stimulator of interferon genes protein [Stylophora pistillata]	GCA_002571385.1	TRADDN-DEDD2
XP_015765336.1	GT4-GTase+TRADDN-DEDD2+UBI+UBI+UBI	1194	LOC107344209	cnidaria	Acropora digitifera	PREDICTED: uncharacterized protein LOC107344209 [Acropora digitifera]	GCF_000222465.1	TRADDN-DEDD2
XP_020617495.1	GT4-GTase+TRADDN-DEDD2+UBI+UBI+UBI+UBI+TRADDN-DEDD2+UBI+UBI+UBI+UBI+UBI+TRADDN-DEDD2+Coiled-coil	1442	LOC110055445	cnidaria	Orbicella faveolata	polyubiquitin-A-like isoform X1 [Orbicella faveolata]	GCF_002042975.1	TRADDN-DEDD2
XP_020617496.1	GT4-GTase+TRADDN-DEDD2+UBI+UBI+UBI+UBI+TRADDN-DEDD2+UBI+UBI+UBI+UBI+UBI+TRADDN-DEDD2+Coiled-coil	1220	LOC110055445	cnidaria	Orbicella faveolata	polyubiquitin-A-like isoform X2 [Orbicella faveolata]	GCF_002042975.1	TRADDN-DEDD2
XP_015778868.1	GT4-GTase+TRADDN-DEDD2+vWA+SERAprotease+ZnR	1221	LOC107356762	cnidaria	Acropora digitifera	PREDICTED: uncharacterized protein LOC107356762 isoform X1 [Acropora digitifera]	GCF_000222465.1	TRADDN-DEDD2
XP_015778871.1	GT4-GTase+TRADDN-DEDD2+vWA+SERAprotease+ZnR	1221	LOC107356762	cnidaria	Acropora digitifera	PREDICTED: uncharacterized protein LOC107356762 isoform X2 [Acropora digitifera]	GCF_000222465.1	TRADDN-DEDD2
XP_015778873.1	GT4-GTase+TRADDN-DEDD2+vWA+SERAprotease+ZnR	1221	LOC107356762	cnidaria	Acropora digitifera	PREDICTED: uncharacterized protein LOC107356762 isoform X3 [Acropora digitifera]	GCF_000222465.1	TRADDN-DEDD2
XP_015778874.1	GT4-GTase+TRADDN-DEDD2+vWA+SERAprotease+ZnR	1218	LOC107356762	cnidaria	Acropora digitifera	PREDICTED: uncharacterized protein LOC107356762 isoform X4 [Acropora digitifera]	GCF_000222465.1	TRADDN-DEDD2
XP_015778875.1	GT4-GTase+TRADDN-DEDD2+vWA+SERAprotease+ZnR	1145	LOC107356762	cnidaria	Acropora digitifera	PREDICTED: uncharacterized protein LOC107356762 isoform X5 [Acropora digitifera]	GCF_000222465.1	TRADDN-DEDD2
XP_015778876.1	GT4-GTase+TRADDN-DEDD2+vWA+SERAprotease+ZnR	1080	LOC107356762	cnidaria	Acropora digitifera	PREDICTED: uncharacterized protein LOC107356762 isoform X6 [Acropora digitifera]	GCF_000222465.1	TRADDN-DEDD2

acc	architecture	len	gen.name	taxend	species	defline	gca	clade
XP_015778880.1	GT4-GTase+TRADDN-DEDD2+vWA+SERAprotease+ZnR	1218	LOC107356768	cnidaria	Acropora digitifera	PREDICTED: uncharacterized protein LOC107356768 isoform X2 [Acropora digitifera]	GCF_000222465.1	TRADDN-DEDD2
XP_015778882.1	GT4-GTase+TRADDN-DEDD2+vWA+SERAprotease+ZnR	1145	LOC107356768	cnidaria	Acropora digitifera	PREDICTED: uncharacterized protein LOC107356768 isoform X3 [Acropora digitifera]	GCF_000222465.1	TRADDN-DEDD2
PFX19725.1	HEPN+Coiled-coil+TRADDN-DEDD2	517	Dzip3	cnidaria	Stylophora pistillata	E3 ubiquitin-protein ligase DZIP3 [Stylophora pistillata]	GCA_002571385.1	TRADDN-DEDD2
PFX24805.1	HEPN+Coiled-coil+TRADDN-DEDD2	535	Dzip3	cnidaria	Stylophora pistillata	E3 ubiquitin-protein ligase DZIP3 [Stylophora pistillata]	GCA_002571385.1	TRADDN-DEDD2
XP_022791936.1	HEPN+Coiled-coil+TRADDN-DEDD2	514	LOC111331144	cnidaria	Stylophora pistillata	uncharacterized protein LOC111331144 [Stylophora pistillata]	GCF_002571385.1	TRADDN-DEDD2
XP_020604217.1	HEPN+Coiled-coil+TRADDN-DEDD2+Ankyrin+LRRs+APGTPase+COR+Pkinase+Beta-propeller	2876	LOC110043138	cnidaria	Orbicella faveolata	uncharacterized protein LOC110043138 isoform X1 [Orbicella faveolata]	GCF_002042975.1	TRADDN-DEDD2
XP_020604218.1	HEPN+Coiled-coil+TRADDN-DEDD2+Ankyrin+LRRs+APGTPase+COR+Pkinase+Beta-propeller	2875	LOC110043138	cnidaria	Orbicella faveolata	uncharacterized protein LOC110043138 isoform X2 [Orbicella faveolata]	GCF_002042975.1	TRADDN-DEDD2
XP_022788761.1	HEPN+Coiled-coil+TRADDN-DEDD2+Coiled-coil+TM	528	LOC111328564	cnidaria	Stylophora pistillata	E3 ubiquitin-protein ligase DZIP3-like [Stylophora pistillata]	GCF_002571385.1	TRADDN-DEDD2
XP_020627088.1	HEPN+Coiled-coil+TRADDN-DEDD2+Dynamin-like_sGTPase+Pkinase	1240	LOC110064381	cnidaria	Orbicella faveolata	uncharacterized protein LOC110064381 [Orbicella faveolata]	GCF_002042975.1	TRADDN-DEDD2
XP_020616738.1	HEPN+Coiled-coil+TRADDN-DEDD2+HEPN+Coiled-coil	911	LOC110054720	cnidaria	Orbicella faveolata	uncharacterized protein LOC110054720 [Orbicella faveolata]	GCF_002042975.1	TRADDN-DEDD2
XP_020616797.1	HEPN+Coiled-coil+TRADDN-DEDD2+KH	549	LOC110054780	cnidaria	Orbicella faveolata	uncharacterized protein LOC110054780 isoform X1 [Orbicella faveolata]	GCF_002042975.1	TRADDN-DEDD2
XP_020632576.1	HEPN+Coiled-coil+TRADDN-DEDD2+KH	580	LOC110069390	cnidaria	Orbicella faveolata	uncharacterized protein LOC110069390 isoform X1 [Orbicella faveolata]	GCF_002042975.1	TRADDN-DEDD2
XP_020632577.1	HEPN+Coiled-coil+TRADDN-DEDD2+KH	563	LOC110069390	cnidaria	Orbicella faveolata	uncharacterized protein LOC110069390 isoform X2 [Orbicella faveolata]	GCF_002042975.1	TRADDN-DEDD2
RMX55969.1	HEPN+Coiled-coil+TRADDN-DEDD2+KH	390	pdam_00023994	cnidaria	Pocillopora damicornis	hypothetical protein pdam_00023994 [Pocillopora damicornis]	GCA_003704095.1	TRADDN-DEDD2
RMX48053.1	HEPN+Coiled-coil+TRADDN-DEDD2+RRM+RRM+KH+KH+KH+KH+KH+KH+KH+Macro+Macro+PARP	2135	pdam_00002642	cnidaria	Pocillopora damicornis	hypothetical protein pdam_00002642 [Pocillopora damicornis]	GCA_003704095.1	TRADDN-DEDD2
XP_022804139.1	HEPN+Coiled-coil+TRADDN-DEDD2+RRM+RRM+KH+KH+KH+KH+KH+KH+KH+Macro+Macro+PARP	2107	LOC111341437	cnidaria	Stylophora pistillata	LOW QUALITY PROTEIN: poly [ADP-ribose] polymerase 14-like [Stylophora pistillata]	GCF_002571385.1	TRADDN-DEDD2
XP_022809699.1	HEPN+Coiled-coil+TRADDN-DEDD2+STAND+wHTH+TPRs	1142	LOC111346660	cnidaria	Stylophora pistillata	uncharacterized protein LOC111346660 [Stylophora pistillata]	GCF_002571385.1	TRADDN-DEDD2
RMX40274.1	HEPN+Coiled-coil+TRADDN-DEDD2+TPRs	829	pdam_00008680	cnidaria	Pocillopora damicornis	hypothetical protein pdam_00008680 [Pocillopora damicornis]	GCA_003704095.1	TRADDN-DEDD2
XP_020617640.1	HEPN+Coiled-coil+TRADDN-DEDD2+UBI+UBI	765	LOC110055589	cnidaria	Orbicella faveolata	uncharacterized protein LOC110055589 [Orbicella faveolata]	GCF_002042975.1	TRADDN-DEDD2

acc	architecture	len	gen.name	taxend	species	defline	gca	clade
PFX19302.1	HEPN+Coiled-coil+TRADDN-DEDD2+UBI+UBI+UBI+UBI+UBI+UBI+UBI+DDE_Tnp_4	1518	UBC	cnidaria	Stylophora pistillata	Polyubiquitin-C [Stylophora pistillata]	GCA_002571385.1	TRADDN-DEDD2
XP_022800602.1	HEPN+Coiled-coil+TRADDN-DEDD2+UBI+UBI+UBI+UBI+UBI+UBI+UBI+UBI	1069	LOC111338383	cnidaria	Stylophora pistillata	uncharacterized protein LOC111338383 [Stylophora pistillata]	GCF_002571385.1	TRADDN-DEDD2
XP_022800615.1	HEPN+Coiled-coil+TRADDN-DEDD2+UBI+UBI+UBI+UBI+UBI+UBI+UBI+UBI	1227	LOC111338392	cnidaria	Stylophora pistillata	LOW QUALITY PROTEIN: polyubiquitin-C-like [Stylophora pistillata]	GCF_002571385.1	TRADDN-DEDD2
XP_020629970.1	HEPN+Coiled-coil+TRADDN-DEDD2+ZU5+ZU5+UPA	1199	LOC110067038	cnidaria	Orbicella faveolata	uncharacterized protein LOC110067038 isoform X1 [Orbicella faveolata]	GCF_002042975.1	TRADDN-DEDD2
XP_020629971.1	HEPN+Coiled-coil+TRADDN-DEDD2+ZU5+ZU5+UPA	1198	LOC110067038	cnidaria	Orbicella faveolata	uncharacterized protein LOC110067038 isoform X2 [Orbicella faveolata]	GCF_002042975.1	TRADDN-DEDD2
XP_020629972.1	HEPN+Coiled-coil+TRADDN-DEDD2+ZU5+ZU5+UPA	1197	LOC110067038	cnidaria	Orbicella faveolata	uncharacterized protein LOC110067038 isoform X3 [Orbicella faveolata]	GCF_002042975.1	TRADDN-DEDD2
XP_020629973.1	HEPN+Coiled-coil+TRADDN-DEDD2+ZU5+ZU5+UPA	1155	LOC110067038	cnidaria	Orbicella faveolata	uncharacterized protein LOC110067038 isoform X4 [Orbicella faveolata]	GCF_002042975.1	TRADDN-DEDD2
KXJ10812.1	HEPN+TRADDN-DEDD2	656	DZIP3	cnidaria	Exaiptasia pallida	E3 ubiquitin-protein ligase DZIP3 [Exaiptasia pallida]	GCA_001417965.1	TRADDN-DEDD2
KXJ12478.1	HEPN+TRADDN-DEDD2	472	AC249_AIPGENE12578	cnidaria	Exaiptasia pallida	hypothetical protein AC249_AIPGENE12578 [Exaiptasia pallida]	GCA_001417965.1	TRADDN-DEDD2
XP_020604617.1	HEPN+TRADDN-DEDD2	415	LOC110043500	cnidaria	Orbicella faveolata	uncharacterized protein LOC110043500 [Orbicella faveolata]	GCF_002042975.1	TRADDN-DEDD2
XP_020628007.1	HEPN+TRADDN-DEDD2	388	LOC110065239	cnidaria	Orbicella faveolata	E3 ubiquitin-protein ligase DZIP3-like [Orbicella faveolata]	GCF_002042975.1	TRADDN-DEDD2
RMX39187.1	HEPN+TRADDN-DEDD2	222	pdam_00013525	cnidaria	Pocillopora damicornis	hypothetical protein pdam_00013525 [Pocillopora damicornis]	GCA_003704095.1	TRADDN-DEDD2
RMX48238.1	HEPN+TRADDN-DEDD2	262	pdam_00005399	cnidaria	Pocillopora damicornis	hypothetical protein pdam_00005399 [Pocillopora damicornis]	GCA_003704095.1	TRADDN-DEDD2
PFX23548.1	HEPN+TRADDN-DEDD2	419	AWC38_SpisGene11915	cnidaria	Stylophora pistillata	hypothetical protein AWC38_SpisGene11915 [Stylophora pistillata]	GCA_002571385.1	TRADDN-DEDD2
XP_022802965.1	HEPN+TRADDN-DEDD2	393	LOC111340387	cnidaria	Stylophora pistillata	uncharacterized protein LOC111340387 [Stylophora pistillata]	GCF_002571385.1	TRADDN-DEDD2
PFX15141.1	HEPN+TRADDN-DEDD2+Ankyrin+ZU5+ZU5+THAP+Coiled-coil+HTH+HTH+DDE_Tnp_4	1450	Dzip3	cnidaria	Stylophora pistillata	E3 ubiquitin-protein ligase DZIP3 [Stylophora pistillata]	GCA_002571385.1	TRADDN-DEDD2
XP_020625600.1	HEPN+TRADDN-DEDD2+CASPASE	726	LOC110062993	cnidaria	Orbicella faveolata	uncharacterized protein LOC110062993 [Orbicella faveolata]	GCF_002042975.1	TRADDN-DEDD2
PFX17858.1	HEPN+TRADDN-DEDD2+Coiled-coil+Pkinase	717	Tnni3k	cnidaria	Stylophora pistillata	Serine/threonine-protein kinase TNNI3K [Stylophora pistillata]	GCA_002571385.1	TRADDN-DEDD2
XP_022791026.1	HEPN+TRADDN-DEDD2+Coiled-coil+TPRs	597	LOC111330432	cnidaria	Stylophora pistillata	uncharacterized protein LOC111330432 [Stylophora pistillata]	GCF_002571385.1	TRADDN-DEDD2
PFX13296.1	HEPN+TRADDN-DEDD2+Filamin+Beta-propeller	1115	trim71	cnidaria	Stylophora pistillata	E3 ubiquitin-protein ligase TRIM71 [Stylophora pistillata]	GCA_002571385.1	TRADDN-DEDD2

acc	architecture	len	gen.name	taxend	species	defline	gca	clade
XP_022799946.1	HEPN+TRADDN-DEDD2+Filamin+Beta-propeller	1041	LOC111337842	cnidaria	Stylophora pistillata	uncharacterized protein LOC111337842 [Stylophora pistillata]	GCF_002571385.1	TRADDN-DEDD2
XP_020906505.1	HEPN+TRADDN-DEDD2+HEPN+TRADDN-DEDD2	937	LOC110244637	cnidaria	Exaiptasia pallida	uncharacterized protein LOC110244637 [Exaiptasia pallida]	GCF_001417965.1	TRADDN-DEDD2
XP_020602264.1	HEPN+TRADDN-DEDD2+P-loop-NTPase	537	LOC110041324	cnidaria	Orbicella faveolata	uncharacterized protein LOC110041324 [Orbicella faveolata]	GCF_002042975.1	TRADDN-DEDD2
PFX17023.1	HEPN+TRADDN-DEDD2+RRM+KH+KH+KH+Macro+Macro+PARP	2052	PARP14	cnidaria	Stylophora pistillata	Poly [ADP-ribose] polymerase 14 [Stylophora pistillata]	GCA_002571385.1	TRADDN-DEDD2
PFX17028.1	HEPN+TRADDN-DEDD2+RRM+KH+KH+KH+Macro+Macro+PARP	1990	PARP14	cnidaria	Stylophora pistillata	Poly [ADP-ribose] polymerase 14 [Stylophora pistillata]	GCA_002571385.1	TRADDN-DEDD2
XP_022804128.1	HEPN+TRADDN-DEDD2+RRM+RRM	694	LOC111341426	cnidaria	Stylophora pistillata	uncharacterized protein LOC111341426 [Stylophora pistillata]	GCF_002571385.1	TRADDN-DEDD2
KXJ06110.1	HEPN+TRADDN-DEDD2+SF2-helicase	767	DDX58	cnidaria	Exaiptasia pallida	putative ATP-dependent RNA helicase DDX58, partial [Exaiptasia pallida]	GCA_001417965.1	TRADDN-DEDD2
XP_020906364.1	HEPN+TRADDN-DEDD2+SF2-helicase	766	LOC110244496	cnidaria	Exaiptasia pallida	probable ATP-dependent RNA helicase DDX58, partial [Exaiptasia pallida]	GCF_001417965.1	TRADDN-DEDD2
XP_015749627.1	HEPN+TRADDN-DEDD2+STAND+wHTH+TPRs	1188	LOC107329454	cnidaria	Acropora digitifera	PREDICTED: uncharacterized protein LOC107329454 [Acropora digitifera]	GCF_000222465.1	TRADDN-DEDD2
KXJ10800.1	HEPN+TRADDN-DEDD2+TPRs	597	DZIP3	cnidaria	Exaiptasia pallida	E3 ubiquitin-protein ligase DZIP3 [Exaiptasia pallida]	GCA_001417965.1	TRADDN-DEDD2
KXJ15695.1	HEPN+TRADDN-DEDD2+TPRs	524	Ttc28	cnidaria	Exaiptasia pallida	Tetratricopeptide repeat protein 28 [Exaiptasia pallida]	GCA_001417965.1	TRADDN-DEDD2
XP_020898253.1	HEPN+TRADDN-DEDD2+TPRs	509	LOC110237022	cnidaria	Exaiptasia pallida	uncharacterized protein LOC110237022 [Exaiptasia pallida]	GCF_001417965.1	TRADDN-DEDD2
PFX19470.1	HEPN+TRADDN-DEDD2+TPRs	1185	nphp3	cnidaria	Stylophora pistillata	Nephrocystin-3 [Stylophora pistillata]	GCA_002571385.1	TRADDN-DEDD2
XP_022810481.1	HEPN+TRADDN-DEDD2+TPRs	558	LOC111347506	cnidaria	Stylophora pistillata	uncharacterized protein LOC111347506, partial [Stylophora pistillata]	GCF_002571385.1	TRADDN-DEDD2
KXJ10818.1	HEPN+TRADDN-DEDD2+TPRs+TM+TM	590	DZIP3	cnidaria	Exaiptasia pallida	E3 ubiquitin-protein ligase DZIP3 [Exaiptasia pallida]	GCA_001417965.1	TRADDN-DEDD2
XP_020628005.1	HEPN+TRADDN-DEDD2+TRADDN-DEDD2	510	LOC110065237	cnidaria	Orbicella faveolata	uncharacterized protein LOC110065237 isoform X1 [Orbicella faveolata]	GCF_002042975.1	TRADDN-DEDD2
XP_020628006.1	HEPN+TRADDN-DEDD2+TRADDN-DEDD2	509	LOC110065237	cnidaria	Orbicella faveolata	uncharacterized protein LOC110065237 isoform X2 [Orbicella faveolata]	GCF_002042975.1	TRADDN-DEDD2
PFX17772.1	HEPN+TRADDN-DEDD2+TRADDN-DEDD2	550	Dzip3	cnidaria	Stylophora pistillata	E3 ubiquitin-protein ligase DZIP3 [Stylophora pistillata]	GCA_002571385.1	TRADDN-DEDD2
XP_020629974.1	HEPN+TRADDN-DEDD2+ZU5+ZU5+UPA	1001	LOC110067038	cnidaria	Orbicella faveolata	uncharacterized protein LOC110067038 isoform X5 [Orbicella faveolata]	GCF_002042975.1	TRADDN-DEDD2
XP_022803160.1	HEPN+TRADDN-DEDD2+ZU5+ZU5+UPA+Death	1141	LOC111340569	cnidaria	Stylophora pistillata	uncharacterized protein LOC111340569 isoform X1 [Stylophora pistillata]	GCF_002571385.1	TRADDN-DEDD2

acc	architecture	len	gen.name	taxend	species	defline	gca	clade
XP_022806853.1	HEPN+TRADDN-DEDD2+ZU5+ZU5+UPA+Death	1232	LOC111343921	cnidaria	Stylophora pistillata	uncharacterized protein LOC111343921 isoform X1 [Stylophora pistillata]	GCF_002571385.1	TRADDN-DEDD2
XP_022806854.1	HEPN+TRADDN-DEDD2+ZU5+ZU5+UPA+Death	1231	LOC111343921	cnidaria	Stylophora pistillata	uncharacterized protein LOC111343921 isoform X2 [Stylophora pistillata]	GCF_002571385.1	TRADDN-DEDD2
XP_022806855.1	HEPN+TRADDN-DEDD2+ZU5+ZU5+UPA+Death	1129	LOC111343921	cnidaria	Stylophora pistillata	uncharacterized protein LOC111343921 isoform X3 [Stylophora pistillata]	GCF_002571385.1	TRADDN-DEDD2
PFX21943.1	Ig+TM+TM+TRADDN-DEDD2+Death	2620	HMCN2	cnidaria	Stylophora pistillata	Hemicentin-2 [Stylophora pistillata]	GCA_002571385.1	TRADDN-DEDD2
PFX14620.1	low-complexity+TRADDN-DEDD2+HEPN+Ankyrin	1215	AWC38_SpisGene21205	cnidaria	Stylophora pistillata	Reticulocyte-binding protein 2-like a [Stylophora pistillata]	GCA_002571385.1	TRADDN-DEDD2
PFX12801.1	LRRs+GT4-GTase+LRRs+GT4-GTase+RVT_1+TRADDN-DEDD2	2516	mshA	cnidaria	Stylophora pistillata	D-inositol 3-phosphate glycosyltransferase [Stylophora pistillata]	GCA_002571385.1	TRADDN-DEDD2
XP_015753893.1	N-ter+TRADDN-Bac+FAM124	433	LOC107333575	cnidaria	Acropora digitifera	PREDICTED: uncharacterized protein LOC107333575 [Acropora digitifera]	GCF_000222465.1	TRADDN-Bacterial-like
EDO38212.1	N-ter+TRADDN-Bac+FAM124	482	NEMVEDRAFT_v1g244535	cnidaria	Nematostella vectensis	predicted protein [Nematostella vectensis]	GCA_000209225.1	TRADDN-Bacterial-like
XP_001630275.1	N-ter+TRADDN-Bac+FAM124	482	NEMVEDRAFT_v1g244535	cnidaria	Nematostella vectensis	predicted protein [Nematostella vectensis]	GCF_000209225.1	TRADDN-Bacterial-like
XP_020612279.1	N-ter+TRADDN-Bac+FAM124	406	LOC110050703	cnidaria	Orbicella faveolata	uncharacterized protein LOC110050703 [Orbicella faveolata]	GCF_002042975.1	TRADDN-Bacterial-like
XP_022780971.1	N-ter+TRADDN-Bac+FAM124	433	LOC111322172	cnidaria	Stylophora pistillata	uncharacterized protein LOC111322172 [Stylophora pistillata]	GCF_002571385.1	TRADDN-Bacterial-like
PFX28323.1	Peptidase_A17+TRADDN-DEDD2+Death+Death+ZU5+ZU5+UPA+Death	2079	AWC38_SpisGene6919	cnidaria	Stylophora pistillata	hypothetical protein AWC38_SpisGene6919 [Stylophora pistillata]	GCA_002571385.1	TRADDN-DEDD2
PFX26308.1	Peptidase_C48+PHD+DED+DED+TRADDN-DEDD2+Coiled-coil+Death+Coiled-coil+domain1	1276	AWC38_SpisGene9039	cnidaria	Stylophora pistillata	hypothetical protein AWC38_SpisGene9039 [Stylophora pistillata]	GCA_002571385.1	TRADDN-DEDD2
XP_015748670.1	PNPase+TRADDN-DEDD2	447	LOC107328451	cnidaria	Acropora digitifera	PREDICTED: uncharacterized protein LOC107328451 [Acropora digitifera]	GCF_000222465.1	TRADDN-DEDD2
XP_015758944.1	PNPase+TRADDN-DEDD2	519	LOC107338220	cnidaria	Acropora digitifera	PREDICTED: uncharacterized protein LOC107338220 [Acropora digitifera]	GCF_000222465.1	TRADDN-DEDD2
XP_015762495.1	PNPase+TRADDN-DEDD2	397	LOC107341564	cnidaria	Acropora digitifera	PREDICTED: uncharacterized protein LOC107341564 [Acropora digitifera]	GCF_000222465.1	TRADDN-DEDD2
XP_020609267.1	PNPase+TRADDN-DEDD2	518	LOC110047864	cnidaria	Orbicella faveolata	uncharacterized protein LOC110047864 isoform X1 [Orbicella faveolata]	GCF_002042975.1	TRADDN-DEDD2
XP_020609268.1	PNPase+TRADDN-DEDD2	517	LOC110047864	cnidaria	Orbicella faveolata	uncharacterized protein LOC110047864 isoform X2 [Orbicella faveolata]	GCF_002042975.1	TRADDN-DEDD2
RMX48369.1	PNPase+TRADDN-DEDD2	284	pdam_00016310	cnidaria	Pocillopora damicornis	hypothetical protein pdam_00016310, partial [Pocillopora damicornis]	GCA_003704095.1	TRADDN-DEDD2

acc	architecture	len	gen.name	taxend	species	defline	gca	clade
XP_022806002.1	PNPase+TRADDN-DEDD2+STAND+wHTH+helical-repeat+LRRs	1382	LOC111343123	cnidaria	Stylophora pistillata	uncharacterized protein LOC111343123, partial [Stylophora pistillata]	GCF_002571385.1	TRADDN-DEDD2
XP_015770836.1	PNPase+TRADDN-DEDD2+STAND+wHTH+LRRs	1754	LOC107349236	cnidaria	Acropora digitifera	PREDICTED: uncharacterized protein LOC107349236 isoform X1 [Acropora digitifera]	GCF_000222465.1	TRADDN-DEDD2
XP_015770837.1	PNPase+TRADDN-DEDD2+STAND+wHTH+LRRs	1691	LOC107349236	cnidaria	Acropora digitifera	PREDICTED: uncharacterized protein LOC107349236 isoform X2 [Acropora digitifera]	GCF_000222465.1	TRADDN-DEDD2
XP_015774318.1	PNPase+TRADDN-DEDD2+TPRs+ZU5+ZU5+Glycosyl_transferase	1563	LOC107352503	cnidaria	Acropora digitifera	PREDICTED: uncharacterized protein LOC107352503 [Acropora digitifera]	GCF_000222465.1	TRADDN-DEDD2
PFX12282.1	RVT_1+TPRs+HEPN+TRADDN-DEDD2	861	Ttc28	cnidaria	Stylophora pistillata	Tetratricopeptide repeat protein 28, partial [Stylophora pistillata]	GCA_002571385.1	TRADDN-DEDD2
PFX11418.1	RVT_1+TRADDN-DEDD2	230	TY3B-I	cnidaria	Stylophora pistillata	Transposon Ty3-I Gag-Pol polyprotein [Stylophora pistillata]	GCA_002571385.1	TRADDN-DEDD2
XP_022778213.1	RVT_1+TRADDN-DEDD2	263	LOC111319737	cnidaria	Stylophora pistillata	uncharacterized protein LOC111319737, partial [Stylophora pistillata]	GCF_002571385.1	TRADDN-DEDD2
XP_022810423.1	RVT_1+TRADDN-DEDD2	448	LOC111347442	cnidaria	Stylophora pistillata	uncharacterized protein LOC111347442 [Stylophora pistillata]	GCF_002571385.1	TRADDN-DEDD2
PFX12330.1	RVT_1+TRADDN-DEDD2+helical-region+STAND+DED+wHTH+helical-region	1264	AWC38_SpisGene23734	cnidaria	Stylophora pistillata	hypothetical protein AWC38_SpisGene23734 [Stylophora pistillata]	GCA_002571385.1	TRADDN-DEDD2
PFX32742.1	RVT_1+TRADDN-DEDD2+TRADDN-DEDD2+Beta-propeller	873	trim71	cnidaria	Stylophora pistillata	E3 ubiquitin-protein ligase TRIM71 [Stylophora pistillata]	GCA_002571385.1	TRADDN-DEDD2
PFX12799.1	STAND+TPRs+TRADDN-DEDD2+APGTPase+COR+LRRs	2176	Nrx-IV	cnidaria	Stylophora pistillata	Neurexin-4 [Stylophora pistillata]	GCA_002571385.1	TRADDN-DEDD2
XP_015780420.1	STAND+TRADDN-DEDD2+TRADDN-DEDD2+LRRs	720	LOC107358323	cnidaria	Acropora digitifera	PREDICTED: uncharacterized protein LOC107358323 [Acropora digitifera]	GCF_000222465.1	TRADDN-DEDD2
XP_022784672.1	THAP+GT4-GTase+TRADDN-DEDD2+TRADDN-DEDD2+PH+GT4-GTase	1075	LOC111325184	cnidaria	Stylophora pistillata	uncharacterized protein LOC111325184 [Stylophora pistillata]	GCF_002571385.1	TRADDN-DEDD2
XP_015751850.1	TM+TM+TRADDN-DEDD2+TM+TM	970	LOC107331748	cnidaria	Acropora digitifera	PREDICTED: uncharacterized protein LOC107331748 isoform X1 [Acropora digitifera]	GCF_000222465.1	TRADDN-DEDD2
XP_015751851.1	TM+TM+TRADDN-DEDD2+TM+TM	969	LOC107331748	cnidaria	Acropora digitifera	PREDICTED: uncharacterized protein LOC107331748 isoform X2 [Acropora digitifera]	GCF_000222465.1	TRADDN-DEDD2
XP_015751852.1	TM+TM+TRADDN-DEDD2+TM+TM	969	LOC107331748	cnidaria	Acropora digitifera	PREDICTED: uncharacterized protein LOC107331748 isoform X3 [Acropora digitifera]	GCF_000222465.1	TRADDN-DEDD2
XP_015751853.1	TM+TM+TRADDN-DEDD2+TM+TM	968	LOC107331748	cnidaria	Acropora digitifera	PREDICTED: uncharacterized protein LOC107331748 isoform X4 [Acropora digitifera]	GCF_000222465.1	TRADDN-DEDD2
XP_015751854.1	TM+TM+TRADDN-DEDD2+TM+TM	956	LOC107331748	cnidaria	Acropora digitifera	PREDICTED: uncharacterized protein LOC107331748 isoform X5 [Acropora digitifera]	GCF_000222465.1	TRADDN-DEDD2
XP_015751855.1	TM+TM+TRADDN-DEDD2+TM+TM	955	LOC107331748	cnidaria	Acropora digitifera	PREDICTED: uncharacterized protein LOC107331748 isoform X6 [Acropora digitifera]	GCF_000222465.1	TRADDN-DEDD2

acc	architecture	len	gen.name	taxend	species	defline	gca	clade
XP_015751856.1	TM+TM+TRADDN-DEDD2+TM+TM	941	LOC107331748	cnidaria	Acropora digitifera	PREDICTED: uncharacterized protein LOC107331748 isoform X7 [Acropora digitifera]	GCF_000222465.1	TRADDN-DEDD2
XP_015751857.1	TM+TM+TRADDN-DEDD2+TM+TM	940	LOC107331748	cnidaria	Acropora digitifera	PREDICTED: uncharacterized protein LOC107331748 isoform X8 [Acropora digitifera]	GCF_000222465.1	TRADDN-DEDD2
XP_015751859.1	TM+TM+TRADDN-DEDD2+TM+TM	937	LOC107331748	cnidaria	Acropora digitifera	PREDICTED: uncharacterized protein LOC107331748 isoform X9 [Acropora digitifera]	GCF_000222465.1	TRADDN-DEDD2
XP_015751861.1	TM+TM+TRADDN-DEDD2+TM+TM	936	LOC107331748	cnidaria	Acropora digitifera	PREDICTED: uncharacterized protein LOC107331748 isoform X10 [Acropora digitifera]	GCF_000222465.1	TRADDN-DEDD2
XP_015751862.1	TM+TM+TRADDN-DEDD2+TM+TM	935	LOC107331748	cnidaria	Acropora digitifera	PREDICTED: uncharacterized protein LOC107331748 isoform X11 [Acropora digitifera]	GCF_000222465.1	TRADDN-DEDD2
XP_015748671.1	TRADDN-DEDD2	122	LOC107328452	cnidaria	Acropora digitifera	PREDICTED: uncharacterized protein LOC107328452 [Acropora digitifera]	GCF_000222465.1	TRADDN-DEDD2
XP_015757833.1	TRADDN-DEDD2	200	LOC107337233	cnidaria	Acropora digitifera	PREDICTED: uncharacterized protein LOC107337233 [Acropora digitifera]	GCF_000222465.1	TRADDN-DEDD2
XP_015758789.1	TRADDN-DEDD2	331	LOC107338054	cnidaria	Acropora digitifera	PREDICTED: uncharacterized protein LOC107338054 [Acropora digitifera]	GCF_000222465.1	TRADDN-DEDD2
XP_015762347.1	TRADDN-DEDD2	470	LOC107341454	cnidaria	Acropora digitifera	PREDICTED: uncharacterized protein LOC107341454 isoform X1 [Acropora digitifera]	GCF_000222465.1	TRADDN-DEDD2
XP_015762348.1	TRADDN-DEDD2	448	LOC107341454	cnidaria	Acropora digitifera	PREDICTED: uncharacterized protein LOC107341454 isoform X2 [Acropora digitifera]	GCF_000222465.1	TRADDN-DEDD2
XP_015762349.1	TRADDN-DEDD2	412	LOC107341454	cnidaria	Acropora digitifera	PREDICTED: uncharacterized protein LOC107341454 isoform X3 [Acropora digitifera]	GCF_000222465.1	TRADDN-DEDD2
XP_015762350.1	TRADDN-DEDD2	412	LOC107341454	cnidaria	Acropora digitifera	PREDICTED: uncharacterized protein LOC107341454 isoform X4 [Acropora digitifera]	GCF_000222465.1	TRADDN-DEDD2
XP_015768941.1	TRADDN-DEDD2	454	LOC107347503	cnidaria	Acropora digitifera	PREDICTED: uncharacterized protein LOC107347503 [Acropora digitifera]	GCF_000222465.1	TRADDN-DEDD2
XP_015770131.1	TRADDN-DEDD2	144	LOC107348586	cnidaria	Acropora digitifera	PREDICTED: uncharacterized protein LOC107348586 isoform X1 [Acropora digitifera]	GCF_000222465.1	TRADDN-DEDD2
XP_015778314.1	TRADDN-DEDD2	161	LOC107356184	cnidaria	Acropora digitifera	PREDICTED: uncharacterized protein LOC107356184 [Acropora digitifera]	GCF_000222465.1	TRADDN-DEDD2
XP_015780424.1	TRADDN-DEDD2	113	LOC107358328	cnidaria	Acropora digitifera	PREDICTED: uncharacterized protein LOC107358328 [Acropora digitifera]	GCF_000222465.1	TRADDN-DEDD2
XP_015780573.1	TRADDN-DEDD2	196	LOC107358484	cnidaria	Acropora digitifera	PREDICTED: uncharacterized protein LOC107358484 [Acropora digitifera]	GCF_000222465.1	TRADDN-DEDD2

acc	architecture	len	gen.name	taxend	species	defline	gca	clade
KXJ16920.1	TRADDN-DEDD2	140	AC249_AIPGENE264	cnidaria	Exaiptasia pallida	hypothetical protein AC249_AIPGENE264 [Exaiptasia pallida]	GCA_001417965.1	TRADDN-DEDD2
KXJ16939.1	TRADDN-DEDD2	136	AC249_AIPGENE269	cnidaria	Exaiptasia pallida	hypothetical protein AC249_AIPGENE269 [Exaiptasia pallida]	GCA_001417965.1	TRADDN-DEDD2
XP_020610443.1	TRADDN-DEDD2	381	LOC110049013	cnidaria	Orbicella faveolata	uncharacterized protein LOC110049013 [Orbicella faveolata]	GCF_002042975.1	TRADDN-DEDD2
XP_020611285.1	TRADDN-DEDD2	214	LOC110049799	cnidaria	Orbicella faveolata	uncharacterized protein LOC110049799 [Orbicella faveolata]	GCF_002042975.1	TRADDN-DEDD2
XP_020611286.1	TRADDN-DEDD2	197	LOC110049801	cnidaria	Orbicella faveolata	uncharacterized protein LOC110049801 [Orbicella faveolata]	GCF_002042975.1	TRADDN-DEDD2
XP_020611948.1	TRADDN-DEDD2	184	LOC110050370	cnidaria	Orbicella faveolata	uncharacterized protein LOC110050370 [Orbicella faveolata]	GCF_002042975.1	TRADDN-DEDD2
XP_020614050.1	TRADDN-DEDD2	399	LOC110052283	cnidaria	Orbicella faveolata	uncharacterized protein LOC110052283 [Orbicella faveolata]	GCF_002042975.1	TRADDN-DEDD2
XP_020616566.1	TRADDN-DEDD2	260	LOC110054561	cnidaria	Orbicella faveolata	uncharacterized protein LOC110054561 [Orbicella faveolata]	GCF_002042975.1	TRADDN-DEDD2
XP_020616567.1	TRADDN-DEDD2	149	LOC110054562	cnidaria	Orbicella faveolata	uncharacterized protein LOC110054562 [Orbicella faveolata]	GCF_002042975.1	TRADDN-DEDD2
XP_020616779.1	TRADDN-DEDD2	186	LOC110054760	cnidaria	Orbicella faveolata	uncharacterized protein LOC110054760 [Orbicella faveolata]	GCF_002042975.1	TRADDN-DEDD2
XP_020617426.1	TRADDN-DEDD2	489	LOC110055381	cnidaria	Orbicella faveolata	protein argonaute 12-like isoform X2 [Orbicella faveolata]	GCF_002042975.1	TRADDN-DEDD2
XP_020621781.1	TRADDN-DEDD2	203	LOC110059425	cnidaria	Orbicella faveolata	uncharacterized protein LOC110059425 [Orbicella faveolata]	GCF_002042975.1	TRADDN-DEDD2
XP_020622242.1	TRADDN-DEDD2	324	LOC110059859	cnidaria	Orbicella faveolata	uncharacterized protein LOC110059859 [Orbicella faveolata]	GCF_002042975.1	TRADDN-DEDD2
XP_020629198.1	TRADDN-DEDD2	527	LOC110066318	cnidaria	Orbicella faveolata	uncharacterized protein LOC110066318 [Orbicella faveolata]	GCF_002042975.1	TRADDN-DEDD2
RMX57216.1	TRADDN-DEDD2	453	pdam_00022805	cnidaria	Pocillopora damicornis	hypothetical protein pdam_00022805 [Pocillopora damicornis]	GCA_003704095.1	TRADDN-DEDD2
RMX57324.1	TRADDN-DEDD2	173	pdam_00021312	cnidaria	Pocillopora damicornis	hypothetical protein pdam_00021312, partial [Pocillopora damicornis]	GCA_003704095.1	TRADDN-DEDD2
PFX11085.1	TRADDN-DEDD2	196	AWC38_SpisGene25448	cnidaria	Stylophora pistillata	hypothetical protein AWC38_SpisGene25448, partial [Stylophora pistillata]	GCA_002571385.1	TRADDN-DEDD2
PFX13352.1	TRADDN-DEDD2	314	AWC38_SpisGene22565	cnidaria	Stylophora pistillata	hypothetical protein AWC38_SpisGene22565 [Stylophora pistillata]	GCA_002571385.1	TRADDN-DEDD2
PFX14439.1	TRADDN-DEDD2	162	AWC38_SpisGene21405	cnidaria	Stylophora pistillata	hypothetical protein AWC38_SpisGene21405 [Stylophora pistillata]	GCA_002571385.1	TRADDN-DEDD2
XP_022777983.1	TRADDN-DEDD2	149	LOC111319487	cnidaria	Stylophora pistillata	uncharacterized protein LOC111319487 [Stylophora pistillata]	GCF_002571385.1	TRADDN-DEDD2
XP_022778046.1	TRADDN-DEDD2	400	LOC111319566	cnidaria	Stylophora pistillata	uncharacterized protein LOC111319566, partial [Stylophora pistillata]	GCF_002571385.1	TRADDN-DEDD2

acc	architecture	len	gen.name	taxend	species	defline	gca	clade
XP_022778193.1	TRADDN-DEDD2	237	LOC111319717	cnidaria	Stylophora pistillata	uncharacterized protein LOC111319717 [Stylophora pistillata]	GCF_002571385.1	TRADDN-DEDD2
XP_022793788.1	TRADDN-DEDD2	167	LOC111332659	cnidaria	Stylophora pistillata	uncharacterized protein LOC111332659 [Stylophora pistillata]	GCF_002571385.1	TRADDN-DEDD2
XP_022800160.1	TRADDN-DEDD2	169	LOC111338019	cnidaria	Stylophora pistillata	uncharacterized protein LOC111338019 [Stylophora pistillata]	GCF_002571385.1	TRADDN-DEDD2
XP_022802996.1	TRADDN-DEDD2	156	LOC111340412	cnidaria	Stylophora pistillata	uncharacterized protein LOC111340412 [Stylophora pistillata]	GCF_002571385.1	TRADDN-DEDD2
XP_022806342.1	TRADDN-DEDD2	245	LOC111343432	cnidaria	Stylophora pistillata	uncharacterized protein LOC111343432 [Stylophora pistillata]	GCF_002571385.1	TRADDN-DEDD2
XP_022806344.1	TRADDN-DEDD2	231	LOC111343434	cnidaria	Stylophora pistillata	uncharacterized protein LOC111343434 [Stylophora pistillata]	GCF_002571385.1	TRADDN-DEDD2
XP_022806851.1	TRADDN-DEDD2	393	LOC111343917	cnidaria	Stylophora pistillata	uncharacterized protein LOC111343917 [Stylophora pistillata]	GCF_002571385.1	TRADDN-DEDD2
XP_022809194.1	TRADDN-DEDD2	124	LOC111346152	cnidaria	Stylophora pistillata	uncharacterized protein LOC111346152 [Stylophora pistillata]	GCF_002571385.1	TRADDN-DEDD2
XP_022809576.1	TRADDN-DEDD2	264	LOC111346561	cnidaria	Stylophora pistillata	uncharacterized protein LOC111346561 [Stylophora pistillata]	GCF_002571385.1	TRADDN-DEDD2
XP_022809800.1	TRADDN-DEDD2	311	LOC111346799	cnidaria	Stylophora pistillata	uncharacterized protein LOC111346799 isoform X1 [Stylophora pistillata]	GCF_002571385.1	TRADDN-DEDD2
XP_022809801.1	TRADDN-DEDD2	274	LOC111346799	cnidaria	Stylophora pistillata	uncharacterized protein LOC111346799 isoform X2 [Stylophora pistillata]	GCF_002571385.1	TRADDN-DEDD2
XP_022810532.1	TRADDN-DEDD2	258	LOC111347564	cnidaria	Stylophora pistillata	uncharacterized protein LOC111347564 [Stylophora pistillata]	GCF_002571385.1	TRADDN-DEDD2
XP_015754290.1	TRADDN-DEDD2+Ankyrin	953	LOC107333937	cnidaria	Acropora digitifera	PREDICTED: ankyrin-3-like isoform X1 [Acropora digitifera]	GCF_000222465.1	TRADDN-DEDD2
XP_015754291.1	TRADDN-DEDD2+Ankyrin	928	LOC107333937	cnidaria	Acropora digitifera	PREDICTED: ankyrin-3-like isoform X2 [Acropora digitifera]	GCF_000222465.1	TRADDN-DEDD2
XP_015768945.1	TRADDN-DEDD2+Ankyrin	763	LOC107347506	cnidaria	Acropora digitifera	PREDICTED: ankyrin-1-like [Acropora digitifera]	GCF_000222465.1	TRADDN-DEDD2
XP_015768950.1	TRADDN-DEDD2+Ankyrin	915	LOC107347512	cnidaria	Acropora digitifera	PREDICTED: ankyrin-3-like isoform X1 [Acropora digitifera]	GCF_000222465.1	TRADDN-DEDD2
XP_015768951.1	TRADDN-DEDD2+Ankyrin	911	LOC107347512	cnidaria	Acropora digitifera	PREDICTED: ankyrin-3-like isoform X2 [Acropora digitifera]	GCF_000222465.1	TRADDN-DEDD2
XP_015768952.1	TRADDN-DEDD2+Ankyrin	898	LOC107347512	cnidaria	Acropora digitifera	PREDICTED: ankyrin-3-like isoform X3 [Acropora digitifera]	GCF_000222465.1	TRADDN-DEDD2
XP_015772599.1	TRADDN-DEDD2+Ankyrin	1350	LOC107350870	cnidaria	Acropora digitifera	PREDICTED: ankyrin-3-like isoform X1 [Acropora digitifera]	GCF_000222465.1	TRADDN-DEDD2
XP_015772601.1	TRADDN-DEDD2+Ankyrin	1350	LOC107350870	cnidaria	Acropora digitifera	PREDICTED: ankyrin-1-like isoform X2 [Acropora digitifera]	GCF_000222465.1	TRADDN-DEDD2

acc	architecture	len	gen.name	taxend	species	defline	gca	clade
XP_015778948.1	TRADDN-DEDD2+Ankyrin	628	LOC107356839	cnidaria	Acropora digitifera	PREDICTED: serine/threonine-protein phosphatase 6 regulatory ankyrin repeat subunit A-like [Acropora digitifera]	GCF_000222465.1	TRADDN-DEDD2
KXJ06959.1	TRADDN-DEDD2+Ankyrin	674	AC249_AIPGENE2190	cnidaria	Exaiptasia pallida	putative ankyrin repeat protein MM-0045, partial [Exaiptasia pallida]	GCA_001417965.1	TRADDN-DEDD2
XP_020902162.1	TRADDN-DEDD2+Ankyrin	780	LOC110240688	cnidaria	Exaiptasia pallida	uncharacterized protein LOC110240688 [Exaiptasia pallida]	-	TRADDN-DEDD2
XP_020914387.1	TRADDN-DEDD2+Ankyrin	637	LOC110251974	cnidaria	Exaiptasia pallida	serine/threonine-protein phosphatase 6 regulatory ankyrin repeat subunit C-like [Exaiptasia pallida]	GCF_001417965.1	TRADDN-DEDD2
XP_020915956.1	TRADDN-DEDD2+Ankyrin	854	LOC110253397	cnidaria	Exaiptasia pallida	serine/threonine-protein phosphatase 6 regulatory ankyrin repeat subunit A-like [Exaiptasia pallida]	-	TRADDN-DEDD2
PFX21728.1	TRADDN-DEDD2+Ankyrin	755	Ankyrin3	cnidaria	Stylophora pistillata	Ankyrinyrin-3 [Stylophora pistillata]	GCA_002571385.1	TRADDN-DEDD2
XP_022796757.1	TRADDN-DEDD2+Ankyrin	863	LOC111335162	cnidaria	Stylophora pistillata	uncharacterized protein LOC111335162 isoform X1 [Stylophora pistillata]	GCF_002571385.1	TRADDN-DEDD2
XP_022796758.1	TRADDN-DEDD2+Ankyrin	855	LOC111335162	cnidaria	Stylophora pistillata	uncharacterized protein LOC111335162 isoform X2 [Stylophora pistillata]	GCF_002571385.1	TRADDN-DEDD2
XP_022796759.1	TRADDN-DEDD2+Ankyrin	612	LOC111335162	cnidaria	Stylophora pistillata	ankyrin-3-like isoform X3 [Stylophora pistillata]	GCF_002571385.1	TRADDN-DEDD2
PFX19726.1	TRADDN-DEDD2+Beta-propeller	364	brat	cnidaria	Stylophora pistillata	Brain tumor protein [Stylophora pistillata]	GCA_002571385.1	TRADDN-DEDD2
XP_022777971.1	TRADDN-DEDD2+Beta-propeller	563	LOC111319469	cnidaria	Stylophora pistillata	E3 ubiquitin-protein ligase TRIM71-like [Stylophora pistillata]	GCF_002571385.1	TRADDN-DEDD2
XP_022778089.1	TRADDN-DEDD2+Beta-propeller	380	LOC111319603	cnidaria	Stylophora pistillata	E3 ubiquitin-protein ligase TRIM71-like [Stylophora pistillata]	GCF_002571385.1	TRADDN-DEDD2
XP_020622142.1	TRADDN-DEDD2+CARD	365	LOC110059758	cnidaria	Orbicella faveolata	uncharacterized protein LOC110059758 isoform X3 [Orbicella faveolata]	GCF_002042975.1	TRADDN-DEDD2
KXJ07117.1	TRADDN-DEDD2+Coiled-coil	840	AC249_AIPGENE15613	cnidaria	Exaiptasia pallida	hypothetical protein AC249_AIPGENE15613 [Exaiptasia pallida]	GCA_001417965.1	TRADDN-DEDD2
XP_020913810.1	TRADDN-DEDD2+Coiled-coil	364	LOC110251434	cnidaria	Exaiptasia pallida	uncharacterized protein LOC110251434, partial [Exaiptasia pallida]	-	TRADDN-DEDD2
XP_022796410.1	TRADDN-DEDD2+Coiled-coil+Death	477	LOC111334859	cnidaria	Stylophora pistillata	uncharacterized protein LOC111334859 [Stylophora pistillata]	GCF_002571385.1	TRADDN-DEDD2
PFX16518.1	TRADDN-DEDD2+Coiled-coil+Death+Coiled-coil+domain1	728	AWC38_SpisGene19208	cnidaria	Stylophora pistillata	hypothetical protein AWC38_SpisGene19208 [Stylophora pistillata]	GCA_002571385.1	TRADDN-DEDD2
XP_022800990.1	TRADDN-DEDD2+Coiled-coil+TPRs	639	LOC111338702	cnidaria	Stylophora pistillata	uncharacterized protein LOC111338702 [Stylophora pistillata]	GCF_002571385.1	TRADDN-DEDD2

acc	architecture	len	gen.name	taxend	species	defline	gca	clade
KXJ06752.1	TRADDN-DEDD2+Coiled-coil+TRADDN-DEDD2+Coiled-coil+TM	1695	AC249_AIPGENE24613	cnidaria	Exaiptasia pallida	hypothetical protein AC249_AIPGENE24613, partial [Exaiptasia pallida]	GCA_001417965.1	TRADDN-DEDD2
PFX14013.1	TRADDN-DEDD2+Death	385	AWC38_SpisGene21870	cnidaria	Stylophora pistillata	hypothetical protein AWC38_SpisGene21870 [Stylophora pistillata]	GCA_002571385.1	TRADDN-DEDD2
XP_022791905.1	TRADDN-DEDD2+Death	381	LOC111331120	cnidaria	Stylophora pistillata	uncharacterized protein LOC111331120 isoform X4 [Stylophora pistillata]	GCF_002571385.1	TRADDN-DEDD2
XP_022808473.1	TRADDN-DEDD2+Death	334	LOC111345454	cnidaria	Stylophora pistillata	uncharacterized protein LOC111345454 [Stylophora pistillata]	GCF_002571385.1	TRADDN-DEDD2
XP_015757179.1	TRADDN-DEDD2+Death+APGTPase+COR+Death+Death	1313	LOC107336623	cnidaria	Acropora digitifera	PREDICTED: uncharacterized protein LOC107336623 isoform X1 [Acropora digitifera]	GCF_000222465.1	TRADDN-DEDD2
XP_015757180.1	TRADDN-DEDD2+Death+APGTPase+COR+Death+Death	1306	LOC107336623	cnidaria	Acropora digitifera	PREDICTED: uncharacterized protein LOC107336623 isoform X2 [Acropora digitifera]	GCF_000222465.1	TRADDN-DEDD2
XP_015757182.1	TRADDN-DEDD2+Death+APGTPase+COR+Death+Death	1284	LOC107336623	cnidaria	Acropora digitifera	PREDICTED: uncharacterized protein LOC107336623 isoform X3 [Acropora digitifera]	GCF_000222465.1	TRADDN-DEDD2
XP_015757183.1	TRADDN-DEDD2+Death+APGTPase+COR+Death+Death	1257	LOC107336623	cnidaria	Acropora digitifera	PREDICTED: uncharacterized protein LOC107336623 isoform X4 [Acropora digitifera]	GCF_000222465.1	TRADDN-DEDD2
XP_015763307.1	TRADDN-DEDD2+Death+Coiled-coil+domain1	534	LOC107342338	cnidaria	Acropora digitifera	PREDICTED: uncharacterized protein LOC107342338 [Acropora digitifera]	GCF_000222465.1	TRADDN-DEDD2
XP_015769816.1	TRADDN-DEDD2+Death+Coiled-coil+domain1	599	LOC107348306	cnidaria	Acropora digitifera	PREDICTED: uncharacterized protein LOC107348306 [Acropora digitifera]	GCF_000222465.1	TRADDN-DEDD2
PFX24827.1	TRADDN-DEDD2+Death+Death	471	AWC38_SpisGene10551	cnidaria	Stylophora pistillata	hypothetical protein AWC38_SpisGene10551 [Stylophora pistillata]	GCA_002571385.1	TRADDN-DEDD2
XP_020604807.1	TRADDN-DEDD2+EF-hand+SH2	458	LOC110043668	cnidaria	Orbicella faveolata	uncharacterized protein LOC110043668 isoform X3 [Orbicella faveolata]	GCF_002042975.1	TRADDN-DEDD2
XP_001622298.1	TRADDN-DEDD2+Exo_endo_phos	330	NEMVEDRAFT_v1g220915	cnidaria	Nematostella vectensis	predicted protein [Nematostella vectensis]	GCF_000209225.1	TRADDN-DEDD2
PFX31376.1	TRADDN-DEDD2+GBP	343	GBP7	cnidaria	Stylophora pistillata	Guanylate-binding protein 7 [Stylophora pistillata]	GCA_002571385.1	TRADDN-DEDD2
XP_015748075.1	TRADDN-DEDD2+GT4-GTase	636	LOC107327853	cnidaria	Acropora digitifera	PREDICTED: uncharacterized protein LOC107327853 [Acropora digitifera]	GCF_000222465.1	TRADDN-DEDD2
PFX28928.1	TRADDN-DEDD2+GT4-GTase+TRADDN-DEDD2	828	mshA	cnidaria	Stylophora pistillata	D-inositol 3-phosphate glycosyltransferase [Stylophora pistillata]	GCA_002571385.1	TRADDN-DEDD2
XP_001621708.1	TRADDN-DEDD2+helical-repeats	575	NEMVEDRAFT_v1g221660	cnidaria	Nematostella vectensis	hypothetical protein NEMVEDRAFT_v1g221660, partial [Nematostella vectensis]	GCF_000209225.1	TRADDN-DEDD2
XP_022778682.1	TRADDN-DEDD2+HEPN	341	LOC111320265	cnidaria	Stylophora pistillata	uncharacterized protein LOC111320265, partial [Stylophora pistillata]	GCF_002571385.1	TRADDN-DEDD2

acc	architecture	len	gen.name	taxend	species	defline	gca	clade
XP_022807529.1	TRADDN-DEDD2+HEPN+Ankyrin	916	LOC111344551	cnidaria	Stylophora pistillata	uncharacterized protein LOC111344551 [Stylophora pistillata]	GCF_002571385.1	TRADDN-DEDD2
XP_015753922.1	TRADDN-DEDD2+HEPN+STAND	413	LOC107333606	cnidaria	Acropora digitifera	PREDICTED: uncharacterized protein LOC107333606 [Acropora digitifera]	GCF_000222465.1	TRADDN-DEDD2
XP_015769096.1	TRADDN-DEDD2+HEPN+STAND+wHTH+helical-region+LRRs+RVT_1	975	LOC107347651	cnidaria	Acropora digitifera	PREDICTED: uncharacterized protein LOC107347651 [Acropora digitifera]	GCF_000222465.1	TRADDN-DEDD2
XP_015770794.1	TRADDN-DEDD2+HSP90-Sacsin	661	LOC107349202	cnidaria	Acropora digitifera	PREDICTED: sacsin-like [Acropora digitifera]	GCF_000222465.1	TRADDN-DEDD2
XP_022789410.1	TRADDN-DEDD2+HSP90-Sacsin	720	LOC111329073	cnidaria	Stylophora pistillata	sacsin-like [Stylophora pistillata]	GCF_002571385.1	TRADDN-DEDD2
XP_022808646.1	TRADDN-DEDD2+HSP90-Sacsin+DUF3684+HSP90-Sacsin+DUF3684+HSP90-Sacsin+DUF3684+DnaJ+HEPN	4675	LOC111345629	cnidaria	Stylophora pistillata	sacsin-like [Stylophora pistillata]	GCF_002571385.1	TRADDN-DEDD2
XP_020604502.1	TRADDN-DEDD2+HTH	305	LOC110043387	cnidaria	Orbicella faveolata	uncharacterized protein LOC110043387 [Orbicella faveolata]	GCF_002042975.1	TRADDN-DEDD2
XP_020622210.1	TRADDN-DEDD2+LRRs	875	LOC110059824	cnidaria	Orbicella faveolata	neurofilament heavy polypeptide-like [Orbicella faveolata]	GCF_002042975.1	TRADDN-DEDD2
XP_020622238.1	TRADDN-DEDD2+LRRs	1040	LOC110059854	cnidaria	Orbicella faveolata	uncharacterized protein LOC110059854 isoform X1 [Orbicella faveolata]	GCF_002042975.1	TRADDN-DEDD2
XP_020622239.1	TRADDN-DEDD2+LRRs	1018	LOC110059854	cnidaria	Orbicella faveolata	uncharacterized protein LOC110059854 isoform X2 [Orbicella faveolata]	GCF_002042975.1	TRADDN-DEDD2
XP_020617793.1	TRADDN-DEDD2+RRM+RRM+KH+KH+KH+KH+KH+KH+KH++Macro+Macro+Macro+WWE	1593	LOC110055733	cnidaria	Orbicella faveolata	poly [ADP-ribose] polymerase 14-like [Orbicella faveolata]	GCF_002042975.1	TRADDN-DEDD2
XP_015766538.1	TRADDN-DEDD2+RRM+RRM+RING+BBOX+BBOX+Coiled-coil	754	LOC107345350	cnidaria	Acropora digitifera	PREDICTED: uncharacterized protein LOC107345350 [Acropora digitifera]	GCF_000222465.1	TRADDN-DEDD2
XP_015777040.1	TRADDN-DEDD2+RRM+RRM+RING+BBOX+Coiled-coil+Filamin+Beta-propeller	1262	LOC107355040	cnidaria	Acropora digitifera	PREDICTED: E3 ubiquitin-protein ligase TRIM71-like [Acropora digitifera]	GCF_000222465.1	TRADDN-DEDD2
PFX15770.1	TRADDN-DEDD2+STAND+wHTH+helical-region+LRRs	1149	Nlrc5	cnidaria	Stylophora pistillata	Protein NLRC5, partial [Stylophora pistillata]	GCA_002571385.1	TRADDN-DEDD2
XP_015754763.1	TRADDN-DEDD2+STAND+wHTH+helical-repeat+LRRs	1363	LOC107334347	cnidaria	Acropora digitifera	PREDICTED: uncharacterized protein LOC107334347 [Acropora digitifera]	GCF_000222465.1	TRADDN-DEDD2
XP_022797605.1	TRADDN-DEDD2+Thioredoxin+PITH	547	LOC111335871	cnidaria	Stylophora pistillata	uncharacterized protein LOC111335871 [Stylophora pistillata]	GCF_002571385.1	TRADDN-DEDD2
XP_022810296.1	TRADDN-DEDD2+TM	380	LOC111347305	cnidaria	Stylophora pistillata	uncharacterized protein LOC111347305 [Stylophora pistillata]	GCF_002571385.1	TRADDN-DEDD2
XP_015761223.1	TRADDN-DEDD2+TM+TM+TM	553	LOC107340384	cnidaria	Acropora digitifera	PREDICTED: uncharacterized protein LOC107340384 [Acropora digitifera]	GCF_000222465.1	TRADDN-DEDD2
XP_015749629.1	TRADDN-DEDD2+TPRs	382	LOC107329456	cnidaria	Acropora digitifera	PREDICTED: uncharacterized protein LOC107329456 [Acropora digitifera]	GCF_000222465.1	TRADDN-DEDD2
XP_015760874.1	TRADDN-DEDD2+TPRs	263	LOC107340046	cnidaria	Acropora digitifera	PREDICTED: tetratricopeptide repeat protein 28-like [Acropora digitifera]	GCF_000222465.1	TRADDN-DEDD2

acc	architecture	len	gen.name	taxend	species	defline	gca	clade
XP_020619807.1	TRADDN-DEDD2+TPRs	537	LOC110057557	cnidaria	Orbicella faveolata	nephrocystin-3-like [Orbicella faveolata]	GCF_002042975.1	TRADDN-DEDD2
XP_020619855.1	TRADDN-DEDD2+TPRs	598	LOC110057601	cnidaria	Orbicella faveolata	kinesin light chain 3-like isoform X3 [Orbicella faveolata]	GCF_002042975.1	TRADDN-DEDD2
XP_022800308.1	TRADDN-DEDD2+TPRs	974	LOC111338144	cnidaria	Stylophora pistillata	tetratricopeptide repeat protein 28-like [Stylophora pistillata]	GCF_002571385.1	TRADDN-DEDD2
PFX35021.1	TRADDN-DEDD2+TPRs+STAND+TPRs	1567	Klc2	cnidaria	Stylophora pistillata	Kinesin light chain 2 [Stylophora pistillata]	GCA_002571385.1	TRADDN-DEDD2
XP_015749168.1	TRADDN-DEDD2+TRADDN-DEDD2	474	LOC107328948	cnidaria	Acropora digitifera	PREDICTED: uncharacterized protein LOC107328948 [Acropora digitifera]	GCF_000222465.1	TRADDN-DEDD2
XP_020622259.1	TRADDN-DEDD2+TRADDN-DEDD2	569	LOC110059882	cnidaria	Orbicella faveolata	uncharacterized protein DDB_G0286299-like isoform X1 [Orbicella faveolata]	GCF_002042975.1	TRADDN-DEDD2
XP_020622260.1	TRADDN-DEDD2+TRADDN-DEDD2	569	LOC110059882	cnidaria	Orbicella faveolata	uncharacterized protein DDB_G0286299-like isoform X2 [Orbicella faveolata]	GCF_002042975.1	TRADDN-DEDD2
XP_020628009.1	TRADDN-DEDD2+TRADDN-DEDD2	290	LOC110065241	cnidaria	Orbicella faveolata	uncharacterized protein LOC110065241 isoform X1 [Orbicella faveolata]	GCF_002042975.1	TRADDN-DEDD2
XP_020628010.1	TRADDN-DEDD2+TRADDN-DEDD2	289	LOC110065241	cnidaria	Orbicella faveolata	uncharacterized protein LOC110065241 isoform X2 [Orbicella faveolata]	GCF_002042975.1	TRADDN-DEDD2
XP_015765569.1	TRADDN-DEDD2+TRADDN-DEDD2+Ankyrin	683	LOC107344436	cnidaria	Acropora digitifera	PREDICTED: uncharacterized protein LOC107344436, partial [Acropora digitifera]	GCF_000222465.1	TRADDN-DEDD2
RMX58800.1	TRADDN-DEDD2+TRADDN-DEDD2+Filamin+TRADDN-DEDD2+TRADDN-DEDD2+Beta-propeller	929	pdam_00021877	cnidaria	Pocillopora damicornis	hypothetical protein pdam_00021877 [Pocillopora damicornis]	GCA_003704095.1	TRADDN-DEDD2
XP_022809254.1	TRADDN-DEDD2+TRADDN-DEDD2+Filamin+TRADDN-DEDD2+TRADDN-DEDD2+Beta-propeller	820	LOC111346217	cnidaria	Stylophora pistillata	tripartite motif-containing protein 2-like [Stylophora pistillata]	GCF_002571385.1	TRADDN-DEDD2
XP_015760896.1	TRADDN-DEDD2+TRADDN-DEDD2+GT4-GTase	652	LOC107340068	cnidaria	Acropora digitifera	PREDICTED: uncharacterized protein LOC107340068 isoform X1 [Acropora digitifera]	GCF_000222465.1	TRADDN-DEDD2
XP_015760897.1	TRADDN-DEDD2+TRADDN-DEDD2+GT4-GTase	651	LOC107340068	cnidaria	Acropora digitifera	PREDICTED: uncharacterized protein LOC107340068 isoform X2 [Acropora digitifera]	GCF_000222465.1	TRADDN-DEDD2
XP_020622197.1	TRADDN-DEDD2+TRADDN-DEDD2+LRRs	1040	LOC110059814	cnidaria	Orbicella faveolata	uncharacterized protein LOC110059814 isoform X1 [Orbicella faveolata]	GCF_002042975.1	TRADDN-DEDD2
XP_020622198.1	TRADDN-DEDD2+TRADDN-DEDD2+LRRs	1037	LOC110059814	cnidaria	Orbicella faveolata	uncharacterized protein LOC110059814 isoform X2 [Orbicella faveolata]	GCF_002042975.1	TRADDN-DEDD2
PFX32737.1	TRADDN-DEDD2+TRADDN-DEDD2+TRADDN-DEDD2+Filamin+Beta-propeller	646	trim71	cnidaria	Stylophora pistillata	E3 ubiquitin-protein ligase TRIM71 [Stylophora pistillata]	GCA_002571385.1	TRADDN-DEDD2
PFX32741.1	TRADDN-DEDD2+TRADDN-DEDD2+TRADDN-DEDD2+Filamin+Beta-propeller	931	Trim71	cnidaria	Stylophora pistillata	E3 ubiquitin-protein ligase TRIM71 [Stylophora pistillata]	GCA_002571385.1	TRADDN-DEDD2
XP_022800589.1	TRADDN-DEDD2+UBI+UBI+UBI	494	LOC111338372	cnidaria	Stylophora pistillata	uncharacterized protein LOC111338372 [Stylophora pistillata]	GCF_002571385.1	TRADDN-DEDD2
PFX19320.1	TRADDN-DEDD2+UBI+UBI+UBI+UBI	1030	UBC	cnidaria	Stylophora pistillata	Polyubiquitin-C [Stylophora pistillata]	GCA_002571385.1	TRADDN-DEDD2

acc	architecture	len	gen.name	taxend	species	defline	gca	clade
PFX23674.1	TRADDN-DEDD2+vWA+Dynamin-like_sGTPase+helical-region	1654	AWC38_SpisGene11766	cnidaria	Stylophora pistillata	hypothetical protein AWC38_SpisGene11766 [Stylophora pistillata]	GCA_002571385.1	TRADDN-DEDD2
XP_022790559.1	TRADDN-DEDD2+zf-C2H2	428	LOC111330036	cnidaria	Stylophora pistillata	uncharacterized protein LOC111330036 [Stylophora pistillata]	GCF_002571385.1	TRADDN-DEDD2
XP_022803161.1	TRADDN-DEDD2+ZU5+ZU5+UPA+Death	791	LOC111340569	cnidaria	Stylophora pistillata	uncharacterized protein LOC111340569 isoform X2 [Stylophora pistillata]	GCF_002571385.1	TRADDN-DEDD2
XP_022806856.1	TRADDN-DEDD2+ZU5+ZU5+UPA+Death	979	LOC111343923	cnidaria	Stylophora pistillata	uncharacterized protein LOC111343923 [Stylophora pistillata]	GCF_002571385.1	TRADDN-DEDD2
XP_015770516.1	TRADDN-NCOA6	919	LOC107348937	cnidaria	Acropora digitifera	PREDICTED: rho GTPase-activating protein gacF-like [Acropora digitifera]	GCF_000222465.1	TRADDN-NCOA6
XP_020902197.1	TRADDN-NCOA6	927	LOC110240720	cnidaria	Exaiptasia pallida	uncharacterized protein LOC110240720 [Exaiptasia pallida]	GCF_001417965.1	TRADDN-NCOA6
XP_001629421.1	TRADDN-NCOA6	1000	NEMVEDRAFT_v1g245030	cnidaria	Nematostella vectensis	predicted protein [Nematostella vectensis]	GCF_000209225.1	TRADDN-NCOA6
XP_020623664.1	TRADDN-NCOA6	1056	LOC110061163	cnidaria	Orbicella faveolata	chitinase-like protein PB1E7.04c [Orbicella faveolata]	GCF_002042975.1	TRADDN-NCOA6
XP_022781542.1	TRADDN-NCOA6	985	LOC111322669	cnidaria	Stylophora pistillata	uncharacterized protein LOC111322669 [Stylophora pistillata]	GCF_002571385.1	TRADDN-NCOA6
PFX25374.1	TRADDN-NCOA6+Ankyrin	1046	FEM1C	cnidaria	Stylophora pistillata	Protein fem-1-like C [Stylophora pistillata]	GCA_002571385.1	TRADDN-NCOA6
XP_020916389.1	TRADDN-NCOA6+vWA+vWA	534	LOC110253783	cnidaria	Exaiptasia pallida	uncharacterized protein LOC110253783, partial [Exaiptasia pallida]	GCF_001417965.1	TRADDN-NCOA6
XP_020616729.1	UBI+UBI+UBI+UBI+UBI+UBI+UBI+UBI+UBI+HEPN+TRADDN-DEDD2+UBI+UBI	1445	LOC110054710	cnidaria	Orbicella faveolata	LOW QUALITY PROTEIN: polyubiquitin-C-like [Orbicella faveolata]	GCF_002042975.1	TRADDN-DEDD2
KZS16547.1	TRADDN-NCOA6	659	APZ42_017697	crustacea	Daphnia magna	Uncharacterized protein APZ42_017697, partial [Daphnia magna]	GCA_001632505.1	TRADDN-NCOA6
XP_023339692.1	TRADDN-NCOA6	1600	LOC111709943	crustacea	Eurytemora affinis	uncharacterized protein LOC111709943 [Eurytemora affinis]	GCF_000591075.1	TRADDN-NCOA6
XP_018010260.1	TRADDN-NCOA6	2647	LOC108667717	crustacea	Hyalella azteca	PREDICTED: mucin-4-like isoform X1 [Hyalella azteca]	GCF_000764305.1	TRADDN-NCOA6
XP_018010261.1	TRADDN-NCOA6	2646	LOC108667717	crustacea	Hyalella azteca	PREDICTED: mucin-4-like isoform X2 [Hyalella azteca]	GCF_000764305.1	TRADDN-NCOA6
PIK36113.1	CARD+Coiled-coil+DED+TRADDN-DEDD2+CARD	1289	BSL78_27056	echinodermata	Apostichopus japonicus	putative up-regulator of cell proliferation-like [Apostichopus japonicus]	GCA_002754855.1	TRADDN-DEDD2
PIK42735.1	CARD+DED+Death+TRADDN-DEDD2	1092	BSL78_20407	echinodermata	Apostichopus japonicus	hypothetical protein BSL78_20407 [Apostichopus japonicus]	GCA_002754855.1	TRADDN-DEDD2
XP_011682936.1	Death+Death+TRADDN-DEDD2	578	LOC105447040	echinodermata	Strongylocentrotus purpuratus	PREDICTED: uncharacterized protein LOC105447040 [Strongylocentrotus purpuratus]	-	TRADDN-DEDD2
XP_011668608.1	Death+Death+TRADDN-DEDD2+SAM	1074	LOC100890193	echinodermata	Strongylocentrotus purpuratus	PREDICTED: uncharacterized protein LOC100890193 [Strongylocentrotus purpuratus]	-	TRADDN-DEDD2

acc	architecture	len	gen.name	taxend	species	defline	gca	clade
XP_011668610.1	Death+Death+TRADDN-DEDD2+SAM+STAND+wHTH+TPRs	1947	LOC100890281	echinodermata	Strongylocentrotus purpuratus	PREDICTED: sterile alpha motif domain-containing protein 9-like [Strongylocentrotus purpuratus]	-	TRADDN-DEDD2
XP_011681878.1	Death+Death+TRADDN-DEDD2+SAM+STAND+wHTH+TPRs	2028	LOC105446577	echinodermata	Strongylocentrotus purpuratus	PREDICTED: sterile alpha motif domain-containing protein 9-like [Strongylocentrotus purpuratus]	-	TRADDN-DEDD2
XP_011664960.1	Death+Death+TRADDN-DEDD2+ZU5+UPA	818	LOC105438624	echinodermata	Strongylocentrotus purpuratus	PREDICTED: uncharacterized protein LOC105438624 isoform X1 [Strongylocentrotus purpuratus]	-	TRADDN-DEDD2
XP_011664961.1	Death+Death+TRADDN-DEDD2+ZU5+UPA	710	LOC105438624	echinodermata	Strongylocentrotus purpuratus	PREDICTED: uncharacterized protein LOC105438624 isoform X2 [Strongylocentrotus purpuratus]	-	TRADDN-DEDD2
XP_011669783.1	Death+TM+TM+TRADDN-DEDD2	491	LOC105440891	echinodermata	Strongylocentrotus purpuratus	PREDICTED: uncharacterized protein LOC105440891 [Strongylocentrotus purpuratus]	-	TRADDN-DEDD2
XP_011683422.1	Death+TRADDN-DEDD2+AP-GTPase	616	LOC105447276	echinodermata	Strongylocentrotus purpuratus	PREDICTED: uncharacterized protein LOC105447276 [Strongylocentrotus purpuratus]	-	TRADDN-DEDD2
XP_022109238.1	Death+TRADDN-DEDD2+MIB_HERC2+ZZ+MIB_HERC2+SH3+Ankyrin	1234	LOC110989278	echinodermata	Acanthaster planci	uncharacterized protein LOC110989278 isoform X1 [Acanthaster planci]	GCF_001949145.1	TRADDN-DEDD2
XP_022109239.1	Death+TRADDN-DEDD2+MIB_HERC2+ZZ+MIB_HERC2+SH3+Ankyrin	1233	LOC110989278	echinodermata	Acanthaster planci	uncharacterized protein LOC110989278 isoform X2 [Acanthaster planci]	GCF_001949145.1	TRADDN-DEDD2
XP_022109240.1	Death+TRADDN-DEDD2+MIB_HERC2+ZZ+MIB_HERC2+SH3+Ankyrin	1186	LOC110989278	echinodermata	Acanthaster planci	uncharacterized protein LOC110989278 isoform X3 [Acanthaster planci]	GCF_001949145.1	TRADDN-DEDD2
XP_022109242.1	Death+TRADDN-DEDD2+MIB_HERC2+ZZ+MIB_HERC2+SH3+Ankyrin	1149	LOC110989278	echinodermata	Acanthaster planci	E3 ubiquitin-protein ligase MIB2-like isoform X4 [Acanthaster planci]	GCF_001949145.1	TRADDN-DEDD2
XP_022109243.1	Death+TRADDN-DEDD2+MIB_HERC2+ZZ+MIB_HERC2+SH3+Ankyrin	1101	LOC110989278	echinodermata	Acanthaster planci	E3 ubiquitin-protein ligase MIB2-like isoform X5 [Acanthaster planci]	GCF_001949145.1	TRADDN-DEDD2
XP_011676328.1	Death+TRADDN-DEDD2+SAM	910	LOC105444162	echinodermata	Strongylocentrotus purpuratus	PREDICTED: uncharacterized protein LOC105444162 [Strongylocentrotus purpuratus]	-	TRADDN-DEDD2
XP_022105930.1	Death+TRADDN-DEDD2+STAND	685	LOC110987482	echinodermata	Acanthaster planci	uncharacterized protein LOC110987482 [Acanthaster planci]	GCF_001949145.1	TRADDN-DEDD2
PIK50631.1	DED+DED+TRADDN-DEDD2	799	BSL78_12477	echinodermata	Apostichopus japonicus	hypothetical protein BSL78_12477 [Apostichopus japonicus]	GCA_002754855.1	TRADDN-DEDD2
PIK43142.1	DED+TRADDN-DEDD2+C2H2-ZF+ZF-CCCH+Pkinase+P-loop-NTPase	1174	BSL78_19998	echinodermata	Apostichopus japonicus	putative serine/threonine-protein kinase/endoribonuclease IRE1-like isoform X4 [Apostichopus japonicus]	GCA_002754855.1	TRADDN-DEDD2
XP_011667260.1	FN3+FN3+FN3+FN3+FN3+RVT_1+helical_bundle+RNaseH+lambda_integrase-N+HTH+TRADDN-DEDD2+SAM+STAND+TPRs	3989	LOC105439689	echinodermata	Strongylocentrotus purpuratus	PREDICTED: uncharacterized protein LOC105439689 [Strongylocentrotus purpuratus]	-	TRADDN-DEDD2
XP_792092.3	low-complexity+DED+TRADDN-DEDD2	371	LOC587260	echinodermata	Strongylocentrotus purpuratus	PREDICTED: death effector domain-containing protein [Strongylocentrotus purpuratus]	GCF_000002235.5	TRADDN-DEDD2
XP_022107075.1	N-ter+TRADDN-Bac+FAM124	407	LOC110988125	echinodermata	Acanthaster planci	uncharacterized protein LOC110988125 [Acanthaster planci]	GCF_001949145.1	TRADDN-Bacterial-like

acc	architecture	len	gen.name	taxend	species	defline	gca	clade
PIK35605.1	N-ter+TRADDN-Bac+FAM124	383	BSL78_27565	echinoder-mata	Apostichopus japonicus	hypothetical protein BSL78_27565 [Apostichopus japonicus]	GCA_002754855.1	TRADDN-Bacterial-like
PIK50692.1	N-ter+TRADDN-Bac+FAM124	383	BSL78_12425	echinoder-mata	Apostichopus japonicus	hypothetical protein BSL78_12425 [Apostichopus japonicus]	GCA_002754855.1	TRADDN-Bacterial-like
XP_003726836.1	N-ter+TRADDN-Bac+FAM124	399	LOC100889127	echinoder-mata	Strongylocentrotus purpuratus	PREDICTED: uncharacterized protein LOC100889127 [Strongylocentrotus purpuratus]	-	TRADDN-Bacterial-like
XP_022106408.1	TRADDN-Bac+TM+TM+TM	383	LOC110987722	echinoder-mata	Acanthaster planci	uncharacterized protein LOC110987722 [Acanthaster planci]	GCF_001949145.1	TRADDN-Bacterial-like
XP_022093016.1	TRADDN-DEDD2	272	LOC110980537	echinoder-mata	Acanthaster planci	uncharacterized protein LOC110980537 isoform X1 [Acanthaster planci]	GCF_001949145.1	TRADDN-DEDD2
XP_022093018.1	TRADDN-DEDD2	271	LOC110980537	echinoder-mata	Acanthaster planci	uncharacterized protein LOC110980537 isoform X2 [Acanthaster planci]	GCF_001949145.1	TRADDN-DEDD2
XP_022093019.1	TRADDN-DEDD2	244	LOC110980537	echinoder-mata	Acanthaster planci	uncharacterized protein LOC110980537 isoform X3 [Acanthaster planci]	GCF_001949145.1	TRADDN-DEDD2
PIK33877.1	TRADDN-DEDD2	286	BSL78_29305	echinoder-mata	Apostichopus japonicus	hypothetical protein BSL78_29305 [Apostichopus japonicus]	GCA_002754855.1	TRADDN-DEDD2
PIK49872.1	TRADDN-DEDD2	257	BSL78_13243	echinoder-mata	Apostichopus japonicus	hypothetical protein BSL78_13243 [Apostichopus japonicus]	GCA_002754855.1	TRADDN-DEDD2
XP_011666569.1	TRADDN-DEDD2+Death	242	LOC105439365	echinoder-mata	Strongylocentrotus purpuratus	PREDICTED: uncharacterized protein LOC105439365 [Strongylocentrotus purpuratus]	-	TRADDN-DEDD2
XP_011679352.1	TRADDN-DEDD2+Death	291	LOC105445475	echinoder-mata	Strongylocentrotus purpuratus	PREDICTED: uncharacterized protein LOC105445475 isoform X1 [Strongylocentrotus purpuratus]	-	TRADDN-DEDD2
XP_011679356.1	TRADDN-DEDD2+Death	290	LOC105445475	echinoder-mata	Strongylocentrotus purpuratus	PREDICTED: uncharacterized protein LOC105445475 isoform X2 [Strongylocentrotus purpuratus]	-	TRADDN-DEDD2
XP_788029.4	TRADDN-DEDD2+RNA-receptor_SF2-helicase+RNA-receptor_SF2-helicase+RIG-I_C-RD	809	LOC583008	echinoder-mata	Strongylocentrotus purpuratus	PREDICTED: probable ATP-dependent RNA helicase DDX58 [Strongylocentrotus purpuratus]	-	TRADDN-DEDD2
XP_011671465.1	TRADDN-DEDD2+SAM	435	LOC105441747	echinoder-mata	Strongylocentrotus purpuratus	PREDICTED: uncharacterized protein LOC105441747 [Strongylocentrotus purpuratus]	-	TRADDN-DEDD2
XP_011671798.1	TRADDN-DEDD2+SAM	585	LOC105441891	echinoder-mata	Strongylocentrotus purpuratus	PREDICTED: uncharacterized protein LOC105441891 [Strongylocentrotus purpuratus]	-	TRADDN-DEDD2
XP_022081709.1	TRADDN-DEDD2+SAM+STAND+wHTH+helical-region	1712	LOC110974409	echinoder-mata	Acanthaster planci	sterile alpha motif domain-containing protein 9-like [Acanthaster planci]	GCF_001949145.1	TRADDN-DEDD2
XP_003727489.1	TRADDN-DEDD2+SAM+STAND+wHTH+helical-region+TPRs	1565	LOC100890659	echinoder-mata	Strongylocentrotus purpuratus	PREDICTED: sterile alpha motif domain-containing protein 9-like [Strongylocentrotus purpuratus]	-	TRADDN-DEDD2
XP_022107499.1	TRADDN-DEDD2+TM+TM+TM+TM	592	LOC110988373	echinoder-mata	Acanthaster planci	uncharacterized protein LOC110988373 [Acanthaster planci]	GCF_001949145.1	TRADDN-DEDD2
XP_022087985.1	TRADDN-NCOA6	2510	LOC110977825	echinoder-mata	Acanthaster planci	mucin-5AC-like isoform X1 [Acanthaster planci]	GCF_001949145.1	TRADDN-NCOA6

acc	architecture	len	gen.name	taxend	species	defline	gca	clade
XP_022088003.1	TRADDN-NCOA6	2506	LOC110977825	echinodermata	Acanthaster planci	mucin-5AC-like isoform X2 [Acanthaster planci]	GCF_001949145.1	TRADDN-NCOA6
PIK35968.1	TRADDN-NCOA6	1498	BSL78_27199	echinodermata	Apostichopus japonicus	hypothetical protein BSL78_27199 [Apostichopus japonicus]	GCA_002754855.1	TRADDN-NCOA6
XP_011673750.1	TRADDN-NCOA6	314	LOC105442838	echinodermata	Strongylocentrotus purpuratus	PREDICTED: uncharacterized protein DDB_G0285291-like [Strongylocentrotus purpuratus]	-	TRADDN-NCOA6
XP_022105938.1	UBI+Death+UBI+Death+Death+TRADDN-DEDD2	1118	LOC110987487	echinodermata	Acanthaster planci	uncharacterized protein LOC110987487 isoform X1 [Acanthaster planci]	GCF_001949145.1	TRADDN-DEDD2
XP_022105942.1	UBI+Death+UBI+Death+Death+TRADDN-DEDD2	1092	LOC110987487	echinodermata	Acanthaster planci	uncharacterized protein LOC110987487 isoform X2 [Acanthaster planci]	GCF_001949145.1	TRADDN-DEDD2
XP_022107080.1	UBI+SAM+TRADDN-DEDD2+SAM+STAND+wHTH+TPRs+Coiled-coil	2106	LOC110988130	echinodermata	Acanthaster planci	sterile alpha motif domain-containing protein 9-like isoform X1 [Acanthaster planci]	GCF_001949145.1	TRADDN-DEDD2
XP_022107081.1	UBI+SAM+TRADDN-DEDD2+SAM+STAND+wHTH+TPRs+Coiled-coil	2102	LOC110988130	echinodermata	Acanthaster planci	sterile alpha motif domain-containing protein 9-like isoform X2 [Acanthaster planci]	GCF_001949145.1	TRADDN-DEDD2
XP_022111951.1	UBI+TRADDN-DEDD2+SAM+STAND+wHTH+TPRs	1886	LOC110991092	echinodermata	Acanthaster planci	uncharacterized protein LOC110991092 [Acanthaster planci]	GCF_001949145.1	TRADDN-DEDD2
XP_022110138.1	UBI+TRADDN-DEDD2+SAM+STAND+wHTH+TPRs+Coiled-coil	2003	LOC110989809	echinodermata	Acanthaster planci	sterile alpha motif domain-containing protein 9-like isoform X1 [Acanthaster planci]	GCF_001949145.1	TRADDN-DEDD2
XP_022110147.1	UBI+TRADDN-DEDD2+SAM+STAND+wHTH+TPRs+Coiled-coil	2002	LOC110989809	echinodermata	Acanthaster planci	sterile alpha motif domain-containing protein 9-like isoform X2 [Acanthaster planci]	GCF_001949145.1	TRADDN-DEDD2
XP_022110598.1	UBI+TRADDN-DEDD2+STAND+wHTH+helical-region+LRRs	1732	LOC110990090	echinodermata	Acanthaster planci	uncharacterized protein LOC110990090 [Acanthaster planci]	GCF_001949145.1	TRADDN-DEDD2
XP_022110603.1	UBI+UBI+Death+TRADDN-DEDD2	933	LOC110990092	echinodermata	Acanthaster planci	uncharacterized protein LOC110990092 [Acanthaster planci]	GCF_001949145.1	TRADDN-DEDD2
XP_022110590.1	UBI+UBI+TRADDN-DEDD2+STAND+LRRs	1792	LOC110990087	echinodermata	Acanthaster planci	uncharacterized protein LOC110990087 isoform X1 [Acanthaster planci]	GCF_001949145.1	TRADDN-DEDD2
XP_022110592.1	UBI+UBI+TRADDN-DEDD2+STAND+LRRs	1739	LOC110990087	echinodermata	Acanthaster planci	uncharacterized protein LOC110990087 isoform X3 [Acanthaster planci]	GCF_001949145.1	TRADDN-DEDD2
XP_022110591.1	UBI+UBI+TRADDN-DEDD2+STAND+wHTH+helical-region+LRRs	1740	LOC110990087	echinodermata	Acanthaster planci	uncharacterized protein LOC110990087 isoform X2 [Acanthaster planci]	GCF_001949145.1	TRADDN-DEDD2
XP_006812311.1	CARD+TRADDN-DEDD2+FN3	977	LOC102807547	hemichordata	Saccoglossus kowalevskii	PREDICTED: uncharacterized protein LOC102807547 [Saccoglossus kowalevskii]	GCF_000003605.2	TRADDN-DEDD2
XP_006825576.1	Coiled-coil+TRADDN-DEDD2+STAND	1051	LOC102800950	hemichordata	Saccoglossus kowalevskii	PREDICTED: uncharacterized protein LOC102800950 [Saccoglossus kowalevskii]	GCF_000003605.2	TRADDN-DEDD2
XP_006825575.1	Coiled-coil+TRADDN-DEDD2+STAND+wHTH+helical-repeat	1282	LOC102800837	hemichordata	Saccoglossus kowalevskii	PREDICTED: uncharacterized protein LOC102800837 [Saccoglossus kowalevskii]	GCF_000003605.2	TRADDN-DEDD2
XP_002731064.1	Death+TRADDN-DEDD2+Coiled-coil	771	LOC100368257	hemichordata	Saccoglossus kowalevskii	PREDICTED: uncharacterized protein LOC100368257 [Saccoglossus kowalevskii]	GCF_000003605.2	TRADDN-DEDD2

acc	architecture	len	gen.name	taxend	species	defline	gca	clade
XP_006820812.1	DED+TRADDN-DEDD2	483	LOC100366565	hemichordata	Saccoglossus kowalevskii	PREDICTED: uncharacterized protein LOC100366565 [Saccoglossus kowalevskii]	GCF_000003605.2	TRADDN-DEDD2
XP_006823454.1	DED+TRADDN-DEDD2+Ankyrin	547	LOC102801342	hemichordata	Saccoglossus kowalevskii	PREDICTED: early endosome antigen 1-like, partial [Saccoglossus kowalevskii]	GCF_000003605.2	TRADDN-DEDD2
XP_006816288.1	DED+TRADDN-DEDD2+Coiled-coil	828	LOC102805649	hemichordata	Saccoglossus kowalevskii	PREDICTED: putative leucine-rich repeat-containing protein DDB_G0290503-like, partial [Saccoglossus kowalevskii]	GCF_000003605.2	TRADDN-DEDD2
XP_006824354.1	DED+TRADDN-DEDD2+Coiled-coil+DED	792	LOC102805146	hemichordata	Saccoglossus kowalevskii	PREDICTED: uncharacterized protein LOC102805146 [Saccoglossus kowalevskii]	GCF_000003605.2	TRADDN-DEDD2
XP_006824651.1	EGF+EGF+EGF+CLECTIN+GT4-GTase+TRADDN-DEDD2	945	LOC102808514	hemichordata	Saccoglossus kowalevskii	PREDICTED: uncharacterized protein LOC102808514, partial [Saccoglossus kowalevskii]	GCF_000003605.2	TRADDN-DEDD2
XP_006814349.1	GT4-GTase+TRADDN-DEDD2	1053	LOC102800835	hemichordata	Saccoglossus kowalevskii	PREDICTED: protein PFC0760c-like [Saccoglossus kowalevskii]	GCF_000003605.2	TRADDN-DEDD2
XP_006811676.1	GT4-GTase+TRADDN-DEDD2+Bcl-2	814	LOC102807814	hemichordata	Saccoglossus kowalevskii	PREDICTED: uncharacterized protein LOC102807814 [Saccoglossus kowalevskii]	GCF_000003605.2	TRADDN-DEDD2
XP_006823290.1	GT4-GTase+TRADDN-DEDD2+TM+LRRs	1015	LOC102801620	hemichordata	Saccoglossus kowalevskii	PREDICTED: uncharacterized protein LOC102801620, partial [Saccoglossus kowalevskii]	GCF_000003605.2	TRADDN-DEDD2
XP_002735384.1	N-ter+TRADDN-Bac+FAM124	412	LOC100374528	hemichordata	Saccoglossus kowalevskii	PREDICTED: uncharacterized protein LOC100374528 [Saccoglossus kowalevskii]	GCF_000003605.2	TRADDN-Bacterial-like
XP_006822589.1	TM+TM+Beta-propeller+GT4-GTase+TRADDN-DEDD2+Ankyrin	3508	LOC102803126	hemichordata	Saccoglossus kowalevskii	PREDICTED: uncharacterized protein LOC102803126 [Saccoglossus kowalevskii]	GCF_000003605.2	TRADDN-DEDD2
XP_006824219.1	TM+TRADDN-DEDD2+TM+TM+TM+TM+TM	615	LOC102808738	hemichordata	Saccoglossus kowalevskii	PREDICTED: uncharacterized protein LOC102808738 [Saccoglossus kowalevskii]	GCF_000003605.2	TRADDN-DEDD2
XP_006821553.1	TRADDN-DEDD2	260	LOC102801950	hemichordata	Saccoglossus kowalevskii	PREDICTED: uncharacterized protein LOC102801950, partial [Saccoglossus kowalevskii]	GCF_000003605.2	TRADDN-DEDD2
XP_006822240.1	TRADDN-DEDD2	1314	LOC100371376	hemichordata	Saccoglossus kowalevskii	PREDICTED: microtubule-associated protein 1B-like [Saccoglossus kowalevskii]	GCF_000003605.2	TRADDN-DEDD2
XP_006823467.1	TRADDN-DEDD2	120	LOC102803591	hemichordata	Saccoglossus kowalevskii	PREDICTED: death effector domain-containing protein-like, partial [Saccoglossus kowalevskii]	GCF_000003605.2	TRADDN-DEDD2
XP_006822047.1	TRADDN-DEDD2+Ankyrin	670	LOC102807037	hemichordata	Saccoglossus kowalevskii	PREDICTED: ankyrin repeat domain-containing protein 50-like [Saccoglossus kowalevskii]	GCF_000003605.2	TRADDN-DEDD2
XP_006822031.1	TRADDN-DEDD2+CDC48_N+Coiled-coil+Classical-AAA+Classical-AAA	1246	LOC100375289	hemichordata	Saccoglossus kowalevskii	PREDICTED: transitional endoplasmic reticulum ATPase-like [Saccoglossus kowalevskii]	GCF_000003605.2	TRADDN-DEDD2
XP_006811155.1	TRADDN-DEDD2+Coiled-coil	362	LOC102809883	hemichordata	Saccoglossus kowalevskii	PREDICTED: uncharacterized protein LOC102809883 [Saccoglossus kowalevskii]	GCF_000003605.2	TRADDN-DEDD2

acc	architecture	len	gen.name	taxend	species	defline	gca	clade
XP_006812069.1	TRADDN-DEDD2+Coiled-coil	337	LOC102807464	hemichordata	Saccoglossus kowalevskii	PREDICTED: uncharacterized protein LOC102807464 [Saccoglossus kowalevskii]	GCF_000003605.2	TRADDN-DEDD2
XP_006819467.1	TRADDN-DEDD2+TM+Coiled-coil	1062	LOC102807364	hemichordata	Saccoglossus kowalevskii	PREDICTED: uncharacterized protein LOC102807364 [Saccoglossus kowalevskii]	GCF_000003605.2	TRADDN-DEDD2
XP_006822792.1	TRADDN-NCOA6	2092	LOC100375987	hemichordata	Saccoglossus kowalevskii	PREDICTED: mediator of RNA polymerase II transcription subunit 12-like [Saccoglossus kowalevskii]	GCF_000003605.2	TRADDN-NCOA6
XP_011062119.1	TRADDN-NCOA6	2294	LOC105150621	hexapoda	Acromyrmex echinatior	PREDICTED: uncharacterized protein LOC105150621 isoform X1 [Acromyrmex echinatior]	GCF_000204515.1	TRADDN-NCOA6
XP_011062120.1	TRADDN-NCOA6	2292	LOC105150621	hexapoda	Acromyrmex echinatior	PREDICTED: uncharacterized protein LOC105150621 isoform X2 [Acromyrmex echinatior]	GCF_000204515.1	TRADDN-NCOA6
XP_011062121.1	TRADDN-NCOA6	2283	LOC105150621	hexapoda	Acromyrmex echinatior	PREDICTED: uncharacterized protein LOC105150621 isoform X3 [Acromyrmex echinatior]	GCF_000204515.1	TRADDN-NCOA6
XP_011062122.1	TRADDN-NCOA6	2247	LOC105150621	hexapoda	Acromyrmex echinatior	PREDICTED: uncharacterized protein LOC105150621 isoform X4 [Acromyrmex echinatior]	GCF_000204515.1	TRADDN-NCOA6
XP_011062123.1	TRADDN-NCOA6	2082	LOC105150621	hexapoda	Acromyrmex echinatior	PREDICTED: bromodomain-containing protein DDB_G0270170-like isoform X5 [Acromyrmex echinatior]	GCF_000204515.1	TRADDN-NCOA6
XP_011062124.1	TRADDN-NCOA6	2080	LOC105150621	hexapoda	Acromyrmex echinatior	PREDICTED: bromodomain-containing protein DDB_G0270170-like isoform X6 [Acromyrmex echinatior]	GCF_000204515.1	TRADDN-NCOA6
XP_011062125.1	TRADDN-NCOA6	2055	LOC105150621	hexapoda	Acromyrmex echinatior	PREDICTED: bromodomain-containing protein DDB_G0270170-like isoform X7 [Acromyrmex echinatior]	GCF_000204515.1	TRADDN-NCOA6
XP_011062126.1	TRADDN-NCOA6	2044	LOC105150621	hexapoda	Acromyrmex echinatior	PREDICTED: bromodomain-containing protein DDB_G0270170-like isoform X8 [Acromyrmex echinatior]	GCF_000204515.1	TRADDN-NCOA6
XP_011062127.1	TRADDN-NCOA6	2033	LOC105150621	hexapoda	Acromyrmex echinatior	PREDICTED: bromodomain-containing protein DDB_G0270170-like isoform X9 [Acromyrmex echinatior]	GCF_000204515.1	TRADDN-NCOA6
XP_008184466.1	TRADDN-NCOA6	1508	LOC100570013	hexapoda	Acyrthosiphon pisum	PREDICTED: uncharacterized protein LOC100570013 isoform X1 [Acyrthosiphon pisum]	GCF_005508785.1	TRADDN-NCOA6
XP_008184467.1	TRADDN-NCOA6	1506	LOC100570013	hexapoda	Acyrthosiphon pisum	PREDICTED: uncharacterized protein LOC100570013 isoform X2 [Acyrthosiphon pisum]	GCF_005508785.1	TRADDN-NCOA6
XP_008184468.1	TRADDN-NCOA6	1472	LOC100570013	hexapoda	Acyrthosiphon pisum	PREDICTED: uncharacterized protein LOC100570013 isoform X3 [Acyrthosiphon pisum]	GCF_005508785.1	TRADDN-NCOA6
EAT38891.1	TRADDN-NCOA6	2378	AaeL_AAEL009265	hexapoda	Aedes aegypti	AAEL009265-PA [Aedes aegypti]	GCA_000004015.3	TRADDN-NCOA6

acc	architecture	len	gen.name	taxend	species	defline	gca	clade
XP_021700734.1	TRADDN-NCOA6	2237	LOC5571731	hexapoda	Aedes aegypti	uncharacterized protein LOC5571731 isoform X1 [Aedes aegypti]	GCF_002204515.2	TRADDN-NCOA6
XP_021700736.1	TRADDN-NCOA6	2195	LOC5571731	hexapoda	Aedes aegypti	uncharacterized protein LOC5571731 isoform X2 [Aedes aegypti]	GCF_002204515.2	TRADDN-NCOA6
KXJ80712.1	TRADDN-NCOA6	2343	RP20_CCG023748	hexapoda	Aedes albopictus	hypothetical protein RP20_CCG023748 [Aedes albopictus]	GCA_001444175.2	TRADDN-NCOA6
XP_019540319.1	TRADDN-NCOA6	2213	LOC109411255	hexapoda	Aedes albopictus	PREDICTED: uncharacterized protein LOC109411255 [Aedes albopictus]	-	TRADDN-NCOA6
XP_019564984.1	TRADDN-NCOA6	2272	LOC109433059	hexapoda	Aedes albopictus	PREDICTED: uncharacterized protein LOC109433059 [Aedes albopictus]	-	TRADDN-NCOA6
XP_018334564.1	TRADDN-NCOA6	1413	LOC108743489	hexapoda	Agrilus planipennis	PREDICTED: uncharacterized protein LOC108743489 [Agrilus planipennis]	-	TRADDN-NCOA6
XP_013191593.1	TRADDN-NCOA6	1576	LOC106135763	hexapoda	Amyelois transitella	PREDICTED: uncharacterized protein LOC106135763 [Amyelois transitella]	GCF_001186105.1	TRADDN-NCOA6
ETN61294.1	TRADDN-NCOA6	3050	AND_007043	hexapoda	Anopheles darlingi	hypothetical protein AND_007043 [Anopheles darlingi]	GCA_000211455.3	TRADDN-NCOA6
XP_001689054.1	TRADDN-NCOA6	2423	AgaP_AGAP007771	hexapoda	Anopheles gambiae str. PEST	AGAP007771-PA, partial [Anopheles gambiae str. PEST]	GCF_000005575.2	TRADDN-NCOA6
KFB46201.1	TRADDN-NCOA6	461	ZHAS_00014205	hexapoda	Anopheles sinensis	AGAP007771-PA-like protein [Anopheles sinensis]	GCA_000441895.2	TRADDN-NCOA6
XP_018568099.1	TRADDN-NCOA6	1508	LOC108908525	hexapoda	Anoplophora glabripennis	uncharacterized protein LOC108908525 [Anoplophora glabripennis]	GCF_000390285.2	TRADDN-NCOA6
XP_016905450.1	TRADDN-NCOA6	2244	LOC107993508	hexapoda	Apis cerana	PREDICTED: putative mediator of RNA polymerase II transcription subunit 26 [Apis cerana]	GCF_001442555.1	TRADDN-NCOA6
PBC29023.1	TRADDN-NCOA6	2416	APICC_03446	hexapoda	Apis cerana cerana	RNA-binding protein pno1 [Apis cerana cerana]	GCA_002290385.1	TRADDN-NCOA6
XP_006618876.1	TRADDN-NCOA6	2252	LOC102680856	hexapoda	Apis dorsata	PREDICTED: LOW QUALITY PROTEIN: putative mediator of RNA polymerase II transcription subunit 26-like [Apis dorsata]	-	TRADDN-NCOA6
XP_016768247.1	TRADDN-NCOA6	2239	LOC725706	hexapoda	Apis mellifera	putative mediator of RNA polymerase II transcription subunit 26 isoform X1 [Apis mellifera]	GCF_003254395.2	TRADDN-NCOA6
XP_016768249.1	TRADDN-NCOA6	2229	LOC725706	hexapoda	Apis mellifera	putative mediator of RNA polymerase II transcription subunit 26 isoform X2 [Apis mellifera]	GCF_003254395.2	TRADDN-NCOA6
XP_016768250.1	TRADDN-NCOA6	2227	LOC725706	hexapoda	Apis mellifera	putative mediator of RNA polymerase II transcription subunit 26 isoform X3 [Apis mellifera]	GCF_003254395.2	TRADDN-NCOA6
XP_016768251.1	TRADDN-NCOA6	2215	LOC725706	hexapoda	Apis mellifera	putative mediator of RNA polymerase II transcription subunit 26 isoform X4 [Apis mellifera]	GCF_003254395.2	TRADDN-NCOA6

acc	architecture	len	gen.name	taxend	species	defline	gca	clade
XP_016768252.1	TRADDN-NCOA6	2080	LOC725706	hexapoda	Apis mellifera	putative mediator of RNA polymerase II transcription subunit 26 isoform X5 [Apis mellifera]	GCF_003254395.2	TRADDN-NCOA6
XP_012257407.1	TRADDN-NCOA6	2226	LOC105686833	hexapoda	Athalia rosae	serine/arginine repetitive matrix protein 2-like [Athalia rosae]	GCF_000344095.2	TRADDN-NCOA6
XP_012062303.1	TRADDN-NCOA6	2279	LOC105625590	hexapoda	Atta cephalotes	PREDICTED: putative mediator of RNA polymerase II transcription subunit 26 [Atta cephalotes]	GCF_000143395.1	TRADDN-NCOA6
KYM77727.1	TRADDN-NCOA6	2250	ALC53_11738	hexapoda	Atta colombica	Nuclear receptor coactivator 6 [Atta colombica]	GCA_001594045.1	TRADDN-NCOA6
XP_018054838.1	TRADDN-NCOA6	2282	LOC108691560	hexapoda	Atta colombica	PREDICTED: serine-rich adhesin for platelets isoform X1 [Atta colombica]	GCF_001594045.1	TRADDN-NCOA6
XP_018054840.1	TRADDN-NCOA6	2271	LOC108691560	hexapoda	Atta colombica	PREDICTED: uncharacterized protein LOC108691560 isoform X2 [Atta colombica]	GCF_001594045.1	TRADDN-NCOA6
XP_018802060.1	TRADDN-NCOA6	2428	LOC108977023	hexapoda	Bactrocera latifrons	PREDICTED: probable serine/threonine-protein kinase DDB_G0282963 isoform X1 [Bactrocera latifrons]	GCF_001853355.1	TRADDN-NCOA6
XP_018802061.1	TRADDN-NCOA6	2427	LOC108977023	hexapoda	Bactrocera latifrons	PREDICTED: probable serine/threonine-protein kinase DDB_G0282963 isoform X2 [Bactrocera latifrons]	GCF_001853355.1	TRADDN-NCOA6
XP_014095526.1	TRADDN-NCOA6	2258	LOC106621276	hexapoda	Bactrocera oleae	PREDICTED: serine-rich adhesin for platelets-like isoform X1 [Bactrocera oleae]	GCF_001188975.1	TRADDN-NCOA6
XP_014095527.1	TRADDN-NCOA6	2257	LOC106621276	hexapoda	Bactrocera oleae	PREDICTED: serine-rich adhesin for platelets-like isoform X2 [Bactrocera oleae]	GCF_001188975.1	TRADDN-NCOA6
XP_014095528.1	TRADDN-NCOA6	2257	LOC106621276	hexapoda	Bactrocera oleae	PREDICTED: serine-rich adhesin for platelets-like isoform X3 [Bactrocera oleae]	GCF_001188975.1	TRADDN-NCOA6
XP_014095529.1	TRADDN-NCOA6	2239	LOC106621276	hexapoda	Bactrocera oleae	PREDICTED: serine-rich adhesin for platelets-like isoform X4 [Bactrocera oleae]	GCF_001188975.1	TRADDN-NCOA6
XP_014095530.1	TRADDN-NCOA6	2231	LOC106621276	hexapoda	Bactrocera oleae	PREDICTED: serine-rich adhesin for platelets-like isoform X5 [Bactrocera oleae]	GCF_001188975.1	TRADDN-NCOA6
XP_018909976.1	TRADDN-NCOA6	2107	LOC109039092	hexapoda	Bemisia tabaci	PREDICTED: uncharacterized protein LOC109039092 [Bemisia tabaci]	GCF_001854935.1	TRADDN-NCOA6
XP_023944256.1	TRADDN-NCOA6	1708	LOC112050261	hexapoda	Bicyclus anynana	uncharacterized protein LOC112050261 [Bicyclus anynana]	GCF_900239965.1	TRADDN-NCOA6
PSN32597.1	TRADDN-NCOA6	2647	C0J52_15181	hexapoda	Blattella germanica	hypothetical protein C0J52_15181 [Blattella germanica]	GCA_003018175.1	TRADDN-NCOA6
XP_012236498.1	TRADDN-NCOA6	2247	LOC100746843	hexapoda	Bombus impatiens	uncharacterized protein LOC100746843 isoform X1 [Bombus impatiens]	GCF_000188095.3	TRADDN-NCOA6
XP_012236500.1	TRADDN-NCOA6	2237	LOC100746843	hexapoda	Bombus impatiens	uncharacterized protein LOC100746843 isoform X2 [Bombus impatiens]	GCF_000188095.3	TRADDN-NCOA6

acc	architecture	len	gen.name	taxend	species	defline	gca	clade
XP_012236501.1	TRADDN-NCOA6	2235	LOC100746843	hexapoda	Bombus impatiens	uncharacterized protein LOC100746843 isoform X3 [Bombus impatiens]	GCF_000188095.3	TRADDN-NCOA6
XP_012236502.1	TRADDN-NCOA6	2223	LOC100746843	hexapoda	Bombus impatiens	uncharacterized protein LOC100746843 isoform X5 [Bombus impatiens]	GCF_000188095.3	TRADDN-NCOA6
XP_012236503.1	TRADDN-NCOA6	2088	LOC100746843	hexapoda	Bombus impatiens	uncharacterized protein LOC100746843 isoform X7 [Bombus impatiens]	GCF_000188095.3	TRADDN-NCOA6
XP_024220777.1	TRADDN-NCOA6	2224	LOC100746843	hexapoda	Bombus impatiens	uncharacterized protein LOC100746843 isoform X4 [Bombus impatiens]	GCF_000188095.3	TRADDN-NCOA6
XP_024220778.1	TRADDN-NCOA6	2214	LOC100746843	hexapoda	Bombus impatiens	uncharacterized protein LOC100746843 isoform X6 [Bombus impatiens]	GCF_000188095.3	TRADDN-NCOA6
XP_003402722.1	TRADDN-NCOA6	2235	LOC100651147	hexapoda	Bombus terrestris	uncharacterized protein LOC100651147 isoform X2 [Bombus terrestris]	GCF_000214255.1	TRADDN-NCOA6
XP_020723554.1	TRADDN-NCOA6	2245	LOC100651147	hexapoda	Bombus terrestris	uncharacterized protein LOC100651147 isoform X1 [Bombus terrestris]	GCF_000214255.1	TRADDN-NCOA6
XP_020723557.1	TRADDN-NCOA6	2233	LOC100651147	hexapoda	Bombus terrestris	uncharacterized protein LOC100651147 isoform X3 [Bombus terrestris]	GCF_000214255.1	TRADDN-NCOA6
XP_020723558.1	TRADDN-NCOA6	2222	LOC100651147	hexapoda	Bombus terrestris	uncharacterized protein LOC100651147 isoform X4 [Bombus terrestris]	GCF_000214255.1	TRADDN-NCOA6
XP_020723559.1	TRADDN-NCOA6	2221	LOC100651147	hexapoda	Bombus terrestris	uncharacterized protein LOC100651147 isoform X5 [Bombus terrestris]	GCF_000214255.1	TRADDN-NCOA6
XP_020723560.1	TRADDN-NCOA6	2212	LOC100651147	hexapoda	Bombus terrestris	uncharacterized protein LOC100651147 isoform X6 [Bombus terrestris]	GCF_000214255.1	TRADDN-NCOA6
XP_020723561.1	TRADDN-NCOA6	2086	LOC100651147	hexapoda	Bombus terrestris	uncharacterized protein LOC100651147 isoform X7 [Bombus terrestris]	GCF_000214255.1	TRADDN-NCOA6
XP_021203661.1	TRADDN-NCOA6	1280	LOC101738211	hexapoda	Bombyx mori	nuclear receptor coactivator 6-like, partial [Bombyx mori]	GCF_000151625.1	TRADDN-NCOA6
EFN61718.1	TRADDN-NCOA6	2139	EAG_05855	hexapoda	Camponotus floridanus	Nuclear receptor coactivator 6 [Camponotus floridanus]	GCA_000147175.1	TRADDN-NCOA6
XP_011265954.1	TRADDN-NCOA6	2288	LOC105257194	hexapoda	Camponotus floridanus	uncharacterized protein LOC105257194 isoform X1 [Camponotus floridanus]	GCF_003227725.1	TRADDN-NCOA6
XP_011265957.1	TRADDN-NCOA6	2277	LOC105257194	hexapoda	Camponotus floridanus	uncharacterized protein LOC105257194 isoform X2 [Camponotus floridanus]	GCF_003227725.1	TRADDN-NCOA6
XP_015586594.1	TRADDN-NCOA6	2273	LOC107263660	hexapoda	Cephus cinctus	uncharacterized protein LOC107263660 isoform X1 [Cephus cinctus]	GCF_000341935.1	TRADDN-NCOA6
XP_015586595.1	TRADDN-NCOA6	2263	LOC107263660	hexapoda	Cephus cinctus	uncharacterized protein LOC107263660 isoform X2 [Cephus cinctus]	GCF_000341935.1	TRADDN-NCOA6

acc	architecture	len	gen.name	taxend	species	defline	gca	clade
XP_015586596.1	TRADDN-NCOA6	2249	LOC107263660	hexapoda	Cephus cinctus	uncharacterized protein LOC107263660 isoform X3 [Cephus cinctus]	GCF_000341935.1	TRADDN-NCOA6
XP_015586597.1	TRADDN-NCOA6	2225	LOC107263660	hexapoda	Cephus cinctus	uncharacterized protein LOC107263660 isoform X4 [Cephus cinctus]	GCF_000341935.1	TRADDN-NCOA6
XP_015586598.1	TRADDN-NCOA6	2158	LOC107263660	hexapoda	Cephus cinctus	uncharacterized protein LOC107263660 isoform X5 [Cephus cinctus]	GCF_000341935.1	TRADDN-NCOA6
XP_017890956.1	TRADDN-NCOA6	2245	LOC108631503	hexapoda	Ceratina calcarata	uncharacterized protein LOC108631503 isoform X1 [Ceratina calcarata]	GCF_001652005.1	TRADDN-NCOA6
XP_017890957.1	TRADDN-NCOA6	2231	LOC108631503	hexapoda	Ceratina calcarata	PREDICTED: uncharacterized protein LOC108631503 isoform X2 [Ceratina calcarata]	-	TRADDN-NCOA6
XP_012156850.1	TRADDN-NCOA6	2498	LOC101451441	hexapoda	Ceratitis capitata	uncharacterized protein LOC101451441 isoform X1 [Ceratitis capitata]	GCF_000347755.3	TRADDN-NCOA6
XP_012156851.1	TRADDN-NCOA6	2497	LOC101451441	hexapoda	Ceratitis capitata	uncharacterized protein LOC101451441 isoform X2 [Ceratitis capitata]	GCF_000347755.3	TRADDN-NCOA6
XP_011501182.1	TRADDN-NCOA6	2507	LOC105364851	hexapoda	Ceratosolen solmsi marchali	PREDICTED: uncharacterized protein LOC105364851 [Ceratosolen solmsi marchali]	GCF_000503995.1	TRADDN-NCOA6
XP_014241680.1	TRADDN-NCOA6	898	LOC106662255	hexapoda	Cimex lectularius	titin-like isoform X2 [Cimex lectularius]	GCF_000648675.2	TRADDN-NCOA6
XP_024081587.1	TRADDN-NCOA6	901	LOC106662255	hexapoda	Cimex lectularius	nuclear receptor coactivator 6-like isoform X1 [Cimex lectularius]	GCF_000648675.2	TRADDN-NCOA6
CRK89745.1	TRADDN-NCOA6	2026	putative,Nuclear,receptor,coactivator,6	hexapoda	Clunio marinus	CLUMA_CG003408, isoform A [Clunio marinus]	GCA_900005825.1	TRADDN-NCOA6
XP_023244951.1	TRADDN-NCOA6	2578	LOC106639766	hexapoda	Copidosoma floridanum	uncharacterized protein LOC106639766 [Copidosoma floridanum]	GCF_000648655.2	TRADDN-NCOA6
XP_023710716.1	TRADDN-NCOA6	2718	LOC111866191	hexapoda	Cryptotermes secundus	uncharacterized protein LOC111866191 isoform X1 [Cryptotermes secundus]	GCF_002891405.2	TRADDN-NCOA6
XP_023711129.1	TRADDN-NCOA6	2717	LOC111866191	hexapoda	Cryptotermes secundus	uncharacterized protein LOC111866191 isoform X2 [Cryptotermes secundus]	GCF_002891405.2	TRADDN-NCOA6
XP_001842144.1	TRADDN-NCOA6	2320	CpipJ_CPIJ000474	hexapoda	Culex quinquefasciatus	conserved hypothetical protein [Culex quinquefasciatus]	GCF_000209185.1	TRADDN-NCOA6
KYN07097.1	TRADDN-NCOA6	2253	ALC62_01905	hexapoda	Cyphomyrmex costatus	Nuclear receptor coactivator 6 [Cyphomyrmex costatus]	GCA_001594065.1	TRADDN-NCOA6
XP_018406241.1	TRADDN-NCOA6	2252	LOC108782459	hexapoda	Cyphomyrmex costatus	PREDICTED: LOW QUALITY PROTEIN: uncharacterized protein LOC108782459 [Cyphomyrmex costatus]	GCF_001594065.1	TRADDN-NCOA6
OWR54636.1	TRADDN-NCOA6	1648	KGM_216157	hexapoda	Danaus plexippus plexippus	hypothetical protein KGM_216157 [Danaus plexippus plexippus]	GCA_000235995.2	TRADDN-NCOA6
XP_019769268.1	TRADDN-NCOA6	1712	LOC109543823	hexapoda	Dendroctonus ponderosae	PREDICTED: uncharacterized protein LOC109543823 isoform X1 [Dendroctonus ponderosae]	GCF_000355655.1	TRADDN-NCOA6

acc	architecture	len	gen.name	taxend	species	defline	gca	clade
XP_019769270.1	TRADDN-NCOA6	1711	LOC109543823	hexapoda	Dendroctonus ponderosae	PREDICTED: uncharacterized protein LOC109543823 isoform X2 [Dendroctonus ponderosae]	GCF_000355655.1	TRADDN-NCOA6
XP_019769271.1	TRADDN-NCOA6	1708	LOC109543823	hexapoda	Dendroctonus ponderosae	PREDICTED: uncharacterized protein LOC109543823 isoform X3 [Dendroctonus ponderosae]	GCF_000355655.1	TRADDN-NCOA6
XP_019769272.1	TRADDN-NCOA6	1707	LOC109543823	hexapoda	Dendroctonus ponderosae	PREDICTED: uncharacterized protein LOC109543823 isoform X4 [Dendroctonus ponderosae]	GCF_000355655.1	TRADDN-NCOA6
XP_015124832.1	TRADDN-NCOA6	2126	LOC107046674	hexapoda	Diachasma alloeum	PREDICTED: uncharacterized protein LOC107046674 [Diachasma alloeum]	GCF_001412515.2	TRADDN-NCOA6
XP_017298161.1	TRADDN-NCOA6	1849	LOC103505989	hexapoda	Diaphorina citri	WASH complex subunit 2 [Diaphorina citri]	GCF_000475195.1	TRADDN-NCOA6
XP_014469461.1	TRADDN-NCOA6	2234	LOC106741710	hexapoda	Dinoponera quadriceps	PREDICTED: uncharacterized protein LOC106741710 isoform X1 [Dinoponera quadriceps]	GCF_001313825.1	TRADDN-NCOA6
XP_014469463.1	TRADDN-NCOA6	2220	LOC106741710	hexapoda	Dinoponera quadriceps	PREDICTED: uncharacterized protein LOC106741710 isoform X2 [Dinoponera quadriceps]	GCF_001313825.1	TRADDN-NCOA6
XP_015380052.1	TRADDN-NCOA6	1493	LOC107173851	hexapoda	Diuraphis noxia	PREDICTED: uncharacterized protein LOC107173851 [Diuraphis noxia]	GCF_001186385.1	TRADDN-NCOA6
XP_001962545.2	TRADDN-NCOA6	2295	dana_GLEANR_16282,GF15516	hexapoda	Drosophila ananassae	uncharacterized protein Dana_GF15516 [Drosophila ananassae]	GCF_000005115.1	TRADDN-NCOA6
XP_017859165.1	TRADDN-NCOA6	2412	LOC108611180	hexapoda	Drosophila arizonae	PREDICTED: putative mediator of RNA polymerase II transcription subunit 26 isoform X1 [Drosophila arizonae]	GCF_001654025.1	TRADDN-NCOA6
XP_017859166.1	TRADDN-NCOA6	2055	LOC108611180	hexapoda	Drosophila arizonae	PREDICTED: putative mediator of RNA polymerase II transcription subunit 26 isoform X2 [Drosophila arizonae]	GCF_001654025.1	TRADDN-NCOA6
XP_016956652.1	TRADDN-NCOA6	2289	LOC108029079	hexapoda	Drosophila biarmipes	PREDICTED: nuclear receptor coactivator 6 isoform X1 [Drosophila biarmipes]	GCF_000233415.1	TRADDN-NCOA6
XP_016956653.1	TRADDN-NCOA6	2288	LOC108029079	hexapoda	Drosophila biarmipes	PREDICTED: nuclear receptor coactivator 6 isoform X2 [Drosophila biarmipes]	GCF_000233415.1	TRADDN-NCOA6
XP_017099792.1	TRADDN-NCOA6	2227	LOC108127313	hexapoda	Drosophila bipectinata	PREDICTED: putative mediator of RNA polymerase II transcription subunit 26 [Drosophila bipectinata]	GCF_000236285.1	TRADDN-NCOA6
ALC38286.1	TRADDN-NCOA6	1954	Dbus_chr2Lg371	hexapoda	Drosophila busckii	Ncoa6 [Drosophila busckii]	GCA_001277935.1	TRADDN-NCOA6
XP_017854487.1	TRADDN-NCOA6	2278	LOC108607892	hexapoda	Drosophila busckii	PREDICTED: nuclear receptor coactivator 6 [Drosophila busckii]	GCF_001277935.1	TRADDN-NCOA6
XP_017132283.1	TRADDN-NCOA6	2341	LOC108149330	hexapoda	Drosophila elegans	PREDICTED: nuclear receptor coactivator 6 isoform X1 [Drosophila elegans]	GCF_000224195.1	TRADDN-NCOA6
XP_017132284.1	TRADDN-NCOA6	2337	LOC108149330	hexapoda	Drosophila elegans	PREDICTED: nuclear receptor coactivator 6 isoform X2 [Drosophila elegans]	GCF_000224195.1	TRADDN-NCOA6

acc	architecture	len	gen.name	taxend	species	defline	gca	clade
XP_017132286.1	TRADDN-NCOA6	2336	LOC108149330	hexapoda	Drosophila elegans	PREDICTED: nuclear receptor coactivator 6 isoform X3 [Drosophila elegans]	GCF_000224195.1	TRADDN-NCOA6
XP_001968791.2	TRADDN-NCOA6	2452	dere_GLEANR_9049,GG24309	hexapoda	Drosophila erecta	LOW QUALITY PROTEIN: uncharacterized protein Dere_GG24309 [Drosophila erecta]	-	TRADDN-NCOA6
XP_017063177.1	TRADDN-NCOA6	2239	LOC108102587	hexapoda	Drosophila eugracilis	PREDICTED: polyhomeotic-proximal chromatin protein isoform X1 [Drosophila eugracilis]	GCF_000236325.1	TRADDN-NCOA6
XP_017063178.1	TRADDN-NCOA6	2238	LOC108102587	hexapoda	Drosophila eugracilis	PREDICTED: polyhomeotic-proximal chromatin protein isoform X2 [Drosophila eugracilis]	GCF_000236325.1	TRADDN-NCOA6
XP_017043850.1	TRADDN-NCOA6	2318	LOC108089891	hexapoda	Drosophila ficusphila	PREDICTED: histone-lysine N-methyltransferase 2D isoform X1 [Drosophila ficusphila]	GCF_000220665.1	TRADDN-NCOA6
XP_017043851.1	TRADDN-NCOA6	2317	LOC108089891	hexapoda	Drosophila ficusphila	PREDICTED: histone-lysine N-methyltransferase 2D isoform X2 [Drosophila ficusphila]	GCF_000220665.1	TRADDN-NCOA6
XP_001992910.1	TRADDN-NCOA6	957	dgri_GLEANR_13444,GH13537	hexapoda	Drosophila grimshawi	GH13537 [Drosophila grimshawi]	GCF_000005155.2	TRADDN-NCOA6
XP_023177608.1	TRADDN-NCOA6	2385	LOC111603995	hexapoda	Drosophila hydei	putative mediator of RNA polymerase II transcription subunit 26 [Drosophila hydei]	-	TRADDN-NCOA6
XP_017032055.1	TRADDN-NCOA6	2266	LOC108081407	hexapoda	Drosophila kikkawai	PREDICTED: AF4/FMR2 family member 4 [Drosophila kikkawai]	GCF_000224215.1	TRADDN-NCOA6
NP_001097088.3	TRADDN-NCOA6	2467	CG14023,Dmel-CG14023,dNCOA6,NcoA6,NCOA6	hexapoda	Drosophila melanogaster	nuclear receptor coactivator 6, isoform F [Drosophila melanogaster]	-	TRADDN-NCOA6
NP_001245884.1	TRADDN-NCOA6	2266	CG14023,Dmel-CG14023,dNCOA6,NcoA6,NCOA6	hexapoda	Drosophila melanogaster	nuclear receptor coactivator 6, isoform D [Drosophila melanogaster]	-	TRADDN-NCOA6
NP_608921.3	TRADDN-NCOA6	2265	CG14023,Dmel-CG14023,dNCOA6,NcoA6,NCOA6	hexapoda	Drosophila melanogaster	nuclear receptor coactivator 6, isoform C [Drosophila melanogaster]	-	TRADDN-NCOA6
XP_017152980.1	TRADDN-NCOA6	2390	LOC108162657	hexapoda	Drosophila miranda	PREDICTED: nuclear receptor coactivator 6-like [Drosophila miranda]	GCF_000269505.1	TRADDN-NCOA6
XP_002002892.2	TRADDN-NCOA6	2370	dmoj_GLEANR_1047,GI10553	hexapoda	Drosophila mojavensis	uncharacterized protein Dmoj_GI10553 [Drosophila mojavensis]	GCF_000005175.2	TRADDN-NCOA6
XP_017954029.1	TRADDN-NCOA6	1557	LOC108649574	hexapoda	Drosophila navojoa	PREDICTED: putative mediator of RNA polymerase II transcription subunit 26 isoform X1 [Drosophila navojoa]	-	TRADDN-NCOA6
XP_017954030.1	TRADDN-NCOA6	1400	LOC108649574	hexapoda	Drosophila navojoa	PREDICTED: putative mediator of RNA polymerase II transcription subunit 26 isoform X2 [Drosophila navojoa]	-	TRADDN-NCOA6
XP_022220919.1	TRADDN-NCOA6	2425	LOC111073094	hexapoda	Drosophila obscura	AF4/FMR2 family member 4 [Drosophila obscura]	GCF_002217835.1	TRADDN-NCOA6
XP_002015177.1	TRADDN-NCOA6	2373	dper_GLEANR_20704,GL18557	hexapoda	Drosophila persimilis	GL18557 [Drosophila persimilis]	-	TRADDN-NCOA6

acc	architecture	len	gen.name	taxend	species	defline	gca	clade
XP_002132783.2	TRADDN-NCOA6	2618	dpse_GLEANR_1839,GA25686	hexapoda	Drosophila pseudoobscura pseudoobscura	LOW QUALITY PROTEIN: uncharacterized protein Dpse_GA25686 [Drosophila pseudoobscura pseudoobscura]	GCF_000001765.3	TRADDN-NCOA6
XP_016968921.1	TRADDN-NCOA6	2276	LOC108036999	hexapoda	Drosophila rhopaloa	PREDICTED: nuclear receptor coactivator 6 isoform X1 [Drosophila rhopaloa]	GCF_000236305.1	TRADDN-NCOA6
XP_016968922.1	TRADDN-NCOA6	2275	LOC108036999	hexapoda	Drosophila rhopaloa	PREDICTED: nuclear receptor coactivator 6 isoform X2 [Drosophila rhopaloa]	GCF_000236305.1	TRADDN-NCOA6
XP_002037942.1	TRADDN-NCOA6	563	dsec_GLEANR_19530,GM18027	hexapoda	Drosophila sechellia	GM18027 [Drosophila sechellia]	GCF_000005215.3	TRADDN-NCOA6
XP_020800132.1	TRADDN-NCOA6	1353	LOC110177660	hexapoda	Drosophila serrata	LOW QUALITY PROTEIN: histone-lysine N-methyltransferase 2D-like, partial [Drosophila serrata]	GCF_002093755.1	TRADDN-NCOA6
XP_020812428.1	TRADDN-NCOA6	2494	LOC110187381	hexapoda	Drosophila serrata	LOW QUALITY PROTEIN: uncharacterized protein LOC110187381 [Drosophila serrata]	GCF_002093755.1	TRADDN-NCOA6
XP_016938638.1	TRADDN-NCOA6	2282	LOC108016483	hexapoda	Drosophila suzukii	PREDICTED: histone-lysine N-methyltransferase 2D isoform X1 [Drosophila suzukii]	GCF_000472105.1	TRADDN-NCOA6
XP_016938646.1	TRADDN-NCOA6	2281	LOC108016483	hexapoda	Drosophila suzukii	PREDICTED: histone-lysine N-methyltransferase 2D isoform X2 [Drosophila suzukii]	GCF_000472105.1	TRADDN-NCOA6
XP_017006613.1	TRADDN-NCOA6	2182	LOC108063861	hexapoda	Drosophila takahashii	PREDICTED: nuclear receptor coactivator 6 isoform X1 [Drosophila takahashii]	GCF_000224235.1	TRADDN-NCOA6
XP_017006614.1	TRADDN-NCOA6	2181	LOC108063861	hexapoda	Drosophila takahashii	PREDICTED: nuclear receptor coactivator 6 isoform X2 [Drosophila takahashii]	GCF_000224235.1	TRADDN-NCOA6
XP_002052423.2	TRADDN-NCOA6	2306	dvir_GLEANR_710,GJ21770	hexapoda	Drosophila virilis	uncharacterized protein Dvir_GJ21770 [Drosophila virilis]	GCF_000005245.1	TRADDN-NCOA6
EDW78155.2	TRADDN-NCOA6	2157	dwil_GLEANR_8984	hexapoda	Drosophila willistoni	LOW QUALITY PROTEIN: uncharacterized protein Dwil_GK24846 [Drosophila willistoni]	GCA_000005925.1	TRADDN-NCOA6
XP_023032478.1	TRADDN-NCOA6	2307	LOC6644292	hexapoda	Drosophila willistoni	putative mediator of RNA polymerase II transcription subunit 26 [Drosophila willistoni]	GCF_000005925.1	TRADDN-NCOA6
XP_002089227.2	TRADDN-NCOA6	2438	dyak_GLEANR_2784,GE19004	hexapoda	Drosophila yakuba	LOW QUALITY PROTEIN: uncharacterized protein Dyak_GE19004 [Drosophila yakuba]	GCF_000005975.2	TRADDN-NCOA6
KZC12186.1	TRADDN-NCOA6	2209	WN55_03914	hexapoda	Dufourea novaeangliae	Nuclear receptor coactivator 6 [Dufourea novaeangliae]	GCA_001272555.1	TRADDN-NCOA6
XP_015434572.1	TRADDN-NCOA6	2226	LOC107190297	hexapoda	Dufourea novaeangliae	PREDICTED: uncharacterized protein LOC107190297 isoform X1 [Dufourea novaeangliae]	GCF_001272555.1	TRADDN-NCOA6
XP_015434574.1	TRADDN-NCOA6	2203	LOC107190297	hexapoda	Dufourea novaeangliae	PREDICTED: uncharacterized protein LOC107190297 isoform X2 [Dufourea novaeangliae]	GCF_001272555.1	TRADDN-NCOA6
XP_015434575.1	TRADDN-NCOA6	2189	LOC107190297	hexapoda	Dufourea novaeangliae	PREDICTED: uncharacterized protein LOC107190297 isoform X3 [Dufourea novaeangliae]	GCF_001272555.1	TRADDN-NCOA6

acc	architecture	len	gen.name	taxend	species	defline	gca	clade
OAD53702.1	TRADDN-NCOA6	2211	WN48_09443	hexapoda	Eufriesea mexicana	Nuclear receptor coactivator 6 [Eufriesea mexicana]	GCA_001483705.1	TRADDN-NCOA6
XP_017762430.1	TRADDN-NCOA6	2249	LOC108552415	hexapoda	Eufriesea mexicana	PREDICTED: uncharacterized protein LOC108552415 isoform X1 [Eufriesea mexicana]	GCF_001483705.1	TRADDN-NCOA6
XP_017762431.1	TRADDN-NCOA6	2239	LOC108552415	hexapoda	Eufriesea mexicana	PREDICTED: uncharacterized protein LOC108552415 isoform X2 [Eufriesea mexicana]	GCF_001483705.1	TRADDN-NCOA6
XP_017762432.1	TRADDN-NCOA6	2225	LOC108552415	hexapoda	Eufriesea mexicana	PREDICTED: uncharacterized protein LOC108552415 isoform X3 [Eufriesea mexicana]	GCF_001483705.1	TRADDN-NCOA6
OXA65226.1	TRADDN-NCOA6	3667	Fcan01_02940	hexapoda	Folsomia candida	Nuclear receptor coactivator 6 [Folsomia candida]	GCA_002217175.1	TRADDN-NCOA6
XP_021945657.1	TRADDN-NCOA6	586	LOC110843905	hexapoda	Folsomia candida	uncharacterized protein DDB_G0271670-like [Folsomia candida]	GCF_002217175.1	TRADDN-NCOA6
XP_011296707.1	TRADDN-NCOA6	2101	LOC105262692	hexapoda	Fopius arisanus	PREDICTED: uncharacterized protein LOC105262692 isoform X1 [Fopius arisanus]	GCF_000806365.1	TRADDN-NCOA6
XP_011296708.1	TRADDN-NCOA6	2100	LOC105262692	hexapoda	Fopius arisanus	PREDICTED: uncharacterized protein LOC105262692 isoform X2 [Fopius arisanus]	GCF_000806365.1	TRADDN-NCOA6
XP_011296709.1	TRADDN-NCOA6	2083	LOC105262692	hexapoda	Fopius arisanus	PREDICTED: uncharacterized protein LOC105262692 isoform X3 [Fopius arisanus]	GCF_000806365.1	TRADDN-NCOA6
XP_011296710.1	TRADDN-NCOA6	1949	LOC105262692	hexapoda	Fopius arisanus	PREDICTED: uncharacterized protein LOC105262692 isoform X4 [Fopius arisanus]	GCF_000806365.1	TRADDN-NCOA6
XP_017793211.1	TRADDN-NCOA6	2241	LOC108575026	hexapoda	Habropoda laboriosa	PREDICTED: putative mediator of RNA polymerase II transcription subunit 26 [Habropoda laboriosa]	GCF_001263275.1	TRADDN-NCOA6
XP_014294306.1	TRADDN-NCOA6	811	LOC106692715	hexapoda	Halyomorpha halys	bromodomain-containing protein 4-like isoform X2 [Halyomorpha halys]	GCF_000696795.2	TRADDN-NCOA6
XP_014294307.1	TRADDN-NCOA6	806	LOC106692715	hexapoda	Halyomorpha halys	uncharacterized protein KIAA1211-like isoform X3 [Halyomorpha halys]	GCF_000696795.2	TRADDN-NCOA6
XP_024219354.1	TRADDN-NCOA6	812	LOC106692715	hexapoda	Halyomorpha halys	uncharacterized protein KIAA1211-like isoform X1 [Halyomorpha halys]	GCF_000696795.2	TRADDN-NCOA6
XP_024219357.1	TRADDN-NCOA6	769	LOC106692715	hexapoda	Halyomorpha halys	bromodomain-containing protein 4-like isoform X4 [Halyomorpha halys]	GCF_000696795.2	TRADDN-NCOA6
EFN81453.1	TRADDN-NCOA6	2218	EAI_16623	hexapoda	Harpegnathos saltator	Nuclear receptor coactivator 6 [Harpegnathos saltator]	GCA_000147195.1	TRADDN-NCOA6
XP_011144197.2	TRADDN-NCOA6	2234	LOC105185988	hexapoda	Harpegnathos saltator	putative mediator of RNA polymerase II transcription subunit 26 isoform X1 [Harpegnathos saltator]	GCF_003227715.1	TRADDN-NCOA6

acc	architecture	len	gen.name	taxend	species	defline	gca	clade
XP_011144200.2	TRADDN-NCOA6	2098	LOC105185988	hexapoda	Harpegnathos saltator	putative mediator of RNA polymerase II transcription subunit 26 isoform X6 [Harpegnathos saltator]	GCF_003227715.1	TRADDN-NCOA6
XP_011144201.2	TRADDN-NCOA6	2098	LOC105185988	hexapoda	Harpegnathos saltator	putative mediator of RNA polymerase II transcription subunit 26 isoform X7 [Harpegnathos saltator]	GCF_003227715.1	TRADDN-NCOA6
XP_019698285.2	TRADDN-NCOA6	2232	LOC105185988	hexapoda	Harpegnathos saltator	putative mediator of RNA polymerase II transcription subunit 26 isoform X2 [Harpegnathos saltator]	GCF_003227715.1	TRADDN-NCOA6
XP_019698286.2	TRADDN-NCOA6	2220	LOC105185988	hexapoda	Harpegnathos saltator	putative mediator of RNA polymerase II transcription subunit 26 isoform X3 [Harpegnathos saltator]	GCF_003227715.1	TRADDN-NCOA6
XP_019698287.2	TRADDN-NCOA6	2096	LOC105185988	hexapoda	Harpegnathos saltator	putative mediator of RNA polymerase II transcription subunit 26 isoform X8 [Harpegnathos saltator]	GCF_003227715.1	TRADDN-NCOA6
XP_025160035.1	TRADDN-NCOA6	2186	LOC105185988	hexapoda	Harpegnathos saltator	putative mediator of RNA polymerase II transcription subunit 26 isoform X4 [Harpegnathos saltator]	GCF_003227715.1	TRADDN-NCOA6
XP_025160036.1	TRADDN-NCOA6	2140	LOC105185988	hexapoda	Harpegnathos saltator	putative mediator of RNA polymerase II transcription subunit 26 isoform X5 [Harpegnathos saltator]	GCF_003227715.1	TRADDN-NCOA6
XP_025160037.1	TRADDN-NCOA6	2059	LOC105185988	hexapoda	Harpegnathos saltator	putative mediator of RNA polymerase II transcription subunit 26 isoform X9 [Harpegnathos saltator]	GCF_003227715.1	TRADDN-NCOA6
XP_025160039.1	TRADDN-NCOA6	2050	LOC105185988	hexapoda	Harpegnathos saltator	putative mediator of RNA polymerase II transcription subunit 26 isoform X10 [Harpegnathos saltator]	GCF_003227715.1	TRADDN-NCOA6
PZC73250.1	TRADDN-NCOA6	1275	HaOG209747	hexapoda	Helicoverpa armigera	hypothetical protein B5X24_HaOG209747 [Helicoverpa armigera]	GCA_002156985.1	TRADDN-NCOA6
XP_021196522.1	TRADDN-NCOA6	1292	LOC110380753	hexapoda	Helicoverpa armigera	lysine-specific demethylase 6B-like [Helicoverpa armigera]	GCF_002156985.1	TRADDN-NCOA6
PCG69216.1	TRADDN-NCOA6	1428	B5V51_4374	hexapoda	Heliothis virescens	hypothetical protein B5V51_4374 [Heliothis virescens]	GCA_002382865.1	TRADDN-NCOA6
XP_023022070.1	TRADDN-NCOA6	1483	LOC111510398	hexapoda	Leptinotarsa decemlineata	uncharacterized protein LOC111510398 [Leptinotarsa decemlineata]	GCF_000500325.1	TRADDN-NCOA6
XP_012218728.1	TRADDN-NCOA6	2335	LOC105670024	hexapoda	Linepithema humile	PREDICTED: uncharacterized protein LOC105670024 isoform X1 [Linepithema humile]	GCF_000217595.1	TRADDN-NCOA6
XP_012218729.1	TRADDN-NCOA6	2333	LOC105670024	hexapoda	Linepithema humile	PREDICTED: uncharacterized protein LOC105670024 isoform X2 [Linepithema humile]	GCF_000217595.1	TRADDN-NCOA6

acc	architecture	len	gen.name	taxend	species	defline	gca	clade
XP_012218730.1	TRADDN-NCOA6	2333	LOC105670024	hexapoda	Linepithema humile	PREDICTED: uncharacterized protein LOC105670024 isoform X3 [Linepithema humile]	GCF_000217595.1	TRADDN-NCOA6
XP_012218731.1	TRADDN-NCOA6	2287	LOC105670024	hexapoda	Linepithema humile	PREDICTED: uncharacterized protein LOC105670024 isoform X4 [Linepithema humile]	GCF_000217595.1	TRADDN-NCOA6
XP_012218732.1	TRADDN-NCOA6	2081	LOC105670024	hexapoda	Linepithema humile	PREDICTED: uncharacterized protein LOC105670024 isoform X5 [Linepithema humile]	GCF_000217595.1	TRADDN-NCOA6
KNC28988.1	TRADDN-NCOA6	3078	FF38_00661	hexapoda	Lucilia cuprina	hypothetical protein FF38_00661 [Lucilia cuprina]	GCA_001187945.1	TRADDN-NCOA6
XP_023298934.1	TRADDN-NCOA6	2719	LOC111681399	hexapoda	Lucilia cuprina	uncharacterized protein LOC111681399 isoform X1 [Lucilia cuprina]	GCF_000699065.1	TRADDN-NCOA6
XP_023298935.1	TRADDN-NCOA6	2718	LOC111681399	hexapoda	Lucilia cuprina	uncharacterized protein LOC111681399 isoform X2 [Lucilia cuprina]	GCF_000699065.1	TRADDN-NCOA6
XP_012146297.1	TRADDN-NCOA6	2229	LOC100875083	hexapoda	Megachile rotundata	PREDICTED: uncharacterized protein LOC100875083 isoform X3 [Megachile rotundata]	GCF_000220905.1	TRADDN-NCOA6
XP_012146298.1	TRADDN-NCOA6	2227	LOC100875083	hexapoda	Megachile rotundata	PREDICTED: uncharacterized protein LOC100875083 isoform X4 [Megachile rotundata]	GCF_000220905.1	TRADDN-NCOA6
XP_012146299.1	TRADDN-NCOA6	2218	LOC100875083	hexapoda	Megachile rotundata	PREDICTED: uncharacterized protein LOC100875083 isoform X5 [Megachile rotundata]	GCF_000220905.1	TRADDN-NCOA6
XP_012146300.1	TRADDN-NCOA6	2215	LOC100875083	hexapoda	Megachile rotundata	PREDICTED: uncharacterized protein LOC100875083 isoform X6 [Megachile rotundata]	GCF_000220905.1	TRADDN-NCOA6
XP_012146301.1	TRADDN-NCOA6	2206	LOC100875083	hexapoda	Megachile rotundata	PREDICTED: uncharacterized protein LOC100875083 isoform X7 [Megachile rotundata]	GCF_000220905.1	TRADDN-NCOA6
XP_012146302.1	TRADDN-NCOA6	2074	LOC100875083	hexapoda	Megachile rotundata	PREDICTED: uncharacterized protein LOC100875083 isoform X8 [Megachile rotundata]	GCF_000220905.1	TRADDN-NCOA6
XP_025201013.1	TRADDN-NCOA6	1507	LOC112598690	hexapoda	Melanaphis sacchari	uncharacterized protein LOC112598690 isoform X1 [Melanaphis sacchari]	GCF_002803265.2	TRADDN-NCOA6
XP_025201014.1	TRADDN-NCOA6	1505	LOC112598690	hexapoda	Melanaphis sacchari	uncharacterized protein LOC112598690 isoform X2 [Melanaphis sacchari]	GCF_002803265.2	TRADDN-NCOA6
XP_025201015.1	TRADDN-NCOA6	1471	LOC112598690	hexapoda	Melanaphis sacchari	uncharacterized protein LOC112598690 isoform X3 [Melanaphis sacchari]	GCF_002803265.2	TRADDN-NCOA6
KOX76309.1	TRADDN-NCOA6	2210	WN51_11640	hexapoda	Melipona quadrifasciata	Nuclear receptor coactivator 6 [Melipona quadrifasciata]	GCA_001276565.1	TRADDN-NCOA6
XP_008546152.1	TRADDN-NCOA6	2440	LOC103570246	hexapoda	Microplitis demolitor	PREDICTED: serine-rich adhesin for platelets [Microplitis demolitor]	GCF_000572035.2	TRADDN-NCOA6
XP_012540715.1	TRADDN-NCOA6	2324	LOC105839161	hexapoda	Monomorium pharaonis	PREDICTED: putative mediator of RNA polymerase II transcription subunit 26 isoform X1 [Monomorium pharaonis]	GCF_003260585.2	TRADDN-NCOA6

acc	architecture	len	gen.name	taxend	species	defline	gca	clade
XP_012540716.1	TRADDN-NCOA6	2313	LOC105839161	hexapoda	Monomorium pharaonis	PREDICTED: putative mediator of RNA polymerase II transcription subunit 26 isoform X2 [Monomorium pharaonis]	GCF_003260585.2	TRADDN-NCOA6
XP_012540717.1	TRADDN-NCOA6	2276	LOC105839161	hexapoda	Monomorium pharaonis	PREDICTED: bromodomain-containing protein DDB_G0270170 isoform X3 [Monomorium pharaonis]	GCF_003260585.2	TRADDN-NCOA6
XP_012540718.1	TRADDN-NCOA6	2117	LOC105839161	hexapoda	Monomorium pharaonis	PREDICTED: putative mediator of RNA polymerase II transcription subunit 26 isoform X4 [Monomorium pharaonis]	GCF_003260585.2	TRADDN-NCOA6
XP_012540720.1	TRADDN-NCOA6	2089	LOC105839161	hexapoda	Monomorium pharaonis	PREDICTED: putative mediator of RNA polymerase II transcription subunit 26 isoform X5 [Monomorium pharaonis]	-	TRADDN-NCOA6
XP_012540721.1	TRADDN-NCOA6	2078	LOC105839161	hexapoda	Monomorium pharaonis	PREDICTED: putative mediator of RNA polymerase II transcription subunit 26 isoform X6 [Monomorium pharaonis]	GCF_003260585.2	TRADDN-NCOA6
XP_011290982.1	TRADDN-NCOA6	2921	LOC101901121	hexapoda	Musca domestica	PREDICTED: uncharacterized protein LOC101901121 isoform X1 [Musca domestica]	GCF_000371365.1	TRADDN-NCOA6
XP_011290985.1	TRADDN-NCOA6	2919	LOC101901121	hexapoda	Musca domestica	PREDICTED: uncharacterized protein LOC101901121 isoform X2 [Musca domestica]	GCF_000371365.1	TRADDN-NCOA6
XP_019891079.1	TRADDN-NCOA6	2918	LOC101901121	hexapoda	Musca domestica	PREDICTED: uncharacterized protein LOC101901121 isoform X3 [Musca domestica]	GCF_000371365.1	TRADDN-NCOA6
XP_022172572.1	TRADDN-NCOA6	1508	LOC111035313	hexapoda	Myzus persicae	uncharacterized protein LOC111035313 isoform X1 [Myzus persicae]	GCF_001856785.1	TRADDN-NCOA6
XP_022172573.1	TRADDN-NCOA6	1506	LOC111035313	hexapoda	Myzus persicae	uncharacterized protein LOC111035313 isoform X2 [Myzus persicae]	GCF_001856785.1	TRADDN-NCOA6
XP_022172574.1	TRADDN-NCOA6	1472	LOC111035313	hexapoda	Myzus persicae	uncharacterized protein LOC111035313 isoform X3 [Myzus persicae]	GCF_001856785.1	TRADDN-NCOA6
XP_008204160.2	TRADDN-NCOA6	2524	LOC100679897	hexapoda	Nasonia vitripennis	PREDICTED: LOW QUALITY PROTEIN: putative mediator of RNA polymerase II transcription subunit 26 [Nasonia vitripennis]	GCF_000002325.3	TRADDN-NCOA6
XP_015524592.1	TRADDN-NCOA6	2227	LOC107227844	hexapoda	Neodiprion lecontei	PREDICTED: uncharacterized protein LOC107227844 [Neodiprion lecontei]	GCF_001263575.1	TRADDN-NCOA6
XP_017771567.1	TRADDN-NCOA6	1402	LOC108558972	hexapoda	Nicrophorus vespilloides	PREDICTED: serine-rich adhesin for platelets [Nicrophorus vespilloides]	GCF_001412225.1	TRADDN-NCOA6
XP_022199641.1	TRADDN-NCOA6	2166	LOC111056571	hexapoda	Nilaparvata lugens	uncharacterized protein LOC111056571 [Nilaparvata lugens]	GCF_000757685.1	TRADDN-NCOA6

acc	architecture	len	gen.name	taxend	species	defline	gca	clade
XP_022904603.1	TRADDN-NCOA6	1207	LOC111416741	hexapoda	Onthophagus taurus	uncharacterized protein LOC111416741 [Onthophagus taurus]	GCF_000648695.1	TRADDN-NCOA6
XP_011335995.1	TRADDN-NCOA6	2363	LOC105278539	hexapoda	Ooceraea biroi	PREDICTED: uncharacterized protein LOC105278539 isoform X1 [Ooceraea biroi]	-	TRADDN-NCOA6
XP_011335997.1	TRADDN-NCOA6	2315	LOC105278539	hexapoda	Ooceraea biroi	PREDICTED: uncharacterized protein LOC105278539 isoform X2 [Ooceraea biroi]	-	TRADDN-NCOA6
XP_011335998.1	TRADDN-NCOA6	2148	LOC105278539	hexapoda	Ooceraea biroi	PREDICTED: uncharacterized protein LOC105278539 isoform X4 [Ooceraea biroi]	-	TRADDN-NCOA6
XP_011335999.1	TRADDN-NCOA6	2100	LOC105278539	hexapoda	Ooceraea biroi	PREDICTED: uncharacterized protein LOC105278539 isoform X6 [Ooceraea biroi]	-	TRADDN-NCOA6
XP_019886915.1	TRADDN-NCOA6	2170	LOC105278539	hexapoda	Ooceraea biroi	PREDICTED: uncharacterized protein LOC105278539 isoform X3 [Ooceraea biroi]	-	TRADDN-NCOA6
XP_019886916.1	TRADDN-NCOA6	2139	LOC105278539	hexapoda	Ooceraea biroi	PREDICTED: uncharacterized protein LOC105278539 isoform X5 [Ooceraea biroi]	-	TRADDN-NCOA6
KOB68708.1	TRADDN-NCOA6	135	OBRU01_17957	hexapoda	Operophtera brumata	Nuclear receptor coactivator 6 [Operophtera brumata]	GCA_001266575.1	TRADDN-NCOA6
XP_012272747.1	TRADDN-NCOA6	2131	LOC105695604	hexapoda	Orussus abietinus	uncharacterized protein LOC105695604 isoform X1 [Orussus abietinus]	GCF_000612105.2	TRADDN-NCOA6
XP_012272756.1	TRADDN-NCOA6	2128	LOC105695604	hexapoda	Orussus abietinus	uncharacterized protein LOC105695604 isoform X2 [Orussus abietinus]	GCF_000612105.2	TRADDN-NCOA6
XP_012272776.1	TRADDN-NCOA6	2121	LOC105695604	hexapoda	Orussus abietinus	uncharacterized protein LOC105695604 isoform X3 [Orussus abietinus]	GCF_000612105.2	TRADDN-NCOA6
XP_012272784.1	TRADDN-NCOA6	2083	LOC105695604	hexapoda	Orussus abietinus	uncharacterized protein LOC105695604 isoform X4 [Orussus abietinus]	GCF_000612105.2	TRADDN-NCOA6
KRT82121.1	TRADDN-NCOA6	1458	AMK59_3368	hexapoda	Oryctes borbonicus	hypothetical protein AMK59_3368 [Oryctes borbonicus]	GCA_001443705.1	TRADDN-NCOA6
KPJ07754.1	TRADDN-NCOA6	1730	RR48_11310	hexapoda	Papilio machaon	Nuclear receptor coactivator 6 [Papilio machaon]	GCA_001298355.1	TRADDN-NCOA6
XP_014368318.1	TRADDN-NCOA6	1744	LOC106718680	hexapoda	Papilio machaon	PREDICTED: uncharacterized protein LOC106718680 [Papilio machaon]	GCF_001298355.1	TRADDN-NCOA6
XP_013137855.1	TRADDN-NCOA6	1388	LOC106102816	hexapoda	Papilio polytes	PREDICTED: uncharacterized protein LOC106102816 [Papilio polytes]	GCF_000836215.1	TRADDN-NCOA6
KPJ04213.1	TRADDN-NCOA6	1637	RR46_07972	hexapoda	Papilio xuthus	Nuclear receptor coactivator 6 [Papilio xuthus]	GCA_001298345.1	TRADDN-NCOA6
XP_013180482.1	TRADDN-NCOA6	1741	LOC106127046	hexapoda	Papilio xuthus	PREDICTED: protein piccolo [Papilio xuthus]	GCF_000836235.1	TRADDN-NCOA6
XP_002431865.1	TRADDN-NCOA6	1870	Phum_PHUM551970	hexapoda	Pediculus humanus corporis	conserved hypothetical protein [Pediculus humanus corporis]	GCF_000006295.1	TRADDN-NCOA6

acc	architecture	len	gen.name	taxend	species	defline	gca	clade
XP_022120368.1	TRADDN-NCOA6	1581	LOC110996820	hexapoda	Pieris rapae	protein piccolo-like isoform X1 [Pieris rapae]	GCF_001856805.1	TRADDN-NCOA6
XP_022120369.1	TRADDN-NCOA6	1579	LOC110996820	hexapoda	Pieris rapae	protein piccolo-like isoform X2 [Pieris rapae]	GCF_001856805.1	TRADDN-NCOA6
XP_011560880.1	TRADDN-NCOA6	920	LOC105391148	hexapoda	Plutella xylostella	PREDICTED: serine/arginine repetitive matrix protein 1-like, partial [Plutella xylostella]	GCF_000330985.1	TRADDN-NCOA6
XP_011645748.1	TRADDN-NCOA6	2321	LOC105432581	hexapoda	Pogonomyrmex barbatus	uncharacterized protein LOC105432581 isoform X1 [Pogonomyrmex barbatus]	GCF_000187915.1	TRADDN-NCOA6
XP_011645750.1	TRADDN-NCOA6	2089	LOC105432581	hexapoda	Pogonomyrmex barbatus	uncharacterized protein LOC105432581 isoform X2 [Pogonomyrmex barbatus]	GCF_000187915.1	TRADDN-NCOA6
XP_014604298.1	TRADDN-NCOA6	2256	LOC106786918	hexapoda	Polistes canadensis	PREDICTED: uncharacterized protein LOC106786918 isoform X1 [Polistes canadensis]	GCF_001313835.1	TRADDN-NCOA6
XP_014604299.1	TRADDN-NCOA6	2242	LOC106786918	hexapoda	Polistes canadensis	PREDICTED: uncharacterized protein LOC106786918 isoform X2 [Polistes canadensis]	GCF_001313835.1	TRADDN-NCOA6
XP_014604300.1	TRADDN-NCOA6	2233	LOC106786918	hexapoda	Polistes canadensis	PREDICTED: uncharacterized protein LOC106786918 isoform X3 [Polistes canadensis]	GCF_001313835.1	TRADDN-NCOA6
XP_014604301.1	TRADDN-NCOA6	2214	LOC106786918	hexapoda	Polistes canadensis	PREDICTED: uncharacterized protein LOC106786918 isoform X4 [Polistes canadensis]	GCF_001313835.1	TRADDN-NCOA6
XP_015186754.1	TRADDN-NCOA6	2278	LOC107071869	hexapoda	Polistes dominula	PREDICTED: putative mediator of RNA polymerase II transcription subunit 26 isoform X1 [Polistes dominula]	GCF_001465965.1	TRADDN-NCOA6
XP_015186755.1	TRADDN-NCOA6	2264	LOC107071869	hexapoda	Polistes dominula	PREDICTED: putative mediator of RNA polymerase II transcription subunit 26 isoform X2 [Polistes dominula]	GCF_001465965.1	TRADDN-NCOA6
XP_015186756.1	TRADDN-NCOA6	2255	LOC107071869	hexapoda	Polistes dominula	PREDICTED: putative mediator of RNA polymerase II transcription subunit 26 isoform X3 [Polistes dominula]	GCF_001465965.1	TRADDN-NCOA6
XP_015186757.1	TRADDN-NCOA6	2236	LOC107071869	hexapoda	Polistes dominula	PREDICTED: putative mediator of RNA polymerase II transcription subunit 26 isoform X4 [Polistes dominula]	GCF_001465965.1	TRADDN-NCOA6
XP_020295483.1	TRADDN-NCOA6	2301	LOC109860653	hexapoda	Pseudomyrmex gracilis	uncharacterized protein LOC109860653 isoform X1 [Pseudomyrmex gracilis]	GCF_002006095.1	TRADDN-NCOA6
XP_020295484.1	TRADDN-NCOA6	2284	LOC109860653	hexapoda	Pseudomyrmex gracilis	uncharacterized protein LOC109860653 isoform X2 [Pseudomyrmex gracilis]	GCF_002006095.1	TRADDN-NCOA6
XP_017467919.1	TRADDN-NCOA6	2337	LOC108360220	hexapoda	Rhagoletis zephyria	PREDICTED: uncharacterized protein LOC108360220 isoform X1 [Rhagoletis zephyria]	GCF_001687245.1	TRADDN-NCOA6

acc	architecture	len	gen.name	taxend	species	defline	gca	clade
XP_017467920.1	TRADDN-NCOA6	2336	LOC108360220	hexapoda	Rhagoletis zephyria	PREDICTED: uncharacterized protein LOC108360220 isoform X2 [Rhagoletis zephyria]	GCF_001687245.1	TRADDN-NCOA6
XP_017467921.1	TRADDN-NCOA6	2298	LOC108360220	hexapoda	Rhagoletis zephyria	PREDICTED: uncharacterized protein LOC108360220 isoform X3 [Rhagoletis zephyria]	GCF_001687245.1	TRADDN-NCOA6
XP_025407016.1	TRADDN-NCOA6	1520	LOC112680973	hexapoda	Sipha flava	uncharacterized protein LOC112680973 isoform X1 [Sipha flava]	GCF_003268045.1	TRADDN-NCOA6
XP_025407019.1	TRADDN-NCOA6	1507	LOC112680973	hexapoda	Sipha flava	uncharacterized protein LOC112680973 isoform X2 [Sipha flava]	GCF_003268045.1	TRADDN-NCOA6
XP_025407020.1	TRADDN-NCOA6	1484	LOC112680973	hexapoda	Sipha flava	uncharacterized protein LOC112680973 isoform X3 [Sipha flava]	GCF_003268045.1	TRADDN-NCOA6
EFZ13556.1	TRADDN-NCOA6	2103	SINV_09701	hexapoda	Solenopsis invicta	hypothetical protein SINV_09701, partial [Solenopsis invicta]	-	TRADDN-NCOA6
XP_011166780.1	TRADDN-NCOA6	2250	LOC105200769	hexapoda	Solenopsis invicta	PREDICTED: putative mediator of RNA polymerase II transcription subunit 26 [Solenopsis invicta]	-	TRADDN-NCOA6
XP_022818643.1	TRADDN-NCOA6	1402	LOC111351105	hexapoda	Spodoptera litura	uncharacterized protein LOC111351105, partial [Spodoptera litura]	GCF_002706865.1	TRADDN-NCOA6
XP_013107027.1	TRADDN-NCOA6	2568	LOC106086772	hexapoda	Stomoxys calcitrans	PREDICTED: putative mediator of RNA polymerase II transcription subunit 26 [Stomoxys calcitrans]	GCF_001015335.1	TRADDN-NCOA6
XP_024876013.1	TRADDN-NCOA6	2302	LOC112457301	hexapoda	Temnothorax curvispinosus	uncharacterized protein LOC112457301 isoform X1 [Temnothorax curvispinosus]	GCF_003070985.1	TRADDN-NCOA6
XP_024876014.1	TRADDN-NCOA6	2300	LOC112457301	hexapoda	Temnothorax curvispinosus	uncharacterized protein LOC112457301 isoform X2 [Temnothorax curvispinosus]	GCF_003070985.1	TRADDN-NCOA6
XP_024876016.1	TRADDN-NCOA6	2291	LOC112457301	hexapoda	Temnothorax curvispinosus	uncharacterized protein LOC112457301 isoform X3 [Temnothorax curvispinosus]	GCF_003070985.1	TRADDN-NCOA6
XP_024876017.1	TRADDN-NCOA6	2254	LOC112457301	hexapoda	Temnothorax curvispinosus	uncharacterized protein LOC112457301 isoform X4 [Temnothorax curvispinosus]	GCF_003070985.1	TRADDN-NCOA6
XP_024876018.1	TRADDN-NCOA6	2102	LOC112457301	hexapoda	Temnothorax curvispinosus	uncharacterized protein LOC112457301 isoform X5 [Temnothorax curvispinosus]	GCF_003070985.1	TRADDN-NCOA6
XP_024876019.1	TRADDN-NCOA6	2063	LOC112457301	hexapoda	Temnothorax curvispinosus	uncharacterized protein LOC112457301 isoform X6 [Temnothorax curvispinosus]	GCF_003070985.1	TRADDN-NCOA6
XP_018359436.1	TRADDN-NCOA6	2308	LOC108758802	hexapoda	Trachymyrmex cornetzi	PREDICTED: serine-rich adhesin for platelets-like isoform X1 [Trachymyrmex cornetzi]	GCF_001594075.1	TRADDN-NCOA6
XP_018359438.1	TRADDN-NCOA6	2297	LOC108758802	hexapoda	Trachymyrmex cornetzi	PREDICTED: serine-rich adhesin for platelets-like isoform X2 [Trachymyrmex cornetzi]	GCF_001594075.1	TRADDN-NCOA6

acc	architecture	len	gen.name	taxend	species	defline	gca	clade
XP_018359440.1	TRADDN-NCOA6	2260	LOC108758802	hexapoda	Trachymyrmex cornetzi	PREDICTED: serine-rich adhesin for platelets-like isoform X3 [Trachymyrmex cornetzi]	GCF_001594075.1	TRADDN-NCOA6
XP_018359441.1	TRADDN-NCOA6	2091	LOC108758802	hexapoda	Trachymyrmex cornetzi	PREDICTED: putative mediator of RNA polymerase II transcription subunit 26 isoform X4 [Trachymyrmex cornetzi]	GCF_001594075.1	TRADDN-NCOA6
KYN32003.1	TRADDN-NCOA6	2254	ALC56_13756	hexapoda	Trachymyrmex septentrionalis	Nuclear receptor coactivator 6 [Trachymyrmex septentrionalis]	GCA_001594115.1	TRADDN-NCOA6
XP_018353001.1	TRADDN-NCOA6	2291	LOC108754868	hexapoda	Trachymyrmex septentrionalis	PREDICTED: serine-rich adhesin for platelets-like isoform X1 [Trachymyrmex septentrionalis]	GCF_001594115.1	TRADDN-NCOA6
XP_018353002.1	TRADDN-NCOA6	2280	LOC108754868	hexapoda	Trachymyrmex septentrionalis	PREDICTED: serine-rich adhesin for platelets-like isoform X2 [Trachymyrmex septentrionalis]	GCF_001594115.1	TRADDN-NCOA6
XP_018353003.1	TRADDN-NCOA6	2243	LOC108754868	hexapoda	Trachymyrmex septentrionalis	PREDICTED: serine-rich adhesin for platelets-like isoform X3 [Trachymyrmex septentrionalis]	GCF_001594115.1	TRADDN-NCOA6
XP_018353004.1	TRADDN-NCOA6	2084	LOC108754868	hexapoda	Trachymyrmex septentrionalis	PREDICTED: putative mediator of RNA polymerase II transcription subunit 26 isoform X4 [Trachymyrmex septentrionalis]	GCF_001594115.1	TRADDN-NCOA6
KYQ49864.1	TRADDN-NCOA6	2203	ALC60_11039	hexapoda	Trachymyrmex zeteki	Nuclear receptor coactivator 6 [Trachymyrmex zeteki]	GCA_001594055.1	TRADDN-NCOA6
XP_018311426.1	TRADDN-NCOA6	2299	LOC108727714	hexapoda	Trachymyrmex zeteki	PREDICTED: serine-rich adhesin for platelets-like isoform X1 [Trachymyrmex zeteki]	GCF_001594055.1	TRADDN-NCOA6
XP_018311428.1	TRADDN-NCOA6	2288	LOC108727714	hexapoda	Trachymyrmex zeteki	PREDICTED: serine-rich adhesin for platelets-like isoform X2 [Trachymyrmex zeteki]	GCF_001594055.1	TRADDN-NCOA6
XP_018311429.1	TRADDN-NCOA6	2252	LOC108727714	hexapoda	Trachymyrmex zeteki	PREDICTED: serine-rich adhesin for platelets-like isoform X3 [Trachymyrmex zeteki]	GCF_001594055.1	TRADDN-NCOA6
XP_018311430.1	TRADDN-NCOA6	2090	LOC108727714	hexapoda	Trachymyrmex zeteki	PREDICTED: putative mediator of RNA polymerase II transcription subunit 26 isoform X4 [Trachymyrmex zeteki]	GCF_001594055.1	TRADDN-NCOA6
XP_008195092.1	TRADDN-NCOA6	1139	LOC656611	hexapoda	Tribolium castaneum	PREDICTED: uncharacterized protein LOC656611 [Tribolium castaneum]	GCF_000002335.3	TRADDN-NCOA6
XP_023315366.1	TRADDN-NCOA6	2477	LOC106648493	hexapoda	Trichogramma pretiosum	uncharacterized protein LOC106648493 [Trichogramma pretiosum]	GCF_000599845.2	TRADDN-NCOA6
OXU20663.1	TRADDN-NCOA6	2706	TSAR_004647	hexapoda	Trichomalopsis sarcophagae	hypothetical protein TSAR_004647 [Trichomalopsis sarcophagae]	GCA_002249905.1	TRADDN-NCOA6
XP_011863875.1	TRADDN-NCOA6	2331	LOC105559864	hexapoda	Vollenhovia emeryi	PREDICTED: putative mediator of RNA polymerase II transcription subunit 26 [Vollenhovia emeryi]	GCF_000949405.1	TRADDN-NCOA6
XP_011694201.1	TRADDN-NCOA6	2310	LOC105453719	hexapoda	Wasmannia auropunctata	PREDICTED: putative mediator of RNA polymerase II transcription subunit 26 isoform X1 [Wasmannia auropunctata]	GCF_000956235.1	TRADDN-NCOA6

acc	architecture	len	gen.name	taxend	species	defline	gca	clade
XP_011694202.1	TRADDN-NCOA6	2307	LOC105453719	hexapoda	Wasmannia auropunctata	PREDICTED: putative mediator of RNA polymerase II transcription subunit 26 isoform X2 [Wasmannia auropunctata]	GCF_000956235.1	TRADDN-NCOA6
XP_011694203.1	TRADDN-NCOA6	2099	LOC105453719	hexapoda	Wasmannia auropunctata	PREDICTED: putative mediator of RNA polymerase II transcription subunit 26 isoform X3 [Wasmannia auropunctata]	GCF_000956235.1	TRADDN-NCOA6
XP_011694204.1	TRADDN-NCOA6	2299	LOC105453719	hexapoda	Wasmannia auropunctata	PREDICTED: putative mediator of RNA polymerase II transcription subunit 26 isoform X4 [Wasmannia auropunctata]	GCF_000956235.1	TRADDN-NCOA6
XP_011194615.1	TRADDN-NCOA6	2434	LOC105219952	hexapoda	Zeugodacus cucurbitae	PREDICTED: nuclear receptor coactivator 6 [Zeugodacus cucurbitae]	GCF_000806345.1	TRADDN-NCOA6
XP_021935501.1	TRADDN-NCOA6	2715	LOC110837561	hexapoda	Zootermopsis nevadensis	mucin-5AC-like [Zootermopsis nevadensis]	GCF_000696155.1	TRADDN-NCOA6
EGI60794.1	TRADDN-NCOA6+KH	2409	G5I_10992	hexapoda	Acromyrmex echinatior	RNA-binding protein pno1 [Acromyrmex echinatior]	GCA_000204515.1	TRADDN-NCOA6
XP_012351037.1	TRADDN-NCOA6+KH	2447	LOC100870488	hexapoda	Apis florea	PREDICTED: LOW QUALITY PROTEIN: uncharacterized protein LOC100870488 [Apis florea]	GCF_000184785.2	TRADDN-NCOA6
KOC62415.1	TRADDN-NCOA6+KH	2448	WH47_03828	hexapoda	Habropoda laboriosa	RNA-binding protein pno1 [Habropoda laboriosa]	GCA_001263275.1	TRADDN-NCOA6
XP_012146292.1	TRADDN-NCOA6+KH	2446	LOC100875083	hexapoda	Megachile rotundata	PREDICTED: uncharacterized protein LOC100875083 isoform X1 [Megachile rotundata]	GCF_000220905.1	TRADDN-NCOA6
XP_012146296.1	TRADDN-NCOA6+KH	2423	LOC100875083	hexapoda	Megachile rotundata	PREDICTED: uncharacterized protein LOC100875083 isoform X2 [Megachile rotundata]	GCF_000220905.1	TRADDN-NCOA6
EZA56195.1	TRADDN-NCOA6+KH	2460	X777_03527	hexapoda	Ooceraea biroi	RNA-binding protein pno1 [Ooceraea biroi]	GCA_000611835.1	TRADDN-NCOA6
KYN23013.1	TRADDN-NCOA6+KH	2468	ALC57_04796	hexapoda	Trachymyrmex cornetzi	RNA-binding protein pno1 [Trachymyrmex cornetzi]	GCA_001594075.1	TRADDN-NCOA6
XP_022287297.1	ATP-synt_F+CARD+CARD+TRADDN-DEDD2+CARD+CARD	867	LOC111100011	mollusca	Crassostrea virginica	uncharacterized protein LOC111100011 isoform X3 [Crassostrea virginica]	GCF_002022765.2	TRADDN-DEDD2
XP_022297006.1	CARD+CARD+CARD+TRADDN-DEDD2+CARD+CARD+CARD+SAM+GTPase-AIG	1088	LOC111106572	mollusca	Crassostrea virginica	uncharacterized protein LOC111106572 [Crassostrea virginica]	GCF_002022765.2	TRADDN-DEDD2
XP_022287285.1	CARD+CARD+TRADDN-DEDD2+CARD+CARD	766	LOC111100011	mollusca	Crassostrea virginica	uncharacterized protein LOC111100011 isoform X1 [Crassostrea virginica]	GCF_002022765.2	TRADDN-DEDD2
XP_022287291.1	CARD+CARD+TRADDN-DEDD2+CARD+CARD	765	LOC111100011	mollusca	Crassostrea virginica	uncharacterized protein LOC111100011 isoform X2 [Crassostrea virginica]	GCF_002022765.2	TRADDN-DEDD2
XP_011437929.1	CARD+CARD+TRADDN-DEDD2+LRRs	1170	LOC105335644	mollusca	Crassostrea gigas	PREDICTED: uncharacterized protein LOC105335644 [Crassostrea gigas]	GCF_000297895.1	TRADDN-DEDD2
XP_022308687.1	CARD+CARD+TRADDN-DEDD2+LRRs	1124	LOC111114601	mollusca	Crassostrea virginica	uncharacterized protein LOC111114601 [Crassostrea virginica]	GCF_002022765.2	TRADDN-DEDD2

acc	architecture	len	gen.name	taxend	species	defline	gca	clade
EKC32250.1	CARD+TRADDN-DEDD2	297	CGI_10026238	mollusca	Crassostrea gigas	Caspase-2, partial [Crassostrea gigas]	-	TRADDN-DEDD2
XP_011416077.1	CARD+TRADDN-DEDD2	480	LOC105320022	mollusca	Crassostrea gigas	PREDICTED: uncharacterized protein LOC105320022 [Crassostrea gigas]	GCF_000297895.1	TRADDN-DEDD2
XP_022312200.1	CARD+TRADDN-DEDD2	476	LOC111117388	mollusca	Crassostrea virginica	uncharacterized protein LOC111117388 [Crassostrea virginica]	GCF_002022765.2	TRADDN-DEDD2
XP_009059501.1	CARD+TRADDN-DEDD2	459	LOTGIDRAFT_234111	mollusca	Lottia gigantea	hypothetical protein LOTGIDRAFT_234111 [Lottia gigantea]	GCF_000327385.1	TRADDN-DEDD2
XP_021350772.1	CARD+TRADDN-DEDD2	544	LOC110448711	mollusca	Mizuhopecten yessoensis	uncharacterized protein LOC110448711 [Mizuhopecten yessoensis]	GCF_002113885.1	TRADDN-DEDD2
KOF75889.1	CARD+TRADDN-DEDD2	391	OCBIM_22034109mg	mollusca	Octopus bimaculoides	hypothetical protein OCBIM_22034109mg [Octopus bimaculoides]	GCA_001194135.1	TRADDN-DEDD2
XP_025082216.1	CARD+TRADDN-DEDD2	517	LOC112556935	mollusca	Pomacea canaliculata	uncharacterized protein LOC112556935 [Pomacea canaliculata]	GCF_003073045.1	TRADDN-DEDD2
XP_014781391.1	CARD+TRADDN-DEDD2+CARD	587	LOC106877111	mollusca	Octopus bimaculoides	PREDICTED: uncharacterized protein LOC106877111, partial [Octopus bimaculoides]	GCF_001194135.1	TRADDN-DEDD2
XP_013075628.1	CARD+TRADDN-DEDD2+Coiled-coil	524	LOC106061941	mollusca	Biomphalaria glabrata	PREDICTED: uncharacterized protein LOC106061941 [Biomphalaria glabrata]	GCF_000457365.1	TRADDN-DEDD2
XP_005105349.2	Coiled-coil+CARD+TRADDN-DEDD2	616	LOC101861459	mollusca	Aplysia californica	PREDICTED: uncharacterized protein LOC101861459 isoform X1 [Aplysia californica]	GCF_000002075.1	TRADDN-DEDD2
XP_012941847.1	Coiled-coil+CARD+TRADDN-DEDD2	569	LOC101861459	mollusca	Aplysia californica	PREDICTED: uncharacterized protein LOC101861459 isoform X2 [Aplysia californica]	GCF_000002075.1	TRADDN-DEDD2
XP_022304237.1	cysteine-rich-region+TM+DED+TRADDN-NCOA6	637	LOC111111512	mollusca	Crassostrea virginica	uncharacterized protein LOC111111512 [Crassostrea virginica]	GCF_002022765.2	TRADDN-NCOA6
XP_022305084.1	cysteine-rich-region+TM+DED+TRADDN-NCOA6	477	LOC111112077	mollusca	Crassostrea virginica	uncharacterized protein LOC111112077 [Crassostrea virginica]	GCF_002022765.2	TRADDN-NCOA6
XP_022303703.1	cysteine-rich-region+TM+TRADDN-NCOA6+GTPase-AIG	762	LOC111111178	mollusca	Crassostrea virginica	uncharacterized protein LOC111111178 [Crassostrea virginica]	GCF_002022765.2	TRADDN-NCOA6
EKC33132.1	Death+TRADDN-DEDD2+Coiled-coil	484	CGI_10016451	mollusca	Crassostrea gigas	hypothetical protein CGI_10016451 [Crassostrea gigas]	-	TRADDN-DEDD2
XP_011450141.1	Death+TRADDN-DEDD2+Coiled-coil	521	LOC105344171	mollusca	Crassostrea gigas	PREDICTED: uncharacterized protein LOC105344171 isoform X1 [Crassostrea gigas]	GCF_000297895.1	TRADDN-DEDD2
XP_011450142.1	Death+TRADDN-DEDD2+Coiled-coil	520	LOC105344171	mollusca	Crassostrea gigas	PREDICTED: uncharacterized protein LOC105344171 isoform X2 [Crassostrea gigas]	GCF_000297895.1	TRADDN-DEDD2
XP_022313640.1	Death+TRADDN-DEDD2+Coiled-coil	512	LOC111118443	mollusca	Crassostrea virginica	uncharacterized protein LOC111118443 [Crassostrea virginica]	GCF_002022765.2	TRADDN-DEDD2

acc	architecture	len	gen.name	taxend	species	defline	gca	clade
XP_021344594.1	Death+TRADDN-DEDD2+Coiled-coil	397	LOC110444548	mollusca	Mizuhopecten yessoensis	uncharacterized protein LOC110444548 [Mizuhopecten yessoensis]	GCF_002113885.1	TRADDN-DEDD2
XP_021346619.1	Death+TRADDN-DEDD2+Coiled-coil	526	LOC110446013	mollusca	Mizuhopecten yessoensis	uncharacterized protein LOC110446013 [Mizuhopecten yessoensis]	GCF_002113885.1	TRADDN-DEDD2
EKC20172.1	DED+TRADDN-DEDD2	282	CGI_10006549	mollusca	Crassostrea gigas	Death effector domain-containing protein [Crassostrea gigas]	-	TRADDN-DEDD2
XP_021353196.1	DED+TRADDN-DEDD2	671	LOC110450202	mollusca	Mizuhopecten yessoensis	polycystic kidney disease protein 1-like 3 [Mizuhopecten yessoensis]	GCF_002113885.1	TRADDN-DEDD2
XP_011415319.2	DED+TRADDN-NCOA6	749	LOC105319464	mollusca	Crassostrea gigas	PREDICTED: uncharacterized protein LOC105319464, partial [Crassostrea gigas]	GCF_000297895.1	TRADDN-NCOA6
XP_011424084.1	DED+TRADDN-NCOA6	254	LOC105325967	mollusca	Crassostrea gigas	PREDICTED: uncharacterized protein LOC105325967, partial [Crassostrea gigas]	GCF_000297895.1	TRADDN-NCOA6
XP_019927893.1	DED+TRADDN-NCOA6	248	LOC105340598	mollusca	Crassostrea gigas	PREDICTED: uncharacterized protein LOC105340598 [Crassostrea gigas]	GCF_000297895.1	TRADDN-NCOA6
OWF50257.1	DED+TRADDN-NCOA6	653	KP79_PYT16096	mollusca	Mizuhopecten yessoensis	hypothetical protein KP79_PYT16096 [Mizuhopecten yessoensis]	GCA_002113885.2	TRADDN-NCOA6
XP_021353940.1	DED+TRADDN-NCOA6	634	LOC110450644	mollusca	Mizuhopecten yessoensis	uncharacterized protein LOC110450644 [Mizuhopecten yessoensis]	GCF_002113885.1	TRADDN-NCOA6
XP_011439450.1	DED+TRADDN-NCOA6+alpha-rich-region	613	LOC105336712	mollusca	Crassostrea gigas	PREDICTED: uncharacterized protein LOC105336712 [Crassostrea gigas]	GCF_000297895.1	TRADDN-NCOA6
XP_011447297.1	DED+TRADDN-NCOA6+alpha-rich-region	559	LOC105342134	mollusca	Crassostrea gigas	PREDICTED: uncharacterized protein LOC105342134 [Crassostrea gigas]	GCF_000297895.1	TRADDN-NCOA6
XP_022330360.1	DED+TRADDN-NCOA6+alpha-rich-region	605	LOC111128796	mollusca	Crassostrea virginica	uncharacterized protein LOC111128796 [Crassostrea virginica]	GCF_002022765.2	TRADDN-NCOA6
XP_022343986.1	DED+TRADDN-NCOA6+alpha-rich-region	627	LOC111137049	mollusca	Crassostrea virginica	uncharacterized protein LOC111137049 [Crassostrea virginica]	GCF_002022765.2	TRADDN-NCOA6
XP_011457018.2	DED+TRADDN-NCOA6+DED+domain1+CARD	859	LOC105349046	mollusca	Crassostrea gigas	PREDICTED: uncharacterized protein LOC105349046 [Crassostrea gigas]	GCF_000297895.1	TRADDN-NCOA6
EKC32942.1	DED+TRADDN-NCOA6+RRM+RRM+RRM+RRM+KH+KH+KH+KH+KH+KH+KH+Macro+PARP	1786	CGI_10024733	mollusca	Crassostrea gigas	Poly [ADP-ribose] polymerase 14 [Crassostrea gigas]	-	TRADDN-NCOA6
XP_011455095.1	DED+TRADDN-NCOA6+RRM+RRM+RRM+RRM+KH+KH+KH+KH+KH+KH+KH+Macro+PARP	1802	LOC105347636	mollusca	Crassostrea gigas	PREDICTED: poly [ADP-ribose] polymerase 14 [Crassostrea gigas]	GCF_000297895.1	TRADDN-NCOA6
XP_022299287.1	DED+TRADDN-NCOA6+RRM+RRM+RRM+RRM+KH+KH+KH+KH+KH+KH+KH+Macro+PARP	1866	LOC111108063	mollusca	Crassostrea virginica	poly [ADP-ribose] polymerase 14-like isoform X1 [Crassostrea virginica]	GCF_002022765.2	TRADDN-NCOA6
XP_022299288.1	DED+TRADDN-NCOA6+RRM+RRM+RRM+RRM+KH+KH+KH+KH+KH+KH+KH+Macro+PARP	1860	LOC111108063	mollusca	Crassostrea virginica	poly [ADP-ribose] polymerase 14-like isoform X2 [Crassostrea virginica]	GCF_002022765.2	TRADDN-NCOA6
XP_022299290.1	DED+TRADDN-NCOA6+RRM+RRM+RRM+RRM+KH+KH+KH+KH+KH+KH+KH+Macro+PARP	1855	LOC111108063	mollusca	Crassostrea virginica	poly [ADP-ribose] polymerase 14-like isoform X3 [Crassostrea virginica]	GCF_002022765.2	TRADDN-NCOA6
XP_022299291.1	DED+TRADDN-NCOA6+RRM+RRM+RRM+KH+KH+KH+KH+KH+KH+KH+Macro+PARP	1725	LOC111108063	mollusca	Crassostrea virginica	poly [ADP-ribose] polymerase 14-like isoform X4 [Crassostrea virginica]	GCF_002022765.2	TRADDN-NCOA6

acc	architecture	len	gen.name	taxend	species	defline	gca	clade
XP_022299593.1	DED+TRADDN-NCOA6+RRM+RRM+RRM+RRM+KH+KH+KH+KH+KH+KH+KH+Macro+PARP	1859	LOC111108206	mollusca	Crassostrea virginica	poly [ADP-ribose] polymerase 14-like isoform X1 [Crassostrea virginica]	GCF_002022765.2	TRADDN-NCOA6
XP_022299594.1	DED+TRADDN-NCOA6+RRM+RRM+RRM+RRM+KH+KH+KH+KH+KH+KH+KH+Macro+PARP	1848	LOC111108206	mollusca	Crassostrea virginica	poly [ADP-ribose] polymerase 14-like isoform X2 [Crassostrea virginica]	GCF_002022765.2	TRADDN-NCOA6
EKC20864.1	DED+TRADDN-NCOA6+Transposase_mutator	959	CGI_10005191	mollusca	Crassostrea gigas	hypothetical protein CGI_10005191 [Crassostrea gigas]	-	TRADDN-NCOA6
XP_011421347.1	HEPN+DED+TRADDN-NCOA6	466	LOC105323978	mollusca	Crassostrea gigas	PREDICTED: uncharacterized protein LOC105323978 [Crassostrea gigas]	GCF_000297895.1	TRADDN-NCOA6
XP_011436933.2	HEPN+Ig+DED+TRADDN-NCOA6	1313	LOC105334978	mollusca	Crassostrea gigas	PREDICTED: uncharacterized protein LOC105334978 [Crassostrea gigas]	GCF_000297895.1	TRADDN-NCOA6
XP_011420321.1	HEPN+Ig+Ig+Ig+Ig+DED+TRADDN-NCOA6	1253	LOC105323062	mollusca	Crassostrea gigas	PREDICTED: uncharacterized protein LOC105323062 [Crassostrea gigas]	GCF_000297895.1	TRADDN-NCOA6
XP_011420322.1	HEPN+Ig+Ig+Ig+Ig+DED+TRADDN-NCOA6	1253	LOC105323062	mollusca	Crassostrea gigas	PREDICTED: uncharacterized protein LOC105323062 [Crassostrea gigas]	GCF_000297895.1	TRADDN-NCOA6
XP_011431936.1	HEPN+Ig+Ig+Ig+Ig+DED+TRADDN-NCOA6	970	LOC105331445	mollusca	Crassostrea gigas	PREDICTED: uncharacterized protein LOC105331445 isoform X8 [Crassostrea gigas]	GCF_000297895.1	TRADDN-NCOA6
XP_011431937.1	HEPN+Ig+Ig+Ig+Ig+DED+TRADDN-NCOA6	970	LOC105331445	mollusca	Crassostrea gigas	PREDICTED: uncharacterized protein LOC105331445 isoform X8 [Crassostrea gigas]	GCF_000297895.1	TRADDN-NCOA6
XP_011431938.1	HEPN+Ig+Ig+Ig+Ig+DED+TRADDN-NCOA6	970	LOC105331445	mollusca	Crassostrea gigas	PREDICTED: uncharacterized protein LOC105331445 isoform X8 [Crassostrea gigas]	GCF_000297895.1	TRADDN-NCOA6
XP_011440659.1	HEPN+Ig+Ig+Ig+Ig+DED+TRADDN-NCOA6	955	LOC105337573	mollusca	Crassostrea gigas	PREDICTED: uncharacterized protein LOC105337573 [Crassostrea gigas]	GCF_000297895.1	TRADDN-NCOA6
XP_011452299.1	HEPN+Ig+Ig+Ig+Ig+DED+TRADDN-NCOA6	821	LOC105345719	mollusca	Crassostrea gigas	PREDICTED: muscle M-line assembly protein unc-89-like [Crassostrea gigas]	GCF_000297895.1	TRADDN-NCOA6
XP_019926764.1	HEPN+Ig+Ig+Ig+Ig+DED+TRADDN-NCOA6	955	LOC105337703	mollusca	Crassostrea gigas	PREDICTED: muscle M-line assembly protein unc-89-like [Crassostrea gigas]	GCF_000297895.1	TRADDN-NCOA6
EKC27565.1	HEPN+Ig+Ig+Ig+Ig+DED+TRADDN-NCOA6+Beta-propeller	957	CGI_10017571	mollusca	Crassostrea gigas	hypothetical protein CGI_10017571 [Crassostrea gigas]	-	TRADDN-NCOA6
XP_011431928.1	HEPN+Ig+Ig+Ig+Ig+DED+TRADDN-NCOA6+Beta-propeller	1511	LOC105331445	mollusca	Crassostrea gigas	PREDICTED: uncharacterized protein LOC105331445 isoform X1 [Crassostrea gigas]	GCF_000297895.1	TRADDN-NCOA6
XP_011431929.1	HEPN+Ig+Ig+Ig+Ig+DED+TRADDN-NCOA6+Beta-propeller	1511	LOC105331445	mollusca	Crassostrea gigas	PREDICTED: uncharacterized protein LOC105331445 isoform X1 [Crassostrea gigas]	GCF_000297895.1	TRADDN-NCOA6
XP_011431930.1	HEPN+Ig+Ig+Ig+Ig+DED+TRADDN-NCOA6+Beta-propeller	1462	LOC105331445	mollusca	Crassostrea gigas	PREDICTED: uncharacterized protein LOC105331445 isoform X2 [Crassostrea gigas]	GCF_000297895.1	TRADDN-NCOA6
XP_011431931.1	HEPN+Ig+Ig+Ig+Ig+DED+TRADDN-NCOA6+Beta-propeller	1462	LOC105331445	mollusca	Crassostrea gigas	PREDICTED: uncharacterized protein LOC105331445 isoform X3 [Crassostrea gigas]	GCF_000297895.1	TRADDN-NCOA6

acc	architecture	len	gen.name	taxend	species	defline	gca	clade
XP_011431932.1	HEPN+Ig+Ig+Ig+Ig+DED+TRADDN-NCOA6+Beta-propeller	1457	LOC105331445	mollusca	Crassostrea gigas	PREDICTED: uncharacterized protein LOC105331445 isoform X4 [Crassostrea gigas]	GCF_000297895.1	TRADDN-NCOA6
XP_011431933.1	HEPN+Ig+Ig+Ig+Ig+DED+TRADDN-NCOA6+Beta-propeller	1457	LOC105331445	mollusca	Crassostrea gigas	PREDICTED: uncharacterized protein LOC105331445 isoform X5 [Crassostrea gigas]	GCF_000297895.1	TRADDN-NCOA6
XP_011431934.1	HEPN+Ig+Ig+Ig+Ig+DED+TRADDN-NCOA6+Beta-propeller	1410	LOC105331445	mollusca	Crassostrea gigas	PREDICTED: uncharacterized protein LOC105331445 isoform X6 [Crassostrea gigas]	GCF_000297895.1	TRADDN-NCOA6
XP_011431935.1	HEPN+Ig+Ig+Ig+Ig+DED+TRADDN-NCOA6+Beta-propeller	1305	LOC105331445	mollusca	Crassostrea gigas	PREDICTED: uncharacterized protein LOC105331445 isoform X7 [Crassostrea gigas]	GCF_000297895.1	TRADDN-NCOA6
XP_011454060.1	HEPN+Ig+Ig+Ig+Ig+DED+TRADDN-NCOA6+Mab-21+TPRs	1647	LOC105346950	mollusca	Crassostrea gigas	PREDICTED: uncharacterized protein LOC105346950 [Crassostrea gigas]	GCF_000297895.1	TRADDN-NCOA6
EKC23269.1	HEPN+Ig+TRADDN-NCOA6	424	CGI_10019635	mollusca	Crassostrea gigas	hypothetical protein CGI_10019635 [Crassostrea gigas]	-	TRADDN-NCOA6
XP_022313827.1	HEPN+TRADDN-DEDD2+CCCH-ZF+CCCH-ZF+CCCH-ZF+C2H2-ZF	1228	LOC111118580	mollusca	Crassostrea virginica	uncharacterized protein LOC111118580 isoform X1 [Crassostrea virginica]	GCF_002022765.2	TRADDN-DEDD2
XP_011421835.1	Ig+DED+TRADDN-NCOA6+BBOX+Beta-propeller	1314	LOC105324463	mollusca	Crassostrea gigas	PREDICTED: uncharacterized protein LOC105324463 [Crassostrea gigas]	GCF_000297895.1	TRADDN-NCOA6
EKC29935.1	Ig+TRADDN-NCOA6	261	CGI_10008535	mollusca	Crassostrea gigas	hypothetical protein CGI_10008535 [Crassostrea gigas]	-	TRADDN-NCOA6
EKC37633.1	Ig+TRADDN-NCOA6	421	CGI_10026917	mollusca	Crassostrea gigas	hypothetical protein CGI_10026917 [Crassostrea gigas]	-	TRADDN-NCOA6
EKC17255.1	Ig+TRADDN-NCOA6+Beta-propeller	526	CGI_10001397	mollusca	Crassostrea gigas	Tripartite motif-containing protein 3 [Crassostrea gigas]	-	TRADDN-NCOA6
XP_011413782.1	low-complexity+DED+TRADDN-DEDD2	319	LOC105318392	mollusca	Crassostrea gigas	PREDICTED: death effector domain-containing protein [Crassostrea gigas]	GCF_000297895.1	TRADDN-DEDD2
XP_022321014.1	low-complexity+DED+TRADDN-DEDD2	319	LOC111123153	mollusca	Crassostrea virginica	LOW QUALITY PROTEIN: death effector domain-containing protein-like [Crassostrea virginica]	GCF_002022765.2	TRADDN-DEDD2
XP_009049201.1	low-complexity+DED+TRADDN-DEDD2	292	LOTGIDRAFT_213215	mollusca	Lottia gigantea	hypothetical protein LOTGIDRAFT_213215 [Lottia gigantea]	GCF_000327385.1	TRADDN-DEDD2
XP_014787580.1	low-complexity+DED+TRADDN-DEDD2	364	LOC106881630	mollusca	Octopus bimaculoides	PREDICTED: death effector domain-containing protein-like [Octopus bimaculoides]	GCF_001194135.1	TRADDN-DEDD2
XP_025103018.1	low-complexity+DED+TRADDN-DEDD2	353	LOC112569446	mollusca	Pomacea canaliculata	death effector domain-containing protein-like [Pomacea canaliculata]	GCF_003073045.1	TRADDN-DEDD2
XP_014785637.1	N-ter+TRADDN-Bac	197	LOC106880279	mollusca	Octopus bimaculoides	PREDICTED: uncharacterized protein LOC106880279, partial [Octopus bimaculoides]	GCF_001194135.1	TRADDN-Bacterial-like
EKC32787.1	PID+PDZ+PDZ+TRADDN-NCOA6+DED+domain1+DED+domain1	1885	CGI_10017177	mollusca	Crassostrea gigas	Protein lin-10 [Crassostrea gigas]	-	TRADDN-NCOA6
XP_011439255.1	TM+cysteine-rich-region+TM+alpha-rich-region+TRADDN-NCOA6	521	LOC105336589	mollusca	Crassostrea gigas	PREDICTED: uncharacterized protein LOC105336589 isoform X2 [Crassostrea gigas]	GCF_000297895.1	TRADDN-NCOA6

acc	architecture	len	gen.name	taxend	species	defline	gca	clade
XP_019926313.1	TM+cysteine-rich-region+TM+alpha-rich-region+TRADDN-NCOA6	533	LOC105336589	mollusca	Crassostrea gigas	PREDICTED: uncharacterized protein LOC105336589 isoform X1 [Crassostrea gigas]	GCF_000297895.1	TRADDN-NCOA6
XP_022296697.1	TM+cysteine-rich-region+TM+alpha-rich-region+TRADDN-NCOA6	549	LOC111106341	mollusca	Crassostrea virginica	uncharacterized protein LOC111106341 isoform X1 [Crassostrea virginica]	GCF_002022765.2	TRADDN-NCOA6
XP_022296698.1	TM+cysteine-rich-region+TM+alpha-rich-region+TRADDN-NCOA6	537	LOC111106341	mollusca	Crassostrea virginica	uncharacterized protein LOC111106341 isoform X2 [Crassostrea virginica]	GCF_002022765.2	TRADDN-NCOA6
XP_022302686.1	TM+cysteine-rich-region+TM+alpha-rich-region+TRADDN-NCOA6+BBOX+BBOX+alpha-rich-region+Beta-propeller+C-terminal-helical-extension	1236	LOC111110476	mollusca	Crassostrea virginica	uncharacterized protein LOC111110476 isoform X1 [Crassostrea virginica]	GCF_002022765.2	TRADDN-NCOA6
XP_022302687.1	TM+cysteine-rich-region+TM+alpha-rich-region+TRADDN-NCOA6+BBOX+BBOX+alpha-rich-region+Beta-propeller+C-terminal-helical-extension	1235	LOC111110476	mollusca	Crassostrea virginica	uncharacterized protein LOC111110476 isoform X2 [Crassostrea virginica]	GCF_002022765.2	TRADDN-NCOA6
XP_022302688.1	TM+cysteine-rich-region+TM+alpha-rich-region+TRADDN-NCOA6+BBOX+BBOX+alpha-rich-region+Beta-propeller+C-terminal-helical-extension	1235	LOC111110476	mollusca	Crassostrea virginica	uncharacterized protein LOC111110476 isoform X3 [Crassostrea virginica]	GCF_002022765.2	TRADDN-NCOA6
XP_022302689.1	TM+cysteine-rich-region+TM+alpha-rich-region+TRADDN-NCOA6+BBOX+BBOX+alpha-rich-region+Beta-propeller+C-terminal-helical-extension	1215	LOC111110476	mollusca	Crassostrea virginica	uncharacterized protein LOC111110476 isoform X4 [Crassostrea virginica]	GCF_002022765.2	TRADDN-NCOA6
XP_022302690.1	TM+cysteine-rich-region+TM+alpha-rich-region+TRADDN-NCOA6+BBOX+BBOX+alpha-rich-region+Beta-propeller+C-terminal-helical-extension	1215	LOC111110476	mollusca	Crassostrea virginica	uncharacterized protein LOC111110476 isoform X5 [Crassostrea virginica]	GCF_002022765.2	TRADDN-NCOA6
XP_022302692.1	TM+cysteine-rich-region+TM+alpha-rich-region+TRADDN-NCOA6+BBOX+BBOX+alpha-rich-region+Beta-propeller+C-terminal-helical-extension	1214	LOC111110476	mollusca	Crassostrea virginica	uncharacterized protein LOC111110476 isoform X6 [Crassostrea virginica]	GCF_002022765.2	TRADDN-NCOA6
XP_022302693.1	TM+cysteine-rich-region+TM+alpha-rich-region+TRADDN-NCOA6+BBOX+BBOX+alpha-rich-region+Beta-propeller+C-terminal-helical-extension	1194	LOC111110476	mollusca	Crassostrea virginica	uncharacterized protein LOC111110476 isoform X7 [Crassostrea virginica]	GCF_002022765.2	TRADDN-NCOA6
XP_022302851.1	TM+cysteine-rich-region+TM+alpha-rich-region+TRADDN-NCOA6+BBOX+BBOX+alpha-rich-region+Beta-propeller+C-terminal-helical-extension	1482	LOC111110585	mollusca	Crassostrea virginica	uncharacterized protein LOC111110585 isoform X1 [Crassostrea virginica]	GCF_002022765.2	TRADDN-NCOA6
XP_022302852.1	TM+cysteine-rich-region+TM+alpha-rich-region+TRADDN-NCOA6+BBOX+BBOX+alpha-rich-region+Beta-propeller+C-terminal-helical-extension	1467	LOC111110585	mollusca	Crassostrea virginica	uncharacterized protein LOC111110585 isoform X2 [Crassostrea virginica]	GCF_002022765.2	TRADDN-NCOA6
XP_022302853.1	TM+cysteine-rich-region+TM+alpha-rich-region+TRADDN-NCOA6+BBOX+BBOX+alpha-rich-region+Beta-propeller+C-terminal-helical-extension	1467	LOC111110585	mollusca	Crassostrea virginica	uncharacterized protein LOC111110585 isoform X3 [Crassostrea virginica]	GCF_002022765.2	TRADDN-NCOA6
XP_022302854.1	TM+cysteine-rich-region+TM+alpha-rich-region+TRADDN-NCOA6+BBOX+BBOX+alpha-rich-region+Beta-propeller+C-terminal-helical-extension	1447	LOC111110585	mollusca	Crassostrea virginica	uncharacterized protein LOC111110585 isoform X4 [Crassostrea virginica]	GCF_002022765.2	TRADDN-NCOA6
XP_022302856.1	TM+cysteine-rich-region+TM+alpha-rich-region+TRADDN-NCOA6+BBOX+BBOX+alpha-rich-region+Beta-propeller+C-terminal-helical-extension	1447	LOC111110585	mollusca	Crassostrea virginica	uncharacterized protein LOC111110585 isoform X5 [Crassostrea virginica]	GCF_002022765.2	TRADDN-NCOA6
XP_022302694.1	TM+cysteine-rich-region+TM+alpha-rich-region+TRADDN-NCOA6+HSP70	998	LOC111110477	mollusca	Crassostrea virginica	uncharacterized protein LOC111110477 isoform X1 [Crassostrea virginica]	GCF_002022765.2	TRADDN-NCOA6
XP_022302695.1	TM+cysteine-rich-region+TM+alpha-rich-region+TRADDN-NCOA6+HSP70	987	LOC111110477	mollusca	Crassostrea virginica	uncharacterized protein LOC111110477 isoform X2 [Crassostrea virginica]	GCF_002022765.2	TRADDN-NCOA6

acc	architecture	len	gen.name	taxend	species	defline	gca	clade
XP_022302696.1	TM+cysteine-rich-region+TM+alpha-rich-region+TRADDN-NCOA6+HSP70	987	LOC111110477	mollusca	Crassostrea virginica	uncharacterized protein LOC111110477 isoform X3 [Crassostrea virginica]	GCF_002022765.2	TRADDN-NCOA6
XP_022302697.1	TM+cysteine-rich-region+TM+alpha-rich-region+TRADDN-NCOA6+HSP70	979	LOC111110477	mollusca	Crassostrea virginica	uncharacterized protein LOC111110477 isoform X4 [Crassostrea virginica]	GCF_002022765.2	TRADDN-NCOA6
XP_022302698.1	TM+cysteine-rich-region+TM+alpha-rich-region+TRADDN-NCOA6+HSP70	976	LOC111110477	mollusca	Crassostrea virginica	uncharacterized protein LOC111110477 isoform X5 [Crassostrea virginica]	GCF_002022765.2	TRADDN-NCOA6
XP_022302699.1	TM+cysteine-rich-region+TM+alpha-rich-region+TRADDN-NCOA6+HSP70	965	LOC111110477	mollusca	Crassostrea virginica	uncharacterized protein LOC111110477 isoform X6 [Crassostrea virginica]	GCF_002022765.2	TRADDN-NCOA6
XP_022302700.1	TM+cysteine-rich-region+TM+alpha-rich-region+TRADDN-NCOA6+HSP70	924	LOC111110477	mollusca	Crassostrea virginica	uncharacterized protein LOC111110477 isoform X7 [Crassostrea virginica]	GCF_002022765.2	TRADDN-NCOA6
XP_022302701.1	TM+cysteine-rich-region+TM+alpha-rich-region+TRADDN-NCOA6+HSP70	904	LOC111110479	mollusca	Crassostrea virginica	uncharacterized protein LOC111110479 [Crassostrea virginica]	GCF_002022765.2	TRADDN-NCOA6
XP_022297444.1	TM+cysteine-rich-region+TM+alpha-rich-region+TRADDN-NCOA6+nSTAND3+Ankyrin	2660	LOC111106874	mollusca	Crassostrea virginica	uncharacterized protein LOC111106874 isoform X1 [Crassostrea virginica]	GCF_002022765.2	TRADDN-NCOA6
XP_022297449.1	TM+cysteine-rich-region+TM+alpha-rich-region+TRADDN-NCOA6+nSTAND3+Ankyrin	2654	LOC111106874	mollusca	Crassostrea virginica	uncharacterized protein LOC111106874 isoform X2 [Crassostrea virginica]	GCF_002022765.2	TRADDN-NCOA6
XP_022297450.1	TM+cysteine-rich-region+TM+alpha-rich-region+TRADDN-NCOA6+nSTAND3+Ankyrin	2653	LOC111106874	mollusca	Crassostrea virginica	uncharacterized protein LOC111106874 isoform X3 [Crassostrea virginica]	GCF_002022765.2	TRADDN-NCOA6
XP_022297451.1	TM+cysteine-rich-region+TM+alpha-rich-region+TRADDN-NCOA6+nSTAND3+Ankyrin	2649	LOC111106874	mollusca	Crassostrea virginica	uncharacterized protein LOC111106874 isoform X4 [Crassostrea virginica]	GCF_002022765.2	TRADDN-NCOA6
XP_022297452.1	TM+cysteine-rich-region+TM+alpha-rich-region+TRADDN-NCOA6+nSTAND3+Ankyrin	2550	LOC111106874	mollusca	Crassostrea virginica	uncharacterized protein LOC111106874 isoform X5 [Crassostrea virginica]	GCF_002022765.2	TRADDN-NCOA6
XP_019920469.1	TM+cysteine-rich-region+TM+DED+TRADDN-NCOA6+DED+domain1+Death+Death	1005	LOC105322444	mollusca	Crassostrea gigas	PREDICTED: uncharacterized protein LOC105322444 isoform X1 [Crassostrea gigas]	GCF_000297895.1	TRADDN-NCOA6
XP_019920470.1	TM+cysteine-rich-region+TM+DED+TRADDN-NCOA6+DED+domain1+Death+Death	998	LOC105322444	mollusca	Crassostrea gigas	PREDICTED: uncharacterized protein LOC105322444 isoform X2 [Crassostrea gigas]	GCF_000297895.1	TRADDN-NCOA6
XP_022332993.1	TM+cysteine-rich-region+TM+DED+TRADDN-NCOA6+DED+domain1+Death+Death	1013	LOC111130339	mollusca	Crassostrea virginica	uncharacterized protein LOC111130339 [Crassostrea virginica]	GCF_002022765.2	TRADDN-NCOA6
XP_022332995.1	TM+cysteine-rich-region+TM+DED+TRADDN-NCOA6+DED+domain1+Death+Death	984	LOC111130340	mollusca	Crassostrea virginica	uncharacterized protein LOC111130340 isoform X1 [Crassostrea virginica]	GCF_002022765.2	TRADDN-NCOA6
XP_022332998.1	TM+cysteine-rich-region+TM+DED+TRADDN-NCOA6+DED+domain1+Death+Death	983	LOC111130340	mollusca	Crassostrea virginica	uncharacterized protein LOC111130340 isoform X2 [Crassostrea virginica]	GCF_002022765.2	TRADDN-NCOA6
XP_022333000.1	TM+cysteine-rich-region+TM+DED+TRADDN-NCOA6+DED+domain1+Death+Death	973	LOC111130340	mollusca	Crassostrea virginica	uncharacterized protein LOC111130340 isoform X3 [Crassostrea virginica]	GCF_002022765.2	TRADDN-NCOA6

acc	architecture	len	gen.name	taxend	species	defline	gca	clade
XP_022333001.1	TM+cysteine-rich-region+TM+DED+TRADDN-NCOA6+DED+domain1+Death+Death	972	LOC111130340	mollusca	Crassostrea virginica	uncharacterized protein LOC111130340 isoform X4 [Crassostrea virginica]	GCF_002022765.2	TRADDN-NCOA6
XP_022334756.1	TM+cysteine-rich-region+TM+DED+TRADDN-NCOA6+DED+domain1+Death+Death	994	LOC111131489	mollusca	Crassostrea virginica	uncharacterized protein LOC111131489 isoform X1 [Crassostrea virginica]	GCF_002022765.2	TRADDN-NCOA6
XP_022334757.1	TM+cysteine-rich-region+TM+DED+TRADDN-NCOA6+DED+domain1+Death+Death	976	LOC111131489	mollusca	Crassostrea virginica	uncharacterized protein LOC111131489 isoform X2 [Crassostrea virginica]	GCF_002022765.2	TRADDN-NCOA6
XP_022334759.1	TM+cysteine-rich-region+TM+DED+TRADDN-NCOA6+DED+domain1+Death+Death	914	LOC111131489	mollusca	Crassostrea virginica	uncharacterized protein LOC111131489 isoform X3 [Crassostrea virginica]	GCF_002022765.2	TRADDN-NCOA6
XP_022334760.1	TM+cysteine-rich-region+TM+DED+TRADDN-NCOA6+DED+domain1+Death+Death	980	LOC111131490	mollusca	Crassostrea virginica	uncharacterized protein LOC111131490 isoform X1 [Crassostrea virginica]	GCF_002022765.2	TRADDN-NCOA6
XP_022334764.1	TM+cysteine-rich-region+TM+DED+TRADDN-NCOA6+DED+domain1+Death+Death	979	LOC111131490	mollusca	Crassostrea virginica	uncharacterized protein LOC111131490 isoform X2 [Crassostrea virginica]	GCF_002022765.2	TRADDN-NCOA6
XP_022300526.1	TM+cysteine-rich-region+TM+DED+TRADDN-NCOA6+EB+EB+GTPase-AIG	1255	LOC111108757	mollusca	Crassostrea virginica	uncharacterized protein LOC111108757 [Crassostrea virginica]	GCF_002022765.2	TRADDN-NCOA6
XP_022306074.1	TM+cysteine-rich-region+TM+TRADDN-NCOA6	741	LOC111112665	mollusca	Crassostrea virginica	uncharacterized protein LOC111112665 isoform X5 [Crassostrea virginica]	GCF_002022765.2	TRADDN-NCOA6
XP_022308757.1	TM+cysteine-rich-region+TM+TRADDN-NCOA6	761	LOC111114647	mollusca	Crassostrea virginica	uncharacterized protein LOC111114647 isoform X4 [Crassostrea virginica]	GCF_002022765.2	TRADDN-NCOA6
XP_022306073.1	TM+cysteine-rich-region+TM+TRADDN-NCOA6+HEPN	927	LOC111112665	mollusca	Crassostrea virginica	uncharacterized protein LOC111112665 isoform X4 [Crassostrea virginica]	GCF_002022765.2	TRADDN-NCOA6
XP_022306070.1	TM+cysteine-rich-region+TM+TRADDN-NCOA6+HEPN+nSTAND3+Ankyrin	1802	LOC111112665	mollusca	Crassostrea virginica	uncharacterized protein LOC111112665 isoform X1 [Crassostrea virginica]	GCF_002022765.2	TRADDN-NCOA6
XP_022306071.1	TM+cysteine-rich-region+TM+TRADDN-NCOA6+HEPN+nSTAND3+Ankyrin	1789	LOC111112665	mollusca	Crassostrea virginica	uncharacterized protein LOC111112665 isoform X2 [Crassostrea virginica]	GCF_002022765.2	TRADDN-NCOA6
XP_022306072.1	TM+cysteine-rich-region+TM+TRADDN-NCOA6+HEPN+nSTAND3+Ankyrin	1682	LOC111112665	mollusca	Crassostrea virginica	uncharacterized protein LOC111112665 isoform X3 [Crassostrea virginica]	GCF_002022765.2	TRADDN-NCOA6
XP_022308753.1	TM+cysteine-rich-region+TM+TRADDN-NCOA6+HEPN+nSTAND3+Ankyrin	2456	LOC111114647	mollusca	Crassostrea virginica	uncharacterized protein LOC111114647 isoform X1 [Crassostrea virginica]	GCF_002022765.2	TRADDN-NCOA6
XP_022308755.1	TM+cysteine-rich-region+TM+TRADDN-NCOA6+HEPN+nSTAND3+Ankyrin	2391	LOC111114647	mollusca	Crassostrea virginica	uncharacterized protein LOC111114647 isoform X2 [Crassostrea virginica]	GCF_002022765.2	TRADDN-NCOA6
XP_022308756.1	TM+cysteine-rich-region+TM+TRADDN-NCOA6+HEPN+nSTAND3+Ankyrin	2376	LOC111114647	mollusca	Crassostrea virginica	uncharacterized protein LOC111114647 isoform X3 [Crassostrea virginica]	GCF_002022765.2	TRADDN-NCOA6
XP_019918422.1	TM+DED+DED+TRADDN-NCOA6+domain1	661	LOC105317437	mollusca	Crassostrea gigas	PREDICTED: uncharacterized protein LOC105317437 [Crassostrea gigas]	GCF_000297895.1	TRADDN-NCOA6

acc	architecture	len	gen.name	taxend	species	defline	gca	clade
XP_011456644.1	TM+TM+TM+TM+TRADDN-DEDD2	289	LOC105348775	mollusca	Crassostrea gigas	PREDICTED: uncharacterized protein LOC105348775 [Crassostrea gigas]	GCF_000297895.1	TRADDN-DEDD2
XP_012942262.1	TRADDN-DEDD2	275	LOC106012799	mollusca	Aplysia californica	PREDICTED: uncharacterized protein LOC106012799 [Aplysia californica]	GCF_000002075.1	TRADDN-DEDD2
XP_013068013.1	TRADDN-DEDD2	168	LOC106056012	mollusca	Biomphalaria glabrata	PREDICTED: uncharacterized protein LOC106056012 [Biomphalaria glabrata]	GCF_000457365.1	TRADDN-DEDD2
EKC22839.1	TRADDN-DEDD2	89	CGI_10001393	mollusca	Crassostrea gigas	Death effector domain-containing protein [Crassostrea gigas]	-	TRADDN-DEDD2
XP_011411881.1	TRADDN-DEDD2	123	LOC105317049	mollusca	Crassostrea gigas	PREDICTED: death effector domain-containing protein, partial [Crassostrea gigas]	GCF_000297895.1	TRADDN-DEDD2
XP_019919482.1	TRADDN-DEDD2	1059	LOC105319902	mollusca	Crassostrea gigas	PREDICTED: uncharacterized protein LOC105319902 [Crassostrea gigas]	GCF_000297895.1	TRADDN-DEDD2
XP_022312839.1	TRADDN-DEDD2	959	LOC111117894	mollusca	Crassostrea virginica	uncharacterized protein LOC111117894 [Crassostrea virginica]	GCF_002022765.2	TRADDN-DEDD2
XP_009061487.1	TRADDN-DEDD2	1132	LOTGIDRAFT_166189	mollusca	Lottia gigantea	hypothetical protein LOTGIDRAFT_166189 [Lottia gigantea]	GCF_000327385.1	TRADDN-DEDD2
XP_021368028.1	TRADDN-DEDD2	1153	LOC110459884	mollusca	Mizuhopecten yessoensis	uncharacterized protein LOC110459884 [Mizuhopecten yessoensis]	GCF_002113885.1	TRADDN-DEDD2
PVD26877.1	TRADDN-DEDD2	256	C0Q70_12025	mollusca	Pomacea canaliculata	hypothetical protein C0Q70_12025 [Pomacea canaliculata]	GCA_003073045.1	TRADDN-DEDD2
PVD36255.1	TRADDN-DEDD2	776	C0Q70_03233	mollusca	Pomacea canaliculata	hypothetical protein C0Q70_03233 [Pomacea canaliculata]	GCA_003073045.1	TRADDN-DEDD2
XP_025081834.1	TRADDN-DEDD2	431	LOC112556745	mollusca	Pomacea canaliculata	uncharacterized protein LOC112556745 isoform X2 [Pomacea canaliculata]	GCF_003073045.1	TRADDN-DEDD2
XP_005100108.1	TRADDN-DEDD2+Coiled-coil	374	LOC101858718	mollusca	Aplysia californica	PREDICTED: uncharacterized protein LOC101858718 isoform X1 [Aplysia californica]	GCF_000002075.1	TRADDN-DEDD2
XP_005100110.1	TRADDN-DEDD2+Coiled-coil	357	LOC101858718	mollusca	Aplysia californica	PREDICTED: uncharacterized protein LOC101858718 isoform X3 [Aplysia californica]	GCF_000002075.1	TRADDN-DEDD2
XP_012938929.1	TRADDN-DEDD2+Coiled-coil	365	LOC101858718	mollusca	Aplysia californica	PREDICTED: uncharacterized protein LOC101858718 isoform X2 [Aplysia californica]	GCF_000002075.1	TRADDN-DEDD2
XP_011445141.1	TRADDN-DEDD2+Coiled-coil	363	LOC105340663	mollusca	Crassostrea gigas	PREDICTED: uncharacterized protein LOC105340663 isoform X1 [Crassostrea gigas]	GCF_000297895.1	TRADDN-DEDD2
XP_011445142.1	TRADDN-DEDD2+Coiled-coil	349	LOC105340663	mollusca	Crassostrea gigas	PREDICTED: uncharacterized protein LOC105340663 isoform X2 [Crassostrea gigas]	GCF_000297895.1	TRADDN-DEDD2
XP_011445144.1	TRADDN-DEDD2+Coiled-coil	308	LOC105340663	mollusca	Crassostrea gigas	PREDICTED: uncharacterized protein LOC105340663 isoform X3 [Crassostrea gigas]	GCF_000297895.1	TRADDN-DEDD2

acc	architecture	len	gen.name	taxend	species	defline	gca	clade
XP_022317278.1	TRADDN-DEDD2+Coiled-coil	363	LOC111120669	mollusca	Crassostrea virginica	uncharacterized protein LOC111120669 isoform X1 [Crassostrea virginica]	GCF_002022765.2	TRADDN-DEDD2
XP_022317286.1	TRADDN-DEDD2+Coiled-coil	349	LOC111120669	mollusca	Crassostrea virginica	uncharacterized protein LOC111120669 isoform X2 [Crassostrea virginica]	GCF_002022765.2	TRADDN-DEDD2
XP_022321425.1	TRADDN-DEDD2+Coiled-coil	363	LOC111123411	mollusca	Crassostrea virginica	uncharacterized protein LOC111123411 isoform X1 [Crassostrea virginica]	GCF_002022765.2	TRADDN-DEDD2
XP_022321434.1	TRADDN-DEDD2+Coiled-coil	349	LOC111123411	mollusca	Crassostrea virginica	uncharacterized protein LOC111123411 isoform X2 [Crassostrea virginica]	GCF_002022765.2	TRADDN-DEDD2
XP_009046635.1	TRADDN-DEDD2+Coiled-coil	357	LOTGIDRAFT_237861	mollusca	Lottia gigantea	hypothetical protein LOTGIDRAFT_237861 [Lottia gigantea]	GCF_000327385.1	TRADDN-DEDD2
XP_021343490.1	TRADDN-DEDD2+Coiled-coil	353	LOC110443545	mollusca	Mizuhopecten yessoensis	uncharacterized protein LOC110443545 [Mizuhopecten yessoensis]	GCF_002113885.1	TRADDN-DEDD2
XP_025081831.1	TRADDN-DEDD2+CTD	572	LOC112556745	mollusca	Pomacea canaliculata	uncharacterized protein LOC112556745 isoform X1 [Pomacea canaliculata]	GCF_003073045.1	TRADDN-DEDD2
OWF39790.1	TRADDN-DEDD2+low-complexity	1112	KP79_PYT15425	mollusca	Mizuhopecten yessoensis	protein FAM47C [Mizuhopecten yessoensis]	GCA_002113885.2	TRADDN-DEDD2
EKC30605.1	TRADDN-DEDD2+LRRs	974	CGI_10012965	mollusca	Crassostrea gigas	Protein NLRC3 [Crassostrea gigas]	-	TRADDN-DEDD2
XP_021375602.1	TRADDN-DEDD2+vWA+Coiled-coil	772	LOC110464614	mollusca	Mizuhopecten yessoensis	uncharacterized protein LOC110464614, partial [Mizuhopecten yessoensis]	GCF_002113885.1	TRADDN-DEDD2
XP_005096372.1	TRADDN-NCOA6	2543	LOC101862496	mollusca	Aplysia californica	PREDICTED: mucin-17-like isoform X2 [Aplysia californica]	GCF_000002075.1	TRADDN-NCOA6
XP_012936779.1	TRADDN-NCOA6	2552	LOC101862496	mollusca	Aplysia californica	PREDICTED: mucin-17-like isoform X1 [Aplysia californica]	GCF_000002075.1	TRADDN-NCOA6
XP_013075031.1	TRADDN-NCOA6	2174	LOC106061427	mollusca	Biomphalaria glabrata	PREDICTED: serine-rich adhesin for platelets-like isoform X1 [Biomphalaria glabrata]	GCF_000457365.1	TRADDN-NCOA6
XP_013075040.1	TRADDN-NCOA6	2154	LOC106061427	mollusca	Biomphalaria glabrata	PREDICTED: serine-rich adhesin for platelets-like isoform X2 [Biomphalaria glabrata]	GCF_000457365.1	TRADDN-NCOA6
XP_013075047.1	TRADDN-NCOA6	2152	LOC106061427	mollusca	Biomphalaria glabrata	PREDICTED: serine-rich adhesin for platelets-like isoform X3 [Biomphalaria glabrata]	GCF_000457365.1	TRADDN-NCOA6
XP_011412379.2	TRADDN-NCOA6	322	LOC105317436	mollusca	Crassostrea gigas	PREDICTED: uncharacterized protein LOC105317436 [Crassostrea gigas]	GCF_000297895.1	TRADDN-NCOA6
XP_011421349.2	TRADDN-NCOA6	158	LOC105323982	mollusca	Crassostrea gigas	PREDICTED: uncharacterized protein LOC105323982, partial [Crassostrea gigas]	GCF_000297895.1	TRADDN-NCOA6
XP_011439656.1	TRADDN-NCOA6	574	LOC105336877	mollusca	Crassostrea gigas	PREDICTED: uncharacterized protein LOC105336877 [Crassostrea gigas]	GCF_000297895.1	TRADDN-NCOA6
XP_011454817.1	TRADDN-NCOA6	1680	LOC105347421	mollusca	Crassostrea gigas	PREDICTED: mucin-12-like isoform X1 [Crassostrea gigas]	GCF_000297895.1	TRADDN-NCOA6

acc	architecture	len	gen.name	taxend	species	defline	gca	clade
XP_011454818.1	TRADDN-NCOA6	1673	LOC105347421	mollusca	Crassostrea gigas	PREDICTED: mucin-12-like isoform X2 [Crassostrea gigas]	GCF_000297895.1	TRADDN-NCOA6
XP_011454819.1	TRADDN-NCOA6	1606	LOC105347421	mollusca	Crassostrea gigas	PREDICTED: uncharacterized protein LOC105347421 isoform X3 [Crassostrea gigas]	GCF_000297895.1	TRADDN-NCOA6
XP_019930063.1	TRADDN-NCOA6	135	LOC105346222	mollusca	Crassostrea gigas	PREDICTED: uncharacterized protein LOC105346222 [Crassostrea gigas]	GCF_000297895.1	TRADDN-NCOA6
XP_022314769.1	TRADDN-NCOA6	1676	LOC111119183	mollusca	Crassostrea virginica	uncharacterized protein LOC111119183 isoform X1 [Crassostrea virginica]	GCF_002022765.2	TRADDN-NCOA6
XP_022314770.1	TRADDN-NCOA6	1669	LOC111119183	mollusca	Crassostrea virginica	uncharacterized protein LOC111119183 isoform X2 [Crassostrea virginica]	GCF_002022765.2	TRADDN-NCOA6
XP_022314771.1	TRADDN-NCOA6	1669	LOC111119183	mollusca	Crassostrea virginica	uncharacterized protein LOC111119183 isoform X3 [Crassostrea virginica]	GCF_002022765.2	TRADDN-NCOA6
XP_022314773.1	TRADDN-NCOA6	1602	LOC111119183	mollusca	Crassostrea virginica	uncharacterized protein LOC111119183 isoform X4 [Crassostrea virginica]	GCF_002022765.2	TRADDN-NCOA6
XP_022314889.1	TRADDN-NCOA6	1676	LOC111119225	mollusca	Crassostrea virginica	uncharacterized protein LOC111119225 isoform X1 [Crassostrea virginica]	GCF_002022765.2	TRADDN-NCOA6
XP_022314890.1	TRADDN-NCOA6	1669	LOC111119225	mollusca	Crassostrea virginica	uncharacterized protein LOC111119225 isoform X2 [Crassostrea virginica]	GCF_002022765.2	TRADDN-NCOA6
XP_022314892.1	TRADDN-NCOA6	1669	LOC111119225	mollusca	Crassostrea virginica	uncharacterized protein LOC111119225 isoform X3 [Crassostrea virginica]	GCF_002022765.2	TRADDN-NCOA6
XP_022314893.1	TRADDN-NCOA6	1602	LOC111119225	mollusca	Crassostrea virginica	uncharacterized protein LOC111119225 isoform X4 [Crassostrea virginica]	GCF_002022765.2	TRADDN-NCOA6
XP_009061142.1	TRADDN-NCOA6	1658	LOTGIDRAFT_234686	mollusca	Lottia gigantea	hypothetical protein LOTGIDRAFT_234686 [Lottia gigantea]	GCF_000327385.1	TRADDN-NCOA6
XP_021343526.1	TRADDN-NCOA6	1999	LOC110443570	mollusca	Mizuhopecten yessoensis	uncharacterized protein LOC110443570 [Mizuhopecten yessoensis]	GCF_002113885.1	TRADDN-NCOA6
XP_014778602.1	TRADDN-NCOA6	2269	LOC106875131	mollusca	Octopus bimaculoides	PREDICTED: serine-rich adhesin for platelets-like isoform X1 [Octopus bimaculoides]	GCF_001194135.1	TRADDN-NCOA6
XP_014778605.1	TRADDN-NCOA6	2268	LOC106875131	mollusca	Octopus bimaculoides	PREDICTED: serine-rich adhesin for platelets-like isoform X2 [Octopus bimaculoides]	GCF_001194135.1	TRADDN-NCOA6
XP_025109424.1	TRADDN-NCOA6	2637	LOC112573353	mollusca	Pomacea canaliculata	uncharacterized protein LOC112573353 isoform X1 [Pomacea canaliculata]	GCF_003073045.1	TRADDN-NCOA6
XP_025109427.1	TRADDN-NCOA6	2631	LOC112573353	mollusca	Pomacea canaliculata	uncharacterized protein LOC112573353 isoform X2 [Pomacea canaliculata]	GCF_003073045.1	TRADDN-NCOA6

acc	architecture	len	gen.name	taxend	species	defline	gca	clade
XP_011454771.2	TRADDN-NCOA6+BBOX+BBOX+Beta-propeller	1171	LOC105347399	mollusca	Crassostrea gigas	PREDICTED: uncharacterized protein LOC105347399 [Crassostrea gigas]	GCF_000297895.1	TRADDN-NCOA6
XP_011448221.2	TRADDN-NCOA6+DED+domain1	467	LOC105342858	mollusca	Crassostrea gigas	PREDICTED: uncharacterized protein LOC105342858, partial [Crassostrea gigas]	GCF_000297895.1	TRADDN-NCOA6
EKC37751.1	TRADDN-NCOA6+ROT	336	CGI_10011599	mollusca	Crassostrea gigas	hypothetical protein CGI_10011599 [Crassostrea gigas]	-	TRADDN-NCOA6
XP_011428649.2	TRADDN-NCOA6+UPF0515(ZBD)	344	LOC105329165	mollusca	Crassostrea gigas	PREDICTED: repressor of yield of DENV protein homolog [Crassostrea gigas]	GCF_000297895.1	TRADDN-NCOA6
KHN84334.1	TRADDN-NCOA6	1626	Tcan_12392	nematoda	Toxocara canis	hypothetical protein Tcan_12392 [Toxocara canis]	GCA_000803305.1	TRADDN-NCOA6
KRY57978.1	TRADDN-NCOA6	600	T03_7673	nematoda	Trichinella britovi	hypothetical protein T03_7673 [Trichinella britovi]	GCA_001447585.1	TRADDN-NCOA6
KRY57979.1	TRADDN-NCOA6	580	T03_7673	nematoda	Trichinella britovi	hypothetical protein T03_7673 [Trichinella britovi]	GCA_001447585.1	TRADDN-NCOA6
KRX50653.1	TRADDN-NCOA6	729	T05_3233	nematoda	Trichinella murrelli	hypothetical protein T05_3233 [Trichinella murrelli]	GCA_001447425.1	TRADDN-NCOA6
KRZ54558.1	TRADDN-NCOA6	659	T02_7117	nematoda	Trichinella nativa	hypothetical protein T02_7117 [Trichinella nativa]	GCA_001447565.1	TRADDN-NCOA6
KRX17925.1	TRADDN-NCOA6	663	T07_14018	nematoda	Trichinella nelsoni	hypothetical protein T07_14018 [Trichinella nelsoni]	GCA_001447455.1	TRADDN-NCOA6
KRZ69554.1	TRADDN-NCOA6	613	T10_4646	nematoda	Trichinella papuae	hypothetical protein T10_4646 [Trichinella papuae]	GCA_001447755.1	TRADDN-NCOA6
KRY13562.1	TRADDN-NCOA6	641	T12_16605	nematoda	Trichinella patagoniensis	hypothetical protein T12_16605 [Trichinella patagoniensis]	GCA_001447655.1	TRADDN-NCOA6
KRX91511.1	TRADDN-NCOA6	621	T4E_7662	nematoda	Trichinella pseudospiralis	hypothetical protein T4E_7662 [Trichinella pseudospiralis]	GCA_001447445.1	TRADDN-NCOA6
KRX91512.1	TRADDN-NCOA6	649	T4E_7662	nematoda	Trichinella pseudospiralis	hypothetical protein T4E_7662 [Trichinella pseudospiralis]	GCA_001447445.1	TRADDN-NCOA6
KRY68453.1	TRADDN-NCOA6	649	T4A_7215	nematoda	Trichinella pseudospiralis	hypothetical protein T4A_7215 [Trichinella pseudospiralis]	GCA_001447675.1	TRADDN-NCOA6
KRY68454.1	TRADDN-NCOA6	649	T4A_7215	nematoda	Trichinella pseudospiralis	hypothetical protein T4A_7215 [Trichinella pseudospiralis]	GCA_001447675.1	TRADDN-NCOA6
KRY86157.1	TRADDN-NCOA6	649	T4D_918	nematoda	Trichinella pseudospiralis	hypothetical protein T4D_918 [Trichinella pseudospiralis]	GCA_001447575.1	TRADDN-NCOA6
KRZ25843.1	TRADDN-NCOA6	648	T4B_5690	nematoda	Trichinella pseudospiralis	hypothetical protein T4B_5690 [Trichinella pseudospiralis]	GCA_001447725.1	TRADDN-NCOA6
KRZ25844.1	TRADDN-NCOA6	648	T4B_5690	nematoda	Trichinella pseudospiralis	hypothetical protein T4B_5690 [Trichinella pseudospiralis]	GCA_001447725.1	TRADDN-NCOA6
KRX75463.1	TRADDN-NCOA6	586	T06_6177	nematoda	Trichinella sp. T6	hypothetical protein T06_6177 [Trichinella sp. T6]	GCA_001447435.1	TRADDN-NCOA6
KRX75464.1	TRADDN-NCOA6	643	T06_6177	nematoda	Trichinella sp. T6	hypothetical protein T06_6177 [Trichinella sp. T6]	GCA_001447435.1	TRADDN-NCOA6
KRZ93493.1	TRADDN-NCOA6	729	T08_6434	nematoda	Trichinella sp. T8	hypothetical protein T08_6434 [Trichinella sp. T8]	GCA_001447745.1	TRADDN-NCOA6
KRX60436.1	TRADDN-NCOA6	725	T09_11053	nematoda	Trichinella sp. T9	hypothetical protein T09_11053 [Trichinella sp. T9]	GCA_001447505.1	TRADDN-NCOA6
KRY31454.1	TRADDN-NCOA6	754	T01_13249	nematoda	Trichinella spiralis	hypothetical protein T01_13249 [Trichinella spiralis]	GCA_001447595.1	TRADDN-NCOA6

acc	architecture	len	gen.name	taxend	species	defline	gca	clade
KRY31455.1	TRADDN-NCOA6	726	T01_13249	nematoda	Trichinella spiralis	hypothetical protein T01_13249 [Trichinella spiralis]	GCA_001447595.1	TRADDN-NCOA6
XP_003371284.1	TRADDN-NCOA6	653	Tsp_09519	nematoda	Trichinella spiralis	hypothetical protein Tsp_09519 [Trichinella spiralis]	GCF_000181795.1	TRADDN-NCOA6
KRZ15489.1	TRADDN-NCOA6	658	T11_7060	nematoda	Trichinella zimbabwensis	hypothetical protein T11_7060 [Trichinella zimbabwensis]	GCA_001447665.1	TRADDN-NCOA6
KRZ15490.1	TRADDN-NCOA6	637	T11_7060	nematoda	Trichinella zimbabwensis	hypothetical protein T11_7060 [Trichinella zimbabwensis]	GCA_001447665.1	TRADDN-NCOA6
KFD53706.1	TRADDN-NCOA6	805	M513_05411	nematoda	Trichuris suis	hypothetical protein M513_05411 [Trichuris suis]	GCA_000701005.1	TRADDN-NCOA6
KFD72418.1	TRADDN-NCOA6	1177	M514_05411	nematoda	Trichuris suis	hypothetical protein M514_05411 [Trichuris suis]	GCA_000701025.1	TRADDN-NCOA6
EDV28184.1	N-ter+TRADDN-Bac+FAM124	401	TRIADDRAFT_53522	placozoa	Trichoplax adhaerens	predicted protein [Trichoplax adhaerens]	GCA_000150275.1	TRADDN-Bacterial-like
XP_002110018.1	N-ter+TRADDN-Bac+FAM124	401	TRIADDRAFT_53522	placozoa	Trichoplax adhaerens	predicted protein [Trichoplax adhaerens]	GCF_000150275.1	TRADDN-Bacterial-like

Gene neighborhoods and domain architectures of the bacterial GT4-like glycosyltransferases described in this study

<table>
<tr><th>acc</th><th>operon</th><th>architecture</th><th>len</th><th>gen.name</th><th>taxend</th><th>species</th><th>defline</th><th>gca</th></tr>
<tr><td>ADU34882.1</td><td>GT4+B12-binding+RADICAL-SAM*→</td><td>GT4+B12-binding+RADICAL-SAM</td><td>1405</td><td>Varpa_0662</td><td>Betaproteobacteria</td><td>Variovorax paradoxus EPS</td><td>Radical SAM domain protein [Variovorax paradoxus EPS]</td><td>GCA_000184745.1</td></tr>
<tr><td>AEV82483.1</td><td>WXG+GT4+GT4+REase*→</td><td>WXG+GT4+GT4+REase</td><td>4143</td><td>inaW</td><td>Actinobacteria</td><td>Actinoplanes sp. SE50/110</td><td>Ice nucleation protein [Actinoplanes sp. SE50/110]</td><td>GCA_000237145.1</td></tr>
<tr><td>AMB84622.1</td><td>APATPase+GT4*→</td><td>APATPase+GT4</td><td>1299</td><td>AWM79_04595</td><td>Gammaproteobacteria</td><td>Pseudomonas agarici</td><td>hypothetical protein AWM79_04595 [Pseudomonas agarici]</td><td>GCA_001543125.1</td></tr>
<tr><td>ANP51773.1</td><td>Caspase+NACHT+GT4*→||||<-?<-?<-?||?→?→||||<-?<-MFS</td><td>Caspase+NACHT+GT4</td><td>1134</td><td>AVL59_21225</td><td>Actinobacteria</td><td>Streptomyces griseochromogenes</td><td>hypothetical protein AVL59_21225 [Streptomyces griseochromogenes]</td><td>GCA_001542625.2</td></tr>
<tr><td>AWZ06155.1</td><td>Caspase+NACHT+GT4*→||||<-?<-?<-?<-?||APATPase→</td><td>Caspase+NACHT+GT4</td><td>1156</td><td>DRB89_17720</td><td>Actinobacteria</td><td>Streptomyces sp. ICC4</td><td>hypothetical protein DRB89_17720 [Streptomyces sp. ICC4]</td><td>GCA_003287915.1</td></tr>
<tr><td>BAO61700.1</td><td>SWACOS+GT4*→?→?→||||<-?<-?||MFS→</td><td>SWACOS+GT4</td><td>1084</td><td>PPC_2353</td><td>Gammaproteobacteria</td><td>Pseudomonas protegens Cab57</td><td>hypothetical protein PPC_2353 [Pseudomonas protegens Cab57]</td><td>GCA_000828695.1</td></tr>
<tr><td>CEL20906.1</td><td>ART+Caspase+GT4*→?→ GT4→</td><td>ART+Caspase+GT4</td><td>1145</td><td>BN1199_RS39095</td><td>Actinobacteria</td><td>Kibdelosporangium sp. MJ126-NF4</td><td>hypothetical protein [Kibdelosporangium sp. MJ126-NF4]</td><td>GCA_000826545.1</td></tr>
<tr><td>CUU59694.1</td><td>TPR+Caspase→ TPR+Caspase→||||<-CATASP||CATRA-N+CATRA-C+GT4*→||||<-?||HTH+APATPase+TPR→</td><td>CATRA-N+CATRA-C+GT4</td><td>904</td><td>Ga0074812_13074</td><td>Actinobacteria</td><td>Frankia irregularis</td><td>Glycosyltransferase involved in cell wall bisynthesis [Frankia irregularis]</td><td>GCA_001536285.1</td></tr>
<tr><td>EIV94947.1</td><td>CATRA-N+CATRA-C+GT4*→||||<-TPR+Caspase<-?<-?<-?<-?||APATPase+TPR→ CATASP→</td><td>CATRA-N+CATRA-C+GT4</td><td>810</td><td>FraQA3DRAFT_4743</td><td>Actinobacteria</td><td>Frankia sp. QA3</td><td>glycosyltransferase [Frankia sp. QA3]</td><td>GCA_000262465.1</td></tr>
</table>

<table>
<thead>
<tr><th>acc</th><th>operon</th><th>architecture</th><th>len</th><th>gen.name</th><th>taxend</th><th>species</th><th>defline</th><th>gca</th></tr>
</thead>
<tbody>
<tr><td>EKX63918.1</td><td>ZetaToxin+GT4+NUDIX+ART+NTase*→?→?→||||<-Caspase<-?||?→?→||||<-NLPC</td><td>ZetaToxin+GT4+NUDIX+ART+NTase</td><td>2156</td><td>STRIP9103_04937</td><td>Actinobacteria</td><td>Streptomyces ipomoeae 91-03</td><td>hydrolase, NUDIX family [Streptomyces ipomoeae 91-03]</td><td>GCA_000317595.1</td></tr>
<tr><td>EWM12074.1</td><td><-CATASP||CATRA-N+CATRA-C+GT4*→||||<-HTH+APATPase+TPR</td><td>CATRA-N+CATRA-C+GT4</td><td>750</td><td>KUTG_02378</td><td>Actinobacteria</td><td>Kutzneria sp. 744</td><td>LigA protein [Kutzneria sp. 744]</td><td>GCA_000568255.1</td></tr>
<tr><td>GBE24927.1</td><td>Asparaginase_II→ Acetyltransf_6→ GT4*→?→?→?→||||<-?<-SIG+TM+TM+TM<-THUMP+ThiI</td><td>GT4</td><td>407</td><td>mshA</td><td>unclassified Bacteria</td><td>bacterium BMS3Bbin02</td><td>D-inositol 3-phosphate glycosyltransferase [bacterium BMS3Bbin02]</td><td>GCA_002898115.1</td></tr>
<tr><td>HBN24878.1</td><td>GT4*→</td><td>GT4</td><td>721</td><td>DD447_07855</td><td>Firmicutes</td><td>Lachnospiraceae bacterium</td><td>TPA: hypothetical protein DD447_07855 [Lachnospiraceae bacterium]</td><td>GCA_003503295.1</td></tr>
<tr><td>HCI14011.1</td><td>GT4+APATPase+TPR*→</td><td>GT4+APATPase+TPR</td><td>1340</td><td>DFK12_08625</td><td>Betaproteobacteria</td><td>Gallionellaceae bacterium</td><td>TPA: hypothetical protein DFK12_08625 [Gallionellaceae bacterium]</td><td>GCA_003521785.1</td></tr>
<tr><td>HCS09332.1</td><td><-MODE-HTH+PBPII||?→||||<-?<-PSE<-PrpF||?→ PSE→ STAND+TPR+GT4*→</td><td>STAND+TPR+GT4</td><td>1081</td><td>DIW67_19725</td><td>Gammaproteobacteria</td><td>Pseudomonas sp.</td><td>TPA: glycosyltransferase [Pseudomonas sp.]</td><td>GCA_003531165.1</td></tr>
<tr><td>HDL41796.1</td><td>GT4*→</td><td>GT4</td><td>345</td><td>ENG98_02120</td><td>Actinobacteria</td><td>Actinobacteria bacterium</td><td>TPA: glycosyltransferase, partial [Actinobacteria bacterium]</td><td>GCA_011041235.1</td></tr>
<tr><td>KDN80799.1</td><td>MNS+GT4*→||||<-?||?→ ABC_tran→</td><td>MNS+GT4</td><td>1137</td><td>KCH_74340</td><td>Actinobacteria</td><td>Kitasatospora cheerisanensis KCTC 2395</td><td>hypothetical protein KCH_74340 [Kitasatospora cheerisanensis KCTC 2395]</td><td>GCA_000696185.1</td></tr>
<tr><td>KNY05162.1</td><td>GT4*→</td><td>GT4</td><td>533</td><td>AKH00_12305</td><td>Actinobacteria</td><td>Microbacterium sp. GCS4</td><td>hypothetical protein AKH00_12305 [Microbacterium sp. GCS4]</td><td>GCA_001262495.1</td></tr>
<tr><td>KOX13006.1</td><td><-ABC_tran||?→?→||||<-?<-TIR+NACHT||HTH+APATPase+TPR+GT4*→</td><td>HTH+APATPase+TPR+GT4</td><td>1158</td><td>ADK67_45665</td><td>Actinobacteria</td><td>Saccharothrix sp. NRRL B-16348</td><td>NTPase [Saccharothrix sp. NRRL B-16348]</td><td>GCA_001280085.1</td></tr>
<tr><td>KQM59620.1</td><td>GT4*→||||<-TRPR-HTH+PBPI<-?<-?<-?<-?||PSE→?→ PfkB→</td><td>GT4</td><td>155</td><td>ASE64_09860</td><td>Actinobacteria</td><td>Agreia sp. Leaf210</td><td>hypothetical protein ASE64_09860 [Agreia sp. Leaf210]</td><td>GCA_001421485.1</td></tr>
</tbody>
</table>

<table>
<tr><th>acc</th><th>operon</th><th>architecture</th><th>len</th><th>gen.name</th><th>taxend</th><th>species</th><th>defline</th><th>gca</th></tr>
<tr><td>KQW27056.1</td><td>STAND+GT4*→</td><td>STAND+GT4</td><td>1005</td><td>ASC85_27055</td><td>Gammaproteobacteria</td><td>Pseudomonas sp. Root401</td><td>hypothetical protein ASC85_27055 [Pseudomonas sp. Root401]</td><td>GCA_001425105.1</td></tr>
<tr><td>KVF32402.1</td><td><-SIG+RCDG1+YidC_periplas+60KD_IMP<-?<-?<-?||?→?→?→ GT4+B12-binding+RADICAL-SAM*→||||<-rve_3</td><td>GT4+B12-binding+RADICAL-SAM</td><td>1400</td><td>WJ08_12515</td><td>Betaproteobacteria</td><td>Burkholderia vietnamiensis</td><td>radical SAM protein [Burkholderia vietnamiensis]</td><td>GCA_001524025.1</td></tr>
<tr><td>KVM90153.1</td><td>GT4+B12-binding+RADICAL-SAM*→</td><td>GT4+B12-binding+RADICAL-SAM</td><td>1404</td><td>WT05_03580</td><td>Betaproteobacteria</td><td>Burkholderia stagnalis</td><td>radical SAM protein [Burkholderia stagnalis]</td><td>GCA_001527545.1</td></tr>
<tr><td>KVM94412.1</td><td>TniB→?→ NLPC→||||<-?||?→ GT4+B12-binding+RADICAL-SAM*→</td><td>GT4+B12-binding+RADICAL-SAM</td><td>1404</td><td>WT07_28140</td><td>Betaproteobacteria</td><td>Burkholderia stagnalis</td><td>radical SAM protein [Burkholderia stagnalis]</td><td>GCA_001527585.1</td></tr>
<tr><td>KVX13946.1</td><td>GT4*→||||<-?||ABhydrolase→||||<-TRANSGLUTAMINASE</td><td>GT4</td><td>282</td><td>WL03_19390</td><td>Betaproteobacteria</td><td>Burkholderia ubonensis</td><td>hypothetical protein WL03_19390 [Burkholderia ubonensis]</td><td>GCA_001539165.1</td></tr>
<tr><td>KVZ11040.1</td><td>GT4+APATPase+TPR+TPR*→</td><td>GT4+APATPase+TPR+TPR</td><td>1278</td><td>WL11_04565</td><td>Betaproteobacteria</td><td>Burkholderia ubonensis</td><td>hypothetical protein WL11_04565 [Burkholderia ubonensis]</td><td>GCA_001530465.1</td></tr>
<tr><td>KWI84975.1</td><td>GT4*→||||<-?||ABhydrolase→||||<-TRANSGLUTAMINASE</td><td>GT4</td><td>325</td><td>WM08_23745</td><td>Betaproteobacteria</td><td>Burkholderia ubonensis</td><td>hypothetical protein WM08_23745 [Burkholderia ubonensis]</td><td>GCA_001534135.1</td></tr>
<tr><td>KWK82218.1</td><td>NACHT+TPR+GT4*→</td><td>NACHT+TPR+GT4</td><td>1024</td><td>WM17_17155</td><td>Betaproteobacteria</td><td>Burkholderia ubonensis</td><td>hypothetical protein WM17_17155 [Burkholderia ubonensis]</td><td>GCA_001533745.1</td></tr>
<tr><td>MBA3007143.1</td><td>APATPase+GT4+TPR+TPR+TPR+TPR+TPR*→</td><td>APATPase+GT4+TPR+TPR+TPR+TPR+TPR</td><td>1488</td><td>FP810_11305</td><td>Deltaproteobacteria</td><td>Desulfocapsa sp.</td><td>tetratricopeptide repeat protein [Desulfocapsa sp.]</td><td>GCA_013792135.1</td></tr>
<tr><td>MBA3548505.1</td><td>GT4*→</td><td>GT4</td><td>125</td><td>H0T76_18640</td><td>Deltaproteobacteria</td><td>Nannocystis sp.</td><td>glycosyltransferase [Nannocystis sp.]</td><td>GCA_013812955.1</td></tr>
<tr><td>MBS82255.1</td><td>NACHT+GT4*→?→?→?→||||<-?<-?<-SIG+rve</td><td>NACHT+GT4</td><td>1659</td><td>CMO32_42310</td><td>Betaproteobacteria</td><td>Variovorax sp.</td><td>hypothetical protein CMO32_42310 [Variovorax sp.]</td><td>GCA_002729445.1</td></tr>
</table>

acc	operon	architecture	len	gen.name	taxend	species	defline	gca
MQY06528.1	REase+SFI-ATPase→?→?→||||<-?||?→||||<-?||GT4+APATPase+TPR+TPR*→	GT4+APATPase+TPR+TPR	1104	mshA_10	Actinobacteria	Actinomadura sp. RB68	D-inositol-3-phosphate glycosyltransferase [Actinomadura sp. RB68]	GCA_009604375.1
MQY24997.1	ESX-1_EspG→||||<-?<-?||?→?→?→ WXG+TOXIN-PL+ART+Caspase+ZetaToxin+GT4*→	WXG+TOXIN-PL+ART+Caspase+ZetaToxin+GT4	3249	mshA_3	Actinobacteria	Nocardia sp. RB56	D-inositol-3-phosphate glycosyltransferase [Nocardia sp. RB56]	GCA_009604425.1
MSV29911.1	GT4+APATPase+BetaPropeller*→	GT4+APATPase+BetaPropeller	1058	EXQ52_14375	Acidobacteria	Bryobacterales bacterium	glycosyltransferase [Bryobacterales bacterium]	GCA_009697455.1
MWJ13606.1	ABC_tran→?→||||<-?||GT4*→||||<-?||?→?→?→||||<-?||tRNA→?→ Pribosyltran_N+Pribosyl_synth→	GT4	267	DOT98_12255	Actinobacteria	Clavibacter michiganensis subsp. michiganensis	hypothetical protein DOT98_12255 [Clavibacter michiganensis subsp. michiganensis]	GCA_009793225.1
NED51435.1	GT4*→	GT4	88	G3I24_11000	Actinobacteria	Micromonospora aurantiaca	glycosyl transferase family 1, partial [Micromonospora aurantiaca]	GCA_010550585.1
NNJ62389.1	DUF4913→?→ GT4*→	GT4	464	HKP61_15900	Actinobacteria	Dactylosporangium sp.	glycosyltransferase family 4 protein [Dactylosporangium sp.]	GCA_013041765.1
NOX21677.1	Asparaginase_II→ Acetyltransf_6→ GT4*→?→?→||||<-?<-SIG+TM+TM+TM<-THUMP+ThiI	GT4	407	GXP36_01090	Actinobacteria	Actinobacteria bacterium	glycosyltransferase [Actinobacteria bacterium]	GCA_013151445.1
NQZ33933.1	GT4+APATPase*→	GT4+APATPase	841	HRU06_21905	Gammaproteobacteria	Oceanospirillaceae bacterium	glycosyltransferase, partial [Oceanospirillaceae bacterium]	GCA_013215375.1
NTC82454.1	<-TniB<-?<-?<-?||?→?→?→ NACHT+GT4*→	NACHT+GT4	1640	G6M81_07325	Alphaproteobacteria	Agrobacterium tumefaciens	glycosyltransferase [Agrobacterium tumefaciens]	GCA_013319045.1
NUQ74496.1	SIR2+TPR+SAVED+GT4*→	SIR2+TPR+SAVED+GT4	1362	HUU21_13150	Deltaproteobacteria	Polyangiaceae bacterium	glycosyltransferase [Polyangiaceae bacterium]	GCA_013360665.1

<table>
<tr><th>acc</th><th>operon</th><th>architecture</th><th>len</th><th>gen.name</th><th>taxend</th><th>species</th><th>defline</th><th>gca</th></tr>
<tr><td>NUS43770.1</td><td>GT4*→</td><td>GT4</td><td>631</td><td>HOQ24_08805</td><td>Actinobacteria</td><td>Mycobacteriaceae bacterium</td><td>glycosyltransferase family 4 protein, partial [Mycobacteriaceae bacterium]</td><td>GCA_013362555.1</td></tr>
<tr><td>NUS72884.1</td><td>SIG+GT4+ZetaToxin*→</td><td>SIG+GT4+ZetaToxin</td><td>750</td><td>HOQ05_05695</td><td>Actinobacteria</td><td>Corynebacteriales bacterium</td><td>glycosyltransferase [Corynebacteriales bacterium]</td><td>GCA_013362805.1</td></tr>
<tr><td>NUT53093.1</td><td>SWACOS+GT4*→</td><td>SWACOS+GT4</td><td>1162</td><td>HOV94_38270</td><td>Actinobacteria</td><td>Saccharothrix sp.</td><td>glycosyltransferase [Saccharothrix sp.]</td><td>GCA_013362745.1</td></tr>
<tr><td>OAI03591.1</td><td>GT4+APATPase+TPR*→</td><td>GT4+APATPase+TPR</td><td>1115</td><td>A1332_15425</td><td>Gammaproteobacteria</td><td>Methylomonas methanica</td><td>hypothetical protein A1332_15425, partial [Methylomonas methanica]</td><td>GCA_001644035.1</td></tr>
<tr><td>ODT28534.1</td><td>GT4*→||||<-?<-?||?→||||<-?<-?||?→ NUDIX→</td><td>GT4</td><td>325</td><td>ABS63_03615</td><td>Actinobacteria</td><td>Microbacterium sp. SCN 70-27</td><td>hypothetical protein ABS63_03615 [Microbacterium sp. SCN 70-27]</td><td>GCA_001724425.1</td></tr>
<tr><td>OGS75014.1</td><td>GT4+APATPase+TPR*→</td><td>GT4+APATPase+TPR</td><td>1314</td><td>A2063_09475</td><td>Betaproteobacteria</td><td>Gallionellales bacterium GWA2_60_142</td><td>hypothetical protein A2063_09475 [Gallionellales bacterium GWA2_60_142]</td><td>GCA_001801085.1</td></tr>
<tr><td>OKJ80919.1</td><td><-TPR+Caspase<-CATASP||CATRA-N+CATRA-C+GT4*→ HTH+APATPase+TPR→?→?→||||<-?<-CATASP</td><td>CATRA-N+CATRA-C+GT4</td><td>694</td><td>AMK32_24710</td><td>Actinobacteria</td><td>Streptomyces sp. CB01883</td><td>hypothetical protein AMK32_24710 [Streptomyces sp. CB01883]</td><td>GCA_001905855.1</td></tr>
<tr><td>OLT27035.1</td><td>ZetaToxin+GT4+NUDIX+ART*→</td><td>ZetaToxin+GT4+NUDIX+ART</td><td>1451</td><td>BJF83_19460</td><td>Actinobacteria</td><td>Nocardiopsis sp. CNR-923</td><td>hypothetical protein BJF83_19460 [Nocardiopsis sp. CNR-923]</td><td>GCA_001942255.1</td></tr>
<tr><td>ONI48735.1</td><td><-TPR+Caspase<-CATASP||CATRA-N+CATRA-C+GT4*→ HTH+APATPase+TPR→||||<-HTH||?→?→?→||||<-?<-CATASP</td><td>CATRA-N+CATRA-C+GT4</td><td>727</td><td>mshA_4</td><td>Actinobacteria</td><td>Streptomyces sp. IB2014 011-1</td><td>D-inositol 3-phosphate glycosyltransferase [Streptomyces sp. IB2014 011-1]</td><td>GCA_001983595.1</td></tr>
<tr><td>OPK01862.1</td><td>GT4+B12-binding+RADICAL-SAM*→</td><td>GT4+B12-binding+RADICAL-SAM</td><td>1407</td><td>BZ164_25460</td><td>Gammaproteobacteria</td><td>Pseudomonas veronii</td><td>hypothetical protein BZ164_25460 [Pseudomonas veronii]</td><td>GCA_002029325.1</td></tr>
</table>

acc	operon	architecture	len	gen.name	taxend	species	defline	gca
OWQ88588.1	GT4+HD*→	GT4+HD	1415	CDN99_17225	Betaproteobacteria	Roseateles aquatilis	hypothetical protein CDN99_17225 [Roseateles aquatilis]	GCA_002205645.1
OXM47905.1	<-TIR+APATPase||HTH+APATPase+TPR+GT4*→	HTH+APATPase+TPR+GT4	1166	CFP75_23295	Actinobacteria	Amycolatopsis alba DSM 44262	NTPase [Amycolatopsis alba DSM 44262]	GCA_002234385.1
OZV80564.1	<-TPR+Caspase||GT4*→?→?→||||<-?<-?<-ABC_tran	GT4	587	CA850_14545	Actinobacteria	Micromonospora echinospora	hypothetical protein CA850_14545 [Micromonospora echinospora]	GCA_002266845.1
PBV03828.1	STAND+GT4*→	STAND+GT4	1006	CJU35_32055	Gammaproteobacteria	Pseudomonas aeruginosa	hypothetical protein CJU35_32055 [Pseudomonas aeruginosa]	GCA_002326115.1
PJI96546.1	GT4+B12-binding+RADICAL-SAM*→?→?→||||<-?<-LexA	GT4+B12-binding+RADICAL-SAM	1404	CLU85_1299	Betaproteobacteria	Acidovorax sp. 69	radical SAM superfamily enzyme YgiQ (UPF0313 family) [Acidovorax sp. 69]	GCA_002797445.1
PJO54021.1	GT4*→	GT4	721	CR156_18515	Gammaproteobacteria	Stenotrophomonas lactitubi	hypothetical protein CR156_18515 [Stenotrophomonas lactitubi]	GCA_002803515.1
PKN17915.1	NACHT+GT4*→	NACHT+GT4	1644	CVU71_10310	Deltaproteobacteria	Deltaproteobacteria bacterium HGW-Deltaproteobacteria-6	hypothetical protein CVU71_10310 [Deltaproteobacteria bacterium HGW-Deltaproteobacteria-6]	GCA_002840435.1
PKV83308.1	HTH→?→?→||||<-?<-?||Caspase+NACHT+GT4*→	Caspase+NACHT+GT4	1229	BX283_0808	Actinobacteria	Streptomyces sp. TLI_146	glycosyltransferase involved in cell wall biosynthesis [Streptomyces sp. TLI_146]	GCA_002846415.1
PNA01938.1	GT4+APATPase+TPR+TPR*→	GT4+APATPase+TPR+TPR	1383	C1X79_03750	Gammaproteobacteria	Pseudomonas sp. FW305-42	hypothetical protein C1X79_03750 [Pseudomonas sp. FW305-42]	GCA_002883975.1

acc	operon	architecture	len	gen.name	taxend	species	defline	gca
PRY58656.1	GT4*→ APATPase+TPR→||||<-?<-TRPR-HTH+PBPI	GT4	412	B0I28_105371	Actinobacteria	Glycomyces artemisiae	glycosyltransferase involved in cell wall biosynthesis [Glycomyces artemisiae]	GCA_003002955.1
PSR67860.1	WXG+MPTase→ WXG+LuxR-HTH+LuxR-HTH+LuxR-HTH+LuxR-HTH+TetR-HTH+TetR-HTH+LuxR-HTH+LuxR-HTH+LexA-HTH+LuxR-HTH+LuxR-HTH+LuxR-HTH+LuxR-HTH+LuxR-HTH+PP2C*→?→ ABhydrolase→	WXG+LuxR-HTH+LuxR-HTH+LuxR-HTH+LuxR-HTH+TetR-HTH+TetR-HTH+LuxR-HTH+LuxR-HTH+LexA-HTH+LuxR-HTH+LuxR-HTH+LuxR-HTH+LuxR-HTH+LuxR-HTH+PP2C	14198	C8258_13415	Actinobacteria	Nocardia sp. MDA0666	hypothetical protein C8258_13415 [Nocardia sp. MDA0666]	GCA_003023875.1
PXX02325.1	GT4+B12-binding+RADICAL-SAM*→	GT4+B12-binding+RADICAL-SAM	1404	C7400_1582	Betaproteobacteria	Paraburkholderia tropica	radical SAM superfamily enzyme YgiQ (UPF0313 family) [Paraburkholderia tropica]	GCA_003201665.1
PZM02119.1	<-MODE-HTH+PBPII||?→||||<-MODE-HTH+PBPII||?→?→?→?→ NACHT+GT4*→	NACHT+GT4	1624	CPJ17_11030	Alphaproteobacteria	Rhizobium tumorigenes	hypothetical protein CPJ17_11030 [Rhizobium tumorigenes]	GCA_003240565.1
REF53875.1	SWACOS+GT4*→||||<-?<-MFS	SWACOS+GT4	1165	BX279_1810	Actinobacteria	Streptomyces sp. Ag82_O1-9	glycosyltransferase involved in cell wall biosynthesis [Streptomyces sp. Ag82_O1-9]	GCA_003386845.1
RII01628.1	STAND→?→?→ DUF4913→||||<-AbiEii<-AbiEi_4||?→ GT4*→?→ AbiEi_4→	GT4	476	NI17_16340	Actinobacteria	Thermobifida halotolerans	hypothetical protein NI17_16340 [Thermobifida halotolerans]	GCA_003574835.1
RKS05362.1	<-APATPase+TPR||?→ GT4*→	GT4	412	DFP74_0957	Actinobacteria	Nocardiopsis sp. Huas11	glycosyltransferase involved in cell wall biosynthesis [Nocardiopsis sp. Huas11]	GCA_003634495.1
ROV69600.1	<-ABC_tran<-?||PSE→ Caspase+NACHT+GT4*→	Caspase+NACHT+GT4	1187	D3105_04960	Actinobacteria	Streptomyces globisporus	glycosyltransferase [Streptomyces globisporus]	GCA_003501885.1
RSM57832.1	cNMP_binding+HTH_Crp_2→?→ GT4*→||||<-ACYC+APATPase+TPR	GT4	411	DMH03_26130	Actinobacteria	Amycolatopsis sp. WAC 01376	hypothetical protein DMH03_26130 [Amycolatopsis sp. WAC 01376]	GCA_003947415.1
RWH25252.1	<-Vsr||?→?→?→||||<-?||GT4+Trypsin+PNPase*→	GT4+Trypsin+PNPase	961	EOQ76_19695	Alphaproteobacteria	Mesorhizobium sp.	glycosyltransferase [Mesorhizobium sp.]	GCA_004019165.1

acc	operon	architecture	len	gen.name	taxend	species	defline	gca
RYG90150.1	GT4*→	GT4	425	EON59_00395	Alphaproteobacteria	Alphaproteobacteria bacterium	glycosyltransferase, partial [Alphaproteobacteria bacterium]	GCA_004145145.1
RYZ67144.1	GT4*→	GT4	290	EOP09_11535	Proteobacteria	Proteobacteria bacterium	glycosyltransferase family 1 protein, partial [Proteobacteria bacterium]	GCA_004174565.1
SCF29539.1	<-TPR+Caspase||GT4*→?→?→||||<-?<-?<-ABC_tran	GT4	549	GA0070618_4943	Actinobacteria	Micromonospora echinospora	Glycosyltransferase involved in cell wall bisynthesis [Micromonospora echinospora]	GCA_900091495.1
SCF46304.1	<-WXG<-?<-?<-?<-?<-TPR+Caspase||GT4*→	GT4	549	GA0070563_114162	Actinobacteria	Micromonospora carbonacea	Glycosyltransferase involved in cell wall bisynthesis [Micromonospora carbonacea]	GCA_900091535.1
SCH34126.1	GT4*→ TIR→	GT4	721	SAMEA3545339_00864	Firmicutes	uncultured Clostridium sp.	glycosyltransferase%2C MSMEG_0565 family [uncultured Clostridium sp.]	GCA_900066565.1
SCL28302.1	<-TPR+Caspase||GT4*→	GT4	545	GA0074694_5072	Actinobacteria	Micromonospora inyonensis	Glycosyltransferase involved in cell wall bisynthesis [Micromonospora inyonensis]	GCA_900091415.1
SCZ75034.1	SWACOS+GT4*→?→||||<-?<-?||MFS→	SWACOS+GT4	1147	SAMN03159460_05304	Gammaproteobacteria	Pseudomonas sp. NFPP17	Glycosyltransferase involved in cell wall bisynthesis [Pseudomonas sp. NFPP17]	GCA_900102935.1
SDU29240.1	rve_3→ GT4*→	GT4	362	SAMN04488548_134376	Actinobacteria	Gordonia westfalica	Glycosyltransferase involved in cell wall bisynthesis [Gordonia westfalica]	GCA_900105725.1
SDX14297.1	LRR+AP-GTPase+COR→ GT4*→	GT4	472	SAMN05216300_1202	Betaproteobacteria	Nitrosomonas oligotropha	Glycosyltransferase involved in cell wall bisynthesis [Nitrosomonas oligotropha]	GCA_900106555.1

<table>
<tr><th>acc</th><th>operon</th><th>architecture</th><th>len</th><th>gen.name</th><th>taxend</th><th>species</th><th>defline</th><th>gca</th></tr>
<tr><td>SEK03344.1</td><td><-Sigma||?→||||<-?<-?<-?||GT4+B12-binding*→</td><td>GT4+B12-binding</td><td>1051</td><td>SAMN05518849_113037</td><td>Alphaproteobacteria</td><td>Sphingobium sp. AP50</td><td>Glycosyltransferase involved in cell wall bisynthesis [Sphingobium sp. AP50]</td><td>GCA_900109095.1</td></tr>
<tr><td>SIO56974.1</td><td>STAND+TPR+GT4*→</td><td>STAND+TPR+GT4</td><td>1125</td><td>SAMN05444165_3787</td><td>Betaproteobacteria</td><td>Paraburkholderia phenazinium</td><td>Glycosyltransferase involved in cell wall bisynthesis [Paraburkholderia phenazinium]</td><td>GCA_900142845.1</td></tr>
<tr><td>SNR45646.1</td><td>HTH_Tnp_1→ TnpB_IS66→?→ NACHT+GT4*→</td><td>NACHT+GT4</td><td>1639</td><td>SAMN06265370_105216</td><td>Alphaproteobacteria</td><td>Puniceibacterium sediminis</td><td>Glycosyltransferase involved in cell wall bisynthesis [Puniceibacterium sediminis]</td><td>GCA_900188035.1</td></tr>
<tr><td>TAK32775.1</td><td>GT4*→</td><td>GT4</td><td>646</td><td>EPO40_01735</td><td>Deltaproteobacteria</td><td>Myxococcaceae bacterium</td><td>glycosyltransferase [Myxococcaceae bacterium]</td><td>GCA_004297725.1</td></tr>
<tr><td>TCO61886.1</td><td>GT4*→||||<-TPR</td><td>GT4</td><td>392</td><td>EV192_10223</td><td>Actinobacteria</td><td>Actinocrispum wychmicini</td><td>glycosyltransferase involved in cell wall biosynthesis [Actinocrispum wychmicini]</td><td>GCA_004345645.1</td></tr>
<tr><td>TDP89614.1</td><td>GT4*→ TIR+APATPase+TPR→ TPR+Caspase→||||<-TPR+Caspase<-CATASP||CATRA-N+CATRA-C+GT4→</td><td>GT4</td><td>328</td><td>EV186_11214</td><td>Actinobacteria</td><td>Labedaea rhizosphaerae</td><td>glycosyltransferase involved in cell wall biosynthesis [Labedaea rhizosphaerae]</td><td>GCA_004362825.1</td></tr>
<tr><td>TGX49078.1</td><td>GT4+NACHT*→</td><td>GT4+NACHT</td><td>1219</td><td>E5A73_19725</td><td>Alphaproteobacteria</td><td>Sphingomonas gei</td><td>glycosyltransferase [Sphingomonas gei]</td><td>GCA_004792685.1</td></tr>
<tr><td>TPG40509.1</td><td>GT4*→</td><td>GT4</td><td>136</td><td>EAH79_11405</td><td>Alphaproteobacteria</td><td>Sphingomonas koreensis</td><td>glycosyltransferase [Sphingomonas koreensis]</td><td>GCA_006438735.1</td></tr>
<tr><td>TQL90659.1</td><td><-DUF4242<-?<-?<-PSE||?→||||<-HTH+APATPase+TPR<-TPR+Caspase<-CATASP||CATRA-N+CATRA-C+GT4*→||||<-?<-?<-?<-?<-?<-REase+SFI-ATPase</td><td>CATRA-N+CATRA-C+GT4</td><td>735</td><td>FB559_7970</td><td>Actinobacteria</td><td>Actinoallomurus bryophytorum</td><td>glycosyltransferase involved in cell wall biosynthesis [Actinoallomurus bryophytorum]</td><td>GCA_006716425.1</td></tr>
</table>

<table>
<thead>
<tr><th>acc</th><th>operon</th><th>architecture</th><th>len</th><th>gen.name</th><th>taxend</th><th>species</th><th>defline</th><th>gca</th></tr>
</thead>
<tbody>
<tr><td>TWH06591.1</td><td><-TPR+Caspase||GT4*→?→?→||||<-?<-?<-ABC_tran</td><td>GT4</td><td>587</td><td>JD80_04174</td><td>Actinobacteria</td><td>Micromonospora echinospora</td><td>glycosyltransferase involved in cell wall biosynthesis [Micromonospora echinospora]</td><td>GCA_007829475.1</td></tr>
<tr><td>TWJ29688.1</td><td><-TPR+Caspase||GT4*→?→||||<-?<-?<-ABC_tran</td><td>GT4</td><td>549</td><td>JD81_03199</td><td>Actinobacteria</td><td>Micromonospora sagamiensis</td><td>glycosyltransferase involved in cell wall biosynthesis [Micromonospora sagamiensis]</td><td>GCA_007829995.1</td></tr>
<tr><td>TXH45940.1</td><td>GT4+nSTAND1+ANK+ANK*→</td><td>GT4+nSTAND1+ANK+ANK</td><td>1838</td><td>E6Q92_02710</td><td>Betaproteobacteria</td><td>Burkholderiaceae bacterium</td><td>hypothetical protein E6Q92_02710 [Burkholderiaceae bacterium]</td><td>GCA_008012415.1</td></tr>
<tr><td>VBB15457.1</td><td>GT4+B12-binding+RADICAL-SAM*→</td><td>GT4+B12-binding+RADICAL-SAM</td><td>1404</td><td>BSTAB16_5653</td><td>Betaproteobacteria</td><td>Burkholderia stabilis</td><td>radical SAM protein [Burkholderia stabilis]</td><td>GCA_900240005.1</td></tr>
<tr><td>VBK75063.1</td><td>GT4+B12-binding+RADICAL-SAM*→</td><td>GT4+B12-binding+RADICAL-SAM</td><td>1404</td><td>SAMEA2064914_0[illegible]</td><td>Betaproteobacteria</td><td>Burkholderia pseudomallei</td><td>Glycogen synthase [Burkholderia pseudomallei]</td><td>GCA_900592755.1</td></tr>
<tr><td>WP_005483680.1</td><td>TRANSGLUTAMINASE→||||<-?<-Zeta_toxin<-?<-?||?→ GT4*→||||<-?<-?<-?<-?<-DUF4913</td><td>GT4</td><td>590</td><td>SBD_RS28370</td><td>Actinobacteria</td><td>Streptomyces</td><td>MULTISPECIES: glycosyltransferase [Streptomyces]</td><td>GCF_000340335.1</td></tr>
<tr><td>WP_007453552.1</td><td>Multi-TM→?→ STAND→?→ DUF4913→||||<-?||?→ GT4*→?→?→?→ NUDIX→||||<-?||?→ PSE→ ABC_tran→</td><td>GT4</td><td>437</td><td>MILUP08_RS00[illegible]</td><td>Actinobacteria</td><td>Micromonospora lupini</td><td>glycosyltransferase family 4 protein [Micromonospora lupini]</td><td>GCF_000297395.2</td></tr>
<tr><td>WP_007734957.1</td><td>STAND+TPR+GT4*→</td><td>STAND+TPR+GT4</td><td>1062</td><td>B5Z53_RS05165</td><td>Betaproteobacteria</td><td>Paraburkholderia hospita</td><td>glycosyltransferase [Paraburkholderia hospita]</td><td>GCF_900167965.1</td></tr>
<tr><td>WP_007954182.1</td><td>SQHop_cyclase_C+Prenyltrans+GT4*→?→||||<-?||?→||||<-?||?→ XylR_N+V4R+Sigma54_activat+HTH_8→</td><td>SQHop_cyclase_C+Prenyltrans+GT4</td><td>779</td><td>JBW_RS18725</td><td>Firmicutes</td><td>Pelosinus fermentans</td><td>glycosyltransferase [Pelosinus fermentans]</td><td>GCF_000271665.2</td></tr>
<tr><td>WP_010223837.1</td><td>GT4+STAND*→ TPR→||||<-MODE-HTH+PBPII</td><td>GT4+STAND</td><td>546</td><td>UW3_RS011698[illegible]</td><td>Gammaproteobacteria</td><td>Pseudomonas donghuensis</td><td>glycosyltransferase [Pseudomonas donghuensis]</td><td>GCF_000259195.1</td></tr>
<tr><td>WP_013226178.1</td><td><-TIR+APATPase||HTH+APATPase+TPR+GT4*→</td><td>HTH+APATPase+TPR+GT4</td><td>1178</td><td>IW14_RS45345</td><td>Actinobacteria</td><td>Amycolatopsis mediterranei</td><td>glycosyltransferase [Amycolatopsis mediterranei]</td><td>GCF_000196835.1</td></tr>
<tr><td>WP_017131143.1</td><td>APATPase+GT4*→</td><td>APATPase+GT4</td><td>1299</td><td>BMX68_RS1184[illegible]</td><td>Gammaproteobacteria</td><td>Pseudomonas agarici</td><td>glycosyltransferase [Pseudomonas agarici]</td><td>GCF_900109755.1</td></tr>
</tbody>
</table>

<table>
<tr><th>acc</th><th>operon</th><th>architecture</th><th>len</th><th>gen.name</th><th>taxend</th><th>species</th><th>defline</th><th>gca</th></tr>
<tr><td>WP_017594508.1</td><td>GT4*→</td><td>GT4</td><td>503</td><td>D459_RS0118565</td><td>Actinobacteria</td><td>Nocardiopsis potens</td><td>glycosyltransferase family 4 protein [Nocardiopsis potens]</td><td>GCF_000341105.1</td></tr>
<tr><td>WP_018327601.1</td><td><-TniB<-?||?→||||<-?<-?||GT4+B12-binding+RADICAL-SAM*→||||<-?<-?||SIG+rve→</td><td>GT4+B12-binding+RADICAL-SAM</td><td>1381</td><td>GGD55_RS15170</td><td>Alphaproteobacteria</td><td>Rhizobium giardinii</td><td>B12-binding domain-containing radical SAM protein [Rhizobium giardinii]</td><td>GCF_014200295.1</td></tr>
<tr><td>WP_018612920.1</td><td>STAND+TPR+GT4*→</td><td>STAND+TPR+GT4</td><td>1063</td><td>CFII68_RS03385</td><td>Gammaproteobacteria</td><td>Pseudomonas sp. CFII68</td><td>glycosyltransferase [Pseudomonas sp. CFII68]</td><td>GCF_000416195.1</td></tr>
<tr><td>WP_019063569.1</td><td>MFS→||||<-?<-TPR+Caspase<-CATASP||CATRA-N+CATRA-C+GT4*→ HTH+APATPase+TPR→||||<-PSE||?→?→||||<-?<-CATASP<-TPR+Caspase</td><td>CATRA-N+CATRA-C+GT4</td><td>720</td><td>SPR02S_RS44725</td><td>Actinobacteria</td><td>Streptomyces prunicolor</td><td>glycosyltransferase family 4 protein [Streptomyces prunicolor]</td><td>GCF_000367365.1</td></tr>
<tr><td>WP_019093712.1</td><td>SWACOS+GT4*→</td><td>SWACOS+GT4</td><td>1138</td><td>BK646_RS17250</td><td>Gammaproteobacteria</td><td>Pseudomonas protegens</td><td>glycosyltransferase [Pseudomonas protegens]</td><td>GCF_003732085.1</td></tr>
<tr><td>WP_019609270.1</td><td><-AbiEii<-AbiEi_4||?→ GT4*→</td><td>GT4</td><td>497</td><td>G011_RS0115485</td><td>Actinobacteria</td><td>Nocardiopsis sp. CNS-639</td><td>glycosyltransferase family 4 protein [Nocardiopsis sp. CNS-639]</td><td>GCF_000381685.1</td></tr>
<tr><td>WP_020647322.1</td><td><-TIR+APATPase||HTH+APATPase+TPR+GT4*→</td><td>HTH+APATPase+TPR+GT4</td><td>1192</td><td>A3CE_RS0148230</td><td>Actinobacteria</td><td>Amycolatopsis balhimycina</td><td>tetratricopeptide repeat protein [Amycolatopsis balhimycina]</td><td>GCF_000384295.1</td></tr>
<tr><td>WP_023267980.1</td><td>GT4+APATPase+TPR+TPR*→</td><td>GT4+APATPase+TPR+TPR</td><td>1323</td><td>SHD_RS15540</td><td>Gammaproteobacteria</td><td>Shewanella decolorationis</td><td>tetratricopeptide repeat protein [Shewanella decolorationis]</td><td>GCF_000485795.1</td></tr>
<tr><td>WP_024631061.1</td><td><-AAA_21||GT4*→</td><td>GT4</td><td>1140</td><td>P364_RS0115745</td><td>Firmicutes</td><td>Paenibacillus sp. MAEPY2</td><td>glycosyltransferase [Paenibacillus sp. MAEPY2]</td><td>GCF_000499205.1</td></tr>
<tr><td>WP_027196515.1</td><td>Cluster214_2clades→ GT4*→</td><td>GT4</td><td>760</td><td>BJG93_RS27460</td><td>Betaproteobacteria</td><td>Paraburkholderia sprentiae</td><td>glycosyltransferase family 4 protein [Paraburkholderia sprentiae]</td><td>GCF_001865575.1</td></tr>
<tr><td>WP_029881581.1</td><td><-AAA_21||GT4*→</td><td>GT4</td><td>1003</td><td>P363_RS0103940</td><td>Firmicutes</td><td>Paenibacillus sp. MAEPY1</td><td>glycosyltransferase, partial [Paenibacillus sp. MAEPY1]</td><td>GCF_000499305.1</td></tr>
</table>

acc	operon	architecture	len	gen.name	taxend	species	defline	gca
WP_031021212.1	ABC_tran→||||<-?<-CATASP||CATRA-N+CATRA-C+GT4*→ HTH+APATPase+TPR→||||<-HTH||?→?→?→||||<-?<-CATASP	CATRA-N+CATRA-C+GT4	705	IF98_RS0120305	Actinobacteria	Streptomyces sp. NRRL S-1314	glycosyltransferase family 4 protein [Streptomyces sp. NRRL S-1314]	GCF_000719785.1
WP_031063336.1	ABC_tran→||||<-?<-CATASP||CATRA-N+CATRA-C+GT4*→ HTH+APATPase+TPR→||||<-HTH||?→?→?→||||<-?<-CATASP	CATRA-N+CATRA-C+GT4	705	IH17_RS0118610	Actinobacteria	Streptomyces sp. NRRL F-5527	glycosyltransferase family 4 protein [Streptomyces sp. NRRL F-5527]	GCF_014649055.1
WP_033524509.1	TRANSGLUTAMINASE→||||<-?||?→||||<-?||GT4+NUDIX+ART+PolB-NTase→*////<-?<-?<-?//?→ GT4*→||||<-?<-?<-?<-?<-DUF4913	GT4+NUDIX+ART+PolB-NTase	589	KS09_RS00195	Actinobacteria	Streptomyces galbus	glycosyltransferase [Streptomyces galbus]	GCF_000772895.1
WP_035284396.1	APATPase+TPR→ GT4*→	GT4	502	UO65_RS36825	Actinobacteria	Actinokineospora spheciospongiae	glycosyltransferase [Actinokineospora spheciospongiae]	GCF_000564855.1
WP_036515011.1	WXG+MPTase→ WXG+SecA+HTH+HTH+HTH+HTH+HTH+ST-Hiskin+PP2C+MPTase+GT4+TetR-HTH+TetR-HTH+TM+TM+TM+TM+TM+TM+LuxR-HTH+LuxR-HTH+LuxR-HTH+LuxR-HTH+LuxR-HTH+LuxR-HTH+LuxR-HTH+GNTR-HTH+GNTR-HTH+GNTR-HTH+GNTR-HTH+GNTR-HTH+GNTR-HTH*→	WXG+SecA+HTH+HTH+HTH+HTH+HTH+ST-Hiskin+PP2C+MPTase+GT4+TetR-HTH+TetR-HTH+TM+TM+TM+TM+TM+TM+LuxR-HTH+LuxR-HTH+LuxR-HTH+LuxR-HTH+LuxR-HTH+LuxR-HTH+LuxR-HTH+GNTR-HTH+GNTR-HTH+GNTR-HTH+GNTR-HTH+GNTR-HTH+GNTR-HTH	12585	NRH01S_RS188[illegible]0	Actinobacteria	Nocardia rhamnosiphila	GntR family transcriptional regulator [Nocardia rhamnosiphila]	GCF_001613485.1
WP_036994826.1	MFS→||||<-?||GT4+APATPase+TPR*→||||<-MODE-HTH+PBPII	GT4+APATPase+TPR	1318	BV82_RS07620	Gammaproteobacteria	Pseudomonas donghuensis	glycosyltransferase [Pseudomonas donghuensis]	GCF_000696345.1
WP_037254756.1	cNMP_binding+HTH_Crp_2→?→ GT4*→||||<-ACYC+APATPase+TPR	GT4	404	DMH04_42005	Actinobacteria	Kibdelosporangium aridum	glycosyltransferase [Kibdelosporangium aridum]	-
WP_039793765.1	<-TIR+APATPase||HTH+APATPase+TPR+GT4*→	HTH+APATPase+TPR+GT4	1175	CFP75_RS23290	Actinobacteria	Amycolatopsis alba	glycosyltransferase [Amycolatopsis alba]	GCF_000384215.1
WP_040866957.1	WXG+MPTase→ WXG+SecA+GT4+TetR-HTH+MPTase+HTH+HTH+HTH+HTH+HTH+HTH+HTH+HTH*→	WXG+SecA+GT4+TetR-HTH+MPTase+HTH+HTH+HTH+HTH+HTH+HTH+HTH+HTH	5345	ON29_RS15345	Actinobacteria	Nocardia exalbida	helix-turn-helix domain-containing protein [Nocardia exalbida]	GCF_000308575.1
WP_041117337.1	SWACOS+GT4*→?→||||<-?<-?||MFS→	SWACOS+GT4	1138	PPC_RS11795	Gammaproteobacteria	Pseudomonas protegens	glycosyltransferase [Pseudomonas protegens]	GCF_000828695.1
WP_042132624.1	GT4+B12-binding+RADICAL-SAM*→	GT4+B12-binding+RADICAL-SAM	1393	BJQ15_RS27760	Gammaproteobacteria	Pseudomonas	MULTISPECIES: B12-binding domain-containing radical SAM protein [Pseudomonas]	GCF_000730605.1

acc	operon	architecture	len	gen.name	taxend	species	defline	gca
WP_043243023.1	Ribosomal_L20→?→?→?→?→ tRNA→?→ GT4+B12-binding+RADICAL-SAM*→	GT4+B12-binding+RADICAL-SAM	1402	TX97_RS10150	Gammaproteobacteria	Pseudomonas alcaligenes	B12-binding domain-containing radical SAM protein [Pseudomonas alcaligenes]	GCF_000474255.1
WP_043510995.1	WXG+GT4+GT4+REase*→	WXG+GT4+GT4+REase	4153	ACSP50_RS07520	Actinobacteria	unclassified Actinoplanes	MULTISPECIES: glycosyltransferase [unclassified Actinoplanes]	GCF_000237145.1
WP_043738635.1	WXG+MPTase→ WXG+SecA+GT4+TetR-HTH+TetR-HTH+MPTase+HTH+HTH+HTH+HTH+HTH+HTH*→	WXG+SecA+GT4+TetR-HTH+TetR-HTH+MPTase+HTH+HTH+HTH+HTH+HTH+HTH	8037	ON27_RS37615	Actinobacteria	Nocardia asiatica	helix-turn-helix domain-containing protein [Nocardia asiatica]	GCF_000308415.1
WP_045583922.1	<-SIG+HTH_23+HTH_32||GT4+TPR*→	GT4+TPR	1241	VY88_RS20945	Alphaproteobacteria	Azospirillum thiophilum	tetratricopeptide repeat protein [Azospirillum thiophilum]	GCF_001305595.1
WP_050363680.1	Multi-TM→?→ STAND→?→ DUF4913→?→ GT4*→?→ Zeta_toxin→||||<-ABC_tran	GT4	555	DC095_RS07800	Actinobacteria	Streptomyces	MULTISPECIES: glycosyltransferase family 4 protein [Streptomyces]	GCF_000700005.2
WP_050791877.1	GT4+B12-binding+RADICAL-SAM*→?→||||<-PSE<-?||?→||||<-?<-?||Sigma→	GT4+B12-binding+RADICAL-SAM	1403	BUBO0001_RS30235	Betaproteobacteria	Burkholderia ubonensis	B12-binding domain-containing radical SAM protein [Burkholderia ubonensis]	GCF_000170335.1
WP_051466857.1	TPR+Caspase→ CATRA-N+CATRA-C+GT4*→ HTH+APATPase+TPR→||||<-TPR+Caspase||CATASP→	CATRA-N+CATRA-C+GT4	812	BCD49_RS04570	Actinobacteria	Frankia	MULTISPECIES: glycosyltransferase family 4 protein [Frankia]	GCF_000235425.2
WP_051653780.1	MNS+GT4*→||||<-?||PSE→ ABC_tran→	MNS+GT4	1132	KCH_RS38295	Actinobacteria	Kitasatospora cheerisanensis	glycosyltransferase [Kitasatospora cheerisanensis]	GCF_000696185.1
WP_051722648.1	Multi-TM→?→ STAND→?→ DUF4913→?→ GT4*→?→ Zeta_toxin→	GT4	561	IF83_RS0111160	Actinobacteria	Streptomyces albus	glycosyltransferase family 4 protein [Streptomyces albus]	GCF_000719865.1
WP_051765847.1	<-CASPASE+FGS<-?<-?<-?||GT4*→	GT4	406	OQ01_RS21365	Actinobacteria	Saccharothrix syringae	glycosyltransferase [Saccharothrix syringae]	GCF_000716755.1
WP_051865326.1	GT4*→	GT4	621	IG99_RS44170	Actinobacteria	Streptomyces griseus	glycosyltransferase [Streptomyces griseus]	GCF_000720255.1

acc	operon	architecture	len	gen.name	taxend	species	defline	gca
WP_052457075.1	<-TPR+Caspase<-CATASP||CATRA-N+CATRA-C+GT4*→ PSE→ PSE→ APATPase+TPR→||||<-HTH||?→||||<-?<-CATASP<-TPR+Caspase	CATRA-N+CATRA-C+GT4	747	HY68_RS39570	Actinobacteria	Streptomyces sp. AcH 505	glycosyltransferase [Streptomyces sp. AcH 505]	GCF_000818175.1
WP_052479793.1	TIR+APATPase+GT4*→	TIR+APATPase+GT4	810	APS67_RS01150	Actinobacteria	unclassified Streptomyces	MULTISPECIES: TIR domain-containing protein [unclassified Streptomyces]	GCF_000829695.1
WP_053258773.1	GT4+B12-binding+RADICAL-SAM*→	GT4+B12-binding+RADICAL-SAM	1401	ALQ35_RS05775	Gammaproteobacteria	Pseudomonas fluorescens	cobalamin B12-binding domain-containing protein [Pseudomonas fluorescens]	GCF_002091595.1
WP_053682326.1	MFS→?→||||<-?<-?||?→||||<-?||MPTase+GT4*→?→?→||||<-?<-?<-?<-ABC_tran	MPTase+GT4	764	ADK55_RS26505	Actinobacteria	Streptomyces sp. WM4235	DUF4157 domain-containing protein [Streptomyces sp. WM4235]	GCF_001279725.1
WP_054048489.1	TPR+Caspase→ TIR+APATPase+TPR→||||<-APATPase+TPR||GT4*→||||<-HTH+APATPase+TPR	GT4	456	BN1701_RS12505	Actinobacteria	Alloactinosynnema sp. L-07	glycosyltransferase family 4 protein [Alloactinosynnema sp. L-07]	GCF_900070365.1
WP_055337289.1	STAND+TPR+GT4*→?→ SIG+HTH_23+HTH_32→||||<-?<-?||DJ-1_PfpI→	STAND+TPR+GT4	1061	HXP36_RS10750	Betaproteobacteria	Ralstonia solanacearum	glycosyltransferase [Ralstonia solanacearum]	GCF_001373335.1
WP_055417577.1	MNS+GT4*→	MNS+GT4	1164	B1H29_RS20255	Actinobacteria	Streptomyces pactum	glycosyltransferase [Streptomyces pactum]	GCF_002005225.1
WP_055618904.1	NACHT+MNS→ MNS+GT4*→	MNS+GT4	754	JHAN_RS00420	Actinobacteria	Streptomyces sp. JHA19	glycosyltransferase [Streptomyces sp. JHA19]	GCF_001417695.1
WP_055624376.1	MNS+GT4*→	MNS+GT4	1149	JHAN_RS28925	Actinobacteria	Streptomyces sp. JHA19	AAA family ATPase [Streptomyces sp. JHA19]	GCF_001417695.1
WP_057006131.1	GT4*→	GT4	773	TU80_RS30870	Gammaproteobacteria	Pseudomonas	MULTISPECIES: glycosyltransferase [Pseudomonas]	GCF_900104875.1
WP_058422527.1	GT4+B12-binding+RADICAL-SAM*→	GT4+B12-binding+RADICAL-SAM	1407	AO063_RS26420	Gammaproteobacteria	Pseudomonas fluorescens	radical SAM protein [Pseudomonas fluorescens]	GCF_001466835.1

<table>
<tr><th>acc</th><th>operon</th><th>architecture</th><th>len</th><th>gen.name</th><th>taxend</th><th>species</th><th>defline</th><th>gca</th></tr>
<tr><td>WP_058721560.1</td><td>STAND+TPR+GT4*→||||<-?<-?<-?<-ABC_tran</td><td>STAND+TPR+GT4</td><td>1063</td><td>AT984_RS19735</td><td>Betaproteobacteria</td><td>Paucibacter sp. KCTC 42545</td><td>glycosyltransferase [Paucibacter sp. KCTC 42545]</td><td>GCF_001477625.1</td></tr>
<tr><td>WP_060944940.1</td><td>MNS+GT4*→</td><td>MNS+GT4</td><td>1149</td><td>AXW62_RS14785</td><td>Actinobacteria</td><td>Streptomyces hygroscopicus</td><td>glycosyltransferase [Streptomyces hygroscopicus]</td><td>GCF_001553455.1</td></tr>
<tr><td>WP_062579281.1</td><td>GT4*→||||<-?||?→?→?→?→ SIG+SnoaL→</td><td>GT4</td><td>372</td><td>ASG42_RS32705</td><td>Alphaproteobacteria</td><td>Rhizobium sp. Leaf391</td><td>glycosyltransferase family 1 protein [Rhizobium sp. Leaf391]</td><td>GCF_001424505.1</td></tr>
<tr><td>WP_062720264.1</td><td><-TPR+Caspase||GT4*→</td><td>GT4</td><td>610</td><td>AQJ67_RS19460</td><td>Actinobacteria</td><td>Streptomyces caeruleatus</td><td>glycosyltransferase [Streptomyces caeruleatus]</td><td>GCF_001514235.1</td></tr>
<tr><td>WP_062723774.1</td><td>MNS+GT4*→</td><td>MNS+GT4</td><td>1219</td><td>AQJ67_RS36455</td><td>Actinobacteria</td><td>Streptomyces caeruleatus</td><td>glycosyltransferase [Streptomyces caeruleatus]</td><td>GCF_001514235.1</td></tr>
<tr><td>WP_062964870.1</td><td>WXG+MPTase→ WXG+SecA+ST-Hiskin+LuxR-HTH+LuxR-HTH+LuxR-HTH+LuxR-HTH+ST-Hiskin+GT4+TetR-HTH+TetR-HTH+Tox-ART-RSE+LuxR-HTH+PP2C+MPTase+MPTase*→?→ ABhydrolase→</td><td>WXG+SecA+ST-Hiskin+LuxR-HTH+LuxR-HTH+LuxR-HTH+LuxR-HTH+ST-Hiskin+GT4+TetR-HTH+TetR-HTH+Tox-ART-RSE+LuxR-HTH+PP2C+MPTase+MPTase</td><td>9117</td><td>NA3_RS18555</td><td>Actinobacteria</td><td>Nocardia africana</td><td>glycosyltransferase [Nocardia africana]</td><td>GCF_001612635.1</td></tr>
<tr><td>WP_063038911.1</td><td>WXG+TM+TM+TM→?→?→?→?→?→ WXG+ZetaToxin+GT4*→</td><td>WXG+ZetaToxin+GT4</td><td>1614</td><td>NP3_RS06870</td><td>Actinobacteria</td><td>Nocardia pseudovaccinii</td><td>zeta toxin family protein [Nocardia pseudovaccinii]</td><td>GCF_001613225.1</td></tr>
<tr><td>WP_063127006.1</td><td>Zeta_toxin→||||<-?||MPTase→?→ WXG+TM+TM+TM+TM+GT4*→</td><td>WXG+TM+TM+TM+TM+GT4</td><td>1018</td><td>NF4_RS09930</td><td>Actinobacteria</td><td>Nocardia fusca</td><td>glycosyltransferase [Nocardia fusca]</td><td>-</td></tr>
<tr><td>WP_063882776.1</td><td>GT4+APATPase+TPR+TPR*→</td><td>GT4+APATPase+TPR+TPR</td><td>1322</td><td>A1L58_RS04155</td><td>Gammaproteobacteria</td><td>Shewanella baltica</td><td>tetratricopeptide repeat protein [Shewanella baltica]</td><td>GCF_001620325.1</td></tr>
<tr><td>WP_063889953.1</td><td>GT4+B12-binding+RADICAL-SAM*→||||<-?||?→||||<-?||?→||||<-?||?→||||<-TRPR-HTH+PBPI</td><td>GT4+B12-binding+RADICAL-SAM</td><td>1415</td><td>WI77_RS33765</td><td>Betaproteobacteria</td><td>Burkholderia ubonensis</td><td>radical SAM protein [Burkholderia ubonensis]</td><td>GCF_001525885.1</td></tr>
<tr><td>WP_064054195.1</td><td><-HTH_3||?→?→||||<-?<-Cluster466_2clades||STAND+GT4*→||||<-?<-Sigma</td><td>STAND+GT4</td><td>1084</td><td>ALQ29_RS11960</td><td>Gammaproteobacteria</td><td>Pseudomonas marginalis</td><td>glycosyltransferase [Pseudomonas marginalis]</td><td>GCF_001645105.1</td></tr>
<tr><td>WP_064273456.1</td><td>GT4*→</td><td>GT4</td><td>135</td><td>A7B19_RS17315</td><td>Actinobacteria</td><td>Streptomyces sp. RTd22</td><td>hypothetical protein [Streptomyces sp. RTd22]</td><td>GCF_001650215.1</td></tr>
</table>

acc	operon	architecture	len	gen.name	taxend	species	defline	gca
WP_065910241.1	GT4+B12-binding+RADICAL-SAM*→	GT4+B12-binding+RADICAL-SAM	1393	C9382_RS17200	Gammaproteobacteria	unclassified Pseudomonas	MULTISPECIES: cobalamin B12-binding domain-containing protein [unclassified Pseudomonas]	GCF_001702265.1
WP_067257069.1	GT4*→	GT4	398	AQJ58_RS33820	Actinobacteria	Streptomyces sp. DSM 15324	glycosyltransferase family 4 protein [Streptomyces sp. DSM 15324]	GCF_001514285.1
WP_067481412.1	HTH→?→||||<-?||NACHT+MNS→ MNS+GT4*→	MNS+GT4	764	AH6_RS18370	Actinobacteria	Actinomadura hibisca	glycosyltransferase [Actinomadura hibisca]	GCF_001552635.1
WP_067904030.1	SWACOS+GT4*→	SWACOS+GT4	1149	ACH01S_RS37935	Actinobacteria	Actinomadura chibensis	glycosyltransferase [Actinomadura chibensis]	GCF_001552135.1
WP_068874779.1	HTH_3→?→||||<-?<-?||?→ MPTase→?→ NACHT+GT4*→||||<-?||?→||||<-?<-?<-PSE<-?||?→ SIG+Lipase_GDSL_2→	NACHT+GT4	1671	AX747_RS22605	Alphaproteobacteria	unclassified Phenylobacterium	MULTISPECIES: glycosyltransferase [unclassified Phenylobacterium]	GCF_001557235.1
WP_069042340.1	NACHT+GT4*→	NACHT+GT4	1642	BSY240_RS11090	Alphaproteobacteria	Agrobacterium sp. RAC06	glycosyltransferase [Agrobacterium sp. RAC06]	GCF_001713475.1
WP_070086299.1	GT4+B12-binding+RADICAL-SAM*→	GT4+B12-binding+RADICAL-SAM	1393	BJQ21_RS10430	Gammaproteobacteria	unclassified Pseudomonas	MULTISPECIES: radical SAM protein [unclassified Pseudomonas]	GCF_001753875.1
WP_071611953.1	GT4*→?→?→?→ PadR→?→||||<-?<-ABC_tran	GT4	724	F7R00_RS19755	Firmicutes	Clostridium estertheticum	glycosyltransferase family 4 protein [Clostridium estertheticum]	GCF_001877035.1
WP_071621050.1	<-AbiEii<-AbiEi_4||?→ GT4*→	GT4	497	CGQ36_RS01685	Actinobacteria	Nocardiopsis dassonvillei	glycosyltransferase family 4 protein [Nocardiopsis dassonvillei]	GCF_001877055.1
WP_072444656.1	SWACOS+GT4*→?→||||<-?<-?||MFS→	SWACOS+GT4	1138	BM382_RS27510	Gammaproteobacteria	unclassified Pseudomonas	MULTISPECIES: glycosyltransferase [unclassified Pseudomonas]	GCF_900102935.1
WP_073746098.1	CASPASE→ tRNA→||||<-?||MNS+GT4*→	MNS+GT4	1169	AMK28_RS33605	Actinobacteria	Streptomyces sp. CB02115	glycosyltransferase [Streptomyces sp. CB02115]	GCF_001905645.1

<table>
<tr><th>acc</th><th>operon</th><th>architecture</th><th>len</th><th>gen.name</th><th>taxend</th><th>species</th><th>defline</th><th>gca</th></tr>
<tr><td>WP_074374750.1</td><td>DctM→?→?→?→?→||||<-?||GT4*→</td><td>GT4</td><td>488</td><td>VSP9026_RS20020</td><td>Gammaproteobacteria</td><td>Vibrio spartinae</td><td>glycosyltransferase family 4 protein [Vibrio spartinae]</td><td>GCF_900149295.1</td></tr>
<tr><td>WP_074848880.1</td><td>GT4*→</td><td>GT4</td><td>397</td><td>BLU62_RS07095</td><td>Actinobacteria</td><td>Gordonia westfalica</td><td>glycosyltransferase [Gordonia westfalica]</td><td>GCF_900105725.1</td></tr>
<tr><td>WP_076845054.1</td><td>WXG+TM+GT4+GT4*→</td><td>WXG+TM+GT4+GT4</td><td>1675</td><td>BL254_RS27435</td><td>Actinobacteria</td><td>Frankia sp. BMG5.30</td><td>hypothetical protein [Frankia sp. BMG5.30]</td><td>GCF_001983005.1</td></tr>
<tr><td>WP_077298179.1</td><td>PrpF→?→||||<-?<-?||?→||||<-?||GT4+nSTAND3+TPR*→||||<-rve+rve_3||?→ tRNA-synt_1b+S4→</td><td>GT4+nSTAND3+TPR</td><td>972</td><td>BXU06_RS07175</td><td>Betaproteobacteria</td><td>Aquaspirillum sp. LM1</td><td>hypothetical protein [Aquaspirillum sp. LM1]</td><td>GCF_002002905.1</td></tr>
<tr><td>WP_078753771.1</td><td>GT4+APATPase+TPR+TPR*→</td><td>GT4+APATPase+TPR+TPR</td><td>1333</td><td>B5D35_RS17215</td><td>Gammaproteobacteria</td><td>Enterovibrio nigricans</td><td>tetratricopeptide repeat protein [Enterovibrio nigricans]</td><td>GCF_900167155.1</td></tr>
<tr><td>WP_078789290.1</td><td>XylR_N+V4R+Sigma54_activat+HTH_8→?→ GT4+APATPase+TPR*→</td><td>GT4+APATPase+TPR</td><td>1307</td><td>B5D39_RS04945</td><td>Deltaproteobacteria</td><td>Geobacter thiogenes</td><td>tetratricopeptide repeat protein [Geobacter thiogenes]</td><td>GCF_900167465.1</td></tr>
<tr><td>WP_078936874.1</td><td>-</td><td>-</td><td>1884</td><td>KS09_RS00220</td><td>Actinobacteria</td><td>Streptomyces galbus</td><td>glycosyltransferase family 4 protein [Streptomyces galbus]</td><td>GCF_000772895.1</td></tr>
<tr><td>WP_078960520.1</td><td>TIR+APATPase+GT4*→</td><td>TIR+APATPase+GT4</td><td>839</td><td>ADL01_RS15000</td><td>Actinobacteria</td><td>Streptomyces sp. NRRL WC-3618</td><td>TIR domain-containing protein [Streptomyces sp. NRRL WC-3618]</td><td>GCF_001280005.1</td></tr>
<tr><td>WP_079101522.1</td><td>GT4*→</td><td>GT4</td><td>542</td><td>AOK16_RS25865</td><td>Actinobacteria</td><td>Streptomyces pathocidini</td><td>glycosyltransferase [Streptomyces pathocidini]</td><td>GCF_001418495.1</td></tr>
<tr><td>WP_080416707.1</td><td>AA_permease→?→ GT4+APATPase+TPR+TPR*→</td><td>GT4+APATPase+TPR+TPR</td><td>1339</td><td>WL41_RS32965</td><td>Betaproteobacteria</td><td>Burkholderia ubonensis</td><td>tetratricopeptide repeat protein [Burkholderia ubonensis]</td><td>GCF_001529565.1</td></tr>
<tr><td>WP_080417742.1</td><td>GT4+APATPase+TPR+TPR*→</td><td>GT4+APATPase+TPR+TPR</td><td>1293</td><td>WJ64_RS17075</td><td>Betaproteobacteria</td><td>Burkholderia ubonensis</td><td>tetratricopeptide repeat protein [Burkholderia ubonensis]</td><td>GCF_001527745.1</td></tr>
<tr><td>WP_080419087.1</td><td>STAND+TPR+GT4*→||||<-?||ABhydrolase→||||<-TRANSGLUTAMINASE</td><td>STAND+TPR+GT4</td><td>1126</td><td>WL82_RS32325</td><td>Betaproteobacteria</td><td>Burkholderia ubonensis</td><td>glycosyltransferase [Burkholderia ubonensis]</td><td>-</td></tr>
<tr><td>WP_080421888.1</td><td>AA_permease→?→ GT4+APATPase+TPR+TPR*→</td><td>GT4+APATPase+TPR+TPR</td><td>1339</td><td>WK71_RS32030</td><td>Betaproteobacteria</td><td>Burkholderia ubonensis</td><td>tetratricopeptide repeat protein [Burkholderia ubonensis]</td><td>GCF_001529155.1</td></tr>
</table>

<table>
<tr><th>acc</th><th>operon</th><th>architecture</th><th>len</th><th>gen.name</th><th>taxend</th><th>species</th><th>defline</th><th>gca</th></tr>
<tr><td>WP_080422913.1</td><td>GT4+APATPase+TPR+TPR*→</td><td>GT4+APATPase+TPR+TPR</td><td>1339</td><td>WL11_RS09715</td><td>Betaproteobacteria</td><td>Burkholderia ubonensis</td><td>tetratricopeptide repeat protein [Burkholderia ubonensis]</td><td>GCF_001530465.1</td></tr>
<tr><td>WP_080436327.1</td><td>NACHT+TPR+GT4*→</td><td>NACHT+TPR+GT4</td><td>1226</td><td>WM18_RS12015</td><td>Betaproteobacteria</td><td>Burkholderia ubonensis</td><td>glycosyltransferase [Burkholderia ubonensis]</td><td>GCF_001533745.1</td></tr>
<tr><td>WP_080492181.1</td><td>AA_permease→?→ GT4+APATPase+TPR+TPR*→</td><td>GT4+APATPase+TPR+TPR</td><td>1293</td><td>BGV55_RS22015</td><td>Betaproteobacteria</td><td>Burkholderia ubonensis</td><td>tetratricopeptide repeat protein [Burkholderia ubonensis]</td><td>GCF_001882545.1</td></tr>
<tr><td>WP_082136219.1</td><td>GT4+APATPase+TPR+TPR*→</td><td>GT4+APATPase+TPR+TPR</td><td>1282</td><td>TR67_RS23240</td><td>Gammaproteobacteria</td><td>Pseudomonas deceptionensis</td><td>glycosyltransferase [Pseudomonas deceptionensis]</td><td>GCF_900106095.1</td></tr>
<tr><td>WP_082297595.1</td><td>GT4*→</td><td>GT4</td><td>599</td><td>AKH00_RS12250</td><td>Actinobacteria</td><td>Microbacterium sp. GCS4</td><td>glycosyltransferase family 4 protein [Microbacterium sp. GCS4]</td><td>GCF_001262495.1</td></tr>
<tr><td>WP_082404817.1</td><td><-ABC_tran||?→?→||||<-?<-TIR+NACHT||HTH+APATPase+TPR+GT4*→</td><td>HTH+APATPase+TPR+GT4</td><td>1223</td><td>ADK67_RS45535</td><td>Actinobacteria</td><td>Saccharothrix sp. NRRL B-16348</td><td>glycosyltransferase [Saccharothrix sp. NRRL B-16348]</td><td>GCF_001280085.1</td></tr>
<tr><td>WP_082466077.1</td><td>GT4*→||||<-TRPR-HTH+PBPI<-?<-?<-?<-?||ABC_tran→</td><td>GT4</td><td>618</td><td>ASE64_RS17155</td><td>Actinobacteria</td><td>Agreia sp. Leaf210</td><td>glycosyltransferase [Agreia sp. Leaf210]</td><td>GCF_001421485.1</td></tr>
<tr><td>WP_083469142.1</td><td>GT4+NUDIX*→</td><td>GT4+NUDIX</td><td>750</td><td>VQ02_RS35520</td><td>Alphaproteobacteria</td><td>Methylobacterium variabile</td><td>glycosyltransferase family 4 protein [Methylobacterium variabile]</td><td>GCF_001043975.1</td></tr>
<tr><td>WP_083731258.1</td><td>Multi-TM→?→ STAND→?→ DUF4913→?→||||<-?||GT4+NUDIX+ART+PolB-NTase*→</td><td>GT4+NUDIX+ART+PolB-NTase</td><td>1854</td><td>BL254_RS25375</td><td>Actinobacteria</td><td>Frankia sp. BMG5.30</td><td>glycosyltransferase [Frankia sp. BMG5.30]</td><td>GCF_001983005.1</td></tr>
<tr><td>WP_083895344.1</td><td>WXG→?→?→ WXG→?→?→?→ WXG+Caspase+VWA+Papain+ZetaToxin+GT4*→</td><td>WXG+Caspase+VWA+Papain+ZetaToxin+GT4</td><td>3035</td><td>ON40_RS01890</td><td>Actinobacteria</td><td>Nocardia jiangxiensis</td><td>zeta toxin family protein [Nocardia jiangxiensis]</td><td>GCF_000308615.1</td></tr>
<tr><td>WP_085710134.1</td><td><-LexA||GT4+APATPase+TPR+TPR*→||||<-?<-?<-ABC_tran</td><td>GT4+APATPase+TPR+TPR</td><td>1389</td><td>CAG24_RS08895</td><td>Gammaproteobacteria</td><td>unclassified Pseudomonas</td><td>MULTISPECIES: glycosyltransferase [unclassified Pseudomonas]</td><td>GCF_002112315.1</td></tr>
<tr><td>WP_086055635.1</td><td>DUF4913→?→||||<-?||GT4+NUDIX+ART+PolB-NTase*→</td><td>GT4+NUDIX+ART+PolB-NTase</td><td>1854</td><td>FrCorBMG51_RS27570</td><td>Actinobacteria</td><td>Frankia coriariae</td><td>glycosyltransferase [Frankia coriariae]</td><td>-</td></tr>
<tr><td>WP_086802688.1</td><td>GT4+NUDIX+ART+PolB-NTase*→</td><td>GT4+NUDIX+ART+PolB-NTase</td><td>1883</td><td>CCN48_RS29200</td><td>Actinobacteria</td><td>Streptomyces scabiei</td><td>glycosyltransferase family 4 protein [Streptomyces scabiei]</td><td>GCF_002155725.1</td></tr>
</table>

<table>
<tr><th>acc</th><th>operon</th><th>architecture</th><th>len</th><th>gen.name</th><th>taxend</th><th>species</th><th>defline</th><th>gca</th></tr>
<tr><td>WP_087651531.1</td><td>GT4+Trypsin+PNPase*→</td><td>GT4+Trypsin+PNPase</td><td>929</td><td>S1001342_RS03745</td><td>Alphaproteobacteria</td><td>Acetobacter pasteurianus</td><td>glycosyltransferase [Acetobacter pasteurianus]</td><td>GCF_002173735.1</td></tr>
<tr><td>WP_088088251.1</td><td>GT4*→</td><td>GT4</td><td>624</td><td>CCK98_RS20350</td><td>Firmicutes</td><td>Bacillus sp. OV166</td><td>glycosyltransferase family 4 protein [Bacillus sp. OV166]</td><td>GCF_900177675.1</td></tr>
<tr><td>WP_088287445.1</td><td>GT4+APATPase+TPR+TPR*→</td><td>GT4+APATPase+TPR+TPR</td><td>1066</td><td>EOIN-JALH_RS30615</td><td>Betaproteobacteria</td><td>Ideonella sp. A 288</td><td>tetratricopeptide repeat protein [Ideonella sp. A 288]</td><td>GCF_002198735.1</td></tr>
<tr><td>WP_090142138.1</td><td>GT4*→</td><td>GT4</td><td>516</td><td>AMM33_RS15110</td><td>Betaproteobacteria</td><td>Limnohabitans sp. DM1</td><td>glycosyltransferase family 4 protein [Limnohabitans sp. DM1]</td><td>GCF_001269385.1</td></tr>
<tr><td>WP_091283948.1</td><td>TPR+Caspase→ TPR+Caspase→||||<-CATASP||CATRA-N+CATRA-C+GT4*→||||<-?||HTH+APATPase+TPR→</td><td>CATRA-N+CATRA-C+GT4</td><td>839</td><td>AWX74_RS30820</td><td>Actinobacteria</td><td>Frankia irregularis</td><td>glycosyltransferase family 4 protein [Frankia irregularis]</td><td>GCF_001536285.1</td></tr>
<tr><td>WP_091296338.1</td><td>cNMP_binding+HTH_Crp_2→?→ GT4*→||||<-ACYC+APATPase+TPR</td><td>GT4</td><td>406</td><td>BLV57_RS34110</td><td>Actinobacteria</td><td>Amycolatopsis xylanica</td><td>glycosyltransferase family 4 protein [Amycolatopsis xylanica]</td><td>GCF_900107045.1</td></tr>
<tr><td>WP_091582937.1</td><td>STAND→?→ DUF4913→||||<-?<-?||?→ GT4*→?→?→?→ NUDIX→||||<-?<-ABC_tran</td><td>GT4</td><td>438</td><td>GA0070622_RS30350</td><td>Actinobacteria</td><td>Micromonospora sediminicola</td><td>glycosyltransferase family 4 protein [Micromonospora sediminicola]</td><td>GCF_900089585.1</td></tr>
<tr><td>WP_092202072.1</td><td>ABC_tran→?→?→?→?→?→||||<-?||STAND+TPR+GT4*→</td><td>STAND+TPR+GT4</td><td>1063</td><td>BLU85_RS18570</td><td>Gammaproteobacteria</td><td>unclassified Pseudomonas</td><td>MULTISPECIES: glycosyltransferase [unclassified Pseudomonas]</td><td>GCF_900103555.1</td></tr>
<tr><td>WP_092992899.1</td><td>GT4+TPR+TPR*→</td><td>GT4+TPR+TPR</td><td>1441</td><td>BLP65_RS04115</td><td>Gammaproteobacteria</td><td>Thiohalomonas denitrificans</td><td>tetratricopeptide repeat protein [Thiohalomonas denitrificans]</td><td>GCF_900102855.1</td></tr>
<tr><td>WP_094198012.1</td><td>GST_N_2+GST_C→?→||||<-?<-?<-?||tRNA→ Cluster214_2clades→ GT4*→||||<-?<-?<-?<-DctM</td><td>GT4</td><td>756</td><td>AFA_RS17265</td><td>Betaproteobacteria</td><td>Alcaligenes faecalis</td><td>glycosyltransferase family 4 protein [Alcaligenes faecalis]</td><td>GCF_002242175.1</td></tr>
<tr><td>WP_096772335.1</td><td>rve+rve_3→||||<-?||?→||||<-?||PSE→||||<-?<-?||GT4+B12-binding+RADICAL-SAM*→</td><td>GT4+B12-binding+RADICAL-SAM</td><td>1313</td><td>GGE61_RS22535</td><td>Alphaproteobacteria</td><td>Rhizobium</td><td>MULTISPECIES: cobalamin B12-binding domain-containing protein [Rhizobium]</td><td>GCF_013087515.1</td></tr>
</table>

<table>
<thead>
<tr><th>acc</th><th>operon</th><th>architecture</th><th>len</th><th>gen.name</th><th>taxend</th><th>species</th><th>defline</th><th>gca</th></tr>
</thead>
<tbody>
<tr><td>WP_096876300.1</td><td>GT4+APATPase*→</td><td>GT4+APATPase</td><td>888</td><td>MKLM6_RS16005</td><td>Gammaproteobacteria</td><td>Methylomonas koyamae</td><td>glycosyltransferase [Methylomonas koyamae]</td><td>GCF_002441955.1</td></tr>
<tr><td>WP_097325277.1</td><td>WXG→?→ GT4*→</td><td>GT4</td><td>626</td><td>CRP55_RS32890</td><td>Actinobacteria</td><td>Actinoplanes atraurantiacus</td><td>glycosyltransferase family 4 protein [Actinoplanes atraurantiacus]</td><td>GCF_900215205.1</td></tr>
<tr><td>WP_097329102.1</td><td>wHTH+TPR+APATPase→?→?→||||<-?||GT4*→||||<-?||?→?→?→?→||||<-?<-WXG+Caspase</td><td>GT4</td><td>510</td><td>CRP55_RS49635</td><td>Actinobacteria</td><td>Actinoplanes atraurantiacus</td><td>glycosyltransferase family 4 protein [Actinoplanes atraurantiacus]</td><td>GCF_900215205.1</td></tr>
<tr><td>WP_097811834.1</td><td>GT4*→</td><td>GT4</td><td>600</td><td>CN930_RS12860</td><td>Firmicutes</td><td>Bacillus cereus</td><td>glycosyltransferase [Bacillus cereus]</td><td>GCF_002574885.1</td></tr>
<tr><td>WP_097931751.1</td><td><-DNA_methylase||?→?→?→?→?→||||<-?||MNS+GT4*→</td><td>MNS+GT4</td><td>1170</td><td>CR483_RS02605</td><td>Actinobacteria</td><td>unclassified Streptomyces</td><td>MULTISPECIES: glycosyltransferase [unclassified Streptomyces]</td><td>GCF_002551215.1</td></tr>
<tr><td>WP_098141735.1</td><td>GT4*→?→?→?→?→?→ NLPC→</td><td>GT4</td><td>541</td><td>CN671_RS19510</td><td>Firmicutes</td><td>Bacillus toyonensis</td><td>glycosyltransferase [Bacillus toyonensis]</td><td>GCF_012395365.1</td></tr>
<tr><td>WP_098743858.1</td><td>STAND→?→ DUF4913→||||<-?<-?||?→ GT4*→?→?→?→ NUDIX→||||<-?<-ABC_tran</td><td>GT4</td><td>438</td><td>COO58_RS03965</td><td>Actinobacteria</td><td>Micromonospora sp. WMMA1996</td><td>glycosyltransferase family 4 protein [Micromonospora sp. WMMA1996]</td><td>GCF_002573675.1</td></tr>
<tr><td>WP_099013704.1</td><td>MNS+GT4*→</td><td>MNS+GT4</td><td>1149</td><td>SMALA_RS12770</td><td>Actinobacteria</td><td>Streptomyces malaysiensis</td><td>AAA family ATPase [Streptomyces malaysiensis]</td><td>GCF_002591335.1</td></tr>
<tr><td>WP_100783315.1</td><td>MFS→||||<-?||GT4+APATPase+TPR*→||||<-MODE-HTH+PBPII</td><td>GT4+APATPase+TPR</td><td>1318</td><td>COO64_RS24545</td><td>Gammaproteobacteria</td><td>Pseudomonas donghuensis</td><td>glycosyltransferase [Pseudomonas donghuensis]</td><td>GCF_002811135.1</td></tr>
<tr><td>WP_101210325.1</td><td>GT4*→ TPR→</td><td>GT4</td><td>541</td><td>AT251_RS15815</td><td>Gammaproteobacteria</td><td>Enterovibrio nigricans</td><td>hypothetical protein [Enterovibrio nigricans]</td><td>-</td></tr>
<tr><td>WP_101386266.1</td><td>-</td><td>-</td><td>1324</td><td>-</td><td>Actinobacteria</td><td>Streptomyces sp. TLI_146</td><td>caspase family protein [Streptomyces sp. TLI_146]</td><td>-</td></tr>
<tr><td>WP_101718466.1</td><td><-ABC_tran<-?<-?<-?<-PSE<-?||?→?→ GT4*→</td><td>GT4</td><td>612</td><td>SGCZBJ_RS13280</td><td>Alphaproteobacteria</td><td>Caulobacter zeae</td><td>glycosyltransferase [Caulobacter zeae]</td><td>GCF_002858925.1</td></tr>
</tbody>
</table>

<table>
<tr><th>acc</th><th>operon</th><th>architecture</th><th>len</th><th>gen.name</th><th>taxend</th><th>species</th><th>defline</th><th>gca</th></tr>
<tr><td>WP_102909088.1</td><td>NUDIX→?→ GT4*→</td><td>GT4</td><td>433</td><td>C1J00_RS12325</td><td>Actinobacteria</td><td>Streptomyces sp. 13K301</td><td>glycosyltransferase family 4 protein [Streptomyces sp. 13K301]</td><td>GCF_002891435.1</td></tr>
<tr><td>WP_103291295.1</td><td>SWACOS+GT4*→?→||||<-?<-?||MFS→</td><td>SWACOS+GT4</td><td>1138</td><td>C1633_RS27595</td><td>Gammaproteobacteria</td><td>Pseudomonas protegens</td><td>glycosyltransferase [Pseudomonas protegens]</td><td>GCF_002899905.1</td></tr>
<tr><td>WP_103660162.1</td><td>NACHT+GT4*→</td><td>NACHT+GT4</td><td>1646</td><td>CPJ18_RS23160</td><td>Alphaproteobacteria</td><td>Agrobacterium rosae</td><td>glycosyltransferase [Agrobacterium rosae]</td><td>GCF_002915175.1</td></tr>
<tr><td>WP_104365810.1</td><td>GT4*→</td><td>GT4</td><td>428</td><td>C5E51_RS10910</td><td>Actinobacteria</td><td>Nocardia nova</td><td>glycosyltransferase family 4 protein [Nocardia nova]</td><td>GCF_002933545.1</td></tr>
<tr><td>WP_105007055.1</td><td>GT4*→?→||||<-PSE||?→?→ Calcineurin→</td><td>GT4</td><td>606</td><td>CUJ84_RS16890</td><td>Alphaproteobacteria</td><td>Rhizobium leguminosarum</td><td>glycosyltransferase family 4 protein [Rhizobium leguminosarum]</td><td>GCF_002953715.1</td></tr>
<tr><td>WP_106119969.1</td><td>Cluster214_2clades→ GT4*→</td><td>GT4</td><td>736</td><td>C7A11_RS23785</td><td>Gammaproteobacteria</td><td>Pseudomonas simiae</td><td>glycosyltransferase family 4 protein [Pseudomonas simiae]</td><td>GCF_003001535.1</td></tr>
<tr><td>WP_107027343.1</td><td>NACHT+GT4*→||||<-?<-?||PSE→ TIR→</td><td>NACHT+GT4</td><td>1646</td><td>C6558_RS13435</td><td>Alphaproteobacteria</td><td>Ensifer sp. NM-2</td><td>glycosyltransferase [Ensifer sp. NM-2]</td><td>GCF_003024455.1</td></tr>
<tr><td>WP_107161461.1</td><td>GT4+NACHT+BetaPropeller*→</td><td>GT4+NACHT+BetaPropeller</td><td>1536</td><td>C8054_RS26470</td><td>Actinobacteria</td><td>Micromonospora sp. RP3T</td><td>glycosyltransferase [Micromonospora sp. RP3T]</td><td>GCF_003027925.1</td></tr>
<tr><td>WP_107259933.1</td><td>nSTAND3→?→?→ GT4*→||||<-?<-?||?→||||<-?||?→ PSE→||||<-NUDIX</td><td>GT4</td><td>596</td><td>C6W10_RS21380</td><td>Actinobacteria</td><td>Plantactinospora sp. BB1</td><td>glycosyltransferase family 4 protein [Plantactinospora sp. BB1]</td><td>GCF_003030385.1</td></tr>
<tr><td>WP_107266333.1</td><td>nSTAND3→?→?→ GT4*→</td><td>GT4</td><td>596</td><td>C6361_RS00275</td><td>Actinobacteria</td><td>Plantactinospora sp. BC1</td><td>glycosyltransferase [Plantactinospora sp. BC1]</td><td>GCF_003030345.1</td></tr>
<tr><td>WP_107402046.1</td><td>Multi-TM→?→ STAND→?→ DUF4913→?→ GT4*→ Zeta_toxin→</td><td>GT4</td><td>374</td><td>AN216_RS27375</td><td>Actinobacteria</td><td>Streptomyces oceani</td><td>glycosyltransferase [Streptomyces oceani]</td><td>GCF_001751245.1</td></tr>
<tr><td>WP_107867044.1</td><td>LRR+LRR+LRR+AP-GTPase+COR+GT4*→</td><td>LRR+LRR+LRR+AP-GTPase+COR+GT4</td><td>1510</td><td>C8N29_RS15840</td><td>Firmicutes</td><td>Agitococcus lubricus</td><td>leucine-rich repeat protein [Agitococcus lubricus]</td><td>GCF_003051055.1</td></tr>
</table>

acc	operon	architecture	len	gen.name	taxend	species	defline	gca
WP_108006164.1	<-HTH+APATPase+TPR||GT4*→?→||||<-?<-?<-?||TPR→	GT4	421	C7821_RS00535	Actinobacteria	Streptomyces sp. VMFN-G11Ma	glycosyltransferase family 4 protein [Streptomyces sp. VMFN-G11Ma]	GCF_003046555.1
WP_108666907.1	NLPC→?→?→||||<-?||GT4*→	GT4	564	CUC05_RS14855	Actinobacteria	Euzebya rosea	glycosyltransferase family 4 protein [Euzebya rosea]	GCF_003073135.1
WP_109448176.1	MPTase+GT4*→	MPTase+GT4	753	CK485_RS28700	Actinobacteria	Streptomyces sp. ICBB 8177	DUF4157 domain-containing protein [Streptomyces sp. ICBB 8177]	GCF_003144095.1
WP_112357576.1	GT4*→	GT4	628	DTY03_RS10805	Alphaproteobacteria	Rhizobiales bacterium	glycosyltransferase family 4 protein [Rhizobiales bacterium]	-
WP_112733881.1	GT4+APATPase+TPR*→	GT4+APATPase+TPR	1361	ONO86_RS28915	Actinobacteria	Micromonospora noduli	glycosyltransferase [Micromonospora noduli]	GCF_003264435.1
WP_113016426.1	HTH+MPTase→||||<-?<-?<-?||GT4+B12-binding+RADICAL-SAM*→	GT4+B12-binding+RADICAL-SAM	1381	DUD68_RS27095	Alphaproteobacteria	Rhizobiales bacterium	radical SAM protein [Rhizobiales bacterium]	-
WP_113272579.1	HTH+MPTase→?→ Helicase_C→ SIG+DUF1998→||||<-?<-?||?→ NACHT+GT4*→	NACHT+GT4	1640	DUJ98_RS17735	Alphaproteobacteria	Rhizobiales bacterium	glycosyltransferase [Rhizobiales bacterium]	-
WP_114055463.1	TIR+APATPase+GT4*→	TIR+APATPase+GT4	775	C0216_RS13195	Actinobacteria	Streptomyces globosus	TIR domain-containing protein [Streptomyces globosus]	GCF_003325375.1
WP_114396907.1	GT4*→	GT4	529	DEF24_RS15180	Actinobacteria	Marinitenerispora sediminis	glycosyltransferase family 4 protein [Marinitenerispora sediminis]	GCF_003336445.1
WP_116211561.1	NACHT→ GT4*→	GT4	406	DXZ73_RS15355	Actinobacteria	Streptomyces olivoreticuli	glycosyltransferase family 4 protein [Streptomyces olivoreticuli]	GCF_003391135.1
WP_116568912.1	GT4*→?→||||<-?<-?||?→||||<-?<-MODE-HTH+PBPII	GT4	612	DDF65_RS17385	Alphaproteobacteria	Caulobacter radicis	glycosyltransferase [Caulobacter radicis]	GCF_003094595.1

acc	operon	architecture	len	gen.name	taxend	species	defline	gca
WP_120733613.1	GT4+B12-binding+RADICAL-SAM*→	GT4+B12-binding+RADICAL-SAM	1400	D7M10_RS18130	Gammaproteobacteria	Pseudomonas fluorescens	radical SAM protein [Pseudomonas fluorescens]	GCF_003626995.1
WP_120967974.1	GT4+APATPase+TPR+TPR*→	GT4+APATPase+TPR+TPR	1425	EAO39_RS12970	Betaproteobacteria	Comamonas sp. lk	tetratricopeptide repeat protein [Comamonas sp. lk]	GCF_900564145.1
WP_121165351.1	Sigma→?→?→?→?→?→ LRR+LRR+LRR+AP-GTPase+COR+GT4*→	LRR+LRR+LRR+AP-GTPase+COR+GT4	1488	B0H84_RS06470	Betaproteobacteria	Nitrosomonas sp. Nm120	leucine-rich repeat domain-containing protein [Nitrosomonas sp. Nm120]	GCF_003664085.1
WP_121799508.1	Multi-TM→?→ STAND→?→ DUF4913→?→ GT4*→	GT4	488	CTZ27_RS14745	Actinobacteria	Streptomyces griseocarneus	glycosyltransferase family 4 protein [Streptomyces griseocarneus]	GCF_003674045.1
WP_122257475.1	GT4+B12-binding+RADICAL-SAM*→	GT4+B12-binding+RADICAL-SAM	1421	ALP97_05253	Gammaproteobacteria	Pseudomonas salomonii	radical SAM protein [Pseudomonas salomonii]	-
WP_122528181.1	STAND+TPR+GT4*→	STAND+TPR+GT4	1067	EE945_RS06325	Gammaproteobacteria	Pseudomonas viridiflava	glycosyltransferase [Pseudomonas viridiflava]	GCF_900580695.1
WP_123299261.1	GT4+B12-binding+RADICAL-SAM*→||||<-?||?→?→||||<-?<-?<-?||MODE-HTH+PBPII→	GT4+B12-binding+RADICAL-SAM	1405	EC845_RS16630	Betaproteobacteria	Comamonas sp. BIGb0124	cobalamin B12-binding domain-containing protein [Comamonas sp. BIGb0124]	GCF_003752175.1
WP_123439297.1	<-TPR+Caspase<-CATASP||CATRA-N+CATRA-C+GT4*→ HTH+APATPase+TPR→||||<-HTH||?→?→?→||||<-?<-CATASP	CATRA-N+CATRA-C+GT4	694	EDD94_RS26400	Actinobacteria	Streptomyces sp. PanSC9	glycosyltransferase family 4 protein [Streptomyces sp. PanSC9]	GCF_003751985.1
WP_124130642.1	STAND+GT4*→	STAND+GT4	1082	CJU35_RS32065	Gammaproteobacteria	Pseudomonas aeruginosa	glycosyltransferase [Pseudomonas aeruginosa]	GCF_901482505.1
WP_124284015.1	NUDIX→?→||||<-?||?→ MNS+GT4*→	MNS+GT4	1151	EES39_RS04745	Actinobacteria	Streptomyces sp. ADI92-24	glycosyltransferase [Streptomyces sp. ADI92-24]	GCF_003846195.1
WP_124458549.1	STAND+TPR+GT4*→||||<-?<-?<-?||Calcineurin→	STAND+TPR+GT4	1054	DBV14_RS07405	Betaproteobacteria	Variovorax sp. KBW07	glycosyltransferase [Variovorax sp. KBW07]	GCF_003852515.1

<table>
<tr><th>acc</th><th>operon</th><th>architecture</th><th>len</th><th>gen.name</th><th>taxend</th><th>species</th><th>defline</th><th>gca</th></tr>
<tr><td>WP_125624986.1</td><td>Multi-TM→?→ STAND→?→ DUF4913→?→ GT4*→?→ Zeta_toxin→||||<-ABC_tran</td><td>GT4</td><td>547</td><td>DMH02_RS17910</td><td>Actinobacteria</td><td>Streptomyces sp. WAC 00631</td><td>glycosyltransferase family 4 protein [Streptomyces sp. WAC 00631]</td><td>GCF_003947265.1</td></tr>
<tr><td>WP_125642507.1</td><td>MNS+GT4*→</td><td>MNS+GT4</td><td>1274</td><td>EF918_07135</td><td>Actinobacteria</td><td>Streptomyces sp. WAC06614</td><td>glycosyltransferase [Streptomyces sp. WAC06614]</td><td>-</td></tr>
<tr><td>WP_125650904.1</td><td>Caspase+NACHT+GT4*→</td><td>Caspase+NACHT+GT4</td><td>1208</td><td>EF918_25550</td><td>Actinobacteria</td><td>Streptomyces sp. WAC06614</td><td>glycosyltransferase [Streptomyces sp. WAC06614]</td><td>-</td></tr>
<tr><td>WP_125870434.1</td><td>GT4+B12-binding+RADICAL-SAM*→</td><td>GT4+B12-binding+RADICAL-SAM</td><td>1426</td><td>EGJ08_07220</td><td>Gammaproteobacteria</td><td>Pseudomonas stutzeri</td><td>radical SAM protein [Pseudomonas stutzeri]</td><td>-</td></tr>
<tr><td>WP_125995743.1</td><td>NACHT+GT4*→</td><td>NACHT+GT4</td><td>1650</td><td>BRX43_RS14145</td><td>Alphaproteobacteria</td><td>Sphingomonas sp. S-NIH.Pt15_0812</td><td>glycosyltransferase [Sphingomonas sp. S-NIH.Pt15_0812]</td><td>GCF_003951315.1</td></tr>
<tr><td>WP_127872272.1</td><td>NACHT+GT4*→||||<-Vsr</td><td>NACHT+GT4</td><td>1637</td><td>EN845_RS20190</td><td>Alphaproteobacteria</td><td>unclassified Mesorhizobium</td><td>MULTISPECIES: glycosyltransferase family 4 protein [unclassified Mesorhizobium]</td><td>-</td></tr>
<tr><td>WP_128145588.1</td><td>MPTase→ WXG+SecA→?→?→ GT4+TetR-HTH+TetR-HTH+Tox-ART-RSE+LuxR-HTH*→?→?→ ABhydrolase→</td><td>GT4+TetR-HTH+TetR-HTH+Tox-ART-RSE+LuxR-HTH</td><td>2725</td><td>NCTC13184_06625</td><td>Actinobacteria</td><td>Nocardia africana</td><td>glycosyltransferase [Nocardia africana]</td><td>-</td></tr>
<tr><td>WP_129557405.1</td><td><-TnpB_IS66<-HTH_Tnp_1||?→ SIG+GT4→ GT4→
tRNA→||||<-?<-PSE||?→||||<-?<-PSE<-?<-?||Calcineurin→||||<-cNMP_binding+HTH_Crp_2</td><td>SIG+GT4</td><td>316</td><td>NK6_RS51120</td><td>Alphaproteobacteria</td><td>Bradyrhizobium diazoefficiens</td><td>glycosyltransferase [Bradyrhizobium diazoefficiens]</td><td>GCF_001549695.1</td></tr>
<tr><td>WP_130174915.1</td><td>GT4+B12-binding+RADICAL-SAM*→</td><td>GT4+B12-binding+RADICAL-SAM</td><td>1409</td><td>EUX58_RS06685</td><td>Gammaproteobacteria</td><td>Pseudomonas</td><td>MULTISPECIES: radical SAM protein [Pseudomonas]</td><td>GCF_005930695.1</td></tr>
<tr><td>WP_130264935.1</td><td>GT4+APATPase+TPR+TPR*→||||<-MODE-HTH+PBPII</td><td>GT4+APATPase+TPR+TPR</td><td>1330</td><td>EXN22_RS15725</td><td>Gammaproteobacteria</td><td>Pseudomonas sp. SNU WT1</td><td>glycosyltransferase family 4 protein [Pseudomonas sp. SNU WT1]</td><td>GCF_004214895.1</td></tr>
<tr><td>WP_130343732.1</td><td>WXG+TM+EDA39C+Papain+Caspase+GT4*→?→ GT4→</td><td>WXG+TM+EDA39C+Papain+Caspase+GT4</td><td>2616</td><td>EV193_RS09710</td><td>Actinobacteria</td><td>Herbihabitans rhizosphaerae</td><td>glycosyltransferase [Herbihabitans rhizosphaerae]</td><td>GCF_004216555.1</td></tr>
<tr><td>WP_130721989.1</td><td>CASPASE→?→?→?→ GT4*→</td><td>GT4</td><td>606</td><td>ELI52_RS15625</td><td>Alphaproteobacteria</td><td>Rhizobium leguminosarum</td><td>glycosyltransferase [Rhizobium leguminosarum]</td><td>GCF_004303135.1</td></tr>
</table>

<table>
<tr><th>acc</th><th>operon</th><th>architecture</th><th>len</th><th>gen.name</th><th>taxend</th><th>species</th><th>defline</th><th>gca</th></tr>
<tr><td>WP_131447141.1</td><td><-Ribosomal_L20<-?<-?<-?<-tRNA||?→||||<-?<-?<-PSE||APATPase+GT4+TPR+TPR+TPR+TPR+TPR*→||||<-?||?→ ABC_tran→</td><td>APATPase+GT4+TPR+TPR+TPR+TPR+TPR</td><td>1789</td><td>EZJ19_RS09965</td><td>Betaproteobacteria</td><td>Thiobacillaceae bacterium LSR1</td><td>tetratricopeptide repeat protein [Thiobacillaceae bacterium LSR1]</td><td>GCF_004337445.1</td></tr>
<tr><td>WP_131728683.1</td><td>GT4+STAND+TPR+TPR*→</td><td>GT4+STAND+TPR+TPR</td><td>1465</td><td>AOZ47_RS33140</td><td>Betaproteobacteria</td><td>Achromobacter xylosoxidans</td><td>tetratricopeptide repeat protein [Achromobacter xylosoxidans]</td><td>GCF_001299055.1</td></tr>
<tr><td>WP_131738585.1</td><td><-VWA+APATPase+TPR+TPR+TPR+TPR<-STAND<-?||GT4*→?→||||<-?||MFS→</td><td>GT4</td><td>696</td><td>E1291_RS15920</td><td>Actinobacteria</td><td>Actinomadura roseirufa</td><td>glycosyltransferase family 4 protein [Actinomadura roseirufa]</td><td>GCF_900659635.1</td></tr>
<tr><td>WP_131761874.1</td><td><-cNMP_binding+HTH_Crp_2||GT4*→||||<-HTH+APATPase+TPR||?→||||<-cNMP_binding+HTH_Crp_2</td><td>GT4</td><td>466</td><td>E1300_RS37495</td><td>Actinobacteria</td><td>Actinomadura fibrosa</td><td>glycosyltransferase family 4 protein [Actinomadura fibrosa]</td><td>GCF_900659615.1</td></tr>
<tr><td>WP_132113254.1</td><td>GT4*→||||<-TPR</td><td>GT4</td><td>404</td><td>EV192_RS08750</td><td>Actinobacteria</td><td>Actinocrispum wychmicini</td><td>glycosyltransferase family 4 protein [Actinocrispum wychmicini]</td><td>GCF_004345645.1</td></tr>
<tr><td>WP_132114377.1</td><td>cNMP_binding+HTH_Crp_2→ GT4*→||||<-ACYC+APATPase+TPR</td><td>GT4</td><td>407</td><td>EV192_RS12015</td><td>Actinobacteria</td><td>Actinocrispum wychmicini</td><td>glycosyltransferase family 4 protein [Actinocrispum wychmicini]</td><td>GCF_004345645.1</td></tr>
<tr><td>WP_132118129.1</td><td>EAD1+fvmYukDl-Nterm+GT4*→</td><td>EAD1+fvmYukDl-Nterm+GT4</td><td>667</td><td>EV192_RS21815</td><td>Actinobacteria</td><td>Actinocrispum wychmicini</td><td>glycosyltransferase family 4 protein [Actinocrispum wychmicini]</td><td>GCF_004345645.1</td></tr>
<tr><td>WP_132175767.1</td><td>GT4*→</td><td>GT4</td><td>453</td><td>E1263_RS36055</td><td>Actinobacteria</td><td>Kribbella antibiotica</td><td>glycosyltransferase family 4 protein [Kribbella antibiotica]</td><td>GCF_004349055.1</td></tr>
<tr><td>WP_132323823.1</td><td>GT4*→</td><td>GT4</td><td>446</td><td>E1218_RS23930</td><td>Actinobacteria</td><td>Kribbella sp. 16K104</td><td>glycosyltransferase family 4 protein [Kribbella sp. 16K104]</td><td>GCF_004348725.1</td></tr>
<tr><td>WP_133190629.1</td><td>GT4+B12-binding+RADICAL-SAM*→</td><td>GT4+B12-binding+RADICAL-SAM</td><td>1398</td><td>E1N52_RS41050</td><td>Betaproteobacteria</td><td>Paraburkholderia guartelaensis</td><td>radical SAM protein [Paraburkholderia guartelaensis]</td><td>GCF_004353905.1</td></tr>
<tr><td>WP_133255578.1</td><td><-MODE-HTH+PBPII||?→||||<-MODE-HTH+PBPII||?→?→?→?→ NACHT+GT4*→</td><td>NACHT+GT4</td><td>1640</td><td>CPJ17_RS11090</td><td>Alphaproteobacteria</td><td>Rhizobium tumorigenes</td><td>glycosyltransferase [Rhizobium tumorigenes]</td><td>GCF_003240565.1</td></tr>
</table>

<table>
<tr><th>acc</th><th>operon</th><th>architecture</th><th>len</th><th>gen.name</th><th>taxend</th><th>species</th><th>defline</th><th>gca</th></tr>
<tr><td>WP_133739590.1</td><td>STAND→?→?→ DUF4913→||||<-AbiEii<-AbiEi_4||?→ GT4*→</td><td>GT4</td><td>493</td><td>EV190_RS01575</td><td>Actinobacteria</td><td>Actinorugispora endophytica</td><td>glycosyltransferase family 4 protein [Actinorugispora endophytica]</td><td>GCF_004363075.1</td></tr>
<tr><td>WP_133854316.1</td><td>Helicase_C→ SIG+DUF1998→||||<-?||?→?→||||<-?||?→ GT4→ TIR+APATPase+TPR→ TPR+Caspase→||||<-TPR+Caspase<-CATASP||CATRA-N+CATRA-C+GT4→</td><td>GT4</td><td>423</td><td>EV186_RS37520</td><td>Actinobacteria</td><td>Labedaea rhizosphaerae</td><td>glycosyltransferase family 4 protein [Labedaea rhizosphaerae]</td><td>GCF_004362825.1</td></tr>
<tr><td>WP_133925636.1</td><td>GT4*→</td><td>GT4</td><td>622</td><td>EV573_RS04015</td><td>Actinobacteria</td><td>Streptomyces sp. BK161</td><td>glycosyltransferase [Streptomyces sp. BK161]</td><td>GCF_004364215.1</td></tr>
<tr><td>WP_133927416.1</td><td>SWACOS+GT4*→||||<-?<-?||?→||||<-?<-?<-ABC_tran</td><td>SWACOS+GT4</td><td>1159</td><td>EV573_RS13335</td><td>Actinobacteria</td><td>Streptomyces sp. BK161</td><td>glycosyltransferase [Streptomyces sp. BK161]</td><td>GCF_004364215.1</td></tr>
<tr><td>WP_133932223.1</td><td><-Sigma<-?<-TPR||GT4*→</td><td>GT4</td><td>410</td><td>EV573_RS39315</td><td>Actinobacteria</td><td>Streptomyces sp. BK161</td><td>glycosyltransferase family 4 protein [Streptomyces sp. BK161]</td><td>GCF_004364215.1</td></tr>
<tr><td>WP_134939719.1</td><td>GT4+B12-binding+RADICAL-SAM*→</td><td>GT4+B12-binding+RADICAL-SAM</td><td>1421</td><td>BZ164_RS25605</td><td>Gammaproteobacteria</td><td>Pseudomonas veronii</td><td>radical SAM protein [Pseudomonas veronii]</td><td>-</td></tr>
<tr><td>WP_135904996.1</td><td><-Vsr||?→?→?→||||<-?<-?||GT4+Trypsin+PNPase*→</td><td>GT4+Trypsin+PNPase</td><td>836</td><td>EN818_RS17550</td><td>Alphaproteobacteria</td><td>unclassified Mesorhizobium</td><td>MULTISPECIES: glycosyltransferase [unclassified Mesorhizobium]</td><td>-</td></tr>
<tr><td>WP_136526013.1</td><td>SWACOS+GT4*→</td><td>SWACOS+GT4</td><td>1144</td><td>E8L22_RS15545</td><td>Deltaproteobacteria</td><td>Geomonas ferrireducens</td><td>glycosyltransferase [Geomonas ferrireducens]</td><td>-</td></tr>
<tr><td>WP_136738577.1</td><td>GT4*→</td><td>GT4</td><td>394</td><td>FCH28_RS05590</td><td>Actinobacteria</td><td>Streptomyces sp. jys28</td><td>glycosyltransferase family 4 protein [Streptomyces sp. jys28]</td><td>GCF_005048155.1</td></tr>
<tr><td>WP_136743126.1</td><td>MNS+GT4*→</td><td>MNS+GT4</td><td>1188</td><td>FCH28_RS29075</td><td>Actinobacteria</td><td>Streptomyces sp. jys28</td><td>glycosyltransferase [Streptomyces sp. jys28]</td><td>GCF_005048155.1</td></tr>
<tr><td>WP_138637396.1</td><td>NUDIX→?→ GT4*→</td><td>GT4</td><td>432</td><td>ETD96_RS16755</td><td>Actinobacteria</td><td>Actinomadura geliboluensis</td><td>glycosyltransferase family 4 protein [Actinomadura geliboluensis]</td><td>GCF_005889745.1</td></tr>
<tr><td>WP_141100843.1</td><td>GT4+HD*→</td><td>GT4+HD</td><td>1421</td><td>CDN99_RS17245</td><td>Betaproteobacteria</td><td>Roseateles aquatilis</td><td>HD domain-containing protein [Roseateles aquatilis]</td><td>GCF_002205645.1</td></tr>
</table>

acc	operon	architecture	len	gen.name	taxend	species	defline	gca
WP_141569577.1	ZetaToxin+GT4+NUDIX+ART+NTase*→?→?→?→||||<-Caspase	ZetaToxin+GT4+NUDIX+ART+NTase	2159	SipoB123_RS12910	Actinobacteria	Streptomyces ipomoeae	zeta toxin family protein [Streptomyces ipomoeae]	GCF_006547165.1
WP_141585352.1	ZetaToxin+GT4+NUDIX+ART+NTase*→?→?→?→||||<-Caspase<-?||?→ PSE→||||<-NLPC	ZetaToxin+GT4+NUDIX+ART+NTase	2156	Sipo7851_RS16610	Actinobacteria	Streptomyces ipomoeae	zeta toxin family protein [Streptomyces ipomoeae]	GCF_006547175.1
WP_141714270.1	<-TPR+Caspase||GT4*→	GT4	542	GA0074694_RS24775	Actinobacteria	Micromonospora inyonensis	glycosyltransferase [Micromonospora inyonensis]	GCF_900091415.1
WP_141749885.1	GT4+NACHT*→||||<-MFS	GT4+NACHT	1197	JAB9_RS27490	Betaproteobacteria	Janthinobacterium sp. HH107	glycosyltransferase family 4 protein [Janthinobacterium sp. HH107]	GCF_001758765.1
WP_141888935.1	MFS→||||<-ABC_tran<-?<-?<-?<-?||GT4*→	GT4	608	FB468_RS16790	Actinobacteria	Leucobacter komagatae	glycosyltransferase [Leucobacter komagatae]	GCF_006716085.1
WP_141962659.1	<-HTH+APATPase+TPR<-TPR+Caspase<-CATASP||CATRA-N+CATRA-C+GT4*→||||<-?<-?<-?<-?<-?<-REase+SFI-ATPase	CATRA-N+CATRA-C+GT4	732	FB559_RS39205	Actinobacteria	Actinoallomurus bryophytorum	glycosyltransferase [Actinoallomurus bryophytorum]	GCF_006716425.1
WP_142260239.1	MNS+GT4*→?→||||<-?<-Sigma<-?||ABC_tran→	MNS+GT4	1152	FHX40_RS15230	Actinobacteria	Thermopolyspora flexuosa	glycosyltransferase [Thermopolyspora flexuosa]	GCF_014647775.1
WP_142588942.1	NACHT+GT4*→	NACHT+GT4	1640	HNP80_RS07130	Alphaproteobacteria	Rhizobium halotolerans	glycosyltransferase [Rhizobium halotolerans]	GCF_902153235.2
WP_143026968.1	LRR+AP-GTPase+COR→ GT4*→	GT4	484	BMY41_RS11080	Betaproteobacteria	Nitrosomonas oligotropha	glycosyltransferase [Nitrosomonas oligotropha]	GCF_900106555.1
WP_143176277.1	AA_permease→?→ GT4+APATPase+TPR+TPR*→	GT4+APATPase+TPR+TPR	1172	BGV47_RS35370	Betaproteobacteria	Burkholderia ubonensis	tetratricopeptide repeat protein, partial [Burkholderia ubonensis]	GCF_001901485.1
WP_143660221.1	<-TPR+Caspase<-CATASP||CATRA-N+CATRA-C+GT4*→ HTH+APATPase+TPR→||||<-HTH||?→?→?→||||<-?<-CATASP	CATRA-N+CATRA-C+GT4	703	DDV98_RS32255	Actinobacteria	unclassified Streptomyces	MULTISPECIES: glycosyltransferase [unclassified Streptomyces]	GCF_001983595.1
WP_143740316.1	<-TPR+Caspase||GT4*→?→?→||||<-?<-?<-ABC_tran	GT4	546	GA0070618_RS24540	Actinobacteria	Micromonospora echinospora	glycosyltransferase [Micromonospora echinospora]	GCF_900091495.1

<table>
<tr><th>acc</th><th>operon</th><th>architecture</th><th>len</th><th>gen.name</th><th>taxend</th><th>species</th><th>defline</th><th>gca</th></tr>
<tr><td>WP_143788398.1</td><td>STAND+TPR+GT4*→</td><td>STAND+TPR+GT4</td><td>1136</td><td>BUS12_RS18575</td><td>Betaproteobacteria</td><td>Paraburkholderia phenazinium</td><td>glycosyltransferase [Paraburkholderia phenazinium]</td><td>GCF_900142845.1</td></tr>
<tr><td>WP_143860247.1</td><td>WXG+MPTase→ WXG+SecA+LuxR-HTH+LuxR-HTH+LuxR-HTH+LuxR-HTH+ST-Hiskin+MPTase+GT4+TetR-HTH+TetR-HTH+Tox-ART-RSE+LuxR-HTH+LuxR-HTH+LuxR-HTH+GNTR-HTH+LuxR-HTH+LuxR-HTH+ST-Hiskin+ST-Hiskin+XPC-C+TM+TM+TM+TM+LuxR-HTH*→</td><td>WXG+SecA+LuxR-HTH+LuxR-HTH+LuxR-HTH+LuxR-HTH+ST-Hiskin+MPTase+GT4+TetR-HTH+TetR-HTH+Tox-ART-RSE+LuxR-HTH+LuxR-HTH+LuxR-HTH+GNTR-HTH+LuxR-HTH+LuxR-HTH+ST-Hiskin+ST-Hiskin+XPC-C+TM+TM+TM+TM+LuxR-HTH</td><td>11649</td><td>B7C42_RS30150</td><td>Actinobacteria</td><td>Nocardia cerradoensis</td><td>MFS transporter, partial [Nocardia cerradoensis]</td><td>GCF_002236815.1</td></tr>
<tr><td>WP_144299077.1</td><td>GT4+B12-binding+RADICAL-SAM*→</td><td>GT4+B12-binding+RADICAL-SAM</td><td>1385</td><td>VARPA_RS03315</td><td>Betaproteobacteria</td><td>Variovorax paradoxus</td><td>radical SAM protein [Variovorax paradoxus]</td><td>GCF_000184745.1</td></tr>
<tr><td>WP_144384941.1</td><td>Multi-TM→?→ STAND→?→ DUF4913→?→ GT4*→</td><td>GT4</td><td>497</td><td>CD790_RS21520</td><td>Actinobacteria</td><td>Streptomyces sp. SAJ15</td><td>glycosyltransferase [Streptomyces sp. SAJ15]</td><td>GCF_007595705.1</td></tr>
<tr><td>WP_145763552.1</td><td>NUDIX→?→ MNS+GT4*→</td><td>MNS+GT4</td><td>1151</td><td>FHX80_RS07940</td><td>Actinobacteria</td><td>Streptomyces brevispora</td><td>glycosyltransferase [Streptomyces brevispora]</td><td>GCF_007829885.1</td></tr>
<tr><td>WP_145809941.1</td><td>MNS+GT4*→?→?→||||<-?||?→ CSD→</td><td>MNS+GT4</td><td>1180</td><td>FHW51_RS26100</td><td>Actinobacteria</td><td>Streptomyces argenteolus</td><td>glycosyltransferase [Streptomyces argenteolus]</td><td>GCF_007829855.1</td></tr>
<tr><td>WP_145818563.1</td><td><-TPR+Caspase||GT4*→?→||||<-?<-?<-ABC_tran</td><td>GT4</td><td>546</td><td>JD81_RS15875</td><td>Actinobacteria</td><td>Micromonospora sagamiensis</td><td>glycosyltransferase [Micromonospora sagamiensis]</td><td>GCF_014680085.1</td></tr>
<tr><td>WP_145835614.1</td><td><-TPR+Caspase||GT4*→</td><td>GT4</td><td>546</td><td>JD80_RS20680</td><td>Actinobacteria</td><td>Micromonospora echinospora</td><td>glycosyltransferase [Micromonospora echinospora]</td><td>GCF_007829475.1</td></tr>
<tr><td>WP_145885265.1</td><td>ABhydrolase→||||<-?<-?||?→||||<-?||?→?→ MNS+GT4*→</td><td>MNS+GT4</td><td>1144</td><td>FB157_RS37885</td><td>Actinobacteria</td><td>Streptomyces sp. BK340</td><td>glycosyltransferase [Streptomyces sp. BK340]</td><td>GCF_007827595.1</td></tr>
<tr><td>WP_145921569.1</td><td>WXG+TM+GT4+GT4*→</td><td>WXG+TM+GT4+GT4</td><td>3484</td><td>FJK98_RS27630</td><td>Actinobacteria</td><td>Micromonospora sp. HM134</td><td>glycosyltransferase [Micromonospora sp. HM134]</td><td>GCF_007833915.1</td></tr>
<tr><td>WP_146161226.1</td><td>WXG+MPTase→ WXG+LuxR-HTH+LuxR-HTH+LuxR-HTH+LuxR-HTH+TetR-HTH+TetR-HTH+LuxR-HTH+LuxR-HTH+LexA-HTH+LuxR-HTH+LuxR-HTH+LuxR-HTH+LuxR-HTH+LuxR-HTH+PP2C*→?→ ABhydrolase→</td><td>WXG+LuxR-HTH+LuxR-HTH+LuxR-HTH+LuxR-HTH+TetR-HTH+TetR-HTH+LuxR-HTH+LuxR-HTH+LexA-HTH+LuxR-HTH+LuxR-HTH+LuxR-HTH+LuxR-HTH+LuxR-HTH+PP2C</td><td>14195</td><td>C8258_RS13420</td><td>Actinobacteria</td><td>Nocardia sp. MDA0666</td><td>MFS transporter [Nocardia sp. MDA0666]</td><td>GCF_003023875.1</td></tr>
<tr><td>WP_146241295.1</td><td>APATPase+TPR→||||<-?||GT4*→?→?→ GT4→</td><td>GT4</td><td>502</td><td>DFQ13_RS33200</td><td>Actinobacteria</td><td>Actinokineospora mzabensis</td><td>glycosyltransferase [Actinokineospora mzabensis]</td><td>-</td></tr>
<tr><td>WP_146349043.1</td><td><-APATPase<-APATPase||?→ NLPC→ cNMP_binding+HTH_Crp_2→?→ GT4*→||||<-ACYC+APATPase+TPR</td><td>GT4</td><td>408</td><td>FKR81_RS01510</td><td>Actinobacteria</td><td>Lentzea sp. FXJ1.1311</td><td>glycosyltransferase [Lentzea sp. FXJ1.1311]</td><td>GCF_007845675.1</td></tr>
</table>

<table>
<thead>
<tr><th>acc</th><th>operon</th><th>architecture</th><th>len</th><th>gen.name</th><th>taxend</th><th>species</th><th>defline</th><th>gca</th></tr>
</thead>
<tbody>
<tr><td>WP_147321781.1</td><td>SWACOS+GT4*→||||<-?<-MFS</td><td>SWACOS+GT4</td><td>1096</td><td>BX279_RS09075</td><td>Actinobacteria</td><td>Streptomyces sp. Ag82_O1-9</td><td>glycosyltransferase [Streptomyces sp. Ag82_O1-9]</td><td>GCF_003386845.1</td></tr>
<tr><td>WP_147416956.1</td><td>STAND→?→?→ DUF4913→||||<-AbiEii<-AbiEi_4||?→ GT4*→ AbiEi_4→</td><td>GT4</td><td>495</td><td>NI17_RS16590</td><td>Actinobacteria</td><td>Thermobifida halotolerans</td><td>glycosyltransferase [Thermobifida halotolerans]</td><td>GCF_003574835.1</td></tr>
<tr><td>WP_147994072.1</td><td>TRPR-HTH+PBPI→?→?→||||<-TPR+Caspase||GT4*→</td><td>GT4</td><td>597</td><td>EAO75_RS06975</td><td>Actinobacteria</td><td>Streptomyces sp. uw30</td><td>glycosyltransferase [Streptomyces sp. uw30]</td><td>GCF_008042075.1</td></tr>
<tr><td>WP_148348287.1</td><td>SWACOS+GT4*→</td><td>SWACOS+GT4</td><td>1238</td><td>FXF65_RS04105</td><td>Actinobacteria</td><td>Actinomadura syzygii</td><td>glycosyltransferase [Actinomadura syzygii]</td><td>-</td></tr>
<tr><td>WP_148640710.1</td><td>MNS+GT4*→</td><td>MNS+GT4</td><td>1142</td><td>EH183_RS01595</td><td>Actinobacteria</td><td>Streptomyces sp. CB01881</td><td>glycosyltransferase [Streptomyces sp. CB01881]</td><td>GCF_002953255.1</td></tr>
<tr><td>WP_148757712.1</td><td>STAND→ VWA+vWA-L+STAND+TPR+GT4*→ Sigma→?→ ABC_tran→</td><td>VWA+vWA-L+STAND+TPR+GT4</td><td>1697</td><td>FXF68_RS05215</td><td>Actinobacteria</td><td>Actinomadura sp. CYP1-5</td><td>glycosyltransferase [Actinomadura sp. CYP1-5]</td><td>GCF_008121305.1</td></tr>
<tr><td>WP_148758378.1</td><td>SWACOS+GT4*→</td><td>SWACOS+GT4</td><td>1147</td><td>FXF68_RS08315</td><td>Actinobacteria</td><td>Actinomadura sp. CYP1-5</td><td>glycosyltransferase [Actinomadura sp. CYP1-5]</td><td>GCF_008121305.1</td></tr>
<tr><td>WP_148759187.1</td><td>GT4*→</td><td>GT4</td><td>581</td><td>FXF68_RS13230</td><td>Actinobacteria</td><td>Actinomadura sp. CYP1-5</td><td>glycosyltransferase [Actinomadura sp. CYP1-5]</td><td>GCF_008121305.1</td></tr>
<tr><td>WP_149263194.1</td><td>LexA→||||<-?||?→||||<-?<-?<-?<-cNMP_binding+HTH_Crp_2||GT4*→||||<-HTH+APATPase+TPR</td><td>GT4</td><td>459</td><td>K4S16_RS34110</td><td>Actinobacteria</td><td>Actinomadura sp. K4S16</td><td>glycosyltransferase [Actinomadura sp. K4S16]</td><td>GCF_008327685.1</td></tr>
<tr><td>WP_149830792.1</td><td><-HTH+APATPase+TPR||GT4*→?→||||<-?<-?<-?<-?||TPR→</td><td>GT4</td><td>419</td><td>F3T56_RS42265</td><td>Actinobacteria</td><td>Streptomyces sp. TRM68348</td><td>glycosyltransferase [Streptomyces sp. TRM68348]</td><td>GCF_008386495.1</td></tr>
<tr><td>WP_149896003.1</td><td>NACHT+GT4*→</td><td>NACHT+GT4</td><td>1646</td><td>DXU01_RS22280</td><td>Alphaproteobacteria</td><td>Agrobacterium rosae</td><td>glycosyltransferase [Agrobacterium rosae]</td><td>GCF_008501885.1</td></tr>
<tr><td>WP_151009920.1</td><td><-cNMP_binding+HTH_Crp_2||GT4*→||||<-HTH+APATPase+TPR</td><td>GT4</td><td>457</td><td>F7P10_RS15075</td><td>Actinobacteria</td><td>Actinomadura sp. WMMB 499</td><td>glycosyltransferase [Actinomadura sp. WMMB 499]</td><td>GCF_008824145.1</td></tr>
<tr><td>WP_153338839.1</td><td>ESX-1_EspG→||||<-?<-?||?→?→?→ WXG+TOXIN-PL+ART+Caspase+ZetaToxin+GT4*→</td><td>WXG+TOXIN-PL+ART+Caspase+ZetaToxin+GT4</td><td>3261</td><td>NRB56_RS02760</td><td>Actinobacteria</td><td>Nocardia sp. RB56</td><td>zeta toxin family protein [Nocardia sp. RB56]</td><td>GCF_009604425.1</td></tr>
</tbody>
</table>

<table>
<thead>
<tr><th>acc</th><th>operon</th><th>architecture</th><th>len</th><th>gen.name</th><th>taxend</th><th>species</th><th>defline</th><th>gca</th></tr>
</thead>
<tbody>
<tr><td>WP_153430492.1</td><td>GT4*→</td><td>GT4</td><td>1126</td><td>GFJ39_RS06035</td><td>Alphaproteobacteria</td><td>Gluconobacter sp. AC10</td><td>glycosyltransferase [Gluconobacter sp. AC10]</td><td>-</td></tr>
<tr><td>WP_153467262.1</td><td>GT4*→</td><td>GT4</td><td>460</td><td>F7Q99_RS30660</td><td>Actinobacteria</td><td>Streptomyces kaniharaensis</td><td>glycosyltransferase [Streptomyces kaniharaensis]</td><td>-</td></tr>
<tr><td>WP_153535837.1</td><td>REase+SFI-ATPase→||||<-?||?→||||<-?||?→ GT4+APATPase+TPR+TPR*→</td><td>GT4+APATPase+TPR+TPR</td><td>1190</td><td>ACRB68_RS23060</td><td>Actinobacteria</td><td>Actinomadura sp. RB68</td><td>tetratricopeptide repeat protein [Actinomadura sp. RB68]</td><td>-</td></tr>
<tr><td>WP_154363881.1</td><td>TniB→?→ STAND+TPR+GT4*→</td><td>STAND+TPR+GT4</td><td>1054</td><td>GJ697_RS09905</td><td>Betaproteobacteria</td><td>Duganella</td><td>MULTISPECIES: glycosyltransferase [Duganella]</td><td>GCF_009674525.1</td></tr>
<tr><td>WP_155055777.1</td><td>TIR+APATPase+GT4*→</td><td>TIR+APATPase+GT4</td><td>772</td><td>GFH49_RS11700</td><td>Actinobacteria</td><td>Streptomyces sp. TRM63209</td><td>TIR domain-containing protein [Streptomyces sp. TRM63209]</td><td>GCF_009709555.1</td></tr>
<tr><td>WP_155097668.1</td><td>GT4*→</td><td>GT4</td><td>609</td><td>GL286_RS21805</td><td>Alphaproteobacteria</td><td>Paracoccus aestuariivivens</td><td>hypothetical protein [Paracoccus aestuariivivens]</td><td>GCF_009711225.1</td></tr>
<tr><td>WP_155252689.1</td><td>Multi-TM→?→ STAND→?→ DUF4913→||||<-?||?→ GT4*→?→?→?→ NUDIX→</td><td>GT4</td><td>438</td><td>B170_RS0111915</td><td>Actinobacteria</td><td>Salinispora pacifica</td><td>glycosyltransferase [Salinispora pacifica]</td><td>GCF_000514515.1</td></tr>
<tr><td>WP_156002545.1</td><td><-AbiEii<-AbiEi_4||AbiEi_4→ AbiEii→||||<-AbiEii<-AbiEi_4||?→ GT4*→||||<-DUF4913<-?<-?<-STAND</td><td>GT4</td><td>494</td><td>FZ103_RS17370</td><td>Actinobacteria</td><td>Streptomonospora sp. PA3</td><td>glycosyltransferase [Streptomonospora sp. PA3]</td><td>GCF_009728995.1</td></tr>
<tr><td>WP_156395661.1</td><td>STAND+GT4*→</td><td>STAND+GT4</td><td>1081</td><td>ASC85_RS19490</td><td>Gammaproteobacteria</td><td>Pseudomonas sp. Root401</td><td>glycosyltransferase [Pseudomonas sp. Root401]</td><td>GCF_001425105.1</td></tr>
<tr><td>WP_156427756.1</td><td>APATPase+TPR+GT4*→</td><td>APATPase+TPR+GT4</td><td>1394</td><td>ML01_RS15555</td><td>Gammaproteobacteria</td><td>Thiohalocapsa sp. ML1</td><td>tetratricopeptide repeat protein [Thiohalocapsa sp. ML1]</td><td>GCF_001469165.1</td></tr>
<tr><td>WP_156551292.1</td><td>NACHT+GT4*→</td><td>NACHT+GT4</td><td>1640</td><td>GOZ95_RS24735</td><td>Alphaproteobacteria</td><td>Agrobacterium vitis</td><td>glycosyltransferase [Agrobacterium vitis]</td><td>GCF_009744055.1</td></tr>
<tr><td>WP_156901549.1</td><td>GT4*→?→||||<-?<-?<-?||?→||||<-MFS</td><td>GT4</td><td>643</td><td>K328_RS25440</td><td>Alphaproteobacteria</td><td>Nisaea denitrificans</td><td>glycosyltransferase, partial [Nisaea denitrificans]</td><td>GCF_000426505.1</td></tr>
</tbody>
</table>

acc	operon	architecture	len	gen.name	taxend	species	defline	gca
WP_157059028.1	GT4*→	GT4	261	AN191_RS16995	Alphaproteobacteria	Loktanella sp. 5RATIMAR09	glycosyltransferase family 4 protein, partial [Loktanella sp. 5RATIMAR09]	GCF_001419985.1
WP_157097111.1	GT4*→?→||||<-?<-?<-?||HTH→	GT4	608	FHS67_RS12310	Alphaproteobacteria	Aminobacter aminovorans	glycosyltransferase [Aminobacter aminovorans]	GCF_001605015.1
WP_157104014.1	WXG+MPTase→ WXG+SecA+ST-Hiskin+HTH+HTH+HTH+HTH+HTH+HTH+HTH+HTH+ST-Hiskin+MPTase+GT4+TetR-HTH+TetR-HTH+LuxR-HTH+LuxR-HTH+LuxR-HTH+GNTR-HTH+LuxR-HTH+LuxR-HTH+ST-Hiskin+ST-Hiskin+LuxR-HTH+TM+TM+TM+TM+MPTase+MPTase*→?→ ABhydrolase→	WXG+SecA+ST-Hiskin+HTH+HTH+HTH+HTH+HTH+HTH+HTH+HTH+ST-Hiskin+MPTase+GT4+TetR-HTH+TetR-HTH+LuxR-HTH+LuxR-HTH+LuxR-HTH+GNTR-HTH+LuxR-HTH+LuxR-HTH+ST-Hiskin+ST-Hiskin+LuxR-HTH+TM+TM+TM+TM+MPTase+MPTase	11529	NK1_RS00230	Actinobacteria	Nocardia kruczakiae	MFS transporter [Nocardia kruczakiae]	GCF_001612965.1
WP_157106745.1	MPTase→ WXG+SecA+GT4+TetR-HTH+TetR-HTH+MPTase+HTH+HTH+HTH+HTH+HTH+CHTH+HTH+HTH*→	WXG+SecA+GT4+TetR-HTH+TetR-HTH+MPTase+HTH+HTH+HTH+HTH+HTH+CHTH+HTH+HTH	5359	NA8_RS16815	Actinobacteria	Nocardia arthritidis	helix-turn-helix domain-containing protein [Nocardia arthritidis]	GCF_001612765.1
WP_157114874.1	<-ABC_tran<-?<-?||?→?→ MPTase→ WXG+SecA+GT4+TetR-HTH+TetR-HTH+HTH+HTH+HTH+HTH+HTH+CHTH+MPTase*→	WXG+SecA+GT4+TetR-HTH+TetR-HTH+HTH+HTH+HTH+HTH+HTH+CHTH+MPTase	6963	NNI01S_RS19995	Actinobacteria	Nocardia niwae	helix-turn-helix domain-containing protein [Nocardia niwae]	GCF_001613465.1
WP_157120634.1	TRPR-HTH+HTH+HTH+HTH+CHTH+HTH+HTH+NUDIX+NUDIX+Tox-MCF+GT4*→?→||||<-ESX-1_EspG<-?<-PSE||?→?→||||<-?||WXG+TM+TM+TM+TM+FN3+MPTase+MPTase+MPTase+MPTase+MPTase+MPTase+MPTase+MPTase+MPTase+MPTase+MPTase+MPTase+MPTase+MPTase+MPTase→	TRPR-HTH+HTH+HTH+HTH+CHTH+HTH+HTH+NUDIX+NUDIX+Tox-MCF+GT4	11280	NF4_RS27240	Actinobacteria	Nocardia fusca	helix-turn-helix domain-containing protein [Nocardia fusca]	GCF_001618425.1
WP_157120699.1	<-MPTase<-?||?→?→ MPTase→ WXG+SecA+GT4+MPTase+LuxR-HTH+Abhydrolase*→?→||||<-?<-?||PSE→?→||||<-?||SIG+SnoaL→	WXG+SecA+GT4+MPTase+LuxR-HTH+Abhydrolase	4137	NF4_RS31035	Actinobacteria	Nocardia fusca	glycosyltransferase [Nocardia fusca]	GCF_001618425.1
WP_157121874.1	WXG+MPTase→ WXG+SecA+ST-Hiskin+LuxR-HTH+LuxR-HTH+LuxR-HTH+LuxR-HTH+GT4+TetR-HTH+TetR-HTH+ART+SIGMA-HTH+LuxR-HTH+LuxR-HTH+LuxR-HTH+GNTR-HTH+LuxR-HTH+LuxR-HTH+ST-Hiskin+ST-Hiskin+XPC-C+LuxR-HTH+LuxR-HTH+MFS(6xTM)+PP2C+STYKinase-like*→?→ ABhydrolase→	WXG+SecA+ST-Hiskin+LuxR-HTH+LuxR-HTH+LuxR-HTH+LuxR-HTH+GT4+TetR-HTH+TetR-HTH+ART+SIGMA-HTH+LuxR-HTH+LuxR-HTH+LuxR-HTH+GNTR-HTH+LuxR-HTH+LuxR-HTH+ST-Hiskin+ST-Hiskin+XPC-C+LuxR-HTH+LuxR-HTH+MFS(6xTM)+PP2C+STYKinase-like	12511	NV5_RS14795	Actinobacteria	Nocardia violaceofusca	MFS transporter [Nocardia violaceofusca]	GCF_001613525.1
WP_157126117.1	WXG+MPTase→ WXG+SecA+ST-Hiskin+HTH+HTH+HTH+HTH+HTH+HTH+HTH+HTH+ST-Hiskin+MPTase+GT4+TetR-HTH+TetR-HTH+LuxR-HTH+LuxR-HTH+LuxR-HTH+LuxR-HTH+GNTR-HTH+LuxR-HTH+LuxR-HTH+PP2C+MPTase+MPTase+8TMR-UT+TM+TM+ST-Hiskin*→?→ ABhydrolase→	WXG+SecA+ST-Hiskin+HTH+HTH+HTH+HTH+HTH+HTH+HTH+HTH+ST-Hiskin+MPTase+GT4+TetR-HTH+TetR-HTH+LuxR-HTH+LuxR-HTH+LuxR-HTH+LuxR-HTH+GNTR-HTH+LuxR-HTH+LuxR-HTH+PP2C+MPTase+MPTase+8TMR-UT+TM+TM+ST-Hiskin	15995	NMI01S_RS10045	Actinobacteria	Nocardia mikamii	MFS transporter [Nocardia mikamii]	GCF_001613505.1
WP_157129222.1	HTH+APATPase+TPR→?→ GT4*→||||<-?<-?<-AAA_21	GT4	601	NA4_RS22535	Actinobacteria	Nocardia amamiensis	glycosyltransferase [Nocardia amamiensis]	GCF_001612745.1
WP_157163478.1	WXG+MPTase→ WXG+SecA+ST-Hiskin+LuxR-HTH+LuxR-HTH+LuxR-HTH+LuxR-HTH+GT4+TetR-HTH+TetR-HTH+ART+SIGMA-HTH+LuxR-HTH+LuxR-HTH+LuxR-HTH+GNTR-HTH+LuxR-HTH+LuxR-HTH+ST-Hiskin+ST-Hiskin+XPC-C+LuxR-HTH+LuxR-HTH+MFS(6xTM)+PP2C+STYKinase-like*→?→ ABhydrolase→	WXG+SecA+ST-Hiskin+LuxR-HTH+LuxR-HTH+LuxR-HTH+LuxR-HTH+GT4+TetR-HTH+TetR-HTH+ART+SIGMA-HTH+LuxR-HTH+LuxR-HTH+LuxR-HTH+GNTR-HTH+LuxR-HTH+LuxR-HTH+ST-Hiskin+ST-Hiskin+XPC-C+LuxR-HTH+LuxR-HTH+MFS(6xTM)+PP2C+STYKinase-like	12510	RJ03_RS22280	Actinobacteria	Nocardia aobensis	MFS transporter [Nocardia aobensis]	GCF_000308375.1
WP_157170272.1	WXG+MPTase→ WXG+SecA+LuxR-HTH+LuxR-HTH+LuxR-HTH+LuxR-HTH+ST-Hiskin+MPTase+GT4+TetR-HTH+TetR-HTH+Tox-ART-RSE+LuxR-HTH+LuxR-HTH+LuxR-HTH+GNTR-HTH+LuxR-HTH+LuxR-HTH+ST-Hiskin+ST-Hiskin+XPC-C+TM+TM+TM+TM+LuxR-HTH*→	WXG+SecA+LuxR-HTH+LuxR-HTH+LuxR-HTH+LuxR-HTH+ST-Hiskin+MPTase+GT4+TetR-HTH+TetR-HTH+Tox-ART-RSE+LuxR-HTH+LuxR-HTH+LuxR-HTH+GNTR-HTH+LuxR-HTH+LuxR-HTH+ST-Hiskin+ST-Hiskin+XPC-C+TM+TM+TM+TM+LuxR-HTH	11647	ON23_RS23640	Actinobacteria	Nocardia cerradoensis	MFS transporter, partial [Nocardia cerradoensis]	GCF_000308535.1
WP_157172575.1	ESX-1_EspG→?→?→?→ WXG+GT4+PP2C+GDPD+Amidohydrolase+Abhydrolase+GT4*→	WXG+GT4+PP2C+GDPD+Amidohydrolase+Abhydrolase+GT4	27919	ON34_RS32865	Actinobacteria	Nocardia pneumoniae	glycosyltransferase [Nocardia pneumoniae]	GCF_000308755.1
WP_157180627.1	WXG+TM+GT4+GT4*→	WXG+TM+GT4+GT4	1681	FrCorBMG51_RS28730	Actinobacteria	Frankia coriariae	hypothetical protein [Frankia coriariae]	GCF_001017755.1

acc	operon	architecture	len	gen.name	taxend	species	defline	gca
WP_157204012.1	GT4+APATPase+TPR*→	GT4+APATPase+TPR	1141	A1332_RS13095	Gammaproteobacteria	Methylomonas methanica	tetratricopeptide repeat protein, partial [Methylomonas methanica]	-
WP_157224231.1	WXG+ZetaToxin+GT4*→?→||||<-WXG+MPTase	WXG+ZetaToxin+GT4	1061	ON32_RS18080	Actinobacteria	Nocardia paucivorans	zeta toxin family protein [Nocardia paucivorans]	GCF_000308675.1
WP_157229180.1	MPTase→ WXG+SecA+GT4+TetR-HTH+TetR-HTH+MFS+MFS(12x)+ABhydrolase+ST-Hiskin*→||||<-?||CSD→	WXG+SecA+GT4+TetR-HTH+TetR-HTH+MFS+MFS(12x)+ABhydrolase+ST-Hiskin	4779	ON20_RS32170	Actinobacteria	Nocardia brevicatena	MFS transporter [Nocardia brevicatena]	GCF_000308495.1
WP_157355048.1	GT4*→	GT4	618	ARA48_RS12920	Bacteroidetes	Parabacteroides distasonis	glycosyltransferase [Parabacteroides distasonis]	GCF_001405935.1
WP_157419626.1	<-cNMP_binding+HTH_Crp_2||GT4*→||||<-HTH+APATPase+TPR	GT4	468	AKI01S_RS02785	Actinobacteria	Actinomadura kijaniata	glycosyltransferase [Actinomadura kijaniata]	GCF_001552175.1
WP_157468665.1	CATRA-N+CATRA-C+GT4*→||||<-TPR+Caspase<-?<-?<-?<-?||CHTH+APATPase+TPR+TPR→ CATASP→	CATRA-N+CATRA-C+GT4	825	FRAQA3DRAFT_RS22440	Actinobacteria	Frankia sp. QA3	glycosyltransferase [Frankia sp. QA3]	GCF_000262465.1
WP_157494479.1	<-CATASP||CATRA-N+CATRA-C+GT4*→||||<-HTH+APATPase+TPR	CATRA-N+CATRA-C+GT4	786	KUTG_RS53310	Actinobacteria	Kutzneria sp. 744	glycosyltransferase [Kutzneria sp. 744]	-
WP_157528945.1	ART+Caspase+GT4*→?→ GT4→	ART+Caspase+GT4	1289	BN1199_RS39095	Actinobacteria	Kibdelosporangium sp. MJ126-NF4	glycosyltransferase [Kibdelosporangium sp. MJ126-NF4]	-
WP_157636396.1	STAND+TPR+GT4*→||||<-?||ABhydrolase→||||<-TRANSGLUTAMINASE	STAND+TPR+GT4	1130	WL03_RS32310	Betaproteobacteria	Burkholderia ubonensis	glycosyltransferase [Burkholderia ubonensis]	GCF_001528155.1
WP_157851161.1	<-TPR+Caspase<-CATASP||CATRA-N+CATRA-C+GT4*→ HTH+APATPase+TPR→||||<-HTH||?→?→?→||||<-?<-CATASP	CATRA-N+CATRA-C+GT4	703	IF17_RS1000000138590	Actinobacteria	Streptomyces cyaneofuscatus	glycosyltransferase [Streptomyces cyaneofuscatus]	GCF_000718135.1
WP_157856282.1	ABC_tran→?→?→ NACHT+MNS→?→ GT4*→	GT4	412	BS72_RS20070	Actinobacteria	Streptomyces yeochonensis	glycosyltransferase [Streptomyces yeochonensis]	-
WP_157978213.1	GT4*→	GT4	199	DAT37_RS00050	Actinobacteria	Nocardia sp. SYSU K10002	hypothetical protein [Nocardia sp. SYSU K10002]	-
WP_158075353.1	<-TPR+Caspase<-CATASP||CATRA-N+CATRA-C+GT4*→ HTH+APATPase+TPR→||||<-HTH||?→?→?→||||<-?<-CATASP	CATRA-N+CATRA-C+GT4	718	AMK32_RS25155	Actinobacteria	Streptomyces sp. CB01883	glycosyltransferase [Streptomyces sp. CB01883]	-

<table>
<tr><th>acc</th><th>operon</th><th>architecture</th><th>len</th><th>gen.name</th><th>taxend</th><th>species</th><th>defline</th><th>gca</th></tr>
<tr><td>WP_158247498.1</td><td>GT4+APATPase+TPR+TPR*→</td><td>GT4+APATPase+TPR+TPR</td><td>1379</td><td>C1X76_RS08190</td><td>Gammaproteobacteria</td><td>unclassified Pseudomonas</td><td>MULTISPECIES: tetratricopeptide repeat protein [unclassified Pseudomonas]</td><td>GCF_017350895.1</td></tr>
<tr><td>WP_158633123.1</td><td>cNMP_binding+HTH_Crp_2→?→ GT4*→||||<-ACYC+APATPase+TPR</td><td>GT4</td><td>391</td><td>DMH03_RS26200</td><td>Actinobacteria</td><td>Amycolatopsis sp. WAC 01376</td><td>glycosyltransferase [Amycolatopsis sp. WAC 01376]</td><td>GCF_003947415.1</td></tr>
<tr><td>WP_158709116.1</td><td><-ABC_tran<-?||PSE→||||<-?||Caspase+NACHT+GT4*→</td><td>Caspase+NACHT+GT4</td><td>1143</td><td>D3105_RS04960</td><td>Actinobacteria</td><td>Streptomyces globisporus</td><td>caspase family protein [Streptomyces globisporus]</td><td>GCF_000718455.1</td></tr>
<tr><td>WP_158893312.1</td><td><-TIR+APATPase||HTH+APATPase+TPR+GT4*→||||<-PSE<-?||?→?→?→ Sigma→</td><td>HTH+APATPase+TPR+GT4</td><td>1166</td><td>FXO23_RS42605</td><td>Actinobacteria</td><td>Amycolatopsis anabasis</td><td>tetratricopeptide repeat protein [Amycolatopsis anabasis]</td><td>GCF_009765355.1</td></tr>
<tr><td>WP_159015082.1</td><td><-TPR+Caspase<-CATASP||CATRA-N+CATRA-C+GT4*→ HTH+APATPase+TPR→||||<-HTH||?→||||<-?<-CATASP<-TPR+Caspase</td><td>CATRA-N+CATRA-C+GT4</td><td>711</td><td>AAY49_RS44335</td><td>Actinobacteria</td><td>Streptomyces europaeiscabiei</td><td>glycosyltransferase [Streptomyces europaeiscabiei]</td><td>GCF_000988945.1</td></tr>
<tr><td>WP_159058466.1</td><td><-CATASP||CATRA-N+CATRA-C+GT4*→ HTH+APATPase→</td><td>CATRA-N+CATRA-C+GT4</td><td>711</td><td>AWZ10_RS35055</td><td>Actinobacteria</td><td>Streptomyces europaeiscabiei</td><td>glycosyltransferase [Streptomyces europaeiscabiei]</td><td>GCF_001550375.1</td></tr>
<tr><td>WP_159073577.1</td><td>GT4*→</td><td>GT4</td><td>122</td><td>A6027_RS50895</td><td>Actinobacteria</td><td>Streptomyces sp. RTd22</td><td>hypothetical protein [Streptomyces sp. RTd22]</td><td>GCF_001640565.1</td></tr>
<tr><td>WP_159388811.1</td><td>MFS→||||<-?||GT4+APATPase+TPR*→||||<-MODE-HTH+PBPII</td><td>GT4+APATPase+TPR</td><td>1318</td><td>PspR32_RS15205</td><td>Gammaproteobacteria</td><td>Pseudomonas sp. R32</td><td>glycosyltransferase [Pseudomonas sp. R32]</td><td>GCF_009866705.1</td></tr>
<tr><td>WP_159399980.1</td><td>Caspase+NACHT+GT4*→||||<-?<-?||PSE→||||<-?||?→||||<-?<-MFS</td><td>Caspase+NACHT+GT4</td><td>1145</td><td>AVL59_RS21225</td><td>Actinobacteria</td><td>Streptomyces griseochromogenes</td><td>caspase family protein [Streptomyces griseochromogenes]</td><td>GCF_001542625.1</td></tr>
<tr><td>WP_160448122.1</td><td>ABC_tran→?→ GT4*→||||<-?||?→?→?→||||<-?||tRNA→?→ Pribosyltran_N+Pribosyl_synth→</td><td>GT4</td><td>643</td><td>DOU06_RS11775</td><td>Actinobacteria</td><td>Clavibacter michiganensis</td><td>glycosyltransferase [Clavibacter michiganensis]</td><td>GCF_009793225.1</td></tr>
<tr><td>WP_161065972.1</td><td>DUF4913→ GT4*→</td><td>GT4</td><td>463</td><td>GR925_RS14975</td><td>Actinobacteria</td><td>Streptomyces sp. HUCO-GS316</td><td>glycosyltransferase [Streptomyces sp. HUCO-GS316]</td><td>GCF_009827025.1</td></tr>
<tr><td>WP_161268609.1</td><td>CATRA-N+CATRA-C+GT4*→</td><td>CATRA-N+CATRA-C+GT4</td><td>703</td><td>GT040_RS36085</td><td>Actinobacteria</td><td>Streptomyces sp. SID2119</td><td>glycosyltransferase [Streptomyces sp. SID2119]</td><td>GCF_009865505.1</td></tr>
</table>

<table>
<tr><th>acc</th><th>operon</th><th>architecture</th><th>len</th><th>gen.name</th><th>taxend</th><th>species</th><th>defline</th><th>gca</th></tr>
<tr><td>WP_161606588.1</td><td>GT4*→</td><td>GT4</td><td>962</td><td>BHS06_RS25085</td><td>Deltaproteobacteria</td><td>Myxococcus xanthus</td><td>glycosyltransferase [Myxococcus xanthus]</td><td>GCF_006401635.1</td></tr>
<tr><td>WP_161684505.1</td><td>Sigma→?→?→||||<-?<-?<-TPR+Caspase||GT4*→||||<-PSE<-?||?→||||<-TRPR-HTH+PBPI</td><td>GT4</td><td>547</td><td>GVV04_RS04065</td><td>Actinobacteria</td><td>Micromonospora sp. NEAU-HG-1</td><td>glycosyltransferase [Micromonospora sp. NEAU-HG-1]</td><td>GCF_009908295.1</td></tr>
<tr><td>WP_161790163.1</td><td>GT4*→||||<-?<-?<-?<-CSD</td><td>GT4</td><td>353</td><td>TR46_RS20005</td><td>Actinobacteria</td><td>Streptacidiphilus carbonis</td><td>glycosyltransferase [Streptacidiphilus carbonis]</td><td>GCF_000787775.1</td></tr>
<tr><td>WP_162672520.1</td><td>GT4+HEPN+TPR+GreAB-C+PIN*→</td><td>GT4+HEPN+TPR+GreAB-C+PIN</td><td>1685</td><td>SOIL9_RS38685</td><td>Planctomycetes</td><td>Gemmata massiliana</td><td>glycosyltransferase [Gemmata massiliana]</td><td>GCF_901538265.1</td></tr>
<tr><td>WP_162688623.1</td><td>Caspase+NACHT+GT4*→||||<-?<-?<-?<-?||APATPase→</td><td>Caspase+NACHT+GT4</td><td>1148</td><td>DRB89_RS17705</td><td>Actinobacteria</td><td>unclassified Streptomyces</td><td>MULTISPECIES: caspase family protein [unclassified Streptomyces]</td><td>GCF_003287915.1</td></tr>
<tr><td>WP_162948626.1</td><td><-CATASP||CATRA-N+CATRA-C+GT4*→ HTH+APATPase→</td><td>CATRA-N+CATRA-C+GT4</td><td>711</td><td>CQR77_RS44880</td><td>Actinobacteria</td><td>Streptomyces europaeiscabiei</td><td>glycosyltransferase [Streptomyces europaeiscabiei]</td><td>GCF_003584825.1</td></tr>
<tr><td>WP_163053769.1</td><td>Calcineurin→?→?→ NACHT+MNS→ MNS+GT4*→</td><td>MNS+GT4</td><td>749</td><td>G3I70_RS06860</td><td>Actinobacteria</td><td>Actinomadura bangladeshensis</td><td>glycosyltransferase [Actinomadura bangladeshensis]</td><td>GCF_010548065.1</td></tr>
<tr><td>WP_164236650.1</td><td><-TPR+Caspase<-CATASP||CATRA-N+CATRA-C+GT4*→ HTH+APATPase+TPR→||||<-HTH||?→?→?→||||<-?<-CATASP</td><td>CATRA-N+CATRA-C+GT4</td><td>703</td><td>G3I52_RS10700</td><td>Actinobacteria</td><td>Streptomyces cyaneofuscatus</td><td>glycosyltransferase [Streptomyces cyaneofuscatus]</td><td>GCF_010548165.1</td></tr>
<tr><td>WP_164336692.1</td><td>Calcineurin→?→?→ Caspase+NACHT+GT4*→</td><td>Caspase+NACHT+GT4</td><td>1126</td><td>G3I38_RS20825</td><td>Actinobacteria</td><td>Streptomyces sp. SID7958</td><td>caspase family protein [Streptomyces sp. SID7958]</td><td>GCF_010550365.1</td></tr>
<tr><td>WP_164374106.1</td><td>NACHT→ GT4→||||<-?<-PSE<-?<-?<-?||HTH+APATPase→ TPR→||||<-GT4</td><td>GT4</td><td>405</td><td>G3I62_RS29240</td><td>Actinobacteria</td><td>Streptomyces sp. SID14446</td><td>glycosyltransferase [Streptomyces sp. SID14446]</td><td>GCF_010548515.1</td></tr>
<tr><td>WP_164374112.1</td><td>-</td><td>-</td><td>402</td><td>G3I62_RS29280</td><td>Actinobacteria</td><td>Streptomyces sp. SID14446</td><td>glycosyltransferase [Streptomyces sp. SID14446]</td><td>GCF_010548515.1</td></tr>
<tr><td>WP_164438832.1</td><td>Calcineurin→?→?→ Caspase+NACHT+GT4*→</td><td>Caspase+NACHT+GT4</td><td>1113</td><td>G3I53_RS25925</td><td>Actinobacteria</td><td>Streptomyces sp. SID14436</td><td>caspase family protein [Streptomyces sp. SID14436]</td><td>GCF_010548465.1</td></tr>
<tr><td>WP_164932131.1</td><td><-HTH+APATPase+TPR||GT4*→?→||||<-?<-?<-?||TPR→</td><td>GT4</td><td>394</td><td>DBB27_RS07160</td><td>Actinobacteria</td><td>unclassified Streptomyces</td><td>MULTISPECIES: glycosyltransferase [unclassified Streptomyces]</td><td>GCF_004104505.1</td></tr>
</table>

acc	operon	architecture	len	gen.name	taxend	species	defline	gca
WP_165781010.1	GT4*→	GT4	631	CR156_RS18515	Gammaproteobacteria	Stenotrophomonas lactitubi	glycosyltransferase [Stenotrophomonas lactitubi]	GCF_014763005.1
WP_166659524.1	-	-	752	EV186_RS37545	Actinobacteria	Labedaea rhizosphaerae	glycosyltransferase family 4 protein [Labedaea rhizosphaerae]	GCF_004362825.1
WP_167375417.1	APATPase+GT4*→	APATPase+GT4	1344	AWM79_RS04595	Gammaproteobacteria	Pseudomonas agarici	glycosyltransferase [Pseudomonas agarici]	GCF_001543125.1
WP_167485160.1	<-ESX-1_EspG<-?<-?<-?||?→?→ WXG+PolB-NTase+GT4+GT4+Caspase*→?→?→ GT4→	WXG+PolB-NTase+GT4+GT4+Caspase	2733	F6W96_RS05200	Actinobacteria	Nocardia terpenica	glycosyltransferase [Nocardia terpenica]	GCF_011801165.1
WP_167531487.1	NACHT+GT4*→||||<-Vsr	NACHT+GT4	1557	EN799_RS48175	Alphaproteobacteria	Mesorhizobium sp. M8A.F.Ca.ET.197.01.1.1	glycosyltransferase [Mesorhizobium sp. M8A.F.Ca.ET.197.01.1.1]	GCF_004791385.1
WP_167631834.1	GT4+APATPase+TPR*→	GT4+APATPase+TPR	1107	HCI87_RS07630	Zetaproteobacteria	Mariprofundus ferrooxydans	tetratricopeptide repeat protein, partial [Mariprofundus ferrooxydans]	GCF_011947535.1
WP_167983001.1	CATRA-N+CATRA-C+GT4*→ HTH+APATPase+TPR→||||<-HTH||?→?→?→||||<-?<-CATASP	CATRA-N+CATRA-C+GT4	845	HCN08_RS12120	Actinobacteria	Streptomyces sp. PRB2-1	glycosyltransferase family 4 protein [Streptomyces sp. PRB2-1]	GCF_012034175.1
WP_167985631.1	GT4*→	GT4	425	HCN08_RS25735	Actinobacteria	Streptomyces sp. PRB2-1	glycosyltransferase [Streptomyces sp. PRB2-1]	GCF_012034175.1
WP_168028895.1	<-ABC_tran<-?<-?<-?<-?||?→ GT4+APATPase*→	GT4+APATPase	988	HC024_RS03995	Gammaproteobacteria	Methylococcaceae bacterium WWC4	glycosyltransferase, partial [Methylococcaceae bacterium WWC4]	-
WP_168031569.1	GT4*→	GT4	644	HC024_RS14290	Gammaproteobacteria	Methylococcaceae bacterium WWC4	glycosyltransferase, partial [Methylococcaceae bacterium WWC4]	-
WP_168435140.1	WXG+MPTase→ WXG+SecA+LuxR-HTH+LuxR-HTH+LuxR-HTH+LuxR-HTH+ST-Hiskin+MPTase+GT4+TetR-HTH+TetR-HTH+Tox-ART-RSE+LuxR-HTH+LuxR-HTH+LuxR-HTH+GNTR-HTH+LuxR-HTH+LuxR-HTH+ST-Hiskin+ST-Hiskin+XPC-C+TM+TM+TM+TM+LuxR-HTH+PP2C+MPTase+MPTase+LuxR-HTH+LuxR-HTH+DOC*→?→ ABhydrolase→	WXG+SecA+LuxR-HTH+LuxR-HTH+LuxR-HTH+LuxR-HTH+ST-Hiskin+MPTase+GT4+TetR-HTH+TetR-HTH+Tox-ART-RSE+LuxR-HTH+LuxR-HTH+LuxR-HTH+GNTR-HTH+LuxR-HTH+LuxR-HTH+ST-Hiskin+ST-Hiskin+XPC-C+TM+TM+TM+TM+LuxR-HTH+PP2C+MPTase+MPTase+LuxR-HTH+LuxR-HTH+DOC	13688	HGB45_RS23560	Actinobacteria	Nocardia cerradoensis	MFS transporter [Nocardia cerradoensis]	GCF_012396065.1
WP_168477813.1	GT4*→?→?→||||<-?||?→||||<-NUDIX	GT4	455	HEP86_RS05755	Actinobacteria	Streptomyces sp. RPA4-5	glycosyltransferase [Streptomyces sp. RPA4-5]	GCF_012273495.1

<table>
<tr><th>acc</th><th>operon</th><th>architecture</th><th>len</th><th>gen.name</th><th>taxend</th><th>species</th><th>defline</th><th>gca</th></tr>
<tr><td>WP_168512294.1</td><td><-ABhydrolase||?→?→ GT4*→||||<-?||TRPR-HTH+PBPI→</td><td>GT4</td><td>650</td><td>HEP87_RS39620</td><td>Actinobacteria</td><td>Streptomyces sp. S1D4-11</td><td>glycosyltransferase [Streptomyces sp. S1D4-11]</td><td>-</td></tr>
<tr><td>WP_169316088.1</td><td>APATPase+GT4*→||||<-?<-?<-?<-?||?→||||<-?<-PSE||GST_N_2+GST_C→</td><td>APATPase+GT4</td><td>1344</td><td>PAGAR_RS28405</td><td>Gammaproteobacteria</td><td>Pseudomonas agarici</td><td>glycosyltransferase [Pseudomonas agarici]</td><td>GCF_000280785.1</td></tr>
<tr><td>WP_169804810.1</td><td>GT4+APATPase*→</td><td>GT4+APATPase</td><td>986</td><td>HTA01S_RS24340</td><td>Betaproteobacteria</td><td>Hydrogenophaga taeniospiralis</td><td>glycosyltransferase, partial [Hydrogenophaga taeniospiralis]</td><td>GCF_001592305.1</td></tr>
<tr><td>WP_169807203.1</td><td>GT4*→</td><td>GT4</td><td>449</td><td>AWW66_RS34115</td><td>Actinobacteria</td><td>Micromonospora rosaria</td><td>glycosyltransferase, partial [Micromonospora rosaria]</td><td>GCF_001567585.1</td></tr>
<tr><td>WP_169850673.1</td><td>GT4+B12-binding+RADICAL-SAM*→</td><td>GT4+B12-binding+RADICAL-SAM</td><td>1115</td><td>HBO43_RS21615</td><td>Gammaproteobacteria</td><td>Pseudomonas veronii</td><td>cobalamin B12-binding domain-containing protein [Pseudomonas veronii]</td><td>GCF_012985315.1</td></tr>
<tr><td>WP_170285467.1</td><td>SWACOS+GT4*→?→||||<-?||?→ PadR→</td><td>SWACOS+GT4</td><td>1175</td><td>FHX75_RS26815</td><td>Actinobacteria</td><td>Micromonospora palomenae</td><td>glycosyltransferase [Micromonospora palomenae]</td><td>GCF_007829925.1</td></tr>
<tr><td>WP_171081495.1</td><td>GT4*→</td><td>GT4</td><td>489</td><td>HG542_RS14775</td><td>Actinobacteria</td><td>Streptomyces morookaense</td><td>glycosyltransferase [Streptomyces morookaense]</td><td>GCF_014656115.1</td></tr>
<tr><td>WP_171579219.1</td><td><-SIG+RCDG1+YidC_periplas+60KD_IMP<-?<-?||?→ tRNA→?→ NACHT+GT4*→?→?→?→?→?→?→ DUF4242→</td><td>NACHT+GT4</td><td>1638</td><td>HCN58_RS10240</td><td>Alphaproteobacteria</td><td>Bradyrhizobium sp. WSM 1791</td><td>glycosyltransferase [Bradyrhizobium sp. WSM 1791]</td><td>GCF_013114825.1</td></tr>
<tr><td>WP_171675029.1</td><td>GT4*→</td><td>GT4</td><td>451</td><td>HNR71_RS02445</td><td>Actinobacteria</td><td>Kribbella sandramycini</td><td>glycosyltransferase [Kribbella sandramycini]</td><td>GCF_013131805.1</td></tr>
<tr><td>WP_171901162.1</td><td>-</td><td>-</td><td>147</td><td>NK6_RS51125</td><td>Alphaproteobacteria</td><td>Bradyrhizobium diazoefficiens</td><td>glycosyltransferase [Bradyrhizobium diazoefficiens]</td><td>GCF_001549695.1</td></tr>
<tr><td>WP_172109703.1</td><td>SWACOS+GT4*→</td><td>SWACOS+GT4</td><td>1148</td><td>HL667_RS06640</td><td>Alphaproteobacteria</td><td>Bradyrhizobium sp. 83012</td><td>glycosyltransferase [Bradyrhizobium sp. 83012]</td><td>GCF_013178925.1</td></tr>
<tr><td>WP_172132695.1</td><td>SWACOS+GT4*→</td><td>SWACOS+GT4</td><td>1148</td><td>HL666_RS23775</td><td>Alphaproteobacteria</td><td>unclassified Bradyrhizobium</td><td>MULTISPECIES: glycosyltransferase [unclassified Bradyrhizobium]</td><td>GCF_013178945.1</td></tr>
</table>

<table>
<tr><th>acc</th><th>operon</th><th>architecture</th><th>len</th><th>gen.name</th><th>taxend</th><th>species</th><th>defline</th><th>gca</th></tr>
<tr><td>WP_172175287.1</td><td>GT4+B12-binding+RADICAL-SAM*→</td><td>GT4+B12-binding+RADICAL-SAM</td><td>1278</td><td>GNZ13_RS39275</td><td>Betaproteobacteria</td><td>Paraburkholderia sp. 5N</td><td>radical SAM protein [Paraburkholderia sp. 5N]</td><td>GCF_013177735.1</td></tr>
<tr><td>WP_173124146.1</td><td><-STAND<-?<-?<-?<-?||cNMP_binding+HTH_Crp_2→?→ GT4*→||||<-ACYC+APATPase+TPR</td><td>GT4</td><td>409</td><td>GC106_RS03330</td><td>Actinobacteria</td><td>Kibdelosporangium sp. 4NS15</td><td>glycosyltransferase [Kibdelosporangium sp. 4NS15]</td><td>GCF_013280595.1</td></tr>
<tr><td>WP_173195298.1</td><td>GT4+APATPase+TPR+TPR*→</td><td>GT4+APATPase+TPR+TPR</td><td>1427</td><td>HR085_RS00175</td><td>Deltaproteobacteria</td><td>Geobacter sp. SVR</td><td>tetratricopeptide repeat protein [Geobacter sp. SVR]</td><td>GCF_016865365.1</td></tr>
<tr><td>WP_173395462.1</td><td>DJ-1_PfpI→ LexA→?→||||<-?<-?<-?<-cNMP_binding+HTH_Crp_2||GT4*→||||<-HTH+APATPase+TPR</td><td>GT4</td><td>438</td><td>GWI34_RS19435</td><td>Actinobacteria</td><td>Actinomadura sp. DSM 109109</td><td>glycosyltransferase [Actinomadura sp. DSM 109109]</td><td>-</td></tr>
<tr><td>WP_173526832.1</td><td>GT4+APATPase+TPR+TPR*→</td><td>GT4+APATPase+TPR+TPR</td><td>1208</td><td>HII36_RS31010</td><td>Actinobacteria</td><td>Nonomuraea sp. NN258</td><td>tetratricopeptide repeat protein [Nonomuraea sp. NN258]</td><td>GCF_013283785.1</td></tr>
<tr><td>WP_173991751.1</td><td>PfkB→?→?→?→ GT4+B12-binding+RADICAL-SAM*→</td><td>GT4+B12-binding+RADICAL-SAM</td><td>1387</td><td>G6M20_RS23615</td><td>Alphaproteobacteria</td><td>Agrobacterium salinitolerans</td><td>cobalamin B12-binding domain-containing protein [Agrobacterium salinitolerans]</td><td>GCF_013317635.1</td></tr>
<tr><td>WP_174156125.1</td><td>HTH+MPTase→?→||||<-?||?→?→ NACHT+GT4*→</td><td>NACHT+GT4</td><td>1640</td><td>G6L88_RS21970</td><td>Alphaproteobacteria</td><td>Rhizobium skierniewicense</td><td>glycosyltransferase [Rhizobium skierniewicense]</td><td>GCF_013320815.1</td></tr>
<tr><td>WP_174295717.1</td><td>GT4*→</td><td>GT4</td><td>613</td><td>G7A66_RS11060</td><td>Alphaproteobacteria</td><td>Altererythrobacter sp. SALINAS58</td><td>glycosyltransferase [Altererythrobacter sp. SALINAS58]</td><td>GCF_013336795.1</td></tr>
<tr><td>WP_174397973.1</td><td>GT4*→</td><td>GT4</td><td>602</td><td>FEG63_RS11180</td><td>Actinobacteria</td><td>Mycolicibacterium sphagni</td><td>glycosyltransferase [Mycolicibacterium sphagni]</td><td>GCF_013337765.1</td></tr>
<tr><td>WP_174527417.1</td><td>STAND→?→ DUF4913→||||<-?||?→ GT4*→?→?→?→ NUDIX→||||<-?<-ABC_tran</td><td>GT4</td><td>438</td><td>HUW36_RS07305</td><td>Actinobacteria</td><td>Micromonospora maritima</td><td>glycosyltransferase [Micromonospora maritima]</td><td>GCF_902825405.1</td></tr>
<tr><td>WP_175264897.1</td><td>NACHT+MNS→ MNS+GT4*→||||<-?<-CASPASE</td><td>MNS+GT4</td><td>752</td><td>HRW23_RS35520</td><td>Actinobacteria</td><td>Streptomyces lunaelactis</td><td>glycosyltransferase [Streptomyces lunaelactis]</td><td>GCF_013357925.1</td></tr>
<tr><td>WP_176052200.1</td><td>STAND+GT4*→?→||||<-Cluster466_2clades<-?<-?<-?||DNA_methylase→||||<-Vsr</td><td>STAND+GT4</td><td>1090</td><td>HWY02_RS01425</td><td>Betaproteobacteria</td><td>Paraburkholderia caribensis</td><td>glycosyltransferase [Paraburkholderia caribensis]</td><td>GCF_902833585.1</td></tr>
</table>

<table>
<thead>
<tr><th>acc</th><th>operon</th><th>architecture</th><th>len</th><th>gen.name</th><th>taxend</th><th>species</th><th>defline</th><th>gca</th></tr>
</thead>
<tbody>
<tr><td>WP_176182010.1</td><td>MNS+GT4*→||||<-?<-?<-AA_permease</td><td>MNS+GT4</td><td>1151</td><td>HUT19_RS21385</td><td>Actinobacteria</td><td>Streptomyces sp. NA02950</td><td>glycosyltransferase [Streptomyces sp. NA02950]</td><td>GCF_013364155.1</td></tr>
<tr><td>WP_176404534.1</td><td><-cNMP_binding+HTH_Crp_2||GT4*→</td><td>GT4</td><td>427</td><td>HUX53_RS18900</td><td>Actinobacteria</td><td>Actinomadura sp. BRA 177</td><td>glycosyltransferase [Actinomadura sp. BRA 177]</td><td>GCF_013372625.1</td></tr>
<tr><td>WP_176437046.1</td><td><-ABC_tran<-?||?→||||<-?<-PSE<-?||GT4+TPR+TPR*→</td><td>GT4+TPR+TPR</td><td>1428</td><td>HUW62_RS36515</td><td>Deltaproteobacteria</td><td>Myxococcus sp. AM011</td><td>tetratricopeptide repeat protein [Myxococcus sp. AM011]</td><td>GCF_013372595.1</td></tr>
<tr><td>WP_176439087.1</td><td>HTH_Tnp_1→ TnpB_IS66→?→ NACHT+GT4*→</td><td>NACHT+GT4</td><td>1580</td><td>CHB75_RS08020</td><td>Alphaproteobacteria</td><td>Puniceibacterium sediminis</td><td>glycosyltransferase [Puniceibacterium sediminis]</td><td>GCF_900188035.1</td></tr>
<tr><td>WP_176652673.1</td><td>GT4+NACHT*→ HTH_Tnp_1→ TnpB_IS66→</td><td>GT4+NACHT</td><td>1759</td><td>FHT31_RS13555</td><td>Betaproteobacteria</td><td>Rugamonas sp. SG757</td><td>glycosyltransferase [Rugamonas sp. SG757]</td><td>GCF_013375995.1</td></tr>
<tr><td>WP_176735010.1</td><td><-TPR+Caspase||GT4*→</td><td>GT4</td><td>546</td><td>GA0070563_RS2[illegible]590</td><td>Actinobacteria</td><td>Micromonospora carbonacea</td><td>glycosyltransferase [Micromonospora carbonacea]</td><td>GCF_900091535.1</td></tr>
<tr><td>WP_177014872.1</td><td>STAND+GT4*→</td><td>STAND+GT4</td><td>1080</td><td>HX791_RS25785</td><td>Gammaproteobacteria</td><td>Pseudomonas costantinii</td><td>glycosyltransferase [Pseudomonas costantinii]</td><td>GCF_013386225.1</td></tr>
<tr><td>WP_177060102.1</td><td><-MFS||?→||||<-?<-?||?→||||<-?||APATPase+GT4*→</td><td>APATPase+GT4</td><td>1336</td><td>HX876_RS19435</td><td>Gammaproteobacteria</td><td>Pseudomonas gingeri</td><td>glycosyltransferase [Pseudomonas gingeri]</td><td>GCF_013385575.1</td></tr>
<tr><td>WP_177062848.1</td><td>GT4+B12-binding+RADICAL-SAM*→</td><td>GT4+B12-binding+RADICAL-SAM</td><td>1404</td><td>HX876_RS14360</td><td>Gammaproteobacteria</td><td>Pseudomonas gingeri</td><td>radical SAM protein [Pseudomonas gingeri]</td><td>GCF_013385575.1</td></tr>
<tr><td>WP_177065618.1</td><td><-MFS||?→||||<-?<-?||?→||||<-?||APATPase+TPR+GT4*→</td><td>APATPase+TPR+GT4</td><td>1336</td><td>HX861_RS09040</td><td>Gammaproteobacteria</td><td>Pseudomonas gingeri</td><td>glycosyltransferase [Pseudomonas gingeri]</td><td>GCF_013385925.1</td></tr>
<tr><td>WP_177072953.1</td><td><-MFS||?→||||<-?<-?||?→||||<-?||APATPase+TPR+GT4*→</td><td>APATPase+TPR+GT4</td><td>1336</td><td>HX800_RS13020</td><td>Gammaproteobacteria</td><td>Pseudomonas gingeri</td><td>glycosyltransferase [Pseudomonas gingeri]</td><td>GCF_013386965.1</td></tr>
<tr><td>WP_177108140.1</td><td><-MFS||?→||||<-?<-?||?→||||<-?||APATPase+GT4*→</td><td>APATPase+GT4</td><td>1299</td><td>HX867_RS22145</td><td>Gammaproteobacteria</td><td>Pseudomonas gingeri</td><td>glycosyltransferase [Pseudomonas gingeri]</td><td>GCF_013386235.1</td></tr>
<tr><td>WP_177126920.1</td><td><-MFS||?→||||<-?<-?||?→||||<-?||APATPase+TPR+GT4*→</td><td>APATPase+TPR+GT4</td><td>1336</td><td>HX831_RS01005</td><td>Gammaproteobacteria</td><td>Pseudomonas gingeri</td><td>glycosyltransferase [Pseudomonas gingeri]</td><td>GCF_013386815.1</td></tr>
</tbody>
</table>

<table>
<tr><th>acc</th><th>operon</th><th>architecture</th><th>len</th><th>gen.name</th><th>taxend</th><th>species</th><th>defline</th><th>gca</th></tr>
<tr><td>WP_177129208.1</td><td><-MFS||?→||||<-?<-?||?→||||<-?||APATPase+TPR+GT4*→</td><td>APATPase+TPR+GT4</td><td>1299</td><td>HX808_RS17340</td><td>Gammaproteobacteria</td><td>Pseudomonas gingeri</td><td>glycosyltransferase [Pseudomonas gingeri]</td><td>GCF_013385905.1</td></tr>
<tr><td>WP_177204219.1</td><td><-Sigma||?→||||<-?<-?<-?||GT4+B12-binding*→</td><td>GT4+B12-binding</td><td>1122</td><td>BMY84_RS26750</td><td>Alphaproteobacteria</td><td>Sphingobium sp. AP50</td><td>glycosyltransferase [Sphingobium sp. AP50]</td><td>GCF_900109095.1</td></tr>
<tr><td>WP_178066132.1</td><td><-TPR+Caspase||GT4*→</td><td>GT4</td><td>546</td><td>HDA31_RS04955</td><td>Actinobacteria</td><td>Micromonospora carbonacea</td><td>glycosyltransferase [Micromonospora carbonacea]</td><td>GCF_013389765.1</td></tr>
<tr><td>WP_178963184.1</td><td>GT4+B12-binding+RADICAL-SAM*→</td><td>GT4+B12-binding+RADICAL-SAM</td><td>1404</td><td>HX895_RS03680</td><td>Gammaproteobacteria</td><td>Pseudomonas gingeri</td><td>radical SAM protein [Pseudomonas gingeri]</td><td>GCF_013387125.1</td></tr>
<tr><td>WP_178963665.1</td><td><-MFS||?→||||<-?<-?||?→||||<-?||APATPase+GT4*→</td><td>APATPase+GT4</td><td>1336</td><td>HX895_RS14805</td><td>Gammaproteobacteria</td><td>Pseudomonas gingeri</td><td>glycosyltransferase [Pseudomonas gingeri]</td><td>GCF_013387125.1</td></tr>
<tr><td>WP_179766782.1</td><td>TRPR-HTH+PBPI→?→ SIG+Lipase_GDSL_2→?→ Zeta_toxin→?→ GT4*→</td><td>GT4</td><td>461</td><td>HNR12_RS07370</td><td>Actinobacteria</td><td>Streptomonospora nanhaiensis</td><td>glycosyltransferase [Streptomonospora nanhaiensis]</td><td>GCF_013410565.1</td></tr>
<tr><td>WP_179767386.1</td><td>GT4*→?→||||<-?<-?<-tRNA-synt_1b+S4</td><td>GT4</td><td>489</td><td>HNR12_RS10915</td><td>Actinobacteria</td><td>Streptomonospora nanhaiensis</td><td>glycosyltransferase [Streptomonospora nanhaiensis]</td><td>GCF_013410565.1</td></tr>
<tr><td>WP_179826407.1</td><td><-APATPase+TPR||?→ GT4*→</td><td>GT4</td><td>524</td><td>HNR10_RS21750</td><td>Actinobacteria</td><td>Nocardiopsis aegyptia</td><td>hypothetical protein [Nocardiopsis aegyptia]</td><td>GCF_013410755.1</td></tr>
<tr><td>WP_179835700.1</td><td>GT4*→||||<-?<-?<-cNMP_binding+HTH_Crp_2</td><td>GT4</td><td>388</td><td>BJ999_RS25940</td><td>Actinobacteria</td><td>Actinomadura citrea</td><td>glycosyltransferase [Actinomadura citrea]</td><td>GCF_014648455.1</td></tr>
<tr><td>WP_180357038.1</td><td>HTH→?→?→||||<-?<-?||Caspase+NACHT+GT4*→</td><td>Caspase+NACHT+GT4</td><td>1165</td><td>BX283_RS03940</td><td>Actinobacteria</td><td>Streptomyces sp. TLI_146</td><td>caspase family protein [Streptomyces sp. TLI_146]</td><td>GCF_002846415.1</td></tr>
<tr><td>WP_180930380.1</td><td>MNS+GT4*→</td><td>MNS+GT4</td><td>1268</td><td>E0L36_RS19290</td><td>Actinobacteria</td><td>Streptomyces sp. AJS327</td><td>glycosyltransferase [Streptomyces sp. AJS327]</td><td>GCF_013450295.1</td></tr>
<tr><td>WP_181043331.1</td><td>MFS→?→?→ WXG→?→ GT4*→?→ GT4→</td><td>GT4</td><td>391</td><td>CLV40_RS37790</td><td>Actinobacteria</td><td>Actinokineospora auranticolor</td><td>glycosyltransferase family 4 protein [Actinokineospora auranticolor]</td><td>GCF_002934265.1</td></tr>
</table>

<table>
<tr><th>acc</th><th>operon</th><th>architecture</th><th>len</th><th>gen.name</th><th>taxend</th><th>species</th><th>defline</th><th>gca</th></tr>
<tr><td>WP_181245803.1</td><td>GT4*→ APATPase+TPR→||||<-?<-TRPR-HTH+PBPI</td><td>GT4</td><td>401</td><td>B0I28_RS15405</td><td>Actinobacteria</td><td>Glycomyces artemisiae</td><td>glycosyltransferase [Glycomyces artemisiae]</td><td>GCF_003002955.1</td></tr>
</table>

Domain architectures of eukaryotic GT4s with a special emphasis on the LSE in Acropora

acc	architecture	len	gen.name	tax	species	GCA
XP_015747238.1	GT4	461	LOC107327009	Eukaryota>Opisthokonta>Metazoa>Cnidaria	Acropora digitifera	GCF_000222465.1
XP_015747313.1	GT4+NACHT	648	LOC107327071	Eukaryota>Opisthokonta>Metazoa>Cnidaria	Acropora digitifera	GCF_000222465.1
XP_015747371.1	GT4	492	LOC107327132	Eukaryota>Opisthokonta>Metazoa>Cnidaria	Acropora digitifera	GCF_000222465.1
XP_015747395.1	GT4+NACHT	628	LOC107327154	Eukaryota>Opisthokonta>Metazoa>Cnidaria	Acropora digitifera	GCF_000222465.1
XP_015747754.1	GT4	430	LOC107327516	Eukaryota>Opisthokonta>Metazoa>Cnidaria	Acropora digitifera	GCF_000222465.1
XP_015747753.1	GT4	437	LOC107327516	Eukaryota>Opisthokonta>Metazoa>Cnidaria	Acropora digitifera	GCF_000222465.1
XP_015747750.1	GT4	437	LOC107327516	Eukaryota>Opisthokonta>Metazoa>Cnidaria	Acropora digitifera	GCF_000222465.1
XP_015747752.1	GT4	437	LOC107327516	Eukaryota>Opisthokonta>Metazoa>Cnidaria	Acropora digitifera	GCF_000222465.1
XP_015747751.1	GT4	437	LOC107327516	Eukaryota>Opisthokonta>Metazoa>Cnidaria	Acropora digitifera	GCF_000222465.1
XP_015747749.1	GT4	451	LOC107327516	Eukaryota>Opisthokonta>Metazoa>Cnidaria	Acropora digitifera	GCF_000222465.1
XP_015748052.1	GT4	595	LOC107327825	Eukaryota>Opisthokonta>Metazoa>Cnidaria	Acropora digitifera	GCF_000222465.1
XP_015748207.1	GT4	424	LOC107328002	Eukaryota>Opisthokonta>Metazoa>Cnidaria	Acropora digitifera	GCF_000222465.1
XP_015748206.1	GT4	424	LOC107328002	Eukaryota>Opisthokonta>Metazoa>Cnidaria	Acropora digitifera	GCF_000222465.1
XP_015748205.1	GT4	429	LOC107328002	Eukaryota>Opisthokonta>Metazoa>Cnidaria	Acropora digitifera	GCF_000222465.1
XP_015748202.1	GT4	430	LOC107328002	Eukaryota>Opisthokonta>Metazoa>Cnidaria	Acropora digitifera	GCF_000222465.1
XP_015748203.1	GT4	430	LOC107328002	Eukaryota>Opisthokonta>Metazoa>Cnidaria	Acropora digitifera	GCF_000222465.1
XP_015748201.1	GT4	437	LOC107328002	Eukaryota>Opisthokonta>Metazoa>Cnidaria	Acropora digitifera	GCF_000222465.1
XP_015748200.1	GT4	442	LOC107328002	Eukaryota>Opisthokonta>Metazoa>Cnidaria	Acropora digitifera	GCF_000222465.1
XP_015748274.1	GT4	440	LOC107328074	Eukaryota>Opisthokonta>Metazoa>Cnidaria	Acropora digitifera	GCF_000222465.1
XP_015748402.1	ZU5+ZU5+GT4	1030	LOC107328179	Eukaryota>Opisthokonta>Metazoa>Cnidaria	Acropora digitifera	GCF_000222465.1
XP_015748412.1	GT4+NACHT	551	LOC107328186	Eukaryota>Opisthokonta>Metazoa>Cnidaria	Acropora digitifera	GCF_000222465.1
XP_015748513.1	SIG+GT4+DEATH	524	LOC107328287	Eukaryota>Opisthokonta>Metazoa>Cnidaria	Acropora digitifera	GCF_000222465.1
XP_015748512.1	SIG+GT4+DEATH	526	LOC107328287	Eukaryota>Opisthokonta>Metazoa>Cnidaria	Acropora digitifera	GCF_000222465.1
XP_015748877.1	GT4	416	LOC107328661	Eukaryota>Opisthokonta>Metazoa>Cnidaria	Acropora digitifera	GCF_000222465.1
XP_015748878.1	GT4+NACHT	578	LOC107328662	Eukaryota>Opisthokonta>Metazoa>Cnidaria	Acropora digitifera	GCF_000222465.1
XP_015748920.1	GT4	389	LOC107328707	Eukaryota>Opisthokonta>Metazoa>Cnidaria	Acropora digitifera	GCF_000222465.1
XP_015748918.1	GT4	391	LOC107328707	Eukaryota>Opisthokonta>Metazoa>Cnidaria	Acropora digitifera	GCF_000222465.1
XP_015748919.1	GT4	391	LOC107328707	Eukaryota>Opisthokonta>Metazoa>Cnidaria	Acropora digitifera	GCF_000222465.1
XP_015749378.1	GT4	610	LOC107329155	Eukaryota>Opisthokonta>Metazoa>Cnidaria	Acropora digitifera	GCF_000222465.1
XP_015749585.1	GT4	400	LOC107329409	Eukaryota>Opisthokonta>Metazoa>Cnidaria	Acropora digitifera	GCF_000222465.1
XP_015749584.1	GT4+TF-AF0608	480	LOC107329409	Eukaryota>Opisthokonta>Metazoa>Cnidaria	Acropora digitifera	GCF_000222465.1
XP_015749790.1	GT4	470	LOC107329632	Eukaryota>Opisthokonta>Metazoa>Cnidaria	Acropora digitifera	GCF_000222465.1
XP_015749876.1	GT4+NACHT	583	LOC107329721	Eukaryota>Opisthokonta>Metazoa>Cnidaria	Acropora digitifera	GCF_000222465.1
XP_015750005.1	GT4	637	LOC107329865	Eukaryota>Opisthokonta>Metazoa>Cnidaria	Acropora digitifera	GCF_000222465.1
XP_015750060.1	GT4	418	LOC107329915	Eukaryota>Opisthokonta>Metazoa>Cnidaria	Acropora digitifera	GCF_000222465.1
XP_015750058.1	GT4	483	LOC107329915	Eukaryota>Opisthokonta>Metazoa>Cnidaria	Acropora digitifera	GCF_000222465.1
XP_015750090.1	GT4	464	LOC107329946	Eukaryota>Opisthokonta>Metazoa>Cnidaria	Acropora digitifera	GCF_000222465.1
XP_015750089.1	GT4	477	LOC107329946	Eukaryota>Opisthokonta>Metazoa>Cnidaria	Acropora digitifera	GCF_000222465.1
XP_015750088.1	GT4	510	LOC107329946	Eukaryota>Opisthokonta>Metazoa>Cnidaria	Acropora digitifera	GCF_000222465.1
XP_015750086.1	GT4	513	LOC107329946	Eukaryota>Opisthokonta>Metazoa>Cnidaria	Acropora digitifera	GCF_000222465.1
XP_015750087.1	GT4	513	LOC107329946	Eukaryota>Opisthokonta>Metazoa>Cnidaria	Acropora digitifera	GCF_000222465.1
XP_015750637.1	GT4	341	LOC107330559	Eukaryota>Opisthokonta>Metazoa>Cnidaria	Acropora digitifera	GCF_000222465.1
XP_015753085.1	SIG+GT4	635	LOC107332846	Eukaryota>Opisthokonta>Metazoa>Cnidaria	Acropora digitifera	GCF_000222465.1

acc	architecture	len	gen.name	tax	species	GCA
XP_015753084.1	SIG+GT4	670	LOC107332846	Eukaryota>Opisthokonta>Metazoa>Cnidaria	Acropora digitifera	GCF_000222465.1
XP_015753082.1	SIG+GT4	715	LOC107332846	Eukaryota>Opisthokonta>Metazoa>Cnidaria	Acropora digitifera	GCF_000222465.1
XP_015753081.1	SIG+GT4	717	LOC107332846	Eukaryota>Opisthokonta>Metazoa>Cnidaria	Acropora digitifera	GCF_000222465.1
XP_015754166.1	GT4	465	LOC107333809	Eukaryota>Opisthokonta>Metazoa>Cnidaria	Acropora digitifera	GCF_000222465.1
XP_015754272.1	GT4	660	LOC107333922	Eukaryota>Opisthokonta>Metazoa>Cnidaria	Acropora digitifera	GCF_000222465.1
XP_015754292.1	GT4	491	LOC107333938	Eukaryota>Opisthokonta>Metazoa>Cnidaria	Acropora digitifera	GCF_000222465.1
XP_015754293.1	GT4	432	LOC107333939	Eukaryota>Opisthokonta>Metazoa>Cnidaria	Acropora digitifera	GCF_000222465.1
XP_015754495.1	GT4	533	LOC107334108	Eukaryota>Opisthokonta>Metazoa>Cnidaria	Acropora digitifera	GCF_000222465.1
XP_015755589.1	ZU5+GT4	822	LOC107335136	Eukaryota>Opisthokonta>Metazoa>Cnidaria	Acropora digitifera	GCF_000222465.1
XP_015755588.1	ZU5+ZU5+GT4	849	LOC107335136	Eukaryota>Opisthokonta>Metazoa>Cnidaria	Acropora digitifera	GCF_000222465.1
XP_015755587.1	ZU5+ZU5+GT4	851	LOC107335136	Eukaryota>Opisthokonta>Metazoa>Cnidaria	Acropora digitifera	GCF_000222465.1
XP_015755586.1	ZU5+ZU5+GT4	857	LOC107335136	Eukaryota>Opisthokonta>Metazoa>Cnidaria	Acropora digitifera	GCF_000222465.1
XP_015757828.1	GT4	557	LOC107337230	Eukaryota>Opisthokonta>Metazoa>Cnidaria	Acropora digitifera	GCF_000222465.1
XP_015757831.1	GT4	448	LOC107337231	Eukaryota>Opisthokonta>Metazoa>Cnidaria	Acropora digitifera	GCF_000222465.1
XP_015757830.1	GT4	510	LOC107337231	Eukaryota>Opisthokonta>Metazoa>Cnidaria	Acropora digitifera	GCF_000222465.1
XP_015757829.1	GT4	528	LOC107337231	Eukaryota>Opisthokonta>Metazoa>Cnidaria	Acropora digitifera	GCF_000222465.1
XP_015758947.1	GT4	493	LOC107338227	Eukaryota>Opisthokonta>Metazoa>Cnidaria	Acropora digitifera	GCF_000222465.1
XP_015759245.1	GT4	347	LOC107338521	Eukaryota>Opisthokonta>Metazoa>Cnidaria	Acropora digitifera	GCF_000222465.1
XP_015759244.1	GT4	348	LOC107338521	Eukaryota>Opisthokonta>Metazoa>Cnidaria	Acropora digitifera	GCF_000222465.1
XP_015759943.1	GT4	403	LOC107339196	Eukaryota>Opisthokonta>Metazoa>Cnidaria	Acropora digitifera	GCF_000222465.1
XP_015760072.1	GT4+DEATH	565	LOC107339308	Eukaryota>Opisthokonta>Metazoa>Cnidaria	Acropora digitifera	GCF_000222465.1
XP_015760071.1	GT4+DEATH	571	LOC107339308	Eukaryota>Opisthokonta>Metazoa>Cnidaria	Acropora digitifera	GCF_000222465.1
XP_015760070.1	GT4+DEATH	595	LOC107339308	Eukaryota>Opisthokonta>Metazoa>Cnidaria	Acropora digitifera	GCF_000222465.1
XP_015760069.1	GT4+DEATH	596	LOC107339308	Eukaryota>Opisthokonta>Metazoa>Cnidaria	Acropora digitifera	GCF_000222465.1
XP_015760068.1	GT4+DEATH	604	LOC107339308	Eukaryota>Opisthokonta>Metazoa>Cnidaria	Acropora digitifera	GCF_000222465.1
XP_015760067.1	GT4+DEATH	610	LOC107339308	Eukaryota>Opisthokonta>Metazoa>Cnidaria	Acropora digitifera	GCF_000222465.1
XP_015760066.1	GT4+DEATH	614	LOC107339308	Eukaryota>Opisthokonta>Metazoa>Cnidaria	Acropora digitifera	GCF_000222465.1
XP_015760065.1	GT4+DEATH	620	LOC107339308	Eukaryota>Opisthokonta>Metazoa>Cnidaria	Acropora digitifera	GCF_000222465.1
XP_015760063.1	GT4+DEATH	627	LOC107339308	Eukaryota>Opisthokonta>Metazoa>Cnidaria	Acropora digitifera	GCF_000222465.1
XP_015760062.1	GT4+DEATH	651	LOC107339308	Eukaryota>Opisthokonta>Metazoa>Cnidaria	Acropora digitifera	GCF_000222465.1
XP_015760060.1	GT4+DEATH	651	LOC107339308	Eukaryota>Opisthokonta>Metazoa>Cnidaria	Acropora digitifera	GCF_000222465.1
XP_015760061.1	GT4+DEATH	651	LOC107339308	Eukaryota>Opisthokonta>Metazoa>Cnidaria	Acropora digitifera	GCF_000222465.1
XP_015760057.1	GT4+DEATH	675	LOC107339308	Eukaryota>Opisthokonta>Metazoa>Cnidaria	Acropora digitifera	GCF_000222465.1
XP_015760059.1	GT4+DEATH	675	LOC107339308	Eukaryota>Opisthokonta>Metazoa>Cnidaria	Acropora digitifera	GCF_000222465.1
XP_015760058.1	GT4+DEATH	675	LOC107339308	Eukaryota>Opisthokonta>Metazoa>Cnidaria	Acropora digitifera	GCF_000222465.1
XP_015760603.1	GT4	399	LOC107339804	Eukaryota>Opisthokonta>Metazoa>Cnidaria	Acropora digitifera	GCF_000222465.1
XP_015760882.1	GT4	399	LOC107340053	Eukaryota>Opisthokonta>Metazoa>Cnidaria	Acropora digitifera	GCF_000222465.1
XP_015764085.1	GT4	446	LOC107343054	Eukaryota>Opisthokonta>Metazoa>Cnidaria	Acropora digitifera	GCF_000222465.1
XP_015764101.1	GT4	523	LOC107343072	Eukaryota>Opisthokonta>Metazoa>Cnidaria	Acropora digitifera	GCF_000222465.1
XP_015764103.1	GT4	468	LOC107343073	Eukaryota>Opisthokonta>Metazoa>Cnidaria	Acropora digitifera	GCF_000222465.1
XP_015764656.1	GT4	312	LOC107343585	Eukaryota>Opisthokonta>Metazoa>Cnidaria	Acropora digitifera	GCF_000222465.1
XP_015764718.1	GT4	435	LOC107343652	Eukaryota>Opisthokonta>Metazoa>Cnidaria	Acropora digitifera	GCF_000222465.1
XP_015764719.1	GT4	435	LOC107343652	Eukaryota>Opisthokonta>Metazoa>Cnidaria	Acropora digitifera	GCF_000222465.1
XP_015764720.1	GT4	435	LOC107343652	Eukaryota>Opisthokonta>Metazoa>Cnidaria	Acropora digitifera	GCF_000222465.1
XP_015764793.1	GT4	386	LOC107343725	Eukaryota>Opisthokonta>Metazoa>Cnidaria	Acropora digitifera	GCF_000222465.1
XP_015764794.1	GT4	386	LOC107343725	Eukaryota>Opisthokonta>Metazoa>Cnidaria	Acropora digitifera	GCF_000222465.1
XP_015764792.1	GT4	391	LOC107343725	Eukaryota>Opisthokonta>Metazoa>Cnidaria	Acropora digitifera	GCF_000222465.1
XP_015764791.1	GT4	443	LOC107343725	Eukaryota>Opisthokonta>Metazoa>Cnidaria	Acropora digitifera	GCF_000222465.1
XP_015764795.1	SIG+GT4	423	LOC107343726	Eukaryota>Opisthokonta>Metazoa>Cnidaria	Acropora digitifera	GCF_000222465.1
XP_015764797.1	GT4	395	LOC107343727	Eukaryota>Opisthokonta>Metazoa>Cnidaria	Acropora digitifera	GCF_000222465.1
XP_015764798.1	GT4	395	LOC107343727	Eukaryota>Opisthokonta>Metazoa>Cnidaria	Acropora digitifera	GCF_000222465.1
XP_015764796.1	GT4	395	LOC107343727	Eukaryota>Opisthokonta>Metazoa>Cnidaria	Acropora digitifera	GCF_000222465.1

acc	architecture	len	gen.name	tax	species	GCA
XP_015764806.1	GT4	469	LOC107343730	Eukaryota>Opisthokonta>Metazoa>Cnidaria	Acropora digitifera	GCF_000222465.1
XP_015764805.1	GT4	472	LOC107343730	Eukaryota>Opisthokonta>Metazoa>Cnidaria	Acropora digitifera	GCF_000222465.1
XP_015764804.1	GT4+DEATH	655	LOC107343730	Eukaryota>Opisthokonta>Metazoa>Cnidaria	Acropora digitifera	GCF_000222465.1
XP_015764802.1	GT4+DEATH	661	LOC107343730	Eukaryota>Opisthokonta>Metazoa>Cnidaria	Acropora digitifera	GCF_000222465.1
XP_015764803.1	GT4+DEATH	661	LOC107343730	Eukaryota>Opisthokonta>Metazoa>Cnidaria	Acropora digitifera	GCF_000222465.1
XP_015764810.1	GT4	455	LOC107343734	Eukaryota>Opisthokonta>Metazoa>Cnidaria	Acropora digitifera	GCF_000222465.1
XP_015766096.1	GT4	346	LOC107344923	Eukaryota>Opisthokonta>Metazoa>Cnidaria	Acropora digitifera	GCF_000222465.1
XP_015766734.1	GT4	492	LOC107345529	Eukaryota>Opisthokonta>Metazoa>Cnidaria	Acropora digitifera	GCF_000222465.1
XP_015766737.1	GT4	492	LOC107345529	Eukaryota>Opisthokonta>Metazoa>Cnidaria	Acropora digitifera	GCF_000222465.1
XP_015766736.1	GT4	492	LOC107345529	Eukaryota>Opisthokonta>Metazoa>Cnidaria	Acropora digitifera	GCF_000222465.1
XP_015767847.1	GT4	341	LOC107346555	Eukaryota>Opisthokonta>Metazoa>Cnidaria	Acropora digitifera	GCF_000222465.1
XP_015767923.1	GT4	456	LOC107346627	Eukaryota>Opisthokonta>Metazoa>Cnidaria	Acropora digitifera	GCF_000222465.1
XP_015767937.1	GT4	425	LOC107346649	Eukaryota>Opisthokonta>Metazoa>Cnidaria	Acropora digitifera	GCF_000222465.1
XP_015767991.1	SIG+GT4	542	LOC107346690	Eukaryota>Opisthokonta>Metazoa>Cnidaria	Acropora digitifera	GCF_000222465.1
XP_015767999.1	SIG+GT4	542	LOC107346690	Eukaryota>Opisthokonta>Metazoa>Cnidaria	Acropora digitifera	GCF_000222465.1
XP_015768000.1	SIG+GT4	542	LOC107346690	Eukaryota>Opisthokonta>Metazoa>Cnidaria	Acropora digitifera	GCF_000222465.1
XP_015767993.1	SIG+GT4	542	LOC107346690	Eukaryota>Opisthokonta>Metazoa>Cnidaria	Acropora digitifera	GCF_000222465.1
XP_015768002.1	SIG+GT4	542	LOC107346690	Eukaryota>Opisthokonta>Metazoa>Cnidaria	Acropora digitifera	GCF_000222465.1
XP_015767994.1	SIG+GT4	542	LOC107346690	Eukaryota>Opisthokonta>Metazoa>Cnidaria	Acropora digitifera	GCF_000222465.1
XP_015767998.1	SIG+GT4	542	LOC107346690	Eukaryota>Opisthokonta>Metazoa>Cnidaria	Acropora digitifera	GCF_000222465.1
XP_015767997.1	SIG+GT4	542	LOC107346690	Eukaryota>Opisthokonta>Metazoa>Cnidaria	Acropora digitifera	GCF_000222465.1
XP_015768005.1	SIG+GT4	542	LOC107346690	Eukaryota>Opisthokonta>Metazoa>Cnidaria	Acropora digitifera	GCF_000222465.1
XP_015767996.1	SIG+GT4	542	LOC107346690	Eukaryota>Opisthokonta>Metazoa>Cnidaria	Acropora digitifera	GCF_000222465.1
XP_015767992.1	SIG+GT4	542	LOC107346690	Eukaryota>Opisthokonta>Metazoa>Cnidaria	Acropora digitifera	GCF_000222465.1
XP_015768003.1	SIG+GT4	542	LOC107346690	Eukaryota>Opisthokonta>Metazoa>Cnidaria	Acropora digitifera	GCF_000222465.1
XP_015767995.1	SIG+GT4	542	LOC107346690	Eukaryota>Opisthokonta>Metazoa>Cnidaria	Acropora digitifera	GCF_000222465.1
XP_015768001.1	SIG+GT4	542	LOC107346690	Eukaryota>Opisthokonta>Metazoa>Cnidaria	Acropora digitifera	GCF_000222465.1
XP_015768432.1	GT4	421	LOC107347079	Eukaryota>Opisthokonta>Metazoa>Cnidaria	Acropora digitifera	GCF_000222465.1
XP_015768793.1	GT4	589	LOC107347415	Eukaryota>Opisthokonta>Metazoa>Cnidaria	Acropora digitifera	GCF_000222465.1
XP_015768813.1	GT4	503	LOC107347417	Eukaryota>Opisthokonta>Metazoa>Cnidaria	Acropora digitifera	GCF_000222465.1
XP_015768812.1	GT4	503	LOC107347417	Eukaryota>Opisthokonta>Metazoa>Cnidaria	Acropora digitifera	GCF_000222465.1
XP_015768821.1	SIG+GT4	448	LOC107347421	Eukaryota>Opisthokonta>Metazoa>Cnidaria	Acropora digitifera	GCF_000222465.1
XP_015768820.1	SIG+GT4	473	LOC107347421	Eukaryota>Opisthokonta>Metazoa>Cnidaria	Acropora digitifera	GCF_000222465.1
XP_015768819.1	SIG+GT4	486	LOC107347421	Eukaryota>Opisthokonta>Metazoa>Cnidaria	Acropora digitifera	GCF_000222465.1
XP_015768846.1	GT4	503	LOC107347433	Eukaryota>Opisthokonta>Metazoa>Cnidaria	Acropora digitifera	GCF_000222465.1
XP_015768847.1	GT4	503	LOC107347433	Eukaryota>Opisthokonta>Metazoa>Cnidaria	Acropora digitifera	GCF_000222465.1
XP_015768850.1	SIG+GT4	470	LOC107347435	Eukaryota>Opisthokonta>Metazoa>Cnidaria	Acropora digitifera	GCF_000222465.1
XP_015768849.1	SIG+GT4	486	LOC107347435	Eukaryota>Opisthokonta>Metazoa>Cnidaria	Acropora digitifera	GCF_000222465.1
XP_015768848.1	SIG+GT4	494	LOC107347435	Eukaryota>Opisthokonta>Metazoa>Cnidaria	Acropora digitifera	GCF_000222465.1
XP_015770124.1	GT4+DEATH	668	LOC107348579	Eukaryota>Opisthokonta>Metazoa>Cnidaria	Acropora digitifera	GCF_000222465.1
XP_015770346.1	ZU5+ZU5+GT4	847	LOC107348784	Eukaryota>Opisthokonta>Metazoa>Cnidaria	Acropora digitifera	GCF_000222465.1
XP_015770345.1	ZU5+ZU5+GT4	853	LOC107348784	Eukaryota>Opisthokonta>Metazoa>Cnidaria	Acropora digitifera	GCF_000222465.1
XP_015770481.1	GT4	445	LOC107348902	Eukaryota>Opisthokonta>Metazoa>Cnidaria	Acropora digitifera	GCF_000222465.1
XP_015770480.1	GT4	463	LOC107348902	Eukaryota>Opisthokonta>Metazoa>Cnidaria	Acropora digitifera	GCF_000222465.1
XP_015770479.1	GT4	470	LOC107348902	Eukaryota>Opisthokonta>Metazoa>Cnidaria	Acropora digitifera	GCF_000222465.1
XP_015770478.1	GT4	517	LOC107348902	Eukaryota>Opisthokonta>Metazoa>Cnidaria	Acropora digitifera	GCF_000222465.1
XP_015770477.1	GT4	549	LOC107348902	Eukaryota>Opisthokonta>Metazoa>Cnidaria	Acropora digitifera	GCF_000222465.1
XP_015771034.1	GT4	411	LOC107349395	Eukaryota>Opisthokonta>Metazoa>Cnidaria	Acropora digitifera	GCF_000222465.1
XP_015771060.1	SIG+GT4	438	LOC107349429	Eukaryota>Opisthokonta>Metazoa>Cnidaria	Acropora digitifera	GCF_000222465.1
XP_015771078.1	GT4	415	LOC107349446	Eukaryota>Opisthokonta>Metazoa>Cnidaria	Acropora digitifera	GCF_000222465.1
XP_015771077.1	SIG+GT4	442	LOC107349446	Eukaryota>Opisthokonta>Metazoa>Cnidaria	Acropora digitifera	GCF_000222465.1
XP_015771076.1	SIG+GT4	442	LOC107349446	Eukaryota>Opisthokonta>Metazoa>Cnidaria	Acropora digitifera	GCF_000222465.1

acc	architecture	len	gen.name	tax	species	GCA
XP_015771922.1	ZU5+ZU5+GT4	956	LOC107350214	Eukaryota>Opisthokonta>Metazoa>Cnidaria	Acropora digitifera	GCF_000222465.1
XP_015774186.1	GT4	423	LOC107352364	Eukaryota>Opisthokonta>Metazoa>Cnidaria	Acropora digitifera	GCF_000222465.1
XP_015774185.1	GT4	423	LOC107352364	Eukaryota>Opisthokonta>Metazoa>Cnidaria	Acropora digitifera	GCF_000222465.1
XP_015774885.1	GT4	570	LOC107353084	Eukaryota>Opisthokonta>Metazoa>Cnidaria	Acropora digitifera	GCF_000222465.1
XP_015774884.1	GT4	606	LOC107353084	Eukaryota>Opisthokonta>Metazoa>Cnidaria	Acropora digitifera	GCF_000222465.1
XP_015774883.1	GT4	620	LOC107353084	Eukaryota>Opisthokonta>Metazoa>Cnidaria	Acropora digitifera	GCF_000222465.1
XP_015775255.1	ZU5+ZU5+GT4	837	LOC107353433	Eukaryota>Opisthokonta>Metazoa>Cnidaria	Acropora digitifera	GCF_000222465.1
XP_015775499.1	GT4	662	LOC107353672	Eukaryota>Opisthokonta>Metazoa>Cnidaria	Acropora digitifera	GCF_000222465.1
XP_015776074.1	GT4	452	LOC107354141	Eukaryota>Opisthokonta>Metazoa>Cnidaria	Acropora digitifera	GCF_000222465.1
XP_015776071.1	GT4	458	LOC107354141	Eukaryota>Opisthokonta>Metazoa>Cnidaria	Acropora digitifera	GCF_000222465.1
XP_015776070.1	GT4	458	LOC107354141	Eukaryota>Opisthokonta>Metazoa>Cnidaria	Acropora digitifera	GCF_000222465.1
XP_015776073.1	GT4	458	LOC107354141	Eukaryota>Opisthokonta>Metazoa>Cnidaria	Acropora digitifera	GCF_000222465.1
XP_015776072.1	GT4	458	LOC107354141	Eukaryota>Opisthokonta>Metazoa>Cnidaria	Acropora digitifera	GCF_000222465.1
XP_015777147.1	GT4	352	LOC107355140	Eukaryota>Opisthokonta>Metazoa>Cnidaria	Acropora digitifera	GCF_000222465.1
XP_015777237.1	ZU5+ZU5+GT4	919	LOC107355217	Eukaryota>Opisthokonta>Metazoa>Cnidaria	Acropora digitifera	GCF_000222465.1
XP_015777240.1	GT4	381	LOC107355218	Eukaryota>Opisthokonta>Metazoa>Cnidaria	Acropora digitifera	GCF_000222465.1
XP_015777238.1	GT4	381	LOC107355218	Eukaryota>Opisthokonta>Metazoa>Cnidaria	Acropora digitifera	GCF_000222465.1
XP_015777306.1	GT4	536	LOC107355277	Eukaryota>Opisthokonta>Metazoa>Cnidaria	Acropora digitifera	GCF_000222465.1
XP_015777305.1	GT4	588	LOC107355277	Eukaryota>Opisthokonta>Metazoa>Cnidaria	Acropora digitifera	GCF_000222465.1
XP_015777303.1	GT4	640	LOC107355277	Eukaryota>Opisthokonta>Metazoa>Cnidaria	Acropora digitifera	GCF_000222465.1
XP_015777304.1	GT4	640	LOC107355277	Eukaryota>Opisthokonta>Metazoa>Cnidaria	Acropora digitifera	GCF_000222465.1
XP_015777355.1	GT4	700	LOC107355327	Eukaryota>Opisthokonta>Metazoa>Cnidaria	Acropora digitifera	GCF_000222465.1
XP_015777501.1	SIG+GT4	422	LOC107355435	Eukaryota>Opisthokonta>Metazoa>Cnidaria	Acropora digitifera	GCF_000222465.1
XP_015777594.1	GT4	404	LOC107355536	Eukaryota>Opisthokonta>Metazoa>Cnidaria	Acropora digitifera	GCF_000222465.1
XP_015777688.1	GT4	508	LOC107355611	Eukaryota>Opisthokonta>Metazoa>Cnidaria	Acropora digitifera	GCF_000222465.1
XP_015777687.1	GT4	522	LOC107355611	Eukaryota>Opisthokonta>Metazoa>Cnidaria	Acropora digitifera	GCF_000222465.1
XP_015777733.1	GT4	409	LOC107355668	Eukaryota>Opisthokonta>Metazoa>Cnidaria	Acropora digitifera	GCF_000222465.1
XP_015778308.1	GT4	422	LOC107356176	Eukaryota>Opisthokonta>Metazoa>Cnidaria	Acropora digitifera	GCF_000222465.1
XP_015778319.1	GT4	359	LOC107356189	Eukaryota>Opisthokonta>Metazoa>Cnidaria	Acropora digitifera	GCF_000222465.1
XP_015778457.1	GT4	501	LOC107356350	Eukaryota>Opisthokonta>Metazoa>Cnidaria	Acropora digitifera	GCF_000222465.1
XP_015778456.1	GT4	501	LOC107356350	Eukaryota>Opisthokonta>Metazoa>Cnidaria	Acropora digitifera	GCF_000222465.1
XP_015778455.1	GT4	501	LOC107356350	Eukaryota>Opisthokonta>Metazoa>Cnidaria	Acropora digitifera	GCF_000222465.1
XP_015778598.1	GT4	344	LOC107356505	Eukaryota>Opisthokonta>Metazoa>Cnidaria	Acropora digitifera	GCF_000222465.1
XP_015778867.1	GT4	504	LOC107356761	Eukaryota>Opisthokonta>Metazoa>Cnidaria	Acropora digitifera	GCF_000222465.1
XP_015778951.1	GT4	574	LOC107356841	Eukaryota>Opisthokonta>Metazoa>Cnidaria	Acropora digitifera	GCF_000222465.1
XP_015778954.1	GT4	528	LOC107356843	Eukaryota>Opisthokonta>Metazoa>Cnidaria	Acropora digitifera	GCF_000222465.1
XP_015778953.1	GT4	551	LOC107356843	Eukaryota>Opisthokonta>Metazoa>Cnidaria	Acropora digitifera	GCF_000222465.1
XP_015778980.1	ZU5+GT4	670	LOC107356864	Eukaryota>Opisthokonta>Metazoa>Cnidaria	Acropora digitifera	GCF_000222465.1
XP_015779015.1	GT4	495	LOC107356898	Eukaryota>Opisthokonta>Metazoa>Cnidaria	Acropora digitifera	GCF_000222465.1
XP_015779020.1	GT4	464	LOC107356906	Eukaryota>Opisthokonta>Metazoa>Cnidaria	Acropora digitifera	GCF_000222465.1
XP_015779021.1	GT4	464	LOC107356906	Eukaryota>Opisthokonta>Metazoa>Cnidaria	Acropora digitifera	GCF_000222465.1
XP_015779237.1	GT4	537	LOC107357099	Eukaryota>Opisthokonta>Metazoa>Cnidaria	Acropora digitifera	GCF_000222465.1
XP_015779238.1	GT4	468	LOC107357101	Eukaryota>Opisthokonta>Metazoa>Cnidaria	Acropora digitifera	GCF_000222465.1
XP_015780640.1	GT4	651	LOC107358547	Eukaryota>Opisthokonta>Metazoa>Cnidaria	Acropora digitifera	GCF_000222465.1
XP_015780642.1	GT4	422	LOC107358548	Eukaryota>Opisthokonta>Metazoa>Cnidaria	Acropora digitifera	GCF_000222465.1
XP_015780885.1	GT4	402	LOC107358821	Eukaryota>Opisthokonta>Metazoa>Cnidaria	Acropora digitifera	GCF_000222465.1
XP_015748198.1	GT4+NACHT	1089	LOC107328000	Eukaryota>Opisthokonta>Metazoa>Cnidaria	Acropora digitifera	GCF_000222465.1
XP_015748252.1	GT4+NACHT	1354	LOC107328050	Eukaryota>Opisthokonta>Metazoa>Cnidaria	Acropora digitifera	GCF_000222465.1
XP_015748251.1	GT4+NACHT	1378	LOC107328050	Eukaryota>Opisthokonta>Metazoa>Cnidaria	Acropora digitifera	GCF_000222465.1
XP_015748250.1	GT4+NACHT	1379	LOC107328050	Eukaryota>Opisthokonta>Metazoa>Cnidaria	Acropora digitifera	GCF_000222465.1
XP_015748253.1	GT4+NACHT	1267	LOC107328051	Eukaryota>Opisthokonta>Metazoa>Cnidaria	Acropora digitifera	GCF_000222465.1
XP_015748270.1	GT4+NACHT	1262	LOC107328071	Eukaryota>Opisthokonta>Metazoa>Cnidaria	Acropora digitifera	GCF_000222465.1

acc	architecture	len	gen.name	tax	species	GCA
XP_015748415.1	GT4+NACHT	785	LOC107328189	Eukaryota>Opisthokonta>Metazoa>Cnidaria	Acropora digitifera	GCF_000222465.1
XP_015749493.1	GT4+NACHT	1051	LOC107329298	Eukaryota>Opisthokonta>Metazoa>Cnidaria	Acropora digitifera	GCF_000222465.1
XP_015756823.1	GT4+NACHT	1117	LOC107336267	Eukaryota>Opisthokonta>Metazoa>Cnidaria	Acropora digitifera	GCF_000222465.1
XP_015757684.1	GT4+NACHT	1102	LOC107337083	Eukaryota>Opisthokonta>Metazoa>Cnidaria	Acropora digitifera	GCF_000222465.1
XP_015757683.1	GT4+NACHT	1214	LOC107337083	Eukaryota>Opisthokonta>Metazoa>Cnidaria	Acropora digitifera	GCF_000222465.1
XP_015759265.1	GT4+NACHT	1127	LOC107338542	Eukaryota>Opisthokonta>Metazoa>Cnidaria	Acropora digitifera	GCF_000222465.1
XP_015760037.1	GT4+NACHT	897	LOC107339286	Eukaryota>Opisthokonta>Metazoa>Cnidaria	Acropora digitifera	GCF_000222465.1
XP_015764733.1	GT4+NACHT	1250	LOC107343665	Eukaryota>Opisthokonta>Metazoa>Cnidaria	Acropora digitifera	GCF_000222465.1
XP_015764740.1	GT4+NACHT	1273	LOC107343669	Eukaryota>Opisthokonta>Metazoa>Cnidaria	Acropora digitifera	GCF_000222465.1
XP_015766114.1	GT4+NACHT	883	LOC107344942	Eukaryota>Opisthokonta>Metazoa>Cnidaria	Acropora digitifera	GCF_000222465.1
XP_015767800.1	GT4+NACHT	1362	LOC107346514	Eukaryota>Opisthokonta>Metazoa>Cnidaria	Acropora digitifera	GCF_000222465.1
XP_015769559.1	GT4+NACHT	1073	LOC107348070	Eukaryota>Opisthokonta>Metazoa>Cnidaria	Acropora digitifera	GCF_000222465.1
XP_015771075.1	GT4+NACHT	1131	LOC107349444	Eukaryota>Opisthokonta>Metazoa>Cnidaria	Acropora digitifera	GCF_000222465.1
XP_015771449.1	GT4+NACHT	1095	LOC107349777	Eukaryota>Opisthokonta>Metazoa>Cnidaria	Acropora digitifera	GCF_000222465.1
XP_015774737.1	GT4+NACHT	1214	LOC107352931	Eukaryota>Opisthokonta>Metazoa>Cnidaria	Acropora digitifera	GCF_000222465.1
XP_015774735.1	GT4+NACHT	1240	LOC107352931	Eukaryota>Opisthokonta>Metazoa>Cnidaria	Acropora digitifera	GCF_000222465.1
XP_015774736.1	GT4+NACHT	1240	LOC107352931	Eukaryota>Opisthokonta>Metazoa>Cnidaria	Acropora digitifera	GCF_000222465.1
XP_015775665.1	GT4+NACHT	1107	LOC107353792	Eukaryota>Opisthokonta>Metazoa>Cnidaria	Acropora digitifera	GCF_000222465.1
XP_015775664.1	GT4+NACHT	1107	LOC107353792	Eukaryota>Opisthokonta>Metazoa>Cnidaria	Acropora digitifera	GCF_000222465.1
XP_015777175.1	GT4+NACHT	1097	LOC107355160	Eukaryota>Opisthokonta>Metazoa>Cnidaria	Acropora digitifera	GCF_000222465.1
XP_015777174.1	GT4+NACHT	1160	LOC107355160	Eukaryota>Opisthokonta>Metazoa>Cnidaria	Acropora digitifera	GCF_000222465.1
XP_015777173.1	GT4+NACHT	1185	LOC107355160	Eukaryota>Opisthokonta>Metazoa>Cnidaria	Acropora digitifera	GCF_000222465.1
XP_015777171.1	GT4+NACHT	1210	LOC107355160	Eukaryota>Opisthokonta>Metazoa>Cnidaria	Acropora digitifera	GCF_000222465.1
XP_015777168.1	GT4+NACHT	1214	LOC107355160	Eukaryota>Opisthokonta>Metazoa>Cnidaria	Acropora digitifera	GCF_000222465.1
XP_015777169.1	GT4+NACHT	1214	LOC107355160	Eukaryota>Opisthokonta>Metazoa>Cnidaria	Acropora digitifera	GCF_000222465.1
XP_015777170.1	GT4+NACHT	1214	LOC107355160	Eukaryota>Opisthokonta>Metazoa>Cnidaria	Acropora digitifera	GCF_000222465.1
XP_015777167.1	GT4+NACHT	1229	LOC107355160	Eukaryota>Opisthokonta>Metazoa>Cnidaria	Acropora digitifera	GCF_000222465.1
XP_015777166.1	GT4+NACHT	1234	LOC107355160	Eukaryota>Opisthokonta>Metazoa>Cnidaria	Acropora digitifera	GCF_000222465.1
XP_015777164.1	GT4+NACHT	1237	LOC107355160	Eukaryota>Opisthokonta>Metazoa>Cnidaria	Acropora digitifera	GCF_000222465.1
XP_015777165.1	GT4+NACHT	1237	LOC107355160	Eukaryota>Opisthokonta>Metazoa>Cnidaria	Acropora digitifera	GCF_000222465.1
XP_015778512.1	GT4+NACHT	1054	LOC107356410	Eukaryota>Opisthokonta>Metazoa>Cnidaria	Acropora digitifera	GCF_000222465.1
XP_015779196.1	GT4+NACHT	1094	LOC107357068	Eukaryota>Opisthokonta>Metazoa>Cnidaria	Acropora digitifera	GCF_000222465.1
XP_015779200.1	GT4+NACHT	980	LOC107357071	Eukaryota>Opisthokonta>Metazoa>Cnidaria	Acropora digitifera	GCF_000222465.1
XP_015779450.1	GT4+NACHT	1180	LOC107357325	Eukaryota>Opisthokonta>Metazoa>Cnidaria	Acropora digitifera	GCF_000222465.1
XP_015779456.1	GT4+NACHT	723	LOC107357328	Eukaryota>Opisthokonta>Metazoa>Cnidaria	Acropora digitifera	GCF_000222465.1
XP_015779455.1	GT4+NACHT	748	LOC107357328	Eukaryota>Opisthokonta>Metazoa>Cnidaria	Acropora digitifera	GCF_000222465.1
XP_015779454.1	GT4+NACHT	750	LOC107357328	Eukaryota>Opisthokonta>Metazoa>Cnidaria	Acropora digitifera	GCF_000222465.1
XP_015779453.1	GT4+NACHT	775	LOC107357328	Eukaryota>Opisthokonta>Metazoa>Cnidaria	Acropora digitifera	GCF_000222465.1
XP_015779585.1	GT4+NACHT	1318	LOC107357460	Eukaryota>Opisthokonta>Metazoa>Cnidaria	Acropora digitifera	GCF_000222465.1
KHO50967.1	GT4	400	QT05_C0047G0016	Archaea	archaeon GW2011_AR13	GCA_000805965.1
EGD71903.1	GT4	378	CSMARM5_0008	Archaea>DPANN group	Candidatus Parvarchaeum acidophilus ARMAN-5_'5-way FS'	GCA_000192615.1
XP_015768804.1	SIG+GT4+DEATH+sGTP+sGTP	2069	LOC107347416	Eukaryota>Opisthokonta>Metazoa>Cnidaria	Acropora digitifera	GCF_000222465.1
XP_015768802.1	SIG+GT4+DEATH+sGTP+sGTP	2089	LOC107347416	Eukaryota>Opisthokonta>Metazoa>Cnidaria	Acropora digitifera	GCF_000222465.1
XP_015768801.1	GT4+DEATH+sGTP+sGTP	2091	LOC107347416	Eukaryota>Opisthokonta>Metazoa>Cnidaria	Acropora digitifera	GCF_000222465.1
XP_015768800.1	GT4+DEATH+sGTP+sGTP	2092	LOC107347416	Eukaryota>Opisthokonta>Metazoa>Cnidaria	Acropora digitifera	GCF_000222465.1
XP_015768799.1	GT4+DEATH+sGTP+sGTP	2092	LOC107347416	Eukaryota>Opisthokonta>Metazoa>Cnidaria	Acropora digitifera	GCF_000222465.1
XP_015768797.1	SIG+GT4+DEATH+sGTP+sGTP	2094	LOC107347416	Eukaryota>Opisthokonta>Metazoa>Cnidaria	Acropora digitifera	GCF_000222465.1
XP_015768795.1	GT4+DEATH+sGTP+sGTP	2094	LOC107347416	Eukaryota>Opisthokonta>Metazoa>Cnidaria	Acropora digitifera	GCF_000222465.1
XP_015768798.1	SIG+GT4+DEATH+sGTP+sGTP	2094	LOC107347416	Eukaryota>Opisthokonta>Metazoa>Cnidaria	Acropora digitifera	GCF_000222465.1
XP_015768796.1	SIG+GT4+DEATH+sGTP+sGTP	2094	LOC107347416	Eukaryota>Opisthokonta>Metazoa>Cnidaria	Acropora digitifera	GCF_000222465.1
XP_015768794.1	GT4+DEATH+sGTP+sGTP	2094	LOC107347416	Eukaryota>Opisthokonta>Metazoa>Cnidaria	Acropora digitifera	GCF_000222465.1

acc	architecture	len	gen.name	tax	species	GCA
XP_015768836.1	SIG+GT4+DEATH+sGTP+sGTP	2069	LOC107347432	Eukaryota>Opisthokonta>Metazoa>Cnidaria	Acropora digitifera	GCF_000222465.1
XP_015768838.1	GT4+DEATH+sGTP+sGTP	2069	LOC107347432	Eukaryota>Opisthokonta>Metazoa>Cnidaria	Acropora digitifera	GCF_000222465.1
XP_015768835.1	SIG+GT4+DEATH+sGTP+sGTP	2089	LOC107347432	Eukaryota>Opisthokonta>Metazoa>Cnidaria	Acropora digitifera	GCF_000222465.1
XP_015768834.1	GT4+DEATH+sGTP+sGTP	2091	LOC107347432	Eukaryota>Opisthokonta>Metazoa>Cnidaria	Acropora digitifera	GCF_000222465.1
XP_015768833.1	GT4+DEATH+sGTP+sGTP	2092	LOC107347432	Eukaryota>Opisthokonta>Metazoa>Cnidaria	Acropora digitifera	GCF_000222465.1
XP_015768832.1	GT4+DEATH+sGTP+sGTP	2092	LOC107347432	Eukaryota>Opisthokonta>Metazoa>Cnidaria	Acropora digitifera	GCF_000222465.1
XP_015768829.1	SIG+GT4+DEATH+sGTP+sGTP	2094	LOC107347432	Eukaryota>Opisthokonta>Metazoa>Cnidaria	Acropora digitifera	GCF_000222465.1
XP_015768830.1	SIG+GT4+DEATH+sGTP+sGTP	2094	LOC107347432	Eukaryota>Opisthokonta>Metazoa>Cnidaria	Acropora digitifera	GCF_000222465.1
XP_015768831.1	SIG+GT4+DEATH+sGTP+sGTP	2094	LOC107347432	Eukaryota>Opisthokonta>Metazoa>Cnidaria	Acropora digitifera	GCF_000222465.1
XP_015768828.1	GT4+DEATH+sGTP+sGTP	2094	LOC107347432	Eukaryota>Opisthokonta>Metazoa>Cnidaria	Acropora digitifera	GCF_000222465.1
XP_015768827.1	GT4+DEATH+sGTP+sGTP	2094	LOC107347432	Eukaryota>Opisthokonta>Metazoa>Cnidaria	Acropora digitifera	GCF_000222465.1
XP_015777374.1	GT4+ANK	1352	LOC107355329	Eukaryota>Opisthokonta>Metazoa>Cnidaria	Acropora digitifera	GCF_000222465.1
XP_015777373.1	GT4+ANK	1356	LOC107355329	Eukaryota>Opisthokonta>Metazoa>Cnidaria	Acropora digitifera	GCF_000222465.1
XP_015777372.1	GT4+ANK	1356	LOC107355329	Eukaryota>Opisthokonta>Metazoa>Cnidaria	Acropora digitifera	GCF_000222465.1
XP_015777371.1	GT4+Immank+ANK	1368	LOC107355329	Eukaryota>Opisthokonta>Metazoa>Cnidaria	Acropora digitifera	GCF_000222465.1
XP_015777369.1	GT4+ANK	1372	LOC107355329	Eukaryota>Opisthokonta>Metazoa>Cnidaria	Acropora digitifera	GCF_000222465.1
XP_015777368.1	GT4+ANK	1372	LOC107355329	Eukaryota>Opisthokonta>Metazoa>Cnidaria	Acropora digitifera	GCF_000222465.1
XP_015777370.1	GT4+ANK	1372	LOC107355329	Eukaryota>Opisthokonta>Metazoa>Cnidaria	Acropora digitifera	GCF_000222465.1
XP_015777367.1	GT4+ANK	1372	LOC107355329	Eukaryota>Opisthokonta>Metazoa>Cnidaria	Acropora digitifera	GCF_000222465.1
XP_015777366.1	GT4+ANK+ANK	1384	LOC107355329	Eukaryota>Opisthokonta>Metazoa>Cnidaria	Acropora digitifera	GCF_000222465.1
XP_015777362.1	GT4+ANK+ANK	1388	LOC107355329	Eukaryota>Opisthokonta>Metazoa>Cnidaria	Acropora digitifera	GCF_000222465.1
XP_015777363.1	GT4+ANK+ANK	1388	LOC107355329	Eukaryota>Opisthokonta>Metazoa>Cnidaria	Acropora digitifera	GCF_000222465.1
XP_015777365.1	GT4+ANK+ANK	1388	LOC107355329	Eukaryota>Opisthokonta>Metazoa>Cnidaria	Acropora digitifera	GCF_000222465.1
XP_015777361.1	GT4+ANK+ANK	1400	LOC107355329	Eukaryota>Opisthokonta>Metazoa>Cnidaria	Acropora digitifera	GCF_000222465.1
XP_015777360.1	GT4+ANK+ANK	1404	LOC107355329	Eukaryota>Opisthokonta>Metazoa>Cnidaria	Acropora digitifera	GCF_000222465.1
XP_015777359.1	GT4+ANK+ANK	1420	LOC107355329	Eukaryota>Opisthokonta>Metazoa>Cnidaria	Acropora digitifera	GCF_000222465.1
XP_015777358.1	GT4+ANK+ANK	1420	LOC107355329	Eukaryota>Opisthokonta>Metazoa>Cnidaria	Acropora digitifera	GCF_000222465.1
XP_015777357.1	GT4+ANK+ANK	1436	LOC107355329	Eukaryota>Opisthokonta>Metazoa>Cnidaria	Acropora digitifera	GCF_000222465.1
XP_015775968.1	ZU5+ZU5+TM+GT4+APATPase	1462	LOC107354065	Eukaryota>Opisthokonta>Metazoa>Cnidaria	Acropora digitifera	GCF_000222465.1
XP_015775965.1	ZU5+ZU5+TM+GT4+APATPase+TPR	1578	LOC107354065	Eukaryota>Opisthokonta>Metazoa>Cnidaria	Acropora digitifera	GCF_000222465.1
XP_015775967.1	ZU5+ZU5+TM+GT4+APATPase+TPR	1578	LOC107354065	Eukaryota>Opisthokonta>Metazoa>Cnidaria	Acropora digitifera	GCF_000222465.1
XP_015775966.1	ZU5+ZU5+TM+GT4+APATPase+TPR	1578	LOC107354065	Eukaryota>Opisthokonta>Metazoa>Cnidaria	Acropora digitifera	GCF_000222465.1
XP_015775964.1	ZU5+ZU5+TM+GT4+APATPase+TPR	1593	LOC107354065	Eukaryota>Opisthokonta>Metazoa>Cnidaria	Acropora digitifera	GCF_000222465.1
XP_015775963.1	ZU5+ZU5+TM+GT4+APATPase+TPR	1626	LOC107354065	Eukaryota>Opisthokonta>Metazoa>Cnidaria	Acropora digitifera	GCF_000222465.1
XP_015775962.1	ZU5+ZU5+TM+GT4+APATPase+TPR	1655	LOC107354065	Eukaryota>Opisthokonta>Metazoa>Cnidaria	Acropora digitifera	GCF_000222465.1
XP_015775961.1	ZU5+ZU5+TM+GT4+APATPase+TPR	1674	LOC107354065	Eukaryota>Opisthokonta>Metazoa>Cnidaria	Acropora digitifera	GCF_000222465.1
XP_015775960.1	ZU5+ZU5+TM+GT4+APATPase+TPR	1689	LOC107354065	Eukaryota>Opisthokonta>Metazoa>Cnidaria	Acropora digitifera	GCF_000222465.1
XP_015775959.1	ZU5+ZU5+TM+GT4+APATPase+TPR	1689	LOC107354065	Eukaryota>Opisthokonta>Metazoa>Cnidaria	Acropora digitifera	GCF_000222465.1
XP_015775958.1	ZU5+ZU5+TM+GT4+APATPase+TPR	1689	LOC107354065	Eukaryota>Opisthokonta>Metazoa>Cnidaria	Acropora digitifera	GCF_000222465.1
XP_015775956.1	ZU5+ZU5+TM+GT4+APATPase+TPR	1690	LOC107354065	Eukaryota>Opisthokonta>Metazoa>Cnidaria	Acropora digitifera	GCF_000222465.1
XP_015775955.1	ZU5+ZU5+TM+GT4+APATPase+TPR	1693	LOC107354065	Eukaryota>Opisthokonta>Metazoa>Cnidaria	Acropora digitifera	GCF_000222465.1
XP_015775954.1	ZU5+ZU5+TM+GT4+APATPase+TPR	1703	LOC107354065	Eukaryota>Opisthokonta>Metazoa>Cnidaria	Acropora digitifera	GCF_000222465.1
XP_015775953.1	ZU5+ZU5+TM+GT4+APATPase+TPR	1703	LOC107354065	Eukaryota>Opisthokonta>Metazoa>Cnidaria	Acropora digitifera	GCF_000222465.1
XP_015768811.1	GT4+sGTP+sGTP	1904	LOC107347416	Eukaryota>Opisthokonta>Metazoa>Cnidaria	Acropora digitifera	GCF_000222465.1
XP_015768810.1	GT4+sGTP+sGTP	1928	LOC107347416	Eukaryota>Opisthokonta>Metazoa>Cnidaria	Acropora digitifera	GCF_000222465.1
XP_015768809.1	GT4+sGTP+sGTP	1928	LOC107347416	Eukaryota>Opisthokonta>Metazoa>Cnidaria	Acropora digitifera	GCF_000222465.1
XP_015768808.1	SIG+GT4+sGTP+sGTP	1929	LOC107347416	Eukaryota>Opisthokonta>Metazoa>Cnidaria	Acropora digitifera	GCF_000222465.1
XP_015768807.1	SIG+GT4+sGTP+sGTP	1930	LOC107347416	Eukaryota>Opisthokonta>Metazoa>Cnidaria	Acropora digitifera	GCF_000222465.1
XP_015768806.1	SIG+GT4+sGTP+sGTP	1951	LOC107347416	Eukaryota>Opisthokonta>Metazoa>Cnidaria	Acropora digitifera	GCF_000222465.1
XP_015768805.1	SIG+GT4+sGTP+sGTP	1953	LOC107347416	Eukaryota>Opisthokonta>Metazoa>Cnidaria	Acropora digitifera	GCF_000222465.1
XP_015768845.1	GT4+sGTP+sGTP	1904	LOC107347432	Eukaryota>Opisthokonta>Metazoa>Cnidaria	Acropora digitifera	GCF_000222465.1
XP_015768843.1	GT4+sGTP+sGTP	1928	LOC107347432	Eukaryota>Opisthokonta>Metazoa>Cnidaria	Acropora digitifera	GCF_000222465.1

acc	architecture	len	gen.name	tax	species	GCA
XP_015768844.1	GT4+sGTP+sGTP	1928	LOC107347432	Eukaryota>Opisthokonta>Metazoa>Cnidaria	Acropora digitifera	GCF_000222465.1
XP_015768842.1	SIG+GT4+sGTP+sGTP	1929	LOC107347432	Eukaryota>Opisthokonta>Metazoa>Cnidaria	Acropora digitifera	GCF_000222465.1
XP_015768841.1	SIG+GT4+sGTP+sGTP	1930	LOC107347432	Eukaryota>Opisthokonta>Metazoa>Cnidaria	Acropora digitifera	GCF_000222465.1
XP_015768840.1	SIG+GT4+sGTP+sGTP	1951	LOC107347432	Eukaryota>Opisthokonta>Metazoa>Cnidaria	Acropora digitifera	GCF_000222465.1
XP_015768839.1	SIG+GT4+sGTP+sGTP	1953	LOC107347432	Eukaryota>Opisthokonta>Metazoa>Cnidaria	Acropora digitifera	GCF_000222465.1
XP_015767989.1	GT4+NACHT	1796	LOC107346689	Eukaryota>Opisthokonta>Metazoa>Cnidaria	Acropora digitifera	GCF_000222465.1
XP_015767988.1	SIG+GT4+NACHT	1806	LOC107346689	Eukaryota>Opisthokonta>Metazoa>Cnidaria	Acropora digitifera	GCF_000222465.1
XP_015776065.1	GT4+NACHT	1674	LOC107354137	Eukaryota>Opisthokonta>Metazoa>Cnidaria	Acropora digitifera	GCF_000222465.1
XP_015776064.1	GT4+NACHT	1675	LOC107354137	Eukaryota>Opisthokonta>Metazoa>Cnidaria	Acropora digitifera	GCF_000222465.1
XP_015776063.1	SIG+GT4+NACHT	1801	LOC107354137	Eukaryota>Opisthokonta>Metazoa>Cnidaria	Acropora digitifera	GCF_000222465.1
XP_015776062.1	SIG+GT4+NACHT	1828	LOC107354137	Eukaryota>Opisthokonta>Metazoa>Cnidaria	Acropora digitifera	GCF_000222465.1
XP_015776061.1	SIG+GT4+NACHT	1838	LOC107354137	Eukaryota>Opisthokonta>Metazoa>Cnidaria	Acropora digitifera	GCF_000222465.1
XP_015776060.1	SIG+GT4+NACHT	1877	LOC107354137	Eukaryota>Opisthokonta>Metazoa>Cnidaria	Acropora digitifera	GCF_000222465.1
XP_015776059.1	SIG+GT4+NACHT	1885	LOC107354137	Eukaryota>Opisthokonta>Metazoa>Cnidaria	Acropora digitifera	GCF_000222465.1
XP_015776058.1	SIG+GT4+NACHT	1914	LOC107354137	Eukaryota>Opisthokonta>Metazoa>Cnidaria	Acropora digitifera	GCF_000222465.1
XP_015776057.1	SIG+GT4+NACHT	1938	LOC107354137	Eukaryota>Opisthokonta>Metazoa>Cnidaria	Acropora digitifera	GCF_000222465.1
XP_015776056.1	SIG+GT4+NACHT	1947	LOC107354137	Eukaryota>Opisthokonta>Metazoa>Cnidaria	Acropora digitifera	GCF_000222465.1
XP_015776055.1	SIG+GT4+NACHT	1951	LOC107354137	Eukaryota>Opisthokonta>Metazoa>Cnidaria	Acropora digitifera	GCF_000222465.1
XP_015776054.1	SIG+GT4+NACHT	1951	LOC107354137	Eukaryota>Opisthokonta>Metazoa>Cnidaria	Acropora digitifera	GCF_000222465.1
XP_015747144.1	GT4+NACHT	1735	LOC107326906	Eukaryota>Opisthokonta>Metazoa>Cnidaria	Acropora digitifera	GCF_000222465.1
XP_015764064.1	GT4+NACHT	1703	LOC107343050	Eukaryota>Opisthokonta>Metazoa>Cnidaria	Acropora digitifera	GCF_000222465.1
XP_015764063.1	GT4+NACHT	1703	LOC107343050	Eukaryota>Opisthokonta>Metazoa>Cnidaria	Acropora digitifera	GCF_000222465.1
XP_015764065.1	GT4+NACHT	1703	LOC107343050	Eukaryota>Opisthokonta>Metazoa>Cnidaria	Acropora digitifera	GCF_000222465.1
XP_015764062.1	GT4+NACHT	1703	LOC107343050	Eukaryota>Opisthokonta>Metazoa>Cnidaria	Acropora digitifera	GCF_000222465.1
XP_015764061.1	GT4+NACHT	1715	LOC107343050	Eukaryota>Opisthokonta>Metazoa>Cnidaria	Acropora digitifera	GCF_000222465.1
XP_015764057.1	GT4+NACHT	1719	LOC107343050	Eukaryota>Opisthokonta>Metazoa>Cnidaria	Acropora digitifera	GCF_000222465.1
XP_015764060.1	GT4+NACHT	1719	LOC107343050	Eukaryota>Opisthokonta>Metazoa>Cnidaria	Acropora digitifera	GCF_000222465.1
XP_015764058.1	GT4+NACHT	1719	LOC107343050	Eukaryota>Opisthokonta>Metazoa>Cnidaria	Acropora digitifera	GCF_000222465.1
XP_015764059.1	GT4+NACHT	1719	LOC107343050	Eukaryota>Opisthokonta>Metazoa>Cnidaria	Acropora digitifera	GCF_000222465.1
XP_015777734.1	GT4+NACHT	1739	LOC107355669	Eukaryota>Opisthokonta>Metazoa>Cnidaria	Acropora digitifera	GCF_000222465.1
XP_015778874.1	SIG+GT4+VWA+SERAprotease	1218	LOC107356762	Eukaryota>Opisthokonta>Metazoa>Cnidaria	Acropora digitifera	GCF_000222465.1
XP_015778871.1	SIG+GT4+VWA+SERAprotease	1221	LOC107356762	Eukaryota>Opisthokonta>Metazoa>Cnidaria	Acropora digitifera	GCF_000222465.1
XP_015778870.1	SIG+GT4+VWA+SERAprotease	1221	LOC107356762	Eukaryota>Opisthokonta>Metazoa>Cnidaria	Acropora digitifera	GCF_000222465.1
XP_015778873.1	SIG+GT4+VWA+SERAprotease	1221	LOC107356762	Eukaryota>Opisthokonta>Metazoa>Cnidaria	Acropora digitifera	GCF_000222465.1
XP_015778868.1	SIG+GT4+VWA+SERAprotease	1221	LOC107356762	Eukaryota>Opisthokonta>Metazoa>Cnidaria	Acropora digitifera	GCF_000222465.1
XP_015778872.1	SIG+GT4+VWA+SERAprotease	1221	LOC107356762	Eukaryota>Opisthokonta>Metazoa>Cnidaria	Acropora digitifera	GCF_000222465.1
XP_015778880.1	SIG+GT4+VWA+SERAprotease	1218	LOC107356768	Eukaryota>Opisthokonta>Metazoa>Cnidaria	Acropora digitifera	GCF_000222465.1
XP_015778878.1	SIG+GT4+VWA+SERAprotease	1221	LOC107356768	Eukaryota>Opisthokonta>Metazoa>Cnidaria	Acropora digitifera	GCF_000222465.1
XP_015778879.1	SIG+GT4+VWA+SERAprotease	1221	LOC107356768	Eukaryota>Opisthokonta>Metazoa>Cnidaria	Acropora digitifera	GCF_000222465.1
XP_015751629.1	DED+TPR+GT4	1894	LOC107331536	Eukaryota>Opisthokonta>Metazoa>Cnidaria	Acropora digitifera	GCF_000222465.1
XP_015751628.1	DED+TPR+GT4	2015	LOC107331536	Eukaryota>Opisthokonta>Metazoa>Cnidaria	Acropora digitifera	GCF_000222465.1
XP_015751627.1	DED+TPR+GT4	2019	LOC107331536	Eukaryota>Opisthokonta>Metazoa>Cnidaria	Acropora digitifera	GCF_000222465.1
XP_015751626.1	DED+TPR+GT4	2020	LOC107331536	Eukaryota>Opisthokonta>Metazoa>Cnidaria	Acropora digitifera	GCF_000222465.1
XP_015758975.1	SIG+TPR+GT4	1867	LOC107338255	Eukaryota>Opisthokonta>Metazoa>Cnidaria	Acropora digitifera	GCF_000222465.1
KXJ17955.1	DED+TPR+GT4	1737	AC249_AIPGENE20062	Eukaryota>Opisthokonta>Metazoa>Cnidaria	Exaiptasia diaphana	GCA_001417965.1
KXJ23466.1	DED+GT4	1693	Samd9l	Eukaryota>Opisthokonta>Metazoa>Cnidaria	Exaiptasia diaphana	GCA_001417965.1
XP_001627530.1	DED+GT4	1802	NEMVEDRAFT_v1g214094	Eukaryota>Opisthokonta>Metazoa>Cnidaria	Nematostella vectensis	-
XP_002586741.1	DED+GT4	538	BRAFLDRAFT_105739	Eukaryota>Opisthokonta>Metazoa>Chordata	Branchiostoma floridae	-
XP_002593761.1	sGTP+GT4	511	BRAFLDRAFT_107710	Eukaryota>Opisthokonta>Metazoa>Chordata	Branchiostoma floridae	-
XP_002585662.1	GT4	514	BRAFLDRAFT_111592	Eukaryota>Opisthokonta>Metazoa>Chordata	Branchiostoma floridae	-
XP_002588370.1	SIG+GT4	424	BRAFLDRAFT_63320	Eukaryota>Opisthokonta>Metazoa>Chordata	Branchiostoma floridae	-
XP_002594621.1	GT4	411	BRAFLDRAFT_77600	Eukaryota>Opisthokonta>Metazoa>Chordata	Branchiostoma floridae	-

acc	architecture	len	gen.name	tax	species	GCA
XP_002594622.1	DEATH+GT4	506	BRAFLDRAFT_77601	Eukaryota>Opisthokonta>Metazoa>Chordata	Branchiostoma floridae	-
XP_002604494.1	GT4	475	BRAFLDRAFT_79207	Eukaryota>Opisthokonta>Metazoa>Chordata	Branchiostoma floridae	-
XP_002731475.1	GT4	433	LOC100372804	Eukaryota>Opisthokonta>Metazoa>Hemichordata	Saccoglossus kowalevskii	GCF_000003605.2
XP_015772447.1	GT4+TPR	596	LOC107350717	Eukaryota>Opisthokonta>Metazoa>Cnidaria	Acropora digitifera	GCF_000222465.1
XP_015772446.1	GT4+TPR	623	LOC107350717	Eukaryota>Opisthokonta>Metazoa>Cnidaria	Acropora digitifera	GCF_000222465.1
XP_015772445.1	GT4+TPR	624	LOC107350717	Eukaryota>Opisthokonta>Metazoa>Cnidaria	Acropora digitifera	GCF_000222465.1
XP_015772444.1	GT4+TPR	642	LOC107350717	Eukaryota>Opisthokonta>Metazoa>Cnidaria	Acropora digitifera	GCF_000222465.1
XP_015772442.1	GT4+TPR	674	LOC107350717	Eukaryota>Opisthokonta>Metazoa>Cnidaria	Acropora digitifera	GCF_000222465.1
XP_015772443.1	GT4+TPR	674	LOC107350717	Eukaryota>Opisthokonta>Metazoa>Cnidaria	Acropora digitifera	GCF_000222465.1
XP_015775035.1	GT4+TPR	635	LOC107353224	Eukaryota>Opisthokonta>Metazoa>Cnidaria	Acropora digitifera	GCF_000222465.1
XP_015770118.1	GT4+sGTP+sGTP+DEATH	1249	LOC107348573	Eukaryota>Opisthokonta>Metazoa>Cnidaria	Acropora digitifera	GCF_000222465.1
XP_015770115.1	GT4+sGTP+sGTP+DEATH	1249	LOC107348573	Eukaryota>Opisthokonta>Metazoa>Cnidaria	Acropora digitifera	GCF_000222465.1
XP_015770116.1	GT4+sGTP+sGTP+DEATH	1249	LOC107348573	Eukaryota>Opisthokonta>Metazoa>Cnidaria	Acropora digitifera	GCF_000222465.1
XP_015770117.1	GT4+sGTP+sGTP+DEATH	1249	LOC107348573	Eukaryota>Opisthokonta>Metazoa>Cnidaria	Acropora digitifera	GCF_000222465.1
XP_015770113.1	GT4+sGTP+sGTP+DEATH	1249	LOC107348573	Eukaryota>Opisthokonta>Metazoa>Cnidaria	Acropora digitifera	GCF_000222465.1
XP_015776051.1	GT4+sGTP	1386	LOC107354135	Eukaryota>Opisthokonta>Metazoa>Cnidaria	Acropora digitifera	GCF_000222465.1
XP_015764075.1	GT4+NACHT	1225	LOC107343052	Eukaryota>Opisthokonta>Metazoa>Cnidaria	Acropora digitifera	GCF_000222465.1
XP_015764074.1	GT4+NACHT	1233	LOC107343052	Eukaryota>Opisthokonta>Metazoa>Cnidaria	Acropora digitifera	GCF_000222465.1
XP_015764072.1	GT4+NACHT	1238	LOC107343052	Eukaryota>Opisthokonta>Metazoa>Cnidaria	Acropora digitifera	GCF_000222465.1
XP_015764073.1	GT4+NACHT	1238	LOC107343052	Eukaryota>Opisthokonta>Metazoa>Cnidaria	Acropora digitifera	GCF_000222465.1
XP_015764071.1	GT4+NACHT	1255	LOC107343052	Eukaryota>Opisthokonta>Metazoa>Cnidaria	Acropora digitifera	GCF_000222465.1
XP_015764070.1	GT4+NACHT	1259	LOC107343052	Eukaryota>Opisthokonta>Metazoa>Cnidaria	Acropora digitifera	GCF_000222465.1
XP_015747310.1	GT4+NACHT	1361	LOC107327069	Eukaryota>Opisthokonta>Metazoa>Cnidaria	Acropora digitifera	GCF_000222465.1
XP_015772577.1	GT4+NACHT	1796	LOC107350842	Eukaryota>Opisthokonta>Metazoa>Cnidaria	Acropora digitifera	GCF_000222465.1
XP_015772579.1	SAP+GT4+NACHT	2199	LOC107350844	Eukaryota>Opisthokonta>Metazoa>Cnidaria	Acropora digitifera	GCF_000222465.1
XP_015776067.1	GT4+NACHT	1600	LOC107354137	Eukaryota>Opisthokonta>Metazoa>Cnidaria	Acropora digitifera	GCF_000222465.1
XP_015776066.1	SIG+GT4+NACHT	1671	LOC107354137	Eukaryota>Opisthokonta>Metazoa>Cnidaria	Acropora digitifera	GCF_000222465.1
XP_015759030.1	sGTP+GT4+TM	1258	LOC107338309	Eukaryota>Opisthokonta>Metazoa>Cnidaria	Acropora digitifera	GCF_000222465.1
XP_015759029.1	sGTP+GT4+TM	1258	LOC107338309	Eukaryota>Opisthokonta>Metazoa>Cnidaria	Acropora digitifera	GCF_000222465.1
XP_015759028.1	sGTP+GT4+TM	1271	LOC107338309	Eukaryota>Opisthokonta>Metazoa>Cnidaria	Acropora digitifera	GCF_000222465.1
XP_015759026.1	sGTP+GT4+TM	1272	LOC107338309	Eukaryota>Opisthokonta>Metazoa>Cnidaria	Acropora digitifera	GCF_000222465.1
XP_015759027.1	sGTP+GT4+TM	1272	LOC107338309	Eukaryota>Opisthokonta>Metazoa>Cnidaria	Acropora digitifera	GCF_000222465.1
XP_015749674.1	GT4+PNPase	965	LOC107329499	Eukaryota>Opisthokonta>Metazoa>Cnidaria	Acropora digitifera	GCF_000222465.1
XP_015749675.1	GT4+PNPase	965	LOC107329499	Eukaryota>Opisthokonta>Metazoa>Cnidaria	Acropora digitifera	GCF_000222465.1
XP_015749860.1	GT4+PNPase+NABD-unk4	933	LOC107329706	Eukaryota>Opisthokonta>Metazoa>Cnidaria	Acropora digitifera	GCF_000222465.1
XP_015749859.1	GT4+PNPase+NABD-unk4	961	LOC107329706	Eukaryota>Opisthokonta>Metazoa>Cnidaria	Acropora digitifera	GCF_000222465.1
XP_015749858.1	GT4+PNPase+NABD-unk4	982	LOC107329706	Eukaryota>Opisthokonta>Metazoa>Cnidaria	Acropora digitifera	GCF_000222465.1
XP_015770482.1	GT4	132	LOC107348905	Eukaryota>Opisthokonta>Metazoa>Cnidaria	Acropora digitifera	GCF_000222465.1
XP_015777289.1	GT4	119	LOC107355256	Eukaryota>Opisthokonta>Metazoa>Cnidaria	Acropora digitifera	GCF_000222465.1
XP_015777288.1	GT4	135	LOC107355256	Eukaryota>Opisthokonta>Metazoa>Cnidaria	Acropora digitifera	GCF_000222465.1
XP_015777287.1	GT4	135	LOC107355256	Eukaryota>Opisthokonta>Metazoa>Cnidaria	Acropora digitifera	GCF_000222465.1
XP_015777286.1	GT4	135	LOC107355256	Eukaryota>Opisthokonta>Metazoa>Cnidaria	Acropora digitifera	GCF_000222465.1
XP_015754287.1	GT4+ANK	1391	LOC107333936	Eukaryota>Opisthokonta>Metazoa>Cnidaria	Acropora digitifera	GCF_000222465.1
XP_015754286.1	GT4+ANK	1391	LOC107333936	Eukaryota>Opisthokonta>Metazoa>Cnidaria	Acropora digitifera	GCF_000222465.1
XP_015754284.1	GT4+ANK	1391	LOC107333936	Eukaryota>Opisthokonta>Metazoa>Cnidaria	Acropora digitifera	GCF_000222465.1
XP_015779234.1	GT4+ANK+Immank+Immank	1392	LOC107357096	Eukaryota>Opisthokonta>Metazoa>Cnidaria	Acropora digitifera	GCF_000222465.1
XP_015780882.1	GT4+DEATH	240	LOC107358816	Eukaryota>Opisthokonta>Metazoa>Cnidaria	Acropora digitifera	GCF_000222465.1
XP_015780881.1	GT4+DEATH	243	LOC107358816	Eukaryota>Opisthokonta>Metazoa>Cnidaria	Acropora digitifera	GCF_000222465.1
XP_015780880.1	GT4+DEATH	247	LOC107358816	Eukaryota>Opisthokonta>Metazoa>Cnidaria	Acropora digitifera	GCF_000222465.1
XP_015780879.1	GT4+DEATH	250	LOC107358816	Eukaryota>Opisthokonta>Metazoa>Cnidaria	Acropora digitifera	GCF_000222465.1
XP_015754283.1	GT4+ANK+DED+APATPase+TPR	1546	LOC107333935	Eukaryota>Opisthokonta>Metazoa>Cnidaria	Acropora digitifera	GCF_000222465.1
XP_015754282.1	GT4+ANK+DED+APATPase+TPR	1560	LOC107333935	Eukaryota>Opisthokonta>Metazoa>Cnidaria	Acropora digitifera	GCF_000222465.1

acc	architecture	len	gen.name	tax	species	GCA
XP_015754281.1	GT4+ANK+DED+APATPase+TPR	1597	LOC107333935	Eukaryota>Opisthokonta>Metazoa>Cnidaria	Acropora digitifera	GCF_000222465.1
XP_015754288.1	GT4+ANK	1351	LOC107333936	Eukaryota>Opisthokonta>Metazoa>Cnidaria	Acropora digitifera	GCF_000222465.1
XP_015778952.1	GT4+ANK+Immank+Immank	1280	LOC107356842	Eukaryota>Opisthokonta>Metazoa>Cnidaria	Acropora digitifera	GCF_000222465.1
XP_015779236.1	GT4+Immank+ANK	1086	LOC107357098	Eukaryota>Opisthokonta>Metazoa>Cnidaria	Acropora digitifera	GCF_000222465.1
XP_015764079.1	GT4+NACHT	1177	LOC107343052	Eukaryota>Opisthokonta>Metazoa>Cnidaria	Acropora digitifera	GCF_000222465.1
XP_015764078.1	GT4+NACHT	1178	LOC107343052	Eukaryota>Opisthokonta>Metazoa>Cnidaria	Acropora digitifera	GCF_000222465.1
XP_015764077.1	GT4+NACHT	1188	LOC107343052	Eukaryota>Opisthokonta>Metazoa>Cnidaria	Acropora digitifera	GCF_000222465.1
XP_015764083.1	GT4+NACHT	1101	LOC107343052	Eukaryota>Opisthokonta>Metazoa>Cnidaria	Acropora digitifera	GCF_000222465.1
XP_015764082.1	GT4+NACHT	1107	LOC107343052	Eukaryota>Opisthokonta>Metazoa>Cnidaria	Acropora digitifera	GCF_000222465.1
XP_015764081.1	GT4+NACHT	1161	LOC107343052	Eukaryota>Opisthokonta>Metazoa>Cnidaria	Acropora digitifera	GCF_000222465.1
XP_015778876.1	SIG+GT4+VWA+SERAprotease	1080	LOC107356762	Eukaryota>Opisthokonta>Metazoa>Cnidaria	Acropora digitifera	GCF_000222465.1
XP_015778875.1	SIG+GT4+VWA+SERAprotease	1145	LOC107356762	Eukaryota>Opisthokonta>Metazoa>Cnidaria	Acropora digitifera	GCF_000222465.1
XP_015778882.1	SIG+GT4+VWA+SERAprotease	1145	LOC107356768	Eukaryota>Opisthokonta>Metazoa>Cnidaria	Acropora digitifera	GCF_000222465.1
XP_015771521.1	SIG+GT4	729	LOC107349844	Eukaryota>Opisthokonta>Metazoa>Cnidaria	Acropora digitifera	GCF_000222465.1
XP_015771522.1	SIG+GT4	729	LOC107349844	Eukaryota>Opisthokonta>Metazoa>Cnidaria	Acropora digitifera	GCF_000222465.1
XP_015771520.1	SIG+GT4	756	LOC107349844	Eukaryota>Opisthokonta>Metazoa>Cnidaria	Acropora digitifera	GCF_000222465.1
XP_015750317.1	GT4+ZU5	721	LOC107330190	Eukaryota>Opisthokonta>Metazoa>Cnidaria	Acropora digitifera	GCF_000222465.1
XP_015770123.1	GT4	704	LOC107348579	Eukaryota>Opisthokonta>Metazoa>Cnidaria	Acropora digitifera	GCF_000222465.1
XP_015770122.1	GT4	705	LOC107348579	Eukaryota>Opisthokonta>Metazoa>Cnidaria	Acropora digitifera	GCF_000222465.1
XP_015752964.1	SIG+GT4	564	LOC107332737	Eukaryota>Opisthokonta>Metazoa>Cnidaria	Acropora digitifera	GCF_000222465.1
XP_015752963.1	SIG+GT4	565	LOC107332737	Eukaryota>Opisthokonta>Metazoa>Cnidaria	Acropora digitifera	GCF_000222465.1
XP_015756764.1	SIG+GT4	570	LOC107336220	Eukaryota>Opisthokonta>Metazoa>Cnidaria	Acropora digitifera	GCF_000222465.1
OLD79042.1	GT4	474	AUF62_00760	Archaea	archaeon 13_1_20CM_52_20	GCA_001919675.1
OLC62495.1	GT4	495	AUH73_04505	Archaea	archaeon 13_1_40CM_4_53_4	GCA_001918315.1
OLB46884.1	GT4	495	AUI07_02860	Archaea	archaeon 13_2_20CM_2_53_6	GCA_001915065.1
XP_015765897.1	GT4	245	LOC107344718	Eukaryota>Opisthokonta>Metazoa>Cnidaria	Acropora digitifera	GCF_000222465.1
XP_015768791.1	GT4	252	LOC107347414	Eukaryota>Opisthokonta>Metazoa>Cnidaria	Acropora digitifera	GCF_000222465.1
XP_015773769.1	GT4	263	LOC107351980	Eukaryota>Opisthokonta>Metazoa>Cnidaria	Acropora digitifera	GCF_000222465.1
XP_015754295.1	GT4	202	LOC107333943	Eukaryota>Opisthokonta>Metazoa>Cnidaria	Acropora digitifera	GCF_000222465.1
XP_015754297.1	GT4	202	LOC107333943	Eukaryota>Opisthokonta>Metazoa>Cnidaria	Acropora digitifera	GCF_000222465.1
XP_015754296.1	GT4	202	LOC107333943	Eukaryota>Opisthokonta>Metazoa>Cnidaria	Acropora digitifera	GCF_000222465.1
XP_015764068.1	GT4+NACHT	1309	LOC107343052	Eukaryota>Opisthokonta>Metazoa>Cnidaria	Acropora digitifera	GCF_000222465.1
XP_015764067.1	GT4+NACHT	1312	LOC107343052	Eukaryota>Opisthokonta>Metazoa>Cnidaria	Acropora digitifera	GCF_000222465.1
XP_015747884.1	RVT+DEATH+ZU5+ZU5+GT4	1232	LOC107327653	Eukaryota>Opisthokonta>Metazoa>Cnidaria	Acropora digitifera	GCF_000222465.1
XP_015747883.1	RVT+DEATH+ZU5+ZU5+GT4	1238	LOC107327653	Eukaryota>Opisthokonta>Metazoa>Cnidaria	Acropora digitifera	GCF_000222465.1
XP_015765549.1	GT4+ANK	1083	LOC107344415	Eukaryota>Opisthokonta>Metazoa>Cnidaria	Acropora digitifera	GCF_000222465.1
XP_015765548.1	GT4+ANK	1084	LOC107344415	Eukaryota>Opisthokonta>Metazoa>Cnidaria	Acropora digitifera	GCF_000222465.1
XP_015780922.1	GT4+GT4	738	LOC107358864	Eukaryota>Opisthokonta>Metazoa>Cnidaria	Acropora digitifera	GCF_000222465.1
XP_015780923.1	GT4+GT4	738	LOC107358864	Eukaryota>Opisthokonta>Metazoa>Cnidaria	Acropora digitifera	GCF_000222465.1
XP_015760897.1	GT4	651	LOC107340068	Eukaryota>Opisthokonta>Metazoa>Cnidaria	Acropora digitifera	GCF_000222465.1
XP_015760896.1	GT4	652	LOC107340068	Eukaryota>Opisthokonta>Metazoa>Cnidaria	Acropora digitifera	GCF_000222465.1
XP_015748510.1	GT4	305	LOC107328284	Eukaryota>Opisthokonta>Metazoa>Cnidaria	Acropora digitifera	GCF_000222465.1
XP_015776097.1	GT4	305	LOC107354170	Eukaryota>Opisthokonta>Metazoa>Cnidaria	Acropora digitifera	GCF_000222465.1
XP_002599631.1	GT4+GTPase-AIG	700	BRAFLDRAFT_102570	Eukaryota>Opisthokonta>Metazoa>Chordata	Branchiostoma floridae	-
XP_002586742.1	GT4	133	BRAFLDRAFT_105740	Eukaryota>Opisthokonta>Metazoa>Chordata	Branchiostoma floridae	-
XP_002606956.1	GT4	718	BRAFLDRAFT_64952	Eukaryota>Opisthokonta>Metazoa>Chordata	Branchiostoma floridae	-
XP_002592892.1	GT4	115	BRAFLDRAFT_65478	Eukaryota>Opisthokonta>Metazoa>Chordata	Branchiostoma floridae	-
XP_002605447.1	GT4	734	BRAFLDRAFT_74262	Eukaryota>Opisthokonta>Metazoa>Chordata	Branchiostoma floridae	-
XP_002598007.1	GT4	253	BRAFLDRAFT_79766	Eukaryota>Opisthokonta>Metazoa>Chordata	Branchiostoma floridae	-
XP_015747221.1	GT4	151	LOC107326989	Eukaryota>Opisthokonta>Metazoa>Cnidaria	Acropora digitifera	GCF_000222465.1
XP_015747315.1	GT4	232	LOC107327073	Eukaryota>Opisthokonta>Metazoa>Cnidaria	Acropora digitifera	GCF_000222465.1
XP_015748075.1	GT4	636	LOC107327853	Eukaryota>Opisthokonta>Metazoa>Cnidaria	Acropora digitifera	GCF_000222465.1

acc	architecture	len	gen.name	tax	species	GCA
XP_015759886.1	GT4	685	LOC107339155	Eukaryota>Opisthokonta>Metazoa>Cnidaria	Acropora digitifera	GCF_000222465.1
XP_015764084.1	GT4+NACHT	901	LOC107343052	Eukaryota>Opisthokonta>Metazoa>Cnidaria	Acropora digitifera	GCF_000222465.1
XP_015767948.1	GT4	201	LOC107346661	Eukaryota>Opisthokonta>Metazoa>Cnidaria	Acropora digitifera	GCF_000222465.1
XP_015774918.1	GT4+NACHT	1264	LOC107353111	Eukaryota>Opisthokonta>Metazoa>Cnidaria	Acropora digitifera	GCF_000222465.1
XP_015777737.1	GT4	925	LOC107355671	Eukaryota>Opisthokonta>Metazoa>Cnidaria	Acropora digitifera	GCF_000222465.1
XP_015748669.1	GT4	245	LOC107328450	Eukaryota>Opisthokonta>Metazoa>Cnidaria	Acropora digitifera	GCF_000222465.1
XP_015750057.1	GT4	520	LOC107329915	Eukaryota>Opisthokonta>Metazoa>Cnidaria	Acropora digitifera	GCF_000222465.1
XP_015754313.1	GT4	241	LOC107333962	Eukaryota>Opisthokonta>Metazoa>Cnidaria	Acropora digitifera	GCF_000222465.1
XP_015761270.1	GT4	712	LOC107340435	Eukaryota>Opisthokonta>Metazoa>Cnidaria	Acropora digitifera	GCF_000222465.1
XP_015770112.1	GT4	253	LOC107348572	Eukaryota>Opisthokonta>Metazoa>Cnidaria	Acropora digitifera	GCF_000222465.1
XP_015771016.1	GT4	390	LOC107349380	Eukaryota>Opisthokonta>Metazoa>Cnidaria	Acropora digitifera	GCF_000222465.1
XP_015771260.1	GT4	491	LOC107349592	Eukaryota>Opisthokonta>Metazoa>Cnidaria	Acropora digitifera	GCF_000222465.1
XP_015772349.1	GT4	256	LOC107350627	Eukaryota>Opisthokonta>Metazoa>Cnidaria	Acropora digitifera	GCF_000222465.1
XP_015777221.1	GT4	205	LOC107355205	Eukaryota>Opisthokonta>Metazoa>Cnidaria	Acropora digitifera	GCF_000222465.1
XP_015778002.1	GT4	437	LOC107355902	Eukaryota>Opisthokonta>Metazoa>Cnidaria	Acropora digitifera	GCF_000222465.1
XP_015779016.1	GT4	186	LOC107356899	Eukaryota>Opisthokonta>Metazoa>Cnidaria	Acropora digitifera	GCF_000222465.1
XP_015780870.1	GT4	274	LOC107358800	Eukaryota>Opisthokonta>Metazoa>Cnidaria	Acropora digitifera	GCF_000222465.1
AFD00853.1	GT4	431	Mtc_2115	Archaea>Euryarchaeota	Methanocella conradii HZ254	GCA_000251105.1
AGB33684.1	GT4	394	Natpe_3933	Archaea>Euryarchaeota	Natrinema pellirubrum DSM 15624	GCA_000230735.3
XP_015751533.1	GT4+NACHT	797	LOC107331457	Eukaryota>Opisthokonta>Metazoa>Cnidaria	Acropora digitifera	GCF_000222465.1
XP_015747435.1	GT4+NACHT	357	LOC107327199	Eukaryota>Opisthokonta>Metazoa>Cnidaria	Acropora digitifera	GCF_000222465.1
XP_015759313.1	GT4+NACHT	988	LOC107338581	Eukaryota>Opisthokonta>Metazoa>Cnidaria	Acropora digitifera	GCF_000222465.1
XP_015759411.1	GT4+NACHT	1346	LOC107338694	Eukaryota>Opisthokonta>Metazoa>Cnidaria	Acropora digitifera	GCF_000222465.1
XP_015769571.1	GT4+NACHT	716	LOC107348075	Eukaryota>Opisthokonta>Metazoa>Cnidaria	Acropora digitifera	GCF_000222465.1
XP_015773770.1	GT4+APATPase	1843	LOC107351981	Eukaryota>Opisthokonta>Metazoa>Cnidaria	Acropora digitifera	GCF_000222465.1
XP_015779460.1	GT4+NACHT	537	LOC107357334	Eukaryota>Opisthokonta>Metazoa>Cnidaria	Acropora digitifera	GCF_000222465.1
XP_015756756.1	GT4+NACHT+NACHT	770	LOC107336211	Eukaryota>Opisthokonta>Metazoa>Cnidaria	Acropora digitifera	GCF_000222465.1
XP_015764080.1	GT4+NACHT	1168	LOC107343052	Eukaryota>Opisthokonta>Metazoa>Cnidaria	Acropora digitifera	GCF_000222465.1
XP_015764069.1	GT4+NACHT	1295	LOC107343052	Eukaryota>Opisthokonta>Metazoa>Cnidaria	Acropora digitifera	GCF_000222465.1
XP_015770110.1	GT4+APATPase+NACHT	1677	LOC107348570	Eukaryota>Opisthokonta>Metazoa>Cnidaria	Acropora digitifera	GCF_000222465.1
XP_015749857.1	GT4+PNPase+NABD-unk4	1031	LOC107329706	Eukaryota>Opisthokonta>Metazoa>Cnidaria	Acropora digitifera	GCF_000222465.1
XP_015778949.1	GT4+GT4	1007	LOC107356840	Eukaryota>Opisthokonta>Metazoa>Cnidaria	Acropora digitifera	GCF_000222465.1
XP_002610411.1	GT4+DEATH	1032	BRAFLDRAFT_72365	Eukaryota>Opisthokonta>Metazoa>Chordata	Branchiostoma floridae	-
XP_015768823.1	GT4+DEATH	402	LOC107347423	Eukaryota>Opisthokonta>Metazoa>Cnidaria	Acropora digitifera	GCF_000222465.1
XP_015761974.1	SIG+GT4	503	LOC107341068	Eukaryota>Opisthokonta>Metazoa>Cnidaria	Acropora digitifera	GCF_000222465.1
XP_002599100.1	GT4	900	BRAFLDRAFT_81765	Eukaryota>Opisthokonta>Metazoa>Chordata	Branchiostoma floridae	-
XP_002601934.1	GT4	652	BRAFLDRAFT_86418	Eukaryota>Opisthokonta>Metazoa>Chordata	Branchiostoma floridae	-
XP_015753083.1	SIG+GT4	678	LOC107332846	Eukaryota>Opisthokonta>Metazoa>Cnidaria	Acropora digitifera	GCF_000222465.1
XP_015770111.1	SIG+GT4+NACHT	1452	LOC107348571	Eukaryota>Opisthokonta>Metazoa>Cnidaria	Acropora digitifera	GCF_000222465.1
XP_015759562.1	GT4	954	LOC107338825	Eukaryota>Opisthokonta>Metazoa>Cnidaria	Acropora digitifera	GCF_000222465.1
XP_015772173.1	GT4	684	LOC107350460	Eukaryota>Opisthokonta>Metazoa>Cnidaria	Acropora digitifera	GCF_000222465.1
XP_015767496.1	GT4+PNPase	423	LOC107346243	Eukaryota>Opisthokonta>Metazoa>Cnidaria	Acropora digitifera	GCF_000222465.1
XP_002610420.1	CARD+sGTP+DEATH+GT4	1671	BRAFLDRAFT_72355	Eukaryota>Opisthokonta>Metazoa>Chordata	Branchiostoma floridae	-
XP_002601932.1	CARD+sGTP+DEATH+GT4+CARD	2070	BRAFLDRAFT_86416	Eukaryota>Opisthokonta>Metazoa>Chordata	Branchiostoma floridae	-
XP_002606064.1	GT4+TIR	594	BRAFLDRAFT_92083	Eukaryota>Opisthokonta>Metazoa>Chordata	Branchiostoma floridae	-
XP_015747743.1	GT4+SidE	864	LOC107327511	Eukaryota>Opisthokonta>Metazoa>Cnidaria	Acropora digitifera	GCF_000222465.1
XP_015771496.1	GT4+TPR+TPR+TPR+RVT	1988	LOC107349827	Eukaryota>Opisthokonta>Metazoa>Cnidaria	Acropora digitifera	GCF_000222465.1
XP_015777375.1	GT4+ANK	878	LOC107355332	Eukaryota>Opisthokonta>Metazoa>Cnidaria	Acropora digitifera	GCF_000222465.1
XP_006812785.1	GT4+PIN+HEPN	648	LOC102807894	Eukaryota>Opisthokonta>Metazoa>Hemichordata	Saccoglossus kowalevskii	GCF_000003605.2
XP_002601187.1	ImmLRR+LRR+sGTP+OTU+GT4	1577	BRAFLDRAFT_75632	Eukaryota>Opisthokonta>Metazoa>Chordata	Branchiostoma floridae	-
XP_015779017.1	GT4+RNA-Helicase+CXC	1910	LOC107356900	Eukaryota>Opisthokonta>Metazoa>Cnidaria	Acropora digitifera	GCF_000222465.1
XP_015767918.1	GT4+NACHT	1211	LOC107346622	Eukaryota>Opisthokonta>Metazoa>Cnidaria	Acropora digitifera	GCF_000222465.1

acc	architecture	len	gen.name	tax	species	GCA
XP_015747744.1	GT4+GT4	1044	LOC107327512	Eukaryota>Opisthokonta>Metazoa>Cnidaria	Acropora digitifera	GCF_000222465.1
XP_006822589.1	TM+TM+BetaPropeller+BetaPropeller+GT4+ANK+Immank+ANK+ANK+Immank+Immank+Immank	3508	LOC102803126	Eukaryota>Opisthokonta>Metazoa>Hemichordata	Saccoglossus kowalevskii	GCF_000003605.2
XP_002608839.1	DED+DED+OTU+GT4+DEATH	1285	BRAFLDRAFT_89707	Eukaryota>Opisthokonta>Metazoa>Chordata	Branchiostoma floridae	-
XP_002602261.1	ANK+DEATH+GT4	983	BRAFLDRAFT_76949	Eukaryota>Opisthokonta>Metazoa>Chordata	Branchiostoma floridae	-
XP_006824651.1	SIG+EGF+EGF+EGF+CLECTIN+GT4	945	LOC102808514	Eukaryota>Opisthokonta>Metazoa>Hemichordata	Saccoglossus kowalevskii	GCF_000003605.2
XP_015754275.1	GT4+GT4+APATPase+TPR	1815	LOC107333926	Eukaryota>Opisthokonta>Metazoa>Cnidaria	Acropora digitifera	GCF_000222465.1
XP_015764809.1	GT4+DEATH	660	LOC107343733	Eukaryota>Opisthokonta>Metazoa>Cnidaria	Acropora digitifera	GCF_000222465.1
XP_015763304.1	GT4+CR-ATPase4+TPR	1073	LOC107342333	Eukaryota>Opisthokonta>Metazoa>Cnidaria	Acropora digitifera	GCF_000222465.1
XP_015765336.1	GT4+UBI+UBI+UBI+UBI	1194	LOC107344209	Eukaryota>Opisthokonta>Metazoa>Cnidaria	Acropora digitifera	GCF_000222465.1
XP_015765496.1	GT4+CASPASE	587	LOC107344359	Eukaryota>Opisthokonta>Metazoa>Cnidaria	Acropora digitifera	GCF_000222465.1
XP_002601970.1	DEATH+GT4	1118	BRAFLDRAFT_94541	Eukaryota>Opisthokonta>Metazoa>Chordata	Branchiostoma floridae	-
XP_015756490.1	GT4+NACHT	1270	LOC107335955	Eukaryota>Opisthokonta>Metazoa>Cnidaria	Acropora digitifera	GCF_000222465.1
XP_015754270.1	GT4+drhyd+NACHT	1358	LOC107333920	Eukaryota>Opisthokonta>Metazoa>Cnidaria	Acropora digitifera	GCF_000222465.1
XP_015780567.1	GT4+ANK	1294	LOC107358478	Eukaryota>Opisthokonta>Metazoa>Cnidaria	Acropora digitifera	GCF_000222465.1
XP_015748771.1	GT4+TM+TM	876	LOC107328563	Eukaryota>Opisthokonta>Metazoa>Cnidaria	Acropora digitifera	GCF_000222465.1
XP_002606060.1	GT4+DED+DED+DED+DED	1082	BRAFLDRAFT_92079	Eukaryota>Opisthokonta>Metazoa>Chordata	Branchiostoma floridae	-
XP_015764066.1	GT4+NACHT	1352	LOC107343052	Eukaryota>Opisthokonta>Metazoa>Cnidaria	Acropora digitifera	GCF_000222465.1
XP_015754289.1	GT4+ANK	1133	LOC107333936	Eukaryota>Opisthokonta>Metazoa>Cnidaria	Acropora digitifera	GCF_000222465.1
XP_002601931.1	DEATH+GT4	1086	BRAFLDRAFT_86415	Eukaryota>Opisthokonta>Metazoa>Chordata	Branchiostoma floridae	-
XP_015765568.1	SIG+ANK+GT4	607	LOC107344435	Eukaryota>Opisthokonta>Metazoa>Cnidaria	Acropora digitifera	GCF_000222465.1
XP_015771899.1	SIG+GT4+NACHT+RVT	942	LOC107350190	Eukaryota>Opisthokonta>Metazoa>Cnidaria	Acropora digitifera	GCF_000222465.1
XP_015759049.1	GT4+TPR+TPR+TPR+sGTP+sGTP	2065	LOC107338329	Eukaryota>Opisthokonta>Metazoa>Cnidaria	Acropora digitifera	GCF_000222465.1
XP_015765567.1	GT4+ANK	1016	LOC107344434	Eukaryota>Opisthokonta>Metazoa>Cnidaria	Acropora digitifera	GCF_000222465.1
XP_002601929.1	CARD+sGTP+DEATH+GT4+sGTP+DEATH+CARD	3425	BRAFLDRAFT_86413	Eukaryota>Opisthokonta>Metazoa>Chordata	Branchiostoma floridae	-
XP_002607644.1	GT4+DEATH+Phytase-like+DEATH+DEATH+DEATH+ZNF+ZNF+CRN-ZnBind.csq+ZNF+ZNF	3703	BRAFLDRAFT_84667	Eukaryota>Opisthokonta>Metazoa>Chordata	Branchiostoma floridae	-
XP_002608763.1	TPR+TPR+TPR+sGTP+DEATH+GT4	2376	BRAFLDRAFT_73982	Eukaryota>Opisthokonta>Metazoa>Chordata	Branchiostoma floridae	-
XP_002606489.1	SIG+ImmLRR+DEATH+GT4	791	BRAFLDRAFT_91930	Eukaryota>Opisthokonta>Metazoa>Chordata	Branchiostoma floridae	-
XP_015759356.1	APATPase+TPR+ZU5+ZU5+GT4	1494	LOC107338634	Eukaryota>Opisthokonta>Metazoa>Cnidaria	Acropora digitifera	GCF_000222465.1
XP_015767926.1	GT4+NACHT	1234	LOC107346634	Eukaryota>Opisthokonta>Metazoa>Cnidaria	Acropora digitifera	GCF_000222465.1
XP_006823290.1	SIG+GT4+TM	1015	LOC102801620	Eukaryota>Opisthokonta>Metazoa>Hemichordata	Saccoglossus kowalevskii	GCF_000003605.2
XP_015777279.1	GT4+sGTP	1178	LOC107355250	Eukaryota>Opisthokonta>Metazoa>Cnidaria	Acropora digitifera	GCF_000222465.1
XP_015771245.1	TPR+TPR+TPR+TPR+TPR+sGTP+GT4	1879	LOC107349580	Eukaryota>Opisthokonta>Metazoa>Cnidaria	Acropora digitifera	GCF_000222465.1
XP_002601933.1	sGTP+DEATH+GT4+sGTP+DEATH+GT4+CARD+sGTP+DEATH	4075	BRAFLDRAFT_86417	Eukaryota>Opisthokonta>Metazoa>Chordata	Branchiostoma floridae	-
XP_002601971.1	SIG+CARD+sGTP+DEATH+GT4+CARD+sGTP+DEATH+DEATH	3440	BRAFLDRAFT_94542	Eukaryota>Opisthokonta>Metazoa>Chordata	Branchiostoma floridae	-
KXJ24237.1	DED+GT4+GT4	2343	AC249_AIPGENE4704	Eukaryota>Opisthokonta>Metazoa>Cnidaria	Exaiptasia diaphana	GCA_001417965.1
XP_006814385.1	DED+TM+GT4	488	LOC102807017	Eukaryota>Opisthokonta>Metazoa>Hemichordata	Saccoglossus kowalevskii	GCF_000003605.2
XP_002592882.1	SIG+ZU5+ZU5+GT4	930	BRAFLDRAFT_117744	Eukaryota>Opisthokonta>Metazoa>Chordata	Branchiostoma floridae	-
XP_015778947.1	GT4+ANK+ANK+ANK	1575	LOC107356838	Eukaryota>Opisthokonta>Metazoa>Cnidaria	Acropora digitifera	GCF_000222465.1
XP_002609384.1	SIG+LRR+sGTP+OTU+GT4+Phytase-like	1519	BRAFLDRAFT_86475	Eukaryota>Opisthokonta>Metazoa>Chordata	Branchiostoma floridae	-
XP_002606063.1	GT4+GTPase-AIG	1278	BRAFLDRAFT_92082	Eukaryota>Opisthokonta>Metazoa>Chordata	Branchiostoma floridae	-
XP_002601968.1	ZNF+ZNF+ZNF+CARD+sGTP+DEATH+GT4	1973	BRAFLDRAFT_94539	Eukaryota>Opisthokonta>Metazoa>Chordata	Branchiostoma floridae	-
XP_015778866.1	GT4+VWA	1117	LOC107356760	Eukaryota>Opisthokonta>Metazoa>Cnidaria	Acropora digitifera	GCF_000222465.1
XP_002592893.1	SIG+DEATH+PspA+GT4	873	BRAFLDRAFT_65479	Eukaryota>Opisthokonta>Metazoa>Chordata	Branchiostoma floridae	-
XP_002610413.1	sGTP+DEATH+GT4+CARD+sGTP+sGTP+DEATH+GT4	4179	BRAFLDRAFT_72363	Eukaryota>Opisthokonta>Metazoa>Chordata	Branchiostoma floridae	-
XP_002599992.1	GT4+TPR+TPR+TPR+TPR	1404	BRAFLDRAFT_74113	Eukaryota>Opisthokonta>Metazoa>Chordata	Branchiostoma floridae	-
XP_002598016.1	DEATH+sGTP+ZU5+GT4	2055	BRAFLDRAFT_79758	Eukaryota>Opisthokonta>Metazoa>Chordata	Branchiostoma floridae	-
XP_015765571.1	GT4+GT4	1248	LOC107344438	Eukaryota>Opisthokonta>Metazoa>Cnidaria	Acropora digitifera	GCF_000222465.1
XP_002608841.1	DED+OTU+sGTP+ZU5+DEATH+DEATH+DED+OTU+CASPASE+sGTP+CASPASE+DED+DEATH+OTU+GT4+sGTP+OTU+GT4+DED+DEATH+CASPASE+sGTP+OTU+DED+DEATH	7154	BRAFLDRAFT_89710	Eukaryota>Opisthokonta>Metazoa>Chordata	Branchiostoma floridae	-
XP_015754687.1	SIG+GT4+CR-ATPase4+TPR	1371	LOC107334268	Eukaryota>Opisthokonta>Metazoa>Cnidaria	Acropora digitifera	GCF_000222465.1
XP_014535703.1	GT4	1299	PDIP_38710	Eukaryota>Opisthokonta>Fungi>Ascomycota	Penicillium digitatum Pd1	GCF_000315645.1
XP_015767848.1	GT4+NACHT	1489	LOC107346556	Eukaryota>Opisthokonta>Metazoa>Cnidaria	Acropora digitifera	GCF_000222465.1

acc	architecture	len	gen.name	tax	species	GCA
XP_002599095.1	DEATH+sGTP+DEATH+GT4	1974	BRAFLDRAFT_81759	Eukaryota>Opisthokonta>Metazoa>Chordata	Branchiostoma floridae	-
XP_015754268.1	GT4+GT4+GT4+IG	2108	LOC107333918	Eukaryota>Opisthokonta>Metazoa>Cnidaria	Acropora digitifera	GCF_000222465.1
XP_015747885.1	RVT+DEATH+ZU5+ZU5+GT4	1158	LOC107327653	Eukaryota>Opisthokonta>Metazoa>Cnidaria	Acropora digitifera	GCF_000222465.1
XP_015756824.1	GT4+APATPase	727	LOC107336267	Eukaryota>Opisthokonta>Metazoa>Cnidaria	Acropora digitifera	GCF_000222465.1
XP_015774399.1	GT4+CASPASE+CASPASE	742	LOC107352599	Eukaryota>Opisthokonta>Metazoa>Cnidaria	Acropora digitifera	GCF_000222465.1
XP_015752969.1	GT4	255	LOC107332740	Eukaryota>Opisthokonta>Metazoa>Cnidaria	Acropora digitifera	GCF_000222465.1
XP_006819467.1	GT4+TM+TM+Snf7	1062	LOC102807364	Eukaryota>Opisthokonta>Metazoa>Hemichordata	Saccoglossus kowalevskii	GCF_000003605.2
XP_002602157.1	DEATH+GT4	1248	BRAFLDRAFT_97971	Eukaryota>Opisthokonta>Metazoa>Chordata	Branchiostoma floridae	-
XP_015774318.1	PNPase+TPR+ZU5+ZU5+GT4	1563	LOC107352503	Eukaryota>Opisthokonta>Metazoa>Cnidaria	Acropora digitifera	GCF_000222465.1
XP_006818589.1	TM+GT4	781	LOC100368229	Eukaryota>Opisthokonta>Metazoa>Hemichordata	Saccoglossus kowalevskii	GCF_000003605.2
XP_015749947.1	GT4+CASPASE+CASPASE	619	LOC107329787	Eukaryota>Opisthokonta>Metazoa>Cnidaria	Acropora digitifera	GCF_000222465.1
XP_002610422.1	sGTP+DEATH+GT4	1881	BRAFLDRAFT_72353	Eukaryota>Opisthokonta>Metazoa>Chordata	Branchiostoma floridae	-
XP_009019645.1	GT4	717	HELRODRAFT_161481	Eukaryota>Opisthokonta>Metazoa>Annelida	Helobdella robusta	GCF_000326865.1
XP_015748178.1	GT4	374	LOC107327971	Eukaryota>Opisthokonta>Metazoa>Cnidaria	Acropora digitifera	GCF_000222465.1
XP_015750171.1	GT4	397	LOC107330034	Eukaryota>Opisthokonta>Metazoa>Cnidaria	Acropora digitifera	GCF_000222465.1
XP_015750733.1	GT4	186	LOC107330680	Eukaryota>Opisthokonta>Metazoa>Cnidaria	Acropora digitifera	GCF_000222465.1
XP_015760591.1	ZU5+GT4	573	LOC107339792	Eukaryota>Opisthokonta>Metazoa>Cnidaria	Acropora digitifera	GCF_000222465.1
XP_002731836.1	GT4	901	LOC100368975	Eukaryota>Opisthokonta>Metazoa>Hemichordata	Saccoglossus kowalevskii	GCF_000003605.2
XP_006814349.1	GT4	1053	LOC102800835	Eukaryota>Opisthokonta>Metazoa>Hemichordata	Saccoglossus kowalevskii	GCF_000003605.2
XP_006811676.1	GT4	814	LOC102807814	Eukaryota>Opisthokonta>Metazoa>Hemichordata	Saccoglossus kowalevskii	GCF_000003605.2

Gene neighborhoods and domain architectures of the MNS-like STAND domains (nSTAND2) that is present in a 2-gene operon system each containing a STAND NTPase

acc	operon	architecture	len	gen.name	taxend	species	defline	gca
AZF56106.1	??+STAND+wHTH→ nSTAND2+wHTH+TPRs+HEPN*→	nSTAND2+wHTH+TPRs+HEPN	621	C4J84_0196	Gammaproteobacteria	Pseudomonas sp. R11-23-07	hypothetical protein C4J84_0196 [Pseudomonas sp. R11-23-07].	GCA_003852295.1
EEV8712171.1	??+STAND+wHTH→ nSTAND2+wHTH+TPRs+HEPN*→	nSTAND2+wHTH+TPRs+HEPN	596	EHU50_23110	Gammaproteobacteria	Escherichia coli	hypothetical protein EHU50_23110, partial [Escherichia coli].	GCA_012057895.1
EEZ9035304.1	??+STAND+wHTH→ nSTAND2+wHTH*→	nSTAND2+wHTH	308	C2D11_004496	Gammaproteobacteria	Escherichia coli O75	hypothetical protein C2D11_004496, partial [Escherichia coli O75].	GCA_012229525.1
KAA9385705.1	nSTAND2+wHTH+TPRs+HEPN*→	nSTAND2+wHTH+TPRs+HEPN	533	F4V88_04125	Alphaproteobacteria	Neorhizobium galegae	hypothetical protein F4V88_04125 [Neorhizobium galegae].	GCA_008728145.1
KPX30733.1	??+STAND+wHTH→ nSTAND2+wHTH+TPRs+HEPN*→	nSTAND2+wHTH+TPRs+HEPN	621	ALO77_200057	Gammaproteobacteria	Pseudomonas coronafaciens pv. garcae	hypothetical protein ALO77_200057 [Pseudomonas coronafaciens pv. garcae].	GCA_001400345.1
KQY48570.1	nSTAND2+wHTH+TPRs+HEPN*→	nSTAND2+wHTH+TPRs+HEPN	547	ASD32_09250	Alphaproteobacteria	Rhizobium sp. Root483D2	hypothetical protein ASD32_09250 [Rhizobium sp. Root483D2].	GCA_001426685.1
KYF49123.1	??+STAND+wHTH→ nSTAND2+wHTH+TPRs+HEPN*→	nSTAND2+wHTH+TPRs+HEPN	636	BE08_43115	Deltaproteobacteria	Sorangium cellulosum	hypothetical protein BE08_43115 [Sorangium cellulosum].	GCA_001589265.1
MBA3548110.1	??+STAND+wHTH→ nSTAND2+wHTH+TPRs+HEPN*→ Integrin_beta(vWA)→	nSTAND2+wHTH+TPRs+HEPN	632	H0T76_16635	Deltaproteobacteria	Nannocystis sp.	hypothetical protein H0T76_16635 [Nannocystis sp.].	GCA_013812955.1
OJX80461.1	nSTAND2+wHTH+TPRs+HEPN*→||<-??	nSTAND2+wHTH+TPRs+HEPN	533	BGO93_00115	Alphaproteobacteria	Mesorhizobium sp. 65-26	hypothetical protein BGO93_00115 [Mesorhizobium sp. 65-26].	GCA_001899205.1
PMZ92294.1	??+STAND+wHTH→ nSTAND2+wHTH+TPRs+HEPN*→ PSE→??→	nSTAND2+wHTH+TPRs+HEPN	621	C1X61_03125	Gammaproteobacteria	Pseudomonas sp. FW215-T2	hypothetical protein C1X61_03125 [Pseudomonas sp. FW215-T2].	GCA_002883935.1
POD52929.1	??+STAND+wHTH→ nSTAND2+wHTH+TPRs+HEPN*→	nSTAND2+wHTH+TPRs+HEPN	590	BKM15_13675	Gammaproteobacteria	Pseudomonas syringae pv. syringae	hypothetical protein BKM15_13675 [Pseudomonas syringae pv. syringae].	GCA_002905935.1
PWI54427.1	nSTAND2+wHTH+TPRs+HEPN*→	nSTAND2+wHTH+TPRs+HEPN	532	B5K03_09585	Alphaproteobacteria	Rhizobium phaseoli	hypothetical protein B5K03_09585 [Rhizobium phaseoli].	GCA_003150695.1
PZX00307.1	??+STAND+wHTH→ nSTAND2+wHTH+TPRs+HEPN*→	nSTAND2+wHTH+TPRs+HEPN	621	DFS28_10221	Gammaproteobacteria	Pseudomonas sp. 478	hypothetical protein DFS28_10221 [Pseudomonas sp. 478].	GCA_003254125.1
RCM87736.1	??+STAND+wHTH→ nSTAND2+wHTH+TPRs+HEPN*→	nSTAND2+wHTH+TPRs+HEPN	588	PA57_04301	Gammaproteobacteria	Pseudomonas aeruginosa	hypothetical protein PA57_04301 [Pseudomonas aeruginosa].	GCA_003332525.1
RMG61681.1	Membrane-bound-b-barrel→ STAND+wHTH*→ Pkinase+cNMP_cyclase→	STAND+wHTH	408	D6715_13175	Calditrichaeota	Calditrichaeota bacterium	ATP-binding protein [Calditrichaeota bacterium].	GCA_003696445.1
RMS07982.1	??+STAND+wHTH→ nSTAND2+wHTH+TPRs+HEPN*→	nSTAND2+wHTH+TPRs+HEPN	621	ALP73_02675	Gammaproteobacteria	Pseudomonas coronafaciens pv. garcae	hypothetical protein ALP73_02675 [Pseudomonas coronafaciens pv. garcae].	GCA_003701555.1
RWN51709.1	??+STAND+wHTH→ nSTAND2+wHTH+TPRs+HEPN*→	nSTAND2+wHTH+TPRs+HEPN	599	EOR98_25445	Alphaproteobacteria	Mesorhizobium sp.	hypothetical protein EOR98_25445 [Mesorhizobium sp.].	GCA_004020775.1
RYG89382.1	??→??+STAND+wHTH→ nSTAND2+wHTH+TPRs+HEPN*→	nSTAND2+wHTH+TPRs+HEPN	629	EON59_01575	Alphaproteobacteria	Alphaproteobacteria bacterium	hypothetical protein EON59_01575 [Alphaproteobacteria bacterium].	GCA_004145145.1
TIN07909.1	??+STAND+wHTH→ nSTAND2+wHTH+TPRs+HEPN*→	nSTAND2+wHTH+TPRs+HEPN	599	E5Y14_22895	Alphaproteobacteria	Mesorhizobium sp.	hypothetical protein E5Y14_22895 [Mesorhizobium sp.].	GCA_004961475.1
TIT02463.1	??+STAND+wHTH→ nSTAND2+wHTH+TPRs+HEPN*→	nSTAND2+wHTH+TPRs+HEPN	596	E5W87_09945	Alphaproteobacteria	Mesorhizobium sp.	hypothetical protein E5W87_09945 [Mesorhizobium sp.].	GCA_004962925.1

acc	operon	architecture	len	gen.name	taxend	species	defline	gca
TJV17958.1	??+STAND+wHTH→ nSTAND2+wHTH+TPRs+HEPN*→	nSTAND2+wHTH+TPRs+HEPN	626	E5Y07_09865	Alphaproteobacteria	Mesorhizobium sp.	hypothetical protein E5Y07_09865 [Mesorhizobium sp.].	GCA_005046125.1
WP_008322742.1	??+STAND+wHTH→ nSTAND2+wHTH+TPRs+HEPN*→||<-??	nSTAND2+wHTH+TPRs+HEPN	613	-	Gammaproteobacteria	Enterobacteriaceae	MULTISPECIES: hypothetical protein [Enterobacteriaceae].	GCF_000277565.1
WP_026230868.1	??+STAND+wHTH→ nSTAND2+wHTH+TPRs+HEPN*→ SF2-helicase+DEAD_assoc→ Calcineurin→	nSTAND2+wHTH+TPRs+HEPN	637	-	Alphaproteobacteria	Rhizobium	MULTISPECIES: hypothetical protein [Rhizobium].	GCF_014138515.1
WP_027196515.1	??+STAND→ nSTAND2+wHTH+GT4*→	nSTAND2+wHTH+GT4	760	-	Betaproteobacteria	Paraburkholderia sprentiae	glycosyltransferase family 4 protein [Paraburkholderia sprentiae].	GCF_001865575.1
WP_048993481.1	??+STAND+wHTH→ nSTAND2+wHTH+TPRs+HEPN*→||<-??	nSTAND2+wHTH+TPRs+HEPN	613	-	Gammaproteobacteria	Klebsiella pneumoniae	hypothetical protein [Klebsiella pneumoniae].	GCF_001065235.1
WP_072160039.1	nSTAND2+wHTH+TPRs+HEPN*→	nSTAND2+wHTH+TPRs+HEPN	613	-	Gammaproteobacteria	Pluralibacter gergoviae	hypothetical protein [Pluralibacter gergoviae].	GCF_001276415.1
WP_088567149.1	??+STAND+wHTH→ nSTAND2+wHTH+TPRs+HEPN*→||<-??	nSTAND2+wHTH+TPRs+HEPN	613	-	Gammaproteobacteria	Enterobacter hormaechei	hypothetical protein [Enterobacter hormaechei].	GCF_002208275.1
WP_089463191.1	??+STAND→ nSTAND2+wHTH+GT4*→	nSTAND2+wHTH+GT4	768	-	Betaproteobacteria	Burkholderia	MULTISPECIES: glycosyltransferase [Burkholderia].	GCF_002924455.1
WP_094198012.1	??+STAND→ nSTAND2+wHTH+GT4*→	nSTAND2+wHTH+GT4	756	-	Betaproteobacteria	Alcaligenes faecalis	glycosyltransferase family 4 protein [Alcaligenes faecalis].	GCF_002242175.1
WP_097401548.1	nSTAND2+wHTH+TPRs+HEPN*→||<-??	nSTAND2+wHTH+TPRs+HEPN	613	-	Gammaproteobacteria	Enterobacteriaceae	MULTISPECIES: hypothetical protein [Enterobacteriaceae].	GCF_002510255.1
WP_106119969.1	??+STAND→ nSTAND2+wHTH+GT4*→	nSTAND2+wHTH+GT4	736	-	Gammaproteobacteria	Pseudomonas simiae	glycosyltransferase family 4 protein [Pseudomonas simiae].	GCF_003001535.1
WP_107112219.1	??+STAND+wHTH→ nSTAND2+wHTH+TPRs+HEPN*→||<-??	nSTAND2+wHTH+TPRs+HEPN	613	-	Gammaproteobacteria	Klebsiella quasipneumoniae	hypothetical protein [Klebsiella quasipneumoniae].	GCF_002854535.1
WP_116343459.1	??+STAND+wHTH→ nSTAND2+wHTH+TPRs+HEPN*→||<-??	nSTAND2+wHTH+TPRs+HEPN	613	-	Gammaproteobacteria	Enterobacter cloacae	hypothetical protein [Enterobacter cloacae].	GCF_003401145.1
WP_122437975.1	??+STAND+wHTH→ nSTAND2+wHTH+TPRs+HEPN*→	nSTAND2+wHTH+TPRs+HEPN	621	-	Gammaproteobacteria	Pseudomonas viridiflava	hypothetical protein [Pseudomonas viridiflava].	GCF_900580835.1
WP_124434788.1	??+STAND+wHTH→ nSTAND2+wHTH+TPRs+HEPN*→	nSTAND2+wHTH+TPRs+HEPN	598	-	Gammaproteobacteria	Pseudomonas sp. R11-23-07	hypothetical protein [Pseudomonas sp. R11-23-07].	GCF_003852295.1
WP_127426060.1	??+STAND+wHTH→ nSTAND2+wHTH+TPRs+HEPN*→	nSTAND2+wHTH+TPRs+HEPN	606	-	Alphaproteobacteria	unclassified Mesorhizobium	MULTISPECIES: hypothetical protein [unclassified Mesorhizobium].	GCF_004021725.2
WP_132834030.1	??+STAND+wHTH→ nSTAND2+wHTH+TPRs+HEPN*→	nSTAND2+wHTH+TPRs+HEPN	598	-	Gammaproteobacteria	unclassified Pseudomonas	MULTISPECIES: hypothetical protein [unclassified Pseudomonas].	GCF_003254125.1
WP_134049903.1	??+STAND+wHTH→ nSTAND2+wHTH+TPRs+HEPN*→	nSTAND2+wHTH+TPRs+HEPN	621	-	Gammaproteobacteria	Pseudomonas sp. OV144	hypothetical protein [Pseudomonas sp. OV144].	GCF_004368835.1
WP_139645958.1	??+STAND+wHTH→ nSTAND2+wHTH+TPRs+HEPN*→	nSTAND2+wHTH+TPRs+HEPN	623	-	Gammaproteobacteria	Pseudomonas sp. ICMP22404	hypothetical protein [Pseudomonas sp. ICMP22404].	GCF_006227205.1
WP_145962680.1	??+STAND+wHTH→ nSTAND2+wHTH+TPRs+HEPN*→	nSTAND2+wHTH+TPRs+HEPN	592	-	Alphaproteobacteria	Rhizobium phaseoli	hypothetical protein [Rhizobium phaseoli].	GCF_003150695.1
WP_146023689.1	??+STAND+wHTH→ nSTAND2+wHTH+TPRs+HEPN*→ PSE→??→	nSTAND2+wHTH+TPRs+HEPN	598	-	Gammaproteobacteria	unclassified Pseudomonas	MULTISPECIES: hypothetical protein [unclassified Pseudomonas].	GCF_002884305.1
WP_147262087.1	??+STAND+wHTH→ nSTAND2+wHTH+TPRs+HEPN*→	nSTAND2+wHTH+TPRs+HEPN	612	-	Gammaproteobacteria	Pseudomonas aeruginosa	hypothetical protein [Pseudomonas aeruginosa].	GCF_003332525.1
WP_147467220.1	nSTAND2+wHTH+TPRs+HEPN*→	nSTAND2+wHTH+TPRs+HEPN	486	-	Gammaproteobacteria	Pseudomonas coronafaciens	hypothetical protein [Pseudomonas coronafaciens].	GCF_003700475.1
WP_147475620.1	nSTAND2+wHTH+TPRs+HEPN*→	nSTAND2+wHTH+TPRs+HEPN	486	-	Gammaproteobacteria	Pseudomonas coronafaciens	hypothetical protein [Pseudomonas coronafaciens].	GCF_001400345.1
WP_153438665.1	??+STAND+wHTH→ nSTAND2+wHTH+TPRs+HEPN*→	nSTAND2+wHTH+TPRs+HEPN	620	-	Gammaproteobacteria	Pseudomonas helleri	hypothetical protein [Pseudomonas helleri].	GCF_009600265.1
WP_156383024.1	??→ STAND+wHTH→ nSTAND2+wHTH+TPRs+HEPN*→	nSTAND2+wHTH+TPRs+HEPN	602	-	Alphaproteobacteria	Rhizobium sp. Root483D2	hypothetical protein [Rhizobium sp. Root483D2].	GCF_001426685.1

acc	operon	architecture	len	gen.name	taxend	species	defline	gca
WP_160320279.1	nSTAND2(fragment)*→	nSTAND2(fragment)	113	-	Alphaproteobacteria	Rhizobium ecuadorense	hypothetical protein, partial [Rhizobium ecuadorense].	GCF_001187535.1
WP_160610812.1	??+STAND+wHTH→ nSTAND2+wHTH+TPRs+HEPN*→	nSTAND2+wHTH+TPRs+HEPN	640	-	Alphaproteobacteria	Altererythrobacter aerius	hypothetical protein [Altererythrobacter aerius].	GCF_009827495.1
WP_163011207.1	??+STAND+wHTH→ nSTAND2+wHTH+TPRs+HEPN*→	nSTAND2+wHTH+TPRs+HEPN	621	-	Gammaproteobacteria	Pseudomonas viridiflava	hypothetical protein [Pseudomonas viridiflava].	GCF_900582025.1
WP_163014645.1	nSTAND2+wHTH+TPRs+HEPN*→	nSTAND2+wHTH+TPRs+HEPN	560	-	Gammaproteobacteria	Pseudomonas viridiflava	hypothetical protein [Pseudomonas viridiflava].	GCF_900582435.1
WP_164708313.1	nSTAND2+wHTH+TPRs+HEPN*→	nSTAND2+wHTH+TPRs+HEPN	534	-	Gammaproteobacteria	Pseudomonas viridiflava	hypothetical protein [Pseudomonas viridiflava].	GCF_900602385.1
WP_169852212.1	??+STAND+wHTH→ nSTAND2+wHTH+TPRs+HEPN*→	nSTAND2+wHTH+TPRs+HEPN	623	-	Gammaproteobacteria	Pseudomonas proteolytica	hypothetical protein [Pseudomonas proteolytica].	GCF_012985975.1

Gene neighborhoods and domain architectures of prokaryotic AP-GTPases described in the text

acc	operon	architecture	len	gen.name	taxend	species	defline	gca
AAU84033.1	pc1599→ Cluster1746_2clades→ Calcineurin→||<-tRNA||LRR-repeats+AP-GTPase+COR+TM+TM*→?→?→ Aminotran_1_2→||<-?<-?<-?||Cluster1483_2clades→	LRR-repeats+AP-GTPase+COR+TM+TM	737	GZ35D7_19	Archaea	uncultured archaeon GZfos35D7	leucine-rich-repeat protein [uncultured archaeon GZfos35D7].	-
AAX07516.1	LRR-repeats+AP-GTPase+COR*→	LRR-repeats+AP-GTPase+COR	1016	-	Planctomycetes	Gemmata sp. Wa1-1	GTP-binding protein [Gemmata sp. Wa1-1].	-
ABD75797.1	HISKIN→ LRR-repeats+AP-GTPase+COR+EAD8*→ HISKIN→?→||<-?||Cluster1512_2clades→ TetR-HTH→?→ Ferredoxin-RRM→	LRR-repeats+AP-GTPase+COR+EAD8	847	-	Bacteria	uncultured bacterium	hypothetical protein [uncultured bacterium].	-
ACC84329.1	<-Arginase<-?<-?<-?||LRR-repeats+AP-GTPase+COR+TIR*→	LRR-repeats+AP-GTPase+COR+TIR	1109	Npun_F6039	Cyanobacteria	Nostoc punctiforme PCC 73102	Miro domain protein [Nostoc punctiforme PCC 73102].	GCA_000020025.1
ADB42932.1	SIG+TM+TM+TM+HISKIN→?→||<-?||CENPB→?→ Cluster2094_2clades→ LRR-repeats+AP-GTPase+COR*→||<-?<-?<-?<-?<-?<-?||ParA-Soj-PloopNTPase→	LRR-repeats+AP-GTPase+COR	925	Slin_6989	Bacteroidetes	Spirosoma linguale DSM 74	small GTP-binding protein (plasmid) [Spirosoma linguale DSM 74].	GCA_000024525.1
AEE53900.1	35exo→||<-?<-?||?→?→?→ LRR-repeats+AP-GTPase+COR+TIR*→||<-?<-?<-ABC-ATPase+APATPase+ABC-ATPase<-?||SIGMA-HTH→	LRR-repeats+AP-GTPase+COR+TIR	998	Halhy_6078	Bacteroidetes	Haliscomenobacter hydrossis DSM 1100	small GTP-binding protein [Haliscomenobacter hydrossis DSM 1100].	GCA_000212735.1
AEE54359.1	LRR-repeats+AP-GTPase+COR+TIR*→ LRR-repeats+AP-GTPase+COR→ LRR-repeats+AP-GTPase+COR→	LRR-repeats+AP-GTPase+COR+TIR	1058	Halhy_6543	Bacteroidetes	Haliscomenobacter hydrossis DSM 1100	Miro domain protein [Haliscomenobacter hydrossis DSM 1100].	GCA_000212735.1
AEE54482.1	LRR-repeats+AP-GTPase+COR+EAD11*→	LRR-repeats+AP-GTPase+COR+EAD11	1141	Halhy_6666	Bacteroidetes	Haliscomenobacter hydrossis DSM 1100	small GTP-binding protein (plasmid) [Haliscomenobacter hydrossis DSM 1100].	GCA_000212735.1
AEE54508.1	MACRODOMAIN→?→ LRR-repeats+AP-GTPase+COR+EAD11*→	LRR-repeats+AP-GTPase+COR+EAD11	1023	Halhy_6692	Bacteroidetes	Haliscomenobacter hydrossis DSM 1100	Miro domain protein (plasmid) [Haliscomenobacter hydrossis DSM 1100].	GCA_000212735.1
AFY40133.1	<-SIG+Trypsin+PDZ<-?||?→?→ LRR-repeats+AP-GTPase+COR+TCAD2*→	LRR-repeats+AP-GTPase+COR+TCAD2	918	Lepto7376_3994	Cyanobacteria	Leptolyngbya sp. PCC 7376	small GTP-binding protein [Leptolyngbya sp. PCC 7376].	GCA_000316605.1
AFY57475.1	EACC2+CASPASE+LRR-repeats+AP-GTPase*→ AP-GTPase+COR→	EACC2+CASPASE+LRR-repeats+AP-GTPase	982	Riv7116_5077	Cyanobacteria	Rivularia sp. PCC 7116	leucine-rich repeat (LRR) protein [Rivularia sp. PCC 7116].	GCA_000316665.1
AKB24843.1	<-HhH-RADC+JAB||?→ NTP_transf_3→ LRR-repeats+AP-GTPase+COR→?→ LRR-repeats+AP-GTPase+COR*→	LRR-repeats+AP-GTPase+COR	599	MSMTP_1374	Euryarchaeota	Methanosarcina sp. MTP4	E3 ubiquitin-protein ligase SspH2 [Methanosarcina sp. MTP4].	GCA_000970045.1
AKB47719.1	LRR-repeats→ LRR-repeats+AP-GTPase+COR+TIR*→?→?→?→?→?→?→||<-TPR-repeats	LRR-repeats+AP-GTPase+COR+TIR	590	MSKOL_1942	Euryarchaeota	Methanosarcina sp. Kolksee	hypothetical protein MSKOL_1942 [Methanosarcina sp. Kolksee].	GCA_000969945.1
AKB51553.1	LRR-repeats+AP-GTPase+COR*→	LRR-repeats+AP-GTPase+COR	880	MSBRW_2300	Euryarchaeota	Methanosarcina barkeri str. Wiesmoor	hypothetical protein MSBRW_2300 [Methanosarcina barkeri str. Wiesmoor].	GCA_000969985.1
AKB56819.1	<-SIG+TPR+TPR+TPR+TPR+TPR+TPR||?→||<-?||?→?→?→?→ LRR-repeats+AP-GTPase+COR*→?→?→ PRTase→	LRR-repeats+AP-GTPase+COR	880	MSBR2_0303	Euryarchaeota	Methanosarcina barkeri 227	hypothetical protein MSBR2_0303 [Methanosarcina barkeri 227].	GCA_000970065.1
AKJ07375.1	Cluster971_2clades→?→||<-?<-?||LRR-repeats+AP-GTPase+COR+TIR*→?→||<-?<-?<-?||?→||<-?<-SIG+Lysozyme	LRR-repeats+AP-GTPase+COR+TIR	873	AA314_09001	Deltaproteobacteria	Archangium gephyra	Chaperone protein DnaK [Archangium gephyra].	GCA_001027285.1
AOY82617.1	REC→||<-?<-HNH||REC→||<-TPR-repeats<-?||LRR-repeats→ LRR-repeats+AP-GTPase+COR*→ AP-GTPase+COR+TM+TM→	LRR-repeats+AP-GTPase+COR	252	BJP36_24575	Cyanobacteria	Moorea producens JHB	hypothetical protein BJP36_24575 [Moorea producens JHB].	GCA_001854205.1
AQR64774.1	LRR-repeats+AP-GTPase+COR+TIR*→||<-?<-?<-MarR-HTH||?→||<-MarR-HTH<-?||Thioredoxin→	LRR-repeats+AP-GTPase+COR+TIR	1295	BXU06_06620	Betaproteobacteria	Aquaspirillum sp. LM1	hypothetical protein BXU06_06620 [Aquaspirillum sp. LM1].	GCA_002002905.1

<table>
<tr><th>acc</th><th>operon</th><th>architecture</th><th>len</th><th>gen.name</th><th>taxend</th><th>species</th><th>defline</th><th>gca</th></tr>
<tr><td>AQR65390.1</td><td>HSP70→ DNAJ→?→?→ LRR-repeats+AP-GTPase+COR+TIR*→</td><td>LRR-repeats+AP-GTPase+COR+TIR</td><td>890</td><td>BXU06_10225</td><td>Betaproteobacteria</td><td>Aquaspirillum sp. LM1</td><td>hypothetical protein BXU06_10225 [Aquaspirillum sp. LM1].</td><td>GCA_002002905.1</td></tr>
<tr><td>AUN33853.1</td><td><-SIG+TM+TM+TM+TM+TM+TM+TM+TM+TM+TM<-?<-?<-?<-?||?→?→ LRR-repeats+AP-GTPase+COR+TIR*→||<-ParB<-ParA-Soj-PloopNTPase</td><td>LRR-repeats+AP-GTPase+COR+TIR</td><td>965</td><td>C0V82_25960</td><td>Alphaproteobacteria</td><td>Niveispirillum cyanobacteriorum</td><td>hypothetical protein C0V82_25960 (plasmid) [Niveispirillum cyanobacteriorum].</td><td>GCA_002868735.1</td></tr>
<tr><td>AVH71003.1</td><td>LRR-repeats+AP-GTPase+COR+DUF4404*→||<-?||?→||<-?||?→?→ MoxR-AAA→</td><td>LRR-repeats+AP-GTPase+COR+DUF4404</td><td>1233</td><td>NLP_2293</td><td>Cyanobacteria</td><td>Nostoc sp. ‘Lobaria pulmonaria (5183) cyanobiont’</td><td>small GTP-binding protein [Nostoc sp. ‘Lobaria pulmonaria (5183) cyanobiont’].</td><td>GCA_002949795.1</td></tr>
<tr><td>AXO38068.1</td><td>LRR-repeats+AP-GTPase+COR*→</td><td>LRR-repeats+AP-GTPase+COR</td><td>942</td><td>MicB006_5810</td><td>Actinobacteria</td><td>Micromonospora sp. B006</td><td>cytoplasmic membrane protein [Micromonospora sp. B006].</td><td>GCA_003408515.1</td></tr>
<tr><td>AYQ35346.1</td><td>FGS→ FGS→ FGS→ LRR-repeats+AP-GTPase+COR+CASPASE*→</td><td>LRR-repeats+AP-GTPase+COR+CASPASE</td><td>998</td><td>DTQ70_25690</td><td>Bacteroidetes</td><td>Runella sp. SP2</td><td>CHAT domain-containing protein [Runella sp. SP2].</td><td>GCA_003711225.1</td></tr>
<tr><td>BAY10551.1</td><td>LRR-repeats→ LRR-repeats+AP-GTPase+COR*→</td><td>LRR-repeats+AP-GTPase+COR</td><td>793</td><td>NIES2098_37230</td><td>Cyanobacteria</td><td>Calothrix sp. NIES-2098</td><td>Miro domain protein [Calothrix sp. NIES-2098].</td><td>GCA_002368175.1</td></tr>
<tr><td>BAY18983.1</td><td>HISKIN→||<-?||LRR-repeats+AP-GTPase+COR*→</td><td>LRR-repeats+AP-GTPase+COR</td><td>946</td><td>NIES21_48420</td><td>Cyanobacteria</td><td>Anabaenopsis circularis NIES-21</td><td>Miro domain-containing protein [Anabaenopsis circularis NIES-21].</td><td>GCA_002367975.1</td></tr>
<tr><td>BAY19588.1</td><td>LRR-repeats+AP-GTPase+COR+TM+TM*→||<-tRNA<-?||?→?→||<-?<-?<-SNF||RnasePH+RnasePH+KH+S1→</td><td>LRR-repeats+AP-GTPase+COR+TM+TM</td><td>739</td><td>NIES21_54510</td><td>Cyanobacteria</td><td>Anabaenopsis circularis NIES-21</td><td>Miro domain protein [Anabaenopsis circularis NIES-21].</td><td>GCA_002367975.1</td></tr>
<tr><td>BAY24777.1</td><td>Pro_CA→||<-?<-?||Chromo-N+CHROMO→||<-?||LRR-repeats+AP-GTPase+COR+TIR+TCAD4+CASPASE*→</td><td>LRR-repeats+AP-GTPase+COR+TIR+TCAD4+CAS-PASE</td><td>1450</td><td>NIES2100_45740</td><td>Cyanobacteria</td><td>Calothrix sp. NIES-2100</td><td>Miro domain-containing protein [Calothrix sp. NIES-2100].</td><td>GCA_002368195.1</td></tr>
<tr><td>BAY74940.1</td><td>LRR-repeats+AP-GTPase+COR*→||<-ClpABN-AAA+ClpABC-AAA<-?<-?||METHYLASE→</td><td>LRR-repeats+AP-GTPase+COR</td><td>1016</td><td>NIES25_13570</td><td>Cyanobacteria</td><td>Nostoc linckia NIES-25</td><td>Miro domain-containing protein [Nostoc linckia NIES-25].</td><td>GCA_002368035.1</td></tr>
<tr><td>BAY88950.1</td><td>LRR-repeats→ LRR-repeats+AP-GTPase+COR+CASPASE*→</td><td>LRR-repeats+AP-GTPase+COR+CASPASE</td><td>963</td><td>NIES3275_09520</td><td>Cyanobacteria</td><td>Fremyella diplosiphon NIES-3275</td><td>Miro domain-containing protein [Fremyella diplosiphon NIES-3275].</td><td>GCA_002368275.1</td></tr>
<tr><td>BAY98896.1</td><td><-REC<-?||Chromo-N+CHROMO→?→||<-?<-?<-?||LRR-repeats+AP-GTPase+COR+CASPASE*→</td><td>LRR-repeats+AP-GTPase+COR+CASPASE</td><td>1138</td><td>NIES37_28740</td><td>Cyanobacteria</td><td>Tolypothrix tenuis PCC 7101</td><td>Miro domain-containing protein [Tolypothrix tenuis PCC 7101].</td><td>GCA_002368295.1</td></tr>
<tr><td>BAZ08233.1</td><td><-ParB<-ParA-Soj-PloopNTPase||?→ LRR-repeats→ LRR-repeats+AP-GTPase+COR+EAD1*→ ParB→||<-NACHT</td><td>LRR-repeats+AP-GTPase+COR+EAD1</td><td>856</td><td>NIES4071_00380</td><td>Cyanobacteria</td><td>Calothrix sp. NIES-4071</td><td>Miro domain-containing protein [Calothrix sp. NIES-4071].</td><td>GCA_002368455.1</td></tr>
<tr><td>BAZ30312.1</td><td><-SIG+GT4<-?||?→ LRR-repeats→ LRR-repeats+AP-GTPase+COR+TM+TM*→?→?→||<-?<-Arginase</td><td>LRR-repeats+AP-GTPase+COR+TM+TM</td><td>638</td><td>NIES4074_27680</td><td>Cyanobacteria</td><td>Cylindrospermum sp. NIES-4074</td><td>small GTP-binding protein [Cylindrospermum sp. NIES-4074].</td><td>GCA_003994795.1</td></tr>
<tr><td>BBD58750.1</td><td><-DSBH<-?<-?<-?<-ParA-Soj-PloopNTPase<-?||LRR-repeats+AP-GTPase+COR+DUF4404*→</td><td>LRR-repeats+AP-GTPase+COR+DUF4404</td><td>1041</td><td>NIES2109_15280</td><td>Cyanobacteria</td><td>Nostoc sp. HK-01</td><td>small GTP-binding protein domain-containing protein [Nostoc sp. HK-01].</td><td>GCA_003990705.1</td></tr>
<tr><td>BBD61760.1</td><td>RelE-ParE→?→||<-?<-?||LRR-repeats+AP-GTPase+COR+TM+TM*→||<-tRNA<-?||?→?→||<-?<-?<-SNF</td><td>LRR-repeats+AP-GTPase+COR+TM+TM</td><td>954</td><td>NIES2109_45950</td><td>Cyanobacteria</td><td>Nostoc sp. HK-01</td><td>Miro domain protein [Nostoc sp. HK-01].</td><td>GCA_003990705.1</td></tr>
<tr><td>BBD62362.1</td><td>HISKIN→ LRR-repeats+AP-GTPase+COR*→</td><td>LRR-repeats+AP-GTPase+COR</td><td>854</td><td>NIES2109_52010</td><td>Cyanobacteria</td><td>Nostoc sp. HK-01</td><td>Miro domain protein [Nostoc sp. HK-01].</td><td>GCA_003990705.1</td></tr>
<tr><td>BBH86778.1</td><td>LRR-repeats+AP-GTPase+COR*→</td><td>LRR-repeats+AP-GTPase+COR</td><td>1152</td><td>KTC_15290</td><td>Chloroflexi</td><td>Thermosporothrix sp. COM3</td><td>hypothetical protein KTC_15290 [Thermosporothrix sp. COM3].</td><td>-</td></tr>
<tr><td>CAA0113265.1</td><td>LRR-repeats+AP-GTPase+COR+DrHyd*→</td><td>LRR-repeats+AP-GTPase+COR+DrHyd</td><td>947</td><td>inlA</td><td>Gammaproteobacteria</td><td>Halioglobus japonicus</td><td>Internalin-A [Halioglobus japonicus].</td><td>GCA_902705825.1</td></tr>
<tr><td>CAA6825421.1</td><td>TM+TM+TM→ LRR-repeats+AP-GTPase+COR+TIR*→</td><td>LRR-repeats+AP-GTPase+COR+TIR</td><td>931</td><td>HELGO_WM44885</td><td>Gammaproteobacteria</td><td>uncultured Thiotrichaceae bacterium</td><td>GTP-binding protein [uncultured Thiotrichaceae bacterium].</td><td>-</td></tr>
<tr><td>CBG70605.1</td><td>PAIREDC-HTH→||<-?||LRR-repeats→ LRR-repeats+AP-GTPase+COR*→||<-?||?→||<-?<-SIGMA-HTH<-?<-SIGMA-HTH</td><td>LRR-repeats+AP-GTPase+COR</td><td>743</td><td>SCAB_35141</td><td>Actinobacteria</td><td>Streptomyces scabiei 87.22</td><td>putative GTPase [Streptomyces scabiei 87.22].</td><td>GCA_000091305.1</td></tr>
<tr><td>CBS87910.1</td><td>LRR-repeats+AP-GTPase+COR+TIR*→||<-?<-?||ABC_membrane+ABC_tran→?→ Cluster968_2clades→</td><td>LRR-repeats+AP-GTPase+COR+TIR</td><td>1026</td><td>AZOLI_2723</td><td>Alphaproteobacteria</td><td>Azospirillum lipoferum 4B</td><td>Conserved protein of unknown function; Leucine-rich repeat domain [Azospirillum lipoferum 4B].</td><td>GCA_000283655.1</td></tr>
<tr><td>CCG09157.1</td><td>LRR-repeats+AP-GTPase+COR+TIR*→||<-?<-?<-?<-?<-?<-?<-REC</td><td>LRR-repeats+AP-GTPase+COR+TIR</td><td>756</td><td>RSPPHO_02531</td><td>Alphaproteobacteria</td><td>Pararhodospirillum photometricum DSM 122</td><td>Leucine-rich repeat [Pararhodospirillum photometricum DSM 122].</td><td>GCA_000284415.2</td></tr>
</table>

<table>
<tr><th>acc</th><th>operon</th><th>architecture</th><th>len</th><th>gen.name</th><th>taxend</th><th>species</th><th>defline</th><th>gca</th></tr>
<tr><td>CCH96195.1</td><td>LRR-repeats+AP-GTPase+COR+TM+TM*→</td><td>LRR-repeats+AP-GTPase+COR+TM+TM</td><td>625</td><td>MICAB_1690001</td><td>Cyanobacteria</td><td>Microcystis aeruginosa PCC 9717</td><td>Leucine-rich-repeat protein (fragment) [Microcystis aeruginosa PCC 9717].</td><td>GCA_000312165.1</td></tr>
<tr><td>CCI18671.1</td><td>LRR-repeats+AP-GTPase+COR*→ AP-GTPase+COR+TM+TM→</td><td>LRR-repeats+AP-GTPase+COR</td><td>302</td><td>MICAF_4270008</td><td>Cyanobacteria</td><td>Microcystis aeruginosa PCC 9807</td><td>Leucine-rich-repeat protein (fragment) [Microcystis aeruginosa PCC 9807].</td><td>GCA_000312225.1</td></tr>
<tr><td>CCQ52177.1</td><td>LRR-repeats+AP-GTPase+COR*→</td><td>LRR-repeats+AP-GTPase+COR</td><td>620</td><td>CWATWH8502_1464</td><td>Cyanobacteria</td><td>Crocosphaera watsonii WH 8502</td><td>Leucine-rich repeat [Crocosphaera watsonii WH 8502].</td><td>GCA_001039555.1</td></tr>
<tr><td>CCQ56427.1</td><td>LRR-repeats+AP-GTPase+COR*→||<-?<-?<-?||RelE→ CHTH→</td><td>LRR-repeats+AP-GTPase+COR</td><td>761</td><td>CWATWH0005_5558</td><td>Cyanobacteria</td><td>Crocosphaera watsonii WH 0005</td><td>Leucine-rich repeat [Crocosphaera watsonii WH 0005].</td><td>GCA_001050835.1</td></tr>
<tr><td>CCQ61794.1</td><td>LRR-repeats+AP-GTPase+COR*→||<-?<-?||RelE→ CHTH→</td><td>LRR-repeats+AP-GTPase+COR</td><td>674</td><td>CWATWH0401_3405</td><td>Cyanobacteria</td><td>Crocosphaera watsonii WH 0401</td><td>Leucine-rich repeat [Crocosphaera watsonii WH 0401].</td><td>GCA_001039615.1</td></tr>
<tr><td>CCQ66314.1</td><td>LRR-repeats+AP-GTPase+COR*→</td><td>LRR-repeats+AP-GTPase+COR</td><td>378</td><td>CWATWH0402_4408</td><td>Cyanobacteria</td><td>Crocosphaera watsonii WH 0402</td><td>Leucine-rich repeat [Crocosphaera watsonii WH 0402].</td><td>GCA_001039635.1</td></tr>
<tr><td>CDA78514.1</td><td>LRR-repeats+AP-GTPase+COR*→</td><td>LRR-repeats+AP-GTPase+COR</td><td>1064</td><td>BN558_01944</td><td>Firmicutes</td><td>Clostridium sp. CAG:242</td><td>miro domain protein [Clostridium sp. CAG:242].</td><td>GCA_000435455.1</td></tr>
<tr><td>CDU15633.1</td><td>LRR-repeats+AP-GTPase+COR*→</td><td>LRR-repeats+AP-GTPase+COR</td><td>1092</td><td>VCR17J2_90139</td><td>Gammaproteobacteria</td><td>Vibrio sp. J2-17</td><td>hypothetical protein VCR17J2_90139 [Vibrio sp. J2-17].</td><td>GCA_001244075.1</td></tr>
<tr><td>CRH07284.1</td><td>LRR-repeats+AP-GTPase+COR*→||<-?||?→?→||<-?<-SIG+Lysozyme</td><td>LRR-repeats+AP-GTPase+COR</td><td>759</td><td>MAGMO_3143</td><td>Alphaproteobacteria</td><td>Candidatus Magnetococcus massalia</td><td>Conserved protein of unknown function. Containing small GTP-binding domain and Leucine-rich repeat (LRR) domain [Candidatus Magnetococcus massalia].</td><td>-</td></tr>
<tr><td>EAM49963.1</td><td>Cluster1136_2clades→?→||<-?||LRR-repeats→ LRR-repeats+AP-GTPase+COR*→</td><td>LRR-repeats+AP-GTPase+COR</td><td>715</td><td>CwatDRAFT_2830</td><td>Cyanobacteria</td><td>Crocosphaera watsonii WH 8501</td><td>Leucine-rich repeat [Crocosphaera watsonii WH 8501].</td><td>GCA_000167195.1</td></tr>
<tr><td>EAY30117.1</td><td>LRR-repeats→?→ LRR-repeats+AP-GTPase+COR*→</td><td>LRR-repeats+AP-GTPase+COR</td><td>1165</td><td>M23134_05450</td><td>Bacteroidetes</td><td>Microscilla marina ATCC 23134</td><td>Rab family protein [Microscilla marina ATCC 23134].</td><td>GCA_000169175.1</td></tr>
<tr><td>EDN67620.1</td><td>LRR-repeats+AP-GTPase+COR*→</td><td>LRR-repeats+AP-GTPase+COR</td><td>602</td><td>BGP_2058</td><td>Gammaproteobacteria</td><td>Beggiatoa sp. PS</td><td>leucine-rich-repeat protein [Beggiatoa sp. PS].</td><td>GCA_000170715.1</td></tr>
<tr><td>EDN68964.1</td><td>LRR-repeats+AP-GTPase+COR*→</td><td>LRR-repeats+AP-GTPase+COR</td><td>833</td><td>BGP_3136</td><td>Gammaproteobacteria</td><td>Beggiatoa sp. PS</td><td>leucine-rich-repeat protein [Beggiatoa sp. PS].</td><td>GCA_000170715.1</td></tr>
<tr><td>EDX78020.1</td><td>Cluster1415_2clades→?→||<-?||LRR-repeats→ LRR-repeats+AP-GTPase+COR+DUF4404*→</td><td>LRR-repeats+AP-GTPase+COR+DUF4404</td><td>1115</td><td>MC7420_7758</td><td>Cyanobacteria</td><td>Coleofasciculus chthonoplastes PCC 7420</td><td>Ras family, putative [Coleofasciculus chthonoplastes PCC 7420].</td><td>GCA_000155555.1</td></tr>
<tr><td>EHJ11819.1</td><td>LRR-repeats+AP-GTPase+COR*→||<-?<-?||RelE→ CHTH→</td><td>LRR-repeats+AP-GTPase+COR</td><td>674</td><td>CWATWH0003_3458</td><td>Cyanobacteria</td><td>Crocosphaera watsonii WH 0003</td><td>Leucine-rich repeat [Crocosphaera watsonii WH 0003].</td><td>GCA_000235665.2</td></tr>
<tr><td>EIG53576.1</td><td>LRR-repeats+AP-GTPase+COR*→?→?→?→ Cluster1496_2clades→</td><td>LRR-repeats+AP-GTPase+COR</td><td>1279</td><td>DesU5LDRAFT_1901</td><td>Deltaproteobacteria</td><td>Desulfovibrio sp. U5L</td><td>small GTP-binding protein domain [Desulfovibrio sp. U5L].</td><td>GCA_000245055.1</td></tr>
<tr><td>ESA34932.1</td><td>LRR-repeats+AP-GTPase+COR+TM+TM*→</td><td>LRR-repeats+AP-GTPase+COR+TM+TM</td><td>1002</td><td>N836_14320</td><td>Cyanobacteria</td><td>Leptolyngbya sp. Heron Island J</td><td>small gtp-binding protein [Leptolyngbya sp. Heron Island J].</td><td>GCA_000482245.1</td></tr>
<tr><td>ESA35951.1</td><td><-NACHT<-?||LRR-repeats+AP-GTPase+COR+TIR*→</td><td>LRR-repeats+AP-GTPase+COR+TIR</td><td>824</td><td>N836_09535</td><td>Cyanobacteria</td><td>Leptolyngbya sp. Heron Island J</td><td>small gtp-binding protein [Leptolyngbya sp. Heron Island J].</td><td>GCA_000482245.1</td></tr>
<tr><td>ESA37051.1</td><td>LRR-repeats+AP-GTPase+COR*→?→?→?→||<-Pentapeptide-repeats</td><td>LRR-repeats+AP-GTPase+COR</td><td>818</td><td>N836_04700</td><td>Cyanobacteria</td><td>Leptolyngbya sp. Heron Island J</td><td>small gtp-binding protein [Leptolyngbya sp. Heron Island J].</td><td>GCA_000482245.1</td></tr>
<tr><td>ESQ09086.1</td><td>LRR-repeats+AP-GTPase+COR+TIR*→</td><td>LRR-repeats+AP-GTPase+COR+TIR</td><td>840</td><td>N838_02740</td><td>Gammaproteobacteria</td><td>Thiohalocapsa sp. PB-PSB1</td><td>hypothetical protein N838_02740 [Thiohalocapsa sp. PB-PSB1].</td><td>GCA_000495615.1</td></tr>
<tr><td>ETR72570.1</td><td>Cluster1438_2clades→?→ LRR-repeats+AP-GTPase+COR+TIR*→ CCTBP→||<-REC</td><td>LRR-repeats+AP-GTPase+COR+TIR</td><td>808</td><td>OMM_01620</td><td>Deltaproteobacteria</td><td>Candidatus Magnetoglobus multicellularis str. Araruama</td><td>leucine-rich repeat-containing protein [Candidatus Magnetoglobus multicellularis str. Araruama].</td><td>GCA_000516475.1</td></tr>
<tr><td>ETW97925.1</td><td>LRR-repeats+AP-GTPase+COR+DUF4404*→?→||<-RNA-Helicase||?→ SIG+BLBD→</td><td>LRR-repeats+AP-GTPase+COR+DUF4404</td><td>767</td><td>ETSY1_20835</td><td>Nitrospinae/Tectomicrobia group</td><td>Candidatus Entotheonella factor</td><td>hypothetical protein ETSY1_20835, partial [Candidatus Entotheonella factor].</td><td>GCA_000522425.1</td></tr>
</table>

<table>
<tr><th>acc</th><th>operon</th><th>architecture</th><th>len</th><th>gen.name</th><th>taxend</th><th>species</th><th>defline</th><th>gca</th></tr>
<tr><td>ETX07308.1</td><td>LRR-repeats+AP-GTPase+COR*→</td><td>LRR-repeats+AP-GTPase+COR</td><td>513</td><td>ETSY2_11950</td><td>Nitrospinae/Tectomicrobia group</td><td>Candidatus Entotheonella gemina</td><td>hypothetical protein ETSY2_11950, partial [Candidatus Entotheonella gemina].</td><td>GCA_000522445.1</td></tr>
<tr><td>GAK50293.1</td><td><-CR-REase5<-?<-Cluster1448_2clades<-Cluster1281_2clades||?→ LRR-repeats+AP-GTPase*→ COR→</td><td>LRR-repeats+AP-GTPase</td><td>420</td><td>U14_01521</td><td>Bacteria</td><td>Candidatus Moduliflexus flocculans</td><td>leucine-rich-repeat protein [Candidatus Moduliflexus flocculans].</td><td>GCA_000739515.1</td></tr>
<tr><td>GBE92899.1</td><td>HISKIN→||<-?||?→ LRR-repeats+AP-GTPase+COR*→</td><td>LRR-repeats+AP-GTPase+COR</td><td>946</td><td>NCWK1_2658</td><td>Cyanobacteria</td><td>Nostoc cycadae WK-1</td><td>leucine rich repeat (LRR)-containing protein [Nostoc cycadae WK-1].</td><td>GCA_002897135.1</td></tr>
<tr><td>GCL40268.1</td><td>LRR-repeats+AP-GTPase+COR*→</td><td>LRR-repeats+AP-GTPase+COR</td><td>261</td><td>SR1949_54040</td><td>Cyanobacteria</td><td>Sphaerospermopsis reniformis</td><td>small GTP-binding protein [Sphaerospermopsis reniformis].</td><td>GCA_005402885.1</td></tr>
<tr><td>GEP41915.1</td><td>SIG+TM→?→ LRR-repeats+AP-GTPase+COR*→</td><td>LRR-repeats+AP-GTPase+COR</td><td>809</td><td>BGE01nite_12060</td><td>Verrucomicrobia</td><td>Brevifollis gellanilyticus</td><td>hypothetical protein BGE01nite_12060 [Brevifollis gellanilyticus].</td><td>GCA_007992435.1</td></tr>
<tr><td>GFZ95281.1</td><td>LRR-repeats+AP-GTPase+COR*→</td><td>LRR-repeats+AP-GTPase+COR</td><td>911</td><td>CYANOKiyG1_06360</td><td>Cyanobacteria</td><td>Okeania sp. KiyG1</td><td>hypothetical protein CYANOKiyG1_06360 [Okeania sp. KiyG1].</td><td>GCA_014132355.1</td></tr>
<tr><td>GFZ95952.1</td><td><-HhH-RADC+JAB<-?||LRR-repeats+AP-GTPase+COR*→</td><td>LRR-repeats+AP-GTPase+COR</td><td>747</td><td>CYANOKiyG1_07050</td><td>Cyanobacteria</td><td>Okeania sp. KiyG1</td><td>hypothetical protein CYANOKiyG1_07050 [Okeania sp. KiyG1].</td><td>GCA_014132355.1</td></tr>
<tr><td>GGA29094.1</td><td><-RADICAL-SAM||?→ REC→?→ LRR-repeats+AP-GTPase+COR*→||<-?<-NACHT+68TM-wHTH<-?||?→||<-TPR-repeats<-HISKIN</td><td>LRR-repeats+AP-GTPase+COR</td><td>1395</td><td>CYANOKiyG1_45460</td><td>Cyanobacteria</td><td>Okeania sp. KiyG1</td><td>hypothetical protein CYANOKiyG1_45460 [Okeania sp. KiyG1].</td><td>GCA_014132355.1</td></tr>
<tr><td>GGA48436.1</td><td>LRR-repeats+AP-GTPase+COR*→?→ COR+TM+TM→||<-?<-?<-NUDIX||TPR-repeats→</td><td>LRR-repeats+AP-GTPase+COR</td><td>405</td><td>CYANOKiyG1_67520</td><td>Cyanobacteria</td><td>Okeania sp. KiyG1</td><td>hypothetical protein CYANOKiyG1_67520 [Okeania sp. KiyG1].</td><td>GCA_014132355.1</td></tr>
<tr><td>GGD73013.1</td><td>LRR-repeats+AP-GTPase+COR+TM+TM*→?→?→||<-?<-Cluster1601_2clades</td><td>LRR-repeats+AP-GTPase+COR+TM+TM</td><td>937</td><td>GCM10011514_41350</td><td>Bacteroidetes</td><td>Emticicia aquatilis</td><td>hypothetical protein GCM10011514_41350 [Emticicia aquatilis].</td><td>GCA_014644295.1</td></tr>
<tr><td>GGU83059.1</td><td>LRR-repeats+AP-GTPase+COR*→||<-?||?→||<-?<-SIGMA-HTH<-?<-SIGMA-HTH</td><td>LRR-repeats+AP-GTPase+COR</td><td>996</td><td>GCM10010260_14720</td><td>Actinobacteria</td><td>Streptomyces filipinensis</td><td>hypothetical protein GCM10010260_14720 [Streptomyces filipinensis].</td><td>GCA_014649495.1</td></tr>
<tr><td>HAA21349.1</td><td>LRR-repeats+AP-GTPase+COR*→</td><td>LRR-repeats+AP-GTPase+COR</td><td>805</td><td>DCP28_22240</td><td>Bacteroidetes</td><td>Cytophagales bacterium</td><td>TPA: hypothetical protein DCP28_22240, partial [Cytophagales bacterium].</td><td>GCA_003444325.1</td></tr>
<tr><td>HAC62039.1</td><td>ABC_membrane+ABC_tran→?→ LRR-repeats→ LRR-repeats+AP-GTPase+COR*→||<-Cluster2488_2clades<-Cluster2227_2clades||ISOFLAVOMETHYLASE-HTH→</td><td>LRR-repeats+AP-GTPase+COR</td><td>852</td><td>DCF68_00525</td><td>Cyanobacteria</td><td>Cyanothece sp. UBA12306</td><td>TPA: GTPase, partial [Cyanothece sp. UBA12306].</td><td>GCA_003448685.1</td></tr>
<tr><td>HAC64827.1</td><td><-METHYLASE<-?||LRR-repeats+AP-GTPase+COR+TM+TM*→?→||<-?<-?<-SWACOS</td><td>LRR-repeats+AP-GTPase+COR+TM+TM</td><td>931</td><td>DCF68_15170</td><td>Cyanobacteria</td><td>Cyanothece sp. UBA12306</td><td>TPA: GTPase [Cyanothece sp. UBA12306].</td><td>GCA_003448685.1</td></tr>
<tr><td>HAG85135.1</td><td>Ferredoxin-RRM→ LRR-repeats+AP-GTPase+COR*→</td><td>LRR-repeats+AP-GTPase+COR</td><td>819</td><td>DCL61_29295</td><td>Cyanobacteria</td><td>Cyanobacteria bacterium UBA12227</td><td>TPA: GTPase [Cyanobacteria bacterium UBA12227].</td><td>GCA_003450835.1</td></tr>
<tr><td>HAP64559.1</td><td>LRR-repeats+AP-GTPase+COR*→</td><td>LRR-repeats+AP-GTPase+COR</td><td>754</td><td>DCR93_35450</td><td>Bacteroidetes</td><td>Cytophagales bacterium</td><td>TPA: hypothetical protein DCR93_35450, partial [Cytophagales bacterium].</td><td>GCA_003454975.1</td></tr>
<tr><td>HAR63371.1</td><td>LRR-repeats+AP-GTPase+COR*→</td><td>LRR-repeats+AP-GTPase+COR</td><td>611</td><td>DCS13_07910</td><td>Terrabacteria group</td><td>Candidatus Margulisbacteria bacterium</td><td>TPA: hypothetical protein DCS13_07910, partial [Candidatus Margulisbacteria bacterium].</td><td>GCA_003475905.1</td></tr>
<tr><td>HAS39197.1</td><td>LRR-repeats+AP-GTPase+COR*→</td><td>LRR-repeats+AP-GTPase+COR</td><td>1145</td><td>DCS93_01900</td><td>Bacteroidetes</td><td>Microscillaceae bacterium</td><td>TPA: hypothetical protein DCS93_01900 [Microscillaceae bacterium].</td><td>GCA_003483885.1</td></tr>
<tr><td>HAS43483.1</td><td>LRR-repeats→?→?→||<-?||LRR-repeats+AP-GTPase+COR+EAD11*→||<-PAS+HISKIN</td><td>LRR-repeats+AP-GTPase+COR+EAD11</td><td>868</td><td>DCS93_23590</td><td>Bacteroidetes</td><td>Microscillaceae bacterium</td><td>TPA: hypothetical protein DCS93_23590 [Microscillaceae bacterium].</td><td>GCA_003483885.1</td></tr>
</table>

<table>
<tr><th>acc</th><th>operon</th><th>architecture</th><th>len</th><th>gen.name</th><th>taxend</th><th>species</th><th>defline</th><th>gca</th></tr>
<tr><td>HAS44199.1</td><td>LRR-repeats+AP-GTPase+COR→//<-?<-?<-?//LRR-repeats+AP-GTPase+COR→</td><td>LRR-repeats+AP-GTPase+COR</td><td>958</td><td>DCS93_27220</td><td>Bacteroidetes</td><td>Microscillaceae bacterium</td><td>TPA: hypothetical protein DCS93_27220 [Microscillaceae bacterium].</td><td>GCA_003483885.1</td></tr>
<tr><td>HAS44203.1</td><td>LRR-repeats+AP-GTPase+COR*→</td><td>LRR-repeats+AP-GTPase+COR</td><td>1037</td><td>DCS93_27240</td><td>Bacteroidetes</td><td>Microscillaceae bacterium</td><td>TPA: hypothetical protein DCS93_27240 [Microscillaceae bacterium].</td><td>GCA_003483885.1</td></tr>
<tr><td>HAS46073.1</td><td><-LRR-repeats<-ABC-ATPase||?→||<-?||?→?→ LRR-repeats+AP-GTPase+COR*→</td><td>LRR-repeats+AP-GTPase+COR</td><td>864</td><td>DCS93_36670</td><td>Bacteroidetes</td><td>Microscillaceae bacterium</td><td>TPA: hypothetical protein DCS93_36670 [Microscillaceae bacterium].</td><td>GCA_003483885.1</td></tr>
<tr><td>HAV28009.1</td><td>Pro_CA→ LRR-repeats+AP-GTPase+COR*→</td><td>LRR-repeats+AP-GTPase+COR</td><td>1019</td><td>DCX23_06795</td><td>Firmicutes</td><td>Lachnospiraceae bacterium</td><td>TPA: hypothetical protein DCX23_06795 [Lachnospiraceae bacterium].</td><td>GCA_003484485.1</td></tr>
<tr><td>HAW32544.1</td><td>LRR-repeats+AP-GTPase+COR*→</td><td>LRR-repeats+AP-GTPase+COR</td><td>1236</td><td>DCY03_31275</td><td>Planctomycetes</td><td>Planctomycetaceae bacterium</td><td>TPA: hypothetical protein DCY03_31275 [Planctomycetaceae bacterium].</td><td>GCA_003487605.1</td></tr>
<tr><td>HAX71152.1</td><td><-Cluster1437_2clades||?→ LRR-repeats→ LRR-repeats+AP-GTPase+COR*→</td><td>LRR-repeats+AP-GTPase+COR</td><td>869</td><td>DCY14_16175</td><td>Chloroflexi</td><td>Anaerolineae bacterium</td><td>TPA: hypothetical protein DCY14_16175, partial [Anaerolineae bacterium].</td><td>GCA_003486425.1</td></tr>
<tr><td>HBE20120.1</td><td>LRR-repeats+AP-GTPase+COR*→</td><td>LRR-repeats+AP-GTPase+COR</td><td>816</td><td>DDW51_21520</td><td>Cyanobacteria</td><td>Cyanobacteria bacterium UBA11367</td><td>TPA: hypothetical protein DDW51_21520 [Cyanobacteria bacterium UBA11367].</td><td>GCA_003501765.1</td></tr>
<tr><td>HBE59255.1</td><td>LRR-repeats→ LRR-repeats+AP-GTPase+COR*→</td><td>LRR-repeats+AP-GTPase+COR</td><td>893</td><td>DDW56_17600</td><td>Cyanobacteria</td><td>Cyanobacteria bacterium UBA11366</td><td>TPA: hypothetical protein DDW56_17600, partial [Cyanobacteria bacterium UBA11366].</td><td>GCA_003501625.1</td></tr>
<tr><td>HBG28935.1</td><td>ABC-ATPase+ABC-ATPase→?→?→ S1COLD→ LRR-repeats+AP-GTPase+COR+TIR*→</td><td>LRR-repeats+AP-GTPase+COR+TIR</td><td>1033</td><td>DDX75_17760</td><td>Planctomycetes</td><td>Phycisphaerales bacterium</td><td>TPA: serine/threonine protein kinase [Phycisphaerales bacterium].</td><td>GCA_003499685.1</td></tr>
<tr><td>HBH73318.1</td><td>LRR-repeats→ LRR-repeats+AP-GTPase+COR+DrHyd*→||<-?<-?<-?||?→?→||<-PRTase</td><td>LRR-repeats+AP-GTPase+COR+DrHyd</td><td>872</td><td>DDY43_07685</td><td>Cyanobacteria</td><td>Synechococcales bacterium UBA10510</td><td>TPA: hypothetical protein DDY43_07685, partial [Synechococcales bacterium UBA10510].</td><td>GCA_003508015.1</td></tr>
<tr><td>HBH73336.1</td><td>LRR-repeats+AP-GTPase+COR+TIR*→</td><td>LRR-repeats+AP-GTPase+COR+TIR</td><td>698</td><td>DDY43_07790</td><td>Cyanobacteria</td><td>Synechococcales bacterium UBA10510</td><td>TPA: hypothetical protein DDY43_07790 [Synechococcales bacterium UBA10510].</td><td>GCA_003508015.1</td></tr>
<tr><td>HBK96125.1</td><td>LRR-repeats+AP-GTPase+COR+TM+TM*→</td><td>LRR-repeats+AP-GTPase+COR+TM+TM</td><td>656</td><td>DD001_01755</td><td>Cyanobacteria</td><td>Microcoleaceae bacterium UBA10368</td><td>TPA: GTPase, partial [Microcoleaceae bacterium UBA10368].</td><td>GCA_003506815.1</td></tr>
<tr><td>HBK98262.1</td><td>GTPase-AIG→?→?→||<-?<-?||LRR-repeats+AP-GTPase+COR+DUF4404*→</td><td>LRR-repeats+AP-GTPase+COR+DUF4404</td><td>1165</td><td>DD001_13585</td><td>Cyanobacteria</td><td>Microcoleaceae bacterium UBA10368</td><td>TPA: GTPase [Microcoleaceae bacterium UBA10368].</td><td>GCA_003506815.1</td></tr>
<tr><td>HBP89205.1</td><td>CHTH→||<-?<-?<-?<-?<-?<-?||LRR-repeats+AP-GTPase+COR+DrHyd*→||<-?<-?<-TM</td><td>LRR-repeats+AP-GTPase+COR+DrHyd</td><td>948</td><td>DD706_16075</td><td>Nitrospirae</td><td>Nitrospiraceae bacterium</td><td>TPA: hypothetical protein DD706_16075 [Nitrospiraceae bacterium].</td><td>GCA_003523945.1</td></tr>
<tr><td>HBU22381.1</td><td>LRR-repeats+AP-GTPase+COR*→?→?→?→?→?→||<-?||ParA-Soj-PloopNTPase→</td><td>LRR-repeats+AP-GTPase+COR</td><td>865</td><td>DEB17_00005</td><td>Chlorobi</td><td>Chlorobaculum sp.</td><td>TPA: hypothetical protein DEB17_00005, partial [Chlorobaculum sp.].</td><td>GCA_003510485.1</td></tr>
<tr><td>HBW31420.1</td><td>LRR-repeats+AP-GTPase+COR+DUF4404*→||<-?<-ABC-ATPase<-?||?→||<-REC</td><td>LRR-repeats+AP-GTPase+COR+DUF4404</td><td>766</td><td>DEF48_15370</td><td>Cyanobacteria</td><td>Nostoc sp. UBA8866</td><td>TPA: Ras family protein, partial [Nostoc sp. UBA8866].</td><td>GCA_003514525.1</td></tr>
<tr><td>HBY87332.1</td><td>LRR-repeats+AP-GTPase+COR*→</td><td>LRR-repeats+AP-GTPase+COR</td><td>930</td><td>DEO86_15815</td><td>Gammaproteobacteria</td><td>Colwellia sp.</td><td>TPA: hypothetical protein DEO86_15815 [Colwellia sp.].</td><td>GCA_003520945.1</td></tr>
</table>

acc	operon	architecture	len	gen.name	taxend	species	defline	gca
HCA94361.1	LRR-repeats→ LRR-repeats+AP-GTPase+COR*→	LRR-repeats+AP-GTPase+COR	892	DEP38_06745	Cyanobacteria	Cyanobacteria bacterium UBA9226	TPA: hypothetical protein DEP38_06745, partial [Cyanobacteria bacterium UBA9226].	GCA_003520045.1
HCF17408.1	LRR-repeats+AP-GTPase+COR+TIR*→ TM+TM+TM→	LRR-repeats+AP-GTPase+COR+TIR	737	DEV96_05085	Alphaproteobacteria	Rhodospirillum rubrum	TPA: hypothetical protein DEV96_05085, partial [Rhodospirillum rubrum].	GCA_003530895.1
HCN30450.1	LRR-repeats+AP-GTPase+COR*→	LRR-repeats+AP-GTPase+COR	1024	DIT64_17260	Verrucomicrobia	Verrucomicrobiales bacterium	TPA: hypothetical protein DIT64_17260 [Verrucomicrobiales bacterium].	GCA_003545395.1
HCO95252.1	LRR-repeats+AP-GTPase+COR*→	LRR-repeats+AP-GTPase+COR	809	DIU00_15100	Planctomycetes	Phycisphaerales bacterium	TPA: hypothetical protein DIU00_15100 [Phycisphaerales bacterium].	GCA_003533825.1
HCQ19950.1	LRR-repeats+AP-GTPase+COR*→?→||<-?<-?||TM→	LRR-repeats+AP-GTPase+COR	754	DIU28_00335	Cyanobacteria	Anabaena sp. UBA12330	TPA: hypothetical protein DIU28_00335, partial [Anabaena sp. UBA12330].	GCA_003525565.1
HCQ20817.1	LRR-repeats→ LRR-repeats+AP-GTPase+COR+TM+TM*→||<-?||?→?→?→?→ ABC-ATPase→	LRR-repeats+AP-GTPase+COR+TM+TM	721	DIU28_04955	Cyanobacteria	Anabaena sp. UBA12330	TPA: hypothetical protein DIU28_04955, partial [Anabaena sp. UBA12330].	GCA_003525565.1
HCR54081.1	LRR-repeats→ LRR-repeats+AP-GTPase+COR*→	LRR-repeats+AP-GTPase+COR	797	DIW27_06670	Bacteroidetes	Cytophagales bacterium	TPA: hypothetical protein DIW27_06670 [Cytophagales bacterium].	GCA_003524675.1
HCS91704.1	LRR-repeats+AP-GTPase+COR+TIR*→	LRR-repeats+AP-GTPase+COR+TIR	749	DIW77_17080	Gammaproteobacteria	Chromatiaceae bacterium	TPA: hypothetical protein DIW77_17080 [Chromatiaceae bacterium].	GCA_003525925.1
HCT83732.1	LRR-repeats+AP-GTPase+COR*→	LRR-repeats+AP-GTPase+COR	585	DF296_00860	Terrabacteria group	Candidatus Margulisbacteria bacterium	TPA: hypothetical protein DF296_00860 [Candidatus Margulisbacteria bacterium].	GCA_003542625.1
HCU95641.1	LRR-repeats+AP-GTPase+COR*→	LRR-repeats+AP-GTPase+COR	896	DHU96_24175	Actinobacteria	Actinobacteria bacterium	TPA: hypothetical protein DHU96_24175 [Actinobacteria bacterium].	GCA_003541285.1
HDN27288.1	LRR-repeats+AP-GTPase+COR*→	LRR-repeats+AP-GTPase+COR	598	ENG03_09385	Gammaproteobacteria	Thioploca sp.	TPA: GTP-binding protein, partial [Thioploca sp.].	GCA_011043325.1
HEC83827.1	LRR-repeats+AP-GTPase+COR*→	LRR-repeats+AP-GTPase+COR	907	ENI48_01075	Gammaproteobacteria	Thioploca sp.	TPA: GTPase [Thioploca sp.].	GCA_011052595.1
HEG42537.1	<-S1COLD||LRR-repeats+AP-GTPase+COR+TIR*→?→||<-SIG+Alba<-?||?→?→?→ REC→	LRR-repeats+AP-GTPase+COR+TIR	1095	ENH94_00660	Planctomycetes	Phycisphaerales bacterium	TPA: TIR domain-containing protein [Phycisphaerales bacterium].	GCA_011053245.1
HER03270.1	LRR-repeats+AP-GTPase+COR+TIR*→||<-?||?→?→ Cluster1306_2clades→?→ SIG+TM→ RuvB-AAA+RuvB-HTH→	LRR-repeats+AP-GTPase+COR+TIR	910	ENR03_13235	Alphaproteobacteria	Rhizobiales bacterium	TPA: TIR domain-containing protein, partial [Rhizobiales bacterium].	GCA_011046675.1
HEU35175.1	Cluster1718_2clades→ LRR-repeats+AP-GTPase+COR*→	LRR-repeats+AP-GTPase+COR	718	ENQ60_27120	unclassified Bacteria	bacterium	TPA: GTP-binding protein, partial [bacterium].	GCA_011047125.1
HFB99816.1	LRR-repeats+AP-GTPase+COR+TIR*→	LRR-repeats+AP-GTPase+COR+TIR	1132	ENJ53_03320	Bacteroidetes	Phaeodactylibacter sp.	TPA: TIR domain-containing protein, partial [Phaeodactylibacter sp.].	GCA_011322485.1
HFS07270.1	Cluster1370_2clades→ LRR-repeats+AP-GTPase+COR+TIR*→||<-?||REase→	LRR-repeats+AP-GTPase+COR+TIR	1009	ENR14_02795	Cyanobacteria	Anabaena sp.	TPA: TIR domain-containing protein [Anabaena sp.].	GCA_011332035.1
HFS07901.1	LRR-repeats+AP-GTPase+COR+TIR*→||<-?<-?<-ParA-Soj-PloopNTPase	LRR-repeats+AP-GTPase+COR+TIR	1021	ENR14_06085	Cyanobacteria	Anabaena sp.	TPA: GTP-binding protein [Anabaena sp.].	GCA_011332035.1
HGZ29232.1	LRR-repeats+AP-GTPase+COR*→	LRR-repeats+AP-GTPase+COR	903	ENR17_03505	Bacteroidetes	Bacteroidetes bacterium	TPA: hypothetical protein ENR17_03505 [Bacteroidetes bacterium].	GCA_011331955.1

<table>
<tr><th>acc</th><th>operon</th><th>architecture</th><th>len</th><th>gen.name</th><th>taxend</th><th>species</th><th>defline</th><th>gca</th></tr>
<tr><td>HGZ31593.1</td><td>LRR-repeats+AP-GTPase+COR+TIR*→</td><td>LRR-repeats+AP-GTPase+COR+TIR</td><td>868</td><td>ENR17_15550</td><td>Bacteroidetes</td><td>Bacteroidetes bacterium</td><td>TPA: TIR domain-containing protein, partial [Bacteroidetes bacterium].</td><td>GCA_011331955.1</td></tr>
<tr><td>HHB79310.1</td><td>FGS→?→ RVT→ LRR-repeats+AP-GTPase+COR*→</td><td>LRR-repeats+AP-GTPase+COR</td><td>458</td><td>ENK85_08770</td><td>Bacteroidetes</td><td>Saprospiraceae bacterium</td><td>TPA: hypothetical protein ENK85_08770, partial [Saprospiraceae bacterium].</td><td>GCA_011371135.1</td></tr>
<tr><td>HHH53255.1</td><td>LRR-repeats+AP-GTPase+COR*→</td><td>LRR-repeats+AP-GTPase+COR</td><td>413</td><td>ENK91_06320</td><td>Bacteroidetes</td><td>Bacteroidetes bacterium</td><td>TPA: GTP-binding protein, partial [Bacteroidetes bacterium].</td><td>GCA_011371025.1</td></tr>
<tr><td>HHV24864.1</td><td>LRR-repeats+AP-GTPase+COR*→</td><td>LRR-repeats+AP-GTPase+COR</td><td>716</td><td>GXX65_10185</td><td>Euryarchaeota</td><td>Methanosarcina sp.</td><td>TPA: GTP-binding protein [Methanosarcina sp.].</td><td>GCA_012839505.1</td></tr>
<tr><td>HIE02480.1</td><td>Cluster1917_2clades→?→?→?→?→||<-?||LRR-repeats+AP-GTPase+COR*→</td><td>LRR-repeats+AP-GTPase+COR</td><td>853</td><td>EYP59_19720</td><td>Gammaproteobacteria</td><td>Thiotrichaceae bacterium</td><td>TPA: GTPase [Thiotrichaceae bacterium].</td><td>GCA_012961805.1</td></tr>
<tr><td>HIE31128.1</td><td>LRR-repeats+AP-GTPase+COR*→</td><td>LRR-repeats+AP-GTPase+COR</td><td>636</td><td>EYP67_01930</td><td>Euryarchaeota</td><td>Methanosarcinales archaeon</td><td>TPA: GTP-binding protein, partial [Methanosarcinales archaeon].</td><td>GCA_012961645.1</td></tr>
<tr><td>HII01375.1</td><td>LRR-repeats+AP-GTPase+COR*→</td><td>LRR-repeats+AP-GTPase+COR</td><td>622</td><td>HA351_06900</td><td>Euryarchaeota</td><td>Methanosarcinaceae archaeon</td><td>TPA: GTP-binding protein [Methanosarcinaceae archaeon].</td><td>GCA_013329415.1</td></tr>
<tr><td>HII91446.1</td><td><-HhH-RADC+JAB||?→?→?→ NTP_transf_3→ LRR-repeats→ LRR-repeats+AP-GTPase+COR*→</td><td>LRR-repeats+AP-GTPase+COR</td><td>672</td><td>HA262_04570</td><td>Euryarchaeota</td><td>Methanosarcina sp.</td><td>TPA: GTP-binding protein, partial [Methanosarcina sp.].</td><td>GCA_013331275.1</td></tr>
<tr><td>HII91684.1</td><td>NTP_transf_3→ LRR-repeats+AP-GTPase+COR*→</td><td>LRR-repeats+AP-GTPase+COR</td><td>735</td><td>HA262_05815</td><td>Euryarchaeota</td><td>Methanosarcina sp.</td><td>TPA: GTP-binding protein [Methanosarcina sp.].</td><td>GCA_013331275.1</td></tr>
<tr><td>KAA3610009.1</td><td>MORC→?→ LRR-repeats→ LRR-repeats+AP-GTPase+COR*→?→||<-?<-?<-BetaPropeller+BetaPropeller</td><td>LRR-repeats+AP-GTPase+COR</td><td>653</td><td>DWQ03_18700</td><td>Calditrichaeota</td><td>Calditrichaeota bacterium</td><td>hypothetical protein DWQ03_18700 [Calditrichaeota bacterium].</td><td>GCA_008501795.1</td></tr>
<tr><td>KAA3656070.1</td><td>LRR-repeats+AP-GTPase+COR+EAD7*→</td><td>LRR-repeats+AP-GTPase+COR+EAD7</td><td>779</td><td>DWQ04_32125</td><td>Chloroflexi</td><td>Chloroflexi bacterium</td><td>GTPase, partial [Chloroflexi bacterium].</td><td>GCA_008501785.1</td></tr>
<tr><td>KAA3659887.1</td><td>LRR-repeats+AP-GTPase+COR*→</td><td>LRR-repeats+AP-GTPase+COR</td><td>842</td><td>DWQ04_20995</td><td>Chloroflexi</td><td>Chloroflexi bacterium</td><td>GTPase [Chloroflexi bacterium].</td><td>GCA_008501785.1</td></tr>
<tr><td>KAB8152691.1</td><td><-HISKIN<-?||?→?→ REC→ LRR-repeats+AP-GTPase+COR+TIR*→||<-RADICAL-SAM</td><td>LRR-repeats+AP-GTPase+COR+TIR</td><td>1007</td><td>EZY14_012780</td><td>Bacteroidetes</td><td>Kordia sp. TARA_039_SRF</td><td>TIR domain-containing protein [Kordia sp. TARA_039_SRF].</td><td>GCA_006383075.2</td></tr>
<tr><td>KAB8153612.1</td><td>LRR-repeats+AP-GTPase+COR+TIR*→||<-?<-?||?→||<-?||TM+RHOMBOID→</td><td>LRR-repeats+AP-GTPase+COR+TIR</td><td>966</td><td>EZY14_010295</td><td>Bacteroidetes</td><td>Kordia sp. TARA_039_SRF</td><td>TIR domain-containing protein [Kordia sp. TARA_039_SRF].</td><td>GCA_006383075.2</td></tr>
<tr><td>KAF0107167.1</td><td><-HISKIN<-REC||?→?→?→?→ LRR-repeats+AP-GTPase+COR*→||<-SIG+GT4</td><td>LRR-repeats+AP-GTPase+COR</td><td>1065</td><td>FD146_2008</td><td>Chloroflexi</td><td>Anaerolineaceae bacterium</td><td>hypothetical protein FD146_2008 [Anaerolineaceae bacterium].</td><td>GCA_009772985.1</td></tr>
<tr><td>KAF5415978.1</td><td>LRR-repeats+AP-GTPase+COR*→</td><td>LRR-repeats+AP-GTPase+COR</td><td>561</td><td>C5S48_04205</td><td>Euryarchaeota</td><td>ANME-2 cluster archaeon</td><td>hypothetical protein C5S48_04205 [ANME-2 cluster archaeon].</td><td>GCA_013374355.1</td></tr>
<tr><td>KAF5419007.1</td><td>LRR-repeats+AP-GTPase+COR*→</td><td>LRR-repeats+AP-GTPase+COR</td><td>865</td><td>C5S49_00980</td><td>Euryarchaeota</td><td>ANME-2 cluster archaeon</td><td>hypothetical protein C5S49_00980 [ANME-2 cluster archaeon].</td><td>GCA_013374365.1</td></tr>
<tr><td>KAF5420469.1</td><td>Cluster1076_2clades→ Cluster1076_2clades→ LRR-repeats+AP-GTPase+COR*→</td><td>LRR-repeats+AP-GTPase+COR</td><td>878</td><td>C5S44_08435</td><td>Euryarchaeota</td><td>ANME-2 cluster archaeon</td><td>hypothetical protein C5S44_08435 [ANME-2 cluster archaeon].</td><td>GCA_013374455.1</td></tr>
<tr><td>KAF5421986.1</td><td>LRR-repeats+AP-GTPase+COR*→</td><td>LRR-repeats+AP-GTPase+COR</td><td>596</td><td>C5S45_02955</td><td>Euryarchaeota</td><td>ANME-2 cluster archaeon</td><td>hypothetical protein C5S45_02955, partial [ANME-2 cluster archaeon].</td><td>GCA_013374465.1</td></tr>
<tr><td>KAF5428813.1</td><td><-CBS<-CBS||?→?→ LRR-repeats+AP-GTPase+COR*→</td><td>LRR-repeats+AP-GTPase+COR</td><td>788</td><td>C5S39_10445</td><td>Euryarchaeota</td><td>Methanophagales archaeon</td><td>Leucine-rich repeat (LRR) protein [Methanophagales archaeon].</td><td>GCA_013374505.1</td></tr>
<tr><td>KAF5432496.1</td><td>LRR-repeats+AP-GTPase+COR*→</td><td>LRR-repeats+AP-GTPase+COR</td><td>599</td><td>C5S39_03480</td><td>Euryarchaeota</td><td>Methanophagales archaeon</td><td>hypothetical protein C5S39_03480 [Methanophagales archaeon].</td><td>GCA_013374505.1</td></tr>
<tr><td>KAF5433375.1</td><td>LRR-repeats+AP-GTPase+COR*→</td><td>LRR-repeats+AP-GTPase+COR</td><td>838</td><td>C5S35_16480</td><td>Euryarchaeota</td><td>Methanophagales archaeon</td><td>hypothetical protein C5S35_16480 [Methanophagales archaeon].</td><td>GCA_013374565.1</td></tr>
<tr><td>KAF5435535.1</td><td>SIG+TM+TM+TM+TM+TM+TM→||<-?<-?||?→?→ Aminotran_1_2→ LRR-repeats+AP-GTPase+COR*→</td><td>LRR-repeats+AP-GTPase+COR</td><td>659</td><td>C5S35_10775</td><td>Euryarchaeota</td><td>Methanophagales archaeon</td><td>hypothetical protein C5S35_10775, partial [Methanophagales archaeon].</td><td>GCA_013374565.1</td></tr>
<tr><td>KAF5438029.1</td><td>LRR-repeats+AP-GTPase+COR*→</td><td>LRR-repeats+AP-GTPase+COR</td><td>787</td><td>C5S35_01055</td><td>Euryarchaeota</td><td>Methanophagales archaeon</td><td>internalin A, partial [Methanophagales archaeon].</td><td>GCA_013374565.1</td></tr>
<tr><td>KFF11476.1</td><td>LRR-repeats+AP-GTPase+COR*→</td><td>LRR-repeats+AP-GTPase+COR</td><td>902</td><td>IW20_19515</td><td>Bacteroidetes</td><td>Flavobacterium hydatis</td><td>hypothetical protein IW20_19515 [Flavobacterium hydatis].</td><td>GCA_000737695.1</td></tr>
</table>

acc	operon	architecture	len	gen.name	taxend	species	defline	gca
KJV08837.1	LRR-repeats+AP-GTPase+COR*→	LRR-repeats+AP-GTPase+COR	553	VZ95_15215	Alphaproteobacteria	Elstera litoralis	hypothetical protein VZ95_15215, partial [Elstera litoralis].	GCA_000963705.1
KOR30146.1	LRR-repeats+AP-GTPase+COR*→ SIG+RHOD-CDC25→	LRR-repeats+AP-GTPase+COR	653	TI04_06910	Gammaproteobacteria	Achromatium sp. WMS2	hypothetical protein TI04_06910 [Achromatium sp. WMS2].	GCA_001276605.1
KPA09194.1	HNH→?→?→?→?→ LRR-repeats+AP-GTPase+COR*→ McrA-NTD→?→ RlaP→ Cluster2261_2clades→	LRR-repeats+AP-GTPase+COR	923	MHK_010613	Deltaproteobacteria	Candidatus Magnetomorum sp. HK-1	Leucine Rich Repeat (LRR)-containing protein [Candidatus Magnetomorum sp. HK-1].	GCA_001292585.1
KPA10003.1	Cluster2271_2clades→ TPR+TPR→?→?→ LRR-repeats+AP-GTPase+COR+DUF4404*→	LRR-repeats+AP-GTPase+COR+DUF4404	883	MHK_009799	Deltaproteobacteria	Candidatus Magnetomorum sp. HK-1	small GTP-binding protein domain protein [Candidatus Magnetomorum sp. HK-1].	GCA_001292585.1
KPA12970.1	LRR-repeats+AP-GTPase+COR*→	LRR-repeats+AP-GTPase+COR	676	MHK_006822	Deltaproteobacteria	Candidatus Magnetomorum sp. HK-1	leucine-rich repeat-containing protein [Candidatus Magnetomorum sp. HK-1].	GCA_001292585.1
KPA13876.1	TIR+LRR-repeats+AP-GTPase+COR+Calcineurin*→	TIR+LRR-repeats+AP-GTPase+COR+Calcineurin	1426	MHK_005915	Deltaproteobacteria	Candidatus Magnetomorum sp. HK-1	small gtp-binding protein [Candidatus Magnetomorum sp. HK-1].	GCA_001292585.1
KPA14771.1	LRR-repeats+AP-GTPase+COR+DrHyd*→	LRR-repeats+AP-GTPase+COR+DrHyd	898	MHK_005023	Deltaproteobacteria	Candidatus Magnetomorum sp. HK-1	leucine-rich repeat-containing protein [Candidatus Magnetomorum sp. HK-1].	GCA_001292585.1
KPA15132.1	LRR-repeats+AP-GTPase+COR+TIR*→	LRR-repeats+AP-GTPase+COR+TIR	1146	MHK_004657	Deltaproteobacteria	Candidatus Magnetomorum sp. HK-1	small GTP-binding domain protein [Candidatus Magnetomorum sp. HK-1].	GCA_001292585.1
KPQ33355.1	ARSR-HTH→ Cluster2335_2clades→||<-?||LRR-repeats→ LRR-repeats+AP-GTPase+COR+TIR*→||<-METHYLASE||?→||<-SIG+TM+TM+TM<-ABC_membrane+ABC_tran	LRR-repeats+AP-GTPase+COR+TIR	887	HLUCCA11_18720	Cyanobacteria	Phormidesmis priestleyi Ana	Leucine Rich repeats (2 copies)/TIR domain/Miro-like protein/Leucine rich repeat [Phormidesmis priestleyi Ana].	GCA_001314865.1
KPQ36660.1	<-Cluster54_3clades<-?||LRR-repeats+AP-GTPase+COR*→	LRR-repeats+AP-GTPase+COR	1200	inlA	Cyanobacteria	Phormidesmis priestleyi Ana	internalin A [Phormidesmis priestleyi Ana].	GCA_001314865.1
KTE25244.1	TetR-HTH→?→||<-?<-?<-?<-?||LRR-repeats+AP-GTPase+COR+TIR*→	LRR-repeats+AP-GTPase+COR+TIR	719	ATE62_22325	Alphaproteobacteria	Sphingopyxis sp. HIX	hypothetical protein ATE62_22325 [Sphingopyxis sp. HIX].	GCA_001468285.1
KTE72816.1	TetR-HTH→?→||<-?<-?<-?<-?||LRR-repeats+AP-GTPase+COR+TIR*→	LRR-repeats+AP-GTPase+COR+TIR	716	ATE72_22070	Alphaproteobacteria	Sphingopyxis sp. HXXIV	hypothetical protein ATE72_22070 [Sphingopyxis sp. HXXIV].	GCA_001468305.1
MAB62252.1	Cluster1163_3clades→?→ Cluster1131_3clades→?→||<-?<-?||?→ LRR-repeats+AP-GTPase+COR+TIR*→?→?→ METHYLASE→ ParA-Soj-PloopNTPase→ ParB→	LRR-repeats+AP-GTPase+COR+TIR	1212	CMK67_03795	Gammaproteobacteria	Pseudoalteromonas sp.	hypothetical protein CMK67_03795 [Pseudoalteromonas sp.].	GCA_002684115.1
MAD04103.1	LRR-repeats+AP-GTPase+COR*→?→||<-?<-?<-?||CNMP+CRP-HTH→	LRR-repeats+AP-GTPase+COR	956	CMK65_10845	Gammaproteobacteria	Pseudoalteromonas sp.	hypothetical protein CMK65_10845 [Pseudoalteromonas sp.].	GCA_002685175.1
MAG37481.1	AP-GTPase+COR+TIR+ACYC→ LRR-repeats+AP-GTPase+COR+DUF4404*→?→||<-?||?→?→?→||<-DOC+CITB-HTH	LRR-repeats+AP-GTPase+COR+DUF4404	884	CL878_14695	Chloroflexi	Dehalococcoidia bacterium	serine/threonine protein kinase [Dehalococcoidia bacterium].	GCA_002688445.1
MAM30487.1	LRR-repeats+AP-GTPase+COR*→||<-?<-tRNA<-?||Nitroreductase-like→||<-FKBP	LRR-repeats+AP-GTPase+COR	839	CMC13_15825	Bacteroidetes	Flavobacteriaceae bacterium	hypothetical protein CMC13_15825 [Flavobacteriaceae bacterium].	GCA_002693005.1
MAS92856.1	<-Cluster1364_2clades||SIG+TM+TM+TM+TM+TM→ TIR+LRR-repeats+AP-GTPase+COR*→||<-?<-?<-?<-SIG+TM+TM+TM	TIR+LRR-repeats+AP-GTPase+COR	1140	CMO55_06625	Verrucomicrobia	Verrucomicrobiales bacterium	hypothetical protein CMO55_06625 [Verrucomicrobiales bacterium].	GCA_002698785.1
MAT45170.1	LRR-repeats+AP-GTPase+COR*→	LRR-repeats+AP-GTPase+COR	1068	CL609_22815	Chloroflexi	Anaerolineaceae bacterium	hypothetical protein CL609_22815 [Anaerolineaceae bacterium].	GCA_002702705.1
MAT70109.1	<-Cluster1140_2clades<-?||?→||<-?<-?||LRR-repeats+AP-GTPase+COR+TIR*→	LRR-repeats+AP-GTPase+COR+TIR	1220	CMJ58_11380	Planctomycetes	Planctomycetaceae bacterium	hypothetical protein CMJ58_11380 [Planctomycetaceae bacterium].	GCA_002702655.1
MAT98393.1	LRR-repeats+AP-GTPase+COR+EAD7*→?→ SIG+PBPB→||<-?||?→||<-SIG+TM+TM+TM+TM+TM+TM+TM+TM+TM+TM<-REC	LRR-repeats+AP-GTPase+COR+EAD7	845	CL608_14715	Chloroflexi	Anaerolineaceae bacterium	hypothetical protein CL608_14715 [Anaerolineaceae bacterium].	GCA_002699125.1
MAY74446.1	ParB→?→||<-?||ClpABN-AAA+ClpABC-AAA→ LRR-repeats+AP-GTPase+COR+TM+TM*→	LRR-repeats+AP-GTPase+COR+TM+TM	1163	CMJ31_06925	Planctomycetes	Phycisphaerae bacterium	hypothetical protein CMJ31_06925 [Phycisphaerae bacterium].	GCA_002706885.1

<table>
<tr><th>acc</th><th>operon</th><th>architecture</th><th>len</th><th>gen.name</th><th>taxend</th><th>species</th><th>defline</th><th>gca</th></tr>
<tr><td>MBA2243669.1</td><td>LRR-repeats+AP-GTPase+COR+TIR*→?→?→||<-?<-?||Cluster1476_2clades→</td><td>LRR-repeats+AP-GTPase+COR+TIR</td><td>748</td><td>H0W11_01850</td><td>Gemmatimonadetes</td><td>Gemmatimonadetes bacterium</td><td>TIR domain-containing protein [Gemmatimonadetes bacterium].</td><td>GCA_013694785.1</td></tr>
<tr><td>MBA2243921.1</td><td>LRR-repeats+AP-GTPase+COR+TIR*→</td><td>LRR-repeats+AP-GTPase+COR+TIR</td><td>693</td><td>H0W11_03125</td><td>Gemmatimonadetes</td><td>Gemmatimonadetes bacterium</td><td>TIR domain-containing protein, partial [Gemmatimonadetes bacterium].</td><td>GCA_013694785.1</td></tr>
<tr><td>MBA2678589.1</td><td>LRR-repeats+AP-GTPase+COR*→?→||<-?<-Cluster2349_2clades</td><td>LRR-repeats+AP-GTPase+COR</td><td>363</td><td>H0U76_09400</td><td>Chloroflexi</td><td>Ktedonobacteraceae bacterium</td><td>leucine-rich repeat domain-containing protein [Ktedonobacteraceae bacterium].</td><td>GCA_013697605.1</td></tr>
<tr><td>MBA2681600.1</td><td>LRR-repeats+AP-GTPase+COR*→</td><td>LRR-repeats+AP-GTPase+COR</td><td>724</td><td>H0U76_24795</td><td>Chloroflexi</td><td>Ktedonobacteraceae bacterium</td><td>leucine-rich repeat domain-containing protein, partial [Ktedonobacteraceae bacterium].</td><td>GCA_013697605.1</td></tr>
<tr><td>MBA3240620.1</td><td>LRR-repeats+AP-GTPase+COR*→</td><td>LRR-repeats+AP-GTPase+COR</td><td>321</td><td>H0T60_05265</td><td>Acidobacteria</td><td>Acidobacteria bacterium</td><td>leucine-rich repeat domain-containing protein, partial [Acidobacteria bacterium].</td><td>GCA_013813375.1</td></tr>
<tr><td>MBA3574619.1</td><td>LRR-repeats+AP-GTPase+COR*→</td><td>LRR-repeats+AP-GTPase+COR</td><td>706</td><td>H0W37_06570</td><td>Actinobacteria</td><td>Pseudonocardiales bacterium</td><td>hypothetical protein H0W37_06570, partial [Pseudonocardiales bacterium].</td><td>GCA_013817435.1</td></tr>
<tr><td>MBA3673339.1</td><td>Trichomonas-DAM→?→ LRR-repeats+AP-GTPase+COR+TIR*→</td><td>LRR-repeats+AP-GTPase+COR+TIR</td><td>1186</td><td>H0W75_00010</td><td>Bacteroidetes</td><td>Chitinophagaceae bacterium</td><td>leucine-rich repeat domain-containing protein [Chitinophagaceae bacterium].</td><td>GCA_013816565.1</td></tr>
<tr><td>MBA3805221.1</td><td>LRR-repeats+AP-GTPase+COR+TIR*→</td><td>LRR-repeats+AP-GTPase+COR+TIR</td><td>805</td><td>H0X14_05855</td><td>Acidobacteria</td><td>Acidobacteria bacterium</td><td>TIR domain-containing protein [Acidobacteria bacterium].</td><td>GCA_013815675.1</td></tr>
<tr><td>MBA3964374.1</td><td>LRR-repeats+AP-GTPase+COR+TIR*→</td><td>LRR-repeats+AP-GTPase+COR+TIR</td><td>1028</td><td>H0X47_01080</td><td>Nitrospirae</td><td>Nitrospirales bacterium</td><td>leucine-rich repeat domain-containing protein [Nitrospirales bacterium].</td><td>GCA_013821375.1</td></tr>
<tr><td>MBA4062951.1</td><td><-NUDIX||?→||<-tRNA||?→?→||<-?||LRR-repeats+AP-GTPase+COR+TIR*→</td><td>LRR-repeats+AP-GTPase+COR+TIR</td><td>867</td><td>C0501_04445</td><td>Planctomycetes</td><td>Isosphaera sp.</td><td>serine/threonine protein kinase [Isosphaera sp.].</td><td>GCA_013822345.1</td></tr>
<tr><td>MBB3841051.1</td><td>TPR-repeats→?→ TPR-repeats→?→ LRR-repeats+AP-GTPase+COR+TIR*→||<-?||?→||<-?||?→||<-?<-?||TIR+TPR+TPR-S→</td><td>LRR-repeats+AP-GTPase+COR+TIR</td><td>794</td><td>FHS57_005072</td><td>Bacteroidetes</td><td>Runella defluvii</td><td>GTPase SAR1 family protein [Runella defluvii].</td><td>GCA_014195535.1</td></tr>
<tr><td>MBB5874185.1</td><td>LRR-repeats+AP-GTPase+COR*→||<-?<-?<-?<-BACTERIALFRINGE+TM+TM</td><td>LRR-repeats+AP-GTPase+COR</td><td>1250</td><td>F4553_007619</td><td>Actinobacteria</td><td>Allocatelliglobosispora scoriae</td><td>Leucine-rich repeat (LRR) protein/GTPase SAR1 family protein [Allocatelliglobosispora scoriae].</td><td>GCA_014204945.1</td></tr>
<tr><td>MBC6434062.1</td><td>LRR-repeats+AP-GTPase+COR*→||<-?<-?||?→||<-?<-PSE<-N-OB</td><td>LRR-repeats+AP-GTPase+COR</td><td>824</td><td>FM036_26905</td><td>Cyanobacteria</td><td>Nostoc sp. HG1</td><td>hypothetical protein FM036_26905, partial [Nostoc sp. HG1].</td><td>GCA_014324315.1</td></tr>
<tr><td>MBC7569567.1</td><td>LRR-repeats+AP-GTPase+COR+TIR*→</td><td>LRR-repeats+AP-GTPase+COR+TIR</td><td>863</td><td>H7319_07530</td><td>Bacteroidetes</td><td>Spirosoma sp.</td><td>leucine-rich repeat domain-containing protein [Spirosoma sp.].</td><td>GCA_014377625.1</td></tr>
<tr><td>MBC7571377.1</td><td>Cluster1281_2clades→||<-?||LRR-repeats+AP-GTPase+COR*→||<-?<-?||?→||<-?<-?<-?<-Cluster1413_2clades</td><td>LRR-repeats+AP-GTPase+COR</td><td>1077</td><td>H7319_16850</td><td>Bacteroidetes</td><td>Spirosoma sp.</td><td>leucine-rich repeat domain-containing protein [Spirosoma sp.].</td><td>GCA_014377625.1</td></tr>
<tr><td>MBC7773965.1</td><td>LRR-repeats+AP-GTPase+COR+TIR*→</td><td>LRR-repeats+AP-GTPase+COR+TIR</td><td>856</td><td>H7246_00900</td><td>Planctomycetes</td><td>Phycisphaerae bacterium</td><td>TIR domain-containing protein [Phycisphaerae bacterium].</td><td>GCA_014379065.1</td></tr>
<tr><td>MBC7775420.1</td><td>LRR-repeats+AP-GTPase+COR*→</td><td>LRR-repeats+AP-GTPase+COR</td><td>616</td><td>H7246_08275</td><td>Planctomycetes</td><td>Phycisphaerae bacterium</td><td>leucine-rich repeat domain-containing protein, partial [Phycisphaerae bacterium].</td><td>GCA_014379065.1</td></tr>
<tr><td>MBC7777197.1</td><td>LRR-repeats+AP-GTPase+COR+TIR*→?→ HNH→</td><td>LRR-repeats+AP-GTPase+COR+TIR</td><td>693</td><td>H7246_17325</td><td>Planctomycetes</td><td>Phycisphaerae bacterium</td><td>GTP-binding protein, partial [Phycisphaerae bacterium].</td><td>GCA_014379065.1</td></tr>
<tr><td>MBC7778407.1</td><td>LRR-repeats+AP-GTPase+COR+TIR*→</td><td>LRR-repeats+AP-GTPase+COR+TIR</td><td>831</td><td>H7246_23450</td><td>Planctomycetes</td><td>Phycisphaerae bacterium</td><td>TIR domain-containing protein [Phycisphaerae bacterium].</td><td>GCA_014379065.1</td></tr>
</table>

<table>
<thead>
<tr><th>acc</th><th>operon</th><th>architecture</th><th>len</th><th>gen.name</th><th>taxend</th><th>species</th><th>defline</th><th>gca</th></tr>
</thead>
<tbody>
<tr><td>MBC7817185.1</td><td>LRR-repeats+AP-GTPase+COR*→</td><td>LRR-repeats+AP-GTPase+COR</td><td>238</td><td>IAG10_09885</td><td>Planctomycetes</td><td>Planctomycetaceae bacterium</td><td>hypothetical protein IAG10_09885, partial [Planctomycetaceae bacterium].</td><td>GCA_014379175.1</td></tr>
<tr><td>MBC7817749.1</td><td><-RHOD-CDC25||LRR-repeats+AP-GTPase+COR+TIR*→</td><td>LRR-repeats+AP-GTPase+COR+TIR</td><td>865</td><td>IAG10_12720</td><td>Planctomycetes</td><td>Planctomycetaceae bacterium</td><td>leucine-rich repeat domain-containing protein [Planctomycetaceae bacterium].</td><td>GCA_014379175.1</td></tr>
<tr><td>MBC7826556.1</td><td>LRR-repeats+AP-GTPase+COR+TIR*→</td><td>LRR-repeats+AP-GTPase+COR+TIR</td><td>752</td><td>H7122_02330</td><td>Bacteroidetes</td><td>Chitinophagaceae bacterium</td><td>TIR domain-containing protein, partial [Chitinophagaceae bacterium].</td><td>GCA_014379345.1</td></tr>
<tr><td>MBC7848891.1</td><td>LRR-repeats+AP-GTPase+COR*→</td><td>LRR-repeats+AP-GTPase+COR</td><td>927</td><td>H7Y31_04105</td><td>Bacteroidetes</td><td>Chitinophagaceae bacterium</td><td>leucine-rich repeat domain-containing protein, partial [Chitinophagaceae bacterium].</td><td>GCA_014379515.1</td></tr>
<tr><td>MBC7877029.1</td><td>RADICAL-SAM→?→?→?→||<-?||?→ LRR-repeats→ LRR-repeats+AP-GTPase+COR*→?→ ABC-ATPase→</td><td>LRR-repeats+AP-GTPase+COR</td><td>718</td><td>H7Y59_07640</td><td>Chloroflexi</td><td>Anaerolineales bacterium</td><td>leucine-rich repeat domain-containing protein [Anaerolineales bacterium].</td><td>GCA_014379595.1</td></tr>
<tr><td>MBC7877562.1</td><td><-TM+RHOMBOID<-?<-?<-?||TM→ LRR-repeats+AP-GTPase+COR*→||<-?||SIGMA-HTH→</td><td>LRR-repeats+AP-GTPase+COR</td><td>1057</td><td>H7Y59_10360</td><td>Chloroflexi</td><td>Anaerolineales bacterium</td><td>hypothetical protein H7Y59_10360 [Anaerolineales bacterium].</td><td>GCA_014379595.1</td></tr>
<tr><td>MBC8029843.1</td><td><-REC||?→?→ Hsp10→?→ LRR-repeats+AP-GTPase+COR+TIR*→||<-?<-?<-?<-?<-ABC-ATPase</td><td>LRR-repeats+AP-GTPase+COR+TIR</td><td>871</td><td>H7Z16_07010</td><td>Acidobacteria</td><td>Pyrinomonadaceae bacterium</td><td>TIR domain-containing protein [Pyrinomonadaceae bacterium].</td><td>GCA_014380365.1</td></tr>
<tr><td>MBC8182613.1</td><td>LRR-repeats+AP-GTPase+COR*→?→?→ RelE-ParE→||<-?<-RelE-ParE<-?<-HISKIN</td><td>LRR-repeats+AP-GTPase+COR</td><td>687</td><td>H8E88_16055</td><td>Bacteria</td><td>candidate division KSB1 bacterium</td><td>hypothetical protein H8E88_16055, partial [candidate division KSB1 bacterium].</td><td>GCA_014381395.1</td></tr>
<tr><td>MBC8235006.1</td><td>LRR-repeats+AP-GTPase+COR+TIR*→</td><td>LRR-repeats+AP-GTPase+COR+TIR</td><td>1069</td><td>H8E77_36130</td><td>unclassified Bacteria</td><td>bacterium</td><td>leucine-rich repeat domain-containing protein [bacterium].</td><td>GCA_014381605.1</td></tr>
<tr><td>MBC8264797.1</td><td>LRR-repeats+AP-GTPase+COR*→</td><td>LRR-repeats+AP-GTPase+COR</td><td>674</td><td>H8E47_11810</td><td>Chloroflexi</td><td>Anaerolineales bacterium</td><td>leucine-rich repeat domain-containing protein [Anaerolineales bacterium].</td><td>GCA_014382145.1</td></tr>
<tr><td>MBC8291013.1</td><td>LRR-repeats+AP-GTPase+COR*→</td><td>LRR-repeats+AP-GTPase+COR</td><td>692</td><td>H8E37_11925</td><td>Planctomycetes</td><td>Planctomycetes bacterium</td><td>leucine-rich repeat domain-containing protein, partial [Planctomycetes bacterium].</td><td>GCA_014382385.1</td></tr>
<tr><td>MBC8374880.1</td><td>Cluster1757_2clades→?→ LRR-repeats+AP-GTPase+COR+DrHyd*→</td><td>LRR-repeats+AP-GTPase+COR+DrHyd</td><td>931</td><td>H8E26_02475</td><td>FCB group</td><td>FCB group bacterium</td><td>50S ribosome-binding GTPase [FCB group bacterium].</td><td>GCA_014382565.1</td></tr>
<tr><td>MBC8468948.1</td><td>LRR-repeats+AP-GTPase+COR*→||<-?||?→||<-?<-?||?→||<-?<-SIGMA-HTH</td><td>LRR-repeats+AP-GTPase+COR</td><td>1003</td><td>H8D56_05705</td><td>Planctomycetes</td><td>Planctomycetes bacterium</td><td>leucine-rich repeat domain-containing protein [Planctomycetes bacterium].</td><td>GCA_014384025.1</td></tr>
<tr><td>MBC9785332.1</td><td>Cluster1542_2clades→ LRR-repeats+AP-GTPase+COR*→</td><td>LRR-repeats+AP-GTPase+COR</td><td>976</td><td>H1S01_12510</td><td>Firmicutes</td><td>Heliobacterium chlorum</td><td>leucine-rich repeat domain-containing protein [Heliobacterium chlorum].</td><td>-</td></tr>
<tr><td>MBP93715.1</td><td>LRR-repeats+AP-GTPase+COR*→||<-?||?→||<-?<-Cluster1995_2clades</td><td>LRR-repeats+AP-GTPase+COR</td><td>894</td><td>CMC55_06315</td><td>Bacteroidetes</td><td>Flavobacteriaceae bacterium</td><td>hypothetical protein CMC55_06315 [Flavobacteriaceae bacterium].</td><td>GCA_002726785.1</td></tr>
<tr><td>MBV11156.1</td><td>APATPase+BetaPropeller_WD40-repeats→?→||<-?||FGS→ FGS→ FGS→ LRR-repeats+AP-GTPase+COR+TIR*→ Cluster1411_2clades→ FGS→ FGS→ FGS→</td><td>LRR-repeats+AP-GTPase+COR+TIR</td><td>1075</td><td>CMN21_18290</td><td>Planctomycetes</td><td>Rubinisphaera sp.</td><td>hypothetical protein CMN21_18290 [Rubinisphaera sp.].</td><td>GCA_002731545.1</td></tr>
<tr><td>MQP68112.1</td><td>LRR-repeats+AP-GTPase+COR+TIR*→||<-ParB<-ParA-Soj-PloopNTPase</td><td>LRR-repeats+AP-GTPase+COR+TIR</td><td>993</td><td>GE253_22605</td><td>Alphaproteobacteria</td><td>Niveispirillum sp. SYP-B3756</td><td>TIR domain-containing protein [Niveispirillum sp. SYP-B3756].</td><td>GCA_009495745.1</td></tr>
<tr><td>MQY15494.1</td><td><-Cluster2127_2clades<-?||?→ LRR-repeats+AP-GTPase+COR*→?→ REC→ HISKIN→</td><td>LRR-repeats+AP-GTPase+COR</td><td>980</td><td>SRB5_56760</td><td>Actinobacteria</td><td>Streptomyces sp. RB5</td><td>hypothetical protein SRB5_56760 [Streptomyces sp. RB5].</td><td>GCA_009604385.1</td></tr>
<tr><td>MSP13971.1</td><td>LRR-repeats+AP-GTPase+COR*→</td><td>LRR-repeats+AP-GTPase+COR</td><td>792</td><td>EXR62_13575</td><td>Chloroflexi</td><td>Chloroflexi bacterium</td><td>hypothetical protein EXR62_13575, partial [Chloroflexi bacterium].</td><td>GCA_009692745.1</td></tr>
<tr><td>MSS71311.1</td><td>Cluster1601_2clades→ LRR-repeats+AP-GTPase+COR*→ COR+TIR→||<-Cluster1913_2clades<-?<-RPN10</td><td>LRR-repeats+AP-GTPase+COR</td><td>556</td><td>EXS64_07460</td><td>FCB group</td><td>Candidatus Latescibacteria bacterium</td><td>hypothetical protein EXS64_07460 [Candidatus Latescibacteria bacterium].</td><td>GCA_009694805.1</td></tr>
</tbody>
</table>

<table>
<tr><th>acc</th><th>operon</th><th>architecture</th><th>len</th><th>gen.name</th><th>taxend</th><th>species</th><th>defline</th><th>gca</th></tr>
<tr><td>NAS89011.1</td><td>POLYSACPOLYMERASE→ LRR-repeats→ LRR-repeats+AP-GTPase+COR*→||<-?<-?||?→?→||<-PIN</td><td>LRR-repeats+AP-GTPase+COR</td><td>604</td><td>C4E24_04665</td><td>Euryarchaeota</td><td>ANME-1 cluster archaeon AG-394-G21</td><td>GTP-binding protein [ANME-1 cluster archaeon AG-394-G21].</td><td>GCA_009903435.1</td></tr>
<tr><td>NAT10404.1</td><td>Cluster1536_2clades→||<-?<-?<-?||POLYSACPOLYMERASE→?→ LRR-repeats+AP-GTPase+COR→ LRR-repeats→ LRR-repeats+AP-GTPase+COR→?→||<-PIN</td><td>LRR-repeats+AP-GTPase+COR</td><td>688</td><td>C4E22_02460</td><td>Euryarchaeota</td><td>ANME-1 cluster archaeon AG-394-G06</td><td>GTP-binding protein [ANME-1 cluster archaeon AG-394-G06].</td><td>GCA_009903405.1</td></tr>
<tr><td>NBC32680.1</td><td>LRR-repeats+AP-GTPase+COR+TM+TM+TM+TM*→||<-?<-?||?→?→?→||<-TM+RHOMBOID</td><td>LRR-repeats+AP-GTPase+COR+TM+TM+TM+TM</td><td>806</td><td>GVY13_08395</td><td>Alphaproteobacteria</td><td>Alphaproteobacteria bacterium</td><td>GTP-binding protein, partial [Alphaproteobacteria bacterium].</td><td>GCA_009909005.1</td></tr>
<tr><td>NCP86657.1</td><td><-XIS-HTH||HTH+HTH→||<-WLM||?→ NACHT+68TM-wHTH→ REase→?→ LRR-repeats+AP-GTPase+COR*→</td><td>LRR-repeats+AP-GTPase+COR</td><td>960</td><td>GW829_04700</td><td>unclassified Bacteria</td><td>bacterium</td><td>hypothetical protein GW829_04700, partial [bacterium].</td><td>GCA_009994085.1</td></tr>
<tr><td>NCP88511.1</td><td>REase→ LRR-repeats+AP-GTPase+COR*→</td><td>LRR-repeats+AP-GTPase+COR</td><td>336</td><td>GW829_14210</td><td>unclassified Bacteria</td><td>bacterium</td><td>hypothetical protein GW829_14210, partial [bacterium].</td><td>GCA_009994085.1</td></tr>
<tr><td>NCP88560.1</td><td>LRR-repeats+AP-GTPase+COR*→</td><td>LRR-repeats+AP-GTPase+COR</td><td>382</td><td>GW829_14460</td><td>unclassified Bacteria</td><td>bacterium</td><td>hypothetical protein GW829_14460, partial [bacterium].</td><td>GCA_009994085.1</td></tr>
<tr><td>NCQ69077.1</td><td>CHTH→ LRR-repeats+AP-GTPase+COR*→</td><td>LRR-repeats+AP-GTPase+COR</td><td>382</td><td>GPI97_07600</td><td>Cyanobacteria</td><td>Microcystis aeruginosa W13-16</td><td>leucine-rich repeat protein, partial [Microcystis aeruginosa W13-16].</td><td>GCA_009995625.1</td></tr>
<tr><td>NCQ78095.1</td><td>CHTH→ LRR-repeats+AP-GTPase+COR*→</td><td>LRR-repeats+AP-GTPase+COR</td><td>359</td><td>GPI99_08005</td><td>Cyanobacteria</td><td>Microcystis aeruginosa W13-15</td><td>GTP-binding protein, partial [Microcystis aeruginosa W13-15].</td><td>GCA_009995645.1</td></tr>
<tr><td>NCQ94522.1</td><td>Ferredoxin-betagrasp→ tRNA→ tRNA→ SAM-methylase→?→||<-?<-SIG+TPM_phosphatase+TM+TM+TM||LRR-repeats+AP-GTPase+COR+TM+TM*→||<-?<-?<-?<-Pro_CA</td><td>LRR-repeats+AP-GTPase+COR+TM+TM</td><td>805</td><td>GPJ33_04160</td><td>Cyanobacteria</td><td>Microcystis aeruginosa W11-03</td><td>leucine-rich repeat protein [Microcystis aeruginosa W11-03].</td><td>GCA_009995775.1</td></tr>
<tr><td>NCR15129.1</td><td>CHTH→ LRR-repeats+AP-GTPase+COR*→</td><td>LRR-repeats+AP-GTPase+COR</td><td>413</td><td>GPJ24_21065</td><td>Cyanobacteria</td><td>Microcystis aeruginosa SX13-11</td><td>GTP-binding protein, partial [Microcystis aeruginosa SX13-11].</td><td>GCA_009995725.1</td></tr>
<tr><td>NCR28927.1</td><td>CHTH→ LRR-repeats+AP-GTPase+COR*→</td><td>LRR-repeats+AP-GTPase+COR</td><td>386</td><td>GPJ25_22505</td><td>Cyanobacteria</td><td>Microcystis aeruginosa LE13-04</td><td>leucine-rich repeat protein, partial [Microcystis aeruginosa LE13-04].</td><td>GCA_009995995.1</td></tr>
<tr><td>NCR34650.1</td><td>LRR-repeats+AP-GTPase+COR+TM+TM*→</td><td>LRR-repeats+AP-GTPase+COR+TM+TM</td><td>789</td><td>GPJ31_03655</td><td>Cyanobacteria</td><td>Microcystis aeruginosa S11-05</td><td>leucine-rich repeat protein [Microcystis aeruginosa S11-05].</td><td>GCA_009995785.1</td></tr>
<tr><td>NCR46978.1</td><td>CHTH→ LRR-repeats+AP-GTPase+COR*→</td><td>LRR-repeats+AP-GTPase+COR</td><td>514</td><td>GPJ09_24595</td><td>Cyanobacteria</td><td>Microcystis aeruginosa SX13-01</td><td>leucine-rich repeat protein, partial [Microcystis aeruginosa SX13-01].</td><td>GCA_009995735.1</td></tr>
<tr><td>NCR55589.1</td><td>LRR-repeats+AP-GTPase+COR+TM+TM*→||<-?<-?<-?<-Pro_CA||?→||<-?<-tRNA<-tRNA<-Ferredoxin-betagrasp</td><td>LRR-repeats+AP-GTPase+COR+TM+TM</td><td>710</td><td>GPJ10_20085</td><td>Cyanobacteria</td><td>Microcystis aeruginosa L211-07</td><td>GTP-binding protein, partial [Microcystis aeruginosa L211-07].</td><td>GCA_009996085.1</td></tr>
<tr><td>NCR60375.1</td><td>CHTH→ LRR-repeats+AP-GTPase+COR*→</td><td>LRR-repeats+AP-GTPase+COR</td><td>478</td><td>GPJ01_22875</td><td>Cyanobacteria</td><td>Microcystis aeruginosa LL13-06</td><td>GTP-binding protein, partial [Microcystis aeruginosa LL13-06].</td><td>GCA_009995895.1</td></tr>
<tr><td>NCR68842.1</td><td>CHTH→ LRR-repeats+AP-GTPase+COR*→</td><td>LRR-repeats+AP-GTPase+COR</td><td>545</td><td>GPJ34_21175</td><td>Cyanobacteria</td><td>Microcystis aeruginosa LL11-07</td><td>GTP-binding protein, partial [Microcystis aeruginosa LL11-07].</td><td>GCA_009995855.1</td></tr>
<tr><td>NCR71934.1</td><td>SAM-methylase→?→||<-?<-SIG+TPM_phosphatase+TM+TM+TM||LRR-repeats+AP-GTPase+COR+TM+TM*→||<-?<-?<-?<-Pro_CA</td><td>LRR-repeats+AP-GTPase+COR+TM+TM</td><td>751</td><td>GPI93_12415</td><td>Cyanobacteria</td><td>Microcystis aeruginosa LG13-12</td><td>leucine-rich repeat protein [Microcystis aeruginosa LG13-12].</td><td>GCA_009995925.1</td></tr>
<tr><td>NCR77025.1</td><td>SIG+TM+TM+TM+TM+TM+TM+TM→||<-?||Mbetalac→ SAM-methylase→?→||<-?<-SIG+TPM_phosphatase+TM+TM+TM||LRR-repeats+AP-GTPase+COR+TM+TM*→</td><td>LRR-repeats+AP-GTPase+COR+TM+TM</td><td>897</td><td>GPI92_16105</td><td>Cyanobacteria</td><td>Microcystis aeruginosa K13-06</td><td>GTP-binding protein [Microcystis aeruginosa K13-06].</td><td>GCA_009996165.1</td></tr>
<tr><td>NCR80564.1</td><td>SIG+TM+TM+TM+TM+TM+TM+TM→||<-?||Mbetalac→ SAM-methylase→?→||<-?<-SIG+TPM_phosphatase+TM+TM+TM||LRR-repeats+AP-GTPase+COR+TM+TM*→</td><td>LRR-repeats+AP-GTPase+COR+TM+TM</td><td>805</td><td>GPI91_11595</td><td>Cyanobacteria</td><td>Microcystis aeruginosa K13-10</td><td>leucine-rich repeat protein [Microcystis aeruginosa K13-10].</td><td>GCA_009996105.1</td></tr>
<tr><td>NCR97058.1</td><td>SIG+TM+TM+TM+TM+TM+TM+TM→||<-?||Mbetalac→ SAM-methylase→?→||<-?<-SIG+TPM_phosphatase+TM+TM+TM||LRR-repeats+AP-GTPase+COR+TM+TM*→||<-?<-?<-?<-Pro_CA||?→||<-?<-tRNA<-tRNA<-Ferredoxin-betagrasp</td><td>LRR-repeats+AP-GTPase+COR+TM+TM</td><td>920</td><td>GPJ32_04180</td><td>Cyanobacteria</td><td>Microcystis aeruginosa L311-01</td><td>GTP-binding protein [Microcystis aeruginosa L311-01].</td><td>GCA_009996025.1</td></tr>
<tr><td>NCS08858.1</td><td>CHTH→ LRR-repeats+AP-GTPase+COR*→</td><td>LRR-repeats+AP-GTPase+COR</td><td>386</td><td>GPJ07_21130</td><td>Cyanobacteria</td><td>Microcystis aeruginosa G13-07</td><td>GTP-binding protein, partial [Microcystis aeruginosa G13-07].</td><td>GCA_009996305.1</td></tr>
<tr><td>NCS11263.1</td><td>CHTH→ LRR-repeats+AP-GTPase+COR+TM+TM*→</td><td>LRR-repeats+AP-GTPase+COR+TM+TM</td><td>852</td><td>GPJ08_08960</td><td>Cyanobacteria</td><td>Microcystis aeruginosa G13-09</td><td>GTP-binding protein [Microcystis aeruginosa G13-09].</td><td>GCA_009996255.1</td></tr>
<tr><td>NCS17812.1</td><td>CHTH→ LRR-repeats+AP-GTPase+COR*→</td><td>LRR-repeats+AP-GTPase+COR</td><td>503</td><td>GPJ02_20805</td><td>Cyanobacteria</td><td>Microcystis aeruginosa G13-12</td><td>leucine-rich repeat protein, partial [Microcystis aeruginosa G13-12].</td><td>GCA_009996205.1</td></tr>
<tr><td>NCS22942.1</td><td>LRR-repeats+AP-GTPase+COR*→</td><td>LRR-repeats+AP-GTPase+COR</td><td>353</td><td>GPJ15_26105</td><td>Cyanobacteria</td><td>Microcystis aeruginosa G11-06</td><td>GTP-binding protein, partial [Microcystis aeruginosa G11-06].</td><td>GCA_009996385.1</td></tr>
<tr><td>NCS24651.1</td><td>LRR-repeats+AP-GTPase+COR+TM+TM*→||<-?<-FGS<-?<-?||?→?→ Aminotran_1_2→</td><td>LRR-repeats+AP-GTPase+COR+TM+TM</td><td>732</td><td>GPI96_09100</td><td>Cyanobacteria</td><td>Microcystis aeruginosa BS13-02</td><td>GTP-binding protein, partial [Microcystis aeruginosa BS13-02].</td><td>GCA_009996485.1</td></tr>
<tr><td>NCS52102.1</td><td>CHTH→ LRR-repeats+AP-GTPase+COR*→</td><td>LRR-repeats+AP-GTPase+COR</td><td>397</td><td>GPJ23_06895</td><td>Cyanobacteria</td><td>Microcystis aeruginosa G13-05</td><td>leucine-rich repeat protein, partial [Microcystis aeruginosa G13-05].</td><td>GCA_009996285.1</td></tr>
</table>

<table>
<tr><th>acc</th><th>operon</th><th>architecture</th><th>len</th><th>gen.name</th><th>taxend</th><th>species</th><th>defline</th><th>gca</th></tr>
<tr><td>NCS59859.1</td><td>LRR-repeats+AP-GTPase+COR*→</td><td>LRR-repeats+AP-GTPase+COR</td><td>339</td><td>GPJ16_25035</td><td>Cyanobacteria</td><td>Microcystis aeruginosa G11-04</td><td>GTP-binding protein, partial [Microcystis aeruginosa G11-04].</td><td>GCA_009996445.1</td></tr>
<tr><td>NCT21185.1</td><td><-XIS-HTH||HTH+HTH→||<-WLM||?→ NACHT+68TM-wHTH→ REase→?→ LRR-repeats+AP-GTPase+COR*→</td><td>LRR-repeats+AP-GTPase+COR</td><td>956</td><td>GW781_08530</td><td>unclassified Bacteria</td><td>bacterium</td><td>hypothetical protein GW781_08530, partial [bacterium].</td><td>GCA_009994975.1</td></tr>
<tr><td>NCT65324.1</td><td>CHTH→ LRR-repeats+AP-GTPase+COR*→</td><td>LRR-repeats+AP-GTPase+COR</td><td>434</td><td>GPJ03_21265</td><td>Cyanobacteria</td><td>Microcystis aeruginosa G13-01</td><td>leucine-rich repeat protein, partial [Microcystis aeruginosa G13-01].</td><td>GCA_009996325.1</td></tr>
<tr><td>NCY16589.1</td><td>LRR-repeats+AP-GTPase+COR+TIR*→</td><td>LRR-repeats+AP-GTPase+COR+TIR</td><td>1058</td><td>EBX39_07430</td><td>Actinobacteria</td><td>Actinobacteria bacterium</td><td>hypothetical protein EBX39_07430 [Actinobacteria bacterium].</td><td>GCA_010021725.1</td></tr>
<tr><td>NEN97779.1</td><td>REC→||<-TPR-repeats||LRR-repeats+AP-GTPase+COR+DUF4404*→</td><td>LRR-repeats+AP-GTPase+COR+DUF4404</td><td>971</td><td>F6K50_20310</td><td>Cyanobacteria</td><td>Moorea sp. SIO3I7</td><td>GTPase [Moorea sp. SIO3I7].</td><td>GCA_010692375.1</td></tr>
<tr><td>NEO07696.1</td><td>SIG+TM+TM+TM+TM+TM+TM+TM→?→ ABC-ATPase→?→ ABC-ATPase→?→ LRR-repeats+AP-GTPase+COR*→</td><td>LRR-repeats+AP-GTPase+COR</td><td>1373</td><td>F6K51_19885</td><td>Cyanobacteria</td><td>Moorea sp. SIO3I8</td><td>leucine-rich repeat protein [Moorea sp. SIO3I8].</td><td>GCA_010692325.1</td></tr>
<tr><td>NEO14597.1</td><td>REC→||<-TPR-repeats<-?||LRR-repeats+AP-GTPase+COR+DUF4404*→</td><td>LRR-repeats+AP-GTPase+COR+DUF4404</td><td>797</td><td>F6K46_20370</td><td>Cyanobacteria</td><td>Moorea sp. SIO3E8</td><td>GTPase [Moorea sp. SIO3E8].</td><td>GCA_010692445.1</td></tr>
<tr><td>NEO18066.1</td><td>ABC-ATPase→||<-?||ABC-ATPase→?→?→||<-?||LRR-repeats+AP-GTPase+COR*→?→||<-?<-HNH<-PSE||?→||<-Cluster5_5clades</td><td>LRR-repeats+AP-GTPase+COR</td><td>852</td><td>F6K57_01150</td><td>Cyanobacteria</td><td>Moorea sp. SIO4A5</td><td>GTPase [Moorea sp. SIO4A5].</td><td>GCA_010672005.1</td></tr>
<tr><td>NEO21732.1</td><td>REC→||<-?||REC→||<-TPR-repeats||LRR-repeats+AP-GTPase+COR+DUF4404*→</td><td>LRR-repeats+AP-GTPase+COR+DUF4404</td><td>949</td><td>F6K57_21060</td><td>Cyanobacteria</td><td>Moorea sp. SIO4A5</td><td>GTPase [Moorea sp. SIO4A5].</td><td>GCA_010672005.1</td></tr>
<tr><td>NEO28667.1</td><td>LRR-repeats+AP-GTPase+COR*→</td><td>LRR-repeats+AP-GTPase+COR</td><td>372</td><td>F6K03_17765</td><td>Cyanobacteria</td><td>Kamptonema sp. SIO4C4</td><td>GTPase, partial [Kamptonema sp. SIO4C4].</td><td>GCA_010692335.1</td></tr>
<tr><td>NEO36773.1</td><td>SIG+TM+TM+TM+TM+TM+TM+TM→ ABC-ATPase→?→ ABC-ATPase→||<-?||?→ LRR-repeats+AP-GTPase+COR*→</td><td>LRR-repeats+AP-GTPase+COR</td><td>847</td><td>F6J90_10815</td><td>Cyanobacteria</td><td>Moorea sp. SIOASIH</td><td>GTPase [Moorea sp. SIOASIH].</td><td>GCA_010671925.1</td></tr>
<tr><td>NEO37599.1</td><td>REC→||<-?<-?||REC→||<-TPR-repeats<-?||?→ LRR-repeats+AP-GTPase+COR+DUF4404*→ PSE→||<-?<-PSE||?→?→?→||<-?||TM→</td><td>LRR-repeats+AP-GTPase+COR+DUF4404</td><td>1099</td><td>F6J90_15180</td><td>Cyanobacteria</td><td>Moorea sp. SIOASIH</td><td>leucine-rich repeat protein [Moorea sp. SIOASIH].</td><td>GCA_010671925.1</td></tr>
<tr><td>NEO43843.1</td><td>SbcC→?→ LRR-repeats+AP-GTPase+COR*→</td><td>LRR-repeats+AP-GTPase+COR</td><td>1256</td><td>F6K55_06775</td><td>Cyanobacteria</td><td>Moorea sp. SIO4A3</td><td>leucine-rich repeat protein [Moorea sp. SIO4A3].</td><td>GCA_010672045.1</td></tr>
<tr><td>NEO48162.1</td><td>REC→||<-TPR-repeats||LRR-repeats+AP-GTPase+COR*→</td><td>LRR-repeats+AP-GTPase+COR</td><td>616</td><td>F6K55_30210</td><td>Cyanobacteria</td><td>Moorea sp. SIO4A3</td><td>leucine-rich repeat protein, partial [Moorea sp. SIO4A3].</td><td>GCA_010672045.1</td></tr>
<tr><td>NEO55671.1</td><td>LRR-repeats→?→ AP-GTPase*→||<-?<-SIG+Trypsin+PDZ</td><td>AP-GTPase</td><td>113</td><td>F6K54_22940</td><td>Cyanobacteria</td><td>Okeania sp. SIO3B5</td><td>hypothetical protein F6K54_22940 [Okeania sp. SIO3B5].</td><td>GCA_010692555.1</td></tr>
<tr><td>NEO56464.1</td><td>LRR-repeats+AP-GTPase+COR*→||<-RelE-ParE</td><td>LRR-repeats+AP-GTPase+COR</td><td>856</td><td>F6K54_27310</td><td>Cyanobacteria</td><td>Okeania sp. SIO3B5</td><td>GTPase [Okeania sp. SIO3B5].</td><td>GCA_010692555.1</td></tr>
<tr><td>NEO57234.1</td><td><-RADICAL-SAM||PSE→ REC→?→ LRR-repeats+AP-GTPase+COR*→</td><td>LRR-repeats+AP-GTPase+COR</td><td>472</td><td>F6K54_31710</td><td>Cyanobacteria</td><td>Okeania sp. SIO3B5</td><td>GTPase, partial [Okeania sp. SIO3B5].</td><td>GCA_010692555.1</td></tr>
<tr><td>NEO68320.1</td><td>SIG+PBPI→?→ SIG+TM+TM+TM+TM+TM+TM+TM→?→ ABC-ATPase→ ABC-ATPase→||<-?||LRR-repeats+AP-GTPase+COR*→</td><td>LRR-repeats+AP-GTPase+COR</td><td>884</td><td>F6K52_02085</td><td>Cyanobacteria</td><td>Moorea sp. SIO3H5</td><td>leucine-rich repeat protein [Moorea sp. SIO3H5].</td><td>GCA_010692395.1</td></tr>
<tr><td>NEO73423.1</td><td>REC→||<-TPR-repeats<-?||LRR-repeats+AP-GTPase+COR+DUF4404*→</td><td>LRR-repeats+AP-GTPase+COR+DUF4404</td><td>933</td><td>F6K52_29165</td><td>Cyanobacteria</td><td>Moorea sp. SIO3H5</td><td>GTPase [Moorea sp. SIO3H5].</td><td>GCA_010692395.1</td></tr>
<tr><td>NEO79069.1</td><td>REC→||<-TPR-repeats||LRR-repeats+AP-GTPase+COR+DUF4404*→</td><td>LRR-repeats+AP-GTPase+COR+DUF4404</td><td>929</td><td>F6J99_23600</td><td>Cyanobacteria</td><td>Moorea sp. SIO4G3</td><td>GTPase [Moorea sp. SIO4G3].</td><td>GCA_010692305.1</td></tr>
<tr><td>NEO83336.1</td><td>Cluster5_5clades→?→ PSE→ LRR-repeats+AP-GTPase+COR*→</td><td>LRR-repeats+AP-GTPase+COR</td><td>1006</td><td>F6J87_03595</td><td>Cyanobacteria</td><td>Spirulina sp. SIO3F2</td><td>leucine-rich repeat protein [Spirulina sp. SIO3F2].</td><td>GCA_010672165.1</td></tr>
<tr><td>NEO85545.1</td><td>MNS-STAND+CR-REase3→||<-?<-?||?→||<-REC<-REC||HISKIN→ LRR-repeats+AP-GTPase+COR*→||<-HISKIN</td><td>LRR-repeats+AP-GTPase+COR</td><td>1127</td><td>F6J87_15045</td><td>Cyanobacteria</td><td>Spirulina sp. SIO3F2</td><td>GTPase [Spirulina sp. SIO3F2].</td><td>GCA_010672165.1</td></tr>
<tr><td>NEO86840.1</td><td>TM+TM+TM+TM+TM+TM+TM+TM→?→||<-?<-Pentapeptide-repeats||?→ LRR-repeats+AP-GTPase+COR+DUF4404*→</td><td>LRR-repeats+AP-GTPase+COR+DUF4404</td><td>1131</td><td>F6J87_21660</td><td>Cyanobacteria</td><td>Spirulina sp. SIO3F2</td><td>GTP-binding protein [Spirulina sp. SIO3F2].</td><td>GCA_010672165.1</td></tr>
<tr><td>NEO88027.1</td><td>LRR-repeats→ LRR-repeats+AP-GTPase+COR*→</td><td>LRR-repeats+AP-GTPase+COR</td><td>800</td><td>F6J87_27790</td><td>Cyanobacteria</td><td>Spirulina sp. SIO3F2</td><td>hypothetical protein F6J87_27790 [Spirulina sp. SIO3F2].</td><td>GCA_010672165.1</td></tr>
<tr><td>NEO88064.1</td><td>Cluster1048_2clades→ LRR-repeats+AP-GTPase+COR*→</td><td>LRR-repeats+AP-GTPase+COR</td><td>1166</td><td>F6J87_27980</td><td>Cyanobacteria</td><td>Spirulina sp. SIO3F2</td><td>GTPase [Spirulina sp. SIO3F2].</td><td>GCA_010672165.1</td></tr>
<tr><td>NEO88189.1</td><td>LRR-repeats+AP-GTPase+COR*→</td><td>LRR-repeats+AP-GTPase+COR</td><td>1134</td><td>F6J87_28620</td><td>Cyanobacteria</td><td>Spirulina sp. SIO3F2</td><td>hypothetical protein F6J87_28620 [Spirulina sp. SIO3F2].</td><td>GCA_010672165.1</td></tr>
<tr><td>NEP02262.1</td><td>LRR-repeats+AP-GTPase+COR*→ ParA-Soj-PloopNTPase→</td><td>LRR-repeats+AP-GTPase+COR</td><td>814</td><td>F6K58_27120</td><td>Cyanobacteria</td><td>Symploca sp. SIO2E9</td><td>leucine-rich repeat protein [Symploca sp. SIO2E9].</td><td>GCA_010692645.1</td></tr>
<tr><td>NEP15532.1</td><td>LRR-repeats+AP-GTPase+COR+TIR*→?→||<-?||?→||<-?<-?<-?||ABhydrolase→</td><td>LRR-repeats+AP-GTPase+COR+TIR</td><td>1041</td><td>F6J97_01370</td><td>Cyanobacteria</td><td>Leptolyngbya sp. SIO4C1</td><td>TIR domain-containing protein [Leptolyngbya sp. SIO4C1].</td><td>GCA_010671975.1</td></tr>
</table>

<table>
<tr><th>acc</th><th>operon</th><th>architecture</th><th>len</th><th>gen.name</th><th>taxend</th><th>species</th><th>defline</th><th>gca</th></tr>
<tr><td>NEP17061.1</td><td>TM+TM+TM+TM+TM→ LRR-repeats+AP-GTPase+COR+DUF4404*→||<-SIG+TM+TM</td><td>LRR-repeats+AP-GTPase+COR+DUF4404</td><td>1026</td><td>F6J97_09150</td><td>Cyanobacteria</td><td>Leptolyngbya sp. SIO4C1</td><td>GTP-binding protein [Leptolyngbya sp. SIO4C1].</td><td>GCA_010671975.1</td></tr>
<tr><td>NEP23180.1</td><td>SIG+TM+TM+TM+TM+TM+TM+TM→?→ ABC-ATPase→ ABC-ATPase→||<-?||?→||<-?||LRR-repeats+AP-GTPase+COR*→?→||<-?<-HNH<-PSE||?→||<-Cluster5_5clades</td><td>LRR-repeats+AP-GTPase+COR</td><td>892</td><td>F6K49_14560</td><td>Cyanobacteria</td><td>Moorea sp. SIO3I6</td><td>GTPase [Moorea sp. SIO3I6].</td><td>GCA_010672095.1</td></tr>
<tr><td>NEP27050.1</td><td>LRR-repeats+AP-GTPase+COR+DUF4404*→</td><td>LRR-repeats+AP-GTPase+COR+DUF4404</td><td>909</td><td>F6K49_35465</td><td>Cyanobacteria</td><td>Moorea sp. SIO3I6</td><td>GTPase [Moorea sp. SIO3I6].</td><td>GCA_010672095.1</td></tr>
<tr><td>NEP34707.1</td><td>LRR-repeats+AP-GTPase+COR+DUF4404*→</td><td>LRR-repeats+AP-GTPase+COR+DUF4404</td><td>924</td><td>F6K38_25730</td><td>Cyanobacteria</td><td>Moorea sp. SIO3B2</td><td>GTPase, partial [Moorea sp. SIO3B2].</td><td>GCA_010692525.1</td></tr>
<tr><td>NEP80618.1</td><td>LRR-repeats+AP-GTPase+COR*→</td><td>LRR-repeats+AP-GTPase+COR</td><td>891</td><td>F6K39_22060</td><td>Cyanobacteria</td><td>Okeania sp. SIO3B3</td><td>GTPase [Okeania sp. SIO3B3].</td><td>GCA_010692535.1</td></tr>
<tr><td>NEP81305.1</td><td>LRR-repeats+AP-GTPase+COR*→</td><td>LRR-repeats+AP-GTPase+COR</td><td>790</td><td>F6K39_25960</td><td>Cyanobacteria</td><td>Okeania sp. SIO3B3</td><td>GTPase [Okeania sp. SIO3B3].</td><td>GCA_010692535.1</td></tr>
<tr><td>NEP82037.1</td><td>LRR-repeats+AP-GTPase+COR*→</td><td>LRR-repeats+AP-GTPase+COR</td><td>664</td><td>F6K39_30180</td><td>Cyanobacteria</td><td>Okeania sp. SIO3B3</td><td>hypothetical protein F6K39_30180 [Okeania sp. SIO3B3].</td><td>GCA_010692535.1</td></tr>
<tr><td>NEP82994.1</td><td>LRR-repeats+AP-GTPase*→</td><td>LRR-repeats+AP-GTPase</td><td>713</td><td>F6K39_35565</td><td>Cyanobacteria</td><td>Okeania sp. SIO3B3</td><td>leucine-rich repeat protein [Okeania sp. SIO3B3].</td><td>GCA_010692535.1</td></tr>
<tr><td>NEP88726.1</td><td><-HhH-RADC+JAB<-?||LRR-repeats+AP-GTPase+COR*→</td><td>LRR-repeats+AP-GTPase+COR</td><td>800</td><td>F6K18_18835</td><td>Cyanobacteria</td><td>Okeania sp. SIO2C2</td><td>GTPase [Okeania sp. SIO2C2].</td><td>GCA_010692625.1</td></tr>
<tr><td>NEQ25590.1</td><td>LRR-repeats+AP-GTPase+COR*→</td><td>LRR-repeats+AP-GTPase+COR</td><td>329</td><td>F6K28_42320</td><td>Cyanobacteria</td><td>Microcoleus sp. SIO2G3</td><td>hypothetical protein F6K28_42320, partial [Microcoleus sp. SIO2G3].</td><td>GCA_010672365.1</td></tr>
<tr><td>NEQ30222.1</td><td>SF2-DUF3427A→||<-PSE||?→ LRR-repeats+AP-GTPase+COR*→</td><td>LRR-repeats+AP-GTPase+COR</td><td>425</td><td>F6K04_04355</td><td>Cyanobacteria</td><td>Leptolyngbya sp. SIO4C5</td><td>GTP-binding protein, partial [Leptolyngbya sp. SIO4C5].</td><td>GCA_010671965.1</td></tr>
<tr><td>NEQ30763.1</td><td>LRR-repeats+AP-GTPase+COR+DrHyd*→||<-SAM-methylase</td><td>LRR-repeats+AP-GTPase+COR+DrHyd</td><td>1168</td><td>F6K04_07140</td><td>Cyanobacteria</td><td>Leptolyngbya sp. SIO4C5</td><td>hypothetical protein F6K04_07140 [Leptolyngbya sp. SIO4C5].</td><td>GCA_010671965.1</td></tr>
<tr><td>NEQ30823.1</td><td>LRR-repeats+AP-GTPase+COR*→</td><td>LRR-repeats+AP-GTPase+COR</td><td>1087</td><td>F6K04_07455</td><td>Cyanobacteria</td><td>Leptolyngbya sp. SIO4C5</td><td>hypothetical protein F6K04_07455 [Leptolyngbya sp. SIO4C5].</td><td>GCA_010671965.1</td></tr>
<tr><td>NEQ33180.1</td><td>LRR-repeats+AP-GTPase+COR+TIR*→||<-Cluster2398_2clades</td><td>LRR-repeats+AP-GTPase+COR+TIR</td><td>974</td><td>F6K04_19645</td><td>Cyanobacteria</td><td>Leptolyngbya sp. SIO4C5</td><td>TIR domain-containing protein [Leptolyngbya sp. SIO4C5].</td><td>GCA_010671965.1</td></tr>
<tr><td>NEQ38623.1</td><td>LRR-repeats→||<-?||?→ LRR-repeats+AP-GTPase+COR*→</td><td>LRR-repeats+AP-GTPase+COR</td><td>778</td><td>F6K40_21075</td><td>Cyanobacteria</td><td>Okeania sp. SIO3I5</td><td>GTPase [Okeania sp. SIO3I5].</td><td>GCA_010672085.1</td></tr>
<tr><td>NEQ39708.1</td><td>LRR-repeats+AP-GTPase+COR*→</td><td>LRR-repeats+AP-GTPase+COR</td><td>641</td><td>F6K40_27040</td><td>Cyanobacteria</td><td>Okeania sp. SIO3I5</td><td>hypothetical protein F6K40_27040 [Okeania sp. SIO3I5].</td><td>GCA_010672085.1</td></tr>
<tr><td>NEQ41322.1</td><td>LRR-repeats+AP-GTPase+COR*→</td><td>LRR-repeats+AP-GTPase+COR</td><td>923</td><td>F6K40_36055</td><td>Cyanobacteria</td><td>Okeania sp. SIO3I5</td><td>GTPase [Okeania sp. SIO3I5].</td><td>GCA_010672085.1</td></tr>
<tr><td>NEQ51480.1</td><td>LRR-repeats+AP-GTPase+COR+DrHyd*→||<-?||?→||<-SF2-DUF3427A</td><td>LRR-repeats+AP-GTPase+COR+DrHyd</td><td>841</td><td>F6K11_15280</td><td>Cyanobacteria</td><td>Leptolyngbya sp. SIO3F4</td><td>hypothetical protein F6K11_15280, partial [Leptolyngbya sp. SIO3F4].</td><td>GCA_010672145.1</td></tr>
<tr><td>NEQ51880.1</td><td>LRR-repeats+AP-GTPase+COR+TM+TM*→</td><td>LRR-repeats+AP-GTPase+COR+TM+TM</td><td>1022</td><td>F6K11_17360</td><td>Cyanobacteria</td><td>Leptolyngbya sp. SIO3F4</td><td>GTP-binding protein [Leptolyngbya sp. SIO3F4].</td><td>GCA_010672145.1</td></tr>
<tr><td>NEQ52141.1</td><td>LRR-repeats+AP-GTPase+COR+TIR*→</td><td>LRR-repeats+AP-GTPase+COR+TIR</td><td>825</td><td>F6K11_18705</td><td>Cyanobacteria</td><td>Leptolyngbya sp. SIO3F4</td><td>GTP-binding protein, partial [Leptolyngbya sp. SIO3F4].</td><td>GCA_010672145.1</td></tr>
<tr><td>NEQ52634.1</td><td>LRR-repeats+AP-GTPase+COR+TIR*→ LRR-repeats→</td><td>LRR-repeats+AP-GTPase+COR+TIR</td><td>827</td><td>F6K11_21260</td><td>Cyanobacteria</td><td>Leptolyngbya sp. SIO3F4</td><td>TIR domain-containing protein, partial [Leptolyngbya sp. SIO3F4].</td><td>GCA_010672145.1</td></tr>
<tr><td>NEQ54121.1</td><td>LRR-repeats+AP-GTPase+COR+TIR*→</td><td>LRR-repeats+AP-GTPase+COR+TIR</td><td>826</td><td>F6K11_28985</td><td>Cyanobacteria</td><td>Leptolyngbya sp. SIO3F4</td><td>TIR domain-containing protein, partial [Leptolyngbya sp. SIO3F4].</td><td>GCA_010672145.1</td></tr>
<tr><td>NEQ54707.1</td><td>LRR-repeats+AP-GTPase+COR*→</td><td>LRR-repeats+AP-GTPase+COR</td><td>547</td><td>F6K11_32030</td><td>Cyanobacteria</td><td>Leptolyngbya sp. SIO3F4</td><td>GTP-binding protein, partial [Leptolyngbya sp. SIO3F4].</td><td>GCA_010672145.1</td></tr>
<tr><td>NEQ55863.1</td><td>LRR-repeats+AP-GTPase+COR*→</td><td>LRR-repeats+AP-GTPase+COR</td><td>335</td><td>F6K11_38165</td><td>Cyanobacteria</td><td>Leptolyngbya sp. SIO3F4</td><td>serine/threonine protein kinase, partial [Leptolyngbya sp. SIO3F4].</td><td>GCA_010672145.1</td></tr>
<tr><td>NEQ58292.1</td><td>SIG+TM+TM+TM+TM+TM+TM+TM→?→ ABC-ATPase→ ABC-ATPase→?→||<-?||LRR-repeats+AP-GTPase+COR*→?→||<-?<-HNH<-PSE||?→||<-Cluster5_5clades</td><td>LRR-repeats+AP-GTPase+COR</td><td>849</td><td>F6K53_13090</td><td>Cyanobacteria</td><td>Moorea sp. SIO4A1</td><td>GTPase [Moorea sp. SIO4A1].</td><td>GCA_010672065.1</td></tr>
<tr><td>NEQ85055.1</td><td>LRR-repeats+AP-GTPase+COR*→</td><td>LRR-repeats+AP-GTPase+COR</td><td>554</td><td>F6K26_34455</td><td>Cyanobacteria</td><td>Moorea sp. SIO2I5</td><td>GTPase, partial [Moorea sp. SIO2I5].</td><td>GCA_010672285.1</td></tr>
<tr><td>NER03418.1</td><td>LRR-repeats+AP-GTPase+COR*→</td><td>LRR-repeats+AP-GTPase+COR</td><td>738</td><td>F6K17_12770</td><td>Cyanobacteria</td><td>Okeania sp. SIO3C4</td><td>GTPase, partial [Okeania sp. SIO3C4].</td><td>GCA_010672215.1</td></tr>
<tr><td>NER25299.1</td><td>TM+TM+TM+TM+TM+TM→||<-?||?→ Pkinase→?→ LRR-repeats+AP-GTPase+COR*→ Cluster5_5clades→</td><td>LRR-repeats+AP-GTPase+COR</td><td>1031</td><td>F6J96_32295</td><td>Cyanobacteria</td><td>Symploca sp. SIO1C2</td><td>hypothetical protein F6J96_32295 [Symploca sp. SIO1C2].</td><td>GCA_010672925.1</td></tr>
</table>

<table>
<tr><th>acc</th><th>operon</th><th>architecture</th><th>len</th><th>gen.name</th><th>taxend</th><th>species</th><th>defline</th><th>gca</th></tr>
<tr><td>NER33110.1</td><td>SIG+TM+TM+TM+TM+TM+TM+TM→?→||<-?||PSE→||<-?||?→ LRR-repeats+AP-GTPase+COR*→?→?→?→ ABC_membrane+ABC_tran→||<-NUDIX</td><td>LRR-repeats+AP-GTPase+COR</td><td>1105</td><td>F6J93_03360</td><td>Cyanobacteria</td><td>Oscillatoria sp. SIO1A7</td><td>hypothetical protein F6J93_03360 [Oscillatoria sp. SIO1A7].</td><td>GCA_010672965.1</td></tr>
<tr><td>NER35832.1</td><td>LRR-repeats+AP-GTPase+COR*→?→ PIN→</td><td>LRR-repeats+AP-GTPase+COR</td><td>1467</td><td>F6J93_17900</td><td>Cyanobacteria</td><td>Oscillatoria sp. SIO1A7</td><td>hypothetical protein F6J93_17900 [Oscillatoria sp. SIO1A7].</td><td>GCA_010672965.1</td></tr>
<tr><td>NER38074.1</td><td><-SIG+TM+TM+TM+TM+TM<-ABC-ATPase||?→?→||<-?||?→?→ LRR-repeats+AP-GTPase+COR*→?→?→?→?→ ABC-ATPase→</td><td>LRR-repeats+AP-GTPase+COR</td><td>1117</td><td>F6J93_29640</td><td>Cyanobacteria</td><td>Oscillatoria sp. SIO1A7</td><td>hypothetical protein F6J93_29640 [Oscillatoria sp. SIO1A7].</td><td>GCA_010672965.1</td></tr>
<tr><td>NER40156.1</td><td>LRR-repeats→ LRR-repeats+AP-GTPase+COR*→</td><td>LRR-repeats+AP-GTPase+COR</td><td>515</td><td>F6J93_40580</td><td>Cyanobacteria</td><td>Oscillatoria sp. SIO1A7</td><td>hypothetical protein F6J93_40580 [Oscillatoria sp. SIO1A7].</td><td>GCA_010672965.1</td></tr>
<tr><td>NER70803.1</td><td>SIG+DM13→ HISKIN→||<-?<-?<-?||?→ Cluster2343_2clades→ LRR-repeats+AP-GTPase+COR*→||<-Cluster1740_2clades<-?<-?<-?<-?<-Cluster1476_2clades</td><td>LRR-repeats+AP-GTPase+COR</td><td>1150</td><td>F6J95_01375</td><td>-</td><td>Leptolyngbya sp. SIO1E4</td><td>GTPase [Leptolyngbya sp. SIO1E4].</td><td>-</td></tr>
<tr><td>NER71871.1</td><td>BACTERIALFRINGE+TM+TM→?→?→ SIG+TM+TM+TM+TM+TM+TM+TM→||<-?<-?<-?||LRR-repeats+AP-GTPase+COR+DUF4404*→||<-?<-TM+TM||NACHT→||<-?<-Cluster5_5clades<-Aminotran_1_2</td><td>LRR-repeats+AP-GTPase+COR+DUF4404</td><td>1283</td><td>F6J95_06845</td><td>-</td><td>Leptolyngbya sp. SIO1E4</td><td>GTP-binding protein [Leptolyngbya sp. SIO1E4].</td><td>-</td></tr>
<tr><td>NER73482.1</td><td>Cluster2482_2clades→?→||<-?||GNTR-HTH→?→ LRR-repeats+AP-GTPase+COR*→ Cluster1404_2clades→?→ Cluster2050_2clades→</td><td>LRR-repeats+AP-GTPase+COR</td><td>891</td><td>F6J95_15105</td><td>-</td><td>Leptolyngbya sp. SIO1E4</td><td>GTPase [Leptolyngbya sp. SIO1E4].</td><td>-</td></tr>
<tr><td>NER74435.1</td><td>LRR-repeats+AP-GTPase+COR+TIR*→ DSBH→?→?→?→||<-?<-?<-ACT</td><td>LRR-repeats+AP-GTPase+COR+TIR</td><td>1183</td><td>F6J95_19985</td><td>-</td><td>Leptolyngbya sp. SIO1E4</td><td>TIR domain-containing protein [Leptolyngbya sp. SIO1E4].</td><td>-</td></tr>
<tr><td>NER74649.1</td><td>Cluster2260_2clades→?→ LRR-repeats+AP-GTPase+COR+DrHyd*→ SIG+FAD-NAD-dep-oxidoreductase→||<-Cluster850_2clades||PSE→ SIG+TM+TM+TM+TM→</td><td>LRR-repeats+AP-GTPase+COR+DrHyd</td><td>1173</td><td>F6J95_21075</td><td>-</td><td>Leptolyngbya sp. SIO1E4</td><td>hypothetical protein F6J95_21075 [Leptolyngbya sp. SIO1E4].</td><td>-</td></tr>
<tr><td>NER78030.1</td><td>Aminotran_1_2→ LRR-repeats+AP-GTPase+COR*→</td><td>LRR-repeats+AP-GTPase+COR</td><td>904</td><td>F6K42_00285</td><td>Cyanobacteria</td><td>Leptolyngbya sp. SIO1D8</td><td>GTP-binding protein [Leptolyngbya sp. SIO1D8].</td><td>GCA_010672895.1</td></tr>
<tr><td>NER81647.1</td><td>LRR-repeats→ LRR-repeats+AP-GTPase+COR+TIR*→</td><td>LRR-repeats+AP-GTPase+COR+TIR</td><td>836</td><td>F6K42_19200</td><td>Cyanobacteria</td><td>Leptolyngbya sp. SIO1D8</td><td>TIR domain-containing protein [Leptolyngbya sp. SIO1D8].</td><td>GCA_010672895.1</td></tr>
<tr><td>NER83464.1</td><td>LRR-repeats+AP-GTPase+COR+TIR*→</td><td>LRR-repeats+AP-GTPase+COR+TIR</td><td>785</td><td>F6K42_28715</td><td>Cyanobacteria</td><td>Leptolyngbya sp. SIO1D8</td><td>TIR domain-containing protein [Leptolyngbya sp. SIO1D8].</td><td>GCA_010672895.1</td></tr>
<tr><td>NER88741.1</td><td>ABC-ATPase→||<-?||ABC-ATPase→?→?→||<-?<-?||LRR-repeats+AP-GTPase+COR*→?→||<-?<-?||?→ PSE→?→||<-Cluster5_5clades</td><td>LRR-repeats+AP-GTPase+COR</td><td>856</td><td>F6K64_17615</td><td>Cyanobacteria</td><td>Moorea sp. SIO3A2</td><td>leucine-rich repeat protein [Moorea sp. SIO3A2].</td><td>GCA_010672265.1</td></tr>
<tr><td>NER98009.1</td><td>TM+TM+TM+TM+TM+TM→||<-?||?→ Pkinase→?→?→ LRR-repeats+AP-GTPase+COR*→ COR→</td><td>LRR-repeats+AP-GTPase+COR</td><td>417</td><td>F6J86_29885</td><td>Cyanobacteria</td><td>Symploca sp. SIO1B1</td><td>hypothetical protein F6J86_29885 [Symploca sp. SIO1B1].</td><td>GCA_010672945.1</td></tr>
<tr><td>NES02436.1</td><td>LRR-repeats+AP-GTPase+COR*→</td><td>LRR-repeats+AP-GTPase+COR</td><td>964</td><td>F6K22_06035</td><td>Cyanobacteria</td><td>Okeania sp. SIO2F4</td><td>leucine-rich repeat protein [Okeania sp. SIO2F4].</td><td>GCA_010672385.1</td></tr>
<tr><td>NES08071.1</td><td>LRR-repeats+AP-GTPase+COR*→</td><td>LRR-repeats+AP-GTPase+COR</td><td>502</td><td>F6K22_38010</td><td>Cyanobacteria</td><td>Okeania sp. SIO2F4</td><td>leucine-rich repeat protein, partial [Okeania sp. SIO2F4].</td><td>GCA_010672385.1</td></tr>
<tr><td>NES23982.1</td><td>LRR-repeats+AP-GTPase+COR*→</td><td>LRR-repeats+AP-GTPase+COR</td><td>230</td><td>F6K41_34995</td><td>Cyanobacteria</td><td>Caldora sp. SIO3E6</td><td>hypothetical protein F6K41_34995, partial [Caldora sp. SIO3E6].</td><td>GCA_010672185.1</td></tr>
<tr><td>NES70611.1</td><td>LRR-repeats→ LRR-repeats+AP-GTPase+COR*→</td><td>LRR-repeats+AP-GTPase+COR</td><td>400</td><td>F6K24_38075</td><td>Cyanobacteria</td><td>Okeania sp. SIO2D1</td><td>GTPase, partial [Okeania sp. SIO2D1].</td><td>GCA_010672395.1</td></tr>
<tr><td>NES87329.1</td><td>LRR-repeats+AP-GTPase+COR+DUF4404*→</td><td>LRR-repeats+AP-GTPase+COR+DUF4404</td><td>855</td><td>F6K10_41780</td><td>Cyanobacteria</td><td>Moorea sp. SIO2B7</td><td>GTPase, partial [Moorea sp. SIO2B7].</td><td>GCA_010672555.1</td></tr>
<tr><td>NES99177.1</td><td><-NUDIX||?→?→ LRR-repeats+AP-GTPase+COR+DUF4404*→ PSE→?→?→?→||<-ABC-ATPase</td><td>LRR-repeats+AP-GTPase+COR+DUF4404</td><td>1012</td><td>F6K61_01110</td><td>Cyanobacteria</td><td>Sphaerospermopsis sp. SIO1G1</td><td>GTP-binding protein [Sphaerospermopsis sp. SIO1G1].</td><td>GCA_010672785.1</td></tr>
<tr><td>NET13009.1</td><td>LRR-repeats+AP-GTPase+COR*→</td><td>LRR-repeats+AP-GTPase+COR</td><td>307</td><td>F6K08_09185</td><td>Cyanobacteria</td><td>Okeania sp. SIO1H6</td><td>GTPase, partial [Okeania sp. SIO1H6].</td><td>GCA_010672645.1</td></tr>
<tr><td>NET29408.1</td><td>LRR-repeats+AP-GTPase+COR*→||<-?<-NACHT+68TM-wHTH||?→||<-?<-PSE<-TPR-repeats<-HISKIN</td><td>LRR-repeats+AP-GTPase+COR</td><td>1247</td><td>F6K01_30010</td><td>Cyanobacteria</td><td>Okeania sp. SIO1I7</td><td>leucine-rich repeat protein [Okeania sp. SIO1I7].</td><td>GCA_010672585.1</td></tr>
<tr><td>NET38765.1</td><td>LRR-repeats+AP-GTPase+COR*→</td><td>LRR-repeats+AP-GTPase+COR</td><td>802</td><td>F6K19_43415</td><td>Cyanobacteria</td><td>Cyanothece sp. SIO1E1</td><td>GTPase [Cyanothece sp. SIO1E1].</td><td>GCA_010672835.1</td></tr>
<tr><td>NET39396.1</td><td>LRR-repeats+AP-GTPase+COR+TIR*→</td><td>LRR-repeats+AP-GTPase+COR+TIR</td><td>803</td><td>F6K19_46760</td><td>Cyanobacteria</td><td>Cyanothece sp. SIO1E1</td><td>TIR domain-containing protein, partial [Cyanothece sp. SIO1E1].</td><td>GCA_010672835.1</td></tr>
<tr><td>NET43146.1</td><td><-RADICAL-SAM||?→ REC→?→||<-PSE||LRR-repeats+AP-GTPase+COR*→||<-?<-NACHT+68TM-wHTH</td><td>LRR-repeats+AP-GTPase+COR</td><td>927</td><td>F6K15_15105</td><td>Cyanobacteria</td><td>Okeania sp. SIO2B3</td><td>GTPase [Okeania sp. SIO2B3].</td><td>GCA_010672525.1</td></tr>
<tr><td>NET44979.1</td><td>LRR-repeats+AP-GTPase+COR*→||<-?<-PSE<-?<-?||?→?→||<-PSE<-PSE<-PSE<-Cluster5_5clades<-ABC_membrane+ABC_tran</td><td>LRR-repeats+AP-GTPase+COR</td><td>1085</td><td>F6K15_25170</td><td>Cyanobacteria</td><td>Okeania sp. SIO2B3</td><td>GTPase [Okeania sp. SIO2B3].</td><td>GCA_010672525.1</td></tr>
<tr><td>NET45691.1</td><td><-HISKIN||?→?→ LRR-repeats+AP-GTPase+COR*→||<-PSE<-?<-?<-NUDIX<-?||?→ TPR-repeats→</td><td>LRR-repeats+AP-GTPase+COR</td><td>856</td><td>F6K15_29075</td><td>Cyanobacteria</td><td>Okeania sp. SIO2B3</td><td>GTPase [Okeania sp. SIO2B3].</td><td>GCA_010672525.1</td></tr>
<tr><td>NET64212.1</td><td>SIG+TM+TM+TM+TM+TM+TM+TM→ ABC-ATPase→||<-?||ABC-ATPase→?→?→||<-?||LRR-repeats+AP-GTPase+COR*→?→||<-?<-?||?→||<-Cluster5_5clades</td><td>LRR-repeats+AP-GTPase+COR</td><td>914</td><td>F6K63_07330</td><td>Cyanobacteria</td><td>Moorea sp. SIO1G6</td><td>leucine-rich repeat protein [Moorea sp. SIO1G6].</td><td>GCA_010672795.1</td></tr>
</table>

acc	operon	architecture	len	gen.name	taxend	species	defline	gca
NET68629.1	REC→||<-TPR-repeats<-?||LRR-repeats+AP-GTPase+COR+DUF4404*→	LRR-repeats+AP-GTPase+COR+DUF4404	938	F6K63_31235	Cyanobacteria	Moorea sp. SIO1G6	GTPase [Moorea sp. SIO1G6].	GCA_010672795.1
NET73474.1	LRR-repeats+AP-GTPase+COR+DUF4404*→	LRR-repeats+AP-GTPase+COR+DUF4404	821	F6K62_21805	Cyanobacteria	Sphaerospermopsis sp. SIO1G2	GTP-binding protein, partial [Sphaerospermopsis sp. SIO1G2].	GCA_010672725.1
NET83099.1	REC→||<-?||REC→||<-TPR-repeats||LRR-repeats+AP-GTPase+COR+DUF4404*→	LRR-repeats+AP-GTPase+COR+DUF4404	946	F6J94_14560	Cyanobacteria	Moorea sp. SIO1F2	GTPase [Moorea sp. SIO1F2].	GCA_010672755.1
NEZ62303.1	PIN→ LRR-repeats→ LRR-repeats+AP-GTPase+COR*→?→ COR+TIR→ SIG+Trypsin+PDZ→	LRR-repeats+AP-GTPase+COR	318	D0962_05850	Cyanobacteria	Leptolyngbyaceae cyanobacterium CCMR0082	hypothetical protein D0962_05850 [Leptolyngbyaceae cyanobacterium CCMR0082].	GCA_011009535.1
NIM11849.1	S1COLD→||<-?<-?<-?||?→ Cluster54_3clades→ Cluster1093_2clades→ LRR-repeats+AP-GTPase+COR*→||<-?<-Cluster2413_2clades	LRR-repeats+AP-GTPase+COR	882	GTO81_07705	Bacteria	Candidatus Aminicenantes bacterium	GTP-binding protein [Candidatus Aminicenantes bacterium].	GCA_011771225.1
NIM14508.1	Aminotran_1_2→||<-?<-Cluster1540_2clades<-?||?→?→ LRR-repeats+AP-GTPase+COR+TIR*→	LRR-repeats+AP-GTPase+COR+TIR	784	GTO81_21225	Bacteria	Candidatus Aminicenantes bacterium	TIR domain-containing protein [Candidatus Aminicenantes bacterium].	GCA_011771225.1
NIM17826.1	LRR-repeats+AP-GTPase+COR*→	LRR-repeats+AP-GTPase+COR	952	GTO81_38090	Bacteria	Candidatus Aminicenantes bacterium	hypothetical protein GTO81_38090 [Candidatus Aminicenantes bacterium].	GCA_011771225.1
NIN06216.1	LRR-repeats+AP-GTPase+COR*→	LRR-repeats+AP-GTPase+COR	781	GTO43_07600	Armatimonadetes	Armatimonadetes bacterium	GTPase, partial [Armatimonadetes bacterium].	GCA_011771945.1
NIP25303.1	<-SIG+TIMbarrel||LRR-repeats+AP-GTPase+COR+TIR*→	LRR-repeats+AP-GTPase+COR+TIR	868	GWO38_16175	Planctomycetes	Phycisphaerae bacterium	TIR domain-containing protein [Phycisphaerae bacterium].	GCA_011774285.1
NIP41904.1	LRR-repeats+AP-GTPase+COR*→	LRR-repeats+AP-GTPase+COR	457	GWO28_04170	FCB group	candidate division Zixibacteria bacterium	GTPase, partial [candidate division Zixibacteria bacterium].	GCA_011774485.1
NIR09229.1	LRR-repeats+AP-GTPase*→ COR→	LRR-repeats+AP-GTPase	251	GTN82_27750	Bacteria	Candidatus Aminicenantes bacterium	GTP-binding protein, partial [Candidatus Aminicenantes bacterium].	GCA_011773265.1
NIU83786.1	LRR-repeats+AP-GTPase+COR*→	LRR-repeats+AP-GTPase+COR	888	GWN64_10000	Asgard group	Candidatus Thorarchaeota archaeon	GTPase [Candidatus Thorarchaeota archaeon].	GCA_011775775.1
NJD78109.1	SAM-methylase→||<-?||?→?→||<-?||?→?→ LRR-repeats+AP-GTPase+COR*→	LRR-repeats+AP-GTPase+COR	844	FIB08_13625	Euryarchaeota	Candidatus Methanoperedens sp.	GTP-binding protein [Candidatus Methanoperedens sp.].	GCA_012026835.1
NJK31611.1	LRR-repeats+AP-GTPase+COR*→	LRR-repeats+AP-GTPase+COR	784	HC927_03900	Deltaproteobacteria	Deltaproteobacteria bacterium	hypothetical protein HC927_03900 [Deltaproteobacteria bacterium].	GCA_012031025.1
NJK68783.1	LRR-repeats+AP-GTPase+COR*→?→?→?→?→||<-Pentapeptide-repeats	LRR-repeats+AP-GTPase+COR	637	HC941_21705	Cyanobacteria	Microcoleus sp. SU_5_3	GTP-binding protein, partial [Microcoleus sp. SU_5_3].	GCA_012030835.1
NJK99654.1	LRR-repeats+AP-GTPase+COR*→	LRR-repeats+AP-GTPase+COR	665	HC910_03475	Cyanobacteria	Spirulinaceae cyanobacterium SM2_1_0	GTP-binding protein [Spirulinaceae cyanobacterium SM2_1_0].	GCA_012031265.1
NJL39962.1	LRR-repeats+AP-GTPase+COR*→	LRR-repeats+AP-GTPase+COR	814	HC899_26815	Cyanobacteria	Leptolyngbyaceae cyanobacterium SM1_4_3	GTP-binding protein [Leptolyngbyaceae cyanobacterium SM1_4_3].	GCA_012031415.1
NJL55090.1	LRR-repeats+AP-GTPase+COR+TIR*→||<-REase-4||?→ Cluster5_5clades→?→||<-Cluster2398_2clades	LRR-repeats+AP-GTPase+COR+TIR	879	HC928_07795	unclassified Bacteria	bacterium	TIR domain-containing protein [bacterium].	GCA_012031555.1
NJL55819.1	Cluster1137_2clades→?→ LRR-repeats+AP-GTPase+COR*→ PSE→?→||<-Cluster1102_3clades	LRR-repeats+AP-GTPase+COR	671	HC928_11965	unclassified Bacteria	bacterium	GTP-binding protein [bacterium].	GCA_012031555.1
NJL58018.1	LRR-repeats+AP-GTPase+COR*→ COR→	LRR-repeats+AP-GTPase+COR	365	HC928_25050	unclassified Bacteria	bacterium	hypothetical protein HC928_25050, partial [bacterium].	GCA_012031555.1
NJL60049.1	LRR-repeats+AP-GTPase+COR*→ COR+TIR→	LRR-repeats+AP-GTPase+COR	389	HC887_10835	Deltaproteobacteria	Desulfobacteraceae bacterium	hypothetical protein HC887_10835 [Desulfobacteraceae bacterium].	GCA_012031825.1
NJL77569.1	LRR-repeats+AP-GTPase+COR*→	LRR-repeats+AP-GTPase+COR	408	HC892_23555	Bacteroidetes	Saprospiraceae bacterium	hypothetical protein HC892_23555, partial [Saprospiraceae bacterium].	GCA_012031785.1
NJL83760.1	LRR-repeats+AP-GTPase+COR*→	LRR-repeats+AP-GTPase+COR	688	HC890_14000	Chloroflexi	Chloroflexaceae bacterium	GTP-binding protein [Chloroflexaceae bacterium].	GCA_012031815.1

acc	operon	architecture	len	gen.name	taxend	species	defline	gca
NJL90622.1	<-RelE-ParE<-?||LRR-repeats+AP-GTPase+COR*→?→ PSE→?→ PSE→ RNA-Helicase→	LRR-repeats+AP-GTPase+COR	1140	HC916_13170	Cyanobacteria	Coleofasciculaceae cyanobacterium SM2_1_6	GTP-binding protein [Coleofasciculaceae cyanobacterium SM2_1_6].	GCA_012031635.1
NJL91916.1	LRR-repeats+AP-GTPase+COR*→?→ HNH→	LRR-repeats+AP-GTPase+COR	640	HC916_20640	Cyanobacteria	Coleofasciculaceae cyanobacterium SM2_1_6	GTPase, partial [Coleofasciculaceae cyanobacterium SM2_1_6].	GCA_012031635.1
NJM60379.1	<-SIG+TM+TM+TM<-?||TPR-repeats→||<-?<-Cluster5_5clades||?→?→ LRR-repeats+AP-GTPase+COR*→	LRR-repeats+AP-GTPase+COR	729	HC849_09565	Cyanobacteria	Oscillatoriales cyanobacterium RU_3_3	hypothetical protein HC849_09565, partial [Oscillatoriales cyanobacterium RU_3_3].	GCA_012032195.1
NJM78344.1	LRR-repeats+AP-GTPase+COR*→	LRR-repeats+AP-GTPase+COR	500	HC852_24405	Cyanobacteria	Acaryochloridaceae cyanobacterium RU_4_10	hypothetical protein HC852_24405 [Acaryochloridaceae cyanobacterium RU_4_10].	GCA_012032135.1
NJM97967.1	LRR-repeats→ LRR-repeats+AP-GTPase+COR*→ AP-GTPase+COR+TM+TM→	LRR-repeats+AP-GTPase+COR	278	HC800_13155	Cyanobacteria	Phormidesmis sp. RL_2_1	hypothetical protein HC800_13155 [Phormidesmis sp. RL_2_1].	GCA_012033015.1
NJN20692.1	LRR-repeats+AP-GTPase+COR*→ TIR→||<-Cluster5_5clades<-Pentapeptide||REC→	LRR-repeats+AP-GTPase+COR	276	HC812_05240	Cyanobacteria	Leptolyngbya sp. RL_3_1	hypothetical protein HC812_05240, partial [Leptolyngbya sp. RL_3_1].	GCA_012032425.1
NJN57594.1	LRR-repeats+AP-GTPase+COR+TIR*→	LRR-repeats+AP-GTPase+COR+TIR	1087	HC879_08885	Cyanobacteria	Leptolyngbyaceae cyanobacterium SL_5_9	TIR domain-containing protein [Leptolyngbyaceae cyanobacterium SL_5_9].	GCA_012032565.1
NJN63246.1	LRR-repeats+AP-GTPase+COR*→	LRR-repeats+AP-GTPase+COR	747	HC795_18555	Cyanobacteria	Coleofasciculaceae cyanobacterium RL_1_1	hypothetical protein HC795_18555, partial [Coleofasciculaceae cyanobacterium RL_1_1].	GCA_012033105.1
NJN72933.1	LRR-repeats+AP-GTPase+COR+DUF4404*→||<-?||?→ SIG+Classical-AAA→	LRR-repeats+AP-GTPase+COR+DUF4404	832	HC799_09060	Cyanobacteria	Limnothrix sp. RL_2_0	GTP-binding protein [Limnothrix sp. RL_2_0].	GCA_012033055.1
NJN75229.1	LRR-repeats+AP-GTPase→ AP-GTPase*→?→ COR+TM+TM+TM→	AP-GTPase	180	HC796_01955	Cyanobacteria	Synechococcaceae cyanobacterium RL_1_2	hypothetical protein HC796_01955 [Synechococcaceae cyanobacterium RL_1_2].	GCA_012033095.1
NJN88810.1	LRR-repeats+AP-GTPase+COR*→	LRR-repeats+AP-GTPase+COR	1016	HC881_24165	Cyanobacteria	Leptolyngbyaceae cyanobacterium SL_7_1	GTP-binding protein [Leptolyngbyaceae cyanobacterium SL_7_1].	GCA_012032525.1
NJN89941.1	LRR-repeats+AP-GTPase+COR*→	LRR-repeats+AP-GTPase+COR	814	HC878_05910	Cyanobacteria	Leptolyngbyaceae cyanobacterium SL_5_14	GTP-binding protein [Leptolyngbyaceae cyanobacterium SL_5_14].	GCA_012032515.1
NJN96139.1	Cluster1841_2clades→?→ LRR-repeats→ LRR-repeats+AP-GTPase+COR*→ Cluster2316_2clades→ Cluster2224_2clades→?→ Cluster1087_2clades→||<-?<-?<-ABC-ATPase	LRR-repeats+AP-GTPase+COR	573	HC875_19545	Chloroflexi	Anaerolineales bacterium	GTP-binding protein [Anaerolineales bacterium].	GCA_012032575.1
NJO03971.1	LRR-repeats+AP-GTPase+COR+EAD7*→	LRR-repeats+AP-GTPase+COR+EAD7	814	HC880_21890	Bacteroidetes	Bacteroidia bacterium	hypothetical protein HC880_21890 [Bacteroidia bacterium].	GCA_012033155.1
NJO04035.1	LRR-repeats+AP-GTPase+COR+EAD7*→	LRR-repeats+AP-GTPase+COR+EAD7	843	HC880_22270	Bacteroidetes	Bacteroidia bacterium	hypothetical protein HC880_22270 [Bacteroidia bacterium].	GCA_012033155.1
NJO50334.1	LRR-repeats+AP-GTPase+COR*→	LRR-repeats+AP-GTPase+COR	510	HC840_13875	Cyanobacteria	Leptolyngbyaceae cyanobacterium RM2_2_4	GTP-binding protein, partial [Leptolyngbyaceae cyanobacterium RM2_2_4].	GCA_012033305.1
NJP08786.1	<-Cluster5_5clades||?→?→?→?→ LRR-repeats+AP-GTPase+COR*→	LRR-repeats+AP-GTPase+COR	841	HC866_04300	Cyanobacteria	Leptolyngbyaceae cyanobacterium RU_5_1	GTPase [Leptolyngbyaceae cyanobacterium RU_5_1].	GCA_012034055.1
NJR23767.1	GTPase-AIG→?→?→ LRR-repeats+AP-GTPase+COR+DUF4404*→	LRR-repeats+AP-GTPase+COR+DUF4404	1061	HC786_17240	Cyanobacteria	Richelia sp. CSU_2_1	GTP-binding protein [Richelia sp. CSU_2_1].	GCA_012034575.1
NJR25667.1	LRR-repeats+AP-GTPase+COR*→	LRR-repeats+AP-GTPase+COR	856	HC786_27780	Cyanobacteria	Richelia sp. CSU_2_1	hypothetical protein HC786_27780, partial [Richelia sp. CSU_2_1].	GCA_012034575.1
NJR41095.1	LRR-repeats+AP-GTPase+COR+TIR*→	LRR-repeats+AP-GTPase+COR+TIR	677	HC781_22475	Cyanobacteria	Leptolyngbyaceae cyanobacterium CSU_1_4	GTP-binding protein [Leptolyngbyaceae cyanobacterium CSU_1_4].	GCA_012034615.1
NJR68835.1	<-SIGMA-HTH<-?<-?<-?||?→ LRR-repeats+AP-GTPase+COR+TCAD1*→?→||<-?<-?<-?||?→||<-TPR-repeats	LRR-repeats+AP-GTPase+COR+TCAD1	892	HC771_09325	Cyanobacteria	Synechococcales cyanobacterium CRU_2_2	GTPase [Synechococcales cyanobacterium CRU_2_2].	GCA_012034805.1

<table>
<tr><th>acc</th><th>operon</th><th>architecture</th><th>len</th><th>gen.name</th><th>taxend</th><th>species</th><th>defline</th><th>gca</th></tr>
<tr><td>NJS09810.1</td><td>GTPase-AIG→?→?→||<-?<-?||LRR-repeats+AP-GTPase+COR*→?→?→?→?→||<-Pentapeptide-repeats</td><td>LRR-repeats+AP-GTPase+COR</td><td>761</td><td>HC789_05185</td><td>Cyanobacteria</td><td>Microcoleus sp. CSU_2_2</td><td>GTP-binding protein [Microcoleus sp. CSU_2_2].</td><td>GCA_012035135.1</td></tr>
<tr><td>NKC15125.1</td><td>LRR-repeats+AP-GTPase+COR+DrHyd*→</td><td>LRR-repeats+AP-GTPase+COR+DrHyd</td><td>1164</td><td>GKR94_23910</td><td>Gammaproteobacteria</td><td>Gammaproteobacteria bacterium</td><td>hypothetical protein GKR94_23910 [Gammaproteobacteria bacterium].</td><td>GCA_012103455.1</td></tr>
<tr><td>NKQ34928.1</td><td>RelE-ParE→?→?→ PSE→ LRR-repeats+AP-GTPase+COR+EAD7*→</td><td>LRR-repeats+AP-GTPase+COR+EAD7</td><td>553</td><td>HF973_04850</td><td>Chloroflexi</td><td>Chloroflexi bacterium</td><td>hypothetical protein HF973_04850 [Chloroflexi bacterium].</td><td>GCA_012329075.1</td></tr>
<tr><td>NLT19133.1</td><td>LRR-repeats+AP-GTPase+COR*→</td><td>LRR-repeats+AP-GTPase+COR</td><td>709</td><td>GXY10_07015</td><td>Firmicutes</td><td>Clostridiales bacterium</td><td>GTP-binding protein, partial [Clostridiales bacterium].</td><td>GCA_012718845.1</td></tr>
<tr><td>NMF86259.1</td><td>RelE→ CHTH→?→||<-?<-?<-?<-?||LRR-repeats+AP-GTPase+COR+TIR*→?→||<-?<-?||?→?→||<-?<-RelE-ParE</td><td>LRR-repeats+AP-GTPase+COR+TIR</td><td>1176</td><td>E1H13_23515</td><td>Cyanobacteria</td><td>Nodosilinea sp. P-1105</td><td>TIR domain-containing protein [Nodosilinea sp. P-1105].</td><td>GCA_012911975.1</td></tr>
<tr><td>NND77029.1</td><td>LRR-repeats+AP-GTPase+COR*→||<-?<-?<-?<-?<-SIG+TM+TM+TM+TM</td><td>LRR-repeats+AP-GTPase+COR</td><td>842</td><td>HKN39_02455</td><td>Bacteroidetes</td><td>Flavobacteriales bacterium</td><td>hypothetical protein HKN39_02455 [Flavobacteriales bacterium].</td><td>GCA_013002285.1</td></tr>
<tr><td>NOG45111.1</td><td>MORC→?→ LRR-repeats→ LRR-repeats+AP-GTPase+COR*→?→||<-?<-?<-BetaPropeller+BetaPropeller</td><td>LRR-repeats+AP-GTPase+COR</td><td>652</td><td>HND50_07765</td><td>Calditrichaeota</td><td>Calditrichaeota bacterium</td><td>hypothetical protein HND50_07765 [Calditrichaeota bacterium].</td><td>GCA_013112635.1</td></tr>
<tr><td>NOH03519.1</td><td>DAGKIN→ Cluster1917_2clades→?→||<-?<-?||LRR-repeats→ PSE→ LRR-repeats+AP-GTPase+COR*→?→||<-PSE||?→||<-?<-GTPase-AIG||Thioredoxin→</td><td>LRR-repeats+AP-GTPase+COR</td><td>345</td><td>HND47_16930</td><td>Chloroflexi</td><td>Chloroflexi bacterium</td><td>hypothetical protein HND47_16930 [Chloroflexi bacterium].</td><td>GCA_013112685.1</td></tr>
<tr><td>NOH03560.1</td><td>REase→ LRR-repeats+AP-GTPase+COR→ LRR-repeats+AP-GTPase+COR→</td><td>LRR-repeats+AP-GTPase+COR</td><td>878</td><td>HND47_17170</td><td>Chloroflexi</td><td>Chloroflexi bacterium</td><td>hypothetical protein HND47_17170 [Chloroflexi bacterium].</td><td>GCA_013112685.1</td></tr>
<tr><td>NOH03561.1</td><td>LRR-repeats+AP-GTPase+COR*→</td><td>LRR-repeats+AP-GTPase+COR</td><td>852</td><td>HND47_17175</td><td>Chloroflexi</td><td>Chloroflexi bacterium</td><td>GTP-binding protein [Chloroflexi bacterium].</td><td>GCA_013112685.1</td></tr>
<tr><td>NOH03734.1</td><td>PIN→ PSE→?→?→ LRR-repeats+AP-GTPase+COR*→?→?→?→ PSE→||<-?<-PSE||?→ Cluster2515_2clades→ Cluster2268_2clades→</td><td>LRR-repeats+AP-GTPase+COR</td><td>973</td><td>HND47_18110</td><td>Chloroflexi</td><td>Chloroflexi bacterium</td><td>hypothetical protein HND47_18110 [Chloroflexi bacterium].</td><td>GCA_013112685.1</td></tr>
<tr><td>NOQ25006.1</td><td>Arginase→ REase→?→?→ LRR-repeats+AP-GTPase+COR+DrHyd*→</td><td>LRR-repeats+AP-GTPase+COR+DrHyd</td><td>1054</td><td>GQ564_06545</td><td>Bacteroidetes</td><td>Bacteroidales bacterium</td><td>GTP-binding protein [Bacteroidales bacterium].</td><td>GCA_013138975.1</td></tr>
<tr><td>NOQ25654.1</td><td>LRR-repeats+AP-GTPase+COR*→ FGS→ PSE→?→||<-?<-?<-REC</td><td>LRR-repeats+AP-GTPase+COR</td><td>1082</td><td>GQ564_09870</td><td>Bacteroidetes</td><td>Bacteroidales bacterium</td><td>GTP-binding protein [Bacteroidales bacterium].</td><td>GCA_013138975.1</td></tr>
<tr><td>NOQ25870.1</td><td>LRR-repeats+AP-GTPase+COR*→ Cluster881_3clades→ Polbeta+HEPN→||<-?<-Cluster998_2clades<-?<-RADICAL-SAM</td><td>LRR-repeats+AP-GTPase+COR</td><td>983</td><td>GQ564_10955</td><td>Bacteroidetes</td><td>Bacteroidales bacterium</td><td>GTP-binding protein [Bacteroidales bacterium].</td><td>GCA_013138975.1</td></tr>
<tr><td>NOQ35071.1</td><td>LRR-repeats+AP-GTPase+COR*→</td><td>LRR-repeats+AP-GTPase+COR</td><td>779</td><td>GQ569_04160</td><td>Gammaproteobacteria</td><td>Methylococcaceae bacterium</td><td>hypothetical protein GQ569_04160 [Methylococcaceae bacterium].</td><td>GCA_013138855.1</td></tr>
<tr><td>NOQ64054.1</td><td>LRR-repeats+AP-GTPase+COR+DrHyd*→</td><td>LRR-repeats+AP-GTPase+COR+DrHyd</td><td>751</td><td>GQ582_06035</td><td>Gammaproteobacteria</td><td>Methyloprofundus sp.</td><td>hypothetical protein GQ582_06035 [Methyloprofundus sp.].</td><td>GCA_013138595.1</td></tr>
<tr><td>NOR78337.1</td><td>LRR-repeats+AP-GTPase+COR*→</td><td>LRR-repeats+AP-GTPase+COR</td><td>537</td><td>GQ523_07950</td><td>Euryarchaeota</td><td>Methanophagales archaeon</td><td>GTP-binding protein, partial [Methanophagales archaeon].</td><td>GCA_013139985.1</td></tr>
<tr><td>NOS87464.1</td><td>LRR-repeats+AP-GTPase+COR+TIR*→</td><td>LRR-repeats+AP-GTPase+COR+TIR</td><td>972</td><td>HOP34_02800</td><td>Gammaproteobacteria</td><td>Methylococcaceae bacterium</td><td>TIR domain-containing protein [Methylococcaceae bacterium].</td><td>GCA_013140465.1</td></tr>
<tr><td>NOT10704.1</td><td>Cluster2349_2clades→ PSE→?→ LRR-repeats+AP-GTPase+COR*→</td><td>LRR-repeats+AP-GTPase+COR</td><td>810</td><td>HOP23_02545</td><td>Gammaproteobacteria</td><td>Methylococcaceae bacterium</td><td>hypothetical protein HOP23_02545 [Methylococcaceae bacterium].</td><td>GCA_013140705.1</td></tr>
<tr><td>NOT87605.1</td><td>PNPase→?→ LRR-repeats+AP-GTPase+COR+TIR*→</td><td>LRR-repeats+AP-GTPase+COR+TIR</td><td>1152</td><td>HOP03_05430</td><td>Gammaproteobacteria</td><td>Lysobacter sp.</td><td>TIR domain-containing protein [Lysobacter sp.].</td><td>GCA_013141175.1</td></tr>
<tr><td>NOU20113.1</td><td>SIGMA-HTH→?→ LRR-repeats+AP-GTPase+COR+DrHyd*→ LRR-repeats+AP-GTPase+COR+DrHyd→</td><td>LRR-repeats+AP-GTPase+COR+DrHyd</td><td>1204</td><td>HOO91_21360</td><td>Bacteroidetes</td><td>Bacteroidales bacterium</td><td>hypothetical protein HOO91_21360 [Bacteroidales bacterium].</td><td>GCA_013141385.1</td></tr>
<tr><td>NOU47191.1</td><td>LRR-repeats+AP-GTPase+COR*→</td><td>LRR-repeats+AP-GTPase+COR</td><td>693</td><td>HOO86_09030</td><td>Bacteroidetes</td><td>Bacteroidales bacterium</td><td>GTP-binding protein [Bacteroidales bacterium].</td><td>GCA_013141455.1</td></tr>
<tr><td>NOZ36014.1</td><td>Cluster1478_2clades→?→?→?→?→ LRR-repeats+AP-GTPase+COR*→||<-?<-?||?→?→ D5ATPase→</td><td>LRR-repeats+AP-GTPase+COR</td><td>842</td><td>GXO80_12040</td><td>Chlorobi</td><td>Chlorobi bacterium</td><td>GTP-binding protein [Chlorobi bacterium].</td><td>GCA_013152535.1</td></tr>
<tr><td>NQT12774.1</td><td>LRR-repeats+AP-GTPase+COR+DUF4404*→</td><td>LRR-repeats+AP-GTPase+COR+DUF4404</td><td>936</td><td>HQ582_08495</td><td>Planctomycetes</td><td>Planctomycetes bacterium</td><td>leucine-rich repeat domain-containing protein, partial [Planctomycetes bacterium].</td><td>GCA_013202485.1</td></tr>
<tr><td>NQT13736.1</td><td>LRR-repeats+AP-GTPase+COR*→ TIR→</td><td>LRR-repeats+AP-GTPase+COR</td><td>583</td><td>HQ582_13365</td><td>Planctomycetes</td><td>Planctomycetes bacterium</td><td>hypothetical protein HQ582_13365, partial [Planctomycetes bacterium].</td><td>GCA_013202485.1</td></tr>
</table>

acc	operon	architecture	len	gen.name	taxend	species	defline	gca
NQT64030.1	TM+TM+TM→?→?→?→?→ SIG+LRR-repeats+AP-GTPase+COR+TIR*→	SIG+LRR-repeats+AP-GTPase+COR+TIR	1188	HQ556_13800	FCB group	Candidatus Marinimicrobia bacterium	TIR domain-containing protein [Candidatus Marinimicrobia bacterium].	GCA_013202495.1
NQT91808.1	LRR-repeats+AP-GTPase+COR+DUF4404*→	LRR-repeats+AP-GTPase+COR+DUF4404	843	HQ559_03535	Lentisphaerae	Lentisphaerae bacterium	serine/threonine protein kinase [Lentisphaerae bacterium].	GCA_013202215.1
NQU06371.1	LRR-repeats+AP-GTPase+COR*→	LRR-repeats+AP-GTPase+COR	749	HQ568_09785	Calditrichaeota	Calditrichaeota bacterium	GTP-binding protein [Calditrichaeota bacterium].	GCA_013202805.1
NQU20766.1	LRR-repeats+AP-GTPase+COR+TIR*→	LRR-repeats+AP-GTPase+COR+TIR	813	HQ567_05735	Bacteria	Candidatus Nealsonbacteria bacterium	TIR domain-containing protein [Candidatus Nealsonbacteria bacterium].	GCA_013202865.1
NQU25297.1	LRR-repeats+AP-GTPase+COR*→ TIR→	LRR-repeats+AP-GTPase+COR	608	HQ567_28770	Bacteria	Candidatus Nealsonbacteria bacterium	hypothetical protein HQ567_28770 [Candidatus Nealsonbacteria bacterium].	GCA_013202865.1
NQU51997.1	LRR-repeats+AP-GTPase+COR+TIR*→	LRR-repeats+AP-GTPase+COR+TIR	946	HQ522_05615	Bacteroidetes	Bacteroidetes bacterium	leucine-rich repeat domain-containing protein [Bacteroidetes bacterium].	GCA_013203755.1
NQU93487.1	RVT→ LRR-repeats+AP-GTPase+COR+TIR*→?→?→ TM+TM+TM→	LRR-repeats+AP-GTPase+COR+TIR	903	HQ540_23735	Bacteroidetes	Bacteroidetes bacterium	TIR domain-containing protein [Bacteroidetes bacterium].	GCA_013203545.1
NQY23053.1	S4→?→?→?→?→ LRR-repeats+AP-GTPase+COR+DrHyd*→||<-?<-Cluster1695_2clades||TraJ-RHH→ RelE-ParE→?→?→ ACET→	LRR-repeats+AP-GTPase+COR+DrHyd	962	HRT41_03420	Epsilonproteobacteria	Campylobacteraceae bacterium	leucine-rich repeat domain-containing protein [Campylobacteraceae bacterium].	GCA_013215945.1
NQZ07155.1	GDSL→ Cluster2260_2clades→ LRR-repeats+AP-GTPase+COR+E59-ABC*→?→ RelE→ CHTH→	LRR-repeats+AP-GTPase+COR+E59-ABC	1302	HRT35_08335	Gammaproteobacteria	Algicola sp.	leucine-rich repeat domain-containing protein [Algicola sp.].	GCA_013216025.1
NQZ07918.1	LRR-repeats+AP-GTPase+COR*→	LRR-repeats+AP-GTPase+COR	815	HRT35_12215	Gammaproteobacteria	Algicola sp.	GTP-binding protein [Algicola sp.].	GCA_013216025.1
NQZ09239.1	<-MNS-STAND+CR-REase3||LRR-repeats+AP-GTPase+COR*→ Cluster5_5clades→	LRR-repeats+AP-GTPase+COR	1075	HRT35_18935	Gammaproteobacteria	Algicola sp.	leucine-rich repeat domain-containing protein [Algicola sp.].	GCA_013216025.1
NQZ10194.1	LRR-repeats+AP-GTPase+COR+TIR*→?→||<-?<-HSP70	LRR-repeats+AP-GTPase+COR+TIR	972	HRT35_23840	Gammaproteobacteria	Algicola sp.	TIR domain-containing protein [Algicola sp.].	GCA_013216025.1
NQZ62063.1	Cluster1136_2clades→?→||<-?||LRR-repeats+AP-GTPase+COR*→	LRR-repeats+AP-GTPase+COR	829	HRT59_07970	Cyanobacteria	Crocosphaera sp.	leucine-rich repeat domain-containing protein [Crocosphaera sp.].	GCA_013215395.1
NQZ63007.1	LRR-repeats+AP-GTPase+COR*→	LRR-repeats+AP-GTPase+COR	689	HRT59_12970	Cyanobacteria	Crocosphaera sp.	leucine-rich repeat domain-containing protein, partial [Crocosphaera sp.].	GCA_013215395.1
NRA27019.1	FGS→?→ FGS→ RVT→ FGS→ LRR-repeats+AP-GTPase+COR*→||<-?<-nSTAND3+REase-DUF4143<-tRNA<-?||Pro_CA→||<-Cluster1292_2clades	LRR-repeats+AP-GTPase+COR	859	HRU10_07210	Verrucomicrobia	Opitutales bacterium	hypothetical protein HRU10_07210 [Opitutales bacterium].	GCA_013215165.1
NRT54298.1	LRR-repeats+AP-GTPase+COR+TIR*→||<-?<-ABC_membrane+ABC_tran||Cyanophycinsyn-ATPgrasp→ Cyanophycinsyn-ATPgrasp→	LRR-repeats+AP-GTPase+COR+TIR	788	HNQ01_000005	Betaproteobacteria	Leptothrix sp. C29	internalin A [Leptothrix sp. C29].	GCA_013294065.1
NTV46488.1	LRR-repeats+AP-GTPase+COR+TIR*→	LRR-repeats+AP-GTPase+COR+TIR	657	HGB11_08225	Chlorobi	Chlorobiales bacterium	TIR domain-containing protein, partial [Chlorobiales bacterium].	GCA_013334725.1
NTV66504.1	LRR-repeats+AP-GTPase+COR+TIR*→?→?→?→ PurT-ATPgrasp→	LRR-repeats+AP-GTPase+COR+TIR	755	HGB06_02250	Chlorobi	Chlorobaculum sp.	TIR domain-containing protein [Chlorobaculum sp.].	GCA_013334855.1
NTV67443.1	REase→||<-?<-SIG+TM||LRR-repeats+AP-GTPase+COR*→	LRR-repeats+AP-GTPase+COR	453	HGB06_07145	Chlorobi	Chlorobaculum sp.	hypothetical protein HGB06_07145 [Chlorobaculum sp.].	GCA_013334855.1
NTW50168.1	LRR-repeats+AP-GTPase+COR+TIR*→	LRR-repeats+AP-GTPase+COR+TIR	839	HGB19_10655	Chlorobi	Chlorobiales bacterium	TIR domain-containing protein [Chlorobiales bacterium].	GCA_013334605.1
NTW54860.1	LRR-repeats+AP-GTPase+COR*→?→?→?→?→?→ Cluster2062_2clades→	LRR-repeats+AP-GTPase+COR	1090	HGB15_08930	Chlorobi	Chlorobaculum sp.	hypothetical protein HGB15_08930 [Chlorobaculum sp.].	GCA_013334655.1
NTW70387.1	DAM→ LRR-repeats+AP-GTPase+COR+TIR*→	LRR-repeats+AP-GTPase+COR+TIR	876	HGB23_11195	Chlorobi	Chlorobiaceae bacterium	TIR domain-containing protein [Chlorobiaceae bacterium].	GCA_013334485.1

acc	operon	architecture	len	gen.name	taxend	species	defline	gca
NTW83729.1	<-NUDIX||?→?→?→ LRR-repeats+AP-GTPase+COR+TIR*→	LRR-repeats+AP-GTPase+COR+TIR	892	HGB36_10245	Chlorobi	Chlorobiaceae bacterium	TIR domain-containing protein [Chlorobiaceae bacterium].	GCA_013334225.1
NUM66201.1	SHS2→ LRR-repeats+AP-GTPase+COR*→?→?→ Cluster1134_2clades→ SNF→	LRR-repeats+AP-GTPase+COR	1051	HUU39_13110	Bacteria	candidate division KSB1 bacterium	hypothetical protein HUU39_13110 [candidate division KSB1 bacterium].	GCA_013359425.1
NUM68318.1	LRR-repeats+AP-GTPase+COR*→?→?→ FGS→	LRR-repeats+AP-GTPase+COR	955	HUU39_24115	Bacteria	candidate division KSB1 bacterium	leucine-rich repeat domain-containing protein [candidate division KSB1 bacterium].	GCA_013359425.1
NUO01599.1	<-S18-HTH<-Cluster1004_3clades<-?<-?<-?||FGS→ LRR-repeats+_AP-GTPase+COR+EAD11+TIR*→ FGS→	LRR-repeats+_AP-GTPase+COR+EAD11+TIR	1085	HUU01_13415	Bacteroidetes	Saprospiraceae bacterium	TIR domain-containing protein [Saprospiraceae bacterium].	GCA_013361075.1
NUO02098.1	LRR-repeats+AP-GTPase+COR+TM+TM+TM*→?→||<-Cluster2427_2clades	LRR-repeats+AP-GTPase+COR+TM+TM+TM	791	HUU01_15950	Bacteroidetes	Saprospiraceae bacterium	hypothetical protein HUU01_15950, partial [Saprospiraceae bacterium].	GCA_013361075.1
NUO79752.1	SIG+7TMR-DISMED1→||<-?<-?||LRR-repeats+AP-GTPase+COR+TIR*→||<-Cluster2261_2clades<-RlaP||SIG+TIMbarrel→	LRR-repeats+AP-GTPase+COR+TIR	968	HUU05_06725	Bacteria	candidate division KSB1 bacterium	leucine-rich repeat domain-containing protein [candidate division KSB1 bacterium].	GCA_013361015.1
NUO80023.1	ABhydrolase→?→ Cluster1134_2clades→?→ LRR-repeats+AP-GTPase+COR*→	LRR-repeats+AP-GTPase+COR	824	HUU05_08095	Bacteria	candidate division KSB1 bacterium	leucine-rich repeat domain-containing protein [candidate division KSB1 bacterium].	GCA_013361015.1
NUO97795.1	LRR-repeats+AP-GTPase+COR*→	LRR-repeats+AP-GTPase+COR	986	HOW59_07675	Actinobacteria	Nonomuraea sp.	hypothetical protein HOW59_07675, partial [Nonomuraea sp.].	GCA_013361365.1
NUP68620.1	LRR-repeats+AP-GTPase+COR*→	LRR-repeats+AP-GTPase+COR	1447	HOW71_41335	Actinobacteria	Nonomuraea sp.	hypothetical protein HOW71_41335 [Nonomuraea sp.].	GCA_013361665.1
NUQ24465.1	FGS→?→||<-?||EAD11+LRR-repeats+S1+S1+AP-GTPase+COR*→	EAD11+LRR-repeats+S1+S1+AP-GTPase+COR	1140	HUU34_10980	Bacteroidetes	Saprospiraceae bacterium	leucine-rich repeat domain-containing protein [Saprospiraceae bacterium].	GCA_013360405.1
NUQ25704.1	PIN→ FGS→?→ RVT→?→ LRR-repeats+AP-GTPase+COR+TIR*→?→?→?→ TM+TM+TM→	LRR-repeats+AP-GTPase+COR+TIR	826	HUU34_17280	Bacteroidetes	Saprospiraceae bacterium	TIR domain-containing protein [Saprospiraceae bacterium].	GCA_013360405.1
NUQ26825.1	LRR-repeats+AP-GTPase+COR+TM*→ FGS→	LRR-repeats+AP-GTPase+COR+TM	924	HUU34_23015	Bacteroidetes	Saprospiraceae bacterium	hypothetical protein HUU34_23015 [Saprospiraceae bacterium].	GCA_013360405.1
NUQ46249.1	LRR-repeats+AP-GTPase+COR*→ ABC_membrane+ABC_tran→	LRR-repeats+AP-GTPase+COR	843	HUU22_09465	Planctomycetes	Phycisphaerae bacterium	leucine-rich repeat domain-containing protein, partial [Phycisphaerae bacterium].	GCA_013360645.1
NUQ73394.1	LRR-repeats+AP-GTPase+COR+TM+TM*→	LRR-repeats+AP-GTPase+COR+TM+TM	869	HUU21_07550	Deltaproteobacteria	Polyangiaceae bacterium	GTP-binding protein [Polyangiaceae bacterium].	GCA_013360665.1
NUQ87064.1	LRR-repeats+AP-GTPase+COR*→	LRR-repeats+AP-GTPase+COR	767	HOQ43_01165	Actinobacteria	Glycomyces artemisiae	hypothetical protein HOQ43_01165 [Glycomyces artemisiae].	GCA_013361895.1
NUR27723.1	REC→?→?→?→ LRR-repeats+AP-GTPase+COR*→	LRR-repeats+AP-GTPase+COR	958	HOV83_18080	Actinobacteria	Catenulispora sp.	hypothetical protein HOV83_18080 [Catenulispora sp.].	GCA_013362085.1
NUS08684.1	LRR-repeats+AP-GTPase+COR*→	LRR-repeats+AP-GTPase+COR	1447	HOV97_39690	Actinobacteria	Nonomuraea sp.	hypothetical protein HOV97_39690 [Nonomuraea sp.].	GCA_013362305.1
NVM16177.1	LRR-repeats+AP-GTPase+COR*→	LRR-repeats+AP-GTPase+COR	1327	HWN80_00570	Asgard group	Candidatus Lokiarchaeota archaeon	leucine-rich repeat protein [Candidatus Lokiarchaeota archaeon].	GCA_013375495.1
NVM57338.1	LRR-repeats+AP-GTPase+COR*→	LRR-repeats+AP-GTPase+COR	588	HWN51_04390	Deltaproteobacteria	Desulfobacterales bacterium	leucine-rich repeat domain-containing protein, partial [Desulfobacterales bacterium].	GCA_013375265.1
NVO09292.1	<-SIG+TM+VWA+TM<-SIG+TM+VWA+TM<-?<-VWA<-?<-MoxR-AAA||?→ LRR-repeats+AP-GTPase+COR*→?→?→?→||<-?<-?<-?||DSBH→	LRR-repeats+AP-GTPase+COR	965	HXX16_04945	Bacteroidetes	Bacteroidales bacterium	leucine-rich repeat domain-containing protein [Bacteroidales bacterium].	GCA_013376985.1

<table>
<thead>
<tr><th>acc</th><th>operon</th><th>architecture</th><th>len</th><th>gen.name</th><th>taxend</th><th>species</th><th>defline</th><th>gca</th></tr>
</thead>
<tbody>
<tr><td>NZD48025.1</td><td>LRR-repeats+AP-GTPase+COR+TIR*→||<-?<-ABC_membrane+ABC_tran||Cyanophycinsyn-ATPgrasp→ Cyanophycinsyn-ATPgrasp→</td><td>LRR-repeats+AP-GTPase+COR+TIR</td><td>885</td><td>HZU84_19525</td><td>Betaproteobacteria</td><td>Sphaerotilus natans subsp. sulfidivorans</td><td>leucine-rich repeat domain-containing protein [Sphaerotilus natans subsp. sulfidivorans].</td><td>GCA_013426975.1</td></tr>
<tr><td>OAB01099.1</td><td>LRR-repeats+AP-GTPase+COR*→||<-?||?→||<-?<-SIGMA-HTH<-SIGMA-HTH</td><td>LRR-repeats+AP-GTPase+COR</td><td>996</td><td>A6P39_08275</td><td>Actinobacteria</td><td>Streptomyces sp. FXJ1.172</td><td>hypothetical protein A6P39_08275 [Streptomyces sp. FXJ1.172].</td><td>GCA_001636945.1</td></tr>
<tr><td>OAI12102.1</td><td>Cluster5_5clades→?→?→ HTH→ LRR-repeats+AP-GTPase+COR+TIR*→ Cluster1019_2clades→?→||<-?<-?<-?||Cluster833_2clades→</td><td>LRR-repeats+AP-GTPase+COR+TIR</td><td>1065</td><td>A1507_19115</td><td>Gammaproteobacteria</td><td>Methylomonas koyamae</td><td>hypothetical protein A1507_19115 [Methylomonas koyamae].</td><td>GCA_001644135.1</td></tr>
<tr><td>OBQ24605.1</td><td>S18-HTH→?→||<-tRNA||Ferredoxin-betagrasp→?→?→ LRR-repeats+AP-GTPase+COR*→?→||<-?||TM→</td><td>LRR-repeats+AP-GTPase+COR</td><td>1095</td><td>AN488_00160</td><td>Cyanobacteria</td><td>Anabaena sp. WA113</td><td>GTPase [Anabaena sp. WA113].</td><td>GCA_001672155.1</td></tr>
<tr><td>OBQ25153.1</td><td>LRR-repeats+AP-GTPase+COR*→?→||<-?||TM→</td><td>LRR-repeats+AP-GTPase+COR</td><td>780</td><td>AN481_11440</td><td>Cyanobacteria</td><td>Aphanizomenon flos-aquae LD13</td><td>hypothetical protein AN481_11440, partial [Aphanizomenon flos-aquae LD13].</td><td>GCA_001672165.1</td></tr>
<tr><td>OBQ31054.1</td><td>S18-HTH→?→||<-tRNA||Ferredoxin-betagrasp→?→?→ LRR-repeats+AP-GTPase+COR*→?→||<-?||TM→</td><td>LRR-repeats+AP-GTPase+COR</td><td>942</td><td>AN483_02530</td><td>Cyanobacteria</td><td>Aphanizomenon flos-aquae MDT14a</td><td>hypothetical protein AN483_02530 [Aphanizomenon flos-aquae MDT14a].</td><td>GCA_001672095.1</td></tr>
<tr><td>OBQ35403.1</td><td>LRR-repeats+AP-GTPase+COR*→||<-?<-sGTP+sGTP||?→||<-REC</td><td>LRR-repeats+AP-GTPase+COR</td><td>799</td><td>AN487_15705</td><td>Cyanobacteria</td><td>Anabaena sp. CRKS33</td><td>hypothetical protein AN487_15705, partial [Anabaena sp. CRKS33].</td><td>GCA_001672075.1</td></tr>
<tr><td>OBQ36155.1</td><td>LRR-repeats+AP-GTPase+COR*→</td><td>LRR-repeats+AP-GTPase+COR</td><td>567</td><td>AN484_25870</td><td>Cyanobacteria</td><td>Aphanizomenon flos-aquae WA102</td><td>GTPase, partial [Aphanizomenon flos-aquae WA102].</td><td>GCA_001672105.1</td></tr>
<tr><td>OCQ90934.1</td><td>LRR-repeats+AP-GTPase+COR→ *LRR-repeats+AP-GTPase+COR*→?→ LRR-repeats+AP-GTPase+COR→</td><td>LRR-repeats+AP-GTPase+COR</td><td>795</td><td>BCR12_04520</td><td>Cyanobacteria</td><td>Limnothrix sp. P13C2</td><td>hypothetical protein BCR12_04520 [Limnothrix sp. P13C2].</td><td>GCA_001698445.1</td></tr>
<tr><td>OCQ90935.1</td><td>LRR-repeats+AP-GTPase+COR*→</td><td>LRR-repeats+AP-GTPase+COR</td><td>952</td><td>BCR12_04525</td><td>Cyanobacteria</td><td>Limnothrix sp. P13C2</td><td>hypothetical protein BCR12_04525 [Limnothrix sp. P13C2].</td><td>GCA_001698445.1</td></tr>
<tr><td>OCQ97014.1</td><td>TPR-repeats→?→?→ CASPASE+TM+TPR→?→?→?→ LRR-repeats+AP-GTPase+COR+DUF4404*→||<-?<-?||?→||<-?||REC→</td><td>LRR-repeats+AP-GTPase+COR+DUF4404</td><td>833</td><td>BCD64_15615</td><td>Cyanobacteria</td><td>Nostoc sp. MBR 210</td><td>GTP-binding protein [Nostoc sp. MBR 210].</td><td>GCA_001698435.1</td></tr>
<tr><td>OCR00628.1</td><td>LRR-repeats+AP-GTPase+COR+TM+TM*→||<-?<-?||?→||<-?||?→||<-PRTase<-SIG+TM+TM+TM+TM</td><td>LRR-repeats+AP-GTPase+COR+TM+TM</td><td>689</td><td>BCD67_11660</td><td>Cyanobacteria</td><td>Oscillatoriales cyanobacterium USR001</td><td>GTPase, partial [Oscillatoriales cyanobacterium USR001].</td><td>GCA_001698425.1</td></tr>
<tr><td>OCW73618.1</td><td>LRR-repeats+AP-GTPase+COR*→</td><td>LRR-repeats+AP-GTPase+COR</td><td>918</td><td>A4G24_00320</td><td>Bacteroidetes</td><td>Elizabethkingia anophelis</td><td>hypothetical protein A4G24_00320 [Elizabethkingia anophelis].</td><td>GCA_001703835.1</td></tr>
<tr><td>ODG98072.1</td><td>LRR-repeats+AP-GTPase+COR+TIR*→||<-CASPASE+TM</td><td>LRR-repeats+AP-GTPase+COR+TIR</td><td>858</td><td>A4S05_10875</td><td>Cyanobacteria</td><td>Nostoc sp. KVJ20</td><td>hypothetical protein A4S05_10875, partial [Nostoc sp. KVJ20].</td><td>GCA_001712795.1</td></tr>
<tr><td>ODS35505.1</td><td>Cluster846_3clades→ LRR-repeats→ LRR-repeats+AP-GTPase+COR*→?→ VWA→</td><td>LRR-repeats+AP-GTPase+COR</td><td>644</td><td>A7316_10690</td><td>DPANN group</td><td>Candidatus Altiarchaeales archaeon WOR_SM1_86-2</td><td>hypothetical protein A7316_10690 [Candidatus Altiarchaeales archaeon WOR_SM1_86-2].</td><td>GCA_001723855.1</td></tr>
<tr><td>ODS40288.1</td><td>LRR-repeats+AP-GTPase+CASPASE*→</td><td>LRR-repeats+AP-GTPase+CASPASE</td><td>811</td><td>A7315_09000</td><td>DPANN group</td><td>Candidatus Altiarchaeales archaeon WOR_SM1_79</td><td>hypothetical protein A7315_09000 [Candidatus Altiarchaeales archaeon WOR_SM1_79].</td><td>GCA_001723835.1</td></tr>
<tr><td>OED06513.1</td><td>LRR-repeats+AP-GTPase+COR*→</td><td>LRR-repeats+AP-GTPase+COR</td><td>666</td><td>A9239_11540</td><td>Euryarchaeota</td><td>Methanosarcina sp. A14</td><td>hypothetical protein A9239_11540 [Methanosarcina sp. A14].</td><td>GCA_001729375.1</td></tr>
<tr><td>OEU63639.1</td><td>LRR-repeats→ LRR-repeats+AP-GTPase+COR*→</td><td>LRR-repeats+AP-GTPase+COR</td><td>623</td><td>BBJ57_05215</td><td>Deltaproteobacteria</td><td>Desulfobacterales bacterium PC51MH44</td><td>hypothetical protein BBJ57_05215 [Desulfobacterales bacterium PC51MH44].</td><td>GCA_001751165.1</td></tr>
<tr><td>OFX48341.1</td><td>ABC-ATPase→?→ LRR-repeats+AP-GTPase+COR*→</td><td>LRR-repeats+AP-GTPase+COR</td><td>720</td><td>A2X10_07060</td><td>Bacteroidetes</td><td>Bacteroidetes bacterium GWA2_33_15</td><td>hypothetical protein A2X10_07060 [Bacteroidetes bacterium GWA2_33_15].</td><td>GCA_001769045.1</td></tr>
<tr><td>OFX79107.1</td><td>LRR-repeats+AP-GTPase+COR+REase7*→</td><td>LRR-repeats+AP-GTPase+COR+REase7</td><td>1101</td><td>A2X12_10435</td><td>Bacteroidetes</td><td>Bacteroidetes bacterium GWE2_29_8</td><td>hypothetical protein A2X12_10435 [Bacteroidetes bacterium GWE2_29_8].</td><td>GCA_001769085.1</td></tr>
<tr><td>OFY84271.1</td><td>LRR-repeats→ LRR-repeats+AP-GTPase+COR+REase7*→</td><td>LRR-repeats+AP-GTPase+COR+REase7</td><td>598</td><td>A3F72_14720</td><td>Bacteroidetes</td><td>Bacteroidetes bacterium RIFC-SPLOWO2_12_FULL_35_15</td><td>hypothetical protein A3F72_14720 [Bacteroidetes bacterium RIFC-SPLOWO2_12_FULL_35_15].</td><td>GCA_001769385.1</td></tr>
</tbody>
</table>

<table>
<thead>
<tr><th>acc</th><th>operon</th><th>architecture</th><th>len</th><th>gen.name</th><th>taxend</th><th>species</th><th>defline</th><th>gca</th></tr>
</thead>
<tbody>
<tr><td>OGC77574.1</td><td>LRR-repeats+AP-GTPase+COR*→?→||<-?<-CCTBP</td><td>LRR-repeats+AP-GTPase+COR</td><td>975</td><td>A2Z27_00115</td><td>FCB group</td><td>candidate division Zixibacteria bacterium RBG_16_50_21</td><td>hypothetical protein A2Z27_00115 [candidate division Zixibacteria bacterium RBG_16_50_21].</td><td>GCA_001773465.1</td></tr>
<tr><td>OGH99179.1</td><td>LRR-repeats+AP-GTPase+COR*→</td><td>LRR-repeats+AP-GTPase+COR</td><td>572</td><td>A2X43_07200</td><td>Terrabacteria group</td><td>Candidatus Margulisbacteria bacterium GWD2_39_127</td><td>hypothetical protein A2X43_07200 [Candidatus Margulisbacteria bacterium GWD2_39_127].</td><td>GCA_001783265.1</td></tr>
<tr><td>OGV70404.1</td><td>Pkinase+LRR-repeats+AP-GTPase+COR+TM+TM*→</td><td>Pkinase+LRR-repeats+AP-GTPase+COR+TM+TM</td><td>1157</td><td>A2283_19020</td><td>Lentisphaerae</td><td>Lentisphaerae bacterium RI-FOXYA12_FULL_48_11</td><td>hypothetical protein A2283_19020 [Lentisphaerae bacterium RIFOXYA12_FULL_48_11].</td><td>GCA_001804885.1</td></tr>
<tr><td>OHB50686.1</td><td>S1COLD→ LRR-repeats+AP-GTPase+COR+TIR*→</td><td>LRR-repeats+AP-GTPase+COR+TIR</td><td>953</td><td>A2Y10_02350</td><td>Planctomycetes</td><td>Planctomycetes bacterium GWF2_41_51</td><td>hypothetical protein A2Y10_02350 [Planctomycetes bacterium GWF2_41_51].</td><td>GCA_001825665.1</td></tr>
<tr><td>OHB52204.1</td><td>LRR-repeats+AP-GTPase+COR*→?→||<-?||SIG+TM→</td><td>LRR-repeats+AP-GTPase+COR</td><td>1147</td><td>A2Y10_11365</td><td>Planctomycetes</td><td>Planctomycetes bacterium GWF2_41_51</td><td>hypothetical protein A2Y10_11365 [Planctomycetes bacterium GWF2_41_51].</td><td>GCA_001825665.1</td></tr>
<tr><td>OHB64560.1</td><td>REC→ TPR-repeats→||<-?||PSE→||<-?<-?<-?||YBAK→ LRR-repeats+AP-GTPase+COR+DUF4404*→</td><td>LRR-repeats+AP-GTPase+COR+DUF4404</td><td>740</td><td>A2Y76_11060</td><td>Planctomycetes</td><td>Planctomycetes bacterium RBG_13_60_9</td><td>hypothetical protein A2Y76_11060 [Planctomycetes bacterium RBG_13_60_9].</td><td>GCA_001824685.1</td></tr>
<tr><td>OHD05988.1</td><td>LRR-repeats+AP-GTPase+COR+TM+TM*→ TraJ-RHH→ RelE-ParE→</td><td>LRR-repeats+AP-GTPase+COR+TM+TM</td><td>1257</td><td>A2Z98_10570</td><td>Spirochaetes</td><td>Spirochaetes bacterium GWB1_27_13</td><td>hypothetical protein A2Z98_10570 [Spirochaetes bacterium GWB1_27_13].</td><td>GCA_001829125.1</td></tr>
<tr><td>OHD18029.1</td><td>MORC→?→?→?→||<-?<-PSE<-?||LRR-repeats+AP-GTPase+COR→//<-?<-?<-?//?→?→?→?→ REC→//<-LRR-repeats+AP-GTPase+COR+DrHyd</td><td>LRR-repeats+AP-GTPase+COR</td><td>1064</td><td>A2Z98_11125</td><td>Spirochaetes</td><td>Spirochaetes bacterium GWB1_27_13</td><td>hypothetical protein A2Z98_11125 [Spirochaetes bacterium GWB1_27_13].</td><td>GCA_001829125.1</td></tr>
<tr><td>OHD18038.1</td><td>LRR-repeats+AP-GTPase+COR+DrHyd*→</td><td>LRR-repeats+AP-GTPase+COR+DrHyd</td><td>986</td><td>A2Z98_11170</td><td>Spirochaetes</td><td>Spirochaetes bacterium GWB1_27_13</td><td>hypothetical protein A2Z98_11170 [Spirochaetes bacterium GWB1_27_13].</td><td>GCA_001829125.1</td></tr>
<tr><td>OHD26231.1</td><td>LRR-repeats+AP-GTPase+COR+TM+TM*→ TraJ-RHH→ RelE-ParE→</td><td>LRR-repeats+AP-GTPase+COR+TM+TM</td><td>1234</td><td>A2Y34_11235</td><td>Spirochaetes</td><td>Spirochaetes bacterium GWC1_27_15</td><td>hypothetical protein A2Y34_11235 [Spirochaetes bacterium GWC1_27_15].</td><td>GCA_001829235.1</td></tr>
<tr><td>OHD42853.1</td><td>MORC→?→?→?→||<-?<-PSE<-?||LRR-repeats+AP-GTPase+COR→//<-?<-?<-?//?→?→?→?→ REC→//<-LRR-repeats+AP-GTPase+COR+DrHyd</td><td>LRR-repeats+AP-GTPase+COR</td><td>1020</td><td>A2086_15855</td><td>Spirochaetes</td><td>Spirochaetes bacterium GWD1_27_9</td><td>hypothetical protein A2086_15855 [Spirochaetes bacterium GWD1_27_9].</td><td>GCA_001830585.1</td></tr>
<tr><td>OIO84219.1</td><td><-XIS-HTH||HTH+HTH→||<-WLM||?→ NACHT+68TM-wHTH→ REase→?→ LRR-repeats+AP-GTPase+COR*→</td><td>LRR-repeats+AP-GTPase+COR</td><td>1004</td><td>AUK01_09955</td><td>Chloroflexi</td><td>Anaerolineae bacterium CG2_30_57_67</td><td>hypothetical protein AUK01_09955, partial [Anaerolineae bacterium CG2_30_57_67].</td><td>GCA_001872455.1</td></tr>
<tr><td>OJJ15121.1</td><td>LRR-repeats+AP-GTPase+COR*→</td><td>LRR-repeats+AP-GTPase+COR</td><td>1037</td><td>BKI52_39320</td><td>Bacteroidetes</td><td>marine bacterium AO1-C</td><td>hypothetical protein BKI52_39320 [marine bacterium AO1-C].</td><td>GCA_001890965.1</td></tr>
<tr><td>OJJ19161.1</td><td>LRR-repeats+AP-GTPase+COR*→</td><td>LRR-repeats+AP-GTPase+COR</td><td>851</td><td>BKI52_20325</td><td>Bacteroidetes</td><td>marine bacterium AO1-C</td><td>hypothetical protein BKI52_20325 [marine bacterium AO1-C].</td><td>GCA_001890965.1</td></tr>
<tr><td>OJJ19619.1</td><td>LRR-repeats+AP-GTPase+COR*→||<-?<-?<-?<-?<-TetR-HTH</td><td>LRR-repeats+AP-GTPase+COR</td><td>723</td><td>BKI52_22705</td><td>Bacteroidetes</td><td>marine bacterium AO1-C</td><td>hypothetical protein BKI52_22705, partial [marine bacterium AO1-C].</td><td>GCA_001890965.1</td></tr>
<tr><td>OJJ19690.1</td><td><-Four-helical-protein<-?<-?<-?<-?||LRR-repeats+AP-GTPase+COR*→</td><td>LRR-repeats+AP-GTPase+COR</td><td>1463</td><td>BKI52_19350</td><td>Bacteroidetes</td><td>marine bacterium AO1-C</td><td>hypothetical protein BKI52_19350 [marine bacterium AO1-C].</td><td>GCA_001890965.1</td></tr>
<tr><td>OJJ20943.1</td><td>Cluster2042_2clades→||<-?<-?||?→?→?→||<-?||LRR-repeats+AP-GTPase+COR+EAD11*→||<-PAS+HISKIN</td><td>LRR-repeats+AP-GTPase+COR+EAD11</td><td>871</td><td>BKI52_10210</td><td>Bacteroidetes</td><td>marine bacterium AO1-C</td><td>hypothetical protein BKI52_10210 [marine bacterium AO1-C].</td><td>GCA_001890965.1</td></tr>
<tr><td>OJJ21241.1</td><td>LRR-repeats+AP-GTPase+COR*→?→||<-?<-cNMPBD</td><td>LRR-repeats+AP-GTPase+COR</td><td>910</td><td>BKI52_11795</td><td>Bacteroidetes</td><td>marine bacterium AO1-C</td><td>hypothetical protein BKI52_11795 [marine bacterium AO1-C].</td><td>GCA_001890965.1</td></tr>
<tr><td>OJJ22385.1</td><td><-SIGMA-HTH||?→?→?→?→?→||<-?||LRR-repeats+AP-GTPase+COR*→?→?→?→||<-?<-?<-?<-SIGMA-HTH</td><td>LRR-repeats+AP-GTPase+COR</td><td>1192</td><td>BKI52_06800</td><td>Bacteroidetes</td><td>marine bacterium AO1-C</td><td>hypothetical protein BKI52_06800 [marine bacterium AO1-C].</td><td>GCA_001890965.1</td></tr>
<tr><td>OJJ23263.1</td><td>D5ATPase→?→ LRR-repeats+AP-GTPase+COR*→</td><td>LRR-repeats+AP-GTPase+COR</td><td>1296</td><td>BKI52_02595</td><td>Bacteroidetes</td><td>marine bacterium AO1-C</td><td>hypothetical protein BKI52_02595 [marine bacterium AO1-C].</td><td>GCA_001890965.1</td></tr>
</tbody>
</table>

<table>
<tr><th>acc</th><th>operon</th><th>architecture</th><th>len</th><th>gen.name</th><th>taxend</th><th>species</th><th>defline</th><th>gca</th></tr>
<tr><td>OJJ23599.1</td><td>SF2-DUF3427A→?→ TRD+TRD→?→?→ LRR-repeats+AP-GTPase+COR*→?→||<-?<-LRR-repeats<-?||cNMPBD→</td><td>LRR-repeats+AP-GTPase+COR</td><td>1001</td><td>BKI52_04350</td><td>Bacteroidetes</td><td>marine bacterium AO1-C</td><td>hypothetical protein BKI52_04350 [marine bacterium AO1-C].</td><td>GCA_001890965.1</td></tr>
<tr><td>OKH11101.1</td><td>LRR-repeats+AP-GTPase+COR+TM+TM*→</td><td>LRR-repeats+AP-GTPase+COR+TM+TM</td><td>944</td><td>NIES208_17750</td><td>Cyanobacteria</td><td>Limnothrix rosea IAM M-220</td><td>hypothetical protein NIES208_17750 [Limnothrix rosea IAM M-220].</td><td>GCA_001904615.1</td></tr>
<tr><td>OKH34496.1</td><td>LRR-repeats+AP-GTPase+COR+TM+TM*→||<-CASPASE+TPR</td><td>LRR-repeats+AP-GTPase+COR+TM+TM</td><td>902</td><td>FACHB389_15645</td><td>Cyanobacteria</td><td>Nostoc calcicola FACHB-389</td><td>GTPase [Nostoc calcicola FACHB-389].</td><td>GCA_001904715.1</td></tr>
<tr><td>OLE55789.1</td><td><-RFC-AAA<-?<-Trichomonas-DAM<-?<-?<-ISOCOT||LRR-repeats+AP-GTPase+COR+TIR*→</td><td>LRR-repeats+AP-GTPase+COR+TIR</td><td>862</td><td>AUG51_01855</td><td>Acidobacteria</td><td>Acidobacteria bacterium 13_1_20CM_3_53_8</td><td>serine/threonine protein kinase [Acidobacteria bacterium 13_1_20CM_3_53_8].</td><td>GCA_001920415.1</td></tr>
<tr><td>OLF12412.1</td><td>LRR-repeats+AP-GTPase+COR*→?→?→?→||<-?<-ABC-ATPase+ABC-ATPase</td><td>LRR-repeats+AP-GTPase+COR</td><td>927</td><td>BLA60_09370</td><td>Actinobacteria</td><td>Actinophytocola xinjiangensis</td><td>hypothetical protein BLA60_09370 [Actinophytocola xinjiangensis].</td><td>GCA_001921215.1</td></tr>
<tr><td>OQX01793.1</td><td>LRR-repeats+AP-GTPase+COR+TIR*→||<-?<-PSE<-?<-PSE<-PSE<-?<-?<-?||PSE→||<-?||ABhydrolase→</td><td>LRR-repeats+AP-GTPase+COR+TIR</td><td>928</td><td>BWK73_44950</td><td>Gammaproteobacteria</td><td>Thiothrix lacustris</td><td>GTP-binding protein [Thiothrix lacustris].</td><td>GCA_002083875.1</td></tr>
<tr><td>OQX02054.1</td><td>nSTAND3+REase-DUF4143→ LRR-repeats+AP-GTPase+COR*→ TIR→?→||<-Thioredoxin</td><td>LRR-repeats+AP-GTPase+COR</td><td>784</td><td>BWK73_43990</td><td>Gammaproteobacteria</td><td>Thiothrix lacustris</td><td>hypothetical protein BWK73_43990 [Thiothrix lacustris].</td><td>GCA_002083875.1</td></tr>
<tr><td>OQX07365.1</td><td>LRR-repeats+AP-GTPase+COR+CASPASE*→</td><td>LRR-repeats+AP-GTPase+COR+CASPASE</td><td>879</td><td>BWK80_49550</td><td>Deltaproteobacteria</td><td>Desulfobacteraceae bacterium IS3</td><td>hypothetical protein BWK80_49550 [Desulfobacteraceae bacterium IS3].</td><td>GCA_002083955.1</td></tr>
<tr><td>OQX24659.1</td><td>LRR-repeats+AP-GTPase+COR→ *Cluster2372_2clades→?→ LRR-repeats+AP-GTPase+COR*→ LRR-repeats+AP-GTPase+COR*→</td><td>LRR-repeats+AP-GTPase+COR</td><td>881</td><td>BWK80_19660</td><td>Deltaproteobacteria</td><td>Desulfobacteraceae bacterium IS3</td><td>hypothetical protein BWK80_19660 [Desulfobacteraceae bacterium IS3].</td><td>GCA_002083955.1</td></tr>
<tr><td>OQX24662.1</td><td>LRR-repeats+AP-GTPase+COR+TM*→</td><td>LRR-repeats+AP-GTPase+COR+TM</td><td>925</td><td>BWK80_19675</td><td>Deltaproteobacteria</td><td>Desulfobacteraceae bacterium IS3</td><td>hypothetical protein BWK80_19675 [Desulfobacteraceae bacterium IS3].</td><td>GCA_002083955.1</td></tr>
<tr><td>OQX24663.1</td><td>LRR-repeats+AP-GTPase+COR*→</td><td>LRR-repeats+AP-GTPase+COR</td><td>870</td><td>BWK80_19680</td><td>Deltaproteobacteria</td><td>Desulfobacteraceae bacterium IS3</td><td>hypothetical protein BWK80_19680 [Desulfobacteraceae bacterium IS3].</td><td>GCA_002083955.1</td></tr>
<tr><td>OQX25920.1</td><td>LRR-repeats+AP-GTPase+COR*→?→?→?→?→ ABC-ATPase→ SIG+TM+TM+TM+TM+TM→||<-Cluster968_2clades</td><td>LRR-repeats+AP-GTPase+COR</td><td>802</td><td>BWK80_13160</td><td>Deltaproteobacteria</td><td>Desulfobacteraceae bacterium IS3</td><td>hypothetical protein BWK80_13160 [Desulfobacteraceae bacterium IS3].</td><td>GCA_002083955.1</td></tr>
<tr><td>OQY27201.1</td><td>LRR-repeats+AP-GTPase+COR*→?→||<-?<-?<-?||TM+TM+TM+TM+TM+TM→</td><td>LRR-repeats+AP-GTPase+COR</td><td>700</td><td>B6244_11350</td><td>FCB group</td><td>Candidatus Cloacimonetes bacterium 4572_55</td><td>hypothetical protein B6244_11350 [Candidatus Cloacimonetes bacterium 4572_55].</td><td>GCA_002084765.1</td></tr>
<tr><td>OQY46926.1</td><td>LRR-repeats+AP-GTPase+COR*→||<-PSE||?→ LRR-repeats→</td><td>LRR-repeats+AP-GTPase+COR</td><td>887</td><td>B6247_27150</td><td>Gammaproteobacteria</td><td>Beggiatoa sp. 4572_84</td><td>GTPase [Beggiatoa sp. 4572_84].</td><td>GCA_002085445.1</td></tr>
<tr><td>OQY52602.1</td><td>SIG+TM+TM+TM+TM+TM+TM+TM→?→?→?→?→ LRR-repeats+AP-GTPase+COR→*?→?→?→ LRR-repeats+AP-GTPase+COR*→</td><td>LRR-repeats+AP-GTPase+COR</td><td>429</td><td>B6245_23480</td><td>Deltaproteobacteria</td><td>Desulfobacteraceae bacterium 4572_88</td><td>hypothetical protein B6245_23480, partial [Desulfobacteraceae bacterium 4572_88].</td><td>GCA_002085455.1</td></tr>
<tr><td>OQY52606.1</td><td>LRR-repeats+AP-GTPase+COR*→</td><td>LRR-repeats+AP-GTPase+COR</td><td>933</td><td>B6245_23500</td><td>Deltaproteobacteria</td><td>Desulfobacteraceae bacterium 4572_88</td><td>hypothetical protein B6245_23500 [Desulfobacteraceae bacterium 4572_88].</td><td>GCA_002085455.1</td></tr>
<tr><td>OQY56003.1</td><td>CBS→ PSE→||<-?||?→?→ LRR-repeats→?→ PSE→ LRR-repeats→ LRR-repeats+AP-GTPase+COR*→</td><td>LRR-repeats+AP-GTPase+COR</td><td>907</td><td>B6247_05655</td><td>Gammaproteobacteria</td><td>Beggiatoa sp. 4572_84</td><td>GTPase [Beggiatoa sp. 4572_84].</td><td>GCA_002085445.1</td></tr>
<tr><td>OQY58390.1</td><td>LRR-repeats+AP-GTPase+COR*→ SIG+DADALIGASE-ATPgrasp→</td><td>LRR-repeats+AP-GTPase+COR</td><td>791</td><td>B6245_12095</td><td>Deltaproteobacteria</td><td>Desulfobacteraceae bacterium 4572_88</td><td>hypothetical protein B6245_12095 [Desulfobacteraceae bacterium 4572_88].</td><td>GCA_002085455.1</td></tr>
<tr><td>OSM01684.1</td><td>SIGMA-HTH→?→||<-?<-?<-?<-?<-?||LRR-repeats+AP-GTPase+COR*→</td><td>LRR-repeats+AP-GTPase+COR</td><td>876</td><td>MAIT1_01702</td><td>Alphaproteobacteria</td><td>Magnetofaba australis IT-1</td><td>putative small GTP-binding protein [Magnetofaba australis IT-1].</td><td>GCA_002109495.1</td></tr>
<tr><td>OUC12119.1</td><td>LRR-repeats+AP-GTPase+COR+DrHyd*→?→?→ FGS→</td><td>LRR-repeats+AP-GTPase+COR+DrHyd</td><td>810</td><td>B0A82_24265</td><td>Cyanobacteria</td><td>Alkalinema sp. CACIAM 70d</td><td>hypothetical protein B0A82_24265, partial [Alkalinema sp. CACIAM 70d].</td><td>GCA_002148405.1</td></tr>
<tr><td>OUC12470.1</td><td><-HISKIN<-?<-?<-PRTase<-?||?→ LRR-repeats+AP-GTPase+COR*→</td><td>LRR-repeats+AP-GTPase+COR</td><td>820</td><td>B0A82_22225</td><td>Cyanobacteria</td><td>Alkalinema sp. CACIAM 70d</td><td>hypothetical protein B0A82_22225 [Alkalinema sp. CACIAM 70d].</td><td>GCA_002148405.1</td></tr>
<tr><td>OUC13078.1</td><td><-TIR+TM+CHASE2+CHASE2+TM+TM+TM<-SIG+RnaseT2<-?||?→?→ LRR-repeats+AP-GTPase+COR*→</td><td>LRR-repeats+AP-GTPase+COR</td><td>919</td><td>B0A82_18905</td><td>Cyanobacteria</td><td>Alkalinema sp. CACIAM 70d</td><td>GTPase [Alkalinema sp. CACIAM 70d].</td><td>GCA_002148405.1</td></tr>
<tr><td>OUC15727.1</td><td>LRR-repeats+AP-GTPase+COR+DUF4404*→?→||<-Cluster2098_2clades</td><td>LRR-repeats+AP-GTPase+COR+DUF4404</td><td>969</td><td>B0A82_05270</td><td>Cyanobacteria</td><td>Alkalinema sp. CACIAM 70d</td><td>hypothetical protein B0A82_05270, partial [Alkalinema sp. CACIAM 70d].</td><td>GCA_002148405.1</td></tr>
</table>

<table>
<tr><th>acc</th><th>operon</th><th>architecture</th><th>len</th><th>gen.name</th><th>taxend</th><th>species</th><th>defline</th><th>gca</th></tr>
<tr><td>OWK46761.1</td><td><-Aminotran_1_2||LRR-repeats+AP-GTPase+COR+TIR*→||<-?<-?<-?<-DSBH+AraC-HTH+AraC-HTH||?→?→ SIG+TM+TM+TM→</td><td>LRR-repeats+AP-GTPase+COR+TIR</td><td>1310</td><td>FRUB_00460</td><td>Planctomycetes</td><td>Fimbriiglobus ruber</td><td>HtrA protease/chaperone protein [Fimbriiglobus ruber].</td><td>GCA_002197845.1</td></tr>
<tr><td>OWK47329.1</td><td><-RelE-ParE<-?||?→ LRR-repeats→||<-?||LRR-repeats+AP-GTPase+COR*→</td><td>LRR-repeats+AP-GTPase+COR</td><td>967</td><td>FRUB_01028</td><td>Planctomycetes</td><td>Fimbriiglobus ruber</td><td>putative internalin [Fimbriiglobus ruber].</td><td>GCA_002197845.1</td></tr>
<tr><td>OYQ36252.1</td><td><-SIG+TM+TM+TM+HISKIN<-?<-?<-?||?→ LRR-repeats+AP-GTPase+COR+TIR*→ ParA-Soj-PloopNTPase→ ParB→</td><td>LRR-repeats+AP-GTPase+COR+TIR</td><td>890</td><td>CHU95_05565</td><td>Alphaproteobacteria</td><td>Niveispirillum lacus</td><td>hypothetical protein CHU95_05565 [Niveispirillum lacus].</td><td>GCA_002251795.1</td></tr>
<tr><td>OYU93091.1</td><td>LRR-repeats+AP-GTPase+COR+TM+TM*→</td><td>LRR-repeats+AP-GTPase+COR+TM+TM</td><td>1040</td><td>CFE21_21825</td><td>Bacteroidetes</td><td>Bacteroidetes bacterium B1(2017)</td><td>hypothetical protein CFE21_21825, partial [Bacteroidetes bacterium B1(2017)].</td><td>GCA_002256395.1</td></tr>
<tr><td>OYW76150.1</td><td>LRR-repeats+AP-GTPase+COR+TIR*→?→||<-?<-WCAK||?→ REC→ REC→</td><td>LRR-repeats+AP-GTPase+COR+TIR</td><td>996</td><td>B7Z37_09950</td><td>Verrucomicrobia</td><td>Verrucomicrobia bacterium 12-59-8</td><td>hypothetical protein B7Z37_09950 [Verrucomicrobia bacterium 12-59-8].</td><td>GCA_002279765.1</td></tr>
<tr><td>OYW77555.1</td><td>SIG+ABhydrolase→?→ LRR-repeats+AP-GTPase+COR+TIR*→</td><td>LRR-repeats+AP-GTPase+COR+TIR</td><td>939</td><td>B7Z37_03775</td><td>Verrucomicrobia</td><td>Verrucomicrobia bacterium 12-59-8</td><td>hypothetical protein B7Z37_03775 [Verrucomicrobia bacterium 12-59-8].</td><td>GCA_002279765.1</td></tr>
<tr><td>OYW78304.1</td><td>FGS→ LRR-repeats+AP-GTPase+COR*→</td><td>LRR-repeats+AP-GTPase+COR</td><td>1063</td><td>B7Z37_00425</td><td>Verrucomicrobia</td><td>Verrucomicrobia bacterium 12-59-8</td><td>hypothetical protein B7Z37_00425 [Verrucomicrobia bacterium 12-59-8].</td><td>GCA_002279765.1</td></tr>
<tr><td>PCI29610.1</td><td>LRR-repeats+RNA-Helicase+AP-GTPase+COR+TIR*→?→||<-AraC-HTH||?→?→||<-Cluster1048_2clades<-POLYSACPOLYMERASE</td><td>LRR-repeats+RNA-Helicase+AP-GTPase+COR+TIR</td><td>1264</td><td>COB67_03805</td><td>Deltaproteobacteria</td><td>SAR324 cluster bacterium</td><td>hypothetical protein COB67_03805 [SAR324 cluster bacterium].</td><td>GCA_002401295.1</td></tr>
<tr><td>PCK09719.1</td><td>LRR-repeats+AP-GTPase+COR*→</td><td>LRR-repeats+AP-GTPase+COR</td><td>908</td><td>COA42_02805</td><td>Gammaproteobacteria</td><td>Alteromonadaceae bacterium</td><td>hypothetical protein COA42_02805 [Alteromonadaceae bacterium].</td><td>GCA_002746115.1</td></tr>
<tr><td>PHM06637.1</td><td>LRR-repeats+AP-GTPase+COR+TM+TM*→</td><td>LRR-repeats+AP-GTPase+COR+TM+TM</td><td>817</td><td>CK516_32280</td><td>Cyanobacteria</td><td>Nostoc sp. ‘Peltigera malacea cyanobiont’ DB3992</td><td>GTPase [Nostoc sp. ‘Peltigera malacea cyanobiont’ DB3992].</td><td>GCA_002631755.1</td></tr>
<tr><td>PHS08569.1</td><td>LRR-repeats+AP-GTPase+COR*→ SIG+TM+TM+TM+TM+TM+TM+TM+TM+TM+TM+TM→</td><td>LRR-repeats+AP-GTPase+COR</td><td>924</td><td>COA88_06290</td><td>Bacteroidetes</td><td>Kordia sp.</td><td>GTP-binding protein [Kordia sp.].</td><td>GCA_002733415.1</td></tr>
<tr><td>PID45108.1</td><td>Cluster1540_2clades→?→?→?→?→?→ tRNA→ LRR-repeats+AP-GTPase+COR*→||<-?<-?||Cluster2057_2clades→</td><td>LRR-repeats+AP-GTPase+COR</td><td>1016</td><td>CSB47_10150</td><td>Proteobacteria</td><td>Proteobacteria bacterium</td><td>hypothetical protein CSB47_10150 [Proteobacteria bacterium].</td><td>GCA_002747455.1</td></tr>
<tr><td>PID49971.1</td><td>SIG+TM+TM+TM+TM+TM+TM+TM+TM+TM+TM+TM→
LRR-repeats+AP-GTPase+COR+TIR*→?→||<-?<-?<-?<-?<-Cluster1063_3clades<-Cluster1067_3clades</td><td>LRR-repeats+AP-GTPase+COR+TIR</td><td>916</td><td>CR991_03995</td><td>Proteobacteria</td><td>Proteobacteria bacterium</td><td>hypothetical protein CR991_03995 [Proteobacteria bacterium].</td><td>GCA_002747435.1</td></tr>
<tr><td>PID58617.1</td><td><-Cluster1581_2clades<-?||NUDIX→ LRR-repeats+AP-GTPase+COR*→</td><td>LRR-repeats+AP-GTPase+COR</td><td>1061</td><td>CSB45_03475</td><td>Bacteria</td><td>candidate division KSB3 bacterium</td><td>GTPase [candidate division KSB3 bacterium].</td><td>GCA_002747525.1</td></tr>
<tr><td>PIE10915.1</td><td>LRR-repeats+AP-GTPase+COR*→||<-?<-tRNA||?→?→ Cluster1995_2clades→</td><td>LRR-repeats+AP-GTPase+COR</td><td>994</td><td>CSA72_05310</td><td>Alphaproteobacteria</td><td>Rhodobacterales bacterium</td><td>hypothetical protein CSA72_05310 [Rhodobacterales bacterium].</td><td>GCA_002748265.1</td></tr>
<tr><td>PJB68563.1</td><td><-XIS-HTH||HTH+HTH→||<-WLM||?→ NACHT+68TM-wHTH→ REase→?→ LRR-repeats+AP-GTPase+COR*→</td><td>LRR-repeats+AP-GTPase+COR</td><td>947</td><td>CO094_00645</td><td>Chloroflexi</td><td>Anaerolineae bacterium CG_4_9_14_3_um_filter_57_17</td><td>hypothetical protein CO094_00645, partial [Anaerolineae bacterium CG_4_9_14_3_um_filter_57_17].</td><td>GCA_002789715.1</td></tr>
<tr><td>PJF39486.1</td><td>LRR-repeats+AP-GTPase+COR+TM+TM*→</td><td>LRR-repeats+AP-GTPase+COR+TM+TM</td><td>751</td><td>CUN54_08620</td><td>Chloroflexi</td><td>Candidatus Thermofonsia Clade 2 bacterium</td><td>GTP-binding protein [Candidatus Thermofonsia Clade 2 bacterium].</td><td>GCA_002794595.1</td></tr>
<tr><td>PKB73283.1</td><td>LRR-repeats+AP-GTPase+COR*→||<-?<-?<-PSE<-SIG+ABhydrolase<-?<-ParA-Soj-PloopNTPase</td><td>LRR-repeats+AP-GTPase+COR</td><td>593</td><td>BZY75_02725</td><td>Chloroflexi</td><td>SAR202 cluster bacterium Io17-Chloro-G7</td><td>hypothetical protein BZY75_02725 [SAR202 cluster bacterium Io17-Chloro-G7].</td><td>GCA_002817055.1</td></tr>
<tr><td>PKD18393.1</td><td>LRR-repeats+AP-GTPase+COR*→</td><td>LRR-repeats+AP-GTPase+COR</td><td>845</td><td>APR41_04380</td><td>Bacteroidetes</td><td>Salegentibacter salinarum</td><td>hypothetical protein APR41_04380 [Salegentibacter salinarum].</td><td>GCA_002833365.1</td></tr>
<tr><td>PKN92928.1</td><td>UvsW-A18→ LRR-repeats+AP-GTPase+COR→ NACHT+68TM-wHTH→ LRR-repeats+AP-GTPase+COR→
LRR-repeats+AP-GTPase+COR+DUF4404→?→?→ Cluster1559_2clades→?→?→||<-?||LRR-repeats+AP-GTPase+COR→</td><td>LRR-repeats+AP-GTPase+COR</td><td>769</td><td>CVU44_12925</td><td>Chloroflexi</td><td>Chloroflexi bacterium HGW-Chloroflexi-6</td><td>GTP-binding protein [Chloroflexi bacterium HGW-Chloroflexi-6].</td><td>GCA_002840685.1</td></tr>
<tr><td>PKN92930.1</td><td>LRR-repeats+AP-GTPase+COR*→</td><td>LRR-repeats+AP-GTPase+COR</td><td>1044</td><td>CVU44_12935</td><td>Chloroflexi</td><td>Chloroflexi bacterium HGW-Chloroflexi-6</td><td>hypothetical protein CVU44_12935 [Chloroflexi bacterium HGW-Chloroflexi-6].</td><td>GCA_002840685.1</td></tr>
<tr><td>PKN92931.1</td><td>LRR-repeats+AP-GTPase+COR+DUF4404*→</td><td>LRR-repeats+AP-GTPase+COR+DUF4404</td><td>812</td><td>CVU44_12940</td><td>Chloroflexi</td><td>Chloroflexi bacterium HGW-Chloroflexi-6</td><td>hypothetical protein CVU44_12940 [Chloroflexi bacterium HGW-Chloroflexi-6].</td><td>GCA_002840685.1</td></tr>
<tr><td>PKN92938.1</td><td>LRR-repeats+AP-GTPase+COR*→</td><td>LRR-repeats+AP-GTPase+COR</td><td>673</td><td>CVU44_12975</td><td>Chloroflexi</td><td>Chloroflexi bacterium HGW-Chloroflexi-6</td><td>GTP-binding protein [Chloroflexi bacterium HGW-Chloroflexi-6].</td><td>GCA_002840685.1</td></tr>
</table>

acc	operon	architecture	len	gen.name	taxend	species	defline	gca
PKP35889.1	REC→ HISKIN→?→ LRR-repeats+AP-GTPase+COR*→	LRR-repeats+AP-GTPase+COR	654	CVU00_00150	Bacteroidetes	Bacteroidetes bacterium HGW-Bacteroidetes-17	hypothetical protein CVU00_00150 [Bacteroidetes bacterium HGW-Bacteroidetes-17].	GCA_002840985.1
PLX96646.1	LRR-repeats+AP-GTPase+COR+DrHyd*→||<-?||?→?→?→||<-?<-?<-SIG+PDZ	LRR-repeats+AP-GTPase+COR+DrHyd	1001	C0622_14350	Deltaproteobacteria	Desulfuromonas sp.	hypothetical protein C0622_14350 [Desulfuromonas sp.].	GCA_002869665.1
PLY10861.1	LRR-repeats+AP-GTPase+COR*→||<-?<-tRNA<-?<-?||Cluster1398_2clades→ ABC-ATPase→	LRR-repeats+AP-GTPase+COR	1225	C0626_04310	Epsilonproteobacteria	Arcobacter sp.	hypothetical protein C0626_04310 [Arcobacter sp.].	GCA_002869535.1
POZ53622.1	REC→||<-?<-?<-?<-?||?→ LRR-repeats+AP-GTPase+COR+DpnII-MboI-NTD+TIR*→	LRR-repeats+AP-GTPase+COR+DpnII-MboI-NTD+TIR	1108	AADEFJLK_00657	Gammaproteobacteria	Methylovulum psychrotolerans	GTP-binding protein [Methylovulum psychrotolerans].	GCA_002923755.1
PPT07796.1	LRR-repeats+AP-GTPase+COR+BactIG*→	LRR-repeats+AP-GTPase+COR+BactIG	726	CKA32_002768	Cyanobacteria	Geitlerinema sp. FC II	Leucine-rich repeat containing protein [Geitlerinema sp. FC II].	GCA_002286845.1
PRQ08092.1	LRR-repeats+AP-GTPase+COR+PNPase*→	LRR-repeats+AP-GTPase+COR+PNPase	933	mtnN	Deltaproteobacteria	Enhygromyxa salina	5'-methylthioadenosine/S-adenosylhomocysteine nucleosidase [Enhygromyxa salina].	GCA_002994635.1
PSB34361.1	<-REC||REC→||<-?<-?<-?<-TIR+TM+CHASE2+CHASE2+TM+TM+TM||PSE→ LRR-repeats+AP-GTPase+COR+TIR*→	LRR-repeats+AP-GTPase+COR+TIR	1113	C7B82_02520	Cyanobacteria	Stenomitos frigidus ULC18	serine/threonine protein kinase [Stenomitos frigidus ULC18].	GCA_003003795.1
PSM30901.1	LRR-repeats+AP-GTPase+COR*→	LRR-repeats+AP-GTPase+COR	431	BVG81_008130	Deltaproteobacteria	Haliangium sp. UPWRP_2	GTPase, partial [Haliangium sp. UPWRP_2].	GCA_002212765.2
PTS96034.1	LRR-repeats+AP-GTPase*→	LRR-repeats+AP-GTPase	357	DBR27_16615	Bacteroidetes	Flavobacterium sp. HMWF030	hypothetical protein DBR27_16615, partial [Flavobacterium sp. HMWF030].	GCA_003061345.1
PWQ95663.1	LRR-repeats+AP-GTPase+COR+TIR*→	LRR-repeats+AP-GTPase+COR+TIR	801	DKT75_11555	Gammaproteobacteria	Leucothrix arctica	GTP-binding protein, partial [Leucothrix arctica].	GCA_003172895.1
PXF61747.1	LRR-repeats+AP-GTPase+COR*→	LRR-repeats+AP-GTPase+COR	978	C4B59_02515	Euryarchaeota	ANME-2 cluster archaeon	hypothetical protein C4B59_02515 [ANME-2 cluster archaeon].	GCA_003194445.1
PXX81924.1	<-ACET<-?<-?<-?||?→ HSP70→ DNAJ→ LRR-repeats+AP-GTPase+COR+TIR*→	LRR-repeats+AP-GTPase+COR+TIR	947	DFR34_101153	Betaproteobacteria	Rivicola pingtungensis	internalin A [Rivicola pingtungensis].	GCA_003201855.1
PYI92098.1	LRR-repeats+AP-GTPase+COR+TIR*→	LRR-repeats+AP-GTPase+COR+TIR	882	DME97_11675	Verrucomicrobia	Verrucomicrobia bacterium	hypothetical protein DME97_11675 [Verrucomicrobia bacterium].	GCA_003218375.1
PYQ65591.1	LRR-repeats+AP-GTPase+COR+TIR*→||<-START	LRR-repeats+AP-GTPase+COR+TIR	985	DMF53_05195	Acidobacteria	Acidobacteria bacterium	hypothetical protein DMF53_05195 [Acidobacteria bacterium].	GCA_003223675.1
PZN70482.1	LRR-repeats+AP-GTPase+COR*→ TIR→	LRR-repeats+AP-GTPase+COR	479	DM484_28490	Gammaproteobacteria	Candidatus Methyloumidiphilus alinensis	hypothetical protein DM484_28490 [Candidatus Methyloumidiphilus alinensis].	GCA_003242955.1
PZN77149.1	LRR-repeats+AP-GTPase+COR+TIR*→	LRR-repeats+AP-GTPase+COR+TIR	1032	DM484_15240	Gammaproteobacteria	Candidatus Methyloumidiphilus alinensis	hypothetical protein DM484_15240 [Candidatus Methyloumidiphilus alinensis].	GCA_003242955.1
PZN84233.1	LRR-repeats+AP-GTPase+COR+TIR*→	LRR-repeats+AP-GTPase+COR+TIR	820	DM484_03135	Gammaproteobacteria	Candidatus Methyloumidiphilus alinensis	GTP-binding protein [Candidatus Methyloumidiphilus alinensis].	GCA_003242955.1
PZO19892.1	<-SIG+TM+TM+TM||?→?→?→ LRR-repeats+AP-GTPase+COR*→	LRR-repeats+AP-GTPase+COR	715	DCF25_07770	Cyanobacteria	Leptolyngbya foveolarum	hypothetical protein DCF25_07770, partial [Leptolyngbya foveolarum].	GCA_003242035.1
PZO41190.1	LRR-repeats+AP-GTPase+COR*→||<-PIN<-PSE||HISKIN→ REC→	LRR-repeats+AP-GTPase+COR	872	DCF19_10445	Cyanobacteria	Pseudanabaena frigida	GTPase [Pseudanabaena frigida].	GCA_003242085.1
PZO43272.1	Cluster53_2clades→ Cluster53_2clades→?→ LRR-repeats+AP-GTPase+COR+CASPASE*→||<-?<-?||?→?→ Cluster944_3clades→||<-tRNA<-NUDIX||REC→	LRR-repeats+AP-GTPase+COR+CASPASE	1158	DCF19_04785	Cyanobacteria	Pseudanabaena frigida	GTPase [Pseudanabaena frigida].	GCA_003242085.1
PZO45083.1	PAS+HISKIN→ REC→ HISKIN→||<-?<-?<-?||LRR-repeats+AP-GTPase+COR*→||<-?||?→||<-?||?→?→?→||<-ABC-ATPase	LRR-repeats+AP-GTPase+COR	1026	DCF19_00905	Cyanobacteria	Pseudanabaena frigida	hypothetical protein DCF19_00905 [Pseudanabaena frigida].	GCA_003242085.1
PZS26024.1	LRR-repeats+AP-GTPase*→	LRR-repeats+AP-GTPase	304	DLM61_19115	Actinobacteria	Pseudonocardiales bacterium	hypothetical protein DLM61_19115 [Pseudonocardiales bacterium].	GCA_003244245.1
PZU97949.1	<-Cluster53_2clades||?→ Cluster53_2clades→ Cluster54_3clades→?→ RelE-ParE→ LRR-repeats+AP-GTPase+COR+DUF4404*→?→?→||<-Cluster1583_2clades<-Cluster1881_2clades<-Cluster1340_2clades	LRR-repeats+AP-GTPase+COR+DUF4404	1125	DCE90_05495	Cyanobacteria	Pseudanabaena sp.	Ras family protein [Pseudanabaena sp.].	GCA_003249015.1

<table>
<tr><th>acc</th><th>operon</th><th>architecture</th><th>len</th><th>gen.name</th><th>taxend</th><th>species</th><th>defline</th><th>gca</th></tr>
<tr><td>PZV15467.1</td><td>Cluster53_2clades→?→ LRR-repeats+AP-GTPase+COR+TM+TM*→?→||<-?<-?<-ABC-ATPase<-?||PSE→?→ ABC_membrane+ABC_tran→</td><td>LRR-repeats+AP-GTPase+COR+TM+TM</td><td>966</td><td>DCF20_10315</td><td>Cyanobacteria</td><td>Pseudanabaena sp.</td><td>GTP-binding protein [Pseudanabaena sp.].</td><td>GCA_003249035.1</td></tr>
<tr><td>PZV19789.1</td><td>LRR-repeats+AP-GTPase+COR*→?→ Cluster2508_2clades→</td><td>LRR-repeats+AP-GTPase+COR</td><td>779</td><td>DCF21_06795</td><td>Cyanobacteria</td><td>Leptolyngbya sp.</td><td>GTP-binding protein [Leptolyngbya sp.].</td><td>GCA_003249155.1</td></tr>
<tr><td>PZV24423.1</td><td>LRR-repeats+AP-GTPase+COR*→</td><td>LRR-repeats+AP-GTPase+COR</td><td>522</td><td>DCF12_17370</td><td>Cyanobacteria</td><td>Snowella sp.</td><td>hypothetical protein DCF12_17370 [Snowella sp.].</td><td>GCA_003249095.1</td></tr>
<tr><td>PZV25744.1</td><td><-REC||?→?→||<-?<-?||?→ LRR-repeats+AP-GTPase+COR*→||<-DAGKIN||?→ Cluster5_5clades→</td><td>LRR-repeats+AP-GTPase+COR</td><td>860</td><td>DCF12_13370</td><td>Cyanobacteria</td><td>Snowella sp.</td><td>GTP-binding protein [Snowella sp.].</td><td>GCA_003249095.1</td></tr>
<tr><td>PZV26136.1</td><td><-Cluster1004_3clades||Cluster1843_2clades→||<-?||Cluster5_5clades→||<-Cluster1306_2clades<-?<-?||LRR-repeats+AP-GTPase+COR*→</td><td>LRR-repeats+AP-GTPase+COR</td><td>885</td><td>DCF12_11170</td><td>Cyanobacteria</td><td>Snowella sp.</td><td>GTPase [Snowella sp.].</td><td>GCA_003249095.1</td></tr>
<tr><td>PZW23552.1</td><td>LRR-repeats→ LRR-repeats+AP-GTPase+COR*→</td><td>LRR-repeats+AP-GTPase+COR</td><td>813</td><td>EI42_04935</td><td>Chloroflexi</td><td>Thermosporothrix hazakensis</td><td>GTPase SAR1 family protein, partial [Thermosporothrix hazakensis].</td><td>GCA_003253565.1</td></tr>
<tr><td>QDV38731.1</td><td>RelE-ParE→ WCAK→?→||<-Cluster2079_2clades||?→?→?→ LRR-repeats+AP-GTPase+COR+DUF4404*→</td><td>LRR-repeats+AP-GTPase+COR+DUF4404</td><td>887</td><td>slrP</td><td>Planctomycetes</td><td>Tautonia plasticadhaerens</td><td>E3 ubiquitin-protein ligase SlrP [Tautonia plasticadhaerens].</td><td>GCA_007752535.1</td></tr>
<tr><td>QEN02468.1</td><td>HTH→?→?→ LRR-repeats+AP-GTPase+COR+TIR*→||<-?<-ABC_membrane+ABC_tran||Cyanophycinsyn-ATPgrasp→ Cyanophycinsyn-ATPgrasp→</td><td>LRR-repeats+AP-GTPase+COR+TIR</td><td>1307</td><td>EWH46_18040</td><td>Betaproteobacteria</td><td>Sphaerotilus natans subsp. sulfidivorans</td><td>TIR domain-containing protein [Sphaerotilus natans subsp. sulfidivorans].</td><td>GCA_008329925.1</td></tr>
<tr><td>QHA06431.1</td><td>LRR-repeats+AP-GTPase+COR*→||<-?||?→||<-?<-SIGMA-HTH<-?<-SIGMA-HTH</td><td>LRR-repeats+AP-GTPase+COR</td><td>1022</td><td>GQF42_26905</td><td>Actinobacteria</td><td>Streptomyces broussonetiae</td><td>GTPase [Streptomyces broussonetiae].</td><td>GCA_009796285.1</td></tr>
<tr><td>QKQ77944.1</td><td>LRR-repeats+AP-GTPase+COR*→?→||<-?||?→||<-?||?→ ClpABN-AAA+ClpABC-AAA→||<-PSE<-SIG+TPR+TPR+TPR+TPR+TPR+TPR</td><td>LRR-repeats+AP-GTPase+COR</td><td>894</td><td>FBB35_26070</td><td>Cyanobacteria</td><td>Nostoc sp. TCL240-02</td><td>GTPase [Nostoc sp. TCL240-02].</td><td>GCA_013343235.1</td></tr>
<tr><td>QLE48778.1</td><td>ABhydrolase→?→||<-?||LRR-repeats+AP-GTPase+COR+TIR*→||<-PSE<-?||?→||<-?<-SIG+NUDIX<-?<-?<-Cluster5_5clades</td><td>LRR-repeats+AP-GTPase+COR+TIR</td><td>1021</td><td>FD724_12065</td><td>Cyanobacteria</td><td>Nostoc sp. C057</td><td>TIR domain-containing protein [Nostoc sp. C057].</td><td>GCA_013393925.1</td></tr>
<tr><td>QLH39529.1</td><td>Cluster850_2clades→?→?→ PRTase→||<-ENDO3+NUDIX<-?||LRR-repeats+AP-GTPase+COR+TIR*→</td><td>LRR-repeats+AP-GTPase+COR+TIR</td><td>816</td><td>HWD60_12020</td><td>Alphaproteobacteria</td><td>Defluviicoccus sp.</td><td>leucine-rich repeat domain-containing protein [Defluviicoccus sp.].</td><td>GCA_013414705.1</td></tr>
<tr><td>RAM50807.1</td><td>LRR-repeats+AP-GTPase+COR+TIR*→?→||<-SIG+TM+TM+TM+TM+TM+TM+TM+TM+TM+TM+TM</td><td>LRR-repeats+AP-GTPase+COR+TIR</td><td>849</td><td>C6Y22_14980</td><td>Cyanobacteria</td><td>Hapalosiphonaceae cyanobacterium JJU2</td><td>hypothetical protein C6Y22_14980 [Hapalosiphonaceae cyanobacterium JJU2].</td><td>GCA_003261315.1</td></tr>
<tr><td>RCJ18019.1</td><td><-Pkinase+Pentapeptide<-?<-?||?→||<-?<-TIR+EAD9<-?||LRR-repeats+AP-GTPase+COR+TIR*→</td><td>LRR-repeats+AP-GTPase+COR+TIR</td><td>986</td><td>A6770_33290</td><td>Cyanobacteria</td><td>Nostoc minutum NIES-26</td><td>GTPase [Nostoc minutum NIES-26].</td><td>GCA_003326215.1</td></tr>
<tr><td>RCJ19389.1</td><td>LRR-repeats+AP-GTPase+COR*→||<-ClpABN-AAA+ClpABC-AAA<-?||METHYLASE→</td><td>LRR-repeats+AP-GTPase+COR</td><td>907</td><td>A6S26_26810</td><td>Cyanobacteria</td><td>Nostoc sp. ATCC 43529</td><td>GTPase [Nostoc sp. ATCC 43529].</td><td>GCA_003326205.1</td></tr>
<tr><td>RCJ25694.1</td><td><-Pkinase+Pentapeptide<-?<-?||?→||<-?||LRR-repeats+AP-GTPase+COR+TIR*→||<-?<-?<-?||?→||<-NUDIX</td><td>LRR-repeats+AP-GTPase+COR+TIR</td><td>962</td><td>A6S26_15250</td><td>Cyanobacteria</td><td>Nostoc sp. ATCC 43529</td><td>GTPase [Nostoc sp. ATCC 43529].</td><td>GCA_003326205.1</td></tr>
<tr><td>RCJ30096.1</td><td>CR-REase5→ LRR-repeats+AP-GTPase+COR*→?→||<-?||?→||<-?||?→ ClpABN-AAA+ClpABC-AAA→</td><td>LRR-repeats+AP-GTPase+COR</td><td>894</td><td>A6770_21505</td><td>Cyanobacteria</td><td>Nostoc minutum NIES-26</td><td>GTPase [Nostoc minutum NIES-26].</td><td>GCA_003326215.1</td></tr>
<tr><td>RCJ30909.1</td><td>Cyanophycinsyn-ATPgrasp→||<-Cluster833_2clades<-?||?→||<-?<-Four-helical-protein<-?||LRR-repeats+AP-GTPase+COR+TIR*→||<-ncRNA<-?<-?<-?||?→||<-?||?→ Thioredoxin→</td><td>LRR-repeats+AP-GTPase+COR+TIR</td><td>928</td><td>A6770_20515</td><td>Cyanobacteria</td><td>Nostoc minutum NIES-26</td><td>hypothetical protein A6770_20515 [Nostoc minutum NIES-26].</td><td>GCA_003326215.1</td></tr>
<tr><td>RCJ31306.1</td><td>METHYLASE→||<-Arginase<-?<-?||LRR-repeats+AP-GTPase+COR+TIR*→?→?→||<-?<-?||?→ Pentapeptide-repeats→</td><td>LRR-repeats+AP-GTPase+COR+TIR</td><td>1103</td><td>A6770_20105</td><td>Cyanobacteria</td><td>Nostoc minutum NIES-26</td><td>GTPase [Nostoc minutum NIES-26].</td><td>GCA_003326215.1</td></tr>
<tr><td>RCJ40793.1</td><td>LRR-repeats+AP-GTPase+COR+TIR*→</td><td>LRR-repeats+AP-GTPase+COR+TIR</td><td>1079</td><td>A6770_10335</td><td>Cyanobacteria</td><td>Nostoc minutum NIES-26</td><td>GTPase [Nostoc minutum NIES-26].</td><td>GCA_003326215.1</td></tr>
<tr><td>RCJ41185.1</td><td><-TM||?→||<-?||?→||<-?||?→ LRR-repeats+AP-GTPase+COR+TM+TM*→</td><td>LRR-repeats+AP-GTPase+COR+TM+TM</td><td>837</td><td>A6770_08860</td><td>Cyanobacteria</td><td>Nostoc minutum NIES-26</td><td>hypothetical protein A6770_08860 [Nostoc minutum NIES-26].</td><td>GCA_003326215.1</td></tr>
<tr><td>RCV66029.1</td><td>LRR-repeats+AP-GTPase+COR→?→||<-?<-?||?→||<-?<-?||LRR-repeats→ LRR-repeats+AP-GTPase+COR→</td><td>LRR-repeats+AP-GTPase+COR</td><td>640</td><td>C5S53_00730</td><td>Euryarchaeota</td><td>Methanophagales archaeon</td><td>GTPase SAR1 family protein [Methanophagales archaeon].</td><td>GCA_003336485.1</td></tr>
<tr><td>RCV66037.1</td><td>LRR-repeats+AP-GTPase+COR*→</td><td>LRR-repeats+AP-GTPase+COR</td><td>730</td><td>C5S53_00775</td><td>Euryarchaeota</td><td>Methanophagales archaeon</td><td>Leucine-rich repeat (LRR) protein [Methanophagales archaeon].</td><td>GCA_003336485.1</td></tr>
<tr><td>REJ39442.1</td><td>SIG+TM+TM+TM+TM+TM+TM+TM→||<-?||Mbetalac→ SAM-methylase→?→||<-?<-SIG+TPM_phosphatase+TM+TM+TM||PSE→ LRR-repeats+AP-GTPase+COR+TM+TM*→||<-?<-?<-?<-Pro_CA||?→||<-?<-tRNA<-tRNA<-Ferredoxin-betagrasp</td><td>LRR-repeats+AP-GTPase+COR+TM+TM</td><td>898</td><td>DWQ54_21980</td><td>Cyanobacteria</td><td>Microcystis flos-aquae TF09</td><td>GTP-binding protein [Microcystis flos-aquae TF09].</td><td>GCA_003390815.1</td></tr>
<tr><td>REJ48778.1</td><td>SIG+TM+TM+TM+TM+TM+TM+TM→||<-?||Mbetalac→ SAM-methylase→?→||<-?<-SIG+TPM_phosphatase+TM+TM+TM||PSE→ LRR-repeats+AP-GTPase+COR+TM+TM*→</td><td>LRR-repeats+AP-GTPase+COR+TM+TM</td><td>852</td><td>DWQ53_05035</td><td>Cyanobacteria</td><td>Microcystis flos-aquae DF17</td><td>GTP-binding protein [Microcystis flos-aquae DF17].</td><td>GCA_003389435.1</td></tr>
<tr><td>REJ60675.1</td><td>SIG+TM+TM+TM+TM+TM+TM+TM→||<-?||Mbetalac→ SAM-methylase→?→||<-?<-SIG+TPM_phosphatase+TM+TM+TM||PSE→ LRR-repeats+AP-GTPase+COR+TM+TM*→||<-?<-?<-?<-Pro_CA||?→||<-?<-tRNA<-tRNA<-Ferredoxin-betagrasp</td><td>LRR-repeats+AP-GTPase+COR+TM+TM</td><td>826</td><td>DWQ56_05670</td><td>Cyanobacteria</td><td>Microcystis aeruginosa DA14</td><td>GTP-binding protein [Microcystis aeruginosa DA14].</td><td>GCA_003388675.1</td></tr>
</table>

acc	operon	architecture	len	gen.name	taxend	species	defline	gca
RFP53135.1	LRR-repeats+AP-GTPase+COR*→||<-?||?→||<-tRNA||?→?→?→ Cluster1088_2clades→	LRR-repeats+AP-GTPase+COR	695	BJG00_017645	Cyanobacteria	Limnothrix sp. CACIAM 69d	hypothetical protein BJG00_017645 [Limnothrix sp. CACIAM 69d].	GCA_001913845.2
RFP63081.1	<-CR-REase5||LRR-repeats+AP-GTPase+COR*→ LRR-repeats→	LRR-repeats+AP-GTPase+COR	886	BJG00_001105	Cyanobacteria	Limnothrix sp. CACIAM 69d	GTPase [Limnothrix sp. CACIAM 69d].	GCA_001913845.2
RIK29270.1	LRR-repeats+AP-GTPase+COR*→||<-?<-?<-?<-Mbetalac<-?<-ABC-ATPase<-TM+TM+TM+TM+TM	LRR-repeats+AP-GTPase+COR	1096	DCC56_13010	Chloroflexi	Anaerolineae bacterium	hypothetical protein DCC56_13010 [Anaerolineae bacterium].	GCA_003577395.1
RJO63533.1	LRR-repeats+AP-GTPase+COR*→	LRR-repeats+AP-GTPase+COR	1030	C4523_20060	Deltaproteobacteria	Myxococcales bacterium	hypothetical protein C4523_20060 [Myxococcales bacterium].	GCA_003598065.1
RJP51705.1	TM+TM+TM+TM+TM+TM+TM+TM→?→?→||<-?||?→ Cluster1070_2clades→ REase→ LRR-repeats+AP-GTPase+COR*→||<-?||?→?→?→||<-Cluster1854_2clades	LRR-repeats+AP-GTPase+COR	984	C4583_08075	Chloroflexi	Anaerolineaceae bacterium	hypothetical protein C4583_08075 [Anaerolineaceae bacterium].	GCA_003598975.1
RKS02636.1	LRR-repeats+AP-GTPase+COR+TM+TM*→	LRR-repeats+AP-GTPase+COR+TM+TM	1044	C8C84_2360	Bacteroidetes	Flavobacterium sp. 102	RocCOR-like putative regulator of kinase activity [Flavobacterium sp. 102].	GCA_003634615.1
RKZ87118.1	LRR-repeats+AP-GTPase+COR*→ AP-GTPase+COR→ COR→	LRR-repeats+AP-GTPase+COR	159	DRR19_14025	Gammaproteobacteria	Gammaproteobacteria bacterium	hypothetical protein DRR19_14025 [Gammaproteobacteria bacterium].	GCA_003646175.1
RKZ92191.1	LRR-repeats+AP-GTPase+COR*→	LRR-repeats+AP-GTPase+COR	778	DRR19_04995	Gammaproteobacteria	Gammaproteobacteria bacterium	hypothetical protein DRR19_04995 [Gammaproteobacteria bacterium].	GCA_003646175.1
RLC02552.1	LRR-repeats+AP-GTPase+COR*→	LRR-repeats+AP-GTPase+COR	868	DRI57_29970	Deltaproteobacteria	Deltaproteobacteria bacterium	hypothetical protein DRI57_29970, partial [Deltaproteobacteria bacterium].	GCA_003647425.1
RLC14341.1	LRR-repeats+AP-GTPase+COR*→	LRR-repeats+AP-GTPase+COR	442	DRI57_14615	Deltaproteobacteria	Deltaproteobacteria bacterium	hypothetical protein DRI57_14615, partial [Deltaproteobacteria bacterium].	GCA_003647425.1
RLC16559.1	LRR-repeats+AP-GTPase+COR*→	LRR-repeats+AP-GTPase+COR	930	DRI57_11050	Deltaproteobacteria	Deltaproteobacteria bacterium	hypothetical protein DRI57_11050 [Deltaproteobacteria bacterium].	GCA_003647425.1
RLD05606.1	UvsW-A18→ LRR-repeats+AP-GTPase+COR+TIR*→	LRR-repeats+AP-GTPase+COR+TIR	759	DRI32_03940	Chloroflexi	Chloroflexi bacterium	GTP-binding protein [Chloroflexi bacterium].	GCA_003648185.1
RLS84177.1	SIG+AP-GTPase+COR+TIR→ *FGS+LRR-repeats+AP-GTPase+COR+TIR→*	SIG+AP-GTPase+COR+TIR	879	DWI04_01885	Planctomycetes	Planctomycetes bacterium	TIR domain-containing protein, partial [Planctomycetes bacterium].	GCA_003669255.1
RLS84178.1	FGS+LRR-repeats+AP-GTPase+COR+TIR*→	FGS+LRR-repeats+AP-GTPase+COR+TIR	1292	DWI04_01890	Planctomycetes	Planctomycetes bacterium	hypothetical protein DWI04_01890, partial [Planctomycetes bacterium].	GCA_003669255.1
RME07294.1	RVT→?→ LRR-repeats+AP-GTPase+COR*→	LRR-repeats+AP-GTPase+COR	780	D6816_06800	Bacteroidetes	Bacteroidetes bacterium	hypothetical protein D6816_06800 [Bacteroidetes bacterium].	GCA_003694615.1
RMG75718.1	LRR-repeats+AP-GTPase+COR*→?→ FGS→	LRR-repeats+AP-GTPase+COR	691	D6722_00510	Bacteroidetes	Bacteroidetes bacterium	hypothetical protein D6722_00510 [Bacteroidetes bacterium].	GCA_003696875.1
RPH24755.1	LRR-repeats+AP-GTPase+COR*→	LRR-repeats+AP-GTPase+COR	627	EHM93_20250	Bacteroidetes	Bacteroidales bacterium	GTP-binding protein, partial [Bacteroidales bacterium].	GCA_003818205.1
RPI17218.1	LRR-repeats+AP-GTPase+COR*→	LRR-repeats+AP-GTPase+COR	944	EHM58_10175	Ignavibacteriae	Ignavibacteriae bacterium	GTP-binding protein [Ignavibacteriae bacterium].	GCA_003820375.1
RPJ19448.1	LRR-repeats+AP-GTPase+COR*→	LRR-repeats+AP-GTPase+COR	750	EHM33_30260	Chloroflexi	Chloroflexi bacterium	hypothetical protein EHM33_30260, partial [Chloroflexi bacterium].	GCA_003820175.1
RPJ72349.1	LRR-repeats+AP-GTPase+COR*→	LRR-repeats+AP-GTPase+COR	496	EHM20_13865	Alphaproteobacteria	Alphaproteobacteria bacterium	GTP-binding protein, partial [Alphaproteobacteria bacterium].	GCA_003820255.1
RQW02493.1	LRR-repeats+AP-GTPase+COR+DrHyd*→?→?→ Cluster1207_2clades→?→ Cluster2271_2clades→	LRR-repeats+AP-GTPase+COR+DrHyd	763	EH223_12385	Bacteria	candidate division KSB1 bacterium	hypothetical protein EH223_12385, partial [candidate division KSB1 bacterium].	GCA_003854975.1
RUR83995.1	<-Cluster5_5clades||LRR-repeats→ LRR-repeats+AP-GTPase+COR+DUF4404*→||<-?<-ABC-ATPase||?→||<-REC	LRR-repeats+AP-GTPase+COR+DUF4404	746	DSM107007_30530	Cyanobacteria	Nostoc sp. PCC 7120 = FACHB-418	hypothetical protein DSM107007_30530 [Nostoc sp. PCC 7120 = FACHB-418].	GCA_003990585.1
RWX44973.1	<-Cluster1731_2clades<-?<-?||?→?→ LRR-repeats+AP-GTPase+COR*→?→?→||<-Cluster1409_2clades	LRR-repeats+AP-GTPase+COR	648	H206_01140	Deltaproteobacteria	Candidatus Electrothrix aarhusiensis	hypothetical protein H206_01140 [Candidatus Electrothrix aarhusiensis].	GCA_004028505.1

acc	operon	architecture	len	gen.name	taxend	species	defline	gca
RWX45664.1	<-S18-HTH<-Cluster1004_3clades<-Cluster2139_2clades<-?<-?<-?||?→ LRR-repeats+AP-GTPase+COR*→	LRR-repeats+AP-GTPase+COR	605	H206_01449	Deltaproteobacteria	Candidatus Electrothrix aarhusiensis	hypothetical protein H206_01449 [Candidatus Electrothrix aarhusiensis].	GCA_004028505.1
RWX46469.1	Aminotran_1_2→||<-ABC-ATPase<-?<-?<-?<-?||LRR-repeats+AP-GTPase+COR*→	LRR-repeats+AP-GTPase+COR	908	H206_00859	Deltaproteobacteria	Candidatus Electrothrix aarhusiensis	internalin A [Candidatus Electrothrix aarhusiensis].	GCA_004028505.1
RWX47043.1	LRR-repeats+AP-GTPase+COR+TM+TM+TM*→ WCAK→	LRR-repeats+AP-GTPase+COR+TM+TM+TM	873	H206_03377	Deltaproteobacteria	Candidatus Electrothrix aarhusiensis	Ras of Complex, Roc, domain of DAPkinase [Candidatus Electrothrix aarhusiensis].	GCA_004028505.1
RWX51844.1	LRR-repeats+AP-GTPase+COR*→	LRR-repeats+AP-GTPase+COR	785	VU01_10733	Deltaproteobacteria	Candidatus Electrothrix marina	hypothetical protein VU01_10733 [Candidatus Electrothrix marina].	GCA_004028495.1
RYD26380.1	LRR-repeats+AP-GTPase+COR*→	LRR-repeats+AP-GTPase+COR	442	EOP86_26045	Verrucomicrobia	Verrucomicrobiaceae bacterium	hypothetical protein EOP86_26045, partial [Verrucomicrobiaceae bacterium].	GCA_004143925.1
RYD41313.1	LRR-repeats+AP-GTPase+COR*→	LRR-repeats+AP-GTPase+COR	546	EOP85_12890	Verrucomicrobia	Verrucomicrobiaceae bacterium	hypothetical protein EOP85_12890, partial [Verrucomicrobiaceae bacterium].	GCA_004144085.1
RYD45970.1	LRR-repeats+AP-GTPase*→	LRR-repeats+AP-GTPase	529	EOP85_08340	Verrucomicrobia	Verrucomicrobiaceae bacterium	hypothetical protein EOP85_08340, partial [Verrucomicrobiaceae bacterium].	GCA_004144085.1
RYD85024.1	LRR-repeats+AP-GTPase+COR+TIR*→	LRR-repeats+AP-GTPase+COR+TIR	722	EOP84_03815	Verrucomicrobia	Verrucomicrobiaceae bacterium	TIR domain-containing protein, partial [Verrucomicrobiaceae bacterium].	GCA_004144095.1
RYE22211.1	LRR-repeats+AP-GTPase+COR*→	LRR-repeats+AP-GTPase+COR	770	EOP45_08535	Bacteroidetes	Sphingobacteriaceae bacterium	GTPase, partial [Sphingobacteriaceae bacterium].	GCA_004144285.1
RYE59584.1	LRR-repeats+AP-GTPase+COR+TIR*→	LRR-repeats+AP-GTPase+COR+TIR	1318	EOP48_00430	Bacteroidetes	Sphingobacteriales bacterium	TIR domain-containing protein [Sphingobacteriales bacterium].	GCA_004144425.1
RYF36188.1	LRR-repeats+AP-GTPase*→	LRR-repeats+AP-GTPase	370	EOO38_28030	Bacteroidetes	Cytophagaceae bacterium	hypothetical protein EOO38_28030, partial [Cytophagaceae bacterium].	GCA_004145185.1
RYY12294.1	LRR-repeats+AP-GTPase*→	LRR-repeats+AP-GTPase	540	EOO04_33805	Bacteroidetes	Chitinophagaceae bacterium	hypothetical protein EOO04_33805, partial [Chitinophagaceae bacterium].	GCA_004172945.1
RYZ86256.1	LRR-repeats→ AP-GTPase+COR*→	AP-GTPase+COR	573	EOP04_14245	Proteobacteria	Proteobacteria bacterium	hypothetical protein EOP04_14245, partial [Proteobacteria bacterium].	GCA_004193275.1
RZK07337.1	LRR-repeats+AP-GTPase*→	LRR-repeats+AP-GTPase	262	EOO43_21605	Bacteroidetes	Flavobacterium sp.	hypothetical protein EOO43_21605, partial [Flavobacterium sp.].	GCA_004211555.1
RZK10466.1	LRR-repeats+AP-GTPase+COR*→	LRR-repeats+AP-GTPase+COR	421	EOO43_20245	Bacteroidetes	Flavobacterium sp.	hypothetical protein EOO43_20245, partial [Flavobacterium sp.].	GCA_004211555.1
RZN33809.1	pc1599→ Cluster1746_2clades→?→?→ Calcineurin→||<-tRNA||LRR-repeats+AP-GTPase+COR+TM+TM*→?→ Aminotran_1_2→||<-?<-?||Cluster1483_2clades→	LRR-repeats+AP-GTPase+COR+TM+TM	832	EF813_10660	Euryarchaeota	Methanosarcinales archaeon	GTP-binding protein [Methanosarcinales archaeon].	GCA_004211975.1
SCD37814.1	LRR-repeats+AP-GTPase+COR*→||<-NUDIX	LRR-repeats+AP-GTPase+COR	1358	GA0115243_1012136	Actinobacteria	Streptomyces sp. ScaeMP-e83	Leucine-rich repeat (LRR) protein, partial [Streptomyces sp. ScaeMP-e83].	GCA_900091775.1
SDD54826.1	Mbetalac→?→ LRR-repeats+AP-GTPase+COR*→||<-?<-ICLR-HTH||?→?→?→ ABhydrolase→	LRR-repeats+AP-GTPase+COR	887	SAMN05660690_4513	Actinobacteria	Geodermatophilus telluris	Leucine rich repeat-containing protein [Geodermatophilus telluris].	GCA_900102745.1
SEA38734.1	LRR-repeats+AP-GTPase+COR+TIR*→?→?→||<-Thioredoxin||YBAK→	LRR-repeats+AP-GTPase+COR+TIR	849	SAMN05660964_01466	Gammaproteobacteria	Thiothrix caldifontis	Leucine Rich repeat-containing protein [Thiothrix caldifontis].	GCA_900107695.1
SEM73063.1	<-ABC-ATPase<-?||?→?→||<-?||LRR-repeats+AP-GTPase+COR*→?→?→?→||<-?<-?<-SIR2	LRR-repeats+AP-GTPase+COR	717	SAMN05660976_05948	Actinobacteria	Nonomuraea pusilla	GTPase SAR1 family protein [Nonomuraea pusilla].	GCA_900109355.1
SFG01605.1	LRR-repeats+AP-GTPase+COR+TIR*→	LRR-repeats+AP-GTPase+COR+TIR	812	SAMN05216175_102423	Gammaproteobacteria	Neptunomonas qingdaonensis	internalin A, partial [Neptunomonas qingdaonensis].	GCA_900113275.1

acc	operon	architecture	len	gen.name	taxend	species	defline	gca
SFL80318.1	LRR-repeats+AP-GTPase+COR+DrHyd*→?→?→?→?→||<-?<-SIG+TM+TM+TM+TM	LRR-repeats+AP-GTPase+COR+DrHyd	738	SAMN05421863_1004142	Betaproteobacteria	Nitrosomonas communis	Ras of Complex, Roc, domain of DAPkinase [Nitrosomonas communis].	GCA_900114745.1
SHH94122.1	LRR-repeats+AP-GTPase+COR+TIR*→	LRR-repeats+AP-GTPase+COR+TIR	756	VA7868_00952	Gammaproteobacteria	Vibrio aerogenes CECT 7868	Ras family protein [Vibrio aerogenes CECT 7868].	GCA_900130105.1
SHI24236.1	LRR-repeats+AP-GTPase+COR+TIR*→	LRR-repeats+AP-GTPase+COR+TIR	954	inlA	Gammaproteobacteria	Vibrio aerogenes CECT 7868	Internalin-A precursor [Vibrio aerogenes CECT 7868].	GCA_900130105.1
SKA94563.1	METHYLASE→?→||<-?||SIG+Trypsin+PDZ→?→ Cluster978_2clades→ LRR-repeats+AP-GTPase+COR+TIR*→?→?→ GDSL→	LRR-repeats+AP-GTPase+COR+TIR	1235	SAMN02745166_02182	Verrucomicrobia	Prosthecobacter debontii	Leucine-rich repeat (LRR) protein [Prosthecobacter debontii].	GCA_900167535.1
SKC31468.1	CI→ CI→?→ LRR-repeats+AP-GTPase+COR*→	LRR-repeats+AP-GTPase+COR	1108	CZ809_00946	Gammaproteobacteria	Photobacterium piscicola	Ras family protein [Photobacterium piscicola].	GCA_900166965.1
SMS01989.1	LRR-repeats+AP-GTPase+COR+TIR*→	LRR-repeats+AP-GTPase+COR+TIR	1190	inlA	Gammaproteobacteria	Vibrio mangrovi	Internalin-A precursor [Vibrio mangrovi].	GCA_900184095.1
SNS86340.1	SIG+Concanavalin-like+SUN→ LRR-repeats+AP-GTPase+COR*→?→?→?→||<-?<-ABC-ATPase	LRR-repeats+AP-GTPase+COR	686	SAMN06264365_12694	Actinobacteria	Actinoplanes regularis	hypothetical protein SAMN06264365_12694 [Actinoplanes regularis].	GCA_900188005.1
SUS04594.1	LRR-repeats+AP-GTPase+COR+TIR*→	LRR-repeats+AP-GTPase+COR+TIR	965	DF3PB_1340002	Alphaproteobacteria	uncultured Defluviicoccus sp.	Leucine-rich repeat (LRR) protein (fragment) [uncultured Defluviicoccus sp.].	GCA_900498315.1
SUS07166.1	LRR-repeats+AP-GTPase+COR+TIR*→	LRR-repeats+AP-GTPase+COR+TIR	1031	DF3PB_390011	Alphaproteobacteria	uncultured Defluviicoccus sp.	conserved hypothetical protein [uncultured Defluviicoccus sp.].	GCA_900498315.1
TAA73809.1	<-Cluster1510_2clades<-?<-?||LRR-repeats+AP-GTPase+COR→ *LRR-repeats+AP-GTPase+COR*→	LRR-repeats+AP-GTPase+COR	571	CDV28_1587	Deltaproteobacteria	Candidatus Electronema sp. GS	hypothetical protein CDV28_1587 [Candidatus Electronema sp. GS].	GCA_004284765.1
TAA73810.1	LRR-repeats+AP-GTPase+COR*→	LRR-repeats+AP-GTPase+COR	678	CDV28_1588	Deltaproteobacteria	Candidatus Electronema sp. GS	hypothetical protein CDV28_1588 [Candidatus Electronema sp. GS].	GCA_004284765.1
TAA74005.1	LRR-repeats+AP-GTPase+COR*→ SIG+TM+TM+TM+TM→	LRR-repeats+AP-GTPase+COR	803	CDV28_14413	Deltaproteobacteria	Candidatus Electronema sp. GS	hypothetical protein CDV28_14413 [Candidatus Electronema sp. GS].	GCA_004284765.1
TAA74213.1	Cluster1147_2clades→?→ LRR-repeats+AP-GTPase+COR*→	LRR-repeats+AP-GTPase+COR	680	CDV28_13522	Deltaproteobacteria	Candidatus Electronema sp. GS	hypothetical protein CDV28_13522 [Candidatus Electronema sp. GS].	GCA_004284765.1
TAA75585.1	Cluster2427_2clades→?→ Cluster1969_2clades→ Thioredoxin→?→?→?→ LRR-repeats+AP-GTPase+COR*→	LRR-repeats+AP-GTPase+COR	694	CDV28_10542	Deltaproteobacteria	Candidatus Electronema sp. GS	hypothetical protein CDV28_10542 [Candidatus Electronema sp. GS].	GCA_004284765.1
TAA76176.1	<-SIG+HSP70<-?<-?<-?<-?||LRR-repeats+AP-GTPase+COR*→||<-?<-?<-?<-?<-RelE-ParE	LRR-repeats+AP-GTPase+COR	627	CDV28_10174	Deltaproteobacteria	Candidatus Electronema sp. GS	hypothetical protein CDV28_10174 [Candidatus Electronema sp. GS].	GCA_004284765.1
TAD75245.1	RelE-ParE→ LRR-repeats+AP-GTPase+COR*→	LRR-repeats+AP-GTPase+COR	952	EA001_14520	Cyanobacteria	Oscillatoriales cyanobacterium	GTPase [Oscillatoriales cyanobacterium].	GCA_004292515.1
TAD78959.1	HISKIN→?→ LRR-repeats+AP-GTPase+COR*→||<-Cluster971_2clades	LRR-repeats+AP-GTPase+COR	1063	EA001_05550	Cyanobacteria	Oscillatoriales cyanobacterium	GTPase [Oscillatoriales cyanobacterium].	GCA_004292515.1
TAE00317.1	<-Cluster1805_2clades||?→?→ LRR-repeats+AP-GTPase+COR*→	LRR-repeats+AP-GTPase+COR	631	EAZ97_06315	Bacteroidetes	Bacteroidetes bacterium	hypothetical protein EAZ97_06315 [Bacteroidetes bacterium].	GCA_004292785.1
TAE06670.1	LRR-repeats→ LRR-repeats+AP-GTPase+COR+DUF4404*→?→?→||<-Pentapeptide-repeats	LRR-repeats+AP-GTPase+COR+DUF4404	900	EAZ94_30300	Cyanobacteria	Oscillatoriales cyanobacterium	GTPase [Oscillatoriales cyanobacterium].	GCA_004292775.1
TAE07007.1	LRR-repeats+AP-GTPase+COR*→	LRR-repeats+AP-GTPase+COR	808	EAZ95_18790	Bacteroidetes	Bacteroidetes bacterium	GTP-binding protein [Bacteroidetes bacterium].	GCA_004292795.1
TAE07511.1	LRR-repeats+AP-GTPase+COR*→	LRR-repeats+AP-GTPase+COR	774	EAZ95_18335	Bacteroidetes	Bacteroidetes bacterium	hypothetical protein EAZ95_18335 [Bacteroidetes bacterium].	GCA_004292795.1
TAE08557.1	LRR-repeats+AP-GTPase+COR*→	LRR-repeats+AP-GTPase+COR	487	EAZ95_17095	Bacteroidetes	Bacteroidetes bacterium	hypothetical protein EAZ95_17095, partial [Bacteroidetes bacterium].	GCA_004292795.1
TAE08925.1	LRR-repeats+AP-GTPase+COR*→	LRR-repeats+AP-GTPase+COR	911	EAZ94_23755	Cyanobacteria	Oscillatoriales cyanobacterium	hypothetical protein EAZ94_23755 [Oscillatoriales cyanobacterium].	GCA_004292775.1
TAE12199.1	LRR-repeats+AP-GTPase+COR*→ SIG+TM+TM+TM+TM+TM→ REase→	LRR-repeats+AP-GTPase+COR	811	EAZ95_12720	Bacteroidetes	Bacteroidetes bacterium	hypothetical protein EAZ95_12720, partial [Bacteroidetes bacterium].	GCA_004292795.1

<table>
<tr><th>acc</th><th>operon</th><th>architecture</th><th>len</th><th>gen.name</th><th>taxend</th><th>species</th><th>defline</th><th>gca</th></tr>
<tr><td>TAE14819.1</td><td><-Pkinase+TM||?→ PIN→ LRR-repeats+AP-GTPase+COR*→</td><td>LRR-repeats+AP-GTPase+COR</td><td>769</td><td>EAZ95_09495</td><td>Bacteroidetes</td><td>Bacteroidetes bacterium</td><td>GTP-binding protein [Bacteroidetes bacterium].</td><td>GCA_004292795.1</td></tr>
<tr><td>TAE15606.1</td><td>LRR-repeats+AP-GTPase+COR*→</td><td>LRR-repeats+AP-GTPase+COR</td><td>866</td><td>EAZ95_08545</td><td>Bacteroidetes</td><td>Bacteroidetes bacterium</td><td>GTP-binding protein [Bacteroidetes bacterium].</td><td>GCA_004292795.1</td></tr>
<tr><td>TAE15646.1</td><td><-CR-REase5<-?<-?||LRR-repeats→?→ PSE→ LRR-repeats+AP-GTPase+COR*→?→?→?→?→||<-?<-PSE<-?||Pentapeptide-repeats→</td><td>LRR-repeats+AP-GTPase+COR</td><td>1106</td><td>EAZ94_03785</td><td>Cyanobacteria</td><td>Oscillatoriales cyanobacterium</td><td>GTP-binding protein [Oscillatoriales cyanobacterium].</td><td>GCA_004292775.1</td></tr>
<tr><td>TAE18080.1</td><td><-ABC_membrane+ABC_tran||?→?→ LRR-repeats+AP-GTPase+COR*→</td><td>LRR-repeats+AP-GTPase+COR</td><td>778</td><td>EAZ95_05200</td><td>Bacteroidetes</td><td>Bacteroidetes bacterium</td><td>hypothetical protein EAZ95_05200 [Bacteroidetes bacterium].</td><td>GCA_004292795.1</td></tr>
<tr><td>TAE30098.1</td><td>LRR-repeats→ LRR-repeats+AP-GTPase+COR+DUF4404*→?→?→||<-Pentapeptide-repeats</td><td>LRR-repeats+AP-GTPase+COR+DUF4404</td><td>1078</td><td>EAZ93_01305</td><td>Cyanobacteria</td><td>Oscillatoriales cyanobacterium</td><td>GTPase [Oscillatoriales cyanobacterium].</td><td>GCA_004292845.1</td></tr>
<tr><td>TAE37012.1</td><td>LRR-repeats+AP-GTPase+COR+DUF4404*→?→?→||<-Pentapeptide-repeats</td><td>LRR-repeats+AP-GTPase+COR+DUF4404</td><td>1282</td><td>EAZ90_27320</td><td>Cyanobacteria</td><td>Oscillatoriales cyanobacterium</td><td>GTPase [Oscillatoriales cyanobacterium].</td><td>GCA_004292895.1</td></tr>
<tr><td>TAE38303.1</td><td>LRR-repeats+AP-GTPase+COR*→?→?→ PSE→?→ PSE→?→||<-?<-PSE<-?||Pentapeptide-repeats→</td><td>LRR-repeats+AP-GTPase+COR</td><td>909</td><td>EAZ90_25170</td><td>Cyanobacteria</td><td>Oscillatoriales cyanobacterium</td><td>GTP-binding protein [Oscillatoriales cyanobacterium].</td><td>GCA_004292895.1</td></tr>
<tr><td>TAE48904.1</td><td>LRR-repeats→?→?→?→ LRR-repeats+AP-GTPase+COR*→</td><td>LRR-repeats+AP-GTPase+COR</td><td>695</td><td>EAZ88_23370</td><td>Cyanobacteria</td><td>Oscillatoriales cyanobacterium</td><td>GTP-binding protein, partial [Oscillatoriales cyanobacterium].</td><td>GCA_004292945.1</td></tr>
<tr><td>TAE51174.1</td><td>LRR-repeats+AP-GTPase+COR*→</td><td>LRR-repeats+AP-GTPase+COR</td><td>790</td><td>EAZ89_10570</td><td>Bacteroidetes</td><td>Bacteroidetes bacterium</td><td>hypothetical protein EAZ89_10570, partial [Bacteroidetes bacterium].</td><td>GCA_004292985.1</td></tr>
<tr><td>TAE53250.1</td><td>LRR-repeats+AP-GTPase+COR*→</td><td>LRR-repeats+AP-GTPase+COR</td><td>912</td><td>EAZ88_12540</td><td>Cyanobacteria</td><td>Oscillatoriales cyanobacterium</td><td>hypothetical protein EAZ88_12540 [Oscillatoriales cyanobacterium].</td><td>GCA_004292945.1</td></tr>
<tr><td>TAE59343.1</td><td>LRR-repeats+AP-GTPase+COR+EAD11*→</td><td>LRR-repeats+AP-GTPase+COR+EAD11</td><td>737</td><td>EAZ89_02575</td><td>Bacteroidetes</td><td>Bacteroidetes bacterium</td><td>hypothetical protein EAZ89_02575 [Bacteroidetes bacterium].</td><td>GCA_004292985.1</td></tr>
<tr><td>TAE66346.1</td><td>LRR-repeats+AP-GTPase+COR*→</td><td>LRR-repeats+AP-GTPase+COR</td><td>742</td><td>EAZ85_15975</td><td>Bacteroidetes</td><td>Bacteroidetes bacterium</td><td>GTP-binding protein, partial [Bacteroidetes bacterium].</td><td>GCA_004293265.1</td></tr>
<tr><td>TAE67608.1</td><td>LRR-repeats+AP-GTPase+COR*→</td><td>LRR-repeats+AP-GTPase+COR</td><td>911</td><td>EAZ86_16170</td><td>Cyanobacteria</td><td>Oscillatoriales cyanobacterium</td><td>hypothetical protein EAZ86_16170 [Oscillatoriales cyanobacterium].</td><td>GCA_004292955.1</td></tr>
<tr><td>TAE67866.1</td><td>FGS→?→ LRR-repeats+AP-GTPase+COR*→</td><td>LRR-repeats+AP-GTPase+COR</td><td>987</td><td>EAZ85_14755</td><td>Bacteroidetes</td><td>Bacteroidetes bacterium</td><td>hypothetical protein EAZ85_14755 [Bacteroidetes bacterium].</td><td>GCA_004293265.1</td></tr>
<tr><td>TAE68409.1</td><td><-CR-REase5<-?||?→||<-?||LRR-repeats→?→?→ LRR-repeats+AP-GTPase+COR*→?→?→?→ PSE→||<-?<-PSE<-?||Pentapeptide-repeats→</td><td>LRR-repeats+AP-GTPase+COR</td><td>1061</td><td>EAZ86_13565</td><td>Cyanobacteria</td><td>Oscillatoriales cyanobacterium</td><td>GTP-binding protein [Oscillatoriales cyanobacterium].</td><td>GCA_004292955.1</td></tr>
<tr><td>TAE73426.1</td><td>LRR-repeats+AP-GTPase+COR*→</td><td>LRR-repeats+AP-GTPase+COR</td><td>1078</td><td>EAZ85_07065</td><td>Bacteroidetes</td><td>Bacteroidetes bacterium</td><td>hypothetical protein EAZ85_07065 [Bacteroidetes bacterium].</td><td>GCA_004293265.1</td></tr>
<tr><td>TAE76344.1</td><td>LRR-repeats+AP-GTPase+COR*→||<-Cluster1921_2clades</td><td>LRR-repeats+AP-GTPase+COR</td><td>919</td><td>EAZ85_00290</td><td>Bacteroidetes</td><td>Bacteroidetes bacterium</td><td>hypothetical protein EAZ85_00290 [Bacteroidetes bacterium].</td><td>GCA_004293265.1</td></tr>
<tr><td>TAE86412.1</td><td>LRR-repeats+AP-GTPase+COR+TIR*→||<-?<-Cluster409_3clades</td><td>LRR-repeats+AP-GTPase+COR+TIR</td><td>1151</td><td>EAY81_05595</td><td>Bacteroidetes</td><td>Bacteroidetes bacterium</td><td>TIR domain-containing protein [Bacteroidetes bacterium].</td><td>GCA_004293295.1</td></tr>
<tr><td>TAF49891.1</td><td>LRR-repeats+AP-GTPase+COR+DUF4404*→</td><td>LRR-repeats+AP-GTPase+COR+DUF4404</td><td>845</td><td>EAZ61_13785</td><td>Cyanobacteria</td><td>Oscillatoriales cyanobacterium</td><td>GTPase, partial [Oscillatoriales cyanobacterium].</td><td>GCA_004293855.1</td></tr>
<tr><td>TAF64535.1</td><td>LRR-repeats+AP-GTPase+COR+EAD11*→</td><td>LRR-repeats+AP-GTPase+COR+EAD11</td><td>1108</td><td>EAZ55_10760</td><td>Bacteroidetes</td><td>Cytophagales bacterium</td><td>hypothetical protein EAZ55_10760 [Cytophagales bacterium].</td><td>GCA_004293905.1</td></tr>
<tr><td>TAF89999.1</td><td>GTPase-AIG→?→?→||<-?<-?||LRR-repeats+AP-GTPase+COR+DUF4404*→?→?→||<-Pentapeptide-repeats</td><td>LRR-repeats+AP-GTPase+COR+DUF4404</td><td>1282</td><td>EAZ49_10560</td><td>Cyanobacteria</td><td>Oscillatoriales cyanobacterium</td><td>GTPase [Oscillatoriales cyanobacterium].</td><td>GCA_004294035.1</td></tr>
<tr><td>TAF91931.1</td><td><-CR-REase5<-?<-?||LRR-repeats→?→?→ LRR-repeats+AP-GTPase+COR*→?→?→?→ PSE→||<-?<-PSE<-?||Pentapeptide-repeats→</td><td>LRR-repeats+AP-GTPase+COR</td><td>952</td><td>EAZ49_03665</td><td>Cyanobacteria</td><td>Oscillatoriales cyanobacterium</td><td>GTP-binding protein [Oscillatoriales cyanobacterium].</td><td>GCA_004294035.1</td></tr>
<tr><td>TAG07081.1</td><td>RVT→?→ LRR-repeats+AP-GTPase+COR+ACYC*→?→||<-?<-?<-HISKIN</td><td>LRR-repeats+AP-GTPase+COR+ACYC</td><td>962</td><td>EAZ44_01180</td><td>Bacteroidetes</td><td>Cytophagia bacterium</td><td>hypothetical protein EAZ44_01180 [Cytophagia bacterium].</td><td>GCA_004294005.1</td></tr>
<tr><td>TAG17458.1</td><td><-CENPB||?→?→||<-?<-?||Aminotran_1_2→ LRR-repeats+AP-GTPase+COR*→</td><td>LRR-repeats+AP-GTPase+COR</td><td>975</td><td>EAZ38_17395</td><td>Bacteroidetes</td><td>Cytophagales bacterium</td><td>hypothetical protein EAZ38_17395 [Cytophagales bacterium].</td><td>GCA_004293585.1</td></tr>
<tr><td>TAG62903.1</td><td>LRR-repeats+AP-GTPase+COR*→</td><td>LRR-repeats+AP-GTPase+COR</td><td>778</td><td>EAZ28_01955</td><td>Cyanobacteria</td><td>Oscillatoriales cyanobacterium</td><td>hypothetical protein EAZ28_01955, partial [Oscillatoriales cyanobacterium].</td><td>GCA_004294275.1</td></tr>
</table>

<table>
<tr><th>acc</th><th>operon</th><th>architecture</th><th>len</th><th>gen.name</th><th>taxend</th><th>species</th><th>defline</th><th>gca</th></tr>
<tr><td>TAG65048.1</td><td>LRR-repeats→?→ PSE→ LRR-repeats+AP-GTPase+COR*→ PSE→?→?→ PSE→||<-?<-PSE<-?||Pentapeptide-repeats→</td><td>LRR-repeats+AP-GTPase+COR</td><td>939</td><td>EAZ25_17645</td><td>Cyanobacteria</td><td>Oscillatoriales cyanobacterium</td><td>GTP-binding protein [Oscillatoriales cyanobacterium].</td><td>GCA_004294345.1</td></tr>
<tr><td>TAG86009.1</td><td>LRR-repeats+AP-GTPase+COR*→</td><td>LRR-repeats+AP-GTPase+COR</td><td>565</td><td>EAZ18_26350</td><td>Cyanobacteria</td><td>Oscillatoriales cyanobacterium</td><td>GTP-binding protein, partial [Oscillatoriales cyanobacterium].</td><td>GCA_004294385.1</td></tr>
<tr><td>TAG86671.1</td><td>LRR-repeats+AP-GTPase+COR*→</td><td>LRR-repeats+AP-GTPase+COR</td><td>892</td><td>EAZ20_12320</td><td>Bacteroidetes</td><td>Bacteroidetes bacterium</td><td>hypothetical protein EAZ20_12320, partial [Bacteroidetes bacterium].</td><td>GCA_004294185.1</td></tr>
<tr><td>TAG91526.1</td><td>FGS→?→ LRR-repeats+AP-GTPase*→</td><td>LRR-repeats+AP-GTPase</td><td>279</td><td>EAZ20_03245</td><td>Bacteroidetes</td><td>Bacteroidetes bacterium</td><td>hypothetical protein EAZ20_03245, partial [Bacteroidetes bacterium].</td><td>GCA_004294185.1</td></tr>
<tr><td>TAG93154.1</td><td>LRR-repeats+AP-GTPase+COR*→</td><td>LRR-repeats+AP-GTPase+COR</td><td>935</td><td>EAZ19_16680</td><td>Cyanobacteria</td><td>Oscillatoriales cyanobacterium</td><td>hypothetical protein EAZ19_16680 [Oscillatoriales cyanobacterium].</td><td>GCA_004293705.1</td></tr>
<tr><td>TAH14728.1</td><td>LRR-repeats+AP-GTPase+COR*→</td><td>LRR-repeats+AP-GTPase+COR</td><td>344</td><td>EAZ09_26195</td><td>Cyanobacteria</td><td>Oscillatoriales cyanobacterium</td><td>GTP-binding protein, partial [Oscillatoriales cyanobacterium].</td><td>GCA_004293745.1</td></tr>
<tr><td>TAH21032.1</td><td>Cluster1569_2clades→ ABhydrolase→||<-?||?→?→?→||<-?||LRR-repeats+AP-GTPase*→</td><td>LRR-repeats+AP-GTPase</td><td>257</td><td>EAZ08_04815</td><td>Bacteroidetes</td><td>Cytophagales bacterium</td><td>hypothetical protein EAZ08_04815 [Cytophagales bacterium].</td><td>GCA_004294445.1</td></tr>
<tr><td>TAK37700.1</td><td>LRR-repeats+AP-GTPase+COR+EAD11*→ Cluster5_5clades→?→ METHYLASE→</td><td>LRR-repeats+AP-GTPase+COR+EAD11</td><td>783</td><td>EPO28_11660</td><td>Bacteroidetes</td><td>Saprospiraceae bacterium</td><td>hypothetical protein EPO28_11660 [Saprospiraceae bacterium].</td><td>GCA_004297645.1</td></tr>
<tr><td>TAK50544.1</td><td>Cluster1854_2clades→?→?→ PSE→?→||<-Cluster5_5clades||LRR-repeats→ LRR-repeats+AP-GTPase+COR+EAD11*→?→?→?→?→ ABhydrolase→||<-Cluster5_5clades</td><td>LRR-repeats+AP-GTPase+COR+EAD11</td><td>588</td><td>EPO28_00120</td><td>Bacteroidetes</td><td>Saprospiraceae bacterium</td><td>hypothetical protein EPO28_00120 [Saprospiraceae bacterium].</td><td>GCA_004297645.1</td></tr>
<tr><td>TAK57982.1</td><td>HhH-RADC+JAB→||<-?||?→ LRR-repeats+AP-GTPase+COR*→</td><td>LRR-repeats+AP-GTPase+COR</td><td>666</td><td>EPO24_09130</td><td>Bacteroidetes</td><td>Bacteriodetes bacterium</td><td>GTP-binding protein, partial [Bacteriodetes bacterium].</td><td>GCA_004322375.1</td></tr>
<tr><td>TAK59812.1</td><td>LRR-repeats+AP-GTPase+COR+TIR*→ tRNA→ tRNA→?→?→ Cluster2248_2clades→</td><td>LRR-repeats+AP-GTPase+COR+TIR</td><td>884</td><td>EPO24_07295</td><td>Bacteroidetes</td><td>Bacteriodetes bacterium</td><td>TIR domain-containing protein [Bacteriodetes bacterium].</td><td>GCA_004322375.1</td></tr>
<tr><td>TAK63714.1</td><td>LRR-repeats+AP-GTPase+COR+TIR*→</td><td>LRR-repeats+AP-GTPase+COR+TIR</td><td>903</td><td>EPO24_03975</td><td>Bacteroidetes</td><td>Bacteriodetes bacterium</td><td>TIR domain-containing protein [Bacteriodetes bacterium].</td><td>GCA_004322375.1</td></tr>
<tr><td>TFV86935.1</td><td>LRR-repeats+AP-GTPase+COR+DrHyd*→</td><td>LRR-repeats+AP-GTPase+COR+DrHyd</td><td>783</td><td>E4K72_22790</td><td>Betaproteobacteria</td><td>Oxalobacteraceae bacterium OM1</td><td>hypothetical protein E4K72_22790, partial [Oxalobacteraceae bacterium OM1].</td><td>GCA_004570315.1</td></tr>
<tr><td>THV28317.1</td><td>TM→ LRR-repeats+AP-GTPase+COR*→</td><td>LRR-repeats+AP-GTPase+COR</td><td>1065</td><td>E9998_11935</td><td>Actinobacteria</td><td>Glycomyces paridis</td><td>hypothetical protein E9998_11935 [Glycomyces paridis].</td><td>GCA_004912155.1</td></tr>
<tr><td>TKJ39119.1</td><td>SIGMA-HTH→?→?→||<-?<-?<-?||LRR-repeats+AP-GTPase+COR*→||<-?<-Ferredoxin-RRM</td><td>LRR-repeats+AP-GTPase+COR</td><td>968</td><td>CEE37_11920</td><td>FCB group</td><td>candidate division LCP-89 bacterium B3_LCP</td><td>GTPase [candidate division LCP-89 bacterium B3_LCP].</td><td>GCA_005223185.1</td></tr>
<tr><td>TKJ40235.1</td><td>HISKIN→?→?→?→?→?→ ACET→ LRR-repeats+AP-GTPase+COR*→?→?→||<-?<-?<-SMPB||?→ Cluster1833_2clades→</td><td>LRR-repeats+AP-GTPase+COR</td><td>925</td><td>CEE37_07880</td><td>FCB group</td><td>candidate division LCP-89 bacterium B3_LCP</td><td>GTPase [candidate division LCP-89 bacterium B3_LCP].</td><td>GCA_005223185.1</td></tr>
<tr><td>TKJ41682.1</td><td>TPR+TPR→?→?→ RPN10→?→?→ LRR-repeats+AP-GTPase+COR*→||<-?||LRR-repeats+AP-GTPase+COR→||<-?<-tRNA||RADICAL-SAM→||<-tRNA<-MerR-HTH</td><td>LRR-repeats+AP-GTPase+COR</td><td>1047</td><td>CEE37_03700</td><td>FCB group</td><td>candidate division LCP-89 bacterium B3_LCP</td><td>hypothetical protein CEE37_03700 [candidate division LCP-89 bacterium B3_LCP].</td><td>GCA_005223185.1</td></tr>
<tr><td>TKZ22725.1</td><td>LRR-repeats+AP-GTPase+COR*→||<-?||COR→</td><td>LRR-repeats+AP-GTPase+COR</td><td>653</td><td>EBE85_34345</td><td>Cyanobacteria</td><td>Hormoscilla sp. GUM007</td><td>hypothetical protein EBE85_34345 [Hormoscilla sp. GUM007].</td><td>-</td></tr>
<tr><td>TKZ31764.1</td><td>Cluster1207_2clades→||<-?<-?<-?<-?<-?<-?||LRR-repeats+AP-GTPase+COR*→ COR→</td><td>LRR-repeats+AP-GTPase+COR</td><td>424</td><td>EBE86_14970</td><td>Cyanobacteria</td><td>Hormoscilla sp. GUM202</td><td>hypothetical protein EBE86_14970, partial [Hormoscilla sp. GUM202].</td><td>-</td></tr>
<tr><td>TLU81885.1</td><td>Cluster1765_2clades→||<-?<-?||InPase→||<-?||TM→ LRR-repeats+AP-GTPase+COR*→?→?→ ACT→||<-?<-?<-PSE<-DNAJ</td><td>LRR-repeats+AP-GTPase+COR</td><td>953</td><td>FDX21_09980</td><td>Chlorobi</td><td>Chlorobium sp.</td><td>hypothetical protein FDX21_09980 [Chlorobium sp.].</td><td>GCA_005862225.1</td></tr>
<tr><td>TLU87350.1</td><td><-FGS<-?||?→||<-?<-?||PSE→?→?→ LRR-repeats+AP-GTPase+COR+TIR*→||<-PSE<-RelE-ParE</td><td>LRR-repeats+AP-GTPase+COR+TIR</td><td>892</td><td>FDX21_00465</td><td>Chlorobi</td><td>Chlorobium sp.</td><td>TIR domain-containing protein [Chlorobium sp.].</td><td>GCA_005862225.1</td></tr>
<tr><td>TMQ13801.1</td><td>LRR-repeats+AP-GTPase+COR*→ COR+TIR→</td><td>LRR-repeats+AP-GTPase+COR</td><td>391</td><td>E6J90_27900</td><td>Deltaproteobacteria</td><td>Deltaproteobacteria bacterium</td><td>GTP-binding protein, partial [Deltaproteobacteria bacterium].</td><td>GCA_005887915.1</td></tr>
<tr><td>TRT70055.1</td><td>LRR-repeats+AP-GTPase+COR+TM+TM*→</td><td>LRR-repeats+AP-GTPase+COR+TM+TM</td><td>710</td><td>EWV84_09945</td><td>Cyanobacteria</td><td>Microcystis sp. M_QC_C_20170808_M3Col</td><td>GTP-binding protein, partial [Microcystis sp. M_QC_C_20170808_M3Col].</td><td>GCA_007095505.1</td></tr>
<tr><td>TRT86788.1</td><td>SIG+TM+TM+TM+TM+TM+TM+TM→||<-?||Mbetalac→ SAM-methylase→?→||<-?<-SIG+TPM_phosphatase+TM+TM+TM||LRR-repeats+AP-GTPase+COR*→</td><td>LRR-repeats+AP-GTPase+COR</td><td>597</td><td>EWV66_15420</td><td>Cyanobacteria</td><td>Microcystis sp. M_OC_Ca_00000000_C217Col</td><td>GTP-binding protein, partial [Microcystis sp. M_OC_Ca_00000000_C217Col].</td><td>GCA_007095585.1</td></tr>
</table>

<table>
<thead>
<tr><th>acc</th><th>operon</th><th>architecture</th><th>len</th><th>gen.name</th><th>taxend</th><th>species</th><th>defline</th><th>gca</th></tr>
</thead>
<tbody>
<tr><td>TRT92075.1</td><td>Mbetalac→ SAM-methylase→?→||<-?||?→||<-SIG+TPM_phosphatase+TM+TM+TM||LRR-repeats+AP-GTPase+COR+TM+TM*→||<-?<-?<-?<-Pro_CA||?→||<-?<-tRNA<-tRNA<-Ferredoxin-betagrasp</td><td>LRR-repeats+AP-GTPase+COR+TM+TM</td><td>874</td><td>EWV65_21090</td><td>Cyanobacteria</td><td>Microcystis flos-aquae Ma_QC_C_20070823_S18D</td><td>GTP-binding protein [Microcystis flos-aquae Ma_QC_C_20070823_S18D].</td><td>GCA_007095605.1</td></tr>
<tr><td>TRU14937.1</td><td>SIG+TM+TM+TM+TM+TM+TM+TM→||<-?||Mbetalac→ SAM-methylase→?→||<-?<-SIG+TPM_phosphatase+TM+TM+TM||LRR-repeats+AP-GTPase+COR+TM+TM*→||<-?<-?<-?<-Pro_CA||?→||<-?<-tRNA<-tRNA<-Ferredoxin-betagrasp</td><td>LRR-repeats+AP-GTPase+COR+TM+TM</td><td>874</td><td>EWV60_01670</td><td>Cyanobacteria</td><td>Microcystis sp. Msp_OC_L_20101000_S702</td><td>GTP-binding protein [Microcystis sp. Msp_OC_L_20101000_S702].</td><td>GCA_007095705.1</td></tr>
<tr><td>TRU25637.1</td><td>LRR-repeats+AP-GTPase+COR+TM+TM*→||<-?<-FGS<-?<-?||?→?→ Aminotran_1_2→</td><td>LRR-repeats+AP-GTPase+COR+TM+TM</td><td>921</td><td>EWV79_07400</td><td>Cyanobacteria</td><td>Microcystis aeruginosa Ma_MB_S_20031200_S102D</td><td>GTP-binding protein [Microcystis aeruginosa Ma_MB_S_20031200_S102D].</td><td>GCA_007095795.1</td></tr>
<tr><td>TRU29987.1</td><td>SIG+TM+TM+TM+TM+TM+TM+TM→||<-?||Mbetalac→ SAM-methylase→?→||<-?<-SIG+TPM_phosphatase+TM+TM+TM||LRR-repeats+AP-GTPase+COR+TM+TM*→||<-?<-?<-?<-?<-Pro_CA</td><td>LRR-repeats+AP-GTPase+COR+TM+TM</td><td>921</td><td>EWV80_03065</td><td>Cyanobacteria</td><td>Microcystis aeruginosa Ma_QC_B_20070730_S2</td><td>GTP-binding protein [Microcystis aeruginosa Ma_QC_B_20070730_S2].</td><td>GCA_007095805.1</td></tr>
<tr><td>TRU36579.1</td><td>SIG+TM+TM+TM+TM+TM+TM+TM→||<-PSE||Mbetalac→ SAM-methylase→?→||<-?<-SIG+TPM_phosphatase+TM+TM+TM||LRR-repeats+AP-GTPase+COR+TM+TM*→||<-?<-?<-?<-Pro_CA||?→||<-?<-tRNA<-tRNA<-Ferredoxin-betagrasp</td><td>LRR-repeats+AP-GTPase+COR+TM+TM</td><td>805</td><td>EWV78_08805</td><td>Cyanobacteria</td><td>Microcystis aeruginosa Ma_MB_F_20061100_S20D</td><td>GTP-binding protein [Microcystis aeruginosa Ma_MB_F_20061100_S20D].</td><td>GCA_007095855.1</td></tr>
<tr><td>TRU47882.1</td><td>CHTH→ LRR-repeats+AP-GTPase+COR+TM+TM*→</td><td>LRR-repeats+AP-GTPase+COR+TM+TM</td><td>874</td><td>EWV49_14300</td><td>Cyanobacteria</td><td>Microcystis aeruginosa Ma_QC_Ch_20071001_S25</td><td>GTP-binding protein [Microcystis aeruginosa Ma_QC_Ch_20071001_S25].</td><td>GCA_007095935.1</td></tr>
<tr><td>TRU57932.1</td><td>CHTH→ LRR-repeats+AP-GTPase+COR*→</td><td>LRR-repeats+AP-GTPase+COR</td><td>559</td><td>EWV56_15605</td><td>Cyanobacteria</td><td>Microcystis aeruginosa Ma_QC_C_20070823_S13D</td><td>GTP-binding protein, partial [Microcystis aeruginosa Ma_QC_C_20070823_S13D].</td><td>GCA_007095965.1</td></tr>
<tr><td>TRU80903.1</td><td>SIG+TM+TM+TM+TM+TM+TM+TM→||<-?||Mbetalac→ SAM-methylase→?→||<-?<-SIG+TPM_phosphatase+TM+TM+TM||LRR-repeats+AP-GTPase+COR+TM+TM*→</td><td>LRR-repeats+AP-GTPase+COR+TM+TM</td><td>806</td><td>EWV76_22185</td><td>Cyanobacteria</td><td>Microcystis novacekii Mn_MB_F_20050700_S1</td><td>GTP-binding protein [Microcystis novacekii Mn_MB_F_20050700_S1].</td><td>GCA_007096045.1</td></tr>
<tr><td>TRU95593.1</td><td>Ferredoxin-betagrasp→ tRNA→ tRNA→ SAM-methylase→?→||<-?<-SIG+TPM_phosphatase+TM+TM+TM||LRR-repeats+AP-GTPase+COR+TM+TM*→||<-?<-?<-?<-Pro_CA</td><td>LRR-repeats+AP-GTPase+COR+TM+TM</td><td>852</td><td>EWV73_20055</td><td>Cyanobacteria</td><td>Microcystis wesenbergii Mw_QC_B_20070930_S4D</td><td>GTP-binding protein [Microcystis wesenbergii Mw_QC_B_20070930_S4D].</td><td>GCA_007096105.1</td></tr>
<tr><td>TRU97707.1</td><td>Ferredoxin-betagrasp→ tRNA→ tRNA→ SAM-methylase→?→||<-?<-SIG+TPM_phosphatase+TM+TM+TM||LRR-repeats+AP-GTPase+COR+TM+TM*→||<-?<-?<-?<-Pro_CA</td><td>LRR-repeats+AP-GTPase+COR+TM+TM</td><td>943</td><td>EWV75_08635</td><td>Cyanobacteria</td><td>Microcystis wesenbergii Mw_QC_S_20081001_S30D</td><td>GTP-binding protein [Microcystis wesenbergii Mw_QC_S_20081001_S30D].</td><td>GCA_007096035.1</td></tr>
<tr><td>TRU98843.1</td><td>Ferredoxin-betagrasp→ tRNA→ tRNA→ SAM-methylase→?→||<-?<-SIG+TPM_phosphatase+TM+TM+TM||LRR-repeats+AP-GTPase+COR+TM+TM*→||<-?<-?<-?<-Pro_CA</td><td>LRR-repeats+AP-GTPase+COR+TM+TM</td><td>920</td><td>EWV74_15160</td><td>Cyanobacteria</td><td>Microcystis wesenbergii Mw_QC_S_20081001_S30</td><td>GTP-binding protein [Microcystis wesenbergii Mw_QC_S_20081001_S30].</td><td>GCA_007096535.1</td></tr>
<tr><td>TRV08313.1</td><td>Ferredoxin-betagrasp→ tRNA→ tRNA→ SAM-methylase→?→||<-?<-SIG+TPM_phosphatase+TM+TM+TM||LRR-repeats+AP-GTPase+COR+TM+TM*→?→||<-?<-?<-?||?→||<-Pro_CA</td><td>LRR-repeats+AP-GTPase+COR+TM+TM</td><td>751</td><td>EWV41_10615</td><td>Cyanobacteria</td><td>Microcystis wesenbergii Mw_MB_S_20031200_S109</td><td>GTP-binding protein [Microcystis wesenbergii Mw_MB_S_20031200_S109].</td><td>GCA_007096585.1</td></tr>
<tr><td>TRV43292.1</td><td>SAM-methylase→?→||<-?<-SIG+TPM_phosphatase+TM+TM+TM||LRR-repeats+AP-GTPase+COR+TM+TM*→||<-?<-?<-?<-Pro_CA||?→||<-?<-tRNA<-tRNA<-Ferredoxin-betagrasp</td><td>LRR-repeats+AP-GTPase+COR+TM+TM</td><td>874</td><td>EWV87_21500</td><td>Cyanobacteria</td><td>Microcystis panniformis Mp_GB_SS_20050300_S99</td><td>GTP-binding protein [Microcystis panniformis Mp_GB_SS_20050300_S99].</td><td>GCA_007096815.1</td></tr>
<tr><td>TVP67596.1</td><td>LRR-repeats+AP-GTPase+COR+TM+TM*→</td><td>LRR-repeats+AP-GTPase+COR+TM+TM</td><td>667</td><td>EA343_00435</td><td>Cyanobacteria</td><td>Nodularia sp. (in: Bacteria)</td><td>GTPase, partial [Nodularia sp. (in: Bacteria)].</td><td>GCA_007692755.1</td></tr>
<tr><td>TVQ58623.1</td><td>LRR-repeats+AP-GTPase+COR*→||<-Cluster2343_2clades</td><td>LRR-repeats+AP-GTPase+COR</td><td>976</td><td>EA366_06380</td><td>Cyanobacteria</td><td>Spirulina sp. DLM2.Bin59</td><td>hypothetical protein EA366_06380 [Spirulina sp. DLM2.Bin59].</td><td>GCA_007693875.1</td></tr>
<tr><td>TVV43679.1</td><td><-MarR-HTH<-?<-Cluster1077_2clades||Aminotran_1_2→ PSE→||<-?<-?||?→ LRR-repeats+AP-GTPase+COR*→</td><td>LRR-repeats+AP-GTPase+COR</td><td>985</td><td>FOT50_08370</td><td>Gammaproteobacteria</td><td>Thalassolituus sp. C2-1</td><td>GTP-binding protein [Thalassolituus sp. C2-1].</td><td>GCA_007785795.1</td></tr>
<tr><td>TXI24418.1</td><td>LRR-repeats+AP-GTPase+COR+DrHyd*→?→?→?→?→?→?→ Cluster1726_2clades→</td><td>LRR-repeats+AP-GTPase+COR+DrHyd</td><td>1061</td><td>E6Q61_04940</td><td>Betaproteobacteria</td><td>Nitrosomonas sp.</td><td>hypothetical protein E6Q61_04940 [Nitrosomonas sp.].</td><td>GCA_008015595.1</td></tr>
<tr><td>TXL81991.1</td><td>LRR-repeats+AP-GTPase+COR+CASPASE*→</td><td>LRR-repeats+AP-GTPase+COR+CASPASE</td><td>1175</td><td>FHP25_02680</td><td>Alphaproteobacteria</td><td>Enhydrobacter sp. CC-CFT640</td><td>CHAT domain-containing protein [Enhydrobacter sp. CC-CFT640].</td><td>GCA_008039615.1</td></tr>
<tr><td>TYC52188.1</td><td>WCAK→?→ LRR-repeats→ LRR-repeats+AP-GTPase+COR*→</td><td>LRR-repeats+AP-GTPase+COR</td><td>860</td><td>FMN50_19470</td><td>Alphaproteobacteria</td><td>Rhodobacterales bacterium</td><td>hypothetical protein FMN50_19470 [Rhodobacterales bacterium].</td><td>GCA_008107755.1</td></tr>
</tbody>
</table>

<table>
<thead>
<tr><th>acc</th><th>operon</th><th>architecture</th><th>len</th><th>gen.name</th><th>taxend</th><th>species</th><th>defline</th><th>gca</th></tr>
</thead>
<tbody>
<tr><td>VFJ48800.1</td><td>RelE-ParE→?→?→ LRR-repeats+AP-GTPase+COR*→</td><td>LRR-repeats+AP-GTPase+COR</td><td>467</td><td>BECKDK2373C_GA0170839_10231</td><td>Gammaproteobacteria</td><td>Candidatus Kentron sp. DK</td><td>Leucine rich repeat-containing protein, partial [Candidatus Kentron sp. DK].</td><td>-</td></tr>
<tr><td>VFJ57569.1</td><td>RelE-ParE→?→ Cluster54_3clades→?→ LRR-repeats+AP-GTPase+COR*→</td><td>LRR-repeats+AP-GTPase+COR</td><td>1027</td><td>BECKFW1821A_GA0114235_107219</td><td>Gammaproteobacteria</td><td>Candidatus Kentron sp. FW</td><td>Leucine rich repeat-containing protein [Candidatus Kentron sp. FW].</td><td>-</td></tr>
<tr><td>VFJ62833.1</td><td>Cluster1147_2clades→?→?→ RelE-ParE→?→?→ LRR-repeats+AP-GTPase+COR*→ Cluster2123_2clades→</td><td>LRR-repeats+AP-GTPase+COR</td><td>702</td><td>BECKDK2373B_GA0170837_11172</td><td>Gammaproteobacteria</td><td>Candidatus Kentron sp. DK</td><td>Leucine rich repeat-containing protein [Candidatus Kentron sp. DK].</td><td>-</td></tr>
<tr><td>VFJ65980.1</td><td>Cluster1147_2clades→?→?→ RelE-ParE→?→?→ LRR-repeats+AP-GTPase+COR*→</td><td>LRR-repeats+AP-GTPase+COR</td><td>470</td><td>BECKDK2373C_GA0170839_113817</td><td>Gammaproteobacteria</td><td>Candidatus Kentron sp. DK</td><td>Leucine rich repeat-containing protein, partial [Candidatus Kentron sp. DK].</td><td>-</td></tr>
<tr><td>VFJ75018.1</td><td>LRR-repeats+AP-GTPase+COR*→</td><td>LRR-repeats+AP-GTPase+COR</td><td>772</td><td>BECKFW1821C_GA0114237_10681</td><td>Gammaproteobacteria</td><td>Candidatus Kentron sp. FW</td><td>Ras of Complex, Roc, domain of DAPkinase [Candidatus Kentron sp. FW].</td><td>-</td></tr>
<tr><td>VFJ87041.1</td><td>RelE-ParE→ LRR-repeats+AP-GTPase+COR*→</td><td>LRR-repeats+AP-GTPase+COR</td><td>1091</td><td>BECKLFY1418B_GA0070995_100545</td><td>Gammaproteobacteria</td><td>Candidatus Kentron sp. LFY</td><td>Leucine rich repeat-containing protein [Candidatus Kentron sp. LFY].</td><td>-</td></tr>
<tr><td>VFJ90339.1</td><td>RelE-ParE→ LRR-repeats+AP-GTPase+COR*→ COR→</td><td>LRR-repeats+AP-GTPase+COR</td><td>380</td><td>BECKLFY1418A_GA0070994_101124</td><td>Gammaproteobacteria</td><td>Candidatus Kentron sp. LFY</td><td>Leucine rich repeat-containing protein [Candidatus Kentron sp. LFY].</td><td>-</td></tr>
<tr><td>VFK39292.1</td><td>RelE-ParE→?→ LRR-repeats+AP-GTPase+COR*→?→?→?→ RPN10→</td><td>LRR-repeats+AP-GTPase+COR</td><td>1088</td><td>BECKTC1821E_GA0114239_100479</td><td>Gammaproteobacteria</td><td>Candidatus Kentron sp. TC</td><td>Leucine rich repeat-containing protein [Candidatus Kentron sp. TC].</td><td>-</td></tr>
<tr><td>VFK43940.1</td><td>Cluster54_3clades→?→ RelE-ParE→ LRR-repeats+AP-GTPase+COR*→?→ Cluster1137_2clades→?→?→ RPN10→</td><td>LRR-repeats+AP-GTPase+COR</td><td>1017</td><td>BECKTC1821D_GA0114238_101924</td><td>Gammaproteobacteria</td><td>Candidatus Kentron sp. TC</td><td>Leucine rich repeat-containing protein [Candidatus Kentron sp. TC].</td><td>-</td></tr>
<tr><td>VFK55324.1</td><td>RelE-ParE→ LRR-repeats+AP-GTPase+COR*→?→ Cluster1137_2clades→?→?→ RPN10→</td><td>LRR-repeats+AP-GTPase+COR</td><td>1013</td><td>BECKTC1821F_GA0114240_100673</td><td>Gammaproteobacteria</td><td>Candidatus Kentron sp. TC</td><td>Leucine rich repeat-containing protein [Candidatus Kentron sp. TC].</td><td>-</td></tr>
<tr><td>VUX45208.1</td><td>LRR-repeats+AP-GTPase+COR+TIR*→</td><td>LRR-repeats+AP-GTPase+COR+TIR</td><td>1031</td><td>DF3PA_100056</td><td>Alphaproteobacteria</td><td>Candidatus Defluviicoccus seviourii</td><td>conserved hypothetical protein [Candidatus Defluviicoccus seviourii].</td><td>GCA_900609035.2</td></tr>
<tr><td>VUX54879.1</td><td><-SIG+Trypsin+PDZ<-?||?→?→||<-?<-?<-?||LRR-repeats+AP-GTPase+COR*→</td><td>LRR-repeats+AP-GTPase+COR</td><td>935</td><td>JTBB02_V1_20008</td><td>Gammaproteobacteria</td><td>uncultured Woeseiaceae bacterium</td><td>protein of unknown function [uncultured Woeseiaceae bacterium].</td><td>GCA_902167415.1</td></tr>
<tr><td>WP_002686189.1</td><td><-Cluster1370_2clades||CNMP+CRP-HTH→?→||<-?||LRR-repeats+AP-GTPase+COR+DUF4404*→||<-?<-?<-?||?→ PurT-ATPgrasp→</td><td>LRR-repeats+AP-GTPase+COR+DUF4404</td><td>1098</td><td>-</td><td>Gammaproteobacteria</td><td>Beggiatoa alba</td><td>leucine-rich repeat domain-containing protein [Beggiatoa alba].</td><td>GCF_000245015.1</td></tr>
<tr><td>WP_002693173.1</td><td><-NUDIX<-?||?→?→ SIG+TM+TM+Calcineurin→||<-?||LRR-repeats+AP-GTPase+COR*→||<-?||?→||<-?||Cluster1843_2clades→</td><td>LRR-repeats+AP-GTPase+COR</td><td>1282</td><td>-</td><td>Bacteroidetes</td><td>Microscilla marina</td><td>leucine-rich repeat protein [Microscilla marina].</td><td>GCF_000169175.1</td></tr>
<tr><td>WP_002693833.1</td><td>LRR-repeats+AP-GTPase+COR+EAD11*→?→||<-PAS+HISKIN</td><td>LRR-repeats+AP-GTPase+COR+EAD11</td><td>847</td><td>-</td><td>Bacteroidetes</td><td>Microscilla marina</td><td>leucine rich repeat protein [Microscilla marina].</td><td>GCF_000169175.1</td></tr>
<tr><td>WP_002694398.1</td><td>NUDIX→||<-?<-?<-?<-?||LRR-repeats+AP-GTPase+COR*→?→?→?→||<-?<-?<-SIGMA-HTH</td><td>LRR-repeats+AP-GTPase+COR</td><td>836</td><td>-</td><td>Bacteroidetes</td><td>Microscilla marina</td><td>leucine-rich repeat domain-containing protein [Microscilla marina].</td><td>GCF_000169175.1</td></tr>
<tr><td>WP_002703871.1</td><td>ABC_membrane+ABC_tran→?→?→ LRR-repeats+AP-GTPase+COR*→?→?→||<-SIG+SecA<-?<-?<-?||SIGMA-HTH→</td><td>LRR-repeats+AP-GTPase+COR</td><td>966</td><td>-</td><td>Bacteroidetes</td><td>Microscilla marina</td><td>leucine-rich repeat domain-containing protein [Microscilla marina].</td><td>GCF_000169175.1</td></tr>
<tr><td>WP_002704578.1</td><td><-DAGKIN<-?||?→ LRR-repeats+AP-GTPase+COR*→||<-?||CNMP+CRP-HTH→||<-?||?→ InPase→</td><td>LRR-repeats+AP-GTPase+COR</td><td>897</td><td>-</td><td>Bacteroidetes</td><td>Microscilla marina</td><td>leucine-rich repeat domain-containing protein [Microscilla marina].</td><td>GCF_000169175.1</td></tr>
</tbody>
</table>

acc	operon	architecture	len	gen.name	taxend	species	defline	gca
WP_002736512.1	SIG+TM+TM+TM+TM+TM+TM+TM→||<-?||Mbetalac→ SAM-methylase→?→||<-?<-SIG+TPM_phosphatase+TM+TM+TM||LRR-repeats+AP-GTPase+COR+TM+TM*→||<-?<-?<-?<-Pro_CA||?→||<-?<-tRNA<-tRNA<-Ferredoxin-betagrasp	LRR-repeats+AP-GTPase+COR+TM+TM	852	-	Cyanobacteria	Microcystis aeruginosa	leucine-rich repeat protein [Microcystis aeruginosa].	GCF_000330925.1
WP_002754362.1	SIG+TM+TM+TM+TM+TM+TM+TM→||<-?||Mbetalac→ SAM-methylase→?→||<-?<-SIG+TPM_phosphatase+TM+TM+TM||LRR-repeats+AP-GTPase+COR+TM+TM*→||<-?<-?<-?<-Pro_CA||?→||<-?<-tRNA<-tRNA<-Ferredoxin-betagrasp	LRR-repeats+AP-GTPase+COR+TM+TM	806	-	Cyanobacteria	Microcystis aeruginosa	leucine-rich repeat domain-containing protein [Microcystis aeruginosa].	GCF_000307995.1
WP_002775884.1	SIG+TM+TM+TM+TM+TM+TM+TM→||<-?||Mbetalac→ SAM-methylase→?→||<-?<-SIG+TPM_phosphatase+TM+TM+TM||LRR-repeats+AP-GTPase+COR+TM+TM*→||<-?<-?<-?<-Pro_CA||?→||<-?<-tRNA<-tRNA<-Ferredoxin-betagrasp	LRR-repeats+AP-GTPase+COR+TM+TM	783	-	Cyanobacteria	Microcystis aeruginosa	leucine-rich repeat protein [Microcystis aeruginosa].	GCF_000312205.1
WP_002792425.1	SIG+TM+TM+TM+TM+TM+TM+TM→||<-?||Mbetalac→ SAM-methylase→?→||<-?<-SIG+TPM_phosphatase+TM+TM+TM||LRR-repeats+AP-GTPase+COR+TM+TM*→||<-?<-?<-?<-Pro_CA||?→||<-PSE<-?<-tRNA<-tRNA<-Ferredoxin-betagrasp	LRR-repeats+AP-GTPase+COR+TM+TM	834	-	Cyanobacteria	Microcystis aeruginosa	leucine-rich repeat domain-containing protein [Microcystis aeruginosa].	GCF_000312245.1
WP_002797004.1	SIG+TM+TM+TM+TM+TM+TM+TM→||<-?||Mbetalac→ SAM-methylase→?→||<-?<-SIG+TPM_phosphatase+TM+TM+TM||LRR-repeats+AP-GTPase+COR+TM+TM*→	LRR-repeats+AP-GTPase+COR+TM+TM	865	-	Cyanobacteria	Microcystis aeruginosa	leucine-rich repeat protein [Microcystis aeruginosa].	GCF_000312265.1
WP_004156960.1	SIG+TM+TM+TM+TM+TM+TM+TM→||<-?||Mbetalac→ SAM-methylase→?→||<-?<-SIG+TPM_phosphatase+TM+TM+TM||LRR-repeats+AP-GTPase+COR+TM+TM*→||<-?<-?<-?<-Pro_CA||?→||<-?<-tRNA<-tRNA<-Ferredoxin-betagrasp	LRR-repeats+AP-GTPase+COR+TM+TM	875	-	Cyanobacteria	Microcystis aeruginosa	leucine-rich repeat protein [Microcystis aeruginosa].	GCF_000332585.1
WP_005857943.1	LRR-repeats+AP-GTPase+COR*→||<-tRNA<-?<-?<-?<-Arginase	LRR-repeats+AP-GTPase+COR	797	-	Alphaproteobacteria	Sagittula stellata	hypothetical protein [Sagittula stellata].	GCF_000169415.1
WP_006099017.1	CR-REase5→ Cluster1847_2clades→?→ SIG+WWE→?→ LRR-repeats+AP-GTPase+COR*→?→?→ Pentapeptide-repeats→	LRR-repeats+AP-GTPase+COR	1117	-	Cyanobacteria	Coleofasciculus chthonoplastes	leucine-rich repeat domain-containing protein [Coleofasciculus chthonoplastes].	GCF_000155555.1
WP_006103609.1	FGS→?→?→?→||<-?||?→ LRR-repeats+AP-GTPase+COR+TIR*→	LRR-repeats+AP-GTPase+COR+TIR	1090	-	Cyanobacteria	Coleofasciculus chthonoplastes	leucine-rich repeat domain-containing protein [Coleofasciculus chthonoplastes].	GCF_000155555.1
WP_006456809.1	<-REC||?→?→ ABC_membrane+ABC_tran→?→||<-?||?→ LRR-repeats+AP-GTPase+COR*→	LRR-repeats+AP-GTPase+COR	1260	-	Cyanobacteria	Synechococcus sp. PCC 7335	leucine-rich repeat domain-containing protein [Synechococcus sp. PCC 7335].	GCF_000155595.1
WP_006507306.1	LRR-repeats+AP-GTPase+COR*→	LRR-repeats+AP-GTPase+COR	705	-	Cyanobacteria	Xenococcus sp. PCC 7305	small G protein, GTPase SAR1, partial [Xenococcus sp. PCC 7305].	-
WP_006512482.1	<-GTPase-AIG||Cluster2189_2clades→||<-?||?→||<-?||LRR-repeats+AP-GTPase+COR*→?→||<-?<-?<-?<-Pentapeptide-repeats	LRR-repeats+AP-GTPase+COR	806	-	Cyanobacteria	Xenococcus sp. PCC 7305	leucine-rich repeat domain-containing protein [Xenococcus sp. PCC 7305].	GCF_000332055.1
WP_006519951.1	Cluster2165_2clades→ Cluster1999_2clades→ LRR-repeats+AP-GTPase+COR*→	LRR-repeats+AP-GTPase+COR	1104	-	Cyanobacteria	Leptolyngbya sp. PCC 7375	hypothetical protein [Leptolyngbya sp. PCC 7375].	GCF_000316115.1
WP_006528821.1	LRR-repeats+AP-GTPase+COR*→	LRR-repeats+AP-GTPase+COR	629	-	Cyanobacteria	Gloeocapsa sp. PCC 73106	leucine-rich repeat domain-containing protein [Gloeocapsa sp. PCC 73106].	GCF_000332035.1
WP_006631836.1	LRR-repeats+AP-GTPase+COR+DUF4404*→||<-?<-?<-?<-?||Ferretin→	LRR-repeats+AP-GTPase+COR+DUF4404	1060	-	Cyanobacteria	Microcoleus vaginatus	leucine-rich repeat domain-containing protein [Microcoleus vaginatus].	GCF_000214075.1
WP_007307526.1	LRR-repeats+AP-GTPase+COR*→	LRR-repeats+AP-GTPase+COR	795	-	Cyanobacteria	Crocosphaera watsonii	leucine-rich repeat domain-containing protein [Crocosphaera watsonii].	GCF_000167195.1
WP_007312599.1	LRR-repeats+AP-GTPase+COR*→	LRR-repeats+AP-GTPase+COR	378	-	Cyanobacteria	Crocosphaera watsonii	hypothetical protein [Crocosphaera watsonii].	GCF_001050835.1
WP_007458231.1	LRR-repeats+AP-GTPase+COR*→||<-?<-?||?→||<-?<-?||SIG+TM→ REC→	LRR-repeats+AP-GTPase+COR	1135	-	Actinobacteria	Micromonospora lupini	hypothetical protein [Micromonospora lupini].	GCF_000297395.2
WP_007460872.1	LRR-repeats+AP-GTPase+COR*→||<-ABC_membrane+ABC_tran	LRR-repeats+AP-GTPase+COR	902	-	Actinobacteria	Micromonospora lupini	leucine-rich repeat domain-containing protein [Micromonospora lupini].	GCF_000297395.2
WP_008188574.1	SIG+TM+TM+TM+TM+TM+TM+TM→ ABC-ATPase→ ABC-ATPase→?→?→||<-?<-?||LRR-repeats+AP-GTPase+COR*→?→||<-?<-?||PSE→||<-Cluster5_5clades	LRR-repeats+AP-GTPase+COR	868	-	Cyanobacteria	Moorea producens	leucine-rich repeat domain-containing protein [Moorea producens].	GCF_000211815.1

<table>
<thead>
<tr><th>acc</th><th>operon</th><th>architecture</th><th>len</th><th>gen.name</th><th>taxend</th><th>species</th><th>defline</th><th>gca</th></tr>
</thead>
<tbody>
<tr><td>WP_008190613.1</td><td>REC→||<-PSE<-HNH<-?||REC→||<-TPR-repeats<-?<-?||LRR-repeats+AP-GTPase+COR+DUF4404*→</td><td>LRR-repeats+AP-GTPase+COR+DUF4404</td><td>948</td><td>-</td><td>Cyanobacteria</td><td>Moorea producens</td><td>leucine-rich repeat domain-containing protein [Moorea producens].</td><td>GCF_000211815.1</td></tr>
<tr><td>WP_008231466.1</td><td><-Cluster1131_3clades<-?<-Cluster1163_3clades||?→?→?→ tRNA→ LRR-repeats+AP-GTPase+COR*→</td><td>LRR-repeats+AP-GTPase+COR</td><td>970</td><td>-</td><td>Alphaproteobacteria</td><td>Roseobacter sp. CCS2</td><td>hypothetical protein [Roseobacter sp. CCS2].</td><td>GCF_000169435.1</td></tr>
<tr><td>WP_008273960.1</td><td>Cluster1550_2clades→?→?→ PIN→ LRR-repeats+AP-GTPase+COR*→</td><td>LRR-repeats+AP-GTPase+COR</td><td>830</td><td>-</td><td>Cyanobacteria</td><td>Crocosphaera chwakensis</td><td>leucine-rich repeat domain-containing protein [Crocosphaera chwakensis].</td><td>GCF_000169335.1</td></tr>
<tr><td>WP_008277167.1</td><td><-REC||SIG+TM+TM+TM+TM→||<-?||?→||<-?||?→ LRR-repeats+AP-GTPase+COR*→</td><td>LRR-repeats+AP-GTPase+COR</td><td>974</td><td>-</td><td>Cyanobacteria</td><td>Crocosphaera chwakensis</td><td>leucine-rich repeat domain-containing protein [Crocosphaera chwakensis].</td><td>GCF_000169335.1</td></tr>
<tr><td>WP_008680517.1</td><td>LRR-repeats+AP-GTPase+COR*→</td><td>LRR-repeats+AP-GTPase+COR</td><td>1157</td><td>-</td><td>Planctomycetes</td><td>Rhodopirellula sallentina</td><td>small GTP-binding protein [Rhodopirellula sallentina].</td><td>GCF_000346505.1</td></tr>
<tr><td>WP_009282559.1</td><td>LRR-repeats+AP-GTPase+COR+TIR*→||<-?<-?||?→||<-?<-?<-?<-TM+TM+TM+TM+TM</td><td>LRR-repeats+AP-GTPase+COR+TIR</td><td>925</td><td>-</td><td>Bacteroidetes</td><td>Fibrisoma limi</td><td>leucine-rich repeat domain-containing protein [Fibrisoma limi].</td><td>GCF_000296815.2</td></tr>
<tr><td>WP_009283610.1</td><td><-McrA-NTD||?→ N-OB→?→?→ LRR-repeats+AP-GTPase+COR+TIR*→ Cluster1088_2clades→?→?→?→||<-SIGMA-HTH</td><td>LRR-repeats+AP-GTPase+COR+TIR</td><td>1023</td><td>-</td><td>Bacteroidetes</td><td>Fibrisoma limi</td><td>leucine-rich repeat protein [Fibrisoma limi].</td><td>GCF_000296815.2</td></tr>
<tr><td>WP_009627973.1</td><td>LRR-repeats+AP-GTPase+COR+TM+TM*→||<-REC</td><td>LRR-repeats+AP-GTPase+COR+TM+TM</td><td>945</td><td>-</td><td>Cyanobacteria</td><td>Pseudanabaena biceps</td><td>leucine-rich repeat domain-containing protein [Pseudanabaena biceps].</td><td>GCF_000332215.1</td></tr>
<tr><td>WP_009964949.1</td><td><-SMS-KaiC<-?<-?<-Cluster1581_2clades||?→?→?→ LRR-repeats+AP-GTPase+COR+TIR*→?→ TIR→?→?→ DrHyd+APATPase+TPR+TPR+TPR→</td><td>LRR-repeats+AP-GTPase+COR+TIR</td><td>961</td><td>-</td><td>Verrucomicrobia</td><td>Verrucomicrobium spinosum</td><td>GTP-binding protein [Verrucomicrobium spinosum].</td><td>GCF_000172155.1</td></tr>
<tr><td>WP_010471910.1</td><td><-REC||?→ LRR-repeats+AP-GTPase+COR*→</td><td>LRR-repeats+AP-GTPase+COR</td><td>1082</td><td>-</td><td>Cyanobacteria</td><td>Acaryochloris sp. CCMEE 5410</td><td>leucine-rich repeat domain-containing protein [Acaryochloris sp. CCMEE 5410].</td><td>GCF_000238775.1</td></tr>
<tr><td>WP_010477835.1</td><td><-ABC_membrane+ABC_tran||?→?→||<-?<-?||?→?→ LRR-repeats+AP-GTPase+COR*→</td><td>LRR-repeats+AP-GTPase+COR</td><td>1235</td><td>-</td><td>Cyanobacteria</td><td>Acaryochloris sp. CCMEE 5410</td><td>leucine-rich repeat domain-containing protein [Acaryochloris sp. CCMEE 5410].</td><td>GCF_000238775.1</td></tr>
<tr><td>WP_010933192.1</td><td><-SIG+TM||LRR-repeats+AP-GTPase+COR*→?→?→?→?→ ParA-Soj-PloopNTPase→</td><td>LRR-repeats+AP-GTPase+COR</td><td>1102</td><td>-</td><td>Chlorobi</td><td>Chlorobaculum tepidum</td><td>leucine-rich repeat domain-containing protein [Chlorobaculum tepidum].</td><td>GCF_000006985.1</td></tr>
<tr><td>WP_010994301.1</td><td><-Cluster5_5clades||LRR-repeats+AP-GTPase+COR+DUF4404*→||<-?<-PSE<-?||?→||<-REC</td><td>LRR-repeats+AP-GTPase+COR+DUF4404</td><td>1119</td><td>-</td><td>Cyanobacteria</td><td>Nostocaceae</td><td>MULTISPECIES: leucine-rich repeat domain-containing protein [Nostocaceae].</td><td>GCF_000009705.1</td></tr>
<tr><td>WP_011022280.1</td><td>PAS+HISKIN→||<-?||?→?→?→?→||<-?||LRR-repeats+AP-GTPase+COR*→</td><td>LRR-repeats+AP-GTPase+COR</td><td>631</td><td>-</td><td>Euryarchaeota</td><td>Methanosarcina acetivorans</td><td>leucine-rich repeat domain-containing protein [Methanosarcina acetivorans].</td><td>-</td></tr>
<tr><td>WP_011307276.1</td><td><-HhH-RADC+JAB||?→ NTP_transf_3→ LRR-repeats+AP-GTPase+COR*→||<-?<-?<-?<-Thioredoxin</td><td>LRR-repeats+AP-GTPase+COR</td><td>863</td><td>-</td><td>Euryarchaeota</td><td>Methanosarcina barkeri</td><td>leucine-rich repeat domain-containing protein [Methanosarcina barkeri].</td><td>GCF_000195895.1</td></tr>
<tr><td>WP_011307410.1</td><td>LRR-repeats+AP-GTPase+COR*→</td><td>LRR-repeats+AP-GTPase+COR</td><td>886</td><td>-</td><td>Euryarchaeota</td><td>Methanosarcina barkeri</td><td>leucine-rich repeat domain-containing protein [Methanosarcina barkeri].</td><td>GCF_000195895.1</td></tr>
<tr><td>WP_011308262.1</td><td><-Mbetalac<-PSE<-PSE||?→||<-?||?→?→?→||<-?||LRR-repeats+AP-GTPase+COR*→||<-?||?→||<-?||?→ CHTH→||<-?||ABC-ATPase→</td><td>LRR-repeats+AP-GTPase+COR</td><td>892</td><td>-</td><td>Euryarchaeota</td><td>Methanosarcina barkeri</td><td>leucine-rich repeat domain-containing protein [Methanosarcina barkeri].</td><td>GCF_000195895.1</td></tr>
<tr><td>WP_011318314.1</td><td><-Cluster5_5clades||LRR-repeats+AP-GTPase+COR+DUF4404*→||<-?<-ABC-ATPase<-?||?→||<-REC</td><td>LRR-repeats+AP-GTPase+COR+DUF4404</td><td>1107</td><td>-</td><td>Cyanobacteria</td><td>Trichormus variabilis</td><td>leucine-rich repeat domain-containing protein [Trichormus variabilis].</td><td>GCF_000204075.1</td></tr>
<tr><td>WP_011361554.1</td><td>BACTERIALFRINGE+TM+TM→?→ LRR-repeats+AP-GTPase+COR*→||<-?<-DAGKIN<-?||?→ Cluster881_3clades→?→ Cluster1718_2clades→</td><td>LRR-repeats+AP-GTPase+COR</td><td>811</td><td>-</td><td>Chlorobi</td><td>Chlorobium chlorochromatii</td><td>Rab family protein [Chlorobium chlorochromatii].</td><td>-</td></tr>
</tbody>
</table>

<table>
<thead>
<tr><th>acc</th><th>operon</th><th>architecture</th><th>len</th><th>gen.name</th><th>taxend</th><th>species</th><th>defline</th><th>gca</th></tr>
</thead>
<tbody>
<tr><td>WP_011388592.1</td><td><-ABC_membrane+ABC_tran<-ABC_membrane+ABC_tran<-?<-?<-?<-?||?→ LRR-repeats+AP-GTPase+COR+TIR*→ TM+TM+TM→</td><td>LRR-repeats+AP-GTPase+COR+TIR</td><td>1085</td><td>-</td><td>Alphaproteobacteria</td><td>Rhodospirillum rubrum</td><td>leucine-rich repeat domain-containing protein [Rhodospirillum rubrum].</td><td>GCF_000013085.1</td></tr>
<tr><td>WP_011613173.1</td><td><-Cluster1413_2clades<-?||TPR-repeats→||<-?<-?<-PSE||LRR-repeats+AP-GTPase+COR*→</td><td>LRR-repeats+AP-GTPase+COR</td><td>1041</td><td>-</td><td>Cyanobacteria</td><td>Trichodesmium erythraeum</td><td>leucine-rich repeat domain-containing protein [Trichodesmium erythraeum].</td><td>GCF_000014265.1</td></tr>
<tr><td>WP_011712156.1</td><td>OmpA→?→||<-?<-?<-?||LRR-repeats+AP-GTPase+COR*→ CNMP+CRP-HTH→||<-?<-Cluster997_2clades<-?<-Thioredoxin<-SIG+RHOD-CDC25<-ARSR-HTH</td><td>LRR-repeats+AP-GTPase+COR</td><td>761</td><td>-</td><td>Alphaproteobacteria</td><td>Magnetococcus marinus</td><td>small GTP-binding protein [Magnetococcus marinus].</td><td>GCF_000014865.1</td></tr>
<tr><td>WP_012162488.1</td><td>Cluster1052_2clades→ NUDIX→ LRR-repeats+AP-GTPase+COR+TIR*→ TM+TM+TM→</td><td>LRR-repeats+AP-GTPase+COR+TIR</td><td>842</td><td>-</td><td>Cyanobacteria</td><td>Acaryochloris marina</td><td>leucine-rich repeat domain-containing protein [Acaryochloris marina].</td><td>GCF_000018105.1</td></tr>
<tr><td>WP_012407958.1</td><td>SIG+PBPB→ HISKIN→ HISKIN→ LRR-repeats+AP-GTPase+COR+TIR*→||<-?||?→?→?→||<-?||?→ Pentapeptide-repeats→</td><td>LRR-repeats+AP-GTPase+COR+TIR</td><td>1185</td><td>-</td><td>Cyanobacteria</td><td>Nostoc punctiforme</td><td>leucine-rich repeat domain-containing protein [Nostoc punctiforme].</td><td>GCF_000020025.1</td></tr>
<tr><td>WP_012412722.1</td><td>LRR-repeats+AP-GTPase+COR*→</td><td>LRR-repeats+AP-GTPase+COR</td><td>1124</td><td>-</td><td>Cyanobacteria</td><td>Nostoc punctiforme</td><td>leucine-rich repeat domain-containing protein [Nostoc punctiforme].</td><td>GCF_000020025.1</td></tr>
<tr><td>WP_012466409.1</td><td><-SF2-DUF3427A+TnsA-REase<-?<-?<-?<-?||?→?→ LRR-repeats+AP-GTPase+COR+TIR*→ TIR→</td><td>LRR-repeats+AP-GTPase+COR+TIR</td><td>998</td><td>-</td><td>Chlorobi</td><td>Chlorobium limicola</td><td>leucine-rich repeat domain-containing protein [Chlorobium limicola].</td><td>GCF_000020465.1</td></tr>
<tr><td>WP_012597192.1</td><td>Cluster1134_2clades→ REase-4→?→?→?→ HAD→ LRR-repeats+AP-GTPase+COR+TM+TM*→</td><td>LRR-repeats+AP-GTPase+COR+TM+TM</td><td>937</td><td>-</td><td>Cyanobacteria</td><td>Rippkaea orientalis</td><td>GTP-binding protein [Rippkaea orientalis].</td><td>GCF_000021805.1</td></tr>
<tr><td>WP_013320928.1</td><td>Cluster5_5clades→ LRR-repeats+AP-GTPase+COR*→?→ SIG+GT4→||<-?<-?||Cluster5_5clades→</td><td>LRR-repeats+AP-GTPase+COR</td><td>876</td><td>-</td><td>Cyanobacteria</td><td>Gloeothece verrucosa</td><td>leucine-rich repeat domain-containing protein [Gloeothece verrucosa].</td><td>GCF_000147335.1</td></tr>
<tr><td>WP_013323827.1</td><td>LRR-repeats+AP-GTPase+COR+TM+TM*→</td><td>LRR-repeats+AP-GTPase+COR+TM+TM</td><td>857</td><td>-</td><td>Cyanobacteria</td><td>Gloeothece verrucosa</td><td>leucine-rich repeat domain-containing protein [Gloeothece verrucosa].</td><td>GCF_000147335.1</td></tr>
<tr><td>WP_013762559.1</td><td>SIGMA-HTH→?→ SIG+TM→ LRR-repeats+AP-GTPase+COR+TIR*→?→ SIGMA-HTH→?→||<-SIG+TM+TM+TM+HISKIN</td><td>LRR-repeats+AP-GTPase+COR+TIR</td><td>995</td><td>-</td><td>Bacteroidetes</td><td>Haliscomenobacter hydrossis</td><td>leucine-rich repeat domain-containing protein [Haliscomenobacter hydrossis].</td><td>GCF_000212735.1</td></tr>
<tr><td>WP_013763674.1</td><td><-ABC_membrane+ABC_tran||FGS→ PSE→ FGS→ LRR-repeats+AP-GTPase+COR+EAD11+CASPASE*→ RNASEII→</td><td>LRR-repeats+AP-GTPase+COR+EAD11+CASPASE</td><td>1108</td><td>-</td><td>Bacteroidetes</td><td>Haliscomenobacter hydrossis</td><td>leucine-rich repeat domain-containing protein [Haliscomenobacter hydrossis].</td><td>GCF_000212735.1</td></tr>
<tr><td>WP_013766755.1</td><td>LRR-repeats+AP-GTPase+COR+EAD11*→</td><td>LRR-repeats+AP-GTPase+COR+EAD11</td><td>1448</td><td>-</td><td>Bacteroidetes</td><td>Haliscomenobacter hydrossis</td><td>leucine-rich repeat protein [Haliscomenobacter hydrossis].</td><td>GCF_000212735.1</td></tr>
<tr><td>WP_013767289.1</td><td><-SIG+ABhydrolase||SIG+7TMR-DISMED1→ FGS→ FGS→ LRR-repeats+AP-GTPase+COR+EAD11*→||<-?<-Cluster1645_2clades</td><td>LRR-repeats+AP-GTPase+COR+EAD11</td><td>840</td><td>-</td><td>Bacteroidetes</td><td>Haliscomenobacter hydrossis</td><td>leucine-rich repeat protein [Haliscomenobacter hydrossis].</td><td>GCF_000212735.1</td></tr>
<tr><td>WP_013768874.1</td><td>LRR-repeats+AP-GTPase+COR+TIR→ LRR-repeats+AP-GTPase+COR→ LRR-repeats+AP-GTPase+COR*→?→?→?→||<-?||?→?→||<-REC</td><td>LRR-repeats+AP-GTPase+COR+TIR</td><td>741</td><td>-</td><td>Bacteroidetes</td><td>Haliscomenobacter hydrossis</td><td>hypothetical protein [Haliscomenobacter hydrossis].</td><td>GCF_000212735.1</td></tr>
<tr><td>WP_013768875.1</td><td>LRR-repeats+AP-GTPase+COR*→</td><td>LRR-repeats+AP-GTPase+COR</td><td>786</td><td>-</td><td>Bacteroidetes</td><td>Haliscomenobacter hydrossis</td><td>hypothetical protein [Haliscomenobacter hydrossis].</td><td>GCF_000212735.1</td></tr>
<tr><td>WP_013818452.1</td><td>LRR-repeats+AP-GTPase+COR+TIR*→</td><td>LRR-repeats+AP-GTPase+COR+TIR</td><td>944</td><td>-</td><td>Gammaproteobacteria</td><td>Methylomonas methanica</td><td>leucine-rich repeat domain-containing protein [Methylomonas methanica].</td><td>GCF_000214665.1</td></tr>
<tr><td>WP_014444162.1</td><td>REC→||<-?<-?||?→||<-?<-?<-?||LRR-repeats+AP-GTPase+COR*→||<-?<-7TMR-DISMED1</td><td>LRR-repeats+AP-GTPase+COR</td><td>979</td><td>-</td><td>Actinobacteria</td><td>Actinoplanes missouriensis</td><td>hypothetical protein [Actinoplanes missouriensis].</td><td>GCF_000284295.1</td></tr>
<tr><td>WP_014798951.1</td><td>LRR-repeats+AP-GTPase+COR*→||<-?<-?<-Pkinase+TM+TPR+TPR</td><td>LRR-repeats+AP-GTPase+COR</td><td>1071</td><td>-</td><td>Bacteroidetes</td><td>Bernardetia litoralis</td><td>leucine-rich repeat domain-containing protein [Bernardetia litoralis].</td><td>GCF_000265505.1</td></tr>
</tbody>
</table>

<table>
<thead>
<tr><th>acc</th><th>operon</th><th>architecture</th><th>len</th><th>gen.name</th><th>taxend</th><th>species</th><th>defline</th><th>gca</th></tr>
</thead>
<tbody>
<tr><td>WP_015112282.1</td><td><-ParA-Soj-PloopNTPase<-?||?→ PIN→ LRR-repeats+AP-GTPase+COR+DUF4404*→||<-?<-?||?→?→||<-?<-?||Thioredoxin→</td><td>LRR-repeats+AP-GTPase+COR+DUF4404</td><td>925</td><td>-</td><td>Cyanobacteria</td><td>Nostoc sp. PCC 7107</td><td>leucine-rich repeat domain-containing protein [Nostoc sp. PCC 7107].</td><td>GCF_000316625.1</td></tr>
<tr><td>WP_015118030.1</td><td>LRR-repeats+AP-GTPase+COR+EAD1*→||<-IG</td><td>LRR-repeats+AP-GTPase+COR+EAD1</td><td>955</td><td>-</td><td>Cyanobacteria</td><td>Rivularia sp. PCC 7116</td><td>leucine-rich repeat domain-containing protein [Rivularia sp. PCC 7116].</td><td>GCF_000316665.1</td></tr>
<tr><td>WP_015120500.1</td><td>LRR-repeats+AP-GTPase+COR+EAD1*→?→?→?→?→?→?→ ABC_membrane+ABC_tran→</td><td>LRR-repeats+AP-GTPase+COR+EAD1</td><td>868</td><td>-</td><td>Cyanobacteria</td><td>Rivularia sp. PCC 7116</td><td>leucine-rich repeat domain-containing protein [Rivularia sp. PCC 7116].</td><td>GCF_000316665.1</td></tr>
<tr><td>WP_015120705.1</td><td><-Cluster1048_2clades<-?||?→||<-?||LRR-repeats+AP-GTPase+COR+EAD1*→||<-?<-?||?→||<-PSE<-?||ABC-ATPase→</td><td>LRR-repeats+AP-GTPase+COR+EAD1</td><td>989</td><td>-</td><td>Cyanobacteria</td><td>Rivularia sp. PCC 7116</td><td>leucine-rich repeat domain-containing protein [Rivularia sp. PCC 7116].</td><td>GCF_000316665.1</td></tr>
<tr><td>WP_015132911.1</td><td>REase→ LRR-repeats+AP-GTPase+COR+DUF4404*→</td><td>LRR-repeats+AP-GTPase+COR+DUF4404</td><td>1183</td><td>-</td><td>Cyanobacteria</td><td>Leptolyngbya sp. PCC 7376</td><td>leucine-rich repeat protein [Leptolyngbya sp. PCC 7376].</td><td>GCF_000316605.1</td></tr>
<tr><td>WP_015135895.1</td><td><-Cluster998_2clades||?→?→||<-?||WCAK→||<-?<-?||LRR-repeats+AP-GTPase+COR+TM+TM*→ ABC-ATPase→ ABC_membrane+ABC_tran→||<-?<-?<-?||ISOFLAVOMETHYLASE-HTH→</td><td>LRR-repeats+AP-GTPase+COR+TM+TM</td><td>925</td><td>-</td><td>Cyanobacteria</td><td>Leptolyngbya sp. PCC 7376</td><td>leucine-rich repeat domain-containing protein [Leptolyngbya sp. PCC 7376].</td><td>GCF_000316605.1</td></tr>
<tr><td>WP_015147146.1</td><td>Pentapeptide-repeats→?→ CBS→ Pentapeptide-repeats→ Pentapeptide-repeats→?→ LRR-repeats+AP-GTPase+COR*→?→?→ Cluster2236_2clades→||<-?<-?<-Hsp10</td><td>LRR-repeats+AP-GTPase+COR</td><td>922</td><td>-</td><td>Cyanobacteria</td><td>Oscillatoria acuminata</td><td>leucine-rich repeat domain-containing protein [Oscillatoria acuminata].</td><td>GCF_000317105.1</td></tr>
<tr><td>WP_015161253.1</td><td>TM→?→||<-?||SF2-DUF3427A→||<-?||LRR-repeats+AP-GTPase+COR*→||<-?||?→?→ TRD+TRD→?→ PIN→</td><td>LRR-repeats+AP-GTPase+COR</td><td>993</td><td>-</td><td>Cyanobacteria</td><td>Chamaesiphon minutus</td><td>leucine-rich repeat domain-containing protein [Chamaesiphon minutus].</td><td>GCF_000317145.1</td></tr>
<tr><td>WP_015196406.1</td><td>HTH→?→||<-?||LRR-repeats+AP-GTPase+COR+DUF4404*→</td><td>LRR-repeats+AP-GTPase+COR+DUF4404</td><td>1034</td><td>-</td><td>Cyanobacteria</td><td>Calothrix parietina</td><td>leucine-rich repeat domain-containing protein [Calothrix parietina].</td><td>GCF_000317435.1</td></tr>
<tr><td>WP_015197432.1</td><td><-Cluster5_5clades<-?<-Cluster5_5clades<-Chelatase-AAA+VWA<-?<-?<-?||LRR-repeats+AP-GTPase+COR+DUF4404*→?→?→ RNASEII→</td><td>LRR-repeats+AP-GTPase+COR+DUF4404</td><td>1408</td><td>-</td><td>Cyanobacteria</td><td>Calothrix parietina</td><td>leucine-rich repeat domain-containing protein [Calothrix parietina].</td><td>GCF_000317435.1</td></tr>
<tr><td>WP_015203756.1</td><td>LRR-repeats+AP-GTPase+COR*→||<-?<-?||TM+TM+TM+TM+TM+TM+TM+TM+TM→</td><td>LRR-repeats+AP-GTPase+COR</td><td>1011</td><td>-</td><td>Cyanobacteria</td><td>Crinalium epipsammum</td><td>leucine-rich repeat domain-containing protein [Crinalium epipsammum].</td><td>GCF_000317495.1</td></tr>
<tr><td>WP_015206852.1</td><td><-HNH<-?||LRR-repeats+AP-GTPase+COR*→||<-CASPASE+TPR</td><td>LRR-repeats+AP-GTPase+COR</td><td>938</td><td>-</td><td>Cyanobacteria</td><td>Cylindrospermum stagnale</td><td>leucine-rich repeat protein [Cylindrospermum stagnale].</td><td>GCF_000317535.1</td></tr>
<tr><td>WP_015280068.1</td><td><-CENPB||LRR-repeats+AP-GTPase+COR+TIR*→||<-?<-?||MoxR-AAA→ VWA→?→ SIG+TM+VWA+TM→ SIG+TM+VWA+TM→</td><td>LRR-repeats+AP-GTPase+COR+TIR</td><td>1283</td><td>-</td><td>Gammaproteobacteria</td><td>Thioflavicoccus mobilis</td><td>leucine-rich repeat domain-containing protein [Thioflavicoccus mobilis].</td><td>GCF_000327045.1</td></tr>
<tr><td>WP_015361283.1</td><td>Cluster2083_2clades→?→?→?→?→||<-?||?→ LRR-repeats+AP-GTPase+COR+TIR*→</td><td>LRR-repeats+AP-GTPase+COR+TIR</td><td>1060</td><td>-</td><td>Bacteroidetes</td><td>Nonlabens dokdonensis</td><td>leucine-rich repeat domain-containing protein [Nonlabens dokdonensis].</td><td>GCF_000332115.1</td></tr>
<tr><td>WP_015785030.1</td><td>REase-4→?→?→?→ HAD→ LRR-repeats+AP-GTPase+COR+TM+TM*→</td><td>LRR-repeats+AP-GTPase+COR+TM+TM</td><td>937</td><td>-</td><td>Cyanobacteria</td><td>Rippkaea orientalis</td><td>GTP-binding protein [Rippkaea orientalis].</td><td>GCF_000024045.1</td></tr>
<tr><td>WP_015948508.1</td><td><-Arginase<-?||?→?→ LRR-repeats→ LRR-repeats+AP-GTPase+COR+ARSR-HTH*→||<-?<-?<-Nitroreductase-like<-PEP-utilisers-C</td><td>LRR-repeats+AP-GTPase+COR+ARSR-HTH</td><td>792</td><td>-</td><td>Deltaproteobacteria</td><td>Desulfatibacillum aliphaticivorans</td><td>leucine-rich repeat domain-containing protein [Desulfatibacillum aliphaticivorans].</td><td>GCF_000021905.1</td></tr>
<tr><td>WP_015955249.1</td><td>LRR-repeats+AP-GTPase+COR*→||<-DAGKIN<-?<-?||SIG+GT4→</td><td>LRR-repeats+AP-GTPase+COR</td><td>867</td><td>-</td><td>Cyanobacteria</td><td>Gloeothece citriformis</td><td>leucine-rich repeat domain-containing protein [Gloeothece citriformis].</td><td>GCF_000021825.1</td></tr>
<tr><td>WP_015956848.1</td><td>REC+LuxR-HTH→||<-tRNA<-?<-?<-?||PolB→ Cluster2355_2clades→ LRR-repeats+AP-GTPase+COR+TIR*→</td><td>LRR-repeats+AP-GTPase+COR+TIR</td><td>1015</td><td>-</td><td>Cyanobacteria</td><td>Gloeothece citriformis</td><td>leucine-rich repeat domain-containing protein [Gloeothece citriformis].</td><td>GCF_000021825.1</td></tr>
</tbody>
</table>

<table>
<tr><th>acc</th><th>operon</th><th>architecture</th><th>len</th><th>gen.name</th><th>taxend</th><th>species</th><th>defline</th><th>gca</th></tr>
<tr><td>WP_016798830.1</td><td>CI→ LRR-repeats+AP-GTPase+COR*→</td><td>LRR-repeats+AP-GTPase+COR</td><td>1080</td><td>-</td><td>Gammaproteobacteria</td><td>Vibrio cyclitrophicus</td><td>GTP-binding protein [Vibrio cyclitrophicus].</td><td>GCF_000256425.1</td></tr>
<tr><td>WP_016860490.1</td><td><-HISKIN||?→?→ LRR-repeats→ LRR-repeats+AP-GTPase+COR+TM+TM*→||<-ABhydrolase</td><td>LRR-repeats+AP-GTPase+COR+TM+TM</td><td>719</td><td>-</td><td>Cyanobacteria</td><td>Fischerella muscicola</td><td>GTP-binding protein [Fischerella muscicola].</td><td>GCF_000317245.1</td></tr>
<tr><td>WP_016952822.1</td><td>LRR-repeats+AP-GTPase+COR*→</td><td>LRR-repeats+AP-GTPase+COR</td><td>829</td><td>-</td><td>Cyanobacteria</td><td>Anabaena sp. PCC 7108</td><td>leucine-rich repeat domain-containing protein [Anabaena sp. PCC 7108].</td><td>GCF_000332135.1</td></tr>
<tr><td>WP_017313212.1</td><td>Cluster1069__2clades→?→?→||<-?||?→?→ LRR-repeats+AP-GTPase+COR*→||<-?||?→?→?→?→ Mbetalac→</td><td>LRR-repeats+AP-GTPase+COR</td><td>1085</td><td>-</td><td>Cyanobacteria</td><td>Fischerella sp. PCC 9339</td><td>leucine-rich repeat protein [Fischerella sp. PCC 9339].</td><td>GCF_000315585.1</td></tr>
<tr><td>WP_017720442.1</td><td>LRR-repeats+AP-GTPase+COR*→</td><td>LRR-repeats+AP-GTPase+COR</td><td>852</td><td>-</td><td>Cyanobacteria</td><td>Oscillatoria sp. PCC 10802</td><td>leucine-rich repeat domain-containing protein [Oscillatoria sp. PCC 10802].</td><td>GCF_000332335.1</td></tr>
<tr><td>WP_017739957.1</td><td>Cluster5__5clades→?→||<-Cluster5__5clades||?→ PSE→||<-?<-PSE||?→ LRR-repeats+AP-GTPase+COR+EAD1*→ PSE→?→?→ Cluster2194__2clades→||<-?||Cluster1640__2clades→</td><td>LRR-repeats+AP-GTPase+COR+EAD1</td><td>965</td><td>-</td><td>Cyanobacteria</td><td>Scytonema hofmannii</td><td>leucine-rich repeat domain-containing protein [Scytonema hofmannii].</td><td>GCF_000346485.2</td></tr>
<tr><td>WP_018619729.1</td><td><-HISKIN<-?||Cluster1877__2clades→ LRR-repeats+AP-GTPase+COR*→</td><td>LRR-repeats+AP-GTPase+COR</td><td>925</td><td>-</td><td>Bacteroidetes</td><td>Spirosoma luteum</td><td>leucine-rich repeat domain-containing protein [Spirosoma luteum].</td><td>GCF_000374065.1</td></tr>
<tr><td>WP_019496026.1</td><td>ParB→||<-?<-TM+TM||?→ SIG+TM+TM+TM+TM+TM+TM+TM+TM+TM+TM+TM→?→ EAD1+LRR-repeats+AP-GTPase+COR*→?→?→?→?→ SIG+GT4→||<-PSE||?→ TPR+nSTAND1+BetaPropeller_WD40+BetaPropeller_WD40→</td><td>EAD1+LRR-repeats+AP-GTPase+COR</td><td>961</td><td>-</td><td>Cyanobacteria</td><td>Calothrix sp. PCC 7103</td><td>leucine-rich repeat domain-containing protein [Calothrix sp. PCC 7103].</td><td>GCF_000331305.1</td></tr>
<tr><td>WP_019496060.1</td><td>Cluster5__5clades→ LRR-repeats+AP-GTPase+COR+EAD1*→ PSE→?→||<-CR-REase5||?→ APATPase+BetaPropeller_WD40-repeats→?→ APATPase+BetaPropeller_WD40-repeats→ APATPase+BetaPropeller_WD40-repeats→</td><td>LRR-repeats+AP-GTPase+COR+EAD1</td><td>912</td><td>-</td><td>Cyanobacteria</td><td>Calothrix sp. PCC 7103</td><td>leucine-rich repeat domain-containing protein [Calothrix sp. PCC 7103].</td><td>GCF_000331305.1</td></tr>
<tr><td>WP_019867072.1</td><td>LRR-repeats+AP-GTPase+COR+TIR*→</td><td>LRR-repeats+AP-GTPase+COR+TIR</td><td>856</td><td>-</td><td>Gammaproteobacteria</td><td>Methylovulum miyakonense</td><td>TIR domain-containing protein [Methylovulum miyakonense].</td><td>GCF_000384075.1</td></tr>
<tr><td>WP_020379418.1</td><td>SF2-DUF3427A+TnsA-REase→ DAM→ LRR-repeats+AP-GTPase+COR+TM+TM*→</td><td>LRR-repeats+AP-GTPase+COR+TM+TM</td><td>1195</td><td>-</td><td>Actinobacteria</td><td>Candidatus Microthrix parvicella</td><td>hypothetical protein [Candidatus Microthrix parvicella].</td><td>GCF_000299415.1</td></tr>
<tr><td>WP_020396599.1</td><td>LRR-repeats+AP-GTPase+COR+TIR*→ TM+TM+TM→</td><td>LRR-repeats+AP-GTPase+COR+TIR</td><td>767</td><td>-</td><td>Gammaproteobacteria</td><td>Thiolinea disciformis</td><td>TIR domain-containing protein, partial [Thiolinea disciformis].</td><td>GCF_000371925.1</td></tr>
<tr><td>WP_020483095.1</td><td>Cluster1270__2clades→||<-?<-?<-?<-?<-?||?→ LRR-repeats+AP-GTPase+COR*→||<-?<-?<-?<-?||?→ SIGMA-HTH→</td><td>LRR-repeats+AP-GTPase+COR</td><td>1111</td><td>-</td><td>Gammaproteobacteria</td><td>Methylomonas sp. MK1</td><td>leucine-rich repeat domain-containing protein [Methylomonas sp. MK1].</td><td>GCF_000365425.1</td></tr>
<tr><td>WP_020496162.1</td><td><-ISOCOT<-?<-?||InPase→||<-?||LRR-repeats+AP-GTPase+COR*→</td><td>LRR-repeats+AP-GTPase+COR</td><td>833</td><td>-</td><td>Actinobacteria</td><td>Sciscionella marina</td><td>hypothetical protein [Sciscionella marina].</td><td>GCF_000379465.1</td></tr>
<tr><td>WP_020559023.1</td><td>Cluster2057__2clades→ LRR-repeats+AP-GTPase+COR+TIR→?→?→?→?→ LRR-repeats+AP-GTPase+COR+TIR→ Cluster54__3clades→ PSE→||<-TIR+TPR+TPR-S</td><td>LRR-repeats+AP-GTPase+COR+TIR</td><td>972</td><td>-</td><td>Gammaproteobacteria</td><td>Thiofilum flexile</td><td>leucine-rich repeat domain-containing protein [Thiofilum flexile].</td><td>GCF_000380185.1</td></tr>
<tr><td>WP_020559030.1</td><td>LRR-repeats+AP-GTPase+COR+TIR*→</td><td>LRR-repeats+AP-GTPase+COR+TIR</td><td>943</td><td>-</td><td>Gammaproteobacteria</td><td>Thiofilum flexile</td><td>leucine-rich repeat domain-containing protein [Thiofilum flexile].</td><td>GCF_000380185.1</td></tr>
<tr><td>WP_020560643.1</td><td>LRR-repeats+AP-GTPase+COR+TIR*→ TM+TM+TM→</td><td>LRR-repeats+AP-GTPase+COR+TIR</td><td>900</td><td>-</td><td>Gammaproteobacteria</td><td>Thiofilum flexile</td><td>TIR domain-containing protein [Thiofilum flexile].</td><td>GCF_000380185.1</td></tr>
<tr><td>WP_020570498.1</td><td><-SIG+ABhydrolase<-?<-?<-?||LRR-repeats+AP-GTPase+COR+CASPASE*→?→ FGS→?→ RVT→?→ FGS→</td><td>LRR-repeats+AP-GTPase+COR+CASPASE</td><td>1039</td><td>-</td><td>Bacteroidetes</td><td>Lewinella persica</td><td>CHAT domain-containing protein [Lewinella persica].</td><td>GCF_000373105.1</td></tr>
<tr><td>WP_020607470.1</td><td>LRR-repeats+AP-GTPase+COR+TIR*→</td><td>LRR-repeats+AP-GTPase+COR+TIR</td><td>954</td><td>-</td><td>Bacteroidetes</td><td>Spirosoma spitsbergense</td><td>leucine-rich repeat domain-containing protein [Spirosoma spitsbergense].</td><td>GCF_000374085.1</td></tr>
<tr><td>WP_021020180.</td><td>Polbeta+HEPN→ LRR-repeats→ LRR-repeats+AP-GTPase+COR+TIR*→?→||<-?||?→?→ SIG+TM+TM+TM+TM+TM+TM+TM+TM+TM+TM+TM→ SIG+TM→</td><td>LRR-repeats+AP-GTPase+COR+TIR</td><td>944</td><td>-</td><td>Gammaproteobacteria</td><td>Vibrio gazogenes</td><td>TIR domain-containing protein [Vibrio gazogenes].</td><td>GCF_000390165.1</td></tr>
<tr><td>WP_021802668.1</td><td>HAD→?→?→?→ Cluster881__3clades→ LRR-repeats+AP-GTPase+COR*→?→?→?→?→?→?→ Cluster1292__2clades→</td><td>LRR-repeats+AP-GTPase+COR</td><td>936</td><td>-</td><td>Firmicutes</td><td>Clostridium intestinale</td><td>GTP-binding protein [Clostridium intestinale].</td><td>GCF_013781885.1</td></tr>
</table>

<table>
<tr><th>acc</th><th>operon</th><th>architecture</th><th>len</th><th>gen.name</th><th>taxend</th><th>species</th><th>defline</th><th>gca</th></tr>
<tr><td>WP_022571049.1</td><td>CI→ LRR-repeats+AP-GTPase+COR*→</td><td>LRR-repeats+AP-GTPase+COR</td><td>1095</td><td>-</td><td>Gammaproteobacteria</td><td>Vibrio cyclitrophicus</td><td>leucine rich repeat protein [Vibrio cyclitrophicus].</td><td>GCF_000473545.1</td></tr>
<tr><td>WP_022589047.1</td><td>ABC-ATPase→?→?→||<-Acylphosphatase||?→?→||<-?||LRR-repeats+AP-GTPase+COR+TIR*→</td><td>LRR-repeats+AP-GTPase+COR+TIR</td><td>1264</td><td>-</td><td>Gammaproteobacteria</td><td>Vibrio nigripulchritudo</td><td>TIR domain-containing protein [Vibrio nigripulchritudo].</td><td>GCF_900067775.1</td></tr>
<tr><td>WP_023074611.1</td><td>LRR-repeats+AP-GTPase+COR+TIR*→||<-?||?→||<-?||Cluster1646_2clades→</td><td>LRR-repeats+AP-GTPase+COR+TIR</td><td>777</td><td>-</td><td>Cyanobacteria</td><td>Leptolyngbya sp. Heron Island J</td><td>leucine-rich repeat domain-containing protein, partial [Leptolyngbya sp. Heron Island J].</td><td>GCF_000482245.1</td></tr>
<tr><td>WP_023076066.1</td><td>Cluster53_2clades→||<-RelE-ParE<-?||LRR-repeats+AP-GTPase+COR+TIR*→||<-?||?→||<-?||?→?→||<-?<-SIG+TM+TM+TM+TM+TM+TM</td><td>LRR-repeats+AP-GTPase+COR+TIR</td><td>1129</td><td>-</td><td>Cyanobacteria</td><td>Leptolyngbya sp. Heron Island J</td><td>leucine-rich repeat domain-containing protein [Leptolyngbya sp. Heron Island J].</td><td>GCF_000482245.1</td></tr>
<tr><td>WP_023571556.1</td><td>LRR-repeats+AP-GTPase+COR+TIR*→</td><td>LRR-repeats+AP-GTPase+COR+TIR</td><td>1142</td><td>-</td><td>Bacteroidetes</td><td>Flavobacterium cauense</td><td>TIR domain-containing protein [Flavobacterium cauense].</td><td>GCF_000498475.1</td></tr>
<tr><td>WP_024594403.1</td><td>LRR-repeats+AP-GTPase+COR*→</td><td>LRR-repeats+AP-GTPase+COR</td><td>982</td><td>-</td><td>Gammaproteobacteria</td><td>Pseudoalteromonas sp. TB13</td><td>GTP-binding protein [Pseudoalteromonas sp. TB13].</td><td>GCF_000497915.1</td></tr>
<tr><td>WP_025517521.1</td><td>CI→ LRR-repeats+AP-GTPase+COR*→</td><td>LRR-repeats+AP-GTPase+COR</td><td>1105</td><td>-</td><td>Gammaproteobacteria</td><td>Vibrio parahaemolyticus</td><td>50S ribosome-binding GTPase [Vibrio parahaemolyticus].</td><td>GCF_000489555.1</td></tr>
<tr><td>WP_025605295.1</td><td>CI→?→ PSE→ LRR-repeats+AP-GTPase+COR+TIR*→</td><td>LRR-repeats+AP-GTPase+COR+TIR</td><td>1265</td><td>-</td><td>Gammaproteobacteria</td><td>Vibrio parahaemolyticus</td><td>TIR domain-containing protein [Vibrio parahaemolyticus].</td><td>GCF_000489075.1</td></tr>
<tr><td>WP_026726296.1</td><td>LRR-repeats+AP-GTPase+COR*→</td><td>LRR-repeats+AP-GTPase+COR</td><td>698</td><td>-</td><td>Bacteroidetes</td><td>Flavobacterium sasangense</td><td>GTP-binding protein [Flavobacterium sasangense].</td><td>GCF_000686885.1</td></tr>
<tr><td>WP_028083535.1</td><td>LRR-repeats+AP-GTPase+COR*→</td><td>LRR-repeats+AP-GTPase+COR</td><td>993</td><td>-</td><td>Cyanobacteria</td><td>Dolichospermum circinale</td><td>leucine-rich repeat domain-containing protein [Dolichospermum circinale].</td><td>GCF_000426925.1</td></tr>
<tr><td>WP_028469928.1</td><td>LRR-repeats+AP-GTPase+COR*→?→?→ GTPase-AIG→</td><td>LRR-repeats+AP-GTPase+COR</td><td>697</td><td>-</td><td>Gammaproteobacteria</td><td>Neptunomonas japonica</td><td>hypothetical protein [Neptunomonas japonica].</td><td>GCF_000422765.1</td></tr>
<tr><td>WP_028667519.1</td><td><-Cluster1063_3clades<-Cluster1067_3clades||?→?→?→?→ LRR-repeats+AP-GTPase+COR*→</td><td>LRR-repeats+AP-GTPase+COR</td><td>798</td><td>-</td><td>Bacteroidetes</td><td>Runella zeae</td><td>GTP-binding protein [Runella zeae].</td><td>GCF_000423565.1</td></tr>
<tr><td>WP_029631472.1</td><td>LRR-repeats+AP-GTPase+COR*→</td><td>LRR-repeats+AP-GTPase+COR</td><td>1051</td><td>-</td><td>Planctomycetes</td><td>Zavarzinella formosa</td><td>leucine-rich repeat domain-containing protein [Zavarzinella formosa].</td><td>GCF_000255705.1</td></tr>
<tr><td>WP_030435343.1</td><td>REC→ PSE→?→?→||<-?||?→ Cluster2103_2clades→||<-?||LRR-repeats+AP-GTPase+COR*→ AP-GTPase+COR+TM+TM→||<-REC<-?||ABC-ATPase→</td><td>LRR-repeats+AP-GTPase+COR</td><td>551</td><td>-</td><td>Actinobacteria</td><td>Actinoplanes subtropicus</td><td>GTP-binding protein [Actinoplanes subtropicus].</td><td>GCF_000721705.1</td></tr>
<tr><td>WP_030488330.1</td><td>LRR-repeats+AP-GTPase+COR*→||<-?<-?||?→?→||<-?||SIG+TM→ REC→</td><td>LRR-repeats+AP-GTPase+COR</td><td>1088</td><td>-</td><td>Actinobacteria</td><td>Micromonospora chokoriensis</td><td>hypothetical protein [Micromonospora chokoriensis].</td><td>GCF_000718555.1</td></tr>
<tr><td>WP_031429699.1</td><td>CI→?→ PSE→ LRR-repeats+AP-GTPase+COR+TIR*→</td><td>LRR-repeats+AP-GTPase+COR+TIR</td><td>1261</td><td>-</td><td>Gammaproteobacteria</td><td>Vibrio parahaemolyticus</td><td>TIR domain-containing protein [Vibrio parahaemolyticus].</td><td>GCF_000736345.1</td></tr>
<tr><td>WP_034058880.1</td><td>S1COLD→?→||<-?||?→?→||<-?||?→ LRR-repeats+AP-GTPase+COR*→</td><td>LRR-repeats+AP-GTPase+COR</td><td>924</td><td>-</td><td>Bacteroidetes</td><td>Lacinutrix jangbogonensis</td><td>leucine-rich repeat domain-containing protein [Lacinutrix jangbogonensis].</td><td>GCF_000797445.1</td></tr>
<tr><td>WP_035121352.1</td><td>LRR-repeats+AP-GTPase+COR+TIR*→||<-?<-?<-?<-ABC-ATPase</td><td>LRR-repeats+AP-GTPase+COR+TIR</td><td>1115</td><td>-</td><td>Cyanobacteria</td><td>Fischerella sp. PCC 9431</td><td>leucine-rich repeat domain-containing protein [Fischerella sp. PCC 9431].</td><td>GCF_000447295.1</td></tr>
<tr><td>WP_035197779.1</td><td>LRR-repeats+AP-GTPase+COR*→</td><td>LRR-repeats+AP-GTPase+COR</td><td>914</td><td>-</td><td>Firmicutes</td><td>Calidifontibacillus azotoformans</td><td>GTP-binding protein [Calidifontibacillus azotoformans].</td><td>GCF_000708505.1</td></tr>
<tr><td>WP_035613546.1</td><td>LRR-repeats+AP-GTPase+COR*→</td><td>LRR-repeats+AP-GTPase+COR</td><td>1058</td><td>-</td><td>Verrucomicrobia</td><td>Haloferula sp. BvORR071</td><td>hypothetical protein [Haloferula sp. BvORR071].</td><td>-</td></tr>
<tr><td>WP_035758421.1</td><td>LRR-repeats+AP-GTPase+COR*→</td><td>LRR-repeats+AP-GTPase+COR</td><td>956</td><td>-</td><td>Bacteroidetes</td><td>Hugenholtzia roseola</td><td>leucine-rich repeat protein, partial [Hugenholtzia roseola].</td><td>GCF_000422585.1</td></tr>
<tr><td>WP_038164534.1</td><td>LRR-repeats+AP-GTPase+COR+TIR*→</td><td>LRR-repeats+AP-GTPase+COR+TIR</td><td>793</td><td>-</td><td>Verrucomicrobia</td><td>Verrucomicrobium sp. BvORR106</td><td>TIR domain-containing protein [Verrucomicrobium sp. BvORR106].</td><td>-</td></tr>
<tr><td>WP_039108669.1</td><td><-Cluster1070_2clades<-ENDO3+NUDIX||HU-IHF→?→ LRR-repeats+AP-GTPase+COR*→</td><td>LRR-repeats+AP-GTPase+COR</td><td>912</td><td>-</td><td>Bacteroidetes</td><td>Flavobacterium sp. AED</td><td>leucine-rich repeat domain-containing protein [Flavobacterium sp. AED].</td><td>GCF_000812945.1</td></tr>
</table>

<table>
<tr><th>acc</th><th>operon</th><th>architecture</th><th>len</th><th>gen.name</th><th>taxend</th><th>species</th><th>defline</th><th>gca</th></tr>
<tr><td>WP_039351391.1</td><td>LRR-repeats+AP-GTPase+COR*→||<-?||?→||<-RnasePH+RnasePH+KH+S1</td><td>LRR-repeats+AP-GTPase+COR</td><td>912</td><td>-</td><td>Bacteroidetes</td><td>Chryseobacterium jeonii</td><td>GTP-binding protein [Chryseobacterium jeonii].</td><td>GCF_000812865.1</td></tr>
<tr><td>WP_039727351.1</td><td>SIG+TM+TM+TM+TM+TM+TM→||<-?||?→?→?→ LRR-repeats+AP-GTPase+COR*→?→ PIN→||<-?<-REC+LuxR-HTH<-?<-?<-Cluster1069_2clades</td><td>LRR-repeats+AP-GTPase+COR</td><td>1075</td><td>-</td><td>Terrabacteria group</td><td>Cyanobacteria</td><td>MULTISPECIES: leucine-rich repeat domain-containing protein [Cyanobacteria].</td><td>GCF_000817775.2</td></tr>
<tr><td>WP_039983671.1</td><td>LRR-repeats+AP-GTPase+COR*→</td><td>LRR-repeats+AP-GTPase+COR</td><td>1101</td><td>-</td><td>Gammaproteobacteria</td><td>Vibrio sagamiensis</td><td>50S ribosome-binding GTPase [Vibrio sagamiensis].</td><td>GCF_000400425.1</td></tr>
<tr><td>WP_040248977.1</td><td>LRR-repeats+AP-GTPase+COR*→||<-?<-?<-?||?→?→?→ SIG+TM+TM→</td><td>LRR-repeats+AP-GTPase+COR</td><td>924</td><td>-</td><td>Bacteroidetes</td><td>Psychroserpens mesophilus</td><td>leucine-rich repeat domain-containing protein [Psychroserpens mesophilus].</td><td>GCF_000826645.1</td></tr>
<tr><td>WP_044220039.1</td><td>FGS→?→ RVT→?→ FGS→?→ LRR-repeats+AP-GTPase+COR+CASPASE*→?→ FGS→</td><td>LRR-repeats+AP-GTPase+COR+CASPASE</td><td>977</td><td>-</td><td>Bacteroidetes</td><td>Phaeodactylibacter xiamenensis</td><td>CHAT domain-containing protein [Phaeodactylibacter xiamenensis].</td><td>GCF_000759025.1</td></tr>
<tr><td>WP_044222080.1</td><td>REC→ FGS→||<-?||RVT→?→ FGS→ LRR-repeats+AP-GTPase+COR+CASPASE*→?→?→?→ FGS→ FGS→ FGS→</td><td>LRR-repeats+AP-GTPase+COR+CASPASE</td><td>931</td><td>-</td><td>Bacteroidetes</td><td>Phaeodactylibacter xiamenensis</td><td>leucine-rich repeat domain-containing protein [Phaeodactylibacter xiamenensis].</td><td>GCF_000759025.1</td></tr>
<tr><td>WP_044448603.1</td><td>LRR-repeats+AP-GTPase+COR+TIR*→</td><td>LRR-repeats+AP-GTPase+COR+TIR</td><td>1176</td><td>-</td><td>Cyanobacteria</td><td>Mastigocladus laminosus</td><td>leucine-rich repeat domain-containing protein [Mastigocladus laminosus].</td><td>-</td></tr>
<tr><td>WP_044448681.1</td><td>LRR-repeats+AP-GTPase+COR+DUF4404*→</td><td>LRR-repeats+AP-GTPase+COR+DUF4404</td><td>806</td><td>-</td><td>Cyanobacteria</td><td>Mastigocladus laminosus</td><td>GTP-binding protein [Mastigocladus laminosus].</td><td>-</td></tr>
<tr><td>WP_045613052.1</td><td><-Cluster1074_2clades||?→?→?→ LRR-repeats+AP-GTPase+COR*→</td><td>LRR-repeats+AP-GTPase+COR</td><td>1105</td><td>-</td><td>Gammaproteobacteria</td><td>Vibrio vulnificus</td><td>50S ribosome-binding GTPase [Vibrio vulnificus].</td><td>GCF_000960125.1</td></tr>
<tr><td>WP_046355878.1</td><td>LRR-repeats+AP-GTPase+COR+TIR*→</td><td>LRR-repeats+AP-GTPase+COR+TIR</td><td>1154</td><td>-</td><td>Gammaproteobacteria</td><td>Pseudoalteromonas luteoviolacea</td><td>leucine-rich repeat domain-containing protein [Pseudoalteromonas luteoviolacea].</td><td>GCF_001625715.1</td></tr>
<tr><td>WP_047776098.1</td><td>LRR-repeats+AP-GTPase+COR+TIR*→</td><td>LRR-repeats+AP-GTPase+COR+TIR</td><td>1055</td><td>-</td><td>Bacteroidetes</td><td>Flavobacterium sp. ABG</td><td>leucine-rich repeat domain-containing protein [Flavobacterium sp. ABG].</td><td>GCF_001027725.1</td></tr>
<tr><td>WP_047863083.1</td><td>Cluster971_2clades→?→||<-?<-?||LRR-repeats+AP-GTPase+COR+TIR*→?→||<-?<-?<-?||?→||<-?<-SIG+Lysozyme</td><td>LRR-repeats+AP-GTPase+COR+TIR</td><td>921</td><td>-</td><td>Deltaproteobacteria</td><td>Archangium gephyra</td><td>GTP-binding protein [Archangium gephyra].</td><td>GCF_001027285.1</td></tr>
<tr><td>WP_048040436.1</td><td><-PIN<-?<-SIG+TM+TM+TM+TM+TM+TM+TM+TM+TM+TM+TM||?→?→ LRR-repeats+AP-GTPase+COR*→</td><td>LRR-repeats+AP-GTPase+COR</td><td>935</td><td>-</td><td>Euryarchaeota</td><td>Methanosarcina mazei</td><td>leucine-rich repeat domain-containing protein [Methanosarcina mazei].</td><td>GCF_000970225.1</td></tr>
<tr><td>WP_048109136.1</td><td><-SIG+TM+VWA+TM<-VWA<-MoxR-AAA||?→ LRR-repeats+AP-GTPase+COR*→?→||<-?||SIG+Calcineurin→?→ PIN→||<-Ferredoxin-RRM</td><td>LRR-repeats+AP-GTPase+COR</td><td>931</td><td>-</td><td>Euryarchaeota</td><td>Methanosarcina barkeri</td><td>leucine-rich repeat domain-containing protein [Methanosarcina barkeri].</td><td>GCF_000970305.1</td></tr>
<tr><td>WP_048669264.1</td><td><-ABC_membrane+ABC_tran<-ABC_membrane+ABC_tran<-?||LRR-repeats+AP-GTPase+COR*→</td><td>LRR-repeats+AP-GTPase+COR</td><td>960</td><td>-</td><td>Gammaproteobacteria</td><td>Vibrio crassostreae</td><td>GTP-binding protein [Vibrio crassostreae].</td><td>GCF_001048475.1</td></tr>
<tr><td>WP_048752807.1</td><td>LRR-repeats+AP-GTPase+COR*→||<-PSE<-?||RelE→ CHTH→</td><td>LRR-repeats+AP-GTPase+COR</td><td>801</td><td>-</td><td>Cyanobacteria</td><td>Crocosphaera watsonii</td><td>leucine-rich repeat domain-containing protein, partial [Crocosphaera watsonii].</td><td>GCF_001050835.1</td></tr>
<tr><td>WP_049769521.1</td><td>LRR-repeats+AP-GTPase+COR*→</td><td>LRR-repeats+AP-GTPase+COR</td><td>662</td><td>-</td><td>Cyanobacteria</td><td>Rippkaea orientalis</td><td>GTP-binding protein [Rippkaea orientalis].</td><td>GCF_000021805.1</td></tr>
<tr><td>WP_049825328.1</td><td>LRR-repeats+AP-GTPase+COR*→</td><td>LRR-repeats+AP-GTPase+COR</td><td>616</td><td>-</td><td>Cyanobacteria</td><td>Rippkaea orientalis</td><td>GTP-binding protein [Rippkaea orientalis].</td><td>GCF_000024045.1</td></tr>
<tr><td>WP_049875725.1</td><td>PAIREDC-HTH→||<-?||LRR-repeats→ LRR-repeats+AP-GTPase+COR*→</td><td>LRR-repeats+AP-GTPase+COR</td><td>772</td><td>-</td><td>Actinobacteria</td><td>Streptomyces scabiei</td><td>hypothetical protein [Streptomyces scabiei].</td><td>GCF_000091305.1</td></tr>
<tr><td>WP_049974475.1</td><td>LRR-repeats+AP-GTPase+COR+TIR*→</td><td>LRR-repeats+AP-GTPase+COR+TIR</td><td>1267</td><td>-</td><td>Alphaproteobacteria</td><td>Azospirillum sp. B4</td><td>leucine-rich repeat domain-containing protein [Azospirillum sp. B4].</td><td>GCF_000333275.1</td></tr>
<tr><td>WP_050652657.1</td><td>LRR-repeats+AP-GTPase+COR*→</td><td>LRR-repeats+AP-GTPase+COR</td><td>1099</td><td>-</td><td>Gammaproteobacteria</td><td>Vibrio sp. J2-17</td><td>hypothetical protein [Vibrio sp. J2-17].</td><td>GCF_001244075.1</td></tr>
</table>

<table>
<tr><th>acc</th><th>operon</th><th>architecture</th><th>len</th><th>gen.name</th><th>taxend</th><th>species</th><th>defline</th><th>gca</th></tr>
<tr><td>WP_050710728.1</td><td>LRR-repeats+AP-GTPase+COR+DrHyd*→</td><td>LRR-repeats+AP-GTPase+COR+DrHyd</td><td>1196</td><td>-</td><td>Bacteroidetes</td><td>Dysgonomonas sp. HGC4</td><td>leucine-rich repeat domain-containing protein [Dysgonomonas sp. HGC4].</td><td>GCF_001261715.1</td></tr>
<tr><td>WP_050857550.1</td><td>LRR-repeats+AP-GTPase+COR*→||<-?<-?<-ABC-ATPase</td><td>LRR-repeats+AP-GTPase+COR</td><td>958</td><td>-</td><td>Cyanobacteria</td><td>Acaryochloris sp. CCMEE 5410</td><td>leucine-rich repeat domain-containing protein [Acaryochloris sp. CCMEE 5410].</td><td>GCF_000238775.1</td></tr>
<tr><td>WP_051210834.1</td><td>SIGMA-HTH→?→?→ LRR-repeats+AP-GTPase+COR*→||<-?<-?<-?<-?<-SIGMA-HTH</td><td>LRR-repeats+AP-GTPase+COR</td><td>1280</td><td>-</td><td>Bacteroidetes</td><td>Runella zeae</td><td>leucine-rich repeat domain-containing protein [Runella zeae].</td><td>GCF_000423565.1</td></tr>
<tr><td>WP_051242709.1</td><td>LRR-repeats+AP-GTPase+COR+TIR*→</td><td>LRR-repeats+AP-GTPase+COR+TIR</td><td>1043</td><td>-</td><td>Alphaproteobacteria</td><td>Stappia stellulata</td><td>leucine-rich repeat domain-containing protein [Stappia stellulata].</td><td>GCF_000423705.1</td></tr>
<tr><td>WP_051289443.1</td><td>LRR-repeats+AP-GTPase+COR+TM+TM*→||<-PSE||?→?→||<-?<-?||?→||<-?<-ABC-ATPase</td><td>LRR-repeats+AP-GTPase+COR+TM+TM</td><td>821</td><td>-</td><td>Cyanobacteria</td><td>Dolichospermum circinale</td><td>leucine-rich repeat domain-containing protein [Dolichospermum circinale].</td><td>GCF_000426925.1</td></tr>
<tr><td>WP_051347554.1</td><td>LRR-repeats+AP-GTPase+COR*→</td><td>LRR-repeats+AP-GTPase+COR</td><td>814</td><td>-</td><td>Cyanobacteria</td><td>Dolichospermum circinale</td><td>hypothetical protein, partial [Dolichospermum circinale].</td><td>GCF_000426905.1</td></tr>
<tr><td>WP_051394619.1</td><td>LRR-repeats+AP-GTPase+COR+TM+TM*→</td><td>LRR-repeats+AP-GTPase+COR+TM+TM</td><td>1004</td><td>-</td><td>Cyanobacteria</td><td>Leptolyngbya sp. Heron Island J</td><td>GTP-binding protein [Leptolyngbya sp. Heron Island J].</td><td>GCF_000482245.1</td></tr>
<tr><td>WP_051793473.1</td><td>LRR-repeats+AP-GTPase+COR*→</td><td>LRR-repeats+AP-GTPase+COR</td><td>946</td><td>-</td><td>Actinobacteria</td><td>Kibdelosporangium aridum</td><td>leucine-rich repeat domain-containing protein [Kibdelosporangium aridum].</td><td>GCF_003947235.1</td></tr>
<tr><td>WP_051898832.1</td><td>InPase→||<-?||LRR-repeats+AP-GTPase+COR*→</td><td>LRR-repeats+AP-GTPase+COR</td><td>833</td><td>-</td><td>Actinobacteria</td><td>Sciscionella sp. SE31</td><td>hypothetical protein [Sciscionella sp. SE31].</td><td>GCF_000737195.2</td></tr>
<tr><td>WP_051926645.1</td><td>LRR-repeats+AP-GTPase+COR*→||<-?||?→||<-?<-SIGMA-HTH<-?<-SIGMA-HTH</td><td>LRR-repeats+AP-GTPase+COR</td><td>996</td><td>-</td><td>Actinobacteria</td><td>Streptomyces durhamensis</td><td>hypothetical protein [Streptomyces durhamensis].</td><td>GCF_000725475.1</td></tr>
<tr><td>WP_051937621.1</td><td>LRR-repeats+AP-GTPase+COR*→</td><td>LRR-repeats+AP-GTPase+COR</td><td>1086</td><td>-</td><td>Actinobacteria</td><td>Streptomyces scabiei</td><td>hypothetical protein [Streptomyces scabiei].</td><td>GCF_000738715.1</td></tr>
<tr><td>WP_051942852.1</td><td>LRR-repeats+AP-GTPase+COR*→||<-?<-?<-Cluster1515_2clades<-?<-ABC-ATPase<-?<-SIG+TM+TM+TM+TM+TM+TM+TM</td><td>LRR-repeats+AP-GTPase+COR</td><td>1022</td><td>-</td><td>Actinobacteria</td><td>Streptacidiphilus rugosus</td><td>hypothetical protein [Streptacidiphilus rugosus].</td><td>GCF_000744655.1</td></tr>
<tr><td>WP_052150025.1</td><td>LRR-repeats+AP-GTPase+COR*→?→?→||<-?||?→?→?→||<-Pentapeptide</td><td>LRR-repeats+AP-GTPase+COR</td><td>949</td><td>-</td><td>Cyanobacteria</td><td>Aphanizomenon flos-aquae</td><td>leucine-rich repeat domain-containing protein [Aphanizomenon flos-aquae].</td><td>GCF_000789435.1</td></tr>
<tr><td>WP_052277881.1</td><td>SIG+TM+TM+TM+TM+TM+TM+TM→||<-?||Mbetalac→ SAM-methylase→?→||<-?<-SIG+TPM_phosphatase+TM+TM+TM||LRR-repeats+AP-GTPase+COR+TM+TM*→</td><td>LRR-repeats+AP-GTPase+COR+TM+TM</td><td>921</td><td>-</td><td>Cyanobacteria</td><td>Microcystis panniformis</td><td>leucine-rich repeat domain-containing protein [Microcystis panniformis].</td><td>-</td></tr>
<tr><td>WP_052288305.1</td><td><-Hsp10||?→ REC→ Ferretin→||<-Cluster1950_2clades||?→ LRR-repeats+AP-GTPase+COR+TIR*→||<-?<-?||?→?→?→?→ SIG+TM+TM+TM+TM+TM+TM→</td><td>LRR-repeats+AP-GTPase+COR+TIR</td><td>848</td><td>-</td><td>Terrabacteria group</td><td>Cyanobacteria</td><td>MULTISPECIES: TIR domain-containing protein [Cyanobacteria].</td><td>GCF_000817775.2</td></tr>
<tr><td>WP_052324486.1</td><td>LRR-repeats→ AP-GTPase+COR*→</td><td>AP-GTPase+COR</td><td>434</td><td>-</td><td>Bacteroidetes</td><td>Haliscomenobacter hydrossis</td><td>50S ribosome-binding GTPase [Haliscomenobacter hydrossis].</td><td>GCF_000212735.1</td></tr>
<tr><td>WP_052478236.1</td><td>LRR-repeats+AP-GTPase+COR*→||<-?||?→||<-?<-?<-Aminotran_1_2</td><td>LRR-repeats+AP-GTPase+COR</td><td>942</td><td>-</td><td>Actinobacteria</td><td>Kibdelosporangium sp. MJ126-NF4</td><td>leucine-rich repeat domain-containing protein [Kibdelosporangium sp. MJ126-NF4].</td><td>GCF_000826545.1</td></tr>
<tr><td>WP_052712873.1</td><td><-HhH-RADC+JAB||?→ NTP_transf_3→ LRR-repeats+AP-GTPase+COR*→||<-?<-?||?→||<-?<-?||SIR2→</td><td>LRR-repeats+AP-GTPase+COR</td><td>926</td><td>-</td><td>Euryarchaeota</td><td>Methanosarcina barkeri</td><td>leucine-rich repeat domain-containing protein [Methanosarcina barkeri].</td><td>GCF_001027005.1</td></tr>
<tr><td>WP_052718309.1</td><td><-HhH-RADC+JAB||?→ NTP_transf_3→ LRR-repeats+AP-GTPase+COR→?→ LRR-repeats+AP-GTPase+COR→</td><td>LRR-repeats+AP-GTPase+COR</td><td>871</td><td>-</td><td>Euryarchaeota</td><td>Methanosarcina sp. MTP4</td><td>leucine-rich repeat domain-containing protein [Methanosarcina sp. MTP4].</td><td>GCF_000970045.1</td></tr>
</table>

acc	operon	architecture	len	gen.name	taxend	species	defline	gca
WP_052718311.1	LRR-repeats+AP-GTPase+COR*→	LRR-repeats+AP-GTPase+COR	999	-	Euryarchaeota	Methanosarcina sp. MTP4	leucine-rich repeat domain-containing protein [Methanosarcina sp. MTP4].	GCF_000970045.1
WP_052718346.1	LRR-repeats+AP-GTPase+COR*→?→||<-?<-SIG+TM+TM+TM+TM+TM<-ABC-ATPase	LRR-repeats+AP-GTPase+COR	891	-	Euryarchaeota	Methanosarcina sp. MTP4	leucine-rich repeat domain-containing protein [Methanosarcina sp. MTP4].	GCF_000970045.1
WP_052721594.1	<-HhH-RADC+JAB||?→ NTP_transf_3→ LRR-repeats+AP-GTPase+COR*→	LRR-repeats+AP-GTPase+COR	890	-	Euryarchaeota	Methanosarcina siciliae	leucine-rich repeat domain-containing protein [Methanosarcina siciliae].	GCF_000970085.1
WP_052725465.1	<-HhH-RADC+JAB||?→ NTP_transf_3→ LRR-repeats+AP-GTPase+COR*→	LRR-repeats+AP-GTPase+COR	886	-	Euryarchaeota	Methanosarcina sp. Kolksee	leucine-rich repeat domain-containing protein [Methanosarcina sp. Kolksee].	GCF_000969945.1
WP_052725699.1	<-HhH-RADC+JAB||?→ NTP_transf_3→ LRR-repeats+AP-GTPase+COR*→||<-?<-?||?→||<-?<-?||SIR2→	LRR-repeats+AP-GTPase+COR	926	-	Euryarchaeota	Methanosarcina barkeri	leucine-rich repeat domain-containing protein [Methanosarcina barkeri].	GCF_000970065.1
WP_052725704.1	<-SIG+TPR+TPR+TPR+TPR+TPR+TPR||?→||<-?||?→?→ PSE→?→ LRR-repeats+AP-GTPase+COR*→?→?→ PRTase→	LRR-repeats+AP-GTPase+COR	886	-	Euryarchaeota	Methanosarcina barkeri	leucine-rich repeat domain-containing protein [Methanosarcina barkeri].	GCF_000970065.1
WP_052727304.1	PAS+HISKIN→||<-?||?→?→?→?→||<-PSE||LRR-repeats+AP-GTPase+COR*→||<-?||?→ PSE→||<-?||Cluster1640_2clades→||<-SIG+HIN-HTH	LRR-repeats+AP-GTPase+COR	1010	-	Euryarchaeota	Methanosarcina siciliae	leucine-rich repeat domain-containing protein [Methanosarcina siciliae].	GCF_000970145.1
WP_052727948.1	LRR-repeats+AP-GTPase+COR+TIR*→?→?→?→?→?→ PSE→||<-TPR-repeats	LRR-repeats+AP-GTPase+COR+TIR	877	-	Euryarchaeota	Methanosarcina vacuolata	leucine-rich repeat domain-containing protein [Methanosarcina vacuolata].	GCF_000969905.1
WP_052727973.1	<-HhH-RADC+JAB||?→ NTP_transf_3→ LRR-repeats+AP-GTPase+COR*→	LRR-repeats+AP-GTPase+COR	886	-	Euryarchaeota	Methanosarcina vacuolata	leucine-rich repeat domain-containing protein [Methanosarcina vacuolata].	GCF_000969905.1
WP_052728059.1	LRR-repeats+AP-GTPase+COR*→	LRR-repeats+AP-GTPase+COR	930	-	Euryarchaeota	Methanosarcina mazei	leucine-rich repeat domain-containing protein [Methanosarcina mazei].	GCF_000970205.1
WP_052728060.1	<-PIN<-?<-SIG+TM+TM+TM+TM+TM+TM+TM+TM+TM+TM+TM||?→?→ LRR-repeats+AP-GTPase+COR*→	LRR-repeats+AP-GTPase+COR	935	-	Euryarchaeota	Methanosarcina mazei	leucine-rich repeat domain-containing protein [Methanosarcina mazei].	GCF_000970205.1
WP_052735402.1	LRR-repeats+AP-GTPase+COR*→	LRR-repeats+AP-GTPase+COR	845	-	Euryarchaeota	Methanosarcina sp. 1.H.A.2.2	leucine-rich repeat domain-containing protein [Methanosarcina sp. 1.H.A.2.2].	GCF_000979975.1
WP_052752245.1	LRR-repeats+AP-GTPase+COR*→ TIR→	LRR-repeats+AP-GTPase+COR	1015	-	Betaproteobacteria	Nitrosomonas communis	leucine-rich repeat domain-containing protein [Nitrosomonas communis].	GCF_001007935.1
WP_054015538.1	<-REC||?→||<-?||?→ LRR-repeats+AP-GTPase+COR+TIR*→	LRR-repeats+AP-GTPase+COR+TIR	1274	-	Gammaproteobacteria	Pseudoalteromonas sp. R3	TIR domain-containing protein [Pseudoalteromonas sp. R3].	GCF_004014715.1
WP_054015917.1	LRR-repeats+AP-GTPase+COR+TIR*→||<-?<-?<-TM	LRR-repeats+AP-GTPase+COR+TIR	1128	-	Gammaproteobacteria	Pseudoalteromonas sp. R3	leucine-rich repeat domain-containing protein [Pseudoalteromonas sp. R3].	GCF_004014715.1
WP_054961809.1	DSBH+AraC-HTH+AraC-HTH→?→ LRR-repeats+AP-GTPase+COR*→||<-ABC_membrane+ABC_tran	LRR-repeats+AP-GTPase+COR	1057	-	Gammaproteobacteria	Vibrio bivalvicida	hypothetical protein [Vibrio bivalvicida].	GCF_001399455.2
WP_055505179.1	<-ABC-ATPase<-?||?→?→||<-?||LRR-repeats+AP-GTPase+COR*→?→?→?→||<-?<-?<-SIR2	LRR-repeats+AP-GTPase+COR	941	-	Actinobacteria	Nonomuraea pusilla	leucine-rich repeat domain-containing protein [Nonomuraea pusilla].	GCF_001417755.1
WP_057935115.1	SIG+Concanavalin-like+SUN→||<-?||?→ FGS→?→?→?→ LRR-repeats+AP-GTPase+COR+TIR*→?→?→||<-?<-?||?→ TIR→	LRR-repeats+AP-GTPase+COR+TIR	1318	-	Bacteroidetes	Pedobacter ginsenosidimutans	leucine-rich repeat domain-containing protein [Pedobacter ginsenosidimutans].	GCF_001442625.1

<table>
<tr><th>acc</th><th>operon</th><th>architecture</th><th>len</th><th>gen.name</th><th>taxend</th><th>species</th><th>defline</th><th>gca</th></tr>
<tr><td>WP_058183692.1</td><td>LRR-repeats+AP-GTPase+COR+CASPASE*→ LRR-repeats→</td><td>LRR-repeats+AP-GTPase+COR+CASPASE</td><td>1194</td><td>-</td><td>Cyanobacteria</td><td>Mastigocoleus testarum</td><td>leucine-rich repeat domain-containing protein [Mastigocoleus testarum].</td><td>GCF_001456025.1</td></tr>
<tr><td>WP_058183771.1</td><td>LRR-repeats+AP-GTPase+COR+EAD1*→</td><td>LRR-repeats+AP-GTPase+COR+EAD1</td><td>1014</td><td>-</td><td>Cyanobacteria</td><td>Mastigocoleus testarum</td><td>leucine-rich repeat domain-containing protein [Mastigocoleus testarum].</td><td>GCF_001456025.1</td></tr>
<tr><td>WP_058554438.1</td><td>LRR-repeats+AP-GTPase+COR+TIR*→</td><td>LRR-repeats+AP-GTPase+COR+TIR</td><td>758</td><td>-</td><td>Gammaproteobacteria</td><td>Thiohalocapsa sp. ML1</td><td>TIR domain-containing protein [Thiohalocapsa sp. ML1].</td><td>GCF_001469165.1</td></tr>
<tr><td>WP_058998621.1</td><td>LRR-repeats+AP-GTPase+COR*→?→?→ HNH→</td><td>LRR-repeats+AP-GTPase+COR</td><td>418</td><td>-</td><td>Cyanobacteria</td><td>Leptolyngbya sp. NIES-2104</td><td>leucine-rich repeat domain-containing protein [Leptolyngbya sp. NIES-2104].</td><td>GCF_001485215.1</td></tr>
<tr><td>WP_059136441.1</td><td>LRR-repeats+AP-GTPase+COR+TIR*→||<-RnasePH+RnasePH+KH+S1</td><td>LRR-repeats+AP-GTPase+COR+TIR</td><td>1173</td><td>-</td><td>Bacteroidetes</td><td>Chryseobacterium greenlandense</td><td>leucine-rich repeat domain-containing protein [Chryseobacterium greenlandense].</td><td>GCF_001507325.1</td></tr>
<tr><td>WP_061431817.1</td><td>SIG+TM+TM+TM+TM+TM+TM+TM→||<-?||PSE→ PSE→||<-?||SAM-methylase→?→||<-?<-SIG+TPM_phosphatase+TM+TM+TM||PSE→ LRR-repeats+AP-GTPase+COR+TM+TM*→||<-?<-?<-?<-?<-?||?→||<-Classical-AAA+Classical-AAA</td><td>LRR-repeats+AP-GTPase+COR+TM+TM</td><td>670</td><td>-</td><td>Cyanobacteria</td><td>Microcystis aeruginosa</td><td>GTP-binding protein, partial [Microcystis aeruginosa].</td><td>GCF_001578075.1</td></tr>
<tr><td>WP_062148002.1</td><td><-TPR-repeats+CASPASE||?→?→ LRR-repeats+AP-GTPase+COR+DUF4404*→</td><td>LRR-repeats+AP-GTPase+COR+DUF4404</td><td>1474</td><td>-</td><td>Gammaproteobacteria</td><td>Beggiatoa leptomitoformis</td><td>leucine-rich repeat domain-containing protein [Beggiatoa leptomitoformis].</td><td>GCF_001305575.3</td></tr>
<tr><td>WP_062292830.1</td><td>LRR-repeats+AP-GTPase+COR*→||<-?<-Aminotran_1_2<-HU-IHF</td><td>LRR-repeats+AP-GTPase+COR</td><td>946</td><td>-</td><td>Cyanobacteria</td><td>Nostoc piscinale</td><td>leucine-rich repeat domain-containing protein [Nostoc piscinale].</td><td>GCF_001298445.1</td></tr>
<tr><td>WP_062689807.1</td><td><-Cluster2368_2clades||?→ ncRNA→?→?→?→?→?→ LRR-repeats+AP-GTPase+COR*→</td><td>LRR-repeats+AP-GTPase+COR</td><td>909</td><td>-</td><td>Gammaproteobacteria</td><td>Photobacterium sanguinicancri</td><td>GTP-binding protein [Photobacterium sanguinicancri].</td><td>GCF_001563765.1</td></tr>
<tr><td>WP_063362501.1</td><td>LRR-repeats+AP-GTPase+COR+TIR*→</td><td>LRR-repeats+AP-GTPase+COR+TIR</td><td>1154</td><td>-</td><td>Gammaproteobacteria</td><td>Pseudoalteromonas luteoviolacea</td><td>leucine-rich repeat domain-containing protein [Pseudoalteromonas luteoviolacea].</td><td>GCF_001625695.1</td></tr>
<tr><td>WP_064232935.1</td><td>LRR-repeats+AP-GTPase+COR*→</td><td>LRR-repeats+AP-GTPase+COR</td><td>699</td><td>-</td><td>Gammaproteobacteria</td><td>Halomonas sp. ALS9</td><td>hypothetical protein [Halomonas sp. ALS9].</td><td>GCF_001651035.1</td></tr>
<tr><td>WP_065709937.1</td><td>LRR-repeats+AP-GTPase+COR*→||<-?<-?||?→ ABC-ATPase→ tRNA→?→||<-?<-Pentapeptide-repeats</td><td>LRR-repeats+AP-GTPase+COR</td><td>963</td><td>-</td><td>Cyanobacteria</td><td>Synechococcus sp. PCC 7117</td><td>leucine-rich repeat domain-containing protein [Synechococcus sp. PCC 7117].</td><td>-</td></tr>
<tr><td>WP_066434724.1</td><td>LRR-repeats+AP-GTPase+COR*→</td><td>LRR-repeats+AP-GTPase+COR</td><td>276</td><td>-</td><td>Bacteroidetes</td><td>Gelidibacter algens</td><td>hypothetical protein [Gelidibacter algens].</td><td>GCF_001678675.1</td></tr>
<tr><td>WP_067315083.1</td><td>SMPB→?→||<-?||?→||<-?||?→?→ LRR-repeats+AP-GTPase+COR*→||<-SIG+TM+TM+TM||?→||<-?||?→ SIGMA-HTH→</td><td>LRR-repeats+AP-GTPase+COR</td><td>968</td><td>-</td><td>Actinobacteria</td><td>Streptomyces griseochromogenes</td><td>hypothetical protein [Streptomyces griseochromogenes].</td><td>GCF_001542625.1</td></tr>
<tr><td>WP_067770734.1</td><td>LRR-repeats+AP-GTPase+COR*→</td><td>LRR-repeats+AP-GTPase+COR</td><td>1123</td><td>-</td><td>Cyanobacteria</td><td>Nostoc sp. NIES-3756</td><td>hypothetical protein [Nostoc sp. NIES-3756].</td><td>GCF_001548375.1</td></tr>
<tr><td>WP_068061695.1</td><td>7TMR-DISMED1→?→ SIG+Concanavalin-like+SUN→?→?→||<-?||?→ LRR-repeats+AP-GTPase+COR+TIR*→ ABhydrolase+ABhydrolase+CASPASE+TPR-S→?→||<-tRNA<-?<-?<-Cluster2189_2clades</td><td>LRR-repeats+AP-GTPase+COR+TIR</td><td>1268</td><td>-</td><td>Gammaproteobacteria</td><td>Rheinheimera sp. SA_1</td><td>TIR domain-containing protein [Rheinheimera sp. SA_1].</td><td>-</td></tr>
<tr><td>WP_068843099.1</td><td>LRR-repeats+AP-GTPase+COR*→||<-?||?→?→||<-cNMPBD</td><td>LRR-repeats+AP-GTPase+COR</td><td>1015</td><td>-</td><td>Bacteroidetes</td><td>Flavobacterium chilense</td><td>GTP-binding protein [Flavobacterium chilense].</td><td>GCF_900142685.1</td></tr>
<tr><td>WP_068887322.1</td><td>LRR-repeats+AP-GTPase+COR+TIR*→ Cluster2230_2clades→?→?→||<-?<-?<-?<-Cluster2322_2clades</td><td>LRR-repeats+AP-GTPase+COR+TIR</td><td>1168</td><td>-</td><td>Bacteroidetes</td><td>Pedobacter panaciterrae</td><td>leucine-rich repeat domain-containing protein [Pedobacter panaciterrae].</td><td>GCF_001636695.1</td></tr>
<tr><td>WP_069069853.1</td><td>LRR-repeats+AP-GTPase+COR+TIR*→</td><td>LRR-repeats+AP-GTPase+COR+TIR</td><td>1138</td><td>-</td><td>Cyanobacteria</td><td>Nostoc sp. KVJ20</td><td>leucine-rich repeat protein [Nostoc sp. KVJ20].</td><td>GCF_001712795.1</td></tr>
<tr><td>WP_069070238.1</td><td>LRR-repeats+AP-GTPase+COR*→||<-SIG+DADALIGASE-ATPgrasp<-?||?→?→||<-?<-?<-CBS</td><td>LRR-repeats+AP-GTPase+COR</td><td>1078</td><td>-</td><td>Cyanobacteria</td><td>Nostoc sp. KVJ20</td><td>leucine-rich repeat domain-containing protein [Nostoc sp. KVJ20].</td><td>GCF_001712795.1</td></tr>
</table>

<table>
<tr><th>acc</th><th>operon</th><th>architecture</th><th>len</th><th>gen.name</th><th>taxend</th><th>species</th><th>defline</th><th>gca</th></tr>
<tr><td>WP_069474666.1</td><td>SIG+TM+TM+TM+TM+TM+TM+TM→||<-?||Mbetalac→ SAM-methylase→?→||<-?<-SIG+TPM_phosphatase+TM+TM+TM||LRR-repeats+AP-GTPase+COR+TM+TM*→||<-?<-?<-?<-Pro_CA||?→||<-?<-tRNA<-tRNA<-Ferredoxin-betagrasp</td><td>LRR-repeats+AP-GTPase+COR+TM+TM</td><td>897</td><td>-</td><td>Cyanobacteria</td><td>Microcystis aeruginosa</td><td>leucine-rich repeat domain-containing protein [Microcystis aeruginosa].</td><td>GCF_001725075.1</td></tr>
<tr><td>WP_070392558.1</td><td>SIG+PBPI→?→?→ SIG+TM+TM+TM+TM+TM+TM+TM→||<-?||ABC-ATPase→ ABC-ATPase→||<-PSE||LRR-repeats+AP-GTPase+COR*→</td><td>LRR-repeats+AP-GTPase+COR</td><td>909</td><td>-</td><td>Cyanobacteria</td><td>Moorea producens</td><td>leucine-rich repeat domain-containing protein [Moorea producens].</td><td>GCF_001767235.1</td></tr>
<tr><td>WP_070393924.1</td><td>REC→||<-TPR-repeats<-?||LRR-repeats+AP-GTPase+COR+DUF4404*→</td><td>LRR-repeats+AP-GTPase+COR+DUF4404</td><td>938</td><td>-</td><td>Cyanobacteria</td><td>Moorea producens</td><td>leucine-rich repeat domain-containing protein [Moorea producens].</td><td>GCF_001767235.1</td></tr>
<tr><td>WP_071837136.1</td><td><-PIN<-?<-PIN<-?<-PIN<-?||LRR-repeats+AP-GTPase+COR+DUF4404*→?→ PSE→||<-Cluster5_5clades<-?<-Cluster2453_2clades</td><td>LRR-repeats+AP-GTPase+COR+DUF4404</td><td>1008</td><td>-</td><td>Cyanobacteria</td><td>Leptolyngbya sp. NIES-3755</td><td>leucine-rich repeat domain-containing protein [Leptolyngbya sp. NIES-3755].</td><td>GCF_001548435.1</td></tr>
<tr><td>WP_071987478.1</td><td>LRR-repeats+AP-GTPase+COR*→||<-?<-?||RelE→ CHTH→</td><td>LRR-repeats+AP-GTPase+COR</td><td>725</td><td>-</td><td>Cyanobacteria</td><td>Crocosphaera watsonii</td><td>leucine-rich repeat domain-containing protein [Crocosphaera watsonii].</td><td>GCF_000235665.1</td></tr>
<tr><td>WP_072015976.1</td><td><-SIG+TM||?→||<-RelE-ParE<-?||LRR-repeats+AP-GTPase+COR*→||<-?||?→?→ COR→</td><td>LRR-repeats+AP-GTPase+COR</td><td>651</td><td>-</td><td>Cyanobacteria</td><td>Leptolyngbya sp. KIOST-1</td><td>hypothetical protein [Leptolyngbya sp. KIOST-1].</td><td>GCF_000763385.1</td></tr>
<tr><td>WP_072022104.1</td><td><-ARSR-HTH<-PSE||?→||<-?||?→||<-SIG+Trypsin+PDZ<-?||?→ LRR-repeats+AP-GTPase+COR*→?→||<-RPN10<-?<-?<-Cluster2290_2clades<-Cluster2428_2clades</td><td>LRR-repeats+AP-GTPase+COR</td><td>838</td><td>-</td><td>Cyanobacteria</td><td>[Scytonema hofmanni] UTEX B 1581</td><td>leucine-rich repeat domain-containing protein [[Scytonema hofmanni] UTEX B 1581].</td><td>GCF_000582685.1</td></tr>
<tr><td>WP_072061087.1</td><td>LRR-repeats+AP-GTPase+COR*→||<-?<-?||RelE→ CHTH→</td><td>LRR-repeats+AP-GTPase+COR</td><td>725</td><td>-</td><td>Cyanobacteria</td><td>Crocosphaera watsonii</td><td>leucine-rich repeat domain-containing protein [Crocosphaera watsonii].</td><td>GCF_001039615.1</td></tr>
<tr><td>WP_072926417.1</td><td>Mbetalac→ SAM-methylase→?→||<-?<-SIG+TPM_phosphatase+TM+TM+TM||LRR-repeats+AP-GTPase+COR+TM+TM*→</td><td>LRR-repeats+AP-GTPase+COR+TM+TM</td><td>828</td><td>-</td><td>Cyanobacteria</td><td>Microcystis aeruginosa</td><td>leucine-rich repeat domain-containing protein [Microcystis aeruginosa].</td><td>GCF_001895325.1</td></tr>
<tr><td>WP_073070049.1</td><td>LRR-repeats+AP-GTPase+COR+TM+TM*→</td><td>LRR-repeats+AP-GTPase+COR+TM+TM</td><td>759</td><td>-</td><td>Cyanobacteria</td><td>Phormidesmis priestleyi</td><td>leucine-rich repeat domain-containing protein [Phormidesmis priestleyi].</td><td>GCF_001895925.1</td></tr>
<tr><td>WP_073584587.1</td><td><-Aminotran_1_2<-TetR-HTH||Cluster2045_2clades→?→ LRR-repeats+AP-GTPase+COR+TIR*→</td><td>LRR-repeats+AP-GTPase+COR+TIR</td><td>844</td><td>-</td><td>Gammaproteobacteria</td><td>Vibrio quintilis</td><td>TIR domain-containing protein [Vibrio quintilis].</td><td>GCF_900143745.1</td></tr>
<tr><td>WP_073604448.1</td><td>LRR-repeats+AP-GTPase+COR+TIR*→</td><td>LRR-repeats+AP-GTPase+COR+TIR</td><td>1052</td><td>-</td><td>Gammaproteobacteria</td><td>Vibrio aerogenes</td><td>TIR domain-containing protein, partial [Vibrio aerogenes].</td><td>GCF_900130105.1</td></tr>
<tr><td>WP_073609490.1</td><td>LRR-repeats+AP-GTPase+COR*→</td><td>LRR-repeats+AP-GTPase+COR</td><td>1026</td><td>-</td><td>Cyanobacteria</td><td>Phormidium tenue</td><td>leucine-rich repeat domain-containing protein [Phormidium tenue].</td><td>GCF_014696675.1</td></tr>
<tr><td>WP_073639373.1</td><td>LRR-repeats+AP-GTPase+COR+TIR*→</td><td>LRR-repeats+AP-GTPase+COR+TIR</td><td>1122</td><td>-</td><td>Cyanobacteria</td><td>Nostoc calcicola</td><td>leucine-rich repeat domain-containing protein [Nostoc calcicola].</td><td>GCF_001904715.1</td></tr>
<tr><td>WP_073640743.1</td><td>LRR-repeats+AP-GTPase+COR+TIR*→?→||<-Arginase</td><td>LRR-repeats+AP-GTPase+COR+TIR</td><td>1070</td><td>-</td><td>Cyanobacteria</td><td>Nostoc calcicola</td><td>leucine-rich repeat domain-containing protein [Nostoc calcicola].</td><td>GCF_014697355.1</td></tr>
<tr><td>WP_073641321.1</td><td><-PIN<-?||?→?→ PIN→ LRR-repeats+AP-GTPase+COR+TIR*→||<-?||?→||<-?||?→?→ RPN10→</td><td>LRR-repeats+AP-GTPase+COR+TIR</td><td>1188</td><td>-</td><td>Cyanobacteria</td><td>Nostoc calcicola</td><td>leucine-rich repeat protein [Nostoc calcicola].</td><td>GCF_001904715.1</td></tr>
<tr><td>WP_074191634.1</td><td>CI→ LRR-repeats+AP-GTPase+COR*→</td><td>LRR-repeats+AP-GTPase+COR</td><td>1105</td><td>-</td><td>Gammaproteobacteria</td><td>Vibrio antiquarius</td><td>50S ribosome-binding GTPase [Vibrio antiquarius].</td><td>GCF_001909305.1</td></tr>
<tr><td>WP_074382488.1</td><td><-Cluster1076_2clades<-?||?→||<-?<-?||?→||<-?||LRR-repeats+AP-GTPase+COR+TM+TM*→</td><td>LRR-repeats+AP-GTPase+COR+TM+TM</td><td>1015</td><td>-</td><td>Cyanobacteria</td><td>Phormidesmis priestleyi</td><td>leucine-rich repeat domain-containing protein [Phormidesmis priestleyi].</td><td>GCF_001650195.1</td></tr>
<tr><td>WP_075071341.1</td><td><-ABC-ATPase<-?||?→?→?→?→?→ LRR-repeats+AP-GTPase+COR*→ SIG+TM+TM+TM+TM→?→?→||<-?||?→ SIG+TM+TM+TM+TM+TM+TM+TM+TM+TM→</td><td>LRR-repeats+AP-GTPase+COR</td><td>692</td><td>-</td><td>Chloroflexi</td><td>Levilinea saccharolytica</td><td>GTP-binding protein [Levilinea saccharolytica].</td><td>GCF_001050255.2</td></tr>
</table>

acc	operon	architecture	len	gen.name	taxend	species	defline	gca
WP_075170580.1	RNA-Helicase→||<-PSE<-?<-?<-?<-?||?→||<-TM+TM||LRR-repeats+AP-GTPase+COR*→||<-?<-Cluster1726_2clades	LRR-repeats+AP-GTPase+COR	960	-	Gammaproteobacteria	Pseudoalteromonas haloplanktis	GTP-binding protein [Pseudoalteromonas haloplanktis].	GCF_001924935.1
WP_075904287.1	ABC-ATPase→ ABC-ATPase→?→?→||<-?||LRR-repeats+AP-GTPase+COR*→	LRR-repeats+AP-GTPase+COR	801	-	Cyanobacteria	Moorea bouillonii	leucine-rich repeat domain-containing protein [Moorea bouillonii].	GCF_001942495.1
WP_076382638.1	<-Polbeta+HEPN<-?||?→ LRR-repeats+AP-GTPase+COR+TIR*→ TIR→ TM+TM+TM→?→?→||<-CNMP+CRP-HTH	LRR-repeats+AP-GTPase+COR+TIR	1349	-	Bacteroidetes	Filimonas lacunae	TIR domain-containing protein [Filimonas lacunae].	GCF_002355595.1
WP_076393053.1	LRR-repeats+AP-GTPase+COR*→	LRR-repeats+AP-GTPase+COR	925	-	Bacteroidetes	Chryseobacterium	MULTISPECIES: leucine-rich repeat domain-containing protein [Chryseobacterium].	GCF_900156825.1
WP_076396200.1	LRR-repeats+AP-GTPase+COR*→||<-?||?→ SIG+NUDIX→	LRR-repeats+AP-GTPase+COR	890	-	Bacteroidetes	Chryseobacterium gambrini	hypothetical protein [Chryseobacterium gambrini].	GCF_900156825.1
WP_076994187.1	<-ABC-ATPase<-?||?→?→||<-?||PSE→||<-?||LRR-repeats+AP-GTPase+COR*→||<-?||?→||<-?<-?<-Aminotran_1_2	LRR-repeats+AP-GTPase+COR	942	-	Actinobacteria	Actinosynnema sp. ALI-1.44	hypothetical protein [Actinosynnema sp. ALI-1.44].	GCF_001984155.1
WP_077025618.1	LRR-repeats+AP-GTPase+COR*→?→||<-?<-?||?→||<-?<-?<-ABC-ATPase	LRR-repeats+AP-GTPase+COR	1153	-	Planctomycetes	Fuerstia marisgermanicae	leucine-rich repeat domain-containing protein [Fuerstia marisgermanicae].	GCF_001983935.1
WP_078402495.1	LRR-repeats+AP-GTPase+COR*→	LRR-repeats+AP-GTPase+COR	901	-	Bacteroidetes	Elizabethkingia ursingii	leucine-rich repeat domain-containing protein [Elizabethkingia ursingii].	GCF_002022125.1
WP_078608910.1	<-RNA-Helicase||?→?→||<-?||CI→ LRR-repeats+AP-GTPase+COR*→	LRR-repeats+AP-GTPase+COR	1012	-	Gammaproteobacteria	Vibrio owensii	GTP-binding protein [Vibrio owensii].	GCF_002021755.1
WP_078814763.1	<-REC||LRR-repeats+AP-GTPase+COR+TIR*→?→?→||<-?||?→||<-?<-NUDIX	LRR-repeats+AP-GTPase+COR+TIR	959	-	Verrucomicrobia	Prosthecobacter debontii	leucine-rich repeat domain-containing protein [Prosthecobacter debontii].	GCF_900167535.1
WP_079184311.1	LRR-repeats+AP-GTPase+COR*→?→||<-?<-SIGMA-HTH	LRR-repeats+AP-GTPase+COR	1056	-	Actinobacteria	Streptomyces uncialis	hypothetical protein [Streptomyces uncialis].	GCF_001905345.1
WP_079712025.1	LRR-repeats+AP-GTPase+COR*→	LRR-repeats+AP-GTPase+COR	882	-	Bacteroidetes	Salegentibacter salinarum	hypothetical protein [Salegentibacter salinarum].	GCF_900168115.1
WP_080603599.1	LRR-repeats+AP-GTPase+COR+TM+TM*→	LRR-repeats+AP-GTPase+COR+TM+TM	799	-	Cyanobacteria	Microcystis aeruginosa	leucine-rich repeat protein [Microcystis aeruginosa].	-
WP_080963556.1	Cluster1140_2clades→?→ LRR-repeats+AP-GTPase+COR+TM+TM*→	LRR-repeats+AP-GTPase+COR+TM+TM	830	-	Gammaproteobacteria	Pseudomonas putida	GTP-binding protein [Pseudomonas putida].	GCF_001027965.1
WP_081155426.1	LRR-repeats+AP-GTPase+COR*→||<-?<-?<-?<-?||?→||<-?<-REC	LRR-repeats+AP-GTPase+COR	1088	-	Bacteroidetes	Niastella vici	hypothetical protein [Niastella vici].	GCF_002077945.1
WP_081336246.1	LRR-repeats+AP-GTPase+COR*→	LRR-repeats+AP-GTPase+COR	1380	-	Actinobacteria	Mycobacteroides chelonae	hypothetical protein [Mycobacteroides chelonae].	GCF_001853965.1
WP_081583858.1	LRR-repeats→ LRR-repeats+AP-GTPase+COR+CASPASE*→	LRR-repeats+AP-GTPase+COR+CASPASE	1375	-	Cyanobacteria	unclassified Tolypothrix	MULTISPECIES: leucine-rich repeat domain-containing protein [unclassified Tolypothrix].	GCF_015207145.1
WP_081656227.1	LRR-repeats+AP-GTPase+COR+TIR*→	LRR-repeats+AP-GTPase+COR+TIR	1354	-	Cyanobacteria	Fischerella sp. PCC 9431	leucine-rich repeat domain-containing protein [Fischerella sp. PCC 9431].	-
WP_081983983.1	Ferredoxin-RRM→?→ Cluster1494_2clades→||<-?<-?||?→?→ LRR-repeats+AP-GTPase+COR+DUF4404*→	LRR-repeats+AP-GTPase+COR+DUF4404	971	-	Betaproteobacteria	Massilia sp. JS1662	GTP-binding protein [Massilia sp. JS1662].	GCF_000759615.1
WP_082167682.1	LRR-repeats+AP-GTPase+COR+TIR*→||<-?||Cluster850_2clades→	LRR-repeats+AP-GTPase+COR+TIR	797	-	Alphaproteobacteria	Methylobacterium aquaticum	TIR domain-containing protein, partial [Methylobacterium aquaticum].	GCF_001043915.1
WP_082209748.1	LRR-repeats+AP-GTPase+COR+TCAD4*→||<-Cluster5_5clades<-?||?→?→ Pro_CA→	LRR-repeats+AP-GTPase+COR+TCAD4	714	-	Cyanobacteria	Fischerella sp. PCC 9605	leucine-rich repeat domain-containing protein [Fischerella sp. PCC 9605].	GCF_000517105.1

<table>
<thead>
<tr><th>acc</th><th>operon</th><th>architecture</th><th>len</th><th>gen.name</th><th>taxend</th><th>species</th><th>defline</th><th>gca</th></tr>
</thead>
<tbody>
<tr><td>WP_082226537.1</td><td>LRR-repeats→?→ LRR-repeats+AP-GTPase+COR*→||<-?<-?||?→||<-?<-?<-?||Aminotran_1_2→</td><td>LRR-repeats+AP-GTPase+COR</td><td>1171</td><td>-</td><td>Bacteroidetes</td><td>Microscilla marina</td><td>leucine-rich repeat domain-containing protein [Microscilla marina].</td><td>GCF_000169175.1</td></tr>
<tr><td>WP_083250213.1</td><td>LRR-repeats+AP-GTPase+COR*→</td><td>LRR-repeats+AP-GTPase+COR</td><td>817</td><td>-</td><td>Euryarchaeota</td><td>Methanosarcina sp. A14</td><td>leucine-rich repeat domain-containing protein, partial [Methanosarcina sp. A14].</td><td>GCF_001729375.1</td></tr>
<tr><td>WP_083398305.1</td><td>LRR-repeats→ LRR-repeats+AP-GTPase+COR+DrHyd*→?→?→?→?→||<-?<-SIG+TM+TM+TM+TM</td><td>LRR-repeats+AP-GTPase+COR+DrHyd</td><td>751</td><td>-</td><td>Betaproteobacteria</td><td>Nitrosomonas communis</td><td>hypothetical protein [Nitrosomonas communis].</td><td>GCF_900114745.1</td></tr>
<tr><td>WP_083620324.1</td><td><-SIG+TM+TM+TM+TM+TM+TM<-?||LRR-repeats+AP-GTPase+COR*→?→||<-HNH||Cluster1548_2clades→?→||<-?||CCTBP→</td><td>LRR-repeats+AP-GTPase+COR</td><td>975</td><td>-</td><td>Cyanobacteria</td><td>Planktothrix paucivesiculata</td><td>leucine-rich repeat domain-containing protein [Planktothrix paucivesiculata].</td><td>GCF_900009265.2</td></tr>
<tr><td>WP_083622583.1</td><td>Cluster1745_2clades→?→?→||<-?||?→ LRR-repeats+AP-GTPase+COR*→</td><td>LRR-repeats+AP-GTPase+COR</td><td>1058</td><td>-</td><td>Cyanobacteria</td><td>Planktothrix paucivesiculata</td><td>leucine-rich repeat domain-containing protein [Planktothrix paucivesiculata].</td><td>GCF_900009265.2</td></tr>
<tr><td>WP_083850172.1</td><td>LRR-repeats+AP-GTPase+COR*→</td><td>LRR-repeats+AP-GTPase+COR</td><td>1286</td><td>-</td><td>Deltaproteobacteria</td><td>Desulfovibrio sp. U5L</td><td>GTP-binding protein [Desulfovibrio sp. U5L].</td><td>-</td></tr>
<tr><td>WP_083886975.1</td><td><-SIG+TM||?→||<-?<-?||LRR-repeats→?→ LRR-repeats+AP-GTPase+COR*→</td><td>LRR-repeats+AP-GTPase+COR</td><td>863</td><td>-</td><td>Cyanobacteria</td><td>Nodosilinea nodulosa</td><td>hypothetical protein [Nodosilinea nodulosa].</td><td>GCF_000309385.1</td></tr>
<tr><td>WP_083887059.1</td><td>RelE→ CHTH→ LRR-repeats→ PSE→||<-?||PSE→ LRR-repeats+AP-GTPase+COR*→||<-?<-?||COR+TIR→||<-REase-4</td><td>LRR-repeats+AP-GTPase+COR</td><td>410</td><td>-</td><td>Cyanobacteria</td><td>Nodosilinea nodulosa</td><td>leucine-rich repeat domain-containing protein [Nodosilinea nodulosa].</td><td>GCF_000309385.1</td></tr>
<tr><td>WP_083887212.1</td><td>Cluster2123_2clades→?→||<-?<-?<-?||PSE→ LRR-repeats→ AP-GTPase*→</td><td>AP-GTPase</td><td>221</td><td>-</td><td>Cyanobacteria</td><td>Nodosilinea nodulosa</td><td>50S ribosome-binding GTPase [Nodosilinea nodulosa].</td><td>GCF_000309385.1</td></tr>
<tr><td>WP_083918254.1</td><td>LRR-repeats+AP-GTPase+COR+TIR*→||<-?<-?||?→||<-?<-?<-SHS2</td><td>LRR-repeats+AP-GTPase+COR+TIR</td><td>954</td><td>-</td><td>Gammaproteobacteria</td><td>Methylosarcina fibrata</td><td>TIR domain-containing protein [Methylosarcina fibrata].</td><td>GCF_000372865.1</td></tr>
<tr><td>WP_084022355.1</td><td><-NUDIX||?→||<-HNH<-?||?→?→?→ LRR-repeats+AP-GTPase+COR+TIR*→</td><td>LRR-repeats+AP-GTPase+COR+TIR</td><td>850</td><td>-</td><td>Chlorobi</td><td>Chlorobaculum limnaeum</td><td>TIR domain-containing protein [Chlorobaculum limnaeum].</td><td>GCF_001747405.1</td></tr>
<tr><td>WP_084172951.1</td><td>LRR-repeats+AP-GTPase+COR*→</td><td>LRR-repeats+AP-GTPase+COR</td><td>639</td><td>-</td><td>Cyanobacteria</td><td>Phormidium tenue</td><td>GTP-binding protein [Phormidium tenue].</td><td>GCF_014696675.1</td></tr>
<tr><td>WP_084177732.1</td><td>LRR-repeats+AP-GTPase+COR+TM+TM*→||<-CASPASE+TM</td><td>LRR-repeats+AP-GTPase+COR+TM+TM</td><td>985</td><td>-</td><td>Cyanobacteria</td><td>Nostoc calcicola</td><td>leucine-rich repeat domain-containing protein [Nostoc calcicola].</td><td>GCF_001904715.1</td></tr>
<tr><td>WP_084227301.1</td><td>LRR-repeats+AP-GTPase+COR+TIR*→</td><td>LRR-repeats+AP-GTPase+COR+TIR</td><td>804</td><td>-</td><td>Cyanobacteria</td><td>Nostoc sp. KVJ20</td><td>leucine-rich repeat domain-containing protein [Nostoc sp. KVJ20].</td><td>GCF_001712795.1</td></tr>
<tr><td>WP_084428809.1</td><td>LRR-repeats+AP-GTPase+COR*→</td><td>LRR-repeats+AP-GTPase+COR</td><td>944</td><td>-</td><td>Actinobacteria</td><td>Kibdelosporangium aridum</td><td>hypothetical protein [Kibdelosporangium aridum].</td><td>GCF_900176515.1</td></tr>
<tr><td>WP_084630566.1</td><td><-HhH-RADC+JAB||?→ NTP_transf_3→ LRR-repeats+AP-GTPase+COR*→||<-?<-?||?→||<-?||?→||<-Cluster1409_2clades</td><td>LRR-repeats+AP-GTPase+COR</td><td>906</td><td>-</td><td>Euryarchaeota</td><td>Methanosarcina lacustris</td><td>leucine-rich repeat domain-containing protein [Methanosarcina lacustris].</td><td>GCF_000970265.1</td></tr>
<tr><td>WP_084713364.1</td><td>LRR-repeats+AP-GTPase+COR*→</td><td>LRR-repeats+AP-GTPase+COR</td><td>1355</td><td>-</td><td>Actinobacteria</td><td>Streptacidiphilus jeojiense</td><td>leucine-rich repeat domain-containing protein [Streptacidiphilus jeojiense].</td><td>-</td></tr>
<tr><td>WP_084763145.1</td><td>Pentapeptide-repeats→||<-TPR+nSTAND1+BetaPropeller_WD40+BetaPropeller_WD40<-?||?→||<-?||?→||<-?||LRR-repeats+AP-GTPase+COR+CASPASE*→||<-RelE-ParE</td><td>LRR-repeats+AP-GTPase+COR+CASPASE</td><td>1441</td><td>-</td><td>Cyanobacteria</td><td>[Scytonema hofmanni] UTEX B 1581</td><td>leucine-rich repeat domain-containing protein [[Scytonema hofmanni] UTEX B 1581].</td><td>GCF_000582685.1</td></tr>
<tr><td>WP_084765096.1</td><td>LRR-repeats+AP-GTPase+COR*→</td><td>LRR-repeats+AP-GTPase+COR</td><td>940</td><td>-</td><td>Firmicutes</td><td>Clostridium intestinale</td><td>GTP-binding protein [Clostridium intestinale].</td><td>-</td></tr>
<tr><td>WP_084967529.1</td><td>Cluster1877_2clades→||<-?||Cluster1140_2clades→?→ LRR-repeats+AP-GTPase+COR+TM+TM*→</td><td>LRR-repeats+AP-GTPase+COR+TM+TM</td><td>830</td><td>-</td><td>Gammaproteobacteria</td><td>Pseudomonas putida</td><td>GTP-binding protein [Pseudomonas putida].</td><td>GCF_002094785.1</td></tr>
</tbody>
</table>

<table>
<tr><th>acc</th><th>operon</th><th>architecture</th><th>len</th><th>gen.name</th><th>taxend</th><th>species</th><th>defline</th><th>gca</th></tr>
<tr><td>WP_086488496.1</td><td>GTPase-AIG→||<-?||?→?→?→?→ PIN→ LRR-repeats+AP-GTPase+COR+TIR*→?→||<-?<-SIG+TM+TM+TM+TM+TM+TM+TM+TM+TM+TM+TM<-?||?→||<-?<-Cluster1501_2clades</td><td>LRR-repeats+AP-GTPase+COR+TIR</td><td>936</td><td>-</td><td>Gammaproteobacteria</td><td>Thioflexothrix psekupsii</td><td>leucine-rich repeat domain-containing protein [Thioflexothrix psekupsii].</td><td>GCF_002149925.1</td></tr>
<tr><td>WP_086489369.1</td><td>LRR-repeats+AP-GTPase+COR+HEPN*→ ABC-ATPase→?→?→?→?→ Cluster1668_2clades→</td><td>LRR-repeats+AP-GTPase+COR+HEPN</td><td>919</td><td>-</td><td>Gammaproteobacteria</td><td>Thioflexothrix psekupsii</td><td>leucine-rich repeat domain-containing protein [Thioflexothrix psekupsii].</td><td>GCF_002149925.1</td></tr>
<tr><td>WP_086489735.1</td><td><-Cluster1597_2clades<-Cluster1052_2clades<-?||?→?→?→ LRR-repeats+AP-GTPase+COR+HEPN*→ Cluster2027_2clades→?→||<-ABC-ATPase<-MoxR-AAA<-HISKIN</td><td>LRR-repeats+AP-GTPase+COR+HEPN</td><td>857</td><td>-</td><td>Gammaproteobacteria</td><td>Thioflexothrix psekupsii</td><td>leucine-rich repeat domain-containing protein [Thioflexothrix psekupsii].</td><td>GCF_002149925.1</td></tr>
<tr><td>WP_086671081.1</td><td>SIG+TM+TM+TM+TM→||<-?<-?<-?||?→||<-Cluster2027_2clades<-?||tRNA→ LRR-repeats+AP-GTPase+COR+TM+TM*→||<-?<-?||PSE→||<-?||?→?→||<-?<-SIG+Calcineurin</td><td>LRR-repeats+AP-GTPase+COR+TM+TM</td><td>937</td><td>-</td><td>Actinobacteria</td><td>Amycolatopsis pretoriensis</td><td>GTP-binding protein [Amycolatopsis pretoriensis].</td><td>GCF_900107925.1</td></tr>
<tr><td>WP_086685880.1</td><td>LRR-repeats+AP-GTPase+COR+DUF4404*→</td><td>LRR-repeats+AP-GTPase+COR+DUF4404</td><td>1074</td><td>-</td><td>Cyanobacteria</td><td>Nostoc sp. T09</td><td>leucine-rich repeat domain-containing protein [Nostoc sp. T09].</td><td>GCF_002154695.1</td></tr>
<tr><td>WP_086688381.1</td><td>LRR-repeats+AP-GTPase+COR+CASPASE*→||<-?<-?||?→ RelE-ParE→||<-?<-?||Cluster1437_2clades→</td><td>LRR-repeats+AP-GTPase+COR+CASPASE</td><td>897</td><td>-</td><td>Cyanobacteria</td><td>Nostoc sp. T09</td><td>CHAT domain-containing protein, partial [Nostoc sp. T09].</td><td>GCF_002154695.1</td></tr>
<tr><td>WP_086688547.1</td><td>LRR-repeats+AP-GTPase+COR+TM+TM*→</td><td>LRR-repeats+AP-GTPase+COR+TM+TM</td><td>883</td><td>-</td><td>Cyanobacteria</td><td>Nostoc sp. T09</td><td>leucine-rich repeat domain-containing protein [Nostoc sp. T09].</td><td>GCF_002154695.1</td></tr>
<tr><td>WP_086759966.1</td><td>LRR-repeats+AP-GTPase+COR+DUF4404*→ TPR-repeats+CASPASE→||<-?||?→ HTH→</td><td>LRR-repeats+AP-GTPase+COR+DUF4404</td><td>1097</td><td>-</td><td>Cyanobacteria</td><td>Nostoc sp. 106C</td><td>leucine-rich repeat domain-containing protein [Nostoc sp. 106C].</td><td>GCF_002154725.1</td></tr>
<tr><td>WP_086760374.1</td><td>LRR-repeats+AP-GTPase+COR*→</td><td>LRR-repeats+AP-GTPase+COR</td><td>1054</td><td>-</td><td>Cyanobacteria</td><td>Nostoc sp. 106C</td><td>leucine-rich repeat domain-containing protein [Nostoc sp. 106C].</td><td>GCF_002154725.1</td></tr>
<tr><td>WP_086768657.1</td><td>RPN10→ Chromo-N+CHROMO→||<-?||LRR-repeats+AP-GTPase+COR+CASPASE*→</td><td>LRR-repeats+AP-GTPase+COR+CASPASE</td><td>1254</td><td>-</td><td>Cyanobacteria</td><td>Nostoc sp. 106C</td><td>leucine-rich repeat domain-containing protein [Nostoc sp. 106C].</td><td>GCF_002154725.1</td></tr>
<tr><td>WP_086834293.1</td><td>LRR-repeats+AP-GTPase+COR+TM+TM*→</td><td>LRR-repeats+AP-GTPase+COR+TM+TM</td><td>884</td><td>-</td><td>Cyanobacteria</td><td>Nostoc sp. RF31YmG</td><td>leucine-rich repeat domain-containing protein [Nostoc sp. RF31YmG].</td><td>GCF_002155185.1</td></tr>
<tr><td>WP_087590341.1</td><td>LRR-repeats+AP-GTPase+COR*→</td><td>LRR-repeats+AP-GTPase+COR</td><td>452</td><td>-</td><td>Cyanobacteria</td><td>unicellular cyanobacterium SU3</td><td>leucine-rich repeat domain-containing protein [unicellular cyanobacterium SU3].</td><td>-</td></tr>
<tr><td>WP_087656332.1</td><td>Cluster1742_2clades→ Cluster1685_2clades→?→?→?→?→?→ LRR-repeats+AP-GTPase+COR*→?→ TIR→</td><td>LRR-repeats+AP-GTPase+COR</td><td>560</td><td>-</td><td>Betaproteobacteria</td><td>Caballeronia terrestris</td><td>hypothetical protein [Caballeronia terrestris].</td><td>GCF_001544515.1</td></tr>
<tr><td>WP_088134442.1</td><td>OmpA→?→ Cluster1633_2clades→||<-?||?→ tRNA→?→ Polbeta+HEPN→ LRR-repeats+AP-GTPase+COR+TIR*→?→||<-?||?→?→ SIG+TM+TM+TM+TM+TM+TM+TM+TM+TM+TM+TM→ SIG+TM→</td><td>LRR-repeats+AP-GTPase+COR+TIR</td><td>1220</td><td>-</td><td>Gammaproteobacteria</td><td>Vibrio gazogenes</td><td>TIR domain-containing protein [Vibrio gazogenes].</td><td>GCF_002196515.1</td></tr>
<tr><td>WP_088620375.1</td><td><-Cluster2094_2clades||LRR-repeats+AP-GTPase+COR*→||<-?<-?<-?<-HISKIN||?→||<-REC</td><td>LRR-repeats+AP-GTPase+COR</td><td>806</td><td>-</td><td>Gammaproteobacteria</td><td>Methylovulum psychrotolerans</td><td>GTP-binding protein [Methylovulum psychrotolerans].</td><td>GCF_002209385.1</td></tr>
<tr><td>WP_088620603.1</td><td><-ABC-ATPase<-SIG+BLBD<-?<-?||REC→ HISKIN→ REase→ LRR-repeats+AP-GTPase+COR+TIR*→?→?→ Ferredoxin-RRM→</td><td>LRR-repeats+AP-GTPase+COR+TIR</td><td>1013</td><td>-</td><td>Gammaproteobacteria</td><td>Methylovulum psychrotolerans</td><td>TIR domain-containing protein [Methylovulum psychrotolerans].</td><td>GCF_002209385.1</td></tr>
<tr><td>WP_088621060.1</td><td>LRR-repeats+AP-GTPase+COR+DpnII-MboI-NTD+TIR*→</td><td>LRR-repeats+AP-GTPase+COR+DpnII-MboI-NTD+TIR</td><td>866</td><td>-</td><td>Gammaproteobacteria</td><td>Methylovulum psychrotolerans</td><td>leucine-rich repeat domain-containing protein [Methylovulum psychrotolerans].</td><td>GCF_002209385.1</td></tr>
<tr><td>WP_088646515.1</td><td>LRR-repeats+AP-GTPase+COR*→||<-?<-?<-?<-?<-Cluster1404_2clades</td><td>LRR-repeats+AP-GTPase+COR</td><td>987</td><td>-</td><td>Actinobacteria</td><td>Micromonospora wenchangensis</td><td>hypothetical protein [Micromonospora wenchangensis].</td><td>GCF_002210435.1</td></tr>
<tr><td>WP_088745883.1</td><td>LRR-repeats+AP-GTPase+COR*→</td><td>LRR-repeats+AP-GTPase+COR</td><td>863</td><td>-</td><td>Bacteroidetes</td><td>unclassified Sphingobacteriales</td><td>MULTISPECIES: GTP-binding protein [unclassified Sphingobacteriales].</td><td>-</td></tr>
</table>

<table>
<tr><th>acc</th><th>operon</th><th>architecture</th><th>len</th><th>gen.name</th><th>taxend</th><th>species</th><th>defline</th><th>gca</th></tr>
<tr><td>WP_088762693.1</td><td>TPR-repeats→?→ SIG+Alba→||<-?<-?<-?<-?||LRR-repeats+AP-GTPase+COR*→</td><td>LRR-repeats+AP-GTPase+COR</td><td>851</td><td>-</td><td>Bacteroidetes</td><td>unclassified Sphingobacteriales</td><td>MULTISPECIES: GTP-binding protein [unclassified Sphingobacteriales].</td><td>-</td></tr>
<tr><td>WP_088764296.1</td><td>LRR-repeats+AP-GTPase+COR+TM+TM*→</td><td>LRR-repeats+AP-GTPase+COR+TM+TM</td><td>969</td><td>-</td><td>Bacteroidetes</td><td>unclassified Sphingobacteriales</td><td>MULTISPECIES: leucine-rich repeat domain-containing protein [unclassified Sphingobacteriales].</td><td>-</td></tr>
<tr><td>WP_089288360.1</td><td><-SIG+TM+TM+TM+TM+TM+TM+TM+TM+TM+TM+TM<-?||?→ Cluster1974_2clades→?→?→||<-?||LRR-repeats+AP-GTPase+COR+TIR*→||<-?<-ParB<-ParA-Soj-PloopNTPase<-?<-Thioredoxin</td><td>LRR-repeats+AP-GTPase+COR+TIR</td><td>1212</td><td>-</td><td>Alphaproteobacteria</td><td>unclassified Azospirillum</td><td>MULTISPECIES: TIR domain-containing protein [unclassified Azospirillum].</td><td>GCF_900188385.1</td></tr>
<tr><td>WP_089298330.1</td><td>SIG+Concanavalin-like+SUN→ LRR-repeats+AP-GTPase+COR*→||<-PSE||?→?→?→||<-?<-ABC-ATPase</td><td>LRR-repeats+AP-GTPase+COR</td><td>908</td><td>-</td><td>Actinobacteria</td><td>Actinoplanes regularis</td><td>GTP-binding protein [Actinoplanes regularis].</td><td>GCF_016862355.1</td></tr>
<tr><td>WP_089681784.1</td><td>LRR-repeats+AP-GTPase+COR*→?→||<-Thioredoxin</td><td>LRR-repeats+AP-GTPase+COR</td><td>787</td><td>-</td><td>Bacteroidetes</td><td>Catalinimonas alkaloidigena</td><td>hypothetical protein [Catalinimonas alkaloidigena].</td><td>GCF_900100765.1</td></tr>
<tr><td>WP_089727137.1</td><td>LRR-repeats+AP-GTPase+COR*→</td><td>LRR-repeats+AP-GTPase+COR</td><td>1009</td><td>-</td><td>Gammaproteobacteria</td><td>Candidatus Thiosymbion oneisti</td><td>leucine-rich repeat domain-containing protein [Candidatus Thiosymbion oneisti].</td><td>GCF_900092655.1</td></tr>
<tr><td>WP_089824678.1</td><td>Cluster1536_2clades→?→||<-tRNA||LRR-repeats+AP-GTPase+COR*→</td><td>LRR-repeats+AP-GTPase+COR</td><td>978</td><td>-</td><td>Firmicutes</td><td>Clostridiales bacterium CHKCI001</td><td>leucine-rich repeat domain-containing protein [Clostridiales bacterium CHKCI001].</td><td>-</td></tr>
<tr><td>WP_089856669.1</td><td>LRR-repeats+AP-GTPase+COR*→</td><td>LRR-repeats+AP-GTPase+COR</td><td>925</td><td>-</td><td>Bacteroidetes</td><td>Chryseobacterium taeanense</td><td>leucine-rich repeat domain-containing protein [Chryseobacterium taeanense].</td><td>GCF_900099685.1</td></tr>
<tr><td>WP_089938297.1</td><td>COR→ LRR-repeats+AP-GTPase+COR*→</td><td>LRR-repeats+AP-GTPase+COR</td><td>705</td><td>-</td><td>Nitrospinae/Tectomicrobia group</td><td>Candidatus Entotheonella palauensis</td><td>hypothetical protein [Candidatus Entotheonella palauensis].</td><td>GCF_900079095.1</td></tr>
<tr><td>WP_089941349.1</td><td><-Aminotran_1_2<-Cluster1597_2clades||?→?→?→?→ LRR-repeats+AP-GTPase+COR*→</td><td>LRR-repeats+AP-GTPase+COR</td><td>514</td><td>-</td><td>Nitrospinae/Tectomicrobia group</td><td>Candidatus Entotheonella palauensis</td><td>leucine-rich repeat domain-containing protein [Candidatus Entotheonella palauensis].</td><td>GCF_900079095.1</td></tr>
<tr><td>WP_090106546.1</td><td>LRR-repeats+AP-GTPase+COR+TIR*→</td><td>LRR-repeats+AP-GTPase+COR+TIR</td><td>1148</td><td>-</td><td>Bacteroidetes</td><td>Chitinophaga sp. CF118</td><td>leucine-rich repeat domain-containing protein [Chitinophaga sp. CF118].</td><td>GCF_900112615.1</td></tr>
<tr><td>WP_090320517.1</td><td>LRR-repeats+AP-GTPase+COR*→?→?→ ParA-Soj-PloopNTPase→</td><td>LRR-repeats+AP-GTPase+COR</td><td>738</td><td>-</td><td>Betaproteobacteria</td><td>Nitrosomonas oligotropha</td><td>leucine-rich repeat domain-containing protein [Nitrosomonas oligotropha].</td><td>GCF_900106555.1</td></tr>
<tr><td>WP_090640679.1</td><td><-RNA-Helicase||LRR-repeats+AP-GTPase+COR+DrHyd*→||<-CBS</td><td>LRR-repeats+AP-GTPase+COR+DrHyd</td><td>1059</td><td>-</td><td>Bacteroidetes</td><td>Mucilaginibacter sp. OK283</td><td>leucine-rich repeat domain-containing protein [Mucilaginibacter sp. OK283].</td><td>GCF_900110415.1</td></tr>
<tr><td>WP_090662001.1</td><td>LRR-repeats+AP-GTPase+COR+TIR*→?→||<-?<-?||?→ SIG+FKBP→</td><td>LRR-repeats+AP-GTPase+COR+TIR</td><td>1182</td><td>-</td><td>Bacteroidetes</td><td>Parafilimonas terrae</td><td>leucine-rich repeat domain-containing protein [Parafilimonas terrae].</td><td>GCF_900115755.1</td></tr>
<tr><td>WP_091029330.1</td><td>TM→ LRR-repeats+AP-GTPase+COR*→</td><td>LRR-repeats+AP-GTPase+COR</td><td>1045</td><td>-</td><td>Actinobacteria</td><td>Glycomyces harbinensis</td><td>hypothetical protein [Glycomyces harbinensis].</td><td>GCF_900101745.1</td></tr>
<tr><td>WP_091054155.1</td><td><-Cluster2137_2clades<-?<-?||?→||<-?||?→?→ LRR-repeats+AP-GTPase+COR*→</td><td>LRR-repeats+AP-GTPase+COR</td><td>1094</td><td>-</td><td>Actinobacteria</td><td>Glycomyces sambucus</td><td>hypothetical protein [Glycomyces sambucus].</td><td>GCF_900102815.1</td></tr>
<tr><td>WP_091103947.1</td><td><-ABC-ATPase<-?||?→?→||<-?||LRR-repeats+AP-GTPase+COR*→?→?→?→||<-?<-?<-SIR2</td><td>LRR-repeats+AP-GTPase+COR</td><td>941</td><td>-</td><td>Actinobacteria</td><td>Nonomuraea pusilla</td><td>leucine-rich repeat domain-containing protein [Nonomuraea pusilla].</td><td>GCF_900109355.1</td></tr>
<tr><td>WP_091133979.1</td><td>LRR-repeats+AP-GTPase+COR+TIR*→</td><td>LRR-repeats+AP-GTPase+COR+TIR</td><td>1190</td><td>-</td><td>Bacteroidetes</td><td>Flavobacterium anhuiense</td><td>TIR domain-containing protein [Flavobacterium anhuiense].</td><td>GCF_900101855.1</td></tr>
</table>

acc	operon	architecture	len	gen.name	taxend	species	defline	gca
WP_091435026.1	<-Cluster1070_2clades<-ENDO3+NUDIX||HU-IHF→?→ LRR-repeats+AP-GTPase+COR*→	LRR-repeats+AP-GTPase+COR	916	-	Bacteroidetes	Flavobacterium degerlachei	leucine-rich repeat domain-containing protein [Flavobacterium degerlachei].	GCF_900106645.1
WP_091506051.1	Cluster944_3clades→||<-?<-?||?→||<-Cluster2045_2clades<-?||LRR-repeats+AP-GTPase+COR+TIR*→	LRR-repeats+AP-GTPase+COR+TIR	997	-	Bacteroidetes	Flexibacter flexilis	leucine-rich repeat domain-containing protein [Flexibacter flexilis].	GCF_900112255.1
WP_091630534.1	<-ABC-ATPase||?→ LRR-repeats+AP-GTPase+COR*→||<-ABC_membrane+ABC_tran	LRR-repeats+AP-GTPase+COR	901	-	Actinobacteria	Micromonospora peucetia	leucine-rich repeat domain-containing protein [Micromonospora peucetia].	GCF_900091625.1
WP_092162518.1	REC→ REC→?→?→?→ LRR-repeats+AP-GTPase+COR*→?→?→ SIG+WWE→?→?→ ABC-ATPase→ ABC-ATPase→	LRR-repeats+AP-GTPase+COR	863	-	Deltaproteobacteria	Maridesulfovibrio ferrireducens	GTP-binding protein [Maridesulfovibrio ferrireducens].	GCF_900101105.1
WP_093066920.1	LRR-repeats+AP-GTPase+COR+TIR*→?→?→||<-Thioredoxin||YBAK→	LRR-repeats+AP-GTPase+COR+TIR	847	-	Gammaproteobacteria	Thiothrix caldifontis	TIR domain-containing protein [Thiothrix caldifontis].	GCF_900107695.1
WP_093070768.1	LRR-repeats+AP-GTPase+COR+TIR*→	LRR-repeats+AP-GTPase+COR+TIR	1327	-	Gammaproteobacteria	Thiothrix caldifontis	leucine-rich repeat domain-containing protein [Thiothrix caldifontis].	GCF_900107695.1
WP_094331224.1	Cluster1074_2clades→?→ LRR-repeats+AP-GTPase+COR+DUF4404*→||<-PSE<-?||?→?→ SAM-methylase→	LRR-repeats+AP-GTPase+COR+DUF4404	1016	-	Cyanobacteria	Nostoc sp. ‘Peltigera membranacea cyanobiont’ 213	leucine-rich repeat domain-containing protein [Nostoc sp. ‘Peltigera membranacea cyanobiont’ 213].	GCF_002245975.1
WP_094342929.1	<-Arginase<-?||?→ LRR-repeats+AP-GTPase+COR+TIR*→?→||<-?<-?<-ABC_membrane+ABC_tran	LRR-repeats+AP-GTPase+COR+TIR	1126	-	Cyanobacteria	Nostoc sp. ‘Peltigera membranacea cyanobiont’ 232	leucine-rich repeat domain-containing protein [Nostoc sp. ‘Peltigera membranacea cyanobiont’ 232].	GCF_002245985.1
WP_094343165.1	LRR-repeats+AP-GTPase+COR+TIR*→?→||<-?<-TIR<-?<-SIG+NUDIX	LRR-repeats+AP-GTPase+COR+TIR	1023	-	Cyanobacteria	Nostoc sp. ‘Peltigera membranacea cyanobiont’ 232	leucine-rich repeat domain-containing protein [Nostoc sp. ‘Peltigera membranacea cyanobiont’ 232].	GCF_002245985.1
WP_094350322.1	LRR-repeats+AP-GTPase+COR*→?→?→||<-ABhydrolase<-Cluster1087_2clades	LRR-repeats+AP-GTPase+COR	1217	-	Cyanobacteria	Nostoc sp. ‘Peltigera membranacea cyanobiont’ 210A	leucine-rich repeat domain-containing protein [Nostoc sp. ‘Peltigera membranacea cyanobiont’ 210A].	GCF_002246015.1
WP_094350573.1	LRR-repeats+AP-GTPase+COR+DUF4404*→ REC→	LRR-repeats+AP-GTPase+COR+DUF4404	1154	-	Cyanobacteria	Nostoc sp. ‘Peltigera membranacea cyanobiont’ 210A	leucine-rich repeat domain-containing protein [Nostoc sp. ‘Peltigera membranacea cyanobiont’ 210A].	GCF_002246015.1
WP_094350736.1	LRR-repeats+AP-GTPase+COR*→	LRR-repeats+AP-GTPase+COR	996	-	Cyanobacteria	Nostoc sp. ‘Peltigera membranacea cyanobiont’ 210A	leucine-rich repeat domain-containing protein [Nostoc sp. ‘Peltigera membranacea cyanobiont’ 210A].	-
WP_094350925.1	<-TPR+TPR<-?||?→?→ ABhydrolase→?→||<-?||LRR-repeats+AP-GTPase+COR+TIR*→||<-?<-?<-?<-?<-SIG+NUDIX	LRR-repeats+AP-GTPase+COR+TIR	1078	-	Cyanobacteria	Nostoc sp. ‘Peltigera membranacea cyanobiont’ 210A	leucine-rich repeat domain-containing protein [Nostoc sp. ‘Peltigera membranacea cyanobiont’ 210A].	GCF_002246015.1
WP_094408510.1	<-ABC-ATPase||ICLR-HTH→ LRR-repeats+AP-GTPase+COR+TIR*→||<-SIG+TM+TM+TM+TM+TM+TM+TM+TM+TM	LRR-repeats+AP-GTPase+COR+TIR	974	-	Alphaproteobacteria	Elstera cyanobacteriorum	leucine-rich repeat domain-containing protein [Elstera cyanobacteriorum].	GCF_014643715.1
WP_094530685.1	LRR-repeats+AP-GTPase+COR*→||<-PSE||?→?→?→||<-ABC-ATPase<-ABC-ATPase<-SIG+TM+TM+TM+TM+TM+TM+TM	LRR-repeats+AP-GTPase+COR	1019	-	Cyanobacteria	Pseudanabaena sp. SR411	leucine-rich repeat domain-containing protein [Pseudanabaena sp. SR411].	GCF_002251945.1

<table>
<tr><th>acc</th><th>operon</th><th>architecture</th><th>len</th><th>gen.name</th><th>taxend</th><th>species</th><th>defline</th><th>gca</th></tr>
<tr><td>WP_094533976.1</td><td>Cluster54_3clades→ Cluster1093_2clades→ LRR-repeats+AP-GTPase+COR+TM+TM*→</td><td>LRR-repeats+AP-GTPase+COR+TM+TM</td><td>1051</td><td>-</td><td>Cyanobacteria</td><td>Pseudanabaena sp. SR411</td><td>leucine-rich repeat domain-containing protein [Pseudanabaena sp. SR411].</td><td>GCF_002251945.1</td></tr>
<tr><td>WP_095644020.1</td><td>LRR-repeats+AP-GTPase+COR*→</td><td>LRR-repeats+AP-GTPase+COR</td><td>774</td><td>-</td><td>Euryarchaeota</td><td>Methanosarcina spelaei</td><td>leucine-rich repeat domain-containing protein, partial [Methanosarcina spelaei].</td><td>GCF_002287235.1</td></tr>
<tr><td>WP_095644584.1</td><td>LRR-repeats+AP-GTPase+COR*→</td><td>LRR-repeats+AP-GTPase+COR</td><td>877</td><td>-</td><td>Euryarchaeota</td><td>Methanosarcina spelaei</td><td>leucine-rich repeat domain-containing protein [Methanosarcina spelaei].</td><td>GCF_002287235.1</td></tr>
<tr><td>WP_096193484.1</td><td>LRR-repeats+AP-GTPase+COR+TIR*→?→||<-FAD-NAD-dep-oxidoreductase<-?<-?<-RNA-Helicase</td><td>LRR-repeats+AP-GTPase+COR+TIR</td><td>1173</td><td>-</td><td>Bacteroidetes</td><td>Cytophagales bacterium TFI 002</td><td>leucine-rich repeat domain-containing protein [Cytophagales bacterium TFI 002].</td><td>-</td></tr>
<tr><td>WP_096535168.1</td><td>LRR-repeats+AP-GTPase+COR*→||<-ClpABN-AAA+ClpABC-AAA<-?||METHYLASE→</td><td>LRR-repeats+AP-GTPase+COR</td><td>1018</td><td>-</td><td>Cyanobacteria</td><td>Nostoc linckia</td><td>leucine-rich repeat domain-containing protein [Nostoc linckia].</td><td>GCF_002368035.1</td></tr>
<tr><td>WP_096538729.1</td><td><-Pkinase+Pentapeptide<-?<-?||?→||<-?||?→ LRR-repeats+AP-GTPase+COR+TIR*→</td><td>LRR-repeats+AP-GTPase+COR+TIR</td><td>1033</td><td>-</td><td>Cyanobacteria</td><td>Nostoc linckia</td><td>leucine-rich repeat domain-containing protein [Nostoc linckia].</td><td>GCF_002368035.1</td></tr>
<tr><td>WP_096538739.1</td><td>LRR-repeats+AP-GTPase+COR+TIR*→?→||<-?||?→?→?→ RPN10→</td><td>LRR-repeats+AP-GTPase+COR+TIR</td><td>1115</td><td>-</td><td>Cyanobacteria</td><td>Nostoc linckia</td><td>leucine-rich repeat domain-containing protein [Nostoc linckia].</td><td>GCF_002368035.1</td></tr>
<tr><td>WP_096551538.1</td><td>LRR-repeats+AP-GTPase+COR*→</td><td>LRR-repeats+AP-GTPase+COR</td><td>818</td><td>-</td><td>Cyanobacteria</td><td>Nostoc sp. NIES-4103</td><td>leucine-rich repeat domain-containing protein [Nostoc sp. NIES-4103].</td><td>GCF_002368335.1</td></tr>
<tr><td>WP_096566542.1</td><td>HNH→ PSE→ LRR-repeats+AP-GTPase+COR*→?→?→?→||<-?||?→||<-?||PAIREDC-HTH→</td><td>LRR-repeats+AP-GTPase+COR</td><td>1268</td><td>-</td><td>Cyanobacteria</td><td>Scytonema sp. NIES-4073</td><td>leucine-rich repeat protein [Scytonema sp. NIES-4073].</td><td>GCF_002368435.1</td></tr>
<tr><td>WP_096579899.1</td><td><-DSBH<-?<-?<-?<-ParA-Soj-PloopNTPase<-?||Cluster5_5clades→ LRR-repeats+AP-GTPase+COR+DUF4404*→</td><td>LRR-repeats+AP-GTPase+COR+DUF4404</td><td>1153</td><td>-</td><td>Cyanobacteria</td><td>Anabaenopsis circularis</td><td>leucine-rich repeat domain-containing protein [Anabaenopsis circularis].</td><td>GCF_002367975.1</td></tr>
<tr><td>WP_096585603.1</td><td>HISKIN→||<-?<-?||LRR-repeats+AP-GTPase+COR*→</td><td>LRR-repeats+AP-GTPase+COR</td><td>937</td><td>-</td><td>Cyanobacteria</td><td>Anabaenopsis circularis</td><td>leucine-rich repeat domain-containing protein [Anabaenopsis circularis].</td><td>GCF_002367975.1</td></tr>
<tr><td>WP_096586956.1</td><td><-ABC-ATPase||?→ Pkinase+TM→ Chromo-N+CHROMO→||<-?||LRR-repeats+AP-GTPase+COR+CASPASE*→||<-?<-?||Aminotran_1_2→</td><td>LRR-repeats+AP-GTPase+COR+CASPASE</td><td>1148</td><td>-</td><td>Cyanobacteria</td><td>Calothrix sp. NIES-2098</td><td>leucine-rich repeat domain-containing protein [Calothrix sp. NIES-2098].</td><td>GCF_002368175.1</td></tr>
<tr><td>WP_096588412.1</td><td>LRR-repeats+AP-GTPase+COR*→ TPR-repeats+CASPASE→</td><td>LRR-repeats+AP-GTPase+COR</td><td>1066</td><td>-</td><td>Cyanobacteria</td><td>Calothrix sp. NIES-2098</td><td>leucine-rich repeat domain-containing protein [Calothrix sp. NIES-2098].</td><td>GCF_002368175.1</td></tr>
<tr><td>WP_096598125.1</td><td>LRR-repeats+AP-GTPase+COR+DUF4404*→?→||<-?||?→||<-RelE-ParE</td><td>LRR-repeats+AP-GTPase+COR+DUF4404</td><td>1134</td><td>-</td><td>Cyanobacteria</td><td>Calothrix sp. NIES-2100</td><td>leucine-rich repeat domain-containing protein [Calothrix sp. NIES-2100].</td><td>GCF_002368195.1</td></tr>
<tr><td>WP_096598883.1</td><td>SIG+TM→?→ tRNA→||<-?||LRR-repeats+AP-GTPase+COR+CASPASE*→||<-PSE<-?||?→?→||<-?<-?<-Acylphosphatase</td><td>LRR-repeats+AP-GTPase+COR+CASPASE</td><td>1108</td><td>-</td><td>Cyanobacteria</td><td>Calothrix sp. NIES-2100</td><td>leucine-rich repeat domain-containing protein [Calothrix sp. NIES-2100].</td><td>GCF_002368195.1</td></tr>
<tr><td>WP_096598950.1</td><td>LRR-repeats+AP-GTPase+COR+TM+TM*→||<-?||?→||<-REC</td><td>LRR-repeats+AP-GTPase+COR+TM+TM</td><td>929</td><td>-</td><td>Cyanobacteria</td><td>Calothrix sp. NIES-2100</td><td>leucine-rich repeat domain-containing protein [Calothrix sp. NIES-2100].</td><td>GCF_002368195.1</td></tr>
<tr><td>WP_096621624.1</td><td>LRR-repeats→ PSE→ LRR-repeats+AP-GTPase+COR+CASPASE*→</td><td>LRR-repeats+AP-GTPase+COR+CASPASE</td><td>981</td><td>-</td><td>Cyanobacteria</td><td>Microchaete diplosiphon</td><td>CHAT domain-containing protein [Microchaete diplosiphon].</td><td>GCF_002368275.1</td></tr>
<tr><td>WP_096658516.1</td><td>LRR-repeats+AP-GTPase+COR*→ PSE→?→||<-?||?→||<-?||?→||<-?<-Pentapeptide-repeats</td><td>LRR-repeats+AP-GTPase+COR</td><td>708</td><td>-</td><td>Cyanobacteria</td><td>Calothrix parasitica</td><td>leucine-rich repeat domain-containing protein [Calothrix parasitica].</td><td>GCF_002368095.1</td></tr>
</table>

<table>
<tr><th>acc</th><th>operon</th><th>architecture</th><th>len</th><th>gen.name</th><th>taxend</th><th>species</th><th>defline</th><th>gca</th></tr>
<tr><td>WP_096683758.1</td><td><-TPR-repeats+CASPASE||?→||<-?<-PSE||?→||<-ParB<-ParA-Soj-PloopNTPase||PSE→ LRR-repeats+AP-GTPase+COR+EAD1*→ ParB→||<-NACHT</td><td>LRR-repeats+AP-GTPase+COR+EAD1</td><td>1112</td><td>-</td><td>Cyanobacteria</td><td>Calothrix sp. NIES-4105</td><td>leucine-rich repeat domain-containing protein [Calothrix sp. NIES-4105].</td><td>GCF_002368415.1</td></tr>
<tr><td>WP_096729037.1</td><td><-TPR-repeats+CASPASE||?→||<-?<-PSE||?→||<-ParB<-ParA-Soj-PloopNTPase||PSE→ LRR-repeats→ LRR-repeats+AP-GTPase+COR+EAD1*→ ParB→||<-NACHT</td><td>LRR-repeats+AP-GTPase+COR+EAD1</td><td>885</td><td>-</td><td>Cyanobacteria</td><td>Calothrix sp. NIES-4071</td><td>leucine-rich repeat domain-containing protein [Calothrix sp. NIES-4071].</td><td>GCF_002368455.1</td></tr>
<tr><td>WP_096789603.1</td><td><-S4<-?||?→?→?→?→?→ LRR-repeats+AP-GTPase+COR*→||<-tRNA<-rRNA<-rRNA<-tRNA<-tRNA<-rRNA<-FAD-NAD-dep-oxidoreductase</td><td>LRR-repeats+AP-GTPase+COR</td><td>972</td><td>-</td><td>Alphaproteobacteria</td><td>Phaeobacter piscinae</td><td>leucine-rich repeat domain-containing protein [Phaeobacter piscinae].</td><td>GCF_002407245.1</td></tr>
<tr><td>WP_096793541.1</td><td><-Cluster2127_2clades||?→||<-?||?→ LRR-repeats+AP-GTPase+COR*→</td><td>LRR-repeats+AP-GTPase+COR</td><td>938</td><td>-</td><td>Gammaproteobacteria</td><td>Pseudoalteromonas sp. 1_2015MBL_MicDiv</td><td>GTP-binding protein [Pseudoalteromonas sp. 1_2015MBL_MicDiv].</td><td>GCF_002407505.1</td></tr>
<tr><td>WP_097126565.1</td><td>LRR-repeats+AP-GTPase+COR*→||<-?||?→||<-?||?→||<-?||?→||<-HISKIN</td><td>LRR-repeats+AP-GTPase+COR</td><td>1103</td><td>-</td><td>Bacteroidetes</td><td>Spirosoma fluviale</td><td>leucine-rich repeat domain-containing protein [Spirosoma fluviale].</td><td>GCF_900230225.1</td></tr>
<tr><td>WP_097298537.1</td><td>LRR-repeats+AP-GTPase+COR*→</td><td>LRR-repeats+AP-GTPase+COR</td><td>802</td><td>-</td><td>Euryarchaeota</td><td>Candidatus Methanoperedens sp. BLZ2</td><td>leucine-rich repeat domain-containing protein [Candidatus Methanoperedens sp. BLZ2].</td><td>GCF_002487355.1</td></tr>
<tr><td>WP_097998439.1</td><td>LRR-repeats+AP-GTPase+COR*→</td><td>LRR-repeats+AP-GTPase+COR</td><td>906</td><td>-</td><td>Firmicutes</td><td>Bacillus cereus</td><td>GTP-binding protein [Bacillus cereus].</td><td>GCF_002575505.1</td></tr>
<tr><td>WP_098908392.1</td><td>LRR-repeats+AP-GTPase+COR*→</td><td>LRR-repeats+AP-GTPase+COR</td><td>934</td><td>-</td><td>Firmicutes</td><td>Bacillus toyonensis</td><td>GTP-binding protein [Bacillus toyonensis].</td><td>GCF_002551725.1</td></tr>
<tr><td>WP_099009377.1</td><td>LRR-repeats+AP-GTPase+COR+EAD11*→</td><td>LRR-repeats+AP-GTPase+COR+EAD11</td><td>813</td><td>-</td><td>Bacteroidetes</td><td>Lewinellaceae bacterium SD302</td><td>leucine-rich repeat domain-containing protein [Lewinellaceae bacterium SD302].</td><td>-</td></tr>
<tr><td>WP_099066006.1</td><td>LRR-repeats+AP-GTPase+COR+TIR*→||<-PSE<-Arginase||?→||<-?||?→?→?→ Pro_CA→</td><td>LRR-repeats+AP-GTPase+COR+TIR</td><td>1138</td><td>-</td><td>Cyanobacteria</td><td>Nostoc linckia</td><td>leucine-rich repeat domain-containing protein [Nostoc linckia].</td><td>GCF_002607965.1</td></tr>
<tr><td>WP_099069019.1</td><td><-Pkinase+Pentapeptide<-?<-?||?→||<-?||LRR-repeats+AP-GTPase+COR+TIR*→?→||<-SIG+TM+TM+TM+TM+TM+TM<-?||?→ Cluster1668_2clades→</td><td>LRR-repeats+AP-GTPase+COR+TIR</td><td>1053</td><td>-</td><td>Cyanobacteria</td><td>Nostoc linckia</td><td>TIR domain-containing protein [Nostoc linckia].</td><td>GCF_002607965.1</td></tr>
<tr><td>WP_099076744.1</td><td>LRR-repeats+AP-GTPase+COR+TIR*→||<-PSE<-Arginase</td><td>LRR-repeats+AP-GTPase+COR+TIR</td><td>1138</td><td>-</td><td>Cyanobacteria</td><td>Nostoc linckia</td><td>leucine-rich repeat domain-containing protein [Nostoc linckia].</td><td>GCF_002608325.1</td></tr>
<tr><td>WP_099103609.1</td><td>LRR-repeats+AP-GTPase+COR+TM+TM*→</td><td>LRR-repeats+AP-GTPase+COR+TM+TM</td><td>825</td><td>-</td><td>Cyanobacteria</td><td>Nostoc sp. ‘Peltigera malacea cyanobiont’ DB3992</td><td>leucine-rich repeat domain-containing protein, partial [Nostoc sp. ‘Peltigera malacea cyanobiont’ DB3992].</td><td>-</td></tr>
<tr><td>WP_099151645.1</td><td>SIG+TM+TM+TM+HISKIN→?→ tRNA→?→?→ LRR-repeats+AP-GTPase+COR+EAD11*→</td><td>LRR-repeats+AP-GTPase+COR+EAD11</td><td>881</td><td>-</td><td>Bacteroidetes</td><td>Flavilitoribacter nigricans</td><td>leucine-rich repeat domain-containing protein [Flavilitoribacter nigricans].</td><td>GCF_002646595.1</td></tr>
<tr><td>WP_099285181.1</td><td>LRR-repeats+AP-GTPase+COR*→?→||<-?<-SIGMA-HTH</td><td>LRR-repeats+AP-GTPase+COR</td><td>1056</td><td>-</td><td>Actinobacteria</td><td>unclassified Streptomyces</td><td>MULTISPECIES: GTPase [unclassified Streptomyces].</td><td>GCF_002705975.1</td></tr>
<tr><td>WP_099426012.1</td><td>TPR-repeats→?→?→ tRNA→?→ HNH→ LRR-repeats+AP-GTPase+COR+DUF4404*→?→?→?→ REC→</td><td>LRR-repeats+AP-GTPase+COR+DUF4404</td><td>1272</td><td>-</td><td>Cyanobacteria</td><td>Tychonema bourrellyi</td><td>leucine-rich repeat domain-containing protein [Tychonema bourrellyi].</td><td>GCF_002412335.2</td></tr>
<tr><td>WP_099426065.1</td><td>GTPase-AIG→?→?→ Cluster53_2clades→?→ LRR-repeats+AP-GTPase+COR+DUF4404*→</td><td>LRR-repeats+AP-GTPase+COR+DUF4404</td><td>959</td><td>-</td><td>Cyanobacteria</td><td>Tychonema bourrellyi</td><td>leucine-rich repeat domain-containing protein [Tychonema bourrellyi].</td><td>GCF_002412335.2</td></tr>
<tr><td>WP_099506658.1</td><td>ABC_membrane+ABC_tran→||<-?||?→||<-?||PSE→||<-PSE||LRR-repeats+AP-GTPase+COR*→</td><td>LRR-repeats+AP-GTPase+COR</td><td>799</td><td>-</td><td>Actinobacteria</td><td>Streptomyces dengpaensis</td><td>hypothetical protein [Streptomyces dengpaensis].</td><td>GCF_002946835.1</td></tr>
</table>

<table>
<tr><th>acc</th><th>operon</th><th>architecture</th><th>len</th><th>gen.name</th><th>taxend</th><th>species</th><th>defline</th><th>gca</th></tr>
<tr><td>WP_099533140.1</td><td>AP-GTPase+COR→?→ LRR-repeats+AP-GTPase+COR*→||<-?||?→||<-tRNA||?→?→ Cluster1088_2clades→||<-?<-tRNA<-BACTERIALFRINGE+TM+TM</td><td>LRR-repeats+AP-GTPase+COR</td><td>826</td><td>-</td><td>Cyanobacteria</td><td>Limnothrix sp. PR1529</td><td>leucine-rich repeat domain-containing protein [Limnothrix sp. PR1529].</td><td>GCF_002742025.1</td></tr>
<tr><td>WP_100899585.1</td><td>LRR-repeats+AP-GTPase+COR+EAD1*→||<-CASPASE+TM</td><td>LRR-repeats+AP-GTPase+COR+EAD1</td><td>945</td><td>-</td><td>Cyanobacteria</td><td>Nostoc flagelliforme</td><td>leucine-rich repeat domain-containing protein [Nostoc flagelliforme].</td><td>GCF_002813575.1</td></tr>
<tr><td>WP_100900079.1</td><td>LRR-repeats+AP-GTPase+COR*→ COR→?→||<-?||?→||<-?||?→ ClpABN-AAA+ClpABC-AAA→</td><td>LRR-repeats+AP-GTPase+COR</td><td>431</td><td>-</td><td>Cyanobacteria</td><td>Nostoc flagelliforme</td><td>leucine-rich repeat domain-containing protein [Nostoc flagelliforme].</td><td>GCF_002813575.1</td></tr>
<tr><td>WP_100917604.1</td><td><-METHYLASE<-?||?→||<-?||Cluster2515_2clades→ Cluster2268_2clades→ LRR-repeats+AP-GTPase+COR+TIR*→</td><td>LRR-repeats+AP-GTPase+COR+TIR</td><td>983</td><td>-</td><td>Gammaproteobacteria</td><td>Candidatus Thiodictyon syntrophicum</td><td>TIR domain-containing protein [Candidatus Thiodictyon syntrophicum].</td><td>GCF_002813775.1</td></tr>
<tr><td>WP_100918672.1</td><td><-Cluster944_3clades<-SIG+PDZ||?→?→||<-ABC-ATPase<-?<-?||LRR-repeats+AP-GTPase+COR+TIR*→||<-ABC-ATPase<-SIG+TM+TM+TM+TM+TM+TM+TM<-?<-SIG+PBPI</td><td>LRR-repeats+AP-GTPase+COR+TIR</td><td>1095</td><td>-</td><td>Gammaproteobacteria</td><td>Candidatus Thiodictyon syntrophicum</td><td>leucine-rich repeat domain-containing protein [Candidatus Thiodictyon syntrophicum].</td><td>GCF_002813775.1</td></tr>
<tr><td>WP_101084187.1</td><td><-Cluster1951_2clades||?→ LRR-repeats+AP-GTPase+COR*→?→?→?→?→ Cluster846_3clades→</td><td>LRR-repeats+AP-GTPase+COR</td><td>1013</td><td>-</td><td>Gammaproteobacteria</td><td>Psychromonas sp. Urea-02u-13</td><td>GTP-binding protein [Psychromonas sp. Urea-02u-13].</td><td>GCF_002835995.1</td></tr>
<tr><td>WP_101221578.1</td><td>SIG+TM+TM+TM+TM+TM+TM+TM→||<-?||?→?→?→ PSE→||<-?<-?||PSE→ LRR-repeats+AP-GTPase+COR*→</td><td>LRR-repeats+AP-GTPase+COR</td><td>897</td><td>-</td><td>Firmicutes</td><td>Bacillus sp. BA3</td><td>GTP-binding protein [Bacillus sp. BA3].</td><td>GCF_002835675.1</td></tr>
<tr><td>WP_101309496.1</td><td>LRR-repeats+AP-GTPase+COR*→</td><td>LRR-repeats+AP-GTPase+COR</td><td>888</td><td>-</td><td>Bacteroidetes</td><td>Labilibaculum manganireducens</td><td>hypothetical protein [Labilibaculum manganireducens].</td><td>GCF_002843385.1</td></tr>
<tr><td>WP_101439075.1</td><td>LRR-repeats+AP-GTPase+COR*→</td><td>LRR-repeats+AP-GTPase+COR</td><td>1694</td><td>-</td><td>Actinobacteria</td><td>Amycolatopsis</td><td>MULTISPECIES: leucine-rich repeat domain-containing protein [Amycolatopsis].</td><td>GCF_014174495.1</td></tr>
<tr><td>WP_101728686.1</td><td>MACRODOMAIN+LRR-repeats+AP-GTPase+COR+TIR*→</td><td>MACRODOMAIN+LRR-repeats+AP-GTPase+COR+TIR</td><td>1357</td><td>-</td><td>Bacteroidetes</td><td>Emticicia sp. TH156</td><td>leucine-rich repeat domain-containing protein [Emticicia sp. TH156].</td><td>GCF_002855535.1</td></tr>
<tr><td>WP_101955079.1</td><td><-TM<-WCAK<-?||?→ LRR-repeats+AP-GTPase+COR+TIR*→</td><td>LRR-repeats+AP-GTPase+COR+TIR</td><td>1144</td><td>-</td><td>Bacteroidetes</td><td>Tenacibaculum finnmarkense</td><td>leucine-rich repeat domain-containing protein [Tenacibaculum finnmarkense].</td><td>GCF_900239185.1</td></tr>
<tr><td>WP_102406975.1</td><td>LRR-repeats+AP-GTPase+COR+DrHyd*→</td><td>LRR-repeats+AP-GTPase+COR+DrHyd</td><td>1169</td><td>-</td><td>Bacteroidetes</td><td>Parabacteroides bouchesdurhonensis</td><td>leucine-rich repeat domain-containing protein [Parabacteroides bouchesdurhonensis].</td><td>GCF_900186615.1</td></tr>
<tr><td>WP_102484140.1</td><td>CI→?→?→ LRR-repeats+AP-GTPase+COR*→?→?→||<-?<-?<-?<-ABC-ATPase</td><td>LRR-repeats+AP-GTPase+COR</td><td>1107</td><td>-</td><td>Gammaproteobacteria</td><td>Vibrio lentus</td><td>GTP-binding protein [Vibrio lentus].</td><td>GCF_002874115.1</td></tr>
<tr><td>WP_102498010.1</td><td>REC→?→?→?→||<-ABC_membrane+ABC_tran<-ABC_membrane+ABC_tran<-?||LRR-repeats+AP-GTPase+COR*→</td><td>LRR-repeats+AP-GTPase+COR</td><td>960</td><td>-</td><td>Gammaproteobacteria</td><td>Vibrio splendidus</td><td>leucine-rich repeat domain-containing protein [Vibrio splendidus].</td><td>GCF_002876045.1</td></tr>
<tr><td>WP_102512513.1</td><td>CI→ LRR-repeats+AP-GTPase+COR*→?→?→||<-PSE<-?<-?<-?<-ABC-ATPase</td><td>LRR-repeats+AP-GTPase+COR</td><td>1108</td><td>-</td><td>Gammaproteobacteria</td><td>Vibrio splendidus</td><td>GTP-binding protein [Vibrio splendidus].</td><td>GCF_002876945.1</td></tr>
<tr><td>WP_102562621.1</td><td>CI→ LRR-repeats+AP-GTPase+COR*→||<-?<-?||?→||<-?<-RPN10</td><td>LRR-repeats+AP-GTPase+COR</td><td>1108</td><td>-</td><td>Gammaproteobacteria</td><td>Vibrio splendidus</td><td>50S ribosome-binding GTPase [Vibrio splendidus].</td><td>GCF_002878555.1</td></tr>
<tr><td>WP_102571188.1</td><td>CI→ LRR-repeats+AP-GTPase+COR*→</td><td>LRR-repeats+AP-GTPase+COR</td><td>1107</td><td>-</td><td>Gammaproteobacteria</td><td>Vibrio splendidus</td><td>50S ribosome-binding GTPase [Vibrio splendidus].</td><td>GCF_002875585.1</td></tr>
<tr><td>WP_102579518.1</td><td>CI→?→ LRR-repeats+AP-GTPase+COR*→?→?→||<-?<-?<-?<-ABC-ATPase</td><td>LRR-repeats+AP-GTPase+COR</td><td>1106</td><td>-</td><td>Gammaproteobacteria</td><td>Vibrio lentus</td><td>GTP-binding protein [Vibrio lentus].</td><td>GCF_002876365.1</td></tr>
<tr><td>WP_102756439.1</td><td>TM+TM+TM+TM+TM→?→ Cluster2372_2clades→||<-?||?→?→?→ LRR-repeats+AP-GTPase+COR+DrHyd*→</td><td>LRR-repeats+AP-GTPase+COR+DrHyd</td><td>1054</td><td>-</td><td>Bacteroidetes</td><td>Flavivirga eckloniae</td><td>leucine-rich repeat domain-containing protein [Flavivirga eckloniae].</td><td>GCF_002886045.1</td></tr>
<tr><td>WP_103111669.1</td><td>SIG+TM+TM+TM+TM+TM+TM+TM→||<-?||Mbetalac→ SAM-methylase→?→||<-?<-SIG+TPM_phosphatase+TM+TM+TM||LRR-repeats+AP-GTPase+COR+TM+TM*→||<-?<-?<-?<-Pro_CA||?→||<-?<-tRNA<-tRNA<-Ferredoxin-betagrasp</td><td>LRR-repeats+AP-GTPase+COR+TM+TM</td><td>851</td><td>-</td><td>Cyanobacteria</td><td>Microcystis aeruginosa</td><td>leucine-rich repeat domain-containing protein [Microcystis aeruginosa].</td><td>GCF_002897275.1</td></tr>
</table>

<table>
<tr><th>acc</th><th>operon</th><th>architecture</th><th>len</th><th>gen.name</th><th>taxend</th><th>species</th><th>defline</th><th>gca</th></tr>
<tr><td>WP_103125147.1</td><td>HISKIN→||<-?||LRR-repeats+AP-GTPase+COR*→</td><td>LRR-repeats+AP-GTPase+COR</td><td>889</td><td>-</td><td>Cyanobacteria</td><td>Nostoc cycadae</td><td>leucine-rich repeat domain-containing protein [Nostoc cycadae].</td><td>GCF_002897135.1</td></tr>
<tr><td>WP_103137712.1</td><td>LRR-repeats+AP-GTPase+COR+TIR*→ Cluster1064_2clades→?→||<-?<-?<-?||Arginase→</td><td>LRR-repeats+AP-GTPase+COR+TIR</td><td>1058</td><td>-</td><td>Cyanobacteria</td><td>Nostoc sp. CENA543</td><td>leucine-rich repeat domain-containing protein [Nostoc sp. CENA543].</td><td>GCF_002896875.1</td></tr>
<tr><td>WP_103666629.1</td><td><-Cluster1740_2clades<-TM<-?<-?||NACHT→||<-?||LRR-repeats+AP-GTPase+COR+TM+TM*→||<-?||?→?→?→ SIG+TM+TM+TM+TM+TM→</td><td>LRR-repeats+AP-GTPase+COR+TM+TM</td><td>1164</td><td>-</td><td>Cyanobacteria</td><td>Pseudanabaena sp. BC1403</td><td>leucine-rich repeat domain-containing protein [Pseudanabaena sp. BC1403].</td><td>GCF_002914585.1</td></tr>
<tr><td>WP_103669449.1</td><td>Cluster54_3clades→?→?→?→ PIN→ LRR-repeats+AP-GTPase+COR+TM+TM*→||<-?<-?||ABC-ATPase→</td><td>LRR-repeats+AP-GTPase+COR+TM+TM</td><td>922</td><td>-</td><td>Cyanobacteria</td><td>Pseudanabaena sp. BC1403</td><td>leucine-rich repeat domain-containing protein [Pseudanabaena sp. BC1403].</td><td>GCF_002914585.1</td></tr>
<tr><td>WP_103972898.1</td><td>LRR-repeatsAP-GTPase+COR+E59-ABC*→</td><td>LRR-repeatsAP-GTPase+COR+E59-ABC</td><td>953</td><td>-</td><td>Gammaproteobacteria</td><td>Methylovulum psychrotolerans</td><td>AAA family ATPase [Methylovulum psychrotolerans].</td><td>GCF_002923755.1</td></tr>
<tr><td>WP_103972982.1</td><td><-ABC-ATPase<-SIG+BLBD<-?||REC→ HISKIN→ REase→ LRR-repeats+AP-GTPase+COR*→?→?→?→ Ferredoxin-RRM→</td><td>LRR-repeats+AP-GTPase+COR</td><td>911</td><td>-</td><td>Gammaproteobacteria</td><td>Methylovulum psychrotolerans</td><td>hypothetical protein [Methylovulum psychrotolerans].</td><td>GCF_002923755.1</td></tr>
<tr><td>WP_103974058.1</td><td><-PurT-ATPgrasp<-?<-?<-?||?→||<-?<-Cluster1542_2clades||LRR-repeats+AP-GTPase+COR+TIR*→ REase→||<-?<-?<-FKBP<-RHOD-CDC25<-Aminotran_1_2||Cluster2179_2clades→</td><td>LRR-repeats+AP-GTPase+COR+TIR</td><td>1203</td><td>-</td><td>Gammaproteobacteria</td><td>Methylovulum psychrotolerans</td><td>TIR domain-containing protein [Methylovulum psychrotolerans].</td><td>GCF_002923755.1</td></tr>
<tr><td>WP_103999297.1</td><td>LRR-repeats+AP-GTPase+COR*→?→||<-?<-?<-ABC-ATPase||Cluster995_2clades→</td><td>LRR-repeats+AP-GTPase+COR</td><td>925</td><td>-</td><td>Bacteroidetes</td><td>Flavobacterium urumqiense</td><td>leucine-rich repeat domain-containing protein [Flavobacterium urumqiense].</td><td>GCF_900108015.1</td></tr>
<tr><td>WP_104395581.1</td><td>SIG+TM+TM+TM+TM+TM+TM+TM→||<-?||Mbetalac→ SAM-methylase→?→||<-?<-SIG+TPM_phosphatase+TM+TM+TM||LRR-repeats+AP-GTPase+COR+TM+TM*→||<-?<-?<-?<-Pro_CA||?→||<-?<-tRNA<-tRNA<-Ferredoxin-betagrasp</td><td>LRR-repeats+AP-GTPase+COR+TM+TM</td><td>874</td><td>-</td><td>Cyanobacteria</td><td>Microcystis aeruginosa</td><td>leucine-rich repeat protein [Microcystis aeruginosa].</td><td>GCF_002933835.1</td></tr>
<tr><td>WP_104424498.1</td><td>Cluster1018_2clades→?→?→?→ PIN→?→ PIN→ LRR-repeats+AP-GTPase+COR+TIR*→||<-?||Cluster1018_2clades→</td><td>LRR-repeats+AP-GTPase+COR+TIR</td><td>999</td><td>-</td><td>Gammaproteobacteria</td><td>Methylobacter tundripaludum</td><td>TIR domain-containing protein [Methylobacter tundripaludum].</td><td>GCF_002934365.1</td></tr>
<tr><td>WP_104900247.1</td><td>Cluster1074_2clades→||<-?<-Cluster53_2clades||LRR-repeats+AP-GTPase+COR*→||<-?<-?||?→?→ SAM-methylase→</td><td>LRR-repeats+AP-GTPase+COR</td><td>1039</td><td>-</td><td>Cyanobacteria</td><td>Nostoc sp. ‘Peltigera membranacea cyanobiont’ N6</td><td>leucine-rich repeat domain-containing protein [Nostoc sp. ‘Peltigera membranacea cyanobiont’ N6].</td><td>GCF_002949735.1</td></tr>
<tr><td>WP_104904652.1</td><td>LRR-repeats+AP-GTPase+COR*→?→?→||<-ABhydrolase<-Cluster1087_2clades</td><td>LRR-repeats+AP-GTPase+COR</td><td>1097</td><td>-</td><td>Cyanobacteria</td><td>Nostoc sp. ‘Lobaria pulmonaria (5183) cyanobiont’</td><td>leucine-rich repeat domain-containing protein [Nostoc sp. ‘Lobaria pulmonaria (5183) cyanobiont’].</td><td>GCF_002949795.1</td></tr>
<tr><td>WP_104909847.1</td><td>LRR-repeats+AP-GTPase+COR+DUF4404*→</td><td>LRR-repeats+AP-GTPase+COR+DUF4404</td><td>1125</td><td>-</td><td>Cyanobacteria</td><td>Nostoc sp. ‘Lobaria pulmonaria (5183) cyanobiont’</td><td>leucine-rich repeat domain-containing protein [Nostoc sp. ‘Lobaria pulmonaria (5183) cyanobiont’].</td><td>-</td></tr>
<tr><td>WP_105041123.1</td><td>LRR-repeats+AP-GTPase+COR*→</td><td>LRR-repeats+AP-GTPase+COR</td><td>925</td><td>-</td><td>Bacteroidetes</td><td>Flavipsychrobacter stenotrophus</td><td>leucine-rich repeat domain-containing protein [Flavipsychrobacter stenotrophus].</td><td>GCF_002954265.1</td></tr>
<tr><td>WP_106089276.1</td><td>LRR-repeats+AP-GTPase+COR+PNPase*→</td><td>LRR-repeats+AP-GTPase+COR+PNPase</td><td>915</td><td>-</td><td>Deltaproteobacteria</td><td>Enhygromyxa salina</td><td>50S ribosome-binding GTPase [Enhygromyxa salina].</td><td>GCF_002994635.1</td></tr>
<tr><td>WP_106150696.1</td><td>LRR-repeats+AP-GTPase+COR+DUF4404*→</td><td>LRR-repeats+AP-GTPase+COR+DUF4404</td><td>779</td><td>-</td><td>Cyanobacteria</td><td>filamentous cyanobacterium Phorm 46</td><td>leucine-rich repeat domain-containing protein, partial [filamentous cyanobacterium Phorm 46].</td><td>-</td></tr>
<tr><td>WP_106254335.1</td><td>LRR-repeats+AP-GTPase+COR+TIR*→</td><td>LRR-repeats+AP-GTPase+COR+TIR</td><td>930</td><td>-</td><td>Cyanobacteria</td><td>Stenomitos frigidus</td><td>leucine-rich repeat domain-containing protein [Stenomitos frigidus].</td><td>GCF_003003795.1</td></tr>
<tr><td>WP_106256414.1</td><td>LRR-repeats+AP-GTPase+COR*→</td><td>LRR-repeats+AP-GTPase+COR</td><td>961</td><td>-</td><td>Cyanobacteria</td><td>Stenomitos frigidus</td><td>GTPase [Stenomitos frigidus].</td><td>GCF_003003795.1</td></tr>
</table>

acc	operon	architecture	len	gen.name	taxend	species	defline	gca
WP_106289057.1	LRR-repeats+AP-GTPase+COR+DUF4404*→	LRR-repeats+AP-GTPase+COR+DUF4404	918	-	Cyanobacteria	Merismopedia glauca	leucine-rich repeat domain-containing protein [Merismopedia glauca].	GCF_003003775.1
WP_106331563.1	TPR-repeats→?→?→?→ tRNA→||<-PSE<-?||Cluster53_2clades→?→ LRR-repeats+AP-GTPase+COR+DUF4404*→	LRR-repeats+AP-GTPase+COR+DUF4404	1048	-	Cyanobacteria	filamentous cyanobacterium Phorm 6	leucine-rich repeat domain-containing protein [filamentous cyanobacterium Phorm 6].	-
WP_106333130.1	LRR-repeats+AP-GTPase+COR+DUF4404*→?→?→||<-?<-?<-?<-?<-RelE-ParE	LRR-repeats+AP-GTPase+COR+DUF4404	786	-	Cyanobacteria	filamentous cyanobacterium Phorm 6	leucine-rich repeat domain-containing protein, partial [filamentous cyanobacterium Phorm 6].	-
WP_106364139.1	<-ABC-ATPase||?→?→ LRR-repeats+AP-GTPase+COR*→	LRR-repeats+AP-GTPase+COR	1146	-	Actinobacteria	Glycomyces artemisiae	hypothetical protein [Glycomyces artemisiae].	GCF_003002955.1
WP_106623442.1	LRR-repeats+AP-GTPase+COR+TM+TM*→||<-ABC-ATPase	LRR-repeats+AP-GTPase+COR+TM+TM	970	-	Bacteroidetes	Sphingobacteriales bacterium UPWRP_1	leucine-rich repeat domain-containing protein [Sphingobacteriales bacterium UPWRP_1].	-
WP_106872506.1	RelE-ParE→ LRR-repeats+AP-GTPase+COR+TIR*→	LRR-repeats+AP-GTPase+COR+TIR	1126	-	Cyanobacteria	unclassified Cyanobacteria	MULTISPECIES: leucine-rich repeat domain-containing protein [unclassified Cyanobacteria].	-
WP_106922429.1	LRR-repeats+AP-GTPase+COR+TIR*→||<-REase-4	LRR-repeats+AP-GTPase+COR+TIR	826	-	Cyanobacteria	filamentous cyanobacterium CCP3	leucine-rich repeat domain-containing protein [filamentous cyanobacterium CCP3].	-
WP_107346265.1	Arginase→?→||<-?||?→ LRR-repeats+AP-GTPase+COR+TIR*→||<-?<-?<-?<-ABC-ATPase||?→ REC→	LRR-repeats+AP-GTPase+COR+TIR	1173	-	Alphaproteobacteria	Rhodopseudomonas palustris	leucine-rich repeat domain-containing protein [Rhodopseudomonas palustris].	GCF_003031265.1
WP_107357189.1	Arginase→?→||<-?||?→ LRR-repeats+AP-GTPase+COR+TIR*→||<-?<-?<-?<-ABC-ATPase||?→ REC→	LRR-repeats+AP-GTPase+COR+TIR	1173	-	Alphaproteobacteria	Rhodopseudomonas palustris	leucine-rich repeat domain-containing protein [Rhodopseudomonas palustris].	GCF_003031245.1
WP_107867044.1	LRR-repeats+AP-GTPase+COR+GT4*→	LRR-repeats+AP-GTPase+COR+GT4	1510	-	Firmicutes	Agitococcus lubricus	leucine-rich repeat protein [Agitococcus lubricus].	GCF_003051055.1
WP_108091428.1	ABC_membrane+ABC_tran→||<-?||CI→ LRR-repeats+AP-GTPase+COR+TIR*→	LRR-repeats+AP-GTPase+COR+TIR	1243	-	Gammaproteobacteria	Vibrio splendidus	TIR domain-containing protein [Vibrio splendidus].	GCF_003050005.1
WP_108099554.1	<-Cluster1756_2clades<-?<-?<-?<-?||?→?→ LRR-repeats+AP-GTPase+COR*→	LRR-repeats+AP-GTPase+COR	1107	-	Gammaproteobacteria	Vibrio splendidus	50S ribosome-binding GTPase [Vibrio splendidus].	GCF_003050165.1
WP_108112883.1	<-HISKIN<-?||LRR-repeats+AP-GTPase+COR+TIR*→||<-HISKIN<-HISKIN	LRR-repeats+AP-GTPase+COR+TIR	973	-	Bacteroidetes	Kordia periserrulae	leucine-rich repeat domain-containing protein [Kordia periserrulae].	GCF_003054265.1
WP_108114870.1	CHTH→||<-?<-?||RNA-Helicase→?→?→||<-?||LRR-repeats+AP-GTPase+COR+TIR*→||<-?<-?<-?||?→||<-TM+TM+TM	LRR-repeats+AP-GTPase+COR+TIR	1012	-	Bacteroidetes	Kordia periserrulae	leucine-rich repeat domain-containing protein [Kordia periserrulae].	GCF_003054265.1
WP_108176265.1	LRR-repeats+AP-GTPase+COR*→?→ ABC_membrane+ABC_tran→ ABC_membrane+ABC_tran→	LRR-repeats+AP-GTPase+COR	1122	-	Gammaproteobacteria	Vibrio splendidus	hypothetical protein [Vibrio splendidus].	GCF_003050125.1
WP_108207948.1	REC→?→?→?→||<-ABC_membrane+ABC_tran<-ABC_membrane+ABC_tran<-?||LRR-repeats+AP-GTPase+COR*→	LRR-repeats+AP-GTPase+COR	960	-	Gammaproteobacteria	Vibrio splendidus	leucine-rich repeat domain-containing protein [Vibrio splendidus].	GCF_003050345.1
WP_108801834.1	<-REC<-?||LRR-repeats+AP-GTPase+COR+TM+TM*→?→?→?→?→||<-?<-SIG+TM+TM+TM+HISKIN	LRR-repeats+AP-GTPase+COR+TM+TM	1086	-	Bacteroidetes	Aquimarina sp. Aq107	leucine-rich repeat domain-containing protein [Aquimarina sp. Aq107].	GCF_900299505.1
WP_109009086.1	ABhydrolase→?→||<-PSE||LRR-repeats+AP-GTPase+COR+TIR*→||<-?<-?<-?<-?<-SIG+NUDIX	LRR-repeats+AP-GTPase+COR+TIR	1078	-	Cyanobacteria	Nostoc commune	leucine-rich repeat domain-containing protein [Nostoc commune].	GCF_003113895.1

<table>
<tr><th>acc</th><th>operon</th><th>architecture</th><th>len</th><th>gen.name</th><th>taxend</th><th>species</th><th>defline</th><th>gca</th></tr>
<tr><td>WP_109322545.1</td><td>LRR-repeats+AP-GTPase+COR*→?→?→?→ Cluster978_2clades→</td><td>LRR-repeats+AP-GTPase+COR</td><td>841</td><td>-</td><td>Bacteroidetes</td><td>Allopseudarcicella aquatilis</td><td>hypothetical protein [Allopseudarcicella aquatilis].</td><td>GCF_003143535.1</td></tr>
<tr><td>WP_109617479.1</td><td>LRR-repeats+AP-GTPase+COR*→</td><td>LRR-repeats+AP-GTPase+COR</td><td>1124</td><td>-</td><td>Bacteroidetes</td><td>Chryseobacterium oncorhynchi</td><td>leucine-rich repeat domain-containing protein [Chryseobacterium oncorhynchi].</td><td>GCF_002899895.1</td></tr>
<tr><td>WP_109694822.1</td><td>LRR-repeats+AP-GTPase+COR+TIR*→</td><td>LRR-repeats+AP-GTPase+COR+TIR</td><td>1143</td><td>-</td><td>Bacteroidetes</td><td>Chitinophaga deserti</td><td>TIR domain-containing protein [Chitinophaga deserti].</td><td>-</td></tr>
<tr><td>WP_110392243.1</td><td>ABC-ATPase→?→ LRR-repeats+AP-GTPase+COR+TIR*→</td><td>LRR-repeats+AP-GTPase+COR+TIR</td><td>1128</td><td>-</td><td>Betaproteobacteria</td><td>Rivicola pingtungensis</td><td>leucine-rich repeat domain-containing protein [Rivicola pingtungensis].</td><td>GCF_003201855.1</td></tr>
<tr><td>WP_110578614.1</td><td>SIG+TM+TM+TM+TM+TM+TM+TM→||<-PSE||Mbetalac→ SAM-methylase→?→||<-?<-SIG+TPM_phosphatase+TM+TM+TM||LRR-repeats+AP-GTPase+COR+TM+TM*→||<-?<-?<-?<-Pro_CA||?→||<-?<-tRNA<-tRNA<-Ferredoxin-betagrasp</td><td>LRR-repeats+AP-GTPase+COR+TM+TM</td><td>865</td><td>-</td><td>Cyanobacteria</td><td>Microcystis aeruginosa</td><td>leucine-rich repeat domain-containing protein [Microcystis aeruginosa].</td><td>GCF_003206555.1</td></tr>
<tr><td>WP_110984619.1</td><td><-Cluster1569_2clades||Mbetalac→||<-?<-?||?→ Mbetalac→||<-?||LRR-repeats+AP-GTPase+COR*→?→ PIN→||<-Cluster1559_2clades</td><td>LRR-repeats+AP-GTPase+COR</td><td>995</td><td>-</td><td>Cyanobacteria</td><td>Acaryochloris sp. RCC1774</td><td>leucine-rich repeat domain-containing protein [Acaryochloris sp. RCC1774].</td><td>-</td></tr>
<tr><td>WP_111129223.1</td><td>LRR-repeats+AP-GTPase+COR*→</td><td>LRR-repeats+AP-GTPase+COR</td><td>894</td><td>-</td><td>Bacteroidetes</td><td>Mesonia sp. K7</td><td>50S ribosome-binding GTPase [Mesonia sp. K7].</td><td>GCF_003233725.1</td></tr>
<tr><td>WP_111283162.1</td><td>LRR-repeats+AP-GTPase+COR*→||<-?||?→?→||<-?||?→?→||<-cNMPBD</td><td>LRR-repeats+AP-GTPase+COR</td><td>1144</td><td>-</td><td>Bacteroidetes</td><td>Flavobacterium ginsenosidimutans</td><td>leucine-rich repeat domain-containing protein [Flavobacterium ginsenosidimutans].</td><td>GCF_003254625.1</td></tr>
<tr><td>WP_111625904.1</td><td>LRR-repeats+AP-GTPase+COR*→</td><td>LRR-repeats+AP-GTPase+COR</td><td>846</td><td>-</td><td>Bacteroidetes</td><td>Gelidibacter algens</td><td>hypothetical protein [Gelidibacter algens].</td><td>GCF_003259265.1</td></tr>
<tr><td>WP_111845807.1</td><td>LRR-repeats+AP-GTPase+COR*→</td><td>LRR-repeats+AP-GTPase+COR</td><td>812</td><td>-</td><td>Bacteroidetes</td><td>Aequorivita antarctica</td><td>hypothetical protein, partial [Aequorivita antarctica].</td><td>-</td></tr>
<tr><td>WP_112086237.1</td><td>LRR-repeats+AP-GTPase+COR*→</td><td>LRR-repeats+AP-GTPase+COR</td><td>847</td><td>-</td><td>Bacteroidetes</td><td>Flavobacterium lacus</td><td>hypothetical protein [Flavobacterium lacus].</td><td>GCF_003268815.1</td></tr>
<tr><td>WP_112134132.1</td><td>LRR-repeats+AP-GTPase+COR*→?→||<-?<-?<-?<-REC<-HISKIN</td><td>LRR-repeats+AP-GTPase+COR</td><td>1186</td><td>-</td><td>Actinobacteria</td><td>Glycomyces dulcitolivorans</td><td>leucine-rich repeat domain-containing protein [Glycomyces dulcitolivorans].</td><td>GCF_003265355.1</td></tr>
<tr><td>WP_112263949.1</td><td>wHTH-4stranded+TPRs+APATPase+TPR-repeats→?→?→?→ ABC_membrane+ABC_tran→?→?→ LRR-repeats+AP-GTPase+COR*→ COR→</td><td>LRR-repeats+AP-GTPase+COR</td><td>608</td><td>-</td><td>Actinobacteria</td><td>Lentzea terrae</td><td>leucine-rich repeat domain-containing protein [Lentzea terrae].</td><td>GCF_003265345.1</td></tr>
<tr><td>WP_114082655.1</td><td>Cluster5_5clades→ LRR-repeats+AP-GTPase+COR+TM+TM*→||<-CASPASE+TM</td><td>LRR-repeats+AP-GTPase+COR+TM+TM</td><td>904</td><td>-</td><td>Cyanobacteria</td><td>Nostoc sp. ATCC 53789</td><td>leucine-rich repeat domain-containing protein [Nostoc sp. ATCC 53789].</td><td>GCF_003326245.1</td></tr>
<tr><td>WP_114084090.1</td><td>Pentapeptide-repeats→||<-?<-?||?→?→||<-Arginase<-?||LRR-repeats+AP-GTPase+COR+TIR*→?→||<-?||?→?→?→?→ APATPase+TPR+TPR+TPR→</td><td>LRR-repeats+AP-GTPase+COR+TIR</td><td>802</td><td>-</td><td>Cyanobacteria</td><td>Nostoc sp. ATCC 53789</td><td>leucine-rich repeat domain-containing protein [Nostoc sp. ATCC 53789].</td><td>GCF_003326245.1</td></tr>
<tr><td>WP_114085324.1</td><td>ABhydrolase→?→||<-?||LRR-repeats+AP-GTPase+COR+TIR*→||<-?<-?<-?<-SIG+NUDIX</td><td>LRR-repeats+AP-GTPase+COR+TIR</td><td>1069</td><td>-</td><td>Cyanobacteria</td><td>Nostoc sp. ATCC 53789</td><td>leucine-rich repeat domain-containing protein [Nostoc sp. ATCC 53789].</td><td>GCF_003326245.1</td></tr>
<tr><td>WP_114085448.1</td><td>LRR-repeats+AP-GTPase+COR+CASPASE*→?→?→?→ ParA-Soj-PloopNTPase→</td><td>LRR-repeats+AP-GTPase+COR+CASPASE</td><td>1512</td><td>-</td><td>Cyanobacteria</td><td>Nostoc sp. ATCC 53789</td><td>leucine-rich repeat domain-containing protein [Nostoc sp. ATCC 53789].</td><td>GCF_003326245.1</td></tr>
<tr><td>WP_114085638.1</td><td><-ParA-Soj-PloopNTPase||LRR-repeats+AP-GTPase+COR*→</td><td>LRR-repeats+AP-GTPase+COR</td><td>881</td><td>-</td><td>Cyanobacteria</td><td>Nostoc sp. ATCC 53789</td><td>hypothetical protein [Nostoc sp. ATCC 53789].</td><td>GCF_009873495.1</td></tr>
<tr><td>WP_114460382.1</td><td>Cluster1398_2clades→?→ LRR-repeats+AP-GTPase*→</td><td>LRR-repeats+AP-GTPase</td><td>477</td><td>-</td><td>Bacteroidetes</td><td>Runella sp. YX9</td><td>leucine-rich repeat domain-containing protein [Runella sp. YX9].</td><td>GCF_003339505.1</td></tr>
<tr><td>WP_114694555.1</td><td>SIGMA-HTH→?→||<-?||?→?→ LRR-repeats+AP-GTPase+COR*→</td><td>LRR-repeats+AP-GTPase+COR</td><td>865</td><td>-</td><td>Gammaproteobacteria</td><td>Motiliproteus coralliicola</td><td>GTP-binding protein [Motiliproteus coralliicola].</td><td>GCF_003345655.1</td></tr>
</table>

<table>
<thead>
<tr><th>acc</th><th>operon</th><th>architecture</th><th>len</th><th>gen.name</th><th>taxend</th><th>species</th><th>defline</th><th>gca</th></tr>
</thead>
<tbody>
<tr><td>WP_114902638.1</td><td>LRR-repeats+AP-GTPase+COR+DrHyd*→</td><td>LRR-repeats+AP-GTPase+COR+DrHyd</td><td>890</td><td>-</td><td>Bacteroidetes</td><td>Kordia sp. SMS9</td><td>leucine-rich repeat domain-containing protein [Kordia sp. SMS9].</td><td>GCF_003352465.1</td></tr>
<tr><td>WP_114902899.1</td><td>REC→||<-?||?→ LRR-repeats+AP-GTPase+COR+TIR*→||<-RADICAL-SAM<-?<-?<-SIGMA-HTH</td><td>LRR-repeats+AP-GTPase+COR+TIR</td><td>972</td><td>-</td><td>Bacteroidetes</td><td>Kordia sp. SMS9</td><td>leucine-rich repeat domain-containing protein [Kordia sp. SMS9].</td><td>GCF_003352465.1</td></tr>
<tr><td>WP_114904001.1</td><td>LRR-repeats+AP-GTPase+COR+TIR*→</td><td>LRR-repeats+AP-GTPase+COR+TIR</td><td>947</td><td>-</td><td>Bacteroidetes</td><td>Kordia sp. SMS9</td><td>leucine-rich repeat domain-containing protein [Kordia sp. SMS9].</td><td>GCF_003352465.1</td></tr>
<tr><td>WP_115919083.1</td><td>LRR-repeats+AP-GTPase+COR+TIR*→?→?→ PSE→?→||<-?<-tRNA||?→ REC→</td><td>LRR-repeats+AP-GTPase+COR+TIR</td><td>1186</td><td>-</td><td>Bacteroidetes</td><td>Chryseobacterium rhizosphaerae</td><td>TIR domain-containing protein [Chryseobacterium rhizosphaerae].</td><td>GCF_007991415.1</td></tr>
<tr><td>WP_116754014.1</td><td>LRR-repeats+AP-GTPase+COR*→||<-?<-Cluster1163_3clades</td><td>LRR-repeats+AP-GTPase+COR</td><td>857</td><td>-</td><td>Bacteroidetes</td><td>Flavobacterium sp. HTF</td><td>leucine-rich repeat domain-containing protein, partial [Flavobacterium sp. HTF].</td><td>GCF_003105115.1</td></tr>
<tr><td>WP_116856563.1</td><td><-Cluster1512_2clades||?→?→?→ LRR-repeats+AP-GTPase+COR+TIR*→?→?→?→||<-?<-?<-cNMPBD</td><td>LRR-repeats+AP-GTPase+COR+TIR</td><td>1040</td><td>-</td><td>Bacteroidetes</td><td>Chitinophaga silvisoli</td><td>TIR domain-containing protein [Chitinophaga silvisoli].</td><td>GCF_003412465.1</td></tr>
<tr><td>WP_116857273.1</td><td>LRR-repeats+AP-GTPase+COR+TIR→ LRR-repeats+AP-GTPase+COR+TIR→||<-?<-?<-SIGMA-HTH<-SIGMA-HTH||?→ LRR-repeats+AP-GTPase+COR+TIR*→||<-SIG+ABhydrolase</td><td>LRR-repeats+AP-GTPase+COR+TIR</td><td>1390</td><td>-</td><td>Bacteroidetes</td><td>Chitinophaga silvisoli</td><td>leucine-rich repeat domain-containing protein [Chitinophaga silvisoli].</td><td>GCF_003412465.1</td></tr>
<tr><td>WP_116857280.1</td><td>LRR-repeats+AP-GTPase+COR_TIR*→</td><td>LRR-repeats+AP-GTPase+COR_TIR</td><td>1378</td><td>-</td><td>Bacteroidetes</td><td>Chitinophaga silvisoli</td><td>leucine-rich repeat domain-containing protein [Chitinophaga silvisoli].</td><td>GCF_003412465.1</td></tr>
<tr><td>WP_116857281.1</td><td>LRR-repeats+AP-GTPase+COR+TIR*→</td><td>LRR-repeats+AP-GTPase+COR+TIR</td><td>1379</td><td>-</td><td>Bacteroidetes</td><td>Chitinophaga silvisoli</td><td>leucine-rich repeat domain-containing protein [Chitinophaga silvisoli].</td><td>GCF_003412465.1</td></tr>
<tr><td>WP_116982533.1</td><td>PNPase→?→?→?→?→?→ LRR-repeats+AP-GTPase+COR+TIR*→</td><td>LRR-repeats+AP-GTPase+COR+TIR</td><td>956</td><td>-</td><td>Bacteroidetes</td><td>Emticicia sp. C21</td><td>leucine-rich repeat domain-containing protein [Emticicia sp. C21].</td><td>GCF_003418935.1</td></tr>
<tr><td>WP_117208282.1</td><td><-Aminotran_1_2<-?||?→||<-?||?→?→ LRR-repeats+AP-GTPase+COR*→||<-?<-?<-?<-?<-Cluster2179_2clades<-SIG+TM+TM+TM+TM+TM+TM+TM+TM+TM+TM+TM</td><td>LRR-repeats+AP-GTPase+COR</td><td>905</td><td>-</td><td>Actinobacteria</td><td>Allorhizocola rhizosphaerae</td><td>hypothetical protein [Allorhizocola rhizosphaerae].</td><td>GCF_003426775.1</td></tr>
<tr><td>WP_117882884.1</td><td>REC→||<-?<-?<-?<-?||MORC→?→ LRR-repeats+AP-GTPase+COR*→?→ Cluster1438_2clades→</td><td>LRR-repeats+AP-GTPase+COR</td><td>797</td><td>-</td><td>Bacteroidetes</td><td>Lutibacter sp. SM1352</td><td>GTP-binding protein [Lutibacter sp. SM1352].</td><td>GCF_003449015.1</td></tr>
<tr><td>WP_118170224.1</td><td>Cluster5_5clades→ Cluster5_5clades→||<-?||LRR-repeats+AP-GTPase+COR+TM+TM*→||<-CASPASE+TM||?→||<-?<-?||?→||<-?||Cluster1756_2clades→</td><td>LRR-repeats+AP-GTPase+COR+TM+TM</td><td>962</td><td>-</td><td>Cyanobacteria</td><td>Nostoc sphaeroides</td><td>leucine-rich repeat domain-containing protein [Nostoc sphaeroides].</td><td>GCF_003443655.1</td></tr>
<tr><td>WP_118317581.1</td><td>LRR-repeats+AP-GTPase+COR+DrHyd*→</td><td>LRR-repeats+AP-GTPase+COR+DrHyd</td><td>1169</td><td>-</td><td>Bacteroidetes</td><td>Bacteroidales</td><td>MULTISPECIES: leucine-rich repeat domain-containing protein [Bacteroidales].</td><td>GCF_902364465.1</td></tr>
<tr><td>WP_118840037.1</td><td>ABC_membrane+ABC_tran→||<-Cluster2290_2clades<-Cluster2428_2clades<-?<-DSBH<-?||ClpABN-AAA+ClpABC-AAA→ LRR-repeats+AP-GTPase+COR*→||<-?<-HISKIN<-REC</td><td>LRR-repeats+AP-GTPase+COR</td><td>1051</td><td>-</td><td>Bacteroidetes</td><td>Salinibacter ruber</td><td>leucine-rich repeat domain-containing protein [Salinibacter ruber].</td><td>GCF_003491185.1</td></tr>
<tr><td>WP_119405939.1</td><td>SIG+TM+TM+TM+HISKIN→?→?→||<-?<-PSE||?→?→ TIR+LRR-repeats+AP-GTPase+TIR*→</td><td>TIR+LRR-repeats+AP-GTPase+TIR</td><td>1046</td><td>-</td><td>Bacteroidetes</td><td>Mucilaginibacter sp. HYN0043</td><td>TIR domain-containing protein [Mucilaginibacter sp. HYN0043].</td><td>GCF_003576455.2</td></tr>
<tr><td>WP_119406030.1</td><td>LRR-repeats+AP-GTPase+COR+TIR*→</td><td>LRR-repeats+AP-GTPase+COR+TIR</td><td>885</td><td>-</td><td>Bacteroidetes</td><td>Mucilaginibacter sp. HYN0043</td><td>TIR domain-containing protein [Mucilaginibacter sp. HYN0043].</td><td>GCF_003576455.2</td></tr>
<tr><td>WP_119756574.1</td><td>LRR-repeats+AP-GTPase+COR*→</td><td>LRR-repeats+AP-GTPase+COR</td><td>792</td><td>-</td><td>Bacteroidetes</td><td>Chryseolinea soli</td><td>GTP-binding protein [Chryseolinea soli].</td><td>GCF_003589925.1</td></tr>
<tr><td>WP_120086745.1</td><td>Cluster2248_2clades→?→?→ LRR-repeats+AP-GTPase+COR*→</td><td>LRR-repeats+AP-GTPase+COR</td><td>952</td><td>-</td><td>Actinobacteria</td><td>Pseudonocardiaceae bacterium YIM PH 21723</td><td>leucine-rich repeat domain-containing protein [Pseudonocardiaceae bacterium YIM PH 21723].</td><td>-</td></tr>
</tbody>
</table>

<table>
<tr><th>acc</th><th>operon</th><th>architecture</th><th>len</th><th>gen.name</th><th>taxend</th><th>species</th><th>defline</th><th>gca</th></tr>
<tr><td>WP_120519107.1</td><td><-SIG+TM+TM+TM<-?||Cluster1612_2clades→?→||<-?||?→?→ LRR-repeats+AP-GTPase+COR+REase7*→?→?→||<-?||ABC_membrane+ABC_tran→</td><td>LRR-repeats+AP-GTPase+COR+REase7</td><td>971</td><td>-</td><td>Bacteroidetes</td><td>Chitinophaga barathri</td><td>leucine-rich repeat domain-containing protein [Chitinophaga barathri].</td><td>GCF_003614855.1</td></tr>
<tr><td>WP_121068083.1</td><td>LRR-repeats+AP-GTPase+COR*→||<-?<-MoxR-AAA</td><td>LRR-repeats+AP-GTPase+COR</td><td>1055</td><td>-</td><td>Bacteroidetes</td><td>Maribacter vaceletii</td><td>50S ribosome-binding GTPase [Maribacter vaceletii].</td><td>GCF_003634105.1</td></tr>
<tr><td>WP_121165351.1</td><td>SIGMA-HTH→?→?→?→?→?→ LRR-repeats+AP-GTPase+COR+GT4*→?→ ParA-Soj-PloopNTPase→||<-?<-?<-?<-?||Cluster1906_2clades→</td><td>LRR-repeats+AP-GTPase+COR+GT4</td><td>1488</td><td>-</td><td>Betaproteobacteria</td><td>Nitrosomonas sp. Nm120</td><td>leucine-rich repeat domain-containing protein [Nitrosomonas sp. Nm120].</td><td>GCF_003664085.1</td></tr>
<tr><td>WP_121968578.1</td><td>LRR-repeats+AP-GTPase+COR*→</td><td>LRR-repeats+AP-GTPase+COR</td><td>985</td><td>-</td><td>Cyanobacteria</td><td>Leptolyngbya sp. BC1307</td><td>GTPase [Leptolyngbya sp. BC1307].</td><td>GCF_002286735.1</td></tr>
<tr><td>WP_122929305.1</td><td>Cluster1633_2clades→ Cluster1019_2clades→?→?→ Cluster1131_3clades→ FGS→ LRR-repeats+AP-GTPase+COR+TIR*→ TM+TM+TM→?→?→?→?→ tRNA→?→||<-Cluster1496_2clades</td><td>LRR-repeats+AP-GTPase+COR+TIR</td><td>943</td><td>-</td><td>Bacteroidetes</td><td>Runella sp. SP2</td><td>TIR domain-containing protein [Runella sp. SP2].</td><td>GCF_003711225.1</td></tr>
<tr><td>WP_123129115.1</td><td>TIR→ LRR-repeats+AP-GTPase+COR+TIR*→</td><td>LRR-repeats+AP-GTPase+COR+TIR</td><td>802</td><td>-</td><td>Bacteroidetes</td><td>Rufibacter latericius</td><td>TIR domain-containing protein [Rufibacter latericius].</td><td>GCF_003721515.1</td></tr>
<tr><td>WP_123230450.1</td><td>SIG+TM+TM+TM+TM+TM+TM+TM→||<-?||Mbetalac→ SAM-methylase→?→||<-?<-SIG+TPM_phosphatase+TM+TM+TM||LRR-repeats+AP-GTPase+COR+TM+TM*→||<-?<-?<-?<-Pro_CA||?→||<-?<-tRNA<-tRNA<-Ferredoxin-betagrasp</td><td>LRR-repeats+AP-GTPase+COR+TM+TM</td><td>875</td><td>-</td><td>Cyanobacteria</td><td>Microcystis aeruginosa</td><td>leucine-rich repeat domain-containing protein [Microcystis aeruginosa].</td><td>GCF_003730145.1</td></tr>
<tr><td>WP_123277931.1</td><td>LRR-repeats+AP-GTPase+COR*→?→||<-?<-?<-RFC-AAA</td><td>LRR-repeats+AP-GTPase+COR</td><td>891</td><td>-</td><td>Bacteroidetes</td><td>Chryseobacterium cucumeris</td><td>hypothetical protein [Chryseobacterium cucumeris].</td><td>GCF_003729985.1</td></tr>
<tr><td>WP_123852487.1</td><td>LRR-repeats+AP-GTPase+COR+TIR*→</td><td>LRR-repeats+AP-GTPase+COR+TIR</td><td>1174</td><td>-</td><td>Bacteroidetes</td><td>Chryseobacterium shandongense</td><td>leucine-rich repeat domain-containing protein [Chryseobacterium shandongense].</td><td>GCF_003815615.1</td></tr>
<tr><td>WP_123904443.1</td><td>LRR-repeats+AP-GTPase+COR+TIR*→ TIR→</td><td>LRR-repeats+AP-GTPase+COR+TIR</td><td>986</td><td>-</td><td>Bacteroidetes</td><td>Chryseobacterium haifense</td><td>leucine-rich repeat domain-containing protein [Chryseobacterium haifense].</td><td>GCF_003816015.1</td></tr>
<tr><td>WP_124155292.1</td><td>LRR-repeats+AP-GTPase+COR*→</td><td>LRR-repeats+AP-GTPase+COR</td><td>723</td><td>-</td><td>Cyanobacteria</td><td>Okeania hirsuta</td><td>leucine-rich repeat domain-containing protein [Okeania hirsuta].</td><td>GCF_003838195.1</td></tr>
<tr><td>WP_124898852.1</td><td>LRR-repeats+AP-GTPase+COR*→</td><td>LRR-repeats+AP-GTPase+COR</td><td>925</td><td>-</td><td>Bacteroidetes</td><td>Flavobacterium viscosus</td><td>GTP-binding protein [Flavobacterium viscosus].</td><td>GCF_003858535.1</td></tr>
<tr><td>WP_125246445.1</td><td>TM→ LRR-repeats+AP-GTPase+COR*→</td><td>LRR-repeats+AP-GTPase+COR</td><td>1040</td><td>-</td><td>Actinobacteria</td><td>Glycomyces terrestris</td><td>hypothetical protein [Glycomyces terrestris].</td><td>GCF_003933745.1</td></tr>
<tr><td>WP_125249833.1</td><td><-Cluster1244_2clades||?→?→?→||<-?<-ABC-ATPase||?→ LRR-repeats+AP-GTPase+COR*→?→||<-?<-REC<-HISKIN</td><td>LRR-repeats+AP-GTPase+COR</td><td>1253</td><td>-</td><td>Actinobacteria</td><td>Glycomyces terrestris</td><td>leucine-rich repeat domain-containing protein [Glycomyces terrestris].</td><td>GCF_003933745.1</td></tr>
<tr><td>WP_126388556.1</td><td>Cluster54_3clades→?→ RelE-ParE→ LRR-repeats+AP-GTPase+COR*→?→?→ Cluster1841_2clades→</td><td>LRR-repeats+AP-GTPase+COR</td><td>906</td><td>-</td><td>Cyanobacteria</td><td>Pseudanabaena sp. ABRG5-3</td><td>leucine-rich repeat domain-containing protein [Pseudanabaena sp. ABRG5-3].</td><td>GCF_003967015.1</td></tr>
<tr><td>WP_126389050.1</td><td>LRR-repeats→?→?→ LRR-repeats+AP-GTPase+COR+CASPASE*→||<-?||TPR+nSTAND1+BetaPropeller_WD40+BetaPropeller_WD40→?→||<-?||Pentapeptide-repeats→</td><td>LRR-repeats+AP-GTPase+COR+CASPASE</td><td>1062</td><td>-</td><td>Cyanobacteria</td><td>Pseudanabaena sp. ABRG5-3</td><td>leucine-rich repeat domain-containing protein [Pseudanabaena sp. ABRG5-3].</td><td>GCF_003967015.1</td></tr>
<tr><td>WP_126391374.1</td><td>Pentapeptide-repeats→||<-Cluster5_5clades||Cluster1009_2clades→ Cluster53_2clades→?→ LRR-repeats+AP-GTPase+COR+TM+TM*→||<-?<-?<-?<-?<-?||REC→</td><td>LRR-repeats+AP-GTPase+COR+TM+TM</td><td>865</td><td>-</td><td>Cyanobacteria</td><td>Pseudanabaena sp. ABRG5-3</td><td>GTPase [Pseudanabaena sp. ABRG5-3].</td><td>GCF_003967015.1</td></tr>
<tr><td>WP_126618238.1</td><td><-RADICAL-SAM<-?||SIGMA-HTH→?→||<-?||?→?→ LRR-repeats+AP-GTPase+COR+TIR*→?→||<-?||SWACOS→||<-Cluster409_3clades||SIG+RnaseT2→?→ Cluster995_2clades→</td><td>LRR-repeats+AP-GTPase+COR+TIR</td><td>913</td><td>-</td><td>Alphaproteobacteria</td><td>Azospirillum sp. L-25-5w-1</td><td>leucine-rich repeat domain-containing protein [Azospirillum sp. L-25-5w-1].</td><td>GCF_003966125.1</td></tr>
<tr><td>WP_127052641.1</td><td>LRR-repeats+AP-GTPase+COR*→</td><td>LRR-repeats+AP-GTPase+COR</td><td>1083</td><td>-</td><td>Cyanobacteria</td><td>Trichormus variabilis</td><td>leucine-rich repeat domain-containing protein [Trichormus variabilis].</td><td>GCF_003991935.1</td></tr>
<tr><td>WP_127087241.1</td><td>LRR-repeats+AP-GTPase+COR+EAD1*→</td><td>LRR-repeats+AP-GTPase+COR+EAD1</td><td>929</td><td>-</td><td>Cyanobacteria</td><td>Calothrix desertica</td><td>GTPase [Calothrix desertica].</td><td>GCF_003991905.1</td></tr>
<tr><td>WP_127498999.1</td><td>LRR-repeats+AP-GTPase+COR+TIR*→?→?→||<-?<-?||?→||<-Cluster1023_3clades</td><td>LRR-repeats+AP-GTPase+COR+TIR</td><td>999</td><td>-</td><td>Actinobacteria</td><td>Actinoplanes sp. LAM7112</td><td>TIR domain-containing protein [Actinoplanes sp. LAM7112].</td><td>GCF_003999975.1</td></tr>
</table>

<table>
<tr><th>acc</th><th>operon</th><th>architecture</th><th>len</th><th>gen.name</th><th>taxend</th><th>species</th><th>defline</th><th>gca</th></tr>
<tr><td>WP_127504182.1</td><td>LRR-repeats+AP-GTPase+COR*→?→ PSE→||<-?||SIGMA-HTH→</td><td>LRR-repeats+AP-GTPase+COR</td><td>1080</td><td>-</td><td>Actinobacteria</td><td>Actinoplanes sp. LAM7112</td><td>hypothetical protein [Actinoplanes sp. LAM7112].</td><td>GCF_003999975.1</td></tr>
<tr><td>WP_127505072.1</td><td>Cluster1009__2clades→||<-Cluster1999__2clades<-Cluster2165__2clades<-?<-?<-?||?→ LRR-repeats+AP-GTPase+COR*→</td><td>LRR-repeats+AP-GTPase+COR</td><td>1680</td><td>-</td><td>Actinobacteria</td><td>Actinoplanes sp. LAM7112</td><td>leucine-rich repeat domain-containing protein [Actinoplanes sp. LAM7112].</td><td>GCF_003999975.1</td></tr>
<tr><td>WP_129758774.1</td><td>MACRODOMAIN+LRR-repeats+AP-GTPase+COR+TIR*→||<-tRNA<-?<-RNASEII</td><td>MACRODOMAIN+LRR-repeats+AP-GTPase+COR+TIR</td><td>1357</td><td>-</td><td>Bacteroidetes</td><td>Flavobacterium sp. TCH3-2</td><td>leucine-rich repeat domain-containing protein [Flavobacterium sp. TCH3-2].</td><td>GCF_004151275.1</td></tr>
<tr><td>WP_129778498.1</td><td>Cluster1906__2clades→?→||<-?<-?<-?||?→ LRR-repeats+AP-GTPase+COR*→||<-?<-?||?→?→?→||<-tRNA||SIGMA-HTH→</td><td>LRR-repeats+AP-GTPase+COR</td><td>1040</td><td>-</td><td>Gammaproteobacteria</td><td>Steroidobacter soli</td><td>hypothetical protein [Steroidobacter soli].</td><td>GCF_004138485.1</td></tr>
<tr><td>WP_130757642.1</td><td>SIG+TM+TM+TM+TM+TM+TM+TM→||<-?||Mbetalac→ SAM-methylase→?→||<-?<-SIG+TPM__phosphatase+TM+TM+TM||LRR-repeats+AP-GTPase+COR+TM+TM*→||<-?<-?<-?<-Pro_CA||?→||<-?<-tRNA<-tRNA<-Ferredoxin-betagrasp</td><td>LRR-repeats+AP-GTPase+COR+TM+TM</td><td>851</td><td>-</td><td>Cyanobacteria</td><td>Microcystis aeruginosa</td><td>leucine-rich repeat domain-containing protein [Microcystis aeruginosa].</td><td>GCF_004305995.1</td></tr>
<tr><td>WP_130938329.1</td><td>AraC-HTH→?→ SIG+TM+TM+TM→?→?→?→?→ LRR-repeats+AP-GTPase+COR*→?→ ABC__membrane+ABC__tran→||<-?<-?<-?<-?<-Trichomonas-DAM</td><td>LRR-repeats+AP-GTPase+COR</td><td>925</td><td>-</td><td>Bacteroidetes</td><td>Hyunsoonleella pacifica</td><td>leucine-rich repeat domain-containing protein [Hyunsoonleella pacifica].</td><td>GCF_014636335.1</td></tr>
<tr><td>WP_131118878.1</td><td>HNH→?→||<-?<-PIN<-?||LRR-repeats+AP-GTPase+COR+TIR*→||<-Cluster1077__2clades</td><td>LRR-repeats+AP-GTPase+COR+TIR</td><td>912</td><td>-</td><td>Cyanobacteria</td><td>Westiellopsis prolifica</td><td>leucine-rich repeat domain-containing protein, partial [Westiellopsis prolifica].</td><td>GCF_004323185.1</td></tr>
<tr><td>WP_131118899.1</td><td>Cluster1069__2clades→?→?→||<-?||?→?→ PSE→ LRR-repeats→ LRR-repeats+AP-GTPase+COR+DUF4404*→||<-?||?→?→?→?→ Mbetalac→</td><td>LRR-repeats+AP-GTPase+COR+DUF4404</td><td>695</td><td>-</td><td>Cyanobacteria</td><td>Westiellopsis prolifica</td><td>GTP-binding protein, partial [Westiellopsis prolifica].</td><td>GCF_004323185.1</td></tr>
<tr><td>WP_132561933.1</td><td><-Classical-AAA||?→ LRR-repeats+AP-GTPase+COR+TIR*→?→||<-?<-tRNA||?→||<-tRNA<-?||?→||<-?||ABC-ATPase→</td><td>LRR-repeats+AP-GTPase+COR+TIR</td><td>1348</td><td>-</td><td>Bacteroidetes</td><td>Niastella sp. CF465</td><td>TIR domain-containing protein [Niastella sp. CF465].</td><td>GCF_004341975.1</td></tr>
<tr><td>WP_132562618.1</td><td>LRR-repeats+AP-GTPase+COR*→</td><td>LRR-repeats+AP-GTPase+COR</td><td>1028</td><td>-</td><td>Bacteroidetes</td><td>Niastella sp. CF465</td><td>GTP-binding protein [Niastella sp. CF465].</td><td>GCF_004341975.1</td></tr>
<tr><td>WP_132566699.1</td><td>SIGMA-HTH→ LRR-repeats+AP-GTPase+COR*→</td><td>LRR-repeats+AP-GTPase+COR</td><td>890</td><td>-</td><td>Bacteroidetes</td><td>Niastella sp. CF465</td><td>leucine-rich repeat domain-containing protein [Niastella sp. CF465].</td><td>GCF_004341975.1</td></tr>
<tr><td>WP_132835660.1</td><td>LRR-repeats+AP-GTPase+COR*→</td><td>LRR-repeats+AP-GTPase+COR</td><td>819</td><td>-</td><td>Actinobacteria</td><td>Streptomyces sp. BK205</td><td>hypothetical protein [Streptomyces sp. BK205].</td><td>GCF_004341025.1</td></tr>
<tr><td>WP_133504241.1</td><td>Cluster2322__2clades→||<-?||PSE→||<-?||LRR-repeats+AP-GTPase+COR+TIR*→</td><td>LRR-repeats+AP-GTPase+COR+TIR</td><td>764</td><td>-</td><td>Gammaproteobacteria</td><td>Marinomonas balearica</td><td>TIR domain-containing protein [Marinomonas balearica].</td><td>GCF_004362145.1</td></tr>
<tr><td>WP_133511071.1</td><td>Cluster2079__2clades→||<-?||?→||<-SIG+TM+TM+TM+TM+TM+TM+TM+TM+TM+TM+TM<-SIG+TM||PSE→ LRR-repeats+AP-GTPase+COR+TIR*→</td><td>LRR-repeats+AP-GTPase+COR+TIR</td><td>986</td><td>-</td><td>Gammaproteobacteria</td><td>Candidatus Thiosymbion oneisti</td><td>TIR domain-containing protein [Candidatus Thiosymbion oneisti].</td><td>GCF_011392785.1</td></tr>
<tr><td>WP_133512972.1</td><td><-PIN<-?||ABC-ATPase→?→?→?→?→ LRR-repeats+AP-GTPase+COR+DrHyd*→</td><td>LRR-repeats+AP-GTPase+COR+DrHyd</td><td>998</td><td>-</td><td>Gammaproteobacteria</td><td>Candidatus Thiosymbion oneisti</td><td>hypothetical protein [Candidatus Thiosymbion oneisti].</td><td>GCF_011392785.1</td></tr>
<tr><td>WP_133793944.1</td><td>LRR-repeats→ LRR-repeats+AP-GTPase+COR+DUF4404*→</td><td>LRR-repeats+AP-GTPase+COR+DUF4404</td><td>768</td><td>-</td><td>Verrucomicrobia</td><td>Prosthecobacter fusiformis</td><td>GTP-binding protein [Prosthecobacter fusiformis].</td><td>GCF_004364345.1</td></tr>
<tr><td>WP_133884327.1</td><td>LRR-repeats+AP-GTPase+COR*→</td><td>LRR-repeats+AP-GTPase+COR</td><td>996</td><td>-</td><td>Actinobacteria</td><td>unclassified Glycomyces</td><td>MULTISPECIES: leucine-rich repeat domain-containing protein [unclassified Glycomyces].</td><td>GCF_000719515.1</td></tr>
<tr><td>WP_133903373.1</td><td>Cluster2083__2clades→||<-?||?→||<-?||?→||<-?||LRR-repeats→ LRR-repeats+AP-GTPase+COR*→</td><td>LRR-repeats+AP-GTPase+COR</td><td>1284</td><td>-</td><td>Actinobacteria</td><td>Actinophytocola oryzae</td><td>leucine-rich repeat domain-containing protein [Actinophytocola oryzae].</td><td>GCF_004364325.1</td></tr>
<tr><td>WP_135106965.1</td><td>LRR-repeats+AP-GTPase+COR+DUF4404*→||<-?||?→?→?→?→?→ Mbetalac→</td><td>LRR-repeats+AP-GTPase+COR+DUF4404</td><td>967</td><td>-</td><td>Cyanobacteria</td><td>Mastigocladus laminosus</td><td>leucine-rich repeat domain-containing protein [Mastigocladus laminosus].</td><td>GCF_001990805.2</td></tr>
<tr><td>WP_135107787.1</td><td><-PIN<-?||LRR-repeats+AP-GTPase+COR+TIR*→||<-Cluster1077__2clades</td><td>LRR-repeats+AP-GTPase+COR+TIR</td><td>1167</td><td>-</td><td>Cyanobacteria</td><td>Mastigocladus laminosus</td><td>leucine-rich repeat domain-containing protein [Mastigocladus laminosus].</td><td>GCF_001990805.2</td></tr>
<tr><td>WP_135191212.1</td><td>LRR-repeats+AP-GTPase+COR+DrHyd*→?→?→?→||<-?<-SIG+Phytase-like</td><td>LRR-repeats+AP-GTPase+COR+DrHyd</td><td>1147</td><td>-</td><td>Betaproteobacteria</td><td>Massilia sp. ONC3</td><td>hypothetical protein [Massilia sp. ONC3].</td><td>GCF_004614195.1</td></tr>
</table>

<table>
<thead>
<tr><th>acc</th><th>operon</th><th>architecture</th><th>len</th><th>gen.name</th><th>taxend</th><th>species</th><th>defline</th><th>gca</th></tr>
</thead>
<tbody>
<tr><td>WP_136836096.1</td><td>LRR-repeats+AP-GTPase+COR+TIR*→</td><td>LRR-repeats+AP-GTPase+COR+TIR</td><td>1357</td><td>-</td><td>Bacteroidetes</td><td>Pedobacter sp. RP-3-15</td><td>leucine-rich repeat domain-containing protein [Pedobacter sp. RP-3-15].</td><td>GCF_005116445.1</td></tr>
<tr><td>WP_136929189.1</td><td>SIG+TM+TM+TM+TM+TM+TM+TM+TM+TM→ LRR-repeats+AP-GTPase+COR*→</td><td>LRR-repeats+AP-GTPase+COR</td><td>961</td><td>-</td><td>Deltaproteobacteria</td><td>Polyangium fumosum</td><td>GTP-binding protein [Polyangium fumosum].</td><td>GCF_005144585.1</td></tr>
<tr><td>WP_137669597.1</td><td>LRR-repeats+AP-GTPase+COR*→</td><td>LRR-repeats+AP-GTPase+COR</td><td>272</td><td>-</td><td>Cyanobacteria</td><td>Sphaerospermopsis reniformis</td><td>GTP-binding protein, partial [Sphaerospermopsis reniformis].</td><td>GCF_005402885.1</td></tr>
<tr><td>WP_137686407.1</td><td>LRR-repeats+AP-GTPase+COR*→</td><td>LRR-repeats+AP-GTPase+COR</td><td>1152</td><td>-</td><td>Chloroflexi</td><td>Thermosporothrix hazakensis</td><td>leucine-rich repeat domain-containing protein [Thermosporothrix hazakensis].</td><td>GCF_005402645.1</td></tr>
<tr><td>WP_137908463.1</td><td>LRR-repeats+AP-GTPase+COR+TM+TM*→||<-PSE||?→?→||<-PSE<-?||CCTBP→</td><td>LRR-repeats+AP-GTPase+COR+TM+TM</td><td>875</td><td>-</td><td>Cyanobacteria</td><td>Dolichospermum planctonicum</td><td>leucine-rich repeat domain-containing protein [Dolichospermum planctonicum].</td><td>GCF_005402965.1</td></tr>
<tr><td>WP_138500060.1</td><td><-Pkinase+Pentapeptide<-?<-?||PSE→||<-?||LRR-repeats+AP-GTPase+COR+TIR*→||<-?<-?<-PSE||?→||<-?||?→?→||<-NUDIX</td><td>LRR-repeats+AP-GTPase+COR+TIR</td><td>920</td><td>-</td><td>Cyanobacteria</td><td>Nostoc sp. PA-18-2419</td><td>leucine-rich repeat domain-containing protein [Nostoc sp. PA-18-2419].</td><td>GCF_005869855.1</td></tr>
<tr><td>WP_138503290.1</td><td><-HhH-RADC+JAB||?→?→||<-?||LRR-repeats→ LRR-repeats+AP-GTPase+COR*→||<-?||?→?→||<-PSE<-?||?→?→ PEP-utilisers-C→</td><td>LRR-repeats+AP-GTPase+COR</td><td>1100</td><td>-</td><td>Cyanobacteria</td><td>Nostoc sp. PA-18-2419</td><td>leucine-rich repeat domain-containing protein [Nostoc sp. PA-18-2419].</td><td>GCF_005869855.1</td></tr>
<tr><td>WP_138504428.1</td><td><-ABC-ATPase<-?<-?<-?||?→ TM+TM+TM→ LRR-repeats+AP-GTPase+COR+TIR*→ PSE→||<-PSE<-?||PSE→?→?→||<-SIG+TM+TM+TM+TM+TM</td><td>LRR-repeats+AP-GTPase+COR+TIR</td><td>1019</td><td>-</td><td>Bacteroidetes</td><td>Spirosoma lacussanchae</td><td>TIR domain-containing protein [Spirosoma lacussanchae].</td><td>GCF_005870035.1</td></tr>
<tr><td>WP_138544765.1</td><td>LRR-repeats+AP-GTPase+COR+TIR*→||<-?<-?<-TM</td><td>LRR-repeats+AP-GTPase+COR+TIR</td><td>1128</td><td>-</td><td>Gammaproteobacteria</td><td>Pseudoalteromonas rubra</td><td>leucine-rich repeat domain-containing protein [Pseudoalteromonas rubra].</td><td>GCF_005887115.1</td></tr>
<tr><td>WP_138544832.1</td><td><-REC<-?||?→ LRR-repeats+AP-GTPase+COR+TIR*→</td><td>LRR-repeats+AP-GTPase+COR+TIR</td><td>1256</td><td>-</td><td>Gammaproteobacteria</td><td>Pseudoalteromonas rubra</td><td>TIR domain-containing protein [Pseudoalteromonas rubra].</td><td>GCF_005887115.1</td></tr>
<tr><td>WP_138553289.1</td><td><-REC<-?||?→ LRR-repeats+AP-GTPase+COR+TIR*→</td><td>LRR-repeats+AP-GTPase+COR+TIR</td><td>1255</td><td>-</td><td>Gammaproteobacteria</td><td>Pseudoalteromonas rubra</td><td>TIR domain-containing protein [Pseudoalteromonas rubra].</td><td>GCF_005887405.1</td></tr>
<tr><td>WP_139281522.1</td><td>LRR-repeats+AP-GTPase+COR+TIR*→</td><td>LRR-repeats+AP-GTPase+COR+TIR</td><td>850</td><td>-</td><td>Gammaproteobacteria</td><td>Vibrio aerogenes</td><td>leucine-rich repeat domain-containing protein, partial [Vibrio aerogenes].</td><td>GCF_900130105.1</td></tr>
<tr><td>WP_139999876.1</td><td>TM+TM+TM→ LRR-repeats+AP-GTPase+COR+TIR*→</td><td>LRR-repeats+AP-GTPase+COR+TIR</td><td>1134</td><td>-</td><td>Bacteroidetes</td><td>Flavobacterium microcysteis</td><td>leucine-rich repeat domain-containing protein [Flavobacterium microcysteis].</td><td>GCF_006385255.1</td></tr>
<tr><td>WP_140058884.1</td><td>LRR-repeats+AP-GTPase+COR+TIR*→</td><td>LRR-repeats+AP-GTPase+COR+TIR</td><td>1265</td><td>-</td><td>Gammaproteobacteria</td><td>Vibrio parahaemolyticus</td><td>TIR domain-containing protein [Vibrio parahaemolyticus].</td><td>GCF_006376835.1</td></tr>
<tr><td>WP_140063885.1</td><td>LRR-repeats+AP-GTPase+COR*→</td><td>LRR-repeats+AP-GTPase+COR</td><td>553</td><td>-</td><td>Gammaproteobacteria</td><td>Vibrio parahaemolyticus</td><td>hypothetical protein, partial [Vibrio parahaemolyticus].</td><td>GCF_006377025.1</td></tr>
<tr><td>WP_140305225.1</td><td>CI→ LRR-repeats+AP-GTPase+COR*→</td><td>LRR-repeats+AP-GTPase+COR</td><td>1113</td><td>-</td><td>Gammaproteobacteria</td><td>Vibrio parahaemolyticus</td><td>GTP-binding protein [Vibrio parahaemolyticus].</td><td>GCF_006374225.1</td></tr>
<tr><td>WP_140384737.1</td><td>LRR-repeats+AP-GTPase+COR+TIR*→</td><td>LRR-repeats+AP-GTPase+COR+TIR</td><td>829</td><td>-</td><td>Bacteroidetes</td><td>Elizabethkingia anophelis</td><td>TIR domain-containing protein, partial [Elizabethkingia anophelis].</td><td>GCF_900156995.1</td></tr>
<tr><td>WP_141211867.1</td><td>LRR-repeats+AP-GTPase+COR+CASPASE*→||<-?||Cluster1731_2clades→?→||<-Cluster2482_2clades</td><td>LRR-repeats+AP-GTPase+COR+CASPASE</td><td>835</td><td>-</td><td>Cyanobacteria</td><td>Pseudanabaena sp. SR411</td><td>leucine-rich repeat domain-containing protein, partial [Pseudanabaena sp. SR411].</td><td>GCF_002251945.1</td></tr>
<tr><td>WP_141954907.1</td><td>LRR-repeats+AP-GTPase+COR*→</td><td>LRR-repeats+AP-GTPase+COR</td><td>1074</td><td>-</td><td>Actinobacteria</td><td>Actinoallomurus bryophytorum</td><td>hypothetical protein [Actinoallomurus bryophytorum].</td><td>GCF_006716425.1</td></tr>
<tr><td>WP_142449077.1</td><td>LRR-repeats+AP-GTPase+COR+TIR*→ TIR→</td><td>LRR-repeats+AP-GTPase+COR+TIR</td><td>700</td><td>-</td><td>Bacteroidetes</td><td>Flavobacterium resistens</td><td>leucine-rich repeat domain-containing protein [Flavobacterium resistens].</td><td>GCF_900182645.1</td></tr>
</tbody>
</table>

acc	operon	architecture	len	gen.name	taxend	species	defline	gca
WP_142602418.1	LRR-repeats+AP-GTPase+COR*→ PSE→ PSE→||<-?||?→||<-?<-?<-Cluster2042_2clades	LRR-repeats+AP-GTPase+COR	718	-	Cyanobacteria	Pseudanabaena sp. UWO310	leucine-rich repeat domain-containing protein [Pseudanabaena sp. UWO310].	GCF_006861605.1
WP_142653260.1	LRR-repeats→ LRR-repeats+AP-GTPase+COR+CASPASE*→?→?→||<-?<-?<-REC	LRR-repeats+AP-GTPase+COR+CASPASE	1028	-	Cyanobacteria	Pseudanabaena sp. UWO311	leucine-rich repeat domain-containing protein [Pseudanabaena sp. UWO311].	GCF_006937785.1
WP_142654307.1	Cluster54_3clades→?→?→?→ LRR-repeats+AP-GTPase+COR+CASPASE*→?→||<-?<-TPR-repeats+CASPASE<-TPR-repeats+CASPASE	LRR-repeats+AP-GTPase+COR+CASPASE	1096	-	Cyanobacteria	Pseudanabaena sp. UWO311	leucine-rich repeat domain-containing protein [Pseudanabaena sp. UWO311].	GCF_006937785.1
WP_142657357.1	ABC-ATPase→?→?→?→?→ Cluster1009_2clades→ Cluster53_2clades→ LRR-repeats+AP-GTPase+COR*→||<-?||?→?→?→ SIG+TM+TM+TM+TM+TM→	LRR-repeats+AP-GTPase+COR	992	-	Cyanobacteria	Pseudanabaena sp. UWO311	leucine-rich repeat domain-containing protein [Pseudanabaena sp. UWO311].	GCF_006937785.1
WP_142891598.1	LRR-repeats+AP-GTPase+COR+DrHyd*→	LRR-repeats+AP-GTPase+COR+DrHyd	1516	-	Gammaproteobacteria	Aliikangiella sp. M105	hypothetical protein [Aliikangiella sp. M105].	GCF_007004725.1
WP_143234350.1	<-REC<-?||?→ LRR-repeats+AP-GTPase+COR+TIR*→	LRR-repeats+AP-GTPase+COR+TIR	888	-	Actinobacteria	Actinoplanes atraurantiacus	GTPase [Actinoplanes atraurantiacus].	GCF_900215205.1
WP_143288104.1	LRR-repeats+AP-GTPase+COR*→||<-SIG+TM+TM+TM+TM	LRR-repeats+AP-GTPase+COR	716	-	Cyanobacteria	Calothrix rhizosoleniae	GTP-binding protein, partial [Calothrix rhizosoleniae].	GCF_900185595.1
WP_143288141.1	LRR-repeats+AP-GTPase+COR+TM+TM*→	LRR-repeats+AP-GTPase+COR+TM+TM	777	-	Cyanobacteria	Calothrix rhizosoleniae	leucine-rich repeat domain-containing protein, partial [Calothrix rhizosoleniae].	GCF_900185595.1
WP_143301191.1	LRR-repeats+AP-GTPase+COR+DUF4404*→?→||<-RNA-Helicase	LRR-repeats+AP-GTPase+COR+DUF4404	650	-	Nitrospinae/Tectomicrobia group	Candidatus Entotheonella palauensis	GTP-binding protein, partial [Candidatus Entotheonella palauensis].	GCF_900079105.1
WP_143674573.1	LRR-repeats+AP-GTPase+COR*→	LRR-repeats+AP-GTPase+COR	802	-	Actinobacteria	Streptomyces sp. HG99	hypothetical protein, partial [Streptomyces sp. HG99].	GCF_002742045.1
WP_143814854.1	SIGMA-HTH→?→||<-?<-?<-?<-?<-?||LRR-repeats+AP-GTPase+COR*→	LRR-repeats+AP-GTPase+COR	861	-	Alphaproteobacteria	Magnetofaba australis	hypothetical protein [Magnetofaba australis].	GCF_002109495.1
WP_144282986.1	LRR-repeats+AP-GTPase+COR+TIR*→?→?→?→?→||<-?<-?<-?<-HISKIN	LRR-repeats+AP-GTPase+COR+TIR	1293	-	Bacteroidetes	Chryseobacterium echinoideorum	leucine-rich repeat domain-containing protein [Chryseobacterium echinoideorum].	GCF_007474535.1
WP_144984133.1	TIR+APATPase+TPR+TPR+TPR→ LRR-repeats+AP-GTPase+COR+TM+TM+TM+TM*→	LRR-repeats+AP-GTPase+COR+TM+TM+TM+TM	1242	-	Planctomycetes	Gimesia aquarii	leucine-rich repeat domain-containing protein [Gimesia aquarii].	GCF_007748195.1
WP_145277465.1	LRR-repeats+AP-GTPase+COR+DUF4404*→	LRR-repeats+AP-GTPase+COR+DUF4404	1013	-	Planctomycetes	Planctomycetes bacterium ElP	leucine-rich repeat domain-containing protein [Planctomycetes bacterium ElP].	-
WP_145532816.1	CI→ LRR-repeats+AP-GTPase+COR*→	LRR-repeats+AP-GTPase+COR	1113	-	Gammaproteobacteria	Vibrio sp. ES.045	GTP-binding protein [Vibrio sp. ES.045].	GCF_007827265.1
WP_145988187.1	<-Classical-AAA<-Trichomonas-DAM||LRR-repeats+AP-GTPase*→||<-?<-Cluster1368_2clades<-Cluster2311_2clades	LRR-repeats+AP-GTPase	621	-	Alphaproteobacteria	Blastochloris tepida	leucine-rich repeat domain-containing protein [Blastochloris tepida].	GCF_003966715.1
WP_146033967.1	LRR-repeats+AP-GTPase+COR+DUF4404*→	LRR-repeats+AP-GTPase+COR+DUF4404	700	-	Cyanobacteria	Nostoc cycadae	GTP-binding protein, partial [Nostoc cycadae].	GCF_002897135.1
WP_146129808.1	LRR-repeats→ LRR-repeats+AP-GTPase+COR*→	LRR-repeats+AP-GTPase+COR	715	-	Bacteroidetes	Muricauda pacifica	hypothetical protein [Muricauda pacifica].	GCF_003001695.1
WP_146156812.1	TPR-repeats→?→ SIG+Alba→||<-?<-?<-?<-?||LRR-repeats+AP-GTPase+COR*→	LRR-repeats+AP-GTPase+COR	857	-	Bacteroidetes	Sphingobacteriales bacterium UPWRP_1	GTP-binding protein [Sphingobacteriales bacterium UPWRP_1].	-
WP_146201360.1	LRR-repeats+AP-GTPase+COR+TIR*→	LRR-repeats+AP-GTPase+COR+TIR	780	-	Gammaproteobacteria	Leucothrix arctica	TIR domain-containing protein [Leucothrix arctica].	GCF_003172895.1

<table>
<thead>
<tr><th>acc</th><th>operon</th><th>architecture</th><th>len</th><th>gen.name</th><th>taxend</th><th>species</th><th>defline</th><th>gca</th></tr>
</thead>
<tbody>
<tr><td>WP_146215520.1</td><td>LRR-repeats+AP-GTPase+COR+TM+TM+TM*→||<-PSE<-?<-?<-?<-?<-Cluster1270_2clades</td><td>LRR-repeats+AP-GTPase+COR+TM+TM+TM</td><td>1504</td><td>-</td><td>Alphaproteobacteria</td><td>Hoeflea marina</td><td>hypothetical protein [Hoeflea marina].</td><td>GCF_003182275.1</td></tr>
<tr><td>WP_146505417.1</td><td>LRR-repeats→ LRR-repeats+AP-GTPase+COR*→</td><td>LRR-repeats+AP-GTPase+COR</td><td>795</td><td>-</td><td>Chloroflexi</td><td>Thermosporothrix hazakensis</td><td>GTPase [Thermosporothrix hazakensis].</td><td>GCF_003253565.1</td></tr>
<tr><td>WP_146848114.1</td><td>LRR-repeats+AP-GTPase+COR*→</td><td>LRR-repeats+AP-GTPase+COR</td><td>846</td><td>-</td><td>Bacteroidetes</td><td>Aequorivita antarctica</td><td>hypothetical protein [Aequorivita antarctica].</td><td>GCF_007997155.1</td></tr>
<tr><td>WP_146849445.1</td><td>SIG+TM→?→ LRR-repeats+AP-GTPase+COR*→</td><td>LRR-repeats+AP-GTPase+COR</td><td>1099</td><td>-</td><td>Verrucomicrobia</td><td>Brevifollis gellanilyticus</td><td>leucine-rich repeat domain-containing protein [Brevifollis gellanilyticus].</td><td>GCF_007992435.1</td></tr>
<tr><td>WP_146856190.1</td><td>LRR-repeats+AP-GTPase+COR+DUF4404*→</td><td>LRR-repeats+AP-GTPase+COR+DUF4404</td><td>944</td><td>-</td><td>Verrucomicrobia</td><td>Brevifollis gellanilyticus</td><td>leucine-rich repeat domain-containing protein [Brevifollis gellanilyticus].</td><td>GCF_007992435.1</td></tr>
<tr><td>WP_147290429.1</td><td><-Aminotran_1_2<-?||?→?→||<-Arginase||?→ LRR-repeats+AP-GTPase+COR+TIR*→||<-?<-PSE<-?<-?<-?||?→||<-Cluster1063_3clades<-Cluster1067_3clades</td><td>LRR-repeats+AP-GTPase+COR+TIR</td><td>1134</td><td>-</td><td>Alphaproteobacteria</td><td>Pannonibacter phragmitetus</td><td>leucine-rich repeat domain-containing protein [Pannonibacter phragmitetus].</td><td>GCF_000382365.1</td></tr>
<tr><td>WP_147864606.1</td><td>LRR-repeats+AP-GTPase+COR+TIR*→</td><td>LRR-repeats+AP-GTPase+COR+TIR</td><td>1297</td><td>-</td><td>Bacteroidetes</td><td>Mesonia sp. K4-1</td><td>leucine-rich repeat domain-containing protein [Mesonia sp. K4-1].</td><td>GCF_008017825.1</td></tr>
<tr><td>WP_148270831.1</td><td>35exo→||<-?<-?||?→?→?→ LRR-repeats+AP-GTPase+COR+TIR*→||<-?<-?<-ABC-ATPase+APATPase+ABC-ATPase<-?||SIGMA-HTH→</td><td>LRR-repeats+AP-GTPase+COR+TIR</td><td>935</td><td>-</td><td>Bacteroidetes</td><td>Haliscomenobacter hydrossis</td><td>leucine-rich repeat domain-containing protein [Haliscomenobacter hydrossis].</td><td>GCF_000212735.1</td></tr>
<tr><td>WP_148270849.1</td><td>LRR-repeats+AP-GTPase+COR+EAD11*→?→?→?→?→?→?→ UvsW-A18→</td><td>LRR-repeats+AP-GTPase+COR+EAD11</td><td>1113</td><td>-</td><td>Bacteroidetes</td><td>Haliscomenobacter hydrossis</td><td>GTP-binding protein [Haliscomenobacter hydrossis].</td><td>GCF_000212735.1</td></tr>
<tr><td>WP_148270854.1</td><td>MACRODOMAIN→?→ LRR-repeats+AP-GTPase+COR+EAD11*→</td><td>LRR-repeats+AP-GTPase+COR+EAD11</td><td>1041</td><td>-</td><td>Bacteroidetes</td><td>Haliscomenobacter hydrossis</td><td>Miro domain-containing protein [Haliscomenobacter hydrossis].</td><td>GCF_000212735.1</td></tr>
<tr><td>WP_148705297.1</td><td><-HhH-RADC+JAB||?→ NTP_transf_3→ LRR-repeats+AP-GTPase+COR*→</td><td>LRR-repeats+AP-GTPase+COR</td><td>1056</td><td>-</td><td>Euryarchaeota</td><td>Methanosarcina siciliae</td><td>leucine-rich repeat domain-containing protein [Methanosarcina siciliae].</td><td>GCF_000970125.1</td></tr>
<tr><td>WP_149986249.1</td><td>SIG+TM+TM+TM+TM+TM+TM+TM→||<-?||Mbetalac→ SAM-methylase→?→||<-?<-SIG+TPM_phosphatase+TM+TM+TM||LRR-repeats+AP-GTPase+COR+TM+TM*→</td><td>LRR-repeats+AP-GTPase+COR+TM+TM</td><td>829</td><td>-</td><td>Cyanobacteria</td><td>Microcystis aeruginosa</td><td>leucine-rich repeat protein [Microcystis aeruginosa].</td><td>GCF_008579325.1</td></tr>
<tr><td>WP_150032603.1</td><td>Cluster1550_2clades→?→?→?→?→||<-Cluster995_2clades||tRNA→ LRR-repeats+AP-GTPase+COR+TIR*→</td><td>LRR-repeats+AP-GTPase+COR+TIR</td><td>1161</td><td>-</td><td>Bacteroidetes</td><td>Taibaiella sp. KVB11</td><td>leucine-rich repeat domain-containing protein [Taibaiella sp. KVB11].</td><td>GCF_008629695.1</td></tr>
<tr><td>WP_150875462.1</td><td><-ABC-ATPase<-?<-?||?→?→ LRR-repeats+AP-GTPase+COR+TIR*→</td><td>LRR-repeats+AP-GTPase+COR+TIR</td><td>976</td><td>-</td><td>Bacteroidetes</td><td>Larkinella sp. MA1</td><td>leucine-rich repeat domain-containing protein [Larkinella sp. MA1].</td><td>GCF_008727875.1</td></tr>
<tr><td>WP_150977944.1</td><td>CHTH→ LRR-repeats+AP-GTPase+COR+TM+TM*→</td><td>LRR-repeats+AP-GTPase+COR+TM+TM</td><td>796</td><td>-</td><td>Cyanobacteria</td><td>Microcystis aeruginosa</td><td>leucine-rich repeat domain-containing protein [Microcystis aeruginosa].</td><td>GCF_008757435.1</td></tr>
<tr><td>WP_151015117.1</td><td>LRR-repeats+AP-GTPase+COR*→</td><td>LRR-repeats+AP-GTPase+COR</td><td>946</td><td>-</td><td>Actinobacteria</td><td>Micromonospora aurantiaca</td><td>leucine-rich repeat domain-containing protein [Micromonospora aurantiaca].</td><td>GCF_008806405.1</td></tr>
<tr><td>WP_151968636.1</td><td>LRR-repeats+AP-GTPase+COR*→</td><td>LRR-repeats+AP-GTPase+COR</td><td>803</td><td>-</td><td>Planctomycetes</td><td>Planctomycetes bacterium SRT547</td><td>leucine-rich repeat domain-containing protein [Planctomycetes bacterium SRT547].</td><td>-</td></tr>
<tr><td>WP_152037914.1</td><td>LRR-repeats+AP-GTPase+COR*→</td><td>LRR-repeats+AP-GTPase+COR</td><td>919</td><td>-</td><td>Actinobacteria</td><td>Micromonospora sp. B006</td><td>leucine-rich repeat domain-containing protein [Micromonospora sp. B006].</td><td>GCF_003408515.1</td></tr>
<tr><td>WP_152532113.1</td><td>LRR-repeats+AP-GTPase+COR*→</td><td>LRR-repeats+AP-GTPase+COR</td><td>814</td><td>-</td><td>Cyanobacteria</td><td>Leptolyngbya sp. Heron Island J</td><td>leucine-rich repeat domain-containing protein [Leptolyngbya sp. Heron Island J].</td><td>-</td></tr>
</tbody>
</table>

<table>
<tr><th>acc</th><th>operon</th><th>architecture</th><th>len</th><th>gen.name</th><th>taxend</th><th>species</th><th>defline</th><th>gca</th></tr>
<tr><td>WP_152589868.1</td><td><-Ferretin<-PSE<-?||?→||<-Ferredoxin-betagrasp<-PSE<-?||LRR-repeats+AP-GTPase+COR*→?→||<-?||?→||<-?||PSE→ PSE→ ClpABN-AAA+ClpABC-AAA→</td><td>LRR-repeats+AP-GTPase+COR</td><td>993</td><td>-</td><td>Cyanobacteria</td><td>Nostoc sphaeroides</td><td>leucine-rich repeat domain-containing protein [Nostoc sphaeroides].</td><td>GCF_009372195.1</td></tr>
<tr><td>WP_152648774.1</td><td>NUDIX→?→?→ LRR-repeats+AP-GTPase+COR*→?→?→||<-?<-?||?→||<-Cluster2230__2clades</td><td>LRR-repeats+AP-GTPase+COR</td><td>954</td><td>-</td><td>Actinobacteria</td><td>Streptacidiphilus anmyonensis</td><td>hypothetical protein [Streptacidiphilus anmyonensis].</td><td>GCF_000787855.1</td></tr>
<tr><td>WP_153009609.1</td><td><-Cluster1102__3clades<-Cluster1102__3clades||?→?→?→||<-Cluster5__5clades<-?||LRR-repeats+AP-GTPase+COR*→</td><td>LRR-repeats+AP-GTPase+COR</td><td>997</td><td>-</td><td>Cyanobacteria</td><td>Mastigocoleus testarum</td><td>leucine-rich repeat domain-containing protein [Mastigocoleus testarum].</td><td>GCF_001456025.1</td></tr>
<tr><td>WP_153023587.1</td><td>TM→ LRR-repeats+AP-GTPase+COR*→</td><td>LRR-repeats+AP-GTPase+COR</td><td>1070</td><td>-</td><td>Actinobacteria</td><td>Glycomyces albidus</td><td>leucine-rich repeat domain-containing protein [Glycomyces albidus].</td><td>-</td></tr>
<tr><td>WP_153228358.1</td><td><-Cluster5__5clades||LRR-repeats+AP-GTPase+COR+DUF4404*→||<-?<-ABC-ATPase<-?||?→||<-REC</td><td>LRR-repeats+AP-GTPase+COR+DUF4404</td><td>992</td><td>-</td><td>Cyanobacteria</td><td>Anabaena sp. YBS01</td><td>leucine-rich repeat domain-containing protein [Anabaena sp. YBS01].</td><td>GCF_009498015.1</td></tr>
<tr><td>WP_153456319.1</td><td>LRR-repeats+AP-GTPase+COR*→</td><td>LRR-repeats+AP-GTPase+COR</td><td>992</td><td>-</td><td>Actinobacteria</td><td>Streptomyces sp. RB5</td><td>GTPase [Streptomyces sp. RB5].</td><td>-</td></tr>
<tr><td>WP_153661567.1</td><td><-cNMPBD<-cNMPBD||?→?→ LRR-repeats+AP-GTPase+COR+TM+TM*→</td><td>LRR-repeats+AP-GTPase+COR+TM+TM</td><td>1103</td><td>-</td><td>Bacteroidetes</td><td>Chitinophaga sp. SYP-B3965</td><td>leucine-rich repeat domain-containing protein [Chitinophaga sp. SYP-B3965].</td><td>GCF_009647655.1</td></tr>
<tr><td>WP_155076197.1</td><td>LRR-repeats+AP-GTPase+COR*→</td><td>LRR-repeats+AP-GTPase+COR</td><td>899</td><td>-</td><td>Bacteroidetes</td><td>Flavobacterium sp. MC2016-06</td><td>GTP-binding protein [Flavobacterium sp. MC2016-06].</td><td>GCF_009711165.1</td></tr>
<tr><td>WP_155705093.1</td><td>SIG+Trypsin+PDZ→?→?→ LRR-repeats+AP-GTPase+COR*→</td><td>LRR-repeats+AP-GTPase+COR</td><td>951</td><td>-</td><td>Firmicutes</td><td>Paenibacillus psychroresistens</td><td>GTP-binding protein [Paenibacillus psychroresistens].</td><td>GCF_009728935.1</td></tr>
<tr><td>WP_155743676.1</td><td>LRR-repeats+AP-GTPase+COR+EAD1*→||<-PSE||Pkinase→ Pkinase→?→||<-Cluster5__5clades</td><td>LRR-repeats+AP-GTPase+COR+EAD1</td><td>1152</td><td>-</td><td>Cyanobacteria</td><td>Scytonema sp. UIC 10036</td><td>leucine-rich repeat domain-containing protein [Scytonema sp. UIC 10036].</td><td>GCF_009725235.1</td></tr>
<tr><td>WP_155746360.1</td><td>LRR-repeats→||<-?<-Cluster5__5clades||LRR-repeats→?→ LRR-repeats+AP-GTPase+COR+EAD1*→?→?→ Cluster5__5clades→</td><td>LRR-repeats+AP-GTPase+COR+EAD1</td><td>988</td><td>-</td><td>Cyanobacteria</td><td>Scytonema sp. UIC 10036</td><td>leucine-rich repeat domain-containing protein [Scytonema sp. UIC 10036].</td><td>GCF_009725235.1</td></tr>
<tr><td>WP_156420697.1</td><td>TetR-HTH→?→||<-?<-?<-?||LRR-repeats+AP-GTPase+COR+TIR*→</td><td>LRR-repeats+AP-GTPase+COR+TIR</td><td>1173</td><td>-</td><td>Alphaproteobacteria</td><td>Sphingopyxis sp. HXXIV</td><td>TIR domain-containing protein [Sphingopyxis sp. HXXIV].</td><td>GCF_001468305.1</td></tr>
<tr><td>WP_157205648.1</td><td>Cluster5__5clades→?→?→ HTH→ LRR-repeats+AP-GTPase+COR+TIR*→ Cluster1019__2clades→?→||<-?<-?<-?||Cluster833__2clades→</td><td>LRR-repeats+AP-GTPase+COR+TIR</td><td>1096</td><td>-</td><td>Gammaproteobacteria</td><td>Methylomonas koyamae</td><td>TIR domain-containing protein [Methylomonas koyamae].</td><td>GCF_001644135.1</td></tr>
<tr><td>WP_157310052.1</td><td>LRR-repeats+AP-GTPase+COR+TIR*→</td><td>LRR-repeats+AP-GTPase+COR+TIR</td><td>917</td><td>-</td><td>Bacteroidetes</td><td>Chitinophaga sp. ysch24</td><td>leucine-rich repeat protein [Chitinophaga sp. ysch24].</td><td>GCF_009758205.1</td></tr>
<tr><td>WP_157453024.1</td><td>LRR-repeats+AP-GTPase+COR+DUF4404*→</td><td>LRR-repeats+AP-GTPase+COR+DUF4404</td><td>1156</td><td>-</td><td>Cyanobacteria</td><td>Coleofasciculus chthonoplastes</td><td>leucine-rich repeat domain-containing protein [Coleofasciculus chthonoplastes].</td><td>-</td></tr>
<tr><td>WP_157546628.1</td><td><-SIG+TM+TM+TM+TM+TM+TM+TM+TM||?→?→||<-?||?→?→ LRR-repeats+AP-GTPase+COR*→</td><td>LRR-repeats+AP-GTPase+COR</td><td>585</td><td>-</td><td>Actinobacteria</td><td>Hamadaea tsunoensis</td><td>leucine-rich repeat domain-containing protein [Hamadaea tsunoensis].</td><td>GCF_000428945.1</td></tr>
<tr><td>WP_157584649.1</td><td>LRR-repeats+AP-GTPase+COR+TIR*→||<-?||ABC_membrane+ABC_tran→</td><td>LRR-repeats+AP-GTPase+COR+TIR</td><td>937</td><td>-</td><td>Bacteroidetes</td><td>Spirosoma sp. HMF4905</td><td>leucine-rich repeat domain-containing protein [Spirosoma sp. HMF4905].</td><td>GCF_009754945.1</td></tr>
<tr><td>WP_157585892.1</td><td>LRR-repeats+AP-GTPase+COR+TIR*→ Cluster1510__2clades→</td><td>LRR-repeats+AP-GTPase+COR+TIR</td><td>1129</td><td>-</td><td>Bacteroidetes</td><td>Spirosoma sp. HMF4905</td><td>leucine-rich repeat domain-containing protein [Spirosoma sp. HMF4905].</td><td>-</td></tr>
<tr><td>WP_157590734.1</td><td>ABhydrolase+ABhydrolase+CASPASE+TPR-S→||<-?||LRR-repeats+AP-GTPase+COR+TIR*→</td><td>LRR-repeats+AP-GTPase+COR+TIR</td><td>1169</td><td>-</td><td>Bacteroidetes</td><td>Spirosoma sp. HMF4905</td><td>leucine-rich repeat domain-containing protein [Spirosoma sp. HMF4905].</td><td>GCF_009754945.1</td></tr>
<tr><td>WP_157879249.1</td><td>LRR-repeats+AP-GTPase+COR+TIR*→||<-PSE<-?<-?<-?<-?<-?<-REC</td><td>LRR-repeats+AP-GTPase+COR+TIR</td><td>656</td><td>-</td><td>Alphaproteobacteria</td><td>Pararhodospirillum photometricum</td><td>hypothetical protein [Pararhodospirillum photometricum].</td><td>GCF_000284415.1</td></tr>
</table>

<table>
<thead>
<tr><th>acc</th><th>operon</th><th>architecture</th><th>len</th><th>gen.name</th><th>taxend</th><th>species</th><th>defline</th><th>gca</th></tr>
</thead>
<tbody>
<tr><td>WP_157881522.1</td><td>Cluster1792_2clades→?→?→||<-?||?→ LRR-repeats+AP-GTPase+COR*→||<-?||?→ SIG+TM+TM→</td><td>LRR-repeats+AP-GTPase+COR</td><td>950</td><td>-</td><td>Actinobacteria</td><td>Streptomyces phaeochromogenes</td><td>hypothetical protein [Streptomyces phaeochromogenes].</td><td>GCF_001418655.1</td></tr>
<tr><td>WP_158043876.1</td><td>LRR-repeats+AP-GTPase+COR_TIR*→</td><td>LRR-repeats+AP-GTPase+COR_TIR</td><td>1167</td><td>-</td><td>Alphaproteobacteria</td><td>Skermanella pratensis</td><td>TIR domain-containing protein [Skermanella pratensis].</td><td>-</td></tr>
<tr><td>WP_158234411.1</td><td>LRR-repeats+AP-GTPase+COR*→ LRR-repeats→ LRR-repeats→</td><td>LRR-repeats+AP-GTPase+COR</td><td>852</td><td>-</td><td>Cyanobacteria</td><td>Limnothrix sp. PR1529</td><td>leucine-rich repeat domain-containing protein [Limnothrix sp. PR1529].</td><td>GCF_002742025.1</td></tr>
<tr><td>WP_158281705.1</td><td><-ACET<-?<-?<-?||?→ HSP70→ DNAJ→ LRR-repeats+AP-GTPase+COR+TIR*→</td><td>LRR-repeats+AP-GTPase+COR+TIR</td><td>944</td><td>-</td><td>Betaproteobacteria</td><td>Rivicola pingtungensis</td><td>TIR domain-containing protein [Rivicola pingtungensis].</td><td>GCF_003201855.1</td></tr>
<tr><td>WP_158299785.1</td><td>TM→ LRR-repeats+AP-GTPase+COR*→</td><td>LRR-repeats+AP-GTPase+COR</td><td>1050</td><td>-</td><td>Actinobacteria</td><td>Glycomyces paridis</td><td>hypothetical protein [Glycomyces paridis].</td><td>GCF_004912155.1</td></tr>
<tr><td>WP_158592567.1</td><td>LRR-repeats+AP-GTPase+COR+TM+TM*→</td><td>LRR-repeats+AP-GTPase+COR+TM+TM</td><td>1002</td><td>-</td><td>Bacteroidetes</td><td>Flavobacterium sp. 102</td><td>50S ribosome-binding GTPase [Flavobacterium sp. 102].</td><td>GCF_003634615.1</td></tr>
<tr><td>WP_158623299.1</td><td>TPR-repeats→ LRR-repeats+AP-GTPase+COR+TIR*→?→?→?→?→||<-LexA-protease</td><td>LRR-repeats+AP-GTPase+COR+TIR</td><td>1083</td><td>-</td><td>Deltaproteobacteria</td><td>Corallococcus sp. CA053C</td><td>TIR domain-containing protein [Corallococcus sp. CA053C].</td><td>GCF_003611675.1</td></tr>
<tr><td>WP_158660235.1</td><td><-SIG+TM+TM+TM+TM+TM+TM+TM+TM+TM+TM<-?<-?<-?<-?||?→?→ LRR-repeats+AP-GTPase+COR+TIR*→||<-ParB<-ParA-Soj-PloopNTPase</td><td>LRR-repeats+AP-GTPase+COR+TIR</td><td>723</td><td>-</td><td>Alphaproteobacteria</td><td>Niveispirillum cyanobacteriorum</td><td>TIR domain-containing protein [Niveispirillum cyanobacteriorum].</td><td>GCF_014640215.1</td></tr>
<tr><td>WP_158690560.1</td><td>LRR-repeats+AP-GTPase+COR*→</td><td>LRR-repeats+AP-GTPase+COR</td><td>1355</td><td>-</td><td>Actinobacteria</td><td>Streptomyces sp. 142MFCol3.1</td><td>hypothetical protein, partial [Streptomyces sp. 142MFCol3.1].</td><td>GCF_000424945.1</td></tr>
<tr><td>WP_158924018.1</td><td>LRR-repeats+AP-GTPase+COR*→||<-?||?→||<-?<-SIGMA-HTH<-?<-SIGMA-HTH</td><td>LRR-repeats+AP-GTPase+COR</td><td>996</td><td>-</td><td>Actinobacteria</td><td>Streptomyces broussonetiae</td><td>GTPase [Streptomyces broussonetiae].</td><td>GCF_009796285.1</td></tr>
<tr><td>WP_159021496.1</td><td>Cluster1757_2clades→||<-?||LRR-repeats+AP-GTPase+COR*→?→?→?→?→||<-PSE<-PSE<-Cluster1695_2clades</td><td>LRR-repeats+AP-GTPase+COR</td><td>704</td><td>-</td><td>Bacteroidetes</td><td>Formosa sp. L2A11</td><td>GTP-binding protein [Formosa sp. L2A11].</td><td>GCF_009796865.1</td></tr>
<tr><td>WP_159170123.1</td><td>LRR-repeats+AP-GTPase+COR+DrHyd*→</td><td>LRR-repeats+AP-GTPase+COR+DrHyd</td><td>855</td><td>-</td><td>Gammaproteobacteria</td><td>Halioglobus japonicus</td><td>hypothetical protein [Halioglobus japonicus].</td><td>GCF_902705825.1</td></tr>
<tr><td>WP_159248779.1</td><td>SIG+TM+TM+TM+TM+TM+TM+TM→||<-?||Mbetalac→ SAM-methylase→?→||<-?<-SIG+TPM_phosphatase+TM+TM+TM||LRR-repeats+AP-GTPase+COR+TM+TM*→||<-?<-?<-?<-Pro_CA||?→||<-?<-tRNA<-tRNA<-Ferredoxin-betagrasp</td><td>LRR-repeats+AP-GTPase+COR+TM+TM</td><td>805</td><td>-</td><td>Cyanobacteria</td><td>Microcystis aeruginosa</td><td>leucine-rich repeat domain-containing protein [Microcystis aeruginosa].</td><td>GCF_009811815.1</td></tr>
<tr><td>WP_159254721.1</td><td>LRR-repeats+AP-GTPase+COR*→?→?→?→||<-?<-ABC-ATPase</td><td>LRR-repeats+AP-GTPase+COR</td><td>923</td><td>-</td><td>Bacteroidetes</td><td>Tenacibaculum maritimum</td><td>leucine-rich repeat domain-containing protein [Tenacibaculum maritimum].</td><td>GCF_902705305.1</td></tr>
<tr><td>WP_159395983.1</td><td>LRR-repeats+AP-GTPase+COR*→||<-?||?→||<-?<-SIGMA-HTH<-?<-SIGMA-HTH</td><td>LRR-repeats+AP-GTPase+COR</td><td>988</td><td>-</td><td>Actinobacteria</td><td>Streptomyces sp. FXJ1.172</td><td>GTPase [Streptomyces sp. FXJ1.172].</td><td>GCF_001636945.1</td></tr>
<tr><td>WP_159402479.1</td><td>ABhydrolase→?→||<-?||LRR-repeats+AP-GTPase+COR+TIR*→||<-?<-?<-?<-SIG+NUDIX</td><td>LRR-repeats+AP-GTPase+COR+TIR</td><td>1069</td><td>-</td><td>Cyanobacteria</td><td>Nostoc sp. ATCC 53789</td><td>leucine-rich repeat domain-containing protein [Nostoc sp. ATCC 53789].</td><td>GCF_009873495.1</td></tr>
<tr><td>WP_159402492.1</td><td>Pentapeptide-repeats→||<-?<-?||?→?→||<-Arginase<-?||LRR-repeats+AP-GTPase+COR+TIR*→</td><td>LRR-repeats+AP-GTPase+COR+TIR</td><td>802</td><td>-</td><td>Cyanobacteria</td><td>Nostoc sp. ATCC 53789</td><td>leucine-rich repeat domain-containing protein [Nostoc sp. ATCC 53789].</td><td>GCF_009873495.1</td></tr>
<tr><td>WP_159402558.1</td><td>Cluster5_5clades→ LRR-repeats+AP-GTPase+COR+TM+TM*→||<-CASPASE+TM</td><td>LRR-repeats+AP-GTPase+COR+TM+TM</td><td>904</td><td>-</td><td>Cyanobacteria</td><td>Nostoc sp. ATCC 53789</td><td>leucine-rich repeat domain-containing protein [Nostoc sp. ATCC 53789].</td><td>GCF_009873495.1</td></tr>
<tr><td>WP_159402710.1</td><td>LRR-repeats+AP-GTPase+COR+CASPASE*→?→?→?→ ParA-Soj-PloopNTPase→</td><td>LRR-repeats+AP-GTPase+COR+CASPASE</td><td>1535</td><td>-</td><td>Cyanobacteria</td><td>Nostoc sp. ATCC 53789</td><td>leucine-rich repeat domain-containing protein [Nostoc sp. ATCC 53789].</td><td>GCF_009873495.1</td></tr>
<tr><td>WP_159447934.1</td><td>CI→ CI→ LRR-repeats+AP-GTPase+COR*→</td><td>LRR-repeats+AP-GTPase+COR</td><td>1103</td><td>-</td><td>Gammaproteobacteria</td><td>Photobacterium piscicola</td><td>GTP-binding protein [Photobacterium piscicola].</td><td>GCF_900166965.1</td></tr>
<tr><td>WP_159457453.1</td><td>LRR-repeats+AP-GTPase+COR+TIR*→</td><td>LRR-repeats+AP-GTPase+COR+TIR</td><td>1186</td><td>-</td><td>Gammaproteobacteria</td><td>Vibrio mangrovi</td><td>leucine-rich repeat domain-containing protein [Vibrio mangrovi].</td><td>GCF_900184095.1</td></tr>
</tbody>
</table>

<table>
<tr><th>acc</th><th>operon</th><th>architecture</th><th>len</th><th>gen.name</th><th>taxend</th><th>species</th><th>defline</th><th>gca</th></tr>
<tr><td>WP_159789529.1</td><td><-Cluster1921_2clades<-?<-?||?→||<-?||?→?→ LRR-repeats+AP-GTPase+COR*→?→||<-?<-REC</td><td>LRR-repeats+AP-GTPase+COR</td><td>771</td><td>-</td><td>Cyanobacteria</td><td>Microcoleus sp. IPPAS B-353</td><td>leucine-rich repeat domain-containing protein [Microcoleus sp. IPPAS B-353].</td><td>GCF_009846485.1</td></tr>
<tr><td>WP_160075537.1</td><td>LRR-repeats+AP-GTPase+COR+TIR*→</td><td>LRR-repeats+AP-GTPase+COR+TIR</td><td>1088</td><td>-</td><td>Bacteroidetes</td><td>Saprospirales bacterium GYS_P2D</td><td>leucine-rich repeat domain-containing protein [Saprospirales bacterium GYS_P2D].</td><td>-</td></tr>
<tr><td>WP_160131147.1</td><td>SIGMA-HTH→?→ RADICAL-SAM→ LRR-repeats+AP-GTPase+COR+TIR*→||<-REC<-?<-?<-?||?→ HISKIN→</td><td>LRR-repeats+AP-GTPase+COR+TIR</td><td>1229</td><td>-</td><td>Bacteroidetes</td><td>Kordia antarctica</td><td>leucine-rich repeat domain-containing protein [Kordia antarctica].</td><td>GCF_009901525.1</td></tr>
<tr><td>WP_160148531.1</td><td><-SIG+Trypsin+PDZ<-?<-?<-?||?→?→ LRR-repeats+AP-GTPase+COR+TCAD2*→</td><td>LRR-repeats+AP-GTPase+COR+TCAD2</td><td>919</td><td>-</td><td>Cyanobacteria</td><td>Leptolyngbya sp. PCC 7376</td><td>leucine-rich repeat domain-containing protein [Leptolyngbya sp. PCC 7376].</td><td>GCF_000316605.1</td></tr>
<tr><td>WP_161144175.1</td><td>Cluster1913_2clades→?→?→?→?→ LRR-repeats+AP-GTPase*→</td><td>LRR-repeats+AP-GTPase</td><td>512</td><td>-</td><td>Firmicutes</td><td>Dorea sp. BIOML-A1</td><td>hypothetical protein [Dorea sp. BIOML-A1].</td><td>GCF_009875615.1</td></tr>
<tr><td>WP_161213661.1</td><td>LRR-repeats+AP-GTPase+COR*→||<-NUDIX</td><td>LRR-repeats+AP-GTPase+COR</td><td>1340</td><td>-</td><td>Actinobacteria</td><td>unclassified Streptomyces</td><td>MULTISPECIES: leucine-rich repeat domain-containing protein [unclassified Streptomyces].</td><td>GCF_900091775.1</td></tr>
<tr><td>WP_161967152.1</td><td><-Aminotran_1_2<-?||LRR-repeats+AP-GTPase+COR+TIR*→||<-?<-?<-?<-DSBH+AraC-HTH+AraC-HTH</td><td>LRR-repeats+AP-GTPase+COR+TIR</td><td>1219</td><td>-</td><td>Planctomycetes</td><td>Fimbriiglobus ruber</td><td>hypothetical protein [Fimbriiglobus ruber].</td><td>GCF_002197845.1</td></tr>
<tr><td>WP_161967198.1</td><td><-RelE-ParE<-?||?→?→ LRR-repeats→||<-?||LRR-repeats+AP-GTPase+COR*→</td><td>LRR-repeats+AP-GTPase+COR</td><td>915</td><td>-</td><td>Planctomycetes</td><td>Fimbriiglobus ruber</td><td>leucine-rich repeat domain-containing protein [Fimbriiglobus ruber].</td><td>GCF_002197845.1</td></tr>
<tr><td>WP_162292932.1</td><td>LRR-repeats+AP-GTPase+COR*→?→?→?→||<-?<-ABC-ATPase+ABC-ATPase</td><td>LRR-repeats+AP-GTPase+COR</td><td>1975</td><td>-</td><td>Actinobacteria</td><td>Actinophytocola xinjiangensis</td><td>leucine-rich repeat domain-containing protein [Actinophytocola xinjiangensis].</td><td>GCF_001921215.1</td></tr>
<tr><td>WP_162399370.1</td><td>LRR-repeats+AP-GTPase+COR*→?→||<-?||?→||<-?<-?||?→ ClpABN-AAA+ClpABC-AAA→</td><td>LRR-repeats+AP-GTPase+COR</td><td>895</td><td>-</td><td>Cyanobacteria</td><td>Nostoc sp. B(2019)</td><td>leucine-rich repeat domain-containing protein, partial [Nostoc sp. B(2019)].</td><td>GCF_010091925.1</td></tr>
<tr><td>WP_162488066.1</td><td>TM+TM→?→||<-?||?→ PSE→||<-?||?→?→ LRR-repeats+AP-GTPase+COR+TIR*→||<-?<-?||ABC_membrane+ABC_tran→?→ Cluster968_2clades→</td><td>LRR-repeats+AP-GTPase+COR+TIR</td><td>1120</td><td>-</td><td>Alphaproteobacteria</td><td>Azospirillum lipoferum</td><td>TIR domain-containing protein [Azospirillum lipoferum].</td><td>GCF_000283655.1</td></tr>
<tr><td>WP_162528069.1</td><td>LRR-repeats+AP-GTPase+COR*→</td><td>LRR-repeats+AP-GTPase+COR</td><td>781</td><td>-</td><td>Alphaproteobacteria</td><td>Rhodobacterales bacterium</td><td>leucine-rich repeat domain-containing protein [Rhodobacterales bacterium].</td><td>-</td></tr>
<tr><td>WP_162667694.1</td><td><-Cluster1291_2clades<-?<-PSE<-?||?→?→?→ LRR-repeats+AP-GTPase*→?→||<-?||?→?→ ParB→</td><td>LRR-repeats+AP-GTPase</td><td>617</td><td>-</td><td>Planctomycetes</td><td>Gemmata massiliana</td><td>hypothetical protein [Gemmata massiliana].</td><td>GCF_901538265.1</td></tr>
<tr><td>WP_162668063.1</td><td>LRR-repeats+AP-GTPase+COR+DUF4404*→||<-?<-?<-?||?→||<-LRR-repeats||REC→</td><td>LRR-repeats+AP-GTPase+COR+DUF4404</td><td>978</td><td>-</td><td>Planctomycetes</td><td>Gemmata massiliana</td><td>leucine-rich repeat domain-containing protein [Gemmata massiliana].</td><td>GCF_901538265.1</td></tr>
<tr><td>WP_163431087.1</td><td>TIR→ LRR-repeats+AP-GTPase+COR+TIR*→ PSE→?→?→||<-?<-SIG+TM+TM+TM+HISKIN</td><td>LRR-repeats+AP-GTPase+COR+TIR</td><td>1384</td><td>-</td><td>Bacteroidetes</td><td>unclassified Flavobacterium</td><td>MULTISPECIES: leucine-rich repeat domain-containing protein [unclassified Flavobacterium].</td><td>GCF_013874595.1</td></tr>
<tr><td>WP_163660644.1</td><td>LRR-repeats+AP-GTPase*→</td><td>LRR-repeats+AP-GTPase</td><td>311</td><td>-</td><td>Cyanobacteria</td><td>Leptolyngbyaceae cyanobacterium CCMR0082</td><td>leucine-rich repeat domain-containing protein [Leptolyngbyaceae cyanobacterium CCMR0082].</td><td>-</td></tr>
<tr><td>WP_163671600.1</td><td>LRR-repeats+AP-GTPase+COR+TIR*→</td><td>LRR-repeats+AP-GTPase+COR+TIR</td><td>855</td><td>-</td><td>Cyanobacteria</td><td>Leptolyngbyaceae cyanobacterium CCMR0082</td><td>leucine-rich repeat domain-containing protein, partial [Leptolyngbyaceae cyanobacterium CCMR0082].</td><td>-</td></tr>
</table>

acc	operon	architecture	len	gen.name	taxend	species	defline	gca
WP_163671893.1	<-NACHT<-?||LRR-repeats+AP-GTPase+COR+TIR*→||<-?||PSE→||<-NACHT+68TM-wHTH<-?||?→?→||<-?||Cluster53_2clades→	LRR-repeats+AP-GTPase+COR+TIR	726	-	Cyanobacteria	Leptolyngbyaceae cyanobacterium CCMR0082	TIR domain-containing protein [Leptolyngbyaceae cyanobacterium CCMR0082].	-
WP_163697061.1	LRR-repeats+AP-GTPase+COR+TIR*→?→||<-?<-?<-?||?→||<-Cluster1829_2clades	LRR-repeats+AP-GTPase+COR+TIR	1274	-	Cyanobacteria	Leptolyngbyaceae cyanobacterium CCMR0081	leucine-rich repeat domain-containing protein [Leptolyngbyaceae cyanobacterium CCMR0081].	-
WP_163702516.1	<-SIG+DM13||?→?→?→?→?→ PIN→ LRR-repeats+AP-GTPase+COR+TIR*→?→ PSE→?→?→?→||<-?<-SIG+TM+TM+Calcineurin	LRR-repeats+AP-GTPase+COR+TIR	993	-	Cyanobacteria	Leptolyngbyaceae cyanobacterium CCMR0081	leucine-rich repeat domain-containing protein [Leptolyngbyaceae cyanobacterium CCMR0081].	-
WP_163703138.1	SIR2→?→?→?→?→?→?→ LRR-repeats+AP-GTPase+COR+TIR*→ PSE→||<-Pentapeptide	LRR-repeats+AP-GTPase+COR+TIR	830	-	Cyanobacteria	Leptolyngbyaceae cyanobacterium CCMR0081	TIR domain-containing protein [Leptolyngbyaceae cyanobacterium CCMR0081].	-
WP_163833956.1	LRR-repeats+AP-GTPase+COR+DUF4404*→||<-?<-CHTH	LRR-repeats+AP-GTPase+COR+DUF4404	1124	-	Gammaproteobacteria	Spartinivicinus ruber	leucine-rich repeat domain-containing protein [Spartinivicinus ruber].	GCF_011009015.1
WP_163929292.1	LRR-repeats+AP-GTPase+COR+TIR*→||<-?<-?<-TIR<-?<-SIG+NUDIX	LRR-repeats+AP-GTPase+COR+TIR	1024	-	Cyanobacteria	Nostoc sp. UIC 10630	leucine-rich repeat domain-containing protein [Nostoc sp. UIC 10630].	GCF_010747425.1
WP_163938989.1	LRR-repeats+AP-GTPase+COR+TIR*→	LRR-repeats+AP-GTPase+COR+TIR	795	-	Cyanobacteria	Nostoc sp. UIC 10630	leucine-rich repeat domain-containing protein, partial [Nostoc sp. UIC 10630].	GCF_010747425.1
WP_164035239.1	Cluster998_2clades→?→||<-?<-?<-?||SAM-methylase→?→ tRNA→ LRR-repeats+AP-GTPase+COR*→	LRR-repeats+AP-GTPase+COR	858	-	Bacteroidetes	Spirosoma agri	hypothetical protein [Spirosoma agri].	GCF_010747415.1
WP_164490209.1	FGS→ FGS→ FGS→ LRR-repeats+AP-GTPase+COR+CASPASE*→	LRR-repeats+AP-GTPase+COR+CASPASE	976	-	Bacteroidetes	Runella sp. SP2	leucine-rich repeat domain-containing protein [Runella sp. SP2].	GCF_003711225.1
WP_164638795.1	LRR-repeats+AP-GTPase+COR+TIR*→||<-?<-?<-?<-ABC-ATPase||?→ REC→	LRR-repeats+AP-GTPase+COR+TIR	1173	-	Alphaproteobacteria	Rhodopseudomonas sp. BR0G17	leucine-rich repeat domain-containing protein [Rhodopseudomonas sp. BR0G17].	GCF_010907025.1
WP_164714180.1	LRR-repeats+AP-GTPase+COR+TIR*→||<-?<-?||?→ cNMPBD→ SIG+TM+TM+TM→	LRR-repeats+AP-GTPase+COR+TIR	1056	-	Bacteroidetes	Chitinophaga rhizosphaerae	leucine-rich repeat domain-containing protein [Chitinophaga rhizosphaerae].	GCF_003958645.1
WP_165253289.1	LRR-repeats+AP-GTPase+COR+TIR*→	LRR-repeats+AP-GTPase+COR+TIR	1053	-	Planctomycetes	Paludisphaera soli	TIR domain-containing protein [Paludisphaera soli].	GCF_011064595.1
WP_165571460.1	LRR-repeats+AP-GTPase+COR*→	LRR-repeats+AP-GTPase+COR	871	-	Bacteroidetes	Flavobacterium hydatis	hypothetical protein [Flavobacterium hydatis].	GCF_000737695.1
WP_165772028.1	<-SIG+TM+TM+TM+HISKIN<-?<-?<-?||?→ LRR-repeats+AP-GTPase+COR+TIR*→ ParA-Soj-PloopNTPase→ ParB→	LRR-repeats+AP-GTPase+COR+TIR	674	-	Alphaproteobacteria	Niveispirillum lacus	TIR domain-containing protein [Niveispirillum lacus].	GCF_002251795.1
WP_166124895.1	<-Cluster1501_2clades||?→?→ LRR-repeats+AP-GTPase+COR*→?→ Cluster1023_3clades→?→||<-?||ClpABN-AAA+ClpABC-AAA→	LRR-repeats+AP-GTPase+COR	925	-	Bacteroidetes	unclassified Flavobacterium	MULTISPECIES: leucine-rich repeat domain-containing protein [unclassified Flavobacterium].	GCF_011305415.1
WP_166235797.1	LRR-repeats+AP-GTPase+COR+TM+TM*→	LRR-repeats+AP-GTPase+COR+TM+TM	954	-	Bacteroidetes	Flavobacterium sp. TWA-26	hypothetical protein [Flavobacterium sp. TWA-26].	GCF_011392075.1
WP_166562074.1	LRR-repeats+AP-GTPase+COR*→	LRR-repeats+AP-GTPase+COR	931	-	Bacteroidetes	Emticicia sp. CRIBPO	leucine-rich repeat domain-containing protein [Emticicia sp. CRIBPO].	GCF_009905725.1
WP_167031741.1	LRR-repeats+AP-GTPase+COR+TIR*→?→?→ TIR→	LRR-repeats+AP-GTPase+COR+TIR	1295	-	Bacteroidetes	Chryseobacterium sp. Tr-659	leucine-rich repeat domain-containing protein [Chryseobacterium sp. Tr-659].	GCF_011752975.1

<table>
<tr><th>acc</th><th>operon</th><th>architecture</th><th>len</th><th>gen.name</th><th>taxend</th><th>species</th><th>defline</th><th>gca</th></tr>
<tr><td>WP_167211330.1</td><td>LRR-repeats+AP-GTPase+COR+TIR*→ TIR→</td><td>LRR-repeats+AP-GTPase+COR+TIR</td><td>1843</td><td>-</td><td>Bacteroidetes</td><td>Spirosoma aureum</td><td>leucine-rich repeat domain-containing protein [Spirosoma aureum].</td><td>GCF_011604685.1</td></tr>
<tr><td>WP_167291529.1</td><td>LRR-repeats+AP-GTPase+COR+TIR*→</td><td>LRR-repeats+AP-GTPase+COR+TIR</td><td>1060</td><td>-</td><td>Bacteroidetes</td><td>Paraflavitale devenefica</td><td>leucine-rich repeat domain-containing protein [Paraflavitale devenefica].</td><td>GCF_011759375.1</td></tr>
<tr><td>WP_167387879.1</td><td>LRR-repeats+AP-GTPase+COR*→</td><td>LRR-repeats+AP-GTPase+COR</td><td>967</td><td>-</td><td>Actinobacteria</td><td>Streptomyces recifensis</td><td>GTPase [Streptomyces recifensis].</td><td>GCF_002154615.1</td></tr>
<tr><td>WP_168204481.1</td><td>LRR-repeats→ LRR-repeats+AP-GTPase+COR+TM+TM*→</td><td>LRR-repeats+AP-GTPase+COR+TM+TM</td><td>574</td><td>-</td><td>Cyanobacteria</td><td>Dolichospermum sp. UHCC 0315A</td><td>hypothetical protein [Dolichospermum sp. UHCC 0315A].</td><td>GCF_008121535.1</td></tr>
<tr><td>WP_168218871.1</td><td>LRR-repeats+AP-GTPase+COR*→?→?→||<-?<-?<-?<-?<-PIN</td><td>LRR-repeats+AP-GTPase+COR</td><td>864</td><td>-</td><td>Planctomycetes</td><td>Limnoglobus roseus</td><td>leucine-rich repeat domain-containing protein [Limnoglobus roseus].</td><td>GCF_008254045.1</td></tr>
<tr><td>WP_168359257.1</td><td>LRR-repeats+AP-GTPase+COR+TM+TM*→</td><td>LRR-repeats+AP-GTPase+COR+TM+TM</td><td>687</td><td>-</td><td>Cyanobacteria</td><td>Dolichospermum planctonicum</td><td>GTP-binding protein, partial [Dolichospermum planctonicum].</td><td>GCF_009712075.1</td></tr>
<tr><td>WP_168360389.1</td><td>Ferredoxin-betagrasp→?→?→ LRR-repeats→ LRR-repeats+AP-GTPase+COR*→</td><td>LRR-repeats+AP-GTPase+COR</td><td>682</td><td>-</td><td>Cyanobacteria</td><td>Dolichospermum planctonicum</td><td>hypothetical protein, partial [Dolichospermum planctonicum].</td><td>GCF_009712075.1</td></tr>
<tr><td>WP_168467214.1</td><td>LRR-repeats+AP-GTPase+COR*→ PSE→?→||<-?||?→?→?→||<-Pentapeptide</td><td>LRR-repeats+AP-GTPase+COR</td><td>692</td><td>-</td><td>Cyanobacteria</td><td>Aphanizomenon sp. UHCC 0183</td><td>GTP-binding protein, partial [Aphanizomenon sp. UHCC 0183].</td><td>GCF_009712065.1</td></tr>
<tr><td>WP_168493001.1</td><td><-HNH<-?||?→ LRR-repeats+AP-GTPase+COR*→</td><td>LRR-repeats+AP-GTPase+COR</td><td>1079</td><td>-</td><td>Cyanobacteria</td><td>Anabaena sp. UHCC 0204</td><td>leucine-rich repeat domain-containing protein [Anabaena sp. UHCC 0204].</td><td>GCF_009711975.1</td></tr>
<tr><td>WP_168511191.1</td><td>NUDIX→?→?→?→ LRR-repeats+AP-GTPase+COR*→</td><td>LRR-repeats+AP-GTPase+COR</td><td>1365</td><td>-</td><td>Actinobacteria</td><td>Streptomyces sp. S1D4-11</td><td>leucine-rich repeat domain-containing protein [Streptomyces sp. S1D4-11].</td><td>-</td></tr>
<tr><td>WP_168644108.1</td><td>LRR-repeats+AP-GTPase+COR+TIR*→?→||<-?<-SIG+NUDIX</td><td>LRR-repeats+AP-GTPase+COR+TIR</td><td>963</td><td>-</td><td>Cyanobacteria</td><td>Dolichospermum sp. UHCC 0259</td><td>leucine-rich repeat domain-containing protein [Dolichospermum sp. UHCC 0259].</td><td>GCF_009711935.1</td></tr>
<tr><td>WP_169190548.1</td><td><-ABC-ATPase<-?<-?<-?<-?||?→ tRNA→?→ LRR-repeats+AP-GTPase+COR+TIR*→</td><td>LRR-repeats+AP-GTPase+COR+TIR</td><td>1190</td><td>-</td><td>Bacteroidetes</td><td>Chitinophaga sp. Ak27</td><td>leucine-rich repeat domain-containing protein [Chitinophaga sp. Ak27].</td><td>GCF_012726295.1</td></tr>
<tr><td>WP_169266449.1</td><td>LRR-repeats+AP-GTPase+COR+TIR*→||<-?||?→||<-?||NUDIX→</td><td>LRR-repeats+AP-GTPase+COR+TIR</td><td>936</td><td>-</td><td>Cyanobacteria</td><td>Brasilonema octagenarum</td><td>leucine-rich repeat domain-containing protein [Brasilonema octagenarum].</td><td>GCF_012912125.1</td></tr>
<tr><td>WP_169315756.1</td><td>LRR-repeats+AP-GTPase+COR+TIR*→</td><td>LRR-repeats+AP-GTPase+COR+TIR</td><td>1002</td><td>-</td><td>Bacteroidetes</td><td>Haliscomenobacter hydrossis</td><td>leucine-rich repeat domain-containing protein [Haliscomenobacter hydrossis].</td><td>GCF_000212735.1</td></tr>
<tr><td>WP_169363874.1</td><td>SIG+HIN-HTH→?→?→ LRR-repeats+AP-GTPase+COR+TM+TM*→</td><td>LRR-repeats+AP-GTPase+COR+TM+TM</td><td>972</td><td>-</td><td>Cyanobacteria</td><td>Pseudanabaena yagii</td><td>leucine-rich repeat domain-containing protein [Pseudanabaena yagii].</td><td>GCF_012863495.1</td></tr>
<tr><td>WP_169364911.1</td><td>TPR-repeats+CASPASE→?→ PSE→ Cluster54_3clades→ Cluster53_2clades→?→ LRR-repeats+AP-GTPase+COR+CASPASE*→||<-?||?→ REC→</td><td>LRR-repeats+AP-GTPase+COR+CASPASE</td><td>1239</td><td>-</td><td>Cyanobacteria</td><td>Pseudanabaena yagii</td><td>leucine-rich repeat domain-containing protein [Pseudanabaena yagii].</td><td>GCF_012863495.1</td></tr>
<tr><td>WP_169365210.1</td><td>Pentapeptide-repeats→?→ PolB→?→ LRR-repeats→ LRR-repeats+AP-GTPase+COR*→?→?→||<-?||?→?→||<-SIG+TM+TM+TM+TM+TM</td><td>LRR-repeats+AP-GTPase+COR</td><td>682</td><td>-</td><td>Cyanobacteria</td><td>Pseudanabaena yagii</td><td>hypothetical protein [Pseudanabaena yagii].</td><td>GCF_012863495.1</td></tr>
<tr><td>WP_169482131.1</td><td>LRR-repeats+AP-GTPase+COR*→||<-?||?→||<-?<-?<-?<-?<-Cluster1745_2clades</td><td>LRR-repeats+AP-GTPase+COR</td><td>965</td><td>-</td><td>Firmicutes</td><td>Paenibacillus sp. SZ31</td><td>GTP-binding protein [Paenibacillus sp. SZ31].</td><td>GCF_012912005.1</td></tr>
<tr><td>WP_169551474.1</td><td>LRR-repeats+AP-GTPase+COR+DrHyd*→||<-?||?→?→||<-TPR-repeats</td><td>LRR-repeats+AP-GTPase+COR+DrHyd</td><td>1047</td><td>-</td><td>Bacteroidetes</td><td>Spirosoma sp. CJU-R4</td><td>hypothetical protein [Spirosoma sp. CJU-R4].</td><td>GCF_012849055.1</td></tr>
<tr><td>WP_169617268.1</td><td>RelE→ CHTH→?→||<-?<-?<-?<-?||LRR-repeats+AP-GTPase+COR+TIR*→?→||<-?<-?||?→?→||<-?<-RelE-ParE</td><td>LRR-repeats+AP-GTPase+COR+TIR</td><td>1174</td><td>-</td><td>Cyanobacteria</td><td>Nodosilinea sp. P-1105</td><td>leucine-rich repeat domain-containing protein [Nodosilinea sp. P-1105].</td><td>GCF_012911975.1</td></tr>
</table>

<table>
<tr><th>acc</th><th>operon</th><th>architecture</th><th>len</th><th>gen.name</th><th>taxend</th><th>species</th><th>defline</th><th>gca</th></tr>
<tr><td>WP_169792421.1</td><td><-ABC-ATPase<-?<-?<-?||?→ LRR-repeats+AP-GTPase+COR*→</td><td>LRR-repeats+AP-GTPase+COR</td><td>867</td><td>-</td><td>Actinobacteria</td><td>Actinoplanes rectilineatus</td><td>GTP-binding protein [Actinoplanes rectilineatus].</td><td>GCF_000962825.1</td></tr>
<tr><td>WP_170065039.1</td><td>REC→||<-?<-?<-?<-?||?→ LRR-repeats+AP-GTPase+COR+DpnII-MboI-NTD+TIR*→</td><td>LRR-repeats+AP-GTPase+COR+DpnII-MboI-NTD+TIR</td><td>992</td><td>-</td><td>Gammaproteobacteria</td><td>Methylovulum psychrotolerans</td><td>leucine-rich repeat domain-containing protein [Methylovulum psychrotolerans].</td><td>GCF_002923755.1</td></tr>
<tr><td>WP_170200769.1</td><td>HNOB→ HISKIN→?→||<-?<-?||LRR-repeats+AP-GTPase+COR+TIR*→</td><td>LRR-repeats+AP-GTPase+COR+TIR</td><td>921</td><td>-</td><td>Betaproteobacteria</td><td>Ideonella azotifigens</td><td>TIR domain-containing protein [Ideonella azotifigens].</td><td>GCF_006519715.1</td></tr>
<tr><td>WP_170318945.1</td><td>LRR-repeats+AP-GTPase+COR*→</td><td>LRR-repeats+AP-GTPase+COR</td><td>514</td><td>-</td><td>Actinobacteria</td><td>Allorhizocola rhizosphaerae</td><td>leucine-rich repeat domain-containing protein, partial [Allorhizocola rhizosphaerae].</td><td>GCF_003426775.1</td></tr>
<tr><td>WP_170321800.1</td><td>LRR-repeats+AP-GTPase+COR*→?→ Phytase-like→||<-?||?→?→ PSE→ ABC-ATPase→ ABC-ATPase→</td><td>LRR-repeats+AP-GTPase+COR</td><td>1057</td><td>-</td><td>Actinobacteria</td><td>Acrocarpospora pleiomorpha</td><td>leucine-rich repeat domain-containing protein [Acrocarpospora pleiomorpha].</td><td>GCF_009687885.1</td></tr>
<tr><td>WP_170944127.1</td><td>LRR-repeats+AP-GTPase+COR*→ COR→?→ RelE-ParE→</td><td>LRR-repeats+AP-GTPase+COR</td><td>216</td><td>-</td><td>Cyanobacteria</td><td>Leptolyngbya sp. BC1307</td><td>leucine-rich repeat domain-containing protein, partial [Leptolyngbya sp. BC1307].</td><td>GCF_002286735.1</td></tr>
<tr><td>WP_171062796.1</td><td>LRR-repeats+AP-GTPase+COR*→</td><td>LRR-repeats+AP-GTPase+COR</td><td>864</td><td>-</td><td>Bacteroidetes</td><td>Larkinella sp. C7</td><td>hypothetical protein [Larkinella sp. C7].</td><td>GCF_006149005.1</td></tr>
<tr><td>WP_171572097.1</td><td>EAD10+VMAP-M1+VMAP-C→?→ VWA+LRR-repeats+AP-GTPase+COR→
Cluster5_5clades→||<-?<-?<-LRR-repeats+AP-GTPase+COR<-?<-CASPASE+EAD10<-?<-tRNA||?→?→ Cluster2368_2clades→ Cluster2098_2clades→</td><td>VWA+LRR-repeats+AP-GTPase+COR</td><td>1320</td><td>-</td><td>Bacteroidetes</td><td>Flavobacterium sp. CLA17</td><td>leucine-rich repeat domain-containing protein [Flavobacterium sp. CLA17].</td><td>GCF_013112255.1</td></tr>
<tr><td>WP_171572101.1</td><td>LRR-repeats+AP-GTPase+COR*→</td><td>LRR-repeats+AP-GTPase+COR</td><td>845</td><td>-</td><td>Bacteroidetes</td><td>Flavobacterium sp. CLA17</td><td>leucine-rich repeat domain-containing protein [Flavobacterium sp. CLA17].</td><td>GCF_013112255.1</td></tr>
<tr><td>WP_171574279.1</td><td>ABC-ATPase→ Ferredoxin-betagrasp→?→?→||<-?||LRR-repeats+AP-GTPase+COR+TM+TM*→ Cluster1136_2clades→
PSE→?→||<-Cluster1782_2clades<-?<-Cluster1583_2clades<-Cluster1881_2clades<-Cluster1340_2clades</td><td>LRR-repeats+AP-GTPase+COR+TM+TM</td><td>1162</td><td>-</td><td>Bacteroidetes</td><td>Flavobacterium sp. CLA17</td><td>leucine-rich repeat domain-containing protein [Flavobacterium sp. CLA17].</td><td>GCF_013112255.1</td></tr>
<tr><td>WP_171574361.1</td><td><-SIG+TM+TM+TM+TM+TM+TM<-?||?→||<-SAM-methylase<-Cluster2335_2clades<-SIG+TM||?→
LRR-repeats+AP-GTPase+COR*→?→?→?→?→||<-ISOFLAVOMETHYLASE-HTH</td><td>LRR-repeats+AP-GTPase+COR</td><td>1103</td><td>-</td><td>Bacteroidetes</td><td>Flavobacterium sp. CLA17</td><td>leucine-rich repeat domain-containing protein [Flavobacterium sp. CLA17].</td><td>GCF_013112255.1</td></tr>
<tr><td>WP_171626408.1</td><td><-Cluster1969_2clades<-?<-?<-?||?→ LRR-repeats+AP-GTPase+COR+TIR*→||<-?||?→?→ METHYLASE→ ParA-Soj-PloopNTPase→ ParB→</td><td>LRR-repeats+AP-GTPase+COR+TIR</td><td>1281</td><td>-</td><td>Gammaproteobacteria</td><td>Pseudoalteromonas caenipelagi</td><td>leucine-rich repeat protein [Pseudoalteromonas caenipelagi].</td><td>GCF_013140855.1</td></tr>
<tr><td>WP_171740721.1</td><td><-SIG+ABhydrolase<-RNA-Helicase||LRR-repeats+AP-GTPase+COR*→</td><td>LRR-repeats+AP-GTPase+COR</td><td>859</td><td>-</td><td>Bacteroidetes</td><td>Spirosoma sp. TS118</td><td>hypothetical protein [Spirosoma sp. TS118].</td><td>GCF_013127955.1</td></tr>
<tr><td>WP_171801965.1</td><td>LRR-repeats+AP-GTPase+COR*→</td><td>LRR-repeats+AP-GTPase+COR</td><td>907</td><td>-</td><td>Gammaproteobacteria</td><td>Vibrio europaeus</td><td>GTP-binding protein [Vibrio europaeus].</td><td>GCF_013154935.1</td></tr>
<tr><td>WP_171971806.1</td><td>LRR-repeats+AP-GTPase+COR+TM+TM*→</td><td>LRR-repeats+AP-GTPase+COR+TM+TM</td><td>850</td><td>-</td><td>Cyanobacteria</td><td>Limnothrix rosea</td><td>leucine-rich repeat domain-containing protein [Limnothrix rosea].</td><td>GCF_001904615.1</td></tr>
<tr><td>WP_171976623.1</td><td>LRR-repeats+AP-GTPase+COR+TIR*→||<-?||?→||<-?||NUDIX→?→||<-Arginase</td><td>LRR-repeats+AP-GTPase+COR+TIR</td><td>1029</td><td>-</td><td>Cyanobacteria</td><td>Brasilonema octagenarum</td><td>leucine-rich repeat domain-containing protein [Brasilonema octagenarum].</td><td>GCF_006968915.1</td></tr>
<tr><td>WP_171977678.1</td><td><-SIG+TM+TM+Calcineurin||?→?→?→ LRR-repeats→ LRR-repeats+AP-GTPase+COR+TIR*→</td><td>LRR-repeats+AP-GTPase+COR+TIR</td><td>817</td><td>-</td><td>Cyanobacteria</td><td>Brasilonema octagenarum</td><td>leucine-rich repeat domain-containing protein [Brasilonema octagenarum].</td><td>GCF_006968915.1</td></tr>
<tr><td>WP_171982135.1</td><td>LRR-repeats+AP-GTPase+COR+TIR*→||<-?<-?<-MarR-HTH||?→||<-MarR-HTH<-?||Thioredoxin→</td><td>LRR-repeats+AP-GTPase+COR+TIR</td><td>1247</td><td>-</td><td>Betaproteobacteria</td><td>Aquaspirillum sp. LM1</td><td>leucine-rich repeat domain-containing protein [Aquaspirillum sp. LM1].</td><td>GCF_002002905.1</td></tr>
<tr><td>WP_171982185.1</td><td><-LexA-protease||?→||<-?<-?||?→ HSP70→ DNAJ→ PSE→ LRR-repeats+AP-GTPase+COR+TIR*→</td><td>LRR-repeats+AP-GTPase+COR+TIR</td><td>916</td><td>-</td><td>Betaproteobacteria</td><td>Aquaspirillum sp. LM1</td><td>leucine-rich repeat domain-containing protein [Aquaspirillum sp. LM1].</td><td>GCF_002002905.1</td></tr>
</table>

<table>
<tr><th>acc</th><th>operon</th><th>architecture</th><th>len</th><th>gen.name</th><th>taxend</th><th>species</th><th>defline</th><th>gca</th></tr>
<tr><td>WP_172187602.1</td><td>Chromo-N+CHROMO→||<-RelE-ParE<-?||?→ LRR-repeats+AP-GTPase+COR*→?→ SIG+TM+TM+TM+TM+TM+TM+TM+TM+TM+TM→</td><td>LRR-repeats+AP-GTPase+COR</td><td>913</td><td>-</td><td>Cyanobacteria</td><td>Microcoleus sp. IPMA8</td><td>leucine-rich repeat domain-containing protein [Microcoleus sp. IPMA8].</td><td>GCF_013179805.1</td></tr>
<tr><td>WP_172195227.1</td><td>LRR-repeats+AP-GTPase+COR+TIR*→||<-?||?→||<-?||NUDIX→?→||<-Arginase</td><td>LRR-repeats+AP-GTPase+COR+TIR</td><td>1029</td><td>-</td><td>Cyanobacteria</td><td>Brasilonema sennae</td><td>leucine-rich repeat domain-containing protein [Brasilonema sennae].</td><td>GCF_006968745.1</td></tr>
<tr><td>WP_172195319.1</td><td><-SIG+TM+TM+Calcineurin||?→?→?→ LRR-repeats+AP-GTPase+COR+TIR*→</td><td>LRR-repeats+AP-GTPase+COR+TIR</td><td>1330</td><td>-</td><td>Cyanobacteria</td><td>Brasilonema sennae</td><td>leucine-rich repeat domain-containing protein [Brasilonema sennae].</td><td>GCF_006968745.1</td></tr>
<tr><td>WP_172621632.1</td><td>LRR-repeats+AP-GTPase+COR+TIR*→||<-?||?→?→?→?→||<-?||cNMPBD→</td><td>LRR-repeats+AP-GTPase+COR+TIR</td><td>975</td><td>-</td><td>Bacteroidetes</td><td>Chitinophaga pinensis</td><td>TIR domain-containing protein [Chitinophaga pinensis].</td><td>GCF_902167305.1</td></tr>
<tr><td>WP_172622229.1</td><td>LRR-repeats+AP-GTPase+COR+TIR*→||<-?||?→?→?→?→||<-?||cNMPBD→</td><td>LRR-repeats+AP-GTPase+COR+TIR</td><td>975</td><td>-</td><td>Bacteroidetes</td><td>Chitinophaga pinensis</td><td>TIR domain-containing protein [Chitinophaga pinensis].</td><td>GCF_902167325.1</td></tr>
<tr><td>WP_172632369.1</td><td>REC→ LRR-repeats+AP-GTPase+COR*→</td><td>LRR-repeats+AP-GTPase+COR</td><td>1102</td><td>-</td><td>Chloroflexi</td><td>Dictyobacter sp. Uno17</td><td>leucine-rich repeat domain-containing protein [Dictyobacter sp. Uno17].</td><td>GCF_008326305.1</td></tr>
<tr><td>WP_173012781.1</td><td>LRR-repeats+AP-GTPase+COR+TIR*→||<-ParB<-ParA-Soj-PloopNTPase</td><td>LRR-repeats+AP-GTPase+COR+TIR</td><td>751</td><td>-</td><td>Alphaproteobacteria</td><td>Niveispirillum sp. SYP-B3756</td><td>TIR domain-containing protein [Niveispirillum sp. SYP-B3756].</td><td>GCF_009495745.1</td></tr>
<tr><td>WP_173037887.1</td><td><-SIG+TM<-?<-ABC-ATPase||?→?→?→?→ LRR-repeats+AP-GTPase+COR*→</td><td>LRR-repeats+AP-GTPase+COR</td><td>981</td><td>-</td><td>Actinobacteria</td><td>Phytohabitans flavus</td><td>hypothetical protein [Phytohabitans flavus].</td><td>GCF_011764545.1</td></tr>
<tr><td>WP_173124261.1</td><td>Cluster1612_2clades→?→?→||<-?<-?||LRR-repeats+AP-GTPase+COR*→</td><td>LRR-repeats+AP-GTPase+COR</td><td>1244</td><td>-</td><td>Actinobacteria</td><td>Kibdelosporangium persicum</td><td>leucine-rich repeat domain-containing protein [Kibdelosporangium persicum].</td><td>GCF_013280595.1</td></tr>
<tr><td>WP_173803276.1</td><td>LRR-repeats+AP-GTPase+COR+TIR*→||<-?<-ABC_membrane+ABC_tran||Cyanophycinsyn-ATPgrasp→ Cyanophycinsyn-ATPgrasp→</td><td>LRR-repeats+AP-GTPase+COR+TIR</td><td>794</td><td>-</td><td>Betaproteobacteria</td><td>Leptothrix sp. C29</td><td>TIR domain-containing protein, partial [Leptothrix sp. C29].</td><td>GCF_013294065.1</td></tr>
<tr><td>WP_174565117.1</td><td>LRR-repeats+AP-GTPase*→</td><td>LRR-repeats+AP-GTPase</td><td>485</td><td>-</td><td>Bacteroidetes</td><td>Flavobacterium sp. A45</td><td>leucine-rich repeat domain-containing protein, partial [Flavobacterium sp. A45].</td><td>GCF_002001005.1</td></tr>
<tr><td>WP_174708182.1</td><td>PAS+HISKIN→ REC→?→?→ LRR-repeats+AP-GTPase+COR+DUF4404*→||<-?||?→?→ ABC_membrane+ABC_tran→ ABC_membrane+ABC_tran→</td><td>LRR-repeats+AP-GTPase+COR+DUF4404</td><td>1122</td><td>-</td><td>Cyanobacteria</td><td>Nostoc sp. TCL240-02</td><td>leucine-rich repeat domain-containing protein [Nostoc sp. TCL240-02].</td><td>GCF_013343235.1</td></tr>
<tr><td>WP_174708278.1</td><td>ABhydrolase→?→||<-?||LRR-repeats→ PSE→ LRR-repeats+AP-GTPase+COR+TIR*→?→||<-?<-SIG+NUDIX</td><td>LRR-repeats+AP-GTPase+COR+TIR</td><td>919</td><td>-</td><td>Cyanobacteria</td><td>Nostoc sp. TCL240-02</td><td>leucine-rich repeat domain-containing protein [Nostoc sp. TCL240-02].</td><td>GCF_013343235.1</td></tr>
<tr><td>WP_174709624.1</td><td><-Arginase<-?||LRR-repeats+AP-GTPase+COR+TIR→ LRR-repeats+AP-GTPase+COR+TIR→?→||<-ABC-ATPase</td><td>LRR-repeats+AP-GTPase+COR+TIR</td><td>1126</td><td>-</td><td>Cyanobacteria</td><td>Nostoc sp. TCL240-02</td><td>leucine-rich repeat domain-containing protein [Nostoc sp. TCL240-02].</td><td>GCF_013343235.1</td></tr>
<tr><td>WP_174709625.1</td><td>LRR-repeats+AP-GTPase+COR+TIR*→</td><td>LRR-repeats+AP-GTPase+COR+TIR</td><td>1123</td><td>-</td><td>Cyanobacteria</td><td>Nostoc sp. TCL240-02</td><td>leucine-rich repeat domain-containing protein [Nostoc sp. TCL240-02].</td><td>GCF_013343235.1</td></tr>
<tr><td>WP_174711091.1</td><td>LRR-repeats+AP-GTPase+COR*→</td><td>LRR-repeats+AP-GTPase+COR</td><td>1189</td><td>-</td><td>Cyanobacteria</td><td>Nostoc sp. TCL240-02</td><td>leucine-rich repeat domain-containing protein [Nostoc sp. TCL240-02].</td><td>GCF_013343235.1</td></tr>
<tr><td>WP_174712042.1</td><td>LRR-repeats+AP-GTPase+COR*→?→||<-?||?→||<-?||?→ ClpABN-AAA+ClpABC-AAA→</td><td>LRR-repeats+AP-GTPase+COR</td><td>967</td><td>-</td><td>Cyanobacteria</td><td>Nostoc sp. TCL240-02</td><td>leucine-rich repeat domain-containing protein [Nostoc sp. TCL240-02].</td><td>GCF_013343235.1</td></tr>
<tr><td>WP_175357678.1</td><td>Cluster54_3clades→?→ RelE-ParE→ Cluster53_2clades→?→ LRR-repeats+AP-GTPase+COR*→?→?→?→||<-?||?→||<-Pentapeptide-repeats</td><td>LRR-repeats+AP-GTPase+COR</td><td>928</td><td>-</td><td>Cyanobacteria</td><td>Pseudanabaena biceps</td><td>leucine-rich repeat domain-containing protein [Pseudanabaena biceps].</td><td>GCF_013361095.1</td></tr>
<tr><td>WP_175471885.1</td><td>Mbetalac→?→ LRR-repeats+AP-GTPase+COR*→||<-?<-ICLR-HTH||?→?→?→ ABhydrolase→</td><td>LRR-repeats+AP-GTPase+COR</td><td>886</td><td>-</td><td>Actinobacteria</td><td>Geodermatophilus telluris</td><td>hypothetical protein [Geodermatophilus telluris].</td><td>GCF_900102745.1</td></tr>
</table>

<table>
<tr><th>acc</th><th>operon</th><th>architecture</th><th>len</th><th>gen.name</th><th>taxend</th><th>species</th><th>defline</th><th>gca</th></tr>
<tr><td>WP_175586865.1</td><td><-HISKIN||?→||<-?||?→?→ PSE→||<-?||PSE→||<-?||LRR-repeats+AP-GTPase+COR*→</td><td>LRR-repeats+AP-GTPase+COR</td><td>868</td><td>-</td><td>Cyanobacteria</td><td>Nostoc sp. UIC 10630</td><td>leucine-rich repeat domain-containing protein, partial [Nostoc sp. UIC 10630].</td><td>GCF_010747425.1</td></tr>
<tr><td>WP_175608776.1</td><td>LRR-repeats+AP-GTPase+COR+TIR*→?→?→?→?→?→ TIR→ TM+TM+TM→</td><td>LRR-repeats+AP-GTPase+COR+TIR</td><td>1128</td><td>-</td><td>Bacteroidetes</td><td>Chryseobacterium gambrini</td><td>leucine-rich repeat domain-containing protein [Chryseobacterium gambrini].</td><td>GCF_900156825.1</td></tr>
<tr><td>WP_176159351.1</td><td>METHYLASE→?→||<-?||SIG+Trypsin+PDZ→?→ Cluster978_2clades→ LRR-repeats+AP-GTPase+COR+TIR*→?→ GDSL→</td><td>LRR-repeats+AP-GTPase+COR+TIR</td><td>813</td><td>-</td><td>Verrucomicrobia</td><td>Prosthecobacter debontii</td><td>TIR domain-containing protein [Prosthecobacter debontii].</td><td>GCF_900167535.1</td></tr>
<tr><td>WP_176159465.1</td><td>SAM-methylase→?→||<-?<-SIG+ABhydrolase||tRNA→?→ Cluster1685_2clades→||<-?||LRR-repeats+AP-GTPase+COR+TIR*→</td><td>LRR-repeats+AP-GTPase+COR+TIR</td><td>949</td><td>-</td><td>Verrucomicrobia</td><td>Prosthecobacter debontii</td><td>leucine-rich repeat domain-containing protein [Prosthecobacter debontii].</td><td>GCF_900167535.1</td></tr>
<tr><td>WP_176722277.1</td><td>LRR-repeats+AP-GTPase+COR*→</td><td>LRR-repeats+AP-GTPase+COR</td><td>881</td><td>-</td><td>Gammaproteobacteria</td><td>Candidatus Thiosymbion oneisti</td><td>leucine-rich repeat domain-containing protein, partial [Candidatus Thiosymbion oneisti].</td><td>GCF_900092655.1</td></tr>
<tr><td>WP_176722477.1</td><td>LRR-repeats+AP-GTPase+COR*→?→ PSE→?→?→?→ ABC-ATPase→||<-HTH</td><td>LRR-repeats+AP-GTPase+COR</td><td>838</td><td>-</td><td>Gammaproteobacteria</td><td>Candidatus Thiosymbion oneisti</td><td>hypothetical protein [Candidatus Thiosymbion oneisti].</td><td>GCF_900092655.1</td></tr>
<tr><td>WP_177187116.1</td><td>LRR-repeats+AP-GTPase+COR*→||<-?||?→||<-RnasePH+RnasePH+KH+S1</td><td>LRR-repeats+AP-GTPase+COR</td><td>911</td><td>-</td><td>Bacteroidetes</td><td>Chryseobacterium jeonii</td><td>GTP-binding protein [Chryseobacterium jeonii].</td><td>GCF_900112115.1</td></tr>
<tr><td>WP_177199908.1</td><td>PRTase→||<-?<-?<-?||LRR-repeats+AP-GTPase+COR+TIR*→?→?→?→?→ Aminotran_1_2→</td><td>LRR-repeats+AP-GTPase+COR+TIR</td><td>987</td><td>-</td><td>Bacteroidetes</td><td>Flexibacter flexilis</td><td>leucine-rich repeat domain-containing protein [Flexibacter flexilis].</td><td>GCF_900112255.1</td></tr>
<tr><td>WP_177201087.1</td><td>LRR-repeats+AP-GTPase+COR+TIR*→</td><td>LRR-repeats+AP-GTPase+COR+TIR</td><td>764</td><td>-</td><td>Gammaproteobacteria</td><td>Neptunomonas qingdaonensis</td><td>TIR domain-containing protein [Neptunomonas qingdaonensis].</td><td>GCF_900113275.1</td></tr>
<tr><td>WP_178133228.1</td><td>LRR-repeats+AP-GTPase+COR+CASPASE*→</td><td>LRR-repeats+AP-GTPase+COR+CASPASE</td><td>1170</td><td>-</td><td>Alphaproteobacteria</td><td>Enhydrobacter sp. CC-CFT640</td><td>CHAT domain-containing protein [Enhydrobacter sp. CC-CFT640].</td><td>GCF_008039615.1</td></tr>
<tr><td>WP_178316998.1</td><td>LRR-repeats+AP-GTPase+COR*→</td><td>LRR-repeats+AP-GTPase+COR</td><td>1450</td><td>-</td><td>Firmicutes</td><td>Lachnospiraceae bacterium</td><td>GTP-binding protein [Lachnospiraceae bacterium].</td><td>-</td></tr>
<tr><td>WP_179047135.1</td><td>ABC-ATPase→||<-?||?→||<-?||LRR-repeats+AP-GTPase+COR+TIR*→?→||<-?<-Cluster1974_2clades||?→?→||<-PSE<-Cluster1515_2clades</td><td>LRR-repeats+AP-GTPase+COR+TIR</td><td>939</td><td>-</td><td>Cyanobacteria</td><td>Nostoc sp. TCL26-01</td><td>leucine-rich repeat domain-containing protein [Nostoc sp. TCL26-01].</td><td>GCF_013393945.1</td></tr>
<tr><td>WP_179049659.1</td><td>Cluster1081_2clades→?→?→||<-?<-PSE<-?||LRR-repeats+AP-GTPase+COR+TIR*→||<-?<-MoxR-AAA||?→ TPR-repeats→||<-SIG+TM+TM+TM+TM+TM+TM+TM+TM+TM+TM+TM<-SIG+TM</td><td>LRR-repeats+AP-GTPase+COR+TIR</td><td>1127</td><td>-</td><td>Cyanobacteria</td><td>Nostoc sp. TCL26-01</td><td>leucine-rich repeat domain-containing protein [Nostoc sp. TCL26-01].</td><td>GCF_013393945.1</td></tr>
<tr><td>WP_179049876.1</td><td><-ACET<-?||Pentapeptide→ Cluster54_3clades→ Cluster1093_2clades→?→||<-?||LRR-repeats+AP-GTPase+COR+TIR*→?→?→ HNH→?→ PSE→||<-?<-?<-Cluster2062_2clades</td><td>LRR-repeats+AP-GTPase+COR+TIR</td><td>1116</td><td>-</td><td>Cyanobacteria</td><td>Nostoc sp. TCL26-01</td><td>leucine-rich repeat domain-containing protein [Nostoc sp. TCL26-01].</td><td>GCF_013393945.1</td></tr>
<tr><td>WP_179050104.1</td><td><-ABC_membrane+ABC_tran<-?<-?||?→||<-?<-?||LRR-repeats+AP-GTPase+COR+TIR*→?→||<-?<-?<-?<-?<-Cluster2103_2clades</td><td>LRR-repeats+AP-GTPase+COR+TIR</td><td>999</td><td>-</td><td>Cyanobacteria</td><td>Nostoc sp. TCL26-01</td><td>leucine-rich repeat domain-containing protein [Nostoc sp. TCL26-01].</td><td>GCF_013393945.1</td></tr>
<tr><td>WP_179067421.1</td><td>REC→ REC→?→||<-?||LRR-repeats+AP-GTPase+COR+DUF4404*→</td><td>LRR-repeats+AP-GTPase+COR+DUF4404</td><td>1124</td><td>-</td><td>Cyanobacteria</td><td>Nostoc sp. C052</td><td>leucine-rich repeat domain-containing protein [Nostoc sp. C052].</td><td>GCF_013393905.1</td></tr>
<tr><td>WP_179068637.1</td><td>Cluster5_5clades→ Cluster5_5clades→ LRR-repeats+AP-GTPase+COR+TM+TM*→||<-?||Pkinase→</td><td>LRR-repeats+AP-GTPase+COR+TM+TM</td><td>904</td><td>-</td><td>Cyanobacteria</td><td>Nostoc sp. C052</td><td>leucine-rich repeat protein [Nostoc sp. C052].</td><td>GCF_013393905.1</td></tr>
<tr><td>WP_179072263.1</td><td>TPR-repeats→?→ LRR-repeats+AP-GTPase+COR*→</td><td>LRR-repeats+AP-GTPase+COR</td><td>1193</td><td>-</td><td>Cyanobacteria</td><td>Nostoc sp. C057</td><td>leucine-rich repeat domain-containing protein [Nostoc sp. C057].</td><td>GCF_013393925.1</td></tr>
<tr><td>WP_179072929.1</td><td><-Arginase<-?||?→ LRR-repeats+AP-GTPase+COR+TIR*→?→||<-?||?→?→?→||<-tRNA||Cluster1742_2clades→</td><td>LRR-repeats+AP-GTPase+COR+TIR</td><td>1009</td><td>-</td><td>Cyanobacteria</td><td>Nostoc sp. C057</td><td>leucine-rich repeat domain-containing protein [Nostoc sp. C057].</td><td>GCF_013393925.1</td></tr>
</table>

acc	operon	architecture	len	gen.name	taxend	species	defline	gca
WP_179074744.1	Cluster5_5clades→ Cluster5_5clades→ LRR-repeats+AP-GTPase+COR+TM+TM*→||<-CASPASE+TM	LRR-repeats+AP-GTPase+COR+TM+TM	865	-	Cyanobacteria	Nostoc sp. C057	leucine-rich repeat domain-containing protein [Nostoc sp. C057].	GCF_013393925.1
WP_179212721.1	LRR-repeats+AP-GTPase+COR*→	LRR-repeats+AP-GTPase+COR	850	-	Bacteroidetes	Sphingobacteriales bacterium TSM_CSS	GTP-binding protein [Sphingobacteriales bacterium TSM_CSS].	-
WP_180284786.1	<-MarR-HTH||?→ SIG+TM+TM+TM+TM+TM+TM+TM→ SIG+TM+TM+TM+TM+TM+TM+TM→ ABC-ATPase→ ABC-ATPase→?→ LRR-repeats+AP-GTPase+COR+TIR*→	LRR-repeats+AP-GTPase+COR+TIR	709	-	Alphaproteobacteria	Azospirillum oleiclasticum	hypothetical protein [Azospirillum oleiclasticum].	GCF_013423495.1
WP_180287677.1	SbcC→?→ LRR-repeats+AP-GTPase+COR+TIR*→	LRR-repeats+AP-GTPase+COR+TIR	722	-	Alphaproteobacteria	Azospirillum oleiclasticum	hypothetical protein [Azospirillum oleiclasticum].	GCF_013423485.1
WP_180693101.1	LRR-repeats+AP-GTPase+COR+TIR*→||<-?<-ABC_membrane+ABC_tran||Cyanophycinsyn-ATPgrasp→ Cyanophycinsyn-ATPgrasp→	LRR-repeats+AP-GTPase+COR+TIR	904	-	Betaproteobacteria	Sphaerotilus natans	leucine-rich repeat domain-containing protein, partial [Sphaerotilus natans].	GCF_013426975.1
WP_181245610.1	TM→ LRR-repeats+AP-GTPase+COR*→	LRR-repeats+AP-GTPase+COR	1035	-	Actinobacteria	Glycomyces artemisiae	leucine-rich repeat domain-containing protein [Glycomyces artemisiae].	GCF_003002955.1
WP_181357317.1	<-REC||REC→||<-?<-?<-?<-TIR+TM+CHASE2+CHASE2+TM+TM+TM||LRR-repeats+AP-GTPase+COR+TIR*→	LRR-repeats+AP-GTPase+COR+TIR	1082	-	Cyanobacteria	Stenomitos frigidus	leucine-rich repeat domain-containing protein [Stenomitos frigidus].	GCF_003003795.1
WP_181842201.1	VWA+vWA-L+LRR-repeats+AP-GTPase+COR+TIR*→	VWA+vWA-L+LRR-repeats+AP-GTPase+COR+TIR	1607	-	Bacteroidetes	Emticicia sp. BO119	leucine-rich repeat domain-containing protein [Emticicia sp. BO119].	GCF_013912375.1
WP_181930367.1	LRR-repeats+AP-GTPase+COR*→	LRR-repeats+AP-GTPase+COR	1084	-	Cyanobacteria	Nostoc edaphicum	leucine-rich repeat domain-containing protein [Nostoc edaphicum].	GCF_014023275.1
WP_181931253.1	<-Pkinase+Pentapeptide<-?<-?<-?||?→ LRR-repeats+AP-GTPase+COR+TIR*→||<-Cluster5_5clades<-Arginase<-?||?→||<-?<-?||Cluster54_3clades→	LRR-repeats+AP-GTPase+COR+TIR	1046	-	Cyanobacteria	Nostoc edaphicum	leucine-rich repeat domain-containing protein [Nostoc edaphicum].	GCF_014023275.1
WP_182043976.1	LRR-repeats+AP-GTPase+COR+TIR*→	LRR-repeats+AP-GTPase+COR+TIR	1134	-	Bacteroidetes	Moheibacter sp. BDHS18	leucine-rich repeat domain-containing protein [Moheibacter sp. BDHS18].	GCF_014042165.1
WP_182891755.1	LRR-repeats+AP-GTPase+COR*→	LRR-repeats+AP-GTPase+COR	1689	-	Actinobacteria	Amycolatopsis sp. DR6-1	hypothetical protein [Amycolatopsis sp. DR6-1].	GCF_014145675.1
WP_183290945.1	<-SIG+ABhydrolase||?→?→?→?→||<-?<-?||LRR-repeats+AP-GTPase+COR+TIR→ LRR-repeats+AP-GTPase+COR+TIR→?→ Cluster1478_2clades→?→ LRR-repeats+AP-GTPase+COR+TIR*→?→ SIGMA-HTH→ SIGMA-HTH→	LRR-repeats+AP-GTPase+COR+TIR	1386	-	Bacteroidetes	Chitinophaga sancti	leucine-rich repeat domain-containing protein [Chitinophaga sancti].	GCF_014190745.1
WP_183290948.1	LRR-repeats+AP-GTPase+COR_TIR*→	LRR-repeats+AP-GTPase+COR_TIR	1378	-	Bacteroidetes	Chitinophaga sancti	TIR domain-containing protein [Chitinophaga sancti].	GCF_014190745.1
WP_183290949.1	LRR-repeats+AP-GTPase+COR+TIR*→	LRR-repeats+AP-GTPase+COR+TIR	1391	-	Bacteroidetes	Chitinophaga sancti	hypothetical protein [Chitinophaga sancti].	GCF_014190745.1
WP_183974348.1	FGS→ FGS→ FGS→ LRR-repeats+AP-GTPase+COR+CASPASE*→	LRR-repeats+AP-GTPase+COR+CASPASE	981	-	Bacteroidetes	Runella defluvii	hypothetical protein [Runella defluvii].	GCF_014195535.1
WP_183975497.1	<-SIGMA-HTH||SIG+ABhydrolase→ LRR-repeats+AP-GTPase+COR*→?→||<-?<-?<-?<-?<-?<-SIGMA-HTH	LRR-repeats+AP-GTPase+COR	999	-	Bacteroidetes	Runella defluvii	leucine-rich repeat domain-containing protein [Runella defluvii].	GCF_014195535.1
WP_183978441.1	TIR+TPR+TPR-S→||<-?<-?||?→||<-?||LRR-repeats+AP-GTPase+COR+TIR→?→ TPR-repeats→?→ TPR-repeats→?→||<-AP-GTPase+COR<-?<-HTH	LRR-repeats+AP-GTPase+COR+TIR	603	-	Bacteroidetes	Runella defluvii	hypothetical protein [Runella defluvii].	GCF_014195535.1
WP_183978452.1	LRR-repeats+AP-GTPase+COR+TIR*→	LRR-repeats+AP-GTPase+COR+TIR	1000	-	Bacteroidetes	Runella defluvii	leucine-rich repeat domain-containing protein [Runella defluvii].	GCF_014195535.1
WP_184494281.1	Cluster1244_2clades→?→ Cluster1291_2clades→ TIR+LRR-repeats+AP-GTPase+TIR*→||<-?<-?<-?||REC→	TIR+LRR-repeats+AP-GTPase+TIR	1102	-	Bacteroidetes	Algoriphagus iocasae	TIR domain-containing protein [Algoriphagus iocasae].	GCF_014206875.1

<table>
<tr><th>acc</th><th>operon</th><th>architecture</th><th>len</th><th>gen.name</th><th>taxend</th><th>species</th><th>defline</th><th>gca</th></tr>
<tr><td>WP_184831163.1</td><td>SIG+TM+TM+TM+TM+TM→||<-?||?→?→?→?→ PSE→ LRR-repeats+AP-GTPase+COR*→||<-?<-?||wHTH-4stranded+TPRs+APATPase+TPR-repeats→</td><td>LRR-repeats+AP-GTPase+COR</td><td>802</td><td>-</td><td>Actinobacteria</td><td>Allocatelliglobosispora scoriae</td><td>hypothetical protein [Allocatelliglobosispora scoriae].</td><td>GCF_014204945.1</td></tr>
<tr><td>WP_184846103.1</td><td>LRR-repeats+AP-GTPase+COR*→||<-?<-?<-?<-BACTERIALFRINGE+TM+TM</td><td>LRR-repeats+AP-GTPase+COR</td><td>1247</td><td>-</td><td>Actinobacteria</td><td>Allocatelliglobosispora scoriae</td><td>leucine-rich repeat domain-containing protein [Allocatelliglobosispora scoriae].</td><td>GCF_014204945.1</td></tr>
<tr><td>WP_184892074.1</td><td>LRR-repeats+AP-GTPase+COR*→?→?→ REC→ HISKIN→</td><td>LRR-repeats+AP-GTPase+COR</td><td>1463</td><td>-</td><td>Actinobacteria</td><td>Streptomyces scabiei</td><td>leucine-rich repeat domain-containing protein [Streptomyces scabiei].</td><td>GCF_014203845.1</td></tr>
<tr><td>WP_184930759.1</td><td>LRR-repeats+AP-GTPase+COR*→</td><td>LRR-repeats+AP-GTPase+COR</td><td>954</td><td>-</td><td>Actinobacteria</td><td>Streptomyces sp. SFB5A</td><td>hypothetical protein [Streptomyces sp. SFB5A].</td><td>GCF_014203895.1</td></tr>
<tr><td>WP_184953474.1</td><td>GNTR-HTH→||<-?||REC→ REC→?→ LRR-repeats+AP-GTPase+COR+TIR*→||<-35exo</td><td>LRR-repeats+AP-GTPase+COR+TIR</td><td>1043</td><td>-</td><td>Actinobacteria</td><td>Actinoplanes abujensis</td><td>TIR domain-containing protein [Actinoplanes abujensis].</td><td>GCF_016861995.1</td></tr>
<tr><td>WP_185097769.1</td><td>LRR-repeats+AP-GTPase+COR*→</td><td>LRR-repeats+AP-GTPase+COR</td><td>901</td><td>-</td><td>Bacteroidetes</td><td>Elizabethkingia anophelis</td><td>leucine-rich repeat domain-containing protein [Elizabethkingia anophelis].</td><td>GCF_001703835.1</td></tr>
<tr><td>WP_185204695.1</td><td>Cluster1364__2clades→?→?→?→?→?→?→ LRR-repeats+AP-GTPase+COR*→||<-Cyanophycinsyn-ATPgrasp</td><td>LRR-repeats+AP-GTPase+COR</td><td>1038</td><td>-</td><td>Bacteroidetes</td><td>Chryseobacterium sp. C3</td><td>leucine-rich repeat protein [Chryseobacterium sp. C3].</td><td>GCF_014218905.1</td></tr>
<tr><td>WP_185236942.1</td><td>LRR-repeats+AP-GTPase+COR+TM+TM*→</td><td>LRR-repeats+AP-GTPase+COR+TM+TM</td><td>828</td><td>-</td><td>Cyanobacteria</td><td>Microcystis aeruginosa</td><td>leucine-rich repeat protein [Microcystis aeruginosa].</td><td>GCF_014218745.1</td></tr>
<tr><td>WP_185240111.1</td><td>SAM-methylase→?→||<-?<-SIG+TPM__phosphatase+TM+TM+TM||LRR-repeats+AP-GTPase+COR+TM+TM*→||<-?<-?<-?||?→||<-Pro_CA</td><td>LRR-repeats+AP-GTPase+COR+TM+TM</td><td>874</td><td>-</td><td>Cyanobacteria</td><td>Microcystis aeruginosa</td><td>leucine-rich repeat protein [Microcystis aeruginosa].</td><td>GCF_014218765.1</td></tr>
<tr><td>WP_185478866.1</td><td><-Cluster5__5clades||LRR-repeats+AP-GTPase+COR+DUF4404*→</td><td>LRR-repeats+AP-GTPase+COR+DUF4404</td><td>1084</td><td>-</td><td>Cyanobacteria</td><td>Trichormus variabilis</td><td>leucine-rich repeat domain-containing protein [Trichormus variabilis].</td><td>GCF_014222125.1</td></tr>
<tr><td>WP_185479669.1</td><td>LRR-repeats+AP-GTPase+COR+DUF4404*→||<-?<-ABC-ATPase<-?||?→||<-REC</td><td>LRR-repeats+AP-GTPase+COR+DUF4404</td><td>866</td><td>-</td><td>Cyanobacteria</td><td>Trichormus variabilis</td><td>leucine-rich repeat domain-containing protein, partial [Trichormus variabilis].</td><td>GCF_014222155.1</td></tr>
<tr><td>WP_185482851.1</td><td><-Cluster5__5clades||LRR-repeats+AP-GTPase+COR+DUF4404*→||<-?<-ABC-ATPase<-?||?→||<-REC</td><td>LRR-repeats+AP-GTPase+COR+DUF4404</td><td>1084</td><td>-</td><td>Cyanobacteria</td><td>Trichormus variabilis</td><td>leucine-rich repeat domain-containing protein [Trichormus variabilis].</td><td>GCF_014222225.1</td></tr>
<tr><td>WP_185499823.1</td><td><-Cluster5__5clades||LRR-repeats+AP-GTPase+COR+DUF4404*→||<-?<-ABC-ATPase<-?||?→||<-REC</td><td>LRR-repeats+AP-GTPase+COR+DUF4404</td><td>1199</td><td>-</td><td>Cyanobacteria</td><td>Trichormus variabilis</td><td>leucine-rich repeat domain-containing protein [Trichormus variabilis].</td><td>GCF_014222145.1</td></tr>
<tr><td>WP_185505742.1</td><td><-Cluster5__5clades||LRR-repeats+AP-GTPase+COR+DUF4404*→||<-?<-ABC-ATPase<-?||?→||<-REC</td><td>LRR-repeats+AP-GTPase+COR+DUF4404</td><td>992</td><td>-</td><td>Cyanobacteria</td><td>Trichormus variabilis</td><td>leucine-rich repeat domain-containing protein [Trichormus variabilis].</td><td>GCF_014222245.1</td></tr>
<tr><td>WP_185539469.1</td><td>LRR-repeats+AP-GTPase+COR+DUF4404*→||<-?<-ABC-ATPase<-?||?→||<-REC</td><td>LRR-repeats+AP-GTPase+COR+DUF4404</td><td>895</td><td>-</td><td>Cyanobacteria</td><td>Trichormus variabilis</td><td>leucine-rich repeat domain-containing protein, partial [Trichormus variabilis].</td><td>GCF_014222135.1</td></tr>
<tr><td>WP_185567106.1</td><td>LRR-repeats+AP-GTPase+COR+TM+TM*→||<-CASPASE+TM</td><td>LRR-repeats+AP-GTPase+COR+TM+TM</td><td>862</td><td>-</td><td>Cyanobacteria</td><td>Nostoc sp. 2RC</td><td>leucine-rich repeat domain-containing protein [Nostoc sp. 2RC].</td><td>GCF_014222165.1</td></tr>
<tr><td>WP_185570121.1</td><td>PIN→ LRR-repeats+AP-GTPase+COR+TIR*→</td><td>LRR-repeats+AP-GTPase+COR+TIR</td><td>1020</td><td>-</td><td>Cyanobacteria</td><td>Nostoc sp. 2RC</td><td>leucine-rich repeat domain-containing protein [Nostoc sp. 2RC].</td><td>GCF_014222165.1</td></tr>
<tr><td>WP_185579937.1</td><td>ABhydrolase→?→||<-?||COR+TIR→ LRR-repeats+AP-GTPase+COR+TIR*→?→||<-PIN<-?<-?<-SIG+NUDIX</td><td>LRR-repeats+AP-GTPase+COR+TIR</td><td>1003</td><td>-</td><td>Cyanobacteria</td><td>unclassified Nostoc</td><td>MULTISPECIES: leucine-rich repeat domain-containing protein [unclassified Nostoc].</td><td>GCF_014222255.1</td></tr>
<tr><td>WP_185580380.1</td><td>Cluster5__5clades→ LRR-repeats+AP-GTPase+COR+TIR*→||<-CASPASE+TM</td><td>LRR-repeats+AP-GTPase+COR+TIR</td><td>893</td><td>-</td><td>Cyanobacteria</td><td>unclassified Nostoc</td><td>MULTISPECIES: leucine-rich repeat domain-containing protein [unclassified Nostoc].</td><td>GCF_014222255.1</td></tr>
</table>

acc	operon	architecture	len	gen.name	taxend	species	defline	gca
WP_185583983.1	<-PIN<-?<-?||?→?→||<-Arginase<-?||LRR-repeats+AP-GTPase+COR*→	LRR-repeats+AP-GTPase+COR	358	-	Cyanobacteria	Nostoc sp. UCD120	leucine-rich repeat domain-containing protein, partial [Nostoc sp. UCD120].	GCF_014222255.1
WP_185584434.1	PAS+HISKIN→ REC→ PSE→?→?→?→ LRR-repeats+AP-GTPase+COR+TIR*→	LRR-repeats+AP-GTPase+COR+TIR	1163	-	Cyanobacteria	Nostoc sp. UCD120	leucine-rich repeat domain-containing protein [Nostoc sp. UCD120].	GCF_014222255.1
WP_185591915.1	<-PIN<-?<-?||?→?→||<-Arginase<-?||LRR-repeats+AP-GTPase+COR+TIR*→	LRR-repeats+AP-GTPase+COR+TIR	802	-	Cyanobacteria	Nostoc sp. UCD122	leucine-rich repeat domain-containing protein [Nostoc sp. UCD122].	GCF_014222275.1
WP_185592013.1	ABhydrolase→?→||<-?||COR+TIR→ LRR-repeats+AP-GTPase+COR+TIR*→?→||<-PIN	LRR-repeats+AP-GTPase+COR+TIR	1003	-	Cyanobacteria	Nostoc sp. UCD122	leucine-rich repeat domain-containing protein [Nostoc sp. UCD122].	GCF_014222275.1
WP_185592050.1	LRR-repeats+AP-GTPase+COR+TIR*→	LRR-repeats+AP-GTPase+COR+TIR	859	-	Cyanobacteria	Nostoc sp. UCD122	leucine-rich repeat domain-containing protein, partial [Nostoc sp. UCD122].	GCF_014222275.1
WP_185592512.1	Cluster5_5clades→ LRR-repeats+AP-GTPase+COR+TIR*→||<-CASPASE+TM	LRR-repeats+AP-GTPase+COR+TIR	893	-	Cyanobacteria	Nostoc sp. UCD122	leucine-rich repeat domain-containing protein [Nostoc sp. UCD122].	GCF_014222275.1
WP_185607149.1	<-PIN<-?<-?||?→?→||<-Arginase<-?||LRR-repeats+AP-GTPase+COR+TIR*→	LRR-repeats+AP-GTPase+COR+TIR	825	-	Cyanobacteria	Nostoc sp. UCD121	leucine-rich repeat domain-containing protein [Nostoc sp. UCD121].	GCF_014222285.1
WP_185607288.1	LRR-repeats+AP-GTPase+COR+TIR*→?→?→?→ ABC_membrane+ABC_tran→ ABC_membrane+ABC_tran→	LRR-repeats+AP-GTPase+COR+TIR	917	-	Cyanobacteria	Nostoc sp. UCD121	leucine-rich repeat domain-containing protein, partial [Nostoc sp. UCD121].	GCF_014222285.1
WP_186435859.1	<-Cluster1077_2clades||Aminotran_1_2→?→||<-?<-?||?→ LRR-repeats+AP-GTPase+COR*→	LRR-repeats+AP-GTPase+COR	980	-	Gammaproteobacteria	Thalassolituus sp. C2-1	GTP-binding protein [Thalassolituus sp. C2-1].	GCF_007785795.1
WP_186890728.1	<-Cluster1951_2clades<-?<-?||?→ SIG+TM+TM+TM+HISKIN→?→||<-?||LRR-repeats+AP-GTPase+COR+DrHyd*→||<-?<-REC	LRR-repeats+AP-GTPase+COR+DrHyd	1036	-	Betaproteobacteria	Undibacterium amnicola	hypothetical protein [Undibacterium amnicola].	GCF_014284275.1
WP_186956531.1	LRR-repeats+AP-GTPase+COR+TIR*→	LRR-repeats+AP-GTPase+COR+TIR	1232	-	Betaproteobacteria	Undibacterium sp. NL8W	leucine-rich repeat domain-containing protein [Undibacterium sp. NL8W].	GCF_014284125.1
WP_187308183.1	LRR-repeats+AP-GTPase+COR+TM+TM*→||<-?<-?||?→?→||<-?<-?<-SNF	LRR-repeats+AP-GTPase+COR+TM+TM	720	-	Cyanobacteria	Nostoc cycadae	leucine-rich repeat domain-containing protein, partial [Nostoc cycadae].	GCF_002897135.1
WP_187315132.1	LRR-repeats+AP-GTPase+COR*→?→?→?→||<-?<-tRNA<-?<-?<-Cluster1829_2clades	LRR-repeats+AP-GTPase+COR	914	-	Bacteroidetes	Hymenobacter sp. BT190	GTP-binding protein [Hymenobacter sp. BT190].	GCF_014333525.1
WP_187466435.1	LRR-repeats+AP-GTPase+COR+EAD11→*?→?→ LRR-repeats+AP-GTPase+COR+EAD11*→	LRR-repeats+AP-GTPase+COR+EAD11	1040	-	Bacteroidetes	Lewinella lacunae	leucine-rich repeat domain-containing protein [Lewinella lacunae].	GCF_014349155.1
WP_187466438.1	LRR-repeats+AP-GTPase+COR+EAD11*→	LRR-repeats+AP-GTPase+COR+EAD11	931	-	Bacteroidetes	Lewinella lacunae	leucine-rich repeat domain-containing protein [Lewinella lacunae].	GCF_014349155.1
WP_187467589.1	GTPase-AIG→||<-?||?→?→||<-?<-?<-?||LRR-repeats+AP-GTPase+COR+CASPASE*→?→ FGS→?→ RVT→?→ FGS→	LRR-repeats+AP-GTPase+COR+CASPASE	1063	-	Bacteroidetes	Lewinella lacunae	CHAT domain-containing protein [Lewinella lacunae].	GCF_014349155.1
WP_187560609.1	SIGMA-HTH→?→ RADICAL-SAM→ LRR-repeats+AP-GTPase+COR+TIR*→?→ HISKIN→	LRR-repeats+AP-GTPase+COR+TIR	970	-	Bacteroidetes	Kordia sp. YSTF-M3	leucine-rich repeat domain-containing protein [Kordia sp. YSTF-M3].	GCF_014397005.1
WP_187775535.1	HTH→?→?→ LRR-repeats+AP-GTPase+COR+TIR*→||<-?<-ABC_membrane+ABC_tran||Cyanophycinsyn-ATPgrasp→ Cyanophycinsyn-ATPgrasp→	LRR-repeats+AP-GTPase+COR+TIR	1298	-	Betaproteobacteria	Sphaerotilus natans	leucine-rich repeat domain-containing protein [Sphaerotilus natans].	GCF_008329925.1

Gene neighborhoods and domain architectures of APGTPases whose LRR domains are either absent or not detected

<table>
<tr><th>acc</th><th>operon</th><th>architecture</th><th>len</th><th>gen.name</th><th>taxend</th><th>species</th><th>defline</th><th>gca</th></tr>
<tr><td>AAP36207.1</td><td>AP-GTPase*→</td><td>AP-GTPase</td><td>220</td><td>-</td><td>-</td><td>synthetic construct</td><td>Homo sapiens RAB3D, member RAS oncogene family, partial [synthetic construct].</td><td>-</td></tr>
<tr><td>AAP36967.1</td><td>AP-GTPase*→</td><td>AP-GTPase</td><td>208</td><td>-</td><td>-</td><td>synthetic construct</td><td>Homo sapiens mel transforming oncogene (derived from cell line NK14)- RAB8 homolog, partial [synthetic construct].</td><td>-</td></tr>
<tr><td>AAV38505.1</td><td>AP-GTPase*→</td><td>AP-GTPase</td><td>204</td><td>-</td><td>-</td><td>synthetic construct</td><td>RAB13, member RAS oncogene family, partial [synthetic construct].</td><td>-</td></tr>
<tr><td>AAX32379.1</td><td>AP-GTPase*→</td><td>AP-GTPase</td><td>207</td><td>MEL</td><td>-</td><td>synthetic construct</td><td>RAB8A [synthetic construct].</td><td>-</td></tr>
<tr><td>AAX36766.1</td><td>AP-GTPase*→</td><td>AP-GTPase</td><td>204</td><td>RAB13</td><td>-</td><td>synthetic construct</td><td>RAB13 member RAS oncogene family, partial [synthetic construct].</td><td>-</td></tr>
<tr><td>AAX41198.1</td><td>AP-GTPase*→</td><td>AP-GTPase</td><td>203</td><td>RAB13</td><td>-</td><td>synthetic construct</td><td>RAB13 member RAS oncogene family [synthetic construct].</td><td>-</td></tr>
<tr><td>AAX42776.1</td><td>AP-GTPase*→</td><td>AP-GTPase</td><td>204</td><td>RAB13</td><td>-</td><td>synthetic construct</td><td>RAB13 member RAS oncogene family, partial [synthetic construct].</td><td>-</td></tr>
<tr><td>ABG50724.1</td><td>vWA-L+AP-GTPase+COR*→</td><td>vWA-L+AP-GTPase+COR</td><td>748</td><td>Tery_1428</td><td>Cyanobacteria</td><td>Trichodesmium erythraeum IMS101</td><td>Miro-like [Trichodesmium erythraeum IMS101].</td><td>GCA_000014265.1</td></tr>
<tr><td>ABW29719.1</td><td>AP-GTPase+ZNR*→</td><td>AP-GTPase+ZNR</td><td>705</td><td>AM1_4747</td><td>Cyanobacteria</td><td>Acaryochloris marina MBIC11017</td><td>conserved hypothetical protein [Acaryochloris marina MBIC11017].</td><td>GCA_000018105.1</td></tr>
<tr><td>ACL85629.1</td><td>AP-GTPase*→</td><td>AP-GTPase</td><td>204</td><td>Rab10</td><td>-</td><td>Drosophila melanogaster</td><td>Rab10-PA, partial [synthetic construct].</td><td>-</td></tr>
<tr><td>ACL86538.1</td><td>AP-GTPase*→</td><td>AP-GTPase</td><td>223</td><td>Rab30</td><td>-</td><td>Drosophila melanogaster</td><td>Rab30-PA, partial [synthetic construct].</td><td>-</td></tr>
<tr><td>ADQ32831.1</td><td>AP-GTPase*→</td><td>AP-GTPase</td><td>219</td><td>RAB3B</td><td>-</td><td>Homo sapiens</td><td>RAB3B, member RAS oncogene family, partial [synthetic construct].</td><td>-</td></tr>
<tr><td>AFZ01290.1</td><td><-EAD10+VMAP-M1+VMAP-C||?→ CASPASE+EAD10→ EAD10+VMAP-M1+VMAP-C→?→ VWA+AP-GTPase+COR*→</td><td>VWA+AP-GTPase+COR</td><td>1196</td><td>Cal6303_2274</td><td>Cyanobacteria</td><td>Calothrix sp. PCC 6303</td><td>Miro domain protein [Calothrix sp. PCC 6303].</td><td>GCA_000317435.1</td></tr>
<tr><td>AIC55004.1</td><td>AP-GTPase*→</td><td>AP-GTPase</td><td>219</td><td>-</td><td>-</td><td>Homo sapiens</td><td>RAB3B, partial [synthetic construct].</td><td>-</td></tr>
<tr><td>AIC55612.1</td><td>AP-GTPase*→</td><td>AP-GTPase</td><td>219</td><td>-</td><td>-</td><td>Homo sapiens</td><td>RAB3D, partial [synthetic construct].</td><td>-</td></tr>
<tr><td>ASF48224.1</td><td>AP-GTPase+COR+DUF4404*→</td><td>AP-GTPase+COR+DUF4404</td><td>879</td><td>CEK71_20365</td><td>Gammaproteobacteria</td><td>Methylovulum psychrotolerans</td><td>hypothetical protein CEK71_20365 [Methylovulum psychrotolerans].</td><td>GCA_002209385.1</td></tr>
<tr><td>ATO96390.1</td><td>AP-GTPase*→</td><td>AP-GTPase</td><td>207</td><td>rab8b</td><td>-</td><td>Xenopus tropicalis</td><td>RAB8B, member RAS oncoprotein family, partial [synthetic construct].</td><td>-</td></tr>
</table>

acc	operon	architecture	len	gen.name	taxend	species	defline	gca
CCQ62557.1	AP-GTPase+COR*→	AP-GTPase+COR	279	CWATWH0401_4507	Cyanobacteria	Crocosphaera watsonii WH 0401	Leucine-rich repeat [Crocosphaera watsonii WH 0401].	GCA_001039615.1
CCZ82707.1	AP-GTPase+COR*→	AP-GTPase+COR	843	BN709_01979	Bacteroidetes	Odoribacter laneus CAG:561	putative uncharacterized protein [Odoribacter laneus CAG:561].	GCA_000432935.1
HAA12500.1	AP-GTPase+COR*→	AP-GTPase+COR	908	DCE41_12685	Bacteroidetes	Cytophagales bacterium	TPA: hypothetical protein DCE41_12685 [Cytophagales bacterium].	GCA_003444355.1
HAA13307.1	AP-GTPase+COR*→	AP-GTPase+COR	878	DCE41_17110	Bacteroidetes	Cytophagales bacterium	TPA: hypothetical protein DCE41_17110 [Cytophagales bacterium].	GCA_003444355.1
HAC65063.1	DNAJ→ AP-GTPase*→ COR+TM+TM→	AP-GTPase	125	DCF68_16430	Cyanobacteria	Cyanothece sp. UBA12306	TPA: GTPase, partial [Cyanothece sp. UBA12306].	GCA_003448685.1
HAK76392.1	AP-GTPase+COR*→	AP-GTPase+COR	733	DCM71_05675	Bacteroidetes	Runella sp.	TPA: hypothetical protein DCM71_05675, partial [Runella sp.].	GCA_003452005.1
HAO21703.1	AP-GTPase*→	AP-GTPase	569	DCQ37_15290	Deltaproteobacteria	Desulfobacteraceae bacterium	TPA: hypothetical protein DCQ37_15290, partial [Desulfobacteraceae bacterium].	GCA_003456735.1
HAP58612.1	AP-GTPase+COR*→	AP-GTPase+COR	696	DCR93_03550	Bacteroidetes	Cytophagales bacterium	TPA: hypothetical protein DCR93_03550, partial [Cytophagales bacterium].	GCA_003454975.1
HAP62400.1	AP-GTPase+COR*→?→ cNMPBD→ SIG+Phytase-like→	AP-GTPase+COR	744	DCR93_23850	Bacteroidetes	Cytophagales bacterium	TPA: hypothetical protein DCR93_23850 [Cytophagales bacterium].	GCA_003454975.1
HCS91914.1	TIR+BetaPropeller+AP-GTPase*→	TIR+BetaPropeller+AP-GTPase	1236	DIW77_18260	Gammaproteobacteria	Chromatiaceae bacterium	TPA: hypothetical protein DIW77_18260 [Chromatiaceae bacterium].	GCA_003525925.1
HEY83669.1	AP-GTPase+COR*→	AP-GTPase+COR	260	G4N96_00950	Chloroflexi	Chloroflexi bacterium	TPA: hypothetical protein G4N96_00950 [Chloroflexi bacterium].	GCA_011192195.1
HEY85168.1	AP-GTPase*→	AP-GTPase	319	G4N96_08685	Chloroflexi	Chloroflexi bacterium	TPA: hypothetical protein G4N96_08685 [Chloroflexi bacterium].	GCA_011192195.1
HFS11412.1	BetaPropeller+AP-GTPase+TIR*→?→||<-?||?→?→||<-SIG+PSBP	BetaPropeller+AP-GTPase+TIR	1179	ENR14_24625	Cyanobacteria	Anabaena sp.	TPA: TIR domain-containing protein [Anabaena sp.].	GCA_011332035.1
HGZ32338.1	AP-GTPase+COR*→	AP-GTPase+COR	503	ENR17_19310	Bacteroidetes	Bacteroidetes bacterium	TPA: GTPase, partial [Bacteroidetes bacterium].	GCA_011331955.1
HHN34802.1	AP-GTPase*→	AP-GTPase	171	ENM07_01585	Bacteroidetes	Bacteroidetes bacterium	TPA: GTP-binding protein [Bacteroidetes bacterium].	GCA_011375975.1
KAB2958401.1	SIG+AP-GTPase+COR*→	SIG+AP-GTPase+COR	688	F9K13_13725	Bacteria	Candidatus Methylomirabilis oxyfera	hypothetical protein F9K13_13725 [Candidatus Methylomirabilis oxyfera].	GCA_009026205.1
KAF0250167.1	AP-GTPase+COR+Calcineurin+FGS*→	AP-GTPase+COR+Calcineurin+FGS	1237	FD167_433	unclassified Bacteria	bacterium	small GTP-binding protein, partial [bacterium].	GCA_009773835.1
KHD09711.1	AP-GTPase+COR*→	AP-GTPase+COR	530	PN36_20855	Gammaproteobacteria	Candidatus Thiomargarita nelsonii	hypothetical protein PN36_20855 [Candidatus Thiomargarita nelsonii].	GCA_000785145.2
MBC6419378.1	AP-GTPase*→ COR→	AP-GTPase	165	GDA44_11670	Cyanobacteria	Prochloron sp. SP5CPC1	hypothetical protein GDA44_11670 [Prochloron sp. SP5CPC1].	GCA_014323965.1

<table>
<tr><th>acc</th><th>operon</th><th>architecture</th><th>len</th><th>gen.name</th><th>taxend</th><th>species</th><th>defline</th><th>gca</th></tr>
<tr><td>NAT10406.1</td><td>AP-GTPase*→</td><td>AP-GTPase</td><td>611</td><td>C4E22_02470</td><td>Euryarchaeota</td><td>ANME-1 cluster archaeon AG-394-G06</td><td>hypothetical protein C4E22_02470 [ANME-1 cluster archaeon AG-394-G06].</td><td>GCA_009903405.1</td></tr>
<tr><td>NBO93394.1</td><td>AP-GTPase+COR*→</td><td>AP-GTPase+COR</td><td>916</td><td>EBV06_13945</td><td>Planctomycetes</td><td>Planctomycetia bacterium</td><td>hypothetical protein EBV06_13945 [Planctomycetia bacterium].</td><td>GCA_009918525.1</td></tr>
<tr><td>NBQ70341.1</td><td>TIR+AP-GTPase+COR*→</td><td>TIR+AP-GTPase+COR</td><td>582</td><td>EBU46_16510</td><td>Betaproteobacteria</td><td>Nitrosomonadaceae bacterium</td><td>TIR domain-containing protein, partial [Nitrosomonadaceae bacterium].</td><td>GCA_009919685.1</td></tr>
<tr><td>NHJ32288.1</td><td>AP-GTPase*→</td><td>AP-GTPase</td><td>230</td><td>FK732_05460</td><td>Asgard group</td><td>Asgard group archaeon</td><td>GTP-binding protein [Asgard group archaeon].</td><td>GCA_011366225.1</td></tr>
<tr><td>NIM12342.1</td><td>TIR+BetaPropeller+AP-GTPase*→</td><td>TIR+BetaPropeller+AP-GTPase</td><td>1000</td><td>GTO81_10200</td><td>Bacteria</td><td>Candidatus Aminicenantes bacterium</td><td>DUF4365 domain-containing protein [Candidatus Aminicenantes bacterium].</td><td>GCA_011771225.1</td></tr>
<tr><td>NIM12946.1</td><td>TIR+BetaPropeller+AP-GTPase*→</td><td>TIR+BetaPropeller+AP-GTPase</td><td>1108</td><td>GTO81_13270</td><td>Bacteria</td><td>Candidatus Aminicenantes bacterium</td><td>TIR domain-containing protein [Candidatus Aminicenantes bacterium].</td><td>GCA_011771225.1</td></tr>
<tr><td>NIR05736.1</td><td>TIR+BetaPropeller+AP-GTPase*→ Cluster2387_2clades→ Cluster2274_2clades→||<-Cluster2216_2clades||Cluster2371_2clades→||<-?<-?<-Cluster2251_2clades</td><td>TIR+BetaPropeller+AP-GTPase</td><td>1004</td><td>GTN82_09930</td><td>Bacteria</td><td>Candidatus Aminicenantes bacterium</td><td>DUF4365 domain-containing protein [Candidatus Aminicenantes bacterium].</td><td>GCA_011773265.1</td></tr>
<tr><td>NJL71192.1</td><td>AP-GTPase*→</td><td>AP-GTPase</td><td>248</td><td>HC888_06030</td><td>Gammaproteobacteria</td><td>Candidatus Competibacteraceae bacterium</td><td>GTP-binding protein [Candidatus Competibacteraceae bacterium].</td><td>GCA_012031515.1</td></tr>
<tr><td>NJO13505.1</td><td>AP-GTPase*→?→ TIR→</td><td>AP-GTPase</td><td>144</td><td>HC872_08630</td><td>Gammaproteobacteria</td><td>Gammaproteobacteria bacterium</td><td>hypothetical protein HC872_08630 [Gammaproteobacteria bacterium].</td><td>GCA_012032615.1</td></tr>
<tr><td>NKB62496.1</td><td>AP-GTPase+COR*→</td><td>AP-GTPase+COR</td><td>274</td><td>GKR95_10365</td><td>Gammaproteobacteria</td><td>Gammaproteobacteria bacterium</td><td>hypothetical protein GKR95_10365 [Gammaproteobacteria bacterium].</td><td>GCA_012103415.1</td></tr>
<tr><td>NLZ73760.1</td><td>AP-GTPase*→</td><td>AP-GTPase</td><td>202</td><td>GX905_08105</td><td>Bacteroidetes</td><td>Bacteroidales bacterium</td><td>hypothetical protein GX905_08105 [Bacteroidales bacterium].</td><td>GCA_012800615.1</td></tr>
<tr><td>NMC05010.1</td><td>AP-GTPase*→</td><td>AP-GTPase</td><td>190</td><td>GYA24_07360</td><td>Asgard group</td><td>Candidatus Lokiarchaeota archaeon</td><td>GTP-binding protein [Candidatus Lokiarchaeota archaeon].</td><td>GCA_012798355.1</td></tr>
<tr><td>NNJ85485.1</td><td>AP-GTPase*→</td><td>AP-GTPase</td><td>345</td><td>HKP13_11240</td><td>Gammaproteobacteria</td><td>Gammaproteobacteria bacterium</td><td>hypothetical protein HKP13_11240, partial [Gammaproteobacteria bacterium].</td><td>GCA_013042035.1</td></tr>
<tr><td>NOQ28062.1</td><td>AP-GTPase+COR*→</td><td>AP-GTPase+COR</td><td>845</td><td>GQ564_22090</td><td>Bacteroidetes</td><td>Bacteroidales bacterium</td><td>hypothetical protein GQ564_22090 [Bacteroidales bacterium].</td><td>GCA_013138975.1</td></tr>
<tr><td>NOS87668.1</td><td>AP-GTPase+COR+DUF4404*→ REase→</td><td>AP-GTPase+COR+DUF4404</td><td>775</td><td>HOP34_03865</td><td>Gammaproteobacteria</td><td>Methylococcaceae bacterium</td><td>hypothetical protein HOP34_03865, partial [Methylococcaceae bacterium].</td><td>GCA_013140465.1</td></tr>
<tr><td>NOT02643.1</td><td>AP-GTPase+COR*→</td><td>AP-GTPase+COR</td><td>1127</td><td>HOP29_18715</td><td>Planctomycetes</td><td>Phycisphaerales bacterium</td><td>hypothetical protein HOP29_18715 [Phycisphaerales bacterium].</td><td>GCA_013140575.1</td></tr>
<tr><td>NOT61891.1</td><td>TIR+BetaPropeller+AP-GTPase*→</td><td>TIR+BetaPropeller+AP-GTPase</td><td>1134</td><td>HOP19_16880</td><td>Acidobacteria</td><td>Acidobacteria bacterium</td><td>TIR domain-containing protein [Acidobacteria bacterium].</td><td>GCA_013140935.1</td></tr>
</table>

acc	operon	architecture	len	gen.name	taxend	species	defline	gca
NOU19042.1	AP-GTPase+COR*→	AP-GTPase+COR	850	HOO91_15910	Bacteroidetes	Bacteroidales bacterium	hypothetical protein HOO91_15910 [Bacteroidales bacterium].	GCA_013141385.1
NOY42283.1	AP-GTPase+COR*→	AP-GTPase+COR	659	GXP26_10655	Planctomycetes	Planctomycetes bacterium	hypothetical protein GXP26_10655 [Planctomycetes bacterium].	GCA_013152205.1
NOY62616.1	AP-GTPase+COR+DUF4404*→	AP-GTPase+COR+DUF4404	555	GXP10_05565	Gammaproteobacteria	Gammaproteobacteria bacterium	hypothetical protein GXP10_05565, partial [Gammaproteobacteria bacterium].	GCA_013152295.1
NPE06693.1	AP-GTPase*→	AP-GTPase	212	GNW80_00285	Asgard group	Asgard group archaeon	GTP-binding protein [Asgard group archaeon].	GCA_013166835.1
NQT13008.1	TIR+BetaPropeller+AP-GTPase*→	TIR+BetaPropeller+AP-GTPase	698	HQ582_09690	Planctomycetes	Planctomycetes bacterium	TIR domain-containing protein, partial [Planctomycetes bacterium].	GCA_013202485.1
NQU22914.1	AP-GTPase*→	AP-GTPase	350	HQ567_16680	Bacteria	Candidatus Nealsonbacteria bacterium	hypothetical protein HQ567_16680 [Candidatus Nealsonbacteria bacterium].	GCA_013202865.1
NQZ10247.1	AP-GTPase+COR*→	AP-GTPase+COR	798	HRT35_24105	Gammaproteobacteria	Algicola sp.	hypothetical protein HRT35_24105 [Algicola sp.].	GCA_013216025.1
NQZ12034.1	AP-GTPase+COR*→	AP-GTPase+COR	875	HRT35_33190	Gammaproteobacteria	Algicola sp.	hypothetical protein HRT35_33190 [Algicola sp.].	GCA_013216025.1
NTV51179.1	SIG+ZetaToxin→ TIR+BetaPropeller+AP-GTPase*→	TIR+BetaPropeller+AP-GTPase	1151	HGA20_16230	Deltaproteobacteria	Geobacteraceae bacterium	TIR domain-containing protein [Geobacteraceae bacterium].	GCA_013336525.1
NUN13817.1	BetaPropeller+AP-GTPase+Calcineurin*→	BetaPropeller+AP-GTPase+Calcineurin	1598	HUU55_09280	Deltaproteobacteria	Myxococcales bacterium	metallophosphoesterase [Myxococcales bacterium].	GCA_013360285.1
NVO09097.1	AP-GTPase+COR*→	AP-GTPase+COR	866	HXX16_03955	Bacteroidetes	Bacteroidales bacterium	50S ribosome-binding GTPase [Bacteroidales bacterium].	GCA_013376985.1
OHD42862.1	AP-GTPase+COR+DrHyd*→	AP-GTPase+COR+DrHyd	671	A2086_15900	Spirochaetes	Spirochaetes bacterium GWD1_27_9	hypothetical protein A2086_15900 [Spirochaetes bacterium GWD1_27_9].	GCA_001830585.1
OLS14677.1	AP-GTPase*→	AP-GTPase	170	RBG13Loki_1719	Asgard group	Candidatus Lokiarchaeota archaeon CR_4	small GTP-binding protein [Candidatus Lokiarchaeota archaeon CR_4].	GCA_001940655.1
OQW99838.1	AP-GTPase+COR*→	AP-GTPase+COR	550	BWK73_49720	Gammaproteobacteria	Thiothrix lacustris	hypothetical protein BWK73_49720 [Thiothrix lacustris].	GCA_002083875.1
OQX07299.1	AP-GTPase*→	AP-GTPase	495	BWK80_49630	Deltaproteobacteria	Desulfobacteraceae bacterium IS3	hypothetical protein BWK80_49630, partial [Desulfobacteraceae bacterium IS3].	GCA_002083955.1
OQX29968.1	AP-GTPase+COR*→	AP-GTPase+COR	659	B0D92_00955	Spirochaetes	Spirochaeta sp. LUC14_002_19_P3	hypothetical protein B0D92_00955, partial [Spirochaeta sp. LUC14_002_19_P3].	GCA_002084135.1
OQY29953.1	AP-GTPase+COR+CASPASE*→	AP-GTPase+COR+CASPASE	1279	B6244_01455	FCB group	Candidatus Cloacimonetes bacterium 4572_55	hypothetical protein B6244_01455 [Candidatus Cloacimonetes bacterium 4572_55].	GCA_002084765.1

acc	operon	architecture	len	gen.name	taxend	species	defline	gca
OYW77642.1	AP-GTPase+COR*→	AP-GTPase+COR	915	B7Z37_03570	Verrucomicrobia	Verrucomicrobia bacterium 12-59-8	hypothetical protein B7Z37_03570, partial [Verrucomicrobia bacterium 12-59-8].	GCA_002279765.1
PID37771.1	AP-GTPase+COR*→	AP-GTPase+COR	901	CR966_00525	Gammaproteobacteria	Pseudomonadales bacterium	hypothetical protein CR966_00525 [Pseudomonadales bacterium].	GCA_002746545.1
PIE42009.1	AP-GTPase+COR+DUF4404*→	AP-GTPase+COR+DUF4404	686	CSA47_02360	Gammaproteobacteria	Gammaproteobacteria bacterium	hypothetical protein CSA47_02360 [Gammaproteobacteria bacterium].	GCA_002748595.1
PKL67687.1	BetaPropeller+AP-GTPase+PrimaseZnR+TIR*→	BetaPropeller+AP-GTPase+PrimaseZnR+TIR	1195	CVV28_05310	Euryarchaeota	Methanobacteriales archaeon HGW-Methanobacteriales-1	hypothetical protein CVV28_05310 [Methanobacteriales archaeon HGW-Methanobacteriales-1].	GCA_002839705.1
POZ51526.1	AP-GTPase+COR+DUF4404*→	AP-GTPase+COR+DUF4404	919	AADEFJLK_02392	Gammaproteobacteria	Methylovulum psychrotolerans	GTP-binding protein [Methylovulum psychrotolerans].	GCA_002923755.1
PPD43153.1	AP-GTPase+COR+DUF4404*→	AP-GTPase+COR+DUF4404	914	CTY16_13990	Gammaproteobacteria	Methylobacter sp.	hypothetical protein CTY16_13990 [Methylobacter sp.].	GCA_002929035.1
PPD50180.1	AP-GTPase+COR+DUF4404*→	AP-GTPase+COR+DUF4404	927	CTY13_02150	Gammaproteobacteria	Methylobacter sp.	hypothetical protein CTY13_02150 [Methylobacter sp.].	GCA_002929095.1
PPD50460.1	PolB→ Cluster2355_2clades→ AP-GTPase+COR+DUF4404*→	AP-GTPase+COR+DUF4404	904	CTY16_01515	Gammaproteobacteria	Methylobacter sp.	hypothetical protein CTY16_01515 [Methylobacter sp.].	GCA_002929035.1
QDT95532.1	TIR+APATPase+TPR+TPR+TPR→?→ AP-GTPase+COR+DUF4404*→	AP-GTPase+COR+DUF4404	683	V144x_09770	Planctomycetes	Gimesia aquarii	Miro-like protein [Gimesia aquarii].	GCA_007748195.1
RCJ23846.1	VWA+AP-GTPase+COR+TIR+TIR*→	VWA+AP-GTPase+COR+TIR+TIR	1359	A6770_28940	Cyanobacteria	Nostoc minutum NIES-26	hypothetical protein A6770_28940 [Nostoc minutum NIES-26].	GCA_003326215.1
RHO76387.1	AP-GTPase*→	AP-GTPase	197	DW061_22950	Firmicutes	Ruminococcus sp. AF42-9BH	hypothetical protein DW061_22950, partial [Ruminococcus sp. AF42-9BH].	GCA_003477585.1
RIK66911.1	SIG+AP-GTPase+COR*→	SIG+AP-GTPase+COR	867	DCC65_08160	Planctomycetes	Planctomycetes bacterium	hypothetical protein DCC65_08160 [Planctomycetes bacterium].	GCA_003576905.1
RKZ52796.1	AP-GTPase+COR*→	AP-GTPase+COR	469	DRR00_06990	Gammaproteobacteria	Gammaproteobacteria bacterium	hypothetical protein DRR00_06990 [Gammaproteobacteria bacterium].	GCA_003645185.1
RLI66235.1	AP-GTPase*→	AP-GTPase	176	DRO88_02330	Asgard group	Candidatus Lokiarchaeota archaeon	GTP-binding protein [Candidatus Lokiarchaeota archaeon].	GCA_003662865.1
RLI69359.1	AP-GTPase*→	AP-GTPase	199	DRP02_10785	Asgard group	Candidatus Heimdallarchaeota archaeon	hypothetical protein DRP02_10785 [Candidatus Heimdallarchaeota archaeon].	GCA_003662935.1
RTZ62858.1	AP-GTPase+COR*→	AP-GTPase+COR	577	DSZ29_07625	Aquificae	Aquificaceae bacterium	hypothetical protein DSZ29_07625 [Aquificaceae bacterium].	GCA_003972955.1

acc	operon	architecture	len	gen.name	taxend	species	defline	gca
RYE19062.1	AP-GTPase+COR*→	AP-GTPase+COR	577	EOP45_13210	Bacteroidetes	Sphingobacteriaceae bacterium	GTP-binding protein, partial [Sphingobacteriaceae bacterium].	GCA_004144285.1
RZL32479.1	SIG+AP-GTPase+COR*→	SIG+AP-GTPase+COR	392	EOP00_33890	Bacteroidetes	Pedobacter sp.	GTP-binding protein, partial [Pedobacter sp.].	GCA_004211475.1
SEM19691.1	SIG+AP-GTPase+COR*→	SIG+AP-GTPase+COR	929	SAMN04489760_10698	Deltaproteobacteria	Syntrophus gentianae	small GTP-binding protein domain-containing protein [Syntrophus gentianae].	GCA_900109885.1
SKA90036.1	AP-GTPase*→	AP-GTPase	120	SAMN02745166_01647	Verrucomicrobia	Prosthecobacter debontii	hypothetical protein SAMN02745166_01647 [Prosthecobacter debontii].	GCA_900167535.1
SNR48942.1	BetaPropeller+AP-GTPase+TIR*→	BetaPropeller+AP-GTPase+TIR	1008	SAMN06264365_102815	Actinobacteria	Actinoplanes regularis	small GTP-binding protein domain-containing protein [Actinoplanes regularis].	GCA_900188005.1
TAA74167.1	AP-GTPase*→	AP-GTPase	415	CDV28_13720	Deltaproteobacteria	Candidatus Electronema sp. GS	Ras of Complex, Roc, domain of DAPkinase [Candidatus Electronema sp. GS].	GCA_004284765.1
TAN66684.1	AP-GTPase+COR+DUF4404*→	AP-GTPase+COR+DUF4404	912	EPN17_13365	Gammaproteobacteria	Methylobacter sp.	hypothetical protein EPN17_13365 [Methylobacter sp.].	GCA_004299305.1
TET30065.1	AP-GTPase*→	AP-GTPase	212	E3J70_05515	Asgard group	Candidatus Heimdallarchaeota archaeon	GTP-binding protein [Candidatus Heimdallarchaeota archaeon].	GCA_004376455.1
TFF85226.1	AP-GTPase*→	AP-GTPase	173	EU518_01555	Asgard group	Candidatus Lokiarchaeota archaeon	GTP-binding protein [Candidatus Lokiarchaeota archaeon].	GCA_004524075.1
TFF94051.1	AP-GTPase*→	AP-GTPase	179	EU544_04990	Asgard group	Candidatus Lokiarchaeota archaeon	GTP-binding protein [Candidatus Lokiarchaeota archaeon].	GCA_004524325.1
TFG01113.1	AP-GTPase*→	AP-GTPase	173	EU542_07480	Asgard group	Candidatus Lokiarchaeota archaeon	GTP-binding protein [Candidatus Lokiarchaeota archaeon].	GCA_004524355.1
TFG18716.1	AP-GTPase*→	AP-GTPase	217	EU530_08380	Asgard group	Candidatus Lokiarchaeota archaeon	GTP-binding protein [Candidatus Lokiarchaeota archaeon].	GCA_004524545.1
TKG00166.1	AP-GTPase+COR*→	AP-GTPase+COR	467	FCV76_15430	Gammaproteobacteria	Vibrio sp. F13	GTP-binding protein [Vibrio sp. F13].	GCA_005146615.1
TKJ20917.1	AP-GTPase*→	AP-GTPase	192	CEE42_14430	Asgard group	Candidatus Lokiarchaeota archaeon Loki_b31	hypothetical protein CEE42_14430 [Candidatus Lokiarchaeota archaeon Loki_b31].	GCA_005222975.1
TLU87712.1	SIG+ZetaToxin→ TIR+BetaPropeller+AP-GTPase*→	TIR+BetaPropeller+AP-GTPase	1149	FDX21_02485	Chlorobi	Chlorobium sp.	TIR domain-containing protein [Chlorobium sp.].	GCA_005862225.1
TOG86811.1	AP-GTPase+COR*→	AP-GTPase+COR	675	CGI91_22345	Gammaproteobacteria	Vibrio parahaemolyticus	hypothetical protein CGI91_22345 [Vibrio parahaemolyticus].	GCA_006372615.1
TPQ26187.1	AP-GTPase+COR+TM+TM*→	AP-GTPase+COR+TM+TM	752	C2U68_12455	Gammaproteobacteria	Methylomonas koyamae	hypothetical protein C2U68_12455 [Methylomonas koyamae].	GCA_006483455.1
TXT62600.1	AP-GTPase*→	AP-GTPase	178	BAJALOKI3v1_540018	Asgard group	Candidatus Lokiarchaeota archaeon	GTP-binding protein Der [Candidatus Lokiarchaeota archaeon].	GCA_008080735.1

acc	operon	architecture	len	gen.name	taxend	species	defline	gca
VFJ44888.1	TIR+BetaPropeller+AP-GTPase*→	TIR+BetaPropeller+AP-GTPase	766	BECKFW1821A_GA0114235_100653	Gammaproteobacteria	Candidatus Kentron sp. FW	WD domain-containing protein, G-beta repeat-containing protein [Candidatus Kentron sp. FW].	-
VFJ45524.1	AP-GTPase+COR+TM+TM*→ AP-GTPase+COR+TM+TM→	AP-GTPase+COR+TM+TM	839	BECKFM1743C_GA0114222_100266	Gammaproteobacteria	Candidatus Kentron sp. FM	Ras of Complex, Roc, domain of DAPkinase [Candidatus Kentron sp. FM].	-
VFJ50707.1	TIR+BetaPropeller+AP-GTPase*→	TIR+BetaPropeller+AP-GTPase	1132	BECKFW1821B_GA0114236_100746	Gammaproteobacteria	Candidatus Kentron sp. FW	WD40 repeat [Candidatus Kentron sp. FW].	-
VFJ53002.1	AP-GTPase+COR+TM+TM*→	AP-GTPase+COR+TM+TM	812	BECKDK2373C_GA0170839_103824	Gammaproteobacteria	Candidatus Kentron sp. DK	Ras of Complex, Roc, domain of DAPkinase [Candidatus Kentron sp. DK].	-
VFJ54602.1	AP-GTPase+COR+TM+TM*→	AP-GTPase+COR+TM+TM	670	BECKDK2373C_GA0170839_10441	Gammaproteobacteria	Candidatus Kentron sp. DK	Ras of Complex, Roc, domain of DAPkinase, partial [Candidatus Kentron sp. DK].	-
VFJ56646.1	AP-GTPase+COR+TM+TM*→	AP-GTPase+COR+TM+TM	841	BECKDK2373B_GA0170837_106011	Gammaproteobacteria	Candidatus Kentron sp. DK	Ras of Complex, Roc, domain of DAPkinase [Candidatus Kentron sp. DK].	-
VFJ57440.1	AP-GTPase*→	AP-GTPase	395	BECKFW1821B_GA0114236_10341	Gammaproteobacteria	Candidatus Kentron sp. FW	Ras of Complex, Roc, domain of DAPkinase, partial [Candidatus Kentron sp. FW].	-
VFJ59594.1	AP-GTPase+COR+CR-ATPase8+CR-REase7+TM+TM+TM*→	AP-GTPase+COR+CR-ATPase8+CR-REase7+TM+TM+TM	1492	BECKFW1821A_GA0114235_109213	Gammaproteobacteria	Candidatus Kentron sp. FW	GTPase SAR1 family protein [Candidatus Kentron sp. FW].	-
VFJ60987.1	AP-GTPase+COR+TM+TM*→	AP-GTPase+COR+TM+TM	889	BECKFM1743C_GA0114222_102852	Gammaproteobacteria	Candidatus Kentron sp. FM	Ras of Complex, Roc, domain of DAPkinase [Candidatus Kentron sp. FM].	-
VFJ61225.1	AP-GTPase+COR+TM+TM*→	AP-GTPase+COR+TM+TM	683	BECKDK2373B_GA0170837_109915	Gammaproteobacteria	Candidatus Kentron sp. DK	Ras of Complex, Roc, domain of DAPkinase, partial [Candidatus Kentron sp. DK].	-
VFJ62038.1	AP-GTPase+COR+TM+TM*→	AP-GTPase+COR+TM+TM	843	BECKFW1821B_GA0114236_106912	Gammaproteobacteria	Candidatus Kentron sp. FW	GTPase SAR1 family protein [Candidatus Kentron sp. FW].	-
VFJ65256.1	AP-GTPase+COR+TM+TM*→	AP-GTPase+COR+TM+TM	523	BECKFW1821A_GA0114235_11961	Gammaproteobacteria	Candidatus Kentron sp. FW	hypothetical protein BECKFW1821A_GA0114235_11961, partial [Candidatus Kentron sp. FW].	-
VFJ68931.1	AP-GTPase+COR+TM+TM*→	AP-GTPase+COR+TM+TM	948	BECKFM1743C_GA0114222_105041	Gammaproteobacteria	Candidatus Kentron sp. FM	Ras of Complex, Roc, domain of DAPkinase [Candidatus Kentron sp. FM].	-
VFJ70290.1	AP-GTPase+COR+TM+TM*→	AP-GTPase+COR+TM+TM	787	BECKFM1743C_GA0114222_105511	Gammaproteobacteria	Candidatus Kentron sp. FM	Ras of Complex, Roc, domain of DAPkinase [Candidatus Kentron sp. FM].	-
VFJ72898.1	AP-GTPase+COR+NACHT*→	AP-GTPase+COR+NACHT	1882	BECKFW1821C_GA0114237_10407	Gammaproteobacteria	Candidatus Kentron sp. FW	Pentapeptide repeat-containing protein [Candidatus Kentron sp. FW].	-
VFJ73192.1	AP-GTPase+COR+TM+TM*→	AP-GTPase+COR+TM+TM	770	BECKFW1821C_GA0114237_104627	Gammaproteobacteria	Candidatus Kentron sp. FW	GTPase SAR1 family protein, partial [Candidatus Kentron sp. FW].	-
VFJ76094.1	AP-GTPase+COR+TM+TM*→	AP-GTPase+COR+TM+TM	834	BECKFW1821C_GA0114237_10987	Gammaproteobacteria	Candidatus Kentron sp. FW	GTPase SAR1 family protein [Candidatus Kentron sp. FW].	-
VFJ95876.1	AP-GTPase+COR+TM+TM*→	AP-GTPase+COR+TM+TM	753	BECKH772A_GA0070896_1009310	Gammaproteobacteria	Candidatus Kentron sp. H	Ras of Complex, Roc, domain of DAPkinase, partial [Candidatus Kentron sp. H].	-

acc	operon	architecture	len	gen.name	taxend	species	defline	gca
VFK01091.1	AP-GTPase+COR*→	AP-GTPase+COR	275	BECKLFY1418B_GA0070995_12271	Gammaproteobacteria	Candidatus Kentron sp. LFY	Ras of Complex, Roc, domain of DAPkinase, partial [Candidatus Kentron sp. LFY].	-
VFK03671.1	AP-GTPase*→	AP-GTPase	118	BECKLFY1418A_GA0070994_13111	Gammaproteobacteria	Candidatus Kentron sp. LFY	Ras of Complex, Roc, domain of DAPkinase, partial [Candidatus Kentron sp. LFY].	-
VFK03687.1	AP-GTPase*→	AP-GTPase	118	BECKLFY1418A_GA0070994_13121	Gammaproteobacteria	Candidatus Kentron sp. LFY	Ras of Complex, Roc, domain of DAPkinase, partial [Candidatus Kentron sp. LFY].	-
VFK04035.1	AP-GTPase+COR+TM+TM*→	AP-GTPase+COR+TM+TM	677	BECKH772B_GA0070898_104021	Gammaproteobacteria	Candidatus Kentron sp. H	Ras of Complex, Roc, domain of DAPkinase, partial [Candidatus Kentron sp. H].	-
VFK07263.1	AP-GTPase+COR+TM+TM*→	AP-GTPase+COR+TM+TM	674	BECKH772C_GA0070978_104041	Gammaproteobacteria	Candidatus Kentron sp. H	Ras of Complex, Roc, domain of DAPkinase, partial [Candidatus Kentron sp. H].	-
VFK11049.1	AP-GTPase+COR+TM+TM*→	AP-GTPase+COR+TM+TM	865	BECKLPF1236A_GA0070988_100486	Gammaproteobacteria	Candidatus Kentron sp. LPFa	Ras of Complex, Roc, domain of DAPkinase [Candidatus Kentron sp. LPFa].	-
VFK19789.1	AP-GTPase+COR+TM+TM*→	AP-GTPase+COR+TM+TM	853	BECKLPF1236B_GA0070989_11862	Gammaproteobacteria	Candidatus Kentron sp. LPFa	Ras of Complex, Roc, domain of DAPkinase [Candidatus Kentron sp. LPFa].	-
VFK19871.1	AP-GTPase*→	AP-GTPase	161	BECKLFY1418C_GA0070996_106423	Gammaproteobacteria	Candidatus Kentron sp. LFY	small GTP-binding protein domain-containing protein, partial [Candidatus Kentron sp. LFY].	-
VFK26330.1	AP-GTPase*→	AP-GTPase	116	BECKLFY1418C_GA0070996_13331	Gammaproteobacteria	Candidatus Kentron sp. LFY	Ras of Complex, Roc, domain of DAPkinase, partial [Candidatus Kentron sp. LFY].	-
VFK27413.1	AP-GTPase+COR+TM+TM*→ CR-ATPase8+CR-REase7→ CR-ATPase8+CR-REase7→ CR-ATPase8+CR-REase7→ CR-ATPase8+CR-REase7→?→||<-?<-ParA-Soj-PloopNTPase	AP-GTPase+COR+TM+TM	893	BECKMB1821I_GA0114274_100324	Gammaproteobacteria	Candidatus Kentron sp. MB	Ras of Complex, Roc, domain of DAPkinase [Candidatus Kentron sp. MB].	-
VFK29755.1	AP-GTPase+COR+TM+TM*→ AP-GTPase+COR+TM+TM→	AP-GTPase+COR+TM+TM	840	BECKMB1821G_GA0114241_10537	Gammaproteobacteria	Candidatus Kentron sp. MB	Ras of Complex, Roc, domain of DAPkinase [Candidatus Kentron sp. MB].	-
VFK30623.1	AP-GTPase+COR+TM+TM*→ CR-ATPase8+CR-REase7→ CR-ATPase8+CR-REase7→ CR-ATPase8+CR-REase7→ CR-ATPase8+CR-REase7→?→||<-?<-ParA-Soj-PloopNTPase	AP-GTPase+COR+TM+TM	718	BECKMB1821G_GA0114241_10681	Gammaproteobacteria	Candidatus Kentron sp. MB	hypothetical protein BECKMB1821G_GA0114241_10681, partial [Candidatus Kentron sp. MB].	-
VFK30872.1	AP-GTPase+COR+TM+TM*→	AP-GTPase+COR+TM+TM	680	BECKMB1821G_GA0114241_10721	Gammaproteobacteria	Candidatus Kentron sp. MB	hypothetical protein BECKMB1821G_GA0114241_10721, partial [Candidatus Kentron sp. MB].	-
VFK31841.1	AP-GTPase*→	AP-GTPase	476	BECKMB1821I_GA0114274_102735	Gammaproteobacteria	Candidatus Kentron sp. MB	Ras of Complex, Roc, domain of DAPkinase [Candidatus Kentron sp. MB].	-
VFK32767.1	AP-GTPase+COR+TM+TM*→	AP-GTPase+COR+TM+TM	875	BECKMB1821I_GA0114274_103604	Gammaproteobacteria	Candidatus Kentron sp. MB	Ras of Complex, Roc, domain of DAPkinase [Candidatus Kentron sp. MB].	-
VFK33354.1	AP-GTPase+COR+TM+TM*→	AP-GTPase+COR+TM+TM	844	BECKMB1821I_GA0114274_104404	Gammaproteobacteria	Candidatus Kentron sp. MB	Ras of Complex, Roc, domain of DAPkinase [Candidatus Kentron sp. MB].	-

acc	operon	architecture	len	gen.name	taxend	species	defline	gca
VFK39913.1	AP-GTPase+COR+TM+TM*→	AP-GTPase+COR+TM+TM	790	BECKTC1821D_GA0114238_100746	Gammaproteobacteria	Candidatus Kentron sp. TC	Ras of Complex, Roc, domain of DAPkinase [Candidatus Kentron sp. TC].	-
VFK39956.1	RADICAL-SAM→ AP-GTPase+COR*→	AP-GTPase+COR	694	BECKSD772F_GA0070984_105016	Gammaproteobacteria	Candidatus Kentron sp. SD	GTPase SAR1 family protein [Candidatus Kentron sp. SD].	-
VFK40684.1	AP-GTPase+COR+TM+TM*→	AP-GTPase+COR+TM+TM	854	BECKSD772F_GA0070984_10665	Gammaproteobacteria	Candidatus Kentron sp. SD	Ras of Complex, Roc, domain of DAPkinase [Candidatus Kentron sp. SD].	-
VFK42825.1	AP-GTPase+COR+TM+TM*→	AP-GTPase+COR+TM+TM	834	BECKTC1821E_GA0114239_102141	Gammaproteobacteria	Candidatus Kentron sp. TC	Ras of Complex, Roc, domain of DAPkinase [Candidatus Kentron sp. TC].	-
VFK53024.1	AP-GTPase+COR+TM+TM*→	AP-GTPase+COR+TM+TM	852	BECK-TUN1418D_GA0071000_101730	Gammaproteobacteria	Candidatus Kentron sp. TUN	Ras of Complex, Roc, domain of DAPkinase [Candidatus Kentron sp. TUN].	-
VFK54740.1	AP-GTPase+COR+TM+TM*→	AP-GTPase+COR+TM+TM	851	BECK-TUN1418F_GA0071002_10532	Gammaproteobacteria	Candidatus Kentron sp. TUN	GTPase SAR1 family protein [Candidatus Kentron sp. TUN].	-
VFK54787.1	CR-ATPase8+CR-REase7+AP-GTPase+COR+TM+TM*→	CR-ATPase8+CR-REase7+AP-GTPase+COR+TM+TM	1117	BECK-TUN1418F_GA0071002_105513	Gammaproteobacteria	Candidatus Kentron sp. TUN	Ras of Complex, Roc, domain of DAPkinase [Candidatus Kentron sp. TUN].	-
VFK55234.1	AP-GTPase+COR+TM+TM*→	AP-GTPase+COR+TM+TM	870	BECK-TUN1418D_GA0071000_102911	Gammaproteobacteria	Candidatus Kentron sp. TUN	GTPase SAR1 family protein [Candidatus Kentron sp. TUN].	-
VFK55404.1	AP-GTPase+COR+TM+TM*→ AP-GTPase+COR+TM+TM→	AP-GTPase+COR+TM+TM	834	BECK-TUN1418E_GA0071001_10293	Gammaproteobacteria	Candidatus Kentron sp. TUN	Ras of Complex, Roc, domain of DAPkinase [Candidatus Kentron sp. TUN].	-
VFK57219.1	AP-GTPase+COR+TM+TM*→	AP-GTPase+COR+TM+TM	799	BECKTC1821F_GA0114240_101546	Gammaproteobacteria	Candidatus Kentron sp. TC	Ras of Complex, Roc, domain of DAPkinase [Candidatus Kentron sp. TC].	-
VFK57233.1	AP-GTPase+COR*→	AP-GTPase+COR	1361	BECK-TUN1418F_GA0071002_11091	Gammaproteobacteria	Candidatus Kentron sp. TUN	Uncharacterized conserved protein, contains ParB-like and HNH nuclease domains [Candidatus Kentron sp. TUN].	-
VFK58480.1	AP-GTPase+COR*→	AP-GTPase+COR	1384	BECK-TUN1418D_GA0071000_10836	Gammaproteobacteria	Candidatus Kentron sp. TUN	Ras of Complex, Roc, domain of DAPkinase [Candidatus Kentron sp. TUN].	-
VFK60758.1	CR-ATPase8+CR-REase7+AP-GTPase*→	CR-ATPase8+CR-REase7+AP-GTPase	877	BECK-UNK1418G_GA0071005_101311	Gammaproteobacteria	Candidatus Kentron sp. UNK	PD-(D/E)XK nuclease superfamily protein [Candidatus Kentron sp. UNK].	-
VFK62743.1	AP-GTPase*→	AP-GTPase	337	BECK-TUN1418D_GA0071000_11856	Gammaproteobacteria	Candidatus Kentron sp. TUN	Ras of Complex, Roc, domain of DAPkinase, partial [Candidatus Kentron sp. TUN].	-
VFK65329.1	AP-GTPase+COR*→	AP-GTPase+COR	1361	BECK-TUN1418E_GA0071001_11051	Gammaproteobacteria	Candidatus Kentron sp. TUN	Uncharacterized conserved protein, contains ParB-like and HNH nuclease domains [Candidatus Kentron sp. TUN].	-
VFK67825.1	BetaPropeller+AP-GTPase*→	BetaPropeller+AP-GTPase	526	BECK-UNK1418G_GA0071005_11685	Gammaproteobacteria	Candidatus Kentron sp. UNK	WD domain-containing protein, G-beta repeat-containing protein [Candidatus Kentron sp. UNK].	-
VFK67855.1	AP-GTPase+COR+TM+TM*→	AP-GTPase+COR+TM+TM	598	BECK-UNK1418G_GA0071005_11705	Gammaproteobacteria	Candidatus Kentron sp. UNK	Ras of Complex, Roc, domain of DAPkinase [Candidatus Kentron sp. UNK].	-

acc	operon	architecture	len	gen.name	taxend	species	defline	gca
VFK77412.1	AP-GTPase+COR+TM+TM*→	AP-GTPase+COR+TM+TM	734	BECKMB1821H_GA0114242_1135[illegible]	Gammaproteobacteria	Candidatus Kentron sp. MB	Ras of Complex, Roc, domain of DAPkinase, partial [Candidatus Kentron sp. MB].	-
VFK80164.1	AP-GTPase+COR+TM+TM*→	AP-GTPase+COR+TM+TM	872	BECKSD772D_GA0070982_10897	Gammaproteobacteria	Candidatus Kentron sp. SD	Ras of Complex, Roc, domain of DAPkinase [Candidatus Kentron sp. SD].	-
VFM99483.1	AP-GTPase+COR+TM+TM*→	AP-GTPase+COR+TM+TM	725	BECKG1743F_GA0114225_10402[illegible]	Gammaproteobacteria	Candidatus Kentron sp. G	GTPase SAR1 family protein [Candidatus Kentron sp. G].	GCA_900696175.1
VFM99853.1	AP-GTPase+COR+TM+TM*→	AP-GTPase+COR+TM+TM	744	BECKG1743F_GA0114225_10433[illegible]	Gammaproteobacteria	Candidatus Kentron sp. G	small GTP-binding protein domain-containing protein [Candidatus Kentron sp. G].	GCA_900696175.1
WP_002684219.1	AP-GTPase+COR+DUF4404*→	AP-GTPase+COR+DUF4404	883	-	Gammaproteobacteria	Beggiatoa alba	Miro-like protein [Beggiatoa alba].	GCF_000245015.1
WP_009138084.1	AP-GTPase+COR*→	AP-GTPase+COR	843	-	Bacteroidetes	Odoribacter laneus	hypothetical protein [Odoribacter laneus].	GCF_902374615.1
WP_012499245.1	AP-GTPase*→	AP-GTPase	394	-	Chlorobi	Chloroherpeton thalassium	Miro domain-containing protein [Chloroherpeton thalassium].	GCF_000020525.1
WP_020559412.1	GTPase-AIG→ ABC-ATPase→ SIG+TM+TM+TM+TM→ AP-GTPase+COR*→	AP-GTPase+COR	833	-	Gammaproteobacteria	Thiofilum flexile	hypothetical protein [Thiofilum flexile].	GCF_000380185.1
WP_027001878.1	AP-GTPase+COR*→	AP-GTPase+COR	754	-	Bacteroidetes	Hugenholtzia roseola	hypothetical protein [Hugenholtzia roseola].	GCF_000422585.1
WP_040612090.1	AP-GTPase+COR*→	AP-GTPase+COR	849	-	Bacteroidetes	Odoribacter laneus	hypothetical protein [Odoribacter laneus].	-
WP_050025079.1	VWA+AP-GTPase+COR+EAD1+Trypsin*→	VWA+AP-GTPase+COR+EAD1+Trypsin	1458	-	Verrucomicrobia	Verrucomicrobium sp. BvORR034	GTP-binding protein [Verrucomicrobium sp. BvORR034].	-
WP_052368940.1	TIR+BetaPropeller+AP-GTPase*→	TIR+BetaPropeller+AP-GTPase	1107	-	Euryarchaeota	Candidatus Methanoperedens nitroreducens	TIR domain-containing protein [Candidatus Methanoperedens nitroreducens].	GCF_000685155.1
WP_071819957.1	TIR+BetaPropeller+AP-GTPase*→||<-?<-?<-?<-?<-?<-RuvB-AAA+RuvB-HTH	TIR+BetaPropeller+AP-GTPase	1449	-	Cyanobacteria	Acaryochloris marina	TIR domain-containing protein [Acaryochloris marina].	GCF_000018105.1
WP_077027722.1	<-Phytase-like||?→?→ tRNA→||<-?<-?<-?||TIR+BetaPropeller+AP-GTPase*→	TIR+BetaPropeller+AP-GTPase	1401	-	Planctomycetes	Fuerstia marisgermanicae	TIR domain-containing protein [Fuerstia marisgermanicae].	GCF_001983935.1
WP_081716878.1	AP-GTPase+COR+TIR*→	AP-GTPase+COR+TIR	722	-	Cyanobacteria	Leptolyngbya sp. Heron Island J	TIR domain-containing protein [Leptolyngbya sp. Heron Island J].	-
WP_081891667.1	AP-GTPase+COR+TIR*→	AP-GTPase+COR+TIR	825	-	Verrucomicrobia	Verrucomicrobium sp. BvORR106	TIR domain-containing protein [Verrucomicrobium sp. BvORR106].	-
WP_083888126.1	AP-GTPase*→	AP-GTPase	116	-	Cyanobacteria	Oscillatoria nigro-viridis	hypothetical protein [Oscillatoria nigro-viridis].	-
WP_093066994.1	AP-GTPase+COR*→	AP-GTPase+COR	857	-	Gammaproteobacteria	Thiothrix caldifontis	hypothetical protein [Thiothrix caldifontis].	GCF_900107695.1
WP_093068668.1	AP-GTPase+COR*→	AP-GTPase+COR	960	-	Gammaproteobacteria	Thiothrix caldifontis	hypothetical protein [Thiothrix caldifontis].	GCF_900107695.1
WP_093882803.1	SIG+AP-GTPase+COR*→	SIG+AP-GTPase+COR	880	-	Deltaproteobacteria	Syntrophus gentianae	hypothetical protein [Syntrophus gentianae].	GCF_900109885.1
WP_103921835.1	HTH→ AP-GTPase+COR*→	AP-GTPase+COR	576	-	Gammaproteobacteria	Thiotrichales bacterium HS_08	hypothetical protein [Thiotrichales bacterium HS_08].	-
WP_129026230.1	AP-GTPase+COR*→	AP-GTPase+COR	431	-	Bacteroidetes	Flavobacterium sp. YO12	hypothetical protein [Flavobacterium sp. YO12].	GCF_004119495.1

acc	operon	architecture	len	gen.name	taxend	species	defline	gca
WP_137245090.1	TM+AP-GTPase*→	TM+AP-GTPase	93	-	Actinobacteria	Herbidospora galbida	hypothetical protein [Herbidospora galbida].	GCF_005233835.1
WP_139373135.1	AP-GTPase*→	AP-GTPase	134	-	Verrucomicrobia	Prosthecobacter debontii	hypothetical protein [Prosthecobacter debontii].	GCF_900167535.1
WP_140311973.1	AP-GTPase+COR*→	AP-GTPase+COR	697	-	Gammaproteobacteria	Vibrio parahaemolyticus	GTP-binding protein, partial [Vibrio parahaemolyticus].	GCF_006372615.1
WP_144982131.1	TIR+APATPase+TPR+TPR+TPR→?→ AP-GTPase+COR+DUF4404*→	AP-GTPase+COR+DUF4404	692	-	Planctomycetes	Gimesia aquarii	hypothetical protein [Gimesia aquarii].	GCF_007748195.1
WP_145384506.1	AP-GTPase+COR*→	AP-GTPase+COR	680	-	Planctomycetes	Stieleria neptunia	50S ribosome-binding GTPase [Stieleria neptunia].	GCF_007754155.1
WP_146054573.1	AP-GTPase+COR+DUF4404*→	AP-GTPase+COR+DUF4404	731	-	Gammaproteobacteria	Methylovulum psychrotolerans	hypothetical protein [Methylovulum psychrotolerans].	GCF_002923755.1
WP_147662502.1	AP-GTPase*→	AP-GTPase	204	-	Asgard group	Candidatus Prometheoarchaeum syntrophicum	GTP-binding protein [Candidatus Prometheoarchaeum syntrophicum].	GCF_008000775.1
WP_157679487.1	AP-GTPase+COR+DUF4404*→	AP-GTPase+COR+DUF4404	911	-	Gammaproteobacteria	Methylovulum psychrotolerans	hypothetical protein [Methylovulum psychrotolerans].	GCF_002209385.1
WP_157817021.1	<-EAD10+VMAP-M1+VMAP-C||?→||<-PSE||BetaPropeller+AP-GTPase+Calcineurin+CASPASE+NACHT*→	BetaPropeller+AP-GTPase+Calcineurin+CASPASE+NACHT	1731	-	Cyanobacteria	Nostoc flagelliforme	NACHT domain-containing protein [Nostoc flagelliforme].	GCF_002813575.1
WP_160572809.1	AP-GTPase+COR+DUF4404*→	AP-GTPase+COR+DUF4404	720	-	unclassified Bacteria	bacterium D16-29	GTP-binding protein [bacterium D16-29].	-
WP_162669385.1	AP-GTPase+COR+DUF4404*→	AP-GTPase+COR+DUF4404	615	-	Planctomycetes	Gemmata massiliana	hypothetical protein [Gemmata massiliana].	GCF_901538265.1
WP_170962814.1	AP-GTPase+COR*→	AP-GTPase+COR	453	-	Gammaproteobacteria	Vibrio sp. F13	GTP-binding protein [Vibrio sp. F13].	GCF_005146615.1
WP_173978580.1	TIR+BetaPropeller+AP-GTPase*→	TIR+BetaPropeller+AP-GTPase	1154	-	Alphaproteobacteria	Magnetospirillum sp. UT-4	TIR domain-containing protein [Magnetospirillum sp. UT-4].	GCF_902729435.1
WP_175453917.1	AP-GTPase+COR*→	AP-GTPase+COR	757	-	Firmicutes	Sarcina sp. DSM 11001	GTP-binding protein, partial [Sarcina sp. DSM 11001].	GCF_900101015.1
WP_177428378.1	AP-GTPase+COR+CR-ATPase9*→	AP-GTPase+COR+CR-ATPase9	880	-	Gammaproteobacteria	Thiotrichales bacterium HS_08	hypothetical protein [Thiotrichales bacterium HS_08].	-
WP_179276996.1	BetaPropeller+AP-GTPase+TIR*→	BetaPropeller+AP-GTPase+TIR	996	-	Actinobacteria	Actinoplanes regularis	TIR domain-containing protein [Actinoplanes regularis].	GCF_016862355.1
WP_180983884.1	AP-GTPase+COR*→	AP-GTPase+COR	676	-	Gammaproteobacteria	Vibrio agarivorans	50S ribosome-binding GTPase, partial [Vibrio agarivorans].	GCF_002894965.1
WP_186289598.1	AP-GTPase+COR+TM+TM*→	AP-GTPase+COR+TM+TM	905	-	Gammaproteobacteria	Methylomonas koyamae	hypothetical protein [Methylomonas koyamae].	GCF_006483455.1

Gene neighborhoods and domain architectures of the bacterial and prophage/phage SPRY domain

acc	operon	architecture	len	gen.name	taxend	species	defline	gca
ACF52192.1	Phage_connect_1→ Phage_H_T_join→ HK97-gp10_like→ DUF3168→ Phage_TTP_11→ Phage_TAC_13→?→ SPRY+X*→?→ Phage-tail-tape→	SPRY+X	655	Smal_2491	Gammaproteobacteria	Stenotrophomonas maltophilia R551-3	hypothetical protein Smal_2491 [Stenotrophomonas maltophilia R551-3].	GCA_000020665.1
ASA22786.1	SUN→ SPRY+SUN*→ SUN→ Laminin_G_3→ Disaggr_repeat→ Phage_holin_4_1→	SPRY+SUN	358	B9T62_19455	Firmicutes	Paenibacillus donghaensis	hypothetical protein B9T62_19455 [Paenibacillus donghaensis].	GCA_002192415.1
CAA2107092.1	TM+PhageMin_Tail→?→?→?→?→ SPRY*→	SPRY	456	VVAX_04063	Betaproteobacteria	Variovorax paradoxus	hypothetical protein VVAX_04063 [Variovorax paradoxus].	-
CAA2108205.1	TM+PhageMin_Tail→?→?→?→?→ SPRY*→	SPRY	455	VVAX_04716	Betaproteobacteria	Variovorax paradoxus	hypothetical protein VVAX_04716 [Variovorax paradoxus].	-
EJP76868.1	Phage_connect_1→ Phage_H_T_join→ HK97-gp10_like→ DUF3168→ Phage_TTP_11→ Phage_TAC_13→?→ SPRY+X*→?→ Phage-tail-tape→ ConA-repeats→	SPRY+X	658	A1OC_01671	Gammaproteobacteria	Stenotrophomonas maltophilia Ab55555	hypothetical protein A1OC_01671 [Stenotrophomonas maltophilia Ab55555].	GCA_000287935.1
HAG44903.1	SPRY*→ SPRY→ SUN→	SPRY	249	DCL31_18225	Firmicutes	Clostridium sp.	TPA: hypothetical protein DCL31_18225 [Clostridium sp.].	GCA_003451415.1
HAU79614.1	Phage_H_T_join→ HK97-gp10_like→ DUF3168→ Phage_TTP_11→ Phage_TAC_13→?→ SPRY+X*→||<-?||?→?→?→ Phage-tail-tape→	SPRY+X	655	DCW96_02920	Gammaproteobacteria	Stenotrophomonas sp.	TPA: hypothetical protein DCW96_02920 [Stenotrophomonas sp.].	GCA_003484865.1
HBC50726.1	Phage_connect_1→ Phage_H_T_join→ HK97-gp10_like→ DUF3168→ Phage_TTP_11→ Phage_TAC_13→?→ SPRY*→	SPRY	169	DC051_05805	Gammaproteobacteria	Stenotrophomonas maltophilia	TPA: hypothetical protein DC051_05805, partial [Stenotrophomonas maltophilia].	GCA_003487985.1
HBJ00556.1	SPRY*→ SUN→	SPRY	221	DDY89_06200	Firmicutes	Lysinibacillus sp.	TPA: hypothetical protein DDY89_06200, partial [Lysinibacillus sp.].	GCA_003507435.1
HBJ00935.1	SPRY*→	SPRY	146	DDY89_08865	Firmicutes	Lysinibacillus sp.	TPA: hypothetical protein DDY89_08865, partial [Lysinibacillus sp.].	GCA_003507435.1

acc	operon	architecture	len	gen.name	taxend	species	defline	gca
HBS54551.1	Phage_connect_1→ Phage_H_T_join→ HK97-gp10_like→ DUF3168→ Phage_TTP_11→ Phage_TAC_13→?→ SPRY+X*→?→ Phage-tail-tape→ ConA-repeats→	SPRY+X	655	DEA38_02315	Gammaproteobacteria	Stenotrophomonas sp.	TPA: hypothetical protein DEA38_02315 [Stenotrophomonas sp.].	GCA_003510825.1
HBT70779.1	DISCOIDIN→ SUN→ SPRY*→	SPRY	169	DEB37_00445	Firmicutes	Lysinibacillus sp.	TPA: hypothetical protein DEB37_00445, partial [Lysinibacillus sp.].	GCA_003510425.1
HDO36693.1	Cluster187_2clades→?→ Cluster187_2clades→?→ SPRY+SPRY*→	SPRY+SPRY	913	ENH07_10435	Nitrospirae	Nitrospirae bacterium	TPA: hypothetical protein ENH07_10435 [Nitrospirae bacterium].	GCA_011041165.1
HHG11560.1	SPRY+LisH*→	SPRY+LisH	821	ENL98_17835	unclassified Bacteria	bacterium	TPA: hypothetical protein ENL98_17835 [bacterium].	GCA_011373825.1
HHG11879.1	SIG+SPRY*→	SIG+SPRY	553	ENL98_19440	unclassified Bacteria	bacterium	TPA: hypothetical protein ENL98_19440 [bacterium].	GCA_011373825.1
KAB2901287.1	SPRY*→	SPRY	854	F9K31_02560	Gammaproteobacteria	Dokdonella sp.	hypothetical protein F9K31_02560 [Dokdonella sp.].	GCA_008933665.1
KAF6631785.1	<-Polbetant||ACET→ Cupin_2→ ABhydrolase→||<-?<-DUF2935||SIG+SPRY*→	SIG+SPRY	323	H6F38_13125	Firmicutes	Paenibacillus sp. EKM208P	hypothetical protein H6F38_13125 [Paenibacillus sp. EKM208P].	GCA_011066295.1
KGM23936.1	DUF3168→ Phage_TTP_11→ Phage_TAC_13→?→ SPRY+X*→?→?→ Phage-tail-tape→ ConA-repeats→	SPRY+X	657	LI87_0109275	Gammaproteobacteria	Stenotrophomonas maltophilia	hypothetical protein LI87_0109275 [Stenotrophomonas maltophilia].	GCA_000758465.1
KHS06610.1	Phage-tail-tape→?→?→?→?→ SPRY+X*→?→ DAM→	SPRY+X	658	RM61_15205	Gammaproteobacteria	Xanthomonas phaseoli pv. phaseoli	hypothetical protein RM61_15205 [Xanthomonas phaseoli pv. phaseoli].	GCA_000807875.2
KKW33547.1	SIG+SPRY*→	SIG+SPRY	838	UY79_C0020G0005	Bacteria	Parcubacteria group bacterium GW2011_GWA2_53_21	Filamentous hemagglutinin-like protein [Parcubacteria group bacterium GW2011_GWA2_53_21].	GCA_001004625.1
KOS00949.1	SPRY*→	SPRY	155	AM598_20185	Firmicutes	Paenibacillus polymyxa	hypothetical protein AM598_20185, partial [Paenibacillus polymyxa].	GCA_001277855.1
KZT13763.1	SPRY*→?→?→?→ DAM→	SPRY	449	A1D30_21880	Betaproteobacteria	Acidovorax sp. GW101-3H11	hypothetical protein A1D30_21880 [Acidovorax sp. GW101-3H11].	GCA_001633105.1
MAE42393.1	SIG+TM+TM+IG*→	SIG+TM+TM+IG	1722	CMO93_01365	DPANN group	Candidatus Woesearchaeota archaeon	hypothetical protein CMO93_01365, partial [Candidatus Woesearchaeota archaeon].	GCA_002686855.1

acc	operon	architecture	len	gen.name	taxend	species	defline	gca
MAH29320.1	Laminin_G_3+SPRY*→ Cluster74_6clades→	Laminin_G_3+SPRY	903	CL959_01385	Euryarchaeota	Euryarchaeota archaeon	hypothetical protein CL959_01385 [Euryarchaeota archaeon].	GCA_002689085.1
MAL42735.1	SPRY*→?→ Cluster388_2clades→	SPRY	473	CME98_01825	Alphaproteobacteria	Hyphomonas sp.	hypothetical protein CME98_01825 [Hyphomonas sp.].	GCA_002692925.1
MAN64718.1	LamG→ Laminin_G_3+SPRY*→ Cluster74_6clades→ Cluster153_2clades→	Laminin_G_3+SPRY	671	CMI60_22525	Alphaproteobacteria	Parvibaculum sp.	hypothetical protein CMI60_22525 [Parvibaculum sp.].	GCA_002695905.1
MAT11680.1	Phage_T7_tail→ Cluster93_5clades→ Laminin_G_3+Laminin_G_3+SPRY*→ Cluster74_6clades→	Laminin_G_3+Laminin_G_3+SPRY	921	CMM02_11805	Planctomycetes	Rhodopirellula sp.	hypothetical protein CMM02_11805 [Rhodopirellula sp.].	GCA_002698965.1
MAU75364.1	Laminin_G_3+SPRY*→ Cluster74_6clades→	Laminin_G_3+SPRY	630	CL831_00645	Bacteroidetes	Crocinitomicaceae bacterium	hypothetical protein CL831_00645 [Crocinitomicaceae bacterium].	GCA_002700285.1
MBA0297283.1	Phage-tail-tape→ ConA-repeats→?→ SPRY+X*→?→?→?→?→ SIG+TM+TM+TM+TM+TM+TM+TM+TM→ ABC-ATPase→	SPRY+X	604	D7Y24_02420	Gammaproteobacteria	Stenotrophomonas maltophilia	hypothetical protein D7Y24_02420 [Stenotrophomonas maltophilia].	GCA_013463875.1
MBA0313726.1	Phage_connect_1→ Phage_H_T_join→ HK97-gp10_like→ DUF3168→ Phage_TTP_11→ Phage_TAC_13→?→ SPRY*→	SPRY	249	D7Y33_22370	Gammaproteobacteria	Stenotrophomonas maltophilia	hypothetical protein D7Y33_22370, partial [Stenotrophomonas maltophilia].	GCA_013464125.1
MBA0313748.1	SPRY*→	SPRY	249	D7Y33_22510	Gammaproteobacteria	Stenotrophomonas maltophilia	hypothetical protein D7Y33_22510, partial [Stenotrophomonas maltophilia].	GCA_013464125.1
MBA0421339.1	Phage-tail-tape→ ConA-repeats→?→ SPRY+X*→?→?→?→?→?→?→||<-Ribonuc_L-PSP	SPRY+X	604	D7Y22_10225	Gammaproteobacteria	Stenotrophomonas maltophilia	hypothetical protein D7Y22_10225 [Stenotrophomonas maltophilia].	GCA_013463895.1
MBB5530851.1	Phage_connect_1→ Phage_H_T_join→ HK97-gp10_like→ DUF3168→ Phage_TTP_11→ Phage_TAC_13→?→ SPRY+X*→?→ Phage-tail-tape→ ConA-repeats→ ConA→?→||<-SRAP	SPRY+X	657	GGD72_001629	Gammaproteobacteria	Stenotrophomonas maltophilia	hypothetical protein GGD72_001629 [Stenotrophomonas maltophilia].	GCA_014200115.1
MBC17709.1	Phage_T7_tail→ SUN+SPRY*→ Phage_tail_APC→	SUN+SPRY	749	CL942_11735	Deltaproteobacteria	Desulfovibrio sp.	hypothetical protein CL942_11735 [Desulfovibrio sp.].	GCA_002711645.1
MBO71803.1	Cluster310_2clades→?→?→?→ Cluster359_2clades→ Laminin_G_3+SPRY+SPRY*→	Laminin_G_3+SPRY+SPRY	973	CL508_05750	Actinobacteria	Actinobacteria bacterium	hypothetical protein CL508_05750, partial [Actinobacteria bacterium].	GCA_002724835.1
MPS35004.1	Phage_connect_1→ Phage_H_T_join→ HK97-gp10_like→ DUF3168→ Phage_TTP_11→ Phage_TAC_13→?→ SPRY+X*→?→ Phage-tail-tape→?→?→ Cluster201_3clades→	SPRY+X	652	E2593_06880	Gammaproteobacteria	Stenotrophomonas sp.	hypothetical protein E2593_06880 [Stenotrophomonas sp.].	GCA_009360425.1
NBO27128.1	Laminin_G_3+SPRY*→ Cluster366_2clades→	Laminin_G_3+SPRY	547	EBU96_10150	Actinobacteria	Actinobacteria bacterium	hypothetical protein EBU96_10150 [Actinobacteria bacterium].	GCA_009918685.1

acc	operon	architecture	len	gen.name	taxend	species	defline	gca
NBO39260.1	IG+IG+IG+LEVANB+SPRY*→	IG+IG+IG+LEVANB+SPRY	2486	EBU99_11835	unclassified Bacteria	bacterium	hypothetical protein EBU99_11835, partial [bacterium].	GCA_009918675.1
NBQ97491.1	Laminin_G_3+SPRY*→?→ Cluster358_2clades→	Laminin_G_3+SPRY	447	EBU12_04555	Actinobacteria	Microbacteriaceae bacterium	hypothetical protein EBU12_04555 [Microbacteriaceae bacterium].	GCA_009920365.1
NBR26007.1	SPRY*→	SPRY	290	EBU08_19945	Actinobacteria	Micrococcales bacterium	hypothetical protein EBU08_19945, partial [Micrococcales bacterium].	GCA_009920425.1
NBR26025.1	Laminin_G_3+SPRY*→	Laminin_G_3+SPRY	450	EBU08_20035	Actinobacteria	Micrococcales bacterium	hypothetical protein EBU08_20035, partial [Micrococcales bacterium].	GCA_009920425.1
NBS68787.1	Laminin_G_3+SPRY*→?→?→ Cluster153_2clades→	Laminin_G_3+SPRY	775	EBT31_07700	unclassified Bacteria	bacterium	hypothetical protein EBT31_07700 [bacterium].	GCA_009921885.1
NBS71002.1	Laminin_G_3+SPRY*→	Laminin_G_3+SPRY	470	EBT31_19160	unclassified Bacteria	bacterium	hypothetical protein EBT31_19160, partial [bacterium].	GCA_009921885.1
NBS80068.1	Laminin_G_3+SPRY*→	Laminin_G_3+SPRY	347	EBS57_09835	unclassified Bacteria	bacterium	hypothetical protein EBS57_09835 [bacterium].	GCA_009923095.1
NBT48002.1	Laminin_G_3+SPRY*→	Laminin_G_3+SPRY	734	EBT07_09340	Actinobacteria	Actinobacteria bacterium	hypothetical protein EBT07_09340, partial [Actinobacteria bacterium].	GCA_009922275.1
NBT76477.1	Laminin_G_3+SPRY*→ Cluster74_6clades→	Laminin_G_3+SPRY	576	EBT15_11040	Betaproteobacteria	Betaproteobacteria bacterium	hypothetical protein EBT15_11040, partial [Betaproteobacteria bacterium].	GCA_009922135.1
NBU64721.1	Cluster359_2clades→?→ Laminin_G_3+SPRY*→	Laminin_G_3+SPRY	351	EBS29_09505	Chloroflexi	Chloroflexia bacterium	hypothetical protein EBS29_09505, partial [Chloroflexia bacterium].	GCA_009923655.1
NBU69993.1	Laminin_G_3+SPRY+Laminin_G_3*→	Laminin_G_3+SPRY+Laminin_G_3	937	EBS53_00865	Bacteroidetes	Bacteroidetes bacterium	hypothetical protein EBS53_00865 [Bacteroidetes bacterium].	GCA_009923185.1
NBU73444.1	Laminin_G_3+SPRY*→	Laminin_G_3+SPRY	374	EBS53_18720	Bacteroidetes	Bacteroidetes bacterium	hypothetical protein EBS53_18720, partial [Bacteroidetes bacterium].	GCA_009923185.1
NBW17394.1	Laminin_G_3+SPRY*→ Cluster214_3clades→	Laminin_G_3+SPRY	581	EBR82_56380	Alphaproteobacteria	Caulobacteraceae bacterium	hypothetical protein EBR82_56380, partial [Caulobacteraceae bacterium].	GCA_009924585.1
NBW17505.1	Laminin_G_3+SPRY*→	Laminin_G_3+SPRY	764	EBR82_56945	Alphaproteobacteria	Caulobacteraceae bacterium	hypothetical protein EBR82_56945, partial [Caulobacteraceae bacterium].	GCA_009924585.1

acc	operon	architecture	len	gen.name	taxend	species	defline	gca
NBW18892.1	Laminin_G_3+SPRY*→	Laminin_G_3+SPRY	722	EBR82_64050	Alphaproteobacteria	Caulobacteraceae bacterium	hypothetical protein EBR82_64050, partial [Caulobacteraceae bacterium].	GCA_009924585.1
NBW20101.1	SPRY*→	SPRY	400	EBR82_70235	Alphaproteobacteria	Caulobacteraceae bacterium	hypothetical protein EBR82_70235, partial [Caulobacteraceae bacterium].	GCA_009924585.1
NBW20127.1	Laminin_G_3+SPRY*→	Laminin_G_3+SPRY	498	EBR82_70370	Alphaproteobacteria	Caulobacteraceae bacterium	hypothetical protein EBR82_70370, partial [Caulobacteraceae bacterium].	GCA_009924585.1
NBW20301.1	Laminin_G_3+SPRY*→	Laminin_G_3+SPRY	508	EBR82_71270	Alphaproteobacteria	Caulobacteraceae bacterium	hypothetical protein EBR82_71270 [Caulobacteraceae bacterium].	GCA_009924585.1
NBW20891.1	Laminin_G_3+SPRY*→	Laminin_G_3+SPRY	494	EBR82_74310	Alphaproteobacteria	Caulobacteraceae bacterium	hypothetical protein EBR82_74310, partial [Caulobacteraceae bacterium].	GCA_009924585.1
NBW21054.1	Laminin_G_3+SPRY*→	Laminin_G_3+SPRY	448	EBR82_75135	Alphaproteobacteria	Caulobacteraceae bacterium	hypothetical protein EBR82_75135, partial [Caulobacteraceae bacterium].	GCA_009924585.1
NBW21679.1	SPRY*→	SPRY	285	EBR82_78355	Alphaproteobacteria	Caulobacteraceae bacterium	hypothetical protein EBR82_78355, partial [Caulobacteraceae bacterium].	GCA_009924585.1
NBW22642.1	SPRY*→	SPRY	394	EBR82_83325	Alphaproteobacteria	Caulobacteraceae bacterium	hypothetical protein EBR82_83325, partial [Caulobacteraceae bacterium].	GCA_009924585.1
NBW23811.1	Laminin_G_3+SPRY*→	Laminin_G_3+SPRY	335	EBR82_89280	Alphaproteobacteria	Caulobacteraceae bacterium	hypothetical protein EBR82_89280, partial [Caulobacteraceae bacterium].	GCA_009924585.1
NBX81523.1	Laminin_G_3+SPRY*→	Laminin_G_3+SPRY	455	EBQ90_00310	unclassified Bacteria	bacterium	hypothetical protein EBQ90_00310, partial [bacterium].	GCA_009927045.1
NBX88437.1	SPRY*→	SPRY	317	EBQ97_07980	Bacteroidetes	Bacteroidetes bacterium	hypothetical protein EBQ97_07980, partial [Bacteroidetes bacterium].	GCA_009926925.1
NDA10477.1	Laminin_G_3+SPRY*→ Cluster214_3clades→	Laminin_G_3+SPRY	692	EBZ07_06485	Verrucomicrobia	Verrucomicrobia bacterium	hypothetical protein EBZ07_06485, partial [Verrucomicrobia bacterium].	GCA_010025475.1
NDB59170.1	Cluster201_3clades→?→?→?→ LamG→ SPRY+TM*→?→ Cluster366_2clades→	SPRY+TM	300	EB001_12060	unclassified Bacteria	bacterium	hypothetical protein EB001_12060 [bacterium].	GCA_010026765.1

acc	operon	architecture	len	gen.name	taxend	species	defline	gca
NDB60922.1	Laminin_G_3+SPRY*→	Laminin_G_3+SPRY	799	EB001_21125	unclassified Bacteria	bacterium	hypothetical protein EB001_21125 [bacterium].	GCA_010026765.1
NDC49301.1	Cluster93_5clades→ Laminin_G_3+SPRY*→ Cluster214_3clades→	Laminin_G_3+SPRY	803	EBZ61_09545	Actinobacteria	Micrococcales bacterium	hypothetical protein EBZ61_09545 [Micrococcales bacterium].	GCA_010027415.1
NDC57204.1	SIG+SPRY*→	SIG+SPRY	239	EBZ69_10460	Alphaproteobacteria	Alphaproteobacteria bacterium	hypothetical protein EBZ69_10460, partial [Alphaproteobacteria bacterium].	GCA_010027225.1
NDD55753.1	Laminin_G_3+SPRY*→	Laminin_G_3+SPRY	430	EBZ39_18125	unclassified Bacteria	bacterium	hypothetical protein EBZ39_18125, partial [bacterium].	GCA_010027825.1
NDD86023.1	SPRY*→	SPRY	370	EBZ38_17325	unclassified Bacteria	bacterium	hypothetical protein EBZ38_17325, partial [bacterium].	GCA_010027835.1
NDG32417.1	Laminin_G_3+SPRY*→	Laminin_G_3+SPRY	801	EB118_20375	unclassified Bacteria	bacterium	hypothetical protein EB118_20375, partial [bacterium].	GCA_010031135.1
NEU24684.1	HTH_20→||<-PSE<-Polbetant<-Polbetant||ACET→ Cupin_2→||<-?||SIG+SPRY*→	SIG+SPRY	355	G3M74_00590	Firmicutes	Paenibacillus polymyxa	hypothetical protein G3M74_00590 [Paenibacillus polymyxa].	GCA_010692785.1
NEZ74825.1	Cluster171_2clades→?→?→ Cluster216_2clades→?→?→ PSE→ SUN→ SPRY*→ SUN→ TM→ Cluster104_2clades→?→?→35exo→ Cluster112_2clades→	SPRY	447	EXM56_05600	Firmicutes	Clostridium botulinum	cell adhesion protein [Clostridium botulinum].	GCA_011010615.1
NFM46879.1	Cluster171_2clades→?→?→ Cluster143_2clades→?→?→ PSE→ SUN→ SPRY*→ SUN→ TM→ Cluster104_2clades→?→?→35exo→ Cluster112_2clades→	SPRY	447	FDB72_12170	Firmicutes	Clostridium botulinum	cell adhesion protein [Clostridium botulinum].	GCA_011017125.1
NFM77397.1	Cluster216_2clades→?→?→?→ SUN→ SPRY*→ SUN→ TM→ Cluster104_2clades→?→?→35exo→ Cluster112_2clades→	SPRY	448	FDC04_00825	Firmicutes	Clostridium botulinum	cell adhesion protein [Clostridium botulinum].	GCA_011017715.1
NIT14181.1	Cluster165_3clades→?→?→ Cluster303_2clades→ SIG+HORMA→ Cluster138_2clades→ Cluster138_2clades→ SPRY*→ ThuA-GATase→	SPRY	831	GTN99_08085	Bacteria	Candidatus Dadabacteria bacterium	hypothetical protein GTN99_08085 [Candidatus Dadabacteria bacterium].	GCA_011772905.1
NSB16447.1	SPRY+Big_2*→ TM→	SPRY+Big_2	376	BCD95_004706	Firmicutes	Clostridium beijerinckii	uncharacterized protein YjdB [Clostridium beijerinckii].	GCA_013297045.1
NUH63603.1	HK97-gp10_like→ DUF3168→ Phage_TTP_11→ Phage_TAC_13→?→ SPRY*→	SPRY	353	HUF69_20240	Gammaproteobacteria	Stenotrophomonas maltophilia	hypothetical protein HUF69_20240, partial [Stenotrophomonas maltophilia].	GCA_013345715.1
OBU49329.1	Phage_connect_1→ Phage_H_T_join→ HK97-gp10_like→ DUF3168→ Phage_TTP_11→ Phage_TAC_13→?→ SPRY+X*→	SPRY+X	657	A9K69_19720	Gammaproteobacteria	Stenotrophomonas maltophilia	hypothetical protein A9K69_19720 [Stenotrophomonas maltophilia].	GCA_001676445.1

<table>
<thead>
<tr><th>acc</th><th>operon</th><th>architecture</th><th>len</th><th>gen.name</th><th>taxend</th><th>species</th><th>defline</th><th>gca</th></tr>
</thead>
<tbody>
<tr><td>ODV27998.1</td><td>SPRY*→</td><td>SPRY</td><td>1123</td><td>ABT19_00375</td><td>Gammaproteobacteria</td><td>Rhodanobacter sp. SCN 68-63</td><td>hypothetical protein ABT19_00375 [Rhodanobacter sp. SCN 68-63].</td><td>GCA_001725485.1</td></tr>
<tr><td>OEU67544.1</td><td>Cluster138_2clades→?→?→ SPRY*→ Cluster135_2clades→?→ ThuA-GATase→</td><td>SPRY</td><td>483</td><td>BA863_12330</td><td>Deltaproteobacteria</td><td>Desulfovibrio sp. S3730MH75</td><td>hypothetical protein BA863_12330 [Desulfovibrio sp. S3730MH75].</td><td>GCA_001750915.1</td></tr>
<tr><td>OEU68515.1</td><td>SIG+PTX*→</td><td>SIG+PTX</td><td>1668</td><td>BBJ57_07495</td><td>Deltaproteobacteria</td><td>Desulfobacterales bacterium PC51MH44</td><td>hypothetical protein BBJ57_07495 [Desulfobacterales bacterium PC51MH44].</td><td>GCA_001751165.1</td></tr>
<tr><td>OEU69113.1</td><td>SPRY*→ Cluster135_2clades→?→?→ ThuA-GATase→</td><td>SPRY</td><td>436</td><td>BA863_04090</td><td>Deltaproteobacteria</td><td>Desulfovibrio sp. S3730MH75</td><td>hypothetical protein BA863_04090 [Desulfovibrio sp. S3730MH75].</td><td>GCA_001750915.1</td></tr>
<tr><td>OUW13744.1</td><td>Laminin_G_3+Laminin_G_3+SPRY*→ Cluster74_6clades→ Cluster388_2clades→</td><td>Laminin_G_3+Laminin_G_3+SPRY</td><td>1163</td><td>CBD24_02790</td><td>Euryarchaeota</td><td>Euryarchaeota archaeon TMED164</td><td>hypothetical protein CBD24_02790 [Euryarchaeota archaeon TMED164].</td><td>GCA_002170935.1</td></tr>
<tr><td>OYV39756.1</td><td>SPRY*→</td><td>SPRY</td><td>137</td><td>B7Z80_06345</td><td>Alphaproteobacteria</td><td>Rhodospirillales bacterium 20-64-7</td><td>hypothetical protein B7Z80_06345 [Rhodospirillales bacterium 20-64-7].</td><td>GCA_002255195.1</td></tr>
<tr><td>PJO52612.1</td><td>Phage-tail-tape→ ConA-repeats→?→ SPRY+X*→?→?→?→?→?→||<-Ribonuc_L-PSP</td><td>SPRY+X</td><td>604</td><td>CR156_10635</td><td>Gammaproteobacteria</td><td>Stenotrophomonas lactitubi</td><td>hypothetical protein CR156_10635 [Stenotrophomonas lactitubi].</td><td>GCA_002803515.1</td></tr>
<tr><td>PKQ92730.1</td><td>SUN→ SUN→ SUN→ SPRY+SUN*→ Laminin_G_3→ Disaggr_repeat→</td><td>SPRY+SUN</td><td>425</td><td>CXK86_01005</td><td>Firmicutes</td><td>Paenibacillus sp. BGI2013</td><td>hypothetical protein CXK86_01005 [Paenibacillus sp. BGI2013].</td><td>GCA_002843485.1</td></tr>
<tr><td>PPC99495.1</td><td>Cluster368_2clades→?→?→ Cluster201_3clades→ TM+TM→ Laminin_G_3+SPRY*→?→?→||<-?<-?<-REC+OmpR-HTH</td><td>Laminin_G_3+SPRY</td><td>541</td><td>CTY35_04040</td><td>Betaproteobacteria</td><td>Methylotenera sp.</td><td>hypothetical protein CTY35_04040 [Methylotenera sp.].</td><td>GCA_002928435.1</td></tr>
<tr><td>PPD25805.1</td><td>SPRY*→</td><td>SPRY</td><td>389</td><td>CTY21_14065</td><td>Gammaproteobacteria</td><td>Methylomonas sp.</td><td>hypothetical protein CTY21_14065, partial [Methylomonas sp.].</td><td>GCA_002928935.1</td></tr>
<tr><td>QDL55294.1</td><td>Phage-tail_3+FN3→ Cluster93_5clades→ Laminin_G_3+SPRY*→?→?→||<-?<-REC+OmpR-HTH</td><td>Laminin_G_3+SPRY</td><td>544</td><td>EXZ61_14585</td><td>Betaproteobacteria</td><td>Rhodoferax sediminis</td><td>hypothetical protein EXZ61_14585 [Rhodoferax sediminis].</td><td>GCA_006974105.1</td></tr>
<tr><td>QDY47412.1</td><td>Phage_connect_1→ Phage_H_T_join→ HK97-gp10_like→ DUF3168→ Phage_TTP_11→ Phage_TAC_13→?→ SPRY+X*→?→?→?→ Phage-tail-tape→ ConA-repeats→</td><td>SPRY+X</td><td>657</td><td>DUW70_02065</td><td>Gammaproteobacteria</td><td>Stenotrophomonas maltophilia</td><td>hypothetical protein DUW70_02065 [Stenotrophomonas maltophilia].</td><td>GCA_007833655.1</td></tr>
<tr><td>QDY50235.1</td><td>Phage-tail-tape→ ConA-repeats→?→ SPRY+X*→</td><td>SPRY+X</td><td>604</td><td>DUW70_17765</td><td>Gammaproteobacteria</td><td>Stenotrophomonas maltophilia</td><td>hypothetical protein DUW70_17765 [Stenotrophomonas maltophilia].</td><td>GCA_007833655.1</td></tr>
</tbody>
</table>

acc	operon	architecture	len	gen.name	taxend	species	defline	gca
QDY50408.1	Phage_connect_1→ Phage_H_T_join→ HK97-gp10_like→ DUF3168→ Phage_TTP_11→ Phage_TAC_13→?→ SPRY+X*→||<-?||?→ Phage-tail-tape→ ConA-repeats→	SPRY+X	655	DUW70_18735	Gammaproteobacteria	Stenotrophomonas maltophilia	hypothetical protein DUW70_18735 [Stenotrophomonas maltophilia].	GCA_007833655.1
QGG50745.1	SUN→ SUN→ SPRY+SUN*→ SUN→ Laminin_G_3→ Disaggr_repeat→?→?→ Phage_holin_1→	SPRY+SUN	444	GDS87_07165	Firmicutes	Lysinibacillus pakistanensis	hypothetical protein GDS87_07165 [Lysinibacillus pakistanensis].	GCA_009649975.1
QIO87716.1	Phage_connect_1→ Phage_H_T_join→ HK97-gp10_like→?→ Phage_TTP_11→?→ SPRY*→?→?→||<-SRAP	SPRY	278	G9274_001401	Gammaproteobacteria	Stenotrophomonas rhizophila	hypothetical protein G9274_001401 [Stenotrophomonas rhizophila].	GCA_011604605.1
RCK36140.1	Baseplate_J→ SPRY*→	SPRY	575	TH9_05700	Alphaproteobacteria	Thalassospira xiamenensis	hypothetical protein TH9_05700 [Thalassospira xiamenensis].	GCA_003326725.1
REJ66078.1	JAB+NLPC→?→ Phage-tail_3→ Cluster93_5clades→ Laminin_G_3+SPRY*→ Cluster74_6clades→	Laminin_G_3+SPRY	1115	DWQ28_08310	Proteobacteria	Proteobacteria bacterium	hypothetical protein DWQ28_08310 [Proteobacteria bacterium].	GCA_003388625.1
REK63903.1	Phage_tail_APC→ TM+TM→ Laminin_G_3+SPRY*→ Cluster74_6clades→?→?→ Phage_tail_APC→	Laminin_G_3+SPRY	797	DWQ49_02055	Bacteroidetes	Bacteroidetes bacterium	hypothetical protein DWQ49_02055 [Bacteroidetes bacterium].	GCA_003389155.1
RLC76825.1	Cluster187_2clades→ SPRY*→ Cluster135_2clades→	SPRY	515	DRJ03_27900	Chloroflexi	Chloroflexi bacterium	hypothetical protein DRJ03_27900 [Chloroflexi bacterium].	GCA_003648075.1
RPI56186.1	Cluster138_2clades→?→?→ SPRY*→ Cluster135_2clades→	SPRY	495	EHM49_00895	Deltaproteobacteria	Deltaproteobacteria bacterium	hypothetical protein EHM49_00895 [Deltaproteobacteria bacterium].	GCA_003819885.1
RRY03816.1	SPRY*→?→ Phage_lysozyme→	SPRY	771	EGJ58_22195	Alphaproteobacteria	Ochrobactrum anthropi	hypothetical protein EGJ58_22195 [Ochrobactrum anthropi].	GCA_003937425.1
RYG67597.1	fn3+SPRY*→	fn3+SPRY	1230	EON64_07085	Archaea	archaeon	hypothetical protein EON64_07085 [archaeon].	GCA_004145555.1
RYH32433.1	SF2-helicase+SPRY*→	SF2-helicase+SPRY	535	EON65_00615	Archaea	archaeon	DEAD/DEAH box helicase [archaeon].	GCA_004146225.1
RYY37588.1	SPRY*→	SPRY	952	EON62_01040	Archaea	archaeon	hypothetical protein EON62_01040, partial [archaeon].	GCA_004173375.1
RYY72402.1	SPRY*→	SPRY	164	EON63_20910	Archaea	archaeon	hypothetical protein EON63_20910 [archaeon].	GCA_004173275.1
TDL67967.1	SUN→ SUN→ SPRY+SUN*→ Laminin_G_3→ Disaggr_repeat→	SPRY+SUN	425	E2R58_01745	Firmicutes	Paenibacillus amylolyticus	hypothetical protein E2R58_01745 [Paenibacillus amylolyticus].	GCA_004358835.1

<table>
<tr><th>acc</th><th>operon</th><th>architecture</th><th>len</th><th>gen.name</th><th>taxend</th><th>species</th><th>defline</th><th>gca</th></tr>
<tr><td>TSC88001.1</td><td>Laminin_G_3+Laminin_G_3+Laminin_G_3+Laminin_G_3→?→?→?→?→?→ SPRY+SUN*→ Laminin_G_3+Laminin_G_3+Laminin_G_3+Laminin_G_3→</td><td>SPRY+SUN</td><td>607</td><td>G01um10145_950</td><td>Bacteria</td><td>Microgenomates group bacterium Gr01-1014_5</td><td>hypothetical protein G01um10145_950 [Microgenomates group bacterium Gr01-1014_5].</td><td>GCA_007376395.1</td></tr>
<tr><td>VUI62768.1</td><td>Phage_connect_1→ Phage_H_T_join→ HK97-gp10_like→ DUF3168→ Phage_TTP_11→ Phage_TAC_13→?→ SPRY+X*→?→ Phage-tail-tape→</td><td>SPRY+X</td><td>655</td><td>GCPJBBEJ_01199</td><td>Gammaproteobacteria</td><td>Stenotrophomonas maltophilia</td><td>hypothetical protein GCPJBBEJ_01199 [Stenotrophomonas maltophilia].</td><td>GCA_902156415.1</td></tr>
<tr><td>VUJ81145.1</td><td>Phage-tail-tape→ ConA-repeats→?→ SPRY+X*→?→?→?→?→ SIG+TM+TM+TM+TM+TM+TM+TM+TM→ ABC-ATPase→</td><td>SPRY+X</td><td>604</td><td>IEEHEPME_02021</td><td>Gammaproteobacteria</td><td>Stenotrophomonas maltophilia</td><td>hypothetical protein IEEHEPME_02021 [Stenotrophomonas maltophilia].</td><td>GCA_902156595.1</td></tr>
<tr><td>VUL57431.1</td><td>Terminase_LS→ SPRY*→?→||<-?||SIG+TM+TM+TM+TM+ABC-ATPase→</td><td>SPRY</td><td>217</td><td>LBGBLGGB_01284</td><td>Gammaproteobacteria</td><td>Stenotrophomonas maltophilia</td><td>hypothetical protein LBGBLGGB_01284 [Stenotrophomonas maltophilia].</td><td>GCA_902156925.1</td></tr>
<tr><td>VUL62146.1</td><td>Phage_connect_1→ Phage_H_T_join→ HK97-gp10_like→ DUF3168→ Phage_TTP_11→ Phage_TAC_13→?→ SPRY+X*→?→?→ Phage-tail-tape→</td><td>SPRY+X</td><td>657</td><td>CCANPDJD_00380</td><td>Gammaproteobacteria</td><td>Stenotrophomonas maltophilia</td><td>hypothetical protein CCANPDJD_00380 [Stenotrophomonas maltophilia].</td><td>GCA_902157015.1</td></tr>
<tr><td>VUL75017.1</td><td>Phage_connect_1→ Phage_H_T_join→ HK97-gp10_like→?→ Phage_TTP_11→ Phage_TAC_13→?→ SPRY+X*→?→?→ Phage-tail-tape→</td><td>SPRY+X</td><td>655</td><td>GEILPBKL_02464</td><td>Gammaproteobacteria</td><td>Stenotrophomonas maltophilia</td><td>hypothetical protein GEILPBKL_02464 [Stenotrophomonas maltophilia].</td><td>GCA_902156945.1</td></tr>
<tr><td>VUM07479.1</td><td>Phage_connect_1→ Phage_H_T_join→ HK97-gp10_like→ DUF3168→ Phage_TTP_11→ Phage_TAC_13→?→ SPRY+X*→?→?→ Phage-tail-tape→ ConA-repeats→?→?→ SRAP→</td><td>SPRY+X</td><td>657</td><td>IKOGNNCB_02002</td><td>Gammaproteobacteria</td><td>Stenotrophomonas maltophilia</td><td>hypothetical protein IKOGNNCB_02002 [Stenotrophomonas maltophilia].</td><td>GCA_902157055.1</td></tr>
<tr><td>VUM45402.1</td><td>ConA-repeats→?→ SPRY+X*→</td><td>SPRY+X</td><td>604</td><td>FMOPOFGM_00942</td><td>Gammaproteobacteria</td><td>Stenotrophomonas maltophilia</td><td>hypothetical protein FMOPOFGM_00942 [Stenotrophomonas maltophilia].</td><td>GCA_902157105.1</td></tr>
<tr><td>VUM59055.1</td><td>Phage_connect_1→ Phage_H_T_join→ HK97-gp10_like→ DUF3168→ Phage_TTP_11→ Phage_TAC_13→?→ SPRY+X*→?→?→ Phage-tail-tape→ ConA-repeats→</td><td>SPRY+X</td><td>657</td><td>FMOPOFGM_03099</td><td>Gammaproteobacteria</td><td>Stenotrophomonas maltophilia</td><td>hypothetical protein FMOPOFGM_03099 [Stenotrophomonas maltophilia].</td><td>GCA_902157105.1</td></tr>
<tr><td>VUN16325.1</td><td>Phage_connect_1→ Phage_H_T_join→ HK97-gp10_like→ DUF3168→ Phage_TTP_11→ Phage_TAC_13→?→ SPRY+X*→||<-?||?→?→ Phage-tail-tape→ ConA-repeats→</td><td>SPRY+X</td><td>657</td><td>CPDIOOCO_00061</td><td>Gammaproteobacteria</td><td>Stenotrophomonas maltophilia</td><td>hypothetical protein CPDIOOCO_00061 [Stenotrophomonas maltophilia].</td><td>GCA_902157315.1</td></tr>
<tr><td>VUN62462.1</td><td>Phage_connect_1→ Phage_H_T_join→ HK97-gp10_like→ DUF3168→ Phage_TTP_11→ Phage_TAC_13→?→ SPRY+X*→?→?→ Phage-tail-tape→ ConA-repeats→ ConA→</td><td>SPRY+X</td><td>655</td><td>FHOPGADK_00135</td><td>Gammaproteobacteria</td><td>Stenotrophomonas maltophilia</td><td>hypothetical protein FHOPGADK_00135 [Stenotrophomonas maltophilia].</td><td>GCA_902157435.1</td></tr>
<tr><td>VUN67178.1</td><td>Phage_H_T_join→ HK97-gp10_like→ DUF3168→ Phage_TTP_11→ Phage_TAC_13→?→?→ SPRY+X*→||<-?||?→ Phage-tail-tape→</td><td>SPRY+X</td><td>601</td><td>JLECIBJP_00444</td><td>Gammaproteobacteria</td><td>Stenotrophomonas maltophilia</td><td>hypothetical protein JLECIBJP_00444 [Stenotrophomonas maltophilia].</td><td>GCA_902157425.1</td></tr>
</table>

acc	operon	architecture	len	gen.name	taxend	species	defline	gca
VUN71522.1	Phage_connect_1→ Phage_H_T_join→ HK97-gp10_like→ DUF3168→ Phage_TTP_11→ Phage_TAC_13→?→ SPRY+X*→?→ Phage-tail-tape→ ConA-repeats→?→?→ SRAP→	SPRY+X	655	IJMOMJOI_00888	Gammaproteobacteria	Stenotrophomonas maltophilia	hypothetical protein IJMOMJOI_00888 [Stenotrophomonas maltophilia].	GCA_902157415.1
VUO23348.1	Phage_connect_1→ Phage_H_T_join→ HK97-gp10_like→ DUF3168→ Phage_TTP_11→ Phage_TAC_13→?→ SPRY+X*→?→?→ Phage-tail-tape→?→?→?→||<-SRAP	SPRY+X	656	JBIDJGIM_02130	Gammaproteobacteria	Stenotrophomonas maltophilia	hypothetical protein JBIDJGIM_02130 [Stenotrophomonas maltophilia].	GCA_902157545.1
VUO59656.1	Phage_connect_1→ Phage_H_T_join→ HK97-gp10_like→ DUF3168→ Phage_TTP_11→ Phage_TAC_13→?→ SPRY+X*→?→?→ Phage-tail-tape→ ConA-repeats→?→?→||<-SRAP	SPRY+X	655	CMGNCPHI_00882	Gammaproteobacteria	Stenotrophomonas maltophilia	hypothetical protein CMGNCPHI_00882 [Stenotrophomonas maltophilia].	GCA_902157585.1
VUP11864.1	Phage_connect_1→ Phage_H_T_join→ HK97-gp10_like→ DUF3168→ Phage_TTP_11→ Phage_TAC_13→?→ SPRY+X*→?→?→ Phage-tail-tape→	SPRY+X	658	ECEFJIBE_02107	Gammaproteobacteria	Stenotrophomonas maltophilia	hypothetical protein ECEFJIBE_02107 [Stenotrophomonas maltophilia].	GCA_902157845.1
VUP33787.1	Phage_connect_1→ Phage_H_T_join→ HK97-gp10_like→ DUF3168→ Phage_TTP_11→ Phage_TAC_13→?→ SPRY+X*→?→?→ Phage-tail-tape→	SPRY+X	657	ECEFJIBE_03296	Gammaproteobacteria	Stenotrophomonas maltophilia	hypothetical protein ECEFJIBE_03296 [Stenotrophomonas maltophilia].	GCA_902157845.1
VUP45028.1	Phage_connect_1→ Phage_H_T_join→ HK97-gp10_like→ DUF3168→ Phage_TTP_11→ Phage_TAC_13→?→ SPRY+X*→?→?→ Phage-tail-tape→ ConA-repeats→?→?→ SRAP→	SPRY+X	657	KEFPIIEB_04064	Gammaproteobacteria	Stenotrophomonas maltophilia	hypothetical protein KEFPIIEB_04064 [Stenotrophomonas maltophilia].	GCA_902157865.1
VUP91253.1	Phage_connect_1→ Phage_H_T_join→ HK97-gp10_like→ DUF3168→ Phage_TTP_11→ Phage_TAC_13→?→ SPRY+X*→||<-?||?→?→ Phage-tail-tape→ ConA-repeats→	SPRY+X	657	BLDAJIEI_03531	Gammaproteobacteria	Stenotrophomonas maltophilia	hypothetical protein BLDAJIEI_03531 [Stenotrophomonas maltophilia].	GCA_902157935.1
VUP99062.1	Phage_connect_1→ Phage_H_T_join→ HK97-gp10_like→ DUF3168→ Phage_TTP_11→ Phage_TAC_13→?→ SPRY+X*→?→?→ Phage-tail-tape→	SPRY+X	655	BIEDFHEJ_01659	Gammaproteobacteria	Stenotrophomonas maltophilia	hypothetical protein BIEDFHEJ_01659 [Stenotrophomonas maltophilia].	GCA_902157995.1
VUQ06316.1	Phage_connect_1→ Phage_H_T_join→ HK97-gp10_like→ DUF3168→ Phage_TTP_11→ Phage_TAC_13→?→ SPRY+X*→?→?→ Phage-tail-tape→	SPRY+X	655	JPICFAGD_01619	Gammaproteobacteria	Stenotrophomonas maltophilia	hypothetical protein JPICFAGD_01619 [Stenotrophomonas maltophilia].	GCA_902158065.1
VUQ08603.1	Phage_connect_1→ Phage_H_T_join→ HK97-gp10_like→ DUF3168→ Phage_TTP_11→ Phage_TAC_13→?→ SPRY+X*→||<-?||?→ Phage-tail-tape→ ConA-repeats→	SPRY+X	655	MKCCKLKB_01148	Gammaproteobacteria	Stenotrophomonas maltophilia	hypothetical protein MKCCKLKB_01148 [Stenotrophomonas maltophilia].	GCA_902158105.1
VUQ16634.1	Phage_connect_1→ Phage_H_T_join→ HK97-gp10_like→ DUF3168→ Phage_TTP_11→ Phage_TAC_13→?→ SPRY+X*→||<-?||?→ Phage-tail-tape→	SPRY+X	657	DOOMHBAD_02908	Gammaproteobacteria	Stenotrophomonas maltophilia	hypothetical protein DOOMHBAD_02908 [Stenotrophomonas maltophilia].	GCA_902158025.1
VUR39033.1	Phage_connect_1→ Phage_H_T_join→ HK97-gp10_like→ DUF3168→ Phage_TTP_11→ Phage_TAC_13→?→ SPRY+X*→?→?→ Phage-tail-tape→ ConA-repeats→?→?→ SRAP→	SPRY+X	657	JHKEGKPK_04271	Gammaproteobacteria	Stenotrophomonas maltophilia	hypothetical protein JHKEGKPK_04271 [Stenotrophomonas maltophilia].	GCA_902158305.1
VUR45784.1	Phage_connect_1→ Phage_H_T_join→ HK97-gp10_like→ DUF3168→ Phage_TTP_11→ Phage_TAC_13→?→ SPRY+X*→?→?→ Phage-tail-tape→ ConA-repeats→?→?→||<-SRAP	SPRY+X	657	PGEAMFKH_03207	Gammaproteobacteria	Stenotrophomonas maltophilia	hypothetical protein PGEAMFKH_03207 [Stenotrophomonas maltophilia].	GCA_902158325.1

<table>
<tr><th>acc</th><th>operon</th><th>architecture</th><th>len</th><th>gen.name</th><th>taxend</th><th>species</th><th>defline</th><th>gca</th></tr>
<tr><td>VUR81206.1</td><td>Phage_connect_1→ Phage_H_T_join→ HK97-gp10_like→ DUF3168→ Phage_TTP_11→ Phage_TAC_13→?→ SPRY+X*→?→?→ Phage-tail-tape→ ConA-repeats→ ConA→?→||<-SRAP</td><td>SPRY+X</td><td>655</td><td>GMKOK-IBD_01185</td><td>Gammaproteobacteria</td><td>Stenotrophomonas maltophilia</td><td>hypothetical protein GMKOKIBD_01185 [Stenotrophomonas maltophilia].</td><td>GCA_902158475.1</td></tr>
<tr><td>WP_007428531.1</td><td><-ABhydrolase<-?||SIG+SPRY→ SIG+SPRY→ SUN→||<-?<-Glycos_transf_2<-?||SUN→</td><td>SIG+SPRY</td><td>322</td><td>WG8_RS02665</td><td>Firmicutes</td><td>Paenibacillus sp. Aloe-11</td><td>hypothetical protein [Paenibacillus sp. Aloe-11].</td><td>GCF_000245715.1</td></tr>
<tr><td>WP_010076376.1</td><td><-SIG+IG<-?||SPRY+Big_2→ SPRY+Big_2→||<-ACET</td><td>SPRY+Big_2</td><td>292</td><td>CLO-CEL_RS05090</td><td>Firmicutes</td><td>Clostridium cellulovorans</td><td>Ig-like domain-containing protein [Clostridium cellulovorans].</td><td>GCF_000145275.1</td></tr>
<tr><td>WP_013308530.1</td><td>HTH_20→||<-ABhydrolase<-Polbetant||ACET→ Cupin_2→||<-DUF2935||SIG+SPRY*→||<-?||?→||<-Glycos_transf_2</td><td>SIG+SPRY</td><td>346</td><td>PPE_RS02405</td><td>Firmicutes</td><td>Paenibacillus polymyxa</td><td>hypothetical protein [Paenibacillus polymyxa].</td><td>GCF_000146875.3</td></tr>
<tr><td>WP_013513300.1</td><td>Phage_T7_tail→ SPRY*→ Phage_tail_APC→</td><td>SPRY</td><td>528</td><td>DAES_RS01675</td><td>Deltaproteobacteria</td><td>Pseudodesulfovibrio aespoeensis</td><td>SPla/RYanodine receptor SPRY domain-containing protein [Pseudodesulfovibrio aespoeensis].</td><td>GCF_000176915.2</td></tr>
<tr><td>WP_019226907.1</td><td>SIG+SPRY*→</td><td>SIG+SPRY</td><td>378</td><td>BN359_RS15990</td><td>Firmicutes</td><td>Dehalobacter</td><td>MULTISPECIES: hypothetical protein [Dehalobacter].</td><td>GCF_000309295.1</td></tr>
<tr><td>WP_019639008.1</td><td>SPRY+SUN→ SUN+SPRY→ Laminin_G_3→ Disaggr_repeat→ Phage_holin_4_1→</td><td>SPRY+SUN</td><td>411</td><td>G358_RS0119880</td><td>Firmicutes</td><td>Paenibacillus fonticola</td><td>hypothetical protein [Paenibacillus fonticola].</td><td>GCF_000381905.1</td></tr>
<tr><td>WP_019639009.1</td><td>-</td><td>-</td><td>413</td><td>G358_RS0119885</td><td>Firmicutes</td><td>Paenibacillus fonticola</td><td>discoidin domain-containing protein [Paenibacillus fonticola].</td><td>GCF_000381905.1</td></tr>
<tr><td>WP_019687775.1</td><td>SUN→ SPRY+Glyco_hydro_106*→ Laminin_G_3→ Disaggr_repeat→ Phage_holin_4_1→</td><td>SPRY+Glyco_hydro_106</td><td>403</td><td>H7A89_RS14065</td><td>Firmicutes</td><td>Paenibacillus polymyxa</td><td>hypothetical protein [Paenibacillus polymyxa].</td><td>GCF_903797665.1</td></tr>
<tr><td>WP_020591223.1</td><td>Phage_T7_tail→ SPRY*→</td><td>SPRY</td><td>1351</td><td>B152_RS0104640</td><td>Alphaproteobacteria</td><td>Kiloniella laminariae</td><td>hypothetical protein [Kiloniella laminariae].</td><td>GCF_000374005.1</td></tr>
<tr><td>WP_021132610.1</td><td>SPRY*→ Phage_tail_APC→</td><td>SPRY</td><td>746</td><td>K678_RS11425</td><td>Alphaproteobacteria</td><td>Phaeospirillum fulvum</td><td>hypothetical protein [Phaeospirillum fulvum].</td><td>GCF_000442515.1</td></tr>
<tr><td>WP_021203812.1</td><td>Phage_connect_1→ Phage_H_T_join→ HK97-gp10_like→ DUF3168→ Phage_TTP_11→ Phage_TAC_13→?→ SPRY+X*→?→?→ Phage-tail-tape→ ConA-repeats→?→?→||<-SRAP</td><td>SPRY+X</td><td>657</td><td>L681_RS129520</td><td>Gammaproteobacteria</td><td>Stenotrophomonas maltophilia</td><td>hypothetical protein [Stenotrophomonas maltophilia].</td><td>GCF_000455685.1</td></tr>
<tr><td>WP_023986763.1</td><td><-Trypsin<-ABhydrolase<-Polbetant||ACET→ Cupin_2→ ABhydrolase→||<-DUF2935||SIG+SPRY*→||<-PSE||?→ SIG+SPRY→ PSE→||<-Glycos_transf_2</td><td>SIG+SPRY</td><td>331</td><td>X809_RS02170</td><td>Firmicutes</td><td>Paenibacillus polymyxa</td><td>hypothetical protein [Paenibacillus polymyxa].</td><td>GCF_000507205.3</td></tr>
<tr><td>WP_024956478.1</td><td>Phage_connect_1→ Phage_H_T_join→ HK97-gp10_like→ DUF3168→ Phage_TTP_11→ Phage_TAC_13→ SPRY+X*→?→?→ Phage-tail-tape→</td><td>SPRY+X</td><td>657</td><td>BMR86_RS03335</td><td>Gammaproteobacteria</td><td>Stenotrophomonas</td><td>MULTISPECIES: hypothetical protein [Stenotrophomonas].</td><td>GCF_001591205.1</td></tr>
<tr><td>WP_025116403.1</td><td>SPRY→?→ SPRY+SUN→</td><td>SPRY</td><td>559</td><td>U863_RS0116605</td><td>Firmicutes</td><td>Lysinibacillus fusiformis</td><td>discoidin domain-containing protein [Lysinibacillus fusiformis].</td><td>GCF_000633275.1</td></tr>
</table>

<table>
<tr><th>acc</th><th>operon</th><th>architecture</th><th>len</th><th>gen.name</th><th>taxend</th><th>species</th><th>defline</th><th>gca</th></tr>
<tr><td>WP_025116405.1</td><td>-</td><td>-</td><td>396</td><td>U863_RS0116615</td><td>Firmicutes</td><td>Lysinibacillus fusiformis</td><td>hypothetical protein [Lysinibacillus fusiformis].</td><td>GCF_000633275.1</td></tr>
<tr><td>WP_025219035.1</td><td>SUN→ SPRY*→ SUN→ SPRY→ Laminin_G_3→ Disaggr_repeat→?→?→ Phage_holin_1→</td><td>SPRY</td><td>281</td><td>T479_RS06935</td><td>Firmicutes</td><td>Lysinibacillus varians</td><td>hypothetical protein [Lysinibacillus varians].</td><td>GCF_000600105.1</td></tr>
<tr><td>WP_025363879.1</td><td>HTH_20→||<-Trypsin<-ABhydrolase||ACET→||<-DUF2935<-?||SIG+SPRY*→</td><td>SIG+SPRY</td><td>332</td><td>LK13_RS15595</td><td>Firmicutes</td><td>Paenibacillus polymyxa</td><td>hypothetical protein [Paenibacillus polymyxa].</td><td>GCF_000597985.1</td></tr>
<tr><td>WP_025676232.1</td><td>HTH_20→||<-Trypsin<-ABhydrolase||ACET→||<-DUF2935<-?||SIG+SPRY*→</td><td>SIG+SPRY</td><td>327</td><td>L695_RS0111830</td><td>Firmicutes</td><td>Paenibacillus polymyxa</td><td>hypothetical protein [Paenibacillus polymyxa].</td><td>GCF_000520795.1</td></tr>
<tr><td>WP_025682581.1</td><td>HTH_20→||<-?||?→||<-DUF2935<-?||SIG+SPRY*→||<-PSE||SUN→||<-?||?→||<-?<-?<-Phage_holin_4_1</td><td>SIG+SPRY</td><td>332</td><td>L696_RS0104535</td><td>Firmicutes</td><td>Paenibacillus maysiensis</td><td>hypothetical protein [Paenibacillus maysiensis].</td><td>GCF_000520815.1</td></tr>
<tr><td>WP_025716755.1</td><td>HTH_20→||<-ABhydrolase<-?||SIG+SPRY*→</td><td>SIG+SPRY</td><td>314</td><td>L693_RS0107595</td><td>Firmicutes</td><td>Paenibacillus sp. 1-18</td><td>hypothetical protein [Paenibacillus sp. 1-18].</td><td>GCF_000520755.1</td></tr>
<tr><td>WP_025721904.1</td><td>HTH_20→||<-Trypsin<-ABhydrolase<-Polbetant||ACET→ Cupin_2→||<-DUF2935||SIG+SPRY*→</td><td>SIG+SPRY</td><td>218</td><td>L694_RS0112470</td><td>Firmicutes</td><td>Paenibacillus polymyxa</td><td>hypothetical protein, partial [Paenibacillus polymyxa].</td><td>GCF_000520775.1</td></tr>
<tr><td>WP_028541175.1</td><td><-ABhydrolase<-Polbetant||ACET→ Cupin_2→ ABhydrolase→||<-DUF2935<-?||SIG+SPRY*→||<-?||?→ SIG+SPRY→||<-Glycos_transf_2</td><td>SIG+SPRY</td><td>320</td><td>BM358_RS15270</td><td>Firmicutes</td><td>unclassified Paenibacillus</td><td>MULTISPECIES: hypothetical protein [unclassified Paenibacillus].</td><td>GCF_900116035.1</td></tr>
<tr><td>WP_028562231.1</td><td>SPRY+SUN*→ SUN→ Laminin_G_3→ Disaggr_repeat→</td><td>SPRY+SUN</td><td>414</td><td>H583_RS35510</td><td>Firmicutes</td><td>Paenibacillus pinihumi</td><td>hypothetical protein [Paenibacillus pinihumi].</td><td>GCF_000422505.1</td></tr>
<tr><td>WP_029516016.1</td><td><-ABhydrolase<-Polbetant||ACET→ Cupin_2→ ABhydrolase→||<-DUF2935||SUN→ SIG+SPRY*→?→||<-Glycos_transf_2</td><td>SIG+SPRY</td><td>322</td><td>B439_RS0108145</td><td>Firmicutes</td><td>Paenibacillus polymyxa</td><td>hypothetical protein [Paenibacillus polymyxa].</td><td>GCF_000463565.1</td></tr>
<tr><td>WP_029518099.1</td><td>SUN→ SUN*→ Laminin_G_3→ Disaggr_repeat→</td><td>SUN</td><td>403</td><td>B439_RS0121250</td><td>Firmicutes</td><td>Paenibacillus polymyxa</td><td>hypothetical protein [Paenibacillus polymyxa].</td><td>GCF_000463565.1</td></tr>
<tr><td>WP_031462749.1</td><td>HTH_20→||<-Trypsin<-ABhydrolase||ACET→||<-DUF2935<-?||SIG+SPRY*→</td><td>SIG+SPRY</td><td>308</td><td>FC01_RS0115670</td><td>Firmicutes</td><td>Paenibacillus polymyxa</td><td>hypothetical protein [Paenibacillus polymyxa].</td><td>GCF_000735775.1</td></tr>
<tr><td>WP_033066158.1</td><td>Cluster143_2clades→?→?→?→ SUN→ SPRY*→ PSE→ PSE→ SUN→ TM→ Cluster104_2clades→?→?→35exo→ Cluster112_2clades→</td><td>SPRY</td><td>447</td><td>N496_RS05195</td><td>Firmicutes</td><td>Clostridium botulinum</td><td>cell adhesion protein [Clostridium botulinum].</td><td>GCF_000710995.1</td></tr>
<tr><td>WP_036124491.1</td><td>SUN→ SUN→?→ SUN→ SUN→ SPRY*→ SUN→ Laminin_G_3→ Disaggr_repeat→?→?→ Phage_holin_1→</td><td>SPRY</td><td>257</td><td>HR49_RS16910</td><td>Firmicutes</td><td>Lysinibacillus</td><td>MULTISPECIES: hypothetical protein [Lysinibacillus].</td><td>GCF_000724775.3</td></tr>
<tr><td>WP_036147628.1</td><td>SUN→ SPRY+SUN→ SPRY+SUN→ SUN→ SUN→ SUN→ Laminin_G_3→ Disaggr_repeat→</td><td>SPRY+SUN</td><td>414</td><td>KQ41_RS11015</td><td>Firmicutes</td><td>Lysinibacillus fusiformis</td><td>discoidin domain-containing protein [Lysinibacillus fusiformis].</td><td>GCF_000755455.1</td></tr>
<tr><td>WP_036147629.1</td><td>-</td><td>-</td><td>433</td><td>KQ41_RS11020</td><td>Firmicutes</td><td>Lysinibacillus fusiformis</td><td>discoidin domain-containing protein [Lysinibacillus fusiformis].</td><td>GCF_000755455.1</td></tr>
</table>

acc	operon	architecture	len	gen.name	taxend	species	defline	gca
WP_036669882.1	SUN→ SPRY+SUN→ *SPRY+SUN*→ Laminin_G_3→ Disaggr_repeat→	SPRY+SUN	395	C161_RS09450	Firmicutes	Paenibacillus sp. FSL R5-192	hypothetical protein [Paenibacillus sp. FSL R5-192].	GCF_000517845.1
WP_040102262.1	HTH_20→||<-Trypsin<-ABhydrolase||ACET→||<-DUF2935<-?||SIG+SPRY*→	SIG+SPRY	319	RE92_RS09150	Firmicutes	Paenibacillus polymyxa	hypothetical protein [Paenibacillus polymyxa].	GCF_000819665.1
WP_044646514.1	<-DUF2935||?→||<-?||SIG+SPRY*→	SIG+SPRY	326	QD47_RS12890	Firmicutes	Paenibacillus terrae	hypothetical protein [Paenibacillus terrae].	GCF_000943545.1
WP_044648488.1	SIG+SPRY*→||<-?<-?<-?<-?<-?<-Phage_holin_4_1	SIG+SPRY	327	QD47_RS23995	Firmicutes	Paenibacillus terrae	hypothetical protein [Paenibacillus terrae].	GCF_000943545.1
WP_044787083.1	HTH_20→||<-Trypsin<-ABhydrolase||ACET→||<-DUF2935<-?||SIG+SPRY*→	SIG+SPRY	357	DS866_RS12660	Firmicutes	Paenibacillus polymyxa	hypothetical protein [Paenibacillus polymyxa].	GCF_000943535.1
WP_046501577.1	Phage_T7_tail→ SPRY*→	SPRY	1158	WH95_RS00475	Alphaproteobacteria	Kiloniella litopenaei	hypothetical protein [Kiloniella litopenaei].	GCF_000982415.1
WP_048035679.1	SUN→ SUN→ SPRY*→?→?→?→||<-?<-?<-?<-ABC-ATPase	SPRY	890	AB432_RS03735	Firmicutes	Brevibacillus brevis	hypothetical protein [Brevibacillus brevis].	GCF_001039275.2
WP_049431026.1	Phage_connect_1→ Phage_H_T_join→ HK97-gp10_like→ DUF3168→ Phage_TTP_11→ Phage_TAC_13→ SPRY+X*→||<-?||?→?→ Phage-tail-tape→ ConA-repeats→	SPRY+X	657	G5A48_RS10580	Gammaproteobacteria	Stenotrophomonas maltophilia	hypothetical protein [Stenotrophomonas maltophilia].	GCF_001069295.1
WP_049432438.1	Phage_connect_1→ Phage_H_T_join→ HK97-gp10_like→ DUF3168→ Phage_TTP_11→ Phage_TAC_13→?→ SPRY+X*→?→?→ Phage-tail-tape→ ConA-repeats→	SPRY+X	655	A7X76_RS05335	Gammaproteobacteria	Stenotrophomonas maltophilia	hypothetical protein [Stenotrophomonas maltophilia].	GCF_001070785.1
WP_049448603.1	Phage_connect_1→ Phage_H_T_join→ HK97-gp10_like→ DUF3168→ Phage_TTP_11→ Phage_TAC_13→?→ SPRY+X*→?→ Phage-tail-tape→ ConA-repeats→	SPRY+X	657	ADH94_RS22020	Gammaproteobacteria	Stenotrophomonas maltophilia	hypothetical protein [Stenotrophomonas maltophilia].	GCF_001071475.1
WP_049450667.1	Phage_connect_1→ Phage_H_T_join→ HK97-gp10_like→ DUF3168→ Phage_TTP_11→ Phage_TAC_13→?→ SPRY+X*→?→ Phage-tail-tape→?→?→?→||<-SRAP	SPRY+X	656	EGS96_RS20790	Gammaproteobacteria	Stenotrophomonas maltophilia	hypothetical protein [Stenotrophomonas maltophilia].	GCF_001071195.1
WP_049454514.1	Phage_connect_1→ Phage_H_T_join→ HK97-gp10_like→ DUF3168→ Phage_TTP_11→ Phage_TAC_13→?→ SPRY+X*→?→?→ Phage-tail-tape→ ConA-repeats→?→?→||<-SRAP	SPRY+X	658	ADF89_RS18240	Gammaproteobacteria	Stenotrophomonas maltophilia	hypothetical protein [Stenotrophomonas maltophilia].	GCF_001069005.1
WP_049459324.1	Phage_connect_1→ Phage_H_T_join→ HK97-gp10_like→ DUF3168→ Phage_TTP_11→ Phage_TAC_13→?→ SPRY+X*→?→?→ Phage-tail-tape→ ConA-repeats→	SPRY+X	657	ADM04_RS16360	Gammaproteobacteria	Stenotrophomonas maltophilia	hypothetical protein [Stenotrophomonas maltophilia].	GCF_001074705.1
WP_049736558.1	SUN→ Laminin_G_3+SPRY*→	Laminin_G_3+SPRY	822	BRE01_RS08970	Firmicutes	Brevibacillus reuszeri	hypothetical protein [Brevibacillus reuszeri].	GCF_006540225.1
WP_051447145.1	SUN→ SPRY+SUN→ *SUN*→ *SPRY+SUN*→ Laminin_G_3→ Disaggr_repeat→	SPRY+SUN	421	C170_RS30045	Firmicutes	Paenibacillus sp. FSL H7-689	discoidin domain-containing protein [Paenibacillus sp. FSL H7-689].	GCF_000517905.1
WP_051447146.1	-	-	399	C170_RS30050	Firmicutes	Paenibacillus sp. FSL H7-689	discoidin domain-containing protein [Paenibacillus sp. FSL H7-689].	GCF_000517905.1
WP_051449672.1	-	-	402	C161_RS31475	Firmicutes	Paenibacillus sp. FSL R5-192	hypothetical protein [Paenibacillus sp. FSL R5-192].	GCF_000517845.1
WP_051626263.1	<-REC+OmpR-HTH<-?<-OBfold<-?<-?||?→||<-?||SIG+SPRY*→	SIG+SPRY	653	EP01_RS18270	Oligoflexia	Bdellovibrio bacteriovorus	hypothetical protein [Bdellovibrio bacteriovorus].	GCF_000691605.1

<table>
<tr><th>acc</th><th>operon</th><th>architecture</th><th>len</th><th>gen.name</th><th>taxend</th><th>species</th><th>defline</th><th>gca</th></tr>
<tr><td>WP_052310845.1</td><td>-</td><td>-</td><td>229</td><td>WG8_RS02660</td><td>Firmicutes</td><td>Paenibacillus sp. Aloe-11</td><td>hypothetical protein, partial [Paenibacillus sp. Aloe-11].</td><td>GCF_000245715.1</td></tr>
<tr><td>WP_053324515.1</td><td><-ABhydrolase<-Polbetant||ACET→ Cupin_2→ ABhydrolase→||<-DUF2935<-?||SIG+SPRY→//<-PSE//?→ SIG+SPRY→||<-Glycos_transf_2</td><td>SIG+SPRY</td><td>373</td><td>BK119_RS14050</td><td>Firmicutes</td><td>Paenibacillus</td><td>MULTISPECIES: hypothetical protein [Paenibacillus].</td><td>GCF_001922145.1</td></tr>
<tr><td>WP_053324516.1</td><td><-ABhydrolase<-Polbetant||ACET→ Cupin_2→ ABhydrolase→||<-DUF2935<-?||SIG+SPRY*→?→ SIG+SPRY→ PSE→||<-Glycos_transf_2</td><td>SIG+SPRY</td><td>328</td><td>ABE82_RS02500</td><td>Firmicutes</td><td>Paenibacillus peoriae</td><td>hypothetical protein [Paenibacillus peoriae].</td><td>GCF_001272655.2</td></tr>
<tr><td>WP_053497216.1</td><td>Phage_connect_1→ Phage_H_T_join→ HK97-gp10_like→ DUF3168→ Phage_TTP_11→ Phage_TAC_13→?→ SPRY+X*→?→?→ Phage-tail-tape→ ConA-repeats→ ConA→?→||<-SRAP</td><td>SPRY+X</td><td>655</td><td>ABW42_RS03390</td><td>Gammaproteobacteria</td><td>Stenotrophomonas maltophilia</td><td>hypothetical protein [Stenotrophomonas maltophilia].</td><td>GCF_001276345.1</td></tr>
<tr><td>WP_053519524.1</td><td>Phage_connect_1→ Phage_H_T_join→ HK97-gp10_like→ DUF3168→ Phage_TTP_11→ Phage_TAC_13→?→ SPRY+X*→?→ Phage-tail-tape→ ConA-repeats→</td><td>SPRY+X</td><td>655</td><td>ABW43_RS14405</td><td>Gammaproteobacteria</td><td>Stenotrophomonas maltophilia</td><td>hypothetical protein [Stenotrophomonas maltophilia].</td><td>GCF_001276355.1</td></tr>
<tr><td>WP_053595626.1</td><td>SUN→ SPRY+SUN*→ SUN→ SUN→ Laminin_G_3→ Disaggr_repeat→</td><td>SPRY+SUN</td><td>413</td><td>AN161_RS17530</td><td>Firmicutes</td><td>Lysinibacillus sp. FJAT-14222</td><td>discoidin domain-containing protein [Lysinibacillus sp. FJAT-14222].</td><td>GCF_001278875.1</td></tr>
<tr><td>WP_054136806.1</td><td><-SIG+IG<-?||?→?→?→||<-?<-?||SUN+SPRY*→</td><td>SUN+SPRY</td><td>466</td><td>IP80_RS13885</td><td>Betaproteobacteria</td><td>unclassified Betaproteobacteria</td><td>MULTISPECIES: tautomerase family protein [unclassified Betaproteobacteria].</td><td>GCF_001295865.1</td></tr>
<tr><td>WP_054704414.1</td><td>SPRY+TM*→ SUN→ SUN→ Laminin_G_3→ Disaggr_repeat→</td><td>SPRY+TM</td><td>211</td><td>JCM16419_RS00705</td><td>Firmicutes</td><td>Paenibacillus pinihumi</td><td>hypothetical protein [Paenibacillus pinihumi].</td><td>GCF_001312305.1</td></tr>
<tr><td>WP_058710989.1</td><td>HTH_20→||<-Trypsin<-ABhydrolase||ACET→||<-DUF2935<-PSE||SIG+SPRY*→</td><td>SIG+SPRY</td><td>306</td><td>NS115_RS07620</td><td>Firmicutes</td><td>Paenibacillus</td><td>MULTISPECIES: hypothetical protein [Paenibacillus].</td><td>GCF_002872435.1</td></tr>
<tr><td>WP_058830612.1</td><td>HTH_20→||<-Trypsin<-ABhydrolase||ACET→||<-DUF2935<-?||SIG+SPRY*→</td><td>SIG+SPRY</td><td>339</td><td>AVK83_RS16520</td><td>Firmicutes</td><td>Paenibacillus polymyxa</td><td>hypothetical protein [Paenibacillus polymyxa].</td><td>GCF_001481575.1</td></tr>
<tr><td>WP_058981995.1</td><td>Phage_connect_1→ Phage_H_T_join→ HK97-gp10_like→ DUF3168→ Phage_TTP_11→ Phage_TAC_13→?→ SPRY+X*→?→?→?→ Phage-tail-tape→ ConA-repeats→</td><td>SPRY+X</td><td>657</td><td>BN1263_RS14155</td><td>Gammaproteobacteria</td><td>Stenotrophomonas maltophilia</td><td>hypothetical protein [Stenotrophomonas maltophilia].</td><td>GCF_001499715.1</td></tr>
<tr><td>WP_059064521.1</td><td>Phage_connect_1→ Phage_H_T_join→ HK97-gp10_like→ DUF3168→ Phage_TTP_11→ Phage_TAC_13→?→ SPRY+X*→?→ Phage-tail-tape→ ConA-repeats→?→ SRAP→</td><td>SPRY+X</td><td>655</td><td>AV528_RS10010</td><td>Gammaproteobacteria</td><td>Stenotrophomonas maltophilia</td><td>hypothetical protein [Stenotrophomonas maltophilia].</td><td>GCF_001499755.1</td></tr>
<tr><td>WP_063211944.1</td><td>HTH_20→||<-ABhydrolase<-?||ACET→ Cupin_2→ SIG+SPRY*→</td><td>SIG+SPRY</td><td>337</td><td>AV545_RS14475</td><td>Firmicutes</td><td>Paenibacillus jamilae</td><td>hypothetical protein [Paenibacillus jamilae].</td><td>GCF_001619755.1</td></tr>
<tr><td>WP_064201871.1</td><td>SPRY*→?→?→?→||<-?<-?<-?<-ABC-ATPase</td><td>SPRY</td><td>894</td><td>A4S11_RS18990</td><td>Firmicutes</td><td>Brevibacillus brevis</td><td>hypothetical protein [Brevibacillus brevis].</td><td>GCF_001649505.1</td></tr>
<tr><td>WP_064796708.1</td><td>HTH_20→||<-ABhydrolase<-Polbetant||ACET→ Cupin_2→||<-?||SIG+SPRY*→</td><td>SIG+SPRY</td><td>314</td><td>A9P44_RS12255</td><td>Firmicutes</td><td>Paenibacillus polymyxa</td><td>hypothetical protein [Paenibacillus polymyxa].</td><td>GCF_001663585.1</td></tr>
<tr><td>WP_065179322.1</td><td>Phage_connect_1→ Phage_H_T_join→ HK97-gp10_like→ DUF3168→ Phage_TTP_11→ Phage_TAC_13→?→ SPRY+X*→?→ Phage-tail-tape→</td><td>SPRY+X</td><td>655</td><td>A9K70_RS20570</td><td>Gammaproteobacteria</td><td>Stenotrophomonas maltophilia</td><td>hypothetical protein [Stenotrophomonas maltophilia].</td><td>GCF_001676395.1</td></tr>
<tr><td>WP_065181897.1</td><td>Phage_connect_1→ Phage_H_T_join→ HK97-gp10_like→ DUF3168→ Phage_TTP_11→ Phage_TAC_13→?→ SPRY+X*→||<-?||?→?→ Phage-tail-tape→ ConA-repeats→</td><td>SPRY+X</td><td>655</td><td>A9K56_RS08050</td><td>Gammaproteobacteria</td><td>Stenotrophomonas maltophilia</td><td>hypothetical protein [Stenotrophomonas maltophilia].</td><td>GCF_001676315.1</td></tr>
</table>

<table>
<tr><th>acc</th><th>operon</th><th>architecture</th><th>len</th><th>gen.name</th><th>taxend</th><th>species</th><th>defline</th><th>gca</th></tr>
<tr><td>WP_065200553.1</td><td>Phage_connect_1→ Phage_H_T_join→ HK97-gp10_like→ DUF3168→ Phage_TTP_11→ Phage_TAC_13→?→ SPRY+X*→||<-?||?→?→?→ Phage-tail-tape→ ConA-repeats→ ConA→</td><td>SPRY+X</td><td>655</td><td>A9K58_RS17510</td><td>Gammaproteobacteria</td><td>Stenotrophomonas maltophilia</td><td>hypothetical protein [Stenotrophomonas maltophilia].</td><td>GCF_001676385.1</td></tr>
<tr><td>WP_068434738.1</td><td>Cluster93_5clades→ Laminin_G_3+SPRY*→?→?→?→||<-?<-Cluster297_2clades<-EMAP</td><td>Laminin_G_3+SPRY</td><td>467</td><td>XM1_RS14675</td><td>Alphaproteobacteria</td><td>Magnetospirillum sp. XM-1</td><td>hypothetical protein [Magnetospirillum sp. XM-1].</td><td>GCF_001511835.1</td></tr>
<tr><td>WP_068499071.1</td><td><-SUN<-?<-?||SIG+SPRY*→</td><td>SIG+SPRY</td><td>378</td><td>BAZ21_RS08685</td><td>Firmicutes</td><td>Paenibacillus kribbensis</td><td>hypothetical protein [Paenibacillus kribbensis].</td><td>GCF_001705305.1</td></tr>
<tr><td>WP_068504121.1</td><td><-ABhydrolase||?→||<-?||SIG+SPRY*→||<-PSE||SUN→</td><td>SIG+SPRY</td><td>308</td><td>BAZ21_RS19160</td><td>Firmicutes</td><td>Paenibacillus kribbensis</td><td>hypothetical protein [Paenibacillus kribbensis].</td><td>GCF_001705305.1</td></tr>
<tr><td>WP_068940847.1</td><td>SUN→ SPRY+SUN→ *SPRY*→ Disaggr_repeat→</td><td>SPRY+SUN</td><td>698</td><td>A7312_RS18605</td><td>Firmicutes</td><td>Paenibacillus polymyxa</td><td>hypothetical protein [Paenibacillus polymyxa].</td><td>GCF_001707685.1</td></tr>
<tr><td>WP_068940849.1</td><td>-</td><td>-</td><td>419</td><td>A7312_RS18610</td><td>Firmicutes</td><td>Paenibacillus polymyxa</td><td>hypothetical protein [Paenibacillus polymyxa].</td><td>GCF_001707685.1</td></tr>
<tr><td>WP_068941209.1</td><td>-</td><td>-</td><td>320</td><td>BK119_RS14035</td><td>Firmicutes</td><td>Paenibacillus</td><td>MULTISPECIES: hypothetical protein [Paenibacillus].</td><td>GCF_001922145.1</td></tr>
<tr><td>WP_069010793.1</td><td>HTH_20→||<-ABhydrolase<-Polbetant||ACET→ Cupin_2→ ABhydrolase→||<-DUF2935||SIG+SPRY*→?→||<-Glycos_transf_2</td><td>SIG+SPRY</td><td>346</td><td>A7311_RS06950</td><td>Firmicutes</td><td>Paenibacillus polymyxa</td><td>hypothetical protein [Paenibacillus polymyxa].</td><td>GCF_001709075.1</td></tr>
<tr><td>WP_069481585.1</td><td>SUN+SPRY→*?*→*?*→*?*→ *SUN*→ *SPRY+SUN*→</td><td>SUN+SPRY</td><td>559</td><td>BG258_RS12185</td><td>Firmicutes</td><td>Lysinibacillus fusiformis</td><td>discoidin domain-containing protein [Lysinibacillus fusiformis].</td><td>GCF_001726065.1</td></tr>
<tr><td>WP_069481589.1</td><td>-</td><td>-</td><td>403</td><td>BG258_RS12205</td><td>Firmicutes</td><td>Lysinibacillus fusiformis</td><td>hypothetical protein [Lysinibacillus fusiformis].</td><td>GCF_001726065.1</td></tr>
<tr><td>WP_071558628.1</td><td><-SUN||SIG+SPRY*→||<-?<-?<-?<-?<-?<-Phage_holin_4_1</td><td>SIG+SPRY</td><td>334</td><td>CG775_RS21655</td><td>Firmicutes</td><td>Paenibacillus polymyxa</td><td>hypothetical protein [Paenibacillus polymyxa].</td><td>GCF_001874425.3</td></tr>
<tr><td>WP_071639359.1</td><td>HTH_20→||<-ABhydrolase<-Polbetant||ACET→ Cupin_2→ ABhydrolase→||<-DUF2935||SIG+SPRY→*?*→ *SIG+SPRY*→ PSE→||<-Glycos_transf_2</td><td>SIG+SPRY</td><td>373</td><td>PPYC1_RS02475</td><td>Firmicutes</td><td>Paenibacillus polymyxa</td><td>hypothetical protein [Paenibacillus polymyxa].</td><td>GCF_001874405.2</td></tr>
<tr><td>WP_071639361.1</td><td>-</td><td>-</td><td>323</td><td>PPYC1_RS02485</td><td>Firmicutes</td><td>Paenibacillus polymyxa</td><td>hypothetical protein [Paenibacillus polymyxa].</td><td>GCF_001874405.2</td></tr>
<tr><td>WP_072733996.1</td><td>SUN→ SUN→ SUN→ TM+SPRY+SUN→ *SPRY*→ Disaggr_repeat→</td><td>TM+SPRY+SUN</td><td>708</td><td>BUA17_RS14040</td><td>Firmicutes</td><td>Paenibacillus sp. ov031</td><td>hypothetical protein [Paenibacillus sp. ov031].</td><td>GCF_900143165.1</td></tr>
<tr><td>WP_072733997.1</td><td>-</td><td>-</td><td>422</td><td>BUA17_RS14045</td><td>Firmicutes</td><td>Paenibacillus sp. ov031</td><td>discoidin domain-containing protein [Paenibacillus sp. ov031].</td><td>GCF_900143165.1</td></tr>
<tr><td>WP_075141582.1</td><td>Cluster216_2clades→?→?→?→ SUN→ SPRY*→ PSE→ SUN→ SUN→ TM→ Cluster104_2clades→?→?→35exo→</td><td>SPRY</td><td>447</td><td>RSJ3_RS04310</td><td>Firmicutes</td><td>Clostridium botulinum</td><td>cell adhesion protein [Clostridium botulinum].</td><td>GCF_001921965.1</td></tr>
<tr><td>WP_076158833.1</td><td><-ABhydrolase<-Polbetant||ACET→ Cupin_2→ ABhydrolase→||<-DUF2935||SIG+SPRY*→||<-PSE||?→ PSE→||<-Glycos_transf_2</td><td>SIG+SPRY</td><td>301</td><td>BK134_RS12705</td><td>Firmicutes</td><td>Paenibacillus peoriae</td><td>hypothetical protein [Paenibacillus peoriae].</td><td>GCF_001956115.1</td></tr>
</table>

<table>
<tr><th>acc</th><th>operon</th><th>architecture</th><th>len</th><th>gen.name</th><th>taxend</th><th>species</th><th>defline</th><th>gca</th></tr>
<tr><td>WP_076172386.1</td><td>Cluster171_2clades→?→?→ Cluster143_2clades→?→?→ PSE→ SUN→ SPRY*→ SUN→ TM→ Cluster104_2clades→?→?→35exo→ Cluster112_2clades→</td><td>SPRY</td><td>465</td><td>N487_RS14900</td><td>Firmicutes</td><td>Clostridium botulinum</td><td>cell adhesion protein [Clostridium botulinum].</td><td>GCF_000710975.1</td></tr>
<tr><td>WP_076252374.1</td><td>SUN→ SPRY+SUN*→ SUN→ SUN→ Laminin_G_3→ Disaggr_repeat→</td><td>SPRY+SUN</td><td>403</td><td>BK136_RS15140</td><td>Firmicutes</td><td>Paenibacillus amylolyticus</td><td>hypothetical protein [Paenibacillus amylolyticus].</td><td>GCF_001956175.1</td></tr>
<tr><td>WP_076265166.1</td><td><-ABhydrolase<-Polbetant||ACET→ Cupin_2→ ABhydrolase→||<-DUF2935<-?||SIG+SPRY*→||<-?||?→ SIG+SPRY→||<-Glycos_transf_2</td><td>SIG+SPRY</td><td>320</td><td>BK143_RS19680</td><td>Firmicutes</td><td>Paenibacillus peoriae</td><td>hypothetical protein [Paenibacillus peoriae].</td><td>GCF_001956225.1</td></tr>
<tr><td>WP_076293278.1</td><td>HTH_20→||<-PSE<-ABhydrolase<-Polbetant||ACET→ Cupin_2→ ABhydrolase→||<-DUF2935||SIG+SPRY*→||<-?||?→ PSE→||<-Glycos_transf_2</td><td>SIG+SPRY</td><td>326</td><td>BK135_RS10440</td><td>Firmicutes</td><td>Paenibacillus peoriae</td><td>hypothetical protein [Paenibacillus peoriae].</td><td>GCF_001956155.1</td></tr>
<tr><td>WP_076295423.1</td><td><-ABhydrolase<-Polbetant||ACET→ Cupin_2→ ABhydrolase→||<-DUF2935<-?||SIG+SPRY→<i>||<-?||?→ SIG+SPRY</i>→||<-Glycos_transf_2</td><td>SIG+SPRY</td><td>331</td><td>BK145_RS14470</td><td>Firmicutes</td><td>Paenibacillus peoriae</td><td>hypothetical protein [Paenibacillus peoriae].</td><td>GCF_001956235.1</td></tr>
<tr><td>WP_076318343.1</td><td>SUN→ SUN→ SUN→ SPRY+SUN*→ Laminin_G_3→ Disaggr_repeat→</td><td>SPRY+SUN</td><td>412</td><td>BK124_RS08255</td><td>Firmicutes</td><td>Paenibacillus amylolyticus</td><td>hypothetical protein [Paenibacillus amylolyticus].</td><td>GCF_001955995.1</td></tr>
<tr><td>WP_076327021.1</td><td>SUN→ SPRY+SUN*→ SUN→ Laminin_G_3→ Disaggr_repeat→</td><td>SPRY+SUN</td><td>414</td><td>BK129_RS03095</td><td>Firmicutes</td><td>Paenibacillus amylolyticus</td><td>discoidin domain-containing protein [Paenibacillus amylolyticus].</td><td>GCF_001956035.1</td></tr>
<tr><td>WP_079564340.1</td><td>SUN→ SPRY+SUN→ SUN→ SUN→ SPRY+SUN→ SUN→ SUN→ SUN→</td><td>SPRY+SUN</td><td>485</td><td>B5X66_RS24790</td><td>Firmicutes</td><td>unclassified Lysinibacillus</td><td>MULTISPECIES: hypothetical protein [unclassified Lysinibacillus].</td><td>GCF_900167885.1</td></tr>
<tr><td>WP_079564349.1</td><td>-</td><td>-</td><td>413</td><td>B5X66_RS24805</td><td>Firmicutes</td><td>unclassified Lysinibacillus</td><td>MULTISPECIES: discoidin domain-containing protein [unclassified Lysinibacillus].</td><td>GCF_900167885.1</td></tr>
<tr><td>WP_079564406.1</td><td>SUN→ SUN→ SUN→ SPRY+SUN*→?→ SUN→ SUN→ SUN→ SUN→ SUN→</td><td>SPRY+SUN</td><td>389</td><td>B5X66_RS24915</td><td>Firmicutes</td><td>unclassified Lysinibacillus</td><td>MULTISPECIES: discoidin domain-containing protein [unclassified Lysinibacillus].</td><td>GCF_900167885.1</td></tr>
<tr><td>WP_080291782.1</td><td>DUF3168→ Phage_TTP_11→ Phage_TAC_13→?→ SPRY+X*→?→?→ Phage-tail-tape→ ConA-repeats→</td><td>SPRY+X</td><td>655</td><td>LI87_RS0109275</td><td>Gammaproteobacteria</td><td>Stenotrophomonas maltophilia</td><td>hypothetical protein [Stenotrophomonas maltophilia].</td><td>GCF_000758465.1</td></tr>
<tr><td>WP_080639352.1</td><td>RHS_repeat+RHS_repeat+RHS_repeat+RHS_repeat+Laminin_G_3+SPRY*→?→?→ PSE→ PSE→35exo→</td><td>RHS_repeat+RHS_repeat+RHS_repeat+RHS_repeat+Laminin_G_3+SPRY</td><td>2000</td><td>YUK_RS0107305</td><td>Gammaproteobacteria</td><td>Teredinibacter turnerae</td><td>hypothetical protein [Teredinibacter turnerae].</td><td>GCF_000379165.1</td></tr>
<tr><td>WP_080974705.1</td><td>Cluster143_2clades→?→?→?→?→?→ SPRY→ SPRY*→ PSE→ TM→ Cluster104_2clades→</td><td>SPRY</td><td>311</td><td>ADC50_RS20320</td><td>Firmicutes</td><td>Clostridium sporogenes</td><td>hypothetical protein [Clostridium sporogenes].</td><td>GCF_001058825.1</td></tr>
<tr><td>WP_081280966.1</td><td>Phage_connect_1→ Phage_H_T_join→ HK97-gp10_like→ DUF3168→ Phage_TTP_11→ Phage_TAC_13→?→ SPRY+X*→?→?→ Phage-tail-tape→</td><td>SPRY+X</td><td>655</td><td>A9K69_RS19730</td><td>Gammaproteobacteria</td><td>Stenotrophomonas maltophilia</td><td>hypothetical protein [Stenotrophomonas maltophilia].</td><td>GCF_001676445.1</td></tr>
<tr><td>WP_084315242.1</td><td>SIG+TM+TM+TM+TM+TM+TM+TM+TM→ SIG+TM+TM+TM+TM+TM+TM+TM+TM→ ABC-ATPase→ SIG+TM+TM+TM+TM+ABC-ATPase→||<-?||?→ SPRY+Big_2*→?→?→ ACET→</td><td>SPRY+Big_2</td><td>535</td><td>BR69_RS21395</td><td>Firmicutes</td><td>Clostridium hydrogeniformans</td><td>Ig domain-containing protein [Clostridium hydrogeniformans].</td><td>GCF_000686705.1</td></tr>
<tr><td>WP_084575678.1</td><td>EMAP→ Cluster297_2clades→?→?→?→ SIG+SPRY*→?→?→?→?→?→ SIG+METHYLASE→</td><td>SIG+SPRY</td><td>349</td><td>B9A22_RS10460</td><td>Firmicutes</td><td>Sporomusa malonica</td><td>hypothetical protein [Sporomusa malonica].</td><td>GCF_900176355.1</td></tr>
</table>

acc	operon	architecture	len	gen.name	taxend	species	defline	gca
WP_085321805.1	Cluster171_2clades→?→?→ Cluster143_2clades→?→?→ PSE→ SUN→ SPRY*→ SUN→ TM→ Cluster104_2clades→?→?→35exo→ Cluster112_2clades→	SPRY	447	B2M06_RS15395	Firmicutes	Clostridium botulinum	cell adhesion protein [Clostridium botulinum].	GCF_002866045.1
WP_085899262.1	Phage_T7_tail→ SPRY*→	SPRY	1359	BKB51_RS01170	Alphaproteobacteria	Kiloniella majae	hypothetical protein [Kiloniella majae].	GCF_002118315.1
WP_087445183.1	SUN→ SPRY+SUN*→ SUN→ Laminin_G_3→ Disaggr_repeat→ Phage_holin_4_1→?→ SIG+ConA+beta-propeller→	SPRY+SUN	430	FLT43_09110	Firmicutes	Paenibacillus thiaminolyticus	hypothetical protein [Paenibacillus thiaminolyticus].	-
WP_087803178.1	Phage_connect_1→ Phage_H_T_join→ HK97-gp10_like→ DUF3168→ Phage_TTP_11→ Phage_TAC_13→?→ SPRY+X*→?→?→ Phage-tail-tape→ ConA-repeats→?→?→||<-SRAP	SPRY+X	657	B7H27_RS11730	Gammaproteobacteria	Stenotrophomonas maltophilia	hypothetical protein [Stenotrophomonas maltophilia].	GCF_002189565.1
WP_088432438.1	Phage_connect_1→ Phage_H_T_join→ HK97-gp10_like→ DUF3168→ Phage_TTP_11→ Phage_TAC_13→?→ SPRY+X*→?→?→ Phage-tail-tape→ ConA-repeats→	SPRY+X	658	CEE60_RS02675	Gammaproteobacteria	Stenotrophomonas maltophilia	hypothetical protein [Stenotrophomonas maltophilia].	GCF_002205165.1
WP_089986629.1	SUN→ SPRY+SUN*→ DISCOIDIN→ SUN→ SUN→ SUN→ Laminin_G_3→ Disaggr_repeat→	SPRY+SUN	433	BLP61_RS17210	Firmicutes	Lysinibacillus fusiformis	discoidin domain-containing protein [Lysinibacillus fusiformis].	GCF_900102355.1
WP_090738921.1	<-SUN<-?<-PSE||SIG+SPRY*→	SIG+SPRY	373	BN1546_RS23290	Firmicutes	Paenibacillus sp. Mc5Re-14	hypothetical protein [Paenibacillus sp. Mc5Re-14].	GCF_001049835.1
WP_090920079.1	SUN→ SUN→ SPRY+SUN*→ Laminin_G_3→ Disaggr_repeat→	SPRY+SUN	412	BLQ13_RS12675	Firmicutes	Paenibacillus polysaccharolyticus	discoidin domain-containing protein [Paenibacillus polysaccharolyticus].	GCF_900102085.1
WP_092074738.1	SPRY+Big_2*→	SPRY+Big_2	341	HSX37_RS17715	Firmicutes	Dendrosporobacter quercicolus	Ig-like domain-containing protein [Dendrosporobacter quercicolus].	GCF_900104455.1
WP_093534303.1	Phage_connect_1→ Phage_H_T_join→ HK97-gp10_like→ DUF3168→ Phage_TTP_11→ Phage_TAC_13→?→ SPRY+X*→?→?→ Phage-tail-tape→	SPRY+X	655	BQ2375_RS10775	Gammaproteobacteria	Stenotrophomonas rhizophila	hypothetical protein [Stenotrophomonas rhizophila].	GCF_900095765.1
WP_094156505.1	<-ABhydrolase<-?||SIG+SPRY→ *SIG+SPRY*→ SUN→||<-PSE<-Glycos_transf_2	SIG+SPRY	358	B4V02_RS22570	Firmicutes	Paenibacillus kribbensis	hypothetical protein [Paenibacillus kribbensis].	GCF_002240415.1
WP_094936690.1	SUN→ SPRY+SUN*→ SUN→ Laminin_G_3→ Disaggr_repeat→	SPRY+SUN	423	CA599_RS04535	Firmicutes	Paenibacillus taichungensis	discoidin domain-containing protein [Paenibacillus taichungensis].	GCF_002264305.1
WP_095523199.1	Cluster180_2clades→?→?→ Cluster242_2clades→?→?→?→ VWA+Phage-tail_3+SPRY*→?→?→ DAM→	VWA+Phage-tail_3+SPRY	1093	BSR46_RS01740	Betaproteobacteria	Candidatus Dactylopiibacterium carminicum	VWA domain-containing protein [Candidatus Dactylopiibacterium carminicum].	GCF_002284975.1
WP_095529535.1	Cluster180_2clades→?→?→ Cluster242_2clades→?→?→?→ VWA+Phage-tail_3+SPRY*→?→?→ DAM→	VWA+Phage-tail_3+SPRY	1093	CGU28_RS03090	Betaproteobacteria	Candidatus Dactylopiibacterium carminicum	VWA domain-containing protein [Candidatus Dactylopiibacterium carminicum].	GCF_002284935.1

acc	operon	architecture	len	gen.name	taxend	species	defline	gca
WP_097012028.1	Phage_T7_tail→ SUN+SPRY*→ Phage_tail_APC→	SUN+SPRY	749	DPRO_RS10715	Deltaproteobacteria	Pseudodesulfovibrio profundus	hypothetical protein [Pseudodesulfovibrio profundus].	GCF_900217235.1
WP_097053084.1	SPRY*→	SPRY	749	CRO34_RS20720	Alphaproteobacteria	Thalassospira xiamenensis	hypothetical protein [Thalassospira xiamenensis].	GCF_900217835.1
WP_099470440.1	Phage_connect_1→ Phage_H_T_join→ HK97-gp10_like→ DUF3168→ Phage_TTP_11→ Phage_TAC_13→?→ SPRY+X*→?→?→ Phage-tail-tape→ ConA-repeats→?→?→ SRAP→	SPRY+X	657	BRB94_RS07965	Gammaproteobacteria	Stenotrophomonas maltophilia	hypothetical protein [Stenotrophomonas maltophilia].	GCF_002739365.1
WP_099473308.1	Phage_connect_1→ Phage_H_T_join→ HK97-gp10_like→ DUF3168→ Phage_TTP_11→ Phage_TAC_13→?→ SPRY+X*→?→?→ Phage-tail-tape→ ConA-repeats→?→?→ SRAP→	SPRY+X	657	BRB96_RS16380	Gammaproteobacteria	Stenotrophomonas maltophilia	hypothetical protein [Stenotrophomonas maltophilia].	GCF_002739315.1
WP_099475740.1	Phage_TAC_13→?→ SPRY+X*→?→ Phage-tail-tape→?→?→?→ SRAP→	SPRY+X	657	BRB49_RS05320	Gammaproteobacteria	Stenotrophomonas maltophilia	hypothetical protein [Stenotrophomonas maltophilia].	GCF_002739555.1
WP_099491760.1	Phage_connect_1→ Phage_H_T_join→ HK97-gp10_like→ DUF3168→ Phage_TTP_11→ Phage_TAC_13→?→ SPRY+X*→?→?→ Phage-tail-tape→ ConA-repeats→?→?→||<-SRAP	SPRY+X	657	BRB88_RS10470	Gammaproteobacteria	Stenotrophomonas maltophilia	hypothetical protein [Stenotrophomonas maltophilia].	GCF_002739275.1
WP_099494832.1	Phage_connect_1→ Phage_H_T_join→ HK97-gp10_like→ DUF3168→ Phage_TTP_11→ Phage_TAC_13→?→ SPRY+X*→?→ Phage-tail-tape→ ConA-repeats→ ConA→	SPRY+X	655	BRC08_RS17670	Gammaproteobacteria	Stenotrophomonas maltophilia	hypothetical protein [Stenotrophomonas maltophilia].	GCF_002739465.1
WP_099529564.1	Phage_connect_1→ Phage_H_T_join→ HK97-gp10_like→ DUF3168→ Phage_TTP_11→ Phage_TAC_13→?→ PSE→ SPRY+X*→?→ Phage-tail-tape→ ConA-repeats→	SPRY+X	657	BRB33_RS00490	Gammaproteobacteria	Stenotrophomonas maltophilia	hypothetical protein [Stenotrophomonas maltophilia].	GCF_002738715.1
WP_099539415.1	Phage_connect_1→ Phage_H_T_join→ HK97-gp10_like→ DUF3168→ Phage_TTP_11→ Phage_TAC_13→?→ SPRY+X*→?→ Phage-tail-tape→ ConA-repeats→	SPRY+X	657	BRB39_RS15215	Gammaproteobacteria	Stenotrophomonas maltophilia	hypothetical protein [Stenotrophomonas maltophilia].	GCF_002738665.1
WP_099539896.1	Phage_connect_1→ Phage_H_T_join→ HK97-gp10_like→ DUF3168→ Phage_TTP_11→ Phage_TAC_13→?→ SPRY+X*→?→ Phage-tail-tape→	SPRY+X	656	BRB48_RS14865	Gammaproteobacteria	Stenotrophomonas maltophilia	hypothetical protein [Stenotrophomonas maltophilia].	GCF_002738885.1
WP_099553167.1	Phage_connect_1→ Phage_H_T_join→ HK97-gp10_like→ DUF3168→ Phage_TTP_11→ Phage_TAC_13→?→ SPRY+X*→?→?→ Phage-tail-tape→ ConA-repeats→?→?→||<-SRAP	SPRY+X	657	BRC04_RS15490	Gammaproteobacteria	Stenotrophomonas maltophilia	hypothetical protein [Stenotrophomonas maltophilia].	GCF_002739455.1
WP_099605638.1	Phage_connect_1→ Phage_H_T_join→ HK97-gp10_like→ DUF3168→ Phage_TTP_11→ Phage_TAC_13→?→ SPRY+X*→?→ Phage-tail-tape→ ConA-repeats→?→?→?→||<-SRAP	SPRY+X	657	BRC06_RS15605	Gammaproteobacteria	Stenotrophomonas maltophilia	hypothetical protein [Stenotrophomonas maltophilia].	GCF_002740185.1
WP_099654222.1	SPRY*→	SPRY	991	BJV55_RS14285	Gammaproteobacteria	Rhodanobacter sp. TND4EH1	hypothetical protein [Rhodanobacter sp. TND4EH1].	GCF_002749725.1
WP_099854778.1	SUN→ SPRY+SUN*→ SUN→ SUN→ SUN→ Laminin_G_3→ Disaggr_repeat→	SPRY+SUN	425	CS562_RS07475	Firmicutes	Paenibacillus sp. LK1	discoidin domain-containing protein [Paenibacillus sp. LK1].	GCF_002750415.1
WP_099858289.1	SPRY+SUN*→ SUN→ Laminin_G_3→ Disaggr_repeat→ Phage_holin_4_1→	SPRY+SUN	410	CS562_RS22615	Firmicutes	Paenibacillus sp. LK1	hypothetical protein [Paenibacillus sp. LK1].	GCF_002750415.1
WP_100317531.1	Phage-tail-tape→?→?→ SPRY+X*→?→ DAM→	SPRY+X	655	CO615_RS10755	Gammaproteobacteria	Xanthomonadaceae bacterium NML75-0749	hypothetical protein [Xanthomonadaceae bacterium NML75-0749].	GCF_002798185.1

<table>
<tr><th>acc</th><th>operon</th><th>architecture</th><th>len</th><th>gen.name</th><th>taxend</th><th>species</th><th>defline</th><th>gca</th></tr>
<tr><td>WP_100438259.1</td><td>Phage_connect_1→ Phage_H_T_join→ HK97-gp10_like→ DUF3168→ Phage_TTP_11→ Phage_TAC_13→?→ SPRY+X*→?→?→ Phage-tail-tape→ ConA-repeats→</td><td>SPRY+X</td><td>658</td><td>B9Y60_RS06960</td><td>Gammaproteobacteria</td><td>Stenotrophomonas maltophilia</td><td>hypothetical protein [Stenotrophomonas maltophilia].</td><td>GCF_002799135.1</td></tr>
<tr><td>WP_100443561.1</td><td>Phage_connect_1→ Phage_H_T_join→ HK97-gp10_like→ DUF3168→ Phage_TTP_11→ Phage_TAC_13→?→ SPRY+X*→?→ Phage-tail-tape→ ConA-repeats→</td><td>SPRY+X</td><td>657</td><td>B9Y57_RS09005</td><td>Gammaproteobacteria</td><td>Stenotrophomonas maltophilia</td><td>hypothetical protein [Stenotrophomonas maltophilia].</td><td>GCF_002798925.1</td></tr>
<tr><td>WP_100474847.1</td><td>Phage_connect_1→ Phage_H_T_join→ HK97-gp10_like→ DUF3168→ Phage_TTP_11→ Phage_TAC_13→?→ SPRY+X*→?→?→ Phage-tail-tape→</td><td>SPRY+X</td><td>657</td><td>B9Y82_RS18595</td><td>Gammaproteobacteria</td><td>Stenotrophomonas maltophilia</td><td>hypothetical protein [Stenotrophomonas maltophilia].</td><td>GCF_002799155.1</td></tr>
<tr><td>WP_102039859.1</td><td>Glyco_hydro_16+SPRY*→</td><td>Glyco_hydro_16+SPRY</td><td>1003</td><td>CXF86_RS19745</td><td>Gammaproteobacteria</td><td>Shewanella sp. GutCb</td><td>DUF1833 family protein [Shewanella sp. GutCb].</td><td>GCF_002836135.1</td></tr>
<tr><td>WP_102945666.1</td><td>Phage_H_T_join→ HK97-gp10_like→ DUF3168→ Phage_TTP_11→ Phage_TAC_13→?→ SPRY+X*→?→ Phage-tail-tape→</td><td>SPRY+X</td><td>655</td><td>C2U58_RS09715</td><td>Gammaproteobacteria</td><td>Stenotrophomonas sp. VV52</td><td>hypothetical protein [Stenotrophomonas sp. VV52].</td><td>GCF_002893095.1</td></tr>
<tr><td>WP_103046829.1</td><td><-Trypsin<-ABhydrolase<-Polbetant||ACET→ Cupin_2→||<-DUF2935<-?||SIG+SPRY*→</td><td>SIG+SPRY</td><td>326</td><td>C1T21_RS00825</td><td>Firmicutes</td><td>Paenibacillus sp. F4</td><td>hypothetical protein [Paenibacillus sp. F4].</td><td>GCF_002894905.1</td></tr>
<tr><td>WP_103744628.1</td><td>-</td><td>-</td><td>320</td><td>CG775_RS10930</td><td>Firmicutes</td><td>Paenibacillus polymyxa</td><td>hypothetical protein [Paenibacillus polymyxa].</td><td>GCF_002916985.1</td></tr>
<tr><td>WP_103977131.1</td><td>SUN→ SUN→ SUN→ SUN+SPRY*→ Disaggr_repeat→?→?→ Phage_holin_1→</td><td>SUN+SPRY</td><td>980</td><td>LYSIN_RS10210</td><td>Firmicutes</td><td>Lysinibacillus sphaericus</td><td>hypothetical protein [Lysinibacillus sphaericus].</td><td>GCF_002923795.1</td></tr>
<tr><td>WP_104497237.1</td><td>HTH_20→||<-ABhydrolase<-Polbetant||ACET→ Cupin_2→||<-DUF2935||SIG+SPRY*→||<-?||?→||<-Glycos_transf_2</td><td>SIG+SPRY</td><td>332</td><td>C5G87_RS19490</td><td>Firmicutes</td><td>Paenibacillus peoriae</td><td>hypothetical protein [Paenibacillus peoriae].</td><td>GCF_002937395.1</td></tr>
<tr><td>WP_105407850.1</td><td>SUN→ SPRY+SUN*→ SUN→ Laminin_G_3→ Disaggr_repeat→</td><td>SPRY+SUN</td><td>419</td><td>C0Q44_RS13525</td><td>Firmicutes</td><td>Paenibacillus sp. PCH8</td><td>hypothetical protein [Paenibacillus sp. PCH8].</td><td>GCF_002968835.1</td></tr>
<tr><td>WP_106549337.1</td><td>Phage_connect_1→ Phage_H_T_join→ HK97-gp10_like→ DUF3168→ Phage_TTP_11→ Phage_TAC_13→ SPRY+X*→||<-?||?→ Phage-tail-tape→ ConA-repeats→</td><td>SPRY+X</td><td>657</td><td>CV100_RS06160</td><td>Gammaproteobacteria</td><td>Stenotrophomonas maltophilia</td><td>hypothetical protein [Stenotrophomonas maltophilia].</td><td>GCF_003015035.1</td></tr>
<tr><td>WP_106891819.1</td><td>SPRY*→?→?→ DAM→</td><td>SPRY</td><td>820</td><td>C7S18_RS12140</td><td>Gammaproteobacteria</td><td>Ahniella affigens</td><td>hypothetical protein [Ahniella affigens].</td><td>GCF_003015185.1</td></tr>
<tr><td>WP_107951039.1</td><td>SUN→ SPRY+SUN→ *SPRY+SUN*→ SUN→ SUN→ Laminin_G_3→ Disaggr_repeat→?→?→ Phage_holin_1→</td><td>SPRY+SUN</td><td>435</td><td>C9J32_RS17875</td><td>Firmicutes</td><td>Lysinibacillus parviboronicapiens</td><td>discoidin domain-containing protein [Lysinibacillus parviboronicapiens].</td><td>GCF_003049605.1</td></tr>
<tr><td>WP_107951040.1</td><td>-</td><td>-</td><td>411</td><td>C9J32_RS17880</td><td>Firmicutes</td><td>Lysinibacillus parviboronicapiens</td><td>discoidin domain-containing protein [Lysinibacillus parviboronicapiens].</td><td>GCF_003049605.1</td></tr>
<tr><td>WP_107981750.1</td><td>SPRY*→</td><td>SPRY</td><td>401</td><td>B5V90_RS01965</td><td>Firmicutes</td><td>Bacillus sporothermodurans</td><td>hypothetical protein [Bacillus sporothermodurans].</td><td>GCF_003055065.1</td></tr>
<tr><td>WP_108753440.1</td><td>Phage_H_T_join→ HK97-gp10_like→ DUF3168→||<-?||Phage_TTP_11→ Phage_TAC_13→?→ SPRY+X*→?→?→ Phage-tail-tape→ ConA-repeats→ ConA→?→||<-SRAP</td><td>SPRY+X</td><td>657</td><td>C1933_RS11930</td><td>Gammaproteobacteria</td><td>Stenotrophomonas sp. ZAC14D2_NAIMI4_6</td><td>hypothetical protein [Stenotrophomonas sp. ZAC14D2_NAIMI4_6].</td><td>GCF_003086875.1</td></tr>
<tr><td>WP_108762304.1</td><td>Phage_connect_1→ Phage_H_T_join→ HK97-gp10_like→ DUF3168→ Phage_TTP_11→ Phage_TAC_13→?→ SPRY+X*→?→?→ Phage-tail-tape→ ConA-repeats→ ConA→</td><td>SPRY+X</td><td>657</td><td>C1932_RS11845</td><td>Gammaproteobacteria</td><td>Stenotrophomonas sp. YAU14D1_LEIMI4_1</td><td>hypothetical protein [Stenotrophomonas sp. YAU14D1_LEIMI4_1].</td><td>GCF_003086815.1</td></tr>
</table>

acc	operon	architecture	len	gen.name	taxend	species	defline	gca
WP_108767941.1	Phage_connect_1→ Phage_H_T_join→ HK97-gp10_like→ DUF3168→ Phage_TTP_11→ Phage_TAC_13→?→ SPRY+X*→?→?→ Phage-tail-tape→ ConA-repeats→?→‖<-SRAP	SPRY+X	655	C1925_RS05020	Gammaproteobacteria	Stenotrophomonas sp. SAU14A_NAIMI4_5	hypothetical protein [Stenotrophomonas sp. SAU14A_NAIMI4_5].	GCF_003086795.1
WP_109073515.1	SIG+SPRY*→ Phage_tail_APC→?→ Phage_lysozyme→	SIG+SPRY	399	TSH20_RS03980	Alphaproteobacteria	unclassified Azospirillum	MULTISPECIES: hypothetical protein [unclassified Azospirillum].	GCF_003115945.1
WP_109150419.1	SIG+SPRY*→ Phage_tail_APC→ Phage_lysozyme→ Cluster368_2clades→	SIG+SPRY	399	TSO5_RS04345	Alphaproteobacteria	Azospirillum sp. TSO5	hypothetical protein [Azospirillum sp. TSO5].	GCF_003116065.1
WP_109813956.1	Phage_connect_1→ Phage_H_T_join→ HK97-gp10_like→ DUF3168→ Phage_TTP_11→ Phage_TAC_13→?→ SPRY+X*→‖<-?‖?→?→ Phage-tail-tape→ ConA-repeats→	SPRY+X	657	DKY64_RS04935	Gammaproteobacteria	Stenotrophomonas maltophilia	hypothetical protein [Stenotrophomonas maltophilia].	GCF_003172845.1
WP_110895171.1	-	-	698	HUB98_RS06520	Firmicutes	Paenibacillus barcinonensis	hypothetical protein [Paenibacillus barcinonensis].	GCF_013347305.1
WP_110932422.1	SPRY+SUN*→ SUN→ Laminin_G_3→ Disaggr_repeat→ Phage_holin_4_1→	SPRY+SUN	419	CZ379_RS15410	Firmicutes	Paenibacillus bouchesdurhonensis	hypothetical protein [Paenibacillus bouchesdurhonensis].	GCF_900155685.1
WP_111008118.1	Phage_connect_1→ Phage_H_T_join→ HK97-gp10_like→ DUF3168→ Phage_TTP_11→ Phage_TAC_13→?→ SPRY+X*→?→?→ Phage-tail-tape→ ConA-repeats→ ConA→	SPRY+X	655	A7X97_RS14925	Gammaproteobacteria	Stenotrophomonas maltophilia	hypothetical protein [Stenotrophomonas maltophilia].	GCF_003244875.1
WP_111096871.1	Phage_connect_1→ Phage_H_T_join→ HK97-gp10_like→ DUF3168→ Phage_TTP_11→ Phage_TAC_13→?→ SPRY+X*→?→?→ Phage-tail-tape→ ConA-repeats→ ConA→?→‖<-SRAP	SPRY+X	655	A7X84_RS18825	Gammaproteobacteria	Stenotrophomonas maltophilia	hypothetical protein [Stenotrophomonas maltophilia].	GCF_003244495.1
WP_111113613.1	Phage_connect_1→ Phage_H_T_join→ HK97-gp10_like→ DUF3168→ Phage_TTP_11→ Phage_TAC_13→?→ SPRY+X*→?→ Phage-tail-tape→ ConA-repeats→ ConA→?→ SRAP→	SPRY+X	655	A7X83_RS21130	Gammaproteobacteria	Stenotrophomonas maltophilia	hypothetical protein [Stenotrophomonas maltophilia].	GCF_003244775.1
WP_111118812.1	Phage_connect_1→ Phage_H_T_join→ HK97-gp10_like→ DUF3168→ Phage_TTP_11→ Phage_TAC_13→?→ SPRY+X*→?→?→ Phage-tail-tape→ ConA-repeats→?→?→‖<-SRAP	SPRY+X	657	A7X74_RS15970	Gammaproteobacteria	Stenotrophomonas maltophilia	hypothetical protein [Stenotrophomonas maltophilia].	GCF_003244465.1
WP_111176598.1	Phage_connect_1→ Phage_H_T_join→ HK97-gp10_like→ DUF3168→ Phage_TTP_11→ Phage_TAC_13→?→ SPRY+X*→?→ Phage-tail-tape→ ConA-repeats→?→‖<-SRAP	SPRY+X	655	A7X87_RS07275	Gammaproteobacteria	Stenotrophomonas maltophilia	hypothetical protein [Stenotrophomonas maltophilia].	GCF_003244795.1
WP_111187870.1	Phage_connect_1→ Phage_H_T_join→ HK97-gp10_like→ DUF3168→ Phage_TTP_11→ Phage_TAC_13→ SPRY+X*→?→ Phage-tail-tape→ ConA-repeats→?→?→ SRAP→	SPRY+X	657	A7X60_RS00295	Gammaproteobacteria	Stenotrophomonas maltophilia	hypothetical protein [Stenotrophomonas maltophilia].	GCF_003244645.1
WP_111203746.1	Phage_connect_1→ Phage_H_T_join→ HK97-gp10_like→ DUF3168→ Phage_TTP_11→ Phage_TAC_13→?→ SPRY+X*→?→?→ Phage-tail-tape→ ConA-repeats→?→?→ SRAP→	SPRY+X	657	A7X75_RS09430	Gammaproteobacteria	Stenotrophomonas maltophilia	hypothetical protein [Stenotrophomonas maltophilia].	GCF_003244735.1
WP_117310047.1	Phage_H_T_join→ HK97-gp10_like→ DUF3168→‖<-?‖Phage_TTP_11→ Phage_TAC_13→?→ SPRY+X*→?→ Phage-tail-tape→ ConA-repeats→?→ SRAP→	SPRY+X	655	D0Y85_RS07625	Gammaproteobacteria	Stenotrophomonas sp. G4	hypothetical protein [Stenotrophomonas sp. G4].	GCF_003428825.1
WP_119554390.1	SPRY*→	SPRY	674	D3H34_RS15355	Betaproteobacteria	Acidovorax cavernicola	hypothetical protein [Acidovorax cavernicola].	GCF_003581445.1
WP_119798183.1	SUN→ SUN→?→ SPRY*→ Disaggr_repeat→?→ Phage_holin_4_1→	SPRY	707	D3P07_RS00690	Firmicutes	Paenibacillus sp. 1011MAR3C5	hypothetical protein [Paenibacillus sp. 1011MAR3C5].	GCF_003591485.1
WP_120033984.1	SPRY*→?→?→ Phage_holin_1→	SPRY	411	CJ483_RS08545	Firmicutes	Bacillus sp. PK3_68	hypothetical protein [Bacillus sp. PK3_68].	GCF_003600835.1

acc	operon	architecture	len	gen.name	taxend	species	defline	gca
WP_120225163.1	Cluster165_3clades→?→||<-?||?→?→ SPRY*→	SPRY	554	CLY44_RS11090	Alphaproteobacteria	Thalassospira sp. DSM 26710	hypothetical protein [Thalassospira sp. DSM 26710].	GCF_003610215.1
WP_120497558.1	Phage_T7_tail→ SPRY*→	SPRY	1175	D9A03_RS12780	Alphaproteobacteria	Kiloniella sp. EL199	hypothetical protein [Kiloniella sp. EL199].	GCF_900313065.1
WP_120498928.1	<-ACET||?→?→?→?→?→?→ Beta-Roll+Beta-Roll+Beta-Roll+Beta-Roll+BetaPropeller+Beta-Roll+CADHERIN+Beta-Roll+CADHERIN+Beta-Roll+Beta-Roll+BetaPropeller+Beta-Roll*→	Beta-Roll+Beta-Roll+Beta-Roll+Beta-Roll+BetaPropeller+Beta-Roll+CADHERIN+Beta-Roll+CADHERIN+Beta-Roll+Beta-Roll+BetaPropeller+Beta-Roll	4942	D9A03_RS19645	Alphaproteobacteria	Kiloniella sp. EL199	hypothetical protein [Kiloniella sp. EL199].	GCF_900313065.1
WP_121219693.1	SPRY+CARDB*→	SPRY+CARDB	601	BCL74_RS10110	Alphaproteobacteria	Oceanibaculum indicum	hypothetical protein [Oceanibaculum indicum].	GCF_003633955.1
WP_122906669.1	SUN→ SPRY+SUN*→ Laminin_G_3→ Disaggr_repeat→?→?→ Phage_holin→	SPRY+SUN	424	EDM57_RS21100	Firmicutes	Brevibacillus gelatini	discoidin domain-containing protein [Brevibacillus gelatini].	GCF_003710935.1
WP_123769372.1	Phage-tail-tape→?→?→?→?→ SPRY+X*→?→ DAM→?→||<-?||?→||<-Redoxin	SPRY+X	660	EDC50_RS05235	Gammaproteobacteria	Vulcaniibacterium tengchongense	hypothetical protein [Vulcaniibacterium tengchongense].	GCF_003814555.1
WP_123796746.1	HTH_20→||<-Trypsin<-ABhydrolase||ACET→||<-DUF2935<-?||SIG+SPRY*→	SIG+SPRY	332	EG487_RS02750	Firmicutes	Paenibacillus polymyxa	hypothetical protein [Paenibacillus polymyxa].	GCF_003813765.1
WP_123958466.1	Phage_connect_1→ Phage_H_T_join→ HK97-gp10_like→ DUF3168→ Phage_TTP_11→ Phage_TAC_13→?→ SPRY+X*→?→ Phage-tail-tape→ ConA-repeats→	SPRY+X	657	EGY09_RS07240	Gammaproteobacteria	Stenotrophomonas maltophilia	hypothetical protein [Stenotrophomonas maltophilia].	GCF_003812985.1
WP_124116383.1	SUN→ SPRY+SUN→ *SPRY*→ Disaggr_repeat→ Phage_holin_4_1→	SPRY+SUN	707	EDO6_RS18680	Firmicutes	Paenibacillus xylanexedens	hypothetical protein [Paenibacillus xylanexedens].	GCF_003833245.1
WP_124116384.1	-	-	413	EDO6_RS18685	Firmicutes	Paenibacillus xylanexedens	discoidin domain-containing protein [Paenibacillus xylanexedens].	GCF_003833245.1
WP_124650057.1	Arm-DNA-bind_3+SPRY*→	Arm-DNA-bind_3+SPRY	469	DIE19_RS04180	Betaproteobacteria	Burkholderia sp. Bp9126	integrase arm-type DNA-binding domain-containing protein [Burkholderia sp. Bp9126].	GCF_003853715.1
WP_125335565.1	SPRY*→?→ Phage_lysozyme→	SPRY	816	EGJ58_RS22040	Alphaproteobacteria	Ochrobactrum anthropi	hypothetical protein [Ochrobactrum anthropi].	GCF_003937425.1
WP_129381357.1	SUN→ SPRY+SUN→ *SPRY+SUN*→ Laminin_G_3→ Disaggr_repeat→ Phage_holin_4_1→	SPRY+SUN	423	EBB07_RS29185	Firmicutes	Paenibacillaceae bacterium	hypothetical protein [Paenibacillaceae bacterium].	GCF_004134985.1
WP_129381358.1	-	-	415	EBB07_RS29190	Firmicutes	Paenibacillaceae bacterium	hypothetical protein [Paenibacillaceae bacterium].	GCF_004134985.1
WP_130767937.1	Phage_connect_1→ Phage_H_T_join→ HK97-gp10_like→ DUF3168→ Phage_TTP_11→ Phage_TAC_13→?→ SPRY+X*→?→ Phage-tail-tape→ ConA-repeats→ ConA→?→ PSE→||<-?||SRAP→	SPRY+X	655	STNY_RS04735	Gammaproteobacteria	Stenotrophomonas sp. Y	hypothetical protein [Stenotrophomonas sp. Y].	GCF_004306035.1
WP_134709562.1	Phage_connect_1→ Phage_H_T_join→ HK97-gp10_like→ DUF3168→ Phage_TTP_11→ Phage_TAC_13→?→ SPRY+X*→?→?→ Phage-tail-tape→ ConA-repeats→ ConA→	SPRY+X	655	EUA01_RS04295	Gammaproteobacteria	Stenotrophomonas maltophilia	hypothetical protein [Stenotrophomonas maltophilia].	GCF_004522395.1

acc	operon	architecture	len	gen.name	taxend	species	defline	gca
WP_134729602.1	Phage_connect_1→ Phage_H_T_join→ HK97-gp10_like→ DUF3168→ Phage_TTP_11→ Phage_TAC_13→?→ SPRY+X*→?→?→ Phage-tail-tape→ ConA-repeats→?→?→||<-SRAP	SPRY+X	657	EUA00_RS03030	Gammaproteobacteria	Stenotrophomonas maltophilia	hypothetical protein [Stenotrophomonas maltophilia].	GCF_004522355.1
WP_136006293.1	Phage_connect_1→ Phage_H_T_join→ HK97-gp10_like→ DUF3168→ Phage_TTP_11→ Phage_TAC_13→?→ SPRY+X*→?→ Phage-tail-tape→	SPRY+X	656	E5352_RS15345	Gammaproteobacteria	Stenotrophomonas maltophilia	hypothetical protein [Stenotrophomonas maltophilia].	GCF_004794075.1
WP_136702221.1	SPRY*→ Phage_tail_APC→	SPRY	539	E6C67_RS08475	Alphaproteobacteria	Azospirillum sp. TSA2s	hypothetical protein [Azospirillum sp. TSA2s].	GCF_004923315.1
WP_136702953.1	SPRY*→?→?→ Cluster153_2clades→	SPRY	708	E6C67_RS14155	Alphaproteobacteria	Azospirillum sp. TSA2s	hypothetical protein [Azospirillum sp. TSA2s].	GCF_004923315.1
WP_137222083.1	Redoxin→||<-tRNA<-?||?→||<-tRNA<-tRNA<-tRNA||?→?→ SIG+SPRY+Cohesin*→	SIG+SPRY+Cohesin	509	PaeCFBP13512_RS06670	Firmicutes	Paenibacillus sp. CFBP13512	hypothetical protein [Paenibacillus sp. CFBP13512].	GCF_005233705.1
WP_140785213.1	SPRY+Laminin_G_3*→	SPRY+Laminin_G_3	473	FJ417_RS24630	Alphaproteobacteria	Mesorhizobium sp. B3-1-7	hypothetical protein [Mesorhizobium sp. B3-1-7].	GCF_006439385.1
WP_140977547.1	SPRY+Clostridium_P47+TM+TM+TM*→	SPRY+Clostridium_P47+TM+TM+TM	750	C2U70_RS08460	Alphaproteobacteria	Bradyrhizobium guangdongense	TULIP family P47-like protein [Bradyrhizobium guangdongense].	GCF_006483445.1
WP_144787026.1	SUN→ SUN→ SUN→ SUN→ SUN→ SPRY+SUN*→ SUN→ Laminin_G_3→ Disaggr_repeat→?→?→ Phage_holin_1→	SPRY+SUN	427	FS727_RS01315	Firmicutes	Lysinibacillus fusiformis	discoidin domain-containing protein [Lysinibacillus fusiformis].	GCF_007678325.1
WP_145413289.1	SUN→ SUN→ SPRY+SUN*→ Laminin_G_3→ Disaggr_repeat→ Phage_holin_4_1→	SPRY+SUN	411	FS823_RS10960	Firmicutes	Paenibacillus xylanexedens	discoidin domain-containing protein [Paenibacillus xylanexedens].	GCF_007679855.1
WP_145958251.1	Phage-tail-tape→ PSE→?→?→?→ SPRY+X*→?→ DAM→	SPRY+X	658	C0575_RS17020	Gammaproteobacteria	Xanthomonas	MULTISPECIES: hypothetical protein [Xanthomonas].	GCF_002759275.1
WP_147429703.1	<-REC+OmpR-HTH<-?<-?<-?<-?||RHS_repeat+RHS_repeat+RHS_repeat+IG+SPRY+SPRY*→	RHS_repeat+RHS_repeat+RHS_repeat+IG+SPRY+SPRY	1116	K256_RS17555	Gammaproteobacteria	Alteromonadaceae bacterium 2052S.S.stab0a.01	hypothetical protein [Alteromonadaceae bacterium 2052S.S.stab0a.01].	GCF_003634075.1
WP_149425237.1	TM+TM→ Laminin_G_3+SPRY*→ Cluster358_2clades→?→?→ Phage_holin→?→?→||<-SRAP	Laminin_G_3+SPRY	714	OTERR_RS06755	Betaproteobacteria	Oryzomicrobium terrae	hypothetical protein [Oryzomicrobium terrae].	GCF_008274805.1
WP_151150764.1	Phage_T7_tail→ SPRY*→ Phage_tail_APC→	SPRY	494	F8A88_RS08730	Deltaproteobacteria	Desulfovibrio senegalensis	hypothetical protein [Desulfovibrio senegalensis].	GCF_008830225.1
WP_152380319.1	HTH_20→||<-ABhydrolase<-?||SIG+SPRY*→	SIG+SPRY	377	GCU48_RS13350	Firmicutes	Paenibacillus brasilensis	hypothetical protein [Paenibacillus brasilensis].	GCF_009363115.1
WP_152906267.1	Phage-tail-tape→ ConA-repeats→?→ SPRY+X*→?→ PSE→||<-?||REC+OmpR-HTH→	SPRY+X	633	ADI69_RS15035	Gammaproteobacteria	Stenotrophomonas maltophilia	hypothetical protein [Stenotrophomonas maltophilia].	GCF_001073045.1
WP_153164331.1	Phage_connect_1→ Phage_H_T_join→ HK97-gp10_like→ DUF3168→ Phage_TTP_11→ Phage_TAC_13→?→ SPRY+X*→?→ Phage-tail-tape→?→?→?→||<-SRAP	SPRY+X	656	GDJ08_RS03810	Gammaproteobacteria	Stenotrophomonas sp. CPCC 101271	hypothetical protein [Stenotrophomonas sp. CPCC 101271].	GCF_009467805.1

acc	operon	architecture	len	gen.name	taxend	species	defline	gca
WP_153794576.1	SIG+SPRY*→	SIG+SPRY	333	BJH92_RS10685	Firmicutes	Paenibacillus polymyxa	hypothetical protein [Paenibacillus polymyxa].	GCF_009650715.1
WP_154081143.1	Phage_connect_1→ Phage_H_T_join→ HK97-gp10_like→ DUF3168→ Phage_TTP_11→ Phage_TAC_13→?→ SPRY+X*→?→?→ Phage-tail-tape→	SPRY+X	655	B861_RS0208195	Gammaproteobacteria	Stenotrophomonas maltophilia	hypothetical protein [Stenotrophomonas maltophilia].	GCF_000295735.1
WP_154263126.1	Phage-tail-tape→ ConA-repeats→?→ SPRY+X*→	SPRY+X	655	FEO89_RS08390	Gammaproteobacteria	Stenotrophomonas maltophilia	hypothetical protein [Stenotrophomonas maltophilia].	GCF_009676565.1
WP_154352257.1	Phage_connect_1→ Phage_H_T_join→ HK97-gp10_like→ DUF3168→ Phage_TTP_11→ Phage_TAC_13→?→ SPRY+X*→?→?→ Phage-tail-tape→ ConA-repeats→	SPRY+X	657	FEO88_RS08425	Gammaproteobacteria	Stenotrophomonas maltophilia	hypothetical protein [Stenotrophomonas maltophilia].	GCF_009676585.1
WP_154965920.1	SIG+SPRY*→?→||<-?<-PSE<-PSE<-?<-?<-?<-Phage_holin	SIG+SPRY	346	F1386_RS02215	Firmicutes	Paenibacillus azotifigens	hypothetical protein [Paenibacillus azotifigens].	GCF_008635805.1
WP_156830543.1	Cluster180_2clades→?→?→?→?→?→?→ SPRY*→	SPRY	830	METMA_RS27135	Gammaproteobacteria	Methylobacter marinus	hypothetical protein [Methylobacter marinus].	GCF_000383855.1
WP_157628481.1	Phage_connect_1→ Phage_H_T_join→ HK97-gp10_like→ DUF3168→ Phage_TTP_11→ Phage_TAC_13→?→ SPRY+X*→?→ Phage-tail-tape→	SPRY+X	657	SMAL_RS12680	Gammaproteobacteria	Stenotrophomonas maltophilia	hypothetical protein [Stenotrophomonas maltophilia].	GCF_000020665.1
WP_157793929.1	SUN→ SPRY+SUN*→ SUN→ Laminin_G_3→ Disaggr_repeat→ Phage_holin_4_1→	SPRY+SUN	409	B9T62_RS19340	Firmicutes	Paenibacillus donghaensis	discoidin domain-containing protein [Paenibacillus donghaensis].	GCF_002192415.1
WP_158702666.1	SUN→ SPRY+SUN*→	SPRY+SUN	405	C8268_RS03870	Firmicutes	Paenibacillus sp. Marseille-P3787	discoidin domain-containing protein [Paenibacillus sp. Marseille-P3787].	GCF_900289175.1
WP_159054946.1	OBfold→?→?→||<-?||?→ ConA-repeats→?→ SPRY+X*→	SPRY+X	655	C9J74_RS07540	Gammaproteobacteria	Stenotrophomonas maltophilia	hypothetical protein [Stenotrophomonas maltophilia].	GCF_003030985.1
WP_160788604.1	HTH_20→||<-PSE<-ABhydrolase<-Polbetant||ACET→ Cupin_2→ ABhydrolase→||<-DUF2935||SIG+SPRY*→?→ PSE→||<-Glycos_transf_2	SIG+SPRY	338	GRP75_RS03415	Firmicutes	Paenibacillus sp. OT2-17	hypothetical protein [Paenibacillus sp. OT2-17].	GCF_009827695.1
WP_161771818.1	Phage_TAC_13→?→ SPRY+X*→?→?→ Phage-tail-tape→	SPRY+X	657	AR275_RS25795	Gammaproteobacteria	Stenotrophomonas maltophilia	hypothetical protein [Stenotrophomonas maltophilia].	GCF_001506105.1
WP_162174107.1	Cluster165_3clades→?→?→ Cluster303_2clades→?→?→ TM+TM→ SPRY*→?→?→?→?→?→?→ SIG+HORMA→	SPRY	125	HMCN_RS19520	Betaproteobacteria	Herminiimonas sp. CN	hypothetical protein [Herminiimonas sp. CN].	GCF_000622895.1
WP_162407361.1	Phage-tail-tape→?→?→ SUN→ SPRY+X*→?→||<-?||DAM→	SPRY+X	654	CSC62_RS14285	Gammaproteobacteria	Pseudoxanthomonas jiangsuensis	hypothetical protein [Pseudoxanthomonas jiangsuensis].	GCF_010093185.1
WP_162815241.1	SPRY*→	SPRY	219	DOU47_RS11710	Actinobacteria	Microbacterium arborescens	hypothetical protein [Microbacterium arborescens].	GCF_003339645.1
WP_163369807.1	SPRY*→	SPRY	262	G7B23_RS04230	Gammaproteobacteria	Endozoicomonas acroporae	hypothetical protein [Endozoicomonas acroporae].	GCF_010994325.1

acc	operon	architecture	len	gen.name	taxend	species	defline	gca
WP_163387923.1	SPRY*→	SPRY	262	EO230_RS13880	Gammaproteobacteria	Endozoicomonas acroporae	hypothetical protein [Endozoicomonas acroporae].	GCF_010994335.1
WP_163679668.1	Laminin_G_3+SPRY+SPRY*→	Laminin_G_3+SPRY+SPRY	704	G4223_RS11800	Alphaproteobacteria	Magnetospirillum aberrantis	hypothetical protein [Magnetospirillum aberrantis].	GCF_011022235.1
WP_164136018.1	Phage_H_T_join→ HK97-gp10_like→ DUF3168→?→ Phage_TTP_11→ Phage_TAC_13→?→ SPRY+X*→?→?→ Phage-tail-tape→ ConA-repeats→ ConA→	SPRY+X	657	G4995_RS01655	Gammaproteobacteria	Stenotrophomonas maltophilia	hypothetical protein [Stenotrophomonas maltophilia].	GCF_010604725.1
WP_164170835.1	Phage_connect_1→ Phage_H_T_join→ HK97-gp10_like→ DUF3168→ Phage_TTP_11→ Phage_TAC_13→?→ SPRY+X*→?→ Phage-tail-tape→ ConA-repeats→	SPRY+X	657	G5A21_RS04165	Gammaproteobacteria	Stenotrophomonas maltophilia	hypothetical protein [Stenotrophomonas maltophilia].	GCF_010605085.1
WP_164171533.1	Phage_connect_1→ Phage_H_T_join→ HK97-gp10_like→ DUF3168→ Phage_TTP_11→ Phage_TAC_13→?→ SPRY+X*→?→?→ Phage-tail-tape→ ConA-repeats→?→?→ SRAP→	SPRY+X	657	G5A20_RS11530	Gammaproteobacteria	Stenotrophomonas maltophilia	hypothetical protein [Stenotrophomonas maltophilia].	GCF_010607635.1
WP_164186354.1	Phage_connect_1→ Phage_H_T_join→ HK97-gp10_like→ DUF3168→ Phage_TTP_11→ Phage_TAC_13→?→ SPRY+X*→?→?→ Phage-tail-tape→ ConA-repeats→?→?→ SRAP→	SPRY+X	657	G4996_RS10295	Gammaproteobacteria	Stenotrophomonas maltophilia	hypothetical protein [Stenotrophomonas maltophilia].	GCF_010608865.1
WP_164222458.1	Phage_connect_1→ Phage_H_T_join→ HK97-gp10_like→ DUF3168→ Phage_TTP_11→ Phage_TAC_13→?→ SPRY+X*→||<-?||?→?→ Phage-tail-tape→ ConA-repeats→	SPRY+X	658	G5A44_RS14400	Gammaproteobacteria	Stenotrophomonas maltophilia	hypothetical protein [Stenotrophomonas maltophilia].	GCF_010608905.1
WP_164227274.1	Phage_connect_1→ Phage_H_T_join→ HK97-gp10_like→ DUF3168→ Phage_TTP_11→ Phage_TAC_13→?→ SPRY+X*→?→ Phage-tail-tape→ ConA-repeats→?→?→||<-PSE||?→ SRAP→	SPRY+X	658	G5A23_RS09720	Gammaproteobacteria	Stenotrophomonas maltophilia	hypothetical protein [Stenotrophomonas maltophilia].	GCF_010605135.1
WP_164262535.1	Phage_connect_1→ Phage_H_T_join→ HK97-gp10_like→ DUF3168→ Phage_TTP_11→ Phage_TAC_13→?→ SPRY+X*→?→?→ Phage-tail-tape→ ConA-repeats→?→||<-SRAP	SPRY+X	655	G5A42_RS09935	Gammaproteobacteria	Stenotrophomonas maltophilia	hypothetical protein [Stenotrophomonas maltophilia].	GCF_010609005.1
WP_164272506.1	Phage_connect_1→ Phage_H_T_join→ HK97-gp10_like→ DUF3168→ Phage_TTP_11→ Phage_TAC_13→?→ SPRY+X*→?→?→ Phage-tail-tape→ ConA-repeats→ ConA→?→||<-SRAP	SPRY+X	657	G4G00_RS08180	Gammaproteobacteria	Stenotrophomonas sp. B1-1	hypothetical protein [Stenotrophomonas sp. B1-1].	GCF_010894375.1
WP_164283369.1	Phage_connect_1→ Phage_H_T_join→ HK97-gp10_like→ DUF3168→ Phage_TTP_11→ Phage_TAC_13→?→ SPRY+X*→?→ Phage-tail-tape→ ConA-repeats→ ConA→?→||<-SRAP	SPRY+X	655	GJV38_RS08100	Gammaproteobacteria	Stenotrophomonas maltophilia	hypothetical protein [Stenotrophomonas maltophilia].	GCF_010994225.1
WP_164687941.1	Phage_connect_1→ Phage_H_T_join→ HK97-gp10_like→ DUF3168→ Phage_TTP_11→ Phage_TAC_13→?→ SPRY+X*→?→?→ Phage-tail-tape→ ConA-repeats→ ConA→?→||<-SRAP	SPRY+X	655	G5C63_RS04670	Gammaproteobacteria	Stenotrophomonas pavanii	hypothetical protein [Stenotrophomonas pavanii].	GCF_011043715.1
WP_164776353.1	SUN→ SPRY+SUN*→ SUN→ Laminin_G_3→ Disaggr_repeat→ Phage_holin_4_1→?→ SIG+ConA+beta-propeller→	SPRY+SUN	423	FLT43_RS09110	Firmicutes	Paenibacillus thiaminolyticus	hypothetical protein [Paenibacillus thiaminolyticus].	GCF_004001005.1
WP_164965245.1	HTH_20→||<-Trypsin<-ABhydrolase||ACET→||<-DUF2935<-?||SIG+SPRY*→	SIG+SPRY	369	G9G57_RS16175	Firmicutes	Paenibacillus sp. EKM211P	hypothetical protein [Paenibacillus sp. EKM211P].	GCF_011066175.1
WP_165083476.1	HTH_20→||<-Trypsin<-ABhydrolase||ACET→||<-DUF2935<-?||SIG+SPRY*→	SIG+SPRY	312	G9G64_RS19615	Firmicutes	unclassified Paenibacillus	MULTISPECIES: hypothetical protein [unclassified Paenibacillus].	GCF_011066085.1
WP_165148029.1	HTH_20→||<-ABhydrolase<-Polbetant||ACET→ Cupin_2→ ABhydrolase→||<-DUF2935||SIG+SPRY*→?→||<-Glycos_transf_2	SIG+SPRY	345	G9G52_RS14675	Firmicutes	unclassified Paenibacillus	MULTISPECIES: hypothetical protein [unclassified Paenibacillus].	GCF_011066155.1

acc	operon	architecture	len	gen.name	taxend	species	defline	gca
WP_165178844.1	HTH_20→||<-ABhydrolase<-Polbetant||ACET→ Cupin_2→||<-DUF2935||SIG+SPRY*→	SIG+SPRY	330	G9G54_RS16165	Firmicutes	Paenibacillus sp. EKM212P	hypothetical protein [Paenibacillus sp. EKM212P].	GCF_011066115.1
WP_165980135.1	SUN→ SUN→ SPRY+SUN*→ Laminin_G_3→ Disaggr_repeat→	SPRY+SUN	412	E2R58_RS01745	Firmicutes	Paenibacillus amylolyticus	hypothetical protein [Paenibacillus amylolyticus].	GCF_004358835.1
WP_167440979.1	HTH_20→||<-Trypsin<-ABhydrolase||ACET→||<-DUF2935<-?||SIG+SPRY*→	SIG+SPRY	332	HFD99_RS19285	Firmicutes	Paenibacillus sp. EKM301P	hypothetical protein [Paenibacillus sp. EKM301P].	GCF_011800295.1
WP_168180444.1	SPRY+SUN*→ SUN→ SUN→ Laminin_G_3→ Disaggr_repeat→ Phage_holin_4_1→	SPRY+SUN	417	HRG14_RS16580	Firmicutes	Paenibacillus dendritiformis	hypothetical protein [Paenibacillus dendritiformis].	GCF_012272915.1
WP_168224778.1	Phage-tail_3+FN3→ Cluster93_5clades→ Laminin_G_3+SPRY*→?→?→||<-?<-REC+OmpR-HTH	Laminin_G_3+SPRY	442	EXZ61_RS14580	Betaproteobacte-ria	Rhodoferax sediminis	hypothetical protein [Rhodoferax sediminis].	GCF_006974105.1
WP_168759630.1	Phage_H_T_join→ HK97-gp10_like→ DUF3168→?→ Phage_TTP_11→ Phage_TAC_13→?→ SPRY+X*→?→ Phage-tail-tape→ ConA-repeats→?→?→||<-PSE||?→ SRAP→	SPRY+X	655	HGN30_RS14120	Gammaproteobac-teria	Stenotrophomonas maltophilia	hypothetical protein [Stenotrophomonas maltophilia].	GCF_012647025.1
WP_170142066.1	Phage-tail_3→?→ SPRY*→?→?→?→?→?→ DAM→	SPRY	223	DFQ59_RS06730	Gammaproteobac-teria	Thioalbus denitrificans	hypothetical protein [Thioalbus denitrificans].	GCF_003337735.1
WP_171365214.1	SUN→ SUN→?→ DISCOIDIN→ SUN→ SPRY+SUN*→ SUN→ Laminin_G_3→ Disaggr_repeat→?→?→ Phage_holin_1→	SPRY+SUN	425	HL291_RS06145	Firmicutes	Lysinibacillus fusiformis	discoidin domain-containing protein [Lysinibacillus fusiformis].	GCF_013112215.1
WP_180361703.1	SUN→ SUN→ SUN→ SPRY+SUN*→ Laminin_G_3→ Disaggr_repeat→	SPRY+SUN	412	CXK86_RS01005	Firmicutes	Paenibacillus sp. BGI2013	hypothetical protein [Paenibacillus sp. BGI2013].	GCF_002843485.1
WP_180832871.1	Phage_connect_1→ Phage_H_T_join→ HK97-gp10_like→ DUF3168→ Phage_TTP_11→ Phage_TAC_13→?→ SPRY+X*→?→?→ Phage-tail-tape→ ConA-repeats→?→?→||<-SRAP	SPRY+X	657	D7U76_RS15765	Gammaproteobac-teria	Stenotrophomonas maltophilia	hypothetical protein [Stenotrophomonas maltophilia].	GCF_013463655.1
WP_180835450.1	Phage_connect_1→ Phage_H_T_join→ HK97-gp10_like→ DUF3168→ Phage_TTP_11→ Phage_TAC_13→?→ SPRY+X*→?→ Phage-tail-tape→?→?→?→||<-PSE||?→||<-SRAP	SPRY+X	657	D7U87_RS15230	Gammaproteobac-teria	Stenotrophomonas maltophilia	hypothetical protein [Stenotrophomonas maltophilia].	GCF_013463795.1
WP_180837803.1	Phage-tail-tape→ ConA-repeats→?→ SPRY+X*→?→?→?→?→?→?→||<-Ribonuc_L-PSP	SPRY+X	633	D7Y22_RS10130	Gammaproteobac-teria	Stenotrophomonas maltophilia	hypothetical protein [Stenotrophomonas maltophilia].	GCF_013463895.1
WP_180844236.1	Phage-tail-tape→ ConA-repeats→?→ SPRY+X*→?→?→?→?→ SIG+TM+TM+TM+TM+TM+TM+TM+TM→ ABC-ATPase→	SPRY+X	655	D7Y24_RS02400	Gammaproteobac-teria	Stenotrophomonas maltophilia	hypothetical protein [Stenotrophomonas maltophilia].	GCF_013463875.1
WP_180874375.1	Phage_connect_1→ Phage_H_T_join→ HK97-gp10_like→ DUF3168→ Phage_TTP_11→ Phage_TAC_13→?→ SPRY+X*→?→?→ Phage-tail-tape→ ConA-repeats→?→?→||<-SRAP	SPRY+X	657	D7Y39_RS15850	Gammaproteobac-teria	Stenotrophomonas maltophilia	hypothetical protein [Stenotrophomonas maltophilia].	GCF_013464195.1
WP_180877176.1	Phage_connect_1→ Phage_H_T_join→ HK97-gp10_like→ DUF3168→ Phage_TTP_11→ Phage_TAC_13→?→ SPRY+X*→?→?→ Phage-tail-tape→ ConA-repeats→?→?→||<-SRAP	SPRY+X	657	D7U95_RS06530	Gammaproteobac-teria	Stenotrophomonas maltophilia	hypothetical protein [Stenotrophomonas maltophilia].	GCF_013463605.1
WP_180890353.1	Phage_connect_1→ Phage_H_T_join→ HK97-gp10_like→ DUF3168→ Phage_TTP_11→ Phage_TAC_13→?→ SPRY+X*→?→?→ Phage-tail-tape→ ConA-repeats→?→?→||<-SRAP	SPRY+X	658	D7Y45_RS11625	Gammaproteobac-teria	Stenotrophomonas maltophilia	hypothetical protein [Stenotrophomonas maltophilia].	GCF_013464305.1
WP_182068260.1	Phage_connect_1→ Phage_H_T_join→ HK97-gp10_like→ DUF3168→ Phage_TTP_11→ Phage_TAC_13→?→ SPRY+X*→?→?→ Phage-tail-tape→ ConA-repeats→ ConA→	SPRY+X	655	H7A80_RS13755	Gammaproteobac-teria	Stenotrophomonas pavanii	hypothetical protein [Stenotrophomonas pavanii].	GCF_903970895.1

acc	operon	architecture	len	gen.name	taxend	species	defline	gca
WP_182680994.1	Phage_connect_1→ Phage_H_T_join→ HK97-gp10_like→ DUF3168→ Phage_TTP_11→ Phage_TAC_13→?→ SPRY+X*→?→?→ Phage-tail-tape→ ConA-repeats→?→?→||<-SRAP	SPRY+X	657	H4O10_RS07755	Gammaproteobacteria	Stenotrophomonas sp. I18B00994	hypothetical protein [Stenotrophomonas sp. I18B00994].	GCF_014145825.1
WP_182852413.1	Cluster242_2clades→?→ PSE→?→ SPRY*→	SPRY	255	H7K70_RS11535	Gammaproteobacteria	Pseudomonas otitidis	hypothetical protein [Pseudomonas otitidis].	GCF_014161995.1
WP_183080221.1	Phage_connect_1→ Phage_H_T_join→ HK97-gp10_like→ DUF3168→ Phage_TTP_11→ Phage_TAC_13→?→ SPRY+X*→?→?→ Phage-tail-tape→ ConA-repeats→?→?→ SRAP→	SPRY+X	657	C7543_RS14050	Gammaproteobacteria	Stenotrophomonas sp. CF319	hypothetical protein [Stenotrophomonas sp. CF319].	GCF_003633905.1

Gene neighborhoods and domain architectures of TPR-GREAB-C-PIN systems

acc	operon	architecture	len	gen.name	taxend	species	defline	gca
-	-	-	NA	NA	NA	NA	NA	GCF_016236915.1
AAQ66293.1	TPR+GreAB-C+PIN*→	TPR+GreAB-C+PIN	1140	PG_1202	Bacteroidetes	Porphyromonas gingivalis W83	hypothetical protein PG_1202 [Porphyromonas gingivalis W83].	GCA_000007585.1
ABA73572.1	REase+TPR+GreAB-C+PIN*→	REase+TPR+GreAB-C+PIN	1113	Pfl01_1829	Gammaproteobacteria	Pseudomonas fluorescens Pf0-1	hypothetical protein Pfl01_1829 [Pseudomonas fluorescens Pf0-1].	GCA_000012445.1
ABP70317.1	TPR+GreAB-C+PIN*→	TPR+GreAB-C+PIN	1188	Rsph17025_1421	Alphaproteobacteria	Rhodobacter sphaeroides ATCC 17025	hypothetical protein Rsph17025_1421 [Rhodobacter sphaeroides ATCC 17025].	GCA_000016405.1
ACB93983.1	TPR+GreAB-C+PIN*→	TPR+GreAB-C+PIN	1100	Bind_0329	Alphaproteobacteria	Beijerinckia indica subsp. indica ATCC 9039	Tetratricopeptide TPR_2 repeat protein [Beijerinckia indica subsp. indica ATCC 9039].	GCA_000019845.1
ACQ93407.1	HetE-N1→ TPR+GreAB-C+PIN*→	TPR+GreAB-C+PIN	1069	Tola_1798	Gammaproteobacteria	Tolumonas auensis DSM 9187	hypothetical protein Tola_1798 [Tolumonas auensis DSM 9187].	GCA_000023065.1
ADX44119.1	REase+TPR+GreAB-C+PIN*→	REase+TPR+GreAB-C+PIN	1383	Acav_0193	Betaproteobacteria	Acidovorax avenae subsp. avenae ATCC 19860	hypothetical protein Acav_0193 [Acidovorax avenae subsp. avenae ATCC 19860].	GCA_000176855.2
AFD27801.1	TPR+GreAB-C+PIN*→	TPR+GreAB-C+PIN	1286	DGo_PC0009	Deinococci	Deinococcus gobiensis I-0	hypothetical protein DGo_PC0009 (plasmid) [Deinococcus gobiensis I-0].	GCA_000252445.1
AFJ56694.1	REase+TPR+GreAB-C+PIN*→	REase+TPR+GreAB-C+PIN	1321	PflA506_2672	Gammaproteobacteria	Pseudomonas fluorescens A506	tetratricopeptide repeat protein [Pseudomonas fluorescens A506].	GCA_000262325.2
AGO88322.1	REase+TPR+GreAB-C+PIN*→?→?→ Calcineurin→ Calcineurin→?→ Calcineurin→	REase+TPR+GreAB-C+PIN	1344	MOC_1p0084	Alphaproteobacteria	Methylobacterium oryzae CBMB20	Tetratricopeptide TPR_2 repeat protein (plasmid) [Methylobacterium oryzae CBMB20].	-
AHC85672.1	HetE-N1→ TPR+TPR+GreAB-C+PIN*→	TPR+TPR+GreAB-C+PIN	1114	X969_07375	Gammaproteobacteria	Pseudomonas monteilii SB3078	hypothetical protein X969_07375 [Pseudomonas monteilii SB3078].	GCA_000510285.1
AHG89833.1	TPR+GreAB-C+PIN*→	TPR+GreAB-C+PIN	1325	J421_2296	Gemmatimonadetes	Gemmatirosa kalamazoonesis	hypothetical protein J421_2296 [Gemmatirosa kalamazoonesis].	GCA_000522985.1
AHZ68657.1	REase+TPR+GreAB-C+PIN*→	REase+TPR+GreAB-C+PIN	1527	OU5_1578	Gammaproteobacteria	Pseudomonas mandelii JR-1	hypothetical protein OU5_1578 [Pseudomonas mandelii JR-1].	GCA_000257545.3
AIO22452.1	REase+TPR+GreAB-C+PIN*→	REase+TPR+GreAB-C+PIN	1474	DM41_7691	Betaproteobacteria	Burkholderia cepacia ATCC 25416	tetratricopeptide repeat family protein (plasmid) [Burkholderia cepacia ATCC 25416].	GCA_003546465.1
AJG23424.1	REase+TPR+GreAB-C+PIN*→	REase+TPR+GreAB-C+PIN	1348	RR42_s1836	Betaproteobacteria	Cupriavidus basilensis	hypothetical protein RR42_s1836 [Cupriavidus basilensis].	GCA_000832305.1
AJG25013.1	NACHT→ REase+TPR+GreAB-C+PIN*→	REase+TPR+GreAB-C+PIN	1361	RR42_s3437	Betaproteobacteria	Cupriavidus basilensis	hypothetical protein RR42_s3437 [Cupriavidus basilensis].	GCA_000832305.1
ALA80840.1	TPR+GreAB-C+PIN*→	TPR+GreAB-C+PIN	706	VN11_01310	Gammaproteobacteria	Stenotrophomonas maltophilia	hypothetical protein VN11_01310 [Stenotrophomonas maltophilia].	GCA_001274595.1
ALK23663.1	REase+TPR+GreAB-C+PIN*→	REase+TPR+GreAB-C+PIN	1346	APZ15_37705	Betaproteobacteria	Burkholderia cepacia ATCC 25416	hypothetical protein APZ15_37705 (plasmid) [Burkholderia cepacia ATCC 25416].	GCA_001411495.1
ALY84300.1	TPR+GreAB-C+PIN*→	TPR+GreAB-C+PIN	808	HV95_15440	Gammaproteobacteria	Pseudomonas aeruginosa	hypothetical protein HV95_15440 [Pseudomonas aeruginosa].	GCA_001516225.1
AMM82949.1	HetE-N1→ TPR+GreAB-C+PIN*→	TPR+GreAB-C+PIN	1090	AW43_10865	Gammaproteobacteria	Pasteurella multocida subsp. multocida PMTB2.1	hypothetical protein AW43_10865 [Pasteurella multocida subsp. multocida PMTB2.1].	GCA_001578435.2
ANP41882.1	REase+TPR+GreAB-C+PIN*→	REase+TPR+GreAB-C+PIN	1247	K529_013985	Alphaproteobacteria	Epibacterium mobile F1926	hypothetical protein K529_013985 [Epibacterium mobile F1926].	GCA_000376545.2
AOE80004.1	TPR+GreAB-C+PIN*→	TPR+GreAB-C+PIN	962	A7318_15795	Gammaproteobacteria	Pseudomonas lurida	hypothetical protein A7318_15795 [Pseudomonas lurida].	GCA_001708485.1

acc	operon	architecture	len	gen.name	taxend	species	defline	gca
APP29939.1	REase+TPR+GreAB-C+PIN*→||||<-?||HTH→	REase+TPR+GreAB-C+PIN	1216	AUO97_03535	Gammaproteobacteria	Acinetobacter baumannii	hypothetical protein AUO97_03535 [Acinetobacter baumannii].	GCA_001593425.2
APT35017.1	REase+TPR+GreAB-C+PIN*→?→?→ Calcineurin→ Calcineurin→?→ Calcineurin→	REase+TPR+GreAB-C+PIN	1340	MCBMB27_05726	Alphaproteobacteria	Methylobacterium phyllosphaerae	hypothetical protein MCBMB27_05726 (plasmid) [Methylobacterium phyllosphaerae].	GCA_001936175.1
APV35618.1	REase+TPR+GreAB-C+PIN*→	REase+TPR+GreAB-C+PIN	1211	BEN76_06135	Gammaproteobacteria	Acinetobacter soli	hypothetical protein BEN76_06135 [Acinetobacter soli].	GCA_001953195.1
ARP63802.1	REase+TPR+GreAB-C+PIN*→	REase+TPR+GreAB-C+PIN	1328	A9K65_010750	Alphaproteobacteria	Mesorhizobium sp. WSM1497	hypothetical protein A9K65_010750 [Mesorhizobium sp. WSM1497].	GCA_001672455.2
ARS41908.1	TPR+GreAB-C+PIN*→	TPR+GreAB-C+PIN	1265	CA265_20515	Bacteroidetes	Sphingobacteriaceae bacterium GW460-11-11-14-LB5	hypothetical protein CA265_20515 [Sphingobacteriaceae bacterium GW460-11-11-14-LB5].	GCA_002151545.1
AWD09427.1	HetE-N1→?→ TPR+GreAB-C+PIN*→	TPR+GreAB-C+PIN	512	C7D56_14785	Gammaproteobacteria	Salmonella enterica subsp. enterica serovar Corvallis	hypothetical protein C7D56_14785 [Salmonella enterica subsp. enterica serovar Corvallis].	GCA_003071545.1
AXQ72363.1	HetE-N1→ TPR+GreAB-C+PIN*→	TPR+GreAB-C+PIN	1090	AWY89_05050	Gammaproteobacteria	Pasteurella multocida subsp. multocida	hypothetical protein AWY89_05050 [Pasteurella multocida subsp. multocida].	GCA_003428945.1
AXW62973.1	REase+TPR+GreAB-C+PIN*→	REase+TPR+GreAB-C+PIN	1325	CJO94_14695	Betaproteobacteria	Ralstonia solanacearum	hypothetical protein CJO94_14695 [Ralstonia solanacearum].	GCA_003515385.1
AYG03885.1	X+PIN*→	X+PIN	1170	D7I44_10295	Actinobacteria	Gryllotalpicola protaetiae	hypothetical protein D7I44_10295 [Gryllotalpicola protaetiae].	GCA_003627055.1
BAE85210.1	REase+TPR+GreAB-C+PIN*→	REase+TPR+GreAB-C+PIN	1306	DSY3421	Firmicutes	Desulfitobacterium hafniense Y51	hypothetical protein DSY3421 [Desulfitobacterium hafniense Y51].	GCA_000010045.1
BBD03377.1	REase+TPR→ TPR→ TPR+GreAB-C+PIN*→	TPR+GreAB-C+PIN	656	YGS_C2P1391	Alphaproteobacteria	Sphingobium sp. YG1	hypothetical protein YGS_C2P1391 [Sphingobium sp. YG1].	GCA_003609795.1
BBZ79150.1	TPR+PIN*→	TPR+PIN	1186	MANY_44870	Actinobacteria	Mycolicibacterium anyangense	hypothetical protein MANY_44870 [Mycolicibacterium anyangense].	GCA_010731855.1
BBZ91069.1	PIN*→	PIN	190	F07S3_09020	Alphaproteobacteria	Bradyrhizobium diazoefficiens	hypothetical protein F07S3_09020 [Bradyrhizobium diazoefficiens].	GCA_014163475.1
CAA9240899.1	TPR+GreAB-C+PIN*→	TPR+GreAB-C+PIN	1079	AVDCRST_MAG77-1676	environmental samples	uncultured Chloroflexi bacterium	MAG: hypothetical protein AVDCRST_MAG77-1676 [uncultured Chloroflexi bacterium].	GCA_902805635.1
CAB3809090.1	PIN*→	PIN	270	LMG28138_06082	Betaproteobacteria	Pararobbsia alpina	hypothetical protein LMG28138_06082 [Pararobbsia alpina].	GCA_902859895.1
CAC9458388.1	TPR+GreAB-C+PIN*→?→||||<-PSE||PSE→ PSE→?→ PSE→ PSE→ PSE→?→?→ TPR→	TPR+GreAB-C+PIN	1164	SMARSOXL104_LOCUS2140	environmental samples	uncultured Gammaproteobacteria bacterium	hypothetical protein [uncultured Gammaproteobacteria bacterium].	GCA_903813475.1
CAC9474194.1	TPR+GreAB-C+PIN*→	TPR+GreAB-C+PIN	1164	SMARSOXL102_LOCUS3344	environmental samples	uncultured Gammaproteobacteria bacterium	hypothetical protein [uncultured Gammaproteobacteria bacterium].	GCA_903813445.1
CAC9479061.1	TPR+GreAB-C+PIN*→	TPR+GreAB-C+PIN	677	SMARSOXL51_LOCUS3828	environmental samples	uncultured Gammaproteobacteria bacterium	hypothetical protein [uncultured Gammaproteobacteria bacterium].	GCA_903813485.1
CAD79080.1	TRD+TRD→?→?→ REase+SNF2→?→ TM+TM+TPR+GreAB-C+PIN*→	TM+TM+TPR+GreAB-C+PIN	1274	RB11367	Planctomycetes	Rhodopirellula baltica SH 1	hypothetical protein RB11367 [Rhodopirellula baltica SH 1].	GCA_000196115.1
CAE7733776.1	IMS+HHH+GreAB+AraC-HTH+AraC-HTH*→	IMS+HHH+GreAB+AraC-HTH+AraC-HTH	771	dinB	Alveolata	Symbiodinium microadriaticum	dinB [Symbiodinium microadriaticum].	GCA_905231925.1
CAG37728.1	HetE-N1→ TPR+GreAB-C+PIN*→	TPR+GreAB-C+PIN	1114	DP2999	Deltaproteobacteria	Desulfotalea psychrophila LSv54	unknown protein [Desulfotalea psychrophila LSv54].	GCA_000025945.1
CCD86698.1	REase+TPR+TPR+GreAB-C+PIN*→	REase+TPR+TPR+GreAB-C+PIN	1326	BRAO285_2000017	Alphaproteobacteria	Bradyrhizobium sp. ORS 285	conserved hypothetical protein [Bradyrhizobium sp. ORS 285].	GCA_000239755.2
CDF45779.1	X+PIN*→?→?→ CITB-HTH+LexA-protease→	X+PIN	1212	BN450_02242	environmental samples	Roseburia sp. CAG:100	uncharacterized protein BN450_02242 [Roseburia sp. CAG:100].	GCA_000436955.1
CDH20863.1	HetE-N1→ TPR+GreAB-C+PIN*→ TPR+GreAB-C+PIN→	TPR+GreAB-C+PIN	846	XBKQ1_2810009	Gammaproteobacteria	Xenorhabdus bovienii str. kraussei Quebec	conserved hypothetical protein [Xenorhabdus bovienii str. kraussei Quebec].	GCA_000736555.1
CEL29322.1	REase+TPR+GreAB-C+PIN*→	REase+TPR+GreAB-C+PIN	1486	SRM1_02673	Gammaproteobacteria	Pseudomonas fluorescens	hypothetical protein SRM1_02673 [Pseudomonas fluorescens].	GCA_000827755.2

acc	operon	architecture	len	gen.name	taxend	species	defline	gca
CFK79862.1	REase+TPR+GreAB-C+PIN*→	REase+TPR+GreAB-C+PIN	1327	ERS012324_00072	Betaproteobacteria	Burkholderia pseudomallei	Uncharacterised protein [Burkholderia pseudomallei].	GCA_001327015.1
CGG41859.1	TPR+GreAB-C+PIN*→	TPR+GreAB-C+PIN	1147	ERS020510_00033	Firmicutes	Streptococcus pneumoniae	putative PEP-CTERM system TPR-repeat lipoprotein [Streptococcus pneumoniae].	GCA_001339955.1
CNG28227.1	HetE-N1→ TPR+GreAB-C+PIN*→	TPR+GreAB-C+PIN	1094	ERS137937_00749	Gammaproteobacteria	Yersinia enterocolitica	Uncharacterised protein [Yersinia enterocolitica].	GCA_001160345.1
COE44409.1	TPR+GreAB-C+PIN*→	TPR+GreAB-C+PIN	1147	ERS020529_01064	Firmicutes	Streptococcus pneumoniae	putative PEP-CTERM system TPR-repeat lipoprotein [Streptococcus pneumoniae].	GCA_002889635.1
CRI57148.1	REase+TPR+GreAB-C+PIN*→	REase+TPR+GreAB-C+PIN	1419	CCOS191_2612	Gammaproteobacteria	Pseudomonas sp. CCOS 191	hypothetical protein CCOS191_2612 [Pseudomonas sp. CCOS 191].	GCA_001007005.1
CTR52279.1	HetE-N1→ TPR+GreAB-C+PIN*→	TPR+GreAB-C+PIN	1093	ERS085432_03851	Gammaproteobacteria	Escherichia coli	Uncharacterised protein [Escherichia coli].	GCA_001277455.1
CTU76757.1	HetE-N1→ TPR+GreAB-C+PIN*→	TPR+GreAB-C+PIN	1093	ERS085409_04356	Gammaproteobacteria	Escherichia coli	Uncharacterised protein [Escherichia coli].	GCA_001284445.1
EAA6823230.1	HetE-N1→ TPR+GreAB-C+PIN*→	TPR+GreAB-C+PIN	839	DRV62_14440	Gammaproteobacteria	Salmonella enterica subsp. enterica serovar Corvallis	hypothetical protein DRV62_14440, partial [Salmonella enterica subsp. enterica serovar Corvallis].	GCA_004191475.1
EAA7528536.1	HetE-N1→ TPR+GreAB-C+PIN*→	TPR+GreAB-C+PIN	997	WB97_04605	Gammaproteobacteria	Salmonella enterica subsp. enterica	hypothetical protein WB97_04605, partial [Salmonella enterica subsp. enterica].	GCA_004218105.1
EAB9718450.1	HetE-N1→ TPR+GreAB-C+PIN*→	TPR+GreAB-C+PIN	1094	EBD60_05220	Gammaproteobacteria	Escherichia coli	hypothetical protein EBD60_05220 [Escherichia coli].	GCA_004268345.1
EAC0684623.1	HetE-N1→ TPR+GreAB-C+PIN*→	TPR+GreAB-C+PIN	1117	ECA66_22430	Gammaproteobacteria	Salmonella enterica subsp. enterica serovar Molade	hypothetical protein ECA66_22430 [Salmonella enterica subsp. enterica serovar Molade].	GCA_004273605.1
EAM8584006.1	HetE-N1→ TPR+GreAB-C+PIN*→?→||||<-Trypsin	TPR+GreAB-C+PIN	1121	BET14_04310	Gammaproteobacteria	Salmonella enterica	hypothetical protein BET14_04310 [Salmonella enterica].	GCA_005437065.1
EAM9325916.1	HetE-N1→ TPR+GreAB-C+PIN*→	TPR+GreAB-C+PIN	1106	DPR23_14355	Gammaproteobacteria	Salmonella enterica	hypothetical protein DPR23_14355 [Salmonella enterica].	GCA_005440115.1
EAN1748525.1	HetE-N1→ TPR+GreAB-C+PIN*→	TPR+GreAB-C+PIN	1107	D8T88_07580	Gammaproteobacteria	Salmonella enterica	hypothetical protein D8T88_07580 [Salmonella enterica].	GCA_005450625.1
EAO3021897.1	HetE-N1→ TPR+GreAB-C+PIN*→	TPR+GreAB-C+PIN	727	E3A68_19440	Gammaproteobacteria	Salmonella enterica	hypothetical protein E3A68_19440, partial [Salmonella enterica].	GCA_005498265.1
EAO5192492.1	HetE-N1→ TPR+GreAB-C+PIN*→	TPR+GreAB-C+PIN	867	E7928_13885	Gammaproteobacteria	Salmonella enterica	hypothetical protein E7928_13885, partial [Salmonella enterica].	GCA_005509025.1
EAO9608739.1	HetE-N1→ TPR+GreAB-C+PIN*→	TPR+GreAB-C+PIN	776	BH019_23310	Gammaproteobacteria	Salmonella enterica	hypothetical protein BH019_23310, partial [Salmonella enterica].	GCA_005533575.1
EAR5961962.1	HetE-N1→ TPR+GreAB-C+PIN*→	TPR+GreAB-C+PIN	1101	ES925_14455	Gammaproteobacteria	Salmonella enterica	hypothetical protein ES925_14455 [Salmonella enterica].	GCA_005654255.1
EAR6368406.1	HetE-N1→ TPR+GreAB-C+PIN*→	TPR+GreAB-C+PIN	1117	EU234_19825	Gammaproteobacteria	Salmonella enterica	hypothetical protein EU234_19825 [Salmonella enterica].	GCA_005655905.1
EAS2553591.1	TPR+GreAB-C+PIN*→	TPR+GreAB-C+PIN	590	DMT50_24325	Gammaproteobacteria	Salmonella enterica	hypothetical protein DMT50_24325, partial [Salmonella enterica].	GCA_005679955.1
EAS5437941.1	TPR+GreAB-C+PIN*→	TPR+GreAB-C+PIN	985	D8Z11_24970	Gammaproteobacteria	Salmonella enterica	hypothetical protein D8Z11_24970 [Salmonella enterica].	GCA_005692135.1
EAT5893226.1	HetE-N1→ TPR+GreAB-C+PIN*→	TPR+GreAB-C+PIN	1106	E3L72_21800	Gammaproteobacteria	Salmonella enterica	hypothetical protein E3L72_21800 [Salmonella enterica].	GCA_005737135.1
EAT6283696.1	HetE-N1→ TPR+GreAB-C+PIN*→	TPR+GreAB-C+PIN	843	E5797_05930	Gammaproteobacteria	Salmonella enterica	hypothetical protein E5797_05930, partial [Salmonella enterica].	GCA_005739275.1
EAV44914.1	REase+TPR+GreAB-C+PIN*→	REase+TPR+GreAB-C+PIN	1295	SIAM614_12903	Alphaproteobacteria	Labrenzia aggregata IAM 12614	hypothetical protein SIAM614_12903 [Labrenzia aggregata IAM 12614].	GCA_000168975.1
EAW0681535.1	HetE-N1→ TPR+GreAB-C+PIN+TM+TM*→	TPR+GreAB-C+PIN+TM+TM	1106	AIB54_02885	Gammaproteobacteria	Salmonella enterica	hypothetical protein AIB54_02885 [Salmonella enterica].	GCA_005896365.1
EAW0902300.1	TPR+GreAB-C+PIN*→	TPR+GreAB-C+PIN	638	B2H02_24145	Gammaproteobacteria	Salmonella enterica	hypothetical protein B2H02_24145, partial [Salmonella enterica].	GCA_005898285.1
EAY5481430.1	HetE-N1→ TPR+GreAB-C+PIN*→	TPR+GreAB-C+PIN	858	MB58_12360	Gammaproteobacteria	Salmonella enterica	hypothetical protein MB58_12360, partial [Salmonella enterica].	GCA_006012385.1

acc	operon	architecture	len	gen.name	taxend	species	defline	gca
EBD0849223.1	HetE-N1→ TPR+GreAB-C+PIN*→	TPR+GreAB-C+PIN	970	FHN97_07895	Gammaproteobacteria	Salmonella enterica	hypothetical protein FHN97_07895, partial [Salmonella enterica].	GCA_006217605.1
EBD3738487.1	HetE-N1→ TPR+GreAB-C+PIN*→	TPR+GreAB-C+PIN	1107	CRZ11_10865	Gammaproteobacteria	Salmonella enterica	hypothetical protein CRZ11_10865 [Salmonella enterica].	GCA_006248335.1
EBD8181391.1	TPR+GreAB-C+PIN*→	TPR+GreAB-C+PIN	606	C6600_23545	Gammaproteobacteria	Salmonella enterica	hypothetical protein C6600_23545 [Salmonella enterica].	GCA_006268595.1
EBF2432475.1	HetE-N1→ TPR+GreAB-C+PIN*→	TPR+GreAB-C+PIN	986	DKG18_08085	Gammaproteobacteria	Salmonella enterica	hypothetical protein DKG18_08085, partial [Salmonella enterica].	GCA_006332345.1
EBF2786917.1	TPR+GreAB-C+PIN*→	TPR+GreAB-C+PIN	738	C2240_24885	Gammaproteobacteria	Salmonella enterica subsp. enterica serovar Corvallis	hypothetical protein C2240_24885, partial [Salmonella enterica subsp. enterica serovar Corvallis].	GCA_006331745.1
EBF4219990.1	HetE-N1→ TPR+GreAB-C+PIN*→	TPR+GreAB-C+PIN	702	FH403_17525	Gammaproteobacteria	Salmonella enterica	hypothetical protein FH403_17525, partial [Salmonella enterica].	GCA_006342775.1
EBG7115752.1	HetE-N1→ TPR+GreAB-C+PIN*→	TPR+GreAB-C+PIN	822	FJA42_18775	Gammaproteobacteria	Salmonella enterica	hypothetical protein FJA42_18775, partial [Salmonella enterica].	GCA_006457615.1
EBI5135933.1	TPR+GreAB-C+PIN*→	TPR+GreAB-C+PIN	642	DQO15_25245	Gammaproteobacteria	Salmonella enterica	hypothetical protein DQO15_25245, partial [Salmonella enterica].	GCA_006559185.1
EBI9233829.1	HetE-N1→ TPR+GreAB-C+PIN*→	TPR+GreAB-C+PIN	670	DLA64_23180	Gammaproteobacteria	Salmonella enterica	hypothetical protein DLA64_23180, partial [Salmonella enterica].	GCA_006576485.1
EBI9496533.1	TPR+GreAB-C+PIN*→	TPR+GreAB-C+PIN	749	DLP94_24755	Gammaproteobacteria	Salmonella enterica	hypothetical protein DLP94_24755, partial [Salmonella enterica].	GCA_006578745.1
EBK5150964.1	HetE-N1→ TPR+GreAB-C+PIN*→	TPR+GreAB-C+PIN	786	DON25_22080	Gammaproteobacteria	Salmonella enterica	hypothetical protein DON25_22080, partial [Salmonella enterica].	GCA_006645025.1
EBL0898297.1	HetE-N1→ TPR+GreAB-C+PIN*→?→||||<-Trypsin	TPR+GreAB-C+PIN	1121	D0R07_07295	Gammaproteobacteria	Salmonella enterica	hypothetical protein D0R07_07295 [Salmonella enterica].	GCA_006670825.1
EBM3541219.1	HetE-N1→ TPR+GreAB-C+PIN*→	TPR+GreAB-C+PIN	1117	DYJ09_14310	Gammaproteobacteria	Salmonella enterica	hypothetical protein DYJ09_14310 [Salmonella enterica].	GCA_006729905.1
EBM4506587.1	TPR+GreAB-C+PIN*→	TPR+GreAB-C+PIN	652	DZ654_25740	Gammaproteobacteria	Salmonella enterica	hypothetical protein DZ654_25740, partial [Salmonella enterica].	GCA_006730405.1
EBM4858227.1	TPR+GreAB-C+PIN*→	TPR+GreAB-C+PIN	648	D0R05_25725	Gammaproteobacteria	Salmonella enterica	hypothetical protein D0R05_25725, partial [Salmonella enterica].	GCA_006733825.1
EBM6537845.1	HetE-N1→ TPR+GreAB-C+PIN*→	TPR+GreAB-C+PIN	1028	D2U20_21570	Gammaproteobacteria	Salmonella enterica	hypothetical protein D2U20_21570, partial [Salmonella enterica].	GCA_006740525.1
EBO1039185.1	TPR+GreAB-C+PIN*→	TPR+GreAB-C+PIN	643	D3T71_25030	Gammaproteobacteria	Salmonella enterica	hypothetical protein D3T71_25030, partial [Salmonella enterica].	GCA_006805285.1
EBQ9134585.1	TPR+GreAB-C+PIN*→	TPR+GreAB-C+PIN	707	DK746_24010	Gammaproteobacteria	Salmonella enterica subsp. enterica serovar Corvallis	hypothetical protein DK746_24010, partial [Salmonella enterica subsp. enterica serovar Corvallis].	GCA_006932075.1
EBR0236973.1	TPR+GreAB-C+PIN*→	TPR+GreAB-C+PIN	757	DNV25_24155	Gammaproteobacteria	Salmonella enterica subsp. enterica serovar Corvallis	hypothetical protein DNV25_24155, partial [Salmonella enterica subsp. enterica serovar Corvallis].	GCA_006936135.1
EBS1151706.1	TPR+GreAB-C+PIN*→	TPR+GreAB-C+PIN	1007	D6P66_23730	Gammaproteobacteria	Salmonella enterica subsp. enterica serovar Corvallis	hypothetical protein D6P66_23730 [Salmonella enterica subsp. enterica serovar Corvallis].	GCA_006989745.1
EBS2228649.1	TPR+GreAB-C+PIN*→	TPR+GreAB-C+PIN	684	DRL12_24325	Gammaproteobacteria	Salmonella enterica subsp. enterica serovar Corvallis	hypothetical protein DRL12_24325, partial [Salmonella enterica subsp. enterica serovar Corvallis].	GCA_006993385.1
EBS6357254.1	HetE-N1→ TPR+GreAB-C+PIN*→	TPR+GreAB-C+PIN	999	D4E88_23815	Gammaproteobacteria	Salmonella enterica subsp. enterica serovar Albany	hypothetical protein D4E88_23815, partial [Salmonella enterica subsp. enterica serovar Albany].	GCA_007016725.1
EBU7006421.1	TPR+GreAB-C+PIN*→	TPR+GreAB-C+PIN	620	DKU11_24275	Gammaproteobacteria	Salmonella enterica subsp. enterica serovar Kintambo	hypothetical protein DKU11_24275, partial [Salmonella enterica subsp. enterica serovar Kintambo].	GCA_007139165.1

acc	operon	architecture	len	gen.name	taxend	species	defline	gca
EBU7167144.1	HetE-N1→ TPR+GreAB-C+PIN*→	TPR+GreAB-C+PIN	1106	DKU92_07615	Gammaproteobacteria	Salmonella enterica subsp. enterica serovar Stockholm	hypothetical protein DKU92_07615 [Salmonella enterica subsp. enterica serovar Stockholm].	GCA_007140245.1
EBV1256929.1	TPR+GreAB-C+PIN*→	TPR+GreAB-C+PIN	719	DNW25_24775	Gammaproteobacteria	Salmonella enterica subsp. enterica serovar Corvallis	hypothetical protein DNW25_24775, partial [Salmonella enterica subsp. enterica serovar Corvallis].	GCA_007158045.1
EBV2191014.1	HetE-N1→ TPR+GreAB-C+PIN*→	TPR+GreAB-C+PIN	1106	DN155_01500	Gammaproteobacteria	Salmonella enterica subsp. enterica serovar Afula	hypothetical protein DN155_01500 [Salmonella enterica subsp. enterica serovar Afula].	GCA_007161245.1
EBV8290347.1	HetE-N1→ TPR+GreAB-C+PIN*→	TPR+GreAB-C+PIN	704	AUE34_22015	Gammaproteobacteria	Salmonella enterica subsp. arizonae serovar 18:z4,z23:-	hypothetical protein AUE34_22015, partial [Salmonella enterica subsp. arizonae serovar 18:z4,z23:-].	GCA_007188075.1
EBV8290357.1	TPR+GreAB-C+PIN*→	TPR+GreAB-C+PIN	385	AUE34_22090	Gammaproteobacteria	Salmonella enterica subsp. arizonae serovar 18:z4,z23:-	hypothetical protein AUE34_22090, partial [Salmonella enterica subsp. arizonae serovar 18:z4,z23:-].	GCA_007188075.1
EBV8849078.1	HetE-N1→ TPR+GreAB-C+PIN*→	TPR+GreAB-C+PIN	1000	AUA06_21700	Gammaproteobacteria	Salmonella enterica subsp. arizonae serovar 18:z4,z23:-	hypothetical protein AUA06_21700, partial [Salmonella enterica subsp. arizonae serovar 18:z4,z23:-].	GCA_007190395.1
EBV8917369.1	HetE-N1→ TPR+GreAB-C+PIN*→	TPR+GreAB-C+PIN	688	ASH63_21975	Gammaproteobacteria	Salmonella enterica subsp. arizonae serovar 18:z4,z23:-	hypothetical protein ASH63_21975, partial [Salmonella enterica subsp. arizonae serovar 18:z4,z23:-].	GCA_007194415.1
EBV9159081.1	TPR+GreAB-C+PIN*→	TPR+GreAB-C+PIN	620	ASA02_25470	Gammaproteobacteria	Salmonella enterica subsp. enterica serovar Alachua	hypothetical protein ASA02_25470, partial [Salmonella enterica subsp. enterica serovar Alachua].	GCA_007191635.1
EBV9391419.1	HetE-N1→ TPR+GreAB-C+PIN*→	TPR+GreAB-C+PIN	665	AUA09_21865	Gammaproteobacteria	Salmonella enterica subsp. arizonae serovar 18:z4,z23:-	hypothetical protein AUA09_21865, partial [Salmonella enterica subsp. arizonae serovar 18:z4,z23:-].	GCA_007194695.1
EBV9454977.1	TPR+GreAB-C*→	TPR+GreAB-C	257	AUA11_22355	Gammaproteobacteria	Salmonella enterica subsp. arizonae serovar 18:z4,z23:-	hypothetical protein AUA11_22355, partial [Salmonella enterica subsp. arizonae serovar 18:z4,z23:-].	GCA_007193095.1
EBV9865076.1	TPR+GreAB-C*→	TPR+GreAB-C	371	AUA58_21825	Gammaproteobacteria	Salmonella enterica subsp. arizonae serovar 18:z4,z23:-	hypothetical protein AUA58_21825, partial [Salmonella enterica subsp. arizonae serovar 18:z4,z23:-].	GCA_007194715.1
EBV9897412.1	TPR+GreAB-C*→	TPR+GreAB-C	379	AUA59_22385	Gammaproteobacteria	Salmonella enterica subsp. arizonae serovar 18:z4,z23:-	hypothetical protein AUA59_22385, partial [Salmonella enterica subsp. arizonae serovar 18:z4,z23:-].	GCA_007194855.1
EBW0217976.1	HetE-N1→ TPR+GreAB-C+PIN*→	TPR+GreAB-C+PIN	1107	AUA78_22040	Gammaproteobacteria	Salmonella enterica subsp. arizonae serovar 18:z4,z23:-	hypothetical protein AUA78_22040 [Salmonella enterica subsp. arizonae serovar 18:z4,z23:-].	GCA_007204575.1
EBW0231167.1	HetE-N1→ TPR+GreAB-C+PIN*→	TPR+GreAB-C+PIN	990	AUA77_22025	Gammaproteobacteria	Salmonella enterica subsp. arizonae serovar 18:z4,z23:-	hypothetical protein AUA77_22025, partial [Salmonella enterica subsp. arizonae serovar 18:z4,z23:-].	GCA_007204595.1
EBW0678507.1	TPR+GreAB-C+PIN*→	TPR+GreAB-C+PIN	861	AR803_21985	Gammaproteobacteria	Salmonella enterica subsp. arizonae serovar 18:z4,z23:-	hypothetical protein AR803_21985, partial [Salmonella enterica subsp. arizonae serovar 18:z4,z23:-].	GCA_007197055.1
EBW5988048.1	TPR+GreAB-C+PIN*→	TPR+GreAB-C+PIN	813	AUB18_21995	Gammaproteobacteria	Salmonella enterica subsp. arizonae serovar 18:z4,z23:-	hypothetical protein AUB18_21995, partial [Salmonella enterica subsp. arizonae serovar 18:z4,z23:-].	GCA_007217995.1
EBW8169800.1	TPR+GreAB-C*→	TPR+GreAB-C	283	AUB36_22265	Gammaproteobacteria	Salmonella enterica subsp. arizonae serovar 18:z4,z23:-	hypothetical protein AUB36_22265, partial [Salmonella enterica subsp. arizonae serovar 18:z4,z23:-].	GCA_007226835.1
EBW9751630.1	HetE-N1→ TPR+GreAB-C+PIN*→	TPR+GreAB-C+PIN	1106	DQR71_22645	Gammaproteobacteria	Salmonella enterica subsp. enterica serovar Kingston	hypothetical protein DQR71_22645 [Salmonella enterica subsp. enterica serovar Kingston].	GCA_007234155.1

acc	operon	architecture	len	gen.name	taxend	species	defline	gca
EBX0426906.1	HetE-N1→ TPR+GreAB-C+PIN*→	TPR+GreAB-C+PIN	864	DP854_08040	Gammaproteobacteria	Salmonella enterica subsp. enterica serovar Corvallis	hypothetical protein DP854_08040, partial [Salmonella enterica subsp. enterica serovar Corvallis].	GCA_007237735.1
EBX5460983.1	HetE-N1→ TPR+GreAB-C+PIN*→	TPR+GreAB-C+PIN	817	DSN29_17980	Gammaproteobacteria	Salmonella enterica subsp. enterica serovar Corvallis	hypothetical protein DSN29_17980, partial [Salmonella enterica subsp. enterica serovar Corvallis].	GCA_007257955.1
EBX5569113.1	HetE-N1→ TPR+GreAB-C+PIN*→	TPR+GreAB-C+PIN	1106	DSN37_03170	Gammaproteobacteria	Salmonella enterica subsp. enterica serovar Kuessel	hypothetical protein DSN37_03170 [Salmonella enterica subsp. enterica serovar Kuessel].	GCA_007259425.1
EBX6009347.1	TPR+GreAB-C+PIN*→	TPR+GreAB-C+PIN	1021	DSR61_24855	Gammaproteobacteria	Salmonella enterica subsp. enterica serovar Corvallis	hypothetical protein DSR61_24855, partial [Salmonella enterica subsp. enterica serovar Corvallis].	GCA_007258725.1
EBY0196060.1	HetE-N1→ TPR+GreAB-C+PIN*→	TPR+GreAB-C+PIN	991	DUP89_08940	Gammaproteobacteria	Salmonella enterica subsp. enterica serovar Corvallis	hypothetical protein DUP89_08940, partial [Salmonella enterica subsp. enterica serovar Corvallis].	GCA_007275795.1
EBY1533788.1	HetE-N1→?→ TPR+GreAB-C+PIN*→	TPR+GreAB-C+PIN	775	DTD21_03800	Gammaproteobacteria	Salmonella enterica subsp. enterica serovar Mgulani	hypothetical protein DTD21_03800 [Salmonella enterica subsp. enterica serovar Mgulani].	GCA_007282305.1
EBY3547778.1	HetE-N1→ TPR+GreAB-C+PIN*→	TPR+GreAB-C+PIN	781	D4F04_22510	Gammaproteobacteria	Salmonella enterica subsp. enterica serovar Corvallis	hypothetical protein D4F04_22510, partial [Salmonella enterica subsp. enterica serovar Corvallis].	GCA_007290715.1
ECB7917957.1	HetE-N1→ TPR+GreAB-C+PIN*→	TPR+GreAB-C+PIN	841	E1365_23450	Gammaproteobacteria	Salmonella enterica subsp. enterica serovar Corvallis	hypothetical protein E1365_23450, partial [Salmonella enterica subsp. enterica serovar Corvallis].	GCA_007440385.1
ECC0701089.1	HetE-N1→?→ TPR+GreAB-C+PIN*→	TPR+GreAB-C+PIN	815	FMV45_11650	Gammaproteobacteria	Salmonella enterica subsp. enterica serovar Mgulani	hypothetical protein FMV45_11650 [Salmonella enterica subsp. enterica serovar Mgulani].	GCA_007457195.1
ECC3303083.1	TPR+GreAB-C+PIN*→	TPR+GreAB-C+PIN	878	ACP91_22675	Gammaproteobacteria	Salmonella enterica subsp. arizonae	hypothetical protein ACP91_22675, partial [Salmonella enterica subsp. arizonae].	GCA_007472415.1
ECC9297450.1	TPR+GreAB-C+PIN*→?→||||<-Trypsin	TPR+GreAB-C+PIN	1011	DRF02_24595	Gammaproteobacteria	Salmonella enterica subsp. salamae	hypothetical protein DRF02_24595 [Salmonella enterica subsp. salamae].	GCA_007500125.1
ECE0143412.1	TPR+GreAB-C+PIN*→	TPR+GreAB-C+PIN	625	NC53_22885	Gammaproteobacteria	Salmonella enterica subsp. arizonae	hypothetical protein NC53_22885, partial [Salmonella enterica subsp. arizonae].	GCA_007547655.1
ECE0541238.1	TPR+GreAB-C+PIN*→	TPR+GreAB-C+PIN	879	ACK19_22505	Gammaproteobacteria	Salmonella enterica subsp. arizonae	hypothetical protein ACK19_22505, partial [Salmonella enterica subsp. arizonae].	GCA_007549055.1
ECE0545534.1	TPR+GreAB-C*→	TPR+GreAB-C	384	ACK84_23000	Gammaproteobacteria	Salmonella enterica subsp. arizonae	hypothetical protein ACK84_23000, partial [Salmonella enterica subsp. arizonae].	GCA_007549175.1
ECE0554408.1	HetE-N1→ TPR+GreAB-C+PIN*→	TPR+GreAB-C+PIN	669	ACK35_22635	Gammaproteobacteria	Salmonella enterica subsp. arizonae	hypothetical protein ACK35_22635, partial [Salmonella enterica subsp. arizonae].	GCA_007549195.1
ECE0554409.1	TPR+GreAB-C+PIN*→	TPR+GreAB-C+PIN	428	ACK35_22640	Gammaproteobacteria	Salmonella enterica subsp. arizonae	hypothetical protein ACK35_22640, partial [Salmonella enterica subsp. arizonae].	GCA_007549195.1
ECE1015908.1	HetE-N1→ TPR+GreAB-C+PIN*→	TPR+GreAB-C+PIN	1116	AHY48_24995	Gammaproteobacteria	Salmonella enterica subsp. enterica	hypothetical protein AHY48_24995 [Salmonella enterica subsp. enterica].	GCA_007551035.1
ECF6862263.1	TPR+GreAB-C+PIN*→	TPR+GreAB-C+PIN	985	C702_18880	Gammaproteobacteria	Salmonella enterica subsp. enterica serovar Labadi	hypothetical protein C702_18880 [Salmonella enterica subsp. enterica serovar Labadi].	GCA_007627255.1
ECF7216075.1	HetE-N1→ TPR+GreAB-C+PIN*→	TPR+GreAB-C+PIN	985	ACL93_22670	Gammaproteobacteria	Salmonella enterica subsp. arizonae	hypothetical protein ACL93_22670, partial [Salmonella enterica subsp. arizonae].	GCA_007627105.1
ECF7535990.1	HetE-N1→ TPR+GreAB-C+PIN*→	TPR+GreAB-C+PIN	866	AF553_23825	Gammaproteobacteria	Salmonella enterica subsp. enterica	hypothetical protein AF553_23825, partial [Salmonella enterica subsp. enterica].	GCA_007629935.1
ECG8314709.1	HetE-N1→ TPR+GreAB-C+PIN*→	TPR+GreAB-C+PIN	967	E1871_22275	Gammaproteobacteria	Salmonella enterica subsp. enterica serovar Mgulani	hypothetical protein E1871_22275 [Salmonella enterica subsp. enterica serovar Mgulani].	GCA_007694365.1
ECH7956768.1	HetE-N1→ TPR+GreAB-C+PIN*→	TPR+GreAB-C+PIN	731	SW47_20185	Gammaproteobacteria	Salmonella enterica subsp. enterica	hypothetical protein SW47_20185, partial [Salmonella enterica subsp. enterica].	GCA_007742835.1
ECH9260474.1	HetE-N1→ TPR+GreAB-C+PIN→ TPR+GreAB-C+PIN*→	TPR+GreAB-C+PIN	654	YR28_14840	Gammaproteobacteria	Salmonella enterica subsp. enterica	hypothetical protein YR28_14840 [Salmonella enterica subsp. enterica].	GCA_007749495.1

acc	operon	architecture	len	gen.name	taxend	species	defline	gca
ECI0362610.1	HetE-N1→ TPR+GreAB-C+PIN*→	TPR+GreAB-C+PIN	969	ACK10_22270	Gammaproteobacteria	Salmonella enterica subsp. arizonae	hypothetical protein ACK10_22270, partial [Salmonella enterica subsp. arizonae].	GCA_007755195.1
ECI0389037.1	TPR+GreAB-C*→	TPR+GreAB-C	376	ACL48_23155	Gammaproteobacteria	Salmonella enterica subsp. arizonae	hypothetical protein ACL48_23155, partial [Salmonella enterica subsp. arizonae].	GCA_007754635.1
ECI1216662.1	TPR+GreAB-C+PIN*→	TPR+GreAB-C+PIN	836	AF582_21855	Gammaproteobacteria	Salmonella enterica subsp. arizonae	hypothetical protein AF582_21855, partial [Salmonella enterica subsp. arizonae].	GCA_007758765.1
ECI5614625.1	HetE-N1→?→ TPR+GreAB-C+PIN*→	TPR+GreAB-C+PIN	655	AHW94_03505	Gammaproteobacteria	Salmonella enterica subsp. enterica	hypothetical protein AHW94_03505, partial [Salmonella enterica subsp. enterica].	GCA_007777085.1
ECM8537065.1	HetE-N1→ TPR+GreAB-C+PIN*→	TPR+GreAB-C+PIN	1117	YI35_19225	Gammaproteobacteria	Salmonella enterica subsp. enterica serovar Corvallis	hypothetical protein YI35_19225 [Salmonella enterica subsp. enterica serovar Corvallis].	GCA_008050935.1
ECS4963358.1	TPR+GreAB-C+PIN*→	TPR+GreAB-C+PIN	874	A9T74_22060	Gammaproteobacteria	Salmonella enterica subsp. arizonae serovar 18:z4,z23:-	hypothetical protein A9T74_22060, partial [Salmonella enterica subsp. arizonae serovar 18:z4,z23:-].	GCA_008426085.1
ECS5089259.1	TPR+GreAB-C+PIN*→	TPR+GreAB-C+PIN	688	A9T03_21360	Gammaproteobacteria	Salmonella enterica subsp. arizonae serovar 18:z4,z23:-	hypothetical protein A9T03_21360, partial [Salmonella enterica subsp. arizonae serovar 18:z4,z23:-].	GCA_008426245.1
ECS5722565.1	HetE-N1→ TPR+GreAB-C+PIN*→	TPR+GreAB-C+PIN	763	BEE21_21925	Gammaproteobacteria	Salmonella enterica	hypothetical protein BEE21_21925, partial [Salmonella enterica].	GCA_008427725.1
ECS7544753.1	HetE-N1→ TPR+GreAB-C+PIN*→?→||||<-Trypsin	TPR+GreAB-C+PIN	1121	CI474_11845	Gammaproteobacteria	Salmonella enterica subsp. enterica serovar Denver	hypothetical protein CI474_11845 [Salmonella enterica subsp. enterica serovar Denver].	GCA_008435705.1
ECU7350952.1	TPR+GreAB-C+PIN*→	TPR+GreAB-C+PIN	816	A9S02_22180	Gammaproteobacteria	Salmonella enterica subsp. enterica serovar Kentucky	hypothetical protein A9S02_22180, partial [Salmonella enterica subsp. enterica serovar Kentucky].	GCA_008522685.1
ECV0014755.1	TPR+GreAB-C+PIN*→	TPR+GreAB-C+PIN	672	C9G23_25520	Gammaproteobacteria	Salmonella enterica	hypothetical protein C9G23_25520 [Salmonella enterica].	GCA_008534115.1
ECX3800078.1	HetE-N1→ TPR+GreAB-C+PIN*→	TPR+GreAB-C+PIN	647	F6B58_20445	Gammaproteobacteria	Salmonella enterica	hypothetical protein F6B58_20445, partial [Salmonella enterica].	GCA_008659475.1
ECY0836069.1	MarR-HTH→||||<-PSE||PSE→||||<-?||?→?→||||<-?||?→ HetE-N1→ TPR+GreAB-C+PIN*→?→||||<-Trypsin	TPR+GreAB-C+PIN	1121	F6Y43_14905	Gammaproteobacteria	Salmonella enterica subsp. enterica	hypothetical protein F6Y43_14905 [Salmonella enterica subsp. enterica].	GCA_008716255.1
ECY8988972.1	HetE-N1→ TPR+GreAB-C+PIN*→	TPR+GreAB-C+PIN	1117	F7L41_18095	Gammaproteobacteria	Salmonella enterica	hypothetical protein F7L41_18095 [Salmonella enterica].	GCA_008760695.1
ECZ2850292.1	TPR+GreAB-C+PIN*→	TPR+GreAB-C+PIN	736	F7T15_22920	Gammaproteobacteria	Salmonella enterica	hypothetical protein F7T15_22920, partial [Salmonella enterica].	GCA_008785865.1
EDA5083812.1	HetE-N1→ TPR+GreAB-C+PIN*→	TPR+GreAB-C+PIN	861	F9F62_19420	Gammaproteobacteria	Salmonella enterica	hypothetical protein F9F62_19420, partial [Salmonella enterica].	GCA_008860845.1
EDB4036569.1	HetE-N1→ TPR+GreAB-C+PIN*→	TPR+GreAB-C+PIN	810	A6J23_15240	Gammaproteobacteria	Salmonella enterica	hypothetical protein A6J23_15240, partial [Salmonella enterica].	GCA_008904195.1
EDB4763898.1	TPR+GreAB-C+PIN*→	TPR+GreAB-C+PIN	985	A8H08_23565	Gammaproteobacteria	Salmonella enterica subsp. enterica serovar Corvallis	hypothetical protein A8H08_23565 [Salmonella enterica subsp. enterica serovar Corvallis].	GCA_008907365.1
EDB6166846.1	HetE-N1→ TPR+GreAB-C+PIN*→	TPR+GreAB-C+PIN	994	BBD73_10015	Gammaproteobacteria	Salmonella enterica subsp. enterica serovar Corvallis	hypothetical protein BBD73_10015, partial [Salmonella enterica subsp. enterica serovar Corvallis].	GCA_008914005.1
EDN4259744.1	HetE-N1→ TPR+GreAB-C+PIN*→	TPR+GreAB-C+PIN	840	Y652_004461	Gammaproteobacteria	Salmonella enterica subsp. enterica	hypothetical protein Y652_004461, partial [Salmonella enterica subsp. enterica].	GCA_009997765.1
EDO1617810.1	HetE-N1→ TPR+GreAB-C+PIN*→	TPR+GreAB-C+PIN	838	CVA64_22730	Gammaproteobacteria	Salmonella enterica subsp. enterica serovar Molade	hypothetical protein CVA64_22730, partial [Salmonella enterica subsp. enterica serovar Molade].	GCA_010125745.1
EDP8916875.1	HetE-N1→ TPR+GreAB-C+PIN*→	TPR+GreAB-C+PIN	775	ZT80_004634	Gammaproteobacteria	Salmonella enterica subsp. enterica serovar Chailey	hypothetical protein ZT80_004634, partial [Salmonella enterica subsp. enterica serovar Chailey].	GCA_010369345.1

<table>
<tr><th>acc</th><th>operon</th><th>architecture</th><th>len</th><th>gen.name</th><th>taxend</th><th>species</th><th>defline</th><th>gca</th></tr>
<tr><td>EDP9779517.1</td><td>HetE-N1→ TPR+GreAB-C+PIN*→</td><td>TPR+GreAB-C+PIN</td><td>844</td><td>A9223_002456</td><td>Gammaproteobacteria</td><td>Salmonella enterica subsp. enterica serovar Alachua</td><td>hypothetical protein A9223_002456, partial [Salmonella enterica subsp. enterica serovar Alachua].</td><td>GCA_010373825.1</td></tr>
<tr><td>EDR2934822.1</td><td>HetE-N1→ TPR+GreAB-C+PIN*→</td><td>TPR+GreAB-C+PIN</td><td>754</td><td>KS39_001997</td><td>Gammaproteobacteria</td><td>Salmonella enterica subsp. enterica serovar Ruiru</td><td>hypothetical protein KS39_001997, partial [Salmonella enterica subsp. enterica serovar Ruiru].</td><td>GCA_010439595.1</td></tr>
<tr><td>EDR3921234.1</td><td><-HTH<-?<-PSE||HetE-N1→ TPR+GreAB-C+PIN*→</td><td>TPR+GreAB-C+PIN</td><td>1101</td><td>BWC65_003323</td><td>Gammaproteobacteria</td><td>Salmonella enterica</td><td>hypothetical protein BWC65_003323 [Salmonella enterica].</td><td>GCA_010444135.1</td></tr>
<tr><td>EDR7156767.1</td><td>HetE-N1→ TPR+GreAB-C+PIN→ TPR+GreAB-C+PIN*→</td><td>TPR+GreAB-C+PIN</td><td>457</td><td>GRJ98_004481</td><td>Gammaproteobacteria</td><td>Salmonella enterica</td><td>hypothetical protein GRJ98_004481 [Salmonella enterica].</td><td>GCA_010460165.1</td></tr>
<tr><td>EDR9691108.1</td><td>TPR+GreAB-C+PIN*→</td><td>TPR+GreAB-C+PIN</td><td>644</td><td>CSL67_004218</td><td>Gammaproteobacteria</td><td>Salmonella enterica subsp. arizonae</td><td>hypothetical protein CSL67_004218 [Salmonella enterica subsp. arizonae].</td><td>GCA_010471185.1</td></tr>
<tr><td>EDT9803200.1</td><td>HetE-N1→ TPR+GreAB-C+PIN*→</td><td>TPR+GreAB-C+PIN</td><td>828</td><td>GQ119_004456</td><td>Gammaproteobacteria</td><td>Salmonella enterica</td><td>hypothetical protein GQ119_004456, partial [Salmonella enterica].</td><td>GCA_010569565.1</td></tr>
<tr><td>EDU6324669.1</td><td>HetE-N1→ TPR+GreAB-C+PIN*→?→||||<-Trypsin</td><td>TPR+GreAB-C+PIN</td><td>1121</td><td>CAE39_004078</td><td>Gammaproteobacteria</td><td>Salmonella enterica subsp. enterica serovar Edinburgh</td><td>hypothetical protein CAE39_004078 [Salmonella enterica subsp. enterica serovar Edinburgh].</td><td>GCA_010619425.1</td></tr>
<tr><td>EDV9361828.1</td><td>HetE-N1→ TPR+GreAB-C+PIN*→</td><td>TPR+GreAB-C+PIN</td><td>870</td><td>QG02_001683</td><td>Gammaproteobacteria</td><td>Salmonella enterica subsp. enterica</td><td>hypothetical protein QG02_001683, partial [Salmonella enterica subsp. enterica].</td><td>GCA_010676285.1</td></tr>
<tr><td>EDW0654491.1</td><td><-HTH<-?<-?||HetE-N1→ TPR+GreAB-C+PIN*→</td><td>TPR+GreAB-C+PIN</td><td>1106</td><td>JU54_004053</td><td>Gammaproteobacteria</td><td>Salmonella enterica subsp. enterica serovar Weslaco</td><td>hypothetical protein JU54_004053 [Salmonella enterica subsp. enterica serovar Weslaco].</td><td>GCA_010695505.1</td></tr>
<tr><td>EDW1854316.1</td><td>TPR+GreAB-C+PIN*→</td><td>TPR+GreAB-C+PIN</td><td>1107</td><td>S887_003116</td><td>Gammaproteobacteria</td><td>Salmonella enterica subsp. diarizonae</td><td>hypothetical protein S887_003116 [Salmonella enterica subsp. diarizonae].</td><td>GCA_010701205.1</td></tr>
<tr><td>EDW7381411.1</td><td>HetE-N1→ TPR+GreAB-C+PIN*→</td><td>TPR+GreAB-C+PIN</td><td>838</td><td>CJF03_003360</td><td>Gammaproteobacteria</td><td>Salmonella enterica subsp. enterica serovar Alachua</td><td>hypothetical protein CJF03_003360, partial [Salmonella enterica subsp. enterica serovar Alachua].</td><td>GCA_010728605.1</td></tr>
<tr><td>EDX0815680.1</td><td>MarR-HTH→||||<-PSE||PSE→||||<-?||?→?→||||<-?||?→ HetE-N1→ TPR+GreAB-C+PIN*→</td><td>TPR+GreAB-C+PIN</td><td>1047</td><td>GTA77_003301</td><td>Gammaproteobacteria</td><td>Salmonella enterica subsp. enterica</td><td>hypothetical protein GTA77_003301, partial [Salmonella enterica subsp. enterica].</td><td>GCA_010745015.1</td></tr>
<tr><td>EDY1906151.1</td><td>MarR-HTH→||||<-PSE||PSE→||||<-?||?→?→||||<-?||?→ HetE-N1→ TPR+GreAB-C+PIN*→</td><td>TPR+GreAB-C+PIN</td><td>1043</td><td>GTA76_003216</td><td>Gammaproteobacteria</td><td>Salmonella enterica subsp. enterica</td><td>hypothetical protein GTA76_003216, partial [Salmonella enterica subsp. enterica].</td><td>GCA_010803285.1</td></tr>
<tr><td>EDY7338112.1</td><td>HetE-N1→ TPR+GreAB-C+PIN*→</td><td>TPR+GreAB-C+PIN</td><td>873</td><td>GRN59_004759</td><td>Gammaproteobacteria</td><td>Salmonella enterica subsp. enterica serovar Alachua</td><td>hypothetical protein GRN59_004759, partial [Salmonella enterica subsp. enterica serovar Alachua].</td><td>GCA_010827805.1</td></tr>
<tr><td>EDZ6443278.1</td><td>HetE-N1→ TPR+GreAB-C+PIN*→</td><td>TPR+GreAB-C+PIN</td><td>813</td><td>GOH01_21475</td><td>Gammaproteobacteria</td><td>Salmonella enterica</td><td>hypothetical protein GOH01_21475, partial [Salmonella enterica].</td><td>GCA_010859905.1</td></tr>
<tr><td>EED4174297.1</td><td>HetE-N1→ TPR+GreAB-C+PIN*→</td><td>TPR+GreAB-C+PIN</td><td>944</td><td>S577_12620</td><td>Gammaproteobacteria</td><td>Salmonella enterica subsp. enterica serovar Rubislaw</td><td>hypothetical protein S577_12620, partial [Salmonella enterica subsp. enterica serovar Rubislaw].</td><td>GCA_011083145.1</td></tr>
<tr><td>EEG2646873.1</td><td>HetE-N1→ TPR+GreAB-C+PIN*→</td><td>TPR+GreAB-C+PIN</td><td>1117</td><td>GXG13_10075</td><td>Gammaproteobacteria</td><td>Salmonella enterica</td><td>hypothetical protein GXG13_10075 [Salmonella enterica].</td><td>GCA_011218655.1</td></tr>
<tr><td>EEH6562392.1</td><td>TPR+GreAB-C+PIN*→</td><td>TPR+GreAB-C+PIN</td><td>561</td><td>G9D83_004649</td><td>Gammaproteobacteria</td><td>Salmonella enterica</td><td>hypothetical protein G9D83_004649, partial [Salmonella enterica].</td><td>GCA_011296115.1</td></tr>
<tr><td>EEH8607740.1</td><td>HetE-N1→ TPR+GreAB-C+PIN*→</td><td>TPR+GreAB-C+PIN</td><td>751</td><td>GXG09_15375</td><td>Gammaproteobacteria</td><td>Salmonella enterica</td><td>hypothetical protein GXG09_15375, partial [Salmonella enterica].</td><td>GCA_011310075.1</td></tr>
<tr><td>EEI7790049.1</td><td>TPR+GreAB-C+PIN*→</td><td>TPR+GreAB-C+PIN</td><td>1003</td><td>G7421_004640</td><td>Gammaproteobacteria</td><td>Salmonella enterica</td><td>hypothetical protein G7421_004640, partial [Salmonella enterica].</td><td>GCA_011384105.1</td></tr>
<tr><td>EEJ3916542.1</td><td>HetE-N1→ TPR+GreAB-C+PIN*→</td><td>TPR+GreAB-C+PIN</td><td>931</td><td>C1D58_004804</td><td>Gammaproteobacteria</td><td>Salmonella enterica subsp. enterica serovar Waral</td><td>hypothetical protein C1D58_004804, partial [Salmonella enterica subsp. enterica serovar Waral].</td><td>GCA_011421105.1</td></tr>
<tr><td>EEJ8590178.1</td><td>HetE-N1→ TPR+GreAB-C+PIN*→</td><td>TPR+GreAB-C+PIN</td><td>1106</td><td>YR33_002856</td><td>Gammaproteobacteria</td><td>Salmonella enterica subsp. enterica</td><td>hypothetical protein YR33_002856 [Salmonella enterica subsp. enterica].</td><td>GCA_011444435.1</td></tr>
</table>

acc	operon	architecture	len	gen.name	taxend	species	defline	gca
EEM3099552.1	HetE-N1→ TPR+GreAB-C+PIN*→	TPR+GreAB-C+PIN	837	C7106_19315	Gammaproteobacteria	Salmonella enterica subsp. enterica serovar Corvallis	hypothetical protein C7106_19315, partial [Salmonella enterica subsp. enterica serovar Corvallis].	GCA_011562745.1
EEN9063089.1	TPR+GreAB-C+PIN*→	TPR+GreAB-C+PIN	1003	G5014_004598	Gammaproteobacteria	Salmonella enterica subsp. enterica	hypothetical protein G5014_004598, partial [Salmonella enterica subsp. enterica].	GCA_011642815.1
EEO4791769.1	GreAB-C*→	GreAB-C	475	GCV36_004805	Gammaproteobacteria	Salmonella enterica	hypothetical protein GCV36_004805, partial [Salmonella enterica].	GCA_011672825.1
EEP6626700.1	TPR+GreAB-C+PIN*→	TPR+GreAB-C+PIN	1098	HB923_004453	Gammaproteobacteria	Salmonella enterica	hypothetical protein HB923_004453, partial [Salmonella enterica].	GCA_011737335.1
EEQ0870631.1	GreAB-C*→	GreAB-C	401	HC115_004664	Gammaproteobacteria	Salmonella enterica subsp. enterica serovar Corvallis	hypothetical protein HC115_004664, partial [Salmonella enterica subsp. enterica serovar Corvallis].	GCA_011766905.1
EER8058271.1	HetE-N1→ TPR+GreAB-C+PIN*→	TPR+GreAB-C+PIN	1094	F0645_003838	Gammaproteobacteria	Escherichia coli	hypothetical protein F0645_003838 [Escherichia coli].	GCA_011871345.1
EES4613658.1	HetE-N1→ TPR+GreAB-C+PIN*→	TPR+GreAB-C+PIN	1094	EZQ25_003302	Gammaproteobacteria	Escherichia coli	hypothetical protein EZQ25_003302 [Escherichia coli].	GCA_011897605.1
EEU6346619.1	TPR+GreAB-C+PIN*→	TPR+GreAB-C+PIN	663	HES36_004810	Gammaproteobacteria	Salmonella enterica	hypothetical protein HES36_004810, partial [Salmonella enterica].	GCA_011985205.1
EEU8409948.1	TPR+GreAB-C+PIN*→	TPR+GreAB-C+PIN	733	HDI19_004538	Gammaproteobacteria	Salmonella enterica	hypothetical protein HDI19_004538, partial [Salmonella enterica].	GCA_012002765.1
EEU8431734.1	TPR+GreAB-C+PIN*→	TPR+GreAB-C+PIN	770	HDI14_004564	Gammaproteobacteria	Salmonella enterica	hypothetical protein HDI14_004564, partial [Salmonella enterica].	GCA_012002965.1
EEU8450206.1	TPR+GreAB-C+PIN*→	TPR+GreAB-C+PIN	686	HDI09_004415	Gammaproteobacteria	Salmonella enterica	hypothetical protein HDI09_004415, partial [Salmonella enterica].	GCA_012003165.1
EEU8461067.1	HetE-N1→ TPR+GreAB-C+PIN*→	TPR+GreAB-C+PIN	777	HDI12_003619	Gammaproteobacteria	Salmonella enterica	hypothetical protein HDI12_003619, partial [Salmonella enterica].	GCA_012003025.1
EEU8472241.1	HetE-N1→ TPR+GreAB-C+PIN*→	TPR+GreAB-C+PIN	832	HDI08_003001	Gammaproteobacteria	Salmonella enterica	hypothetical protein HDI08_003001, partial [Salmonella enterica].	GCA_012003185.1
EEU8646489.1	HetE-N1→ TPR+GreAB-C+PIN*→	TPR+GreAB-C+PIN	746	HDI02_002414	Gammaproteobacteria	Salmonella enterica	hypothetical protein HDI02_002414, partial [Salmonella enterica].	GCA_012004065.1
EEV5892122.1	HetE-N1→ TPR+GreAB-C+PIN*→	TPR+GreAB-C+PIN	1094	ELJ91_00185	Gammaproteobacteria	Escherichia coli	hypothetical protein ELJ91_00185 [Escherichia coli].	GCA_012046565.1
EEW2250301.1	TPR+GreAB-C+PIN*→	TPR+GreAB-C+PIN	649	D9F29_22745	Gammaproteobacteria	Escherichia coli	hypothetical protein D9F29_22745, partial [Escherichia coli].	GCA_012074905.1
EEW6232025.1	TPR+GreAB-C+PIN*→	TPR+GreAB-C+PIN	1025	D7V76_23725	Gammaproteobacteria	Escherichia coli	hypothetical protein D7V76_23725, partial [Escherichia coli].	GCA_012087875.1
EEZ4464859.1	HetE-N1→ TPR+GreAB-C+PIN*→	TPR+GreAB-C+PIN	1009	DED50_002664	Gammaproteobacteria	Escherichia coli	hypothetical protein DED50_002664, partial [Escherichia coli].	GCA_012207825.1
EFA5448049.1	GreAB-C*→	GreAB-C	270	EZF43_26870	Gammaproteobacteria	Escherichia coli	hypothetical protein EZF43_26870, partial [Escherichia coli].	GCA_012252965.1
EFB4141030.1	TPR+GreAB-C+PIN*→	TPR+GreAB-C+PIN	803	C0X77_005316	Gammaproteobacteria	Escherichia coli O88:H1	hypothetical protein C0X77_005316, partial [Escherichia coli O88:H1].	GCA_012307025.1
EFB7592548.1	TPR+GreAB-C+PIN*→	TPR+GreAB-C+PIN	871	E4J78_25685	Gammaproteobacteria	Escherichia coli	hypothetical protein E4J78_25685, partial [Escherichia coli].	GCA_012318985.1
EFD6843528.1	HetE-N1→ TPR+GreAB-C+PIN*→	TPR+GreAB-C+PIN	785	HG348_004580	Gammaproteobacteria	Salmonella enterica subsp. enterica serovar Corvallis	hypothetical protein HG348_004580, partial [Salmonella enterica subsp. enterica serovar Corvallis].	GCA_012407905.1
EFD8840406.1	TPR+GreAB-C+PIN*→	TPR+GreAB-C+PIN	342	CO273_005362	Gammaproteobacteria	Escherichia coli	hypothetical protein CO273_005362, partial [Escherichia coli].	GCA_012416985.1
EFE7110517.1	TPR+GreAB-C+PIN*→	TPR+GreAB-C+PIN	631	GCY86_26975	Gammaproteobacteria	Escherichia coli	hypothetical protein GCY86_26975, partial [Escherichia coli].	GCA_012449745.1

acc	operon	architecture	len	gen.name	taxend	species	defline	gca
EFE7435055.1	HetE-N1→ TPR+GreAB-C+PIN*→	TPR+GreAB-C+PIN	917	F9S89_16525	Gammaproteobacteria	Escherichia coli	hypothetical protein F9S89_16525, partial [Escherichia coli].	GCA_012452135.1
EFE7908555.1	TPR+GreAB-C+PIN*→	TPR+GreAB-C+PIN	1007	F3644_26165	Gammaproteobacteria	Escherichia coli	hypothetical protein F3644_26165, partial [Escherichia coli].	GCA_012454005.1
EFF2139424.1	TPR+GreAB-C+PIN*→	TPR+GreAB-C+PIN	639	B9T00_004906	Gammaproteobacteria	Escherichia coli	hypothetical protein B9T00_004906, partial [Escherichia coli].	GCA_012475085.1
EFG3018646.1	TPR+GreAB-C+PIN*→	TPR+GreAB-C+PIN	644	BOZ88_000259	Gammaproteobacteria	Escherichia coli	hypothetical protein BOZ88_000259, partial [Escherichia coli].	GCA_012551215.1
EFG6909290.1	HetE-N1→ TPR+GreAB-C+PIN*→	TPR+GreAB-C+PIN	747	BMM73_004701	Gammaproteobacteria	Escherichia coli	hypothetical protein BMM73_004701, partial [Escherichia coli].	GCA_012567505.1
EFH5729117.1	GreAB-C*→	GreAB-C	499	GPH08_24725	Gammaproteobacteria	Escherichia coli	hypothetical protein GPH08_24725, partial [Escherichia coli].	GCA_012607235.1
EFK2051151.1	HetE-N1→ TPR+GreAB-C+PIN*→	TPR+GreAB-C+PIN	1094	A8650_000222	Gammaproteobacteria	Escherichia coli	hypothetical protein A8650_000222 [Escherichia coli].	GCA_012745335.1
EFK2630094.1	HetE-N1→ TPR+GreAB-C+PIN*→	TPR+GreAB-C+PIN	1094	A8611_000403	Gammaproteobacteria	Escherichia coli	hypothetical protein A8611_000403 [Escherichia coli].	GCA_012750705.1
EFK4277858.1	HetE-N1→ TPR+GreAB-C+PIN*→	TPR+GreAB-C+PIN	1094	A8W36_000742	Gammaproteobacteria	Escherichia coli	hypothetical protein A8W36_000742 [Escherichia coli].	GCA_012757595.1
EFK5336626.1	GreAB-C*→	GreAB-C	296	G5Y50_005070	Gammaproteobacteria	Escherichia coli	hypothetical protein G5Y50_005070, partial [Escherichia coli].	GCA_012762195.1
EFM2410662.1	TPR+GreAB-C+PIN*→	TPR+GreAB-C+PIN	1064	HCP88_003534	Gammaproteobacteria	Escherichia coli	hypothetical protein HCP88_003534, partial [Escherichia coli].	GCA_012855135.1
EFM6245585.1	HetE-N1→ TPR+GreAB-C+PIN*→	TPR+GreAB-C+PIN	1094	G8G69_000691	Gammaproteobacteria	Escherichia coli	hypothetical protein G8G69_000691 [Escherichia coli].	GCA_012890895.1
EFN7425919.1	HetE-N1→ HetE-N1+TPR+GreAB-C+PIN*→	HetE-N1+TPR+GreAB-C+PIN	1094	EHR20_13780	Gammaproteobacteria	Escherichia coli	hypothetical protein EHR20_13780 [Escherichia coli].	GCA_013040925.1
EFO0890621.1	GreAB-C*→	GreAB-C	328	DYJ49_27190	Gammaproteobacteria	Escherichia coli	hypothetical protein DYJ49_27190, partial [Escherichia coli].	GCA_013061045.1
EFO1176558.1	TPR+GreAB-C+PIN*→	TPR+GreAB-C+PIN	1073	DW520_17505	Gammaproteobacteria	Escherichia coli	hypothetical protein DW520_17505, partial [Escherichia coli].	GCA_013062025.1
EFS2240967.1	HetE-N1→ TPR+GreAB-C+PIN*→	TPR+GreAB-C+PIN	1094	GTJ56_01995	Gammaproteobacteria	Shigella sonnei	hypothetical protein GTJ56_01995 [Shigella sonnei].	GCA_013627075.1
EFT8083545.1	<-HTH<-PSE||HetE-N1→ TPR+GreAB-C+PIN*→	TPR+GreAB-C+PIN	1107	HVC06_002423	Gammaproteobacteria	Salmonella enterica	hypothetical protein HVC06_002423 [Salmonella enterica].	GCA_013717865.1
EFU7164914.1	TPR+GreAB-C+PIN*→	TPR+GreAB-C+PIN	1012	HTZ39_004610	Gammaproteobacteria	Escherichia coli	hypothetical protein HTZ39_004610, partial [Escherichia coli].	GCA_013766175.1
EFW2864166.1	TPR+GreAB-C*→	TPR+GreAB-C	440	H3T30_004508	Gammaproteobacteria	Salmonella enterica	hypothetical protein H3T30_004508, partial [Salmonella enterica].	GCA_013859415.1
EFZ7349264.1	HetE-N1→ TPR+GreAB-C+PIN*→	TPR+GreAB-C+PIN	1094	A8I24_003489	Gammaproteobacteria	Shigella sonnei	hypothetical protein A8I24_003489 [Shigella sonnei].	GCA_014033075.1
EGB9339943.1	TPR+GreAB-C+PIN*→	TPR+GreAB-C+PIN	694	H8Q22_004544	Gammaproteobacteria	Salmonella enterica	hypothetical protein H8Q22_004544, partial [Salmonella enterica].	GCA_014249275.1
EGF6340579.1	GreAB-C*→	GreAB-C	417	IBT07_004591	Gammaproteobacteria	Salmonella enterica subsp. enterica serovar Molade	hypothetical protein IBT07_004591, partial [Salmonella enterica subsp. enterica serovar Molade].	GCA_014515725.1
EGG4494193.1	TPR+GreAB-C+PIN*→	TPR+GreAB-C+PIN	990	HKL61_004648	Gammaproteobacteria	Salmonella enterica	hypothetical protein HKL61_004648, partial [Salmonella enterica].	GCA_014566815.1
EGG4638401.1	HetE-N1→ TPR+GreAB-C+PIN*→	TPR+GreAB-C+PIN	777	HKL37_003624	Gammaproteobacteria	Salmonella enterica	hypothetical protein HKL37_003624, partial [Salmonella enterica].	GCA_014566995.1
EGG4725781.1	HetE-N1→ TPR+GreAB-C+PIN*→	TPR+GreAB-C+PIN	855	HKE56_003805	Gammaproteobacteria	Salmonella enterica	hypothetical protein HKE56_003805, partial [Salmonella enterica].	GCA_014566275.1

acc	operon	architecture	len	gen.name	taxend	species	defline	gca
EGI5600308.1	HetE-N1→ TPR+GreAB-C+PIN*→	TPR+GreAB-C+PIN	1070	VS45_000308	Gammaproteobacteria	Salmonella enterica subsp. enterica serovar Augustenborg	hypothetical protein VS45_000308, partial [Salmonella enterica subsp. enterica serovar Augustenborg].	GCA_014756925.1
EGI6298009.1	HetE-N1→ TPR+GreAB-C+PIN*→	TPR+GreAB-C+PIN	1117	WB72_004704	Gammaproteobacteria	Salmonella enterica subsp. enterica serovar Molade	hypothetical protein WB72_004704 [Salmonella enterica subsp. enterica serovar Molade].	GCA_014759725.1
EGJ1631486.1	<-HTH<-?<-?||HetE-N1→ TPR+GreAB-C+PIN*→	TPR+GreAB-C+PIN	915	IHG43_004265	Gammaproteobacteria	Salmonella enterica	hypothetical protein IHG43_004265, partial [Salmonella enterica].	GCA_014806485.1
EGJ1647992.1	<-HTH<-?<-?||HetE-N1→ TPR+GreAB-C+PIN*→	TPR+GreAB-C+PIN	1107	IHG42_002561	Gammaproteobacteria	Salmonella enterica	hypothetical protein IHG42_002561 [Salmonella enterica].	GCA_014806725.1
EGK4803692.1	TPR+GreAB-C+PIN*→	TPR+GreAB-C+PIN	879	IOX27_004405	Gammaproteobacteria	Salmonella enterica	hypothetical protein IOX27_004405, partial [Salmonella enterica].	GCA_014937925.1
EGQ7796229.1	HetE-N1→ TPR+GreAB-C+PIN*→	TPR+GreAB-C+PIN	1130	I6Z01_004778	Gammaproteobacteria	Vibrio parahaemolyticus	hypothetical protein I6Z01_004778 [Vibrio parahaemolyticus].	GCA_015745865.1
EGQ8097028.1	TPR+GreAB-C+PIN*→	TPR+GreAB-C+PIN	450	G4T96_003617	Gammaproteobacteria	Vibrio cholerae	hypothetical protein G4T96_003617, partial [Vibrio cholerae].	GCA_015960365.1
EGQ8394401.1	HetE-N1→ TPR+GreAB-C+PIN*→	TPR+GreAB-C+PIN	1089	GTW12_14360	Gammaproteobacteria	Vibrio cholerae	hypothetical protein GTW12_14360 [Vibrio cholerae].	GCA_015775695.1
EGR2511155.1	HetE-N1→ HetE-N1+TPR+GreAB-C+PIN*→	HetE-N1+TPR+GreAB-C+PIN	1088	DYC71_07390	Gammaproteobacteria	Vibrio cholerae	hypothetical protein DYC71_07390 [Vibrio cholerae].	GCA_015803535.1
EGR2571797.1	HetE-N1→ TPR+GreAB-C+PIN*→	TPR+GreAB-C+PIN	1089	DXI14_15890	Gammaproteobacteria	Vibrio cholerae	hypothetical protein DXI14_15890 [Vibrio cholerae].	GCA_015804695.1
EGR4199274.1	HetE-N1→ HetE-N1+TPR+GreAB-C+PIN*→	HetE-N1+TPR+GreAB-C+PIN	1088	DDN79_15180	Gammaproteobacteria	Vibrio cholerae	hypothetical protein DDN79_15180 [Vibrio cholerae].	GCA_015810035.1
EGR4999920.1	HetE-N1→ HetE-N1+TPR+GreAB-C+PIN*→	HetE-N1+TPR+GreAB-C+PIN	1091	C4G32_21475	Gammaproteobacteria	Vibrio parahaemolyticus	hypothetical protein C4G32_21475 [Vibrio parahaemolyticus].	GCA_015814505.1
EGS2470077.1	HetE-N1→ TPR+GreAB-C+PIN*→	TPR+GreAB-C+PIN	1117	I8K59_003334	Gammaproteobacteria	Salmonella enterica	hypothetical protein I8K59_003334 [Salmonella enterica].	GCA_015867645.1
EGS6048781.1	HetE-N1→ TPR+GreAB-C+PIN*→	TPR+GreAB-C+PIN	998	I4Y87_004789	Gammaproteobacteria	Salmonella enterica subsp. enterica serovar Alachua	hypothetical protein I4Y87_004789, partial [Salmonella enterica subsp. enterica serovar Alachua].	GCA_015906765.1
EGT0637485.1	GreAB-C*→	GreAB-C	478	JAG24_004773	Gammaproteobacteria	Citrobacter freundii	hypothetical protein JAG24_004773, partial [Citrobacter freundii].	GCA_015943305.1
EGT3640898.1	TPR+GreAB-C+PIN*→	TPR+GreAB-C+PIN	1067	DFW15_03700	Firmicutes	Clostridioides difficile	hypothetical protein DFW15_03700 [Clostridioides difficile].	GCA_016071215.1
EGT5618809.1	TPR+GreAB-C+PIN*→	TPR+GreAB-C+PIN	1233	BUL45_06960	Firmicutes	Clostridium perfringens	tetratricopeptide repeat protein [Clostridium perfringens].	GCA_016085335.1
EGY6095294.1	REase+TPR+GreAB-C+PIN*→||||<-?||HTH→	REase+TPR+GreAB-C+PIN	1216	A1Z38_19500	Gammaproteobacteria	Acinetobacter baumannii	hypothetical protein A1Z38_19500 [Acinetobacter baumannii].	GCA_016522135.1
EGY8392823.1	REase+TPR+GreAB-C+PIN*→||||<-?||HTH→	REase+TPR+GreAB-C+PIN	1216	AXE44_13945	Gammaproteobacteria	Acinetobacter baumannii	hypothetical protein AXE44_13945 [Acinetobacter baumannii].	GCA_016539145.1
EHA1126835.1	HetE-N1→ TPR+GreAB-C+PIN*→	TPR+GreAB-C+PIN	1114	FG475_17175	Gammaproteobacteria	Vibrio navarrensis	hypothetical protein FG475_17175 [Vibrio navarrensis].	GCA_016611225.1
EHB5819355.1	HetE-N1→ TPR+GreAB-C+PIN*→	TPR+GreAB-C+PIN	1106	JXL86_002072	-	Enterobacter hormaechei	hypothetical protein JXL86_002072 [Enterobacter hormaechei].	GCA_016985635.1
EHB7585965.1	HetE-N1→ TPR+GreAB-C+PIN*→	TPR+GreAB-C+PIN	759	JV429_000621	Gammaproteobacteria	Escherichia coli	hypothetical protein JV429_000621, partial [Escherichia coli].	GCA_017003995.1
EHB7691780.1	TPR+GreAB-C+PIN*→	TPR+GreAB-C+PIN	745	JV466_004427	Gammaproteobacteria	Escherichia coli	hypothetical protein JV466_004427, partial [Escherichia coli].	GCA_017004675.1
EHC1615065.1	TPR+GreAB-C+PIN*→	TPR+GreAB-C+PIN	732	JT622_005238	Gammaproteobacteria	Escherichia coli	hypothetical protein JT622_005238, partial [Escherichia coli].	GCA_017029715.1
EHD4655635.1	HetE-N1→ TPR+GreAB-C+PIN*→	TPR+GreAB-C+PIN	807	JRB69_003203	Gammaproteobacteria	Salmonella enterica	hypothetical protein JRB69_003203, partial [Salmonella enterica].	GCA_017143955.1

acc	operon	architecture	len	gen.name	taxend	species	defline	gca
EHD4703897.1	GreAB-C*→	GreAB-C	419	JRB68_004676	Gammaproteobacteria	Salmonella enterica	hypothetical protein JRB68_004676, partial [Salmonella enterica].	GCA_017144295.1
EHD5811550.1	TPR+GreAB-C+PIN*→	TPR+GreAB-C+PIN	628	JQZ13_004415	Gammaproteobacteria	Escherichia coli	hypothetical protein JQZ13_004415, partial [Escherichia coli].	GCA_017158335.1
EHD6029608.1	HetE-N1→ TPR+GreAB-C+PIN*→	TPR+GreAB-C+PIN	1085	JQ325_002365	Gammaproteobacteria	Vibrio parahaemolyticus	hypothetical protein JQ325_002365 [Vibrio parahaemolyticus].	GCA_017160295.1
EHF0016324.1	?*→	?	690	IFX66_005031	Gammaproteobacteria	Escherichia coli	hypothetical protein IFX66_005031, partial [Escherichia coli].	GCA_017278275.1
EHG1523212.1	TPR+GreAB-C+PIN*→	TPR+GreAB-C+PIN	1001	J1L78_003684	Gammaproteobacteria	Salmonella enterica subsp. enterica serovar Corvallis	hypothetical protein J1L78_003684, partial [Salmonella enterica subsp. enterica serovar Corvallis].	GCA_017373035.1
EHG6154572.1	HetE-N1→ TPR+GreAB-C+PIN*→	TPR+GreAB-C+PIN	1094	J5T29_002362	Gammaproteobacteria	Escherichia fergusonii	hypothetical protein J5T29_002362 [Escherichia fergusonii].	GCA_017528445.1
EHG8446233.1	HetE-N1→ TPR+GreAB-C+PIN*→	TPR+GreAB-C+PIN	1106	J6D68_002379	Gammaproteobacteria	Salmonella enterica subsp. enterica	hypothetical protein J6D68_002379 [Salmonella enterica subsp. enterica].	GCA_017573985.1
EHH5222655.1	TPR+GreAB-C+PIN*→	TPR+GreAB-C+PIN	630	J6876_004216	Gammaproteobacteria	Escherichia coli	hypothetical protein J6876_004216, partial [Escherichia coli].	GCA_017685995.1
EHH5972888.1	TPR+GreAB-C+PIN*→	TPR+GreAB-C+PIN	1039	J8O14_004858	Gammaproteobacteria	Salmonella enterica subsp. enterica serovar Alachua	hypothetical protein J8O14_004858, partial [Salmonella enterica subsp. enterica serovar Alachua].	GCA_017690295.1
EHH6676921.1	HetE-N1→ TPR+GreAB-C+PIN*→	TPR+GreAB-C+PIN	1094	J6H48_001187	Gammaproteobacteria	Escherichia coli	hypothetical protein J6H48_001187 [Escherichia coli].	GCA_017723775.1
EHH7564006.1	TPR+GreAB-C+PIN*→	TPR+GreAB-C+PIN	1015	J5J30_004554	Gammaproteobacteria	Escherichia coli	hypothetical protein J5J30_004554, partial [Escherichia coli].	GCA_017727775.1
EHH7810630.1	GreAB-C*→	GreAB-C	318	J5B41_005221	Gammaproteobacteria	Escherichia coli	hypothetical protein J5B41_005221, partial [Escherichia coli].	GCA_017733975.1
EHI0937798.1	TPR+GreAB-C+PIN*→	TPR+GreAB-C+PIN	636	J1A00_004749	Gammaproteobacteria	Escherichia coli	hypothetical protein J1A00_004749, partial [Escherichia coli].	GCA_017780845.1
EHI2008363.1	TPR+GreAB-C+PIN*→	TPR+GreAB-C+PIN	694	J9U59_004724	Gammaproteobacteria	Salmonella enterica	hypothetical protein J9U59_004724, partial [Salmonella enterica].	GCA_017791685.1
EHI3384480.1	TPR+GreAB-C+PIN*→	TPR+GreAB-C+PIN	747	J9U04_004755	Gammaproteobacteria	Salmonella enterica	hypothetical protein J9U04_004755, partial [Salmonella enterica].	GCA_017798465.1
EHI3712656.1	TPR+GreAB-C+PIN*→	TPR+GreAB-C+PIN	723	J9T76_004561	Gammaproteobacteria	Salmonella enterica	hypothetical protein J9T76_004561, partial [Salmonella enterica].	GCA_017800075.1
EHI5184709.1	TPR+GreAB-C+PIN*→	TPR+GreAB-C+PIN	999	J9014_004542	Gammaproteobacteria	Salmonella enterica	hypothetical protein J9014_004542, partial [Salmonella enterica].	GCA_017806615.1
EHI6070991.1	HetE-N1→ TPR+GreAB-C+PIN*→	TPR+GreAB-C+PIN	984	J9J70_003931	Gammaproteobacteria	Salmonella enterica	hypothetical protein J9J70_003931, partial [Salmonella enterica].	GCA_017810795.1
EHI8244355.1	HetE-N1→ TPR+GreAB-C+PIN*→	TPR+GreAB-C+PIN	784	J9M94_004032	Gammaproteobacteria	Salmonella enterica	hypothetical protein J9M94_004032, partial [Salmonella enterica].	GCA_017828575.1
EHI9713257.1	TPR+GreAB-C+PIN*→	TPR+GreAB-C+PIN	735	J9T07_004736	Gammaproteobacteria	Salmonella enterica	hypothetical protein J9T07_004736, partial [Salmonella enterica].	GCA_017836875.1
EHJ6284301.1	<-HTH<-?<-PSE||HetE-N1→ TPR+GreAB-C+PIN*→	TPR+GreAB-C+PIN	1106	KAM53_001182	Gammaproteobacteria	Salmonella enterica	hypothetical protein KAM53_001182 [Salmonella enterica].	GCA_017922215.1
EHJ8153220.1	TPR+GreAB-C+PIN*→	TPR+GreAB-C+PIN	707	KB264_004740	Gammaproteobacteria	Escherichia coli	hypothetical protein KB264_004740, partial [Escherichia coli].	GCA_017943665.1
EHJ9993052.1	HetE-N1→ TPR+GreAB-C+PIN*→	TPR+GreAB-C+PIN	1085	KB972_002278	Gammaproteobacteria	Vibrio parahaemolyticus	hypothetical protein KB972_002278 [Vibrio parahaemolyticus].	GCA_017968025.1
EHK2882579.1	HetE-N1→ HetE-N1+TPR+GreAB-C+PIN*→	HetE-N1+TPR+GreAB-C+PIN	1091	J7H88_002175	Gammaproteobacteria	Vibrio parahaemolyticus	hypothetical protein J7H88_002175 [Vibrio parahaemolyticus].	GCA_018017275.1
EHL5831134.1	HetE-N1→ TPR+GreAB-C+PIN*→	TPR+GreAB-C+PIN	1117	KEB97_001561	Gammaproteobacteria	Salmonella enterica	hypothetical protein KEB97_001561 [Salmonella enterica].	GCA_018132225.1

<table>
<tr><th>acc</th><th>operon</th><th>architecture</th><th>len</th><th>gen.name</th><th>taxend</th><th>species</th><th>defline</th><th>gca</th></tr>
<tr><td>EKD28835.1</td><td>TPR+GreAB-C+PIN*→</td><td>TPR+GreAB-C+PIN</td><td>1282</td><td>ACD_79C00188G0006</td><td>environmental samples</td><td>uncultured bacterium</td><td>MAG: hypothetical protein ACD_79C00188G0006 [uncultured bacterium].</td><td>GCA_000299275.1</td></tr>
<tr><td>EKM26807.1</td><td>HetE-N1→ TPR+GreAB-C+PIN*→</td><td>TPR+GreAB-C+PIN</td><td>1091</td><td>VCHENC03_4410</td><td>Gammaproteobacteria</td><td>Vibrio sp. HENC-03</td><td>hypothetical protein VCHENC03_4410 [Vibrio sp. HENC-03].</td><td>GCA_000305755.2</td></tr>
<tr><td>ENE18425.1</td><td>GreAB-C*→</td><td>GreAB-C</td><td>334</td><td>ECP03047993_5652</td><td>Gammaproteobacteria</td><td>Escherichia coli P0304799.3</td><td>hypothetical protein ECP03047993_5652, partial [Escherichia coli P0304799.3].</td><td>GCA_000357685.2</td></tr>
<tr><td>ENV19736.1</td><td>HetE-N1→?→?→ GreAB-C+PIN*→</td><td>GreAB-C+PIN</td><td>553</td><td>F963_04374</td><td>Gammaproteobacteria</td><td>Acinetobacter bereziniae NIPH 3</td><td>hypothetical protein F963_04374 [Acinetobacter bereziniae NIPH 3].</td><td>GCA_000368505.1</td></tr>
<tr><td>EOS47428.1</td><td>HNH→?→?→ TPR+GreAB-C+PIN*→</td><td>TPR+GreAB-C+PIN</td><td>1071</td><td>C809_02304</td><td>Firmicutes</td><td>Lachnospiraceae bacterium MD335</td><td>hypothetical protein C809_02304 [Lachnospiraceae bacterium MD335].</td><td>GCA_000403335.2</td></tr>
<tr><td>ERS81691.1</td><td>TPR+GreAB-C+PIN*→</td><td>TPR+GreAB-C+PIN</td><td>480</td><td>Q672_10295</td><td>Gammaproteobacteria</td><td>Marinobacter sp. EVN1</td><td>hypothetical protein Q672_10295 [Marinobacter sp. EVN1].</td><td>GCA_000475375.1</td></tr>
<tr><td>ERU60319.1</td><td>DOC+HTH→ REase+TOPC→?→ HetE-N1→ HetE-N1+TPR+GreAB-C+PIN*→</td><td>HetE-N1+TPR+GreAB-C+PIN</td><td>1126</td><td>Q088_04776</td><td>Gammaproteobacteria</td><td>Pseudomonas aeruginosa C41</td><td>hypothetical protein Q088_04776 [Pseudomonas aeruginosa C41].</td><td>GCA_000480455.1</td></tr>
<tr><td>ETK41841.1</td><td>REase+TPR+GreAB-C+PIN*→</td><td>REase+TPR+GreAB-C+PIN</td><td>1486</td><td>H098_09645</td><td>Gammaproteobacteria</td><td>Pseudomonas fluorescens FH5</td><td>hypothetical protein H098_09645 [Pseudomonas fluorescens FH5].</td><td>GCA_000511155.2</td></tr>
<tr><td>EWS54147.1</td><td>REase+TPR→ TPR+GreAB-C+PIN*→</td><td>TPR+GreAB-C+PIN</td><td>1054</td><td>X551_03051</td><td>Betaproteobacteria</td><td>Methylibium sp. T29</td><td>hypothetical protein X551_03051 [Methylibium sp. T29].</td><td>GCA_000576205.1</td></tr>
<tr><td>EXI84113.1</td><td>REase+TPR+GreAB-C+PIN*→</td><td>REase+TPR+GreAB-C+PIN</td><td>1363</td><td>AW11_03978</td><td>Betaproteobacteria</td><td>Candidatus Accumulibacter sp. BA-93</td><td>MAG: hypothetical protein AW11_03978 [Candidatus Accumulibacter sp. BA-93].</td><td>GCA_000585075.1</td></tr>
<tr><td>EXU80592.1</td><td>REase+TPR+GreAB-C+PIN*→</td><td>REase+TPR+GreAB-C+PIN</td><td>1391</td><td>AX13_15135</td><td>Betaproteobacteria</td><td>Comamonas aquatica DA1877</td><td>hypothetical protein AX13_15135 [Comamonas aquatica DA1877].</td><td>GCA_000590985.1</td></tr>
<tr><td>GAC55419.1</td><td>TPR+PIN*→</td><td>TPR+PIN</td><td>1183</td><td>GOAMI_53_00020</td><td>Actinobacteria</td><td>Gordonia amicalis NBRC 100051 = JCM 11271</td><td>hypothetical protein GOAMI_53_00020 [Gordonia amicalis NBRC 100051 = JCM 11271].</td><td>GCA_000332995.1</td></tr>
<tr><td>GAF05793.1</td><td>TPR+GreAB-C+PIN*→</td><td>TPR+GreAB-C+PIN</td><td>524</td><td>JCM21142_104546</td><td>Bacteroidetes</td><td>Saccharicrinis fermentans DSM 9555 = JCM 21142</td><td>hypothetical protein JCM21142_104546 [Saccharicrinis fermentans DSM 9555 = JCM 21142].</td><td>GCA_000583675.1</td></tr>
<tr><td>GBF59066.1</td><td>REase+TPR+GreAB-C+PIN*→</td><td>REase+TPR+GreAB-C+PIN</td><td>1327</td><td>bepA_7</td><td>Alphaproteobacteria</td><td>Candidatus Phycosocius bacilliformis</td><td>beta-barrel assembly-enhancing protease [Candidatus Phycosocius bacilliformis].</td><td>GCA_003112735.1</td></tr>
<tr><td>GBH08795.1</td><td>HetE-N1→||||<-?||HetE-N1+TPR+GreAB-C+PIN→||||<-?<-?||TPR+GreAB-C+PIN*→</td><td>TPR+GreAB-C+PIN</td><td>953</td><td>KPSA1_02176</td><td>Gammaproteobacteria</td><td>Pseudomonas syringae pv. actinidiae</td><td>ATP-dependent exoDNAse beta subunit [Pseudomonas syringae pv. actinidiae].</td><td>GCA_003202975.1</td></tr>
<tr><td>GBH17945.1</td><td>REase+TPR+GreAB-C+PIN*→?→ REase+nSTAND3→ REase+nSTAND3→</td><td>REase+TPR+GreAB-C+PIN</td><td>1491</td><td>KPSA3_03922</td><td>Gammaproteobacteria</td><td>Pseudomonas syringae pv. actinidiae</td><td>Tetratricopeptide repeat [Pseudomonas syringae pv. actinidiae].</td><td>GCA_003202995.1</td></tr>
<tr><td>GBU16512.1</td><td>TPR+GreAB-C+PIN*→</td><td>TPR+GreAB-C+PIN</td><td>1107</td><td>AwMethylo_07270</td><td>Alphaproteobacteria</td><td>Methylobacterium sp.</td><td>hypothetical protein AwMethylo_07270 [Methylobacterium sp.].</td><td>GCA_003864615.1</td></tr>
<tr><td>GCL65539.1</td><td>REase+TPR+GreAB-C+PIN*→</td><td>REase+TPR+GreAB-C+PIN</td><td>1455</td><td>AQPW35_46200</td><td>Betaproteobacteria</td><td>Aquabacterium pictum</td><td>hypothetical protein AQPW35_46200 [Aquabacterium pictum].</td><td>GCA_005403045.1</td></tr>
<tr><td>GCM29814.1</td><td>TPR+GreAB-C+PIN*→</td><td>TPR+GreAB-C+PIN</td><td>622</td><td>ExPECSC050_01644</td><td>Gammaproteobacteria</td><td>Escherichia coli</td><td>hypothetical protein ExPECSC050_01644 [Escherichia coli].</td><td>GCA_005383045.1</td></tr>
<tr><td>GFJ77328.1</td><td>TM+TPR+PIN*→ REase→</td><td>TM+TPR+PIN</td><td>1362</td><td>Phou_015080</td><td>Actinobacteria</td><td>Phytohabitans houttuyneae</td><td>hypothetical protein Phou_015080 [Phytohabitans houttuyneae].</td><td>GCA_011764425.1</td></tr>
<tr><td>GGJ71040.1</td><td>TPR+PIN*→</td><td>TPR+PIN</td><td>1223</td><td>GCM10012282_79870</td><td>Actinobacteria</td><td>Streptomyces lacrimifluminis</td><td>hypothetical protein GCM10012282_79870 [Streptomyces lacrimifluminis].</td><td>GCA_014646095.1</td></tr>
<tr><td>GGK48292.1</td><td>HetE-N1→ TPR+GreAB-C+PIN*→</td><td>TPR+GreAB-C+PIN</td><td>1120</td><td>GCM10009103_48460</td><td>Gammaproteobacteria</td><td>Pseudomonas koreensis</td><td>hypothetical protein GCM10009103_48460 [Pseudomonas koreensis].</td><td>GCA_014646955.1</td></tr>
<tr><td>GGR17482.1</td><td>REase+TPR+GreAB-C+PIN*→</td><td>REase+TPR+GreAB-C+PIN</td><td>1357</td><td>GCM10008957_32760</td><td>Deinococci</td><td>Deinococcus ruber</td><td>hypothetical protein GCM10008957_32760 [Deinococcus ruber].</td><td>GCA_014648095.1</td></tr>
<tr><td>GGZ02836.1</td><td>HetE-N1→?→ GreAB-C*→</td><td>GreAB-C</td><td>451</td><td>GCM10007169_19550</td><td>Gammaproteobacteria</td><td>Shewanella fodinae</td><td>hypothetical protein GCM10007169_19550 [Shewanella fodinae].</td><td>GCA_014651955.1</td></tr>
<tr><td>GHB08256.1</td><td>HetE-N1→ TPR+GreAB-C+PIN*→</td><td>TPR+GreAB-C+PIN</td><td>1025</td><td>GCM10007107_21780</td><td>Gammaproteobacteria</td><td>Shewanella indica</td><td>hypothetical protein GCM10007107_21780 [Shewanella indica].</td><td>GCA_014652115.1</td></tr>
</table>

acc	operon	architecture	len	gen.name	taxend	species	defline	gca
GIE51569.1	TPR+PIN→?→?→ PIN*→||||<-?<-?||AbiJ-NTD3+REase→	PIN	177	Ani05nite_51030	Actinobacteria	Actinoplanes nipponensis	hypothetical protein Ani05nite_51030 [Actinoplanes nipponensis].	GCA_016862275.1
GIG67348.1	TM+TPR+PIN*→	TM+TPR+PIN	1072	Pen01_36430	Actinobacteria	Phytomonospora endophytica	hypothetical protein Pen01_36430 [Phytomonospora endophytica].	GCA_016862855.1
GIJ10564.1	REase+TPR+GreAB-C+PIN*→	REase+TPR+GreAB-C+PIN	1377	Van01_37780	Actinobacteria	Micromonospora andamanensis	hypothetical protein Van01_37780 [Micromonospora andamanensis].	GCA_016863495.1
GIM80122.1	REase+TPR+GreAB-C+PIN*→	REase+TPR+GreAB-C+PIN	1253	Aau02nite_89080	Actinobacteria	Actinoplanes auranticolor	hypothetical protein Aau02nite_89080 [Actinoplanes auranticolor].	GCA_018332655.1
GIM97804.1	TPR+PIN*→	TPR+PIN	1137	Ato02nite_095970	Actinobacteria	Actinoplanes toevensis	hypothetical protein Ato02nite_095970 [Actinoplanes toevensis].	GCA_018332695.1
HAF5476147.1	HetE-N1→ TPR+GreAB-C+PIN*→	TPR+GreAB-C+PIN	1117	G8A91_001303	Gammaproteobacteria	Salmonella enterica	TPA: hypothetical protein G8A91_001303 [Salmonella enterica].	GCA_011304215.1
HAH9826030.1	HetE-N1→ TPR+GreAB-C+PIN*→	TPR+GreAB-C+PIN	986	HIF24_22945	Gammaproteobacteria	Escherichia coli	TPA: hypothetical protein HIF24_22945, partial [Escherichia coli].	GCA_012753875.1
HAK4911912.1	HetE-N1→ TPR+GreAB-C+PIN*→	TPR+GreAB-C+PIN	1117	H2B38_003821	Gammaproteobacteria	Salmonella enterica	TPA: hypothetical protein H2B38_003821 [Salmonella enterica].	GCA_013572415.1
HAK9873068.1	TPR+GreAB-C+PIN*→	TPR+GreAB-C+PIN	1028	H3L74_004566	Gammaproteobacteria	Escherichia coli	TPA: hypothetical protein H3L74_004566, partial [Escherichia coli].	GCA_013794255.1
HAL9130497.1	HetE-N1→ TPR+GreAB-C+PIN*→	TPR+GreAB-C+PIN	1094	H7U58_003240	Gammaproteobacteria	Escherichia coli	TPA: hypothetical protein H7U58_003240 [Escherichia coli].	GCA_014184975.1
HAL9453557.1	GreAB-C*→	GreAB-C	337	H7V29_005028	Gammaproteobacteria	Escherichia coli	TPA: hypothetical protein H7V29_005028, partial [Escherichia coli].	GCA_014186535.1
HAM4053647.1	HetE-N1→ TPR+GreAB-C+PIN*→	TPR+GreAB-C+PIN	1094	IBV36_002404	Gammaproteobacteria	Escherichia coli	TPA: hypothetical protein IBV36_002404 [Escherichia coli].	GCA_014461175.1
HAM8943229.1	HetE-N1→ TPR+GreAB-C+PIN*→	TPR+GreAB-C+PIN	618	IDX96_004717	Gammaproteobacteria	Escherichia coli	TPA: hypothetical protein IDX96_004717, partial [Escherichia coli].	GCA_014571125.1
HAM8943373.1	TPR+GreAB-C+PIN*→	TPR+GreAB-C+PIN	474	IDX96_004882	Gammaproteobacteria	Escherichia coli	TPA: hypothetical protein IDX96_004882, partial [Escherichia coli].	GCA_014571125.1
HAM8966916.1	HetE-N1→ TPR+GreAB-C+PIN*→	TPR+GreAB-C+PIN	1094	IDX85_003830	Gammaproteobacteria	Escherichia coli	TPA: hypothetical protein IDX85_003830 [Escherichia coli].	GCA_014573095.1
HAM8991159.1	HetE-N1→ TPR+GreAB-C+PIN*→	TPR+GreAB-C+PIN	1094	IDX80_003853	Gammaproteobacteria	Escherichia coli	TPA: hypothetical protein IDX80_003853 [Escherichia coli].	GCA_014573135.1
HAN3697638.1	TPR+GreAB-C+PIN*→	TPR+GreAB-C+PIN	1050	IE973_004718	Gammaproteobacteria	Escherichia coli	TPA: hypothetical protein IE973_004718, partial [Escherichia coli].	GCA_014687295.1
HAN4834938.1	HetE-N1→ TPR+GreAB-C+PIN*→	TPR+GreAB-C+PIN	1094	IFD90_003495	Gammaproteobacteria	Escherichia coli	TPA: hypothetical protein IFD90_003495 [Escherichia coli].	GCA_014683685.1
HAN6118733.1	HetE-N1→ TPR+GreAB-C+PIN*→	TPR+GreAB-C+PIN	1094	IFZ61_002838	Gammaproteobacteria	Escherichia coli	TPA: hypothetical protein IFZ61_002838 [Escherichia coli].	GCA_014708655.1
HAN7634134.1	GreAB-C*→	GreAB-C	447	IF647_005249	Gammaproteobacteria	Escherichia coli	TPA: hypothetical protein IF647_005249, partial [Escherichia coli].	GCA_014724455.1
HAO1938999.1	HetE-N1→ TPR+GreAB-C+PIN*→	TPR+GreAB-C+PIN	1094	IHJ42_000411	Gammaproteobacteria	Escherichia coli	TPA: hypothetical protein IHJ42_000411 [Escherichia coli].	GCA_014815885.1
HAS1413650.1	TPR+GreAB-C+PIN*→	TPR+GreAB-C+PIN	845	I4D28_09180	Gammaproteobacteria	Enterobacter hormaechei	TPA: hypothetical protein I4D28_09180 [Enterobacter hormaechei].	-
HAT2286137.1	HetE-N1→ TPR+GreAB-C+PIN+TM+TM*→	TPR+GreAB-C+PIN+TM+TM	1106	I8156_002891	Gammaproteobacteria	Citrobacter freundii	TPA: hypothetical protein I8156_002891 [Citrobacter freundii].	GCA_015897485.1
HAT3687084.1	HetE-N1→ TPR+GreAB-C+PIN*→	TPR+GreAB-C+PIN	958	I8574_002665	Gammaproteobacteria	Citrobacter freundii	TPA: hypothetical protein I8574_002665, partial [Citrobacter freundii].	GCA_015902705.1
HAT3994325.1	TPR+GreAB-C+PIN*→	TPR+GreAB-C+PIN	1131	I9Y74_003743	Bacteroidetes	Elizabethkingia anophelis	TPA: hypothetical protein I9Y74_003743 [Elizabethkingia anophelis].	GCA_015930145.1
HAT8488811.1	HetE-N1→ HetE-N1+TPR+GreAB-C+PIN*→	HetE-N1+TPR+GreAB-C+PIN	1088	I7187_21710	Gammaproteobacteria	Vibrio vulnificus	TPA: hypothetical protein I7187_21710 [Vibrio vulnificus].	GCA_015968635.1

acc	operon	architecture	len	gen.name	taxend	species	defline	gca
HAU8300026.1	HetE-N1→ TPR+GreAB-C+PIN*→	TPR+GreAB-C+PIN	1130	I7159_21955	Gammaproteobacteria	Vibrio vulnificus	TPA: hypothetical protein I7159_21955 [Vibrio vulnificus].	GCA_016110185.1
HAU9261412.1	HetE-N1→ TPR+GreAB-C+PIN*→	TPR+GreAB-C+PIN	1094	JEX66_004253	Gammaproteobacteria	Escherichia coli	TPA: hypothetical protein JEX66_004253 [Escherichia coli].	GCA_016116905.1
HAV13598.1	GreAB*→	GreAB	620	DCX06_08945	Verrucomicrobia	Opitutae bacterium	MAG TPA: transcription elongation factor GreAB [Opitutae bacterium].	GCA_003485085.1
HAV1378302.1	HetE-N1→ NACHT+GreAB-C+PIN*→	NACHT+GreAB-C+PIN	1085	JG804_003376	Gammaproteobacteria	Vibrio parahaemolyticus	TPA: hypothetical protein JG804_003376 [Vibrio parahaemolyticus].	GCA_016429865.1
HAV1873259.1	TPR+GreAB-C+PIN*→	TPR+GreAB-C+PIN	742	JG951_004564	Gammaproteobacteria	Enterobacter hormaechei subsp. steigerwaltii	TPA: hypothetical protein JG951_004564, partial [Enterobacter hormaechei subsp. steigerwaltii].	GCA_016431145.1
HAV7695504.1	HetE-N1→ TPR+GreAB-C+PIN*→	TPR+GreAB-C+PIN	1094	JK751_001166	Gammaproteobacteria	Escherichia coli	TPA: hypothetical protein JK751_001166 [Escherichia coli].	GCA_016656125.1
HAV9376548.1	TPR+GreAB-C+PIN*→	TPR+GreAB-C+PIN	575	JLF84_000973	Gammaproteobacteria	Escherichia coli	TPA: hypothetical protein JLF84_000973 [Escherichia coli].	GCA_016665225.1
HAW2926691.1	HetE-N1→ TPR+GreAB-C+PIN*→	TPR+GreAB-C+PIN	1094	JLR28_003506	Gammaproteobacteria	Escherichia coli	TPA: hypothetical protein JLR28_003506 [Escherichia coli].	GCA_016681635.1
HAW5283885.1	HetE-N1→ TPR+GreAB-C+PIN*→	TPR+GreAB-C+PIN	1094	JLY51_001282	Gammaproteobacteria	Escherichia coli	TPA: hypothetical protein JLY51_001282 [Escherichia coli].	GCA_016693495.1
HAW6997627.1	REase+TPR+GreAB-C+PIN*→	REase+TPR+GreAB-C+PIN	1250	JIR16_12805	Gammaproteobacteria	Acinetobacter baumannii	TPA: hypothetical protein JIR16_12805 [Acinetobacter baumannii].	GCA_016844125.1
HAY34439.1	TPR+GreAB-C+PIN*→	TPR+GreAB-C+PIN	1108	DCY06_09910	Bacteroidetes	Bacteroidetes bacterium	MAG TPA: hypothetical protein DCY06_09910 [Bacteroidetes bacterium].	GCA_003486905.1
HBA3776861.1	TPR+GreAB-C+PIN*→	TPR+GreAB-C+PIN	1117	J5F89_004850	Gammaproteobacteria	Escherichia coli	TPA: hypothetical protein J5F89_004850 [Escherichia coli].	GCA_017730375.1
HBC0128974.1	TPR+GreAB-C+PIN*→	TPR+GreAB-C+PIN	661	JES91_004961	Gammaproteobacteria	Salmonella enterica subsp. enterica	TPA: hypothetical protein JES91_004961, partial [Salmonella enterica subsp. enterica].	GCA_018032925.1
HBC3987585.1	HetE-N1→ TPR+GreAB-C+PIN*→	TPR+GreAB-C+PIN	1130	KD867_004179	Gammaproteobacteria	Vibrio parahaemolyticus	TPA: hypothetical protein KD867_004179 [Vibrio parahaemolyticus].	GCA_018126825.1
HBJ33554.1	TPR+GreAB-C+PIN*→	TPR+GreAB-C+PIN	857	DDZ51_02100	Planctomycetes	Planctomycetaceae bacterium	MAG TPA: hypothetical protein DDZ51_02100, partial [Planctomycetaceae bacterium].	GCA_003506095.1
HBX77020.1	X+PIN*→	X+PIN	940	DEG43_05200	Actinobacteria	Acidimicrobiaceae bacterium	MAG TPA: hypothetical protein DEG43_05200 [Acidimicrobiaceae bacterium].	GCA_003514205.1
HCD05045.1	HetE-N1→?→ TPR+GreAB-C+PIN*→||||<-REase+UvrD	TPR+GreAB-C+PIN	841	DEQ60_06940	Gammaproteobacteria	Methylophaga sp.	MAG TPA: hypothetical protein DEQ60_06940, partial [Methylophaga sp.].	GCA_003517125.1
HCG03150.1	REase+TPR+GreAB-C+PIN*→	REase+TPR+GreAB-C+PIN	1325	DEV93_21745	Chloroflexi	Chloroflexi bacterium	MAG TPA: hypothetical protein DEV93_21745 [Chloroflexi bacterium].	GCA_003530725.1
HCN10799.1	HTH→?→?→?→?→?→ TPR+GreAB-C+PIN*→	TPR+GreAB-C+PIN	1128	DIS75_00410	Bacteroidetes	Chryseobacterium sp.	MAG TPA: hypothetical protein DIS75_00410 [Chryseobacterium sp.].	GCA_003536155.1
HEE61010.1	GreAB*→	GreAB	217	ENP71_13075	Chloroflexi	Chloroflexi bacterium	MAG TPA: transcription elongation factor GreA [Chloroflexi bacterium].	GCA_011051445.1
HEN27530.1	PIN*→	PIN	194	ENQ77_02470	Bacteria	candidate division WOR-3 bacterium	MAG TPA: RNA ligase partner protein [candidate division WOR-3 bacterium].	GCA_011046955.1
HFX16357.1	REase+TPR+GreAB-C+PIN*→	REase+TPR+GreAB-C+PIN	1269	ENW10_10035	unclassified Bacteria	bacterium	MAG TPA: hypothetical protein ENW10_10035 [bacterium].	GCA_011334465.1
HGB35656.1	PIN*→	PIN	194	ENV38_01955	Bacteria	candidate division WOR-3 bacterium	MAG TPA: RNA ligase partner protein [candidate division WOR-3 bacterium].	GCA_011338235.1
HGZ37950.1	Calcineurin+TPR+GreAB-C+PIN*→	Calcineurin+TPR+GreAB-C+PIN	1423	ENR19_05950	Nitrospirae	Nitrospirae bacterium	MAG TPA: hypothetical protein ENR19_05950, partial [Nitrospirae bacterium].	GCA_011331925.1
HHC78382.1	TPR+GreAB-C+PIN*→	TPR+GreAB-C+PIN	1134	ENK46_00760	Bacteroidetes	Flavobacteriia bacterium	MAG TPA: hypothetical protein ENK46_00760 [Flavobacteriia bacterium].	GCA_011330545.1
HIB63801.1	TPR+GreAB-C+PIN*→	TPR+GreAB-C+PIN	1486	EYO33_01435	Planctomycetes	Phycisphaerales bacterium	MAG TPA: hypothetical protein EYO33_01435 [Phycisphaerales bacterium].	GCA_012964585.1

acc	operon	architecture	len	gen.name	taxend	species	defline	gca
HIP26815.1	TPR+GreAB-C+PIN*→	TPR+GreAB-C+PIN	1038	EYG80_04080	Bacteroidetes	Flavobacteriaceae bacterium	MAG TPA: tetratricopeptide repeat protein [Flavobacteriaceae bacterium].	GCA_015662995.1
KAA0086698.1	TPR+PIN*→	TPR+PIN	1159	CIW52_01945	Actinobacteria	Mycolicibacterium sp. P9-64	hypothetical protein CIW52_01945 [Mycolicibacterium sp. P9-64].	GCA_008329605.1
KAB1070169.1	TPR+GreAB-C+PIN*→	TPR+GreAB-C+PIN	641	F6X53_30340	Alphaproteobacteria	Methylobacterium soli	tetratricopeptide repeat protein [Methylobacterium soli].	GCA_008806385.1
KCZ93824.1	TPR+GreAB-C+PIN*→	TPR+GreAB-C+PIN	1142	HJO_00570	Alphaproteobacteria	Hyphomonas johnsonii MHS-2	hypothetical protein HJO_00570 [Hyphomonas johnsonii MHS-2].	GCA_000685275.1
KGF68042.1	REase+TPR+GreAB-C+PIN*→	REase+TPR+GreAB-C+PIN	1283	LL06_18825	Alphaproteobacteria	Hoeflea sp. BAL378	hypothetical protein LL06_18825 [Hoeflea sp. BAL378].	GCA_000759435.1
KGM47444.1	REase+TPR+GreAB-C+PIN*→	REase+TPR+GreAB-C+PIN	1329	ATO9_17580	Alphaproteobacteria	Pseudooceanicola atlanticus	hypothetical protein ATO9_17580 [Pseudooceanicola atlanticus].	GCA_000768315.1
KGS12485.1	GreAB-C+PIN*→	GreAB-C+PIN	509	OA77_21590	Gammaproteobacteria	Pseudomonas coronafaciens	hypothetical protein OA77_21590, partial [Pseudomonas coronafaciens].	GCA_000773135.1
KHO23731.1	X+PIN*→	X+PIN	762	QQ44_16085	Actinobacteria	Mycolicibacterium setense	hypothetical protein QQ44_16085 [Mycolicibacterium setense].	GCA_000805375.1
KIF65994.1	REase+TPR+TPR+GreAB-C+PIN*→	REase+TPR+TPR+GreAB-C+PIN	1274	HY68_38205	Actinobacteria	Streptomyces sp. AcH 505	hypothetical protein HY68_38205 [Streptomyces sp. AcH 505].	GCA_000818175.1
KIU53353.1	REase+TPR+TPR+GreAB-C+PIN*→	REase+TPR+TPR+GreAB-C+PIN	1254	QU41_01355	Alphaproteobacteria	Bradyrhizobium elkanii	hypothetical protein QU41_01355 [Bradyrhizobium elkanii].	GCA_000878305.1
KJS28984.1	TPR+GreAB-C+PIN*→	TPR+GreAB-C+PIN	1318	VR64_22320	Deltaproteobacteria	Desulfatitalea sp. BRH_c12	MAG: hypothetical protein VR64_22320 [Desulfatitalea sp. BRH_c12].	GCA_000961655.1
KOY46643.1	HetE-N1→ TPR+GreAB-C+PIN*→	TPR+GreAB-C+PIN	1085	ACX03_05530	Gammaproteobacteria	Vibrio parahaemolyticus	hypothetical protein ACX03_05530 [Vibrio parahaemolyticus].	GCA_001280625.1
KPC32725.1	PIN*→	PIN	407	ABJ99_1577	Gammaproteobacteria	Pseudomonas syringae pv. cilantro	Tetratricopeptide repeat protein [Pseudomonas syringae pv. cilantro].	GCA_001293775.1
KPH04186.1	GreAB-C+PIN*→	GreAB-C+PIN	532	AOG23_34735	Alphaproteobacteria	Rhizobium acidisoli	hypothetical protein AOG23_34735 [Rhizobium acidisoli].	GCA_001297245.1
KPW31206.1	REase+TPR+GreAB-C+PIN*→	REase+TPR+GreAB-C+PIN	1491	ALO87_03437	Gammaproteobacteria	Pseudomonas syringae pv. apii	Uncharacterized protein ALO87_03437 [Pseudomonas syringae pv. apii].	GCA_001401385.1
KPW71831.1	PIN*→	PIN	419	ALO76_102575	Gammaproteobacteria	Pseudomonas syringae pv. coriandricola	hypothetical protein ALO76_102575 [Pseudomonas syringae pv. coriandricola].	GCA_001400185.1
KPX07181.1	REase→ TPR+GreAB-C+PIN*→	TPR+GreAB-C+PIN	882	ALO74_05417	Gammaproteobacteria	Pseudomonas syringae pv. cunninghamiae	Uncharacterized protein ALO74_05417, partial [Pseudomonas syringae pv. cunninghamiae].	GCA_001400795.1
KQR06792.1	TPR+PIN*→	TPR+PIN	1157	ASF74_05425	Actinobacteria	Arthrobacter sp. Leaf145	hypothetical protein ASF74_05425 [Arthrobacter sp. Leaf145].	GCA_001423565.1
KQY47975.1	HTH+PNPase+TPR+GreAB-C+PIN*→	HTH+PNPase+TPR+GreAB-C+PIN	1492	ASD18_12170	Actinobacteria	Cellulomonas sp. Root137	hypothetical protein ASD18_12170 [Cellulomonas sp. Root137].	GCA_001426725.1
KRK09263.1	X+PIN*→	X+PIN	1209	FD11_GL001067	Firmicutes	Lactobacillus pobuzihii E100301 = KCTC 13174	hypothetical protein FD11_GL001067 [Lactobacillus pobuzihii E100301 = KCTC 13174].	GCA_001433815.1
KSV64106.1	REase+TPR+TPR+GreAB-C+PIN*→	REase+TPR+TPR+GreAB-C+PIN	1352	N182_36590	Alphaproteobacteria	Sinorhizobium sp. GL2	hypothetical protein N182_36590 [Sinorhizobium sp. GL2].	GCA_001461715.1
KTR52035.1	PIN*→	PIN	473	NS359_08065	Actinobacteria	Curtobacterium oceanosedimentum	hypothetical protein NS359_08065 [Curtobacterium oceanosedimentum].	GCA_001476135.1
KVD10066.1	REase+TPR+GreAB-C+PIN*→	REase+TPR+GreAB-C+PIN	1276	WI81_28275	Betaproteobacteria	Burkholderia ubonensis	hypothetical protein WI81_28275 [Burkholderia ubonensis].	GCA_001523425.1
KVK86408.1	REase+TPR+GreAB-C+PIN*→	REase+TPR+GreAB-C+PIN	1305	WJ44_35230	Betaproteobacteria	Burkholderia ubonensis	hypothetical protein WJ44_35230 [Burkholderia ubonensis].	GCA_001526225.1
KVP54755.1	PIN*→	PIN	270	WJ90_06090	Betaproteobacteria	Burkholderia ubonensis	hypothetical protein WJ90_06090 [Burkholderia ubonensis].	GCA_001527145.1
KVR94766.1	REase+TPR+GreAB-C+PIN*→	REase+TPR+GreAB-C+PIN	1283	WK29_06535	Betaproteobacteria	Burkholderia vietnamiensis	hypothetical protein WK29_06535 [Burkholderia vietnamiensis].	GCA_001528565.1

acc	operon	architecture	len	gen.name	taxend	species	defline	gca
KVS41438.1	REase+TPR+GreAB-C+PIN*→	REase+TPR+GreAB-C+PIN	1273	WK37_19925	Betaproteobacteria	Burkholderia ubonensis	hypothetical protein WK37_19925 [Burkholderia ubonensis].	GCA_001529725.1
KWO74502.1	REase+TPR+GreAB-C+PIN*→	REase+TPR+GreAB-C+PIN	1308	WM31_08005	Betaproteobacteria	Burkholderia ubonensis	hypothetical protein WM31_08005 [Burkholderia ubonensis].	GCA_001534425.1
KWW10220.1	HetE-N1→ TPR+GreAB-C+PIN→ TPR+GreAB-C+PIN*→	TPR+GreAB-C+PIN	695	VM82_06380	Gammaproteobacteria	Pasteurella multocida	hypothetical protein VM82_06380 [Pasteurella multocida].	GCA_001542975.1
KXC04457.1	X+PIN*→?→ Calcineurin→	X+PIN	1058	MhomT_16215	Actinobacteria	Microbacterium hominis	hypothetical protein MhomT_16215 [Microbacterium hominis].	GCA_001553805.1
KXF54879.1	HTH+PNPase+TPR+GreAB-C+PIN*→	HTH+PNPase+TPR+GreAB-C+PIN	1496	AXA44_39745	Actinobacteria	Rhodococcus sp. SC4	hypothetical protein AXA44_39745 [Rhodococcus sp. SC4].	GCA_001555475.1
KYK33744.1	TPR+GreAB-C+PIN*→	TPR+GreAB-C+PIN	1243	AYK18_14685	Euryarchaeota	Theionarchaea archaeon DG-70	MAG: hypothetical protein AYK18_14685 [Theionarchaea archaeon DG-70].	GCA_001595795.1
KZM34260.1	X+PIN*→	X+PIN	802	OJAG_30920	Actinobacteria	Oerskovia enterophila	hypothetical protein OJAG_30920 [Oerskovia enterophila].	GCA_001624335.1
KZM57325.1	TPR+PIN*→	TPR+PIN	1172	A3Q36_16940	Firmicutes	Geobacillus stearothermophilus	hypothetical protein A3Q36_16940 [Geobacillus stearothermophilus].	GCA_001624615.1
MAK67575.1	HetE-N1→ TPR+GreAB-C+PIN*→||||<-REase+UvrD	TPR+GreAB-C+PIN	1111	CMH19_11360	Gammaproteobacteria	Methylophaga sp.	MAG: hypothetical protein CMH19_11360 [Methylophaga sp.].	GCA_002692615.1
MAK82682.1	REase+TPR+GreAB-C+PIN*→	REase+TPR+GreAB-C+PIN	1322	CMJ17_12550	Alphaproteobacteria	Phenylobacterium sp.	MAG: hypothetical protein CMJ17_12550 [Phenylobacterium sp.].	GCA_002693985.1
MAM61427.1	REase+TOPC→||||<-?||REase+TPR+GreAB-C+PIN*→	REase+TPR+GreAB-C+PIN	1313	CMH11_08050	Alphaproteobacteria	Maritimibacter sp.	MAG: hypothetical protein CMH11_08050 [Maritimibacter sp.].	GCA_002695005.1
MAQ47157.1	TPR+GreAB-C+PIN*→	TPR+GreAB-C+PIN	928	CL812_15115	Alphaproteobacteria	Actibacterium sp.	MAG: hypothetical protein CL812_15115 [Actibacterium sp.].	GCA_002701285.1
MAX35781.1	REase+TPR+GreAB-C+PIN*→	REase+TPR+GreAB-C+PIN	1254	CME33_04335	Planctomycetes	Gimesia sp.	MAG: hypothetical protein CME33_04335 [Gimesia sp.].	GCA_002706765.1
MAX78311.1	REase+TPR+GreAB-C+PIN*→	REase+TPR+GreAB-C+PIN	1318	CMN12_16605	Alphaproteobacteria	Roseobacter sp.	MAG: hypothetical protein CMN12_16605 [Roseobacter sp.].	GCA_002706785.1
MBA2669970.1	TPR+GreAB-C+PIN*→	TPR+GreAB-C+PIN	581	H0U67_06335	Gemmatimonadetes	Gemmatimonadetes bacterium	MAG: hypothetical protein H0U67_06335 [Gemmatimonadetes bacterium].	GCA_013697765.1
MBA3005285.1	TPR+GreAB-C+PIN*→	TPR+GreAB-C+PIN	1285	FP810_01590	Deltaproteobacteria	Desulfocapsa sp.	MAG: hypothetical protein FP810_01590 [Desulfocapsa sp.].	GCA_013792135.1
MBA3178888.1	HetE-N1→ TPR+GreAB-C+PIN*→?→||||<-Trypsin	TPR+GreAB-C+PIN	1121	CBX59_021210	Gammaproteobacteria	Salmonella enterica	hypothetical protein CBX59_021210 [Salmonella enterica].	GCA_006112745.2
MBA3341438.1	REase+TPR+GreAB-C+PIN*→	REase+TPR+GreAB-C+PIN	1397	H0T48_06340	Gemmatimonadetes	Gemmatimonadaceae bacterium	MAG: hypothetical protein H0T48_06340 [Gemmatimonadaceae bacterium].	GCA_013813715.1
MBA3557151.1	TPR+GreAB-C+PIN*→	TPR+GreAB-C+PIN	877	H0W30_00995	Gemmatimonadetes	Gemmatimonadaceae bacterium	MAG: hypothetical protein H0W30_00995 [Gemmatimonadaceae bacterium].	GCA_013817545.1
MBA3619565.1	REase→ PIN*→	PIN	572	H0W56_08245	Actinobacteria	Acidothermales bacterium	MAG: hypothetical protein H0W56_08245, partial [Acidothermales bacterium].	GCA_013817005.1
MBA3973802.1	TPR+GreAB-C+PIN*→	TPR+GreAB-C+PIN	1263	C0504_06240	Acidobacteria	Candidatus Solibacter sp.	MAG: hypothetical protein C0504_06240 [Candidatus Solibacter sp.].	GCA_013822245.1
MBA4090929.1	REase+TPR+GreAB-C+PIN*→	REase+TPR+GreAB-C+PIN	1315	C0494_10085	Alphaproteobacteria	Sphingobium sp.	MAG: hypothetical protein C0494_10085 [Sphingobium sp.].	GCA_013822485.1
MBA4148880.1	TPR+PIN*→	TPR+PIN	1103	H0X66_12260	Verrucomicrobia	Verrucomicrobia bacterium	MAG: hypothetical protein H0X66_12260 [Verrucomicrobia bacterium].	GCA_013820875.1
MBB1073428.1	REase+TPR+GreAB-C+PIN*→	REase+TPR+GreAB-C+PIN	1321	HUU62_03265	Betaproteobacteria	Rhodoferax jenense	DUF4365 domain-containing protein [Rhodoferax jenense].	GCA_014145235.1
MBB1121889.1	DOC+HTH→ REase+TOPC→ HetE-N1→?→ TPR+GreAB-C+PIN*→	TPR+GreAB-C+PIN	992	H4983_26725	Gammaproteobacteria	Pseudomonas aeruginosa	hypothetical protein H4983_26725 [Pseudomonas aeruginosa].	GCA_014145725.1
MBB4577222.1	REase+TPR+GreAB-C+PIN*→	REase+TPR+GreAB-C+PIN	1268	GGE58_005472	Alphaproteobacteria	Rhizobium lentis	tetratricopeptide (TPR) repeat protein [Rhizobium lentis].	GCA_014198785.1

acc	operon	architecture	len	gen.name	taxend	species	defline	gca
MBB5991665.1	REase→ TPR+GreAB-C+PIN*→?→?→ HNH→	TPR+GreAB-C+PIN	1242	HNP04_003690	Gammaproteobacteria	Pseudomonas viridiflava	tetratricopeptide (TPR) repeat protein/transcription elongation GreA/GreB family factor [Pseudomonas viridiflava].	GCA_014204055.1
MBB6509406.1	TPR+GreAB-C+PIN*→	TPR+GreAB-C+PIN	780	F4695_002763	Alphaproteobacteria	Rhizobium soli	putative nucleic acid-binding protein [Rhizobium soli].	GCA_014207075.1
MBB9310955.1	TPR+GreAB-C+PIN*→	TPR+GreAB-C+PIN	575	FSF17_011690	Gammaproteobacteria	Escherichia coli	hypothetical protein FSF17_011690 [Escherichia coli].	GCA_012385615.2
MBC6414865.1	REase+TPR+GreAB-C+PIN*→	REase+TPR+GreAB-C+PIN	1326	GDA46_00495	Oligoflexia	Bdellovibrionales bacterium	MAG: hypothetical protein GDA46_00495 [Bdellovibrionales bacterium].	GCA_014323935.1
MBC70602.1	REase+TPR+GreAB-C+PIN*→	REase+TPR+GreAB-C+PIN	1253	CL492_17300	Gammaproteobacteria	Acinetobacter sp.	MAG: hypothetical protein CL492_17300 [Acinetobacter sp.].	GCA_002711945.1
MBC7145404.1	REase+TPR+GreAB-C+PIN*→	REase+TPR+GreAB-C+PIN	1332	H5U24_08360	Alphaproteobacteria	Thioclava marina	MAG: hypothetical protein H5U24_08360 [Thioclava marina].	GCA_014359625.1
MBC7391182.1	TPR+GreAB-C+PIN*→	TPR+GreAB-C+PIN	1270	H7329_18405	Verrucomicrobia	Cytophagales bacterium	MAG: tetratricopeptide repeat protein [Opitutaceae bacterium].	GCA_014376535.1
MBC74230.1	TPR+GreAB-C+PIN*→	TPR+GreAB-C+PIN	1131	CMH47_18575	Bacteroidetes	Muricauda sp.	MAG: hypothetical protein CMH47_18575 [Muricauda sp.].	GCA_002711825.1
MBD0373736.1	TPR+GreAB-C*→	TPR+GreAB-C	885	ICV60_23090	Acidobacteria	Pyrinomonadaceae bacterium	MAG: tetratricopeptide repeat protein, partial [Pyrinomonadaceae bacterium].	GCA_014534425.1
MBD3293793.1	TPR+PIN*→	TPR+PIN	1505	GF393_12790	Armatimonadetes	Armatimonadia bacterium	MAG: hypothetical protein GF393_12790 [Armatimonadia bacterium].	GCA_014728405.1
MBD3811358.1	REase+TPR+GreAB-C+PIN*→	REase+TPR+GreAB-C+PIN	1374	IE917_03935	Betaproteobacteria	Betaproteobacteria bacterium	MAG: hypothetical protein IE917_03935 [Betaproteobacteria bacterium].	GCA_014764035.1
MBD8566197.1	TPR+GreAB-C+PIN*→	TPR+GreAB-C+PIN	1235	IFU01_18215	Betaproteobacteria	Oxalobacteraceae sp. CFBP 8763	hypothetical protein IFU01_18215 [Oxalobacteraceae sp. CFBP 8763].	GCA_014841525.1
MBD8722144.1	REase+TPR+GreAB-C+PIN*→	REase+TPR+GreAB-C+PIN	1511	IFT43_02050	Betaproteobacteria	Oxalobacteraceae sp. CFBP 13708	hypothetical protein IFT43_02050 [Oxalobacteraceae sp. CFBP 13708].	GCA_014842315.1
MBE2259083.1	REase+TPR+GreAB-C+PIN*→	REase+TPR+GreAB-C+PIN	1335	IAE88_09505	Alphaproteobacteria	Rhodobacteraceae bacterium	MAG: hypothetical protein IAE88_09505 [Rhodobacteraceae bacterium].	GCA_014879755.1
MBE3996728.1	HetE-N1→?→ TPR+GreAB-C+PIN*→	TPR+GreAB-C+PIN	910	HJ133_08285	Gammaproteobacteria	Vibrio parahaemolyticus	hypothetical protein HJ133_08285 [Vibrio parahaemolyticus].	GCA_014919735.1
MBE7465263.1	REase+TPR+GreAB-C+PIN*→	REase+TPR+GreAB-C+PIN	1253	HS116_17430	Planctomycetes	Planctomycetes bacterium	MAG: hypothetical protein HS116_17430 [Planctomycetes bacterium].	GCA_015075375.1
MBE8523541.1	TPR+PIN*→	TPR+PIN	1310	ILP97_39665	Actinobacteria	Amycolatopsis sp. H6(2020)	hypothetical protein ILP97_39665 [Amycolatopsis sp. H6(2020)].	GCA_015163035.1
MBE8960063.1	GreAB-C*→	GreAB-C	307	IH766_22715	Gammaproteobacteria	Escherichia coli	hypothetical protein IH766_22715, partial [Escherichia coli].	GCA_015206795.1
MBE9468467.1	TPR+GreAB-C+PIN*→	TPR+GreAB-C+PIN	1221	IMY72_09155	Bacteroidetes	Bacteroidetes bacterium	MAG: hypothetical protein IMY72_09155 [Bacteroidetes bacterium].	GCA_015222055.1
MBF0231519.1	TPR+GreAB-C+PIN*→	TPR+GreAB-C+PIN	1223	HQK65_00575	Deltaproteobacteria	Desulfamplus sp.	MAG: hypothetical protein HQK65_00575 [Desulfamplus sp.].	GCA_015231525.1
MBF0529118.1	TPR+GreAB-C+PIN*→	TPR+GreAB-C+PIN	678	HQK55_07585	Deltaproteobacteria	Deltaproteobacteria bacterium	MAG: hypothetical protein HQK55_07585 [Deltaproteobacteria bacterium].	GCA_015233515.1
MBF3699451.1	TPR+GreAB-C+PIN*→||||<-HTH+HTH	TPR+GreAB-C+PIN	872	ISF48_14710	Betaproteobacteria	Burkholderia pseudomallei	hypothetical protein ISF48_14710 [Burkholderia pseudomallei].	GCA_015319855.1
MBF3842067.1	TPR+GreAB-C+PIN*→||||<-HTH+HTH	TPR+GreAB-C+PIN	872	ISF68_21105	Betaproteobacteria	Burkholderia pseudomallei	hypothetical protein ISF68_21105 [Burkholderia pseudomallei].	GCA_015320325.1
MBF4269202.1	TPR+GreAB-C*→	TPR+GreAB-C	686	EAY71_20240	Gammaproteobacteria	Vibrio anguillarum	hypothetical protein EAY71_20240, partial [Vibrio anguillarum].	GCA_015343035.1
MBF9243682.1	REase+TPR+GreAB-C+PIN*→	REase+TPR+GreAB-C+PIN	1438	I4I80_05390	Gammaproteobacteria	Pseudomonas syringae pv. tomato	hypothetical protein I4I80_05390 [Pseudomonas syringae pv. tomato].	GCA_015694365.1

acc	operon	architecture	len	gen.name	taxend	species	defline	gca
MBF9246381.1	REase+TPR+GreAB-C+PIN*→	REase+TPR+GreAB-C+PIN	1320	I4I80_19980	Gammaproteobacteria	Pseudomonas syringae pv. tomato	DUF4365 domain-containing protein [Pseudomonas syringae pv. tomato].	GCA_015694365.1
MBG2992436.1	PAIREDC-HTH→ PSE→ HetE-N1→ TPR+GreAB-C+PIN*→	TPR+GreAB-C+PIN	1092	I4607_12420	Gammaproteobacteria	Proteus mirabilis	hypothetical protein I4607_12420 [Proteus mirabilis].	GCA_015723275.1
MBG6040926.1	PAIREDC-HTH→ PSE→ HetE-N1→ TPR+GreAB-C+PIN*→	TPR+GreAB-C+PIN	1092	I5F94_05610	Gammaproteobacteria	Proteus mirabilis	hypothetical protein I5F94_05610 [Proteus mirabilis].	GCA_015740165.1
MBG6251627.1	TPR+GreAB-C+PIN*→	TPR+GreAB-C+PIN	1140	I5I20_11010	Gammaproteobacteria	Pseudomonas aeruginosa	hypothetical protein I5I20_11010 [Pseudomonas aeruginosa].	GCA_015762745.1
MBG7090225.1	TPR+GreAB-C+PIN*→	TPR+GreAB-C+PIN	1170	I5G28_04925	Gammaproteobacteria	Pseudomonas aeruginosa	hypothetical protein I5G28_04925 [Pseudomonas aeruginosa].	GCA_015765435.1
MBH04121.1	REase+TPR+GreAB-C+PIN*→	REase+TPR+GreAB-C+PIN	1338	CMP08_08385	Gammaproteobacteria	Xanthomonadales bacterium	MAG: hypothetical protein CMP08_08385 [Xanthomonadales bacterium].	GCA_002715985.1
MBH1476826.1	REase+TPR+GreAB-C+PIN*→	REase+TPR+GreAB-C+PIN	1243	I5U61_05170	Gammaproteobacteria	Stenotrophomonas maltophilia	CDC27 family protein [Stenotrophomonas maltophilia].	GCA_015997065.1
MBH7847781.1	TPR+GreAB-C+PIN*→	TPR+GreAB-C+PIN	1071	I6A75_20240	Firmicutes	Clostridioides difficile	hypothetical protein I6A75_20240 [Clostridioides difficile].	GCA_016052935.1
MBI1182363.1	REase+TPR+TPR+GreAB-C+PIN*→	REase+TPR+TPR+GreAB-C+PIN	1295	GC201_17615	Alphaproteobacteria	Alphaproteobacteria bacterium	MAG: hypothetical protein GC201_17615 [Alphaproteobacteria bacterium].	GCA_016124315.1
MBI1904375.1	TPR+GreAB-C+PIN*→	TPR+GreAB-C+PIN	1292	HYS13_25040	Planctomycetes	Planctomycetia bacterium	MAG: hypothetical protein HYS13_25040 [Planctomycetia bacterium].	GCA_016177915.1
MBI3888209.1	TPR+PIN*→	TPR+PIN	1190	HY311_00230	Bacteria	Candidatus Nomurabacteria bacterium	MAG: tetratricopeptide repeat protein [Candidatus Nomurabacteria bacterium].	GCA_016199805.1
MBI4633825.1	REase+TPR+GreAB-C+PIN*→	REase+TPR+GreAB-C+PIN	1294	HY742_08030	Deltaproteobacteria	Deltaproteobacteria bacterium	MAG: hypothetical protein HY742_08030 [Deltaproteobacteria bacterium].	GCA_016209105.1
MBI4696055.1	Cas_Cas1→ REase+TPR+GreAB-C+PIN*→	REase+TPR+GreAB-C+PIN	1313	HY749_18755	Gammaproteobacteria	Gammaproteobacteria bacterium	MAG: DUF4365 domain-containing protein [Gammaproteobacteria bacterium].	GCA_016209015.1
MBI4846278.1	TM+SIR2+TPR+PIN+PIN+TM*→	TM+SIR2+TPR+PIN+PIN+TM	2304	HY810_07390	PVC group	Candidatus Omnitrophica bacterium	MAG: SIR2 family protein [Candidatus Omnitrophica bacterium].	GCA_016213925.1
MBI5207389.1	HTH+REase+PIN*→	HTH+REase+PIN	808	HY934_06310	Bacteria	Candidatus Firestonebacteria bacterium	MAG: helix-turn-helix domain-containing protein [Candidatus Firestonebacteria bacterium].	GCA_016212735.1
MBI5354508.1	TPR+GreAB-C+PIN*→	TPR+GreAB-C+PIN	1282	HZB50_17850	Chloroflexi	Chloroflexi bacterium	MAG: hypothetical protein HZB50_17850 [Chloroflexi bacterium].	GCA_016219545.1
MBI5356660.1	TPR+PIN*→||||<-?<-?||HTH→	TPR+PIN	1074	HZB78_03530	Bacteria	Candidatus Collierbacteria bacterium	MAG: hypothetical protein HZB78_03530 [Candidatus Collierbacteria bacterium].	GCA_016219325.1
MBI5458930.1	HEPN→ REase+SNF2→ REase+TPR+GreAB-C+PIN*→	REase+TPR+GreAB-C+PIN	1256	HY987_04275	Euryarchaeota	Methanobacterium sp.	MAG: hypothetical protein HY987_04275 [Methanobacterium sp.].	GCA_016217785.1
MBI5516335.1	GreAB*→	GreAB	172	HY909_21300	Deltaproteobacteria	Deltaproteobacteria bacterium	MAG: GreA/GreB family elongation factor [Deltaproteobacteria bacterium].	GCA_016218725.1
MBI5711428.1	GreAB*→	GreAB	903	HZC42_14175	Bacteria	Candidatus Eisenbacteria bacterium	MAG: GreA/GreB family elongation factor [Candidatus Eisenbacteria bacterium].	GCA_016223045.1
MBI5910925.1	REase+TPR+GreAB-C+PIN*→	REase+TPR+GreAB-C+PIN	1318	HY848_13355	Betaproteobacteria	Betaproteobacteria bacterium	MAG: hypothetical protein HY848_13355 [Betaproteobacteria bacterium].	GCA_016234705.1
MBI5925874.1	REase+TPR+GreAB-C+PIN*→	REase+TPR+GreAB-C+PIN	1369	HY836_09780	Betaproteobacteria	Aquabacterium sp.	MAG: hypothetical protein HY836_09780 [Aquabacterium sp.].	GCA_016234775.1
MBI6873434.1	TPR+PIN*→	TPR+PIN	1232	I6U51_12055	Firmicutes	Clostridium aciditolerans	hypothetical protein I6U51_12055 [Clostridium aciditolerans].	GCA_016316925.1
MBJ3588954.1	TPR+GreAB-C+PIN*→	TPR+GreAB-C+PIN	924	JGC49_23910	Gammaproteobacteria	Salmonella enterica subsp. enterica serovar London	hypothetical protein JGC49_23910, partial [Salmonella enterica subsp. enterica serovar London].	GCA_016412075.1
MBJ7510055.1	REase+TPR+TPR+GreAB-C+PIN*→?→ HNH→	REase+TPR+TPR+GreAB-C+PIN	1324	JHC97_02180	Alphaproteobacteria	Brevundimonas sp.	MAG: hypothetical protein JHC97_02180 [Brevundimonas sp.].	GCA_016463795.1

acc	operon	architecture	len	gen.name	taxend	species	defline	gca
MBK4592903.1	TPR+GreAB-C+PIN*→	TPR+GreAB-C+PIN	444	JJQ23_24565	Gammaproteobacteria	Enterobacter hormaechei	hypothetical protein JJQ23_24565, partial [Enterobacter hormaechei].	GCA_016633275.1
MBK4660580.1	TPR+GreAB-C*→	TPR+GreAB-C	362	JJQ36_24785	Gammaproteobacteria	Enterobacter hormaechei	hypothetical protein JJQ36_24785, partial [Enterobacter hormaechei].	GCA_016633665.1
MBK4769022.1	HetE-N1→ TPR+GreAB-C+PIN*→	TPR+GreAB-C+PIN	1118	FT726_04985	Gammaproteobacteria	Pantoea sp. Morm	MAG: hypothetical protein FT726_04985 [Pantoea sp. Morm].	GCA_016643525.1
MBK5650341.1	REase+TPR+TPR+GreAB-C+PIN*→	REase+TPR+TPR+GreAB-C+PIN	1254	I4N50_01815	Alphaproteobacteria	Rhizobium sp.	MAG: hypothetical protein I4N50_01815, partial [Rhizobium sp.].	GCA_016652855.1
MBK7033605.1	TPR+GreAB-C+PIN*→	TPR+GreAB-C+PIN	852	IPH49_10150	Ignavibacteriae	Ignavibacteria bacterium	MAG: hypothetical protein IPH49_10150 [Ignavibacteria bacterium].	GCA_016708405.1
MBK7130254.1	TPR+GreAB-C+PIN*→	TPR+GreAB-C+PIN	1238	IPH66_12960	Bacteroidetes	Crocinitomicaceae bacterium	MAG: hypothetical protein IPH66_12960 [Crocinitomicaceae bacterium].	GCA_016708105.1
MBK7149038.1	TPR+GreAB-C+PIN*→?→?→?→ TRD+TRD→	TPR+GreAB-C+PIN	1255	IPH78_09510	Bacteroidetes	Bacteroidetes bacterium	MAG: tetratricopeptide repeat protein [Bacteroidetes bacterium].	GCA_016707905.1
MBK7953772.1	REase+TPR+GreAB-C+PIN*→	REase+TPR+GreAB-C+PIN	1351	IPK02_07330	Betaproteobacteria	Candidatus Accumulibacter sp.	MAG: hypothetical protein IPK02_07330 [Candidatus Accumulibacter sp.].	GCA_016713625.1
MBK8138001.1	TPR+GreAB-C+PIN*→	TPR+GreAB-C+PIN	749	IPK52_19665	Chloroflexi	Chloroflexi bacterium	MAG: hypothetical protein IPK52_19665 [Chloroflexi bacterium].	GCA_016712885.1
MBK8143858.1	TPR+PIN*→	TPR+PIN	641	IPK62_02070	Bacteroidetes	Bacteroidetes bacterium	MAG: hypothetical protein IPK62_02070 [Bacteroidetes bacterium].	GCA_016712665.1
MBK8927388.1	TPR+GreAB-C+PIN*→	TPR+GreAB-C+PIN	1238	IPM74_16180	Bacteroidetes	Crocinitomicaceae bacterium	MAG: hypothetical protein IPM74_16180 [Crocinitomicaceae bacterium].	GCA_016720025.1
MBK9927324.1	TPR+GreAB-C+PIN*→	TPR+GreAB-C+PIN	1144	IPP66_18800	Chloroflexi	Anaerolineales bacterium	MAG: tetratricopeptide repeat protein [Anaerolineales bacterium].	GCA_016721115.1
MBL0869327.1	TPR+GreAB-C+PIN*→	TPR+GreAB-C+PIN	1510	IBJ18_01985	Planctomycetes	Phycisphaerales bacterium	MAG: SIR2 family protein [Phycisphaerales bacterium].	GCA_016741915.1
MBL0919126.1	REase+TPR+GreAB-C+PIN*→	REase+TPR+GreAB-C+PIN	1320	IBJ14_10515	Betaproteobacteria	Hydrogenophaga sp.	MAG: DUF4365 domain-containing protein [Hydrogenophaga sp.].	GCA_016741995.1
MBL1279106.1	TPR+PIN*→	TPR+PIN	1234	COA33_002470	Bacteroidetes	Fluviicola sp.	MAG: hypothetical protein COA33_002470 [Fluviicola sp.].	GCA_002734065.2
MBL4609540.1	HetE-N1→ TPR+GreAB-C+PIN*→	TPR+GreAB-C+PIN	1114	JKY58_02600	Gammaproteobacteria	Pseudomonas sp.	MAG: hypothetical protein JKY58_02600 [Pseudomonas sp.].	GCA_016763285.1
MBL7760873.1	TPR+GreAB-C+PIN*→	TPR+GreAB-C+PIN	1264	JNK08_09270	Bacteroidetes	Sediminibacterium sp.	MAG: hypothetical protein JNK08_09270 [Sediminibacterium sp.].	GCA_016786865.1
MBL7768787.1	TPR+GreAB-C+PIN*→	TPR+GreAB-C+PIN	1258	JNK20_07425	Bacteroidetes	Flavipsychrobacter sp.	MAG: hypothetical protein JNK20_07425 [Flavipsychrobacter sp.].	GCA_016787265.1
MBL7772244.1	TPR+GreAB-C+PIN*→	TPR+GreAB-C+PIN	1151	JNM95_05270	Bacteroidetes	Chitinophagaceae bacterium	MAG: hypothetical protein JNM95_05270 [Chitinophagaceae bacterium].	GCA_016787065.1
MBL7856049.1	TPR+GreAB-C+PIN*→	TPR+GreAB-C+PIN	1173	JNM57_00050	Bacteroidetes	Cyclobacteriaceae bacterium	MAG: hypothetical protein JNM57_00050 [Cyclobacteriaceae bacterium].	GCA_016787565.1
MBL8263961.1	REase+TPR+GreAB-C+PIN*→	REase+TPR+GreAB-C+PIN	1239	JNM58_16225	Gammaproteobacteria	Xanthomonadaceae bacterium	MAG: hypothetical protein JNM58_16225 [Xanthomonadaceae bacterium].	GCA_016789405.1
MBL8602392.1	<-SIG+SM-MscS<-?<-?||GreAB*→	GreAB	172	JNK72_10760	Deltaproteobacteria	Myxococcales bacterium	MAG: GreA/GreB family elongation factor [Myxococcales bacterium].	GCA_016792845.1
MBL8821106.1	REase→?→ REase+TPR+GreAB-C+PIN*→	REase+TPR+GreAB-C+PIN	1262	JNJ77_00865	Planctomycetes	Planctomycetia bacterium	MAG: hypothetical protein JNJ77_00865 [Planctomycetia bacterium].	GCA_016795155.1
MBL8873833.1	MNS+TPR+PIN*→	MNS+TPR+PIN	2502	JNK90_28920	Planctomycetes	Planctomycetaceae bacterium	MAG: tetratricopeptide repeat protein [Planctomycetaceae bacterium].	GCA_016792705.1
MBL8874044.1	TPR+GreAB-C+PIN*→	TPR+GreAB-C+PIN	1287	JNK90_29995	Planctomycetes	Planctomycetaceae bacterium	MAG: hypothetical protein JNK90_29995 [Planctomycetaceae bacterium].	GCA_016792705.1
MBL9102506.1	Calcineurin+TPR+GreAB-C+PIN*→	Calcineurin+TPR+GreAB-C+PIN	1429	JNL82_16210	Deltaproteobacteria	Myxococcales bacterium	MAG: metallophosphoesterase [Myxococcales bacterium].	GCA_016793465.1

acc	operon	architecture	len	gen.name	taxend	species	defline	gca
MBM0224540.1	TPR+PIN*→	TPR+PIN	990	JNW87_02235	Actinobacteria	Micromonospora sp. ATA51	hypothetical protein JNW87_02235 [Micromonospora sp. ATA51].	GCA_016802855.1
MBM0258116.1	TM+TPR+PIN*→	TM+TPR+PIN	1165	JNW89_16135	Actinobacteria	Micromonospora sp. 4G55	hypothetical protein JNW89_16135 [Micromonospora sp. 4G55].	GCA_016803375.1
MBM4070384.1	TPR+GreAB-C*→	TPR+GreAB-C	409	FJ271_15725	Planctomycetes	Planctomycetes bacterium	MAG: hypothetical protein FJ271_15725 [Planctomycetes bacterium].	GCA_016873095.1
MBM4845996.1	HetE-N1→ HetE-N1+TPR+GreAB-C+PIN*→	HetE-N1+TPR+GreAB-C+PIN	1091	HYO58_20990	Gammaproteobacteria	Vibrio parahaemolyticus	hypothetical protein HYO58_20990 [Vibrio parahaemolyticus].	GCA_016876175.1
MBN1214554.1	TPR+PIN*→	TPR+PIN	1151	JXA99_03840	Asgard group	Candidatus Lokiarchaeota archaeon	MAG: hypothetical protein JXA99_03840 [Candidatus Lokiarchaeota archaeon].	GCA_016926735.1
MBN1453691.1	TPR+GreAB-C+PIN*→	TPR+GreAB-C+PIN	1287	JW963_21935	Chloroflexi	Anaerolineales bacterium	MAG: hypothetical protein JW963_21935 [Anaerolineales bacterium].	GCA_016928525.1
MBN2019675.1	TPR+PIN*→	TPR+PIN	1063	JW749_05560	Planctomycetes	Sedimentisphaerales bacterium	MAG: tetratricopeptide repeat protein [Sedimentisphaerales bacterium].	GCA_016932095.1
MBN2455312.1	TPR+GreAB-C+PIN*→	TPR+GreAB-C+PIN	958	JXB29_02055	Planctomycetes	Sedimentisphaerales bacterium	MAG: hypothetical protein JXB29_02055 [Sedimentisphaerales bacterium].	GCA_016935195.1
MBN4052768.1	TPR+GreAB-C+PIN*→	TPR+GreAB-C+PIN	1150	JYU07_00650	Chloroflexi	Roseiflexus sp. AH-315-K22	hypothetical protein JYU07_00650, partial [Roseiflexus sp. AH-315-K22].	GCA_017114345.1
MBN5297052.1	HetE-N1→ TPR+GreAB-C+PIN*→	TPR+GreAB-C+PIN	1090	JY477_14370	Gammaproteobacteria	Serratia marcescens	hypothetical protein JY477_14370 [Serratia marcescens].	GCA_017152845.1
MBN8213566.1	REase+TPR+GreAB-C+PIN*→	REase+TPR+GreAB-C+PIN	1240	J0M09_11660	Gammaproteobacteria	Xanthomonadales bacterium	MAG: hypothetical protein J0M09_11660 [Xanthomonadales bacterium].	GCA_017302135.1
MBN8506452.1	REase+TPR+GreAB-C+PIN*→	REase+TPR+GreAB-C+PIN	1310	J0L58_18450	Betaproteobacteria	Burkholderiales bacterium	MAG: DUF4365 domain-containing protein [Burkholderiales bacterium].	GCA_017302655.1
MBN8549455.1	<-HNH<-?<-?||?→||||<-?||?→?→ REase+TPR+GreAB-C+PIN*→	REase+TPR+GreAB-C+PIN	1254	J0M12_09085	Deltaproteobacteria	Deltaproteobacteria bacterium	MAG: hypothetical protein J0M12_09085 [Deltaproteobacteria bacterium].	GCA_017302875.1
MBN8618419.1	TPR+PIN*→	TPR+PIN	1277	J0L63_05915	Chloroflexi	Anaerolineae bacterium	MAG: hypothetical protein J0L63_05915 [Anaerolineae bacterium].	GCA_017303775.1
MBN8865737.1	TPR+GreAB-C+PIN*→||||<-REase	TPR+GreAB-C+PIN	435	J0H92_20355	Bacteroidetes	Sphingobacteriales bacterium	MAG: hypothetical protein J0H92_20355 [Sphingobacteriales bacterium].	GCA_017304765.1
MBN8904954.1	TPR+GreAB-C+PIN*→ REase→	TPR+GreAB-C+PIN	679	J0H57_28395	Alphaproteobacteria	Rhodospirillales bacterium	MAG: hypothetical protein J0H57_28395, partial [Rhodospirillales bacterium].	GCA_017305035.1
MBN8956855.1	REase+TPR+TPR+GreAB-C+PIN*→	REase+TPR+TPR+GreAB-C+PIN	1322	J0H17_09800	Alphaproteobacteria	Rhizobiales bacterium	MAG: GreA/GreB family elongation factor [Rhizobiales bacterium].	GCA_017304555.1
MBN8979284.1	TPR+GreAB-C+PIN*→	TPR+GreAB-C+PIN	1129	J0I08_22735	Alphaproteobacteria	Rhizobiales bacterium	MAG: hypothetical protein J0I08_22735 [Rhizobiales bacterium].	GCA_017305555.1
MBN9409395.1	REase+TPR+GreAB-C+PIN*→	REase+TPR+GreAB-C+PIN	1520	J0H69_09625	Betaproteobacteria	Burkholderiales bacterium	MAG: hypothetical protein J0H69_09625 [Burkholderiales bacterium].	GCA_017307855.1
MBN9598737.1	TIR→?→?→ TPR+GreAB-C+PIN*→	TPR+GreAB-C+PIN	1117	J0G28_03540	Alphaproteobacteria	Afipia sp.	MAG: hypothetical protein J0G28_03540 [Afipia sp.].	GCA_017308625.1
MBO1224632.1	TPR+GreAB-C+PIN*→	TPR+GreAB-C+PIN	1279	JYX80_09380	Planctomycetes	Candidatus Scalindua sediminis	MAG: hypothetical protein JYX80_09380 [Candidatus Scalindua sediminis].	GCA_017368835.1
MBO5183033.1	TPR+GreAB-C+PIN*→	TPR+GreAB-C+PIN	1061	J6B64_01405	Firmicutes	Bacilli bacterium	MAG: hypothetical protein J6B64_01405 [Bacilli bacterium].	GCA_017623135.1
MBO5920175.1	TPR+GreAB-C+PIN*→	TPR+GreAB-C+PIN	507	J6Q34_06770	Bacteroidetes	Bacteroidales bacterium	MAG: hypothetical protein J6Q34_06770, partial [Bacteroidales bacterium].	GCA_017631435.1
MBO6496597.1	TPR+GreAB-C+PIN*→	TPR+GreAB-C+PIN	1166	JJ978_13575	Bacteroidetes	Roseivirga sp.	MAG: hypothetical protein JJ978_13575 [Roseivirga sp.].	GCA_017640405.1
MBO6761342.1	TPR+GreAB-C+PIN*→	TPR+GreAB-C+PIN	1095	JJ909_10290	Bacteroidetes	Roseivirga sp.	MAG: hypothetical protein JJ909_10290, partial [Roseivirga sp.].	GCA_017643085.1
MBO7636147.1	TPR+GreAB-C+PIN*→	TPR+GreAB-C+PIN	1171	J6S89_06175	Bacteroidetes	Paludibacteraceae bacterium	MAG: hypothetical protein J6S89_06175 [Paludibacteraceae bacterium].	GCA_017651825.1

acc	operon	architecture	len	gen.name	taxend	species	defline	gca
MBP8894107.1	TPR+GreAB-C+PIN*→	TPR+GreAB-C+PIN	985	KBH09_17290	Bacteroidetes	Saprospiraceae bacterium	MAG: hypothetical protein KBH09_17290 [Saprospiraceae bacterium].	GCA_018055665.1
MBR7522021.1	HetE-N1→ TPR+GreAB-C+PIN*→	TPR+GreAB-C+PIN	1105	KC233_15980	Gammaproteobacteria	Pseudomonas juntendi	hypothetical protein KC233_15980 [Pseudomonas juntendi].	GCA_018138545.1
MHJ03184.1	TPR+GreAB-C+PIN*→	TPR+GreAB-C+PIN	523	EEN62_25195	Gammaproteobacteria	Salmonella enterica	hypothetical protein EEN62_25195 [Salmonella enterica].	GCA_003786665.1
MKC77438.1	TPR+GreAB-C+PIN*→?→||||<-Trypsin	TPR+GreAB-C+PIN	1057	D1800_22940	Gammaproteobacteria	Salmonella enterica subsp. enterica serovar Denver	hypothetical protein D1800_22940, partial [Salmonella enterica subsp. enterica serovar Denver].	GCA_003891855.1
MPS34312.1	REase+TPR+GreAB-C+PIN*→	REase+TPR+GreAB-C+PIN	1237	E2593_03230	Gammaproteobacteria	Stenotrophomonas sp.	MAG: hypothetical protein E2593_03230 [Stenotrophomonas sp.].	GCA_009360425.1
MSW50706.1	TPR+PIN*→	TPR+PIN	1289	F2817_07440	Actinobacteria	Actinobacteria bacterium	MAG: hypothetical protein F2817_07440 [Actinobacteria bacterium].	GCA_009699505.1
MTI67851.1	TPR+GreAB-C+PIN*→	TPR+GreAB-C+PIN	1274	FH753_14795	Firmicutes	Firmicutes bacterium	MAG: hypothetical protein FH753_14795 [Firmicutes bacterium].	GCA_009711765.1
MVM42293.1	-	-	400	GO730_39050	Bacteroidetes	Spirosoma sp. HMF3257	hypothetical protein GO730_39050 [Spirosoma sp. HMF3257].	GCA_009754885.1
MXP30641.1	REase→?→ REase+TPR+GreAB-C+PIN*→	REase+TPR+GreAB-C+PIN	1265	GRI94_02255	Alphaproteobacteria	Parerythrobacter jejuensis	hypothetical protein GRI94_02255 [Parerythrobacter jejuensis].	GCA_009827995.1
MXW27298.1	REase+TPR+GREAB-C+PIN*→	REase+TPR+GREAB-C+PIN	1302	F4Z80_00100	Chloroflexi	Chloroflexi bacterium	MAG: hypothetical protein F4Z80_00100 [Chloroflexi bacterium].	GCA_009835825.1
MXX46821.1	TPR+GreAB-C+PIN*→	TPR+GreAB-C+PIN	1267	F4Z38_00735	Chloroflexi	Chloroflexi bacterium	MAG: tetratricopeptide repeat protein [Chloroflexi bacterium].	GCA_009836695.1
MXX90812.1	SFI-Helicase+REase+TPR+GreAB-C+PIN*→	SFI-Helicase+REase+TPR+GreAB-C+PIN	1846	F4Y68_16490	Alphaproteobacteria	Boseongicola sp. SB0665_bin_10	MAG: DUF4011 domain-containing protein [Boseongicola sp. SB0665_bin_10].	GCA_009838075.1
MXX91940.1	GreAB*→?→?→ DAGKIN→	GreAB	297	F4Y63_00605	Chloroflexi	Chloroflexi bacterium	MAG: transcription elongation factor GreA [Chloroflexi bacterium].	GCA_009838175.1
MXX97366.1	REase+TPR+GreAB-C+PIN*→	REase+TPR+GreAB-C+PIN	1278	F4Y64_07095	Bacteroidetes	Rhodothermaceae bacterium	MAG: hypothetical protein F4Y64_07095 [Rhodothermaceae bacterium].	GCA_009838195.1
MXZ48148.1	REase+TPR+GreAB-C+PIN*→	REase+TPR+GreAB-C+PIN	1281	F4Z13_02660	Bacteria	Candidatus Dadabacteria bacterium	MAG: hypothetical protein F4Z13_02660 [Candidatus Dadabacteria bacterium].	GCA_009837205.1
MXZ55165.1	REase+TPR+GreAB-C+PIN*→	REase+TPR+GreAB-C+PIN	1297	F4Z14_03240	Gammaproteobacteria	Gammaproteobacteria bacterium	MAG: hypothetical protein F4Z14_03240 [Gammaproteobacteria bacterium].	GCA_009837185.1
MXZ61096.1	REase+TPR+GreAB-C+PIN*→	REase+TPR+GreAB-C+PIN	1297	F4Z12_09585	Acidobacteria	Acidobacteria bacterium	MAG: hypothetical protein F4Z12_09585 [Acidobacteria bacterium].	GCA_009837215.1
MYA00894.1	TPR+GreAB-C+PIN*→	TPR+GreAB-C+PIN	1267	F4Y35_03835	Chloroflexi	Chloroflexi bacterium	MAG: tetratricopeptide repeat protein [Chloroflexi bacterium].	GCA_009838735.1
MYA68860.1	TPR+GreAB-C+PIN*→	TPR+GreAB-C+PIN	1308	F4Y19_01960	Bacteria	Candidatus Poribacteria bacterium	MAG: tetratricopeptide repeat protein [Candidatus Poribacteria bacterium].	GCA_009839085.1
MYB15443.1	TPR+GreAB-C+PIN*→	TPR+GreAB-C+PIN	1263	F4X41_00185	Chloroflexi	Chloroflexi bacterium	MAG: hypothetical protein F4X41_00185 [Chloroflexi bacterium].	GCA_009840625.1
MYB42498.1	TPR+GreAB-C+PIN*→	TPR+GreAB-C+PIN	1283	F4X76_09995	Chloroflexi	Chloroflexi bacterium	MAG: tetratricopeptide repeat protein [Chloroflexi bacterium].	GCA_009839925.1
MYB43588.1	TPR+PIN*→	TPR+PIN	1200	F4X74_01405	Actinobacteria	Acidimicrobiia bacterium	MAG: hypothetical protein F4X74_01405 [Acidimicrobiia bacterium].	GCA_009839965.1
MYB75895.1	TPR+GreAB-C+PIN*→	TPR+GreAB-C+PIN	1300	F4X83_02110	Chloroflexi	Chloroflexi bacterium	MAG: hypothetical protein F4X83_02110 [Chloroflexi bacterium].	GCA_009839775.1
MYC64541.1	TPR+GreAB-C+PIN*→	TPR+GreAB-C+PIN	1258	F4X16_17195	Chloroflexi	Caldilineaceae bacterium SB0661_bin_34	MAG: tetratricopeptide repeat protein [Caldilineaceae bacterium SB0661_bin_34].	GCA_009841145.1
MYD93275.1	REase+TPR+GreAB-C+PIN*→	REase+TPR+GreAB-C+PIN	1302	F4Y02_06215	Chloroflexi	Chloroflexi bacterium	MAG: hypothetical protein F4Y02_06215 [Chloroflexi bacterium].	GCA_009839425.1

acc	operon	architecture	len	gen.name	taxend	species	defline	gca
MYE61432.1	TPR+GreAB-C+PIN*→	TPR+GreAB-C+PIN	638	F4235_05210	Bacteria	Candidatus Dadabacteria bacterium	MAG: GreA/GreB family elongation factor [Candidatus Dadabacteria bacterium].	GCA_009841925.1
MYF79095.1	TPR+GreAB-C+PIN*→	TPR+GreAB-C+PIN	716	F4180_04215	Chloroflexi	Chloroflexi bacterium	MAG: tetratricopeptide repeat protein, partial [Chloroflexi bacterium].	GCA_009843025.1
MYH48359.1	TPR+GreAB-C+PIN*→	TPR+GreAB-C+PIN	1248	F4151_02205	Gammaproteobacteria	Gammaproteobacteria bacterium	MAG: hypothetical protein F4151_02205 [Gammaproteobacteria bacterium].	GCA_009843625.1
MYK18704.1	TPR+GreAB-C+PIN*→	TPR+GreAB-C+PIN	911	F4055_11145	Bacteria	Candidatus Poribacteria bacterium	MAG: tetratricopeptide repeat protein, partial [Candidatus Poribacteria bacterium].	GCA_009845505.1
MYK96751.1	TPR+GreAB-C+PIN*→	TPR+GreAB-C+PIN	470	F4009_22610	Bacteria	Candidatus Poribacteria bacterium	MAG: hypothetical protein F4009_22610 [Candidatus Poribacteria bacterium].	GCA_009846415.1
NAT27530.1	TPR+GreAB-C+PIN*→	TPR+GreAB-C+PIN	692	CVE22_06655	Gammaproteobacteria	Pseudomonas syringae pv. actinidiae	hypothetical protein CVE22_06655, partial [Pseudomonas syringae pv. actinidiae].	GCA_009903115.1
NBW14976.1	REase+TPR+GreAB-C+PIN*→	REase+TPR+GreAB-C+PIN	1102	EBR82_43945	Alphaproteobacteria	Caulobacteraceae bacterium	MAG: hypothetical protein EBR82_43945, partial [Caulobacteraceae bacterium].	GCA_009924585.1
NCC04365.1	REase+TPR+GreAB-C+PIN*→	REase+TPR+GreAB-C+PIN	1308	EOM37_10050	Proteobacteria	Proteobacteria bacterium	MAG: tetratricopeptide repeat protein [Proteobacteria bacterium].	GCA_009929575.1
NCN59296.1	<-CITB-HTH+LexA-protease||TPR+GreAB-C+PIN*→	TPR+GreAB-C+PIN	1274	GW940_05255	Bacteria	Candidatus Microgenomates bacterium	MAG: hypothetical protein GW940_05255 [Candidatus Microgenomates bacterium].	GCA_009992065.1
NCO24709.1	TPR+GreAB-C+PIN*→	TPR+GreAB-C+PIN	415	GW897_06315	unclassified Bacteria	bacterium	MAG: hypothetical protein GW897_06315 [bacterium].	GCA_009992775.1
NEZ58684.1	TPR+TPR→ TPR+TPR→ TPR+TPR→ TPR+TPR→ TPR+TPR→ TPR→ TPR→ PNPase+TPR+GreAB-C+PIN*→?→||||<-TPR+TPR	PNPase+TPR+GreAB-C+PIN	1392	DXZ20_24195	Cyanobacteria	Leptolyngbyaceae cyanobacterium CCMR0081	hypothetical protein DXZ20_24195 [Leptolyngbyaceae cyanobacterium CCMR0081].	GCA_011009555.1
NEZ64367.1	TPR+TPR→ TPR+TPR→ TPR+TPR→ TPR+TPR→ TPR+TPR→ TPR+TPR→ PNPase+TPR+GreAB-C+PIN*→?→||||<-TPR+TPR<-TPR+TPR<-?<-TIR	PNPase+TPR+GreAB-C+PIN	1392	D0962_16475	Cyanobacteria	Leptolyngbyaceae cyanobacterium CCMR0082	hypothetical protein D0962_16475 [Leptolyngbyaceae cyanobacterium CCMR0082].	GCA_011009535.1
NHI06604.1	PIN*→	PIN	211	STPH2_1967	Actinobacteria	Streptomyces sp. KO7888	hypothetical protein STPH2_1967 [Streptomyces sp. KO7888].	GCA_011317205.1
NHK99992.1	TPR+GreAB-C+PIN*→	TPR+GreAB-C+PIN	911	G7087_16540	Betaproteobacteria	Rubrivivax benzoatilyticus	hypothetical protein G7087_16540 [Rubrivivax benzoatilyticus].	GCA_011365705.1
NJN11600.1	TPR+GreAB-C+PIN*→	TPR+GreAB-C+PIN	298	HC815_27960	Cyanobacteria	Richelia sp. RM1_1_1	MAG: hypothetical protein HC815_27960 [Richelia sp. RM1_1_1].	GCA_012032385.1
NJO54209.1	REase+TPR+TPR+GreAB-C+PIN*→	REase+TPR+TPR+GreAB-C+PIN	1338	HC829_04580	Bacteroidetes	Bacteroidales bacterium	MAG: hypothetical protein HC829_04580 [Bacteroidales bacterium].	GCA_012033435.1
NKI94745.1	REase+TPR+GreAB-C+PIN*→	REase+TPR+GreAB-C+PIN	1340	FHT26_002685	Betaproteobacteria	Rhizobacter sp. SG703	tetratricopeptide (TPR) repeat protein [Rhizobacter sp. SG703].	GCA_012275445.1
NLD36189.1	REase+TPR+PIN*→	REase+TPR+PIN	1360	GX654_04895	Deltaproteobacteria	Desulfatiglans sp.	MAG: tetratricopeptide repeat protein [Desulfatiglans sp.].	GCA_012513605.1
NLD38959.1	TPR+PIN*→	TPR+PIN	1143	GX654_19035	Deltaproteobacteria	Desulfatiglans sp.	MAG: tetratricopeptide repeat protein [Desulfatiglans sp.].	GCA_012513605.1
NLD90744.1	PNPase+TPR+GreAB-C+PIN*→	PNPase+TPR+GreAB-C+PIN	1613	GX634_10460	Lentisphaerae	Lentisphaerae bacterium	MAG: hypothetical protein GX634_10460 [Lentisphaerae bacterium].	GCA_012514035.1
NLE27049.1	SIR2+TPR+GreAB-C+PIN*→	SIR2+TPR+GreAB-C+PIN	1448	GX625_17245	Firmicutes	Clostridiaceae bacterium	MAG: hypothetical protein GX625_17245 [Clostridiaceae bacterium].	GCA_012514215.1
NLE28033.1	TPR+GreAB-C+PIN*→	TPR+GreAB-C+PIN	758	GX625_22395	Firmicutes	Clostridiaceae bacterium	MAG: hypothetical protein GX625_22395, partial [Clostridiaceae bacterium].	GCA_012514215.1
NLG15547.1	TPR+GreAB-C+PIN*→	TPR+GreAB-C+PIN	1341	GX561_15255	Lentisphaerae	Lentisphaerae bacterium	MAG: hypothetical protein GX561_15255 [Lentisphaerae bacterium].	GCA_012515425.1
NLX50682.1	REase+TPR+GreAB-C+PIN*→ PSE→?→?→ REase+SNF2→	REase+TPR+GreAB-C+PIN	1299	GXY72_01105	Deltaproteobacteria	Deltaproteobacteria bacterium	MAG: hypothetical protein GXY72_01105 [Deltaproteobacteria bacterium].	GCA_012729055.1
NMQ30291.1	REase+TPR+GreAB-C+PIN*→	REase+TPR+GreAB-C+PIN	1072	E4Q23_22545	Betaproteobacteria	Candidatus Accumulibacter phosphatis	hypothetical protein E4Q23_22545, partial [Candidatus Accumulibacter phosphatis].	GCA_012939955.1
NOT34484.1	GreAB*→	GreAB	904	HOP12_09975	Bacteria	Candidatus Eisenbacteria bacterium	MAG: hypothetical protein HOP12_09975 [Candidatus Eisenbacteria bacterium].	GCA_013140805.1

acc	operon	architecture	len	gen.name	taxend	species	defline	gca
NOU31123.1	TPR+GreAB-C+PIN*→	TPR+GreAB-C+PIN	536	HOO96_24755	Deltaproteobacteria	Polyangiaceae bacterium	MAG: hypothetical protein HOO96_24755 [Polyangiaceae bacterium].	GCA_013141295.1
NQE54029.1	REase+TPR+GreAB-C+PIN*→	REase+TPR+GreAB-C+PIN	1291	lapB_1	Euryarchaeota	ANME-1 cluster archaeon GoMg3.2	Lipopolysaccharide assembly protein B [ANME-1 cluster archaeon GoMg3.2].	GCA_013180565.1
NQV22738.1	REase+TPR+GreAB-C+PIN*→	REase+TPR+GreAB-C+PIN	1245	HQ518_00080	Planctomycetes	Rhodopirellula sp.	MAG: hypothetical protein HQ518_00080 [Rhodopirellula sp.].	GCA_013203845.1
NQY79025.1	TPR+PIN*→	TPR+PIN	1272	HRT47_01810	Terrabacteria group	Candidatus Caenarcaniphilales bacterium	MAG: hypothetical protein HRT47_01810 [Candidatus Caenarcaniphilales bacterium].	GCA_013216135.1
NQZ35237.1	TPR+GreAB-C+PIN*→	TPR+GreAB-C+PIN	1069	HRT58_06210	Bacteroidetes	Crocinitomicaceae bacterium	MAG: hypothetical protein HRT58_06210 [Crocinitomicaceae bacterium].	GCA_013214785.1
NQZ40471.1	HetE-N1→ TPR+GreAB-C+PIN*→	TPR+GreAB-C+PIN	1088	HRT96_10385	Gammaproteobacteria	Moritella sp.	MAG: hypothetical protein HRT96_10385 [Moritella sp.].	GCA_013215705.1
NSZ07544.1	TPR+GreAB-C+PIN*→	TPR+GreAB-C+PIN	1180	G6M14_14320	Alphaproteobacteria	Agrobacterium tumefaciens	hypothetical protein G6M14_14320 [Agrobacterium tumefaciens].	GCA_013318315.1
NTV22000.1	TPR+PIN*→	TPR+PIN	942	HGB03_00315	Bacteria	Candidatus Yonathbacteria bacterium	MAG: hypothetical protein HGB03_00315 [Candidatus Yonathbacteria bacterium].	GCA_013334825.1
NTW26872.1	REase→ REase+TPR+GreAB-C+PIN*→	REase+TPR+GreAB-C+PIN	1310	HGA36_00950	Bacteria	Candidatus Moranbacteria bacterium	MAG: hypothetical protein HGA36_00950 [Candidatus Moranbacteria bacterium].	GCA_013336225.1
NTZ52560.1	HetE-N1→?→ TPR+GreAB-C+PIN*→	TPR+GreAB-C+PIN	513	FCH32_20020	Gammaproteobacteria	Citrobacter gillenii	hypothetical protein FCH32_20020 [Citrobacter gillenii].	GCA_013337685.1
NWE92806.1	PIN*→	PIN	344	HX893_32300	Gammaproteobacteria	Pseudomonas reactans	hypothetical protein HX893_32300, partial [Pseudomonas reactans].	GCA_013386215.1
NWO07476.1	HetE-N1→ TPR+GreAB-C+PIN*→	TPR+GreAB-C+PIN	1130	HLX50_17830	Gammaproteobacteria	Alteromonadaceae bacterium	MAG: hypothetical protein HLX50_17830 [Alteromonadaceae bacterium].	GCA_013393545.1
OBH13398.1	X+PIN*→	X+PIN	755	A5695_13915	Actinobacteria	Mycobacterium sp. E1747	hypothetical protein A5695_13915 [Mycobacterium sp. E1747].	GCA_001667775.1
OBR53101.1	REase+TPR+GreAB-C+PIN*→	REase+TPR+GreAB-C+PIN	1277	A6456_09045	Betaproteobacteria	Paraburkholderia tropica	hypothetical protein A6456_09045 [Paraburkholderia tropica].	GCA_001673675.1
OCX17561.1	REase+TPR+TPR+GreAB-C+PIN*→	REase+TPR+TPR+GreAB-C+PIN	1207	QV13_12440	Alphaproteobacteria	Mesorhizobium hungaricum	hypothetical protein QV13_12440 [Mesorhizobium hungaricum].	GCA_001705425.1
ODM45291.1	TPR+GreAB-C+PIN*→	TPR+GreAB-C+PIN	1187	A9320_11670	Alphaproteobacteria	Ruegeria sp. PBVC088	hypothetical protein A9320_11670 [Ruegeria sp. PBVC088].	GCA_001717995.1
ODS63626.1	GreAB-C+PIN*→	GreAB-C+PIN	516	ABS37_11570	Betaproteobacteria	Acidovorax sp. SCN 65-108	MAG: hypothetical protein ABS37_11570 [Acidovorax sp. SCN 65-108].	GCA_001724055.1
ODT73806.1	REase+TPR+TPR+GreAB-C+PIN*→	REase+TPR+TPR+GreAB-C+PIN	1296	ABS76_38455	Alphaproteobacteria	Pelagibacterium sp. SCN 64-44	MAG: hypothetical protein ABS76_38455 [Pelagibacterium sp. SCN 64-44].	GCA_001724955.1
OEJ21925.1	REase+PIN*→	REase+PIN	1260	AR457_38995	Actinobacteria	Streptomyces agglomeratus	hypothetical protein AR457_38995 [Streptomyces agglomeratus].	GCA_001746465.1
OFX87846.1	TPR+GreAB-C+PIN*→	TPR+GreAB-C+PIN	1236	A2X00_15495	Bacteroidetes	Bacteroidetes bacterium GWE2_32_14	MAG: hypothetical protein A2X00_15495 [Bacteroidetes bacterium GWE2_32_14].	GCA_001768405.1
OFY15991.1	TPR+GreAB-C+PIN*→	TPR+GreAB-C+PIN	1237	A2X02_04985	Bacteroidetes	Bacteroidetes bacterium GWF2_29_10	MAG: hypothetical protein A2X02_04985 [Bacteroidetes bacterium GWF2_29_10].	GCA_001768555.1
OGC02499.1	PIN*→	PIN	178	A2V82_02050	Bacteria	candidate division KSB1 bacterium RBG_16_48_16	MAG: hypothetical protein A2V82_02050 [candidate division KSB1 bacterium RBG_16_48_16].	GCA_001771235.1
OGH88116.1	TPR+GreAB-C+PIN*→	TPR+GreAB-C+PIN	1191	A3J93_00010	Bacteria	Candidatus Magasanikbacteria bacterium RIFOXYC2_FULL_42_28	MAG: hypothetical protein A3J93_00010 [Candidatus Magasanikbacteria bacterium RIFOXYC2_FULL_42_28].	GCA_001784435.1
OGI36016.1	TRD+TRD→?→?→?→ TPR+PIN*→	TPR+PIN	1186	A2259_01680	Bacteria	Candidatus Moranbacteria bacterium RIFOXYA2_FULL_43_15	MAG: hypothetical protein A2259_01680 [Candidatus Moranbacteria bacterium RIFOXYA2_FULL_43_15].	GCA_001784835.1

acc	operon	architecture	len	gen.name	taxend	species	defline	gca
OGS08035.1	TPR+GreAB-C+PIN*→	TPR+GreAB-C+PIN	1270	A2270_03040	Elusimicrobia	Elusimicrobia bacterium RIFOXYA12_FULL_51_18	MAG: hypothetical protein A2270_03040 [Elusimicrobia bacterium RIFOXYA12_FULL_51_18].	GCA_001800245.1
OGU66359.1	REase+TPR+GreAB-C+PIN*→	REase+TPR+GreAB-C+PIN	1303	A2W30_02620	Ignavibacteriae	Ignavibacteria bacterium RBG_16_36_9	MAG: hypothetical protein A2W30_02620 [Ignavibacteria bacterium RBG_16_36_9].	GCA_001802895.1
OGU77131.1	PIN*→	PIN	573	A2W11_11280	Ignavibacteriae	Ignavibacteria bacterium RBG_16_35_7	MAG: hypothetical protein A2W11_11280 [Ignavibacteria bacterium RBG_16_35_7].	GCA_001802885.1
OIP66815.1	TPR+GreAB-C+PIN*→	TPR+GreAB-C+PIN	402	AUK42_07760	Bacteria	Candidatus Atribacteria bacterium CG2_30_33_13	MAG: hypothetical protein AUK42_07760 [Candidatus Atribacteria bacterium CG2_30_33_13].	GCA_001873345.1
OJV65414.1	HetE-N1→ HetE-N1+TPR+GreAB-C+PIN*→	HetE-N1+TPR+GreAB-C+PIN	1117	BGO35_21125	Betaproteobacteria	Burkholderiales bacterium 64-34	MAG: hypothetical protein BGO35_21125 [Burkholderiales bacterium 64-34].	GCA_001897585.1
OJW35018.1	TPR+GreAB-C+PIN*→||||<-REase	TPR+GreAB-C+PIN	1246	BGO54_02365	Bacteroidetes	Sphingobacteriales bacterium 46-32	MAG: hypothetical protein BGO54_02365 [Sphingobacteriales bacterium 46-32].	GCA_001898485.1
OJW61617.1	TIR→?→?→ TPR+GreAB-C+PIN*→	TPR+GreAB-C+PIN	1114	BGO65_09490	Alphaproteobacteria	Afipia sp. 64-13	MAG: hypothetical protein BGO65_09490 [Afipia sp. 64-13].	GCA_001897755.1
OMR67119.1	TPR+GreAB-C+PIN*→||||<-HTH+HTH	TPR+GreAB-C+PIN	879	AQ727_25970	Betaproteobacteria	Burkholderia pseudomallei	hypothetical protein AQ727_25970 [Burkholderia pseudomallei].	GCA_001976575.1
OSN14318.1	REase+TPR+GreAB-C+PIN*→	REase+TPR+GreAB-C+PIN	1486	BV340_04459	Gammaproteobacteria	Pseudomonas syringae pv. actinidiae	hypothetical protein BV340_04459 [Pseudomonas syringae pv. actinidiae].	GCA_002111665.1
OSN30099.1	REase+TPR+GreAB-C+PIN*→	REase+TPR+GreAB-C+PIN	1336	BV342_05184	Gammaproteobacteria	Pseudomonas syringae pv. actinidiae	hypothetical protein BV342_05184 [Pseudomonas syringae pv. actinidiae].	GCA_002111745.1
OUC14796.1	TPR+TPR+PNPase→ PNPase+TPR+GreAB-C+PIN*→	PNPase+TPR+GreAB-C+PIN	1396	B0A82_10750	Cyanobacteria	Alkalinema sp. CACIAM 70d	MAG: hypothetical protein B0A82_10750 [Alkalinema sp. CACIAM 70d].	GCA_002148405.1
OUR78422.1	TPR+GreAB-C+PIN*→	TPR+GreAB-C+PIN	1191	A9Q83_07770	Alphaproteobacteria	Alphaproteobacteria bacterium 46_93_T64	hypothetical protein A9Q83_07770 [Alphaproteobacteria bacterium 46_93_T64].	GCA_002163135.1
OWZ89184.1	REase+TPR+TPR+GreAB-C+PIN*→	REase+TPR+TPR+GreAB-C+PIN	1352	B9J07_34245	Alphaproteobacteria	Sinorhizobium sp. LM21	hypothetical protein B9J07_34245 [Sinorhizobium sp. LM21].	GCA_002216665.1
OYD66813.1	REase+TPR+GreAB-C+PIN*→?→ Trypsin→	REase+TPR+GreAB-C+PIN	1243	BDB13_0310	Actinobacteria	Rhodococcus sp. OK302	uncharacterized protein DUF4365 [Rhodococcus sp. OK302].	GCA_002245895.1
OYW86349.1	TPR+GreAB-C+PIN*→	TPR+GreAB-C+PIN	629	B7Z22_06790	Alphaproteobacteria	Hyphomonas sp. 32-62-5	MAG: hypothetical protein B7Z22_06790, partial [Hyphomonas sp. 32-62-5].	GCA_002280065.1
OYX15563.1	REase+TPR+GreAB-C+PIN*→	REase+TPR+GreAB-C+PIN	1211	B7Z15_00100	Alphaproteobacteria	Rhizobiales bacterium 32-66-8	MAG: hypothetical protein B7Z15_00100 [Rhizobiales bacterium 32-66-8].	GCA_002280205.1
OZB15488.1	REase+TPR+GreAB-C+PIN*→	REase+TPR+GreAB-C+PIN	1294	B7X53_11340	Alphaproteobacteria	Hyphomonas sp. 34-62-18	MAG: hypothetical protein B7X53_11340 [Hyphomonas sp. 34-62-18].	GCA_002282565.1
PBC66280.1	X+PIN*→	X+PIN	685	BX265_8343	Actinobacteria	Streptomyces sp. TLI_235	hypothetical protein BX265_8343 [Streptomyces sp. TLI_235].	GCA_002300355.1
PCJ29538.1	REase+TPR+PIN*→	REase+TPR+PIN	1292	COA99_19375	Gammaproteobacteria	Moraxellaceae bacterium	MAG: hypothetical protein COA99_19375 [Moraxellaceae bacterium].	GCA_002402125.1
PDQ17661.1	REase+TPR+TPR+GreAB-C+PIN*→	REase+TPR+TPR+GreAB-C+PIN	1265	CN311_28810	Alphaproteobacteria	Mesorhizobium sanjuanii	hypothetical protein CN311_28810 [Mesorhizobium sanjuanii].	GCA_002529485.1
PGS40120.1	TPR+PIN*→	TPR+PIN	1006	COC58_18130	Firmicutes	Bacillus cereus	hypothetical protein COC58_18130 [Bacillus cereus].	GCA_002577165.1
PIG41510.1	TPR+PIN*→	TPR+PIN	1214	CLZ78_0083	Actinobacteria	Streptomyces sp. 61	hypothetical protein CLZ78_0083 [Streptomyces sp. 61].	GCA_002754535.1
PIP62053.1	<-CITB-HTH+LexA-protease||TPR+GreAB-C+PIN*→	TPR+GreAB-C+PIN	1272	COW99_00715	Bacteria	Candidatus Roizmanbacteria bacterium CG22_combo_CG10-13_8_21_14_all_38_20	MAG: hypothetical protein COW99_00715 [Candidatus Roizmanbacteria bacterium CG22_combo_CG10-13_8_21_14_all_38_20].	GCA_002786635.1
PIU06549.1	TPR+GreAB-C+PIN*→	TPR+GreAB-C+PIN	1132	COT56_09580	Alphaproteobacteria	Methylobacterium sp. CG09_land_8_20_14_0_10_71_15	MAG: hypothetical protein COT56_09580 [Methylobacterium sp. CG09_land_8_20_14_0_10_71_15].	GCA_002778835.1

acc	operon	architecture	len	gen.name	taxend	species	defline	gca
PKM50370.1	TPR+PIN*→	TPR+PIN	1214	CVV02_11355	Firmicutes	Firmicutes bacterium HGW-Firmicutes-7	MAG: hypothetical protein CVV02_11355 [Firmicutes bacterium HGW-Firmicutes-7].	GCA_002841375.1
PKN75416.1	TPR+GreAB-C+PIN*→	TPR+GreAB-C+PIN	1116	CVU49_03915	FCB group	Candidatus Cloacimonetes bacterium HGW-Cloacimonetes-2	MAG: hypothetical protein CVU49_03915 [Candidatus Cloacimonetes bacterium HGW-Cloacimonetes-2].	GCA_002842035.1
PKO82710.1	HetE-N1→ HetE-N1+TPR+GreAB-C+PIN*→	HetE-N1+TPR+GreAB-C+PIN	1126	CVU19_00260	Betaproteobacteria	Betaproteobacteria bacterium HGW-Betaproteobacteria-13	MAG: hypothetical protein CVU19_00260 [Betaproteobacteria bacterium HGW-Betaproteobacteria-13].	GCA_002842325.1
PLP59030.1	REase+TPR+TPR+GreAB-C+PIN*→	REase+TPR+TPR+GreAB-C+PIN	1226	CYK37_11065	Alphaproteobacteria	Mesorhizobium loti	hypothetical protein CYK37_11065 [Mesorhizobium loti].	GCA_002858745.1
PLX86308.1	TPR+GreAB-C+PIN*→	TPR+GreAB-C+PIN	1333	C0617_01645	Deltaproteobacteria	Desulfuromonas sp.	MAG: hypothetical protein C0617_01645 [Desulfuromonas sp.].	GCA_002868845.1
PLY41243.1	TPR+GreAB-C+PIN*→	TPR+GreAB-C+PIN	1106	CSZ94_17070	Betaproteobacteria	Janthinobacterium sp. ROICE36	hypothetical protein CSZ94_17070 [Janthinobacterium sp. ROICE36].	GCA_002869965.1
PMJ73057.1	HetE-N1→?→ GreAB-C*→	GreAB-C	461	BCU23_16040	Gammaproteobacteria	Vibrio splendidus	hypothetical protein BCU23_16040 [Vibrio splendidus].	GCA_002877525.1
POU10505.1	PIN*→	PIN	385	C3420_16980	Gammaproteobacteria	Acinetobacter sp. ACNIH3	hypothetical protein C3420_16980 [Acinetobacter sp. ACNIH3].	GCA_002918965.1
PQZ99140.1	TPR+PIN*→	TPR+PIN	932	CQ019_16410	Actinobacteria	Arthrobacter sp. MYb229	hypothetical protein CQ019_16410 [Arthrobacter sp. MYb229].	GCA_002975405.1
PRY64434.1	HetE-N1→ TPR+GreAB-C+PIN*→	TPR+GreAB-C+PIN	1090	B0H98_10595	Gammaproteobacteria	Halomonas songnenensis	hypothetical protein B0H98_10595 [Halomonas songnenensis].	GCA_003002925.1
PSL26440.1	HNH+TPR+GreAB-C+PIN*→	HNH+TPR+GreAB-C+PIN	1234	CLV42_111154	Bacteroidetes	Chitinophaga ginsengisoli	HNH endonuclease [Chitinophaga ginsengisoli].	GCA_003014595.1
PTH81074.1	TPR+GreAB-C+PIN*→	TPR+GreAB-C+PIN	851	DAA48_11040	Gammaproteobacteria	Aeromonas veronii	hypothetical protein DAA48_11040 [Aeromonas veronii].	GCA_003036425.1
PTL60251.1	X+PIN*→	X+PIN	1319	C7Y72_11685	Actinobacteria	Conexibacter sp. Seoho-28	hypothetical protein C7Y72_11685 [Conexibacter sp. Seoho-28].	GCA_003044185.1
PTX57421.1	TPR+GreAB-C+PIN*→	TPR+GreAB-C+PIN	1184	C8N43_2089	Alphaproteobacteria	Litoreibacter ponti	hypothetical protein C8N43_2089 [Litoreibacter ponti].	GCA_003054285.1
PWM31885.1	GreAB*→	GreAB	675	DBX55_01895	Verrucomicrobia	Verrucomicrobia bacterium	MAG: transcription elongation factor GreAB [Verrucomicrobia bacterium].	GCA_003343565.1
PWM77364.1	NACHT→?→ X+PIN*→ HTH→	X+PIN	733	DBY32_08445	Firmicutes	Phascolarctobacterium sp.	MAG: hypothetical protein DBY32_08445 [Phascolarctobacterium sp.].	GCA_003150755.1
PWM80532.1	TPR+GreAB-C+PIN*→	TPR+GreAB-C+PIN	1185	DBY41_05620	Firmicutes	Clostridium sp.	MAG: hypothetical protein DBY41_05620 [Clostridium sp.].	GCA_003150745.1
PZR80846.1	BirA-HTH→ HEPN→ REase+TPR+TPR+GreAB-C+PIN*→	REase+TPR+TPR+GreAB-C+PIN	1131	DI537_39015	Gammaproteobacteria	Pseudomonas stutzeri	MAG: hypothetical protein DI537_39015, partial [Pseudomonas stutzeri].	GCA_003243385.1
PZR82088.1	TPR+GreAB-C+PIN*→	TPR+GreAB-C+PIN	1139	DI537_37240	Gammaproteobacteria	Pseudomonas stutzeri	MAG: hypothetical protein DI537_37240 [Pseudomonas stutzeri].	GCA_003243385.1
PZU06315.1	REase+TPR+GreAB-C+PIN*→	REase+TPR+GreAB-C+PIN	1323	DI605_19340	Alphaproteobacteria	Sphingomonas sp.	MAG: hypothetical protein DI605_19340 [Sphingomonas sp.].	GCA_003248515.1
PZU12119.1	TPR+GreAB-C+PIN*→	TPR+GreAB-C+PIN	859	DI622_15520	Bacteroidetes	Chryseobacterium sp.	MAG: hypothetical protein DI622_15520, partial [Chryseobacterium sp.].	GCA_003248465.1
QDS92471.1	REase+TPR+GreAB-C+PIN*→	REase+TPR+GreAB-C+PIN	1250	FF011L_12140	Planctomycetes	Planctomycetes bacterium FF011L	hypothetical protein FF011L_12140 [Planctomycetes bacterium FF011L].	GCA_007741495.1
QDT50511.1	REase+TPR+GreAB-C+PIN→ TPR+GreAB-C+PIN*→	TPR+GreAB-C+PIN	495	Pan258_45900	Planctomycetes	Symmachiella dynata	hypothetical protein Pan258_45900 [Symmachiella dynata].	GCA_007744975.1
QDV12361.1	REase+TPR+GreAB-C+PIN*→	REase+TPR+GreAB-C+PIN	1246	CA51_22440	Planctomycetes	Rosistilla oblonga	Tetratricopeptide repeat protein [Rosistilla oblonga].	GCA_007751715.1
QDV84971.1	TPR+GreAB-C+PIN*→	TPR+GreAB-C+PIN	954	TBK1r_39230	Planctomycetes	Planctomycetes bacterium TBK1r	hypothetical protein TBK1r_39230 [Planctomycetes bacterium TBK1r].	GCA_007753675.1

acc	operon	architecture	len	gen.name	taxend	species	defline	gca
QFS96076.1	TPR+GreAB-C+PIN*→	TPR+GreAB-C+PIN	1184	FIV06_01510	Alphaproteobacteria	Labrenzia sp. THAF191b	hypothetical protein FIV06_01510 [Labrenzia sp. THAF191b].	GCA_009363315.1
QHB84489.1	HetE-N1→ TPR+GreAB-C+PIN*→	TPR+GreAB-C+PIN	1094	GIS01_21340	Gammaproteobacteria	Aeromonas veronii	hypothetical protein GIS01_21340 [Aeromonas veronii].	GCA_009833025.1
QIC10441.1	HetE-N1→ TPR+GreAB-C+PIN*→	TPR+GreAB-C+PIN	1069	PmVP161_2026	Gammaproteobacteria	Pasteurella multocida	hypothetical protein PmVP161_2026 [Pasteurella multocida].	GCA_011390865.1
QND19257.1	REase+TPR+TPR+GreAB-C+PIN*→	REase+TPR+TPR+GreAB-C+PIN	1348	HB774_02700	Alphaproteobacteria	Rhizobium leguminosarum bv. viciae	hypothetical protein HB774_02700 [Rhizobium leguminosarum bv. viciae].	GCA_014189655.1
QPL52208.1	HetE-N1→ TPR+GreAB-C+PIN*→	TPR+GreAB-C+PIN	1130	I3X05_08770	Gammaproteobacteria	Vibrio navarrensis	hypothetical protein I3X05_08770 [Vibrio navarrensis].	GCA_015767675.1
QQO85647.1	HetE-N1→ TPR+GreAB-C+PIN*→	TPR+GreAB-C+PIN	1089	D7032_21665	Gammaproteobacteria	Shewanella algae	hypothetical protein D7032_21665 [Shewanella algae].	-
QQR55448.1	X+PIN*→	X+PIN	1237	IPG41_02740	Bacteria	Candidatus Peregrinibacteria bacterium	MAG: hypothetical protein IPG41_02740 [Candidatus Peregrinibacteria bacterium].	GCA_016699145.1
RAZ30556.1	TPR+GreAB-C+PIN*→	TPR+GreAB-C+PIN	1208	DO944_13495	Actinobacteria	Microbacterium sp. SMR1	hypothetical protein DO944_13495 [Microbacterium sp. SMR1].	GCA_003289625.1
RBN51622.1	TPR+GreAB-C+PIN*→	TPR+GreAB-C+PIN	1135	DR980_00180	Bacteroidetes	Flavobacterium psychrolimnae	hypothetical protein DR980_00180 [Flavobacterium psychrolimnae].	GCA_003312425.1
RBP77372.1	TPR+GreAB-C+PIN*→	TPR+GreAB-C+PIN	1175	DFO80_1601	Alphaproteobacteria	Rhodobacter sp. 140A	hypothetical protein DFO80_1601, partial [Rhodobacter sp. 140A].	GCA_003315475.1
RCI79301.1	TPR+GreAB-C+PIN*→	TPR+GreAB-C+PIN	630	DNK03_13895	Alphaproteobacteria	Brucella anthropi	hypothetical protein DNK03_13895 [Brucella anthropi].	GCA_003325675.1
REJ78752.1	TPR+GreAB-C+PIN*→||||<-?<-?<-?<-REase+SNF2	TPR+GreAB-C+PIN	1257	DWQ32_08270	Acidobacteria	Acidobacteria bacterium	MAG: hypothetical protein DWQ32_08270 [Acidobacteria bacterium].	GCA_003385635.1
REJ87566.1	TPR+PIN*→	TPR+PIN	2085	DWQ34_24785	Planctomycetes	Planctomycetes bacterium	MAG: hypothetical protein DWQ34_24785 [Planctomycetes bacterium].	GCA_003388575.1
RGJ64387.1	X+PIN*→ HTH→?→?→ HTH→	X+PIN	1176	DXD50_08885	Firmicutes	Dorea formicigenerans	hypothetical protein DXD50_08885 [Dorea formicigenerans].	GCA_003437395.1
RGN88412.1	X+PIN*→ HTH→?→?→ HTH→	X+PIN	1176	DXB36_13695	Firmicutes	Dorea formicigenerans	hypothetical protein DXB36_13695 [Dorea formicigenerans].	GCA_003439255.1
RGR97298.1	TM+TM+TPR+PIN*→	TM+TM+TPR+PIN	1206	DWY20_07060	Bacteroidetes	Phocaeicola coprocola	hypothetical protein DWY20_07060 [Phocaeicola coprocola].	GCA_003458565.1
RGS66838.1	X+PIN*→ HTH→?→?→ HTH→	X+PIN	766	DWX78_14950	Firmicutes	Dorea formicigenerans	hypothetical protein DWX78_14950 [Dorea formicigenerans].	GCA_003458845.1
RGY02630.1	TPR+GreAB-C+PIN*→	TPR+GreAB-C+PIN	410	DXA56_15985	Firmicutes	Blautia obeum	hypothetical protein DXA56_15985 [Blautia obeum].	GCA_003463085.1
RGZ63692.1	Mbetalac→ Trypsin→?→ TPR+PIN*→	TPR+PIN	1371	DW979_12370	Firmicutes	Eubacterium sp. AM49-13BH	hypothetical protein DW979_12370, partial [Eubacterium sp. AM49-13BH].	GCA_003464165.1
RHN14952.1	X+PIN*→ HTH→?→?→ HTH→	X+PIN	1175	DWZ24_11335	Firmicutes	Dorea formicigenerans	hypothetical protein DWZ24_11335 [Dorea formicigenerans].	GCA_003475555.1
RHV44852.1	X+PIN*→	X+PIN	1229	DXB47_09745	Firmicutes	Firmicutes bacterium OM04-13BH	hypothetical protein DXB47_09745 [Firmicutes bacterium OM04-13BH].	GCA_003481745.1
RHZ93582.1	TPR+GreAB-C+PIN*→	TPR+GreAB-C+PIN	924	D1920_20895	Alphaproteobacteria	Rhodopseudomonas palustris	hypothetical protein D1920_20895 [Rhodopseudomonas palustris].	GCA_003547145.1
RJT21930.1	TPR+GreAB-C*→	TPR+GreAB-C	282	D5I55_15915	Alphaproteobacteria	Aestuariisphingobium litorale	hypothetical protein D5I55_15915 [Aestuariisphingobium litorale].	GCA_003602075.1
RKE07817.1	TPR+PIN*→	TPR+PIN	806	C8E86_2653	Actinobacteria	Catellatospora citrea	hypothetical protein C8E86_2653 [Catellatospora citrea].	GCA_003610235.1
RKG98095.1	TPR+GreAB-C+PIN*→	TPR+GreAB-C+PIN	461	D7X32_30660	Deltaproteobacteria	Corallococcus carmarthensis	hypothetical protein D7X32_30660 [Corallococcus carmarthensis].	GCA_003611695.1
RKS06034.1	TPR+PIN*→ PAIREDC-HTH→	TPR+PIN	1209	DFP74_1655	Actinobacteria	Nocardiopsis sp. Huas11	hypothetical protein DFP74_1655 [Nocardiopsis sp. Huas11].	GCA_003634495.1

acc	operon	architecture	len	gen.name	taxend	species	defline	gca
RKU09109.1	TPR+GreAB-C+PIN*→	TPR+GreAB-C+PIN	1315	C6502_13840	Bacteria	Candidatus Poribacteria bacterium	MAG: hypothetical protein C6502_13840 [Candidatus Poribacteria bacterium].	GCA_003635265.1
RKW53732.1	X+PIN*→	X+PIN	1207	D8H95_11955	Firmicutes	Lachnospiraceae bacterium	MAG: hypothetical protein D8H95_11955 [Lachnospiraceae bacterium].	GCA_003640285.1
RKY60125.1	PIN*→	PIN	174	DRP94_01435	FCB group	Candidatus Latescibacteria bacterium	MAG: DUF3368 domain-containing protein [Candidatus Latescibacteria bacterium].	GCA_003645885.1
RMB87410.1	TPR+PIN*→	TPR+PIN	746	CTZ28_00010	Actinobacteria	Streptomyces shenzhenensis	hypothetical protein CTZ28_00010 [Streptomyces shenzhenensis].	GCA_003688995.1
RMQ50424.1	REase+TPR+GreAB-C+PIN*→	REase+TPR+GreAB-C+PIN	1489	ALQ04_05433	Gammaproteobacteria	Pseudomonas cichorii	hypothetical protein ALQ04_05433 [Pseudomonas cichorii].	GCA_003700275.1
RMV01228.1	REase+TPR+GreAB-C+PIN*→	REase+TPR+GreAB-C+PIN	1486	ALP20_200053	Gammaproteobacteria	Pseudomonas coronafaciens pv. coronafaciens	hypothetical protein ALP20_200053 [Pseudomonas coronafaciens pv. coronafaciens].	GCA_003702885.1
RMV06369.1	TPR+GreAB-C+PIN*→	TPR+GreAB-C+PIN	904	ALP17_05452	Gammaproteobacteria	Pseudomonas savastanoi	hypothetical protein ALP17_05452 [Pseudomonas savastanoi].	GCA_003702965.1
RMV08832.1	REase→ TPR+GreAB-C+PIN*→	TPR+GreAB-C+PIN	881	ALP16_05399	Gammaproteobacteria	Pseudomonas savastanoi	hypothetical protein ALP16_05399, partial [Pseudomonas savastanoi].	GCA_003702985.1
RNA68542.1	<-REase||TPR+PIN*→	TPR+PIN	999	EBO34_00790	Firmicutes	Bacillus sp. KQ-3	hypothetical protein EBO34_00790 [Bacillus sp. KQ-3].	GCA_003710255.1
RND30553.1	HetE-N1→ HetE-N1+TPR+GreAB-C+PIN*→	HetE-N1+TPR+GreAB-C+PIN	1088	EC575_19215	Gammaproteobacteria	Vibrio cholerae	hypothetical protein EC575_19215 [Vibrio cholerae].	GCA_003712005.1
ROZ42670.1	HTH+PNPase+TPR+GreAB-C+PIN*→	HTH+PNPase+TPR+GreAB-C+PIN	1571	EEB13_29810	Actinobacteria	Rhodococcus sp. WS3	hypothetical protein EEB13_29810 [Rhodococcus sp. WS3].	GCA_003797085.1
RPV62916.1	TPR+GreAB-C+PIN*→	TPR+GreAB-C+PIN	808	IPC830_03545	Gammaproteobacteria	Pseudomonas aeruginosa	hypothetical protein IPC830_03545 [Pseudomonas aeruginosa].	GCA_003836445.1
RPX47920.1	DOC+HTH→ REase+TOPC→?→ HetE-N1→ HetE-N1+TPR+GreAB-C+PIN*→	HetE-N1+TPR+GreAB-C+PIN	1126	IPC713_13275	Gammaproteobacteria	Pseudomonas aeruginosa	hypothetical protein IPC713_13275 [Pseudomonas aeruginosa].	GCA_003836815.1
RQR65213.1	REase+TPR+GreAB-C+PIN*→	REase+TPR+GreAB-C+PIN	1346	DIE12_31765	Betaproteobacteria	Burkholderia sp. Bp9015	tetratricopeptide repeat protein [Burkholderia sp. Bp9015].	GCA_003853505.1
RQY09920.1	REase+TPR+GreAB-C+PIN*→	REase+TPR+GreAB-C+PIN	1135	DF117_34925	Betaproteobacteria	Burkholderia stagnalis	tetratricopeptide repeat protein [Burkholderia stagnalis].	GCA_003857615.1
RSM21562.1	TPR+GreAB-C+PIN*→	TPR+GreAB-C+PIN	851	C5B76_20720	Gammaproteobacteria	Aeromonas salmonicida	hypothetical protein C5B76_20720 [Aeromonas salmonicida].	GCA_003947355.1
RSM25744.1	MNS-Npun2340+TPR+GreAB-C+PIN*→	MNS-Npun2340+TPR+GreAB-C+PIN	851	C5B77_18585	Gammaproteobacteria	Aeromonas salmonicida	hypothetical protein C5B77_18585 [Aeromonas salmonicida].	GCA_003947375.1
RWE64146.1	REase+TPR+GreAB-C+PIN*→	REase+TPR+GreAB-C+PIN	1329	EOS62_30690	Alphaproteobacteria	Mesorhizobium sp.	MAG: hypothetical protein EOS62_30690 [Mesorhizobium sp.].	GCA_004018195.1
RWI50090.1	REase+TPR+GreAB-C+PIN*→	REase+TPR+GreAB-C+PIN	1328	EOR16_31040	Alphaproteobacteria	Mesorhizobium sp.	MAG: hypothetical protein EOR16_31040 [Mesorhizobium sp.].	GCA_004019035.1
RWN29279.1	REase+TPR+TPR+GreAB-C+PIN*→	REase+TPR+TPR+GreAB-C+PIN	1333	EOR95_21980	Alphaproteobacteria	Mesorhizobium sp.	MAG: hypothetical protein EOR95_21980 [Mesorhizobium sp.].	GCA_004020785.1
RWN66659.1	REase+TPR+TPR+GreAB-C+PIN*→	REase+TPR+TPR+GreAB-C+PIN	1333	EOR99_16025	Alphaproteobacteria	Mesorhizobium sp.	MAG: hypothetical protein EOR99_16025 [Mesorhizobium sp.].	GCA_004020565.1
RWQ61473.1	REase+TPR+TPR+GreAB-C+PIN*→	REase+TPR+TPR+GreAB-C+PIN	1333	EOS86_33015	Alphaproteobacteria	Mesorhizobium sp.	MAG: hypothetical protein EOS86_33015 [Mesorhizobium sp.].	GCA_004021745.1
RWQ70448.1	REase+TPR+TPR+GreAB-C+PIN*→	REase+TPR+TPR+GreAB-C+PIN	1002	EOS85_27065	Alphaproteobacteria	Mesorhizobium sp.	MAG: hypothetical protein EOS85_27065 [Mesorhizobium sp.].	GCA_004021925.1
RWU17258.1	REase+TPR+GreAB-C+PIN*→	REase+TPR+GreAB-C+PIN	1447	DM813_28290	Gammaproteobacteria	Pseudomonas alkylphenolica	hypothetical protein DM813_28290 [Pseudomonas alkylphenolica].	GCA_004025535.1
RXH58244.1	REase+TPR+GreAB-C+PIN*→	REase+TPR+GreAB-C+PIN	1185	GRAN_1554	Acidobacteria	Granulicella sibirica	hypothetical protein GRAN_1554 [Granulicella sibirica].	GCA_004115155.1
RXK05389.1	REase+TPR+GreAB-C+PIN*→	REase+TPR+GreAB-C+PIN	1249	CRU97_08585	Epsilonproteobacteria	Halarcobacter bivalviorum	hypothetical protein CRU97_08585 [Halarcobacter bivalviorum].	GCA_004116705.1

acc	operon	architecture	len	gen.name	taxend	species	defline	gca
RXO70745.1	HetE-N1→?→ TPR+GreAB-C+PIN*→	TPR+GreAB-C+PIN	686	D0516_07920	Gammaproteobacteria	Salmonella enterica	hypothetical protein D0516_07920 [Salmonella enterica].	GCA_004120835.1
RYE55646.1	PIN*→	PIN	344	EOP48_09765	Bacteroidetes	Sphingobacteriales bacterium	MAG: hypothetical protein EOP48_09765 [Sphingobacteriales bacterium].	GCA_004144425.1
RYG99407.1	REase+TPR+GreAB-C+PIN*→	REase+TPR+GreAB-C+PIN	1330	EON58_04040	Alphaproteobacteria	Alphaproteobacteria bacterium	MAG: hypothetical protein EON58_04040 [Alphaproteobacteria bacterium].	GCA_004145575.1
RYX84272.1	TPR+GreAB-C+PIN*→	TPR+GreAB-C+PIN	1192	EON83_11290	unclassified Bacteria	bacterium	MAG: hypothetical protein EON83_11290 [bacterium].	GCA_004172935.1
RYY90115.1	REase+TPR+GreAB-C+PIN*→	REase+TPR+GreAB-C+PIN	1333	EOO15_03925	Bacteroidetes	Chitinophagaceae bacterium	MAG: hypothetical protein EOO15_03925 [Chitinophagaceae bacterium].	GCA_004173495.1
RZJ58585.1	TPR+PIN*→	TPR+PIN	1309	EOO55_00010	Bacteroidetes	Hymenobacter sp.	MAG: hypothetical protein EOO55_00010 [Hymenobacter sp.].	GCA_004211105.1
RZJ92304.1	TPR+GreAB-C+PIN*→	TPR+GreAB-C+PIN	1267	EOO20_02310	Bacteroidetes	Chryseobacterium sp.	MAG: hypothetical protein EOO20_02310 [Chryseobacterium sp.].	GCA_004210875.1
RZJ92386.1	REase+nSTAND3→ TPR+GreAB-C+PIN*→	TPR+GreAB-C+PIN	1237	EOO20_01975	Bacteroidetes	Chryseobacterium sp.	MAG: tetratricopeptide repeat protein [Chryseobacterium sp.].	GCA_004210875.1
RZL63110.1	PIN*→	PIN	307	EOO93_08085	Bacteroidetes	Pedobacter sp.	MAG: hypothetical protein EOO93_08085 [Pedobacter sp.].	GCA_004295955.1
RZL84003.1	REase+TPR+GreAB-C+PIN*→	REase+TPR+GreAB-C+PIN	1323	EOP66_03300	Alphaproteobacteria	Sphingomonas sp.	MAG: tetratricopeptide repeat protein [Sphingomonas sp.].	GCA_004296035.1
SCB54124.1	REase+TPR+GreAB-C+PIN*→	REase+TPR+GreAB-C+PIN	1171	GA0061098_102379	Alphaproteobacteria	Bradyrhizobium shewense	Transcription elongation factor, GreA/GreB family [Bradyrhizobium shewense].	GCA_900094605.1
SCE01180.1	TPR+PIN*→||||<-NUDIX	TPR+PIN	1134	GA0115241_108994	Actinobacteria	Streptomyces sp. DpondAA-D4	hypothetical protein GA0115241_108994 [Streptomyces sp. DpondAA-D4].	GCA_900091745.1
SCF05211.1	REase+TPR+GreAB-C+PIN*→	REase+TPR+GreAB-C+PIN	1358	GA0070215_1072	Actinobacteria	Micromonospora marina	hypothetical protein GA0070215_1072 [Micromonospora marina].	GCA_900091565.1
SCF05420.1	SAD+HNH→ X+PIN*→	X+PIN	1233	GA0070562_5649	Actinobacteria	Micromonospora tulbaghiae	hypothetical protein GA0070562_5649 [Micromonospora tulbaghiae].	GCA_900091605.1
SCL17674.1	REase+TPR+GreAB-C+PIN*→	REase+TPR+GreAB-C+PIN	1264	GA0074694_2093	Actinobacteria	Micromonospora inyonensis	Tfp pilus assembly protein PilF [Micromonospora inyonensis].	GCA_900091415.1
SCQ18794.1	CHC2+Toprim-DNAG→?→?→?→ HTH→ SIG+TM→ TPR+GreAB-C+PIN*→	TPR+GreAB-C+PIN	1126	TFUB4_00594	Bacteroidetes	Tannerella forsythia	hypothetical protein TFUB4_00594 [Tannerella forsythia].	GCA_900096725.1
SCQ19220.1	CHC2+Toprim-DNAG→?→?→?→ HTH→ SIG+TM→ TPR+GreAB-C+PIN*→	TPR+GreAB-C+PIN	1126	TFUB20_00631	Bacteroidetes	Tannerella forsythia	hypothetical protein TFUB20_00631 [Tannerella forsythia].	GCA_900096735.1
SEP47521.1	TPR+GreAB-C+PIN*→	TPR+GreAB-C+PIN	975	SAMN02787149_1275	Gammaproteobacteria	Pseudomonas sp. Snoq117.2	hypothetical protein SAMN02787149_1275 [Pseudomonas sp. Snoq117.2].	GCA_900110545.1
SFD04359.1	<-KAP_NTPase||TPR+PIN*→	TPR+PIN	1216	SAMN04487968_1267	Actinobacteria	Nocardioides terrae	hypothetical protein SAMN04487968_1267 [Nocardioides terrae].	GCA_900112345.1
SFF03357.1	<-Cas_Cas1<-?||?→?→?→?→ REase+TPR+GreAB-C+PIN*→	REase+TPR+GreAB-C+PIN	1349	SAMN05428977_10446	Betaproteobacteria	Nitrosomonas sp. Nm166	hypothetical protein SAMN05428977_10446 [Nitrosomonas sp. Nm166].	GCA_900112825.1
SHH05937.1	REase+TPR+GreAB-C+PIN*→	REase+TPR+GreAB-C+PIN	1445	SAMN05428948_2561	Betaproteobacteria	Massilia sp. CF038	hypothetical protein SAMN05428948_2561 [Massilia sp. CF038].	GCA_900129765.1
SHN30683.1	PAIREDC-HTH→ REase+TPR+GreAB-C+PIN*→	REase+TPR+GreAB-C+PIN	1588	SAMN02787076_04976	Betaproteobacteria	Rhizobacter sp. OV335	hypothetical protein SAMN02787076_04976 [Rhizobacter sp. OV335].	GCA_900142965.1
SIO63359.1	REase+TPR+GreAB-C+PIN*→	REase+TPR+GreAB-C+PIN	1291	SAMN05444166_7323	Planctomycetes	Singulisphaera sp. GP187	Transcription elongation factor, GreA/GreB family [Singulisphaera sp. GP187].	GCA_900129635.1
SOZ22209.1	REase+TPR+GreAB-C+PIN→?→?→ PIN*→	PIN	271	CBM2604_U10003	Betaproteobacteria	Cupriavidus taiwanensis	hypothetical protein CBM2604_U10003 [Cupriavidus taiwanensis].	GCA_900249855.1
SPT59210.1	TPR+GreAB-C+PIN*→	TPR+GreAB-C+PIN	1218	NCTC11373_05764	Actinobacteria	Actinomadura madurae	Flp pilus assembly protein TadD, contains TPR repeats [Actinomadura madurae].	GCA_900445005.1
SPY99988.1	TPR+GreAB-C+PIN*→	TPR+GreAB-C+PIN	1146	NCTC11842_00133	Gammaproteobacteria	Pseudomonas luteola	Uncharacterised protein [Pseudomonas luteola].	GCA_900455515.1

acc	operon	architecture	len	gen.name	taxend	species	defline	gca
SQK85244.1	DOC+HTH→ REase+TOPC→ HetE-N1→ TPR+GreAB-C+PIN*→	TPR+GreAB-C+PIN	1088	NCTC9433_00599	Gammaproteobacteria	Pseudomonas aeruginosa	Uncharacterised protein [Pseudomonas aeruginosa].	GCA_900478745.1
SSC09307.1	TPR+GreAB-C+PIN*→	TPR+GreAB-C+PIN	1137	BTURTLESOX_2492	unclassified Bacteria	bacterium endosymbiont of Bathymodiolus sp. 5 South	hypothetical protein BTURTLESOX_2492 [bacterium endosymbiont of Bathymodiolus sp. 5 South].	GCA_900127295.1
STJ38438.1	HetE-N1→ TPR+GreAB-C+PIN*→	TPR+GreAB-C+PIN	1093	NCTC9110_01467	Gammaproteobacteria	Escherichia coli	Uncharacterised protein [Escherichia coli].	GCA_900449425.1
STR27356.1	REase+TPR+GreAB-C+PIN*→	REase+TPR+GreAB-C+PIN	1303	NCTC8661_05000	Betaproteobacteria	Janthinobacterium lividum	Putative Zn-dependent protease, contains TPR repeats [Janthinobacterium lividum].	GCA_900451225.1
SUJ03282.1	PIN*→	PIN	223	NCTC4822_01389	Firmicutes	Sporosarcina pasteurii	Uncharacterised protein [Sporosarcina pasteurii].	GCA_900457495.1
TAA79883.1	HetE-N1→ TPR+GreAB-C+PIN*→	TPR+GreAB-C+PIN	1069	PMCND_08490	Gammaproteobacteria	Pasteurella multocida	hypothetical protein PMCND_08490 [Pasteurella multocida].	GCA_004286935.1
TAJ47852.1	TPR+PIN*→	TPR+PIN	1186	EPO52_06520	Actinobacteria	Herbiconiux sp.	MAG: hypothetical protein EPO52_06520 [Herbiconiux sp.].	GCA_004297105.1
TAM51231.1	REase+TPR+GreAB-C+PIN*→	REase+TPR+GreAB-C+PIN	1301	EPN57_18445	Betaproteobacteria	Paraburkholderia sp.	MAG: hypothetical protein EPN57_18445 [Paraburkholderia sp.].	GCA_004322045.1
TAM55201.1	REase+TPR+GreAB-C+PIN*→||||<-?||AbiJ-NTD3+REase→	REase+TPR+GreAB-C+PIN	1335	EPN57_04160	Betaproteobacteria	Paraburkholderia sp.	MAG: hypothetical protein EPN57_04160 [Paraburkholderia sp.].	GCA_004322045.1
TCU73661.1	TPR+GreAB-C+PIN*→	TPR+GreAB-C+PIN	1313	EDE10_104327	Alphaproteobacteria	Bradyrhizobium sp. Y-H1	tetratricopeptide repeat protein [Bradyrhizobium sp. Y-H1].	GCA_004346395.1
TDM08142.1	REase+TPR+GreAB-C+PIN*→	REase+TPR+GreAB-C+PIN	1202	C4K60_01155	Betaproteobacteria	Ideonella sp. MAG2	MAG: hypothetical protein C4K60_01155 [Ideonella sp. MAG2].	GCA_004359425.1
TDZ78257.1	TPR+PIN*→	TPR+PIN	1185	DE4585_04094	Actinobacteria	Mycobacteroides salmoniphilum	hypothetical protein DE4585_04094 [Mycobacteroides salmoniphilum].	GCA_004366855.1
TIN45883.1	TPR+GreAB-C+PIN*→ TPR→	TPR+GreAB-C+PIN	539	E5Y32_12205	Alphaproteobacteria	Mesorhizobium sp.	MAG: GreA/GreB family elongation factor [Mesorhizobium sp.].	GCA_004961865.1
TIT34473.1	TPR+GreAB-C+PIN*→	TPR+GreAB-C+PIN	502	E5W78_10605	Alphaproteobacteria	Mesorhizobium sp.	MAG: hypothetical protein E5W78_10605, partial [Mesorhizobium sp.].	GCA_004965505.1
TIX43905.1	TPR+GreAB-C+PIN*→	TPR+GreAB-C+PIN	708	E5V40_01360	Alphaproteobacteria	Mesorhizobium sp.	MAG: GreA/GreB family elongation factor [Mesorhizobium sp.].	GCA_004964855.1
TLM83646.1	TPR+PIN*→	TPR+PIN	1179	FDW83_09305	Actinobacteria	Pseudarthrobacter sp. NamE2	tetratricopeptide repeat protein [Pseudarthrobacter sp. NamE2].	GCA_005796205.1
TMA89552.1	REase+TPR+GreAB-C+PIN*→	REase+TPR+GreAB-C+PIN	1299	E6J74_25025	Deltaproteobacteria	Deltaproteobacteria bacterium	MAG: hypothetical protein E6J74_25025 [Deltaproteobacteria bacterium].	GCA_005879255.1
TMJ19619.1	REase+TPR+GreAB-C+PIN*→	REase+TPR+GreAB-C+PIN	1331	E6G92_07565	Alphaproteobacteria	Alphaproteobacteria bacterium	MAG: hypothetical protein E6G92_07565 [Alphaproteobacteria bacterium].	GCA_005883305.1
TOF36973.1	TPR+GreAB-C+PIN*→	TPR+GreAB-C+PIN	785	CGJ28_20670	Gammaproteobacteria	Vibrio parahaemolyticus	hypothetical protein CGJ28_20670 [Vibrio parahaemolyticus].	GCA_006371865.1
TOH27190.1	GreAB-C*→	GreAB-C	599	CGI84_22675	Gammaproteobacteria	Vibrio parahaemolyticus	hypothetical protein CGI84_22675, partial [Vibrio parahaemolyticus].	GCA_006372765.1
TQE99579.1	REase+SNF2→ REase+TPR+GreAB-C+PIN*→	REase+TPR+GreAB-C+PIN	1230	FKY71_07895	Gammaproteobacteria	Spiribacter salinus	MAG: hypothetical protein FKY71_07895 [Spiribacter salinus].	GCA_006569195.1
TQS42859.1	REase+TPR+GreAB-C+PIN*→	REase+TPR+GreAB-C+PIN	1252	FL583_22685	Actinobacteria	Cryptosporangium phraense	DUF4365 domain-containing protein [Cryptosporangium phraense].	GCA_006912135.1
TSP11291.1	REase+TPR+GreAB-C+PIN*→	REase+TPR+GreAB-C+PIN	1314	FGG12_18885	Betaproteobacteria	Cupriavidus campinensis	DUF4365 domain-containing protein [Cupriavidus campinensis].	GCA_007572485.1
TVP63446.1	EAD10+SWACOS→?→ EAD10+TPR+GreAB-C+PIN*→	EAD10+TPR+GreAB-C+PIN	1213	EA343_08005	Cyanobacteria	Nodularia sp. (in: Bacteria)	MAG: hypothetical protein EA343_08005 [Nodularia sp. (in: Bacteria)].	GCA_007692755.1
TWD39045.1	HetE-N1→ TPR+GreAB-C+PIN*→	TPR+GreAB-C+PIN	1117	FBY13_107200	Gammaproteobacteria	Pantoea sp. SJZ147	hypothetical protein FBY13_107200 [Pantoea sp. SJZ147].	GCA_007828605.1
TWI95027.1	TPR+GreAB-C+PIN→ PIN*→	PIN	240	JN11_04456	Bacteroidetes	Mucilaginibacter frigoritolerans	hypothetical protein JN11_04456 [Mucilaginibacter frigoritolerans].	GCA_007830615.1

acc	operon	architecture	len	gen.name	taxend	species	defline	gca
TXH46669.1	REase+TPR+GreAB-C+PIN*→	REase+TPR+GreAB-C+PIN	1345	E6Q92_01630	Betaproteobacteria	Burkholderiaceae bacterium	MAG: tetratricopeptide repeat protein [Burkholderiaceae bacterium].	GCA_008012415.1
TXJ01161.1	X+PIN*→	X+PIN	1259	E6Q32_04910	Betaproteobacteria	Neisseriales bacterium	MAG: hypothetical protein E6Q32_04910 [Neisseriales bacterium].	GCA_008017145.1
VBE91451.1	REase+TPR+GreAB-C+PIN*→	REase+TPR+GreAB-C+PIN	1327	SAMEA2066717_00371	Betaproteobacteria	Burkholderia pseudomallei	Uncharacterised protein [Burkholderia pseudomallei].	GCA_900591745.1
VBP17170.1	REase+TPR+GreAB-C+PIN*→	REase+TPR+GreAB-C+PIN	1327	SAMEA2065229_01356	Betaproteobacteria	Burkholderia pseudomallei	Uncharacterised protein [Burkholderia pseudomallei].	GCA_900593515.1
VBP73370.1	REase+TPR+GreAB-C+PIN*→	REase+TPR+GreAB-C+PIN	1327	SAMEA2068240_03247	Betaproteobacteria	Burkholderia pseudomallei	Uncharacterised protein [Burkholderia pseudomallei].	GCA_900593615.1
VEA49048.1	HetE-N1→ TPR+GreAB-C+PIN*→	TPR+GreAB-C+PIN	1107	NCTC7306_04489	Gammaproteobacteria	Salmonella enterica subsp. arizonae	Uncharacterised protein [Salmonella enterica subsp. arizonae].	GCA_900635595.1
VTU02511.1	REase+TPR+GreAB-C+PIN*→	REase+TPR+GreAB-C+PIN	2231	GCJUQL4_63830	Planctomycetes	Gemmataceae bacterium	atpase aaa : Uncharacterized protein OS=delta proteobacterium MLMS-1 GN=MldDRAFT_0410 PE=4 SV=1: TPR_9 [Gemmataceae bacterium].	GCA_901538445.1
VUO82246.1	REase+TPR+GreAB-C+PIN*→	REase+TPR+GreAB-C+PIN	1284	MFJNGALL_01363	Gammaproteobacteria	Stenotrophomonas maltophilia	hypothetical protein MFJNGALL_01363 [Stenotrophomonas maltophilia].	GCA_902157665.1
VUP91206.1	REase+TPR→ TPR+PIN*→	TPR+PIN	777	MKCCKLKB_00143	Gammaproteobacteria	Stenotrophomonas maltophilia	hypothetical protein MKCCKLKB_00143 [Stenotrophomonas maltophilia].	GCA_902158105.1
VVH66851.1	TPR+GreAB-C+PIN*→	TPR+GreAB-C+PIN	1163	BSPLISOX_572	environmental samples	uncultured Gammaproteobacteria bacterium	hypothetical protein BSPLISOX_572 [uncultured Gammaproteobacteria bacterium].	GCA_902497535.1
VVM25671.1	TPR→ TPR+GreAB-C+PIN*→	TPR+GreAB-C+PIN	622	BSPWISOXPB_7707	environmental samples	uncultured Gammaproteobacteria bacterium	hypothetical protein BSPWISOXPB_7707 [uncultured Gammaproteobacteria bacterium].	GCA_902497575.1
WP_000012642.1	TPR+PIN*→	TPR+PIN	1218	-	Firmicutes	Bacillus cereus	hypothetical protein [Bacillus cereus].	GCF_000291235.1
WP_000989353.1	HetE-N1→ TPR+GreAB-C+PIN*→	TPR+GreAB-C+PIN	1094	-	Gammaproteobacteria	Escherichia	MULTISPECIES: hypothetical protein [Escherichia].	GCF_000503375.1
WP_002163366.1	TPR+PIN*→	TPR+PIN	1214	-	Firmicutes	Bacillus paranthracis	hypothetical protein [Bacillus paranthracis].	GCF_900176925.1
WP_002203277.1	TPR+PIN*→	TPR+PIN	1158	-	Firmicutes	Bacillus cereus	hypothetical protein [Bacillus cereus].	GCF_000293545.1
WP_002981151.1	TPR+GreAB-C+PIN*→	TPR+GreAB-C+PIN	1137	-	Bacteroidetes	Chryseobacterium	MULTISPECIES: hypothetical protein [Chryseobacterium].	GCF_003815675.1
WP_003193440.1	TPR+PIN*→	TPR+PIN	1179	-	Firmicutes	Bacillus mycoides	hypothetical protein [Bacillus mycoides].	GCF_000003925.1
WP_003320554.1	TPR+GreAB-C+PIN*→	TPR+GreAB-C+PIN	1148	-	Firmicutes	Alkalihalobacillus alcalophilus	hypothetical protein [Alkalihalobacillus alcalophilus].	GCF_000292245.2
WP_003365555.1	TPR+GreAB-C+PIN*→	TPR+GreAB-C+PIN	1052	-	Firmicutes	Clostridium botulinum	hypothetical protein [Clostridium botulinum].	GCF_000171095.1
WP_004107781.1	REase+TPR+TPR+GreAB-C+PIN*→	REase+TPR+TPR+GreAB-C+PIN	1363	-	Alphaproteobacteria	Rhizobium freirei	restriction endonuclease [Rhizobium freirei].	GCF_000359745.1
WP_004552328.1	REase+TPR+GreAB-C+PIN*→	REase+TPR+GreAB-C+PIN	1345	-	Betaproteobacteria	Burkholderia pseudomallei	hypothetical protein [Burkholderia pseudomallei].	GCF_000259815.1
WP_005336021.1	X+PIN*→ HTH→?→?→ HTH→	X+PIN	1180	-	Firmicutes	Dorea formicigenerans	hypothetical protein [Dorea formicigenerans].	GCF_000169235.1
WP_005619555.1	REase+TPR+GreAB-C+PIN*→	REase+TPR+GreAB-C+PIN	1313	-	Alphaproteobacteria	Epibacterium mobile	tetratricopeptide repeat protein [Epibacterium mobile].	GCF_000376545.2
WP_005875010.1	HTH→ SIG+TM→ TPR+GreAB-C+PIN*→	TPR+GreAB-C+PIN	1125	-	Bacteroidetes	Porphyromonas gingivalis	hypothetical protein [Porphyromonas gingivalis].	GCF_000007585.1
WP_007114289.1	TPR+GreAB-C+PIN*→	TPR+GreAB-C+PIN	1116	-	Gammaproteobacteria	Halomonas boliviensis	hypothetical protein [Halomonas boliviensis].	GCF_000236035.1
WP_007575774.1	REase+TPR+GreAB-C+PIN*→	REase+TPR+GreAB-C+PIN	1298	-	Actinobacteria	Patulibacter medicamentivorans	hypothetical protein [Patulibacter medicamentivorans].	GCF_000240225.1
WP_008074062.1	HetE-N1→ TPR+GreAB-C+PIN*→	TPR+GreAB-C+PIN	1116	-	Gammaproteobacteria	Vibrio sinaloensis	hypothetical protein [Vibrio sinaloensis].	GCF_000189275.1
WP_008371053.1	TPR+GreAB-C+PIN*→	TPR+GreAB-C+PIN	916	-	Gammaproteobacteria	Pseudomonas sp. M47T1	hypothetical protein [Pseudomonas sp. M47T1].	GCF_000263855.1
WP_008676251.1	REase+TPR+GreAB-C+PIN*→	REase+TPR+GreAB-C+PIN	1224	-	Planctomycetes	Rhodopirellula sallentina	phosphorylase family 1 protein [Rhodopirellula sallentina].	GCF_000346505.1
WP_008891423.1	REase+TPR+GreAB-C+PIN*→	REase+TPR+GreAB-C+PIN	1287	-	Alphaproteobacteria	Thalassospira profundimaris	tetratricopeptide repeat protein [Thalassospira profundimaris].	GCF_000300275.1
WP_009135826.1	X+PIN*→	X+PIN	1248	-	Bacteroidetes	Odoribacter laneus	hypothetical protein [Odoribacter laneus].	GCF_902374615.1
WP_009204273.1	X+PIN*→	X+PIN	1224	-	Firmicutes	Anaerostipes hadrus	hypothetical protein [Anaerostipes hadrus].	GCF_000332875.2

acc	operon	architecture	len	gen.name	taxend	species	defline	gca
WP_009805247.1	REase+TPR+GreAB-C+PIN*→	REase+TPR+GreAB-C+PIN	1334	-	Alphaproteobacteria	Pseudooceanicola batsensis	restriction endonuclease [Pseudooceanicola batsensis].	GCF_000152725.1
WP_009857784.1	REase+TPR+GreAB-C+PIN*→	REase+TPR+GreAB-C+PIN	1374	-	Betaproteobacteria	Rubrivivax benzoatilyticus	restriction endonuclease [Rubrivivax benzoatilyticus].	GCF_000190375.1
WP_009899558.1	TPR+GreAB-C+PIN*→	TPR+GreAB-C+PIN	1069	-	Firmicutes	Clostridioides difficile	hypothetical protein [Clostridioides difficile].	GCF_000211235.1
WP_010516915.1	REase+TPR+GreAB-C+PIN*→	REase+TPR+GreAB-C+PIN	1323	-	Alphaproteobacteria	Komagataeibacter oboediens	tetratricopeptide repeat protein [Komagataeibacter oboediens].	GCF_000227565.1
WP_010555470.1	HetE-N1→ TPR+GreAB-C+PIN*→	TPR+GreAB-C+PIN	1100	-	Gammaproteobacteria	Pseudoalteromonas arctica	hypothetical protein [Pseudoalteromonas arctica].	GCF_000238395.3
WP_011204815.1	REase+TPR+GreAB-C+PIN*→||||<-HTH+HTH	REase+TPR+GreAB-C+PIN	1344	-	Betaproteobacteria	Burkholderia pseudomallei	hypothetical protein [Burkholderia pseudomallei].	GCF_000350505.1
WP_011691689.1	TPR+PIN*→	TPR+PIN	1180	-	Actinobacteria	Arthrobacter sp. FB24	tetratricopeptide repeat protein [Arthrobacter sp. FB24].	GCF_000196235.1
WP_012187551.1	TPR+PIN*→	TPR+PIN	1280	-	Gammaproteobacteria	Fluoribacter dumoffii	hypothetical protein [Fluoribacter dumoffii].	-
WP_012304478.1	HetE-N1→ TPR+GreAB-C+PIN*→	TPR+GreAB-C+PIN	1090	-	Gammaproteobacteria	Yersinia pseudotuberculosis	hypothetical protein [Yersinia pseudotuberculosis].	GCF_000019465.1
WP_012365289.1	REase+TPR+GreAB-C+PIN*→	REase+TPR+GreAB-C+PIN	1351	-	Betaproteobacteria	Burkholderia ambifaria	hypothetical protein [Burkholderia ambifaria].	GCF_000019925.1
WP_012655030.1	REase+TPR+GreAB-C+PIN*→	REase+TPR+GreAB-C+PIN	1290	-	Alphaproteobacteria	Agrobacterium vitis	tetratricopeptide repeat protein [Agrobacterium vitis].	GCF_000016285.1
WP_013320645.1	<-TIR+CASPASE<-?<-?||PNPase+TPR+GreAB-C+PIN*→	PNPase+TPR+GreAB-C+PIN	1389	-	Cyanobacteria	Gloeothece verrucosa	purine or other phosphorylase family 1 [Gloeothece verrucosa].	GCF_000147335.1
WP_013724217.1	HetE-N1→ MNS-Npun2340+TPR+GreAB-C+PIN*→	MNS-Npun2340+TPR+GreAB-C+PIN	1094	-	Gammaproteobacteria	Aeromonas veronii	hypothetical protein [Aeromonas veronii].	GCF_000204115.1
WP_013846680.1	REase+TPR+GreAB-C+PIN*→	REase+TPR+GreAB-C+PIN	1311	-	Alphaproteobacteria	Sphingobium chlorophenolicum	hypothetical protein [Sphingobium chlorophenolicum].	GCF_000147835.2
WP_013921742.1	Mbetalac→ Trypsin+TPR+GreAB-C+PIN*→	Trypsin+TPR+GreAB-C+PIN	1363	-	Bacteroidetes	Runella slithyformis	trypsin-like peptidase domain-containing protein [Runella slithyformis].	GCF_000218895.1
WP_014399772.1	TPR+GreAB-C+PIN*→	TPR+GreAB-C+PIN	1160	-	Deltaproteobacteria	Corallococcus coralloides	GreA/GreB family elongation factor [Corallococcus coralloides].	GCF_000255295.1
WP_015949235.1	REase+TPR+GreAB-C+PIN*→	REase+TPR+GreAB-C+PIN	1112	-	Deltaproteobacteria	Desulfatibacillum aliphaticivorans	hypothetical protein [Desulfatibacillum aliphaticivorans].	GCF_000021905.1
WP_016083038.1	TPR+PIN*→	TPR+PIN	1154	-	Firmicutes	Bacillus cereus	hypothetical protein [Bacillus cereus].	GCF_000399005.1
WP_016796932.1	TPR+GreAB-C+PIN*→	TPR+GreAB-C+PIN	1236	-	Gammaproteobacteria	Vibrio cyclitrophicus	hypothetical protein [Vibrio cyclitrophicus].	GCF_000256385.2
WP_016981456.1	REase+TPR+GreAB-C+PIN*→	REase+TPR+GreAB-C+PIN	1113	-	Gammaproteobacteria	Pseudomonas amygdali	hypothetical protein [Pseudomonas amygdali].	GCF_000275945.1
WP_017010147.1	TPR+GreAB-C+PIN*→	TPR+GreAB-C+PIN	1114	-	Gammaproteobacteria	Enterovibrio norvegicus	hypothetical protein [Enterovibrio norvegicus].	GCF_000286875.2
WP_017056082.1	HetE-N1→ TPR+GreAB-C+PIN*→	TPR+GreAB-C+PIN	1088	-	Gammaproteobacteria	Vibrio kanaloae	hypothetical protein [Vibrio kanaloae].	GCF_000272165.2
WP_017278805.1	TPR+GreAB-C+PIN*→	TPR+GreAB-C+PIN	1158	-	Gammaproteobacteria	Pseudomonas syringae	hypothetical protein [Pseudomonas syringae].	GCF_001913215.1
WP_017307658.1	EAD10→?→?→ EAD10+TPR+GreAB-C+PIN*→	EAD10+TPR+GreAB-C+PIN	1214	-	Cyanobacteria	Fischerella sp. PCC 9339	hypothetical protein [Fischerella sp. PCC 9339].	GCF_000315585.1
WP_017684618.1	REase+TPR+GreAB-C+PIN*→	REase+TPR+GreAB-C+PIN	1438	-	Gammaproteobacteria	Pseudomonas syringae	hypothetical protein [Pseudomonas syringae].	GCF_000233815.1
WP_017867504.1	X+PIN*→	X+PIN	1207	-	Firmicutes	Ligilactobacillus pobuzihii	DUF4365 domain-containing protein [Ligilactobacillus pobuzihii].	GCF_001433815.1
WP_018464656.1	TPR+PIN*→	TPR+PIN	1200	-	Bacteroidetes	Prevotella paludivivens	hypothetical protein [Prevotella paludivivens].	GCF_000613605.1
WP_019086277.1	REase+TPR+GreAB-C+PIN*→	REase+TPR+GreAB-C+PIN	1323	-	Alphaproteobacteria	Komagataeibacter europaeus	tetratricopeptide repeat protein [Komagataeibacter europaeus].	GCF_000285295.1
WP_019092358.1	REase+TPR+GreAB-C+PIN*→	REase+TPR+GreAB-C+PIN	1323	-	Alphaproteobacteria	Acetobacteraceae	MULTISPECIES: tetratricopeptide repeat protein [Acetobacteraceae].	GCF_003850965.1
WP_019865944.1	HetE-N1→ TPR+GreAB-C+PIN*→	TPR+GreAB-C+PIN	1111	-	Gammaproteobacteria	Methylovulum miyakonense	hypothetical protein [Methylovulum miyakonense].	GCF_000384075.1
WP_019949723.1	TPR+GreAB-C+PIN*→	TPR+GreAB-C+PIN	909	-	Bacteroidetes	Hymenobacter aerophilus	hypothetical protein [Hymenobacter aerophilus].	GCF_000382225.1
WP_020099407.1	TPR+PIN*→	TPR+PIN	1185	-	Actinobacteria	Mycobacterium sp. 360MFTsu5.1	hypothetical protein [Mycobacterium sp. 360MFTsu5.1].	GCF_000383495.1
WP_020795266.1	HetE-N1→ TPR+GreAB-C+PIN*→	TPR+GreAB-C+PIN	1112	-	Gammaproteobacteria	Pseudomonas sp. G5(2012)	hypothetical protein [Pseudomonas sp. G5(2012)].	GCF_000408945.1
WP_021554497.1	HetE-N1→ TPR+GreAB-C+PIN*→	TPR+GreAB-C+PIN	1093	-	Gammaproteobacteria	Enterobacteriaceae	MULTISPECIES: hypothetical protein [Enterobacteriaceae].	GCF_002110225.1
WP_021876213.1	PIN*→	PIN	1097	-	Firmicutes	Clostridium chauvoei	tetratricopeptide repeat protein [Clostridium chauvoei].	GCF_002327205.1

acc	operon	architecture	len	gen.name	taxend	species	defline	gca
WP_022388983.1	X+PIN*→	X+PIN	1200	-	Firmicutes	Blautia obeum	hypothetical protein [Blautia obeum].	GCF_013299585.1
WP_022619607.1	TPR+GreAB-C+PIN*→	TPR+GreAB-C+PIN	1069	-	Firmicutes	Clostridioides difficile	hypothetical protein [Clostridioides difficile].	GCF_000530295.1
WP_022674357.1	REase→?→ REase+TPR+GreAB-C+PIN*→	REase+TPR+GreAB-C+PIN	1290	-	Alphaproteobacteria	Novosphingopyxis baekryungensis	hypothetical protein [Novosphingopyxis baekryungensis].	GCF_000420305.1
WP_024552132.1	HNH→?→?→ HetE-N1→ TPR+GreAB-C+PIN*→	TPR+GreAB-C+PIN	1092	-	Gammaproteobacteria	Franconibacter helveticus	hypothetical protein [Franconibacter helveticus].	GCF_000485945.1
WP_024678816.1	REase+TPR+GreAB-C+PIN*→	REase+TPR+GreAB-C+PIN	1317	-	Gammaproteobacteria	Pseudomonas syringae	DUF4365 domain-containing protein [Pseudomonas syringae].	GCF_000452685.1
WP_024698051.1	REase+TPR+GreAB-C+PIN*→	REase+TPR+GreAB-C+PIN	1438	-	Gammaproteobacteria	Pseudomonas avellanae	hypothetical protein [Pseudomonas avellanae].	GCF_000452845.1
WP_024925158.1	REase+TPR+TPR+GreAB-C+PIN*→	REase+TPR+TPR+GreAB-C+PIN	1315	-	Alphaproteobacteria	Mesorhizobium	MULTISPECIES: GreA/GreB family elongation factor [Mesorhizobium].	GCF_000568635.1
WP_025240246.1	REase+TOPC→ HetE-N1→ HetE-N1+TPR+GreAB-C+PIN*→	HetE-N1+TPR+GreAB-C+PIN	952	-	Gammaproteobacteria	Pseudomonas stutzeri	hypothetical protein [Pseudomonas stutzeri].	GCF_000590475.1
WP_026105277.1	TPR+GreAB-C+PIN*→	TPR+GreAB-C+PIN	611	-	Alphaproteobacteria	Methylobacterium sp. MB200	hypothetical protein [Methylobacterium sp. MB200].	GCF_000333655.1
WP_026508802.1	HEPN+SNF→?→?→?→ X+PIN*→?→ CITB-HTH+LexA-protease→	X+PIN	1212	-	Firmicutes	Butyrivibrio sp. MC2013	hypothetical protein [Butyrivibrio sp. MC2013].	GCF_000424585.1
WP_026811785.1	TPR+PIN*→	TPR+PIN	1261	-	Bacteroidetes	Arenibacter certesii	hypothetical protein [Arenibacter certesii].	GCF_000429545.1
WP_027471277.1	TPR+GreAB-C+PIN*→	TPR+GreAB-C+PIN	1073	-	Bacteroidetes	Saccharicrinis fermentans	hypothetical protein [Saccharicrinis fermentans].	GCF_000517085.1
WP_028048816.1	HTH+PNPase+TPR+GreAB-C+PIN*→	HTH+PNPase+TPR+GreAB-C+PIN	1518	-	Actinobacteria	Cellulomonas sp. URHD0024	helix-turn-helix domain-containing protein [Cellulomonas sp. URHD0024].	GCF_000426185.1
WP_029071033.1	X+PIN*→	X+PIN	1212	-	Firmicutes	Kandleria vitulina	hypothetical protein [Kandleria vitulina].	GCF_000622125.1
WP_029379391.1	REase+TPR+GreAB-C+PIN*→	REase+TPR+GreAB-C+PIN	1438	-	Gammaproteobacteria	Pseudomonas fuscovaginae	hypothetical protein [Pseudomonas fuscovaginae].	GCF_000251185.1
WP_031437352.1	REase+TPR+GreAB-C+PIN*→	REase+TPR+GreAB-C+PIN	1323	-	Gammaproteobacteria	Methylobacter tundripaludum	DUF4365 domain-containing protein [Methylobacter tundripaludum].	GCF_000733835.1
WP_031445473.1	TPR+GreAB-C+PIN*→	TPR+GreAB-C+PIN	1244	-	Bacteroidetes	Arenibacter algicola	hypothetical protein [Arenibacter algicola].	GCF_000733925.1
WP_031548868.1	REase→ X+PIN*→?→?→?→?→?→?→?→ MACRODOMAIN→	X+PIN	1209	-	Firmicutes	Oribacterium sp. FC2011	hypothetical protein [Oribacterium sp. FC2011].	GCF_000701645.1
WP_032661435.1	TPR+GreAB-C+PIN*→	TPR+GreAB-C+PIN	1139	-	Gammaproteobacteria	Pseudomonas syringae	hypothetical protein [Pseudomonas syringae].	GCF_000800685.1
WP_032902625.1	REase+TPR+GreAB-C+PIN*→	REase+TPR+GreAB-C+PIN	1438	-	Gammaproteobacteria	Pseudomonas	MULTISPECIES: hypothetical protein [Pseudomonas].	GCF_000511155.2
WP_033132679.1	HetE-N1→ TPR+GreAB-C+PIN*→	TPR+GreAB-C+PIN	1074	-	Gammaproteobacteria	Acinetobacter sp. MN12	hypothetical protein [Acinetobacter sp. MN12].	GCF_000764915.1
WP_033469897.1	REase+TPR+GreAB-C+PIN*→	REase+TPR+GreAB-C+PIN	1282	-	Betaproteobacteria	Bordetella bronchiseptica	hypothetical protein [Bordetella bronchiseptica].	GCF_000690175.1
WP_033736921.1	HetE-N1→?→ TPR+GreAB-C+PIN*→	TPR+GreAB-C+PIN	760	-	Gammaproteobacteria	Pantoea sp. Sc1	hypothetical protein [Pantoea sp. Sc1].	GCF_000255315.1
WP_034127539.1	TPR+GreAB-C+PIN*→	TPR+GreAB-C+PIN	1158	-	Gammaproteobacteria	Pseudomonas fluorescens	hypothetical protein [Pseudomonas fluorescens].	GCF_000801795.1
WP_034129364.1	REase+TPR+GreAB-C+PIN*→	REase+TPR+GreAB-C+PIN	1244	-	Gammaproteobacteria	Acinetobacter baumannii	hypothetical protein [Acinetobacter baumannii].	GCF_000802915.1
WP_034392332.1	REase+TPR+GreAB-C+PIN*→	REase+TPR+GreAB-C+PIN	1413	-	Betaproteobacteria	Delftia acidovorans	hypothetical protein [Delftia acidovorans].	GCF_001598795.1
WP_034708052.1	HetE-N1→?→?→ GreAB-C+PIN*→	GreAB-C+PIN	575	-	Gammaproteobacteria	Acinetobacter bereziniae	hypothetical protein [Acinetobacter bereziniae].	GCF_000368505.1
WP_035460854.1	HetE-N1→ TPR+GreAB-C+PIN*→	TPR+GreAB-C+PIN	1104	-	Gammaproteobacteria	unclassified Alcanivorax	MULTISPECIES: hypothetical protein [unclassified Alcanivorax].	GCF_000524665.1
WP_035530189.1	REase+TPR+GreAB-C+PIN*→	REase+TPR+GreAB-C+PIN	1332	-	Alphaproteobacteria	Hoeflea sp. BAL378	hypothetical protein [Hoeflea sp. BAL378].	GCF_000759435.1
WP_035646445.1	REase+TPR+TPR+GreAB-C+PIN*→	REase+TPR+TPR+GreAB-C+PIN	1316	-	Alphaproteobacteria	Bradyrhizobium sp. ORS 285	hypothetical protein [Bradyrhizobium sp. ORS 285].	GCF_000239755.1
WP_036748977.1	HetE-N1→ TPR+GreAB-C+PIN*→	TPR+GreAB-C+PIN	1130	-	Gammaproteobacteria	Photobacterium galatheae	hypothetical protein [Photobacterium galatheae].	GCF_000695255.1
WP_036783497.1	HetE-N1→ TPR+GreAB-C+PIN*→	TPR+GreAB-C+PIN	1104	-	Gammaproteobacteria	Photorhabdus aegyptia	hypothetical protein [Photorhabdus aegyptia].	GCF_000612035.1
WP_038397119.1	<-HTH<-?<-?||HetE-N1→ TPR+GreAB-C+PIN*→	TPR+GreAB-C+PIN	1107	-	Gammaproteobacteria	Salmonella enterica	hypothetical protein [Salmonella enterica].	GCF_000756465.1
WP_039292971.1	TPR+PIN*→	TPR+PIN	1180	-	Firmicutes	Paenibacillus sp. IHB B 3415	hypothetical protein [Paenibacillus sp. IHB B 3415].	GCF_000802655.2
WP_039419134.1	CHC2+Toprim-DNAG→?→?→?→ HTH→ SIG+TM→ TPR+GreAB-C+PIN*→	TPR+GreAB-C+PIN	1140	-	Bacteroidetes	Porphyromonas gulae	hypothetical protein [Porphyromonas gulae].	GCF_000765945.1
WP_039490314.1	HetE-N1→ TPR+GreAB-C+PIN*→	TPR+GreAB-C+PIN	1093	-	Gammaproteobacteria	Pectobacterium odoriferum	hypothetical protein [Pectobacterium odoriferum].	GCF_000754765.1
WP_039768974.1	REase+TPR+GreAB-C+PIN*→	REase+TPR+GreAB-C+PIN	1438	-	Gammaproteobacteria	Pseudomonas fluorescens	hypothetical protein [Pseudomonas fluorescens].	GCF_000817905.1
WP_040182639.1	REase+TPR+GreAB-C+PIN*→	REase+TPR+GreAB-C+PIN	1319	-	Alphaproteobacteria	Phaeobacter sp. S60	hypothetical protein [Phaeobacter sp. S60].	GCF_000826845.1
WP_040439475.1	REase+TPR+GreAB-C+PIN*→	REase+TPR+GreAB-C+PIN	1318	-	Alphaproteobacteria	Labrenzia aggregata	hypothetical protein [Labrenzia aggregata].	GCF_000168975.1
WP_041272554.1	REase+TPR+GreAB-C+PIN*→	REase+TPR+GreAB-C+PIN	1294	-	Firmicutes	Desulfitobacterium hafniense	tetratricopeptide repeat protein [Desulfitobacterium hafniense].	GCF_000010045.1

acc	operon	architecture	len	gen.name	taxend	species	defline	gca
WP_041475188.1	REase+TPR+GreAB-C+PIN*→	REase+TPR+GreAB-C+PIN	1321	-	Gammaproteobacteria	Pseudomonas	MULTISPECIES: DUF4365 domain-containing protein [Pseudomonas].	GCF_000012445.1
WP_041609504.1	HetE-N1→ TPR+GreAB-C+PIN*→	TPR+GreAB-C+PIN	1086	-	Gammaproteobacteria	Tolumonas auensis	hypothetical protein [Tolumonas auensis].	GCF_000023065.1
WP_042089608.1	REase+TPR+GreAB-C+PIN*→	REase+TPR+GreAB-C+PIN	1245	-	Gammaproteobacteria	Acinetobacter bereziniae	hypothetical protein [Acinetobacter bereziniae].	GCF_000825165.1
WP_042562078.1	REase+TPR+GreAB-C+PIN*→	REase+TPR+GreAB-C+PIN	1438	-	Gammaproteobacteria	Pseudomonas fluorescens	hypothetical protein [Pseudomonas fluorescens].	GCF_000827755.1
WP_043355224.1	REase+TPR+GreAB-C+PIN*→	REase+TPR+GreAB-C+PIN	1332	-	Betaproteobacteria	Cupriavidus	MULTISPECIES: hypothetical protein [Cupriavidus].	GCF_001652915.1
WP_043626766.1	REase+TPR+TPR+GreAB-C+PIN*→	REase+TPR+TPR+GreAB-C+PIN	1357	-	Alphaproteobacteria	Ensifer sp. ZNC0028	hypothetical protein [Ensifer sp. ZNC0028].	GCF_000799055.1
WP_043752287.1	REase+TPR+GreAB-C+PIN*→	REase+TPR+GreAB-C+PIN	1318	-	Alphaproteobacteria	Pseudooceanicola atlanticus	restriction endonuclease [Pseudooceanicola atlanticus].	GCF_000768315.1
WP_044036524.1	TPR→ TPR→?→?→ TPR+GreAB-C+PIN*→	TPR+GreAB-C+PIN	473	-	Alphaproteobacteria	Octadecabacter arcticus	hypothetical protein [Octadecabacter arcticus].	GCF_000155735.2
WP_044439030.1	X+PIN*→	X+PIN	1051	-	Actinobacteria	Agreia bicolorata	hypothetical protein [Agreia bicolorata].	GCF_000938265.1
WP_045324066.1	TPR+PIN*→	TPR+PIN	1167	-	Actinobacteria	Streptomyces sp. NRRL F-4428	hypothetical protein [Streptomyces sp. NRRL F-4428].	GCF_000956015.1
WP_045495160.1	HetE-N1→ TPR+GreAB-C+PIN*→	TPR+GreAB-C+PIN	1086	-	Gammaproteobacteria	Vibrio hyugaensis	hypothetical protein [Vibrio hyugaensis].	GCF_000818475.1
WP_046103485.1	REase+TPR+GreAB-C+PIN*→	REase+TPR+GreAB-C+PIN	1314	-	Alphaproteobacteria	Devosia chinhatensis	hypothetical protein [Devosia chinhatensis].	GCF_000969445.1
WP_047008502.1	TPR+GreAB-C+PIN*→	TPR+GreAB-C+PIN	1236	-	-	Vibrio diabolicus	hypothetical protein [Vibrio diabolicus].	GCF_001010935.1
WP_047057492.1	HetE-N1→ TPR+GreAB-C+PIN*→	TPR+GreAB-C+PIN	1106	-	Gammaproteobacteria	Enterobacter hormaechei	hypothetical protein [Enterobacter hormaechei].	GCF_001011695.1
WP_047274590.1	REase+TPR+GreAB-C+PIN*→	REase+TPR+GreAB-C+PIN	1321	-	Gammaproteobacteria	Pseudomonas	MULTISPECIES: DUF4365 domain-containing protein [Pseudomonas].	GCF_012986325.1
WP_047583000.1	HetE-N1→ TPR+GreAB-C+PIN*→	TPR+GreAB-C+PIN	1105	-	Gammaproteobacteria	unclassified Pseudomonas	MULTISPECIES: hypothetical protein [unclassified Pseudomonas].	GCF_000763225.1
WP_047736827.1	HetE-N1→ HetE-N1+TPR+GreAB-C+PIN*→	HetE-N1+TPR+GreAB-C+PIN	1122	-	Gammaproteobacteria	Pseudomonas chlororaphis	hypothetical protein [Pseudomonas chlororaphis].	GCF_001023535.1
WP_047738516.1	HetE-N1→ TPR+GreAB-C+PIN*→	TPR+GreAB-C+PIN	1111	-	Gammaproteobacteria	Pseudomonas chlororaphis	hypothetical protein [Pseudomonas chlororaphis].	GCF_001023535.1
WP_047741525.1	HetE-N1→ TPR+GreAB-C+PIN*→	TPR+GreAB-C+PIN	1106	-	Gammaproteobacteria	Enterobacter cloacae complex	MULTISPECIES: hypothetical protein [Enterobacter cloacae complex].	GCF_014982105.1
WP_047744683.1	HetE-N1→ HetE-N1+TPR+GreAB-C+PIN*→	HetE-N1+TPR+GreAB-C+PIN	1092	-	Gammaproteobacteria	Enterobacter ludwigii	hypothetical protein [Enterobacter ludwigii].	GCF_001022445.2
WP_048126291.1	TPR+GreAB-C+PIN*→	TPR+GreAB-C+PIN	1292	-	Euryarchaeota	Methanosarcina lacustris	hypothetical protein [Methanosarcina lacustris].	GCF_000970265.1
WP_048377212.1	REase+TPR+GreAB-C+PIN*→	REase+TPR+GreAB-C+PIN	1321	-	Gammaproteobacteria	Pseudomonas	MULTISPECIES: DUF4365 domain-containing protein [Pseudomonas].	GCF_012986365.1
WP_048411963.1	TPR+GreAB-C+PIN*→	TPR+GreAB-C+PIN	1163	-	Betaproteobacteria	Chromobacterium sp. LK1	hypothetical protein [Chromobacterium sp. LK1].	GCF_001043555.1
WP_049042149.1	HetE-N1→ HetE-N1+TPR+GreAB-C+PIN*→	HetE-N1+TPR+GreAB-C+PIN	1094	-	Gammaproteobacteria	Citrobacter braakii	hypothetical protein [Citrobacter braakii].	GCF_001059745.1
WP_049582258.1	HetE-N1→ TPR+GreAB-C+PIN*→	TPR+GreAB-C+PIN	1098	-	Gammaproteobacteria	Photorhabdus luminescens	hypothetical protein [Photorhabdus luminescens].	GCF_900102985.1
WP_050091697.1	HetE-N1→ TPR+GreAB-C+PIN*→	TPR+GreAB-C+PIN	1096	-	Gammaproteobacteria	Yersinia pseudotuberculosis	hypothetical protein [Yersinia pseudotuberculosis].	GCF_001123345.1
WP_050298251.1	HetE-N1→ TPR+GreAB-C+PIN*→	TPR+GreAB-C+PIN	1095	-	Gammaproteobacteria	Yersinia enterocolitica	hypothetical protein [Yersinia enterocolitica].	GCF_001160345.1
WP_050323220.1	HetE-N1→ TPR+GreAB-C+PIN*→	TPR+GreAB-C+PIN	1121	-	Gammaproteobacteria	Yersinia enterocolitica	hypothetical protein [Yersinia enterocolitica].	GCF_001182365.1
WP_050376143.1	TPR+GreAB-C+PIN*→||||<-HTH+HTH	TPR+GreAB-C+PIN	872	-	Betaproteobacteria	Burkholderia pseudomallei	hypothetical protein [Burkholderia pseudomallei].	GCF_006538545.1
WP_050590144.1	REase+TPR+GreAB-C+PIN*→	REase+TPR+GreAB-C+PIN	1423	-	Gammaproteobacteria	Pseudomonas sp. URMO17WK12:I12	hypothetical protein [Pseudomonas sp. URMO17WK12:I12].	GCF_000514395.1
WP_051097719.1	TPR+GreAB-C+PIN*→	TPR+GreAB-C+PIN	1140	-	Gammaproteobacteria	Pseudomonas sp. 10-1B	hypothetical protein [Pseudomonas sp. 10-1B].	GCF_000935045.1
WP_051560695.1	HetE-N1→ TPR+GreAB-C+PIN*→	TPR+GreAB-C+PIN	1098	-	Gammaproteobacteria	Photorhabdus aegyptia	hypothetical protein [Photorhabdus aegyptia].	GCF_000612035.1
WP_051570263.1	REase+TPR+GreAB-C+PIN*→	REase+TPR+GreAB-C+PIN	1248	-	Actinobacteria	Cryptosporangium arvum	DUF4365 domain-containing protein [Cryptosporangium arvum].	GCF_000585375.1
WP_052241169.1	REase+TPR+GreAB-C+PIN*→	REase+TPR+GreAB-C+PIN	1236	-	Actinobacteria	Microbacterium hominis	DUF4365 domain-containing protein [Microbacterium hominis].	GCF_000813805.1
WP_052515067.1	PNPase+TPR+GreAB-C+PIN*→	PNPase+TPR+GreAB-C+PIN	1617	-	Deltaproteobacteria	Dethiosulfatarculus sandiegensis	hypothetical protein [Dethiosulfatarculus sandiegensis].	GCF_000931935.2
WP_052917795.1	HetE-N1→ TPR+GreAB-C+PIN*→	TPR+GreAB-C+PIN	1094	-	Gammaproteobacteria	Escherichia coli	hypothetical protein [Escherichia coli].	GCF_001265885.1
WP_053052653.1	HetE-N1→ TPR+GreAB-C+PIN*→	TPR+GreAB-C+PIN	1129	-	Gammaproteobacteria	Vibrio harveyi	hypothetical protein [Vibrio harveyi].	GCF_001262735.1
WP_053269091.1	REase+TPR+GreAB-C+PIN*→	REase+TPR+GreAB-C+PIN	1438	-	Gammaproteobacteria	Pseudomonas chlororaphis	hypothetical protein [Pseudomonas chlororaphis].	GCF_001269595.1
WP_053316591.1	TPR+GreAB-C+PIN*→	TPR+GreAB-C+PIN	982	-	Gammaproteobacteria	Vibrio parahaemolyticus	hypothetical protein [Vibrio parahaemolyticus].	GCF_001268015.1
WP_053446146.1	TPR+PIN*→	TPR+PIN	1160	-	Firmicutes	Bacillus toyonensis	hypothetical protein [Bacillus toyonensis].	GCF_001275045.2

acc	operon	architecture	len	gen.name	taxend	species	defline	gca
WP_053882512.1	HetE-N1→ TPR+GreAB-C+PIN*→	TPR+GreAB-C+PIN	1094	-	Gammaproteobacteria	Escherichia coli	hypothetical protein [Escherichia coli].	GCF_001284445.1
WP_054070021.1	TPR+GreAB-C+PIN*→	TPR+GreAB-C+PIN	1140	-	Gammaproteobacteria	Pseudomonas syringae group genomosp. 3	hypothetical protein [Pseudomonas syringae group genomosp. 3].	GCF_016803175.1
WP_054211402.1	BirA-HTH→ HEPN→ REase+TPR+TPR+GreAB-C+PIN*→	REase+TPR+TPR+GreAB-C+PIN	1333	-	Alphaproteobacteria	Bosea vaviloviae	hypothetical protein [Bosea vaviloviae].	GCF_001298265.1
WP_054464024.1	HetE-N1→ TPR+GreAB-C+PIN*→	TPR+GreAB-C+PIN	1094	-	Gammaproteobacteria	Escherichia coli	hypothetical protein [Escherichia coli].	GCF_001277455.1
WP_055843189.1	REase+TPR+GreAB-C+PIN*→	REase+TPR+GreAB-C+PIN	1293	-	Alphaproteobacteria	Sphingomonas sp. Leaf343	tetratricopeptide repeat protein [Sphingomonas sp. Leaf343].	GCF_001423765.1
WP_056497005.1	TPR+GreAB-C+PIN*→	TPR+GreAB-C+PIN	1110	-	Alphaproteobacteria	Methylobacterium sp. Leaf111	hypothetical protein [Methylobacterium sp. Leaf111].	GCF_001423285.1
WP_056734772.1	TPR+PIN*→	TPR+PIN	1204	-	Actinobacteria	Agromyces sp. Soil535	hypothetical protein [Agromyces sp. Soil535].	GCF_001428255.1
WP_056836493.1	TPR+PIN*→	TPR+PIN	1174	-	Firmicutes	Paenibacillus sp. Soil787	hypothetical protein [Paenibacillus sp. Soil787].	GCF_001429545.1
WP_057430028.1	REase+TPR+GreAB-C+PIN*→	REase+TPR+GreAB-C+PIN	1438	-	Gammaproteobacteria	Pseudomonas syringae group genomosp. 3	hypothetical protein [Pseudomonas syringae group genomosp. 3].	GCF_001401385.1
WP_057580842.1	TPR+GreAB-C+PIN*→	TPR+GreAB-C+PIN	1233	-	Firmicutes	Paeniclostridium sordellii	hypothetical protein [Paeniclostridium sordellii].	GCF_001373055.1
WP_057657430.1	REase+TPR+GreAB-C+PIN*→	REase+TPR+GreAB-C+PIN	1287	-	Gammaproteobacteria	Pseudoxanthomonas dokdonensis	tetratricopeptide repeat protein [Pseudoxanthomonas dokdonensis].	GCF_001431405.1
WP_057831910.1	HetE-N1→ TPR+GreAB-C+PIN*→	TPR+GreAB-C+PIN	1091	-	Gammaproteobacteria	Colwellia sp. TT2012	hypothetical protein [Colwellia sp. TT2012].	GCF_001440345.1
WP_057932289.1	TPR+GreAB-C+PIN*→	TPR+GreAB-C+PIN	1276	-	Bacteroidetes	Pedobacter ginsenosidimutans	tetratricopeptide repeat protein [Pedobacter ginsenosidimutans].	GCF_001442625.1
WP_058039540.1	TPR+GreAB-C+PIN*→||||<-HTH+HTH	TPR+GreAB-C+PIN	872	-	Betaproteobacteria	Burkholderia pseudomallei	hypothetical protein [Burkholderia pseudomallei].	GCF_001445875.1
WP_058297418.1	TPR+PIN*→	TPR+PIN	1225	-	Firmicutes	Bacillus enclensis	hypothetical protein [Bacillus enclensis].	GCF_900094975.1
WP_058461037.1	TPR+PIN*→	TPR+PIN	1277	-	Gammaproteobacteria	Fluoribacter bozemanae	hypothetical protein [Fluoribacter bozemanae].	GCF_900640135.1
WP_058587024.1	<-MarR-HTH||?→?→?→||||<-?||?→ HetE-N1→ TPR+GreAB-C+PIN*→	TPR+GreAB-C+PIN	1117	-	Gammaproteobacteria	Citrobacter amalonaticus	hypothetical protein [Citrobacter amalonaticus].	GCF_001471655.2
WP_058721158.1	REase+TPR+GreAB-C+PIN*→	REase+TPR+GreAB-C+PIN	1509	-	Betaproteobacteria	Paucibacter sp. KCTC 42545	hypothetical protein [Paucibacter sp. KCTC 42545].	GCF_001477625.1
WP_058751880.1	REase+TPR+GreAB-C+PIN*→	REase+TPR+GreAB-C+PIN	1317	-	Alphaproteobacteria	Sphingomonas sanguinis	tetratricopeptide repeat protein [Sphingomonas sanguinis].	GCF_001476905.1
WP_058820070.1	<-HTH<-?<-?||HetE-N1→ TPR+GreAB-C+PIN*→	TPR+GreAB-C+PIN	1107	-	Gammaproteobacteria	Salmonella enterica	hypothetical protein [Salmonella enterica].	GCF_001952125.1
WP_059294982.1	HetE-N1→ TPR+GreAB-C+PIN*→	TPR+GreAB-C+PIN	1106	-	Gammaproteobacteria	Enterobacter hormaechei	hypothetical protein [Enterobacter hormaechei].	GCF_001518595.1
WP_059458986.1	REase+TPR+GreAB-C+PIN*→	REase+TPR+GreAB-C+PIN	1335	-	Betaproteobacteria	Burkholderia vietnamiensis	hypothetical protein [Burkholderia vietnamiensis].	GCF_001523785.1
WP_059543415.1	TPR+GreAB-C+PIN*→||||<-HTH+HTH	TPR+GreAB-C+PIN	872	-	Betaproteobacteria	Burkholderia	MULTISPECIES: hypothetical protein [Burkholderia].	GCF_001523065.1
WP_059687512.1	REase+TPR+GreAB-C+PIN*→	REase+TPR+GreAB-C+PIN	1355	-	Betaproteobacteria	Burkholderia cepacia	hypothetical protein [Burkholderia cepacia].	GCF_001524765.1
WP_059702921.1	REase+TPR→ TPR+GreAB-C+PIN*→	TPR+GreAB-C+PIN	954	-	Betaproteobacteria	Burkholderia sp. RF2-non_BP3	hypothetical protein [Burkholderia sp. RF2-non_BP3].	GCF_001522185.1
WP_059764594.1	REase+TPR+GreAB-C+PIN*→	REase+TPR+GreAB-C+PIN	1321	-	Gammaproteobacteria	unclassified Pseudomonas	MULTISPECIES: DUF4365 domain-containing protein [unclassified Pseudomonas].	GCF_001529305.1
WP_059837999.1	TPR+GreAB-C+PIN*→||||<-HTH+HTH	TPR+GreAB-C+PIN	872	-	Betaproteobacteria	Burkholderia ubonensis	hypothetical protein [Burkholderia ubonensis].	GCF_001528175.1
WP_059992407.1	REase+TPR+GreAB-C+PIN*→	REase+TPR+GreAB-C+PIN	1345	-	Betaproteobacteria	Burkholderia ubonensis	hypothetical protein [Burkholderia ubonensis].	GCF_001527095.1
WP_060002797.1	TPR+GreAB-C+PIN*→	TPR+GreAB-C+PIN	872	-	Betaproteobacteria	Burkholderia gladioli	hypothetical protein [Burkholderia gladioli].	GCF_902831175.1
WP_060217774.1	REase+TPR+GreAB-C+PIN*→	REase+TPR+GreAB-C+PIN	1355	-	Betaproteobacteria	Burkholderia cepacia	hypothetical protein [Burkholderia cepacia].	GCF_001530905.1
WP_060322122.1	REase+TPR+GreAB-C+PIN*→	REase+TPR+GreAB-C+PIN	1351	-	Betaproteobacteria	Burkholderia ubonensis	hypothetical protein [Burkholderia ubonensis].	GCF_001718975.1
WP_060322181.1	REase+TPR+GreAB-C+PIN*→||||<-HEPN	REase+TPR+GreAB-C+PIN	1345	-	Betaproteobacteria	Burkholderia diffusa	hypothetical protein [Burkholderia diffusa].	GCF_001533185.1
WP_060360485.1	REase+TPR+GreAB-C+PIN*→||||<-HEPN	REase+TPR+GreAB-C+PIN	1351	-	Betaproteobacteria	Burkholderia territorii	hypothetical protein [Burkholderia territorii].	GCF_001533385.1
WP_061510730.1	REase+TPR+GreAB-C+PIN*→	REase+TPR+GreAB-C+PIN	1304	-	Alphaproteobacteria	Gluconobacter thailandicus	hypothetical protein [Gluconobacter thailandicus].	GCF_001580835.1
WP_061928567.1	REase+TPR+GreAB-C+PIN*→	REase+TPR+GreAB-C+PIN	1323	-	Alphaproteobacteria	Gluconobacter japonicus	hypothetical protein [Gluconobacter japonicus].	GCF_001580865.1
WP_062265952.1	REase+TPR+GreAB-C+PIN*→	REase+TPR+GreAB-C+PIN	1319	-	Euryarchaeota	Methanoculleus bourgensis	tetratricopeptide repeat protein [Methanoculleus bourgensis].	GCF_900036045.1
WP_062316067.1	TPR+PIN*→	TPR+PIN	1207	-	Actinobacteria	Demequina maris	hypothetical protein [Demequina maris].	GCF_000971375.1
WP_062477592.1	HetE-N1→ TPR+GreAB-C+PIN*→	TPR+GreAB-C+PIN	1109	-	Gammaproteobacteria	Lacimicrobium alkaliphilum	hypothetical protein [Lacimicrobium alkaliphilum].	GCF_001466725.1
WP_062692005.1	HetE-N1→ TPR+GreAB-C+PIN*→	TPR+GreAB-C+PIN	941	-	Gammaproteobacteria	Photobacterium sanguinicancri	hypothetical protein [Photobacterium sanguinicancri].	GCF_001563765.1

acc	operon	architecture	len	gen.name	taxend	species	defline	gca
WP_062794399.1	REase+TPR+GreAB-C+PIN*→	REase+TPR+GreAB-C+PIN	1127	-	Betaproteobacteria	Cupriavidus metallidurans	hypothetical protein, partial [Cupriavidus metallidurans].	GCF_001598775.1
WP_063146823.1	HetE-N1→ TPR+GreAB-C+PIN*→	TPR+GreAB-C+PIN	1106	-	Gammaproteobacteria	Enterobacter cloacae complex	MULTISPECIES: hypothetical protein [Enterobacter cloacae complex].	GCF_013635495.1
WP_063496996.1	TIR+DnaB→ REase+TPR+GreAB-C+PIN*→	REase+TPR+GreAB-C+PIN	1364	-	Betaproteobacteria	Paraburkholderia phytofirmans	hypothetical protein [Paraburkholderia phytofirmans].	GCF_001634365.1
WP_063800929.1	REase+TPR+GreAB-C+PIN*→	REase+TPR+GreAB-C+PIN	1351	-	Betaproteobacteria	Burkholderia ubonensis	hypothetical protein [Burkholderia ubonensis].	GCF_001526225.1
WP_063809712.1	HetE-N1→ TPR+GreAB-C+PIN*→	TPR+GreAB-C+PIN	1117	-	Gammaproteobacteria	Salmonella enterica	hypothetical protein [Salmonella enterica].	GCF_008727555.1
WP_063897359.1	REase+TPR+GreAB-C+PIN*→	REase+TPR+GreAB-C+PIN	1351	-	Betaproteobacteria	Burkholderia ubonensis	hypothetical protein [Burkholderia ubonensis].	GCF_001529725.1
WP_063906337.1	REase+TPR+GreAB-C+PIN*→	REase+TPR+GreAB-C+PIN	1354	-	Betaproteobacteria	Burkholderia ubonensis	hypothetical protein [Burkholderia ubonensis].	GCF_001534425.1
WP_063932878.1	HetE-N1→ TPR+GreAB-C+PIN*→	TPR+GreAB-C+PIN	1034	-	Gammaproteobacteria	Enterobacter hormaechei	hypothetical protein [Enterobacter hormaechei].	GCF_900076145.1
WP_064273010.1	REase+TPR+GreAB-C+PIN*→	REase+TPR+GreAB-C+PIN	1328	-	Alphaproteobacteria	Gluconobacter cerinus	tetratricopeptide repeat protein [Gluconobacter cerinus].	GCF_001645165.1
WP_064374215.1	HetE-N1→ TPR+GreAB-C+PIN*→	TPR+GreAB-C+PIN	1116	-	Gammaproteobacteria	Pantoea ananatis	hypothetical protein [Pantoea ananatis].	GCF_001543055.1
WP_064477497.1	TPR+GreAB-C+PIN*→	TPR+GreAB-C+PIN	879	-	Betaproteobacteria	Ralstonia	MULTISPECIES: hypothetical protein [Ralstonia].	GCF_014884725.1
WP_065176860.1	HetE-N1→ GreAB-C*→	GreAB-C	1123	-	Gammaproteobacteria	Photobacterium aquimaris	hypothetical protein [Photobacterium aquimaris].	GCF_001676065.1
WP_065192964.1	HetE-N1→ GreAB-C*→	GreAB-C	1128	-	Gammaproteobacteria	Photobacterium phosphoreum	hypothetical protein [Photobacterium phosphoreum].	GCF_001676135.1
WP_065645553.1	HetE-N1→ TPR+GreAB-C+PIN*→	TPR+GreAB-C+PIN	1085	-	Gammaproteobacteria	Vibrio diabolicus	hypothetical protein [Vibrio diabolicus].	GCF_001691185.1
WP_065663920.1	BirA-HTH→ HEPN→ REase+TPR+TPR+GreAB-C+PIN*→	REase+TPR+TPR+GreAB-C+PIN	1333	-	Alphaproteobacteria	Agrobacterium sp. B131/95	GreA/GreB family elongation factor [Agrobacterium sp. B131/95].	GCF_001692155.1
WP_065996373.1	REase+TPR+GreAB-C+PIN*→	REase+TPR+GreAB-C+PIN	1244	-	Gammaproteobacteria	Acinetobacter baumannii	hypothetical protein [Acinetobacter baumannii].	GCF_001704675.1
WP_066036869.1	TPR+PIN*→	TPR+PIN	1175	-	Firmicutes	Lysinibacillus sp. AR18-8	hypothetical protein [Lysinibacillus sp. AR18-8].	GCF_001705465.1
WP_066542710.1	REase+TPR+GreAB-C+PIN*→	REase+TPR+GreAB-C+PIN	1122	-	Alphaproteobacteria	Sphingomonas sp. CCH18-B1	tetratricopeptide repeat protein [Sphingomonas sp. CCH18-B1].	GCF_001555895.1
WP_066945120.1	HTH+PNPase+PIN*→	HTH+PNPase+PIN	1527	-	Actinobacteria	Streptomyces lushanensis	5'-methylthioadenosine/S-adenosylhomocysteine nucleosidase [Streptomyces lushanensis].	GCF_001700515.1
WP_067386574.1	REase+TPR+GreAB-C+PIN*→	REase+TPR+GreAB-C+PIN	1361	-	Gammaproteobacteria	Marinobacterium aestuarii	hypothetical protein [Marinobacterium aestuarii].	GCF_001651805.1
WP_067584887.1	GreAB-C*→	GreAB-C	1154	-	Gammaproteobacteria	Endozoicomonas ascidiicola	hypothetical protein [Endozoicomonas ascidiicola].	GCF_001646955.1
WP_067776912.1	PNPase+TPR+GreAB-C+PIN*→	PNPase+TPR+GreAB-C+PIN	1386	-	Cyanobacteria	Nostoc sp. NIES-3756	hypothetical protein [Nostoc sp. NIES-3756].	GCF_001548375.1
WP_067990948.1	TPR+PIN*→||||<-KAP_NTPase	TPR+PIN	1216	-	Actinobacteria	Mycobacterium sp. YC-RL4	hypothetical protein [Mycobacterium sp. YC-RL4].	GCF_001644575.1
WP_068215362.1	TPR+GreAB-C+PIN*→	TPR+GreAB-C+PIN	1166	-	Bacteroidetes	Roseivirga spongicola	hypothetical protein [Roseivirga spongicola].	GCF_001592965.1
WP_069047857.1	REase+TPR+GreAB-C+PIN*→	REase+TPR+GreAB-C+PIN	1383	-	Betaproteobacteria	Hydrogenophaga sp. RAC07	hypothetical protein [Hydrogenophaga sp. RAC07].	GCF_001713375.1
WP_069206296.1	REase+TPR+GreAB-C+PIN*→	REase+TPR+GreAB-C+PIN	1323	-	Alphaproteobacteria	Sphingomonas panacis	hypothetical protein [Sphingomonas panacis].	GCF_001717955.1
WP_069604736.1	TPR+GreAB-C+PIN*→	TPR+GreAB-C+PIN	1147	-	Firmicutes	Bacillus cereus group	MULTISPECIES: tetratricopeptide repeat protein [Bacillus cereus group].	GCF_001729295.1
WP_069859257.1	TPR+GreAB-C+PIN*→	TPR+GreAB-C+PIN	1244	-	Bacteroidetes	Arenibacter sp. NBRC 103722	hypothetical protein [Arenibacter sp. NBRC 103722].	GCF_001748465.2
WP_069931472.1	REase+PIN*→	REase+PIN	1282	-	Actinobacteria	Streptomyces agglomeratus	DUF4365 domain-containing protein [Streptomyces agglomeratus].	GCF_001746465.1
WP_070715046.1	HetE-N1→ TPR+GreAB-C+PIN*→	TPR+GreAB-C+PIN	1094	-	Gammaproteobacteria	Hafnia sp. HMSC23F03	hypothetical protein [Hafnia sp. HMSC23F03].	GCF_001816025.1
WP_070991866.1	HetE-N1→ TPR+GreAB-C+PIN*→	TPR+GreAB-C+PIN	1106	-	Gammaproteobacteria	Pseudoalteromonas byunsanensis	hypothetical protein [Pseudoalteromonas byunsanensis].	GCF_001854475.1
WP_071234936.1	HetE-N1→ TPR+GreAB-C+PIN*→	TPR+GreAB-C+PIN	1111	-	Gammaproteobacteria	Vibrio rotiferianus	hypothetical protein [Vibrio rotiferianus].	GCF_001858245.1
WP_071684330.1	HetE-N1→ HetE-N1+TPR+GreAB-C+PIN*→	HetE-N1+TPR+GreAB-C+PIN	1094	-	Gammaproteobacteria	Citrobacter freundii	hypothetical protein [Citrobacter freundii].	GCF_014901875.1
WP_071741873.1	TPR+PIN*→	TPR+PIN	1160	-	Firmicutes	Bacillus paranthracis	hypothetical protein [Bacillus paranthracis].	GCF_001883995.1
WP_071769547.1	PIN*→	PIN	341	-	Betaproteobacteria	Burkholderia ubonensis	hypothetical protein [Burkholderia ubonensis].	GCF_001882755.1
WP_071890709.1	TPR+GreAB-C+PIN*→||||<-HTH	TPR+GreAB-C+PIN	1157	-	Gammaproteobacteria	Pseudomonas putida	tetratricopeptide repeat protein [Pseudomonas putida].	GCF_001886975.1
WP_072702162.1	TPR+GreAB-C+PIN*→	TPR+GreAB-C+PIN	1125	-	Alphaproteobacteria	Donghicola eburneus	hypothetical protein [Donghicola eburneus].	GCF_900095785.1

acc	operon	architecture	len	gen.name	taxend	species	defline	gca
WP_073552134.1	TPR+GreAB-C+PIN*→	TPR+GreAB-C+PIN	1131	-	Bacteroidetes	Elizabethkingia meningoseptica	hypothetical protein [Elizabethkingia meningoseptica].	GCF_900143665.1
WP_073772512.1	REase+PIN*→	REase+PIN	1292	-	Actinobacteria	Streptomyces sp. MJM1172	DUF4365 domain-containing protein [Streptomyces sp. MJM1172].	GCF_001905425.1
WP_074449514.1	CHC2+Toprim-DNAG→?→?→?→ HTH→ SIG+TM→ TPR+GreAB-C+PIN*→	TPR+GreAB-C+PIN	1141	-	Bacteroidetes	Tannerella forsythia	hypothetical protein [Tannerella forsythia].	GCF_900096735.1
WP_074452850.1	CHC2+Toprim-DNAG→?→ PSE→?→ HTH→ SIG+TM→ TPR+GreAB-C+PIN*→	TPR+GreAB-C+PIN	1141	-	Bacteroidetes	Bacteroidales	MULTISPECIES: hypothetical protein [Bacteroidales].	GCF_900096725.1
WP_074647048.1	TPR+GreAB-C+PIN*→	TPR+GreAB-C+PIN	1184	-	Alphaproteobacteria	Celeribacter baekdonensis	hypothetical protein [Celeribacter baekdonensis].	GCF_900102315.1
WP_074661038.1	TPR+GreAB-C+PIN*→	TPR+GreAB-C+PIN	1127	-	Bacteroidetes	Flavobacterium frigidimaris	hypothetical protein [Flavobacterium frigidimaris].	GCF_002217275.1
WP_074804411.1	REase+TPR+GreAB-C+PIN*→	REase+TPR+GreAB-C+PIN	1335	-	Betaproteobacteria	Burkholderia cenocepacia	hypothetical protein [Burkholderia cenocepacia].	GCF_900100915.1
WP_074854446.1	TPR+GreAB-C+PIN*→	TPR+GreAB-C+PIN	1161	-	Gammaproteobacteria	Pseudomonas salomonii	hypothetical protein [Pseudomonas salomonii].	GCF_900107155.1
WP_075042282.1	REase+TPR+TPR+GreAB-C+PIN*→	REase+TPR+TPR+GreAB-C+PIN	1332	-	Alphaproteobacteria	Brucella intermedia	GreA/GreB family elongation factor [Brucella intermedia].	GCF_001917355.1
WP_075481088.1	TPR+GreAB-C+PIN*→	TPR+GreAB-C+PIN	1236	-	Gammaproteobacteria	Moritella viscosa	hypothetical protein [Moritella viscosa].	GCF_900120145.1
WP_075594884.1	REase+TPR+GreAB-C+PIN*→	REase+TPR+GreAB-C+PIN	1086	-	Alphaproteobacteria	Komagataeibacter hansenii	hypothetical protein [Komagataeibacter hansenii].	GCF_001938745.1
WP_076004144.1	REase+TPR+GreAB-C+PIN*→	REase+TPR+GreAB-C+PIN	1301	-	Chloroflexi	Dehalogenimonas formicexedens	hypothetical protein [Dehalogenimonas formicexedens].	GCF_001953175.1
WP_076215124.1	TPR+PIN*→	TPR+PIN	1179	-	Firmicutes	Paenibacillus odorifer	tetratricopeptide repeat protein [Paenibacillus odorifer].	GCF_001954655.1
WP_076272073.1	TPR+PIN*→	TPR+PIN	1180	-	Firmicutes	Paenibacillus odorifer	hypothetical protein [Paenibacillus odorifer].	GCF_001954285.1
WP_076387298.1	TPR+GreAB-C+PIN*→	TPR+GreAB-C+PIN	1130	-	Bacteroidetes	Kaistella chaponensis	hypothetical protein [Kaistella chaponensis].	GCF_900156725.1
WP_076506278.1	TPR+GreAB-C+PIN*→	TPR+GreAB-C+PIN	1137	-	Bacteroidetes	Chryseobacterium shigense	hypothetical protein [Chryseobacterium shigense].	GCF_900156575.1
WP_076822916.1	REase+TPR+GreAB-C+PIN*→	REase+TPR+GreAB-C+PIN	1162	-	Actinobacteria	Frankia asymbiotica	hypothetical protein [Frankia asymbiotica].	GCF_001983105.1
WP_076832171.1	TPR+GreAB-C+PIN*→	TPR+GreAB-C+PIN	1129	-	Alphaproteobacteria	Bradyrhizobium sp. UFLA 03-321	hypothetical protein [Bradyrhizobium sp. UFLA 03-321].	GCF_001969825.1
WP_076835387.1	REase+TPR+GreAB-C+PIN*→	REase+TPR+GreAB-C+PIN	1345	-	Betaproteobacteria	Burkholderia pseudomallei	hypothetical protein [Burkholderia pseudomallei].	GCF_001980695.1
WP_078005484.1	TPR+GreAB-C+PIN*→	TPR+GreAB-C+PIN	1131	-	Bacteroidetes	Flavobacterium sp. KBS0721	hypothetical protein [Flavobacterium sp. KBS0721].	GCF_002007065.3
WP_078350655.1	TPR+GreAB-C+PIN*→	TPR+GreAB-C+PIN	1286	-	Bacteroidetes	Mucilaginibacter pedocola	hypothetical protein [Mucilaginibacter pedocola].	GCF_002013915.1
WP_078403924.1	TPR+GreAB-C+PIN*→	TPR+GreAB-C+PIN	1140	-	Bacteroidetes	Elizabethkingia ursingii	hypothetical protein [Elizabethkingia ursingii].	GCF_002022125.1
WP_078946818.1	REase+PIN*→	REase+PIN	1277	-	Actinobacteria	Streptomyces sp. NRRL B-3648	DUF4365 domain-containing protein [Streptomyces sp. NRRL B-3648].	GCF_001279545.1
WP_079454673.1	REase+TPR+GreAB-C+PIN*→||||<-?||HTH→	REase+TPR+GreAB-C+PIN	1250	-	Gammaproteobacteria	Acinetobacter baumannii	GreA/GreB family elongation factor [Acinetobacter baumannii].	GCF_902728005.1
WP_079469502.1	TPR+GreAB-C+PIN*→	TPR+GreAB-C+PIN	1234	-	Bacteroidetes	Chitinophaga ginsengisegetis	hypothetical protein [Chitinophaga ginsengisegetis].	GCF_900168065.1
WP_079579807.1	X+PIN*→	X+PIN	1007	-	Epsilonproteobacteria	Malaciobacter marinus	hypothetical protein [Malaciobacter marinus].	GCF_900168035.1
WP_079820569.1	HetE-N1→ TPR+GreAB-C+PIN*→	TPR+GreAB-C+PIN	1117	-	Gammaproteobacteria	Salmonella enterica	hypothetical protein [Salmonella enterica].	GCF_016071195.1
WP_079943789.1	<-HTH<-?<-?||HetE-N1→ TPR+GreAB-C+PIN*→	TPR+GreAB-C+PIN	998	-	Gammaproteobacteria	Salmonella enterica	hypothetical protein [Salmonella enterica].	GCF_002045995.1
WP_079951728.1	<-HTH<-?<-?||HetE-N1→ TPR+GreAB-C+PIN*→	TPR+GreAB-C+PIN	993	-	Gammaproteobacteria	Salmonella enterica	hypothetical protein [Salmonella enterica].	GCF_002052255.1
WP_079954008.1	<-HTH<-?<-?||HetE-N1→ TPR+GreAB-C+PIN*→	TPR+GreAB-C+PIN	998	-	Gammaproteobacteria	Salmonella enterica	hypothetical protein [Salmonella enterica].	GCF_002045095.1
WP_079959318.1	HetE-N1→ TPR+GreAB-C+PIN*→	TPR+GreAB-C+PIN	1119	-	Gammaproteobacteria	Salmonella enterica	hypothetical protein [Salmonella enterica].	GCF_002046115.1
WP_079973127.1	<-HTH<-?<-?||HetE-N1→ TPR+GreAB-C+PIN*→	TPR+GreAB-C+PIN	1106	-	Gammaproteobacteria	Salmonella enterica	hypothetical protein [Salmonella enterica].	GCF_002046215.1
WP_080170163.1	HetE-N1→ TPR+GreAB-C+PIN*→	TPR+GreAB-C+PIN	1106	-	Gammaproteobacteria	Salmonella enterica	hypothetical protein [Salmonella enterica].	GCF_016863875.1
WP_080323117.1	REase+TPR+GreAB-C+PIN*→	REase+TPR+GreAB-C+PIN	1334	-	Betaproteobacteria	Burkholderia cenocepacia	hypothetical protein [Burkholderia cenocepacia].	GCF_002071875.1
WP_080865316.1	PIN+GNAT→?→?→ REase+TPR+GreAB-C+PIN*→	REase+TPR+GreAB-C+PIN	1290	-	Alphaproteobacteria	Agrobacterium tumefaciens	hypothetical protein [Agrobacterium tumefaciens].	GCF_900012605.1
WP_081143626.1	TPR+PIN*→	TPR+PIN	1157	-	Firmicutes	Bacillus cereus	hypothetical protein [Bacillus cereus].	GCF_002078195.1
WP_082055264.1	NACHT→ REase+TPR+GreAB-C+PIN*→	REase+TPR+GreAB-C+PIN	1329	-	Betaproteobacteria	Cupriavidus basilensis	DUF4365 domain-containing protein [Cupriavidus basilensis].	GCF_000832305.1
WP_082573822.1	HTH+PNPase+TPR+GreAB-C+PIN*→	HTH+PNPase+TPR+GreAB-C+PIN	1517	-	Actinobacteria	Cellulomonas sp. Root137	hypothetical protein [Cellulomonas sp. Root137].	GCF_001426725.1

acc	operon	architecture	len	gen.name	taxend	species	defline	gca
WP_082991752.1	REase+TPR+GreAB-C+PIN*→	REase+TPR+GreAB-C+PIN	1320	-	Betaproteobacteria	Paraburkholderia tropica	DUF4365 domain-containing protein [Paraburkholderia tropica].	GCF_001673675.1
WP_083024260.1	TPR+GreAB-C*→	TPR+GreAB-C	833	-	Gammaproteobacteria	Halomonas lionensis	hypothetical protein [Halomonas lionensis].	GCF_002087295.1
WP_083366146.1	HetE-N1→ TPR+GreAB-C+PIN*→	TPR+GreAB-C+PIN	1112	-	Gammaproteobacteria	Pseudomonas koreensis	hypothetical protein [Pseudomonas koreensis].	GCF_014646955.1
WP_083417785.1	REase+TPR+GreAB-C+PIN*→||||<-TIR	REase+TPR+GreAB-C+PIN	1329	-	Betaproteobacteria	Burkholderia contaminans	DUF4365 domain-containing protein [Burkholderia contaminans].	GCF_001865715.1
WP_083925131.1	TPR+GreAB-C+PIN*→	TPR+GreAB-C+PIN	1307	-	Gammaproteobacteria	Thioalkalivibrio sp. ALJ15	GreA/GreB family elongation factor [Thioalkalivibrio sp. ALJ15].	GCF_000383695.1
WP_084371210.1	X+PIN*→	X+PIN	1158	-	Bacteroidetes	Reichenbachiella faecimaris	hypothetical protein [Reichenbachiella faecimaris].	GCF_900176375.1
WP_084886264.1	TPR+GreAB-C+PIN*→	TPR+GreAB-C+PIN	999	-	Gammaproteobacteria	Pantoea septica	hypothetical protein [Pantoea septica].	GCF_002095575.1
WP_085383443.1	REase+TPR+GreAB-C+PIN*→	REase+TPR+GreAB-C+PIN	1301	-	Alphaproteobacteria	Bradyrhizobium canariense	hypothetical protein [Bradyrhizobium canariense].	GCF_002108905.1
WP_085905967.1	TPR+GreAB-C+PIN*→	TPR+GreAB-C+PIN	1176	-	Alphaproteobacteria	Kiloniella majae	hypothetical protein [Kiloniella majae].	GCF_002118275.1
WP_086630532.1	HetE-N1→ TPR+GreAB-C+PIN+TM+TM*→	TPR+GreAB-C+PIN+TM+TM	1106	-	Gammaproteobacteria	Klebsiella aerogenes	hypothetical protein [Klebsiella aerogenes].	GCF_002152925.1
WP_086759874.1	APATPase+BetaPropeller→ PNPase+TPR+GreAB-C+PIN*→	PNPase+TPR+GreAB-C+PIN	1399	-	Cyanobacteria	Nostoc sp. 106C	tetratricopeptide repeat protein [Nostoc sp. 106C].	GCF_002154725.1
WP_086979350.1	HetE-N1→ TPR+GreAB-C+PIN*→	TPR+GreAB-C+PIN	940	-	Gammaproteobacteria	Pseudomonas putida	hypothetical protein [Pseudomonas putida].	GCF_002157515.1
WP_087258361.1	TPR+GreAB-C+PIN*→	TPR+GreAB-C+PIN	1189	-	Firmicutes	[Clostridium] spiroforme	hypothetical protein [[Clostridium] spiroforme].	GCF_002160695.1
WP_087391814.1	TPR+GreAB-C+PIN*→	TPR+GreAB-C+PIN	1189	-	Firmicutes	[Clostridium] spiroforme	hypothetical protein [[Clostridium] spiroforme].	GCF_002160645.1
WP_088070184.1	TPR+PIN*→	TPR+PIN	1157	-	Firmicutes	Bacillus thuringiensis	hypothetical protein [Bacillus thuringiensis].	GCF_002148155.1
WP_088082523.1	TPR+PIN*→	TPR+PIN	1179	-	Firmicutes	Bacillus thuringiensis	hypothetical protein [Bacillus thuringiensis].	GCF_002147935.1
WP_088087080.1	TPR+PIN*→	TPR+PIN	1176	-	Firmicutes	Bacillus sp. OV166	hypothetical protein [Bacillus sp. OV166].	GCF_900177675.1
WP_088476259.1	REase+TPR+GreAB-C+PIN*→	REase+TPR+GreAB-C+PIN	1284	-	Gammaproteobacteria	Stenotrophomonas pavanii	GreA/GreB family elongation factor [Stenotrophomonas pavanii].	GCF_002205885.1
WP_089172847.1	BirA-HTH→ HEPN→ REase+TPR+TPR+GreAB-C+PIN*→	REase+TPR+TPR+GreAB-C+PIN	1332	-	Alphaproteobacteria	unclassified Bosea	MULTISPECIES: hypothetical protein [unclassified Bosea].	GCF_002220095.1
WP_090371407.1	REase→ TPR+GreAB-C+PIN*→	TPR+GreAB-C+PIN	1155	-	Betaproteobacteria	Nitrosospira sp. Nl5	hypothetical protein [Nitrosospira sp. Nl5].	GCF_900102495.1
WP_090455730.1	HetE-N1→ TPR+GreAB-C+PIN*→||||<-TIR	TPR+GreAB-C+PIN	1112	-	Gammaproteobacteria	Pseudomonas jessenii	hypothetical protein [Pseudomonas jessenii].	GCF_900104905.1
WP_090543864.1	TIR+TPR+GreAB-C+PIN*→	TIR+TPR+GreAB-C+PIN	1348	-	Betaproteobacteria	Nitrosomonas sp. Nm132	toll/interleukin-1 receptor domain-containing protein [Nitrosomonas sp. Nm132].	GCF_900100485.1
WP_090721286.1	<-Cas_Cas1<-?||?→?→?→?→ REase+TPR+GreAB-C+PIN*→	REase+TPR+GreAB-C+PIN	1380	-	Betaproteobacteria	Nitrosomonas sp. Nm166	hypothetical protein [Nitrosomonas sp. Nm166].	GCF_900112825.1
WP_091343709.1	HetE-N1→ TPR+GreAB-C+PIN*→	TPR+GreAB-C+PIN	1098	-	Gammaproteobacteria	Alkalimonas amylolytica	hypothetical protein [Alkalimonas amylolytica].	GCF_900107845.1
WP_091456057.1	REase+TPR+GreAB-C+PIN*→	REase+TPR+GreAB-C+PIN	1259	-	Actinobacteria	Micromonospora inyonensis	hypothetical protein [Micromonospora inyonensis].	GCF_900091415.1
WP_091965577.1	TPR+GreAB-C+PIN*→	TPR+GreAB-C+PIN	1229	-	Alphaproteobacteria	Bradyrhizobium shewense	hypothetical protein [Bradyrhizobium shewense].	GCF_900094605.1
WP_092226352.1	TPR+GreAB-C+PIN*→	TPR+GreAB-C+PIN	1306	-	Alphaproteobacteria	Bradyrhizobium sp. Gha	tetratricopeptide repeat protein [Bradyrhizobium sp. Gha].	GCF_900113735.1
WP_092234200.1	REase+TPR+GreAB-C+PIN*→	REase+TPR+GreAB-C+PIN	1321	-	Gammaproteobacteria	Pseudomonas proteolytica	DUF4365 domain-containing protein [Pseudomonas proteolytica].	GCF_900105955.1
WP_092689872.1	HTH→ HetE-N1→ TPR+GreAB-C+PIN*→	TPR+GreAB-C+PIN	1077	-	Gammaproteobacteria	Acinetobacter kyonggiensis	hypothetical protein [Acinetobacter kyonggiensis].	GCF_900107285.1
WP_093257662.1	HetE-N1→ HetE-N1+TPR+GreAB-C+PIN*→	HetE-N1+TPR+GreAB-C+PIN	1120	-	Gammaproteobacteria	Pseudomonas	MULTISPECIES: hypothetical protein [Pseudomonas].	GCF_900110545.1
WP_093289087.1	REase+TPR+GreAB-C+PIN*→	REase+TPR+GreAB-C+PIN	1291	-	Gammaproteobacteria	Pseudoxanthomonas sp. CF125	hypothetical protein [Pseudoxanthomonas sp. CF125].	GCF_900104085.1
WP_095032727.1	HetE-N1→ TPR+GreAB-C+PIN*→	TPR+GreAB-C+PIN	1104	-	Gammaproteobacteria	Klebsiella quasivariicola	hypothetical protein [Klebsiella quasivariicola].	GCF_002269255.1
WP_095064594.1	HetE-N1→ TPR+GreAB-C+PIN*→	TPR+GreAB-C+PIN	1105	-	Gammaproteobacteria	Pseudomonas sp. Irchel s3f19	hypothetical protein [Pseudomonas sp. Irchel s3f19].	GCF_900187555.1
WP_095490577.1	HetE-N1→ HetE-N1+TPR+GreAB-C+PIN*→	HetE-N1+TPR+GreAB-C+PIN	1088	-	Gammaproteobacteria	Vibrio cholerae	hypothetical protein [Vibrio cholerae].	GCF_002284425.1
WP_095570744.1	HetE-N1→ TPR+GreAB-C+PIN*→	TPR+GreAB-C+PIN	1086	-	Gammaproteobacteria	Vibrio coralliilyticus	hypothetical protein [Vibrio coralliilyticus].	GCF_002286405.1
WP_095743432.1	REase+TPR+GreAB-C+PIN*→	REase+TPR+GreAB-C+PIN	1300	-	Betaproteobacteria	Variovorax boronicumulans	hypothetical protein [Variovorax boronicumulans].	GCF_002291405.1
WP_096138380.1	REase+TPR+GreAB-C+PIN*→	REase+TPR+GreAB-C+PIN	1321	-	Gammaproteobacteria	Pseudomonas syringae	DUF4365 domain-containing protein [Pseudomonas syringae].	GCF_002318715.1
WP_096145332.1	PIN*→	PIN	1088	-	Firmicutes	Clostridium chauvoei	tetratricopeptide repeat protein [Clostridium chauvoei].	GCF_002327185.1
WP_096360261.1	REase+TPR+GreAB-C+PIN*→	REase+TPR+GreAB-C+PIN	2100	-	Gammaproteobacteria	Sulfuricaulis limicola	hypothetical protein [Sulfuricaulis limicola].	GCF_002355735.1

acc	operon	architecture	len	gen.name	taxend	species	defline	gca
WP_096444074.1	HetE-N1→ TPR+GreAB-C+PIN*→	TPR+GreAB-C+PIN	1088	-	Gammaproteobacteria	Vibrio mediterranei	hypothetical protein [Vibrio mediterranei].	GCF_002995655.1
WP_096819326.1	REase+TPR+GreAB-C+PIN*→	REase+TPR+GreAB-C+PIN	1424	-	Gammaproteobacteria	Pseudomonas fluorescens	tetratricopeptide repeat protein [Pseudomonas fluorescens].	GCF_002417665.1
WP_096985931.1	HetE-N1→ TPR+GreAB-C+PIN*→	TPR+GreAB-C+PIN	1094	-	Gammaproteobacteria	Escherichia coli	hypothetical protein [Escherichia coli].	GCF_013372365.1
WP_097373330.1	PIN*→	PIN	245	-	Alphaproteobacteria	Celeribacter manganoxidans	hypothetical protein [Celeribacter manganoxidans].	GCF_002504165.1
WP_097730210.1	HetE-N1→ TPR+GreAB-C+PIN*→	TPR+GreAB-C+PIN	1094	-	Gammaproteobacteria	Escherichia coli	hypothetical protein [Escherichia coli].	GCF_002535535.1
WP_097759664.1	HetE-N1→ TPR+GreAB-C+PIN*→	TPR+GreAB-C+PIN	1094	-	Gammaproteobacteria	Escherichia coli	hypothetical protein [Escherichia coli].	GCF_002538945.1
WP_097979402.1	TPR+PIN*→	TPR+PIN	1217	-	Firmicutes	Bacillus toyonensis	hypothetical protein [Bacillus toyonensis].	GCF_002552775.1
WP_098090392.1	HetE-N1→ TPR+GreAB-C+PIN*→	TPR+GreAB-C+PIN	1101	-	Gammaproteobacteria	Pseudomonas putida	hypothetical protein [Pseudomonas putida].	GCF_002554535.1
WP_098280847.1	TPR+PIN*→	TPR+PIN	1183	-	Firmicutes	Bacillus cereus	hypothetical protein [Bacillus cereus].	GCF_002560075.1
WP_098365372.1	TPR+PIN*→	TPR+PIN	1217	-	Firmicutes	Bacillus cereus	hypothetical protein [Bacillus cereus].	GCF_002561575.1
WP_098420086.1	TPR+PIN*→	TPR+PIN	1183	-	Firmicutes	Bacillus cereus	hypothetical protein [Bacillus cereus].	GCF_002562505.1
WP_098433845.1	TPR+PIN*→	TPR+PIN	1184	-	Firmicutes	Priestia megaterium	hypothetical protein [Priestia megaterium].	GCF_002564705.1
WP_098573825.1	TPR+GreAB-C+PIN*→	TPR+GreAB-C+PIN	1147	-	Firmicutes	Bacillus wiedmannii	tetratricopeptide repeat protein [Bacillus wiedmannii].	GCF_002568785.1
WP_098687609.1	TPR+PIN*→	TPR+PIN	1157	-	Firmicutes	Bacillus toyonensis	hypothetical protein [Bacillus toyonensis].	GCF_002571615.1
WP_099262235.1	REase+TPR+GreAB-C+PIN*→	REase+TPR+GreAB-C+PIN	1243	-	Planctomycetes	Rhodopirellula bahusiensis	GreA/GreB family elongation factor [Rhodopirellula bahusiensis].	GCF_002727185.1
WP_099438436.1	TPR+GreAB-C+PIN*→	TPR+GreAB-C+PIN	1252	-	Bacteroidetes	Pedobacter ginsengisoli	hypothetical protein [Pedobacter ginsengisoli].	GCF_002736205.1
WP_100140263.1	HetE-N1→ TPR+GreAB-C+PIN*→	TPR+GreAB-C+PIN	1066	-	Betaproteobacteria	Snodgrassella alvi	hypothetical protein [Snodgrassella alvi].	GCF_002777775.1
WP_100151648.1	HetE-N1→ TPR+GreAB-C+PIN*→	TPR+GreAB-C+PIN	1066	-	Betaproteobacteria	Snodgrassella alvi	hypothetical protein [Snodgrassella alvi].	GCF_002777655.1
WP_100344325.1	REase+TPR+GreAB-C+PIN*→	REase+TPR+GreAB-C+PIN	1215	-	Actinobacteria	Compostimonas suwonensis	DUF4365 domain-containing protein [Compostimonas suwonensis].	GCF_002797855.1
WP_100632757.1	REase+TPR+GreAB-C+PIN*→	REase+TPR+GreAB-C+PIN	1321	-	Gammaproteobacteria	Pseudomonas qingdaonensis	DUF4365 domain-containing protein [Pseudomonas qingdaonensis].	GCF_002806685.1
WP_101291505.1	HetE-N1→ TPR+GreAB-C+PIN*→	TPR+GreAB-C+PIN	1094	-	Gammaproteobacteria	Escherichia coli	hypothetical protein [Escherichia coli].	GCF_002843705.1
WP_102155042.1	REase+TPR+GreAB-C+PIN*→	REase+TPR+GreAB-C+PIN	1270	-	Alphaproteobacteria	Tsuneonella flava	GreA/GreB family elongation factor [Tsuneonella flava].	GCF_002870965.1
WP_102488850.1	HetE-N1→ HetE-N1+TPR+GreAB-C+PIN*→	HetE-N1+TPR+GreAB-C+PIN	1088	-	Gammaproteobacteria	Vibrio splendidus	hypothetical protein [Vibrio splendidus].	GCF_002875305.1
WP_102520861.1	HetE-N1→ TPR+GreAB-C+PIN*→	TPR+GreAB-C+PIN	1088	-	Gammaproteobacteria	Shewanella sp. 10N.286.48.A6	hypothetical protein [Shewanella sp. 10N.286.48.A6].	GCF_002873135.1
WP_102570991.1	HetE-N1→ TPR+GreAB-C+PIN*→	TPR+GreAB-C+PIN	1088	-	Gammaproteobacteria	Vibrio splendidus	hypothetical protein [Vibrio splendidus].	GCF_002875585.1
WP_102574526.1	HetE-N1→ TPR+GreAB-C+PIN*→	TPR+GreAB-C+PIN	1116	-	Gammaproteobacteria	Vibrio splendidus	hypothetical protein [Vibrio splendidus].	GCF_002877195.1
WP_102576920.1	HetE-N1→ HetE-N1+TPR+GreAB-C+PIN*→	HetE-N1+TPR+GreAB-C+PIN	1091	-	Gammaproteobacteria	Vibrio splendidus	hypothetical protein [Vibrio splendidus].	GCF_002877345.1
WP_103206664.1	TPR+GreAB-C+PIN*→	TPR+GreAB-C+PIN	1213	-	Actinobacteria	Microbacterium testaceum	hypothetical protein [Microbacterium testaceum].	GCF_002899925.1
WP_103448257.1	REase+TPR+GreAB-C+PIN*→	REase+TPR+GreAB-C+PIN	1421	-	Gammaproteobacteria	Pseudomonas	MULTISPECIES: hypothetical protein [Pseudomonas].	GCF_015680835.1
WP_103712798.1	REase+TPR+GreAB-C+PIN*→||||<-KAP_NTPase	REase+TPR+GreAB-C+PIN	1321	-	Gammaproteobacteria	Pseudomonas syringae	DUF4365 domain-containing protein [Pseudomonas syringae].	GCF_002916275.1
WP_103713374.1	HetE-N1→ TPR+GreAB-C+PIN*→	TPR+GreAB-C+PIN	1107	-	Gammaproteobacteria	Pseudomonas syringae	hypothetical protein [Pseudomonas syringae].	GCF_002917155.1
WP_103715293.1	TPR+GreAB-C+PIN*→	TPR+GreAB-C+PIN	1131	-	Bacteroidetes	Flavobacterium columnare	hypothetical protein [Flavobacterium columnare].	GCF_002916795.1
WP_103949560.1	HetE-N1→ HetE-N1+TPR+GreAB-C+PIN*→	HetE-N1+TPR+GreAB-C+PIN	1094	-	Gammaproteobacteria	Lelliottia	MULTISPECIES: hypothetical protein [Lelliottia].	GCF_002922995.1
WP_104189366.1	HetE-N1→ TPR+GreAB-C+PIN*→	TPR+GreAB-C+PIN	1110	-	Gammaproteobacteria	unclassified Pantoea	MULTISPECIES: hypothetical protein [unclassified Pantoea].	GCF_002928355.1
WP_104723186.1	REase+TPR+GreAB-C+PIN*→	REase+TPR+GreAB-C+PIN	1321	-	Gammaproteobacteria	Pseudomonas syringae	DUF4365 domain-containing protein [Pseudomonas syringae].	GCF_900235865.1
WP_104925436.1	HetE-N1→ TPR+GreAB-C+PIN*→	TPR+GreAB-C+PIN	1118	-	Gammaproteobacteria	Rahnella sp. ERMR1:05	hypothetical protein [Rahnella sp. ERMR1:05].	GCF_002951615.1
WP_105080712.1	HetE-N1→ TPR+GreAB-C+PIN*→	TPR+GreAB-C+PIN	1111	-	Gammaproteobacteria	Pantoea	MULTISPECIES: hypothetical protein [Pantoea].	GCF_017920435.1
WP_105696567.1	REase+TPR+GreAB-C+PIN*→	REase+TPR+GreAB-C+PIN	1321	-	Gammaproteobacteria	Pseudomonas poae	DUF4365 domain-containing protein [Pseudomonas poae].	GCF_002980135.1
WP_105730942.1	REase+TPR+GreAB-C+PIN*→	REase+TPR+GreAB-C+PIN	1344	-	Betaproteobacteria	Malikia spinosa	hypothetical protein [Malikia spinosa].	GCF_002980625.1
WP_105731559.1	REase+TPR+GreAB-C+PIN*→	REase+TPR+GreAB-C+PIN	1278	-	Alphaproteobacteria	Ochrobactrum sp. MYb29	hypothetical protein [Ochrobactrum sp. MYb29].	GCF_002979345.1

<table>
<tr><th>acc</th><th>operon</th><th>architecture</th><th>len</th><th>gen.name</th><th>taxend</th><th>species</th><th>defline</th><th>gca</th></tr>
<tr><td>WP_105794455.1</td><td>REase+TPR+GreAB-C+PIN*→</td><td>REase+TPR+GreAB-C+PIN</td><td>1351</td><td>-</td><td>Betaproteobacteria</td><td>Burkholderia multivorans</td><td>hypothetical protein [Burkholderia multivorans].</td><td>GCF_002981485.1</td></tr>
<tr><td>WP_106098487.1</td><td>SIG+TM→ TPR+GreAB-C+PIN*→</td><td>TPR+GreAB-C+PIN</td><td>1129</td><td>-</td><td>Bacteroidetes</td><td>Capnocytophaga sp. oral taxon 878</td><td>hypothetical protein [Capnocytophaga sp. oral taxon 878].</td><td>GCF_002999135.1</td></tr>
<tr><td>WP_106374900.1</td><td>HetE-N1→ TPR+GreAB-C+PIN*→</td><td>TPR+GreAB-C+PIN</td><td>1145</td><td>-</td><td>Gammaproteobacteria</td><td>Halomonas songnenensis</td><td>hypothetical protein [Halomonas songnenensis].</td><td>GCF_003002925.1</td></tr>
<tr><td>WP_106946809.1</td><td>TPR+GreAB-C+PIN*→</td><td>TPR+GreAB-C+PIN</td><td>1155</td><td>-</td><td>Alphaproteobacteria</td><td>Bradyrhizobium sp. MOS002</td><td>hypothetical protein [Bradyrhizobium sp. MOS002].</td><td>GCF_003020115.1</td></tr>
<tr><td>WP_107027340.1</td><td>REase+TPR+TPR+GreAB-C+PIN*→</td><td>REase+TPR+TPR+GreAB-C+PIN</td><td>1357</td><td>-</td><td>Alphaproteobacteria</td><td>Ensifer sp. NM-2</td><td>hypothetical protein [Ensifer sp. NM-2].</td><td>GCF_003024455.1</td></tr>
<tr><td>WP_107248740.1</td><td>HetE-N1→ GreAB-C*→</td><td>GreAB-C</td><td>1128</td><td>-</td><td>Gammaproteobacteria</td><td>Photobacterium kishitanii</td><td>hypothetical protein [Photobacterium kishitanii].</td><td>GCF_003025535.1</td></tr>
<tr><td>WP_107289369.1</td><td>HetE-N1→ GreAB-C*→</td><td>GreAB-C</td><td>1164</td><td>-</td><td>Gammaproteobacteria</td><td>Photobacterium kishitanii</td><td>hypothetical protein [Photobacterium kishitanii].</td><td>GCF_003025915.1</td></tr>
<tr><td>WP_107901223.1</td><td>TPR+PIN*→</td><td>TPR+PIN</td><td>1216</td><td>-</td><td>Firmicutes</td><td>Bacillus sp. OV186</td><td>hypothetical protein [Bacillus sp. OV186].</td><td>GCF_003053645.1</td></tr>
<tr><td>WP_108393929.1</td><td>HetE-N1→ TPR+GreAB-C+PIN*→</td><td>TPR+GreAB-C+PIN</td><td>1106</td><td>-</td><td>Gammaproteobacteria</td><td>Enterobacter hormaechei</td><td>hypothetical protein [Enterobacter hormaechei].</td><td>GCF_003056805.1</td></tr>
<tr><td>WP_108462916.1</td><td>REase+TPR+TPR+GreAB-C+PIN*→</td><td>REase+TPR+TPR+GreAB-C+PIN</td><td>1323</td><td>-</td><td>Alphaproteobacteria</td><td>Devosia naphthalenivorans</td><td>GreA/GreB family elongation factor [Devosia naphthalenivorans].</td><td>GCF_003056355.1</td></tr>
<tr><td>WP_108677990.1</td><td>HetE-N1→ HetE-N1+TPR+GreAB-C+PIN*→</td><td>HetE-N1+TPR+GreAB-C+PIN</td><td>1088</td><td>-</td><td>Gammaproteobacteria</td><td>Vibrio vulnificus</td><td>hypothetical protein [Vibrio vulnificus].</td><td>GCF_011754395.1</td></tr>
<tr><td>WP_108816955.1</td><td>REase+TPR+GreAB-C+PIN*→</td><td>REase+TPR+GreAB-C+PIN</td><td>1311</td><td>-</td><td>Alphaproteobacteria</td><td>Loktanella sp. Alg231-35</td><td>hypothetical protein [Loktanella sp. Alg231-35].</td><td>GCF_900143545.1</td></tr>
<tr><td>WP_109158749.1</td><td>X+PIN*→</td><td>X+PIN</td><td>1041</td><td>-</td><td>Epsilonproteobacteria</td><td>Aliarcobacter skirrowii</td><td>hypothetical protein [Aliarcobacter skirrowii].</td><td>GCF_003122145.1</td></tr>
<tr><td>WP_109211325.1</td><td>REase+TPR+GreAB-C+PIN*→</td><td>REase+TPR+GreAB-C+PIN</td><td>1211</td><td>-</td><td>Actinobacteria</td><td>Microbacterium</td><td>MULTISPECIES: DUF4365 domain-containing protein [Microbacterium].</td><td>GCF_015278315.1</td></tr>
<tr><td>WP_109426752.1</td><td>TPR→ TPR+GreAB-C+PIN*→||||<-?||HTH→</td><td>TPR+GreAB-C+PIN</td><td>494</td><td>-</td><td>Gammaproteobacteria</td><td>Acinetobacter baumannii</td><td>GreA/GreB family elongation factor [Acinetobacter baumannii].</td><td>GCF_013416255.1</td></tr>
<tr><td>WP_109802449.1</td><td>TPR+PIN*→</td><td>TPR+PIN</td><td>1203</td><td>-</td><td>Actinobacteria</td><td>Micromonospora sp. 4G51</td><td>hypothetical protein [Micromonospora sp. 4G51].</td><td>GCF_003172935.1</td></tr>
<tr><td>WP_109937286.1</td><td>HetE-N1→ HetE-N1+TPR+GreAB-C+PIN*→</td><td>HetE-N1+TPR+GreAB-C+PIN</td><td>1123</td><td>-</td><td>Gammaproteobacteria</td><td>Pseudomonas sp. RW407</td><td>hypothetical protein [Pseudomonas sp. RW407].</td><td>GCF_003176655.1</td></tr>
<tr><td>WP_109950172.1</td><td>REase+TPR+GreAB-C+PIN*→</td><td>REase+TPR+GreAB-C+PIN</td><td>1332</td><td>-</td><td>-</td><td>Methylobacterium sp. 17Sr1-43</td><td>hypothetical protein [Methylobacterium sp. 17Sr1-43].</td><td>GCF_003173735.1</td></tr>
<tr><td>WP_109967732.1</td><td>PNPase+TPR+GreAB-C+PIN*→</td><td>PNPase+TPR+GreAB-C+PIN</td><td>1415</td><td>-</td><td>Euryarchaeota</td><td>Methanospirillum lacunae</td><td>hypothetical protein [Methanospirillum lacunae].</td><td>GCF_003173355.1</td></tr>
<tr><td>WP_110317339.1</td><td>HTH+PNPase+TPR+GreAB-C+PIN*→</td><td>HTH+PNPase+TPR+GreAB-C+PIN</td><td>1518</td><td>-</td><td>Actinobacteria</td><td>Mycolicibacterium moriokaense</td><td>hypothetical protein [Mycolicibacterium moriokaense].</td><td>GCF_003201655.1</td></tr>
<tr><td>WP_110434863.1</td><td>HetE-N1→ TPR+GreAB-C+PIN*→</td><td>TPR+GreAB-C+PIN</td><td>950</td><td>-</td><td>Gammaproteobacteria</td><td>Gilliamella apicola</td><td>hypothetical protein [Gilliamella apicola].</td><td>GCF_003202655.1</td></tr>
<tr><td>WP_110455267.1</td><td>HetE-N1→ TPR+GreAB-C+PIN*→</td><td>TPR+GreAB-C+PIN</td><td>1126</td><td>-</td><td>Gammaproteobacteria</td><td>Gilliamella apicola</td><td>hypothetical protein [Gilliamella apicola].</td><td>GCF_003202815.1</td></tr>
<tr><td>WP_110459582.1</td><td>HetE-N1→ HetE-N1+TPR+GreAB-C+PIN*→</td><td>HetE-N1+TPR+GreAB-C+PIN</td><td>1107</td><td>-</td><td>Gammaproteobacteria</td><td>Pseudomonas syringae</td><td>hypothetical protein [Pseudomonas syringae].</td><td>GCF_003202975.1</td></tr>
<tr><td>WP_110575159.1</td><td>HetE-N1→ HetE-N1+TPR+GreAB-C+PIN*→?→ MACRODOMAIN→</td><td>HetE-N1+TPR+GreAB-C+PIN</td><td>1099</td><td>-</td><td>Gammaproteobacteria</td><td>Marinomonas alcarazii</td><td>hypothetical protein [Marinomonas alcarazii].</td><td>GCF_003208215.1</td></tr>
<tr><td>WP_110766563.1</td><td>REase+TPR+GreAB-C+PIN*→||||<-KAP_NTPase</td><td>REase+TPR+GreAB-C+PIN</td><td>1321</td><td>-</td><td>Gammaproteobacteria</td><td>Pseudomonas syringae</td><td>DUF4365 domain-containing protein [Pseudomonas syringae].</td><td>GCF_003205895.1</td></tr>
<tr><td>WP_110818130.1</td><td>TPR+GreAB-C+PIN*→</td><td>TPR+GreAB-C+PIN</td><td>1140</td><td>-</td><td>Gammaproteobacteria</td><td>Pseudomonas syringae</td><td>hypothetical protein [Pseudomonas syringae].</td><td>GCF_003205965.1</td></tr>
<tr><td>WP_111087282.1</td><td>REase+TPR+GreAB-C+PIN*→</td><td>REase+TPR+GreAB-C+PIN</td><td>1208</td><td>-</td><td>Actinobacteria</td><td>Curtobacterium sp. MCLR17_055</td><td>DUF4365 domain-containing protein [Curtobacterium sp. MCLR17_055].</td><td>GCF_003234305.1</td></tr>
<tr><td>WP_112353670.1</td><td>TPR+GreAB-C+PIN*→</td><td>TPR+GreAB-C+PIN</td><td>1235</td><td>-</td><td>Gammaproteobacteria</td><td>Shewanella benthica</td><td>hypothetical protein [Shewanella benthica].</td><td>GCF_900476435.1</td></tr>
<tr><td>WP_112921055.1</td><td>HNH→?→?→ PSE→ TPR+GreAB-C+PIN*→</td><td>TPR+GreAB-C+PIN</td><td>1092</td><td>-</td><td>Gammaproteobacteria</td><td>Escherichia coli</td><td>hypothetical protein [Escherichia coli].</td><td>GCF_003292515.1</td></tr>
<tr><td>WP_114150230.1</td><td>TPR+GreAB-C+PIN*→?→||||<-?||?→ PAIREDC-HTH→</td><td>TPR+GreAB-C+PIN</td><td>900</td><td>-</td><td>Gammaproteobacteria</td><td>Acinetobacter baumannii</td><td>GreA/GreB family elongation factor [Acinetobacter baumannii].</td><td>GCF_017726575.1</td></tr>
<tr><td>WP_114915524.1</td><td>TPR+PIN*→</td><td>TPR+PIN</td><td>1187</td><td>-</td><td>Firmicutes</td><td>Oceanobacillus zhaokaii</td><td>hypothetical protein [Oceanobacillus zhaokaii].</td><td>GCF_003352005.1</td></tr>
<tr><td>WP_115015610.1</td><td>HetE-N1→ TPR+GreAB-C+PIN*→</td><td>TPR+GreAB-C+PIN</td><td>1087</td><td>-</td><td>Gammaproteobacteria</td><td>Shewanella putrefaciens</td><td>hypothetical protein [Shewanella putrefaciens].</td><td>GCF_900457065.1</td></tr>
<tr><td>WP_115596495.1</td><td>TPR+GreAB-C+PIN*→?→||||<-?||?→ PAIREDC-HTH→</td><td>TPR+GreAB-C+PIN</td><td>756</td><td>-</td><td>Gammaproteobacteria</td><td>Acinetobacter baumannii</td><td>GreA/GreB family elongation factor [Acinetobacter baumannii].</td><td>GCF_900444745.1</td></tr>
<tr><td>WP_116227219.1</td><td>HetE-N1→ TPR+GreAB-C+PIN*→</td><td>TPR+GreAB-C+PIN</td><td>1121</td><td>-</td><td>Gammaproteobacteria</td><td>Pectobacterium aquaticum</td><td>hypothetical protein [Pectobacterium aquaticum].</td><td>GCF_003382595.2</td></tr>
<tr><td>WP_116600438.1</td><td>REase+TPR+GreAB-C+PIN*→</td><td>REase+TPR+GreAB-C+PIN</td><td>1438</td><td>-</td><td>Gammaproteobacteria</td><td>Pseudomonas sp. HMWF011</td><td>hypothetical protein [Pseudomonas sp. HMWF011].</td><td>GCF_003094675.1</td></tr>
<tr><td>WP_116893902.1</td><td>TPR+GreAB-C+PIN*→</td><td>TPR+GreAB-C+PIN</td><td>1158</td><td>-</td><td>Gammaproteobacteria</td><td>Pseudomonas syringae</td><td>hypothetical protein [Pseudomonas syringae].</td><td>GCF_003412595.1</td></tr>
<tr><td>WP_116994473.1</td><td>REase+TPR+GreAB-C+PIN*→</td><td>REase+TPR+GreAB-C+PIN</td><td>1327</td><td>-</td><td>Betaproteobacteria</td><td>Duganella sp. BJB476</td><td>hypothetical protein [Duganella sp. BJB476].</td><td>GCF_003416895.1</td></tr>
<tr><td>WP_117157406.1</td><td>HetE-N1→ TPR+GreAB-C+PIN*→</td><td>TPR+GreAB-C+PIN</td><td>1117</td><td>-</td><td>Gammaproteobacteria</td><td>Enterobacteriaceae</td><td>MULTISPECIES: hypothetical protein [Enterobacteriaceae].</td><td>GCF_017814635.1</td></tr>
<tr><td>WP_117181470.1</td><td>REase+TPR+GreAB-C+PIN*→</td><td>REase+TPR+GreAB-C+PIN</td><td>1438</td><td>-</td><td>Gammaproteobacteria</td><td>Pseudomonas cichorii</td><td>hypothetical protein [Pseudomonas cichorii].</td><td>GCF_003416755.1</td></tr>
</table>

acc	operon	architecture	len	gen.name	taxend	species	defline	gca
WP_117831329.1	HEPN+SNF→?→?→?→ X+PIN*→	X+PIN	1212	-	Firmicutes	Roseburia sp. TF10-5	hypothetical protein [Roseburia sp. TF10-5].	GCF_003462365.1
WP_118235377.1	X+PIN*→	X+PIN	1200	-	Firmicutes	Blautia obeum	hypothetical protein [Blautia obeum].	GCF_003470555.1
WP_118282562.1	X+PIN*→?→ CITB-HTH+LexA-protease→	X+PIN	1212	-	Firmicutes	Roseburia sp. AM16-25	hypothetical protein [Roseburia sp. AM16-25].	GCF_003473825.1
WP_118371145.1	X+PIN*→	X+PIN	1200	-	Firmicutes	Lachnospira eligens	hypothetical protein [Lachnospira eligens].	GCF_003474735.1
WP_118468213.1	Mbetalac→ Trypsin+TPR+PIN*→	Trypsin+TPR+PIN	1933	-	Firmicutes	unclassified Clostridium	MULTISPECIES: trypsin-like peptidase domain-containing protein [unclassified Clostridium].	GCF_003477925.1
WP_118891177.1	REase+TPR+GreAB-C+PIN*→	REase+TPR+GreAB-C+PIN	1327	-	Betaproteobacteria	Ralstonia solanacearum	DUF4365 domain-containing protein [Ralstonia solanacearum].	GCF_003515285.1
WP_118897359.1	X+PIN*→	X+PIN	1033	-	Epsilonproteobacteria	Malaciobacter marinus	hypothetical protein [Malaciobacter marinus].	GCF_003544855.1
WP_118994196.1	HetE-N1→ HetE-N1+TPR+GreAB-C+PIN*→	HetE-N1+TPR+GreAB-C+PIN	1104	-	Betaproteobacteria	Pusillimonas	MULTISPECIES: hypothetical protein [Pusillimonas].	GCF_004022565.1
WP_119088708.1	REase+TPR+GreAB-C+PIN*→	REase+TPR+GreAB-C+PIN	1271	-	Bacteria	Candidatus Cryosericum terrychapinii	hypothetical protein [Candidatus Cryosericum terrychapinii].	GCF_003570925.1
WP_119196537.1	X+PIN*→ HTH→?→?→ HTH→	X+PIN	1109	-	Firmicutes	Dorea formicigenerans	hypothetical protein [Dorea formicigenerans].	GCF_003465045.1
WP_119369914.1	REase+TPR+GreAB-C+PIN*→||||<-?<-TPR	REase+TPR+GreAB-C+PIN	1436	-	Gammaproteobacteria	Pseudomonas monteilii	GreA/GreB family elongation factor [Pseudomonas monteilii].	GCF_003576045.1
WP_119587627.1	REase+TPR+GreAB-C+PIN*→	REase+TPR+GreAB-C+PIN	1290	-	Alphaproteobacteria	Aurantiacibacter zhengii	hypothetical protein [Aurantiacibacter zhengii].	GCF_003584125.1
WP_119956833.1	REase+TPR+GreAB-C+PIN*→	REase+TPR+GreAB-C+PIN	1511	-	Gammaproteobacteria	Pseudomonas sp. K1S02-6	tetratricopeptide repeat protein [Pseudomonas sp. K1S02-6].	GCF_003596405.1
WP_119992948.1	TM+TM+TPR+PIN*→	TM+TM+TPR+PIN	1208	-	Bacteroidetes	Bacteroides sp. AM41-16	hypothetical protein [Bacteroides sp. AM41-16].	GCF_003603495.1
WP_120172805.1	TM+TM+TPR+PIN*→	TM+TM+TPR+PIN	1208	-	Bacteroidetes	Bacteroides sp. AF20-13LB	hypothetical protein [Bacteroides sp. AF20-13LB].	GCF_003603065.1
WP_120777109.1	TPR+GreAB-C+PIN*→	TPR+GreAB-C+PIN	1192	-	Actinobacteria	Microbacterium sp. CGR2	hypothetical protein [Microbacterium sp. CGR2].	GCF_003626735.1
WP_121482750.1	REase+TPR+GreAB-C+PIN*→	REase+TPR+GreAB-C+PIN	1233	-	Alphaproteobacteria	Brevundimonas naejangsanensis	hypothetical protein [Brevundimonas naejangsanensis].	GCF_003627995.1
WP_122071999.1	PAIREDC-HTH→?→?→?→ GreAB-C+PIN*→	GreAB-C+PIN	683	-	Gammaproteobacteria	Acinetobacter wuhouensis	hypothetical protein [Acinetobacter wuhouensis].	GCF_002165345.2
WP_122252741.1	TPR+GreAB-C+PIN*→	TPR+GreAB-C+PIN	1158	-	Gammaproteobacteria	Pseudomonas savastanoi	hypothetical protein [Pseudomonas savastanoi].	GCF_003703035.1
WP_122265719.1	TPR+GreAB-C+PIN*→	TPR+GreAB-C+PIN	1140	-	Gammaproteobacteria	Pseudomonas syringae	hypothetical protein [Pseudomonas syringae].	GCF_003700575.1
WP_122311960.1	TPR+GreAB-C+PIN*→	TPR+GreAB-C+PIN	1143	-	Gammaproteobacteria	Pseudomonas coronafaciens	hypothetical protein [Pseudomonas coronafaciens].	GCF_003699785.1
WP_122314252.1	REase+TPR+GreAB-C+PIN*→	REase+TPR+GreAB-C+PIN	1436	-	Gammaproteobacteria	Pseudomonas cichorii	hypothetical protein [Pseudomonas cichorii].	GCF_003700275.1
WP_122364448.1	REase+TPR+GreAB-C+PIN*→	REase+TPR+GreAB-C+PIN	1438	-	Gammaproteobacteria	Pseudomonas	MULTISPECIES: hypothetical protein [Pseudomonas].	GCF_014842015.1
WP_122365799.1	TPR+GreAB-C+PIN*→	TPR+GreAB-C+PIN	1140	-	Gammaproteobacteria	Pseudomonas syringae group genomosp. 3	hypothetical protein [Pseudomonas syringae group genomosp. 3].	GCF_003702755.1
WP_122471279.1	HetE-N1→ TPR+GreAB-C+PIN*→	TPR+GreAB-C+PIN	1100	-	Gammaproteobacteria	Pseudomonas viridiflava	hypothetical protein [Pseudomonas viridiflava].	GCF_900582365.1
WP_122509345.1	TPR+GreAB-C+PIN*→	TPR+GreAB-C+PIN	1140	-	Gammaproteobacteria	Pseudomonas viridiflava	hypothetical protein [Pseudomonas viridiflava].	GCF_900581635.1
WP_122559800.1	REase+TPR+GreAB-C+PIN*→	REase+TPR+GreAB-C+PIN	1438	-	Gammaproteobacteria	Pseudomonas viridiflava	hypothetical protein [Pseudomonas viridiflava].	GCF_900580935.1
WP_122732542.1	HetE-N1→ HetE-N1+TPR+GreAB-C+PIN*→	HetE-N1+TPR+GreAB-C+PIN	1106	-	Gammaproteobacteria	Pseudomonas viridiflava	hypothetical protein [Pseudomonas viridiflava].	GCF_900591195.1
WP_122848883.1	REase+TPR+GreAB-C+PIN*→	REase+TPR+GreAB-C+PIN	1321	-	Gammaproteobacteria	Pseudomonas viridiflava	DUF4365 domain-containing protein [Pseudomonas viridiflava].	GCF_900585905.1
WP_122887609.1	REase+TPR+GreAB-C+PIN*→	REase+TPR+GreAB-C+PIN	1345	-	Betaproteobacteria	Burkholderia pseudomallei	hypothetical protein [Burkholderia pseudomallei].	GCF_900593895.1
WP_123094036.1	HetE-N1→ TPR+GreAB-C+PIN*→?→ MACRODOMAIN→	TPR+GreAB-C+PIN	1099	-	Gammaproteobacteria	Marinomonas hwangdonensis	hypothetical protein [Marinomonas hwangdonensis].	GCF_003721245.1
WP_123338621.1	REase+TPR+GreAB-C+PIN*→	REase+TPR+GreAB-C+PIN	1210	-	Actinobacteria	Curtobacterium sp. PhB171	DUF4365 domain-containing protein [Curtobacterium sp. PhB171].	GCF_003752665.1
WP_123718714.1	HetE-N1→ TPR+GreAB-C+PIN*→	TPR+GreAB-C+PIN	1112	-	Gammaproteobacteria	Pseudomonas poae	hypothetical protein [Pseudomonas poae].	GCF_003731985.1
WP_123788903.1	REase+TPR+GreAB-C+PIN*→	REase+TPR+GreAB-C+PIN	1351	-	Betaproteobacteria	Burkholderia multivorans	hypothetical protein [Burkholderia multivorans].	GCF_003812365.1
WP_123901658.1	TPR+GreAB-C+PIN→ GreAB-C+PIN*→	GreAB-C+PIN	535	-	Bacteroidetes	Chryseobacterium bernardetii	hypothetical protein [Chryseobacterium bernardetii].	GCF_003815955.1
WP_124017047.1	TPR+GreAB-C+PIN*→	TPR+GreAB-C+PIN	838	-	Gammaproteobacteria	Shewanella vesiculosa	hypothetical protein [Shewanella vesiculosa].	GCF_003797885.1
WP_124131211.1	DOC+HTH→ REase+TOPC→?→ HetE-N1→ HetE-N1+TPR+GreAB-C+PIN*→	HetE-N1+TPR+GreAB-C+PIN	1124	-	Gammaproteobacteria	Pseudomonas aeruginosa	hypothetical protein [Pseudomonas aeruginosa].	GCF_003836815.1
WP_124132190.1	TPR+GreAB-C+PIN*→	TPR+GreAB-C+PIN	1140	-	Gammaproteobacteria	Pseudomonas aeruginosa	hypothetical protein [Pseudomonas aeruginosa].	GCF_003836445.1

acc	operon	architecture	len	gen.name	taxend	species	defline	gca
WP_124135313.1	TPR+GreAB-C+PIN*→	TPR+GreAB-C+PIN	1158	-	Gammaproteobacteria	Pseudomonas aeruginosa	hypothetical protein [Pseudomonas aeruginosa].	GCF_003836905.1
WP_124172921.1	DOC+HTH→ REase+TOPC→?→ HetE-N1→ HetE-N1+TPR+GreAB-C+PIN*→	HetE-N1+TPR+GreAB-C+PIN	1124	-	Gammaproteobacteria	Pseudomonas aeruginosa	hypothetical protein [Pseudomonas aeruginosa].	GCF_003839465.1
WP_124325287.1	TPR+GreAB-C+PIN*→	TPR+GreAB-C+PIN	1139	-	Gammaproteobacteria	Pseudomonas chlororaphis	hypothetical protein [Pseudomonas chlororaphis].	GCF_003850385.1
WP_124410055.1	HetE-N1→ TPR+GreAB-C+PIN*→	TPR+GreAB-C+PIN	1110	-	Gammaproteobacteria	Pseudomonas sp. R4-34-07	hypothetical protein [Pseudomonas sp. R4-34-07].	GCF_003852375.1
WP_124421918.1	TPR+GreAB-C+PIN*→	TPR+GreAB-C+PIN	1157	-	Gammaproteobacteria	Pseudomonas orientalis	hypothetical protein [Pseudomonas orientalis].	GCF_003851585.1
WP_124432744.1	REase+TPR+GreAB-C+PIN*→	REase+TPR+GreAB-C+PIN	1321	-	Gammaproteobacteria	Pseudomonas orientalis	DUF4365 domain-containing protein [Pseudomonas orientalis].	GCF_003852045.1
WP_124433851.1	REase+TPR+GreAB-C+PIN*→	REase+TPR+GreAB-C+PIN	1321	-	Gammaproteobacteria	Pseudomonas orientalis	DUF4365 domain-containing protein [Pseudomonas orientalis].	GCF_003852045.1
WP_124551445.1	HEPN→?→?→ REase+TPR+GreAB-C+PIN*→	REase+TPR+GreAB-C+PIN	1498	-	Betaproteobacteria	Burkholderia sp. Bp9015	tetratricopeptide repeat protein [Burkholderia sp. Bp9015].	GCF_003853505.1
WP_124582885.1	TPR+GreAB-C+PIN*→	TPR+GreAB-C+PIN	1248	-	Bacteroidetes	Pedobacter sp. KBW06	hypothetical protein [Pedobacter sp. KBW06].	GCF_003852525.1
WP_124686982.1	REase+TPR+GreAB-C+PIN*→	REase+TPR+GreAB-C+PIN	1344	-	Betaproteobacteria	Cupriavidus pauculus	tetratricopeptide repeat protein [Cupriavidus pauculus].	GCF_003854935.1
WP_124885098.1	REase+TPR+GreAB-C+PIN*→	REase+TPR+GreAB-C+PIN	1351	-	Betaproteobacteria	Burkholderia stagnalis	hypothetical protein [Burkholderia stagnalis].	GCF_003857545.1
WP_124919377.1	REase+TPR+GreAB-C+PIN*→	REase+TPR+GreAB-C+PIN	1500	-	Betaproteobacteria	Burkholderia	MULTISPECIES: tetratricopeptide repeat protein [Burkholderia].	GCF_001528845.1
WP_124920354.1	HEPN→?→?→ REase+TPR+GreAB-C+PIN*→	REase+TPR+GreAB-C+PIN	1289	-	Betaproteobacteria	Burkholderia stagnalis	tetratricopeptide repeat protein [Burkholderia stagnalis].	GCF_003857615.1
WP_125127612.1	X+PIN*→?→?→?→?→ MACRODOMAIN→	X+PIN	1232	-	Firmicutes	Schaedlerella arabinosiphila	hypothetical protein [Schaedlerella arabinosiphila].	GCF_003885045.1
WP_125255381.1	REase+TPR+GreAB-C+PIN*→	REase+TPR+GreAB-C+PIN	1327	-	Alphaproteobacteria	Brevundimonas fluminis	DUF4365 domain-containing protein [Brevundimonas fluminis].	GCF_003934285.1
WP_125429200.1	REase+TPR+GreAB-C+PIN*→	REase+TPR+GreAB-C+PIN	1284	-	Gammaproteobacteria	Stenotrophomonas maltophilia	GreA/GreB family elongation factor [Stenotrophomonas maltophilia].	GCF_003935615.1
WP_125440526.1	<-REase+nSTAND3||TPR+GreAB-C+PIN*→	TPR+GreAB-C+PIN	1282	-	Bacteroidetes	Hymenobacter perfusus	hypothetical protein [Hymenobacter perfusus].	GCF_003944765.1
WP_125601225.1	HetE-N1→ MNS-Npun2340+TPR+GreAB-C+PIN*→	MNS-Npun2340+TPR+GreAB-C+PIN	1094	-	Gammaproteobacteria	Aeromonas salmonicida	hypothetical protein [Aeromonas salmonicida].	GCF_003947375.1
WP_125730033.1	HetE-N1→ TPR+GreAB-C+PIN*→	TPR+GreAB-C+PIN	1094	-	Gammaproteobacteria	Aeromonas salmonicida	hypothetical protein [Aeromonas salmonicida].	GCF_003947355.1
WP_126243019.1	REase+TPR+GreAB-C+PIN*→	REase+TPR+GreAB-C+PIN	1333	-	Betaproteobacteria	Burkholderia gladioli	hypothetical protein [Burkholderia gladioli].	GCF_902829535.1
WP_126550729.1	TPR+GreAB-C+PIN*→	TPR+GreAB-C+PIN	1140	-	Gammaproteobacteria	Pseudomonas aeruginosa	hypothetical protein [Pseudomonas aeruginosa].	GCF_001516225.2
WP_126566690.1	TPR+GreAB-C+PIN*→	TPR+GreAB-C+PIN	1043	-	Gammaproteobacteria	Pseudomonas aeruginosa	hypothetical protein [Pseudomonas aeruginosa].	GCF_003968275.1
WP_126570139.1	DOC+HTH→ REase+TOPC→?→ HetE-N1→ HetE-N1→ GreAB-C+PIN*→	GreAB-C+PIN	523	-	Gammaproteobacteria	Pseudomonas aeruginosa	hypothetical protein [Pseudomonas aeruginosa].	GCF_003969065.1
WP_127030837.1	HetE-N1→ TPR+GreAB-C+PIN*→	TPR+GreAB-C+PIN	1093	-	Gammaproteobacteria	Pectobacterium versatile	hypothetical protein [Pectobacterium versatile].	GCF_003990505.1
WP_127257681.1	REase+TPR+GreAB-C+PIN*→	REase+TPR+GreAB-C+PIN	1287	-	Alphaproteobacteria	Mesorhizobium sp. M7A.F.Ca.US.008.03.1.1	hypothetical protein [Mesorhizobium sp. M7A.F.Ca.US.008.03.1.1].	GCF_003997495.1
WP_127294273.1	REase+TPR+TPR+GreAB-C+PIN*→	REase+TPR+TPR+GreAB-C+PIN	1333	-	Alphaproteobacteria	Mesorhizobium sp. M6A.T.Ce.TU.016.01.1.1	GreA/GreB family elongation factor [Mesorhizobium sp. M6A.T.Ce.TU.016.01.1.1].	GCF_003997065.1
WP_127581798.1	BirA-HTH→?→ REase+TPR+TPR+GreAB-C+PIN*→	REase+TPR+TPR+GreAB-C+PIN	1322	-	Alphaproteobacteria	Sinorhizobium medicae	hypothetical protein [Sinorhizobium medicae].	GCF_004002295.1
WP_128106456.1	REase+TPR+GreAB-C+PIN*→	REase+TPR+GreAB-C+PIN	1323	-	Alphaproteobacteria	Acetobacter oryzoeni	tetratricopeptide repeat protein [Acetobacter oryzoeni].	GCF_004014775.2
WP_128141476.1	REase+TPR+GreAB-C+PIN*→	REase+TPR+GreAB-C+PIN	1616	-	Betaproteobacteria	Janthinobacterium lividum	hypothetical protein [Janthinobacterium lividum].	GCF_900451225.1
WP_128142627.1	REase+TPR+GreAB-C+PIN*→	REase+TPR+GreAB-C+PIN	1269	-	Betaproteobacteria	Janthinobacterium lividum	hypothetical protein [Janthinobacterium lividum].	GCF_900451225.1
WP_128181279.1	REase+TPR+TPR+GreAB-C+PIN*→	REase+TPR+TPR+GreAB-C+PIN	1330	-	Alphaproteobacteria	Sinirhodobacter populi	hypothetical protein [Sinirhodobacter populi].	GCF_004022225.1
WP_128291227.1	REase+TPR+GreAB-C+PIN*→	REase+TPR+GreAB-C+PIN	1320	-	Alphaproteobacteria	Afifella aestuarii	hypothetical protein [Afifella aestuarii].	GCF_004023665.1
WP_128326676.1	REase+TPR+GreAB-C+PIN*→	REase+TPR+GreAB-C+PIN	1493	-	Gammaproteobacteria	Pseudomonas alkylphenolica	hypothetical protein [Pseudomonas alkylphenolica].	GCF_004025535.1
WP_128384334.1	X+PIN*→	X+PIN	1055	-	Gammaproteobacteria	Hydrogenovibrio thermophilus	hypothetical protein [Hydrogenovibrio thermophilus].	GCF_004028275.1
WP_128449906.1	REase+TPR+TPR+GreAB-C+PIN*→	REase+TPR+TPR+GreAB-C+PIN	1385	-	Alphaproteobacteria	Rhizobium leguminosarum	hypothetical protein [Rhizobium leguminosarum].	GCF_004054135.1
WP_128549293.1	TPR+GreAB-C+PIN*→	TPR+GreAB-C+PIN	1187	-	Alphaproteobacteria	Salipiger	MULTISPECIES: tetratricopeptide repeat protein [Salipiger].	GCF_014637265.1
WP_128552168.1	DOC+HTH→ REase+TOPC→ HetE-N1→ HetE-N1+TPR+GreAB-C+PIN*→	HetE-N1+TPR+GreAB-C+PIN	1123	-	Gammaproteobacteria	Pseudomonas aeruginosa	hypothetical protein [Pseudomonas aeruginosa].	GCF_003698765.1

acc	operon	architecture	len	gen.name	taxend	species	defline	gca
WP_128664039.1	DOC+HTH→ REase+TOPC→?→ HetE-N1→ HetE-N1+TPR+GreAB-C+PIN*→	HetE-N1+TPR+GreAB-C+PIN	1124	-	Gammaproteobacteria	Pseudomonas	MULTISPECIES: hypothetical protein [Pseudomonas].	GCF_016624455.1
WP_128715623.1	SIG+HIN-HTH→?→ GreAB-C+PIN*→	GreAB-C+PIN	540	-	Alphaproteobacteria	Rhizobium acidisoli	hypothetical protein [Rhizobium acidisoli].	GCF_002531755.2
WP_128816805.1	REase→ TPR+PIN*→	TPR+PIN	875	-	Actinobacteria	Streptomyces sp. S063	tetratricopeptide repeat protein [Streptomyces sp. S063].	GCF_002832675.1
WP_128912272.1	REase+TPR+GreAB-C+PIN*→	REase+TPR+GreAB-C+PIN	1277	-	Acidobacteria	Granulicella sibirica	hypothetical protein [Granulicella sibirica].	GCF_004115155.1
WP_129013963.1	TPR+GreAB-C+PIN*→?→?→?→?→?→?→ TIR+TPR+TPR→	TPR+GreAB-C+PIN	1046	-	Epsilonproteobacteria	Arcobacter cloacae	hypothetical protein [Arcobacter cloacae].	GCF_013201935.1
WP_129107976.1	REase+TPR+GreAB-C+PIN*→	REase+TPR+GreAB-C+PIN	1200	-	Epsilonproteobacteria	Halarcobacter bivalviorum	hypothetical protein [Halarcobacter bivalviorum].	GCF_004116705.1
WP_129330071.1	HetE-N1→?→ TPR+GreAB-C+PIN*→	TPR+GreAB-C+PIN	739	-	Gammaproteobacteria	Salmonella enterica	hypothetical protein [Salmonella enterica].	GCF_004120835.1
WP_129368690.1	HetE-N1→?→ TPR+GreAB-C+PIN*→	TPR+GreAB-C+PIN	855	-	Gammaproteobacteria	Salmonella enterica	hypothetical protein [Salmonella enterica].	GCF_004120585.1
WP_129411798.1	REase+TPR+GreAB-C+PIN*→	REase+TPR+GreAB-C+PIN	1323	-	Alphaproteobacteria	Mesorhizobium sp. Pch-S	hypothetical protein [Mesorhizobium sp. Pch-S].	GCF_004136315.1
WP_129485462.1	REase+TPR+GreAB-C+PIN*→	REase+TPR+GreAB-C+PIN	1468	-	Gammaproteobacteria	Pseudomonas syringae	hypothetical protein [Pseudomonas syringae].	GCF_004124725.1
WP_129893847.1	REase+TPR+GreAB-C+PIN*→	REase+TPR+GreAB-C+PIN	1309	-	Chloroflexi	Ktedonosporobacter rubrisoli	tetratricopeptide repeat protein [Ktedonosporobacter rubrisoli].	GCF_004208415.1
WP_130007120.1	HetE-N1→ HetE-N1+TPR+GreAB-C+PIN*→	HetE-N1+TPR+GreAB-C+PIN	1104	-	Betaproteobacteria	Pusillimonas ginsengisoli	hypothetical protein [Pusillimonas ginsengisoli].	GCF_004153455.1
WP_130044922.1	HetE-N1→ TPR+GreAB-C+PIN*→	TPR+GreAB-C+PIN	1116	-	Gammaproteobacteria	Aliivibrio finisterrensis	hypothetical protein [Aliivibrio finisterrensis].	GCF_004167675.1
WP_130132480.1	REase+TPR+GreAB-C+PIN*→	REase+TPR+GreAB-C+PIN	1245	-	Gammaproteobacteria	Acinetobacter wuhouensis	hypothetical protein [Acinetobacter wuhouensis].	GCF_004209325.1
WP_130204115.1	HetE-N1→ TPR+GreAB-C+PIN*→	TPR+GreAB-C+PIN	1113	-	Gammaproteobacteria	Pseudomonas moorei	hypothetical protein [Pseudomonas moorei].	GCF_004212425.1
WP_130252287.1	HetE-N1→ TPR+GreAB-C+PIN*→	TPR+GreAB-C+PIN	1114	-	Gammaproteobacteria	Vibrio vulnificus	hypothetical protein [Vibrio vulnificus].	GCF_004214575.1
WP_130472343.1	REase+TPR+GreAB-C+PIN*→	REase+TPR+GreAB-C+PIN	1348	-	Alphaproteobacteria	Candidatus Magnetaquicoccus inordinatus	hypothetical protein [Candidatus Magnetaquicoccus inordinatus].	GCF_004217665.1
WP_130525307.1	HetE-N1→ TPR+GreAB-C+PIN*→	TPR+GreAB-C+PIN	1086	-	Gammaproteobacteria	Pasteurella multocida	hypothetical protein [Pasteurella multocida].	GCF_003428945.1
WP_130555679.1	HetE-N1→ TPR+GreAB-C+PIN*→	TPR+GreAB-C+PIN	1086	-	Gammaproteobacteria	Pasteurella multocida	hypothetical protein [Pasteurella multocida].	GCF_001578435.2
WP_130710767.1	REase+TPR+TPR+GreAB-C+PIN*→	REase+TPR+TPR+GreAB-C+PIN	1343	-	Alphaproteobacteria	Rhizobium leguminosarum	hypothetical protein [Rhizobium leguminosarum].	GCF_004304355.1
WP_130725527.1	REase+TPR+GreAB-C+PIN*→	REase+TPR+GreAB-C+PIN	1255	-	Alphaproteobacteria	Rhizobium leguminosarum	hypothetical protein [Rhizobium leguminosarum].	GCF_004303985.1
WP_130928792.1	REase+TPR+GreAB-C+PIN*→	REase+TPR+GreAB-C+PIN	1438	-	Gammaproteobacteria	Pseudomonas sp. Sample_20	hypothetical protein [Pseudomonas sp. Sample_20].	GCF_004307585.1
WP_131049955.1	HetE-N1→ TPR+GreAB-C+PIN*→	TPR+GreAB-C+PIN	1092	-	Gammaproteobacteria	Raoultella ornithinolytica	hypothetical protein [Raoultella ornithinolytica].	GCF_004312065.1
WP_131189445.1	HetE-N1→ HetE-N1+TPR+GreAB-C+PIN*→	HetE-N1+TPR+GreAB-C+PIN	1125	-	Gammaproteobacteria	Pseudomonas kirkiae	hypothetical protein [Pseudomonas kirkiae].	GCF_004327175.1
WP_131590605.1	TPR+PIN*→	TPR+PIN	1153	-	Actinobacteria	Mycolicibacterium setense	hypothetical protein [Mycolicibacterium setense].	GCF_000805375.1
WP_131623255.1	REase+TPR+TPR+GreAB-C+PIN*→	REase+TPR+TPR+GreAB-C+PIN	1361	-	Alphaproteobacteria	Rhizobium leguminosarum	hypothetical protein [Rhizobium leguminosarum].	GCF_004330075.1
WP_131714066.1	REase+TPR+TPR+GreAB-C+PIN*→	REase+TPR+TPR+GreAB-C+PIN	1337	-	Alphaproteobacteria	Rhizobium leguminosarum	hypothetical protein [Rhizobium leguminosarum].	GCF_004330185.1
WP_131718233.1	TPR+GreAB-C+PIN*→	TPR+GreAB-C+PIN	556	-	Alphaproteobacteria	Rhizobium leguminosarum	hypothetical protein [Rhizobium leguminosarum].	GCF_004330625.1
WP_131814603.1	TPR+GreAB-C+PIN*→	TPR+GreAB-C+PIN	595	-	Alphaproteobacteria	Cognatishimia maritima	hypothetical protein [Cognatishimia maritima].	GCF_900129685.1
WP_131869160.1	TPR+GreAB-C+PIN*→	TPR+GreAB-C+PIN	1306	-	Alphaproteobacteria	unclassified Bradyrhizobium	MULTISPECIES: tetratricopeptide repeat protein [unclassified Bradyrhizobium].	GCF_004346395.1
WP_132139369.1	REase+TPR+GreAB-C+PIN*→	REase+TPR+GreAB-C+PIN	1368	-	Betaproteobacteria	Massilia sp. GV090	hypothetical protein [Massilia sp. GV090].	GCF_004341465.1
WP_132251347.1	TPR+GreAB-C+PIN*→	TPR+GreAB-C+PIN	1114	-	Alphaproteobacteria	Methylobacterium segetis	tetratricopeptide repeat protein [Methylobacterium segetis].	GCF_004348265.1
WP_132403375.1	TPR+GreAB-C+PIN*→	TPR+GreAB-C+PIN	1266	-	Bacteroidetes	Pedobacter sp. CF074	hypothetical protein [Pedobacter sp. CF074].	GCF_004342125.1
WP_132537157.1	BirA-HTH→ HEPN→ REase+TPR+TPR+GreAB-C+PIN*→	REase+TPR+TPR+GreAB-C+PIN	1332	-	Alphaproteobacteria	Rhizobium sp. PP-F2F-G48	GreA/GreB family elongation factor [Rhizobium sp. PP-F2F-G48].	GCF_004342335.1
WP_132665856.1	REase+TPR+GreAB-C+PIN*→	REase+TPR+GreAB-C+PIN	1297	-	Alphaproteobacteria	Rhizobium sp. PP-CC-3G-465	tetratricopeptide repeat protein [Rhizobium sp. PP-CC-3G-465].	GCF_004343445.1
WP_132831667.1	TPR+GreAB-C+PIN*→	TPR+GreAB-C+PIN	1188	-	Alphaproteobacteria	Antarcticimicrobium sediminis	hypothetical protein [Antarcticimicrobium sediminis].	GCF_004348975.1
WP_132850706.1	TPR+PIN*→	TPR+PIN	1135	-	Actinobacteria	Streptomyces sp. BK308	tetratricopeptide repeat protein [Streptomyces sp. BK308].	GCF_004346005.1
WP_133023868.1	TPR+PIN*→	TPR+PIN	1216	-	Actinobacteria	Streptomyces sp. BK329	hypothetical protein [Streptomyces sp. BK329].	GCF_004341055.1
WP_133030467.1	REase+TPR+GreAB-C+PIN*→	REase+TPR+GreAB-C+PIN	1317	-	Alphaproteobacteria	Sphingomonas sp. PP-CE-1G-424	tetratricopeptide repeat protein [Sphingomonas sp. PP-CE-1G-424].	GCF_004341085.1

acc	operon	architecture	len	gen.name	taxend	species	defline	gca
WP_133031998.1	REase+TPR+GreAB-C+PIN*→	REase+TPR+GreAB-C+PIN	1323	-	Alphaproteobacteria	Sphingomonas sp. PP-CE-1G-424	hypothetical protein [Sphingomonas sp. PP-CE-1G-424].	GCF_004341085.1
WP_133117283.1	REase+TPR+TPR+GreAB-C+PIN*→	REase+TPR+TPR+GreAB-C+PIN	1332	-	Alphaproteobacteria	Mesorhizobium sanjuanii	hypothetical protein [Mesorhizobium sanjuanii].	GCF_002529485.1
WP_133245819.1	REase+TPR+GreAB-C+PIN*→	REase+TPR+GreAB-C+PIN	1322	-	Alphaproteobacteria	Candidatus Phycosocius bacilliformis	hypothetical protein [Candidatus Phycosocius bacilliformis].	GCF_003112735.1
WP_133303262.1	TPR+GreAB-C+PIN*→	TPR+GreAB-C+PIN	1082	-	Alphaproteobacteria	Rhodopseudomonas palustris	hypothetical protein [Rhodopseudomonas palustris].	GCF_013415845.1
WP_133404970.1	REase+TPR+GreAB-C+PIN*→	REase+TPR+GreAB-C+PIN	1260	-	Actinobacteria	Arthrobacter crusticola	DUF4365 domain-containing protein [Arthrobacter crusticola].	GCF_004357995.1
WP_133633736.1	REase+TPR+GreAB-C+PIN*→	REase+TPR+GreAB-C+PIN	1321	-	Gammaproteobacteria	Pseudomonas sp. OV184	DUF4365 domain-containing protein [Pseudomonas sp. OV184].	GCF_004363115.1
WP_133936590.1	REase+TPR+GreAB-C+PIN*→	REase+TPR+GreAB-C+PIN	1489	-	Betaproteobacteria	Comamonas kerstersii	tetratricopeptide repeat protein [Comamonas kerstersii].	GCF_002002425.1
WP_134016909.1	REase+TPR+GreAB-C+PIN*→	REase+TPR+GreAB-C+PIN	1313	-	Alphaproteobacteria	Litoreibacter halocynthiae	hypothetical protein [Litoreibacter halocynthiae].	GCF_004365635.1
WP_134268494.1	PIN*→	PIN	186	-	Firmicutes	Sporosarcina pasteurii	hypothetical protein [Sporosarcina pasteurii].	GCF_004379295.1
WP_134281591.1	TPR+GreAB-C+PIN*→	TPR+GreAB-C+PIN	1159	-	Gammaproteobacteria	Pseudomonas	MULTISPECIES: hypothetical protein [Pseudomonas].	GCF_013522725.1
WP_135194680.1	REase+TPR+GreAB-C+PIN*→	REase+TPR+GreAB-C+PIN	1327	-	Alphaproteobacteria	Brevundimonas intermedia	DUF4365 domain-containing protein [Brevundimonas intermedia].	GCF_004614235.1
WP_135269295.1	TPR+PIN*→	TPR+PIN	1211	-	Actinobacteria	Nocardioides seonyuensis	hypothetical protein [Nocardioides seonyuensis].	GCF_004683965.1
WP_135300409.1	HetE-N1→ TPR+GreAB-C+PIN*→	TPR+GreAB-C+PIN	1130	-	Gammaproteobacteria	Photobacterium damselae	hypothetical protein [Photobacterium damselae].	GCF_004683985.2
WP_135312567.1	REase+TPR+GreAB-C+PIN→ PIN*→	PIN	479	-	Alphaproteobacteria	Paracoccus liaowanqingii	hypothetical protein [Paracoccus liaowanqingii].	GCF_004683865.2
WP_135953001.1	HetE-N1→ TPR+GreAB-C+PIN*→	TPR+GreAB-C+PIN	987	-	Gammaproteobacteria	Pasteurella multocida	hypothetical protein [Pasteurella multocida].	GCF_004792575.1
WP_135961458.1	HetE-N1→ TPR+GreAB-C+PIN*→	TPR+GreAB-C+PIN	1086	-	Gammaproteobacteria	Pasteurella multocida	hypothetical protein [Pasteurella multocida].	GCF_004792555.1
WP_136247578.1	HetE-N1→ TPR+GreAB-C+PIN*→	TPR+GreAB-C+PIN	1103	-	Gammaproteobacteria	Halomonas borealis	hypothetical protein [Halomonas borealis].	GCF_004798905.1
WP_136878408.1	TPR+GreAB-C+PIN*→	TPR+GreAB-C+PIN	1248	-	Bacteroidetes	Pedobacter sp. AR-2-6	hypothetical protein [Pedobacter sp. AR-2-6].	GCF_005116475.1
WP_137004448.1	REase+TPR+GreAB-C+PIN*→	REase+TPR+GreAB-C+PIN	1290	-	Alphaproteobacteria	Agrobacterium tumefaciens	hypothetical protein [Agrobacterium tumefaciens].	GCF_005221405.1
WP_137059419.1	TPR+PIN*→	TPR+PIN	1157	-	Firmicutes	Bacillus mycoides	hypothetical protein [Bacillus mycoides].	GCF_005217805.1
WP_137213367.1	TPR+GreAB-C+PIN*→	TPR+GreAB-C+PIN	1139	-	Gammaproteobacteria	Pseudomonas sp. CFBP13508	hypothetical protein [Pseudomonas sp. CFBP13508].	GCF_005233515.1
WP_137383131.1	HetE-N1→ TPR+GreAB-C+PIN*→	TPR+GreAB-C+PIN	1091	-	Gammaproteobacteria	Citrobacter sp. wls619	hypothetical protein [Citrobacter sp. wls619].	GCF_005281345.1
WP_137387523.1	HetE-N1→ TPR+GreAB-C+PIN*→	TPR+GreAB-C+PIN	1101	-	Gammaproteobacteria	Pantoea sp. SO10	hypothetical protein [Pantoea sp. SO10].	GCF_005281435.1
WP_137434419.1	REase+TPR+GreAB-C+PIN*→	REase+TPR+GreAB-C+PIN	1288	-	Gammaproteobacteria	Marinobacter sp. PJ-16	hypothetical protein [Marinobacter sp. PJ-16].	GCF_005298175.1
WP_137735239.1	REase+TPR+GreAB-C+PIN*→	REase+TPR+GreAB-C+PIN	1437	-	Betaproteobacteria	Aquabacterium pictum	hypothetical protein [Aquabacterium pictum].	GCF_005403045.1
WP_137905827.1	TPR+GreAB-C+PIN*→	TPR+GreAB-C+PIN	1130	-	Bacteroidetes	Chryseobacterium sp. 2VB	hypothetical protein [Chryseobacterium sp. 2VB].	GCF_005503635.1
WP_138242320.1	HetE-N1→ TPR+GreAB-C+PIN*→	TPR+GreAB-C+PIN	1130	-	Gammaproteobacteria	Photobacterium damselae	hypothetical protein [Photobacterium damselae].	GCF_005819845.1
WP_138262872.1	X+PIN*→	X+PIN	1204	-	Firmicutes	Enterocloster clostridioformis	hypothetical protein [Enterocloster clostridioformis].	GCF_015548065.1
WP_138402485.1	TPR+GreAB-C+PIN*→	TPR+GreAB-C+PIN	1137	-	Bacteroidetes	Chryseobacterium indologenes	hypothetical protein [Chryseobacterium indologenes].	GCF_005862425.1
WP_138437829.1	HetE-N1→ GreAB-C*→	GreAB-C	802	-	Gammaproteobacteria	Marinobacter	MULTISPECIES: hypothetical protein [Marinobacter].	GCF_005871095.1
WP_138616099.1	HetE-N1→ TPR+GreAB-C+PIN*→	TPR+GreAB-C+PIN	1106	-	Gammaproteobacteria	Pseudoalteromonas sp. S2721	hypothetical protein [Pseudoalteromonas sp. S2721].	GCF_005887425.1
WP_139051161.1	TPR+GreAB-C+PIN*→	TPR+GreAB-C+PIN	1158	-	Gammaproteobacteria	unclassified Pseudomonas	MULTISPECIES: hypothetical protein [unclassified Pseudomonas].	GCF_901007645.1
WP_139100594.1	TPR+PIN*→	TPR+PIN	1197	-	Actinobacteria	Amycolatopsis alkalitolerans	hypothetical protein [Amycolatopsis alkalitolerans].	GCF_006152065.1
WP_139135497.1	X+PIN*→	X+PIN	1093	-	Actinobacteria	Micromonospora tulbaghiae	hypothetical protein [Micromonospora tulbaghiae].	GCF_900091605.1
WP_139156465.1	HetE-N1→?→ TPR+GreAB-C+PIN*→	TPR+GreAB-C+PIN	523	-	Gammaproteobacteria	Salmonella enterica	hypothetical protein [Salmonella enterica].	GCF_003071545.1
WP_139230170.1	TPR+PIN*→	TPR+PIN	1206	-	Actinobacteria	Nocardioides terrae	hypothetical protein [Nocardioides terrae].	GCF_900112345.1
WP_139231027.1	TPR+GreAB-C+PIN*→	TPR+GreAB-C+PIN	1155	-	Gammaproteobacteria	Pseudomonas sagittaria	hypothetical protein [Pseudomonas sagittaria].	GCF_900115715.1

acc	operon	architecture	len	gen.name	taxend	species	defline	gca
WP_139231685.1	REase+TPR+GreAB-C+PIN*→?→?→ Calcineurin→ Calcineurin→?→ Calcineurin→	REase+TPR+GreAB-C+PIN	1314	-	Alphaproteobacteria	Methylobacterium phyllosphaerae	hypothetical protein [Methylobacterium phyllosphaerae].	GCF_001936175.1
WP_139585558.1	TPR+PIN*→	TPR+PIN	1173	-	Actinobacteria	Micromonospora orduensis	hypothetical protein [Micromonospora orduensis].	GCF_006228125.1
WP_139699848.1	TPR+GreAB-C+PIN*→	TPR+GreAB-C+PIN	1163	-	unclassified Bacteria	bacterium endosymbiont of Bathymodiolus sp. 5 South	tetratricopeptide repeat protein [bacterium endosymbiont of Bathymodiolus sp. 5 South].	GCF_900127295.1
WP_139711990.1	TPR+PIN*→	TPR+PIN	816	-	Actinobacteria	Streptomyces sp. NP160	hypothetical protein [Streptomyces sp. NP160].	GCF_006335115.1
WP_139741421.1	HetE-N1→ TPR+GreAB-C+PIN*→	TPR+GreAB-C+PIN	1086	-	Gammaproteobacteria	Aeromonas veronii	hypothetical protein [Aeromonas veronii].	GCF_006243415.1
WP_139832542.1	REase+TPR+GreAB-C+PIN*→	REase+TPR+GreAB-C+PIN	1427	-	Gammaproteobacteria	Pseudomonas sp. B26(2017)	hypothetical protein [Pseudomonas sp. B26(2017)].	GCF_002113045.1
WP_139834254.1	HetE-N1→?→ TPR+GreAB-C+PIN*→	TPR+GreAB-C+PIN	686	-	Gammaproteobacteria	Pseudomonas sp. B11(2017)	hypothetical protein [Pseudomonas sp. B11(2017)].	GCF_002113285.1
WP_140034365.1	HetE-N1→ TPR+GreAB-C+PIN*→	TPR+GreAB-C+PIN	1111	-	Gammaproteobacteria	Pantoea vagans	hypothetical protein [Pantoea vagans].	GCF_006385385.1
WP_140089969.1	GreAB-C*→	GreAB-C	561	-	Gammaproteobacteria	Vibrio parahaemolyticus	hypothetical protein [Vibrio parahaemolyticus].	GCF_006372765.1
WP_140136560.1	TPR+GreAB-C+PIN*→	TPR+GreAB-C+PIN	381	-	Gammaproteobacteria	Vibrio parahaemolyticus	hypothetical protein, partial [Vibrio parahaemolyticus].	GCF_006369095.1
WP_140150658.1	HetE-N1→ TPR+GreAB-C+PIN*→	TPR+GreAB-C+PIN	1100	-	Gammaproteobacteria	Vibrio parahaemolyticus	hypothetical protein [Vibrio parahaemolyticus].	GCF_006375025.1
WP_140184851.1	HetE-N1→ TPR+GreAB-C+PIN*→	TPR+GreAB-C+PIN	1133	-	Gammaproteobacteria	Vibrio parahaemolyticus	hypothetical protein [Vibrio parahaemolyticus].	GCF_006375315.1
WP_140247351.1	HetE-N1→ TPR+GreAB-C+PIN*→	TPR+GreAB-C+PIN	1114	-	Gammaproteobacteria	Vibrio parahaemolyticus	hypothetical protein [Vibrio parahaemolyticus].	GCF_006371495.1
WP_140523105.1	REase+TPR+GreAB-C+PIN*→	REase+TPR+GreAB-C+PIN	1330	-	Alphaproteobacteria	unclassified Mesorhizobium	MULTISPECIES: tetratricopeptide repeat protein [unclassified Mesorhizobium].	GCF_006439485.1
WP_140649671.1	REase+TPR+GreAB-C+PIN*→	REase+TPR+GreAB-C+PIN	1330	-	Alphaproteobacteria	Mesorhizobium sp. B2-4-10	tetratricopeptide repeat protein [Mesorhizobium sp. B2-4-10].	GCF_006442675.1
WP_140682745.1	HetE-N1→ TPR+GreAB-C+PIN*→	TPR+GreAB-C+PIN	1113	-	Gammaproteobacteria	Pseudomonas mandelii	hypothetical protein [Pseudomonas mandelii].	GCF_006438925.1
WP_140739373.1	REase+TPR+GreAB-C+PIN*→	REase+TPR+GreAB-C+PIN	994	-	Alphaproteobacteria	unclassified Mesorhizobium	MULTISPECIES: hypothetical protein [unclassified Mesorhizobium].	GCF_006440845.1
WP_141097119.1	TPR+GreAB-C+PIN*→	TPR+GreAB-C+PIN	1209	-	Actinobacteria	Microbacterium sp. AISO3	hypothetical protein [Microbacterium sp. AISO3].	GCF_002204305.1
WP_141144329.1	X+PIN*→	X+PIN	1189	-	Actinobacteria	Schumannella sp. 10F1B-5-1	hypothetical protein [Schumannella sp. 10F1B-5-1].	GCF_006517535.1
WP_141202816.1	TPR+PIN*→	TPR+PIN	1217	-	Actinobacteria	Streptomyces griseorubiginosus	hypothetical protein [Streptomyces griseorubiginosus].	GCF_006516935.1
WP_141414632.1	HetE-N1→ TPR+GreAB-C+PIN*→	TPR+GreAB-C+PIN	1106	-	Gammaproteobacteria	Enterobacter hormaechei	hypothetical protein [Enterobacter hormaechei].	GCF_006546545.1
WP_141566570.1	TPR+PIN*→	TPR+PIN	1237	-	-	Pseudonocardia sp. N23	hypothetical protein [Pseudonocardia sp. N23].	GCF_002583555.1
WP_141708179.1	REase+TPR+GreAB-C+PIN*→	REase+TPR+GreAB-C+PIN	1338	-	Actinobacteria	Micromonospora marina	hypothetical protein [Micromonospora marina].	GCF_900091565.1
WP_141709791.1	TPR+PIN*→||||<-NUDIX	TPR+PIN	1088	-	Actinobacteria	Streptomyces	MULTISPECIES: hypothetical protein [Streptomyces].	GCF_000514055.1
WP_142342557.1	TPR+PIN*→	TPR+PIN	1016	-	Firmicutes	Bacillus cereus	hypothetical protein [Bacillus cereus].	GCF_002577165.1
WP_142621974.1	TPR+GreAB-C+PIN*→	TPR+GreAB-C+PIN	456	-	Gammaproteobacteria	Vibrio cholerae	hypothetical protein [Vibrio cholerae].	GCF_006802685.1
WP_142996755.1	<-REase||TPR+PIN*→	TPR+PIN	993	-	Firmicutes	Bacillus sp. KQ-3	hypothetical protein [Bacillus sp. KQ-3].	GCF_003710255.1
WP_143042354.1	REase+TPR+GreAB-C+PIN*→	REase+TPR+GreAB-C+PIN	1329	-	Alphaproteobacteria	Citreimonas salinaria	restriction endonuclease [Citreimonas salinaria].	GCF_900107235.1
WP_143064837.1	TPR+PIN*→	TPR+PIN	1134	-	Actinobacteria	Streptomyces sp. PAN_FS17	hypothetical protein [Streptomyces sp. PAN_FS17].	GCF_900105465.1
WP_143167506.1	REase+TPR+GreAB-C+PIN*→	REase+TPR+GreAB-C+PIN	1482	-	Betaproteobacteria	Massilia sp. CF038	hypothetical protein [Massilia sp. CF038].	GCF_900129765.1
WP_143171741.1	PAIREDC-HTH→ REase+TPR+GreAB-C+PIN*→	REase+TPR+GreAB-C+PIN	1539	-	Betaproteobacteria	Rhizobacter sp. OV335	hypothetical protein [Rhizobacter sp. OV335].	GCF_900142965.1
WP_143206624.1	REase+TPR+GreAB-C+PIN*→	REase+TPR+GreAB-C+PIN	1273	-	Planctomycetes	Singulisphaera sp. GP187	hypothetical protein [Singulisphaera sp. GP187].	GCF_900129635.1
WP_143268820.1	REase+TPR+GreAB-C+PIN→ PIN*→	PIN	279	-	Actinobacteria	Amycolatopsis vastitatis	hypothetical protein [Amycolatopsis vastitatis].	GCF_002234595.1
WP_143494315.1	DOC+HTH→ REase+TOPC→ HetE-N1→ HetE-N1+TPR+GreAB-C+PIN*→	HetE-N1+TPR+GreAB-C+PIN	1124	-	Gammaproteobacteria	Pseudomonas putida	hypothetical protein [Pseudomonas putida].	GCF_007049805.1
WP_143685467.1	TPR+PIN*→	TPR+PIN	1135	-	Actinobacteria	Streptomyces sp. 1222.2	hypothetical protein [Streptomyces sp. 1222.2].	GCF_900215595.1
WP_143686526.1	TPR+PIN*→	TPR+PIN	1189	-	Actinobacteria	Streptomyces sp. 61	hypothetical protein [Streptomyces sp. 61].	GCF_002754535.1
WP_143775988.1	REase+TPR+GreAB-C+PIN*→	REase+TPR+GreAB-C+PIN	1312	-	Alphaproteobacteria	Sphingorhabdus contaminans	hypothetical protein [Sphingorhabdus contaminans].	GCF_007280415.1
WP_144044861.1	HetE-N1→ GreAB-C*→ TPR+GreAB-C+PIN→	GreAB-C	779	-	Gammaproteobacteria	Shewanella sp. YLB-06	GreA/GreB family elongation factor [Shewanella sp. YLB-06].	GCF_007197555.1

acc	operon	architecture	len	gen.name	taxend	species	defline	gca
WP_144084411.1	HetE-N1→ TPR+TPR+GreAB-C+PIN*→	TPR+TPR+GreAB-C+PIN	1108	-	Gammaproteobacteria	Pseudomonas monteilii	hypothetical protein [Pseudomonas monteilii].	GCF_000510285.1
WP_144190111.1	HetE-N1→ TPR+GreAB-C+PIN*→	TPR+GreAB-C+PIN	1126	-	Gammaproteobacteria	Gilliamella apicola	hypothetical protein [Gilliamella apicola].	GCF_007559165.1
WP_144217154.1	TPR+GreAB-C+PIN*→	TPR+GreAB-C+PIN	1236	-	Gammaproteobacteria	Vibrio cholerae	hypothetical protein [Vibrio cholerae].	GCF_007623805.1
WP_144226215.1	HetE-N1→ TPR+GreAB-C+PIN*→	TPR+GreAB-C+PIN	1101	-	Gammaproteobacteria	Shewanella algae	hypothetical protein [Shewanella algae].	GCF_007595165.1
WP_144269797.1	REase+TPR+GreAB-C+PIN*→	REase+TPR+GreAB-C+PIN	1288	-	Gammaproteobacteria	Pseudomonas fluorescens	DUF4365 domain-containing protein [Pseudomonas fluorescens].	GCF_000262325.2
WP_144298906.1	X+PIN*→	X+PIN	676	-	Actinobacteria	Streptomyces sp. TLI_235	hypothetical protein [Streptomyces sp. TLI_235].	GCF_002300355.1
WP_144431187.1	REase+TPR+GreAB-C+PIN*→	REase+TPR+GreAB-C+PIN	1441	-	Gammaproteobacteria	Pseudomonas syringae pv. coryli	hypothetical protein [Pseudomonas syringae pv. coryli].	GCF_000972175.1
WP_144762711.1	X+PIN*→	X+PIN	1177	-	Actinobacteria	Curtobacterium sp. 9128	hypothetical protein [Curtobacterium sp. 9128].	GCF_007673775.1
WP_144916164.1	TPR+GreAB-C+PIN→ PIN*→	PIN	238	-	Bacteroidetes	Mucilaginibacter frigoritolerans	hypothetical protein [Mucilaginibacter frigoritolerans].	GCF_007830615.1
WP_145052948.1	TPR+PIN*→	TPR+PIN	1410	-	Planctomycetes	Lignipirellula cremea	hypothetical protein [Lignipirellula cremea].	GCF_007751035.1
WP_145376920.1	TPR+PIN*→	TPR+PIN	2081	-	Planctomycetes	Symmachiella dynata	hypothetical protein [Symmachiella dynata].	GCF_007747995.1
WP_145427683.1	REase+TPR+GreAB-C+PIN→ TPR+GreAB-C+PIN*→	TPR+GreAB-C+PIN	514	-	Planctomycetes	Symmachiella dynata	hypothetical protein [Symmachiella dynata].	GCF_007744975.1
WP_145441613.1	TPR+PIN*→	TPR+PIN	2077	-	Planctomycetes	Gimesia chilikensis	hypothetical protein [Gimesia chilikensis].	GCF_007746535.1
WP_145577942.1	HetE-N1→ TPR+GreAB-C+PIN*→	TPR+GreAB-C+PIN	1084	-	Gammaproteobacteria	Vibrio crassostreae	hypothetical protein [Vibrio crassostreae].	GCF_017916675.1
WP_145860820.1	TPR+GreAB-C+PIN→ PIN*→	PIN	347	-	Bacteroidetes	Pedobacter suwonensis	hypothetical protein [Pedobacter suwonensis].	GCF_007833675.1
WP_145895823.1	REase+TPR+GreAB-C+PIN*→	REase+TPR+GreAB-C+PIN	1508	-	Betaproteobacteria	Caenimonas sp. HX-9-20	hypothetical protein [Caenimonas sp. HX-9-20].	GCF_007833165.1
WP_145923949.1	TPR+GreAB-C+PIN*→	TPR+GreAB-C+PIN	1170	-	Gammaproteobacteria	Pseudomonas citronellolis	hypothetical protein [Pseudomonas citronellolis].	GCF_001586155.1
WP_145975538.1	REase+TPR+GreAB-C+PIN*→	REase+TPR+GreAB-C+PIN	1365	-	Deinococci	Deinococcus gobiensis	hypothetical protein [Deinococcus gobiensis].	GCF_000252445.1
WP_145980369.1	HetE-N1→ TPR+GreAB-C+PIN*→	TPR+GreAB-C+PIN	1113	-	Gammaproteobacteria	Pseudomonas lurida	hypothetical protein [Pseudomonas lurida].	GCF_001708485.1
WP_145994348.1	REase+TPR+TPR+GreAB-C+PIN*→	REase+TPR+TPR+GreAB-C+PIN	1339	-	Alphaproteobacteria	Mesorhizobium loti	hypothetical protein [Mesorhizobium loti].	GCF_002858745.1
WP_146048182.1	PIN*→	PIN	371	-	Gammaproteobacteria	Acinetobacter sp. ACNIH3	hypothetical protein [Acinetobacter sp. ACNIH3].	GCF_002918965.1
WP_146113387.1	TPR+PIN*→	TPR+PIN	1210	-	Actinobacteria	unclassified Arthrobacter	MULTISPECIES: hypothetical protein [unclassified Arthrobacter].	GCF_002975405.1
WP_146162727.1	TPR+GreAB-C+PIN*→	TPR+GreAB-C+PIN	1197	-	Gammaproteobacteria	Vibrio splendidus	hypothetical protein [Vibrio splendidus].	GCF_003050425.1
WP_146163497.1	HetE-N1→ TPR+GreAB-C+PIN*→	TPR+GreAB-C+PIN	1094	-	Gammaproteobacteria	Aeromonas veronii	hypothetical protein [Aeromonas veronii].	GCF_003036425.1
WP_146169670.1	REase+TPR+GreAB-C+PIN*→	REase+TPR+GreAB-C+PIN	1355	-	Actinobacteria	Actinoplanes italicus	hypothetical protein [Actinoplanes italicus].	GCF_016862235.1
WP_146174202.1	TPR+GreAB-C+PIN*→	TPR+GreAB-C+PIN	1185	-	Alphaproteobacteria	Litoreibacter ponti	hypothetical protein [Litoreibacter ponti].	GCF_003054285.1
WP_146175347.1	X+PIN*→	X+PIN	1323	-	Actinobacteria	Conexibacter sp. Seoho-28	hypothetical protein [Conexibacter sp. Seoho-28].	GCF_003044185.1
WP_146181743.1	HetE-N1→ TPR+GreAB-C+PIN*→	TPR+GreAB-C+PIN	1094	-	Gammaproteobacteria	Aeromonas sp. HMWF014	hypothetical protein [Aeromonas sp. HMWF014].	GCF_003061085.1
WP_146562359.1	TPR+GreAB-C+PIN*→	TPR+GreAB-C+PIN	1275	-	Planctomycetes	Posidoniimonas corsicana	hypothetical protein [Posidoniimonas corsicana].	GCF_007859765.1
WP_146571406.1	REase+TPR+GreAB-C+PIN*→	REase+TPR+GreAB-C+PIN	1140	-	Planctomycetes	Botrimarina hoheduenensis	hypothetical protein [Botrimarina hoheduenensis].	GCF_007859815.1
WP_146752056.1	TPR+GreAB-C+PIN*→	TPR+GreAB-C+PIN	1230	-	Actinobacteria	Microbacterium sp. SMR1	hypothetical protein [Microbacterium sp. SMR1].	GCF_003289625.1
WP_146766012.1	TPR+GreAB-C+PIN*→	TPR+GreAB-C+PIN	1147	-	Gammaproteobacteria	Pseudomonas	MULTISPECIES: hypothetical protein [Pseudomonas].	GCF_013522795.1
WP_146779203.1	TPR+GreAB-C+PIN*→	TPR+GreAB-C+PIN	1133	-	Bacteroidetes	Flavobacterium psychrolimnae	hypothetical protein [Flavobacterium psychrolimnae].	GCF_003312425.1
WP_146877508.1	TPR+GreAB-C+PIN*→	TPR+GreAB-C+PIN	1076	-	Actinobacteria	Actinomadura madurae	hypothetical protein [Actinomadura madurae].	GCF_900445005.1
WP_146915636.1	TIR→ TPR+GreAB-C+PIN*→||||<-HTH	TPR+GreAB-C+PIN	1255	-	Bacteroidetes	Algoriphagus aquimarinus	hypothetical protein [Algoriphagus aquimarinus].	GCF_007997215.1
WP_146943293.1	TPR+GreAB-C+PIN*→	TPR+GreAB-C+PIN	1126	-	Bacteroidetes	Chryseobacterium hagamense	hypothetical protein [Chryseobacterium hagamense].	GCF_007991455.1
WP_146990354.1	REase+TPR+TPR+GreAB-C+PIN*→	REase+TPR+TPR+GreAB-C+PIN	1315	-	Alphaproteobacteria	Bradyrhizobium macuxiense	hypothetical protein [Bradyrhizobium macuxiense].	GCF_007993935.1
WP_147282746.1	HetE-N1→ TPR+GreAB-C+PIN*→	TPR+GreAB-C+PIN	1118	-	Betaproteobacteria	Comamonas sp. AG1104	hypothetical protein [Comamonas sp. AG1104].	GCF_003350425.1
WP_147289649.1	HetE-N1→ TPR+GreAB-C+PIN*→	TPR+GreAB-C+PIN	1110	-	Gammaproteobacteria	Pantoea agglomerans	hypothetical protein [Pantoea agglomerans].	GCF_900454505.1
WP_147309007.1	REase+TPR+GreAB-C+PIN→?→ PIN*→	PIN	256	-	Betaproteobacteria	Cupriavidus taiwanensis	hypothetical protein [Cupriavidus taiwanensis].	GCF_900249855.1
WP_147316401.1	TPR+GreAB-C+PIN*→	TPR+GreAB-C+PIN	1139	-	Gammaproteobacteria	Pseudomonas aeruginosa	hypothetical protein [Pseudomonas aeruginosa].	GCF_003412045.2
WP_147329285.1	X+PIN*→ HTH→?→?→ HTH→	X+PIN	1180	-	Firmicutes	Dorea formicigenerans	hypothetical protein [Dorea formicigenerans].	GCF_003437395.1
WP_147334977.1	TM+TM+TPR+PIN*→	TM+TM+TPR+PIN	1154	-	Bacteroidetes	Phocaeicola coprocola	hypothetical protein [Phocaeicola coprocola].	GCF_003458565.1

<table>
<thead>
<tr><th>acc</th><th>operon</th><th>architecture</th><th>len</th><th>gen.name</th><th>taxend</th><th>species</th><th>defline</th><th>gca</th></tr>
</thead>
<tbody>
<tr><td>WP_147344604.1</td><td>Mbetalac→ Trypsin→?→ TPR+PIN*→</td><td>TPR+PIN</td><td>1394</td><td>-</td><td>Firmicutes</td><td>Eubacterium sp. AM49-13BH</td><td>hypothetical protein, partial [Eubacterium sp. AM49-13BH].</td><td>GCF_003464165.1</td></tr>
<tr><td>WP_147352805.1</td><td>Mbetalac→ Trypsin→ TPR+PIN*→</td><td>TPR+PIN</td><td>1645</td><td>-</td><td>Firmicutes</td><td>Clostridium sp. AM48-13</td><td>hypothetical protein [Clostridium sp. AM48-13].</td><td>GCF_003478455.1</td></tr>
<tr><td>WP_147353595.1</td><td>Mbetalac→ Trypsin+TPR+PIN*→</td><td>Trypsin+TPR+PIN</td><td>1710</td><td>-</td><td>Firmicutes</td><td>Clostridium sp. AF37-7</td><td>trypsin-like peptidase domain-containing protein, partial [Clostridium sp. AF37-7].</td><td>GCF_003477595.1</td></tr>
<tr><td>WP_147364185.1</td><td>X+PIN*→ HTH→?→?→ HTH→</td><td>X+PIN</td><td>1179</td><td>-</td><td>Firmicutes</td><td>Dorea formicigenerans</td><td>hypothetical protein [Dorea formicigenerans].</td><td>GCF_003475555.1</td></tr>
<tr><td>WP_147402393.1</td><td>TPR+GreAB-C+PIN*→</td><td>TPR+GreAB-C+PIN</td><td>1139</td><td>-</td><td>Gammaproteobacteria</td><td>Pseudomonas aeruginosa</td><td>hypothetical protein [Pseudomonas aeruginosa].</td><td>GCF_003585235.1</td></tr>
<tr><td>WP_147402585.1</td><td>DOC+HTH→ REase+TOPC→ HetE-N1→ HetE-N1+TPR+GreAB-C+PIN*→</td><td>HetE-N1+TPR+GreAB-C+PIN</td><td>1123</td><td>-</td><td>Gammaproteobacteria</td><td>Pseudomonas aeruginosa</td><td>hypothetical protein [Pseudomonas aeruginosa].</td><td>GCF_003585225.1</td></tr>
<tr><td>WP_147432801.1</td><td>REase+TPR+GreAB-C+PIN*→</td><td>REase+TPR+GreAB-C+PIN</td><td>1375</td><td>-</td><td>Actinobacteria</td><td>Catellatospora citrea</td><td>hypothetical protein [Catellatospora citrea].</td><td>GCF_016862615.1</td></tr>
<tr><td>WP_147432945.1</td><td><-TIR<-?||REase+TPR+GreAB-C+PIN*→</td><td>REase+TPR+GreAB-C+PIN</td><td>1245</td><td>-</td><td>Actinobacteria</td><td>Catellatospora citrea</td><td>DUF4365 domain-containing protein [Catellatospora citrea].</td><td>GCF_016862615.1</td></tr>
<tr><td>WP_147450330.1</td><td>REase+TPR+GreAB-C+PIN*→</td><td>REase+TPR+GreAB-C+PIN</td><td>1306</td><td>-</td><td>Deltaproteobacteria</td><td>Corallococcus carmarthensis</td><td>hypothetical protein [Corallococcus carmarthensis].</td><td>GCF_003611695.1</td></tr>
<tr><td>WP_147453837.1</td><td>TPR+PIN*→ PAIREDC-HTH→</td><td>TPR+PIN</td><td>1206</td><td>-</td><td>Actinobacteria</td><td>Nocardiopsis sp. Huas11</td><td>hypothetical protein [Nocardiopsis sp. Huas11].</td><td>GCF_003634495.1</td></tr>
<tr><td>WP_147481098.1</td><td>TPR+PIN*→</td><td>TPR+PIN</td><td>1134</td><td>-</td><td>Actinobacteria</td><td>Streptomyces shenzhenensis</td><td>tetratricopeptide repeat protein [Streptomyces shenzhenensis].</td><td>GCF_003688995.1</td></tr>
<tr><td>WP_147627327.1</td><td>REase+TPR+GreAB-C+PIN→ SIG+HIN-HTH→?→ PIN*→</td><td>PIN</td><td>507</td><td>-</td><td>Gammaproteobacteria</td><td>Rhodanobacter glycinis</td><td>hypothetical protein [Rhodanobacter glycinis].</td><td>GCF_008000795.1</td></tr>
<tr><td>WP_147831354.1</td><td>TPR+GreAB-C+PIN*→</td><td>TPR+GreAB-C+PIN</td><td>1107</td><td>-</td><td>Alphaproteobacteria</td><td>Methylobacterium sp. WL19</td><td>hypothetical protein [Methylobacterium sp. WL19].</td><td>GCF_008040005.1</td></tr>
<tr><td>WP_147893054.1</td><td>X+PIN*→</td><td>X+PIN</td><td>1120</td><td>-</td><td>Actinobacteria</td><td>Microbacterium hatanonis</td><td>hypothetical protein [Microbacterium hatanonis].</td><td>GCF_008017415.1</td></tr>
<tr><td>WP_148210527.1</td><td>TPR+GreAB-C+PIN*→</td><td>TPR+GreAB-C+PIN</td><td>1107</td><td>-</td><td>Alphaproteobacteria</td><td>Beijerinckia indica</td><td>hypothetical protein [Beijerinckia indica].</td><td>GCF_000019845.1</td></tr>
<tr><td>WP_148306266.1</td><td>TPR+GreAB-C+PIN*→</td><td>TPR+GreAB-C+PIN</td><td>1259</td><td>-</td><td>Gemmatimonadetes</td><td>Gemmatirosa kalamazoonesis</td><td>hypothetical protein [Gemmatirosa kalamazoonesis].</td><td>GCF_000522985.1</td></tr>
<tr><td>WP_148421250.1</td><td><-MarR-HTH||?→?→?→||||<-?||?→ HetE-N1→ TPR+GreAB-C+PIN*→</td><td>TPR+GreAB-C+PIN</td><td>1117</td><td>-</td><td>Gammaproteobacteria</td><td>Enterobacter cloacae</td><td>hypothetical protein [Enterobacter cloacae].</td><td>GCF_013376835.1</td></tr>
<tr><td>WP_148564915.1</td><td>REase+TPR+GreAB-C+PIN*→</td><td>REase+TPR+GreAB-C+PIN</td><td>1243</td><td>-</td><td>Gammaproteobacteria</td><td>Stenotrophomonas maltophilia</td><td>hypothetical protein [Stenotrophomonas maltophilia].</td><td>GCF_001274595.1</td></tr>
<tr><td>WP_149326491.1</td><td>HetE-N1→ TPR+GreAB-C+PIN*→ PhospholipaseD→</td><td>TPR+GreAB-C+PIN</td><td>1103</td><td>-</td><td>Gammaproteobacteria</td><td>Halomonas sp. L5</td><td>hypothetical protein [Halomonas sp. L5].</td><td>GCF_008297955.1</td></tr>
<tr><td>WP_149369557.1</td><td>TPR+PIN*→</td><td>TPR+PIN</td><td>1189</td><td>-</td><td>Actinobacteria</td><td>Mycolicibacterium sp. P9-64</td><td>hypothetical protein [Mycolicibacterium sp. P9-64].</td><td>GCF_008329605.1</td></tr>
<tr><td>WP_150024336.1</td><td>TPR+GreAB-C+PIN*→||||<-?||HTH→</td><td>TPR+GreAB-C+PIN</td><td>804</td><td>-</td><td>Gammaproteobacteria</td><td>Acinetobacter baumannii</td><td>GreA/GreB family elongation factor [Acinetobacter baumannii].</td><td>GCF_008630895.1</td></tr>
<tr><td>WP_150359822.1</td><td>REase+TPR+GreAB-C+PIN*→</td><td>REase+TPR+GreAB-C+PIN</td><td>1240</td><td>-</td><td>Gammaproteobacteria</td><td>Stenotrophomonas maltophilia</td><td>hypothetical protein [Stenotrophomonas maltophilia].</td><td>GCF_008693985.1</td></tr>
<tr><td>WP_150409130.1</td><td>REase+TPR+GreAB-C+PIN*→</td><td>REase+TPR+GreAB-C+PIN</td><td>1284</td><td>-</td><td>Gammaproteobacteria</td><td>Xanthomonas sacchari</td><td>GreA/GreB family elongation factor [Xanthomonas sacchari].</td><td>GCF_008705275.1</td></tr>
<tr><td>WP_150412179.1</td><td>REase+TPR+GreAB-C+PIN*→</td><td>REase+TPR+GreAB-C+PIN</td><td>1314</td><td>-</td><td>Alphaproteobacteria</td><td>Sphingobium limneticum</td><td>hypothetical protein [Sphingobium limneticum].</td><td>GCF_008710155.1</td></tr>
<tr><td>WP_150784121.1</td><td>REase+TPR+GreAB-C+PIN*→</td><td>REase+TPR+GreAB-C+PIN</td><td>1318</td><td>-</td><td>Gammaproteobacteria</td><td>Pseudomonas fluorescens</td><td>DUF4365 domain-containing protein [Pseudomonas fluorescens].</td><td>GCF_902497995.1</td></tr>
<tr><td>WP_150981548.1</td><td>REase+TPR+GreAB-C+PIN*→</td><td>REase+TPR+GreAB-C+PIN</td><td>1329</td><td>-</td><td>Betaproteobacteria</td><td>Cupriavidus pauculus</td><td>GreA/GreB family elongation factor [Cupriavidus pauculus].</td><td>GCF_008801835.1</td></tr>
<tr><td>WP_151013632.1</td><td>TPR+PIN*→</td><td>TPR+PIN</td><td>1145</td><td>-</td><td>Actinobacteria</td><td>Micromonospora aurantiaca</td><td>hypothetical protein [Micromonospora aurantiaca].</td><td>GCF_008806405.1</td></tr>
<tr><td>WP_151024479.1</td><td>REase+TPR+GreAB-C+PIN*→</td><td>REase+TPR+GreAB-C+PIN</td><td>1499</td><td>-</td><td>Betaproteobacteria</td><td>Castellaniella defragrans</td><td>hypothetical protein [Castellaniella defragrans].</td><td>GCF_014203015.1</td></tr>
<tr><td>WP_151868021.1</td><td>TPR+GreAB-C+PIN*→</td><td>TPR+GreAB-C+PIN</td><td>463</td><td>-</td><td>Gammaproteobacteria</td><td>Acinetobacter soli</td><td>hypothetical protein, partial [Acinetobacter soli].</td><td>GCF_009013695.1</td></tr>
<tr><td>WP_152091999.1</td><td>HetE-N1→ TPR+GreAB-C+PIN*→</td><td>TPR+GreAB-C+PIN</td><td>1107</td><td>-</td><td>Gammaproteobacteria</td><td>Pseudomonas sp. KUIN-1</td><td>hypothetical protein [Pseudomonas sp. KUIN-1].</td><td>GCF_009176725.1</td></tr>
<tr><td>WP_152099374.1</td><td>TPR+GreAB-C+PIN*→</td><td>TPR+GreAB-C+PIN</td><td>1188</td><td>-</td><td>Planctomycetes</td><td>Lacipirellula parvula</td><td>tetratricopeptide repeat protein [Lacipirellula parvula].</td><td>GCF_009177095.1</td></tr>
<tr><td>WP_152217660.1</td><td>REase+TPR+TPR+GreAB-C+PIN*→</td><td>REase+TPR+TPR+GreAB-C+PIN</td><td>1333</td><td>-</td><td>Alphaproteobacteria</td><td>Parvibaculum sedimenti</td><td>hypothetical protein [Parvibaculum sedimenti].</td><td>GCF_009184905.1</td></tr>
<tr><td>WP_152508433.1</td><td>TPR+GreAB-C+PIN*→</td><td>TPR+GreAB-C+PIN</td><td>1208</td><td>-</td><td>Alphaproteobacteria</td><td>unclassified Labrenzia</td><td>MULTISPECIES: hypothetical protein [unclassified Labrenzia].</td><td>GCF_009363315.1</td></tr>
<tr><td>WP_152533063.1</td><td>REase+TPR+GreAB-C+PIN*→</td><td>REase+TPR+GreAB-C+PIN</td><td>1484</td><td>-</td><td>Gammaproteobacteria</td><td>Pseudomonas syringae</td><td>hypothetical protein [Pseudomonas syringae].</td><td>GCF_000452465.1</td></tr>
<tr><td>WP_152541917.1</td><td>TPR+GreAB-C+PIN*→</td><td>TPR+GreAB-C+PIN</td><td>529</td><td>-</td><td>Bacteroidetes</td><td>Saccharicrinis fermentans</td><td>hypothetical protein [Saccharicrinis fermentans].</td><td>GCF_000583675.1</td></tr>
<tr><td>WP_152640028.1</td><td>TPR+GreAB-C+PIN*→</td><td>TPR+GreAB-C+PIN</td><td>395</td><td>-</td><td>Firmicutes</td><td>Blautia obeum</td><td>hypothetical protein [Blautia obeum].</td><td>GCF_003463085.1</td></tr>
</tbody>
</table>

acc	operon	architecture	len	gen.name	taxend	species	defline	gca
WP_152648143.1	REase+TPR+TPR+GreAB-C+PIN*→	REase+TPR+TPR+GreAB-C+PIN	1321	-	Alphaproteobacteria	Bradyrhizobium elkanii	hypothetical protein [Bradyrhizobium elkanii].	GCF_000878305.1
WP_152803033.1	REase+TPR+GreAB-C+PIN*→||||<-HTH	REase+TPR+GreAB-C+PIN	1328	-	Betaproteobacteria	Rugamonas sp. FT103W	hypothetical protein [Rugamonas sp. FT103W].	GCF_009380165.1
WP_152993715.1	TPR+GreAB-C+PIN*→	TPR+GreAB-C+PIN	677	-	Gammaproteobacteria	Pseudomonas aeruginosa	hypothetical protein [Pseudomonas aeruginosa].	GCF_001451965.1
WP_153001774.1	PIN*→	PIN	483	-	Actinobacteria	Curtobacterium oceanosedimentum	hypothetical protein [Curtobacterium oceanosedimentum].	GCF_001476135.1
WP_153041348.1	TPR+GreAB-C+PIN*→	TPR+GreAB-C+PIN	1322	-	Alphaproteobacteria	Pseudovibrio sp. Ad5	hypothetical protein [Pseudovibrio sp. Ad5].	GCF_001623095.1
WP_153065368.1	REase→ TPR+GreAB-C+PIN*→	TPR+GreAB-C+PIN	1111	-	Gammaproteobacteria	Xanthomonas arboricola	hypothetical protein [Xanthomonas arboricola].	GCF_003352905.1
WP_153226839.1	TPR+PIN*→	TPR+PIN	1140	-	Actinobacteria	Mycobacteroides salmoniphilum	hypothetical protein [Mycobacteroides salmoniphilum].	GCF_004366855.1
WP_153278476.1	TM+TPR+PIN*→	TM+TPR+PIN	1138	-	Actinobacteria	Saccharothrix syringae	hypothetical protein [Saccharothrix syringae].	GCF_009498035.1
WP_153574636.1	TPR+GreAB-C+PIN*→	TPR+GreAB-C+PIN	1139	-	Gammaproteobacteria	Pseudomonas aeruginosa	hypothetical protein [Pseudomonas aeruginosa].	GCF_000791765.1
WP_153576067.1	TPR+GreAB-C+PIN*→	TPR+GreAB-C+PIN	1170	-	Gammaproteobacteria	Pseudomonas aeruginosa	hypothetical protein [Pseudomonas aeruginosa].	GCF_000795585.1
WP_153576415.1	TPR+GreAB-C+PIN*→	TPR+GreAB-C+PIN	1158	-	Gammaproteobacteria	Pseudomonas aeruginosa	hypothetical protein [Pseudomonas aeruginosa].	GCF_000796465.1
WP_153786096.1	TPR+GreAB-C+PIN*→	TPR+GreAB-C+PIN	1139	-	Gammaproteobacteria	Pseudomonas sp. EMN2	hypothetical protein [Pseudomonas sp. EMN2].	GCF_009650555.1
WP_154691510.1	REase+TPR+GreAB-C+PIN*→	REase+TPR+GreAB-C+PIN	1325	-	Alphaproteobacteria	Novosphingobium sp. Gsoil 351	hypothetical protein [Novosphingobium sp. Gsoil 351].	GCF_009707465.1
WP_154808640.1	TPR+GreAB-C+PIN*→||||<-?<-?<-?<-?||?→?→ Cas_Cas1→	TPR+GreAB-C+PIN	1207	-	Euryarchaeota	Methanolobus vulcani	GreA/GreB family elongation factor [Methanolobus vulcani].	GCF_006546655.1
WP_154898518.1	TPR+GreAB-C+PIN*→	TPR+GreAB-C+PIN	970	-	Planctomycetes	Gimesia maris	GreA/GreB family elongation factor [Gimesia maris].	GCF_007747015.1
WP_155001003.1	TPR+GreAB-C+PIN*→	TPR+GreAB-C+PIN	1157	-	Gammaproteobacteria	Pseudomonas fluorescens	hypothetical protein [Pseudomonas fluorescens].	GCF_902497965.1
WP_155247385.1	REase+TPR+GreAB-C+PIN*→	REase+TPR+GreAB-C+PIN	1356	-	Actinobacteria	Salinispora arenicola	hypothetical protein [Salinispora arenicola].	GCF_000375205.1
WP_155347963.1	REase+TPR+GreAB-C+PIN*→	REase+TPR+GreAB-C+PIN	1228	-	Actinobacteria	Acrocarpospora pleiomorpha	DUF4365 domain-containing protein [Acrocarpospora pleiomorpha].	GCF_009687885.1
WP_155468027.1	REase+TPR+GreAB-C+PIN*→	REase+TPR+GreAB-C+PIN	1410	-	Betaproteobacteria	Duganella radicis	DUF4365 domain-containing protein [Duganella radicis].	GCF_009720825.1
WP_155543363.1	REase+TPR+GreAB-C+PIN*→	REase+TPR+GreAB-C+PIN	1179	-	Actinobacteria	Amycolatopsis sp. A23	DUF4365 domain-containing protein [Amycolatopsis sp. A23].	GCF_902497555.1
WP_155546438.1	REase+TPR+GreAB-C+PIN*→	REase+TPR+GreAB-C+PIN	1244	-	Actinobacteria	Amycolatopsis sp. A23	DUF4365 domain-containing protein [Amycolatopsis sp. A23].	GCF_902497555.1
WP_155620995.1	TPR+PIN*→	TPR+PIN	1179	-	Firmicutes	Paenibacillus macerans	hypothetical protein [Paenibacillus macerans].	GCF_009725145.1
WP_155672395.1	HetE-N1→ TPR+GreAB-C+PIN*→	TPR+GreAB-C+PIN	1088	-	Gammaproteobacteria	Aliivibrio fischeri	hypothetical protein [Aliivibrio fischeri].	GCF_009727805.1
WP_155681246.1	DOC+HTH→ REase+TOPC→ HetE-N1→ HetE-N1+TPR+GreAB-C+PIN*→	HetE-N1+TPR+GreAB-C+PIN	1123	-	Gammaproteobacteria	Pseudomonas aeruginosa	hypothetical protein [Pseudomonas aeruginosa].	GCF_009727245.1
WP_155728442.1	HetE-N1→ MNS-Npun2340+TPR+GreAB-C+PIN*→	MNS-Npun2340+TPR+GreAB-C+PIN	1094	-	Gammaproteobacteria	Aeromonas veronii	hypothetical protein [Aeromonas veronii].	GCF_001593245.1
WP_155754272.1	REase+TPR+GreAB-C+PIN*→	REase+TPR+GreAB-C+PIN	1265	-	Betaproteobacteria	Burkholderia vietnamiensis	hypothetical protein [Burkholderia vietnamiensis].	GCF_001528565.1
WP_155849001.1	TPR+PIN*→	TPR+PIN	1211	-	Actinobacteria	Amycolatopsis vancoresmycina	hypothetical protein [Amycolatopsis vancoresmycina].	GCF_000388135.1
WP_155854387.1	TPR+PIN*→	TPR+PIN	1219	-	Actinobacteria	Arthrobacter sp. MA-N2	hypothetical protein [Arthrobacter sp. MA-N2].	GCF_000517125.1
WP_155858633.1	REase+TPR+GreAB-C+PIN*→	REase+TPR+GreAB-C+PIN	1245	-	Gammaproteobacteria	Acinetobacter soli	hypothetical protein [Acinetobacter soli].	GCF_001953195.1
WP_155950759.1	HetE-N1→ TPR+TPR+GreAB-C+PIN*→	TPR+TPR+GreAB-C+PIN	1108	-	Gammaproteobacteria	Pseudomonas taiwanensis	hypothetical protein [Pseudomonas taiwanensis].	GCF_000425785.1
WP_155952204.1	REase+TPR+GreAB-C+PIN*→	REase+TPR+GreAB-C+PIN	1443	-	Gammaproteobacteria	Pseudomonas sp. CHM02	hypothetical protein [Pseudomonas sp. CHM02].	GCF_000612585.1
WP_155956985.1	HTH+PNPase+TPR+GreAB-C+PIN*→	HTH+PNPase+TPR+GreAB-C+PIN	1520	-	Actinobacteria	Rhodococcus sp. UNC23MFCrub1.1	helix-turn-helix domain-containing protein [Rhodococcus sp. UNC23MFCrub1.1].	GCF_000686025.1
WP_156093738.1	REase+TPR+GreAB-C+PIN*→	REase+TPR+GreAB-C+PIN	1179	-	Actinobacteria	Lentzea aerocolonigenes	DUF4365 domain-containing protein [Lentzea aerocolonigenes].	GCF_000974445.1
WP_156124401.1	HetE-N1→ HetE-N1+TPR+GreAB-C+PIN*→	HetE-N1+TPR+GreAB-C+PIN	1122	-	Betaproteobacteria	Achromobacter sp. RTa	hypothetical protein [Achromobacter sp. RTa].	GCF_000757485.1
WP_156177573.1	REase+TPR+GreAB-C+PIN*→	REase+TPR+GreAB-C+PIN	1471	-	Gammaproteobacteria	Pseudomonas sp. CCOS 191	hypothetical protein [Pseudomonas sp. CCOS 191].	GCF_001007005.1
WP_156289500.1	TPR+PIN*→	TPR+PIN	1076	-	Firmicutes	Oceanobacillus salinisoli	hypothetical protein [Oceanobacillus salinisoli].	GCF_009733865.1
WP_156340841.1	REase+TPR+GreAB-C+PIN*→	REase+TPR+GreAB-C+PIN	1470	-	Gammaproteobacteria	Pseudomonas sp. NBRC 111124	hypothetical protein [Pseudomonas sp. NBRC 111124].	GCF_001320245.1

acc	operon	architecture	len	gen.name	taxend	species	defline	gca
WP_156343172.1	REase+TPR+GreAB-C+PIN*→	REase+TPR+GreAB-C+PIN	1368	-	Gammaproteobacteria	Pseudomonas sp. NBRC 111134	hypothetical protein [Pseudomonas sp. NBRC 111134].	GCF_001320655.1
WP_156369768.1	TPR+PIN*→	TPR+PIN	1065	-	Actinobacteria	Arthrobacter sp. Leaf145	hypothetical protein [Arthrobacter sp. Leaf145].	GCF_001423565.1
WP_156457128.1	TPR+GreAB-C+PIN*→	TPR+GreAB-C+PIN	1119	-	Alphaproteobacteria	Bradyrhizobium sp. CCH5-F6	hypothetical protein [Bradyrhizobium sp. CCH5-F6].	GCF_001556045.1
WP_156523151.1	REase+TPR+GreAB-C+PIN*→	REase+TPR+GreAB-C+PIN	1289	-	Betaproteobacteria	Bordetella ansorpii	hypothetical protein [Bordetella ansorpii].	GCF_900078315.1
WP_156681909.1	-	-	1775	-	Actinobacteria	Rhodococcus sp. SC4	helix-turn-helix domain-containing protein [Rhodococcus sp. SC4].	-
WP_156749419.1	TPR+PIN*→	TPR+PIN	1166	-	Actinobacteria	Mycobacterium sp. E1747	hypothetical protein [Mycobacterium sp. E1747].	GCF_001667775.1
WP_156792330.1	HetE-N1→ TPR+GreAB-C+PIN*→	TPR+GreAB-C+PIN	1110	-	Deltaproteobacteria	Desulfotalea psychrophila	hypothetical protein [Desulfotalea psychrophila].	GCF_000025945.1
WP_156831299.1	REase+TPR+GreAB-C+PIN*→	REase+TPR+GreAB-C+PIN	1304	-	Gammaproteobacteria	Arhodomonas aquaeolei	hypothetical protein [Arhodomonas aquaeolei].	GCF_000374645.1
WP_156924526.1	REase+TPR+GreAB-C+PIN*→	REase+TPR+GreAB-C+PIN	1326	-	Betaproteobacteria	Derxia gummosa	hypothetical protein [Derxia gummosa].	GCF_000482785.1
WP_156945361.1	REase+TPR+GreAB-C+PIN*→	REase+TPR+GreAB-C+PIN	1305	-	Alphaproteobacteria	Hyphomonas johnsonii	hypothetical protein [Hyphomonas johnsonii].	GCF_000685275.1
WP_156971043.1	TPR+GreAB-C+PIN*→	TPR+GreAB-C+PIN	1141	-	Alphaproteobacteria	Beijerinckia mobilis	tetratricopeptide repeat protein [Beijerinckia mobilis].	GCF_000745425.1
WP_157119081.1	REase+TPR+GreAB-C+PIN*→	REase+TPR+GreAB-C+PIN	1521	-	Betaproteobacteria	Azohydromonas lata	hypothetical protein [Azohydromonas lata].	GCF_001571085.1
WP_157228354.1	TPR+PIN*→	TPR+PIN	1070	-	Actinobacteria	Gordonia amicalis	hypothetical protein [Gordonia amicalis].	GCF_000332995.1
WP_157237724.1	TPR+GreAB-C+PIN*→	TPR+GreAB-C+PIN	801	-	Deltaproteobacteria	Desulfobacter curvatus	hypothetical protein [Desulfobacter curvatus].	GCF_000373985.1
WP_157376943.1	REase+TPR+GreAB-C+PIN*→	REase+TPR+GreAB-C+PIN	1354	-	Betaproteobacteria	Burkholderia ubonensis	hypothetical protein [Burkholderia ubonensis].	GCF_001523425.1
WP_157516442.1	TPR+PIN*→	TPR+PIN	1239	-	Actinobacteria	Oerskovia enterophila	hypothetical protein [Oerskovia enterophila].	GCF_001624335.1
WP_157553798.1	X+PIN*→?→ Calcineurin→	X+PIN	1140	-	Actinobacteria	Microbacterium hominis	hypothetical protein [Microbacterium hominis].	GCF_001592125.1
WP_157561005.1	TPR+PIN*→	TPR+PIN	758	-	Actinobacteria	Humibacter albus	hypothetical protein [Humibacter albus].	GCF_000421825.1
WP_157621026.1	HetE-N1→ TPR+GreAB-C+PIN*→	TPR+GreAB-C+PIN	1092	-	Gammaproteobacteria	Vibrio sp. HENC-03	hypothetical protein [Vibrio sp. HENC-03].	GCF_000305755.2
WP_157678465.1	REase+TPR+GreAB-C+PIN*→	REase+TPR+GreAB-C+PIN	1297	-	Alphaproteobacteria	Mesorhizobium sp. WSM1497	hypothetical protein [Mesorhizobium sp. WSM1497].	GCF_001672455.2
WP_157721532.1	REase+TPR+GreAB-C+PIN*→	REase+TPR+GreAB-C+PIN	1240	-	Gammaproteobacteria	Stenotrophomonas sp. WZN-1	hypothetical protein [Stenotrophomonas sp. WZN-1].	GCF_002192255.1
WP_157768703.1	REase+TPR+GreAB-C+PIN*→	REase+TPR+GreAB-C+PIN	1380	-	Betaproteobacteria	Acidovorax avenae	hypothetical protein [Acidovorax avenae].	GCF_000176855.2
WP_157788679.1	REase+TPR+GreAB-C+PIN*→	REase+TPR+GreAB-C+PIN	1382	-	Gammaproteobacteria	Pseudomonas fluorescens	GreA/GreB family elongation factor [Pseudomonas fluorescens].	GCF_000308175.1
WP_157822505.1	TPR+GreAB-C+PIN*→	TPR+GreAB-C+PIN	769	-	Gammaproteobacteria	Psychromonas sp. Urea-02u-13	hypothetical protein, partial [Psychromonas sp. Urea-02u-13].	GCF_002835995.1
WP_157852789.1	TPR+PIN*→	TPR+PIN	1134	-	Actinobacteria	Streptomyces iakyrus	hypothetical protein [Streptomyces iakyrus].	GCF_000717055.1
WP_157874592.1	REase+TPR+TPR+GreAB-C+PIN*→	REase+TPR+TPR+GreAB-C+PIN	1330	-	Actinobacteria	Streptomyces sp. AcH 505	hypothetical protein [Streptomyces sp. AcH 505].	GCF_000818175.1
WP_157937504.1	TPR+GreAB-C+PIN*→	TPR+GreAB-C+PIN	964	-	Gammaproteobacteria	Vibrio splendidus	hypothetical protein, partial [Vibrio splendidus].	GCF_002874795.1
WP_157972098.1	TPR+GreAB-C+PIN*→	TPR+GreAB-C+PIN	1234	-	Bacteroidetes	Negadavirga sp. SW125	hypothetical protein [Negadavirga sp. SW125].	GCF_003347495.1
WP_158764527.1	TPR+GreAB-C+PIN*→	TPR+GreAB-C+PIN	761	-	Alphaproteobacteria	Terricaulis silvestris	hypothetical protein [Terricaulis silvestris].	GCF_009792355.1
WP_158793614.1	REase+TPR+GreAB-C+PIN*→	REase+TPR+GreAB-C+PIN	1358	-	Acidobacteria	Granulicella sp. L60	hypothetical protein [Granulicella sp. L60].	GCF_009765825.1
WP_158941358.1	REase+TPR+GreAB-C+PIN*→	REase+TPR+GreAB-C+PIN	1279	-	Acidobacteria	Granulicella sp. S190	hypothetical protein [Granulicella sp. S190].	GCF_009766095.1
WP_159057317.1	TPR+PIN*→	TPR+PIN	1196	-	Actinobacteria	Streptomyces scabiei	hypothetical protein [Streptomyces scabiei].	GCF_001550225.1
WP_159073521.1	TPR+PIN*→	TPR+PIN	1075	-	Actinobacteria	Streptomyces sp. RTd22	hypothetical protein [Streptomyces sp. RTd22].	GCF_001650215.1
WP_159104186.1	TPR+PIN*→	TPR+PIN	1211	-	Actinobacteria	Streptomyces sp. CdTB01	hypothetical protein [Streptomyces sp. CdTB01].	GCF_001484565.1
WP_159389385.1	REase+TPR+GreAB-C+PIN*→	REase+TPR+GreAB-C+PIN	1423	-	Gammaproteobacteria	Pseudomonas sp. R32	hypothetical protein [Pseudomonas sp. R32].	GCF_009866705.1
WP_159401920.1	TPR+PIN*→	TPR+PIN	1075	-	Actinobacteria	Streptomyces hygroscopicus	hypothetical protein [Streptomyces hygroscopicus].	GCF_001705785.1
WP_159477578.1	Mbetalac→ Trypsin+TPR+GreAB-C+PIN*→	Trypsin+TPR+GreAB-C+PIN	1359	-	Bacteroidetes	Dyadobacter sp. 3J3	hypothetical protein [Dyadobacter sp. 3J3].	GCF_009801115.1
WP_159617138.1	PIN*→	PIN	499	-	Actinobacteria	Arthrobacter zhaoguopingii	hypothetical protein [Arthrobacter zhaoguopingii].	GCF_009828605.1
WP_159719775.1	TPR+PIN*→	TPR+PIN	1170	-	Firmicutes	Anoxybacillus sp. PDR2	hypothetical protein [Anoxybacillus sp. PDR2].	GCF_009834045.1
WP_159964006.1	REase+TPR+GreAB-C+PIN*→	REase+TPR+GreAB-C+PIN	1113	-	Alphaproteobacteria	Profundibacterium mesophilum	hypothetical protein [Profundibacterium mesophilum].	GCF_009835145.1
WP_160086901.1	BTLCP+SFI-helicase+REase→?→ REase+TPR+GreAB-C+PIN*→	REase+TPR+GreAB-C+PIN	1492	-	Gammaproteobacteria	Pseudomonas sp. 9AZ	GreA/GreB family elongation factor [Pseudomonas sp. 9AZ].	GCF_902506525.1

<table>
<tr><th>acc</th><th>operon</th><th>architecture</th><th>len</th><th>gen.name</th><th>taxend</th><th>species</th><th>defline</th><th>gca</th></tr>
<tr><td>WP_160308988.1</td><td>TPR+GreAB-C+PIN*→</td><td>TPR+GreAB-C+PIN</td><td>1249</td><td>-</td><td>Deltaproteobacteria</td><td>Anaeromyxobacter sp. PSR-1</td><td>GreA/GreB family elongation factor [Anaeromyxobacter sp. PSR-1].</td><td>GCF_000964525.1</td></tr>
<tr><td>WP_160382728.1</td><td>REase+TPR+GreAB-C+PIN*→</td><td>REase+TPR+GreAB-C+PIN</td><td>1329</td><td>-</td><td>Alphaproteobacteria</td><td>Pseudooceanicola pacificus</td><td>hypothetical protein [Pseudooceanicola pacificus].</td><td>GCF_009789075.1</td></tr>
<tr><td>WP_160512238.1</td><td>HetE-N1→ TPR+GreAB-C+PIN*→</td><td>TPR+GreAB-C+PIN</td><td>1094</td><td>-</td><td>Gammaproteobacteria</td><td>Escherichia coli</td><td>hypothetical protein [Escherichia coli].</td><td>GCF_009820085.1</td></tr>
<tr><td>WP_160657313.1</td><td>HetE-N1→ TPR+GreAB-C+PIN*→</td><td>TPR+GreAB-C+PIN</td><td>1114</td><td>-</td><td>Gammaproteobacteria</td><td>Photobacterium alginatilyticum</td><td>hypothetical protein [Photobacterium alginatilyticum].</td><td>GCF_009910675.1</td></tr>
<tr><td>WP_160663512.1</td><td>TPR+PIN*→</td><td>TPR+PIN</td><td>1180</td><td>-</td><td>Actinobacteria</td><td>Pseudarthrobacter sp. ATCC 49987</td><td>hypothetical protein [Pseudarthrobacter sp. ATCC 49987].</td><td>GCF_009928425.1</td></tr>
<tr><td>WP_160778164.1</td><td>REase→ PSE→ REase+TPR+GreAB-C+PIN*→</td><td>REase+TPR+GreAB-C+PIN</td><td>1252</td><td>-</td><td>Alphaproteobacteria</td><td>Parerythrobacter jejuensis</td><td>hypothetical protein [Parerythrobacter jejuensis].</td><td>GCF_009827995.1</td></tr>
<tr><td>WP_160856107.1</td><td>REase+TPR+GreAB-C+PIN*→</td><td>REase+TPR+GreAB-C+PIN</td><td>1263</td><td>-</td><td>Betaproteobacteria</td><td>Delftia sp. CH05</td><td>hypothetical protein [Delftia sp. CH05].</td><td>GCF_009827015.1</td></tr>
<tr><td>WP_160933674.1</td><td>REase+TPR+GreAB-C+PIN*→</td><td>REase+TPR+GreAB-C+PIN</td><td>1351</td><td>-</td><td>Betaproteobacteria</td><td>unclassified Burkholderia</td><td>MULTISPECIES: hypothetical protein [unclassified Burkholderia].</td><td>GCF_009826925.1</td></tr>
<tr><td>WP_161036960.1</td><td>REase+TPR+GreAB-C+PIN*→</td><td>REase+TPR+GreAB-C+PIN</td><td>1075</td><td>-</td><td>Betaproteobacteria</td><td>Duganella fentianensis</td><td>hypothetical protein [Duganella fentianensis].</td><td>GCF_009857835.1</td></tr>
<tr><td>WP_161077003.1</td><td>REase+TPR+GreAB-C+PIN*→</td><td>REase+TPR+GreAB-C+PIN</td><td>1292</td><td>-</td><td>Betaproteobacteria</td><td>Duganella sp. CY15W</td><td>restriction endonuclease [Duganella sp. CY15W].</td><td>GCF_009857555.1</td></tr>
<tr><td>WP_161352699.1</td><td>REase+TPR+GreAB-C+PIN*→</td><td>REase+TPR+GreAB-C+PIN</td><td>1351</td><td>-</td><td>Alphaproteobacteria</td><td>Maritimibacter sp. DP07</td><td>hypothetical protein [Maritimibacter sp. DP07].</td><td>GCF_009882975.1</td></tr>
<tr><td>WP_161505253.1</td><td>TPR+PIN*→?→?→||||<-TIR</td><td>TPR+PIN</td><td>1268</td><td>-</td><td>Gammaproteobacteria</td><td>Legionella pneumophila</td><td>hypothetical protein [Legionella pneumophila].</td><td>GCF_009933055.1</td></tr>
<tr><td>WP_161571911.1</td><td>HetE-N1→ TPR+GreAB-C+PIN*→</td><td>TPR+GreAB-C+PIN</td><td>1117</td><td>-</td><td>Gammaproteobacteria</td><td>Cronobacter malonaticus</td><td>hypothetical protein [Cronobacter malonaticus].</td><td>GCF_009938405.1</td></tr>
<tr><td>WP_161629377.1</td><td>TPR+GreAB-C+PIN*→</td><td>TPR+GreAB-C+PIN</td><td>488</td><td>-</td><td>Gammaproteobacteria</td><td>Marinobacter sp. EVN1</td><td>hypothetical protein, partial [Marinobacter sp. EVN1].</td><td>GCF_000475375.1</td></tr>
<tr><td>WP_161722347.1</td><td>TPR+GreAB-C+PIN*→</td><td>TPR+GreAB-C+PIN</td><td>1135</td><td>-</td><td>Alphaproteobacteria</td><td>unclassified Microvirga</td><td>MULTISPECIES: tetratricopeptide repeat protein [unclassified Microvirga].</td><td>GCF_009910725.1</td></tr>
<tr><td>WP_161796830.1</td><td>REase+TPR+GreAB-C+PIN*→</td><td>REase+TPR+GreAB-C+PIN</td><td>1210</td><td>-</td><td>Alphaproteobacteria</td><td>Devosia soli</td><td>GreA/GreB family elongation factor [Devosia soli].</td><td>GCF_000970455.1</td></tr>
<tr><td>WP_161897930.1</td><td>HetE-N1→ TPR+GreAB-C+PIN*→</td><td>TPR+GreAB-C+PIN</td><td>1110</td><td>-</td><td>Gammaproteobacteria</td><td>Pseudomonas sp. MY50</td><td>hypothetical protein [Pseudomonas sp. MY50].</td><td>GCF_009932725.1</td></tr>
<tr><td>WP_161916726.1</td><td>X+PIN*→</td><td>X+PIN</td><td>1235</td><td>-</td><td>Actinobacteria</td><td>Cellulomonas sp. APG4</td><td>hypothetical protein [Cellulomonas sp. APG4].</td><td>GCF_009996735.1</td></tr>
<tr><td>WP_161984907.1</td><td>HTH→ HetE-N1→ HetE-N1+GreAB-C+PIN*→</td><td>HetE-N1+GreAB-C+PIN</td><td>1077</td><td>-</td><td>Gammaproteobacteria</td><td>Acinetobacter haemolyticus</td><td>hypothetical protein [Acinetobacter haemolyticus].</td><td>GCF_010014695.1</td></tr>
<tr><td>WP_162386033.1</td><td>Mbetalac→ Trypsin+TPR+GreAB-C+PIN*→</td><td>Trypsin+TPR+GreAB-C+PIN</td><td>1366</td><td>-</td><td>Bacteroidetes</td><td>Spirosoma endbachense</td><td>hypothetical protein [Spirosoma endbachense].</td><td>GCF_010233585.1</td></tr>
<tr><td>WP_162486659.1</td><td>REase+TPR+GreAB-C+PIN*→||||<-HTH+HTH</td><td>REase+TPR+GreAB-C+PIN</td><td>1334</td><td>-</td><td>Betaproteobacteria</td><td>Burkholderia pseudomallei</td><td>hypothetical protein [Burkholderia pseudomallei].</td><td>GCF_000011545.1</td></tr>
<tr><td>WP_162599154.1</td><td>HetE-N1→ TPR+GreAB-C+PIN*→</td><td>TPR+GreAB-C+PIN</td><td>1027</td><td>-</td><td>Gammaproteobacteria</td><td>Shewanella algidipiscicola</td><td>hypothetical protein [Shewanella algidipiscicola].</td><td>GCF_900380485.1</td></tr>
<tr><td>WP_162599389.1</td><td>TPR+GreAB-C+PIN*→</td><td>TPR+GreAB-C+PIN</td><td>1158</td><td>-</td><td>Gammaproteobacteria</td><td>Pseudomonas aeruginosa</td><td>hypothetical protein [Pseudomonas aeruginosa].</td><td>GCF_900406905.1</td></tr>
<tr><td>WP_162672520.1</td><td>HTH→ GT4+HEPN+TPR+GreAB-C+PIN*→</td><td>GT4+HEPN+TPR+GreAB-C+PIN</td><td>1685</td><td>-</td><td>Planctomycetes</td><td>Gemmata massiliana</td><td>glycosyltransferase [Gemmata massiliana].</td><td>GCF_901538265.1</td></tr>
<tr><td>WP_162916214.1</td><td>PIN*→</td><td>PIN</td><td>348</td><td>-</td><td>Betaproteobacteria</td><td>Burkholderia sp. PAMC 26561</td><td>hypothetical protein [Burkholderia sp. PAMC 26561].</td><td>GCF_001557535.2</td></tr>
<tr><td>WP_162940198.1</td><td>X+PIN*→</td><td>X+PIN</td><td>1169</td><td>-</td><td>Actinobacteria</td><td>Gryllotalpicola protaetiae</td><td>hypothetical protein [Gryllotalpicola protaetiae].</td><td>GCF_003627055.1</td></tr>
<tr><td>WP_162960625.1</td><td>DOC+HTH→ REase+TOPC→?→ HetE-N1→ HetE-N1+TPR+GreAB-C+PIN*→</td><td>HetE-N1+TPR+GreAB-C+PIN</td><td>770</td><td>-</td><td>Gammaproteobacteria</td><td>Pseudomonas aeruginosa</td><td>hypothetical protein, partial [Pseudomonas aeruginosa].</td><td>GCF_003630455.1</td></tr>
<tr><td>WP_163210080.1</td><td>TM+TM+TPR+PIN*→</td><td>TM+TM+TPR+PIN</td><td>1195</td><td>-</td><td>Bacteroidetes</td><td>Bacteroides sp. 519</td><td>hypothetical protein [Bacteroides sp. 519].</td><td>GCF_010500955.1</td></tr>
<tr><td>WP_163325035.1</td><td>TPR+GreAB-C+PIN*→</td><td>TPR+GreAB-C+PIN</td><td>1227</td><td>-</td><td>Bacteroidetes</td><td>Draconibacterium sp. GM2-18</td><td>hypothetical protein [Draconibacterium sp. GM2-18].</td><td>GCF_010586825.1</td></tr>
<tr><td>WP_163385713.1</td><td>TPR+GreAB-C+PIN*→</td><td>TPR+GreAB-C+PIN</td><td>509</td><td>-</td><td>Gammaproteobacteria</td><td>Enterobacter hormaechei</td><td>hypothetical protein, partial [Enterobacter hormaechei].</td><td>GCF_011006815.1</td></tr>
<tr><td>WP_163511264.1</td><td>REase+TPR+GreAB-C+PIN*→</td><td>REase+TPR+GreAB-C+PIN</td><td>1231</td><td>-</td><td>Actinobacteria</td><td>Fodinicola acaciae</td><td>DUF4365 domain-containing protein [Fodinicola acaciae].</td><td>GCF_010993745.1</td></tr>
<tr><td>WP_163806192.1</td><td>TPR+PIN*→</td><td>TPR+PIN</td><td>1223</td><td>-</td><td>Actinobacteria</td><td>Mycolicibacterium anyangense</td><td>hypothetical protein [Mycolicibacterium anyangense].</td><td>GCF_010731855.1</td></tr>
<tr><td>WP_163887020.1</td><td>REase+TPR+GreAB-C+PIN*→</td><td>REase+TPR+GreAB-C+PIN</td><td>1290</td><td>-</td><td>Alphaproteobacteria</td><td>Pseudoruegeria sp. M32A2M</td><td>tetratricopeptide repeat protein [Pseudoruegeria sp. M32A2M].</td><td>GCF_010374725.1</td></tr>
<tr><td>WP_164141894.1</td><td>TPR+PIN*→</td><td>TPR+PIN</td><td>1215</td><td>-</td><td>Actinobacteria</td><td>Streptomyces coelicoflavus</td><td>hypothetical protein [Streptomyces coelicoflavus].</td><td>GCF_010548435.1</td></tr>
<tr><td>WP_164363703.1</td><td>TPR+PIN*→</td><td>TPR+PIN</td><td>1135</td><td>-</td><td>Actinobacteria</td><td>Streptomyces griseus</td><td>hypothetical protein [Streptomyces griseus].</td><td>GCF_010548565.1</td></tr>
<tr><td>WP_164366948.1</td><td>TPR+PIN*→</td><td>TPR+PIN</td><td>1215</td><td>-</td><td>Actinobacteria</td><td>Streptomyces diastaticus</td><td>hypothetical protein [Streptomyces diastaticus].</td><td>GCF_010548605.1</td></tr>
<tr><td>WP_164922417.1</td><td>TRD+TRD→?→?→ REase+SNF2→?→ TPR+GreAB-C+PIN*→</td><td>TPR+GreAB-C+PIN</td><td>1273</td><td>-</td><td>Planctomycetes</td><td>Rhodopirellula baltica</td><td>hypothetical protein [Rhodopirellula baltica].</td><td>GCF_000196115.1</td></tr>
<tr><td>WP_165069081.1</td><td>TPR+PIN*→</td><td>TPR+PIN</td><td>1123</td><td>-</td><td>Actinobacteria</td><td>Marisediminicola senii</td><td>hypothetical protein [Marisediminicola senii].</td><td>GCF_011057645.1</td></tr>
<tr><td>WP_165087560.1</td><td>REase+TPR+GreAB-C+PIN*→</td><td>REase+TPR+GreAB-C+PIN</td><td>1326</td><td>-</td><td>Betaproteobacteria</td><td>Caballeronia sp. SBC2</td><td>hypothetical protein [Caballeronia sp. SBC2].</td><td>GCF_011039955.1</td></tr>
</table>

acc	operon	architecture	len	gen.name	taxend	species	defline	gca
WP_165198604.1	TPR+GreAB-C+PIN*→	TPR+GreAB-C+PIN	1240	-	Alphaproteobacteria	Pseudohalocynthiibacter aestuariivivens	hypothetical protein [Pseudohalocynthiibacter aestuariivivens].	GCF_011040495.1
WP_165399219.1	REase+TPR+GreAB-C+PIN*→	REase+TPR+GreAB-C+PIN	1316	-	Alphaproteobacteria	Komagataeibacter xylinus	GreA/GreB family elongation factor [Komagataeibacter xylinus].	GCF_004302915.1
WP_165684855.1	TPR+PIN*→	TPR+PIN	1088	-	Actinobacteria	Streptomyces sp. KO7888	hypothetical protein [Streptomyces sp. KO7888].	GCF_011317205.1
WP_165714007.1	TPR+GreAB-C+PIN*→	TPR+GreAB-C+PIN	924	-	Betaproteobacteria	Rubrivivax benzoatilyticus	hypothetical protein, partial [Rubrivivax benzoatilyticus].	GCF_011365705.1
WP_165715394.1	HetE-N1→?→?→ TPR+GreAB-C+PIN*→	TPR+GreAB-C+PIN	776	-	Gammaproteobacteria	Salmonella enterica	hypothetical protein [Salmonella enterica].	GCF_011368495.1
WP_165722314.1	HetE-N1→ TPR+GreAB-C+PIN*→	TPR+GreAB-C+PIN	1113	-	Gammaproteobacteria	unclassified Pseudoalteromonas	MULTISPECIES: hypothetical protein [unclassified Pseudoalteromonas].	GCF_011378665.1
WP_165919434.1	REase+TPR+GreAB-C+PIN*→ TPR→	REase+TPR+GreAB-C+PIN	1252	-	Actinobacteria	Promicromonospora sp. CF082	DUF4365 domain-containing protein [Promicromonospora sp. CF082].	GCF_004341335.1
WP_166221458.1	HTH→ HetE-N1→ TPR+GreAB-C+PIN*→	TPR+GreAB-C+PIN	1076	-	Gammaproteobacteria	Acinetobacter shaoyimingii	hypothetical protein [Acinetobacter shaoyimingii].	GCF_011578045.1
WP_166315930.1	X+PIN*→	X+PIN	1081	-	Actinobacteria	Microbacterium excoecariae	hypothetical protein [Microbacterium excoecariae].	GCF_011326725.1
WP_166694175.1	Mbetalac→ Trypsin+TPR+GreAB-C+PIN*→	Trypsin+TPR+GreAB-C+PIN	1365	-	Bacteroidetes	Fibrella aestuarina	trypsin-like serine protease [Fibrella aestuarina].	GCF_011742925.1
WP_166748348.1	REase+TPR+GreAB-C+PIN*→	REase+TPR+GreAB-C+PIN	1323	-	Alphaproteobacteria	Sphingomonas aerolata	hypothetical protein [Sphingomonas aerolata].	GCF_011759485.1
WP_166760128.1	X+PIN*→	X+PIN	1124	-	Actinobacteria	Frigoribacterium faeni	hypothetical protein [Frigoribacterium faeni].	GCF_011761235.1
WP_166908249.1	TPR+PIN*→	TPR+PIN	1129	-	Actinobacteria	Mycobacterium sp. DL440	hypothetical protein [Mycobacterium sp. DL440].	GCF_011745145.1
WP_167081447.1	REase+TPR+GreAB-C+PIN*→	REase+TPR+GreAB-C+PIN	1510	-	Betaproteobacteria	'Massilia aquatica' Holochova et al. 2020	hypothetical protein ['Massilia aquatica' Holochova et al. 2020].	GCF_011682045.1
WP_167302996.1	REase+TPR+GreAB-C+PIN*→	REase+TPR+GreAB-C+PIN	1316	-	Alphaproteobacteria	Sphingobium vermicomposti	hypothetical protein [Sphingobium vermicomposti].	GCF_011762025.1
WP_167344464.1	TPR+PIN*→||||<-NUDIX	TPR+PIN	1134	-	Actinobacteria	Streptomyces griseolus	hypothetical protein [Streptomyces griseolus].	GCF_000721185.1
WP_167432534.1	HetE-N1→ TPR+GreAB-C+PIN*→	TPR+GreAB-C+PIN	1111	-	Gammaproteobacteria	Pantoea	MULTISPECIES: hypothetical protein [Pantoea].	GCF_011800365.1
WP_168003144.1	TM+TPR+PIN*→	TM+TPR+PIN	1126	-	Actinobacteria	Micromonospora sp. HSS6-12	hypothetical protein, partial [Micromonospora sp. HSS6-12].	GCF_012034245.1
WP_168341898.1	REase+TPR+TPR+GreAB-C+PIN*→	REase+TPR+TPR+GreAB-C+PIN	1233	-	Alphaproteobacteria	Rhizobium leguminosarum	hypothetical protein [Rhizobium leguminosarum].	GCF_012276075.1
WP_168426515.1	TPR+GreAB-C+PIN*→	TPR+GreAB-C+PIN	461	-	Gammaproteobacteria	Pseudomonas sp. SST3	hypothetical protein, partial [Pseudomonas sp. SST3].	GCF_003325755.1
WP_168560628.1	REase+TPR+GreAB-C+PIN*→	REase+TPR+GreAB-C+PIN	1337	-	Betaproteobacteria	Rhizobacter sp. SG703	hypothetical protein [Rhizobacter sp. SG703].	GCF_012275445.1
WP_168617642.1	TPR+PIN*→	TPR+PIN	1210	-	Actinobacteria	Nocardioides sp. JQ2195	hypothetical protein [Nocardioides sp. JQ2195].	GCF_012272695.1
WP_168725201.1	TPR+PIN*→	TPR+PIN	1218	-	Actinobacteria	Streptomyces galbus	hypothetical protein [Streptomyces galbus].	GCF_014650535.1
WP_168927382.1	TPR+GreAB-C+PIN*→	TPR+GreAB-C+PIN	546	-	Gammaproteobacteria	Enterobacter hormaechei	hypothetical protein, partial [Enterobacter hormaechei].	GCF_902166805.1
WP_169321903.1	TPR+GreAB-C+PIN*→	TPR+GreAB-C+PIN	1137	-	Bacteroidetes	Chryseobacterium	MULTISPECIES: hypothetical protein [Chryseobacterium].	GCF_012952015.1
WP_169448013.1	REase+TPR+GreAB-C+PIN*→	REase+TPR+GreAB-C+PIN	1243	-	Gammaproteobacteria	Stenotrophomonas maltophilia	hypothetical protein [Stenotrophomonas maltophilia].	GCF_012971765.1
WP_169574578.1	REase+TPR+GreAB-C+PIN→ TPR+GreAB-C+PIN*→	TPR+GreAB-C+PIN	628	-	Alphaproteobacteria	Sphingobium psychrophilum	hypothetical protein [Sphingobium psychrophilum].	GCF_012927105.1
WP_169902587.1	REase+TPR+GreAB-C+PIN*→	REase+TPR+GreAB-C+PIN	1321	-	Gammaproteobacteria	Pseudomonas rhodesiae	DUF4365 domain-containing protein [Pseudomonas rhodesiae].	GCF_012985855.1
WP_169912953.1	TPR+GreAB-C+PIN*→	TPR+GreAB-C+PIN	1140	-	Gammaproteobacteria	Pseudomonas chlororaphis	hypothetical protein [Pseudomonas chlororaphis].	GCF_012986925.1
WP_169922112.1	TPR+GreAB-C+PIN*→	TPR+GreAB-C+PIN	1148	-	Gammaproteobacteria	Pseudomonas sp. WS 5503	hypothetical protein [Pseudomonas sp. WS 5503].	GCF_012985385.1
WP_170029824.1	REase+TPR+GreAB-C+PIN*→	REase+TPR+GreAB-C+PIN	1470	-	Gammaproteobacteria	Pseudomonas sp. SK	hypothetical protein [Pseudomonas sp. SK].	GCF_012975065.1
WP_170117623.1	HNH+TPR+GreAB-C+PIN*→	HNH+TPR+GreAB-C+PIN	1207	-	Bacteroidetes	Chitinophaga ginsengisoli	hypothetical protein [Chitinophaga ginsengisoli].	GCF_003014595.1
WP_170323822.1	REase+TPR+GreAB-C+PIN*→	REase+TPR+GreAB-C+PIN	1251	-	Actinobacteria	Cryptosporangium phraense	DUF4365 domain-containing protein [Cryptosporangium phraense].	GCF_006912135.1
WP_170431548.1	REase+TPR+GreAB-C+PIN*→?→||||<-?<-?<-BTLCP+SFI-helicase+REase	REase+TPR+GreAB-C+PIN	1310	-	Alphaproteobacteria	Ruegeria arenilitoris	tetratricopeptide repeat protein [Ruegeria arenilitoris].	GCF_013032225.1
WP_171027511.1	TPR+PIN*→	TPR+PIN	973	-	Actinobacteria	Pseudarthrobacter sp. NamE2	tetratricopeptide repeat protein [Pseudarthrobacter sp. NamE2].	GCF_005796205.1

acc	operon	architecture	len	gen.name	taxend	species	defline	gca
WP_171071268.1	REase+TPR+GreAB-C+PIN*→	REase+TPR+GreAB-C+PIN	1155	-	Gammaproteobacteria	Acinetobacter baumannii	GreA/GreB family elongation factor, partial [Acinetobacter baumannii].	GCF_000580875.1
WP_171275163.1	HetE-N1→ TPR+GreAB-C+PIN*→	TPR+GreAB-C+PIN	1094	-	Gammaproteobacteria	Aeromonas media	hypothetical protein [Aeromonas media].	GCF_013085485.1
WP_171434497.1	DOC+HTH→ REase+TOPC→?→ HetE-N1→ HetE-N1+TPR+GreAB-C+PIN*→	HetE-N1+TPR+GreAB-C+PIN	1124	-	Gammaproteobacteria	Pseudomonas aeruginosa	hypothetical protein [Pseudomonas aeruginosa].	GCF_013115755.1
WP_171451709.1	DOC+HTH→ REase+TOPC→?→ HetE-N1→ HetE-N1→ GreAB-C+PIN*→	GreAB-C+PIN	523	-	Gammaproteobacteria	Pseudomonas aeruginosa	hypothetical protein [Pseudomonas aeruginosa].	GCF_013115805.1
WP_171477953.1	TPR+GreAB-C+PIN*→||||<-?||HTH→	TPR+GreAB-C+PIN	652	-	Gammaproteobacteria	Acinetobacter baumannii	GreA/GreB family elongation factor, partial [Acinetobacter baumannii].	GCF_006491975.1
WP_171493638.1	TPR+GreAB-C+PIN*→	TPR+GreAB-C+PIN	816	-	Gammaproteobacteria	Acinetobacter soli	hypothetical protein, partial [Acinetobacter soli].	GCF_008987205.1
WP_171564538.1	TPR+PIN*→	TPR+PIN	1172	-	Firmicutes	Brevibacillus sp. MCWH	hypothetical protein [Brevibacillus sp. MCWH].	GCF_018195675.1
WP_171662370.1	REase+TPR+GreAB-C+PIN*→	REase+TPR+GreAB-C+PIN	1496	-	Betaproteobacteria	Achromobacter deleyi	hypothetical protein [Achromobacter deleyi].	GCF_013116765.1
WP_171694982.1	HetE-N1→ TPR+GreAB-C+PIN*→	TPR+GreAB-C+PIN	1120	-	Gammaproteobacteria	Methylomonas sp. ZR1	hypothetical protein [Methylomonas sp. ZR1].	GCF_013141865.1
WP_171798091.1	REase+TPR+GreAB-C+PIN*→	REase+TPR+GreAB-C+PIN	1323	-	Alphaproteobacteria	unclassified Novosphingobium	MULTISPECIES: hypothetical protein [unclassified Novosphingobium].	GCF_013149315.1
WP_172598885.1	TPR+GreAB-C+PIN*→	TPR+GreAB-C+PIN	709	-	Alphaproteobacteria	Sphingobium sp. YG1	hypothetical protein [Sphingobium sp. YG1].	GCF_003609795.1
WP_172685391.1	REase+TPR+GreAB-C+PIN*→?→?→ Calcineurin→ Calcineurin→?→ Calcineurin→	REase+TPR+GreAB-C+PIN	1311	-	Alphaproteobacteria	Methylobacterium oryzae	hypothetical protein [Methylobacterium oryzae].	-
WP_172806748.1	TPR+GreAB-C+PIN*→	TPR+GreAB-C+PIN	766	-	Gammaproteobacteria	Endozoicomonas arenosclerae	hypothetical protein [Endozoicomonas arenosclerae].	GCF_001562015.1
WP_172870236.1	TPR+GreAB-C*→	TPR+GreAB-C	233	-	Firmicutes	Bacillus velezensis	hypothetical protein, partial [Bacillus velezensis].	GCF_013267055.1
WP_172883554.1	TPR+GreAB-C+PIN*→	TPR+GreAB-C+PIN	1159	-	Gammaproteobacteria	Pseudomonas stutzeri	hypothetical protein [Pseudomonas stutzeri].	GCF_013267085.1
WP_173054693.1	TM+TPR+PIN*→ REase→	TM+TPR+PIN	1322	-	Actinobacteria	Phytohabitans houttuyneae	hypothetical protein [Phytohabitans houttuyneae].	GCF_011764425.1
WP_173520689.1	REase+TPR+TPR+GreAB-C+PIN*→	REase+TPR+TPR+GreAB-C+PIN	1357	-	Alphaproteobacteria	Ensifer sesbaniae	hypothetical protein [Ensifer sesbaniae].	GCF_013283665.1
WP_173522752.1	REase+TPR+GreAB-C+PIN*→	REase+TPR+GreAB-C+PIN	1313	-	Actinobacteria	Nonomuraea sp. NN258	hypothetical protein [Nonomuraea sp. NN258].	GCF_013283785.1
WP_173794050.1	NACHT→ X+PIN*→ HTH→?→?→ HTH→	X+PIN	1179	-	Firmicutes	Dorea formicigenerans	hypothetical protein [Dorea formicigenerans].	GCF_013301385.1
WP_173821757.1	X+PIN*→ HTH→?→?→ HTH→	X+PIN	1178	-	Firmicutes	Dorea formicigenerans	hypothetical protein [Dorea formicigenerans].	GCF_013301365.1
WP_173853982.1	TPR+GreAB-C+PIN→ PIN*→	PIN	248	-	Bacteroidetes	Flavobacterium sp. 28A	hypothetical protein [Flavobacterium sp. 28A].	GCF_013294005.1
WP_173968533.1	GreAB-C+PIN*→	GreAB-C+PIN	552	-	Bacteroidetes	Flavobacterium collinsii	hypothetical protein, partial [Flavobacterium collinsii].	GCF_902804485.1
WP_173994169.1	TPR+GreAB-C+PIN*→	TPR+GreAB-C+PIN	1181	-	Alphaproteobacteria	Agrobacterium fabrum	hypothetical protein [Agrobacterium fabrum].	GCF_013318355.1
WP_174011044.1	HEPN→ REase+TPR+TPR+GreAB-C+PIN*→	REase+TPR+TPR+GreAB-C+PIN	1333	-	Alphaproteobacteria	Agrobacterium rhizogenes	GreA/GreB family elongation factor [Agrobacterium rhizogenes].	GCF_013321105.1
WP_174166670.1	BirA-HTH→ HEPN→ REase+TPR+TPR+GreAB-C+PIN*→	REase+TPR+TPR+GreAB-C+PIN	1341	-	Alphaproteobacteria	Agrobacterium rhizogenes	hypothetical protein [Agrobacterium rhizogenes].	GCF_013322015.1
WP_174168420.1	BirA-HTH→ HEPN→ REase+TPR+TPR+GreAB-C+PIN*→	REase+TPR+TPR+GreAB-C+PIN	1333	-	Alphaproteobacteria	Agrobacterium rhizogenes	GreA/GreB family elongation factor [Agrobacterium rhizogenes].	GCF_013321875.1
WP_174361404.1	HetE-N1→?→ TPR+GreAB-C+PIN*→	TPR+GreAB-C+PIN	512	-	Gammaproteobacteria	Citrobacter gillenii	hypothetical protein [Citrobacter gillenii].	GCF_013337685.1
WP_175386063.1	PIN*→	PIN	434	-	Gammaproteobacteria	Pseudomonas corrugata	hypothetical protein [Pseudomonas corrugata].	GCF_013359545.1
WP_175666066.1	REase+TPR+GreAB-C+PIN*→	REase+TPR+GreAB-C+PIN	1351	-	Betaproteobacteria	Burkholderia ambifaria	hypothetical protein [Burkholderia ambifaria].	GCF_902829825.1
WP_175722904.1	REase+TPR+GreAB-C+PIN*→	REase+TPR+GreAB-C+PIN	1351	-	Betaproteobacteria	Burkholderia ambifaria	hypothetical protein [Burkholderia ambifaria].	GCF_902829925.1
WP_175818825.1	REase+TPR+GreAB-C+PIN*→	REase+TPR+GreAB-C+PIN	1335	-	Betaproteobacteria	Burkholderia sp. BCC0419	hypothetical protein [Burkholderia sp. BCC0419].	GCF_902830285.1
WP_175896466.1	TPR+GreAB-C+PIN*→||||<-HTH+HTH	TPR+GreAB-C+PIN	872	-	Betaproteobacteria	Burkholderia cepacia	hypothetical protein [Burkholderia cepacia].	GCF_902830675.1
WP_176170943.1	REase+TPR+GreAB-C+PIN*→	REase+TPR+GreAB-C+PIN	1244	-	Actinobacteria	Amycolatopsis sp. Hca4	DUF4365 domain-containing protein [Amycolatopsis sp. Hca4].	GCF_013364075.1
WP_176203105.1	TPR+GreAB-C+PIN*→	TPR+GreAB-C+PIN	681	-	Gammaproteobacteria	Salmonella enterica	hypothetical protein, partial [Salmonella enterica].	GCF_002044735.1
WP_176308009.1	REase+TPR+GreAB-C+PIN*→	REase+TPR+GreAB-C+PIN	1351	-	Betaproteobacteria	Burkholderia ambifaria	hypothetical protein [Burkholderia ambifaria].	GCF_902830205.1
WP_176329154.1	REase+TPR+GreAB-C+PIN*→	REase+TPR+GreAB-C+PIN	1335	-	Betaproteobacteria	Burkholderia vietnamiensis	hypothetical protein [Burkholderia vietnamiensis].	GCF_902833435.1
WP_176389972.1	REase+TPR+GreAB-C+PIN*→	REase+TPR+GreAB-C+PIN	1374	-	Betaproteobacteria	Janthinobacterium sp. BJB401	hypothetical protein [Janthinobacterium sp. BJB401].	GCF_013372745.1
WP_176422167.1	REase+TPR+TPR+GreAB-C+PIN*→	REase+TPR+TPR+GreAB-C+PIN	1325	-	Alphaproteobacteria	Bradyrhizobium sp. 2S1	hypothetical protein [Bradyrhizobium sp. 2S1].	GCF_011516625.2
WP_176457673.1	REase+TPR+GreAB-C+PIN*→	REase+TPR+GreAB-C+PIN	1516	-	Gammaproteobacteria	Pseudomonas mandelii	hypothetical protein [Pseudomonas mandelii].	GCF_000257545.3
WP_176459502.1	REase+TPR+GreAB-C+PIN*→?→ Trypsin→	REase+TPR+GreAB-C+PIN	1228	-	Actinobacteria	Rhodococcus sp. OK302	DUF4365 domain-containing protein [Rhodococcus sp. OK302].	GCF_002245895.1
WP_176508867.1	REase+TPR+GreAB-C+PIN*→	REase+TPR+GreAB-C+PIN	1548	-	Gammaproteobacteria	Pseudomonas sp. BML-PP042	hypothetical protein [Pseudomonas sp. BML-PP042].	GCF_013373915.1

<table>
<tr><th>acc</th><th>operon</th><th>architecture</th><th>len</th><th>gen.name</th><th>taxend</th><th>species</th><th>defline</th><th>gca</th></tr>
<tr><td>WP_177062188.1</td><td>REase+TPR+GreAB-C+PIN*→</td><td>REase+TPR+GreAB-C+PIN</td><td>1424</td><td>-</td><td>Gammaproteobacteria</td><td>Pseudomonas reactans</td><td>hypothetical protein [Pseudomonas reactans].</td><td>GCF_013386305.1</td></tr>
<tr><td>WP_177327801.1</td><td>REase+TPR+GreAB-C+PIN*→</td><td>REase+TPR+GreAB-C+PIN</td><td>1404</td><td>-</td><td>Gammaproteobacteria</td><td>Pseudomonas sp. 5</td><td>GreA/GreB family elongation factor [Pseudomonas sp. 5].</td><td>GCF_000955815.1</td></tr>
<tr><td>WP_179038946.1</td><td>REase+TPR+TPR+GreAB-C+PIN*→</td><td>REase+TPR+TPR+GreAB-C+PIN</td><td>1350</td><td>-</td><td>Alphaproteobacteria</td><td>Rhizobium leguminosarum</td><td>hypothetical protein [Rhizobium leguminosarum].</td><td>GCF_013391705.1</td></tr>
<tr><td>WP_179093684.1</td><td>TPR+GreAB-C+PIN*→||||<-HTH+HTH</td><td>TPR+GreAB-C+PIN</td><td>872</td><td>-</td><td>Betaproteobacteria</td><td>Burkholderia pseudomallei</td><td>hypothetical protein [Burkholderia pseudomallei].</td><td>GCF_002880985.1</td></tr>
<tr><td>WP_179103158.1</td><td>TPR+GreAB-C+PIN*→||||<-HTH+HTH</td><td>TPR+GreAB-C+PIN</td><td>872</td><td>-</td><td>Betaproteobacteria</td><td>Burkholderia pseudomallei</td><td>hypothetical protein [Burkholderia pseudomallei].</td><td>GCF_001981185.1</td></tr>
<tr><td>WP_179448733.1</td><td>-</td><td>-</td><td>914</td><td>-</td><td>Gammaproteobacteria</td><td>Pseudomonas yamanorum</td><td>hypothetical protein [Pseudomonas yamanorum].</td><td>GCF_013403585.1</td></tr>
<tr><td>WP_179622762.1</td><td>DOC+HTH→ REase+TOPC→?→ HetE-N1→ HetE-N1+TPR+GreAB-C+PIN*→</td><td>HetE-N1+TPR+GreAB-C+PIN</td><td>1124</td><td>-</td><td>Gammaproteobacteria</td><td>Pseudomonas mendocina</td><td>hypothetical protein [Pseudomonas mendocina].</td><td>GCF_013410555.1</td></tr>
<tr><td>WP_179654574.1</td><td>REase+TPR+TPR+GreAB-C+PIN*→ REase→</td><td>REase+TPR+TPR+GreAB-C+PIN</td><td>1329</td><td>-</td><td>Alphaproteobacteria</td><td>Mesorhizobium sp. YL-MPnR-2016</td><td>hypothetical protein [Mesorhizobium sp. YL-MPnR-2016].</td><td>GCF_013408905.1</td></tr>
<tr><td>WP_179833445.1</td><td>REase+TPR+PIN*→</td><td>REase+TPR+PIN</td><td>1241</td><td>-</td><td>Actinobacteria</td><td>Actinomadura citrea</td><td>DUF4365 domain-containing protein [Actinomadura citrea].</td><td>GCF_014648455.1</td></tr>
<tr><td>WP_179902819.1</td><td>HetE-N1→ TPR+GreAB-C+PIN*→</td><td>TPR+GreAB-C+PIN</td><td>1094</td><td>-</td><td>Gammaproteobacteria</td><td>Aeromonas veronii</td><td>hypothetical protein [Aeromonas veronii].</td><td>GCF_013415825.1</td></tr>
<tr><td>WP_180191279.1</td><td>HTH→ HetE-N1→ HetE-N1+GreAB-C+PIN*→</td><td>HetE-N1+GreAB-C+PIN</td><td>1077</td><td>-</td><td>Gammaproteobacteria</td><td>Acinetobacter sp. YH01006</td><td>hypothetical protein [Acinetobacter sp. YH01006].</td><td>GCF_013420445.1</td></tr>
<tr><td>WP_180269129.1</td><td>TPR+GreAB-C+PIN*→</td><td>TPR+GreAB-C+PIN</td><td>1231</td><td>-</td><td>Bacteroidetes</td><td>Sphingobacterium sp. 1.A.5</td><td>tetratricopeptide repeat protein [Sphingobacterium sp. 1.A.5].</td><td>GCF_002734235.1</td></tr>
<tr><td>WP_180491971.1</td><td>HetE-N1→ TPR+GreAB-C+PIN*→</td><td>TPR+GreAB-C+PIN</td><td>1106</td><td>-</td><td>Gammaproteobacteria</td><td>Escherichia fergusonii</td><td>hypothetical protein [Escherichia fergusonii].</td><td>GCF_903932165.1</td></tr>
<tr><td>WP_180778225.1</td><td>HetE-N1→ TPR+GreAB-C+PIN*→</td><td>TPR+GreAB-C+PIN</td><td>1117</td><td>-</td><td>Gammaproteobacteria</td><td>Pectobacterium aroidearum</td><td>hypothetical protein [Pectobacterium aroidearum].</td><td>GCF_013449715.1</td></tr>
<tr><td>WP_180799798.1</td><td>TPR+GreAB-C+PIN*→</td><td>TPR+GreAB-C+PIN</td><td>796</td><td>-</td><td>Gammaproteobacteria</td><td>Vibrio parahaemolyticus</td><td>hypothetical protein, partial [Vibrio parahaemolyticus].</td><td>GCF_006371865.1</td></tr>
<tr><td>WP_180830729.1</td><td>TPR+GreAB-C+PIN*→</td><td>TPR+GreAB-C+PIN</td><td>764</td><td>-</td><td>Gammaproteobacteria</td><td>Vibrio parahaemolyticus</td><td>hypothetical protein, partial [Vibrio parahaemolyticus].</td><td>GCF_006375165.1</td></tr>
<tr><td>WP_180977515.1</td><td>TPR+GreAB-C+PIN*→</td><td>TPR+GreAB-C+PIN</td><td>1154</td><td>-</td><td>Betaproteobacteria</td><td>Janthinobacterium sp. ROICE36</td><td>hypothetical protein, partial [Janthinobacterium sp. ROICE36].</td><td>GCF_002869965.1</td></tr>
<tr><td>WP_181464939.1</td><td>TPR+GreAB-C+PIN*→</td><td>TPR+GreAB-C+PIN</td><td>762</td><td>-</td><td>Betaproteobacteria</td><td>Herbaspirillum rubrisubalbicans</td><td>tetratricopeptide repeat protein [Herbaspirillum rubrisubalbicans].</td><td>GCF_003263475.1</td></tr>
<tr><td>WP_181641518.1</td><td>TPR+PIN*→</td><td>TPR+PIN</td><td>1210</td><td>-</td><td>Actinobacteria</td><td>Nocardioides massiliensis</td><td>hypothetical protein [Nocardioides massiliensis].</td><td>GCF_900067705.2</td></tr>
<tr><td>WP_181834083.1</td><td>TPR+GreAB-C+PIN*→</td><td>TPR+GreAB-C+PIN</td><td>678</td><td>-</td><td>Alphaproteobacteria</td><td>Brucella anthropi</td><td>hypothetical protein, partial [Brucella anthropi].</td><td>GCF_003325675.1</td></tr>
<tr><td>WP_182064218.1</td><td>TPR+PIN*→</td><td>TPR+PIN</td><td>1177</td><td>-</td><td>Actinobacteria</td><td>Curtobacterium sp. ME12</td><td>hypothetical protein [Curtobacterium sp. ME12].</td><td>GCF_903970855.1</td></tr>
<tr><td>WP_182166026.1</td><td>REase+TPR+GreAB-C+PIN*→||||<-HTH</td><td>REase+TPR+GreAB-C+PIN</td><td>1304</td><td>-</td><td>Betaproteobacteria</td><td>Duganella sp. LX20W</td><td>hypothetical protein [Duganella sp. LX20W].</td><td>GCF_014042345.1</td></tr>
<tr><td>WP_182270399.1</td><td>HetE-N1→ TPR+GreAB-C+PIN*→</td><td>TPR+GreAB-C+PIN</td><td>1092</td><td>-</td><td>Gammaproteobacteria</td><td>Citrobacter freundii</td><td>hypothetical protein [Citrobacter freundii].</td><td>GCF_014101305.1</td></tr>
<tr><td>WP_182650750.1</td><td>GreAB-C+PIN*→</td><td>GreAB-C+PIN</td><td>566</td><td>-</td><td>Bacteroidetes</td><td>Flavobacterium sp. SOK18b</td><td>hypothetical protein, partial [Flavobacterium sp. SOK18b].</td><td>GCF_014156605.1</td></tr>
<tr><td>WP_182869571.1</td><td>PIN*→</td><td>PIN</td><td>188</td><td>-</td><td>Alphaproteobacteria</td><td>Bradyrhizobium diazoefficiens</td><td>hypothetical protein [Bradyrhizobium diazoefficiens].</td><td>GCF_014163475.1</td></tr>
<tr><td>WP_182925384.1</td><td>REase+TPR+GreAB-C+PIN*→||||<-?||HTH→</td><td>REase+TPR+GreAB-C+PIN</td><td>1250</td><td>-</td><td>Gammaproteobacteria</td><td>Acinetobacter baumannii</td><td>GreA/GreB family elongation factor [Acinetobacter baumannii].</td><td>GCF_014169655.1</td></tr>
<tr><td>WP_183061688.1</td><td>CARF+PIN*→</td><td>CARF+PIN</td><td>1208</td><td>-</td><td>Actinobacteria</td><td>Motilibacter peucedani</td><td>hypothetical protein [Motilibacter peucedani].</td><td>GCF_003634695.1</td></tr>
<tr><td>WP_183296812.1</td><td>REase+TPR+GreAB-C+PIN*→</td><td>REase+TPR+GreAB-C+PIN</td><td>1234</td><td>-</td><td>Actinobacteria</td><td>Cellulomonas cellasea</td><td>DUF4365 domain-containing protein [Cellulomonas cellasea].</td><td>GCF_014190965.1</td></tr>
<tr><td>WP_183420610.1</td><td>REase+TPR+GreAB-C+PIN*→</td><td>REase+TPR+GreAB-C+PIN</td><td>1219</td><td>-</td><td>Actinobacteria</td><td>Microbacterium proteolyticum</td><td>DUF4365 domain-containing protein [Microbacterium proteolyticum].</td><td>GCF_014192415.1</td></tr>
<tr><td>WP_183438886.1</td><td>TPR+GreAB-C+PIN*→</td><td>TPR+GreAB-C+PIN</td><td>1161</td><td>-</td><td>Alphaproteobacteria</td><td>Methylobacterium sp. R2-1</td><td>hypothetical protein [Methylobacterium sp. R2-1].</td><td>GCF_014191355.1</td></tr>
<tr><td>WP_183694785.1</td><td>REase+TPR+TPR+GreAB-C+PIN*→</td><td>REase+TPR+TPR+GreAB-C+PIN</td><td>1363</td><td>-</td><td>Alphaproteobacteria</td><td>Rhizobium lusitanum</td><td>restriction endonuclease [Rhizobium lusitanum].</td><td>GCF_014189535.1</td></tr>
<tr><td>WP_183714092.1</td><td>TIR→?→ TPR+GreAB-C+PIN*→</td><td>TPR+GreAB-C+PIN</td><td>1131</td><td>-</td><td>Alphaproteobacteria</td><td>Bradyrhizobium sp. ERR14</td><td>hypothetical protein [Bradyrhizobium sp. ERR14].</td><td>GCF_014198235.1</td></tr>
<tr><td>WP_183806882.1</td><td>REase+TPR+TPR+GreAB-C+PIN*→</td><td>REase+TPR+TPR+GreAB-C+PIN</td><td>1345</td><td>-</td><td>Alphaproteobacteria</td><td>Rhizobium azooxidifex</td><td>tetratricopeptide repeat protein [Rhizobium azooxidifex].</td><td>GCF_014196765.1</td></tr>
<tr><td>WP_183919890.1</td><td>REase+TPR+GreAB-C+PIN*→</td><td>REase+TPR+GreAB-C+PIN</td><td>1290</td><td>-</td><td>Alphaproteobacteria</td><td>Rhizobium lentis</td><td>hypothetical protein [Rhizobium lentis].</td><td>GCF_014200135.1</td></tr>
<tr><td>WP_183998882.1</td><td>REase+TPR+GreAB-C+PIN*→</td><td>REase+TPR+GreAB-C+PIN</td><td>1313</td><td>-</td><td>Alphaproteobacteria</td><td>Sphingomonas kyeonggiensis</td><td>hypothetical protein [Sphingomonas kyeonggiensis].</td><td>GCF_014196745.1</td></tr>
<tr><td>WP_184249301.1</td><td>REase+TPR+GreAB-C+PIN*→</td><td>REase+TPR+GreAB-C+PIN</td><td>1318</td><td>-</td><td>Betaproteobacteria</td><td>Paraburkholderia atlantica</td><td>DUF4365 domain-containing protein [Paraburkholderia atlantica].</td><td>GCF_014200895.1</td></tr>
</table>

<table>
<tr><th>acc</th><th>operon</th><th>architecture</th><th>len</th><th>gen.name</th><th>taxend</th><th>species</th><th>defline</th><th>gca</th></tr>
<tr><td>WP_184301652.1</td><td>REase+TPR+GreAB-C+PIN*→?→?→ URI+RAMA→</td><td>REase+TPR+GreAB-C+PIN</td><td>1255</td><td>-</td><td>Planctomycetes</td><td>Rhodopirellula rubra</td><td>hypothetical protein [Rhodopirellula rubra].</td><td>GCF_014192335.1</td></tr>
<tr><td>WP_184659486.1</td><td>TPR+GreAB-C+PIN*→</td><td>TPR+GreAB-C+PIN</td><td>1265</td><td>-</td><td>Bacteroidetes</td><td>Pedobacter cryoconitis</td><td>hypothetical protein [Pedobacter cryoconitis].</td><td>GCF_014207145.1</td></tr>
<tr><td>WP_184790984.1</td><td>TM+TPR+PIN*→</td><td>TM+TPR+PIN</td><td>1158</td><td>-</td><td>Actinobacteria</td><td>Phytomonospora endophytica</td><td>hypothetical protein [Phytomonospora endophytica].</td><td>GCF_016862855.1</td></tr>
<tr><td>WP_184920984.1</td><td>REase+PIN*→</td><td>REase+PIN</td><td>1270</td><td>-</td><td>Actinobacteria</td><td>Saccharothrix ecbatanensis</td><td>DUF4365 domain-containing protein [Saccharothrix ecbatanensis].</td><td>GCF_014205015.1</td></tr>
<tr><td>WP_185002860.1</td><td>-</td><td>-</td><td>1209</td><td>-</td><td>Actinobacteria</td><td>unclassified Curtobacterium</td><td>MULTISPECIES: DUF4365 domain-containing protein [unclassified Curtobacterium].</td><td>GCF_003752345.1</td></tr>
<tr><td>WP_186065300.1</td><td>REase+TPR+GreAB-C+PIN*→</td><td>REase+TPR+GreAB-C+PIN</td><td>1351</td><td>-</td><td>Betaproteobacteria</td><td>Burkholderia gladioli</td><td>hypothetical protein [Burkholderia gladioli].</td><td>GCF_902831285.1</td></tr>
<tr><td>WP_186083524.1</td><td>REase+TPR+GreAB-C+PIN*→||||<-?<-?<-HEPN</td><td>REase+TPR+GreAB-C+PIN</td><td>1333</td><td>-</td><td>Betaproteobacteria</td><td>Burkholderia gladioli</td><td>hypothetical protein [Burkholderia gladioli].</td><td>GCF_902831185.1</td></tr>
<tr><td>WP_186117779.1</td><td>HEPN→?→?→ REase+TPR+GreAB-C+PIN*→</td><td>REase+TPR+GreAB-C+PIN</td><td>1498</td><td>-</td><td>Betaproteobacteria</td><td>Burkholderia gladioli</td><td>tetratricopeptide repeat protein [Burkholderia gladioli].</td><td>GCF_902831505.1</td></tr>
<tr><td>WP_186135768.1</td><td>REase+TPR+GreAB-C+PIN*→||||<-HEPN</td><td>REase+TPR+GreAB-C+PIN</td><td>1351</td><td>-</td><td>Betaproteobacteria</td><td>Burkholderia gladioli</td><td>hypothetical protein [Burkholderia gladioli].</td><td>GCF_902831685.1</td></tr>
<tr><td>WP_186296902.1</td><td>REase+TPR+GreAB-C+PIN*→</td><td>REase+TPR+GreAB-C+PIN</td><td>1297</td><td>-</td><td>Betaproteobacteria</td><td>Cupriavidus campinensis</td><td>DUF4365 domain-containing protein [Cupriavidus campinensis].</td><td>GCF_007572485.1</td></tr>
<tr><td>WP_186384760.1</td><td>HetE-N1→ TPR+GreAB-C+PIN*→</td><td>TPR+GreAB-C+PIN</td><td>1117</td><td>-</td><td>Gammaproteobacteria</td><td>Klebsiella sp. Kpp</td><td>hypothetical protein [Klebsiella sp. Kpp].</td><td>GCF_014267405.1</td></tr>
<tr><td>WP_186426553.1</td><td>TPR+GreAB-C+PIN*→</td><td>TPR+GreAB-C+PIN</td><td>1039</td><td>-</td><td>Betaproteobacteria</td><td>Cupriavidus metallidurans</td><td>hypothetical protein [Cupriavidus metallidurans].</td><td>GCF_900185755.1</td></tr>
<tr><td>WP_187028576.1</td><td>TPR+GreAB-C+PIN*→</td><td>TPR+GreAB-C+PIN</td><td>1238</td><td>-</td><td>Bacteroidetes</td><td>Pontibacter sp. KCTC 32443</td><td>hypothetical protein [Pontibacter sp. KCTC 32443].</td><td>GCF_014297265.1</td></tr>
<tr><td>WP_187110408.1</td><td>REase+TPR+GreAB-C+PIN*→</td><td>REase+TPR+GreAB-C+PIN</td><td>1301</td><td>-</td><td>Betaproteobacteria</td><td>Variovorax sp. PAMC26660</td><td>hypothetical protein [Variovorax sp. PAMC26660].</td><td>GCF_014302995.1</td></tr>
<tr><td>WP_187305744.1</td><td>X+PIN*→ HTH→?→?→ HTH→</td><td>X+PIN</td><td>762</td><td>-</td><td>Firmicutes</td><td>Dorea formicigenerans</td><td>hypothetical protein [Dorea formicigenerans].</td><td>GCF_003458845.1</td></tr>
<tr><td>WP_187328643.1</td><td>HTH+PNPase+TPR+GreAB-C+PIN*→</td><td>HTH+PNPase+TPR+GreAB-C+PIN</td><td>1523</td><td>-</td><td>Actinobacteria</td><td>Rhodococcus sp. WS3</td><td>hypothetical protein [Rhodococcus sp. WS3].</td><td>GCF_003797085.1</td></tr>
<tr><td>WP_187494340.1</td><td>HetE-N1→ TPR+GreAB-C+PIN*→</td><td>TPR+GreAB-C+PIN</td><td>1094</td><td>-</td><td>Gammaproteobacteria</td><td>Pantoea agglomerans</td><td>hypothetical protein [Pantoea agglomerans].</td><td>GCF_014353705.1</td></tr>
<tr><td>WP_187598162.1</td><td>REase+TPR+GreAB-C+PIN*→</td><td>REase+TPR+GreAB-C+PIN</td><td>1341</td><td>-</td><td>Betaproteobacteria</td><td>Diaphorobacter ruginosibacter</td><td>hypothetical protein [Diaphorobacter ruginosibacter].</td><td>GCF_014395975.1</td></tr>
<tr><td>WP_187723864.1</td><td>TPR+GreAB-C+PIN*→</td><td>TPR+GreAB-C+PIN</td><td>545</td><td>-</td><td>Betaproteobacteria</td><td>Diaphorobacter aerolatus</td><td>hypothetical protein [Diaphorobacter aerolatus].</td><td>GCF_014489535.1</td></tr>
<tr><td>WP_187993242.1</td><td>TPR+GreAB-C+PIN*→</td><td>TPR+GreAB-C+PIN</td><td>557</td><td>-</td><td>Gammaproteobacteria</td><td>Vibrio harveyi</td><td>hypothetical protein [Vibrio harveyi].</td><td>GCF_014525195.1</td></tr>
<tr><td>WP_188104481.1</td><td>REase+TPR+GreAB-C+PIN*→</td><td>REase+TPR+GreAB-C+PIN</td><td>1312</td><td>-</td><td>Alphaproteobacteria</td><td>Bradyrhizobium campsiandrae</td><td>tetratricopeptide repeat protein [Bradyrhizobium campsiandrae].</td><td>GCF_014529705.1</td></tr>
<tr><td>WP_188533557.1</td><td>TPR+PIN*→</td><td>TPR+PIN</td><td>1228</td><td>-</td><td>Firmicutes</td><td>Paenibacillus abyssi</td><td>hypothetical protein [Paenibacillus abyssi].</td><td>GCF_014640295.1</td></tr>
<tr><td>WP_189032568.1</td><td>TPR+PIN*→</td><td>TPR+PIN</td><td>531</td><td>-</td><td>Firmicutes</td><td>Paenibacillus albidus</td><td>hypothetical protein [Paenibacillus albidus].</td><td>GCF_014644435.1</td></tr>
<tr><td>WP_189091600.1</td><td>REase+TPR+GreAB-C+PIN*→</td><td>REase+TPR+GreAB-C+PIN</td><td>1377</td><td>-</td><td>Deinococci</td><td>Deinococcus ruber</td><td>tetratricopeptide repeat protein [Deinococcus ruber].</td><td>GCF_014648095.1</td></tr>
<tr><td>WP_189152287.1</td><td>TPR+PIN*→</td><td>TPR+PIN</td><td>1218</td><td>-</td><td>Actinobacteria</td><td>Streptomyces lacrimifluminis</td><td>hypothetical protein [Streptomyces lacrimifluminis].</td><td>GCF_014646095.1</td></tr>
<tr><td>WP_189484308.1</td><td>HetE-N1→ TPR+GreAB-C+PIN*→</td><td>TPR+GreAB-C+PIN</td><td>1026</td><td>-</td><td>Gammaproteobacteria</td><td>Shewanella indica</td><td>hypothetical protein [Shewanella indica].</td><td>GCF_014652115.1</td></tr>
<tr><td>WP_189568857.1</td><td>Mbetalac→ TPR+GreAB-C+PIN*→</td><td>TPR+GreAB-C+PIN</td><td>1342</td><td>-</td><td>Bacteroidetes</td><td>Persicitalea jodogahamensis</td><td>hypothetical protein [Persicitalea jodogahamensis].</td><td>GCF_014651615.1</td></tr>
<tr><td>WP_189617689.1</td><td>HetE-N1→?→ GreAB-C*→</td><td>GreAB-C</td><td>498</td><td>-</td><td>Gammaproteobacteria</td><td>Shewanella fodinae</td><td>hypothetical protein [Shewanella fodinae].</td><td>GCF_014651955.1</td></tr>
<tr><td>WP_189635482.1</td><td>REase+TPR+GreAB-C+PIN*→</td><td>REase+TPR+GreAB-C+PIN</td><td>1307</td><td>-</td><td>Alphaproteobacteria</td><td>Gemmobacter lanyuensis</td><td>restriction endonuclease [Gemmobacter lanyuensis].</td><td>GCF_014652355.1</td></tr>
<tr><td>WP_189677864.1</td><td>REase+TPR+GreAB-C+PIN*→</td><td>REase+TPR+GreAB-C+PIN</td><td>1323</td><td>-</td><td>Alphaproteobacteria</td><td>Sphingomonas glacialis</td><td>tetratricopeptide repeat protein [Sphingomonas glacialis].</td><td>GCF_014653575.1</td></tr>
<tr><td>WP_189835257.1</td><td>TPR+PIN*→</td><td>TPR+PIN</td><td>1215</td><td>-</td><td>Actinobacteria</td><td>Streptomyces zaomyceticus</td><td>hypothetical protein [Streptomyces zaomyceticus].</td><td>GCF_014656215.1</td></tr>
<tr><td>WP_190462433.1</td><td>PNPase+TPR+GreAB-C+PIN*→ TPR+TPR→ TPR+TPR→</td><td>PNPase+TPR+GreAB-C+PIN</td><td>1385</td><td>-</td><td>Cyanobacteria</td><td>unclassified Planktothrix</td><td>MULTISPECIES: hypothetical protein [unclassified Planktothrix].</td><td>GCF_014696265.1</td></tr>
<tr><td>WP_190659344.1</td><td>BTLCP+NACHT→ APATPase+BetaPropeller→ APATPase+BetaPropeller→ PNPase+TPR+GreAB-C+PIN*→</td><td>PNPase+TPR+GreAB-C+PIN</td><td>1399</td><td>-</td><td>Cyanobacteria</td><td>Nostoc linckia</td><td>tetratricopeptide repeat protein [Nostoc linckia].</td><td>GCF_014696615.1</td></tr>
<tr><td>WP_190786133.1</td><td><-REase+nSTAND3||TPR+GreAB-C+PIN*→</td><td>TPR+GreAB-C+PIN</td><td>1282</td><td>-</td><td>Bacteroidetes</td><td>Hymenobacter sp. BT646</td><td>hypothetical protein [Hymenobacter sp. BT646].</td><td>GCF_014698995.1</td></tr>
<tr><td>WP_191117766.1</td><td>HetE-N1→ TPR+GreAB-C+PIN*→</td><td>TPR+GreAB-C+PIN</td><td>1091</td><td>-</td><td>Gammaproteobacteria</td><td>Vibrio campbellii</td><td>hypothetical protein [Vibrio campbellii].</td><td>GCF_014769105.1</td></tr>
<tr><td>WP_191340685.1</td><td>REase+SNF2→ URI+RAMA→ TPR+GreAB-C+PIN*→</td><td>TPR+GreAB-C+PIN</td><td>1182</td><td>-</td><td>Firmicutes</td><td>Massilimicrobiota sp. An134</td><td>hypothetical protein [Massilimicrobiota sp. An134].</td><td>GCF_904393125.1</td></tr>
<tr><td>WP_191375963.1</td><td>TPR+GreAB-C+PIN*→</td><td>TPR+GreAB-C+PIN</td><td>879</td><td>-</td><td>Firmicutes</td><td>[Clostridium] spiroforme</td><td>hypothetical protein [[Clostridium] spiroforme].</td><td>GCF_904395875.1</td></tr>
</table>

acc	operon	architecture	len	gen.name	taxend	species	defline	gca
WP_191791215.1	TPR+PIN*→	TPR+PIN	1225	-	Actinobacteria	Oerskovia sp. Sa2CUA8	hypothetical protein [Oerskovia sp. Sa2CUA8].	GCF_014836745.1
WP_191917190.1	HetE-N1→ TPR+GreAB-C+PIN*→	TPR+GreAB-C+PIN	1094	-	Gammaproteobacteria	Pantoea agglomerans	hypothetical protein [Pantoea agglomerans].	GCF_014839545.1
WP_191940182.1	X+PIN*→	X+PIN	1078	-	Actinobacteria	Plantibacter sp. CFBP 8775	hypothetical protein [Plantibacter sp. CFBP 8775].	GCF_014839505.1
WP_191950701.1	REase+TPR+GreAB-C+PIN*→	REase+TPR+GreAB-C+PIN	1485	-	Gammaproteobacteria	Pseudomonas fluorescens	hypothetical protein [Pseudomonas fluorescens].	GCF_014839025.1
WP_191971068.1	TPR+GreAB-C+PIN*→	TPR+GreAB-C+PIN	525	-	Alphaproteobacteria	Methylobacterium soli	hypothetical protein [Methylobacterium soli].	GCF_008806385.1
WP_191999584.1	REase+TPR+GreAB-C+PIN*→||||<-?<-EAD2	REase+TPR+GreAB-C+PIN	1036	-	Gammaproteobacteria	Pseudomonas coleopterorum	hypothetical protein [Pseudomonas coleopterorum].	GCF_014841305.1
WP_192069209.1	HetE-N1→ TPR+GreAB-C+PIN*→	TPR+GreAB-C+PIN	1105	-	Gammaproteobacteria	Pseudomonas coleopterorum	hypothetical protein [Pseudomonas coleopterorum].	GCF_014842375.1
WP_192163653.1	REase+TPR+GreAB-C+PIN*→	REase+TPR+GreAB-C+PIN	1250	-	Gammaproteobacteria	Rhodanobacter sp. DHG33	hypothetical protein [Rhodanobacter sp. DHG33].	GCF_014843005.1
WP_192260260.1	REase+TPR+GreAB-C+PIN*→	REase+TPR+GreAB-C+PIN	1441	-	Gammaproteobacteria	Pseudomonas sp. Pdm06	hypothetical protein [Pseudomonas sp. Pdm06].	GCF_014851795.1
WP_192264655.1	REase+TPR+GreAB-C+PIN*→	REase+TPR+GreAB-C+PIN	1316	-	Alphaproteobacteria	Komagataeibacter hansenii	hypothetical protein [Komagataeibacter hansenii].	GCF_014843995.1
WP_192393207.1	HetE-N1→ TPR+GreAB-C+PIN*→	TPR+GreAB-C+PIN	1117	-	Gammaproteobacteria	Methylomonas sp. EbB	hypothetical protein [Methylomonas sp. EbB].	GCF_903064685.1
WP_192433772.1	HEPN→ REase+TPR+TPR+GreAB-C+PIN*→	REase+TPR+TPR+GreAB-C+PIN	1339	-	Alphaproteobacteria	Ensifer sp. ENS11	hypothetical protein [Ensifer sp. ENS11].	GCF_014851895.1
WP_192504527.1	HetE-N1→ TPR+GreAB-C+PIN*→	TPR+GreAB-C+PIN	1119	-	Gammaproteobacteria	Rouxiella badensis	hypothetical protein [Rouxiella badensis].	GCF_014859005.1
WP_192836185.1	HetE-N1→ TPR+GreAB-C+PIN*→	TPR+GreAB-C+PIN	1092	-	Gammaproteobacteria	Acinetobacter oleivorans	hypothetical protein [Acinetobacter oleivorans].	GCF_014876825.1
WP_193132365.1	HetE-N1→ TPR+GreAB-C+PIN+TM+TM*→	TPR+GreAB-C+PIN+TM+TM	1086	-	Gammaproteobacteria	Enterobacter hormaechei	hypothetical protein, partial [Enterobacter hormaechei].	GCF_014901495.1
WP_193343548.1	HetE-N1→ TPR+GreAB-C+PIN*→?→ PhospholipaseD→	TPR+GreAB-C+PIN	1103	-	Gammaproteobacteria	Halomonas elongata	hypothetical protein [Halomonas elongata].	GCF_014926355.1
WP_193426153.1	TPR+PIN*→	TPR+PIN	1217	-	Firmicutes	Priestia aryabhattai	hypothetical protein [Priestia aryabhattai].	GCF_014932885.1
WP_193843312.1	HetE-N1→ TPR+GreAB-C+PIN*→	TPR+GreAB-C+PIN	1090	-	Gammaproteobacteria	Serratia marcescens	hypothetical protein [Serratia marcescens].	GCF_015160915.1
WP_194030396.1	PNPase+TPR+GreAB-C+PIN*→	PNPase+TPR+GreAB-C+PIN	1392	-	Cyanobacteria	Lusitaniella coriacea	hypothetical protein [Lusitaniella coriacea].	GCF_015207425.1
WP_194552914.1	TPR+GreAB-C+PIN*→	TPR+GreAB-C+PIN	1236	-	Gammaproteobacteria	Vibrio anguillarum	hypothetical protein [Vibrio anguillarum].	GCF_015343355.1
WP_194640651.1	TPR+GreAB-C+PIN*→	TPR+GreAB-C+PIN	1123	-	Bacteroidetes	Flavobacterium sp. HJJ	hypothetical protein [Flavobacterium sp. HJJ].	GCF_015351475.1
WP_194689942.1	REase+TPR+GreAB-C+PIN*→	REase+TPR+GreAB-C+PIN	1323	-	Alphaproteobacteria	unclassified Gluconobacter	MULTISPECIES: hypothetical protein [unclassified Gluconobacter].	GCF_015353155.1
WP_194782158.1	TPR+PIN*→	TPR+PIN	1183	-	Actinobacteria	Arthrobacter gandavensis	hypothetical protein [Arthrobacter gandavensis].	GCF_015355785.1
WP_194849029.1	HetE-N1→ TPR+GreAB-C+PIN*→	TPR+GreAB-C+PIN	1094	-	Gammaproteobacteria	Escherichia coli	hypothetical protein [Escherichia coli].	GCF_015356625.1
WP_194908878.1	TPR+PIN*→	TPR+PIN	1322	-	Actinobacteria	Catenulispora rubra	hypothetical protein [Catenulispora rubra].	GCF_015356825.1
WP_195550768.1	TM+TM+TPR+PIN*→	TM+TM+TPR+PIN	1183	-	Bacteroidetes	Bacteroides uniformis	hypothetical protein [Bacteroides uniformis].	GCF_015557045.1
WP_195789843.1	REase+TPR+GreAB-C+PIN*→	REase+TPR+GreAB-C+PIN	1310	-	Alphaproteobacteria	Bradyrhizobium genosp. L	hypothetical protein [Bradyrhizobium genosp. L].	GCF_015624485.1
WP_196103949.1	TPR+GreAB-C+PIN*→	TPR+GreAB-C+PIN	468	-	Alphaproteobacteria	Pontivivens sp. MT2928	hypothetical protein [Pontivivens sp. MT2928].	GCF_015679265.1
WP_196165874.1	TPR+GreAB-C+PIN*→	TPR+GreAB-C+PIN	1139	-	Gammaproteobacteria	Pseudomonas monteilii	hypothetical protein [Pseudomonas monteilii].	GCF_015680735.1
WP_196184162.1	REase+TPR+GreAB-C+PIN*→	REase+TPR+GreAB-C+PIN	1545	-	Gammaproteobacteria	Pseudomonas asiatica	hypothetical protein [Pseudomonas asiatica].	GCF_015680805.1
WP_196343881.1	HetE-N1→ TPR+GreAB-C+PIN*→	TPR+GreAB-C+PIN	1106	-	Gammaproteobacteria	Enterobacter hormaechei	hypothetical protein [Enterobacter hormaechei].	GCF_015704145.1
WP_196402364.1	REase+TPR+TPR+GreAB-C+PIN*→	REase+TPR+TPR+GreAB-C+PIN	1304	-	Alphaproteobacteria	Methylocystis sp. H62	hypothetical protein [Methylocystis sp. H62].	GCF_015709515.1
WP_197022618.1	REase+TPR+GreAB-C+PIN*→	REase+TPR+GreAB-C+PIN	1388	-	Betaproteobacteria	Comamonas aquatica	hypothetical protein [Comamonas aquatica].	GCF_000590985.1
WP_197265512.1	TPR+PIN*→	TPR+PIN	1231	-	Firmicutes	Fictibacillus sp. 23RED33	hypothetical protein [Fictibacillus sp. 23RED33].	GCF_015903925.1
WP_197283942.1	GreAB-C+PIN*→	GreAB-C+PIN	553	-	Alphaproteobacteria	Rhizobium acidisoli	hypothetical protein, partial [Rhizobium acidisoli].	GCF_001297245.1
WP_197310646.1	REase+TPR+TPR+GreAB-C+PIN*→	REase+TPR+TPR+GreAB-C+PIN	1326	-	Alphaproteobacteria	Methylobrevis sp. L22	hypothetical protein [Methylobrevis sp. L22].	GCF_015904235.1
WP_197451745.1	REase+TPR+GreAB-C+PIN*→	REase+TPR+GreAB-C+PIN	1195	-	Planctomycetes	Rosistilla oblonga	GreA/GreB family elongation factor [Rosistilla oblonga].	GCF_007751715.1
WP_197873110.1	TPR+GreAB-C+PIN*→	TPR+GreAB-C+PIN	1139	-	Gammaproteobacteria	Pseudomonas lutea	hypothetical protein [Pseudomonas lutea].	GCF_016009245.1
WP_198113276.1	TPR+GreAB-C+PIN*→||||<-HTH+HTH	TPR+GreAB-C+PIN	872	-	Betaproteobacteria	Burkholderia contaminans	hypothetical protein [Burkholderia contaminans].	GCF_016092105.1
WP_198215548.1	HetE-N1→ TPR+GreAB-C+PIN*→	TPR+GreAB-C+PIN	1126	-	Gammaproteobacteria	Gilliamella sp. M0320	hypothetical protein [Gilliamella sp. M0320].	GCF_016101635.1
WP_198575560.1	REase+TPR+GreAB-C+PIN*→	REase+TPR+GreAB-C+PIN	1332	-	Alphaproteobacteria	Caulobacter hibisci	DUF4365 domain-containing protein [Caulobacter hibisci].	GCF_016135805.1
WP_198597141.1	HetE-N1→?→ GreAB-C*→	GreAB-C	475	-	Gammaproteobacteria	Vibrio splendidus	hypothetical protein [Vibrio splendidus].	GCF_002877525.1
WP_198628691.1	TPR+GreAB-C+PIN*→	TPR+GreAB-C+PIN	1231	-	Bacteroidetes	Salegentibacter sp. F63223	hypothetical protein [Salegentibacter sp. F63223].	GCF_016236915.1
WP_198694995.1	REase+TPR+GreAB-C+PIN*→	REase+TPR+GreAB-C+PIN	1436	-	Gammaproteobacteria	Pseudomonas viridiflava	hypothetical protein [Pseudomonas viridiflava].	GCF_016308365.1

acc	operon	architecture	len	gen.name	taxend	species	defline	gca
WP_198710862.1	REase+TPR+GreAB-C+PIN*→	REase+TPR+GreAB-C+PIN	1321	-	Gammaproteobacteria	Pseudomonas syringae	DUF4365 domain-containing protein [Pseudomonas syringae].	GCF_016308175.1
WP_198739586.1	HetE-N1→ TPR+GreAB-C+PIN*→	TPR+GreAB-C+PIN	1106	-	Gammaproteobacteria	Enterobacter hormaechei	hypothetical protein [Enterobacter hormaechei].	GCF_016342225.1
WP_198742183.1	HetE-N1→ TPR+GreAB-C+PIN*→	TPR+GreAB-C+PIN	1106	-	Gammaproteobacteria	Enterobacter hormaechei	hypothetical protein [Enterobacter hormaechei].	GCF_016342135.1
WP_198830693.1	REase+TPR+GreAB-C+PIN*→	REase+TPR+GreAB-C+PIN	1441	-	Gammaproteobacteria	Pseudomonas sp. MF5691	hypothetical protein [Pseudomonas sp. MF5691].	GCF_016405645.1
WP_198862330.1	REase+TPR+GreAB-C+PIN*→	REase+TPR+GreAB-C+PIN	1324	-	Gammaproteobacteria	Pseudomonas carnis	DUF4365 domain-containing protein [Pseudomonas carnis].	GCF_016405215.1
WP_199041247.1	REase+TPR+GreAB-C+PIN*→	REase+TPR+GreAB-C+PIN	1250	-	Gammaproteobacteria	Dyella sp. ASV24	hypothetical protein [Dyella sp. ASV24].	GCF_016428905.1
WP_199059400.1	REase+TPR+TPR+GreAB-C+PIN*→	REase+TPR+TPR+GreAB-C+PIN	1334	-	Alphaproteobacteria	Brevundimonas sp. ASV9	hypothetical protein [Brevundimonas sp. ASV9].	GCF_016429195.1
WP_199059891.1	REase+TPR+GreAB-C+PIN*→	REase+TPR+GreAB-C+PIN	1302	-	Alphaproteobacteria	Brevundimonas sp. ASV9	hypothetical protein [Brevundimonas sp. ASV9].	GCF_016429195.1
WP_199133123.1	TPR+GreAB-C+PIN*→	TPR+GreAB-C+PIN	1258	-	Bacteroidetes	Pedobacter sp. ASV17	hypothetical protein [Pedobacter sp. ASV17].	GCF_016429065.1
WP_199161798.1	TPR+GreAB-C+PIN*→	TPR+GreAB-C+PIN	1140	-	Bacteroidetes	Elizabethkingia sp. ASV34	hypothetical protein [Elizabethkingia sp. ASV34].	GCF_016428745.1
WP_199216499.1	TPR+PIN*→	TPR+PIN	1215	-	Actinobacteria	Streptomyces sp. I5	hypothetical protein [Streptomyces sp. I5].	GCF_016432735.1
WP_199272510.1	REase+TPR+GreAB-C+PIN*→	REase+TPR+GreAB-C+PIN	1359	-	Betaproteobacteria	Paraburkholderia acidisoli	hypothetical protein [Paraburkholderia acidisoli].	GCF_009789675.1
WP_199515964.1	TM+TPR+PIN*→	TM+TPR+PIN	1145	-	Actinobacteria	Pseudosporangium sp. NEAU-24	tetratricopeptide repeat protein [Pseudosporangium sp. NEAU-24].	GCF_016464385.1
WP_199591321.1	REase+TPR+GreAB-C+PIN*→	REase+TPR+GreAB-C+PIN	1505	-	Betaproteobacteria	Pelomonas sp. OAE585	hypothetical protein [Pelomonas sp. OAE585].	GCF_016467315.1
WP_199660563.1	TPR+GreAB-C+PIN*→	TPR+GreAB-C+PIN	1035	-	Gammaproteobacteria	Klebsiella quasipneumoniae	hypothetical protein, partial [Klebsiella quasipneumoniae].	GCF_016491635.1
WP_199707330.1	REase→ PIN*→	PIN	932	-	Actinobacteria	Antrihabitans sp. YC3-6	hypothetical protein [Antrihabitans sp. YC3-6].	GCF_016482825.1
WP_199727385.1	REase+TPR+GreAB-C+PIN*→	REase+TPR+GreAB-C+PIN	1488	-	Gammaproteobacteria	Pseudomonas syringae group genomosp. 7	hypothetical protein [Pseudomonas syringae group genomosp. 7].	GCF_003703295.1
WP_199743158.1	REase+TPR+GreAB-C+PIN*→	REase+TPR+GreAB-C+PIN	1009	-	Alphaproteobacteria	Sphingomonas ginsenosidivorax	tetratricopeptide repeat protein [Sphingomonas ginsenosidivorax].	GCF_007995065.1
WP_199871858.1	HetE-N1→ HetE-N1+TPR+GreAB-C+PIN*→	HetE-N1+TPR+GreAB-C+PIN	1088	-	Gammaproteobacteria	Vibrio anguillarum	hypothetical protein [Vibrio anguillarum].	GCF_015350415.1
WP_200204777.1	HetE-N1→ TPR+GreAB-C+PIN*→	TPR+GreAB-C+PIN	1116	-	Gammaproteobacteria	Marinobacter sp. 1-3A	hypothetical protein [Marinobacter sp. 1-3A].	GCF_016597775.1
WP_200282902.1	TPR+PIN*→	TPR+PIN	1263	-	Verrucomicrobia	Haloferula rosea	hypothetical protein [Haloferula rosea].	GCF_016595525.1
WP_200597934.1	HetE-N1→ TPR+GreAB-C+PIN*→	TPR+GreAB-C+PIN	1112	-	Gammaproteobacteria	Pseudomonas stutzeri	hypothetical protein [Pseudomonas stutzeri].	GCF_016622345.1
WP_200602419.1	REase+TPR+GreAB-C+PIN*→	REase+TPR+GreAB-C+PIN	1441	-	Gammaproteobacteria	Pseudomonas sp. S44	hypothetical protein [Pseudomonas sp. S44].	GCF_016624805.1
WP_201009307.1	REase+PIN*→	REase+PIN	1247	-	Actinobacteria	Rhodococcus sp. NKCM2511	DUF4365 domain-containing protein [Rhodococcus sp. NKCM2511].	GCF_014905595.1
WP_201090676.1	HetE-N1→ TPR+GreAB-C+PIN*→	TPR+GreAB-C+PIN	1083	-	Gammaproteobacteria	Entomomonas sp. F2A	hypothetical protein [Entomomonas sp. F2A].	GCF_016653615.1
WP_201420192.1	REase+TPR+GreAB-C+PIN*→	REase+TPR+GreAB-C+PIN	1442	-	Gammaproteobacteria	Pseudomonas syringae	hypothetical protein [Pseudomonas syringae].	GCF_016694755.2
WP_201621921.1	REase+TPR+GreAB-C+PIN*→	REase+TPR+GreAB-C+PIN	1244	-	Gammaproteobacteria	Psychrobacter urativorans	hypothetical protein [Psychrobacter urativorans].	GCF_904846695.1
WP_201915404.1	REase+TPR+GreAB-C+PIN*→	REase+TPR+GreAB-C+PIN	1321	-	Gammaproteobacteria	Pseudomonas fluorescens	DUF4365 domain-containing protein [Pseudomonas fluorescens].	GCF_016728065.1
WP_201921162.1	HetE-N1→ TPR+GreAB-C+PIN*→	TPR+GreAB-C+PIN	1094	-	Gammaproteobacteria	Aeromonas caviae	hypothetical protein [Aeromonas caviae].	GCF_016729435.1
WP_201996904.1	HetE-N1→ MNS-Npun2340+TPR+GreAB-C+PIN*→	MNS-Npun2340+TPR+GreAB-C+PIN	1094	-	Gammaproteobacteria	Aeromonas caviae	hypothetical protein [Aeromonas caviae].	GCF_016728955.1
WP_202685167.1	REase+TPR+GreAB-C+PIN*→	REase+TPR+GreAB-C+PIN	1019	-	Alphaproteobacteria	Skermanella mucosa	hypothetical protein, partial [Skermanella mucosa].	GCF_016765655.1
WP_202722978.1	HetE-N1→ TPR+GreAB-C+PIN*→	TPR+GreAB-C+PIN	1141	-	Gammaproteobacteria	Shewanella schlegeliana	hypothetical protein [Shewanella schlegeliana].	GCF_016765635.1
WP_202727723.1	HetE-N1→ TPR+GreAB-C+PIN*→	TPR+GreAB-C+PIN	1094	-	Gammaproteobacteria	Serratia fonticola	hypothetical protein [Serratia fonticola].	GCF_016770915.1
WP_203042134.1	HetE-N1→ TPR+GreAB-C+PIN*→	TPR+GreAB-C+PIN	1106	-	Gammaproteobacteria	Enterobacter hormaechei	hypothetical protein [Enterobacter hormaechei].	GCF_016791625.1
WP_203341318.1	TPR+PIN*→	TPR+PIN	1141	-	Firmicutes	Planomicrobium sp. REN14	hypothetical protein [Planomicrobium sp. REN14].	GCF_016820615.1
WP_203380482.1	REase+TPR+GreAB-C+PIN*→	REase+TPR+GreAB-C+PIN	1231	-	Actinobacteria	Actinoplanes sp. LDG1-06	DUF4365 domain-containing protein [Actinoplanes sp. LDG1-06].	GCF_016834655.1
WP_203735920.1	TPR+PIN*→	TPR+PIN	1176	-	Actinobacteria	Actinoplanes durhamensis	hypothetical protein [Actinoplanes durhamensis].	GCF_016862175.1
WP_203841861.1	REase+TPR+GreAB-C+PIN*→	REase+TPR+GreAB-C+PIN	1259	-	Actinobacteria	Actinoplanes humidus	DUF4365 domain-containing protein [Actinoplanes humidus].	GCF_016862215.1
WP_204008964.1	REase+TPR+GreAB-C+PIN*→	REase+TPR+GreAB-C+PIN	1356	-	Actinobacteria	Micromonospora andamanensis	hypothetical protein [Micromonospora andamanensis].	GCF_016863495.1
WP_204028926.1	REase+TPR+GreAB-C+PIN*→	REase+TPR+GreAB-C+PIN	1338	-	Actinobacteria	Sinosporangium siamense	hypothetical protein [Sinosporangium siamense].	GCF_016863435.1

<table>
<tr><th>acc</th><th>operon</th><th>architecture</th><th>len</th><th>gen.name</th><th>taxend</th><th>species</th><th>defline</th><th>gca</th></tr>
<tr><td>WP_204127404.1</td><td>HetE-N1→ TPR+GreAB-C+PIN*→||||<-HNH</td><td>TPR+GreAB-C+PIN</td><td>1110</td><td>-</td><td>Gammaproteobacteria</td><td>Pseudomonas sp. RDP1</td><td>hypothetical protein [Pseudomonas sp. RDP1].</td><td>GCF_016887885.1</td></tr>
<tr><td>WP_204370319.1</td><td>TPR+GreAB-C+PIN*→</td><td>TPR+GreAB-C+PIN</td><td>860</td><td>-</td><td>Betaproteobacteria</td><td>Burkholderia cepacia</td><td>tetratricopeptide repeat protein, partial [Burkholderia cepacia].</td><td>GCF_001052915.1</td></tr>
<tr><td>WP_204511064.1</td><td>REase+TPR+GreAB-C+PIN*→</td><td>REase+TPR+GreAB-C+PIN</td><td>1349</td><td>-</td><td>Alphaproteobacteria</td><td>Bradyrhizobium canariense</td><td>tetratricopeptide repeat protein [Bradyrhizobium canariense].</td><td>GCF_016907215.1</td></tr>
<tr><td>WP_204648469.1</td><td>TPR+PIN*→</td><td>TPR+PIN</td><td>1235</td><td>-</td><td>Firmicutes</td><td>Gemmiger formicilis</td><td>hypothetical protein [Gemmiger formicilis].</td><td>GCF_016900095.1</td></tr>
<tr><td>WP_204732305.1</td><td>REase+TPR+GreAB-C+PIN*→</td><td>REase+TPR+GreAB-C+PIN</td><td>1447</td><td>-</td><td>Betaproteobacteria</td><td>Hydrogenophaga laconesensis</td><td>hypothetical protein [Hydrogenophaga laconesensis].</td><td>GCF_016909585.1</td></tr>
<tr><td>WP_204856598.1</td><td>REase+PIN*→</td><td>REase+PIN</td><td>1247</td><td>-</td><td>Actinobacteria</td><td>Rhodococcus fascians</td><td>DUF4365 domain-containing protein [Rhodococcus fascians].</td><td>GCF_016909505.1</td></tr>
<tr><td>WP_204934318.1</td><td>REase+TPR+GreAB-C+PIN*→</td><td>REase+TPR+GreAB-C+PIN</td><td>1321</td><td>-</td><td>Gammaproteobacteria</td><td>Pseudomonas stutzeri</td><td>DUF4365 domain-containing protein [Pseudomonas stutzeri].</td><td>GCF_016909445.1</td></tr>
<tr><td>WP_204944661.1</td><td>TPR+PIN*→</td><td>TPR+PIN</td><td>1203</td><td>-</td><td>Actinobacteria</td><td>Micromonospora luteifusca</td><td>hypothetical protein [Micromonospora luteifusca].</td><td>GCF_016907275.1</td></tr>
<tr><td>WP_205045164.1</td><td>TPR+PIN*→</td><td>TPR+PIN</td><td>1168</td><td>-</td><td>Actinobacteria</td><td>Streptomyces bryophytorum</td><td>hypothetical protein [Streptomyces bryophytorum].</td><td>GCF_017164735.1</td></tr>
<tr><td>WP_205483425.1</td><td>REase+TPR+GreAB-C+PIN→ TPR→||||<-?||PIN*→</td><td>PIN</td><td>365</td><td>-</td><td>Gammaproteobacteria</td><td>Pseudomonas sp. 15A4</td><td>hypothetical protein [Pseudomonas sp. 15A4].</td><td>GCF_016925575.1</td></tr>
<tr><td>WP_206013307.1</td><td>HetE-N1→ TPR+GreAB-C+PIN*→</td><td>TPR+GreAB-C+PIN</td><td>1090</td><td>-</td><td>Gammaproteobacteria</td><td>Pseudoalteromonas sp. Z9A6</td><td>hypothetical protein [Pseudoalteromonas sp. Z9A6].</td><td>GCF_011378805.1</td></tr>
<tr><td>WP_206497574.1</td><td>REase+PIN*→</td><td>REase+PIN</td><td>1021</td><td>-</td><td>Actinobacteria</td><td>Rhodococcus sp. KRD175</td><td>hypothetical protein [Rhodococcus sp. KRD175].</td><td>GCF_017168275.1</td></tr>
<tr><td>WP_206556849.1</td><td>HetE-N1→ TPR+GreAB-C+PIN*→</td><td>TPR+GreAB-C+PIN</td><td>1116</td><td>-</td><td>Gammaproteobacteria</td><td>Marinobacter daepoensis</td><td>hypothetical protein [Marinobacter daepoensis].</td><td>GCF_017255165.1</td></tr>
<tr><td>WP_206628468.1</td><td>HetE-N1→ TPR+GreAB-C+PIN*→</td><td>TPR+GreAB-C+PIN</td><td>1116</td><td>-</td><td>Gammaproteobacteria</td><td>Vibrio vulnificus</td><td>hypothetical protein [Vibrio vulnificus].</td><td>GCF_017291135.1</td></tr>
<tr><td>WP_206676035.1</td><td>HTH→ SIG+TM→ TPR+GreAB-C+PIN*→</td><td>TPR+GreAB-C+PIN</td><td>1125</td><td>-</td><td>Bacteroidetes</td><td>Capnocytophaga canis</td><td>hypothetical protein [Capnocytophaga canis].</td><td>GCF_013276255.1</td></tr>
<tr><td>WP_206820216.1</td><td>REase+TPR+GreAB-C+PIN*→</td><td>REase+TPR+GreAB-C+PIN</td><td>1215</td><td>-</td><td>Actinobacteria</td><td>Microbacterium esteraromaticum</td><td>DUF4365 domain-containing protein [Microbacterium esteraromaticum].</td><td>GCF_017303135.1</td></tr>
<tr><td>WP_206852091.1</td><td>REase+TPR+GreAB-C+PIN*→</td><td>REase+TPR+GreAB-C+PIN</td><td>1289</td><td>-</td><td>Gammaproteobacteria</td><td>Marinobacter hydrocarbonoclasticus</td><td>hypothetical protein [Marinobacter hydrocarbonoclasticus].</td><td>GCF_017303195.1</td></tr>
<tr><td>WP_207418840.1</td><td>TPR+GreAB-C+PIN*→||||<-HTH+HTH</td><td>TPR+GreAB-C+PIN</td><td>872</td><td>-</td><td>Betaproteobacteria</td><td>Burkholderia pseudomallei</td><td>hypothetical protein [Burkholderia pseudomallei].</td><td>GCF_017356705.1</td></tr>
<tr><td>WP_207505725.1</td><td>Mbetalac→ Trypsin+TPR+GreAB-C+PIN*→</td><td>Trypsin+TPR+GreAB-C+PIN</td><td>1362</td><td>-</td><td>Bacteroidetes</td><td>Telluribacter humicola</td><td>serine protease [Telluribacter humicola].</td><td>GCF_017355935.1</td></tr>
<tr><td>WP_207509935.1</td><td>Mbetalac→ TPR+GreAB-C+PIN*→</td><td>TPR+GreAB-C+PIN</td><td>1342</td><td>-</td><td>Bacteroidetes</td><td>Telluribacter humicola</td><td>hypothetical protein [Telluribacter humicola].</td><td>GCF_017355935.1</td></tr>
<tr><td>WP_207967591.1</td><td>HetE-N1→ TPR+GreAB-C+PIN*→</td><td>TPR+GreAB-C+PIN</td><td>1090</td><td>-</td><td>Gammaproteobacteria</td><td>Yersinia pseudotuberculosis</td><td>hypothetical protein [Yersinia pseudotuberculosis].</td><td>GCF_017498805.1</td></tr>
<tr><td>WP_207993663.1</td><td>HetE-N1→ TPR+GreAB-C+PIN*→</td><td>TPR+GreAB-C+PIN</td><td>1122</td><td>-</td><td>Gammaproteobacteria</td><td>Yersinia pseudotuberculosis</td><td>hypothetical protein [Yersinia pseudotuberculosis].</td><td>GCF_017498865.1</td></tr>
<tr><td>WP_208133228.1</td><td>TPR+GreAB-C+PIN*→</td><td>TPR+GreAB-C+PIN</td><td>1306</td><td>-</td><td>Bacteroidetes</td><td>Siccationidurans ginsengisoli</td><td>hypothetical protein [Siccationidurans ginsengisoli].</td><td>GCF_017571525.1</td></tr>
<tr><td>WP_208209475.1</td><td>REase+TPR+GreAB-C+PIN*→</td><td>REase+TPR+GreAB-C+PIN</td><td>1543</td><td>-</td><td>Gammaproteobacteria</td><td>Pseudomonas asiatica</td><td>hypothetical protein [Pseudomonas asiatica].</td><td>GCF_017582485.1</td></tr>
<tr><td>WP_208467197.1</td><td>TPR+PIN*→</td><td>TPR+PIN</td><td>1140</td><td>-</td><td>Actinobacteria</td><td>Actinoplanes sp. NEAU-H7</td><td>hypothetical protein [Actinoplanes sp. NEAU-H7].</td><td>GCF_017592555.1</td></tr>
<tr><td>WP_208622113.1</td><td>HetE-N1→ TPR+GreAB-C+PIN*→</td><td>TPR+GreAB-C+PIN</td><td>1022</td><td>-</td><td>Gammaproteobacteria</td><td>Shewanella indica</td><td>hypothetical protein, partial [Shewanella indica].</td><td>GCF_002836975.1</td></tr>
<tr><td>WP_208660584.1</td><td>HetE-N1→ TPR+GreAB-C+PIN*→</td><td>TPR+GreAB-C+PIN</td><td>1087</td><td>-</td><td>Gammaproteobacteria</td><td>Shewanella decolorationis</td><td>hypothetical protein [Shewanella decolorationis].</td><td>GCF_007923045.1</td></tr>
<tr><td>WP_208757658.1</td><td>TPR+PIN*→</td><td>TPR+PIN</td><td>1184</td><td>-</td><td>Firmicutes</td><td>Priestia megaterium</td><td>hypothetical protein [Priestia megaterium].</td><td>GCF_002872495.1</td></tr>
<tr><td>WP_209187630.1</td><td>REase+TPR+GreAB-C+PIN→ *PIN*→</td><td>REase+TPR+GreAB-C+PIN</td><td>359</td><td>-</td><td>Alphaproteobacteria</td><td>Rhizobium sp. L245/93</td><td>hypothetical protein [Rhizobium sp. L245/93].</td><td>GCF_017741845.1</td></tr>
<tr><td>WP_209187631.1</td><td>-</td><td>-</td><td>978</td><td>-</td><td>Alphaproteobacteria</td><td>Rhizobium sp. L245/93</td><td>hypothetical protein [Rhizobium sp. L245/93].</td><td>GCF_017741845.1</td></tr>
<tr><td>WP_209302601.1</td><td>X+PIN*→</td><td>X+PIN</td><td>1221</td><td>-</td><td>Firmicutes</td><td>Anaerostipes hadrus</td><td>hypothetical protein [Anaerostipes hadrus].</td><td>GCF_017776445.1</td></tr>
<tr><td>WP_209570872.1</td><td>TPR+GreAB-C+PIN*→?→||||<-TPR</td><td>TPR+GreAB-C+PIN</td><td>1131</td><td>-</td><td>Bacteroidetes</td><td>Flavobacterium sp. 1750</td><td>hypothetical protein [Flavobacterium sp. 1750].</td><td>GCF_017833855.1</td></tr>
<tr><td>WP_209629149.1</td><td><-Calcineurin<-?||?→?→?→ TPR+PIN*→</td><td>TPR+PIN</td><td>1016</td><td>-</td><td>Euryarchaeota</td><td>Methanofollis sp. W23</td><td>hypothetical protein [Methanofollis sp. W23].</td><td>GCF_017875325.1</td></tr>
<tr><td>WP_209829315.1</td><td>TPR+PIN*→</td><td>TPR+PIN</td><td>1242</td><td>-</td><td>Actinobacteria</td><td>Cellulosimicrobium sp. BE325</td><td>hypothetical protein [Cellulosimicrobium sp. BE325].</td><td>GCF_017834095.1</td></tr>
<tr><td>WP_210277108.1</td><td>REase+TPR+TPR+GreAB-C+PIN*→</td><td>REase+TPR+TPR+GreAB-C+PIN</td><td>1346</td><td>-</td><td>Alphaproteobacteria</td><td>Rhizobium leguminosarum</td><td>hypothetical protein [Rhizobium leguminosarum].</td><td>GCF_014138515.1</td></tr>
<tr><td>WP_210311799.1</td><td>TPR+GreAB-C+PIN*→</td><td>TPR+GreAB-C+PIN</td><td>812</td><td>-</td><td>Alphaproteobacteria</td><td>Rhizobium soli</td><td>hypothetical protein, partial [Rhizobium soli].</td><td>GCF_014207075.1</td></tr>
[truncated: 497 more chars]
