# Supplementary material for: Bacterial death and TRADD-N domains help define novel apoptosis and immunity mechanisms shared by prokaryotes and metazoans
Source: eLife. 2021 Jun 1;10:e70394. doi: 10.7554/eLife.70394 (PMC8195603; doi:10.7554/eLife.70394)
Supplement: Figure 4—source data 2. [file elife-70394-fig4-data2.pdf]

Non-redundant counts of TRADD-N domains in eukaryotic lineages

GCA	taxonomy	count
Amphimedon queenslandica	GCF_000090795.1	307
Branchiostoma floridae	GCF_000003815.1	113
Stylophora pistillata	GCF_002571385.1	86
Acropora digitifera	GCF_000222465.1	83
Branchiostoma belcheri	GCF_001625305.1	77
Branchiostoma floridae	GCA_000003815.1	65
Orbicella faveolata	GCF_002042975.1	61
Stylophora pistillata	GCA_002571385.1	52
Crassostrea gigas	GCF_000297895.1	35
Crassostrea virginica	GCF_002022765.2	32
Lingula anatina	GCF_001039355.2	31
Saccoglossus kowalevskii	GCF_000003605.2	24
Exaiptasia pallida	GCA_001417965.1	17
Pocillopora damicornis	GCA_003704095.1	17
Acanthaster planci	GCF_001949145.1	15
Exaiptasia pallida	GCF_001417965.1	12
Apostichopus japonicus	GCA_002754855.1	9
Mizuhopecten yessoensis	GCF_002113885.1	9
Capitella teleta	GCA_000328365.1	8
Carassius auratus	GCF_003368295.1	8
Oncorhynchus kisutch	GCF_002021735.1	8
Oncorhynchus mykiss	GCF_002163495.1	8
Salmo salar	GCF_000233375.1	8
Sinocyclocheilus anshuiensis	GCF_001515605.1	8
Sinocyclocheilus rhinocerous	GCF_001515625.1	8
Nematostella vectensis	GCF_000209225.1	8
Limulus polyphemus	GCF_000517525.1	7
Xenopus laevis	GCF_001663975.1	7
Cyprinus carpio	GCA_001270105.1	7
Salvelinus alpinus	GCF_002910315.2	7
Sinocyclocheilus grahami	GCF_001515645.1	7
Cyprinus carpio	GCF_000951615.1	6
Heterocephalus glaber	GCA_000230445.1	5
Microtus ochrogaster	GCF_000317375.1	5

GCA	taxonomy	count
Odocoileus virginianus texanus	GCF_002102435.1	5
Phascolarctos cinereus	GCF_002099425.1	5
Rhincodon typus	GCF_001642345.1	5
Oncorhynchus tshawytscha	GCF_002872995.1	5
Paramormyrops kingsleyae	GCF_002872115.1	5
Lottia gigantea	GCF_000327385.1	5
Pogona vitticeps	GCF_900067755.1	4
Theropithecus gelada	GCF_003255815.1	4
Ailuropoda melanoleuca	GCF_000004335.2	4
Alligator mississippiensis	GCA_000281125.4	4
Alligator sinensis	GCF_000455745.1	4
Anolis carolinensis	GCF_000090745.1	4
Apteryx australis mantelli	GCF_001039765.1	4
Balaenoptera acutorostrata scammoni	GCF_000493695.1	4
Bos taurus	GCF_002263795.1	4
Bubalus bubalis	GCF_003121395.1	4
Calidris pugnax	GCF_001431845.1	4
Callithrix jacchus	GCF_000004665.1	4
Camelus ferus	GCF_000311805.1	4
Canis lupus familiaris	GCF_000002285.3	4
Cariama cristata	GCA_000690535.1	4
Carlito syrichta	GCF_000164805.1	4
Castor canadensis	GCF_001984765.1	4
Cavia porcellus	GCF_000151735.1	4
Cebus capucinus imitator	GCF_001604975.1	4
Cercocebus atys	GCF_000955945.1	4
Chaetura pelagica	GCF_000747805.1	4
Chinchilla lanigera	GCF_000276665.1	4
Chrysemys picta bellii	GCF_000241765.3	4
Chrysochloris asiatica	GCF_000296735.1	4
Dasypus novemcinctus	GCF_000208655.1	4
Desmodus rotundus	GCF_002940915.1	4
Dipodomys ordii	GCF_000151885.1	4
Echinops telfairi	GCF_000313985.2	4

GCA	taxonomy	count
Elephantulus edwardii	GCF_000299155.1	4
Erinaceus europaeus	GCF_000296755.1	4
Gekko japonicus	GCF_001447785.1	4
Heterocephalus glaber	GCF_000247695.1	4
Hipposideros armiger	GCF_001890085.1	4
Homo sapiens	GCF_000001405.39	4
Ictidomys tridecemlineatus	GCF_000236235.1	4
Jaculus jaculus	GCF_000280705.1	4
Latimeria chalumnae	GCF_000225785.1	4
Leptonychotes weddellii	GCF_000349705.1	4
Lipotes vexillifer	GCF_000442215.1	4
Loxodonta africana	GCF_000001905.1	4
Manis javanica	GCF_001685135.1	4
Marmota marmota marmota	GCF_001458135.1	4
Meriones unguiculatus	GCF_002204375.1	4
Mesocricetus auratus	GCF_000349665.1	4
Miniopterus natalensis	GCF_001595765.1	4
Monodelphis domestica	GCF_000002295.2	4
Mus caroli	GCF_900094665.1	4
Mus musculus	GCF_000001635.26	4
Mustela putorius furo	GCF_000215625.1	4
Myotis brandtii	GCF_000412655.1	4
Myotis davidii	GCF_000327345.1	4
Myotis lucifugus	GCF_000147115.1	4
Nannospalax galili	GCF_000622305.1	4
Nanorana parkeri	GCF_000935625.1	4
Neotoma lepida	GCA_001675575.1	4
Nipponia nippon	GCF_000708225.1	4
Ochotona princeps	GCF_000292845.1	4
Octodon degus	GCF_000260255.1	4
Odobenus rosmarus divergens	GCF_000321225.1	4
Orcinus orca	GCF_000331955.2	4
Orycteropus afer afer	GCF_000298275.1	4
Otolemur garnettii	GCF_000181295.1	4

GCA	taxonomy	count
Panthera pardus	GCF_001857705.1	4
Panthera tigris altaica	GCF_000464555.1	4
Pelodiscus sinensis	GCF_000230535.1	4
Peromyscus maniculatus bairdii	GCF_000500345.1	4
Pongo abelii	GCF_002880775.1	4
Protobothrops mucrosquamatus	GCF_001527695.2	4
Pseudopodoces humilis	GCF_000331425.1	4
Pteropus alecto	GCF_000325575.1	4
Python bivittatus	GCF_000186305.1	4
Rattus norvegicus	GCF_000002265.2	4
Rousettus aegyptiacus	GCF_001466805.2	4
Sorex araneus	GCF_000181275.1	4
Sus scrofa	GCF_000003025.6	4
Thamnophis sirtalis	GCF_001077635.1	4
Trichechus manatus latirostris	GCF_000243295.1	4
Tupaia chinensis	GCF_000334495.1	4
Ursus maritimus	GCF_000687225.1	4
Xenopus tropicalis	GCF_000004195.3	4
Acanthochromis polyacanthus	GCF_002109545.1	4
Amphiprion ocellaris	GCF_002776465.1	4
Austrofundulus limnaeus	GCF_001266775.1	4
Boleophthalmus pectinirostris	GCF_000788275.1	4
Clupea harengus	GCF_000966335.1	4
Cynoglossus semilaevis	GCF_000523025.1	4
Cyprinodon variegatus	GCF_000732505.1	4
Danio rerio	GCF_000002035.6	4
Esox lucius	GCF_004634155.1	4
Fundulus heteroclitus	GCF_000826765.1	4
Gambusia affinis	GCA_003097735.1	4
Hippocampus comes	GCF_001891065.1	4
Ictalurus punctatus	GCF_001660625.1	4
Kryptolebias marmoratus	GCF_001649575.1	4
Lates calcarifer	GCF_001640805.1	4
Lepisosteus oculatus	GCF_000242695.1	4

GCA	taxonomy	count
Maylandia zebra	GCF_000238955.4	4
Monopterus albus	GCF_001952655.1	4
Neolamprologus brichardi	GCF_000239395.1	4
Nothobranchius furzeri	GCF_001465895.1	4
Notothenia coriiceps	GCF_000735185.1	4
Oncorhynchus mykiss	GCA_900005705.1	4
Oreochromis niloticus	GCF_001858045.2	4
Oryzias latipes	GCF_002234675.1	4
Oryzias melastigma	GCF_002922805.1	4
Paralichthys olivaceus	GCF_001970005.1	4
Poecilia formosa	GCF_000485575.1	4
Poecilia reticulata	GCF_000633615.1	4
Pundamilia nyererei	GCF_000239375.1	4
Pygocentrus nattereri	GCF_001682695.1	4
Scophthalmus maximus	GCA_003186165.1	4
Seriola dumerili	GCF_002260705.1	4
Seriola lalandi dorsalis	GCF_002814215.1	4
Stegastes partitus	GCF_000690725.1	4
Xiphophorus maculatus	GCF_002775205.1	4
Pomacea canaliculata	GCF_003073045.1	4
Octopus bimaculoides	GCF_001194135.1	4
Aplysia californica	GCF_000002075.1	4
Cervus elaphus hippelaphus	GCA_002197005.1	3
Hirundo rustica rustica	GCA_003692655.1	3
Chelonia mydas	GCA_000344595.1	3
Alligator mississippiensis	GCF_000281125.3	3
Amazona aestiva	GCA_001420675.1	3
Aotus nancymaae	GCF_000952055.2	3
Aptenodytes forsteri	GCF_000699145.1	3
Bos indicus	GCF_000247795.1	3
Bos mutus	GCA_000298355.1	3
Bos mutus	GCF_000298355.1	3
Callipepla squamata	GCA_002218305.1	3
Callorhinchus milii	GCF_000165045.1	3

GCA	taxonomy	count
Callorhinus ursinus	GCF_003265705.1	3
Camelus ferus	GCA_000311805.2	3
Capra hircus	GCF_001704415.1	3
Cariama cristata	GCF_000690535.1	3
Ceratotherium simum simum	GCF_000283155.1	3
Charadrius vociferus	GCA_000708025.2	3
Charadrius vociferus	GCF_000708025.1	3
Columba livia	GCF_000337935.1	3
Condylura cristata	GCF_000260355.1	3
Coturnix japonica	GCF_001577835.1	3
Crocodylus porosus	GCF_001723895.1	3
Cuculus canorus	GCF_000709325.1	3
Delphinapterus leucas	GCF_002288925.2	3
Egretta garzetta	GCF_000687185.1	3
Enhydra lutris kenyoni	GCF_002288905.1	3
Eptesicus fuscus	GCF_000308155.1	3
Equus asinus	GCF_001305755.1	3
Equus caballus	GCF_002863925.1	3
Felis catus	GCF_000181335.3	3
Fukomys damarensis	GCF_000743615.1	3
Galeopterus variegatus	GCF_000696425.1	3
Gallus gallus	GCF_000002315.5	3
Macaca fascicularis	GCA_000230815.1	3
Meleagris gallopavo	GCF_000146605.2	3
Neomonachus schauinslandi	GCF_002201575.1	3
Neophocaena asiaeorientalis asiaeorientalis	GCF_003031525.1	3
Notechis scutatus	GCF_900518725.1	3
Numida meleagris	GCF_002078875.1	3
Oryctolagus cuniculus	GCF_000003625.3	3
Pan paniscus	GCF_000258655.2	3
Papio anubis	GCF_000264685.3	3
Phaethon lepturus	GCF_000687285.1	3
Piliocolobus tephrosceles	GCF_002776525.2	3
Pongo abelii	GCA_002880775.3	3

GCA	taxonomy	count
Propithecus coquereli	GCF_000956105.1	3
Pseudonaja textilis	GCF_900518735.1	3
Pteropus vampyrus	GCF_000151845.1	3
Sarcophilus harrisii	GCF_000189315.1	3
Struthio camelus australis	GCA_000698965.1	3
Struthio camelus australis	GCF_000698965.1	3
Sturnus vulgaris	GCF_001447265.1	3
Xenopus laevis	GCA_001663975.1	3
Acanthisitta chloris	GCF_000695815.1	3
Antrostomus carolinensis	GCF_000700745.1	3
Bambusicola thoracicus	GCA_002909625.1	3
Bison bison bison	GCF_000754665.1	3
Colius striatus	GCF_000690715.1	3
Colobus angolensis palliatus	GCF_000951035.1	3
Corvus brachyrhynchos	GCA_000691975.1	3
Corvus brachyrhynchos	GCF_000691975.1	3
Cyanistes caeruleus	GCF_002901205.1	3
Ficedula albicollis	GCF_000247815.1	3
Lepidothrix coronata	GCF_001604755.1	3
Parus major	GCF_001522545.3	3
Picoides pubescens	GCF_000699005.1	3
Tinamus guttatus	GCF_000705375.1	3
Anabas testudineus	GCF_900324465.1	3
Astyanax mexicanus	GCF_000372685.2	3
Electrophorus electricus	GCF_003665695.1	3
Haplochromis burtoni	GCF_000239415.1	3
Labrus bergylta	GCF_900080235.1	3
Larimichthys crocea	GCF_000972845.2	3
Mastacembelus armatus	GCF_900324485.1	3
Pangasianodon hypophthalmus	GCF_003671635.1	3
Poecilia latipinna	GCF_001443285.1	3
Scleropages formosus	GCA_001005745.2	3
Tetraodon nigroviridis	GCA_000180735.1	3
Biomphalaria glabrata	GCF_000457365.1	3

GCA	taxonomy	count
Macrostomum lignano	GCA_002269645.1	2
Centruroides sculpturatus	GCF_000671375.1	2
Parasteatoda tepidariorum	GCF_000365465.2	2
Ciona intestinalis	GCF_000224145.3	2
Acinonyx jubatus	GCF_003709585.1	2
Aptenodytes forsteri	GCA_000699145.1	2
Chaetura pelagica	GCA_000747805.1	2
Colinus virginianus	GCA_000599465.2	2
Cricetulus griseus	GCA_000448345.1	2
Cuculus canorus	GCA_000709325.1	2
Egretta garzetta	GCA_000687185.1	2
Falco peregrinus	GCF_000337955.1	2
Gorilla gorilla gorilla	GCF_008122165.1	2
Haliaeetus leucocephalus	GCF_000737465.1	2
Macaca mulatta	GCA_000230795.1	2
Macaca mulatta	GCF_003339765.1	2
Mandrillus leucophaeus	GCF_000951045.1	2
Melopsittacus undulatus	GCF_000238935.1	2
Nothoprocta perdicaria	GCF_003342845.1	2
Pan troglodytes	GCA_002880755.3	2
Physeter catodon	GCF_002837175.2	2
Puma concolor	GCF_003327715.1	2
Rhinopithecus roxellana	GCF_007565055.1	2
Tupaia chinensis	GCA_000334495.1	2
Vicugna pacos	GCF_000164845.2	2
Vulpes vulpes	GCF_003160815.1	2
Zonotrichia albicollis	GCF_000385455.1	2
Acanthisitta chloris	GCA_000695815.1	2
Apteryx rowi	GCF_003343035.1	2
Phalacrocorax carbo	GCF_000708925.1	2
Rana catesbeiana	GCA_002284835.2	2
Xenopus tropicalis	GCA_000004195.3	2
Anas platyrhynchos	GCA_000355885.1	2
Anas platyrhynchos	GCF_003850225.1	2

GCA	taxonomy	count
Anser cygnoides domesticus	GCF_000971095.1	2
Antrostomus carolinensis	GCA_000700745.1	2
Apaloderma vittatum	GCA_000703405.1	2
Apaloderma vittatum	GCF_000703405.1	2
Aquila chrysaetos canadensis	GCF_000766835.1	2
Balearica regulorum gibbericeps	GCA_000709895.1	2
Balearica regulorum gibbericeps	GCF_000709895.1	2
Buceros rhinoceros silvestris	GCA_000710305.1	2
Buceros rhinoceros silvestris	GCF_000710305.1	2
Camelus bactrianus	GCF_000767855.1	2
Cathartes aura	GCA_000699945.1	2
Chlamydotis macqueenii	GCA_000695195.1	2
Chlorocebus sabaeus	GCF_000409795.2	2
Colius striatus	GCA_000690715.1	2
Cricetulus griseus	GCF_000223135.1	2
Equus przewalskii	GCF_000696695.1	2
Eurypyga helias	GCA_000690775.1	2
Fulmarus glacialis	GCA_000690835.1	2
Gavia stellata	GCA_000690875.1	2
Gavialis gangeticus	GCF_001723915.1	2
Haliaeetus albicilla	GCF_000691405.1	2
Leptosomus discolor	GCA_000691785.1	2
Lonchura striata domestica	GCA_002197715.1	2
Macaca fascicularis	GCF_000364345.1	2
Merops nubicus	GCA_000691845.1	2
Merops nubicus	GCF_000691845.1	2
Mesitornis unicolor	GCA_000695765.1	2
Microcebus murinus	GCF_000165445.2	2
Mus pahari	GCF_900095145.1	2
Myotis davidii	GCA_000327345.1	2
Nestor notabilis	GCF_000696875.1	2
Nipponia nippon	GCA_000708225.1	2
Nomascus leucogenys	GCF_006542625.1	2
Opisthocomus hoazin	GCF_000692075.1	2

GCA	taxonomy	count
Pelecanus crispus	GCA_000687375.1	2
Phaethon lepturus	GCA_000687285.1	2
Phalacrocorax carbo	GCA_000708925.1	2
Phoenicopterus ruber ruber	GCA_000687265.1	2
Podiceps cristatus	GCA_000699545.1	2
Pterocles gutturalis	GCA_000699245.1	2
Pterocles gutturalis	GCF_000699245.1	2
Pygoscelis adeliae	GCF_000699105.1	2
Rattus norvegicus	GCF_000001895.5	2
Rhinopithecus bieti	GCF_001698545.1	2
Saimiri boliviensis boliviensis	GCF_000235385.1	2
Tauraco erythrolophus	GCA_000709365.1	2
Tauraco erythrolophus	GCF_000709365.1	2
Tursiops truncatus	GCF_001922835.1	2
Tyto alba	GCA_000687205.1	2
Astatotilapia calliptera	GCF_900246225.1	2
Poecilia mexicana	GCF_001443325.1	2
Scleropages formosus	GCF_900964775.1	2
Takifugu rubripes	GCF_901000725.2	2
Drosophila serrata	GCF_002093755.1	2
Mizuhopecten yessoensis	GCA_002113885.2	2
Pomacea canaliculata	GCA_003073045.1	2
Clonorchis sinensis	GCA_003604175.1	1
Echinococcus granulosus	GCF_000524195.1	1
Echinococcus multilocularis	GCA_000469725.3	1
Hymenolepis microstoma	GCA_000469805.2	1
Opisthorchis viverrini	GCF_000715545.1	1
Schistosoma mansoni	GCF_000237925.1	1
Stegodyphus mimosarum	GCA_000611955.2	1
Nephila clavipes	GCA_002102615.1	1
Euroglyphus maynei	GCA_002135145.1	1
Galendromus occidentalis	GCF_000255335.1	1
Priapulus caudatus	GCF_000485595.1	1
Sarcoptes scabiei	GCA_000828355.1	1

GCA	taxonomy	count
Tetranychus urticae	GCF_000239435.1	1
Varroa destructor	GCF_002443255.1	1
Varroa jacobsoni	GCF_002532875.1	1
Helobdella robusta	GCF_000326865.1	1
Canis lupus dingo	GCF_003254725.1	1
Chelonia mydas	GCF_000344595.1	1
Columba livia	GCA_000337935.2	1
Lonchura striata domestica	GCF_005870125.1	1
Mus musculus	GCA_000002165.1	1
Cricetulus griseus	GCA_003668045.1	1
Ailuropoda melanoleuca	GCA_000004335.1	1
Falco cherrug	GCF_000337975.1	1
Fukomys damarensis	GCA_000743615.1	1
Limosa lapponica baueri	GCA_002844005.1	1
Macaca nemestrina	GCF_000956065.1	1
Myotis brandtii	GCA_000412655.1	1
Pan troglodytes	GCF_002880755.1	1
Patagioenas fasciata monilis	GCA_002029285.1	1
Pteropus alecto	GCA_000325575.1	1
Vicugna pacos	GCF_000164845.1	1
Ophiophagus hannah	GCA_000516915.1	1
Gulo gulo	GCA_900006375.2	1
Athene cunicularia	GCF_003259725.1	1
Calypte anna	GCA_000699085.1	1
Chlamydotis macqueenii	GCF_000695195.1	1
Corvus cornix cornix	GCF_000738735.2	1
Cricetulus griseus	GCA_000223135.1	1
Dromaius novaehollandiae	GCF_003342905.1	1
Erythrura gouldiae	GCA_003676055.1	1
Eurypyga helias	GCF_000690775.1	1
Fulmarus glacialis	GCF_000690835.1	1
Gavia stellata	GCF_000690875.1	1
Geospiza fortis	GCF_000277835.1	1
Haliaeetus albicilla	GCA_000691405.1	1

GCA	taxonomy	count
Leptosomus discolor	GCF_000691785.1	1
Manacus vitellinus	GCA_000692015.2	1
Manacus vitellinus	GCF_001715985.3	1
Mesitornis unicolor	GCF_000695765.1	1
Nestor notabilis	GCA_000696875.1	1
Opisthocomus hoazin	GCA_000692075.1	1
Pelecanus crispus	GCF_000687375.1	1
Picoides pubescens	GCA_000699005.1	1
Pygoscelis adeliae	GCA_000699105.1	1
Serinus canaria	GCF_007115625.1	1
Terrapene mexicana triunguis	GCF_002925995.2	1
Tinamus guttatus	GCA_000705375.2	1
Tyto alba	GCF_000687205.1	1
Urocitellus parryii	GCF_003426925.1	1
Ursus arctos horribilis	GCF_003584765.1	1
Anabarilius grahami	GCA_003731715.1	1
Tachysurus fulvidraco	GCF_003724035.1	1
Nematostella vectensis	GCA_000209225.1	1
Daphnia magna	GCA_001632505.1	1
Eurytemora affinis	GCF_000591075.1	1
Hyalella azteca	GCF_000764305.1	1
Strongylocentrotus purpuratus	GCF_000002235.5	1
Acromyrmex echinatior	GCF_000204515.1	1
Acyrthosiphon pisum	GCF_005508785.1	1
Aedes aegypti	GCA_000004015.3	1
Aedes aegypti	GCF_002204515.2	1
Aedes albopictus	GCA_001444175.2	1
Amyelois transitella	GCF_001186105.1	1
Anopheles darlingi	GCA_000211455.3	1
Anopheles gambiae str. PEST	GCF_000005575.2	1
Anopheles sinensis	GCA_000441895.2	1
Anoplophora glabripennis	GCF_000390285.2	1
Apis cerana	GCF_001442555.1	1
Apis cerana cerana	GCA_002290385.1	1

GCA	taxonomy	count
Apis mellifera	GCF_003254395.2	1
Athalia rosae	GCF_000344095.2	1
Atta cephalotes	GCF_000143395.1	1
Atta colombica	GCA_001594045.1	1
Atta colombica	GCF_001594045.1	1
Bactrocera latifrons	GCF_001853355.1	1
Bactrocera oleae	GCF_001188975.1	1
Bemisia tabaci	GCF_001854935.1	1
Bicyclus anynana	GCF_900239965.1	1
Blattella germanica	GCA_003018175.1	1
Bombus impatiens	GCF_000188095.3	1
Bombus terrestris	GCF_000214255.1	1
Bombyx mori	GCF_000151625.1	1
Camponotus floridanus	GCA_000147175.1	1
Camponotus floridanus	GCF_003227725.1	1
Cephus cinctus	GCF_000341935.1	1
Ceratina calcarata	GCF_001652005.1	1
Ceratitis capitata	GCF_000347755.3	1
Ceratosolen solmsi marchali	GCF_000503995.1	1
Cimex lectularius	GCF_000648675.2	1
Clunio marinus	GCA_900005825.1	1
Copidosoma floridanum	GCF_000648655.2	1
Cryptotermes secundus	GCF_002891405.2	1
Culex quinquefasciatus	GCF_000209185.1	1
Cyphomyrmex costatus	GCA_001594065.1	1
Cyphomyrmex costatus	GCF_001594065.1	1
Danaus plexippus plexippus	GCA_000235995.2	1
Dendroctonus ponderosae	GCF_000355655.1	1
Diachasma alloeum	GCF_001412515.2	1
Diaphorina citri	GCF_000475195.1	1
Dinoponera quadriceps	GCF_001313825.1	1
Diuraphis noxia	GCF_001186385.1	1
Drosophila ananassae	GCF_000005115.1	1
Drosophila arizonae	GCF_001654025.1	1

GCA	taxonomy	count
Drosophila biarmipes	GCF_000233415.1	1
Drosophila bipectinata	GCF_000236285.1	1
Drosophila busckii	GCA_001277935.1	1
Drosophila busckii	GCF_001277935.1	1
Drosophila elegans	GCF_000224195.1	1
Drosophila eugracilis	GCF_000236325.1	1
Drosophila ficusphila	GCF_000220665.1	1
Drosophila grimshawi	GCF_000005155.2	1
Drosophila kikkawai	GCF_000224215.1	1
Drosophila miranda	GCF_000269505.1	1
Drosophila mojavensis	GCF_000005175.2	1
Drosophila obscura	GCF_002217835.1	1
Drosophila pseudoobscura pseudoobscura	GCF_000001765.3	1
Drosophila rhopaloa	GCF_000236305.1	1
Drosophila sechellia	GCF_000005215.3	1
Drosophila suzukii	GCF_000472105.1	1
Drosophila takahashii	GCF_000224235.1	1
Drosophila virilis	GCF_000005245.1	1
Drosophila willistoni	GCA_000005925.1	1
Drosophila willistoni	GCF_000005925.1	1
Drosophila yakuba	GCF_000005975.2	1
Dufourea novaeangliae	GCA_001272555.1	1
Dufourea novaeangliae	GCF_001272555.1	1
Eufriesea mexicana	GCA_001483705.1	1
Eufriesea mexicana	GCF_001483705.1	1
Folsomia candida	GCA_002217175.1	1
Folsomia candida	GCF_002217175.1	1
Fopius arisanus	GCF_000806365.1	1
Habropoda laboriosa	GCF_001263275.1	1
Halyomorpha halys	GCF_000696795.2	1
Harpegnathos saltator	GCA_000147195.1	1
Harpegnathos saltator	GCF_003227715.1	1
Helicoverpa armigera	GCA_002156985.1	1
Helicoverpa armigera	GCF_002156985.1	1

GCA	taxonomy	count
Heliothis virescens	GCA_002382865.1	1
Leptinotarsa decemlineata	GCF_000500325.1	1
Linepithema humile	GCF_000217595.1	1
Lucilia cuprina	GCA_001187945.1	1
Lucilia cuprina	GCF_000699065.1	1
Megachile rotundata	GCF_000220905.1	1
Melanaphis sacchari	GCF_002803265.2	1
Melipona quadrifasciata	GCA_001276565.1	1
Microplitis demolitor	GCF_000572035.2	1
Monomorium pharaonis	GCF_003260585.2	1
Musca domestica	GCF_000371365.1	1
Myzus persicae	GCF_001856785.1	1
Nasonia vitripennis	GCF_000002325.3	1
Neodiprion lecontei	GCF_001263575.1	1
Nicrophorus vespilloides	GCF_001412225.1	1
Nilaparvata lugens	GCF_000757685.1	1
Onthophagus taurus	GCF_000648695.1	1
Operophtera brumata	GCA_001266575.1	1
Orussus abietinus	GCF_000612105.2	1
Oryctes borbonicus	GCA_001443705.1	1
Papilio machaon	GCA_001298355.1	1
Papilio machaon	GCF_001298355.1	1
Papilio polytes	GCF_000836215.1	1
Papilio xuthus	GCA_001298345.1	1
Papilio xuthus	GCF_000836235.1	1
Pediculus humanus corporis	GCF_000006295.1	1
Pieris rapae	GCF_001856805.1	1
Plutella xylostella	GCF_000330985.1	1
Pogonomyrmex barbatus	GCF_000187915.1	1
Polistes canadensis	GCF_001313835.1	1
Polistes dominula	GCF_001465965.1	1
Pseudomyrmex gracilis	GCF_002006095.1	1
Rhagoletis zephyria	GCF_001687245.1	1
Sipha flava	GCF_003268045.1	1

GCA	taxonomy	count
Spodoptera litura	GCF_002706865.1	1
Stomoxys calcitrans	GCF_001015335.1	1
Temnothorax curvispinosus	GCF_003070985.1	1
Trachymyrmex cornetzi	GCF_001594075.1	1
Trachymyrmex septentrionalis	GCA_001594115.1	1
Trachymyrmex septentrionalis	GCF_001594115.1	1
Trachymyrmex zeteki	GCA_001594055.1	1
Trachymyrmex zeteki	GCF_001594055.1	1
Tribolium castaneum	GCF_000002335.3	1
Trichogramma pretiosum	GCF_000599845.2	1
Trichomalopsis sarcophagae	GCA_002249905.1	1
Vollenhovia emeryi	GCF_000949405.1	1
Wasmannia auropunctata	GCF_000956235.1	1
Zeugodacus cucurbitae	GCF_000806345.1	1
Zootermopsis nevadensis	GCF_000696155.1	1
Acromyrmex echinatior	GCA_000204515.1	1
Apis florea	GCF_000184785.2	1
Habropoda laboriosa	GCA_001263275.1	1
Ooceraea biroi	GCA_000611835.1	1
Trachymyrmex cornetzi	GCA_001594075.1	1
Octopus bimaculoides	GCA_001194135.1	1
Toxocara canis	GCA_000803305.1	1
Trichinella britovi	GCA_001447585.1	1
Trichinella murrelli	GCA_001447425.1	1
Trichinella nativa	GCA_001447565.1	1
Trichinella nelsoni	GCA_001447455.1	1
Trichinella papuae	GCA_001447755.1	1
Trichinella patagoniensis	GCA_001447655.1	1
Trichinella pseudospiralis	GCA_001447445.1	1
Trichinella pseudospiralis	GCA_001447675.1	1
Trichinella pseudospiralis	GCA_001447575.1	1
Trichinella pseudospiralis	GCA_001447725.1	1
Trichinella sp. T6	GCA_001447435.1	1
Trichinella sp. T8	GCA_001447745.1	1

GCA	taxonomy	count
Trichinella sp. T9	GCA_001447505.1	1
Trichinella spiralis	GCA_001447595.1	1
Trichinella spiralis	GCF_000181795.1	1
Trichinella zimbabwensis	GCA_001447665.1	1
Trichuris suis	GCA_000701005.1	1
Trichuris suis	GCA_000701025.1	1
Trichoplax adhaerens	GCA_000150275.1	1
Trichoplax adhaerens	GCF_000150275.1	1
