# Supplementary material for: Bacterial death and TRADD-N domains help define novel apoptosis and immunity mechanisms shared by prokaryotes and metazoans
Source: eLife. 2021 Jun 1;10:e70394. doi: 10.7554/eLife.70394 (PMC8195603; doi:10.7554/eLife.70394)
Supplement: Figure 6—source data 1. [file elife-70394-fig6-data1.pdf]

Supplementary Material

Bacterial Death and TRADD-N domains help define novel apoptosis and immunity mechanisms shared by prokaryotes and metazoans

Gurmeet Kaur†, Lakshminarayan M. Iyer†, A. Maxwell Burroughs, L. Aravind*

Computational Biology Branch, National Center for Biotechnology Information, National Library of Medicine, National Institutes of Health, Bethesda, MD 20894, USA

†These authors contributed equally to the manuscript

Abstract

Several homologous domains are shared by eukaryotic immunity and programmed cell-death systems and poorly understood bacterial proteins. Recent studies show these to be components of a network of highly regulated systems connecting apoptotic processes to counter-invader immunity, in prokaryotes with a multicellular habit. However, the provenance of key adaptor domains, namely those of the Death-like and TRADD-N superfamilies, a quintessential feature of metazoan apoptotic systems, remained murky. Here, we use sensitive sequence analysis and comparative genomics methods to identify unambiguous bacterial homologs of the Death-like and TRADD-N superfamilies. We show the former to have arisen as part of a radiation of effector-associated α-helical adaptor domains that likely mediate homotypic interactions bringing together diverse effector and signaling domains in predicted bacterial apoptosis- and counter-invader systems. Similarly, we show that the TRADD-N domain defines a key, widespread signaling bridge that links effector deployment to invader-sensing in multicellular bacterial and metazoan counter-invader systems. TRADD-N domains are expanded in aggregating marine invertebrates and point to distinctive diversifying immune strategies probably directed both at RNA and retro- viruses and cellular pathogens that might infect such communities. These TRADD-N and Death-like domains helped identify several new bacterial and metazoan counter-invader systems featuring under-appreciated, common functional principles: the use of intracellular invader-sensing lectin-like (NPCBM and FGS), transcription elongation GreA/B-C, glycosyltransferase-4 family, inactive NTPase (serving as nucleic-acid-receptors) and invader-sensing GTPase switch domains. Finally, these findings point to the possibility of multicellular bacteria-stem metazoan symbiosis in the emergence of the immune/apoptotic systems of the latter.

Supplementary Material

Bacterial Death and TRADD-N domains help define novel apoptosis and immunity mechanisms shared by prokaryotes and metazoans

Figure 6-Source data 1

Gurmeet Kaur†, Lakshminarayan M. Iyer†, A. Maxwell Burroughs, L. Aravind*

Computational Biology Branch, National Center for Biotechnology Information, National Library of Medicine, National Institutes of Health, Bethesda, MD 20894, USA

†These authors contributed equally to the manuscript

Index.

1. Source data. Gene neighborhoods and domain architectures of the bacterial GT4-like glycosyltransferases described in this study
2. Source data. Domain architectures of eukaryotic GT4s with a special emphasis on the LSE in Acropora.
3. Source data. Gene neighborhoods and domain architectures of the MNS-like STAND domains (nSTAND2) that is present in a 2-gene operon system each containing a STAND NTPase
4. Source data. Gene neighborhoods and domain architectures of prokaryotic AP-GTPases described in this study

Source data. Gene neighborhoods and domain architectures of the bacterial GT4-like glycosyltransferases described in this study

<table>
<tr><th>acc</th><th>operon</th><th>architecture</th><th>len</th><th>gen.name</th><th>taxend</th><th>species</th><th>defline</th><th>gca</th></tr>
<tr><td>ADU34882.1</td><td>GT4+B12-binding+RADICAL-SAM*→</td><td>GT4+B12-binding+RADICAL-SAM</td><td>1405</td><td>Varpa_0662</td><td>Betaproteobacteria</td><td>Variovorax paradoxus EPS</td><td>Radical SAM domain protein [Variovorax paradoxus EPS]</td><td>GCA_000184745.1</td></tr>
<tr><td>AEV82483.1</td><td>WXG+GT4+GT4+REase*→</td><td>WXG+GT4+GT4+REase</td><td>4143</td><td>inaW</td><td>Actinobacteria</td><td>Actinoplanes sp. SE50/110</td><td>Ice nucleation protein [Actinoplanes sp. SE50/110]</td><td>GCA_000237145.1</td></tr>
<tr><td>AMB84622.1</td><td>APATPase+GT4*→</td><td>APATPase+GT4</td><td>1299</td><td>AWM79_04595</td><td>Gammaproteobacteria</td><td>Pseudomonas agarici</td><td>hypothetical protein AWM79_04595 [Pseudomonas agarici]</td><td>GCA_001543125.1</td></tr>
<tr><td>ANP51773.1</td><td>Caspase+NACHT+GT4*→||||<-?<-?<-?||?→?→||||<-?<-MFS</td><td>Caspase+NACHT+GT4</td><td>1134</td><td>AVL59_21225</td><td>Actinobacteria</td><td>Streptomyces griseochromogenes</td><td>hypothetical protein AVL59_21225 [Streptomyces griseochromogenes]</td><td>GCA_001542625.2</td></tr>
<tr><td>AWZ06155.1</td><td>Caspase+NACHT+GT4*→||||<-?<-?<-?<-?||APATPase→</td><td>Caspase+NACHT+GT4</td><td>1156</td><td>DRB89_17720</td><td>Actinobacteria</td><td>Streptomyces sp. ICC4</td><td>hypothetical protein DRB89_17720 [Streptomyces sp. ICC4]</td><td>GCA_003287915.1</td></tr>
<tr><td>BAO61700.1</td><td>SWACOS+GT4*→?→?→||||<-?<-?||MFS→</td><td>SWACOS+GT4</td><td>1084</td><td>PPC_2353</td><td>Gammaproteobacteria</td><td>Pseudomonas protegens Cab57</td><td>hypothetical protein PPC_2353 [Pseudomonas protegens Cab57]</td><td>GCA_000828695.1</td></tr>
<tr><td>CEL20906.1</td><td>ART+Caspase+GT4*→?→ GT4→</td><td>ART+Caspase+GT4</td><td>1145</td><td>BN1199_RS39095</td><td>Actinobacteria</td><td>Kibdelosporangium sp. MJ126-NF4</td><td>hypothetical protein [Kibdelosporangium sp. MJ126-NF4]</td><td>GCA_000826545.1</td></tr>
<tr><td>CUU59694.1</td><td>TPR+Caspase→ TPR+Caspase→||||<-CATASP||CATRA-N+CATRA-C+GT4*→||||<-?||HTH+APATPase+TPR→</td><td>CATRA-N+CATRA-C+GT4</td><td>904</td><td>Ga0074812_13074</td><td>Actinobacteria</td><td>Frankia irregularis</td><td>Glycosyltransferase involved in cell wall bisynthesis [Frankia irregularis]</td><td>GCA_001536285.1</td></tr>
<tr><td>EIV94947.1</td><td>CATRA-N+CATRA-C+GT4*→||||<-TPR+Caspase<-?<-?<-?<-?||APATPase+TPR→ CATASP→</td><td>CATRA-N+CATRA-C+GT4</td><td>810</td><td>FraQA3DRAFT_4743</td><td>Actinobacteria</td><td>Frankia sp. QA3</td><td>glycosyltransferase [Frankia sp. QA3]</td><td>GCA_000262465.1</td></tr>
</table>

<table>
<thead>
<tr><th>acc</th><th>operon</th><th>architecture</th><th>len</th><th>gen.name</th><th>taxend</th><th>species</th><th>defline</th><th>gca</th></tr>
</thead>
<tbody>
<tr><td>EKX63918.1</td><td>ZetaToxin+GT4+NUDIX+ART+NTase*→?→?→||||<-Caspase<-?||?→?→||||<-NLPC</td><td>ZetaToxin+GT4+NUDIX+ART+NTase</td><td>2156</td><td>STRIP9103_04937</td><td>Actinobacteria</td><td>Streptomyces ipomoeae 91-03</td><td>hydrolase, NUDIX family [Streptomyces ipomoeae 91-03]</td><td>GCA_000317595.1</td></tr>
<tr><td>EWM12074.1</td><td><-CATASP||CATRA-N+CATRA-C+GT4*→||||<-HTH+APATPase+TPR</td><td>CATRA-N+CATRA-C+GT4</td><td>750</td><td>KUTG_02378</td><td>Actinobacteria</td><td>Kutzneria sp. 744</td><td>LigA protein [Kutzneria sp. 744]</td><td>GCA_000568255.1</td></tr>
<tr><td>GBE24927.1</td><td>Asparaginase_II→ Acetyltransf_6→ GT4*→?→?→?→||||<-?<-SIG+TM+TM+TM<-THUMP+ThiI</td><td>GT4</td><td>407</td><td>mshA</td><td>unclassified Bacteria</td><td>bacterium BMS3Bbin02</td><td>D-inositol 3-phosphate glycosyltransferase [bacterium BMS3Bbin02]</td><td>GCA_002898115.1</td></tr>
<tr><td>HBN24878.1</td><td>GT4*→</td><td>GT4</td><td>721</td><td>DD447_07855</td><td>Firmicutes</td><td>Lachnospiraceae bacterium</td><td>TPA: hypothetical protein DD447_07855 [Lachnospiraceae bacterium]</td><td>GCA_003503295.1</td></tr>
<tr><td>HCI14011.1</td><td>GT4+APATPase+TPR*→</td><td>GT4+APATPase+TPR</td><td>1340</td><td>DFK12_08625</td><td>Betaproteobacteria</td><td>Gallionellaceae bacterium</td><td>TPA: hypothetical protein DFK12_08625 [Gallionellaceae bacterium]</td><td>GCA_003521785.1</td></tr>
<tr><td>HCS09332.1</td><td><-MODE-HTH+PBPII||?→||||<-?<-PSE<-PrpF||?→ PSE→ STAND+TPR+GT4*→</td><td>STAND+TPR+GT4</td><td>1081</td><td>DIW67_19725</td><td>Gammaproteobacteria</td><td>Pseudomonas sp.</td><td>TPA: glycosyltransferase [Pseudomonas sp.]</td><td>GCA_003531165.1</td></tr>
<tr><td>HDL41796.1</td><td>GT4*→</td><td>GT4</td><td>345</td><td>ENG98_02120</td><td>Actinobacteria</td><td>Actinobacteria bacterium</td><td>TPA: glycosyltransferase, partial [Actinobacteria bacterium]</td><td>GCA_011041235.1</td></tr>
<tr><td>KDN80799.1</td><td>MNS+GT4*→||||<-?||?→ ABC_tran→</td><td>MNS+GT4</td><td>1137</td><td>KCH_74340</td><td>Actinobacteria</td><td>Kitasatospora cheerisanensis KCTC 2395</td><td>hypothetical protein KCH_74340 [Kitasatospora cheerisanensis KCTC 2395]</td><td>GCA_000696185.1</td></tr>
<tr><td>KNY05162.1</td><td>GT4*→</td><td>GT4</td><td>533</td><td>AKH00_12305</td><td>Actinobacteria</td><td>Microbacterium sp. GCS4</td><td>hypothetical protein AKH00_12305 [Microbacterium sp. GCS4]</td><td>GCA_001262495.1</td></tr>
<tr><td>KOX13006.1</td><td><-ABC_tran||?→?→||||<-?<-TIR+NACHT||HTH+APATPase+TPR+GT4*→</td><td>HTH+APATPase+TPR+GT4</td><td>1158</td><td>ADK67_45665</td><td>Actinobacteria</td><td>Saccharothrix sp. NRRL B-16348</td><td>NTPase [Saccharothrix sp. NRRL B-16348]</td><td>GCA_001280085.1</td></tr>
<tr><td>KQM59620.1</td><td>GT4*→||||<-TRPR-HTH+PBPI<-?<-?<-?<-?||PSE→?→ PfkB→</td><td>GT4</td><td>155</td><td>ASE64_09860</td><td>Actinobacteria</td><td>Agreia sp. Leaf210</td><td>hypothetical protein ASE64_09860 [Agreia sp. Leaf210]</td><td>GCA_001421485.1</td></tr>
</tbody>
</table>

<table>
<tr><th>acc</th><th>operon</th><th>architecture</th><th>len</th><th>gen.name</th><th>taxend</th><th>species</th><th>defline</th><th>gca</th></tr>
<tr><td>KQW27056.1</td><td>STAND+GT4*→</td><td>STAND+GT4</td><td>1005</td><td>ASC85_27055</td><td>Gammaproteobacteria</td><td>Pseudomonas sp. Root401</td><td>hypothetical protein ASC85_27055 [Pseudomonas sp. Root401]</td><td>GCA_001425105.1</td></tr>
<tr><td>KVF32402.1</td><td><-SIG+RCDG1+YidC__periplas+60KD_IMP<-?<-?<-?||?→?→?→ GT4+B12-binding+RADICAL-SAM*→||||<-rve_3</td><td>GT4+B12-binding+RADICAL-SAM</td><td>1400</td><td>WJ08_12515</td><td>Betaproteobacteria</td><td>Burkholderia vietnamiensis</td><td>radical SAM protein [Burkholderia vietnamiensis]</td><td>GCA_001524025.1</td></tr>
<tr><td>KVM90153.1</td><td>GT4+B12-binding+RADICAL-SAM*→</td><td>GT4+B12-binding+RADICAL-SAM</td><td>1404</td><td>WT05_03580</td><td>Betaproteobacteria</td><td>Burkholderia stagnalis</td><td>radical SAM protein [Burkholderia stagnalis]</td><td>GCA_001527545.1</td></tr>
<tr><td>KVM94412.1</td><td>TniB→?→ NLPC→||||<-?||?→ GT4+B12-binding+RADICAL-SAM*→</td><td>GT4+B12-binding+RADICAL-SAM</td><td>1404</td><td>WT07_28140</td><td>Betaproteobacteria</td><td>Burkholderia stagnalis</td><td>radical SAM protein [Burkholderia stagnalis]</td><td>GCA_001527585.1</td></tr>
<tr><td>KVX13946.1</td><td>GT4*→||||<-?||ABhydrolase→||||<-TRANSGLUTAMINASE</td><td>GT4</td><td>282</td><td>WL03_19390</td><td>Betaproteobacteria</td><td>Burkholderia ubonensis</td><td>hypothetical protein WL03_19390 [Burkholderia ubonensis]</td><td>GCA_001539165.1</td></tr>
<tr><td>KVZ11040.1</td><td>GT4+APATPase+TPR+TPR*→</td><td>GT4+APATPase+TPR+TPR</td><td>1278</td><td>WL11_04565</td><td>Betaproteobacteria</td><td>Burkholderia ubonensis</td><td>hypothetical protein WL11_04565 [Burkholderia ubonensis]</td><td>GCA_001530465.1</td></tr>
<tr><td>KWI84975.1</td><td>GT4*→||||<-?||ABhydrolase→||||<-TRANSGLUTAMINASE</td><td>GT4</td><td>325</td><td>WM08_23745</td><td>Betaproteobacteria</td><td>Burkholderia ubonensis</td><td>hypothetical protein WM08_23745 [Burkholderia ubonensis]</td><td>GCA_001534135.1</td></tr>
<tr><td>KWK82218.1</td><td>NACHT+TPR+GT4*→</td><td>NACHT+TPR+GT4</td><td>1024</td><td>WM17_17155</td><td>Betaproteobacteria</td><td>Burkholderia ubonensis</td><td>hypothetical protein WM17_17155 [Burkholderia ubonensis]</td><td>GCA_001533745.1</td></tr>
<tr><td>MBA3007143.1</td><td>APATPase+GT4+TPR+TPR+TPR+TPR+TPR*→</td><td>APATPase+GT4+TPR+TPR+TPR+TPR+TPR</td><td>1488</td><td>FP810_11305</td><td>Deltaproteobacteria</td><td>Desulfocapsa sp.</td><td>tetratricopeptide repeat protein [Desulfocapsa sp.]</td><td>GCA_013792135.1</td></tr>
<tr><td>MBA3548505.1</td><td>GT4*→</td><td>GT4</td><td>125</td><td>H0T76_18640</td><td>Deltaproteobacteria</td><td>Nannocystis sp.</td><td>glycosyltransferase [Nannocystis sp.]</td><td>GCA_013812955.1</td></tr>
<tr><td>MBS82255.1</td><td>NACHT+GT4*→?→?→?→||||<-?<-?<-SIG+rve</td><td>NACHT+GT4</td><td>1659</td><td>CMO32_42310</td><td>Betaproteobacteria</td><td>Variovorax sp.</td><td>hypothetical protein CMO32_42310 [Variovorax sp.]</td><td>GCA_002729445.1</td></tr>
</table>

acc	operon	architecture	len	gen.name	taxend	species	defline	gca
MQY06528.1	REase+SFI-ATPase→?→?→||||<-?||?→||||<-?||GT4+APATPase+TPR+TPR*→	GT4+APATPase+TPR+TPR	1104	mshA_10	Actinobacteria	Actinomadura sp. RB68	D-inositol-3-phosphate glycosyltransferase [Actinomadura sp. RB68]	GCA_009604375.1
MQY24997.1	ESX-1_EspG→||||<-?<-?||?→?→?→ WXG+TOXIN-PL+ART+Caspase+ZetaToxin+GT4*→	WXG+TOXIN-PL+ART+Caspase+ZetaToxin+GT4	3249	mshA_3	Actinobacteria	Nocardia sp. RB56	D-inositol-3-phosphate glycosyltransferase [Nocardia sp. RB56]	GCA_009604425.1
MSV29911.1	GT4+APATPase+BetaPropeller*→	GT4+APATPase+BetaPropeller	1058	EXQ52_14375	Acidobacteria	Bryobacterales bacterium	glycosyltransferase [Bryobacterales bacterium]	GCA_009697455.1
MWJ13606.1	ABC_tran→?→||||<-?||GT4*→||||<-?||?→?→?→||||<-?||tRNA→?→ Pribosyltran_N+Pribosyl_synth→	GT4	267	DOT98_12255	Actinobacteria	Clavibacter michiganensis subsp. michiganensis	hypothetical protein DOT98_12255 [Clavibacter michiganensis subsp. michiganensis]	GCA_009793225.1
NED51435.1	GT4*→	GT4	88	G3I24_11000	Actinobacteria	Micromonospora aurantiaca	glycosyl transferase family 1, partial [Micromonospora aurantiaca]	GCA_010550585.1
NNJ62389.1	DUF4913→?→ GT4*→	GT4	464	HKP61_15900	Actinobacteria	Dactylosporangium sp.	glycosyltransferase family 4 protein [Dactylosporangium sp.]	GCA_013041765.1
NOX21677.1	Asparaginase_II→ Acetyltransf_6→ GT4*→?→?→||||<-?<-SIG+TM+TM+TM<-THUMP+ThiI	GT4	407	GXP36_01090	Actinobacteria	Actinobacteria bacterium	glycosyltransferase [Actinobacteria bacterium]	GCA_013151445.1
NQZ33933.1	GT4+APATPase*→	GT4+APATPase	841	HRU06_21905	Gammaproteobacteria	Oceanospirillaceae bacterium	glycosyltransferase, partial [Oceanospirillaceae bacterium]	GCA_013215375.1
NTC82454.1	<-TniB<-?<-?<-?||?→?→?→ NACHT+GT4*→	NACHT+GT4	1640	G6M81_07325	Alphaproteobacteria	Agrobacterium tumefaciens	glycosyltransferase [Agrobacterium tumefaciens]	GCA_013319045.1
NUQ74496.1	SIR2+TPR+SAVED+GT4*→	SIR2+TPR+SAVED+GT4	1362	HUU21_13150	Deltaproteobacteria	Polyangiaceae bacterium	glycosyltransferase [Polyangiaceae bacterium]	GCA_013360665.1

<table>
<tr><th>acc</th><th>operon</th><th>architecture</th><th>len</th><th>gen.name</th><th>taxend</th><th>species</th><th>defline</th><th>gca</th></tr>
<tr><td>NUS43770.1</td><td>GT4*→</td><td>GT4</td><td>631</td><td>HOQ24_08805</td><td>Actinobacteria</td><td>Mycobacteriaceae bacterium</td><td>glycosyltransferase family 4 protein, partial [Mycobacteriaceae bacterium]</td><td>GCA_013362555.1</td></tr>
<tr><td>NUS72884.1</td><td>SIG+GT4+ZetaToxin*→</td><td>SIG+GT4+ZetaToxin</td><td>750</td><td>HOQ05_05695</td><td>Actinobacteria</td><td>Corynebacteriales bacterium</td><td>glycosyltransferase [Corynebacteriales bacterium]</td><td>GCA_013362805.1</td></tr>
<tr><td>NUT53093.1</td><td>SWACOS+GT4*→</td><td>SWACOS+GT4</td><td>1162</td><td>HOV94_38270</td><td>Actinobacteria</td><td>Saccharothrix sp.</td><td>glycosyltransferase [Saccharothrix sp.]</td><td>GCA_013362745.1</td></tr>
<tr><td>OAI03591.1</td><td>GT4+APATPase+TPR*→</td><td>GT4+APATPase+TPR</td><td>1115</td><td>A1332_15425</td><td>Gammaproteobacteria</td><td>Methylomonas methanica</td><td>hypothetical protein A1332_15425, partial [Methylomonas methanica]</td><td>GCA_001644035.1</td></tr>
<tr><td>ODT28534.1</td><td>GT4*→||||<-?<-?||?→||||<-?<-?||?→ NUDIX→</td><td>GT4</td><td>325</td><td>ABS63_03615</td><td>Actinobacteria</td><td>Microbacterium sp. SCN 70-27</td><td>hypothetical protein ABS63_03615 [Microbacterium sp. SCN 70-27]</td><td>GCA_001724425.1</td></tr>
<tr><td>OGS75014.1</td><td>GT4+APATPase+TPR*→</td><td>GT4+APATPase+TPR</td><td>1314</td><td>A2063_09475</td><td>Betaproteobacteria</td><td>Gallionellales bacterium GWA2_60_142</td><td>hypothetical protein A2063_09475 [Gallionellales bacterium GWA2_60_142]</td><td>GCA_001801085.1</td></tr>
<tr><td>OKJ80919.1</td><td><-TPR+Caspase<-CATASP||CATRA-N+CATRA-C+GT4*→ HTH+APATPase+TPR→?→?→||||<-?<-CATASP</td><td>CATRA-N+CATRA-C+GT4</td><td>694</td><td>AMK32_24710</td><td>Actinobacteria</td><td>Streptomyces sp. CB01883</td><td>hypothetical protein AMK32_24710 [Streptomyces sp. CB01883]</td><td>GCA_001905855.1</td></tr>
<tr><td>OLT27035.1</td><td>ZetaToxin+GT4+NUDIX+ART*→</td><td>ZetaToxin+GT4+NUDIX+ART</td><td>1451</td><td>BJF83_19460</td><td>Actinobacteria</td><td>Nocardiopsis sp. CNR-923</td><td>hypothetical protein BJF83_19460 [Nocardiopsis sp. CNR-923]</td><td>GCA_001942255.1</td></tr>
<tr><td>ONI48735.1</td><td><-TPR+Caspase<-CATASP||CATRA-N+CATRA-C+GT4*→ HTH+APATPase+TPR→||||<-HTH||?→?→?→||||<-?<-CATASP</td><td>CATRA-N+CATRA-C+GT4</td><td>727</td><td>mshA_4</td><td>Actinobacteria</td><td>Streptomyces sp. IB2014 011-1</td><td>D-inositol 3-phosphate glycosyltransferase [Streptomyces sp. IB2014 011-1]</td><td>GCA_001983595.1</td></tr>
<tr><td>OPK01862.1</td><td>GT4+B12-binding+RADICAL-SAM*→</td><td>GT4+B12-binding+RADICAL-SAM</td><td>1407</td><td>BZ164_25460</td><td>Gammaproteobacteria</td><td>Pseudomonas veronii</td><td>hypothetical protein BZ164_25460 [Pseudomonas veronii]</td><td>GCA_002029325.1</td></tr>
</table>

acc	operon	architecture	len	gen.name	taxend	species	defline	gca
OWQ88588.1	GT4+HD*→	GT4+HD	1415	CDN99_17225	Betaproteobacteria	Roseateles aquatilis	hypothetical protein CDN99_17225 [Roseateles aquatilis]	GCA_002205645.1
OXM47905.1	<-TIR+APATPase||HTH+APATPase+TPR+GT4*→	HTH+APATPase+TPR+GT4	1166	CFP75_23295	Actinobacteria	Amycolatopsis alba DSM 44262	NTPase [Amycolatopsis alba DSM 44262]	GCA_002234385.1
OZV80564.1	<-TPR+Caspase||GT4*→?→?→||||<-?<-?<-ABC_tran	GT4	587	CA850_14545	Actinobacteria	Micromonospora echinospora	hypothetical protein CA850_14545 [Micromonospora echinospora]	GCA_002266845.1
PBV03828.1	STAND+GT4*→	STAND+GT4	1006	CJU35_32055	Gammaproteobacteria	Pseudomonas aeruginosa	hypothetical protein CJU35_32055 [Pseudomonas aeruginosa]	GCA_002326115.1
PJI96546.1	GT4+B12-binding+RADICAL-SAM*→?→?→||||<-?<-LexA	GT4+B12-binding+RADICAL-SAM	1404	CLU85_1299	Betaproteobacteria	Acidovorax sp. 69	radical SAM superfamily enzyme YgiQ (UPF0313 family) [Acidovorax sp. 69]	GCA_002797445.1
PJO54021.1	GT4*→	GT4	721	CR156_18515	Gammaproteobacteria	Stenotrophomonas lactitubi	hypothetical protein CR156_18515 [Stenotrophomonas lactitubi]	GCA_002803515.1
PKN17915.1	NACHT+GT4*→	NACHT+GT4	1644	CVU71_10310	Deltaproteobacteria	Deltaproteobacteria bacterium HGW-Deltaproteobacteria-6	hypothetical protein CVU71_10310 [Deltaproteobacteria bacterium HGW-Deltaproteobacteria-6]	GCA_002840435.1
PKV83308.1	HTH→?→?→||||<-?<-?||Caspase+NACHT+GT4*→	Caspase+NACHT+GT4	1229	BX283_0808	Actinobacteria	Streptomyces sp. TLI_146	glycosyltransferase involved in cell wall biosynthesis [Streptomyces sp. TLI_146]	GCA_002846415.1
PNA01938.1	GT4+APATPase+TPR+TPR*→	GT4+APATPase+TPR+TPR	1383	C1X79_03750	Gammaproteobacteria	Pseudomonas sp. FW305-42	hypothetical protein C1X79_03750 [Pseudomonas sp. FW305-42]	GCA_002883975.1

<table>
<tr><th>acc</th><th>operon</th><th>architecture</th><th>len</th><th>gen.name</th><th>taxend</th><th>species</th><th>defline</th><th>gca</th></tr>
<tr><td>PRY58656.1</td><td>GT4*→ APATPase+TPR→||||<-?<-TRPR-HTH+PBPI</td><td>GT4</td><td>412</td><td>B0I28_105371</td><td>Actinobacteria</td><td>Glycomyces artemisiae</td><td>glycosyltransferase involved in cell wall biosynthesis [Glycomyces artemisiae]</td><td>GCA_003002955.1</td></tr>
<tr><td>PSR67860.1</td><td>WXG+MPTase→ WXG+LuxR-HTH+LuxR-HTH+LuxR-HTH+LuxR-HTH+TetR-HTH+TetR-HTH+LuxR-HTH+LuxR-HTH+LexA-HTH+LuxR-HTH+LuxR-HTH+LuxR-HTH+LuxR-HTH+LuxR-HTH+PP2C*→?→ ABhydrolase→</td><td>WXG+LuxR-HTH+LuxR-HTH+LuxR-HTH+LuxR-HTH+TetR-HTH+TetR-HTH+LuxR-HTH+LuxR-HTH+LexA-HTH+LuxR-HTH+LuxR-HTH+LuxR-HTH+LuxR-HTH+LuxR-HTH+PP2C</td><td>14198</td><td>C8258_13415</td><td>Actinobacteria</td><td>Nocardia sp. MDA0666</td><td>hypothetical protein C8258_13415 [Nocardia sp. MDA0666]</td><td>GCA_003023875.1</td></tr>
<tr><td>PXX02325.1</td><td>GT4+B12-binding+RADICAL-SAM*→</td><td>GT4+B12-binding+RADICAL-SAM</td><td>1404</td><td>C7400_1582</td><td>Betaproteobacteria</td><td>Paraburkholderia tropica</td><td>radical SAM superfamily enzyme YgiQ (UPF0313 family) [Paraburkholderia tropica]</td><td>GCA_003201665.1</td></tr>
<tr><td>PZM02119.1</td><td><-MODE-HTH+PBPII||?→||||<-MODE-HTH+PBPII||?→?→?→?→ NACHT+GT4*→</td><td>NACHT+GT4</td><td>1624</td><td>CPJ17_11030</td><td>Alphaproteobacteria</td><td>Rhizobium tumorigenes</td><td>hypothetical protein CPJ17_11030 [Rhizobium tumorigenes]</td><td>GCA_003240565.1</td></tr>
<tr><td>REF53875.1</td><td>SWACOS+GT4*→||||<-?<-MFS</td><td>SWACOS+GT4</td><td>1165</td><td>BX279_1810</td><td>Actinobacteria</td><td>Streptomyces sp. Ag82_O1-9</td><td>glycosyltransferase involved in cell wall biosynthesis [Streptomyces sp. Ag82_O1-9]</td><td>GCA_003386845.1</td></tr>
<tr><td>RII01628.1</td><td>STAND→?→?→ DUF4913→||||<-AbiEii<-AbiEi_4||?→ GT4*→?→ AbiEi_4→</td><td>GT4</td><td>476</td><td>NI17_16340</td><td>Actinobacteria</td><td>Thermobifida halotolerans</td><td>hypothetical protein NI17_16340 [Thermobifida halotolerans]</td><td>GCA_003574835.1</td></tr>
<tr><td>RKS05362.1</td><td><-APATPase+TPR||?→ GT4*→</td><td>GT4</td><td>412</td><td>DFP74_0957</td><td>Actinobacteria</td><td>Nocardiopsis sp. Huas11</td><td>glycosyltransferase involved in cell wall biosynthesis [Nocardiopsis sp. Huas11]</td><td>GCA_003634495.1</td></tr>
<tr><td>ROV69600.1</td><td><-ABC_tran<-?||PSE→ Caspase+NACHT+GT4*→</td><td>Caspase+NACHT+GT4</td><td>1187</td><td>D3105_04960</td><td>Actinobacteria</td><td>Streptomyces globisporus</td><td>glycosyltransferase [Streptomyces globisporus]</td><td>GCA_003501885.1</td></tr>
<tr><td>RSM57832.1</td><td>cNMP_binding+HTH_Crp_2→?→ GT4*→||||<-ACYC+APATPase+TPR</td><td>GT4</td><td>411</td><td>DMH03_26130</td><td>Actinobacteria</td><td>Amycolatopsis sp. WAC 01376</td><td>hypothetical protein DMH03_26130 [Amycolatopsis sp. WAC 01376]</td><td>GCA_003947415.1</td></tr>
<tr><td>RWH25252.1</td><td><-Vsr||?→?→?→||||<-?||GT4+Trypsin+PNPase*→</td><td>GT4+Trypsin+PNPase</td><td>961</td><td>EOQ76_19695</td><td>Alphaproteobacteria</td><td>Mesorhizobium sp.</td><td>glycosyltransferase [Mesorhizobium sp.]</td><td>GCA_004019165.1</td></tr>
</table>

acc	operon	architecture	len	gen.name	taxend	species	defline	gca
RYG90150.1	GT4*→	GT4	425	EON59_00395	Alphaproteobacteria	Alphaproteobacteria bacterium	glycosyltransferase, partial [Alphaproteobacteria bacterium]	GCA_004145145.1
RYZ67144.1	GT4*→	GT4	290	EOP09_11535	Proteobacteria	Proteobacteria bacterium	glycosyltransferase family 1 protein, partial [Proteobacteria bacterium]	GCA_004174565.1
SCF29539.1	<-TPR+Caspase||GT4*→?→?→||||<-?<-?<-ABC_tran	GT4	549	GA0070618_4943	Actinobacteria	Micromonospora echinospora	Glycosyltransferase involved in cell wall bisynthesis [Micromonospora echinospora]	GCA_900091495.1
SCF46304.1	<-WXG<-?<-?<-?<-?<-TPR+Caspase||GT4*→	GT4	549	GA0070563_114162	Actinobacteria	Micromonospora carbonacea	Glycosyltransferase involved in cell wall bisynthesis [Micromonospora carbonacea]	GCA_900091535.1
SCH34126.1	GT4*→ TIR→	GT4	721	SAMEA3545339_00864	Firmicutes	uncultured Clostridium sp.	glycosyltransferase%2C MSMEG_0565 family [uncultured Clostridium sp.]	GCA_900066565.1
SCL28302.1	<-TPR+Caspase||GT4*→	GT4	545	GA0074694_5072	Actinobacteria	Micromonospora inyonensis	Glycosyltransferase involved in cell wall bisynthesis [Micromonospora inyonensis]	GCA_900091415.1
SCZ75034.1	SWACOS+GT4*→?→||||<-?<-?||MFS→	SWACOS+GT4	1147	SAMN03159460_05301	Gammaproteobacteria	Pseudomonas sp. NFPP17	Glycosyltransferase involved in cell wall bisynthesis [Pseudomonas sp. NFPP17]	GCA_900102935.1
SDU29240.1	rve_3→ GT4*→	GT4	362	SAMN04488548_134376	Actinobacteria	Gordonia westfalica	Glycosyltransferase involved in cell wall bisynthesis [Gordonia westfalica]	GCA_900105725.1
SDX14297.1	LRR+AP-GTPase+COR→ GT4*→	GT4	472	SAMN05216300_1202	Betaproteobacteria	Nitrosomonas oligotropha	Glycosyltransferase involved in cell wall bisynthesis [Nitrosomonas oligotropha]	GCA_900106555.1

<table>
<tr><th>acc</th><th>operon</th><th>architecture</th><th>len</th><th>gen.name</th><th>taxend</th><th>species</th><th>defline</th><th>gca</th></tr>
<tr><td>SEK03344.1</td><td><-Sigma||?→||||<-?<-?<-?||GT4+B12-binding*→</td><td>GT4+B12-binding</td><td>1051</td><td>SAMN05518849_113037</td><td>Alphaproteobacteria</td><td>Sphingobium sp. AP50</td><td>Glycosyltransferase involved in cell wall bisynthesis [Sphingobium sp. AP50]</td><td>GCA_900109095.1</td></tr>
<tr><td>SIO56974.1</td><td>STAND+TPR+GT4*→</td><td>STAND+TPR+GT4</td><td>1125</td><td>SAMN05444165_3787</td><td>Betaproteobacteria</td><td>Paraburkholderia phenazinium</td><td>Glycosyltransferase involved in cell wall bisynthesis [Paraburkholderia phenazinium]</td><td>GCA_900142845.1</td></tr>
<tr><td>SNR45646.1</td><td>HTH_Tnp_1→ TnpB_IS66→?→ NACHT+GT4*→</td><td>NACHT+GT4</td><td>1639</td><td>SAMN06265370_105216</td><td>Alphaproteobacteria</td><td>Puniceibacterium sediminis</td><td>Glycosyltransferase involved in cell wall bisynthesis [Puniceibacterium sediminis]</td><td>GCA_900188035.1</td></tr>
<tr><td>TAK32775.1</td><td>GT4*→</td><td>GT4</td><td>646</td><td>EPO40_01735</td><td>Deltaproteobacteria</td><td>Myxococcaceae bacterium</td><td>glycosyltransferase [Myxococcaceae bacterium]</td><td>GCA_004297725.1</td></tr>
<tr><td>TCO61886.1</td><td>GT4*→||||<-TPR</td><td>GT4</td><td>392</td><td>EV192_10223</td><td>Actinobacteria</td><td>Actinocrispum wychmicini</td><td>glycosyltransferase involved in cell wall biosynthesis [Actinocrispum wychmicini]</td><td>GCA_004345645.1</td></tr>
<tr><td>TDP89614.1</td><td>GT4*→ TIR+APATPase+TPR→ TPR+Caspase→||||<-TPR+Caspase<-CATASP||CATRA-N+CATRA-C+GT4→</td><td>GT4</td><td>328</td><td>EV186_11214</td><td>Actinobacteria</td><td>Labedaea rhizosphaerae</td><td>glycosyltransferase involved in cell wall biosynthesis [Labedaea rhizosphaerae]</td><td>GCA_004362825.1</td></tr>
<tr><td>TGX49078.1</td><td>GT4+NACHT*→</td><td>GT4+NACHT</td><td>1219</td><td>E5A73_19725</td><td>Alphaproteobacteria</td><td>Sphingomonas gei</td><td>glycosyltransferase [Sphingomonas gei]</td><td>GCA_004792685.1</td></tr>
<tr><td>TPG40509.1</td><td>GT4*→</td><td>GT4</td><td>136</td><td>EAH79_11405</td><td>Alphaproteobacteria</td><td>Sphingomonas koreensis</td><td>glycosyltransferase [Sphingomonas koreensis]</td><td>GCA_006438735.1</td></tr>
<tr><td>TQL90659.1</td><td><-DUF4242<-?<-?<-PSE||?→||||<-HTH+APATPase+TPR<-TPR+Caspase<-CATASP||CATRA-N+CATRA-C+GT4*→||||<-?<-?<-?<-?<-?<-REase+SFI-ATPase</td><td>CATRA-N+CATRA-C+GT4</td><td>735</td><td>FB559_7970</td><td>Actinobacteria</td><td>Actinoallomurus bryophytorum</td><td>glycosyltransferase involved in cell wall biosynthesis [Actinoallomurus bryophytorum]</td><td>GCA_006716425.1</td></tr>
</table>

<table>
<tr><th>acc</th><th>operon</th><th>architecture</th><th>len</th><th>gen.name</th><th>taxend</th><th>species</th><th>defline</th><th>gca</th></tr>
<tr><td>TWH06591.1</td><td><-TPR+Caspase||GT4*→?→?→||||<-?<-?<-ABC_tran</td><td>GT4</td><td>587</td><td>JD80_04174</td><td>Actinobacteria</td><td>Micromonospora echinospora</td><td>glycosyltransferase involved in cell wall biosynthesis [Micromonospora echinospora]</td><td>GCA_007829475.1</td></tr>
<tr><td>TWJ29688.1</td><td><-TPR+Caspase||GT4*→?→||||<-?<-?<-ABC_tran</td><td>GT4</td><td>549</td><td>JD81_03199</td><td>Actinobacteria</td><td>Micromonospora sagamiensis</td><td>glycosyltransferase involved in cell wall biosynthesis [Micromonospora sagamiensis]</td><td>GCA_007829995.1</td></tr>
<tr><td>TXH45940.1</td><td>GT4+nSTAND1+ANK+ANK*→</td><td>GT4+nSTAND1+ANK+ANK</td><td>1838</td><td>E6Q92_02710</td><td>Betaproteobacteria</td><td>Burkholderiaceae bacterium</td><td>hypothetical protein E6Q92_02710 [Burkholderiaceae bacterium]</td><td>GCA_008012415.1</td></tr>
<tr><td>VBB15457.1</td><td>GT4+B12-binding+RADICAL-SAM*→</td><td>GT4+B12-binding+RADICAL-SAM</td><td>1404</td><td>BSTAB16_5653</td><td>Betaproteobacteria</td><td>Burkholderia stabilis</td><td>radical SAM protein [Burkholderia stabilis]</td><td>GCA_900240005.1</td></tr>
<tr><td>VBK75063.1</td><td>GT4+B12-binding+RADICAL-SAM*→</td><td>GT4+B12-binding+RADICAL-SAM</td><td>1404</td><td>SAMEA2064914_03874</td><td>Betaproteobacteria</td><td>Burkholderia pseudomallei</td><td>Glycogen synthase [Burkholderia pseudomallei]</td><td>GCA_900592755.1</td></tr>
<tr><td>WP_005483680.1</td><td>TRANSGLUTAMINASE→||||<-?<-Zeta_toxin<-?<-?||?→ GT4*→||||<-?<-?<-?<-?<-DUF4913</td><td>GT4</td><td>590</td><td>SBD_RS28370</td><td>Actinobacteria</td><td>Streptomyces</td><td>MULTISPECIES: glycosyltransferase [Streptomyces]</td><td>GCF_000340335.1</td></tr>
<tr><td>WP_007453552.1</td><td>Multi-TM→?→ STAND→?→ DUF4913→||||<-?||?→ GT4*→?→?→?→ NUDIX→||||<-?||?→ PSE→ ABC_tran→</td><td>GT4</td><td>437</td><td>MILUP08_RS00205</td><td>Actinobacteria</td><td>Micromonospora lupini</td><td>glycosyltransferase family 4 protein [Micromonospora lupini]</td><td>GCF_000297395.2</td></tr>
<tr><td>WP_007734957.1</td><td>STAND+TPR+GT4*→</td><td>STAND+TPR+GT4</td><td>1062</td><td>B5Z53_RS05165</td><td>Betaproteobacteria</td><td>Paraburkholderia hospita</td><td>glycosyltransferase [Paraburkholderia hospita]</td><td>GCF_900167965.1</td></tr>
<tr><td>WP_007954182.1</td><td>SQHop_cyclase_C+Prenyltrans+GT4*→?→||||<-?||?→||||<-?||?→ XylR_N+V4R+Sigma54_activat+HTH_8→</td><td>SQHop_cyclase_C+Prenyltrans+GT4</td><td>779</td><td>JBW_RS18725</td><td>Firmicutes</td><td>Pelosinus fermentans</td><td>glycosyltransferase [Pelosinus fermentans]</td><td>GCF_000271665.2</td></tr>
<tr><td>WP_010223837.1</td><td>GT4+STAND*→ TPR→||||<-MODE-HTH+PBPII</td><td>GT4+STAND</td><td>546</td><td>UW3_RS011698[illegible]</td><td>Gammaproteobacteria</td><td>Pseudomonas donghuensis</td><td>glycosyltransferase [Pseudomonas donghuensis]</td><td>GCF_000259195.1</td></tr>
<tr><td>WP_013226178.1</td><td><-TIR+APATPase||HTH+APATPase+TPR+GT4*→</td><td>HTH+APATPase+TPR+GT4</td><td>1178</td><td>IW14_RS45345</td><td>Actinobacteria</td><td>Amycolatopsis mediterranei</td><td>glycosyltransferase [Amycolatopsis mediterranei]</td><td>GCF_000196835.1</td></tr>
<tr><td>WP_017131143.1</td><td>APATPase+GT4*→</td><td>APATPase+GT4</td><td>1299</td><td>BMX68_RS1184[illegible]</td><td>Gammaproteobacteria</td><td>Pseudomonas agarici</td><td>glycosyltransferase [Pseudomonas agarici]</td><td>GCF_900109755.1</td></tr>
</table>

<table>
<tr><th>acc</th><th>operon</th><th>architecture</th><th>len</th><th>gen.name</th><th>taxend</th><th>species</th><th>defline</th><th>gca</th></tr>
<tr><td>WP_017594508.1</td><td>GT4*→</td><td>GT4</td><td>503</td><td>D459_RS0118565</td><td>Actinobacteria</td><td>Nocardiopsis potens</td><td>glycosyltransferase family 4 protein [Nocardiopsis potens]</td><td>GCF_000341105.1</td></tr>
<tr><td>WP_018327601.1</td><td><-TniB<-?||?→||||<-?<-?||GT4+B12-binding+RADICAL-SAM*→||||<-?<-?||SIG+rve→</td><td>GT4+B12-binding+RADICAL-SAM</td><td>1381</td><td>GGD55_RS15170</td><td>Alphaproteobacteria</td><td>Rhizobium giardinii</td><td>B12-binding domain-containing radical SAM protein [Rhizobium giardinii]</td><td>GCF_014200295.1</td></tr>
<tr><td>WP_018612920.1</td><td>STAND+TPR+GT4*→</td><td>STAND+TPR+GT4</td><td>1063</td><td>CFII68_RS03385</td><td>Gammaproteobacteria</td><td>Pseudomonas sp. CFII68</td><td>glycosyltransferase [Pseudomonas sp. CFII68]</td><td>GCF_000416195.1</td></tr>
<tr><td>WP_019063569.1</td><td>MFS→||||<-?<-TPR+Caspase<-CATASP||CATRA-N+CATRA-C+GT4*→
HTH+APATPase+TPR→||||<-PSE||?→?→||||<-?<-CATASP<-TPR+Caspase</td><td>CATRA-N+CATRA-C+GT4</td><td>720</td><td>SPR02S_RS44725</td><td>Actinobacteria</td><td>Streptomyces prunicolor</td><td>glycosyltransferase family 4 protein [Streptomyces prunicolor]</td><td>GCF_000367365.1</td></tr>
<tr><td>WP_019093712.1</td><td>SWACOS+GT4*→</td><td>SWACOS+GT4</td><td>1138</td><td>BK646_RS17250</td><td>Gammaproteobacteria</td><td>Pseudomonas protegens</td><td>glycosyltransferase [Pseudomonas protegens]</td><td>GCF_003732085.1</td></tr>
<tr><td>WP_019609270.1</td><td><-AbiEii<-AbiEi_4||?→ GT4*→</td><td>GT4</td><td>497</td><td>G011_RS0115485</td><td>Actinobacteria</td><td>Nocardiopsis sp. CNS-639</td><td>glycosyltransferase family 4 protein [Nocardiopsis sp. CNS-639]</td><td>GCF_000381685.1</td></tr>
<tr><td>WP_020647322.1</td><td><-TIR+APATPase||HTH+APATPase+TPR+GT4*→</td><td>HTH+APATPase+TPR+GT4</td><td>1192</td><td>A3CE_RS0148230</td><td>Actinobacteria</td><td>Amycolatopsis balhimycina</td><td>tetratricopeptide repeat protein [Amycolatopsis balhimycina]</td><td>GCF_000384295.1</td></tr>
<tr><td>WP_023267980.1</td><td>GT4+APATPase+TPR+TPR*→</td><td>GT4+APATPase+TPR+TPR</td><td>1323</td><td>SHD_RS15540</td><td>Gammaproteobacteria</td><td>Shewanella decolorationis</td><td>tetratricopeptide repeat protein [Shewanella decolorationis]</td><td>GCF_000485795.1</td></tr>
<tr><td>WP_024631061.1</td><td><-AAA_21||GT4*→</td><td>GT4</td><td>1140</td><td>P364_RS0115745</td><td>Firmicutes</td><td>Paenibacillus sp. MAEPY2</td><td>glycosyltransferase [Paenibacillus sp. MAEPY2]</td><td>GCF_000499205.1</td></tr>
<tr><td>WP_027196515.1</td><td>Cluster214_2clades→ GT4*→</td><td>GT4</td><td>760</td><td>BJG93_RS27460</td><td>Betaproteobacteria</td><td>Paraburkholderia sprentiae</td><td>glycosyltransferase family 4 protein [Paraburkholderia sprentiae]</td><td>GCF_001865575.1</td></tr>
<tr><td>WP_029881581.1</td><td><-AAA_21||GT4*→</td><td>GT4</td><td>1003</td><td>P363_RS0103940</td><td>Firmicutes</td><td>Paenibacillus sp. MAEPY1</td><td>glycosyltransferase, partial [Paenibacillus sp. MAEPY1]</td><td>GCF_000499305.1</td></tr>
</table>

<table>
<tr><th>acc</th><th>operon</th><th>architecture</th><th>len</th><th>gen.name</th><th>taxend</th><th>species</th><th>defline</th><th>gca</th></tr>
<tr><td>WP_031021212.1</td><td>ABC_tran→||||<-?<-CATASP||CATRA-N+CATRA-C+GT4*→ HTH+APATPase+TPR→||||<-HTH||?→?→?→||||<-?<-CATASP</td><td>CATRA-N+CATRA-C+GT4</td><td>705</td><td>IF98_RS0120305</td><td>Actinobacteria</td><td>Streptomyces sp. NRRL S-1314</td><td>glycosyltransferase family 4 protein [Streptomyces sp. NRRL S-1314]</td><td>GCF_000719785.1</td></tr>
<tr><td>WP_031063336.1</td><td>ABC_tran→||||<-?<-CATASP||CATRA-N+CATRA-C+GT4*→ HTH+APATPase+TPR→||||<-HTH||?→?→?→||||<-?<-CATASP</td><td>CATRA-N+CATRA-C+GT4</td><td>705</td><td>IH17_RS0118610</td><td>Actinobacteria</td><td>Streptomyces sp. NRRL F-5527</td><td>glycosyltransferase family 4 protein [Streptomyces sp. NRRL F-5527]</td><td>GCF_014649055.1</td></tr>
<tr><td>WP_03352450[illegible].1</td><td>TRANSGLUTAMINASE→||||<-?||?→||||<-?||GT4+NUDIX+ART+PolB-NTase→////<-?<-?<-?//?→ GT4→||||<-?<-?<-?<-?<-DUF4913</td><td>GT4+NUDIX+ART+PolB-NTase</td><td>589</td><td>KS09_RS00195</td><td>Actinobacteria</td><td>Streptomyces galbus</td><td>glycosyltransferase [Streptomyces galbus]</td><td>GCF_000772895.1</td></tr>
<tr><td>WP_035284396.1</td><td>APATPase+TPR→ GT4*→</td><td>GT4</td><td>502</td><td>UO65_RS36825</td><td>Actinobacteria</td><td>Actinokineospora spheciospongiae</td><td>glycosyltransferase [Actinokineospora spheciospongiae]</td><td>GCF_000564855.1</td></tr>
<tr><td>WP_036515011.1</td><td>WXG+MPTase→ WXG+SecA+HTH+HTH+HTH+HTH+HTH+ST-Hiskin+PP2C+MPTase+GT4+TetR-HTH+TetR-HTH+TM+TM+TM+TM+TM+TM+LuxR-HTH+LuxR-HTH+LuxR-HTH+LuxR-HTH+LuxR-HTH+LuxR-HTH+LuxR-HTH+GNTR-HTH+GNTR-HTH+GNTR-HTH+GNTR-HTH+GNTR-HTH+GNTR-HTH*→</td><td>WXG+SecA+HTH+HTH+HTH+HTH+HTH+ST-Hiskin+PP2C+MPTase+GT4+TetR-HTH+TetR-HTH+TM+TM+TM+TM+TM+TM+LuxR-HTH+LuxR-HTH+LuxR-HTH+LuxR-HTH+LuxR-HTH+LuxR-HTH+LuxR-HTH+GNTR-HTH+GNTR-HTH+GNTR-HTH+GNTR-HTH+GNTR-HTH+GNTR-HTH</td><td>12585</td><td>NRH01S_RS18840</td><td>Actinobacteria</td><td>Nocardia rhamnosiphila</td><td>GntR family transcriptional regulator [Nocardia rhamnosiphila]</td><td>GCF_001613485.1</td></tr>
<tr><td>WP_036994826.1</td><td>MFS→||||<-?||GT4+APATPase+TPR*→||||<-MODE-HTH+PBPII</td><td>GT4+APATPase+TPR</td><td>1318</td><td>BV82_RS07620</td><td>Gammaproteobacteria</td><td>Pseudomonas donghuensis</td><td>glycosyltransferase [Pseudomonas donghuensis]</td><td>GCF_000696345.1</td></tr>
<tr><td>WP_037254756.1</td><td>cNMP_binding+HTH_Crp_2→?→ GT4*→||||<-ACYC+APATPase+TPR</td><td>GT4</td><td>404</td><td>DMH04_42005</td><td>Actinobacteria</td><td>Kibdelosporangium aridum</td><td>glycosyltransferase [Kibdelosporangium aridum]</td><td>-</td></tr>
<tr><td>WP_039793765.1</td><td><-TIR+APATPase||HTH+APATPase+TPR+GT4*→</td><td>HTH+APATPase+TPR+GT4</td><td>1175</td><td>CFP75_RS23290</td><td>Actinobacteria</td><td>Amycolatopsis alba</td><td>glycosyltransferase [Amycolatopsis alba]</td><td>GCF_000384215.1</td></tr>
<tr><td>WP_040866957.1</td><td>WXG+MPTase→ WXG+SecA+GT4+TetR-HTH+MPTase+HTH+HTH+HTH+HTH+HTH+HTH+HTH+HTH*→</td><td>WXG+SecA+GT4+TetR-HTH+MPTase+HTH+HTH+HTH+HTH+HTH+HTH+HTH+HTH</td><td>5345</td><td>ON29_RS15345</td><td>Actinobacteria</td><td>Nocardia exalbida</td><td>helix-turn-helix domain-containing protein [Nocardia exalbida]</td><td>GCF_000308575.1</td></tr>
<tr><td>WP_041117337.1</td><td>SWACOS+GT4*→?→||||<-?<-?||MFS→</td><td>SWACOS+GT4</td><td>1138</td><td>PPC_RS11795</td><td>Gammaproteobacteria</td><td>Pseudomonas protegens</td><td>glycosyltransferase [Pseudomonas protegens]</td><td>GCF_000828695.1</td></tr>
<tr><td>WP_042132624.1</td><td>GT4+B12-binding+RADICAL-SAM*→</td><td>GT4+B12-binding+RADICAL-SAM</td><td>1393</td><td>BJQ15_RS27760</td><td>Gammaproteobacteria</td><td>Pseudomonas</td><td>MULTISPECIES: B12-binding domain-containing radical SAM protein [Pseudomonas]</td><td>GCF_000730605.1</td></tr>
</table>

<table>
<tr><th>acc</th><th>operon</th><th>architecture</th><th>len</th><th>gen.name</th><th>taxend</th><th>species</th><th>defline</th><th>gca</th></tr>
<tr><td>WP_043243023.1</td><td>Ribosomal_L20→?→?→?→?→ tRNA→?→ GT4+B12-binding+RADICAL-SAM*→</td><td>GT4+B12-binding+RADICAL-SAM</td><td>1402</td><td>TX97_RS10150</td><td>Gammaproteobacteria</td><td>Pseudomonas alcaligenes</td><td>B12-binding domain-containing radical SAM protein [Pseudomonas alcaligenes]</td><td>GCF_000474255.1</td></tr>
<tr><td>WP_043510995.1</td><td>WXG+GT4+GT4+REase*→</td><td>WXG+GT4+GT4+REase</td><td>4153</td><td>ACSP50_RS07520</td><td>Actinobacteria</td><td>unclassified Actinoplanes</td><td>MULTISPECIES: glycosyltransferase [unclassified Actinoplanes]</td><td>GCF_000237145.1</td></tr>
<tr><td>WP_043738635.1</td><td>WXG+MPTase→ WXG+SecA+GT4+TetR-HTH+TetR-HTH+MPTase+HTH+HTH+HTH+HTH+HTH+HTH*→</td><td>WXG+SecA+GT4+TetR-HTH+TetR-HTH+MPTase+HTH+HTH+HTH+HTH+HTH+HTH</td><td>8037</td><td>ON27_RS37615</td><td>Actinobacteria</td><td>Nocardia asiatica</td><td>helix-turn-helix domain-containing protein [Nocardia asiatica]</td><td>GCF_000308415.1</td></tr>
<tr><td>WP_045583922.1</td><td><-SIG+HTH_23+HTH_32||GT4+TPR*→</td><td>GT4+TPR</td><td>1241</td><td>VY88_RS20945</td><td>Alphaproteobacteria</td><td>Azospirillum thiophilum</td><td>tetratricopeptide repeat protein [Azospirillum thiophilum]</td><td>GCF_001305595.1</td></tr>
<tr><td>WP_050363680.1</td><td>Multi-TM→?→ STAND→?→ DUF4913→?→ GT4*→?→ Zeta_toxin→||||<-ABC_tran</td><td>GT4</td><td>555</td><td>DC095_RS07800</td><td>Actinobacteria</td><td>Streptomyces</td><td>MULTISPECIES: glycosyltransferase family 4 protein [Streptomyces]</td><td>GCF_000700005.2</td></tr>
<tr><td>WP_050791877.1</td><td>GT4+B12-binding+RADICAL-SAM*→?→||||<-PSE<-?||?→||||<-?<-?||Sigma→</td><td>GT4+B12-binding+RADICAL-SAM</td><td>1403</td><td>BUBO0001_RS30235</td><td>Betaproteobacteria</td><td>Burkholderia ubonensis</td><td>B12-binding domain-containing radical SAM protein [Burkholderia ubonensis]</td><td>GCF_000170335.1</td></tr>
<tr><td>WP_051466857.1</td><td>TPR+Caspase→ CATRA-N+CATRA-C+GT4*→ HTH+APATPase+TPR→||||<-TPR+Caspase||CATASP→</td><td>CATRA-N+CATRA-C+GT4</td><td>812</td><td>BCD49_RS04570</td><td>Actinobacteria</td><td>Frankia</td><td>MULTISPECIES: glycosyltransferase family 4 protein [Frankia]</td><td>GCF_000235425.2</td></tr>
<tr><td>WP_051653780.1</td><td>MNS+GT4*→||||<-?||PSE→ ABC_tran→</td><td>MNS+GT4</td><td>1132</td><td>KCH_RS38295</td><td>Actinobacteria</td><td>Kitasatospora cheerisanensis</td><td>glycosyltransferase [Kitasatospora cheerisanensis]</td><td>GCF_000696185.1</td></tr>
<tr><td>WP_051722648.1</td><td>Multi-TM→?→ STAND→?→ DUF4913→?→ GT4*→?→ Zeta_toxin→</td><td>GT4</td><td>561</td><td>IF83_RS0111160</td><td>Actinobacteria</td><td>Streptomyces albus</td><td>glycosyltransferase family 4 protein [Streptomyces albus]</td><td>GCF_000719865.1</td></tr>
<tr><td>WP_051765847.1</td><td><-CASPASE+FGS<-?<-?<-?||GT4*→</td><td>GT4</td><td>406</td><td>OQ01_RS21365</td><td>Actinobacteria</td><td>Saccharothrix syringae</td><td>glycosyltransferase [Saccharothrix syringae]</td><td>GCF_000716755.1</td></tr>
<tr><td>WP_051865326.1</td><td>GT4*→</td><td>GT4</td><td>621</td><td>IG99_RS44170</td><td>Actinobacteria</td><td>Streptomyces griseus</td><td>glycosyltransferase [Streptomyces griseus]</td><td>GCF_000720255.1</td></tr>
</table>

<table>
<tr><th>acc</th><th>operon</th><th>architecture</th><th>len</th><th>gen.name</th><th>taxend</th><th>species</th><th>defline</th><th>gca</th></tr>
<tr><td>WP_052457075.1</td><td><-TPR+Caspase<-CATASP||CATRA-N+CATRA-C+GT4*→ PSE→ PSE→ APATPase+TPR→||||<-HTH||?→||||<-?<-CATASP<-TPR+Caspase</td><td>CATRA-N+CATRA-C+GT4</td><td>747</td><td>HY68_RS39570</td><td>Actinobacteria</td><td>Streptomyces sp. AcH 505</td><td>glycosyltransferase [Streptomyces sp. AcH 505]</td><td>GCF_000818175.1</td></tr>
<tr><td>WP_052479793.1</td><td>TIR+APATPase+GT4*→</td><td>TIR+APATPase+GT4</td><td>810</td><td>APS67_RS01150</td><td>Actinobacteria</td><td>unclassified Streptomyces</td><td>MULTISPECIES: TIR domain-containing protein [unclassified Streptomyces]</td><td>GCF_000829695.1</td></tr>
<tr><td>WP_053258773.1</td><td>GT4+B12-binding+RADICAL-SAM*→</td><td>GT4+B12-binding+RADICAL-SAM</td><td>1401</td><td>ALQ35_RS05775</td><td>Gammaproteobacteria</td><td>Pseudomonas fluorescens</td><td>cobalamin B12-binding domain-containing protein [Pseudomonas fluorescens]</td><td>GCF_002091595.1</td></tr>
<tr><td>WP_053682326.1</td><td>MFS→?→||||<-?<-?||?→||||<-?||MPTase+GT4*→?→?→||||<-?<-?<-?<-ABC_tran</td><td>MPTase+GT4</td><td>764</td><td>ADK55_RS26505</td><td>Actinobacteria</td><td>Streptomyces sp. WM4235</td><td>DUF4157 domain-containing protein [Streptomyces sp. WM4235]</td><td>GCF_001279725.1</td></tr>
<tr><td>WP_054048489.1</td><td>TPR+Caspase→ TIR+APATPase+TPR→||||<-APATPase+TPR||GT4*→||||<-HTH+APATPase+TPR</td><td>GT4</td><td>456</td><td>BN1701_RS12505</td><td>Actinobacteria</td><td>Alloactinosynnema sp. L-07</td><td>glycosyltransferase family 4 protein [Alloactinosynnema sp. L-07]</td><td>GCF_900070365.1</td></tr>
<tr><td>WP_055337289.1</td><td>STAND+TPR+GT4*→?→ SIG+HTH_23+HTH_32→||||<-?<-?||DJ-1_PfpI→</td><td>STAND+TPR+GT4</td><td>1061</td><td>HXP36_RS10750</td><td>Betaproteobacteria</td><td>Ralstonia solanacearum</td><td>glycosyltransferase [Ralstonia solanacearum]</td><td>GCF_001373335.1</td></tr>
<tr><td>WP_055417577.1</td><td>MNS+GT4*→</td><td>MNS+GT4</td><td>1164</td><td>B1H29_RS20255</td><td>Actinobacteria</td><td>Streptomyces pactum</td><td>glycosyltransferase [Streptomyces pactum]</td><td>GCF_002005225.1</td></tr>
<tr><td>WP_055618904.1</td><td>NACHT+MNS→ MNS+GT4*→</td><td>MNS+GT4</td><td>754</td><td>JHAN_RS00420</td><td>Actinobacteria</td><td>Streptomyces sp. JHA19</td><td>glycosyltransferase [Streptomyces sp. JHA19]</td><td>GCF_001417695.1</td></tr>
<tr><td>WP_055624376.1</td><td>MNS+GT4*→</td><td>MNS+GT4</td><td>1149</td><td>JHAN_RS28925</td><td>Actinobacteria</td><td>Streptomyces sp. JHA19</td><td>AAA family ATPase [Streptomyces sp. JHA19]</td><td>GCF_001417695.1</td></tr>
<tr><td>WP_057006131.1</td><td>GT4*→</td><td>GT4</td><td>773</td><td>TU80_RS30870</td><td>Gammaproteobacteria</td><td>Pseudomonas</td><td>MULTISPECIES: glycosyltransferase [Pseudomonas]</td><td>GCF_900104875.1</td></tr>
<tr><td>WP_058422527.1</td><td>GT4+B12-binding+RADICAL-SAM*→</td><td>GT4+B12-binding+RADICAL-SAM</td><td>1407</td><td>AO063_RS26420</td><td>Gammaproteobacteria</td><td>Pseudomonas fluorescens</td><td>radical SAM protein [Pseudomonas fluorescens]</td><td>GCF_001466835.1</td></tr>
</table>

<table>
<tr><th>acc</th><th>operon</th><th>architecture</th><th>len</th><th>gen.name</th><th>taxend</th><th>species</th><th>defline</th><th>gca</th></tr>
<tr><td>WP_058721560.1</td><td>STAND+TPR+GT4*→||||<-?<-?<-?<-ABC_tran</td><td>STAND+TPR+GT4</td><td>1063</td><td>AT984_RS19735</td><td>Betaproteobacteria</td><td>Paucibacter sp. KCTC 42545</td><td>glycosyltransferase [Paucibacter sp. KCTC 42545]</td><td>GCF_001477625.1</td></tr>
<tr><td>WP_060944940.1</td><td>MNS+GT4*→</td><td>MNS+GT4</td><td>1149</td><td>AXW62_RS14785</td><td>Actinobacteria</td><td>Streptomyces hygroscopicus</td><td>glycosyltransferase [Streptomyces hygroscopicus]</td><td>GCF_001553455.1</td></tr>
<tr><td>WP_062579281.1</td><td>GT4*→||||<-?||?→?→?→?→ SIG+SnoaL→</td><td>GT4</td><td>372</td><td>ASG42_RS32705</td><td>Alphaproteobacteria</td><td>Rhizobium sp. Leaf391</td><td>glycosyltransferase family 1 protein [Rhizobium sp. Leaf391]</td><td>GCF_001424505.1</td></tr>
<tr><td>WP_062720264.1</td><td><-TPR+Caspase||GT4*→</td><td>GT4</td><td>610</td><td>AQJ67_RS19460</td><td>Actinobacteria</td><td>Streptomyces caeruleatus</td><td>glycosyltransferase [Streptomyces caeruleatus]</td><td>GCF_001514235.1</td></tr>
<tr><td>WP_062723774.1</td><td>MNS+GT4*→</td><td>MNS+GT4</td><td>1219</td><td>AQJ67_RS36455</td><td>Actinobacteria</td><td>Streptomyces caeruleatus</td><td>glycosyltransferase [Streptomyces caeruleatus]</td><td>GCF_001514235.1</td></tr>
<tr><td>WP_062964870.1</td><td>WXG+MPTase→ WXG+SecA+ST-Hiskin+LuxR-HTH+LuxR-HTH+LuxR-HTH+LuxR-HTH+ST-Hiskin+GT4+TetR-HTH+TetR-HTH+Tox-ART-RSE+LuxR-HTH+PP2C+MPTase+MPTase*→?→ ABhydrolase→</td><td>WXG+SecA+ST-Hiskin+LuxR-HTH+LuxR-HTH+LuxR-HTH+LuxR-HTH+ST-Hiskin+GT4+TetR-HTH+TetR-HTH+Tox-ART-RSE+LuxR-HTH+PP2C+MPTase+MPTase</td><td>9117</td><td>NA3_RS18555</td><td>Actinobacteria</td><td>Nocardia africana</td><td>glycosyltransferase [Nocardia africana]</td><td>GCF_001612635.1</td></tr>
<tr><td>WP_063038911.1</td><td>WXG+TM+TM+TM→?→?→?→?→?→?→ WXG+ZetaToxin+GT4*→</td><td>WXG+ZetaToxin+GT4</td><td>1614</td><td>NP3_RS06870</td><td>Actinobacteria</td><td>Nocardia pseudovaccinii</td><td>zeta toxin family protein [Nocardia pseudovaccinii]</td><td>GCF_001613225.1</td></tr>
<tr><td>WP_063127006.1</td><td>Zeta_toxin→||||<-?||MPTase→?→ WXG+TM+TM+TM+TM+GT4*→</td><td>WXG+TM+TM+TM+TM+GT4</td><td>1018</td><td>NF4_RS09930</td><td>Actinobacteria</td><td>Nocardia fusca</td><td>glycosyltransferase [Nocardia fusca]</td><td>-</td></tr>
<tr><td>WP_063882776.1</td><td>GT4+APATPase+TPR+TPR*→</td><td>GT4+APATPase+TPR+TPR</td><td>1322</td><td>A1L58_RS04155</td><td>Gammaproteobacteria</td><td>Shewanella baltica</td><td>tetratricopeptide repeat protein [Shewanella baltica]</td><td>GCF_001620325.1</td></tr>
<tr><td>WP_063889953.1</td><td>GT4+B12-binding+RADICAL-SAM*→||||<-?||?→||||<-?||?→||||<-?||?→||||<-TRPR-HTH+PBPI</td><td>GT4+B12-binding+RADICAL-SAM</td><td>1415</td><td>WI77_RS33765</td><td>Betaproteobacteria</td><td>Burkholderia ubonensis</td><td>radical SAM protein [Burkholderia ubonensis]</td><td>GCF_001525885.1</td></tr>
<tr><td>WP_064054195.1</td><td><-HTH_3||?→?→||||<-?<-Cluster466_2clades||STAND+GT4*→||||<-?<-Sigma</td><td>STAND+GT4</td><td>1084</td><td>ALQ29_RS11960</td><td>Gammaproteobacteria</td><td>Pseudomonas marginalis</td><td>glycosyltransferase [Pseudomonas marginalis]</td><td>GCF_001645105.1</td></tr>
<tr><td>WP_064273456.1</td><td>GT4*→</td><td>GT4</td><td>135</td><td>A7B19_RS17315</td><td>Actinobacteria</td><td>Streptomyces sp. RTd22</td><td>hypothetical protein [Streptomyces sp. RTd22]</td><td>GCF_001650215.1</td></tr>
</table>

acc	operon	architecture	len	gen.name	taxend	species	defline	gca
WP_065910241.1	GT4+B12-binding+RADICAL-SAM*→	GT4+B12-binding+RADICAL-SAM	1393	C9382_RS17200	Gammaproteobacteria	unclassified Pseudomonas	MULTISPECIES: cobalamin B12-binding domain-containing protein [unclassified Pseudomonas]	GCF_001702265.1
WP_067257069.1	GT4*→	GT4	398	AQJ58_RS33820	Actinobacteria	Streptomyces sp. DSM 15324	glycosyltransferase family 4 protein [Streptomyces sp. DSM 15324]	GCF_001514285.1
WP_067481412.1	HTH→?→||||<-?||NACHT+MNS→ MNS+GT4*→	MNS+GT4	764	AH6_RS18370	Actinobacteria	Actinomadura hibisca	glycosyltransferase [Actinomadura hibisca]	GCF_001552635.1
WP_067904030.1	SWACOS+GT4*→	SWACOS+GT4	1149	ACH01S_RS37935	Actinobacteria	Actinomadura chibensis	glycosyltransferase [Actinomadura chibensis]	GCF_001552135.1
WP_068874779.1	HTH_3→?→||||<-?<-?||?→ MPTase→?→ NACHT+GT4*→||||<-?||?→||||<-?<-?<-PSE<-?||?→ SIG+Lipase_GDSL_2→	NACHT+GT4	1671	AX747_RS22605	Alphaproteobacteria	unclassified Phenylobacterium	MULTISPECIES: glycosyltransferase [unclassified Phenylobacterium]	GCF_001557235.1
WP_069042340.1	NACHT+GT4*→	NACHT+GT4	1642	BSY240_RS11090	Alphaproteobacteria	Agrobacterium sp. RAC06	glycosyltransferase [Agrobacterium sp. RAC06]	GCF_001713475.1
WP_070086299.1	GT4+B12-binding+RADICAL-SAM*→	GT4+B12-binding+RADICAL-SAM	1393	BJQ21_RS10430	Gammaproteobacteria	unclassified Pseudomonas	MULTISPECIES: radical SAM protein [unclassified Pseudomonas]	GCF_001753875.1
WP_071611953.1	GT4*→?→?→?→ PadR→?→||||<-?<-ABC_tran	GT4	724	F7R00_RS19755	Firmicutes	Clostridium estertheticum	glycosyltransferase family 4 protein [Clostridium estertheticum]	GCF_001877035.1
WP_071621050.1	<-AbiEii<-AbiEi_4||?→ GT4*→	GT4	497	CGQ36_RS01685	Actinobacteria	Nocardiopsis dassonvillei	glycosyltransferase family 4 protein [Nocardiopsis dassonvillei]	GCF_001877055.1
WP_072444656.1	SWACOS+GT4*→?→||||<-?<-?||MFS→	SWACOS+GT4	1138	BM382_RS27510	Gammaproteobacteria	unclassified Pseudomonas	MULTISPECIES: glycosyltransferase [unclassified Pseudomonas]	GCF_900102935.1
WP_073746098.1	CASPASE→ tRNA→||||<-?||MNS+GT4*→	MNS+GT4	1169	AMK28_RS33605	Actinobacteria	Streptomyces sp. CB02115	glycosyltransferase [Streptomyces sp. CB02115]	GCF_001905645.1

acc	operon	architecture	len	gen.name	taxend	species	defline	gca
WP_074374750.1	DctM→?→?→?→?→||||<-?||GT4*→	GT4	488	VSP9026_RS20020	Gammaproteobacteria	Vibrio spartinae	glycosyltransferase family 4 protein [Vibrio spartinae]	GCF_900149295.1
WP_074848880.1	GT4*→	GT4	397	BLU62_RS07095	Actinobacteria	Gordonia westfalica	glycosyltransferase [Gordonia westfalica]	GCF_900105725.1
WP_076845054.1	WXG+TM+GT4+GT4*→	WXG+TM+GT4+GT4	1675	BL254_RS27435	Actinobacteria	Frankia sp. BMG5.30	hypothetical protein [Frankia sp. BMG5.30]	GCF_001983005.1
WP_077298179.1	PrpF→?→||||<-?<-?||?→||||<-?||GT4+nSTAND3+TPR*→||||<-rve+rve_3||?→ tRNA-synt_1b+S4→	GT4+nSTAND3+TPR	972	BXU06_RS07175	Betaproteobacteria	Aquaspirillum sp. LM1	hypothetical protein [Aquaspirillum sp. LM1]	GCF_002002905.1
WP_078753771.1	GT4+APATPase+TPR+TPR*→	GT4+APATPase+TPR+TPR	1333	B5D35_RS17215	Gammaproteobacteria	Enterovibrio nigricans	tetratricopeptide repeat protein [Enterovibrio nigricans]	GCF_900167155.1
WP_078789290.1	XylR_N+V4R+Sigma54_activat+HTH_8→?→ GT4+APATPase+TPR*→	GT4+APATPase+TPR	1307	B5D39_RS04945	Deltaproteobacteria	Geobacter thiogenes	tetratricopeptide repeat protein [Geobacter thiogenes]	GCF_900167465.1
WP_078936874.1	-	-	1884	KS09_RS00220	Actinobacteria	Streptomyces galbus	glycosyltransferase family 4 protein [Streptomyces galbus]	GCF_000772895.1
WP_078960520.1	TIR+APATPase+GT4*→	TIR+APATPase+GT4	839	ADL01_RS15000	Actinobacteria	Streptomyces sp. NRRL WC-3618	TIR domain-containing protein [Streptomyces sp. NRRL WC-3618]	GCF_001280005.1
WP_079101522.1	GT4*→	GT4	542	AOK16_RS25865	Actinobacteria	Streptomyces pathocidini	glycosyltransferase [Streptomyces pathocidini]	GCF_001418495.1
WP_080416707.1	AA_permease→?→ GT4+APATPase+TPR+TPR*→	GT4+APATPase+TPR+TPR	1339	WL41_RS32965	Betaproteobacteria	Burkholderia ubonensis	tetratricopeptide repeat protein [Burkholderia ubonensis]	GCF_001529565.1
WP_080417742.1	GT4+APATPase+TPR+TPR*→	GT4+APATPase+TPR+TPR	1293	WJ64_RS17075	Betaproteobacteria	Burkholderia ubonensis	tetratricopeptide repeat protein [Burkholderia ubonensis]	GCF_001527745.1
WP_080419087.1	STAND+TPR+GT4*→||||<-?||ABhydrolase→||||<-TRANSGLUTAMINASE	STAND+TPR+GT4	1126	WL82_RS32325	Betaproteobacteria	Burkholderia ubonensis	glycosyltransferase [Burkholderia ubonensis]	-
WP_080421888.1	AA_permease→?→ GT4+APATPase+TPR+TPR*→	GT4+APATPase+TPR+TPR	1339	WK71_RS32030	Betaproteobacteria	Burkholderia ubonensis	tetratricopeptide repeat protein [Burkholderia ubonensis]	GCF_001529155.1

<table>
<tr><th>acc</th><th>operon</th><th>architecture</th><th>len</th><th>gen.name</th><th>taxend</th><th>species</th><th>defline</th><th>gca</th></tr>
<tr><td>WP_080422913.1</td><td>GT4+APATPase+TPR+TPR*→</td><td>GT4+APATPase+TPR+TPR</td><td>1339</td><td>WL11_RS09715</td><td>Betaproteobacteria</td><td>Burkholderia ubonensis</td><td>tetratricopeptide repeat protein [Burkholderia ubonensis]</td><td>GCF_001530465.1</td></tr>
<tr><td>WP_080436327.1</td><td>NACHT+TPR+GT4*→</td><td>NACHT+TPR+GT4</td><td>1226</td><td>WM18_RS12015</td><td>Betaproteobacteria</td><td>Burkholderia ubonensis</td><td>glycosyltransferase [Burkholderia ubonensis]</td><td>GCF_001533745.1</td></tr>
<tr><td>WP_080492181.1</td><td>AA_permease→?→ GT4+APATPase+TPR+TPR*→</td><td>GT4+APATPase+TPR+TPR</td><td>1293</td><td>BGV55_RS22015</td><td>Betaproteobacteria</td><td>Burkholderia ubonensis</td><td>tetratricopeptide repeat protein [Burkholderia ubonensis]</td><td>GCF_001882545.1</td></tr>
<tr><td>WP_082136219.1</td><td>GT4+APATPase+TPR+TPR*→</td><td>GT4+APATPase+TPR+TPR</td><td>1282</td><td>TR67_RS23240</td><td>Gammaproteobacteria</td><td>Pseudomonas deceptionensis</td><td>glycosyltransferase [Pseudomonas deceptionensis]</td><td>GCF_900106095.1</td></tr>
<tr><td>WP_082297595.1</td><td>GT4*→</td><td>GT4</td><td>599</td><td>AKH00_RS12250</td><td>Actinobacteria</td><td>Microbacterium sp. GCS4</td><td>glycosyltransferase family 4 protein [Microbacterium sp. GCS4]</td><td>GCF_001262495.1</td></tr>
<tr><td>WP_082404817.1</td><td><-ABC_tran||?→?→||||<-?<-TIR+NACHT||HTH+APATPase+TPR+GT4*→</td><td>HTH+APATPase+TPR+GT4</td><td>1223</td><td>ADK67_RS45535</td><td>Actinobacteria</td><td>Saccharothrix sp. NRRL B-16348</td><td>glycosyltransferase [Saccharothrix sp. NRRL B-16348]</td><td>GCF_001280085.1</td></tr>
<tr><td>WP_082466077.1</td><td>GT4*→||||<-TRPR-HTH+PBPI<-?<-?<-?<-?||ABC_tran→</td><td>GT4</td><td>618</td><td>ASE64_RS17155</td><td>Actinobacteria</td><td>Agreia sp. Leaf210</td><td>glycosyltransferase [Agreia sp. Leaf210]</td><td>GCF_001421485.1</td></tr>
<tr><td>WP_083469142.1</td><td>GT4+NUDIX*→</td><td>GT4+NUDIX</td><td>750</td><td>VQ02_RS35520</td><td>Alphaproteobacteria</td><td>Methylobacterium variabile</td><td>glycosyltransferase family 4 protein [Methylobacterium variabile]</td><td>GCF_001043975.1</td></tr>
<tr><td>WP_083731258.1</td><td>Multi-TM→?→ STAND→?→ DUF4913→?→||||<-?||GT4+NUDIX+ART+PolB-NTase*→</td><td>GT4+NUDIX+ART+PolB-NTase</td><td>1854</td><td>BL254_RS25375</td><td>Actinobacteria</td><td>Frankia sp. BMG5.30</td><td>glycosyltransferase [Frankia sp. BMG5.30]</td><td>GCF_001983005.1</td></tr>
<tr><td>WP_083895344.1</td><td>WXG→?→?→ WXG→?→?→?→ WXG+Caspase+VWA+Papain+ZetaToxin+GT4*→</td><td>WXG+Caspase+VWA+Papain+ZetaToxin+GT4</td><td>3035</td><td>ON40_RS01890</td><td>Actinobacteria</td><td>Nocardia jiangxiensis</td><td>zeta toxin family protein [Nocardia jiangxiensis]</td><td>GCF_000308615.1</td></tr>
<tr><td>WP_085710134.1</td><td><-LexA||GT4+APATPase+TPR+TPR*→||||<-?<-?<-ABC_tran</td><td>GT4+APATPase+TPR+TPR</td><td>1389</td><td>CAG24_RS08895</td><td>Gammaproteobacteria</td><td>unclassified Pseudomonas</td><td>MULTISPECIES: glycosyltransferase [unclassified Pseudomonas]</td><td>GCF_002112315.1</td></tr>
<tr><td>WP_086055635.1</td><td>DUF4913→?→||||<-?||GT4+NUDIX+ART+PolB-NTase*→</td><td>GT4+NUDIX+ART+PolB-NTase</td><td>1854</td><td>FrCorBMG51_RS27570</td><td>Actinobacteria</td><td>Frankia coriariae</td><td>glycosyltransferase [Frankia coriariae]</td><td>-</td></tr>
<tr><td>WP_086802688.1</td><td>GT4+NUDIX+ART+PolB-NTase*→</td><td>GT4+NUDIX+ART+PolB-NTase</td><td>1883</td><td>CCN48_RS29200</td><td>Actinobacteria</td><td>Streptomyces scabiei</td><td>glycosyltransferase family 4 protein [Streptomyces scabiei]</td><td>GCF_002155725.1</td></tr>
</table>

acc	operon	architecture	len	gen.name	taxend	species	defline	gca
WP_087651531.1	GT4+Trypsin+PNPase*→	GT4+Trypsin+PNPase	929	S1001342_RS03745	Alphaproteobacteria	Acetobacter pasteurianus	glycosyltransferase [Acetobacter pasteurianus]	GCF_002173735.1
WP_088088251.1	GT4*→	GT4	624	CCK98_RS20350	Firmicutes	Bacillus sp. OV166	glycosyltransferase family 4 protein [Bacillus sp. OV166]	GCF_900177675.1
WP_088287445.1	GT4+APATPase+TPR+TPR*→	GT4+APATPase+TPR+TPR	1066	EOINJALH_RS30615	Betaproteobacteria	Ideonella sp. A 288	tetratricopeptide repeat protein [Ideonella sp. A 288]	GCF_002198735.1
WP_090142138.1	GT4*→	GT4	516	AMM33_RS15110	Betaproteobacteria	Limnohabitans sp. DM1	glycosyltransferase family 4 protein [Limnohabitans sp. DM1]	GCF_001269385.1
WP_091283948.1	TPR+Caspase→ TPR+Caspase→||||<-CATASP||CATRA-N+CATRA-C+GT4*→||||<-?||HTH+APATPase+TPR→	CATRA-N+CATRA-C+GT4	839	AWX74_RS30820	Actinobacteria	Frankia irregularis	glycosyltransferase family 4 protein [Frankia irregularis]	GCF_001536285.1
WP_091296338.1	cNMP_binding+HTH_Crp_2→?→ GT4*→||||<-ACYC+APATPase+TPR	GT4	406	BLV57_RS34110	Actinobacteria	Amycolatopsis xylanica	glycosyltransferase family 4 protein [Amycolatopsis xylanica]	GCF_900107045.1
WP_091582937.1	STAND→?→ DUF4913→||||<-?<-?||?→ GT4*→?→?→?→ NUDIX→||||<-?<-ABC_tran	GT4	438	GA0070622_RS30350	Actinobacteria	Micromonospora sediminicola	glycosyltransferase family 4 protein [Micromonospora sediminicola]	GCF_900089585.1
WP_092202072.1	ABC_tran→?→?→?→?→?→||||<-?||STAND+TPR+GT4*→	STAND+TPR+GT4	1063	BLU85_RS18570	Gammaproteobacteria	unclassified Pseudomonas	MULTISPECIES: glycosyltransferase [unclassified Pseudomonas]	GCF_900103555.1
WP_092992899.1	GT4+TPR+TPR*→	GT4+TPR+TPR	1441	BLP65_RS04115	Gammaproteobacteria	Thiohalomonas denitrificans	tetratricopeptide repeat protein [Thiohalomonas denitrificans]	GCF_900102855.1
WP_094198012.1	GST_N_2+GST_C→?→||||<-?<-?<-?||tRNA→ Cluster214_2clades→ GT4*→||||<-?<-?<-?<-DctM	GT4	756	AFA_RS17265	Betaproteobacteria	Alcaligenes faecalis	glycosyltransferase family 4 protein [Alcaligenes faecalis]	GCF_002242175.1
WP_096772335.1	rve+rve_3→||||<-?||?→||||<-?||PSE→||||<-?<-?||GT4+B12-binding+RADICAL-SAM*→	GT4+B12-binding+RADICAL-SAM	1313	GGE61_RS22535	Alphaproteobacteria	Rhizobium	MULTISPECIES: cobalamin B12-binding domain-containing protein [Rhizobium]	GCF_013087515.1

acc	operon	architecture	len	gen.name	taxend	species	defline	gca
WP_096876300.1	GT4+APATPase*→	GT4+APATPase	888	MKLM6_RS16005	Gammaproteobacteria	Methylomonas koyamae	glycosyltransferase [Methylomonas koyamae]	GCF_002441955.1
WP_097325277.1	WXG→?→ GT4*→	GT4	626	CRP55_RS32890	Actinobacteria	Actinoplanes atraurantiacus	glycosyltransferase family 4 protein [Actinoplanes atraurantiacus]	GCF_900215205.1
WP_097329102.1	wHTH+TPR+APATPase→?→?→||||<-?||GT4*→||||<-?||?→?→?→?→||||<-?<-WXG+Caspase	GT4	510	CRP55_RS49635	Actinobacteria	Actinoplanes atraurantiacus	glycosyltransferase family 4 protein [Actinoplanes atraurantiacus]	GCF_900215205.1
WP_097811834.1	GT4*→	GT4	600	CN930_RS12860	Firmicutes	Bacillus cereus	glycosyltransferase [Bacillus cereus]	GCF_002574885.1
WP_097931751.1	<-DNA_methylase||?→?→?→?→?→||||<-?||MNS+GT4*→	MNS+GT4	1170	CR483_RS02605	Actinobacteria	unclassified Streptomyces	MULTISPECIES: glycosyltransferase [unclassified Streptomyces]	GCF_002551215.1
WP_098141735.1	GT4*→?→?→?→?→?→ NLPC→	GT4	541	CN671_RS19510	Firmicutes	Bacillus toyonensis	glycosyltransferase [Bacillus toyonensis]	GCF_012395365.1
WP_098743858.1	STAND→?→ DUF4913→||||<-?<-?||?→ GT4*→?→?→?→ NUDIX→||||<-?<-ABC_tran	GT4	438	COO58_RS03965	Actinobacteria	Micromonospora sp. WMMA1996	glycosyltransferase family 4 protein [Micromonospora sp. WMMA1996]	GCF_002573675.1
WP_099013704.1	MNS+GT4*→	MNS+GT4	1149	SMALA_RS12770	Actinobacteria	Streptomyces malaysiensis	AAA family ATPase [Streptomyces malaysiensis]	GCF_002591335.1
WP_100783315.1	MFS→||||<-?||GT4+APATPase+TPR*→||||<-MODE-HTH+PBPII	GT4+APATPase+TPR	1318	COO64_RS24545	Gammaproteobacteria	Pseudomonas donghuensis	glycosyltransferase [Pseudomonas donghuensis]	GCF_002811135.1
WP_101210325.1	GT4*→ TPR→	GT4	541	AT251_RS15815	Gammaproteobacteria	Enterovibrio nigricans	hypothetical protein [Enterovibrio nigricans]	-
WP_101386266.1	-	-	1324	-	Actinobacteria	Streptomyces sp. TLI_146	caspase family protein [Streptomyces sp. TLI_146]	-
WP_101718466.1	<-ABC_tran<-?<-?<-?<-PSE<-?||?→?→ GT4*→	GT4	612	SGCZBJ_RS13280	Alphaproteobacteria	Caulobacter zeae	glycosyltransferase [Caulobacter zeae]	GCF_002858925.1

<table>
<tr><th>acc</th><th>operon</th><th>architecture</th><th>len</th><th>gen.name</th><th>taxend</th><th>species</th><th>defline</th><th>gca</th></tr>
<tr><td>WP_102909088.1</td><td>NUDIX→?→ GT4*→</td><td>GT4</td><td>433</td><td>C1J00_RS12325</td><td>Actinobacteria</td><td>Streptomyces sp. 13K301</td><td>glycosyltransferase family 4 protein [Streptomyces sp. 13K301]</td><td>GCF_002891435.1</td></tr>
<tr><td>WP_103291295.1</td><td>SWACOS+GT4*→?→||||<-?<-?||MFS→</td><td>SWACOS+GT4</td><td>1138</td><td>C1633_RS27595</td><td>Gammaproteobacteria</td><td>Pseudomonas protegens</td><td>glycosyltransferase [Pseudomonas protegens]</td><td>GCF_002899905.1</td></tr>
<tr><td>WP_103660162.1</td><td>NACHT+GT4*→</td><td>NACHT+GT4</td><td>1646</td><td>CPJ18_RS23160</td><td>Alphaproteobacteria</td><td>Agrobacterium rosae</td><td>glycosyltransferase [Agrobacterium rosae]</td><td>GCF_002915175.1</td></tr>
<tr><td>WP_104365810.1</td><td>GT4*→</td><td>GT4</td><td>428</td><td>C5E51_RS10910</td><td>Actinobacteria</td><td>Nocardia nova</td><td>glycosyltransferase family 4 protein [Nocardia nova]</td><td>GCF_002933545.1</td></tr>
<tr><td>WP_105007055.1</td><td>GT4*→?→||||<-PSE||?→?→ Calcineurin→</td><td>GT4</td><td>606</td><td>CUJ84_RS16890</td><td>Alphaproteobacteria</td><td>Rhizobium leguminosarum</td><td>glycosyltransferase family 4 protein [Rhizobium leguminosarum]</td><td>GCF_002953715.1</td></tr>
<tr><td>WP_106119969.1</td><td>Cluster214_2clades→ GT4*→</td><td>GT4</td><td>736</td><td>C7A11_RS23785</td><td>Gammaproteobacteria</td><td>Pseudomonas simiae</td><td>glycosyltransferase family 4 protein [Pseudomonas simiae]</td><td>GCF_003001535.1</td></tr>
<tr><td>WP_107027343.1</td><td>NACHT+GT4*→||||<-?<-?||PSE→ TIR→</td><td>NACHT+GT4</td><td>1646</td><td>C6558_RS13435</td><td>Alphaproteobacteria</td><td>Ensifer sp. NM-2</td><td>glycosyltransferase [Ensifer sp. NM-2]</td><td>GCF_003024455.1</td></tr>
<tr><td>WP_107161461.1</td><td>GT4+NACHT+BetaPropeller*→</td><td>GT4+NACHT+BetaPropeller</td><td>1536</td><td>C8054_RS26470</td><td>Actinobacteria</td><td>Micromonospora sp. RP3T</td><td>glycosyltransferase [Micromonospora sp. RP3T]</td><td>GCF_003027925.1</td></tr>
<tr><td>WP_107259933.1</td><td>nSTAND3→?→?→ GT4*→||||<-?<-?||?→||||<-?||?→ PSE→||||<-NUDIX</td><td>GT4</td><td>596</td><td>C6W10_RS21380</td><td>Actinobacteria</td><td>Plantactinospora sp. BB1</td><td>glycosyltransferase family 4 protein [Plantactinospora sp. BB1]</td><td>GCF_003030385.1</td></tr>
<tr><td>WP_107266333.1</td><td>nSTAND3→?→?→ GT4*→</td><td>GT4</td><td>596</td><td>C6361_RS00275</td><td>Actinobacteria</td><td>Plantactinospora sp. BC1</td><td>glycosyltransferase [Plantactinospora sp. BC1]</td><td>GCF_003030345.1</td></tr>
<tr><td>WP_107402046.1</td><td>Multi-TM→?→ STAND→?→ DUF4913→?→ GT4*→ Zeta_toxin→</td><td>GT4</td><td>374</td><td>AN216_RS27375</td><td>Actinobacteria</td><td>Streptomyces oceani</td><td>glycosyltransferase [Streptomyces oceani]</td><td>GCF_001751245.1</td></tr>
<tr><td>WP_107867044.1</td><td>LRR+LRR+LRR+AP-GTPase+COR+GT4*→</td><td>LRR+LRR+LRR+AP-GTPase+COR+GT4</td><td>1510</td><td>C8N29_RS15840</td><td>Firmicutes</td><td>Agitococcus lubricus</td><td>leucine-rich repeat protein [Agitococcus lubricus]</td><td>GCF_003051055.1</td></tr>
</table>

acc	operon	architecture	len	gen.name	taxend	species	defline	gca
WP_108006164.1	<-HTH+APATPase+TPR||GT4*→?→||||<-?<-?<-?||TPR→	GT4	421	C7821_RS00535	Actinobacteria	Streptomyces sp. VMFN-G11Ma	glycosyltransferase family 4 protein [Streptomyces sp. VMFN-G11Ma]	GCF_003046555.1
WP_108666907.1	NLPC→?→?→||||<-?||GT4*→	GT4	564	CUC05_RS14855	Actinobacteria	Euzebya rosea	glycosyltransferase family 4 protein [Euzebya rosea]	GCF_003073135.1
WP_109448176.1	MPTase+GT4*→	MPTase+GT4	753	CK485_RS28700	Actinobacteria	Streptomyces sp. ICBB 8177	DUF4157 domain-containing protein [Streptomyces sp. ICBB 8177]	GCF_003144095.1
WP_112357576.1	GT4*→	GT4	628	DTY03_RS10805	Alphaproteobacteria	Rhizobiales bacterium	glycosyltransferase family 4 protein [Rhizobiales bacterium]	-
WP_112733881.1	GT4+APATPase+TPR*→	GT4+APATPase+TPR	1361	ONO86_RS28915	Actinobacteria	Micromonospora noduli	glycosyltransferase [Micromonospora noduli]	GCF_003264435.1
WP_113016426.1	HTH+MPTase→||||<-?<-?<-?||GT4+B12-binding+RADICAL-SAM*→	GT4+B12-binding+RADICAL-SAM	1381	DUD68_RS27095	Alphaproteobacteria	Rhizobiales bacterium	radical SAM protein [Rhizobiales bacterium]	-
WP_113272579.1	HTH+MPTase→?→ Helicase_C→ SIG+DUF1998→||||<-?<-?||?→ NACHT+GT4*→	NACHT+GT4	1640	DUJ98_RS17735	Alphaproteobacteria	Rhizobiales bacterium	glycosyltransferase [Rhizobiales bacterium]	-
WP_114055463.1	TIR+APATPase+GT4*→	TIR+APATPase+GT4	775	C0216_RS13195	Actinobacteria	Streptomyces globosus	TIR domain-containing protein [Streptomyces globosus]	GCF_003325375.1
WP_114396907.1	GT4*→	GT4	529	DEF24_RS15180	Actinobacteria	Marinitenerispora sediminis	glycosyltransferase family 4 protein [Marinitenerispora sediminis]	GCF_003336445.1
WP_116211561.1	NACHT→ GT4*→	GT4	406	DXZ73_RS15355	Actinobacteria	Streptomyces olivoreticuli	glycosyltransferase family 4 protein [Streptomyces olivoreticuli]	GCF_003391135.1
WP_116568912.1	GT4*→?→||||<-?<-?||?→||||<-?<-MODE-HTH+PBPII	GT4	612	DDF65_RS17385	Alphaproteobacteria	Caulobacter radicis	glycosyltransferase [Caulobacter radicis]	GCF_003094595.1

<table>
<tr><th>acc</th><th>operon</th><th>architecture</th><th>len</th><th>gen.name</th><th>taxend</th><th>species</th><th>defline</th><th>gca</th></tr>
<tr><td>WP_120733613.1</td><td>GT4+B12-binding+RADICAL-SAM*→</td><td>GT4+B12-binding+RADICAL-SAM</td><td>1400</td><td>D7M10_RS18130</td><td>Gammaproteobacteria</td><td>Pseudomonas fluorescens</td><td>radical SAM protein [Pseudomonas fluorescens]</td><td>GCF_003626995.1</td></tr>
<tr><td>WP_120967974.1</td><td>GT4+APATPase+TPR+TPR*→</td><td>GT4+APATPase+TPR+TPR</td><td>1425</td><td>EAO39_RS12970</td><td>Betaproteobacteria</td><td>Comamonas sp. lk</td><td>tetratricopeptide repeat protein [Comamonas sp. lk]</td><td>GCF_900564145.1</td></tr>
<tr><td>WP_121165351.1</td><td>Sigma→?→?→?→?→?→ LRR+LRR+LRR+AP-GTPase+COR+GT4*→</td><td>LRR+LRR+LRR+AP-GTPase+COR+GT4</td><td>1488</td><td>B0H84_RS06470</td><td>Betaproteobacteria</td><td>Nitrosomonas sp. Nm120</td><td>leucine-rich repeat domain-containing protein [Nitrosomonas sp. Nm120]</td><td>GCF_003664085.1</td></tr>
<tr><td>WP_121799508.1</td><td>Multi-TM→?→ STAND→?→ DUF4913→?→ GT4*→</td><td>GT4</td><td>488</td><td>CTZ27_RS14745</td><td>Actinobacteria</td><td>Streptomyces griseocarneus</td><td>glycosyltransferase family 4 protein [Streptomyces griseocarneus]</td><td>GCF_003674045.1</td></tr>
<tr><td>WP_122257475.1</td><td>GT4+B12-binding+RADICAL-SAM*→</td><td>GT4+B12-binding+RADICAL-SAM</td><td>1421</td><td>ALP97_05253</td><td>Gammaproteobacteria</td><td>Pseudomonas salomonii</td><td>radical SAM protein [Pseudomonas salomonii]</td><td>-</td></tr>
<tr><td>WP_122528181.1</td><td>STAND+TPR+GT4*→</td><td>STAND+TPR+GT4</td><td>1067</td><td>EE945_RS06325</td><td>Gammaproteobacteria</td><td>Pseudomonas viridiflava</td><td>glycosyltransferase [Pseudomonas viridiflava]</td><td>GCF_900580695.1</td></tr>
<tr><td>WP_123299261.1</td><td>GT4+B12-binding+RADICAL-SAM*→||||<-?||?→?→||||<-?<-?<-?||MODE-HTH+PBPII→</td><td>GT4+B12-binding+RADICAL-SAM</td><td>1405</td><td>EC845_RS16630</td><td>Betaproteobacteria</td><td>Comamonas sp. BIGb0124</td><td>cobalamin B12-binding domain-containing protein [Comamonas sp. BIGb0124]</td><td>GCF_003752175.1</td></tr>
<tr><td>WP_123439297.1</td><td><-TPR+Caspase<-CATASP||CATRA-N+CATRA-C+GT4*→ HTH+APATPase+TPR→||||<-HTH||?→?→?→||||<-?<-CATASP</td><td>CATRA-N+CATRA-C+GT4</td><td>694</td><td>EDD94_RS26400</td><td>Actinobacteria</td><td>Streptomyces sp. PanSC9</td><td>glycosyltransferase family 4 protein [Streptomyces sp. PanSC9]</td><td>GCF_003751985.1</td></tr>
<tr><td>WP_124130642.1</td><td>STAND+GT4*→</td><td>STAND+GT4</td><td>1082</td><td>CJU35_RS32065</td><td>Gammaproteobacteria</td><td>Pseudomonas aeruginosa</td><td>glycosyltransferase [Pseudomonas aeruginosa]</td><td>GCF_901482505.1</td></tr>
<tr><td>WP_124284015.1</td><td>NUDIX→?→||||<-?||?→ MNS+GT4*→</td><td>MNS+GT4</td><td>1151</td><td>EES39_RS04745</td><td>Actinobacteria</td><td>Streptomyces sp. ADI92-24</td><td>glycosyltransferase [Streptomyces sp. ADI92-24]</td><td>GCF_003846195.1</td></tr>
<tr><td>WP_124458549.1</td><td>STAND+TPR+GT4*→||||<-?<-?<-?||Calcineurin→</td><td>STAND+TPR+GT4</td><td>1054</td><td>DBV14_RS07405</td><td>Betaproteobacteria</td><td>Variovorax sp. KBW07</td><td>glycosyltransferase [Variovorax sp. KBW07]</td><td>GCF_003852515.1</td></tr>
</table>

<table>
<tr><th>acc</th><th>operon</th><th>architecture</th><th>len</th><th>gen.name</th><th>taxend</th><th>species</th><th>defline</th><th>gca</th></tr>
<tr><td>WP_125624986.1</td><td>Multi-TM→?→ STAND→?→ DUF4913→?→ GT4*→?→ Zeta_toxin→||||<-ABC_tran</td><td>GT4</td><td>547</td><td>DMH02_RS17910</td><td>Actinobacteria</td><td>Streptomyces sp. WAC 00631</td><td>glycosyltransferase family 4 protein [Streptomyces sp. WAC 00631]</td><td>GCF_003947265.1</td></tr>
<tr><td>WP_125642507.1</td><td>MNS+GT4*→</td><td>MNS+GT4</td><td>1274</td><td>EF918_07135</td><td>Actinobacteria</td><td>Streptomyces sp. WAC06614</td><td>glycosyltransferase [Streptomyces sp. WAC06614]</td><td>-</td></tr>
<tr><td>WP_125650904.1</td><td>Caspase+NACHT+GT4*→</td><td>Caspase+NACHT+GT4</td><td>1208</td><td>EF918_25550</td><td>Actinobacteria</td><td>Streptomyces sp. WAC06614</td><td>glycosyltransferase [Streptomyces sp. WAC06614]</td><td>-</td></tr>
<tr><td>WP_125870434.1</td><td>GT4+B12-binding+RADICAL-SAM*→</td><td>GT4+B12-binding+RADICAL-SAM</td><td>1426</td><td>EGJ08_07220</td><td>Gammaproteobacteria</td><td>Pseudomonas stutzeri</td><td>radical SAM protein [Pseudomonas stutzeri]</td><td>-</td></tr>
<tr><td>WP_125995743.1</td><td>NACHT+GT4*→</td><td>NACHT+GT4</td><td>1650</td><td>BRX43_RS14145</td><td>Alphaproteobacteria</td><td>Sphingomonas sp. S-NIH.Pt15_0812</td><td>glycosyltransferase [Sphingomonas sp. S-NIH.Pt15_0812]</td><td>GCF_003951315.1</td></tr>
<tr><td>WP_127872272.1</td><td>NACHT+GT4*→||||<-Vsr</td><td>NACHT+GT4</td><td>1637</td><td>EN845_RS20190</td><td>Alphaproteobacteria</td><td>unclassified Mesorhizobium</td><td>MULTISPECIES: glycosyltransferase family 4 protein [unclassified Mesorhizobium]</td><td>-</td></tr>
<tr><td>WP_128145588.1</td><td>MPTase→ WXG+SecA→?→?→ GT4+TetR-HTH+TetR-HTH+Tox-ART-RSE+LuxR-HTH*→?→?→ ABhydrolase→</td><td>GT4+TetR-HTH+TetR-HTH+Tox-ART-RSE+LuxR-HTH</td><td>2725</td><td>NCTC13184_06625</td><td>Actinobacteria</td><td>Nocardia africana</td><td>glycosyltransferase [Nocardia africana]</td><td>-</td></tr>
<tr><td>WP_129557405.1</td><td><-TnpB_IS66<-HTH_Tnp_1||?→ SIG+GT4→ GT4→ tRNA→||||<-?<-PSE||?→||||<-?<-PSE<-?<-?||Calcineurin→||||<-cNMP_binding+HTH_Crp_2</td><td>SIG+GT4</td><td>316</td><td>NK6_RS51120</td><td>Alphaproteobacteria</td><td>Bradyrhizobium diazoefficiens</td><td>glycosyltransferase [Bradyrhizobium diazoefficiens]</td><td>GCF_001549695.1</td></tr>
<tr><td>WP_130174915.1</td><td>GT4+B12-binding+RADICAL-SAM*→</td><td>GT4+B12-binding+RADICAL-SAM</td><td>1409</td><td>EUX58_RS06685</td><td>Gammaproteobacteria</td><td>Pseudomonas</td><td>MULTISPECIES: radical SAM protein [Pseudomonas]</td><td>GCF_005930695.1</td></tr>
<tr><td>WP_130264935.1</td><td>GT4+APATPase+TPR+TPR*→||||<-MODE-HTH+PBPII</td><td>GT4+APATPase+TPR+TPR</td><td>1330</td><td>EXN22_RS15725</td><td>Gammaproteobacteria</td><td>Pseudomonas sp. SNU WT1</td><td>glycosyltransferase family 4 protein [Pseudomonas sp. SNU WT1]</td><td>GCF_004214895.1</td></tr>
<tr><td>WP_130343732.1</td><td>WXG+TM+EDA39C+Papain+Caspase+GT4*→?→ GT4→</td><td>WXG+TM+EDA39C+Papain+Caspase+GT4</td><td>2616</td><td>EV193_RS09710</td><td>Actinobacteria</td><td>Herbihabitans rhizosphaerae</td><td>glycosyltransferase [Herbihabitans rhizosphaerae]</td><td>GCF_004216555.1</td></tr>
<tr><td>WP_130721989.1</td><td>CASPASE→?→?→?→ GT4*→</td><td>GT4</td><td>606</td><td>ELI52_RS15625</td><td>Alphaproteobacteria</td><td>Rhizobium leguminosarum</td><td>glycosyltransferase [Rhizobium leguminosarum]</td><td>GCF_004303135.1</td></tr>
</table>

acc	operon	architecture	len	gen.name	taxend	species	defline	gca
WP_131447141.1	<-Ribosomal_L20<-?<-?<-?<-tRNA||?→||||<-?<-?<-PSE||APATPase+GT4+TPR+TPR+TPR+TPR+TPR*→||||<-?||?→ ABC_tran→	APATPase+GT4+TPR+TPR+TPR+TPR+TPR	1789	EZJ19_RS09965	Betaproteobacteria	Thiobacillaceae bacterium LSR1	tetratricopeptide repeat protein [Thiobacillaceae bacterium LSR1]	GCF_004337445.1
WP_131728683.1	GT4+STAND+TPR+TPR*→	GT4+STAND+TPR+TPR	1465	AOZ47_RS33140	Betaproteobacteria	Achromobacter xylosoxidans	tetratricopeptide repeat protein [Achromobacter xylosoxidans]	GCF_001299055.1
WP_131738585.1	<-VWA+APATPase+TPR+TPR+TPR+TPR<-STAND<-?||GT4*→?→||||<-?||MFS→	GT4	696	E1291_RS15920	Actinobacteria	Actinomadura roseirufa	glycosyltransferase family 4 protein [Actinomadura roseirufa]	GCF_900659635.1
WP_131761874.1	<-cNMP_binding+HTH_Crp_2||GT4*→||||<-HTH+APATPase+TPR||?→||||<-cNMP_binding+HTH_Crp_2	GT4	466	E1300_RS37495	Actinobacteria	Actinomadura fibrosa	glycosyltransferase family 4 protein [Actinomadura fibrosa]	GCF_900659615.1
WP_132113254.1	GT4*→||||<-TPR	GT4	404	EV192_RS08750	Actinobacteria	Actinocrispum wychmicini	glycosyltransferase family 4 protein [Actinocrispum wychmicini]	GCF_004345645.1
WP_132114377.1	cNMP_binding+HTH_Crp_2→ GT4*→||||<-ACYC+APATPase+TPR	GT4	407	EV192_RS12015	Actinobacteria	Actinocrispum wychmicini	glycosyltransferase family 4 protein [Actinocrispum wychmicini]	GCF_004345645.1
WP_132118129.1	EAD1+fvmYukDl-Nterm+GT4*→	EAD1+fvmYukDl-Nterm+GT4	667	EV192_RS21815	Actinobacteria	Actinocrispum wychmicini	glycosyltransferase family 4 protein [Actinocrispum wychmicini]	GCF_004345645.1
WP_132175767.1	GT4*→	GT4	453	E1263_RS36055	Actinobacteria	Kribbella antibiotica	glycosyltransferase family 4 protein [Kribbella antibiotica]	GCF_004349055.1
WP_132323823.1	GT4*→	GT4	446	E1218_RS23930	Actinobacteria	Kribbella sp. 16K104	glycosyltransferase family 4 protein [Kribbella sp. 16K104]	GCF_004348725.1
WP_133190629.1	GT4+B12-binding+RADICAL-SAM*→	GT4+B12-binding+RADICAL-SAM	1398	E1N52_RS41050	Betaproteobacteria	Paraburkholderia guartelaensis	radical SAM protein [Paraburkholderia guartelaensis]	GCF_004353905.1
WP_133255578.1	<-MODE-HTH+PBPII||?→||||<-MODE-HTH+PBPII||?→?→?→?→ NACHT+GT4*→	NACHT+GT4	1640	CPJ17_RS11090	Alphaproteobacteria	Rhizobium tumorigenes	glycosyltransferase [Rhizobium tumorigenes]	GCF_003240565.1

<table>
<thead>
<tr><th>acc</th><th>operon</th><th>architecture</th><th>len</th><th>gen.name</th><th>taxend</th><th>species</th><th>defline</th><th>gca</th></tr>
</thead>
<tbody>
<tr><td>WP_133739590.1</td><td>STAND→?→?→ DUF4913→||||<-AbiEii<-AbiEi_4||?→ GT4*→</td><td>GT4</td><td>493</td><td>EV190_RS01575</td><td>Actinobacteria</td><td>Actinorugispora endophytica</td><td>glycosyltransferase family 4 protein [Actinorugispora endophytica]</td><td>GCF_004363075.1</td></tr>
<tr><td>WP_133854316.1</td><td>Helicase_C→ SIG+DUF1998→||||<-?||?→?→||||<-?||?→ GT4→ TIR+APATPase+TPR→ TPR+Caspase→||||<-TPR+Caspase<-CATASP||CATRA-N+CATRA-C+GT4→</td><td>GT4</td><td>423</td><td>EV186_RS37520</td><td>Actinobacteria</td><td>Labedaea rhizosphaerae</td><td>glycosyltransferase family 4 protein [Labedaea rhizosphaerae]</td><td>GCF_004362825.1</td></tr>
<tr><td>WP_133925636.1</td><td>GT4*→</td><td>GT4</td><td>622</td><td>EV573_RS04015</td><td>Actinobacteria</td><td>Streptomyces sp. BK161</td><td>glycosyltransferase [Streptomyces sp. BK161]</td><td>GCF_004364215.1</td></tr>
<tr><td>WP_133927416.1</td><td>SWACOS+GT4*→||||<-?<-?||?→||||<-?<-?<-ABC_tran</td><td>SWACOS+GT4</td><td>1159</td><td>EV573_RS13335</td><td>Actinobacteria</td><td>Streptomyces sp. BK161</td><td>glycosyltransferase [Streptomyces sp. BK161]</td><td>GCF_004364215.1</td></tr>
<tr><td>WP_133932223.1</td><td><-Sigma<-?<-TPR||GT4*→</td><td>GT4</td><td>410</td><td>EV573_RS39315</td><td>Actinobacteria</td><td>Streptomyces sp. BK161</td><td>glycosyltransferase family 4 protein [Streptomyces sp. BK161]</td><td>GCF_004364215.1</td></tr>
<tr><td>WP_134939719.1</td><td>GT4+B12-binding+RADICAL-SAM*→</td><td>GT4+B12-binding+RADICAL-SAM</td><td>1421</td><td>BZ164_RS25605</td><td>Gammaproteobacteria</td><td>Pseudomonas veronii</td><td>radical SAM protein [Pseudomonas veronii]</td><td>-</td></tr>
<tr><td>WP_135904996.1</td><td><-Vsr||?→?→?→||||<-?<-?||GT4+Trypsin+PNPase*→</td><td>GT4+Trypsin+PNPase</td><td>836</td><td>EN818_RS17550</td><td>Alphaproteobacteria</td><td>unclassified Mesorhizobium</td><td>MULTISPECIES: glycosyltransferase [unclassified Mesorhizobium]</td><td>-</td></tr>
<tr><td>WP_136526013.1</td><td>SWACOS+GT4*→</td><td>SWACOS+GT4</td><td>1144</td><td>E8L22_RS15545</td><td>Deltaproteobacteria</td><td>Geomonas ferrireducens</td><td>glycosyltransferase [Geomonas ferrireducens]</td><td>-</td></tr>
<tr><td>WP_136738577.1</td><td>GT4*→</td><td>GT4</td><td>394</td><td>FCH28_RS05590</td><td>Actinobacteria</td><td>Streptomyces sp. jys28</td><td>glycosyltransferase family 4 protein [Streptomyces sp. jys28]</td><td>GCF_005048155.1</td></tr>
<tr><td>WP_136743126.1</td><td>MNS+GT4*→</td><td>MNS+GT4</td><td>1188</td><td>FCH28_RS29075</td><td>Actinobacteria</td><td>Streptomyces sp. jys28</td><td>glycosyltransferase [Streptomyces sp. jys28]</td><td>GCF_005048155.1</td></tr>
<tr><td>WP_138637396.1</td><td>NUDIX→?→ GT4*→</td><td>GT4</td><td>432</td><td>ETD96_RS16755</td><td>Actinobacteria</td><td>Actinomadura geliboluensis</td><td>glycosyltransferase family 4 protein [Actinomadura geliboluensis]</td><td>GCF_005889745.1</td></tr>
<tr><td>WP_141100843.1</td><td>GT4+HD*→</td><td>GT4+HD</td><td>1421</td><td>CDN99_RS17245</td><td>Betaproteobacteria</td><td>Roseateles aquatilis</td><td>HD domain-containing protein [Roseateles aquatilis]</td><td>GCF_002205645.1</td></tr>
</tbody>
</table>

acc	operon	architecture	len	gen.name	taxend	species	defline	gca
WP_141569577.1	ZetaToxin+GT4+NUDIX+ART+NTase*→?→?→?→||||<-Caspase	ZetaToxin+GT4+NUDIX+ART+NTase	2159	SipoB123_RS12910	Actinobacteria	Streptomyces ipomoeae	zeta toxin family protein [Streptomyces ipomoeae]	GCF_006547165.1
WP_141585352.1	ZetaToxin+GT4+NUDIX+ART+NTase*→?→?→?→||||<-Caspase<-?||?→ PSE→||||<-NLPC	ZetaToxin+GT4+NUDIX+ART+NTase	2156	Sipo7851_RS16610	Actinobacteria	Streptomyces ipomoeae	zeta toxin family protein [Streptomyces ipomoeae]	GCF_006547175.1
WP_141714270.1	<-TPR+Caspase||GT4*→	GT4	542	GA0074694_RS24775	Actinobacteria	Micromonospora inyonensis	glycosyltransferase [Micromonospora inyonensis]	GCF_900091415.1
WP_141749885.1	GT4+NACHT*→||||<-MFS	GT4+NACHT	1197	JAB9_RS27490	Betaproteobacteria	Janthinobacterium sp. HH107	glycosyltransferase family 4 protein [Janthinobacterium sp. HH107]	GCF_001758765.1
WP_141888935.1	MFS→||||<-ABC_tran<-?<-?<-?<-?||GT4*→	GT4	608	FB468_RS16790	Actinobacteria	Leucobacter komagatae	glycosyltransferase [Leucobacter komagatae]	GCF_006716085.1
WP_141962659.1	<-HTH+APATPase+TPR<-TPR+Caspase<-CATASP||CATRA-N+CATRA-C+GT4*→||||<-?<-?<-?<-?<-?<-REase+SFI-ATPase	CATRA-N+CATRA-C+GT4	732	FB559_RS39205	Actinobacteria	Actinoallomurus bryophytorum	glycosyltransferase [Actinoallomurus bryophytorum]	GCF_006716425.1
WP_142260239.1	MNS+GT4*→?→||||<-?<-Sigma<-?||ABC_tran→	MNS+GT4	1152	FHX40_RS15230	Actinobacteria	Thermopolyspora flexuosa	glycosyltransferase [Thermopolyspora flexuosa]	GCF_014647775.1
WP_142588942.1	NACHT+GT4*→	NACHT+GT4	1640	HNP80_RS07130	Alphaproteobacteria	Rhizobium halotolerans	glycosyltransferase [Rhizobium halotolerans]	GCF_902153235.2
WP_143026968.1	LRR+AP-GTPase+COR→ GT4*→	GT4	484	BMY41_RS11080	Betaproteobacteria	Nitrosomonas oligotropha	glycosyltransferase [Nitrosomonas oligotropha]	GCF_900106555.1
WP_143176277.1	AA_permease→?→ GT4+APATPase+TPR+TPR*→	GT4+APATPase+TPR+TPR	1172	BGV47_RS35370	Betaproteobacteria	Burkholderia ubonensis	tetratricopeptide repeat protein, partial [Burkholderia ubonensis]	GCF_001901485.1
WP_143660221.1	<-TPR+Caspase<-CATASP||CATRA-N+CATRA-C+GT4*→ HTH+APATPase+TPR→||||<-HTH||?→?→?→||||<-?<-CATASP	CATRA-N+CATRA-C+GT4	703	DDV98_RS32255	Actinobacteria	unclassified Streptomyces	MULTISPECIES: glycosyltransferase [unclassified Streptomyces]	GCF_001983595.1
WP_143740316.1	<-TPR+Caspase||GT4*→?→?→||||<-?<-?<-ABC_tran	GT4	546	GA0070618_RS24540	Actinobacteria	Micromonospora echinospora	glycosyltransferase [Micromonospora echinospora]	GCF_900091495.1

<table>
<thead>
<tr><th>acc</th><th>operon</th><th>architecture</th><th>len</th><th>gen.name</th><th>taxend</th><th>species</th><th>defline</th><th>gca</th></tr>
</thead>
<tbody>
<tr><td>WP_143788398.1</td><td>STAND+TPR+GT4*→</td><td>STAND+TPR+GT4</td><td>1136</td><td>BUS12_RS18575</td><td>Betaproteobacteria</td><td>Paraburkholderia phenazinium</td><td>glycosyltransferase [Paraburkholderia phenazinium]</td><td>GCF_900142845.1</td></tr>
<tr><td>WP_143860247.1</td><td>WXG+MPTase→ WXG+SecA+LuxR-HTH+LuxR-HTH+LuxR-HTH+LuxR-HTH+ST-Hiskin+MPTase+GT4+TetR-HTH+TetR-HTH+Tox-ART-RSE+LuxR-HTH+LuxR-HTH+LuxR-HTH+GNTR-HTH+LuxR-HTH+LuxR-HTH+ST-Hiskin+ST-Hiskin+XPC-C+TM+TM+TM+TM+LuxR-HTH*→</td><td>WXG+SecA+LuxR-HTH+LuxR-HTH+LuxR-HTH+LuxR-HTH+ST-Hiskin+MPTase+GT4+TetR-HTH+TetR-HTH+Tox-ART-RSE+LuxR-HTH+LuxR-HTH+LuxR-HTH+GNTR-HTH+LuxR-HTH+LuxR-HTH+ST-Hiskin+ST-Hiskin+XPC-C+TM+TM+TM+TM+LuxR-HTH</td><td>11649</td><td>B7C42_RS30150</td><td>Actinobacteria</td><td>Nocardia cerradoensis</td><td>MFS transporter, partial [Nocardia cerradoensis]</td><td>GCF_002236815.1</td></tr>
<tr><td>WP_144299077.1</td><td>GT4+B12-binding+RADICAL-SAM*→</td><td>GT4+B12-binding+RADICAL-SAM</td><td>1385</td><td>VARPA_RS03315</td><td>Betaproteobacteria</td><td>Variovorax paradoxus</td><td>radical SAM protein [Variovorax paradoxus]</td><td>GCF_000184745.1</td></tr>
<tr><td>WP_144384941.1</td><td>Multi-TM→?→ STAND→?→ DUF4913→?→ GT4*→</td><td>GT4</td><td>497</td><td>CD790_RS21520</td><td>Actinobacteria</td><td>Streptomyces sp. SAJ15</td><td>glycosyltransferase [Streptomyces sp. SAJ15]</td><td>GCF_007595705.1</td></tr>
<tr><td>WP_145763552.1</td><td>NUDIX→?→ MNS+GT4*→</td><td>MNS+GT4</td><td>1151</td><td>FHX80_RS07940</td><td>Actinobacteria</td><td>Streptomyces brevispora</td><td>glycosyltransferase [Streptomyces brevispora]</td><td>GCF_007829885.1</td></tr>
<tr><td>WP_145809941.1</td><td>MNS+GT4*→?→?→||||<-?||?→ CSD→</td><td>MNS+GT4</td><td>1180</td><td>FHW51_RS26100</td><td>Actinobacteria</td><td>Streptomyces argenteolus</td><td>glycosyltransferase [Streptomyces argenteolus]</td><td>GCF_007829855.1</td></tr>
<tr><td>WP_145818563.1</td><td><-TPR+Caspase||GT4*→?→||||<-?<-?<-ABC_tran</td><td>GT4</td><td>546</td><td>JD81_RS15875</td><td>Actinobacteria</td><td>Micromonospora sagamiensis</td><td>glycosyltransferase [Micromonospora sagamiensis]</td><td>GCF_014680085.1</td></tr>
<tr><td>WP_145835614.1</td><td><-TPR+Caspase||GT4*→</td><td>GT4</td><td>546</td><td>JD80_RS20680</td><td>Actinobacteria</td><td>Micromonospora echinospora</td><td>glycosyltransferase [Micromonospora echinospora]</td><td>GCF_007829475.1</td></tr>
<tr><td>WP_145885265.1</td><td>ABhydrolase→||||<-?<-?||?→||||<-?||?→?→ MNS+GT4*→</td><td>MNS+GT4</td><td>1144</td><td>FB157_RS37885</td><td>Actinobacteria</td><td>Streptomyces sp. BK340</td><td>glycosyltransferase [Streptomyces sp. BK340]</td><td>GCF_007827595.1</td></tr>
<tr><td>WP_145921569.1</td><td>WXG+TM+GT4+GT4*→</td><td>WXG+TM+GT4+GT4</td><td>3484</td><td>FJK98_RS27630</td><td>Actinobacteria</td><td>Micromonospora sp. HM134</td><td>glycosyltransferase [Micromonospora sp. HM134]</td><td>GCF_007833915.1</td></tr>
<tr><td>WP_146161220.1</td><td>WXG+MPTase→ WXG+LuxR-HTH+LuxR-HTH+LuxR-HTH+LuxR-HTH+TetR-HTH+TetR-HTH+LuxR-HTH+LuxR-HTH+LexA-HTH+LuxR-HTH+LuxR-HTH+LuxR-HTH+LuxR-HTH+LuxR-HTH+PP2C*→?→ ABhydrolase→</td><td>WXG+LuxR-HTH+LuxR-HTH+LuxR-HTH+LuxR-HTH+TetR-HTH+TetR-HTH+LuxR-HTH+LuxR-HTH+LexA-HTH+LuxR-HTH+LuxR-HTH+LuxR-HTH+LuxR-HTH+LuxR-HTH+PP2C</td><td>14195</td><td>C8258_RS13420</td><td>Actinobacteria</td><td>Nocardia sp. MDA0666</td><td>MFS transporter [Nocardia sp. MDA0666]</td><td>GCF_003023875.1</td></tr>
<tr><td>WP_146241295.1</td><td>APATPase+TPR→||||<-?||GT4*→?→?→ GT4→</td><td>GT4</td><td>502</td><td>DFQ13_RS33200</td><td>Actinobacteria</td><td>Actinokineospora mzabensis</td><td>glycosyltransferase [Actinokineospora mzabensis]</td><td>-</td></tr>
<tr><td>WP_146349043.1</td><td><-APATPase<-APATPase||?→ NLPC→ cNMP__binding+HTH_Crp_2→?→ GT4*→||||<-ACYC+APATPase+TPR</td><td>GT4</td><td>408</td><td>FKR81_RS01510</td><td>Actinobacteria</td><td>Lentzea sp. FXJ1.1311</td><td>glycosyltransferase [Lentzea sp. FXJ1.1311]</td><td>GCF_007845675.1</td></tr>
</tbody>
</table>

<table>
<tr><th>acc</th><th>operon</th><th>architecture</th><th>len</th><th>gen.name</th><th>taxend</th><th>species</th><th>defline</th><th>gca</th></tr>
<tr><td>WP_147321781.1</td><td>SWACOS+GT4*→||||<-?<-MFS</td><td>SWACOS+GT4</td><td>1096</td><td>BX279_RS09075</td><td>Actinobacteria</td><td>Streptomyces sp. Ag82_O1-9</td><td>glycosyltransferase [Streptomyces sp. Ag82_O1-9]</td><td>GCF_003386845.1</td></tr>
<tr><td>WP_147416956.1</td><td>STAND→?→?→ DUF4913→||||<-AbiEii<-AbiEi_4||?→ GT4*→ AbiEi_4→</td><td>GT4</td><td>495</td><td>NI17_RS16590</td><td>Actinobacteria</td><td>Thermobifida halotolerans</td><td>glycosyltransferase [Thermobifida halotolerans]</td><td>GCF_003574835.1</td></tr>
<tr><td>WP_147994072.1</td><td>TRPR-HTH+PBPI→?→?→||||<-TPR+Caspase||GT4*→</td><td>GT4</td><td>597</td><td>EAO75_RS06975</td><td>Actinobacteria</td><td>Streptomyces sp. uw30</td><td>glycosyltransferase [Streptomyces sp. uw30]</td><td>GCF_008042075.1</td></tr>
<tr><td>WP_148348287.1</td><td>SWACOS+GT4*→</td><td>SWACOS+GT4</td><td>1238</td><td>FXF65_RS04105</td><td>Actinobacteria</td><td>Actinomadura syzygii</td><td>glycosyltransferase [Actinomadura syzygii]</td><td>-</td></tr>
<tr><td>WP_148640710.1</td><td>MNS+GT4*→</td><td>MNS+GT4</td><td>1142</td><td>EH183_RS01595</td><td>Actinobacteria</td><td>Streptomyces sp. CB01881</td><td>glycosyltransferase [Streptomyces sp. CB01881]</td><td>GCF_002953255.1</td></tr>
<tr><td>WP_148757712.1</td><td>STAND→ VWA+vWA-L+STAND+TPR+GT4*→ Sigma→?→ ABC_tran→</td><td>VWA+vWA-L+STAND+TPR+GT4</td><td>1697</td><td>FXF68_RS05215</td><td>Actinobacteria</td><td>Actinomadura sp. CYP1-5</td><td>glycosyltransferase [Actinomadura sp. CYP1-5]</td><td>GCF_008121305.1</td></tr>
<tr><td>WP_148758378.1</td><td>SWACOS+GT4*→</td><td>SWACOS+GT4</td><td>1147</td><td>FXF68_RS08315</td><td>Actinobacteria</td><td>Actinomadura sp. CYP1-5</td><td>glycosyltransferase [Actinomadura sp. CYP1-5]</td><td>GCF_008121305.1</td></tr>
<tr><td>WP_148759187.1</td><td>GT4*→</td><td>GT4</td><td>581</td><td>FXF68_RS13230</td><td>Actinobacteria</td><td>Actinomadura sp. CYP1-5</td><td>glycosyltransferase [Actinomadura sp. CYP1-5]</td><td>GCF_008121305.1</td></tr>
<tr><td>WP_149263194.1</td><td>LexA→||||<-?||?→||||<-?<-?<-?<-cNMP_binding+HTH_Crp_2||GT4*→||||<-HTH+APATPase+TPR</td><td>GT4</td><td>459</td><td>K4S16_RS34110</td><td>Actinobacteria</td><td>Actinomadura sp. K4S16</td><td>glycosyltransferase [Actinomadura sp. K4S16]</td><td>GCF_008327685.1</td></tr>
<tr><td>WP_149830792.1</td><td><-HTH+APATPase+TPR||GT4*→?→||||<-?<-?<-?<-?||TPR→</td><td>GT4</td><td>419</td><td>F3T56_RS42265</td><td>Actinobacteria</td><td>Streptomyces sp. TRM68348</td><td>glycosyltransferase [Streptomyces sp. TRM68348]</td><td>GCF_008386495.1</td></tr>
<tr><td>WP_149896003.1</td><td>NACHT+GT4*→</td><td>NACHT+GT4</td><td>1646</td><td>DXU01_RS22280</td><td>Alphaproteobacteria</td><td>Agrobacterium rosae</td><td>glycosyltransferase [Agrobacterium rosae]</td><td>GCF_008501885.1</td></tr>
<tr><td>WP_151009920.1</td><td><-cNMP_binding+HTH_Crp_2||GT4*→||||<-HTH+APATPase+TPR</td><td>GT4</td><td>457</td><td>F7P10_RS15075</td><td>Actinobacteria</td><td>Actinomadura sp. WMMB 499</td><td>glycosyltransferase [Actinomadura sp. WMMB 499]</td><td>GCF_008824145.1</td></tr>
<tr><td>WP_153338839.1</td><td>ESX-1_EspG→||||<-?<-?||?→?→?→ WXG+TOXIN-PL+ART+Caspase+ZetaToxin+GT4*→</td><td>WXG+TOXIN-PL+ART+Caspase+ZetaToxin+GT4</td><td>3261</td><td>NRB56_RS02760</td><td>Actinobacteria</td><td>Nocardia sp. RB56</td><td>zeta toxin family protein [Nocardia sp. RB56]</td><td>GCF_009604425.1</td></tr>
</table>

<table>
<tr><th>acc</th><th>operon</th><th>architecture</th><th>len</th><th>gen.name</th><th>taxend</th><th>species</th><th>defline</th><th>gca</th></tr>
<tr><td>WP_153430492.1</td><td>GT4*→</td><td>GT4</td><td>1126</td><td>GFJ39_RS06035</td><td>Alphaproteobacteria</td><td>Gluconobacter sp. AC10</td><td>glycosyltransferase [Gluconobacter sp. AC10]</td><td>-</td></tr>
<tr><td>WP_153467262.1</td><td>GT4*→</td><td>GT4</td><td>460</td><td>F7Q99_RS30660</td><td>Actinobacteria</td><td>Streptomyces kaniharaensis</td><td>glycosyltransferase [Streptomyces kaniharaensis]</td><td>-</td></tr>
<tr><td>WP_153535837.1</td><td>REase+SFI-ATPase→||||<-?||?→||||<-?||?→ GT4+APATPase+TPR+TPR*→</td><td>GT4+APATPase+TPR+TPR</td><td>1190</td><td>ACRB68_RS23060</td><td>Actinobacteria</td><td>Actinomadura sp. RB68</td><td>tetratricopeptide repeat protein [Actinomadura sp. RB68]</td><td>-</td></tr>
<tr><td>WP_154363881.1</td><td>TniB→?→ STAND+TPR+GT4*→</td><td>STAND+TPR+GT4</td><td>1054</td><td>GJ697_RS09905</td><td>Betaproteobacteria</td><td>Duganella</td><td>MULTISPECIES: glycosyltransferase [Duganella]</td><td>GCF_009674525.1</td></tr>
<tr><td>WP_155055777.1</td><td>TIR+APATPase+GT4*→</td><td>TIR+APATPase+GT4</td><td>772</td><td>GFH49_RS11700</td><td>Actinobacteria</td><td>Streptomyces sp. TRM63209</td><td>TIR domain-containing protein [Streptomyces sp. TRM63209]</td><td>GCF_009709555.1</td></tr>
<tr><td>WP_155097668.1</td><td>GT4*→</td><td>GT4</td><td>609</td><td>GL286_RS21805</td><td>Alphaproteobacteria</td><td>Paracoccus aestuariivivens</td><td>hypothetical protein [Paracoccus aestuariivivens]</td><td>GCF_009711225.1</td></tr>
<tr><td>WP_155252689.1</td><td>Multi-TM→?→ STAND→?→ DUF4913→||||<-?||?→ GT4*→?→?→?→ NUDIX→</td><td>GT4</td><td>438</td><td>B170_RS0111915</td><td>Actinobacteria</td><td>Salinispora pacifica</td><td>glycosyltransferase [Salinispora pacifica]</td><td>GCF_000514515.1</td></tr>
<tr><td>WP_156002545.1</td><td><-AbiEii<-AbiEi_4||AbiEi_4→ AbiEii→||||<-AbiEii<-AbiEi_4||?→ GT4*→||||<-DUF4913<-?<-?<-STAND</td><td>GT4</td><td>494</td><td>FZ103_RS17370</td><td>Actinobacteria</td><td>Streptomonospora sp. PA3</td><td>glycosyltransferase [Streptomonospora sp. PA3]</td><td>GCF_009728995.1</td></tr>
<tr><td>WP_156395661.1</td><td>STAND+GT4*→</td><td>STAND+GT4</td><td>1081</td><td>ASC85_RS19490</td><td>Gammaproteobacteria</td><td>Pseudomonas sp. Root401</td><td>glycosyltransferase [Pseudomonas sp. Root401]</td><td>GCF_001425105.1</td></tr>
<tr><td>WP_156427756.1</td><td>APATPase+TPR+GT4*→</td><td>APATPase+TPR+GT4</td><td>1394</td><td>ML01_RS15555</td><td>Gammaproteobacteria</td><td>Thiohalocapsa sp. ML1</td><td>tetratricopeptide repeat protein [Thiohalocapsa sp. ML1]</td><td>GCF_001469165.1</td></tr>
<tr><td>WP_156551292.1</td><td>NACHT+GT4*→</td><td>NACHT+GT4</td><td>1640</td><td>GOZ95_RS24735</td><td>Alphaproteobacteria</td><td>Agrobacterium vitis</td><td>glycosyltransferase [Agrobacterium vitis]</td><td>GCF_009744055.1</td></tr>
<tr><td>WP_156901549.1</td><td>GT4*→?→||||<-?<-?<-?||?→||||<-MFS</td><td>GT4</td><td>643</td><td>K328_RS25440</td><td>Alphaproteobacteria</td><td>Nisaea denitrificans</td><td>glycosyltransferase, partial [Nisaea denitrificans]</td><td>GCF_000426505.1</td></tr>
</table>

<table>
<tr><th>acc</th><th>operon</th><th>architecture</th><th>len</th><th>gen.name</th><th>taxend</th><th>species</th><th>defline</th><th>gca</th></tr>
<tr><td>WP_157059028.1</td><td>GT4*→</td><td>GT4</td><td>261</td><td>AN191_RS16995</td><td>Alphaproteobacteria</td><td>Loktanella sp. 5RATIMAR09</td><td>glycosyltransferase family 4 protein, partial [Loktanella sp. 5RATIMAR09]</td><td>GCF_001419985.1</td></tr>
<tr><td>WP_157097111.1</td><td>GT4*→?→||||<-?<-?<-?||HTH→</td><td>GT4</td><td>608</td><td>FHS67_RS12310</td><td>Alphaproteobacteria</td><td>Aminobacter aminovorans</td><td>glycosyltransferase [Aminobacter aminovorans]</td><td>GCF_001605015.1</td></tr>
<tr><td>WP_157104014.1</td><td>WXG+MPTase→ WXG+SecA+ST-Hiskin+HTH+HTH+HTH+HTH+HTH+HTH+HTH+HTH+ST-Hiskin+MPTase+GT4+TetR-HTH+TetR-HTH+LuxR-HTH+LuxR-HTH+LuxR-HTH+GNTR-HTH+LuxR-HTH+LuxR-HTH+ST-Hiskin+ST-Hiskin+LuxR-HTH+TM+TM+TM+TM+MPTase+MPTase*→?→ ABhydrolase→</td><td>WXG+SecA+ST-Hiskin+HTH+HTH+HTH+HTH+HTH+HTH+HTH+HTH+ST-Hiskin+MPTase+GT4+TetR-HTH+TetR-HTH+LuxR-HTH+LuxR-HTH+LuxR-HTH+GNTR-HTH+LuxR-HTH+LuxR-HTH+ST-Hiskin+ST-Hiskin+LuxR-HTH+TM+TM+TM+TM+MPTase+MPTase</td><td>11529</td><td>NK1_RS00230</td><td>Actinobacteria</td><td>Nocardia kruczakiae</td><td>MFS transporter [Nocardia kruczakiae]</td><td>GCF_001612965.1</td></tr>
<tr><td>WP_157106745.1</td><td>MPTase→ WXG+SecA+GT4+TetR-HTH+TetR-HTH+MPTase+HTH+HTH+HTH+HTH+HTH+CHTH+HTH+HTH*→</td><td>WXG+SecA+GT4+TetR-HTH+TetR-HTH+MPTase+HTH+HTH+HTH+HTH+HTH+CHTH+HTH+HTH</td><td>5359</td><td>NA8_RS16815</td><td>Actinobacteria</td><td>Nocardia arthritidis</td><td>helix-turn-helix domain-containing protein [Nocardia arthritidis]</td><td>GCF_001612765.1</td></tr>
<tr><td>WP_157114874.1</td><td><-ABC_tran<-?<-?||?→?→ MPTase→ WXG+SecA+GT4+TetR-HTH+TetR-HTH+HTH+HTH+HTH+HTH+HTH+CHTH+MPTase*→</td><td>WXG+SecA+GT4+TetR-HTH+TetR-HTH+HTH+HTH+HTH+HTH+HTH+CHTH+MPTase</td><td>6963</td><td>NNI01S_RS1999[illegible]</td><td>Actinobacteria</td><td>Nocardia niwae</td><td>helix-turn-helix domain-containing protein [Nocardia niwae]</td><td>GCF_001613465.1</td></tr>
<tr><td>WP_1571206[illegible].1</td><td>TRPR-HTH+HTH+HTH+HTH+CHTH+HTH+HTH+NUDIX+NUDIX+Tox-MCF+GT4*→?→||||<-ESX-1_EspG<-?<-PSE||?→?→||||<-?||WXG+TM+TM+TM+TM+FN3+MPTase+MPTase+MPTase+MPTase+MPTase+MPTase+MPTase+MPTase+MPTase+MPTase+MPTase+MPTase+MPTase+MPTase+MPTase→</td><td>TRPR-HTH+HTH+HTH+HTH+CHTH+HTH+HTH+NUDIX+NUDIX+Tox-MCF+GT4</td><td>11280</td><td>NF4_RS27240</td><td>Actinobacteria</td><td>Nocardia fusca</td><td>helix-turn-helix domain-containing protein [Nocardia fusca]</td><td>GCF_001618425.1</td></tr>
<tr><td>WP_157120699.1</td><td><-MPTase<-?||?→?→ MPTase→ WXG+SecA+GT4+MPTase+LuxR-HTH+Abhydrolase*→?→||||<-?<-?||PSE→?→||||<-?||SIG+SnoaL→</td><td>WXG+SecA+GT4+MPTase+LuxR-HTH+Abhydrolase</td><td>4137</td><td>NF4_RS31035</td><td>Actinobacteria</td><td>Nocardia fusca</td><td>glycosyltransferase [Nocardia fusca]</td><td>GCF_001618425.1</td></tr>
<tr><td>WP_157121874.1</td><td>WXG+MPTase→ WXG+SecA+ST-Hiskin+LuxR-HTH+LuxR-HTH+LuxR-HTH+LuxR-HTH+GT4+TetR-HTH+TetR-HTH+ART+SIGMA-HTH+LuxR-HTH+LuxR-HTH+LuxR-HTH+GNTR-HTH+LuxR-HTH+LuxR-HTH+ST-Hiskin+ST-Hiskin+XPC-C+LuxR-HTH+LuxR-HTH+MFS(6xTM)+PP2C+STYKinase-like*→?→ ABhydrolase→</td><td>WXG+SecA+ST-Hiskin+LuxR-HTH+LuxR-HTH+LuxR-HTH+LuxR-HTH+GT4+TetR-HTH+TetR-HTH+ART+SIGMA-HTH+LuxR-HTH+LuxR-HTH+LuxR-HTH+GNTR-HTH+LuxR-HTH+LuxR-HTH+ST-Hiskin+ST-Hiskin+XPC-C+LuxR-HTH+LuxR-HTH+MFS(6xTM)+PP2C+STYKinase-like</td><td>12511</td><td>NV5_RS14795</td><td>Actinobacteria</td><td>Nocardia violaceofusca</td><td>MFS transporter [Nocardia violaceofusca]</td><td>GCF_001613525.1</td></tr>
<tr><td>WP_157126117.1</td><td>WXG+MPTase→ WXG+SecA+ST-Hiskin+HTH+HTH+HTH+HTH+HTH+HTH+HTH+HTH+ST-Hiskin+MPTase+GT4+TetR-HTH+TetR-HTH+LuxR-HTH+LuxR-HTH+LuxR-HTH+LuxR-HTH+GNTR-HTH+LuxR-HTH+LuxR-HTH+PP2C+MPTase+MPTase+8TMR-UT+TM+TM+ST-Hiskin*→?→ ABhydrolase→</td><td>WXG+SecA+ST-Hiskin+HTH+HTH+HTH+HTH+HTH+HTH+HTH+HTH+ST-Hiskin+MPTase+GT4+TetR-HTH+TetR-HTH+LuxR-HTH+LuxR-HTH+LuxR-HTH+LuxR-HTH+GNTR-HTH+LuxR-HTH+LuxR-HTH+PP2C+MPTase+MPTase+8TMR-UT+TM+TM+ST-Hiskin</td><td>15995</td><td>NMI01S_RS1004[illegible]</td><td>Actinobacteria</td><td>Nocardia mikamii</td><td>MFS transporter [Nocardia mikamii]</td><td>GCF_001613505.1</td></tr>
<tr><td>WP_157129222.1</td><td>HTH+APATPase+TPR→?→ GT4*→||||<-?<-?<-AAA_21</td><td>GT4</td><td>601</td><td>NA4_RS22535</td><td>Actinobacteria</td><td>Nocardia amamiensis</td><td>glycosyltransferase [Nocardia amamiensis]</td><td>GCF_001612745.1</td></tr>
<tr><td>WP_157163478.1</td><td>WXG+MPTase→ WXG+SecA+ST-Hiskin+LuxR-HTH+LuxR-HTH+LuxR-HTH+LuxR-HTH+GT4+TetR-HTH+TetR-HTH+ART+SIGMA-HTH+LuxR-HTH+LuxR-HTH+LuxR-HTH+GNTR-HTH+LuxR-HTH+LuxR-HTH+ST-Hiskin+ST-Hiskin+XPC-C+LuxR-HTH+LuxR-HTH+MFS(6xTM)+PP2C+STYKinase-like*→?→ ABhydrolase→</td><td>WXG+SecA+ST-Hiskin+LuxR-HTH+LuxR-HTH+LuxR-HTH+LuxR-HTH+GT4+TetR-HTH+TetR-HTH+ART+SIGMA-HTH+LuxR-HTH+LuxR-HTH+LuxR-HTH+GNTR-HTH+LuxR-HTH+LuxR-HTH+ST-Hiskin+ST-Hiskin+XPC-C+LuxR-HTH+LuxR-HTH+MFS(6xTM)+PP2C+STYKinase-like</td><td>12510</td><td>RJ03_RS22280</td><td>Actinobacteria</td><td>Nocardia aobensis</td><td>MFS transporter [Nocardia aobensis]</td><td>GCF_000308375.1</td></tr>
<tr><td>WP_157170272.1</td><td>WXG+MPTase→ WXG+SecA+LuxR-HTH+LuxR-HTH+LuxR-HTH+LuxR-HTH+ST-Hiskin+MPTase+GT4+TetR-HTH+TetR-HTH+Tox-ART-RSE+LuxR-HTH+LuxR-HTH+LuxR-HTH+GNTR-HTH+LuxR-HTH+LuxR-HTH+ST-Hiskin+ST-Hiskin+XPC-C+TM+TM+TM+TM+LuxR-HTH*→</td><td>WXG+SecA+LuxR-HTH+LuxR-HTH+LuxR-HTH+LuxR-HTH+ST-Hiskin+MPTase+GT4+TetR-HTH+TetR-HTH+Tox-ART-RSE+LuxR-HTH+LuxR-HTH+LuxR-HTH+GNTR-HTH+LuxR-HTH+LuxR-HTH+ST-Hiskin+ST-Hiskin+XPC-C+TM+TM+TM+TM+LuxR-HTH</td><td>11647</td><td>ON23_RS23640</td><td>Actinobacteria</td><td>Nocardia cerradoensis</td><td>MFS transporter, partial [Nocardia cerradoensis]</td><td>GCF_000308535.1</td></tr>
<tr><td>WP_157172575.1</td><td>ESX-1_EspG→?→?→?→ WXG+GT4+PP2C+GDPD+Amidohydrolase+Abhydrolase+GT4*→</td><td>WXG+GT4+PP2C+GDPD+Amidohydrolase+Abhydrolase+GT4</td><td>27919</td><td>ON34_RS32865</td><td>Actinobacteria</td><td>Nocardia pneumoniae</td><td>glycosyltransferase [Nocardia pneumoniae]</td><td>GCF_000308755.1</td></tr>
<tr><td>WP_157180627.1</td><td>WXG+TM+GT4+GT4*→</td><td>WXG+TM+GT4+GT4</td><td>1681</td><td>FrCorBMG51_RS[illegible]</td><td>Actinobacteria</td><td>Frankia coriariae</td><td>hypothetical protein [Frankia coriariae]</td><td>GCF_001017755.1</td></tr>
</table>

acc	operon	architecture	len	gen.name	taxend	species	defline	gca
WP_157204012.1	GT4+APATPase+TPR*→	GT4+APATPase+TPR	1141	A1332_RS13095	Gammaproteobacteria	Methylomonas methanica	tetratricopeptide repeat protein, partial [Methylomonas methanica]	-
WP_157224231.1	WXG+ZetaToxin+GT4*→?→||||<-WXG+MPTase	WXG+ZetaToxin+GT4	1061	ON32_RS18080	Actinobacteria	Nocardia paucivorans	zeta toxin family protein [Nocardia paucivorans]	GCF_000308675.1
WP_157229180.1	MPTase→ WXG+SecA+GT4+TetR-HTH+TetR-HTH+MFS+MFS(12x)+ABhydrolase+ST-Hiskin*→||||<-?||CSD→	WXG+SecA+GT4+TetR-HTH+TetR-HTH+MFS+MFS(12x)+ABhydrolase+ST-Hiskin	4779	ON20_RS32170	Actinobacteria	Nocardia brevicatena	MFS transporter [Nocardia brevicatena]	GCF_000308495.1
WP_157355048.1	GT4*→	GT4	618	ARA48_RS12920	Bacteroidetes	Parabacteroides distasonis	glycosyltransferase [Parabacteroides distasonis]	GCF_001405935.1
WP_157419626.1	<-cNMP_binding+HTH_Crp_2||GT4*→||||<-HTH+APATPase+TPR	GT4	468	AKI01S_RS02785	Actinobacteria	Actinomadura kijaniata	glycosyltransferase [Actinomadura kijaniata]	GCF_001552175.1
WP_157468665.1	CATRA-N+CATRA-C+GT4*→||||<-TPR+Caspase<-?<-?<-?<-?||CHTH+APATPase+TPR+TPR→ CATASP→	CATRA-N+CATRA-C+GT4	825	FRAQA3DRAFT_RS22440	Actinobacteria	Frankia sp. QA3	glycosyltransferase [Frankia sp. QA3]	GCF_000262465.1
WP_157494479.1	<-CATASP||CATRA-N+CATRA-C+GT4*→||||<-HTH+APATPase+TPR	CATRA-N+CATRA-C+GT4	786	KUTG_RS53310	Actinobacteria	Kutzneria sp. 744	glycosyltransferase [Kutzneria sp. 744]	-
WP_157528945.1	ART+Caspase+GT4*→?→ GT4→	ART+Caspase+GT4	1289	BN1199_RS39095	Actinobacteria	Kibdelosporangium sp. MJ126-NF4	glycosyltransferase [Kibdelosporangium sp. MJ126-NF4]	-
WP_157636396.1	STAND+TPR+GT4*→||||<-?||ABhydrolase→||||<-TRANSGLUTAMINASE	STAND+TPR+GT4	1130	WL03_RS32310	Betaproteobacteria	Burkholderia ubonensis	glycosyltransferase [Burkholderia ubonensis]	GCF_001528155.1
WP_157851161.1	<-TPR+Caspase<-CATASP||CATRA-N+CATRA-C+GT4*→ HTH+APATPase+TPR→||||<-HTH||?→?→?→||||<-?<-CATASP	CATRA-N+CATRA-C+GT4	703	IF17_RS1000000138590	Actinobacteria	Streptomyces cyaneofuscatus	glycosyltransferase [Streptomyces cyaneofuscatus]	GCF_000718135.1
WP_157856282.1	ABC_tran→?→?→ NACHT+MNS→?→ GT4*→	GT4	412	BS72_RS20070	Actinobacteria	Streptomyces yeochonensis	glycosyltransferase [Streptomyces yeochonensis]	-
WP_157978213.1	GT4*→	GT4	199	DAT37_RS00050	Actinobacteria	Nocardia sp. SYSU K10002	hypothetical protein [Nocardia sp. SYSU K10002]	-
WP_158075353.1	<-TPR+Caspase<-CATASP||CATRA-N+CATRA-C+GT4*→ HTH+APATPase+TPR→||||<-HTH||?→?→?→||||<-?<-CATASP	CATRA-N+CATRA-C+GT4	718	AMK32_RS25155	Actinobacteria	Streptomyces sp. CB01883	glycosyltransferase [Streptomyces sp. CB01883]	-

<table>
<thead>
<tr><th>acc</th><th>operon</th><th>architecture</th><th>len</th><th>gen.name</th><th>taxend</th><th>species</th><th>defline</th><th>gca</th></tr>
</thead>
<tbody>
<tr><td>WP_158247498.1</td><td>GT4+APATPase+TPR+TPR*→</td><td>GT4+APATPase+TPR+TPR</td><td>1379</td><td>C1X76_RS08190</td><td>Gammaproteobacteria</td><td>unclassified Pseudomonas</td><td>MULTISPECIES: tetratricopeptide repeat protein [unclassified Pseudomonas]</td><td>GCF_017350895.1</td></tr>
<tr><td>WP_158633123.1</td><td>cNMP_binding+HTH_Crp_2→?→ GT4*→||||<-ACYC+APATPase+TPR</td><td>GT4</td><td>391</td><td>DMH03_RS26200</td><td>Actinobacteria</td><td>Amycolatopsis sp. WAC 01376</td><td>glycosyltransferase [Amycolatopsis sp. WAC 01376]</td><td>GCF_003947415.1</td></tr>
<tr><td>WP_158709116.1</td><td><-ABC_tran<-?||PSE→||||<-?||Caspase+NACHT+GT4*→</td><td>Caspase+NACHT+GT4</td><td>1143</td><td>D3105_RS04960</td><td>Actinobacteria</td><td>Streptomyces globisporus</td><td>caspase family protein [Streptomyces globisporus]</td><td>GCF_000718455.1</td></tr>
<tr><td>WP_158893312.1</td><td><-TIR+APATPase||HTH+APATPase+TPR+GT4*→||||<-PSE<-?||?→?→?→ Sigma→</td><td>HTH+APATPase+TPR+GT4</td><td>1166</td><td>FXO23_RS42605</td><td>Actinobacteria</td><td>Amycolatopsis anabasis</td><td>tetratricopeptide repeat protein [Amycolatopsis anabasis]</td><td>GCF_009765355.1</td></tr>
<tr><td>WP_159015082.1</td><td><-TPR+Caspase<-CATASP||CATRA-N+CATRA-C+GT4*→ HTH+APATPase+TPR→||||<-HTH||?→||||<-?<-CATASP<-TPR+Caspase</td><td>CATRA-N+CATRA-C+GT4</td><td>711</td><td>AAY49_RS44335</td><td>Actinobacteria</td><td>Streptomyces europaeiscabiei</td><td>glycosyltransferase [Streptomyces europaeiscabiei]</td><td>GCF_000988945.1</td></tr>
<tr><td>WP_159058466.1</td><td><-CATASP||CATRA-N+CATRA-C+GT4*→ HTH+APATPase→</td><td>CATRA-N+CATRA-C+GT4</td><td>711</td><td>AWZ10_RS35055</td><td>Actinobacteria</td><td>Streptomyces europaeiscabiei</td><td>glycosyltransferase [Streptomyces europaeiscabiei]</td><td>GCF_001550375.1</td></tr>
<tr><td>WP_159073577.1</td><td>GT4*→</td><td>GT4</td><td>122</td><td>A6027_RS50895</td><td>Actinobacteria</td><td>Streptomyces sp. RTd22</td><td>hypothetical protein [Streptomyces sp. RTd22]</td><td>GCF_001640565.1</td></tr>
<tr><td>WP_159388811.1</td><td>MFS→||||<-?||GT4+APATPase+TPR*→||||<-MODE-HTH+PBPII</td><td>GT4+APATPase+TPR</td><td>1318</td><td>PspR32_RS15205</td><td>Gammaproteobacteria</td><td>Pseudomonas sp. R32</td><td>glycosyltransferase [Pseudomonas sp. R32]</td><td>GCF_009866705.1</td></tr>
<tr><td>WP_159399980.1</td><td>Caspase+NACHT+GT4*→||||<-?<-?||PSE→||||<-?||?→||||<-?<-MFS</td><td>Caspase+NACHT+GT4</td><td>1145</td><td>AVL59_RS21225</td><td>Actinobacteria</td><td>Streptomyces griseochromogenes</td><td>caspase family protein [Streptomyces griseochromogenes]</td><td>GCF_001542625.1</td></tr>
<tr><td>WP_160448122.1</td><td>ABC_tran→?→ GT4*→||||<-?||?→?→?→||||<-?||tRNA→?→ Pribosyltran_N+Pribosyl_synth→</td><td>GT4</td><td>643</td><td>DOU06_RS11775</td><td>Actinobacteria</td><td>Clavibacter michiganensis</td><td>glycosyltransferase [Clavibacter michiganensis]</td><td>GCF_009793225.1</td></tr>
<tr><td>WP_161065972.1</td><td>DUF4913→ GT4*→</td><td>GT4</td><td>463</td><td>GR925_RS14975</td><td>Actinobacteria</td><td>Streptomyces sp. HUCO-GS316</td><td>glycosyltransferase [Streptomyces sp. HUCO-GS316]</td><td>GCF_009827025.1</td></tr>
<tr><td>WP_161268609.1</td><td>CATRA-N+CATRA-C+GT4*→</td><td>CATRA-N+CATRA-C+GT4</td><td>703</td><td>GT040_RS36085</td><td>Actinobacteria</td><td>Streptomyces sp. SID2119</td><td>glycosyltransferase [Streptomyces sp. SID2119]</td><td>GCF_009865505.1</td></tr>
</tbody>
</table>

<table>
<thead>
<tr><th>acc</th><th>operon</th><th>architecture</th><th>len</th><th>gen.name</th><th>taxend</th><th>species</th><th>defline</th><th>gca</th></tr>
</thead>
<tbody>
<tr><td>WP_161606588.1</td><td>GT4*→</td><td>GT4</td><td>962</td><td>BHS06_RS25085</td><td>Deltaproteobacteria</td><td>Myxococcus xanthus</td><td>glycosyltransferase [Myxococcus xanthus]</td><td>GCF_006401635.1</td></tr>
<tr><td>WP_161684505.1</td><td>Sigma→?→?→||||<-?<-?<-TPR+Caspase||GT4*→||||<-PSE<-?||?→||||<-TRPR-HTH+PBPI</td><td>GT4</td><td>547</td><td>GVV04_RS04065</td><td>Actinobacteria</td><td>Micromonospora sp. NEAU-HG-1</td><td>glycosyltransferase [Micromonospora sp. NEAU-HG-1]</td><td>GCF_009908295.1</td></tr>
<tr><td>WP_161790163.1</td><td>GT4*→||||<-?<-?<-?<-CSD</td><td>GT4</td><td>353</td><td>TR46_RS20005</td><td>Actinobacteria</td><td>Streptacidiphilus carbonis</td><td>glycosyltransferase [Streptacidiphilus carbonis]</td><td>GCF_000787775.1</td></tr>
<tr><td>WP_162672520.1</td><td>GT4+HEPN+TPR+GreAB-C+PIN*→</td><td>GT4+HEPN+TPR+GreAB-C+PIN</td><td>1685</td><td>SOIL9_RS38685</td><td>Planctomycetes</td><td>Gemmata massiliana</td><td>glycosyltransferase [Gemmata massiliana]</td><td>GCF_901538265.1</td></tr>
<tr><td>WP_162688623.1</td><td>Caspase+NACHT+GT4*→||||<-?<-?<-?<-?||APATPase→</td><td>Caspase+NACHT+GT4</td><td>1148</td><td>DRB89_RS17705</td><td>Actinobacteria</td><td>unclassified Streptomyces</td><td>MULTISPECIES: caspase family protein [unclassified Streptomyces]</td><td>GCF_003287915.1</td></tr>
<tr><td>WP_162948626.1</td><td><-CATASP||CATRA-N+CATRA-C+GT4*→ HTH+APATPase→</td><td>CATRA-N+CATRA-C+GT4</td><td>711</td><td>CQR77_RS44880</td><td>Actinobacteria</td><td>Streptomyces europaeiscabiei</td><td>glycosyltransferase [Streptomyces europaeiscabiei]</td><td>GCF_003584825.1</td></tr>
<tr><td>WP_163053769.1</td><td>Calcineurin→?→?→ NACHT+MNS→ MNS+GT4*→</td><td>MNS+GT4</td><td>749</td><td>G3I70_RS06860</td><td>Actinobacteria</td><td>Actinomadura bangladeshensis</td><td>glycosyltransferase [Actinomadura bangladeshensis]</td><td>GCF_010548065.1</td></tr>
<tr><td>WP_164236650.1</td><td><-TPR+Caspase<-CATASP||CATRA-N+CATRA-C+GT4*→ HTH+APATPase+TPR→||||<-HTH||?→?→?→||||<-?<-CATASP</td><td>CATRA-N+CATRA-C+GT4</td><td>703</td><td>G3I52_RS10700</td><td>Actinobacteria</td><td>Streptomyces cyaneofuscatus</td><td>glycosyltransferase [Streptomyces cyaneofuscatus]</td><td>GCF_010548165.1</td></tr>
<tr><td>WP_164336692.1</td><td>Calcineurin→?→?→ Caspase+NACHT+GT4*→</td><td>Caspase+NACHT+GT4</td><td>1126</td><td>G3I38_RS20825</td><td>Actinobacteria</td><td>Streptomyces sp. SID7958</td><td>caspase family protein [Streptomyces sp. SID7958]</td><td>GCF_010550365.1</td></tr>
<tr><td>WP_164374106.1</td><td>NACHT→ GT4→||||<-?<-PSE<-?<-?<-?||HTH+APATPase→ TPR→||||<-GT4</td><td>GT4</td><td>405</td><td>G3I62_RS29240</td><td>Actinobacteria</td><td>Streptomyces sp. SID14446</td><td>glycosyltransferase [Streptomyces sp. SID14446]</td><td>GCF_010548515.1</td></tr>
<tr><td>WP_164374112.1</td><td>-</td><td>-</td><td>402</td><td>G3I62_RS29280</td><td>Actinobacteria</td><td>Streptomyces sp. SID14446</td><td>glycosyltransferase [Streptomyces sp. SID14446]</td><td>GCF_010548515.1</td></tr>
<tr><td>WP_164438832.1</td><td>Calcineurin→?→?→ Caspase+NACHT+GT4*→</td><td>Caspase+NACHT+GT4</td><td>1113</td><td>G3I53_RS25925</td><td>Actinobacteria</td><td>Streptomyces sp. SID14436</td><td>caspase family protein [Streptomyces sp. SID14436]</td><td>GCF_010548465.1</td></tr>
<tr><td>WP_164932131.1</td><td><-HTH+APATPase+TPR||GT4*→?→||||<-?<-?<-?||TPR→</td><td>GT4</td><td>394</td><td>DBB27_RS07160</td><td>Actinobacteria</td><td>unclassified Streptomyces</td><td>MULTISPECIES: glycosyltransferase [unclassified Streptomyces]</td><td>GCF_004104505.1</td></tr>
</tbody>
</table>

<table>
<tr><th>acc</th><th>operon</th><th>architecture</th><th>len</th><th>gen.name</th><th>taxend</th><th>species</th><th>defline</th><th>gca</th></tr>
<tr><td>WP_165781010.1</td><td>GT4*→</td><td>GT4</td><td>631</td><td>CR156_RS18515</td><td>Gammaproteobacteria</td><td>Stenotrophomonas lactitubi</td><td>glycosyltransferase [Stenotrophomonas lactitubi]</td><td>GCF_014763005.1</td></tr>
<tr><td>WP_166659524.1</td><td>-</td><td>-</td><td>752</td><td>EV186_RS37545</td><td>Actinobacteria</td><td>Labedaea rhizosphaerae</td><td>glycosyltransferase family 4 protein [Labedaea rhizosphaerae]</td><td>GCF_004362825.1</td></tr>
<tr><td>WP_167375417.1</td><td>APATPase+GT4*→</td><td>APATPase+GT4</td><td>1344</td><td>AWM79_RS04595</td><td>Gammaproteobacteria</td><td>Pseudomonas agarici</td><td>glycosyltransferase [Pseudomonas agarici]</td><td>GCF_001543125.1</td></tr>
<tr><td>WP_167485160.1</td><td><-ESX-1_EspG<-?<-?<-?||?→?→ WXG+PolB-NTase+GT4+GT4+Caspase*→?→?→ GT4→</td><td>WXG+PolB-NTase+GT4+GT4+Caspase</td><td>2733</td><td>F6W96_RS05200</td><td>Actinobacteria</td><td>Nocardia terpenica</td><td>glycosyltransferase [Nocardia terpenica]</td><td>GCF_011801165.1</td></tr>
<tr><td>WP_167531487.1</td><td>NACHT+GT4*→||||<-Vsr</td><td>NACHT+GT4</td><td>1557</td><td>EN799_RS48175</td><td>Alphaproteobacteria</td><td>Mesorhizobium sp. M8A.F.Ca.ET.197.01.1.1</td><td>glycosyltransferase [Mesorhizobium sp. M8A.F.Ca.ET.197.01.1.1]</td><td>GCF_004791385.1</td></tr>
<tr><td>WP_167631834.1</td><td>GT4+APATPase+TPR*→</td><td>GT4+APATPase+TPR</td><td>1107</td><td>HCI87_RS07630</td><td>Zetaproteobacteria</td><td>Mariprofundus ferrooxydans</td><td>tetratricopeptide repeat protein, partial [Mariprofundus ferrooxydans]</td><td>GCF_011947535.1</td></tr>
<tr><td>WP_167983001.1</td><td>CATRA-N+CATRA-C+GT4*→ HTH+APATPase+TPR→||||<-HTH||?→?→?→||||<-?<-CATASP</td><td>CATRA-N+CATRA-C+GT4</td><td>845</td><td>HCN08_RS12120</td><td>Actinobacteria</td><td>Streptomyces sp. PRB2-1</td><td>glycosyltransferase family 4 protein [Streptomyces sp. PRB2-1]</td><td>GCF_012034175.1</td></tr>
<tr><td>WP_167985631.1</td><td>GT4*→</td><td>GT4</td><td>425</td><td>HCN08_RS25735</td><td>Actinobacteria</td><td>Streptomyces sp. PRB2-1</td><td>glycosyltransferase [Streptomyces sp. PRB2-1]</td><td>GCF_012034175.1</td></tr>
<tr><td>WP_168028895.1</td><td><-ABC_tran<-?<-?<-?<-?||?→ GT4+APATPase*→</td><td>GT4+APATPase</td><td>988</td><td>HC024_RS03995</td><td>Gammaproteobacteria</td><td>Methylococcaceae bacterium WWC4</td><td>glycosyltransferase, partial [Methylococcaceae bacterium WWC4]</td><td>-</td></tr>
<tr><td>WP_168031569.1</td><td>GT4*→</td><td>GT4</td><td>644</td><td>HC024_RS14290</td><td>Gammaproteobacteria</td><td>Methylococcaceae bacterium WWC4</td><td>glycosyltransferase, partial [Methylococcaceae bacterium WWC4]</td><td>-</td></tr>
<tr><td>WP_168435140.1</td><td>WXG+MPTase→ WXG+SecA+LuxR-HTH+LuxR-HTH+LuxR-HTH+LuxR-HTH+ST-Hiskin+MPTase+GT4+TetR-HTH+TetR-HTH+Tox-ART-RSE+LuxR-HTH+LuxR-HTH+LuxR-HTH+GNTR-HTH+LuxR-HTH+LuxR-HTH+ST-Hiskin+ST-Hiskin+XPC-C+TM+TM+TM+TM+LuxR-HTH+PP2C+MPTase+MPTase+LuxR-HTH+LuxR-HTH+DOC*→?→ ABhydrolase→</td><td>WXG+SecA+LuxR-HTH+LuxR-HTH+LuxR-HTH+LuxR-HTH+ST-Hiskin+MPTase+GT4+TetR-HTH+TetR-HTH+Tox-ART-RSE+LuxR-HTH+LuxR-HTH+LuxR-HTH+GNTR-HTH+LuxR-HTH+LuxR-HTH+ST-Hiskin+ST-Hiskin+XPC-C+TM+TM+TM+TM+LuxR-HTH+PP2C+MPTase+MPTase+LuxR-HTH+LuxR-HTH+DOC</td><td>13688</td><td>HGB45_RS23560</td><td>Actinobacteria</td><td>Nocardia cerradoensis</td><td>MFS transporter [Nocardia cerradoensis]</td><td>GCF_012396065.1</td></tr>
<tr><td>WP_168477813.1</td><td>GT4*→?→?→||||<-?||?→||||<-NUDIX</td><td>GT4</td><td>455</td><td>HEP86_RS05755</td><td>Actinobacteria</td><td>Streptomyces sp. RPA4-5</td><td>glycosyltransferase [Streptomyces sp. RPA4-5]</td><td>GCF_012273495.1</td></tr>
</table>

<table>
<tr><th>acc</th><th>operon</th><th>architecture</th><th>len</th><th>gen.name</th><th>taxend</th><th>species</th><th>defline</th><th>gca</th></tr>
<tr><td>WP_168512294.1</td><td><-ABhydrolase||?→?→ GT4*→||||<-?||TRPR-HTH+PBPI→</td><td>GT4</td><td>650</td><td>HEP87_RS39620</td><td>Actinobacteria</td><td>Streptomyces sp. S1D4-11</td><td>glycosyltransferase [Streptomyces sp. S1D4-11]</td><td>-</td></tr>
<tr><td>WP_169316088.1</td><td>APATPase+GT4*→||||<-?<-?<-?<-?||?→||||<-?<-PSE||GST_N_2+GST_C→</td><td>APATPase+GT4</td><td>1344</td><td>PAGAR_RS28405</td><td>Gammaproteobacteria</td><td>Pseudomonas agarici</td><td>glycosyltransferase [Pseudomonas agarici]</td><td>GCF_000280785.1</td></tr>
<tr><td>WP_169804810.1</td><td>GT4+APATPase*→</td><td>GT4+APATPase</td><td>986</td><td>HTA01S_RS24340</td><td>Betaproteobacteria</td><td>Hydrogenophaga taeniospiralis</td><td>glycosyltransferase, partial [Hydrogenophaga taeniospiralis]</td><td>GCF_001592305.1</td></tr>
<tr><td>WP_169807203.1</td><td>GT4*→</td><td>GT4</td><td>449</td><td>AWW66_RS34115</td><td>Actinobacteria</td><td>Micromonospora rosaria</td><td>glycosyltransferase, partial [Micromonospora rosaria]</td><td>GCF_001567585.1</td></tr>
<tr><td>WP_169850673.1</td><td>GT4+B12-binding+RADICAL-SAM*→</td><td>GT4+B12-binding+RADICAL-SAM</td><td>1115</td><td>HBO43_RS21615</td><td>Gammaproteobacteria</td><td>Pseudomonas veronii</td><td>cobalamin B12-binding domain-containing protein [Pseudomonas veronii]</td><td>GCF_012985315.1</td></tr>
<tr><td>WP_170285467.1</td><td>SWACOS+GT4*→?→||||<-?||?→ PadR→</td><td>SWACOS+GT4</td><td>1175</td><td>FHX75_RS26815</td><td>Actinobacteria</td><td>Micromonospora palomenae</td><td>glycosyltransferase [Micromonospora palomenae]</td><td>GCF_007829925.1</td></tr>
<tr><td>WP_171081495.1</td><td>GT4*→</td><td>GT4</td><td>489</td><td>HG542_RS14775</td><td>Actinobacteria</td><td>Streptomyces morookaense</td><td>glycosyltransferase [Streptomyces morookaense]</td><td>GCF_014656115.1</td></tr>
<tr><td>WP_171579219.1</td><td><-SIG+RCDG1+YidC_periplas+60KD_IMP<-?<-?||?→ tRNA→?→ NACHT+GT4*→?→?→?→?→?→?→ DUF4242→</td><td>NACHT+GT4</td><td>1638</td><td>HCN58_RS10240</td><td>Alphaproteobacteria</td><td>Bradyrhizobium sp. WSM 1791</td><td>glycosyltransferase [Bradyrhizobium sp. WSM 1791]</td><td>GCF_013114825.1</td></tr>
<tr><td>WP_171675029.1</td><td>GT4*→</td><td>GT4</td><td>451</td><td>HNR71_RS02445</td><td>Actinobacteria</td><td>Kribbella sandramycini</td><td>glycosyltransferase [Kribbella sandramycini]</td><td>GCF_013131805.1</td></tr>
<tr><td>WP_171901162.1</td><td>-</td><td>-</td><td>147</td><td>NK6_RS51125</td><td>Alphaproteobacteria</td><td>Bradyrhizobium diazoefficiens</td><td>glycosyltransferase [Bradyrhizobium diazoefficiens]</td><td>GCF_001549695.1</td></tr>
<tr><td>WP_172109703.1</td><td>SWACOS+GT4*→</td><td>SWACOS+GT4</td><td>1148</td><td>HL667_RS06640</td><td>Alphaproteobacteria</td><td>Bradyrhizobium sp. 83012</td><td>glycosyltransferase [Bradyrhizobium sp. 83012]</td><td>GCF_013178925.1</td></tr>
<tr><td>WP_172132695.1</td><td>SWACOS+GT4*→</td><td>SWACOS+GT4</td><td>1148</td><td>HL666_RS23775</td><td>Alphaproteobacteria</td><td>unclassified Bradyrhizobium</td><td>MULTISPECIES: glycosyltransferase [unclassified Bradyrhizobium]</td><td>GCF_013178945.1</td></tr>
</table>

acc	operon	architecture	len	gen.name	taxend	species	defline	gca
WP_172175287.1	GT4+B12-binding+RADICAL-SAM*→	GT4+B12-binding+RADICAL-SAM	1278	GNZ13_RS39275	Betaproteobacteria	Paraburkholderia sp. 5N	radical SAM protein [Paraburkholderia sp. 5N]	GCF_013177735.1
WP_173124146.1	<-STAND<-?<-?<-?<-?||cNMP_binding+HTH_Crp_2→?→ GT4*→||||<-ACYC+APATPase+TPR	GT4	409	GC106_RS03330	Actinobacteria	Kibdelosporangium sp. 4NS15	glycosyltransferase [Kibdelosporangium sp. 4NS15]	GCF_013280595.1
WP_173195298.1	GT4+APATPase+TPR+TPR*→	GT4+APATPase+TPR+TPR	1427	HR085_RS00175	Deltaproteobacteria	Geobacter sp. SVR	tetratricopeptide repeat protein [Geobacter sp. SVR]	GCF_016865365.1
WP_173395462.1	DJ-1_PfpI→ LexA→?→||||<-?<-?<-?<-cNMP_binding+HTH_Crp_2||GT4*→||||<-HTH+APATPase+TPR	GT4	438	GWI34_RS19435	Actinobacteria	Actinomadura sp. DSM 109109	glycosyltransferase [Actinomadura sp. DSM 109109]	-
WP_173526832.1	GT4+APATPase+TPR+TPR*→	GT4+APATPase+TPR+TPR	1208	HII36_RS31010	Actinobacteria	Nonomuraea sp. NN258	tetratricopeptide repeat protein [Nonomuraea sp. NN258]	GCF_013283785.1
WP_173991751.1	PfkB→?→?→?→ GT4+B12-binding+RADICAL-SAM*→	GT4+B12-binding+RADICAL-SAM	1387	G6M20_RS23615	Alphaproteobacteria	Agrobacterium salinitolerans	cobalamin B12-binding domain-containing protein [Agrobacterium salinitolerans]	GCF_013317635.1
WP_174156125.1	HTH+MPTase→?→||||<-?||?→?→ NACHT+GT4*→	NACHT+GT4	1640	G6L88_RS21970	Alphaproteobacteria	Rhizobium skierniewicense	glycosyltransferase [Rhizobium skierniewicense]	GCF_013320815.1
WP_174295717.1	GT4*→	GT4	613	G7A66_RS11060	Alphaproteobacteria	Altererythrobacter sp. SALINAS58	glycosyltransferase [Altererythrobacter sp. SALINAS58]	GCF_013336795.1
WP_174397973.1	GT4*→	GT4	602	FEG63_RS11180	Actinobacteria	Mycolicibacterium sphagni	glycosyltransferase [Mycolicibacterium sphagni]	GCF_013337765.1
WP_174527417.1	STAND→?→ DUF4913→||||<-?||?→ GT4*→?→?→?→ NUDIX→||||<-?<-ABC_tran	GT4	438	HUW36_RS07305	Actinobacteria	Micromonospora maritima	glycosyltransferase [Micromonospora maritima]	GCF_902825405.1
WP_175264897.1	NACHT+MNS→ MNS+GT4*→||||<-?<-CASPASE	MNS+GT4	752	HRW23_RS35520	Actinobacteria	Streptomyces lunaelactis	glycosyltransferase [Streptomyces lunaelactis]	GCF_013357925.1
WP_176052200.1	STAND+GT4*→?→||||<-Cluster466_2clades<-?<-?<-?||DNA_methylase→||||<-Vsr	STAND+GT4	1090	HWY02_RS01425	Betaproteobacteria	Paraburkholderia caribensis	glycosyltransferase [Paraburkholderia caribensis]	GCF_902833585.1

acc	operon	architecture	len	gen.name	taxend	species	defline	gca
WP_176182010.1	MNS+GT4*→||||<-?<-?<-AA_permease	MNS+GT4	1151	HUT19_RS21385	Actinobacteria	Streptomyces sp. NA02950	glycosyltransferase [Streptomyces sp. NA02950]	GCF_013364155.1
WP_176404534.1	<-cNMP_binding+HTH_Crp_2||GT4*→	GT4	427	HUX53_RS18900	Actinobacteria	Actinomadura sp. BRA 177	glycosyltransferase [Actinomadura sp. BRA 177]	GCF_013372625.1
WP_176437046.1	<-ABC_tran<-?||?→||||<-?<-PSE<-?||GT4+TPR+TPR*→	GT4+TPR+TPR	1428	HUW62_RS36515	Deltaproteobacteria	Myxococcus sp. AM011	tetratricopeptide repeat protein [Myxococcus sp. AM011]	GCF_013372595.1
WP_176439087.1	HTH_Tnp_1→ TnpB_IS66→?→ NACHT+GT4*→	NACHT+GT4	1580	CHB75_RS08020	Alphaproteobacteria	Puniceibacterium sediminis	glycosyltransferase [Puniceibacterium sediminis]	GCF_900188035.1
WP_176652673.1	GT4+NACHT*→ HTH_Tnp_1→ TnpB_IS66→	GT4+NACHT	1759	FHT31_RS13555	Betaproteobacteria	Rugamonas sp. SG757	glycosyltransferase [Rugamonas sp. SG757]	GCF_013375995.1
WP_176735010.1	<-TPR+Caspase||GT4*→	GT4	546	GA0070563_RS2[illegible]	Actinobacteria	Micromonospora carbonacea	glycosyltransferase [Micromonospora carbonacea]	GCF_900091535.1
WP_177014872.1	STAND+GT4*→	STAND+GT4	1080	HX791_RS25785	Gammaproteobacteria	Pseudomonas costantinii	glycosyltransferase [Pseudomonas costantinii]	GCF_013386225.1
WP_177060102.1	<-MFS||?→||||<-?<-?||?→||||<-?||APATPase+GT4*→	APATPase+GT4	1336	HX876_RS19435	Gammaproteobacteria	Pseudomonas gingeri	glycosyltransferase [Pseudomonas gingeri]	GCF_013385575.1
WP_177062848.1	GT4+B12-binding+RADICAL-SAM*→	GT4+B12-binding+RADICAL-SAM	1404	HX876_RS14360	Gammaproteobacteria	Pseudomonas gingeri	radical SAM protein [Pseudomonas gingeri]	GCF_013385575.1
WP_177065618.1	<-MFS||?→||||<-?<-?||?→||||<-?||APATPase+TPR+GT4*→	APATPase+TPR+GT4	1336	HX861_RS09040	Gammaproteobacteria	Pseudomonas gingeri	glycosyltransferase [Pseudomonas gingeri]	GCF_013385925.1
WP_177072953.1	<-MFS||?→||||<-?<-?||?→||||<-?||APATPase+TPR+GT4*→	APATPase+TPR+GT4	1336	HX800_RS13020	Gammaproteobacteria	Pseudomonas gingeri	glycosyltransferase [Pseudomonas gingeri]	GCF_013386965.1
WP_177108140.1	<-MFS||?→||||<-?<-?||?→||||<-?||APATPase+GT4*→	APATPase+GT4	1299	HX867_RS22145	Gammaproteobacteria	Pseudomonas gingeri	glycosyltransferase [Pseudomonas gingeri]	GCF_013386235.1
WP_177126920.1	<-MFS||?→||||<-?<-?||?→||||<-?||APATPase+TPR+GT4*→	APATPase+TPR+GT4	1336	HX831_RS01005	Gammaproteobacteria	Pseudomonas gingeri	glycosyltransferase [Pseudomonas gingeri]	GCF_013386815.1

<table>
<tr><th>acc</th><th>operon</th><th>architecture</th><th>len</th><th>gen.name</th><th>taxend</th><th>species</th><th>defline</th><th>gca</th></tr>
<tr><td>WP_177129208.1</td><td><-MFS||?→||||<-?<-?||?→||||<-?||APATPase+TPR+GT4*→</td><td>APATPase+TPR+GT4</td><td>1299</td><td>HX808_RS17340</td><td>Gammaproteobacteria</td><td>Pseudomonas gingeri</td><td>glycosyltransferase [Pseudomonas gingeri]</td><td>GCF_013385905.1</td></tr>
<tr><td>WP_177204219.1</td><td><-Sigma||?→||||<-?<-?<-?||GT4+B12-binding*→</td><td>GT4+B12-binding</td><td>1122</td><td>BMY84_RS26750</td><td>Alphaproteobacteria</td><td>Sphingobium sp. AP50</td><td>glycosyltransferase [Sphingobium sp. AP50]</td><td>GCF_900109095.1</td></tr>
<tr><td>WP_178066132.1</td><td><-TPR+Caspase||GT4*→</td><td>GT4</td><td>546</td><td>HDA31_RS04955</td><td>Actinobacteria</td><td>Micromonospora carbonacea</td><td>glycosyltransferase [Micromonospora carbonacea]</td><td>GCF_013389765.1</td></tr>
<tr><td>WP_178963184.1</td><td>GT4+B12-binding+RADICAL-SAM*→</td><td>GT4+B12-binding+RADICAL-SAM</td><td>1404</td><td>HX895_RS03680</td><td>Gammaproteobacteria</td><td>Pseudomonas gingeri</td><td>radical SAM protein [Pseudomonas gingeri]</td><td>GCF_013387125.1</td></tr>
<tr><td>WP_178963665.1</td><td><-MFS||?→||||<-?<-?||?→||||<-?||APATPase+GT4*→</td><td>APATPase+GT4</td><td>1336</td><td>HX895_RS14805</td><td>Gammaproteobacteria</td><td>Pseudomonas gingeri</td><td>glycosyltransferase [Pseudomonas gingeri]</td><td>GCF_013387125.1</td></tr>
<tr><td>WP_179766782.1</td><td>TRPR-HTH+PBPI→?→ SIG+Lipase_GDSL_2→?→ Zeta_toxin→?→ GT4*→</td><td>GT4</td><td>461</td><td>HNR12_RS07370</td><td>Actinobacteria</td><td>Streptomonospora nanhaiensis</td><td>glycosyltransferase [Streptomonospora nanhaiensis]</td><td>GCF_013410565.1</td></tr>
<tr><td>WP_179767386.1</td><td>GT4*→?→||||<-?<-?<-tRNA-synt_1b+S4</td><td>GT4</td><td>489</td><td>HNR12_RS10915</td><td>Actinobacteria</td><td>Streptomonospora nanhaiensis</td><td>glycosyltransferase [Streptomonospora nanhaiensis]</td><td>GCF_013410565.1</td></tr>
<tr><td>WP_179826407.1</td><td><-APATPase+TPR||?→ GT4*→</td><td>GT4</td><td>524</td><td>HNR10_RS21750</td><td>Actinobacteria</td><td>Nocardiopsis aegyptia</td><td>hypothetical protein [Nocardiopsis aegyptia]</td><td>GCF_013410755.1</td></tr>
<tr><td>WP_179835700.1</td><td>GT4*→||||<-?<-?<-cNMP_binding+HTH_Crp_2</td><td>GT4</td><td>388</td><td>BJ999_RS25940</td><td>Actinobacteria</td><td>Actinomadura citrea</td><td>glycosyltransferase [Actinomadura citrea]</td><td>GCF_014648455.1</td></tr>
<tr><td>WP_180357038.1</td><td>HTH→?→?→||||<-?<-?||Caspase+NACHT+GT4*→</td><td>Caspase+NACHT+GT4</td><td>1165</td><td>BX283_RS03940</td><td>Actinobacteria</td><td>Streptomyces sp. TLI_146</td><td>caspase family protein [Streptomyces sp. TLI_146]</td><td>GCF_002846415.1</td></tr>
<tr><td>WP_180930380.1</td><td>MNS+GT4*→</td><td>MNS+GT4</td><td>1268</td><td>E0L36_RS19290</td><td>Actinobacteria</td><td>Streptomyces sp. AJS327</td><td>glycosyltransferase [Streptomyces sp. AJS327]</td><td>GCF_013450295.1</td></tr>
<tr><td>WP_181043331.1</td><td>MFS→?→?→ WXG→?→ GT4*→?→ GT4→</td><td>GT4</td><td>391</td><td>CLV40_RS37790</td><td>Actinobacteria</td><td>Actinokineospora auranticolor</td><td>glycosyltransferase family 4 protein [Actinokineospora auranticolor]</td><td>GCF_002934265.1</td></tr>
</table>

acc	operon	architecture	len	gen.name	taxend	species	defline	gca
WP_181245803.1	GT4*→ APATPase+TPR→||||<-?<-TRPR-HTH+PBPI	GT4	401	B0I28_RS15405	Actinobacteria	Glycomyces artemisiae	glycosyltransferase [Glycomyces artemisiae]	GCF_003002955.1

Source data. Domain architectures of eukaryotic GT4s with a special emphasis on the LSE in Acropora

**acc**	**architecture**	**len**	**gen.name**	**tax**	**species**	**GCA**
XP_015747238.1	GT4	461	LOC107327009	Eukaryota>Opisthokonta>Metazoa>Cnidaria	Acropora digitifera	GCF_000222465.1
XP_015747313.1	GT4+NACHT	648	LOC107327071	Eukaryota>Opisthokonta>Metazoa>Cnidaria	Acropora digitifera	GCF_000222465.1
XP_015747371.1	GT4	492	LOC107327132	Eukaryota>Opisthokonta>Metazoa>Cnidaria	Acropora digitifera	GCF_000222465.1
XP_015747395.1	GT4+NACHT	628	LOC107327154	Eukaryota>Opisthokonta>Metazoa>Cnidaria	Acropora digitifera	GCF_000222465.1
XP_015747754.1	GT4	430	LOC107327516	Eukaryota>Opisthokonta>Metazoa>Cnidaria	Acropora digitifera	GCF_000222465.1
XP_015747753.1	GT4	437	LOC107327516	Eukaryota>Opisthokonta>Metazoa>Cnidaria	Acropora digitifera	GCF_000222465.1
XP_015747750.1	GT4	437	LOC107327516	Eukaryota>Opisthokonta>Metazoa>Cnidaria	Acropora digitifera	GCF_000222465.1
XP_015747752.1	GT4	437	LOC107327516	Eukaryota>Opisthokonta>Metazoa>Cnidaria	Acropora digitifera	GCF_000222465.1
XP_015747751.1	GT4	437	LOC107327516	Eukaryota>Opisthokonta>Metazoa>Cnidaria	Acropora digitifera	GCF_000222465.1
XP_015747749.1	GT4	451	LOC107327516	Eukaryota>Opisthokonta>Metazoa>Cnidaria	Acropora digitifera	GCF_000222465.1
XP_015748052.1	GT4	595	LOC107327825	Eukaryota>Opisthokonta>Metazoa>Cnidaria	Acropora digitifera	GCF_000222465.1
XP_015748207.1	GT4	424	LOC107328002	Eukaryota>Opisthokonta>Metazoa>Cnidaria	Acropora digitifera	GCF_000222465.1
XP_015748206.1	GT4	424	LOC107328002	Eukaryota>Opisthokonta>Metazoa>Cnidaria	Acropora digitifera	GCF_000222465.1
XP_015748205.1	GT4	429	LOC107328002	Eukaryota>Opisthokonta>Metazoa>Cnidaria	Acropora digitifera	GCF_000222465.1
XP_015748202.1	GT4	430	LOC107328002	Eukaryota>Opisthokonta>Metazoa>Cnidaria	Acropora digitifera	GCF_000222465.1
XP_015748203.1	GT4	430	LOC107328002	Eukaryota>Opisthokonta>Metazoa>Cnidaria	Acropora digitifera	GCF_000222465.1
XP_015748201.1	GT4	437	LOC107328002	Eukaryota>Opisthokonta>Metazoa>Cnidaria	Acropora digitifera	GCF_000222465.1
XP_015748200.1	GT4	442	LOC107328002	Eukaryota>Opisthokonta>Metazoa>Cnidaria	Acropora digitifera	GCF_000222465.1
XP_015748274.1	GT4	440	LOC107328074	Eukaryota>Opisthokonta>Metazoa>Cnidaria	Acropora digitifera	GCF_000222465.1
XP_015748402.1	ZU5+ZU5+GT4	1030	LOC107328179	Eukaryota>Opisthokonta>Metazoa>Cnidaria	Acropora digitifera	GCF_000222465.1
XP_015748412.1	GT4+NACHT	551	LOC107328186	Eukaryota>Opisthokonta>Metazoa>Cnidaria	Acropora digitifera	GCF_000222465.1
XP_015748513.1	SIG+GT4+DEATH	524	LOC107328287	Eukaryota>Opisthokonta>Metazoa>Cnidaria	Acropora digitifera	GCF_000222465.1
XP_015748512.1	SIG+GT4+DEATH	526	LOC107328287	Eukaryota>Opisthokonta>Metazoa>Cnidaria	Acropora digitifera	GCF_000222465.1
XP_015748877.1	GT4	416	LOC107328661	Eukaryota>Opisthokonta>Metazoa>Cnidaria	Acropora digitifera	GCF_000222465.1
XP_015748878.1	GT4+NACHT	578	LOC107328662	Eukaryota>Opisthokonta>Metazoa>Cnidaria	Acropora digitifera	GCF_000222465.1
XP_015748920.1	GT4	389	LOC107328707	Eukaryota>Opisthokonta>Metazoa>Cnidaria	Acropora digitifera	GCF_000222465.1
XP_015748918.1	GT4	391	LOC107328707	Eukaryota>Opisthokonta>Metazoa>Cnidaria	Acropora digitifera	GCF_000222465.1
XP_015748919.1	GT4	391	LOC107328707	Eukaryota>Opisthokonta>Metazoa>Cnidaria	Acropora digitifera	GCF_000222465.1
XP_015749378.1	GT4	610	LOC107329155	Eukaryota>Opisthokonta>Metazoa>Cnidaria	Acropora digitifera	GCF_000222465.1
XP_015749585.1	GT4	400	LOC107329409	Eukaryota>Opisthokonta>Metazoa>Cnidaria	Acropora digitifera	GCF_000222465.1
XP_015749584.1	GT4+TF-AF0608	480	LOC107329409	Eukaryota>Opisthokonta>Metazoa>Cnidaria	Acropora digitifera	GCF_000222465.1
XP_015749790.1	GT4	470	LOC107329632	Eukaryota>Opisthokonta>Metazoa>Cnidaria	Acropora digitifera	GCF_000222465.1
XP_015749876.1	GT4+NACHT	583	LOC107329721	Eukaryota>Opisthokonta>Metazoa>Cnidaria	Acropora digitifera	GCF_000222465.1
XP_015750005.1	GT4	637	LOC107329865	Eukaryota>Opisthokonta>Metazoa>Cnidaria	Acropora digitifera	GCF_000222465.1
XP_015750060.1	GT4	418	LOC107329915	Eukaryota>Opisthokonta>Metazoa>Cnidaria	Acropora digitifera	GCF_000222465.1
XP_015750058.1	GT4	483	LOC107329915	Eukaryota>Opisthokonta>Metazoa>Cnidaria	Acropora digitifera	GCF_000222465.1
XP_015750090.1	GT4	464	LOC107329946	Eukaryota>Opisthokonta>Metazoa>Cnidaria	Acropora digitifera	GCF_000222465.1
XP_015750089.1	GT4	477	LOC107329946	Eukaryota>Opisthokonta>Metazoa>Cnidaria	Acropora digitifera	GCF_000222465.1
XP_015750088.1	GT4	510	LOC107329946	Eukaryota>Opisthokonta>Metazoa>Cnidaria	Acropora digitifera	GCF_000222465.1
XP_015750086.1	GT4	513	LOC107329946	Eukaryota>Opisthokonta>Metazoa>Cnidaria	Acropora digitifera	GCF_000222465.1
XP_015750087.1	GT4	513	LOC107329946	Eukaryota>Opisthokonta>Metazoa>Cnidaria	Acropora digitifera	GCF_000222465.1
XP_015750637.1	GT4	341	LOC107330559	Eukaryota>Opisthokonta>Metazoa>Cnidaria	Acropora digitifera	GCF_000222465.1
XP_015753085.1	SIG+GT4	635	LOC107332846	Eukaryota>Opisthokonta>Metazoa>Cnidaria	Acropora digitifera	GCF_000222465.1

acc	architecture	len	gen.name	tax	species	GCA
XP_015753084.1	SIG+GT4	670	LOC107332846	Eukaryota>Opisthokonta>Metazoa>Cnidaria	Acropora digitifera	GCF_000222465.1
XP_015753082.1	SIG+GT4	715	LOC107332846	Eukaryota>Opisthokonta>Metazoa>Cnidaria	Acropora digitifera	GCF_000222465.1
XP_015753081.1	SIG+GT4	717	LOC107332846	Eukaryota>Opisthokonta>Metazoa>Cnidaria	Acropora digitifera	GCF_000222465.1
XP_015754166.1	GT4	465	LOC107333809	Eukaryota>Opisthokonta>Metazoa>Cnidaria	Acropora digitifera	GCF_000222465.1
XP_015754272.1	GT4	660	LOC107333922	Eukaryota>Opisthokonta>Metazoa>Cnidaria	Acropora digitifera	GCF_000222465.1
XP_015754292.1	GT4	491	LOC107333938	Eukaryota>Opisthokonta>Metazoa>Cnidaria	Acropora digitifera	GCF_000222465.1
XP_015754293.1	GT4	432	LOC107333939	Eukaryota>Opisthokonta>Metazoa>Cnidaria	Acropora digitifera	GCF_000222465.1
XP_015754495.1	GT4	533	LOC107334108	Eukaryota>Opisthokonta>Metazoa>Cnidaria	Acropora digitifera	GCF_000222465.1
XP_015755589.1	ZU5+GT4	822	LOC107335136	Eukaryota>Opisthokonta>Metazoa>Cnidaria	Acropora digitifera	GCF_000222465.1
XP_015755588.1	ZU5+ZU5+GT4	849	LOC107335136	Eukaryota>Opisthokonta>Metazoa>Cnidaria	Acropora digitifera	GCF_000222465.1
XP_015755587.1	ZU5+ZU5+GT4	851	LOC107335136	Eukaryota>Opisthokonta>Metazoa>Cnidaria	Acropora digitifera	GCF_000222465.1
XP_015755586.1	ZU5+ZU5+GT4	857	LOC107335136	Eukaryota>Opisthokonta>Metazoa>Cnidaria	Acropora digitifera	GCF_000222465.1
XP_015757828.1	GT4	557	LOC107337230	Eukaryota>Opisthokonta>Metazoa>Cnidaria	Acropora digitifera	GCF_000222465.1
XP_015757831.1	GT4	448	LOC107337231	Eukaryota>Opisthokonta>Metazoa>Cnidaria	Acropora digitifera	GCF_000222465.1
XP_015757830.1	GT4	510	LOC107337231	Eukaryota>Opisthokonta>Metazoa>Cnidaria	Acropora digitifera	GCF_000222465.1
XP_015757829.1	GT4	528	LOC107337231	Eukaryota>Opisthokonta>Metazoa>Cnidaria	Acropora digitifera	GCF_000222465.1
XP_015758947.1	GT4	493	LOC107338227	Eukaryota>Opisthokonta>Metazoa>Cnidaria	Acropora digitifera	GCF_000222465.1
XP_015759245.1	GT4	347	LOC107338521	Eukaryota>Opisthokonta>Metazoa>Cnidaria	Acropora digitifera	GCF_000222465.1
XP_015759244.1	GT4	348	LOC107338521	Eukaryota>Opisthokonta>Metazoa>Cnidaria	Acropora digitifera	GCF_000222465.1
XP_015759943.1	GT4	403	LOC107339196	Eukaryota>Opisthokonta>Metazoa>Cnidaria	Acropora digitifera	GCF_000222465.1
XP_015760072.1	GT4+DEATH	565	LOC107339308	Eukaryota>Opisthokonta>Metazoa>Cnidaria	Acropora digitifera	GCF_000222465.1
XP_015760071.1	GT4+DEATH	571	LOC107339308	Eukaryota>Opisthokonta>Metazoa>Cnidaria	Acropora digitifera	GCF_000222465.1
XP_015760070.1	GT4+DEATH	595	LOC107339308	Eukaryota>Opisthokonta>Metazoa>Cnidaria	Acropora digitifera	GCF_000222465.1
XP_015760069.1	GT4+DEATH	596	LOC107339308	Eukaryota>Opisthokonta>Metazoa>Cnidaria	Acropora digitifera	GCF_000222465.1
XP_015760068.1	GT4+DEATH	604	LOC107339308	Eukaryota>Opisthokonta>Metazoa>Cnidaria	Acropora digitifera	GCF_000222465.1
XP_015760067.1	GT4+DEATH	610	LOC107339308	Eukaryota>Opisthokonta>Metazoa>Cnidaria	Acropora digitifera	GCF_000222465.1
XP_015760066.1	GT4+DEATH	614	LOC107339308	Eukaryota>Opisthokonta>Metazoa>Cnidaria	Acropora digitifera	GCF_000222465.1
XP_015760065.1	GT4+DEATH	620	LOC107339308	Eukaryota>Opisthokonta>Metazoa>Cnidaria	Acropora digitifera	GCF_000222465.1
XP_015760063.1	GT4+DEATH	627	LOC107339308	Eukaryota>Opisthokonta>Metazoa>Cnidaria	Acropora digitifera	GCF_000222465.1
XP_015760062.1	GT4+DEATH	651	LOC107339308	Eukaryota>Opisthokonta>Metazoa>Cnidaria	Acropora digitifera	GCF_000222465.1
XP_015760060.1	GT4+DEATH	651	LOC107339308	Eukaryota>Opisthokonta>Metazoa>Cnidaria	Acropora digitifera	GCF_000222465.1
XP_015760061.1	GT4+DEATH	651	LOC107339308	Eukaryota>Opisthokonta>Metazoa>Cnidaria	Acropora digitifera	GCF_000222465.1
XP_015760057.1	GT4+DEATH	675	LOC107339308	Eukaryota>Opisthokonta>Metazoa>Cnidaria	Acropora digitifera	GCF_000222465.1
XP_015760059.1	GT4+DEATH	675	LOC107339308	Eukaryota>Opisthokonta>Metazoa>Cnidaria	Acropora digitifera	GCF_000222465.1
XP_015760058.1	GT4+DEATH	675	LOC107339308	Eukaryota>Opisthokonta>Metazoa>Cnidaria	Acropora digitifera	GCF_000222465.1
XP_015760603.1	GT4	399	LOC107339804	Eukaryota>Opisthokonta>Metazoa>Cnidaria	Acropora digitifera	GCF_000222465.1
XP_015760882.1	GT4	399	LOC107340053	Eukaryota>Opisthokonta>Metazoa>Cnidaria	Acropora digitifera	GCF_000222465.1
XP_015764085.1	GT4	446	LOC107343054	Eukaryota>Opisthokonta>Metazoa>Cnidaria	Acropora digitifera	GCF_000222465.1
XP_015764101.1	GT4	523	LOC107343072	Eukaryota>Opisthokonta>Metazoa>Cnidaria	Acropora digitifera	GCF_000222465.1
XP_015764103.1	GT4	468	LOC107343073	Eukaryota>Opisthokonta>Metazoa>Cnidaria	Acropora digitifera	GCF_000222465.1
XP_015764656.1	GT4	312	LOC107343585	Eukaryota>Opisthokonta>Metazoa>Cnidaria	Acropora digitifera	GCF_000222465.1
XP_015764718.1	GT4	435	LOC107343652	Eukaryota>Opisthokonta>Metazoa>Cnidaria	Acropora digitifera	GCF_000222465.1
XP_015764719.1	GT4	435	LOC107343652	Eukaryota>Opisthokonta>Metazoa>Cnidaria	Acropora digitifera	GCF_000222465.1
XP_015764720.1	GT4	435	LOC107343652	Eukaryota>Opisthokonta>Metazoa>Cnidaria	Acropora digitifera	GCF_000222465.1
XP_015764793.1	GT4	386	LOC107343725	Eukaryota>Opisthokonta>Metazoa>Cnidaria	Acropora digitifera	GCF_000222465.1
XP_015764794.1	GT4	386	LOC107343725	Eukaryota>Opisthokonta>Metazoa>Cnidaria	Acropora digitifera	GCF_000222465.1
XP_015764792.1	GT4	391	LOC107343725	Eukaryota>Opisthokonta>Metazoa>Cnidaria	Acropora digitifera	GCF_000222465.1
XP_015764791.1	GT4	443	LOC107343725	Eukaryota>Opisthokonta>Metazoa>Cnidaria	Acropora digitifera	GCF_000222465.1
XP_015764795.1	SIG+GT4	423	LOC107343726	Eukaryota>Opisthokonta>Metazoa>Cnidaria	Acropora digitifera	GCF_000222465.1
XP_015764797.1	GT4	395	LOC107343727	Eukaryota>Opisthokonta>Metazoa>Cnidaria	Acropora digitifera	GCF_000222465.1
XP_015764798.1	GT4	395	LOC107343727	Eukaryota>Opisthokonta>Metazoa>Cnidaria	Acropora digitifera	GCF_000222465.1
XP_015764796.1	GT4	395	LOC107343727	Eukaryota>Opisthokonta>Metazoa>Cnidaria	Acropora digitifera	GCF_000222465.1

acc	architecture	len	gen.name	tax	species	GCA
XP_015764806.1	GT4	469	LOC107343730	Eukaryota>Opisthokonta>Metazoa>Cnidaria	Acropora digitifera	GCF_000222465.1
XP_015764805.1	GT4	472	LOC107343730	Eukaryota>Opisthokonta>Metazoa>Cnidaria	Acropora digitifera	GCF_000222465.1
XP_015764804.1	GT4+DEATH	655	LOC107343730	Eukaryota>Opisthokonta>Metazoa>Cnidaria	Acropora digitifera	GCF_000222465.1
XP_015764802.1	GT4+DEATH	661	LOC107343730	Eukaryota>Opisthokonta>Metazoa>Cnidaria	Acropora digitifera	GCF_000222465.1
XP_015764803.1	GT4+DEATH	661	LOC107343730	Eukaryota>Opisthokonta>Metazoa>Cnidaria	Acropora digitifera	GCF_000222465.1
XP_015764810.1	GT4	455	LOC107343734	Eukaryota>Opisthokonta>Metazoa>Cnidaria	Acropora digitifera	GCF_000222465.1
XP_015766096.1	GT4	346	LOC107344923	Eukaryota>Opisthokonta>Metazoa>Cnidaria	Acropora digitifera	GCF_000222465.1
XP_015766734.1	GT4	492	LOC107345529	Eukaryota>Opisthokonta>Metazoa>Cnidaria	Acropora digitifera	GCF_000222465.1
XP_015766737.1	GT4	492	LOC107345529	Eukaryota>Opisthokonta>Metazoa>Cnidaria	Acropora digitifera	GCF_000222465.1
XP_015766736.1	GT4	492	LOC107345529	Eukaryota>Opisthokonta>Metazoa>Cnidaria	Acropora digitifera	GCF_000222465.1
XP_015767847.1	GT4	341	LOC107346555	Eukaryota>Opisthokonta>Metazoa>Cnidaria	Acropora digitifera	GCF_000222465.1
XP_015767923.1	GT4	456	LOC107346627	Eukaryota>Opisthokonta>Metazoa>Cnidaria	Acropora digitifera	GCF_000222465.1
XP_015767937.1	GT4	425	LOC107346649	Eukaryota>Opisthokonta>Metazoa>Cnidaria	Acropora digitifera	GCF_000222465.1
XP_015767991.1	SIG+GT4	542	LOC107346690	Eukaryota>Opisthokonta>Metazoa>Cnidaria	Acropora digitifera	GCF_000222465.1
XP_015767999.1	SIG+GT4	542	LOC107346690	Eukaryota>Opisthokonta>Metazoa>Cnidaria	Acropora digitifera	GCF_000222465.1
XP_015768000.1	SIG+GT4	542	LOC107346690	Eukaryota>Opisthokonta>Metazoa>Cnidaria	Acropora digitifera	GCF_000222465.1
XP_015767993.1	SIG+GT4	542	LOC107346690	Eukaryota>Opisthokonta>Metazoa>Cnidaria	Acropora digitifera	GCF_000222465.1
XP_015768002.1	SIG+GT4	542	LOC107346690	Eukaryota>Opisthokonta>Metazoa>Cnidaria	Acropora digitifera	GCF_000222465.1
XP_015767994.1	SIG+GT4	542	LOC107346690	Eukaryota>Opisthokonta>Metazoa>Cnidaria	Acropora digitifera	GCF_000222465.1
XP_015767998.1	SIG+GT4	542	LOC107346690	Eukaryota>Opisthokonta>Metazoa>Cnidaria	Acropora digitifera	GCF_000222465.1
XP_015767997.1	SIG+GT4	542	LOC107346690	Eukaryota>Opisthokonta>Metazoa>Cnidaria	Acropora digitifera	GCF_000222465.1
XP_015768005.1	SIG+GT4	542	LOC107346690	Eukaryota>Opisthokonta>Metazoa>Cnidaria	Acropora digitifera	GCF_000222465.1
XP_015767996.1	SIG+GT4	542	LOC107346690	Eukaryota>Opisthokonta>Metazoa>Cnidaria	Acropora digitifera	GCF_000222465.1
XP_015767992.1	SIG+GT4	542	LOC107346690	Eukaryota>Opisthokonta>Metazoa>Cnidaria	Acropora digitifera	GCF_000222465.1
XP_015768003.1	SIG+GT4	542	LOC107346690	Eukaryota>Opisthokonta>Metazoa>Cnidaria	Acropora digitifera	GCF_000222465.1
XP_015767995.1	SIG+GT4	542	LOC107346690	Eukaryota>Opisthokonta>Metazoa>Cnidaria	Acropora digitifera	GCF_000222465.1
XP_015768001.1	SIG+GT4	542	LOC107346690	Eukaryota>Opisthokonta>Metazoa>Cnidaria	Acropora digitifera	GCF_000222465.1
XP_015768432.1	GT4	421	LOC107347079	Eukaryota>Opisthokonta>Metazoa>Cnidaria	Acropora digitifera	GCF_000222465.1
XP_015768793.1	GT4	589	LOC107347415	Eukaryota>Opisthokonta>Metazoa>Cnidaria	Acropora digitifera	GCF_000222465.1
XP_015768813.1	GT4	503	LOC107347417	Eukaryota>Opisthokonta>Metazoa>Cnidaria	Acropora digitifera	GCF_000222465.1
XP_015768812.1	GT4	503	LOC107347417	Eukaryota>Opisthokonta>Metazoa>Cnidaria	Acropora digitifera	GCF_000222465.1
XP_015768821.1	SIG+GT4	448	LOC107347421	Eukaryota>Opisthokonta>Metazoa>Cnidaria	Acropora digitifera	GCF_000222465.1
XP_015768820.1	SIG+GT4	473	LOC107347421	Eukaryota>Opisthokonta>Metazoa>Cnidaria	Acropora digitifera	GCF_000222465.1
XP_015768819.1	SIG+GT4	486	LOC107347421	Eukaryota>Opisthokonta>Metazoa>Cnidaria	Acropora digitifera	GCF_000222465.1
XP_015768846.1	GT4	503	LOC107347433	Eukaryota>Opisthokonta>Metazoa>Cnidaria	Acropora digitifera	GCF_000222465.1
XP_015768847.1	GT4	503	LOC107347433	Eukaryota>Opisthokonta>Metazoa>Cnidaria	Acropora digitifera	GCF_000222465.1
XP_015768850.1	SIG+GT4	470	LOC107347435	Eukaryota>Opisthokonta>Metazoa>Cnidaria	Acropora digitifera	GCF_000222465.1
XP_015768849.1	SIG+GT4	486	LOC107347435	Eukaryota>Opisthokonta>Metazoa>Cnidaria	Acropora digitifera	GCF_000222465.1
XP_015768848.1	SIG+GT4	494	LOC107347435	Eukaryota>Opisthokonta>Metazoa>Cnidaria	Acropora digitifera	GCF_000222465.1
XP_015770124.1	GT4+DEATH	668	LOC107348579	Eukaryota>Opisthokonta>Metazoa>Cnidaria	Acropora digitifera	GCF_000222465.1
XP_015770346.1	ZU5+ZU5+GT4	847	LOC107348784	Eukaryota>Opisthokonta>Metazoa>Cnidaria	Acropora digitifera	GCF_000222465.1
XP_015770345.1	ZU5+ZU5+GT4	853	LOC107348784	Eukaryota>Opisthokonta>Metazoa>Cnidaria	Acropora digitifera	GCF_000222465.1
XP_015770481.1	GT4	445	LOC107348902	Eukaryota>Opisthokonta>Metazoa>Cnidaria	Acropora digitifera	GCF_000222465.1
XP_015770480.1	GT4	463	LOC107348902	Eukaryota>Opisthokonta>Metazoa>Cnidaria	Acropora digitifera	GCF_000222465.1
XP_015770479.1	GT4	470	LOC107348902	Eukaryota>Opisthokonta>Metazoa>Cnidaria	Acropora digitifera	GCF_000222465.1
XP_015770478.1	GT4	517	LOC107348902	Eukaryota>Opisthokonta>Metazoa>Cnidaria	Acropora digitifera	GCF_000222465.1
XP_015770477.1	GT4	549	LOC107348902	Eukaryota>Opisthokonta>Metazoa>Cnidaria	Acropora digitifera	GCF_000222465.1
XP_015771034.1	GT4	411	LOC107349395	Eukaryota>Opisthokonta>Metazoa>Cnidaria	Acropora digitifera	GCF_000222465.1
XP_015771060.1	SIG+GT4	438	LOC107349429	Eukaryota>Opisthokonta>Metazoa>Cnidaria	Acropora digitifera	GCF_000222465.1
XP_015771078.1	GT4	415	LOC107349446	Eukaryota>Opisthokonta>Metazoa>Cnidaria	Acropora digitifera	GCF_000222465.1
XP_015771077.1	SIG+GT4	442	LOC107349446	Eukaryota>Opisthokonta>Metazoa>Cnidaria	Acropora digitifera	GCF_000222465.1
XP_015771076.1	SIG+GT4	442	LOC107349446	Eukaryota>Opisthokonta>Metazoa>Cnidaria	Acropora digitifera	GCF_000222465.1

acc	architecture	len	gen.name	tax	species	GCA
XP_015771922.1	ZU5+ZU5+GT4	956	LOC107350214	Eukaryota>Opisthokonta>Metazoa>Cnidaria	Acropora digitifera	GCF_000222465.1
XP_015774186.1	GT4	423	LOC107352364	Eukaryota>Opisthokonta>Metazoa>Cnidaria	Acropora digitifera	GCF_000222465.1
XP_015774185.1	GT4	423	LOC107352364	Eukaryota>Opisthokonta>Metazoa>Cnidaria	Acropora digitifera	GCF_000222465.1
XP_015774885.1	GT4	570	LOC107353084	Eukaryota>Opisthokonta>Metazoa>Cnidaria	Acropora digitifera	GCF_000222465.1
XP_015774884.1	GT4	606	LOC107353084	Eukaryota>Opisthokonta>Metazoa>Cnidaria	Acropora digitifera	GCF_000222465.1
XP_015774883.1	GT4	620	LOC107353084	Eukaryota>Opisthokonta>Metazoa>Cnidaria	Acropora digitifera	GCF_000222465.1
XP_015775255.1	ZU5+ZU5+GT4	837	LOC107353433	Eukaryota>Opisthokonta>Metazoa>Cnidaria	Acropora digitifera	GCF_000222465.1
XP_015775499.1	GT4	662	LOC107353672	Eukaryota>Opisthokonta>Metazoa>Cnidaria	Acropora digitifera	GCF_000222465.1
XP_015776074.1	GT4	452	LOC107354141	Eukaryota>Opisthokonta>Metazoa>Cnidaria	Acropora digitifera	GCF_000222465.1
XP_015776071.1	GT4	458	LOC107354141	Eukaryota>Opisthokonta>Metazoa>Cnidaria	Acropora digitifera	GCF_000222465.1
XP_015776070.1	GT4	458	LOC107354141	Eukaryota>Opisthokonta>Metazoa>Cnidaria	Acropora digitifera	GCF_000222465.1
XP_015776073.1	GT4	458	LOC107354141	Eukaryota>Opisthokonta>Metazoa>Cnidaria	Acropora digitifera	GCF_000222465.1
XP_015776072.1	GT4	458	LOC107354141	Eukaryota>Opisthokonta>Metazoa>Cnidaria	Acropora digitifera	GCF_000222465.1
XP_015777147.1	GT4	352	LOC107355140	Eukaryota>Opisthokonta>Metazoa>Cnidaria	Acropora digitifera	GCF_000222465.1
XP_015777237.1	ZU5+ZU5+GT4	919	LOC107355217	Eukaryota>Opisthokonta>Metazoa>Cnidaria	Acropora digitifera	GCF_000222465.1
XP_015777240.1	GT4	381	LOC107355218	Eukaryota>Opisthokonta>Metazoa>Cnidaria	Acropora digitifera	GCF_000222465.1
XP_015777238.1	GT4	381	LOC107355218	Eukaryota>Opisthokonta>Metazoa>Cnidaria	Acropora digitifera	GCF_000222465.1
XP_015777306.1	GT4	536	LOC107355277	Eukaryota>Opisthokonta>Metazoa>Cnidaria	Acropora digitifera	GCF_000222465.1
XP_015777305.1	GT4	588	LOC107355277	Eukaryota>Opisthokonta>Metazoa>Cnidaria	Acropora digitifera	GCF_000222465.1
XP_015777303.1	GT4	640	LOC107355277	Eukaryota>Opisthokonta>Metazoa>Cnidaria	Acropora digitifera	GCF_000222465.1
XP_015777304.1	GT4	640	LOC107355277	Eukaryota>Opisthokonta>Metazoa>Cnidaria	Acropora digitifera	GCF_000222465.1
XP_015777355.1	GT4	700	LOC107355327	Eukaryota>Opisthokonta>Metazoa>Cnidaria	Acropora digitifera	GCF_000222465.1
XP_015777501.1	SIG+GT4	422	LOC107355435	Eukaryota>Opisthokonta>Metazoa>Cnidaria	Acropora digitifera	GCF_000222465.1
XP_015777594.1	GT4	404	LOC107355536	Eukaryota>Opisthokonta>Metazoa>Cnidaria	Acropora digitifera	GCF_000222465.1
XP_015777688.1	GT4	508	LOC107355611	Eukaryota>Opisthokonta>Metazoa>Cnidaria	Acropora digitifera	GCF_000222465.1
XP_015777687.1	GT4	522	LOC107355611	Eukaryota>Opisthokonta>Metazoa>Cnidaria	Acropora digitifera	GCF_000222465.1
XP_015777733.1	GT4	409	LOC107355668	Eukaryota>Opisthokonta>Metazoa>Cnidaria	Acropora digitifera	GCF_000222465.1
XP_015778308.1	GT4	422	LOC107356176	Eukaryota>Opisthokonta>Metazoa>Cnidaria	Acropora digitifera	GCF_000222465.1
XP_015778319.1	GT4	359	LOC107356189	Eukaryota>Opisthokonta>Metazoa>Cnidaria	Acropora digitifera	GCF_000222465.1
XP_015778457.1	GT4	501	LOC107356350	Eukaryota>Opisthokonta>Metazoa>Cnidaria	Acropora digitifera	GCF_000222465.1
XP_015778456.1	GT4	501	LOC107356350	Eukaryota>Opisthokonta>Metazoa>Cnidaria	Acropora digitifera	GCF_000222465.1
XP_015778455.1	GT4	501	LOC107356350	Eukaryota>Opisthokonta>Metazoa>Cnidaria	Acropora digitifera	GCF_000222465.1
XP_015778598.1	GT4	344	LOC107356505	Eukaryota>Opisthokonta>Metazoa>Cnidaria	Acropora digitifera	GCF_000222465.1
XP_015778867.1	GT4	504	LOC107356761	Eukaryota>Opisthokonta>Metazoa>Cnidaria	Acropora digitifera	GCF_000222465.1
XP_015778951.1	GT4	574	LOC107356841	Eukaryota>Opisthokonta>Metazoa>Cnidaria	Acropora digitifera	GCF_000222465.1
XP_015778954.1	GT4	528	LOC107356843	Eukaryota>Opisthokonta>Metazoa>Cnidaria	Acropora digitifera	GCF_000222465.1
XP_015778953.1	GT4	551	LOC107356843	Eukaryota>Opisthokonta>Metazoa>Cnidaria	Acropora digitifera	GCF_000222465.1
XP_015778980.1	ZU5+GT4	670	LOC107356864	Eukaryota>Opisthokonta>Metazoa>Cnidaria	Acropora digitifera	GCF_000222465.1
XP_015779015.1	GT4	495	LOC107356898	Eukaryota>Opisthokonta>Metazoa>Cnidaria	Acropora digitifera	GCF_000222465.1
XP_015779020.1	GT4	464	LOC107356906	Eukaryota>Opisthokonta>Metazoa>Cnidaria	Acropora digitifera	GCF_000222465.1
XP_015779021.1	GT4	464	LOC107356906	Eukaryota>Opisthokonta>Metazoa>Cnidaria	Acropora digitifera	GCF_000222465.1
XP_015779237.1	GT4	537	LOC107357099	Eukaryota>Opisthokonta>Metazoa>Cnidaria	Acropora digitifera	GCF_000222465.1
XP_015779238.1	GT4	468	LOC107357101	Eukaryota>Opisthokonta>Metazoa>Cnidaria	Acropora digitifera	GCF_000222465.1
XP_015780640.1	GT4	651	LOC107358547	Eukaryota>Opisthokonta>Metazoa>Cnidaria	Acropora digitifera	GCF_000222465.1
XP_015780642.1	GT4	422	LOC107358548	Eukaryota>Opisthokonta>Metazoa>Cnidaria	Acropora digitifera	GCF_000222465.1
XP_015780885.1	GT4	402	LOC107358821	Eukaryota>Opisthokonta>Metazoa>Cnidaria	Acropora digitifera	GCF_000222465.1
XP_015748198.1	GT4+NACHT	1089	LOC107328000	Eukaryota>Opisthokonta>Metazoa>Cnidaria	Acropora digitifera	GCF_000222465.1
XP_015748252.1	GT4+NACHT	1354	LOC107328050	Eukaryota>Opisthokonta>Metazoa>Cnidaria	Acropora digitifera	GCF_000222465.1
XP_015748251.1	GT4+NACHT	1378	LOC107328050	Eukaryota>Opisthokonta>Metazoa>Cnidaria	Acropora digitifera	GCF_000222465.1
XP_015748250.1	GT4+NACHT	1379	LOC107328050	Eukaryota>Opisthokonta>Metazoa>Cnidaria	Acropora digitifera	GCF_000222465.1
XP_015748253.1	GT4+NACHT	1267	LOC107328051	Eukaryota>Opisthokonta>Metazoa>Cnidaria	Acropora digitifera	GCF_000222465.1
XP_015748270.1	GT4+NACHT	1262	LOC107328071	Eukaryota>Opisthokonta>Metazoa>Cnidaria	Acropora digitifera	GCF_000222465.1

acc	architecture	len	gen.name	tax	species	GCA
XP_015748415.1	GT4+NACHT	785	LOC107328189	Eukaryota>Opisthokonta>Metazoa>Cnidaria	Acropora digitifera	GCF_000222465.1
XP_015749493.1	GT4+NACHT	1051	LOC107329298	Eukaryota>Opisthokonta>Metazoa>Cnidaria	Acropora digitifera	GCF_000222465.1
XP_015756823.1	GT4+NACHT	1117	LOC107336267	Eukaryota>Opisthokonta>Metazoa>Cnidaria	Acropora digitifera	GCF_000222465.1
XP_015757684.1	GT4+NACHT	1102	LOC107337083	Eukaryota>Opisthokonta>Metazoa>Cnidaria	Acropora digitifera	GCF_000222465.1
XP_015757683.1	GT4+NACHT	1214	LOC107337083	Eukaryota>Opisthokonta>Metazoa>Cnidaria	Acropora digitifera	GCF_000222465.1
XP_015759265.1	GT4+NACHT	1127	LOC107338542	Eukaryota>Opisthokonta>Metazoa>Cnidaria	Acropora digitifera	GCF_000222465.1
XP_015760037.1	GT4+NACHT	897	LOC107339286	Eukaryota>Opisthokonta>Metazoa>Cnidaria	Acropora digitifera	GCF_000222465.1
XP_015764733.1	GT4+NACHT	1250	LOC107343665	Eukaryota>Opisthokonta>Metazoa>Cnidaria	Acropora digitifera	GCF_000222465.1
XP_015764740.1	GT4+NACHT	1273	LOC107343669	Eukaryota>Opisthokonta>Metazoa>Cnidaria	Acropora digitifera	GCF_000222465.1
XP_015766114.1	GT4+NACHT	883	LOC107344942	Eukaryota>Opisthokonta>Metazoa>Cnidaria	Acropora digitifera	GCF_000222465.1
XP_015767800.1	GT4+NACHT	1362	LOC107346514	Eukaryota>Opisthokonta>Metazoa>Cnidaria	Acropora digitifera	GCF_000222465.1
XP_015769559.1	GT4+NACHT	1073	LOC107348070	Eukaryota>Opisthokonta>Metazoa>Cnidaria	Acropora digitifera	GCF_000222465.1
XP_015771075.1	GT4+NACHT	1131	LOC107349444	Eukaryota>Opisthokonta>Metazoa>Cnidaria	Acropora digitifera	GCF_000222465.1
XP_015771449.1	GT4+NACHT	1095	LOC107349777	Eukaryota>Opisthokonta>Metazoa>Cnidaria	Acropora digitifera	GCF_000222465.1
XP_015774737.1	GT4+NACHT	1214	LOC107352931	Eukaryota>Opisthokonta>Metazoa>Cnidaria	Acropora digitifera	GCF_000222465.1
XP_015774735.1	GT4+NACHT	1240	LOC107352931	Eukaryota>Opisthokonta>Metazoa>Cnidaria	Acropora digitifera	GCF_000222465.1
XP_015774736.1	GT4+NACHT	1240	LOC107352931	Eukaryota>Opisthokonta>Metazoa>Cnidaria	Acropora digitifera	GCF_000222465.1
XP_015775665.1	GT4+NACHT	1107	LOC107353792	Eukaryota>Opisthokonta>Metazoa>Cnidaria	Acropora digitifera	GCF_000222465.1
XP_015775664.1	GT4+NACHT	1107	LOC107353792	Eukaryota>Opisthokonta>Metazoa>Cnidaria	Acropora digitifera	GCF_000222465.1
XP_015777175.1	GT4+NACHT	1097	LOC107355160	Eukaryota>Opisthokonta>Metazoa>Cnidaria	Acropora digitifera	GCF_000222465.1
XP_015777174.1	GT4+NACHT	1160	LOC107355160	Eukaryota>Opisthokonta>Metazoa>Cnidaria	Acropora digitifera	GCF_000222465.1
XP_015777173.1	GT4+NACHT	1185	LOC107355160	Eukaryota>Opisthokonta>Metazoa>Cnidaria	Acropora digitifera	GCF_000222465.1
XP_015777171.1	GT4+NACHT	1210	LOC107355160	Eukaryota>Opisthokonta>Metazoa>Cnidaria	Acropora digitifera	GCF_000222465.1
XP_015777168.1	GT4+NACHT	1214	LOC107355160	Eukaryota>Opisthokonta>Metazoa>Cnidaria	Acropora digitifera	GCF_000222465.1
XP_015777169.1	GT4+NACHT	1214	LOC107355160	Eukaryota>Opisthokonta>Metazoa>Cnidaria	Acropora digitifera	GCF_000222465.1
XP_015777170.1	GT4+NACHT	1214	LOC107355160	Eukaryota>Opisthokonta>Metazoa>Cnidaria	Acropora digitifera	GCF_000222465.1
XP_015777167.1	GT4+NACHT	1229	LOC107355160	Eukaryota>Opisthokonta>Metazoa>Cnidaria	Acropora digitifera	GCF_000222465.1
XP_015777166.1	GT4+NACHT	1234	LOC107355160	Eukaryota>Opisthokonta>Metazoa>Cnidaria	Acropora digitifera	GCF_000222465.1
XP_015777164.1	GT4+NACHT	1237	LOC107355160	Eukaryota>Opisthokonta>Metazoa>Cnidaria	Acropora digitifera	GCF_000222465.1
XP_015777165.1	GT4+NACHT	1237	LOC107355160	Eukaryota>Opisthokonta>Metazoa>Cnidaria	Acropora digitifera	GCF_000222465.1
XP_015778512.1	GT4+NACHT	1054	LOC107356410	Eukaryota>Opisthokonta>Metazoa>Cnidaria	Acropora digitifera	GCF_000222465.1
XP_015779196.1	GT4+NACHT	1094	LOC107357068	Eukaryota>Opisthokonta>Metazoa>Cnidaria	Acropora digitifera	GCF_000222465.1
XP_015779200.1	GT4+NACHT	980	LOC107357071	Eukaryota>Opisthokonta>Metazoa>Cnidaria	Acropora digitifera	GCF_000222465.1
XP_015779450.1	GT4+NACHT	1180	LOC107357325	Eukaryota>Opisthokonta>Metazoa>Cnidaria	Acropora digitifera	GCF_000222465.1
XP_015779456.1	GT4+NACHT	723	LOC107357328	Eukaryota>Opisthokonta>Metazoa>Cnidaria	Acropora digitifera	GCF_000222465.1
XP_015779455.1	GT4+NACHT	748	LOC107357328	Eukaryota>Opisthokonta>Metazoa>Cnidaria	Acropora digitifera	GCF_000222465.1
XP_015779454.1	GT4+NACHT	750	LOC107357328	Eukaryota>Opisthokonta>Metazoa>Cnidaria	Acropora digitifera	GCF_000222465.1
XP_015779453.1	GT4+NACHT	775	LOC107357328	Eukaryota>Opisthokonta>Metazoa>Cnidaria	Acropora digitifera	GCF_000222465.1
XP_015779585.1	GT4+NACHT	1318	LOC107357460	Eukaryota>Opisthokonta>Metazoa>Cnidaria	Acropora digitifera	GCF_000222465.1
KHO50967.1	GT4	400	QT05_C0047G0016	Archaea	archaeon GW2011_AR13	GCA_000805965.1
EGD71903.1	GT4	378	CSMARM5_0008	Archaea>DPANN group	Candidatus Parvarchaeum acidophilus ARMAN-5_‘5-way FS’	GCA_000192615.1
XP_015768804.1	SIG+GT4+DEATH+sGTP+sGTP	2069	LOC107347416	Eukaryota>Opisthokonta>Metazoa>Cnidaria	Acropora digitifera	GCF_000222465.1
XP_015768802.1	SIG+GT4+DEATH+sGTP+sGTP	2089	LOC107347416	Eukaryota>Opisthokonta>Metazoa>Cnidaria	Acropora digitifera	GCF_000222465.1
XP_015768801.1	GT4+DEATH+sGTP+sGTP	2091	LOC107347416	Eukaryota>Opisthokonta>Metazoa>Cnidaria	Acropora digitifera	GCF_000222465.1
XP_015768800.1	GT4+DEATH+sGTP+sGTP	2092	LOC107347416	Eukaryota>Opisthokonta>Metazoa>Cnidaria	Acropora digitifera	GCF_000222465.1
XP_015768799.1	GT4+DEATH+sGTP+sGTP	2092	LOC107347416	Eukaryota>Opisthokonta>Metazoa>Cnidaria	Acropora digitifera	GCF_000222465.1
XP_015768797.1	SIG+GT4+DEATH+sGTP+sGTP	2094	LOC107347416	Eukaryota>Opisthokonta>Metazoa>Cnidaria	Acropora digitifera	GCF_000222465.1
XP_015768795.1	GT4+DEATH+sGTP+sGTP	2094	LOC107347416	Eukaryota>Opisthokonta>Metazoa>Cnidaria	Acropora digitifera	GCF_000222465.1
XP_015768798.1	SIG+GT4+DEATH+sGTP+sGTP	2094	LOC107347416	Eukaryota>Opisthokonta>Metazoa>Cnidaria	Acropora digitifera	GCF_000222465.1
XP_015768796.1	SIG+GT4+DEATH+sGTP+sGTP	2094	LOC107347416	Eukaryota>Opisthokonta>Metazoa>Cnidaria	Acropora digitifera	GCF_000222465.1
XP_015768794.1	GT4+DEATH+sGTP+sGTP	2094	LOC107347416	Eukaryota>Opisthokonta>Metazoa>Cnidaria	Acropora digitifera	GCF_000222465.1

acc	architecture	len	gen.name	tax	species	GCA
XP_015768836.1	SIG+GT4+DEATH+sGTP+sGTP	2069	LOC107347432	Eukaryota>Opisthokonta>Metazoa>Cnidaria	Acropora digitifera	GCF_000222465.1
XP_015768838.1	GT4+DEATH+sGTP+sGTP	2069	LOC107347432	Eukaryota>Opisthokonta>Metazoa>Cnidaria	Acropora digitifera	GCF_000222465.1
XP_015768835.1	SIG+GT4+DEATH+sGTP+sGTP	2089	LOC107347432	Eukaryota>Opisthokonta>Metazoa>Cnidaria	Acropora digitifera	GCF_000222465.1
XP_015768834.1	GT4+DEATH+sGTP+sGTP	2091	LOC107347432	Eukaryota>Opisthokonta>Metazoa>Cnidaria	Acropora digitifera	GCF_000222465.1
XP_015768833.1	GT4+DEATH+sGTP+sGTP	2092	LOC107347432	Eukaryota>Opisthokonta>Metazoa>Cnidaria	Acropora digitifera	GCF_000222465.1
XP_015768832.1	GT4+DEATH+sGTP+sGTP	2092	LOC107347432	Eukaryota>Opisthokonta>Metazoa>Cnidaria	Acropora digitifera	GCF_000222465.1
XP_015768829.1	SIG+GT4+DEATH+sGTP+sGTP	2094	LOC107347432	Eukaryota>Opisthokonta>Metazoa>Cnidaria	Acropora digitifera	GCF_000222465.1
XP_015768830.1	SIG+GT4+DEATH+sGTP+sGTP	2094	LOC107347432	Eukaryota>Opisthokonta>Metazoa>Cnidaria	Acropora digitifera	GCF_000222465.1
XP_015768831.1	SIG+GT4+DEATH+sGTP+sGTP	2094	LOC107347432	Eukaryota>Opisthokonta>Metazoa>Cnidaria	Acropora digitifera	GCF_000222465.1
XP_015768828.1	GT4+DEATH+sGTP+sGTP	2094	LOC107347432	Eukaryota>Opisthokonta>Metazoa>Cnidaria	Acropora digitifera	GCF_000222465.1
XP_015768827.1	GT4+DEATH+sGTP+sGTP	2094	LOC107347432	Eukaryota>Opisthokonta>Metazoa>Cnidaria	Acropora digitifera	GCF_000222465.1
XP_015777374.1	GT4+ANK	1352	LOC107355329	Eukaryota>Opisthokonta>Metazoa>Cnidaria	Acropora digitifera	GCF_000222465.1
XP_015777373.1	GT4+ANK	1356	LOC107355329	Eukaryota>Opisthokonta>Metazoa>Cnidaria	Acropora digitifera	GCF_000222465.1
XP_015777372.1	GT4+ANK	1356	LOC107355329	Eukaryota>Opisthokonta>Metazoa>Cnidaria	Acropora digitifera	GCF_000222465.1
XP_015777371.1	GT4+Immank+ANK	1368	LOC107355329	Eukaryota>Opisthokonta>Metazoa>Cnidaria	Acropora digitifera	GCF_000222465.1
XP_015777369.1	GT4+ANK	1372	LOC107355329	Eukaryota>Opisthokonta>Metazoa>Cnidaria	Acropora digitifera	GCF_000222465.1
XP_015777368.1	GT4+ANK	1372	LOC107355329	Eukaryota>Opisthokonta>Metazoa>Cnidaria	Acropora digitifera	GCF_000222465.1
XP_015777370.1	GT4+ANK	1372	LOC107355329	Eukaryota>Opisthokonta>Metazoa>Cnidaria	Acropora digitifera	GCF_000222465.1
XP_015777367.1	GT4+ANK	1372	LOC107355329	Eukaryota>Opisthokonta>Metazoa>Cnidaria	Acropora digitifera	GCF_000222465.1
XP_015777366.1	GT4+ANK+ANK	1384	LOC107355329	Eukaryota>Opisthokonta>Metazoa>Cnidaria	Acropora digitifera	GCF_000222465.1
XP_015777362.1	GT4+ANK+ANK	1388	LOC107355329	Eukaryota>Opisthokonta>Metazoa>Cnidaria	Acropora digitifera	GCF_000222465.1
XP_015777363.1	GT4+ANK+ANK	1388	LOC107355329	Eukaryota>Opisthokonta>Metazoa>Cnidaria	Acropora digitifera	GCF_000222465.1
XP_015777365.1	GT4+ANK+ANK	1388	LOC107355329	Eukaryota>Opisthokonta>Metazoa>Cnidaria	Acropora digitifera	GCF_000222465.1
XP_015777361.1	GT4+ANK+ANK	1400	LOC107355329	Eukaryota>Opisthokonta>Metazoa>Cnidaria	Acropora digitifera	GCF_000222465.1
XP_015777360.1	GT4+ANK+ANK	1404	LOC107355329	Eukaryota>Opisthokonta>Metazoa>Cnidaria	Acropora digitifera	GCF_000222465.1
XP_015777359.1	GT4+ANK+ANK	1420	LOC107355329	Eukaryota>Opisthokonta>Metazoa>Cnidaria	Acropora digitifera	GCF_000222465.1
XP_015777358.1	GT4+ANK+ANK	1420	LOC107355329	Eukaryota>Opisthokonta>Metazoa>Cnidaria	Acropora digitifera	GCF_000222465.1
XP_015777357.1	GT4+ANK+ANK	1436	LOC107355329	Eukaryota>Opisthokonta>Metazoa>Cnidaria	Acropora digitifera	GCF_000222465.1
XP_015775968.1	ZU5+ZU5+TM+GT4+APATPase	1462	LOC107354065	Eukaryota>Opisthokonta>Metazoa>Cnidaria	Acropora digitifera	GCF_000222465.1
XP_015775965.1	ZU5+ZU5+TM+GT4+APATPase+TPR	1578	LOC107354065	Eukaryota>Opisthokonta>Metazoa>Cnidaria	Acropora digitifera	GCF_000222465.1
XP_015775967.1	ZU5+ZU5+TM+GT4+APATPase+TPR	1578	LOC107354065	Eukaryota>Opisthokonta>Metazoa>Cnidaria	Acropora digitifera	GCF_000222465.1
XP_015775966.1	ZU5+ZU5+TM+GT4+APATPase+TPR	1578	LOC107354065	Eukaryota>Opisthokonta>Metazoa>Cnidaria	Acropora digitifera	GCF_000222465.1
XP_015775964.1	ZU5+ZU5+TM+GT4+APATPase+TPR	1593	LOC107354065	Eukaryota>Opisthokonta>Metazoa>Cnidaria	Acropora digitifera	GCF_000222465.1
XP_015775963.1	ZU5+ZU5+TM+GT4+APATPase+TPR	1626	LOC107354065	Eukaryota>Opisthokonta>Metazoa>Cnidaria	Acropora digitifera	GCF_000222465.1
XP_015775962.1	ZU5+ZU5+TM+GT4+APATPase+TPR	1655	LOC107354065	Eukaryota>Opisthokonta>Metazoa>Cnidaria	Acropora digitifera	GCF_000222465.1
XP_015775961.1	ZU5+ZU5+TM+GT4+APATPase+TPR	1674	LOC107354065	Eukaryota>Opisthokonta>Metazoa>Cnidaria	Acropora digitifera	GCF_000222465.1
XP_015775960.1	ZU5+ZU5+TM+GT4+APATPase+TPR	1689	LOC107354065	Eukaryota>Opisthokonta>Metazoa>Cnidaria	Acropora digitifera	GCF_000222465.1
XP_015775959.1	ZU5+ZU5+TM+GT4+APATPase+TPR	1689	LOC107354065	Eukaryota>Opisthokonta>Metazoa>Cnidaria	Acropora digitifera	GCF_000222465.1
XP_015775958.1	ZU5+ZU5+TM+GT4+APATPase+TPR	1689	LOC107354065	Eukaryota>Opisthokonta>Metazoa>Cnidaria	Acropora digitifera	GCF_000222465.1
XP_015775956.1	ZU5+ZU5+TM+GT4+APATPase+TPR	1690	LOC107354065	Eukaryota>Opisthokonta>Metazoa>Cnidaria	Acropora digitifera	GCF_000222465.1
XP_015775955.1	ZU5+ZU5+TM+GT4+APATPase+TPR	1693	LOC107354065	Eukaryota>Opisthokonta>Metazoa>Cnidaria	Acropora digitifera	GCF_000222465.1
XP_015775954.1	ZU5+ZU5+TM+GT4+APATPase+TPR	1703	LOC107354065	Eukaryota>Opisthokonta>Metazoa>Cnidaria	Acropora digitifera	GCF_000222465.1
XP_015775953.1	ZU5+ZU5+TM+GT4+APATPase+TPR	1703	LOC107354065	Eukaryota>Opisthokonta>Metazoa>Cnidaria	Acropora digitifera	GCF_000222465.1
XP_015768811.1	GT4+sGTP+sGTP	1904	LOC107347416	Eukaryota>Opisthokonta>Metazoa>Cnidaria	Acropora digitifera	GCF_000222465.1
XP_015768810.1	GT4+sGTP+sGTP	1928	LOC107347416	Eukaryota>Opisthokonta>Metazoa>Cnidaria	Acropora digitifera	GCF_000222465.1
XP_015768809.1	GT4+sGTP+sGTP	1928	LOC107347416	Eukaryota>Opisthokonta>Metazoa>Cnidaria	Acropora digitifera	GCF_000222465.1
XP_015768808.1	SIG+GT4+sGTP+sGTP	1929	LOC107347416	Eukaryota>Opisthokonta>Metazoa>Cnidaria	Acropora digitifera	GCF_000222465.1
XP_015768807.1	SIG+GT4+sGTP+sGTP	1930	LOC107347416	Eukaryota>Opisthokonta>Metazoa>Cnidaria	Acropora digitifera	GCF_000222465.1
XP_015768806.1	SIG+GT4+sGTP+sGTP	1951	LOC107347416	Eukaryota>Opisthokonta>Metazoa>Cnidaria	Acropora digitifera	GCF_000222465.1
XP_015768805.1	SIG+GT4+sGTP+sGTP	1953	LOC107347416	Eukaryota>Opisthokonta>Metazoa>Cnidaria	Acropora digitifera	GCF_000222465.1
XP_015768845.1	GT4+sGTP+sGTP	1904	LOC107347432	Eukaryota>Opisthokonta>Metazoa>Cnidaria	Acropora digitifera	GCF_000222465.1
XP_015768843.1	GT4+sGTP+sGTP	1928	LOC107347432	Eukaryota>Opisthokonta>Metazoa>Cnidaria	Acropora digitifera	GCF_000222465.1

acc	architecture	len	gen.name	tax	species	GCA
XP_015768844.1	GT4+sGTP+sGTP	1928	LOC107347432	Eukaryota>Opisthokonta>Metazoa>Cnidaria	Acropora digitifera	GCF_000222465.1
XP_015768842.1	SIG+GT4+sGTP+sGTP	1929	LOC107347432	Eukaryota>Opisthokonta>Metazoa>Cnidaria	Acropora digitifera	GCF_000222465.1
XP_015768841.1	SIG+GT4+sGTP+sGTP	1930	LOC107347432	Eukaryota>Opisthokonta>Metazoa>Cnidaria	Acropora digitifera	GCF_000222465.1
XP_015768840.1	SIG+GT4+sGTP+sGTP	1951	LOC107347432	Eukaryota>Opisthokonta>Metazoa>Cnidaria	Acropora digitifera	GCF_000222465.1
XP_015768839.1	SIG+GT4+sGTP+sGTP	1953	LOC107347432	Eukaryota>Opisthokonta>Metazoa>Cnidaria	Acropora digitifera	GCF_000222465.1
XP_015767989.1	GT4+NACHT	1796	LOC107346689	Eukaryota>Opisthokonta>Metazoa>Cnidaria	Acropora digitifera	GCF_000222465.1
XP_015767988.1	SIG+GT4+NACHT	1806	LOC107346689	Eukaryota>Opisthokonta>Metazoa>Cnidaria	Acropora digitifera	GCF_000222465.1
XP_015776065.1	GT4+NACHT	1674	LOC107354137	Eukaryota>Opisthokonta>Metazoa>Cnidaria	Acropora digitifera	GCF_000222465.1
XP_015776064.1	GT4+NACHT	1675	LOC107354137	Eukaryota>Opisthokonta>Metazoa>Cnidaria	Acropora digitifera	GCF_000222465.1
XP_015776063.1	SIG+GT4+NACHT	1801	LOC107354137	Eukaryota>Opisthokonta>Metazoa>Cnidaria	Acropora digitifera	GCF_000222465.1
XP_015776062.1	SIG+GT4+NACHT	1828	LOC107354137	Eukaryota>Opisthokonta>Metazoa>Cnidaria	Acropora digitifera	GCF_000222465.1
XP_015776061.1	SIG+GT4+NACHT	1838	LOC107354137	Eukaryota>Opisthokonta>Metazoa>Cnidaria	Acropora digitifera	GCF_000222465.1
XP_015776060.1	SIG+GT4+NACHT	1877	LOC107354137	Eukaryota>Opisthokonta>Metazoa>Cnidaria	Acropora digitifera	GCF_000222465.1
XP_015776059.1	SIG+GT4+NACHT	1885	LOC107354137	Eukaryota>Opisthokonta>Metazoa>Cnidaria	Acropora digitifera	GCF_000222465.1
XP_015776058.1	SIG+GT4+NACHT	1914	LOC107354137	Eukaryota>Opisthokonta>Metazoa>Cnidaria	Acropora digitifera	GCF_000222465.1
XP_015776057.1	SIG+GT4+NACHT	1938	LOC107354137	Eukaryota>Opisthokonta>Metazoa>Cnidaria	Acropora digitifera	GCF_000222465.1
XP_015776056.1	SIG+GT4+NACHT	1947	LOC107354137	Eukaryota>Opisthokonta>Metazoa>Cnidaria	Acropora digitifera	GCF_000222465.1
XP_015776055.1	SIG+GT4+NACHT	1951	LOC107354137	Eukaryota>Opisthokonta>Metazoa>Cnidaria	Acropora digitifera	GCF_000222465.1
XP_015776054.1	SIG+GT4+NACHT	1951	LOC107354137	Eukaryota>Opisthokonta>Metazoa>Cnidaria	Acropora digitifera	GCF_000222465.1
XP_015747144.1	GT4+NACHT	1735	LOC107326906	Eukaryota>Opisthokonta>Metazoa>Cnidaria	Acropora digitifera	GCF_000222465.1
XP_015764064.1	GT4+NACHT	1703	LOC107343050	Eukaryota>Opisthokonta>Metazoa>Cnidaria	Acropora digitifera	GCF_000222465.1
XP_015764063.1	GT4+NACHT	1703	LOC107343050	Eukaryota>Opisthokonta>Metazoa>Cnidaria	Acropora digitifera	GCF_000222465.1
XP_015764065.1	GT4+NACHT	1703	LOC107343050	Eukaryota>Opisthokonta>Metazoa>Cnidaria	Acropora digitifera	GCF_000222465.1
XP_015764062.1	GT4+NACHT	1703	LOC107343050	Eukaryota>Opisthokonta>Metazoa>Cnidaria	Acropora digitifera	GCF_000222465.1
XP_015764061.1	GT4+NACHT	1715	LOC107343050	Eukaryota>Opisthokonta>Metazoa>Cnidaria	Acropora digitifera	GCF_000222465.1
XP_015764057.1	GT4+NACHT	1719	LOC107343050	Eukaryota>Opisthokonta>Metazoa>Cnidaria	Acropora digitifera	GCF_000222465.1
XP_015764060.1	GT4+NACHT	1719	LOC107343050	Eukaryota>Opisthokonta>Metazoa>Cnidaria	Acropora digitifera	GCF_000222465.1
XP_015764058.1	GT4+NACHT	1719	LOC107343050	Eukaryota>Opisthokonta>Metazoa>Cnidaria	Acropora digitifera	GCF_000222465.1
XP_015764059.1	GT4+NACHT	1719	LOC107343050	Eukaryota>Opisthokonta>Metazoa>Cnidaria	Acropora digitifera	GCF_000222465.1
XP_015777734.1	GT4+NACHT	1739	LOC107355669	Eukaryota>Opisthokonta>Metazoa>Cnidaria	Acropora digitifera	GCF_000222465.1
XP_015778874.1	SIG+GT4+VWA+SERAprotease	1218	LOC107356762	Eukaryota>Opisthokonta>Metazoa>Cnidaria	Acropora digitifera	GCF_000222465.1
XP_015778871.1	SIG+GT4+VWA+SERAprotease	1221	LOC107356762	Eukaryota>Opisthokonta>Metazoa>Cnidaria	Acropora digitifera	GCF_000222465.1
XP_015778870.1	SIG+GT4+VWA+SERAprotease	1221	LOC107356762	Eukaryota>Opisthokonta>Metazoa>Cnidaria	Acropora digitifera	GCF_000222465.1
XP_015778873.1	SIG+GT4+VWA+SERAprotease	1221	LOC107356762	Eukaryota>Opisthokonta>Metazoa>Cnidaria	Acropora digitifera	GCF_000222465.1
XP_015778868.1	SIG+GT4+VWA+SERAprotease	1221	LOC107356762	Eukaryota>Opisthokonta>Metazoa>Cnidaria	Acropora digitifera	GCF_000222465.1
XP_015778872.1	SIG+GT4+VWA+SERAprotease	1221	LOC107356762	Eukaryota>Opisthokonta>Metazoa>Cnidaria	Acropora digitifera	GCF_000222465.1
XP_015778880.1	SIG+GT4+VWA+SERAprotease	1218	LOC107356768	Eukaryota>Opisthokonta>Metazoa>Cnidaria	Acropora digitifera	GCF_000222465.1
XP_015778878.1	SIG+GT4+VWA+SERAprotease	1221	LOC107356768	Eukaryota>Opisthokonta>Metazoa>Cnidaria	Acropora digitifera	GCF_000222465.1
XP_015778879.1	SIG+GT4+VWA+SERAprotease	1221	LOC107356768	Eukaryota>Opisthokonta>Metazoa>Cnidaria	Acropora digitifera	GCF_000222465.1
XP_015751629.1	DED+TPR+GT4	1894	LOC107331536	Eukaryota>Opisthokonta>Metazoa>Cnidaria	Acropora digitifera	GCF_000222465.1
XP_015751628.1	DED+TPR+GT4	2015	LOC107331536	Eukaryota>Opisthokonta>Metazoa>Cnidaria	Acropora digitifera	GCF_000222465.1
XP_015751627.1	DED+TPR+GT4	2019	LOC107331536	Eukaryota>Opisthokonta>Metazoa>Cnidaria	Acropora digitifera	GCF_000222465.1
XP_015751626.1	DED+TPR+GT4	2020	LOC107331536	Eukaryota>Opisthokonta>Metazoa>Cnidaria	Acropora digitifera	GCF_000222465.1
XP_015758975.1	SIG+TPR+GT4	1867	LOC107338255	Eukaryota>Opisthokonta>Metazoa>Cnidaria	Acropora digitifera	GCF_000222465.1
KXJ17955.1	DED+TPR+GT4	1737	AC249_AIPGENE20062	Eukaryota>Opisthokonta>Metazoa>Cnidaria	Exaiptasia diaphana	GCA_001417965.1
KXJ23466.1	DED+GT4	1693	Samd9l	Eukaryota>Opisthokonta>Metazoa>Cnidaria	Exaiptasia diaphana	GCA_001417965.1
XP_001627530.1	DED+GT4	1802	NEMVEDRAFT_v1g214094	Eukaryota>Opisthokonta>Metazoa>Cnidaria	Nematostella vectensis	-
XP_002586741.1	DED+GT4	538	BRAFLDRAFT_105739	Eukaryota>Opisthokonta>Metazoa>Chordata	Branchiostoma floridae	-
XP_002593761.1	sGTP+GT4	511	BRAFLDRAFT_107710	Eukaryota>Opisthokonta>Metazoa>Chordata	Branchiostoma floridae	-
XP_002585662.1	GT4	514	BRAFLDRAFT_111592	Eukaryota>Opisthokonta>Metazoa>Chordata	Branchiostoma floridae	-
XP_002588370.1	SIG+GT4	424	BRAFLDRAFT_63320	Eukaryota>Opisthokonta>Metazoa>Chordata	Branchiostoma floridae	-
XP_002594621.1	GT4	411	BRAFLDRAFT_77600	Eukaryota>Opisthokonta>Metazoa>Chordata	Branchiostoma floridae	-

acc	architecture	len	gen.name	tax	species	GCA
XP_002594622.1	DEATH+GT4	506	BRAFLDRAFT_77601	Eukaryota>Opisthokonta>Metazoa>Chordata	Branchiostoma floridae	-
XP_002604494.1	GT4	475	BRAFLDRAFT_79207	Eukaryota>Opisthokonta>Metazoa>Chordata	Branchiostoma floridae	-
XP_002731475.1	GT4	433	LOC100372804	Eukaryota>Opisthokonta>Metazoa>Hemichordata	Saccoglossus kowalevskii	GCF_000003605.2
XP_015772447.1	GT4+TPR	596	LOC107350717	Eukaryota>Opisthokonta>Metazoa>Cnidaria	Acropora digitifera	GCF_000222465.1
XP_015772446.1	GT4+TPR	623	LOC107350717	Eukaryota>Opisthokonta>Metazoa>Cnidaria	Acropora digitifera	GCF_000222465.1
XP_015772445.1	GT4+TPR	624	LOC107350717	Eukaryota>Opisthokonta>Metazoa>Cnidaria	Acropora digitifera	GCF_000222465.1
XP_015772444.1	GT4+TPR	642	LOC107350717	Eukaryota>Opisthokonta>Metazoa>Cnidaria	Acropora digitifera	GCF_000222465.1
XP_015772442.1	GT4+TPR	674	LOC107350717	Eukaryota>Opisthokonta>Metazoa>Cnidaria	Acropora digitifera	GCF_000222465.1
XP_015772443.1	GT4+TPR	674	LOC107350717	Eukaryota>Opisthokonta>Metazoa>Cnidaria	Acropora digitifera	GCF_000222465.1
XP_015775035.1	GT4+TPR	635	LOC107353224	Eukaryota>Opisthokonta>Metazoa>Cnidaria	Acropora digitifera	GCF_000222465.1
XP_015770118.1	GT4+sGTP+sGTP+DEATH	1249	LOC107348573	Eukaryota>Opisthokonta>Metazoa>Cnidaria	Acropora digitifera	GCF_000222465.1
XP_015770115.1	GT4+sGTP+sGTP+DEATH	1249	LOC107348573	Eukaryota>Opisthokonta>Metazoa>Cnidaria	Acropora digitifera	GCF_000222465.1
XP_015770116.1	GT4+sGTP+sGTP+DEATH	1249	LOC107348573	Eukaryota>Opisthokonta>Metazoa>Cnidaria	Acropora digitifera	GCF_000222465.1
XP_015770117.1	GT4+sGTP+sGTP+DEATH	1249	LOC107348573	Eukaryota>Opisthokonta>Metazoa>Cnidaria	Acropora digitifera	GCF_000222465.1
XP_015770113.1	GT4+sGTP+sGTP+DEATH	1249	LOC107348573	Eukaryota>Opisthokonta>Metazoa>Cnidaria	Acropora digitifera	GCF_000222465.1
XP_015776051.1	GT4+sGTP	1386	LOC107354135	Eukaryota>Opisthokonta>Metazoa>Cnidaria	Acropora digitifera	GCF_000222465.1
XP_015764075.1	GT4+NACHT	1225	LOC107343052	Eukaryota>Opisthokonta>Metazoa>Cnidaria	Acropora digitifera	GCF_000222465.1
XP_015764074.1	GT4+NACHT	1233	LOC107343052	Eukaryota>Opisthokonta>Metazoa>Cnidaria	Acropora digitifera	GCF_000222465.1
XP_015764072.1	GT4+NACHT	1238	LOC107343052	Eukaryota>Opisthokonta>Metazoa>Cnidaria	Acropora digitifera	GCF_000222465.1
XP_015764073.1	GT4+NACHT	1238	LOC107343052	Eukaryota>Opisthokonta>Metazoa>Cnidaria	Acropora digitifera	GCF_000222465.1
XP_015764071.1	GT4+NACHT	1255	LOC107343052	Eukaryota>Opisthokonta>Metazoa>Cnidaria	Acropora digitifera	GCF_000222465.1
XP_015764070.1	GT4+NACHT	1259	LOC107343052	Eukaryota>Opisthokonta>Metazoa>Cnidaria	Acropora digitifera	GCF_000222465.1
XP_015747310.1	GT4+NACHT	1361	LOC107327069	Eukaryota>Opisthokonta>Metazoa>Cnidaria	Acropora digitifera	GCF_000222465.1
XP_015772577.1	GT4+NACHT	1796	LOC107350842	Eukaryota>Opisthokonta>Metazoa>Cnidaria	Acropora digitifera	GCF_000222465.1
XP_015772579.1	SAP+GT4+NACHT	2199	LOC107350844	Eukaryota>Opisthokonta>Metazoa>Cnidaria	Acropora digitifera	GCF_000222465.1
XP_015776067.1	GT4+NACHT	1600	LOC107354137	Eukaryota>Opisthokonta>Metazoa>Cnidaria	Acropora digitifera	GCF_000222465.1
XP_015776066.1	SIG+GT4+NACHT	1671	LOC107354137	Eukaryota>Opisthokonta>Metazoa>Cnidaria	Acropora digitifera	GCF_000222465.1
XP_015759030.1	sGTP+GT4+TM	1258	LOC107338309	Eukaryota>Opisthokonta>Metazoa>Cnidaria	Acropora digitifera	GCF_000222465.1
XP_015759029.1	sGTP+GT4+TM	1258	LOC107338309	Eukaryota>Opisthokonta>Metazoa>Cnidaria	Acropora digitifera	GCF_000222465.1
XP_015759028.1	sGTP+GT4+TM	1271	LOC107338309	Eukaryota>Opisthokonta>Metazoa>Cnidaria	Acropora digitifera	GCF_000222465.1
XP_015759026.1	sGTP+GT4+TM	1272	LOC107338309	Eukaryota>Opisthokonta>Metazoa>Cnidaria	Acropora digitifera	GCF_000222465.1
XP_015759027.1	sGTP+GT4+TM	1272	LOC107338309	Eukaryota>Opisthokonta>Metazoa>Cnidaria	Acropora digitifera	GCF_000222465.1
XP_015749674.1	GT4+PNPase	965	LOC107329499	Eukaryota>Opisthokonta>Metazoa>Cnidaria	Acropora digitifera	GCF_000222465.1
XP_015749675.1	GT4+PNPase	965	LOC107329499	Eukaryota>Opisthokonta>Metazoa>Cnidaria	Acropora digitifera	GCF_000222465.1
XP_015749860.1	GT4+PNPase+NABD-unk4	933	LOC107329706	Eukaryota>Opisthokonta>Metazoa>Cnidaria	Acropora digitifera	GCF_000222465.1
XP_015749859.1	GT4+PNPase+NABD-unk4	961	LOC107329706	Eukaryota>Opisthokonta>Metazoa>Cnidaria	Acropora digitifera	GCF_000222465.1
XP_015749858.1	GT4+PNPase+NABD-unk4	982	LOC107329706	Eukaryota>Opisthokonta>Metazoa>Cnidaria	Acropora digitifera	GCF_000222465.1
XP_015770482.1	GT4	132	LOC107348905	Eukaryota>Opisthokonta>Metazoa>Cnidaria	Acropora digitifera	GCF_000222465.1
XP_015777289.1	GT4	119	LOC107355256	Eukaryota>Opisthokonta>Metazoa>Cnidaria	Acropora digitifera	GCF_000222465.1
XP_015777288.1	GT4	135	LOC107355256	Eukaryota>Opisthokonta>Metazoa>Cnidaria	Acropora digitifera	GCF_000222465.1
XP_015777287.1	GT4	135	LOC107355256	Eukaryota>Opisthokonta>Metazoa>Cnidaria	Acropora digitifera	GCF_000222465.1
XP_015777286.1	GT4	135	LOC107355256	Eukaryota>Opisthokonta>Metazoa>Cnidaria	Acropora digitifera	GCF_000222465.1
XP_015754287.1	GT4+ANK	1391	LOC107333936	Eukaryota>Opisthokonta>Metazoa>Cnidaria	Acropora digitifera	GCF_000222465.1
XP_015754286.1	GT4+ANK	1391	LOC107333936	Eukaryota>Opisthokonta>Metazoa>Cnidaria	Acropora digitifera	GCF_000222465.1
XP_015754284.1	GT4+ANK	1391	LOC107333936	Eukaryota>Opisthokonta>Metazoa>Cnidaria	Acropora digitifera	GCF_000222465.1
XP_015779234.1	GT4+ANK+Immank+Immank	1392	LOC107357096	Eukaryota>Opisthokonta>Metazoa>Cnidaria	Acropora digitifera	GCF_000222465.1
XP_015780882.1	GT4+DEATH	240	LOC107358816	Eukaryota>Opisthokonta>Metazoa>Cnidaria	Acropora digitifera	GCF_000222465.1
XP_015780881.1	GT4+DEATH	243	LOC107358816	Eukaryota>Opisthokonta>Metazoa>Cnidaria	Acropora digitifera	GCF_000222465.1
XP_015780880.1	GT4+DEATH	247	LOC107358816	Eukaryota>Opisthokonta>Metazoa>Cnidaria	Acropora digitifera	GCF_000222465.1
XP_015780879.1	GT4+DEATH	250	LOC107358816	Eukaryota>Opisthokonta>Metazoa>Cnidaria	Acropora digitifera	GCF_000222465.1
XP_015754283.1	GT4+ANK+DED+APATPase+TPR	1546	LOC107333935	Eukaryota>Opisthokonta>Metazoa>Cnidaria	Acropora digitifera	GCF_000222465.1
XP_015754282.1	GT4+ANK+DED+APATPase+TPR	1560	LOC107333935	Eukaryota>Opisthokonta>Metazoa>Cnidaria	Acropora digitifera	GCF_000222465.1

acc	architecture	len	gen.name	tax	species	GCA
XP_015754281.1	GT4+ANK+DED+APATPase+TPR	1597	LOC107333935	Eukaryota>Opisthokonta>Metazoa>Cnidaria	Acropora digitifera	GCF_000222465.1
XP_015754288.1	GT4+ANK	1351	LOC107333936	Eukaryota>Opisthokonta>Metazoa>Cnidaria	Acropora digitifera	GCF_000222465.1
XP_015778952.1	GT4+ANK+Immank+Immank	1280	LOC107356842	Eukaryota>Opisthokonta>Metazoa>Cnidaria	Acropora digitifera	GCF_000222465.1
XP_015779236.1	GT4+Immank+ANK	1086	LOC107357098	Eukaryota>Opisthokonta>Metazoa>Cnidaria	Acropora digitifera	GCF_000222465.1
XP_015764079.1	GT4+NACHT	1177	LOC107343052	Eukaryota>Opisthokonta>Metazoa>Cnidaria	Acropora digitifera	GCF_000222465.1
XP_015764078.1	GT4+NACHT	1178	LOC107343052	Eukaryota>Opisthokonta>Metazoa>Cnidaria	Acropora digitifera	GCF_000222465.1
XP_015764077.1	GT4+NACHT	1188	LOC107343052	Eukaryota>Opisthokonta>Metazoa>Cnidaria	Acropora digitifera	GCF_000222465.1
XP_015764083.1	GT4+NACHT	1101	LOC107343052	Eukaryota>Opisthokonta>Metazoa>Cnidaria	Acropora digitifera	GCF_000222465.1
XP_015764082.1	GT4+NACHT	1107	LOC107343052	Eukaryota>Opisthokonta>Metazoa>Cnidaria	Acropora digitifera	GCF_000222465.1
XP_015764081.1	GT4+NACHT	1161	LOC107343052	Eukaryota>Opisthokonta>Metazoa>Cnidaria	Acropora digitifera	GCF_000222465.1
XP_015778876.1	SIG+GT4+VWA+SERAprotease	1080	LOC107356762	Eukaryota>Opisthokonta>Metazoa>Cnidaria	Acropora digitifera	GCF_000222465.1
XP_015778875.1	SIG+GT4+VWA+SERAprotease	1145	LOC107356762	Eukaryota>Opisthokonta>Metazoa>Cnidaria	Acropora digitifera	GCF_000222465.1
XP_015778882.1	SIG+GT4+VWA+SERAprotease	1145	LOC107356768	Eukaryota>Opisthokonta>Metazoa>Cnidaria	Acropora digitifera	GCF_000222465.1
XP_015771521.1	SIG+GT4	729	LOC107349844	Eukaryota>Opisthokonta>Metazoa>Cnidaria	Acropora digitifera	GCF_000222465.1
XP_015771522.1	SIG+GT4	729	LOC107349844	Eukaryota>Opisthokonta>Metazoa>Cnidaria	Acropora digitifera	GCF_000222465.1
XP_015771520.1	SIG+GT4	756	LOC107349844	Eukaryota>Opisthokonta>Metazoa>Cnidaria	Acropora digitifera	GCF_000222465.1
XP_015750317.1	GT4+ZU5	721	LOC107330190	Eukaryota>Opisthokonta>Metazoa>Cnidaria	Acropora digitifera	GCF_000222465.1
XP_015770123.1	GT4	704	LOC107348579	Eukaryota>Opisthokonta>Metazoa>Cnidaria	Acropora digitifera	GCF_000222465.1
XP_015770122.1	GT4	705	LOC107348579	Eukaryota>Opisthokonta>Metazoa>Cnidaria	Acropora digitifera	GCF_000222465.1
XP_015752964.1	SIG+GT4	564	LOC107332737	Eukaryota>Opisthokonta>Metazoa>Cnidaria	Acropora digitifera	GCF_000222465.1
XP_015752963.1	SIG+GT4	565	LOC107332737	Eukaryota>Opisthokonta>Metazoa>Cnidaria	Acropora digitifera	GCF_000222465.1
XP_015756764.1	SIG+GT4	570	LOC107336220	Eukaryota>Opisthokonta>Metazoa>Cnidaria	Acropora digitifera	GCF_000222465.1
OLD79042.1	GT4	474	AUF62_00760	Archaea	archaeon 13_1_20CM_52_20	GCA_001919675.1
OLC62495.1	GT4	495	AUH73_04505	Archaea	archaeon 13_1_40CM_4_53_4	GCA_001918315.1
OLB46884.1	GT4	495	AUI07_02860	Archaea	archaeon 13_2_20CM_2_53_6	GCA_001915065.1
XP_015765897.1	GT4	245	LOC107344718	Eukaryota>Opisthokonta>Metazoa>Cnidaria	Acropora digitifera	GCF_000222465.1
XP_015768791.1	GT4	252	LOC107347414	Eukaryota>Opisthokonta>Metazoa>Cnidaria	Acropora digitifera	GCF_000222465.1
XP_015773769.1	GT4	263	LOC107351980	Eukaryota>Opisthokonta>Metazoa>Cnidaria	Acropora digitifera	GCF_000222465.1
XP_015754295.1	GT4	202	LOC107333943	Eukaryota>Opisthokonta>Metazoa>Cnidaria	Acropora digitifera	GCF_000222465.1
XP_015754297.1	GT4	202	LOC107333943	Eukaryota>Opisthokonta>Metazoa>Cnidaria	Acropora digitifera	GCF_000222465.1
XP_015754296.1	GT4	202	LOC107333943	Eukaryota>Opisthokonta>Metazoa>Cnidaria	Acropora digitifera	GCF_000222465.1
XP_015764068.1	GT4+NACHT	1309	LOC107343052	Eukaryota>Opisthokonta>Metazoa>Cnidaria	Acropora digitifera	GCF_000222465.1
XP_015764067.1	GT4+NACHT	1312	LOC107343052	Eukaryota>Opisthokonta>Metazoa>Cnidaria	Acropora digitifera	GCF_000222465.1
XP_015747884.1	RVT+DEATH+ZU5+ZU5+GT4	1232	LOC107327653	Eukaryota>Opisthokonta>Metazoa>Cnidaria	Acropora digitifera	GCF_000222465.1
XP_015747883.1	RVT+DEATH+ZU5+ZU5+GT4	1238	LOC107327653	Eukaryota>Opisthokonta>Metazoa>Cnidaria	Acropora digitifera	GCF_000222465.1
XP_015765549.1	GT4+ANK	1083	LOC107344415	Eukaryota>Opisthokonta>Metazoa>Cnidaria	Acropora digitifera	GCF_000222465.1
XP_015765548.1	GT4+ANK	1084	LOC107344415	Eukaryota>Opisthokonta>Metazoa>Cnidaria	Acropora digitifera	GCF_000222465.1
XP_015780922.1	GT4+GT4	738	LOC107358864	Eukaryota>Opisthokonta>Metazoa>Cnidaria	Acropora digitifera	GCF_000222465.1
XP_015780923.1	GT4+GT4	738	LOC107358864	Eukaryota>Opisthokonta>Metazoa>Cnidaria	Acropora digitifera	GCF_000222465.1
XP_015760897.1	GT4	651	LOC107340068	Eukaryota>Opisthokonta>Metazoa>Cnidaria	Acropora digitifera	GCF_000222465.1
XP_015760896.1	GT4	652	LOC107340068	Eukaryota>Opisthokonta>Metazoa>Cnidaria	Acropora digitifera	GCF_000222465.1
XP_015748510.1	GT4	305	LOC107328284	Eukaryota>Opisthokonta>Metazoa>Cnidaria	Acropora digitifera	GCF_000222465.1
XP_015776097.1	GT4	305	LOC107354170	Eukaryota>Opisthokonta>Metazoa>Cnidaria	Acropora digitifera	GCF_000222465.1
XP_002599631.1	GT4+GTPase-AIG	700	BRAFLDRAFT_102570	Eukaryota>Opisthokonta>Metazoa>Chordata	Branchiostoma floridae	-
XP_002586742.1	GT4	133	BRAFLDRAFT_105740	Eukaryota>Opisthokonta>Metazoa>Chordata	Branchiostoma floridae	-
XP_002606956.1	GT4	718	BRAFLDRAFT_64952	Eukaryota>Opisthokonta>Metazoa>Chordata	Branchiostoma floridae	-
XP_002592892.1	GT4	115	BRAFLDRAFT_65478	Eukaryota>Opisthokonta>Metazoa>Chordata	Branchiostoma floridae	-
XP_002605447.1	GT4	734	BRAFLDRAFT_74262	Eukaryota>Opisthokonta>Metazoa>Chordata	Branchiostoma floridae	-
XP_002598007.1	GT4	253	BRAFLDRAFT_79766	Eukaryota>Opisthokonta>Metazoa>Chordata	Branchiostoma floridae	-
XP_015747221.1	GT4	151	LOC107326989	Eukaryota>Opisthokonta>Metazoa>Cnidaria	Acropora digitifera	GCF_000222465.1
XP_015747315.1	GT4	232	LOC107327073	Eukaryota>Opisthokonta>Metazoa>Cnidaria	Acropora digitifera	GCF_000222465.1
XP_015748075.1	GT4	636	LOC107327853	Eukaryota>Opisthokonta>Metazoa>Cnidaria	Acropora digitifera	GCF_000222465.1

acc	architecture	len	gen.name	tax	species	GCA
XP_015759886.1	GT4	685	LOC107339155	Eukaryota>Opisthokonta>Metazoa>Cnidaria	Acropora digitifera	GCF_000222465.1
XP_015764084.1	GT4+NACHT	901	LOC107343052	Eukaryota>Opisthokonta>Metazoa>Cnidaria	Acropora digitifera	GCF_000222465.1
XP_015767948.1	GT4	201	LOC107346661	Eukaryota>Opisthokonta>Metazoa>Cnidaria	Acropora digitifera	GCF_000222465.1
XP_015774918.1	GT4+NACHT	1264	LOC107353111	Eukaryota>Opisthokonta>Metazoa>Cnidaria	Acropora digitifera	GCF_000222465.1
XP_015777737.1	GT4	925	LOC107355671	Eukaryota>Opisthokonta>Metazoa>Cnidaria	Acropora digitifera	GCF_000222465.1
XP_015748669.1	GT4	245	LOC107328450	Eukaryota>Opisthokonta>Metazoa>Cnidaria	Acropora digitifera	GCF_000222465.1
XP_015750057.1	GT4	520	LOC107329915	Eukaryota>Opisthokonta>Metazoa>Cnidaria	Acropora digitifera	GCF_000222465.1
XP_015754313.1	GT4	241	LOC107333962	Eukaryota>Opisthokonta>Metazoa>Cnidaria	Acropora digitifera	GCF_000222465.1
XP_015761270.1	GT4	712	LOC107340435	Eukaryota>Opisthokonta>Metazoa>Cnidaria	Acropora digitifera	GCF_000222465.1
XP_015770112.1	GT4	253	LOC107348572	Eukaryota>Opisthokonta>Metazoa>Cnidaria	Acropora digitifera	GCF_000222465.1
XP_015771016.1	GT4	390	LOC107349380	Eukaryota>Opisthokonta>Metazoa>Cnidaria	Acropora digitifera	GCF_000222465.1
XP_015771260.1	GT4	491	LOC107349592	Eukaryota>Opisthokonta>Metazoa>Cnidaria	Acropora digitifera	GCF_000222465.1
XP_015772349.1	GT4	256	LOC107350627	Eukaryota>Opisthokonta>Metazoa>Cnidaria	Acropora digitifera	GCF_000222465.1
XP_015777221.1	GT4	205	LOC107355205	Eukaryota>Opisthokonta>Metazoa>Cnidaria	Acropora digitifera	GCF_000222465.1
XP_015778002.1	GT4	437	LOC107355902	Eukaryota>Opisthokonta>Metazoa>Cnidaria	Acropora digitifera	GCF_000222465.1
XP_015779016.1	GT4	186	LOC107356899	Eukaryota>Opisthokonta>Metazoa>Cnidaria	Acropora digitifera	GCF_000222465.1
XP_015780870.1	GT4	274	LOC107358800	Eukaryota>Opisthokonta>Metazoa>Cnidaria	Acropora digitifera	GCF_000222465.1
AFD00853.1	GT4	431	Mtc_2115	Archaea>Euryarchaeota	Methanocella conradii HZ254	GCA_000251105.1
AGB33684.1	GT4	394	Natpe_3933	Archaea>Euryarchaeota	Natrinema pellirubrum DSM 15624	GCA_000230735.3
XP_015751533.1	GT4+NACHT	797	LOC107331457	Eukaryota>Opisthokonta>Metazoa>Cnidaria	Acropora digitifera	GCF_000222465.1
XP_015747435.1	GT4+NACHT	357	LOC107327199	Eukaryota>Opisthokonta>Metazoa>Cnidaria	Acropora digitifera	GCF_000222465.1
XP_015759313.1	GT4+NACHT	988	LOC107338581	Eukaryota>Opisthokonta>Metazoa>Cnidaria	Acropora digitifera	GCF_000222465.1
XP_015759411.1	GT4+NACHT	1346	LOC107338694	Eukaryota>Opisthokonta>Metazoa>Cnidaria	Acropora digitifera	GCF_000222465.1
XP_015769571.1	GT4+NACHT	716	LOC107348075	Eukaryota>Opisthokonta>Metazoa>Cnidaria	Acropora digitifera	GCF_000222465.1
XP_015773770.1	GT4+APATPase	1843	LOC107351981	Eukaryota>Opisthokonta>Metazoa>Cnidaria	Acropora digitifera	GCF_000222465.1
XP_015779460.1	GT4+NACHT	537	LOC107357334	Eukaryota>Opisthokonta>Metazoa>Cnidaria	Acropora digitifera	GCF_000222465.1
XP_015756756.1	GT4+NACHT+NACHT	770	LOC107336211	Eukaryota>Opisthokonta>Metazoa>Cnidaria	Acropora digitifera	GCF_000222465.1
XP_015764080.1	GT4+NACHT	1168	LOC107343052	Eukaryota>Opisthokonta>Metazoa>Cnidaria	Acropora digitifera	GCF_000222465.1
XP_015764069.1	GT4+NACHT	1295	LOC107343052	Eukaryota>Opisthokonta>Metazoa>Cnidaria	Acropora digitifera	GCF_000222465.1
XP_015770110.1	GT4+APATPase+NACHT	1677	LOC107348570	Eukaryota>Opisthokonta>Metazoa>Cnidaria	Acropora digitifera	GCF_000222465.1
XP_015749857.1	GT4+PNPase+NABD-unk4	1031	LOC107329706	Eukaryota>Opisthokonta>Metazoa>Cnidaria	Acropora digitifera	GCF_000222465.1
XP_015778949.1	GT4+GT4	1007	LOC107356840	Eukaryota>Opisthokonta>Metazoa>Cnidaria	Acropora digitifera	GCF_000222465.1
XP_002610411.1	GT4+DEATH	1032	BRAFLDRAFT_72365	Eukaryota>Opisthokonta>Metazoa>Chordata	Branchiostoma floridae	-
XP_015768823.1	GT4+DEATH	402	LOC107347423	Eukaryota>Opisthokonta>Metazoa>Cnidaria	Acropora digitifera	GCF_000222465.1
XP_015761974.1	SIG+GT4	503	LOC107341068	Eukaryota>Opisthokonta>Metazoa>Cnidaria	Acropora digitifera	GCF_000222465.1
XP_002599100.1	GT4	900	BRAFLDRAFT_81765	Eukaryota>Opisthokonta>Metazoa>Chordata	Branchiostoma floridae	-
XP_002601934.1	GT4	652	BRAFLDRAFT_86418	Eukaryota>Opisthokonta>Metazoa>Chordata	Branchiostoma floridae	-
XP_015753083.1	SIG+GT4	678	LOC107332846	Eukaryota>Opisthokonta>Metazoa>Cnidaria	Acropora digitifera	GCF_000222465.1
XP_015770111.1	SIG+GT4+NACHT	1452	LOC107348571	Eukaryota>Opisthokonta>Metazoa>Cnidaria	Acropora digitifera	GCF_000222465.1
XP_015759562.1	GT4	954	LOC107338825	Eukaryota>Opisthokonta>Metazoa>Cnidaria	Acropora digitifera	GCF_000222465.1
XP_015772173.1	GT4	684	LOC107350460	Eukaryota>Opisthokonta>Metazoa>Cnidaria	Acropora digitifera	GCF_000222465.1
XP_015767496.1	GT4+PNPase	423	LOC107346243	Eukaryota>Opisthokonta>Metazoa>Cnidaria	Acropora digitifera	GCF_000222465.1
XP_002610420.1	CARD+sGTP+DEATH+GT4	1671	BRAFLDRAFT_72355	Eukaryota>Opisthokonta>Metazoa>Chordata	Branchiostoma floridae	-
XP_002601932.1	CARD+sGTP+DEATH+GT4+CARD	2070	BRAFLDRAFT_86416	Eukaryota>Opisthokonta>Metazoa>Chordata	Branchiostoma floridae	-
XP_002606064.1	GT4+TIR	594	BRAFLDRAFT_92083	Eukaryota>Opisthokonta>Metazoa>Chordata	Branchiostoma floridae	-
XP_015747743.1	GT4+SidE	864	LOC107327511	Eukaryota>Opisthokonta>Metazoa>Cnidaria	Acropora digitifera	GCF_000222465.1
XP_015771496.1	GT4+TPR+TPR+TPR+RVT	1988	LOC107349827	Eukaryota>Opisthokonta>Metazoa>Cnidaria	Acropora digitifera	GCF_000222465.1
XP_015777375.1	GT4+ANK	878	LOC107355332	Eukaryota>Opisthokonta>Metazoa>Cnidaria	Acropora digitifera	GCF_000222465.1
XP_006812785.1	GT4+PIN+HEPN	648	LOC102807894	Eukaryota>Opisthokonta>Metazoa>Hemichordata	Saccoglossus kowalevskii	GCF_000003605.2
XP_002601187.1	ImmLRR+LRR+sGTP+OTU+GT4	1577	BRAFLDRAFT_75632	Eukaryota>Opisthokonta>Metazoa>Chordata	Branchiostoma floridae	-
XP_015779017.1	GT4+RNA-Helicase+CXC	1910	LOC107356900	Eukaryota>Opisthokonta>Metazoa>Cnidaria	Acropora digitifera	GCF_000222465.1
XP_015767918.1	GT4+NACHT	1211	LOC107346622	Eukaryota>Opisthokonta>Metazoa>Cnidaria	Acropora digitifera	GCF_000222465.1

acc	architecture	len	gen.name	tax	species	GCA
XP_015747744.1	GT4+GT4	1044	LOC107327512	Eukaryota>Opisthokonta>Metazoa>Cnidaria	Acropora digitifera	GCF_000222465.1
XP_006822589.1	TM+TM+BetaPropeller+BetaPropeller+GT4+ANK+Immank+ANK+ANK+Immank+Immank+Immank	3508	LOC102803126	Eukaryota>Opisthokonta>Metazoa>Hemichordata	Saccoglossus kowalevskii	GCF_000003605.2
XP_002608839.1	DED+DED+OTU+GT4+DEATH	1285	BRAFLDRAFT_89707	Eukaryota>Opisthokonta>Metazoa>Chordata	Branchiostoma floridae	-
XP_002602261.1	ANK+DEATH+GT4	983	BRAFLDRAFT_76949	Eukaryota>Opisthokonta>Metazoa>Chordata	Branchiostoma floridae	-
XP_006824651.1	SIG+EGF+EGF+EGF+CLECTIN+GT4	945	LOC102808514	Eukaryota>Opisthokonta>Metazoa>Hemichordata	Saccoglossus kowalevskii	GCF_000003605.2
XP_015754275.1	GT4+GT4+APATPase+TPR	1815	LOC107333926	Eukaryota>Opisthokonta>Metazoa>Cnidaria	Acropora digitifera	GCF_000222465.1
XP_015764809.1	GT4+DEATH	660	LOC107343733	Eukaryota>Opisthokonta>Metazoa>Cnidaria	Acropora digitifera	GCF_000222465.1
XP_015763304.1	GT4+CR-ATPase4+TPR	1073	LOC107342333	Eukaryota>Opisthokonta>Metazoa>Cnidaria	Acropora digitifera	GCF_000222465.1
XP_015765336.1	GT4+UBI+UBI+UBI+UBI	1194	LOC107344209	Eukaryota>Opisthokonta>Metazoa>Cnidaria	Acropora digitifera	GCF_000222465.1
XP_015765496.1	GT4+CASPASE	587	LOC107344359	Eukaryota>Opisthokonta>Metazoa>Cnidaria	Acropora digitifera	GCF_000222465.1
XP_002601970.1	DEATH+GT4	1118	BRAFLDRAFT_94541	Eukaryota>Opisthokonta>Metazoa>Chordata	Branchiostoma floridae	-
XP_015756490.1	GT4+NACHT	1270	LOC107335955	Eukaryota>Opisthokonta>Metazoa>Cnidaria	Acropora digitifera	GCF_000222465.1
XP_015754270.1	GT4+drhyd+NACHT	1358	LOC107333920	Eukaryota>Opisthokonta>Metazoa>Cnidaria	Acropora digitifera	GCF_000222465.1
XP_015780567.1	GT4+ANK	1294	LOC107358478	Eukaryota>Opisthokonta>Metazoa>Cnidaria	Acropora digitifera	GCF_000222465.1
XP_015748771.1	GT4+TM+TM	876	LOC107328563	Eukaryota>Opisthokonta>Metazoa>Cnidaria	Acropora digitifera	GCF_000222465.1
XP_002606060.1	GT4+DED+DED+DED+DED	1082	BRAFLDRAFT_92079	Eukaryota>Opisthokonta>Metazoa>Chordata	Branchiostoma floridae	-
XP_015764066.1	GT4+NACHT	1352	LOC107343052	Eukaryota>Opisthokonta>Metazoa>Cnidaria	Acropora digitifera	GCF_000222465.1
XP_015754289.1	GT4+ANK	1133	LOC107333936	Eukaryota>Opisthokonta>Metazoa>Cnidaria	Acropora digitifera	GCF_000222465.1
XP_002601931.1	DEATH+GT4	1086	BRAFLDRAFT_86415	Eukaryota>Opisthokonta>Metazoa>Chordata	Branchiostoma floridae	-
XP_015765568.1	SIG+ANK+GT4	607	LOC107344435	Eukaryota>Opisthokonta>Metazoa>Cnidaria	Acropora digitifera	GCF_000222465.1
XP_015771899.1	SIG+GT4+NACHT+RVT	942	LOC107350190	Eukaryota>Opisthokonta>Metazoa>Cnidaria	Acropora digitifera	GCF_000222465.1
XP_015759049.1	GT4+TPR+TPR+TPR+sGTP+sGTP	2065	LOC107338329	Eukaryota>Opisthokonta>Metazoa>Cnidaria	Acropora digitifera	GCF_000222465.1
XP_015765567.1	GT4+ANK	1016	LOC107344434	Eukaryota>Opisthokonta>Metazoa>Cnidaria	Acropora digitifera	GCF_000222465.1
XP_002601929.1	CARD+sGTP+DEATH+GT4+sGTP+DEATH+CARD	3425	BRAFLDRAFT_86413	Eukaryota>Opisthokonta>Metazoa>Chordata	Branchiostoma floridae	-
XP_002607644.1	GT4+DEATH+Phytase-like+DEATH+DEATH+DEATH+ZNF+ZNF+CRN-ZnBind.csq+ZNF+ZNF	3703	BRAFLDRAFT_84667	Eukaryota>Opisthokonta>Metazoa>Chordata	Branchiostoma floridae	-
XP_002608763.1	TPR+TPR+TPR+sGTP+DEATH+GT4	2376	BRAFLDRAFT_73982	Eukaryota>Opisthokonta>Metazoa>Chordata	Branchiostoma floridae	-
XP_002606489.1	SIG+ImmLRR+DEATH+GT4	791	BRAFLDRAFT_91930	Eukaryota>Opisthokonta>Metazoa>Chordata	Branchiostoma floridae	-
XP_015759356.1	APATPase+TPR+ZU5+ZU5+GT4	1494	LOC107338634	Eukaryota>Opisthokonta>Metazoa>Cnidaria	Acropora digitifera	GCF_000222465.1
XP_015767926.1	GT4+NACHT	1234	LOC107346634	Eukaryota>Opisthokonta>Metazoa>Cnidaria	Acropora digitifera	GCF_000222465.1
XP_006823290.1	SIG+GT4+TM	1015	LOC102801620	Eukaryota>Opisthokonta>Metazoa>Hemichordata	Saccoglossus kowalevskii	GCF_000003605.2
XP_015777279.1	GT4+sGTP	1178	LOC107355250	Eukaryota>Opisthokonta>Metazoa>Cnidaria	Acropora digitifera	GCF_000222465.1
XP_015771245.1	TPR+TPR+TPR+TPR+TPR+sGTP+GT4	1879	LOC107349580	Eukaryota>Opisthokonta>Metazoa>Cnidaria	Acropora digitifera	GCF_000222465.1
XP_002601933.1	sGTP+DEATH+GT4+sGTP+DEATH+GT4+CARD+sGTP+DEATH	4075	BRAFLDRAFT_86417	Eukaryota>Opisthokonta>Metazoa>Chordata	Branchiostoma floridae	-
XP_002601971.1	SIG+CARD+sGTP+DEATH+GT4+CARD+sGTP+DEATH+DEATH	3440	BRAFLDRAFT_94542	Eukaryota>Opisthokonta>Metazoa>Chordata	Branchiostoma floridae	-
KXJ24237.1	DED+GT4+GT4	2343	AC249_AIPGENE4704	Eukaryota>Opisthokonta>Metazoa>Cnidaria	Exaiptasia diaphana	GCA_001417965.1
XP_006814385.1	DED+TM+GT4	488	LOC102807017	Eukaryota>Opisthokonta>Metazoa>Hemichordata	Saccoglossus kowalevskii	GCF_000003605.2
XP_002592882.1	SIG+ZU5+ZU5+GT4	930	BRAFLDRAFT_117744	Eukaryota>Opisthokonta>Metazoa>Chordata	Branchiostoma floridae	-
XP_015778947.1	GT4+ANK+ANK+ANK	1575	LOC107356838	Eukaryota>Opisthokonta>Metazoa>Cnidaria	Acropora digitifera	GCF_000222465.1
XP_002609384.1	SIG+LRR+sGTP+OTU+GT4+Phytase-like	1519	BRAFLDRAFT_86475	Eukaryota>Opisthokonta>Metazoa>Chordata	Branchiostoma floridae	-
XP_002606063.1	GT4+GTPase-AIG	1278	BRAFLDRAFT_92082	Eukaryota>Opisthokonta>Metazoa>Chordata	Branchiostoma floridae	-
XP_002601968.1	ZNF+ZNF+ZNF+CARD+sGTP+DEATH+GT4	1973	BRAFLDRAFT_94539	Eukaryota>Opisthokonta>Metazoa>Chordata	Branchiostoma floridae	-
XP_015778866.1	GT4+VWA	1117	LOC107356760	Eukaryota>Opisthokonta>Metazoa>Cnidaria	Acropora digitifera	GCF_000222465.1
XP_002592893.1	SIG+DEATH+PspA+GT4	873	BRAFLDRAFT_65479	Eukaryota>Opisthokonta>Metazoa>Chordata	Branchiostoma floridae	-
XP_002610413.1	sGTP+DEATH+GT4+CARD+sGTP+sGTP+DEATH+GT4	4179	BRAFLDRAFT_72363	Eukaryota>Opisthokonta>Metazoa>Chordata	Branchiostoma floridae	-
XP_002599992.1	GT4+TPR+TPR+TPR+TPR	1404	BRAFLDRAFT_74113	Eukaryota>Opisthokonta>Metazoa>Chordata	Branchiostoma floridae	-
XP_002598016.1	DEATH+sGTP+ZU5+GT4	2055	BRAFLDRAFT_79758	Eukaryota>Opisthokonta>Metazoa>Chordata	Branchiostoma floridae	-
XP_015765571.1	GT4+GT4	1248	LOC107344438	Eukaryota>Opisthokonta>Metazoa>Cnidaria	Acropora digitifera	GCF_000222465.1
XP_002608841.1	DED+OTU+sGTP+ZU5+DEATH+DEATH+DED+OTU+CASPASE+sGTP+CASPASE+DED+DEATH+OTU+GT4+sGTP+OTU+GT4+DED+DEATH+CAS-PASE+sGTP+OTU+DED+DEATH	7154	BRAFLDRAFT_89710	Eukaryota>Opisthokonta>Metazoa>Chordata	Branchiostoma floridae	-
XP_015754687.1	SIG+GT4+CR-ATPase4+TPR	1371	LOC107334268	Eukaryota>Opisthokonta>Metazoa>Cnidaria	Acropora digitifera	GCF_000222465.1
XP_014535703.1	GT4	1299	PDIP_38710	Eukaryota>Opisthokonta>Fungi>Ascomycota	Penicillium digitatum Pd1	GCF_000315645.1
XP_015767848.1	GT4+NACHT	1489	LOC107346556	Eukaryota>Opisthokonta>Metazoa>Cnidaria	Acropora digitifera	GCF_000222465.1

acc	architecture	len	gen.name	tax	species	GCA
XP_002599095.1	DEATH+sGTP+DEATH+GT4	1974	BRAFLDRAFT_81759	Eukaryota>Opisthokonta>Metazoa>Chordata	Branchiostoma floridae	-
XP_015754268.1	GT4+GT4+GT4+IG	2108	LOC107333918	Eukaryota>Opisthokonta>Metazoa>Cnidaria	Acropora digitifera	GCF_000222465.1
XP_015747885.1	RVT+DEATH+ZU5+ZU5+GT4	1158	LOC107327653	Eukaryota>Opisthokonta>Metazoa>Cnidaria	Acropora digitifera	GCF_000222465.1
XP_015756824.1	GT4+APATPase	727	LOC107336267	Eukaryota>Opisthokonta>Metazoa>Cnidaria	Acropora digitifera	GCF_000222465.1
XP_015774399.1	GT4+CASPASE+CASPASE	742	LOC107352599	Eukaryota>Opisthokonta>Metazoa>Cnidaria	Acropora digitifera	GCF_000222465.1
XP_015752969.1	GT4	255	LOC107332740	Eukaryota>Opisthokonta>Metazoa>Cnidaria	Acropora digitifera	GCF_000222465.1
XP_006819467.1	GT4+TM+TM+Snf7	1062	LOC102807364	Eukaryota>Opisthokonta>Metazoa>Hemichordata	Saccoglossus kowalevskii	GCF_000003605.2
XP_002602157.1	DEATH+GT4	1248	BRAFLDRAFT_97971	Eukaryota>Opisthokonta>Metazoa>Chordata	Branchiostoma floridae	-
XP_015774318.1	PNPase+TPR+ZU5+ZU5+GT4	1563	LOC107352503	Eukaryota>Opisthokonta>Metazoa>Cnidaria	Acropora digitifera	GCF_000222465.1
XP_006818589.1	TM+GT4	781	LOC100368229	Eukaryota>Opisthokonta>Metazoa>Hemichordata	Saccoglossus kowalevskii	GCF_000003605.2
XP_015749947.1	GT4+CASPASE+CASPASE	619	LOC107329787	Eukaryota>Opisthokonta>Metazoa>Cnidaria	Acropora digitifera	GCF_000222465.1
XP_002610422.1	sGTP+DEATH+GT4	1881	BRAFLDRAFT_72353	Eukaryota>Opisthokonta>Metazoa>Chordata	Branchiostoma floridae	-
XP_009019645.1	GT4	717	HELRODRAFT_161481	Eukaryota>Opisthokonta>Metazoa>Annelida	Helobdella robusta	GCF_000326865.1
XP_015748178.1	GT4	374	LOC107327971	Eukaryota>Opisthokonta>Metazoa>Cnidaria	Acropora digitifera	GCF_000222465.1
XP_015750171.1	GT4	397	LOC107330034	Eukaryota>Opisthokonta>Metazoa>Cnidaria	Acropora digitifera	GCF_000222465.1
XP_015750733.1	GT4	186	LOC107330680	Eukaryota>Opisthokonta>Metazoa>Cnidaria	Acropora digitifera	GCF_000222465.1
XP_015760591.1	ZU5+GT4	573	LOC107339792	Eukaryota>Opisthokonta>Metazoa>Cnidaria	Acropora digitifera	GCF_000222465.1
XP_002731836.1	GT4	901	LOC100368975	Eukaryota>Opisthokonta>Metazoa>Hemichordata	Saccoglossus kowalevskii	GCF_000003605.2
XP_006814349.1	GT4	1053	LOC102800835	Eukaryota>Opisthokonta>Metazoa>Hemichordata	Saccoglossus kowalevskii	GCF_000003605.2
XP_006811676.1	GT4	814	LOC102807814	Eukaryota>Opisthokonta>Metazoa>Hemichordata	Saccoglossus kowalevskii	GCF_000003605.2

Source data. Gene neighborhoods and domain architectures of the MNS-like STAND domains (nSTAND2) that is present in a 2-gene operon system each containing a STAND NTPase

acc	operon	architecture	len	gen.name	taxend	species	defline	gca
AZF56106.1	??+STAND+wHTH→ nSTAND2+wHTH+TPRs+HEPN*→	nSTAND2+wHTH+TPRs+HEPN	621	C4J84_0196	Gammaproteobacteria	Pseudomonas sp. R11-23-07	hypothetical protein C4J84_0196 [Pseudomonas sp. R11-23-07].	GCA_003852295.1
EEV8712171.1	??+STAND+wHTH→ nSTAND2+wHTH+TPRs+HEPN*→	nSTAND2+wHTH+TPRs+HEPN	596	EHU50_23110	Gammaproteobacteria	Escherichia coli	hypothetical protein EHU50_23110, partial [Escherichia coli].	GCA_012057895.1
EEZ9035304.1	??+STAND+wHTH→ nSTAND2+wHTH*→	nSTAND2+wHTH	308	C2D11_004496	Gammaproteobacteria	Escherichia coli O75	hypothetical protein C2D11_004496, partial [Escherichia coli O75].	GCA_012229525.1
KAA9385705.1	nSTAND2+wHTH+TPRs+HEPN*→	nSTAND2+wHTH+TPRs+HEPN	533	F4V88_04125	Alphaproteobacteria	Neorhizobium galegae	hypothetical protein F4V88_04125 [Neorhizobium galegae].	GCA_008728145.1
KPX30733.1	??+STAND+wHTH→ nSTAND2+wHTH+TPRs+HEPN*→	nSTAND2+wHTH+TPRs+HEPN	621	ALO77_200057	Gammaproteobacteria	Pseudomonas coronafaciens pv. garcae	hypothetical protein ALO77_200057 [Pseudomonas coronafaciens pv. garcae].	GCA_001400345.1
KQY48570.1	nSTAND2+wHTH+TPRs+HEPN*→	nSTAND2+wHTH+TPRs+HEPN	547	ASD32_09250	Alphaproteobacteria	Rhizobium sp. Root483D2	hypothetical protein ASD32_09250 [Rhizobium sp. Root483D2].	GCA_001426685.1
KYF49123.1	??+STAND+wHTH→ nSTAND2+wHTH+TPRs+HEPN*→	nSTAND2+wHTH+TPRs+HEPN	636	BE08_43115	Deltaproteobacteria	Sorangium cellulosum	hypothetical protein BE08_43115 [Sorangium cellulosum].	GCA_001589265.1
MBA3548110.1	??+STAND+wHTH→ nSTAND2+wHTH+TPRs+HEPN*→ Integrin_beta(vWA)→	nSTAND2+wHTH+TPRs+HEPN	632	H0T76_16635	Deltaproteobacteria	Nannocystis sp.	hypothetical protein H0T76_16635 [Nannocystis sp.].	GCA_013812955.1
OJX80461.1	nSTAND2+wHTH+TPRs+HEPN*→||<-??	nSTAND2+wHTH+TPRs+HEPN	533	BGO93_00115	Alphaproteobacteria	Mesorhizobium sp. 65-26	hypothetical protein BGO93_00115 [Mesorhizobium sp. 65-26].	GCA_001899205.1
PMZ92294.1	??+STAND+wHTH→ nSTAND2+wHTH+TPRs+HEPN*→ PSE→??→	nSTAND2+wHTH+TPRs+HEPN	621	C1X61_03125	Gammaproteobacteria	Pseudomonas sp. FW215-T2	hypothetical protein C1X61_03125 [Pseudomonas sp. FW215-T2].	GCA_002883935.1
POD52929.1	??+STAND+wHTH→ nSTAND2+wHTH+TPRs+HEPN*→	nSTAND2+wHTH+TPRs+HEPN	590	BKM15_13675	Gammaproteobacteria	Pseudomonas syringae pv. syringae	hypothetical protein BKM15_13675 [Pseudomonas syringae pv. syringae].	GCA_002905935.1
PWI54427.1	nSTAND2+wHTH+TPRs+HEPN*→	nSTAND2+wHTH+TPRs+HEPN	532	B5K03_09585	Alphaproteobacteria	Rhizobium phaseoli	hypothetical protein B5K03_09585 [Rhizobium phaseoli].	GCA_003150695.1
PZX00307.1	??+STAND+wHTH→ nSTAND2+wHTH+TPRs+HEPN*→	nSTAND2+wHTH+TPRs+HEPN	621	DFS28_10221	Gammaproteobacteria	Pseudomonas sp. 478	hypothetical protein DFS28_10221 [Pseudomonas sp. 478].	GCA_003254125.1
RCM87736.1	??+STAND+wHTH→ nSTAND2+wHTH+TPRs+HEPN*→	nSTAND2+wHTH+TPRs+HEPN	588	PA57_04301	Gammaproteobacteria	Pseudomonas aeruginosa	hypothetical protein PA57_04301 [Pseudomonas aeruginosa].	GCA_003332525.1
RMG61681.1	Membrane-bound-b-barrel→ STAND+wHTH*→ Pkinase+cNMP_cyclase→	STAND+wHTH	408	D6715_13175	Calditrichaeota	Calditrichaeota bacterium	ATP-binding protein [Calditrichaeota bacterium].	GCA_003696445.1
RMS07982.1	??+STAND+wHTH→ nSTAND2+wHTH+TPRs+HEPN*→	nSTAND2+wHTH+TPRs+HEPN	621	ALP73_02675	Gammaproteobacteria	Pseudomonas coronafaciens pv. garcae	hypothetical protein ALP73_02675 [Pseudomonas coronafaciens pv. garcae].	GCA_003701555.1
RWN51709.1	??+STAND+wHTH→ nSTAND2+wHTH+TPRs+HEPN*→	nSTAND2+wHTH+TPRs+HEPN	599	EOR98_25445	Alphaproteobacteria	Mesorhizobium sp.	hypothetical protein EOR98_25445 [Mesorhizobium sp.].	GCA_004020775.1
RYG89382.1	??→??+STAND+wHTH→ nSTAND2+wHTH+TPRs+HEPN*→	nSTAND2+wHTH+TPRs+HEPN	629	EON59_01575	Alphaproteobacteria	Alphaproteobacteria bacterium	hypothetical protein EON59_01575 [Alphaproteobacteria bacterium].	GCA_004145145.1
TIN07909.1	??+STAND+wHTH→ nSTAND2+wHTH+TPRs+HEPN*→	nSTAND2+wHTH+TPRs+HEPN	599	E5Y14_22895	Alphaproteobacteria	Mesorhizobium sp.	hypothetical protein E5Y14_22895 [Mesorhizobium sp.].	GCA_004961475.1
TIT02463.1	??+STAND+wHTH→ nSTAND2+wHTH+TPRs+HEPN*→	nSTAND2+wHTH+TPRs+HEPN	596	E5W87_09945	Alphaproteobacteria	Mesorhizobium sp.	hypothetical protein E5W87_09945 [Mesorhizobium sp.].	GCA_004962925.1

acc	operon	architecture	len	gen.name	taxend	species	defline	gca
TJV17958.1	??+STAND+wHTH→ nSTAND2+wHTH+TPRs+HEPN*→	nSTAND2+wHTH+TPRs+HEPN	626	E5Y07_09865	Alphaproteobacteria	Mesorhizobium sp.	hypothetical protein E5Y07_09865 [Mesorhizobium sp.].	GCA_005046125.1
WP_008322742.1	??+STAND+wHTH→ nSTAND2+wHTH+TPRs+HEPN*→||<-??	nSTAND2+wHTH+TPRs+HEPN	613	-	Gammaproteobacteria	Enterobacteriaceae	MULTISPECIES: hypothetical protein [Enterobacteriaceae].	GCF_000277565.1
WP_026230868.1	??+STAND+wHTH→ nSTAND2+wHTH+TPRs+HEPN*→ SF2-helicase+DEAD_assoc→ Calcineurin→	nSTAND2+wHTH+TPRs+HEPN	637	-	Alphaproteobacteria	Rhizobium	MULTISPECIES: hypothetical protein [Rhizobium].	GCF_014138515.1
WP_027196515.1	??+STAND→ nSTAND2+wHTH+GT4*→	nSTAND2+wHTH+GT4	760	-	Betaproteobacteria	Paraburkholderia sprentiae	glycosyltransferase family 4 protein [Paraburkholderia sprentiae].	GCF_001865575.1
WP_048993481.1	??+STAND+wHTH→ nSTAND2+wHTH+TPRs+HEPN*→||<-??	nSTAND2+wHTH+TPRs+HEPN	613	-	Gammaproteobacteria	Klebsiella pneumoniae	hypothetical protein [Klebsiella pneumoniae].	GCF_001065235.1
WP_072160039.1	nSTAND2+wHTH+TPRs+HEPN*→	nSTAND2+wHTH+TPRs+HEPN	613	-	Gammaproteobacteria	Pluralibacter gergoviae	hypothetical protein [Pluralibacter gergoviae].	GCF_001276415.1
WP_088567149.1	??+STAND+wHTH→ nSTAND2+wHTH+TPRs+HEPN*→||<-??	nSTAND2+wHTH+TPRs+HEPN	613	-	Gammaproteobacteria	Enterobacter hormaechei	hypothetical protein [Enterobacter hormaechei].	GCF_002208275.1
WP_089463191.1	??+STAND→ nSTAND2+wHTH+GT4*→	nSTAND2+wHTH+GT4	768	-	Betaproteobacteria	Burkholderia	MULTISPECIES: glycosyltransferase [Burkholderia].	GCF_002924455.1
WP_094198012.1	??+STAND→ nSTAND2+wHTH+GT4*→	nSTAND2+wHTH+GT4	756	-	Betaproteobacteria	Alcaligenes faecalis	glycosyltransferase family 4 protein [Alcaligenes faecalis].	GCF_002242175.1
WP_097401548.1	nSTAND2+wHTH+TPRs+HEPN*→||<-??	nSTAND2+wHTH+TPRs+HEPN	613	-	Gammaproteobacteria	Enterobacteriaceae	MULTISPECIES: hypothetical protein [Enterobacteriaceae].	GCF_002510255.1
WP_106119969.1	??+STAND→ nSTAND2+wHTH+GT4*→	nSTAND2+wHTH+GT4	736	-	Gammaproteobacteria	Pseudomonas simiae	glycosyltransferase family 4 protein [Pseudomonas simiae].	GCF_003001535.1
WP_107112219.1	??+STAND+wHTH→ nSTAND2+wHTH+TPRs+HEPN*→||<-??	nSTAND2+wHTH+TPRs+HEPN	613	-	Gammaproteobacteria	Klebsiella quasipneumoniae	hypothetical protein [Klebsiella quasipneumoniae].	GCF_002854535.1
WP_116343459.1	??+STAND+wHTH→ nSTAND2+wHTH+TPRs+HEPN*→||<-??	nSTAND2+wHTH+TPRs+HEPN	613	-	Gammaproteobacteria	Enterobacter cloacae	hypothetical protein [Enterobacter cloacae].	GCF_003401145.1
WP_122437975.1	??+STAND+wHTH→ nSTAND2+wHTH+TPRs+HEPN*→	nSTAND2+wHTH+TPRs+HEPN	621	-	Gammaproteobacteria	Pseudomonas viridiflava	hypothetical protein [Pseudomonas viridiflava].	GCF_900580835.1
WP_124434788.1	??+STAND+wHTH→ nSTAND2+wHTH+TPRs+HEPN*→	nSTAND2+wHTH+TPRs+HEPN	598	-	Gammaproteobacteria	Pseudomonas sp. R11-23-07	hypothetical protein [Pseudomonas sp. R11-23-07].	GCF_003852295.1
WP_127426060.1	??+STAND+wHTH→ nSTAND2+wHTH+TPRs+HEPN*→	nSTAND2+wHTH+TPRs+HEPN	606	-	Alphaproteobacteria	unclassified Mesorhizobium	MULTISPECIES: hypothetical protein [unclassified Mesorhizobium].	GCF_004021725.2
WP_132834030.1	??+STAND+wHTH→ nSTAND2+wHTH+TPRs+HEPN*→	nSTAND2+wHTH+TPRs+HEPN	598	-	Gammaproteobacteria	unclassified Pseudomonas	MULTISPECIES: hypothetical protein [unclassified Pseudomonas].	GCF_003254125.1
WP_134049903.1	??+STAND+wHTH→ nSTAND2+wHTH+TPRs+HEPN*→	nSTAND2+wHTH+TPRs+HEPN	621	-	Gammaproteobacteria	Pseudomonas sp. OV144	hypothetical protein [Pseudomonas sp. OV144].	GCF_004368835.1
WP_139645958.1	??+STAND+wHTH→ nSTAND2+wHTH+TPRs+HEPN*→	nSTAND2+wHTH+TPRs+HEPN	623	-	Gammaproteobacteria	Pseudomonas sp. ICMP22404	hypothetical protein [Pseudomonas sp. ICMP22404].	GCF_006227205.1
WP_145962680.1	??+STAND+wHTH→ nSTAND2+wHTH+TPRs+HEPN*→	nSTAND2+wHTH+TPRs+HEPN	592	-	Alphaproteobacteria	Rhizobium phaseoli	hypothetical protein [Rhizobium phaseoli].	GCF_003150695.1
WP_146023689.1	??+STAND+wHTH→ nSTAND2+wHTH+TPRs+HEPN*→ PSE→??→	nSTAND2+wHTH+TPRs+HEPN	598	-	Gammaproteobacteria	unclassified Pseudomonas	MULTISPECIES: hypothetical protein [unclassified Pseudomonas].	GCF_002884305.1
WP_147262087.1	??+STAND+wHTH→ nSTAND2+wHTH+TPRs+HEPN*→	nSTAND2+wHTH+TPRs+HEPN	612	-	Gammaproteobacteria	Pseudomonas aeruginosa	hypothetical protein [Pseudomonas aeruginosa].	GCF_003332525.1
WP_147467220.1	nSTAND2+wHTH+TPRs+HEPN*→	nSTAND2+wHTH+TPRs+HEPN	486	-	Gammaproteobacteria	Pseudomonas coronafaciens	hypothetical protein [Pseudomonas coronafaciens].	GCF_003700475.1
WP_147475620.1	nSTAND2+wHTH+TPRs+HEPN*→	nSTAND2+wHTH+TPRs+HEPN	486	-	Gammaproteobacteria	Pseudomonas coronafaciens	hypothetical protein [Pseudomonas coronafaciens].	GCF_001400345.1
WP_153438665.1	??+STAND+wHTH→ nSTAND2+wHTH+TPRs+HEPN*→	nSTAND2+wHTH+TPRs+HEPN	620	-	Gammaproteobacteria	Pseudomonas helleri	hypothetical protein [Pseudomonas helleri].	GCF_009600265.1
WP_156383024.1	??→ STAND+wHTH→ nSTAND2+wHTH+TPRs+HEPN*→	nSTAND2+wHTH+TPRs+HEPN	602	-	Alphaproteobacteria	Rhizobium sp. Root483D2	hypothetical protein [Rhizobium sp. Root483D2].	GCF_001426685.1

acc	operon	architecture	len	gen.name	taxend	species	defline	gca
WP_160320279.1	nSTAND2(fragment)*→	nSTAND2(fragment)	113	-	Alphaproteobacteria	Rhizobium ecuadorense	hypothetical protein, partial [Rhizobium ecuadorense].	GCF_001187535.1
WP_160610812.1	??+STAND+wHTH→ nSTAND2+wHTH+TPRs+HEPN*→	nSTAND2+wHTH+TPRs+HEPN	640	-	Alphaproteobacteria	Altererythrobacter aerius	hypothetical protein [Altererythrobacter aerius].	GCF_009827495.1
WP_163011207.1	??+STAND+wHTH→ nSTAND2+wHTH+TPRs+HEPN*→	nSTAND2+wHTH+TPRs+HEPN	621	-	Gammaproteobacteria	Pseudomonas viridiflava	hypothetical protein [Pseudomonas viridiflava].	GCF_900582025.1
WP_163014645.1	nSTAND2+wHTH+TPRs+HEPN*→	nSTAND2+wHTH+TPRs+HEPN	560	-	Gammaproteobacteria	Pseudomonas viridiflava	hypothetical protein [Pseudomonas viridiflava].	GCF_900582435.1
WP_164708313.1	nSTAND2+wHTH+TPRs+HEPN*→	nSTAND2+wHTH+TPRs+HEPN	534	-	Gammaproteobacteria	Pseudomonas viridiflava	hypothetical protein [Pseudomonas viridiflava].	GCF_900602385.1
WP_169852212.1	??+STAND+wHTH→ nSTAND2+wHTH+TPRs+HEPN*→	nSTAND2+wHTH+TPRs+HEPN	623	-	Gammaproteobacteria	Pseudomonas proteolytica	hypothetical protein [Pseudomonas proteolytica].	GCF_012985975.1

Source data. Gene neighborhoods and domain architectures of prokaryotic AP-GTPases described in the text

<table>
<tr><th>acc</th><th>operon</th><th>architecture</th><th>len</th><th>gen.name</th><th>taxend</th><th>species</th><th>defline</th><th>gca</th></tr>
<tr><td>AAU84033.1</td><td>pc1599→ Cluster1746_2clades→ Calcineurin→||<-tRNA||LRR-repeats+AP-GTPase+COR+TM+TM*→?→?→ Aminotran_1_2→||<-?<-?<-?||Cluster1483_2clades→</td><td>LRR-repeats+AP-GTPase+COR+TM+TM</td><td>737</td><td>GZ35D7_19</td><td>Archaea</td><td>uncultured archaeon GZfos35D7</td><td>leucine-rich-repeat protein [uncultured archaeon GZfos35D7].</td><td>-</td></tr>
<tr><td>AAX07516.1</td><td>LRR-repeats+AP-GTPase+COR*→</td><td>LRR-repeats+AP-GTPase+COR</td><td>1016</td><td>-</td><td>Planctomycetes</td><td>Gemmata sp. Wa1-1</td><td>GTP-binding protein [Gemmata sp. Wa1-1].</td><td>-</td></tr>
<tr><td>ABD75797.1</td><td>HISKIN→ LRR-repeats+AP-GTPase+COR+EAD8*→ HISKIN→?→||<-?||Cluster1512_2clades→ TetR-HTH→?→ Ferredoxin-RRM→</td><td>LRR-repeats+AP-GTPase+COR+EAD8</td><td>847</td><td>-</td><td>Bacteria</td><td>uncultured bacterium</td><td>hypothetical protein [uncultured bacterium].</td><td>-</td></tr>
<tr><td>ACC84329.1</td><td><-Arginase<-?<-?<-?||LRR-repeats+AP-GTPase+COR+TIR*→</td><td>LRR-repeats+AP-GTPase+COR+TIR</td><td>1109</td><td>Npun_F6039</td><td>Cyanobacteria</td><td>Nostoc punctiforme PCC 73102</td><td>Miro domain protein [Nostoc punctiforme PCC 73102].</td><td>GCA_000020025.1</td></tr>
<tr><td>ADB42932.1</td><td>SIG+TM+TM+TM+HISKIN→?→||<-?||CENPB→?→ Cluster2094_2clades→ LRR-repeats+AP-GTPase+COR*→||<-?<-?<-?<-?<-?<-?||ParA-Soj-PloopNTPase→</td><td>LRR-repeats+AP-GTPase+COR</td><td>925</td><td>Slin_6989</td><td>Bacteroidetes</td><td>Spirosoma linguale DSM 74</td><td>small GTP-binding protein (plasmid) [Spirosoma linguale DSM 74].</td><td>GCA_000024525.1</td></tr>
<tr><td>AEE53900.1</td><td>35exo→||<-?<-?||?→?→?→ LRR-repeats+AP-GTPase+COR+TIR*→||<-?<-?<-ABC-ATPase+APATPase+ABC-ATPase<-?||SIGMA-HTH→</td><td>LRR-repeats+AP-GTPase+COR+TIR</td><td>998</td><td>Halhy_6078</td><td>Bacteroidetes</td><td>Haliscomenobacter hydrossis DSM 1100</td><td>small GTP-binding protein [Haliscomenobacter hydrossis DSM 1100].</td><td>GCA_000212735.1</td></tr>
<tr><td>AEE54359.1</td><td>LRR-repeats+AP-GTPase+COR+TIR*→ LRR-repeats+AP-GTPase+COR→ LRR-repeats+AP-GTPase+COR→</td><td>LRR-repeats+AP-GTPase+COR+TIR</td><td>1058</td><td>Halhy_6543</td><td>Bacteroidetes</td><td>Haliscomenobacter hydrossis DSM 1100</td><td>Miro domain protein [Haliscomenobacter hydrossis DSM 1100].</td><td>GCA_000212735.1</td></tr>
<tr><td>AEE54482.1</td><td>LRR-repeats+AP-GTPase+COR+EAD11*→</td><td>LRR-repeats+AP-GTPase+COR+EAD11</td><td>1141</td><td>Halhy_6666</td><td>Bacteroidetes</td><td>Haliscomenobacter hydrossis DSM 1100</td><td>small GTP-binding protein (plasmid) [Haliscomenobacter hydrossis DSM 1100].</td><td>GCA_000212735.1</td></tr>
<tr><td>AEE54508.1</td><td>MACRODOMAIN→?→ LRR-repeats+AP-GTPase+COR+EAD11*→</td><td>LRR-repeats+AP-GTPase+COR+EAD11</td><td>1023</td><td>Halhy_6692</td><td>Bacteroidetes</td><td>Haliscomenobacter hydrossis DSM 1100</td><td>Miro domain protein (plasmid) [Haliscomenobacter hydrossis DSM 1100].</td><td>GCA_000212735.1</td></tr>
<tr><td>AFY40133.1</td><td><-SIG+Trypsin+PDZ<-?||?→?→ LRR-repeats+AP-GTPase+COR+TCAD2*→</td><td>LRR-repeats+AP-GTPase+COR+TCAD2</td><td>918</td><td>Lepto7376_3994</td><td>Cyanobacteria</td><td>Leptolyngbya sp. PCC 7376</td><td>small GTP-binding protein [Leptolyngbya sp. PCC 7376].</td><td>GCA_000316605.1</td></tr>
<tr><td>AFY57475.1</td><td>EACC2+CASPASE+LRR-repeats+AP-GTPase*→ AP-GTPase+COR→</td><td>EACC2+CASPASE+LRR-repeats+AP-GTPase</td><td>982</td><td>Riv7116_5077</td><td>Cyanobacteria</td><td>Rivularia sp. PCC 7116</td><td>leucine-rich repeat (LRR) protein [Rivularia sp. PCC 7116].</td><td>GCA_000316665.1</td></tr>
<tr><td>AKB24843.1</td><td><-HhH-RADC+JAB||?→ NTP_transf_3→ LRR-repeats+AP-GTPase+COR→?→ LRR-repeats+AP-GTPase+COR*→</td><td>LRR-repeats+AP-GTPase+COR</td><td>599</td><td>MSMTP_1374</td><td>Euryarchaeota</td><td>Methanosarcina sp. MTP4</td><td>E3 ubiquitin-protein ligase SspH2 [Methanosarcina sp. MTP4].</td><td>GCA_000970045.1</td></tr>
<tr><td>AKB47719.1</td><td>LRR-repeats→ LRR-repeats+AP-GTPase+COR+TIR*→?→?→?→?→?→?→||<-TPR-repeats</td><td>LRR-repeats+AP-GTPase+COR+TIR</td><td>590</td><td>MSKOL_1942</td><td>Euryarchaeota</td><td>Methanosarcina sp. Kolksee</td><td>hypothetical protein MSKOL_1942 [Methanosarcina sp. Kolksee].</td><td>GCA_000969945.1</td></tr>
<tr><td>AKB51553.1</td><td>LRR-repeats+AP-GTPase+COR*→</td><td>LRR-repeats+AP-GTPase+COR</td><td>880</td><td>MSBRW_2300</td><td>Euryarchaeota</td><td>Methanosarcina barkeri str. Wiesmoor</td><td>hypothetical protein MSBRW_2300 [Methanosarcina barkeri str. Wiesmoor].</td><td>GCA_000969985.1</td></tr>
<tr><td>AKB56819.1</td><td><-SIG+TPR+TPR+TPR+TPR+TPR+TPR||?→||<-?||?→?→?→?→ LRR-repeats+AP-GTPase+COR*→?→?→ PRTase→</td><td>LRR-repeats+AP-GTPase+COR</td><td>880</td><td>MSBR2_0303</td><td>Euryarchaeota</td><td>Methanosarcina barkeri 227</td><td>hypothetical protein MSBR2_0303 [Methanosarcina barkeri 227].</td><td>GCA_000970065.1</td></tr>
<tr><td>AKJ07375.1</td><td>Cluster971_2clades→?→||<-?<-?||LRR-repeats+AP-GTPase+COR+TIR*→?→||<-?<-?<-?||?→||<-?<-SIG+Lysozyme</td><td>LRR-repeats+AP-GTPase+COR+TIR</td><td>873</td><td>AA314_09001</td><td>Deltaproteobacteria</td><td>Archangium gephyra</td><td>Chaperone protein DnaK [Archangium gephyra].</td><td>GCA_001027285.1</td></tr>
<tr><td>AOY82617.1</td><td>REC→||<-?<-HNH||REC→||<-TPR-repeats<-?||LRR-repeats→ LRR-repeats+AP-GTPase+COR*→ AP-GTPase+COR+TM+TM→</td><td>LRR-repeats+AP-GTPase+COR</td><td>252</td><td>BJP36_24575</td><td>Cyanobacteria</td><td>Moorea producens JHB</td><td>hypothetical protein BJP36_24575 [Moorea producens JHB].</td><td>GCA_001854205.1</td></tr>
<tr><td>AQR64774.1</td><td>LRR-repeats+AP-GTPase+COR+TIR*→||<-?<-?<-MarR-HTH||?→||<-MarR-HTH<-?||Thioredoxin→</td><td>LRR-repeats+AP-GTPase+COR+TIR</td><td>1295</td><td>BXU06_06620</td><td>Betaproteobacteria</td><td>Aquaspirillum sp. LM1</td><td>hypothetical protein BXU06_06620 [Aquaspirillum sp. LM1].</td><td>GCA_002002905.1</td></tr>
</table>

<table>
<tr><th>acc</th><th>operon</th><th>architecture</th><th>len</th><th>gen.name</th><th>taxend</th><th>species</th><th>defline</th><th>gca</th></tr>
<tr><td>AQR65390.1</td><td>HSP70→ DNAJ→?→?→ LRR-repeats+AP-GTPase+COR+TIR*→</td><td>LRR-repeats+AP-GTPase+COR+TIR</td><td>890</td><td>BXU06_10225</td><td>Betaproteobacteria</td><td>Aquaspirillum sp. LM1</td><td>hypothetical protein BXU06_10225 [Aquaspirillum sp. LM1].</td><td>GCA_002002905.1</td></tr>
<tr><td>AUN33853.1</td><td><-SIG+TM+TM+TM+TM+TM+TM+TM+TM+TM+TM<-?<-?<-?<-?||?→?→ LRR-repeats+AP-GTPase+COR+TIR*→||<-ParB<-ParA-Soj-PloopNTPase</td><td>LRR-repeats+AP-GTPase+COR+TIR</td><td>965</td><td>C0V82_25960</td><td>Alphaproteobacteria</td><td>Niveispirillum cyanobacteriorum</td><td>hypothetical protein C0V82_25960 (plasmid) [Niveispirillum cyanobacteriorum].</td><td>GCA_002868735.1</td></tr>
<tr><td>AVH71003.1</td><td>LRR-repeats+AP-GTPase+COR+DUF4404*→||<-?||?→||<-?||?→?→ MoxR-AAA→</td><td>LRR-repeats+AP-GTPase+COR+DUF4404</td><td>1233</td><td>NLP_2293</td><td>Cyanobacteria</td><td>Nostoc sp. 'Lobaria pulmonaria (5183) cyanobiont'</td><td>small GTP-binding protein [Nostoc sp. 'Lobaria pulmonaria (5183) cyanobiont'].</td><td>GCA_002949795.1</td></tr>
<tr><td>AXO38068.1</td><td>LRR-repeats+AP-GTPase+COR*→</td><td>LRR-repeats+AP-GTPase+COR</td><td>942</td><td>MicB006_5810</td><td>Actinobacteria</td><td>Micromonospora sp. B006</td><td>cytoplasmic membrane protein [Micromonospora sp. B006].</td><td>GCA_003408515.1</td></tr>
<tr><td>AYQ35346.1</td><td>FGS→ FGS→ FGS→ LRR-repeats+AP-GTPase+COR+CASPASE*→</td><td>LRR-repeats+AP-GTPase+COR+CASPASE</td><td>998</td><td>DTQ70_25690</td><td>Bacteroidetes</td><td>Runella sp. SP2</td><td>CHAT domain-containing protein [Runella sp. SP2].</td><td>GCA_003711225.1</td></tr>
<tr><td>BAY10551.1</td><td>LRR-repeats→ LRR-repeats+AP-GTPase+COR*→</td><td>LRR-repeats+AP-GTPase+COR</td><td>793</td><td>NIES2098_37230</td><td>Cyanobacteria</td><td>Calothrix sp. NIES-2098</td><td>Miro domain protein [Calothrix sp. NIES-2098].</td><td>GCA_002368175.1</td></tr>
<tr><td>BAY18983.1</td><td>HISKIN→||<-?||LRR-repeats+AP-GTPase+COR*→</td><td>LRR-repeats+AP-GTPase+COR</td><td>946</td><td>NIES21_48420</td><td>Cyanobacteria</td><td>Anabaenopsis circularis NIES-21</td><td>Miro domain-containing protein [Anabaenopsis circularis NIES-21].</td><td>GCA_002367975.1</td></tr>
<tr><td>BAY19588.1</td><td>LRR-repeats+AP-GTPase+COR+TM+TM*→||<-tRNA<-?||?→?→||<-?<-?<-SNF||RnasePH+RnasePH+KH+S1→</td><td>LRR-repeats+AP-GTPase+COR+TM+TM</td><td>739</td><td>NIES21_54510</td><td>Cyanobacteria</td><td>Anabaenopsis circularis NIES-21</td><td>Miro domain protein [Anabaenopsis circularis NIES-21].</td><td>GCA_002367975.1</td></tr>
<tr><td>BAY24777.1</td><td>Pro_CA→||<-?<-?||Chromo-N+CHROMO→||<-?||LRR-repeats+AP-GTPase+COR+TIR+TCAD4+CASPASE*→</td><td>LRR-repeats+AP-GTPase+COR+TIR+TCAD4+CASPASE</td><td>1450</td><td>NIES2100_45740</td><td>Cyanobacteria</td><td>Calothrix sp. NIES-2100</td><td>Miro domain-containing protein [Calothrix sp. NIES-2100].</td><td>GCA_002368195.1</td></tr>
<tr><td>BAY74940.1</td><td>LRR-repeats+AP-GTPase+COR*→||<-ClpABN-AAA+ClpABC-AAA<-?<-?||METHYLASE→</td><td>LRR-repeats+AP-GTPase+COR</td><td>1016</td><td>NIES25_13570</td><td>Cyanobacteria</td><td>Nostoc linckia NIES-25</td><td>Miro domain-containing protein [Nostoc linckia NIES-25].</td><td>GCA_002368035.1</td></tr>
<tr><td>BAY88950.1</td><td>LRR-repeats→ LRR-repeats+AP-GTPase+COR+CASPASE*→</td><td>LRR-repeats+AP-GTPase+COR+CASPASE</td><td>963</td><td>NIES3275_09520</td><td>Cyanobacteria</td><td>Fremyella diplosiphon NIES-3275</td><td>Miro domain-containing protein [Fremyella diplosiphon NIES-3275].</td><td>GCA_002368275.1</td></tr>
<tr><td>BAY98896.1</td><td><-REC<-?||Chromo-N+CHROMO→?→||<-?<-?<-?||LRR-repeats+AP-GTPase+COR+CASPASE*→</td><td>LRR-repeats+AP-GTPase+COR+CASPASE</td><td>1138</td><td>NIES37_28740</td><td>Cyanobacteria</td><td>Tolypothrix tenuis PCC 7101</td><td>Miro domain-containing protein [Tolypothrix tenuis PCC 7101].</td><td>GCA_002368295.1</td></tr>
<tr><td>BAZ08233.1</td><td><-ParB<-ParA-Soj-PloopNTPase||?→ LRR-repeats→ LRR-repeats+AP-GTPase+COR+EAD1*→ ParB→||<-NACHT</td><td>LRR-repeats+AP-GTPase+COR+EAD1</td><td>856</td><td>NIES4071_00380</td><td>Cyanobacteria</td><td>Calothrix sp. NIES-4071</td><td>Miro domain-containing protein [Calothrix sp. NIES-4071].</td><td>GCA_002368455.1</td></tr>
<tr><td>BAZ30312.1</td><td><-SIG+GT4<-?||?→ LRR-repeats→ LRR-repeats+AP-GTPase+COR+TM+TM*→?→?→||<-?<-Arginase</td><td>LRR-repeats+AP-GTPase+COR+TM+TM</td><td>638</td><td>NIES4074_27680</td><td>Cyanobacteria</td><td>Cylindrospermum sp. NIES-4074</td><td>small GTP-binding protein [Cylindrospermum sp. NIES-4074].</td><td>GCA_003994795.1</td></tr>
<tr><td>BBD58750.1</td><td><-DSBH<-?<-?<-?<-ParA-Soj-PloopNTPase<-?||LRR-repeats+AP-GTPase+COR+DUF4404*→</td><td>LRR-repeats+AP-GTPase+COR+DUF4404</td><td>1041</td><td>NIES2109_15280</td><td>Cyanobacteria</td><td>Nostoc sp. HK-01</td><td>small GTP-binding protein domain-containing protein [Nostoc sp. HK-01].</td><td>GCA_003990705.1</td></tr>
<tr><td>BBD61760.1</td><td>RelE-ParE→?→||<-?<-?||LRR-repeats+AP-GTPase+COR+TM+TM*→||<-tRNA<-?||?→?→||<-?<-?<-SNF</td><td>LRR-repeats+AP-GTPase+COR+TM+TM</td><td>954</td><td>NIES2109_45950</td><td>Cyanobacteria</td><td>Nostoc sp. HK-01</td><td>Miro domain protein [Nostoc sp. HK-01].</td><td>GCA_003990705.1</td></tr>
<tr><td>BBD62362.1</td><td>HISKIN→ LRR-repeats+AP-GTPase+COR*→</td><td>LRR-repeats+AP-GTPase+COR</td><td>854</td><td>NIES2109_52010</td><td>Cyanobacteria</td><td>Nostoc sp. HK-01</td><td>Miro domain protein [Nostoc sp. HK-01].</td><td>GCA_003990705.1</td></tr>
<tr><td>BBH86778.1</td><td>LRR-repeats+AP-GTPase+COR*→</td><td>LRR-repeats+AP-GTPase+COR</td><td>1152</td><td>KTC_15290</td><td>Chloroflexi</td><td>Thermosporothrix sp. COM3</td><td>hypothetical protein KTC_15290 [Thermosporothrix sp. COM3].</td><td>-</td></tr>
<tr><td>CAA0113265.1</td><td>LRR-repeats+AP-GTPase+COR+DrHyd*→</td><td>LRR-repeats+AP-GTPase+COR+DrHyd</td><td>947</td><td>inlA</td><td>Gammaproteobacteria</td><td>Halioglobus japonicus</td><td>Internalin-A [Halioglobus japonicus].</td><td>GCA_902705825.1</td></tr>
<tr><td>CAA6825421.1</td><td>TM+TM+TM→ LRR-repeats+AP-GTPase+COR+TIR*→</td><td>LRR-repeats+AP-GTPase+COR+TIR</td><td>931</td><td>HELGO_WM44885</td><td>Gammaproteobacteria</td><td>uncultured Thiotrichaceae bacterium</td><td>GTP-binding protein [uncultured Thiotrichaceae bacterium].</td><td>-</td></tr>
<tr><td>CBG70605.1</td><td>PAIREDC-HTH→||<-?||LRR-repeats→ LRR-repeats+AP-GTPase+COR*→||<-?||?→||<-?<-SIGMA-HTH<-?<-SIGMA-HTH</td><td>LRR-repeats+AP-GTPase+COR</td><td>743</td><td>SCAB_35141</td><td>Actinobacteria</td><td>Streptomyces scabiei 87.22</td><td>putative GTPase [Streptomyces scabiei 87.22].</td><td>GCA_000091305.1</td></tr>
<tr><td>CBS87910.1</td><td>LRR-repeats+AP-GTPase+COR+TIR*→||<-?<-?||ABC_membrane+ABC_tran→?→ Cluster968_2clades→</td><td>LRR-repeats+AP-GTPase+COR+TIR</td><td>1026</td><td>AZOLI_2723</td><td>Alphaproteobacteria</td><td>Azospirillum lipoferum 4B</td><td>Conserved protein of unknown function; Leucine-rich repeat domain [Azospirillum lipoferum 4B].</td><td>GCA_000283655.1</td></tr>
<tr><td>CCG09157.1</td><td>LRR-repeats+AP-GTPase+COR+TIR*→||<-?<-?<-?<-?<-?<-?<-REC</td><td>LRR-repeats+AP-GTPase+COR+TIR</td><td>756</td><td>RSPPHO_02531</td><td>Alphaproteobacteria</td><td>Pararhodospirillum photometricum DSM 122</td><td>Leucine-rich repeat [Pararhodospirillum photometricum DSM 122].</td><td>GCA_000284415.2</td></tr>
</table>

<table>
<tr><th>acc</th><th>operon</th><th>architecture</th><th>len</th><th>gen.name</th><th>taxend</th><th>species</th><th>defline</th><th>gca</th></tr>
<tr><td>CCH96195.1</td><td>LRR-repeats+AP-GTPase+COR+TM+TM*→</td><td>LRR-repeats+AP-GTPase+COR+TM+TM</td><td>625</td><td>MICAB_1690001</td><td>Cyanobacteria</td><td>Microcystis aeruginosa PCC 9717</td><td>Leucine-rich-repeat protein (fragment) [Microcystis aeruginosa PCC 9717].</td><td>GCA_000312165.1</td></tr>
<tr><td>CCI18671.1</td><td>LRR-repeats+AP-GTPase+COR*→ AP-GTPase+COR+TM+TM→</td><td>LRR-repeats+AP-GTPase+COR</td><td>302</td><td>MICAF_4270008</td><td>Cyanobacteria</td><td>Microcystis aeruginosa PCC 9807</td><td>Leucine-rich-repeat protein (fragment) [Microcystis aeruginosa PCC 9807].</td><td>GCA_000312225.1</td></tr>
<tr><td>CCQ52177.1</td><td>LRR-repeats+AP-GTPase+COR*→</td><td>LRR-repeats+AP-GTPase+COR</td><td>620</td><td>CWATWH8502_1464</td><td>Cyanobacteria</td><td>Crocosphaera watsonii WH 8502</td><td>Leucine-rich repeat [Crocosphaera watsonii WH 8502].</td><td>GCA_001039555.1</td></tr>
<tr><td>CCQ56427.1</td><td>LRR-repeats+AP-GTPase+COR*→||<-?<-?<-?||RelE→ CHTH→</td><td>LRR-repeats+AP-GTPase+COR</td><td>761</td><td>CWATWH0005_5558</td><td>Cyanobacteria</td><td>Crocosphaera watsonii WH 0005</td><td>Leucine-rich repeat [Crocosphaera watsonii WH 0005].</td><td>GCA_001050835.1</td></tr>
<tr><td>CCQ61794.1</td><td>LRR-repeats+AP-GTPase+COR*→||<-?<-?||RelE→ CHTH→</td><td>LRR-repeats+AP-GTPase+COR</td><td>674</td><td>CWATWH0401_3405</td><td>Cyanobacteria</td><td>Crocosphaera watsonii WH 0401</td><td>Leucine-rich repeat [Crocosphaera watsonii WH 0401].</td><td>GCA_001039615.1</td></tr>
<tr><td>CCQ66314.1</td><td>LRR-repeats+AP-GTPase+COR*→</td><td>LRR-repeats+AP-GTPase+COR</td><td>378</td><td>CWATWH0402_4408</td><td>Cyanobacteria</td><td>Crocosphaera watsonii WH 0402</td><td>Leucine-rich repeat [Crocosphaera watsonii WH 0402].</td><td>GCA_001039635.1</td></tr>
<tr><td>CDA78514.1</td><td>LRR-repeats+AP-GTPase+COR*→</td><td>LRR-repeats+AP-GTPase+COR</td><td>1064</td><td>BN558_01944</td><td>Firmicutes</td><td>Clostridium sp. CAG:242</td><td>miro domain protein [Clostridium sp. CAG:242].</td><td>GCA_000435455.1</td></tr>
<tr><td>CDU15633.1</td><td>LRR-repeats+AP-GTPase+COR*→</td><td>LRR-repeats+AP-GTPase+COR</td><td>1092</td><td>VCR17J2_90139</td><td>Gammaproteobacteria</td><td>Vibrio sp. J2-17</td><td>hypothetical protein VCR17J2_90139 [Vibrio sp. J2-17].</td><td>GCA_001244075.1</td></tr>
<tr><td>CRH07284.1</td><td>LRR-repeats+AP-GTPase+COR*→||<-?||?→?→||<-?<-SIG+Lysozyme</td><td>LRR-repeats+AP-GTPase+COR</td><td>759</td><td>MAGMO_3143</td><td>Alphaproteobacteria</td><td>Candidatus Magnetococcus massalia</td><td>Conserved protein of unknown function. Containing small GTP-binding domain and Leucine-rich repeat (LRR) domain [Candidatus Magnetococcus massalia].</td><td>-</td></tr>
<tr><td>EAM49963.1</td><td>Cluster1136_2clades→?→||<-?||LRR-repeats→ LRR-repeats+AP-GTPase+COR*→</td><td>LRR-repeats+AP-GTPase+COR</td><td>715</td><td>CwatDRAFT_2830</td><td>Cyanobacteria</td><td>Crocosphaera watsonii WH 8501</td><td>Leucine-rich repeat [Crocosphaera watsonii WH 8501].</td><td>GCA_000167195.1</td></tr>
<tr><td>EAY30117.1</td><td>LRR-repeats→?→ LRR-repeats+AP-GTPase+COR*→</td><td>LRR-repeats+AP-GTPase+COR</td><td>1165</td><td>M23134_05450</td><td>Bacteroidetes</td><td>Microscilla marina ATCC 23134</td><td>Rab family protein [Microscilla marina ATCC 23134].</td><td>GCA_000169175.1</td></tr>
<tr><td>EDN67620.1</td><td>LRR-repeats+AP-GTPase+COR*→</td><td>LRR-repeats+AP-GTPase+COR</td><td>602</td><td>BGP_2058</td><td>Gammaproteobacteria</td><td>Beggiatoa sp. PS</td><td>leucine-rich-repeat protein [Beggiatoa sp. PS].</td><td>GCA_000170715.1</td></tr>
<tr><td>EDN68964.1</td><td>LRR-repeats+AP-GTPase+COR*→</td><td>LRR-repeats+AP-GTPase+COR</td><td>833</td><td>BGP_3136</td><td>Gammaproteobacteria</td><td>Beggiatoa sp. PS</td><td>leucine-rich-repeat protein [Beggiatoa sp. PS].</td><td>GCA_000170715.1</td></tr>
<tr><td>EDX78020.1</td><td>Cluster1415_2clades→?→||<-?||LRR-repeats→ LRR-repeats+AP-GTPase+COR+DUF4404*→</td><td>LRR-repeats+AP-GTPase+COR+DUF4404</td><td>1115</td><td>MC7420_7758</td><td>Cyanobacteria</td><td>Coleofasciculus chthonoplastes PCC 7420</td><td>Ras family, putative [Coleofasciculus chthonoplastes PCC 7420].</td><td>GCA_000155555.1</td></tr>
<tr><td>EHJ11819.1</td><td>LRR-repeats+AP-GTPase+COR*→||<-?<-?||RelE→ CHTH→</td><td>LRR-repeats+AP-GTPase+COR</td><td>674</td><td>CWATWH0003_3458</td><td>Cyanobacteria</td><td>Crocosphaera watsonii WH 0003</td><td>Leucine-rich repeat [Crocosphaera watsonii WH 0003].</td><td>GCA_000235665.2</td></tr>
<tr><td>EIG53576.1</td><td>LRR-repeats+AP-GTPase+COR*→?→?→?→ Cluster1496_2clades→</td><td>LRR-repeats+AP-GTPase+COR</td><td>1279</td><td>DesU5LDRAFT_1901</td><td>Deltaproteobacteria</td><td>Desulfovibrio sp. U5L</td><td>small GTP-binding protein domain [Desulfovibrio sp. U5L].</td><td>GCA_000245055.1</td></tr>
<tr><td>ESA34932.1</td><td>LRR-repeats+AP-GTPase+COR+TM+TM*→</td><td>LRR-repeats+AP-GTPase+COR+TM+TM</td><td>1002</td><td>N836_14320</td><td>Cyanobacteria</td><td>Leptolyngbya sp. Heron Island J</td><td>small gtp-binding protein [Leptolyngbya sp. Heron Island J].</td><td>GCA_000482245.1</td></tr>
<tr><td>ESA35951.1</td><td><-NACHT<-?||LRR-repeats+AP-GTPase+COR+TIR*→</td><td>LRR-repeats+AP-GTPase+COR+TIR</td><td>824</td><td>N836_09535</td><td>Cyanobacteria</td><td>Leptolyngbya sp. Heron Island J</td><td>small gtp-binding protein [Leptolyngbya sp. Heron Island J].</td><td>GCA_000482245.1</td></tr>
<tr><td>ESA37051.1</td><td>LRR-repeats+AP-GTPase+COR*→?→?→?→||<-Pentapeptide-repeats</td><td>LRR-repeats+AP-GTPase+COR</td><td>818</td><td>N836_04700</td><td>Cyanobacteria</td><td>Leptolyngbya sp. Heron Island J</td><td>small gtp-binding protein [Leptolyngbya sp. Heron Island J].</td><td>GCA_000482245.1</td></tr>
<tr><td>ESQ09086.1</td><td>LRR-repeats+AP-GTPase+COR+TIR*→</td><td>LRR-repeats+AP-GTPase+COR+TIR</td><td>840</td><td>N838_02740</td><td>Gammaproteobacteria</td><td>Thiohalocapsa sp. PB-PSB1</td><td>hypothetical protein N838_02740 [Thiohalocapsa sp. PB-PSB1].</td><td>GCA_000495615.1</td></tr>
<tr><td>ETR72570.1</td><td>Cluster1438_2clades→?→ LRR-repeats+AP-GTPase+COR+TIR*→ CCTBP→||<-REC</td><td>LRR-repeats+AP-GTPase+COR+TIR</td><td>808</td><td>OMM_01620</td><td>Deltaproteobacteria</td><td>Candidatus Magnetoglobus multicellularis str. Araruama</td><td>leucine-rich repeat-containing protein [Candidatus Magnetoglobus multicellularis str. Araruama].</td><td>GCA_000516475.1</td></tr>
<tr><td>ETW97925.1</td><td>LRR-repeats+AP-GTPase+COR+DUF4404*→?→||<-RNA-Helicase||?→ SIG+BLBD→</td><td>LRR-repeats+AP-GTPase+COR+DUF4404</td><td>767</td><td>ETSY1_20835</td><td>Nitrospinae/Tectomicrobia group</td><td>Candidatus Entotheonella factor</td><td>hypothetical protein ETSY1_20835, partial [Candidatus Entotheonella factor].</td><td>GCA_000522425.1</td></tr>
</table>

acc	operon	architecture	len	gen.name	taxend	species	defline	gca
ETX07308.1	LRR-repeats+AP-GTPase+COR*→	LRR-repeats+AP-GTPase+COR	513	ETSY2_11950	Nitrospinae/Tectomicrobia group	Candidatus Entotheonella gemina	hypothetical protein ETSY2_11950, partial [Candidatus Entotheonella gemina].	GCA_000522445.1
GAK50293.1	<-CR-REase5<-?<-Cluster1448_2clades<-Cluster1281_2clades||?→ LRR-repeats+AP-GTPase*→ COR→	LRR-repeats+AP-GTPase	420	U14_01521	Bacteria	Candidatus Moduliflexus flocculans	leucine-rich-repeat protein [Candidatus Moduliflexus flocculans].	GCA_000739515.1
GBE92899.1	HISKIN→||<-?||?→ LRR-repeats+AP-GTPase+COR*→	LRR-repeats+AP-GTPase+COR	946	NCWK1_2658	Cyanobacteria	Nostoc cycadae WK-1	leucine rich repeat (LRR)-containing protein [Nostoc cycadae WK-1].	GCA_002897135.1
GCL40268.1	LRR-repeats+AP-GTPase+COR*→	LRR-repeats+AP-GTPase+COR	261	SR1949_54040	Cyanobacteria	Sphaerospermopsis reniformis	small GTP-binding protein [Sphaerospermopsis reniformis].	GCA_005402885.1
GEP41915.1	SIG+TM→?→ LRR-repeats+AP-GTPase+COR*→	LRR-repeats+AP-GTPase+COR	809	BGE01nite_12060	Verrucomicrobia	Brevifollis gellanilyticus	hypothetical protein BGE01nite_12060 [Brevifollis gellanilyticus].	GCA_007992435.1
GFZ95281.1	LRR-repeats+AP-GTPase+COR*→	LRR-repeats+AP-GTPase+COR	911	CYANOKiyG1_06360	Cyanobacteria	Okeania sp. KiyG1	hypothetical protein CYANOKiyG1_06360 [Okeania sp. KiyG1].	GCA_014132355.1
GFZ95952.1	<-HhH-RADC+JAB<-?||LRR-repeats+AP-GTPase+COR*→	LRR-repeats+AP-GTPase+COR	747	CYANOKiyG1_07050	Cyanobacteria	Okeania sp. KiyG1	hypothetical protein CYANOKiyG1_07050 [Okeania sp. KiyG1].	GCA_014132355.1
GGA29094.1	<-RADICAL-SAM||?→ REC→?→ LRR-repeats+AP-GTPase+COR*→||<-?<-NACHT+68TM-wHTH<-?||?→||<-TPR-repeats<-HISKIN	LRR-repeats+AP-GTPase+COR	1395	CYANOKiyG1_45460	Cyanobacteria	Okeania sp. KiyG1	hypothetical protein CYANOKiyG1_45460 [Okeania sp. KiyG1].	GCA_014132355.1
GGA48436.1	LRR-repeats+AP-GTPase+COR*→?→ COR+TM+TM→||<-?<-?<-NUDIX||TPR-repeats→	LRR-repeats+AP-GTPase+COR	405	CYANOKiyG1_67520	Cyanobacteria	Okeania sp. KiyG1	hypothetical protein CYANOKiyG1_67520 [Okeania sp. KiyG1].	GCA_014132355.1
GGD73013.1	LRR-repeats+AP-GTPase+COR+TM+TM*→?→?→||<-?<-Cluster1601_2clades	LRR-repeats+AP-GTPase+COR+TM+TM	937	GCM10011514_41350	Bacteroidetes	Emticicia aquatilis	hypothetical protein GCM10011514_41350 [Emticicia aquatilis].	GCA_014644295.1
GGU83059.1	LRR-repeats+AP-GTPase+COR*→||<-?||?→||<-?<-SIGMA-HTH<-?<-SIGMA-HTH	LRR-repeats+AP-GTPase+COR	996	GCM10010260_14720	Actinobacteria	Streptomyces filipinensis	hypothetical protein GCM10010260_14720 [Streptomyces filipinensis].	GCA_014649495.1
HAA21349.1	LRR-repeats+AP-GTPase+COR*→	LRR-repeats+AP-GTPase+COR	805	DCP28_22240	Bacteroidetes	Cytophagales bacterium	TPA: hypothetical protein DCP28_22240, partial [Cytophagales bacterium].	GCA_003444325.1
HAC62039.1	ABC_membrane+ABC_tran→?→ LRR-repeats→ LRR-repeats+AP-GTPase+COR*→||<-Cluster2488_2clades<-Cluster2227_2clades||ISOFLAVOMETHYLASE-HTH→	LRR-repeats+AP-GTPase+COR	852	DCF68_00525	Cyanobacteria	Cyanothece sp. UBA12306	TPA: GTPase, partial [Cyanothece sp. UBA12306].	GCA_003448685.1
HAC64827.1	<-METHYLASE<-?||LRR-repeats+AP-GTPase+COR+TM+TM*→?→||<-?<-?<-SWACOS	LRR-repeats+AP-GTPase+COR+TM+TM	931	DCF68_15170	Cyanobacteria	Cyanothece sp. UBA12306	TPA: GTPase [Cyanothece sp. UBA12306].	GCA_003448685.1
HAG85135.1	Ferredoxin-RRM→ LRR-repeats+AP-GTPase+COR*→	LRR-repeats+AP-GTPase+COR	819	DCL61_29295	Cyanobacteria	Cyanobacteria bacterium UBA12227	TPA: GTPase [Cyanobacteria bacterium UBA12227].	GCA_003450835.1
HAP64559.1	LRR-repeats+AP-GTPase+COR*→	LRR-repeats+AP-GTPase+COR	754	DCR93_35450	Bacteroidetes	Cytophagales bacterium	TPA: hypothetical protein DCR93_35450, partial [Cytophagales bacterium].	GCA_003454975.1
HAR63371.1	LRR-repeats+AP-GTPase+COR*→	LRR-repeats+AP-GTPase+COR	611	DCS13_07910	Terrabacteria group	Candidatus Margulisbacteria bacterium	TPA: hypothetical protein DCS13_07910, partial [Candidatus Margulisbacteria bacterium].	GCA_003475905.1
HAS39197.1	LRR-repeats+AP-GTPase+COR*→	LRR-repeats+AP-GTPase+COR	1145	DCS93_01900	Bacteroidetes	Microscillaceae bacterium	TPA: hypothetical protein DCS93_01900 [Microscillaceae bacterium].	GCA_003483885.1
HAS43483.1	LRR-repeats→?→?→||<-?||LRR-repeats+AP-GTPase+COR+EAD11*→||<-PAS+HISKIN	LRR-repeats+AP-GTPase+COR+EAD11	868	DCS93_23590	Bacteroidetes	Microscillaceae bacterium	TPA: hypothetical protein DCS93_23590 [Microscillaceae bacterium].	GCA_003483885.1

<table>
<tr><th>acc</th><th>operon</th><th>architecture</th><th>len</th><th>gen.name</th><th>taxend</th><th>species</th><th>defline</th><th>gca</th></tr>
<tr><td>HAS44199.1</td><td>LRR-repeats+AP-GTPase+COR→//<-?<-?<-?//LRR-repeats+AP-GTPase+COR→</td><td>LRR-repeats+AP-GTPase+COR</td><td>958</td><td>DCS93_27220</td><td>Bacteroidetes</td><td>Microscillaceae bacterium</td><td>TPA: hypothetical protein DCS93_27220 [Microscillaceae bacterium].</td><td>GCA_003483885.1</td></tr>
<tr><td>HAS44203.1</td><td>LRR-repeats+AP-GTPase+COR*→</td><td>LRR-repeats+AP-GTPase+COR</td><td>1037</td><td>DCS93_27240</td><td>Bacteroidetes</td><td>Microscillaceae bacterium</td><td>TPA: hypothetical protein DCS93_27240 [Microscillaceae bacterium].</td><td>GCA_003483885.1</td></tr>
<tr><td>HAS46073.1</td><td><-LRR-repeats<-ABC-ATPase||?→||<-?||?→?→ LRR-repeats+AP-GTPase+COR*→</td><td>LRR-repeats+AP-GTPase+COR</td><td>864</td><td>DCS93_36670</td><td>Bacteroidetes</td><td>Microscillaceae bacterium</td><td>TPA: hypothetical protein DCS93_36670 [Microscillaceae bacterium].</td><td>GCA_003483885.1</td></tr>
<tr><td>HAV28009.1</td><td>Pro_CA→ LRR-repeats+AP-GTPase+COR*→</td><td>LRR-repeats+AP-GTPase+COR</td><td>1019</td><td>DCX23_06795</td><td>Firmicutes</td><td>Lachnospiraceae bacterium</td><td>TPA: hypothetical protein DCX23_06795 [Lachnospiraceae bacterium].</td><td>GCA_003484485.1</td></tr>
<tr><td>HAW32544.1</td><td>LRR-repeats+AP-GTPase+COR*→</td><td>LRR-repeats+AP-GTPase+COR</td><td>1236</td><td>DCY03_31275</td><td>Planctomycetes</td><td>Planctomycetaceae bacterium</td><td>TPA: hypothetical protein DCY03_31275 [Planctomycetaceae bacterium].</td><td>GCA_003487605.1</td></tr>
<tr><td>HAX71152.1</td><td><-Cluster1437_2clades||?→ LRR-repeats→ LRR-repeats+AP-GTPase+COR*→</td><td>LRR-repeats+AP-GTPase+COR</td><td>869</td><td>DCY14_16175</td><td>Chloroflexi</td><td>Anaerolineae bacterium</td><td>TPA: hypothetical protein DCY14_16175, partial [Anaerolineae bacterium].</td><td>GCA_003486425.1</td></tr>
<tr><td>HBE20120.1</td><td>LRR-repeats+AP-GTPase+COR*→</td><td>LRR-repeats+AP-GTPase+COR</td><td>816</td><td>DDW51_21520</td><td>Cyanobacteria</td><td>Cyanobacteria bacterium UBA11367</td><td>TPA: hypothetical protein DDW51_21520 [Cyanobacteria bacterium UBA11367].</td><td>GCA_003501765.1</td></tr>
<tr><td>HBE59255.1</td><td>LRR-repeats→ LRR-repeats+AP-GTPase+COR*→</td><td>LRR-repeats+AP-GTPase+COR</td><td>893</td><td>DDW56_17600</td><td>Cyanobacteria</td><td>Cyanobacteria bacterium UBA11366</td><td>TPA: hypothetical protein DDW56_17600, partial [Cyanobacteria bacterium UBA11366].</td><td>GCA_003501625.1</td></tr>
<tr><td>HBG28935.1</td><td>ABC-ATPase+ABC-ATPase→?→?→ S1COLD→ LRR-repeats+AP-GTPase+COR+TIR*→</td><td>LRR-repeats+AP-GTPase+COR+TIR</td><td>1033</td><td>DDX75_17760</td><td>Planctomycetes</td><td>Phycisphaerales bacterium</td><td>TPA: serine/threonine protein kinase [Phycisphaerales bacterium].</td><td>GCA_003499685.1</td></tr>
<tr><td>HBH73318.1</td><td>LRR-repeats→ LRR-repeats+AP-GTPase+COR+DrHyd*→||<-?<-?<-?||?→?→||<-PRTase</td><td>LRR-repeats+AP-GTPase+COR+DrHyd</td><td>872</td><td>DDY43_07685</td><td>Cyanobacteria</td><td>Synechococcales bacterium UBA10510</td><td>TPA: hypothetical protein DDY43_07685, partial [Synechococcales bacterium UBA10510].</td><td>GCA_003508015.1</td></tr>
<tr><td>HBH73336.1</td><td>LRR-repeats+AP-GTPase+COR+TIR*→</td><td>LRR-repeats+AP-GTPase+COR+TIR</td><td>698</td><td>DDY43_07790</td><td>Cyanobacteria</td><td>Synechococcales bacterium UBA10510</td><td>TPA: hypothetical protein DDY43_07790 [Synechococcales bacterium UBA10510].</td><td>GCA_003508015.1</td></tr>
<tr><td>HBK96125.1</td><td>LRR-repeats+AP-GTPase+COR+TM+TM*→</td><td>LRR-repeats+AP-GTPase+COR+TM+TM</td><td>656</td><td>DD001_01755</td><td>Cyanobacteria</td><td>Microcoleaceae bacterium UBA10368</td><td>TPA: GTPase, partial [Microcoleaceae bacterium UBA10368].</td><td>GCA_003506815.1</td></tr>
<tr><td>HBK98262.1</td><td>GTPase-AIG→?→?→||<-?<-?||LRR-repeats+AP-GTPase+COR+DUF4404*→</td><td>LRR-repeats+AP-GTPase+COR+DUF4404</td><td>1165</td><td>DD001_13585</td><td>Cyanobacteria</td><td>Microcoleaceae bacterium UBA10368</td><td>TPA: GTPase [Microcoleaceae bacterium UBA10368].</td><td>GCA_003506815.1</td></tr>
<tr><td>HBP89205.1</td><td>CHTH→||<-?<-?<-?<-?<-?<-?||LRR-repeats+AP-GTPase+COR+DrHyd*→||<-?<-?<-TM</td><td>LRR-repeats+AP-GTPase+COR+DrHyd</td><td>948</td><td>DD706_16075</td><td>Nitrospirae</td><td>Nitrospiraceae bacterium</td><td>TPA: hypothetical protein DD706_16075 [Nitrospiraceae bacterium].</td><td>GCA_003523945.1</td></tr>
<tr><td>HBU22381.1</td><td>LRR-repeats+AP-GTPase+COR*→?→?→?→?→?→||<-?||ParA-Soj-PloopNTPase→</td><td>LRR-repeats+AP-GTPase+COR</td><td>865</td><td>DEB17_00005</td><td>Chlorobi</td><td>Chlorobaculum sp.</td><td>TPA: hypothetical protein DEB17_00005, partial [Chlorobaculum sp.].</td><td>GCA_003510485.1</td></tr>
<tr><td>HBW31420.1</td><td>LRR-repeats+AP-GTPase+COR+DUF4404*→||<-?<-ABC-ATPase<-?||?→||<-REC</td><td>LRR-repeats+AP-GTPase+COR+DUF4404</td><td>766</td><td>DEF48_15370</td><td>Cyanobacteria</td><td>Nostoc sp. UBA8866</td><td>TPA: Ras family protein, partial [Nostoc sp. UBA8866].</td><td>GCA_003514525.1</td></tr>
<tr><td>HBY87332.1</td><td>LRR-repeats+AP-GTPase+COR*→</td><td>LRR-repeats+AP-GTPase+COR</td><td>930</td><td>DEO86_15815</td><td>Gammaproteobacteria</td><td>Colwellia sp.</td><td>TPA: hypothetical protein DEO86_15815 [Colwellia sp.].</td><td>GCA_003520945.1</td></tr>
</table>

acc	operon	architecture	len	gen.name	taxend	species	defline	gca
HCA94361.1	LRR-repeats→ LRR-repeats+AP-GTPase+COR*→	LRR-repeats+AP-GTPase+COR	892	DEP38_06745	Cyanobacteria	Cyanobacteria bacterium UBA9226	TPA: hypothetical protein DEP38_06745, partial [Cyanobacteria bacterium UBA9226].	GCA_003520045.1
HCF17408.1	LRR-repeats+AP-GTPase+COR+TIR*→ TM+TM+TM→	LRR-repeats+AP-GTPase+COR+TIR	737	DEV96_05085	Alphaproteobacteria	Rhodospirillum rubrum	TPA: hypothetical protein DEV96_05085, partial [Rhodospirillum rubrum].	GCA_003530895.1
HCN30450.1	LRR-repeats+AP-GTPase+COR*→	LRR-repeats+AP-GTPase+COR	1024	DIT64_17260	Verrucomicrobia	Verrucomicrobiales bacterium	TPA: hypothetical protein DIT64_17260 [Verrucomicrobiales bacterium].	GCA_003545395.1
HCO95252.1	LRR-repeats+AP-GTPase+COR*→	LRR-repeats+AP-GTPase+COR	809	DIU00_15100	Planctomycetes	Phycisphaerales bacterium	TPA: hypothetical protein DIU00_15100 [Phycisphaerales bacterium].	GCA_003533825.1
HCQ19950.1	LRR-repeats+AP-GTPase+COR*→?→||<-?<-?||TM→	LRR-repeats+AP-GTPase+COR	754	DIU28_00335	Cyanobacteria	Anabaena sp. UBA12330	TPA: hypothetical protein DIU28_00335, partial [Anabaena sp. UBA12330].	GCA_003525565.1
HCQ20817.1	LRR-repeats→ LRR-repeats+AP-GTPase+COR+TM+TM*→||<-?||?→?→?→?→ ABC-ATPase→	LRR-repeats+AP-GTPase+COR+TM+TM	721	DIU28_04955	Cyanobacteria	Anabaena sp. UBA12330	TPA: hypothetical protein DIU28_04955, partial [Anabaena sp. UBA12330].	GCA_003525565.1
HCR54081.1	LRR-repeats→ LRR-repeats+AP-GTPase+COR*→	LRR-repeats+AP-GTPase+COR	797	DIW27_06670	Bacteroidetes	Cytophagales bacterium	TPA: hypothetical protein DIW27_06670 [Cytophagales bacterium].	GCA_003524675.1
HCS91704.1	LRR-repeats+AP-GTPase+COR+TIR*→	LRR-repeats+AP-GTPase+COR+TIR	749	DIW77_17080	Gammaproteobacteria	Chromatiaceae bacterium	TPA: hypothetical protein DIW77_17080 [Chromatiaceae bacterium].	GCA_003525925.1
HCT83732.1	LRR-repeats+AP-GTPase+COR*→	LRR-repeats+AP-GTPase+COR	585	DF296_00860	Terrabacteria group	Candidatus Margulisbacteria bacterium	TPA: hypothetical protein DF296_00860 [Candidatus Margulisbacteria bacterium].	GCA_003542625.1
HCU95641.1	LRR-repeats+AP-GTPase+COR*→	LRR-repeats+AP-GTPase+COR	896	DHU96_24175	Actinobacteria	Actinobacteria bacterium	TPA: hypothetical protein DHU96_24175 [Actinobacteria bacterium].	GCA_003541285.1
HDN27288.1	LRR-repeats+AP-GTPase+COR*→	LRR-repeats+AP-GTPase+COR	598	ENG03_09385	Gammaproteobacteria	Thioploca sp.	TPA: GTP-binding protein, partial [Thioploca sp.].	GCA_011043325.1
HEC83827.1	LRR-repeats+AP-GTPase+COR*→	LRR-repeats+AP-GTPase+COR	907	ENI48_01075	Gammaproteobacteria	Thioploca sp.	TPA: GTPase [Thioploca sp.].	GCA_011052595.1
HEG42537.1	<-S1COLD||LRR-repeats+AP-GTPase+COR+TIR*→?→||<-SIG+Alba<-?||?→?→?→ REC→	LRR-repeats+AP-GTPase+COR+TIR	1095	ENH94_00660	Planctomycetes	Phycisphaerales bacterium	TPA: TIR domain-containing protein [Phycisphaerales bacterium].	GCA_011053245.1
HER03270.1	LRR-repeats+AP-GTPase+COR+TIR*→||<-?||?→?→ Cluster1306_2clades→?→ SIG+TM→ RuvB-AAA+RuvB-HTH→	LRR-repeats+AP-GTPase+COR+TIR	910	ENR03_13235	Alphaproteobacteria	Rhizobiales bacterium	TPA: TIR domain-containing protein, partial [Rhizobiales bacterium].	GCA_011046675.1
HEU35175.1	Cluster1718_2clades→ LRR-repeats+AP-GTPase+COR*→	LRR-repeats+AP-GTPase+COR	718	ENQ60_27120	unclassified Bacteria	bacterium	TPA: GTP-binding protein, partial [bacterium].	GCA_011047125.1
HFB99816.1	LRR-repeats+AP-GTPase+COR+TIR*→	LRR-repeats+AP-GTPase+COR+TIR	1132	ENJ53_03320	Bacteroidetes	Phaeodactylibacter sp.	TPA: TIR domain-containing protein, partial [Phaeodactylibacter sp.].	GCA_011322485.1
HFS07270.1	Cluster1370_2clades→ LRR-repeats+AP-GTPase+COR+TIR*→||<-?||REase→	LRR-repeats+AP-GTPase+COR+TIR	1009	ENR14_02795	Cyanobacteria	Anabaena sp.	TPA: TIR domain-containing protein [Anabaena sp.].	GCA_011332035.1
HFS07901.1	LRR-repeats+AP-GTPase+COR+TIR*→||<-?<-?<-ParA-Soj-PloopNTPase	LRR-repeats+AP-GTPase+COR+TIR	1021	ENR14_06085	Cyanobacteria	Anabaena sp.	TPA: GTP-binding protein [Anabaena sp.].	GCA_011332035.1
HGZ29232.1	LRR-repeats+AP-GTPase+COR*→	LRR-repeats+AP-GTPase+COR	903	ENR17_03505	Bacteroidetes	Bacteroidetes bacterium	TPA: hypothetical protein ENR17_03505 [Bacteroidetes bacterium].	GCA_011331955.1

<table>
<tr><th>acc</th><th>operon</th><th>architecture</th><th>len</th><th>gen.name</th><th>taxend</th><th>species</th><th>defline</th><th>gca</th></tr>
<tr><td>HGZ31593.1</td><td>LRR-repeats+AP-GTPase+COR+TIR*→</td><td>LRR-repeats+AP-GTPase+COR+TIR</td><td>868</td><td>ENR17_15550</td><td>Bacteroidetes</td><td>Bacteroidetes bacterium</td><td>TPA: TIR domain-containing protein, partial [Bacteroidetes bacterium].</td><td>GCA_011331955.1</td></tr>
<tr><td>HHB79310.1</td><td>FGS→?→ RVT→ LRR-repeats+AP-GTPase+COR*→</td><td>LRR-repeats+AP-GTPase+COR</td><td>458</td><td>ENK85_08770</td><td>Bacteroidetes</td><td>Saprospiraceae bacterium</td><td>TPA: hypothetical protein ENK85_08770, partial [Saprospiraceae bacterium].</td><td>GCA_011371135.1</td></tr>
<tr><td>HHH53255.1</td><td>LRR-repeats+AP-GTPase+COR*→</td><td>LRR-repeats+AP-GTPase+COR</td><td>413</td><td>ENK91_06320</td><td>Bacteroidetes</td><td>Bacteroidetes bacterium</td><td>TPA: GTP-binding protein, partial [Bacteroidetes bacterium].</td><td>GCA_011371025.1</td></tr>
<tr><td>HHV24864.1</td><td>LRR-repeats+AP-GTPase+COR*→</td><td>LRR-repeats+AP-GTPase+COR</td><td>716</td><td>GXX65_10185</td><td>Euryarchaeota</td><td>Methanosarcina sp.</td><td>TPA: GTP-binding protein [Methanosarcina sp.].</td><td>GCA_012839505.1</td></tr>
<tr><td>HIE02480.1</td><td>Cluster1917_2clades→?→?→?→?→||<-?||LRR-repeats+AP-GTPase+COR*→</td><td>LRR-repeats+AP-GTPase+COR</td><td>853</td><td>EYP59_19720</td><td>Gammaproteobacteria</td><td>Thiotrichaceae bacterium</td><td>TPA: GTPase [Thiotrichaceae bacterium].</td><td>GCA_012961805.1</td></tr>
<tr><td>HIE31128.1</td><td>LRR-repeats+AP-GTPase+COR*→</td><td>LRR-repeats+AP-GTPase+COR</td><td>636</td><td>EYP67_01930</td><td>Euryarchaeota</td><td>Methanosarcinales archaeon</td><td>TPA: GTP-binding protein, partial [Methanosarcinales archaeon].</td><td>GCA_012961645.1</td></tr>
<tr><td>HII01375.1</td><td>LRR-repeats+AP-GTPase+COR*→</td><td>LRR-repeats+AP-GTPase+COR</td><td>622</td><td>HA351_06900</td><td>Euryarchaeota</td><td>Methanosarcinaceae archaeon</td><td>TPA: GTP-binding protein [Methanosarcinaceae archaeon].</td><td>GCA_013329415.1</td></tr>
<tr><td>HII91446.1</td><td><-HhH-RADC+JAB||?→?→?→ NTP_transf_3→ LRR-repeats→ LRR-repeats+AP-GTPase+COR*→</td><td>LRR-repeats+AP-GTPase+COR</td><td>672</td><td>HA262_04570</td><td>Euryarchaeota</td><td>Methanosarcina sp.</td><td>TPA: GTP-binding protein, partial [Methanosarcina sp.].</td><td>GCA_013331275.1</td></tr>
<tr><td>HII91684.1</td><td>NTP_transf_3→ LRR-repeats+AP-GTPase+COR*→</td><td>LRR-repeats+AP-GTPase+COR</td><td>735</td><td>HA262_05815</td><td>Euryarchaeota</td><td>Methanosarcina sp.</td><td>TPA: GTP-binding protein [Methanosarcina sp.].</td><td>GCA_013331275.1</td></tr>
<tr><td>KAA3610009.1</td><td>MORC→?→ LRR-repeats→ LRR-repeats+AP-GTPase+COR*→?→||<-?<-?<-BetaPropeller+BetaPropeller</td><td>LRR-repeats+AP-GTPase+COR</td><td>653</td><td>DWQ03_18700</td><td>Calditrichaeota</td><td>Calditrichaeota bacterium</td><td>hypothetical protein DWQ03_18700 [Calditrichaeota bacterium].</td><td>GCA_008501795.1</td></tr>
<tr><td>KAA3656070.1</td><td>LRR-repeats+AP-GTPase+COR+EAD7*→</td><td>LRR-repeats+AP-GTPase+COR+EAD7</td><td>779</td><td>DWQ04_32125</td><td>Chloroflexi</td><td>Chloroflexi bacterium</td><td>GTPase, partial [Chloroflexi bacterium].</td><td>GCA_008501785.1</td></tr>
<tr><td>KAA3659887.1</td><td>LRR-repeats+AP-GTPase+COR*→</td><td>LRR-repeats+AP-GTPase+COR</td><td>842</td><td>DWQ04_20995</td><td>Chloroflexi</td><td>Chloroflexi bacterium</td><td>GTPase [Chloroflexi bacterium].</td><td>GCA_008501785.1</td></tr>
<tr><td>KAB8152691.1</td><td><-HISKIN<-?||?→?→ REC→ LRR-repeats+AP-GTPase+COR+TIR*→||<-RADICAL-SAM</td><td>LRR-repeats+AP-GTPase+COR+TIR</td><td>1007</td><td>EZY14_012780</td><td>Bacteroidetes</td><td>Kordia sp. TARA_039_SRF</td><td>TIR domain-containing protein [Kordia sp. TARA_039_SRF].</td><td>GCA_006383075.2</td></tr>
<tr><td>KAB8153612.1</td><td>LRR-repeats+AP-GTPase+COR+TIR*→||<-?<-?||?→||<-?||TM+RHOMBOID→</td><td>LRR-repeats+AP-GTPase+COR+TIR</td><td>966</td><td>EZY14_010295</td><td>Bacteroidetes</td><td>Kordia sp. TARA_039_SRF</td><td>TIR domain-containing protein [Kordia sp. TARA_039_SRF].</td><td>GCA_006383075.2</td></tr>
<tr><td>KAF0107167.1</td><td><-HISKIN<-REC||?→?→?→?→ LRR-repeats+AP-GTPase+COR*→||<-SIG+GT4</td><td>LRR-repeats+AP-GTPase+COR</td><td>1065</td><td>FD146_2008</td><td>Chloroflexi</td><td>Anaerolineaceae bacterium</td><td>hypothetical protein FD146_2008 [Anaerolineaceae bacterium].</td><td>GCA_009772985.1</td></tr>
<tr><td>KAF5415978.1</td><td>LRR-repeats+AP-GTPase+COR*→</td><td>LRR-repeats+AP-GTPase+COR</td><td>561</td><td>C5S48_04205</td><td>Euryarchaeota</td><td>ANME-2 cluster archaeon</td><td>hypothetical protein C5S48_04205 [ANME-2 cluster archaeon].</td><td>GCA_013374355.1</td></tr>
<tr><td>KAF5419007.1</td><td>LRR-repeats+AP-GTPase+COR*→</td><td>LRR-repeats+AP-GTPase+COR</td><td>865</td><td>C5S49_00980</td><td>Euryarchaeota</td><td>ANME-2 cluster archaeon</td><td>hypothetical protein C5S49_00980 [ANME-2 cluster archaeon].</td><td>GCA_013374365.1</td></tr>
<tr><td>KAF5420469.1</td><td>Cluster1076_2clades→ Cluster1076_2clades→ LRR-repeats+AP-GTPase+COR*→</td><td>LRR-repeats+AP-GTPase+COR</td><td>878</td><td>C5S44_08435</td><td>Euryarchaeota</td><td>ANME-2 cluster archaeon</td><td>hypothetical protein C5S44_08435 [ANME-2 cluster archaeon].</td><td>GCA_013374455.1</td></tr>
<tr><td>KAF5421986.1</td><td>LRR-repeats+AP-GTPase+COR*→</td><td>LRR-repeats+AP-GTPase+COR</td><td>596</td><td>C5S45_02955</td><td>Euryarchaeota</td><td>ANME-2 cluster archaeon</td><td>hypothetical protein C5S45_02955, partial [ANME-2 cluster archaeon].</td><td>GCA_013374465.1</td></tr>
<tr><td>KAF5428813.1</td><td><-CBS<-CBS||?→?→ LRR-repeats+AP-GTPase+COR*→</td><td>LRR-repeats+AP-GTPase+COR</td><td>788</td><td>C5S39_10445</td><td>Euryarchaeota</td><td>Methanophagales archaeon</td><td>Leucine-rich repeat (LRR) protein [Methanophagales archaeon].</td><td>GCA_013374505.1</td></tr>
<tr><td>KAF5432496.1</td><td>LRR-repeats+AP-GTPase+COR*→</td><td>LRR-repeats+AP-GTPase+COR</td><td>599</td><td>C5S39_03480</td><td>Euryarchaeota</td><td>Methanophagales archaeon</td><td>hypothetical protein C5S39_03480 [Methanophagales archaeon].</td><td>GCA_013374505.1</td></tr>
<tr><td>KAF5433375.1</td><td>LRR-repeats+AP-GTPase+COR*→</td><td>LRR-repeats+AP-GTPase+COR</td><td>838</td><td>C5S35_16480</td><td>Euryarchaeota</td><td>Methanophagales archaeon</td><td>hypothetical protein C5S35_16480 [Methanophagales archaeon].</td><td>GCA_013374565.1</td></tr>
<tr><td>KAF5435535.1</td><td>SIG+TM+TM+TM+TM+TM+TM→||<-?<-?||?→?→ Aminotran_1_2→ LRR-repeats+AP-GTPase+COR*→</td><td>LRR-repeats+AP-GTPase+COR</td><td>659</td><td>C5S35_10775</td><td>Euryarchaeota</td><td>Methanophagales archaeon</td><td>hypothetical protein C5S35_10775, partial [Methanophagales archaeon].</td><td>GCA_013374565.1</td></tr>
<tr><td>KAF5438029.1</td><td>LRR-repeats+AP-GTPase+COR*→</td><td>LRR-repeats+AP-GTPase+COR</td><td>787</td><td>C5S35_01055</td><td>Euryarchaeota</td><td>Methanophagales archaeon</td><td>internalin A, partial [Methanophagales archaeon].</td><td>GCA_013374565.1</td></tr>
<tr><td>KFF11476.1</td><td>LRR-repeats+AP-GTPase+COR*→</td><td>LRR-repeats+AP-GTPase+COR</td><td>902</td><td>IW20_19515</td><td>Bacteroidetes</td><td>Flavobacterium hydatis</td><td>hypothetical protein IW20_19515 [Flavobacterium hydatis].</td><td>GCA_000737695.1</td></tr>
</table>

acc	operon	architecture	len	gen.name	taxend	species	defline	gca
KJV08837.1	LRR-repeats+AP-GTPase+COR*→	LRR-repeats+AP-GTPase+COR	553	VZ95_15215	Alphaproteobacteria	Elstera litoralis	hypothetical protein VZ95_15215, partial [Elstera litoralis].	GCA_000963705.1
KOR30146.1	LRR-repeats+AP-GTPase+COR*→ SIG+RHOD-CDC25→	LRR-repeats+AP-GTPase+COR	653	TI04_06910	Gammaproteobacteria	Achromatium sp. WMS2	hypothetical protein TI04_06910 [Achromatium sp. WMS2].	GCA_001276605.1
KPA09194.1	HNH→?→?→?→?→ LRR-repeats+AP-GTPase+COR*→ McrA-NTD→?→ RlaP→ Cluster2261_2clades→	LRR-repeats+AP-GTPase+COR	923	MHK_010613	Deltaproteobacteria	Candidatus Magnetomorum sp. HK-1	Leucine Rich Repeat (LRR)-containing protein [Candidatus Magnetomorum sp. HK-1].	GCA_001292585.1
KPA10003.1	Cluster2271_2clades→ TPR+TPR→?→?→ LRR-repeats+AP-GTPase+COR+DUF4404*→	LRR-repeats+AP-GTPase+COR+DUF4404	883	MHK_009799	Deltaproteobacteria	Candidatus Magnetomorum sp. HK-1	small GTP-binding protein domain protein [Candidatus Magnetomorum sp. HK-1].	GCA_001292585.1
KPA12970.1	LRR-repeats+AP-GTPase+COR*→	LRR-repeats+AP-GTPase+COR	676	MHK_006822	Deltaproteobacteria	Candidatus Magnetomorum sp. HK-1	leucine-rich repeat-containing protein [Candidatus Magnetomorum sp. HK-1].	GCA_001292585.1
KPA13876.1	TIR+LRR-repeats+AP-GTPase+COR+Calcineurin*→	TIR+LRR-repeats+AP-GTPase+COR+Calcineurin	1426	MHK_005915	Deltaproteobacteria	Candidatus Magnetomorum sp. HK-1	small gtp-binding protein [Candidatus Magnetomorum sp. HK-1].	GCA_001292585.1
KPA14771.1	LRR-repeats+AP-GTPase+COR+DrHyd*→	LRR-repeats+AP-GTPase+COR+DrHyd	898	MHK_005023	Deltaproteobacteria	Candidatus Magnetomorum sp. HK-1	leucine-rich repeat-containing protein [Candidatus Magnetomorum sp. HK-1].	GCA_001292585.1
KPA15132.1	LRR-repeats+AP-GTPase+COR+TIR*→	LRR-repeats+AP-GTPase+COR+TIR	1146	MHK_004657	Deltaproteobacteria	Candidatus Magnetomorum sp. HK-1	small GTP-binding domain protein [Candidatus Magnetomorum sp. HK-1].	GCA_001292585.1
KPQ33355.1	ARSR-HTH→ Cluster2335_2clades→||<-?||LRR-repeats→ LRR-repeats+AP-GTPase+COR+TIR*→||<-METHYLASE||?→||<-SIG+TM+TM+TM<-ABC_membrane+ABC_tran	LRR-repeats+AP-GTPase+COR+TIR	887	HLUCCA11_18720	Cyanobacteria	Phormidesmis priestleyi Ana	Leucine Rich repeats (2 copies)/TIR domain/Miro-like protein/Leucine rich repeat [Phormidesmis priestleyi Ana].	GCA_001314865.1
KPQ36660.1	<-Cluster54_3clades<-?||LRR-repeats+AP-GTPase+COR*→	LRR-repeats+AP-GTPase+COR	1200	inlA	Cyanobacteria	Phormidesmis priestleyi Ana	internalin A [Phormidesmis priestleyi Ana].	GCA_001314865.1
KTE25244.1	TetR-HTH→?→||<-?<-?<-?<-?||LRR-repeats+AP-GTPase+COR+TIR*→	LRR-repeats+AP-GTPase+COR+TIR	719	ATE62_22325	Alphaproteobacteria	Sphingopyxis sp. HIX	hypothetical protein ATE62_22325 [Sphingopyxis sp. HIX].	GCA_001468285.1
KTE72816.1	TetR-HTH→?→||<-?<-?<-?<-?||LRR-repeats+AP-GTPase+COR+TIR*→	LRR-repeats+AP-GTPase+COR+TIR	716	ATE72_22070	Alphaproteobacteria	Sphingopyxis sp. HXXIV	hypothetical protein ATE72_22070 [Sphingopyxis sp. HXXIV].	GCA_001468305.1
MAB62252.1	Cluster1163_3clades→?→ Cluster1131_3clades→?→||<-?<-?||?→ LRR-repeats+AP-GTPase+COR+TIR*→?→?→ METHYLASE→ ParA-Soj-PloopNTPase→ ParB→	LRR-repeats+AP-GTPase+COR+TIR	1212	CMK67_03795	Gammaproteobacteria	Pseudoalteromonas sp.	hypothetical protein CMK67_03795 [Pseudoalteromonas sp.].	GCA_002684115.1
MAD04103.1	LRR-repeats+AP-GTPase+COR*→?→||<-?<-?<-?||CNMP+CRP-HTH→	LRR-repeats+AP-GTPase+COR	956	CMK65_10845	Gammaproteobacteria	Pseudoalteromonas sp.	hypothetical protein CMK65_10845 [Pseudoalteromonas sp.].	GCA_002685175.1
MAG37481.1	AP-GTPase+COR+TIR+ACYC→ LRR-repeats+AP-GTPase+COR+DUF4404*→?→||<-?||?→?→?→||<-DOC+CITB-HTH	LRR-repeats+AP-GTPase+COR+DUF4404	884	CL878_14695	Chloroflexi	Dehalococcoidia bacterium	serine/threonine protein kinase [Dehalococcoidia bacterium].	GCA_002688445.1
MAM30487.1	LRR-repeats+AP-GTPase+COR*→||<-?<-tRNA<-?||Nitroreductase-like→||<-FKBP	LRR-repeats+AP-GTPase+COR	839	CMC13_15825	Bacteroidetes	Flavobacteriaceae bacterium	hypothetical protein CMC13_15825 [Flavobacteriaceae bacterium].	GCA_002693005.1
MAS92856.1	<-Cluster1364_2clades||SIG+TM+TM+TM+TM+TM→ TIR+LRR-repeats+AP-GTPase+COR*→||<-?<-?<-?<-SIG+TM+TM+TM	TIR+LRR-repeats+AP-GTPase+COR	1140	CMO55_06625	Verrucomicrobia	Verrucomicrobiales bacterium	hypothetical protein CMO55_06625 [Verrucomicrobiales bacterium].	GCA_002698785.1
MAT45170.1	LRR-repeats+AP-GTPase+COR*→	LRR-repeats+AP-GTPase+COR	1068	CL609_22815	Chloroflexi	Anaerolineaceae bacterium	hypothetical protein CL609_22815 [Anaerolineaceae bacterium].	GCA_002702705.1
MAT70109.1	<-Cluster1140_2clades<-?||?→||<-?<-?||LRR-repeats+AP-GTPase+COR+TIR*→	LRR-repeats+AP-GTPase+COR+TIR	1220	CMJ58_11380	Planctomycetes	Planctomycetaceae bacterium	hypothetical protein CMJ58_11380 [Planctomycetaceae bacterium].	GCA_002702655.1
MAT98393.1	LRR-repeats+AP-GTPase+COR+EAD7*→?→ SIG+PBPB→||<-?||?→||<-SIG+TM+TM+TM+TM+TM+TM+TM+TM+TM+TM<-REC	LRR-repeats+AP-GTPase+COR+EAD7	845	CL608_14715	Chloroflexi	Anaerolineaceae bacterium	hypothetical protein CL608_14715 [Anaerolineaceae bacterium].	GCA_002699125.1
MAY74446.1	ParB→?→||<-?||ClpABN-AAA+ClpABC-AAA→ LRR-repeats+AP-GTPase+COR+TM+TM*→	LRR-repeats+AP-GTPase+COR+TM+TM	1163	CMJ31_06925	Planctomycetes	Phycisphaerae bacterium	hypothetical protein CMJ31_06925 [Phycisphaerae bacterium].	GCA_002706885.1

<table>
<tr><th>acc</th><th>operon</th><th>architecture</th><th>len</th><th>gen.name</th><th>taxend</th><th>species</th><th>defline</th><th>gca</th></tr>
<tr><td>MBA2243669.1</td><td>LRR-repeats+AP-GTPase+COR+TIR*→?→?→||<-?<-?||Cluster1476_2clades→</td><td>LRR-repeats+AP-GTPase+COR+TIR</td><td>748</td><td>H0W11_01850</td><td>Gemmatimonadetes</td><td>Gemmatimonadetes bacterium</td><td>TIR domain-containing protein [Gemmatimonadetes bacterium].</td><td>GCA_013694785.1</td></tr>
<tr><td>MBA2243921.1</td><td>LRR-repeats+AP-GTPase+COR+TIR*→</td><td>LRR-repeats+AP-GTPase+COR+TIR</td><td>693</td><td>H0W11_03125</td><td>Gemmatimonadetes</td><td>Gemmatimonadetes bacterium</td><td>TIR domain-containing protein, partial [Gemmatimonadetes bacterium].</td><td>GCA_013694785.1</td></tr>
<tr><td>MBA2678589.1</td><td>LRR-repeats+AP-GTPase+COR*→?→||<-?<-Cluster2349_2clades</td><td>LRR-repeats+AP-GTPase+COR</td><td>363</td><td>H0U76_09400</td><td>Chloroflexi</td><td>Ktedonobacteraceae bacterium</td><td>leucine-rich repeat domain-containing protein [Ktedonobacteraceae bacterium].</td><td>GCA_013697605.1</td></tr>
<tr><td>MBA2681600.1</td><td>LRR-repeats+AP-GTPase+COR*→</td><td>LRR-repeats+AP-GTPase+COR</td><td>724</td><td>H0U76_24795</td><td>Chloroflexi</td><td>Ktedonobacteraceae bacterium</td><td>leucine-rich repeat domain-containing protein, partial [Ktedonobacteraceae bacterium].</td><td>GCA_013697605.1</td></tr>
<tr><td>MBA3240620.1</td><td>LRR-repeats+AP-GTPase+COR*→</td><td>LRR-repeats+AP-GTPase+COR</td><td>321</td><td>H0T60_05265</td><td>Acidobacteria</td><td>Acidobacteria bacterium</td><td>leucine-rich repeat domain-containing protein, partial [Acidobacteria bacterium].</td><td>GCA_013813375.1</td></tr>
<tr><td>MBA3574619.1</td><td>LRR-repeats+AP-GTPase+COR*→</td><td>LRR-repeats+AP-GTPase+COR</td><td>706</td><td>H0W37_06570</td><td>Actinobacteria</td><td>Pseudonocardiales bacterium</td><td>hypothetical protein H0W37_06570, partial [Pseudonocardiales bacterium].</td><td>GCA_013817435.1</td></tr>
<tr><td>MBA3673339.1</td><td>Trichomonas-DAM→?→ LRR-repeats+AP-GTPase+COR+TIR*→</td><td>LRR-repeats+AP-GTPase+COR+TIR</td><td>1186</td><td>H0W75_00010</td><td>Bacteroidetes</td><td>Chitinophagaceae bacterium</td><td>leucine-rich repeat domain-containing protein [Chitinophagaceae bacterium].</td><td>GCA_013816565.1</td></tr>
<tr><td>MBA3805221.1</td><td>LRR-repeats+AP-GTPase+COR+TIR*→</td><td>LRR-repeats+AP-GTPase+COR+TIR</td><td>805</td><td>H0X14_05855</td><td>Acidobacteria</td><td>Acidobacteria bacterium</td><td>TIR domain-containing protein [Acidobacteria bacterium].</td><td>GCA_013815675.1</td></tr>
<tr><td>MBA3964374.1</td><td>LRR-repeats+AP-GTPase+COR+TIR*→</td><td>LRR-repeats+AP-GTPase+COR+TIR</td><td>1028</td><td>H0X47_01080</td><td>Nitrospirae</td><td>Nitrospirales bacterium</td><td>leucine-rich repeat domain-containing protein [Nitrospirales bacterium].</td><td>GCA_013821375.1</td></tr>
<tr><td>MBA4062951.1</td><td><-NUDIX||?→||<-tRNA||?→?→||<-?||LRR-repeats+AP-GTPase+COR+TIR*→</td><td>LRR-repeats+AP-GTPase+COR+TIR</td><td>867</td><td>C0501_04445</td><td>Planctomycetes</td><td>Isosphaera sp.</td><td>serine/threonine protein kinase [Isosphaera sp.].</td><td>GCA_013822345.1</td></tr>
<tr><td>MBB3841051.1</td><td>TPR-repeats→?→ TPR-repeats→?→ LRR-repeats+AP-GTPase+COR+TIR*→||<-?||?→||<-?||?→||<-?<-?||TIR+TPR+TPR-S→</td><td>LRR-repeats+AP-GTPase+COR+TIR</td><td>794</td><td>FHS57_005072</td><td>Bacteroidetes</td><td>Runella defluvii</td><td>GTPase SAR1 family protein [Runella defluvii].</td><td>GCA_014195535.1</td></tr>
<tr><td>MBB5874185.1</td><td>LRR-repeats+AP-GTPase+COR*→||<-?<-?<-?<-BACTERIALFRINGE+TM+TM</td><td>LRR-repeats+AP-GTPase+COR</td><td>1250</td><td>F4553_007619</td><td>Actinobacteria</td><td>Allocatelliglobosispora scoriae</td><td>Leucine-rich repeat (LRR) protein/GTPase SAR1 family protein [Allocatelliglobosispora scoriae].</td><td>GCA_014204945.1</td></tr>
<tr><td>MBC6434062.1</td><td>LRR-repeats+AP-GTPase+COR*→||<-?<-?||?→||<-?<-PSE<-N-OB</td><td>LRR-repeats+AP-GTPase+COR</td><td>824</td><td>FM036_26905</td><td>Cyanobacteria</td><td>Nostoc sp. HG1</td><td>hypothetical protein FM036_26905, partial [Nostoc sp. HG1].</td><td>GCA_014324315.1</td></tr>
<tr><td>MBC7569567.1</td><td>LRR-repeats+AP-GTPase+COR+TIR*→</td><td>LRR-repeats+AP-GTPase+COR+TIR</td><td>863</td><td>H7319_07530</td><td>Bacteroidetes</td><td>Spirosoma sp.</td><td>leucine-rich repeat domain-containing protein [Spirosoma sp.].</td><td>GCA_014377625.1</td></tr>
<tr><td>MBC7571377.1</td><td>Cluster1281_2clades→||<-?||LRR-repeats+AP-GTPase+COR*→||<-?<-?||?→||<-?<-?<-?<-Cluster1413_2clades</td><td>LRR-repeats+AP-GTPase+COR</td><td>1077</td><td>H7319_16850</td><td>Bacteroidetes</td><td>Spirosoma sp.</td><td>leucine-rich repeat domain-containing protein [Spirosoma sp.].</td><td>GCA_014377625.1</td></tr>
<tr><td>MBC7773965.1</td><td>LRR-repeats+AP-GTPase+COR+TIR*→</td><td>LRR-repeats+AP-GTPase+COR+TIR</td><td>856</td><td>H7246_00900</td><td>Planctomycetes</td><td>Phycisphaerae bacterium</td><td>TIR domain-containing protein [Phycisphaerae bacterium].</td><td>GCA_014379065.1</td></tr>
<tr><td>MBC7775420.1</td><td>LRR-repeats+AP-GTPase+COR*→</td><td>LRR-repeats+AP-GTPase+COR</td><td>616</td><td>H7246_08275</td><td>Planctomycetes</td><td>Phycisphaerae bacterium</td><td>leucine-rich repeat domain-containing protein, partial [Phycisphaerae bacterium].</td><td>GCA_014379065.1</td></tr>
<tr><td>MBC7777197.1</td><td>LRR-repeats+AP-GTPase+COR+TIR*→?→ HNH→</td><td>LRR-repeats+AP-GTPase+COR+TIR</td><td>693</td><td>H7246_17325</td><td>Planctomycetes</td><td>Phycisphaerae bacterium</td><td>GTP-binding protein, partial [Phycisphaerae bacterium].</td><td>GCA_014379065.1</td></tr>
<tr><td>MBC7778407.1</td><td>LRR-repeats+AP-GTPase+COR+TIR*→</td><td>LRR-repeats+AP-GTPase+COR+TIR</td><td>831</td><td>H7246_23450</td><td>Planctomycetes</td><td>Phycisphaerae bacterium</td><td>TIR domain-containing protein [Phycisphaerae bacterium].</td><td>GCA_014379065.1</td></tr>
</table>

acc	operon	architecture	len	gen.name	taxend	species	defline	gca
MBC7817185.1	LRR-repeats+AP-GTPase+COR*→	LRR-repeats+AP-GTPase+COR	238	IAG10_09885	Planctomycetes	Planctomycetaceae bacterium	hypothetical protein IAG10_09885, partial [Planctomycetaceae bacterium].	GCA_014379175.1
MBC7817749.1	<-RHOD-CDC25||LRR-repeats+AP-GTPase+COR+TIR*→	LRR-repeats+AP-GTPase+COR+TIR	865	IAG10_12720	Planctomycetes	Planctomycetaceae bacterium	leucine-rich repeat domain-containing protein [Planctomycetaceae bacterium].	GCA_014379175.1
MBC7826556.1	LRR-repeats+AP-GTPase+COR+TIR*→	LRR-repeats+AP-GTPase+COR+TIR	752	H7122_02330	Bacteroidetes	Chitinophagaceae bacterium	TIR domain-containing protein, partial [Chitinophagaceae bacterium].	GCA_014379345.1
MBC7848891.1	LRR-repeats+AP-GTPase+COR*→	LRR-repeats+AP-GTPase+COR	927	H7Y31_04105	Bacteroidetes	Chitinophagaceae bacterium	leucine-rich repeat domain-containing protein, partial [Chitinophagaceae bacterium].	GCA_014379515.1
MBC7877029.1	RADICAL-SAM→?→?→?→||<-?||?→ LRR-repeats→ LRR-repeats+AP-GTPase+COR*→?→ ABC-ATPase→	LRR-repeats+AP-GTPase+COR	718	H7Y59_07640	Chloroflexi	Anaerolineales bacterium	leucine-rich repeat domain-containing protein [Anaerolineales bacterium].	GCA_014379595.1
MBC7877562.1	<-TM+RHOMBOID<-?<-?<-?||TM→ LRR-repeats+AP-GTPase+COR*→||<-?||SIGMA-HTH→	LRR-repeats+AP-GTPase+COR	1057	H7Y59_10360	Chloroflexi	Anaerolineales bacterium	hypothetical protein H7Y59_10360 [Anaerolineales bacterium].	GCA_014379595.1
MBC8029843.1	<-REC||?→?→ Hsp10→?→ LRR-repeats+AP-GTPase+COR+TIR*→||<-?<-?<-?<-?<-ABC-ATPase	LRR-repeats+AP-GTPase+COR+TIR	871	H7Z16_07010	Acidobacteria	Pyrinomonadaceae bacterium	TIR domain-containing protein [Pyrinomonadaceae bacterium].	GCA_014380365.1
MBC8182613.1	LRR-repeats+AP-GTPase+COR*→?→?→ RelE-ParE→||<-?<-RelE-ParE<-?<-HISKIN	LRR-repeats+AP-GTPase+COR	687	H8E88_16055	Bacteria	candidate division KSB1 bacterium	hypothetical protein H8E88_16055, partial [candidate division KSB1 bacterium].	GCA_014381395.1
MBC8235006.1	LRR-repeats+AP-GTPase+COR+TIR*→	LRR-repeats+AP-GTPase+COR+TIR	1069	H8E77_36130	unclassified Bacteria	bacterium	leucine-rich repeat domain-containing protein [bacterium].	GCA_014381605.1
MBC8264797.1	LRR-repeats+AP-GTPase+COR*→	LRR-repeats+AP-GTPase+COR	674	H8E47_11810	Chloroflexi	Anaerolineales bacterium	leucine-rich repeat domain-containing protein [Anaerolineales bacterium].	GCA_014382145.1
MBC8291013.1	LRR-repeats+AP-GTPase+COR*→	LRR-repeats+AP-GTPase+COR	692	H8E37_11925	Planctomycetes	Planctomycetes bacterium	leucine-rich repeat domain-containing protein, partial [Planctomycetes bacterium].	GCA_014382385.1
MBC8374880.1	Cluster1757_2clades→?→ LRR-repeats+AP-GTPase+COR+DrHyd*→	LRR-repeats+AP-GTPase+COR+DrHyd	931	H8E26_02475	FCB group	FCB group bacterium	50S ribosome-binding GTPase [FCB group bacterium].	GCA_014382565.1
MBC8468948.1	LRR-repeats+AP-GTPase+COR*→||<-?||?→||<-?<-?||?→||<-?<-SIGMA-HTH	LRR-repeats+AP-GTPase+COR	1003	H8D56_05705	Planctomycetes	Planctomycetes bacterium	leucine-rich repeat domain-containing protein [Planctomycetes bacterium].	GCA_014384025.1
MBC9785332.1	Cluster1542_2clades→ LRR-repeats+AP-GTPase+COR*→	LRR-repeats+AP-GTPase+COR	976	H1S01_12510	Firmicutes	Heliobacterium chlorum	leucine-rich repeat domain-containing protein [Heliobacterium chlorum].	-
MBP93715.1	LRR-repeats+AP-GTPase+COR*→||<-?||?→||<-?<-Cluster1995_2clades	LRR-repeats+AP-GTPase+COR	894	CMC55_06315	Bacteroidetes	Flavobacteriaceae bacterium	hypothetical protein CMC55_06315 [Flavobacteriaceae bacterium].	GCA_002726785.1
MBV11156.1	APATPase+BetaPropeller_WD40-repeats→?→||<-?||FGS→ FGS→ FGS→ LRR-repeats+AP-GTPase+COR+TIR*→ Cluster1411_2clades→ FGS→ FGS→ FGS→	LRR-repeats+AP-GTPase+COR+TIR	1075	CMN21_18290	Planctomycetes	Rubinisphaera sp.	hypothetical protein CMN21_18290 [Rubinisphaera sp.].	GCA_002731545.1
MQP68112.1	LRR-repeats+AP-GTPase+COR+TIR*→||<-ParB<-ParA-Soj-PloopNTPase	LRR-repeats+AP-GTPase+COR+TIR	993	GE253_22605	Alphaproteobacteria	Niveispirillum sp. SYP-B3756	TIR domain-containing protein [Niveispirillum sp. SYP-B3756].	GCA_009495745.1
MQY15494.1	<-Cluster2127_2clades<-?||?→ LRR-repeats+AP-GTPase+COR*→?→ REC→ HISKIN→	LRR-repeats+AP-GTPase+COR	980	SRB5_56760	Actinobacteria	Streptomyces sp. RB5	hypothetical protein SRB5_56760 [Streptomyces sp. RB5].	GCA_009604385.1
MSP13971.1	LRR-repeats+AP-GTPase+COR*→	LRR-repeats+AP-GTPase+COR	792	EXR62_13575	Chloroflexi	Chloroflexi bacterium	hypothetical protein EXR62_13575, partial [Chloroflexi bacterium].	GCA_009692745.1
MSS71311.1	Cluster1601_2clades→ LRR-repeats+AP-GTPase+COR*→ COR+TIR→||<-Cluster1913_2clades<-?<-RPN10	LRR-repeats+AP-GTPase+COR	556	EXS64_07460	FCB group	Candidatus Latescibacteria bacterium	hypothetical protein EXS64_07460 [Candidatus Latescibacteria bacterium].	GCA_009694805.1

acc	operon	architecture	len	gen.name	taxend	species	defline	gca
NAS89011.1	POLYSACPOLYMERASE→ LRR-repeats→ LRR-repeats+AP-GTPase+COR*→||<-?<-?||?→?→||<-PIN	LRR-repeats+AP-GTPase+COR	604	C4E24_04665	Euryarchaeota	ANME-1 cluster archaeon AG-394-G21	GTP-binding protein [ANME-1 cluster archaeon AG-394-G21].	GCA_009903435.1
NAT10404.1	Cluster1536_2clades→||<-?<-?<-?||POLYSACPOLYMERASE→?→ LRR-repeats+AP-GTPase+COR→ *LRR-repeats→ LRR-repeats+AP-GTPase+COR→*?→||<-PIN	LRR-repeats+AP-GTPase+COR	688	C4E22_02460	Euryarchaeota	ANME-1 cluster archaeon AG-394-G06	GTP-binding protein [ANME-1 cluster archaeon AG-394-G06].	GCA_009903405.1
NBC32680.1	LRR-repeats+AP-GTPase+COR+TM+TM+TM+TM*→||<-?<-?||?→?→?→||<-TM+RHOMBOID	LRR-repeats+AP-GTPase+COR+TM+TM+TM+TM	806	GVY13_08395	Alphaproteobacteria	Alphaproteobacteria bacterium	GTP-binding protein, partial [Alphaproteobacteria bacterium].	GCA_009909005.1
NCP86657.1	<-XIS-HTH||HTH+HTH→||<-WLM||?→ NACHT+68TM-wHTH→ REase→?→ LRR-repeats+AP-GTPase+COR*→	LRR-repeats+AP-GTPase+COR	960	GW829_04700	unclassified Bacteria	bacterium	hypothetical protein GW829_04700, partial [bacterium].	GCA_009994085.1
NCP88511.1	REase→ LRR-repeats+AP-GTPase+COR*→	LRR-repeats+AP-GTPase+COR	336	GW829_14210	unclassified Bacteria	bacterium	hypothetical protein GW829_14210, partial [bacterium].	GCA_009994085.1
NCP88560.1	LRR-repeats+AP-GTPase+COR*→	LRR-repeats+AP-GTPase+COR	382	GW829_14460	unclassified Bacteria	bacterium	hypothetical protein GW829_14460, partial [bacterium].	GCA_009994085.1
NCQ69077.1	CHTH→ LRR-repeats+AP-GTPase+COR*→	LRR-repeats+AP-GTPase+COR	382	GPI97_07600	Cyanobacteria	Microcystis aeruginosa W13-16	leucine-rich repeat protein, partial [Microcystis aeruginosa W13-16].	GCA_009995625.1
NCQ78095.1	CHTH→ LRR-repeats+AP-GTPase+COR*→	LRR-repeats+AP-GTPase+COR	359	GPI99_08005	Cyanobacteria	Microcystis aeruginosa W13-15	GTP-binding protein, partial [Microcystis aeruginosa W13-15].	GCA_009995645.1
NCQ94522.1	Ferredoxin-betagrasp→ tRNA→ tRNA→ SAM-methylase→?→||<-?<-SIG+TPM_phosphatase+TM+TM+TM||LRR-repeats+AP-GTPase+COR+TM+TM*→||<-?<-?<-?<-Pro_CA	LRR-repeats+AP-GTPase+COR+TM+TM	805	GPJ33_04160	Cyanobacteria	Microcystis aeruginosa W11-03	leucine-rich repeat protein [Microcystis aeruginosa W11-03].	GCA_009995775.1
NCR15129.1	CHTH→ LRR-repeats+AP-GTPase+COR*→	LRR-repeats+AP-GTPase+COR	413	GPJ24_21065	Cyanobacteria	Microcystis aeruginosa SX13-11	GTP-binding protein, partial [Microcystis aeruginosa SX13-11].	GCA_009995725.1
NCR28927.1	CHTH→ LRR-repeats+AP-GTPase+COR*→	LRR-repeats+AP-GTPase+COR	386	GPJ25_22505	Cyanobacteria	Microcystis aeruginosa LE13-04	leucine-rich repeat protein, partial [Microcystis aeruginosa LE13-04].	GCA_009995995.1
NCR34650.1	LRR-repeats+AP-GTPase+COR+TM+TM*→	LRR-repeats+AP-GTPase+COR+TM+TM	789	GPJ31_03655	Cyanobacteria	Microcystis aeruginosa S11-05	leucine-rich repeat protein [Microcystis aeruginosa S11-05].	GCA_009995785.1
NCR46978.1	CHTH→ LRR-repeats+AP-GTPase+COR*→	LRR-repeats+AP-GTPase+COR	514	GPJ09_24595	Cyanobacteria	Microcystis aeruginosa SX13-01	leucine-rich repeat protein, partial [Microcystis aeruginosa SX13-01].	GCA_009995735.1
NCR55589.1	LRR-repeats+AP-GTPase+COR+TM+TM*→||<-?<-?<-?<-Pro_CA||?→||<-?<-tRNA<-tRNA<-Ferredoxin-betagrasp	LRR-repeats+AP-GTPase+COR+TM+TM	710	GPJ10_20085	Cyanobacteria	Microcystis aeruginosa L211-07	GTP-binding protein, partial [Microcystis aeruginosa L211-07].	GCA_009996085.1
NCR60375.1	CHTH→ LRR-repeats+AP-GTPase+COR*→	LRR-repeats+AP-GTPase+COR	478	GPJ01_22875	Cyanobacteria	Microcystis aeruginosa LL13-06	GTP-binding protein, partial [Microcystis aeruginosa LL13-06].	GCA_009995895.1
NCR68842.1	CHTH→ LRR-repeats+AP-GTPase+COR*→	LRR-repeats+AP-GTPase+COR	545	GPJ34_21175	Cyanobacteria	Microcystis aeruginosa LL11-07	GTP-binding protein, partial [Microcystis aeruginosa LL11-07].	GCA_009995855.1
NCR71934.1	SAM-methylase→?→||<-?<-SIG+TPM_phosphatase+TM+TM+TM||LRR-repeats+AP-GTPase+COR+TM+TM*→||<-?<-?<-?<-Pro_CA	LRR-repeats+AP-GTPase+COR+TM+TM	751	GPI93_12415	Cyanobacteria	Microcystis aeruginosa LG13-12	leucine-rich repeat protein [Microcystis aeruginosa LG13-12].	GCA_009995925.1
NCR77025.1	SIG+TM+TM+TM+TM+TM+TM+TM→||<-?||Mbetalac→ SAM-methylase→?→||<-?<-SIG+TPM_phosphatase+TM+TM+TM||LRR-repeats+AP-GTPase+COR+TM+TM*→	LRR-repeats+AP-GTPase+COR+TM+TM	897	GPI92_16105	Cyanobacteria	Microcystis aeruginosa K13-06	GTP-binding protein [Microcystis aeruginosa K13-06].	GCA_009996165.1
NCR80564.1	SIG+TM+TM+TM+TM+TM+TM+TM→||<-?||Mbetalac→ SAM-methylase→?→||<-?<-SIG+TPM_phosphatase+TM+TM+TM||LRR-repeats+AP-GTPase+COR+TM+TM*→	LRR-repeats+AP-GTPase+COR+TM+TM	805	GPI91_11595	Cyanobacteria	Microcystis aeruginosa K13-10	leucine-rich repeat protein [Microcystis aeruginosa K13-10].	GCA_009996105.1
NCR97058.1	SIG+TM+TM+TM+TM+TM+TM+TM→||<-?||Mbetalac→ SAM-methylase→?→||<-?<-SIG+TPM_phosphatase+TM+TM+TM||LRR-repeats+AP-GTPase+COR+TM+TM*→||<-?<-?<-?<-Pro_CA||?→||<-?<-tRNA<-tRNA<-Ferredoxin-betagrasp	LRR-repeats+AP-GTPase+COR+TM+TM	920	GPJ32_04180	Cyanobacteria	Microcystis aeruginosa L311-01	GTP-binding protein [Microcystis aeruginosa L311-01].	GCA_009996025.1
NCS08858.1	CHTH→ LRR-repeats+AP-GTPase+COR*→	LRR-repeats+AP-GTPase+COR	386	GPJ07_21130	Cyanobacteria	Microcystis aeruginosa G13-07	GTP-binding protein, partial [Microcystis aeruginosa G13-07].	GCA_009996305.1
NCS11263.1	CHTH→ LRR-repeats+AP-GTPase+COR+TM+TM*→	LRR-repeats+AP-GTPase+COR+TM+TM	852	GPJ08_08960	Cyanobacteria	Microcystis aeruginosa G13-09	GTP-binding protein [Microcystis aeruginosa G13-09].	GCA_009996255.1
NCS17812.1	CHTH→ LRR-repeats+AP-GTPase+COR*→	LRR-repeats+AP-GTPase+COR	503	GPJ02_20805	Cyanobacteria	Microcystis aeruginosa G13-12	leucine-rich repeat protein, partial [Microcystis aeruginosa G13-12].	GCA_009996205.1
NCS22942.1	LRR-repeats+AP-GTPase+COR*→	LRR-repeats+AP-GTPase+COR	353	GPJ15_26105	Cyanobacteria	Microcystis aeruginosa G11-06	GTP-binding protein, partial [Microcystis aeruginosa G11-06].	GCA_009996385.1
NCS24651.1	LRR-repeats+AP-GTPase+COR+TM+TM*→||<-?<-FGS<-?<-?||?→?→ Aminotran_1_2→	LRR-repeats+AP-GTPase+COR+TM+TM	732	GPI96_09100	Cyanobacteria	Microcystis aeruginosa BS13-02	GTP-binding protein, partial [Microcystis aeruginosa BS13-02].	GCA_009996485.1
NCS52102.1	CHTH→ LRR-repeats+AP-GTPase+COR*→	LRR-repeats+AP-GTPase+COR	397	GPJ23_06895	Cyanobacteria	Microcystis aeruginosa G13-05	leucine-rich repeat protein, partial [Microcystis aeruginosa G13-05].	GCA_009996285.1

<table>
<tr><th>acc</th><th>operon</th><th>architecture</th><th>len</th><th>gen.name</th><th>taxend</th><th>species</th><th>defline</th><th>gca</th></tr>
<tr><td>NCS59859.1</td><td>LRR-repeats+AP-GTPase+COR*→</td><td>LRR-repeats+AP-GTPase+COR</td><td>339</td><td>GPJ16_25035</td><td>Cyanobacteria</td><td>Microcystis aeruginosa G11-04</td><td>GTP-binding protein, partial [Microcystis aeruginosa G11-04].</td><td>GCA_009996445.1</td></tr>
<tr><td>NCT21185.1</td><td><-XIS-HTH||HTH+HTH→||<-WLM||?→ NACHT+68TM-wHTH→ REase→?→ LRR-repeats+AP-GTPase+COR*→</td><td>LRR-repeats+AP-GTPase+COR</td><td>956</td><td>GW781_08530</td><td>unclassified Bacteria</td><td>bacterium</td><td>hypothetical protein GW781_08530, partial [bacterium].</td><td>GCA_009994975.1</td></tr>
<tr><td>NCT65324.1</td><td>CHTH→ LRR-repeats+AP-GTPase+COR*→</td><td>LRR-repeats+AP-GTPase+COR</td><td>434</td><td>GPJ03_21265</td><td>Cyanobacteria</td><td>Microcystis aeruginosa G13-01</td><td>leucine-rich repeat protein, partial [Microcystis aeruginosa G13-01].</td><td>GCA_009996325.1</td></tr>
<tr><td>NCY16589.1</td><td>LRR-repeats+AP-GTPase+COR+TIR*→</td><td>LRR-repeats+AP-GTPase+COR+TIR</td><td>1058</td><td>EBX39_07430</td><td>Actinobacteria</td><td>Actinobacteria bacterium</td><td>hypothetical protein EBX39_07430 [Actinobacteria bacterium].</td><td>GCA_010021725.1</td></tr>
<tr><td>NEN97779.1</td><td>REC→||<-TPR-repeats||LRR-repeats+AP-GTPase+COR+DUF4404*→</td><td>LRR-repeats+AP-GTPase+COR+DUF4404</td><td>971</td><td>F6K50_20310</td><td>Cyanobacteria</td><td>Moorea sp. SIO3I7</td><td>GTPase [Moorea sp. SIO3I7].</td><td>GCA_010692375.1</td></tr>
<tr><td>NEO07696.1</td><td>SIG+TM+TM+TM+TM+TM+TM+TM→?→ ABC-ATPase→?→ ABC-ATPase→?→ LRR-repeats+AP-GTPase+COR*→</td><td>LRR-repeats+AP-GTPase+COR</td><td>1373</td><td>F6K51_19885</td><td>Cyanobacteria</td><td>Moorea sp. SIO3I8</td><td>leucine-rich repeat protein [Moorea sp. SIO3I8].</td><td>GCA_010692325.1</td></tr>
<tr><td>NEO14597.1</td><td>REC→||<-TPR-repeats<-?||LRR-repeats+AP-GTPase+COR+DUF4404*→</td><td>LRR-repeats+AP-GTPase+COR+DUF4404</td><td>797</td><td>F6K46_20370</td><td>Cyanobacteria</td><td>Moorea sp. SIO3E8</td><td>GTPase [Moorea sp. SIO3E8].</td><td>GCA_010692445.1</td></tr>
<tr><td>NEO18066.1</td><td>ABC-ATPase→||<-?||ABC-ATPase→?→?→||<-?||LRR-repeats+AP-GTPase+COR*→?→||<-?<-HNH<-PSE||?→||<-Cluster5_5clades</td><td>LRR-repeats+AP-GTPase+COR</td><td>852</td><td>F6K57_01150</td><td>Cyanobacteria</td><td>Moorea sp. SIO4A5</td><td>GTPase [Moorea sp. SIO4A5].</td><td>GCA_010672005.1</td></tr>
<tr><td>NEO21732.1</td><td>REC→||<-?||REC→||<-TPR-repeats||LRR-repeats+AP-GTPase+COR+DUF4404*→</td><td>LRR-repeats+AP-GTPase+COR+DUF4404</td><td>949</td><td>F6K57_21060</td><td>Cyanobacteria</td><td>Moorea sp. SIO4A5</td><td>GTPase [Moorea sp. SIO4A5].</td><td>GCA_010672005.1</td></tr>
<tr><td>NEO28667.1</td><td>LRR-repeats+AP-GTPase+COR*→</td><td>LRR-repeats+AP-GTPase+COR</td><td>372</td><td>F6K03_17765</td><td>Cyanobacteria</td><td>Kamptonema sp. SIO4C4</td><td>GTPase, partial [Kamptonema sp. SIO4C4].</td><td>GCA_010692335.1</td></tr>
<tr><td>NEO36773.1</td><td>SIG+TM+TM+TM+TM+TM+TM+TM→ ABC-ATPase→?→ ABC-ATPase→||<-?||?→ LRR-repeats+AP-GTPase+COR*→</td><td>LRR-repeats+AP-GTPase+COR</td><td>847</td><td>F6J90_10815</td><td>Cyanobacteria</td><td>Moorea sp. SIOASIH</td><td>GTPase [Moorea sp. SIOASIH].</td><td>GCA_010671925.1</td></tr>
<tr><td>NEO37599.1</td><td>REC→||<-?<-?||REC→||<-TPR-repeats<-?||?→ LRR-repeats+AP-GTPase+COR+DUF4404*→ PSE→||<-?<-PSE||?→?→?→||<-?||TM→</td><td>LRR-repeats+AP-GTPase+COR+DUF4404</td><td>1099</td><td>F6J90_15180</td><td>Cyanobacteria</td><td>Moorea sp. SIOASIH</td><td>leucine-rich repeat protein [Moorea sp. SIOASIH].</td><td>GCA_010671925.1</td></tr>
<tr><td>NEO43843.1</td><td>SbcC→?→ LRR-repeats+AP-GTPase+COR*→</td><td>LRR-repeats+AP-GTPase+COR</td><td>1256</td><td>F6K55_06775</td><td>Cyanobacteria</td><td>Moorea sp. SIO4A3</td><td>leucine-rich repeat protein [Moorea sp. SIO4A3].</td><td>GCA_010672045.1</td></tr>
<tr><td>NEO48162.1</td><td>REC→||<-TPR-repeats||LRR-repeats+AP-GTPase+COR*→</td><td>LRR-repeats+AP-GTPase+COR</td><td>616</td><td>F6K55_30210</td><td>Cyanobacteria</td><td>Moorea sp. SIO4A3</td><td>leucine-rich repeat protein, partial [Moorea sp. SIO4A3].</td><td>GCA_010672045.1</td></tr>
<tr><td>NEO55671.1</td><td>LRR-repeats→?→ AP-GTPase*→||<-?<-SIG+Trypsin+PDZ</td><td>AP-GTPase</td><td>113</td><td>F6K54_22940</td><td>Cyanobacteria</td><td>Okeania sp. SIO3B5</td><td>hypothetical protein F6K54_22940 [Okeania sp. SIO3B5].</td><td>GCA_010692555.1</td></tr>
<tr><td>NEO56464.1</td><td>LRR-repeats+AP-GTPase+COR*→||<-RelE-ParE</td><td>LRR-repeats+AP-GTPase+COR</td><td>856</td><td>F6K54_27310</td><td>Cyanobacteria</td><td>Okeania sp. SIO3B5</td><td>GTPase [Okeania sp. SIO3B5].</td><td>GCA_010692555.1</td></tr>
<tr><td>NEO57234.1</td><td><-RADICAL-SAM||PSE→ REC→?→ LRR-repeats+AP-GTPase+COR*→</td><td>LRR-repeats+AP-GTPase+COR</td><td>472</td><td>F6K54_31710</td><td>Cyanobacteria</td><td>Okeania sp. SIO3B5</td><td>GTPase, partial [Okeania sp. SIO3B5].</td><td>GCA_010692555.1</td></tr>
<tr><td>NEO68320.1</td><td>SIG+PBPI→?→ SIG+TM+TM+TM+TM+TM+TM+TM→?→ ABC-ATPase→ ABC-ATPase→||<-?||LRR-repeats+AP-GTPase+COR*→</td><td>LRR-repeats+AP-GTPase+COR</td><td>884</td><td>F6K52_02085</td><td>Cyanobacteria</td><td>Moorea sp. SIO3H5</td><td>leucine-rich repeat protein [Moorea sp. SIO3H5].</td><td>GCA_010692395.1</td></tr>
<tr><td>NEO73423.1</td><td>REC→||<-TPR-repeats<-?||LRR-repeats+AP-GTPase+COR+DUF4404*→</td><td>LRR-repeats+AP-GTPase+COR+DUF4404</td><td>933</td><td>F6K52_29165</td><td>Cyanobacteria</td><td>Moorea sp. SIO3H5</td><td>GTPase [Moorea sp. SIO3H5].</td><td>GCA_010692395.1</td></tr>
<tr><td>NEO79069.1</td><td>REC→||<-TPR-repeats||LRR-repeats+AP-GTPase+COR+DUF4404*→</td><td>LRR-repeats+AP-GTPase+COR+DUF4404</td><td>929</td><td>F6J99_23600</td><td>Cyanobacteria</td><td>Moorea sp. SIO4G3</td><td>GTPase [Moorea sp. SIO4G3].</td><td>GCA_010692305.1</td></tr>
<tr><td>NEO83336.1</td><td>Cluster5_5clades→?→ PSE→ LRR-repeats+AP-GTPase+COR*→</td><td>LRR-repeats+AP-GTPase+COR</td><td>1006</td><td>F6J87_03595</td><td>Cyanobacteria</td><td>Spirulina sp. SIO3F2</td><td>leucine-rich repeat protein [Spirulina sp. SIO3F2].</td><td>GCA_010672165.1</td></tr>
<tr><td>NEO85545.1</td><td>MNS-STAND+CR-REase3→||<-?<-?||?→||<-REC<-REC||HISKIN→ LRR-repeats+AP-GTPase+COR*→||<-HISKIN</td><td>LRR-repeats+AP-GTPase+COR</td><td>1127</td><td>F6J87_15045</td><td>Cyanobacteria</td><td>Spirulina sp. SIO3F2</td><td>GTPase [Spirulina sp. SIO3F2].</td><td>GCA_010672165.1</td></tr>
<tr><td>NEO86840.1</td><td>TM+TM+TM+TM+TM+TM+TM+TM→?→||<-?<-Pentapeptide-repeats||?→ LRR-repeats+AP-GTPase+COR+DUF4404*→</td><td>LRR-repeats+AP-GTPase+COR+DUF4404</td><td>1131</td><td>F6J87_21660</td><td>Cyanobacteria</td><td>Spirulina sp. SIO3F2</td><td>GTP-binding protein [Spirulina sp. SIO3F2].</td><td>GCA_010672165.1</td></tr>
<tr><td>NEO88027.1</td><td>LRR-repeats→ LRR-repeats+AP-GTPase+COR*→</td><td>LRR-repeats+AP-GTPase+COR</td><td>800</td><td>F6J87_27790</td><td>Cyanobacteria</td><td>Spirulina sp. SIO3F2</td><td>hypothetical protein F6J87_27790 [Spirulina sp. SIO3F2].</td><td>GCA_010672165.1</td></tr>
<tr><td>NEO88064.1</td><td>Cluster1048_2clades→ LRR-repeats+AP-GTPase+COR*→</td><td>LRR-repeats+AP-GTPase+COR</td><td>1166</td><td>F6J87_27980</td><td>Cyanobacteria</td><td>Spirulina sp. SIO3F2</td><td>GTPase [Spirulina sp. SIO3F2].</td><td>GCA_010672165.1</td></tr>
<tr><td>NEO88189.1</td><td>LRR-repeats+AP-GTPase+COR*→</td><td>LRR-repeats+AP-GTPase+COR</td><td>1134</td><td>F6J87_28620</td><td>Cyanobacteria</td><td>Spirulina sp. SIO3F2</td><td>hypothetical protein F6J87_28620 [Spirulina sp. SIO3F2].</td><td>GCA_010672165.1</td></tr>
<tr><td>NEP02262.1</td><td>LRR-repeats+AP-GTPase+COR*→ ParA-Soj-PloopNTPase→</td><td>LRR-repeats+AP-GTPase+COR</td><td>814</td><td>F6K58_27120</td><td>Cyanobacteria</td><td>Symploca sp. SIO2E9</td><td>leucine-rich repeat protein [Symploca sp. SIO2E9].</td><td>GCA_010692645.1</td></tr>
<tr><td>NEP15532.1</td><td>LRR-repeats+AP-GTPase+COR+TIR*→?→||<-?||?→||<-?<-?<-?||ABhydrolase→</td><td>LRR-repeats+AP-GTPase+COR+TIR</td><td>1041</td><td>F6J97_01370</td><td>Cyanobacteria</td><td>Leptolyngbya sp. SIO4C1</td><td>TIR domain-containing protein [Leptolyngbya sp. SIO4C1].</td><td>GCA_010671975.1</td></tr>
</table>

<table>
<tr><th>acc</th><th>operon</th><th>architecture</th><th>len</th><th>gen.name</th><th>taxend</th><th>species</th><th>defline</th><th>gca</th></tr>
<tr><td>NEP17061.1</td><td>TM+TM+TM+TM+TM→ LRR-repeats+AP-GTPase+COR+DUF4404*→||<-SIG+TM+TM</td><td>LRR-repeats+AP-GTPase+COR+DUF4404</td><td>1026</td><td>F6J97_09150</td><td>Cyanobacteria</td><td>Leptolyngbya sp. SIO4C1</td><td>GTP-binding protein [Leptolyngbya sp. SIO4C1].</td><td>GCA_010671975.1</td></tr>
<tr><td>NEP23180.1</td><td>SIG+TM+TM+TM+TM+TM+TM+TM→?→ ABC-ATPase→ ABC-ATPase→||<-?||?→||<-?||LRR-repeats+AP-GTPase+COR*→?→||<-?<-HNH<-PSE||?→||<-Cluster5_5clades</td><td>LRR-repeats+AP-GTPase+COR</td><td>892</td><td>F6K49_14560</td><td>Cyanobacteria</td><td>Moorea sp. SIO3I6</td><td>GTPase [Moorea sp. SIO3I6].</td><td>GCA_010672095.1</td></tr>
<tr><td>NEP27050.1</td><td>LRR-repeats+AP-GTPase+COR+DUF4404*→</td><td>LRR-repeats+AP-GTPase+COR+DUF4404</td><td>909</td><td>F6K49_35465</td><td>Cyanobacteria</td><td>Moorea sp. SIO3I6</td><td>GTPase [Moorea sp. SIO3I6].</td><td>GCA_010672095.1</td></tr>
<tr><td>NEP34707.1</td><td>LRR-repeats+AP-GTPase+COR+DUF4404*→</td><td>LRR-repeats+AP-GTPase+COR+DUF4404</td><td>924</td><td>F6K38_25730</td><td>Cyanobacteria</td><td>Moorea sp. SIO3B2</td><td>GTPase, partial [Moorea sp. SIO3B2].</td><td>GCA_010692525.1</td></tr>
<tr><td>NEP80618.1</td><td>LRR-repeats+AP-GTPase+COR*→</td><td>LRR-repeats+AP-GTPase+COR</td><td>891</td><td>F6K39_22060</td><td>Cyanobacteria</td><td>Okeania sp. SIO3B3</td><td>GTPase [Okeania sp. SIO3B3].</td><td>GCA_010692535.1</td></tr>
<tr><td>NEP81305.1</td><td>LRR-repeats+AP-GTPase+COR*→</td><td>LRR-repeats+AP-GTPase+COR</td><td>790</td><td>F6K39_25960</td><td>Cyanobacteria</td><td>Okeania sp. SIO3B3</td><td>GTPase [Okeania sp. SIO3B3].</td><td>GCA_010692535.1</td></tr>
<tr><td>NEP82037.1</td><td>LRR-repeats+AP-GTPase+COR*→</td><td>LRR-repeats+AP-GTPase+COR</td><td>664</td><td>F6K39_30180</td><td>Cyanobacteria</td><td>Okeania sp. SIO3B3</td><td>hypothetical protein F6K39_30180 [Okeania sp. SIO3B3].</td><td>GCA_010692535.1</td></tr>
<tr><td>NEP82994.1</td><td>LRR-repeats+AP-GTPase*→</td><td>LRR-repeats+AP-GTPase</td><td>713</td><td>F6K39_35565</td><td>Cyanobacteria</td><td>Okeania sp. SIO3B3</td><td>leucine-rich repeat protein [Okeania sp. SIO3B3].</td><td>GCA_010692535.1</td></tr>
<tr><td>NEP88726.1</td><td><-HhH-RADC+JAB<-?||LRR-repeats+AP-GTPase+COR*→</td><td>LRR-repeats+AP-GTPase+COR</td><td>800</td><td>F6K18_18835</td><td>Cyanobacteria</td><td>Okeania sp. SIO2C2</td><td>GTPase [Okeania sp. SIO2C2].</td><td>GCA_010692625.1</td></tr>
<tr><td>NEQ25590.1</td><td>LRR-repeats+AP-GTPase+COR*→</td><td>LRR-repeats+AP-GTPase+COR</td><td>329</td><td>F6K28_42320</td><td>Cyanobacteria</td><td>Microcoleus sp. SIO2G3</td><td>hypothetical protein F6K28_42320, partial [Microcoleus sp. SIO2G3].</td><td>GCA_010672365.1</td></tr>
<tr><td>NEQ30222.1</td><td>SF2-DUF3427A→||<-PSE||?→ LRR-repeats+AP-GTPase+COR*→</td><td>LRR-repeats+AP-GTPase+COR</td><td>425</td><td>F6K04_04355</td><td>Cyanobacteria</td><td>Leptolyngbya sp. SIO4C5</td><td>GTP-binding protein, partial [Leptolyngbya sp. SIO4C5].</td><td>GCA_010671965.1</td></tr>
<tr><td>NEQ30763.1</td><td>LRR-repeats+AP-GTPase+COR+DrHyd*→||<-SAM-methylase</td><td>LRR-repeats+AP-GTPase+COR+DrHyd</td><td>1168</td><td>F6K04_07140</td><td>Cyanobacteria</td><td>Leptolyngbya sp. SIO4C5</td><td>hypothetical protein F6K04_07140 [Leptolyngbya sp. SIO4C5].</td><td>GCA_010671965.1</td></tr>
<tr><td>NEQ30823.1</td><td>LRR-repeats+AP-GTPase+COR*→</td><td>LRR-repeats+AP-GTPase+COR</td><td>1087</td><td>F6K04_07455</td><td>Cyanobacteria</td><td>Leptolyngbya sp. SIO4C5</td><td>hypothetical protein F6K04_07455 [Leptolyngbya sp. SIO4C5].</td><td>GCA_010671965.1</td></tr>
<tr><td>NEQ33180.1</td><td>LRR-repeats+AP-GTPase+COR+TIR*→||<-Cluster2398_2clades</td><td>LRR-repeats+AP-GTPase+COR+TIR</td><td>974</td><td>F6K04_19645</td><td>Cyanobacteria</td><td>Leptolyngbya sp. SIO4C5</td><td>TIR domain-containing protein [Leptolyngbya sp. SIO4C5].</td><td>GCA_010671965.1</td></tr>
<tr><td>NEQ38623.1</td><td>LRR-repeats→||<-?||?→ LRR-repeats+AP-GTPase+COR*→</td><td>LRR-repeats+AP-GTPase+COR</td><td>778</td><td>F6K40_21075</td><td>Cyanobacteria</td><td>Okeania sp. SIO3I5</td><td>GTPase [Okeania sp. SIO3I5].</td><td>GCA_010672085.1</td></tr>
<tr><td>NEQ39708.1</td><td>LRR-repeats+AP-GTPase+COR*→</td><td>LRR-repeats+AP-GTPase+COR</td><td>641</td><td>F6K40_27040</td><td>Cyanobacteria</td><td>Okeania sp. SIO3I5</td><td>hypothetical protein F6K40_27040 [Okeania sp. SIO3I5].</td><td>GCA_010672085.1</td></tr>
<tr><td>NEQ41322.1</td><td>LRR-repeats+AP-GTPase+COR*→</td><td>LRR-repeats+AP-GTPase+COR</td><td>923</td><td>F6K40_36055</td><td>Cyanobacteria</td><td>Okeania sp. SIO3I5</td><td>GTPase [Okeania sp. SIO3I5].</td><td>GCA_010672085.1</td></tr>
<tr><td>NEQ51480.1</td><td>LRR-repeats+AP-GTPase+COR+DrHyd*→||<-?||?→||<-SF2-DUF3427A</td><td>LRR-repeats+AP-GTPase+COR+DrHyd</td><td>841</td><td>F6K11_15280</td><td>Cyanobacteria</td><td>Leptolyngbya sp. SIO3F4</td><td>hypothetical protein F6K11_15280, partial [Leptolyngbya sp. SIO3F4].</td><td>GCA_010672145.1</td></tr>
<tr><td>NEQ51880.1</td><td>LRR-repeats+AP-GTPase+COR+TM+TM*→</td><td>LRR-repeats+AP-GTPase+COR+TM+TM</td><td>1022</td><td>F6K11_17360</td><td>Cyanobacteria</td><td>Leptolyngbya sp. SIO3F4</td><td>GTP-binding protein [Leptolyngbya sp. SIO3F4].</td><td>GCA_010672145.1</td></tr>
<tr><td>NEQ52141.1</td><td>LRR-repeats+AP-GTPase+COR+TIR*→</td><td>LRR-repeats+AP-GTPase+COR+TIR</td><td>825</td><td>F6K11_18705</td><td>Cyanobacteria</td><td>Leptolyngbya sp. SIO3F4</td><td>GTP-binding protein, partial [Leptolyngbya sp. SIO3F4].</td><td>GCA_010672145.1</td></tr>
<tr><td>NEQ52634.1</td><td>LRR-repeats+AP-GTPase+COR+TIR*→ LRR-repeats→</td><td>LRR-repeats+AP-GTPase+COR+TIR</td><td>827</td><td>F6K11_21260</td><td>Cyanobacteria</td><td>Leptolyngbya sp. SIO3F4</td><td>TIR domain-containing protein, partial [Leptolyngbya sp. SIO3F4].</td><td>GCA_010672145.1</td></tr>
<tr><td>NEQ54121.1</td><td>LRR-repeats+AP-GTPase+COR+TIR*→</td><td>LRR-repeats+AP-GTPase+COR+TIR</td><td>826</td><td>F6K11_28985</td><td>Cyanobacteria</td><td>Leptolyngbya sp. SIO3F4</td><td>TIR domain-containing protein, partial [Leptolyngbya sp. SIO3F4].</td><td>GCA_010672145.1</td></tr>
<tr><td>NEQ54707.1</td><td>LRR-repeats+AP-GTPase+COR*→</td><td>LRR-repeats+AP-GTPase+COR</td><td>547</td><td>F6K11_32030</td><td>Cyanobacteria</td><td>Leptolyngbya sp. SIO3F4</td><td>GTP-binding protein, partial [Leptolyngbya sp. SIO3F4].</td><td>GCA_010672145.1</td></tr>
<tr><td>NEQ55863.1</td><td>LRR-repeats+AP-GTPase+COR*→</td><td>LRR-repeats+AP-GTPase+COR</td><td>335</td><td>F6K11_38165</td><td>Cyanobacteria</td><td>Leptolyngbya sp. SIO3F4</td><td>serine/threonine protein kinase, partial [Leptolyngbya sp. SIO3F4].</td><td>GCA_010672145.1</td></tr>
<tr><td>NEQ58292.1</td><td>SIG+TM+TM+TM+TM+TM+TM+TM→?→ ABC-ATPase→ ABC-ATPase→?→||<-?||LRR-repeats+AP-GTPase+COR*→?→||<-?<-HNH<-PSE||?→||<-Cluster5_5clades</td><td>LRR-repeats+AP-GTPase+COR</td><td>849</td><td>F6K53_13090</td><td>Cyanobacteria</td><td>Moorea sp. SIO4A1</td><td>GTPase [Moorea sp. SIO4A1].</td><td>GCA_010672065.1</td></tr>
<tr><td>NEQ85055.1</td><td>LRR-repeats+AP-GTPase+COR*→</td><td>LRR-repeats+AP-GTPase+COR</td><td>554</td><td>F6K26_34455</td><td>Cyanobacteria</td><td>Moorea sp. SIO2I5</td><td>GTPase, partial [Moorea sp. SIO2I5].</td><td>GCA_010672285.1</td></tr>
<tr><td>NER03418.1</td><td>LRR-repeats+AP-GTPase+COR*→</td><td>LRR-repeats+AP-GTPase+COR</td><td>738</td><td>F6K17_12770</td><td>Cyanobacteria</td><td>Okeania sp. SIO3C4</td><td>GTPase, partial [Okeania sp. SIO3C4].</td><td>GCA_010672215.1</td></tr>
<tr><td>NER25299.1</td><td>TM+TM+TM+TM+TM+TM→||<-?||?→ Pkinase→?→ LRR-repeats+AP-GTPase+COR*→ Cluster5_5clades→</td><td>LRR-repeats+AP-GTPase+COR</td><td>1031</td><td>F6J96_32295</td><td>Cyanobacteria</td><td>Symploca sp. SIO1C2</td><td>hypothetical protein F6J96_32295 [Symploca sp. SIO1C2].</td><td>GCA_010672925.1</td></tr>
</table>

<table>
<tr><th>acc</th><th>operon</th><th>architecture</th><th>len</th><th>gen.name</th><th>taxend</th><th>species</th><th>defline</th><th>gca</th></tr>
<tr><td>NER33110.1</td><td>SIG+TM+TM+TM+TM+TM+TM+TM→?→||<-?||PSE→||<-?||?→ LRR-repeats+AP-GTPase+COR*→?→?→?→ ABC_membrane+ABC_tran→||<-NUDIX</td><td>LRR-repeats+AP-GTPase+COR</td><td>1105</td><td>F6J93_03360</td><td>Cyanobacteria</td><td>Oscillatoria sp. SIO1A7</td><td>hypothetical protein F6J93_03360 [Oscillatoria sp. SIO1A7].</td><td>GCA_010672965.1</td></tr>
<tr><td>NER35832.1</td><td>LRR-repeats+AP-GTPase+COR*→?→ PIN→</td><td>LRR-repeats+AP-GTPase+COR</td><td>1467</td><td>F6J93_17900</td><td>Cyanobacteria</td><td>Oscillatoria sp. SIO1A7</td><td>hypothetical protein F6J93_17900 [Oscillatoria sp. SIO1A7].</td><td>GCA_010672965.1</td></tr>
<tr><td>NER38074.1</td><td><-SIG+TM+TM+TM+TM+TM<-ABC-ATPase||?→?→||<-?||?→?→ LRR-repeats+AP-GTPase+COR*→?→?→?→?→ ABC-ATPase→</td><td>LRR-repeats+AP-GTPase+COR</td><td>1117</td><td>F6J93_29640</td><td>Cyanobacteria</td><td>Oscillatoria sp. SIO1A7</td><td>hypothetical protein F6J93_29640 [Oscillatoria sp. SIO1A7].</td><td>GCA_010672965.1</td></tr>
<tr><td>NER40156.1</td><td>LRR-repeats→ LRR-repeats+AP-GTPase+COR*→</td><td>LRR-repeats+AP-GTPase+COR</td><td>515</td><td>F6J93_40580</td><td>Cyanobacteria</td><td>Oscillatoria sp. SIO1A7</td><td>hypothetical protein F6J93_40580 [Oscillatoria sp. SIO1A7].</td><td>GCA_010672965.1</td></tr>
<tr><td>NER70803.1</td><td>SIG+DM13→ HISKIN→||<-?<-?<-?||?→ Cluster2343_2clades→ LRR-repeats+AP-GTPase+COR*→||<-Cluster1740_2clades<-?<-?<-?<-?<-Cluster1476_2clades</td><td>LRR-repeats+AP-GTPase+COR</td><td>1150</td><td>F6J95_01375</td><td>-</td><td>Leptolyngbya sp. SIO1E4</td><td>GTPase [Leptolyngbya sp. SIO1E4].</td><td>-</td></tr>
<tr><td>NER71871.1</td><td>BACTERIALFRINGE+TM+TM→?→?→ SIG+TM+TM+TM+TM+TM+TM+TM→||<-?<-?<-?||LRR-repeats+AP-GTPase+COR+DUF4404*→||<-?<-TM+TM||NACHT→||<-?<-Cluster5_5clades<-Aminotran_1_2</td><td>LRR-repeats+AP-GTPase+COR+DUF4404</td><td>1283</td><td>F6J95_06845</td><td>-</td><td>Leptolyngbya sp. SIO1E4</td><td>GTP-binding protein [Leptolyngbya sp. SIO1E4].</td><td>-</td></tr>
<tr><td>NER73482.1</td><td>Cluster2482_2clades→?→||<-?||GNTR-HTH→?→ LRR-repeats+AP-GTPase+COR*→ Cluster1404_2clades→?→ Cluster2050_2clades→</td><td>LRR-repeats+AP-GTPase+COR</td><td>891</td><td>F6J95_15105</td><td>-</td><td>Leptolyngbya sp. SIO1E4</td><td>GTPase [Leptolyngbya sp. SIO1E4].</td><td>-</td></tr>
<tr><td>NER74435.1</td><td>LRR-repeats+AP-GTPase+COR+TIR*→ DSBH→?→?→?→||<-?<-?<-ACT</td><td>LRR-repeats+AP-GTPase+COR+TIR</td><td>1183</td><td>F6J95_19985</td><td>-</td><td>Leptolyngbya sp. SIO1E4</td><td>TIR domain-containing protein [Leptolyngbya sp. SIO1E4].</td><td>-</td></tr>
<tr><td>NER74649.1</td><td>Cluster2260_2clades→?→ LRR-repeats+AP-GTPase+COR+DrHyd*→ SIG+FAD-NAD-dep-oxidoreductase→||<-Cluster850_2clades||PSE→ SIG+TM+TM+TM+TM→</td><td>LRR-repeats+AP-GTPase+COR+DrHyd</td><td>1173</td><td>F6J95_21075</td><td>-</td><td>Leptolyngbya sp. SIO1E4</td><td>hypothetical protein F6J95_21075 [Leptolyngbya sp. SIO1E4].</td><td>-</td></tr>
<tr><td>NER78030.1</td><td>Aminotran_1_2→ LRR-repeats+AP-GTPase+COR*→</td><td>LRR-repeats+AP-GTPase+COR</td><td>904</td><td>F6K42_00285</td><td>Cyanobacteria</td><td>Leptolyngbya sp. SIO1D8</td><td>GTP-binding protein [Leptolyngbya sp. SIO1D8].</td><td>GCA_010672895.1</td></tr>
<tr><td>NER81647.1</td><td>LRR-repeats→ LRR-repeats+AP-GTPase+COR+TIR*→</td><td>LRR-repeats+AP-GTPase+COR+TIR</td><td>836</td><td>F6K42_19200</td><td>Cyanobacteria</td><td>Leptolyngbya sp. SIO1D8</td><td>TIR domain-containing protein [Leptolyngbya sp. SIO1D8].</td><td>GCA_010672895.1</td></tr>
<tr><td>NER83464.1</td><td>LRR-repeats+AP-GTPase+COR+TIR*→</td><td>LRR-repeats+AP-GTPase+COR+TIR</td><td>785</td><td>F6K42_28715</td><td>Cyanobacteria</td><td>Leptolyngbya sp. SIO1D8</td><td>TIR domain-containing protein [Leptolyngbya sp. SIO1D8].</td><td>GCA_010672895.1</td></tr>
<tr><td>NER88741.1</td><td>ABC-ATPase→||<-?||ABC-ATPase→?→?→||<-?<-?||LRR-repeats+AP-GTPase+COR*→?→||<-?<-?||?→ PSE→?→||<-Cluster5_5clades</td><td>LRR-repeats+AP-GTPase+COR</td><td>856</td><td>F6K64_17615</td><td>Cyanobacteria</td><td>Moorea sp. SIO3A2</td><td>leucine-rich repeat protein [Moorea sp. SIO3A2].</td><td>GCA_010672265.1</td></tr>
<tr><td>NER98009.1</td><td>TM+TM+TM+TM+TM+TM→||<-?||?→ Pkinase→?→?→ LRR-repeats+AP-GTPase+COR*→ COR→</td><td>LRR-repeats+AP-GTPase+COR</td><td>417</td><td>F6J86_29885</td><td>Cyanobacteria</td><td>Symploca sp. SIO1B1</td><td>hypothetical protein F6J86_29885 [Symploca sp. SIO1B1].</td><td>GCA_010672945.1</td></tr>
<tr><td>NES02436.1</td><td>LRR-repeats+AP-GTPase+COR*→</td><td>LRR-repeats+AP-GTPase+COR</td><td>964</td><td>F6K22_06035</td><td>Cyanobacteria</td><td>Okeania sp. SIO2F4</td><td>leucine-rich repeat protein [Okeania sp. SIO2F4].</td><td>GCA_010672385.1</td></tr>
<tr><td>NES08071.1</td><td>LRR-repeats+AP-GTPase+COR*→</td><td>LRR-repeats+AP-GTPase+COR</td><td>502</td><td>F6K22_38010</td><td>Cyanobacteria</td><td>Okeania sp. SIO2F4</td><td>leucine-rich repeat protein, partial [Okeania sp. SIO2F4].</td><td>GCA_010672385.1</td></tr>
<tr><td>NES23982.1</td><td>LRR-repeats+AP-GTPase+COR*→</td><td>LRR-repeats+AP-GTPase+COR</td><td>230</td><td>F6K41_34995</td><td>Cyanobacteria</td><td>Caldora sp. SIO3E6</td><td>hypothetical protein F6K41_34995, partial [Caldora sp. SIO3E6].</td><td>GCA_010672185.1</td></tr>
<tr><td>NES70611.1</td><td>LRR-repeats→ LRR-repeats+AP-GTPase+COR*→</td><td>LRR-repeats+AP-GTPase+COR</td><td>400</td><td>F6K24_38075</td><td>Cyanobacteria</td><td>Okeania sp. SIO2D1</td><td>GTPase, partial [Okeania sp. SIO2D1].</td><td>GCA_010672395.1</td></tr>
<tr><td>NES87329.1</td><td>LRR-repeats+AP-GTPase+COR+DUF4404*→</td><td>LRR-repeats+AP-GTPase+COR+DUF4404</td><td>855</td><td>F6K10_41780</td><td>Cyanobacteria</td><td>Moorea sp. SIO2B7</td><td>GTPase, partial [Moorea sp. SIO2B7].</td><td>GCA_010672555.1</td></tr>
<tr><td>NES99177.1</td><td><-NUDIX||?→?→ LRR-repeats+AP-GTPase+COR+DUF4404*→ PSE→?→?→?→||<-ABC-ATPase</td><td>LRR-repeats+AP-GTPase+COR+DUF4404</td><td>1012</td><td>F6K61_01110</td><td>Cyanobacteria</td><td>Sphaerospermopsis sp. SIO1G1</td><td>GTP-binding protein [Sphaerospermopsis sp. SIO1G1].</td><td>GCA_010672785.1</td></tr>
<tr><td>NET13009.1</td><td>LRR-repeats+AP-GTPase+COR*→</td><td>LRR-repeats+AP-GTPase+COR</td><td>307</td><td>F6K08_09185</td><td>Cyanobacteria</td><td>Okeania sp. SIO1H6</td><td>GTPase, partial [Okeania sp. SIO1H6].</td><td>GCA_010672645.1</td></tr>
<tr><td>NET29408.1</td><td>LRR-repeats+AP-GTPase+COR*→||<-?<-NACHT+68TM-wHTH||?→||<-?<-PSE<-TPR-repeats<-HISKIN</td><td>LRR-repeats+AP-GTPase+COR</td><td>1247</td><td>F6K01_30010</td><td>Cyanobacteria</td><td>Okeania sp. SIO1I7</td><td>leucine-rich repeat protein [Okeania sp. SIO1I7].</td><td>GCA_010672585.1</td></tr>
<tr><td>NET38765.1</td><td>LRR-repeats+AP-GTPase+COR*→</td><td>LRR-repeats+AP-GTPase+COR</td><td>802</td><td>F6K19_43415</td><td>Cyanobacteria</td><td>Cyanothece sp. SIO1E1</td><td>GTPase [Cyanothece sp. SIO1E1].</td><td>GCA_010672835.1</td></tr>
<tr><td>NET39396.1</td><td>LRR-repeats+AP-GTPase+COR+TIR*→</td><td>LRR-repeats+AP-GTPase+COR+TIR</td><td>803</td><td>F6K19_46760</td><td>Cyanobacteria</td><td>Cyanothece sp. SIO1E1</td><td>TIR domain-containing protein, partial [Cyanothece sp. SIO1E1].</td><td>GCA_010672835.1</td></tr>
<tr><td>NET43146.1</td><td><-RADICAL-SAM||?→ REC→?→||<-PSE||LRR-repeats+AP-GTPase+COR*→||<-?<-NACHT+68TM-wHTH</td><td>LRR-repeats+AP-GTPase+COR</td><td>927</td><td>F6K15_15105</td><td>Cyanobacteria</td><td>Okeania sp. SIO2B3</td><td>GTPase [Okeania sp. SIO2B3].</td><td>GCA_010672525.1</td></tr>
<tr><td>NET44979.1</td><td>LRR-repeats+AP-GTPase+COR*→||<-?<-PSE<-?<-?||?→?→||<-PSE<-PSE<-PSE<-Cluster5_5clades<-ABC_membrane+ABC_tran</td><td>LRR-repeats+AP-GTPase+COR</td><td>1085</td><td>F6K15_25170</td><td>Cyanobacteria</td><td>Okeania sp. SIO2B3</td><td>GTPase [Okeania sp. SIO2B3].</td><td>GCA_010672525.1</td></tr>
<tr><td>NET45691.1</td><td><-HISKIN||?→?→ LRR-repeats+AP-GTPase+COR*→||<-PSE<-?<-?<-NUDIX<-?||?→ TPR-repeats→</td><td>LRR-repeats+AP-GTPase+COR</td><td>856</td><td>F6K15_29075</td><td>Cyanobacteria</td><td>Okeania sp. SIO2B3</td><td>GTPase [Okeania sp. SIO2B3].</td><td>GCA_010672525.1</td></tr>
<tr><td>NET64212.1</td><td>SIG+TM+TM+TM+TM+TM+TM→ ABC-ATPase→||<-?||ABC-ATPase→?→?→||<-?||LRR-repeats+AP-GTPase+COR*→?→||<-?<-?||?→||<-Cluster5_5clades</td><td>LRR-repeats+AP-GTPase+COR</td><td>914</td><td>F6K63_07330</td><td>Cyanobacteria</td><td>Moorea sp. SIO1G6</td><td>leucine-rich repeat protein [Moorea sp. SIO1G6].</td><td>GCA_010672795.1</td></tr>
</table>

<table>
<thead>
<tr><th>acc</th><th>operon</th><th>architecture</th><th>len</th><th>gen.name</th><th>taxend</th><th>species</th><th>defline</th><th>gca</th></tr>
</thead>
<tbody>
<tr><td>NET68629.1</td><td>REC→||<-TPR-repeats<-?||LRR-repeats+AP-GTPase+COR+DUF4404*→</td><td>LRR-repeats+AP-GTPase+COR+DUF4404</td><td>938</td><td>F6K63_31235</td><td>Cyanobacteria</td><td>Moorea sp. SIO1G6</td><td>GTPase [Moorea sp. SIO1G6].</td><td>GCA_010672795.1</td></tr>
<tr><td>NET73474.1</td><td>LRR-repeats+AP-GTPase+COR+DUF4404*→</td><td>LRR-repeats+AP-GTPase+COR+DUF4404</td><td>821</td><td>F6K62_21805</td><td>Cyanobacteria</td><td>Sphaerospermopsis sp. SIO1G2</td><td>GTP-binding protein, partial [Sphaerospermopsis sp. SIO1G2].</td><td>GCA_010672725.1</td></tr>
<tr><td>NET83099.1</td><td>REC→||<-?||REC→||<-TPR-repeats||LRR-repeats+AP-GTPase+COR+DUF4404*→</td><td>LRR-repeats+AP-GTPase+COR+DUF4404</td><td>946</td><td>F6J94_14560</td><td>Cyanobacteria</td><td>Moorea sp. SIO1F2</td><td>GTPase [Moorea sp. SIO1F2].</td><td>GCA_010672755.1</td></tr>
<tr><td>NEZ62303.1</td><td>PIN→ LRR-repeats→ LRR-repeats+AP-GTPase+COR*→?→ COR+TIR→ SIG+Trypsin+PDZ→</td><td>LRR-repeats+AP-GTPase+COR</td><td>318</td><td>D0962_05850</td><td>Cyanobacteria</td><td>Leptolyngbyaceae cyanobacterium CCMR0082</td><td>hypothetical protein D0962_05850 [Leptolyngbyaceae cyanobacterium CCMR0082].</td><td>GCA_011009535.1</td></tr>
<tr><td>NIM11849.1</td><td>S1COLD→||<-?<-?<-?||?→ Cluster54_3clades→ Cluster1093_2clades→ LRR-repeats+AP-GTPase+COR*→||<-?<-Cluster2413_2clades</td><td>LRR-repeats+AP-GTPase+COR</td><td>882</td><td>GTO81_07705</td><td>Bacteria</td><td>Candidatus Aminicenantes bacterium</td><td>GTP-binding protein [Candidatus Aminicenantes bacterium].</td><td>GCA_011771225.1</td></tr>
<tr><td>NIM14508.1</td><td>Aminotran_1_2→||<-?<-Cluster1540_2clades<-?||?→?→ LRR-repeats+AP-GTPase+COR+TIR*→</td><td>LRR-repeats+AP-GTPase+COR+TIR</td><td>784</td><td>GTO81_21225</td><td>Bacteria</td><td>Candidatus Aminicenantes bacterium</td><td>TIR domain-containing protein [Candidatus Aminicenantes bacterium].</td><td>GCA_011771225.1</td></tr>
<tr><td>NIM17826.1</td><td>LRR-repeats+AP-GTPase+COR*→</td><td>LRR-repeats+AP-GTPase+COR</td><td>952</td><td>GTO81_38090</td><td>Bacteria</td><td>Candidatus Aminicenantes bacterium</td><td>hypothetical protein GTO81_38090 [Candidatus Aminicenantes bacterium].</td><td>GCA_011771225.1</td></tr>
<tr><td>NIN06216.1</td><td>LRR-repeats+AP-GTPase+COR*→</td><td>LRR-repeats+AP-GTPase+COR</td><td>781</td><td>GTO43_07600</td><td>Armatimonadetes</td><td>Armatimonadetes bacterium</td><td>GTPase, partial [Armatimonadetes bacterium].</td><td>GCA_011771945.1</td></tr>
<tr><td>NIP25303.1</td><td><-SIG+TIMbarrel||LRR-repeats+AP-GTPase+COR+TIR*→</td><td>LRR-repeats+AP-GTPase+COR+TIR</td><td>868</td><td>GWO38_16175</td><td>Planctomycetes</td><td>Phycisphaerae bacterium</td><td>TIR domain-containing protein [Phycisphaerae bacterium].</td><td>GCA_011774285.1</td></tr>
<tr><td>NIP41904.1</td><td>LRR-repeats+AP-GTPase+COR*→</td><td>LRR-repeats+AP-GTPase+COR</td><td>457</td><td>GWO28_04170</td><td>FCB group</td><td>candidate division Zixibacteria bacterium</td><td>GTPase, partial [candidate division Zixibacteria bacterium].</td><td>GCA_011774485.1</td></tr>
<tr><td>NIR09229.1</td><td>LRR-repeats+AP-GTPase*→ COR→</td><td>LRR-repeats+AP-GTPase</td><td>251</td><td>GTN82_27750</td><td>Bacteria</td><td>Candidatus Aminicenantes bacterium</td><td>GTP-binding protein, partial [Candidatus Aminicenantes bacterium].</td><td>GCA_011773265.1</td></tr>
<tr><td>NIU83786.1</td><td>LRR-repeats+AP-GTPase+COR*→</td><td>LRR-repeats+AP-GTPase+COR</td><td>888</td><td>GWN64_10000</td><td>Asgard group</td><td>Candidatus Thorarchaeota archaeon</td><td>GTPase [Candidatus Thorarchaeota archaeon].</td><td>GCA_011775775.1</td></tr>
<tr><td>NJD78109.1</td><td>SAM-methylase→||<-?||?→?→||<-?||?→?→ LRR-repeats+AP-GTPase+COR*→</td><td>LRR-repeats+AP-GTPase+COR</td><td>844</td><td>FIB08_13625</td><td>Euryarchaeota</td><td>Candidatus Methanoperedens sp.</td><td>GTP-binding protein [Candidatus Methanoperedens sp.].</td><td>GCA_012026835.1</td></tr>
<tr><td>NJK31611.1</td><td>LRR-repeats+AP-GTPase+COR*→</td><td>LRR-repeats+AP-GTPase+COR</td><td>784</td><td>HC927_03900</td><td>Deltaproteobacteria</td><td>Deltaproteobacteria bacterium</td><td>hypothetical protein HC927_03900 [Deltaproteobacteria bacterium].</td><td>GCA_012031025.1</td></tr>
<tr><td>NJK68783.1</td><td>LRR-repeats+AP-GTPase+COR*→?→?→?→?→||<-Pentapeptide-repeats</td><td>LRR-repeats+AP-GTPase+COR</td><td>637</td><td>HC941_21705</td><td>Cyanobacteria</td><td>Microcoleus sp. SU_5_3</td><td>GTP-binding protein, partial [Microcoleus sp. SU_5_3].</td><td>GCA_012030835.1</td></tr>
<tr><td>NJK99654.1</td><td>LRR-repeats+AP-GTPase+COR*→</td><td>LRR-repeats+AP-GTPase+COR</td><td>665</td><td>HC910_03475</td><td>Cyanobacteria</td><td>Spirulinaceae cyanobacterium SM2_1_0</td><td>GTP-binding protein [Spirulinaceae cyanobacterium SM2_1_0].</td><td>GCA_012031265.1</td></tr>
<tr><td>NJL39962.1</td><td>LRR-repeats+AP-GTPase+COR*→</td><td>LRR-repeats+AP-GTPase+COR</td><td>814</td><td>HC899_26815</td><td>Cyanobacteria</td><td>Leptolyngbyaceae cyanobacterium SM1_4_3</td><td>GTP-binding protein [Leptolyngbyaceae cyanobacterium SM1_4_3].</td><td>GCA_012031415.1</td></tr>
<tr><td>NJL55090.1</td><td>LRR-repeats+AP-GTPase+COR+TIR*→||<-REase-4||?→ Cluster5_5clades→?→||<-Cluster2398_2clades</td><td>LRR-repeats+AP-GTPase+COR+TIR</td><td>879</td><td>HC928_07795</td><td>unclassified Bacteria</td><td>bacterium</td><td>TIR domain-containing protein [bacterium].</td><td>GCA_012031555.1</td></tr>
<tr><td>NJL55819.1</td><td>Cluster1137_2clades→?→ LRR-repeats+AP-GTPase+COR*→ PSE→?→||<-Cluster1102_3clades</td><td>LRR-repeats+AP-GTPase+COR</td><td>671</td><td>HC928_11965</td><td>unclassified Bacteria</td><td>bacterium</td><td>GTP-binding protein [bacterium].</td><td>GCA_012031555.1</td></tr>
<tr><td>NJL58018.1</td><td>LRR-repeats+AP-GTPase+COR*→ COR→</td><td>LRR-repeats+AP-GTPase+COR</td><td>365</td><td>HC928_25050</td><td>unclassified Bacteria</td><td>bacterium</td><td>hypothetical protein HC928_25050, partial [bacterium].</td><td>GCA_012031555.1</td></tr>
<tr><td>NJL60049.1</td><td>LRR-repeats+AP-GTPase+COR*→ COR+TIR→</td><td>LRR-repeats+AP-GTPase+COR</td><td>389</td><td>HC887_10835</td><td>Deltaproteobacteria</td><td>Desulfobacteraceae bacterium</td><td>hypothetical protein HC887_10835 [Desulfobacteraceae bacterium].</td><td>GCA_012031825.1</td></tr>
<tr><td>NJL77569.1</td><td>LRR-repeats+AP-GTPase+COR*→</td><td>LRR-repeats+AP-GTPase+COR</td><td>408</td><td>HC892_23555</td><td>Bacteroidetes</td><td>Saprospiraceae bacterium</td><td>hypothetical protein HC892_23555, partial [Saprospiraceae bacterium].</td><td>GCA_012031785.1</td></tr>
<tr><td>NJL83760.1</td><td>LRR-repeats+AP-GTPase+COR*→</td><td>LRR-repeats+AP-GTPase+COR</td><td>688</td><td>HC890_14000</td><td>Chloroflexi</td><td>Chloroflexaceae bacterium</td><td>GTP-binding protein [Chloroflexaceae bacterium].</td><td>GCA_012031815.1</td></tr>
</tbody>
</table>

acc	operon	architecture	len	gen.name	taxend	species	defline	gca
NJL90622.1	<-RelE-ParE<-?||LRR-repeats+AP-GTPase+COR*→?→ PSE→?→ PSE→ RNA-Helicase→	LRR-repeats+AP-GTPase+COR	1140	HC916_13170	Cyanobacteria	Coleofasciculaceae cyanobacterium SM2_1_6	GTP-binding protein [Coleofasciculaceae cyanobacterium SM2_1_6].	GCA_012031635.1
NJL91916.1	LRR-repeats+AP-GTPase+COR*→?→ HNH→	LRR-repeats+AP-GTPase+COR	640	HC916_20640	Cyanobacteria	Coleofasciculaceae cyanobacterium SM2_1_6	GTPase, partial [Coleofasciculaceae cyanobacterium SM2_1_6].	GCA_012031635.1
NJM60379.1	<-SIG+TM+TM+TM<-?||TPR-repeats→||<-?<-Cluster5_5clades||?→?→ LRR-repeats+AP-GTPase+COR*→	LRR-repeats+AP-GTPase+COR	729	HC849_09565	Cyanobacteria	Oscillatoriales cyanobacterium RU_3_3	hypothetical protein HC849_09565, partial [Oscillatoriales cyanobacterium RU_3_3].	GCA_012032195.1
NJM78344.1	LRR-repeats+AP-GTPase+COR*→	LRR-repeats+AP-GTPase+COR	500	HC852_24405	Cyanobacteria	Acaryochloridaceae cyanobacterium RU_4_10	hypothetical protein HC852_24405 [Acaryochloridaceae cyanobacterium RU_4_10].	GCA_012032135.1
NJM97967.1	LRR-repeats→ LRR-repeats+AP-GTPase+COR*→ AP-GTPase+COR+TM+TM→	LRR-repeats+AP-GTPase+COR	278	HC800_13155	Cyanobacteria	Phormidesmis sp. RL_2_1	hypothetical protein HC800_13155 [Phormidesmis sp. RL_2_1].	GCA_012033015.1
NJN20692.1	LRR-repeats+AP-GTPase+COR*→ TIR→||<-Cluster5_5clades<-Pentapeptide||REC→	LRR-repeats+AP-GTPase+COR	276	HC812_05240	Cyanobacteria	Leptolyngbya sp. RL_3_1	hypothetical protein HC812_05240, partial [Leptolyngbya sp. RL_3_1].	GCA_012032425.1
NJN57594.1	LRR-repeats+AP-GTPase+COR+TIR*→	LRR-repeats+AP-GTPase+COR+TIR	1087	HC879_08885	Cyanobacteria	Leptolyngbyaceae cyanobacterium SL_5_9	TIR domain-containing protein [Leptolyngbyaceae cyanobacterium SL_5_9].	GCA_012032565.1
NJN63246.1	LRR-repeats+AP-GTPase+COR*→	LRR-repeats+AP-GTPase+COR	747	HC795_18555	Cyanobacteria	Coleofasciculaceae cyanobacterium RL_1_1	hypothetical protein HC795_18555, partial [Coleofasciculaceae cyanobacterium RL_1_1].	GCA_012033105.1
NJN72933.1	LRR-repeats+AP-GTPase+COR+DUF4404*→||<-?||?→ SIG+Classical-AAA→	LRR-repeats+AP-GTPase+COR+DUF4404	832	HC799_09060	Cyanobacteria	Limnothrix sp. RL_2_0	GTP-binding protein [Limnothrix sp. RL_2_0].	GCA_012033055.1
NJN75229.1	LRR-repeats+AP-GTPase→ AP-GTPase*→?→ COR+TM+TM+TM→	AP-GTPase	180	HC796_01955	Cyanobacteria	Synechococcaceae cyanobacterium RL_1_2	hypothetical protein HC796_01955 [Synechococcaceae cyanobacterium RL_1_2].	GCA_012033095.1
NJN88810.1	LRR-repeats+AP-GTPase+COR*→	LRR-repeats+AP-GTPase+COR	1016	HC881_24165	Cyanobacteria	Leptolyngbyaceae cyanobacterium SL_7_1	GTP-binding protein [Leptolyngbyaceae cyanobacterium SL_7_1].	GCA_012032525.1
NJN89941.1	LRR-repeats+AP-GTPase+COR*→	LRR-repeats+AP-GTPase+COR	814	HC878_05910	Cyanobacteria	Leptolyngbyaceae cyanobacterium SL_5_14	GTP-binding protein [Leptolyngbyaceae cyanobacterium SL_5_14].	GCA_012032515.1
NJN96139.1	Cluster1841_2clades→?→ LRR-repeats→ LRR-repeats+AP-GTPase+COR*→ Cluster2316_2clades→ Cluster2224_2clades→?→ Cluster1087_2clades→||<-?<-?<-ABC-ATPase	LRR-repeats+AP-GTPase+COR	573	HC875_19545	Chloroflexi	Anaerolineales bacterium	GTP-binding protein [Anaerolineales bacterium].	GCA_012032575.1
NJO03971.1	LRR-repeats+AP-GTPase+COR+EAD7*→	LRR-repeats+AP-GTPase+COR+EAD7	814	HC880_21890	Bacteroidetes	Bacteroidia bacterium	hypothetical protein HC880_21890 [Bacteroidia bacterium].	GCA_012033155.1
NJO04035.1	LRR-repeats+AP-GTPase+COR+EAD7*→	LRR-repeats+AP-GTPase+COR+EAD7	843	HC880_22270	Bacteroidetes	Bacteroidia bacterium	hypothetical protein HC880_22270 [Bacteroidia bacterium].	GCA_012033155.1
NJO50334.1	LRR-repeats+AP-GTPase+COR*→	LRR-repeats+AP-GTPase+COR	510	HC840_13875	Cyanobacteria	Leptolyngbyaceae cyanobacterium RM2_2_4	GTP-binding protein, partial [Leptolyngbyaceae cyanobacterium RM2_2_4].	GCA_012033305.1
NJP08786.1	<-Cluster5_5clades||?→?→?→?→ LRR-repeats+AP-GTPase+COR*→	LRR-repeats+AP-GTPase+COR	841	HC866_04300	Cyanobacteria	Leptolyngbyaceae cyanobacterium RU_5_1	GTPase [Leptolyngbyaceae cyanobacterium RU_5_1].	GCA_012034055.1
NJR23767.1	GTPase-AIG→?→?→ LRR-repeats+AP-GTPase+COR+DUF4404*→	LRR-repeats+AP-GTPase+COR+DUF4404	1061	HC786_17240	Cyanobacteria	Richelia sp. CSU_2_1	GTP-binding protein [Richelia sp. CSU_2_1].	GCA_012034575.1
NJR25667.1	LRR-repeats+AP-GTPase+COR*→	LRR-repeats+AP-GTPase+COR	856	HC786_27780	Cyanobacteria	Richelia sp. CSU_2_1	hypothetical protein HC786_27780, partial [Richelia sp. CSU_2_1].	GCA_012034575.1
NJR41095.1	LRR-repeats+AP-GTPase+COR+TIR*→	LRR-repeats+AP-GTPase+COR+TIR	677	HC781_22475	Cyanobacteria	Leptolyngbyaceae cyanobacterium CSU_1_4	GTP-binding protein [Leptolyngbyaceae cyanobacterium CSU_1_4].	GCA_012034615.1
NJR68835.1	<-SIGMA-HTH<-?<-?<-?||?→ LRR-repeats+AP-GTPase+COR+TCAD1*→?→||<-?<-?<-?||?→||<-TPR-repeats	LRR-repeats+AP-GTPase+COR+TCAD1	892	HC771_09325	Cyanobacteria	Synechococcales cyanobacterium CRU_2_2	GTPase [Synechococcales cyanobacterium CRU_2_2].	GCA_012034805.1

<table>
<tr><th>acc</th><th>operon</th><th>architecture</th><th>len</th><th>gen.name</th><th>taxend</th><th>species</th><th>defline</th><th>gca</th></tr>
<tr><td>NJS09810.1</td><td>GTPase-AIG→?→?→||<-?<-?||LRR-repeats+AP-GTPase+COR*→?→?→?→?→||<-Pentapeptide-repeats</td><td>LRR-repeats+AP-GTPase+COR</td><td>761</td><td>HC789_05185</td><td>Cyanobacteria</td><td>Microcoleus sp. CSU_2_2</td><td>GTP-binding protein [Microcoleus sp. CSU_2_2].</td><td>GCA_012035135.1</td></tr>
<tr><td>NKC15125.1</td><td>LRR-repeats+AP-GTPase+COR+DrHyd*→</td><td>LRR-repeats+AP-GTPase+COR+DrHyd</td><td>1164</td><td>GKR94_23910</td><td>Gammaproteobacteria</td><td>Gammaproteobacteria bacterium</td><td>hypothetical protein GKR94_23910 [Gammaproteobacteria bacterium].</td><td>GCA_012103455.1</td></tr>
<tr><td>NKQ34928.1</td><td>RelE-ParE→?→?→ PSE→ LRR-repeats+AP-GTPase+COR+EAD7*→</td><td>LRR-repeats+AP-GTPase+COR+EAD7</td><td>553</td><td>HF973_04850</td><td>Chloroflexi</td><td>Chloroflexi bacterium</td><td>hypothetical protein HF973_04850 [Chloroflexi bacterium].</td><td>GCA_012329075.1</td></tr>
<tr><td>NLT19133.1</td><td>LRR-repeats+AP-GTPase+COR*→</td><td>LRR-repeats+AP-GTPase+COR</td><td>709</td><td>GXY10_07015</td><td>Firmicutes</td><td>Clostridiales bacterium</td><td>GTP-binding protein, partial [Clostridiales bacterium].</td><td>GCA_012718845.1</td></tr>
<tr><td>NMF86259.1</td><td>RelE→ CHTH→?→||<-?<-?<-?<-?||LRR-repeats+AP-GTPase+COR+TIR*→?→||<-?<-?||?→?→||<-?<-RelE-ParE</td><td>LRR-repeats+AP-GTPase+COR+TIR</td><td>1176</td><td>E1H13_23515</td><td>Cyanobacteria</td><td>Nodosilinea sp. P-1105</td><td>TIR domain-containing protein [Nodosilinea sp. P-1105].</td><td>GCA_012911975.1</td></tr>
<tr><td>NND77029.1</td><td>LRR-repeats+AP-GTPase+COR*→||<-?<-?<-?<-?<-SIG+TM+TM+TM+TM</td><td>LRR-repeats+AP-GTPase+COR</td><td>842</td><td>HKN39_02455</td><td>Bacteroidetes</td><td>Flavobacteriales bacterium</td><td>hypothetical protein HKN39_02455 [Flavobacteriales bacterium].</td><td>GCA_013002285.1</td></tr>
<tr><td>NOG45111.1</td><td>MORC→?→ LRR-repeats→ LRR-repeats+AP-GTPase+COR*→?→||<-?<-?<-BetaPropeller+BetaPropeller</td><td>LRR-repeats+AP-GTPase+COR</td><td>652</td><td>HND50_07765</td><td>Calditrichaeota</td><td>Calditrichaeota bacterium</td><td>hypothetical protein HND50_07765 [Calditrichaeota bacterium].</td><td>GCA_013112635.1</td></tr>
<tr><td>NOH03519.1</td><td>DAGKIN→ Cluster1917_2clades→?→||<-?<-?||LRR-repeats→ PSE→ LRR-repeats+AP-GTPase+COR*→?→||<-PSE||?→||<-?<-GTPase-AIG||Thioredoxin→</td><td>LRR-repeats+AP-GTPase+COR</td><td>345</td><td>HND47_16930</td><td>Chloroflexi</td><td>Chloroflexi bacterium</td><td>hypothetical protein HND47_16930 [Chloroflexi bacterium].</td><td>GCA_013112685.1</td></tr>
<tr><td>NOH03560.1</td><td>REase→ LRR-repeats+AP-GTPase+COR→ LRR-repeats+AP-GTPase+COR→</td><td>LRR-repeats+AP-GTPase+COR</td><td>878</td><td>HND47_17170</td><td>Chloroflexi</td><td>Chloroflexi bacterium</td><td>hypothetical protein HND47_17170 [Chloroflexi bacterium].</td><td>GCA_013112685.1</td></tr>
<tr><td>NOH03561.1</td><td>LRR-repeats+AP-GTPase+COR*→</td><td>LRR-repeats+AP-GTPase+COR</td><td>852</td><td>HND47_17175</td><td>Chloroflexi</td><td>Chloroflexi bacterium</td><td>GTP-binding protein [Chloroflexi bacterium].</td><td>GCA_013112685.1</td></tr>
<tr><td>NOH03734.1</td><td>PIN→ PSE→?→?→ LRR-repeats+AP-GTPase+COR*→?→?→?→ PSE→||<-?<-PSE||?→ Cluster2515_2clades→ Cluster2268_2clades→</td><td>LRR-repeats+AP-GTPase+COR</td><td>973</td><td>HND47_18110</td><td>Chloroflexi</td><td>Chloroflexi bacterium</td><td>hypothetical protein HND47_18110 [Chloroflexi bacterium].</td><td>GCA_013112685.1</td></tr>
<tr><td>NOQ25006.1</td><td>Arginase→ REase→?→?→ LRR-repeats+AP-GTPase+COR+DrHyd*→</td><td>LRR-repeats+AP-GTPase+COR+DrHyd</td><td>1054</td><td>GQ564_06545</td><td>Bacteroidetes</td><td>Bacteroidales bacterium</td><td>GTP-binding protein [Bacteroidales bacterium].</td><td>GCA_013138975.1</td></tr>
<tr><td>NOQ25654.1</td><td>LRR-repeats+AP-GTPase+COR*→ FGS→ PSE→?→||<-?<-?<-REC</td><td>LRR-repeats+AP-GTPase+COR</td><td>1082</td><td>GQ564_09870</td><td>Bacteroidetes</td><td>Bacteroidales bacterium</td><td>GTP-binding protein [Bacteroidales bacterium].</td><td>GCA_013138975.1</td></tr>
<tr><td>NOQ25870.1</td><td>LRR-repeats+AP-GTPase+COR*→ Cluster881_3clades→ Polbeta+HEPN→||<-?<-Cluster998_2clades<-?<-RADICAL-SAM</td><td>LRR-repeats+AP-GTPase+COR</td><td>983</td><td>GQ564_10955</td><td>Bacteroidetes</td><td>Bacteroidales bacterium</td><td>GTP-binding protein [Bacteroidales bacterium].</td><td>GCA_013138975.1</td></tr>
<tr><td>NOQ35071.1</td><td>LRR-repeats+AP-GTPase+COR*→</td><td>LRR-repeats+AP-GTPase+COR</td><td>779</td><td>GQ569_04160</td><td>Gammaproteobacteria</td><td>Methylococcaceae bacterium</td><td>hypothetical protein GQ569_04160 [Methylococcaceae bacterium].</td><td>GCA_013138855.1</td></tr>
<tr><td>NOQ64054.1</td><td>LRR-repeats+AP-GTPase+COR+DrHyd*→</td><td>LRR-repeats+AP-GTPase+COR+DrHyd</td><td>751</td><td>GQ582_06035</td><td>Gammaproteobacteria</td><td>Methyloprofundus sp.</td><td>hypothetical protein GQ582_06035 [Methyloprofundus sp.].</td><td>GCA_013138595.1</td></tr>
<tr><td>NOR78337.1</td><td>LRR-repeats+AP-GTPase+COR*→</td><td>LRR-repeats+AP-GTPase+COR</td><td>537</td><td>GQ523_07950</td><td>Euryarchaeota</td><td>Methanophagales archaeon</td><td>GTP-binding protein, partial [Methanophagales archaeon].</td><td>GCA_013139985.1</td></tr>
<tr><td>NOS87464.1</td><td>LRR-repeats+AP-GTPase+COR+TIR*→</td><td>LRR-repeats+AP-GTPase+COR+TIR</td><td>972</td><td>HOP34_02800</td><td>Gammaproteobacteria</td><td>Methylococcaceae bacterium</td><td>TIR domain-containing protein [Methylococcaceae bacterium].</td><td>GCA_013140465.1</td></tr>
<tr><td>NOT10704.1</td><td>Cluster2349_2clades→ PSE→?→ LRR-repeats+AP-GTPase+COR*→</td><td>LRR-repeats+AP-GTPase+COR</td><td>810</td><td>HOP23_02545</td><td>Gammaproteobacteria</td><td>Methylococcaceae bacterium</td><td>hypothetical protein HOP23_02545 [Methylococcaceae bacterium].</td><td>GCA_013140705.1</td></tr>
<tr><td>NOT87605.1</td><td>PNPase→?→ LRR-repeats+AP-GTPase+COR+TIR*→</td><td>LRR-repeats+AP-GTPase+COR+TIR</td><td>1152</td><td>HOP03_05430</td><td>Gammaproteobacteria</td><td>Lysobacter sp.</td><td>TIR domain-containing protein [Lysobacter sp.].</td><td>GCA_013141175.1</td></tr>
<tr><td>NOU20113.1</td><td>SIGMA-HTH→?→ LRR-repeats+AP-GTPase+COR+DrHyd*→ LRR-repeats+AP-GTPase+COR+DrHyd→</td><td>LRR-repeats+AP-GTPase+COR+DrHyd</td><td>1204</td><td>HOO91_21360</td><td>Bacteroidetes</td><td>Bacteroidales bacterium</td><td>hypothetical protein HOO91_21360 [Bacteroidales bacterium].</td><td>GCA_013141385.1</td></tr>
<tr><td>NOU47191.1</td><td>LRR-repeats+AP-GTPase+COR*→</td><td>LRR-repeats+AP-GTPase+COR</td><td>693</td><td>HOO86_09030</td><td>Bacteroidetes</td><td>Bacteroidales bacterium</td><td>GTP-binding protein [Bacteroidales bacterium].</td><td>GCA_013141455.1</td></tr>
<tr><td>NOZ36014.1</td><td>Cluster1478_2clades→?→?→?→?→ LRR-repeats+AP-GTPase+COR*→||<-?<-?||?→?→ D5ATPase→</td><td>LRR-repeats+AP-GTPase+COR</td><td>842</td><td>GXO80_12040</td><td>Chlorobi</td><td>Chlorobi bacterium</td><td>GTP-binding protein [Chlorobi bacterium].</td><td>GCA_013152535.1</td></tr>
<tr><td>NQT12774.1</td><td>LRR-repeats+AP-GTPase+COR+DUF4404*→</td><td>LRR-repeats+AP-GTPase+COR+DUF4404</td><td>936</td><td>HQ582_08495</td><td>Planctomycetes</td><td>Planctomycetes bacterium</td><td>leucine-rich repeat domain-containing protein, partial [Planctomycetes bacterium].</td><td>GCA_013202485.1</td></tr>
<tr><td>NQT13736.1</td><td>LRR-repeats+AP-GTPase+COR*→ TIR→</td><td>LRR-repeats+AP-GTPase+COR</td><td>583</td><td>HQ582_13365</td><td>Planctomycetes</td><td>Planctomycetes bacterium</td><td>hypothetical protein HQ582_13365, partial [Planctomycetes bacterium].</td><td>GCA_013202485.1</td></tr>
</table>

<table>
<tr><th>acc</th><th>operon</th><th>architecture</th><th>len</th><th>gen.name</th><th>taxend</th><th>species</th><th>defline</th><th>gca</th></tr>
<tr><td>NQT64030.1</td><td>TM+TM+TM→?→?→?→?→ SIG+LRR-repeats+AP-GTPase+COR+TIR*→</td><td>SIG+LRR-repeats+AP-GTPase+COR+TIR</td><td>1188</td><td>HQ556_13800</td><td>FCB group</td><td>Candidatus Marinimicrobia bacterium</td><td>TIR domain-containing protein [Candidatus Marinimicrobia bacterium].</td><td>GCA_013202495.1</td></tr>
<tr><td>NQT91808.1</td><td>LRR-repeats+AP-GTPase+COR+DUF4404*→</td><td>LRR-repeats+AP-GTPase+COR+DUF4404</td><td>843</td><td>HQ559_03535</td><td>Lentisphaerae</td><td>Lentisphaerae bacterium</td><td>serine/threonine protein kinase [Lentisphaerae bacterium].</td><td>GCA_013202215.1</td></tr>
<tr><td>NQU06371.1</td><td>LRR-repeats+AP-GTPase+COR*→</td><td>LRR-repeats+AP-GTPase+COR</td><td>749</td><td>HQ568_09785</td><td>Calditrichaeota</td><td>Calditrichaeota bacterium</td><td>GTP-binding protein [Calditrichaeota bacterium].</td><td>GCA_013202805.1</td></tr>
<tr><td>NQU20766.1</td><td>LRR-repeats+AP-GTPase+COR+TIR*→</td><td>LRR-repeats+AP-GTPase+COR+TIR</td><td>813</td><td>HQ567_05735</td><td>Bacteria</td><td>Candidatus Nealsonbacteria bacterium</td><td>TIR domain-containing protein [Candidatus Nealsonbacteria bacterium].</td><td>GCA_013202865.1</td></tr>
<tr><td>NQU25297.1</td><td>LRR-repeats+AP-GTPase+COR*→ TIR→</td><td>LRR-repeats+AP-GTPase+COR</td><td>608</td><td>HQ567_28770</td><td>Bacteria</td><td>Candidatus Nealsonbacteria bacterium</td><td>hypothetical protein HQ567_28770 [Candidatus Nealsonbacteria bacterium].</td><td>GCA_013202865.1</td></tr>
<tr><td>NQU51997.1</td><td>LRR-repeats+AP-GTPase+COR+TIR*→</td><td>LRR-repeats+AP-GTPase+COR+TIR</td><td>946</td><td>HQ522_05615</td><td>Bacteroidetes</td><td>Bacteroidetes bacterium</td><td>leucine-rich repeat domain-containing protein [Bacteroidetes bacterium].</td><td>GCA_013203755.1</td></tr>
<tr><td>NQU93487.1</td><td>RVT→ LRR-repeats+AP-GTPase+COR+TIR*→?→?→ TM+TM+TM→</td><td>LRR-repeats+AP-GTPase+COR+TIR</td><td>903</td><td>HQ540_23735</td><td>Bacteroidetes</td><td>Bacteroidetes bacterium</td><td>TIR domain-containing protein [Bacteroidetes bacterium].</td><td>GCA_013203545.1</td></tr>
<tr><td>NQY23053.1</td><td>S4→?→?→?→?→ LRR-repeats+AP-GTPase+COR+DrHyd*→||<-?<-Cluster1695_2clades||TraJ-RHH→ RelE-ParE→?→?→ ACET→</td><td>LRR-repeats+AP-GTPase+COR+DrHyd</td><td>962</td><td>HRT41_03420</td><td>Epsilonproteobacteria</td><td>Campylobacteraceae bacterium</td><td>leucine-rich repeat domain-containing protein [Campylobacteraceae bacterium].</td><td>GCA_013215945.1</td></tr>
<tr><td>NQZ07155.1</td><td>GDSL→ Cluster2260_2clades→ LRR-repeats+AP-GTPase+COR+E59-ABC*→?→ RelE→ CHTH→</td><td>LRR-repeats+AP-GTPase+COR+E59-ABC</td><td>1302</td><td>HRT35_08335</td><td>Gammaproteobacteria</td><td>Algicola sp.</td><td>leucine-rich repeat domain-containing protein [Algicola sp.].</td><td>GCA_013216025.1</td></tr>
<tr><td>NQZ07918.1</td><td>LRR-repeats+AP-GTPase+COR*→</td><td>LRR-repeats+AP-GTPase+COR</td><td>815</td><td>HRT35_12215</td><td>Gammaproteobacteria</td><td>Algicola sp.</td><td>GTP-binding protein [Algicola sp.].</td><td>GCA_013216025.1</td></tr>
<tr><td>NQZ09239.1</td><td><-MNS-STAND+CR-REase3||LRR-repeats+AP-GTPase+COR*→ Cluster5_5clades→</td><td>LRR-repeats+AP-GTPase+COR</td><td>1075</td><td>HRT35_18935</td><td>Gammaproteobacteria</td><td>Algicola sp.</td><td>leucine-rich repeat domain-containing protein [Algicola sp.].</td><td>GCA_013216025.1</td></tr>
<tr><td>NQZ10194.1</td><td>LRR-repeats+AP-GTPase+COR+TIR*→?→||<-?<-HSP70</td><td>LRR-repeats+AP-GTPase+COR+TIR</td><td>972</td><td>HRT35_23840</td><td>Gammaproteobacteria</td><td>Algicola sp.</td><td>TIR domain-containing protein [Algicola sp.].</td><td>GCA_013216025.1</td></tr>
<tr><td>NQZ62063.1</td><td>Cluster1136_2clades→?→||<-?||LRR-repeats+AP-GTPase+COR*→</td><td>LRR-repeats+AP-GTPase+COR</td><td>829</td><td>HRT59_07970</td><td>Cyanobacteria</td><td>Crocosphaera sp.</td><td>leucine-rich repeat domain-containing protein [Crocosphaera sp.].</td><td>GCA_013215395.1</td></tr>
<tr><td>NQZ63007.1</td><td>LRR-repeats+AP-GTPase+COR*→</td><td>LRR-repeats+AP-GTPase+COR</td><td>689</td><td>HRT59_12970</td><td>Cyanobacteria</td><td>Crocosphaera sp.</td><td>leucine-rich repeat domain-containing protein, partial [Crocosphaera sp.].</td><td>GCA_013215395.1</td></tr>
<tr><td>NRA27019.1</td><td>FGS→?→ FGS→ RVT→ FGS→ LRR-repeats+AP-GTPase+COR*→||<-?<-nSTAND3+REase-DUF4143<-tRNA<-?||Pro_CA→||<-Cluster1292_2clades</td><td>LRR-repeats+AP-GTPase+COR</td><td>859</td><td>HRU10_07210</td><td>Verrucomicrobia</td><td>Opitutales bacterium</td><td>hypothetical protein HRU10_07210 [Opitutales bacterium].</td><td>GCA_013215165.1</td></tr>
<tr><td>NRT54298.1</td><td>LRR-repeats+AP-GTPase+COR+TIR*→||<-?<-ABC_membrane+ABC_tran||Cyanophycinsyn-ATPgrasp→ Cyanophycinsyn-ATPgrasp→</td><td>LRR-repeats+AP-GTPase+COR+TIR</td><td>788</td><td>HNQ01_000005</td><td>Betaproteobacteria</td><td>Leptothrix sp. C29</td><td>internalin A [Leptothrix sp. C29].</td><td>GCA_013294065.1</td></tr>
<tr><td>NTV46488.1</td><td>LRR-repeats+AP-GTPase+COR+TIR*→</td><td>LRR-repeats+AP-GTPase+COR+TIR</td><td>657</td><td>HGB11_08225</td><td>Chlorobi</td><td>Chlorobiales bacterium</td><td>TIR domain-containing protein, partial [Chlorobiales bacterium].</td><td>GCA_013334725.1</td></tr>
<tr><td>NTV66504.1</td><td>LRR-repeats+AP-GTPase+COR+TIR*→?→?→?→ PurT-ATPgrasp→</td><td>LRR-repeats+AP-GTPase+COR+TIR</td><td>755</td><td>HGB06_02250</td><td>Chlorobi</td><td>Chlorobaculum sp.</td><td>TIR domain-containing protein [Chlorobaculum sp.].</td><td>GCA_013334855.1</td></tr>
<tr><td>NTV67443.1</td><td>REase→||<-?<-SIG+TM||LRR-repeats+AP-GTPase+COR*→</td><td>LRR-repeats+AP-GTPase+COR</td><td>453</td><td>HGB06_07145</td><td>Chlorobi</td><td>Chlorobaculum sp.</td><td>hypothetical protein HGB06_07145 [Chlorobaculum sp.].</td><td>GCA_013334855.1</td></tr>
<tr><td>NTW50168.1</td><td>LRR-repeats+AP-GTPase+COR+TIR*→</td><td>LRR-repeats+AP-GTPase+COR+TIR</td><td>839</td><td>HGB19_10655</td><td>Chlorobi</td><td>Chlorobiales bacterium</td><td>TIR domain-containing protein [Chlorobiales bacterium].</td><td>GCA_013334605.1</td></tr>
<tr><td>NTW54860.1</td><td>LRR-repeats+AP-GTPase+COR*→?→?→?→?→?→?→ Cluster2062_2clades→</td><td>LRR-repeats+AP-GTPase+COR</td><td>1090</td><td>HGB15_08930</td><td>Chlorobi</td><td>Chlorobaculum sp.</td><td>hypothetical protein HGB15_08930 [Chlorobaculum sp.].</td><td>GCA_013334655.1</td></tr>
<tr><td>NTW70387.1</td><td>DAM→ LRR-repeats+AP-GTPase+COR+TIR*→</td><td>LRR-repeats+AP-GTPase+COR+TIR</td><td>876</td><td>HGB23_11195</td><td>Chlorobi</td><td>Chlorobiaceae bacterium</td><td>TIR domain-containing protein [Chlorobiaceae bacterium].</td><td>GCA_013334485.1</td></tr>
</table>

<table>
<tr><th>acc</th><th>operon</th><th>architecture</th><th>len</th><th>gen.name</th><th>taxend</th><th>species</th><th>defline</th><th>gca</th></tr>
<tr><td>NTW83729.1</td><td><-NUDIX||?→?→?→ LRR-repeats+AP-GTPase+COR+TIR*→</td><td>LRR-repeats+AP-GTPase+COR+TIR</td><td>892</td><td>HGB36_10245</td><td>Chlorobi</td><td>Chlorobiaceae bacterium</td><td>TIR domain-containing protein [Chlorobiaceae bacterium].</td><td>GCA_013334225.1</td></tr>
<tr><td>NUM66201.1</td><td>SHS2→ LRR-repeats+AP-GTPase+COR*→?→?→ Cluster1134_2clades→ SNF→</td><td>LRR-repeats+AP-GTPase+COR</td><td>1051</td><td>HUU39_13110</td><td>Bacteria</td><td>candidate division KSB1 bacterium</td><td>hypothetical protein HUU39_13110 [candidate division KSB1 bacterium].</td><td>GCA_013359425.1</td></tr>
<tr><td>NUM68318.1</td><td>LRR-repeats+AP-GTPase+COR*→?→?→ FGS→</td><td>LRR-repeats+AP-GTPase+COR</td><td>955</td><td>HUU39_24115</td><td>Bacteria</td><td>candidate division KSB1 bacterium</td><td>leucine-rich repeat domain-containing protein [candidate division KSB1 bacterium].</td><td>GCA_013359425.1</td></tr>
<tr><td>NUO01599.1</td><td><-S18-HTH<-Cluster1004_3clades<-?<-?<-?||FGS→ LRR-repeats+_AP-GTPase+COR+EAD11+TIR*→ FGS→</td><td>LRR-repeats+_AP-GTPase+COR+EAD11+TIR</td><td>1085</td><td>HUU01_13415</td><td>Bacteroidetes</td><td>Saprospiraceae bacterium</td><td>TIR domain-containing protein [Saprospiraceae bacterium].</td><td>GCA_013361075.1</td></tr>
<tr><td>NUO02098.1</td><td>LRR-repeats+AP-GTPase+COR+TM+TM+TM*→?→||<-Cluster2427_2clades</td><td>LRR-repeats+AP-GTPase+COR+TM+TM+TM</td><td>791</td><td>HUU01_15950</td><td>Bacteroidetes</td><td>Saprospiraceae bacterium</td><td>hypothetical protein HUU01_15950, partial [Saprospiraceae bacterium].</td><td>GCA_013361075.1</td></tr>
<tr><td>NUO79752.1</td><td>SIG+7TMR-DISMED1→||<-?<-?||LRR-repeats+AP-GTPase+COR+TIR*→||<-Cluster2261_2clades<-RlaP||SIG+TIMbarrel→</td><td>LRR-repeats+AP-GTPase+COR+TIR</td><td>968</td><td>HUU05_06725</td><td>Bacteria</td><td>candidate division KSB1 bacterium</td><td>leucine-rich repeat domain-containing protein [candidate division KSB1 bacterium].</td><td>GCA_013361015.1</td></tr>
<tr><td>NUO80023.1</td><td>ABhydrolase→?→ Cluster1134_2clades→?→ LRR-repeats+AP-GTPase+COR*→</td><td>LRR-repeats+AP-GTPase+COR</td><td>824</td><td>HUU05_08095</td><td>Bacteria</td><td>candidate division KSB1 bacterium</td><td>leucine-rich repeat domain-containing protein [candidate division KSB1 bacterium].</td><td>GCA_013361015.1</td></tr>
<tr><td>NUO97795.1</td><td>LRR-repeats+AP-GTPase+COR*→</td><td>LRR-repeats+AP-GTPase+COR</td><td>986</td><td>HOW59_07675</td><td>Actinobacteria</td><td>Nonomuraea sp.</td><td>hypothetical protein HOW59_07675, partial [Nonomuraea sp.].</td><td>GCA_013361365.1</td></tr>
<tr><td>NUP68620.1</td><td>LRR-repeats+AP-GTPase+COR*→</td><td>LRR-repeats+AP-GTPase+COR</td><td>1447</td><td>HOW71_41335</td><td>Actinobacteria</td><td>Nonomuraea sp.</td><td>hypothetical protein HOW71_41335 [Nonomuraea sp.].</td><td>GCA_013361665.1</td></tr>
<tr><td>NUQ24465.1</td><td>FGS→?→||<-?||EAD11+LRR-repeats+S1+S1+AP-GTPase+COR*→</td><td>EAD11+LRR-repeats+S1+S1+AP-GTPase+COR</td><td>1140</td><td>HUU34_10980</td><td>Bacteroidetes</td><td>Saprospiraceae bacterium</td><td>leucine-rich repeat domain-containing protein [Saprospiraceae bacterium].</td><td>GCA_013360405.1</td></tr>
<tr><td>NUQ25704.1</td><td>PIN→ FGS→?→ RVT→?→ LRR-repeats+AP-GTPase+COR+TIR*→?→?→?→ TM+TM+TM→</td><td>LRR-repeats+AP-GTPase+COR+TIR</td><td>826</td><td>HUU34_17280</td><td>Bacteroidetes</td><td>Saprospiraceae bacterium</td><td>TIR domain-containing protein [Saprospiraceae bacterium].</td><td>GCA_013360405.1</td></tr>
<tr><td>NUQ26825.1</td><td>LRR-repeats+AP-GTPase+COR+TM*→ FGS→</td><td>LRR-repeats+AP-GTPase+COR+TM</td><td>924</td><td>HUU34_23015</td><td>Bacteroidetes</td><td>Saprospiraceae bacterium</td><td>hypothetical protein HUU34_23015 [Saprospiraceae bacterium].</td><td>GCA_013360405.1</td></tr>
<tr><td>NUQ46249.1</td><td>LRR-repeats+AP-GTPase+COR*→ ABC_membrane+ABC_tran→</td><td>LRR-repeats+AP-GTPase+COR</td><td>843</td><td>HUU22_09465</td><td>Planctomycetes</td><td>Phycisphaerae bacterium</td><td>leucine-rich repeat domain-containing protein, partial [Phycisphaerae bacterium].</td><td>GCA_013360645.1</td></tr>
<tr><td>NUQ73394.1</td><td>LRR-repeats+AP-GTPase+COR+TM+TM*→</td><td>LRR-repeats+AP-GTPase+COR+TM+TM</td><td>869</td><td>HUU21_07550</td><td>Deltaproteobacteria</td><td>Polyangiaceae bacterium</td><td>GTP-binding protein [Polyangiaceae bacterium].</td><td>GCA_013360665.1</td></tr>
<tr><td>NUQ87064.1</td><td>LRR-repeats+AP-GTPase+COR*→</td><td>LRR-repeats+AP-GTPase+COR</td><td>767</td><td>HOQ43_01165</td><td>Actinobacteria</td><td>Glycomyces artemisiae</td><td>hypothetical protein HOQ43_01165 [Glycomyces artemisiae].</td><td>GCA_013361895.1</td></tr>
<tr><td>NUR27723.1</td><td>REC→?→?→?→ LRR-repeats+AP-GTPase+COR*→</td><td>LRR-repeats+AP-GTPase+COR</td><td>958</td><td>HOV83_18080</td><td>Actinobacteria</td><td>Catenulispora sp.</td><td>hypothetical protein HOV83_18080 [Catenulispora sp.].</td><td>GCA_013362085.1</td></tr>
<tr><td>NUS08684.1</td><td>LRR-repeats+AP-GTPase+COR*→</td><td>LRR-repeats+AP-GTPase+COR</td><td>1447</td><td>HOV97_39690</td><td>Actinobacteria</td><td>Nonomuraea sp.</td><td>hypothetical protein HOV97_39690 [Nonomuraea sp.].</td><td>GCA_013362305.1</td></tr>
<tr><td>NVM16177.1</td><td>LRR-repeats+AP-GTPase+COR*→</td><td>LRR-repeats+AP-GTPase+COR</td><td>1327</td><td>HWN80_00570</td><td>Asgard group</td><td>Candidatus Lokiarchaeota archaeon</td><td>leucine-rich repeat protein [Candidatus Lokiarchaeota archaeon].</td><td>GCA_013375495.1</td></tr>
<tr><td>NVM57338.1</td><td>LRR-repeats+AP-GTPase+COR*→</td><td>LRR-repeats+AP-GTPase+COR</td><td>588</td><td>HWN51_04390</td><td>Deltaproteobacteria</td><td>Desulfobacterales bacterium</td><td>leucine-rich repeat domain-containing protein, partial [Desulfobacterales bacterium].</td><td>GCA_013375265.1</td></tr>
<tr><td>NVO09292.1</td><td><-SIG+TM+VWA+TM<-SIG+TM+VWA+TM<-?<-VWA<-?<-MoxR-AAA||?→ LRR-repeats+AP-GTPase+COR*→?→?→?→||<-?<-?<-?||DSBH→</td><td>LRR-repeats+AP-GTPase+COR</td><td>965</td><td>HXX16_04945</td><td>Bacteroidetes</td><td>Bacteroidales bacterium</td><td>leucine-rich repeat domain-containing protein [Bacteroidales bacterium].</td><td>GCA_013376985.1</td></tr>
</table>

acc	operon	architecture	len	gen.name	taxend	species	defline	gca
NZD48025.1	LRR-repeats+AP-GTPase+COR+TIR*→||<-?<-ABC_membrane+ABC_tran||Cyanophycinsyn-ATPgrasp→ Cyanophycinsyn-ATPgrasp→	LRR-repeats+AP-GTPase+COR+TIR	885	HZU84_19525	Betaproteobacteria	Sphaerotilus natans subsp. sulfidivorans	leucine-rich repeat domain-containing protein [Sphaerotilus natans subsp. sulfidivorans].	GCA_013426975.1
OAB01099.1	LRR-repeats+AP-GTPase+COR*→||<-?||?→||<-?<-SIGMA-HTH<-SIGMA-HTH	LRR-repeats+AP-GTPase+COR	996	A6P39_08275	Actinobacteria	Streptomyces sp. FXJ1.172	hypothetical protein A6P39_08275 [Streptomyces sp. FXJ1.172].	GCA_001636945.1
OAI12102.1	Cluster5_5clades→?→?→ HTH→ LRR-repeats+AP-GTPase+COR+TIR*→ Cluster1019_2clades→?→||<-?<-?<-?||Cluster833_2clades→	LRR-repeats+AP-GTPase+COR+TIR	1065	A1507_19115	Gammaproteobacteria	Methylomonas koyamae	hypothetical protein A1507_19115 [Methylomonas koyamae].	GCA_001644135.1
OBQ24605.1	S18-HTH→?→||<-tRNA||Ferredoxin-betagrasp→?→?→ LRR-repeats+AP-GTPase+COR*→?→||<-?||TM→	LRR-repeats+AP-GTPase+COR	1095	AN488_00160	Cyanobacteria	Anabaena sp. WA113	GTPase [Anabaena sp. WA113].	GCA_001672155.1
OBQ25153.1	LRR-repeats+AP-GTPase+COR*→?→||<-?||TM→	LRR-repeats+AP-GTPase+COR	780	AN481_11440	Cyanobacteria	Aphanizomenon flos-aquae LD13	hypothetical protein AN481_11440, partial [Aphanizomenon flos-aquae LD13].	GCA_001672165.1
OBQ31054.1	S18-HTH→?→||<-tRNA||Ferredoxin-betagrasp→?→?→ LRR-repeats+AP-GTPase+COR*→?→||<-?||TM→	LRR-repeats+AP-GTPase+COR	942	AN483_02530	Cyanobacteria	Aphanizomenon flos-aquae MDT14a	hypothetical protein AN483_02530 [Aphanizomenon flos-aquae MDT14a].	GCA_001672095.1
OBQ35403.1	LRR-repeats+AP-GTPase+COR*→||<-?<-sGTP+sGTP||?→||<-REC	LRR-repeats+AP-GTPase+COR	799	AN487_15705	Cyanobacteria	Anabaena sp. CRKS33	hypothetical protein AN487_15705, partial [Anabaena sp. CRKS33].	GCA_001672075.1
OBQ36155.1	LRR-repeats+AP-GTPase+COR*→	LRR-repeats+AP-GTPase+COR	567	AN484_25870	Cyanobacteria	Aphanizomenon flos-aquae WA102	GTPase, partial [Aphanizomenon flos-aquae WA102].	GCA_001672105.1
OCQ90934.1	LRR-repeats+AP-GTPase+COR→ *LRR-repeats+AP-GTPase+COR*→?→ LRR-repeats+AP-GTPase+COR→	LRR-repeats+AP-GTPase+COR	795	BCR12_04520	Cyanobacteria	Limnothrix sp. P13C2	hypothetical protein BCR12_04520 [Limnothrix sp. P13C2].	GCA_001698445.1
OCQ90935.1	LRR-repeats+AP-GTPase+COR*→	LRR-repeats+AP-GTPase+COR	952	BCR12_04525	Cyanobacteria	Limnothrix sp. P13C2	hypothetical protein BCR12_04525 [Limnothrix sp. P13C2].	GCA_001698445.1
OCQ97014.1	TPR-repeats→?→?→ CASPASE+TM+TPR→?→?→?→ LRR-repeats+AP-GTPase+COR+DUF4404*→||<-?<-?||?→||<-?||REC→	LRR-repeats+AP-GTPase+COR+DUF4404	833	BCD64_15615	Cyanobacteria	Nostoc sp. MBR 210	GTP-binding protein [Nostoc sp. MBR 210].	GCA_001698435.1
OCR00628.1	LRR-repeats+AP-GTPase+COR+TM+TM*→||<-?<-?||?→||<-?||?→||<-PRTase<-SIG+TM+TM+TM+TM	LRR-repeats+AP-GTPase+COR+TM+TM	689	BCD67_11660	Cyanobacteria	Oscillatoriales cyanobacterium USR001	GTPase, partial [Oscillatoriales cyanobacterium USR001].	GCA_001698425.1
OCW73618.1	LRR-repeats+AP-GTPase+COR*→	LRR-repeats+AP-GTPase+COR	918	A4G24_00320	Bacteroidetes	Elizabethkingia anophelis	hypothetical protein A4G24_00320 [Elizabethkingia anophelis].	GCA_001703835.1
ODG98072.1	LRR-repeats+AP-GTPase+COR+TIR*→||<-CASPASE+TM	LRR-repeats+AP-GTPase+COR+TIR	858	A4S05_10875	Cyanobacteria	Nostoc sp. KVJ20	hypothetical protein A4S05_10875, partial [Nostoc sp. KVJ20].	GCA_001712795.1
ODS35505.1	Cluster846_3clades→ LRR-repeats→ LRR-repeats+AP-GTPase+COR*→?→ VWA→	LRR-repeats+AP-GTPase+COR	644	A7316_10690	DPANN group	Candidatus Altiarchaeales archaeon WOR_SM1_86-2	hypothetical protein A7316_10690 [Candidatus Altiarchaeales archaeon WOR_SM1_86-2].	GCA_001723855.1
ODS40288.1	LRR-repeats+AP-GTPase+CASPASE*→	LRR-repeats+AP-GTPase+CASPASE	811	A7315_09000	DPANN group	Candidatus Altiarchaeales archaeon WOR_SM1_79	hypothetical protein A7315_09000 [Candidatus Altiarchaeales archaeon WOR_SM1_79].	GCA_001723835.1
OED06513.1	LRR-repeats+AP-GTPase+COR*→	LRR-repeats+AP-GTPase+COR	666	A9239_11540	Euryarchaeota	Methanosarcina sp. A14	hypothetical protein A9239_11540 [Methanosarcina sp. A14].	GCA_001729375.1
OEU63639.1	LRR-repeats→ LRR-repeats+AP-GTPase+COR*→	LRR-repeats+AP-GTPase+COR	623	BBJ57_05215	Deltaproteobacteria	Desulfobacterales bacterium PC51MH44	hypothetical protein BBJ57_05215 [Desulfobacterales bacterium PC51MH44].	GCA_001751165.1
OFX48341.1	ABC-ATPase→?→ LRR-repeats+AP-GTPase+COR*→	LRR-repeats+AP-GTPase+COR	720	A2X10_07060	Bacteroidetes	Bacteroidetes bacterium GWA2_33_15	hypothetical protein A2X10_07060 [Bacteroidetes bacterium GWA2_33_15].	GCA_001769045.1
OFX79107.1	LRR-repeats+AP-GTPase+COR+REase7*→	LRR-repeats+AP-GTPase+COR+REase7	1101	A2X12_10435	Bacteroidetes	Bacteroidetes bacterium GWE2_29_8	hypothetical protein A2X12_10435 [Bacteroidetes bacterium GWE2_29_8].	GCA_001769085.1
OFY84271.1	LRR-repeats→ LRR-repeats+AP-GTPase+COR+REase7*→	LRR-repeats+AP-GTPase+COR+REase7	598	A3F72_14720	Bacteroidetes	Bacteroidetes bacterium RIFC-SPLOWO2_12_FULL_35_15	hypothetical protein A3F72_14720 [Bacteroidetes bacterium RIFC-SPLOWO2_12_FULL_35_15].	GCA_001769385.1

<table>
<tr><th>acc</th><th>operon</th><th>architecture</th><th>len</th><th>gen.name</th><th>taxend</th><th>species</th><th>defline</th><th>gca</th></tr>
<tr><td>OGC77574.1</td><td>LRR-repeats+AP-GTPase+COR*→?→||<-?<-CCTBP</td><td>LRR-repeats+AP-GTPase+COR</td><td>975</td><td>A2Z27_00115</td><td>FCB group</td><td>candidate division Zixibacteria bacterium RBG_16_50_21</td><td>hypothetical protein A2Z27_00115 [candidate division Zixibacteria bacterium RBG_16_50_21].</td><td>GCA_001773465.1</td></tr>
<tr><td>OGH99179.1</td><td>LRR-repeats+AP-GTPase+COR*→</td><td>LRR-repeats+AP-GTPase+COR</td><td>572</td><td>A2X43_07200</td><td>Terrabacteria group</td><td>Candidatus Margulisbacteria bacterium GWD2_39_127</td><td>hypothetical protein A2X43_07200 [Candidatus Margulisbacteria bacterium GWD2_39_127].</td><td>GCA_001783265.1</td></tr>
<tr><td>OGV70404.1</td><td>Pkinase+LRR-repeats+AP-GTPase+COR+TM+TM*→</td><td>Pkinase+LRR-repeats+AP-GTPase+COR+TM+TM</td><td>1157</td><td>A2283_19020</td><td>Lentisphaerae</td><td>Lentisphaerae bacterium RI-FOXYA12_FULL_48_11</td><td>hypothetical protein A2283_19020 [Lentisphaerae bacterium RIFOXYA12_FULL_48_11].</td><td>GCA_001804885.1</td></tr>
<tr><td>OHB50686.1</td><td>S1COLD→ LRR-repeats+AP-GTPase+COR+TIR*→</td><td>LRR-repeats+AP-GTPase+COR+TIR</td><td>953</td><td>A2Y10_02350</td><td>Planctomycetes</td><td>Planctomycetes bacterium GWF2_41_51</td><td>hypothetical protein A2Y10_02350 [Planctomycetes bacterium GWF2_41_51].</td><td>GCA_001825665.1</td></tr>
<tr><td>OHB52204.1</td><td>LRR-repeats+AP-GTPase+COR*→?→||<-?||SIG+TM→</td><td>LRR-repeats+AP-GTPase+COR</td><td>1147</td><td>A2Y10_11365</td><td>Planctomycetes</td><td>Planctomycetes bacterium GWF2_41_51</td><td>hypothetical protein A2Y10_11365 [Planctomycetes bacterium GWF2_41_51].</td><td>GCA_001825665.1</td></tr>
<tr><td>OHB64560.1</td><td>REC→ TPR-repeats→||<-?||PSE→||<-?<-?<-?||YBAK→ LRR-repeats+AP-GTPase+COR+DUF4404*→</td><td>LRR-repeats+AP-GTPase+COR+DUF4404</td><td>740</td><td>A2Y76_11060</td><td>Planctomycetes</td><td>Planctomycetes bacterium RBG_13_60_9</td><td>hypothetical protein A2Y76_11060 [Planctomycetes bacterium RBG_13_60_9].</td><td>GCA_001824685.1</td></tr>
<tr><td>OHD05988.1</td><td>LRR-repeats+AP-GTPase+COR+TM+TM*→ TraJ-RHH→ RelE-ParE→</td><td>LRR-repeats+AP-GTPase+COR+TM+TM</td><td>1257</td><td>A2Z98_10570</td><td>Spirochaetes</td><td>Spirochaetes bacterium GWB1_27_13</td><td>hypothetical protein A2Z98_10570 [Spirochaetes bacterium GWB1_27_13].</td><td>GCA_001829125.1</td></tr>
<tr><td>OHD18029.1</td><td>MORC→?→?→?→||<-?<-PSE<-?||LRR-repeats+AP-GTPase+COR→//<-?<-?<-?//?→?→?→?→ REC→//<-LRR-repeats+AP-GTPase+COR+DrHyd</td><td>LRR-repeats+AP-GTPase+COR</td><td>1064</td><td>A2Z98_11125</td><td>Spirochaetes</td><td>Spirochaetes bacterium GWB1_27_13</td><td>hypothetical protein A2Z98_11125 [Spirochaetes bacterium GWB1_27_13].</td><td>GCA_001829125.1</td></tr>
<tr><td>OHD18038.1</td><td>LRR-repeats+AP-GTPase+COR+DrHyd*→</td><td>LRR-repeats+AP-GTPase+COR+DrHyd</td><td>986</td><td>A2Z98_11170</td><td>Spirochaetes</td><td>Spirochaetes bacterium GWB1_27_13</td><td>hypothetical protein A2Z98_11170 [Spirochaetes bacterium GWB1_27_13].</td><td>GCA_001829125.1</td></tr>
<tr><td>OHD26231.1</td><td>LRR-repeats+AP-GTPase+COR+TM+TM*→ TraJ-RHH→ RelE-ParE→</td><td>LRR-repeats+AP-GTPase+COR+TM+TM</td><td>1234</td><td>A2Y34_11235</td><td>Spirochaetes</td><td>Spirochaetes bacterium GWC1_27_15</td><td>hypothetical protein A2Y34_11235 [Spirochaetes bacterium GWC1_27_15].</td><td>GCA_001829235.1</td></tr>
<tr><td>OHD42853.1</td><td>MORC→?→?→?→||<-?<-PSE<-?||LRR-repeats+AP-GTPase+COR→//<-?<-?<-?//?→?→?→?→ REC→//<-LRR-repeats+AP-GTPase+COR+DrHyd</td><td>LRR-repeats+AP-GTPase+COR</td><td>1020</td><td>A2086_15855</td><td>Spirochaetes</td><td>Spirochaetes bacterium GWD1_27_9</td><td>hypothetical protein A2086_15855 [Spirochaetes bacterium GWD1_27_9].</td><td>GCA_001830585.1</td></tr>
<tr><td>OIO84219.1</td><td><-XIS-HTH||HTH+HTH→||<-WLM||?→ NACHT+68TM-wHTH→ REase→?→ LRR-repeats+AP-GTPase+COR*→</td><td>LRR-repeats+AP-GTPase+COR</td><td>1004</td><td>AUK01_09955</td><td>Chloroflexi</td><td>Anaerolineae bacterium CG2_30_57_67</td><td>hypothetical protein AUK01_09955, partial [Anaerolineae bacterium CG2_30_57_67].</td><td>GCA_001872455.1</td></tr>
<tr><td>OJJ15121.1</td><td>LRR-repeats+AP-GTPase+COR*→</td><td>LRR-repeats+AP-GTPase+COR</td><td>1037</td><td>BKI52_39320</td><td>Bacteroidetes</td><td>marine bacterium AO1-C</td><td>hypothetical protein BKI52_39320 [marine bacterium AO1-C].</td><td>GCA_001890965.1</td></tr>
<tr><td>OJJ19161.1</td><td>LRR-repeats+AP-GTPase+COR*→</td><td>LRR-repeats+AP-GTPase+COR</td><td>851</td><td>BKI52_20325</td><td>Bacteroidetes</td><td>marine bacterium AO1-C</td><td>hypothetical protein BKI52_20325 [marine bacterium AO1-C].</td><td>GCA_001890965.1</td></tr>
<tr><td>OJJ19619.1</td><td>LRR-repeats+AP-GTPase+COR*→||<-?<-?<-?<-?<-TetR-HTH</td><td>LRR-repeats+AP-GTPase+COR</td><td>723</td><td>BKI52_22705</td><td>Bacteroidetes</td><td>marine bacterium AO1-C</td><td>hypothetical protein BKI52_22705, partial [marine bacterium AO1-C].</td><td>GCA_001890965.1</td></tr>
<tr><td>OJJ19690.1</td><td><-Four-helical-protein<-?<-?<-?<-?||LRR-repeats+AP-GTPase+COR*→</td><td>LRR-repeats+AP-GTPase+COR</td><td>1463</td><td>BKI52_19350</td><td>Bacteroidetes</td><td>marine bacterium AO1-C</td><td>hypothetical protein BKI52_19350 [marine bacterium AO1-C].</td><td>GCA_001890965.1</td></tr>
<tr><td>OJJ20943.1</td><td>Cluster2042_2clades→||<-?<-?||?→?→?→||<-?||LRR-repeats+AP-GTPase+COR+EAD11*→||<-PAS+HISKIN</td><td>LRR-repeats+AP-GTPase+COR+EAD11</td><td>871</td><td>BKI52_10210</td><td>Bacteroidetes</td><td>marine bacterium AO1-C</td><td>hypothetical protein BKI52_10210 [marine bacterium AO1-C].</td><td>GCA_001890965.1</td></tr>
<tr><td>OJJ21241.1</td><td>LRR-repeats+AP-GTPase+COR*→?→||<-?<-cNMPBD</td><td>LRR-repeats+AP-GTPase+COR</td><td>910</td><td>BKI52_11795</td><td>Bacteroidetes</td><td>marine bacterium AO1-C</td><td>hypothetical protein BKI52_11795 [marine bacterium AO1-C].</td><td>GCA_001890965.1</td></tr>
<tr><td>OJJ22385.1</td><td><-SIGMA-HTH||?→?→?→?→?→||<-?||LRR-repeats+AP-GTPase+COR*→?→?→?→||<-?<-?<-?<-SIGMA-HTH</td><td>LRR-repeats+AP-GTPase+COR</td><td>1192</td><td>BKI52_06800</td><td>Bacteroidetes</td><td>marine bacterium AO1-C</td><td>hypothetical protein BKI52_06800 [marine bacterium AO1-C].</td><td>GCA_001890965.1</td></tr>
<tr><td>OJJ23263.1</td><td>D5ATPase→?→ LRR-repeats+AP-GTPase+COR*→</td><td>LRR-repeats+AP-GTPase+COR</td><td>1296</td><td>BKI52_02595</td><td>Bacteroidetes</td><td>marine bacterium AO1-C</td><td>hypothetical protein BKI52_02595 [marine bacterium AO1-C].</td><td>GCA_001890965.1</td></tr>
</table>

acc	operon	architecture	len	gen.name	taxend	species	defline	gca
OJJ23599.1	SF2-DUF3427A→?→ TRD+TRD→?→?→ LRR-repeats+AP-GTPase+COR*→?→||<-?<-LRR-repeats<-?||cNMPBD→	LRR-repeats+AP-GTPase+COR	1001	BKI52_04350	Bacteroidetes	marine bacterium AO1-C	hypothetical protein BKI52_04350 [marine bacterium AO1-C].	GCA_001890965.1
OKH11101.1	LRR-repeats+AP-GTPase+COR+TM+TM*→	LRR-repeats+AP-GTPase+COR+TM+TM	944	NIES208_17750	Cyanobacteria	Limnothrix rosea IAM M-220	hypothetical protein NIES208_17750 [Limnothrix rosea IAM M-220].	GCA_001904615.1
OKH34496.1	LRR-repeats+AP-GTPase+COR+TM+TM*→||<-CASPASE+TPR	LRR-repeats+AP-GTPase+COR+TM+TM	902	FACHB389_15645	Cyanobacteria	Nostoc calcicola FACHB-389	GTPase [Nostoc calcicola FACHB-389].	GCA_001904715.1
OLE55789.1	<-RFC-AAA<-?<-Trichomonas-DAM<-?<-?<-ISOCOT||LRR-repeats+AP-GTPase+COR+TIR*→	LRR-repeats+AP-GTPase+COR+TIR	862	AUG51_01855	Acidobacteria	Acidobacteria bacterium 13_1_20CM_3_53_8	serine/threonine protein kinase [Acidobacteria bacterium 13_1_20CM_3_53_8].	GCA_001920415.1
OLF12412.1	LRR-repeats+AP-GTPase+COR*→?→?→?→||<-?<-ABC-ATPase+ABC-ATPase	LRR-repeats+AP-GTPase+COR	927	BLA60_09370	Actinobacteria	Actinophytocola xinjiangensis	hypothetical protein BLA60_09370 [Actinophytocola xinjiangensis].	GCA_001921215.1
OQX01793.1	LRR-repeats+AP-GTPase+COR+TIR*→||<-?<-PSE<-?<-PSE<-PSE<-?<-?<-?||PSE→||<-?||ABhydrolase→	LRR-repeats+AP-GTPase+COR+TIR	928	BWK73_44950	Gammaproteobacteria	Thiothrix lacustris	GTP-binding protein [Thiothrix lacustris].	GCA_002083875.1
OQX02054.1	nSTAND3+REase-DUF4143→ LRR-repeats+AP-GTPase+COR*→ TIR→?→||<-Thioredoxin	LRR-repeats+AP-GTPase+COR	784	BWK73_43990	Gammaproteobacteria	Thiothrix lacustris	hypothetical protein BWK73_43990 [Thiothrix lacustris].	GCA_002083875.1
OQX07365.1	LRR-repeats+AP-GTPase+COR+CASPASE*→	LRR-repeats+AP-GTPase+COR+CASPASE	879	BWK80_49550	Deltaproteobacteria	Desulfobacteraceae bacterium IS3	hypothetical protein BWK80_49550 [Desulfobacteraceae bacterium IS3].	GCA_002083955.1
OQX24659.1	LRR-repeats+AP-GTPase+COR→ *Cluster2372_2clades→?→ LRR-repeats+AP-GTPase+COR*→ LRR-repeats+AP-GTPase+COR*→	LRR-repeats+AP-GTPase+COR	881	BWK80_19660	Deltaproteobacteria	Desulfobacteraceae bacterium IS3	hypothetical protein BWK80_19660 [Desulfobacteraceae bacterium IS3].	GCA_002083955.1
OQX24662.1	LRR-repeats+AP-GTPase+COR+TM*→	LRR-repeats+AP-GTPase+COR+TM	925	BWK80_19675	Deltaproteobacteria	Desulfobacteraceae bacterium IS3	hypothetical protein BWK80_19675 [Desulfobacteraceae bacterium IS3].	GCA_002083955.1
OQX24663.1	LRR-repeats+AP-GTPase+COR*→	LRR-repeats+AP-GTPase+COR	870	BWK80_19680	Deltaproteobacteria	Desulfobacteraceae bacterium IS3	hypothetical protein BWK80_19680 [Desulfobacteraceae bacterium IS3].	GCA_002083955.1
OQX25920.1	LRR-repeats+AP-GTPase+COR*→?→?→?→?→ ABC-ATPase→ SIG+TM+TM+TM+TM+TM→||<-Cluster968_2clades	LRR-repeats+AP-GTPase+COR	802	BWK80_13160	Deltaproteobacteria	Desulfobacteraceae bacterium IS3	hypothetical protein BWK80_13160 [Desulfobacteraceae bacterium IS3].	GCA_002083955.1
OQY27201.1	LRR-repeats+AP-GTPase+COR*→?→||<-?<-?<-?||TM+TM+TM+TM+TM+TM→	LRR-repeats+AP-GTPase+COR	700	B6244_11350	FCB group	Candidatus Cloacimonetes bacterium 4572_55	hypothetical protein B6244_11350 [Candidatus Cloacimonetes bacterium 4572_55].	GCA_002084765.1
OQY46926.1	LRR-repeats+AP-GTPase+COR*→||<-PSE||?→ LRR-repeats→	LRR-repeats+AP-GTPase+COR	887	B6247_27150	Gammaproteobacteria	Beggiatoa sp. 4572_84	GTPase [Beggiatoa sp. 4572_84].	GCA_002085445.1
OQY52602.1	SIG+TM+TM+TM+TM+TM+TM+TM→?→?→?→?→ LRR-repeats+AP-GTPase+COR→*?→?→?→ LRR-repeats+AP-GTPase+COR*→	LRR-repeats+AP-GTPase+COR	429	B6245_23480	Deltaproteobacteria	Desulfobacteraceae bacterium 4572_88	hypothetical protein B6245_23480, partial [Desulfobacteraceae bacterium 4572_88].	GCA_002085455.1
OQY52606.1	LRR-repeats+AP-GTPase+COR*→	LRR-repeats+AP-GTPase+COR	933	B6245_23500	Deltaproteobacteria	Desulfobacteraceae bacterium 4572_88	hypothetical protein B6245_23500 [Desulfobacteraceae bacterium 4572_88].	GCA_002085455.1
OQY56003.1	CBS→ PSE→||<-?||?→?→ LRR-repeats→?→ PSE→ LRR-repeats→ LRR-repeats+AP-GTPase+COR*→	LRR-repeats+AP-GTPase+COR	907	B6247_05655	Gammaproteobacteria	Beggiatoa sp. 4572_84	GTPase [Beggiatoa sp. 4572_84].	GCA_002085445.1
OQY58390.1	LRR-repeats+AP-GTPase+COR*→ SIG+DADALIGASE-ATPgrasp→	LRR-repeats+AP-GTPase+COR	791	B6245_12095	Deltaproteobacteria	Desulfobacteraceae bacterium 4572_88	hypothetical protein B6245_12095 [Desulfobacteraceae bacterium 4572_88].	GCA_002085455.1
OSM01684.1	SIGMA-HTH→?→||<-?<-?<-?<-?<-?||LRR-repeats+AP-GTPase+COR*→	LRR-repeats+AP-GTPase+COR	876	MAIT1_01702	Alphaproteobacteria	Magnetofaba australis IT-1	putative small GTP-binding protein [Magnetofaba australis IT-1].	GCA_002109495.1
OUC12119.1	LRR-repeats+AP-GTPase+COR+DrHyd*→?→?→ FGS→	LRR-repeats+AP-GTPase+COR+DrHyd	810	B0A82_24265	Cyanobacteria	Alkalinema sp. CACIAM 70d	hypothetical protein B0A82_24265, partial [Alkalinema sp. CACIAM 70d].	GCA_002148405.1
OUC12470.1	<-HISKIN<-?<-?<-PRTase<-?||?→ LRR-repeats+AP-GTPase+COR*→	LRR-repeats+AP-GTPase+COR	820	B0A82_22225	Cyanobacteria	Alkalinema sp. CACIAM 70d	hypothetical protein B0A82_22225 [Alkalinema sp. CACIAM 70d].	GCA_002148405.1
OUC13078.1	<-TIR+TM+CHASE2+CHASE2+TM+TM+TM<-SIG+RnaseT2<-?||?→?→ LRR-repeats+AP-GTPase+COR*→	LRR-repeats+AP-GTPase+COR	919	B0A82_18905	Cyanobacteria	Alkalinema sp. CACIAM 70d	GTPase [Alkalinema sp. CACIAM 70d].	GCA_002148405.1
OUC15727.1	LRR-repeats+AP-GTPase+COR+DUF4404*→?→||<-Cluster2098_2clades	LRR-repeats+AP-GTPase+COR+DUF4404	969	B0A82_05270	Cyanobacteria	Alkalinema sp. CACIAM 70d	hypothetical protein B0A82_05270, partial [Alkalinema sp. CACIAM 70d].	GCA_002148405.1

<table>
<tr><th>acc</th><th>operon</th><th>architecture</th><th>len</th><th>gen.name</th><th>taxend</th><th>species</th><th>defline</th><th>gca</th></tr>
<tr><td>OWK46761.1</td><td><-Aminotran_1_2||LRR-repeats+AP-GTPase+COR+TIR*→||<-?<-?<-?<-DSBH+AraC-HTH+AraC-HTH||?→?→ SIG+TM+TM+TM→</td><td>LRR-repeats+AP-GTPase+COR+TIR</td><td>1310</td><td>FRUB_00460</td><td>Planctomycetes</td><td>Fimbriiglobus ruber</td><td>HtrA protease/chaperone protein [Fimbriiglobus ruber].</td><td>GCA_002197845.1</td></tr>
<tr><td>OWK47329.1</td><td><-RelE-ParE<-?||?→ LRR-repeats→||<-?||LRR-repeats+AP-GTPase+COR*→</td><td>LRR-repeats+AP-GTPase+COR</td><td>967</td><td>FRUB_01028</td><td>Planctomycetes</td><td>Fimbriiglobus ruber</td><td>putative internalin [Fimbriiglobus ruber].</td><td>GCA_002197845.1</td></tr>
<tr><td>OYQ36252.1</td><td><-SIG+TM+TM+TM+HISKIN<-?<-?<-?||?→ LRR-repeats+AP-GTPase+COR+TIR*→ ParA-Soj-PloopNTPase→ ParB→</td><td>LRR-repeats+AP-GTPase+COR+TIR</td><td>890</td><td>CHU95_05565</td><td>Alphaproteobacteria</td><td>Niveispirillum lacus</td><td>hypothetical protein CHU95_05565 [Niveispirillum lacus].</td><td>GCA_002251795.1</td></tr>
<tr><td>OYU93091.1</td><td>LRR-repeats+AP-GTPase+COR+TM+TM*→</td><td>LRR-repeats+AP-GTPase+COR+TM+TM</td><td>1040</td><td>CFE21_21825</td><td>Bacteroidetes</td><td>Bacteroidetes bacterium B1(2017)</td><td>hypothetical protein CFE21_21825, partial [Bacteroidetes bacterium B1(2017)].</td><td>GCA_002256395.1</td></tr>
<tr><td>OYW76150.1</td><td>LRR-repeats+AP-GTPase+COR+TIR*→?→||<-?<-WCAK||?→ REC→ REC→</td><td>LRR-repeats+AP-GTPase+COR+TIR</td><td>996</td><td>B7Z37_09950</td><td>Verrucomicrobia</td><td>Verrucomicrobia bacterium 12-59-8</td><td>hypothetical protein B7Z37_09950 [Verrucomicrobia bacterium 12-59-8].</td><td>GCA_002279765.1</td></tr>
<tr><td>OYW77555.1</td><td>SIG+ABhydrolase→?→ LRR-repeats+AP-GTPase+COR+TIR*→</td><td>LRR-repeats+AP-GTPase+COR+TIR</td><td>939</td><td>B7Z37_03775</td><td>Verrucomicrobia</td><td>Verrucomicrobia bacterium 12-59-8</td><td>hypothetical protein B7Z37_03775 [Verrucomicrobia bacterium 12-59-8].</td><td>GCA_002279765.1</td></tr>
<tr><td>OYW78304.1</td><td>FGS→ LRR-repeats+AP-GTPase+COR*→</td><td>LRR-repeats+AP-GTPase+COR</td><td>1063</td><td>B7Z37_00425</td><td>Verrucomicrobia</td><td>Verrucomicrobia bacterium 12-59-8</td><td>hypothetical protein B7Z37_00425 [Verrucomicrobia bacterium 12-59-8].</td><td>GCA_002279765.1</td></tr>
<tr><td>PCI29610.1</td><td>LRR-repeats+RNA-Helicase+AP-GTPase+COR+TIR*→?→||<-AraC-HTH||?→?→||<-Cluster1048_2clades<-POLYSACPOLYMERASE</td><td>LRR-repeats+RNA-Helicase+AP-GTPase+COR+TIR</td><td>1264</td><td>COB67_03805</td><td>Deltaproteobacteria</td><td>SAR324 cluster bacterium</td><td>hypothetical protein COB67_03805 [SAR324 cluster bacterium].</td><td>GCA_002401295.1</td></tr>
<tr><td>PCK09719.1</td><td>LRR-repeats+AP-GTPase+COR*→</td><td>LRR-repeats+AP-GTPase+COR</td><td>908</td><td>COA42_02805</td><td>Gammaproteobacteria</td><td>Alteromonadaceae bacterium</td><td>hypothetical protein COA42_02805 [Alteromonadaceae bacterium].</td><td>GCA_002746115.1</td></tr>
<tr><td>PHM06637.1</td><td>LRR-repeats+AP-GTPase+COR+TM+TM*→</td><td>LRR-repeats+AP-GTPase+COR+TM+TM</td><td>817</td><td>CK516_32280</td><td>Cyanobacteria</td><td>Nostoc sp. ‘Peltigera malacea cyanobiont’ DB3992</td><td>GTPase [Nostoc sp. ‘Peltigera malacea cyanobiont’ DB3992].</td><td>GCA_002631755.1</td></tr>
<tr><td>PHS08569.1</td><td>LRR-repeats+AP-GTPase+COR*→ SIG+TM+TM+TM+TM+TM+TM+TM+TM+TM+TM+TM→</td><td>LRR-repeats+AP-GTPase+COR</td><td>924</td><td>COA88_06290</td><td>Bacteroidetes</td><td>Kordia sp.</td><td>GTP-binding protein [Kordia sp.].</td><td>GCA_002733415.1</td></tr>
<tr><td>PID45108.1</td><td>Cluster1540_2clades→?→?→?→?→?→ tRNA→ LRR-repeats+AP-GTPase+COR*→||<-?<-?||Cluster2057_2clades→</td><td>LRR-repeats+AP-GTPase+COR</td><td>1016</td><td>CSB47_10150</td><td>Proteobacteria</td><td>Proteobacteria bacterium</td><td>hypothetical protein CSB47_10150 [Proteobacteria bacterium].</td><td>GCA_002747455.1</td></tr>
<tr><td>PID49971.1</td><td>SIG+TM+TM+TM+TM+TM+TM+TM+TM+TM+TM+TM→ LRR-repeats+AP-GTPase+COR+TIR*→?→||<-?<-?<-?<-?<-Cluster1063_3clades<-Cluster1067_3clades</td><td>LRR-repeats+AP-GTPase+COR+TIR</td><td>916</td><td>CR991_03995</td><td>Proteobacteria</td><td>Proteobacteria bacterium</td><td>hypothetical protein CR991_03995 [Proteobacteria bacterium].</td><td>GCA_002747435.1</td></tr>
<tr><td>PID58617.1</td><td><-Cluster1581_2clades<-?||NUDIX→ LRR-repeats+AP-GTPase+COR*→</td><td>LRR-repeats+AP-GTPase+COR</td><td>1061</td><td>CSB45_03475</td><td>Bacteria</td><td>candidate division KSB3 bacterium</td><td>GTPase [candidate division KSB3 bacterium].</td><td>GCA_002747525.1</td></tr>
<tr><td>PIE10915.1</td><td>LRR-repeats+AP-GTPase+COR*→||<-?<-tRNA||?→?→ Cluster1995_2clades→</td><td>LRR-repeats+AP-GTPase+COR</td><td>994</td><td>CSA72_05310</td><td>Alphaproteobacteria</td><td>Rhodobacterales bacterium</td><td>hypothetical protein CSA72_05310 [Rhodobacterales bacterium].</td><td>GCA_002748265.1</td></tr>
<tr><td>PJB68563.1</td><td><-XIS-HTH||HTH+HTH→||<-WLM||?→ NACHT+68TM-wHTH→ REase→?→ LRR-repeats+AP-GTPase+COR*→</td><td>LRR-repeats+AP-GTPase+COR</td><td>947</td><td>CO094_00645</td><td>Chloroflexi</td><td>Anaerolineae bacterium CG_4_9_14_3_um_filter_57_17</td><td>hypothetical protein CO094_00645, partial [Anaerolineae bacterium CG_4_9_14_3_um_filter_57_17].</td><td>GCA_002789715.1</td></tr>
<tr><td>PJF39486.1</td><td>LRR-repeats+AP-GTPase+COR+TM+TM*→</td><td>LRR-repeats+AP-GTPase+COR+TM+TM</td><td>751</td><td>CUN54_08620</td><td>Chloroflexi</td><td>Candidatus Thermofonsia Clade 2 bacterium</td><td>GTP-binding protein [Candidatus Thermofonsia Clade 2 bacterium].</td><td>GCA_002794595.1</td></tr>
<tr><td>PKB73283.1</td><td>LRR-repeats+AP-GTPase+COR*→||<-?<-?<-PSE<-SIG+ABhydrolase<-?<-ParA-Soj-PloopNTPase</td><td>LRR-repeats+AP-GTPase+COR</td><td>593</td><td>BZY75_02725</td><td>Chloroflexi</td><td>SAR202 cluster bacterium Io17-Chloro-G7</td><td>hypothetical protein BZY75_02725 [SAR202 cluster bacterium Io17-Chloro-G7].</td><td>GCA_002817055.1</td></tr>
<tr><td>PKD18393.1</td><td>LRR-repeats+AP-GTPase+COR*→</td><td>LRR-repeats+AP-GTPase+COR</td><td>845</td><td>APR41_04380</td><td>Bacteroidetes</td><td>Salegentibacter salinarum</td><td>hypothetical protein APR41_04380 [Salegentibacter salinarum].</td><td>GCA_002833365.1</td></tr>
<tr><td>PKN92928.1</td><td>UvsW-A18→ LRR-repeats+AP-GTPase+COR→ NACHT+68TM-wHTH→ LRR-repeats+AP-GTPase+COR→ LRR-repeats+AP-GTPase+COR+DUF4404→?→?→ Cluster1559_2clades→?→?→||<-?||LRR-repeats+AP-GTPase+COR→</td><td>LRR-repeats+AP-GTPase+COR</td><td>769</td><td>CVU44_12925</td><td>Chloroflexi</td><td>Chloroflexi bacterium HGW-Chloroflexi-6</td><td>GTP-binding protein [Chloroflexi bacterium HGW-Chloroflexi-6].</td><td>GCA_002840685.1</td></tr>
<tr><td>PKN92930.1</td><td>LRR-repeats+AP-GTPase+COR*→</td><td>LRR-repeats+AP-GTPase+COR</td><td>1044</td><td>CVU44_12935</td><td>Chloroflexi</td><td>Chloroflexi bacterium HGW-Chloroflexi-6</td><td>hypothetical protein CVU44_12935 [Chloroflexi bacterium HGW-Chloroflexi-6].</td><td>GCA_002840685.1</td></tr>
<tr><td>PKN92931.1</td><td>LRR-repeats+AP-GTPase+COR+DUF4404*→</td><td>LRR-repeats+AP-GTPase+COR+DUF4404</td><td>812</td><td>CVU44_12940</td><td>Chloroflexi</td><td>Chloroflexi bacterium HGW-Chloroflexi-6</td><td>hypothetical protein CVU44_12940 [Chloroflexi bacterium HGW-Chloroflexi-6].</td><td>GCA_002840685.1</td></tr>
<tr><td>PKN92938.1</td><td>LRR-repeats+AP-GTPase+COR*→</td><td>LRR-repeats+AP-GTPase+COR</td><td>673</td><td>CVU44_12975</td><td>Chloroflexi</td><td>Chloroflexi bacterium HGW-Chloroflexi-6</td><td>GTP-binding protein [Chloroflexi bacterium HGW-Chloroflexi-6].</td><td>GCA_002840685.1</td></tr>
</table>

<table>
<tr><th>acc</th><th>operon</th><th>architecture</th><th>len</th><th>gen.name</th><th>taxend</th><th>species</th><th>defline</th><th>gca</th></tr>
<tr><td>PKP35889.1</td><td>REC→ HISKIN→?→ LRR-repeats+AP-GTPase+COR*→</td><td>LRR-repeats+AP-GTPase+COR</td><td>654</td><td>CVU00_00150</td><td>Bacteroidetes</td><td>Bacteroidetes bacterium HGW-Bacteroidetes-17</td><td>hypothetical protein CVU00_00150 [Bacteroidetes bacterium HGW-Bacteroidetes-17].</td><td>GCA_002840985.1</td></tr>
<tr><td>PLX96646.1</td><td>LRR-repeats+AP-GTPase+COR+DrHyd*→||<-?||?→?→?→||<-?<-?<-SIG+PDZ</td><td>LRR-repeats+AP-GTPase+COR+DrHyd</td><td>1001</td><td>C0622_14350</td><td>Deltaproteobacteria</td><td>Desulfuromonas sp.</td><td>hypothetical protein C0622_14350 [Desulfuromonas sp.].</td><td>GCA_002869665.1</td></tr>
<tr><td>PLY10861.1</td><td>LRR-repeats+AP-GTPase+COR*→||<-?<-tRNA<-?<-?||Cluster1398_2clades→ ABC-ATPase→</td><td>LRR-repeats+AP-GTPase+COR</td><td>1225</td><td>C0626_04310</td><td>Epsilonproteobacteria</td><td>Arcobacter sp.</td><td>hypothetical protein C0626_04310 [Arcobacter sp.].</td><td>GCA_002869535.1</td></tr>
<tr><td>POZ53622.1</td><td>REC→||<-?<-?<-?<-?||?→ LRR-repeats+AP-GTPase+COR+DpnII-MboI-NTD+TIR*→</td><td>LRR-repeats+AP-GTPase+COR+DpnII-MboI-NTD+TIR</td><td>1108</td><td>AADEFJLK_00657</td><td>Gammaproteobacteria</td><td>Methylovulum psychrotolerans</td><td>GTP-binding protein [Methylovulum psychrotolerans].</td><td>GCA_002923755.1</td></tr>
<tr><td>PPT07796.1</td><td>LRR-repeats+AP-GTPase+COR+BactIG*→</td><td>LRR-repeats+AP-GTPase+COR+BactIG</td><td>726</td><td>CKA32_002768</td><td>Cyanobacteria</td><td>Geitlerinema sp. FC II</td><td>Leucine-rich repeat containing protein [Geitlerinema sp. FC II].</td><td>GCA_002286845.1</td></tr>
<tr><td>PRQ08092.1</td><td>LRR-repeats+AP-GTPase+COR+PNPase*→</td><td>LRR-repeats+AP-GTPase+COR+PNPase</td><td>933</td><td>mtnN</td><td>Deltaproteobacteria</td><td>Enhygromyxa salina</td><td>5'-methylthioadenosine/S-adenosylhomocysteine nucleosidase [Enhygromyxa salina].</td><td>GCA_002994635.1</td></tr>
<tr><td>PSB34361.1</td><td><-REC||REC→||<-?<-?<-?<-TIR+TM+CHASE2+CHASE2+TM+TM+TM||PSE→ LRR-repeats+AP-GTPase+COR+TIR*→</td><td>LRR-repeats+AP-GTPase+COR+TIR</td><td>1113</td><td>C7B82_02520</td><td>Cyanobacteria</td><td>Stenomitos frigidus ULC18</td><td>serine/threonine protein kinase [Stenomitos frigidus ULC18].</td><td>GCA_003003795.1</td></tr>
<tr><td>PSM30901.1</td><td>LRR-repeats+AP-GTPase+COR*→</td><td>LRR-repeats+AP-GTPase+COR</td><td>431</td><td>BVG81_008130</td><td>Deltaproteobacteria</td><td>Haliangium sp. UPWRP_2</td><td>GTPase, partial [Haliangium sp. UPWRP_2].</td><td>GCA_002212765.2</td></tr>
<tr><td>PTS96034.1</td><td>LRR-repeats+AP-GTPase*→</td><td>LRR-repeats+AP-GTPase</td><td>357</td><td>DBR27_16615</td><td>Bacteroidetes</td><td>Flavobacterium sp. HMWF030</td><td>hypothetical protein DBR27_16615, partial [Flavobacterium sp. HMWF030].</td><td>GCA_003061345.1</td></tr>
<tr><td>PWQ95663.1</td><td>LRR-repeats+AP-GTPase+COR+TIR*→</td><td>LRR-repeats+AP-GTPase+COR+TIR</td><td>801</td><td>DKT75_11555</td><td>Gammaproteobacteria</td><td>Leucothrix arctica</td><td>GTP-binding protein, partial [Leucothrix arctica].</td><td>GCA_003172895.1</td></tr>
<tr><td>PXF61747.1</td><td>LRR-repeats+AP-GTPase+COR*→</td><td>LRR-repeats+AP-GTPase+COR</td><td>978</td><td>C4B59_02515</td><td>Euryarchaeota</td><td>ANME-2 cluster archaeon</td><td>hypothetical protein C4B59_02515 [ANME-2 cluster archaeon].</td><td>GCA_003194445.1</td></tr>
<tr><td>PXX81924.1</td><td><-ACET<-?<-?<-?||?→ HSP70→ DNAJ→ LRR-repeats+AP-GTPase+COR+TIR*→</td><td>LRR-repeats+AP-GTPase+COR+TIR</td><td>947</td><td>DFR34_101153</td><td>Betaproteobacteria</td><td>Rivicola pingtungensis</td><td>internalin A [Rivicola pingtungensis].</td><td>GCA_003201855.1</td></tr>
<tr><td>PYI92098.1</td><td>LRR-repeats+AP-GTPase+COR+TIR*→</td><td>LRR-repeats+AP-GTPase+COR+TIR</td><td>882</td><td>DME97_11675</td><td>Verrucomicrobia</td><td>Verrucomicrobia bacterium</td><td>hypothetical protein DME97_11675 [Verrucomicrobia bacterium].</td><td>GCA_003218375.1</td></tr>
<tr><td>PYQ65591.1</td><td>LRR-repeats+AP-GTPase+COR+TIR*→||<-START</td><td>LRR-repeats+AP-GTPase+COR+TIR</td><td>985</td><td>DMF53_05195</td><td>Acidobacteria</td><td>Acidobacteria bacterium</td><td>hypothetical protein DMF53_05195 [Acidobacteria bacterium].</td><td>GCA_003223675.1</td></tr>
<tr><td>PZN70482.1</td><td>LRR-repeats+AP-GTPase+COR*→ TIR→</td><td>LRR-repeats+AP-GTPase+COR</td><td>479</td><td>DM484_28490</td><td>Gammaproteobacteria</td><td>Candidatus Methyloumidiphilus alinensis</td><td>hypothetical protein DM484_28490 [Candidatus Methyloumidiphilus alinensis].</td><td>GCA_003242955.1</td></tr>
<tr><td>PZN77149.1</td><td>LRR-repeats+AP-GTPase+COR+TIR*→</td><td>LRR-repeats+AP-GTPase+COR+TIR</td><td>1032</td><td>DM484_15240</td><td>Gammaproteobacteria</td><td>Candidatus Methyloumidiphilus alinensis</td><td>hypothetical protein DM484_15240 [Candidatus Methyloumidiphilus alinensis].</td><td>GCA_003242955.1</td></tr>
<tr><td>PZN84233.1</td><td>LRR-repeats+AP-GTPase+COR+TIR*→</td><td>LRR-repeats+AP-GTPase+COR+TIR</td><td>820</td><td>DM484_03135</td><td>Gammaproteobacteria</td><td>Candidatus Methyloumidiphilus alinensis</td><td>GTP-binding protein [Candidatus Methyloumidiphilus alinensis].</td><td>GCA_003242955.1</td></tr>
<tr><td>PZO19892.1</td><td><-SIG+TM+TM+TM||?→?→?→ LRR-repeats+AP-GTPase+COR*→</td><td>LRR-repeats+AP-GTPase+COR</td><td>715</td><td>DCF25_07770</td><td>Cyanobacteria</td><td>Leptolyngbya foveolarum</td><td>hypothetical protein DCF25_07770, partial [Leptolyngbya foveolarum].</td><td>GCA_003242035.1</td></tr>
<tr><td>PZO41190.1</td><td>LRR-repeats+AP-GTPase+COR*→||<-PIN<-PSE||HISKIN→ REC→</td><td>LRR-repeats+AP-GTPase+COR</td><td>872</td><td>DCF19_10445</td><td>Cyanobacteria</td><td>Pseudanabaena frigida</td><td>GTPase [Pseudanabaena frigida].</td><td>GCA_003242085.1</td></tr>
<tr><td>PZO43272.1</td><td>Cluster53_2clades→ Cluster53_2clades→?→ LRR-repeats+AP-GTPase+COR+CASPASE*→||<-?<-?||?→?→ Cluster944_3clades→||<-tRNA<-NUDIX||REC→</td><td>LRR-repeats+AP-GTPase+COR+CASPASE</td><td>1158</td><td>DCF19_04785</td><td>Cyanobacteria</td><td>Pseudanabaena frigida</td><td>GTPase [Pseudanabaena frigida].</td><td>GCA_003242085.1</td></tr>
<tr><td>PZO45083.1</td><td>PAS+HISKIN→ REC→ HISKIN→||<-?<-?<-?||LRR-repeats+AP-GTPase+COR*→||<-?||?→||<-?||?→?→?→||<-ABC-ATPase</td><td>LRR-repeats+AP-GTPase+COR</td><td>1026</td><td>DCF19_00905</td><td>Cyanobacteria</td><td>Pseudanabaena frigida</td><td>hypothetical protein DCF19_00905 [Pseudanabaena frigida].</td><td>GCA_003242085.1</td></tr>
<tr><td>PZS26024.1</td><td>LRR-repeats+AP-GTPase*→</td><td>LRR-repeats+AP-GTPase</td><td>304</td><td>DLM61_19115</td><td>Actinobacteria</td><td>Pseudonocardiales bacterium</td><td>hypothetical protein DLM61_19115 [Pseudonocardiales bacterium].</td><td>GCA_003244245.1</td></tr>
<tr><td>PZU97949.1</td><td><-Cluster53_2clades||?→ Cluster53_2clades→ Cluster54_3clades→?→ RelE-ParE→ LRR-repeats+AP-GTPase+COR+DUF4404*→?→?→||<-Cluster1583_2clades<-Cluster1881_2clades<-Cluster1340_2clades</td><td>LRR-repeats+AP-GTPase+COR+DUF4404</td><td>1125</td><td>DCE90_05495</td><td>Cyanobacteria</td><td>Pseudanabaena sp.</td><td>Ras family protein [Pseudanabaena sp.].</td><td>GCA_003249015.1</td></tr>
</table>

<table>
<tr><th>acc</th><th>operon</th><th>architecture</th><th>len</th><th>gen.name</th><th>taxend</th><th>species</th><th>defline</th><th>gca</th></tr>
<tr><td>PZV15467.1</td><td>Cluster53_2clades→?→ LRR-repeats+AP-GTPase+COR+TM+TM*→?→||<-?<-?<-ABC-ATPase<-?||PSE→?→ ABC_membrane+ABC_tran→</td><td>LRR-repeats+AP-GTPase+COR+TM+TM</td><td>966</td><td>DCF20_10315</td><td>Cyanobacteria</td><td>Pseudanabaena sp.</td><td>GTP-binding protein [Pseudanabaena sp.].</td><td>GCA_003249035.1</td></tr>
<tr><td>PZV19789.1</td><td>LRR-repeats+AP-GTPase+COR*→?→ Cluster2508_2clades→</td><td>LRR-repeats+AP-GTPase+COR</td><td>779</td><td>DCF21_06795</td><td>Cyanobacteria</td><td>Leptolyngbya sp.</td><td>GTP-binding protein [Leptolyngbya sp.].</td><td>GCA_003249155.1</td></tr>
<tr><td>PZV24423.1</td><td>LRR-repeats+AP-GTPase+COR*→</td><td>LRR-repeats+AP-GTPase+COR</td><td>522</td><td>DCF12_17370</td><td>Cyanobacteria</td><td>Snowella sp.</td><td>hypothetical protein DCF12_17370 [Snowella sp.].</td><td>GCA_003249095.1</td></tr>
<tr><td>PZV25744.1</td><td><-REC||?→?→||<-?<-?||?→ LRR-repeats+AP-GTPase+COR*→||<-DAGKIN||?→ Cluster5_5clades→</td><td>LRR-repeats+AP-GTPase+COR</td><td>860</td><td>DCF12_13370</td><td>Cyanobacteria</td><td>Snowella sp.</td><td>GTP-binding protein [Snowella sp.].</td><td>GCA_003249095.1</td></tr>
<tr><td>PZV26136.1</td><td><-Cluster1004_3clades||Cluster1843_2clades→||<-?||Cluster5_5clades→||<-Cluster1306_2clades<-?<-?||LRR-repeats+AP-GTPase+COR*→</td><td>LRR-repeats+AP-GTPase+COR</td><td>885</td><td>DCF12_11170</td><td>Cyanobacteria</td><td>Snowella sp.</td><td>GTPase [Snowella sp.].</td><td>GCA_003249095.1</td></tr>
<tr><td>PZW23552.1</td><td>LRR-repeats→ LRR-repeats+AP-GTPase+COR*→</td><td>LRR-repeats+AP-GTPase+COR</td><td>813</td><td>EI42_04935</td><td>Chloroflexi</td><td>Thermosporothrix hazakensis</td><td>GTPase SAR1 family protein, partial [Thermosporothrix hazakensis].</td><td>GCA_003253565.1</td></tr>
<tr><td>QDV38731.1</td><td>RelE-ParE→ WCAK→?→||<-Cluster2079_2clades||?→?→?→ LRR-repeats+AP-GTPase+COR+DUF4404*→</td><td>LRR-repeats+AP-GTPase+COR+DUF4404</td><td>887</td><td>slrP</td><td>Planctomycetes</td><td>Tautonia plasticadhaerens</td><td>E3 ubiquitin-protein ligase SlrP [Tautonia plasticadhaerens].</td><td>GCA_007752535.1</td></tr>
<tr><td>QEN02468.1</td><td>HTH→?→?→ LRR-repeats+AP-GTPase+COR+TIR*→||<-?<-ABC_membrane+ABC_tran||Cyanophycinsyn-ATPgrasp→ Cyanophycinsyn-ATPgrasp→</td><td>LRR-repeats+AP-GTPase+COR+TIR</td><td>1307</td><td>EWH46_18040</td><td>Betaproteobacteria</td><td>Sphaerotilus natans subsp. sulfidivorans</td><td>TIR domain-containing protein [Sphaerotilus natans subsp. sulfidivorans].</td><td>GCA_008329925.1</td></tr>
<tr><td>QHA06431.1</td><td>LRR-repeats+AP-GTPase+COR*→||<-?||?→||<-?<-SIGMA-HTH<-?<-SIGMA-HTH</td><td>LRR-repeats+AP-GTPase+COR</td><td>1022</td><td>GQF42_26905</td><td>Actinobacteria</td><td>Streptomyces broussonetiae</td><td>GTPase [Streptomyces broussonetiae].</td><td>GCA_009796285.1</td></tr>
<tr><td>QKQ77944.1</td><td>LRR-repeats+AP-GTPase+COR*→?→||<-?||?→||<-?||?→ ClpABN-AAA+ClpABC-AAA→||<-PSE<-SIG+TPR+TPR+TPR+TPR+TPR+TPR</td><td>LRR-repeats+AP-GTPase+COR</td><td>894</td><td>FBB35_26070</td><td>Cyanobacteria</td><td>Nostoc sp. TCL240-02</td><td>GTPase [Nostoc sp. TCL240-02].</td><td>GCA_013343235.1</td></tr>
<tr><td>QLE48778.1</td><td>ABhydrolase→?→||<-?||LRR-repeats+AP-GTPase+COR+TIR*→||<-PSE<-?||?→||<-?<-SIG+NUDIX<-?<-?<-Cluster5_5clades</td><td>LRR-repeats+AP-GTPase+COR+TIR</td><td>1021</td><td>FD724_12065</td><td>Cyanobacteria</td><td>Nostoc sp. C057</td><td>TIR domain-containing protein [Nostoc sp. C057].</td><td>GCA_013393925.1</td></tr>
<tr><td>QLH39529.1</td><td>Cluster850_2clades→?→?→ PRTase→||<-ENDO3+NUDIX<-?||LRR-repeats+AP-GTPase+COR+TIR*→</td><td>LRR-repeats+AP-GTPase+COR+TIR</td><td>816</td><td>HWD60_12020</td><td>Alphaproteobacteria</td><td>Defluviicoccus sp.</td><td>leucine-rich repeat domain-containing protein [Defluviicoccus sp.].</td><td>GCA_013414705.1</td></tr>
<tr><td>RAM50807.1</td><td>LRR-repeats+AP-GTPase+COR+TIR*→?→||<-SIG+TM+TM+TM+TM+TM+TM+TM+TM+TM+TM+TM</td><td>LRR-repeats+AP-GTPase+COR+TIR</td><td>849</td><td>C6Y22_14980</td><td>Cyanobacteria</td><td>Hapalosiphonaceae cyanobacterium JJU2</td><td>hypothetical protein C6Y22_14980 [Hapalosiphonaceae cyanobacterium JJU2].</td><td>GCA_003261315.1</td></tr>
<tr><td>RCJ18019.1</td><td><-Pkinase+Pentapeptide<-?<-?||?→||<-?<-TIR+EAD9<-?||LRR-repeats+AP-GTPase+COR+TIR*→</td><td>LRR-repeats+AP-GTPase+COR+TIR</td><td>986</td><td>A6770_33290</td><td>Cyanobacteria</td><td>Nostoc minutum NIES-26</td><td>GTPase [Nostoc minutum NIES-26].</td><td>GCA_003326215.1</td></tr>
<tr><td>RCJ19389.1</td><td>LRR-repeats+AP-GTPase+COR*→||<-ClpABN-AAA+ClpABC-AAA<-?||METHYLASE→</td><td>LRR-repeats+AP-GTPase+COR</td><td>907</td><td>A6S26_26810</td><td>Cyanobacteria</td><td>Nostoc sp. ATCC 43529</td><td>GTPase [Nostoc sp. ATCC 43529].</td><td>GCA_003326205.1</td></tr>
<tr><td>RCJ25694.1</td><td><-Pkinase+Pentapeptide<-?<-?||?→||<-?||LRR-repeats+AP-GTPase+COR+TIR*→||<-?<-?<-?||?→||<-NUDIX</td><td>LRR-repeats+AP-GTPase+COR+TIR</td><td>962</td><td>A6S26_15250</td><td>Cyanobacteria</td><td>Nostoc sp. ATCC 43529</td><td>GTPase [Nostoc sp. ATCC 43529].</td><td>GCA_003326205.1</td></tr>
<tr><td>RCJ30096.1</td><td>CR-REase5→ LRR-repeats+AP-GTPase+COR*→?→||<-?||?→||<-?||?→ ClpABN-AAA+ClpABC-AAA→</td><td>LRR-repeats+AP-GTPase+COR</td><td>894</td><td>A6770_21505</td><td>Cyanobacteria</td><td>Nostoc minutum NIES-26</td><td>GTPase [Nostoc minutum NIES-26].</td><td>GCA_003326215.1</td></tr>
<tr><td>RCJ30909.1</td><td>Cyanophycinsyn-ATPgrasp→||<-Cluster833_2clades<-?||?→||<-?<-Four-helical-protein<-?||LRR-repeats+AP-GTPase+COR+TIR*→||<-ncRNA<-?<-?<-?||?→||<-?||?→
Thioredoxin→</td><td>LRR-repeats+AP-GTPase+COR+TIR</td><td>928</td><td>A6770_20515</td><td>Cyanobacteria</td><td>Nostoc minutum NIES-26</td><td>hypothetical protein A6770_20515 [Nostoc minutum NIES-26].</td><td>GCA_003326215.1</td></tr>
<tr><td>RCJ31306.1</td><td>METHYLASE→||<-Arginase<-?<-?||LRR-repeats+AP-GTPase+COR+TIR*→?→?→||<-?<-?||?→ Pentapeptide-repeats→</td><td>LRR-repeats+AP-GTPase+COR+TIR</td><td>1103</td><td>A6770_20105</td><td>Cyanobacteria</td><td>Nostoc minutum NIES-26</td><td>GTPase [Nostoc minutum NIES-26].</td><td>GCA_003326215.1</td></tr>
<tr><td>RCJ40793.1</td><td>LRR-repeats+AP-GTPase+COR+TIR*→</td><td>LRR-repeats+AP-GTPase+COR+TIR</td><td>1079</td><td>A6770_10335</td><td>Cyanobacteria</td><td>Nostoc minutum NIES-26</td><td>GTPase [Nostoc minutum NIES-26].</td><td>GCA_003326215.1</td></tr>
<tr><td>RCJ41185.1</td><td><-TM||?→||<-?||?→||<-?||?→ LRR-repeats+AP-GTPase+COR+TM+TM*→</td><td>LRR-repeats+AP-GTPase+COR+TM+TM</td><td>837</td><td>A6770_08860</td><td>Cyanobacteria</td><td>Nostoc minutum NIES-26</td><td>hypothetical protein A6770_08860 [Nostoc minutum NIES-26].</td><td>GCA_003326215.1</td></tr>
<tr><td>RCV66029.1</td><td>LRR-repeats+AP-GTPase+COR→?→||<-?<-?||?→||<-?<-?||LRR-repeats→ LRR-repeats+AP-GTPase+COR→</td><td>LRR-repeats+AP-GTPase+COR</td><td>640</td><td>C5S53_00730</td><td>Euryarchaeota</td><td>Methanophagales archaeon</td><td>GTPase SAR1 family protein [Methanophagales archaeon].</td><td>GCA_003336485.1</td></tr>
<tr><td>RCV66037.1</td><td>LRR-repeats+AP-GTPase+COR*→</td><td>LRR-repeats+AP-GTPase+COR</td><td>730</td><td>C5S53_00775</td><td>Euryarchaeota</td><td>Methanophagales archaeon</td><td>Leucine-rich repeat (LRR) protein [Methanophagales archaeon].</td><td>GCA_003336485.1</td></tr>
<tr><td>REJ39442.1</td><td>SIG+TM+TM+TM+TM+TM+TM+TM→||<-?||Mbetalac→ SAM-methylase→?→||<-?<-SIG+TPM_phosphatase+TM+TM+TM||PSE→
LRR-repeats+AP-GTPase+COR+TM+TM*→||<-?<-?<-?<-Pro_CA||?→||<-?<-tRNA<-tRNA<-Ferredoxin-betagrasp</td><td>LRR-repeats+AP-GTPase+COR+TM+TM</td><td>898</td><td>DWQ54_21980</td><td>Cyanobacteria</td><td>Microcystis flos-aquae TF09</td><td>GTP-binding protein [Microcystis flos-aquae TF09].</td><td>GCA_003390815.1</td></tr>
<tr><td>REJ48778.1</td><td>SIG+TM+TM+TM+TM+TM+TM+TM→||<-?||Mbetalac→ SAM-methylase→?→||<-?<-SIG+TPM_phosphatase+TM+TM+TM||PSE→
LRR-repeats+AP-GTPase+COR+TM+TM*→</td><td>LRR-repeats+AP-GTPase+COR+TM+TM</td><td>852</td><td>DWQ53_05035</td><td>Cyanobacteria</td><td>Microcystis flos-aquae DF17</td><td>GTP-binding protein [Microcystis flos-aquae DF17].</td><td>GCA_003389435.1</td></tr>
<tr><td>REJ60675.1</td><td>SIG+TM+TM+TM+TM+TM+TM+TM→||<-?||Mbetalac→ SAM-methylase→?→||<-?<-SIG+TPM_phosphatase+TM+TM+TM||PSE→
LRR-repeats+AP-GTPase+COR+TM+TM*→||<-?<-?<-?<-Pro_CA||?→||<-?<-tRNA<-tRNA<-Ferredoxin-betagrasp</td><td>LRR-repeats+AP-GTPase+COR+TM+TM</td><td>826</td><td>DWQ56_05670</td><td>Cyanobacteria</td><td>Microcystis aeruginosa DA14</td><td>GTP-binding protein [Microcystis aeruginosa DA14].</td><td>GCA_003388675.1</td></tr>
</table>

<table>
<tr><th>acc</th><th>operon</th><th>architecture</th><th>len</th><th>gen.name</th><th>taxend</th><th>species</th><th>defline</th><th>gca</th></tr>
<tr><td>RFP53135.1</td><td>LRR-repeats+AP-GTPase+COR*→||<-?||?→||<-tRNA||?→?→?→ Cluster1088_2clades→</td><td>LRR-repeats+AP-GTPase+COR</td><td>695</td><td>BJG00_017645</td><td>Cyanobacteria</td><td>Limnothrix sp. CACIAM 69d</td><td>hypothetical protein BJG00_017645 [Limnothrix sp. CACIAM 69d].</td><td>GCA_001913845.2</td></tr>
<tr><td>RFP63081.1</td><td><-CR-REase5||LRR-repeats+AP-GTPase+COR*→ LRR-repeats→</td><td>LRR-repeats+AP-GTPase+COR</td><td>886</td><td>BJG00_001105</td><td>Cyanobacteria</td><td>Limnothrix sp. CACIAM 69d</td><td>GTPase [Limnothrix sp. CACIAM 69d].</td><td>GCA_001913845.2</td></tr>
<tr><td>RIK29270.1</td><td>LRR-repeats+AP-GTPase+COR*→||<-?<-?<-?<-Mbetalac<-?<-ABC-ATPase<-TM+TM+TM+TM+TM</td><td>LRR-repeats+AP-GTPase+COR</td><td>1096</td><td>DCC56_13010</td><td>Chloroflexi</td><td>Anaerolineae bacterium</td><td>hypothetical protein DCC56_13010 [Anaerolineae bacterium].</td><td>GCA_003577395.1</td></tr>
<tr><td>RJO63533.1</td><td>LRR-repeats+AP-GTPase+COR*→</td><td>LRR-repeats+AP-GTPase+COR</td><td>1030</td><td>C4523_20060</td><td>Deltaproteobacteria</td><td>Myxococcales bacterium</td><td>hypothetical protein C4523_20060 [Myxococcales bacterium].</td><td>GCA_003598065.1</td></tr>
<tr><td>RJP51705.1</td><td>TM+TM+TM+TM+TM+TM+TM+TM→?→?→||<-?||?→ Cluster1070_2clades→ REase→ LRR-repeats+AP-GTPase+COR*→||<-?||?→?→?→||<-Cluster1854_2clades</td><td>LRR-repeats+AP-GTPase+COR</td><td>984</td><td>C4583_08075</td><td>Chloroflexi</td><td>Anaerolineaceae bacterium</td><td>hypothetical protein C4583_08075 [Anaerolineaceae bacterium].</td><td>GCA_003598975.1</td></tr>
<tr><td>RKS02636.1</td><td>LRR-repeats+AP-GTPase+COR+TM+TM*→</td><td>LRR-repeats+AP-GTPase+COR+TM+TM</td><td>1044</td><td>C8C84_2360</td><td>Bacteroidetes</td><td>Flavobacterium sp. 102</td><td>RocCOR-like putative regulator of kinase activity [Flavobacterium sp. 102].</td><td>GCA_003634615.1</td></tr>
<tr><td>RKZ87118.1</td><td>LRR-repeats+AP-GTPase+COR*→ AP-GTPase+COR→ COR→</td><td>LRR-repeats+AP-GTPase+COR</td><td>159</td><td>DRR19_14025</td><td>Gammaproteobacteria</td><td>Gammaproteobacteria bacterium</td><td>hypothetical protein DRR19_14025 [Gammaproteobacteria bacterium].</td><td>GCA_003646175.1</td></tr>
<tr><td>RKZ92191.1</td><td>LRR-repeats+AP-GTPase+COR*→</td><td>LRR-repeats+AP-GTPase+COR</td><td>778</td><td>DRR19_04995</td><td>Gammaproteobacteria</td><td>Gammaproteobacteria bacterium</td><td>hypothetical protein DRR19_04995 [Gammaproteobacteria bacterium].</td><td>GCA_003646175.1</td></tr>
<tr><td>RLC02552.1</td><td>LRR-repeats+AP-GTPase+COR*→</td><td>LRR-repeats+AP-GTPase+COR</td><td>868</td><td>DRI57_29970</td><td>Deltaproteobacteria</td><td>Deltaproteobacteria bacterium</td><td>hypothetical protein DRI57_29970, partial [Deltaproteobacteria bacterium].</td><td>GCA_003647425.1</td></tr>
<tr><td>RLC14341.1</td><td>LRR-repeats+AP-GTPase+COR*→</td><td>LRR-repeats+AP-GTPase+COR</td><td>442</td><td>DRI57_14615</td><td>Deltaproteobacteria</td><td>Deltaproteobacteria bacterium</td><td>hypothetical protein DRI57_14615, partial [Deltaproteobacteria bacterium].</td><td>GCA_003647425.1</td></tr>
<tr><td>RLC16559.1</td><td>LRR-repeats+AP-GTPase+COR*→</td><td>LRR-repeats+AP-GTPase+COR</td><td>930</td><td>DRI57_11050</td><td>Deltaproteobacteria</td><td>Deltaproteobacteria bacterium</td><td>hypothetical protein DRI57_11050 [Deltaproteobacteria bacterium].</td><td>GCA_003647425.1</td></tr>
<tr><td>RLD05606.1</td><td>UvsW-A18→ LRR-repeats+AP-GTPase+COR+TIR*→</td><td>LRR-repeats+AP-GTPase+COR+TIR</td><td>759</td><td>DRI32_03940</td><td>Chloroflexi</td><td>Chloroflexi bacterium</td><td>GTP-binding protein [Chloroflexi bacterium].</td><td>GCA_003648185.1</td></tr>
<tr><td>RLS84177.1</td><td>SIG+AP-GTPase+COR+TIR→ *FGS+LRR-repeats+AP-GTPase+COR+TIR→*</td><td>SIG+AP-GTPase+COR+TIR</td><td>879</td><td>DWI04_01885</td><td>Planctomycetes</td><td>Planctomycetes bacterium</td><td>TIR domain-containing protein, partial [Planctomycetes bacterium].</td><td>GCA_003669255.1</td></tr>
<tr><td>RLS84178.1</td><td>FGS+LRR-repeats+AP-GTPase+COR+TIR*→</td><td>FGS+LRR-repeats+AP-GTPase+COR+TIR</td><td>1292</td><td>DWI04_01890</td><td>Planctomycetes</td><td>Planctomycetes bacterium</td><td>hypothetical protein DWI04_01890, partial [Planctomycetes bacterium].</td><td>GCA_003669255.1</td></tr>
<tr><td>RME07294.1</td><td>RVT→?→ LRR-repeats+AP-GTPase+COR*→</td><td>LRR-repeats+AP-GTPase+COR</td><td>780</td><td>D6816_06800</td><td>Bacteroidetes</td><td>Bacteroidetes bacterium</td><td>hypothetical protein D6816_06800 [Bacteroidetes bacterium].</td><td>GCA_003694615.1</td></tr>
<tr><td>RMG75718.1</td><td>LRR-repeats+AP-GTPase+COR*→?→ FGS→</td><td>LRR-repeats+AP-GTPase+COR</td><td>691</td><td>D6722_00510</td><td>Bacteroidetes</td><td>Bacteroidetes bacterium</td><td>hypothetical protein D6722_00510 [Bacteroidetes bacterium].</td><td>GCA_003696875.1</td></tr>
<tr><td>RPH24755.1</td><td>LRR-repeats+AP-GTPase+COR*→</td><td>LRR-repeats+AP-GTPase+COR</td><td>627</td><td>EHM93_20250</td><td>Bacteroidetes</td><td>Bacteroidales bacterium</td><td>GTP-binding protein, partial [Bacteroidales bacterium].</td><td>GCA_003818205.1</td></tr>
<tr><td>RPI17218.1</td><td>LRR-repeats+AP-GTPase+COR*→</td><td>LRR-repeats+AP-GTPase+COR</td><td>944</td><td>EHM58_10175</td><td>Ignavibacteriae</td><td>Ignavibacteriae bacterium</td><td>GTP-binding protein [Ignavibacteriae bacterium].</td><td>GCA_003820375.1</td></tr>
<tr><td>RPJ19448.1</td><td>LRR-repeats+AP-GTPase+COR*→</td><td>LRR-repeats+AP-GTPase+COR</td><td>750</td><td>EHM33_30260</td><td>Chloroflexi</td><td>Chloroflexi bacterium</td><td>hypothetical protein EHM33_30260, partial [Chloroflexi bacterium].</td><td>GCA_003820175.1</td></tr>
<tr><td>RPJ72349.1</td><td>LRR-repeats+AP-GTPase+COR*→</td><td>LRR-repeats+AP-GTPase+COR</td><td>496</td><td>EHM20_13865</td><td>Alphaproteobacteria</td><td>Alphaproteobacteria bacterium</td><td>GTP-binding protein, partial [Alphaproteobacteria bacterium].</td><td>GCA_003820255.1</td></tr>
<tr><td>RQW02493.1</td><td>LRR-repeats+AP-GTPase+COR+DrHyd*→?→?→ Cluster1207_2clades→?→ Cluster2271_2clades→</td><td>LRR-repeats+AP-GTPase+COR+DrHyd</td><td>763</td><td>EH223_12385</td><td>Bacteria</td><td>candidate division KSB1 bacterium</td><td>hypothetical protein EH223_12385, partial [candidate division KSB1 bacterium].</td><td>GCA_003854975.1</td></tr>
<tr><td>RUR83995.1</td><td><-Cluster5_5clades||LRR-repeats→ LRR-repeats+AP-GTPase+COR+DUF4404*→||<-?<-ABC-ATPase||?→||<-REC</td><td>LRR-repeats+AP-GTPase+COR+DUF4404</td><td>746</td><td>DSM107007_30530</td><td>Cyanobacteria</td><td>Nostoc sp. PCC 7120 = FACHB-418</td><td>hypothetical protein DSM107007_30530 [Nostoc sp. PCC 7120 = FACHB-418].</td><td>GCA_003990585.1</td></tr>
<tr><td>RWX44973.1</td><td><-Cluster1731_2clades<-?<-?||?→?→ LRR-repeats+AP-GTPase+COR*→?→?→||<-Cluster1409_2clades</td><td>LRR-repeats+AP-GTPase+COR</td><td>648</td><td>H206_01140</td><td>Deltaproteobacteria</td><td>Candidatus Electrothrix aarhusiensis</td><td>hypothetical protein H206_01140 [Candidatus Electrothrix aarhusiensis].</td><td>GCA_004028505.1</td></tr>
</table>

<table>
<tr><th>acc</th><th>operon</th><th>architecture</th><th>len</th><th>gen.name</th><th>taxend</th><th>species</th><th>defline</th><th>gca</th></tr>
<tr><td>RWX45664.1</td><td><-S18-HTH<-Cluster1004_3clades<-Cluster2139_2clades<-?<-?<-?||?→ LRR-repeats+AP-GTPase+COR*→</td><td>LRR-repeats+AP-GTPase+COR</td><td>605</td><td>H206_01449</td><td>Deltaproteobacteria</td><td>Candidatus Electrothrix aarhusiensis</td><td>hypothetical protein H206_01449 [Candidatus Electrothrix aarhusiensis].</td><td>GCA_004028505.1</td></tr>
<tr><td>RWX46469.1</td><td>Aminotran_1_2→||<-ABC-ATPase<-?<-?<-?<-?||LRR-repeats+AP-GTPase+COR*→</td><td>LRR-repeats+AP-GTPase+COR</td><td>908</td><td>H206_00859</td><td>Deltaproteobacteria</td><td>Candidatus Electrothrix aarhusiensis</td><td>internalin A [Candidatus Electrothrix aarhusiensis].</td><td>GCA_004028505.1</td></tr>
<tr><td>RWX47043.1</td><td>LRR-repeats+AP-GTPase+COR+TM+TM+TM*→ WCAK→</td><td>LRR-repeats+AP-GTPase+COR+TM+TM+TM</td><td>873</td><td>H206_03377</td><td>Deltaproteobacteria</td><td>Candidatus Electrothrix aarhusiensis</td><td>Ras of Complex, Roc, domain of DAPkinase [Candidatus Electrothrix aarhusiensis].</td><td>GCA_004028505.1</td></tr>
<tr><td>RWX51844.1</td><td>LRR-repeats+AP-GTPase+COR*→</td><td>LRR-repeats+AP-GTPase+COR</td><td>785</td><td>VU01_10733</td><td>Deltaproteobacteria</td><td>Candidatus Electrothrix marina</td><td>hypothetical protein VU01_10733 [Candidatus Electrothrix marina].</td><td>GCA_004028495.1</td></tr>
<tr><td>RYD26380.1</td><td>LRR-repeats+AP-GTPase+COR*→</td><td>LRR-repeats+AP-GTPase+COR</td><td>442</td><td>EOP86_26045</td><td>Verrucomicrobia</td><td>Verrucomicrobiaceae bacterium</td><td>hypothetical protein EOP86_26045, partial [Verrucomicrobiaceae bacterium].</td><td>GCA_004143925.1</td></tr>
<tr><td>RYD41313.1</td><td>LRR-repeats+AP-GTPase+COR*→</td><td>LRR-repeats+AP-GTPase+COR</td><td>546</td><td>EOP85_12890</td><td>Verrucomicrobia</td><td>Verrucomicrobiaceae bacterium</td><td>hypothetical protein EOP85_12890, partial [Verrucomicrobiaceae bacterium].</td><td>GCA_004144085.1</td></tr>
<tr><td>RYD45970.1</td><td>LRR-repeats+AP-GTPase*→</td><td>LRR-repeats+AP-GTPase</td><td>529</td><td>EOP85_08340</td><td>Verrucomicrobia</td><td>Verrucomicrobiaceae bacterium</td><td>hypothetical protein EOP85_08340, partial [Verrucomicrobiaceae bacterium].</td><td>GCA_004144085.1</td></tr>
<tr><td>RYD85024.1</td><td>LRR-repeats+AP-GTPase+COR+TIR*→</td><td>LRR-repeats+AP-GTPase+COR+TIR</td><td>722</td><td>EOP84_03815</td><td>Verrucomicrobia</td><td>Verrucomicrobiaceae bacterium</td><td>TIR domain-containing protein, partial [Verrucomicrobiaceae bacterium].</td><td>GCA_004144095.1</td></tr>
<tr><td>RYE22211.1</td><td>LRR-repeats+AP-GTPase+COR*→</td><td>LRR-repeats+AP-GTPase+COR</td><td>770</td><td>EOP45_08535</td><td>Bacteroidetes</td><td>Sphingobacteriaceae bacterium</td><td>GTPase, partial [Sphingobacteriaceae bacterium].</td><td>GCA_004144285.1</td></tr>
<tr><td>RYE59584.1</td><td>LRR-repeats+AP-GTPase+COR+TIR*→</td><td>LRR-repeats+AP-GTPase+COR+TIR</td><td>1318</td><td>EOP48_00430</td><td>Bacteroidetes</td><td>Sphingobacteriales bacterium</td><td>TIR domain-containing protein [Sphingobacteriales bacterium].</td><td>GCA_004144425.1</td></tr>
<tr><td>RYF36188.1</td><td>LRR-repeats+AP-GTPase*→</td><td>LRR-repeats+AP-GTPase</td><td>370</td><td>EOO38_28030</td><td>Bacteroidetes</td><td>Cytophagaceae bacterium</td><td>hypothetical protein EOO38_28030, partial [Cytophagaceae bacterium].</td><td>GCA_004145185.1</td></tr>
<tr><td>RYY12294.1</td><td>LRR-repeats+AP-GTPase*→</td><td>LRR-repeats+AP-GTPase</td><td>540</td><td>EOO04_33805</td><td>Bacteroidetes</td><td>Chitinophagaceae bacterium</td><td>hypothetical protein EOO04_33805, partial [Chitinophagaceae bacterium].</td><td>GCA_004172945.1</td></tr>
<tr><td>RYZ86256.1</td><td>LRR-repeats→ AP-GTPase+COR*→</td><td>AP-GTPase+COR</td><td>573</td><td>EOP04_14245</td><td>Proteobacteria</td><td>Proteobacteria bacterium</td><td>hypothetical protein EOP04_14245, partial [Proteobacteria bacterium].</td><td>GCA_004193275.1</td></tr>
<tr><td>RZK07337.1</td><td>LRR-repeats+AP-GTPase*→</td><td>LRR-repeats+AP-GTPase</td><td>262</td><td>EOO43_21605</td><td>Bacteroidetes</td><td>Flavobacterium sp.</td><td>hypothetical protein EOO43_21605, partial [Flavobacterium sp.].</td><td>GCA_004211555.1</td></tr>
<tr><td>RZK10466.1</td><td>LRR-repeats+AP-GTPase+COR*→</td><td>LRR-repeats+AP-GTPase+COR</td><td>421</td><td>EOO43_20245</td><td>Bacteroidetes</td><td>Flavobacterium sp.</td><td>hypothetical protein EOO43_20245, partial [Flavobacterium sp.].</td><td>GCA_004211555.1</td></tr>
<tr><td>RZN33809.1</td><td>pc1599→ Cluster1746_2clades→?→?→ Calcineurin→||<-tRNA||LRR-repeats+AP-GTPase+COR+TM+TM*→?→ Aminotran_1_2→||<-?<-?||Cluster1483_2clades→</td><td>LRR-repeats+AP-GTPase+COR+TM+TM</td><td>832</td><td>EF813_10660</td><td>Euryarchaeota</td><td>Methanosarcinales archaeon</td><td>GTP-binding protein [Methanosarcinales archaeon].</td><td>GCA_004211975.1</td></tr>
<tr><td>SCD37814.1</td><td>LRR-repeats+AP-GTPase+COR*→||<-NUDIX</td><td>LRR-repeats+AP-GTPase+COR</td><td>1358</td><td>GA0115243_1012136</td><td>Actinobacteria</td><td>Streptomyces sp. ScaeMP-e83</td><td>Leucine-rich repeat (LRR) protein, partial [Streptomyces sp. ScaeMP-e83].</td><td>GCA_900091775.1</td></tr>
<tr><td>SDD54826.1</td><td>Mbetalac→?→ LRR-repeats+AP-GTPase+COR*→||<-?<-ICLR-HTH||?→?→?→ ABhydrolase→</td><td>LRR-repeats+AP-GTPase+COR</td><td>887</td><td>SAMN05660690_4513</td><td>Actinobacteria</td><td>Geodermatophilus telluris</td><td>Leucine rich repeat-containing protein [Geodermatophilus telluris].</td><td>GCA_900102745.1</td></tr>
<tr><td>SEA38734.1</td><td>LRR-repeats+AP-GTPase+COR+TIR*→?→?→||<-Thioredoxin||YBAK→</td><td>LRR-repeats+AP-GTPase+COR+TIR</td><td>849</td><td>SAMN05660964_01466</td><td>Gammaproteobacteria</td><td>Thiothrix caldifontis</td><td>Leucine Rich repeat-containing protein [Thiothrix caldifontis].</td><td>GCA_900107695.1</td></tr>
<tr><td>SEM73063.1</td><td><-ABC-ATPase<-?||?→?→||<-?||LRR-repeats+AP-GTPase+COR*→?→?→?→||<-?<-?<-SIR2</td><td>LRR-repeats+AP-GTPase+COR</td><td>717</td><td>SAMN05660976_05948</td><td>Actinobacteria</td><td>Nonomuraea pusilla</td><td>GTPase SAR1 family protein [Nonomuraea pusilla].</td><td>GCA_900109355.1</td></tr>
<tr><td>SFG01605.1</td><td>LRR-repeats+AP-GTPase+COR+TIR*→</td><td>LRR-repeats+AP-GTPase+COR+TIR</td><td>812</td><td>SAMN05216175_102423</td><td>Gammaproteobacteria</td><td>Neptunomonas qingdaonensis</td><td>internalin A, partial [Neptunomonas qingdaonensis].</td><td>GCA_900113275.1</td></tr>
</table>

acc	operon	architecture	len	gen.name	taxend	species	defline	gca
SFL80318.1	LRR-repeats+AP-GTPase+COR+DrHyd*→?→?→?→?→||<-?<-SIG+TM+TM+TM+TM	LRR-repeats+AP-GTPase+COR+DrHyd	738	SAMN05421863_1004142	Betaproteobacteria	Nitrosomonas communis	Ras of Complex, Roc, domain of DAPkinase [Nitrosomonas communis].	GCA_900114745.1
SHH94122.1	LRR-repeats+AP-GTPase+COR+TIR*→	LRR-repeats+AP-GTPase+COR+TIR	756	VA7868_00952	Gammaproteobacteria	Vibrio aerogenes CECT 7868	Ras family protein [Vibrio aerogenes CECT 7868].	GCA_900130105.1
SHI24236.1	LRR-repeats+AP-GTPase+COR+TIR*→	LRR-repeats+AP-GTPase+COR+TIR	954	inlA	Gammaproteobacteria	Vibrio aerogenes CECT 7868	Internalin-A precursor [Vibrio aerogenes CECT 7868].	GCA_900130105.1
SKA94563.1	METHYLASE→?→||<-?||SIG+Trypsin+PDZ→?→ Cluster978_2clades→ LRR-repeats+AP-GTPase+COR+TIR*→?→?→ GDSL→	LRR-repeats+AP-GTPase+COR+TIR	1235	SAMN02745166_02182	Verrucomicrobia	Prosthecobacter debontii	Leucine-rich repeat (LRR) protein [Prosthecobacter debontii].	GCA_900167535.1
SKC31468.1	CI→ CI→?→ LRR-repeats+AP-GTPase+COR*→	LRR-repeats+AP-GTPase+COR	1108	CZ809_00946	Gammaproteobacteria	Photobacterium piscicola	Ras family protein [Photobacterium piscicola].	GCA_900166965.1
SMS01989.1	LRR-repeats+AP-GTPase+COR+TIR*→	LRR-repeats+AP-GTPase+COR+TIR	1190	inlA	Gammaproteobacteria	Vibrio mangrovi	Internalin-A precursor [Vibrio mangrovi].	GCA_900184095.1
SNS86340.1	SIG+Concanavalin-like+SUN→ LRR-repeats+AP-GTPase+COR*→?→?→?→||<-?<-ABC-ATPase	LRR-repeats+AP-GTPase+COR	686	SAMN06264365_12694	Actinobacteria	Actinoplanes regularis	hypothetical protein SAMN06264365_12694 [Actinoplanes regularis].	GCA_900188005.1
SUS04594.1	LRR-repeats+AP-GTPase+COR+TIR*→	LRR-repeats+AP-GTPase+COR+TIR	965	DF3PB_1340002	Alphaproteobacteria	uncultured Defluviicoccus sp.	Leucine-rich repeat (LRR) protein (fragment) [uncultured Defluviicoccus sp.].	GCA_900498315.1
SUS07166.1	LRR-repeats+AP-GTPase+COR+TIR*→	LRR-repeats+AP-GTPase+COR+TIR	1031	DF3PB_390011	Alphaproteobacteria	uncultured Defluviicoccus sp.	conserved hypothetical protein [uncultured Defluviicoccus sp.].	GCA_900498315.1
TAA73809.1	<-Cluster1510_2clades<-?<-?||LRR-repeats+AP-GTPase+COR→ *LRR-repeats+AP-GTPase+COR*→	LRR-repeats+AP-GTPase+COR	571	CDV28_1587	Deltaproteobacteria	Candidatus Electronema sp. GS	hypothetical protein CDV28_1587 [Candidatus Electronema sp. GS].	GCA_004284765.1
TAA73810.1	LRR-repeats+AP-GTPase+COR*→	LRR-repeats+AP-GTPase+COR	678	CDV28_1588	Deltaproteobacteria	Candidatus Electronema sp. GS	hypothetical protein CDV28_1588 [Candidatus Electronema sp. GS].	GCA_004284765.1
TAA74005.1	LRR-repeats+AP-GTPase+COR*→ SIG+TM+TM+TM+TM→	LRR-repeats+AP-GTPase+COR	803	CDV28_14413	Deltaproteobacteria	Candidatus Electronema sp. GS	hypothetical protein CDV28_14413 [Candidatus Electronema sp. GS].	GCA_004284765.1
TAA74213.1	Cluster1147_2clades→?→ LRR-repeats+AP-GTPase+COR*→	LRR-repeats+AP-GTPase+COR	680	CDV28_13522	Deltaproteobacteria	Candidatus Electronema sp. GS	hypothetical protein CDV28_13522 [Candidatus Electronema sp. GS].	GCA_004284765.1
TAA75585.1	Cluster2427_2clades→?→ Cluster1969_2clades→ Thioredoxin→?→?→?→ LRR-repeats+AP-GTPase+COR*→	LRR-repeats+AP-GTPase+COR	694	CDV28_10542	Deltaproteobacteria	Candidatus Electronema sp. GS	hypothetical protein CDV28_10542 [Candidatus Electronema sp. GS].	GCA_004284765.1
TAA76176.1	<-SIG+HSP70<-?<-?<-?<-?||LRR-repeats+AP-GTPase+COR*→||<-?<-?<-?<-?<-RelE-ParE	LRR-repeats+AP-GTPase+COR	627	CDV28_10174	Deltaproteobacteria	Candidatus Electronema sp. GS	hypothetical protein CDV28_10174 [Candidatus Electronema sp. GS].	GCA_004284765.1
TAD75245.1	RelE-ParE→ LRR-repeats+AP-GTPase+COR*→	LRR-repeats+AP-GTPase+COR	952	EA001_14520	Cyanobacteria	Oscillatoriales cyanobacterium	GTPase [Oscillatoriales cyanobacterium].	GCA_004292515.1
TAD78959.1	HISKIN→?→ LRR-repeats+AP-GTPase+COR*→||<-Cluster971_2clades	LRR-repeats+AP-GTPase+COR	1063	EA001_05550	Cyanobacteria	Oscillatoriales cyanobacterium	GTPase [Oscillatoriales cyanobacterium].	GCA_004292515.1
TAE00317.1	<-Cluster1805_2clades||?→?→ LRR-repeats+AP-GTPase+COR*→	LRR-repeats+AP-GTPase+COR	631	EAZ97_06315	Bacteroidetes	Bacteroidetes bacterium	hypothetical protein EAZ97_06315 [Bacteroidetes bacterium].	GCA_004292785.1
TAE06670.1	LRR-repeats→ LRR-repeats+AP-GTPase+COR+DUF4404*→?→?→||<-Pentapeptide-repeats	LRR-repeats+AP-GTPase+COR+DUF4404	900	EAZ94_30300	Cyanobacteria	Oscillatoriales cyanobacterium	GTPase [Oscillatoriales cyanobacterium].	GCA_004292775.1
TAE07007.1	LRR-repeats+AP-GTPase+COR*→	LRR-repeats+AP-GTPase+COR	808	EAZ95_18790	Bacteroidetes	Bacteroidetes bacterium	GTP-binding protein [Bacteroidetes bacterium].	GCA_004292795.1
TAE07511.1	LRR-repeats+AP-GTPase+COR*→	LRR-repeats+AP-GTPase+COR	774	EAZ95_18335	Bacteroidetes	Bacteroidetes bacterium	hypothetical protein EAZ95_18335 [Bacteroidetes bacterium].	GCA_004292795.1
TAE08557.1	LRR-repeats+AP-GTPase+COR*→	LRR-repeats+AP-GTPase+COR	487	EAZ95_17095	Bacteroidetes	Bacteroidetes bacterium	hypothetical protein EAZ95_17095, partial [Bacteroidetes bacterium].	GCA_004292795.1
TAE08925.1	LRR-repeats+AP-GTPase+COR*→	LRR-repeats+AP-GTPase+COR	911	EAZ94_23755	Cyanobacteria	Oscillatoriales cyanobacterium	hypothetical protein EAZ94_23755 [Oscillatoriales cyanobacterium].	GCA_004292775.1
TAE12199.1	LRR-repeats+AP-GTPase+COR*→ SIG+TM+TM+TM+TM+TM→ REase→	LRR-repeats+AP-GTPase+COR	811	EAZ95_12720	Bacteroidetes	Bacteroidetes bacterium	hypothetical protein EAZ95_12720, partial [Bacteroidetes bacterium].	GCA_004292795.1

acc	operon	architecture	len	gen.name	taxend	species	defline	gca
TAE14819.1	<-Pkinase+TM||?→ PIN→ LRR-repeats+AP-GTPase+COR*→	LRR-repeats+AP-GTPase+COR	769	EAZ95_09495	Bacteroidetes	Bacteroidetes bacterium	GTP-binding protein [Bacteroidetes bacterium].	GCA_004292795.1
TAE15606.1	LRR-repeats+AP-GTPase+COR*→	LRR-repeats+AP-GTPase+COR	866	EAZ95_08545	Bacteroidetes	Bacteroidetes bacterium	GTP-binding protein [Bacteroidetes bacterium].	GCA_004292795.1
TAE15646.1	<-CR-REase5<-?<-?||LRR-repeats→?→ PSE→ LRR-repeats+AP-GTPase+COR*→?→?→?→?→||<-?<-PSE<-?||Pentapeptide-repeats→	LRR-repeats+AP-GTPase+COR	1106	EAZ94_03785	Cyanobacteria	Oscillatoriales cyanobacterium	GTP-binding protein [Oscillatoriales cyanobacterium].	GCA_004292775.1
TAE18080.1	<-ABC_membrane+ABC_tran||?→?→ LRR-repeats+AP-GTPase+COR*→	LRR-repeats+AP-GTPase+COR	778	EAZ95_05200	Bacteroidetes	Bacteroidetes bacterium	hypothetical protein EAZ95_05200 [Bacteroidetes bacterium].	GCA_004292795.1
TAE30098.1	LRR-repeats→ LRR-repeats+AP-GTPase+COR+DUF4404*→?→?→||<-Pentapeptide-repeats	LRR-repeats+AP-GTPase+COR+DUF4404	1078	EAZ93_01305	Cyanobacteria	Oscillatoriales cyanobacterium	GTPase [Oscillatoriales cyanobacterium].	GCA_004292845.1
TAE37012.1	LRR-repeats+AP-GTPase+COR+DUF4404*→?→?→||<-Pentapeptide-repeats	LRR-repeats+AP-GTPase+COR+DUF4404	1282	EAZ90_27320	Cyanobacteria	Oscillatoriales cyanobacterium	GTPase [Oscillatoriales cyanobacterium].	GCA_004292895.1
TAE38303.1	LRR-repeats+AP-GTPase+COR*→?→?→ PSE→?→ PSE→?→||<-?<-PSE<-?||Pentapeptide-repeats→	LRR-repeats+AP-GTPase+COR	909	EAZ90_25170	Cyanobacteria	Oscillatoriales cyanobacterium	GTP-binding protein [Oscillatoriales cyanobacterium].	GCA_004292895.1
TAE48904.1	LRR-repeats→?→?→?→ LRR-repeats+AP-GTPase+COR*→	LRR-repeats+AP-GTPase+COR	695	EAZ88_23370	Cyanobacteria	Oscillatoriales cyanobacterium	GTP-binding protein, partial [Oscillatoriales cyanobacterium].	GCA_004292945.1
TAE51174.1	LRR-repeats+AP-GTPase+COR*→	LRR-repeats+AP-GTPase+COR	790	EAZ89_10570	Bacteroidetes	Bacteroidetes bacterium	hypothetical protein EAZ89_10570, partial [Bacteroidetes bacterium].	GCA_004292985.1
TAE53250.1	LRR-repeats+AP-GTPase+COR*→	LRR-repeats+AP-GTPase+COR	912	EAZ88_12540	Cyanobacteria	Oscillatoriales cyanobacterium	hypothetical protein EAZ88_12540 [Oscillatoriales cyanobacterium].	GCA_004292945.1
TAE59343.1	LRR-repeats+AP-GTPase+COR+EAD11*→	LRR-repeats+AP-GTPase+COR+EAD11	737	EAZ89_02575	Bacteroidetes	Bacteroidetes bacterium	hypothetical protein EAZ89_02575 [Bacteroidetes bacterium].	GCA_004292985.1
TAE66346.1	LRR-repeats+AP-GTPase+COR*→	LRR-repeats+AP-GTPase+COR	742	EAZ85_15975	Bacteroidetes	Bacteroidetes bacterium	GTP-binding protein, partial [Bacteroidetes bacterium].	GCA_004293265.1
TAE67608.1	LRR-repeats+AP-GTPase+COR*→	LRR-repeats+AP-GTPase+COR	911	EAZ86_16170	Cyanobacteria	Oscillatoriales cyanobacterium	hypothetical protein EAZ86_16170 [Oscillatoriales cyanobacterium].	GCA_004292955.1
TAE67866.1	FGS→?→ LRR-repeats+AP-GTPase+COR*→	LRR-repeats+AP-GTPase+COR	987	EAZ85_14755	Bacteroidetes	Bacteroidetes bacterium	hypothetical protein EAZ85_14755 [Bacteroidetes bacterium].	GCA_004293265.1
TAE68409.1	<-CR-REase5<-?||?→||<-?||LRR-repeats→?→?→ LRR-repeats+AP-GTPase+COR*→?→?→?→ PSE→||<-?<-PSE<-?||Pentapeptide-repeats→	LRR-repeats+AP-GTPase+COR	1061	EAZ86_13565	Cyanobacteria	Oscillatoriales cyanobacterium	GTP-binding protein [Oscillatoriales cyanobacterium].	GCA_004292955.1
TAE73426.1	LRR-repeats+AP-GTPase+COR*→	LRR-repeats+AP-GTPase+COR	1078	EAZ85_07065	Bacteroidetes	Bacteroidetes bacterium	hypothetical protein EAZ85_07065 [Bacteroidetes bacterium].	GCA_004293265.1
TAE76344.1	LRR-repeats+AP-GTPase+COR*→||<-Cluster1921_2clades	LRR-repeats+AP-GTPase+COR	919	EAZ85_00290	Bacteroidetes	Bacteroidetes bacterium	hypothetical protein EAZ85_00290 [Bacteroidetes bacterium].	GCA_004293265.1
TAE86412.1	LRR-repeats+AP-GTPase+COR+TIR*→||<-?<-Cluster409_3clades	LRR-repeats+AP-GTPase+COR+TIR	1151	EAY81_05595	Bacteroidetes	Bacteroidetes bacterium	TIR domain-containing protein [Bacteroidetes bacterium].	GCA_004293295.1
TAF49891.1	LRR-repeats+AP-GTPase+COR+DUF4404*→	LRR-repeats+AP-GTPase+COR+DUF4404	845	EAZ61_13785	Cyanobacteria	Oscillatoriales cyanobacterium	GTPase, partial [Oscillatoriales cyanobacterium].	GCA_004293855.1
TAF64535.1	LRR-repeats+AP-GTPase+COR+EAD11*→	LRR-repeats+AP-GTPase+COR+EAD11	1108	EAZ55_10760	Bacteroidetes	Cytophagales bacterium	hypothetical protein EAZ55_10760 [Cytophagales bacterium].	GCA_004293905.1
TAF89999.1	GTPase-AIG→?→?→||<-?<-?||LRR-repeats+AP-GTPase+COR+DUF4404*→?→?→||<-Pentapeptide-repeats	LRR-repeats+AP-GTPase+COR+DUF4404	1282	EAZ49_10560	Cyanobacteria	Oscillatoriales cyanobacterium	GTPase [Oscillatoriales cyanobacterium].	GCA_004294035.1
TAF91931.1	<-CR-REase5<-?<-?||LRR-repeats→?→?→ LRR-repeats+AP-GTPase+COR*→?→?→?→ PSE→||<-?<-PSE<-?||Pentapeptide-repeats→	LRR-repeats+AP-GTPase+COR	952	EAZ49_03665	Cyanobacteria	Oscillatoriales cyanobacterium	GTP-binding protein [Oscillatoriales cyanobacterium].	GCA_004294035.1
TAG07081.1	RVT→?→ LRR-repeats+AP-GTPase+COR+ACYC*→?→||<-?<-?<-HISKIN	LRR-repeats+AP-GTPase+COR+ACYC	962	EAZ44_01180	Bacteroidetes	Cytophagia bacterium	hypothetical protein EAZ44_01180 [Cytophagia bacterium].	GCA_004294005.1
TAG17458.1	<-CENPB||?→?→||<-?<-?||Aminotran_1_2→ LRR-repeats+AP-GTPase+COR*→	LRR-repeats+AP-GTPase+COR	975	EAZ38_17395	Bacteroidetes	Cytophagales bacterium	hypothetical protein EAZ38_17395 [Cytophagales bacterium].	GCA_004293585.1
TAG62903.1	LRR-repeats+AP-GTPase+COR*→	LRR-repeats+AP-GTPase+COR	778	EAZ28_01955	Cyanobacteria	Oscillatoriales cyanobacterium	hypothetical protein EAZ28_01955, partial [Oscillatoriales cyanobacterium].	GCA_004294275.1

<table>
<tr><th>acc</th><th>operon</th><th>architecture</th><th>len</th><th>gen.name</th><th>taxend</th><th>species</th><th>defline</th><th>gca</th></tr>
<tr><td>TAG65048.1</td><td>LRR-repeats→?→ PSE→ LRR-repeats+AP-GTPase+COR*→ PSE→?→?→ PSE→||<-?<-PSE<-?||Pentapeptide-repeats→</td><td>LRR-repeats+AP-GTPase+COR</td><td>939</td><td>EAZ25_17645</td><td>Cyanobacteria</td><td>Oscillatoriales cyanobacterium</td><td>GTP-binding protein [Oscillatoriales cyanobacterium].</td><td>GCA_004294345.1</td></tr>
<tr><td>TAG86009.1</td><td>LRR-repeats+AP-GTPase+COR*→</td><td>LRR-repeats+AP-GTPase+COR</td><td>565</td><td>EAZ18_26350</td><td>Cyanobacteria</td><td>Oscillatoriales cyanobacterium</td><td>GTP-binding protein, partial [Oscillatoriales cyanobacterium].</td><td>GCA_004294385.1</td></tr>
<tr><td>TAG86671.1</td><td>LRR-repeats+AP-GTPase+COR*→</td><td>LRR-repeats+AP-GTPase+COR</td><td>892</td><td>EAZ20_12320</td><td>Bacteroidetes</td><td>Bacteroidetes bacterium</td><td>hypothetical protein EAZ20_12320, partial [Bacteroidetes bacterium].</td><td>GCA_004294185.1</td></tr>
<tr><td>TAG91526.1</td><td>FGS→?→ LRR-repeats+AP-GTPase*→</td><td>LRR-repeats+AP-GTPase</td><td>279</td><td>EAZ20_03245</td><td>Bacteroidetes</td><td>Bacteroidetes bacterium</td><td>hypothetical protein EAZ20_03245, partial [Bacteroidetes bacterium].</td><td>GCA_004294185.1</td></tr>
<tr><td>TAG93154.1</td><td>LRR-repeats+AP-GTPase+COR*→</td><td>LRR-repeats+AP-GTPase+COR</td><td>935</td><td>EAZ19_16680</td><td>Cyanobacteria</td><td>Oscillatoriales cyanobacterium</td><td>hypothetical protein EAZ19_16680 [Oscillatoriales cyanobacterium].</td><td>GCA_004293705.1</td></tr>
<tr><td>TAH14728.1</td><td>LRR-repeats+AP-GTPase+COR*→</td><td>LRR-repeats+AP-GTPase+COR</td><td>344</td><td>EAZ09_26195</td><td>Cyanobacteria</td><td>Oscillatoriales cyanobacterium</td><td>GTP-binding protein, partial [Oscillatoriales cyanobacterium].</td><td>GCA_004293745.1</td></tr>
<tr><td>TAH21032.1</td><td>Cluster1569_2clades→ ABhydrolase→||<-?||?→?→?→||<-?||LRR-repeats+AP-GTPase*→</td><td>LRR-repeats+AP-GTPase</td><td>257</td><td>EAZ08_04815</td><td>Bacteroidetes</td><td>Cytophagales bacterium</td><td>hypothetical protein EAZ08_04815 [Cytophagales bacterium].</td><td>GCA_004294445.1</td></tr>
<tr><td>TAK37700.1</td><td>LRR-repeats+AP-GTPase+COR+EAD11*→ Cluster5_5clades→?→ METHYLASE→</td><td>LRR-repeats+AP-GTPase+COR+EAD11</td><td>783</td><td>EPO28_11660</td><td>Bacteroidetes</td><td>Saprospiraceae bacterium</td><td>hypothetical protein EPO28_11660 [Saprospiraceae bacterium].</td><td>GCA_004297645.1</td></tr>
<tr><td>TAK50544.1</td><td>Cluster1854_2clades→?→?→ PSE→?→||<-Cluster5_5clades||LRR-repeats→ LRR-repeats+AP-GTPase+COR+EAD11*→?→?→?→?→ ABhydrolase→||<-Cluster5_5clades</td><td>LRR-repeats+AP-GTPase+COR+EAD11</td><td>588</td><td>EPO28_00120</td><td>Bacteroidetes</td><td>Saprospiraceae bacterium</td><td>hypothetical protein EPO28_00120 [Saprospiraceae bacterium].</td><td>GCA_004297645.1</td></tr>
<tr><td>TAK57982.1</td><td>HhH-RADC+JAB→||<-?||?→ LRR-repeats+AP-GTPase+COR*→</td><td>LRR-repeats+AP-GTPase+COR</td><td>666</td><td>EPO24_09130</td><td>Bacteroidetes</td><td>Bacteriodetes bacterium</td><td>GTP-binding protein, partial [Bacteriodetes bacterium].</td><td>GCA_004322375.1</td></tr>
<tr><td>TAK59812.1</td><td>LRR-repeats+AP-GTPase+COR+TIR*→ tRNA→ tRNA→?→?→ Cluster2248_2clades→</td><td>LRR-repeats+AP-GTPase+COR+TIR</td><td>884</td><td>EPO24_07295</td><td>Bacteroidetes</td><td>Bacteriodetes bacterium</td><td>TIR domain-containing protein [Bacteriodetes bacterium].</td><td>GCA_004322375.1</td></tr>
<tr><td>TAK63714.1</td><td>LRR-repeats+AP-GTPase+COR+TIR*→</td><td>LRR-repeats+AP-GTPase+COR+TIR</td><td>903</td><td>EPO24_03975</td><td>Bacteroidetes</td><td>Bacteriodetes bacterium</td><td>TIR domain-containing protein [Bacteriodetes bacterium].</td><td>GCA_004322375.1</td></tr>
<tr><td>TFV86935.1</td><td>LRR-repeats+AP-GTPase+COR+DrHyd*→</td><td>LRR-repeats+AP-GTPase+COR+DrHyd</td><td>783</td><td>E4K72_22790</td><td>Betaproteobacteria</td><td>Oxalobacteraceae bacterium OM1</td><td>hypothetical protein E4K72_22790, partial [Oxalobacteraceae bacterium OM1].</td><td>GCA_004570315.1</td></tr>
<tr><td>THV28317.1</td><td>TM→ LRR-repeats+AP-GTPase+COR*→</td><td>LRR-repeats+AP-GTPase+COR</td><td>1065</td><td>E9998_11935</td><td>Actinobacteria</td><td>Glycomyces paridis</td><td>hypothetical protein E9998_11935 [Glycomyces paridis].</td><td>GCA_004912155.1</td></tr>
<tr><td>TKJ39119.1</td><td>SIGMA-HTH→?→?→||<-?<-?<-?||LRR-repeats+AP-GTPase+COR*→||<-?<-Ferredoxin-RRM</td><td>LRR-repeats+AP-GTPase+COR</td><td>968</td><td>CEE37_11920</td><td>FCB group</td><td>candidate division LCP-89 bacterium B3_LCP</td><td>GTPase [candidate division LCP-89 bacterium B3_LCP].</td><td>GCA_005223185.1</td></tr>
<tr><td>TKJ40235.1</td><td>HISKIN→?→?→?→?→?→ ACET→ LRR-repeats+AP-GTPase+COR*→?→?→||<-?<-?<-SMPB||?→ Cluster1833_2clades→</td><td>LRR-repeats+AP-GTPase+COR</td><td>925</td><td>CEE37_07880</td><td>FCB group</td><td>candidate division LCP-89 bacterium B3_LCP</td><td>GTPase [candidate division LCP-89 bacterium B3_LCP].</td><td>GCA_005223185.1</td></tr>
<tr><td>TKJ41682.1</td><td>TPR+TPR→?→?→ RPN10→?→?→ LRR-repeats+AP-GTPase+COR*→||<-?||LRR-repeats+AP-GTPase+COR→||<-?<-tRNA||RADICAL-SAM→||<-tRNA<-MerR-HTH</td><td>LRR-repeats+AP-GTPase+COR</td><td>1047</td><td>CEE37_03700</td><td>FCB group</td><td>candidate division LCP-89 bacterium B3_LCP</td><td>hypothetical protein CEE37_03700 [candidate division LCP-89 bacterium B3_LCP].</td><td>GCA_005223185.1</td></tr>
<tr><td>TKZ22725.1</td><td>LRR-repeats+AP-GTPase+COR*→||<-?||COR→</td><td>LRR-repeats+AP-GTPase+COR</td><td>653</td><td>EBE85_34345</td><td>Cyanobacteria</td><td>Hormoscilla sp. GUM007</td><td>hypothetical protein EBE85_34345 [Hormoscilla sp. GUM007].</td><td>-</td></tr>
<tr><td>TKZ31764.1</td><td>Cluster1207_2clades→||<-?<-?<-?<-?<-?<-?||LRR-repeats+AP-GTPase+COR*→ COR→</td><td>LRR-repeats+AP-GTPase+COR</td><td>424</td><td>EBE86_14970</td><td>Cyanobacteria</td><td>Hormoscilla sp. GUM202</td><td>hypothetical protein EBE86_14970, partial [Hormoscilla sp. GUM202].</td><td>-</td></tr>
<tr><td>TLU81885.1</td><td>Cluster1765_2clades→||<-?<-?||InPase→||<-?||TM→ LRR-repeats+AP-GTPase+COR*→?→?→ ACT→||<-?<-?<-PSE<-DNAJ</td><td>LRR-repeats+AP-GTPase+COR</td><td>953</td><td>FDX21_09980</td><td>Chlorobi</td><td>Chlorobium sp.</td><td>hypothetical protein FDX21_09980 [Chlorobium sp.].</td><td>GCA_005862225.1</td></tr>
<tr><td>TLU87350.1</td><td><-FGS<-?||?→||<-?<-?||PSE→?→?→ LRR-repeats+AP-GTPase+COR+TIR*→||<-PSE<-RelE-ParE</td><td>LRR-repeats+AP-GTPase+COR+TIR</td><td>892</td><td>FDX21_00465</td><td>Chlorobi</td><td>Chlorobium sp.</td><td>TIR domain-containing protein [Chlorobium sp.].</td><td>GCA_005862225.1</td></tr>
<tr><td>TMQ13801.1</td><td>LRR-repeats+AP-GTPase+COR*→ COR+TIR→</td><td>LRR-repeats+AP-GTPase+COR</td><td>391</td><td>E6J90_27900</td><td>Deltaproteobacteria</td><td>Deltaproteobacteria bacterium</td><td>GTP-binding protein, partial [Deltaproteobacteria bacterium].</td><td>GCA_005887915.1</td></tr>
<tr><td>TRT70055.1</td><td>LRR-repeats+AP-GTPase+COR+TM+TM*→</td><td>LRR-repeats+AP-GTPase+COR+TM+TM</td><td>710</td><td>EWV84_09945</td><td>Cyanobacteria</td><td>Microcystis sp. M_QC_C_20170808_M3Col</td><td>GTP-binding protein, partial [Microcystis sp. M_QC_C_20170808_M3Col].</td><td>GCA_007095505.1</td></tr>
<tr><td>TRT86788.1</td><td>SIG+TM+TM+TM+TM+TM+TM+TM→||<-?||Mbetalac→ SAM-methylase→?→||<-?<-SIG+TPM_phosphatase+TM+TM+TM||LRR-repeats+AP-GTPase+COR*→</td><td>LRR-repeats+AP-GTPase+COR</td><td>597</td><td>EWV66_15420</td><td>Cyanobacteria</td><td>Microcystis sp. M_OC_Ca_00000000_C217Col</td><td>GTP-binding protein, partial [Microcystis sp. M_OC_Ca_00000000_C217Col].</td><td>GCA_007095585.1</td></tr>
</table>

<table>
<thead>
<tr><th>acc</th><th>operon</th><th>architecture</th><th>len</th><th>gen.name</th><th>taxend</th><th>species</th><th>defline</th><th>gca</th></tr>
</thead>
<tbody>
<tr><td>TRT92075.1</td><td>Mbetalac→ SAM-methylase→?→||<-?||?→||<-SIG+TPM_phosphatase+TM+TM+TM||LRR-repeats+AP-GTPase+COR+TM+TM*→||<-?<-?<-?<-Pro_CA||?→||<-?<-tRNA<-tRNA<-Ferredoxin-betagrasp</td><td>LRR-repeats+AP-GTPase+COR+TM+TM</td><td>874</td><td>EWV65_21090</td><td>Cyanobacteria</td><td>Microcystis flos-aquae Ma_QC_C_20070823_S18D</td><td>GTP-binding protein [Microcystis flos-aquae Ma_QC_C_20070823_S18D].</td><td>GCA_007095605.1</td></tr>
<tr><td>TRU14937.1</td><td>SIG+TM+TM+TM+TM+TM+TM+TM→||<-?||Mbetalac→ SAM-methylase→?→||<-?<-SIG+TPM_phosphatase+TM+TM+TM||LRR-repeats+AP-GTPase+COR+TM+TM*→||<-?<-?<-?<-Pro_CA||?→||<-?<-tRNA<-tRNA<-Ferredoxin-betagrasp</td><td>LRR-repeats+AP-GTPase+COR+TM+TM</td><td>874</td><td>EWV60_01670</td><td>Cyanobacteria</td><td>Microcystis sp. Msp_OC_L_20101000_S702</td><td>GTP-binding protein [Microcystis sp. Msp_OC_L_20101000_S702].</td><td>GCA_007095705.1</td></tr>
<tr><td>TRU25637.1</td><td>LRR-repeats+AP-GTPase+COR+TM+TM*→||<-?<-FGS<-?<-?||?→?→ Aminotran_1_2→</td><td>LRR-repeats+AP-GTPase+COR+TM+TM</td><td>921</td><td>EWV79_07400</td><td>Cyanobacteria</td><td>Microcystis aeruginosa Ma_MB_S_20031200_S102D</td><td>GTP-binding protein [Microcystis aeruginosa Ma_MB_S_20031200_S102D].</td><td>GCA_007095795.1</td></tr>
<tr><td>TRU29987.1</td><td>SIG+TM+TM+TM+TM+TM+TM+TM→||<-?||Mbetalac→ SAM-methylase→?→||<-?<-SIG+TPM_phosphatase+TM+TM+TM||LRR-repeats+AP-GTPase+COR+TM+TM*→||<-?<-?<-?<-?<-Pro_CA</td><td>LRR-repeats+AP-GTPase+COR+TM+TM</td><td>921</td><td>EWV80_03065</td><td>Cyanobacteria</td><td>Microcystis aeruginosa Ma_QC_B_20070730_S2</td><td>GTP-binding protein [Microcystis aeruginosa Ma_QC_B_20070730_S2].</td><td>GCA_007095805.1</td></tr>
<tr><td>TRU36579.1</td><td>SIG+TM+TM+TM+TM+TM+TM+TM→||<-PSE||Mbetalac→ SAM-methylase→?→||<-?<-SIG+TPM_phosphatase+TM+TM+TM||LRR-repeats+AP-GTPase+COR+TM+TM*→||<-?<-?<-?<-Pro_CA||?→||<-?<-tRNA<-tRNA<-Ferredoxin-betagrasp</td><td>LRR-repeats+AP-GTPase+COR+TM+TM</td><td>805</td><td>EWV78_08805</td><td>Cyanobacteria</td><td>Microcystis aeruginosa Ma_MB_F_20061100_S20D</td><td>GTP-binding protein [Microcystis aeruginosa Ma_MB_F_20061100_S20D].</td><td>GCA_007095855.1</td></tr>
<tr><td>TRU47882.1</td><td>CHTH→ LRR-repeats+AP-GTPase+COR+TM+TM*→</td><td>LRR-repeats+AP-GTPase+COR+TM+TM</td><td>874</td><td>EWV49_14300</td><td>Cyanobacteria</td><td>Microcystis aeruginosa Ma_QC_Ch_20071001_S25</td><td>GTP-binding protein [Microcystis aeruginosa Ma_QC_Ch_20071001_S25].</td><td>GCA_007095935.1</td></tr>
<tr><td>TRU57932.1</td><td>CHTH→ LRR-repeats+AP-GTPase+COR*→</td><td>LRR-repeats+AP-GTPase+COR</td><td>559</td><td>EWV56_15605</td><td>Cyanobacteria</td><td>Microcystis aeruginosa Ma_QC_C_20070823_S13D</td><td>GTP-binding protein, partial [Microcystis aeruginosa Ma_QC_C_20070823_S13D].</td><td>GCA_007095965.1</td></tr>
<tr><td>TRU80903.1</td><td>SIG+TM+TM+TM+TM+TM+TM+TM→||<-?||Mbetalac→ SAM-methylase→?→||<-?<-SIG+TPM_phosphatase+TM+TM+TM||LRR-repeats+AP-GTPase+COR+TM+TM*→</td><td>LRR-repeats+AP-GTPase+COR+TM+TM</td><td>806</td><td>EWV76_22185</td><td>Cyanobacteria</td><td>Microcystis novacekii Mn_MB_F_20050700_S1</td><td>GTP-binding protein [Microcystis novacekii Mn_MB_F_20050700_S1].</td><td>GCA_007096045.1</td></tr>
<tr><td>TRU95593.1</td><td>Ferredoxin-betagrasp→ tRNA→ tRNA→ SAM-methylase→?→||<-?<-SIG+TPM_phosphatase+TM+TM+TM||LRR-repeats+AP-GTPase+COR+TM+TM*→||<-?<-?<-?<-Pro_CA</td><td>LRR-repeats+AP-GTPase+COR+TM+TM</td><td>852</td><td>EWV73_20055</td><td>Cyanobacteria</td><td>Microcystis wesenbergii Mw_QC_B_20070930_S4D</td><td>GTP-binding protein [Microcystis wesenbergii Mw_QC_B_20070930_S4D].</td><td>GCA_007096105.1</td></tr>
<tr><td>TRU97707.1</td><td>Ferredoxin-betagrasp→ tRNA→ tRNA→ SAM-methylase→?→||<-?<-SIG+TPM_phosphatase+TM+TM+TM||LRR-repeats+AP-GTPase+COR+TM+TM*→||<-?<-?<-?<-Pro_CA</td><td>LRR-repeats+AP-GTPase+COR+TM+TM</td><td>943</td><td>EWV75_08635</td><td>Cyanobacteria</td><td>Microcystis wesenbergii Mw_QC_S_20081001_S30D</td><td>GTP-binding protein [Microcystis wesenbergii Mw_QC_S_20081001_S30D].</td><td>GCA_007096035.1</td></tr>
<tr><td>TRU98843.1</td><td>Ferredoxin-betagrasp→ tRNA→ tRNA→ SAM-methylase→?→||<-?<-SIG+TPM_phosphatase+TM+TM+TM||LRR-repeats+AP-GTPase+COR+TM+TM*→||<-?<-?<-?<-Pro_CA</td><td>LRR-repeats+AP-GTPase+COR+TM+TM</td><td>920</td><td>EWV74_15160</td><td>Cyanobacteria</td><td>Microcystis wesenbergii Mw_QC_S_20081001_S30</td><td>GTP-binding protein [Microcystis wesenbergii Mw_QC_S_20081001_S30].</td><td>GCA_007096535.1</td></tr>
<tr><td>TRV08313.1</td><td>Ferredoxin-betagrasp→ tRNA→ tRNA→ SAM-methylase→?→||<-?<-SIG+TPM_phosphatase+TM+TM+TM||LRR-repeats+AP-GTPase+COR+TM+TM*→?→||<-?<-?<-?||?→||<-Pro_CA</td><td>LRR-repeats+AP-GTPase+COR+TM+TM</td><td>751</td><td>EWV41_10615</td><td>Cyanobacteria</td><td>Microcystis wesenbergii Mw_MB_S_20031200_S109</td><td>GTP-binding protein [Microcystis wesenbergii Mw_MB_S_20031200_S109].</td><td>GCA_007096585.1</td></tr>
<tr><td>TRV43292.1</td><td>SAM-methylase→?→||<-?<-SIG+TPM_phosphatase+TM+TM+TM||LRR-repeats+AP-GTPase+COR+TM+TM*→||<-?<-?<-?<-Pro_CA||?→||<-?<-tRNA<-tRNA<-Ferredoxin-betagrasp</td><td>LRR-repeats+AP-GTPase+COR+TM+TM</td><td>874</td><td>EWV87_21500</td><td>Cyanobacteria</td><td>Microcystis panniformis Mp_GB_SS_20050300_S99</td><td>GTP-binding protein [Microcystis panniformis Mp_GB_SS_20050300_S99].</td><td>GCA_007096815.1</td></tr>
<tr><td>TVP67596.1</td><td>LRR-repeats+AP-GTPase+COR+TM+TM*→</td><td>LRR-repeats+AP-GTPase+COR+TM+TM</td><td>667</td><td>EA343_00435</td><td>Cyanobacteria</td><td>Nodularia sp. (in: Bacteria)</td><td>GTPase, partial [Nodularia sp. (in: Bacteria)].</td><td>GCA_007692755.1</td></tr>
<tr><td>TVQ58623.1</td><td>LRR-repeats+AP-GTPase+COR*→||<-Cluster2343_2clades</td><td>LRR-repeats+AP-GTPase+COR</td><td>976</td><td>EA366_06380</td><td>Cyanobacteria</td><td>Spirulina sp. DLM2.Bin59</td><td>hypothetical protein EA366_06380 [Spirulina sp. DLM2.Bin59].</td><td>GCA_007693875.1</td></tr>
<tr><td>TVV43679.1</td><td><-MarR-HTH<-?<-Cluster1077_2clades||Aminotran_1_2→ PSE→||<-?<-?||?→ LRR-repeats+AP-GTPase+COR*→</td><td>LRR-repeats+AP-GTPase+COR</td><td>985</td><td>FOT50_08370</td><td>Gammaproteobacteria</td><td>Thalassolituus sp. C2-1</td><td>GTP-binding protein [Thalassolituus sp. C2-1].</td><td>GCA_007785795.1</td></tr>
<tr><td>TXI24418.1</td><td>LRR-repeats+AP-GTPase+COR+DrHyd*→?→?→?→?→?→?→ Cluster1726_2clades→</td><td>LRR-repeats+AP-GTPase+COR+DrHyd</td><td>1061</td><td>E6Q61_04940</td><td>Betaproteobacteria</td><td>Nitrosomonas sp.</td><td>hypothetical protein E6Q61_04940 [Nitrosomonas sp.].</td><td>GCA_008015595.1</td></tr>
<tr><td>TXL81991.1</td><td>LRR-repeats+AP-GTPase+COR+CASPASE*→</td><td>LRR-repeats+AP-GTPase+COR+CASPASE</td><td>1175</td><td>FHP25_02680</td><td>Alphaproteobacteria</td><td>Enhydrobacter sp. CC-CFT640</td><td>CHAT domain-containing protein [Enhydrobacter sp. CC-CFT640].</td><td>GCA_008039615.1</td></tr>
<tr><td>TYC52188.1</td><td>WCAK→?→ LRR-repeats→ LRR-repeats+AP-GTPase+COR*→</td><td>LRR-repeats+AP-GTPase+COR</td><td>860</td><td>FMN50_19470</td><td>Alphaproteobacteria</td><td>Rhodobacterales bacterium</td><td>hypothetical protein FMN50_19470 [Rhodobacterales bacterium].</td><td>GCA_008107755.1</td></tr>
</tbody>
</table>

<table>
<tr><th>acc</th><th>operon</th><th>architecture</th><th>len</th><th>gen.name</th><th>taxend</th><th>species</th><th>defline</th><th>gca</th></tr>
<tr><td>VFJ48800.1</td><td>RelE-ParE→?→?→ LRR-repeats+AP-GTPase+COR*→</td><td>LRR-repeats+AP-GTPase+COR</td><td>467</td><td>BECKDK2373C_GA0170839_10231</td><td>Gammaproteobacteria</td><td>Candidatus Kentron sp. DK</td><td>Leucine rich repeat-containing protein, partial [Candidatus Kentron sp. DK].</td><td>-</td></tr>
<tr><td>VFJ57569.1</td><td>RelE-ParE→?→ Cluster54_3clades→?→ LRR-repeats+AP-GTPase+COR*→</td><td>LRR-repeats+AP-GTPase+COR</td><td>1027</td><td>BECKFW1821A_GA0114235_107219</td><td>Gammaproteobacteria</td><td>Candidatus Kentron sp. FW</td><td>Leucine rich repeat-containing protein [Candidatus Kentron sp. FW].</td><td>-</td></tr>
<tr><td>VFJ62833.1</td><td>Cluster1147_2clades→?→?→ RelE-ParE→?→?→ LRR-repeats+AP-GTPase+COR*→ Cluster2123_2clades→</td><td>LRR-repeats+AP-GTPase+COR</td><td>702</td><td>BECKDK2373B_GA0170837_11172</td><td>Gammaproteobacteria</td><td>Candidatus Kentron sp. DK</td><td>Leucine rich repeat-containing protein [Candidatus Kentron sp. DK].</td><td>-</td></tr>
<tr><td>VFJ65980.1</td><td>Cluster1147_2clades→?→?→ RelE-ParE→?→?→ LRR-repeats+AP-GTPase+COR*→</td><td>LRR-repeats+AP-GTPase+COR</td><td>470</td><td>BECKDK2373C_GA0170839_113817</td><td>Gammaproteobacteria</td><td>Candidatus Kentron sp. DK</td><td>Leucine rich repeat-containing protein, partial [Candidatus Kentron sp. DK].</td><td>-</td></tr>
<tr><td>VFJ75018.1</td><td>LRR-repeats+AP-GTPase+COR*→</td><td>LRR-repeats+AP-GTPase+COR</td><td>772</td><td>BECKFW1821C_GA0114237_10681</td><td>Gammaproteobacteria</td><td>Candidatus Kentron sp. FW</td><td>Ras of Complex, Roc, domain of DAPkinase [Candidatus Kentron sp. FW].</td><td>-</td></tr>
<tr><td>VFJ87041.1</td><td>RelE-ParE→ LRR-repeats+AP-GTPase+COR*→</td><td>LRR-repeats+AP-GTPase+COR</td><td>1091</td><td>BECKLFY1418B_GA0070995_100545</td><td>Gammaproteobacteria</td><td>Candidatus Kentron sp. LFY</td><td>Leucine rich repeat-containing protein [Candidatus Kentron sp. LFY].</td><td>-</td></tr>
<tr><td>VFJ90339.1</td><td>RelE-ParE→ LRR-repeats+AP-GTPase+COR*→ COR→</td><td>LRR-repeats+AP-GTPase+COR</td><td>380</td><td>BECKLFY1418A_GA0070994_101124</td><td>Gammaproteobacteria</td><td>Candidatus Kentron sp. LFY</td><td>Leucine rich repeat-containing protein [Candidatus Kentron sp. LFY].</td><td>-</td></tr>
<tr><td>VFK39292.1</td><td>RelE-ParE→?→ LRR-repeats+AP-GTPase+COR*→?→?→?→ RPN10→</td><td>LRR-repeats+AP-GTPase+COR</td><td>1088</td><td>BECKTC1821E_GA0114239_100479</td><td>Gammaproteobacteria</td><td>Candidatus Kentron sp. TC</td><td>Leucine rich repeat-containing protein [Candidatus Kentron sp. TC].</td><td>-</td></tr>
<tr><td>VFK43940.1</td><td>Cluster54_3clades→?→ RelE-ParE→ LRR-repeats+AP-GTPase+COR*→?→ Cluster1137_2clades→?→?→ RPN10→</td><td>LRR-repeats+AP-GTPase+COR</td><td>1017</td><td>BECKTC1821D_GA0114238_101924</td><td>Gammaproteobacteria</td><td>Candidatus Kentron sp. TC</td><td>Leucine rich repeat-containing protein [Candidatus Kentron sp. TC].</td><td>-</td></tr>
<tr><td>VFK55324.1</td><td>RelE-ParE→ LRR-repeats+AP-GTPase+COR*→?→ Cluster1137_2clades→?→?→ RPN10→</td><td>LRR-repeats+AP-GTPase+COR</td><td>1013</td><td>BECKTC1821F_GA0114240_100673</td><td>Gammaproteobacteria</td><td>Candidatus Kentron sp. TC</td><td>Leucine rich repeat-containing protein [Candidatus Kentron sp. TC].</td><td>-</td></tr>
<tr><td>VUX45208.1</td><td>LRR-repeats+AP-GTPase+COR+TIR*→</td><td>LRR-repeats+AP-GTPase+COR+TIR</td><td>1031</td><td>DF3PA_100056</td><td>Alphaproteobacteria</td><td>Candidatus Defluviicoccus seviourii</td><td>conserved hypothetical protein [Candidatus Defluviicoccus seviourii].</td><td>GCA_900609035.2</td></tr>
<tr><td>VUX54879.1</td><td><-SIG+Trypsin+PDZ<-?||?→?→||<-?<-?<-?||LRR-repeats+AP-GTPase+COR*→</td><td>LRR-repeats+AP-GTPase+COR</td><td>935</td><td>JTBB02_V1_20008</td><td>Gammaproteobacteria</td><td>uncultured Woeseiaceae bacterium</td><td>protein of unknown function [uncultured Woeseiaceae bacterium].</td><td>GCA_902167415.1</td></tr>
<tr><td>WP_002686189.1</td><td><-Cluster1370_2clades||CNMP+CRP-HTH→?→||<-?||LRR-repeats+AP-GTPase+COR+DUF4404*→||<-?<-?<-?||?→ PurT-ATPgrasp→</td><td>LRR-repeats+AP-GTPase+COR+DUF4404</td><td>1098</td><td>-</td><td>Gammaproteobacteria</td><td>Beggiatoa alba</td><td>leucine-rich repeat domain-containing protein [Beggiatoa alba].</td><td>GCF_000245015.1</td></tr>
<tr><td>WP_002693173.1</td><td><-NUDIX<-?||?→?→ SIG+TM+TM+Calcineurin→||<-?||LRR-repeats+AP-GTPase+COR*→||<-?||?→||<-?||Cluster1843_2clades→</td><td>LRR-repeats+AP-GTPase+COR</td><td>1282</td><td>-</td><td>Bacteroidetes</td><td>Microscilla marina</td><td>leucine-rich repeat protein [Microscilla marina].</td><td>GCF_000169175.1</td></tr>
<tr><td>WP_002693833.1</td><td>LRR-repeats+AP-GTPase+COR+EAD11*→?→||<-PAS+HISKIN</td><td>LRR-repeats+AP-GTPase+COR+EAD11</td><td>847</td><td>-</td><td>Bacteroidetes</td><td>Microscilla marina</td><td>leucine rich repeat protein [Microscilla marina].</td><td>GCF_000169175.1</td></tr>
<tr><td>WP_002694398.1</td><td>NUDIX→||<-?<-?<-?<-?||LRR-repeats+AP-GTPase+COR*→?→?→?→||<-?<-?<-SIGMA-HTH</td><td>LRR-repeats+AP-GTPase+COR</td><td>836</td><td>-</td><td>Bacteroidetes</td><td>Microscilla marina</td><td>leucine-rich repeat domain-containing protein [Microscilla marina].</td><td>GCF_000169175.1</td></tr>
<tr><td>WP_002703871.1</td><td>ABC_membrane+ABC_tran→?→?→ LRR-repeats+AP-GTPase+COR*→?→?→||<-SIG+SecA<-?<-?<-?||SIGMA-HTH→</td><td>LRR-repeats+AP-GTPase+COR</td><td>966</td><td>-</td><td>Bacteroidetes</td><td>Microscilla marina</td><td>leucine-rich repeat domain-containing protein [Microscilla marina].</td><td>GCF_000169175.1</td></tr>
<tr><td>WP_002704578.1</td><td><-DAGKIN<-?||?→ LRR-repeats+AP-GTPase+COR*→||<-?||CNMP+CRP-HTH→||<-?||?→ InPase→</td><td>LRR-repeats+AP-GTPase+COR</td><td>897</td><td>-</td><td>Bacteroidetes</td><td>Microscilla marina</td><td>leucine-rich repeat domain-containing protein [Microscilla marina].</td><td>GCF_000169175.1</td></tr>
</table>

<table>
<tr><th>acc</th><th>operon</th><th>architecture</th><th>len</th><th>gen.name</th><th>taxend</th><th>species</th><th>defline</th><th>gca</th></tr>
<tr><td>WP_002736512.1</td><td>SIG+TM+TM+TM+TM+TM+TM+TM→||<-?||Mbetalac→ SAM-methylase→?→||<-?<-SIG+TPM_phosphatase+TM+TM+TM||LRR-repeats+AP-GTPase+COR+TM+TM*→||<-?<-?<-?<-Pro_CA||?→||<-?<-tRNA<-tRNA<-Ferredoxin-betagrasp</td><td>LRR-repeats+AP-GTPase+COR+TM+TM</td><td>852</td><td>-</td><td>Cyanobacteria</td><td>Microcystis aeruginosa</td><td>leucine-rich repeat protein [Microcystis aeruginosa].</td><td>GCF_000330925.1</td></tr>
<tr><td>WP_002754362.1</td><td>SIG+TM+TM+TM+TM+TM+TM+TM→||<-?||Mbetalac→ SAM-methylase→?→||<-?<-SIG+TPM_phosphatase+TM+TM+TM||LRR-repeats+AP-GTPase+COR+TM+TM*→||<-?<-?<-?<-Pro_CA||?→||<-?<-tRNA<-tRNA<-Ferredoxin-betagrasp</td><td>LRR-repeats+AP-GTPase+COR+TM+TM</td><td>806</td><td>-</td><td>Cyanobacteria</td><td>Microcystis aeruginosa</td><td>leucine-rich repeat domain-containing protein [Microcystis aeruginosa].</td><td>GCF_000307995.1</td></tr>
<tr><td>WP_002775884.1</td><td>SIG+TM+TM+TM+TM+TM+TM+TM→||<-?||Mbetalac→ SAM-methylase→?→||<-?<-SIG+TPM_phosphatase+TM+TM+TM||LRR-repeats+AP-GTPase+COR+TM+TM*→||<-?<-?<-?<-Pro_CA||?→||<-?<-tRNA<-tRNA<-Ferredoxin-betagrasp</td><td>LRR-repeats+AP-GTPase+COR+TM+TM</td><td>783</td><td>-</td><td>Cyanobacteria</td><td>Microcystis aeruginosa</td><td>leucine-rich repeat protein [Microcystis aeruginosa].</td><td>GCF_000312205.1</td></tr>
<tr><td>WP_002792425.1</td><td>SIG+TM+TM+TM+TM+TM+TM+TM→||<-?||Mbetalac→ SAM-methylase→?→||<-?<-SIG+TPM_phosphatase+TM+TM+TM||LRR-repeats+AP-GTPase+COR+TM+TM*→||<-?<-?<-?<-Pro_CA||?→||<-PSE<-?<-tRNA<-tRNA<-Ferredoxin-betagrasp</td><td>LRR-repeats+AP-GTPase+COR+TM+TM</td><td>834</td><td>-</td><td>Cyanobacteria</td><td>Microcystis aeruginosa</td><td>leucine-rich repeat domain-containing protein [Microcystis aeruginosa].</td><td>GCF_000312245.1</td></tr>
<tr><td>WP_002797004.1</td><td>SIG+TM+TM+TM+TM+TM+TM+TM→||<-?||Mbetalac→ SAM-methylase→?→||<-?<-SIG+TPM_phosphatase+TM+TM+TM||LRR-repeats+AP-GTPase+COR+TM+TM*→</td><td>LRR-repeats+AP-GTPase+COR+TM+TM</td><td>865</td><td>-</td><td>Cyanobacteria</td><td>Microcystis aeruginosa</td><td>leucine-rich repeat protein [Microcystis aeruginosa].</td><td>GCF_000312265.1</td></tr>
<tr><td>WP_004156960.1</td><td>SIG+TM+TM+TM+TM+TM+TM+TM→||<-?||Mbetalac→ SAM-methylase→?→||<-?<-SIG+TPM_phosphatase+TM+TM+TM||LRR-repeats+AP-GTPase+COR+TM+TM*→||<-?<-?<-?<-Pro_CA||?→||<-?<-tRNA<-tRNA<-Ferredoxin-betagrasp</td><td>LRR-repeats+AP-GTPase+COR+TM+TM</td><td>875</td><td>-</td><td>Cyanobacteria</td><td>Microcystis aeruginosa</td><td>leucine-rich repeat protein [Microcystis aeruginosa].</td><td>GCF_000332585.1</td></tr>
<tr><td>WP_005857943.1</td><td>LRR-repeats+AP-GTPase+COR*→||<-tRNA<-?<-?<-?<-Arginase</td><td>LRR-repeats+AP-GTPase+COR</td><td>797</td><td>-</td><td>Alphaproteobacteria</td><td>Sagittula stellata</td><td>hypothetical protein [Sagittula stellata].</td><td>GCF_000169415.1</td></tr>
<tr><td>WP_006099017.1</td><td>CR-REase5→ Cluster1847_2clades→?→ SIG+WWE→?→ LRR-repeats+AP-GTPase+COR*→?→?→ Pentapeptide-repeats→</td><td>LRR-repeats+AP-GTPase+COR</td><td>1117</td><td>-</td><td>Cyanobacteria</td><td>Coleofasciculus chthonoplastes</td><td>leucine-rich repeat domain-containing protein [Coleofasciculus chthonoplastes].</td><td>GCF_000155555.1</td></tr>
<tr><td>WP_006103609.1</td><td>FGS→?→?→?→||<-?||?→ LRR-repeats+AP-GTPase+COR+TIR*→</td><td>LRR-repeats+AP-GTPase+COR+TIR</td><td>1090</td><td>-</td><td>Cyanobacteria</td><td>Coleofasciculus chthonoplastes</td><td>leucine-rich repeat domain-containing protein [Coleofasciculus chthonoplastes].</td><td>GCF_000155555.1</td></tr>
<tr><td>WP_006456809.1</td><td><-REC||?→?→ ABC_membrane+ABC_tran→?→||<-?||?→ LRR-repeats+AP-GTPase+COR*→</td><td>LRR-repeats+AP-GTPase+COR</td><td>1260</td><td>-</td><td>Cyanobacteria</td><td>Synechococcus sp. PCC 7335</td><td>leucine-rich repeat domain-containing protein [Synechococcus sp. PCC 7335].</td><td>GCF_000155595.1</td></tr>
<tr><td>WP_006507306.1</td><td>LRR-repeats+AP-GTPase+COR*→</td><td>LRR-repeats+AP-GTPase+COR</td><td>705</td><td>-</td><td>Cyanobacteria</td><td>Xenococcus sp. PCC 7305</td><td>small G protein, GTPase SAR1, partial [Xenococcus sp. PCC 7305].</td><td>-</td></tr>
<tr><td>WP_006512482.1</td><td><-GTPase-AIG||Cluster2189_2clades→||<-?||?→||<-?||LRR-repeats+AP-GTPase+COR*→?→||<-?<-?<-?<-Pentapeptide-repeats</td><td>LRR-repeats+AP-GTPase+COR</td><td>806</td><td>-</td><td>Cyanobacteria</td><td>Xenococcus sp. PCC 7305</td><td>leucine-rich repeat domain-containing protein [Xenococcus sp. PCC 7305].</td><td>GCF_000332055.1</td></tr>
<tr><td>WP_006519951.1</td><td>Cluster2165_2clades→ Cluster1999_2clades→ LRR-repeats+AP-GTPase+COR*→</td><td>LRR-repeats+AP-GTPase+COR</td><td>1104</td><td>-</td><td>Cyanobacteria</td><td>Leptolyngbya sp. PCC 7375</td><td>hypothetical protein [Leptolyngbya sp. PCC 7375].</td><td>GCF_000316115.1</td></tr>
<tr><td>WP_006528821.1</td><td>LRR-repeats+AP-GTPase+COR*→</td><td>LRR-repeats+AP-GTPase+COR</td><td>629</td><td>-</td><td>Cyanobacteria</td><td>Gloeocapsa sp. PCC 73106</td><td>leucine-rich repeat domain-containing protein [Gloeocapsa sp. PCC 73106].</td><td>GCF_000332035.1</td></tr>
<tr><td>WP_006631836.1</td><td>LRR-repeats+AP-GTPase+COR+DUF4404*→||<-?<-?<-?<-?||Ferretin→</td><td>LRR-repeats+AP-GTPase+COR+DUF4404</td><td>1060</td><td>-</td><td>Cyanobacteria</td><td>Microcoleus vaginatus</td><td>leucine-rich repeat domain-containing protein [Microcoleus vaginatus].</td><td>GCF_000214075.1</td></tr>
<tr><td>WP_007307526.1</td><td>LRR-repeats+AP-GTPase+COR*→</td><td>LRR-repeats+AP-GTPase+COR</td><td>795</td><td>-</td><td>Cyanobacteria</td><td>Crocosphaera watsonii</td><td>leucine-rich repeat domain-containing protein [Crocosphaera watsonii].</td><td>GCF_000167195.1</td></tr>
<tr><td>WP_007312599.1</td><td>LRR-repeats+AP-GTPase+COR*→</td><td>LRR-repeats+AP-GTPase+COR</td><td>378</td><td>-</td><td>Cyanobacteria</td><td>Crocosphaera watsonii</td><td>hypothetical protein [Crocosphaera watsonii].</td><td>GCF_001050835.1</td></tr>
<tr><td>WP_007458231.1</td><td>LRR-repeats+AP-GTPase+COR*→||<-?<-?||?→||<-?<-?||SIG+TM→ REC→</td><td>LRR-repeats+AP-GTPase+COR</td><td>1135</td><td>-</td><td>Actinobacteria</td><td>Micromonospora lupini</td><td>hypothetical protein [Micromonospora lupini].</td><td>GCF_000297395.2</td></tr>
<tr><td>WP_007460872.1</td><td>LRR-repeats+AP-GTPase+COR*→||<-ABC_membrane+ABC_tran</td><td>LRR-repeats+AP-GTPase+COR</td><td>902</td><td>-</td><td>Actinobacteria</td><td>Micromonospora lupini</td><td>leucine-rich repeat domain-containing protein [Micromonospora lupini].</td><td>GCF_000297395.2</td></tr>
<tr><td>WP_008188574.1</td><td>SIG+TM+TM+TM+TM+TM+TM+TM→ ABC-ATPase→ ABC-ATPase→?→?→||<-?<-?||LRR-repeats+AP-GTPase+COR*→?→||<-?<-?||PSE→||<-Cluster5_5clades</td><td>LRR-repeats+AP-GTPase+COR</td><td>868</td><td>-</td><td>Cyanobacteria</td><td>Moorea producens</td><td>leucine-rich repeat domain-containing protein [Moorea producens].</td><td>GCF_000211815.1</td></tr>
</table>

acc	operon	architecture	len	gen.name	taxend	species	defline	gca
WP_008190613.1	REC→||<-PSE<-HNH<-?||REC→||<-TPR-repeats<-?<-?||LRR-repeats+AP-GTPase+COR+DUF4404*→	LRR-repeats+AP-GTPase+COR+DUF4404	948	-	Cyanobacteria	Moorea producens	leucine-rich repeat domain-containing protein [Moorea producens].	GCF_000211815.1
WP_008231466.1	<-Cluster1131__3clades<-?<-Cluster1163__3clades||?→?→?→ tRNA→ LRR-repeats+AP-GTPase+COR*→	LRR-repeats+AP-GTPase+COR	970	-	Alphaproteobacteria	Roseobacter sp. CCS2	hypothetical protein [Roseobacter sp. CCS2].	GCF_000169435.1
WP_008273960.1	Cluster1550__2clades→?→?→ PIN→ LRR-repeats+AP-GTPase+COR*→	LRR-repeats+AP-GTPase+COR	830	-	Cyanobacteria	Crocosphaera chwakensis	leucine-rich repeat domain-containing protein [Crocosphaera chwakensis].	GCF_000169335.1
WP_008277167.1	<-REC||SIG+TM+TM+TM+TM→||<-?||?→||<-?||?→ LRR-repeats+AP-GTPase+COR*→	LRR-repeats+AP-GTPase+COR	974	-	Cyanobacteria	Crocosphaera chwakensis	leucine-rich repeat domain-containing protein [Crocosphaera chwakensis].	GCF_000169335.1
WP_008680517.1	LRR-repeats+AP-GTPase+COR*→	LRR-repeats+AP-GTPase+COR	1157	-	Planctomycetes	Rhodopirellula sallentina	small GTP-binding protein [Rhodopirellula sallentina].	GCF_000346505.1
WP_009282559.1	LRR-repeats+AP-GTPase+COR+TIR*→||<-?<-?||?→||<-?<-?<-?<-TM+TM+TM+TM+TM	LRR-repeats+AP-GTPase+COR+TIR	925	-	Bacteroidetes	Fibrisoma limi	leucine-rich repeat domain-containing protein [Fibrisoma limi].	GCF_000296815.2
WP_009283610.1	<-McrA-NTD||?→ N-OB→?→?→ LRR-repeats+AP-GTPase+COR+TIR*→ Cluster1088__2clades→?→?→?→||<-SIGMA-HTH	LRR-repeats+AP-GTPase+COR+TIR	1023	-	Bacteroidetes	Fibrisoma limi	leucine-rich repeat protein [Fibrisoma limi].	GCF_000296815.2
WP_009627973.1	LRR-repeats+AP-GTPase+COR+TM+TM*→||<-REC	LRR-repeats+AP-GTPase+COR+TM+TM	945	-	Cyanobacteria	Pseudanabaena biceps	leucine-rich repeat domain-containing protein [Pseudanabaena biceps].	GCF_000332215.1
WP_009964949.1	<-SMS-KaiC<-?<-?<-Cluster1581__2clades||?→?→?→ LRR-repeats+AP-GTPase+COR+TIR*→?→ TIR→?→?→ DrHyd+APATPase+TPR+TPR+TPR→	LRR-repeats+AP-GTPase+COR+TIR	961	-	Verrucomicrobia	Verrucomicrobium spinosum	GTP-binding protein [Verrucomicrobium spinosum].	GCF_000172155.1
WP_010471910.1	<-REC||?→ LRR-repeats+AP-GTPase+COR*→	LRR-repeats+AP-GTPase+COR	1082	-	Cyanobacteria	Acaryochloris sp. CCMEE 5410	leucine-rich repeat domain-containing protein [Acaryochloris sp. CCMEE 5410].	GCF_000238775.1
WP_010477835.1	<-ABC__membrane+ABC__tran||?→?→||<-?<-?||?→?→ LRR-repeats+AP-GTPase+COR*→	LRR-repeats+AP-GTPase+COR	1235	-	Cyanobacteria	Acaryochloris sp. CCMEE 5410	leucine-rich repeat domain-containing protein [Acaryochloris sp. CCMEE 5410].	GCF_000238775.1
WP_010933192.1	<-SIG+TM||LRR-repeats+AP-GTPase+COR*→?→?→?→?→ ParA-Soj-PloopNTPase→	LRR-repeats+AP-GTPase+COR	1102	-	Chlorobi	Chlorobaculum tepidum	leucine-rich repeat domain-containing protein [Chlorobaculum tepidum].	GCF_000006985.1
WP_010994301.1	<-Cluster5__5clades||LRR-repeats+AP-GTPase+COR+DUF4404*→||<-?<-PSE<-?||?→||<-REC	LRR-repeats+AP-GTPase+COR+DUF4404	1119	-	Cyanobacteria	Nostocaceae	MULTISPECIES: leucine-rich repeat domain-containing protein [Nostocaceae].	GCF_000009705.1
WP_011022280.1	PAS+HISKIN→||<-?||?→?→?→?→||<-?||LRR-repeats+AP-GTPase+COR*→	LRR-repeats+AP-GTPase+COR	631	-	Euryarchaeota	Methanosarcina acetivorans	leucine-rich repeat domain-containing protein [Methanosarcina acetivorans].	-
WP_011307276.1	<-HhH-RADC+JAB||?→ NTP__transf__3→ LRR-repeats+AP-GTPase+COR*→||<-?<-?<-?<-Thioredoxin	LRR-repeats+AP-GTPase+COR	863	-	Euryarchaeota	Methanosarcina barkeri	leucine-rich repeat domain-containing protein [Methanosarcina barkeri].	GCF_000195895.1
WP_011307410.1	LRR-repeats+AP-GTPase+COR*→	LRR-repeats+AP-GTPase+COR	886	-	Euryarchaeota	Methanosarcina barkeri	leucine-rich repeat domain-containing protein [Methanosarcina barkeri].	GCF_000195895.1
WP_011308262.1	<-Mbetalac<-PSE<-PSE||?→||<-?||?→?→?→||<-?||LRR-repeats+AP-GTPase+COR*→||<-?||?→||<-?||?→ CHTH→||<-?||ABC-ATPase→	LRR-repeats+AP-GTPase+COR	892	-	Euryarchaeota	Methanosarcina barkeri	leucine-rich repeat domain-containing protein [Methanosarcina barkeri].	GCF_000195895.1
WP_011318314.1	<-Cluster5__5clades||LRR-repeats+AP-GTPase+COR+DUF4404*→||<-?<-ABC-ATPase<-?||?→||<-REC	LRR-repeats+AP-GTPase+COR+DUF4404	1107	-	Cyanobacteria	Trichormus variabilis	leucine-rich repeat domain-containing protein [Trichormus variabilis].	GCF_000204075.1
WP_011361554.1	BACTERIALFRINGE+TM+TM→?→ LRR-repeats+AP-GTPase+COR*→||<-?<-DAGKIN<-?||?→ Cluster881__3clades→?→ Cluster1718__2clades→	LRR-repeats+AP-GTPase+COR	811	-	Chlorobi	Chlorobium chlorochromatii	Rab family protein [Chlorobium chlorochromatii].	-

<table>
<thead>
<tr><th>acc</th><th>operon</th><th>architecture</th><th>len</th><th>gen.name</th><th>taxend</th><th>species</th><th>defline</th><th>gca</th></tr>
</thead>
<tbody>
<tr><td>WP_011388592.1</td><td><-ABC_membrane+ABC_tran<-ABC_membrane+ABC_tran<-?<-?<-?<-?||?→ LRR-repeats+AP-GTPase+COR+TIR*→ TM+TM+TM→</td><td>LRR-repeats+AP-GTPase+COR+TIR</td><td>1085</td><td>-</td><td>Alphaproteobacteria</td><td>Rhodospirillum rubrum</td><td>leucine-rich repeat domain-containing protein [Rhodospirillum rubrum].</td><td>GCF_000013085.1</td></tr>
<tr><td>WP_011613173.1</td><td><-Cluster1413_2clades<-?||TPR-repeats→||<-?<-?<-PSE||LRR-repeats+AP-GTPase+COR*→</td><td>LRR-repeats+AP-GTPase+COR</td><td>1041</td><td>-</td><td>Cyanobacteria</td><td>Trichodesmium erythraeum</td><td>leucine-rich repeat domain-containing protein [Trichodesmium erythraeum].</td><td>GCF_000014265.1</td></tr>
<tr><td>WP_011712156.1</td><td>OmpA→?→||<-?<-?<-?||LRR-repeats+AP-GTPase+COR*→ CNMP+CRP-HTH→||<-?<-Cluster997_2clades<-?<-Thioredoxin<-SIG+RHOD-CDC25<-ARSR-HTH</td><td>LRR-repeats+AP-GTPase+COR</td><td>761</td><td>-</td><td>Alphaproteobacteria</td><td>Magnetococcus marinus</td><td>small GTP-binding protein [Magnetococcus marinus].</td><td>GCF_000014865.1</td></tr>
<tr><td>WP_012162488.1</td><td>Cluster1052_2clades→ NUDIX→ LRR-repeats+AP-GTPase+COR+TIR*→ TM+TM+TM→</td><td>LRR-repeats+AP-GTPase+COR+TIR</td><td>842</td><td>-</td><td>Cyanobacteria</td><td>Acaryochloris marina</td><td>leucine-rich repeat domain-containing protein [Acaryochloris marina].</td><td>GCF_000018105.1</td></tr>
<tr><td>WP_012407958.1</td><td>SIG+PBPB→ HISKIN→ HISKIN→ LRR-repeats+AP-GTPase+COR+TIR*→||<-?||?→?→?→||<-?||?→ Pentapeptide-repeats→</td><td>LRR-repeats+AP-GTPase+COR+TIR</td><td>1185</td><td>-</td><td>Cyanobacteria</td><td>Nostoc punctiforme</td><td>leucine-rich repeat domain-containing protein [Nostoc punctiforme].</td><td>GCF_000020025.1</td></tr>
<tr><td>WP_012412722.1</td><td>LRR-repeats+AP-GTPase+COR*→</td><td>LRR-repeats+AP-GTPase+COR</td><td>1124</td><td>-</td><td>Cyanobacteria</td><td>Nostoc punctiforme</td><td>leucine-rich repeat domain-containing protein [Nostoc punctiforme].</td><td>GCF_000020025.1</td></tr>
<tr><td>WP_012466409.1</td><td><-SF2-DUF3427A+TnsA-REase<-?<-?<-?<-?||?→?→ LRR-repeats+AP-GTPase+COR+TIR*→ TIR→</td><td>LRR-repeats+AP-GTPase+COR+TIR</td><td>998</td><td>-</td><td>Chlorobi</td><td>Chlorobium limicola</td><td>leucine-rich repeat domain-containing protein [Chlorobium limicola].</td><td>GCF_000020465.1</td></tr>
<tr><td>WP_012597192.1</td><td>Cluster1134_2clades→ REase-4→?→?→?→ HAD→ LRR-repeats+AP-GTPase+COR+TM+TM*→</td><td>LRR-repeats+AP-GTPase+COR+TM+TM</td><td>937</td><td>-</td><td>Cyanobacteria</td><td>Rippkaea orientalis</td><td>GTP-binding protein [Rippkaea orientalis].</td><td>GCF_000021805.1</td></tr>
<tr><td>WP_013320928.1</td><td>Cluster5_5clades→ LRR-repeats+AP-GTPase+COR*→?→ SIG+GT4→||<-?<-?||Cluster5_5clades→</td><td>LRR-repeats+AP-GTPase+COR</td><td>876</td><td>-</td><td>Cyanobacteria</td><td>Gloeothece verrucosa</td><td>leucine-rich repeat domain-containing protein [Gloeothece verrucosa].</td><td>GCF_000147335.1</td></tr>
<tr><td>WP_013323827.1</td><td>LRR-repeats+AP-GTPase+COR+TM+TM*→</td><td>LRR-repeats+AP-GTPase+COR+TM+TM</td><td>857</td><td>-</td><td>Cyanobacteria</td><td>Gloeothece verrucosa</td><td>leucine-rich repeat domain-containing protein [Gloeothece verrucosa].</td><td>GCF_000147335.1</td></tr>
<tr><td>WP_013762559.1</td><td>SIGMA-HTH→?→ SIG+TM→ LRR-repeats+AP-GTPase+COR+TIR*→?→ SIGMA-HTH→?→||<-SIG+TM+TM+TM+HISKIN</td><td>LRR-repeats+AP-GTPase+COR+TIR</td><td>995</td><td>-</td><td>Bacteroidetes</td><td>Haliscomenobacter hydrossis</td><td>leucine-rich repeat domain-containing protein [Haliscomenobacter hydrossis].</td><td>GCF_000212735.1</td></tr>
<tr><td>WP_013763674.1</td><td><-ABC_membrane+ABC_tran||FGS→ PSE→ FGS→ LRR-repeats+AP-GTPase+COR+EAD11+CASPASE*→ RNASEII→</td><td>LRR-repeats+AP-GTPase+COR+EAD11+CASPASE</td><td>1108</td><td>-</td><td>Bacteroidetes</td><td>Haliscomenobacter hydrossis</td><td>leucine-rich repeat domain-containing protein [Haliscomenobacter hydrossis].</td><td>GCF_000212735.1</td></tr>
<tr><td>WP_013766755.1</td><td>LRR-repeats+AP-GTPase+COR+EAD11*→</td><td>LRR-repeats+AP-GTPase+COR+EAD11</td><td>1448</td><td>-</td><td>Bacteroidetes</td><td>Haliscomenobacter hydrossis</td><td>leucine-rich repeat protein [Haliscomenobacter hydrossis].</td><td>GCF_000212735.1</td></tr>
<tr><td>WP_013767289.1</td><td><-SIG+ABhydrolase||SIG+7TMR-DISMED1→ FGS→ FGS→ LRR-repeats+AP-GTPase+COR+EAD11*→||<-?<-Cluster1645_2clades</td><td>LRR-repeats+AP-GTPase+COR+EAD11</td><td>840</td><td>-</td><td>Bacteroidetes</td><td>Haliscomenobacter hydrossis</td><td>leucine-rich repeat protein [Haliscomenobacter hydrossis].</td><td>GCF_000212735.1</td></tr>
<tr><td>WP_013768874.1</td><td>LRR-repeats+AP-GTPase+COR+TIR→ *LRR-repeats+AP-GTPase+COR*→ LRR-repeats+AP-GTPase+COR*→?→?→?→||<-?||?→?→||<-REC</td><td>LRR-repeats+AP-GTPase+COR+TIR</td><td>741</td><td>-</td><td>Bacteroidetes</td><td>Haliscomenobacter hydrossis</td><td>hypothetical protein [Haliscomenobacter hydrossis].</td><td>GCF_000212735.1</td></tr>
<tr><td>WP_013768875.1</td><td>LRR-repeats+AP-GTPase+COR*→</td><td>LRR-repeats+AP-GTPase+COR</td><td>786</td><td>-</td><td>Bacteroidetes</td><td>Haliscomenobacter hydrossis</td><td>hypothetical protein [Haliscomenobacter hydrossis].</td><td>GCF_000212735.1</td></tr>
<tr><td>WP_013818452.1</td><td>LRR-repeats+AP-GTPase+COR+TIR*→</td><td>LRR-repeats+AP-GTPase+COR+TIR</td><td>944</td><td>-</td><td>Gammaproteobacteria</td><td>Methylomonas methanica</td><td>leucine-rich repeat domain-containing protein [Methylomonas methanica].</td><td>GCF_000214665.1</td></tr>
<tr><td>WP_014444162.1</td><td>REC→||<-?<-?||?→||<-?<-?<-?||LRR-repeats+AP-GTPase+COR*→||<-?<-7TMR-DISMED1</td><td>LRR-repeats+AP-GTPase+COR</td><td>979</td><td>-</td><td>Actinobacteria</td><td>Actinoplanes missouriensis</td><td>hypothetical protein [Actinoplanes missouriensis].</td><td>GCF_000284295.1</td></tr>
<tr><td>WP_014798951.1</td><td>LRR-repeats+AP-GTPase+COR*→||<-?<-?<-Pkinase+TM+TPR+TPR</td><td>LRR-repeats+AP-GTPase+COR</td><td>1071</td><td>-</td><td>Bacteroidetes</td><td>Bernardetia litoralis</td><td>leucine-rich repeat domain-containing protein [Bernardetia litoralis].</td><td>GCF_000265505.1</td></tr>
</tbody>
</table>

acc	operon	architecture	len	gen.name	taxend	species	defline	gca
WP_015112282.1	<-ParA-Soj-PloopNTPase<-?||?→ PIN→ LRR-repeats+AP-GTPase+COR+DUF4404*→||<-?<-?||?→?→||<-?<-?||Thioredoxin→	LRR-repeats+AP-GTPase+COR+DUF4404	925	-	Cyanobacteria	Nostoc sp. PCC 7107	leucine-rich repeat domain-containing protein [Nostoc sp. PCC 7107].	GCF_000316625.1
WP_015118030.1	LRR-repeats+AP-GTPase+COR+EAD1*→||<-IG	LRR-repeats+AP-GTPase+COR+EAD1	955	-	Cyanobacteria	Rivularia sp. PCC 7116	leucine-rich repeat domain-containing protein [Rivularia sp. PCC 7116].	GCF_000316665.1
WP_015120500.1	LRR-repeats+AP-GTPase+COR+EAD1*→?→?→?→?→?→?→ ABC_membrane+ABC_tran→	LRR-repeats+AP-GTPase+COR+EAD1	868	-	Cyanobacteria	Rivularia sp. PCC 7116	leucine-rich repeat domain-containing protein [Rivularia sp. PCC 7116].	GCF_000316665.1
WP_015120705.1	<-Cluster1048_2clades<-?||?→||<-?||LRR-repeats+AP-GTPase+COR+EAD1*→||<-?<-?||?→||<-PSE<-?||ABC-ATPase→	LRR-repeats+AP-GTPase+COR+EAD1	989	-	Cyanobacteria	Rivularia sp. PCC 7116	leucine-rich repeat domain-containing protein [Rivularia sp. PCC 7116].	GCF_000316665.1
WP_015132911.1	REase→ LRR-repeats+AP-GTPase+COR+DUF4404*→	LRR-repeats+AP-GTPase+COR+DUF4404	1183	-	Cyanobacteria	Leptolyngbya sp. PCC 7376	leucine-rich repeat protein [Leptolyngbya sp. PCC 7376].	GCF_000316605.1
WP_015135895.1	<-Cluster998_2clades||?→?→||<-?||WCAK→||<-?<-?||LRR-repeats+AP-GTPase+COR+TM+TM*→ ABC-ATPase→ ABC_membrane+ABC_tran→||<-?<-?<-?||ISOFLAVOMETHYLASE-HTH→	LRR-repeats+AP-GTPase+COR+TM+TM	925	-	Cyanobacteria	Leptolyngbya sp. PCC 7376	leucine-rich repeat domain-containing protein [Leptolyngbya sp. PCC 7376].	GCF_000316605.1
WP_015147146.1	Pentapeptide-repeats→?→ CBS→ Pentapeptide-repeats→ Pentapeptide-repeats→?→ LRR-repeats+AP-GTPase+COR*→?→?→ Cluster2236_2clades→||<-?<-?<-Hsp10	LRR-repeats+AP-GTPase+COR	922	-	Cyanobacteria	Oscillatoria acuminata	leucine-rich repeat domain-containing protein [Oscillatoria acuminata].	GCF_000317105.1
WP_015161253.1	TM→?→||<-?||SF2-DUF3427A→||<-?||LRR-repeats+AP-GTPase+COR*→||<-?||?→?→ TRD+TRD→?→ PIN→	LRR-repeats+AP-GTPase+COR	993	-	Cyanobacteria	Chamaesiphon minutus	leucine-rich repeat domain-containing protein [Chamaesiphon minutus].	GCF_000317145.1
WP_015196406.1	HTH→?→||<-?||LRR-repeats+AP-GTPase+COR+DUF4404*→	LRR-repeats+AP-GTPase+COR+DUF4404	1034	-	Cyanobacteria	Calothrix parietina	leucine-rich repeat domain-containing protein [Calothrix parietina].	GCF_000317435.1
WP_015197432.1	<-Cluster5_5clades<-?<-Cluster5_5clades<-Chelatase-AAA+VWA<-?<-?<-?||LRR-repeats+AP-GTPase+COR+DUF4404*→?→?→ RNASEII→	LRR-repeats+AP-GTPase+COR+DUF4404	1408	-	Cyanobacteria	Calothrix parietina	leucine-rich repeat domain-containing protein [Calothrix parietina].	GCF_000317435.1
WP_015203756.1	LRR-repeats+AP-GTPase+COR*→||<-?<-?||TM+TM+TM+TM+TM+TM+TM+TM+TM→	LRR-repeats+AP-GTPase+COR	1011	-	Cyanobacteria	Crinalium epipsammum	leucine-rich repeat domain-containing protein [Crinalium epipsammum].	GCF_000317495.1
WP_015206852.1	<-HNH<-?||LRR-repeats+AP-GTPase+COR*→||<-CASPASE+TPR	LRR-repeats+AP-GTPase+COR	938	-	Cyanobacteria	Cylindrospermum stagnale	leucine-rich repeat protein [Cylindrospermum stagnale].	GCF_000317535.1
WP_015280068.1	<-CENPB||LRR-repeats+AP-GTPase+COR+TIR*→||<-?<-?||MoxR-AAA→ VWA→?→ SIG+TM+VWA+TM→ SIG+TM+VWA+TM→	LRR-repeats+AP-GTPase+COR+TIR	1283	-	Gammaproteobacteria	Thioflavicoccus mobilis	leucine-rich repeat domain-containing protein [Thioflavicoccus mobilis].	GCF_000327045.1
WP_015361283.1	Cluster2083_2clades→?→?→?→?→||<-?||?→ LRR-repeats+AP-GTPase+COR+TIR*→	LRR-repeats+AP-GTPase+COR+TIR	1060	-	Bacteroidetes	Nonlabens dokdonensis	leucine-rich repeat domain-containing protein [Nonlabens dokdonensis].	GCF_000332115.1
WP_015785030.1	REase-4→?→?→?→ HAD→ LRR-repeats+AP-GTPase+COR+TM+TM*→	LRR-repeats+AP-GTPase+COR+TM+TM	937	-	Cyanobacteria	Rippkaea orientalis	GTP-binding protein [Rippkaea orientalis].	GCF_000024045.1
WP_015948508.1	<-Arginase<-?||?→?→ LRR-repeats→ LRR-repeats+AP-GTPase+COR+ARSR-HTH*→||<-?<-?<-Nitroreductase-like<-PEP-utilisers-C	LRR-repeats+AP-GTPase+COR+ARSR-HTH	792	-	Deltaproteobacteria	Desulfatibacillum aliphaticivorans	leucine-rich repeat domain-containing protein [Desulfatibacillum aliphaticivorans].	GCF_000021905.1
WP_015955249.1	LRR-repeats+AP-GTPase+COR*→||<-DAGKIN<-?<-?||SIG+GT4→	LRR-repeats+AP-GTPase+COR	867	-	Cyanobacteria	Gloeothece citriformis	leucine-rich repeat domain-containing protein [Gloeothece citriformis].	GCF_000021825.1
WP_015956848.1	REC+LuxR-HTH→||<-tRNA<-?<-?<-?||PolB→ Cluster2355_2clades→ LRR-repeats+AP-GTPase+COR+TIR*→	LRR-repeats+AP-GTPase+COR+TIR	1015	-	Cyanobacteria	Gloeothece citriformis	leucine-rich repeat domain-containing protein [Gloeothece citriformis].	GCF_000021825.1

<table>
<tr><th>acc</th><th>operon</th><th>architecture</th><th>len</th><th>gen.name</th><th>taxend</th><th>species</th><th>defline</th><th>gca</th></tr>
<tr><td>WP_016798830.1</td><td>CI→ LRR-repeats+AP-GTPase+COR*→</td><td>LRR-repeats+AP-GTPase+COR</td><td>1080</td><td>-</td><td>Gammaproteobacteria</td><td>Vibrio cyclitrophicus</td><td>GTP-binding protein [Vibrio cyclitrophicus].</td><td>GCF_000256425.1</td></tr>
<tr><td>WP_016860490.1</td><td><-HISKIN||?→?→ LRR-repeats→ LRR-repeats+AP-GTPase+COR+TM+TM*→||<-ABhydrolase</td><td>LRR-repeats+AP-GTPase+COR+TM+TM</td><td>719</td><td>-</td><td>Cyanobacteria</td><td>Fischerella muscicola</td><td>GTP-binding protein [Fischerella muscicola].</td><td>GCF_000317245.1</td></tr>
<tr><td>WP_016952822.1</td><td>LRR-repeats+AP-GTPase+COR*→</td><td>LRR-repeats+AP-GTPase+COR</td><td>829</td><td>-</td><td>Cyanobacteria</td><td>Anabaena sp. PCC 7108</td><td>leucine-rich repeat domain-containing protein [Anabaena sp. PCC 7108].</td><td>GCF_000332135.1</td></tr>
<tr><td>WP_017313212.1</td><td>Cluster1069__2clades→?→?→||<-?||?→?→ LRR-repeats+AP-GTPase+COR*→||<-?||?→?→?→?→ Mbetalac→</td><td>LRR-repeats+AP-GTPase+COR</td><td>1085</td><td>-</td><td>Cyanobacteria</td><td>Fischerella sp. PCC 9339</td><td>leucine-rich repeat protein [Fischerella sp. PCC 9339].</td><td>GCF_000315585.1</td></tr>
<tr><td>WP_017720442.1</td><td>LRR-repeats+AP-GTPase+COR*→</td><td>LRR-repeats+AP-GTPase+COR</td><td>852</td><td>-</td><td>Cyanobacteria</td><td>Oscillatoria sp. PCC 10802</td><td>leucine-rich repeat domain-containing protein [Oscillatoria sp. PCC 10802].</td><td>GCF_000332335.1</td></tr>
<tr><td>WP_017739957.1</td><td>Cluster5__5clades→?→||<-Cluster5__5clades||?→ PSE→||<-?<-PSE||?→ LRR-repeats+AP-GTPase+COR+EAD1*→ PSE→?→?→ Cluster2194__2clades→||<-?||Cluster1640__2clades→</td><td>LRR-repeats+AP-GTPase+COR+EAD1</td><td>965</td><td>-</td><td>Cyanobacteria</td><td>Scytonema hofmannii</td><td>leucine-rich repeat domain-containing protein [Scytonema hofmannii].</td><td>GCF_000346485.2</td></tr>
<tr><td>WP_018619729.1</td><td><-HISKIN<-?||Cluster1877__2clades→ LRR-repeats+AP-GTPase+COR*→</td><td>LRR-repeats+AP-GTPase+COR</td><td>925</td><td>-</td><td>Bacteroidetes</td><td>Spirosoma luteum</td><td>leucine-rich repeat domain-containing protein [Spirosoma luteum].</td><td>GCF_000374065.1</td></tr>
<tr><td>WP_019496026.1</td><td>ParB→||<-?<-TM+TM||?→ SIG+TM+TM+TM+TM+TM+TM+TM+TM+TM+TM→?→ EAD1+LRR-repeats+AP-GTPase+COR*→?→?→?→?→ SIG+GT4→||<-PSE||?→ TPR+nSTAND1+BetaPropeller_WD40+BetaPropeller_WD40→</td><td>EAD1+LRR-repeats+AP-GTPase+COR</td><td>961</td><td>-</td><td>Cyanobacteria</td><td>Calothrix sp. PCC 7103</td><td>leucine-rich repeat domain-containing protein [Calothrix sp. PCC 7103].</td><td>GCF_000331305.1</td></tr>
<tr><td>WP_019496060.1</td><td>Cluster5__5clades→ LRR-repeats+AP-GTPase+COR+EAD1*→ PSE→?→||<-CR-REase5||?→ APATPase+BetaPropeller_WD40-repeats→?→ APATPase+BetaPropeller_WD40-repeats→ APATPase+BetaPropeller_WD40-repeats→</td><td>LRR-repeats+AP-GTPase+COR+EAD1</td><td>912</td><td>-</td><td>Cyanobacteria</td><td>Calothrix sp. PCC 7103</td><td>leucine-rich repeat domain-containing protein [Calothrix sp. PCC 7103].</td><td>GCF_000331305.1</td></tr>
<tr><td>WP_019867072.1</td><td>LRR-repeats+AP-GTPase+COR+TIR*→</td><td>LRR-repeats+AP-GTPase+COR+TIR</td><td>856</td><td>-</td><td>Gammaproteobacteria</td><td>Methylovulum miyakonense</td><td>TIR domain-containing protein [Methylovulum miyakonense].</td><td>GCF_000384075.1</td></tr>
<tr><td>WP_020379418.1</td><td>SF2-DUF3427A+TnsA-REase→ DAM→ LRR-repeats+AP-GTPase+COR+TM+TM*→</td><td>LRR-repeats+AP-GTPase+COR+TM+TM</td><td>1195</td><td>-</td><td>Actinobacteria</td><td>Candidatus Microthrix parvicella</td><td>hypothetical protein [Candidatus Microthrix parvicella].</td><td>GCF_000299415.1</td></tr>
<tr><td>WP_020396599.1</td><td>LRR-repeats+AP-GTPase+COR+TIR*→ TM+TM+TM→</td><td>LRR-repeats+AP-GTPase+COR+TIR</td><td>767</td><td>-</td><td>Gammaproteobacteria</td><td>Thiolinea disciformis</td><td>TIR domain-containing protein, partial [Thiolinea disciformis].</td><td>GCF_000371925.1</td></tr>
<tr><td>WP_020483095.1</td><td>Cluster1270__2clades→||<-?<-?<-?<-?<-?||?→ LRR-repeats+AP-GTPase+COR*→||<-?<-?<-?<-?||?→ SIGMA-HTH→</td><td>LRR-repeats+AP-GTPase+COR</td><td>1111</td><td>-</td><td>Gammaproteobacteria</td><td>Methylomonas sp. MK1</td><td>leucine-rich repeat domain-containing protein [Methylomonas sp. MK1].</td><td>GCF_000365425.1</td></tr>
<tr><td>WP_020496162.1</td><td><-ISOCOT<-?<-?||InPase→||<-?||LRR-repeats+AP-GTPase+COR*→</td><td>LRR-repeats+AP-GTPase+COR</td><td>833</td><td>-</td><td>Actinobacteria</td><td>Sciscionella marina</td><td>hypothetical protein [Sciscionella marina].</td><td>GCF_000379465.1</td></tr>
<tr><td>WP_020559023.1</td><td>Cluster2057__2clades→ LRR-repeats+AP-GTPase+COR+TIR→?→?→?→?→?→ LRR-repeats+AP-GTPase+COR+TIR→ Cluster54__3clades→ PSE→||<-TIR+TPR+TPR-S</td><td>LRR-repeats+AP-GTPase+COR+TIR</td><td>972</td><td>-</td><td>Gammaproteobacteria</td><td>Thiofilum flexile</td><td>leucine-rich repeat domain-containing protein [Thiofilum flexile].</td><td>GCF_000380185.1</td></tr>
<tr><td>WP_020559030.1</td><td>LRR-repeats+AP-GTPase+COR+TIR*→</td><td>LRR-repeats+AP-GTPase+COR+TIR</td><td>943</td><td>-</td><td>Gammaproteobacteria</td><td>Thiofilum flexile</td><td>leucine-rich repeat domain-containing protein [Thiofilum flexile].</td><td>GCF_000380185.1</td></tr>
<tr><td>WP_020560643.1</td><td>LRR-repeats+AP-GTPase+COR+TIR*→ TM+TM+TM→</td><td>LRR-repeats+AP-GTPase+COR+TIR</td><td>900</td><td>-</td><td>Gammaproteobacteria</td><td>Thiofilum flexile</td><td>TIR domain-containing protein [Thiofilum flexile].</td><td>GCF_000380185.1</td></tr>
<tr><td>WP_020570498.1</td><td><-SIG+ABhydrolase<-?<-?<-?||LRR-repeats+AP-GTPase+COR+CASPASE*→?→ FGS→?→ RVT→?→ FGS→</td><td>LRR-repeats+AP-GTPase+COR+CASPASE</td><td>1039</td><td>-</td><td>Bacteroidetes</td><td>Lewinella persica</td><td>CHAT domain-containing protein [Lewinella persica].</td><td>GCF_000373105.1</td></tr>
<tr><td>WP_020607470.1</td><td>LRR-repeats+AP-GTPase+COR+TIR*→</td><td>LRR-repeats+AP-GTPase+COR+TIR</td><td>954</td><td>-</td><td>Bacteroidetes</td><td>Spirosoma spitsbergense</td><td>leucine-rich repeat domain-containing protein [Spirosoma spitsbergense].</td><td>GCF_000374085.1</td></tr>
<tr><td>WP_021020180.</td><td>Polbeta+HEPN→ LRR-repeats→ LRR-repeats+AP-GTPase+COR+TIR*→?→||<-?||?→?→ SIG+TM+TM+TM+TM+TM+TM+TM+TM+TM+TM+TM→ SIG+TM→</td><td>LRR-repeats+AP-GTPase+COR+TIR</td><td>944</td><td>-</td><td>Gammaproteobacteria</td><td>Vibrio gazogenes</td><td>TIR domain-containing protein [Vibrio gazogenes].</td><td>GCF_000390165.1</td></tr>
<tr><td>WP_021802668.1</td><td>HAD→?→?→?→ Cluster881__3clades→ LRR-repeats+AP-GTPase+COR*→?→?→?→?→?→?→ Cluster1292__2clades→</td><td>LRR-repeats+AP-GTPase+COR</td><td>936</td><td>-</td><td>Firmicutes</td><td>Clostridium intestinale</td><td>GTP-binding protein [Clostridium intestinale].</td><td>GCF_013781885.1</td></tr>
</table>

acc	operon	architecture	len	gen.name	taxend	species	defline	gca
WP_022571049.1	CI→ LRR-repeats+AP-GTPase+COR*→	LRR-repeats+AP-GTPase+COR	1095	-	Gammaproteobacteria	Vibrio cyclitrophicus	leucine rich repeat protein [Vibrio cyclitrophicus].	GCF_000473545.1
WP_022589047.1	ABC-ATPase→?→?→||<-Acylphosphatase||?→?→||<-?||LRR-repeats+AP-GTPase+COR+TIR*→	LRR-repeats+AP-GTPase+COR+TIR	1264	-	Gammaproteobacteria	Vibrio nigripulchritudo	TIR domain-containing protein [Vibrio nigripulchritudo].	GCF_900067775.1
WP_023074611.1	LRR-repeats+AP-GTPase+COR+TIR*→||<-?||?→||<-?||Cluster1646__2clades→	LRR-repeats+AP-GTPase+COR+TIR	777	-	Cyanobacteria	Leptolyngbya sp. Heron Island J	leucine-rich repeat domain-containing protein, partial [Leptolyngbya sp. Heron Island J].	GCF_000482245.1
WP_023076066.1	Cluster53__2clades→||<-RelE-ParE<-?||LRR-repeats+AP-GTPase+COR+TIR*→||<-?||?→||<-?||?→?→||<-?<-SIG+TM+TM+TM+TM+TM+TM	LRR-repeats+AP-GTPase+COR+TIR	1129	-	Cyanobacteria	Leptolyngbya sp. Heron Island J	leucine-rich repeat domain-containing protein [Leptolyngbya sp. Heron Island J].	GCF_000482245.1
WP_023571556.1	LRR-repeats+AP-GTPase+COR+TIR*→	LRR-repeats+AP-GTPase+COR+TIR	1142	-	Bacteroidetes	Flavobacterium cauense	TIR domain-containing protein [Flavobacterium cauense].	GCF_000498475.1
WP_024594403.1	LRR-repeats+AP-GTPase+COR*→	LRR-repeats+AP-GTPase+COR	982	-	Gammaproteobacteria	Pseudoalteromonas sp. TB13	GTP-binding protein [Pseudoalteromonas sp. TB13].	GCF_000497915.1
WP_025517521.1	CI→ LRR-repeats+AP-GTPase+COR*→	LRR-repeats+AP-GTPase+COR	1105	-	Gammaproteobacteria	Vibrio parahaemolyticus	50S ribosome-binding GTPase [Vibrio parahaemolyticus].	GCF_000489555.1
WP_025605295.1	CI→?→ PSE→ LRR-repeats+AP-GTPase+COR+TIR*→	LRR-repeats+AP-GTPase+COR+TIR	1265	-	Gammaproteobacteria	Vibrio parahaemolyticus	TIR domain-containing protein [Vibrio parahaemolyticus].	GCF_000489075.1
WP_026726296.1	LRR-repeats+AP-GTPase+COR*→	LRR-repeats+AP-GTPase+COR	698	-	Bacteroidetes	Flavobacterium sasangense	GTP-binding protein [Flavobacterium sasangense].	GCF_000686885.1
WP_028083535.1	LRR-repeats+AP-GTPase+COR*→	LRR-repeats+AP-GTPase+COR	993	-	Cyanobacteria	Dolichospermum circinale	leucine-rich repeat domain-containing protein [Dolichospermum circinale].	GCF_000426925.1
WP_028469928.1	LRR-repeats+AP-GTPase+COR*→?→?→ GTPase-AIG→	LRR-repeats+AP-GTPase+COR	697	-	Gammaproteobacteria	Neptunomonas japonica	hypothetical protein [Neptunomonas japonica].	GCF_000422765.1
WP_028667519.1	<-Cluster1063__3clades<-Cluster1067__3clades||?→?→?→?→ LRR-repeats+AP-GTPase+COR*→	LRR-repeats+AP-GTPase+COR	798	-	Bacteroidetes	Runella zeae	GTP-binding protein [Runella zeae].	GCF_000423565.1
WP_029631472.1	LRR-repeats+AP-GTPase+COR*→	LRR-repeats+AP-GTPase+COR	1051	-	Planctomycetes	Zavarzinella formosa	leucine-rich repeat domain-containing protein [Zavarzinella formosa].	GCF_000255705.1
WP_030435343.1	REC→ PSE→?→?→||<-?||?→ Cluster2103__2clades→||<-?||LRR-repeats+AP-GTPase+COR*→ AP-GTPase+COR+TM+TM→||<-REC<-?||ABC-ATPase→	LRR-repeats+AP-GTPase+COR	551	-	Actinobacteria	Actinoplanes subtropicus	GTP-binding protein [Actinoplanes subtropicus].	GCF_000721705.1
WP_030488330.1	LRR-repeats+AP-GTPase+COR*→||<-?<-?||?→?→||<-?||SIG+TM→ REC→	LRR-repeats+AP-GTPase+COR	1088	-	Actinobacteria	Micromonospora chokoriensis	hypothetical protein [Micromonospora chokoriensis].	GCF_000718555.1
WP_031429699.1	CI→?→ PSE→ LRR-repeats+AP-GTPase+COR+TIR*→	LRR-repeats+AP-GTPase+COR+TIR	1261	-	Gammaproteobacteria	Vibrio parahaemolyticus	TIR domain-containing protein [Vibrio parahaemolyticus].	GCF_000736345.1
WP_034058880.1	S1COLD→?→||<-?||?→?→||<-?||?→ LRR-repeats+AP-GTPase+COR*→	LRR-repeats+AP-GTPase+COR	924	-	Bacteroidetes	Lacinutrix jangbogonensis	leucine-rich repeat domain-containing protein [Lacinutrix jangbogonensis].	GCF_000797445.1
WP_035121352.1	LRR-repeats+AP-GTPase+COR+TIR*→||<-?<-?<-?<-ABC-ATPase	LRR-repeats+AP-GTPase+COR+TIR	1115	-	Cyanobacteria	Fischerella sp. PCC 9431	leucine-rich repeat domain-containing protein [Fischerella sp. PCC 9431].	GCF_000447295.1
WP_035197779.1	LRR-repeats+AP-GTPase+COR*→	LRR-repeats+AP-GTPase+COR	914	-	Firmicutes	Calidifontibacillus azotoformans	GTP-binding protein [Calidifontibacillus azotoformans].	GCF_000708505.1
WP_035613546.1	LRR-repeats+AP-GTPase+COR*→	LRR-repeats+AP-GTPase+COR	1058	-	Verrucomicrobia	Haloferula sp. BvORR071	hypothetical protein [Haloferula sp. BvORR071].	-
WP_035758421.1	LRR-repeats+AP-GTPase+COR*→	LRR-repeats+AP-GTPase+COR	956	-	Bacteroidetes	Hugenholtzia roseola	leucine-rich repeat protein, partial [Hugenholtzia roseola].	GCF_000422585.1
WP_038164534.1	LRR-repeats+AP-GTPase+COR+TIR*→	LRR-repeats+AP-GTPase+COR+TIR	793	-	Verrucomicrobia	Verrucomicrobium sp. BvORR106	TIR domain-containing protein [Verrucomicrobium sp. BvORR106].	-
WP_039108669.1	<-Cluster1070__2clades<-ENDO3+NUDIX||HU-IHF→?→ LRR-repeats+AP-GTPase+COR*→	LRR-repeats+AP-GTPase+COR	912	-	Bacteroidetes	Flavobacterium sp. AED	leucine-rich repeat domain-containing protein [Flavobacterium sp. AED].	GCF_000812945.1

<table>
<thead>
<tr><th>acc</th><th>operon</th><th>architecture</th><th>len</th><th>gen.name</th><th>taxend</th><th>species</th><th>defline</th><th>gca</th></tr>
</thead>
<tbody>
<tr><td>WP_039351391.1</td><td>LRR-repeats+AP-GTPase+COR*→||<-?||?→||<-RnasePH+RnasePH+KH+S1</td><td>LRR-repeats+AP-GTPase+COR</td><td>912</td><td>-</td><td>Bacteroidetes</td><td>Chryseobacterium jeonii</td><td>GTP-binding protein [Chryseobacterium jeonii].</td><td>GCF_000812865.1</td></tr>
<tr><td>WP_039727351.1</td><td>SIG+TM+TM+TM+TM+TM+TM→||<-?||?→?→?→ LRR-repeats+AP-GTPase+COR*→?→ PIN→||<-?<-REC+LuxR-HTH<-?<-?<-Cluster1069_2clades</td><td>LRR-repeats+AP-GTPase+COR</td><td>1075</td><td>-</td><td>Terrabacteria group</td><td>Cyanobacteria</td><td>MULTISPECIES: leucine-rich repeat domain-containing protein [Cyanobacteria].</td><td>GCF_000817775.2</td></tr>
<tr><td>WP_039983671.1</td><td>LRR-repeats+AP-GTPase+COR*→</td><td>LRR-repeats+AP-GTPase+COR</td><td>1101</td><td>-</td><td>Gammaproteobacteria</td><td>Vibrio sagamiensis</td><td>50S ribosome-binding GTPase [Vibrio sagamiensis].</td><td>GCF_000400425.1</td></tr>
<tr><td>WP_040248977.1</td><td>LRR-repeats+AP-GTPase+COR*→||<-?<-?<-?||?→?→?→ SIG+TM+TM→</td><td>LRR-repeats+AP-GTPase+COR</td><td>924</td><td>-</td><td>Bacteroidetes</td><td>Psychroserpens mesophilus</td><td>leucine-rich repeat domain-containing protein [Psychroserpens mesophilus].</td><td>GCF_000826645.1</td></tr>
<tr><td>WP_044220039.1</td><td>FGS→?→ RVT→?→ FGS→?→ LRR-repeats+AP-GTPase+COR+CASPASE*→?→ FGS→</td><td>LRR-repeats+AP-GTPase+COR+CASPASE</td><td>977</td><td>-</td><td>Bacteroidetes</td><td>Phaeodactylibacter xiamenensis</td><td>CHAT domain-containing protein [Phaeodactylibacter xiamenensis].</td><td>GCF_000759025.1</td></tr>
<tr><td>WP_044222080.1</td><td>REC→ FGS→||<-?||RVT→?→ FGS→ LRR-repeats+AP-GTPase+COR+CASPASE*→?→?→?→ FGS→ FGS→ FGS→</td><td>LRR-repeats+AP-GTPase+COR+CASPASE</td><td>931</td><td>-</td><td>Bacteroidetes</td><td>Phaeodactylibacter xiamenensis</td><td>leucine-rich repeat domain-containing protein [Phaeodactylibacter xiamenensis].</td><td>GCF_000759025.1</td></tr>
<tr><td>WP_044448603.1</td><td>LRR-repeats+AP-GTPase+COR+TIR*→</td><td>LRR-repeats+AP-GTPase+COR+TIR</td><td>1176</td><td>-</td><td>Cyanobacteria</td><td>Mastigocladus laminosus</td><td>leucine-rich repeat domain-containing protein [Mastigocladus laminosus].</td><td>-</td></tr>
<tr><td>WP_044448681.1</td><td>LRR-repeats+AP-GTPase+COR+DUF4404*→</td><td>LRR-repeats+AP-GTPase+COR+DUF4404</td><td>806</td><td>-</td><td>Cyanobacteria</td><td>Mastigocladus laminosus</td><td>GTP-binding protein [Mastigocladus laminosus].</td><td>-</td></tr>
<tr><td>WP_045613052.1</td><td><-Cluster1074_2clades||?→?→?→ LRR-repeats+AP-GTPase+COR*→</td><td>LRR-repeats+AP-GTPase+COR</td><td>1105</td><td>-</td><td>Gammaproteobacteria</td><td>Vibrio vulnificus</td><td>50S ribosome-binding GTPase [Vibrio vulnificus].</td><td>GCF_000960125.1</td></tr>
<tr><td>WP_046355878.1</td><td>LRR-repeats+AP-GTPase+COR+TIR*→</td><td>LRR-repeats+AP-GTPase+COR+TIR</td><td>1154</td><td>-</td><td>Gammaproteobacteria</td><td>Pseudoalteromonas luteoviolacea</td><td>leucine-rich repeat domain-containing protein [Pseudoalteromonas luteoviolacea].</td><td>GCF_001625715.1</td></tr>
<tr><td>WP_047776098.1</td><td>LRR-repeats+AP-GTPase+COR+TIR*→</td><td>LRR-repeats+AP-GTPase+COR+TIR</td><td>1055</td><td>-</td><td>Bacteroidetes</td><td>Flavobacterium sp. ABG</td><td>leucine-rich repeat domain-containing protein [Flavobacterium sp. ABG].</td><td>GCF_001027725.1</td></tr>
<tr><td>WP_047863083.1</td><td>Cluster971_2clades→?→||<-?<-?||LRR-repeats+AP-GTPase+COR+TIR*→?→||<-?<-?<-?||?→||<-?<-SIG+Lysozyme</td><td>LRR-repeats+AP-GTPase+COR+TIR</td><td>921</td><td>-</td><td>Deltaproteobacteria</td><td>Archangium gephyra</td><td>GTP-binding protein [Archangium gephyra].</td><td>GCF_001027285.1</td></tr>
<tr><td>WP_048040436.1</td><td><-PIN<-?<-SIG+TM+TM+TM+TM+TM+TM+TM+TM+TM+TM+TM||?→?→ LRR-repeats+AP-GTPase+COR*→</td><td>LRR-repeats+AP-GTPase+COR</td><td>935</td><td>-</td><td>Euryarchaeota</td><td>Methanosarcina mazei</td><td>leucine-rich repeat domain-containing protein [Methanosarcina mazei].</td><td>GCF_000970225.1</td></tr>
<tr><td>WP_048109136.1</td><td><-SIG+TM+VWA+TM<-VWA<-MoxR-AAA||?→ LRR-repeats+AP-GTPase+COR*→?→||<-?||SIG+Calcineurin→?→ PIN→||<-Ferredoxin-RRM</td><td>LRR-repeats+AP-GTPase+COR</td><td>931</td><td>-</td><td>Euryarchaeota</td><td>Methanosarcina barkeri</td><td>leucine-rich repeat domain-containing protein [Methanosarcina barkeri].</td><td>GCF_000970305.1</td></tr>
<tr><td>WP_048669264.1</td><td><-ABC_membrane+ABC_tran<-ABC_membrane+ABC_tran<-?||LRR-repeats+AP-GTPase+COR*→</td><td>LRR-repeats+AP-GTPase+COR</td><td>960</td><td>-</td><td>Gammaproteobacteria</td><td>Vibrio crassostreae</td><td>GTP-binding protein [Vibrio crassostreae].</td><td>GCF_001048475.1</td></tr>
<tr><td>WP_048752807.1</td><td>LRR-repeats+AP-GTPase+COR*→||<-PSE<-?||RelE→ CHTH→</td><td>LRR-repeats+AP-GTPase+COR</td><td>801</td><td>-</td><td>Cyanobacteria</td><td>Crocosphaera watsonii</td><td>leucine-rich repeat domain-containing protein, partial [Crocosphaera watsonii].</td><td>GCF_001050835.1</td></tr>
<tr><td>WP_049769521.1</td><td>LRR-repeats+AP-GTPase+COR*→</td><td>LRR-repeats+AP-GTPase+COR</td><td>662</td><td>-</td><td>Cyanobacteria</td><td>Rippkaea orientalis</td><td>GTP-binding protein [Rippkaea orientalis].</td><td>GCF_000021805.1</td></tr>
<tr><td>WP_049825328.1</td><td>LRR-repeats+AP-GTPase+COR*→</td><td>LRR-repeats+AP-GTPase+COR</td><td>616</td><td>-</td><td>Cyanobacteria</td><td>Rippkaea orientalis</td><td>GTP-binding protein [Rippkaea orientalis].</td><td>GCF_000024045.1</td></tr>
<tr><td>WP_049875725.1</td><td>PAIREDC-HTH→||<-?||LRR-repeats→ LRR-repeats+AP-GTPase+COR*→</td><td>LRR-repeats+AP-GTPase+COR</td><td>772</td><td>-</td><td>Actinobacteria</td><td>Streptomyces scabiei</td><td>hypothetical protein [Streptomyces scabiei].</td><td>GCF_000091305.1</td></tr>
<tr><td>WP_049974475.1</td><td>LRR-repeats+AP-GTPase+COR+TIR*→</td><td>LRR-repeats+AP-GTPase+COR+TIR</td><td>1267</td><td>-</td><td>Alphaproteobacteria</td><td>Azospirillum sp. B4</td><td>leucine-rich repeat domain-containing protein [Azospirillum sp. B4].</td><td>GCF_000333275.1</td></tr>
<tr><td>WP_050652657.1</td><td>LRR-repeats+AP-GTPase+COR*→</td><td>LRR-repeats+AP-GTPase+COR</td><td>1099</td><td>-</td><td>Gammaproteobacteria</td><td>Vibrio sp. J2-17</td><td>hypothetical protein [Vibrio sp. J2-17].</td><td>GCF_001244075.1</td></tr>
</tbody>
</table>

<table>
<tr><th>acc</th><th>operon</th><th>architecture</th><th>len</th><th>gen.name</th><th>taxend</th><th>species</th><th>defline</th><th>gca</th></tr>
<tr><td>WP_050710728.1</td><td>LRR-repeats+AP-GTPase+COR+DrHyd*→</td><td>LRR-repeats+AP-GTPase+COR+DrHyd</td><td>1196</td><td>-</td><td>Bacteroidetes</td><td>Dysgonomonas sp. HGC4</td><td>leucine-rich repeat domain-containing protein [Dysgonomonas sp. HGC4].</td><td>GCF_001261715.1</td></tr>
<tr><td>WP_050857550.1</td><td>LRR-repeats+AP-GTPase+COR*→||<-?<-?<-ABC-ATPase</td><td>LRR-repeats+AP-GTPase+COR</td><td>958</td><td>-</td><td>Cyanobacteria</td><td>Acaryochloris sp. CCMEE 5410</td><td>leucine-rich repeat domain-containing protein [Acaryochloris sp. CCMEE 5410].</td><td>GCF_000238775.1</td></tr>
<tr><td>WP_051210834.1</td><td>SIGMA-HTH→?→?→ LRR-repeats+AP-GTPase+COR*→||<-?<-?<-?<-?<-SIGMA-HTH</td><td>LRR-repeats+AP-GTPase+COR</td><td>1280</td><td>-</td><td>Bacteroidetes</td><td>Runella zeae</td><td>leucine-rich repeat domain-containing protein [Runella zeae].</td><td>GCF_000423565.1</td></tr>
<tr><td>WP_051242709.1</td><td>LRR-repeats+AP-GTPase+COR+TIR*→</td><td>LRR-repeats+AP-GTPase+COR+TIR</td><td>1043</td><td>-</td><td>Alphaproteobacteria</td><td>Stappia stellulata</td><td>leucine-rich repeat domain-containing protein [Stappia stellulata].</td><td>GCF_000423705.1</td></tr>
<tr><td>WP_051289443.1</td><td>LRR-repeats+AP-GTPase+COR+TM+TM*→||<-PSE||?→?→||<-?<-?||?→||<-?<-ABC-ATPase</td><td>LRR-repeats+AP-GTPase+COR+TM+TM</td><td>821</td><td>-</td><td>Cyanobacteria</td><td>Dolichospermum circinale</td><td>leucine-rich repeat domain-containing protein [Dolichospermum circinale].</td><td>GCF_000426925.1</td></tr>
<tr><td>WP_051347554.1</td><td>LRR-repeats+AP-GTPase+COR*→</td><td>LRR-repeats+AP-GTPase+COR</td><td>814</td><td>-</td><td>Cyanobacteria</td><td>Dolichospermum circinale</td><td>hypothetical protein, partial [Dolichospermum circinale].</td><td>GCF_000426905.1</td></tr>
<tr><td>WP_051394619.1</td><td>LRR-repeats+AP-GTPase+COR+TM+TM*→</td><td>LRR-repeats+AP-GTPase+COR+TM+TM</td><td>1004</td><td>-</td><td>Cyanobacteria</td><td>Leptolyngbya sp. Heron Island J</td><td>GTP-binding protein [Leptolyngbya sp. Heron Island J].</td><td>GCF_000482245.1</td></tr>
<tr><td>WP_051793473.1</td><td>LRR-repeats+AP-GTPase+COR*→</td><td>LRR-repeats+AP-GTPase+COR</td><td>946</td><td>-</td><td>Actinobacteria</td><td>Kibdelosporangium aridum</td><td>leucine-rich repeat domain-containing protein [Kibdelosporangium aridum].</td><td>GCF_003947235.1</td></tr>
<tr><td>WP_051898832.1</td><td>InPase→||<-?||LRR-repeats+AP-GTPase+COR*→</td><td>LRR-repeats+AP-GTPase+COR</td><td>833</td><td>-</td><td>Actinobacteria</td><td>Sciscionella sp. SE31</td><td>hypothetical protein [Sciscionella sp. SE31].</td><td>GCF_000737195.2</td></tr>
<tr><td>WP_051926645.1</td><td>LRR-repeats+AP-GTPase+COR*→||<-?||?→||<-?<-SIGMA-HTH<-?<-SIGMA-HTH</td><td>LRR-repeats+AP-GTPase+COR</td><td>996</td><td>-</td><td>Actinobacteria</td><td>Streptomyces durhamensis</td><td>hypothetical protein [Streptomyces durhamensis].</td><td>GCF_000725475.1</td></tr>
<tr><td>WP_051937621.1</td><td>LRR-repeats+AP-GTPase+COR*→</td><td>LRR-repeats+AP-GTPase+COR</td><td>1086</td><td>-</td><td>Actinobacteria</td><td>Streptomyces scabiei</td><td>hypothetical protein [Streptomyces scabiei].</td><td>GCF_000738715.1</td></tr>
<tr><td>WP_051942852.1</td><td>LRR-repeats+AP-GTPase+COR*→||<-?<-?<-Cluster1515_2clades<-?<-ABC-ATPase<-?<-SIG+TM+TM+TM+TM+TM+TM+TM</td><td>LRR-repeats+AP-GTPase+COR</td><td>1022</td><td>-</td><td>Actinobacteria</td><td>Streptacidiphilus rugosus</td><td>hypothetical protein [Streptacidiphilus rugosus].</td><td>GCF_000744655.1</td></tr>
<tr><td>WP_052150025.1</td><td>LRR-repeats+AP-GTPase+COR*→?→?→||<-?||?→?→?→||<-Pentapeptide</td><td>LRR-repeats+AP-GTPase+COR</td><td>949</td><td>-</td><td>Cyanobacteria</td><td>Aphanizomenon flos-aquae</td><td>leucine-rich repeat domain-containing protein [Aphanizomenon flos-aquae].</td><td>GCF_000789435.1</td></tr>
<tr><td>WP_052277881.1</td><td>SIG+TM+TM+TM+TM+TM+TM+TM→||<-?||Mbetalac→ SAM-methylase→?→||<-?<-SIG+TPM_phosphatase+TM+TM+TM||LRR-repeats+AP-GTPase+COR+TM+TM*→</td><td>LRR-repeats+AP-GTPase+COR+TM+TM</td><td>921</td><td>-</td><td>Cyanobacteria</td><td>Microcystis panniformis</td><td>leucine-rich repeat domain-containing protein [Microcystis panniformis].</td><td>-</td></tr>
<tr><td>WP_052288305.1</td><td><-Hsp10||?→ REC→ Ferretin→||<-Cluster1950_2clades||?→ LRR-repeats+AP-GTPase+COR+TIR*→||<-?<-?||?→?→?→?→ SIG+TM+TM+TM+TM+TM→</td><td>LRR-repeats+AP-GTPase+COR+TIR</td><td>848</td><td>-</td><td>Terrabacteria group</td><td>Cyanobacteria</td><td>MULTISPECIES: TIR domain-containing protein [Cyanobacteria].</td><td>GCF_000817775.2</td></tr>
<tr><td>WP_052324486.1</td><td>LRR-repeats→ AP-GTPase+COR*→</td><td>AP-GTPase+COR</td><td>434</td><td>-</td><td>Bacteroidetes</td><td>Haliscomenobacter hydrossis</td><td>50S ribosome-binding GTPase [Haliscomenobacter hydrossis].</td><td>GCF_000212735.1</td></tr>
<tr><td>WP_052478236.1</td><td>LRR-repeats+AP-GTPase+COR*→||<-?||?→||<-?<-?<-Aminotran_1_2</td><td>LRR-repeats+AP-GTPase+COR</td><td>942</td><td>-</td><td>Actinobacteria</td><td>Kibdelosporangium sp. MJ126-NF4</td><td>leucine-rich repeat domain-containing protein [Kibdelosporangium sp. MJ126-NF4].</td><td>GCF_000826545.1</td></tr>
<tr><td>WP_052712873.1</td><td><-HhH-RADC+JAB||?→ NTP_transf_3→ LRR-repeats+AP-GTPase+COR*→||<-?<-?||?→||<-?<-?||SIR2→</td><td>LRR-repeats+AP-GTPase+COR</td><td>926</td><td>-</td><td>Euryarchaeota</td><td>Methanosarcina barkeri</td><td>leucine-rich repeat domain-containing protein [Methanosarcina barkeri].</td><td>GCF_001027005.1</td></tr>
<tr><td>WP_052718309.1</td><td><-HhH-RADC+JAB||?→ NTP_transf_3→ LRR-repeats+AP-GTPase+COR→*?→ LRR-repeats+AP-GTPase+COR→*</td><td>LRR-repeats+AP-GTPase+COR</td><td>871</td><td>-</td><td>Euryarchaeota</td><td>Methanosarcina sp. MTP4</td><td>leucine-rich repeat domain-containing protein [Methanosarcina sp. MTP4].</td><td>GCF_000970045.1</td></tr>
</table>

acc	operon	architecture	len	gen.name	taxend	species	defline	gca
WP_052718311.1	LRR-repeats+AP-GTPase+COR*→	LRR-repeats+AP-GTPase+COR	999	-	Euryarchaeota	Methanosarcina sp. MTP4	leucine-rich repeat domain-containing protein [Methanosarcina sp. MTP4].	GCF_000970045.1
WP_052718346.1	LRR-repeats+AP-GTPase+COR*→?→||<-?<-SIG+TM+TM+TM+TM+TM<-ABC-ATPase	LRR-repeats+AP-GTPase+COR	891	-	Euryarchaeota	Methanosarcina sp. MTP4	leucine-rich repeat domain-containing protein [Methanosarcina sp. MTP4].	GCF_000970045.1
WP_052721594.1	<-HhH-RADC+JAB||?→ NTP_transf_3→ LRR-repeats+AP-GTPase+COR*→	LRR-repeats+AP-GTPase+COR	890	-	Euryarchaeota	Methanosarcina siciliae	leucine-rich repeat domain-containing protein [Methanosarcina siciliae].	GCF_000970085.1
WP_052725465.1	<-HhH-RADC+JAB||?→ NTP_transf_3→ LRR-repeats+AP-GTPase+COR*→	LRR-repeats+AP-GTPase+COR	886	-	Euryarchaeota	Methanosarcina sp. Kolksee	leucine-rich repeat domain-containing protein [Methanosarcina sp. Kolksee].	GCF_000969945.1
WP_052725699.1	<-HhH-RADC+JAB||?→ NTP_transf_3→ LRR-repeats+AP-GTPase+COR*→||<-?<-?||?→||<-?<-?||SIR2→	LRR-repeats+AP-GTPase+COR	926	-	Euryarchaeota	Methanosarcina barkeri	leucine-rich repeat domain-containing protein [Methanosarcina barkeri].	GCF_000970065.1
WP_052725704.1	<-SIG+TPR+TPR+TPR+TPR+TPR+TPR||?→||<-?||?→?→ PSE→?→ LRR-repeats+AP-GTPase+COR*→?→?→ PRTase→	LRR-repeats+AP-GTPase+COR	886	-	Euryarchaeota	Methanosarcina barkeri	leucine-rich repeat domain-containing protein [Methanosarcina barkeri].	GCF_000970065.1
WP_052727304.1	PAS+HISKIN→||<-?||?→?→?→?→||<-PSE||LRR-repeats+AP-GTPase+COR*→||<-?||?→ PSE→||<-?||Cluster1640_2clades→||<-SIG+HIN-HTH	LRR-repeats+AP-GTPase+COR	1010	-	Euryarchaeota	Methanosarcina siciliae	leucine-rich repeat domain-containing protein [Methanosarcina siciliae].	GCF_000970145.1
WP_052727948.1	LRR-repeats+AP-GTPase+COR+TIR*→?→?→?→?→?→ PSE→||<-TPR-repeats	LRR-repeats+AP-GTPase+COR+TIR	877	-	Euryarchaeota	Methanosarcina vacuolata	leucine-rich repeat domain-containing protein [Methanosarcina vacuolata].	GCF_000969905.1
WP_052727973.1	<-HhH-RADC+JAB||?→ NTP_transf_3→ LRR-repeats+AP-GTPase+COR*→	LRR-repeats+AP-GTPase+COR	886	-	Euryarchaeota	Methanosarcina vacuolata	leucine-rich repeat domain-containing protein [Methanosarcina vacuolata].	GCF_000969905.1
WP_052728059.1	LRR-repeats+AP-GTPase+COR*→	LRR-repeats+AP-GTPase+COR	930	-	Euryarchaeota	Methanosarcina mazei	leucine-rich repeat domain-containing protein [Methanosarcina mazei].	GCF_000970205.1
WP_052728060.1	<-PIN<-?<-SIG+TM+TM+TM+TM+TM+TM+TM+TM+TM+TM+TM||?→?→ LRR-repeats+AP-GTPase+COR*→	LRR-repeats+AP-GTPase+COR	935	-	Euryarchaeota	Methanosarcina mazei	leucine-rich repeat domain-containing protein [Methanosarcina mazei].	GCF_000970205.1
WP_052735402.1	LRR-repeats+AP-GTPase+COR*→	LRR-repeats+AP-GTPase+COR	845	-	Euryarchaeota	Methanosarcina sp. 1.H.A.2.2	leucine-rich repeat domain-containing protein [Methanosarcina sp. 1.H.A.2.2].	GCF_000979975.1
WP_052752245.1	LRR-repeats+AP-GTPase+COR*→ TIR→	LRR-repeats+AP-GTPase+COR	1015	-	Betaproteobacteria	Nitrosomonas communis	leucine-rich repeat domain-containing protein [Nitrosomonas communis].	GCF_001007935.1
WP_054015538.1	<-REC||?→||<-?||?→ LRR-repeats+AP-GTPase+COR+TIR*→	LRR-repeats+AP-GTPase+COR+TIR	1274	-	Gammaproteobacteria	Pseudoalteromonas sp. R3	TIR domain-containing protein [Pseudoalteromonas sp. R3].	GCF_004014715.1
WP_054015917.1	LRR-repeats+AP-GTPase+COR+TIR*→||<-?<-?<-TM	LRR-repeats+AP-GTPase+COR+TIR	1128	-	Gammaproteobacteria	Pseudoalteromonas sp. R3	leucine-rich repeat domain-containing protein [Pseudoalteromonas sp. R3].	GCF_004014715.1
WP_054961809.1	DSBH+AraC-HTH+AraC-HTH→?→ LRR-repeats+AP-GTPase+COR*→||<-ABC_membrane+ABC_tran	LRR-repeats+AP-GTPase+COR	1057	-	Gammaproteobacteria	Vibrio bivalvicida	hypothetical protein [Vibrio bivalvicida].	GCF_001399455.2
WP_055505179.1	<-ABC-ATPase<-?||?→?→||<-?||LRR-repeats+AP-GTPase+COR*→?→?→?→||<-?<-?<-SIR2	LRR-repeats+AP-GTPase+COR	941	-	Actinobacteria	Nonomuraea pusilla	leucine-rich repeat domain-containing protein [Nonomuraea pusilla].	GCF_001417755.1
WP_057935115.1	SIG+Concanavalin-like+SUN→||<-?||?→ FGS→?→?→?→ LRR-repeats+AP-GTPase+COR+TIR*→?→?→||<-?<-?||?→ TIR→	LRR-repeats+AP-GTPase+COR+TIR	1318	-	Bacteroidetes	Pedobacter ginsenosidimutans	leucine-rich repeat domain-containing protein [Pedobacter ginsenosidimutans].	GCF_001442625.1

<table>
<tr><th>acc</th><th>operon</th><th>architecture</th><th>len</th><th>gen.name</th><th>taxend</th><th>species</th><th>defline</th><th>gca</th></tr>
<tr><td>WP_058183692.1</td><td>LRR-repeats+AP-GTPase+COR+CASPASE*→ LRR-repeats→</td><td>LRR-repeats+AP-GTPase+COR+CASPASE</td><td>1194</td><td>-</td><td>Cyanobacteria</td><td>Mastigocoleus testarum</td><td>leucine-rich repeat domain-containing protein [Mastigocoleus testarum].</td><td>GCF_001456025.1</td></tr>
<tr><td>WP_058183771.1</td><td>LRR-repeats+AP-GTPase+COR+EAD1*→</td><td>LRR-repeats+AP-GTPase+COR+EAD1</td><td>1014</td><td>-</td><td>Cyanobacteria</td><td>Mastigocoleus testarum</td><td>leucine-rich repeat domain-containing protein [Mastigocoleus testarum].</td><td>GCF_001456025.1</td></tr>
<tr><td>WP_058554438.1</td><td>LRR-repeats+AP-GTPase+COR+TIR*→</td><td>LRR-repeats+AP-GTPase+COR+TIR</td><td>758</td><td>-</td><td>Gammaproteobacteria</td><td>Thiohalocapsa sp. ML1</td><td>TIR domain-containing protein [Thiohalocapsa sp. ML1].</td><td>GCF_001469165.1</td></tr>
<tr><td>WP_058998621.1</td><td>LRR-repeats+AP-GTPase+COR*→?→?→ HNH→</td><td>LRR-repeats+AP-GTPase+COR</td><td>418</td><td>-</td><td>Cyanobacteria</td><td>Leptolyngbya sp. NIES-2104</td><td>leucine-rich repeat domain-containing protein [Leptolyngbya sp. NIES-2104].</td><td>GCF_001485215.1</td></tr>
<tr><td>WP_059136441.1</td><td>LRR-repeats+AP-GTPase+COR+TIR*→||<-RnasePH+RnasePH+KH+S1</td><td>LRR-repeats+AP-GTPase+COR+TIR</td><td>1173</td><td>-</td><td>Bacteroidetes</td><td>Chryseobacterium greenlandense</td><td>leucine-rich repeat domain-containing protein [Chryseobacterium greenlandense].</td><td>GCF_001507325.1</td></tr>
<tr><td>WP_061431817.1</td><td>SIG+TM+TM+TM+TM+TM+TM+TM→||<-?||PSE→ PSE→||<-?||SAM-methylase→?→||<-?<-SIG+TPM_phosphatase+TM+TM+TM||PSE→ LRR-repeats+AP-GTPase+COR+TM+TM*→||<-?<-?<-?<-?<-?||?→||<-Classical-AAA+Classical-AAA</td><td>LRR-repeats+AP-GTPase+COR+TM+TM</td><td>670</td><td>-</td><td>Cyanobacteria</td><td>Microcystis aeruginosa</td><td>GTP-binding protein, partial [Microcystis aeruginosa].</td><td>GCF_001578075.1</td></tr>
<tr><td>WP_062148002.1</td><td><-TPR-repeats+CASPASE||?→?→ LRR-repeats+AP-GTPase+COR+DUF4404*→</td><td>LRR-repeats+AP-GTPase+COR+DUF4404</td><td>1474</td><td>-</td><td>Gammaproteobacteria</td><td>Beggiatoa leptomitoformis</td><td>leucine-rich repeat domain-containing protein [Beggiatoa leptomitoformis].</td><td>GCF_001305575.3</td></tr>
<tr><td>WP_062292830.1</td><td>LRR-repeats+AP-GTPase+COR*→||<-?<-Aminotran_1_2<-HU-IHF</td><td>LRR-repeats+AP-GTPase+COR</td><td>946</td><td>-</td><td>Cyanobacteria</td><td>Nostoc piscinale</td><td>leucine-rich repeat domain-containing protein [Nostoc piscinale].</td><td>GCF_001298445.1</td></tr>
<tr><td>WP_062689807.1</td><td><-Cluster2368_2clades||?→ ncRNA→?→?→?→?→?→ LRR-repeats+AP-GTPase+COR*→</td><td>LRR-repeats+AP-GTPase+COR</td><td>909</td><td>-</td><td>Gammaproteobacteria</td><td>Photobacterium sanguinicancri</td><td>GTP-binding protein [Photobacterium sanguinicancri].</td><td>GCF_001563765.1</td></tr>
<tr><td>WP_063362501.1</td><td>LRR-repeats+AP-GTPase+COR+TIR*→</td><td>LRR-repeats+AP-GTPase+COR+TIR</td><td>1154</td><td>-</td><td>Gammaproteobacteria</td><td>Pseudoalteromonas luteoviolacea</td><td>leucine-rich repeat domain-containing protein [Pseudoalteromonas luteoviolacea].</td><td>GCF_001625695.1</td></tr>
<tr><td>WP_064232935.1</td><td>LRR-repeats+AP-GTPase+COR*→</td><td>LRR-repeats+AP-GTPase+COR</td><td>699</td><td>-</td><td>Gammaproteobacteria</td><td>Halomonas sp. ALS9</td><td>hypothetical protein [Halomonas sp. ALS9].</td><td>GCF_001651035.1</td></tr>
<tr><td>WP_065709937.1</td><td>LRR-repeats+AP-GTPase+COR*→||<-?<-?||?→ ABC-ATPase→ tRNA→?→||<-?<-Pentapeptide-repeats</td><td>LRR-repeats+AP-GTPase+COR</td><td>963</td><td>-</td><td>Cyanobacteria</td><td>Synechococcus sp. PCC 7117</td><td>leucine-rich repeat domain-containing protein [Synechococcus sp. PCC 7117].</td><td>-</td></tr>
<tr><td>WP_066434724.1</td><td>LRR-repeats+AP-GTPase+COR*→</td><td>LRR-repeats+AP-GTPase+COR</td><td>276</td><td>-</td><td>Bacteroidetes</td><td>Gelidibacter algens</td><td>hypothetical protein [Gelidibacter algens].</td><td>GCF_001678675.1</td></tr>
<tr><td>WP_067315083.1</td><td>SMPB→?→||<-?||?→||<-?||?→?→ LRR-repeats+AP-GTPase+COR*→||<-SIG+TM+TM+TM||?→||<-?||?→ SIGMA-HTH→</td><td>LRR-repeats+AP-GTPase+COR</td><td>968</td><td>-</td><td>Actinobacteria</td><td>Streptomyces griseochromogenes</td><td>hypothetical protein [Streptomyces griseochromogenes].</td><td>GCF_001542625.1</td></tr>
<tr><td>WP_067770734.1</td><td>LRR-repeats+AP-GTPase+COR*→</td><td>LRR-repeats+AP-GTPase+COR</td><td>1123</td><td>-</td><td>Cyanobacteria</td><td>Nostoc sp. NIES-3756</td><td>hypothetical protein [Nostoc sp. NIES-3756].</td><td>GCF_001548375.1</td></tr>
<tr><td>WP_068061695.1</td><td>7TMR-DISMED1→?→ SIG+Concanavalin-like+SUN→?→?→||<-?||?→ LRR-repeats+AP-GTPase+COR+TIR*→ ABhydrolase+ABhydrolase+CASPASE+TPR-S→?→||<-tRNA<-?<-?<-Cluster2189_2clades</td><td>LRR-repeats+AP-GTPase+COR+TIR</td><td>1268</td><td>-</td><td>Gammaproteobacteria</td><td>Rheinheimera sp. SA_1</td><td>TIR domain-containing protein [Rheinheimera sp. SA_1].</td><td>-</td></tr>
<tr><td>WP_068843099.1</td><td>LRR-repeats+AP-GTPase+COR*→||<-?||?→?→||<-cNMPBD</td><td>LRR-repeats+AP-GTPase+COR</td><td>1015</td><td>-</td><td>Bacteroidetes</td><td>Flavobacterium chilense</td><td>GTP-binding protein [Flavobacterium chilense].</td><td>GCF_900142685.1</td></tr>
<tr><td>WP_068887322.1</td><td>LRR-repeats+AP-GTPase+COR+TIR*→ Cluster2230_2clades→?→?→||<-?<-?<-?<-Cluster2322_2clades</td><td>LRR-repeats+AP-GTPase+COR+TIR</td><td>1168</td><td>-</td><td>Bacteroidetes</td><td>Pedobacter panaciterrae</td><td>leucine-rich repeat domain-containing protein [Pedobacter panaciterrae].</td><td>GCF_001636695.1</td></tr>
<tr><td>WP_069069853.1</td><td>LRR-repeats+AP-GTPase+COR+TIR*→</td><td>LRR-repeats+AP-GTPase+COR+TIR</td><td>1138</td><td>-</td><td>Cyanobacteria</td><td>Nostoc sp. KVJ20</td><td>leucine-rich repeat protein [Nostoc sp. KVJ20].</td><td>GCF_001712795.1</td></tr>
<tr><td>WP_069070238.1</td><td>LRR-repeats+AP-GTPase+COR*→||<-SIG+DADALIGASE-ATPgrasp<-?||?→?→||<-?<-?<-CBS</td><td>LRR-repeats+AP-GTPase+COR</td><td>1078</td><td>-</td><td>Cyanobacteria</td><td>Nostoc sp. KVJ20</td><td>leucine-rich repeat domain-containing protein [Nostoc sp. KVJ20].</td><td>GCF_001712795.1</td></tr>
</table>

<table>
<tr><th>acc</th><th>operon</th><th>architecture</th><th>len</th><th>gen.name</th><th>taxend</th><th>species</th><th>defline</th><th>gca</th></tr>
<tr><td>WP_069474666.1</td><td>SIG+TM+TM+TM+TM+TM+TM+TM→||<-?||Mbetalac→ SAM-methylase→?→||<-?<-SIG+TPM_phosphatase+TM+TM+TM||LRR-repeats+AP-GTPase+COR+TM+TM*→||<-?<-?<-?<-Pro_CA||?→||<-?<-tRNA<-tRNA<-Ferredoxin-betagrasp</td><td>LRR-repeats+AP-GTPase+COR+TM+TM</td><td>897</td><td>-</td><td>Cyanobacteria</td><td>Microcystis aeruginosa</td><td>leucine-rich repeat domain-containing protein [Microcystis aeruginosa].</td><td>GCF_001725075.1</td></tr>
<tr><td>WP_070392558.1</td><td>SIG+PBPI→?→?→ SIG+TM+TM+TM+TM+TM+TM+TM→||<-?||ABC-ATPase→ ABC-ATPase→||<-PSE||LRR-repeats+AP-GTPase+COR*→</td><td>LRR-repeats+AP-GTPase+COR</td><td>909</td><td>-</td><td>Cyanobacteria</td><td>Moorea producens</td><td>leucine-rich repeat domain-containing protein [Moorea producens].</td><td>GCF_001767235.1</td></tr>
<tr><td>WP_070393924.1</td><td>REC→||<-TPR-repeats<-?||LRR-repeats+AP-GTPase+COR+DUF4404*→</td><td>LRR-repeats+AP-GTPase+COR+DUF4404</td><td>938</td><td>-</td><td>Cyanobacteria</td><td>Moorea producens</td><td>leucine-rich repeat domain-containing protein [Moorea producens].</td><td>GCF_001767235.1</td></tr>
<tr><td>WP_071837136.1</td><td><-PIN<-?<-PIN<-?<-PIN<-?||LRR-repeats+AP-GTPase+COR+DUF4404*→?→ PSE→||<-Cluster5_5clades<-?<-Cluster2453_2clades</td><td>LRR-repeats+AP-GTPase+COR+DUF4404</td><td>1008</td><td>-</td><td>Cyanobacteria</td><td>Leptolyngbya sp. NIES-3755</td><td>leucine-rich repeat domain-containing protein [Leptolyngbya sp. NIES-3755].</td><td>GCF_001548435.1</td></tr>
<tr><td>WP_071987478.1</td><td>LRR-repeats+AP-GTPase+COR*→||<-?<-?||RelE→ CHTH→</td><td>LRR-repeats+AP-GTPase+COR</td><td>725</td><td>-</td><td>Cyanobacteria</td><td>Crocosphaera watsonii</td><td>leucine-rich repeat domain-containing protein [Crocosphaera watsonii].</td><td>GCF_000235665.1</td></tr>
<tr><td>WP_072015976.1</td><td><-SIG+TM||?→||<-RelE-ParE<-?||LRR-repeats+AP-GTPase+COR*→||<-?||?→?→ COR→</td><td>LRR-repeats+AP-GTPase+COR</td><td>651</td><td>-</td><td>Cyanobacteria</td><td>Leptolyngbya sp. KIOST-1</td><td>hypothetical protein [Leptolyngbya sp. KIOST-1].</td><td>GCF_000763385.1</td></tr>
<tr><td>WP_072022104.1</td><td><-ARSR-HTH<-PSE||?→||<-?||?→||<-SIG+Trypsin+PDZ<-?||?→ LRR-repeats+AP-GTPase+COR*→?→||<-RPN10<-?<-?<-Cluster2290_2clades<-Cluster2428_2clades</td><td>LRR-repeats+AP-GTPase+COR</td><td>838</td><td>-</td><td>Cyanobacteria</td><td>[Scytonema hofmanni] UTEX B 1581</td><td>leucine-rich repeat domain-containing protein [[Scytonema hofmanni] UTEX B 1581].</td><td>GCF_000582685.1</td></tr>
<tr><td>WP_072061087.1</td><td>LRR-repeats+AP-GTPase+COR*→||<-?<-?||RelE→ CHTH→</td><td>LRR-repeats+AP-GTPase+COR</td><td>725</td><td>-</td><td>Cyanobacteria</td><td>Crocosphaera watsonii</td><td>leucine-rich repeat domain-containing protein [Crocosphaera watsonii].</td><td>GCF_001039615.1</td></tr>
<tr><td>WP_072926417.1</td><td>Mbetalac→ SAM-methylase→?→||<-?<-SIG+TPM_phosphatase+TM+TM+TM||LRR-repeats+AP-GTPase+COR+TM+TM*→</td><td>LRR-repeats+AP-GTPase+COR+TM+TM</td><td>828</td><td>-</td><td>Cyanobacteria</td><td>Microcystis aeruginosa</td><td>leucine-rich repeat domain-containing protein [Microcystis aeruginosa].</td><td>GCF_001895325.1</td></tr>
<tr><td>WP_073070049.1</td><td>LRR-repeats+AP-GTPase+COR+TM+TM*→</td><td>LRR-repeats+AP-GTPase+COR+TM+TM</td><td>759</td><td>-</td><td>Cyanobacteria</td><td>Phormidesmis priestleyi</td><td>leucine-rich repeat domain-containing protein [Phormidesmis priestleyi].</td><td>GCF_001895925.1</td></tr>
<tr><td>WP_073584587.1</td><td><-Aminotran_1_2<-TetR-HTH||Cluster2045_2clades→?→ LRR-repeats+AP-GTPase+COR+TIR*→</td><td>LRR-repeats+AP-GTPase+COR+TIR</td><td>844</td><td>-</td><td>Gammaproteobacteria</td><td>Vibrio quintilis</td><td>TIR domain-containing protein [Vibrio quintilis].</td><td>GCF_900143745.1</td></tr>
<tr><td>WP_073604448.1</td><td>LRR-repeats+AP-GTPase+COR+TIR*→</td><td>LRR-repeats+AP-GTPase+COR+TIR</td><td>1052</td><td>-</td><td>Gammaproteobacteria</td><td>Vibrio aerogenes</td><td>TIR domain-containing protein, partial [Vibrio aerogenes].</td><td>GCF_900130105.1</td></tr>
<tr><td>WP_073609490.1</td><td>LRR-repeats+AP-GTPase+COR*→</td><td>LRR-repeats+AP-GTPase+COR</td><td>1026</td><td>-</td><td>Cyanobacteria</td><td>Phormidium tenue</td><td>leucine-rich repeat domain-containing protein [Phormidium tenue].</td><td>GCF_014696675.1</td></tr>
<tr><td>WP_073639373.1</td><td>LRR-repeats+AP-GTPase+COR+TIR*→</td><td>LRR-repeats+AP-GTPase+COR+TIR</td><td>1122</td><td>-</td><td>Cyanobacteria</td><td>Nostoc calcicola</td><td>leucine-rich repeat domain-containing protein [Nostoc calcicola].</td><td>GCF_001904715.1</td></tr>
<tr><td>WP_073640743.1</td><td>LRR-repeats+AP-GTPase+COR+TIR*→?→||<-Arginase</td><td>LRR-repeats+AP-GTPase+COR+TIR</td><td>1070</td><td>-</td><td>Cyanobacteria</td><td>Nostoc calcicola</td><td>leucine-rich repeat domain-containing protein [Nostoc calcicola].</td><td>GCF_014697355.1</td></tr>
<tr><td>WP_073641321.1</td><td><-PIN<-?||?→?→ PIN→ LRR-repeats+AP-GTPase+COR+TIR*→||<-?||?→||<-?||?→?→ RPN10→</td><td>LRR-repeats+AP-GTPase+COR+TIR</td><td>1188</td><td>-</td><td>Cyanobacteria</td><td>Nostoc calcicola</td><td>leucine-rich repeat protein [Nostoc calcicola].</td><td>GCF_001904715.1</td></tr>
<tr><td>WP_074191634.1</td><td>CI→ LRR-repeats+AP-GTPase+COR*→</td><td>LRR-repeats+AP-GTPase+COR</td><td>1105</td><td>-</td><td>Gammaproteobacteria</td><td>Vibrio antiquarius</td><td>50S ribosome-binding GTPase [Vibrio antiquarius].</td><td>GCF_001909305.1</td></tr>
<tr><td>WP_074382488.1</td><td><-Cluster1076_2clades<-?||?→||<-?<-?||?→||<-?||LRR-repeats+AP-GTPase+COR+TM+TM*→</td><td>LRR-repeats+AP-GTPase+COR+TM+TM</td><td>1015</td><td>-</td><td>Cyanobacteria</td><td>Phormidesmis priestleyi</td><td>leucine-rich repeat domain-containing protein [Phormidesmis priestleyi].</td><td>GCF_001650195.1</td></tr>
<tr><td>WP_075071341.1</td><td><-ABC-ATPase<-?||?→?→?→?→?→ LRR-repeats+AP-GTPase+COR*→ SIG+TM+TM+TM+TM→?→?→||<-?||?→ SIG+TM+TM+TM+TM+TM+TM+TM+TM+TM→</td><td>LRR-repeats+AP-GTPase+COR</td><td>692</td><td>-</td><td>Chloroflexi</td><td>Levilinea saccharolytica</td><td>GTP-binding protein [Levilinea saccharolytica].</td><td>GCF_001050255.2</td></tr>
</table>

<table>
<tr><th>acc</th><th>operon</th><th>architecture</th><th>len</th><th>gen.name</th><th>taxend</th><th>species</th><th>defline</th><th>gca</th></tr>
<tr><td>WP_075170580.1</td><td>RNA-Helicase→||<-PSE<-?<-?<-?<-?||?→||<-TM+TM||LRR-repeats+AP-GTPase+COR*→||<-?<-Cluster1726_2clades</td><td>LRR-repeats+AP-GTPase+COR</td><td>960</td><td>-</td><td>Gammaproteobacteria</td><td>Pseudoalteromonas haloplanktis</td><td>GTP-binding protein [Pseudoalteromonas haloplanktis].</td><td>GCF_001924935.1</td></tr>
<tr><td>WP_075904287.1</td><td>ABC-ATPase→ ABC-ATPase→?→?→||<-?||LRR-repeats+AP-GTPase+COR*→</td><td>LRR-repeats+AP-GTPase+COR</td><td>801</td><td>-</td><td>Cyanobacteria</td><td>Moorea bouillonii</td><td>leucine-rich repeat domain-containing protein [Moorea bouillonii].</td><td>GCF_001942495.1</td></tr>
<tr><td>WP_076382638.1</td><td><-Polbeta+HEPN<-?||?→ LRR-repeats+AP-GTPase+COR+TIR*→ TIR→ TM+TM+TM→?→?→||<-CNMP+CRP-HTH</td><td>LRR-repeats+AP-GTPase+COR+TIR</td><td>1349</td><td>-</td><td>Bacteroidetes</td><td>Filimonas lacunae</td><td>TIR domain-containing protein [Filimonas lacunae].</td><td>GCF_002355595.1</td></tr>
<tr><td>WP_076393053.1</td><td>LRR-repeats+AP-GTPase+COR*→</td><td>LRR-repeats+AP-GTPase+COR</td><td>925</td><td>-</td><td>Bacteroidetes</td><td>Chryseobacterium</td><td>MULTISPECIES: leucine-rich repeat domain-containing protein [Chryseobacterium].</td><td>GCF_900156825.1</td></tr>
<tr><td>WP_076396200.1</td><td>LRR-repeats+AP-GTPase+COR*→||<-?||?→ SIG+NUDIX→</td><td>LRR-repeats+AP-GTPase+COR</td><td>890</td><td>-</td><td>Bacteroidetes</td><td>Chryseobacterium gambrini</td><td>hypothetical protein [Chryseobacterium gambrini].</td><td>GCF_900156825.1</td></tr>
<tr><td>WP_076994187.1</td><td><-ABC-ATPase<-?||?→?→||<-?||PSE→||<-?||LRR-repeats+AP-GTPase+COR*→||<-?||?→||<-?<-?<-Aminotran_1_2</td><td>LRR-repeats+AP-GTPase+COR</td><td>942</td><td>-</td><td>Actinobacteria</td><td>Actinosynnema sp. ALI-1.44</td><td>hypothetical protein [Actinosynnema sp. ALI-1.44].</td><td>GCF_001984155.1</td></tr>
<tr><td>WP_077025618.1</td><td>LRR-repeats+AP-GTPase+COR*→?→||<-?<-?||?→||<-?<-?<-ABC-ATPase</td><td>LRR-repeats+AP-GTPase+COR</td><td>1153</td><td>-</td><td>Planctomycetes</td><td>Fuerstia marisgermanicae</td><td>leucine-rich repeat domain-containing protein [Fuerstia marisgermanicae].</td><td>GCF_001983935.1</td></tr>
<tr><td>WP_078402495.1</td><td>LRR-repeats+AP-GTPase+COR*→</td><td>LRR-repeats+AP-GTPase+COR</td><td>901</td><td>-</td><td>Bacteroidetes</td><td>Elizabethkingia ursingii</td><td>leucine-rich repeat domain-containing protein [Elizabethkingia ursingii].</td><td>GCF_002022125.1</td></tr>
<tr><td>WP_078608910.1</td><td><-RNA-Helicase||?→?→||<-?||CI→ LRR-repeats+AP-GTPase+COR*→</td><td>LRR-repeats+AP-GTPase+COR</td><td>1012</td><td>-</td><td>Gammaproteobacteria</td><td>Vibrio owensii</td><td>GTP-binding protein [Vibrio owensii].</td><td>GCF_002021755.1</td></tr>
<tr><td>WP_078814763.1</td><td><-REC||LRR-repeats+AP-GTPase+COR+TIR*→?→?→||<-?||?→||<-?<-NUDIX</td><td>LRR-repeats+AP-GTPase+COR+TIR</td><td>959</td><td>-</td><td>Verrucomicrobia</td><td>Prosthecobacter debontii</td><td>leucine-rich repeat domain-containing protein [Prosthecobacter debontii].</td><td>GCF_900167535.1</td></tr>
<tr><td>WP_079184311.1</td><td>LRR-repeats+AP-GTPase+COR*→?→||<-?<-SIGMA-HTH</td><td>LRR-repeats+AP-GTPase+COR</td><td>1056</td><td>-</td><td>Actinobacteria</td><td>Streptomyces uncialis</td><td>hypothetical protein [Streptomyces uncialis].</td><td>GCF_001905345.1</td></tr>
<tr><td>WP_079712025.1</td><td>LRR-repeats+AP-GTPase+COR*→</td><td>LRR-repeats+AP-GTPase+COR</td><td>882</td><td>-</td><td>Bacteroidetes</td><td>Salegentibacter salinarum</td><td>hypothetical protein [Salegentibacter salinarum].</td><td>GCF_900168115.1</td></tr>
<tr><td>WP_080603599.1</td><td>LRR-repeats+AP-GTPase+COR+TM+TM*→</td><td>LRR-repeats+AP-GTPase+COR+TM+TM</td><td>799</td><td>-</td><td>Cyanobacteria</td><td>Microcystis aeruginosa</td><td>leucine-rich repeat protein [Microcystis aeruginosa].</td><td>-</td></tr>
<tr><td>WP_080963556.1</td><td>Cluster1140_2clades→?→ LRR-repeats+AP-GTPase+COR+TM+TM*→</td><td>LRR-repeats+AP-GTPase+COR+TM+TM</td><td>830</td><td>-</td><td>Gammaproteobacteria</td><td>Pseudomonas putida</td><td>GTP-binding protein [Pseudomonas putida].</td><td>GCF_001027965.1</td></tr>
<tr><td>WP_081155426.1</td><td>LRR-repeats+AP-GTPase+COR*→||<-?<-?<-?<-?||?→||<-?<-REC</td><td>LRR-repeats+AP-GTPase+COR</td><td>1088</td><td>-</td><td>Bacteroidetes</td><td>Niastella vici</td><td>hypothetical protein [Niastella vici].</td><td>GCF_002077945.1</td></tr>
<tr><td>WP_081336246.1</td><td>LRR-repeats+AP-GTPase+COR*→</td><td>LRR-repeats+AP-GTPase+COR</td><td>1380</td><td>-</td><td>Actinobacteria</td><td>Mycobacteroides chelonae</td><td>hypothetical protein [Mycobacteroides chelonae].</td><td>GCF_001853965.1</td></tr>
<tr><td>WP_081583858.1</td><td>LRR-repeats→ LRR-repeats+AP-GTPase+COR+CASPASE*→</td><td>LRR-repeats+AP-GTPase+COR+CASPASE</td><td>1375</td><td>-</td><td>Cyanobacteria</td><td>unclassified Tolypothrix</td><td>MULTISPECIES: leucine-rich repeat domain-containing protein [unclassified Tolypothrix].</td><td>GCF_015207145.1</td></tr>
<tr><td>WP_081656227.1</td><td>LRR-repeats+AP-GTPase+COR+TIR*→</td><td>LRR-repeats+AP-GTPase+COR+TIR</td><td>1354</td><td>-</td><td>Cyanobacteria</td><td>Fischerella sp. PCC 9431</td><td>leucine-rich repeat domain-containing protein [Fischerella sp. PCC 9431].</td><td>-</td></tr>
<tr><td>WP_081983983.1</td><td>Ferredoxin-RRM→?→ Cluster1494_2clades→||<-?<-?||?→?→ LRR-repeats+AP-GTPase+COR+DUF4404*→</td><td>LRR-repeats+AP-GTPase+COR+DUF4404</td><td>971</td><td>-</td><td>Betaproteobacteria</td><td>Massilia sp. JS1662</td><td>GTP-binding protein [Massilia sp. JS1662].</td><td>GCF_000759615.1</td></tr>
<tr><td>WP_082167682.1</td><td>LRR-repeats+AP-GTPase+COR+TIR*→||<-?||Cluster850_2clades→</td><td>LRR-repeats+AP-GTPase+COR+TIR</td><td>797</td><td>-</td><td>Alphaproteobacteria</td><td>Methylobacterium aquaticum</td><td>TIR domain-containing protein, partial [Methylobacterium aquaticum].</td><td>GCF_001043915.1</td></tr>
<tr><td>WP_082209748.1</td><td>LRR-repeats+AP-GTPase+COR+TCAD4*→||<-Cluster5_5clades<-?||?→?→ Pro_CA→</td><td>LRR-repeats+AP-GTPase+COR+TCAD4</td><td>714</td><td>-</td><td>Cyanobacteria</td><td>Fischerella sp. PCC 9605</td><td>leucine-rich repeat domain-containing protein [Fischerella sp. PCC 9605].</td><td>GCF_000517105.1</td></tr>
</table>

<table>
<tr><th>acc</th><th>operon</th><th>architecture</th><th>len</th><th>gen.name</th><th>taxend</th><th>species</th><th>defline</th><th>gca</th></tr>
<tr><td>WP_082226537.1</td><td>LRR-repeats→?→ LRR-repeats+AP-GTPase+COR*→||<-?<-?||?→||<-?<-?<-?||Aminotran_1_2→</td><td>LRR-repeats+AP-GTPase+COR</td><td>1171</td><td>-</td><td>Bacteroidetes</td><td>Microscilla marina</td><td>leucine-rich repeat domain-containing protein [Microscilla marina].</td><td>GCF_000169175.1</td></tr>
<tr><td>WP_083250213.1</td><td>LRR-repeats+AP-GTPase+COR*→</td><td>LRR-repeats+AP-GTPase+COR</td><td>817</td><td>-</td><td>Euryarchaeota</td><td>Methanosarcina sp. A14</td><td>leucine-rich repeat domain-containing protein, partial [Methanosarcina sp. A14].</td><td>GCF_001729375.1</td></tr>
<tr><td>WP_083398305.1</td><td>LRR-repeats→ LRR-repeats+AP-GTPase+COR+DrHyd*→?→?→?→?→||<-?<-SIG+TM+TM+TM+TM</td><td>LRR-repeats+AP-GTPase+COR+DrHyd</td><td>751</td><td>-</td><td>Betaproteobacteria</td><td>Nitrosomonas communis</td><td>hypothetical protein [Nitrosomonas communis].</td><td>GCF_900114745.1</td></tr>
<tr><td>WP_083620324.1</td><td><-SIG+TM+TM+TM+TM+TM+TM<-?||LRR-repeats+AP-GTPase+COR*→?→||<-HNH||Cluster1548_2clades→?→||<-?||CCTBP→</td><td>LRR-repeats+AP-GTPase+COR</td><td>975</td><td>-</td><td>Cyanobacteria</td><td>Planktothrix paucivesiculata</td><td>leucine-rich repeat domain-containing protein [Planktothrix paucivesiculata].</td><td>GCF_900009265.2</td></tr>
<tr><td>WP_083622583.1</td><td>Cluster1745_2clades→?→?→||<-?||?→ LRR-repeats+AP-GTPase+COR*→</td><td>LRR-repeats+AP-GTPase+COR</td><td>1058</td><td>-</td><td>Cyanobacteria</td><td>Planktothrix paucivesiculata</td><td>leucine-rich repeat domain-containing protein [Planktothrix paucivesiculata].</td><td>GCF_900009265.2</td></tr>
<tr><td>WP_083850172.1</td><td>LRR-repeats+AP-GTPase+COR*→</td><td>LRR-repeats+AP-GTPase+COR</td><td>1286</td><td>-</td><td>Deltaproteobacteria</td><td>Desulfovibrio sp. U5L</td><td>GTP-binding protein [Desulfovibrio sp. U5L].</td><td>-</td></tr>
<tr><td>WP_083886975.1</td><td><-SIG+TM||?→||<-?<-?||LRR-repeats→?→ LRR-repeats+AP-GTPase+COR*→</td><td>LRR-repeats+AP-GTPase+COR</td><td>863</td><td>-</td><td>Cyanobacteria</td><td>Nodosilinea nodulosa</td><td>hypothetical protein [Nodosilinea nodulosa].</td><td>GCF_000309385.1</td></tr>
<tr><td>WP_083887059.1</td><td>RelE→ CHTH→ LRR-repeats→ PSE→||<-?||PSE→ LRR-repeats+AP-GTPase+COR*→||<-?<-?||COR+TIR→||<-REase-4</td><td>LRR-repeats+AP-GTPase+COR</td><td>410</td><td>-</td><td>Cyanobacteria</td><td>Nodosilinea nodulosa</td><td>leucine-rich repeat domain-containing protein [Nodosilinea nodulosa].</td><td>GCF_000309385.1</td></tr>
<tr><td>WP_083887212.1</td><td>Cluster2123_2clades→?→||<-?<-?<-?||PSE→ LRR-repeats→ AP-GTPase*→</td><td>AP-GTPase</td><td>221</td><td>-</td><td>Cyanobacteria</td><td>Nodosilinea nodulosa</td><td>50S ribosome-binding GTPase [Nodosilinea nodulosa].</td><td>GCF_000309385.1</td></tr>
<tr><td>WP_083918254.1</td><td>LRR-repeats+AP-GTPase+COR+TIR*→||<-?<-?||?→||<-?<-?<-SHS2</td><td>LRR-repeats+AP-GTPase+COR+TIR</td><td>954</td><td>-</td><td>Gammaproteobacteria</td><td>Methylosarcina fibrata</td><td>TIR domain-containing protein [Methylosarcina fibrata].</td><td>GCF_000372865.1</td></tr>
<tr><td>WP_084022355.1</td><td><-NUDIX||?→||<-HNH<-?||?→?→?→ LRR-repeats+AP-GTPase+COR+TIR*→</td><td>LRR-repeats+AP-GTPase+COR+TIR</td><td>850</td><td>-</td><td>Chlorobi</td><td>Chlorobaculum limnaeum</td><td>TIR domain-containing protein [Chlorobaculum limnaeum].</td><td>GCF_001747405.1</td></tr>
<tr><td>WP_084172951.1</td><td>LRR-repeats+AP-GTPase+COR*→</td><td>LRR-repeats+AP-GTPase+COR</td><td>639</td><td>-</td><td>Cyanobacteria</td><td>Phormidium tenue</td><td>GTP-binding protein [Phormidium tenue].</td><td>GCF_014696675.1</td></tr>
<tr><td>WP_084177732.1</td><td>LRR-repeats+AP-GTPase+COR+TM+TM*→||<-CASPASE+TM</td><td>LRR-repeats+AP-GTPase+COR+TM+TM</td><td>985</td><td>-</td><td>Cyanobacteria</td><td>Nostoc calcicola</td><td>leucine-rich repeat domain-containing protein [Nostoc calcicola].</td><td>GCF_001904715.1</td></tr>
<tr><td>WP_084227301.1</td><td>LRR-repeats+AP-GTPase+COR+TIR*→</td><td>LRR-repeats+AP-GTPase+COR+TIR</td><td>804</td><td>-</td><td>Cyanobacteria</td><td>Nostoc sp. KVJ20</td><td>leucine-rich repeat domain-containing protein [Nostoc sp. KVJ20].</td><td>GCF_001712795.1</td></tr>
<tr><td>WP_084428809.1</td><td>LRR-repeats+AP-GTPase+COR*→</td><td>LRR-repeats+AP-GTPase+COR</td><td>944</td><td>-</td><td>Actinobacteria</td><td>Kibdelosporangium aridum</td><td>hypothetical protein [Kibdelosporangium aridum].</td><td>GCF_900176515.1</td></tr>
<tr><td>WP_084630566.1</td><td><-HhH-RADC+JAB||?→ NTP_transf_3→ LRR-repeats+AP-GTPase+COR*→||<-?<-?||?→||<-?||?→||<-Cluster1409_2clades</td><td>LRR-repeats+AP-GTPase+COR</td><td>906</td><td>-</td><td>Euryarchaeota</td><td>Methanosarcina lacustris</td><td>leucine-rich repeat domain-containing protein [Methanosarcina lacustris].</td><td>GCF_000970265.1</td></tr>
<tr><td>WP_084713364.1</td><td>LRR-repeats+AP-GTPase+COR*→</td><td>LRR-repeats+AP-GTPase+COR</td><td>1355</td><td>-</td><td>Actinobacteria</td><td>Streptacidiphilus jeojiense</td><td>leucine-rich repeat domain-containing protein [Streptacidiphilus jeojiense].</td><td>-</td></tr>
<tr><td>WP_084763145.1</td><td>Pentapeptide-repeats→||<-TPR+nSTAND1+BetaPropeller_WD40+BetaPropeller_WD40<-?||?→||<-?||?→||<-?||LRR-repeats+AP-GTPase+COR+CASPASE*→||<-RelE-ParE</td><td>LRR-repeats+AP-GTPase+COR+CASPASE</td><td>1441</td><td>-</td><td>Cyanobacteria</td><td>[Scytonema hofmanni] UTEX B 1581</td><td>leucine-rich repeat domain-containing protein [[Scytonema hofmanni] UTEX B 1581].</td><td>GCF_000582685.1</td></tr>
<tr><td>WP_084765096.1</td><td>LRR-repeats+AP-GTPase+COR*→</td><td>LRR-repeats+AP-GTPase+COR</td><td>940</td><td>-</td><td>Firmicutes</td><td>Clostridium intestinale</td><td>GTP-binding protein [Clostridium intestinale].</td><td>-</td></tr>
<tr><td>WP_084967529.1</td><td>Cluster1877_2clades→||<-?||Cluster1140_2clades→?→ LRR-repeats+AP-GTPase+COR+TM+TM*→</td><td>LRR-repeats+AP-GTPase+COR+TM+TM</td><td>830</td><td>-</td><td>Gammaproteobacteria</td><td>Pseudomonas putida</td><td>GTP-binding protein [Pseudomonas putida].</td><td>GCF_002094785.1</td></tr>
</table>

<table>
<tr><th>acc</th><th>operon</th><th>architecture</th><th>len</th><th>gen.name</th><th>taxend</th><th>species</th><th>defline</th><th>gca</th></tr>
<tr><td>WP_086488496.1</td><td>GTPase-AIG→||<-?||?→?→?→?→ PIN→ LRR-repeats+AP-GTPase+COR+TIR*→?→||<-?<-SIG+TM+TM+TM+TM+TM+TM+TM+TM+TM+TM+TM<-?||?→||<-?<-Cluster1501_2clades</td><td>LRR-repeats+AP-GTPase+COR+TIR</td><td>936</td><td>-</td><td>Gammaproteobacteria</td><td>Thioflexothrix psekupsii</td><td>leucine-rich repeat domain-containing protein [Thioflexothrix psekupsii].</td><td>GCF_002149925.1</td></tr>
<tr><td>WP_086489369.1</td><td>LRR-repeats+AP-GTPase+COR+HEPN*→ ABC-ATPase→?→?→?→?→ Cluster1668_2clades→</td><td>LRR-repeats+AP-GTPase+COR+HEPN</td><td>919</td><td>-</td><td>Gammaproteobacteria</td><td>Thioflexothrix psekupsii</td><td>leucine-rich repeat domain-containing protein [Thioflexothrix psekupsii].</td><td>GCF_002149925.1</td></tr>
<tr><td>WP_086489735.1</td><td><-Cluster1597_2clades<-Cluster1052_2clades<-?||?→?→?→ LRR-repeats+AP-GTPase+COR+HEPN*→ Cluster2027_2clades→?→||<-ABC-ATPase<-MoxR-AAA<-HISKIN</td><td>LRR-repeats+AP-GTPase+COR+HEPN</td><td>857</td><td>-</td><td>Gammaproteobacteria</td><td>Thioflexothrix psekupsii</td><td>leucine-rich repeat domain-containing protein [Thioflexothrix psekupsii].</td><td>GCF_002149925.1</td></tr>
<tr><td>WP_086671081.1</td><td>SIG+TM+TM+TM+TM→||<-?<-?<-?||?→||<-Cluster2027_2clades<-?||tRNA→ LRR-repeats+AP-GTPase+COR+TM+TM*→||<-?<-?||PSE→||<-?||?→?→||<-?<-SIG+Calcineurin</td><td>LRR-repeats+AP-GTPase+COR+TM+TM</td><td>937</td><td>-</td><td>Actinobacteria</td><td>Amycolatopsis pretoriensis</td><td>GTP-binding protein [Amycolatopsis pretoriensis].</td><td>GCF_900107925.1</td></tr>
<tr><td>WP_086685880.1</td><td>LRR-repeats+AP-GTPase+COR+DUF4404*→</td><td>LRR-repeats+AP-GTPase+COR+DUF4404</td><td>1074</td><td>-</td><td>Cyanobacteria</td><td>Nostoc sp. T09</td><td>leucine-rich repeat domain-containing protein [Nostoc sp. T09].</td><td>GCF_002154695.1</td></tr>
<tr><td>WP_086688381.1</td><td>LRR-repeats+AP-GTPase+COR+CASPASE*→||<-?<-?||?→ RelE-ParE→||<-?<-?||Cluster1437_2clades→</td><td>LRR-repeats+AP-GTPase+COR+CASPASE</td><td>897</td><td>-</td><td>Cyanobacteria</td><td>Nostoc sp. T09</td><td>CHAT domain-containing protein, partial [Nostoc sp. T09].</td><td>GCF_002154695.1</td></tr>
<tr><td>WP_086688547.1</td><td>LRR-repeats+AP-GTPase+COR+TM+TM*→</td><td>LRR-repeats+AP-GTPase+COR+TM+TM</td><td>883</td><td>-</td><td>Cyanobacteria</td><td>Nostoc sp. T09</td><td>leucine-rich repeat domain-containing protein [Nostoc sp. T09].</td><td>GCF_002154695.1</td></tr>
<tr><td>WP_086759966.1</td><td>LRR-repeats+AP-GTPase+COR+DUF4404*→ TPR-repeats+CASPASE→||<-?||?→ HTH→</td><td>LRR-repeats+AP-GTPase+COR+DUF4404</td><td>1097</td><td>-</td><td>Cyanobacteria</td><td>Nostoc sp. 106C</td><td>leucine-rich repeat domain-containing protein [Nostoc sp. 106C].</td><td>GCF_002154725.1</td></tr>
<tr><td>WP_086760374.1</td><td>LRR-repeats+AP-GTPase+COR*→</td><td>LRR-repeats+AP-GTPase+COR</td><td>1054</td><td>-</td><td>Cyanobacteria</td><td>Nostoc sp. 106C</td><td>leucine-rich repeat domain-containing protein [Nostoc sp. 106C].</td><td>GCF_002154725.1</td></tr>
<tr><td>WP_086768657.1</td><td>RPN10→ Chromo-N+CHROMO→||<-?||LRR-repeats+AP-GTPase+COR+CASPASE*→</td><td>LRR-repeats+AP-GTPase+COR+CASPASE</td><td>1254</td><td>-</td><td>Cyanobacteria</td><td>Nostoc sp. 106C</td><td>leucine-rich repeat domain-containing protein [Nostoc sp. 106C].</td><td>GCF_002154725.1</td></tr>
<tr><td>WP_086834293.1</td><td>LRR-repeats+AP-GTPase+COR+TM+TM*→</td><td>LRR-repeats+AP-GTPase+COR+TM+TM</td><td>884</td><td>-</td><td>Cyanobacteria</td><td>Nostoc sp. RF31YmG</td><td>leucine-rich repeat domain-containing protein [Nostoc sp. RF31YmG].</td><td>GCF_002155185.1</td></tr>
<tr><td>WP_087590341.1</td><td>LRR-repeats+AP-GTPase+COR*→</td><td>LRR-repeats+AP-GTPase+COR</td><td>452</td><td>-</td><td>Cyanobacteria</td><td>unicellular cyanobacterium SU3</td><td>leucine-rich repeat domain-containing protein [unicellular cyanobacterium SU3].</td><td>-</td></tr>
<tr><td>WP_087656332.1</td><td>Cluster1742_2clades→ Cluster1685_2clades→?→?→?→?→?→ LRR-repeats+AP-GTPase+COR*→?→ TIR→</td><td>LRR-repeats+AP-GTPase+COR</td><td>560</td><td>-</td><td>Betaproteobacteria</td><td>Caballeronia terrestris</td><td>hypothetical protein [Caballeronia terrestris].</td><td>GCF_001544515.1</td></tr>
<tr><td>WP_088134442.1</td><td>OmpA→?→ Cluster1633_2clades→||<-?||?→ tRNA→?→ Polbeta+HEPN→ LRR-repeats+AP-GTPase+COR+TIR*→?→||<-?||?→?→ SIG+TM+TM+TM+TM+TM+TM+TM+TM+TM+TM→ SIG+TM→</td><td>LRR-repeats+AP-GTPase+COR+TIR</td><td>1220</td><td>-</td><td>Gammaproteobacteria</td><td>Vibrio gazogenes</td><td>TIR domain-containing protein [Vibrio gazogenes].</td><td>GCF_002196515.1</td></tr>
<tr><td>WP_088620375.1</td><td><-Cluster2094_2clades||LRR-repeats+AP-GTPase+COR*→||<-?<-?<-?<-HISKIN||?→||<-REC</td><td>LRR-repeats+AP-GTPase+COR</td><td>806</td><td>-</td><td>Gammaproteobacteria</td><td>Methylovulum psychrotolerans</td><td>GTP-binding protein [Methylovulum psychrotolerans].</td><td>GCF_002209385.1</td></tr>
<tr><td>WP_088620603.1</td><td><-ABC-ATPase<-SIG+BLBD<-?<-?||REC→ HISKIN→ REase→ LRR-repeats+AP-GTPase+COR+TIR*→?→?→ Ferredoxin-RRM→</td><td>LRR-repeats+AP-GTPase+COR+TIR</td><td>1013</td><td>-</td><td>Gammaproteobacteria</td><td>Methylovulum psychrotolerans</td><td>TIR domain-containing protein [Methylovulum psychrotolerans].</td><td>GCF_002209385.1</td></tr>
<tr><td>WP_088621060.1</td><td>LRR-repeats+AP-GTPase+COR+DpnII-MboI-NTD+TIR*→</td><td>LRR-repeats+AP-GTPase+COR+DpnII-MboI-NTD+TIR</td><td>866</td><td>-</td><td>Gammaproteobacteria</td><td>Methylovulum psychrotolerans</td><td>leucine-rich repeat domain-containing protein [Methylovulum psychrotolerans].</td><td>GCF_002209385.1</td></tr>
<tr><td>WP_088646515.1</td><td>LRR-repeats+AP-GTPase+COR*→||<-?<-?<-?<-?<-Cluster1404_2clades</td><td>LRR-repeats+AP-GTPase+COR</td><td>987</td><td>-</td><td>Actinobacteria</td><td>Micromonospora wenchangensis</td><td>hypothetical protein [Micromonospora wenchangensis].</td><td>GCF_002210435.1</td></tr>
<tr><td>WP_088745883.1</td><td>LRR-repeats+AP-GTPase+COR*→</td><td>LRR-repeats+AP-GTPase+COR</td><td>863</td><td>-</td><td>Bacteroidetes</td><td>unclassified Sphingobacteriales</td><td>MULTISPECIES: GTP-binding protein [unclassified Sphingobacteriales].</td><td>-</td></tr>
</table>

acc	operon	architecture	len	gen.name	taxend	species	defline	gca
WP_088762693.1	TPR-repeats→?→ SIG+Alba→||<-?<-?<-?<-?||LRR-repeats+AP-GTPase+COR*→	LRR-repeats+AP-GTPase+COR	851	-	Bacteroidetes	unclassified Sphingobacteriales	MULTISPECIES: GTP-binding protein [unclassified Sphingobacteriales].	-
WP_088764296.1	LRR-repeats+AP-GTPase+COR+TM+TM*→	LRR-repeats+AP-GTPase+COR+TM+TM	969	-	Bacteroidetes	unclassified Sphingobacteriales	MULTISPECIES: leucine-rich repeat domain-containing protein [unclassified Sphingobacteriales].	-
WP_089288360.1	<-SIG+TM+TM+TM+TM+TM+TM+TM+TM+TM+TM<-?||?→ Cluster1974__2clades→?→?→||<-?||LRR-repeats+AP-GTPase+COR+TIR*→||<-?<-ParB<-ParA-Soj-PloopNTPase<-?<-Thioredoxin	LRR-repeats+AP-GTPase+COR+TIR	1212	-	Alphaproteobacteria	unclassified Azospirillum	MULTISPECIES: TIR domain-containing protein [unclassified Azospirillum].	GCF_900188385.1
WP_089298330.1	SIG+Concanavalin-like+SUN→ LRR-repeats+AP-GTPase+COR*→||<-PSE||?→?→?→||<-?<-ABC-ATPase	LRR-repeats+AP-GTPase+COR	908	-	Actinobacteria	Actinoplanes regularis	GTP-binding protein [Actinoplanes regularis].	GCF_016862355.1
WP_089681784.1	LRR-repeats+AP-GTPase+COR*→?→||<-Thioredoxin	LRR-repeats+AP-GTPase+COR	787	-	Bacteroidetes	Catalinimonas alkaloidigena	hypothetical protein [Catalinimonas alkaloidigena].	GCF_900100765.1
WP_089727137.1	LRR-repeats+AP-GTPase+COR*→	LRR-repeats+AP-GTPase+COR	1009	-	Gammaproteobacteria	Candidatus Thiosymbion oneisti	leucine-rich repeat domain-containing protein [Candidatus Thiosymbion oneisti].	GCF_900092655.1
WP_089824678.1	Cluster1536__2clades→?→||<-tRNA||LRR-repeats+AP-GTPase+COR*→	LRR-repeats+AP-GTPase+COR	978	-	Firmicutes	Clostridiales bacterium CHKCI001	leucine-rich repeat domain-containing protein [Clostridiales bacterium CHKCI001].	-
WP_089856669.1	LRR-repeats+AP-GTPase+COR*→	LRR-repeats+AP-GTPase+COR	925	-	Bacteroidetes	Chryseobacterium taeanense	leucine-rich repeat domain-containing protein [Chryseobacterium taeanense].	GCF_900099685.1
WP_089938297.1	COR→ LRR-repeats+AP-GTPase+COR*→	LRR-repeats+AP-GTPase+COR	705	-	Nitrospinae/Tectomicrobia group	Candidatus Entotheonella palauensis	hypothetical protein [Candidatus Entotheonella palauensis].	GCF_900079095.1
WP_089941349.1	<-Aminotran_1_2<-Cluster1597__2clades||?→?→?→?→ LRR-repeats+AP-GTPase+COR*→	LRR-repeats+AP-GTPase+COR	514	-	Nitrospinae/Tectomicrobia group	Candidatus Entotheonella palauensis	leucine-rich repeat domain-containing protein [Candidatus Entotheonella palauensis].	GCF_900079095.1
WP_090106546.1	LRR-repeats+AP-GTPase+COR+TIR*→	LRR-repeats+AP-GTPase+COR+TIR	1148	-	Bacteroidetes	Chitinophaga sp. CF118	leucine-rich repeat domain-containing protein [Chitinophaga sp. CF118].	GCF_900112615.1
WP_090320517.1	LRR-repeats+AP-GTPase+COR*→?→?→ ParA-Soj-PloopNTPase→	LRR-repeats+AP-GTPase+COR	738	-	Betaproteobacteria	Nitrosomonas oligotropha	leucine-rich repeat domain-containing protein [Nitrosomonas oligotropha].	GCF_900106555.1
WP_090640679.1	<-RNA-Helicase||LRR-repeats+AP-GTPase+COR+DrHyd*→||<-CBS	LRR-repeats+AP-GTPase+COR+DrHyd	1059	-	Bacteroidetes	Mucilaginibacter sp. OK283	leucine-rich repeat domain-containing protein [Mucilaginibacter sp. OK283].	GCF_900110415.1
WP_090662001.1	LRR-repeats+AP-GTPase+COR+TIR*→?→||<-?<-?||?→ SIG+FKBP→	LRR-repeats+AP-GTPase+COR+TIR	1182	-	Bacteroidetes	Parafilimonas terrae	leucine-rich repeat domain-containing protein [Parafilimonas terrae].	GCF_900115755.1
WP_091029330.1	TM→ LRR-repeats+AP-GTPase+COR*→	LRR-repeats+AP-GTPase+COR	1045	-	Actinobacteria	Glycomyces harbinensis	hypothetical protein [Glycomyces harbinensis].	GCF_900101745.1
WP_091054155.1	<-Cluster2137__2clades<-?<-?||?→||<-?||?→?→ LRR-repeats+AP-GTPase+COR*→	LRR-repeats+AP-GTPase+COR	1094	-	Actinobacteria	Glycomyces sambucus	hypothetical protein [Glycomyces sambucus].	GCF_900102815.1
WP_091103947.1	<-ABC-ATPase<-?||?→?→||<-?||LRR-repeats+AP-GTPase+COR*→?→?→?→||<-?<-?<-SIR2	LRR-repeats+AP-GTPase+COR	941	-	Actinobacteria	Nonomuraea pusilla	leucine-rich repeat domain-containing protein [Nonomuraea pusilla].	GCF_900109355.1
WP_091133979.1	LRR-repeats+AP-GTPase+COR+TIR*→	LRR-repeats+AP-GTPase+COR+TIR	1190	-	Bacteroidetes	Flavobacterium anhuiense	TIR domain-containing protein [Flavobacterium anhuiense].	GCF_900101855.1

acc	operon	architecture	len	gen.name	taxend	species	defline	gca
WP_091435026.1	<-Cluster1070_2clades<-ENDO3+NUDIX||HU-IHF→?→ LRR-repeats+AP-GTPase+COR*→	LRR-repeats+AP-GTPase+COR	916	-	Bacteroidetes	Flavobacterium degerlachei	leucine-rich repeat domain-containing protein [Flavobacterium degerlachei].	GCF_900106645.1
WP_091506051.1	Cluster944_3clades→||<-?<-?||?→||<-Cluster2045_2clades<-?||LRR-repeats+AP-GTPase+COR+TIR*→	LRR-repeats+AP-GTPase+COR+TIR	997	-	Bacteroidetes	Flexibacter flexilis	leucine-rich repeat domain-containing protein [Flexibacter flexilis].	GCF_900112255.1
WP_091630534.1	<-ABC-ATPase||?→ LRR-repeats+AP-GTPase+COR*→||<-ABC_membrane+ABC_tran	LRR-repeats+AP-GTPase+COR	901	-	Actinobacteria	Micromonospora peucetia	leucine-rich repeat domain-containing protein [Micromonospora peucetia].	GCF_900091625.1
WP_092162518.1	REC→ REC→?→?→?→ LRR-repeats+AP-GTPase+COR*→?→?→ SIG+WWE→?→?→ ABC-ATPase→ ABC-ATPase→	LRR-repeats+AP-GTPase+COR	863	-	Deltaproteobacteria	Maridesulfovibrio ferrireducens	GTP-binding protein [Maridesulfovibrio ferrireducens].	GCF_900101105.1
WP_093066920.1	LRR-repeats+AP-GTPase+COR+TIR*→?→?→||<-Thioredoxin||YBAK→	LRR-repeats+AP-GTPase+COR+TIR	847	-	Gammaproteobacteria	Thiothrix caldifontis	TIR domain-containing protein [Thiothrix caldifontis].	GCF_900107695.1
WP_093070768.1	LRR-repeats+AP-GTPase+COR+TIR*→	LRR-repeats+AP-GTPase+COR+TIR	1327	-	Gammaproteobacteria	Thiothrix caldifontis	leucine-rich repeat domain-containing protein [Thiothrix caldifontis].	GCF_900107695.1
WP_094331224.1	Cluster1074_2clades→?→ LRR-repeats+AP-GTPase+COR+DUF4404*→||<-PSE<-?||?→?→ SAM-methylase→	LRR-repeats+AP-GTPase+COR+DUF4404	1016	-	Cyanobacteria	Nostoc sp. 'Peltigera membranacea cyanobiont' 213	leucine-rich repeat domain-containing protein [Nostoc sp. 'Peltigera membranacea cyanobiont' 213].	GCF_002245975.1
WP_094342929.1	<-Arginase<-?||?→ LRR-repeats+AP-GTPase+COR+TIR*→?→||<-?<-?<-ABC_membrane+ABC_tran	LRR-repeats+AP-GTPase+COR+TIR	1126	-	Cyanobacteria	Nostoc sp. 'Peltigera membranacea cyanobiont' 232	leucine-rich repeat domain-containing protein [Nostoc sp. 'Peltigera membranacea cyanobiont' 232].	GCF_002245985.1
WP_094343165.1	LRR-repeats+AP-GTPase+COR+TIR*→?→||<-?<-TIR<-?<-SIG+NUDIX	LRR-repeats+AP-GTPase+COR+TIR	1023	-	Cyanobacteria	Nostoc sp. 'Peltigera membranacea cyanobiont' 232	leucine-rich repeat domain-containing protein [Nostoc sp. 'Peltigera membranacea cyanobiont' 232].	GCF_002245985.1
WP_094350322.1	LRR-repeats+AP-GTPase+COR*→?→?→||<-ABhydrolase<-Cluster1087_2clades	LRR-repeats+AP-GTPase+COR	1217	-	Cyanobacteria	Nostoc sp. 'Peltigera membranacea cyanobiont' 210A	leucine-rich repeat domain-containing protein [Nostoc sp. 'Peltigera membranacea cyanobiont' 210A].	GCF_002246015.1
WP_094350573.1	LRR-repeats+AP-GTPase+COR+DUF4404*→ REC→	LRR-repeats+AP-GTPase+COR+DUF4404	1154	-	Cyanobacteria	Nostoc sp. 'Peltigera membranacea cyanobiont' 210A	leucine-rich repeat domain-containing protein [Nostoc sp. 'Peltigera membranacea cyanobiont' 210A].	GCF_002246015.1
WP_094350736.1	LRR-repeats+AP-GTPase+COR*→	LRR-repeats+AP-GTPase+COR	996	-	Cyanobacteria	Nostoc sp. 'Peltigera membranacea cyanobiont' 210A	leucine-rich repeat domain-containing protein [Nostoc sp. 'Peltigera membranacea cyanobiont' 210A].	-
WP_094350925.1	<-TPR+TPR<-?||?→?→ ABhydrolase→?→||<-?||LRR-repeats+AP-GTPase+COR+TIR*→||<-?<-?<-?<-?<-SIG+NUDIX	LRR-repeats+AP-GTPase+COR+TIR	1078	-	Cyanobacteria	Nostoc sp. 'Peltigera membranacea cyanobiont' 210A	leucine-rich repeat domain-containing protein [Nostoc sp. 'Peltigera membranacea cyanobiont' 210A].	GCF_002246015.1
WP_094408510.1	<-ABC-ATPase||ICLR-HTH→ LRR-repeats+AP-GTPase+COR+TIR*→||<-SIG+TM+TM+TM+TM+TM+TM+TM+TM+TM	LRR-repeats+AP-GTPase+COR+TIR	974	-	Alphaproteobacteria	Elstera cyanobacteriorum	leucine-rich repeat domain-containing protein [Elstera cyanobacteriorum].	GCF_014643715.1
WP_094530685.1	LRR-repeats+AP-GTPase+COR*→||<-PSE||?→?→?→||<-ABC-ATPase<-ABC-ATPase<-SIG+TM+TM+TM+TM+TM+TM+TM	LRR-repeats+AP-GTPase+COR	1019	-	Cyanobacteria	Pseudanabaena sp. SR411	leucine-rich repeat domain-containing protein [Pseudanabaena sp. SR411].	GCF_002251945.1

<table>
<tr><th>acc</th><th>operon</th><th>architecture</th><th>len</th><th>gen.name</th><th>taxend</th><th>species</th><th>defline</th><th>gca</th></tr>
<tr><td>WP_094533976.1</td><td>Cluster54_3clades→ Cluster1093_2clades→ LRR-repeats+AP-GTPase+COR+TM+TM*→</td><td>LRR-repeats+AP-GTPase+COR+TM+TM</td><td>1051</td><td>-</td><td>Cyanobacteria</td><td>Pseudanabaena sp. SR411</td><td>leucine-rich repeat domain-containing protein [Pseudanabaena sp. SR411].</td><td>GCF_002251945.1</td></tr>
<tr><td>WP_095644020.1</td><td>LRR-repeats+AP-GTPase+COR*→</td><td>LRR-repeats+AP-GTPase+COR</td><td>774</td><td>-</td><td>Euryarchaeota</td><td>Methanosarcina spelaei</td><td>leucine-rich repeat domain-containing protein, partial [Methanosarcina spelaei].</td><td>GCF_002287235.1</td></tr>
<tr><td>WP_095644584.1</td><td>LRR-repeats+AP-GTPase+COR*→</td><td>LRR-repeats+AP-GTPase+COR</td><td>877</td><td>-</td><td>Euryarchaeota</td><td>Methanosarcina spelaei</td><td>leucine-rich repeat domain-containing protein [Methanosarcina spelaei].</td><td>GCF_002287235.1</td></tr>
<tr><td>WP_096193484.1</td><td>LRR-repeats+AP-GTPase+COR+TIR*→?→||<-FAD-NAD-dep-oxidoreductase<-?<-?<-RNA-Helicase</td><td>LRR-repeats+AP-GTPase+COR+TIR</td><td>1173</td><td>-</td><td>Bacteroidetes</td><td>Cytophagales bacterium TFI 002</td><td>leucine-rich repeat domain-containing protein [Cytophagales bacterium TFI 002].</td><td>-</td></tr>
<tr><td>WP_096535168.1</td><td>LRR-repeats+AP-GTPase+COR*→||<-ClpABN-AAA+ClpABC-AAA<-?||METHYLASE→</td><td>LRR-repeats+AP-GTPase+COR</td><td>1018</td><td>-</td><td>Cyanobacteria</td><td>Nostoc linckia</td><td>leucine-rich repeat domain-containing protein [Nostoc linckia].</td><td>GCF_002368035.1</td></tr>
<tr><td>WP_096538729.1</td><td><-Pkinase+Pentapeptide<-?<-?||?→||<-?||?→ LRR-repeats+AP-GTPase+COR+TIR*→</td><td>LRR-repeats+AP-GTPase+COR+TIR</td><td>1033</td><td>-</td><td>Cyanobacteria</td><td>Nostoc linckia</td><td>leucine-rich repeat domain-containing protein [Nostoc linckia].</td><td>GCF_002368035.1</td></tr>
<tr><td>WP_096538739.1</td><td>LRR-repeats+AP-GTPase+COR+TIR*→?→||<-?||?→?→?→ RPN10→</td><td>LRR-repeats+AP-GTPase+COR+TIR</td><td>1115</td><td>-</td><td>Cyanobacteria</td><td>Nostoc linckia</td><td>leucine-rich repeat domain-containing protein [Nostoc linckia].</td><td>GCF_002368035.1</td></tr>
<tr><td>WP_096551538.1</td><td>LRR-repeats+AP-GTPase+COR*→</td><td>LRR-repeats+AP-GTPase+COR</td><td>818</td><td>-</td><td>Cyanobacteria</td><td>Nostoc sp. NIES-4103</td><td>leucine-rich repeat domain-containing protein [Nostoc sp. NIES-4103].</td><td>GCF_002368335.1</td></tr>
<tr><td>WP_096566542.1</td><td>HNH→ PSE→ LRR-repeats+AP-GTPase+COR*→?→?→?→||<-?||?→||<-?||PAIREDC-HTH→</td><td>LRR-repeats+AP-GTPase+COR</td><td>1268</td><td>-</td><td>Cyanobacteria</td><td>Scytonema sp. NIES-4073</td><td>leucine-rich repeat protein [Scytonema sp. NIES-4073].</td><td>GCF_002368435.1</td></tr>
<tr><td>WP_096579899.1</td><td><-DSBH<-?<-?<-?<-ParA-Soj-PloopNTPase<-?||Cluster5_5clades→ LRR-repeats+AP-GTPase+COR+DUF4404*→</td><td>LRR-repeats+AP-GTPase+COR+DUF4404</td><td>1153</td><td>-</td><td>Cyanobacteria</td><td>Anabaenopsis circularis</td><td>leucine-rich repeat domain-containing protein [Anabaenopsis circularis].</td><td>GCF_002367975.1</td></tr>
<tr><td>WP_096585603.1</td><td>HISKIN→||<-?<-?||LRR-repeats+AP-GTPase+COR*→</td><td>LRR-repeats+AP-GTPase+COR</td><td>937</td><td>-</td><td>Cyanobacteria</td><td>Anabaenopsis circularis</td><td>leucine-rich repeat domain-containing protein [Anabaenopsis circularis].</td><td>GCF_002367975.1</td></tr>
<tr><td>WP_096586956.1</td><td><-ABC-ATPase||?→ Pkinase+TM→ Chromo-N+CHROMO→||<-?||LRR-repeats+AP-GTPase+COR+CASPASE*→||<-?<-?||Aminotran_1_2→</td><td>LRR-repeats+AP-GTPase+COR+CASPASE</td><td>1148</td><td>-</td><td>Cyanobacteria</td><td>Calothrix sp. NIES-2098</td><td>leucine-rich repeat domain-containing protein [Calothrix sp. NIES-2098].</td><td>GCF_002368175.1</td></tr>
<tr><td>WP_096588412.1</td><td>LRR-repeats+AP-GTPase+COR*→ TPR-repeats+CASPASE→</td><td>LRR-repeats+AP-GTPase+COR</td><td>1066</td><td>-</td><td>Cyanobacteria</td><td>Calothrix sp. NIES-2098</td><td>leucine-rich repeat domain-containing protein [Calothrix sp. NIES-2098].</td><td>GCF_002368175.1</td></tr>
<tr><td>WP_096598125.1</td><td>LRR-repeats+AP-GTPase+COR+DUF4404*→?→||<-?||?→||<-RelE-ParE</td><td>LRR-repeats+AP-GTPase+COR+DUF4404</td><td>1134</td><td>-</td><td>Cyanobacteria</td><td>Calothrix sp. NIES-2100</td><td>leucine-rich repeat domain-containing protein [Calothrix sp. NIES-2100].</td><td>GCF_002368195.1</td></tr>
<tr><td>WP_096598883.1</td><td>SIG+TM→?→ tRNA→||<-?||LRR-repeats+AP-GTPase+COR+CASPASE*→||<-PSE<-?||?→?→||<-?<-?<-Acylphosphatase</td><td>LRR-repeats+AP-GTPase+COR+CASPASE</td><td>1108</td><td>-</td><td>Cyanobacteria</td><td>Calothrix sp. NIES-2100</td><td>leucine-rich repeat domain-containing protein [Calothrix sp. NIES-2100].</td><td>GCF_002368195.1</td></tr>
<tr><td>WP_096598950.1</td><td>LRR-repeats+AP-GTPase+COR+TM+TM*→||<-?||?→||<-REC</td><td>LRR-repeats+AP-GTPase+COR+TM+TM</td><td>929</td><td>-</td><td>Cyanobacteria</td><td>Calothrix sp. NIES-2100</td><td>leucine-rich repeat domain-containing protein [Calothrix sp. NIES-2100].</td><td>GCF_002368195.1</td></tr>
<tr><td>WP_096621624.1</td><td>LRR-repeats→ PSE→ LRR-repeats+AP-GTPase+COR+CASPASE*→</td><td>LRR-repeats+AP-GTPase+COR+CASPASE</td><td>981</td><td>-</td><td>Cyanobacteria</td><td>Microchaete diplosiphon</td><td>CHAT domain-containing protein [Microchaete diplosiphon].</td><td>GCF_002368275.1</td></tr>
<tr><td>WP_096658516.1</td><td>LRR-repeats+AP-GTPase+COR*→ PSE→?→||<-?||?→||<-?||?→||<-?<-Pentapeptide-repeats</td><td>LRR-repeats+AP-GTPase+COR</td><td>708</td><td>-</td><td>Cyanobacteria</td><td>Calothrix parasitica</td><td>leucine-rich repeat domain-containing protein [Calothrix parasitica].</td><td>GCF_002368095.1</td></tr>
</table>

<table>
<thead>
<tr><th>acc</th><th>operon</th><th>architecture</th><th>len</th><th>gen.name</th><th>taxend</th><th>species</th><th>defline</th><th>gca</th></tr>
</thead>
<tbody>
<tr><td>WP_096683758.1</td><td><-TPR-repeats+CASPASE||?→||<-?<-PSE||?→||<-ParB<-ParA-Soj-PloopNTPase||PSE→ LRR-repeats+AP-GTPase+COR+EAD1*→ ParB→||<-NACHT</td><td>LRR-repeats+AP-GTPase+COR+EAD1</td><td>1112</td><td>-</td><td>Cyanobacteria</td><td>Calothrix sp. NIES-4105</td><td>leucine-rich repeat domain-containing protein [Calothrix sp. NIES-4105].</td><td>GCF_002368415.1</td></tr>
<tr><td>WP_096729037.1</td><td><-TPR-repeats+CASPASE||?→||<-?<-PSE||?→||<-ParB<-ParA-Soj-PloopNTPase||PSE→ LRR-repeats→ LRR-repeats+AP-GTPase+COR+EAD1*→ ParB→||<-NACHT</td><td>LRR-repeats+AP-GTPase+COR+EAD1</td><td>885</td><td>-</td><td>Cyanobacteria</td><td>Calothrix sp. NIES-4071</td><td>leucine-rich repeat domain-containing protein [Calothrix sp. NIES-4071].</td><td>GCF_002368455.1</td></tr>
<tr><td>WP_096789603.1</td><td><-S4<-?||?→?→?→?→?→ LRR-repeats+AP-GTPase+COR*→||<-tRNA<-rRNA<-rRNA<-tRNA<-tRNA<-rRNA<-FAD-NAD-dep-oxidoreductase</td><td>LRR-repeats+AP-GTPase+COR</td><td>972</td><td>-</td><td>Alphaproteobacteria</td><td>Phaeobacter piscinae</td><td>leucine-rich repeat domain-containing protein [Phaeobacter piscinae].</td><td>GCF_002407245.1</td></tr>
<tr><td>WP_096793541.1</td><td><-Cluster2127_2clades||?→||<-?||?→ LRR-repeats+AP-GTPase+COR*→</td><td>LRR-repeats+AP-GTPase+COR</td><td>938</td><td>-</td><td>Gammaproteobacteria</td><td>Pseudoalteromonas sp. 1_2015MBL_MicDiv</td><td>GTP-binding protein [Pseudoalteromonas sp. 1_2015MBL_MicDiv].</td><td>GCF_002407505.1</td></tr>
<tr><td>WP_097126565.1</td><td>LRR-repeats+AP-GTPase+COR*→||<-?||?→||<-?||?→||<-?||?→||<-HISKIN</td><td>LRR-repeats+AP-GTPase+COR</td><td>1103</td><td>-</td><td>Bacteroidetes</td><td>Spirosoma fluviale</td><td>leucine-rich repeat domain-containing protein [Spirosoma fluviale].</td><td>GCF_900230225.1</td></tr>
<tr><td>WP_097298537.1</td><td>LRR-repeats+AP-GTPase+COR*→</td><td>LRR-repeats+AP-GTPase+COR</td><td>802</td><td>-</td><td>Euryarchaeota</td><td>Candidatus Methanoperedens sp. BLZ2</td><td>leucine-rich repeat domain-containing protein [Candidatus Methanoperedens sp. BLZ2].</td><td>GCF_002487355.1</td></tr>
<tr><td>WP_097998439.1</td><td>LRR-repeats+AP-GTPase+COR*→</td><td>LRR-repeats+AP-GTPase+COR</td><td>906</td><td>-</td><td>Firmicutes</td><td>Bacillus cereus</td><td>GTP-binding protein [Bacillus cereus].</td><td>GCF_002575505.1</td></tr>
<tr><td>WP_098908392.1</td><td>LRR-repeats+AP-GTPase+COR*→</td><td>LRR-repeats+AP-GTPase+COR</td><td>934</td><td>-</td><td>Firmicutes</td><td>Bacillus toyonensis</td><td>GTP-binding protein [Bacillus toyonensis].</td><td>GCF_002551725.1</td></tr>
<tr><td>WP_099009377.1</td><td>LRR-repeats+AP-GTPase+COR+EAD11*→</td><td>LRR-repeats+AP-GTPase+COR+EAD11</td><td>813</td><td>-</td><td>Bacteroidetes</td><td>Lewinellaceae bacterium SD302</td><td>leucine-rich repeat domain-containing protein [Lewinellaceae bacterium SD302].</td><td>-</td></tr>
<tr><td>WP_099066006.1</td><td>LRR-repeats+AP-GTPase+COR+TIR*→||<-PSE<-Arginase||?→||<-?||?→?→?→ Pro_CA→</td><td>LRR-repeats+AP-GTPase+COR+TIR</td><td>1138</td><td>-</td><td>Cyanobacteria</td><td>Nostoc linckia</td><td>leucine-rich repeat domain-containing protein [Nostoc linckia].</td><td>GCF_002607965.1</td></tr>
<tr><td>WP_099069019.1</td><td><-Pkinase+Pentapeptide<-?<-?||?→||<-?||LRR-repeats+AP-GTPase+COR+TIR*→?→||<-SIG+TM+TM+TM+TM+TM+TM<-?||?→ Cluster1668_2clades→</td><td>LRR-repeats+AP-GTPase+COR+TIR</td><td>1053</td><td>-</td><td>Cyanobacteria</td><td>Nostoc linckia</td><td>TIR domain-containing protein [Nostoc linckia].</td><td>GCF_002607965.1</td></tr>
<tr><td>WP_099076744.1</td><td>LRR-repeats+AP-GTPase+COR+TIR*→||<-PSE<-Arginase</td><td>LRR-repeats+AP-GTPase+COR+TIR</td><td>1138</td><td>-</td><td>Cyanobacteria</td><td>Nostoc linckia</td><td>leucine-rich repeat domain-containing protein [Nostoc linckia].</td><td>GCF_002608325.1</td></tr>
<tr><td>WP_099103609.1</td><td>LRR-repeats+AP-GTPase+COR+TM+TM*→</td><td>LRR-repeats+AP-GTPase+COR+TM+TM</td><td>825</td><td>-</td><td>Cyanobacteria</td><td>Nostoc sp. ‘Peltigera malacea cyanobiont’ DB3992</td><td>leucine-rich repeat domain-containing protein, partial [Nostoc sp. ‘Peltigera malacea cyanobiont’ DB3992].</td><td>-</td></tr>
<tr><td>WP_099151645.1</td><td>SIG+TM+TM+TM+HISKIN→?→ tRNA→?→?→ LRR-repeats+AP-GTPase+COR+EAD11*→</td><td>LRR-repeats+AP-GTPase+COR+EAD11</td><td>881</td><td>-</td><td>Bacteroidetes</td><td>Flavilitoribacter nigricans</td><td>leucine-rich repeat domain-containing protein [Flavilitoribacter nigricans].</td><td>GCF_002646595.1</td></tr>
<tr><td>WP_099285181.1</td><td>LRR-repeats+AP-GTPase+COR*→?→||<-?<-SIGMA-HTH</td><td>LRR-repeats+AP-GTPase+COR</td><td>1056</td><td>-</td><td>Actinobacteria</td><td>unclassified Streptomyces</td><td>MULTISPECIES: GTPase [unclassified Streptomyces].</td><td>GCF_002705975.1</td></tr>
<tr><td>WP_099426012.1</td><td>TPR-repeats→?→?→ tRNA→?→ HNH→ LRR-repeats+AP-GTPase+COR+DUF4404*→?→?→?→ REC→</td><td>LRR-repeats+AP-GTPase+COR+DUF4404</td><td>1272</td><td>-</td><td>Cyanobacteria</td><td>Tychonema bourrellyi</td><td>leucine-rich repeat domain-containing protein [Tychonema bourrellyi].</td><td>GCF_002412335.2</td></tr>
<tr><td>WP_099426065.1</td><td>GTPase-AIG→?→?→ Cluster53_2clades→?→ LRR-repeats+AP-GTPase+COR+DUF4404*→</td><td>LRR-repeats+AP-GTPase+COR+DUF4404</td><td>959</td><td>-</td><td>Cyanobacteria</td><td>Tychonema bourrellyi</td><td>leucine-rich repeat domain-containing protein [Tychonema bourrellyi].</td><td>GCF_002412335.2</td></tr>
<tr><td>WP_099506658.1</td><td>ABC_membrane+ABC_tran→||<-?||?→||<-?||PSE→||<-PSE||LRR-repeats+AP-GTPase+COR*→</td><td>LRR-repeats+AP-GTPase+COR</td><td>799</td><td>-</td><td>Actinobacteria</td><td>Streptomyces dengpaensis</td><td>hypothetical protein [Streptomyces dengpaensis].</td><td>GCF_002946835.1</td></tr>
</tbody>
</table>

acc	operon	architecture	len	gen.name	taxend	species	defline	gca
WP_099533140.1	AP-GTPase+COR→?→ LRR-repeats+AP-GTPase+COR*→||<-?||?→||<-tRNA||?→?→ Cluster1088_2clades→||<-?<-tRNA<-BACTERIALFRINGE+TM+TM	LRR-repeats+AP-GTPase+COR	826	-	Cyanobacteria	Limnothrix sp. PR1529	leucine-rich repeat domain-containing protein [Limnothrix sp. PR1529].	GCF_002742025.1
WP_100899585.1	LRR-repeats+AP-GTPase+COR+EAD1*→||<-CASPASE+TM	LRR-repeats+AP-GTPase+COR+EAD1	945	-	Cyanobacteria	Nostoc flagelliforme	leucine-rich repeat domain-containing protein [Nostoc flagelliforme].	GCF_002813575.1
WP_100900079.1	LRR-repeats+AP-GTPase+COR*→ COR→?→||<-?||?→||<-?||?→ ClpABN-AAA+ClpABC-AAA→	LRR-repeats+AP-GTPase+COR	431	-	Cyanobacteria	Nostoc flagelliforme	leucine-rich repeat domain-containing protein [Nostoc flagelliforme].	GCF_002813575.1
WP_100917604.1	<-METHYLASE<-?||?→||<-?||Cluster2515_2clades→ Cluster2268_2clades→ LRR-repeats+AP-GTPase+COR+TIR*→	LRR-repeats+AP-GTPase+COR+TIR	983	-	Gammaproteobacteria	Candidatus Thiodictyon syntrophicum	TIR domain-containing protein [Candidatus Thiodictyon syntrophicum].	GCF_002813775.1
WP_100918672.1	<-Cluster944_3clades<-SIG+PDZ||?→?→||<-ABC-ATPase<-?<-?||LRR-repeats+AP-GTPase+COR+TIR*→||<-ABC-ATPase<-SIG+TM+TM+TM+TM+TM+TM+TM<-?<-SIG+PBPI	LRR-repeats+AP-GTPase+COR+TIR	1095	-	Gammaproteobacteria	Candidatus Thiodictyon syntrophicum	leucine-rich repeat domain-containing protein [Candidatus Thiodictyon syntrophicum].	GCF_002813775.1
WP_101084187.1	<-Cluster1951_2clades||?→ LRR-repeats+AP-GTPase+COR*→?→?→?→?→ Cluster846_3clades→	LRR-repeats+AP-GTPase+COR	1013	-	Gammaproteobacteria	Psychromonas sp. Urea-02u-13	GTP-binding protein [Psychromonas sp. Urea-02u-13].	GCF_002835995.1
WP_101221578.1	SIG+TM+TM+TM+TM+TM+TM+TM→||<-?||?→?→?→ PSE→||<-?<-?||PSE→ LRR-repeats+AP-GTPase+COR*→	LRR-repeats+AP-GTPase+COR	897	-	Firmicutes	Bacillus sp. BA3	GTP-binding protein [Bacillus sp. BA3].	GCF_002835675.1
WP_101309496.1	LRR-repeats+AP-GTPase+COR*→	LRR-repeats+AP-GTPase+COR	888	-	Bacteroidetes	Labilibaculum manganireducens	hypothetical protein [Labilibaculum manganireducens].	GCF_002843385.1
WP_101439075.1	LRR-repeats+AP-GTPase+COR*→	LRR-repeats+AP-GTPase+COR	1694	-	Actinobacteria	Amycolatopsis	MULTISPECIES: leucine-rich repeat domain-containing protein [Amycolatopsis].	GCF_014174495.1
WP_101728686.1	MACRODOMAIN+LRR-repeats+AP-GTPase+COR+TIR*→	MACRODOMAIN+LRR-repeats+AP-GTPase+COR+TIR	1357	-	Bacteroidetes	Emticicia sp. TH156	leucine-rich repeat domain-containing protein [Emticicia sp. TH156].	GCF_002855535.1
WP_101955079.1	<-TM<-WCAK<-?||?→ LRR-repeats+AP-GTPase+COR+TIR*→	LRR-repeats+AP-GTPase+COR+TIR	1144	-	Bacteroidetes	Tenacibaculum finnmarkense	leucine-rich repeat domain-containing protein [Tenacibaculum finnmarkense].	GCF_900239185.1
WP_102406975.1	LRR-repeats+AP-GTPase+COR+DrHyd*→	LRR-repeats+AP-GTPase+COR+DrHyd	1169	-	Bacteroidetes	Parabacteroides bouchesdurhonensis	leucine-rich repeat domain-containing protein [Parabacteroides bouchesdurhonensis].	GCF_900186615.1
WP_102484140.1	CI→?→?→ LRR-repeats+AP-GTPase+COR*→?→?→||<-?<-?<-?<-ABC-ATPase	LRR-repeats+AP-GTPase+COR	1107	-	Gammaproteobacteria	Vibrio lentus	GTP-binding protein [Vibrio lentus].	GCF_002874115.1
WP_102498010.1	REC→?→?→?→||<-ABC_membrane+ABC_tran<-ABC_membrane+ABC_tran<-?||LRR-repeats+AP-GTPase+COR*→	LRR-repeats+AP-GTPase+COR	960	-	Gammaproteobacteria	Vibrio splendidus	leucine-rich repeat domain-containing protein [Vibrio splendidus].	GCF_002876045.1
WP_102512513.1	CI→ LRR-repeats+AP-GTPase+COR*→?→?→||<-PSE<-?<-?<-?<-ABC-ATPase	LRR-repeats+AP-GTPase+COR	1108	-	Gammaproteobacteria	Vibrio splendidus	GTP-binding protein [Vibrio splendidus].	GCF_002876945.1
WP_102562621.1	CI→ LRR-repeats+AP-GTPase+COR*→||<-?<-?||?→||<-?<-RPN10	LRR-repeats+AP-GTPase+COR	1108	-	Gammaproteobacteria	Vibrio splendidus	50S ribosome-binding GTPase [Vibrio splendidus].	GCF_002878555.1
WP_102571188.1	CI→ LRR-repeats+AP-GTPase+COR*→	LRR-repeats+AP-GTPase+COR	1107	-	Gammaproteobacteria	Vibrio splendidus	50S ribosome-binding GTPase [Vibrio splendidus].	GCF_002875585.1
WP_102579518.1	CI→?→ LRR-repeats+AP-GTPase+COR*→?→?→||<-?<-?<-?<-ABC-ATPase	LRR-repeats+AP-GTPase+COR	1106	-	Gammaproteobacteria	Vibrio lentus	GTP-binding protein [Vibrio lentus].	GCF_002876365.1
WP_102756439.1	TM+TM+TM+TM+TM→?→ Cluster2372_2clades→||<-?||?→?→?→ LRR-repeats+AP-GTPase+COR+DrHyd*→	LRR-repeats+AP-GTPase+COR+DrHyd	1054	-	Bacteroidetes	Flavivirga eckloniae	leucine-rich repeat domain-containing protein [Flavivirga eckloniae].	GCF_002886045.1
WP_103111669.1	SIG+TM+TM+TM+TM+TM+TM+TM→||<-?||Mbetalac→ SAM-methylase→?→||<-?<-SIG+TPM_phosphatase+TM+TM+TM||LRR-repeats+AP-GTPase+COR+TM+TM*→||<-?<-?<-?<-Pro_CA||?→||<-?<-tRNA<-tRNA<-Ferredoxin-betagrasp	LRR-repeats+AP-GTPase+COR+TM+TM	851	-	Cyanobacteria	Microcystis aeruginosa	leucine-rich repeat domain-containing protein [Microcystis aeruginosa].	GCF_002897275.1

<table>
<tr><th>acc</th><th>operon</th><th>architecture</th><th>len</th><th>gen.name</th><th>taxend</th><th>species</th><th>defline</th><th>gca</th></tr>
<tr><td>WP_103125147.1</td><td>HISKIN→||<-?||LRR-repeats+AP-GTPase+COR*→</td><td>LRR-repeats+AP-GTPase+COR</td><td>889</td><td>-</td><td>Cyanobacteria</td><td>Nostoc cycadae</td><td>leucine-rich repeat domain-containing protein [Nostoc cycadae].</td><td>GCF_002897135.1</td></tr>
<tr><td>WP_103137712.1</td><td>LRR-repeats+AP-GTPase+COR+TIR*→ Cluster1064_2clades→?→||<-?<-?<-?||Arginase→</td><td>LRR-repeats+AP-GTPase+COR+TIR</td><td>1058</td><td>-</td><td>Cyanobacteria</td><td>Nostoc sp. CENA543</td><td>leucine-rich repeat domain-containing protein [Nostoc sp. CENA543].</td><td>GCF_002896875.1</td></tr>
<tr><td>WP_103666629.1</td><td><-Cluster1740_2clades<-TM<-?<-?||NACHT→||<-?||LRR-repeats+AP-GTPase+COR+TM+TM*→||<-?||?→?→?→ SIG+TM+TM+TM+TM+TM→</td><td>LRR-repeats+AP-GTPase+COR+TM+TM</td><td>1164</td><td>-</td><td>Cyanobacteria</td><td>Pseudanabaena sp. BC1403</td><td>leucine-rich repeat domain-containing protein [Pseudanabaena sp. BC1403].</td><td>GCF_002914585.1</td></tr>
<tr><td>WP_103669449.1</td><td>Cluster54_3clades→?→?→?→ PIN→ LRR-repeats+AP-GTPase+COR+TM+TM*→||<-?<-?||ABC-ATPase→</td><td>LRR-repeats+AP-GTPase+COR+TM+TM</td><td>922</td><td>-</td><td>Cyanobacteria</td><td>Pseudanabaena sp. BC1403</td><td>leucine-rich repeat domain-containing protein [Pseudanabaena sp. BC1403].</td><td>GCF_002914585.1</td></tr>
<tr><td>WP_103972898.1</td><td>LRR-repeatsAP-GTPase+COR+E59-ABC*→</td><td>LRR-repeatsAP-GTPase+COR+E59-ABC</td><td>953</td><td>-</td><td>Gammaproteobacteria</td><td>Methylovulum psychrotolerans</td><td>AAA family ATPase [Methylovulum psychrotolerans].</td><td>GCF_002923755.1</td></tr>
<tr><td>WP_103972982.1</td><td><-ABC-ATPase<-SIG+BLBD<-?||REC→ HISKIN→ REase→ LRR-repeats+AP-GTPase+COR*→?→?→?→ Ferredoxin-RRM→</td><td>LRR-repeats+AP-GTPase+COR</td><td>911</td><td>-</td><td>Gammaproteobacteria</td><td>Methylovulum psychrotolerans</td><td>hypothetical protein [Methylovulum psychrotolerans].</td><td>GCF_002923755.1</td></tr>
<tr><td>WP_103974058.1</td><td><-PurT-ATPgrasp<-?<-?<-?||?→||<-?<-Cluster1542_2clades||LRR-repeats+AP-GTPase+COR+TIR*→ REase→||<-?<-?<-FKBP<-RHOD-CDC25<-Aminotran_1_2||Cluster2179_2clades→</td><td>LRR-repeats+AP-GTPase+COR+TIR</td><td>1203</td><td>-</td><td>Gammaproteobacteria</td><td>Methylovulum psychrotolerans</td><td>TIR domain-containing protein [Methylovulum psychrotolerans].</td><td>GCF_002923755.1</td></tr>
<tr><td>WP_103999297.1</td><td>LRR-repeats+AP-GTPase+COR*→?→||<-?<-?<-ABC-ATPase||Cluster995_2clades→</td><td>LRR-repeats+AP-GTPase+COR</td><td>925</td><td>-</td><td>Bacteroidetes</td><td>Flavobacterium urumqiense</td><td>leucine-rich repeat domain-containing protein [Flavobacterium urumqiense].</td><td>GCF_900108015.1</td></tr>
<tr><td>WP_104395581.1</td><td>SIG+TM+TM+TM+TM+TM+TM+TM→||<-?||Mbetalac→ SAM-methylase→?→||<-?<-SIG+TPM_phosphatase+TM+TM+TM||LRR-repeats+AP-GTPase+COR+TM+TM*→||<-?<-?<-?<-Pro_CA||?→||<-?<-tRNA<-tRNA<-Ferredoxin-betagrasp</td><td>LRR-repeats+AP-GTPase+COR+TM+TM</td><td>874</td><td>-</td><td>Cyanobacteria</td><td>Microcystis aeruginosa</td><td>leucine-rich repeat protein [Microcystis aeruginosa].</td><td>GCF_002933835.1</td></tr>
<tr><td>WP_104424498.1</td><td>Cluster1018_2clades→?→?→?→ PIN→?→ PIN→ LRR-repeats+AP-GTPase+COR+TIR*→||<-?||Cluster1018_2clades→</td><td>LRR-repeats+AP-GTPase+COR+TIR</td><td>999</td><td>-</td><td>Gammaproteobacteria</td><td>Methylobacter tundripaludum</td><td>TIR domain-containing protein [Methylobacter tundripaludum].</td><td>GCF_002934365.1</td></tr>
<tr><td>WP_104900247.1</td><td>Cluster1074_2clades→||<-?<-Cluster53_2clades||LRR-repeats+AP-GTPase+COR*→||<-?<-?||?→?→ SAM-methylase→</td><td>LRR-repeats+AP-GTPase+COR</td><td>1039</td><td>-</td><td>Cyanobacteria</td><td>Nostoc sp. ‘Peltigera membranacea cyanobiont’ N6</td><td>leucine-rich repeat domain-containing protein [Nostoc sp. ‘Peltigera membranacea cyanobiont’ N6].</td><td>GCF_002949735.1</td></tr>
<tr><td>WP_104904652.1</td><td>LRR-repeats+AP-GTPase+COR*→?→?→||<-ABhydrolase<-Cluster1087_2clades</td><td>LRR-repeats+AP-GTPase+COR</td><td>1097</td><td>-</td><td>Cyanobacteria</td><td>Nostoc sp. ‘Lobaria pulmonaria (5183) cyanobiont’</td><td>leucine-rich repeat domain-containing protein [Nostoc sp. ‘Lobaria pulmonaria (5183) cyanobiont’].</td><td>GCF_002949795.1</td></tr>
<tr><td>WP_104909847.1</td><td>LRR-repeats+AP-GTPase+COR+DUF4404*→</td><td>LRR-repeats+AP-GTPase+COR+DUF4404</td><td>1125</td><td>-</td><td>Cyanobacteria</td><td>Nostoc sp. ‘Lobaria pulmonaria (5183) cyanobiont’</td><td>leucine-rich repeat domain-containing protein [Nostoc sp. ‘Lobaria pulmonaria (5183) cyanobiont’].</td><td>-</td></tr>
<tr><td>WP_105041123.1</td><td>LRR-repeats+AP-GTPase+COR*→</td><td>LRR-repeats+AP-GTPase+COR</td><td>925</td><td>-</td><td>Bacteroidetes</td><td>Flavipsychrobacter stenotrophus</td><td>leucine-rich repeat domain-containing protein [Flavipsychrobacter stenotrophus].</td><td>GCF_002954265.1</td></tr>
<tr><td>WP_106089276.1</td><td>LRR-repeats+AP-GTPase+COR+PNPase*→</td><td>LRR-repeats+AP-GTPase+COR+PNPase</td><td>915</td><td>-</td><td>Deltaproteobacteria</td><td>Enhygromyxa salina</td><td>50S ribosome-binding GTPase [Enhygromyxa salina].</td><td>GCF_002994635.1</td></tr>
<tr><td>WP_106150696.1</td><td>LRR-repeats+AP-GTPase+COR+DUF4404*→</td><td>LRR-repeats+AP-GTPase+COR+DUF4404</td><td>779</td><td>-</td><td>Cyanobacteria</td><td>filamentous cyanobacterium Phorm 46</td><td>leucine-rich repeat domain-containing protein, partial [filamentous cyanobacterium Phorm 46].</td><td>-</td></tr>
<tr><td>WP_106254335.1</td><td>LRR-repeats+AP-GTPase+COR+TIR*→</td><td>LRR-repeats+AP-GTPase+COR+TIR</td><td>930</td><td>-</td><td>Cyanobacteria</td><td>Stenomitos frigidus</td><td>leucine-rich repeat domain-containing protein [Stenomitos frigidus].</td><td>GCF_003003795.1</td></tr>
<tr><td>WP_106256414.1</td><td>LRR-repeats+AP-GTPase+COR*→</td><td>LRR-repeats+AP-GTPase+COR</td><td>961</td><td>-</td><td>Cyanobacteria</td><td>Stenomitos frigidus</td><td>GTPase [Stenomitos frigidus].</td><td>GCF_003003795.1</td></tr>
</table>

acc	operon	architecture	len	gen.name	taxend	species	defline	gca
WP_106289057.1	LRR-repeats+AP-GTPase+COR+DUF4404*→	LRR-repeats+AP-GTPase+COR+DUF4404	918	-	Cyanobacteria	Merismopedia glauca	leucine-rich repeat domain-containing protein [Merismopedia glauca].	GCF_003003775.1
WP_106331563.1	TPR-repeats→?→?→?→ tRNA→||<-PSE<-?||Cluster53_2clades→?→ LRR-repeats+AP-GTPase+COR+DUF4404*→	LRR-repeats+AP-GTPase+COR+DUF4404	1048	-	Cyanobacteria	filamentous cyanobacterium Phorm 6	leucine-rich repeat domain-containing protein [filamentous cyanobacterium Phorm 6].	-
WP_106333130.1	LRR-repeats+AP-GTPase+COR+DUF4404*→?→?→||<-?<-?<-?<-?<-RelE-ParE	LRR-repeats+AP-GTPase+COR+DUF4404	786	-	Cyanobacteria	filamentous cyanobacterium Phorm 6	leucine-rich repeat domain-containing protein, partial [filamentous cyanobacterium Phorm 6].	-
WP_106364139.1	<-ABC-ATPase||?→?→ LRR-repeats+AP-GTPase+COR*→	LRR-repeats+AP-GTPase+COR	1146	-	Actinobacteria	Glycomyces artemisiae	hypothetical protein [Glycomyces artemisiae].	GCF_003002955.1
WP_106623442.1	LRR-repeats+AP-GTPase+COR+TM+TM*→||<-ABC-ATPase	LRR-repeats+AP-GTPase+COR+TM+TM	970	-	Bacteroidetes	Sphingobacteriales bacterium UPWRP_1	leucine-rich repeat domain-containing protein [Sphingobacteriales bacterium UPWRP_1].	-
WP_106872506.1	RelE-ParE→ LRR-repeats+AP-GTPase+COR+TIR*→	LRR-repeats+AP-GTPase+COR+TIR	1126	-	Cyanobacteria	unclassified Cyanobacteria	MULTISPECIES: leucine-rich repeat domain-containing protein [unclassified Cyanobacteria].	-
WP_106922429.1	LRR-repeats+AP-GTPase+COR+TIR*→||<-REase-4	LRR-repeats+AP-GTPase+COR+TIR	826	-	Cyanobacteria	filamentous cyanobacterium CCP3	leucine-rich repeat domain-containing protein [filamentous cyanobacterium CCP3].	-
WP_107346265.1	Arginase→?→||<-?||?→ LRR-repeats+AP-GTPase+COR+TIR*→||<-?<-?<-?<-ABC-ATPase||?→ REC→	LRR-repeats+AP-GTPase+COR+TIR	1173	-	Alphaproteobacteria	Rhodopseudomonas palustris	leucine-rich repeat domain-containing protein [Rhodopseudomonas palustris].	GCF_003031265.1
WP_107357189.1	Arginase→?→||<-?||?→ LRR-repeats+AP-GTPase+COR+TIR*→||<-?<-?<-?<-ABC-ATPase||?→ REC→	LRR-repeats+AP-GTPase+COR+TIR	1173	-	Alphaproteobacteria	Rhodopseudomonas palustris	leucine-rich repeat domain-containing protein [Rhodopseudomonas palustris].	GCF_003031245.1
WP_107867044.1	LRR-repeats+AP-GTPase+COR+GT4*→	LRR-repeats+AP-GTPase+COR+GT4	1510	-	Firmicutes	Agitococcus lubricus	leucine-rich repeat protein [Agitococcus lubricus].	GCF_003051055.1
WP_108091428.1	ABC_membrane+ABC_tran→||<-?||CI→ LRR-repeats+AP-GTPase+COR+TIR*→	LRR-repeats+AP-GTPase+COR+TIR	1243	-	Gammaproteobacteria	Vibrio splendidus	TIR domain-containing protein [Vibrio splendidus].	GCF_003050005.1
WP_108099554.1	<-Cluster1756_2clades<-?<-?<-?<-?||?→?→ LRR-repeats+AP-GTPase+COR*→	LRR-repeats+AP-GTPase+COR	1107	-	Gammaproteobacteria	Vibrio splendidus	50S ribosome-binding GTPase [Vibrio splendidus].	GCF_003050165.1
WP_108112883.1	<-HISKIN<-?||LRR-repeats+AP-GTPase+COR+TIR*→||<-HISKIN<-HISKIN	LRR-repeats+AP-GTPase+COR+TIR	973	-	Bacteroidetes	Kordia periserrulae	leucine-rich repeat domain-containing protein [Kordia periserrulae].	GCF_003054265.1
WP_108114870.1	CHTH→||<-?<-?||RNA-Helicase→?→?→||<-?||LRR-repeats+AP-GTPase+COR+TIR*→||<-?<-?<-?||?→||<-TM+TM+TM	LRR-repeats+AP-GTPase+COR+TIR	1012	-	Bacteroidetes	Kordia periserrulae	leucine-rich repeat domain-containing protein [Kordia periserrulae].	GCF_003054265.1
WP_108176265.1	LRR-repeats+AP-GTPase+COR*→?→ ABC_membrane+ABC_tran→ ABC_membrane+ABC_tran→	LRR-repeats+AP-GTPase+COR	1122	-	Gammaproteobacteria	Vibrio splendidus	hypothetical protein [Vibrio splendidus].	GCF_003050125.1
WP_108207948.1	REC→?→?→?→||<-ABC_membrane+ABC_tran<-ABC_membrane+ABC_tran<-?||LRR-repeats+AP-GTPase+COR*→	LRR-repeats+AP-GTPase+COR	960	-	Gammaproteobacteria	Vibrio splendidus	leucine-rich repeat domain-containing protein [Vibrio splendidus].	GCF_003050345.1
WP_108801834.1	<-REC<-?||LRR-repeats+AP-GTPase+COR+TM+TM*→?→?→?→?→||<-?<-SIG+TM+TM+TM+HISKIN	LRR-repeats+AP-GTPase+COR+TM+TM	1086	-	Bacteroidetes	Aquimarina sp. Aq107	leucine-rich repeat domain-containing protein [Aquimarina sp. Aq107].	GCF_900299505.1
WP_109009086.1	ABhydrolase→?→||<-PSE||LRR-repeats+AP-GTPase+COR+TIR*→||<-?<-?<-?<-?<-SIG+NUDIX	LRR-repeats+AP-GTPase+COR+TIR	1078	-	Cyanobacteria	Nostoc commune	leucine-rich repeat domain-containing protein [Nostoc commune].	GCF_003113895.1

acc	operon	architecture	len	gen.name	taxend	species	defline	gca
WP_109322545.1	LRR-repeats+AP-GTPase+COR*→?→?→?→ Cluster978_2clades→	LRR-repeats+AP-GTPase+COR	841	-	Bacteroidetes	Allopseudarcicella aquatilis	hypothetical protein [Allopseudarcicella aquatilis].	GCF_003143535.1
WP_109617479.1	LRR-repeats+AP-GTPase+COR*→	LRR-repeats+AP-GTPase+COR	1124	-	Bacteroidetes	Chryseobacterium oncorhynchi	leucine-rich repeat domain-containing protein [Chryseobacterium oncorhynchi].	GCF_002899895.1
WP_109694822.1	LRR-repeats+AP-GTPase+COR+TIR*→	LRR-repeats+AP-GTPase+COR+TIR	1143	-	Bacteroidetes	Chitinophaga deserti	TIR domain-containing protein [Chitinophaga deserti].	-
WP_110392243.1	ABC-ATPase→?→ LRR-repeats+AP-GTPase+COR+TIR*→	LRR-repeats+AP-GTPase+COR+TIR	1128	-	Betaproteobacteria	Rivicola pingtungensis	leucine-rich repeat domain-containing protein [Rivicola pingtungensis].	GCF_003201855.1
WP_110578614.1	SIG+TM+TM+TM+TM+TM+TM+TM→||<-PSE||Mbetalac→ SAM-methylase→?→||<-?<-SIG+TPM_phosphatase+TM+TM+TM||LRR-repeats+AP-GTPase+COR+TM+TM*→||<-?<-?<-?<-Pro_CA||?→||<-?<-tRNA<-tRNA<-Ferredoxin-betagrasp	LRR-repeats+AP-GTPase+COR+TM+TM	865	-	Cyanobacteria	Microcystis aeruginosa	leucine-rich repeat domain-containing protein [Microcystis aeruginosa].	GCF_003206555.1
WP_110984619.1	<-Cluster1569_2clades||Mbetalac→||<-?<-?||?→ Mbetalac→||<-?||LRR-repeats+AP-GTPase+COR*→?→ PIN→||<-Cluster1559_2clades	LRR-repeats+AP-GTPase+COR	995	-	Cyanobacteria	Acaryochloris sp. RCC1774	leucine-rich repeat domain-containing protein [Acaryochloris sp. RCC1774].	-
WP_111129223.1	LRR-repeats+AP-GTPase+COR*→	LRR-repeats+AP-GTPase+COR	894	-	Bacteroidetes	Mesonia sp. K7	50S ribosome-binding GTPase [Mesonia sp. K7].	GCF_003233725.1
WP_111283162.1	LRR-repeats+AP-GTPase+COR*→||<-?||?→?→||<-?||?→?→||<-cNMPBD	LRR-repeats+AP-GTPase+COR	1144	-	Bacteroidetes	Flavobacterium ginsenosidimutans	leucine-rich repeat domain-containing protein [Flavobacterium ginsenosidimutans].	GCF_003254625.1
WP_111625904.1	LRR-repeats+AP-GTPase+COR*→	LRR-repeats+AP-GTPase+COR	846	-	Bacteroidetes	Gelidibacter algens	hypothetical protein [Gelidibacter algens].	GCF_003259265.1
WP_111845807.1	LRR-repeats+AP-GTPase+COR*→	LRR-repeats+AP-GTPase+COR	812	-	Bacteroidetes	Aequorivita antarctica	hypothetical protein, partial [Aequorivita antarctica].	-
WP_112086237.1	LRR-repeats+AP-GTPase+COR*→	LRR-repeats+AP-GTPase+COR	847	-	Bacteroidetes	Flavobacterium lacus	hypothetical protein [Flavobacterium lacus].	GCF_003268815.1
WP_112134132.1	LRR-repeats+AP-GTPase+COR*→?→||<-?<-?<-?<-REC<-HISKIN	LRR-repeats+AP-GTPase+COR	1186	-	Actinobacteria	Glycomyces dulcitolivorans	leucine-rich repeat domain-containing protein [Glycomyces dulcitolivorans].	GCF_003265355.1
WP_112263949.1	wHTH-4stranded+TPRs+APATPase+TPR-repeats→?→?→?→ ABC_membrane+ABC_tran→?→?→ LRR-repeats+AP-GTPase+COR*→ COR→	LRR-repeats+AP-GTPase+COR	608	-	Actinobacteria	Lentzea terrae	leucine-rich repeat domain-containing protein [Lentzea terrae].	GCF_003265345.1
WP_114082655.1	Cluster5_5clades→ LRR-repeats+AP-GTPase+COR+TM+TM*→||<-CASPASE+TM	LRR-repeats+AP-GTPase+COR+TM+TM	904	-	Cyanobacteria	Nostoc sp. ATCC 53789	leucine-rich repeat domain-containing protein [Nostoc sp. ATCC 53789].	GCF_003326245.1
WP_114084090.1	Pentapeptide-repeats→||<-?<-?||?→?→||<-Arginase<-?||LRR-repeats+AP-GTPase+COR+TIR*→?→||<-?||?→?→?→?→ APATPase+TPR+TPR+TPR→	LRR-repeats+AP-GTPase+COR+TIR	802	-	Cyanobacteria	Nostoc sp. ATCC 53789	leucine-rich repeat domain-containing protein [Nostoc sp. ATCC 53789].	GCF_003326245.1
WP_114085324.1	ABhydrolase→?→||<-?||LRR-repeats+AP-GTPase+COR+TIR*→||<-?<-?<-?<-SIG+NUDIX	LRR-repeats+AP-GTPase+COR+TIR	1069	-	Cyanobacteria	Nostoc sp. ATCC 53789	leucine-rich repeat domain-containing protein [Nostoc sp. ATCC 53789].	GCF_003326245.1
WP_114085448.1	LRR-repeats+AP-GTPase+COR+CASPASE*→?→?→?→ ParA-Soj-PloopNTPase→	LRR-repeats+AP-GTPase+COR+CASPASE	1512	-	Cyanobacteria	Nostoc sp. ATCC 53789	leucine-rich repeat domain-containing protein [Nostoc sp. ATCC 53789].	GCF_003326245.1
WP_114085638.1	<-ParA-Soj-PloopNTPase||LRR-repeats+AP-GTPase+COR*→	LRR-repeats+AP-GTPase+COR	881	-	Cyanobacteria	Nostoc sp. ATCC 53789	hypothetical protein [Nostoc sp. ATCC 53789].	GCF_009873495.1
WP_114460382.1	Cluster1398_2clades→?→ LRR-repeats+AP-GTPase*→	LRR-repeats+AP-GTPase	477	-	Bacteroidetes	Runella sp. YX9	leucine-rich repeat domain-containing protein [Runella sp. YX9].	GCF_003339505.1
WP_114694555.1	SIGMA-HTH→?→||<-?||?→?→ LRR-repeats+AP-GTPase+COR*→	LRR-repeats+AP-GTPase+COR	865	-	Gammaproteobacteria	Motiliproteus coralliicola	GTP-binding protein [Motiliproteus coralliicola].	GCF_003345655.1

<table>
<tr><th>acc</th><th>operon</th><th>architecture</th><th>len</th><th>gen.name</th><th>taxend</th><th>species</th><th>defline</th><th>gca</th></tr>
<tr><td>WP_114902638.1</td><td>LRR-repeats+AP-GTPase+COR+DrHyd*→</td><td>LRR-repeats+AP-GTPase+COR+DrHyd</td><td>890</td><td>-</td><td>Bacteroidetes</td><td>Kordia sp. SMS9</td><td>leucine-rich repeat domain-containing protein [Kordia sp. SMS9].</td><td>GCF_003352465.1</td></tr>
<tr><td>WP_114902899.1</td><td>REC→||<-?||?→ LRR-repeats+AP-GTPase+COR+TIR*→||<-RADICAL-SAM<-?<-?<-SIGMA-HTH</td><td>LRR-repeats+AP-GTPase+COR+TIR</td><td>972</td><td>-</td><td>Bacteroidetes</td><td>Kordia sp. SMS9</td><td>leucine-rich repeat domain-containing protein [Kordia sp. SMS9].</td><td>GCF_003352465.1</td></tr>
<tr><td>WP_114904001.1</td><td>LRR-repeats+AP-GTPase+COR+TIR*→</td><td>LRR-repeats+AP-GTPase+COR+TIR</td><td>947</td><td>-</td><td>Bacteroidetes</td><td>Kordia sp. SMS9</td><td>leucine-rich repeat domain-containing protein [Kordia sp. SMS9].</td><td>GCF_003352465.1</td></tr>
<tr><td>WP_115919083.1</td><td>LRR-repeats+AP-GTPase+COR+TIR*→?→?→ PSE→?→||<-?<-tRNA||?→ REC→</td><td>LRR-repeats+AP-GTPase+COR+TIR</td><td>1186</td><td>-</td><td>Bacteroidetes</td><td>Chryseobacterium rhizosphaerae</td><td>TIR domain-containing protein [Chryseobacterium rhizosphaerae].</td><td>GCF_007991415.1</td></tr>
<tr><td>WP_116754014.1</td><td>LRR-repeats+AP-GTPase+COR*→||<-?<-Cluster1163_3clades</td><td>LRR-repeats+AP-GTPase+COR</td><td>857</td><td>-</td><td>Bacteroidetes</td><td>Flavobacterium sp. HTF</td><td>leucine-rich repeat domain-containing protein, partial [Flavobacterium sp. HTF].</td><td>GCF_003105115.1</td></tr>
<tr><td>WP_116856563.1</td><td><-Cluster1512_2clades||?→?→?→ LRR-repeats+AP-GTPase+COR+TIR*→?→?→?→||<-?<-?<-cNMPBD</td><td>LRR-repeats+AP-GTPase+COR+TIR</td><td>1040</td><td>-</td><td>Bacteroidetes</td><td>Chitinophaga silvisoli</td><td>TIR domain-containing protein [Chitinophaga silvisoli].</td><td>GCF_003412465.1</td></tr>
<tr><td>WP_116857273.1</td><td>LRR-repeats+AP-GTPase+COR+TIR→ *LRR-repeats+AP-GTPase+COR+TIR*→||<-?<-?<-SIGMA-HTH<-SIGMA-HTH||?→ LRR-repeats+AP-GTPase+COR+TIR*→||<-SIG+ABhydrolase</td><td>LRR-repeats+AP-GTPase+COR+TIR</td><td>1390</td><td>-</td><td>Bacteroidetes</td><td>Chitinophaga silvisoli</td><td>leucine-rich repeat domain-containing protein [Chitinophaga silvisoli].</td><td>GCF_003412465.1</td></tr>
<tr><td>WP_116857280.1</td><td>LRR-repeats+AP-GTPase+COR_TIR*→</td><td>LRR-repeats+AP-GTPase+COR_TIR</td><td>1378</td><td>-</td><td>Bacteroidetes</td><td>Chitinophaga silvisoli</td><td>leucine-rich repeat domain-containing protein [Chitinophaga silvisoli].</td><td>GCF_003412465.1</td></tr>
<tr><td>WP_116857281.1</td><td>LRR-repeats+AP-GTPase+COR+TIR*→</td><td>LRR-repeats+AP-GTPase+COR+TIR</td><td>1379</td><td>-</td><td>Bacteroidetes</td><td>Chitinophaga silvisoli</td><td>leucine-rich repeat domain-containing protein [Chitinophaga silvisoli].</td><td>GCF_003412465.1</td></tr>
<tr><td>WP_116982533.1</td><td>PNPase→?→?→?→?→?→ LRR-repeats+AP-GTPase+COR+TIR*→</td><td>LRR-repeats+AP-GTPase+COR+TIR</td><td>956</td><td>-</td><td>Bacteroidetes</td><td>Emticicia sp. C21</td><td>leucine-rich repeat domain-containing protein [Emticicia sp. C21].</td><td>GCF_003418935.1</td></tr>
<tr><td>WP_117208282.1</td><td><-Aminotran_1_2<-?||?→||<-?||?→?→ LRR-repeats+AP-GTPase+COR*→||<-?<-?<-?<-?<-Cluster2179_2clades<-SIG+TM+TM+TM+TM+TM+TM+TM+TM+TM+TM+TM</td><td>LRR-repeats+AP-GTPase+COR</td><td>905</td><td>-</td><td>Actinobacteria</td><td>Allorhizocola rhizosphaerae</td><td>hypothetical protein [Allorhizocola rhizosphaerae].</td><td>GCF_003426775.1</td></tr>
<tr><td>WP_117882884.1</td><td>REC→||<-?<-?<-?<-?||MORC→?→ LRR-repeats+AP-GTPase+COR*→?→ Cluster1438_2clades→</td><td>LRR-repeats+AP-GTPase+COR</td><td>797</td><td>-</td><td>Bacteroidetes</td><td>Lutibacter sp. SM1352</td><td>GTP-binding protein [Lutibacter sp. SM1352].</td><td>GCF_003449015.1</td></tr>
<tr><td>WP_118170224.1</td><td>Cluster5_5clades→ Cluster5_5clades→||<-?||LRR-repeats+AP-GTPase+COR+TM+TM*→||<-CASPASE+TM||?→||<-?<-?||?→||<-?||Cluster1756_2clades→</td><td>LRR-repeats+AP-GTPase+COR+TM+TM</td><td>962</td><td>-</td><td>Cyanobacteria</td><td>Nostoc sphaeroides</td><td>leucine-rich repeat domain-containing protein [Nostoc sphaeroides].</td><td>GCF_003443655.1</td></tr>
<tr><td>WP_118317581.1</td><td>LRR-repeats+AP-GTPase+COR+DrHyd*→</td><td>LRR-repeats+AP-GTPase+COR+DrHyd</td><td>1169</td><td>-</td><td>Bacteroidetes</td><td>Bacteroidales</td><td>MULTISPECIES: leucine-rich repeat domain-containing protein [Bacteroidales].</td><td>GCF_902364465.1</td></tr>
<tr><td>WP_118840037.1</td><td>ABC_membrane+ABC_tran→||<-Cluster2290_2clades<-Cluster2428_2clades<-?<-DSBH<-?||ClpABN-AAA+ClpABC-AAA→ LRR-repeats+AP-GTPase+COR*→||<-?<-HISKIN<-REC</td><td>LRR-repeats+AP-GTPase+COR</td><td>1051</td><td>-</td><td>Bacteroidetes</td><td>Salinibacter ruber</td><td>leucine-rich repeat domain-containing protein [Salinibacter ruber].</td><td>GCF_003491185.1</td></tr>
<tr><td>WP_119405939.1</td><td>SIG+TM+TM+TM+HISKIN→?→?→||<-?<-PSE||?→?→ TIR+LRR-repeats+AP-GTPase+TIR*→</td><td>TIR+LRR-repeats+AP-GTPase+TIR</td><td>1046</td><td>-</td><td>Bacteroidetes</td><td>Mucilaginibacter sp. HYN0043</td><td>TIR domain-containing protein [Mucilaginibacter sp. HYN0043].</td><td>GCF_003576455.2</td></tr>
<tr><td>WP_119406030.1</td><td>LRR-repeats+AP-GTPase+COR+TIR*→</td><td>LRR-repeats+AP-GTPase+COR+TIR</td><td>885</td><td>-</td><td>Bacteroidetes</td><td>Mucilaginibacter sp. HYN0043</td><td>TIR domain-containing protein [Mucilaginibacter sp. HYN0043].</td><td>GCF_003576455.2</td></tr>
<tr><td>WP_119756574.1</td><td>LRR-repeats+AP-GTPase+COR*→</td><td>LRR-repeats+AP-GTPase+COR</td><td>792</td><td>-</td><td>Bacteroidetes</td><td>Chryseolinea soli</td><td>GTP-binding protein [Chryseolinea soli].</td><td>GCF_003589925.1</td></tr>
<tr><td>WP_120086745.1</td><td>Cluster2248_2clades→?→?→ LRR-repeats+AP-GTPase+COR*→</td><td>LRR-repeats+AP-GTPase+COR</td><td>952</td><td>-</td><td>Actinobacteria</td><td>Pseudonocardiaceae bacterium YIM PH 21723</td><td>leucine-rich repeat domain-containing protein [Pseudonocardiaceae bacterium YIM PH 21723].</td><td>-</td></tr>
</table>

<table>
<thead>
<tr><th>acc</th><th>operon</th><th>architecture</th><th>len</th><th>gen.name</th><th>taxend</th><th>species</th><th>defline</th><th>gca</th></tr>
</thead>
<tbody>
<tr><td>WP_120519107.1</td><td><-SIG+TM+TM+TM<-?||Cluster1612_2clades→?→||<-?||?→?→ LRR-repeats+AP-GTPase+COR+REase7*→?→?→||<-?||ABC_membrane+ABC_tran→</td><td>LRR-repeats+AP-GTPase+COR+REase7</td><td>971</td><td>-</td><td>Bacteroidetes</td><td>Chitinophaga barathri</td><td>leucine-rich repeat domain-containing protein [Chitinophaga barathri].</td><td>GCF_003614855.1</td></tr>
<tr><td>WP_121068083.1</td><td>LRR-repeats+AP-GTPase+COR*→||<-?<-MoxR-AAA</td><td>LRR-repeats+AP-GTPase+COR</td><td>1055</td><td>-</td><td>Bacteroidetes</td><td>Maribacter vaceletii</td><td>50S ribosome-binding GTPase [Maribacter vaceletii].</td><td>GCF_003634105.1</td></tr>
<tr><td>WP_121165351.1</td><td>SIGMA-HTH→?→?→?→?→?→ LRR-repeats+AP-GTPase+COR+GT4*→?→ ParA-Soj-PloopNTPase→||<-?<-?<-?<-?||Cluster1906_2clades→</td><td>LRR-repeats+AP-GTPase+COR+GT4</td><td>1488</td><td>-</td><td>Betaproteobacteria</td><td>Nitrosomonas sp. Nm120</td><td>leucine-rich repeat domain-containing protein [Nitrosomonas sp. Nm120].</td><td>GCF_003664085.1</td></tr>
<tr><td>WP_121968578.1</td><td>LRR-repeats+AP-GTPase+COR*→</td><td>LRR-repeats+AP-GTPase+COR</td><td>985</td><td>-</td><td>Cyanobacteria</td><td>Leptolyngbya sp. BC1307</td><td>GTPase [Leptolyngbya sp. BC1307].</td><td>GCF_002286735.1</td></tr>
<tr><td>WP_122929305.1</td><td>Cluster1633_2clades→ Cluster1019_2clades→?→?→ Cluster1131_3clades→ FGS→ LRR-repeats+AP-GTPase+COR+TIR*→ TM+TM+TM→?→?→?→?→ tRNA→?→||<-Cluster1496_2clades</td><td>LRR-repeats+AP-GTPase+COR+TIR</td><td>943</td><td>-</td><td>Bacteroidetes</td><td>Runella sp. SP2</td><td>TIR domain-containing protein [Runella sp. SP2].</td><td>GCF_003711225.1</td></tr>
<tr><td>WP_123129115.1</td><td>TIR→ LRR-repeats+AP-GTPase+COR+TIR*→</td><td>LRR-repeats+AP-GTPase+COR+TIR</td><td>802</td><td>-</td><td>Bacteroidetes</td><td>Rufibacter latericius</td><td>TIR domain-containing protein [Rufibacter latericius].</td><td>GCF_003721515.1</td></tr>
<tr><td>WP_123230450.1</td><td>SIG+TM+TM+TM+TM+TM+TM+TM→||<-?||Mbetalac→ SAM-methylase→?→||<-?<-SIG+TPM_phosphatase+TM+TM+TM||LRR-repeats+AP-GTPase+COR+TM+TM*→||<-?<-?<-?<-Pro_CA||?→||<-?<-tRNA<-tRNA<-Ferredoxin-betagrasp</td><td>LRR-repeats+AP-GTPase+COR+TM+TM</td><td>875</td><td>-</td><td>Cyanobacteria</td><td>Microcystis aeruginosa</td><td>leucine-rich repeat domain-containing protein [Microcystis aeruginosa].</td><td>GCF_003730145.1</td></tr>
<tr><td>WP_123277931.1</td><td>LRR-repeats+AP-GTPase+COR*→?→||<-?<-?<-RFC-AAA</td><td>LRR-repeats+AP-GTPase+COR</td><td>891</td><td>-</td><td>Bacteroidetes</td><td>Chryseobacterium cucumeris</td><td>hypothetical protein [Chryseobacterium cucumeris].</td><td>GCF_003729985.1</td></tr>
<tr><td>WP_123852487.1</td><td>LRR-repeats+AP-GTPase+COR+TIR*→</td><td>LRR-repeats+AP-GTPase+COR+TIR</td><td>1174</td><td>-</td><td>Bacteroidetes</td><td>Chryseobacterium shandongense</td><td>leucine-rich repeat domain-containing protein [Chryseobacterium shandongense].</td><td>GCF_003815615.1</td></tr>
<tr><td>WP_123904443.1</td><td>LRR-repeats+AP-GTPase+COR+TIR*→ TIR→</td><td>LRR-repeats+AP-GTPase+COR+TIR</td><td>986</td><td>-</td><td>Bacteroidetes</td><td>Chryseobacterium haifense</td><td>leucine-rich repeat domain-containing protein [Chryseobacterium haifense].</td><td>GCF_003816015.1</td></tr>
<tr><td>WP_124155292.1</td><td>LRR-repeats+AP-GTPase+COR*→</td><td>LRR-repeats+AP-GTPase+COR</td><td>723</td><td>-</td><td>Cyanobacteria</td><td>Okeania hirsuta</td><td>leucine-rich repeat domain-containing protein [Okeania hirsuta].</td><td>GCF_003838195.1</td></tr>
<tr><td>WP_124898852.1</td><td>LRR-repeats+AP-GTPase+COR*→</td><td>LRR-repeats+AP-GTPase+COR</td><td>925</td><td>-</td><td>Bacteroidetes</td><td>Flavobacterium viscosus</td><td>GTP-binding protein [Flavobacterium viscosus].</td><td>GCF_003858535.1</td></tr>
<tr><td>WP_125246445.1</td><td>TM→ LRR-repeats+AP-GTPase+COR*→</td><td>LRR-repeats+AP-GTPase+COR</td><td>1040</td><td>-</td><td>Actinobacteria</td><td>Glycomyces terrestris</td><td>hypothetical protein [Glycomyces terrestris].</td><td>GCF_003933745.1</td></tr>
<tr><td>WP_125249833.1</td><td><-Cluster1244_2clades||?→?→?→||<-?<-ABC-ATPase||?→ LRR-repeats+AP-GTPase+COR*→?→||<-?<-REC<-HISKIN</td><td>LRR-repeats+AP-GTPase+COR</td><td>1253</td><td>-</td><td>Actinobacteria</td><td>Glycomyces terrestris</td><td>leucine-rich repeat domain-containing protein [Glycomyces terrestris].</td><td>GCF_003933745.1</td></tr>
<tr><td>WP_126388556.1</td><td>Cluster54_3clades→?→ RelE-ParE→ LRR-repeats+AP-GTPase+COR*→?→?→ Cluster1841_2clades→</td><td>LRR-repeats+AP-GTPase+COR</td><td>906</td><td>-</td><td>Cyanobacteria</td><td>Pseudanabaena sp. ABRG5-3</td><td>leucine-rich repeat domain-containing protein [Pseudanabaena sp. ABRG5-3].</td><td>GCF_003967015.1</td></tr>
<tr><td>WP_126389050.1</td><td>LRR-repeats→?→?→ LRR-repeats+AP-GTPase+COR+CASPASE*→||<-?||TPR+nSTAND1+BetaPropeller_WD40+BetaPropeller_WD40→?→||<-?||Pentapeptide-repeats→</td><td>LRR-repeats+AP-GTPase+COR+CASPASE</td><td>1062</td><td>-</td><td>Cyanobacteria</td><td>Pseudanabaena sp. ABRG5-3</td><td>leucine-rich repeat domain-containing protein [Pseudanabaena sp. ABRG5-3].</td><td>GCF_003967015.1</td></tr>
<tr><td>WP_126391374.1</td><td>Pentapeptide-repeats→||<-Cluster5_5clades||Cluster1009_2clades→ Cluster53_2clades→?→ LRR-repeats+AP-GTPase+COR+TM+TM*→||<-?<-?<-?<-?<-?||REC→</td><td>LRR-repeats+AP-GTPase+COR+TM+TM</td><td>865</td><td>-</td><td>Cyanobacteria</td><td>Pseudanabaena sp. ABRG5-3</td><td>GTPase [Pseudanabaena sp. ABRG5-3].</td><td>GCF_003967015.1</td></tr>
<tr><td>WP_126618238.1</td><td><-RADICAL-SAM<-?||SIGMA-HTH→?→||<-?||?→?→ LRR-repeats+AP-GTPase+COR+TIR*→?→||<-?||SWACOS→||<-Cluster409_3clades||SIG+RnaseT2→?→ Cluster995_2clades→</td><td>LRR-repeats+AP-GTPase+COR+TIR</td><td>913</td><td>-</td><td>Alphaproteobacteria</td><td>Azospirillum sp. L-25-5w-1</td><td>leucine-rich repeat domain-containing protein [Azospirillum sp. L-25-5w-1].</td><td>GCF_003966125.1</td></tr>
<tr><td>WP_127052641.1</td><td>LRR-repeats+AP-GTPase+COR*→</td><td>LRR-repeats+AP-GTPase+COR</td><td>1083</td><td>-</td><td>Cyanobacteria</td><td>Trichormus variabilis</td><td>leucine-rich repeat domain-containing protein [Trichormus variabilis].</td><td>GCF_003991935.1</td></tr>
<tr><td>WP_127087241.1</td><td>LRR-repeats+AP-GTPase+COR+EAD1*→</td><td>LRR-repeats+AP-GTPase+COR+EAD1</td><td>929</td><td>-</td><td>Cyanobacteria</td><td>Calothrix desertica</td><td>GTPase [Calothrix desertica].</td><td>GCF_003991905.1</td></tr>
<tr><td>WP_127498999.1</td><td>LRR-repeats+AP-GTPase+COR+TIR*→?→?→||<-?<-?||?→||<-Cluster1023_3clades</td><td>LRR-repeats+AP-GTPase+COR+TIR</td><td>999</td><td>-</td><td>Actinobacteria</td><td>Actinoplanes sp. LAM7112</td><td>TIR domain-containing protein [Actinoplanes sp. LAM7112].</td><td>GCF_003999975.1</td></tr>
</tbody>
</table>

<table>
<thead>
<tr><th>acc</th><th>operon</th><th>architecture</th><th>len</th><th>gen.name</th><th>taxend</th><th>species</th><th>defline</th><th>gca</th></tr>
</thead>
<tbody>
<tr><td>WP_127504182.1</td><td>LRR-repeats+AP-GTPase+COR*→?→ PSE→||<-?||SIGMA-HTH→</td><td>LRR-repeats+AP-GTPase+COR</td><td>1080</td><td>-</td><td>Actinobacteria</td><td>Actinoplanes sp. LAM7112</td><td>hypothetical protein [Actinoplanes sp. LAM7112].</td><td>GCF_003999975.1</td></tr>
<tr><td>WP_127505072.1</td><td>Cluster1009_2clades→||<-Cluster1999_2clades<-Cluster2165_2clades<-?<-?<-?||?→ LRR-repeats+AP-GTPase+COR*→</td><td>LRR-repeats+AP-GTPase+COR</td><td>1680</td><td>-</td><td>Actinobacteria</td><td>Actinoplanes sp. LAM7112</td><td>leucine-rich repeat domain-containing protein [Actinoplanes sp. LAM7112].</td><td>GCF_003999975.1</td></tr>
<tr><td>WP_129758774.1</td><td>MACRODOMAIN+LRR-repeats+AP-GTPase+COR+TIR*→||<-tRNA<-?<-RNASEII</td><td>MACRODOMAIN+LRR-repeats+AP-GTPase+COR+TIR</td><td>1357</td><td>-</td><td>Bacteroidetes</td><td>Flavobacterium sp. TCH3-2</td><td>leucine-rich repeat domain-containing protein [Flavobacterium sp. TCH3-2].</td><td>GCF_004151275.1</td></tr>
<tr><td>WP_129778498.1</td><td>Cluster1906_2clades→?→||<-?<-?<-?||?→ LRR-repeats+AP-GTPase+COR*→||<-?<-?||?→?→?→||<-tRNA||SIGMA-HTH→</td><td>LRR-repeats+AP-GTPase+COR</td><td>1040</td><td>-</td><td>Gammaproteobacteria</td><td>Steroidobacter soli</td><td>hypothetical protein [Steroidobacter soli].</td><td>GCF_004138485.1</td></tr>
<tr><td>WP_130757642.1</td><td>SIG+TM+TM+TM+TM+TM+TM+TM→||<-?||Mbetalac→ SAM-methylase→?→||<-?<-SIG+TPM_phosphatase+TM+TM+TM||LRR-repeats+AP-GTPase+COR+TM+TM*→||<-?<-?<-?<-Pro_CA||?→||<-?<-tRNA<-tRNA<-Ferredoxin-betagrasp</td><td>LRR-repeats+AP-GTPase+COR+TM+TM</td><td>851</td><td>-</td><td>Cyanobacteria</td><td>Microcystis aeruginosa</td><td>leucine-rich repeat domain-containing protein [Microcystis aeruginosa].</td><td>GCF_004305995.1</td></tr>
<tr><td>WP_130938329.1</td><td>AraC-HTH→?→ SIG+TM+TM+TM→?→?→?→?→ LRR-repeats+AP-GTPase+COR*→?→ ABC_membrane+ABC_tran→||<-?<-?<-?<-?<-Trichomonas-DAM</td><td>LRR-repeats+AP-GTPase+COR</td><td>925</td><td>-</td><td>Bacteroidetes</td><td>Hyunsoonleella pacifica</td><td>leucine-rich repeat domain-containing protein [Hyunsoonleella pacifica].</td><td>GCF_014636335.1</td></tr>
<tr><td>WP_131118878.1</td><td>HNH→?→||<-?<-PIN<-?||LRR-repeats+AP-GTPase+COR+TIR*→||<-Cluster1077_2clades</td><td>LRR-repeats+AP-GTPase+COR+TIR</td><td>912</td><td>-</td><td>Cyanobacteria</td><td>Westiellopsis prolifica</td><td>leucine-rich repeat domain-containing protein, partial [Westiellopsis prolifica].</td><td>GCF_004323185.1</td></tr>
<tr><td>WP_131118899.1</td><td>Cluster1069_2clades→?→?→||<-?||?→?→ PSE→ LRR-repeats→ LRR-repeats+AP-GTPase+COR+DUF4404*→||<-?||?→?→?→?→ Mbetalac→</td><td>LRR-repeats+AP-GTPase+COR+DUF4404</td><td>695</td><td>-</td><td>Cyanobacteria</td><td>Westiellopsis prolifica</td><td>GTP-binding protein, partial [Westiellopsis prolifica].</td><td>GCF_004323185.1</td></tr>
<tr><td>WP_132561933.1</td><td><-Classical-AAA||?→ LRR-repeats+AP-GTPase+COR+TIR*→?→||<-?<-tRNA||?→||<-tRNA<-?||?→||<-?||ABC-ATPase→</td><td>LRR-repeats+AP-GTPase+COR+TIR</td><td>1348</td><td>-</td><td>Bacteroidetes</td><td>Niastella sp. CF465</td><td>TIR domain-containing protein [Niastella sp. CF465].</td><td>GCF_004341975.1</td></tr>
<tr><td>WP_132562618.1</td><td>LRR-repeats+AP-GTPase+COR*→</td><td>LRR-repeats+AP-GTPase+COR</td><td>1028</td><td>-</td><td>Bacteroidetes</td><td>Niastella sp. CF465</td><td>GTP-binding protein [Niastella sp. CF465].</td><td>GCF_004341975.1</td></tr>
<tr><td>WP_132566699.1</td><td>SIGMA-HTH→ LRR-repeats+AP-GTPase+COR*→</td><td>LRR-repeats+AP-GTPase+COR</td><td>890</td><td>-</td><td>Bacteroidetes</td><td>Niastella sp. CF465</td><td>leucine-rich repeat domain-containing protein [Niastella sp. CF465].</td><td>GCF_004341975.1</td></tr>
<tr><td>WP_132835660.1</td><td>LRR-repeats+AP-GTPase+COR*→</td><td>LRR-repeats+AP-GTPase+COR</td><td>819</td><td>-</td><td>Actinobacteria</td><td>Streptomyces sp. BK205</td><td>hypothetical protein [Streptomyces sp. BK205].</td><td>GCF_004341025.1</td></tr>
<tr><td>WP_133504241.1</td><td>Cluster2322_2clades→||<-?||PSE→||<-?||LRR-repeats+AP-GTPase+COR+TIR*→</td><td>LRR-repeats+AP-GTPase+COR+TIR</td><td>764</td><td>-</td><td>Gammaproteobacteria</td><td>Marinomonas balearica</td><td>TIR domain-containing protein [Marinomonas balearica].</td><td>GCF_004362145.1</td></tr>
<tr><td>WP_133511071.1</td><td>Cluster2079_2clades→||<-?||?→||<-SIG+TM+TM+TM+TM+TM+TM+TM+TM+TM+TM+TM<-SIG+TM||PSE→ LRR-repeats+AP-GTPase+COR+TIR*→</td><td>LRR-repeats+AP-GTPase+COR+TIR</td><td>986</td><td>-</td><td>Gammaproteobacteria</td><td>Candidatus Thiosymbion oneisti</td><td>TIR domain-containing protein [Candidatus Thiosymbion oneisti].</td><td>GCF_011392785.1</td></tr>
<tr><td>WP_133512972.1</td><td><-PIN<-?||ABC-ATPase→?→?→?→?→ LRR-repeats+AP-GTPase+COR+DrHyd*→</td><td>LRR-repeats+AP-GTPase+COR+DrHyd</td><td>998</td><td>-</td><td>Gammaproteobacteria</td><td>Candidatus Thiosymbion oneisti</td><td>hypothetical protein [Candidatus Thiosymbion oneisti].</td><td>GCF_011392785.1</td></tr>
<tr><td>WP_133793944.1</td><td>LRR-repeats→ LRR-repeats+AP-GTPase+COR+DUF4404*→</td><td>LRR-repeats+AP-GTPase+COR+DUF4404</td><td>768</td><td>-</td><td>Verrucomicrobia</td><td>Prosthecobacter fusiformis</td><td>GTP-binding protein [Prosthecobacter fusiformis].</td><td>GCF_004364345.1</td></tr>
<tr><td>WP_133884327.1</td><td>LRR-repeats+AP-GTPase+COR*→</td><td>LRR-repeats+AP-GTPase+COR</td><td>996</td><td>-</td><td>Actinobacteria</td><td>unclassified Glycomyces</td><td>MULTISPECIES: leucine-rich repeat domain-containing protein [unclassified Glycomyces].</td><td>GCF_000719515.1</td></tr>
<tr><td>WP_133903373.1</td><td>Cluster2083_2clades→||<-?||?→||<-?||?→||<-?||LRR-repeats→ LRR-repeats+AP-GTPase+COR*→</td><td>LRR-repeats+AP-GTPase+COR</td><td>1284</td><td>-</td><td>Actinobacteria</td><td>Actinophytocola oryzae</td><td>leucine-rich repeat domain-containing protein [Actinophytocola oryzae].</td><td>GCF_004364325.1</td></tr>
<tr><td>WP_135106965.1</td><td>LRR-repeats+AP-GTPase+COR+DUF4404*→||<-?||?→?→?→?→?→ Mbetalac→</td><td>LRR-repeats+AP-GTPase+COR+DUF4404</td><td>967</td><td>-</td><td>Cyanobacteria</td><td>Mastigocladus laminosus</td><td>leucine-rich repeat domain-containing protein [Mastigocladus laminosus].</td><td>GCF_001990805.2</td></tr>
<tr><td>WP_135107787.1</td><td><-PIN<-?||LRR-repeats+AP-GTPase+COR+TIR*→||<-Cluster1077_2clades</td><td>LRR-repeats+AP-GTPase+COR+TIR</td><td>1167</td><td>-</td><td>Cyanobacteria</td><td>Mastigocladus laminosus</td><td>leucine-rich repeat domain-containing protein [Mastigocladus laminosus].</td><td>GCF_001990805.2</td></tr>
<tr><td>WP_135191212.1</td><td>LRR-repeats+AP-GTPase+COR+DrHyd*→?→?→?→||<-?<-SIG+Phytase-like</td><td>LRR-repeats+AP-GTPase+COR+DrHyd</td><td>1147</td><td>-</td><td>Betaproteobacteria</td><td>Massilia sp. ONC3</td><td>hypothetical protein [Massilia sp. ONC3].</td><td>GCF_004614195.1</td></tr>
</tbody>
</table>

<table>
<tr><th>acc</th><th>operon</th><th>architecture</th><th>len</th><th>gen.name</th><th>taxend</th><th>species</th><th>defline</th><th>gca</th></tr>
<tr><td>WP_136836096.1</td><td>LRR-repeats+AP-GTPase+COR+TIR*→</td><td>LRR-repeats+AP-GTPase+COR+TIR</td><td>1357</td><td>-</td><td>Bacteroidetes</td><td>Pedobacter sp. RP-3-15</td><td>leucine-rich repeat domain-containing protein [Pedobacter sp. RP-3-15].</td><td>GCF_005116445.1</td></tr>
<tr><td>WP_136929189.1</td><td>SIG+TM+TM+TM+TM+TM+TM+TM+TM+TM→ LRR-repeats+AP-GTPase+COR*→</td><td>LRR-repeats+AP-GTPase+COR</td><td>961</td><td>-</td><td>Deltaproteobacteria</td><td>Polyangium fumosum</td><td>GTP-binding protein [Polyangium fumosum].</td><td>GCF_005144585.1</td></tr>
<tr><td>WP_137669597.1</td><td>LRR-repeats+AP-GTPase+COR*→</td><td>LRR-repeats+AP-GTPase+COR</td><td>272</td><td>-</td><td>Cyanobacteria</td><td>Sphaerospermopsis reniformis</td><td>GTP-binding protein, partial [Sphaerospermopsis reniformis].</td><td>GCF_005402885.1</td></tr>
<tr><td>WP_137686407.1</td><td>LRR-repeats+AP-GTPase+COR*→</td><td>LRR-repeats+AP-GTPase+COR</td><td>1152</td><td>-</td><td>Chloroflexi</td><td>Thermosporothrix hazakensis</td><td>leucine-rich repeat domain-containing protein [Thermosporothrix hazakensis].</td><td>GCF_005402645.1</td></tr>
<tr><td>WP_137908463.1</td><td>LRR-repeats+AP-GTPase+COR+TM+TM*→||<-PSE||?→?→||<-PSE<-?||CCTBP→</td><td>LRR-repeats+AP-GTPase+COR+TM+TM</td><td>875</td><td>-</td><td>Cyanobacteria</td><td>Dolichospermum planctonicum</td><td>leucine-rich repeat domain-containing protein [Dolichospermum planctonicum].</td><td>GCF_005402965.1</td></tr>
<tr><td>WP_138500060.1</td><td><-Pkinase+Pentapeptide<-?<-?||PSE→||<-?||LRR-repeats+AP-GTPase+COR+TIR*→||<-?<-?<-PSE||?→||<-?||?→?→||<-NUDIX</td><td>LRR-repeats+AP-GTPase+COR+TIR</td><td>920</td><td>-</td><td>Cyanobacteria</td><td>Nostoc sp. PA-18-2419</td><td>leucine-rich repeat domain-containing protein [Nostoc sp. PA-18-2419].</td><td>GCF_005869855.1</td></tr>
<tr><td>WP_138503290.1</td><td><-HhH-RADC+JAB||?→?→||<-?||LRR-repeats→ LRR-repeats+AP-GTPase+COR*→||<-?||?→?→||<-PSE<-?||?→?→ PEP-utilisers-C→</td><td>LRR-repeats+AP-GTPase+COR</td><td>1100</td><td>-</td><td>Cyanobacteria</td><td>Nostoc sp. PA-18-2419</td><td>leucine-rich repeat domain-containing protein [Nostoc sp. PA-18-2419].</td><td>GCF_005869855.1</td></tr>
<tr><td>WP_138504428.1</td><td><-ABC-ATPase<-?<-?<-?||?→ TM+TM+TM→ LRR-repeats+AP-GTPase+COR+TIR*→ PSE→||<-PSE<-?||PSE→?→?→||<-SIG+TM+TM+TM+TM+TM</td><td>LRR-repeats+AP-GTPase+COR+TIR</td><td>1019</td><td>-</td><td>Bacteroidetes</td><td>Spirosoma lacussanchae</td><td>TIR domain-containing protein [Spirosoma lacussanchae].</td><td>GCF_005870035.1</td></tr>
<tr><td>WP_138544765.1</td><td>LRR-repeats+AP-GTPase+COR+TIR*→||<-?<-?<-TM</td><td>LRR-repeats+AP-GTPase+COR+TIR</td><td>1128</td><td>-</td><td>Gammaproteobacteria</td><td>Pseudoalteromonas rubra</td><td>leucine-rich repeat domain-containing protein [Pseudoalteromonas rubra].</td><td>GCF_005887115.1</td></tr>
<tr><td>WP_138544832.1</td><td><-REC<-?||?→ LRR-repeats+AP-GTPase+COR+TIR*→</td><td>LRR-repeats+AP-GTPase+COR+TIR</td><td>1256</td><td>-</td><td>Gammaproteobacteria</td><td>Pseudoalteromonas rubra</td><td>TIR domain-containing protein [Pseudoalteromonas rubra].</td><td>GCF_005887115.1</td></tr>
<tr><td>WP_138553289.1</td><td><-REC<-?||?→ LRR-repeats+AP-GTPase+COR+TIR*→</td><td>LRR-repeats+AP-GTPase+COR+TIR</td><td>1255</td><td>-</td><td>Gammaproteobacteria</td><td>Pseudoalteromonas rubra</td><td>TIR domain-containing protein [Pseudoalteromonas rubra].</td><td>GCF_005887405.1</td></tr>
<tr><td>WP_139281522.1</td><td>LRR-repeats+AP-GTPase+COR+TIR*→</td><td>LRR-repeats+AP-GTPase+COR+TIR</td><td>850</td><td>-</td><td>Gammaproteobacteria</td><td>Vibrio aerogenes</td><td>leucine-rich repeat domain-containing protein, partial [Vibrio aerogenes].</td><td>GCF_900130105.1</td></tr>
<tr><td>WP_139999876.1</td><td>TM+TM+TM→ LRR-repeats+AP-GTPase+COR+TIR*→</td><td>LRR-repeats+AP-GTPase+COR+TIR</td><td>1134</td><td>-</td><td>Bacteroidetes</td><td>Flavobacterium microcysteis</td><td>leucine-rich repeat domain-containing protein [Flavobacterium microcysteis].</td><td>GCF_006385255.1</td></tr>
<tr><td>WP_140058884.1</td><td>LRR-repeats+AP-GTPase+COR+TIR*→</td><td>LRR-repeats+AP-GTPase+COR+TIR</td><td>1265</td><td>-</td><td>Gammaproteobacteria</td><td>Vibrio parahaemolyticus</td><td>TIR domain-containing protein [Vibrio parahaemolyticus].</td><td>GCF_006376835.1</td></tr>
<tr><td>WP_140063885.1</td><td>LRR-repeats+AP-GTPase+COR*→</td><td>LRR-repeats+AP-GTPase+COR</td><td>553</td><td>-</td><td>Gammaproteobacteria</td><td>Vibrio parahaemolyticus</td><td>hypothetical protein, partial [Vibrio parahaemolyticus].</td><td>GCF_006377025.1</td></tr>
<tr><td>WP_140305225.1</td><td>CI→ LRR-repeats+AP-GTPase+COR*→</td><td>LRR-repeats+AP-GTPase+COR</td><td>1113</td><td>-</td><td>Gammaproteobacteria</td><td>Vibrio parahaemolyticus</td><td>GTP-binding protein [Vibrio parahaemolyticus].</td><td>GCF_006374225.1</td></tr>
<tr><td>WP_140384737.1</td><td>LRR-repeats+AP-GTPase+COR+TIR*→</td><td>LRR-repeats+AP-GTPase+COR+TIR</td><td>829</td><td>-</td><td>Bacteroidetes</td><td>Elizabethkingia anophelis</td><td>TIR domain-containing protein, partial [Elizabethkingia anophelis].</td><td>GCF_900156995.1</td></tr>
<tr><td>WP_141211867.1</td><td>LRR-repeats+AP-GTPase+COR+CASPASE*→||<-?||Cluster1731_2clades→?→||<-Cluster2482_2clades</td><td>LRR-repeats+AP-GTPase+COR+CASPASE</td><td>835</td><td>-</td><td>Cyanobacteria</td><td>Pseudanabaena sp. SR411</td><td>leucine-rich repeat domain-containing protein, partial [Pseudanabaena sp. SR411].</td><td>GCF_002251945.1</td></tr>
<tr><td>WP_141954907.1</td><td>LRR-repeats+AP-GTPase+COR*→</td><td>LRR-repeats+AP-GTPase+COR</td><td>1074</td><td>-</td><td>Actinobacteria</td><td>Actinoallomurus bryophytorum</td><td>hypothetical protein [Actinoallomurus bryophytorum].</td><td>GCF_006716425.1</td></tr>
<tr><td>WP_142449077.1</td><td>LRR-repeats+AP-GTPase+COR+TIR*→ TIR→</td><td>LRR-repeats+AP-GTPase+COR+TIR</td><td>700</td><td>-</td><td>Bacteroidetes</td><td>Flavobacterium resistens</td><td>leucine-rich repeat domain-containing protein [Flavobacterium resistens].</td><td>GCF_900182645.1</td></tr>
</table>

<table>
<tr><th>acc</th><th>operon</th><th>architecture</th><th>len</th><th>gen.name</th><th>taxend</th><th>species</th><th>defline</th><th>gca</th></tr>
<tr><td>WP_142602418.1</td><td>LRR-repeats+AP-GTPase+COR*→ PSE→ PSE→||<-?||?→||<-?<-?<-Cluster2042_2clades</td><td>LRR-repeats+AP-GTPase+COR</td><td>718</td><td>-</td><td>Cyanobacteria</td><td>Pseudanabaena sp. UWO310</td><td>leucine-rich repeat domain-containing protein [Pseudanabaena sp. UWO310].</td><td>GCF_006861605.1</td></tr>
<tr><td>WP_142653260.1</td><td>LRR-repeats→ LRR-repeats+AP-GTPase+COR+CASPASE*→?→?→||<-?<-?<-REC</td><td>LRR-repeats+AP-GTPase+COR+CASPASE</td><td>1028</td><td>-</td><td>Cyanobacteria</td><td>Pseudanabaena sp. UWO311</td><td>leucine-rich repeat domain-containing protein [Pseudanabaena sp. UWO311].</td><td>GCF_006937785.1</td></tr>
<tr><td>WP_142654307.1</td><td>Cluster54_3clades→?→?→?→ LRR-repeats+AP-GTPase+COR+CASPASE*→?→||<-?<-TPR-repeats+CASPASE<-TPR-repeats+CASPASE</td><td>LRR-repeats+AP-GTPase+COR+CASPASE</td><td>1096</td><td>-</td><td>Cyanobacteria</td><td>Pseudanabaena sp. UWO311</td><td>leucine-rich repeat domain-containing protein [Pseudanabaena sp. UWO311].</td><td>GCF_006937785.1</td></tr>
<tr><td>WP_142657357.1</td><td>ABC-ATPase→?→?→?→?→ Cluster1009_2clades→ Cluster53_2clades→ LRR-repeats+AP-GTPase+COR*→||<-?||?→?→?→ SIG+TM+TM+TM+TM+TM→</td><td>LRR-repeats+AP-GTPase+COR</td><td>992</td><td>-</td><td>Cyanobacteria</td><td>Pseudanabaena sp. UWO311</td><td>leucine-rich repeat domain-containing protein [Pseudanabaena sp. UWO311].</td><td>GCF_006937785.1</td></tr>
<tr><td>WP_142891598.1</td><td>LRR-repeats+AP-GTPase+COR+DrHyd*→</td><td>LRR-repeats+AP-GTPase+COR+DrHyd</td><td>1516</td><td>-</td><td>Gammaproteobacteria</td><td>Aliikangiella sp. M105</td><td>hypothetical protein [Aliikangiella sp. M105].</td><td>GCF_007004725.1</td></tr>
<tr><td>WP_143234350.1</td><td><-REC<-?||?→ LRR-repeats+AP-GTPase+COR+TIR*→</td><td>LRR-repeats+AP-GTPase+COR+TIR</td><td>888</td><td>-</td><td>Actinobacteria</td><td>Actinoplanes atraurantiacus</td><td>GTPase [Actinoplanes atraurantiacus].</td><td>GCF_900215205.1</td></tr>
<tr><td>WP_143288104.1</td><td>LRR-repeats+AP-GTPase+COR*→||<-SIG+TM+TM+TM+TM</td><td>LRR-repeats+AP-GTPase+COR</td><td>716</td><td>-</td><td>Cyanobacteria</td><td>Calothrix rhizosoleniae</td><td>GTP-binding protein, partial [Calothrix rhizosoleniae].</td><td>GCF_900185595.1</td></tr>
<tr><td>WP_143288141.1</td><td>LRR-repeats+AP-GTPase+COR+TM+TM*→</td><td>LRR-repeats+AP-GTPase+COR+TM+TM</td><td>777</td><td>-</td><td>Cyanobacteria</td><td>Calothrix rhizosoleniae</td><td>leucine-rich repeat domain-containing protein, partial [Calothrix rhizosoleniae].</td><td>GCF_900185595.1</td></tr>
<tr><td>WP_143301191.1</td><td>LRR-repeats+AP-GTPase+COR+DUF4404*→?→||<-RNA-Helicase</td><td>LRR-repeats+AP-GTPase+COR+DUF4404</td><td>650</td><td>-</td><td>Nitrospinae/Tectomicrobia group</td><td>Candidatus Entotheonella palauensis</td><td>GTP-binding protein, partial [Candidatus Entotheonella palauensis].</td><td>GCF_900079105.1</td></tr>
<tr><td>WP_143674573.1</td><td>LRR-repeats+AP-GTPase+COR*→</td><td>LRR-repeats+AP-GTPase+COR</td><td>802</td><td>-</td><td>Actinobacteria</td><td>Streptomyces sp. HG99</td><td>hypothetical protein, partial [Streptomyces sp. HG99].</td><td>GCF_002742045.1</td></tr>
<tr><td>WP_143814854.1</td><td>SIGMA-HTH→?→||<-?<-?<-?<-?<-?||LRR-repeats+AP-GTPase+COR*→</td><td>LRR-repeats+AP-GTPase+COR</td><td>861</td><td>-</td><td>Alphaproteobacteria</td><td>Magnetofaba australis</td><td>hypothetical protein [Magnetofaba australis].</td><td>GCF_002109495.1</td></tr>
<tr><td>WP_144282986.1</td><td>LRR-repeats+AP-GTPase+COR+TIR*→?→?→?→||<-?<-?<-?<-HISKIN</td><td>LRR-repeats+AP-GTPase+COR+TIR</td><td>1293</td><td>-</td><td>Bacteroidetes</td><td>Chryseobacterium echinoideorum</td><td>leucine-rich repeat domain-containing protein [Chryseobacterium echinoideorum].</td><td>GCF_007474535.1</td></tr>
<tr><td>WP_144984133.1</td><td>TIR+APATPase+TPR+TPR+TPR→ LRR-repeats+AP-GTPase+COR+TM+TM+TM+TM*→</td><td>LRR-repeats+AP-GTPase+COR+TM+TM+TM+TM</td><td>1242</td><td>-</td><td>Planctomycetes</td><td>Gimesia aquarii</td><td>leucine-rich repeat domain-containing protein [Gimesia aquarii].</td><td>GCF_007748195.1</td></tr>
<tr><td>WP_145277465.1</td><td>LRR-repeats+AP-GTPase+COR+DUF4404*→</td><td>LRR-repeats+AP-GTPase+COR+DUF4404</td><td>1013</td><td>-</td><td>Planctomycetes</td><td>Planctomycetes bacterium ElP</td><td>leucine-rich repeat domain-containing protein [Planctomycetes bacterium ElP].</td><td>-</td></tr>
<tr><td>WP_145532816.1</td><td>CI→ LRR-repeats+AP-GTPase+COR*→</td><td>LRR-repeats+AP-GTPase+COR</td><td>1113</td><td>-</td><td>Gammaproteobacteria</td><td>Vibrio sp. ES.045</td><td>GTP-binding protein [Vibrio sp. ES.045].</td><td>GCF_007827265.1</td></tr>
<tr><td>WP_145988187.1</td><td><-Classical-AAA<-Trichomonas-DAM||LRR-repeats+AP-GTPase*→||<-?<-Cluster1368_2clades<-Cluster2311_2clades</td><td>LRR-repeats+AP-GTPase</td><td>621</td><td>-</td><td>Alphaproteobacteria</td><td>Blastochloris tepida</td><td>leucine-rich repeat domain-containing protein [Blastochloris tepida].</td><td>GCF_003966715.1</td></tr>
<tr><td>WP_146033967.1</td><td>LRR-repeats+AP-GTPase+COR+DUF4404*→</td><td>LRR-repeats+AP-GTPase+COR+DUF4404</td><td>700</td><td>-</td><td>Cyanobacteria</td><td>Nostoc cycadae</td><td>GTP-binding protein, partial [Nostoc cycadae].</td><td>GCF_002897135.1</td></tr>
<tr><td>WP_146129808.1</td><td>LRR-repeats→ LRR-repeats+AP-GTPase+COR*→</td><td>LRR-repeats+AP-GTPase+COR</td><td>715</td><td>-</td><td>Bacteroidetes</td><td>Muricauda pacifica</td><td>hypothetical protein [Muricauda pacifica].</td><td>GCF_003001695.1</td></tr>
<tr><td>WP_146156812.1</td><td>TPR-repeats→?→ SIG+Alba→||<-?<-?<-?<-?||LRR-repeats+AP-GTPase+COR*→</td><td>LRR-repeats+AP-GTPase+COR</td><td>857</td><td>-</td><td>Bacteroidetes</td><td>Sphingobacteriales bacterium UPWRP_1</td><td>GTP-binding protein [Sphingobacteriales bacterium UPWRP_1].</td><td>-</td></tr>
<tr><td>WP_146201360.1</td><td>LRR-repeats+AP-GTPase+COR+TIR*→</td><td>LRR-repeats+AP-GTPase+COR+TIR</td><td>780</td><td>-</td><td>Gammaproteobacteria</td><td>Leucothrix arctica</td><td>TIR domain-containing protein [Leucothrix arctica].</td><td>GCF_003172895.1</td></tr>
</table>

<table>
<tr><th>acc</th><th>operon</th><th>architecture</th><th>len</th><th>gen.name</th><th>taxend</th><th>species</th><th>defline</th><th>gca</th></tr>
<tr><td>WP_146215520.1</td><td>LRR-repeats+AP-GTPase+COR+TM+TM+TM*→||<-PSE<-?<-?<-?<-?<-Cluster1270_2clades</td><td>LRR-repeats+AP-GTPase+COR+TM+TM+TM</td><td>1504</td><td>-</td><td>Alphaproteobacteria</td><td>Hoeflea marina</td><td>hypothetical protein [Hoeflea marina].</td><td>GCF_003182275.1</td></tr>
<tr><td>WP_146505417.1</td><td>LRR-repeats→ LRR-repeats+AP-GTPase+COR*→</td><td>LRR-repeats+AP-GTPase+COR</td><td>795</td><td>-</td><td>Chloroflexi</td><td>Thermosporothrix hazakensis</td><td>GTPase [Thermosporothrix hazakensis].</td><td>GCF_003253565.1</td></tr>
<tr><td>WP_146848114.1</td><td>LRR-repeats+AP-GTPase+COR*→</td><td>LRR-repeats+AP-GTPase+COR</td><td>846</td><td>-</td><td>Bacteroidetes</td><td>Aequorivita antarctica</td><td>hypothetical protein [Aequorivita antarctica].</td><td>GCF_007997155.1</td></tr>
<tr><td>WP_146849445.1</td><td>SIG+TM→?→ LRR-repeats+AP-GTPase+COR*→</td><td>LRR-repeats+AP-GTPase+COR</td><td>1099</td><td>-</td><td>Verrucomicrobia</td><td>Brevifollis gellanilyticus</td><td>leucine-rich repeat domain-containing protein [Brevifollis gellanilyticus].</td><td>GCF_007992435.1</td></tr>
<tr><td>WP_146856190.1</td><td>LRR-repeats+AP-GTPase+COR+DUF4404*→</td><td>LRR-repeats+AP-GTPase+COR+DUF4404</td><td>944</td><td>-</td><td>Verrucomicrobia</td><td>Brevifollis gellanilyticus</td><td>leucine-rich repeat domain-containing protein [Brevifollis gellanilyticus].</td><td>GCF_007992435.1</td></tr>
<tr><td>WP_147290429.1</td><td><-Aminotran_1_2<-?||?→?→||<-Arginase||?→
LRR-repeats+AP-GTPase+COR+TIR*→||<-?<-PSE<-?<-?<-?||?→||<-Cluster1063_3clades<-Cluster1067_3clades</td><td>LRR-repeats+AP-GTPase+COR+TIR</td><td>1134</td><td>-</td><td>Alphaproteobacteria</td><td>Pannonibacter phragmitetus</td><td>leucine-rich repeat domain-containing protein [Pannonibacter phragmitetus].</td><td>GCF_000382365.1</td></tr>
<tr><td>WP_147864606.1</td><td>LRR-repeats+AP-GTPase+COR+TIR*→</td><td>LRR-repeats+AP-GTPase+COR+TIR</td><td>1297</td><td>-</td><td>Bacteroidetes</td><td>Mesonia sp. K4-1</td><td>leucine-rich repeat domain-containing protein [Mesonia sp. K4-1].</td><td>GCF_008017825.1</td></tr>
<tr><td>WP_148270831.1</td><td>35exo→||<-?<-?||?→?→?→ LRR-repeats+AP-GTPase+COR+TIR*→||<-?<-?<-ABC-ATPase+APATPase+ABC-ATPase<-?||SIGMA-HTH→</td><td>LRR-repeats+AP-GTPase+COR+TIR</td><td>935</td><td>-</td><td>Bacteroidetes</td><td>Haliscomenobacter hydrossis</td><td>leucine-rich repeat domain-containing protein [Haliscomenobacter hydrossis].</td><td>GCF_000212735.1</td></tr>
<tr><td>WP_148270849.1</td><td>LRR-repeats+AP-GTPase+COR+EAD11*→?→?→?→?→?→?→ UvsW-A18→</td><td>LRR-repeats+AP-GTPase+COR+EAD11</td><td>1113</td><td>-</td><td>Bacteroidetes</td><td>Haliscomenobacter hydrossis</td><td>GTP-binding protein [Haliscomenobacter hydrossis].</td><td>GCF_000212735.1</td></tr>
<tr><td>WP_148270854.1</td><td>MACRODOMAIN→?→ LRR-repeats+AP-GTPase+COR+EAD11*→</td><td>LRR-repeats+AP-GTPase+COR+EAD11</td><td>1041</td><td>-</td><td>Bacteroidetes</td><td>Haliscomenobacter hydrossis</td><td>Miro domain-containing protein [Haliscomenobacter hydrossis].</td><td>GCF_000212735.1</td></tr>
<tr><td>WP_148705297.1</td><td><-HhH-RADC+JAB||?→ NTP_transf_3→ LRR-repeats+AP-GTPase+COR*→</td><td>LRR-repeats+AP-GTPase+COR</td><td>1056</td><td>-</td><td>Euryarchaeota</td><td>Methanosarcina siciliae</td><td>leucine-rich repeat domain-containing protein [Methanosarcina siciliae].</td><td>GCF_000970125.1</td></tr>
<tr><td>WP_149986249.1</td><td>SIG+TM+TM+TM+TM+TM+TM+TM→||<-?||Mbetalac→
SAM-methylase→?→||<-?<-SIG+TPM_phosphatase+TM+TM+TM||LRR-repeats+AP-GTPase+COR+TM+TM*→</td><td>LRR-repeats+AP-GTPase+COR+TM+TM</td><td>829</td><td>-</td><td>Cyanobacteria</td><td>Microcystis aeruginosa</td><td>leucine-rich repeat protein [Microcystis aeruginosa].</td><td>GCF_008579325.1</td></tr>
<tr><td>WP_150032603.1</td><td>Cluster1550_2clades→?→?→?→?→||<-Cluster995_2clades||tRNA→ LRR-repeats+AP-GTPase+COR+TIR*→</td><td>LRR-repeats+AP-GTPase+COR+TIR</td><td>1161</td><td>-</td><td>Bacteroidetes</td><td>Taibaiella sp. KVB11</td><td>leucine-rich repeat domain-containing protein [Taibaiella sp. KVB11].</td><td>GCF_008629695.1</td></tr>
<tr><td>WP_150875462.1</td><td><-ABC-ATPase<-?<-?||?→?→ LRR-repeats+AP-GTPase+COR+TIR*→</td><td>LRR-repeats+AP-GTPase+COR+TIR</td><td>976</td><td>-</td><td>Bacteroidetes</td><td>Larkinella sp. MA1</td><td>leucine-rich repeat domain-containing protein [Larkinella sp. MA1].</td><td>GCF_008727875.1</td></tr>
<tr><td>WP_150977944.1</td><td>CHTH→ LRR-repeats+AP-GTPase+COR+TM+TM*→</td><td>LRR-repeats+AP-GTPase+COR+TM+TM</td><td>796</td><td>-</td><td>Cyanobacteria</td><td>Microcystis aeruginosa</td><td>leucine-rich repeat domain-containing protein [Microcystis aeruginosa].</td><td>GCF_008757435.1</td></tr>
<tr><td>WP_151015117.1</td><td>LRR-repeats+AP-GTPase+COR*→</td><td>LRR-repeats+AP-GTPase+COR</td><td>946</td><td>-</td><td>Actinobacteria</td><td>Micromonospora aurantiaca</td><td>leucine-rich repeat domain-containing protein [Micromonospora aurantiaca].</td><td>GCF_008806405.1</td></tr>
<tr><td>WP_151968636.1</td><td>LRR-repeats+AP-GTPase+COR*→</td><td>LRR-repeats+AP-GTPase+COR</td><td>803</td><td>-</td><td>Planctomycetes</td><td>Planctomycetes bacterium SRT547</td><td>leucine-rich repeat domain-containing protein [Planctomycetes bacterium SRT547].</td><td>-</td></tr>
<tr><td>WP_152037914.1</td><td>LRR-repeats+AP-GTPase+COR*→</td><td>LRR-repeats+AP-GTPase+COR</td><td>919</td><td>-</td><td>Actinobacteria</td><td>Micromonospora sp. B006</td><td>leucine-rich repeat domain-containing protein [Micromonospora sp. B006].</td><td>GCF_003408515.1</td></tr>
<tr><td>WP_152532113.1</td><td>LRR-repeats+AP-GTPase+COR*→</td><td>LRR-repeats+AP-GTPase+COR</td><td>814</td><td>-</td><td>Cyanobacteria</td><td>Leptolyngbya sp. Heron Island J</td><td>leucine-rich repeat domain-containing protein [Leptolyngbya sp. Heron Island J].</td><td>-</td></tr>
</table>

<table>
<thead>
<tr><th>acc</th><th>operon</th><th>architecture</th><th>len</th><th>gen.name</th><th>taxend</th><th>species</th><th>defline</th><th>gca</th></tr>
</thead>
<tbody>
<tr><td>WP_152589868.1</td><td><-Ferretin<-PSE<-?||?→||<-Ferredoxin-betagrasp<-PSE<-?||LRR-repeats+AP-GTPase+COR*→?→||<-?||?→||<-?||PSE→ PSE→ ClpABN-AAA+ClpABC-AAA→</td><td>LRR-repeats+AP-GTPase+COR</td><td>993</td><td>-</td><td>Cyanobacteria</td><td>Nostoc sphaeroides</td><td>leucine-rich repeat domain-containing protein [Nostoc sphaeroides].</td><td>GCF_009372195.1</td></tr>
<tr><td>WP_152648774.1</td><td>NUDIX→?→?→ LRR-repeats+AP-GTPase+COR*→?→?→||<-?<-?||?→||<-Cluster2230__2clades</td><td>LRR-repeats+AP-GTPase+COR</td><td>954</td><td>-</td><td>Actinobacteria</td><td>Streptacidiphilus anmyonensis</td><td>hypothetical protein [Streptacidiphilus anmyonensis].</td><td>GCF_000787855.1</td></tr>
<tr><td>WP_153009609.1</td><td><-Cluster1102__3clades<-Cluster1102__3clades||?→?→?→||<-Cluster5__5clades<-?||LRR-repeats+AP-GTPase+COR*→</td><td>LRR-repeats+AP-GTPase+COR</td><td>997</td><td>-</td><td>Cyanobacteria</td><td>Mastigocoleus testarum</td><td>leucine-rich repeat domain-containing protein [Mastigocoleus testarum].</td><td>GCF_001456025.1</td></tr>
<tr><td>WP_153023587.1</td><td>TM→ LRR-repeats+AP-GTPase+COR*→</td><td>LRR-repeats+AP-GTPase+COR</td><td>1070</td><td>-</td><td>Actinobacteria</td><td>Glycomyces albidus</td><td>leucine-rich repeat domain-containing protein [Glycomyces albidus].</td><td>-</td></tr>
<tr><td>WP_153228358.1</td><td><-Cluster5__5clades||LRR-repeats+AP-GTPase+COR+DUF4404*→||<-?<-ABC-ATPase<-?||?→||<-REC</td><td>LRR-repeats+AP-GTPase+COR+DUF4404</td><td>992</td><td>-</td><td>Cyanobacteria</td><td>Anabaena sp. YBS01</td><td>leucine-rich repeat domain-containing protein [Anabaena sp. YBS01].</td><td>GCF_009498015.1</td></tr>
<tr><td>WP_153456319.1</td><td>LRR-repeats+AP-GTPase+COR*→</td><td>LRR-repeats+AP-GTPase+COR</td><td>992</td><td>-</td><td>Actinobacteria</td><td>Streptomyces sp. RB5</td><td>GTPase [Streptomyces sp. RB5].</td><td>-</td></tr>
<tr><td>WP_153661567.1</td><td><-cNMPBD<-cNMPBD||?→?→ LRR-repeats+AP-GTPase+COR+TM+TM*→</td><td>LRR-repeats+AP-GTPase+COR+TM+TM</td><td>1103</td><td>-</td><td>Bacteroidetes</td><td>Chitinophaga sp. SYP-B3965</td><td>leucine-rich repeat domain-containing protein [Chitinophaga sp. SYP-B3965].</td><td>GCF_009647655.1</td></tr>
<tr><td>WP_155076197.1</td><td>LRR-repeats+AP-GTPase+COR*→</td><td>LRR-repeats+AP-GTPase+COR</td><td>899</td><td>-</td><td>Bacteroidetes</td><td>Flavobacterium sp. MC2016-06</td><td>GTP-binding protein [Flavobacterium sp. MC2016-06].</td><td>GCF_009711165.1</td></tr>
<tr><td>WP_155705093.1</td><td>SIG+Trypsin+PDZ→?→?→ LRR-repeats+AP-GTPase+COR*→</td><td>LRR-repeats+AP-GTPase+COR</td><td>951</td><td>-</td><td>Firmicutes</td><td>Paenibacillus psychroresistens</td><td>GTP-binding protein [Paenibacillus psychroresistens].</td><td>GCF_009728935.1</td></tr>
<tr><td>WP_155743676.1</td><td>LRR-repeats+AP-GTPase+COR+EAD1*→||<-PSE||Pkinase→ Pkinase→?→||<-Cluster5__5clades</td><td>LRR-repeats+AP-GTPase+COR+EAD1</td><td>1152</td><td>-</td><td>Cyanobacteria</td><td>Scytonema sp. UIC 10036</td><td>leucine-rich repeat domain-containing protein [Scytonema sp. UIC 10036].</td><td>GCF_009725235.1</td></tr>
<tr><td>WP_155746360.1</td><td>LRR-repeats→||<-?<-Cluster5__5clades||LRR-repeats→?→ LRR-repeats+AP-GTPase+COR+EAD1*→?→?→ Cluster5__5clades→</td><td>LRR-repeats+AP-GTPase+COR+EAD1</td><td>988</td><td>-</td><td>Cyanobacteria</td><td>Scytonema sp. UIC 10036</td><td>leucine-rich repeat domain-containing protein [Scytonema sp. UIC 10036].</td><td>GCF_009725235.1</td></tr>
<tr><td>WP_156420697.1</td><td>TetR-HTH→?→||<-?<-?<-?||LRR-repeats+AP-GTPase+COR+TIR*→</td><td>LRR-repeats+AP-GTPase+COR+TIR</td><td>1173</td><td>-</td><td>Alphaproteobacteria</td><td>Sphingopyxis sp. HXXIV</td><td>TIR domain-containing protein [Sphingopyxis sp. HXXIV].</td><td>GCF_001468305.1</td></tr>
<tr><td>WP_157205648.1</td><td>Cluster5__5clades→?→?→ HTH→ LRR-repeats+AP-GTPase+COR+TIR*→ Cluster1019__2clades→?→||<-?<-?<-?||Cluster833__2clades→</td><td>LRR-repeats+AP-GTPase+COR+TIR</td><td>1096</td><td>-</td><td>Gammaproteobacteria</td><td>Methylomonas koyamae</td><td>TIR domain-containing protein [Methylomonas koyamae].</td><td>GCF_001644135.1</td></tr>
<tr><td>WP_157310052.1</td><td>LRR-repeats+AP-GTPase+COR+TIR*→</td><td>LRR-repeats+AP-GTPase+COR+TIR</td><td>917</td><td>-</td><td>Bacteroidetes</td><td>Chitinophaga sp. ysch24</td><td>leucine-rich repeat protein [Chitinophaga sp. ysch24].</td><td>GCF_009758205.1</td></tr>
<tr><td>WP_157453024.1</td><td>LRR-repeats+AP-GTPase+COR+DUF4404*→</td><td>LRR-repeats+AP-GTPase+COR+DUF4404</td><td>1156</td><td>-</td><td>Cyanobacteria</td><td>Coleofasciculus chthonoplastes</td><td>leucine-rich repeat domain-containing protein [Coleofasciculus chthonoplastes].</td><td>-</td></tr>
<tr><td>WP_157546628.1</td><td><-SIG+TM+TM+TM+TM+TM+TM+TM+TM||?→?→||<-?||?→?→ LRR-repeats+AP-GTPase+COR*→</td><td>LRR-repeats+AP-GTPase+COR</td><td>585</td><td>-</td><td>Actinobacteria</td><td>Hamadaea tsunoensis</td><td>leucine-rich repeat domain-containing protein [Hamadaea tsunoensis].</td><td>GCF_000428945.1</td></tr>
<tr><td>WP_157584649.1</td><td>LRR-repeats+AP-GTPase+COR+TIR*→||<-?||ABC__membrane+ABC__tran→</td><td>LRR-repeats+AP-GTPase+COR+TIR</td><td>937</td><td>-</td><td>Bacteroidetes</td><td>Spirosoma sp. HMF4905</td><td>leucine-rich repeat domain-containing protein [Spirosoma sp. HMF4905].</td><td>GCF_009754945.1</td></tr>
<tr><td>WP_157585892.1</td><td>LRR-repeats+AP-GTPase+COR+TIR*→ Cluster1510__2clades→</td><td>LRR-repeats+AP-GTPase+COR+TIR</td><td>1129</td><td>-</td><td>Bacteroidetes</td><td>Spirosoma sp. HMF4905</td><td>leucine-rich repeat domain-containing protein [Spirosoma sp. HMF4905].</td><td>-</td></tr>
<tr><td>WP_157590734.1</td><td>ABhydrolase+ABhydrolase+CASPASE+TPR-S→||<-?||LRR-repeats+AP-GTPase+COR+TIR*→</td><td>LRR-repeats+AP-GTPase+COR+TIR</td><td>1169</td><td>-</td><td>Bacteroidetes</td><td>Spirosoma sp. HMF4905</td><td>leucine-rich repeat domain-containing protein [Spirosoma sp. HMF4905].</td><td>GCF_009754945.1</td></tr>
<tr><td>WP_157879249.1</td><td>LRR-repeats+AP-GTPase+COR+TIR*→||<-PSE<-?<-?<-?<-?<-?<-REC</td><td>LRR-repeats+AP-GTPase+COR+TIR</td><td>656</td><td>-</td><td>Alphaproteobacteria</td><td>Pararhodospirillum photometricum</td><td>hypothetical protein [Pararhodospirillum photometricum].</td><td>GCF_000284415.1</td></tr>
</tbody>
</table>

<table>
<tr><th>acc</th><th>operon</th><th>architecture</th><th>len</th><th>gen.name</th><th>taxend</th><th>species</th><th>defline</th><th>gca</th></tr>
<tr><td>WP_157881522.1</td><td>Cluster1792__2clades→?→?→||<-?||?→ LRR-repeats+AP-GTPase+COR*→||<-?||?→ SIG+TM+TM→</td><td>LRR-repeats+AP-GTPase+COR</td><td>950</td><td>-</td><td>Actinobacteria</td><td>Streptomyces phaeochromogenes</td><td>hypothetical protein [Streptomyces phaeochromogenes].</td><td>GCF_001418655.1</td></tr>
<tr><td>WP_158043876.1</td><td>LRR-repeats+AP-GTPase+COR__TIR*→</td><td>LRR-repeats+AP-GTPase+COR__TIR</td><td>1167</td><td>-</td><td>Alphaproteobacteria</td><td>Skermanella pratensis</td><td>TIR domain-containing protein [Skermanella pratensis].</td><td>-</td></tr>
<tr><td>WP_158234411.1</td><td>LRR-repeats+AP-GTPase+COR*→ LRR-repeats→ LRR-repeats→</td><td>LRR-repeats+AP-GTPase+COR</td><td>852</td><td>-</td><td>Cyanobacteria</td><td>Limnothrix sp. PR1529</td><td>leucine-rich repeat domain-containing protein [Limnothrix sp. PR1529].</td><td>GCF_002742025.1</td></tr>
<tr><td>WP_158281705.1</td><td><-ACET<-?<-?<-?||?→ HSP70→ DNAJ→ LRR-repeats+AP-GTPase+COR+TIR*→</td><td>LRR-repeats+AP-GTPase+COR+TIR</td><td>944</td><td>-</td><td>Betaproteobacteria</td><td>Rivicola pingtungensis</td><td>TIR domain-containing protein [Rivicola pingtungensis].</td><td>GCF_003201855.1</td></tr>
<tr><td>WP_158299785.1</td><td>TM→ LRR-repeats+AP-GTPase+COR*→</td><td>LRR-repeats+AP-GTPase+COR</td><td>1050</td><td>-</td><td>Actinobacteria</td><td>Glycomyces paridis</td><td>hypothetical protein [Glycomyces paridis].</td><td>GCF_004912155.1</td></tr>
<tr><td>WP_158592567.1</td><td>LRR-repeats+AP-GTPase+COR+TM+TM*→</td><td>LRR-repeats+AP-GTPase+COR+TM+TM</td><td>1002</td><td>-</td><td>Bacteroidetes</td><td>Flavobacterium sp. 102</td><td>50S ribosome-binding GTPase [Flavobacterium sp. 102].</td><td>GCF_003634615.1</td></tr>
<tr><td>WP_158623299.1</td><td>TPR-repeats→ LRR-repeats+AP-GTPase+COR+TIR*→?→?→?→?→||<-LexA-protease</td><td>LRR-repeats+AP-GTPase+COR+TIR</td><td>1083</td><td>-</td><td>Deltaproteobacteria</td><td>Corallococcus sp. CA053C</td><td>TIR domain-containing protein [Corallococcus sp. CA053C].</td><td>GCF_003611675.1</td></tr>
<tr><td>WP_158660235.1</td><td><-SIG+TM+TM+TM+TM+TM+TM+TM+TM+TM+TM<-?<-?<-?<-?||?→?→ LRR-repeats+AP-GTPase+COR+TIR*→||<-ParB<-ParA-Soj-PloopNTPase</td><td>LRR-repeats+AP-GTPase+COR+TIR</td><td>723</td><td>-</td><td>Alphaproteobacteria</td><td>Niveispirillum cyanobacteriorum</td><td>TIR domain-containing protein [Niveispirillum cyanobacteriorum].</td><td>GCF_014640215.1</td></tr>
<tr><td>WP_158690560.1</td><td>LRR-repeats+AP-GTPase+COR*→</td><td>LRR-repeats+AP-GTPase+COR</td><td>1355</td><td>-</td><td>Actinobacteria</td><td>Streptomyces sp. 142MFCol3.1</td><td>hypothetical protein, partial [Streptomyces sp. 142MFCol3.1].</td><td>GCF_000424945.1</td></tr>
<tr><td>WP_158924018.1</td><td>LRR-repeats+AP-GTPase+COR*→||<-?||?→||<-?<-SIGMA-HTH<-?<-SIGMA-HTH</td><td>LRR-repeats+AP-GTPase+COR</td><td>996</td><td>-</td><td>Actinobacteria</td><td>Streptomyces broussonetiae</td><td>GTPase [Streptomyces broussonetiae].</td><td>GCF_009796285.1</td></tr>
<tr><td>WP_159021496.1</td><td>Cluster1757__2clades→||<-?||LRR-repeats+AP-GTPase+COR*→?→?→?→?→||<-PSE<-PSE<-Cluster1695__2clades</td><td>LRR-repeats+AP-GTPase+COR</td><td>704</td><td>-</td><td>Bacteroidetes</td><td>Formosa sp. L2A11</td><td>GTP-binding protein [Formosa sp. L2A11].</td><td>GCF_009796865.1</td></tr>
<tr><td>WP_159170123.1</td><td>LRR-repeats+AP-GTPase+COR+DrHyd*→</td><td>LRR-repeats+AP-GTPase+COR+DrHyd</td><td>855</td><td>-</td><td>Gammaproteobacteria</td><td>Halioglobus japonicus</td><td>hypothetical protein [Halioglobus japonicus].</td><td>GCF_902705825.1</td></tr>
<tr><td>WP_159248779.1</td><td>SIG+TM+TM+TM+TM+TM+TM+TM→||<-?||Mbetalac→ SAM-methylase→?→||<-?<-SIG+TPM__phosphatase+TM+TM+TM||LRR-repeats+AP-GTPase+COR+TM+TM*→||<-?<-?<-?<-Pro__CA||?→||<-?<-tRNA<-tRNA<-Ferredoxin-betagrasp</td><td>LRR-repeats+AP-GTPase+COR+TM+TM</td><td>805</td><td>-</td><td>Cyanobacteria</td><td>Microcystis aeruginosa</td><td>leucine-rich repeat domain-containing protein [Microcystis aeruginosa].</td><td>GCF_009811815.1</td></tr>
<tr><td>WP_159254721.1</td><td>LRR-repeats+AP-GTPase+COR*→?→?→?→||<-?<-ABC-ATPase</td><td>LRR-repeats+AP-GTPase+COR</td><td>923</td><td>-</td><td>Bacteroidetes</td><td>Tenacibaculum maritimum</td><td>leucine-rich repeat domain-containing protein [Tenacibaculum maritimum].</td><td>GCF_902705305.1</td></tr>
<tr><td>WP_159395983.1</td><td>LRR-repeats+AP-GTPase+COR*→||<-?||?→||<-?<-SIGMA-HTH<-?<-SIGMA-HTH</td><td>LRR-repeats+AP-GTPase+COR</td><td>988</td><td>-</td><td>Actinobacteria</td><td>Streptomyces sp. FXJ1.172</td><td>GTPase [Streptomyces sp. FXJ1.172].</td><td>GCF_001636945.1</td></tr>
<tr><td>WP_159402479.1</td><td>ABhydrolase→?→||<-?||LRR-repeats+AP-GTPase+COR+TIR*→||<-?<-?<-?<-SIG+NUDIX</td><td>LRR-repeats+AP-GTPase+COR+TIR</td><td>1069</td><td>-</td><td>Cyanobacteria</td><td>Nostoc sp. ATCC 53789</td><td>leucine-rich repeat domain-containing protein [Nostoc sp. ATCC 53789].</td><td>GCF_009873495.1</td></tr>
<tr><td>WP_159402492.1</td><td>Pentapeptide-repeats→||<-?<-?||?→?→||<-Arginase<-?||LRR-repeats+AP-GTPase+COR+TIR*→</td><td>LRR-repeats+AP-GTPase+COR+TIR</td><td>802</td><td>-</td><td>Cyanobacteria</td><td>Nostoc sp. ATCC 53789</td><td>leucine-rich repeat domain-containing protein [Nostoc sp. ATCC 53789].</td><td>GCF_009873495.1</td></tr>
<tr><td>WP_159402558.1</td><td>Cluster5__5clades→ LRR-repeats+AP-GTPase+COR+TM+TM*→||<-CASPASE+TM</td><td>LRR-repeats+AP-GTPase+COR+TM+TM</td><td>904</td><td>-</td><td>Cyanobacteria</td><td>Nostoc sp. ATCC 53789</td><td>leucine-rich repeat domain-containing protein [Nostoc sp. ATCC 53789].</td><td>GCF_009873495.1</td></tr>
<tr><td>WP_159402710.1</td><td>LRR-repeats+AP-GTPase+COR+CASPASE*→?→?→?→ ParA-Soj-PloopNTPase→</td><td>LRR-repeats+AP-GTPase+COR+CASPASE</td><td>1535</td><td>-</td><td>Cyanobacteria</td><td>Nostoc sp. ATCC 53789</td><td>leucine-rich repeat domain-containing protein [Nostoc sp. ATCC 53789].</td><td>GCF_009873495.1</td></tr>
<tr><td>WP_159447934.1</td><td>CI→ CI→ LRR-repeats+AP-GTPase+COR*→</td><td>LRR-repeats+AP-GTPase+COR</td><td>1103</td><td>-</td><td>Gammaproteobacteria</td><td>Photobacterium piscicola</td><td>GTP-binding protein [Photobacterium piscicola].</td><td>GCF_900166965.1</td></tr>
<tr><td>WP_159457453.1</td><td>LRR-repeats+AP-GTPase+COR+TIR*→</td><td>LRR-repeats+AP-GTPase+COR+TIR</td><td>1186</td><td>-</td><td>Gammaproteobacteria</td><td>Vibrio mangrovi</td><td>leucine-rich repeat domain-containing protein [Vibrio mangrovi].</td><td>GCF_900184095.1</td></tr>
</table>

<table>
<tr><th>acc</th><th>operon</th><th>architecture</th><th>len</th><th>gen.name</th><th>taxend</th><th>species</th><th>defline</th><th>gca</th></tr>
<tr><td>WP_159789529.1</td><td><-Cluster1921_2clades<-?<-?||?→||<-?||?→?→ LRR-repeats+AP-GTPase+COR*→?→||<-?<-REC</td><td>LRR-repeats+AP-GTPase+COR</td><td>771</td><td>-</td><td>Cyanobacteria</td><td>Microcoleus sp. IPPAS B-353</td><td>leucine-rich repeat domain-containing protein [Microcoleus sp. IPPAS B-353].</td><td>GCF_009846485.1</td></tr>
<tr><td>WP_160075537.1</td><td>LRR-repeats+AP-GTPase+COR+TIR*→</td><td>LRR-repeats+AP-GTPase+COR+TIR</td><td>1088</td><td>-</td><td>Bacteroidetes</td><td>Saprospirales bacterium GYS_P2D</td><td>leucine-rich repeat domain-containing protein [Saprospirales bacterium GYS_P2D].</td><td>-</td></tr>
<tr><td>WP_160131147.1</td><td>SIGMA-HTH→?→ RADICAL-SAM→ LRR-repeats+AP-GTPase+COR+TIR*→||<-REC<-?<-?<-?||?→ HISKIN→</td><td>LRR-repeats+AP-GTPase+COR+TIR</td><td>1229</td><td>-</td><td>Bacteroidetes</td><td>Kordia antarctica</td><td>leucine-rich repeat domain-containing protein [Kordia antarctica].</td><td>GCF_009901525.1</td></tr>
<tr><td>WP_160148531.1</td><td><-SIG+Trypsin+PDZ<-?<-?<-?||?→?→ LRR-repeats+AP-GTPase+COR+TCAD2*→</td><td>LRR-repeats+AP-GTPase+COR+TCAD2</td><td>919</td><td>-</td><td>Cyanobacteria</td><td>Leptolyngbya sp. PCC 7376</td><td>leucine-rich repeat domain-containing protein [Leptolyngbya sp. PCC 7376].</td><td>GCF_000316605.1</td></tr>
<tr><td>WP_161144175.1</td><td>Cluster1913_2clades→?→?→?→?→ LRR-repeats+AP-GTPase*→</td><td>LRR-repeats+AP-GTPase</td><td>512</td><td>-</td><td>Firmicutes</td><td>Dorea sp. BIOML-A1</td><td>hypothetical protein [Dorea sp. BIOML-A1].</td><td>GCF_009875615.1</td></tr>
<tr><td>WP_161213661.1</td><td>LRR-repeats+AP-GTPase+COR*→||<-NUDIX</td><td>LRR-repeats+AP-GTPase+COR</td><td>1340</td><td>-</td><td>Actinobacteria</td><td>unclassified Streptomyces</td><td>MULTISPECIES: leucine-rich repeat domain-containing protein [unclassified Streptomyces].</td><td>GCF_900091775.1</td></tr>
<tr><td>WP_161967152.1</td><td><-Aminotran_1_2<-?||LRR-repeats+AP-GTPase+COR+TIR*→||<-?<-?<-?<-DSBH+AraC-HTH+AraC-HTH</td><td>LRR-repeats+AP-GTPase+COR+TIR</td><td>1219</td><td>-</td><td>Planctomycetes</td><td>Fimbriiglobus ruber</td><td>hypothetical protein [Fimbriiglobus ruber].</td><td>GCF_002197845.1</td></tr>
<tr><td>WP_161967198.1</td><td><-RelE-ParE<-?||?→?→ LRR-repeats→||<-?||LRR-repeats+AP-GTPase+COR*→</td><td>LRR-repeats+AP-GTPase+COR</td><td>915</td><td>-</td><td>Planctomycetes</td><td>Fimbriiglobus ruber</td><td>leucine-rich repeat domain-containing protein [Fimbriiglobus ruber].</td><td>GCF_002197845.1</td></tr>
<tr><td>WP_162292932.1</td><td>LRR-repeats+AP-GTPase+COR*→?→?→?→||<-?<-ABC-ATPase+ABC-ATPase</td><td>LRR-repeats+AP-GTPase+COR</td><td>1975</td><td>-</td><td>Actinobacteria</td><td>Actinophytocola xinjiangensis</td><td>leucine-rich repeat domain-containing protein [Actinophytocola xinjiangensis].</td><td>GCF_001921215.1</td></tr>
<tr><td>WP_162399370.1</td><td>LRR-repeats+AP-GTPase+COR*→?→||<-?||?→||<-?<-?||?→ ClpABN-AAA+ClpABC-AAA→</td><td>LRR-repeats+AP-GTPase+COR</td><td>895</td><td>-</td><td>Cyanobacteria</td><td>Nostoc sp. B(2019)</td><td>leucine-rich repeat domain-containing protein, partial [Nostoc sp. B(2019)].</td><td>GCF_010091925.1</td></tr>
<tr><td>WP_162488066.1</td><td>TM+TM→?→||<-?||?→ PSE→||<-?||?→?→ LRR-repeats+AP-GTPase+COR+TIR*→||<-?<-?||ABC_membrane+ABC_tran→?→ Cluster968_2clades→</td><td>LRR-repeats+AP-GTPase+COR+TIR</td><td>1120</td><td>-</td><td>Alphaproteobacteria</td><td>Azospirillum lipoferum</td><td>TIR domain-containing protein [Azospirillum lipoferum].</td><td>GCF_000283655.1</td></tr>
<tr><td>WP_162528069.1</td><td>LRR-repeats+AP-GTPase+COR*→</td><td>LRR-repeats+AP-GTPase+COR</td><td>781</td><td>-</td><td>Alphaproteobacteria</td><td>Rhodobacterales bacterium</td><td>leucine-rich repeat domain-containing protein [Rhodobacterales bacterium].</td><td>-</td></tr>
<tr><td>WP_162667694.1</td><td><-Cluster1291_2clades<-?<-PSE<-?||?→?→?→ LRR-repeats+AP-GTPase*→?→||<-?||?→?→ ParB→</td><td>LRR-repeats+AP-GTPase</td><td>617</td><td>-</td><td>Planctomycetes</td><td>Gemmata massiliana</td><td>hypothetical protein [Gemmata massiliana].</td><td>GCF_901538265.1</td></tr>
<tr><td>WP_162668063.1</td><td>LRR-repeats+AP-GTPase+COR+DUF4404*→||<-?<-?<-?||?→||<-LRR-repeats||REC→</td><td>LRR-repeats+AP-GTPase+COR+DUF4404</td><td>978</td><td>-</td><td>Planctomycetes</td><td>Gemmata massiliana</td><td>leucine-rich repeat domain-containing protein [Gemmata massiliana].</td><td>GCF_901538265.1</td></tr>
<tr><td>WP_163431087.1</td><td>TIR→ LRR-repeats+AP-GTPase+COR+TIR*→ PSE→?→?→||<-?<-SIG+TM+TM+TM+HISKIN</td><td>LRR-repeats+AP-GTPase+COR+TIR</td><td>1384</td><td>-</td><td>Bacteroidetes</td><td>unclassified Flavobacterium</td><td>MULTISPECIES: leucine-rich repeat domain-containing protein [unclassified Flavobacterium].</td><td>GCF_013874595.1</td></tr>
<tr><td>WP_163660644.1</td><td>LRR-repeats+AP-GTPase*→</td><td>LRR-repeats+AP-GTPase</td><td>311</td><td>-</td><td>Cyanobacteria</td><td>Leptolyngbyaceae cyanobacterium CCMR0082</td><td>leucine-rich repeat domain-containing protein [Leptolyngbyaceae cyanobacterium CCMR0082].</td><td>-</td></tr>
<tr><td>WP_163671600.1</td><td>LRR-repeats+AP-GTPase+COR+TIR*→</td><td>LRR-repeats+AP-GTPase+COR+TIR</td><td>855</td><td>-</td><td>Cyanobacteria</td><td>Leptolyngbyaceae cyanobacterium CCMR0082</td><td>leucine-rich repeat domain-containing protein, partial [Leptolyngbyaceae cyanobacterium CCMR0082].</td><td>-</td></tr>
</table>

<table>
<thead>
<tr><th>acc</th><th>operon</th><th>architecture</th><th>len</th><th>gen.name</th><th>taxend</th><th>species</th><th>defline</th><th>gca</th></tr>
</thead>
<tbody>
<tr><td>WP_163671893.1</td><td><-NACHT<-?||LRR-repeats+AP-GTPase+COR+TIR*→||<-?||PSE→||<-NACHT+68TM-wHTH<-?||?→?→||<-?||Cluster53_2clades→</td><td>LRR-repeats+AP-GTPase+COR+TIR</td><td>726</td><td>-</td><td>Cyanobacteria</td><td>Leptolyngbyaceae cyanobacterium CCMR0082</td><td>TIR domain-containing protein [Leptolyngbyaceae cyanobacterium CCMR0082].</td><td>-</td></tr>
<tr><td>WP_163697061.1</td><td>LRR-repeats+AP-GTPase+COR+TIR*→?→||<-?<-?<-?||?→||<-Cluster1829_2clades</td><td>LRR-repeats+AP-GTPase+COR+TIR</td><td>1274</td><td>-</td><td>Cyanobacteria</td><td>Leptolyngbyaceae cyanobacterium CCMR0081</td><td>leucine-rich repeat domain-containing protein [Leptolyngbyaceae cyanobacterium CCMR0081].</td><td>-</td></tr>
<tr><td>WP_163702516.1</td><td><-SIG+DM13||?→?→?→?→?→ PIN→ LRR-repeats+AP-GTPase+COR+TIR*→?→ PSE→?→?→?→||<-?<-SIG+TM+TM+Calcineurin</td><td>LRR-repeats+AP-GTPase+COR+TIR</td><td>993</td><td>-</td><td>Cyanobacteria</td><td>Leptolyngbyaceae cyanobacterium CCMR0081</td><td>leucine-rich repeat domain-containing protein [Leptolyngbyaceae cyanobacterium CCMR0081].</td><td>-</td></tr>
<tr><td>WP_163703138.1</td><td>SIR2→?→?→?→?→?→?→ LRR-repeats+AP-GTPase+COR+TIR*→ PSE→||<-Pentapeptide</td><td>LRR-repeats+AP-GTPase+COR+TIR</td><td>830</td><td>-</td><td>Cyanobacteria</td><td>Leptolyngbyaceae cyanobacterium CCMR0081</td><td>TIR domain-containing protein [Leptolyngbyaceae cyanobacterium CCMR0081].</td><td>-</td></tr>
<tr><td>WP_163833956.1</td><td>LRR-repeats+AP-GTPase+COR+DUF4404*→||<-?<-CHTH</td><td>LRR-repeats+AP-GTPase+COR+DUF4404</td><td>1124</td><td>-</td><td>Gammaproteobacteria</td><td>Spartinivicinus ruber</td><td>leucine-rich repeat domain-containing protein [Spartinivicinus ruber].</td><td>GCF_011009015.1</td></tr>
<tr><td>WP_163929292.1</td><td>LRR-repeats+AP-GTPase+COR+TIR*→||<-?<-?<-TIR<-?<-SIG+NUDIX</td><td>LRR-repeats+AP-GTPase+COR+TIR</td><td>1024</td><td>-</td><td>Cyanobacteria</td><td>Nostoc sp. UIC 10630</td><td>leucine-rich repeat domain-containing protein [Nostoc sp. UIC 10630].</td><td>GCF_010747425.1</td></tr>
<tr><td>WP_163938989.1</td><td>LRR-repeats+AP-GTPase+COR+TIR*→</td><td>LRR-repeats+AP-GTPase+COR+TIR</td><td>795</td><td>-</td><td>Cyanobacteria</td><td>Nostoc sp. UIC 10630</td><td>leucine-rich repeat domain-containing protein, partial [Nostoc sp. UIC 10630].</td><td>GCF_010747425.1</td></tr>
<tr><td>WP_164035239.1</td><td>Cluster998_2clades→?→||<-?<-?<-?||SAM-methylase→?→ tRNA→ LRR-repeats+AP-GTPase+COR*→</td><td>LRR-repeats+AP-GTPase+COR</td><td>858</td><td>-</td><td>Bacteroidetes</td><td>Spirosoma agri</td><td>hypothetical protein [Spirosoma agri].</td><td>GCF_010747415.1</td></tr>
<tr><td>WP_164490209.1</td><td>FGS→ FGS→ FGS→ LRR-repeats+AP-GTPase+COR+CASPASE*→</td><td>LRR-repeats+AP-GTPase+COR+CASPASE</td><td>976</td><td>-</td><td>Bacteroidetes</td><td>Runella sp. SP2</td><td>leucine-rich repeat domain-containing protein [Runella sp. SP2].</td><td>GCF_003711225.1</td></tr>
<tr><td>WP_164638795.1</td><td>LRR-repeats+AP-GTPase+COR+TIR*→||<-?<-?<-?<-ABC-ATPase||?→ REC→</td><td>LRR-repeats+AP-GTPase+COR+TIR</td><td>1173</td><td>-</td><td>Alphaproteobacteria</td><td>Rhodopseudomonas sp. BR0G17</td><td>leucine-rich repeat domain-containing protein [Rhodopseudomonas sp. BR0G17].</td><td>GCF_010907025.1</td></tr>
<tr><td>WP_164714180.1</td><td>LRR-repeats+AP-GTPase+COR+TIR*→||<-?<-?||?→ cNMPBD→ SIG+TM+TM+TM→</td><td>LRR-repeats+AP-GTPase+COR+TIR</td><td>1056</td><td>-</td><td>Bacteroidetes</td><td>Chitinophaga rhizosphaerae</td><td>leucine-rich repeat domain-containing protein [Chitinophaga rhizosphaerae].</td><td>GCF_003958645.1</td></tr>
<tr><td>WP_165253289.1</td><td>LRR-repeats+AP-GTPase+COR+TIR*→</td><td>LRR-repeats+AP-GTPase+COR+TIR</td><td>1053</td><td>-</td><td>Planctomycetes</td><td>Paludisphaera soli</td><td>TIR domain-containing protein [Paludisphaera soli].</td><td>GCF_011064595.1</td></tr>
<tr><td>WP_165571460.1</td><td>LRR-repeats+AP-GTPase+COR*→</td><td>LRR-repeats+AP-GTPase+COR</td><td>871</td><td>-</td><td>Bacteroidetes</td><td>Flavobacterium hydatis</td><td>hypothetical protein [Flavobacterium hydatis].</td><td>GCF_000737695.1</td></tr>
<tr><td>WP_165772028.1</td><td><-SIG+TM+TM+TM+HISKIN<-?<-?<-?||?→ LRR-repeats+AP-GTPase+COR+TIR*→ ParA-Soj-PloopNTPase→ ParB→</td><td>LRR-repeats+AP-GTPase+COR+TIR</td><td>674</td><td>-</td><td>Alphaproteobacteria</td><td>Niveispirillum lacus</td><td>TIR domain-containing protein [Niveispirillum lacus].</td><td>GCF_002251795.1</td></tr>
<tr><td>WP_166124895.1</td><td><-Cluster1501_2clades||?→?→ LRR-repeats+AP-GTPase+COR*→?→ Cluster1023_3clades→?→||<-?||ClpABN-AAA+ClpABC-AAA→</td><td>LRR-repeats+AP-GTPase+COR</td><td>925</td><td>-</td><td>Bacteroidetes</td><td>unclassified Flavobacterium</td><td>MULTISPECIES: leucine-rich repeat domain-containing protein [unclassified Flavobacterium].</td><td>GCF_011305415.1</td></tr>
<tr><td>WP_166235797.1</td><td>LRR-repeats+AP-GTPase+COR+TM+TM*→</td><td>LRR-repeats+AP-GTPase+COR+TM+TM</td><td>954</td><td>-</td><td>Bacteroidetes</td><td>Flavobacterium sp. TWA-26</td><td>hypothetical protein [Flavobacterium sp. TWA-26].</td><td>GCF_011392075.1</td></tr>
<tr><td>WP_166562074.1</td><td>LRR-repeats+AP-GTPase+COR*→</td><td>LRR-repeats+AP-GTPase+COR</td><td>931</td><td>-</td><td>Bacteroidetes</td><td>Emticicia sp. CRIBPO</td><td>leucine-rich repeat domain-containing protein [Emticicia sp. CRIBPO].</td><td>GCF_009905725.1</td></tr>
<tr><td>WP_167031741.1</td><td>LRR-repeats+AP-GTPase+COR+TIR*→?→?→ TIR→</td><td>LRR-repeats+AP-GTPase+COR+TIR</td><td>1295</td><td>-</td><td>Bacteroidetes</td><td>Chryseobacterium sp. Tr-659</td><td>leucine-rich repeat domain-containing protein [Chryseobacterium sp. Tr-659].</td><td>GCF_011752975.1</td></tr>
</tbody>
</table>

<table>
<tr><th>acc</th><th>operon</th><th>architecture</th><th>len</th><th>gen.name</th><th>taxend</th><th>species</th><th>defline</th><th>gca</th></tr>
<tr><td>WP_167211330.1</td><td>LRR-repeats+AP-GTPase+COR+TIR*→ TIR→</td><td>LRR-repeats+AP-GTPase+COR+TIR</td><td>1843</td><td>-</td><td>Bacteroidetes</td><td>Spirosoma aureum</td><td>leucine-rich repeat domain-containing protein [Spirosoma aureum].</td><td>GCF_011604685.1</td></tr>
<tr><td>WP_167291529.1</td><td>LRR-repeats+AP-GTPase+COR+TIR*→</td><td>LRR-repeats+AP-GTPase+COR+TIR</td><td>1060</td><td>-</td><td>Bacteroidetes</td><td>Paraflavitale devenefica</td><td>leucine-rich repeat domain-containing protein [Paraflavitale devenefica].</td><td>GCF_011759375.1</td></tr>
<tr><td>WP_167387879.1</td><td>LRR-repeats+AP-GTPase+COR*→</td><td>LRR-repeats+AP-GTPase+COR</td><td>967</td><td>-</td><td>Actinobacteria</td><td>Streptomyces recifensis</td><td>GTPase [Streptomyces recifensis].</td><td>GCF_002154615.1</td></tr>
<tr><td>WP_168204481.1</td><td>LRR-repeats→ LRR-repeats+AP-GTPase+COR+TM+TM*→</td><td>LRR-repeats+AP-GTPase+COR+TM+TM</td><td>574</td><td>-</td><td>Cyanobacteria</td><td>Dolichospermum sp. UHCC 0315A</td><td>hypothetical protein [Dolichospermum sp. UHCC 0315A].</td><td>GCF_008121535.1</td></tr>
<tr><td>WP_168218871.1</td><td>LRR-repeats+AP-GTPase+COR*→?→?→||<-?<-?<-?<-?<-PIN</td><td>LRR-repeats+AP-GTPase+COR</td><td>864</td><td>-</td><td>Planctomycetes</td><td>Limnoglobus roseus</td><td>leucine-rich repeat domain-containing protein [Limnoglobus roseus].</td><td>GCF_008254045.1</td></tr>
<tr><td>WP_168359257.1</td><td>LRR-repeats+AP-GTPase+COR+TM+TM*→</td><td>LRR-repeats+AP-GTPase+COR+TM+TM</td><td>687</td><td>-</td><td>Cyanobacteria</td><td>Dolichospermum planctonicum</td><td>GTP-binding protein, partial [Dolichospermum planctonicum].</td><td>GCF_009712075.1</td></tr>
<tr><td>WP_168360389.1</td><td>Ferredoxin-betagrasp→?→?→ LRR-repeats→ LRR-repeats+AP-GTPase+COR*→</td><td>LRR-repeats+AP-GTPase+COR</td><td>682</td><td>-</td><td>Cyanobacteria</td><td>Dolichospermum planctonicum</td><td>hypothetical protein, partial [Dolichospermum planctonicum].</td><td>GCF_009712075.1</td></tr>
<tr><td>WP_168467214.1</td><td>LRR-repeats+AP-GTPase+COR*→ PSE→?→||<-?||?→?→?→||<-Pentapeptide</td><td>LRR-repeats+AP-GTPase+COR</td><td>692</td><td>-</td><td>Cyanobacteria</td><td>Aphanizomenon sp. UHCC 0183</td><td>GTP-binding protein, partial [Aphanizomenon sp. UHCC 0183].</td><td>GCF_009712065.1</td></tr>
<tr><td>WP_168493001.1</td><td><-HNH<-?||?→ LRR-repeats+AP-GTPase+COR*→</td><td>LRR-repeats+AP-GTPase+COR</td><td>1079</td><td>-</td><td>Cyanobacteria</td><td>Anabaena sp. UHCC 0204</td><td>leucine-rich repeat domain-containing protein [Anabaena sp. UHCC 0204].</td><td>GCF_009711975.1</td></tr>
<tr><td>WP_168511191.1</td><td>NUDIX→?→?→?→ LRR-repeats+AP-GTPase+COR*→</td><td>LRR-repeats+AP-GTPase+COR</td><td>1365</td><td>-</td><td>Actinobacteria</td><td>Streptomyces sp. S1D4-11</td><td>leucine-rich repeat domain-containing protein [Streptomyces sp. S1D4-11].</td><td>-</td></tr>
<tr><td>WP_168644108.1</td><td>LRR-repeats+AP-GTPase+COR+TIR*→?→||<-?<-SIG+NUDIX</td><td>LRR-repeats+AP-GTPase+COR+TIR</td><td>963</td><td>-</td><td>Cyanobacteria</td><td>Dolichospermum sp. UHCC 0259</td><td>leucine-rich repeat domain-containing protein [Dolichospermum sp. UHCC 0259].</td><td>GCF_009711935.1</td></tr>
<tr><td>WP_169190548.1</td><td><-ABC-ATPase<-?<-?<-?<-?||?→ tRNA→?→ LRR-repeats+AP-GTPase+COR+TIR*→</td><td>LRR-repeats+AP-GTPase+COR+TIR</td><td>1190</td><td>-</td><td>Bacteroidetes</td><td>Chitinophaga sp. Ak27</td><td>leucine-rich repeat domain-containing protein [Chitinophaga sp. Ak27].</td><td>GCF_012726295.1</td></tr>
<tr><td>WP_169266449.1</td><td>LRR-repeats+AP-GTPase+COR+TIR*→||<-?||?→||<-?||NUDIX→</td><td>LRR-repeats+AP-GTPase+COR+TIR</td><td>936</td><td>-</td><td>Cyanobacteria</td><td>Brasilonema octagenarum</td><td>leucine-rich repeat domain-containing protein [Brasilonema octagenarum].</td><td>GCF_012912125.1</td></tr>
<tr><td>WP_169315756.1</td><td>LRR-repeats+AP-GTPase+COR+TIR*→</td><td>LRR-repeats+AP-GTPase+COR+TIR</td><td>1002</td><td>-</td><td>Bacteroidetes</td><td>Haliscomenobacter hydrossis</td><td>leucine-rich repeat domain-containing protein [Haliscomenobacter hydrossis].</td><td>GCF_000212735.1</td></tr>
<tr><td>WP_169363874.1</td><td>SIG+HIN-HTH→?→?→ LRR-repeats+AP-GTPase+COR+TM+TM*→</td><td>LRR-repeats+AP-GTPase+COR+TM+TM</td><td>972</td><td>-</td><td>Cyanobacteria</td><td>Pseudanabaena yagii</td><td>leucine-rich repeat domain-containing protein [Pseudanabaena yagii].</td><td>GCF_012863495.1</td></tr>
<tr><td>WP_169364911.1</td><td>TPR-repeats+CASPASE→?→ PSE→ Cluster54_3clades→ Cluster53_2clades→?→ LRR-repeats+AP-GTPase+COR+CASPASE*→||<-?||?→ REC→</td><td>LRR-repeats+AP-GTPase+COR+CASPASE</td><td>1239</td><td>-</td><td>Cyanobacteria</td><td>Pseudanabaena yagii</td><td>leucine-rich repeat domain-containing protein [Pseudanabaena yagii].</td><td>GCF_012863495.1</td></tr>
<tr><td>WP_169365210.1</td><td>Pentapeptide-repeats→?→ PolB→?→ LRR-repeats→ LRR-repeats+AP-GTPase+COR*→?→?→||<-?||?→?→||<-SIG+TM+TM+TM+TM+TM</td><td>LRR-repeats+AP-GTPase+COR</td><td>682</td><td>-</td><td>Cyanobacteria</td><td>Pseudanabaena yagii</td><td>hypothetical protein [Pseudanabaena yagii].</td><td>GCF_012863495.1</td></tr>
<tr><td>WP_169482131.1</td><td>LRR-repeats+AP-GTPase+COR*→||<-?||?→||<-?<-?<-?<-?<-Cluster1745_2clades</td><td>LRR-repeats+AP-GTPase+COR</td><td>965</td><td>-</td><td>Firmicutes</td><td>Paenibacillus sp. SZ31</td><td>GTP-binding protein [Paenibacillus sp. SZ31].</td><td>GCF_012912005.1</td></tr>
<tr><td>WP_169551474.1</td><td>LRR-repeats+AP-GTPase+COR+DrHyd*→||<-?||?→?→||<-TPR-repeats</td><td>LRR-repeats+AP-GTPase+COR+DrHyd</td><td>1047</td><td>-</td><td>Bacteroidetes</td><td>Spirosoma sp. CJU-R4</td><td>hypothetical protein [Spirosoma sp. CJU-R4].</td><td>GCF_012849055.1</td></tr>
<tr><td>WP_169617268.1</td><td>RelE→ CHTH→?→||<-?<-?<-?<-?||LRR-repeats+AP-GTPase+COR+TIR*→?→||<-?<-?||?→?→||<-?<-RelE-ParE</td><td>LRR-repeats+AP-GTPase+COR+TIR</td><td>1174</td><td>-</td><td>Cyanobacteria</td><td>Nodosilinea sp. P-1105</td><td>leucine-rich repeat domain-containing protein [Nodosilinea sp. P-1105].</td><td>GCF_012911975.1</td></tr>
</table>

<table>
<tr><th>acc</th><th>operon</th><th>architecture</th><th>len</th><th>gen.name</th><th>taxend</th><th>species</th><th>defline</th><th>gca</th></tr>
<tr><td>WP_169792421.1</td><td><-ABC-ATPase<-?<-?<-?||?→ LRR-repeats+AP-GTPase+COR*→</td><td>LRR-repeats+AP-GTPase+COR</td><td>867</td><td>-</td><td>Actinobacteria</td><td>Actinoplanes rectilineatus</td><td>GTP-binding protein [Actinoplanes rectilineatus].</td><td>GCF_000962825.1</td></tr>
<tr><td>WP_170065039.1</td><td>REC→||<-?<-?<-?<-?||?→ LRR-repeats+AP-GTPase+COR+DpnII-MboI-NTD+TIR*→</td><td>LRR-repeats+AP-GTPase+COR+DpnII-MboI-NTD+TIR</td><td>992</td><td>-</td><td>Gammaproteobacteria</td><td>Methylovulum psychrotolerans</td><td>leucine-rich repeat domain-containing protein [Methylovulum psychrotolerans].</td><td>GCF_002923755.1</td></tr>
<tr><td>WP_170200769.1</td><td>HNOB→ HISKIN→?→||<-?<-?||LRR-repeats+AP-GTPase+COR+TIR*→</td><td>LRR-repeats+AP-GTPase+COR+TIR</td><td>921</td><td>-</td><td>Betaproteobacteria</td><td>Ideonella azotifigens</td><td>TIR domain-containing protein [Ideonella azotifigens].</td><td>GCF_006519715.1</td></tr>
<tr><td>WP_170318945.1</td><td>LRR-repeats+AP-GTPase+COR*→</td><td>LRR-repeats+AP-GTPase+COR</td><td>514</td><td>-</td><td>Actinobacteria</td><td>Allorhizocola rhizosphaerae</td><td>leucine-rich repeat domain-containing protein, partial [Allorhizocola rhizosphaerae].</td><td>GCF_003426775.1</td></tr>
<tr><td>WP_170321800.1</td><td>LRR-repeats+AP-GTPase+COR*→?→ Phytase-like→||<-?||?→?→ PSE→ ABC-ATPase→ ABC-ATPase→</td><td>LRR-repeats+AP-GTPase+COR</td><td>1057</td><td>-</td><td>Actinobacteria</td><td>Acrocarpospora pleiomorpha</td><td>leucine-rich repeat domain-containing protein [Acrocarpospora pleiomorpha].</td><td>GCF_009687885.1</td></tr>
<tr><td>WP_170944127.1</td><td>LRR-repeats+AP-GTPase+COR*→ COR→?→ RelE-ParE→</td><td>LRR-repeats+AP-GTPase+COR</td><td>216</td><td>-</td><td>Cyanobacteria</td><td>Leptolyngbya sp. BC1307</td><td>leucine-rich repeat domain-containing protein, partial [Leptolyngbya sp. BC1307].</td><td>GCF_002286735.1</td></tr>
<tr><td>WP_171062796.1</td><td>LRR-repeats+AP-GTPase+COR*→</td><td>LRR-repeats+AP-GTPase+COR</td><td>864</td><td>-</td><td>Bacteroidetes</td><td>Larkinella sp. C7</td><td>hypothetical protein [Larkinella sp. C7].</td><td>GCF_006149005.1</td></tr>
<tr><td>WP_171572097.1</td><td>EAD10+VMAP-M1+VMAP-C→?→ VWA+LRR-repeats+AP-GTPase+COR→
Cluster5_5clades→||<-?<-?<-LRR-repeats+AP-GTPase+COR<-?<-CASPASE+EAD10<-?<-tRNA||?→?→ Cluster2368_2clades→ Cluster2098_2clades→</td><td>VWA+LRR-repeats+AP-GTPase+COR</td><td>1320</td><td>-</td><td>Bacteroidetes</td><td>Flavobacterium sp. CLA17</td><td>leucine-rich repeat domain-containing protein [Flavobacterium sp. CLA17].</td><td>GCF_013112255.1</td></tr>
<tr><td>WP_171572101.1</td><td>LRR-repeats+AP-GTPase+COR*→</td><td>LRR-repeats+AP-GTPase+COR</td><td>845</td><td>-</td><td>Bacteroidetes</td><td>Flavobacterium sp. CLA17</td><td>leucine-rich repeat domain-containing protein [Flavobacterium sp. CLA17].</td><td>GCF_013112255.1</td></tr>
<tr><td>WP_171574279.1</td><td>ABC-ATPase→ Ferredoxin-betagrasp→?→?→||<-?||LRR-repeats+AP-GTPase+COR+TM+TM*→ Cluster1136_2clades→
PSE→?→||<-Cluster1782_2clades<-?<-Cluster1583_2clades<-Cluster1881_2clades<-Cluster1340_2clades</td><td>LRR-repeats+AP-GTPase+COR+TM+TM</td><td>1162</td><td>-</td><td>Bacteroidetes</td><td>Flavobacterium sp. CLA17</td><td>leucine-rich repeat domain-containing protein [Flavobacterium sp. CLA17].</td><td>GCF_013112255.1</td></tr>
<tr><td>WP_171574361.1</td><td><-SIG+TM+TM+TM+TM+TM+TM<-?||?→||<-SAM-methylase<-Cluster2335_2clades<-SIG+TM||?→
LRR-repeats+AP-GTPase+COR*→?→?→?→?→||<-ISOFLAVOMETHYLASE-HTH</td><td>LRR-repeats+AP-GTPase+COR</td><td>1103</td><td>-</td><td>Bacteroidetes</td><td>Flavobacterium sp. CLA17</td><td>leucine-rich repeat domain-containing protein [Flavobacterium sp. CLA17].</td><td>GCF_013112255.1</td></tr>
<tr><td>WP_171626408.1</td><td><-Cluster1969_2clades<-?<-?<-?||?→ LRR-repeats+AP-GTPase+COR+TIR*→||<-?||?→?→ METHYLASE→ ParA-Soj-PloopNTPase→ ParB→</td><td>LRR-repeats+AP-GTPase+COR+TIR</td><td>1281</td><td>-</td><td>Gammaproteobacteria</td><td>Pseudoalteromonas caenipelagi</td><td>leucine-rich repeat protein [Pseudoalteromonas caenipelagi].</td><td>GCF_013140855.1</td></tr>
<tr><td>WP_171740721.1</td><td><-SIG+ABhydrolase<-RNA-Helicase||LRR-repeats+AP-GTPase+COR*→</td><td>LRR-repeats+AP-GTPase+COR</td><td>859</td><td>-</td><td>Bacteroidetes</td><td>Spirosoma sp. TS118</td><td>hypothetical protein [Spirosoma sp. TS118].</td><td>GCF_013127955.1</td></tr>
<tr><td>WP_171801965.1</td><td>LRR-repeats+AP-GTPase+COR*→</td><td>LRR-repeats+AP-GTPase+COR</td><td>907</td><td>-</td><td>Gammaproteobacteria</td><td>Vibrio europaeus</td><td>GTP-binding protein [Vibrio europaeus].</td><td>GCF_013154935.1</td></tr>
<tr><td>WP_171971806.1</td><td>LRR-repeats+AP-GTPase+COR+TM+TM*→</td><td>LRR-repeats+AP-GTPase+COR+TM+TM</td><td>850</td><td>-</td><td>Cyanobacteria</td><td>Limnothrix rosea</td><td>leucine-rich repeat domain-containing protein [Limnothrix rosea].</td><td>GCF_001904615.1</td></tr>
<tr><td>WP_171976623.1</td><td>LRR-repeats+AP-GTPase+COR+TIR*→||<-?||?→||<-?||NUDIX→?→||<-Arginase</td><td>LRR-repeats+AP-GTPase+COR+TIR</td><td>1029</td><td>-</td><td>Cyanobacteria</td><td>Brasilonema octagenarum</td><td>leucine-rich repeat domain-containing protein [Brasilonema octagenarum].</td><td>GCF_006968915.1</td></tr>
<tr><td>WP_171977678.1</td><td><-SIG+TM+TM+Calcineurin||?→?→?→ LRR-repeats→ LRR-repeats+AP-GTPase+COR+TIR*→</td><td>LRR-repeats+AP-GTPase+COR+TIR</td><td>817</td><td>-</td><td>Cyanobacteria</td><td>Brasilonema octagenarum</td><td>leucine-rich repeat domain-containing protein [Brasilonema octagenarum].</td><td>GCF_006968915.1</td></tr>
<tr><td>WP_171982135.1</td><td>LRR-repeats+AP-GTPase+COR+TIR*→||<-?<-?<-MarR-HTH||?→||<-MarR-HTH<-?||Thioredoxin→</td><td>LRR-repeats+AP-GTPase+COR+TIR</td><td>1247</td><td>-</td><td>Betaproteobacteria</td><td>Aquaspirillum sp. LM1</td><td>leucine-rich repeat domain-containing protein [Aquaspirillum sp. LM1].</td><td>GCF_002002905.1</td></tr>
<tr><td>WP_171982185.1</td><td><-LexA-protease||?→||<-?<-?||?→ HSP70→ DNAJ→ PSE→ LRR-repeats+AP-GTPase+COR+TIR*→</td><td>LRR-repeats+AP-GTPase+COR+TIR</td><td>916</td><td>-</td><td>Betaproteobacteria</td><td>Aquaspirillum sp. LM1</td><td>leucine-rich repeat domain-containing protein [Aquaspirillum sp. LM1].</td><td>GCF_002002905.1</td></tr>
</table>

<table>
<tr><th>acc</th><th>operon</th><th>architecture</th><th>len</th><th>gen.name</th><th>taxend</th><th>species</th><th>defline</th><th>gca</th></tr>
<tr><td>WP_172187602.1</td><td>Chromo-N+CHROMO→||<-RelE-ParE<-?||?→ LRR-repeats+AP-GTPase+COR*→?→ SIG+TM+TM+TM+TM+TM+TM+TM+TM+TM+TM+TM→</td><td>LRR-repeats+AP-GTPase+COR</td><td>913</td><td>-</td><td>Cyanobacteria</td><td>Microcoleus sp. IPMA8</td><td>leucine-rich repeat domain-containing protein [Microcoleus sp. IPMA8].</td><td>GCF_013179805.1</td></tr>
<tr><td>WP_172195227.1</td><td>LRR-repeats+AP-GTPase+COR+TIR*→||<-?||?→||<-?||NUDIX→?→||<-Arginase</td><td>LRR-repeats+AP-GTPase+COR+TIR</td><td>1029</td><td>-</td><td>Cyanobacteria</td><td>Brasilonema sennae</td><td>leucine-rich repeat domain-containing protein [Brasilonema sennae].</td><td>GCF_006968745.1</td></tr>
<tr><td>WP_172195319.1</td><td><-SIG+TM+TM+Calcineurin||?→?→?→ LRR-repeats+AP-GTPase+COR+TIR*→</td><td>LRR-repeats+AP-GTPase+COR+TIR</td><td>1330</td><td>-</td><td>Cyanobacteria</td><td>Brasilonema sennae</td><td>leucine-rich repeat domain-containing protein [Brasilonema sennae].</td><td>GCF_006968745.1</td></tr>
<tr><td>WP_172621632.1</td><td>LRR-repeats+AP-GTPase+COR+TIR*→||<-?||?→?→?→?→||<-?||cNMPBD→</td><td>LRR-repeats+AP-GTPase+COR+TIR</td><td>975</td><td>-</td><td>Bacteroidetes</td><td>Chitinophaga pinensis</td><td>TIR domain-containing protein [Chitinophaga pinensis].</td><td>GCF_902167305.1</td></tr>
<tr><td>WP_172622229.1</td><td>LRR-repeats+AP-GTPase+COR+TIR*→||<-?||?→?→?→?→||<-?||cNMPBD→</td><td>LRR-repeats+AP-GTPase+COR+TIR</td><td>975</td><td>-</td><td>Bacteroidetes</td><td>Chitinophaga pinensis</td><td>TIR domain-containing protein [Chitinophaga pinensis].</td><td>GCF_902167325.1</td></tr>
<tr><td>WP_172632369.1</td><td>REC→ LRR-repeats+AP-GTPase+COR*→</td><td>LRR-repeats+AP-GTPase+COR</td><td>1102</td><td>-</td><td>Chloroflexi</td><td>Dictyobacter sp. Uno17</td><td>leucine-rich repeat domain-containing protein [Dictyobacter sp. Uno17].</td><td>GCF_008326305.1</td></tr>
<tr><td>WP_173012781.1</td><td>LRR-repeats+AP-GTPase+COR+TIR*→||<-ParB<-ParA-Soj-PloopNTPase</td><td>LRR-repeats+AP-GTPase+COR+TIR</td><td>751</td><td>-</td><td>Alphaproteobacteria</td><td>Niveispirillum sp. SYP-B3756</td><td>TIR domain-containing protein [Niveispirillum sp. SYP-B3756].</td><td>GCF_009495745.1</td></tr>
<tr><td>WP_173037887.1</td><td><-SIG+TM<-?<-ABC-ATPase||?→?→?→?→ LRR-repeats+AP-GTPase+COR*→</td><td>LRR-repeats+AP-GTPase+COR</td><td>981</td><td>-</td><td>Actinobacteria</td><td>Phytohabitans flavus</td><td>hypothetical protein [Phytohabitans flavus].</td><td>GCF_011764545.1</td></tr>
<tr><td>WP_173124261.1</td><td>Cluster1612_2clades→?→?→||<-?<-?||LRR-repeats+AP-GTPase+COR*→</td><td>LRR-repeats+AP-GTPase+COR</td><td>1244</td><td>-</td><td>Actinobacteria</td><td>Kibdelosporangium persicum</td><td>leucine-rich repeat domain-containing protein [Kibdelosporangium persicum].</td><td>GCF_013280595.1</td></tr>
<tr><td>WP_173803276.1</td><td>LRR-repeats+AP-GTPase+COR+TIR*→||<-?<-ABC_membrane+ABC_tran||Cyanophycinsyn-ATPgrasp→ Cyanophycinsyn-ATPgrasp→</td><td>LRR-repeats+AP-GTPase+COR+TIR</td><td>794</td><td>-</td><td>Betaproteobacteria</td><td>Leptothrix sp. C29</td><td>TIR domain-containing protein, partial [Leptothrix sp. C29].</td><td>GCF_013294065.1</td></tr>
<tr><td>WP_174565117.1</td><td>LRR-repeats+AP-GTPase*→</td><td>LRR-repeats+AP-GTPase</td><td>485</td><td>-</td><td>Bacteroidetes</td><td>Flavobacterium sp. A45</td><td>leucine-rich repeat domain-containing protein, partial [Flavobacterium sp. A45].</td><td>GCF_002001005.1</td></tr>
<tr><td>WP_174708182.1</td><td>PAS+HISKIN→ REC→?→?→ LRR-repeats+AP-GTPase+COR+DUF4404*→||<-?||?→?→ ABC_membrane+ABC_tran→ ABC_membrane+ABC_tran→</td><td>LRR-repeats+AP-GTPase+COR+DUF4404</td><td>1122</td><td>-</td><td>Cyanobacteria</td><td>Nostoc sp. TCL240-02</td><td>leucine-rich repeat domain-containing protein [Nostoc sp. TCL240-02].</td><td>GCF_013343235.1</td></tr>
<tr><td>WP_174708278.1</td><td>ABhydrolase→?→||<-?||LRR-repeats→ PSE→ LRR-repeats+AP-GTPase+COR+TIR*→?→||<-?<-SIG+NUDIX</td><td>LRR-repeats+AP-GTPase+COR+TIR</td><td>919</td><td>-</td><td>Cyanobacteria</td><td>Nostoc sp. TCL240-02</td><td>leucine-rich repeat domain-containing protein [Nostoc sp. TCL240-02].</td><td>GCF_013343235.1</td></tr>
<tr><td>WP_174709624.1</td><td><-Arginase<-?||LRR-repeats+AP-GTPase+COR+TIR→ LRR-repeats+AP-GTPase+COR+TIR→?→||<-ABC-ATPase</td><td>LRR-repeats+AP-GTPase+COR+TIR</td><td>1126</td><td>-</td><td>Cyanobacteria</td><td>Nostoc sp. TCL240-02</td><td>leucine-rich repeat domain-containing protein [Nostoc sp. TCL240-02].</td><td>GCF_013343235.1</td></tr>
<tr><td>WP_174709625.1</td><td>LRR-repeats+AP-GTPase+COR+TIR*→</td><td>LRR-repeats+AP-GTPase+COR+TIR</td><td>1123</td><td>-</td><td>Cyanobacteria</td><td>Nostoc sp. TCL240-02</td><td>leucine-rich repeat domain-containing protein [Nostoc sp. TCL240-02].</td><td>GCF_013343235.1</td></tr>
<tr><td>WP_174711091.1</td><td>LRR-repeats+AP-GTPase+COR*→</td><td>LRR-repeats+AP-GTPase+COR</td><td>1189</td><td>-</td><td>Cyanobacteria</td><td>Nostoc sp. TCL240-02</td><td>leucine-rich repeat domain-containing protein [Nostoc sp. TCL240-02].</td><td>GCF_013343235.1</td></tr>
<tr><td>WP_174712042.1</td><td>LRR-repeats+AP-GTPase+COR*→?→||<-?||?→||<-?||?→ ClpABN-AAA+ClpABC-AAA→</td><td>LRR-repeats+AP-GTPase+COR</td><td>967</td><td>-</td><td>Cyanobacteria</td><td>Nostoc sp. TCL240-02</td><td>leucine-rich repeat domain-containing protein [Nostoc sp. TCL240-02].</td><td>GCF_013343235.1</td></tr>
<tr><td>WP_175357678.1</td><td>Cluster54_3clades→?→ RelE-ParE→ Cluster53_2clades→?→ LRR-repeats+AP-GTPase+COR*→?→?→?→||<-?||?→||<-Pentapeptide-repeats</td><td>LRR-repeats+AP-GTPase+COR</td><td>928</td><td>-</td><td>Cyanobacteria</td><td>Pseudanabaena biceps</td><td>leucine-rich repeat domain-containing protein [Pseudanabaena biceps].</td><td>GCF_013361095.1</td></tr>
<tr><td>WP_175471885.1</td><td>Mbetalac→?→ LRR-repeats+AP-GTPase+COR*→||<-?<-ICLR-HTH||?→?→?→ ABhydrolase→</td><td>LRR-repeats+AP-GTPase+COR</td><td>886</td><td>-</td><td>Actinobacteria</td><td>Geodermatophilus telluris</td><td>hypothetical protein [Geodermatophilus telluris].</td><td>GCF_900102745.1</td></tr>
</table>

<table>
<tr><th>acc</th><th>operon</th><th>architecture</th><th>len</th><th>gen.name</th><th>taxend</th><th>species</th><th>defline</th><th>gca</th></tr>
<tr><td>WP_175586865.1</td><td><-HISKIN||?→||<-?||?→?→ PSE→||<-?||PSE→||<-?||LRR-repeats+AP-GTPase+COR*→</td><td>LRR-repeats+AP-GTPase+COR</td><td>868</td><td>-</td><td>Cyanobacteria</td><td>Nostoc sp. UIC 10630</td><td>leucine-rich repeat domain-containing protein, partial [Nostoc sp. UIC 10630].</td><td>GCF_010747425.1</td></tr>
<tr><td>WP_175608776.1</td><td>LRR-repeats+AP-GTPase+COR+TIR*→?→?→?→?→?→ TIR→ TM+TM+TM→</td><td>LRR-repeats+AP-GTPase+COR+TIR</td><td>1128</td><td>-</td><td>Bacteroidetes</td><td>Chryseobacterium gambrini</td><td>leucine-rich repeat domain-containing protein [Chryseobacterium gambrini].</td><td>GCF_900156825.1</td></tr>
<tr><td>WP_176159351.1</td><td>METHYLASE→?→||<-?||SIG+Trypsin+PDZ→?→ Cluster978_2clades→ LRR-repeats+AP-GTPase+COR+TIR*→?→ GDSL→</td><td>LRR-repeats+AP-GTPase+COR+TIR</td><td>813</td><td>-</td><td>Verrucomicrobia</td><td>Prosthecobacter debontii</td><td>TIR domain-containing protein [Prosthecobacter debontii].</td><td>GCF_900167535.1</td></tr>
<tr><td>WP_176159465.1</td><td>SAM-methylase→?→||<-?<-SIG+ABhydrolase||tRNA→?→ Cluster1685_2clades→||<-?||LRR-repeats+AP-GTPase+COR+TIR*→</td><td>LRR-repeats+AP-GTPase+COR+TIR</td><td>949</td><td>-</td><td>Verrucomicrobia</td><td>Prosthecobacter debontii</td><td>leucine-rich repeat domain-containing protein [Prosthecobacter debontii].</td><td>GCF_900167535.1</td></tr>
<tr><td>WP_176722277.1</td><td>LRR-repeats+AP-GTPase+COR*→</td><td>LRR-repeats+AP-GTPase+COR</td><td>881</td><td>-</td><td>Gammaproteobacteria</td><td>Candidatus Thiosymbion oneisti</td><td>leucine-rich repeat domain-containing protein, partial [Candidatus Thiosymbion oneisti].</td><td>GCF_900092655.1</td></tr>
<tr><td>WP_176722477.1</td><td>LRR-repeats+AP-GTPase+COR*→?→ PSE→?→?→?→ ABC-ATPase→||<-HTH</td><td>LRR-repeats+AP-GTPase+COR</td><td>838</td><td>-</td><td>Gammaproteobacteria</td><td>Candidatus Thiosymbion oneisti</td><td>hypothetical protein [Candidatus Thiosymbion oneisti].</td><td>GCF_900092655.1</td></tr>
<tr><td>WP_177187116.1</td><td>LRR-repeats+AP-GTPase+COR*→||<-?||?→||<-RnasePH+RnasePH+KH+S1</td><td>LRR-repeats+AP-GTPase+COR</td><td>911</td><td>-</td><td>Bacteroidetes</td><td>Chryseobacterium jeonii</td><td>GTP-binding protein [Chryseobacterium jeonii].</td><td>GCF_900112115.1</td></tr>
<tr><td>WP_177199908.1</td><td>PRTase→||<-?<-?<-?||LRR-repeats+AP-GTPase+COR+TIR*→?→?→?→?→ Aminotran_1_2→</td><td>LRR-repeats+AP-GTPase+COR+TIR</td><td>987</td><td>-</td><td>Bacteroidetes</td><td>Flexibacter flexilis</td><td>leucine-rich repeat domain-containing protein [Flexibacter flexilis].</td><td>GCF_900112255.1</td></tr>
<tr><td>WP_177201087.1</td><td>LRR-repeats+AP-GTPase+COR+TIR*→</td><td>LRR-repeats+AP-GTPase+COR+TIR</td><td>764</td><td>-</td><td>Gammaproteobacteria</td><td>Neptunomonas qingdaonensis</td><td>TIR domain-containing protein [Neptunomonas qingdaonensis].</td><td>GCF_900113275.1</td></tr>
<tr><td>WP_178133228.1</td><td>LRR-repeats+AP-GTPase+COR+CASPASE*→</td><td>LRR-repeats+AP-GTPase+COR+CASPASE</td><td>1170</td><td>-</td><td>Alphaproteobacteria</td><td>Enhydrobacter sp. CC-CFT640</td><td>CHAT domain-containing protein [Enhydrobacter sp. CC-CFT640].</td><td>GCF_008039615.1</td></tr>
<tr><td>WP_178316998.1</td><td>LRR-repeats+AP-GTPase+COR*→</td><td>LRR-repeats+AP-GTPase+COR</td><td>1450</td><td>-</td><td>Firmicutes</td><td>Lachnospiraceae bacterium</td><td>GTP-binding protein [Lachnospiraceae bacterium].</td><td>-</td></tr>
<tr><td>WP_179047135.1</td><td>ABC-ATPase→||<-?||?→||<-?||LRR-repeats+AP-GTPase+COR+TIR*→?→||<-?<-Cluster1974_2clades||?→?→||<-PSE<-Cluster1515_2clades</td><td>LRR-repeats+AP-GTPase+COR+TIR</td><td>939</td><td>-</td><td>Cyanobacteria</td><td>Nostoc sp. TCL26-01</td><td>leucine-rich repeat domain-containing protein [Nostoc sp. TCL26-01].</td><td>GCF_013393945.1</td></tr>
<tr><td>WP_179049659.1</td><td>Cluster1081_2clades→?→?→||<-?<-PSE<-?||LRR-repeats+AP-GTPase+COR+TIR*→||<-?<-MoxR-AAA||?→ TPR-repeats→||<-SIG+TM+TM+TM+TM+TM+TM+TM+TM+TM+TM+TM<-SIG+TM</td><td>LRR-repeats+AP-GTPase+COR+TIR</td><td>1127</td><td>-</td><td>Cyanobacteria</td><td>Nostoc sp. TCL26-01</td><td>leucine-rich repeat domain-containing protein [Nostoc sp. TCL26-01].</td><td>GCF_013393945.1</td></tr>
<tr><td>WP_179049876.1</td><td><-ACET<-?||Pentapeptide→ Cluster54_3clades→ Cluster1093_2clades→?→||<-?||LRR-repeats+AP-GTPase+COR+TIR*→?→?→ HNH→?→ PSE→||<-?<-?<-Cluster2062_2clades</td><td>LRR-repeats+AP-GTPase+COR+TIR</td><td>1116</td><td>-</td><td>Cyanobacteria</td><td>Nostoc sp. TCL26-01</td><td>leucine-rich repeat domain-containing protein [Nostoc sp. TCL26-01].</td><td>GCF_013393945.1</td></tr>
<tr><td>WP_179050104.1</td><td><-ABC_membrane+ABC_tran<-?<-?||?→||<-?<-?||LRR-repeats+AP-GTPase+COR+TIR*→?→||<-?<-?<-?<-?<-Cluster2103_2clades</td><td>LRR-repeats+AP-GTPase+COR+TIR</td><td>999</td><td>-</td><td>Cyanobacteria</td><td>Nostoc sp. TCL26-01</td><td>leucine-rich repeat domain-containing protein [Nostoc sp. TCL26-01].</td><td>GCF_013393945.1</td></tr>
<tr><td>WP_179067421.1</td><td>REC→ REC→?→||<-?||LRR-repeats+AP-GTPase+COR+DUF4404*→</td><td>LRR-repeats+AP-GTPase+COR+DUF4404</td><td>1124</td><td>-</td><td>Cyanobacteria</td><td>Nostoc sp. C052</td><td>leucine-rich repeat domain-containing protein [Nostoc sp. C052].</td><td>GCF_013393905.1</td></tr>
<tr><td>WP_179068637.1</td><td>Cluster5_5clades→ Cluster5_5clades→ LRR-repeats+AP-GTPase+COR+TM+TM*→||<-?||Pkinase→</td><td>LRR-repeats+AP-GTPase+COR+TM+TM</td><td>904</td><td>-</td><td>Cyanobacteria</td><td>Nostoc sp. C052</td><td>leucine-rich repeat protein [Nostoc sp. C052].</td><td>GCF_013393905.1</td></tr>
<tr><td>WP_179072263.1</td><td>TPR-repeats→?→ LRR-repeats+AP-GTPase+COR*→</td><td>LRR-repeats+AP-GTPase+COR</td><td>1193</td><td>-</td><td>Cyanobacteria</td><td>Nostoc sp. C057</td><td>leucine-rich repeat domain-containing protein [Nostoc sp. C057].</td><td>GCF_013393925.1</td></tr>
<tr><td>WP_179072929.1</td><td><-Arginase<-?||?→ LRR-repeats+AP-GTPase+COR+TIR*→?→||<-?||?→?→?→||<-tRNA||Cluster1742_2clades→</td><td>LRR-repeats+AP-GTPase+COR+TIR</td><td>1009</td><td>-</td><td>Cyanobacteria</td><td>Nostoc sp. C057</td><td>leucine-rich repeat domain-containing protein [Nostoc sp. C057].</td><td>GCF_013393925.1</td></tr>
</table>

<table>
<tr><th>acc</th><th>operon</th><th>architecture</th><th>len</th><th>gen.name</th><th>taxend</th><th>species</th><th>defline</th><th>gca</th></tr>
<tr><td>WP_179074744.1</td><td>Cluster5_5clades→ Cluster5_5clades→ LRR-repeats+AP-GTPase+COR+TM+TM*→||<-CASPASE+TM</td><td>LRR-repeats+AP-GTPase+COR+TM+TM</td><td>865</td><td>-</td><td>Cyanobacteria</td><td>Nostoc sp. C057</td><td>leucine-rich repeat domain-containing protein [Nostoc sp. C057].</td><td>GCF_013393925.1</td></tr>
<tr><td>WP_179212721.1</td><td>LRR-repeats+AP-GTPase+COR*→</td><td>LRR-repeats+AP-GTPase+COR</td><td>850</td><td>-</td><td>Bacteroidetes</td><td>Sphingobacteriales bacterium TSM_CSS</td><td>GTP-binding protein [Sphingobacteriales bacterium TSM_CSS].</td><td>-</td></tr>
<tr><td>WP_180284786.1</td><td><-MarR-HTH||?→ SIG+TM+TM+TM+TM+TM+TM+TM→ SIG+TM+TM+TM+TM+TM+TM+TM→ ABC-ATPase→ ABC-ATPase→?→ LRR-repeats+AP-GTPase+COR+TIR*→</td><td>LRR-repeats+AP-GTPase+COR+TIR</td><td>709</td><td>-</td><td>Alphaproteobacteria</td><td>Azospirillum oleiclasticum</td><td>hypothetical protein [Azospirillum oleiclasticum].</td><td>GCF_013423495.1</td></tr>
<tr><td>WP_180287677.1</td><td>SbcC→?→ LRR-repeats+AP-GTPase+COR+TIR*→</td><td>LRR-repeats+AP-GTPase+COR+TIR</td><td>722</td><td>-</td><td>Alphaproteobacteria</td><td>Azospirillum oleiclasticum</td><td>hypothetical protein [Azospirillum oleiclasticum].</td><td>GCF_013423485.1</td></tr>
<tr><td>WP_180693101.1</td><td>LRR-repeats+AP-GTPase+COR+TIR*→||<-?<-ABC_membrane+ABC_tran||Cyanophycinsyn-ATPgrasp→ Cyanophycinsyn-ATPgrasp→</td><td>LRR-repeats+AP-GTPase+COR+TIR</td><td>904</td><td>-</td><td>Betaproteobacteria</td><td>Sphaerotilus natans</td><td>leucine-rich repeat domain-containing protein, partial [Sphaerotilus natans].</td><td>GCF_013426975.1</td></tr>
<tr><td>WP_181245610.1</td><td>TM→ LRR-repeats+AP-GTPase+COR*→</td><td>LRR-repeats+AP-GTPase+COR</td><td>1035</td><td>-</td><td>Actinobacteria</td><td>Glycomyces artemisiae</td><td>leucine-rich repeat domain-containing protein [Glycomyces artemisiae].</td><td>GCF_003002955.1</td></tr>
<tr><td>WP_181357317.1</td><td><-REC||REC→||<-?<-?<-?<-TIR+TM+CHASE2+CHASE2+TM+TM+TM||LRR-repeats+AP-GTPase+COR+TIR*→</td><td>LRR-repeats+AP-GTPase+COR+TIR</td><td>1082</td><td>-</td><td>Cyanobacteria</td><td>Stenomitos frigidus</td><td>leucine-rich repeat domain-containing protein [Stenomitos frigidus].</td><td>GCF_003003795.1</td></tr>
<tr><td>WP_181842201.1</td><td>VWA+vWA-L+LRR-repeats+AP-GTPase+COR+TIR*→</td><td>VWA+vWA-L+LRR-repeats+AP-GTPase+COR+TIR</td><td>1607</td><td>-</td><td>Bacteroidetes</td><td>Emticicia sp. BO119</td><td>leucine-rich repeat domain-containing protein [Emticicia sp. BO119].</td><td>GCF_013912375.1</td></tr>
<tr><td>WP_181930367.1</td><td>LRR-repeats+AP-GTPase+COR*→</td><td>LRR-repeats+AP-GTPase+COR</td><td>1084</td><td>-</td><td>Cyanobacteria</td><td>Nostoc edaphicum</td><td>leucine-rich repeat domain-containing protein [Nostoc edaphicum].</td><td>GCF_014023275.1</td></tr>
<tr><td>WP_181931253.1</td><td><-Pkinase+Pentapeptide<-?<-?<-?||?→ LRR-repeats+AP-GTPase+COR+TIR*→||<-Cluster5_5clades<-Arginase<-?||?→||<-?<-?||Cluster54_3clades→</td><td>LRR-repeats+AP-GTPase+COR+TIR</td><td>1046</td><td>-</td><td>Cyanobacteria</td><td>Nostoc edaphicum</td><td>leucine-rich repeat domain-containing protein [Nostoc edaphicum].</td><td>GCF_014023275.1</td></tr>
<tr><td>WP_182043976.1</td><td>LRR-repeats+AP-GTPase+COR+TIR*→</td><td>LRR-repeats+AP-GTPase+COR+TIR</td><td>1134</td><td>-</td><td>Bacteroidetes</td><td>Moheibacter sp. BDHS18</td><td>leucine-rich repeat domain-containing protein [Moheibacter sp. BDHS18].</td><td>GCF_014042165.1</td></tr>
<tr><td>WP_182891755.1</td><td>LRR-repeats+AP-GTPase+COR*→</td><td>LRR-repeats+AP-GTPase+COR</td><td>1689</td><td>-</td><td>Actinobacteria</td><td>Amycolatopsis sp. DR6-1</td><td>hypothetical protein [Amycolatopsis sp. DR6-1].</td><td>GCF_014145675.1</td></tr>
<tr><td>WP_183290945.1</td><td><-SIG+ABhydrolase||?→?→?→?→||<-?<-?||LRR-repeats+AP-GTPase+COR+TIR→ LRR-repeats+AP-GTPase+COR+TIR→?→ Cluster1478_2clades→?→ LRR-repeats+AP-GTPase+COR+TIR*→?→ SIGMA-HTH→ SIGMA-HTH→</td><td>LRR-repeats+AP-GTPase+COR+TIR</td><td>1386</td><td>-</td><td>Bacteroidetes</td><td>Chitinophaga sancti</td><td>leucine-rich repeat domain-containing protein [Chitinophaga sancti].</td><td>GCF_014190745.1</td></tr>
<tr><td>WP_183290948.1</td><td>LRR-repeats+AP-GTPase+COR_TIR*→</td><td>LRR-repeats+AP-GTPase+COR_TIR</td><td>1378</td><td>-</td><td>Bacteroidetes</td><td>Chitinophaga sancti</td><td>TIR domain-containing protein [Chitinophaga sancti].</td><td>GCF_014190745.1</td></tr>
<tr><td>WP_183290949.1</td><td>LRR-repeats+AP-GTPase+COR+TIR*→</td><td>LRR-repeats+AP-GTPase+COR+TIR</td><td>1391</td><td>-</td><td>Bacteroidetes</td><td>Chitinophaga sancti</td><td>hypothetical protein [Chitinophaga sancti].</td><td>GCF_014190745.1</td></tr>
<tr><td>WP_183974348.1</td><td>FGS→ FGS→ FGS→ LRR-repeats+AP-GTPase+COR+CASPASE*→</td><td>LRR-repeats+AP-GTPase+COR+CASPASE</td><td>981</td><td>-</td><td>Bacteroidetes</td><td>Runella defluvii</td><td>hypothetical protein [Runella defluvii].</td><td>GCF_014195535.1</td></tr>
<tr><td>WP_183975497.1</td><td><-SIGMA-HTH||SIG+ABhydrolase→ LRR-repeats+AP-GTPase+COR*→?→||<-?<-?<-?<-?<-?<-SIGMA-HTH</td><td>LRR-repeats+AP-GTPase+COR</td><td>999</td><td>-</td><td>Bacteroidetes</td><td>Runella defluvii</td><td>leucine-rich repeat domain-containing protein [Runella defluvii].</td><td>GCF_014195535.1</td></tr>
<tr><td>WP_183978441.1</td><td>TIR+TPR+TPR-S→||<-?<-?||?→||<-?||LRR-repeats+AP-GTPase+COR+TIR→<i>?→ TPR-repeats→?→ TPR-repeats→?→||<-AP-GTPase+COR</i><-?<-HTH</td><td>LRR-repeats+AP-GTPase+COR+TIR</td><td>603</td><td>-</td><td>Bacteroidetes</td><td>Runella defluvii</td><td>hypothetical protein [Runella defluvii].</td><td>GCF_014195535.1</td></tr>
<tr><td>WP_183978452.1</td><td>LRR-repeats+AP-GTPase+COR+TIR*→</td><td>LRR-repeats+AP-GTPase+COR+TIR</td><td>1000</td><td>-</td><td>Bacteroidetes</td><td>Runella defluvii</td><td>leucine-rich repeat domain-containing protein [Runella defluvii].</td><td>GCF_014195535.1</td></tr>
<tr><td>WP_184494281.1</td><td>Cluster1244_2clades→?→ Cluster1291_2clades→ TIR+LRR-repeats+AP-GTPase+TIR*→||<-?<-?<-?||REC→</td><td>TIR+LRR-repeats+AP-GTPase+TIR</td><td>1102</td><td>-</td><td>Bacteroidetes</td><td>Algoriphagus iocasae</td><td>TIR domain-containing protein [Algoriphagus iocasae].</td><td>GCF_014206875.1</td></tr>
</table>

<table>
<tr><th>acc</th><th>operon</th><th>architecture</th><th>len</th><th>gen.name</th><th>taxend</th><th>species</th><th>defline</th><th>gca</th></tr>
<tr><td>WP_184831163.1</td><td>SIG+TM+TM+TM+TM+TM→||<-?||?→?→?→?→ PSE→ LRR-repeats+AP-GTPase+COR*→||<-?<-?||wHTH-4stranded+TPRs+APATPase+TPR-repeats→</td><td>LRR-repeats+AP-GTPase+COR</td><td>802</td><td>-</td><td>Actinobacteria</td><td>Allocatelliglobosispora scoriae</td><td>hypothetical protein [Allocatelliglobosispora scoriae].</td><td>GCF_014204945.1</td></tr>
<tr><td>WP_184846103.1</td><td>LRR-repeats+AP-GTPase+COR*→||<-?<-?<-?<-BACTERIALFRINGE+TM+TM</td><td>LRR-repeats+AP-GTPase+COR</td><td>1247</td><td>-</td><td>Actinobacteria</td><td>Allocatelliglobosispora scoriae</td><td>leucine-rich repeat domain-containing protein [Allocatelliglobosispora scoriae].</td><td>GCF_014204945.1</td></tr>
<tr><td>WP_184892074.1</td><td>LRR-repeats+AP-GTPase+COR*→?→?→ REC→ HISKIN→</td><td>LRR-repeats+AP-GTPase+COR</td><td>1463</td><td>-</td><td>Actinobacteria</td><td>Streptomyces scabiei</td><td>leucine-rich repeat domain-containing protein [Streptomyces scabiei].</td><td>GCF_014203845.1</td></tr>
<tr><td>WP_184930759.1</td><td>LRR-repeats+AP-GTPase+COR*→</td><td>LRR-repeats+AP-GTPase+COR</td><td>954</td><td>-</td><td>Actinobacteria</td><td>Streptomyces sp. SFB5A</td><td>hypothetical protein [Streptomyces sp. SFB5A].</td><td>GCF_014203895.1</td></tr>
<tr><td>WP_184953474.1</td><td>GNTR-HTH→||<-?||REC→ REC→?→ LRR-repeats+AP-GTPase+COR+TIR*→||<-35exo</td><td>LRR-repeats+AP-GTPase+COR+TIR</td><td>1043</td><td>-</td><td>Actinobacteria</td><td>Actinoplanes abujensis</td><td>TIR domain-containing protein [Actinoplanes abujensis].</td><td>GCF_016861995.1</td></tr>
<tr><td>WP_185097769.1</td><td>LRR-repeats+AP-GTPase+COR*→</td><td>LRR-repeats+AP-GTPase+COR</td><td>901</td><td>-</td><td>Bacteroidetes</td><td>Elizabethkingia anophelis</td><td>leucine-rich repeat domain-containing protein [Elizabethkingia anophelis].</td><td>GCF_001703835.1</td></tr>
<tr><td>WP_185204695.1</td><td>Cluster1364_2clades→?→?→?→?→?→?→ LRR-repeats+AP-GTPase+COR*→||<-Cyanophycinsyn-ATPgrasp</td><td>LRR-repeats+AP-GTPase+COR</td><td>1038</td><td>-</td><td>Bacteroidetes</td><td>Chryseobacterium sp. C3</td><td>leucine-rich repeat protein [Chryseobacterium sp. C3].</td><td>GCF_014218905.1</td></tr>
<tr><td>WP_185236942.1</td><td>LRR-repeats+AP-GTPase+COR+TM+TM*→</td><td>LRR-repeats+AP-GTPase+COR+TM+TM</td><td>828</td><td>-</td><td>Cyanobacteria</td><td>Microcystis aeruginosa</td><td>leucine-rich repeat protein [Microcystis aeruginosa].</td><td>GCF_014218745.1</td></tr>
<tr><td>WP_185240111.1</td><td>SAM-methylase→?→||<-?<-SIG+TPM_phosphatase+TM+TM+TM||LRR-repeats+AP-GTPase+COR+TM+TM*→||<-?<-?<-?||?→||<-Pro_CA</td><td>LRR-repeats+AP-GTPase+COR+TM+TM</td><td>874</td><td>-</td><td>Cyanobacteria</td><td>Microcystis aeruginosa</td><td>leucine-rich repeat protein [Microcystis aeruginosa].</td><td>GCF_014218765.1</td></tr>
<tr><td>WP_185478866.1</td><td><-Cluster5_5clades||LRR-repeats+AP-GTPase+COR+DUF4404*→</td><td>LRR-repeats+AP-GTPase+COR+DUF4404</td><td>1084</td><td>-</td><td>Cyanobacteria</td><td>Trichormus variabilis</td><td>leucine-rich repeat domain-containing protein [Trichormus variabilis].</td><td>GCF_014222125.1</td></tr>
<tr><td>WP_185479669.1</td><td>LRR-repeats+AP-GTPase+COR+DUF4404*→||<-?<-ABC-ATPase<-?||?→||<-REC</td><td>LRR-repeats+AP-GTPase+COR+DUF4404</td><td>866</td><td>-</td><td>Cyanobacteria</td><td>Trichormus variabilis</td><td>leucine-rich repeat domain-containing protein, partial [Trichormus variabilis].</td><td>GCF_014222155.1</td></tr>
<tr><td>WP_185482851.1</td><td><-Cluster5_5clades||LRR-repeats+AP-GTPase+COR+DUF4404*→||<-?<-ABC-ATPase<-?||?→||<-REC</td><td>LRR-repeats+AP-GTPase+COR+DUF4404</td><td>1084</td><td>-</td><td>Cyanobacteria</td><td>Trichormus variabilis</td><td>leucine-rich repeat domain-containing protein [Trichormus variabilis].</td><td>GCF_014222225.1</td></tr>
<tr><td>WP_185499823.1</td><td><-Cluster5_5clades||LRR-repeats+AP-GTPase+COR+DUF4404*→||<-?<-ABC-ATPase<-?||?→||<-REC</td><td>LRR-repeats+AP-GTPase+COR+DUF4404</td><td>1199</td><td>-</td><td>Cyanobacteria</td><td>Trichormus variabilis</td><td>leucine-rich repeat domain-containing protein [Trichormus variabilis].</td><td>GCF_014222145.1</td></tr>
<tr><td>WP_185505742.1</td><td><-Cluster5_5clades||LRR-repeats+AP-GTPase+COR+DUF4404*→||<-?<-ABC-ATPase<-?||?→||<-REC</td><td>LRR-repeats+AP-GTPase+COR+DUF4404</td><td>992</td><td>-</td><td>Cyanobacteria</td><td>Trichormus variabilis</td><td>leucine-rich repeat domain-containing protein [Trichormus variabilis].</td><td>GCF_014222245.1</td></tr>
<tr><td>WP_185539469.1</td><td>LRR-repeats+AP-GTPase+COR+DUF4404*→||<-?<-ABC-ATPase<-?||?→||<-REC</td><td>LRR-repeats+AP-GTPase+COR+DUF4404</td><td>895</td><td>-</td><td>Cyanobacteria</td><td>Trichormus variabilis</td><td>leucine-rich repeat domain-containing protein, partial [Trichormus variabilis].</td><td>GCF_014222135.1</td></tr>
<tr><td>WP_185567106.1</td><td>LRR-repeats+AP-GTPase+COR+TM+TM*→||<-CASPASE+TM</td><td>LRR-repeats+AP-GTPase+COR+TM+TM</td><td>862</td><td>-</td><td>Cyanobacteria</td><td>Nostoc sp. 2RC</td><td>leucine-rich repeat domain-containing protein [Nostoc sp. 2RC].</td><td>GCF_014222165.1</td></tr>
<tr><td>WP_185570121.1</td><td>PIN→ LRR-repeats+AP-GTPase+COR+TIR*→</td><td>LRR-repeats+AP-GTPase+COR+TIR</td><td>1020</td><td>-</td><td>Cyanobacteria</td><td>Nostoc sp. 2RC</td><td>leucine-rich repeat domain-containing protein [Nostoc sp. 2RC].</td><td>GCF_014222165.1</td></tr>
<tr><td>WP_185579937.1</td><td>ABhydrolase→?→||<-?||COR+TIR→ LRR-repeats+AP-GTPase+COR+TIR*→?→||<-PIN<-?<-?<-SIG+NUDIX</td><td>LRR-repeats+AP-GTPase+COR+TIR</td><td>1003</td><td>-</td><td>Cyanobacteria</td><td>unclassified Nostoc</td><td>MULTISPECIES: leucine-rich repeat domain-containing protein [unclassified Nostoc].</td><td>GCF_014222255.1</td></tr>
<tr><td>WP_185580380.1</td><td>Cluster5_5clades→ LRR-repeats+AP-GTPase+COR+TIR*→||<-CASPASE+TM</td><td>LRR-repeats+AP-GTPase+COR+TIR</td><td>893</td><td>-</td><td>Cyanobacteria</td><td>unclassified Nostoc</td><td>MULTISPECIES: leucine-rich repeat domain-containing protein [unclassified Nostoc].</td><td>GCF_014222255.1</td></tr>
</table>

<table>
<thead>
<tr><th>acc</th><th>operon</th><th>architecture</th><th>len</th><th>gen.name</th><th>taxend</th><th>species</th><th>defline</th><th>gca</th></tr>
</thead>
<tbody>
<tr><td>WP_185583983.1</td><td><-PIN<-?<-?||?→?→||<-Arginase<-?||LRR-repeats+AP-GTPase+COR*→</td><td>LRR-repeats+AP-GTPase+COR</td><td>358</td><td>-</td><td>Cyanobacteria</td><td>Nostoc sp. UCD120</td><td>leucine-rich repeat domain-containing protein, partial [Nostoc sp. UCD120].</td><td>GCF_014222255.1</td></tr>
<tr><td>WP_185584434.1</td><td>PAS+HISKIN→ REC→ PSE→?→?→?→ LRR-repeats+AP-GTPase+COR+TIR*→</td><td>LRR-repeats+AP-GTPase+COR+TIR</td><td>1163</td><td>-</td><td>Cyanobacteria</td><td>Nostoc sp. UCD120</td><td>leucine-rich repeat domain-containing protein [Nostoc sp. UCD120].</td><td>GCF_014222255.1</td></tr>
<tr><td>WP_185591915.1</td><td><-PIN<-?<-?||?→?→||<-Arginase<-?||LRR-repeats+AP-GTPase+COR+TIR*→</td><td>LRR-repeats+AP-GTPase+COR+TIR</td><td>802</td><td>-</td><td>Cyanobacteria</td><td>Nostoc sp. UCD122</td><td>leucine-rich repeat domain-containing protein [Nostoc sp. UCD122].</td><td>GCF_014222275.1</td></tr>
<tr><td>WP_185592013.1</td><td>ABhydrolase→?→||<-?||COR+TIR→ LRR-repeats+AP-GTPase+COR+TIR*→?→||<-PIN</td><td>LRR-repeats+AP-GTPase+COR+TIR</td><td>1003</td><td>-</td><td>Cyanobacteria</td><td>Nostoc sp. UCD122</td><td>leucine-rich repeat domain-containing protein [Nostoc sp. UCD122].</td><td>GCF_014222275.1</td></tr>
<tr><td>WP_185592050.1</td><td>LRR-repeats+AP-GTPase+COR+TIR*→</td><td>LRR-repeats+AP-GTPase+COR+TIR</td><td>859</td><td>-</td><td>Cyanobacteria</td><td>Nostoc sp. UCD122</td><td>leucine-rich repeat domain-containing protein, partial [Nostoc sp. UCD122].</td><td>GCF_014222275.1</td></tr>
<tr><td>WP_185592512.1</td><td>Cluster5_5clades→ LRR-repeats+AP-GTPase+COR+TIR*→||<-CASPASE+TM</td><td>LRR-repeats+AP-GTPase+COR+TIR</td><td>893</td><td>-</td><td>Cyanobacteria</td><td>Nostoc sp. UCD122</td><td>leucine-rich repeat domain-containing protein [Nostoc sp. UCD122].</td><td>GCF_014222275.1</td></tr>
<tr><td>WP_185607149.1</td><td><-PIN<-?<-?||?→?→||<-Arginase<-?||LRR-repeats+AP-GTPase+COR+TIR*→</td><td>LRR-repeats+AP-GTPase+COR+TIR</td><td>825</td><td>-</td><td>Cyanobacteria</td><td>Nostoc sp. UCD121</td><td>leucine-rich repeat domain-containing protein [Nostoc sp. UCD121].</td><td>GCF_014222285.1</td></tr>
<tr><td>WP_185607288.1</td><td>LRR-repeats+AP-GTPase+COR+TIR*→?→?→?→ ABC_membrane+ABC_tran→ ABC_membrane+ABC_tran→</td><td>LRR-repeats+AP-GTPase+COR+TIR</td><td>917</td><td>-</td><td>Cyanobacteria</td><td>Nostoc sp. UCD121</td><td>leucine-rich repeat domain-containing protein, partial [Nostoc sp. UCD121].</td><td>GCF_014222285.1</td></tr>
<tr><td>WP_186435859.1</td><td><-Cluster1077_2clades||Aminotran_1_2→?→||<-?<-?||?→ LRR-repeats+AP-GTPase+COR*→</td><td>LRR-repeats+AP-GTPase+COR</td><td>980</td><td>-</td><td>Gammaproteobacteria</td><td>Thalassolituus sp. C2-1</td><td>GTP-binding protein [Thalassolituus sp. C2-1].</td><td>GCF_007785795.1</td></tr>
<tr><td>WP_186890728.1</td><td><-Cluster1951_2clades<-?<-?||?→ SIG+TM+TM+TM+HISKIN→?→||<-?||LRR-repeats+AP-GTPase+COR+DrHyd*→||<-?<-REC</td><td>LRR-repeats+AP-GTPase+COR+DrHyd</td><td>1036</td><td>-</td><td>Betaproteobacteria</td><td>Undibacterium amnicola</td><td>hypothetical protein [Undibacterium amnicola].</td><td>GCF_014284275.1</td></tr>
<tr><td>WP_186956531.1</td><td>LRR-repeats+AP-GTPase+COR+TIR*→</td><td>LRR-repeats+AP-GTPase+COR+TIR</td><td>1232</td><td>-</td><td>Betaproteobacteria</td><td>Undibacterium sp. NL8W</td><td>leucine-rich repeat domain-containing protein [Undibacterium sp. NL8W].</td><td>GCF_014284125.1</td></tr>
<tr><td>WP_187308183.1</td><td>LRR-repeats+AP-GTPase+COR+TM+TM*→||<-?<-?||?→?→||<-?<-?<-SNF</td><td>LRR-repeats+AP-GTPase+COR+TM+TM</td><td>720</td><td>-</td><td>Cyanobacteria</td><td>Nostoc cycadae</td><td>leucine-rich repeat domain-containing protein, partial [Nostoc cycadae].</td><td>GCF_002897135.1</td></tr>
<tr><td>WP_187315132.1</td><td>LRR-repeats+AP-GTPase+COR*→?→?→?→||<-?<-tRNA<-?<-?<-Cluster1829_2clades</td><td>LRR-repeats+AP-GTPase+COR</td><td>914</td><td>-</td><td>Bacteroidetes</td><td>Hymenobacter sp. BT190</td><td>GTP-binding protein [Hymenobacter sp. BT190].</td><td>GCF_014333525.1</td></tr>
<tr><td>WP_187466435.1</td><td>LRR-repeats+AP-GTPase+COR+EAD11→?→?→ LRR-repeats+AP-GTPase+COR+EAD11→</td><td>LRR-repeats+AP-GTPase+COR+EAD11</td><td>1040</td><td>-</td><td>Bacteroidetes</td><td>Lewinella lacunae</td><td>leucine-rich repeat domain-containing protein [Lewinella lacunae].</td><td>GCF_014349155.1</td></tr>
<tr><td>WP_187466438.1</td><td>LRR-repeats+AP-GTPase+COR+EAD11*→</td><td>LRR-repeats+AP-GTPase+COR+EAD11</td><td>931</td><td>-</td><td>Bacteroidetes</td><td>Lewinella lacunae</td><td>leucine-rich repeat domain-containing protein [Lewinella lacunae].</td><td>GCF_014349155.1</td></tr>
<tr><td>WP_187467589.1</td><td>GTPase-AIG→||<-?||?→?→||<-?<-?<-?||LRR-repeats+AP-GTPase+COR+CASPASE*→?→ FGS→?→ RVT→?→ FGS→</td><td>LRR-repeats+AP-GTPase+COR+CASPASE</td><td>1063</td><td>-</td><td>Bacteroidetes</td><td>Lewinella lacunae</td><td>CHAT domain-containing protein [Lewinella lacunae].</td><td>GCF_014349155.1</td></tr>
<tr><td>WP_187560609.1</td><td>SIGMA-HTH→?→ RADICAL-SAM→ LRR-repeats+AP-GTPase+COR+TIR*→?→ HISKIN→</td><td>LRR-repeats+AP-GTPase+COR+TIR</td><td>970</td><td>-</td><td>Bacteroidetes</td><td>Kordia sp. YSTF-M3</td><td>leucine-rich repeat domain-containing protein [Kordia sp. YSTF-M3].</td><td>GCF_014397005.1</td></tr>
<tr><td>WP_187775535.1</td><td>HTH→?→?→ LRR-repeats+AP-GTPase+COR+TIR*→||<-?<-ABC_membrane+ABC_tran||Cyanophycinsyn-ATPgrasp→ Cyanophycinsyn-ATPgrasp→</td><td>LRR-repeats+AP-GTPase+COR+TIR</td><td>1298</td><td>-</td><td>Betaproteobacteria</td><td>Sphaerotilus natans</td><td>leucine-rich repeat domain-containing protein [Sphaerotilus natans].</td><td>GCF_008329925.1</td></tr>
</tbody>
</table>

Gene neighborhoods and domain architectures of APGTPases whose LRR domains are either absent or not detected

acc	operon	architecture	len	gen.name	taxend	species	defline	gca
AAP36207.1	AP-GTPase*→	AP-GTPase	220	-	-	synthetic construct	Homo sapiens RAB3D, member RAS oncogene family, partial [synthetic construct].	-
AAP36967.1	AP-GTPase*→	AP-GTPase	208	-	-	synthetic construct	Homo sapiens mel transforming oncogene (derived from cell line NK14)- RAB8 homolog, partial [synthetic construct].	-
AAV38505.1	AP-GTPase*→	AP-GTPase	204	-	-	synthetic construct	RAB13, member RAS oncogene family, partial [synthetic construct].	-
AAX32379.1	AP-GTPase*→	AP-GTPase	207	MEL	-	synthetic construct	RAB8A [synthetic construct].	-
AAX36766.1	AP-GTPase*→	AP-GTPase	204	RAB13	-	synthetic construct	RAB13 member RAS oncogene family, partial [synthetic construct].	-
AAX41198.1	AP-GTPase*→	AP-GTPase	203	RAB13	-	synthetic construct	RAB13 member RAS oncogene family [synthetic construct].	-
AAX42776.1	AP-GTPase*→	AP-GTPase	204	RAB13	-	synthetic construct	RAB13 member RAS oncogene family, partial [synthetic construct].	-
ABG50724.1	vWA-L+AP-GTPase+COR*→	vWA-L+AP-GTPase+COR	748	Tery_1428	Cyanobacteria	Trichodesmium erythraeum IMS101	Miro-like [Trichodesmium erythraeum IMS101].	GCA_000014265.1
ABW29719.1	AP-GTPase+ZNR*→	AP-GTPase+ZNR	705	AM1_4747	Cyanobacteria	Acaryochloris marina MBIC11017	conserved hypothetical protein [Acaryochloris marina MBIC11017].	GCA_000018105.1
ACL85629.1	AP-GTPase*→	AP-GTPase	204	Rab10	-	Drosophila melanogaster	Rab10-PA, partial [synthetic construct].	-
ACL86538.1	AP-GTPase*→	AP-GTPase	223	Rab30	-	Drosophila melanogaster	Rab30-PA, partial [synthetic construct].	-
ADQ32831.1	AP-GTPase*→	AP-GTPase	219	RAB3B	-	Homo sapiens	RAB3B, member RAS oncogene family, partial [synthetic construct].	-
AFZ01290.1	<-EAD10+VMAP-M1+VMAP-C‖?→ CASPASE+EAD10→ EAD10+VMAP-M1+VMAP-C→?→ VWA+AP-GTPase+COR*→	VWA+AP-GTPase+COR	1196	Cal6303_2274	Cyanobacteria	Calothrix sp. PCC 6303	Miro domain protein [Calothrix sp. PCC 6303].	GCA_000317435.1
AIC55004.1	AP-GTPase*→	AP-GTPase	219	-	-	Homo sapiens	RAB3B, partial [synthetic construct].	-
AIC55612.1	AP-GTPase*→	AP-GTPase	219	-	-	Homo sapiens	RAB3D, partial [synthetic construct].	-
ASF48224.1	AP-GTPase+COR+DUF4404*→	AP-GTPase+COR+DUF4404	879	CEK71_20365	Gammaproteobacteria	Methylovulum psychrotolerans	hypothetical protein CEK71_20365 [Methylovulum psychrotolerans].	GCA_002209385.1
ATO96390.1	AP-GTPase*→	AP-GTPase	207	rab8b	-	Xenopus tropicalis	RAB8B, member RAS oncoprotein family, partial [synthetic construct].	-

acc	operon	architecture	len	gen.name	taxend	species	defline	gca
CCQ62557.1	AP-GTPase+COR*→	AP-GTPase+COR	279	CWATWH0401_4507	Cyanobacteria	Crocosphaera watsonii WH 0401	Leucine-rich repeat [Crocosphaera watsonii WH 0401].	GCA_001039615.1
CCZ82707.1	AP-GTPase+COR*→	AP-GTPase+COR	843	BN709_01979	Bacteroidetes	Odoribacter laneus CAG:561	putative uncharacterized protein [Odoribacter laneus CAG:561].	GCA_000432935.1
HAA12500.1	AP-GTPase+COR*→	AP-GTPase+COR	908	DCE41_12685	Bacteroidetes	Cytophagales bacterium	TPA: hypothetical protein DCE41_12685 [Cytophagales bacterium].	GCA_003444355.1
HAA13307.1	AP-GTPase+COR*→	AP-GTPase+COR	878	DCE41_17110	Bacteroidetes	Cytophagales bacterium	TPA: hypothetical protein DCE41_17110 [Cytophagales bacterium].	GCA_003444355.1
HAC65063.1	DNAJ→ AP-GTPase*→ COR+TM+TM→	AP-GTPase	125	DCF68_16430	Cyanobacteria	Cyanothece sp. UBA12306	TPA: GTPase, partial [Cyanothece sp. UBA12306].	GCA_003448685.1
HAK76392.1	AP-GTPase+COR*→	AP-GTPase+COR	733	DCM71_05675	Bacteroidetes	Runella sp.	TPA: hypothetical protein DCM71_05675, partial [Runella sp.].	GCA_003452005.1
HAO21703.1	AP-GTPase*→	AP-GTPase	569	DCQ37_15290	Deltaproteobacteria	Desulfobacteraceae bacterium	TPA: hypothetical protein DCQ37_15290, partial [Desulfobacteraceae bacterium].	GCA_003456735.1
HAP58612.1	AP-GTPase+COR*→	AP-GTPase+COR	696	DCR93_03550	Bacteroidetes	Cytophagales bacterium	TPA: hypothetical protein DCR93_03550, partial [Cytophagales bacterium].	GCA_003454975.1
HAP62400.1	AP-GTPase+COR*→?→ cNMPBD→ SIG+Phytase-like→	AP-GTPase+COR	744	DCR93_23850	Bacteroidetes	Cytophagales bacterium	TPA: hypothetical protein DCR93_23850 [Cytophagales bacterium].	GCA_003454975.1
HCS91914.1	TIR+BetaPropeller+AP-GTPase*→	TIR+BetaPropeller+AP-GTPase	1236	DIW77_18260	Gammaproteobacteria	Chromatiaceae bacterium	TPA: hypothetical protein DIW77_18260 [Chromatiaceae bacterium].	GCA_003525925.1
HEY83669.1	AP-GTPase+COR*→	AP-GTPase+COR	260	G4N96_00950	Chloroflexi	Chloroflexi bacterium	TPA: hypothetical protein G4N96_00950 [Chloroflexi bacterium].	GCA_011192195.1
HEY85168.1	AP-GTPase*→	AP-GTPase	319	G4N96_08685	Chloroflexi	Chloroflexi bacterium	TPA: hypothetical protein G4N96_08685 [Chloroflexi bacterium].	GCA_011192195.1
HFS11412.1	BetaPropeller+AP-GTPase+TIR*→?→||<-?||?→?→||<-SIG+PSBP	BetaPropeller+AP-GTPase+TIR	1179	ENR14_24625	Cyanobacteria	Anabaena sp.	TPA: TIR domain-containing protein [Anabaena sp.].	GCA_011332035.1
HGZ32338.1	AP-GTPase+COR*→	AP-GTPase+COR	503	ENR17_19310	Bacteroidetes	Bacteroidetes bacterium	TPA: GTPase, partial [Bacteroidetes bacterium].	GCA_011331955.1
HHN34802.1	AP-GTPase*→	AP-GTPase	171	ENM07_01585	Bacteroidetes	Bacteroidetes bacterium	TPA: GTP-binding protein [Bacteroidetes bacterium].	GCA_011375975.1
KAB2958401.1	SIG+AP-GTPase+COR*→	SIG+AP-GTPase+COR	688	F9K13_13725	Bacteria	Candidatus Methylomirabilis oxyfera	hypothetical protein F9K13_13725 [Candidatus Methylomirabilis oxyfera].	GCA_009026205.1
KAF0250167.1	AP-GTPase+COR+Calcineurin+FGS*→	AP-GTPase+COR+Calcineurin+FGS	1237	FD167_433	unclassified Bacteria	bacterium	small GTP-binding protein, partial [bacterium].	GCA_009773835.1
KHD09711.1	AP-GTPase+COR*→	AP-GTPase+COR	530	PN36_20855	Gammaproteobacteria	Candidatus Thiomargarita nelsonii	hypothetical protein PN36_20855 [Candidatus Thiomargarita nelsonii].	GCA_000785145.2
MBC6419378.1	AP-GTPase*→ COR→	AP-GTPase	165	GDA44_11670	Cyanobacteria	Prochloron sp. SP5CPC1	hypothetical protein GDA44_11670 [Prochloron sp. SP5CPC1].	GCA_014323965.1

acc	operon	architecture	len	gen.name	taxend	species	defline	gca
NAT10406.1	AP-GTPase*→	AP-GTPase	611	C4E22_02470	Euryarchaeota	ANME-1 cluster archaeon AG-394-G06	hypothetical protein C4E22_02470 [ANME-1 cluster archaeon AG-394-G06].	GCA_009903405.1
NBO93394.1	AP-GTPase+COR*→	AP-GTPase+COR	916	EBV06_13945	Planctomycetes	Planctomycetia bacterium	hypothetical protein EBV06_13945 [Planctomycetia bacterium].	GCA_009918525.1
NBQ70341.1	TIR+AP-GTPase+COR*→	TIR+AP-GTPase+COR	582	EBU46_16510	Betaproteobacteria	Nitrosomonadaceae bacterium	TIR domain-containing protein, partial [Nitrosomonadaceae bacterium].	GCA_009919685.1
NHJ32288.1	AP-GTPase*→	AP-GTPase	230	FK732_05460	Asgard group	Asgard group archaeon	GTP-binding protein [Asgard group archaeon].	GCA_011366225.1
NIM12342.1	TIR+BetaPropeller+AP-GTPase*→	TIR+BetaPropeller+AP-GTPase	1000	GTO81_10200	Bacteria	Candidatus Aminicenantes bacterium	DUF4365 domain-containing protein [Candidatus Aminicenantes bacterium].	GCA_011771225.1
NIM12946.1	TIR+BetaPropeller+AP-GTPase*→	TIR+BetaPropeller+AP-GTPase	1108	GTO81_13270	Bacteria	Candidatus Aminicenantes bacterium	TIR domain-containing protein [Candidatus Aminicenantes bacterium].	GCA_011771225.1
NIR05736.1	TIR+BetaPropeller+AP-GTPase*→ Cluster2387_2clades→ Cluster2274_2clades→||<-Cluster2216_2clades||Cluster2371_2clades→||<-?<-?<-Cluster2251_2clades	TIR+BetaPropeller+AP-GTPase	1004	GTN82_09930	Bacteria	Candidatus Aminicenantes bacterium	DUF4365 domain-containing protein [Candidatus Aminicenantes bacterium].	GCA_011773265.1
NJL71192.1	AP-GTPase*→	AP-GTPase	248	HC888_06030	Gammaproteobacteria	Candidatus Competibacteraceae bacterium	GTP-binding protein [Candidatus Competibacteraceae bacterium].	GCA_012031515.1
NJO13505.1	AP-GTPase*→?→ TIR→	AP-GTPase	144	HC872_08630	Gammaproteobacteria	Gammaproteobacteria bacterium	hypothetical protein HC872_08630 [Gammaproteobacteria bacterium].	GCA_012032615.1
NKB62496.1	AP-GTPase+COR*→	AP-GTPase+COR	274	GKR95_10365	Gammaproteobacteria	Gammaproteobacteria bacterium	hypothetical protein GKR95_10365 [Gammaproteobacteria bacterium].	GCA_012103415.1
NLZ73760.1	AP-GTPase*→	AP-GTPase	202	GX905_08105	Bacteroidetes	Bacteroidales bacterium	hypothetical protein GX905_08105 [Bacteroidales bacterium].	GCA_012800615.1
NMC05010.1	AP-GTPase*→	AP-GTPase	190	GYA24_07360	Asgard group	Candidatus Lokiarchaeota archaeon	GTP-binding protein [Candidatus Lokiarchaeota archaeon].	GCA_012798355.1
NNJ85485.1	AP-GTPase*→	AP-GTPase	345	HKP13_11240	Gammaproteobacteria	Gammaproteobacteria bacterium	hypothetical protein HKP13_11240, partial [Gammaproteobacteria bacterium].	GCA_013042035.1
NOQ28062.1	AP-GTPase+COR*→	AP-GTPase+COR	845	GQ564_22090	Bacteroidetes	Bacteroidales bacterium	hypothetical protein GQ564_22090 [Bacteroidales bacterium].	GCA_013138975.1
NOS87668.1	AP-GTPase+COR+DUF4404*→ REase→	AP-GTPase+COR+DUF4404	775	HOP34_03865	Gammaproteobacteria	Methylococcaceae bacterium	hypothetical protein HOP34_03865, partial [Methylococcaceae bacterium].	GCA_013140465.1
NOT02643.1	AP-GTPase+COR*→	AP-GTPase+COR	1127	HOP29_18715	Planctomycetes	Phycisphaerales bacterium	hypothetical protein HOP29_18715 [Phycisphaerales bacterium].	GCA_013140575.1
NOT61891.1	TIR+BetaPropeller+AP-GTPase*→	TIR+BetaPropeller+AP-GTPase	1134	HOP19_16880	Acidobacteria	Acidobacteria bacterium	TIR domain-containing protein [Acidobacteria bacterium].	GCA_013140935.1

acc	operon	architecture	len	gen.name	taxend	species	defline	gca
NOU19042.1	AP-GTPase+COR*→	AP-GTPase+COR	850	HOO91_15910	Bacteroidetes	Bacteroidales bacterium	hypothetical protein HOO91_15910 [Bacteroidales bacterium].	GCA_013141385.1
NOY42283.1	AP-GTPase+COR*→	AP-GTPase+COR	659	GXP26_10655	Planctomycetes	Planctomycetes bacterium	hypothetical protein GXP26_10655 [Planctomycetes bacterium].	GCA_013152205.1
NOY62616.1	AP-GTPase+COR+DUF4404*→	AP-GTPase+COR+DUF4404	555	GXP10_05565	Gammaproteobacteria	Gammaproteobacteria bacterium	hypothetical protein GXP10_05565, partial [Gammaproteobacteria bacterium].	GCA_013152295.1
NPE06693.1	AP-GTPase*→	AP-GTPase	212	GNW80_00285	Asgard group	Asgard group archaeon	GTP-binding protein [Asgard group archaeon].	GCA_013166835.1
NQT13008.1	TIR+BetaPropeller+AP-GTPase*→	TIR+BetaPropeller+AP-GTPase	698	HQ582_09690	Planctomycetes	Planctomycetes bacterium	TIR domain-containing protein, partial [Planctomycetes bacterium].	GCA_013202485.1
NQU22914.1	AP-GTPase*→	AP-GTPase	350	HQ567_16680	Bacteria	Candidatus Nealsonbacteria bacterium	hypothetical protein HQ567_16680 [Candidatus Nealsonbacteria bacterium].	GCA_013202865.1
NQZ10247.1	AP-GTPase+COR*→	AP-GTPase+COR	798	HRT35_24105	Gammaproteobacteria	Algicola sp.	hypothetical protein HRT35_24105 [Algicola sp.].	GCA_013216025.1
NQZ12034.1	AP-GTPase+COR*→	AP-GTPase+COR	875	HRT35_33190	Gammaproteobacteria	Algicola sp.	hypothetical protein HRT35_33190 [Algicola sp.].	GCA_013216025.1
NTV51179.1	SIG+ZetaToxin→ TIR+BetaPropeller+AP-GTPase*→	TIR+BetaPropeller+AP-GTPase	1151	HGA20_16230	Deltaproteobacteria	Geobacteraceae bacterium	TIR domain-containing protein [Geobacteraceae bacterium].	GCA_013336525.1
NUN13817.1	BetaPropeller+AP-GTPase+Calcineurin*→	BetaPropeller+AP-GTPase+Calcineurin	1598	HUU55_09280	Deltaproteobacteria	Myxococcales bacterium	metallophosphoesterase [Myxococcales bacterium].	GCA_013360285.1
NVO09097.1	AP-GTPase+COR*→	AP-GTPase+COR	866	HXX16_03955	Bacteroidetes	Bacteroidales bacterium	50S ribosome-binding GTPase [Bacteroidales bacterium].	GCA_013376985.1
OHD42862.1	AP-GTPase+COR+DrHyd*→	AP-GTPase+COR+DrHyd	671	A2086_15900	Spirochaetes	Spirochaetes bacterium GWD1_27_9	hypothetical protein A2086_15900 [Spirochaetes bacterium GWD1_27_9].	GCA_001830585.1
OLS14677.1	AP-GTPase*→	AP-GTPase	170	RBG13Loki_1719	Asgard group	Candidatus Lokiarchaeota archaeon CR_4	small GTP-binding protein [Candidatus Lokiarchaeota archaeon CR_4].	GCA_001940655.1
OQW99838.1	AP-GTPase+COR*→	AP-GTPase+COR	550	BWK73_49720	Gammaproteobacteria	Thiothrix lacustris	hypothetical protein BWK73_49720 [Thiothrix lacustris].	GCA_002083875.1
OQX07299.1	AP-GTPase*→	AP-GTPase	495	BWK80_49630	Deltaproteobacteria	Desulfobacteraceae bacterium IS3	hypothetical protein BWK80_49630, partial [Desulfobacteraceae bacterium IS3].	GCA_002083955.1
OQX29968.1	AP-GTPase+COR*→	AP-GTPase+COR	659	B0D92_00955	Spirochaetes	Spirochaeta sp. LUC14_002_19_P3	hypothetical protein B0D92_00955, partial [Spirochaeta sp. LUC14_002_19_P3].	GCA_002084135.1
OQY29953.1	AP-GTPase+COR+CASPASE*→	AP-GTPase+COR+CASPASE	1279	B6244_01455	FCB group	Candidatus Cloacimonetes bacterium 4572_55	hypothetical protein B6244_01455 [Candidatus Cloacimonetes bacterium 4572_55].	GCA_002084765.1

acc	operon	architecture	len	gen.name	taxend	species	defline	gca
OYW77642.1	AP-GTPase+COR*→	AP-GTPase+COR	915	B7Z37_03570	Verrucomicrobia	Verrucomicrobia bacterium 12-59-8	hypothetical protein B7Z37_03570, partial [Verrucomicrobia bacterium 12-59-8].	GCA_002279765.1
PID37771.1	AP-GTPase+COR*→	AP-GTPase+COR	901	CR966_00525	Gammaproteobacteria	Pseudomonadales bacterium	hypothetical protein CR966_00525 [Pseudomonadales bacterium].	GCA_002746545.1
PIE42009.1	AP-GTPase+COR+DUF4404*→	AP-GTPase+COR+DUF4404	686	CSA47_02360	Gammaproteobacteria	Gammaproteobacteria bacterium	hypothetical protein CSA47_02360 [Gammaproteobacteria bacterium].	GCA_002748595.1
PKL67687.1	BetaPropeller+AP-GTPase+PrimaseZnR+TIR*→	BetaPropeller+AP-GTPase+PrimaseZnR+TIR	1195	CVV28_05310	Euryarchaeota	Methanobacteriales archaeon HGW-Methanobacteriales-1	hypothetical protein CVV28_05310 [Methanobacteriales archaeon HGW-Methanobacteriales-1].	GCA_002839705.1
POZ51526.1	AP-GTPase+COR+DUF4404*→	AP-GTPase+COR+DUF4404	919	AADEFJLK_02392	Gammaproteobacteria	Methylovulum psychrotolerans	GTP-binding protein [Methylovulum psychrotolerans].	GCA_002923755.1
PPD43153.1	AP-GTPase+COR+DUF4404*→	AP-GTPase+COR+DUF4404	914	CTY16_13990	Gammaproteobacteria	Methylobacter sp.	hypothetical protein CTY16_13990 [Methylobacter sp.].	GCA_002929035.1
PPD50180.1	AP-GTPase+COR+DUF4404*→	AP-GTPase+COR+DUF4404	927	CTY13_02150	Gammaproteobacteria	Methylobacter sp.	hypothetical protein CTY13_02150 [Methylobacter sp.].	GCA_002929095.1
PPD50460.1	PolB→ Cluster2355_2clades→ AP-GTPase+COR+DUF4404*→	AP-GTPase+COR+DUF4404	904	CTY16_01515	Gammaproteobacteria	Methylobacter sp.	hypothetical protein CTY16_01515 [Methylobacter sp.].	GCA_002929035.1
QDT95532.1	TIR+APATPase+TPR+TPR+TPR→?→ AP-GTPase+COR+DUF4404*→	AP-GTPase+COR+DUF4404	683	V144x_09770	Planctomycetes	Gimesia aquarii	Miro-like protein [Gimesia aquarii].	GCA_007748195.1
RCJ23846.1	VWA+AP-GTPase+COR+TIR+TIR*→	VWA+AP-GTPase+COR+TIR+TIR	1359	A6770_28940	Cyanobacteria	Nostoc minutum NIES-26	hypothetical protein A6770_28940 [Nostoc minutum NIES-26].	GCA_003326215.1
RHO76387.1	AP-GTPase*→	AP-GTPase	197	DW061_22950	Firmicutes	Ruminococcus sp. AF42-9BH	hypothetical protein DW061_22950, partial [Ruminococcus sp. AF42-9BH].	GCA_003477585.1
RIK66911.1	SIG+AP-GTPase+COR*→	SIG+AP-GTPase+COR	867	DCC65_08160	Planctomycetes	Planctomycetes bacterium	hypothetical protein DCC65_08160 [Planctomycetes bacterium].	GCA_003576905.1
RKZ52796.1	AP-GTPase+COR*→	AP-GTPase+COR	469	DRR00_06990	Gammaproteobacteria	Gammaproteobacteria bacterium	hypothetical protein DRR00_06990 [Gammaproteobacteria bacterium].	GCA_003645185.1
RLI66235.1	AP-GTPase*→	AP-GTPase	176	DRO88_02330	Asgard group	Candidatus Lokiarchaeota archaeon	GTP-binding protein [Candidatus Lokiarchaeota archaeon].	GCA_003662865.1
RLI69359.1	AP-GTPase*→	AP-GTPase	199	DRP02_10785	Asgard group	Candidatus Heimdallarchaeota archaeon	hypothetical protein DRP02_10785 [Candidatus Heimdallarchaeota archaeon].	GCA_003662935.1
RTZ62858.1	AP-GTPase+COR*→	AP-GTPase+COR	577	DSZ29_07625	Aquificae	Aquificaceae bacterium	hypothetical protein DSZ29_07625 [Aquificaceae bacterium].	GCA_003972955.1

acc	operon	architecture	len	gen.name	taxend	species	defline	gca
RYE19062.1	AP-GTPase+COR*→	AP-GTPase+COR	577	EOP45_13210	Bacteroidetes	Sphingobacteriaceae bacterium	GTP-binding protein, partial [Sphingobacteriaceae bacterium].	GCA_004144285.1
RZL32479.1	SIG+AP-GTPase+COR*→	SIG+AP-GTPase+COR	392	EOP00_33890	Bacteroidetes	Pedobacter sp.	GTP-binding protein, partial [Pedobacter sp.].	GCA_004211475.1
SEM19691.1	SIG+AP-GTPase+COR*→	SIG+AP-GTPase+COR	929	SAMN04489760_10698	Deltaproteobacteria	Syntrophus gentianae	small GTP-binding protein domain-containing protein [Syntrophus gentianae].	GCA_900109885.1
SKA90036.1	AP-GTPase*→	AP-GTPase	120	SAMN02745166_01647	Verrucomicrobia	Prosthecobacter debontii	hypothetical protein SAMN02745166_01647 [Prosthecobacter debontii].	GCA_900167535.1
SNR48942.1	BetaPropeller+AP-GTPase+TIR*→	BetaPropeller+AP-GTPase+TIR	1008	SAMN06264365_102815	Actinobacteria	Actinoplanes regularis	small GTP-binding protein domain-containing protein [Actinoplanes regularis].	GCA_900188005.1
TAA74167.1	AP-GTPase*→	AP-GTPase	415	CDV28_13720	Deltaproteobacteria	Candidatus Electronema sp. GS	Ras of Complex, Roc, domain of DAPkinase [Candidatus Electronema sp. GS].	GCA_004284765.1
TAN66684.1	AP-GTPase+COR+DUF4404*→	AP-GTPase+COR+DUF4404	912	EPN17_13365	Gammaproteobacteria	Methylobacter sp.	hypothetical protein EPN17_13365 [Methylobacter sp.].	GCA_004299305.1
TET30065.1	AP-GTPase*→	AP-GTPase	212	E3J70_05515	Asgard group	Candidatus Heimdallarchaeota archaeon	GTP-binding protein [Candidatus Heimdallarchaeota archaeon].	GCA_004376455.1
TFF85226.1	AP-GTPase*→	AP-GTPase	173	EU518_01555	Asgard group	Candidatus Lokiarchaeota archaeon	GTP-binding protein [Candidatus Lokiarchaeota archaeon].	GCA_004524075.1
TFF94051.1	AP-GTPase*→	AP-GTPase	179	EU544_04990	Asgard group	Candidatus Lokiarchaeota archaeon	GTP-binding protein [Candidatus Lokiarchaeota archaeon].	GCA_004524325.1
TFG01113.1	AP-GTPase*→	AP-GTPase	173	EU542_07480	Asgard group	Candidatus Lokiarchaeota archaeon	GTP-binding protein [Candidatus Lokiarchaeota archaeon].	GCA_004524355.1
TFG18716.1	AP-GTPase*→	AP-GTPase	217	EU530_08380	Asgard group	Candidatus Lokiarchaeota archaeon	GTP-binding protein [Candidatus Lokiarchaeota archaeon].	GCA_004524545.1
TKG00166.1	AP-GTPase+COR*→	AP-GTPase+COR	467	FCV76_15430	Gammaproteobacteria	Vibrio sp. F13	GTP-binding protein [Vibrio sp. F13].	GCA_005146615.1
TKJ20917.1	AP-GTPase*→	AP-GTPase	192	CEE42_14430	Asgard group	Candidatus Lokiarchaeota archaeon Loki_b31	hypothetical protein CEE42_14430 [Candidatus Lokiarchaeota archaeon Loki_b31].	GCA_005222975.1
TLU87712.1	SIG+ZetaToxin→ TIR+BetaPropeller+AP-GTPase*→	TIR+BetaPropeller+AP-GTPase	1149	FDX21_02485	Chlorobi	Chlorobium sp.	TIR domain-containing protein [Chlorobium sp.].	GCA_005862225.1
TOG86811.1	AP-GTPase+COR*→	AP-GTPase+COR	675	CGI91_22345	Gammaproteobacteria	Vibrio parahaemolyticus	hypothetical protein CGI91_22345 [Vibrio parahaemolyticus].	GCA_006372615.1
TPQ26187.1	AP-GTPase+COR+TM+TM*→	AP-GTPase+COR+TM+TM	752	C2U68_12455	Gammaproteobacteria	Methylomonas koyamae	hypothetical protein C2U68_12455 [Methylomonas koyamae].	GCA_006483455.1
TXT62600.1	AP-GTPase*→	AP-GTPase	178	BAJALOKI3v1_540018	Asgard group	Candidatus Lokiarchaeota archaeon	GTP-binding protein Der [Candidatus Lokiarchaeota archaeon].	GCA_008080735.1

acc	operon	architecture	len	gen.name	taxend	species	defline	gca
VFJ44888.1	TIR+BetaPropeller+AP-GTPase*→	TIR+BetaPropeller+AP-GTPase	766	BECKFW1821A_GA0114235_100653	Gammaproteobacteria	Candidatus Kentron sp. FW	WD domain-containing protein, G-beta repeat-containing protein [Candidatus Kentron sp. FW].	-
VFJ45524.1	AP-GTPase+COR+TM+TM*→ AP-GTPase+COR+TM+TM→	AP-GTPase+COR+TM+TM	839	BECKFM1743C_GA0114222_100266	Gammaproteobacteria	Candidatus Kentron sp. FM	Ras of Complex, Roc, domain of DAPkinase [Candidatus Kentron sp. FM].	-
VFJ50707.1	TIR+BetaPropeller+AP-GTPase*→	TIR+BetaPropeller+AP-GTPase	1132	BECKFW1821B_GA0114236_100746	Gammaproteobacteria	Candidatus Kentron sp. FW	WD40 repeat [Candidatus Kentron sp. FW].	-
VFJ53002.1	AP-GTPase+COR+TM+TM*→	AP-GTPase+COR+TM+TM	812	BECKDK2373C_GA0170839_103824	Gammaproteobacteria	Candidatus Kentron sp. DK	Ras of Complex, Roc, domain of DAPkinase [Candidatus Kentron sp. DK].	-
VFJ54602.1	AP-GTPase+COR+TM+TM*→	AP-GTPase+COR+TM+TM	670	BECKDK2373C_GA0170839_10441	Gammaproteobacteria	Candidatus Kentron sp. DK	Ras of Complex, Roc, domain of DAPkinase, partial [Candidatus Kentron sp. DK].	-
VFJ56646.1	AP-GTPase+COR+TM+TM*→	AP-GTPase+COR+TM+TM	841	BECKDK2373B_GA0170837_106011	Gammaproteobacteria	Candidatus Kentron sp. DK	Ras of Complex, Roc, domain of DAPkinase [Candidatus Kentron sp. DK].	-
VFJ57440.1	AP-GTPase*→	AP-GTPase	395	BECKFW1821B_GA0114236_10341	Gammaproteobacteria	Candidatus Kentron sp. FW	Ras of Complex, Roc, domain of DAPkinase, partial [Candidatus Kentron sp. FW].	-
VFJ59594.1	AP-GTPase+COR+CR-ATPase8+CR-REase7+TM+TM+TM*→	AP-GTPase+COR+CR-ATPase8+CR-REase7+TM+TM+TM	1492	BECKFW1821A_GA0114235_109213	Gammaproteobacteria	Candidatus Kentron sp. FW	GTPase SAR1 family protein [Candidatus Kentron sp. FW].	-
VFJ60987.1	AP-GTPase+COR+TM+TM*→	AP-GTPase+COR+TM+TM	889	BECKFM1743C_GA0114222_102852	Gammaproteobacteria	Candidatus Kentron sp. FM	Ras of Complex, Roc, domain of DAPkinase [Candidatus Kentron sp. FM].	-
VFJ61225.1	AP-GTPase+COR+TM+TM*→	AP-GTPase+COR+TM+TM	683	BECKDK2373B_GA0170837_109915	Gammaproteobacteria	Candidatus Kentron sp. DK	Ras of Complex, Roc, domain of DAPkinase, partial [Candidatus Kentron sp. DK].	-
VFJ62038.1	AP-GTPase+COR+TM+TM*→	AP-GTPase+COR+TM+TM	843	BECKFW1821B_GA0114236_106912	Gammaproteobacteria	Candidatus Kentron sp. FW	GTPase SAR1 family protein [Candidatus Kentron sp. FW].	-
VFJ65256.1	AP-GTPase+COR+TM+TM*→	AP-GTPase+COR+TM+TM	523	BECKFW1821A_GA0114235_11961	Gammaproteobacteria	Candidatus Kentron sp. FW	hypothetical protein BECKFW1821A_GA0114235_11961, partial [Candidatus Kentron sp. FW].	-
VFJ68931.1	AP-GTPase+COR+TM+TM*→	AP-GTPase+COR+TM+TM	948	BECKFM1743C_GA0114222_105041	Gammaproteobacteria	Candidatus Kentron sp. FM	Ras of Complex, Roc, domain of DAPkinase [Candidatus Kentron sp. FM].	-
VFJ70290.1	AP-GTPase+COR+TM+TM*→	AP-GTPase+COR+TM+TM	787	BECKFM1743C_GA0114222_105511	Gammaproteobacteria	Candidatus Kentron sp. FM	Ras of Complex, Roc, domain of DAPkinase [Candidatus Kentron sp. FM].	-
VFJ72898.1	AP-GTPase+COR+NACHT*→	AP-GTPase+COR+NACHT	1882	BECKFW1821C_GA0114237_10407	Gammaproteobacteria	Candidatus Kentron sp. FW	Pentapeptide repeat-containing protein [Candidatus Kentron sp. FW].	-
VFJ73192.1	AP-GTPase+COR+TM+TM*→	AP-GTPase+COR+TM+TM	770	BECKFW1821C_GA0114237_104627	Gammaproteobacteria	Candidatus Kentron sp. FW	GTPase SAR1 family protein, partial [Candidatus Kentron sp. FW].	-
VFJ76094.1	AP-GTPase+COR+TM+TM*→	AP-GTPase+COR+TM+TM	834	BECKFW1821C_GA0114237_10987	Gammaproteobacteria	Candidatus Kentron sp. FW	GTPase SAR1 family protein [Candidatus Kentron sp. FW].	-
VFJ95876.1	AP-GTPase+COR+TM+TM*→	AP-GTPase+COR+TM+TM	753	BECKH772A_GA0070896_1009310	Gammaproteobacteria	Candidatus Kentron sp. H	Ras of Complex, Roc, domain of DAPkinase, partial [Candidatus Kentron sp. H].	-

acc	operon	architecture	len	gen.name	taxend	species	defline	gca
VFK01091.1	AP-GTPase+COR*→	AP-GTPase+COR	275	BECKLFY1418B_GA0070995_12271	Gammaproteobacteria	Candidatus Kentron sp. LFY	Ras of Complex, Roc, domain of DAPkinase, partial [Candidatus Kentron sp. LFY].	-
VFK03671.1	AP-GTPase*→	AP-GTPase	118	BECKLFY1418A_GA0070994_13111	Gammaproteobacteria	Candidatus Kentron sp. LFY	Ras of Complex, Roc, domain of DAPkinase, partial [Candidatus Kentron sp. LFY].	-
VFK03687.1	AP-GTPase*→	AP-GTPase	118	BECKLFY1418A_GA0070994_13121	Gammaproteobacteria	Candidatus Kentron sp. LFY	Ras of Complex, Roc, domain of DAPkinase, partial [Candidatus Kentron sp. LFY].	-
VFK04035.1	AP-GTPase+COR+TM+TM*→	AP-GTPase+COR+TM+TM	677	BECKH772B_GA0070898_104021	Gammaproteobacteria	Candidatus Kentron sp. H	Ras of Complex, Roc, domain of DAPkinase, partial [Candidatus Kentron sp. H].	-
VFK07263.1	AP-GTPase+COR+TM+TM*→	AP-GTPase+COR+TM+TM	674	BECKH772C_GA0070978_104041	Gammaproteobacteria	Candidatus Kentron sp. H	Ras of Complex, Roc, domain of DAPkinase, partial [Candidatus Kentron sp. H].	-
VFK11049.1	AP-GTPase+COR+TM+TM*→	AP-GTPase+COR+TM+TM	865	BECKLPF1236A_GA0070988_100486	Gammaproteobacteria	Candidatus Kentron sp. LPFa	Ras of Complex, Roc, domain of DAPkinase [Candidatus Kentron sp. LPFa].	-
VFK19789.1	AP-GTPase+COR+TM+TM*→	AP-GTPase+COR+TM+TM	853	BECKLPF1236B_GA0070989_11862	Gammaproteobacteria	Candidatus Kentron sp. LPFa	Ras of Complex, Roc, domain of DAPkinase [Candidatus Kentron sp. LPFa].	-
VFK19871.1	AP-GTPase*→	AP-GTPase	161	BECKLFY1418C_GA0070996_106423	Gammaproteobacteria	Candidatus Kentron sp. LFY	small GTP-binding protein domain-containing protein, partial [Candidatus Kentron sp. LFY].	-
VFK26330.1	AP-GTPase*→	AP-GTPase	116	BECKLFY1418C_GA0070996_13331	Gammaproteobacteria	Candidatus Kentron sp. LFY	Ras of Complex, Roc, domain of DAPkinase, partial [Candidatus Kentron sp. LFY].	-
VFK27413.1	AP-GTPase+COR+TM+TM*→ CR-ATPase8+CR-REase7→ CR-ATPase8+CR-REase7→ CR-ATPase8+CR-REase7→ CR-ATPase8+CR-REase7→?→||<-?<-ParA-Soj-PloopNTPase	AP-GTPase+COR+TM+TM	893	BECKMB1821I_GA0114274_100324	Gammaproteobacteria	Candidatus Kentron sp. MB	Ras of Complex, Roc, domain of DAPkinase [Candidatus Kentron sp. MB].	-
VFK29755.1	AP-GTPase+COR+TM+TM*→ AP-GTPase+COR+TM+TM→	AP-GTPase+COR+TM+TM	840	BECKMB1821G_GA0114241_10537	Gammaproteobacteria	Candidatus Kentron sp. MB	Ras of Complex, Roc, domain of DAPkinase [Candidatus Kentron sp. MB].	-
VFK30623.1	AP-GTPase+COR+TM+TM*→ CR-ATPase8+CR-REase7→ CR-ATPase8+CR-REase7→ CR-ATPase8+CR-REase7→ CR-ATPase8+CR-REase7→?→||<-?<-ParA-Soj-PloopNTPase	AP-GTPase+COR+TM+TM	718	BECKMB1821G_GA0114241_10681	Gammaproteobacteria	Candidatus Kentron sp. MB	hypothetical protein BECKMB1821G_GA0114241_10681, partial [Candidatus Kentron sp. MB].	-
VFK30872.1	AP-GTPase+COR+TM+TM*→	AP-GTPase+COR+TM+TM	680	BECKMB1821G_GA0114241_10721	Gammaproteobacteria	Candidatus Kentron sp. MB	hypothetical protein BECKMB1821G_GA0114241_10721, partial [Candidatus Kentron sp. MB].	-
VFK31841.1	AP-GTPase*→	AP-GTPase	476	BECKMB1821I_GA0114274_102765	Gammaproteobacteria	Candidatus Kentron sp. MB	Ras of Complex, Roc, domain of DAPkinase [Candidatus Kentron sp. MB].	-
VFK32767.1	AP-GTPase+COR+TM+TM*→	AP-GTPase+COR+TM+TM	875	BECKMB1821I_GA0114274_103604	Gammaproteobacteria	Candidatus Kentron sp. MB	Ras of Complex, Roc, domain of DAPkinase [Candidatus Kentron sp. MB].	-
VFK33354.1	AP-GTPase+COR+TM+TM*→	AP-GTPase+COR+TM+TM	844	BECKMB1821I_GA0114274_104404	Gammaproteobacteria	Candidatus Kentron sp. MB	Ras of Complex, Roc, domain of DAPkinase [Candidatus Kentron sp. MB].	-

acc	operon	architecture	len	gen.name	taxend	species	defline	gca
VFK39913.1	AP-GTPase+COR+TM+TM*→	AP-GTPase+COR+TM+TM	790	BECKTC1821D_GA0114238_100746	Gammaproteobacteria	Candidatus Kentron sp. TC	Ras of Complex, Roc, domain of DAPkinase [Candidatus Kentron sp. TC].	-
VFK39956.1	RADICAL-SAM→ AP-GTPase+COR*→	AP-GTPase+COR	694	BECKSD772F_GA0070984_105016	Gammaproteobacteria	Candidatus Kentron sp. SD	GTPase SAR1 family protein [Candidatus Kentron sp. SD].	-
VFK40684.1	AP-GTPase+COR+TM+TM*→	AP-GTPase+COR+TM+TM	854	BECKSD772F_GA0070984_10665	Gammaproteobacteria	Candidatus Kentron sp. SD	Ras of Complex, Roc, domain of DAPkinase [Candidatus Kentron sp. SD].	-
VFK42825.1	AP-GTPase+COR+TM+TM*→	AP-GTPase+COR+TM+TM	834	BECKTC1821E_GA0114239_102141	Gammaproteobacteria	Candidatus Kentron sp. TC	Ras of Complex, Roc, domain of DAPkinase [Candidatus Kentron sp. TC].	-
VFK53024.1	AP-GTPase+COR+TM+TM*→	AP-GTPase+COR+TM+TM	852	BECK-TUN1418D_GA0071000_101730	Gammaproteobacteria	Candidatus Kentron sp. TUN	Ras of Complex, Roc, domain of DAPkinase [Candidatus Kentron sp. TUN].	-
VFK54740.1	AP-GTPase+COR+TM+TM*→	AP-GTPase+COR+TM+TM	851	BECK-TUN1418F_GA0071002_10532	Gammaproteobacteria	Candidatus Kentron sp. TUN	GTPase SAR1 family protein [Candidatus Kentron sp. TUN].	-
VFK54787.1	CR-ATPase8+CR-REase7+AP-GTPase+COR+TM+TM*→	CR-ATPase8+CR-REase7+AP-GTPase+COR+TM+TM	1117	BECK-TUN1418F_GA0071002_105513	Gammaproteobacteria	Candidatus Kentron sp. TUN	Ras of Complex, Roc, domain of DAPkinase [Candidatus Kentron sp. TUN].	-
VFK55234.1	AP-GTPase+COR+TM+TM*→	AP-GTPase+COR+TM+TM	870	BECK-TUN1418D_GA0071000_102911	Gammaproteobacteria	Candidatus Kentron sp. TUN	GTPase SAR1 family protein [Candidatus Kentron sp. TUN].	-
VFK55404.1	AP-GTPase+COR+TM+TM*→ AP-GTPase+COR+TM+TM→	AP-GTPase+COR+TM+TM	834	BECK-TUN1418E_GA0071001_10293	Gammaproteobacteria	Candidatus Kentron sp. TUN	Ras of Complex, Roc, domain of DAPkinase [Candidatus Kentron sp. TUN].	-
VFK57219.1	AP-GTPase+COR+TM+TM*→	AP-GTPase+COR+TM+TM	799	BECKTC1821F_GA0114240_101546	Gammaproteobacteria	Candidatus Kentron sp. TC	Ras of Complex, Roc, domain of DAPkinase [Candidatus Kentron sp. TC].	-
VFK57233.1	AP-GTPase+COR*→	AP-GTPase+COR	1361	BECK-TUN1418F_GA0071002_11091	Gammaproteobacteria	Candidatus Kentron sp. TUN	Uncharacterized conserved protein, contains ParB-like and HNH nuclease domains [Candidatus Kentron sp. TUN].	-
VFK58480.1	AP-GTPase+COR*→	AP-GTPase+COR	1384	BECK-TUN1418D_GA0071000_10836	Gammaproteobacteria	Candidatus Kentron sp. TUN	Ras of Complex, Roc, domain of DAPkinase [Candidatus Kentron sp. TUN].	-
VFK60758.1	CR-ATPase8+CR-REase7+AP-GTPase*→	CR-ATPase8+CR-REase7+AP-GTPase	877	BECK-UNK1418G_GA0071005_101311	Gammaproteobacteria	Candidatus Kentron sp. UNK	PD-(D/E)XK nuclease superfamily protein [Candidatus Kentron sp. UNK].	-
VFK62743.1	AP-GTPase*→	AP-GTPase	337	BECK-TUN1418D_GA0071000_11856	Gammaproteobacteria	Candidatus Kentron sp. TUN	Ras of Complex, Roc, domain of DAPkinase, partial [Candidatus Kentron sp. TUN].	-
VFK65329.1	AP-GTPase+COR*→	AP-GTPase+COR	1361	BECK-TUN1418E_GA0071001_11051	Gammaproteobacteria	Candidatus Kentron sp. TUN	Uncharacterized conserved protein, contains ParB-like and HNH nuclease domains [Candidatus Kentron sp. TUN].	-
VFK67825.1	BetaPropeller+AP-GTPase*→	BetaPropeller+AP-GTPase	526	BECK-UNK1418G_GA0071005_11685	Gammaproteobacteria	Candidatus Kentron sp. UNK	WD domain-containing protein, G-beta repeat-containing protein [Candidatus Kentron sp. UNK].	-
VFK67855.1	AP-GTPase+COR+TM+TM*→	AP-GTPase+COR+TM+TM	598	BECK-UNK1418G_GA0071005_11705	Gammaproteobacteria	Candidatus Kentron sp. UNK	Ras of Complex, Roc, domain of DAPkinase [Candidatus Kentron sp. UNK].	-

acc	operon	architecture	len	gen.name	taxend	species	defline	gca
VFK77412.1	AP-GTPase+COR+TM+TM*→	AP-GTPase+COR+TM+TM	734	BECKMB1821H_GA0114242_1135[illegible]	Gammaproteobacteria	Candidatus Kentron sp. MB	Ras of Complex, Roc, domain of DAPkinase, partial [Candidatus Kentron sp. MB].	-
VFK80164.1	AP-GTPase+COR+TM+TM*→	AP-GTPase+COR+TM+TM	872	BECKSD772D_GA0070982_10897	Gammaproteobacteria	Candidatus Kentron sp. SD	Ras of Complex, Roc, domain of DAPkinase [Candidatus Kentron sp. SD].	-
VFM99483.1	AP-GTPase+COR+TM+TM*→	AP-GTPase+COR+TM+TM	725	BECKG1743F_GA0114225_10402	Gammaproteobacteria	Candidatus Kentron sp. G	GTPase SAR1 family protein [Candidatus Kentron sp. G].	GCA_900696175.1
VFM99853.1	AP-GTPase+COR+TM+TM*→	AP-GTPase+COR+TM+TM	744	BECKG1743F_GA0114225_10433[illegible]	Gammaproteobacteria	Candidatus Kentron sp. G	small GTP-binding protein domain-containing protein [Candidatus Kentron sp. G].	GCA_900696175.1
WP_002684219.1	AP-GTPase+COR+DUF4404*→	AP-GTPase+COR+DUF4404	883	-	Gammaproteobacteria	Beggiatoa alba	Miro-like protein [Beggiatoa alba].	GCF_000245015.1
WP_009138084.1	AP-GTPase+COR*→	AP-GTPase+COR	843	-	Bacteroidetes	Odoribacter laneus	hypothetical protein [Odoribacter laneus].	GCF_902374615.1
WP_012499245.1	AP-GTPase*→	AP-GTPase	394	-	Chlorobi	Chloroherpeton thalassium	Miro domain-containing protein [Chloroherpeton thalassium].	GCF_000020525.1
WP_020559412.1	GTPase-AIG→ ABC-ATPase→ SIG+TM+TM+TM+TM→ AP-GTPase+COR*→	AP-GTPase+COR	833	-	Gammaproteobacteria	Thiofilum flexile	hypothetical protein [Thiofilum flexile].	GCF_000380185.1
WP_027001878.1	AP-GTPase+COR*→	AP-GTPase+COR	754	-	Bacteroidetes	Hugenholtzia roseola	hypothetical protein [Hugenholtzia roseola].	GCF_000422585.1
WP_040612090.1	AP-GTPase+COR*→	AP-GTPase+COR	849	-	Bacteroidetes	Odoribacter laneus	hypothetical protein [Odoribacter laneus].	-
WP_050025079.1	VWA+AP-GTPase+COR+EAD1+Trypsin*→	VWA+AP-GTPase+COR+EAD1+Trypsin	1458	-	Verrucomicrobia	Verrucomicrobium sp. BvORR034	GTP-binding protein [Verrucomicrobium sp. BvORR034].	-
WP_052368940.1	TIR+BetaPropeller+AP-GTPase*→	TIR+BetaPropeller+AP-GTPase	1107	-	Euryarchaeota	Candidatus Methanoperedens nitroreducens	TIR domain-containing protein [Candidatus Methanoperedens nitroreducens].	GCF_000685155.1
WP_071819957.1	TIR+BetaPropeller+AP-GTPase*→||<-?<-?<-?<-?<-?<-RuvB-AAA+RuvB-HTH	TIR+BetaPropeller+AP-GTPase	1449	-	Cyanobacteria	Acaryochloris marina	TIR domain-containing protein [Acaryochloris marina].	GCF_000018105.1
WP_077027722.1	<-Phytase-like||?→?→ tRNA→||<-?<-?<-?||TIR+BetaPropeller+AP-GTPase*→	TIR+BetaPropeller+AP-GTPase	1401	-	Planctomycetes	Fuerstia marisgermanicae	TIR domain-containing protein [Fuerstia marisgermanicae].	GCF_001983935.1
WP_081716878.1	AP-GTPase+COR+TIR*→	AP-GTPase+COR+TIR	722	-	Cyanobacteria	Leptolyngbya sp. Heron Island J	TIR domain-containing protein [Leptolyngbya sp. Heron Island J].	-
WP_081891667.1	AP-GTPase+COR+TIR*→	AP-GTPase+COR+TIR	825	-	Verrucomicrobia	Verrucomicrobium sp. BvORR106	TIR domain-containing protein [Verrucomicrobium sp. BvORR106].	-
WP_083888126.1	AP-GTPase*→	AP-GTPase	116	-	Cyanobacteria	Oscillatoria nigro-viridis	hypothetical protein [Oscillatoria nigro-viridis].	-
WP_093066994.1	AP-GTPase+COR*→	AP-GTPase+COR	857	-	Gammaproteobacteria	Thiothrix caldifontis	hypothetical protein [Thiothrix caldifontis].	GCF_900107695.1
WP_093068668.1	AP-GTPase+COR*→	AP-GTPase+COR	960	-	Gammaproteobacteria	Thiothrix caldifontis	hypothetical protein [Thiothrix caldifontis].	GCF_900107695.1
WP_093882803.1	SIG+AP-GTPase+COR*→	SIG+AP-GTPase+COR	880	-	Deltaproteobacteria	Syntrophus gentianae	hypothetical protein [Syntrophus gentianae].	GCF_900109885.1
WP_103921835.1	HTH→ AP-GTPase+COR*→	AP-GTPase+COR	576	-	Gammaproteobacteria	Thiotrichales bacterium HS_08	hypothetical protein [Thiotrichales bacterium HS_08].	-
WP_129026230.1	AP-GTPase+COR*→	AP-GTPase+COR	431	-	Bacteroidetes	Flavobacterium sp. YO12	hypothetical protein [Flavobacterium sp. YO12].	GCF_004119495.1

acc	operon	architecture	len	gen.name	taxend	species	defline	gca
WP_137245090.1	TM+AP-GTPase*→	TM+AP-GTPase	93	-	Actinobacteria	Herbidospora galbida	hypothetical protein [Herbidospora galbida].	GCF_005233835.1
WP_139373135.1	AP-GTPase*→	AP-GTPase	134	-	Verrucomicrobia	Prosthecobacter debontii	hypothetical protein [Prosthecobacter debontii].	GCF_900167535.1
WP_140311973.1	AP-GTPase+COR*→	AP-GTPase+COR	697	-	Gammaproteobacteria	Vibrio parahaemolyticus	GTP-binding protein, partial [Vibrio parahaemolyticus].	GCF_006372615.1
WP_144982131.1	TIR+APATPase+TPR+TPR+TPR→?→ AP-GTPase+COR+DUF4404*→	AP-GTPase+COR+DUF4404	692	-	Planctomycetes	Gimesia aquarii	hypothetical protein [Gimesia aquarii].	GCF_007748195.1
WP_145384506.1	AP-GTPase+COR*→	AP-GTPase+COR	680	-	Planctomycetes	Stieleria neptunia	50S ribosome-binding GTPase [Stieleria neptunia].	GCF_007754155.1
WP_146054573.1	AP-GTPase+COR+DUF4404*→	AP-GTPase+COR+DUF4404	731	-	Gammaproteobacteria	Methylovulum psychrotolerans	hypothetical protein [Methylovulum psychrotolerans].	GCF_002923755.1
WP_147662502.1	AP-GTPase*→	AP-GTPase	204	-	Asgard group	Candidatus Prometheoarchaeum syntrophicum	GTP-binding protein [Candidatus Prometheoarchaeum syntrophicum].	GCF_008000775.1
WP_157679487.1	AP-GTPase+COR+DUF4404*→	AP-GTPase+COR+DUF4404	911	-	Gammaproteobacteria	Methylovulum psychrotolerans	hypothetical protein [Methylovulum psychrotolerans].	GCF_002209385.1
WP_157817021.1	<-EAD10+VMAP-M1+VMAP-C||?→||<-PSE||BetaPropeller+AP-GTPase+Calcineurin+CASPASE+NACHT*→	BetaPropeller+AP-GTPase+Calcineurin+CASPASE+NACHT	1731	-	Cyanobacteria	Nostoc flagelliforme	NACHT domain-containing protein [Nostoc flagelliforme].	GCF_002813575.1
WP_160572809.1	AP-GTPase+COR+DUF4404*→	AP-GTPase+COR+DUF4404	720	-	unclassified Bacteria	bacterium D16-29	GTP-binding protein [bacterium D16-29].	-
WP_162669385.1	AP-GTPase+COR+DUF4404*→	AP-GTPase+COR+DUF4404	615	-	Planctomycetes	Gemmata massiliana	hypothetical protein [Gemmata massiliana].	GCF_901538265.1
WP_170962814.1	AP-GTPase+COR*→	AP-GTPase+COR	453	-	Gammaproteobacteria	Vibrio sp. F13	GTP-binding protein [Vibrio sp. F13].	GCF_005146615.1
WP_173978580.1	TIR+BetaPropeller+AP-GTPase*→	TIR+BetaPropeller+AP-GTPase	1154	-	Alphaproteobacteria	Magnetospirillum sp. UT-4	TIR domain-containing protein [Magnetospirillum sp. UT-4].	GCF_902729435.1
WP_175453917.1	AP-GTPase+COR*→	AP-GTPase+COR	757	-	Firmicutes	Sarcina sp. DSM 11001	GTP-binding protein, partial [Sarcina sp. DSM 11001].	GCF_900101015.1
WP_177428378.1	AP-GTPase+COR+CR-ATPase9*→	AP-GTPase+COR+CR-ATPase9	880	-	Gammaproteobacteria	Thiotrichales bacterium HS_08	hypothetical protein [Thiotrichales bacterium HS_08].	-
WP_179276996.1	BetaPropeller+AP-GTPase+TIR*→	BetaPropeller+AP-GTPase+TIR	996	-	Actinobacteria	Actinoplanes regularis	TIR domain-containing protein [Actinoplanes regularis].	GCF_016862355.1
WP_180983884.1	AP-GTPase+COR*→	AP-GTPase+COR	676	-	Gammaproteobacteria	Vibrio agarivorans	50S ribosome-binding GTPase, partial [Vibrio agarivorans].	GCF_002894965.1
WP_186289598.1	AP-GTPase+COR+TM+TM*→	AP-GTPase+COR+TM+TM	905	-	Gammaproteobacteria	Methylomonas koyamae	hypothetical protein [Methylomonas koyamae].	GCF_006483455.1
